# Environmental Pseudomonads Inhibit Cystic Fibrosis Patient-Derived Pseudomonas aeruginosa

**DOI:** 10.1128/AEM.02701-16

**Published:** 2016-12-30

**Authors:** Payel Chatterjee, Elizabeth Davis, Fengan Yu, Sarah James, Julia H. Wildschutte, Daniel D. Wiegmann, David H. Sherman, Robert M. McKay, John J. LiPuma, Hans Wildschutte

**Affiliations:** a Department of Biological Sciences, Bowling Green State University, Bowling Green, Ohio, USA; b Life Sciences Institute and Departments of Medicinal Chemistry, Chemistry and Microbiology & Immunology, University of Michigan, Ann Arbor, Michigan, USA; c Department of Pediatrics, University of Michigan Medical School, Ann Arbor, Michigan, USA; University of Manchester

**Keywords:** Pseudomonas aeruginosa, antagonistic, kynurenine, pseudomonads, siderophore, thioquinolobactin

## Abstract

Pseudomonas aeruginosa is an opportunistic pathogen which is evolving resistance to many currently used antibiotics. While much research has been devoted to the roles of pathogenic P. aeruginosa in cystic fibrosis (CF) patients, less is known of its ecological properties. P. aeruginosa dominates the lungs during chronic infection in CF patients, yet its abundance in some environments is less than that of other diverse groups of pseudomonads. Here, we sought to determine if clinical isolates of P. aeruginosa are vulnerable to environmental pseudomonads that dominate soil and water habitats in one-to-one competitions which may provide a source of inhibitory factors. We isolated a total of 330 pseudomonads from diverse habitats of soil and freshwater ecosystems and competed these strains against one another to determine their capacity for antagonistic activity. Over 900 individual inhibitory events were observed. Extending the analysis to P. aeruginosa isolates revealed that clinical isolates, including ones with increased alginate production, were susceptible to competition by multiple environmental strains. We performed transposon mutagenesis on one isolate and identified an ∼14.8-kb locus involved in antagonistic activity. Only two other environmental isolates were observed to carry the locus, suggesting the presence of additional unique compounds or interactions among other isolates involved in outcompeting P. aeruginosa. This collection of strains represents a source of compounds that are active against multiple pathogenic strains. With the evolution of resistance of P. aeruginosa to currently used antibiotics, these environmental strains provide opportunities for novel compound discovery against drug-resistant clinical strains.

**IMPORTANCE** We demonstrate that clinical CF-derived isolates of P. aeruginosa are susceptible to competition in the presence of environmental pseudomonads. We observed that many diverse environmental strains exhibited varied antagonistic profiles against a panel of clinical P. aeruginosa isolates, suggesting the presence of distinct mechanisms of inhibition among these ecological strains. Understanding the properties of these antagonistic events offers the potential for discoveries of antimicrobial compounds or metabolic pathways important to the development of novel treatments for P. aeruginosa infections.

## INTRODUCTION

Pseudomonas aeruginosa is a Gram-negative opportunistic pathogen responsible for a range of infections (1, 2). Among cystic fibrosis (CF) patients, P. aeruginosa is the dominant pathogen that causes chronic lung infections and life-threatening disease (3–6). Within the CF lung, P. aeruginosa rapidly adapts to the human host (7, 8) through mutations that inactivate regulators controlling quorum sensing (5, 6), motility (9), and the production of outer membrane structures, such as flagella (10, 11) and the O antigen (12, 13), thus evading the immune system and contributing to the survival of this pathogen in the CF patient (4, 5, 14, 15); these are all traits that otherwise benefit bacteria in a native ecological habitat outside the human host. However, the rapid adaptability of P. aeruginosa to the CF lung may come at a fitness cost in the absence of the human host, in turn negatively affecting competitiveness in other environments. Although CF patients can be infected with P. aeruginosa at any age, the majority of children who become infected carry unique genotypes of P. aeruginosa, suggesting that acquisition originates from the environment (16), particularly from nosocomial water sources (17, 18). Thus, understanding the natural life cycle of P. aeruginosa outside the human host may provide insight into important aspects contributing to its survival in the environment.

Pseudomonas spp. are ubiquitous in the environment and capable of persisting in diverse habitats, including soil and in association with plants (19–21) or within freshwater ecosystems (22–24). Environmentally derived pseudomonads are notable for extensive genetic diversity, with reported genomes ranging in size from ∼5 to 6.5 Mbp (21, 25–27). Reflecting such genomic diversity is the collective capability to produce a widely diverse repertoire of secondary metabolites, including nonribosomal peptides, bacteriocins, and quinolones, that have been utilized for important roles ranging from bioremediation or biocontrol (see references 25 and 26 for a review) to the key precursor for the FDA-approved semisynthetic anticancer drug ET-743 (28). For example, pseudomonads have been shown to breakdown refractory recalcitrant compounds, such as chloroanilines (29), insecticides (30), and chitin (31), and they inhibit the growth of plant-pathogenic fungi (32–34), exhibit antitumor activity (35, 36), and inhibit the growth of a wide range of bacteria, including the human-pathogenic pathogens methicillin-resistant Staphylococcus aureus (37, 38) and Mycobacterium tuberculosis (39). Thus, unique selective pressures within soil and water environments likely contribute to the evolution of genomic diversity and the production of diverse natural metabolites that affect bacterial fitness and abundance in such habitats.

Although P. aeruginosa is ubiquitous in the environment and has been isolated from terrestrial (40–42) and aquatic habitats (17, 43, 44), we observe this group to exist in low abundance within soil and water environments. From our sampling efforts, we rarely identify P. aeruginosa when sampling for pseudomonads, which is reflected in other studies that specifically probe for P. aeruginosa using selective media (43–45) or molecular techniques (46–48); this suggests that this species represents a minority of community-derived Pseudomonas populations (i.e., closely related isolates that cluster as distinct phylogenetic groups). Instead, we observe these environments to be dominated by non-aeruginosa Pseudomonas groups (22, 26, 27, 46) whose abundances suggest the possession of traits that are advantageous to survival in that ecological habitat. Traits that could contribute to such fitness effects include the ability to outcompete nearby bacteria through the production of inhibitory factors. In native environments where multiple groups of closely related pseudomonads are in sustained populations, we predict that antagonistic strains may provide a source of novel antibiotics. Thus, we sought to identify if clinical isolates of P. aeruginosa are susceptible to environmental strains that dominate soil and water habitats and probed their activity for inhibitory compounds.

In this study, we investigated the outcome patterns of one-to-one competitions between environmental Pseudomonas strains (env-Ps) and CF-derived clinical Pseudomonas aeruginosa isolates (CF-Ps) as a means to identify potential producers of inhibitory factors to CF-Ps. We first analyzed the population structure of a collection of 330 wild pseudomonads isolated from freshwater and soil based on the *gyrB* gene sequence and then assessed the ability of individuals to inhibit other isolates. Extending the analysis to a panel of 33 unrelated CF-Ps revealed several such env-Ps that displayed the ability to inhibit both environmental and clinical stains. Transposon (Tn) mutagenesis and genome sequencing were performed on one environmental isolate that inhibited multiple CF-Ps. A loss-of-inhibition mutant led to the identification of an ∼14.8-kb genomic region that has been shown to be involved in tryptophan catabolism and the production of thioquinolobactin (TQB) (49), a compound that exhibits antifungal and oomycete activity (50). Further PCR screens of TQB metabolic gene products indicated that just 3 closely related antagonistic strains encoded this product. These data suggest such that wild pseudomonads may represent a potential source of diverse compounds that are capable of inhibiting clinical CF-Ps.

## RESULTS

### Population-level diversity of wild pseudomonads.

We explored the diversity of naturally occurring pseudomonads sampled across different seasons and from soil and freshwater ecosystem habitats. Given the unique physical state of these distinct habitats, we reasoned that bacteria isolated from different environments should encode unique metabolic pathways capable of producing diverse secondary metabolites. As a warm monomictic lake, Lake Erie is thermally stratified during the summer but undergoes complete mixing in the fall and throughout the winter (51), increasing the potential for competitive interactions between diverse microbes, for example, in gaining access to resources and nutrients. Pseudomonads adapted to such a freshwater environment may maintain a collection of diverse secreted compounds in comparison to pseudomonads that are otherwise adapted to a warmer solid-state soil environment in which the diffusion of secreted products and local interactions are decreased.

**FIG 1:**
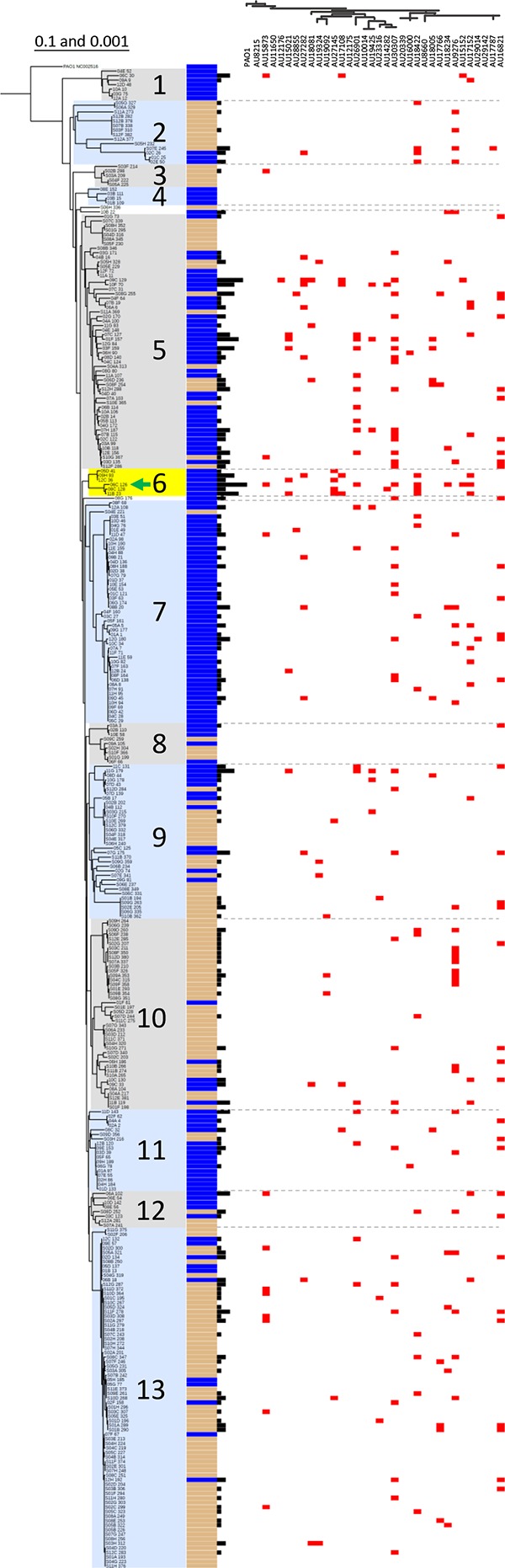
Phylogenetic analysis and antagonistic activity among environmental pseudomonads. Population structure for 330 environmental pseudomonads by neighbor-joining analysis of the *gyrB* sequence, overlain with data for habitat (inner columns: cream color, soil; blue, water) and antagonistic activity (outer bars: black). The magnitude of antagonism is indicated by bar height. Populations are shaded and numbered. Population 6 (highlighted in yellow) is enriched with strains that antagonize multiple environmental and CF-derived isolates. Strain 06C 126 was used for transposon mutagenesis (indicated by the green arrow). Red squares indicate inhibition of CF-derived P. aeruginosa. Dotted gray lines delineate population boundaries. The top *x* axis consists of the pathogenic P. aeruginosa strain phylogeny based on the *gyrB* gene sequence. Both trees were rooted by P. aeruginosa PAO1. The 0.1 and 0.001 represent bar scale values of diversity among environmental and clinical strains, respectively.

As an initial examination of Pseudomonas population-level diversity, a neighbor-joining analysis of the *gyrB* housekeeping gene was performed to determine the genetic variability of the strains isolated from freshwater and soil habitats. Each of 330 env-Ps, consisting of 163 and 167 water- and soil-derived strains, respectively, was partitioned according to *gyrB* gene sequence, and a phylogenetic tree was constructed and rooted using P. aeruginosa PAO1 to visualize the collective population structure, as indicated by number in Fig. 1. Thirteen populations consisting of three or more strains were identified based on nucleotide divergence and branching patterns. To investigate the ecological distribution of natural Pseudomonas isolates by population, data corresponding to derived habitats were superimposed onto the *gyrB*-based phylogeny (Fig. 1). We observed that isolates from populations 2, 3, 10, and 13 derived primarily from the soil, while populations 1, 4, 6, 7, and 11 included isolates almost exclusively derived from water, suggesting that distinct groups tend to be found in one of either habitat. We also observed strains isolated from different habitats that were closely related, for example, the presence of both water- and soil-derived strains in populations 5, 8, 9, and 12. Taken together, these results confirm that genetic diversity exists among wild pseudomonads. No P. aeruginosa strains were isolated in our sampling efforts.

### Environmental pseudomonads exhibit antagonistic activity.

**FIG 2:**
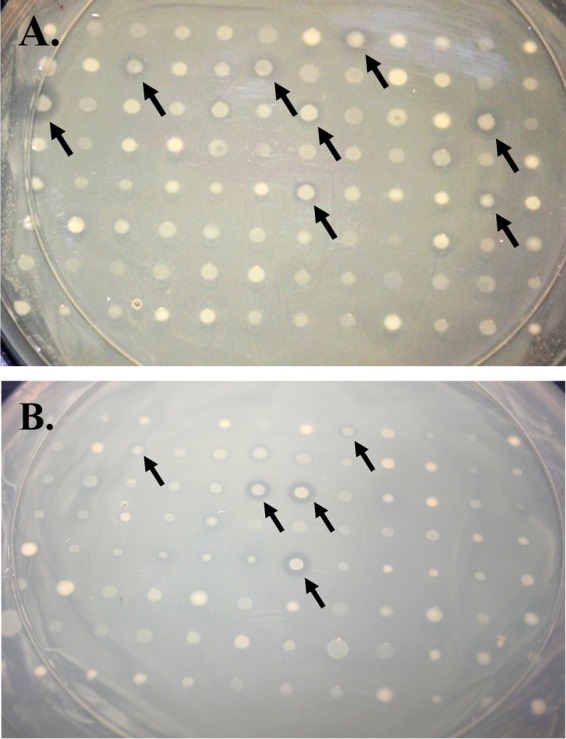
Competition plate assay used to determine antagonistic activity among isolates. One strain was spread plated, and 96 other isolates were replicated to it. Strains were cogrown and observed for competitive interactions indicated by a zone of clearing (indicated by arrows). (A and B) Inhibition by water-derived isolates on an environmental (A) or pathogenic (B) P. aeruginosa strain.

As a means to assess competition, we utilized a plate-based assay in which env-Ps are cogrown in one-to-one competition. In this assay, antagonistic activity exerted by one strain is inferred by a zone of clearing that extends at least 1 mm from the colony edge (Fig. 2A). Individual strains from either water or soil habitat were tested against one another, as those isolates would most likely face competition from strains within their respective habitats. Thus, all 167 soil-derived strains were competed against each other in a pairwise fashion using a high-throughput plate assay resulting in 27,889 one-to-one interactions, and all 163 strains isolated from freshwater were competed against each other for 26,569 interactions.

**FIG 3:**
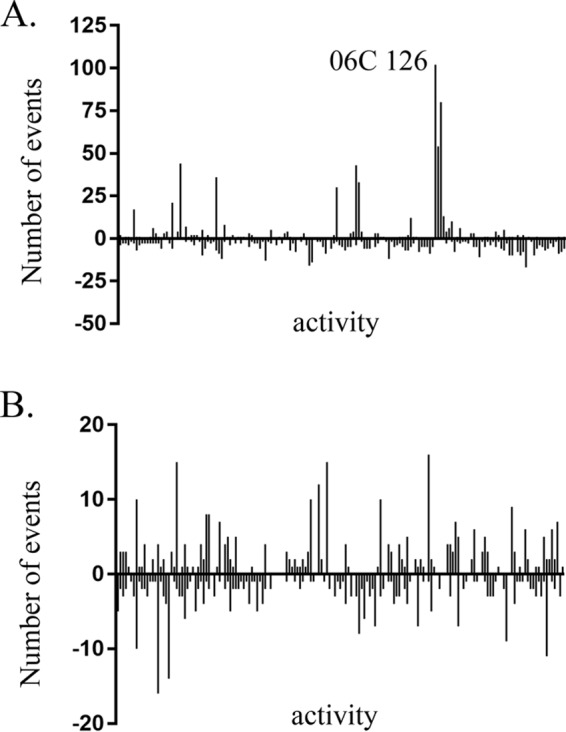
Isolates from water and soil environments were competed against intrahabitat strains in a pairwise fashion, as shown in Fig. 2A. (A) A total of 691 antagonistic events (positive events) were observed among 82 water isolates; strain 06C 126 from population 6 antagonized 102 other strains. From all antagonistic events, 146 strains were susceptible to killing (negative events). The mean number of antagonistic events per strains was 4.0. (B) Among soil-derived strains, 295 antagonistic events were observed among 88 isolates (positive events), and 116 were susceptible to killing (negative events). The mean number of antagonistic events per strains was 2.1.

From all possible interactions, a total of 691 and 295 antagonistic events were observed from 82 water-derived and 88 soil-derived strains, respectively (Fig. 3 and Table 1). Among strains isolated from water, 50 (∼61%) strains were observed to inhibit at least two other strains. Nine of these strains inhibited the growth of >20 other isolates, of which 3 were notably capable of inhibiting 236 (∼72%) of the tested water-derived strains (Fig. 3). From the soil habitat, 61 strains (∼69.3%) were able to inhibit at least two other isolates, and eight strains had antagonistic activity toward >10 other strains (Fig. 3). The mean numbers of strains antagonized by water and soil isolates were 4.0 and 2.1, respectively. These results suggest that env-Ps express the ability to engage in competitive interactions. From both soil and water environments, strains were shown to exhibit antagonistic activity with specific (a single natural isolate that antagonizes one other strain) and broad (one strain antagonizing multiple isolates) host ranges. Specific activity may result from bacteriocin production (52), while a broad range in activity may result from nonribosomal peptides, phenazines, and quinolones, which Pseudomonas are known to produce (25). From these antagonistic events, 146 and 116 water- and soil-derived strains were inhibited, respectively (Fig. 3). No significant difference was observed in a comparison of the distribution of water and soil antagonism profiles (Fig. S1); however, a difference was observed (*P* < 0.0001) in the number of inhibitory events, suggesting that water isolates are more susceptible to inhibition (Fig. S2). Thus, given the population-level genetic diversity and range in antagonistic phenotypes and inhibition, our data suggest that a variety of factors are expressed in this strain collection. We next tested the hypothesis that environmental pseudomonads inhibit P. aeruginosa strains.

**TABLE 1:** Antagonistic events observed from Pseudomonas

Population (no. of strains)	No. of strains by habitat	No. of antagonistic events against strains by type	
		Environmental	Pathogenic P. aeruginosa
1 (7)	7 water	1	4
	0 soil		
2 (14)	3 water	45	4
	11 soil	10	6
3 (5)	0 water		
	5 soil	12	1
4 (4)	4 water	3	
	0 soil		
5 (56)	38 water	153	54
	18 soil	35	16
6 (6)	6 water	270	22
	0 soil		
7 (49)	48 water	151	37
	1 soil		
8 (9)	5 water	2	1
	4 soil	4	
9 (34)	12 water	19	12
	22 soil	63	10
10 (42)	6 water	9	8
	36 soil	43	20
11 (18)	16 water	4	11
	2 soil	15	1
12 (8)	5 water	3	4
	3 soil		2
13 (75)	11 water	27	8
	64 soil	113	38
Undefined (3)	2 water	4	4
	1 soil		
Total (330)	163 water	691	169
	167 soil	295	94
	Combined	986	263

### Environmental pseudomonads inhibit pathogenic P. aeruginosa.

**FIG 4:**
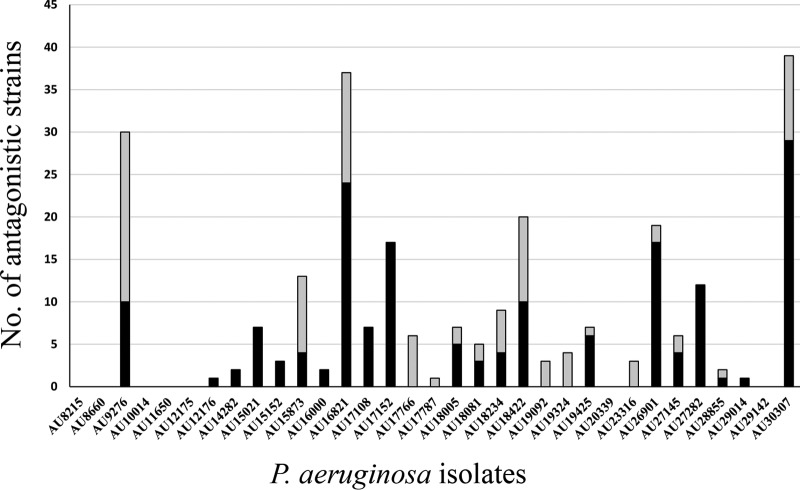
Pathogenic P. aeruginosa isolates antagonized by environmental strains. The competition plate assay was used to determine antagonistic activity. In all, 263 inhibitory events were observed from 88 and 67 water- and soil-derived strains, respectively. One hundred sixty-nine and 94 events were from water-derived (black bars) and soil-derived (gray bars) strains, respectively. Twenty-six out of 33 P. aeruginosa isolates were inhibited by at least one strain, and 23 P. aeruginosa isolates were inhibited by more than one environmental strain. The mean numbers of inhibitory events from water and soil strains were 5.1 and 2.8, respectively.

We competed all 330 env-Ps against a panel of 33 CF-Ps to determine if natural isolates could inhibit P. aeruginosa (Fig. 2B). For these purposes, we utilized a collection of 33 pathogenic strains that were isolated from 32 unrelated patients in 12 cities in the United States between 2004 and 2014. From 10,890 one-to-one competitions, we observed a range of susceptibility and antagonistic effects toward CF-Ps. A total of 263 antagonistic activities were collectively observed from 156 env-Ps (97/163 [∼60.0%] water derived; 68/167 [∼40.7%] soil derived) that directly inhibited the growth of 26/33 (∼78.8%) CF-Ps (antagonistic events are plotted in red between individuals in Fig. 1). Of the 263 antagonistic activities, 169 and 94 inhibitory events to CF-Ps were from water and soil strains (Fig. 4), respectively. This CF-Ps susceptibility reflects the intrahabitat vulnerability observed among env-Ps, whereby CF-Ps are significantly more susceptible to water than soil-derived env-Ps (*P* < 0.0001, Fig. S2). The majority of individual CF-Ps susceptibility was from multiple environmental strains. For example, strains AU9276, AU16821, and AU30307 were inhibited by 30, 37, and 39 env-Ps from both habitats, respectively (Fig. 4), while seven and five CF-Ps were inhibited by water-derived and soil-derived env-Ps alone, respectively. The mean number of env-Ps that inhibited a CF-P was 4.0 from water strains and 2.1 from soil isolates. The variability observed in the susceptibility of both CF-Ps to inhibition by these environmental strains and the differential ability of env-Ps to exert antagonistic effects suggest the presence of distinct mechanisms involved in these interactions. Consistent with these observations, 7 of the 33 clinical isolates were not inhibited by any environmental strains from this study, indicating resistance mechanisms in those isolates to inhibitory factors from the tested environmental strains.

### Identification of a locus involved in antagonistic activity.

**FIG 5:**
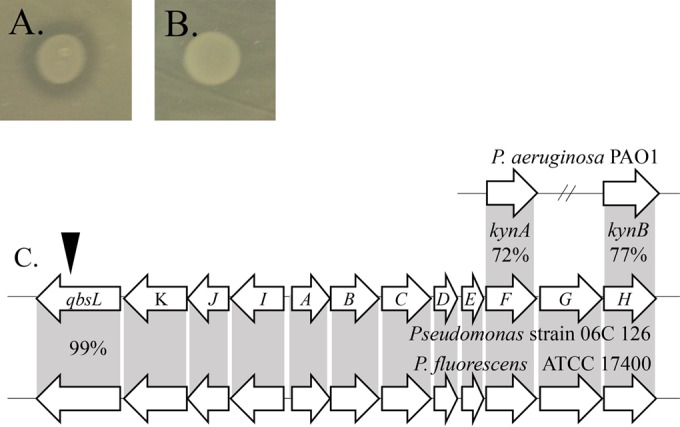
Loss of antagonistic phenotype by transposon insertion in the quinolobactin gene region in Pseudomonas strain 06C 126. (A and B) Wild-type (A) and mutant (B) strains showing a loss-of-inhibition phenotype. (C) The position of the transposon insertion in the *qbsL* gene is indicated by the arrow. The *qbs* locus nucleotide sequence is 99% similar to the region found in P. fluorescens ATCC 17400. The *qbsF* and *qbsH* genes that are involved in the kynurenine pathway are homologous to *kynA* and *kynB* in P. aeruginosa PAO1, with 72% and 77% nucleotide similarity, respectively.

Population 6 (Fig. 1, shaded in yellow) consisted of water-derived env-Ps that were collectively able to inhibit 270 environmental and 22 pathogenic strains (Table 1), suggesting that this group encodes effective mechanisms involved in antagonizing other pseudomonads. In order to identify and begin to characterize putative genetic loci involved in antagonistic abilities toward CF-Ps, we selected one environmental isolate from population 6, strain 06C 126, for its ability to exert antagonistic effects against 102 environmental (Fig. 3) and 7 clinical strains (Fig. 1, green arrow). We optimized a Tn mutagenesis assay for this strain and screened for a loss of antagonistic effect in the presence of the susceptible P. aeruginosa strain AU18234 (Fig. 5A). In screening ∼10,000 mutants, a mutant was identified that lost the ability to inhibit growth of the P. aeruginosa AU18234 (Fig. 5A and B). Through linker-mediated PCR of the Tn flanking region coupled with genome sequencing of strain 06C 126, we confirmed insertion of the Tn within the ∼14.8-kb *qbs* locus, specifically, within the *qbsL* gene that encodes an AMP ligase within that cluster (Fig. 5C). All genes in the *qbs* locus of 06C 126 were 99% similar at the nucleotide level to the Pseudomonas fluorescens ATCC 17400 *qbs* gene cluster that has been shown to encode thioquinolobactin (TQB), a siderophore with antifungal and oomycete activities (49, 50). Because Pseudomonas strains are known for their ability to produce siderophores, we reasoned that other strains might contain the *qbs* gene cluster. To determine population-level presence of this locus, primers were designed utilizing conserved nucleotide sequences from strain 06C 126 and P. fluorescens ATCC 17400 to amplify the *qbsL* and *qbsB* (kynurenine transaminase [KTM]) genes within the *qbs* gene cluster, and all 330 environmental and pathogenic P. aeruginosa strains were subjected to PCR screening of the locus. Positive PCRs were obtained for three of the 330 isolates tested and confirmed by sequencing, suggesting the *qbs* gene cluster is present in these isolates. Interestingly, the three strains were found to cluster within the population 6 clade with strain 06C 126 (Fig. 1) and exhibited overlapping, but not identical, killing profiles against CF-derived pathogens. This observation in similar antagonistic profiles is not unexpected, as diverse activity among closely related strains has previously been observed in other model systems (53–56). These data indicate the involvement of additional factors or resistance mechanisms in the antagonistic activity observed from these strains. Further investigation is required to determine the extent to which the *qbs* gene cluster and its products are involved in antagonistic activity.

## DISCUSSION

Interactions among env-Ps and P. aeruginosa isolates are not well characterized. Here, we investigate if interactions among env-Ps may produce antagonistic factors that inhibit P. aeruginosa. In order for natural populations of pseudomonads to be a source of diverse inhibitory factors, we would predict env-Ps to (i) consist of genetically diverse populations in ecological habitats, (ii) exhibit dissimilar antagonistic properties, and (iii) inhibit P. aeruginosa. To address these points, we performed a population-level analysis of env-Ps in which strains were isolated from diverse environments, genetically characterized, and tested for their ability to inhibit a panel of pathogenic CF-Ps. We observed genetic diversity across environmental pseudomonads as inferred through diversity of the *gyrB* gene, from which strains were clustered into 13 distinct populations (Fig. 1). The diversity observed among these isolates is similar to that observed in other bacterial groups, such as marine Vibrio spp., for which population diversity not only exists, but certain groups also persist over years (57, 58) and are found in association with particular habitats, facilitating adaptation to a niche (59, 60). Thus, we reasoned that env-Ps from a broad context of water and soil ecosystems should be better adapted to native conditions and produce diverse compounds that facilitate their persistence in such habitats.

As a measure of between-strain competition, we first assessed the abilities of environmental pseudomonads to exhibit antagonistic activity against other environmental strains. Our results show that, collectively, 986 antagonistic events occurred from strains within all populations (Table 1). There was no significant difference in intrahabitat antagonistic activity (Fig. S1), and we observed both single events of antagonistic display (i.e., specific) and strains exerting activity to multiple susceptible strains (i.e., broad-range interactions). Most activity was observed from water-derived strains within populations 5, 6, and 7, which contributed to 574 out of 986 events observed (Fig. 1 and Table 1). Notably, population 6 consisted of just 6 strains and produced 270 inhibitory events. Given such interactions and variability in antagonistic activity, these observations suggest that inhibitory factors are produced among pseudomonads in an environment. We then extended the analysis to CF-Ps for two reasons. First, in our repeated sampling efforts, non-aeruginosa Pseudomonas populations are recovered in an extreme majority, consistent with increased fitness of these groups in ecological habitats. Second, our approach offers the potential for a source of discovery of natural antimicrobial products, raising the possibility of alternative treatments for cystic fibrosis. For these purposes, a panel of 33 CF-Ps was subjected to one-to-one competition against all env-Ps.

In all, 263 antagonistic events were observed against CF-Ps from 169 and 94 interactions with water- and soil-derived isolates, respectively (Table 1 and Fig. 1). Water isolates from populations 5, 7, and 10 contributed to the most antagonistic activity, with 115 out of 169 observed events against pathogens (Fig. 1). Thirty-eight antagonistic events were observed from within population 13, which consisted of the largest group and mostly of soil isolates (Table 1 and Fig. 1). Within all populations, except 4, which does not inhibit any P. aeruginosa, diverse antagonistic profiles are observed, suggesting that dissimilar interactions or unique compounds are produced by env-Ps. Inhibition was also observed against P. aeruginosa strains AU9276, AU27145, and AU30307, which express a robust alginate phenotype, a characteristic hallmark of P. aeruginosa pathogens isolated from CF patients that contributes to increasing antibiotic resistance. Interestingly, AU9276 and AU30307 were two of the most susceptible strains by competition and were antagonized by 30 and 39 environmental strains, respectively (Fig. 4), indicating that various environmental isolates produce factors that inhibit these biofilm-producing CF-Ps. However, CF-Ps were significantly more susceptible to water than soil strains, suggesting that env-Ps in the water column (Fig. S2) may express more active compounds against clinical isolates than soil env-Ps under the conditions we tested. Given our observations of antagonistic activity among env-Ps (Fig. 3), and that CF-Ps are susceptible to a host range of multiple env-Ps (Fig. 4), we predict that diverse compounds are produced among these isolates that have the remarkable ability to inhibit clinically derived CF-Ps.

We identified one compound with clear evidence of involvement in antagonistic activity in env-P strain 06C 126 that inhibited multiple env-Ps and CF-Ps (Fig. 5C). Based on Tn mutagenesis for a loss-of-phenotype mutant coupled with genome sequencing of the wild-type strain, an ∼14.8-kb locus was identified whose gene cluster is homologous to the *qbs* locus in P. fluorescens ATCC 17400 that produces thioquinolobactin (TQB), a low-affinity siderophore that inhibits fungal and oomycete plant pathogens (50). TQB is derived from the kynurenine pathway that breaks down tryptophan through the intermediate xanthurenic acid (49). Our results suggest that TQB also acts as an inhibitor of CF-Ps and offer two possible explanations for competition involving the *qbs* gene locus. First, during chronic lung infections, P. aeruginosa has shown to downregulate siderophore production and modify the flux of extracellular heme (61). These changes may increase fitness in the CF lung but confer a cost against environmental strains that efficiently bind iron. Second, although P. aeruginosa encodes the kynurenine pathway, tryptophan metabolism is facilitated through anthranilic acid, which is further converted to Krebs cycle intermediates (38, 62) and signaling factors, such as the Pseudomonas quinolone signal (PQS) molecule. PQS is involved in virulence gene regulation (63–65) during infection (66), implying that this pathway may contribute to the fitness of P. aeruginosa in the CF lung. Thus, adaptation of P. aeruginosa to a CF host environment may confer a cost of vulnerability to env-Ps.

Based on the genetic diversity of the env-Ps and their wide range in antagonistic activities against CF-Ps, our data suggest that pseudomonads produce a variety of antagonistic factors. Some env-Ps exhibit strain-specific activity against CF-Ps, possibly resulting from the production of bacteriocins or encoded prophages that exhibit a narrow range of host activity; alternatively, other strains, such as 06C 126, produce factors that have a wide range of effects and can inhibit multiple CF-Ps, including increased alginate producers. Thus, this strain collection represents a source of potentially unique and unexplored compounds that have the ability to inhibit CF-Ps. Because P. aeruginosa and other pathogens are evolving resistance to many currently used antibiotics, environmental bacteria should provide a potential source of antibiotic discovery. Uncovering the mechanisms of such inhibition should impact future applications to CF treatment, for example, from novel antibiotic discovery or alternative treatments involving phage and bacterial therapies. A comprehensive investigation into the mechanisms and products involved these antagonistic events is being pursued to not only understand ecological contexts but to uncover alternative treatments for CF patients and a better understanding of P. aeruginosa.

## MATERIALS AND METHODS

### Strain isolation and growth conditions.

The Lake Erie Central Basin was sampled during surveys aboard the Canadian Coast Guard Ship (CCGS) *Griffon* during February 2011 and 2012. Water samples were obtained from the Central Basin station EC 880 in February 2012. During occupation of station EC 880 (41°55′00″N, 81°38′00″W), samples were collected from the photic zone at a depth of 1 m and a temperature of 1.5°C using a 10-liter Niskin bottle on a metered winch. Bacteria were concentrated on Supor 200 0.2-μm-pore 47-mm single-wrapped filters (Pall Corporation) and then cultured on cetrimide agar (Fluka Analytical) at 23°C and 37°C to select for pseudomonads. Soil samples were obtained from Bowling Green, OH (41°22′9.8652″N, 83°39′5.8212″W), in April 2012. Samples were obtained from the H horizon at a depth of 1 in. and a temperature of 63°C. One gram of soil was resuspended in 10 ml of sterile water, homogenized, and passed over 0.2-μm-pore filters. To purify strains, colonies were picked and successively restreaked two times to sterile Nutrient broth (NB) solid medium (BD Difco) with 1.5% agar (BD Difco). This isolation and sequencing strategy yielded 163 and 167 water- and soil-derived isolates, respectively, per sampling effort, for a collection of 330 culturable isolates of env-Ps used in this study. Pseudomonas strain 06C 126 was a water-derived strain isolated from Lake Erie station EC 880 in February 2012 and used for genomic sequencing and Tn mutagenesis.

CF-Ps were from obtained from the strain collection of the Burkholderia cepacia Research Laboratory and Repository (BcRLR) at the University of Michigan. These strains had been sent to the BcRLR after being identified as P. aeruginosa by referring clinical microbiology laboratories in the United States. At the BcRLR, all were confirmed as P. aeruginosa by using a 16S rRNA gene-targeted species-specific PCR assay, as previously described (67). Environmental or clinical Pseudomonas strains were grown at 23°C or 37°C, respectively, in liquid or on agar NB medium. For Tn mutagenesis (described further below), Pseudomonas was grown in NB, Escherichia coli helper strain HB101 was grown in lysate broth (LB) liquid medium with 150 μg/ml ampicillin (Ap), and strain CC118 carrying pBAM1 was grown in LB with 50 μg/ml kanamycin (Km) and 30 μg/ml chloramphenicol (Cm), as previously described (68). E. coli strains were incubated at 37°C.

### Gene sequencing and phylogenetic analysis.

For gene sequencing, bacterial strains were grown in liquid culture for 2 days in NB at 23°C with shaking. A 10-μl sample was treated with Lyse-N-Go (Thermo Fisher Scientific) in order to extract and prepare genomic DNA as a template for PCR. Primers targeting the *gyrB* gene (*gyrB* 271 forward [5′-TCB GCR GCV GAR GTS ATC ATG AC-3′], and *gyrB* 1022 reverse [5′-TTG TCY TTG GTC TGS GAG CTG AA-3′]) were used to amplify and sequence a 751-bp region. The PCR conditions were 30 cycles of 92°C denaturing for 120 s, 65°C annealing for 30 s, and elongation at 72°C for 90 s. A nucleotide alignment was generated from 656 bp of the *gyrB* gene, and a neighbor-joining tree was then constructed using Jukes-Cantor nucleotide distance measurement in CLC Main Workbench (CLC bio, Qiagen). Bootstrapping was performed in 100 replicates. The iTOL program was used to view the tree and overlay data corresponding to antagonistic activity (69). For TQB gene screening, primers targeting the *qbsL* gene (*qbsL* 2176542 forward [5′-AGA ATC TGG CTC ATG ATC AC-3′] and *qbsL* 2177569 reverse [5′-GTG AGT TGG AAA TGC TCT TG-3′]) and *qbsB* (*qbsB* 2185019 forward [5′-ACT TCT TTA GAA ACA GCC AG-3′] and *qbsB* 2185875 reverse [5′-CAT GGA ATG TCC CGT GAT TG-3′]) were used to amplify and sequence a 1,027- and 856-bp region, respectively. The PCR conditions for both *qbs* genes were 34 cycles of 94°C denaturing for 120 s, 54°C annealing for 30 s, and elongation at 72°C for 60 s.

### Antagonistic activity.

env-Ps were cultured overnight for 20 h in NB medium prior to the assay. To generate a bacterial lawn, 50 μl of a single culture was spread on NB agar plates. Subsequently, a 1-μl aliquot of each environmental strain culture was transferred to the lawn from 96-deep-well plates using a replicator (Boekel Microplate Replicator). Antagonistic activity was assessed by the presence and magnitude of zones of clearing and was recorded 20 h after incubation at 24°C. To test for false-positive results, all inhibitory strains were selected and the assay replicated at least three times against all pathogens. Antagonistic assays involving CF-Ps were performed similarly, with the exception that after 24 h of incubation at 24°C, the temperature of the plates was shifted to 37°C, and the plates were incubated for 48 h. The two-sample Kolmogorov-Smirnov test was used to determine if there were differences in the distribution of env-Ps antagonistic events and susceptibility of env-Ps and CF-Ps.

### Genome sequencing of strain 06C 126.

Genomic DNA was extracted using the Wizard genomic DNA purification kit (Promega). DNA samples were sheared to approximately 500 nucleotides (nt) average fragment size, and then Illumina-compatible sequencing libraries were prepared from those fragments on an Apollo 324 robotic workstation (WaferGen Biosystems), using the Kapa high-throughput plate (HTP) library preparation kit (Kapa Biosystems), according to the manufacturer's protocols. Subsequent libraries were sequenced on an Illumina HiSeq 2000, obtaining paired-end sequence data with 100-nt reads at each end, as per the recommended protocols from Illumina, Inc. The genome was assembled *de novo* using Velvet 1.2.10 (70), with parameters using a k-mer of 71, and then assembled with coverage cutoff of 10 and expected coverage of 20.

### Transposon mutagenesis.

Triparental mating was used to deliver the Tn*5* minitransposon from E. coli strain CC118 with helper strain HB101 to Pseudomonas strain 06C 126 (68). E. coli and Pseudomonas strains were cultured overnight, as described above. Cells were washed with 1 ml of 10 mM MgSO_4_ to remove any traces of antibiotics. The optical density at 600 nm (OD_600_) of the three cultures was then adjusted to 0.03, and the cells were mixed in a 1:1:1 ratio into 5 ml of 10 mM MgSO_4_. The mating mixture was then vortexed and the resuspension passed over a 0.45-μm-pore filter disk with a 47-mm diameter (Whatman). The filter was placed onto solid NB agar and incubated overnight at 30°C. Following incubation, the filter was transferred to a culture tube in 5.0 ml of 10 mM MgSO_4_ solution and vortexed to resuspension, and 100 μl was then plated onto solid cetrimide agar with 50 μg/ml Km to select for Pseudomonas transconjugants. Transconjugants were replica plated onto a sensitive P. aeruginosa strain and screened for mutants exhibiting a loss-of-antagonism phenotype.

### Mutant DNA extraction and linker-mediated PCR.

Genomic DNA was extracted from the 06C 126 mutant using the Wizard genomic DNA purification kit (Promega). Two micrograms of genomic DNA was digested using restriction enzymes PvuII, ScaI, SmaI, and SspI from New England BioLabs, according to their protocols. The fragmented products were purified using the NucleoSpin gel and PCR Clean-Up kit (Macherey-Nagel). The resulting purified digested DNA was ligated to 4 μM annealed linker PCR primers BPHI (5′-CAA GGA AGG ACG CTG TCT GTC GAA GGT AAG GAA CGG ACG AGA GAA GGG AGA G-3′) and BPHII (5′-CTC TCC CTT TCG AAT CGT AAC CGT TCG TAC GAG AAT CGC TGT CCT CTC CTT G-3′) using T4 DNA ligase. Purification of the ligation was done according to the manufacturer's protocol (Macherey-Nagel). Linker-mediated PCR (LM-PCR) was performed in two cycles. LM-PCR I was performed using 2 μl of ligated DNA and 5 μM primers 224 (5′-CGA ATC GTA CCG TTC GTA CGA GAA TCG CT-3′) and Tn primer 1 pBAM1 3424 Rev (5′-ATC CAT GTT GCT GTT CAG AC-3′). The PCR conditions for the bridge PCR (BPCR) I reaction were 19 cycles of 92°C denaturing for 10 s, 50°C annealing for 60 s, and elongation at 72°C for 90 s. One microliter of LM-PCR I products was used as the template for LM-PCR II using primer 224 (5′-CGA ATC GTA CCG TTC GTA CGA GAA TCG CT-3′) and Tn primer 2 pBAM1 3373 Rev (5′-ATG GCT CAT AAC ACC CCT TG-3′). The PCR conditions for the LM-PCR II reactions were 34 cycles of 92°C for 10 s, 55°C for 30 s, and 72°C for 90 s. Sequencing was performed using primers 224 and pBAM1 3373 Rev at the University of Chicago Comprehensive Cancer Center DNA Sequencing and Genotyping facility.

### Accession number(s).

The NCBI accession number for the wild-type 06C 126 strain is MIFU00000000.1.

## Supplementary Material

Supplemental material
