# Supplementary material for: Environmental Pseudomonads Inhibit Cystic Fibrosis Patient-Derived Pseudomonas aeruginosa
Source: Appl Environ Microbiol. 2016 Dec 30;83(2):e02701-16. doi: 10.1128/AEM.02701-16 (PMC5203635; doi:10.1128/AEM.02701-16)
Supplement: Supplemental material [file supp_83_2_e02701-16__index.html]

Environmental Pseudomonads Inhibit Cystic Fibrosis Patient-Derived Pseudomonas aeruginosa — Supplemental material 

# Environmental Pseudomonads Inhibit Cystic Fibrosis Patient-Derived Pseudomonas aeruginosa

## Supplemental material

- Supplemental file 1 -

  The antagonistic distributions for env-Ps (Fig. S1), susceptibility distributions for env-Ps and CF-Ps (Fig. S2), and gene and contig sequences.

  PDF, 2.8M
