# Supplementary material for: Environmental Pseudomonads Inhibit Cystic Fibrosis Patient-Derived Pseudomonas aeruginosa
Source: Appl Environ Microbiol. 2016 Dec 30;83(2):e02701-16. doi: 10.1128/AEM.02701-16 (PMC5203635; doi:10.1128/AEM.02701-16)
Supplement: Supplemental material [file AEM.02701-16_zam999117603s1.pdf]

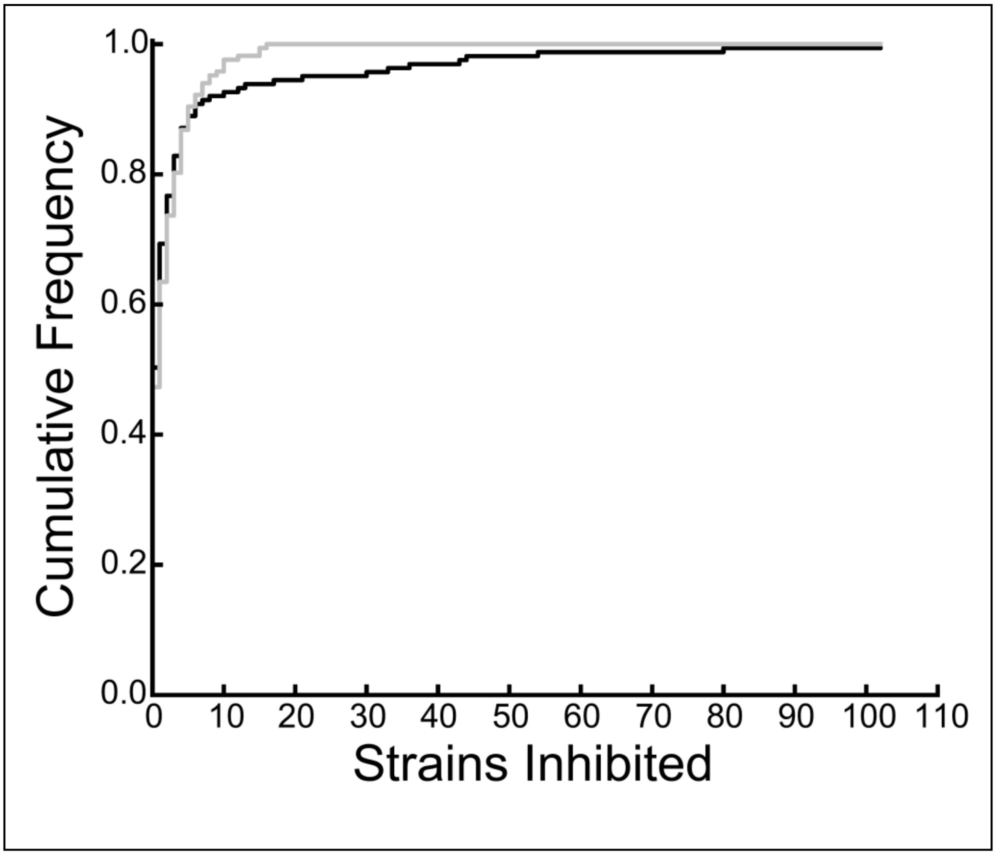

Supplementary Figure 1. The antagonistic distributions for env-Ps. A two-sample Kolmogorov-Smirnov test was used to determine if the antagonistic distributions were significantly different. Based on antagonistic activity in Figure 3, there is no significant difference in distributions among soil (grey) and water (black) derived strains. Although three water-derived strains exhibit high antagonistic activity, the two distributions are similar. An alpha of 0.01 was used for all tests was to control for Type I error.

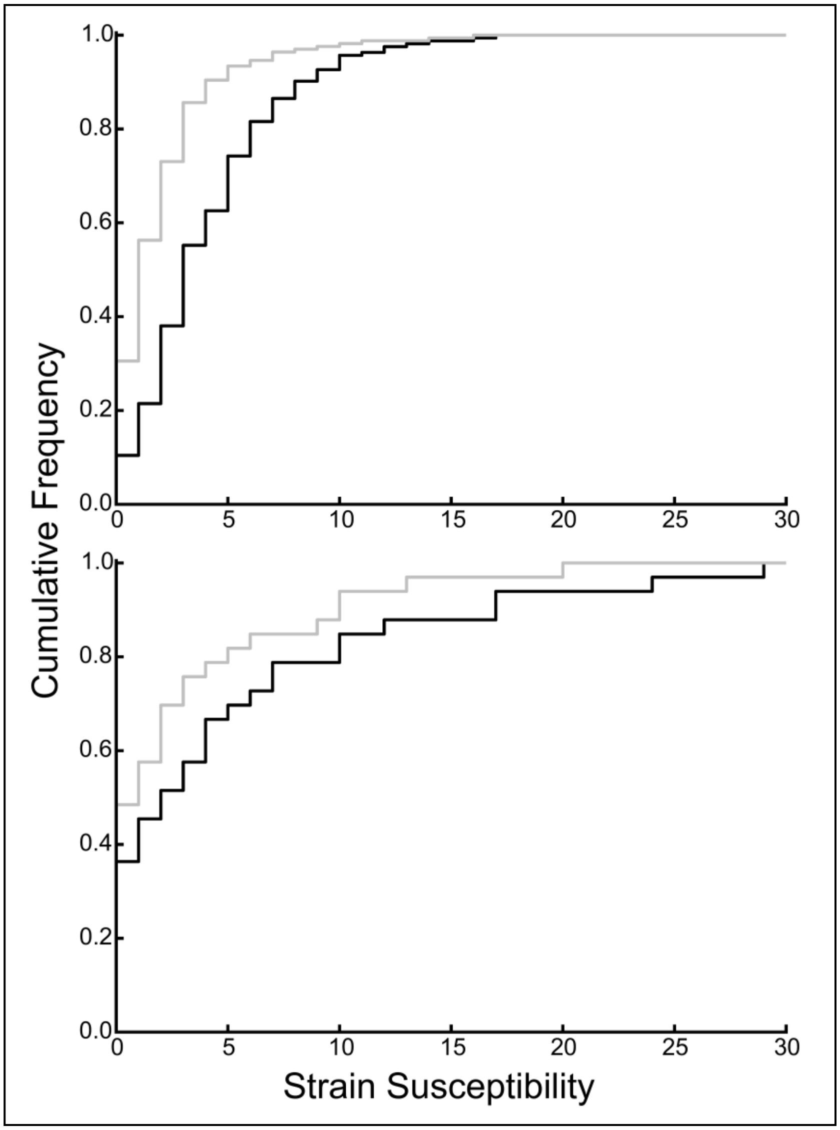

Supplementary Figure 2. The susceptibility distributions for env-Ps and CF-Ps. A two-sample Kolmogorov-Smirnov test was used to determine if the distributions of susceptibility from antagonistic activity were significantly different among strains. From the susceptibility results of env-Ps in Figure 3, there is a significant difference between susceptibility of soil and water strains; water strains are more susceptibility to antagonistic activity (top plot, $P<0.0001$). From the susceptibility of CF-Ps to env-Ps shown in Figure 4, CF-Ps are more susceptible to water strains (bottom plot, $P<0.0001$); black step function, water; grey step function, soil. An alpha of 0.01 was used for all tests was to control for Type I error.

The *gyrB* nucleotide sequences of environmental strains

```
>S02D 204
CTGCACGGTGTAGGTGTGTCGGTAGTGAATGCGCTGTCCAAAGAACTGATCCTGACTGTTCGCCGTAGCGGCAAGAT
CTGGGAACAAACTTACATCCACGGTGTTCCGCAAGAGCCGATGAAAATCGTTGGCGAGAGCGAAACCACGGGTACGC
AGATTCACTTCAAGCCTTCGGCTGAAACCTTCAAGAACATCCACTTCAGCTGGGACATCCTGGCCAAGCGGATTCGT
GAACTGTCCTTCCTGAACTCCGGTGTCGGCATCGTCCTCAAGGATGAGCGCAGCGGCAAGGAAGAGCTGTTCAAGTA
CGAAGGTGGTTTGCGTGCGTTCGTTGAATACCTGAACACCAACAAGACTGCGGTCAACCAGGTGTTCCACTTCAATA
TCCAGCGTGAAGACGGCATTGGCGTGGAAATCGCCCTGCAGTGGAACGACAGCTTCAACGAGAACCTGTTGTGCTTC
ACCAACAACATTCCTCAGCGCGATGGCGGTACTCACCTGGTGGGTTTCCGTTCCGCACTGACGCGTAACCTGAACAC
CTACATCGAAGCTGAAGGCCTGGCGAAGAAACACAAAGTCGCCACCACCGGCGACGACGCCCGCGAAGGCCTGACCG
CGATTATCTCGGTCAAGGTTCCGGATCCGAAGTTCAGCTC
>S04D 220
CTACACGGTGTAGGTGTGTCGGTAGTGAATGCGCTGTCCAAAGAACTGATCCTGACTGTTCGCCGTAGCGGCAAGAT
CTGGGAACAAACTTACATCCACGGTGTTCCGCAAGAGCCGATGAAAATCGTTGGCGAGAGCGAAACCACGGGTACGC
AGATTCACTTCAAGCCTTCGGCTGAAACCTTCAAGAACATCCACTTCAGCTGGGACATCCTGGCCAAGCGGATTCGT
GAACTGTCCTTCCTGAACTCCGGTGTCGGCATCGTCCTCAAGGATGAGCGCAGCGGCAAGGAAGAGCTGTTCAAGTA
CGAAGGTGGTTTGCGTGCGTTCGTTGAATACCTGAACACCAACAAGACTGCGGTCAACCAGGTGTTCCACTTCAATA
TCCAGCGTGAAGACGGCATTGGCGTGGAAATCGCCCTGCAGTGGAACGACAGCTTCAACGAGAACCTGTTGTGCTTC
ACCAACAACATTCCTCAGCGCGATGGCGGTACTCACCTGGTGGGTTTCCGTTCCGCACTGACGCGTAACCTGAACAC
CTACATCGAAGCTGAAGGCCTGGCGAAGAAACACAAAGTCGCCACCACCGGCGACGACGCCCGCGAAGGCCTGACCG
CGATTATCTCGGTCAAGGTTCCGGATCCGAAGTTCAGCTC
>S12C 283
CTACACGGTGTAGGTGTGTCGGTAGTGAATGCGCTGTCCAAAGAACTGATCCTGACTGTTCGCCGTAGCGGCAAGAT
CTGGGAACAAACTTACATCCACGGTGTTCCGCAAGAGCCGATGAAAATCGTTGGCGAGAGCGAAACCACGGGTACGC
AGATTCACTTCAAGCCTTCGGCTGAAACCTTCAAGAACATCCACTTCAGCTGGGACATCCTGGCCAAGCGGATTCGT
GAACTGTCCTTCCTGAACTCCGGTGTCGGCATCGTCCTCAAGGATGAGCGCAGCGGCAAGGAAGAGCTGTTCAAGTA
CGAAGGTGGTTTGCGTGCGTTCGTTGAATACCTGAACACCAACAAGACTGCGGTCAACCAGGTGTTCCACTTCAATA
TCCAGCGTGAAGACGGCATTGGCGTGGAAATCGCCCTGCAGTGGAACGACAGCTTCAACGAGAACCTGTTGTGCTTC
ACCAACAACATTCCTCAGCGCGATGGCGGTACTCACCTGGTGGGTTTCCGTTCCGCACTGACGCGTAACCTGAACAC
CTACATCGAAGCTGAAGGCCTGGCGAAGAAACACAAAGTCGCCACCACCGGCGACGACGCCCGCGAAGGCCTGACCG
CGATTATCTCGGTCAAGGTTCCGGATCCGAAGTTCAGCTC
>S04B 314
CTGCACGGTGTAGGTGTGTCGGTAGTGAATGCGCTGTCCAAAGAACTGATCCTGACTGTTCGCCGTAGCGGCAAGAT
CTGGGAACAAACTTACATCCATGGTGTTCCGCAAGAGCCGATGAAAATCGTTGGCGAGAGCGAAACCACGGGTACGC
AGATTCACTTCAAGCCTTCGGCTGAAACCTTCAAGAACATCCACTTCAGCTGGGACATCCTGGCCAAGCGGATTCGT
GAACTGTCCTTCCTGAACTCCGGTGTCGGCATCGTCCTCAAGGATGAGCGCAGCGGCAAGGAAGAGCTGTTCAAGTA
CGAAGGTGGTTTGCGTGCGTTCGTTGAATACCTGAACACCAACAAGACTGCGGTCAACCAGGTGTTCCACTTCAACA
TCCAGCGTGAAGACGGCATTGGCGTGGAAATCGCCCTGCAGTGGAACGACAGCTTCAACGAGAACCTGTTGTGCTTC
ACCAACAACATTCCTCAGCGCGATGGCGGTACTCACCTGGTGGGTTTCCGTTCCGCACTGACGCGTAACCTGAACAC
CTACATCGAAGCTGAAGGCCTGGCGAAGAAACACAAAGTCGCCACCACCGGCGACGACGCCCGCGAAGGCCTGACCG
CGATTATCTCGGTCAAGGTTCCGGATCCGAAGTTCAGCTC
>S03B 306
CTGCACGGTGTAGGTGTGTCGGTAGTGAATGCGCTGTCCAAAGAACTGATCCTGACTGTTCGCCGTAGCGGCAAGAT
CTGGGAACAAACTTACATCCACGGTGTTCCGCAAGAGCCGATGAAAATCGTTGGCGAGAGCGAAACCACGGGTACGC
AGATTCACTTCAAGCCTTCGGCTGAAACCTTCAAGAACATCCACTTCAGCTGGGACATCCTGGCCAAGCGGATTCGT
GAACTGTCCTTCCTGAACTCCGGTGTCGGCATCGTCCTCAAGGATGAGCGCAGCGGCAAGGAAGAGCTGTTCAAGTA
CGAAGGTGGTTTGCGTGCGTTCGTTGAATACCTGAACACCAACAAGACTGCGGTCAACCAGGTGTTCCACTTCAATA
TCCAGCGTGAAGACGGCATTGGCGTGGAAATCGCCCTGCAGTGGAACGACAGCTTCAACGAGAACCTGTTGTGCTTC
ACCAACAACATTCCTCAGCGCGATGGCGGTACTCACCTGGTGGGTTTCCGTTCCGCACTGACGCGTAACCTGAACAC
CTACATCGAAGCTGAAGGCCTGGCGAAGAAACACAAAGTCGCCACCACCGGCGACGACGCCCGCGAAGGCCTGACCG
CGATTATCTCGGTCAAGGTTCCGGATCCGAAGTTCAGCTC
>S01F 294
CTGCACGGTGTAGGTGTGTCGGTAGTGAATGCGCTGTCCAAAGAACTGATCCTGACTGTTCGCCGTAGCGGCAAGAT
CTGGGAACAAACTTACATCCACGGTGTTCCGCAAGAGCCGATGAAAATCGTTGGCGAGAGCGAAACCACGGGTACGC
```

AGATTCACTTCAAGCCTTCGGCTGAAACCTTCAAGAACATCCACTTCAGCTGGGACATCCTGGCCAAGCGGATTCGT
GAACTGTCCTTCCTGAACTCCGGTGTCGGCATCGTCCTCAAGGATGAGCGCAGCGGCAAGGAAGAGCTGTTCAAGTA
CGAAGGTGGTTTGCGTGCGTTCGTTGAATACCTGAACACCAACAAGACTGCGGTCAACCAGGTGTTCCACTTCAATA
TCCAGCGTGAAGACGGCATTGGCGTGGAAATCGCCCTGCAGTGGAACGACAGCTTCAACGAGAACCTGTTGTGCTTC
ACCAACAACATTCCTCAGCGCGATGGCGGTACTCACCTGGTGGGTTTCCGTTCCGCACTGACGCGTAACCTGAACAC
CTACATCGAAGCTGAAGGCCTGGCGAAGAAACACAAAGTCGCCACCACCGGCGACGACGCCCGCGAAGGCCTGACCG
CGATTATCTCGGTCAAGGTTCCGGATCCGAAGTTCAGCTC
>S01A 193
CTACACGGTGTAGGTGTGTCGGTAGTGAATGCGCTGTCCAAAGAACTGATCCTGACTGTTCGCCGTAGCGGCAAGAT
CTGGGAACAAACTTACATCCACGGTGTTCCGCAAGAGCCGATGAAAATCGTTGGCGAGAGCGAAACCACGGGTACGC
AGATTCACTTCAAGCCTTCGGCTGAAACCTTCAAGAACATCCACTTCAGCTGGGACATCCTGGCCAAGCGGATTCGT
GAACTGTCCTTCCTGAACTCCGGTGTCGGCATCGTCCTCAAGGATGAGCGCAGCGGCAAGGAAGAGCTGTTCAAGTA
CGAAGGTGGTTTGCGTGCGTTCGTTGAATACCTGAACACCAACAAGACTGCGGTCAACCAGGTGTTCCACTTCAATA
TCCAGCGTGAAGACGGCATTGGCGTGGAAATCGCCCTGCAGTGGAACGACAGCTTCAACGAGAACCTGTTGTGCTTC
ACCAACAACATTCCTCAGCGCGATGGCGGTACTCACCTGGTGGGTTTCCGTTCCGCACTGACGCGTAACCTGAACAC
CTACATCGAAGCTGAAGGCCTGGCGAAGAAACACAAAGTCGCCACCACCGGCGACGACGCCCGCGAAGGCCTGACCG
CGATTATCTCGGTCAAGGTTCCGGATCCGAAGTTCAGCTC
>S08C 251
CTGCACGGTGTAGGTGTGTCGGTAGTGAATGCGCTGTCCAAAGAACTGATCCTGACTGTTCGCCGTAGCGGCAAGAT
CTGGGAACAAACTTACATCCACGGTGTTCCGCAAGAGCCGATGAAAATCGTTGGCGAGAGCGAAACCACGGGTACGC
AGATTCACTTCAAGCCTTCGGCTGAAACCTTCAAGAACATCCACTTCAGCTGGGACATCCTGGCCAAGCGGATTCGT
GAACTGTCCTTCCTGAACTCCGGTGTCGGCATCGTCCTCAAGGATGAGCGCAGCGGCAAGGAAGAGCTGTTCAAGTA
CGAAGGTGGTTTGCGTGCGTTCGTTGAATACCTGAACACCAACAAGACTGCGGTCAACCAGGTGTTCCACTTCAATA
TCCAGCGTGAAGACGGCATTGGCGTGGAAATCGCCCTGCAGTGGAACGACAGCTTCAACGAGAACCTGTTGTGCTTC
ACCAACAACATTCCTCAGCGCGATGGCGGTACTCACCTGGTGGGTTTCCGTTCCGCACTGACGCGTAACCTGAACAC
CTACATCGAAGCTGAAGGCCTGGCGAAAAAACACAAAGTCGCCACCACCGGCGACGACGCCCGCGAAGGCCTGACCG
CGATTATCTCGGTCAAGGTTCCGGATCCGAAGTTCAGCTC
>S11H 280
CTGCACGGTGTAGGTGTGTCGGTAGTGAATGCGCTGTCCAAAGAACTGATCCTGACTGTTCGCCGTAGCGGCAAGAT
CTGGGAACAAACTTACATCCACGGTGTTCCGCAAGAGCCGATGAAAATCGTTGGCGAGAGCGAAACCACGGGTACGC
AGATTCACTTCAAGCCTTCGGCTGAAACCTTCAAGAACATCCACTTCAGCTGGGACATCCTGGCCAAGCGGATTCGT
GAACTGTCCTTCCTGAACTCCGGTGTCGGCATCGTCCTCAAGGATGAGCGCAGCGGCAAGGAAGAGCTGTTCAAGTA
CGAAGGTGGTTTGCGTGCGTTCGTTGAATACCTGAACACCAACAAGACTGCGGTCAACCAGGTGTTCCACTTCAATA
TCCAGCGTGAAGACGGCATTGGCGTGGAAATCGCCCTGCAGTGGAACGACAGCTTCAACGAGAACCTGTTGTGCTTC
ACCAACAACATTCCTCAGCGCGATGGCGGTACTCACCTGGTGGGTTTCCGTTCCGCACTGACGCGTAACCTGAACAC
CTACATCGAAGCTGAAGGCCTGGCGAAGAAACACAAAGTCGCCACCACCGGCGACGACGCCCGCGAAGGCCTGACCG
CGATTATCTCGGTCAAGGTTCCGGATCCGAAGTTCAGCTC
>S02G 303
CTGCACGGTGTAGGTGTGTCGGTAGTGAATGCGCTGTCCAAAGAACTGATCCTGACTGTTCGCCGTAGCGGCAAGAT
CTGGGAACAAACTTACATCCACGGTGTTCCGCAAGAGCCGATGAAAATCGTTGGCGAGAGCGAAACCACGGGTACGC
AGATTCACTTCAAGCCTTCGGCTGAAACCTTCAAGAACATCCACTTCAGCTGGGACATCCTGGCCAAGCGGATTCGT
GAACTGTCCTTCCTGAACTCCGGTGTCGGCATCGTCCTCAAGGATGAGCGCAGCGGCAAGGAAGAGCTGTTCAAGTA
CGAAGGTGGTTTGCGTGCGTTCGTTGAATACCTGAACACCAACAAGACTGCGGTCAACCAGGTGTTCCACTTCAATA
TCCAGCGTGAAGACGGCATTGGCGTGGAAATCGCCCTGCAGTGGAACGACAGCTTCAACGAGAACCTGTTGTGCTTC
ACCAACAACATTCCTCAGCGCGATGGCGGTACTCACCTGGTGGGTTTCCGTTCCGCACTGACGCGTAACCTGAACAC
CTACATCGAAGCTGAAGGCCTGGCGAAGAAACACAAAGTCGCCACCACCGGCGACGACGCCCGCGAAGGCCTGACCG
CGATTATCTCGGTCAAGGTTCCGGATCCGAAGTTCAGCTC
>S02C 299
CTGCACGGTGTAGGTGTGTCGGTAGTGAATGCGCTGTCCAAAGAACTGATCCTGACTGTTCGCCGTAGCGGCAAGAT
CTGGGAACAAACTTACATCCACGGTGTTCCGCAAGAGCCGATGAAAATCGTTGGCGAGAGCGAAACCACGGGTACGC
AGATTCACTTCAAGCCTTCGGCTGAAACCTTCAAGAACATCCACTTCAGCTGGGACATCCTGGCCAAGCGGATTCGT
GAACTGTCCTTCCTGAACTCCGGTGTCGGCATCGTCCTCAAGGATGAGCGCAGCGGCAAGGAAGAGCTGTTCAAGTA
CGAAGGTGGTTTGCGTGCGTTCGTTGAATACCTGAACACCAACAAGACTGCGGTCAACCAGGTGTTCCACTTCAATA
TCCAGCGTGAAGACGGCATTGGCGTGGAAATCGCCCTGCAGTGGAACGACAGCTTCAACGAGAACCTGTTGTGCTTC
ACCAACAACATTCCTCAGCGCGATGGCGGTACTCACCTGGTGGGTTTCCGTTCCGCACTGACGCGTAACCTGAACAC
CTACATCGAAGCTGAAGGCCTGGCGAAGAAACACAAAGTCGCCACCACCGGCGACGACGCCCGCGAAGGCCTGACCG
CGATTATCTCGGTCAAGGTTCCGGATCCGAAGTTCAGCTC

>S05C 323
CTGCACGGTGTAGGTGTGTCGGTAGTGAATGCGCTGTCCAAAGAACTGATCCTGACTGTTCGCCGTAGCGGCAAGAT
CTGGGAACAAACTTACATCCACGGTGTTCCGCAAGAGCCGATGAAAATCGTTGGCGAGAGCGAAACCACGGGTACGC
AGATTCACTTCAAGCCTTCGGCTGAAACCTTCAAGAACATCCACTTCAGCTGGGACATCCTGGCCAAGCGGATTCGT
GAACTGTCCTTCCTGAACTCCGGTGTCGGCATCGTCCTCAAGGATGAGCGCAGCGGCAAGGAAGAGCTGTTCAAGTA
CGAAGGTGGTTTGCGTGCGTTCGTTGAATACCTGAACACCAACAAGACTGCGGTCAACCAGGTGTTCCACTTCAATA
TCCAGCGTGAAGACGGCATTGGCGTGGAAATCGCCCTGCAGTGGAACGACAGCTTCAACGAGAACCTGTTGTGCTTC
ACCAACAACATTCCTCAGCGCGATGGCGGTACTCACCTGGTGGGTTTCCGTTCCGCACTGACGCGTAACCTGAACAC
CTACATCGAAGCTGAAGGCCTGGCGAAGAAACACAAAGTCGCCACCACCGGCGACGACGCCCGCGAAGGCCTGACCG
CGATTATCTCGGTCAAGGTTCCGGATCCGAAGTTCAGCTC
>S11F 374
CTGCACGGTGTAGGTGTGTCGGTAGTGAATGCGCTGTCCAAAGAACTGATCCTGACTGTTCGCCGTAGCGGCAAGAT
CTGGGAACAAACTTACATCCACGGTGTTCCGCAAGAGCCGATGAAAATCGTTGGCGAGAGCGAAACCACGGGTACGC
AGATTCACTTCAAGCCTTCGGCTGAAACCTTCAAGAACATCCACTTCAGCTGGGACATCCTGGCCAAGCGGATTCGT
GAACTGTCCTTCCTGAACTCCGGTGTCGGCATCGTCCTCAAGGATGAGCGCAGCGGCAAGGAAGAGCTGTTCAAGTA
CGAAGGTGGTTTGCGTGCGTTCGTTGAATACCTGAACACCAACAAGACTGCGGTTAACCAGGTTTTCCACTTCAACA
TCCAGCGTGAAGACGGCATTGGCGTGGAAATCGCCCTGCAGTGGAACGACAGCTTCAACGAGAACCTGTTGTGCTTC
ACCAACAACATTCCTCAGCGCGATGGCGGTACTCACCTGGTGGGTTTCCGTTCCGCACTGACGCGTAACCTGAACAC
CTACATCGAAGCTGAAGGCCTGGCGAAGAAACACAAAGTCGCCACCACCGGCGACGACGCCCGCGAAGGCCTGACCG
CGATTATCTCGGTCAAGGTTCCGGATCCGAAGTTCAGCTC
>S11H 376
CTACACGGTGTAGGTGTGTCGGTAGTGAATGCGCTGTCCAAAGAACTGATCCTGACTGTTCGCCGTAGCGGCAAGAT
CTGGGAACAAACTTACATCCACGGTGTTCCGCAAGAGCCGATGAAAATCGTTGGCGAGAGCGAAACCACGGGTACGC
AGATTCACTTCAAGCCTTCGGCTGAAACCTTCAAGAACATCCACTTCAGCTGGGACATCCTGGCCAAGCGGATTCGT
GAACTGTCCTTCCTGAACTCCGGTGTCGGCATCGTCCTCAAGGATGAGCGCAGCGGCAAGGAAGAGCTGTTCAAGTA
CGAAGGTGGTTTGCGTGCGTTCGTTGAATACCTGAACACCAACAAGACTGCGGTCAACCAGGTGTTCCACTTCAATA
TCCAGCGTGAAGACGGCATTGGCGTGGAAATCGCCCTGCAGTGGAACGACAGCTTCAACGAGAACCTGTTGTGCTTC
ACCAACAACATTCCTCAGCGCGATGGCGGTACTCACCTGGTGGGTTTCCGTTCCGCACTGACGCGTAACCTGAACAC
CTACATCGAAGCTGAAGGCCTGGCGAAGAAACACAAAGTCGCCACCACCGGCGACGACGCCCGCGAAGGCCTGACCG
CGATTATCTCGGTCAAGGTTCCGGATCCGAAGTTCAGCTC
>S08A 249
CTGCACGGTGTAGGTGTGTCGGTAGTGAATGCGCTGTCCAAAGAACTGATCCTGACTGTTCGCCGTAGCGGCAAGAT
CTGGGAACAAACTTACATCCACGGTGTTCCGCAAGAGCCGATGAAAATCGTTGGCGAGAGCGAAACCACGGGTACGC
AGATTCACTTCAAGCCTTCGGCTGAAACCTTCAAGAACATCCACTTCAGCTGGGACATCCTGGCCAAGCGGATTCGT
GAACTGTCCTTCCTGAACTCCGGTGTCGGCATCGTCCTCAAGGATGAGCGCAGCGGCAAGGAAGAGCTGTTCAAGTA
CGAAGGTGGTTTGCGTGCGTTCGTTGAATACCTGAACACCAACAAGACTGCGGTCAACCAGGTGTTCCACTTCAATA
TCCAGCGTGAAGACGGCATTGGCGTGGAAATCGCCCTGCAGTGGAACGACAGCTTCAACGAGAACCTGTTGTGCTTC
ACCAACAACATTCCTCAGCGCGATGGCGGTACTCACCTGGTGGGTTTCCGTTCCGCACTGACGCGTAACCTGAACAC
CTACATCGAAGCTGAAGGCCTGGCGAAGAAACACAAAGTCGCCACCACCGGCGACGACGCCCGCGAAGGCCTGACCG
CGATTATCTCGGTCAAGGTTCCGGATCCGAAGTTCAGCTC
>S08E 253
CTGCACGGTGTAGGTGTGTCGGTAGTGAATGCGCTGTCCAAAGAACTGATCCTGACTGTTCGCCGTAGCGGCAAGAT
CTGGGAACAAACTTACATCCACGGTGTTCCGCAAGAGCCGATGAAAATCGTTGGCGAGAGCGAAACCACGGGTACGC
AGATTCACTTCAAGCCTTCGGCTGAAACCTTCAAGAACATCCACTTCAGCTGGGACATCCTGGCCAAGCGGATTCGT
GAACTGTCCTTCCTGAACTCCGGTGTCGGCATCGTCCTCAAGGATGAGCGCAGCGGCAAGGAAGAGCTGTTCAAGTA
CGAAGGTGGTTTGCGTGCGTTCGTTGAATACCTGAACACCAACAAGACTGCGGTCAACCAGGTGTTCCACTTCAATA
TCCAGCGTGAAGACGGCATTGGCGTGGAAATCGCCCTGCAGTGGAACGACAGCTTCAACGAGAACCTGTTGTGCTTC
ACCAACAACATTCCTCAGCGCGATGGCGGTACTCACCTGGTGGGTTTCCGTTCCGCACTGACGCGTAACCTGAACAC
CTACATCGAAGCTGAAGGCCTGGCGAAGAAACACAAAGTCGCCACCACCGGCGACGACGCCCGCGAAGGCCTGACCG
CGATTATCTCGGTCAAGGTTCCGGATCCGAAGTTCAGCTC
>S05B 322
CTGCACGGTGTAGGTGTGTCGGTAGTGAATGCGCTGTCCAAAGAACTGATCCTGACTGTTCGCCGTAGCGGCAAGAT
CTGGGAACAAACTTACATCCACGGTGTTCCGCAAGAGCCGATGAAAATCGTTGGCGAGAGCGAAACCACGGGTACGC
AGATTCACTTCAAGCCTTCGGCTGAAACCTTCAAGAACATCCACTTCAGCTGGGACATCCTGGCCAAGCGGATTCGT
GAACTGTCCTTCCTGAACTCCGGTGTCGGCATCGTCCTCAAGGATGAGCGCAGCGGCAAGGAAGAGCTGTTCAAGTA
CGAAGGTGGTTTGCGTGCGTTCGTTGAATACCTGAACACCAACAAGACTGCGGTCAACCAGGTGTTCCACTTCAATA
TCCAGCGTGAAGACGGCATTGGCGTGGAAATCGCCCTGCAGTGGAACGACAGCTTCAACGAGAACCTGTTGTGCTTC

ACCAACAACATTCCTCAGCGCGATGGCGGTACTCACCTGGTGGGTTTCCGTTCCGCACTGACGCGTAACCTGAACAC
CTACATCGAAGCTGAAGGCCTGGCGAAGAAACACAAAGTCGCCACCACCGGCGACGACGCCCGCGAAGGCCTGACCG
CGATTATCTCGGTCAAGGTTCCGGATCCGAAGTTCAGCTC
>S05B 226
CTGCACGGTGTAGGTGTGTCGGTAGTGAATGCGCTGTCCAAAGAACTGATCCTGACTGTTCGCCGTAGCGGCAAGAT
CTGGGAACAAACTTACATCCACGGTGTTCCGCAAGAGCCGATGAAAATCGTTGGCGAGAGCGAAACCACGGGTACGC
AGATTCACTTCAAGCCTTCGGCTGAAACCTTCAAGAACATCCACTTCAGCTGGGACATCCTGGCCAAGCGGATTCGT
GAACTGTCCTTCCTGAACTCCGGTGTCGGCATCGTCCTCAAGGATGAGCGCAGCGGCAAGGAAGAGCTGTTCAAGTA
CGAAGGTGGTTTGCGTGCGTTCGTTGAATACCTGAACACCAACAAGACTGCGGTCAACCAGGTGTTCCACTTCAATA
TCCAGCGTGAAGACGGCATTGGCGTGGAAATCGCCCTGCAGTGGAACGACAGCTTCAACGAGAACCTGTTGTGCTTC
ACCAACAACATTCCTCAGCGCGATGGCGGTACTCACCTGGTGGGTTTCCGTTCCGCACTGACGCGTAACCTGAACAC
CTACATCGAAGCTGAAGGCCTGGCGAAGAAACACAAAGTCGCCACCACCGGCGACGACGCCCGCGAAGGCCTGACCG
CGATTATCTCGGTCAAGGTTCCGGATCCGAAGTTCAGCTC
>07F 67
CTGCACGGTGTAGGTGTGTCGGTAGTGAATGCGCTGTCCAAAGAACTGATCCTGACTGTTCGCCGTAGCGGCAAGAT
CTGGGAACAAACTTACATCCACGGTGTTCCGCAAGAGCCGATGAAAATCGTTGGCGAGAGCGAAACCACGGGTACGC
AGATTCACTTCAAGCCTTCGGCTGAAACCTTCAAGAACATCCACTTCAGCTGGGACATCCTGGCCAAGCGGATTCGT
GAACTGTCCTTCCTGAACTCCGGTGTCGGCATCGTCCTCAAGGATGAGCGCAGCGGCAAGGAAGAGCTGTTCAAGTA
CGAAGGTGGTTTGCGTGCGTTCGTTGAATACCTGAACACCAACAAGACTGCGGTCAACCAGGTGTTCCACTTCAACA
TCCAGCGTGAAGACGGCATTGGCGTGGAAATCGCCCTGCAGTGGAACGACAGCTTCAACGAGAACCTGTTGTGCTTC
ACCAACAACATTCCTCAGCGCGATGGCGGTACTCACCTGGTGGGTTTCCGTTCCGCACTGACGCGTAACCTGAACAC
CTACATCGAAGCTGAAGGCCTGGCGAAGAAACACAAAGTCGCCACCACCGGCGACGACGCCCGCGAAGGCCTGACTG
CGATTATCTCGGTCAAGGTTCCGGATCCGAAGTTCAGCTC
>S04G 223
CTACACGGTGTAGGTGTGTCGGTAGTGAATGCGCTGTCCAAAGAACTGATCCTGACTGTTCGCCGTAGCGGCAAGAT
CTGGGAACAAACTTACATCCACGGTGTTCCGCAAGAGCCGATGAAAATCGTTGGCGAGAGCGAAACCACGGGTACGC
AGATTCACTTCAAGCCTTCGGCTGAAACCTTCAAGAACATCCACTTCAGCTGGGACATCCTGGCCAAGCGGATTCGT
GAACTGTCCTTCCTGAACTCCGGTGTCGGCATCGTCCTCAAGGATGAGCGCAGCGGCAAGGAAGAGCTGTTCAAGTA
CGAAGGTGGTTTGCGTGCGTTCGTTGAATACCTGAACACCAACAAGACTGCGGTCAACCAGGTGTTCCACTTCAATA
TCCAGCGTGAAGACGGCATTGGCGTGGAAATCGCCCTGCAGTGGAACGACAGCTTCAACGAGAACCTGTTGTGCTTC
ACCAACAACATTCCTCAGCGCGATGGCGGTACTCACCTGGTGGGTTTCCGTTCCGCACTGACGCGTAACCTGAACAC
CTACATCGAAGCTGAAGGCCTGGCGAAGAAACACAAAGTCGCCACCACCGGCGACGACGCCCGCGAAGGCCTGACCG
CGATTATCTCGGTCAAGGTTCCGGATCCGAAGTTCAGCTC
>S01C 195
CTGCACGGTGTAGGTGTGTCGGTAGTGAATGCGCTGTCCAAAGAACTGATCCTGACCGTTCGCCGTAGCGGCAAGAT
CTGGGAACAAACTTACATCCACGGTGTTCCGCAAGAGCCGATGAAAATCGTTGGCGAGAGCGAAACCACGGGTACGC
AGATTCACTTCAAGCCTTCGGCTGAAACCTTCAAGAACATCCACTTCAGCTGGGACATCCTGGCCAAGCGGATTCGT
GAACTGTCCTTCCTGAACTCCGGTGTCGGCATCGTCCTCAAGGATGAGCGCAGCGGCAAGGAAGAGCTGTTCAAGTA
TGAAGGTGGTTTGCGTGCGTTCGTTGAATACCTGAACACCAACAAGACTGCGGTCAACCAGGTGTTCCACTTCAACA
TCCAGCGTGAAGACGGCATCGGCGTGGAAATCGCCCTGCAGTGGAACGACAGCTTCAACGAGAACCTGTTGTGCTTC
ACCAACAACATTCCTCAGCGCGATGGCGGTACTCACCTGGTGGGTTTCCGTTCCGCACTGACGCGTAACCTGAACAC
CTACATCGAAGCTGAAGGCCTGGCGAAAAAACACAAAGTCGCCACCACCGGCGACGACGCCCGCGAAGGCCTGACCG
CGATTATTTCGGTCAAGGTTCCGGATCCGAAGTTCAGCTC
>S02A 201
CTGCACGGTGTAGGTGTGTCGGTAGTGAATGCGCTGTCCAAAGAACTGATCCTGACCGTTCGCCGTAGCGGCAAGAT
CTGGGAACAAACTTACATCCACGGTGTTCCGCAAGAGCCGATGAAAATCGTTGGCGAGAGCGAAACCACGGGTACGC
AGATTCACTTCAAGCCTTCGGCTGAAACCTTCAAGAACATCCACTTCAGCTGGGACATCCTGGCCAAGCGGATTCGT
GAACTGTCCTTCCTGAACTCCGGTGTCGGCATCGTCCTCAAGGATGAGCGCAGCGGCAAGGAAGAGCTGTTCAAGTA
CGAAGGTGGTTTGCGTGCGTTCGTTGAATACCTGAACACCAACAAGACTGCGGTCAACCAGGTGTTCCACTTCAACA
TCCAGCGTGAAGACGGCATCGGCGTGGAAATCGCCCTGCAGTGGAACGACAGCTTCAACGAGAACCTGTTGTGCTTC
ACCAACAACATTCCTCAGCGCGATGGCGGTACTCACCTGGTGGGTTTCCGTTCCGCACTGACGCGTAACCTGAACAC
CTACATCGAAGCTGAAGGCCTGGCGAAGAAACACAAAGTCGCCACCACCGGCGACGACGCCCGCGAAGGCCTGACCG
CGATTATCTCGGTCAAGGTTCCGGATCCGAAGTTCAGCTC
>S05C 227
CTGCACGGTGTAGGTGTGTCGGTAGTGAATGCGCTGTCCAAAGAACTGATCCTGACTGTTCGCCGTAGCGGCAAGAT
CTGGGAACAAACTTACATCCATGGTGTTCCGCAAGAGCCGATGAAAATCGTTGGCGAGAGCGAAACCACGGGTACGC
AGATTCACTTCAAGCCTTCGGCTGAAACCTTCAAGAACATCCACTTCAGCTGGGACATCCTGGCCAAGCGGATTCGT

GAACTGTCCTTCCTGAACTCCGGTGTCGGCATCGTCCTCAAGGATGAGCGCAGCGGCAAGGAAGAGCTGTTCAAGTA
CGAAGGTGGTTTGCGTGCGTTCGTTGAATACCTGAACACCAACAAGACTGCGGTCAACCAGGTGTTCCACTTCAACA
TCCAGCGTGAAGACGGCATTGGCGTGGAAATCGCCCTGCAGTGGAACGACAGCTTCAACGAGAACCTGTTGTGCTTC
ACCAACAACATTCCTCAGCGCGATGGCGGTACTCACCTGGTGGGTTTCCGTTCCGCACTGACGCGTAACCTGAACAC
CTACATCGAAGCTGAAGGCCTGGCGAAGAAACACAAAGTCGCCACCACCGGCGACGACGCCCGCGAAGGCCTGACCG
CGATTATCTCGGTCAAGGTTCCGGATCCGAAGTTCAGCTC
>S07G 247
CTGCACGGTGTAGGTGTGTCGGTAGTGAATGCGCTGTCCAAAGAACTGATCCTGACTGTTCGCCGTAGCGGCAAGAT
CTGGGAACAAACTTACATCCACGGTGTTCCGCAAGAGCCGATGAAAATCGTTGGCGAGAGCGAAACCACGGGTACGC
AGATTCACTTCAAGCCTTCGGCTGAAACCTTCAAGAACATCCACTTCAGCTGGGACATCCTGGCCAAGCGGATTCGT
GAACTGTCCTTCCTGAACTCCGGTGTCGGCATCGTCCTCAAGGATGAGCGCAGCGGCAAGGAAGAGCTGTTCAAGTA
CGAAGGTGGTTTGCGTGCGTTCGTTGAATACCTGAACACCAACAAGACTGCGGTCAACCAGGTGTTCCACTTCAATA
TCCAGCGTGAAGACGGCATTGGCGTGGAAATCGCCCTGCAGTGGAACGACAGCTTCAACGAGAACCTGTTGTGCTTC
ACCAACAACATTCCTCAGCGCGATGGCGGTACTCACCTGGTGGGTTTCCGTTCCGCACTGACGCGTAACCTGAACAC
CTACATCGAAGCTGAAGGCCTGGCGAAGAAACACAAAGTCGCCACCACCGGCGACGACGCCCGCGAAGGCCTGACCG
CGATTATCTCGGTCAAGGTTCCGGATCCGAAGTTCAGCTC
>S10C 267
CTGCACGGTGTAGGTGTGTCGGTAGTGAATGCGCTGTCCAAAGAACTGATCCTGACCGTTCGCCGTAGCGGCAAGAT
CTGGGAACAAACTTACATCCACGGTGTTCCGCAAGAGCCGATGAAAATCGTTGGCGAGAGCGAAACCACGGGTACGC
AGATTCACTTCAAGCCTTCGGCTGAAACCTTCAAGAACATCCACTTCAGCTGGGACATCCTGGCCAAGCGGATTCGT
GAACTGTCCTTCCTGAACTCCGGTGTCGGCATCGTCCTCAAGGATGAGCGCAGCGGCAAGGAAGAGCTGTTCAAGTA
TGAAGGTGGTTTGCGTGCGTTCGTTGAATACCTGAACACCAACAAGACTGCGGTCAACCAGGTGTTCCACTTCAACA
TCCAGCGTGAAGACGGCATCGGCGTGGAAATCGCCCTGCAGTGGAACGACAGCTTCAACGAGAACCTGTTGTGCTTC
ACCAACAACATTCCTCAGCGCGATGGCGGTACTCACCTGGTGGGTTTCCGTTCCGCACTGACGCGTAACCTGAACAC
CTACATCGAAGCTGAAGGCCTGGCGAAAAAACACAAAGTCGCCACCACCGGCGACGACGCCCGCGAAGGCCTGACCG
CGATTATTTCGGTCAAGGTTCCGGATCCGAAGTTCAGCTC
>S05D 324
CTGCACGGTGTAGGTGTGTCGGTAGTGAATGCGCTGTCCAAAGAACTGATCCTGACCGTTCGCCGTAGCGGCAAGAT
CTGGGAACAAACTTACATCCACGGTGTTCCGCAAGAGCCGATGAAAATCGTTGGCGAGAGCGAAACCACGGGTACGC
AGATTCACTTCAAGCCTTCGGCTGAAACCTTCAAGAACATCCACTTCAGCTGGGACATCCTGGCCAAGCGGATTCGT
GAACTGTCCTTCCTGAACTCCGGTGTCGGCATCGTCCTCAAGGATGAGCGCAGCGGCAAGGAAGAGCTGTTCAAGTA
TGAAGGTGGTTTGCGTGCGTTCGTTGAATACCTGAACACCAACAAGACTGCGGTCAACCAGGTGTTCCACTTCAACA
TCCAGCGTGAAGACGGCATCGGCGTGGAAATCGCCCTGCAGTGGAACGACAGCTTCAACGAGAACCTGTTGTGCTTC
ACCAACAACATTCCTCAGCGCGATGGCGGTACTCACCTGGTGGGTTTCCGTTCCGCACTGACGCGTAACCTGAACAC
CTACATCGAAGCTGAAGGCCTGGCGAAAAAACACAAAGTCGCCACCACCGGCGACGACGCCCGCGAAGGCCTGACCG
CGATTATTTCGGTCAAGGTTCCGGATCCGAAGTTCAGCTC
>S11F 278
CTGCACGGTGTAGGTGTGTCGGTAGTGAATGCGCTGTCCAAAGAACTGATCCTGACCGTTCGCCGTAGCGGCAAGAT
CTGGGAACAAACTTACATCCACGGTGTTCCGCAAGAGCCGATGAAAATCGTTGGCGAGAGCGAAACCACGGGTACGC
AGATTCACTTCAAGCCTTCGGCTGAAACCTTCAAGAACATCCACTTCAGCTGGGACATCCTGGCCAAGCGGATTCGT
GAACTGTCCTTCCTGAACTCCGGTGTCGGCATCGTCCTCAAGGATGAGCGCAGCGGCAAGGAAGAGCTGTTCAAGTA
TGAAGGTGGTTTGCGTGCGTTCGTTGAATACCTGAACACCAACAAGACTGCGGTCAACCAGGTGTTCCACTTCAACA
TCCAGCGTGAAGACGGCATCGGCGTGGAAATCGCCCTGCAGTGGAACGACAGCTTCAACGAGAACCTGTTGTGCTTC
ACCAACAACATTCCTCAGCGCGATGGCGGTACTCACCTGGTGGGTTTCCGTTCCGCACTGACGCGTAACCTGAACAC
CTACATCGAAGCTGAAGGCCTGGCGAAAAAACACAAAGTCGCCACCACCGGCGACGACGCCCGCGAAGGCCTGACCG
CGATTATTTCGGTCAAGGTTCCGGATCCGAAGTTCAGCTC
>12C 132
CTGCACGGTGTAGGTGTGTCGGTAGTGAACGCACTGTCCAAAGAACTGATCCTGACTGTTCGCCGTAGCGGCAAGAT
CTGGGAACAAACTTACATCCACGGTGTTCCGCAAGAGCCGATGAAAATCGTTGGCGAGAGCGAAACCACGGGTACGC
AGATTCACTTCAAGCCTTCGGCTGAAACCTTCAAGAACATCCACTTCAGCTGGGACATCCTGGCCAAGCGGATTCGT
GAACTGTCCTTCCTGAACTCCGGTGTCGGCATCGTCCTCAAGGATGAGCGCAGCGGCAAGGAAGAGCTGTTCAAGTA
CGAAGGTGGTTTGCGTGCGTTCGTTGAATACCTGAACACCAACAAGACTGCGGTCAACCAGGTGTTCCACTTCAACA
TCCAGCGTGAAGACGGCATCGGCGTGGAAATCGCCCTGCAGTGGAACGACAGCTTCAACGAGAACCTGTTGTGCTTC
ACCAACAACATTCCTCAGCGCGATGGCGGTACTCACCTGGTGGGTTTCCGTTCCGCACTGACGCGTAACCTGAACAC
CTACATCGAAGCTGAAGGCCTGGCGAAAAAACACAAAGTCGCCACCACCGGCGACGACGCCCGCGAAGGCCTGACCG
CGATTATTTCGGTCAAGGTTCCGGATCCGAAGTTCAGCTC

>09E 57
CTGCACGGTGTAGGTGTGTCGGTAGTGAACGCACTGTCCAAAGAACTGATCCTGACTGTTCGCCGTAGCGGCAAGAT
CTGGGAACAAACTTACATCCACGGTGTTCCGCAAGAGCCGATGAAAATCGTTGGCGAGAGCGAAACCACGGGTACGC
AGATTCACTTCAAGCCTTCGGCTGAAACCTTCAAGAACATCCACTTCAGCTGGGACATCCTGGCCAAGCGGATTCGT
GAACTGTCCTTCCTGAACTCCGGTGTCGGCATCGTCCTCAAGGATGAGCGCAGCGGCAAGGAAGAGCTGTTCAAGTA
CGAAGGTGGTTTGCGTGCGTTCGTTGAATACCTGAACACCAACAAGACTGCGGTCAACCAGGTGTTCCACTTCAACA
TCCAGCGTGAAGACGGCATCGGCGTGGAAATCGCCCTGCAGTGGAACGACAGCTTCAACGAGAACCTGTTGTGCTTC
ACCAACAACATTCCTCAGCGCGATGGCGGTACTCACCTGGTGGGTTTCCGTTCCGCACTGACGCGTAACCTGAACAC
CTACATCGAAGCTGAAGGCCTGGCGAAAAAACACAAAGTCGCCACCACCGGCGACGACGCCCGCGAAGGCCTGACCG
CGATTATTTCGGTCAAGGTTCCGGATCCGAAGTTCAGCTC
>S02D 300
CTGCACGGTGTAGGTGTGTCGGTAGTGAACGCACTGTCCAAAGAACTGATCCTGACTGTTCGCCGTAGCGGCAAGAT
CTGGGAACAAACTTACATCCACGGTGTTCCGCAAGAGCCGATGAAAATCGTTGGCGAGAGCGAAACCACGGGTACGC
AGATTCACTTCAAGCCTTCGGCTGAAACCTTCAAGAACATCCACTTCAGCTGGGACATCCTGGCCAAGCGGATTCGT
GAACTGTCCTTCCTGAACTCCGGTGTCGGCATCGTCCTCAAGGATGAGCGCAGCGGCAAGGAAGAGCTGTTCAAGTA
CGAAGGTGGTTTGCGTGCGTTCGTTGAATACCTGAACACCAACAAGACTGCGGTCAACCAGGTGTTCCACTTCAACA
TCCAGCGTGAAGACGGCATCGGCGTGGAAATCGCCCTGCAGTGGAACGACAGCTTCAACGAGAACCTGTTGTGCTTC
ACCAACAACATTCCTCAGCGCGATGGCGGTACTCACCTGGTGGGTTTCCGTTCCGCACTGACGCGTAACCTGAACAC
CTACATCGAAGCTGAAGGCCTGGCGAAAAAACACAAAGTCGCCACCACCGGCGACGACGCCCGCGAAGGCCTGACCG
CGATTATTTCGGTCAAGGTTCCGGATCCGAAGTTCAGCTC
>S05A 321
CTGCACGGTGTAGGTGTGTCGGTAGTGAACGCACTGTCCAAAGAACTGATCCTGACTGTTCGCCGTAGCGGCAAGAT
CTGGGAACAAACTTACATCCACGGTGTTCCGCAAGAGCCGATGAAAATCGTTGGCGAGAGCGAAACCACGGGTACGC
AGATTCACTTCAAGCCTTCGGCTGAAACCTTCAAGAACATCCACTTCAGCTGGGACATCCTGGCCAAGCGGATTCGT
GAACTGTCCTTCCTGAACTCCGGTGTCGGCATCGTCCTCAAGGATGAGCGCAGCGGCAAGGAAGAGCTGTTCAAGTA
CGAAGGTGGTTTGCGTGCGTTCGTTGAATACCTGAACACCAACAAGACTGCGGTCAACCAGGTGTTCCACTTCAACA
TCCAGCGTGAAGACGGCATCGGCGTGGAAATCGCCCTGCAGTGGAACGACAGCTTCAACGAGAACCTGTTGTGCTTC
ACCAACAACATTCCTCAGCGCGATGGCGGTACTCACCTGGTGGGTTTCCGTTCCGCACTGACGCGTAACCTGAACAC
CTACATCGAAGCTGAAGGCCTGGCGAAAAAACACAAAGTCGCCACCACCGGCGACGACGCCCGCGAAGGCCTGACCG
CGATTATTTCGGTCAAGGTTCCGGATCCGAAGTTCAGCTC
>02D 134
CTGCACGGTGTAGGTGTGTCGGTAGTGAACGCACTGTCCAAAGAACTGATCCTGACTGTTCGCCGTAGCGGCAAGAT
CTGGGAACAAACTTACATCCACGGTGTTCCGCAAGAGCCGATGAAAATCGTTGGCGAGAGCGAAACCACGGGTACGC
AGATTCACTTCAAGCCTTCGGCTGAAACCTTCAAGAACATCCACTTCAGCTGGGACATCCTGGCCAAGCGGATTCGT
GAACTGTCCTTCCTGAACTCCGGTGTCGGCATCGTCCTCAAGGATGAGCGCAGCGGCAAGGAAGAGCTGTTCAAGTA
CGAAGGTGGTTTGCGTGCGTTCGTTGAATACCTGAACACCAACAAGACTGCGGTCAACCAGGTGTTCCACTTCAACA
TCCAGCGTGAAGACGGCATCGGCGTGGAAATCGCCCTGCAGTGGAACGACAGCTTCAACGAGAACCTGTTGTGCTTC
ACCAACAACATTCCTCAGCGCGATGGCGGTACTCACCTGGTGGGTTTCCGTTCCGCACTGACGCGTAACCTGAACAC
CTACATCGAAGCTGAAGGCCTGGCGAAAAAACACAAAGTCGCCACCACCGGCGACGACGCCCGCGAAGGCCTGACCG
CGATTATTTCGGTCAAGGTTCCGGATCCGAAGTTCAGCTC
>S08B 250
CTGCACGGTGTAGGTGTGTCGGTAGTGAACGCACTGTCCAAAGAACTGATCCTGACTGTTCGCCGTAGCGGCAAGAT
CTGGGAACAAACTTACATCCACGGTGTTCCGCAAGAGCCGATGAAAATCGTTGGCGAGAGCGAAACCACGGGTACGC
AGATTCACTTCAAGCCTTCGGCTGAAACCTTCAAGAACATCCACTTCAGCTGGGACATCCTGGCCAAGCGGATTCGT
GAACTGTCCTTCCTGAACTCCGGTGTCGGCATCGTCCTCAAGGATGAGCGCAGCGGCAAGGAAGAGCTGTTCAAGTA
CGAAGGTGGTTTGCGTGCGTTCGTTGAATACCTGAACACCAACAAGACTGCGGTCAACCAGGTGTTCCACTTCAACA
TCCAGCGTGAAGACGGCATCGGCGTGGAAATCGCCCTGCAGTGGAACGACAGCTTCAACGAGAACCTGTTGTGCTTC
ACCAACAACATTCCTCAGCGCGATGGCGGTACTCACCTGGTGGGTTTCCGTTCCGCACTGACGCGTAACCTGAACAC
CTACATCGAAGCTGAAGGCCTGGCGAAAAAACACAAAGTCGCCACCACCGGCGACGACGCCCGCGAAGGCCTGACCG
CGATTATTTCGGTCAAGGTTCCGGATCCGAAGTTCAGCTC
>05D 137
CTGCACGGTGTAGGTGTGTCGGTAGTGAACGCACTGTCCAAAGAACTGATCCTGACTGTTCGCCGTAGCGGCAAGAT
CTGGGAACAAACTTACATCCACGGTGTTCCGCAAGAGCCGATGAAAATCGTTGGCGAGAGCGAAACCACGGGTACGC
AGATTCACTTCAAGCCTTCGGCTGAAACCTTCAAGAACATCCACTTCAGCTGGGACATCCTGGCCAAGCGGATTCGT
GAACTGTCCTTCCTGAACTCCGGTGTCGGCATCGTCCTCAAGGATGAGCGCAGCGGCAAGGAAGAGCTGTTCAAGTA
CGAAGGTGGTTTGCGTGCGTTCGTTGAATACCTGAACACCAACAAGACTGCGGTCAACCAGGTGTTCCACTTCAACA
TCCAGCGTGAAGACGGCATCGGCGTGGAAATCGCCCTGCAGTGGAACGACAGCTTCAACGAGAACCTGTTGTGCTTC

ACCAACAACATTCCTCAGCGCGATGGCGGTACTCACCTGGTGGGTTTCCGTTCCGCACTGACGCGTAACCTGAACAC
CTACATCGAAGCTGAAGGCCTGGCGAAAAAACACAAAGTCGCCACCACCGGCGACGACGCCCGCGAAGGCCTGACCG
CGATTATTTCGGTCAAGGTTCCGGATCCGAAGTTCAGCTC
>S10D 364
CTGCACGGTGTAGGTGTGTCGGTAGTGAATGCGCTGTCCAAAGAACTGATCCTGACTGTTCGCCGTAGCGGCAAGAT
CTGGGAACAAACTTACATCCACGGTGTTCCGCAAGAGCCGATGAAAATCGTTGGCGAGAGCGAAACCACGGGTACGC
AGATTCACTTCAAGCCTTCGGCTGAAACCTTCAAGAACATCCACTTCAGCTGGGACATCCTGGCCAAGCGGATTCGT
GAACTGTCCTTCCTGAACTCCGGTGTCGGCATCGTCCTCAAGGATGAGCGCAGCGGCAAGGAAGAGCTGTTCAAGTA
TGAAGGTGGTTTGCGTGCGTTCGTTGAATACCTGAACACCAACAAGACTGCGGTCAACCAGGTGTTCCACTTCAACA
TCCAGCGTGAAGACGGCATCGGCGTGGAAATCGCCCTGCAGTGGAACGACAGCTTCAACGAGAACCTGTTGTGCTTC
ACCAACAACATTCCTCAGCGCGATGGCGGTACTCACCTGGTGGGTTTCCGTTCCGCACTGACGCGTAACCTGAACAC
CTACATCGAAGCTGAAGGCCTGGCGAAAAAACACAAAGTCGCCACCACCGGCGACGACGCCCGCGAAGGCCTGACCG
CGATTATTTCGGTCAAGGTTCCGGATCCGAAGTTCAGCTC
>S11D 372
CTGCACGGTGTAGGTGTGTCGGTAGTGAATGCGCTGTCCAAAGAACTGATCCTGACTGTTCGCCGTAGCGGCAAGAT
CTGGGAACAAACTTACATCCACGGTGTTCCGCAAGAGCCGATGAAAATCGTTGGCGAGAGCGAAACCACGGGTACGC
AGATTCACTTCAAGCCTTCGGCTGAAACCTTCAAGAACATCCACTTCAGCTGGGACATCCTGGCCAAGCGGATTCGT
GAACTGTCCTTCCTGAACTCCGGTGTCGGCATCGTCCTCAAGGATGAGCGCAGCGGCAAGGAAGAGCTGTTCAAGTA
TGAAGGTGGTTTGCGTGCGTTCGTTGAATACCTGAACACCAACAAGACTGCGGTCAACCAGGTGTTCCACTTCAACA
TCCAGCGTGAAGACGGCATCGGCGTGGAAATCGCCCTGCAGTGGAACGACAGCTTCAACGAGAACCTGTTGTGCTTC
ACCAACAACATTCCTCAGCGCGATGGCGGTACTCACCTGGTGGGTTTCCGTTCCGCACTGACGCGTAACCTGAACAC
CTACATCGAAGCTGAAGGCCTGGCGAAAAAACACAAAGTCGCCACCACCGGCGACGACGCCCGCGAAGGCCTGACCG
CGATTATTTCGGTCAAGGTTCCGGATCCGAAGTTCAGCTC
>S03D 308
CTGCACGGTGTAGGTGTGTCGGTAGTGAATGCGCTGTCCAAAGAACTGATCCTGACCGTTCGCCGTAGCGGCAAGAT
CTGGGAACAAACTTACATCCACGGTGTTCCGCAAGAGCCGATGAAAATCGTTGGCGAGAGCGAAACCACGGGTACGC
AGATTCACTTCAAGCCTTCGGCTGAAACCTTCAAGAACATCCACTTCAGCTGGGACATCCTGGCCAAGCGGATTCGT
GAACTGTCCTTCCTGAACTCCGGTGTCGGCATCGTCCTCAAGGATGAGCGCAGCGGCAAGGAAGAGCTGTTCAAGTA
TGAAGGTGGTTTGCGTGCGTTCGTTGAATACCTGAACACCAACAAGACTGCGGTCAACCAGGTGTTCCACTTCAACA
TCCAGCGTGAAGACGGCATCGGCGTGGAAATCGCCCTGCAGTGGAACGACAGCTTCAACGAGAACCTGTTGTGCTTC
ACCAACAACATTCCTCAGCGCGATGGCGGTACTCACCTGGTGGGTTTCCGTTCCGCACTGACGCGTAACCTGAACAC
CTACATCGAAGCTGAAGGCCTGGCGAAAAAACACAAAGTCGCCACCACCGGCGACGACGCCCGCGAAGGCCTGACCG
CGATTATTTCGGTCAAGGTTCCGGATCCGAAGTTCAGCTC
>S02A 297
CTGCACGGTGTAGGTGTGTCGGTAGTGAATGCGCTGTCCAAAGAACTGATCCTGACCGTTCGCCGTAGCGGCAAGAT
CTGGGAACAAACTTACATCCACGGTGTTCCGCAAGAGCCGATGAAAATCGTTGGCGAGAGCGAAACCACGGGTACGC
AGATTCACTTCAAGCCTTCGGCTGAAACCTTCAAGAACATCCACTTCAGCTGGGACATCCTGGCCAAGCGGATTCGT
GAACTGTCCTTCCTGAACTCCGGTGTCGGCATCGTCCTCAAGGATGAGCGCAGCGGCAAGGAAGAGCTGTTCAAGTA
TGAAGGTGGTTTGCGTGCGTTCGTTGAATACCTGAACACCAACAAGACTGCGGTCAACCAGGTGTTCCACTTCAACA
TCCAGCGTGAAGACGGCATCGGCGTGGAAATCGCCCTGCAGTGGAACGACAGCTTCAACGAGAACCTGTTGTGCTTC
ACCAACAACATTCCTCAGCGCGATGGCGGTACTCACCTGGTGGGTTTCCGTTCCGCACTGACGCGTAACCTGAACAC
CTACATCGAAGCTGAAGGCCTGGCGAAAAAACACAAAGTCGCCACCACCGGCGACGACGCCCGCGAAGGCCTGACCG
CGATTATTTCGGTCAAGGTTCCGGATCCGAAGTTCAGCTC
>S07H 248
CTGCACGGTGTAGGTGTGTCGGTAGTGAATGCGCTGTCCAAAGAACTGATCCTGACTGTTCGCCGTAGCGGCAAGAT
CTGGGAACAAACTTACATCCACGGTGTTCCGCAAGAGCCGATGAAAATCGTTGGCGAGAGCGAAACCACGGGTACGC
AGATTCACTTCAAGCCTTCGGCTGAAACCTTCAAGAACATCCACTTCAGCTGGGACATCCTGGCCAAGCGGATTCGT
GAACTGTCCTTCCTGAACTCCGGTGTCGGCATCGTCCTCAAGGATGAGCGCAGCGGCAAGGAAGAGCTGTTCAAGTA
CGAAGGTGGTTTGCGTGCGTTCGTTGAATACCTGAACACCAACAAGACTGCGGTTAACCAGGTTTTCCACTTCAACA
TCCAGCGTGAAGACGGCATTGGCGTGGAAATCGCCCTGCAGTGGAACGACAGCTTCAACGAGAACCTGTTGTGCTTC
ACCAACAACATTCCTCAGCGCGATGGCGGTACTCACCTGGTGGGTTTCCGTTCCGCACTGACGCGTAACCTGAACAC
CTACATCGAAGCTGAAGGCCTGGCGAAGAAACACAAAGTCGCCACCACCGGCGACGACGCCCGCGAAGGCCTGACCG
CGATTATCTCGGTCAAGGTTCCGGATCCGAAGTTCAGCTC
>05H 185
CTGCACGGTGTAGGTGTGTCGGTAGTGAATGCGCTGTCCAAAGAACTGATCCTGACTGTTCGCCGTAGCGGCAAGAT
CTGGGAACAAACTTACGTACACGGTGTTCCGCAAGAGCCGATGAAAATCGTTGGCGAGAGCGAAACCACGGGTACGC
AGATTCACTTCAAGCCTTCGGCTGAAACCTTCAAGAACATCCACTTCAGCTGGGACATCCTGGCCAAGCGGATTCGT

GAACTGTCGTTCCTGAACTCCGGTGTCGGCATCGTCCTCAAGGATGAGCGCAGCGGCAAGGAAGAGCTGTTCAAGTA
CGAAGGTGGTTTGCGTGCGTTCGTTGAATACCTGAACACCAACAAGACTGCGGTCAACCAGGTGTTCCACTTCAACA
TCCAGCGTGAAGACGGCATCGGCGTGGAAATCGCCCTGCAGTGGAACGACAGCTTCAACGAGAACCTGTTGTGCTTC
ACCAACAACATTCCTCAGCGCGATGGCGGTACTCACCTGGTGGGTTTCCGTTCCGCACTGACGCGTAACCTGAACAC
CTACATCGAAGCTGAAGGCCTGGCGAAGAAACACAAAGTCGCCACCACCGGCGACGACGCCCGCGAAGGCCTGACCG
CTATTATCTCGGTCAAGGTTCCGGATCCGAAGTTCAGCTC
>05G 77
CTGCACGGTGTAGGTGTGTCGGTAGTGAATGCGCTGTCCAAAGAACTGATCCTGACTGTTCGCCGTAGCGGCAAGAT
CTGGGAACAAACTTACGTACACGGTGTTCCGCAAGAGCCGATGAAAATCGTTGGCGAGAGCGAAACCACGGGTACGC
AGATTCACTTCAAGCCTTCGGCTGAAACCTTCAAGAACATCCACTTCAGCTGGGACATCCTGGCCAAGCGGATTCGT
GAACTGTCGTTCCTGAACTCCGGTGTCGGCATCGTCCTCAAGGATGAGCGCAGCGGCAAGGAAGAGCTGTTCAAGTA
CGAAGGTGGTTTGCGTGCGTTCGTTGAATACCTGAACACCAACAAGACTGCGGTCAACCAGGTGTTCCACTTCAACA
TCCAGCGTGAAGACGGCATCGGCGTGGAAATCGCCCTGCAGTGGAACGACAGCTTCAACGAGAACCTGTTGTGCTTC
ACCAACAACATTCCTCAGCGCGATGGCGGTACTCACCTGGTGGGTTTCCGTTCCGCACTGACGCGTAACCTGAACAC
CTACATCGAAGCTGAAGGCCTGGCGAAGAAACACAAAGTCGCCACCACCGGCGACGACGCCCGCGAAGGCCTGACCG
CTATTATCTCGGTCAAGGTTCCGGATCCGAAGTTCAGCTC
>S11E 373
CTGCACGGTGTAGGTGTGTCGGTAGTGAATGCGCTGTCCAAAGAACTGATCCTGACTGTTCGCCGTAGCGGCAAGAT
CTGGGAACAAACTTACGTACACGGTGTTCCGCAAGAGCCGATGAAAATCGTTGGCGAGAGCGAAACCACGGGTACGC
AGATTCACTTCAAGCCTTCGGCTGAAACCTTCAAGAACATCCACTTCAGCTGGGACATCCTGGCCAAGCGGATTCGT
GAACTGTCGTTCCTGAACTCCGGTGTCGGCATCGTCCTCAAGGATGAGCGCAGCGGCAAGGAAGAGCTGTTCAAGTA
CGAAGGTGGTTTGCGTGCGTTCGTTGAATACCTGAACACCAACAAGACTGCGGTCAACCAGGTGTTCCACTTCAACA
TCCAGCGTGAAGACGGCATCGGCGTGGAAATCGCCCTGCAGTGGAACGACAGCTTCAACGAGAACCTGTTGTGCTTC
ACCAACAACATTCCTCAGCGCGATGGCGGTACTCACCTGGTGGGTTTCCGTTCCGCACTGACGCGTAACCTGAACAC
CTACATCGAAGCTGAAGGCCTGGCGAAGAAACACAAAGTCGCCACCACCGGCGACGACGCCCGCGAAGGCCTGACCG
CTATTATCTCGGTCAAGGTTCCGGATCCGAAGTTCAGCTC
>S10D 268
CTGCACGGTGTAGGTGTGTCGGTAGTGAATGCGCTGTCCAAAGAACTGATCCTGACTGTTCGCCGTAGCGGCAAGAT
CTGGGAACAAACTTACGTACACGGTGTTCCGCAAGAGCCGATGAAAATCGTTGGCGAGAGCGAAACCACGGGTACGC
AGATTCACTTCAAGCCTTCGGCTGAAACCTTCAAGAACATCCACTTCAGCTGGGACATCCTGGCCAAGCGGATTCGT
GAACTGTCGTTCCTGAACTCCGGTGTCGGCATCGTCCTCAAGGATGAGCGCAGCGGCAAGGAAGAGCTGTTCAAGTA
CGAAGGTGGTTTGCGTGCGTTCGTTGAATACCTGAACACCAACAAGACTGCGGTCAACCAGGTGTTCCACTTCAACA
TCCAGCGTGAAGACGGCATCGGCGTGGAAATCGCCCTGCAGTGGAACGACAGCTTCAACGAGAACCTGTTGTGCTTC
ACCAACAACATTCCTCAGCGCGATGGCGGTACTCACCTGGTGGGTTTCCGTTCCGCACTGACGCGTAACCTGAACAC
CTACATCGAAGCTGAAGGCCTGGCGAAGAAACACAAAGTCGCCACCACCGGCGACGACGCCCGCGAAGGCCTGACCG
CTATTATCTCGGTCAAGGTTCCGGATCCGAAGTTCAGCTC
>S09E 261
CTGCACGGTGTAGGTGTGTCGGTAGTGAATGCGCTGTCCAAAGAACTGATCCTGACTGTTCGCCGTAGCGGCAAGAT
CTGGGAACAAACTTACGTACACGGTGTTCCGCAAGAGCCGATGAAAATCGTTGGCGAGAGCGAAACCACGGGTACGC
AGATTCACTTCAAGCCTTCGGCTGAAACCTTCAAGAACATCCACTTCAGCTGGGACATCCTGGCCAAGCGGATTCGT
GAACTGTCGTTCCTGAACTCCGGTGTCGGCATCGTCCTCAAGGATGAGCGCAGCGGCAAGGAAGAGCTGTTCAAGTA
CGAAGGTGGTTTGCGTGCGTTCGTTGAATACCTGAACACCAACAAGACTGCGGTCAACCAGGTGTTCCACTTCAACA
TCCAGCGTGAAGACGGCATCGGCGTGGAAATCGCCCTGCAGTGGAACGACAGCTTCAACGAGAACCTGTTGTGCTTC
ACCAACAACATTCCTCAGCGCGATGGCGGTACTCACCTGGTGGGTTTCCGTTCCGCACTGACGCGTAACCTGAACAC
CTACATCGAAGCTGAAGGCCTGGCGAAGAAACACAAAGTCGCCACCACCGGCGACGACGCCCGCGAAGGCCTGACCG
CTATTATCTCGGTCAAGGTTCCGGATCCGAAGTTCAGCTC
>S07B 242
CTGCACGGTGTAGGTGTGTCGGTAGTGAATGCGCTGTCCAAAGAACTGATCCTGACTGTTCGCCGTAGCGGCAAGAT
CTGGGAACAAACTTACGTACACGGTGTTCCGCAAGAGCCGATGAAAATCGTTGGCGAGAGCGAAACCACGGGTACGC
AGATTCACTTCAAGCCTTCGGCTGAAACCTTCAAGAACATCCACTTCAGCTGGGACATCCTGGCCAAGCGGATTCGT
GAACTGTCGTTCCTGAACTCCGGTGTCGGCATCGTCCTCAAGGATGAGCGCAGCGGCAAGGAAGAGCTGTTCAAGTA
CGAAGGTGGTTTGCGTGCGTTCGTTGAATACCTGAACACCAACAAGACTGCGGTCAACCAGGTGTTCCACTTCAACA
TTCAGCGTGAAGACGGCATCGGCGTGGAAATCGCCCTGCAGTGGAACGACAGCTTCAACGAGAACCTGTTGTGCTTC
ACCAACAACATTCCTCAGCGCGATGGCGGTACTCACCTGGTGGGTTTCCGTTCCGCACTGACGCGTAACCTGAACAC
CTACATCGAAGCTGAAGGCCTGGCGAAGAAACACAAAGTCGCCACCACCGGCGACGACGCCCGCGAAGGCCTGACCG
CGATTATCTCGGTCAAGGTTCCGGATCCGAAGTTCAGCTC

>01B 13
CTGCACGGTGTAGGTGTGTCGGTAGTGAACGCACTGTCCAAAGAACTGATCCTGACTGTTCGCCGTAGCGGCAAGAT
CTGGGAACAAACTTACATCCACGGTGTTCCGCAAGAGCCGATGAAAATCGTTGGCGAGAGCGAAACCACGGGTACGC
AGATTCACTTCAAGCCTTCGGCTGAAACCTTCAAGAACATCCACTTCAGCTGGGACATCCTGGCCAAGCGGATTCGT
GAACTGTCCTTCCTGAACTCCGGTGTCGGCATCGTCCTCAAGGATGAGCGCAGCGGCAAGGAAGAGCTGTTCAAGTA
CGAAGGTGGTTTGCGTGCGTTCGTTGAATACCTGAACACCAACAAGACTGCGGTCAACCAGGTGTTCCACTTCAACA
TCCAGCGTGAAGACGGCATCGGCGTGGAAATCGCCCTGCAGTGGAACGACAGCTTCAACGAGAACCTGTTGTGCTTC
ACCAACAACATTCCTCAGCGCGATGGCGGTACTCACCTGGTGGGTTTCCGTTCCGCACTGACGCGTAACCTGAACAC
CTACATCGAAGCTGAAGGCCTGGCGAAAAAACACAAAGTCGCCACCACCGGCGACGACGCCCGCGAAGGCCTGACCG
CGATTATTTCGGTCAAGGTTCCGGATCCGAAGTTCAGCTC
>06B 18
CTGCACGGTGTAGGTGTGTCGGTAGTGAACGCACTGTCCAAAGAACTGATCCTGACTGTTCGCCGTAGCGGCAAGAT
CTGGGAACAAACTTACATCCACGGTGTTCCGCAAGAGCCGATGAAAATCGTTGGCGAGAGCGAAACCACGGGTACGC
AGATTCACTTCAAGCCTTCGGCTGAAACCTTCAAGAACATCCACTTCAGCTGGGACATCCTGGCCAAGCGGATTCGT
GAACTGTCCTTCCTGAACTCCGGTGTCGGCATCGTCCTCAAGGATGAGCGCAGCGGCAAGGAAGAGCTGTTCAAGTA
CGAAGGTGGTTTGCGTGCGTTCGTTGAATACCTGAACACCAACAAGACTGCGGTCAACCAGGTGTTCCACTTCAACA
TCCAGCGTGAAGACGGCATCGGCGTGGAAATCGCCCTGCAGTGGAACGACAGCTTCAACGAGAACCTGTTGTGCTTC
ACCAACAACATTCCTCAGCGCGATGGCGGTACTCACCTGGTGGGTTTCCGTTCCGCACTGACGCGTAACCTGAACAC
CTACATCGAAGCTGAAGGCCTGGCGAAAAAACACAAAGTCGCCACCACCGGCGACGACGCCCGCGAAGGCCTGACCG
CGATTATTTCGGTCAAGGTTCCGGATCCGAAGTTCAGCTC
>S04C 219
CTGCACGGTGTAGGTGTGTCGGTAGTGAATGCGCTGTCCAAAGAACTGATCCTGACTGTTCGCCGTAGCGGCAAGAT
CTGGGAACAAACTTACATCCACGGTGTTCCGCAAGAGCCGATGAAAATCGTTGGCGAGAGCGAAACCACGGGTACGC
AGATTCACTTCAAGCCTTCGGCTGAAACCTTCAAGAACATCCACTTCAGCTGGGACATCCTGGCCAAGCGGATTCGT
GAACTGTCCTTCCTGAACTCCGGTGTCGGCATCGTCCTCAAGGATGAGCGCAGCGGCAAGGAAGAGCTGTTCAAGTA
CGAAGGTGGTTTGCGTGCGTTCGTTGAATACCTGAACACCAACAAGACTGCGGTCAACCAGGTGTTCCACTTCAACA
TCCAGCGTGAAGACGGCATTGGCGTGGAAATCGCCCTGCAGTGGAACGACAGCTTCAACGAGAACCTGTTGTGCTTC
ACCAACAACATTCCTCAGCGCGATGGCGGGACTCACCTGGTGGGTTTCCGTTCCGCACTGACGCGTAACCTGAACAC
CTACATCGAAGCTGAAGGCCTGGCGAAGAAACACAAAGTCGCCACCACCGGCGACGACGCCCGCGAAGGCCTGACTG
CGATTATCTCGGTCAAGGTTCCGGATCCGAAGTTCAGCTC
>S03E 213
CTGCACGGTGTAGGTGTGTCGGTAGTGAATGCGCTGTCCAAAGAACTGATCCTGACTGTTCGCCGTAGCGGCAAGAT
CTGGGAACAAACTTACATCCACGGTGTTCCGCAAGAGCCGATGAAAATCGTTGGCGAGAGCGAAACCACGGGTACGC
AGATTCACTTCAAGCCTTCGGCTGAAACCTTCAAGAACATCCACTTCAGCTGGGACATCCTGGCCAAGCGGATTCGT
GAACTGTCCTTCCTGAACTCCGGTGTCGGCATCGTCCTCAAGGATGAGCGCAGCGGCAAGGAAGAGCTGTTCAAGTA
CGAAGGTGGTTTGCGTGCGTTCGTTGAATACCTGAACACCAACAAGACTGCGGTCAACCAGGTGTTCCACTTCAACA
TCCAGCGTGAAGACGGCATTGGCGTGGAAATCGCCCTGCAGTGGAACGACAGCTTCAACGAGAACCTGTTGTGCTTC
ACCAACAACATTCCTCAGCGCGATGGCGGTACTCACCTGGTGGGTTTCCGTTCCGCACTGACGCGTAACCTGAACAC
CTACATCGAAGCTGAAGGCCTGGCGAAGAAACACAAAGTCGCCACCACCGGCGACGACGCCCGCGAAGGCCTGACTG
CGATTATCTCGGTCAAGGTTCCGGATCCGAAGTTCAGCTC
>S08H 256
CTGCACGGTGTAGGTGTGTCGGTAGTGAATGCGCTGTCCAAAGAACTGATCCTGACTGTTCGCCGTAGCGGCAAGAT
CTGGGAACAAACTTACATCCACGGTGTTCCGCAAGAGCCGATGAAAATCGTTGGCGAGAGCGAAACCACGGGTACGC
AGATTCACTTCAAGCCTTCGGCTGAAACCTTCAAGAACATCCACTTCAGCTGGGACATCCTGGCCAAGCGGATTCGT
GAACTGTCCTTCCTGAACTCCGGTGTCGGCATCGTCCTCAAGGATGAGCGCAGCGGCAAGGAAGAGCTGTTCAAGTA
CGAAGGTGGTTTGCGTGCGTTCGTTGAATACCTGAACACCAACAAGACTGCGGTCAACCAGGTGTTCCACTTCAATA
TCCAGCGTGAAGACGGCATTGGCGTGGAAATCGCCCTGCAGTGGAACGACAGCTTCAACGAGAACCTGTTGTGCTTC
ACCAACAACATTCCTCAGCGCGATGGCGGTACTCACCTGGTGGGTTTCCGTTCCGCACTGACGCGTAACCTGAACAC
CTACATCGAAGCTGAAGGCCTGGCGAAGAAACACAAAGTCGCCACCACCGGCGACGACGCCCGCGAAGGCCTGACCG
CGATTATCTCGGTCAAGGTTCCGGATCCGAAGTTCAGCTC
>S03A 305
CTGCACGGTGTAGGTGTGTCCGTAGTGAATGCGCTGTCCAAAGAACTGATCCTGACTGTTCGCCGTAGCGGCAAGAT
CTGGGAACAAACTTACGTCCACGGTGTTCCGCAAGAGCCGATGAAAATCGTTGGCGAGAGCGAAACCACGGGTACGC
AGATTCACTTCAAGCCTTCGGCTGAAACCTTCAAGAACATCCACTTCAGCTGGGACATCCTGGCCAAGCGGATTCGT
GAACTGTCCTTCCTGAACTCCGGTGTCGGCATCGTCCTCAAGGATGAGCGCAGCGGCAAGGAAGAGCTGTTCAAGTA
CGAAGGTGGTTTGCGTGCGTTCGTTGAATACCTGAATACCAACAAGACTGCGGTCAACCAGGTGTTCCACTTCAACA
TCCAGCGTGAAGACGGCATCGGCGTGGAAATCGCCCTGCAGTGGAACGACAGCTTCAACGAGAACCTGTTGTGCTTC

ACCAACAACATTCCTCAGCGCGATGGCGGTACTCACCTGGTGGGTTTCCGTTCCGCACTGACGCGTAACCTGAACAC
CTACATCGAAGCTGAAGGCCTGGCGAAGAAACACAAAGTCGCCACCACCGGCGACGACGCCCGCGAAGGCCTGACCG
CGATTATCTCGGTCAAGGTTCCGGATCCGAAGTTCAGCTC
>S07F 246
CTGCACGGTGTAGGTGTGTCCGTAGTGAATGCGCTGTCCAAAGAACTGATCCTGACTGTTCGCCGTAGCGGCAAGAT
CTGGGAACAAACTTACGTCCACGGTGTTCCGCAAGAGCCGATGAAAATCGTTGGCGAGAGCGAAACCACGGGTACGC
AGATTCACTTCAAGCCTTCGGCTGAAACCTTCAAGAACATCCACTTCAGCTGGGACATCCTGGCCAAGCGGATTCGT
GAACTGTCCTTCCTGAACTCCGGTGTCGGCATCGTCCTCAAGGATGAGCGCAGCGGCAAGGAAGAGCTGTTCAAGTA
CGAAGGTGGTTTGCGTGCGTTCGTTGAATACCTGAACACCAACAAGACTGCGGTCAACCAGGTGTTCCACTTCAACA
TCCAGCGTGAAGACGGCATCGGCGTGGAAATCGCCCTGCAGTGGAACGACAGCTTCAACGAGAACCTGTTGTGCTTC
ACCAACAACATTCCTCAGCGCGATGGCGGTACTCACCTGGTGGGTTTCCGTTCCGCACTGACGCGTAACCTGAACAC
CTACATCGAAGCTGAAGGCCTGGCGAAGAAACACAAAGTCGCCACCACCGGCGACGACGCCCGCGAAGGCCTGACCG
CGATTATCTCGGTCAAGGTTCCGGATCCGAAGTTCAGCTC
>S11G 279
CTGCACGGTGTAGGTGTGTCGGTAGTGAATGCGCTGTCCAAAGAACTGATCCTGACCGTTCGCCGTAGCGGCAAGAT
CTGGGAACAAACTTACATCCACGGTGTTCCGCAAGAGCCGATGAAAATCGTTGGCGAGAGCGAAACCACGGGTACGC
AGATTCACTTCAAGCCTTCGGCTGAAACCTTCAAGAACATCCACTTCAGCTGGGACATCCTGGCCAAGCGGATTCGT
GAACTGTCCTTCCTGAACTCCGGTGTCGGCATCGTCCTCAAGGATGAGCGCAGCGGCAAGGAAGAGCTGTTCAAGTA
TGAAGGTGGTTTGCGTGCGTTCGTTGAATACCTGAACACCAACAAGACTGCGGTCAACCAGGTGTTCCACTTCAACA
TCCAGCGTGAAGACGGCATCGGCGTGGAAATCGCCCTGCAGTGGAACGACAGCTTCAACGAGAACCTGTTGTGCTTC
ACCAACAACATTCCTCAGCGCGATGGCGGTACTCACCTGGTGGGTTTCCGTTCCGCACTGACGCGTAACCTGAACAC
CTACATCGAAGCTGAAGGCCTGGCGAAAAAACACAAAGTCGCCACCACCGGCGACGACGCCCGCGAAGGCCTGACCG
CGATTATTTCGGTCAAGGTTCCGGATCCGAAGTTCAGCTC
>S04B 218
CTGCACGGTGTAGGTGTGTCGGTAGTGAATGCGCTGTCCAAAGAACTGATCCTGACCGTTCGCCGTAGCGGCAAGAT
CTGGGAACAAACTTACATCCACGGTGTTCCGCAAGAGCCGATGAAAATCGTTGGCGAGAGCGAAACCACGGGTACGC
AGATTCACTTCAAGCCTTCGGCTGAAACCTTCAAGAACATCCACTTCAGCTGGGACATCCTGGCCAAGCGGATTCGT
GAACTGTCCTTCCTGAACTCCGGTGTCGGCATCGTCCTCAAGGATGAGCGCAGCGGCAAGGAAGAGCTGTTCAAGTA
TGAAGGTGGTTTGCGTGCGTTCGTTGAATACCTGAACACCAACAAGACTGCGGTCAACCAGGTGTTCCACTTCAACA
TCCAGCGTGAAGACGGCATCGGCGTGGAAATCGCCCTGCAGTGGAACGACAGCTTCAACGAGAACCTGTTGTGCTTC
ACCAACAACATTCCTCAGCGCGATGGCGGTACTCACCTGGTGGGTTTCCGTTCCGCACTGACGCGTAACCTGAACAC
CTACATCGAAGCTGAAGGCCTGGCGAAAAAACACAAAGTCGCCACCACCGGCGACGACGCCCGCGAAGGCCTGACCG
CGATTATTTCGGTCAAGGTTCCGGATCCGAAGTTCAGCTC
>S07C 243
CTGCACGGTGTAGGTGTGTCGGTAGTGAATGCGCTGTCCAAAGAACTGATCCTGACCGTTCGCCGTAGCGGCAAGAT
CTGGGAACAAACTTACATCCACGGTGTTCCGCAAGAGCCGATGAAAATCGTTGGCGAGAGCGAAACCACGGGTACGC
AGATTCACTTCAAGCCTTCGGCTGAAACCTTCAAGAACATCCACTTCAGCTGGGACATCCTGGCCAAGCGGATTCGT
GAACTGTCCTTCCTGAACTCCGGTGTCGGCATCGTCCTCAAGGATGAGCGCAGCGGCAAGGAAGAGCTGTTCAAGTA
TGAAGGTGGTTTGCGTGCGTTCGTTGAATACCTGAACACCAACAAGACTGCGGTCAACCAGGTGTTCCACTTCAACA
TCCAGCGTGAAGACGGCATCGGCGTGGAAATCGCCCTGCAGTGGAACGACAGCTTCAACGAGAACCTGTTGTGCTTC
ACCAACAACATTCCTCAGCGCGATGGCGGTACTCACCTGGTGGGTTTCCGTTCCGCACTGACGCGTAACCTGAACAC
CTACATCGAAGCTGAAGGCCTGGCGAAAAAACACAAAGTCGCCACCACCGGCGACGACGCCCGCGAAGGCCTGACCG
CGATTATTTCGGTCAAGGTTCCGGATCCGAAGTTCAGCTC
>S02H 208
CTGCACGGTGTAGGTGTGTCGGTAGTGAATGCGCTGTCCAAAGAACTGATCCTGACCGTTCGCCGTAGCGGCAAGAT
CTGGGAACAAACTTACATCCACGGTGTTCCGCAAGAGCCGATGAAAATCGTTGGCGAGAGCGAAACCACGGGTACGC
AGATTCACTTCAAGCCTTCGGCTGAAACCTTCAAGAACATCCACTTCAGCTGGGACATCCTGGCCAAGCGGATTCGT
GAACTGTCCTTCCTGAACTCCGGTGTCGGCATCGTCCTCAAGGATGAGCGCAGCGGCAAGGAAGAGCTGTTCAAGTA
TGAAGGTGGTTTGCGTGCGTTCGTTGAATACCTGAACACCAACAAGACTGCGGTCAACCAGGTGTTCCACTTCAACA
TCCAGCGTGAAGACGGCATCGGCGTGGAAATCGCCCTGCAGTGGAACGACAGCTTCAACGAGAACCTGTTGTGCTTC
ACCAACAACATTCCTCAGCGCGATGGCGGTACTCACCTGGTGGGTTTCCGTTCCGCACTGACGCGTAACCTGAACAC
CTACATCGAAGCTGAAGGCCTGGCGAAAAAACACAAAGTCGCCACCACCGGCGACGACGCCCGCGAAGGCCTGACCG
CGATTATTTCGGTCAAGGTTCCGGATCCGAAGTTCAGCTC
>S04H 224
CTGCACGGTGTAGGTGTGTCGGTAGTGAATGCGCTGTCCAAAGAACTGATCCTGACTGTTCGCCGTAGCGGCAAGAT
CTGGGAACAAACTTACATCCACGGTGTTCCGCAAGAGCCGATGAAAATCGTTGGCGAGAGCGAAACCACGGGTACGC
AGATTCACTTCAAGCCTTCGGCTGAAACCTTCAAGAACATCCACTTCAGCTGGGACATCCTGGCCAAGCGGATTCGT

GAACTGTCCTTCCTGAACTCCGGTGTCGGCATCGTCCTCAAGGATGAGCGCAGCGGCAAGGAAGAGCTGTTCAAGTA
CGAAGGTGGTTTGCGTGCGTTCGTTGAATACCTGAACACCAACAAGACTGCGGTCAACCAGGTGTTCCACTTCAACA
TCCAGCGTGAAGACGGCATTGGCGTGGAAATCGCCCTGCAGTGGAACGACAGCTTCAACGAGAACCTGTTGTGCTTC
ACCAACAACATTCCTCAGCGCGATGGCGGTACTCACCTGGTGGGTTTCCGTTCCGCACTGACGCGTAACCTGAACAC
CTACATCGAAGCTGAAGGCCTGGCGAAGAAACACAAAGTCGCCACCACCGGCGACGACGCCCGCGAAGGCCTGACTG
CGATTATCTCGGTCAAGGTTCCGGATCCGAAGTTCAGCTC
>S07H 344
CTGCACGGTGTAGGTGTGTCGGTAGTGAATGCGCTGTCCAAAGAACTGATCCTGACCGTTCGCCGTAGCGGCAAGAT
CTGGGAACAAACTTACATCCACGGTGTTCCGCAAGAGCCGATGAAAATCGTTGGCGAGAGCGAAACCACGGGTACGC
AGATTCACTTCAAGCCTTCGGCTGAAACCTTCAAGAACATCCACTTCAGCTGGGACATCCTGGCCAAGCGGATTCGT
GAACTGTCCTTCCTGAACTCCGGTGTCGGCATCGTCCTCAAGGATGAGCGCAGCGGCAAGGAAGAGCTGTTCAAGTA
TGAAGGTGGTTTGCGTGCGTTCGTTGAATACCTGAACACCAACAAGACTGCGGTCAACCAGGTGTTCCACTTCAACA
TCCAGCGTGAAGACGGCATCGGCGTGGAAATCGCCCTGCAGTGGAACGACAGCTTCAACGAGAACCTGTTGTGCTTC
ACCAACAACATTCCTCAGCGCGATGGCGGTACTCACCTGGTGGGTTTCCGTTCCGCACTGACGCGTAACCTGAACAC
CTACATCGAAGCTGAAGGCCTGGCGAAAAAACACAAAGTCGCCACCACCGGCGACGACGCCCGCGAAGGCCTGACCG
CGATTATTTCGGTCAAGGTTCCGGATCCGAAGTTCAGCTC
>S03H 312
CTGCACGGTGTAGGTGTGTCGGTAGTGAATGCGCTGTCCAAAGAACTGATCCTGACTGTTCGCCGTAGCGGCAAGAT
CTGGGAACAAACTTACATCCACGGTGTTCCGCAAGAGCCGATGAAAATCGTTGGCGAGAGCGAAACCACGGGTACGC
AGATTCACTTCAAGCCTTCGGCTGAAACCTTCAAGAACATCCACTTCAGCTGGGACATCCTGGCCAAGCGGATTCGT
GAACTGTCCTTCCTGAACTCCGGTGTCGGCATCGTCCTCAAGGATGAGCGCAGCGGCAAGGAAGAGCTGTTCAAGTA
CGAAGGTGGTTTGCGTGCGTTCGTTGAATACCTGAACACCAACAAGACTGCGGTCAACCAGGTGTTCCACTTCAATA
TCCAGCGTGAAGACGGCATTGGCGTGGAAATCGCCCTGCAGTGGAACGACAGCTTCAACGAGAACCTGTTGTGCTTC
ACCAACAACATTCCTCAGCGCGATGGCGGTACTCACCTGGTGGGTTTCCGTTCCGCACTGACGCGTAACCTGAACAC
CTACATCGAAGCTGAAGGCCTGGCGAAGAAACACAAAGTCGCCACCACCGGCGACGACGCCCGCGAAGGCCTGACCG
CGATTATCTCGGTCAAGGTTCCGGATCCGAAGTTCAGCTC
>S08C 347
CTGCACGGTGTAGGTGTATCCGTAGTGAATGCGCTGTCCAAAGAACTGATCCTGACTGTTCGCCGTAGCGGCAAGAT
CTGGGAACAAACTTACGTCCACGGTGTTCCGCAAGAGCCGATGAAAATCGTTGGCGAGAGCGAAACCACGGGTACGC
AGATTCACTTCAAGCCTTCGGCTGAAACCTTCAAGAACATCCACTTCAGCTGGGACATCCTGGCCAAGCGGATTCGT
GAACTGTCCTTCCTGAACTCCGGTGTCGGCATCGTCCTCAAGGATGAGCGCAGCGGTAAGGAAGAGCTGTTCAAGTA
CGAAGGTGGTTTGCGTGCGTTCGTTGAATACCTGAACACCAACAAGACTGCGGTCAACCAGGTGTTCCACTTCAACA
TCCAGCGTGAAGACGGCATTGGCGTGGAAATCGCCCTGCAGTGGAACGACAGCTTCAACGAGAACCTGTTGTGCTTC
ACCAACAACATTCCTCAGCGCGATGGCGGTACTCACCTGGTGGGTTTCCGTTCCGCACTGACGCGTAACCTGAACAC
CTACATCGAAGCTGAAGGCCTGGCGAAGAAACACAAAGTCGCCACCACCGGCGACGACGCCCGCGAAGGCCTGACCG
CGATTATCTCGGTCAAGGTTCCGGATCCGAAGTTCAGCTC
>S05G 231
CTGCACGGTGTAGGTGTGTCGGTAGTGAATGCGCTGTCCAAAGAACTGATCCTGACTGTTCGCCGTAGCGGCAAGAT
CTGGGAACAAACTTACGTACACGGTGTTCCGCAAGAGCCGATGAAAATCGTTGGCGAGAGCGAAACCACGGGTACGC
AGATTCACTTCAAGCCTTCGGCTGAAACCTTCAAGAACATCCACTTCAGCTGGGACATCCTGGCCAAGCGGATTCGT
GAACTGTCCTTCCTGAACTCCGGTGTCGGCATCGTCCTCAAGGATGAGCGCAGCGGCAAGGAAGAGCTGTTCAAGTA
CGAAGGTGGTTTGCGTGCGTTCGTTGAATACCTGAATACCAACAAGACTGCGGTCAACCAGGTGTTCCACTTCAACA
TCCAGCGTGAAGACGGCATCGGCGTGGAAATCGCCCTGCAGTGGAACGACAGCTTCAACGAGAACCTGTTGTGCTTC
ACCAACAACATTCCTCAGCGCGATGGCGGTACTCACCTGGTGGGTTTCCGTTCCGCACTGACGCGTAACCTGAACAC
CTACATCGAAGCTGAAGGCCTGGCGAAGAAACACAAAGTCGCCACCACCGGCGACGACGCCCGCGAAGGCCTGACCG
CGATTATCTCGGTCAAGGTTCCGGATCCGAAGTTCAGCTC
>S01H 296
CTGCACGGTGTAGGTGTGTCCGTAGTGAATGCGCTGTCCAAAGAACTGATCCTGACTGTTCGCCGTAGCGGCAAGAT
CTGGGAACAAACTTACGTCCACGGTGTTCCGCAAGAGCCGATGAAAATCGTTGGCGAGAGCGAAACCACGGGGACGC
AGATTCACTTCAAGCCTTCGGCTGAAACCTTCAAGAACATCCACTTCAGCTGGGACATCCTGGCCAAGCGGATTCGT
GAACTGTCGTTCCTGAACTCCGGTGTCGGCATCGTCCTCAAGGATGAGCGCAGCGGTAAGGAAGAGCTGTTCAAGTA
CGAAGGTGGTTTGCGTGCGTTCGTTGAATACCTGAACACCAACAAGACTGCGGTCAACCAGGTGTTCCACTTCAACA
TCCAGCGTGAAGACGGCATCGGCGTGGAAATCGCCCTGCAGTGGAACGACAGCTTCAACGAGAACCTGTTGTGCTTC
ACCAACAACATTCCTCAGCGCGATGGCGGTACTCACCTGGTGGGTTTCCGTTCCGCACTGACGCGTAACCTGAACAC
CTACATCGAAGCTGAAGGCCTGGCGAAGAAACACAAAGTCGCCACCACCGGCGACGACGCCCGCGAAGGCCTGACCG
CGATTATCTCGGTCAAGGTTCCGGATCCGAAGTTCAGCTC

>S05E 325
CTGCACGGTGTAGGTGTGTCCGTAGTGAATGCGCTGTCCAAAGAACTGATCCTGACTGTTCGCCGTAGCGGCAAGAT
CTGGGAACAAACTTACGTCCACGGTGTTCCGCAAGAGCCGATGAAAATCGTTGGCGAGAGCGAAACCACGGGGACGC
AGATTCACTTCAAGCCTTCGGCTGAAACCTTCAAGAACATCCACTTCAGCTGGGACATCCTGGCCAAGCGGATTCGT
GAACTGTCGTTCCTGAACTCCGGTGTCGGCATCGTCCTCAAGGATGAGCGCAGCGGTAAGGAAGAGCTGTTCAAGTA
CGAAGGTGGTTTGCGTGCGTTCGTTGAATACCTGAACACCAACAAGACTGCGGTCAACCAGGTGTTCCACTTCAACA
TCCAGCGTGAAGACGGCATCGGCGTGGAAATCGCCCTGCAGTGGAACGACAGCTTCAACGAGAACCTGTTGTGCTTC
ACCAACAACATTCCTCAGCGCGATGGCGGTACTCACCTGGTGGGTTTCCGTTCCGCACTGACGCGTAACCTGAACAC
CTACATCGAAGCTGAAGGCCTGGCGAAGAAACACAAAGTCGCCACCACCGGCGACGACGCCCGCGAAGGCCTGACCG
CGATTATCTCGGTCAAGGTTCCGGATCCGAAGTTCAGCTC
>02F 158
CTGCACGGTGTAGGTGTGTCGGTAGTGAATGCGCTGTCCAAAGAACTGATCCTGACTGTTCGCCGTAGCGGCAAGAT
CTGGGAACAAACTTACGTCCACGGTGTTCCGCAAGAGCCGATGAAAATCGTTGGCGAGAGCGAAACCACGGGGACGC
AGATTCACTTCAAGCCTTCGGCTGAAACCTTCAAGAACATCCACTTCAGCTGGGACATCCTGGCCAAGCGGATTCGT
GAACTGTCGTTCCTGAACTCCGGTGTCGGCATCGTCCTCAAGGATGAGCGCAGCGGTAAGGAAGAGCTGTTCAAGTA
CGAAGGTGGTTTGCGTGCGTTCGTTGAATACCTGAACACCAACAAGACTGCGGTCAACCAGGTGTTCCACTTCAACA
TCCAGCGTGAAGACGGCATCGGCGTGGAAATCGCCCTGCAGTGGAACGACAGCTTCAACGAGAACCTGTTGTGCTTC
ACCAACAACATTCCTCAGCGCGATGGCGGTACTCACCTGGTGGGTTTCCGTTCCGCACTGACGCGTAACCTGAACAC
CTACATCGAAGCTGAAGGCCTGGCGAAGAAACACAAAGTCGCCACCACCGGCGACGACGCCCGCGAAGGCCTGACCG
CGATTATCTCGGTCAAGGTTCCGGATCCGAAGTTCAGCTC
>S01D 196
CTGCACGGTGTAGGTGTGTCCGTAGTGAATGCGCTGTCCAAAGAACTGATCCTGACTGTTCGCCGTAGCGGCAAGAT
CTGGGAACAAACTTACGTCCACGGTGTTCCGCAAGAGCCGATGAAAATCGTTGGCGAGAGCGAAACCACGGGGACGC
AGATTCACTTCAAGCCTTCGGCTGAAACCTTCAAGAACATCCACTTCAGCTGGGACATCCTGGCCAAGCGGATTCGT
GAACTGTCGTTCCTGAACTCCGGTGTCGGCATCGTCCTCAAGGATGAGCGCAGCGGTAAGGAAGAGCTGTTCAAGTA
CGAAGGTGGTTTGCGTGCGTTCGTTGAATACCTGAACACCAACAAGACTGCGGTCAACCAGGTGTTCCACTTCAACA
TTCAGCGTGAAGACGGCATCGGCGTGGAAATCGCCCTGCAGTGGAACGACAGCTTCAACGAGAACCTGTTGTGCTTC
ACCAACAACATTCCTCAGCGCGATGGCGGTACTCACCTGGTGGGTTTCCGTTCCGCACTGACGCGTAACCTGAACAC
CTACATCGAAGCTGAAGGCCTGGCGAAGAAACACAAAGTCGCCACCACCGGCGACGACGCCCGCGAAGGCCTGACCG
CGATTATCTCGGTCAAGGTTCCGGATCCGAAGTTCAGCTC
>S01B 290
CTGCACGGTGTAGGTGTGTCCGTAGTGAATGCGCTGTCCAAAGAACTGATCCTGACTGTTCGCCGTAGCGGCAAGAT
CTGGGAACAAACTTACGTCCACGGTGTTCCGCAAGAGCCGATGAAAATCGTTGGCGAGAGCGAAACCACGGGGACGC
AGATTCACTTCAAGCCTTCGGCTGAAACCTTCAAGAACATCCACTTCAGCTGGGACATCCTGGCCAAGCGGATTCGT
GAACTGTCGTTCCTGAACTCCGGTGTCGGCATCGTCCTCAAGGATGAGCGCAGCGGTAAGGAAGAGCTGTTCAAGTA
CGAAGGTGGTTTGCGTGCGTTCGTTGAATACCTGAACACCAACAAGACTGCGGTCAACCAGGTGTTCCACTTCAACA
TTCAGCGTGAAGACGGCATCGGCGTGGAAATCGCCCTGCAGTGGAACGACAGCTTCAACGAGAACCTGTTGTGCTTC
ACCAACAACATTCCTCAGCGCGATGGCGGTACTCACCTGGTGGGTTTCCGTTCCGCACTGACGCGTAACCTGAACAC
CTACATCGAAGCTGAAGGCCTGGCGAAGAAACACAAAGTCGCCACCACCGGCGACGACGCCCGCGAAGGCCTGACCG
CGATTATCTCGGTCAAGGTTCCGGATCCGAAGTTCAGCTC
>S01A 289
CTGCACGGTGTAGGTGTGTCCGTAGTGAATGCGCTGTCCAAAGAACTGATCCTGACTGTTCGCCGTAGCGGCAAGAT
CTGGGAACAAACTTACGTCCACGGTGTTCCGCAAGAGCCGATGAAAATCGTTGGCGAGAGCGAAACCACGGGGACGC
AGATTCACTTCAAGCCTTCGGCTGAAACCTTCAAGAACATCCACTTCAGCTGGGACATCCTGGCCAAGCGGATTCGT
GAACTGTCGTTCCTGAACTCCGGTGTCGGCATCGTCCTCAAGGATGAGCGCAGCGGTAAGGAAGAGCTGTTCAAGTA
CGAAGGTGGTTTGCGTGCGTTCGTTGAATACCTGAACACCAACAAGACTGCGGTCAACCAGGTGTTCCACTTCAACA
TTCAGCGTGAAGACGGCATCGGCGTGGAAATCGCCCTGCAGTGGAACGACAGCTTCAACGAGAACCTGTTGTGCTTC
ACCAACAACATTCCTCAGCGCGATGGCGGTACTCACCTGGTGGGTTTCCGTTCCGCACTGACGCGTAACCTGAACAC
CTACATCGAAGCTGAAGGCCTGGCGAAGAAACACAAAGTCGCCACCACCGGCGACGACGCCCGCGAAGGCCTGACCG
CGATTATCTCGGTCAAGGTTCCGGATCCGAAGTTCAGCTC
>S03C 307
CTGCACGGTGTAGGTGTGTCCGTAGTGAATGCGCTGTCCAAAGAACTGATCCTGACTGTTCGCCGTAGCGGCAAGAT
CTGGGAACAAACTTACGTCCACGGTGTTCCGCAAGAGCCGATGAAAATCGTTGGCGAGAGCGAAACCACGGGGACGC
AGATTCACTTCAAGCCTTCGGCTGAAACCTTCAAGAACATCCACTTCAGCTGGGACATCCTGGCCAAGCGGATTCGT
GAACTGTCGTTCCTGAACTCCGGTGTCGGCATCGTCCTCAAGGATGAGCGCAGCGGTAAGGAAGAGCTGTTCAAGTA
CGAAGGTGGTTTGCGTGCGTTCGTTGAATACCTGAACACCAACAAGACTGCGGTCAACCAGGTGTTCCACTTCAACA
TCCAGCGTGAAGACGGCATCGGCGTGGAAATCGCCCTGCAGTGGAACGACAGCTTCAACGAGAACCTGTTGTGCTTC

ACCAACAACATTCCTCAGCGCGATGGCGGTACTCACCTGGTGGGTTTCCGTTCCGCACTGACGCGTAACCTGAACAC
CTACATCGAAGCTGAAGGCCTGGCGAAGAAACACAAAGTCGCCACCACCGGCGACGACGCCCGCGAAGGCCTGACCG
CGATTATCTCGGTCAAGGTTCCGGATCCGAAGTTCAGCTC
>12H 192
CTGCACGGTGTAGGTGTGTCGGTAGTGAATGCGCTGTCCAAAGAACTGATCCTGACTGTTCGCCGTAGCGGCAAGAT
CTGGGAACAAACTTACATCCACGGTGTTCCGCAAGAGCCGATGAAAATCGTTGGCGAGAGCGAAACCACGGGTACGC
AGATTCACTTCAAGCCTTCGGCTGAAACCTTCAAGAACATCCACTTCAGCTGGGACATCCTGGCCAAGCGGATTCGT
GAACTGTCCTTCCTGAACTCCGGTGTCGGCATCGTCCTCAAGGATGAGCGCAGCGGCAAGGAAGAGCTGTTCAAGTA
CGAAGGTGGTTTGCGTGCGTTCGTTGAATACCTGAACACCAACAAGACTGCGGTCAACCAGGTGTTCCACTTCAATA
TCCAGCGTGAAGACGGCATTGGCGTGGAAATCGCCCTGCAGTGGAACGACAGCTTCAACGAGAACCTGTTGTGCTTC
ACCAACAACATTCCTCAGCGCGATGGCGGTACTCACCTGGTGGGTTTCCGTTCCGCACTGACGCGTAACCTGAACAC
CTACATCGAAGCTGAAGGCCTGGCGAAGAAACACAAAGTCGCCACCACCGGCGACGACGCCCGCGAAGGCCTGACCG
CGATTATCTCGGTCAAGGTTCCGGATCCGAAGTTCAGCTC
>S04G 319
CTGCACGGTGTAGGTGTGTCGGTAGTGAACGCACTGTCCAAAGAACTGATCCTGACTGTTCGCCGTAGCGGCAAGAT
CTGGGAACAAACTTACATCCACGGTGTTCCGCAAGAGCCGATGAAAATCGTTGGCGAGAGCGAAACCACGGGTACGC
AGATTCACTTCAAGCCTTCGGCTGAAACCTTCAAGAACATCCACTTCAGCTGGGACATCCTGGCCAAGCGGATTCGT
GAACTGTCCTTCCTGAACTCCGGTGTCGGCATCGTCCTCAAGGATGAGCGCAGCGGCAAGGAAGAGCTGTTCAAGTA
CGAAGGTGGTTTGCGTGCGTTCGTTGAATACCTGAACACCAACAAGACTGCGGTCAACCAGGTGTTCCACTTCAACA
TCCAGCGTGAAGACGGCATCGGCGTGGAAATCGCCCTGCAGTGGAACGACAGCTTCAACGAGAACCTGTTGTGCTTC
ACCAACAACATTCCTCAGCGCGATGGCGGTACTCACGTGGTGGGTTTCCGTTCCGCACTGACGCGTAACCTGAACAC
CTACATCGAAGCTGAAGGCCTGGCGAAAAAACACAAAGTCGCCACCACCGGCGACGACGCCCGCGAAGGCCTGACCG
CGATTATTTCGGTCAAGGTTCCGGATCCGAAGTTCAGCTC
>S12G 287
CTGCACGGTGTAGGTGTGTCGGTAGTGAATGCGCTGTCCAAAGAACTGATCCTGACTGTTCGCCGTAGCGGCAAGAT
CTGGGAACAAACTTACATCCACGGTGTTCCGCAAGAGCCGATGAAAATCGTTGGCGAGAGCGAAACCACGGGTACGC
AGATTCACTTCAAGCCTTCGGCTGAAACCTTCAAGAACATCCACTTCAGCTGGGACATCCTGGCCAAGCGGATTCGT
GAACTGTCCTTCCTGAACTCCGGTGTCGGCATCGTCCTCAAGGATGAGCGCAGCGGCAAGGAAGAGCTGTTCAAGTA
CGAAGGTGGTTTGCGTGCGTTCGTTGAATACCTGAACACCAACAAGACTGCGGTCAACCAGGTGTTCCACTTCAACA
TCCAGCGTGAAGACGGCATCGGCGTGGAAATCGCCCTGCAGTGGAACGACAGCTTCAACGAGAACCTGTTGTGCTTC
ACCAACAACATTCCTCAGCGCGATGGCGGTACTCACCTGGTGGGTTTCCGTTCCGCACTGACGCGTAACCTGAACAC
CTACATCGAAGCTGAAGGCCTGGCGAAAAAACACAAAGTCGCCACCACCGGCGACGACGCCCGCGAAGGCCTGACCG
CGATTATTTCGGTCAAGGTTCCGGATCCGAAGTTCAGCTC
>S10H 272
CTGCACGGTGTAGGTGTGTCGGTAGTGAATGCGCTGTCCAAAGAACTGATCCTGACCGTTCGCCGTAGCGGCAAGAT
CTGGGAACAAACTTACATCCACGGTGTTCCGCAAGAGCCGATGAAAATCGTTGGCGAGAGCGAAACCACGGGTACGC
AGATTCACTTCAAGCCTTCGGCTGAAACCTTCAAGAACATCCACTTCAGCTGGGACATCCTGGCCAAGCGGATTCGT
GAACTGTCCTTCCTGAACTCCGGTGTCGGCATCGTCCTCAAGGATGAGCGCAGCGGCAAGGAAGAGCTGTTCAAGTA
TGAAGGTGGTTTGCGTGCGTTCGTTGAATACCTGAACACCAACAAGACTGCGGTCAACCAGGTGTTCCACTTCAACA
TCCAGCGTGAAGACGGCATCGGCGTGGAAATCGCCCTGCAGTGGAACGACAGCTTCAACGAGAACCTGTTGTGCTTC
ACCAACAACATTCCTCAGCGCGATGGCGGTACTCACCTGGTGGGTTTCCGTTCCGCACTGACGCGTAACCTGAACAC
CTACATCGAAGCTGAAGGCCTGGCGAAAAAACACAAAGTCGCCACCACCGGCGACGACGCCCGCGAAGGCCTGACCG
CGATTATTTCGGTCAAGGTTCCGGATCCGAAGTTCAGCTC
>S02E 301
CTGCACGGTGTAGGTGTGTCGGTAGTGAATGCGCTGTCCAAAGAACTGATCCTGACTGTTCGCCGTAGCGGCAAGAT
CTGGGAACAAACTTACATCCACGGTGTTCCGCAAGAGCCGATGAAAATCGTTGGCGAGAGCGAAACCACGGGTACGC
AGATTCACTTCAAGCCTTCGGCTGAAACCTTCAAGAACATCCACTTCAGCTGGGACATCCTGGCCAAGCGGATTCGT
GAACTGTCCTTCCTGAACTCCGGTGTCGGCATCGTCCTCAAGGATGAGCGCAGCGGCAAGGAAGAGCTGTTCAAGTA
CGAAGGTGGTTTGCGTGCGTTCGTTGAATACCTGAACACCAACAAGACTGCGGTTAACCAGGTTTTCCACTTCAACA
TCCAGCGTGAAGACGGCATTGGCGTGGAAATCGCCCTGCAGTGGAACGACAGCTTCAACGAGAACCTGTTGTGCTTC
ACCAACAACATTCCTCAGCGCGATGGCGGTACTCACCTGGTGGGTTTCCGTTCCGCACTGACGCGTAACCTGAACAC
CTACATCGAAGCTGAAGGCCTGGCGAAGAAACACAAAGTCGCCACCACCGGCGACGACGCCCGCGAAGGCCTGACCG
CGATTATCTCGGTCAAGGTTCCGGATCCGAAGTTCAGCTC
>12B 120
CTGCACGGTGTAGGTGTGTCGGTAGTGAACGCACTGTCCAAAGAACTGATCCTGACCGTTCGCCGCAGCGGCAAGAT
CTGGGAACAGACTTACGTCCACGGTGTTCCGCAAGAGCCGATGAAAATCGTTGGCGAGAGCGAAACCACGGGCACCC
AGATCCACTTCAAGCCATCGGCTGAAACCTTCAAGAACATCCACTTCAGCTGGGACATCCTGGCCAAGCGGATTCGT

GAACTGTCATTCCTCAACTCCGGTGTCGGCATCGTCCTCAAGGATGAGCGCAGCGGCAAGGAAGAGCTGTTCAAGTA
CGAAGGCGGCCTGCGTGCGTTCGTTGAATACCTGAACACCAACAAGACTGCGGTCAACCAGGTGTTCCACTTCAACA
TCCAGCGTGAAGACGGCATCGGCGTTGAAATCGCCCTGCAGTGGAACGACAGCTTCAACGAGAACCTGTTGTGCTTC
ACCAACAACATTCCACAGCGCGATGGCGGTACTCACCTGGTGGGTTTCCGTTCCGCACTGACGCGTAACCTGAACAC
CTACATCGAAGCTGAAGGTCTGGCGAAGAAGCATAAAGTCGCGACCACCGGTGACGATGCCCGTGAAGGCCTGACCG
CGATTATTTCGGTGAAAGTACCGGATCCGAAGTTCAGCTC
>09E 153
CTGCACGGTGTAGGTGTGTCGGTAGTGAACGCACTGTCCAAAGAACTGATCCTGACCGTTCGCCGCAGCGGCAAGAT
CTGGGAACAGACTTACGTCCACGGTGTTCCGCAAGAGCCGATGAAAATCGTTGGCGAGAGCGAAACCACGGGCACCC
AGATCCACTTCAAGCCATCGGCTGAAACCTTCAAGAACATCCACTTCAGCTGGGACATCCTGGCCAAGCGGATTCGT
GAACTGTCATTCCTCAACTCCGGTGTCGGCATCGTCCTCAAGGATGAGCGCAGCGGCAAGGAAGAGCTGTTCAAGTA
CGAAGGCGGCCTGCGTGCGTTCGTTGAATACCTGAACACCAACAAGACTGCGGTCAACCAGGTGTTCCACTTCAACA
TCCAGCGTGAAGACGGCATCGGCGTTGAAATCGCCCTGCAGTGGAACGACAGCTTCAACGAGAACCTGTTGTGCTTC
ACCAACAACATTCCACAGCGCGATGGCGGTACTCACCTGGTGGGTTTCCGTTCCGCACTGACGCGTAACCTGAACAC
CTACATCGAAGCTGAAGGTCTGGCGAAGAAGCATAAAGTCGCGACCACCGGTGACGATGCCCGTGAAGGCCTGACCG
CGATTATTTCGGTGAAAGTACCGGATCCGAAGTTCAGCTC
>03D 39
CTGCACGGTGTAGGTGTGTCGGTAGTGAACGCACTGTCCAAAGAACTGATCCTGACCGTTCGCCGCAGCGGCAAGAT
CTGGGAACAGACTTACGTCCACGGTGTTCCGCAAGAGCCGATGAAAATCGTTGGCGAGAGCGAAACCACGGGCACCC
AGATCCACTTCAAGCCATCGGCTGAAACCTTCAAGAACATCCACTTCAGCTGGGACATCCTGGCCAAGCGGATTCGT
GAACTGTCATTCCTCAACTCCGGTGTCGGCATCGTCCTCAAGGATGAGCGCAGCGGCAAGGAAGAGCTGTTCAAGTA
CGAAGGCGGCCTGCGTGCGTTCGTTGAATACCTGAACACCAACAAGACTGCGGTCAACCAGGTGTTCCACTTCAACA
TCCAGCGTGAAGACGGCATCGGCGTTGAAATCGCCCTGCAGTGGAACGACAGCTTCAACGAGAACCTGTTGTGCTTC
ACCAACAACATTCCACAGCGCGATGGCGGTACTCACCTGGTGGGTTTCCGTTCCGCACTGACGCGTAACCTGAACAC
CTACATCGAAGCTGAAGGTCTGGCGAAGAAGCATAAAGTCGCGACCACCGGTGACGATGCCCGTGAAGGCCTGACCG
CGATTATTTCGGTGAAAGTACCGGATCCGAAGTTCAGCTC
>05F 65
CTGCACGGTGTAGGTGTGTCGGTAGTGAACGCACTGTCCAAAGAACTGATCCTGACCGTTCGCCGCAGCGGCAAGAT
CTGGGAACAGACTTACGTCCACGGTGTTCCGCAAGAGCCGATGAAAATCGTTGGCGAGAGCGAAACCACGGGCACCC
AGATCCACTTCAAGCCATCGGCTGAAACCTTCAAGAACATCCACTTCAGCTGGGACATCCTGGCCAAGCGGATTCGT
GAACTGTCATTCCTCAACTCCGGTGTCGGCATCGTCCTCAAGGATGAGCGCAGCGGCAAGGAAGAGCTGTTCAAGTA
CGAAGGCGGCCTGCGTGCGTTCGTTGAATACCTGAACACCAACAAGACTGCGGTCAACCAGGTGTTCCACTTCAACA
TCCAGCGTGAAGACGGCATCGGCGTTGAAATCGCCCTGCAGTGGAACGACAGCTTCAACGAGAACCTGTTGTGCTTC
ACCAACAACATTCCACAGCGCGATGGCGGTACTCACCTGGTGGGTTTCCGTTCCGCACTGACGCGTAACCTGAACAC
CTACATCGAAGCTGAAGGTCTGGCGAAGAAGCATAAAGTCGCGACCACCGGTGACGATGCCCGTGAAGGCCTGACCG
CGATTATTTCGGTGAAAGTACCGGATCCGAAGTTCAGCTC
>06G 78
CTGCACGGTGTAGGTGTGTCGGTAGTGAACGCACTGTCCAAAGTACTGATCCTGACCGTTCGCCGCAGCGGCAAGAT
CTGGGAACAGACTTACGTCCACGGTGTTCCGCAAGAGCCGATGAAAATCGTTGGCGAGAGCGAAACCACGGGCACCC
AGATCCACTTCAAGCCATCGGCTGAAACCTTCAAGAACATCCACTTCAGCTGGGACATCCTGGCCAAGCGGATTCGT
GAACTGTCATTCCTCAACTCCGGTGTCGGCATCGTCCTCAAGGATGAGCGCAGCGGCAAGGAAGAGCTGTTCAAGTA
CGAAGGCGGCCTGCGTGCGTTCGTTGAATACCTGAACACCAACAAGACTGCGGTCAACCAGGTGTTCCACTTCAACA
TCCAGCGTGAAGACGGCATCGGCGTTGAAATCGCCCTGCAGTGGAACGACAGCTTCAACGAGAACCTGTTGTGCTTC
ACCAACAACATTCCACAGCGCGATGGCGGTACTCACCTGGTGGGTTTCCGTTCCGCACTGACGCGTAACCTGAACAC
CTACATCGAAGCTGAAGGTCTGGCGAAGAAGCATAAAGTCGCGACCACCGGTGACGATGCCCGTGAAGGCCTGACCG
CGATTATTTCGGTGAAAGTACCGGATCCGAAGTTCAGCTC
>09H 189
CTGCACGGTGTAGGTGTGTCGGTAGTGAACGCACTGTCCAAAGAACTGATCCTGACCGTTCGCCGCAGCGGCAAGAT
CTGGGAACAGACTTACGTCCACGGTGTTCCGCAAGAGCCGATGAAAATCGTTGGCGAGAGCGAAACCACGGGCACCC
AGATCCACTTCAAGCCATCGGCTGAAACCTTCAAGAACATCCACTTCAGCTGGGACATCCTGGCCAAGCGGATTCGT
GAACTGTCATTCCTCAACTCCGGTGTCGGCATCGTCCTCAAGGATGAGCGCAGCGGCAAGGAAGAGCTGTTCAAGTA
CGAAGGCGGCCTGCGTGCGTTCGTTGAATACCTGAACACCAACAAGACTGCGGTCAACCAGGTGTTCCACTTCAACA
TCCAGCGTGAAGACGGCATCGGCGTTGAAATCGCCCTGCAGTGGAACGACAGCTTCAACGAGAACCTGTTGTGCTTC
ACCAACAACATTCCACAGCGCGATGGCGGTACTCACCTGGTGGGTTTCCGTTCCGCACTGACGCGTAACCTGAACAC
CTACATCGAAGCTGAAGGTCTGGCGAAGAAGCATAAAGTCGCGACCACCGGTGACGATGCCCGTGAAGGCCTGACCG
CGATTATTTCGGTGAAAGTACCGGATCCGAAGTTCAGCTC

>01A 97
CTGCACGGTGTAGGTGTGTCGGTAGTGAACGCACTGTCCAAAGAACTGATCCTGACCGTTCGCCGCAGCGGCAAGAT
CTGGGAACAGACTTACGTCCACGGTGTTCCGCAAGAGCCGATGAAAATCGTTGGCGAGAGCGAAACCACGGGCACCC
AGATCCACTTCAAGCCATCGGCTGAAACCTTCAAGAACATCCACTTCAGCTGGGATATCCTGGCCAAGCGGATTCGT
GAACTGTCATTTCTCAACTCCGGTGTCGGCATCGTCCTCAAGGATGAGCGCAGCGGCAAGGAAGAGCTGTTCAAGTA
CGAAGGCGGCCTGCGTGCGTTCGTTGAATACCTGAACACCAACAAGACTGCGGTCAACCAGGTGTTCCACTTCAACA
TCCAGCGTGAAGACGGCATCGGCGTTGAAATCGCCCTGCAGTGGAACGACAGCTTCAACGAGAACCTGTTGTGCTTC
ACCAACAACATTCCACAGCGCGATGGCGGTACTCACCTGGTGGGTTTCCGTTCCGCACTGACGCGTAACCTGAACAC
CTACATCGAAGCTGAAGGTCTGGCGAAGAAGCATAAAGTCGCGACCACCGGTGACGATGCCCGTGAAGGCCTGACCG
CGATTATTTCGGTGAAAGTACCGGATCCGAAGTTCAGCTC
>07E 55
CTGCACGGTGTAGGTGTGTCGGTAGTGAACGCACTGTCCAAAGAACTGATCCTGACCGTTCGCCGCAGCGGCAAGAT
CTGGGAACAGACTTACGTCCACGGTGTTCCGCAAGAGCCGATGAAAATCGTTGGCGAGAGCGAAACCACGGGCACCC
AGATCCACTTCAAGCCATCGGCTGAAACCTTCAAGAACATCCACTTCAGCTGGGATATCCTGGCCAAGCGGATTCGT
GAACTGTCATTTCTCAACTCCGGTGTCGGCATCGTCCTCAAGGATGAGCGCAGCGGCAAGGAAGAGCTGTTCAAGTA
CGAAGGCGGCCTGCGTGCGTTCGTTGAATACCTGAACACCAACAAGACTGCGGTCAACCAGGTGTTCCACTTCAACA
TCCAGCGTGAAGACGGCATCGGCGTTGAAATCGCCCTGCAGTGGAACGACAGCTTCAACGAGAACCTGTTGTGCTTC
ACCAACAACATTCCACAGCGCGATGGCGGTACTCACCTGGTGGGTTTCCGTTCCGCACTGACGCGTAACCTGAACAC
CTACATCGAAGCTGAAGGTCTGGCGAAGAAGCATAAAGTCGCGACCACCGGTGACGATGCCCGTGAAGGCCTGACCG
CGATTATTTCGGTGAAAGTACCGGATCCGAAGTTCAGCTC
>01D 133
CTGCACGGTGTAGGTGTGTCGGTAGTGAACGCACTGTCCAAAGAACTGATCCTGACCGTTCGCCGCAGCGGCAAGAT
CTGGGAACAGACTTACGTCCACGGTGTTCCGCAAGAGCCGATGAAAATCGTTGGCGAGAGCGAAACCACGGGCACCC
AGATCCACTTCAAGCCATCGGCTGAAACCTTCAAGAACATCCACTTCAGCTGGGATATCCTGGCCAAGCGGATTCGT
GAACTGTCATTTCTCAACTCCGGTGTCGGCATCGTCCTCAAGGATGAGCGCAGCGGCAAGGAAGAGCTGTTCAAGTA
CGAAGGCGGCCTGCGTGCGTTCGTTGAATACCTGAACACCAACAAGACTGCGGTCAACCAGGTGTTCCACTTCAACA
TCCAGCGTGAAGACGGCATCGGCGTTGAAATCGCCCTGCAGTGGAACGACAGCTTTAACGAGAACCTGTTGTGCTTC
ACCAACAACATTCCACAGCGCGATGGCGGTACTCACCTGGTGGGTTTCCGTTCCGCACTGACGCGTAACCTGAACAC
CTACATCGAAGCTGAAGGTCTGGCGAAGAAGCATAAAGTCGCGACCACCGGTGACGATGCCCGTGAAGGCCTGACCG
CGATTATTTCGGTGAAAGTACCGGATCCGAAGTTCAGCTC
>02H 86
CTGCACGGTGTAGGTGTGTCGGTAGTGAACGCACTGTCCAAAGAACTGATCCTGACCGTTCGCCGCAGCGGCAAGAT
CTGGGAACAGACTTACGTCCACGGTGTTCCGCAAGAGCCGATGAAAATCGTTGGCGAGAGCGAAACCACGGGCACCC
AGATCCACTTCAAGCCATCGGCTGAAACCTTCAAGAACATCCACTTCAGCTGGGATATCCTGGCCAAGCGGATTCGT
GAACTGTCATTTCTCAACTCCGGTGTCGGCATCGTCCTCAAGGATGAGCGCAGCGGCAAGGAAGAGCTGTTCAAGTA
CGAAGGCGGCCTGCGTGCGTTCGTTGAATACCTGAACACCAACAAGACTGCGGTCAACCAGGTGTTCCACTTCAACA
TCCAGCGTGAAGACGGCATCGGCGTTGAAATCGCCCTGCAGTGGAACGACAGCTTCAACGAGAACCTGTTGTGCTTC
ACCAACAACATTCCACAGCGCGATGGCGGTACTCACCTGGTGGGTTTCCGTTCCGCACTGACGCGTAACCTGAACAC
CTACATCGAAGCTGAAGGTCTGGCGAAGAAGCATAAAGTCGCGACCACCGGTGACGATGCCCGTGAAGGCCTGACCG
CGATTATTTCGGTGAAAGTACCGGATCCGAAGTTCAGCTC
>04H 184
CTGCACGGTGTAGGTGTGTCGGTAGTGAACGCACTGTCCAAAGAACTGATCCTGACCGTTCGCCGCAGCGGCAAGAT
CTGGGAACAGACTTACGTCCACGGTGTTCCGCAAGAGCCGATGAAAATCGTTGGCGAGAGCGAAACCACGGGCACCC
AGATCCACTTCAAGCCATCGGCTGAAACCTTCAAGAACATCCACTTCAGCTGGGATATCCTGGCCAAGCGGATTCGT
GAACTGTCATTTCTCAACTCCGGTGTCGGCATCGTCCTCAAGGATGAGCGCAGCGGCAAGGAAGAGCTGTTCAAGTA
CGAAGGCGGCCTGCGTGCGTTCGTTGAATACCTGAACACCAACAAGACTGCGGTCAACCAGGTGTTCCACTTCAACA
TCCAGCGTGAAGACGGCATCGGCGTTGAAATCGCCCTGCAGTGGAACGACAGCTTCAACGAGAACCTGTTGTGCTTC
ACCAACAACATTCCACAGCGCGATGGCGGTACTCACCTGGTGGGTTTCCGTTCCGCACTGACGCGTAACCTGAACAC
CTACATCGAAGCTGAAGGTCTGGCGAAGAAGCATAAAGTCGCGACCACCGGTGACGATGCCCGTGAAGGCCTGACCG
CGATTATTTCGGTGAAAGTACCGGATCCGAAGTTCAGCTC
>11D 143
CTGCACGGTGTAGGTGTTTCGGTAGTGAACGCACTGTCCAAGGAACTGGTCCTGACCGTTCGCCGCAGCGGCAAGAT
CTGGGAACAGACTTACGTCCATGGCGTCCCGCAAGAGCCGATGAAAATCGTTGGTGAGAGCGAAACCACGGGCACAC
AGATTCACTTCAAGCCATCGGCTGAAACCTTCAAGAACATCCACTTCAGCTGGGACATCCTGGCCAAGCGGATTCGT
GAACTGTCCTTCCTCAACTCCGGTGTCGGCATCGTCCTCAAGGATGAGCGCAGCGGCAAGGAAGAGCTGTTCAAGTA
CGAAGGCGGCCTGCGTGCGTTCGTTGAATACCTGAACACCAACAAGACTGCGGTCAACCAGGTGTTCCACTTCAACA
TCCAGCGTGAAGACGGCATCGGCGTGGAAATCGCCCTGCAGTGGAACGACAGCTTCAACGAGAACCTGTTGTGCTTC

ACCAACAACATCCCGCAGCGTGATGGCGGTACTCACCTGGTGGGTTTCCGTTCCGCACTGACGCGTAACCTGAACAC
CTACATCGAAGCTGAAGGCCTGGCCAAGAAGCACAAGGTTGCCACCACCGGTGATGACGCCCGTGAAGGCTTGACCG
CGATCATCTCGGTGAAGGTGCCGGATCCGAAGTTCAGCTC
>S03H 216
CTGCACGGTGTAGGTGTGTCGGTGGTAAACGCACTGTCCAAAGAACTGATCCTGACCGTTCGCCGCAGCGGCAAGAT
CTGGGAACAGACGTACGTCCACGGTGTTCCGCAAGAGCCGATGAAGATCGTTGGCGAGAGCGAAACCACAGGCACCC
AGATTCACTTCAAGCCTTCGGCTGAAACTTTCAAGAACATCCACTTCAGCTGGGACATCCTGGCCAAGCGGATTCGC
GAACTGTCCTTCCTCAACTCCGGTGTCGGCATCGTCCTCAAGGATGAACGCAGCGGTAAGGAAGAGCTGTTCAAGTA
TGAAGGCGGCCTGCGTGCGTTCGTTGAATACCTGAACACCAACAAGACTGCGGTCAACCAGGTGTTCCACTTCAACA
TTCAGCGTGAAGACGGCATCGGCGTAGAAATCGCCCTGCAGTGGAACGACAGCTTCAACGAGAACCTGTTGTGCTTC
ACCAACAACATTCCACAACGTGATGGCGGTACTCACCTGGTGGGTTTCCGTTCCGCGCTGACGCGTAACCTGAACAC
CTACATCGAAGCTGAAGGCCTGGCGAAGAAGCACAAAGTCGCGACCACCGGTGACGATGCCCGTGAAGGCCTGACCG
CGATCATTTCGGTGAAAGTGCCGGATCCGAAGTTCAGCTC
>S07A 241
CTGCACGGTGTAGGTGTGTCGGTGGTGAACGCACTGTCCAAAGAACTGATCCTGACCGTTCGCCGCAGCGGAAAAAT
CTGGGAACAGACTTATGTCCACGGTGTTCCGCAAGAGCCGATGAAAATCGTTGGCGAAAGCGAAACGACTGGCACCC
AGATCCACTTCAAGCCTTCGGCTGAAACCTTCAAGAACATCCACTTCAGCTGGGACATCCTGGCCAAGCGGATTCGC
GAACTGTCCTTCCTCAACTCCGGTGTCGGCATCGTCCTCAAGGACGAGCGCAGCGGCAAGGAAGAGTTGTTCAAGTA
CGAAGGTGGTCTGCGTGCGTTCGTTGAATACCTGAACACCAACAAGACTGCGGTCAACCAGGTGTTCCACTTCAACA
TCCAGCGTGAAGACGGCATCGGCGTGGAAATCGCCCTGCAGTGGAACGACAGCTTCAACGAGAACCTGTTGTGCTTC
ACCAACAACATTCCACAGCGCGATGGCGGTACTCACCTGGTGGGTTTCCGTTCCGCGCTGACGCGTAACCTGAACAC
CTACATCGAAGCTGAAGGTCTGGCGAAGAAGCACAAAGTTGCCACCACCGGTGACGATGCCCGCGAAGGCCTGACAG
CGATCATTTCGGTAAAAGTGCCGGATCCGAAATTCAGCTC
>S12A 281
CTGCACGGTGTAGGTGTGTCGGTGGTGAACGCACTGTCCAAAGAACTGATCCTGACCGTTCGCCGCAGCGGAAAAAT
CTGGGAACAGACTTATGTCCACGGTGTTCCGCAAGAGCCGATGAAAATCGTTGGCGAAAGCGAAACCACTGGCACCC
AGATCCACTTCAAGCCTTCGGCTGAAACCTTCAAGAACATCCACTTCAGCTGGGACATCCTGGCCAAGCGGATTCGC
GAACTGTCCTTCCTCAACTCCGGTGTCGGCATCGTCCTCAAGGATGAGCGCAGCGGCAAGGAAGAGTTGTTCAAGTA
CGAAGGTGGTCTGCGTGCGTTCGTTGAATACCTGAACACCAACAAGACTGCGGTCAACCAGGTGTTCCACTTCAACA
TCCAGCGTGAAGACGGCATCGGCGTTGAAATCGCCCTGCAGTGGAACGACAGCTTCAACGAGAACCTGTTGTGCTTC
ACCAACAACATTCCACAGCGCGATGGCGGTACTCACCTGGTGGGTTTCCGTTCCGCACTGACGCGTAACCTGAACAC
CTACATCGAAGCTGAAGGTCTGGCGAAGAAGCACAAAGTTGCCACCACCGGTGACGATGCCCGCGAAGGCCTGACAG
CGATCATTTCGGTAAAAGTGCCGGATCCGAAGTTCAGCTC
>S08D 252
CTGCACGGTGTAGGTGTGTCGGTGGTGAACGCACTGTCCAAAGAACTGATCCTGACCGTTCGCCGCAGCGGCAAGAT
CTGGGAACAGACTTATGTCCACGGTGTTCCGCAAGAGCCGATGAAAATCGTTGGCGAGAGCGAAACCACTGGTACCC
AGATTCACTTCAAGCCTTCGGCTGAAACCTTCAAGAACATCCATTTCAGCTGGGACATCCTGGCCAAGCGGATTCGC
GAACTGTCTTTCCTCAACTCCGGTGTCGGCATCGTCCTCAAGGACGAGCGCAGCGGCAAGGAAGAGTTGTTCAAGTA
CGAAGGCGGTCTGCGTGCGTTCGTTGAATACCTGAACACCAACAAGACTGCGGTCAACCAGGTGTTCCACTTCAACA
TCCAGCGTGAAGACGGCATCGGCGTGGAAATCGCCCTGCAGTGGAACGACAGCTTCAACGAGAACCTGTTGTGCTTC
ACCAACAACATTCCGCAGCGCGATGGCGGTACTCACCTGGTGGGTTTCCGTTCCGCATTGACGCGTAACCTGAATAC
CTACATCGAAGCTGAAGGTCTGGCGAAGAAGCACAAAGTTGCCACCACCGGTGACGACGCCCGCGAGGGCCTGACCG
CGATCATTTCGGTAAAAGTGCCGGATCCGAAGTTCAGCTC
>03C 123
CTGCACGGTGTAGGTGTGTCGGTGGTGAACGCACTGTCCAAAGAACTGATCCTGACCGTTCGCCGCAGCGGCAAGAT
CTGGGAACAGACTTACGTCCACGGTGTTCCGCAAGAGCCGATGAAGATCGTTGGCGAAAGCGAAACCACCGGCACCC
AGATCCACTTCAAGCCTTCGGCTGAAACCTTCAAGAACATACACTTCAGCTGGGACATTCTGGCCAAGCGGATTCGT
GAACTGTCCTTCCTCAACTCCGGTGTCGGCATCGTCCTCAAGGACGAGCGCAGCGGCAAGGAAGAGTTGTTCAAGTA
TGAAGGTGGCCTGCGTGCGTTCGTTGAATACCTGAACACCAACAAGACTGCGGTCAATCAGGTGTTCCACTTCAACA
TCCAGCGTGAAGATGGCATCGGCGTGGAAATCGCCCTGCAGTGGAACGATAGCTTCAACGAGAATCTGTTGTGCTTC
ACCAACAACATCCCTCAGCGCGATGGCGGTACTCACCTGGTGGGTTTCCGTTCCGCACTGACGCGTAACCTGAACAC
TTACATCGAAGCTGAAGGTCTGGCGAAGAAGCACAAAGTTGCCACCACCGGTGACGATGCCCGCGAAGGCCTGACAG
CGATCATTTCGGTAAAAGTGCCGGATCCGAAGTTCAGCTC
>S09D 356
CTGCACGGTGTAGGTGTGTCGGTGGTGAACGCACTGTCCAAAGAACTGATCCTGACCGTTCGCCGCAGCGGCAAGAT
CTGGGAACAGACTTATATCCACGGTGTTCCGCAAGAGCCGATGAAGATCGTTGGCGAGAGCGAAACCACGGGTACCC
AGATTCACTTCAAACCATCGGCTGAAACCTTCAAGAACATCCACTTCAGCTGGGACATTCTGGCCAAGCGGATTCGT

GAACTGTCCTTCCTCAACTCCGGTGTCGGCATCGTCCTCAAGGATGAGCGCAGCGGCAAGGAAGAGCTGTTCAAATA
CGAAGGGGGCCTGCGTGCGTTCGTTGAATACCTGAACACCAACAAGACTGCGGTCAACCAGGTGTTCCACTTCAACA
TTCAGCGTGAAGACGGCATTGGCGTGGAAATCGCCCTGCAGTGGAACGACAGCTTCAACGAGAACCTGTTGTGCTTC
ACCAACAACATTCCTCAGCGCGATGGCGGTACTCACTTGGTGGGTTTCCGTTCCGCACTGACGCGTAACCTGAACAC
CTACATCGAAGCTGAAGGCCTGGCGAAGAAGCACAAAGTCGCGACCACCGGTGACGATGCCCGTGAAGGTCTGACCG
CGATCATTTCGGTGAAAGTGCCGGATCCCAAGTTCAGCTC
>08C 32
CTGCACGGTGTAGGTGTGTCGGTGGTGAACGCCCTGTCCAAAGAACTGGTCCTGACCGTTCGCCGCAGTGGCAAGAT
CTGGGAACAGACTTACATCCATGGTGTTCCGAAAGAGCCGATGAAGATCGTTGGCGAAAGCGAATCCACTGGTACCC
AGATTCACTTCAAGCCTTCAGAAGACACCTTCAAGAACATCCACTTCAGCTGGGACATCCTGGCCAAGCGGATTCGT
GAACTGTCCTTCCTCAACTCCGGTGTCGGCATCGTCCTCAAGGACGAGCGCAGCGGCAAGGAAGAGCTGTTCAAGTA
CGAAGGCGGCCTGCGTGCATTCGTTGAATACCTGAACACCAACAAGACTGCGGTCAACCAGGTGTTCCACTTCAACA
TCCAGCGTGAAGACGGCATTGGCGTGGAAATCGCCCTGCAGTGGAACGACAGCTTCAACGAGAACCTGTTGTGCTTC
ACCAACAACATTCCACAGCGCGATGGCGGTACTCACTTGGTGGGTTTCCGTTCCGCACTGACGCGTAACCTGAACAC
CTACATCGAAGCTGAAGGTCTGGCGAAGAAGCATAAAGTCGCGACCACCGGTGACGACGCCCGTGAAGGCCTGACCG
CGATCATTTCGGTGAAAGTACCGGATCCGAAGTTCAGCTC
>09A 105
CTGCACGGTGTAGGTGTGTCGGTAGTGAACGCACTGTCCAAAGAGCTTGTTCTTACGGTGCGCCGCAGCGGCAAAAT
CTGGGAACAGACTTACGTCCACGGTGTTCCACAAGAACCGATGAAAATCGTTGGTGAGAGTGAAACCACCGGCACCC
AGATTCACTTCAAGCCATCGGCCGAGACGTTCAAGAACATCCACTTCAGCTGGGACATCCTGGCCAAGCGGATTCGT
GAACTGTCCTTCCTCAACTCCGGTGTCGGCATCGTCCTCAAGGATGAGCGCAGCGGCAAGGAAGAGTTGTTCAAGTA
CGAAGGCGGCCTGCGAGCGTTCGTTGAATACCTGAACACCAACAAGACTGCGGTCAACCAGGTGTTTCACTTCAATA
TTCAGCGCGAAGACGGCATCGGCGTGGAAATCGCCTTGCAGTGGAACGACAGCTTCAACGAGAACCTGTTGTGTTTC
ACCAACAACATTCCACAGCGCGATGGCGGTACTCACCTGGTGGGTTTCCGTTCCGCACTGACGCGTAACCTGAATAC
CTATATCGAAGCCGAAGGTCTGGCGAAGAAGCACAAAGTCGCGACCACCGGTGACGATGCCCGTGAAGGCCTGACCG
CGATCATTTCGGTGAAAGTACCGGATCCGAAGTTCAGCTC
>S10F 366
CTGCACGGTGTAGGTGTGTCGGTGGTGAACGCACTGTCCAAAGAGCTTGTTCTTACGGTGCGCCGCAGCGGCAAAAT
CTGGGAACAGACTTACGTCCACGGTGTTCCACAAGAGCCGATGAAAATCGTTGGTGAGAGTGAAACCACTGGCACCC
AGATTCACTTCAAGCCATCGGCCGAGACGTTCAAGAACATCCACTTCAGCTGGGACATCCTGGCCAAGCGGATTCGT
GAACTGTCCTTCCTCAACTCCGGTGTCGGCATCGTCCTCAAGGATGAGCGCAGCGGCAAGGAAGAGTTGTTCAAGTA
CGAAGGCGGCCTGCGAGCGTTCGTTGAATACCTGAACACCAACAAGACTGCGGTCAACCAGGTGTTTCACTTCAACA
TTCAGCGCGAAGACGGCATTGGCGTGGAAATCGCCCTGCAGTGGAACGACAGTTTCAACGAGAACCTGTTGTGTTTC
ACCAACAACATTCCACAGCGCGATGGCGGTACTCACCTGGTGGGTTTCCGTTCCGCACTGACGCGTAACCTGAATAC
CTACATCGAAGCCGAAGGTCTGGCGAAGAAACACAAAGTCGCGACCACCGGTGACGATGCCCGTGAAGGCCTGACCG
CGATCATTTCGGTGAAAGTACCGGATCCGAAGTTCAGCTC
>S02H 304
CTCCACGGTGTAGGTGTGTCGGTGGTGAACGCACTGTCCAAAGAGCTTGTTCTTACGGTGCGCCGCAGCGGCAAAAT
CTGGGAACAGACTTACGTCCACGGTGTTCCACAAGAGCCGATGAAAATCGTTGGTGAGAGTGAAACCACTGGCACCC
AGATTCACTTCAAACCATCGGCCGAGACGTTCAAGAACATCCACTTCAGCTGGGACATCCTGGCCAAGCGGATTCGT
GAACTGTCCTTCCTCAACTCCGGTGTCGGCATCGTCCTCAAGGATGAGCGCAGCGGCAAGGAAGAGTTGTTCAAGTA
CGAAGGCGGCCTGCGAGCGTTCGTTGAATACCTGAACACCAACAAGACTGCGGTCAACCAGGTGTTTCACTTCAACA
TTCAGCGCGAAGACGGCATCGGCGTGGAAATCGCCCTGCAGTGGAACGACAGCTTCAACGAGAACCTGTTGTGTTTC
ACCAACAACATTCCACAGCGCGATGGCGGTACTCACCTGGTGGGTTTCCGTTCCGCACTGACGCGTAACCTGAATAC
CTACATCGAAGCCGAAGGTCTGGCGAAGAAGCACAAAGTCGCGACCACCGGTGACGATGCCCGTGAAGGCCTGACCG
CGATCATTTCGGTGAAAGTACCGGATCCGAAGTTCAGCTC
>06F 66
CTGCACGGTGTAGGTGTGTCGGTGGTGAACGCACTGTCCAAAGAGCTTGTTCTTACGGTGCGCCGCAGCGGCAAAAT
CTGGGAACAGACTTACGTCCACGGTGTTCCACAAGAGCCGATGAAAATCGTTGGTGAGAGTGAAACCACCGGCACCC
AGATTCATTTCAAGCCATCGGCCGAGACGTTCAAGAACATCCACTTCAGCTGGGACATCCTGGCCAAGCGGATTCGT
GAACTGTCCTTCCTCAACTCCGGTGTTGGCATCGTCCTCAAGGATGAGCGCAGCGGCAAGGAAGAGTTGTTCAAGTA
CGAAGGCGGCCTGCGAGCGTTTGTTGAATACCTGAACACCAACAAGACTGCGGTCAACCAGGTGTTTCACTTCAACA
TTCAGCGCGAAGACGGCATCGGCGTGGAAATCGCCCTGCAGTGGAACGACAGCTTCAACGAGAACCTGTTGTGTTTC
ACCAACAACATTCCACAGCGCGATGGCGGTACTCACCTGGTGGGTTTCCGTTCCGCACTGACGCGTAACCTGAATAC
CTACATCGAAGCCGAAGGTCTGGCGAAGAAGCACAAAGTCGCGACCACCGGTGACGATGCCCGTGAAGGCCTGACCG
CGATCATTTCGGTGAAAGTACCGGATCCGAAGTTCAGCTC

>S01G 199
CTGCACGGTGTAGGTGTGTCGGTGGTGAACGCACTGTCCAAAGAGCTTGTTCTTACGGTGCGCCGCAGCGGCAAAAT
CTGGGAACAGACTTACGTCCACGGTGTTCCACAAGAGCCGATGAAAATCGTTGGTGAGAGTGAAACCACCGGCACCC
AGATTCATTTCAAGCCATCGGCCGAGACGTTCAAGAACATCCACTTCAGCTGGGACATCCTGGCCAAGCGGATTCGT
GAACTGTCCTTCCTCAACTCCGGTGTTGGCATCGTCCTCAAGGATGAGCGCAGCGGCAAGGAAGAGTTGTTCAAGTA
CGAAGGCGGCCTGCGAGCGTTTGTTGAATACCTGAACACCAACAAGACTGCGGTCAACCAGGTGTTTCACTTCAACA
TTCAGCGCGAAGACGGCATCGGCGTGGAAATCGCCCTGCAGTGGAACGACAGCTTCAACGAGAACCTGTTGTGTTTC
ACCAACAACATTCCACAGCGCGATGGCGGTACTCACCTGGTGGGTTTCCGTTCCGCACTGACGCGTAACCTGAATAC
CTACATCGAAGCCGAAGGTCTGGCGAAGAAGCACAAAGTCGCGACCACCGGTGACGATGCCCGTGAAGGCCTGACCG
CGATCATTTCGGTGAAAGTACCGGATCCGAAGTTCAGCTC
>S09C 259
CTGCACGGTGTAGGTGTGTCGGTGGTGAACGCACTGTCCAAAGAACTGGTCCTGACCGTTCGCCGCAGCGGCAAAAT
CTGGGAACAGACTTACGTCCACGGTGTTCCACAAGAGCCGATGAAAATCGTTGGTGAGAGTGAAACCACCGGCACCC
AGATTCACTTCAAGCCATCGGCCGAGACGTTCAAGAACATCCACTTCAGCTGGGACATCCTGGCCAAGCGGATTCGT
GAACTGTCCTTCCTCAACTCCGGTGTCGGCATCGTCCTCAAGGATGAGCGCAGCGGCAAGGAAGAGTTGTTCAAGTA
CGAAGGCGGCCTGCGAGCGTTTGTTGAATACCTGAACACCAACAAGACTGCGGTCAACCAGGTGTTTCACTTCAACA
TTCAGCGCGAAGACGGCATCGGCGTGGAAATCGCCCTGCAGTGGAACGACAGCTTCAACGAGAACCTGTTGTGTTTC
ACCAACAACATTCCACAGCGCGATGGCGGTACTCACCTGGTGGGTTTCCGTTCCGCACTGACGCGTAACCTGAATAC
CTACATCGAAGCCGAAGGTCTGGCGAAGAAGCACAAGGTCGCCACCACCGGTGACGATGCCCGTGAAGGCCTGACCG
CGATCATTTCGGTGAAAGTACCGGATCCGAAGTTCAGCTC
>08E 56
CTGCACGGTGTAGGTGTATCGGTGGTTAACGCCCTGTCCGAAGAACTGGTCCTGACCGTTCGCCGCAGCGGCAAGAT
CTGGGAACAGACCTACGTCCACGGTGTTCCGCAAGAACCGATGAAAATCGTTGGCGACAGCGAATCCACCGGTACCC
AGATTCACTTCAAGCCTTCGGCTGACACCTTCAAGAACATCCACTTCAGCTGGGACATCCTGGCCAAGCGGATTCGC
GAACTGTCCTTCCTCAACTCCGGTGTTGGCATCGTCCTCAAGGACGAGCGCAGCGGCAAGGAAGAGTTGTTCAAGTA
CGAAGGTGGTCTGCGTGCATTCGTTGAATACCTGAACACCAACAAGACTGCGGTCAACCAGGTGTTCCACTTCAACA
TCCAGCGTGAAGACGGCATCGGCGTGGAAATCGCCCTGCAGTGGAACGACAGCTTCAACGAGAACCTGTTGTGCTTC
ACCAACAACATTCCGCAGCGCGATGGCGGTACTCACCTGGTGGGTTTCCGTTCGGCACTGACGCGTAACCTGAACAC
CTACATCGAAGCTGAAGGCCTGGCGAAGAAGCACAAAGTCGCCACCACCGGTGACGATGCCCGCGAAGGCCTGACGG
CGATCATTTCGGTGAAAGTGCCGGATCCGAAGTTCAGCTC
>10D 142
CTGCACGGTGTAGGTGTATCGGTGGTTAACGCCCTGTCCGAAGAACTGGTCCTGACCGTTCGCCGCAGCGGCAAGAT
CTGGGAACAGACCTACGTCCACGGTGTTCCGCAAGAACCGATGAAAATCGTTGGCGACAGCGAATCCACCGGTACCC
AGATTCACTTCAAGCCTTCGGCTGACACCTTCAAGAACATCCACTTCAGCTGGGACATCCTGGCCAAGCGGATTCGC
GAACTGTCCTTCCTCAACTCCGGTGTTGGCATCGTCCTCAAGGACGAGCGCAGCGGCAAGGAAGAGTTGTTCAAGTA
CGAAGGTGGTCTGCGTGCATTCGTTGAATACCTGAACACCAACAAGACTGCGGTCAACCAGGTGTTCCACTTCAACA
TCCAGCGTGAAGACGGCATCGGCGTGGAAATCGCCCTGCAGTGGAACGACAGCTTCAACGAGAACCTGTTGTGCTTC
ACCAACAACATTCCGCAGCGCGATGGCGGTACTCACCTGGTGGGTTTCCGTTCGGCACTGACGCGTAACCTGAACAC
CTACATCGAAGCTGAAGGCCTGGCGAAGAAGCACAAAGTCGCCACCACCGGTGACGATGCCCGCGAAGGCCTGACGG
CGATCATTTCGGTGAAAGTGCCGGATCCGAAGTTCAGCTC
>06E 54
CTGCACGGTGTAGGTGTGTCGGTGGTGAACGCACTGTCCAAAGAGCTTGTTCTTACAGTGCGCCGCAGCGGCAAGAT
CTGGGAACAGACTTACGTCCACGGTGTTCCGCAAGAACCGATGAAAATCGTCGGCGAGAGCGAGACAACCGGTACCC
AGATTCACTTTAAGCCATCGGCTGAAACCTTCAAGAATATCCACTTCAGCTGGGACATTCTGGCCAAGCGGATTCGC
GAACTGTCCTTCCTCAACTCCGGTGTCGGCATCGTCCTCAAGGACGAGCGCAGCGGCAAGGAAGAGCTGTTCAAGTA
CGAAGGTGGTCTGCGTGCGTTCGTTGAATACCTGAACACCAACAAGACTGCGGTCAACCAGGTGTTCCACTTCAACA
TCCAGCGTGAAGACGGCATCGGCGTGGAAATCGCCCTGCAGTGGAACGACAGCTTCAACGAGAACCTGTTGTGCTTC
ACCAACAACATTCCGCAGCGCGATGGCGGTACTCACCTGGTGGGTTTCCGTTCCGCCCTGACGCGTAACCTGAACAC
CTACATCGAAGCTGAAGGTCTGGCGAAGAAGCACAAAGTCGCCACCACCGGTGACGATGCCCGTGAAGGCCTGACGG
CGATCATTTCGGTAAAAGTGCCGGATCCGAAGTTCAGCTC
>06A 102
CTGCACGGTGTGGGTGTATCGGTTGTGAACGCCCTGTCCGAAGAGCTGATCCTGACTGTTCGCCGTAGCGGCAAAAT
CTGGGAACAGACTTATGTCCACGGAGTTCCGCAAGAGCCGATGAAAATCGTTGGCGACAGCGAATCTACCGGTACGC
AGATTCACTTCAAGCCGTCGGCTGACACCTTCAAGAACATTCACTTCAGCTGGGACATCCTGGCCAAGCGGATTCGC
GAACTGTCCTTCCTCAACTCCGGTGTCGGCATCGTCCTCAAGGATGAGCGCAGCGGCAAGGAAGAGTTGTTCAAGTA
CGAAGGTGGTCTGCGTGCGTTCGTTGAGTACCTGAACACCAACAAGACTGCGGTCAACCAGGTGTTCCACTTCAACG
TCCAGCGTGAAGACGGCATCGGCGTGGAAATCGCCCTGCAGTGGAACGACAGCTTCAACGAGAACCTGTTGTGCTTC

ACCAACAACATTCCACAGCGCGATGGCGGTACTCACTTGGTGGGTTTCCGTTCCGCACTGACGCGTAACCTGAACAC
CTACATCGAAGCTGAAGGTCTGGCGAAGAAGCACAAAGTCGCCACCACCGGTGACGACGCCCGTGAAGGCCTGACGG
CGATCATTTCGGTAAAAGTGCCGGATCCGAAGTTCAGCTC
>S02F 206
CTGCACGGTGTAGGTGTGTCGGTAGTGAACGCACTGTCCAAAGAACTGATCCTGACTGTTCGCCGTAGCGGCAAGAT
CTGGGAACAAACATACGTCCACGGTGTTCCGCAAGAGCCGATGAAAATCGTTGGTGAGAGCGAAACCACAGGCACTC
AGATTCACTTCAAGCCTTCGGCTGAAACCTTCAAGAACATCCACTTCAGCTGGGACATCCTGGCCAAGCGGATTCGT
GAATTGTCCTTCCTTAACTCCGGTGTCGGCATCGTCCTCAAGGATGAACGCAGCGGCAAGGAAGAGCTGTTCAAGTA
TGAAGGCGGCCTCCGTGCGTTTGTTGAATACCTGAACACCAACAAGACAGCGGTCAACCAGGTGTTCCACTTCAACA
TTCAGCGTGAAGACGGCATCGGCGTGGAAATCGCCTTGCAGTGGAACGACAGCTTCAACGAGAACCTGTTGTGCTTC
ACCAACAACATTCCGCAGCGCGATGGCGGTACTCACCTGGTGGGTTTCCGTTCCGCTCTGACGCGTAACCTGAACAC
CTACATCGAAGCCGAAGGTCTGGCGAAGAAACACAAAGTCGCGACCACCGGTGACGATGCCCGCGAAGGCTTGACCG
CGATCATTTCGGTGAAAGTACCGGATCCGAAGTTCAGCTC
>S11G 375
CTGCACGGTGTAGGTGTGTCGGTAGTGAACGCACTGTCCAAAGAACTGATCCTGACTGTTCGCCGTAGCGGCAAGAT
CTGGGAACAAACATACGTCCACGGTGTTCCGCAAGAGCCGATGAAAATCGTTGGCGAGAGCGAAACCACAGGCACTC
AGATTCACTTCAAGCCTTCGGCTGAAACCTTCAAGAACATCCACTTCAGCTGGGACATCCTGGCCAAGCGGATTCGT
GAATTGTCCTTCCTTAACTCCGGTGTCGGCATCGTCCTCAAGGATGAACGCAGCGGCAAGGAAGAGCTGTTCAAGTA
TGAAGGCGGCCTCCGTGCGTTTGTTGAATACCTGAACACCAACAAGACAGCGGTCAACCAGGTGTTCCACTTCAACA
TTCAGCGTGAAGACGGCATCGGCGTGGAAATCGCCTTGCAGTGGAACGACAGCTTCAACGAGAACCTGTTGTGCTTC
ACCAACAACATTCCGCAGCGCGATGGCGGTACTCACCTGGTGGGTTTCCGTTCCGCTCTGACGCGTAACCTGAACAC
CTACATCGAAGCCGAAGGTCTGGCGAAGAAACACAAAGTCGCAACCACCGGTGACGATGCCCGCGAAGGCTTGACCG
CGATCATTTCGGTGAAAGTACCGGATCCGAAGTTCAGCTC
>07H 91
CTGCACGGTGTAGGTGTGTCGGTTGTGAACGCCCTGTCCGAAGAACTGGTCTTGACCGTTCGCCGCAGCGGCAAAAT
CTGGGAACAGACCTACATTCACGGTGTTCCAAAAGAGCCGATGAAAATCGTCGGTGAGAGTGAAACCACCGGCACCC
AGATCCACTTCAAACCGTCCGACCTGACCTTCAAGAACATCCATTTCAGCTGGGACATCCTGGCCAAGCGGATTCGT
GAACTGTCCTTCCTCAACTCCGGTGTCGGCATCGTCCTCAAGGATGAGCGCAGCGGCAAGGAAGAGCTGTTCAAGTA
CGAGGGCGGCCTGCGTGCGTTCGTTGAATACCTGAACACCAACAAGACCCCGGTCAACCAGGTGTTCCACTTCAACA
TCCAGCGCGAAGACGGCATCGGCGTGGAAATCGCCTTGCAGTGGAACGACAGCTTCAACGAGAACCTGTTGTGCTTC
ACCAACAACATTCCACAGCGCGATGGCGGTACTCACCTGGTGGGTTTCCGTTCCGCGCTGACGCGCAACCTGAACAC
CTACATCGAAGCCGAAGGTCTGGCGAAGAAGCACAAAGTCGCGACCACCGGTGACGATGCCCGTGAAGGCCTGACCG
CGATTATCTCGGTAAAAGTGCCGGATCCGAAGTTCAGCTC
>11H 95
CTGCACGGTGTAGGTGTGTCGGTTGTGAACGCCCTGTCCGAAGAACTGGTCTTGACCGTTCGCCGCAGCGGCAAAAT
CTGGGAACAGACCTACATTCACGGTGTTCCAAAAGAGCCGATGAAAATCGTCGGTGAGAGTGAAACCACCGGCACCC
AGATCCACTTCAAACCGTCCGACCTGACCTTCAAGAACATCCATTTCAGCTGGGACATCCTGGCCAAGCGGATTCGT
GAACTGTCCTTCCTCAACTCCGGTGTCGGCATCGTCCTCAAGGATGAGCGCAGCGGCAAGGAAGAGCTGTTCAAGTA
CGAGGGCGGCCTGCGTGCGTTCGTTGAATACCTGAACACCAACAAGACCCCGGTCAACCAGGTGTTCCACTTCAACA
TCCAGCGCGAAGACGGCATCGGCGTGGAAATCGCCTTGCAGTGGAACGACAGCTTCAACGAGAACCTGTTGTGCTTC
ACCAACAACATTCCACAGCGCGATGGCGGTACTCACCTGGTGGGTTTCCGTTCCGCGCTGACGCGCAACCTGAACAC
CTACATCGAAGCCGAAGGTCTGGCGAAGAAGCACAAAGTCGCGACCACCGGTGACGATGCCCGTGAAGGCCTGACCG
CGATTATCTCGGTAAAAGTGCCGGATCCGAAGTTCAGCTC
>09D 45
CTGCACGGTGTAGGTGTGTCGGTTGTGAACGCCCTGTCCGAAGAACTGGTCTTGACCGTTCGCCGCAGCGGCAAAAT
CTGGGAACAGACCTACATTCACGGTGTTCCAAAAGAGCCGATGAAAATCGTCGGTGAGAGTGAAACCACCGGCACCC
AGATCCACTTCAAACCGTCCGACCTGACCTTCAAGAACATCCATTTCAGCTGGGACATCCTGGCCAAGCGGATTCGT
GAACTGTCCTTCCTCAACTCCGGTGTCGGCATCGTCCTCAAGGATGAGCGCAGCGGCAAGGAAGAGCTGTTCAAGTA
CGAGGGCGGCCTGCGTGCGTTCGTTGAATACCTGAACACCAACAAGACCCCGGTCAACCAGGTGTTCCACTTCAACA
TCCAGCGCGAAGACGGCATCGGCGTGGAAATCGCCTTGCAGTGGAACGACAGCTTCAACGAGAACCTGTTGTGCTTC
ACCAACAACATTCCACAGCGCGATGGCGGTACTCACCTGGTGGGTTTCCGTTCCGCGCTGACGCGCAACCTGAACAC
CTACATCGAAGCCGAAGGTCTGGCGAAGAAGCACAAAGTCGCGACCACCGGTGACGATGCCCGTGAAGGCCTGACCG
CGATTATCTCGGTAAAAGTGCCGGATCCGAAGTTCAGCTC
>10H 94
CTGCACGGTGTAGGTGTGTCGGTTGTGAACGCCCTGTCCGAAGAACTGGTCTTGACCGTTCGCCGCAGCGGCAAAAT
CTGGGAACAGACCTACATTCACGGTGTTCCAAAAGAGCCGATGAAAATCGTCGGTGAGAGTGAAACCACCGGCACCC
AGATCCACTTCAAACCGTCCGACCTGACCTTCAAGAACATCCATTTCAGCTGGGACATCCTGGCCAAGCGGATTCGT

GAACTGTCCTTCCTCAACTCCGGTGTCGGCATCGTCCTCAAGGATGAGCGCAGCGGCAAGGAAGAGCTGTTCAAGTA
CGAGGGCGGCCTGCGTGCGTTCGTTGAATACCTGAACACCAACAAGACCCCGGTCAACCAGGTGTTCCACTTCAACA
TCCAGCGCGAAGACGGCATCGGCGTGGAAATCGCCTTGCAGTGGAACGACAGCTTCAACGAGAACCTGTTGTGCTTC
ACCAACAACATTCCACAGCGCGATGGCGGTACTCACCTGGTGGGTTTCCGTTCCGCGCTGACGCGCAACCTGAACAC
CTACATCGAAGCCGAAGGTCTGGCGAAGAAGCACAAAGTCGCGACCACCGGTGACGATGCCCGTGAAGGCCTGACCG
CGATTATCTCGGTAAAAGTGCCGGATCCGAAGTTCAGCTC
>09F 69
CTGCACGGTGTAGGTGTGTCGGTTGTGAACGCCCTGTCCGAAGAACTGGTCTTGACCGTTCGCCGCAGCGGCAAAAT
CTGGGAACAGACCTACATTCACGGTGTTCCAAAAGAGCCGATGAAAATCGTCGGTGAGAGTGAAACCACCGGCACCC
AGATCCACTTCAAACCGTCCGACCTGACCTTCAAGAACATCCATTTCAGCTGGGACATCCTGGCCAAGCGGATTCGT
GAACTGTCCTTCCTCAACTCCGGTGTCGGCATCGTCCTCAAGGATGAGCGCAGCGGCAAGGAAGAGCTGTTCAAGTA
CGAGGGCGGCCTGCGTGCGTTCGTTGAATACCTGAACACCAACAAGACCCCGGTCAACCAGGTGTTCCACTTCAACA
TCCAGCGCGAAGACGGCATCGGCGTGGAAATCGCCTTGCAGTGGAACGACAGCTTCAACGAGAACCTGTTGTGCTTC
ACCAACAACATTCCACAGCGCGATGGCGGTACTCACCTGGTGGGTTTCCGTTCCGCGCTGACGCGCAACCTGAACAC
CTACATCGAAGCCGAAGGTCTGGCGAAGAAGCACAAAGTCGCGACCACCGGTGACGATGCCCGTGAAGGCCTGACCG
CGATTATCTCGGTAAAAGTGCCGGATCCGAAGTTCAGCTC
>10G 82
CTGCACGGTGTAGGTGTGTCGGTTGTGAACGCCCTGTCCGAAGAACTGGTCTTGACCGTTCGCCGCAGCGGCAAAAT
CTGGGAACAGACCTACATTCACGGTGTTCCAAAAGAGCCGATGAAAATCGTCGGTGAGAGTGAAACCACCGGCACCC
AGATCCACTTCAAACCGTCCGACCTGACCTTCAAGAACATCCATTTCAGCTGGGACATCCTGGCCAAGCGGATTCGT
GAACTGTCCTTCCTCAACTCCGGTGTCGGCATCGTCCTCAAGGATGAGCGCAGCGGCAAGGAAGAGTTGTTCAAGTA
CGAGGGCGGCCTGCGTGCGTTCGTTGAATACCTGAACACCAACAAGACCCCGGTCAACCAGGTGTTCCACTTCAATA
TCCAGCGCGAAGACGGCATCGGCGTGGAAATCGCCTTGCAGTGGAACGACAGCTTCAACGAGAACCTGTTGTGCTTC
ACCAACAACATTCCACAGCGCGATGGCGGTACTCACCTGGTGGGTTTCCGTTCCGCGCTGACGCGCAACCTGAACAC
CTACATCGAAGCCGAAGGTCTGGCGAAGAAGCACAAAGTCGCGACCACCGGTGACGATGCCCGTGAAGGCCTGACCG
CGATTATCTCGGTAAAAGTGCCGGATCCAAAGTTCAGCTC
>06D 42
CTGCACGGTGTAGGTGTGTCGGTTGTGAACGCCCTGTCCGAAGAACTGGTCTTGACCGTTCGCCGCAGCGGCAAAAT
CTGGGAACAGACCTACATTCACGGTGTTCCAAAAGAGCCGATGAAAATCGTCGGTGAGAGTGAAACCACCGGCACCC
AGATCCACTTCAAACCGTCCGACCTGACCTTCAAGAACATCCATTTCAGCTGGGACATCCTGGCCAAGCGGATTCGT
GAACTGTCCTTCCTCAACTCCGGTGTCGGCATCGTCCTCAAGGATGAGCGCAGCGGCAAGGAAGAGCTGTTCAAGTA
CGAGGGCGGCCTGCGTGCGTTCGTTGAATACCTGAACACCAACAAGACCCCGGTCAACCAGGTGTTCCACTTCAACA
TCCAGCGCGAAGACGGCATCGGCGTGGAAATCGCCTTGCAGTGGAACGACAGCTTCAACGAGAACCTGTTGTGCTTC
ACCAACAACATTCCACAGCGCGATGGCGGTACTCACCTGGTGGGTTTCCGTTCCGCGCTGACGCGCAACCTGAACAC
CTACATCGAAGCCGAAGGTCTGGCGAAGAAGCACAAAGTCGCGACCACCGGTGACGATGCCCGTGAAGGCCTGACCG
CGATTATCTCGGTAAAAGTGCCGGATCCGAAGTTCAGCTC
>05C 29
CTGCACGGTGTAGGTGTGTCGGTTGTGAACGCCCTGTCCGAAGAACTGGTCTTGACCGTTCGCCGCAGCGGCAAAAT
CTGGGAACAGACCTACATTCACGGTGTTCCAAAAGAGCCGATGAAAATCGTCGGTGAGAGTGAAACCACCGGCACCC
AGATCCACTTCAAACCGTCCGACCTGACCTTCAAGAACATCCATTTCAGCTGGGACATCCTGGCCAAGCGGATTCGT
GAACTGTCCTTCCTCAACTCCGGTGTCGGCATCGTCCTCAAGGATGAGCGCAGCGGCAAGGAAGAGCTGTTCAAGTA
CGAGGGCGGCCTGCGTGCGTTCGTTGAATACCTGAACACCAACAAGACCCCGGTCAACCAGGTGTTCCACTTCAACA
TCCAGCGCGAAGACGGCATCGGCGTGGAAATCGCCTTGCAGTGGAACGACAGCTTCAACGAGAACCTGTTGTGCTTC
ACCAACAACATTCCACAGCGCGATGGCGGTACTCACCTGGTGGGTTTCCGTTCCGCGCTGACGCGCAACCTGAACAC
CTACATCGAAGCCGAAGGTCTGGCGAAGAAGCACAAAGTCGCGACCACCGGTGACGATGCCCGTGAAGGCCTGACCG
CGATTATCTCGGTAAAAGTGCCGGATCCGAAGTTCAGCTC
>11F 71
CTGCACGGTGTAGGTGTGTCGGTTGTGAACGCCCTGTCCGAAGAACTGGTCTTGACCGTTCGCCGCAGCGGCAAAAT
CTGGGAACAGACCTACATTCACGGTGTTCCAAAAGAGCCGATGAAAATCGTCGGTGAGAGTGAAACCACCGGCACCC
AGATCCACTTCAAACCGTCCGACCTGACCTTCAAGAACATCCATTTCAGCTGGGACATCCTGGCCAAGCGGATTCGT
GAACTGTCCTTCCTCAACTCCGGTGTCGGCATCGTCCTCAAGGATGAGCGCAGCGGCAAGGAAGAGCTGTTCAAGTA
CGAGGGCGGCCTGCGTGCGTTCGTTGAATACCTGAACACCAACAAGACCCCGGTCAACCAGGTGTTCCACTTCAACA
TCCAGCGCGAAGACGGCATCGGCGTGGAAATCGCCTTGCAGTGGAACGACAGCTTCAACGAGAACCTGTTGTGCTTC
ACCAACAACATTCCACAGCGCGATGGCGGTACTCACCTGGTGGGTTTCCGTTCCGCGCTGACGCGCAACCTGAACAC
CTACATCGAAGCCGAAGGTCTGGCGAAGAAGCATAAAGTCGCGACCACCGGTGACGATGCCCGTGAAGGCCTGACCG
CGATTATCTCGGTAAAAGTGCCGGATCCGAAGTTCAGCTC

>08A 8
CTGCACGGTGTAGGTGTGTCGGTTGTGAACGCCCTGTCCGAAGAACTGGTCTTGACCGTTCGCCGCAGCGGCAAAAT
CTGGGAACAGACCTACATTCACGGTGTTCCAAAAGAGCCGATGAAAATCGTCGGTGAGAGTGAAACCACCGGCACCC
AGATCCACTTCAAACCGTCCGACCTGACCTTCAAGAACATCCATTTCAGCTGGGACATCCTGGCCAAGCGGATTCGT
GAACTGTCCTTCCTCAACTCCGGTGTCGGCATCGTCCTCAAGGATGAGCGCAGCGGCAAGGAAGAGTTGTTCAAGTA
CGAGGGCGGCCTGCGTGCGTTCGTTGAATACCTGAACACCAACAAGACCCCGGTCAACCAGGTGTTCCACTTCAACA
TCCAGCGCGAAGACGGCATCGGCGTGGAAATCGCCTTGCAGTGGAACGACAGCTTCAACGAGAACCTGTTGTGCTTC
ACCAACAACATTCCACAGCGCGATGGCGGTACTCACCTGGTGGGTTTCCGTTCCGCGCTGACGCGCAACCTGAACAC
CTACATCGAAGCCGAAGGTCTGGCGAAGAAGCACAAAGTCGCGACCACCGGTGACGATGCCCGTGAAGGCCTGACCG
CGATTATCTCGGTAAAAGTGCCGGATCCGAAGTTCAGCTC
>04C 28
CTGCACGGTGTAGGTGTGTCGGTTGTGAACGCCCTGTCCGAAGAACTGGTCTTGACCGTTCGCCGCAGCGGCAAAAT
CTGGGAACAGACCTACATTCACGGTGTTCCAAAAGAGCCGATGAAAATCGTCGGTGAGAGTGAAACCACCGGCACCC
AGATCCACTTCAAACCGTCCGACCTGACCTTCAAGAACATCCATTTCAGCTGGGACATCCTGGCCAAGCGGATTCGT
GAACTGTCCTTCCTCAACTCCGGTGTCGGCATCGTCCTCAAGGATGAGCGCAGCGGCAAGGAAGAGCTGTTCAAGTA
CGAGGGCGGCCTGCGTGCGTTCGTTGAATACCTGAACACCAACAAGACCCCGGTCAACCAGGTGTTCCACTTCAACA
TCCAGCGCGAAGACGGCATCGGCGTGGAAATCGCCTTGCAGTGGAACGACAGCTTCAACGAGAACCTGTTGTGCTTC
ACCAACAACATTCCACAGCGCGATGGCGGTACTCACCTGGTGGGTTTCCGTTCCGCGCTGACGCGCAACCTGAACAC
CTACATCGAAGCCGAAGGTCTGGCGAAGAAGCACAAAGTCGCGACCACCGGTGACGATGCCCGTGAAGGCCTGACCG
CGATTATCTCGGTAAAAGTGCCGGATCCGAAGTTCAGCTC
>07F 163
CTGCACGGTGTAGGTGTGTCGGTTGTGAACGCCCTGTCCGAAGAACTGGTCTTGACCGTTCGCCGCAGCGGCAAAAT
CTGGGAACAGACCTACATTCACGGTGTTCCAAAAGAGCCGATGAAAATCGTCGGTGAGAGTGAAACCACCGGCACCC
AGATCCACTTCAAACCGTCCGACCTGACCTTCAAGAACATCCATTTCAGCTGGGACATCCTGGCCAAGCGGATTCGT
GAACTGTCCTTCCTCAACTCCGGTGTCGGCATCGTCCTCAAGGATGAGCGCAGCGGCAAGGAAGAGTTGTTCAAGTA
CGAGGGCGGCCTGCGTGCGTTCGTTGAATACCTGAACACCAACAAGACCCCGGTCAACCAGGTGTTCCACTTCAATA
TCCAGCGCGAAGACGGCATCGGCGTGGAAATCGCCTTGCAGTGGAACGACAGCTTCAACGAGAACCTGTTGTGCTTC
ACCAACAACATTCCACAGCGCGATGGCGGTACTCACCTGGTGGGTTTCCGTTCCGCGCTGACGCGCAACCTGAACAC
CTACATCGAAGCCGAAGGTCTGGCGAAGAAGCATAAAGTCGCGACCACCGGTGACGATGCCCGTGAAGGCCTGACCG
CGATTATCTCGGTAAAAGTGCCGGATCCAAAGTTCAGCTC
>07A 7
CTGCACGGTGTAGGTGTGTCGGTTGTGAACGCCCTGTCCGAAGAACTGGTCTTGACCGTTCGCCGCAGCGGCAAAAT
CTGGGAACAGACCTACATTCACGGTGTTCCAAAAGAGCCGATGAAAATCGTCGGTGAGAGTGAAACCACCGGCACCC
AGATCCACTTCAAACCGTCCGACCTGACCTTCAAGAACATCCATTTCAGCTGGGACATCCTGGCCAAGCGGATTCGT
GAACTGTCCTTCCTCAACTCCGGTGTCGGCATCGTCCTCAAGGATGAGCGCAGCGGCAAGGAAGAGCTGTTCAAGTA
CGAGGGCGGCCTGCGTGCGTTCGTTGAATACCTGAACACCAACAAGACCCCGGTCAACCAGGTGTTCCACTTCAACA
TCCAGCGCGAAGACGGCATCGGCGTGGAAATCGCCTTGCAGTGGAACGACAGCTTCAACGAGAACCTGTTGTGCTTC
ACCAACAACATTCCACAGCGCGATGGCGGTACTCACCTGGTGGGTTTCCGTTCCGCGCTGACGCGCAACCTGAACAC
CTACATCGAAGCCGAAGGTCTGGCGAAGAAGCATAAAGTCGCGACCACCGGTGACGATGCCCGTGAAGGCCTGACCG
CGATTATCTCGGTAAAAGTGCCGGATCCAAAGTTCAGCTC
>12B 24
CTGCACGGTGTAGGTGTGTCGGTTGTGAACGCCCTGTCCGAAGAACTGGTCTTGACCGTTCGCCGCAGCGGCAAAAT
CTGGGAACAGACCTACATTCACGGTGTTCCAAAAGAGCCGATGAAAATCGTCGGTGAGAGTGAAACCACCGGCACCC
AGATCCACTTCAAACCGTCCGACCTGACCTTCAAGAACATCCATTTCAGCTGGGACATCCTGGCCAAGCGGATTCGT
GAACTGTCCTTCCTCAACTCCGGTGTCGGCATCGTCCTCAAGGATGAGCGCAGCGGCAAGGAAGAGTTGTTCAAGTA
CGAGGGCGGCCTGCGTGCGTTCGTTGAATACCTGAACACCAACAAGACCCCGGTCAACCAGGTGTTCCACTTCAATA
TCCAGCGCGAAGACGGCATCGGCGTGGAAATCGCCTTGCAGTGGAACGACAGCTTCAACGAGAACCTGTTGTGCTTC
ACCAACAACATTCCACAGCGCGATGGCGGTACTCACCTGGTGGGTTTCCGTTCCGCGCTGACGCGCAACCTGAACAC
CTACATCGAAGCCGAAGGTCTGGCGAAGAAGCATAAAGTCGCGACCACCGGTGACGATGCCCGTGAAGGCCTGACCG
CGATTATCTCGGTAAAAGTGCCGGATCCAAAGTTCAGCTC
>06D 138
CTGCACGGTGTAGGTGTGTCGGTTGTGAACGCCCTGTCCGAAGAACTGGTCTTGACCGTTCGCCGCAGCGGCAAAAT
CTGGGAACAGACCTACATTCACGGTGTTCCAAAAGAGCCGATGAAAATCGTCGGTGAGAGTGAAACCACCGGCACCC
AGATCCACTTCAAACCGTCCGACCTGACCTTCAAGAACATCCATTTCAGCTGGGACATCCTGGCCAAGCGGATTCGT
GAACTGTCCTTCCTCAACTCCGGTGTCGGCATCGTCCTCAAGGATGAGCGCAGCGGCAAGGAAGAGTTGTTCAAGTA
CGAGGGCGGCCTGCGTGCGTTCGTTGAATACCTGAACACCAACAAGACCCCGGTCAACCAGGTGTTCCACTTCAATA
TCCAGCGCGAAGACGGCATCGGCGTGGAAATCGCCTTGCAGTGGAACGACAGCTTCAACGAGAACCTGTTGTGCTTC

ACCAACAACATTCCACAGCGCGATGGCGGTACTCACCTGGTGGGTTTCCGTTCCGCGCTGACGCGCAACCTGAACAC
CTACATCGAAGCCGAAGGTCTGGCGAAGAAGCATAAAGTCGCGACCACCGGTGACGATGCCCGTGAAGGCCTGACCG
CGATTATCTCGGTAAAAGTGCCGGATCCAAAGTTCAGCTC
>08F 164
CTGCACGGTGTAGGTGTGTCGGTTGTGAACGCCCTGTCCGAAGAACTGGTCTTGACCGTTCGCCGCAGCGGCAAAAT
CTGGGAACAGACCTACATTCACGGTGTTCCAAAAGAGCCGATGAAAATCGTCGGTGAGAGTGAAACCACCGGCACCC
AGATCCACTTCAAACCGTCCGACCTGACCTTCAAGAACATCCATTTCAGCTGGGACATCCTGGCCAAGCGGATTCGT
GAACTGTCCTTCCTCAACTCCGGTGTCGGCATCGTCCTCAAGGATGAGCGCAGCGGCAAGGAAGAGTTGTTCAAGTA
CGAGGGCGGCCTGCGTGCGTTCGTTGAATACCTGAACACCAACAAGACCCCGGTCAACCAGGTGTTCCACTTCAATA
TCCAGCGCGAAGACGGCATCGGCGTGGAAATCGCCTTGCAGTGGAACGACAGCTTCAACGAGAACCTGTTGTGCTTC
ACCAACAACATTCCACAGCGCGATGGCGGTACTCACCTGGTGGGTTTCCGTTCCGCGCTGACGCGCAACCTGAACAC
CTACATCGAAGCCGAAGGTCTGGCGAAGAAGCATAAAGTCGCGACCACCGGTGACGATGCCCGTGAAGGCCTGACCG
CGATTATCTCGGTAAAAGTGCCGGATCCAAAGTTCAGCTC
>10C 34
CTGCACGGTGTAGGCGTCTCGGTTGTGAACGCCCTGTCCGAAGAACTGGTCTTGACCGTTCGCCGCAGCGGCAAAAT
CTGGGAACAGACCTACATTCACGGTGTTCCAAAAGAGCCGATGAAAATCGTCGGCGAGAGTGAAACCACCGGCACCC
AGATCCACTTCAAACCGTCCGACCTGACCTTCAAGAACATCCACTTCAGCTGGGACATCCTGGCCAAGCGGATTCGT
GAACTGTCCTTCCTCAACTCCGGTGTCGGCATCGTCCTCAAGGATGAGCGCAGTGGCAAGGAAGAGCTGTTCAAGTA
CGAGGGCGGCCTGCGTGCGTTCGTTGAATACCTGAACACCAACAAGACCCCGGTCAACCAGGTGTTCCACTTCAACA
TCCAGCGCGAAGACGGCATCGGCGTGGAAATCGCCTTGCAGTGGAACGACAGCTTCAACGAGAACCTGTTGTGCTTC
ACCAACAACATTCCACAGCGCGATGGCGGTACTCACCTGGTGGGTTTCCGTTCCGCGCTGACGCGTAACCTGAACAC
CTACATCGAAGCTGAAGGTTTGGCGAAGAAGCACAAAGTCGCGACCACCGGTGACGATGCCCGTGAAGGCCTGACCG
CGATTATCTCGGTAAAAGTGCCGGATCCGAAGTTCAGCTC
>12G 180
CTGCACGGTGTAGGCGTCTCGGTTGTGAACGCCCTGTCCGAAGAACTGGTCTTGACCGTTCGCCGCAGCGGCAAAAT
CTGGGAACAGACCTACATTCACGGTGTTCCAAAAGAGCCGATGAAAATCGTCGGCGAGAGTGAAACCACCGGCACCC
AGATCCACTTCAAACCGTCCGACCTGACCTTCAAGAACATCCACTTCAGTTGGGACATCCTGGCCAAGCGGATTCGT
GAACTGTCCTTCCTCAACTCCGGTGTCGGCATCGTCCTCAAGGATGAGCGCAGTGGCAAGGAAGAGCTGTTCAAGTA
CGAGGGCGGCCTGCGTGCGTTCGTTGAATACCTGAACACCAACAAGACCCCGGTCAACCAGGTGTTCCACTTCAACA
TCCAGCGCGAAGACGGCATCGGCGTGGAAATCGCCTTGCAGTGGAACGACAGCTTCAACGAGAACCTGTTGTGCTTC
ACCAACAACATTCCACAGCGCGATGGCGGTACTCACCTGGTGGGTTTCCGTTCCGCGCTGACGCGTAACCTGAACAC
CTACATCGAAGCTGAAGGTCTGGCGAAGAAGCACAAAGTCGCGACCACCGGTGACGATGCCCGTGAAGGCCTGACCG
CGATCATCTCGGTGAAAGTGCCGGATCCGAAGTTCAGCTC
>01A 1
CTGCACGGTGTAGGTGTGTCGGTTGTGAACGCCTTGTCCGAAGAGCTGGTCTTGACCGTTCGCCGCAGCGGCAAAAT
CTGGGAACAGACCTACATTCACGGTGTTCCAAAAGAACCGATGAAAATCGTCGGTGAGAGTGAAACCACCGGCACCC
AGATCCACTTCAAACCGTCCGACCTGACGTTCAAGAACATCCACTTCAGCTGGGACATCCTGGCCAAGCGGATTCGT
GAACTGTCCTTCCTCAACTCCGGTGTCGGCATCGTCCTCAAGGATGAGCGCAGCGGCAAGGAAGAGTTGTTCAAGTA
CGAGGGTGGCCTGCGTGCGTTCGTTGAATACCTGAACACCAACAAGACCCCGGTCAACCAGGTGTTCCACTTCAACA
TCCAGCGCGAAGACGGCATCGGTGTGGAAATCGCCTTGCAGTGGAACGACAGCTTCAACGAGAACCTGTTGTGCTTC
ACCAACAACATTCCACAGCGCGATGGCGGTACTCACCTGGTGGGTTTCCGTTCCGCTCTGACGCGTAACCTGAACAC
CTACATCGAAGCCGAAGGTCTGGCGAAGAAGCACAAAGTCGCGACCACCGGTGACGATGCCCGTGAAGGCCTGACCG
CGATCATCTCGGTAAAAGTGCCGGATCCGAAGTTCAGCTC
>09G 177
CTGCACGGTGTAGGTGTGTCGGTTGTGAACGCCCTGTCCGAAGAGCTGGTCTTGACCGTTCGCCGCAGCGGCAAAAT
CTGGGAACAGACCTACATTCACGGTGTTCCAAAAGAACCGATGAAAATCGTCGGTGAGAGTGAAACCACCGGCACCC
AGATCCACTTCAAACCGTCCGACCTGACGTTCAAGAACATCCACTTCAGCTGGGACATCCTGGCCAAGCGGATTCGT
GAACTGTCCTTCCTCAACTCCGGTGTCGGCATCGTCCTCAAGGATGAGCGCAGCGGCAAGGAAGAGTTGTTCAAGTA
CGAGGGTGGCCTGCGTGCGTTCGTTGAATACCTGAACACCAACAAGACCCCGGTCAACCAGGTGTTCCACTTCAACA
TCCAGCGCGAAGACGGCATCGGTGTGGAAATCGCCTTGCAGTGGAACGACAGCTTCAACGAGAACCTGTTGTGCTTC
ACCAACAACATTCCACAGCGCGATGGCGGTACTCACCTGGTGGGTTTCCGTTCCGCTCTGACGCGTAACCTGAACAC
CTACATCGAAGCCGAAGGTCTGGCGAAGAAGCACAAAGTCGCGACCACCGGTGACGATGCCCGTGAAGGCCTGACCG
CGATCATCTCGGTAAAAGTGCCGGATCCGAAGTTCAGCTC
>05A 5
CTGCACGGTGTAGGTGTGTCGGTTGTGAACGCCTTGTCCGAAGAGCTGGTCTTGACCGTTCGCCGCAGCGGCAAAAT
CTGGGAACAGACCTACATTCACGGTGTTCCAAAAGAACCGATGAAAATCGTCGGTGAGAGTGAAACCACCGGCACCC
AGATCCACTTCAAACCGTCCGACCTGACGTTCAAGAACATCCACTTCAGCTGGGACATCCTGGCCAAGCGGATTCGT

GAACTGTCCTTCCTCAACTCCGGTGTCGGCATCGTCCTCAAGGATGAGCGCAGCGGCAAGGAAGAGCTATTCAAGTA
CGAGGGTGGCCTGCGTGCTTTCGTTGAATACCTGAACACCAACAAGACTCCGGTCAACCAGGTGTTCCACTTCAACA
TCCAACGCGAAGACGGCATTGGCGTGGAAATCGCCTTGCAGTGGAACGACAGCTTCAACGAGAACCTGTTGTGCTTC
ACCAACAACATTCCACAGCGCGATGGCGGTACTCACCTGGTGGGTTTCCGTTCCGCTCTGACGCGTAACCTGAACAC
CTACATCGAGGCCGAAGGTCTGGCGAAGAAGCACAAAGTCGCGACCACCGGTGACGATGCCCGTGAAGGCCTGACCG
CGATCATCTCGGTAAAAGTGCCGGATCCGAAGTTCAGCTC
>05F 161
CTGCACGGTGTAGGTGTGTCGGTTGTGAATGCCTTGTCCGAAGAGCTGGTCTTGACCGTTCGCCGCAGCGGCAAAAT
CTGGGAACAGACCTACATTCACGGTGTTCCAAAAGAACCGATGAAAATCGTCGGCGAAAGCGAAACCACCGGCACCC
AGATCCACTTCAAACCGTCCGACCTGACGTTCAAGAACATCCACTTCAGCTGGGACATCCTGGCCAAGCGGATTCGT
GAACTGTCCTTCCTCAACTCCGGTGTCGGCATCGTCCTCAAGGATGAGCGCAGCGGCAAGGAAGAGCTGTTCAAGTA
CGAGGGCGGCCTGCGTGCGTTCGTTGAATACCTGAACACCAACAAGACTCCGGTCAACCAGGTGTTCCACTTCAACA
TCCAGCGTGAAGACGGCATCGGCGTGGAAATCGCCTTGCAGTGGAACGACAGCTTCAACGAGAACCTGTTGTGCTTC
ACCAACAACATTCCACAGCGCGATGGCGGTACTCACCTGGTGGGTTTCCGTTCCGCGCTGACGCGTAACCTGAACAC
CTACATCGAAGCCGAAGGTCTGGCGAAGAAGCACAAAGTCGCGACCACCGGTGACGATGCCCGTGAAGGCCTGACCG
CGATCATCTCGGTAAAAGTGCCGGATCCGAAGTTCAGCTC
>03C 27
CTGCACGGTGTAGGTGTGTCGGTTGTGAACGCCTTGTCCGAAGAGCTCGTGCTGACTGTTCGCCGCAGCGGCAAGAT
ATGGGAACAGACCTACATCCACGGTGTTCCGAAAGAGCCGATGAAAATCGTCGGCGAAAGCGAAACCACGGGTACCC
AGATTCACTTCAAGCCGTCCGACCTGACCTTCAAGAACATCCACTTCAGCTGGGACATCCTGGCCAAGCGGATTCGT
GAACTGTCCTTCCTCAACTCCGGTGTCGGCATCGTCCTCAAGGATGAGCGCAGCGGCAAGGAAGAGCTGTTCAAGTA
CGAAGGCGGCCTGCGTGCGTTTGTTGAATACCTGAACACCAACAAGACCCCGGTCAACCAGGTGTTCCACTTCAACA
TCCAGCGTGAAGACGGCATCGGCGTGGAAATCGCCCTGCAGTGGAACGACAGCTTCAACGAGAACCTGTTGTGCTTC
ACCAACAACATTCCACAGCGCGATGGCGGTACTCACCTGGTGGGTTTCCGTTCCGCGCTGACGCGTAACCTGAACAC
CTACATCGAAGCCGAAGGTCTGGCGAAGAAGCACAAAGTCGCGACCACCGGTGACGATGCCCGTGAAGGCCTGACCG
CGATCATCTCGGTAAAAGTGCCGGATCCGAAGTTCAGCTC
>04F 160
CTGCACGGTGTAGGTGTGTCGGTTGTGAACGCCTTGTCCGAAGAGCTCGTGCTGACTGTTCGCCGCAGCGGCAAGAT
CTGGGAACAGACCTACATCCACGGTGTTCCGAAAGAGCCGATGAAAATCGTCGGCGAAAGCGAAACCACGGGTACCC
AGATTCACTTCAAGCCGTCCGACCTGACCTTCAAGAACATCCACTTCAGCTGGGACATCCTGGCCAAGCGGATTCGT
GAACTGTCCTTCCTCAACTCCGGTGTCGGCATCGTCCTCAAGGATGAGCGCAGCGGCAAGGAAGAGCTGTTCAAGTA
CGAAGGCGGCCTGCGTGCGTTTGTTGAATACCTGAACACCAACAAGACCCCGGTCAACCAGGTGTTCCACTTCAACA
TCCAGCGTGAAGACGGCATCGGCGTGGAAATCGCCCTGCAGTGGAACGACAGCTTCAACGAGAACCTGTTGTGCTTC
ACCAACAACATTCCACAGCGCGATGGCGGTACTCACCTGGTGGGTTTCCGTTCTGCTCTGACGCGTAACCTGAACAC
CTACATCGAAGCCGAAGGTCTGGCGAAGAAGCACAAAGTCGCGACCACCGGTGACGATGCCCGTGAAGGCCTGACCG
CGATCATCTCGGTAAAAGTGCCGGATCCGAAGTTCAGCTC
>S04E 221
CTGCACGGCGTAGGTGTGTCGGTAGTGAACGCCCTGTCGGAAGAGCTGGTCCTGACCGTTCGCCGCAGCGGCAAGAT
CTGGGAACAGACTTACATCCACGGTGTTCCGAAAGAGCCGATGAAAATCGTTGGTGAGAGCGAAACCACCGGTACCC
AGATCCACTTCAAGCCATCCGACCTGACCTTCAAGAACATCCATTTCAGCTGGGACATCCTGGCCAAGCGGATTCGT
GAATTGTCCTTCCTCAACTCCGGCGTCGGCATCGTCCTCAAGGATGAACGCAGCGGCAAGGAAGAGCTGTTCAAATA
CGAAGGCGGCCTGCGTGCGTTCGTTGAATACCTGAACACCAACAAGACCCCGGTCAACCAGGTGTTCCATTTCAATA
TCCAGCGTGAAGACGGCATCGGTGTGGAAATCGCCCTGCAGTGGAACGACAGCTTCAACGAGAACCTGTTGTGCTTC
ACCAACAACATTCCACAGCGCGATGGCGGTACTCACCTAGTGGGTTTCCGTTCCGCACTGACGCGTAACCTGAACAC
CTACATCGAAGCTGAAGGCCTGGCGAAGAAACACAAAGTCGCCACCACCGGTGATGACGCCCGTGAAGGCTTGACCG
CGATTATTTCGGTGAAAGTCCCGGATCCGAAGTTCAGCTC
>S04A 217
CTGCACGGTGTGGGTGTGTCGGTCGTTAATGCCCTGTCGGAAGAGCTGGTCCTGACCGTTCGCCGTAGTGGCAAGAT
CTGGGAACAGACTTACGTCCACGGTGTTCCGCAAGAGCCGATGAAAATCGTTGGCGACTCCGAAACCACCGGTACGC
AGATCCACTTCAAGGCTTCCAGCGAAACCTTCAAGAACATCCACTTCAGCTGGGACATCCTGGCCAAGCGGATTCGT
GAACTGTCTTTCCTCAACTCCGGTGTCGGTATCGTCCTCAAGGATGAGCGCAGCGGCAAGGAAGAGCTGTTCAAGTA
TGAAGGCGGCCTGCGTGCGTTCGTTGAATACCTGAACACCAACAAGACCCCGGTCAACCAGGTTTTCCACTTCAACA
TCCAGCGTGAAGACGGCATCGGCGTGGAAATCGCCTTGCAGTGGAACGACAGCTTCAACGAGAACCTGTTGTGCTTC
ACCAACAACATTCCACAGCGCGACGGCGGCACTCACCTGGTGGGTTTCCGTTCCGCACTGACGCGTAACCTGAACAC
CTACATCGAAGCAGAAGGCCTGGCGAAGAAGCACAAGGTTGCCACTACCGGTGACGATGCCCGTGAAGGCCTGACCG
CGATCATTTCGGTGAAGGTACCGGATCCGAAGTTCAGCTC

>S12E 381
CTGCACGGTGTGGGTGTGTCGGTCGTTAATGCCCTGTCGGAAGAGCTGGTCCTGACCGTTCGCCGTAGTGGCAAGAT
CTGGGAACAGACTTACGTCCACGGTGTTCCGCAAGAGCCGATGAAAATCGTTGGCGACTCCGAAACCACCGGTACGC
AGATCCACTTCAAGGCTTCCAGCGAAACCTTCAAGAACATCCACTTCAGCTGGGACATCCTGGCCAAGCGGATTCGT
GAACTGTCTTTCCTCAACTCCGGTGTCGGTATCGTCCTCAAGGATGAGCGCAGCGGCAAGGAAGAGCTGTTCAAGTA
TGAAGGCGGCCTGCGTGCGTTCGTTGAATACCTGAACACCAACAAGACCCCGGTCAACCAGGTTTTCCACTTCAACA
TCCAGCGTGAAGACGGCATCGGCGTGGAAATCGCCTTGCAGTGGAACGACAGCTTCAACGAGAACCTGTTGTGCTTC
ACCAACAACATTCCACAGCGCGACGGCGGCACTCACCTGGTGGGTTTCCGTTCCGCACTGACGCGTAACCTGAACAC
CTACATCGAAGCAGAAGGCCTGGCGAAGAAGCACAAGGTTGCCACTACCGGTGACGATGCCCGTGAAGGCCTGACCG
CGATCATTTCGGTGAAGGTACCGGATCCGAAGTTCAGCTC
>S01F 198
CTGCACGGTGTGGGTGTGTCGGTCGTTAATGCCCTGTCGGAAGAGCTGGTCCTGACCGTTCGCCGTAGTGGCAAGAT
CTGGGAACAGACTTACGTCCACGGTGTTCCGCAAGAGCCGATGAAAATCGTTGGCGACTCCGAAACCACCGGTACGC
AGATCCACTTCAAGGCTTCCAGCGAAACCTTCAAGAACATCCACTTCAGCTGGGACATCCTGGCCAAGCGGATTCGT
GAACTGTCTTTCCTCAACTCCGGTGTCGGTATCGTCCTCAAGGATGAGCGCAGCGGCAAGGAAGAGCTGTTCAAGTA
TGAAGGCGGCCTGCGTGCGTTCGTTGAATACCTGAACACCAACAAGACCCCGGTCAACCAGGTTTTCCACTTCAACA
TCCAGCGTGAAGACGGCATCGGCGTGGAAATCGCCTTGCAGTGGAACGACAGCTTCAACGAGAACCTGTTGTGCTTC
ACCAACAACATTCCACAGCGCGACGGCGGCACTCACCTGGTGGGTTTCCGTTCCGCACTGACGCGTAACCTGAACAC
CTACATCGAAGCAGAAGGCCTGGCGAAGAAGCACAAGGTTGCCACTACCGGTGACGATGCCCGTGAAGGCCTGACCG
CGATCATTTCGGTGAAGGTACCGGATCCGAAGTTCAGCTC
>11B 119
CTGCACGGTGTGGGTGTGTCGGTCGTTAATGCCCTGTCGGAAGAGCTGGTCCTGACCGTTCGCCGTAGTGGCAAGAT
CTGGGAACAGACTTACGTCCACGGTGTTCCGCAAGAGCCGATGAAAATCGTTGGCGACTCCGAAACCACCGGTACGC
AGATCCACTTCAAGGCTTCCAGCGAAACCTTCAAGAACATCCACTTCAGCTGGGACATCCTGGCCAAGCGGATTCGT
GAACTGTCTTTCCTCAACTCCGGTGTCGGTATCGTCCTCAAGGATGAGCGCAGCGGCAAGGAAGAGCTGTTCAAGTA
TGAAGGCGGCCTGCGTGCGTTCGTTGAATACCTGAACACCAACAAGACCCCGGTCAACCAGGTTTTCCACTTCAACA
TCCAGCGTGAAGACGGCATCGGCGTGGAAATCGCCTTGCAGTGGAACGACAGCTTCAACGAGAACCTGTTGTGCTTC
ACCAACAACATTCCACAGCGCGACGGCGGCACTCACCTGGTGGGTTTCCGTTCCGCACTGACGCGTAACCTGAACAC
CTACATCGAAGCAGAAGGCCTGGCGAAGAAGCACAAGGTTGCCACTACCGGTGACGATGCCCGTGAAGGCCTGACCG
CGATCATTTCGGTGAAGGTACCGGATCCGAAGTTCAGCTC
>08A 104
CTGCACGGTGTGGGTGTGTCGGTCGTGAACGCCCTCTCGGAAGAGCTGGTCCTGACCGTTCGCCGTAGTGGCAAGAT
CTGGGAGCAGACTTACGTCCACGGTGTTCCGCAAGAGCCGATGAAAATCGTTGGCGACTCCGAAACCACCGGTACGC
AGATCCACTTCAAGGCTTCCAGCGAAACCTTCAAGAACATTCACTTCAGCTGGGACATCCTGGCCAAGCGGATTCGT
GAACTGTCTTTCCTCAACTCCGGTGTCGGTATCGTCCTCAAGGATGAGCGCAGCGGCAAGGAAGAGCTGTTCAAGTA
TGAAGGCGGCCTGCGTGCGTTCGTTGAATACCTGAACACCAACAAGACTCCGGTCAACCAGGTGTTCCACTTCAACA
TCCAGCGTGAAGACGGCATCGGCGTGGAAATCGCCTTGCAGTGGAACGACAGCTTCAACGAGAACCTGTTGTGCTTC
ACCAACAACATTCCACAGCGCGACGGCGGCACTCACCTGGTGGGTTTCCGTTCCGCACTGACGCGTAACCTGAACAC
CTATATCGAAGCAGAAGGCCTGGCGAAGAAGCACAAGGTCGCCACTACCGGTGACGATGCCCGTGAAGGCCTGACCG
CGATCATTTCGGTGAAGGTACCGGATCCGAAGTTCAGCTC
>09C 33
CTGCACGGTGTGGGTGTGTCGGTCGTGAACGCCCTGTCGGAAGAGTTGGTTCTGACCGTTCGCCGTAGTGGCAAGAT
CTGGGAACAGACTTACGTCCACGGTGTTCCGCAAGAGCCGATGAAAATCGTTGGCGACTCCGAAACCACCGGCACGC
AAATCCACTTCAAGGCTTCCAGCGAAACTTTCAAGAACATCCACTTCAGCTGGGACATCCTGGCCAAGCGGATTCGT
GAACTGTCCTTCCTCAACTCCGGTGTCGGCATCGTCCTCAAGGACGAGCGCAGCGGCAAGGAAGAGCTGTTCAAGTA
CGAAGGCGGCCTGCGTGCGTTCGTTGAATACCTGAACACCAACAAGACTCCGGTCAACCAGGTGTTCCACTTCAACA
TCCAGCGTGAAGACGGCATCGGCGTGGAAATCGCCCTGCAGTGGAACGACAGCTTCAACGAGAACCTGTTGTGCTTC
ACCAACAACATTCCACAGCGCGACGGCGGCACTCACCTGGTGGGTTTCCGTTCTGCACTGACGCGTAACCTGAATAC
CTACATCGAAGCAGAAGGCCTGGCGAAGAAGCACAAAGTCGCCACTACCGGTGACGATGCCCGTGAAGGCCTGACCG
CGATCATTTCGGTGAAGGTACCCGATCCGAAGTTCAGCTC
>10C 130
CTGCACGGTGTGGGTGTGTCGGTCGTGAACGCCCTGTCGGAAGAGTTGGTTCTGACCGTTCGCCGTAGTGGCAAGAT
CTGGGAACAGACTTACGTCCACGGTGTTCCGCAAGAGCCGATGAAAATCGTTGGCGACTCCGAAACCACCGGCACGC
AGATCCACTTCAAGGCTTCCAGCGAAACTTTCAAGAACATCCACTTCAGCTGGGACATCCTGGCCAAGCGGATTCGT
GAACTGTCCTTCCTCAACTCCGGTGTCGGCATCGTCCTCAAGGACGAGCGCAGCGGCAAGGAAGAGCTGTTCAAGTA
CGAAGGCGGCCTGCGTGCGTTCGTTGAATACCTGAACACCAACAAGACTCCGGTCAACCAGGTGTTCCACTTCAACA
TCCAGCGTGAAGACGGCATCGGCGTGGAAATCGCCCTGCAGTGGAACGACAGCTTCAACGAGAACCTGTTGTGCTTC

ACCAACAACATTCCACAGCGCGACGGCGGCACTCACCTGGTGGGTTTCCGTTCTGCACTGACGCGTAACCTGAATAC
CTACATCGAAGCAGAAGGCCTGGCGAAGAAGCACAAAGTCGCCACTACCGGTGACGATGCCCGTGAAGGCCTGACCG
CGATCATTTCGGTGAAGGTACCCGATCCGAAGTTCAGCTC
>S10B 266
CTGCACGGCGTGGGTGTGTCGGTGGTTAACGCCCTGTCGGAAGAACTGGTCCTGACCGTTCGCCGTAGCGGCAAGAT
CTGGGAACAGACTTACGTCCACGGTGTTCCGCAAGAGCCGATGAAAATCGTTGGCGACAGCGAAACCACCGGTACGC
AGATCCACTTCAAGGCTTCCAGCGAAACCTTCAAGAACATCCATTTCAGCTGGGACATCCTGGCCAAGCGGATTCGT
GAACTGTCCTTCCTCAACTCCGGTGTCGGCATCGTCCTCAAGGACGAGCGCAGCGGCAAGGAAGAGCTGTTCAAATA
CGAAGGCGGCCTGCGTGCATTCGTTGAATACCTGAACACCAACAAGACCCCGGTCAACCAGGTGTTCCACTTCAACA
TCCAGCGTGAAGACGGCATCGGCGTGGAAATCGCCCTGCAGTGGAACGACAGCTTCAACGAGAACCTGTTGTGTTTC
ACCAACAACATCCCGCAACGCGATGGCGGTACTCACCTGGTGGGTTTCCGTTCCGCGCTGACGCGTAACCTGAACAC
CTACATCGAAGCTGAAGGCCTGGCGAAGAAGCACAAGGTCGCCACCACCGGTGACGATGCCCGTGAAGGCCTGACCG
CGATCATTTCGGTAAAAGTGCCGGATCCGAAGTTCAGCTC
>S10A 265
CTGCACGGCGTGGGTGTGTCGGTGGTTAACGCCCTGTCGGAAGAACTGGTCCTGACCGTTCGCCGTAGCGGCAAGAT
CTGGGAACAGACTTACGTCCACGGTGTTCCGCAAGAGCCGATGAAAATCGTTGGCGACAGCGAAACCACCGGTACGC
AGATCCACTTCAAGGCTTCCAGCGAAACCTTCAAGAACATCCATTTCAGCTGGGACATCCTGGCCAAGCGGATTCGT
GAACTGTCCTTCCTCAACTCCGGTGTCGGCATCGTCCTCAAGGACGAGCGCAGCGGCAAGGAAGAGCTGTTCAAATA
CGAAGGCGGCCTGCGTGCATTCGTTGAATACCTGAACACCAACAAGACCCCGGTCAACCAGGTGTTCCACTTCAACA
TCCAGCGTGAAGACGGCATCGGCGTGGAAATCGCCCTGCAGTGGAACGACAGCTTCAACGAGAACCTGTTGTGTTTC
ACCAACAACATCCCGCAACGCGATGGCGGTACTCACCTGGTGGGTTTCCGTTCCGCGCTGACGCGTAACCTGAACAC
CTACATCGAAGCTGAAGGCCTGGCGAAGAAGCACAAGGTCGCCACCACCGGTGACGATGCCCGTGAAGGCCTGACCG
CGATCATTTCGGTAAAAGTGCCGGATCCGAAGTTCAGCTC
>S11B 274
CTGCACGGCGTGGGTGTGTCGGTGGTTAACGCCCTGTCGGAAGAACTGGTCCTGACCGTTCGCCGTAGCGGCAAGAT
CTGGGAACAGACTTACGTCCACGGTGTTCCGCAAGAGCCGATGAAAATCGTTGGCGACAGCGAAACCACCGGTACGC
AGATCCACTTCAAGGCTTCCAGCGAAACCTTCAAGAACATCCATTTCAGCTGGGACATCCTGGCCAAGCGGATTCGT
GAACTGTCCTTCCTCAACTCCGGTGTCGGCATCGTCCTCAAGGACGAGCGCAGCGGCAAGGAAGAGCTGTTCAAATA
CGAAGGCGGCCTGCGTGCATTCGTTGAATACCTGAACACCAACAAGACCCCGGTCAACCAGGTGTTCCACTTCAACA
TCCAGCGTGAAGACGGCATCGGCGTGGAAATAGCCCTGCAGTGGAACGACAGCTTCAACGAGAACCTGTTGTGTTTC
ACCAACAACATCCCGCAACGCGATGGCGGTACTCACCTGGTGGGTTTCCGTTCCGCGCTGACGCGTAACCTGAACAC
CTACATCGAAGCTGAAGGCCTGGCGAAGAAGCACAAGGTCGCCACCACCGGTGACGATGCCCGTGAAGGCCTGACCG
CGATCATTTCGGTAAAAGTGCCGGATCCGAAGTTCAGCTC
>06H 186
CTGCACGGCGTGGGTGTGTCGGTCGTTAACGCCCTGTCGGAACAGCTGGTCCTGACCGTTCGCCGTAGCGGCAAGAT
CTGGGAACAGACTTACGTCCACGGTGTTCCGCAAGAACCGATGAGAATCGTTGGCGACAGCGAAACCACCGGTACGC
AGATCCACTTCAAGGCTTCCAGCGAAACCTTCAAGAACATCCATTTCAGCTGGGACATCCTGGCCAAGCGGATCCGT
GAACTGTCCTTCCTCAACTCCGGTGTCGGCATCGTCCTCAAGGACGAGCGCAGCGGCAAGGAAGAGCTGTTCAAATA
CGAAGGCGGCCTGCGTGCGTTCGTTGAATACCTGAACACCAACAAGACCCCGGTCAACCAGGTGTTCCACTTCAACA
TCCAGCGTGAAGACGGCATCGGCGTGGAAATCGCCCTGCAGTGGAACGATAGCTTCAACGAGAACCTGTTGTGTTTC
ACCAACAACATTCCACAGCGCGATGGCGGTACTCACCTGGTGGGTTTCCGTTCCGCACTGACGCGTAACCTGAATAC
CTACATCGAAGCCGAAGGTCTGGCGAAGAAGCACAAAGTCGCGACCACCGGTGACGATGCCCGTGAAGGCCTGACCG
CGATCATTTCGGTGAAAGTACCGGATCCGAAGTTCAGCTC
>S02C 203
CTGCACGGTGTGGGTGTGTCGGTAGTTAACGCCCTGTCGGAAGAGCTCGTCCTGACCGTTCGCCGTAGCGGCAAGAT
CTGGGAACAGACTTACGTCCACGGTGTTCCGCAAGAGCCGATGAAAATCGTTGGCGACAGCGAAACCACCGGTACAC
AGATCCACTTCAAGGCTTCCAGCGAAACCTTCAAGAACATCCACTTCAGCTGGGACATCCTGGCCAAGCGGATTCGT
GAACTGTCCTTCCTCAACTCCGGTGTCGGTATCGTCCTCAAGGACGAGCGCAGCGGCAAGGAAGAACTGTTCAAATA
CGAAGGCGGCCTGCGTGCGTTCGTTGAATATCTGAACACCAACAAGACCCCGGTCAACCAGGTGTTCCACTTCAACG
TCCAGCGTGAAGACGGCATCGGCGTGGAAATTGCCCTGCAGTGGAACGACAGCTTCAACGAGAACCTGTTGTGCTTC
ACCAACAACATTCCACAGCGCGACGGCGGTACTCACTTGGTGGGTTTCCGTTCCGCACTGACGCGTAACCTGAACAC
CTACATCGAAGCAGAAGGCCTGGCGAAGAAGCACAAAGTCGCCACTACCGGTGACGATGCCCGTGAAGGCCTGACCG
CGATCATTTCGGTAAAAGTGCCGGATCCGAAGTTCAGCTC
>S07D 340
CTGCACGGTGTGGGTGTGTCGGTAGTTAACGCCCTGTCGGAAGAGCTCGTCCTGACCGTTCGCCGTAGCGGCAAGAT
CTGGGAACAGACTTACGTCCACGGTGTTCCGCAAGAGCCGATGAAAATCGTTGGCGACAGCGAAACCACCGGTACGC
AGATCCACTTCAAGGCTTCCAGCGAAACCTTCAAGAACATCCACTTCAGCTGGGACATCCTGGCCAAGCGGATTCGT

GAACTGTCCTTCCTCAACTCCGGTGTCGGTATCGTCCTCAAGGACGAGCGCAGCGGCAAGGAAGAACTGTTCAAATA
CGAAGGCGGCCTGCGTGCGTTCGTTGAATACCTGAACACCAACAAGACCCCGGTCAATCAGGTGTTCCACTTCAACG
TCCAACGTGAAGACGGCATCGGTGTGGAAATTGCCCTGCAGTGGAACGACAGCTTCAACGAGAACCTGTTGTGCTTC
ACCAACAACATTCCACAGCGCGACGGCGGTACTCACCTGGTGGGTTTCCGTTCCGCACTGACGCGTAACCTGAACAC
CTACATCGAAGCAGAAGGCCTGGCGAAGAAGCACAAAGTCGCCACCACCGGTGACGATGCCCGTGAAGGCCTGACCG
CGATCATTTCGGTGAAGGTGCCGGATCCGAAGTTCAGCTC
>12E 156
CTGCACGGTGTAGGTGTGTCGGTAGTGAACGCACTGTCCGAAGAGCTGGTTCTGACTGTTCGCCGCAGCGGCAAGAT
CTGGGAACAGACCTACGTCCACGGCGTACCTCAGGCACCGATGGCCATCGTTGGCGACAGCGAAACCACCGGCACCC
AGATCCACTTCAAGGCTTCCAGCGAAACCTTCAAGAACATTCACTTCAGCTGGGACATCCTGGCCAAGCGCATTCGT
GAACTGTCCTTCCTCAACTCCGGTGTCGGCATCGTCCTCAAGGATGAGCGCAGCGGCAAGGAAGAGCTGTTCAAGTA
CGAAGGCGGCCTGCGTGCGTTCGTTGAATACCTGAACACCAACAAGACCGCGGTCAACCAGGTGTTCCACTTCAACA
TCCAGCGTGAAGACGGCATCGGCGTGGAAATCGCCCTGCAGTGGAACGACAGCTTCAACGAGAACCTGTTGTGCTTC
ACCAACAACATTCCGCAGCGCGACGGCGGTACCCACCTGGTGGGCTTCCGTTCGGCACTGACGCGTAACCTGAACAA
CTACATTGAGCAGGAAGGTCTGGCGAAGAAGCACAAAGTCGCCACCACCGGTGACGATGCCCGTGAAGGCCTGACTG
CGATCATCTCGGTGAAAGTGCCGGATCCGAAGTTCAGCTC
>S12F 286
CTGCACGGTGTAGGTGTGTCGGTAGTGAACGCACTGTCCGAAGAGCTGGTTCTGACTGTTCGCCGCAGCGGCAAGAT
CTGGGAACAGACCTACGTCCACGGCGTACCTCAGGCACCGATGGCCATCGTTGGCGACAGCGAAACCACCGGCACCC
AGATCCACTTCAAGGCTTCCAGCGAAACCTTCAAGAACATTCACTTCAGCTGGGACATCCTGGCCAAGCGCATTCGT
GAACTGTCCTTCCTCAACTCCGGTGTCGGCATCGTCCTCAAGGATGAGCGCAGCGGCAAGGAAGAGCTGTTCAAGTA
CGAAGGCGGCCTGCGTGCGTTCGTTGAATACCTGAACACCAACAAGACTGCGGTCAACCAGGTGTTCCACTTCAACA
TCCAGCGTGAAGACGGCATCGGCGTGGAAATCGCTCTGCAGTGGAACGACAGCTTCAACGAGAACCTGTTGTGCTTC
ACCAACAACATTCCGCAGCGCGACGGCGGTACCCACCTGGTGGGCTTCCGTTCGGCACTGACGCGTAACCTGAACAA
CTACATTGAGCAGGAAGGTCTGGCGAAGAAGCACAAAGTCGCCACCACCGGTGACGATGCCCGTGAAGGCCTGACTG
CGATCATCTCGGTGAAAGTGCCGGATCCGAAGTTCAGCTC
>10B 118
CTGCACGGTGTAGGTGTGTCGGTAGTGAACGCACTGTCCGAAGAGCTGGTTCTGACTGTTCGCCGCAGCGGCAAGAT
CTGGGAACAGACCTACGTCCACGGCGTACCTCAGGCACCGATGGCCATCGTTGGCGACAGCGAAACCACCGGCACCC
AGATCCACTTCAAGGCTTCCAGCGAAACCTTCAAGAACATTCACTTCAGCTGGGACATCCTGGCCAAGCGCATTCGT
GAACTGTCCTTCCTCAACTCCGGTGTCGGCATCGTCCTCAAGGATGAGCGCAGCGGCAAGGAAGAGCTGTTCAAGTA
CGAAGGCGGCCTGCGTGCGTTCGTTGAATACCTGAACACCAACAAGACCGCGGTCAACCAGGTGTTCCACTTCAACA
TCCAGCGTGAAGACGGCATCGGCGTGGAAATCGCCCTGCAGTGGAACGACAGCTTCAACGAGAACCTGTTGTGCTTC
ACCAACAACATTCCGCAGCGCGACGGCGGTACCCACCTGGTGGGCTTCCGTTCGGCACTGACGCGTAACCTGAACAA
CTACATTGAGCAGGAAGGTCTGGCGAAGAAGCACAAAGTCGCCACCACCGGTGACGATGCCCGTGAAGGCCTGACTG
CGATCATCTCGGTGAAAGTGCCGGATCCGAAGTTCAGCTC
>03D 135
CTGCACGGTGTAGGTGTGTCGGTAGTGAACGCACTGTCCGAAGAGCTGGTTCTGACTGTTCGCCGCAGCGGCAAGAT
CTGGGAACAGACCTACGTCCACGGCGTACCTCAGGCACCGATGGCCATCGTTGGCGACAGCGAAACCACCGGCACCC
AGATCCACTTCAAGGCTTCCAGCGAAACCTTCAAGAACATTCACTTCAGTTGGGACATCCTGGCCAAGCGCATTCGT
GAACTGTCCTTCCTCAACTCCGGTGTCGGCATCGTCCTCAAGGATGAGCGCAGCGGCAAGGAAGAGCTGTTCAAGTA
CGAAGGCGGCCTGCGTGCGTTCGTTGAATACCTGAACACCAACAAGACTGCGGTCAACCAGGTGTTCCACTTCAACA
TCCAGCGTGAAGACGGCATCGGCGTGGAAATCGCTCTGCAGTGGAACGACAGCTTCAACGAGAACCTGTTGTGCTTC
ACCAACAACATTCCGCAGCGCGACGGCGGTACCCACCTGGTGGGCTTCCGTTCGGCACTGACGCGTAACCTGAACAA
CTACATTGAGCAGGAAGGTCTGGCGAAGAAGCACAAAGTCGCCACCACCGGTGACGATGCCCGTGAAGGCCTGACTG
CGATCATCTCGGTGAAAGTGCCGGATCCGAAGTTCAGCTC
>03A 99
CTGCACGGTGTAGGTGTGTCGGTAGTGAACGCACTGTCCGAAGAGCTGGTTCTGACTGTTCGCCGCAGCGGCAAGAT
CTGGGAACAGACCTACGTCCACGGCGTACCTCAGGCACCGATGGCCATCGTTGGCGACAGCGAAACCACCGGCACCC
AGATCCACTTCAAGGCTTCCAGCGAAACCTTCAAGAACATTCACTTCAGCTGGGACATCCTGGCCAAGCGCATTCGT
GAACTGTCCTTCCTCAACTCCGGTGTCGGCATCGTCCTCAAGGATGAGCGCAGCGGCAAGGAAGAGCTGTTCAAGTA
CGAAGGCGGCCTGCGTGCGTTCGTTGAATACCTGAACACCAACAAGACTGCGGTCAACCAGGTGTTCCACTTCAACA
TCCAGCGTGAAGACGGCATCGGCGTGGAAATCGCTCTGCAGTGGAACGACAGCTTCAACGAGAACCTGTTGTGCTTC
ACCAACAACATTCCGCAGCGCGACGGCGGTACCCACCTGGTGGGCTTCCGTTCGGCACTGACGCGTAACCTGAACAA
CTACATCGAGCAGGAAGGTCTGGCGAAGAAGCACAAAGTCGCCACCACCGGTGACGATGCCCGTGAAGGCCTGACTG
CGATCATCTCGGTGAAAGTGCCGGATCCGAAGTTCAGCTC

>02C 122
CTGCACGGTGTAGGTGTGTCGGTAGTGAACGCACTGTCCGAAGAGCTGGTTCTGACTGTTCGCCGCAGCGGCAAGAT
CTGGGAACAGACCTACGTCCACGGCGTACCTCAGGCACCGATGGCCATCGTTGGCGACAGCGAAACCACCGGCACCC
AGATCCACTTCAAGGCTTCCAGCGAAACCTTCAAGAACATTCACTTCAGCTGGGACATCCTGGCCAAGCGCATTCGT
GAACTGTCCTTCCTCAACTCCGGTGTCGGCATCGTCCTCAAGGATGAGCGCAGCGGCAAGGAAGAGCTGTTCAAGTA
CGAAGGCGGCCTGCGTGCGTTCGTTGAATACCTGAACACCAACAAGACTGCGGTCAACCAGGTGTTCCACTTCAACA
TCCAGCGTGAAGACGGCATCGGCGTGGAAATCGCCCTGCAGTGGAACGACAGCTTCAACGAGAACCTGTTGTGCTTC
ACCAACAACATTCCGCAGCGCGACGGCGGTACTCACCTGGTGGGTTTCCGTTCGGCGCTGACGCGTAACCTGAACAA
CTACATCGAGCAGGAAGGTCTGGCGAAGAAGCACAAAGTCGCCACCACCGGTGACGATGCCCGTGAAGGCCTGACTG
CGATCATCTCGGTGAAAGTGCCGGATCCGAAGTTCAGCTC
>S10G 367
CTGCACGGTGTAGGTGTGTCGGTAGTGAACGCACTGTCCGAAGAGCTGGTTCTGACTGTTCGCCGCAGCGGCAAGAT
CTGGGAACAGACCTACGTCCACGGCGTACCTCAGGCACCGATGGCCATCGTTGGCGACAGCGAAACCACCGGCACCC
AGATCCACTTCAAGGCTTCCAGCGAAACCTTCAAGAACATTCACTTCAGCTGGGACATCCTGGCCAAGCGCATTCGT
GAACTGTCCTTCCTCAACTCCGGTGTCGGCATCGTCCTCAAGGATGAGCGCAGCGGCAAGGAAGAGCTGTTCAAGTA
CGAAGGCGGCCTGCGTGCGTTCGTTGAATACCTGAACACCAACAAGACTGCGGTCAACCAGGTGTTCCACTTCAACA
TCCAGCGTGAAGACGGCATCGGCGTGGAAATCGCCCTGCAGTGGAACGACAGCTTCAACGAGAACCTGTTGTGCTTC
ACCAACAACATTCCGCAGCGCGACGGCGGTACCCACCTGGTGGGCTTCCGTTCGGCACTGACGCGTAACCTGAACAA
TTACATTGAGCAGGAAGGTCTGGCGAAGAAGCACAAAGTCGCCACCACCGGTGACGATGCCCGTGAAGGCCTGACTG
CGATCATCTCGGTGAAAGTGCCGGATCCGAAGTTCAGCTC
>07H 187
CTGCACGGTGTAGGTGTGTCGGTAGTGAACGCACTGTCCGAAGAACTGGTTCTGACTGTTCGCCGCAGCGGCAAGAT
CTGGGAACAGACCTACGTCCACGGTGTACCTCAGGCACCGATGGCCATCGTTGGCGACAGCGAAACCACCGGTACCC
AGATCCACTTCAAGGCTTCCAGCGAAACCTTCAAGAACATTCACTTCAGCTGGGACATCCTGGCCAAGCGCATTCGT
GAACTGTCCTTCCTCAACTCCGGTGTCGGCATTGTCCTCAAGGACGAGCGCAGCGGCAAGGAAGAGCTGTTCAAGTA
CGAAGGCGGCCTGCGTGCGTTCGTTGAATACCTGAACACCAACAAGACTGCGGTCAACCAGGTGTTCCACTTCAACA
TCCAGCGTGAAGACGGAATCGGCGTGGAAATCGCCCTGCAGTGGAACGACAGCTTCAACGAGAACCTGTTGTGCTTC
ACCAACAACATTCCGCAGCGCGACGGCGGTACTCACCTGGTGGGTTTCCGTTCGGCGCTGACGCGTAACCTGAACAA
CTACATCGAGCAGGAAGGTCTGGCGAAGAAGCACAAAGTCGCCACCACCGGTGACGATGCCCGTGAAGGCCTGACTG
CGATCATCTCGGTGAAGGTGCCGGATCCGAAGTTCAGCTC
>02B 14
CTGCACGGTGTAGGTGTGTCGGTAGTGAACGCACTGTCCGAAGAGCTGGTTCTGACTGTTCGCCGCAGCGGCAAGAT
CTGGGAACAGACCTACGTCCACGGCGTACCTCAGGCACCGATGGCCATCGTTGGCGACAGCGAAACCACCGGCACCC
AGATCCACTTCAAGGCTTCCAGCGAAACCTTCAAGAACATTCACTTCAGCTGGGACATCCTCGCCAAGCGTATTCGT
GAACTGTCCTTCCTCAACTCCGGTGTCGGCATCGTCCTCAAGGACGAGCGCAGTGGCAAGGAAGAGCTGTTCAAGTA
CGAAGGCGGCCTGCGTGCGTTCGTTGAATACCTGAACACCAACAAGACTGCGGTCAACCAGGTGTTCCACTTCAACA
TCCAGCGTGAAGACGGCATCGGCGTGGAAATCGCCCTGCAGTGGAACGACAGCTTCAACGAGAACCTGTTGTGCTTC
ACCAACAACATTCCGCAGCGCGACGGCGGTACTCACCTGGTGGGTTTCCGTTCGGCACTGACGCGTAACCTGAACAA
CTACATCGAGCAGGAAGGTCTGGCGAAGAAGCACAAAGTCGCCACCACCGGTGACGATGCCCGTGAAGGCCTGACCG
CGATCATCTCGGTGAAAGTGCCGGATCCGAAGTTCAGCTC
>04D 40
CTGCACGGTGTAGGTGTGTCGGTAGTGAACGCACTGTCCGAAGAATTGGTACTGACCGTTCGCCGCTGCGGCAAGAT
CTGGGAACAGACCTACGTCCACGGCGTACCTCAGGCACCGATGGCCATCGTTGGCGACAGCGAAACCACTGGTACCC
AGATCCACTTCAAGGCTTCCAGCGAAACCTTCAAGAACATTCACTTCAGCTGGGACATCCTGGCCAAGCGCATTCGT
GAACTGTCCTTCCTCAACTCCGGTGTCGGCATCGTCCTCAAGGATGAGCGCAGCGGCAAGGAAGAACTGTTCAAGTA
CGAAGGCGGCCTGCGTGCGTTCGTTGAATACCTGAACACCAACAAGACTGCGGTCAACCAGGTGTTCCACTTCAACA
TCCAGCGTGAAGACGGCATCGGCGTGGAAATCGCCCTGCAGTGGAACGACAGCTTCAACGAGAACCTGTTGTGCTTC
ACCAACAACATTCCGCAGCGCGACGGCGGTACTCACCTGGTGGGCTTCCGTTCGGCACTGACGCGTAACCTGAACAA
CTACATCGAGCAGGAAGGTCTGGCGAAGAAGCACAAAGTCGCCACCACCGGTGACGATGCCCGTGAAGGCCTGACCG
CGATCATTTCGGTGAAAGTGCCGGATCCGAAGTTCAGCTC
>S12H 288
CTGCACGGTGTAGGTGTGTCGGTAGTGAACGCACTGTCCGAAGAATTGGTACTGACCGTTCGCCGCAGCGGAAAGAT
CTGGGAACAGACCTACGTCCACGGCGTACCTCAGGCACCGATGGCCATCGTTGGCGACAGCGAAACCACTGGTACCC
AGATCCACTTCAAGGCTTCCAGCGAAACCTTCAAGAACATTCACTTCAGCTGGGACATCCTGGCCAAGCGCATTCGT
GAACTGTCCTTCCTCAACTCCGGTGTCGGCATCGTCCTCAAGGATGAGCGCAGCGGCAAGGAAGAACTGTTCAAGTA
CGAAGGCGGCCTGCGTGCGTTCGTTGAATACCTGAACACCAACAAGACTGCGGTCAACCAGGTGTTCCACTTCAACA
TCCAGCGTGAAGACGGCATCGGCGTGGAAATCGCCCTGCAGTGGAACGACAGCTTCAACGAGAACCTGTTGTGCTTC

ACCAACAACATTCCGCAGCGCGACGGCGGTACTCACCTGGTGGGCTTCCGTTCGGCACTGACGCGTAACCTGAACAA
CTACATCGAGCAGGAAGGTCTGGCGAAGAAGCACAAAGTCGCCACCACCGGTGACGATGCCCGTGAAGGCCTGACCG
CGATCATTTCGGTGAAAGTGCCGGATCCGAAGTTCAGCTC
>S06D 236
CTGCACGGTGTAGGTGTGTCGGTAGTGAACGCACTGTCCGAAGAGCTGGTACTCACCGTGCGCCGCAGCGGCAAGAT
CTGGGAACAGACCTACGTCCACGGCGTACCTCAGGCACCGATGGCCATCGTTGGCGACAGCGAAACCACCGGTACCC
AGATCCACTTCAAGGCTTCCAGCGAAACCTTCAAGAACATTCACTTCAGCTGGGACATCCTGGCCAAGCGCATTCGT
GAGCTGTCCTTCCTCAACTCCGGTGTCGGCATCGTCCTCAAGGATGAGCGCAGCGGCAAGGAAGAGCTGTTCAAGTA
CGAAGGCGGCCTGCGTGCGTTCGTTGAATACCTGAACACCAACAAGACTGCGGTCAACCAGGTATTCCACTTCAACA
TCCAGCGTGAAGACGGCATCGGCGTGGAAATCGCCCTGCAGTGGAACGACAGCTTCAACGAGAACCTGTTGTGCTTC
ACCAACAATATTCCGCAGCGCGACGGCGGTACTCACCTGGTGGGCTTCCGTTCGGCACTGACGCGTAACCTGAACAA
CTATATCGAGCAGGAAGGTCTGGCGAAGAAGCACAAAGTCGCCACCACCGGTGACGATGCCCGTGAAGGCCTGACCG
CGATCATCTCGGTGAAAGTGCCGGATCCGAAGTTCAGCTC
>04G 172
CTGCACGGTGTAGGTGTGTCGGTAGTGAACGCACTGTCCGAAGAGCTGGTTCTGACTGTTCGCCGCAGCGGCAAGAT
CTGGGAACAGACCTACGTCCACGGCGTACCTCAGGCGCCGATGGCCATCGTTGGTGACAGCGAAACCACCGGTACCC
AGATCCACTTCAAGGCTTCCAGCGAAACCTTCAAGAACATTCACTTCAGCTGGGACATCCTGGCCAAGCGTATTCGT
GAACTGTCCTTCCTCAACTCCGGTGTCGGCATCGTCCTCAAGGACGAGCGCAGTGGCAAGGAAGAGCTGTTCAAGTA
CGAAGGCGGCCTGCGTGCGTTCGTTGAATACCTGAACACCAACAAGACTGCGGTCAACCAGGTGTTCCACTTCAACA
TCCAGCGTGAAGACGGCATCGGCGTGGAAATCGCCCTGCAGTGGAACGACAGCTTCAACGAGAACCTGTTGTGCTTC
ACCAACAACATTCCGCAGCGCGACGGCGGTACTCACCTGGTGGGTTTCCGTTCGGCACTGACGCGTAACCTGAACAA
CTACATCGAGCAGGAAGGTCTGGCGAAGAAGCACAAAGTCGCCACCACCGGTGACGATGCCCGTGAAGGCCTGACCG
CGATCATCTCGGTGAAAGTGCCGGATCCGAAGTTCAGCTC
>S10E 365
CTGCACGGTGTAGGTGTGTCGGTAGTGAACGCACTGTCCGAAGAGCTGGTTCTGACTGTTCGCCGCAGCGGCAAGAT
CTGGGAACAGACCTATGTCCACGGCGTACCTCAGGCGCCGATGGCCATCGTTGGCGACAGCGAAACCACCGGTACCC
AGATCCACTTCAAGGCTTCCAGTGAAACCTTCAAGAACATTCACTTCAGCTGGGACATCCTGGCCAAGCGTATTCGT
GAACTGTCCTTCCTCAACTCCGGTGTCGGCATCGTCCTCAAGGATGAGCGCAGCGGCAAGGAAGAGTTGTTCAAGTA
CGAAGGCGGCTTGCGTGCATTCGTTGAATACCTGAACACCAACAAGACCGCGGTCAACCAGGTGTTCCACTTCAACA
TTCAGCGTGAAGACGGCATCGGCGTGGAAATCGCCCTGCAGTGGAACGACAGCTTCAACGAGAACCTGTTGTGCTTC
ACCAACAACATTCCGCAGCGCGACGGTGGCACCCACTTGGTGGGCTTCCGATCGGCGCTGACGCGTAACCTGAACAA
CTACATCGAGCAGGAAGGTCTGGCGAAGAAGCACAAAGTTGCCACCACCGGTGACGATGCCCGTGAAGGCCTGACCG
CGATCATCTCGGTGAAAGTGCCGGATCCGAAGTTCAGCTC
>10A 106
CTGCACGGTGTAGGTGTGTCGGTAGTGAACGCACTGTCCGAAGAGCTGGTACTGACCGTTCGCCGCAGCGGCAAGAT
CTGGGAACAGACCTACGTCCACGGCGTACCTCAGGCTCCGATGGCCATCGTTGGCGACAGCGAAACCACCGGTACCC
AGATCCACTTCAAGGCTTCCAGCGAAACCTTCAAGAACATTCACTTCAGCTGGGACATCCTCGCCAAGCGTATTCGT
GAACTGTCCTTCCTCAACTCCGGTGTCGGCATCGTCCTCAAGGACGAGCGCAGTGGCAAGGAAGAGCTGTTCAAGTA
CGAAGGCGGCCTGCGTGCGTTCGTTGAATACCTGAACACCAACAAGACTGCGGTCAACCAGGTGTTCCACTTCAACA
TCCAGCGTGAAGACGGCATCGGCGTGGAAATCGCCCTGCAGTGGAACGACAGCTTCAACGAGAACCTGTTGTGCTTC
ACCAACAACATTCCGCAGCGCGACGGCGGTACTCACCTGGTGGGTTTCCGTTCTGCACTGACGCGTAACCTGAACAA
CTACATCGAGCAGGAAGGTCTGGCGAAGAAGCACAAAGTCGCCACCACCGGTGACGATGCCCGTGAAGGTCTGACCG
CGATCATCTCGGTGAAAGTGCCGGATCCGAAGTTCAGCTC
>S08F 254
CTGCACGGTGTGGGTGTGTCGGTAGTGAACGCACTGTCCGAAGAATTGGTTCTGACCGTTCGCCGCAGCGGCAAGAT
CTGGGAACAGACCTACGTCCACGGCGTACCTCAGGCACCGATGGCCATCGTTGGCGACAGCGAAACCACCGGTACCC
AGATACACTTCAAGGCTTCCAGCGAAACCTTTAAGAACATTCACTTCAGCTGGGACATCCTCGCCAAGCGCATTCGT
GAGCTGTCCTTCCTCAACTCCGGTGTCGGCATCGTCCTCAAGGATGAGCGCAGCGGCAAGGAAGAACTGTTCAAGTA
CGAAGGCGGCTTGCGTGCGTTCGTTGAATACCTGAACACCAACAAGACTGCGGTCAACCAGGTGTTCCACTTCAACA
TCCAGCGTGAAGACGGCATCGGCGTAGAGATCGCCCTGCAGTGGAACGACAGCTTCAACGAGAACCTGTTGTGCTTC
ACCAACAATATTCCGCAGCGCGACGGCGGTACTCACCTGGTGGGCTTCCGTTCGGCACTGACGCGTAACCTGAACAA
CTACATCGAGCAGGAAGGTCTGGCGAAGAAGCACAAAGTCGCCACCACCGGTGACGATGCCCGTGAAGGCCTGACCG
CGATCATTTCGGTGAAAGTGCCGGATCCGAAGTTCAGCTC
>11G 83
CTGCACGGTGTAGGTGTGTCGGTCGTGAACGCCCTGTCCGAAGAGCTGGTACTGACCGTACGCCGCAGCGGCAAGAT
CTGGGAACAGACCTACGTCCACGGTGTACCTCAGGCACCGATGGCGATCGTTGGCGACAGCGAAACCACTGGCACCC
AGATCCATTTCAAGGCTTCCAGCGAAACCTTCAAGAACATTCACTTCAGCTGGGACATCCTGGCCAAACGCATTCGT

GAACTGTCCTTCCTCAACTCCGGTGTCGGCATCGTCCTCAAGGATGAGCGCAGCGGCAAGGAAGAGCTGTTCAAGTA
CGAAGGCGGCCTGCGTGCGTTCGTTGAATACCTGAACACCAACAAGACTGCGGTCAACCAGGTGTTCCACTTCAACA
TCCAGCGTGAAGACGGCATCGGCGTCGAAATCGCCCTGCAATGGAACGACAGCTTCAACGAGAACCTGTTGTGCTTC
ACCAACAACATTCCGCAGCGCGACGGCGGTACTCACCTGGTGGGTTTCCGTTCCGCACTGACGCGTAACCTGAACAA
CTATATCGAGCAGGAAGGTCTGGCGAAGAAGCACAAAGTCGCGACCACCGGTGACGATGCCCGTGAAGGCCTGACCG
CGATCATCTCGGTAAAAGTGCCGGATCCGAAGTTCAGCTC
>S11A 369
CTGCACGGTGTAGGTGTGTCGGTAGTGAACGCACTGTCCGAAGAACTGGTACTGACCGTTCGCCGCAGCGGCAAGAT
CTGGGAACAGACCTACGTCCATGGCGTACCTCAGGCACCGATGGCCATCGTTGGCGACAGCGAAACCACCGGTACCC
AGATCCACTTCAAGGCTTCCAGCGAAACCTTCAAGAACATTCACTTCAGCTGGGACATCCTGGCCAAGCGCATTCGT
GAACTGTCCTTCCTCAACTCCGGTGTCGGCATCGTCCTCAAGGACGAGCGCAGCGGCAAGGAAGAACTGTTCAAGTA
CGAAGGCGGCCTGCGTGCGTTCGTTGAATACCTGAACACCAACAAGACTCCGGTCAACCAGGTGTTCCACTTCAACA
TCCAGCGTGAAGACGGCATCGGCGTGGAAATCGCCCTGCAGTGGAACGACAGCTTCAACGAGAACCTGTTGTGCTTC
ACCAACAACATTCCGCAGCGCGACGGCGGTACCCACTTGGTGGGCTTCCGTTCTGCACTGACGCGTAACCTGAATAC
CTATATCGAAGCTGAAGGCCTGGCGAAGAAGCACAAAGTCGCCACCACCGGTGACGATGCCCGTGAAGGCCTGACCG
CGATCATCTCGGTGAAAGTGCCGGATCCGAAGTTCAGCTC
>11A 107
CTGCACGGTGTAGGTGTGTCGGTAGTGAACGCACTGTCCGAAGAGCTGGTCCTTACCGTTCGCCGCAGTGGCAAGAT
CTGGGAACAGACCTACGTCCACGGTGTGCCTCAGGCACCGATGGCCATCGTTGGCGACAGCGAAACCACCGGTACCC
AGATCCACTTCAAGGCTTCCAGCGAAACCTTCAAGAACATTCACTTCAGCTGGGACATCCTCGCCAAGCGCATTCGT
GAACTGTCCTTCCTCAACTCCGGTGTCGGCATCGTCCTCAAGGACGAGCGCAGCGGCAAGGAAGAGCTGTTCAAGTA
CGAAGGCGGCCTGCGTGCGTTCGTTGAATACCTGAACACCAACAAGACTGCGGTCAACCAAGTGTTCCACTTCAACA
TCCAGCGTGAAGACGGGATCGGCGTGGAAATCGCCCTGCAGTGGAACGACAGCTTCAACGAGAACCTGTTGTGCTTC
ACCAACAACATTCCGCAGCGCGACGGCGGCACCCACCTGGTGGGCTTCCGCTCGGCACTGACGCGTAACCTGAACAA
CTACATCGAGCAGGAAGGTCTGGCGAAGAAGCACAAAGTCGCCACCACCGGTGACGATGCCCGTGAAGGCCTGACCG
CAATCATCTCGGTGAAAGTGCCGGATCCGAAATTCAGCTC
>01F 157
CTGCACGGTGTAGGTGTTTCGGTCGTGAACGCGCTGTCTGAAGAACTGGTCCTGACCGTGCGCCGCAGCGGCAAGAT
CTGGGAACAGACCTACGTCCACGGCGTGCCTCAGGCACCGATGGCGATCGTTGGCGACAGCGAAACCACTGGCACCC
AGATTCACTTCAAGGCTTCCAGCGAAACCTTCAAGAACATTCACTTCAGCTGGGATATCCTGGCCAAGCGCATTCGT
GAACTGTCTTTCCTCAACTCCGGTGTAGGCATCGTCCTCAAGGACGAGCGCAGCGGCAAGGAAGAACTGTTCAAGTA
CGAAGGCGGCCTGCGTGCGTTCGTTGAATACCTGAACACCAACAAGACTGCGGTCAACCAGGTGTTCCACTTCAACA
TCCAGCGTGAAGACGGCATCGGCGTGGAAATCGCCCTGCAGTGGAACGACAGCTTCAACGAGAACCTGTTGTGCTTC
ACCAACAACATTCCGCAGCGCGACGGTGGCACTCACCTGGTGGGCTTCCGCTCGGCACTGACGCGTAACCTGAACAA
CTACATCGAGCAGGAAGGCCTGGCGAAGAAGCACAAAGTCGCCACCACCGGTGACGATGCCCGTGAAGGCCTGACCG
CGATCATCTCGGTGAAGGTGCCGGATCCGAAGTTCAGCTC
>07C 127
CTGCACGGTGTAGGTGTTTCGGTCGTGAACGCGCTGTCTGAAGAACTGGTCCTGACCGTGCGCCGCAGCGGCAAGAT
CTGGGAACAGACCTACGTCCACGGCGTGCCTCAGGCACCGATGGCGATCGTTGGCGACAGCGAAACCACTGGCACCC
AGATTCACTTCAAGGCTTCCAGCGAAACCTTCAAGAACATTCACTTCAGCTGGGATATCCTGGCCAAGCGCATTCGT
GAACTGTCTTTCCTCAACTCCGGTGTAGGCATCGTCCTCAAGGACGAGCGCAGCGGCAAGGAAGAACTGTTCAAGTA
CGAAGGCGGCCTGCGTGCGTTCGTTGAATACCTGAACACCAACAAGACTGCGGTCAACCAGGTGTTCCACTTCAACA
TCCAGCGTGAAGACGGCATCGGCGTGGAAATCGCCCTGCAGTGGAACGACAGCTTCAACGAGAACCTGTTGTGCTTC
ACCAACAACATTCCGCAGCGCGACGGCGGCACCCACCTGGTGGGCTTCCGTTCCGCACTGACGCGTAACCTGAACAA
CTACATCGAGCAGGAAGGTCTGGCGAAGAAGCACAAAGTCGCCACCACCGGTGACGATGCCCGTGAAGGCCTGACCG
CGATCATCTCGGTGAAGGTGCCGGATCCGAAGTTCAGCTC
>12G 84
CTGCACGGTGTAGGTGTTTCGGTTGTGAACGCGCTGTCCGAAGAGCTGGTCCTGACCGTTCGCCGCAGCGGCAAGAT
CTGGGAACAGACCTACGTCCACGGCGTACCTCAGGCACCGATGGCGATCGTTGGCGACAGCGAAACCACCGGCACCC
AGATTCACTTCAAGGCTTCCAGCGAAACCTTCAAGAACATTCACTTCAGCTGGGACATCCTGGCCAAGCGCATTCGT
GAACTGTCCTTCCTCAACTCCGGTGTCGGCATCGTCCTCAAGGATGAGCGCAGCGGCAAGGAAGAGCTGTTCAAATA
CGAAGGCGGCCTGCGTGCGTTCGTTGAATACCTGAACACCAACAAGACTGCGGTCAACCAGGTGTTCCACTTCAACA
TCCAGCGTGAAGACGGCATCGGCGTGGAAATCGCCCTGCAGTGGAACGACAGCTTCAACGAGAACCTGTTGTGCTTC
ACCAACAACATTCCGCAGCGCGACGGCGGCACCCATCTGGTGGGCTTCCGTTCCGCACTGACGCGTAACCTGAACAA
CTACATCGAGCAGGAAGGCCTGGCGAAGAAGCACAAAGTCGCCACCACCGGTGACGATGCCCGTGAAGGCCTGACCG
CGATCATCTCGGTGAAGGTGCCTGATCCGAAGTTCAGCTC

>04C 124
CTGCACGGTGTAGGTGTTTCTGTTGTGAACGCGCTGTCCGAAGAGCTGGTACTGACCGTTCGCCGCAGCGGCAAGAT
CTGGGAACAGACCTACGTTCACGGCGTGCCTCAGGCACCGATGGCGATCGTTGGCGACAGCGAAACCACCGGTACCC
AGATTCACTTCAAGGCTTCCAGCGAAACCTTCAAGAACATTCACTTCAGCTGGGACATCCTGGCCAAGCGCATTCGT
GAACTGTCCTTCCTAAACTCCGGTGTCGGCATCGTCCTCAAGGATGAGCGCAGCGGAAAGGAAGAGCTGTTCAAGTA
CGAAGGCGGCCTGCGTGCGTTCGTTGAATACCTGAACACCAACAAGACTGCGGTCAACCAGGTGTTCCACTTCAACA
TCCAGCGTGAAGACGGCATCGGCGTGGAAATCGCCCTGCAGTGGAACGACAGTTTCAACGAGAACCTGTTGTGCTTC
ACCAACAACATTCCGCAGCGCGACGGCGGCACCCACCTGGTGGGCTTCCGTTCCGCACTGACGCGTAACCTGAACAA
CTACATCGAGCAGGAAGGTCTGGCGAAGAAGCACAAAGTCGCCACCACCGGTGACGATGCCCGTGAAGGCCTGACCG
CGATCATCTCGGTGAAGGTGCCGGATCCGAAGTTCAGCTC
>08D 140
CTGCACGGTGTAGGTGTTTCTGTTGTGAACGCGCTGTCCGAAGAGCTGGTACTGACCGTTCGCCGCAGCGGCAAGAT
CTGGGAACAGACCTACGTTCACGGCGTGCCTCAGGCACCGATGGCGATCGTTGGCGACAGCGAAACCACCGGTACCC
AGATTCACTTCAAGGCTTCCAGCGAAACCTTCAAGAACATTCACTTCAGCTGGGACATCCTGGCCAAGCGCATTCGT
GAACTGTCCTTCCTCAACTCCGGTGTCGGCATCGTCCTCAAGGATGAGCGCAGCGGAAAGGAAGAGCTGTTCAAGTA
CGAAGGCGGCCTGCGTGCGTTCGTTGAATACCTGAACACCAACAAGACTGCGGTCAACCAGGTGTTCCACTTCAACA
TCCAGCGTGAAGACGGCATCGGCGTGGAAATCGCCCTGCAGTGGAACGACAGTTTCAACGAGAACCTGTTGTGCTTC
ACCAACAACATTCCGCAGCGCGACGGCGGCACCCACCTGGTGGGGTTCCGTTCCGCACTGACGCGTAACCTGAACAA
CTACATCGAGCAGGAAGGTCTGGCGAAGAAGCACAAAGTCGCCACCACCGGTGACGATGCCCGTGAAGGCTTGACCG
CGATCATCTCGGTGAAGGTGCCGGATCCGAAGTTCAGCTC
>03F 159
CTGCACGGTGTAGGTGTTTCTGTTGTGAACGCGCTGTCCGAAGAACTGGTCCTGACCGTTCGCCGCAGCGGCAAAAT
CTGGGAACAGACCTACGTCCACGGCGTACCTCAGGCACCGATGGCGATCGTTGGCGACAGCGAAACCACCGGTACCC
AGATTCACTTCAAGGCTTCCAGCGAAACCTTCAAGAACATTCACTTCAGCTGGGACATCCTGGCCAAGCGCATTCGT
GAACTGTCTTTCCTCAACTCCGGTGTCGGCATCGTCCTCAAGGATGAGCGCAGCGGCAAGGAAGAGCTGTTCAAGTA
CGAAGGCGGCCTGCGTGCGTTCGTTGAATACCTGAACACCAACAAGACTGCGGTCAACCAGGTGTTTCACTTCAACA
TCCAGCGTGAAGACGGCATCGGCGTGGAAATCGCCCTGCAGTGGAACGACAGCTTCAACGAGAACCTGTTGTGCTTC
ACCAACAACATCCCGCAGCGCGACGGCGGCACTCACCTGGTGGGCTTCCGTTCCGCACTGACGCGTAACCTGAACAA
CTACATCGAGCAGGAAGGTCTGGCGAAGAAGCACAAAGTCGCCACCACCGGTGACGATGCCCGTGAAGGCCTGACCG
CGATCATCTCGGTGAAGGTGCCGGATCCGAAGTTCAGCTC
>06H 90
CTGCACGGTGTAGGTGTTTCGGTTGTGAACGCGCTGTCCGAAGAACTGGTTCTGACCGTCCGCCGCAGTGGAAAGAT
CTGGGAACAGACCTACGTTCACGGCGTGCCTCAGGCACCGATGGCGATCGTTGGCGACAGCGAAACCACCGGTACCC
AGATTCACTTCAAGGCTTCCAGCGAAACCTTCAAGAACATTCACTTCAGCTGGGACATCCTGGCCAAGCGCATTCGT
GAACTGTCCTTCCTCAACTCCGGTGTCGGCATCGTCCTCAAGGATGAGCGCAGCGGCAAGGAAGAGCTGTTCAAGTA
CGAAGGCGGCCTGCGTGCGTTCGTTGAATACCTGAACACCAACAAGACTGCGGTCAACCAGGTGTTCCACTTCAACA
TCCAGCGTGAAGACGGCATCGGCGTGGAAATCGCCCTGCAGTGGAACGACAGCTTCAACGAGAACCTGTTGTGCTTC
ACCAACAACATTCCGCAGCGCGATGGCGGCACCCACCTGGTGGGCTTCCGTTCCGCACTGACGCGTAACCTGAACAA
CTACATCGAGCAGGAAGGTCTGGCGAAGAAGCACAAAGTCGCCACCACCGGTGACGATGCCCGTGAAGGCCTGACCG
CGATCATCTCGGTGAAGGTGCCGGATCCGAAGTTCAGCTC
>08G 80
CTGCACGGCGTAGGTGTATCGGTAGTGAACGCACTGTCCGAAGAACTGGTCCTGACCGTTCGCCGCAGCGGCAAGAT
CTGGGAACAGACCTACGTCCACGGCGTACCTCAGGCACCGATGGCCATCGTTGGCGACAGCGAAACCACCGGTACCC
AGATCCACTTCAAGGCTTCCAGCGAAACCTTCAAGAACATTCACTTCAGCTGGGACATCCTGGCCAAGCGCATTCGT
GAACTGTCCTTCCTCAACTCCGGTGTCGGCATCGTCCTCAAGGATGAGCGCAGCGGCAAGGAAGAGCTGTTCAAATA
CGAAGGTGGCCTGCGTGCGTTCGTTGAATACCTGAACACCAACAAGACTGCGGTCAACCAGGTGTTCCACTTCAACA
TCCAGCGTGAAGACGGCATCGGCGTGGAAATCGCCCTGCAGTGGAACGACAGCTTCAACGAGAACCTGTTGTGCTTC
ACCAACAACATTCCGCAGCGCGACGGCGGTACCCACCTGGTGGGCTTCCGCTCGGCCCTGACGCGTAACCTGAACAA
CTACATCGAGCAGGAAGGTCTGGCGAAGAAGCACAAAGTCGCCACCACCGGCGACGATGCCCGTGAAGGCCTGACCG
CGATCATTTCGGTGAAAGTGCCGGATCCGAAGTTCAGCTC
>S05E 229
CTGCACGGTGTAGGTGTGTCGGTGGTGAACGCACTGTCCGAAGAATTGGTTCTGACCGTTCGCCGTAGCGGCAAGAT
CTGGGAACAGACTTACGTCCACGGCGTACCTCAGGCGCCGATGGCGATCGTTGGCGACAGCGAAACCACCGGTACCC
AGATCCACTTCAAGCCTTCGGCTGAAACTTTCAAGAATATCCACTTCAGCTGGGACATCCTGGCCAAGCGTATTCGT
GAACTGTCCTTCCTCAACTCCGGTGTCGGCATCGTCCTCAAGGACGAGCGCAGTGGTAAGGAAGAGCTGTTCAAGTA
TGAAGGCGGTCTGCGTGCGTTCGTTGAATACCTGAACACCAACAAGACTGCGGTCAACCAGGTGTTCCACTTCAACA
TTCAGCGTGAAGACGGCATCGGCGTGGAAATCGCCCTGCAGTGGAACGACAGCTTCAACGAGAACCTGTTGTGCTTC

ACCAACAACATTCCGCAGCGCGACGGCGGTACTCACCTGGTGGGCTTCCGTTCGGCACTGACGCGTAACCTGAACAA
CTACATCGAGCAGGAAGGTCTGGCGAAGAAGCACAAAGTCGCCACCACCGGTGACGATGCCCGTGAAGGCCTGACTG
CGATCATCTCGGTGAAAGTGCCGGATCCGAAGTTCAGCTC
>S05H 328
CTGCACGGTGTAGGTGTGTCGGTGGTGAACGCACTGTCCGAAGAATTGGTTCTGACCGTTCGCCGTAGCGGCAAGAT
CTGGGAACAGACCTACGTCCACGGCGTACCTCAGGCGCCGATGGCGATCGTTGGCGACAGCGAAACCACCGGTACCC
AGATCCACTTCAAGCCTTCGGCTGAAACTTTCAAGAATATCCACTTCAGCTGGGACATCCTGGCCAAGCGTATTCGT
GAACTGTCCTTCCTCAACTCCGGTGTCGGCATCGTCCTCAAGGACGAGCGCAGTGGTAAGGAAGAGCTGTTCAAGTA
CGAGGGCGGTCTGCGTGCGTTCGTTGAATACCTGAACACCAACAAGACTGCGGTCAACCAGGTGTTCCACTTCAACA
TTCAGCGTGAAGACGGCATCGGCGTGGAAATCGCCCTGCAGTGGAACGACAGCTTCAACGAGAACCTGTTGTGCTTC
ACCAACAACATTCCGCAGCGCGACGGCGGTACTCACCTGGTGGGCTTCCGTTCGGCACTGACGCGTAACCTGAACAA
CTACATCGAGCAGGAAGGTCTGGCGAAGAAGCACAAAGTCGCCACCACCGGTGACGATGCCCGTGAAGGCCTGACTG
CGATCATCTCGGTGAAAGTGCCGGATCCGAAGTTCAGCTC
>11A 11
CTGCACGGTGTAGGTGTGTCGGTGGTGAACGCACTGTCCGAAGAACTGGTTCTGACCGTTCGGCGTAGCGGCAAGAT
CTGGGAACAGACTTACGTCCACGGCGTACCTCAGGCGCCGATGGCGATCGTTGGCGACAGCGAAACCACCGGTACCC
AGATCCACTTCAAGCCTTCGGCTGAAACTTTCAAGAATATCCACTTCAGCTGGGACATCCTGGCCAAGCGTATTCGT
GAACTGTCCTTCCTCAACTCCGGTGTCGGCATCGTCCTCAAGGACGAGCGCAGTGGTAAGGAAGAGCTGTTCAAGTA
TGAAGGCGGTCTGCGTGCGTTCGTTGAATACCTGAACACCAACAAGACTGCGGTCAACCAGGTGTTCCACTTCAACA
TTCAGCGTGAAGACGGCATCGGCGTGGAAATCGCCCTGCAGTGGAACGACAGCTTCAACGAGAACCTGTTGTGCTTC
ACCAACAACATTCCGCAGCGCGACGGCGGTACTCACCTGGTGGGCTTCCGTTCGGCACTGACGCGTAACCTGAACAA
CTACATCGAGCAGGAAGGTCTGGCGAAGAAGCACAAAGTCGCCACCACCGGTGACGATGCCCGTGAAGGCCTGACTG
CGATCATCTCGGTGAAAGTGCCGGATCCGAAGTTCAGCTC
>12F 72
CTGCACGGTGTAGGTGTGTCGGTGGTGAACGCACTGTCCGAAGAACTGGTTCTGACCGTTCGGCGTAGCGGCAAGAT
CTGGGAACAGACTTACGTCCACGGCGTACCTCAGGCGCCGATGGCGATCGTTGGCGACAGCGAAACCACCGGTACCC
AGATCCACTTCAAGCCTTCGGCTGAAACTTTCAAGAATATCCACTTCAGCTGGGACATCCTGGCCAAGCGTATTCGT
GAACTGTCCTTCCTCAACTCCGGTGTCGGCATCGTCCTCAAGGACGAGCGCAGTGGTAAGGAAGAGCTGTTCAAGTA
CGAAGGCGGTCTGCGTGCTTTCGTTGAATACCTGAACACCAACAAGACTGCGGTCAACCAGGTGTTCCACTTCAACA
TTCAGCGTGAAGACGGCATCGGCGTGGAAATCGCCCTGCAGTGGAACGACAGCTTCAACGAGAACCTGTTGTGCTTC
ACCAACAACATTCCGCAGCGCGACGGCGGTACTCACCTGGTGGGCTTCCGTTCGGCACTGACGCGTAACCTGAACAA
CTACATCGAGCAGGAAGGTCTGGCGAAGAAGCACAAAGTCGCCACCACCGGTGACGATGCCCGTGAAGGCCTGACTG
CGATCATCTCGGTGAAAGTGCCGGATCCGAAGTTCAGCTC
>04E 148
CTGCACGGTGTAGGTGTTTCGGTAGTGAACGCGCTGTCCGAAGAGCTGGTCCTGACCGTTCGCCGCAGCGGCAAGAT
CTGGGAACAGACCTACGTCCACGGTGTACCTCAGGCACCGATGGCGATCGTTGGCGACAGCGAAACCACCGGCACCC
AGATTCACTTCAAGGCTTCCAGCGAAACCTTCAAGAACATTCACTTCAGCTGGGACATCCTGGCCAAGCGCATTCGT
GAACTGTCCTTCCTCAACTCCGGTGTCGGTATCGTCCTCAAGGACGAGCGCAGCGGCAAGGAAGAACTGTTCAAGTA
CGAAGGCGGTCTGCGTGCGTTCGTTGAATACCTGAACACCAACAAGACTGCGGTCAACCAGGTGTTCCACTTCAACA
TCCAGCGTGAAGACGGCATCGGCGTGGAAATTGCCCTGCAGTGGAACGACAGCTTCAACGAGAACCTGTTGTGCTTC
ACCAACAACATTCCGCAGCGCGACGGCGGCACCCACCTGGTGGGCTTCCGTTCTGCACTGACGCGTAACCTGAACAA
CTACATCGAGCAAGAAGGTCTGGCGAAGAAACACAAAGTCGCCACCACCGGTGACGATGCCCGTGAAGGCCTGACCG
CGATCATCTCGGTGAAGGTGCCGGATCCGAAGTTCAGCTC
>06B 114
CTGCACGGTGTAGGTGTGTCGGTAGTGAACGCGCTGTCCGAAGAGCTGGTACTGACTGTTCGCCGCAGCGGCAAGAT
CTGGGAACAGACCTACGTCCACGGCGTACCTCAGGCGCCGATGGCCATCGTTGGCGATAGTGAAACCACCGGTACCC
AGATCCACTTCAAGGCTTCCAGCGAAACCTTCAAGAACATTCACTTCAGCTGGGACATCCTCGCCAAGCGTATTCGT
GAACTGTCCTTCCTCAACTCCGGTGTCGGCATCGTCCTCAAGGACGAGCGCAGTGGCAAGGAAGAGCTGTTCAAGTA
CGAAGGCGGCCTGCGTGCGTTCGTTGAATACCTGAACACCAACAAGACTGCGGTCAACCAGGTGTTCCACTTCAACA
TCCANCGTGAAGACGGCATCGGCGTGGAAATCGCCCTGCAGTGGAACGACAGCTTCAACGANAACCTGTTGTGCTTC
ACCAACAACATTCCGCAGCGCGACGGCGGNACTCACCTGGTGGGTTTCCGTTCTGCACTGACGCGTAACCTGAACAA
CTACATCGAGCAGGAAGGTCTGGCGAAGAAGCACAAAGTCGCCACCACCGGTGACGATGCCCGTGAAGGTCTGACCG
CGATCATCTCGGTGAAAGTGCCGGATCCGAAGTTCAGCTC
>S04A 313
CTGCACGGTGTAGGTGTGTCGGTAGTGAACGCGCTGTCCGAAGAGCTGGTTCTGACCGTTCGCCGTAGCGGCAAGAT
CTGGGAACAGACCTACGTCCACGGCGTACCTCAGGCGCCAATGGCCATCGTTGGCGACAGCGAGACCACCGGTACGC
AGATTCACTTCAAGGCTTCCAGCGAAACCTTCAAGAACATTCACTTCAGCTGGGACATCCTCGCCAAGCGCATTCGT

GAACTGTCCTTCCTCAACTCCGGTGTCGGCATCGTCCTCAAGGATGAGCGCAGCGGCAAGGAAGAGCTGTTCAAGTA
CGAAGGCGGCCTGCGTGCATTCGTTGAATATCTGAACACCAACAAGACTGCGGTCAATCAGGTGTTCCACTTCAATA
TCCAGCGTGAAGACGGCATCGGCGTGGAAATCGCCCTGCAGTGGAACGACAGCTTCAATGAGAACCTGTTGTGCTTC
ACCAACAACATTCCGCAGCGAGACGGCGGTACTCACCTGGTGGGCTTCCGTTCGGCACTGACCCGTAACCTGAACAA
CTACATCGAGCAGGAAGGTCTGGCGAAGAAGCACAAAGTCGCCACAACCGGTGACGATGCCCGTGAAGGCCTGACCG
CAATCATCTCGGTGAAAGTGCCGGATCCGAAGTTCAGCTC
>04B 16
CTGCACGGTGTAGGTGTTTCGGTAGTGAACGCGCTGTCCGAAGAACTGGTACTGACCGTTCGCCGCAGCGGCAAGAT
CTGGGAACAGACCTACGTCCACGGTGTGCCTCAGGCACCGATGGCTATCGTTGGCGACAGCGAAACCACCGGCACCC
AGATTCACTTCAAGCCGTCGGCGGACACCTTCAAGAATATCCACTTCAGCTGGGACATCCTGGCCAAGCGTATTCGT
GAACTGTCCTTCCTCAACTCCGGTGTCGGCATCGTCCTCAAGGACGAGCGCAGCGGCAAGGAAGAGCTGTTCAAGTA
CGAAGGCGGCCTGCGTGCGTTCGTTGAGTACCTGAACACCAACAAGACTGCGGTCAACCAGGTGTTCCACTTCAACA
TCCAGCGTGAAGACGGCATCGGCGTGGAAATCGCCCTGCAGTGGAACGACAGCTTCAACGAGAACCTGTTGTGCTTC
ACCAACAACATTCCGCAGCGCGACGGCGGCACCCACCTGGTGGGCTTCCGTTCGGCACTGACGCGTAACCTGAACAC
CTACATCGAAGCCGAAGGCCTGGCGAAGAAGCACAAAGTCGCCACCACCGGTGACGATGCCCGCGAAGGCCTGACCG
CGATCATTTCGGTGAAGGTGCCGGATCCGAAGTTCAGCTC
>03G 171
CTGCACGGTGTAGGTGTTTCGGTAGTGAACGCGCTGTCCGAAGAACTGGTACTAACCGTTCGCCGCAGCGGCAAGAT
CTGGGAACAGACCTACGTCCACGGTGTGCCTCAGGCACCGATGGCCATCGTTGGCGACAGCGAAACCACCGGCACCC
AGATTCACTTCAAGCCGTCGGCGGACACCTTCAAGAATATCCACTTCAGCTGGGACATCCTGGCCAAGCGTATTCGT
GAACTGTCCTTCCTCAACTCCGGTGTCGGCATCGTCCTCAAGGACGAACGCAGCGGTAAGGAAGAGCTGTTCAAATA
CGAAGGCGGCCTGCGTGCGTTCGTTGAATACCTGAACACCAATAAGACTGCGGTCAACCAGGTGTTCCACTTCAACA
TCCAGCGTGAAGACGGCATCGGCGTGGAAATCGCCCTGCAGTGGAACGACAGCTTCAACGAGAACCTGTTGTGCTTC
ACCAACAACATTCCGCAGCGCGACGGCGGCACCCACCTGGTGGGCTTCCGTTCGGCACTGACGCGTAACCTGAACAC
CTACATCGAAGCCGAAGGCCTGGCGAAGAAGCACAAAGTCGCCACCACCGGTGACGATGCCCGTGAAGGCCTGACTG
CGATCATCTCGGTGAAGGTGCCGGATCCGAAGTTCAGCTC
>S08B 346
CTGCACGGTGTAGGTGTTTCGGTAGTGAACGCGCTGTCCGAAGAACTGGTCCTGACCGTTCGCCGCAGCGGCAAGAT
CTGGGAACAGACCTACGTCCACGGCGTACCTCAGGCACCGATGGCCATCGTTGGCGACAGCGAAACCACCGGCACCC
AGATTCACTTCAAGCCGTCGGCGGACACCTTCAAGAATATCCACTTCAGCTGGGACATCCTGGCCAAGCGTATTCGT
GAACTGTCTTTCCTCAACTCCGGTGTCGGCATCGTCCTCAAGGATGAGCGCAGCGGCAAGGAAGAGCTGTTCAAGTA
CGAAGGCGGCCTGCGTGCGTTCGTTGAATACCTGAACACCAACAAGACTGCGGTCAATCAGGTGTTCCACTTCAACA
TTCAGCGTGAAGACGGCATCGGCGTGGAAATCGCCCTGCAGTGGAACGACAGCTTCAACGAGAACCTGTTGTGCTTC
ACCAACAACATTCCACAGCGCGACGGCGGCACTCACCTGGTGGGCTTCCGTTCGGCACTGACGCGTAACCTGAACAC
CTACATCGAAGCCGAAGGCCTGGCGAAGAAGCACAAAGTCGCCACCACCGGTGACGATGCCCGTGAAGGCCTGACCG
CGATCATCTCGGTGAAAGTGCCGGATCCAAAGTTCAGCTC
>06A 6
CTGCACGGCGTAGGTGTATCGGTAGTGAACGCGCTGTCCGAACAACTGGTCCTGACCGTTCGCCGCAGCGGCAAGAT
CTGGGAGCAGACCTACATCCACGGCGTGCCCCAGGCACCGATGGCGATCGTTGGCGACAGCGAAAGCACAGGCACCC
AGATCCACTTCAAGGCTTCGAGCGAAACCTTCAAGAACATCCACTTCAGCTGGGACATCCTGGCCAAGCGGATTCGT
GAACTGTCCTTCCTCAACTCCGGTGTCGGCATCGTCCTCAAGGACGAGCGCAGCGGCAAGGAAGAGCTGTTCAAGTA
CGAAGGCGGCCTGCGTGCGTTCGTTGAGTACCTGAACACCAACAAGACCCCGGTCAACCAGGTGTTCCATTTCAACG
TCCAGCGTGAAGACGGCATCGGCGTGGAAATCGCCCTGCAGTGGAACGACAGCTTCAACGAGAACCTGTTGTGCTTC
ACCAACAACATCCCGCAGCGCGACGGCGGTACCCACCTGGTGGGCTTCCGTTCGGCGCTGACCCGTAACCTGAACAA
CTACATCGAACAGGAAGGCCTGGCGAAGAAGCATAAAGTCGCCACCACCGGTGACGATGCCCGTGAAGGCCTGACCG
CGATTATCTCGGTGAAGGTGCCGGATCCGAAGTTCAGCTC
>07B 19
CTGCACGGCGTAGGTGTATCGGTAGTGAACGCGCTGTCCGAACAACTGGTCCTGACCGTTCGCCGCAGCGGCAAGAT
CTGGGAGCAGACCTACATCCACGGCGTGCCCCAGGCACCGATGGCGATCGTTGGCGACAGCGAAAGCACAGGCACCC
AGATCCACTTCAAGGCTTCGAGCGAAACCTTCAAGAACATCCACTTCAGCTGGGACATCCTGGCCAAGCGGATTCGT
GAACTGTCCTTCCTCAACTCCGGTGTCGGCATCGTCCTCAAGGACGAGCGCAGCGGCAAGGAAGAGCTGTTCAAGTA
CGAAGGCGGCCTGCGTGCGTTCGTTGAGTACCTGAACACCAACAAGACCCCGGTCAACCAGGTGTTCCATTTCAACG
TCCAGCGTGAAGACGGCATCGGCGTGGAAATCGCCCTGCAGTGGAACGACAGCTTCAACGAGAACCTGTTGTGCTTC
ACCAACAACATCCCGCAGCGCGACGGCGGTACCCACCTGGTGGGCTTCCGTTCGGCGCTGACCCGTAACCTGAACAA
CTACATCGAACAGGAAGGCCTGGCGAAGAAGCATAAAGTCGCCACCACCGGTGACGATGCCCGTGAAGGCCTGACCG
CGATTATCTCGGTGAAGGTGCCGGATCCGAAGTTCAGCTC

>04F 64
CTGCACGGTGTAGGTGTTTCGGTGGTGAATGCGCTGTCCGAAGAACTGGTCCTGACCGTTCGCCGCAGCGGCAAGAT
CTGGGAGCAGACCTACATCCACGGCGTGCCCCAGGCACCCATGGCGATCGTTGGCGACAGCGAAAGCACGGGCACCC
AGATCCACTTCAAGGCTTCGAGCGAAACCTTCAAGAACATCCACTTCAGCTGGGACATCCTGGCCAAGCGGATTCGT
GAGCTGTCCTTCCTCAACTCCGGTGTCGGCATCGTCCTCAAGGACGAGCGCAGTGGCAAGGAAGAGCTATTCAAATA
TGAAGGTGGCTTGCGTGCGTTCGTTGAGTACCTGAACACCAACAAGACCCCGGTCAACCAGGTGTTCCACTTCAACG
TCCAGCGTGAAGACGGCATCGGCGTGGAAATCGCCCTTCAGTGGAACGACAGCTTCAACGAGAACCTGTTGTGCTTC
ACCAACAACATCCCGCAGCGCGACGGCGGTACTCACCTGGTGGGCTTCCGCTCGGCACTGACCCGCAACCTGAACAA
CTACATCGAACAGGAAGGCCTGGCGAAGAAGCATAAAGTCGCCACCACCGGTGACGATGCCCGTGAAGGCCTTACCG
CGATCATCTCGGTGAAGGTGCCGGATCCGAAGTTCAGCTC
>04A 100
CTGCACGGTGTAGGTGTGTCGGTAGTGAACGCACTGTCCGAGGAGCTGGTACTGACTGTTCGCCGCTGCGGCCAGAT
CTGGGAACAGACCTACGTCCACGGCGTACCTCAGGATCCGATGGCCATCGTTGGCGACAGCGAAACCACCGGTACCC
AGATCCACTTCAAGGCTTCGAGCGAAACCTTCAAGAACATTCACTTCAGCTGGGACATCCTGGCCAAGCGGATTCGT
GAACTGTCCTTCCTCAACTCCGGTGTCGGCATCGTCCTCAAGGATGAGCGCAGCGGCAAGGAAGAGCTGTTCAAGTA
CGAAGGCGGCCTGCGTGCGTTCGTTGAATACCTGAACACCAACAAGACTGCGGTCAACCAGGTGTTCCACTTCAACA
TCCAGCGTGAAGACGGCATCGGCGTGGAAATCGCCCTGCAGTGGAACGACAGCTTCAACGAGAACCTGTTGTGCTTC
ACCAACAACATTCCGCAGCGCGACGGCGGTACCCACCTGGTGGGCTTCCGTTCGGCCCTGACGCGTAACCTGAACAA
CTACATCGAGCAGGAAGGTCTGGCGAAGAAGCATAAAGTCGCCACCACCGGTGATGATGCCCGTGAAGGCCTGACCG
CGATCATCTCGGTAAAAGTGCCGGATCCGAAGTTCAGCTC
>S11C 275
CTGCACGGCGTAGGCGTGTCGGTAGTGAACGCACTGTCTGAAGAGCTTGTTCTGACGGTGCGCCGTAGTGGCAAGAT
CTGGGAACAGACCTACGTACACGGTGTTCCTCAGGAACCGATGAAGATCGTTGGCGACAGCGAAACCACGGGTACCC
AGATCCACTTCAAGGCTTCCAGCGAAACTTTCAAGAATATCCACTTCAGCTGGGACATCCTGGCCAAGCGGATCCGT
GAACTGTCCTTCCTCAACTCCGGTGTCGGCATCGTCCTCAAGGATGAGCGCAGCGGCAAGGAAGAGTTGTTCAAGTA
CGAAGGCGGCCTGCGTGCGTTCGTTGAATACCTGAACACTAACAAGACCCCGGTCAACCAGGTGTTCCACTTCAACA
TCCAGCGTGAAGACGGCATCGGCGTGGAAATCGCCCTGCAGTGGAACGACAGCTTCAACGAGAACCTGTTGTGCTTC
ACCAACAACATTCCGCAGCGCGACGGCGGTACCCACCTGGTGGGCTTCCGTTCGGCCCTTACGCGTAACCTCAACAC
CTATATCGAAGCTGAAGGCCTGGCGAAGAAGCACAAAGTCGCCACCACTGGTGACGATGCCCGTGAAGGCCTGACCG
CGATCATTTCGGTGAAGGTGCCGGATCCGAAGTTCAGCTC
>S05D 228
CTGCACGGCGTAGGCGTGTCGGTAGTGAACGCACTGTCTGAAGAGCTTGTTCTGACGGTGCGCCGTAGTGGCAAGAT
CTGGGAACAGACCTACGTACACGGTGTTCCTCAGGAACCGATGAAGATCGTTGGCGACAGCGAAACCACGGGTACCC
AGATCCACTTCAAGGCTTCCAGCGAAACCTTCAAGAATATCCACTTCAGCTGGGACATCCTGGCCAAGCGGATTCGT
GAATTGTCCTTCCTCAACTCCGGTGTCGGCATCGTCCTCAAGGATGAGCGCAGCGGCAAGGAAGAGCTGTTCAAGTA
CGAAGGCGGCCTGCGTGCGTTCGTTGAATACCTGAACACCAACAAGACCCCGGTCAACCAGGTGTTCCACTTCAACA
TCCAGCGTGAAGACGGCATCGGCGTGGAAATCGCCCTGCAGTGGAACGACAGCTTCAACGAGAACCTGTTGTGCTTC
ACCAACAACATTCCGCAGCGCGACGGCGGTACCCACCTGGTGGGCTTCCGTTCGGCCCTTACGCGTAACCTCAACAC
CTATATCGAAGCTGAAGGCCTGGCGAAGAAGCACAAAGTCGCCACCACTGGTGACGATGCCCGTGAAGGCCTGACCG
CGATCATTTCGGTGAAGGTGCCGGATCCGAAGTTCAGCTC
>S07D 244
CTGCACGGCGTAGGCGTGTCGGTAGTGAACGCACTGTCTGAAGAGCTTGTTCTGACGGTGCGCCGTAGCGGCAAGAT
CTGGGAACAGACCTACGTCCACGGTGTTCCCCAGGAACCGATGAAGATCGTTGGCGACAGCGAAACCACGGGTACCC
AGATCCACTTCAAGGCTTCCAGCGAAACTTTCAAGAATATCCACTTCAGCTGGGACATCCTGGCCAAGCGGATTCGT
GAACTGTCCTTCCTCAACTCCGGTGTCGGCATCGTCCTCAAGGATGAGCGCAGCGGCAAGGAAGAGTTGTTCAAGTA
CGAAGGCGGCCTGCGTGCGTTCGTTGAATACCTGAACACTAACAAGACCCCGGTCAACCAGGTGTTCCACTTTAACA
TCCAGCGTGAAGACGGCATCGGCGTGGAAATCGCCCTGCAGTGGAACGACAGCTTCAACGAGAACCTGTTGTGCTTC
ACCAACAACATTCCGCAGCGCGACGGCGGTACCCACCTGGTGGGCTTCCGTTCGGCCCTTACGCGTAACCTCAACAC
CTATATCGAAGCTGAAGGCCTGGCGAAGAAGCACAAAGTCGCCACCACTGGTGACGATGCCCGTGAAGGCCTGACCG
CGATCATTTCGGTGAAGGTGCCGGATCCGAAGTTCAGCTC
>S01E 197
CTGCACGGCGTAGGCGTGTCGGTAGTGAACGCACTGTCTGAAGAGCTGGTTCTCACTGTGCGCCGTAGTGGCAAGAT
CTGGGAACAGACCTACGTACACGGTGTTCCTCAGGAACCGATGAAGATCGTTGGCGACAGCGAAACCACGGGTACCC
AGATCCATTTCAAGGCTTCCAGCGAAACCTTCAAGAATATTCACTTCAGCTGGGACATCCTGGCCAAGCGGATCCGT
GAACTGTCCTTCCTCAACTCCGGTGTCGGCATCGTCCTTAAGGATGAGCGCAGCGGCAAGGAAGAGTTGTTCAAGTA
CGAAGGCGGCCTGCGTGCGTTCGTTGAATACCTGAACACCAACAAGACCCCGGTCAACCAGGTGTTTCACTTCAATA
TCCAGCGTGAAGACGGCATCGGCGTGGAAATCGCCCTGCAGTGGAACGACAGCTTCAACGAGAACCTGTTGTGCTTC

ACCAACAACATTCCGCAGCGCGACGGCGGTACTCACCTGGTGGGCTTCCGTTCCGCACTGACGCGTAACCTCAACAC
CTACATCGAAGCCGAAGGCCTGGCGAAGAAGCACAAGGTTGCCACCACCGGTGACGACGCCCGTGAAGGCCTGACCG
CGATCATTTCGGTGAAGGTGCCGGATCCGAAGTTCAGCTC
>02A 2
CTGCACGGTGTAGGTGTGTCGGTTGTTAACGCACTCTCCAAAGAACTGATTCTGACCGTTCGCCGCAGTGGCAAGAT
CTGGGAACAGACCTACATCCATGGTGTGCCGCAAGAGCCGATGAAGATCGTTGGCGAGAGCGAAACCACAGGTACCC
AGATTCACTTCAAGCCATCGGCTGAGACGTTCAAGAACATCCACTTCAGTTGGGACATCCTGGCCAAGCGGATTCGT
GAACTGTCCTTCCTCAACTCCGGTGTCGGCATCGTCCTCAAGGATGAGCGCAGCGGCAAGGAAGAGCTGTTCAAGTA
CGAAGGTGGCCTGCGTGCGTTCGTTGAATACCTGAACACCAACAAGACCCCGGTCAATCAGGTGTTCCACTTCAACA
TCCAGCGCGAAGACGGCATCGGCGTGGAAATCGCCCTGCAGTGGAACGACAGCTTCAACGAGAACCTGTTGTGCTTC
ACCAACAACATTCCGCAGCGCGACGGCGGTACTCACCTGGTGGGTTTCCGTTCCGCACTGACGCGTAACCTCAACAC
CTACATCGAAGCTGAAGGTCTGGCAAAGAAGCATAAAGTTGCCACCACCGGTGATGATGCGCGTGAAGGCCTGACCG
CGATTATCTCGGTAAAAGTGCCGGATCCGAAGTTCAGCTC
>04A 4
CTGCACGGTGTAGGTGTGTCGGTTGTTAACGCACTCTCCAAAGAACTGATTCTGACCGTTCGCCGCAGTGGCAAGAT
CTGGGAACAGACCTACATCCATGGTGTGCCGCAAGAGCCGATGAAGATCGTTGGCGAGAGCGAAACCACAGGTACCC
AGATTCACTTCAAGCCATCGGCTGAGACGTTCAAGAACATCCACTTCAGTTGGGACATCCTGGCCAAGCGGATTCGT
GAACTGTCCTTCCTCAACTCCGGTGTCGGCATCGTCCTCAAGGATGAGCGCAGCGGCAAGGAAGAGCTGTTCAAGTA
CGAAGGTGGCCTGCGTGCGTTCGTTGAATACCTGAACACCAACAAGACCCCGGTCAATCAGGTGTTCCACTTCAACA
TCCAGCGCGAAGACGGCATCGGCGTGGAAATCGCCCTGCAGTGGAACGACAGCTTCAACGAGAACCTGTTGTGCTTC
ACCAACAACATTCCGCAGCGCGACGGCGGTACTCACCTGGTGGGTTTCCGTTCCGCACTGACGCGTAACCTCAACAC
CTACATCGAAGCTGAAGGTCTGGCAAAGAAGCATAAAGTTGCCACCACCGGTGATGATGCGCGTGAAGGCCTGACCG
CGATTATCTCGGTAAAAGTGCCGGATCCGAAGTTCAGCTC
>02F 62
CTGCACGGTGTAGGTGTGTCGGTTGTTAACGCACTGTCCAAAGAACTGATTCTGACCGTTCGCCGCAGTGGCAAGAT
CTGGGAACAGACTTACATCCATGGTGTGCCGCAAGAGCCGATGAAGATCGTTGGCGAGAGCGAAACCACAGGTACCC
AGATTCACTTCAAGCCATCGGCTGAGACGTTCAAGAACATCCACTTCAGTTGGGACATCCTGGCCAAGCGGATTCGT
GAACTGTCCTTCCTCAACTCCGGTGTCGGCATCGTCCTCAAGGATGAGCGCAGCGGCAAGGAAGAGCTGTTCAAGTA
CGAAGGTGGCCTGCGTGCGTTCGTTGAATACCTGAACACCAACAAGACCCCGGTCAATCAGGTGTTCCACTTCAACA
TCCAGCGCGAAGACGGCATCGGCGTGGAAATCGCCCTGCAGTGGAACGACAGCTTCAACGAGAACCTGTTGTGCTTC
ACCAACAACATTCCGCAGCGCGACGGCGGTACTCACCTGGTGGGTTTCCGTTCCGCACTGACGCGTAACCTCAACAC
CTACATCGAAGCTGAAGGTCTGGCAAAGAAGCATAAAGTTGCCACCACCGGTGATGATGCGCGTGAAGGCCTGACCG
CGATTATCTCGGTAAAAGTGCCGGATCCGAAGTTCAGCTC
>S06A 233
CTGCACGGTGTAGGCGTGTCGGTAGTGAACGCGCTGTCCGAAGAGCTGGTACTGACGGTTCGCCGTAGCGGCAAGAT
CTGGGAACAGACTTACGTCCACGGTGTTCCACAAGAACCGATGAAGATCGTTGGCGACAGTGAAACCACCGGTACTC
AGATCCACTTCAAGGCTTCCAGCGAAACCTTCAAGAACATCCACTTCAGCTGGGACATCCTGGCCAAGCGGATTCGT
GAACTGTCCTTCCTGAACTCCGGTGTCGGCATCGTCCTCAAGGATGAGCGCAGCGGCAAGGAAGAACTGTTCAAATA
CGAAGGCGGCCTGCGTGCGTTCGTTGAATACCTGAACACCAACAAGACCCCGGTCAACCAGGTGTTCCATTTCAACA
TCCAGCGTGAAGACGGCATCGGCGTGGAAATCGCCCTGCAGTGGAACGACAGCTTCAACGAGAACCTGTTGTGCTTC
ACCAACAACATTCCGCAGCGCGATGGCGGTACTCACCTGGTGGGTTTCCGTTCTGCACTGACGCGTAACCTCAACAC
CTACATCGAAGCTGAAGGCCTGGCGAAGAAGCACAAAGTCGCCACCACCGGTGACGACGCGCGTGAAGGCCTGACCG
CGATCATCTCGGTAAAAGTACCGGATCCGAAGTTCAGCTC
>S03D 212
CTGCACGGTGTAGGCGTGTCGGTAGTGAACGCGCTGTCCGAAGAGCTGGTACTGACGGTTCGCCGTAGCGGCAAGAT
CTGGGAACAGACTTACGTCCACGGTGTTCCACAAGAACCGATGAAGATCGTTGGCGACAGTGAAACCACCGGTACTC
AGATCCACTTCAAGGCTTCCAGCGAAACCTTCAAGAACATCCACTTCAGCTGGGACATCCTGGCCAAGCGGATTCGT
GAACTGTCCTTCCTGAACTCCGGTGTCGGCATCGTCCTCAAGGATGAGCGCAGCGGCAAGGAAGAACTGTTCAAATA
CGAAGGCGGCCTGCGTGCGTTCGTTGAATACCTGAACACCAACAAGACCCCGGTCAACCAGGTGTTCCATTTCAACA
TCCAGCGTGAAGACGGCATCGGCGTGGAAATCGCCCTGCAGTGGAACGACAGCTTCAACGAGAACCTGTTGTGCTTC
ACCAACAACATTCCGCAGCGCGATGGCGGTACTCACCTGGTGGGTTTCCGTTCTGCACTGACGCGTAACCTCAACAC
CTACATCGAAGCTGAAGGCCTGGCGAAGAAGCACAAAGTCGCCACCACCGGTGACGACGCGCGTGAAGGCCTGACCG
CGATCATCTCGGTAAAAGTACCGGATCCGAAGTTCAGCTC
>S11C 371
CTGCACGGTGTAGGCGTGTCGGTAGTGAACGCGCTGTCCGAAGAGCTGGTACTGACGGTTCGCCGTAGCGGCAAGAT
CTGGGAACAGACTTACGTCCACGGTGTTCCACAAGAACCGATGAAGATCGTTGGCGACAGTGAAACCACCGGTACTC
AGATCCACTTCAAGGCTTCCAGCGAAACCTTCAAGAACATCCACTTCAGCTGGGACATCCTGGCCAAGCGGATTCGT

GAACTGTCCTTCCTGAACTCCGGTGTCGGCATCGTCCTCAAGGATGAGCGCAGCGGCAAGGAAGAACTGTTCAAATA
CGAAGGCGGCCTGCGTGCGTTCGTTGAATACCTGAACACCAACAAGACCCCGGTCAACCAGGTGTTCCATTTCAACA
TCCAGCGTGAAGACGGCATCGGCGTGGAAATCGCCCTGCAGTGGAACGACAGCTTCAACGAGAACCTGTTGTGCTTC
ACCAACAACATTCCGCAGCGCGATGGCGGTACTCACCTGGTGGGTTTCCGTTCTGCACTGACGCGTAACCTCAACAC
CTACATCGAAGCTGAAGGCCTGGCGAAGAAGCACAAAGTCGCCACCACCGGTGACGACGCGCGTGAAGGCCTGACCG
CGATCATCTCGGTAAAAGTACCGGATCCGAAGTTCAGCTC
>S10G 271
CTGCACGGTGTAGGCGTGTCGGTAGTGAACGCGCTGTCCGAAGAGCTGGTACTGACGGTTCGCCGTAGCGGCAAGAT
CTGGGAACAGACTTACGTCCACGGTGTTCCACAAGAACCGATGAAGATCGTTGGCGACAGTGAAACCACCGGTACTC
AGATCCACTTCAAGGCTTCCAGCGAAACCTTCAAGAACATCCACTTCAGCTGGGACATCCTGGCCAAGCGGATTCGT
GAACTGTCCTTCCTGAACTCCGGTGTCGGCATCGTCCTTAAGGATGAGCGCAGCGGCAAGGAAGAACTGTTCAAATA
CGAAGGCGGCCTGCGTGCGTTCGTTGAATACCTGAACACCAACAAGACCCCGGTCAACCAGGTGTTCCATTTCAACA
TCCAGCGTGAAGACGGCATCGGCGTGGAAATCGCCCTGCAGTGGAACGACAGCTTCAACGAGAACCTGTTGTGCTTC
ACCAACAACATTCCGCAGCGCGATGGCGGTACTCACCTGGTGGGTTTCCGTTCTGCACTGACGCGTAACCTCAACAC
CTACATCGAAGCTGAAGGCCTGGCGAAGAAGCACAAAGTCGCCACCACCGGTGACGACGCGCGTGAAGGCCTGACCG
CGATCATCTCGGTAAAAGTACCGGATCCGAAGTTCAGCTC
>S04H 320
CTGCACGGTGTAGGCGTGTCGGTAGTGAACGCGCTGTCCGAAGAGCTGGTACTGACGGTTCGCCGTAGCGGCAAGAT
CTGGGAACAGACTTACGTCCACGGTGTTCCACAAGAACCGATGAAGATCGTTGGCGACAGTGAAACCACCGGTACTC
AGATCCACTTCAAGGCTTCCAGCGAAACCTTCAAGAACATCCACTTCAGCTGGGACATCCTGGCCAAGCGGATTCGT
GAACTGTCCTTCCTGAACTCCGGTGTCGGCATCGTCCTTAAGGATGAGCGCAGCGGCAAGGAAGAACTGTTCAAATA
CGAAGGCGGCCTGCGTGCGTTCGTTGAATACCTGAACACCAACAAGACCCCGGTCAACCAGGTGTTCCATTTCAACA
TCCAGCGTGAAGACGGCATCGGCGTGGAAATCGCCCTGCAGTGGAACGACAGCTTCAACGAGAACCTGTTGTGCTTC
ACCAACAACATTCCGCAGCGCGATGGCGGTACTCACCTGGTGGGTTTCCGTTCTGCACTGACGCGTAACCTCAACAC
CTACATCGAAGCTGAAGGCCTGGCGAAGAAGCACAAAGTCGCCACCACCGGTGACGACGCGCGTGAAGGCCTGACCG
CGATCATCTCGGTAAAAGTACCGGATCCGAAGTTCAGCTC
>S07G 343
CTGCACGGTGTAGGCGTGTCGGTAGTGAACGCGCTGTCCGAAGAGCTGGTACTGACGGTTCGCCGTAGCGGCAAGAT
CTGGGAACAGACTTACGTCCACGGTGTTCCACAAGAACCGATGAAGATCGTTGGCGACAGTGAAACCACCGGTACTC
AGATCCACTTCAAGGCTTCCAGCGAAACCTTCAAGAACATCCACTTCAGCTGGGACATCCTGGCCAAACGGATTCGT
GAACTGTCCTTCCTGAACTCCGGTGTTGGCATCGTCCTCAAGGATGAGCGCAGCGGCAAGGAAGAACTGTTCAAGTA
CGAAGGCGGCCTGCGTGCGTTCGTTGAATACCTGAACACCAACAAGACCCCGGTCAACCAGGTGTTCCATTTCAACA
TCCAGCGTGAAGACGGCATCGGCGTGGAAATCGCCCTGCAGTGGAACGACAGCTTCAACGAGAACCTGTTGTGCTTC
ACCAACAACATTCCGCAGCGCGATGGCGGTACTCACCTGGTGGGTTTCCGTTCTGCACTGACGCGTAACCTCAACAC
CTACATCGAAGCTGAAGGCCTGGCGAAGAAGCACAAAGTCGCCACCACCGGTGACGACGCGCGTGAAGGCCTGACCG
CGATCATCTCGGTAAAAGTACCGGATCCGAAGTTCAGCTC
>11E 155
CTGCACGGTGTAGGTGTTTCGGTGGTCAACGCCCTGTCCGAAGAGCTGGTCCTGACCGTTCGCCGCAGCGGCAAGAT
CTGGGAACAGACTTACATTCACGGCGTTCCGAAAGAGCCGATGAAAATTGTTGGTGAGAGCGAAACCACCGGCACCC
AGATCCACTTCAAACCGTCCGATCTGACCTTCAAGAATATCCACTTCAGCTGGGACATCCTGGCCAAGCGGATTCGT
GAACTGTCCTTCCTCAACTCCGGTGTCGGTATCGTCCTCAAGGATGAGCGCAGCGGCAAGGAAGAGCTGTTCAAGTA
TGAAGGTGGCCTGCGTGCGTTCGTTGAATACCTGAACACCAACAAGACTCCGGTCAACCAGGTGTTCCACTTCAACA
TCCAGCGCGAAGACGGCATCGGCGTGGAAATCGCCCTGCAGTGGAACGACAGCTTCAACGAGAACCTGTTGTGCTTC
ACCAACAACATTCCACAGCGCGATGGCGGTACTCACTTGGTGGGTTTCCGCTCCGCACTGACGCGTAACTTGAACAC
CTACATCGAAGCGGAAGGCCTGGCGAAGAAGCACAAGGTCGCGACCACCGGTGACGATGCCCGTGAAGGCCTGACCG
CAATCATTTCGGTGAAAGTCCCGGATCCGAAGTTCAGCTC
>04H 88
CTGCACGGTGTAGGTGTTTCGGTGGTCAACGCCCTGTCCGAAGAGCTGGTCCTGACCGTTCGCCGCAGCGGCAAGAT
CTGGGAACAGACGTACATTCACGGCGTTCCGAAAGAGCCGATGAAAATTGTTGGTGAGAGCGAAACCACCGGCACCC
AGATCCACTTCAAACCGTCCGATCTGACCTTCAAGAATATCCACTTCAGCTGGGACATCCTGGCCAAGCGGATTCGT
GAACTGTCCTTCCTCAACTCCGGTGTCGGTATCGTCCTCAAGGATGAGCGCAGCGGCAAGGAAGAGCTGTTCAAGTA
TGAAGGTGGCCTGCGTGCGTTCGTTGAATACCTGAACACCAACAAGACTCCGGTCAACCAGGTGTTCCACTTCAACA
TCCAGCGCGAAGACGGCATCGGCGTGGAAATCGCCCTGCAGTGGAACGACAGCTTCAACGAGAACCTGTTGTGCTTC
ACCAACAACATTCCACAGCGCGATGGCGGTACTCACTTGGTGGGTTTCCGCTCCGCACTGACGCGTAACTTGAACAC
CTACATCGAAGCGGAAGGCCTGGCGAAGAAGCACAAGGTCGCGACCACCGGTGACGATGCCCGTGAAGGCCTGACCG
CAATCATTTCGGTGAAAGTCCCGGATCCGAAGTTCAGCTC

>10H 190
CTGCACGGTGTAGGTGTTTCGGTGGTCAACGCCCTGTCCGAAGAGCTGGTCCTGACCGTTCGCCGCAGCGGCAAGAT
CTGGGAACAGACTTACATTCACGGCGTTCCGAAAGAGCCGATGAAAATTGTTGGTGAGAGCGAAACCACCGGCACCC
AGATCCACTTCAAACCGTCCGATCTGACCTTCAAGAATATCCACTTCAGCTGGGACATCCTGGCCAAGCGGATTCGT
GAACTGTCCTTCCTCAACTCCGGTGTCGGTATCGTCCTCAAGGATGAGCGCAGCGGCAAGGAAGAGCTGTTCAAGTA
TGAAGGTGGCCTGCGTGCGTTCGTTGAATACCTGAACACCAACAAGACTCCGGTCAACCAGGTGTTCCACTTCAACA
TCCAGCGCGAAGACGGCATCGGCGTGGAAATCGCCCTGCAGTGGAACGACAGCTTCAACGAGAACCTGTTGTGCTTC
ACCAACAACATTCCACAGCGCGATGGCGGTACTCACTTGGTGGGTTTCCGCTCCGCACTGACGCGTAACTTGAACAC
CTACATCGAAGCGGAAGGCCTGGCGAAGAAGCACAAGGTCGCGACCACCGGTGACGATGCCCGTGAAGGCCTGACCG
CAATCATTTCGGTGAAAGTCCCGGATCCGAAGTTCAGCTC
>10E 154
CTGCACGGTGTAGGTGTTTCGGTGGTCAACGCCCTGTCCGAAGAGCTGGTCCTGACCGTTCGCCGCAGCGGCAAGAT
TTGGGAACAGACGTACATTCACGGCGTTCCGAAAGAGCCGATGAAAATTGTTGGTGAGAGCGAAACCACCGGCACCC
AGATCCACTTCAAACCGTCCGATCTGACCTTCAAGAATATCCACTTCAGCTGGGACATCCTGGCCAAGCGGATTCGT
GAACTGTCCTTCCTCAACTCCGGTGTCGGTATCGTCCTCAAGGATGAGCGCAGCGGCAAGGAAGAGCTGTTCAAGTA
TGAAGGTGGCCTGCGTGCGTTCGTTGAATACCTGAACACCAACAAGACTCCGGTCAACCAGGTGTTCCACTTCAACA
TCCAGCGCGAAGACGGCATCGGCGTGGAAATCGCCCTGCAGTGGAACGACAGCTTCAACGAGAACCTGTTGTGCTTC
ACCAACAACATTCCACAGCGCGATGGCGGTACTCACTTGGTGGGTTTCCGCTCCGCACTGACGCGTAACTTGAACAC
CTACATCGAAGCGGAAGGTCTGGCGAAGAAGCACAAGGTCGCGACCACCGGTGACGATGCCCGTGAAGGCCTGACCG
CAATCATTTCGGTGAAAGTCCCGGATCCGAAGTTCAGCTC
>07G 79
CTGCACGGTGTAGGTGTTTCGGTGGTCAACGCCCTGTCCGAAGAGCTGGTCCTGACCGTTCGCCGCAGCGGCAAGAT
TTGGGAACAGACGTACATTCACGGCGTTCCGAAAGAGCCGATGAAAATTGTTGGTGAGAGCGAAACCACCGGCACCC
AGATCCACTTCAAACCGTCCGATCTGACCTTCAAGAATATCCACTTCAGCTGGGACATCCTGGCCAAGCGGATTCGT
GAACTGTCCTTCCTCAACTCCGGTGTCGGTATCGTCCTCAAGGATGAGCGCAGCGGCAAGGAAGAGCTGTTCAAGTA
TGAAGGTGGCCTGCGTGCGTTCGTTGAATACCTGAACACCAACAAGACTCCGGTCAACCAGGTGTTCCACTTCAACA
TCCAGCGCGAAGACGGCATCGGCGTGGAAATCGCCCTGCAGTGGAACGACAGCTTTAACGAGAACCTGTTGTGCTTC
ACCAACAACATTCCACAGCGCGATGGCGGTACTCACTTGGTGGGTTTCCGCTCCGCACTGACGCGTAACTTGAACAC
CTACATCGAAGCGGAAGGCCTGGCGAAGAAGCACAAGGTCGCGACCACCGGTGACGATGCCCGTGAAGGCTTGACCG
CAATCATTTCGGTGAAAGTCCCGGATCCGAAGTTCAGCTC
>09B 21
CTGCACGGTGTAGGTGTTTCGGTGGTCAACGCCCTGTCCGAAGAGCTGGTCCTGACCGTTCGCCGCAGCGGCAAGAT
CTGGGAACAGACGTACATTCACGGCGTTCCGAAAGAGCCGATGAAAATTGTTGGTGAGAGCGAAACCACCGGCACCC
AGATCCACTTCAAACCGTCCGATCTGACCTTCAAGAATATCCACTTCAGCTGGGACATCCTGGCCAAGCGGATTCGT
GAACTGTCCTTCCTCAACTCCGGTGTCGGTATCGTCCTCAAGGATGAGCGCAGCGGCAAGGAAGAGCTGTTCAAGTA
TGAAGGTGGCCTGCGTGCGTTCGTTGAATACCTGAACACCAACAAGACTCCGGTCAACCAGGTGTTCCACTTCAACA
TCCAGCGCGAAGACGGCATCGGCGTGGAAATCGCCCTGCAGTGGAACGACAGCTTCAACGAGAACCTGTTGTGCTTC
ACCAACAACATTCCACAGCGCGATGGCGGTACTCACTTGGTGGGTTTCCGCTCCGCACTGACGCGTAACTTGAACAC
CTACATCGAAGCGGAAGGCCTGGCGAAGAAGCACAAGGTCGCGACCACCGGTGACGATGCCCGTGAAGGCCTGACCG
CAATCATTTCGGTGAAAGTCCCGGATCCGAAGTTCAGCTC
>03F 63
CTGCACGGTGTAGGTGTTTCGGTGGTCAACGCCCTGTCCGAAGAGCTGGTCCTGACCGTTCGCCGCAGCGGCAAGAT
CTGGGAACAGACGTACATTCACGGCGTTCCGAAAGAGCCGATGAAAATTGTTGGTGAGAGCGAAACCACCGGCACCC
AGATCCACTTCAAACCGTCCGATCTGACCTTCAAGAATATCCACTTCAGCTGGGACATCCTGGCCAAGCGGATTCGT
GAACTGTCCTTCCTCAACTCCGGTGTCGGTATCGTCCTCAAGGATGAGCGCAGCGGCAAGGAAGAGCTGTTCAAGTA
TGAAGGTGGCCTGCGTGCGTTCGTTGAATACCTGAACACCAACAAGACTCCGGTCAACCAGGTGTTCCACTTCAACA
TCCAGCGCGAAGACGGCATCGGCGTGGAAATCGCCCTGCAGTGGAACGACAGCTTCAACGAGAACCTGTTGTGCTTC
ACCAACAACATTCCACAGCGCGATGGCGGTACTCACTTGGTGGGTTTCCGCTCCGCACTGACGCGTAACTTGAACAC
CTACATCGAAGCGGAAGGTCTGGCGAAGAAGCACAAGGTCGCGACCACCGGTGACGATGCCCGTGAAGGTCTGACCG
CAATCATTTCGGTGAAAGTCCCGGATCCGAAGTTCAGCTC
>08B 20
CTGCACGGTGTAGGTGTTTCGGTGGTCAACGCCCTGTCCGAAGAGCTGGTCCTGACCGTTCGCCGCAGCGGCAAGAT
CTGGGAACAGACGTACATTCACGGCGTTCCGAAAGAGCCGATGAAAATTGTTGGTGAGAGCGAAACCACCGGCACCC
AGATCCACTTCAAACCGTCCGATCTGACCTTCAAGAATATCCACTTCAGCTGGGACATCCTGGCCAAGCGGATTCGT
GAACTGTCCTTCCTCAACTCCGGTGTCGGTATCGTCCTCAAGGATGAGCGCAGCGGCAAGGAAGAGCTGTTCAAGTA
TGAAGGTGGCCTGCGTGCGTTCGTTGAATACCTGAACACCAACAAGACTCCGGTCAACCAGGTGTTCCACTTCAACA
TCCAGCGCGAAGACGGCATCGGCGTGGAAATCGCCCTGCAGTGGAACGACAGCTTCAACGAGAACCTGTTGTGCTTC

ACCAACAACATTCCACAGCGCGATGGCGGTACTCACTTGGTGGGTTTCCGCTCCGCACTGACGCGTAACTTGAACAC
CTACATCGAAGCGGAAGGTCTGGCGAAGAAGCACAAGGTCGCGACCACCGGTGACGATGCCCGTGAAGGTCTGACCG
CAATCATTTCGGTGAAAGTCCCGGATCCGAAGTTCAGCTC
>01C 121
CTGCACGGTGTAGGTGTTTCGGTGGTCAACGCCCTGTCCGAAGAGCTGGTCCTGACCGTTCGCCGCAGCGGCAAGAT
CTGGGAACAGACGTACATTCACGGCGTTCCGAAAGAGCCGATGAAAATTGTTGGTGAGAGCGAAACCACCGGCACCC
AGATCCACTTCAAACCGTCCGATCTGACCTTCAAGAATATCCACTTCAGCTGGGACATCCTGGCCAAGCGGATTCGC
GAACTGTCCTTCCTCAACTCCGGTGTCGGTATCGTCCTCAAGGATGAGCGCAGCGGCAAGGAAGAGCTGTTCAAGTA
TGAAGGTGGCCTGCGTGCGTTCGTTGAATACCTGAACACCAACAAGACTCCGGTCAACCAGGTGTTCCACTTCAACA
TCCAGCGCGAAGACGGCATCGGCGTGGAAATCGCCCTGCAGTGGAACGACAGCTTCAACGAGAACCTGTTGTGCTTC
ACCAACAACATTCCACAGCGCGATGGCGGTACTCACTTGGTGGGTTTCCGCTCCGCACTGACGCGTAACTTGAACAC
CTACATCGAAGCGGAAGGTCTGGCGAAGAAGCACAAGGTCGCGACCACCGGTGACGATGCCCGTGAAGGTCTGACCG
CAATCATTTCGGTGAAAGTCCCGGATCCGAAGTTCAGCTC
>06G 174
CTGCACGGTGTAGGTGTTTCGGTGGTCAACGCCCTGTCCGAAGAGCTGGTCCTGACCGTTCGCCGCAGCGGCAAGAT
CTGGGAACAGACGTACATTCACGGCGTTCCGAAAGAGCCGATGAAAATTGTTGGTGAGAGCGAAACCACCGGCACCC
AGATCCACTTCAAACCGTCCGATCTGACCTTCAAGAATATCCACTTCAGCTGGGACATCCTGGCCAAGCGGATTCGT
GAACTGTCCTTCCTCAACTCCGGTGTCGGTATCGTCCTCAAGGATGAGCGCAGCGGCAAGGAAGAGCTGTTCAAGTA
TGAAGGTGGCCTGCGTGCGTTCGTTGAATACCTGAACACCAACAAGACTCCGGTCAACCAGGTGTTCCACTTCAACA
TCCAGCGCGAAGACGGCATCGGCGTGGAAATCGCCCTGCAGTGGAACGACAGCTTCAACGAGAACCTGTTGTGCTTC
ACCAACAACATTCCACAGCGCGATGGCGGTACTCACTTGGTGGGTTTCCGCTCCGCACTGACGCGTAACTTGAACAC
CTACATCGAAGCGGAAGGTCTGGCGAAGAAGCACAAGGTCGCGACCACCGGTGACGATGCCCGTGAAGGTCTGACCG
CAATCATTTCGGTGAAAGTCCCGGATCCGAAGTTCAGCTC
>08H 188
CTGCACGGTGTAGGTGTTTCGGTGGTCAACGCCCTGTCCGAAGAGCTGGTCCTGACCGTTCGCCGCAGCGGCAAGAT
CTGGGAACAGACGTACATTCACGGCGTTCCGAAAGAGCCGATGAAAATTGTTGGTGAGAGCGAAACCACCGGCACCC
AGATCCACTTCAAACCGTCCGATCTGACCTTCAAGAATATCCACTTCAGCTGGGACATCCTGGCCAAGCGGATTCGT
GAACTGTCCTTCCTCAACTCCGGTGTCGGTATCGTCCTCAAGGATGAGCGCAGCGGCAAGGAAGAGCTGTTCAAGTA
TGAAGGTGGCCTGCGTGCGTTCGTTGAATACCTGAACACCAACAAGACTCCGGTCAACCAGGTGTTCCACTTCAACA
TCCAGCGCGAAGACGGCATCGGCGTGGAAATCGCCCTGCAGTGGAACGACAGCTTTAACGAGAACCTGTTGTGCTTC
ACCAACAACATTCCACAGCGCGATGGCGGTACTCACTTGGTGGGTTTCCGCTCCGCACTGACGCGTAACTTGAACAC
CTACATCGAAGCGGAAGGCCTGGCGAAGAAGCACAAGGTCGCGACCACCGGTGACGATGCCCGTGAAGGCTTGACCG
CAATCATTTCGGTGAAAGTCCCGGATCCGAAGTTCAGCTC
>01D 37
CTGCACGGTGTAGGTGTTTCGGTGGTCAACGCCCTGTCCGAAGAGCTGGTCCTGACCGTTCGCCGCAGCGGCAAGAT
TTGGGAACAGACGTACATTCACGGCGTTCCGAAAGAGCCGATGAAAATTGTTGGTGAGAGCGAAACCACCGGCACCC
AGATCCACTTCAAACCGTCCGATCTGACCTTCAAGAATATCCACTTCAGCTGGGACATCCTGGCCAAGCGGATTCGT
GAACTGTCCTTCCTCAACTCCGGTGTCGGTATCGTCCTCAAGGATGAGCGCAGCGGCAAGGAAGAGCTGTTCAAGTA
TGAAGGTGGCCTGCGTGCGTTCGTTGAATACCTGAACACCAACAAGACTCCGGTCAACCAGGTGTTCCACTTCAACA
TCCAGCGCGAAGACGGCATCGGCGTGGAAATCGCCCTGCAGTGGAACGACAGCTTCAACGAGAACCTGTTGTGCTTC
ACCAACAACATTCCACAGCGCGATGGCGGTACTCACTTGGTGGGTTTCCGCTCCGCACTGACGCGTAACTTGAACAC
CTACATCGAAGCGGAAGGCCTGGCGAAGAAGCACAAGGTCGCGACCACCGGTGACGATGCCCGTGAAGGCCTGACCG
CAATCATTTCGGTGAAAGTCCCGGATCCGAAGTTCAGCTC
>05E 53
CTGCACGGTGTAGGTGTTTCGGTGGTCAACGCCCTGTCCGAAGAGCTGGTCCTGACCGTTCGCCGCAGCGGCAAGAT
CTGGGAACAGACGTACATTCACGGCGTTCCGAAAGAGCCGATGAAAATTGTTGGTGAGAGCGAAACCACCGGCACCC
AGATCCACTTCAAACCGTCCGATCTGACCTTCAAGAATATCCACTTCAGCTGGGACATCCTGGCCAAGCGGATTCGC
GAACTGTCCTTCCTCAACTCCGGTGTCGGTATCGTCCTCAAGGATGAGCGCAGCGGCAAGGAAGAGCTGTTCAAGTA
TGAAGGTGGCCTGCGTGCGTTCGTTGAATACCTGAACACCAACAAGACTCCGGTCAACCAGGTGTTCCACTTCAACA
TCCAGCGCGAAGACGGCATCGGCGTGGAAATCGCCCTGCAGTGGAACGACAGCTTCAACGAGAACCTGTTGTGCTTC
ACCAACAACATTCCACAGCGCGATGGCGGTACTCACTTGGTGGGTTTCCGCTCCGCACTGACGCGTAACTTGAACAC
CTACATCGAAGCGGAAGGCCTGGCGAAGAAGCACAAGGTCGCGACCACCGGTGACGATGCCCGTGAAGGCCTGACCG
CAATCATTTCGGTGAAAGTCCCGGATCCGAAGTTCAGCTC
>02A 98
CTGCACGGTGTAGGTGTTTCGGTGGTGAACGCCCTGTCCGAAGAGCTGGTCCTGACCGTTCGCCGCAGCGGCAAGAT
CTGGGAACAGACGTACATTCACGGCGTTCCGAAAGAGCCGATGAAAATTGTTGGTGAGAGCGAAACCACCGGCACCC
AGATCCACTTCAAACCGTCCGATCTGACCTTCAAGAATATCCACTTCAGCTGGGACATCCTGGCCAAGCGGATTCGT

GAACTGTCCTTCCTCAACTCCGGTGTCGGTATCGTCCTCAAGGATGAGCGCAGCGGCAAGGAAGAGCTGTTCAAGTA
TGAAGGTGGCCTGCGTGCGTTCGTTGAATACCTGAACACCAACAAGACTCCGGTCAACCAGGTGTTCCACTTCAACA
TCCAGCGCGAAGATGGCATCGGCGTGGAAATCGCCCTGCAGTGGAACGACAGCTTCAACGAGAACCTGTTGTGCTTC
ACCAACAACATTCCACAGCGCGATGGCGGTACTCACTTGGTGGGTTTCCGCTCCGCACTGACGCGTAACTTGAACAC
CTACATCGAAGCGGAAGGTCTGGCGAAGAAGCACAAGGTCGCGACCACCGGTGACGATGCCCGTGAAGGCCTGACCG
CAATCATTTCGGTGAAAGTCCCAGATCCGAAGTTCAGCTC
>04D 136
CTGCACGGTGTAGGTGTTTCAGTGGTCAACGCCCTGTCCGAAGAGCTGGTCCTGACCGTTCGCCGCAGCGGCAAGAT
CTGGGAACAGACGTACATTCACGGCGTTCCGAAAGAGCCGATGAAAATTGTTGGTGAGAGCGAAACCACCGGCACCC
AGATCCACTTCAAACCGTCCGATCTGACCTTCAAGAATATCCACTTCAGCTGGGACATCCTGGCCAAGCGGATTCGT
GAACTGTCCTTCCTCAACTCCGGTGTCGGTATCGTCCTCAAGGATGAGCGCAGCGGCAAGGAAGAGCTGTTCAAGTA
TGAAGGTGGCCTGCGTGCGTTCGTTGAATACCTGAACACCAACAAGACTCCGGTCAACCAGGTGTTCCACTTCAACA
TCCAGCGCGAAGACGGCATCGGCGTGGAAATCGCCCTGCAGTGGAACGACAGCTTCAACGAGAACCTGTTGTGCTTC
ACCAACAACATTCCACAGCGCGATGGCGGTACTCACTTGGTGGGTTTCCGCTCCGCACTGACGCGTAACTTGAACAC
CTACATCGAAGCGGAAGGCCTGGCGAAGAAGCACAAGGTCGCGACCACCGGTGACGATGCCCGTGAAGGCTTGACCG
CAATCATTTCGGTGAAAGTCCCGGATCCGAAGTTCAGCTC
>10D 46
CTGCACGGTGTAGGTGTTTCGGTGGTCAACGCCCTGTCCGAAGAGCTGGTCCTGACCGTTCGCCGCAGCGGCAAGAT
CTGGGAACAGACGTACATTCACGGCGTTCCGAAAGAGCCGATGAAAATTGTCGGTGAGAGCGAAACCACCGGCACCC
AGATCCACTTCAAACCGTCCGATCTGACCTTCAAGAACATCCACTTCAGTTGGGACATCCTGGCCAAGCGGATTCGT
GAACTGTCCTTCCTCAACTCCGGTGTCGGTATCGTCCTCAAGGATGAGCGCAGCGGCAAGGAAGAGCTGTTCAAGTA
TGAAGGTGGCCTGCGTGCGTTCGTTGAATACCTGAACACCAACAAGACTCCGGTCAACCAGGTGTTCCACTTCAACA
TCCAGCGCGAAGACGGCATTGGCGTGGAAATCGCCTTGCAGTGGAACGACAGCTTCAACGAGAACCTGTTGTGCTTT
ACCAACAACATTCCACAGCGCGATGGCGGTACTCACCTGGTGGGTTTTCGCTCCGCATTGACGCGTAACTTGAACAC
CTACATCGAAGCGGAAGGCCTGGCGAAGAAGCACAAAGTCGCGACCACCGGTGACGATGCCCGTGAAGGCCTGACCG
CGATCATTTCGGTGAAAGTCCCGGATCCGAAGTTCAGCTC
>04G 76
CTGCACGGTGTAGGTGTTTCGGTGGTCAACGCCCTGTCCGAAGAGCTGGTCCTGACCGTTCGCCGCAGCGGCAAGAT
CTGGGAACAGACGTACATTCACGGCGTTCCGAAAGAGCCGATGAAAATTGTCGGTGAGAGCGAAACCACCGGCACCC
AGATCCACTTCAAACCGTCCGATCTGACCTTCAAGAACATCCACTTCAGTTGGGACATCCTGGCCAAGCGGATTCGT
GAACTGTCCTTCCTCAACTCCGGTGTCGGTATCGTCCTCAAGGATGAGCGCAGCGGCAAGGAAGAGCTGTTCAAGTA
TGAAGGTGGCCTGCGTGCGTTCGTTGAATACCTGAACACCAACAAGACTCCGGTCAACCAGGTGTTCCACTTCAACA
TCCAGCGCGAAGACGGCATTGGCGTGGAAATCGCCTTGCAGTGGAACGACAGCTTCAACGAGAACCTGTTGTGCTTT
ACCAACAACATTCCACAGCGCGATGGCGGTACTCACCTGGTGGGTTTTCGCTCCGCATTGACGCGTAACTTGAACAC
CTACATCGAAGCGGAAGGCCTGGCGAAGAAGCACAAAGTCGCGACCACCGGTGACGATGCCCGTGAAGGCCTGACCG
CGATCATTTCGGTGAAAGTCCCGGATCCGAAGTTCAGCTC
>11D 47
CTGCACGGTGTAGGTGTTTCGGTGGTCAACGCCCTGTCCGAAGAGCTGGTCCTGACCGTTCGCCGCAGCGGCAAGAT
CTGGGAACAGACGTACATTCACGGCGTTCCGAAAGAGCCGATGAAAATTGTCGGTGAGAGCGAAACCACCGGCACCC
AGATCCACTTCAAACCGTCCGATCTGACCTTCAAGAACATCCACTTCAGTTGGGACATCCTGGCCAAGCGGATTCGT
GAACTGTCCTTCCTCAACTCCGGTGTCGGTATCGTCCTCAAGGATGAGCGCAGCGGCAAGGAAGAGCTGTTCAAGTA
TGAAGGTGGCCTGCGTGCGTTCGTTGAATACCTGAACACCAACAAGACTCCGGTCAACCAGGTGTTCCACTTCAACA
TCCAGCGCGAAGACGGCATTGGCGTGGAAATCGCCTTGCAGTGGAACGACAGCTTCAACGAGAACCTGTTGTGCTTT
ACCAACAACATTCCACAGCGCGATGGCGGTACTCACCTGGTGGGTTTTCGCTCCGCATTGACGCGTAACTTGAACAC
CTACATCGAAGCGGAAGGCCTGGCGAAGAAGCACAAAGTCGCGACCACCGGTGACGATGCCCGTGAAGGCCTGACCG
CGATCATTTCGGTGAAAGTCCCGGATCCGAAGTTCAGCTC
>01E 49
CTGCACGGTGTAGGTGTTTCGGTGGTCAACGCCCTGTCCGAAGAGCTGGTCCTGACCGTTCGCCGCAGCGGCAAGAT
CTGGGAACAGACGTACATTCACGGCGTTCCGAAAGAGCCGATGAAAATTGTCGGTGAGAGCGAAACCACCGGCACCC
AGATCCACTTCAAACCGTCCGATCTGACCTTCAAGAACATCCACTTCAGTTGGGACATCCTGGCCAAGCGGATTCGT
GAACTGTCCTTCCTCAACTCCGGTGTCGGTATCGTCCTCAAGGATGAGCGCAGCGGCAAGGAAGAGCTGTTCAAGTA
TGAAGGTGGCCTGCGTGCGTTCGTTGAATACCTGAACACCAACAAGACTCCGGTCAACCAGGTGTTCCACTTCAACA
TCCAGCGCGAAGACGGCATTGGCGTGGAAATCGCCTTGCAGTGGAACGACAGCTTCAACGAGAACCTGTTGTGCTTT
ACCAACAACATTCCACAGCGCGATGGCGGTACTCACCTGGTGGGTTTTCGCTCCGCATTGACGCGTAACTTGAACAC
CTACATCGAAGCGGAAGGCCTGGCGAAGAAGCACAAAGTCGCGACCACCGGTGACGATGCCCGTGAAGGCCTGACCG
CGATCATTTCGGTGAAAGTCCCGGATCCGAAGTTCAGCTC

>03E 51
CTGCACGGTGTAGGTGTTTCGGTGGTCAACGCCCTGTCCGAAGAGCTGGTCCTGACCGTTCGCCGCAGCGGCAAGAT
CTGGGAACAGACGTACATTCACGGCGTTCCGAAAGAGCCGATGAAAATTGTCGGTGAGAGCGAAACCACCGGCACCC
AGATCCACTTCAAACCGTCCGATCTGACCTTCAAGAACATCCACTTCAGTTGGGACATCCTGGCCAAGCGGATTCGT
GAACTGTCCTTCCTCAACTCCGGTGTCGGTATCGTCCTCAAGGATGAGCGCAGCGGCAAGGAAGAGCTGTTCAAGTA
TGAAGGTGGCCTGCGTGCGTTCGTTGAATACCTGAACACCAACAAGACTCCGGTCAACCAGGTGTTCCACTTCAACA
TCCAGCGCGAAGACGGCATTGGCGTGGAAATCGCCTTGCAGTGGAACGACAGCTTCAACGAGAACCTGTTGTGCTTT
ACCAACAACATTCCACAGCGCGATGGCGGTACTCACCTGGTGGGTTTTCGCTCCNCATTGACGCGTAACTTGAACAC
CTACATCGAAGCGGAAGGCCTGGCGAAGAAGCACAAAGTCGCGACCACCGGTGACGATGCCCGTGAAGGCCTGACCG
CGATCATTTCGGTGAAAGTCCCGGATCCGAAGTTCAGCTC
>02D 38
CTGCACGGTGTAGGTGTTTCGGTGGTCAACGCCCTGTCCGAAGAGCTGGTCCTGACCGTTCGCCGCAGCGGCAAGAT
CTGGGAACAGACGTACATTCACGGCGTTCCGAAAGAGCCGATGAAAATTGTTGGTGAGAGCGAAACCACCGGCACCC
AGATCCACTTCAAACCGTCCGATCTGACCTTCAAGAATATCCACTTCAGCTGGGACATCCTGGCCAAGCGGATTCGT
GAACTGTCCTTCCTCAACTCCGGTGTCGGTATCGTCCTCAAGGATGAGCGCAGCGGCAAGGAAGAGCTGTTCAAGTA
TGAAGGTGGCCTGCGTGCGTTCGTTGAATACCTGAACACCAACAAGACTCCGGTCAACCAGGTGTTCCACTTCAACA
TCCAGCGCGAAGACGGCATCGGCGTGGAAATCGCCCTGCAGTGGAACGACAGCTTTAACGAGAACCTGTTGTGCTTC
ACCAACAACATTCCACAGCGCGATGGCGGTACTCACTTGGTGGGTTTCCGCTCCGCACTGACGCGTAACTTGAACAC
CTACATCGAAGCGGAAGGCCTGGCGAAGAAGCACAAGGTCGCGACCACCGGTGACGATGCCCGTGAAGGCTTGACCG
CAATCATTTCGGTGAAAGTCCCGGATCCGAAGTTCAGCTC
>04B 112
CTGCACGGTGTAGGTGTGTCGGTAGTGAACGCACTGTCTGAAGAACTGATCCTGACCGTTCGCCGTAGCGGCAAGAT
CTGGGAACAGACCTACGTGCACGGCGTTCCGCAGGAGCCGATGAAGATCGTTGGCGAAAGCGAGACCACCGGTACCC
AGATCCACTTCAAGCCATCGGCTGAAACCTTCAAGAACATCCACTTCAGCTGGGACATCCTGGCCAAGCGGATTCGT
GAACTGTCGTTCCTCAACTCCGGTGTGGGTATCGTCCTCAAGGATGAGCGCAGCGGCAAGGAAGAGCTGTTCAAGTA
CGAAGGCGGCCTGCGTGCATTCGTTGAATACCTGAACACCAACAAGACTGCGGTCAACCAGGTGTTCCACTTCAACA
TCCAGCGTGAAGACGGCATTGGCGTGGAAATCGCCCTGCAGTGGAACGACAGCTTCAACGAGAACCTGTTGTGCTTC
ACCAACAACATTCCCCAGCGCGATGGCGGCACTCACCTGGTGGGTTTCCGCTCCGCGCTGACGCGTAACCTGAACAC
CTACATCGAAGCCGAAGGCTTGGCCAAGAAGCACAAAGTCGCCACCACTGGTGACGATGCGCGTGAAGGCCTGACCG
CAATCATTTCGGTGAAAGTGCCGGATCCGAAGTTCAGCTC
>S02B 202
CTGCACGGTGTAGGTGTGTCGGTAGTGAACGCACTGTCTGAAGAACTGATCCTGACCGTTCGCCGTAGCGGCAAGAT
CTGGGAACAGACCTACGTGCACGGCGTTCCGCAGGAGCCGATGAAGATCGTTGGCGAAAGCGAGACCACCGGTACCC
AGATCCACTTCAAGCCATCGGCTGAAACCTTCAAGAACATCCACTTCAGCTGGGACATCCTGGCCAAGCGGATTCGT
GAACTGTCGTTCCTCAACTCCGGTGTGGGTATCGTCCTCAAGGATGAGCGCAGCGGCAAGGAAGAGCTGTTCAAGTA
CGAAGGCGGCCTGCGTGCATTCGTTGAATACCTGAACACCAACAAGACTGCGGTCAACCAGGTGTTCCACTTCAACA
TCCAGCGTGAAGACGGCATTGGCGTGGAAATCGCCCTGCAGTGGAACGACAGCTTCAACGAGAACCTGTTGTGCTTC
ACCAACAACATTCCCCAGCGCGATGGCGGCACTCACCTGGTGGGTTTCCGCTCCGCGCTGACGCGTAACCTGAACAC
CTACATCGAAGCCGAAGGCTTGGCCAAGAAGCACAAAGTCGCCACCACTGGTGACGATGCGCGTGAAGGCCTGACCG
CAATCATTTCGGTGAAAGTGCCGGATCCGAAGTTCAGCTC
>S10F 270
CTGCACGGTGTAGGTGTGTCGGTAGTGAACGCACTGTCTGAAGAACTGATCCTGACCGTTCGCCGTAGCGGCAAGAT
CTGGGAACAGACCTACGTGCACGGCGTTCCGCAGGAGCCGATGAAGATCGTTGGCGAAAGCGAGACCACCGGTACCC
AGATCCACTTCAAGCCATCGGCTGAAACCTTCAAGAACATCCACTTCAGCTGGGACATCCTGGCCAAGCGGATTCGT
GAACTGTCGTTCCTCAACTCCGGTGTGGGTATCGTCCTCAAGGACGAGCGCAGCGGCAAGGAAGAGCTGTTCAAGTA
CGAAGGCGGCCTGCGTGCATTCGTTGAATACCTGAACACCAACAAGACTGCGGTCAACCAGGTGTTCCACTTCAACA
TCCAGCGTGAAGACGGCATTGGCGTGGAAATCGCCCTGCAGTGGAACGACAGCTTCAACGAGAACCTGTTGTGCTTC
ACCAACAACATTCCCCAGCGCGATGGCGGCACTCACCTGGTGGGTTTCCGCTCCGCGCTGACGCGTAACCTGAACAC
CTACATCGAAGCCGAAGGCTTGGCCAAGAAGCACAAAGTCGCCACCACTGGTGACGATGCGCGTGAAGGCCTGACCG
CAATCATTTCGGTGAAAGTGCCGGATCCGAAGTTCAGCTC
>S03G 215
CTGCACGGTGTAGGTGTGTCGGTAGTGAACGCACTGTCTGAAGAACTGATCCTGACCGTTCGCCGTAGCGGCAAGAT
CTGGGAACAGACCTACGTGCACGGCGTTCCGCAGGAGCCGATGAAGATCGTTGGCGAAAGCGAGACCACCGGTACCC
AGATCCACTTCAAGCCATCGGCTGAAACCTTCAAGAACATCCACTTCAGCTGGGACATCCTGGCCAAGCGGATTCGT
GAACTGTCGTTCCTCAACTCCGGTGTGGGTATCGTCCTCAAGGACGAGCGCAGCGGCAAGGAAGAGCTGTTCAAGTA
CGAAGGCGGCCTGCGTGCATTCGTTGAATACCTGAACACCAACAAGACTGCGGTCAACCAGGTGTTCCACTTCAACA
TCCAGCGTGAAGACGGCATTGGCGTGGAAATCGCCCTGCAGTGGAACGACAGCTTCAACGAGAACCTGTTGTGCTTC

ACCAACAACATTCCCCAGCGCGATGGCGGCACTCACCTGGTGGGTTTCCGCTCCGCGCTGACGCGTAACCTGAACAC
CTACATCGAAGCCGAAGGCTTGGCCAAGAAGCACAAAGTCGCCACCACTGGTGACGATGCGCGTGAAGGCCTGACCG
CAATCATTTCGGTGAAAGTGCCGGATCCGAAGTTCAGCTC
>S10E 269
CTGCACGGTGTAGGTGTGTCGGTAGTGAACGCACTGTCTGAAGAACTGATCCTGACCGTTCGCCGTAGCGGCAAGAT
CTGGGAACAGACCTACGTGCACGGCGTTCCGCAGGAGCCGATGAAGATCGTTGGCGAAAGCGAGACCACCGGTACCC
AGATCCACTTCAAGCCTTCGGCTGAAACCTTCAAGAACATCCACTTCAGCTGGGACATCCTGGCCAAGCGGATTCGT
GAACTGTCGTTCCTCAACTCCGGTGTGGGTATCGTCCTCAAGGACGAGCGCAGCGGCAAGGAAGAGCTGTTCAAGTA
CGAAGGCGGCCTGCGTGCGTTCGTTGAATACCTGAACACCAACAAGACTGCGGTCAACCAGGTGTTCCACTTCAACA
TCCAGCGTGAAGACGGCATTGGCGTGGAAATCGCCCTGCAGTGGAACGACAGCTTCAACGAGAACCTGTTGTGCTTC
ACCAACAACATTCCCCAGCGCGATGGCGGCACCCACCTGGTGGGTTTCCGCTCCGCGCTGACGCGTAACCTGAACAC
CTACATCGAAGCCGAAGGCTTGGCCAAGAAGCACAAAGTCGCCACCACTGGTGACGATGCGCGTGAAGGCCTGACCG
CAATCATTTCGGTGAAAGTGCCGGATCCGAAGTTCAGCTC
>S12C 379
CTGCACGGTGTAGGTGTGTCGGTAGTGAACGCACTGTCTGAAGAACTGATCCTGACCGTTCGCCGTAGCGGCAAGAT
CTGGGAACAGACCTACGTGCACGGCGTTCCGCAGGAGCCGATGAAGATCGTTGGCGAAAGCGAGACCACCGGTACCC
AGATCCACTTCAAGCCTTCGGCTGAAACCTTCAAGAACATCCACTTCAGCTGGGACATCCTGGCCAAGCGGATTCGT
GAACTGTCGTTCCTCAACTCCGGTGTGGGTATCGTCCTCAAGGACGAGCGCAGCGGCAAGGAAGAGCTGTTCAAGTA
CGAAGGCGGCCTGCGTGCGTTCGTTGAATACCTGAACACCAACAAGACTGCGGTCAACCAGGTGTTCCACTTCAACA
TCCAGCGTGAAGACGGCATTGGCGTGGAAATCGCCCTGCAGTGGAACGACAGCTTCAACGAGAACCTGTTGTGCTTC
ACCAACAACATTCCCCAGCGCGATGGCGGCACCCACCTGGTGGGTTTCCGCTCCGCGCTGACGCGTAACCTGAACAC
CTACATCGAAGCCGAAGGCTTGGCCAAGAAGCACAAAGTCGCCACCACTGGTGACGATGCGCGTGAAGGCCTGACCG
CAATCATTTCGGTGAAAGTGCCGGATCCGAAGTTCAGCTC
>S06D 332
CTGCACGGTGTAGGTGTGTCGGTAGTGAACGCACTGTCTGAAGAACTGATCCTGACCGTTCGCCGTAGCGGCAAGAT
CTGGGAACAGACCTACGTGCACGGCGTTCCGCAGGAGCCGATGAAGATCGTTGGCGAAAGCGAGACCACCGGTACCC
AGATCCACTTCAAGCCTTCGGCTGAAACCTTCAAGAACATCCACTTCAGCTGGGACATCCTGGCCAAGCGGATTCGT
GAACTGTCGTTCCTCAACTCCGGTGTGGGTATCGTCCTCAAGGACGAGCGCAGCGGCAAGGAAGAGCTGTTCAAGTA
CGAAGGCGGCCTGCGTGCGTTCGTTGAATACCTGAACACCAACAAGACTGCGGTCAACCAGGTGTTCCACTTCAACA
TCCAGCGTGAAGACGGCATTGGCGTGGAAATCGCCCTGCAGTGGAACGACAGCTTCAACGAGAACCTGTTGTGCTTC
ACCAACAACATTCCCCAGCGCGATGGCGGCACCCACCTGGTGGGTTTCCGCTCCGCGCTGACGCGTAACCTGAACAC
CTACATCGAAGCCGAAGGCTTGGCCAAGAAGCACAAAGTCGCCACCACTGGTGACGATGCGCGTGAAGGCCTGACCG
CAATCATTTCGGTGAAAGTGCCGGATCCGAAGTTCAGCTC
>S04F 318
CTGCACGGTGTAGGTGTGTCGGTAGTGAACGCACTGTCTGAAGAACTGATCCTGACCGTTCGCCGTAGCGGCAAGAT
CTGGGAACAGACCTACGTGCACGGCGTTCCGCAGGAGCCGATGAAGATCGTTGGCGAAAGCGAGACCACCGGTACCC
AGATCCACTTCAAGCCTTCGGCTGAAACCTTCAAGAACATCCACTTCAGCTGGGACATCCTGGCCAAGCGGATTCGT
GAACTGTCGTTCCTCAACTCCGGTGTGGGTATCGTCCTCAAGGACGAGCGCAGCGGCAAGGAAGAGCTGTTCAAGTA
CGAAGGCGGCCTGCGTGCGTTCGTTGAATACCTGAACACCAACAAGACTGCGGTCAACCAGGTGTTCCACTTCAACA
TCCAGCGTGAAGACGGCATTGGCGTGGAAATCGCCCTGCAGTGGAACGACAGCTTCAACGAGAACCTGTTGTGCTTC
ACCAACAACATTCCCCAGCGCGATGGCGGCACCCACCTGGTGGGTTTCCGCTCCGCGCTGACGCGTAACCTGAACAC
CTACATCGAAGCCGAAGGCTTGGCCAAGAAGCACAAAGTCGCCACCACTGGTGACGATGCGCGTGAAGGCCTGACCG
CAATCATTTCGGTGAAAGTGCCGGATCCGAAGTTCAGCTC
>S06H 240
CTGCACGGTGTAGGTGTGTCGGTAGTGAACGCACTGTCTGAAGAACTGATCCTGACCGTTCGCCGTAGCGGCAAGAT
CTGGGAACAGACCTACGTGCACGGCGTTCCGCAGGAGCCGATGAAGATCGTTGGCGAAAGCGAGACCACCGGTACCC
AGATCCACTTCAAGCCTTCGGCTGAAACCTTCAAGAACATCCACTTCAGCTGGGACATCCTGGCCAAGCGGATTCGT
GAACTGTCGTTCCTCAACTCCGGTGTGGGTATCGTCCTCAAGGACGAGCGCAGCGGCAAGGAAGAGCTGTTCAAGTA
CGAAGGCGGCCTGCGTGCGTTCGTTGAATACCTGAACACCAACAAGACTGCGGTCAACCAGGTGTTCCACTTCAACA
TCCAGCGTGAAGACGGCATTGGCGTGGAAATCGCCCTGCAGTGGAACGACAGCTTCAACGAGAACCTGTTGTGCTTC
ACCAACAACATTCCCCAGCGCGATGGCGGCACCCACCTGGTGGGTTTCCGCTCCGCGCTGACGCGTAACCTGAACAC
CTACATCGAAGCCGAAGGCTTGGCCAAGAAGCACAAAGTCGCCACCACTGGTGACGATGCGCGTGAAGGCCTGACCG
CAATCATTTCGGTGAAAGTGCCGGATCCGAAGTTCAGCTC
>S04E 317
CTGCACGGTGTAGGTGTGTCGGTAGTGAACGCACTGTCTGAAGAACTGATCCTGACCGTTCGCCGTAGCGGCAAGAT
CTGGGAACAGACCTACGTGCACGGCGTTCCGCAGGAGCCGATGAAGATCGTTGGCGAAAGCGAGACCACCGGTACCC
AGATCCACTTCAAGCCTTCGGCTGAAACCTTCAAGAACATCCACTTCAGCTGGGACATCCTGGCCAAGCGGATTCGT

GAACTGTCGTTCCTCAACTCCGGTGTGGGTATCGTCCTCAAGGACGAGCGCAGCGGCAAGGAAGAGCTGTTCAAGTA
CGAAGGCGGCCTGCGTGCGTTCGTTGAATACCTGAACACCAACAAGACTGCGGTCAACCAGGTGTTCCACTTCAACA
TCCAGCGTGAAGACGGCATTGGCGTGGAAATCGCCCTGCAGTGGAACGACAGCTTCAACGAGAACCTGTTGTGCTTC
ACCAACAACATTCCCCAGCGCGATGGCGGCACCCACCTGGTGGGTTTCCGCTCCGCGCTGACGCGTAACCTGAACAC
CTACATCGAAGCCGAAGGCTTGGCCAAGAAGCACAAAGTCGCCACCACTGGTGACGATGCGCGTGAAGGCCTGACCG
CAATCATTTCGGTGAAAGTGCCGGATCCGAAGTTCAGCTC
>05B 17
CTGCACGGTGTAGGTGTGTCGGTAGTGAACGCACTGTCTGAAGAACTGATCCTGACCGTTCGCCGTAGCGGCAAGAT
CTGGGAACAGACCTACGTCCACGGTGTTCCGCAGGAGCCGATGAAGATCGTTGGCGAGAGCGAGACGACCGGTACCC
AGATCCACTTCAAGCCTTCGGCTGAAACCTTCAAGAACATCCACTTCAGCTGGGACATCCTGGCCAAGCGGATTCGC
GAACTGTCGTTCCTCAACTCCGGTGTGGGTATCGTCCTCAAGGACGAGCGCAGCGGCAAGGAAGAGCTGTTCAAGTA
CGAAGGCGGCCTGCGTGCGTTCGTTGAATACCTGAACACCAACAAGACTGCGGTCAACCAGGTGTTCCACTTCAACA
TCCAGCGTGAAGACGGCATCGGCGTTGAAATCGCCCTGCAGTGGAACGACAGCTTCAACGAGAACCTGTTGTGCTTC
ACCAACAACATTCCCCAACGCGATGGCGGCACCCACCTGGTGGGCTTCCGTTCCGCGCTGACGCGTAACCTGAACAC
CTACATCGAAGCTGAAGGCCTGGCGAAGAAGCACAAGGTCGCCACCACCGGTGACGACGCCCGTGAAGGCCTGACGG
CGATTATCTCGGTAAAAGTGCCGGATCCGAAGTTCAGCTC
>07G 175
TTGCACGGTGTAGGTGTGTCGGTAGTGAACGCCCTGTCCGAAGAGCTGGTTCTGACCGTTCGCCGCAGCGGCAAGAT
CTGGGAGCAGACCTACGTCCACGGCGTACCACAAGAGCCGATGAAGATCGTTGGCGACAGTGAGTCAACCGGTACCC
AGATCCACTTCAAGCCTTCGGCTGAAACCTTCAAGAATATCCACTTCAGCTGGGACATCCTGGCCAAGCGGATTCGT
GAACTGTCCTTCCTCAACTCCGGTGTCGGCATCGTCCTCAAGGATGAGCGCAGCGGCAAGGAAGAGTTGTTCAAGTA
CGAAGGCGGCCTGCGTGCGTTCGTTGAATACCTGAACACCAACAAGACTGCGGTCAACCAGGTGTTCCACTTCAACA
TCCAGCGTGAAGACGGCATCGGTGTGGAAATCGCCCTGCAGTGGAACGACAGCTTCAACGAGAACCTGTTGTGCTTC
ACCAACAACATTCCTCAGCGCGATGGCGGTACTCACCTGGTGGGTTTCCGTTCCGCACTGACGCGTAACCTGAACAC
CTACATCGAAGCCGAAGGCTTGGCCAAGAAGCACAAAGTCGCCACCACGGGTGACGATGCGCGTGAAGGCCTGACCG
CGATCATTTCGGTGAAAGTGCCGGATCCCAAGTTCAGCTC
>01F 61
CTTCACGGCGTAGGTGTGTCGGTAGTGAACGCACTGTCTGAAGAGCTTGTTCTTACTGTCCGCCGTAGCGGCAAGAT
CTGGGAACAGACCTACGTCCACGGTGTGCCACAGGAGCCGATGAAGATCGTTGGCGACAGCGAAACTACCGGTACCC
AGATCCACTTCAAGGCTTCCAGCGAAACCTTCAAGAACATCCACTTCAGCTGGGACATCCTGGCCAAGCGGATTCGT
GAACTGTCCTTCCTCAACTCCGGTGTTGGCATCGTTCTCAAGGATGAGCGCAGCGGCAAGGAAGAGTTGTTCAAGTA
CGAAGGTGGCCTGCGCGCGTTTGTTGAATACCTGAACACCAACAAAACCCCGGTCAATCAGGTGTTCCACTTCAACA
TCCAGCGTGAAGACGGCATCGGCGTGGAAATCGCCCTGCAGTGGAACGACAGCTTCAACGAGAACCTGTTGTGCTTC
ACCAACAACATTCCGCAGCGCGACGGCGGTACCCACCTGGTGGGCTTCCGTTCCGCACTGACGCGTAACCTCAACAC
CTACATCGAAGCCGAAGGCCTGGCCAAGAAGCACAAGGTTGCAACCACCGGTGACGACGCCCGTGAAGGCCTGACCG
CGATCATTTCGGTAAAGGTTCCGGATCCGAAGTTCAGCTC
>12C 36
TTGCACGGTGTAGGTGTGTCGGTGGTAAACGCCCTGTCGGAAGAACTGATCCTCACCGTTCGCCGCAGCGGCAAGAT
CTGGGAACAGACCTACGTGCATGGCGTGCCTCAGGCACCGATGGCAATCGTCGGTGATAGCGAAACCACCGGTACCC
AGATTCACTTCAAGCCCTCGGCTGACACGTTCAAAAATATCCACTTCAGCTGGGACATCCTGGCCAAGCGGATTCGT
GAACTGTCCTTCCTCAACTCCGGTGTGGGTATCGTCCTCAAGGATGAGCGCAGCGGCAAGGAAGAGCTGTTCAAGTA
CGAAGGTGGCCTGCGTGCGTTCGTTGAATACCTGAACACCAACAAGACTGCGGTCAACCAGGTGTTCCACTTCAACA
TCCAGCGTGAAGACGGCATCGGCGTGGAAATCGCCCTGCAGTGGAACGACAGCTTCAACGAGAACCTGTTGTGCTTC
ACCAACAACATTCCTCAGCGCGATGGCGGTACTCACCTGGTGGGTTTCCGTTCCGCACTGACGCGTAACCTGAACAC
CTACATCGAAGCTGAAGGCTTGGCGAAGAAGCACAAAGTCGCCACCACCGGTGACGATGCCCGTGAAGGCCTGACCG
CGATTATTTCGGTCAAGGTTCCGGATCCGAAGTTCAGCTC
>09H 93
TTGCACGGTGTAGGTGTGTCGGTGGTAAACGCCCTGTCGGAAGAACTGATCCTCACCGTTCGCCGCAGCGGCAAGAT
CTGGGAACAGACCTACGTGCATGGCGTGCCTCAGGCACCGATGGCAATCGTCGGTGATAGCGAAACCACCGGTACCC
AGATTCACTTCAAGCCCTCGGCTGACACGTTCAAAAATATCCACTTCAGTTGGGACATCCTGGCCAAGCGGATTCGT
GAACTGTCCTTCCTCAACTCCGGTGTGGGTATCGTCCTCAAGGATGAGCGCAGCGGCAAGGAAGAGCTGTTCAAGTA
CGAAGGTGGCCTGCGTGCGTTCGTTGAATACCTGAACACCAACAAGACTGCGGTCAACCAGGTGTTCCACTTCAACA
TCCAGCGTGAAGACGGCATCGGCGTGGAAATCGCCCTGCAGTGGAACGACAGCTTCAACGAGAACCTGTTGTGCTTC
ACCAACAACATTCCTCAGCGCGATGGCGGTACTCACCTGGTGGGTTTCCGTTCCGCACTGACGCGTAACCTGAACAC
CTACATCGAAGCTGAAGGCTTGGCGAAGAAGCACAAAGTCGCCACCACCGGTGACGATGCCCGTGAAGGCCTGACCG
CGATTATTTCGGTCAAGGTTCCGGATCCGAAGTTCAGCTC

>05D 41
TTGCACGGTGTAGGTGTGTCGGTGGTAAACGCCTTGTCGGAAGAGCTGATCCTCACCGTTCGCCGCAGCGGCAAGAT
CTGGGAACAGACCTACGTGCATGGCGTGCCTCAGGCACCGATGGCAATCGTCGGTGATAGCGAAACCACCGGTACCC
AGATTCACTTCAAGCCCTCGGCTGACACGTTCAAAAATATCCACTTCAGCTGGGACATCCTGGCCAAGCGGATTCGT
GAACTGTCCTTCCTCAACTCCGGTGTGGGTATCGTCCTCAAGGATGAGCGCAGCGGCAAGGAAGAGCTGTTCAAGTA
CGAAGGTGGCCTGCGTGCGTTCGTTGAATACCTGAACACCAACAAGACTGCGGTCAACCAGGTGTTCCACTTCAACA
TCCAGCGTGAAGATGGCATCGGCGTGGAAATCGCCCTGCAGTGGAACGACAGCTTCAACGAGAACCTGTTGTGCTTC
ACCAACAACATTCCTCAGCGCGATGGCGGTACTCACCTGGTGGGTTTCCGTTCCGCACTGACGCGTAACCTGAACAC
CTACATCGAAGCTGAAGGCTTGGCGAAGAAGCACAAAGTCGCCACCACTGGTGACGATGCCCGTGAAGGCCTGACCG
CGATTATTTCGGTCAAGGTTCCGGATCCGAAGTTCAGCTC
>10F 70
CTGCACGGTGTGGGTGTGTCGGTAGTGAACGCCCTGTCCGAAGAACTGGTCCTGACCGTTCGCCGCAGTGGCAAGAT
CTGGGAACAGACCTACGTTCACGGTGTGCCTCAGGCACCTATGGCGATCGTCGGTGACAGCGAAACCACCGGTACCC
AGATTCACTTCAAGGCTTCCAGCGAGACCTTCAAGAACATCCACTTCAGCTGGGACATCCTGGCCAAGCGGATTCGT
GAACTGTCCTTCCTCAACTCCGGTGTCGGTATCGTTCTGAAGGACGAGCGCAGCGGCAAGGAAGAGCTGTTCAAGTA
CGAAGGCGGCTTGCGTGCGTTCGTTGAATACCTGAACACCAACAAGACTGCGGTCAACCAGGTGTTCCACTTCAATG
TGCAGCGTGAAGACGGCATCGGCGTGGAAATCGCCCTGCAGTGGAACGACAGCTTCAACGAGAACCTGCAGTGCTTC
ACCAACAACATTCCGCAGCGCGACGGCGGCACCCACCTGGTGGGCTTCCGTTCGGCGCTGACGCGTAACCTGAACAA
CTACATCGAGCAGGAAGGCCTGGCGAAGAAGCACAAGGTCGCCACCACCGGTGACGATGCCCGCGAAGGCCTGACTG
CGATCATTTCGGTCAAGGTGCCGGATCCGAAGTTCAGCTC
>09C 129
CTGCACGGTGTGGGTGTGTCGGTAGTGAACGCCCTGTCCGAAGAACTGGTCCTGACCGTTCGCCGCAGTGGCAAGAT
CTGGGAACAGACCTACGTTCACGGTGTGCCTCAGGCGCCTATGGCTATCGTCGGTGACAGCGAAACCACCGGTACCC
AGATTCACTTCAAGGCTTCCAGCGAGACCTTCAAGAACATCCACTTTAGCTGGGACATCCTGGCCAAGCGGATTCGT
GAACTGTCCTTCCTCAACTCCGGTGTCGGTATCGTTCTGAAGGACGAGCGCAGCGGCAAGGAAGAACTGTTCAAGTA
CGAAGGCGGCTTGCGTGCGTTCGTTGAATACCTGAACACCAACAAGACTGCGGTCAACCAGGTGTTCCACTTCAATG
TGCAGCGTGAAGACGGCATCGGCGTGGAAATCGCCCTGCAGTGGAACGACAGCTTCAACGAGAACCTGCAGTGCTTC
ACCAACAACATTCCGCAGCGCGACGGTGGTACCCACCTGGTGGGCTTCCGTTCGGCGCTGACGCGTAACCTGAACAA
CTACATCGAACAGGAAGGCCTGGCGAAGAAGCACAAGGTCGCCACCACCGGTGACGATGCCCGCGAAGGCCTGACCG
CGATCATTTCGGTCAAGGTGCCGGATCCGAAGTTCAGCTC
>08D 44
CTGCACGGCGTAGGTGTGTCGGTGGTGAACGCTCTGTCTGAAGAGCTGATCCTGACCGTGCGCCGTAGCGGCAAGAT
CTGGGAACAGACCTACGTTCACGGTGTGCCGCAAGAGCGGATGAAGATCGTTGGCGACAGCGAATCCACCGGTACTC
AGATTCACTTCAAGCCTTCGGCTGAAACCTTCAAGAACATCCACTTCAGCTGGGACATCCTGGCCAAGCGGATTCGT
GAACTGTCTTTCCTCAACTCCGGTGTCGGCATCGTCCTCAAGGACGAGCGCAGTGGCAAGGAAGAGCTGTTCAAGTA
CGAAGGCGGCCTGCGTGCATTCGTTGAATACCTGAACACCAACAAGACTGCGGTCAACCAGGTGTTCCACTTCAACA
TCCAGCGTGAAGACGGCATCGGCGTGGAAATCGCCCTGCAGTGGAACGACAGCTTCAACGAGAACCTGTTGTGCTTC
ACCAACAACATCCCGCAGCGCGACGGCGGTACTCACTTGGTGGGTTTCCGTTCCGCACTGACCCGTAACCTGAATAC
CTACATCGAAGCCGAAGGCCTGGCCAAGAAGCACAAGGTCGCCACCACCGGTGACGACGCTCGTGAAGGTCTGACCG
CGATCATTTCGGTGAAGGTGCCGGATCCGAAGTTCAGCTC
>11G 179
CTGCACGGCGTAGGTGTGTCGGTGGTGAACGCTCTGTCTGAAGAGCTGATCCTGACCGTGCGCCGTAGCGGCAAGAT
CTGGGAACAGACCTACGTTCACGGTGTGCCGCAAGAGCGGATGAAGATCGTTGGCGACAGCGAATCCACCGGTACTC
AGATTCACTTCAAGCCTTCGGCTGAAACCTTCAAGAACATCCACTTCAGCTGGGACATCCTGGCCAAGCGGATTCGT
GAACTGTCTTTCCTCAACTCCGGTGTCGGCATCGTCCTCAAGGACGAGCGCAGTGGCAAGGAAGAGCTGTTCAAGTA
CGAAGGCGGCCTGCGTGCATTCGTTGAATACCTGAACACCAACAAGACTGCGGTCAACCAGGTGTTCCACTTCAACA
TCCAGCGTGAAGACGGCATCGGCGTGGAAATCGCCCTGCAGTGGAACGACAGCTTCAACGAGAACCTGTTGTGCTTC
ACCAACAACATCCCGCAGCGCGACGGCGGTACTCACTTGGTGGGTTTCCGTTCCGCACTGACCCGTAACCTGAATAC
CTACATCGAAGCCGAAGGCCTGGCCAAGAAGCACAAGGTCGCCACCACCGGTGACGACGCTCGTGAAGGCCTGACCG
CGATCATTTCGGTGAAGGTGCCGGATCCGAAGTTCAGCTC
>07D 43
CTGCACGGCGTAGGTGTGTCGGTGGTGAACGCTCTGTCTGAAGAGCTGATCCTGACCGTGCGCCGTAGCGGCAAGAT
CTGGGAACAGACCTACGTTCACGGTGTGCCGCAAGAGCGGATGAAGATCGTTGGCGACAGCGAATCCACCGGTACTC
AGATTCACTTCAAGCCTTCGGCTGAAACCTTCAAGAACATCCACTTCAGCTGGGACATCCTGGCCAAGCGGATTCGT
GAACTGTCCTTCCTCAACTCCGGTGTCGGCATCGTCCTCAAGGACGAGCGCAGCGGCAAGGAAGAGCTGTTCAAGTA
CGAAGGCGGCCTGCGTGCATTCGTTGAATACCTGAACACCAACAAGACTGCGGTCAACCAGGTGTTCCACTTCAACA
TCCAGCGTGAAGACGGCATCGGCGTCGAAATCGCCCTGCAGTGGAACGACAGCTTCAACGAGAACCTGTTGTGCTTC

ACCAACAACATCCCGCAGCGCGACGGCGGTACTCACTTGGTGGGTTTCCGTTCCGCACTGACCCGTAACCTGAATAC
CTACATCGAAGCCGAAGGCCTGGCCAAGAAGCACAAGGTCGCCACCACCGGTGACGACGCTCGTGAAGGTCTGACCG
CGATCATTTCGGTGAAGGTGCCGGATCCGAAGTTCAGCTC
>07D 139
CTGCACGGCGTAGGTGTGTCGGTGGTGAATGCTCTGTCTGAAGAGCTGATTCTGACCGTGCGCCGTAGCGGCAAGAT
CTGGGAACAGACCTACGTTCACGGTGTGCCGCAAGAGCGGATGAAGATCGTTGGCGACAGCGAATCCACCGGTACTC
AGATTCACTTCAAGCCTTCGGCTGAAACCTTCAAGAACATCCACTTCAGCTGGGACATCCTGGCCAAGCGGATTCGT
GAACTGTCCTTCCTCAACTCCGGTGTCGGCATCGTCCTCAAGGACGAGCGCAGCGGCAAGGAAGAGCTGTTCAAGTA
CGAAGGCGGCCTGCGTGCATTCGTTGAATACCTGAACACCAACAAGACTGCGGTCAACCAGGTGTTCCACTTCAACA
TCCAGCGTGAAGACGGCATCGGCGTCGAAATCGCCCTGCAGTGGAACGACAGCTTCAACGAGAACCTGTTGTGCTTC
ACCAACAACATCCCGCAGCGCGACGGCGGTACTCACTTGGTGGGTTTCCGTTCCGCACTGACCCGTAACCTGAATAC
CTACATCGAAGCCGAAGGCCTGGCCAAGAAGCACAAGGTCGCCACCACCGGTGACGACGCTCGTGAAGGCCTGACCG
CGATCATTTCGGTGAAGGTGCCGGATCCGAAGTTCAGCTC
>S12D 284
CTGCACGGCGTAGGTGTGTCGGTGGTGAATGCTCTGTCTGAAGAGCTGATTCTGACCGTGCGCCGTAGCGGCAAGAT
CTGGGAACAGACCTACGTTCACGGTGTGCCGCAAGAGCGGATGAAGATCGTTGGCGACAGCGAATCCACCGGTACTC
AGATTCACTTCAAGCCTTCGGCTGAAACCTTCAAGAACATCCACTTCAGCTGGGACATCCTGGCCAAGCGGATTCGT
GAACTGTCCTTCCTCAACTCCGGTGTCGGCATCGTCCTCAAGGACGAGCGCAGCGGCAAGGAAGAGCTGTTCAAGTA
CGAAGGCGGCCTGCGTGCATTCGTTGAATACCTGAACACCAACAAGACTGCGGTCAACCAGGTGTTCCACTTCAACA
TCCAGCGTGAAGACGGCATCGGCGTCGAAATCGCCCTGCAGTGGAACGACAGCTTCAACGAGAACCTGTTGTGCTTC
ACCAACAACATCCCGCAGCGCGACGGCGGTACTCACTTGGTGGGTTTCCGTTCCGCACTGACCCGTAACCTGAATAC
CTACATCGAAGCCGAAGGCCTGGCCAAGAAGCACAAGGTCGCCACCACCGGTGACGACGCTCGTGAAGGCCTGACCG
CGATCATTTCGGTGAAGGTGCCGGATCCGAAGTTCAGCTC
>10G 178
CTGCACGGCGTAGGTGTGTCGGTGGTGAACGCTCTGTCTGAAGAGCTGATCCTGACCGTGCGCCGTAGCGGCAAGAT
CTGGGAACAGACCTACGTTCACGGTGTGCCGCAAGAGCGGATGAAGATCGTTGGCGACAGCGAATCCACCGGTACTC
AGATTCACTTCAAGCCTTCGGCTGAAACCTTCAAGAACATCCACTTCAGCTGGGACATCCTGGCCAAGCGGATTCGT
GAACTGTCCTTCCTCAACTCCGGTGTCGGCATCGTCCTCAAGGACGAGCGCAGCGGCAAGGAAGAGCTTTTCAAGTA
CGAAGGCGGCCTGCGTGCATTCGTTGAATACCTGAACACCAACAAGACTGCGGTCAACCAGGTGTTCCACTTCAACA
TCCAGCGTGAAGACGGCATCGGCGTCGAAATCGCCCTGCAGTGGAACGACAGCTTCAACGAGAACCTGTTGTGCTTC
ACCAACAACATCCCGCAGCGCGACGGCGGTACTCACTTGGTGGGTTTCCGTTCCGCACTGACCCGTAACCTGAATAC
CTACATCGAAGCCGAAGGCCTGGCCAAGAAGCACAAGGTCGCCACCACCGGTGACGACGCTCGTGAAGGTCTGACCG
CGATCATTTCGGTGAAGGTGCCGGATCCGAAGTTCAGCTC
>02G 170
CTGCACGGTGTAGGTGTGTCGGTAGTGAACGCACTGTCCGAGGAGCTGGTACTGACCGTTCGCCGTAGCGGCCAGAT
CTGGGAACAGACCTACCTCGACGGTGTACCTCAAGATCCGATGGACATCGTTGGCGAAAGCGAAACCACCGGTACCC
AGATCCACTTCGAGGCTTCGAGCGAAACCTTCAAGAACATTCACTTCAGCTGGGACATCCTGGCCAAGCGGATTCGT
GAACTGTCCTTCCTCAACTCCGGTGTCGGCATCGTCCTCAAGGATGAGCGCAGCGGCAAGGAAGAGCTGTTCAAGTA
CGAAGGCGGCCTGCGTGCGTTCGTTGAATACCTGAACACCAACAAGACTGCGGTCAACCAGGTGTTCCACTTCAATA
TCCAGCGTGAAGACGGCATCGGCGTGGAAATCGCCCTGCAGTGGAACGACAGCTTCAACGAGAACCTGTTGTGCTTC
ACCAACAACATTCCGCAGCGCGACGGCGGTACCCACCTGGTGGGCTTCCGTTCGGCCCTGACGCGTAACCTGAACAA
CTACATCGAGCAGGAAGGTCTGGCGAAGAAGCATAAAGTCGCCACCACCGGTGATGATGCCCGTGAAGGCCTGACCG
CGATCATCTCGGTGAAAGTACCGGATCCGAAGTTCAGCTC
>S09A 353
CTGCACGGTGTAGGTGTTTCGGTAGTTAACGCTCTTTCCGAACAACTGGTCCTGACGGTTCGTCGTAGCGGCAAGAT
CTGGGAACAGACCTACGTACACGGTGTTCCGCAAGAGCCGATGAAAATCGTTGGCGACAGCGAAACCACTGGTACCC
AGATTCACTTCAAGGCTTCCAGCGAAACCTTCAAGAATATCCACTTCAGCTGGGACATCCTGGCCAAGCGGATTCGT
GAGCTGTCCTTCCTCAACTCCGGTGTCGGTATCGTCCTCAAGGACGAGCGCAGCGGCAAGGAAGAGCTGTTCAAGTA
CGAAGGTGGTCTGCGTGCGTTCGTTGAATACCTGAACACTAACAAGACTCCAGTCAACCAGGTGTTCCACTTCAACA
TCCAGCGCGAAGACGGCATTGGCGTGGAAATCGCCCTGCAGTGGAACGACAGCTTCAACGAGAATCTGTTGTGCTTC
ACCAACAACATTCCGCAGCGCGACGGCGGTACTCACCTGGTCGGTTTCCGTTCCGCTCTGACGCGTAACCTGAACAC
CTACATCGAAGCTGAAGGTCTGGCGAAGAAGCACAAAGTCGCGACTACCGGTGACGATGCCCGTGAAGGCCTGACCG
CGATCATTTCGGTAAAGGTGCCGGATCCGAAGTTCAGCTC
>S09F 358
CTGCACGGTGTAGGTGTTTCGGTAGTTAACGCTCTTTCCGAACAACTGGTCCTGACGGTTCGTCGTAGCGGCAAGAT
CTGGGAACAGACCTACGTACACGGTGTTCCGCAAGAGCCGATGAAAATCGTTGGCGACAGCGAAACCACTGGTACCC
AGATTCACTTCAAGGCTTCCAGCGAAACCTTCAAGAATATCCACTTCAGCTGGGACATCCTGGCCAAGCGGATTCGT

GAGCTGTCCTTCCTCAACTCCGGTGTCGGTATCGTCCTCAAGGACGAGCGCAGCGGCAAGGAAGAGCTGTTCAAGTA
CGAAGGTGGTCTGCGTGCGTTCGTTGAATACCTGAACACTAACAAGACTCCAGTCAACCAGGTGTTCCACTTCAACA
TCCAGCGCGAAGACGGCATTGGCGTGGAAATCGCCCTGCAGTGGAACGACAGCTTCAACGAGAATCTGTTGTGCTTC
ACCAACAACATTCCGCAGCGCGACGGCGGTACTCACCTGGTCGGTTTCCGTTCCGCTCTGACGCGTAACCTGAACAC
CTACATCGAAGCTGAAGGTCTGGCGAAGAAGCACAAAGTCGCGACTACCGGTGACGATGCCCGTGAAGGCCTGACCG
CGATCATTTCGGTAAAGGTGCCGGATCCGAAGTTCAGCTC
>S04C 315
CTGCACGGTGTAGGTGTTTCGGTAGTTAACGCTCTTTCCGAACAACTGGTCCTGACGGTTCGTCGTAGCGGCAAGAT
CTGGGAACAGACCTACGTACACGGTGTTCCGCAAGAGCCGATGAAAATCGTTGGCGACAGCGAAACCACTGGTACCC
AGATTCACTTCAAGGCTTCCAGCGAAACCTTCAAGAATATCCACTTCAGCTGGGACATCCTGGCCAAGCGGATTCGT
GAGCTGTCCTTCCTCAACTCCGGTGTCGGTATCGTCCTCAAGGACGAGCGCAGCGGCAAGGAAGAGCTGTTCAAGTA
CGAAGGTGGTCTGCGTGCGTTCGTTGAATACCTGAACACTAACAAGACTCCAGTCAACCAGGTGTTCCACTTCAACA
TCCAGCGCGAAGACGGCATTGGCGTGGAAATCGCCCTGCAGTGGAACGACAGCTTCAACGAGAATCTGTTGTGCTTC
ACCAACAACATTCCGCAGCGCGACGGCGGTACTCACCTGGTCGGTTTCCGTTCCGCTCTGACGCGTAACCTGAACAC
CTACATCGAAGCTGAAGGTCTGGCGAAGAAGCACAAAGTCGCGACTACCGGTGACGATGCCCGTGAAGGCCTGACCG
CGATCATTTCGGTAAAGGTGCCGGATCCGAAGTTCAGCTC
>S05F 326
CTGCACGGTGTAGGTGTTTCGGTAGTTAACGCTCTTTCCGAACAACTGGTCCTGACGGTTCGTCGTAGCGGCAAGAT
CTGGGAACAGACCTACGTACACGGTGTTCCGCAAGAGCCGATGAAAATCGTTGGCGACAGCGAAACCACTGGTACCC
AGATTCACTTCAAGGCTTCCAGCGAAACCTTCAAGAATATCCACTTCAGCTGGGACATCCTGGCCAAGCGGATTCGT
GAGCTGTCCTTCCTCAACTCCGGTGTCGGTATCGTCCTCAAGGACGAGCGCAGCGGCAAGGAAGAGCTGTTCAAGTA
CGAAGGTGGTCTGCGTGCGTTCGTTGAATACCTGAACACTAACAAGACTCCAGTCAACCAGGTGTTCCACTTCAACA
TCCAGCGCGAAGACGGCATTGGCGTGGAAATCGCCCTGCAGTGGAACGACAGCTTCAACGAGAATCTGTTGTGCTTC
ACCAACAACATTCCGCAGCGCGACGGCGGTACTCACCTGGTGGGTTTCCGTTCCGCTCTGACGCGTAACCTGAACAC
CTACATCGAAGCTGAAGGTCTGGCGAAGAAGCACAAAGTCGCCACTACCGGTGACGATGCCCGTGAAGGCCTGACCG
CGATCATTTCGGTAAAGGTGCCGGATCCGAAGTTCAGCTC
>S01E 293
CTGCACGGTGTAGGTGTTTCGGTAGTTAACGCTCTTTCCGAACAACTGGTCTTGACGGTTCGTCGTAGCGGCAAGAT
CTGGGAACAGACCTACGTACACGGTGTTCCGCAAGAGCCGATGAAAATCGTTGGCGACAGCGAAACCACTGGTACCC
AGATTCACTTCAAGGCTTCCAGCGAAACCTTCAAGAATATCCACTTCAGCTGGGACATCCTGGCCAAGCGGATTCGT
GAGCTGTCCTTCCTCAACTCCGGTGTCGGTATCGTCCTCAAGGACGAGCGCAGCGGCAAGGAAGAGCTGTTCAAGTA
CGAAGGTGGTCTGCGTGCGTTCGTTGAATACCTGAACACTAACAAGACTCCAGTCAACCAGGTGTTCCACTTCAACA
TCCAGCGCGAAGACGGCATTGGCGTGGAAATCGCCCTGCAGTGGAACGACAGCTTCAACGAGAATCTGTTGTGCTTC
ACCAACAACATTCCGCAGCGCGACGGCGGTACTCACCTGGTGGGTTTCCGTTCCGCTCTGACGCGTAACCTGAACAC
CTACATCGAAGCTGAAGGTCTGGCGAAGAAGCACAAAGTCGCGACTACCGGTGACGATGCCCGTGAAGGCCTGACCG
CGATCATTTCGGTAAAGGTGCCGGATCCGAAGTTCAGCTC
>S08G 351
CTGCACGGTGTAGGTGTTTCGGTAGTTAACGCTCTTTCCGAACAACTGGTCTTGACGGTTCGTCGTAGCGGCAAGAT
CTGGGAACAGACCTACGTACACGGTGTTCCGCAAGAGCCGATGAAAATCGTTGGCGACAGCGAAACCACTGGTACCC
AGATTCACTTCAAGGCTTCCAGCGAAACCTTCAAGAATATCCACTTCAGCTGGGACATCCTGGCCAAGCGGATTCGT
GAGCTGTCCTTCCTCAACTCCGGTGTCGGTATCGTCCTCAAGGACGAGCGCAGCGGCAAGGAAGAGCTGTTCAAGTA
CGAAGGTGGTCTGCGTGCGTTCGTTGAATACCTGAACACTAACAAGACTCCAGTCAACCAGGTGTTCCACTTCAACA
TCCAGCGCGAAGACGGCATTGGCGTGGAAATCGCCCTGCAGTGGAACGACAGCTTCAACGAGAATCTGTTGTGCTTC
ACCAACAACATTCCGCAGCGCGACGGCGGTACTCACCTGGTGGGTTTCCGTTCCGCTCTGACGCGTAACCTGAACAC
CTACATCGAAGCTGAAGGTCTGGCGAAGAAGCACAAAGTCGCGACTACCGGTGACGATGCCCGTGAAGGCCTGACCG
CGATCATTTCGGTAAAGGTGCCGGATCCGAAGTTCAGCTC
>S03B 210
CTGCACGGTGTAGGTGTTTCGGTAGTTAACGCTCTTTCCGAACAACTGGTCCTGACGGTTCGTCGTAGCGGCAAGAT
CTGGGAACAGACCTACGTACACGGTGTTCCGCAAGAGCCGATGAAAATCGTTGGCGACAGCGAAACCACTGGTACCC
AGATTCACTTCAAGGCTTCCAGCGAAACCTTCAAGAATATCCACTTCAGCTGGGACATCCTGGCCAAGCGGATTCGT
GAGCTGTCCTTCCTCAACTCCGGTGTCGGTATCGTCCTCAAGGACGAGCGCAGCGGCAAGGAAGAGCTGTTCAAGTA
CGAAGGTGGTCTGCGTGCGTTCGTTGAATACCTGAACACTAACAAGACTCCAGTCAACCAGGTGTTCCACTTCAACA
TCCAGCGCGAAGACGGCATTGGCGTGGAAATCGCCCTGCAGTGGAACGACAGCTTCAACGAGAATCTGTTGTGCTTC
ACCAACAACATTCCGCAGCGCGACGGCGGTACTCACCTGGTGGGTTTCCGTTCCGCTCTGACGCGTAACCTGAACAC
CTACATCGAAGCTGAAGGACTGGCGAAGAAGCACAAAGTCGCCACTACCGGTGACGATGCCCGTGAAGGCCTGACCG
CGATCATTTCGGTAAAGGTGCCGGATCCGAAGTTCAGCTC

>S03C 211
CTGCACGGTGTAGGTGTTTCGGTAGTTAACGCTCTTTCCGAACAACTGGTCCTGACGGTTCGTCGTAGCGGCAAGAT
CTGGGAACAGACCTACGTACACGGTGTTCCGCAAGAGCCGATGAAAATCGTTGGCGACAGCGAAACCACTGGTACCC
AGATTCACTTCAAGGCTTCCAGCGAAACCTTCAAGAACATCCACTTCAGCTGGGACATTTTGGCCAAGCGGATTCGT
GAACTGTCCTTCCTCAACTCCGGTGTCGGTATCGTCCTCAAGGACGAGCGCAGCGGCAAGGAAGAGCTGTTCAAGTA
CGAAGGTGGTCTGCGTGCGTTCGTTGAATACCTGAACACTAACAAGACTCCAGTCAACCAGGTGTTCCACTTCAACA
TCCAGCGCGAAGACGGCATTGGCGTGGAAATCGCCCTGCAGTGGAACGACAGCTTCAACGAGAATCTGTTGTGCTTC
ACCAACAACATTCCGCAGCGCGACGGCGGTACTCACCTGGTGGGTTTCCGTTCCGCTCTGACGCGTAACCTGAACAC
CTACATCGAAGCTGAAGGTCTGGCGAAGAAGCACAAAGTCGCCACTACCGGTGACGATGCCCGTGAAGGCCTGACCG
CGATCATTTCGGTAAAGGTGCCGGATCCGAAGTTCAGCTC
>S08F 350
CTGCACGGTGTAGGTGTTTCGGTAGTTAACGCTCTTTCCGAACAACTGGTCCTGACGGTTCGTCGTAGCGGCAAGAT
CTGGGAACAGACCTACGTACACGGTGTTCCGCAAGAGCCGATGAAAATCGTTGGCGACAGCGAAACCACTGGTACCC
AGATTCACTTCAAGGCTTCCAGCGAAACCTTCAAGAACATCCACTTCAGCTGGGACATTTTGGCCAAGCGGATTCGT
GAACTGTCCTTCCTCAACTCCGGTGTCGGTATCGTCCTCAAGGACGAGCGCAGCGGCAAGGAAGAGCTGTTCAAGTA
CGAAGGTGGTCTGCGTGCGTTCGTTGAATACCTGAACACTAACAAGACTCCAGTCAACCAGGTGTTCCACTTCAACA
TCCAGCGCGAAGACGGCATTGGCGTGGAAATCGCCCTGCAGTGGAACGACAGCTTCAACGAGAATCTGTTGTGCTTC
ACCAACAACATTCCGCAGCGCGACGGCGGTACTCACCTGGTGGGTTTCCGTTCCGCTCTGACGCGTAACCTGAACAC
CTACATCGAAGCTGAAGGTCTGGCGAAGAAGCACAAAGTCGCCACTACCGGTGACGATGCCCGTGAAGGCCTGACCG
CGATCATTTCGGTAAAGGTGCCGGATCCGAAGTTCAGCTC
>S07A 337
CTGCACGGTGTAGGTGTTTCGGTAGTTAACGCTCTTTCCGAACAACTGGTCCTGACGGTTCGTCGTAGCGGCAAGAT
CTGGGAACAGACCTACGTACACGGTGTTCCGCAAGAGCCGATGAAAATCGTTGGCGACAGCGAAACCACTGGTACCC
AGATTCACTTCAAGGCTTCCAGCGAAACCTTCAAGAACATCCACTTCAGCTGGGACATTTTGGCCAAGCGGATTCGT
GAACTGTCCTTCCTCAACTCCGGTGTCGGTATCGTCCTCAAGGACGAGCGCAGCGGCAAGGAAGAGCTGTTCAAGTA
CGAAGGTGGTCTGCGTGCGTTCGTTGAATACCTGAACACTAACAAGACTCCAGTCAACCAGGTGTTCCACTTCAACA
TCCAGCGCGAAGACGGCATTGGCGTGGAAATCGCCCTGCAGTGGAACGACAGCTTCAACGAGAATCTGTTGTGCTTC
ACCAACAACATTCCGCAGCGCGACGGCGGTACTCACCTGGTGGGTTTCCGTTCCGCTCTGACGCGTAACCTGAACAC
CTACATCGAAGCTGAAGGTCTGGCGAAGAAGCACAAAGTCGCCACTACCGGTGACGATGCCCGTGAAGGCCTGACCG
CGATCATTTCGGTAAAGGTGCCGGATCCGAAGTTCAGCTC
>S12D 380
CTGCACGGTGTAGGTGTTTCGGTAGTTAACGCTCTTTCCGAACAACTGGTCCTGACGGTTCGTCGTAGCGGCAAGAT
CTGGGAACAGACCTACGTACACGGTGTTCCGCAAGAGCCGATGAAAATCGTTGGCGACAGCGAAACCACTGGTACCC
AGATTCACTTCAAGGCTTCCAGCGAAACCTTCAAGAACATCCACTTCAGCTGGGACATTTTGGCCAAGCGGATTCGT
GAACTGTCCTTCCTCAACTCCGGTGTCGGTATCGTCCTCAAGGACGAGCGCAGCGGCAAGGAAGAGCTGTTCAAGTA
CGAAGGTGGTCTGCGTGCGTTCGTTGAATACCTGAACACTAACAAGACTCCAGTCAACCAGGTGTTCCACTTCAACA
TCCAGCGCGAAGACGGCATTGGCGTGGAAATCGCCCTGCAGTGGAACGACAGCTTCAACGAGAATCTGTTGTGCTTC
ACCAACAACATTCCGCAGCGCGACGGCGGTACTCACCTGGTGGGTTTCCGTTCCGCTCTGACGCGTAACCTGAACAC
CTACATCGAAGCTGAAGGTCTGGCGAAGAAGCACAAAGTCGCCACTACCGGTGACGATGCCCGTGAAGGCCTGACCG
CGATCATTTCGGTAAAGGTGCCGGATCCGAAGTTCAGCTC
>S09B 354
CTGCACGGTGTAGGTGTTTCGGTAGTTAACGCTCTTTCCGAACAACTGGTCTTGACGGTTCGTCGTAGCGGCAAGAT
CTGGGAACAGACCTACGTACACGGTGTTCCGCAAGAGCCGATGAAAATCGTTGGCGACAGCGAAACCACTGGTACCC
AGATTCACTTCAAGGCTTCCAGCGAAACCTTCAAGAATATCCACTTCAGCTGGGACATCCTGGCCAAGCGGATTCGT
GAGCTGTCCTTCCTCAACTCCGGTGTCGGTATCGTCCTCAAGGACGAGCGCAGCGGCAAGGAAGAGCTGTTCAAGTA
CGAAGGTGGTCTGCGTGCGTTCGTTGAATACCTGAACACTAACAAGACTCCAGTCAACCAGGTGTTCCACTTCAACA
TCCAGCGCGAAGACGGCATTGGCGTGGAAATCGCCCTGCAGTGGAACGACAGCTTCAACGAGAATCTGTTGTGCTTC
ACCAACAACATTCCGCAGCGCGACGGCGGTACTCACCTGGTGGGTTTCCGTTCCGCTCTGACGCGTAACCTGAACAC
CTACATCGAAGCTGAAGGTCTGGCGAAGAAGCACAAAGTCGCGACTACCGGTGACGATGCCCGTGAAGGCCTGACCG
CGATCATTTCGGTAAAGGTGCCGGATCCGAAGTTCAGCTC
>S06F 238
CTGCACGGTGTAGGTGTTTCGGTAGTTAACGCTCTTTCCGAACAACTGGTCCTGACGGTTCGTCGTAGCGGCAAGAT
CTGGGAACAGACCTACGTACACGGTGTTCCGCAAGAGCCGATGAAAATCGTTGGCGACAGCGAAACCACTGGTACCC
AGATTCACTTCAAGGCTTCCAGCGAAACCTTCAAGAATATCCACTTCAGCTGGGACATCCTGGCCAAGCGGATTCGT
GAGCTGTCCTTCCTCAACTCCGGTGTCGGTATCGTCCTCAAGGACGAGCGCAGCGGCAAGGAAGAGCTGTTCAAGTA
CGAAGGTGGTCTGCGTGCGTTCGTTGAATACCTGAACACTAACAAGACTCCGGTCAACCAGGTGTTTCACTTCAACA
TCCAGCGCGAAGACGGCATCGGCGTGGAAATCGCCCTGCAGTGGAACGACAGCTTCAACGAGAATCTGTTGTGCTTC

ACCAACAACATTCCGCAGCGCGACGGCGGTACTCACCTGGTGGGTTTCCGTTCTGCACTGACGCGTAACCTGAACAC
CTACATCGAAGCTGAAGGTCTGGCGAAGAAGCACAAAGTCGCCACTACCGGTGACGATGCCCGTGAAGGCCTGACCG
CAATCATTTCGGTGAAAGTGCCGGATCCGAAGTTCAGCTC
>S02G 207
CTGCACGGTGTAGGTGTTTCGGTAGTTAACGCTCTTTCCGAACAACTGGTCCTGACGGTTCGTCGTAGCGGCAAGAT
CTGGGAACAGACCTACGTACACGGTGTTCCGCAAGAGCCGATGAAAATCGTTGGCGACAGCGAAACCACTGGTACCC
AGATTCACTTCAAGGCTTCCAGCGAAACCTTCAAGAATATCCACTTCAGCTGGGACATCCTGGCCAAGCGGATTCGT
GAGCTGTCCTTCCTCAACTCCGGTGTCGGTATCGTCCTCAAGGACGAGCGCAGCGGCAAGGAAGAGCTGTTCAAGTA
CGAAGGTGGTCTGCGTGCGTTCGTTGAATACCTGAACACTAACAAGACTCCGGTCAACCAGGTGTTTCACTTCAACA
TCCAGCGCGAAGACGGCATCGGCGTGGAAATCGCCCTGCAGTGGAACGACAGCTTCAACGAGAATCTGTTGTGCTTC
ACCAACAACATTCCGCAGCGCGACGGCGGTACTCACCTGGTGGGTTTCCGTTCTGCACTGACGCGTAACCTGAACAC
CTACATCGAAGCTGAAGGTCTGGCGAAGAAGCACAAAGTCGCCACTACCGGTGACGATGCCCGTGAAGGCCTGACCG
CAATCATTTCGGTGAAAGTGCCGGATCCGAAGTTCAGCTC
>S09D 260
CTGCACGGTGTAGGTGTTTCGGTAGTTAACGCTCTTTCCGAACAACTGGTCCTGACGGTTCGTCGTAGCGGCAAGAT
CTGGGAACAGACCTACGTACACGGTGTTCCGCAAGAGCCGATGAAAATCGTTGGCGACAGCGAAACCACTGGTACCC
AGATTCACTTCAAGGCTTCCAGCGAAACCTTCAAGAACATCCACTTCAGCTGGGACATCCTGGCCAAGCGGATTCGT
GAGCTGTCCTTCCTCAACTCCGGTGTCGGTATCGTCCTCAAGGACGAGCGCAGCGGCAAGGAAGAGCTGTTCAAGTA
CGAAGGTGGTCTGCGTGCGTTCGTTGAATACCTGAACACTAACAAGACTCCGGTCAACCAGGTGTTTCACTTCAACA
TCCAGCGCGAAGACGGCATCGGCGTGGAAATCGCCCTGCAGTGGAACGACAGCTTCAACGAGAATCTGTTGTGCTTC
ACCAACAACATTCCGCAGCGCGACGGCGGTACTCACCTGGTGGGTTTCCGTTCTGCACTGACGCGTAACCTGAACAC
CTACATCGAAGCTGAAGGTCTGGCGAAGAAGCACAAAGTCGCCACTACCGGTGACGATGCCCGTGAAGGCCTGACCG
CAATCATTTCGGTGAAAGTGCCGGATCCGAAGTTCAGCTC
>S12E 285
CTGCACGGTGTAGGTGTTTCGGTAGTTAACGCTCTTTCCGAACAACTGGTCCTGACGGTTCGTCGTAGCGGCAAGAT
CTGGGAACAGACCTACGTACACGGTGTTCCGCAAGAGCCGATGAAAATCGTTGGCGACAGCGAAACCACTGGTACCC
AGATTCACTTCAAGGCTTCCAGCGAAACCTTCAAGAATATCCACTTCAGCTGGGACATCCTGGCCAAGCGGATTCGT
GAGCTGTCCTTCCTCAACTCCGGTGTCGGTATCGTCCTCAAGGACGAGCGCAGCGGCAAGGAAGAGCTGTTCAAGTA
CGAAGGTGGTCTGCGTGCGTTCGTTGAATACCTGAACACTAACAAGACTCCGGTCAACCAGGTGTTTCACTTCAACA
TCCAGCGCGAAGACGGCATCGGCGTGGAAATCGCCCTGCAGTGGAACGACAGCTTCAACGAGAATCTGTTGTGCTTC
ACCAACAACATTCCGCAGCGCGACGGCGGTACTCACCTGGTGGGTTTCCGTTCTGCACTGACGCGTAACCTGAACAC
CTACATCGAAGCTGAAGGTCTGGCGAAGAAGCACAAAGTCGCCACTACCGGTGACGATGCCCGTGAAGGCCTGACCG
CAATCATTTCGGTGAAAGTGCCGGATCCGAAGTTCAGCTC
>S06G 239
CTGCACGGTGTAGGTGTTTCGGTAGTTAACGCTCTTTCCGAACAACTGGTCCTGACGGTTCGTCGTAGCGGCAAGAT
CTGGGAACAGACCTATGTTCACGGTGTTCCGCAAGAGCCGATGAAAATCGTTGGCGACAGCGAAACCACCGGTACCC
AGATTCACTTCAAGGCTTCCAGCGAAACCTTCAAGAATATCCACTTCAGCTGGGACATCCTGGCCAAGCGGATTCGT
GAACTGTCCTTCCTCAACTCCGGTGTCGGTATCGTCCTCAAGGACGAGCGCAGCGGCAAGGAAGAGCTGTTCAAGTA
CGAAGGTGGTCTGCGTGCGTTCGTTGAATATCTGAACACCAACAAGACTCCGGTCAACCAGGTGTTTCACTTCAACA
TCCAGCGTGAAGACGGCATCGGCGTGGAAATCGCCCTGCAGTGGAACGACAGCTTCAACGAGAATCTGTTGTGCTTC
ACCAACAACATTCCGCAGCGCGACGGCGGTACTCACCTGGTCGGTTTCCGTTCCGCTCTGACGCGTAACCTGAACAC
CTACATCGAAGCTGAAGGTCTGGCGAAGAAGCACAAAGTCGCCACTACCGGTGACGATGCCCGTGAAGGCCTGACCG
CAATCATTTCGGTTAAGGTGCCGGATCCGAAGTTCAGCTC
>S09H 264
CTGCACGGTGTAGGTGTTTCGGTAGTTAACGCTCTTTCCGAACAACTGGTCCTGACGGTTCGTCGTAGCGGCAAGAT
CTGGGAACAGACCTATGTTCACGGTGTTCCGCAAGAGCCGATGAAAATCGTTGGCGACAGCGAAACCACCGGTACCC
AGATTCACTTCAAGGCTTCCAGCGAAACCTTCAAGAATATCCACTTCAGCTGGGACATCCTGGCCAAGCGGATTCGT
GAACTGTCCTTCCTCAACTCCGGTGTCGGTATCGTCCTCAAGGACGAGCGCAGCGGCAAGGAAGAGCTGTTCAAGTA
CGAAGGTGGTCTGCGTGCGTTCGTTGAATATCTGAACACCAACAAGACTCCGGTCAACCAGGTGTTTCACTTCAACA
TCCAGCGTGAAGACGGCATCGGCGTGGAAATCGCCCTGCAGTGGAACGACAGCTTCAACGAGAATCTGTTGTGCTTC
ACCAACAACATTCCGCAGCGCGACGGCGGTACTCACCTGGTCGGTTTCCGTTCCGCTCTGACGCGTAACCTGAACAC
CTACATCGAAGCTGAAGGTCTGGCGAAGAAGCACAAAGTCGCCACTACCGGTGACGATGCCCGTGAAGGCCTGACCG
CAATCATTTCGGTTAAGGTGCCGGATCCGAAGTTCAGCTC
>01G 73
CTGCACGGCGTAGGTGTGTCGGTCGTGAACGCCTTGTCCGAAGAACTGGTCCTGACCGTTCGCCGCAGCGGCAAGAT
CTGGGAACAGACCTACGTTCACGGAGTGCCGCAGGCACCGATGGCCATCGTTGGCGACAGCGACACCACGGGCACCC
AGATCCACTTCAAGGCTTCCAGCGAAACCTTCAAGAACATTCACTTCAGCTGGGACATCCTGGCCAAGCGAATTCGT

GAGCTGTCCTTCCTCAACTCCGGTGTCGGCATCGTCCTCAAGGATGAGCGCTCGGGCAAGGAAGAGCTGTTCAAATA
CGAAGGCGGTTTGCGTGCGTTCGTTGAATACCTGAACACCAACAAGACTGCGGTCAACCAGGTGTTCCACTTCAACA
TCCAGCGTGAAGACGGCATCGGCGTGGAAATCGCCCTGCAGTGGAACGACAGCTTCAACGAGAATCTGTTGTGCTTC
ACCAACAACATTCCGCAGCGCGATGGCGGTACTCACCTGGTGGGTTTCCGTTCTGCGCTGACGCGTAACCTCAACAC
CTACATCGAAGCGGAAGGTCTGGCCAAGAAACACAAGGTCGCGACCACCGGTGACGATGCCCGTGAAGGCCTGACCG
CGATCATCTCGGTGAAGGTGCCGGATCCGAAGTTCAGCTC
>S08G 255
CTGCACGGCGTAGGTGTATCGGTGGTGAACGCGCTGTCCGAACAGCTGGTTCTCACGGTGCGCCGCAGTGGCAAGAT
TTGGGAACAGACTTACGTCCATGGCGTGCCTCAGGCACCCATGGCGATCGTTGGTGACAGCGAGTCCACCGGCACCC
AGATTCACTTCAAGGCGTCGAGCGAAACCTTCAAGAATATCCACTTCAGCTGGGACATCCTGGCCAAGCGGATTCGC
GAACTGTCCTTCCTCAACTCCGGTGTCGGCATCGTCCTCAAGGACGAGCGCAGTGGCAAGGAAGAGCTGTTCAAGTA
TGAAGGTGGCCTGCGGGCGTTCGTTGAGTACCTGAACACCAACAAGACCCCGGTCAACCAGGTGTTCCACTTCAACA
TCCAGCGTGAAGACGGCATCGGCGTGGAAATCGCCCTGCAGTGGAACGACAGCTTCAACGAGAACCTGTTGTGCTTC
ACCAACAACATTCCGCAGCGCGACGGCGGTACCCACCTGGTGGGCTTCCGTTCGGCGCTGACCCGTAACCTGAACAA
CTACATCGAACAGGAAGGCCTGGCGAAGAAGCATAAAGTCGCCACTACCGGTGACGATGCCCGCGAAGGCCTGACAG
CGATCATTTCGGTGAAGGTGCCGGATCCGAAGTTCAGCTC
>07A 103
CTGCACGGTGTGGGTGTGTCGGTGGTGAACGCACTGTCCGAAGAACTGGTTCTGACTGTTCGCCGCAGCGGCAAGAT
CTGAGAACAGACCTACGTCCACGGAGTACCTCAGGCACCGATGGCCATCGTTGGTGACAGCGAAACCACCGGCACCC
AGATCCACTTCAAGGCTTCCAGCGAAACCTTCAAGAACATTCACTTCAGCTGGGACATCCTCGCCAAGCGCATTCGT
GAACTGTCCTTCCTCAACTCCGGTGTTGGCATCGTCCTCAAGGATGAGCGCAGCGGCAAGGAAGAGCTGTTCAAGTA
CGAAGGCGGTTTGCGTGCGTTCGTTGAGTACCTGAACACCAACAAGACCGCGGTCAACCAGGTGTTCCACTTCAACA
TTCAGCGTGAAGACGGCATCGGCGTGGAAATCGCCCTGCAGTGGAACGACAGCTTCAACGAGAACCTGTTGTGCTTC
ACCAACAACATTCCGCAGCGCGACGGCGGTACTCACCTTGTGGGCTTCCGTTCGGCACTGACGCGTAACCTGAACAA
CTACATCGAGCAGGAAGGTCTGGCGAAGAAGCACAAAGTCGCCACCACCGGTGACGATGCCCGTGAAGGCCTGACCG
CGATCATCTCGGTGAAAGTGCCGGATCCGAAGTTCAGCTC
>05B 113
CTGCACGGTGTAGGTGTGTCGGTAGTGAACGCACTGTCCGAAGAGCTGGTTCTGACTGTTCGCCGCAGCGGCAAGAT
CTGGGAACAGACCTACGTCCACGGCGTACCTCAGGCGCCGATGGCCATCGTTGGTGACAGCGAAACCACCGGTACCC
AGATCCACTTCAAGGCTTCCGGCGAAACCTTCAAGAACATTCACTTCAGCTGGGACATCCTGGCCAAGCGTATTCGT
GAACTGTCCTTCCTCAACTCCGGTGTCGGCATCGTCCTCAAGGACGAGCGCAGTGGCAAGGAAGAGCTGTTCAAGTA
CGAAGGCGGCCTGCGTGCGTTCGTTGAATACCTGAACACCAACAAGACTGCGGTCAACCAGGTGTTCCACTTCAACA
TCCACCGTGAAGACGGCATCGGCGTGGAAATCGCCCTGCAGTGGAACGACAGCTTCAACGAGAACCTGTTGTGCTTC
ACCAACAACATTCCGCAGCGCGACGGCGGTACTCACCTGGTGGGTTTCCGTTCGGCACTGACGCGTAACCTGAACAA
CTACATCGAGCAGGAAGGTCTGGCGAAGAAGCACAAAGTCGCCACCACCGGTGACGATGCCCGTGAAGGCCTGACCG
CGATCATCTCGGTGAAAGTGCCGGATCCGAAGTTCAGCTC
>11B 23
TTGCACGGTGTAGGCGTGTCGGTAGTAAACGCCCTGTCAGAAGAGTTGATCCTGACTGTTCGCCGTAGCGGCAAGAT
CTGGGAACAGACTTACGTGCATGGTGTACCTCAGGCACCCATGGCGATCGTCGGTGACAGCGAAACCACCGGCACCC
AGATTCACTTCAAGCCTTCGGCTGACACCTTCAAGAATATTCACTTCAGCTGGGACATCCTGGCCAAGCGGATTCGT
GAACTGTCGTTCCTCAACTCCGGTGTGGGTATCGTCCTCAAGGACGAACGCAGCGGCAAGGAAGAGCTGTTCAAGTA
CGAAGGTGGTCTGCGTGCATTCGTTGAATACCTGAACACCAACAAGACTGCGGTCAACCAGGTGTTCCACTTCAACA
TCCAGCGTGAAGACGGCATCGGCGTGGAAATCGCCCTGCAATGGAACGACAGCTTCAACGAGAACCTGTTGTGCTTC
ACCAACAACATTCCCCAGCGCGACGGCGGTACTCACTTGGTGGGTTTCCGTTCTGCACTGACGCGCAACCTGAACAC
CTACATCGAAGCCGAAGGCCTGGCCAAGAAGCACAAGGTCGCCACCACCGGTGACGATGCCCGTGAAGGCCTGACTG
CGATTATTTCGGTCAAGGTCCCGGATCCGAAGTTCAGCTC
>08C 128
TTGCACGGTGTAGGCGTGTCGGTAGTAAACGCCCTGTCAGAAGAGTTGATCCTGACCGTTCGCCGTAGCGGCAAGAT
CTGGGAACAGACTTACGTGCATGGCGTACCTCAGGCACCCATGGCGATCGTCGGTGACAGCGAAACCACCGGCACCC
AGATTCACTTCAAGCCTTCGGCTGACACCTTCAAGAATATTCACTTCAGCTGGGACATCCTGGCCAAGCGGATTCGT
GAACTGTCGTTCCTCAACTCCGGTGTGGGTATCGTCCTCAAGGACGAACGCAGCGGCAAGGAAGAGCTGTTCAAGTA
CGAAGGTGGTCTGCGTGCATTCGTTGAATACCTGAACACCAACAAGACTGCGGTCAACCAGGTGTTCCACTTCAACA
TCCAGCGTGAAGACGGCATCGGCGTGGAAATCGCCCTGCAATGGAACGACAGCTTCAACGAGAACCTGTTGTGCTTC
ACCAACAACATTCCCCAGCGCGACGGCGGTACTCACTTGGTGGGTTTCCGTTCTGCACTGACGCGCAACCTGAACAC
CTACATCGAAGCCGAAGGCCTGGCCAAGAAGCACAAGGTAGCCACCACCGGTGACGATGCCCGTGAAGGCCTGACTG
CGATTATTTCGGTCAAGGTCCCGGATCCGAAGTTCAGCTC

>06C 126
TTGCACGGTGTAGGCGTGTCGGTAGTAAACGCCCTGTCAGAAGAGTTGATCCTGACCGTTCGCCGTAGCGGCAAGAT
CTGGGAACAGACTTACGTGCATGGCGTACCTCAGGCACCCATGGCGATCGTCGGTGACAGCGAAACCACCGGCACCC
AGATTCACTTCAAGCCTTCGGCCGAGACTTTCAAGAATATCCATTTCAGCTGGGACATCCTGGCCAAGCGGATTCGT
GAACTGTCGTTCCTCAACTCCGGTGTGGGTATCGTCCTCAAGGACGAACGCAGCGGCAAGGAAGAGCTGTTCAAGTA
CGAAGGTGGTCTGCGTGCATTCGTTGAATACCTGAACACCAACAAGACTGCGGTCAACCAGGTGTTCCACTTCAACA
TCCAGCGTGAAGACGGCATCGGCGTGGAGATCGCCCTGCAATGGAACGACAGCTTCAACGAGAACCTGTTGTGCTTC
ACCAACAACATTCCCCAGCGCGACGGCGGTACTCACTTGGTGGGTTTCCGTTCTGCACTGACGCGCAACCTGAACAC
CTATATCGAAGCCGAAGGCCTGGCCAAGAAGCACAAGGTCGCCACCACCGGTGACGATGCCCGTGAAGGCCTGACTG
CGATTATTTCGGTCAAGGTTCCGGATCCCAAGTTCAGCTC
>S01G 295
CTGCACGGTGTAGGTGTATCGGTAGTGAACGCCCTCTCCGAGGAGCTGGTCCTGACCGTACGCCGCAGCGGCAAGAT
CTGGGAGCAGACCTACGTCCACGGCGTTCCACAGGCTCCGATGGCCATCGTTGGCGACAGCGAAACCACCGGCACCC
AGATCCACTTCAAGGCCTCGAGCGAAACCTTCAAGAACATCCACTTCAGCTGGGACATTCTGGCCAAGCGGATTCGT
GAACTGTCCTTCCTCAACTCCGGTGTCGGTATCGTCCTCAAGGATGAGCGCAGTGGCAAGGAGGAACTGTTCAAATA
CGAAGGTGGCTTGCGTGCGTTCGTTGAATACCTGAACACCAACAAGACTGCGGTCAACCAGGTGTTTCACTTCAACA
TCCAGCGTGAAGACGGCATCGGCGTAGAAATCGCCCTGCAGTGGAACGACAGCTTCAACGAGAATCTGTTGTGCTTC
ACCAACAACATTCCACAGCGCGATGGCGGTACTCACCTGGTGGGTTTCCGTTCCGCACTGACCCGTAACCTGAACAC
CTACATCGAAGCCGAAGGCCTGGCGAAGAAGCACAAGGTCGCCACTACCGGTGATGATGCTCGTGAAGGCCTGACGG
CGATTATTTCGGTGAAAGTCCCGGATCCGAAGTTCAGCTC
>S04D 316
CTGCACGGTGTAGGTGTATCGGTAGTGAACGCCCTCTCCGAGGAGCTGGTCCTGACCGTACGCCGCAGCGGCAAGAT
CTGGGAGCAGACCTACGTCCACGGCGTTCCACAGGCTCCGATGGCCATCGTTGGCGACAGCGAAACCACCGGCACCC
AGATCCACTTCAAGGCCTCGAGCGAAACCTTCAAGAACATCCACTTCAGCTGGGACATTCTGGCCAAGCGGATTCGT
GAACTGTCCTTCCTCAACTCCGGTGTCGGCATCGTCCTCAAGGATGAGCGCAGTGGCAAGGAGGAGCTGTTCAAATA
CGAAGGTGGCTTGCGTGCGTTCGTTGAATACCTGAACACCAACAAGACTGCGGTCAACCAGGTGTTTCACTTCAACA
TCCAGCGTGAAGACGGCATCGGCGTAGAAATCGCCCTGCAGTGGAACGACAGCTTCAACGAGAATCTGTTGTGCTTC
ACCAACAACATTCCACAGCGCGATGGCGGTACTCACCTGGTGGGTTTCCGTTCCGCACTGACCCGTAACCTGAACAC
CTACATCGAAGCCGAAGGCCTGGCGAAGAAGCACAAGGTCGCCACTACCGGTGATGATGCTCGTGAAGGCCTGACCG
CGATTATTTCGGTGAAAGTCCCGGATCCGAAGTTCAGCTC
>S05F 230
CTGCACGGTGTAGGTGTATCGGTAGTGAACGCCCTCTCCGAGGAGCTGGTCCTGACCGTACGCCGCAGCGGCAAGAT
CTGGGAGCAGACCTACGTCCACGGCGTTCCACAGGCTCCGATGGCCATCGTTGGCGACAGCGAAACCACCGGCACCC
AGATCCACTTCAAGGCCTCGAGCGAAACCTTCAAGAACATCCACTTCAGCTGGGACATTCTGGCCAAGCGGATTCGT
GAACTGTCCTTCCTCAACTCCGGTGTCGGCATCGTCCTCAAGGATGAGCGCAGTGGCAAGGAGGAGCTGTTCAAATA
CGAAGGTGGCTTGCGTGCGTTCGTTGAATACCTGAACACCAACAAGACTGCGGTCAACCAGGTGTTTCACTTCAACA
TCCAGCGTGAAGACGGCATCGGCGTAGAAATCGCCCTGCAGTGGAACGACAGCTTCAACGAGAATCTGTTGTGCTTC
ACCAACAACATTCCACAGCGCGATGGCGGTACTCACCTGGTGGGTTTCCGTTCCGCACTGACCCGTAACCTGAACAC
CTACATCGAAGCCGAAGGCCTGGCGAAGAAGCACAAGGTCGCCACTACCGGTGATGATGCTCGTGAAGGCCTGACCG
CGATTATTTCGGTGAAAGTCCCGGATCCGAAGTTCAGCTC
>S08A 345
CTGCACGGTGTAGGTGTATCGGTAGTGAACGCCCTCTCCGAGGAGCTGGTCCTGACCGTACGCCGCAGCGGCAAGAT
CTGGGAGCAGACCTACGTCCACGGCGTTCCACAGGCTCCGATGGCCATCGTTGGCGACAGCGAAACCACCGGCACCC
AGATCCACTTCAAGGCCTCGAGCGAAACCTTCAAGAACATCCACTTCAGCTGGGACATTCTGGCCAAGCGGATTCGT
GAACTGTCCTTCCTCAACTCCGGTGTCGGCATCGTCCTCAAGGATGAGCGCAGTGGCAAGGAGGAGCTGTTCAAATA
CGAAGGTGGCTTGCGTGCGTTCGTTGAATACCTGAACACCAACAAGACTGCGGTCAACCAGGTGTTTCACTTCAACA
TCCAGCGTGAAGACGGCATCGGCGTAGAAATCGCCCTGCAGTGGAACGACAGCTTCAACGAGAATCTGTTGTGCTTC
ACCAACAACATTCCACAGCGCGATGGCGGTACTCACCTGGTGGGTTTCCGTTCCGCACTGACCCGTAACCTGAACAC
CTACATCGAAGCCGAAGGCCTGGCGAAGAAGCACAAGGTCGCCACTACCGGTGATGATGCTCGTGAAGGCCTGACCG
CGATTATTTCGGTGAAAGTCCCGGATCCGAAGTTCAGCTC
>S08H 352
CTGCACGGTGTAGGTGTATCGGTAGTGAACGCCCTCTCCGAGGAGCTGGTCCTGACCGTACGCCGCAGCGGCAAGAT
CTGGGAGCAGACCTACGTCCACGGCGTTCCACAGGCTCCGATGGCCATCGTTGGCGACAGCGAAACCACCGGCACCC
AGATCCACTTCAAGGCCTCGAGCGAAACCTTCAAGAACATCCACTTCAGCTGGGACATTCTGGCCAAGCGGATTCGT
GAACTGTCCTTCCTCAACTCCGGTGTCGGTATCGTCCTCAAGGATGAGCGCAGTGGCAAGGAGGAACTGTTCAAATA
CGAAGGTGGCTTGCGTGCGTTCGTTGAATACCTGAACACCAACAAGACTGCGGTCAACCAGGTGTTTCACTTCAACA
TCCAGCGTGAAGACGGCATCGGCGTAGAAATCGCCCTGCAGTGGAACGACAGCTTCAACGAGAATCTGTTGTGCTTC

ACCAACAACATTCCACAGCGCGATGGCGGTACTCACCTGGTGGGTTTCCGTTCCGCACTGACCCGTAACCTGAACAC
CTACATCGAAGCCGAAGGCCTGGCGAAGAAGCACAAGGTCGCCACTACCGGTGATGATGCTCGTGAAGGCCTGACGG
CGATTATTTCGGTGAAAGTCCCGGATCCGAAGTTCAGCTC
>S07C 339
CTGCACGGTGTAAGTGTGTCGGTAGTGAACGCCCTCTCCGAAGAGCTGGTACTGACCGTTCGCCGCAGCGGCAAGAT
CTGGGAACAGACCTACGTCCACGGCGTGCCACAGGCTCCTATGGCCATCGTTGGCGACAGCGAAACCACCGGCACCC
AGATCCACTTCAAGGCCTCCAGCGAGACCTTCAAGAACATCCATTTCAGCTGGGACATCCTGGCCAAACGGATCCGT
GAGCTGTCCTTCCTCAACTCCGGTGTCGGCATCGTCCTCAAGGATGAGCGCAGCGGCAAGGAAGAGCTGTTCAAGTA
CGAAGGTGGCCTGCGTGCGTTCGTTGAATACCTGAACACCAACAAGACGGCGGTCAACCAGGTGTTCCACTTCAACA
TCCAGCGTGAAGACGGCATCGGCGTGGAAATCGCCCTGCAGTGGAACGACAGCTTCAACGAGAACCTGTTGTGCTTC
ACCAACAACATTCCACAGCGCGATGGCGGTACTCACCTGGTGGGTTTCCGTTCCGCACTGACGCGTAACCTCAACAC
CTACATCGAAGCAGAAGGCCTGGCGAAGAAACACAAGGTCGCCACCACCGGTGATGATGCTCGTGAAGGCCTGACCG
CGATTATCTCGGTGAAAGTCCCGGATCCGAAGTTCAGCTC
>S09G 359
TTGCACGGTGTAGGTGTGTCGGTGGTGAACGCTCTGTCCGAAGAGCTGATCCTCACGGTGCGCCGTAGTGGCAAGAT
CTGGGAACAGACCTACGTCCACGGTGTTCCACAAGAACCGATGAAAATCGTTGGCGACAGTGAGTCCACCGGTACGC
AGATCCACTTCAAGCCATCGGCTGAAACCTTCAAGAACATCCACTTCAGCTGGGACATCCTGGCCAAGCGGATTCGT
GAGCTGTCCTTCCTCAACTCCGGTGTTGGTATCGTCCTCAAGGACGAGCGCAGCGGCAAGGAAGAACTGTTCAAGTA
CGAAGGTGGCTTGCGCGCATTCGTTGAATACCTGAACACCAACAAGACTGCGGTCAACCAGGTGTTCCACTTCAACA
TCCAGCGTGAAGACGGCATCGGCGTGGAAATCGCCCTGCAGTGGAACGACAGCTTCAACGAGAACCTGTTGTGCTTC
ACCAACAACATTCCACAACGCGATGGCGGTACTCACCTGGTGGGTTTCCGTTCCGCGTTGACGCGTAACCTGAACAC
CTACATCGAAGCCGAGGGCCTGGCCAAAAAGCACAAAGTTGCCACCACCGGTGACGATGCGCGTGAAGGCCTGACGG
CGATTATTTCGGTGAAAGTGCCGGACCCGAAATTCAGCTC
>S11B 370
TTGCACGGTGTAGGTGTGTCGGTGGTGAACGCTCTGTCTGAAGAGCTGATCCTGACCGTACGCCGTAGCGGCAAGAT
CTGGGAACAGACCTACGTGCACGGTGTTCCGCAAGAACCGATGAAAATCGTTGGTGACAGTGAATCCACTGGTACGC
AGATCCATTTCAAGCCCTCGGCTGAAACCTTCAAGAACATCCACTTCAGCTGGGACATCCTCGCCAAGCGGATTCGT
GAACTGTCCTTCCTCAACTCCGGTGTGGGTATCGTCCTCAAGGACGAGCGCAGCGGCAAGGAAGAGCTGTTCAAGTA
CGAAGGCGGCCTGCGTGCGTTCGTTGAATACCTGAACACCAACAAGACTGCGGTCAACCAGGTGTTCCACTTCAATA
TCCAGCGTGAAGACGGCATTGGCGTTGAGATCGCCCTGCAGTGGAACGACAGCTTCAACGAGAACCTGTTGTGCTTC
ACCAACAACATTCCACAGCGCGATGGCGGTACTCACCTGGTGGGCTTCCGTTCCGCGTTGACGCGTAACCTGAACAC
CTACATCGAAGCCGAAGGCCTGGCCAAAAAGCATAAAGTTGCCACCACCGGTGACGACGCGCGCGAAGGCCTGACCG
CGATTATTTCGGTGAAAGTGCCCGACCCGAAATTCAGCTC
>07B 115
CTGCACGGTGTAGGTGTGTCGGTAGTGAACGCACTGTCCGAAGAGCTGGTTCTGACTGTTCGCCGCAGCGGCAAGAT
CTGGGAACAGACCTACGTCCACGGCGTACCTCAGGCACCGATGGCCATCGTTGGCGACAGCGAAACCACCGGCACCC
AGATCCACTTCAAGGNTTCCGGCGAAACCTTCAAGAACATTCACTTCAGCTGGGACATCCTGGCCAAGCGCATTCGT
GAACTGTCCTTCCTCAACTCCGGTGTCGGCATCGTCCTCAAGGATGAGCGCAGCGGCAAGGAAGAGCTGTTCAAGTA
CGAAGGCGGCCTGCGTGCGTTCGTTGAATACCTGAACACCAACAAGACTGCGGTCAACCAGGTGTTCCACTTCAACA
TCCAGCGTGAAGACGGCATCGGCGTGGAAATCGCCCTGCAGTGGAACGACAGCTTCAACGAGAACCTGTTGTGCTTC
ACCAACAACATTCCACAGCGCGACGGCGGTACTCACCTGGTGGGTTTCCGTTCGGCGCTGACGCGTAACCTGAACAA
CTACATCGAGCAGGAAGGTCTGGCGAAGAAGCACAAAGGCGCCACCACCGGTGACGATGCGCGTGAAGGCCTGACTG
CGATCATCTCGGTGAAAGTGCCGGATCCGAAGTTCAGCTC
>05C 125
TTGCACGGTGTGGGCGTGTCGGTGGTGAACGCACTGTCCGAAGAGCTGATCCTGACCGTTCGCCGCAGTGGCAAGAT
CTGGGAACAGACCTATGTCCATGGTGTACCGAAAGAGCCGATGAAGATCGTTGGCGACAGCGAATCGACCGGTACTC
AGATCCACTTCAAGCCATCGGCTGAAACCTTCAAGAACATCCATTTCAGCTGGGACATCCTGGCCAAGCGGATTCGT
GAATTGTCCTTCCTCAACTCCGGTGTGGGTATCGTCCTCAAGGACGAGCGCAGCGGCAAGGAAGAGCTGTTCAAGTA
CGAAGGCGGCCTGCGTGCGTTTGTTGAATACCTGAACACCAACAAGACTGCGGTCAACCAGGTGTTCCATTTCAACA
TCCAGCGCGAAGACGGCATCGGCGTGGAAATCGCCCTGCAGTGGAACGACAGCTTCAACGAGAACCTGTTGTGCTTC
ACCAACAACATTCCTCAGCGCGACGGTGGTACTCACCTCGTGGGTTTCCGTTCCGCACTGACGCGTAACCTGAACAC
CTACATCGAAGCCGAAGGCTTGGCAAAGAAGCACAAAGTTGCCACTACCGGTGACGATGCGCGCGAAGGCCTGACCG
CGATTATCTCGGTGAAAGTGCCGGATCCAAAGTTCAGCTC
>02G 74
CTGCACGGTGTGGGCGTTTCGGTAGTGAACGCTCTTTCTGAAGAGTTGATCCTGACTGTTCGCCGCAGTGGCAAGAT
CTGGGAACAGACCTACGTTCACGGTGTTCCAAAAGAACCGATGAAAATCGTCGGTGACAGTGAAACCACCGGTACGC
AGATCCACTTCAAGCCATCGGCTGAGACCTTCAAGAATATCCACTTTAGCTGGGACATCCTGGCCAAGCGTATTCGT

GAACTGTCCTTCCTGAACTCCGGTGTGGGTATCGTCCTCAAGGATGAGCGCAGCGGTAAGGAAGAGCTGTTCAAGTA
TGAAGGTGGCCTGCGCGCATTCGTTGAATACCTGAACACCAACAAGACCGCGGTCAACCAGGTGTTCCACTTCAACA
TTCAGCGTGAAGACGGCATCGGCGTGGAAATCGCTCTGCAGTGGAACGACAGCTTCAACGAGAACCTGTTGTGCTTC
ACCAACAACATTCCTCAGCGCGACGGTGGTACTCACCTGGTGGGTTTCCGTTCCGCACTGACGCGTAACCTGAACAC
CTACATTGAAGCCGAAGGCCTGGCCAAAAAGCACAAAGTCGCCACCACCGGTGACGATGCCCGTGAAGGCCTGACCG
CGATTATCTCGGTAAAAGTACCTGATCCGAAGTTCAGCTC
>S06B 234
CTGCACGGTGTGGGTGTGTCGGTAGTGAACGCGCTGTCCGAAGAGCTGATCCTCACTGTCCGTCGCAGCGGCAAGAT
CTGGGAACAGACCTACGTCCACGGTGTTCCACAGGAACCGATGAAAATCGTCGGCGACAGTGAAACCACCGGTACGC
AGATCCATTTCAAGCCATCGGCTGAAACCTTCAAGAATATCCACTTCAGCTGGGACATCCTGGCCAAGCGTATTCGT
GAACTGTCCTTCCTCAACTCCGGCGTCGGTATCGTCCTCAAGGATGAGCGCAGCGGTAAGGAAGAGCTGTTCAAGTA
TGAAGGCGGCTTGCGTGCATTCGTTGAATACCTGAACACCAACAAGACTGCGGTCAACCAGGTGTTCCACTTCAACA
TCCAGCGTGAAGACGGCATCGGCGTGGAAATCGCCCTGCAGTGGAACGACAGCTTCAACGAGAACCTGTTGTGCTTC
ACCAACAACATTCCTCAGCGTGACGGCGGTACTCACCTGGTGGGTTTCCGCTCCGCACTGACGCGTAACCTGAACAC
CTATATCGAAGCCGAAGGTCTGGCCAAAAAGCATAAAGTTGCCACCACCGGTGACGATGCCCGTGAAGGCCTCACCG
CGATTATCTCGGTGAAGGTGCCGGATCCGAAGTTCAGCTC
>S07E 341
TTGCACGGTGTGGGCGTTTCGGTAGTGAACGCACTGTCCGAAGAGCTGATCCTGACGGTGCGTCGCAGCGGCAAGAT
CTGGGAACAGACCTACGTTCACGGCGTTCCACAAGAACCGATGAAAATCGTCGGCGACAGTGAAACCACCGGCACCC
AGATTCACTTCAAGCCATCGGCTGAAACCTTCAAGAATATTCACTTCAGCTGGGACATCCTGGCCAAGCGTATTCGT
GAACTGTCGTTCCTTAACTCCGGCGTGGGTATCGTCCTCAAGGACGAGCGCAGCGGCAAGGAAGAGCTGTTCAAGTA
CGAAGGCGGCTTGCGTGCGTTCGTTGAATACCTGAACACCAACAAGACTGCGGTCAACCAGGTGTTCCACTTCAACA
TCCAGCGTGAAGACGGCATTGGCGTAGAAATCGCCCTGCAGTGGAACGACAGCTTCAACGAGAACCTGTTGTGCTTC
ACCAACAACATTCCTCAGCGCGATGGCGGTACTCACCTGGTGGGTTTCCGTTCGGCACTGACGCGTAACCTGAACAC
CTACATCGAAGCCGAAGGCCTGGCGAAGAAGCATAAAGTTGCCACCACCGGTGACGATGCCCGCGAAGGCTTGACCG
CGATTATCTCGGTGAAAGTACCGGACCCGAAGTTCAGCTC
>10E 58
CTGCACGGTGTTGGTGTGTCGGTGGTGAACGCACTGTCCAAAGAGCTGATTCTAACAGTGCGCCGTAGCGGCAAAAT
CTGGGAACAGACCTACATCCATGGTGTTCCACAAGAACCGATGAGGATCGTTGGCGAGAGTGAATCTACCGGTACGC
AGATCCACTTCAAGCCATCGGCTGAAACCTTCAAGAACATCCACTTTAGCTGGGACATCCTGGCCAAGCGGATTCGT
GAACTGTCCTTCCTCAACTCCGGTGTTGGCATCGTCCTCAAGGATGAGCGCAGCGGCAAGGAAGAGCTGTTCAAATA
CGAAGGCGGCCTGCGTGCGTTCGTTGAATACCTGAACACCAACAAGACTGCGGTCAACCAGGTGTTTCACTTCAATG
TTCAGCGTGAAGACGGCATCGGCGTGGAAATCGCCCTGCAGTGGAACGACAGCTTCAACGAGAACCTGTTGTGCTTC
ACCAACAACATTCCACAGCGCGACGGCGGTACTCACCTGGTGGGTTTCCGTTCCGCACTGACGCGTAACCTGAACAC
CTATATCGAAGCCGAAGGCCTGGCCAAGAAGCATAAAGTCGCCACCACCGGTGACGATGCCCGTGAAGGTCTGACTG
CGATTATTTCGGTGAAAGTACCGGACCCCAAGTTCAGCTC
>03A 3
CTGCACGGTGTTGGTGTGTCGGTGGTGAACGCACTGTCCAAAGAGCTGATTCTAACAGTGCGCCGTAGCGGCAAAAT
CTGGGAACAGACCTACATCCATGGTGTTCCACAAGAACCGATGAGGATCGTTGGCGAGAGTGAATCTACCGGTACGC
AGATCCACTTCAAGCCATCGGCTGAAACCTTCAAGAACATCCACTTTAGCTGGGACATCCTGGCCAAGCGGATTCGT
GAACTGTCCTTCCTCAACTCCGGTGTTGGCATCGTCCTCAAGGATGAGCGCAGCGGCAAGGAAGAGCTGTTCAAATA
CGAAGGCGGTCTGCGTGCGTTCGTTGAATACCTGAACACCAACAAGACTGCGGTCAACCAGGTGTTCCACTTCAATG
TTCAGCGTGAAGACGGCATCGGCGTGGAAATCGCCCTGCAGTGGAACGACAGCTTCAACGAGAACCTGTTGTGCTTC
ACCAACAACATTCCACAGCGCGACGGCGGTACGCACCTGGTGGGTTTCCGTTCCGCACTGACGCGTAACCTGAACAC
CTACATCGAAGCCGAAGGCCTGGCCAAGAAGCATAAAGTCGCCACCACCGGTGACGATGCCCGTGAAGGTCTGACTG
CGATTATTTCGGTGAAAGTACCGGACCCCAAGTTCAGCTC
>02B 110
CTGCACGGTGTTGGTGTGTCGGTGGTGAACGCACTGTCCAAAGAGCTGATTCTAACAGTGCGCCGTAGCGGCAAAAT
CTGGGAACAGACCTACATCCATGGTGTTCCACAAGAACCGATGAGGATCGTTGGCGAGAGTGAATCTACCGGTACGC
AGATCCACTTCAAGCCATCGGCTGAAACCTTCAAGAACATCCACTTTAGCTGGGACATCCTGGCCAAGCGGATTCGT
GAACTGTCCTTCCTCAACTCCGGTGTTGGCATCGTCCTCAAGGATGAGCGCAGCGGCAAGGAAGAGCTGTTCAAATA
CGAAGGCGGCCTGCGTGCGTTCGTTGAATACCTGAACACCAACAAGACTGCGGTCAACCAGGTGTTTCACTTCAATG
TTCAGCGTGAAGACGGCATCGGCGTGGAAATCGCCCTGCAGTGGAACGACAGCTTCAACGAGAACCTGTTGTGCTTC
ACCAACAACATTCCACAGCGCGACGGCGGTACTCACCTGGTGGGTTTCCGTTCTGCACTGACGCGTAACCTGAACAC
CTACATCGAAGCCGAAGGCCTGGCCAAGAAGCATAAAGTCGCCACCACCGGTGACGATGCCCGTGAAGGTCTGACTG
CGATTATTTCGGTGAAAGTACCGGACCCCAAGTTCAGCTC

>S06C 331
TTGCACGGCGTAGGTGTTTCGGTAGTGAACGCGCTGTCCGAAGAGCTGATTCTGACCGTCCGCCGTAGCGGCAAAAT
CTGGGAACAGACCTACGTCCACGGTGTTCCACAAGAACCGATGAAAATCGTCGGTGACAGTGAAACTACCGGCACGC
AGATTCACTTCAAACCATCGGCTGAAACCTTCAAGAATATTCACTTTAGCTGGGACATCCTGGCCAAGCGTATTCGT
GAGCTGTCGTTCCTCAACTCCGGTGTGGGTATCGTCCTCAAGGACGAGCGCAGCGGCAAGGAAGAGCTGTTCAAGTA
CGAAGGCGGCCTGCGTGCGTTCGTTGAATACCTGAACACCAACAAGACTGCGGTCAACCAGGTATTCCACTTCAACA
TCCAGCGCGAAGACGGCATTGGCGTAGAGATCGCCCTGCAGTGGAACGACAGCTTCAACGAGAACCTGTTGTGCTTC
ACCAACAACATTCCACAGCGCGACGGCGGTACTCACTTGGTGGGTTTCCGTTCGGCATTGACGCGTAACCTCAATAC
CTACATCGAAGCCGAAGGCTTGGCCAAGAAGCACAAGGTCGCCACCACCGGTGACGATGCGCGTGAAGGTTTGACCG
CGATTATCTCGGTGAAAGTGCCAGACCCCAAGTTCAGCTC
>S08E 349
TTGCACGGTGTAGGTGTTTCGGTAGTGAACGCACTGTCTGAAGAGCTGATCCTGACTGTGCGCCGTAGCGGCAAGAT
CTGGGAACAGACCTACGTCCACGGTGTTCCACAAGAGCCGATGAAAATCGTCGGTGACAGTGAGACTACTGGTACGC
AGATTCACTTCAAGCCATCGGCTGAAACTTTCAAGAATATTCACTTCAGCTGGGACATCCTGGCCAAGCGTATTCGT
GAGCTTTCCTTCCTCAACTCCGGTGTGGGTATCGTCCTCAAGGACGAACGCAGCGGCAAGGAAGAGCTGTTCAAGTA
CGAAGGCGGCCTGCGTGCGTTTGTTGAATACCTGAACACCAACAAGACCGCGGTCAACCAGGTGTTCCACTTCAATA
TCCAGCGTGAAGACGGCATCGGCGTAGAAATCGCCCTGCAGTGGAACGACAGCTTCAACGAGAACCTGTTGTGCTTC
ACCAACAACATTCCTCAGCGCGACGGCGGCACTCACTTGGTGGGTTTCCGTTCCGCACTGACGCGTAACCTGAACAC
CTATATCGAAGCCGAAGGTTTGGCCAAGAAGCACAAGGTCGCCACTACCGGTGACGACGCGCGTGAAGGCTTGACCG
CAATTATCTCGGTAAAAGTGCCGGACCCCAAGTTCAGCTC
>10B 22
TTGCACGGCGTAGGTGTTTCCGTAGTAAACGCCCTCTCCGAATTGCTGGTGCTGACCGTGCGTCGCAGCGGCAAGAT
TTGGGAGCAGACCTACGTTCACGGTGTTCCGCAGGAACCGATGAAGGTTGTTGGCGAAAGCGAAACTACCGGTACCC
AGATTCACTTCCGTCCTTCTGACGAAACCTTCAAGAACATCCACTTCAGCTGGGACATTCTGGCCAAGCGGATTCGT
GAACTGTCCTTCCTCAACTCCGGTGTCGGCATCGTCCTCAAGGATGAGCGCAGCGGCAAGGAAGAGCTGTTCAAGTA
CGAAGGCGGCCTGCGTGCGTTCGTTGAATACCTGAACACCAACAAGACTCCGGTCAACCAGGTGTTCCACTTCAACG
TCCAGCGTGAAGACGGCATCGGCGTGGAAATCGCCCTGCAGTGGAACGACAGCTTCAACGAGAACCTGTTGTGCTTC
ACCAACAACATTCCGCAGCGCGACGGCGGTACTCACCTGGTGGGTTTCCGTTCCGCGCTGACGCGTAACCTGAACAC
CTACATCGAAGCCGAAGGCCTGGCCAAGAAGCACAAAGTTGCCACCACCGGTGACGATGCCCGTGAAGGTCTGACTG
CGATTATTTCGGTGAAAGTACCGGACCCTAAGTTCAGCTC
>S06E 237
TTGCACGGTGTAGGTGTGTCGGTGGTGAACGCGCTGTCCGAAGAGCTGATCCTGACCGTTCGCCGTAGCGGCAAGAT
CTGGGAACAGACCTATGTCCACGGTGTTCCACAAGAACCGATGAAAATCGTCGGTGACAGTGAAGCCACGGGCACCC
AGATTCACTTCAAACCATCGGCCGATACCTTCAAGAATATCCACTTCAGCTGGGACATCCTGGCCAAGCGTATTCGT
GAGCTGTCTTTCCTTAACTCCGGTGTGGGTATCGTCCTCAAGGACGAGCGCAGCGGCAAGGAAGAGCTGTTCAAGTA
CGAAGGCGGTCTGCGTGCGTTCGTTGAGTACCTGAACACCAACAAGACTGCGGTCAACCAGGTGTTCCACTTCAACA
TCCAGCGTGAAGATGGCATCGGCGTAGAAATCGCTTTGCAGTGGAACGACAGCTTCAACGAGAACCTGTTGTGCTTC
ACCAACAATATTCCGCAGCGCGACGGCGGTACTCACTTGGTGGGTTTCCGTTCCGCACTGACGCGTAACCTGAACAC
CTACATCGAAGCCGAAGGTCTGGCCAAGAAGCATAAAGTCGCCACCACCGGTGACGATGCGCGTGAAGGCCTGACTG
CGATCATCTCGGTGAAAGTGCCGGACCCTAAGTTCAGCTC
>11E 59
CTGCACGGTGTAGGTGTGTCGGTTGTGAACGCCCTGTCCGAACAACTGGTCTTGACCGTTCGCCGCATCGGCAAAAT
CTGGGAACAGACCTACGTTCACGGTGTTCCAAAAGAGCCGATGAAAATCGTCGGTGAGAGTGAAACCACCGGCACCC
ATATCCACTTCAAACCGTCGGATCTGACCTTCAAGAACATCCATTTCAGCTGGGACATCCTGGCCAAACGGATTCAT
GAACTGTCCTTCCTCAACTCCGGTGTCGGCATCGTCCTCAAGGATGAGCGCGACGAAAAGGAAGAGTTGTTCAAGTA
CGAGGGCGGCCTGCGTGCGTTCGTTGAATACCTGAACACCAACAAGACCCCGGTCAACCAGGTGTTCCACTTCAATA
TCCAGCGCGAAGACGGCATCGGCGTGGAAATCGCCTTGCAGTGGAACGACAGCTTCAACGAGAACCTGTTGTGCTTC
ACCAACAACATTCCACAGCGCGATGGCGGTACTCACCTGGTGGGTTTCCGTTCCGCGCTGACGCGCAACCTGAACAC
CTACATCGAAGCCGAAGGTCTGGCGAAGAAGCATAAAGTCGCGACCACCGGTGACGATGCCCGTGAAGGCCTGACCG
CGATTATCTCGGTAAAAGTGCCGGATCCAAAGTTCAGCTC
>11C 131
CTGCACGGCGTAGGTGTATCGGTAGTGAACGCCCTGTCCGAGGAGCTGATCCTCACCGTGCGCCGTAGCGGCAAGAT
CTGGGAACAGACCTATGTCCACGGTGTTCCGCAAGAGCGGATGAAGATCGTTGGCGACAGCGAAACCACCGGTACCC
AGATCCACTTCAAGCCTTCGGCTGAAACCTTCAAGAACATCCACTTCAGCTGGGACGTCCTGGCCAAGCGGATCCGT
GAACTGTCCTTCCTCAACTCCGGTGTCGGCATCGTCCTCAAGGACGAGCGCAGCGGCAAGGAAGAGCTGTTCAAGTA
CGAAGGCGGCCTGCGGGCATTCGTTGAATACCTGAACACCAACAAGACTGCGGTCAACCAGGTGTTCCACTTCAACA
TCCAGCGTGAAGACGGCATCGGCGTGGAAATCGCCTTGCAGTGGAACGACAGCTTCAACGAGAACCTGTTGTGCTTC

ACCAACAACATTCCTCAGCGCGACGGCGGTACCCACCTGGTGGGCTTCCGTTCGGCCCTGACCCGTAACCTGAACAA
CTACATCGAGCAGGAAGGCCTGGCCAAGAAACACAAGGTCGCCACCACTGGTGACGATGCTCGTGAAGGCCTGACCG
CGATCATCTCGGTGAAGGTGCCGGATTCGAAGTTCAGCTC
>12A 108
CTTCACGGTGTAGGTGTGTCGGTGGTTAACGCCCTGTCCGAAGAGCTTATTCTCACGGTGCGACGCAGCGGCAAGAT
CTGGGAACAGACGTATGTCCACGGTGTTCCTCAAGAGCCGATGAGAATCGTTGGCGAGAGCGAAACAACCGGTACAC
AGATCCACTTCAAACCGTCGGACCAGACGTTCAAAAACATCCACTTCAGCTGGGACATCCTGGCCAAGCGGATTCGT
GAACTGTCCTTCCTCAACTCCGGTGTCGGCATCGTCCTCAAAGACGAACGCAGCGGGAAGGAAGAGCTGTTCAAGTA
TGAAGGTGGCCTGCGTGCATTCGTTGAATACCTGAACACCAACAAGACTCCGGTCAACCAGGTGTTCCACTTCAACA
TCCAGCGTGAAGACGGTATCGGCGTAGAAATCGCCCTGCAGTGGAACGACAGCTTCAACGAGAACCTGTTGTGCTTC
ACCAACAACATTCCACAGCGCGATGGCGGTACTCACCTGGTGGGTTTCCGTTCCGCACTGACGCGTAACCTGAACAC
TTACATCGAAGCTGAAGGCCTGGCGAAGAAGCACAAGGTTGCCACCACCGGTGACGACGCTCGTGAAGGCCTGACTG
CGATCATTTCGGTAAAGGTTCCGGATCCGAAGTTCAGCTC
>08F 68
CTTCACGGTGTAGGTGTGTCGGTGGTTAACGCCCTGTCCGAAGAGCTTATTCTCACGGTGCGACGCAGCGGCAAGAT
CTGGGAACAGACGTATGTCCACGGTGTTCCTCAAGAGCCGATGAGAATCGTTGGCGAGAGCGAAACAACCGGTACAC
AGATCCACTTCAAACCGTCGGATCAAACGTTCAAAAACATCCACTTCAGCTGGGACATCCTGGCCAAGCGGATTCGT
GAACTGTCCTTCCTCAACTCCGGTGTCGGCATCGTCCTCAAAGACGAACGCAGCGGGAAGGAAGAGCTGTTCAAGTA
TGAAGGTGGCCTGCGTGCATTCGTTGAATACCTGAACACCAACAAGACTCCGGTCAACCAGGTGTTCCACTTCAACA
TCCAGCGTGAAGACGGTATCGGCGTAGAAATCGCCCTGCAGTGGAACGACAGCTTCAACGAGAACCTGTTGTGCTTC
ACCAACAACATTCCACAGCGCGATGGCGGTACTCACCTGGTGGGTTTCCGTTCCGCACTGACGCGTAACCTGAACAC
TTACATCGAAGCTGAAGGCCTGGCGAAGAAGCACAAGGTTGCCACCACCGGTGACGACGCTTGTGAAGGCCTGACTG
CGATCATTTCGGTAAAGGTTCCGGATCCCAAGTTCAGCTC
>01B 109
TTGCACGGTGTAGGTGTTTCGGTGGTAAACGCCTTGTCCGAACTGCTGGTCCTGACGGTACGTCGCAGCGGCAAGAT
CTGGGAACAGACCTACGTCCACGGTGTTCCTCAGGCGCCTATGGCTATTGTGGGTGAAAGCGAAACCACGGGTACGC
AGATCCACTTCAAGCCTTCGGCTGAAACCTTCAAGAACATCCACTTCAGCTGGGACATCCTGGCCAAGCGGATTCGT
GAACTGTCCTTCCTCAACTCCGGTGTAGGTATCGTCCTCAAGGACGAGCGCAGCGGCAAGGAAGAGCTGTTCAAGTA
CGAAGGCGGCCTGCGTGCATTCGTTGATTACCTGAACACCAACAAGAACCCTGTGAACCAGGTGTTCCACTTCAATG
TTCAGCGTGAAGACGGCATCGGCGTAGAAATCGCCCTGCAGTGGAACGACAGCTTCAACGAGAACCTGTTGTGCTTC
ACCAACAACATTCCACAGCGCGATGGCGGCACCCACCTGGTGGGCTTCCGCTCTGCCCTGACGCGAAACCTCAACAC
CTACATCGAAGCTGAAGGCCTGGCCAAAAAGCACAAGGTTGCCACCACCGGTGATGACGCGCGTGAAGGCCTGACTG
CAATCATCTCGGTAAAAGTACCGGATCCGAAGTTCAGCTC
>03B 15
TTGCACGGTGTAGGTGTTTCGGTGGTAAACGCCTTGTCCGAACTGCTGGTCCTGACGGTACGTCGCAGCGGCAAGAT
CTGGGAACAGACCTACGTCCACGGTGTTCCTCAGGCGCCTATGGCTATTGTGGGTGAAAGCGAAACCACGGGTACGC
AGATCCACTTCAAGCCTTCGGCTGAAACCTTCAAGAACATCCACTTCAGCTGGGACATCCTGGCCAAGCGGATTCGT
GAACTGTCCTTCCTCAACTCCGGTGTAGGTATCGTCCTCAAGGACGAGCGCAGCGGCAAGGAAGAGCTGTTCAAGTA
CGAAGGCGGCCTGCGTGCATTCGTTGATTACCTGAACACCAACAAGAACCCTGTGAACCAGGTGTTCCACTTCAATG
TTCAGCGTGAAGACGGCATCGGCGTAGAAATCGCCCTGCAGTGGAACGACAGCTTCAACGAGAACCTGTTGTGCTTC
ACCAACAACATTCCACAGCGCGATGGCGGCACCCACCTGGTGGGCTTCCGCTCTGCCCTGACGCGAAACCTCAACAC
CTACATCGAAGCTGAAGGCCTGGCCAAAAAGCACAAGGTTGCCACCACCGGTGATGACGCGCGTGAAGGCCTGACTG
CAATCATCTCGGTGAAAGTACCGGATCCGAAGTTCAGCTC
>03B 111
TTGCACGGTGTAGGTGTTTCGGTGGTAAACGCCCTGTCCGAACTGCTGGTCCTGACTGTACGCCGCAGCGGCAAGAT
CTGGGAACAGACCTACGTCCACGGTGTTCCTCAGGCGCCTATGGCTATTGTGGGTGAAAGCGAAACCACGGGTACGC
AGATCCACTTCAAGCCTTCGGCTGAAACATTCAAGAATATCCACTTTAGCTGGGACATCCTGGCCAAGCGGATTCGT
GAACTGTCCTTCCTGAACTCCGGTGTGGGTATCGTCCTCAAGGACGAGCGCAGCGGCAAGGAGGAGCTGTTCAAGTA
CGAAGGTGGCCTGCGTGCATTCGTTGATTACCTGAACACCAACAAGAACCCTGTGAACCAGGTGTTCCACTTCAATG
TTCAGCGTGAAGACGGCATCGGCGTGGAAATCGCCCTGCAGTGGAACGACAGCTTCAACGAGAACCTGTTGTGCTTC
ACCAACAACATTCCACAGCGCGATGGTGGCACGCACTTGGTGGGCTTCCGCTCTGCCCTGACGCGTAACCTCAACAC
GTACATCGAAGCTGAAGGCCTGGCCAAGAAGCACAAGGTCGCCACCACCGGTGATGACGCCCGTGAAGGCTTGACCG
CGATCATCTCGGTGAAAGTGCCGGATCCAAAGTTCAGCTC
>08E 152
CTGCACGGTGTAGGTGTTTCGGTGGTTAACGCCCTGTCCGAACTGCTGGTGCTGACCGTGCGTCGCAGCGGCAAGAT
CTGGGAACAGACCTATGTCCACGGTGTTCCTCAGGCCCCGATGGCTATCGTAGGTGAAAGCGAAACCACCGGTACCC
AGATCCACTTCAAGCCTTCGGCTGAAACCTTCAAGAACATCCACTTCAGCTGGGACATCCTGGCCAAGCGGATTCGT

GAACTGTCGTTCCTTAACTCCGGTGTCGGCATCGTCCTCAAGGACGAGCGCAGCGGCAAGGAAGAGCTGTTCAAGTA
CGAAGGCGGCCTGCGTGCATTCGTTGATTACCTGAACACCAACAAGAACCCTGTAAACGAGGTGTTCCACTTCAATG
TCCAGCGTGAAGACGGCATTGGCGTAGAAATCGCCCTGCAGTGGAACGACAGCTTCAACGAGAACCTGTTGTGCTTC
ACCAACAACATTCCGCAGCGCGATGGCGGTACGCACCTGGTGGGCTTCCGCTCTGCCCTGACGCGTAACCTCAACAC
CTACATCGAAGCTGAAGGCCTGGCCAAGAAGCACAAAGTGGCCACCACCGGTGACGATGCCCGTGAAGGCCTGACGG
CGATCATTTCGGTGAAAGTGCCGGATCCGAAGTTCAGCTC
>09G 81
TTGCACGGCGTAGGTGTGTCGGTCGTGAACGCGCTGTCCGAAGAGCTGATCCTGACTGTTCGCCGTAGCGGCAAGAT
CTGGGAACAGACCTACGTGCACGGTGTTCCACAAGAACCGATGAAAATTGTCGGCGACAGCGAAACTACCGGTACGC
AGATTCACTTCAAACCGTCTGCCGAAACCTTCAAGAATATCCACTTCAGCTGGGACATCCTGGCCAAGCGTATTCGC
GAGTTGTCCTTCCTTAACTCCGGTGTGGGTATCGTCCTCAAGGACGAGCGCAGCGGCAAGGAAGAGCTGTTCAAATA
CGAAGGCGGTCTGCGTGCGTTCGTTGAATACCTGAACACCAACAAGACTGCGGTCAACCAGGTGTTCCACTTCAACA
TCCAGCGTGAAGACGGCATCGGCGTGGAAATCGCCCTGCAGTGGAACGACAGCTTCAACGAGAACCTGTTGTGCTTC
ACCAACAACATTCCACAGCGCGACGGTGGTACTCACTTGGTGGGTTTCCGTTCCGCACTGACGCGTAACCTCAACAC
CTATATCGAAGCCGAAGGTTTGGCCAAGAAGCATAAAGTCGCCACGACCGGTGACGATGCTCGCGAAGGCCTGACGG
CGATCATTTCGGTGAAAGTGCCGGACCCTAAGTTCAGCTC
>S01B 194
TTGCACGGTGTAGGCGTCTCGGTAGTGAATGCTTTGTCTGAAGAGCTGATCCTGACCGTTCGCCGCAGCGGCAAGAT
CTGGGAACAAACCTATGTCCATGGCGTTCCACAAGAACCGATGAAAATCGTCGGCGAAAGTGAAACCACCGGTACGC
AGATCCACTTCAAACCATCGGCTGATACGTTCAAGAATATCCACTTCAGCTGGGACATCCTGGCCAAGCGTATTCGT
GAACTTTCGTTCCTCAACTCCGGTGTGGGTATCGTCCTTAAGGACGAGCGCAGCGGCAAGGAAGAACTGTTCAAGTA
CGAAGGCGGCTTGCGTGCATTCGTTGAATACCTGAACACCAACAAGACTGCGGTCAACCAGGTGTTCCACTTCAACA
TCCAGCGTGAAGACGGCATCGGCGTGGAAATCGCCTTGCAGTGGAACGACAGTTTCAACGAGAACCTGTTGTGCTTC
ACCAACAACATCCCGCAGCGCGACGGCGGTACTCACCTGGTGGGTTTTCGCTCCGCGCTGACACGTAACCTGAACAC
CTACATCGAAGCTGAAGGCTTGGCCAAGAAGCACAAAGTTGCCACTACTGGTGATGACGCGCGCGAAGGCCTGACTG
CGATTATTTCGGTAAAAGTGCCGGATCCGAAGTTCAGCTC
>S09G 263
TTGCACGGTGTAGGCGTCTCGGTAGTGAATGCTTTGTCTGAAGAGCTGATCCTGACCGTTCGCCGCAGCGGCAAGAT
CTGGGAACAAACCTATGTCCATGGCGTTCCACAAGAACCGATGAAAATCGTCGGCGAAAGTGAAACCACCGGTACGC
AGATCCACTTCAAACCATCGGCTGATACGTTCAAGAATATCCACTTCAGCTGGGACATCCTGGCCAAGCGTATTCGT
GAACTTTCGTTCCTCAACTCCGGTGTGGGTATCGTCCTTAAGGACGAGCGCAGCGGCAAGGAAGAACTGTTCAAGTA
CGAAGGCGGCTTGCGTGCATTCGTTGAATACCTGAACACCAACAAGACTGCGGTCAACCAGGTGTTCCACTTCAACA
TCCAGCGTGAAGACGGCATCGGCGTGGAAATCGCCTTGCAGTGGAACGACAGTTTCAACGAGAACCTGTTGTGCTTC
ACCAACAACATCCCGCAGCGCGACGGCGGTACTCACCTGGTGGGTTTTCGCTCCGCGCTGACACGTAACCTGAACAC
CTACATCGAAGCTGAAGGCTTGGCCAAGAAGCACAAAGTTGCCACTACTGGTGATGACGCGCGCGAAGGCCTGACTG
CGATTATTTCGGTAAAAGTGCCGGATCCGAAGTTCAGCTC
>S02E 205
TTGCACGGTGTAGGCGTCTCGGTAGTGAATGCTTTGTCTGAAGAGCTGATCCTGACCGTTCGCCGCAGCGGCAAGAT
CTGGGAACAAACCTATGTCCATGGCGTTCCACAAGAACCGATGAAAATCGTCGGCGAAAGTGAAACCACCGGTACGC
AGATCCACTTCAAACCATCGGCTGATACGTTCAAGAATATCCACTTCAGCTGGGACATCCTGGCCAAGCGTATTCGT
GAACTTTCGTTCCTCAACTCCGGTGTGGGTATCGTCCTTAAGGACGAGCGCAGCGGCAAGGAAGAACTGTTCAAGTA
CGAAGGCGGCTTGCGTGCATTCGTTGAATACCTGAACACCAACAAGACTGCGGTCAACCAGGTGTTCCACTTCAACA
TCCAGCGTGAAGACGGCATCGGCGTGGAAATCGCCTTGCAGTGGAACGACAGTTTCAACGAGAACCTGTTGTGCTTC
ACCAACAACATCCCGCAGCGCGACGGCGGTACTCACCTGGTGGGTTTTCGCTCCGCGCTGACACGTAACCTGAACAC
CTACATCGAAGCTGAAGGCTTGGCCAAGAAGCACAAAGTTGCCACTACTGGTGATGACGCGCGCGAAGGCCTGACTG
CGATTATTTCGGTAAAAGTGCCGGATCCGAAGTTCAGCTC
>S10B 362
TTGCACGGTGTAGGCGTCTCGGTAGTGAATGCTTTGTCTGAAGAGCTGATCCTGACCGTTCGCCGCAGCGGCAAGAT
CTGGGAACAAACCTATGTCCATGGCGTTCCACAAGAACCGATGAAAATCGTCGGCGAAAGTGAAACCACCGGTACGC
AGATCCACTTCAAACCATCGGCTGATACGTTCAAGAATATCCACTTCAGCTGGGACATCCTGGCCAAGCGTATTCGT
GAACTTTCGTTCCTCAACTCCGGTGTGGGTATCGTCCTTAAGGACGAGCGCAGCGGCAAGGAAGAACTGTTCAAGTA
CGAAGGCGGCTTGCGTGCATTCGTTGAATACCTGAACACCAACAAGACTGCGGTCAACCAGGTGTTCCACTTCAACA
TCCAGCGTGAAGACGGCATCGGCGTGGAAATCGCCTTGCAGTGGAACGACAGTTTCAACGAGAACCTGTTGTGCTTC
ACCAACAACATCCCGCAGCGCGACGGCGGTACTCACCTGGTGGGTTTTCGCTCCGCGCTGACACGTAACCTGAACAC
CTACATCGAAGCTGAAGGCTTGGCCAAGAAGCACAAAGTTGCCACTACTGGTGATGACGCGCGCGAAGGCCTGACTG
CGATTATTTCGGTAAAAGTGCCGGATCCGAAGTTCAGCTC

>S06G 335
TTGCACGGTGTAGGCGTCTCGGTAGTGAATGCTTTGTCTGAAGAGCTGATCCTGACCGTTCGCCGCAGCGGCAAGAT
CTGGGAACAAACCTATGTCCATGGCGTTCCACAAGAACCGATGAAAATCGTCGGCGAAAGTGAAACCACCGGTACGC
AGATCCACTTCAAACCATCGGCTGATACGTTCAAGAATATCCACTTCAGCTGGGACATCCTGGCCAAGCGTATTCGT
GAACTTTCGTTCCTCAACTCCGGTGTGGGTATCGTCCTTAAGGACGAGCGCAGCGGCAAGGAAGAACTGTTCAAGTA
CGAAGGCGGCTTGCGTGCATTCGTTGAATACCTGAACACCAACAAGACTGCGGTCAACCAGGTGTTCCACTTCAACA
TCCAGCGTGAAGACGGCATCGGCGTGGAAATCGCCTTGCAGTGGAACGACAGTTTCAACGAGAACCTGTTGTGCTTC
ACCAACAACATCCCGCAGCGCGACGGCGGTACTCACCTGGTGGGTTTTCGCTCCGCGCTGACACGTAACCTGAACAC
CTACATCGAAGCTGAAGGCTTGGCCAAGAAGCACAAAGTTGCCACTACTGGTGATGACGCGCGCGAAGGCCTGACTG
CGATTATTTCGGTAAAAGTGCCGGATCCGAAGTTCAGCTC
>S06H 336
TTGCACGGCGTGGGCGTCTCGGTGGTCAACGCACTGTCCGAACTGCTGGTGCTGACCGTGCGTCGCAGCGGCAAGAT
CTGGGAACAGACCTACGTTCACGGTGTGCCTCAGGAACCGATGAAAATCGTCGGCGAGAGCGAAACCACCGGTACCC
AGATTCATTTCAAGGCTTCCGGCGAAACCTTCAAGAATATCCATTTCAGCTGGGACATCCTGGCCAAGCGGATCCGT
GAACTGTCCTTCCTCAACTCCGGTGTCGGCATCGTCCTCAAGGACGAGCGCAGCGGCAAGGAAGAACTGTTCAAGTA
CGAAGGCGGTCTGCGTGCGTTCGTTGAATACCTGAACACCAACAAGACGCCGGTCAACCAGGTGTTCCACTTCAACG
TCCAGCGTGAAGACGGCATCGGCGTGGAAATCGCCCTGCAGTGGAACGACAGCTTCAACGAGAACCTGTTGTGCTTC
ACCAACAACATCCCGCAGCGCGACGGCGGTACTCACCTGGTGGGTTTCCGCTCCGCACTGACGCGTAACCTGAATAC
CTACATCGAAGCGGAAGGCCTGGCGAAAAAGCACAAGGTCGCCACCACCGGTGACGATGCCCGTGAAGGCCTGACAG
CGATCATTTCGGTGAAAGTGCCGGATCCCAAGTTCAGCTC
>08G 176
TTGCACGGTGTGGGTGTTTCGGTGGTAAACGCCCTGTCCGAAGAGCTGATCCTCACCGTCCGCCGCAGCGGCAAAAT
CTGGGAACAGACCTATATCCACGGTGTGCCAAAAGAGCCGATGCGCATCGTCGGTGAAAGCGAAACCACCGGGACCC
AGATCCACTTCAAGCCCTCGCCTGAAACCTTCAAGAACATCCACTTCAGCTGGGACATCCTGGCCAAGCGGATTCGT
GAACTGTCCTTCCTCAACTCCGGTGTCGGCATCGTCCTCAAGGATGAGCGCAGCGGCAAGGAAGAACTGTTCAAGTA
CGAAGGTGGCCTGCGCGCATTCGTTGAATACCTGAATACGAACCGCACCCCGGTCAACCAGGTGTTCCACTTCAATA
TCCAGCGCGAAGACGGCATCGGCGTTGAAATCGCTCTGCAGTGGAACGACAGCTTCAACGAAAACCTGCTGTGCTTC
ACCAACAACATTCCGCAGCGCGATGGCGGTACTCACCTGGTGGGCTTCCGTTCGGCGCTGACGCGTAACCTGAACAC
CTACATCGAAGCCGAAGGCCTGGCGAAGAAGCACAAAGTCGCCACCACCGGTGACGACGCCCGCGAAGGCCTGACCG
CCATTATCTCGGTCAAGGTGCCGGACCCTAAGTTCAGCTC
>S05A 225
CTGCACGGTGTAGGTGTTTCTGTTGTTAACGCTTTGTCCGAACTGTTGCTGCTGACTGTACGTCGCAGCGGCAAAAT
CTGGGAGCAGACTTACGTTCATGGTGTTCCACAGGAACCTATGCGTATCGTCGGCGAAAGCGATACCACGGGCACCG
AGATCCACTTCAAGCCTTCTGCTGAAACCTTCAAGAACATCCACTTCAGCTGGGACATCCTGGCCAAGCGGATTCGT
GAACTGTCCTTCCTGAACTCCGGTGTTGGTATCGTCCTGAAAGACGAGCGCAGCGGCAAGGAAGAGCTGTTCAAATA
CGAAGGCGGCTTGCGTGCGTTCGTGGAATACCTGAACACCAACAAGACTCCGGTGAACCAGGTGTTCCACTTCAACA
TTCAGCGTGATGATGGCATTGGCGTCGAAATCGCTCTGCAGTGGAACGACAGCTTCAACGAGAACCTGTTGTGCTTC
ACCAACAACATTCCTCAGCGCGATGGCGGTACTCACCTGGTGGGTTTCCGTTCCGCACTGACGCGTAACCTGAACAC
TTACATCGAGCAGGAAGGTTTGGCCAAGAAGCACAAGGTCGCGACTACTGGTGACGATGCACGTGAAGGTCTGACCG
CAATCATCTCGGTAAAAGTGCCTGATCCGAAGTTCAGCTC
>S04F 222
CTGCACGGTGTAGGTGTTTCTGTTGTTAACGCTTTGTCCGAACTGTTGCTGCTGACTGTACGTCGCAGCGGCAAAAT
CTGGGAGCAGACTTACGTTCATGGTGTTCCACAGGAACCTATGCGTATCGTCGGCGAAAGCGATACCACGGGCACCG
AGATCCACTTCAAGCCTTCTGCTGAAACCTTCAAGAACATCCACTTCAGCTGGGACATCCTGGCCAAGCGGATTCGT
GAACTGTCCTTCCTGAACTCCGGTGTTGGTATCGTCCTGAAAGACGAGCGCAGCGGCAAGGAAGAGCTGTTCAAATA
CGAAGGCGGCTTGCGTGCGTTCGTGGAATACCTGAACACCAACAAGACTCCGGTGAACCAGGTGTTCCACTTCAACA
TTCAGCGTGATGATGGCATTGGCGTCGAAATCGCTCTGCAGTGGAACGACAGCTTCAACGAGAACCTGTTGTGCTTC
ACCAACAACATTCCTCAGCGCGATGGCGGTACTCACCTGGTGGGTTTCCGTTCCGCACTGACGCGTAACCTGAACAC
TTACATCGAGCAGGAAGGTTTGGCCAAGAAGCACAAGGTCGCGACTACTGGTGACGATGCACGTGAAGGTCTGACCG
CAATCATCTCGGTAAAAGTGCCTGATCCGAAGTTCAGCTC
>S03A 209
CTGCACGGTGTAGGTGTTTCTGTTGTTAACGCTTTGTCCGAACTGTTGCTGCTGACTGTACGTCGCAGCGGCAAAAT
CTGGGAGCAGACTTACGTTCATGGTGTTCCACAGGAACCTATGCGTATCGTCGGCGAAAGCGATACCACGGGCACCG
AGATCCACTTCAAGCCTTCTGCTGAAACCTTCAAGAACATCCACTTCAGCTGGGACATCCTCGCCAAGCGGATTCGT
GAACTGTCCTTCCTGAACTCCGGTGTTGGTATCGTCCTTAAAGACGAACGCAGCGGCAAGGAAGAGCTGTTCAAGTA
CGAAGGCGGCTTGCGTGCGTTCGTTGAATACCTGAACACCAACAAGACTCCGGTGAACCAGGTGTTCCACTTCAACA
TTCAGCGTGATGACGGCATTGGCGTCGAAATCGCTCTGCAGTGGAACGACAGCTTCAACGAGAACCTGTTGTGCTTC

ACCAACAACATTCCTCAGCGCGATGGCGGTACTCACCTGGTGGGGTTCCGTTCCGCACTGACGCGTAACCTGAACAC
CTACATCGAGCAGGAAGGTCTGGCCAAGAAGCACAAGGTCGCGACTACCGGTGACGATGCACGTGAAGGTCTGACCG
CGATCATTTCGGTGAAAGTGCCTGATCCGAAGTTCAGCTC
>S02B 298
CTGCACGGTGTAGGTGTTTCTGTTGTTAACGCTTTGTCCGAACTGTTGCTGCTGACTGTACGTCGCAGCGGCAAAAT
CTGGGAGCAGACTTACGTTCATGGTGTTCCACAGGAACCTATGCGTATCGTCGGCGAAAGCGATACCACGGGCACCG
AGATCCACTTCAAGCCTTCTGCTGAAACCTTCAAGAACATCCACTTCAGCTGGGACATCCTGGCCAAGCGGATTCGT
GAACTGTCCTTCCTGAACTCCGGTGTTGGTATCGTCCTTAAAGACGAACGCAGCGGCAAGGAAGAGCTGTTCAAGTA
CGAAGGCGGCTTGCGTGCGTTCGTTGAATACCTGAACACCAACAAGACTCCGGTGAACCAGGTGTTCCACTTCAACA
TTCAGCGTGATGACGGCATTGGCGTCGAAATCGCTCTGCAGTGGAACGACAGCTTCAACGAGAACCTGTTGTGCTTC
ACCAACAACATTCCTCAGCGCGATGGCGGTACTCACCTGGTGGGGTTCCGTTCCGCACTGACGCGTAACCTGAACAC
CTACATCGAGCAGGAAGGTCTGGCCAAGAAGCACAAGGTCGCGACTACCGGTGACGATGCACGTGAAGGTCTGACCG
CGATCATTTCGGTGAAAGTGCCTGATCCGAAGTTCAGCTC
>S03F 214
TTGCACGGTGTAGGTGTTTCCGTTGTTAACGCTTTGTCCGAACTGTTGCTGCTGACTGTACGTCGCAGCGGCAAGAT
CTGGGAGCAGACATACGTACATGGTGTTCCACAGGAACCTATGCGCATTGTCGGCGAAAGCGACACTACCGGCACTG
AGATCCATTTCAAGCCGTCTGCTGAAACCTTCAAGAATATTCACTTCAGCTGGGACATCCTGGCCAAGCGGATTCGT
GAACTGTCCTTCCTGAACTCGGGTGTCGGCATCGTCCTCAAGGACGAGCGCAGCGGTAAGGAAGAACTGTTCAAATA
CGAAGGCGGCTTGCGTGCGTTCGTTGAATACCTGAACACCAACAAGACTCCGGTGAATCAGGTGTTCCACTTCAACA
TCCAGAGAGATGACGGCATTGGTGTCGAAATCGCTCTTCAGTGGAACGACAGCTTCAACGAAAACCTGCTGTGCTTC
ACCAACAACATTCCGCAGCGAGATGGCGGTACTCACCTGGTGGGTTTCCGTTCTGCATTGACGCGTAACCTGAACAC
CTACATCGAGCAGGAAGGTCTGGCCAAGAAGCACAAGGTCGCGACTACCGGTGACGATGCACGTGAGGGTCTGACTG
CGATCATTTCGGTGAAAGTGCCTGATCCTAAGTTCAGCTC
>07C 31
TTGCACGGTGTAGGTGTCTCGGTGGTGAACGCGCTGTCCGAGGAACTGGTTCTGACGGTTCGCCGCAGCGGCAAGAT
CTGGGAACAGACCTATGTCCACGGGGTGCCTCAGGAACCGATGGAAATCGTCGGCGACAGCGAGACCACCGGCACGC
AGATCCACTTCAAGGCTTCGAGCGAAACCTTCAAGAACATCCACTTCAGCTGGGACATCCTGGCCAAGCGGATTCGT
GAACTGTCCTTCCTCAACTCCGGTGTCGGCATCGTCCTCAAGGACGAGCGCAGCGGCAAGGAGGAGCTATTCAAATA
CGAAGGTGGCCTGCGTGCGTTCGTTGAGTACCTGAACACCAACAAGACCCCGGTCAACCAAGTGTTCCACTTCAACG
TCCAGCGTGAAGACGGCATCGGCGTGGAAATCGCCCTGCAGTGGAACGACAGCTTCAACGAGAACCTGTTGTGCTTC
ACCAACAACATTCCACAGCGTGATGGCGGTACCCACCTGGTGGGCTTCCGTTCGGCGCTGACCCGTAACTTGAACAA
CTACATCGAACAGGAAGGCCTGGCGAAGAAGCACAAAGTCGCCACTACCGGTGACGATGCCCGTGAAGGCCTGACCG
CGATCATTTCGGTGAAGGTGCCGGATCCGAAGTTCAGCTC
>S12B 282
CTGCACGGCGTGGGCGTATCGGTTGTGAACGCACTGTCCGAAGAGCTGATCCTCACCGTTCGCCGCAGCGGCAAGAT
CTGGGAACAGACCTACGTGCATGGTGTGCCTCAAGCCCCGATGAAAATCGTCGGCGACAGCGAAACCACCGGCACCC
AGATCCATTTCAAACCGTCGGCTGAAACCTTCAAGAACATCCACTTCAGCTGGGACATCCTGGCCAAGCGGATTCGT
GAGCTGTCGTTCCTGAACTCCGGCGTGGGCATCCTGTTGAAGGACGAGCGCTCGGGCAAGGAAGAAGAGTTCAAGTA
CGAAGGCGGCCTGCGTGCGTTCGTCGAGTACCTCAACACCAACAAGACCCCGGTGAACGAAGTGTTCCACTTCAATG
TTCAGCGTGAAGACGGTGTGGGTGTGGAGATCGCGTTGCAGTGGAACGACAGCTTCAACGAGAACCTGTTGTGCTTC
ACCAACAACATTCCACAACGCGACGGCGGCACCCACCTGGTGGGCTTCCGTTCTGCGCTGACGCGTAACCTGAACAA
CTACATCGAGCAGGAAGGCCTGGCCAAGAAGAACAAGGTCGCGACCACCGGTGATGATGCCCGTGAAGGTCTGACCG
CGATCATCTCGGTAAAGGTACCCGACCCCAAGTTCAGCTC
>S12B 378
CTGCACGGCGTGGGCGTATCGGTTGTGAACGCACTGTCCGAAGAGCTGATCCTCACCGTTCGCCGCAGCGGCAAGAT
CTGGGAACAGACCTACGTGCATGGTGTGCCTCAAGCCCCGATGAAAATCGTCGGCGACAGCGAAACCACCGGCACCC
AGATCCATTTCAAACCGTCGGCTGAAACCTTCAAGAACATCCACTTCAGCTGGGACATCCTGGCCAAGCGGATTCGT
GAGCTGTCGTTCCTGAACTCCGGCGTGGGCATCCTGTTGAAGGACGAGCGCTCGGGCAAGGAAGAAGAGTTCAAGTA
CGAAGGCGGCCTGCGTGCGTTCGTCGAGTACCTCAACACCAACAAGACCCCGGTGAACGAAGTGTTCCACTTCAATG
TTCAGCGTGAAGACGGTGTGGGTGTGGAGATCGCGTTGCAGTGGAACGACAGCTTCAACGAGAACCTGTTGTGCTTC
ACCAACAACATTCCACAACGCGACGGCGGCACCCACCTGGTGGGCTTCCGTTCTGCGCTGACGCGTAACCTGAACAA
CTACATCGAGCAGGAAGGCCTGGCCAAGAAGAACAAGGTCGCGACCACCGGTGATGATGCCCGTGAAGGTCTGACCG
CGATCATCTCGGTAAAGGTACCCGACCCCAAGTTCAGCTC
>S07B 338
CTGCACGGCGTGGGCGTATCGGTTGTGAACGCACTGTCCGAAGAGCTGATCCTCACCGTTCGCCGCAGCGGCAAGAT
CTGGGAACAGACCTACGTGCATGGTGTGCCTCAAGCCCCGATGAAAATCGTCGGCGACAGCGAAACCACCGGCACCC
AGATCCATTTCAAACCGTCGGCTGAAACCTTCAAGAACATCCACTTCAGCTGGGACATCCTGGCCAAGCGGATTCGT

GAGCTGTCGTTCCTGAACTCCGGCGTGGGCATCCTGTTGAAGGACGAGCGCTCGGGCAAGGAAGAAGAGTTCAAGTA
CGAAGGCGGCCTGCGTGCGTTCGTCGAGTACCTCAACACCAACAAGACCCCGGTGAACGAAGTGTTCCACTTCAATG
TTCAGCGTGAAGACGGTGTGGGTGTGGAGATCGCGTTGCAGTGGAACGACAGCTTCAACGAGAACCTGTTGTGCTTC
ACCAACAACATTCCACAACGCGACGGCGGCACCCACCTGGTGGGCTTCCGTTCTGCGCTGACGCGTAACCTGAACAA
CTACATCGAGCAGGAAGGCCTGGCCAAGAAGAACAAGGTCGCGACCACCGGTGATGATGCCCGTGAAGGTCTGACCG
CGATCATCTCGGTAAAGGTACCCGACCCCAAGTTCAGCTC
>S12F 382
CTGCACGGCGTGGGCGTATCGGTTGTGAACGCACTGTCCGAAGAGCTGATCCTCACCGTTCGCCGCAGCGGCAAGAT
CTGGGAACAGACCTACGTGCATGGTGTGCCTCAAGCCCCGATGAAAATCGTCGGCGACAGCGAAACCACCGGCACCC
AGATCCATTTCAAACCGTCGGCTGAAACCTTCAAGAACATCCACTTCAGCTGGGACATCCTGGCCAAGCGGATTCGT
GAGCTGTCGTTCCTGAACTCCGGCGTGGGCATCCTGTTGAAGGACGAGCGCTCGGGCAAGGAAGAAGAGTTCAAGTA
CGAAGGCGGCCTGCGTGCGTTCGTCGAGTACCTCAACACCAACAAGACCCCGGTGAACGAAGTGTTCCACTTCAATG
TTCAGCGTGAAGACGGTGTGGGTGTGGAGATCGCGTTGCAGTGGAACGACAGCTTCAACGAGAACCTGTTGTGCTTC
ACCAACAACATTCCACAACGCGACGGCGGCACCCACCTGGTGGGCTTCCGTTCTGCGCTGACGCGTAACCTGAACAA
CTACATCGAGCAGGAAGGCCTGGCCAAGAAGAACAAGGTCGCGACCACCGGTGATGATGCCCGTGAAGGTCTGACCG
CGATCATCTCGGTAAAGGTACCCGACCCCAAGTTCAGCTC
>S03F 310
CTGCACGGCGTGGGCGTATCGGTTGTGAACGCACTGTCCGAAGAGCTGATCCTCACCGTTCGCCGCAGCGGCAAGAT
CTGGGAACAGACCTACGTGCATGGTGTGCCTCAAGCCCCGATGAAAATCGTCGGCGACAGCGAAACCACCGGCACCC
AGATCCATTTCAAACCGTCGGCTGAAACCTTCAAGAACATCCACTTCAGCTGGGACATCCTGGCCAAGCGGATTCGT
GAGCTGTCGTTCCTGAACTCCGGCGTGGGCATCCTGTTGAAGGACGAGCGCTCGGGCAAGGAAGAAGAGTTCAAGTA
CGAAGGCGGCCTGCGTGCGTTCGTCGAGTACCTCAACACCAACAAGACCCCGGTGAACGAAGTGTTCCACTTCAATG
TTCAGCGTGAAGACGGTGTGGGTGTGGAGATCGCGTTGCAGTGGAACGACAGCTTCAACGAGAACCTGTTGTGCTTC
ACCAACAACATTCCACAACGCGACGGCGGCACCCACCTGGTGGGCTTCCGTTCTGCGCTGACGCGTAACCTGAACAA
CTACATCGAGCAGGAAGGCCTGGCCAAGAAGAACAAGGTCGCGACCACCGGTGATGATGCCCGTGAAGGTCTGACCG
CGATCATCTCGGTAAAGGTACCCGACCCCAAGTTCAGCTC
>S12A 377
CTGCACGGCGTTGGTGTTTCGGTAGTAAACGCCCTGTCCGAACAACTGATCCTGACTGTTCGCCGTAGCGGCAAAGT
CTGGGAGCAGACCTACGTGCACGGTGTGCCACAGGCACCGATGGCGGTTGTCGGCGAAAGCGAAAGCACCGGTACCC
ACATCCATTTCAAGCCTTCGGCGGAAACCTTCAAGAACATCCACTTCAGCTGGGACATCCTGGCCAAGCGGATTCGT
GAACTGTCCTTCCTCAACTCCGGCGTCGGCATCCTGCTCAAGGACGAACGCAGCGGCAAAGAGGAGTACTTCAAGTA
CGAAGGTGGCCTGCGCGCGTTCGTTGAATACCTGAACACCAACAAGACCCCGGTCAATCAGGTGTTCCACTTCAACA
TTCAGCGTGAAGACGGTGTCGGTGTCGAAGTCGCTCTGCAGTGGAACGACAGCTTCAACGAAAACCTGCTGTGCTTC
ACCAACAACATTCCGCAGCGCGACGGTGGTACTCACCTGGTCGGCTTCCGTTCTTCGCTGACCCGCAGCCTCAATGC
TTACATCGAGCAAGAAGGCCTGGCCAAGAAGAACAAGGTGGCGACCACTGGCGACGACGCCCGTGAAGGCCTGACCG
CGATCATCTCGGTCAAGGTACCGGACCCGAAGTTCAGCTC
>S11A 273
CTGCACGGTGTAGGTGTGTCTGTAGTTAACGCCCTCTCTGAACAGCTGATTCTGACTGTTCGCCGCAGTGGCAAGAT
TTGGGAACAGACTTACGTTCATGGTGTTCCACAAGCCCCGATGAAAATCGTTGGTGACAGTGAAACCACCGGTACCC
ATATCCATTTCAAGCCGTCGTCTGAGACCTTCAAGAATATCCACTTCAGCTGGGACATCTTGGCCAAGCGGATTCGT
GAACTGTCTTTCCTTAACTCTGGTGTTGGCATCCTGCTGAAGGATGAGCGCTCGGGCAAGGAAGAGCACTTCAAGTA
CGAAGGGGGCCTGCGTGCCTTCGTTGAATACCTGAACACCAACAAGACTCCGGTCAACCAGGTATTCCACTTCAGCG
TTCAACGCGAAGACGGTGTGGGCGTAGAAATCGCCCTGCAGTGGAACGACAGTTTCAACGAGAACCTGTTGTGCTTC
ACCAACAACATTCCGCAGCGTGACGGTGGTACTCACCTGGTTGGTTTCCGCTCGGCATTGACCCGTAACCTGAACAA
CTACATTGAGCAGGAAGGCCTGGCCAAGAAGAACAAGGTATCGACCACGGGTGACGACGCCCGTGAAGGTCTGACCG
CGATCATCTCGGTGAAGGTTCCAGATCCTAAGTTCAGCTC
>S06A 329
CTGCACGGCGTTGGTGTTTCGGTGGTGAACGCCTTGTCCGAGCTGTTGGTACTGACTGTACGTCGCAGCGGCAAGAT
ATGGGAACAGACTTACGTGCACGGCGTACCGCAGGAGCCGATGAAAATCGTTGGCGAAAGCGAAACCACCGGTACCC
AGATCCATTTCAAGCCGTCGGCTGAAACCTTCAAGAATATCCACTTCAGCTGGGACATTCTGGCCAAGCGAATTCGC
GAACTGTCGTTCCTCAACTCCGGTGTGGGTATCTTCCTGAAGGATGAGCGCGCGGGCAAGGACGAAACATTCAAGTA
CGAGGGTGGCTTGCGTGCCTTCGTTGAGTACCTGAACACCAACAAGACTGCCGTGAACGAAGTGTTCCACTTCAACG
TGCAGCGCGATGACGGTGTGGGCGTAGAGATTGCCCTGCAGTGGAACGACAGCTTCAACGAAAACCTGTTGTGCTTC
ACCAACAACATTCCGCAGCGCGACGGCGGTACCCACCTGGTAGGATTCCGTTCCGCGCTGACGCGCAACCTGAACAC
CTACATCGAGCAGGAAGGCCTGGCCAAGAAACACAAGGTCGCGACAACTGGCGACGATGCCCGTGAAGGCCTCACGG
CGATTATTTCGGTAAAAGTGCCAGATCCGAAGTTCAGCTC

>S05G 327
CTGCACGGCGTTGGTGTTTCGGTGGTGAACGCCTTGTCCGAGCTGTTGGTACTGACTGTACGTCGCAGCGGCAAGAT
ATGGGAACAGACTTACGTGCACGGCGTACCGCAGGAGCCGATGAAAATCGTTGGCGAAAGCGAAACCACCGGTACCC
AGATCCATTTCAAGCCGTCGGCTGAAACCTTCAAGAATATCCACTTCAGCTGGGACATTCTGGCCAAGCGAATTCGC
GAACTGTCGTTCCTCAACTCCGGTGTGGGTATCTTCCTGAAGGATGAGCGCGCGGGCAAGGACGAAACATTCAAGTA
CGAGGGTGGCTTGCGTGCCTTCGTTGAGTACCTGAACACCAACAAGACTGCCGTGAACGAAGTGTTCCACTTCAACG
TGCAGCGCGATGACGGTGTGGGCGTAGAGATTGCCCTGCAGTGGAACGACAGCTTCAACGAAAACCTGTTGTGCTTC
ACCAACAACATTCCGCAGCGCGACGGCGGTACCCACCTGGTAGGATTCCGTTCCGCGCTGACGCGCAACCTGAACAC
CTACATCGAGCAGGAAGGCCTGGCCAAGAAACACAAGGTCGCGACAACTGGCGACGATGCCCGTGAAGGCCTCACGG
CGATTATTTCGGTAAAAGTGCCAGATCCGAAGTTCAGCTC
>S05H 232
CTGCACGGTGTTGGTGTTTCCGTGGTGAACGCCCTGTCCGAAAAGCTGGTACTGACGGTACGTCGTAGCGGCAAGAT
CTGGGAGCAAACCTACGTTCATGGTGTCCCTCAAGCGCCGATGGCGATTGTAGGTGACAGCGAAACGACGGGTACCC
ACATCCACTTCAAGCCTTCGGCTGAAACGTTCAAGAACATCCACTTCAGCTGGGACATCCTGGCCAAGCGGATCCGT
GAGCTGTCGTTCCTTAACTCGGGCGTCGGCATTCTGCTCAAGGATGAACGCAGCGGTAAGGAAGAGTTCTTCAAGTA
CGAAGGCGGCCTGCGGGCTTTCGTCGAGTACCTGAACACCAACAAGACCCCCGTGAACAACCAGGTGTTCCACTTCA
ACGTCCAGCGTGACGATGGTGTGGGCGTTGAAGTCGCTCTGCAGTGGAACGACAGCTTCAACGAGAACCTGCTGTGC
TTCACCAACAACATTCCGCAGCGCGATGGCGGCACTCACCTGGTAGGCTTCCGTTCTTCGCTGACCCGTAGCCTGAA
CAGCTACATCGAGCAGGAAGGCCTGGCCAAGAAGAACAAGGTCGCGACCACGGGCGACGACGCCCGTGAAGGCCTGA
CCGCGATCATCTCGGTCAAAGTCCCCGATCCCAAGTTCAGCTC
>12A 12
TTGCACGGTGTTGGCGTTTCGGTAGTTAACGCCCTGTCCGAATTACTGCTGCTGACGGTGCGTCGTAGCGGGCATAT
TTGGGAGCAGACCTACATCCACGGTGTACCGCAAGAGCCGATGCGCATCGTTGGCGACAGTGATAGCACCGGCACCC
AGATCCACTTTAAACCCTCGCCTTTGACCTTCAAAAACATCCACTTCAGCTGGGAGATTTTGGCTAAGCGGTTGCGC
GAACTGTCCTTCCTCAACTCCGGTGTTGGCATCGTGTTGAAGGATGAGCGCAGCGGTAAGGAAGAGCTGTTCAAGTA
CGAGGGTGGCTTGCGTGCCTTCGTTGAATACCTGAACACCAACAAGACCGTGGTTAACCAGGTTTTCCACTTCAACG
TGCAGCGCGATGATGGCGTCGGCGTTGAGGTTGCCTTGCAGTGGAACGACAGCTTCAACGAGAACCTGTTGTGCTTC
ACCAACAACATTCCACAACGCGATGGCGGCGCGCATTTGGCCGGTTTCCGTTCGGCGTTAACGCGCAACCTGAACAC
CTACATCGAACAAGAAGGCTTGGCCAAGAAACACAAGATCGCCACCACTGGCGACGATGCCCGCGAAGGCTTGACCG
CGATTATCTCGGTCAAAGTGCCGGACCCTAAGTTCAGCTC
>03G 75
TTGCACGGTGTTGGCGTTTCGGTAGTTAACGCCCTGTCCGAATTACTGCTGCTGACGGTGCGTCGTAGCGGGCATAT
TTGGGAGCAGACCTACATCCACGGTGTACCGCAAGAGCCGATGCGCATCGTTGGCGACAGTGATAGCACCGGCACCC
AGATCCACTTTAAACCCTCGCCTTTGACCTTCAAAAACATCCACTTCAGCTGGGAGATTTTGGCTAAGCGGTTGCGC
GAACTGTCCTTCCTCAACTCCGGTGTTGGCATCGTGTTGAAGGATGAGCGCAGCGGTAAGGAAGAGCTGTTCAAGTA
CGAGGGTGGCTTGCGTGCCTTCGTTGAATACCTGAACACCAACAAGACCGTGGTTAACCAGGTTTTCCACTTCAACG
TGCAGCGCGATGATGGCGTCGGCGTTGAGGTTGCCTTGCAGTGGAACGACAGCTTCAACGAGAACCTGTTGTGCTTC
ACCAACAACATTCCACAACGCGATGGCGGCGCGCATTTGGCCGGTTTCCGTTCGGCGTTAACGCGCAACCTGAACAC
CTACATCGAACAAGAAGGCTTGGCCAAGAAACACAAGATCGCCACCACTGGCGACGATGCCCGCGAAGGCTTGACCG
CGATTATCTCGGTCAAAGTGCCGGACCCTAAGTTCAGCTC
>10A 10
TTGCACGGTGTGGGCGTCTCGGTAGTTAACGCCCTGTCCGAATTACTGCTGCTGACGGTGCGTCGTAGCGGGCATAT
TTGGGAGCAGACCTACATCCACGGTGTACCGCAAGAGCCGATGCGCATCGTTGGCGACAGTGATAGCACCGGCACCC
AGATCCACTTTAAACCCTCGCCTTTGACCTTCAAAAACATCCACTTCAGCTGGGAGATTTTGGCTAAGCGGTTGCGC
GAACTGTCCTTCCTCAACTCCGGTGTTGGCATCGTGTTGAAGGATGAGCGCAGCGGTAAGGAAGAGCTGTTCAAGTA
CGAGGGTGGCTTGCGTGCCTTCGTTGAATACCTGAACACCAACAAGACCGTGGTTAACCAGGTTTTCCACTTCAACG
TGCAGCGCGATGATGGCGTCGGCGTTGAGGTTGCCTTGCAGTGGAACGACAGCTTCAACGAGAACCTGTTGTGCTTC
ACCAACAACATTCCACAGCGCGATGGCGGCGCGCATTTGGCCGGTTTCCGTTCGGCGTTAACGCGCAACCTGAACAC
CTACATCGAACAAGAAGGCTTGGCCAAGAAACACAAGATCGCCACCACTGGCGACGATGCCCGCGAAGGCTTGACCG
CGATTATCTCGGTCAAGGTTCCAGATCCTAAGTTCAGCTC
>12D 48
TTGCACGGTGTGGGCGTCTCGGTGGTTAACGCCCTGTCCGAATTACTGCTGCTGACGGTGCGTCGTAGCGGGCATAT
TTGGGAGCAGACCTACATCCACGGCGTACCGCAAGAGCCGATGCGCATCGTTGGCGACAGCGACTCCACCGGCACCC
AGATCCACTTCAAACCCTCGCCTTTGACCTTTAAAAACATCCACTTCAGCTGGGAAATTCTGGCTAAGCGCTTGCGT
GAATTGTCCTTCCTCAACTCCGGTGTTGGCATCGTGTTGAAGGATGAGCGCAGCGGTAAGGAAGAGTTGTTCAAGTA
CGAGGGCGGTTTGCGGGCGTTCGTTGAATACCTGAACACCAACAAGACCGTGGTTAACCAGATTTTCCACTTCAACG
TGCAGCGCGATGATGGCGTTGGCGTTGAGGTTGCCCTGCAGTGGAACGACAGCTTCAACGAGAACCTGTTGTGCTTC

ACCAACAACATTCCACAACGCGATGGCGGCGCGCATTTGGCCGGTTTCCGTTCGGCGTTAACGCGCAACCTGAACAC
CTACATCGAACAAGAAGGCTTGGCCAAAAAGCACAAGATCGCCACCACTGGCGACGATGCCCGCGAAGGCTTGACCG
CTATCATTTCGGTCAAGGTGCCAGACCCTAAGTTCAGCTC
>06C 30
TTGCACGGTGTTGGCGTCTCGGTGGTTAATGCCCTGTCTGAATTACTGCTGCTGACTGTGCGTCGTAGCGGGCATAT
TTGGGAACAGACCTACATCCACGGCGTACCGCAAGAGCCGATGCGCATCGTTGGTGATAGCGACACTACCGGCACCC
AGATTCACTTTAAACCCTCGCCTTTGACCTTCAAAAACATCCACTTCAGCTGGGAAATTCTGGCTAAGCGCTTGCGC
GAACTGTCCTTCCTCAACTCCGGTGTTGGCATCGTGTTGAAGGATGAGCGCAGCGGTAAGGAAGAATTGTTCAAATA
CGAGGGCGGCTTGCGGGCGTTCGTTGAATACCTGAACACCAACAAGACCGTGGTTAACCAGATTTTCCACTTCAACG
TGCAGCGCGATGATGGCGTTGGCGTTGAGGTTGCCCTGCAGTGGAACGACAGCTTCAACGAGAACCTGTTGTGCTTC
ACCAACAACATTCCACAACGTGATGGCGGCGCGCATTTGGCCGGTTTCCGTTCGGCGTTAACGCGCAACCTGAACAC
CTACATCGAACAAGAAGGTTTGGCCAAAAAGCACAAGATCGCCACCACTGGCGACGATGCGCGCGAAGGCTTGACCG
CGATTATCTCGGTCAAGGTGCCGGACCCTAAGTTCAGCTC
>09A 9
TTGCACGGTGTTGGTGTTTCGGTGGTTAACGCCCTGTCCGAATTGCTGCTGCTGACTGTGCGTCGTAGCGGGCATAT
TTGGGAGCAGACCTACATCCACGGCGTACCGCAAGAACCGATGCGCATCGTTGGTGACAGCGACTCCACTGGCACTC
AGATCCACTTTAAACCCTCGCCTTTGACCTTCAAAAACATCCACTTCAGCTGGGAGATTTTGGCTAAGCGATTGCGC
GAACTGTCCTTCCTCAACTCCGGTGTAGGCATCGTGTTGAAGGATGAGCGCAGCGGTAAGGAAGAGTTGTTCAAGTA
CGAGGGCGGCTTGCGGGCGTTCGTTGAATACCTGAACACCAACAAGACCGTGGTTAACCAGATTTTCCACTTCAACG
TGCAGCGTGATGATGGCGTCGGCGTTGAGGTTGCCCTGCAGTGGAACGACAGCTTCAACGAGAACCTGTTGTGCTTC
ACCAACAACATTCCACAACGCGATGGTGGCGCGCATTTGGCCGGTTTCCGTTCGGCGTTAACGCGCAACCTGAACAC
CTACATCGAACAAGAAGGCTTGGCCAAAAAGCACAAGATCGCCACCACGGGCGACGATGCTCGCGAAGGCTTGACCG
CTATCATTTCGGTCAAGGTGCCAGACCCTAAGTTCAGCTC
>04E 52
TTGCACGGTGTTGGCGTCTCGGTGGTTAACGCCCTGTCCGAATTACTGCTGCTGACTGTGCGTCGTAGCGGGCATAT
TTGGGAGCAGACCTACATCCACGGCGTTCCGCAAGAGCCGATGCGCATCGTTGGCGACAGTGATAGCACCGGCACCC
AGATCCACTTCAAACCCTCGCCTTTGACCTTCAAAAACATCCACTTCAGCTGGGAGATTTTGGCTAAGCGGTTGCGC
GAGCTGTCCTTCCTCAACTCCGGTGTAGGCATCGTGTTGAAGGATGAGCGCAGCGGTAAGGAAGAATTGTTCAAATA
CGAGGGCGGCTTGCGGGCGTTCGTTGAATACCTGAACACCAACAAGACCGTGGTTAACCAAATTTTCCACTTCAACG
TGCAGCGCGATGATGGCGTCGGCGTTGAGGTTGCCCTGCAGTGGAACGACAGTTTTAACGAGAACCTGTTGTGCTTC
ACCAACAACATTCCGCAACGTGACGGCGGCGCGCATTTGGCCGGTTTCCGTTCCGCGTTAACGCGTAACCTGAACAA
CTACATCGAACAAGAAGGTCTGGCCAAGAAGCACAAGATCGCCACCACCGGTGACGATGCCCGTGAAGGTTTGACCG
CGATTATCTCGGTCAAAGTACCGGACCCTAAGTTCAGCTC
>05B 898
TTGCACGGTGTGGGCGTCTCGGTGGTGAACGCGCTGTCCCATGAACTGCGCCTGACCATCCGTCGCCACAACAAGGT
CTGGGAACAGGTCTACCACCACGGCGTTCCGCAGTTCCCACTGCGCGAAGTGGGCGAGACCGATGGCTCCGGCACCG
AAGTTCACTTCAAGCCGTCCCCGGAGACCTTCAGCAACATCCACTTCAGTTGGGACATCCTGGCCAAGCGCATCCGC
GAGCTGTCCTTCCTCAACTCCGGCGTCGGCATCCTGCTGCGCGACGAGCGTACCGGCAAGGAGGAGCTGTTCAAGTA
CGAAGGCGGTCTGAAGGCCTTCGTCGAGTACCTGAACACCAACAAGACCGCGGTGAACGAGGTATTCCACTTCAACG
TCCAGCGTGAAGAGGACGGCGTGGGTGTGGAAGTCGCCTTGCAGTGGAACGACAGCTTCAACGAGAACCTGCTCTGC
TTCACCAACAACATCCCGCAGCGTGATGGCGGCACCCACCTGGCCGGTTTCCGTTCGGCGCTGACGCGTAACCTGAA
CAACTACATCGAGGCCGAAGGCCTGGCGAAGAAATTCAAGATCGCCACCACCGGCGACGATGCCCGCGAAGGCCTCA
CCGCGATCATCTCGGTGAAGGTACCGGACCCGAAGTTCAGCTC
>11D 948
TTGCACGGTGTGGGCGTCTCGGTGGTGAACGCGCTGTCCCATGAACTGCGCCTGACCATCCGTCGCCACAACAAGGT
CTGGGAACAGGTCTACCACCACGGCGTTCCGCAGTTCCCACTGCGCGAAGTGGGCGAGACCGATGGCTCCGGCACCG
AAGTTCACTTCAAGCCGTCCCCGGAGACCTTCAGCAACATCCACTTCAGTTGGGACATCCTGGCCAAGCGCATCCGC
GAGCTGTCCTTCCTCAACTCCGGCGTCGGCATCCTGCTGCGCGACGAGCGTACCGGCAAGGAGGAGCTGTTCAAGTA
CGAAGGCGGTCTGAAGGCCTTCGTCGAGTACCTGAACACCAACAAGACCGCGGTGAACGAGGTATTCCACTTCAACG
TCCAGCGTGAAGAGGACGGCGTGGGTGTGGAAGTCGCCTTGCAGTGGAACGACAGCTTCAACGAGAACCTGCTCTGC
TTCACCAACAACATCCCGCAGCGTGATGGCGGCACCCACCTGGCCGGTTTCCGTTCGGCGCTGACGCGTAACCTGAA
CAACTACATCGAGGCCGAAGGCCTGGCGAAGAAATTCAAGATCGCCACCACCGGCGACGATGCCCGCGAAGGCCTCA
CCGCGATCATCTCGGTGAAGGTACCGGACCCGAAGTTCAGCTC
>03C 883
TTGCACGGTGTGGGCGTCTCGGTGGTGAACGCGCTGTCCCATGAACTGCGCCTGACCATCCGTCGCCACAACAAGGT
CTGGGAACAGGTCTACCACCACGGCGTTCCGCAGTTCCCACTGCGCGAAGTGGGCGAGACCGATGGCTCCGGCACCG
AAGTTCACTTCAAGCCGTCCCCGGAGACCTTCAGCAACATCCACTTCAGTTGGGACATCCTGGCCAAGCGCATCCGC

GAGCTGTCCTTCCTCAACTCCGGCGTCGGCATCCTGCTGCGCGACGAGCGTACCGGCAAGGAGGAGCTGTTCAAGTA
CGAAGGCGGTCTGAAGGCCTTCGTCGAGTACCTGAACACCAACAAGACCGCGGTGAACGAGGTATTCCACTTCAACG
TCCAGCGTGAAGAGGACGGCGTGGGTGTGGAAGTCGCCTTGCAGTGGAACGACAGCTTCAACGAGAACCTGCTCTGC
TTCACCAACAACATCCCGCAGCGTGATGGCGGCACCCACCTGGCCGGTTTCCGTTCGGCGCTGACGCGTAACCTGAA
CAACTACATCGAGGCCGAAGGCCTGGCGAAGAAATTCAAGATCGCCACCACCGGCGACGATGCCCGCGAAGGCCTCA
CCGCGATCATCTCGGTGAAGGTACCGGACCCGAAGTTCAGCTC
>08D MC252
TTGCACGGTGTGGGCGTCTCGGTGGTGAACGCGCTGTCCCATGAACTGCGCCTGACCATCCGTCGCCACAACAAGGT
CTGGGAACAGGTCTACCACCACGGCGTTCCGCAGTTCCCACTGCGCGAAGTGGGCGAGACCGATGGCTCCGGCACCG
AAGTTCACTTCAAGCCGTCCCCGGAGACCTTCAGCAACATCCACTTCAGTTGGGACATCCTGGCCAAGCGCATCCGC
GAGCTGTCCTTCCTCAACTCCGGCGTCGGCATCCTGCTGCGCGACGAGCGTACCGGCAAGGAGGAGCTGTTCAAGTA
CGAAGGCGGTCTGAAGGCCTTCGTCGAGTACCTGAACACCAACAAGACCGCGGTGAACGAGGTATTCCACTTCAACG
TCCAGCGTGAAGAGGACGGCGTGGGTGTGGAAGTCGCCTTGCAGTGGAACGACAGCTTCAACGAGAACCTGCTCTGC
TTCACCAACAACATCCCGCAGCGTGATGGCGGCACCCACCTGGCCGGTTTCCGTTCGGCGCTGACGCGTAACCTGAA
CAACTACATCGAGGCCGAAGGCCTGGCGAAGAAATTCAAGATCGCCACCACCGGCGACGATGCCCGCGAAGGCCTCA
CCGCGATCATCTCGGTGAAGGTACCGGACCCGAAGTTCAGCTC
>03D 884
TTGCACGGTGTGGGCGTCTCGGTGGTGAACGCGCTGTCCCATGAACTGCGCCTGACCATCCGTCGCCACAACAAGGT
CTGGGAACAGGTCTACCACCACGGCGTTCCGCAGTTCCCACTGCGCGAAGTGGGCGAGACCGATGGCTCCGGCACCG
AAGTTCACTTCAAGCCGTCCCCGGAGACCTTCAGCAACATCCACTTCAGTTGGGACATCCTGGCCAAGCGCATCCGC
GAGCTGTCCTTCCTCAACTCCGGCGTCGGCATCCTGCTGCGCGACGAGCGTACCGGCAAGGAGGAGCTGTTCAAGTA
CGAAGGCGGTCTGAAGGCCTTCGTCGAGTACCTGAACACCAACAAGACCGCGGTGAACGAGGTATTCCACTTCAACG
TCCAGCGTGAAGAGGACGGCGTGGGTGTGGAAGTCGCCTTGCAGTGGAACGACAGCTTCAACGAGAACCTGCTCTGC
TTCACCAACAACATCCCGCAGCGTGATGGCGGCACCCACCTGGCCGGTTTCCGTTCGGCGCTGACGCGTAACCTGAA
CAACTACATCGAGGCCGAAGGCCTGGCGAAGAAATTCAAGATCGCCACCACCGGCGACGATGCCCGCGAAGGCCTCA
CCGCGATCATCTCGGTGAAGGTACCGGACCCGAAGTTCAGCTC
>05G MC231
TTGCACGGTGTGGGCGTCTCGGTGGTGAACGCGCTGTCCCATGAACTGCGCCTGACCATCCGTCGCCACAACAAGGT
CTGGGAACAGGTCTACCACCACGGCGTTCCGCAGTTCCCACTGCGCGAAGTGGGCGAGACCGATGGCTCCGGCACCG
AAGTTCACTTCAAGCCGTCCCCGGAGACCTTCAGCAACATCCACTTCAGTTGGGACATCCTGGCCAAGCGCATCCGC
GAGCTGTCCTTCCTCAACTCCGGCGTCGGCATCCTGCTGCGCGACGAGCGTACCGGCAAGGAGGAGCTGTTCAAGTA
CGAAGGCGGTCTGAAGGCCTTCGTCGAGTACCTGAACACCAACAAGACCGCGGTGAACGAGGTATTCCACTTCAACG
TCCAGCGTGAAGAGGACGGCGTGGGTGTGGAAGTCGCCTTGCAGTGGAACGACAGCTTCAACGAGAACCTGCTCTGC
TTCACCAACAACATCCCGCAGCGTGATGGCGGCACCCACCTGGCCGGTTTCCGTTCGGCGCTGACGCGTAACCTGAA
CAACTACATCGAGGCCGAAGGCCTGGCGAAGAAATTCAAGATCGCCACCACCGGCGACGATGCCCGCGAAGGCCTCA
CCGCGATCATCTCGGTGAAGGTACCGGACCCGAAGTTCAGCTC
>05C 899
TTGCACGGTGTGGGCGTCTCGGTGGTGAACGCGCTGTCCCATGAACTGCGCCTGACCATCCGTCGCCACAACAAGGT
CTGGGAACAGGTCTACCACCACGGCGTTCCGCAGTTCCCACTGCGCGAAGTGGGCGAGACCGATGGCTCCGGCACCG
AAGTTCACTTCAAGCCGTCCCCGGAGACCTTCAGCAACATCCACTTCAGTTGGGACATCCTGGCCAAGCGCATCCGC
GAGCTGTCCTTCCTCAACTCCGGCGTCGGCATCCTGCTGCGCGACGAGCGTACCGGCAAGGAGGAGCTGTTCAAGTA
CGAAGGCGGTCTGAAGGCCTTCGTCGAGTACCTGAACACCAACAAGACCGCGGTGAACGAGGTATTCCACTTCAACG
TCCAGCGTGAAGAGGACGGCGTGGGTGTGGAAGTCGCCTTGCAGTGGAACGACAGCTTCAACGAGAACCTGCTCTGC
TTCACCAACAACATCCCGCAGCGTGATGGCGGCACCCACCTGGCCGGTTTCCGTTCGGCGCTGACGCGTAACCTGAA
CAACTACATCGAGGCCGAAGGCCTGGCGAAGAAATTCAAGATCGCCACCACCGGCGACGATGCCCGCGAAGGCCTCA
CCGCGATCATCTCGGTGAAGGTACCGGACCCGAAGTTCAGCTC
>11H 952
TTGCACGGTGTGGGCGTCTCGGTGGTGAACGCGCTGTCCCATGAACTGCGCCTGACCATCCGTCGCCACAACAAGGT
CTGGGAACAGGTCTACCACCACGGCGTTCCGCAGTTCCCACTGCGCGAAGTGGGCGAGACCGATGGCTCCGGCACCG
AAGTTCACTTCAAGCCGTCCCCGGAGACCTTCAGCAACATCCACTTCAGTTGGGACATCCTGGCCAAGCGCATCCGC
GAGCTGTCCTTCCTCAACTCCGGCGTCGGCATCCTGCTGCGCGACGAGCGTACCGGCAAGGAGGAGCTGTTCAAGTA
CGAAGGCGGTCTGAAGGCCTTCGTCGAGTACCTGAACACCAACAAGACCGCGGTGAACGAGGTATTCCACTTCAACG
TCCAGCGTGAAGAGGACGGCGTGGGTGTGGAAGTCGCCTTGCAGTGGAACGACAGCTTCAACGAGAACCTGCTCTGC
TTCACCAACAACATCCCGCAGCGTGATGGCGGCACCCACCTGGCCGGTTTCCGTTCGGCGCTGACGCGTAACCTGAA
CAACTACATCGAGGCCGAAGGCCTGGCGAAGAAATTCAAGATCGCCACCACCGGCGACGATGCCCGCGAAGGCCTCA
CCGCGATCATCTCGGTGAAGGTACCGGACCCGAAGTTCAGCTC

>12F MC286
TTGCACGGTGTGGGCGTCTCGGTGGTGAACGCGCTGTCCCATGAACTGCGCCTGACCATCCGTCGCCACAACAAGGT
CTGGGAACAGGTCTACCACCACGGCGTTCCGCAGTTCCCACTGCGCGAAGTGGGCGAGACCGATGGCTCCGGCACCG
AAGTTCACTTCAAGCCGTCCCCGGAGACCTTCAGCAACATCCACTTCAGTTGGGACATCCTGGCCAAGCGCATCCGC
GAGCTGTCCTTCCTCAACTCCGGCGTCGGCATCCTGCTGCGCGACGAGCGTACCGGCAAGGAGGAGCTGTTCAAGTA
CGAAGGCGGTCTGAAGGCCTTCGTCGAGTACCTGAACACCAACAAGACCGCGGTGAACGAGGTATTCCACTTCAACG
TCCAGCGTGAAGAGGACGGCGTGGGTGTGGAAGTCGCCTTGCAGTGGAACGACAGCTTCAACGAGAACCTGCTCTGC
TTCACCAACAACATCCCGCAGCGTGATGGCGGCACCCACCTGGCCGGTTTCCGTTCGGCGCTGACGCGTAACCTGAA
CAACTACATCGAGGCCGAAGGCCTGGCGAAGAAATTCAAGATCGCCACCACCGGCGACGATGCCCGCGAAGGCCTCA
CCGCGATCATCTCGGTGAAGGTACCGGACCCGAAGTTCAGCTC
>PAO1 NC002
TTGCACGGTGTGGGCGTCTCGGTGGTGAACGCGCTGTCCCATGAACTACGCCTGACCATCCGTCGCCACAACAAGGT
CTGGGAACAGGTCTACCACCACGGCGTTCCGCAGTTCCCACTGCGCGAAGTGGGCGAGACCGATGGCTCCGGCACCG
AAGTTCACTTCAAGCCGTCCCCGGAGACCTTCAGCAACATCCACTTCAGTTGGGACATCCTGGCCAAGCGCATCCGC
GAGCTGTCCTTCCTCAACTCCGGCGTCGGCATCCTGCTGCGCGACGAGCGTACCGGCAAGGAGGAGCTGTTCAAGTA
CGAAGGCGGTCTGAAGGCCTTCGTCGAGTACCTGAACACCAACAAGACCGCGGTGAACGAGGTATTCCACTTCAACG
TCCAGCGTGAAGAGGACGGCGTGGGTGTGGAAGTCGCCTTGCAGTGGAACGACAGCTTCAACGAGAACCTGCTCTGC
TTCACCAACAACATCCCGCAGCGTGACGGCGGCACCCACCTGGCCGGTTTCCGTTCGGCGCTGACGCGTAACCTGAA
CAACTACATCGAGGCCGAAGGCCTGGCGAAGAAGTTCAAGATCGCCACCACCGGCGACGATGCCCGCGAAGGCCTCA
CCGCGATCATCTCGGTGAAGGTACCGGACCCGAAGTTCAGCTC
>02C 26
CTGCACGGTGTTGGCGTTTCGGTAGTGAACGCCCTTTCTGAGCAACTCATTCTGACCGTACGCCGTAGTGGCAAGGT
CTGGGAACAGACTTACGTCCACGGCGTACCGCAAGCGCCTATGGCGGTTGTTGGCGAAAGCGACAGCACCGGTACCC
ATATCCACTTCAAGCCGTCTGCTGACACCTTCAAGAATATCCACTTCAGCTGGGACATTCTGGCCAAGCGTATTCGC
GAGTTGTCGTTCCTGAACTCGGGTGTTGGCATTCTGTTGAAGGATGAGCGCAGTGGCAAAGAAGAGCTCTTCAAATA
TGAAGGCGGCCTGCGCGCATTTGTCGAGTATCTGAACACCAACAAGACGCCGGTCAACAGCCAGGTCTTCCACTTCA
GCACCCAGCGTGAAGATGGCGTCGGTGTTGAAGTTGCCCTGCAGTGGAACGACAGCTTCAACGAAAACCTGTTGTGC
TTCACCAACAACATTCCGCAGCGCGATGGTGGTACTCACCTGGTCGGCTTCCGTTCTTCGTTGACCCGCAGCCTGAA
TGCTTACATCGAGCAAGAAGGCCTGGCCAAGAAGAACAAGGTTGCAACCACGGGTGACGACGCCCGGGAAGGCCTGA
CCGCGATCATCTCGGTGAAGGTACCGGATCCGAAGTTCAGCTC
>S07E 245
CTGCACGGTGTTGGCGTTTCGGTAGTGAACGCCCTTTCTGAGCAACTCATTCTGACCGTACGCCGTAGTGGCAAGGT
CTGGGAACAGACTTACGTCCACGGCGTACCGCAAGCGCCTATGGCGGTTGTTGGCGAAAGCGACAGCACCGGTACCC
ATATCCACTTCAAGCCGTCTGCTGACACCTTCAAGAATATCCACTTCAGCTGGGACATTCTGGCCAAGCGTATTCGC
GAGTTGTCGTTCCTGAACTCGGGTGTTGGCATTCTGTTGAAGGATGAGCGCAGTGGCAAAGAAGAGCTCTTCAAATA
TGAAGGCGGCCTGCGCGCATTTGTCGAGTATCTGAACACCAACAAGACGCCGGTCAACAGCCAGGTCTTCCACTTCA
GCACCCAGCGTGAAGATGGCGTCGGTGTTGAAGTTGCCCTGCAGTGGAACGACAGCTTCAACGAAAACCTGTTGTGC
TTCACCAACAACATTCCGCAGCGCGATGGTGGTACTCACCTGGTCGGCTTCCGTTCTTCGTTGACCCGCAGCCTGAA
TGCTTACATCGAGCAAGAAGGCCTGGCCAAGAAGAACAAGGTTGCAACCACGGGTGACGACGCCCGGGAAGGCCTGA
CCGCGATCATCTCGGTGAAGGTGCCGGACCCGAAGTTCAGCTC
>02E 50
CTGCACGGTGTTGGCGTTTCGGTAGTGAACGCCCTTTCTGAGCAACTCATTCTGACCGTACGCCGTAGTGGCAAGGT
CTGGGAACAGACTTACGTCCACGGCGTACCGCAAGCGCCTATGGCGGTTGTTGGTGAAAGCGACAGCACCGGTACCC
ATATCCACTTCAAGCCGTCTGCTGACACCTTCAAGAATATCCACTTCAGCTGGGACATTCTGGCCAAGCGTATTCGC
GAGTTGTCGTTCTTGAACTCGGGTGTTGGCATTCTGTTGAAGGATGAGCGCAGTGGCAAAGAAGAGCTCTTCAAATA
TGAAGGCGGCCTGCGCGCATTTGTCGAGTATCTGAACACCAACAAGACGCCGGTCAACAGCCAGGTCTTCCACTTCA
GCACCCAGCGTGAAGATGGCGTCGGTGTTGAAGTTGCCCTGCAGTGGAACGACAGCTTCAACGAAAACCTGTTGTGC
TTTACCAATAACATTCCGCAGCGCGATGGTGGTACTCACCTGGTCGGCTTCCGTTCTTCGTTGACCCGCAGCCTGAA
TGCTTACATTGAGCAAGAAGGCCTGGCCAAGAAGAACAAGGTTGCAACCACGGGTGACGACGCCCGGGAAGGCCTGA
CCGCGATCATCTCGGTGAAGGTACCGGATCCGAAGTTCAGCTC
>01C 25
CTGCACGGTGTTGGCGTTTCGGTAGTGAACGCCCTTTCTGAGCAACTCATTCTGACCGTACGCAGTAGTGGCAAGGT
CTGGGAACAGACTTACGTCCACGGCGTACCGCAAGCGCCTATGGCGGTTGTTGGTGAAAGCGACAGCACCGGTACCC
ATATCCACTTCAAGCCGTCTGCTGACACCTTCAAGAATATCCACTTCAGCTGGGACATTCTGGCCAAGCGTATTCGC
GAGTTGTCGTTCTTGAACTCGGGTGTTGGCATTCTGTTGAAGGATGAGCGCAGTGGCAAAGAAGAGCTCTTCAAATA
TGAAGGCGGCCTGCGCGCATTTGTCGAGTATCTGAACACCAACAAGACGCCGGTCAACAGCCAGGTCTTCCACTTCA
GCACCCAGCGTGAAGATGGCGTCGGTGTTGAAGTTGCCCTGCAGTGGAACGACAGCTTCAACGAAAACCTGTTGTGC

TTTACCAATAACATTCCGCAGCGCGATGGTGGTACTCACCTGGTCGGCTTCCGTTCTTCGTTGACCCGCAGCCTGAA
TGCTTACATTGAGCAAGAAGGCCTGGCCAAGAAGAACAAGGTTGCAACCACGGGTGACGACGCCCGGGAAGGCCTGA
CCGCGATCATCTCGGTGAAGGTACCGGATCCGAAGTTCAGCTC

The *gyrB* nucleotide sequences of *P. aeruginosa* strains

>AU17152
TTGCACGGTGTGGGCGTCTCGGTGGTGAACGCGCTGTCCCATGAACTACGCCTGACCATCCGTCGCCACAACAAGGT
CTGGGAACAGGTCTACCACCACGGCGTTCCGCAGTTCCCACTGCGCGAAGTGGGCGAGACCGATGGCTCCGGCACCG
AAGTTCACTTCAAGCCGTCCCCGGAGACCTTCAGCAACATCCACTTCAGTTGGGACATCCTGGCCAAGCGCATCCGC
GAGCTGTCCTTCCTCAACTCCGGCGTCGGCATCCTGCTGCGCGACGAGCGTACCGGCAAGGAGGAGCTGTTCAAGTA
CGAAGGCGGTCTGAAGGCCTTCGTCGAGTACCTGAACACCAACAAGACCGCGGTGAACGAGGTATTCCACTTCAACG
TCCAGCGTGAAGAGGACGGCGTGGGTGTGGAAGTCGCCTTGCAGTGGAACGACAGCTTCAACGAGAACCTGCTCTGC
TTCACCAACAACATCCCGCAGCGTGACGGCGGCACCCACCTGGCCGGTTTCCGTTCGGCGCTGACGCGTAACCTGAA
CAACTACATCGAGGCCGAAGGCCTGGCGAAGAAGTTCAAGATCGCCACCACCGGCGACGATGCCCGCGAAGGCCTCA
CCGCGATCATCTCGGTGAAGGTACCGGACCCGAAGTTCAGCTC
>AU27282
TTGCACGGTGTGGGCGTCTCGGTGGTGAACGCGCTGTCCCATGAACTGCGCCTGACCATCCGTCGCCACAACAAGGT
CTGGGAACAGGTCTACCACCACGGCGTTCCGCAGTTCCCACTGCGCGAAGTGGGCGAGACCGATGGCTCCGGCACCG
AAGTTCACTTCAAGCCGTCCCCGGAGACCTTCAGCAACATCCACTTCAGTTGGGACATCCTGGCCAAGCGCATCCGC
GAGCTGTCCTTCCTCAACTCCGGCGTCGGCATCCTGCTGCGCGACGAGCGTACCGGCAAGGAGGAGCTGTTCAAGTA
CGAAGGCGGTCTGAAGGCCTTCGTCGAGTACCTGAACACCAACAAGACCGCGGTGAACGAGGTATTCCACTTCAACG
TCCAGCGTGAAGAGGACGGCGTGGGTGTGGAAGTCGCCTTGCAGTGGAACGACAGCTTCAACGAGAACCTGCTCTGC
TTCACCAACAACATCCCGCAGCGTGACGGCGGCACCCACCTGGCCGGTTTCCGTTCGGCGCTGACGCGTAACCTGAA
CAACTACATCGAGGCCGAAGGCCTGGCGAAGAAGTTCAAGATCGCCACCACCGGCGACGATGCCCGCGAAGGCCTCA
CCGCGATCATCTCGGTGAAGGTACCGGACCCGAAGTTCAGCTC
>AU28855
TTGCACGGTGTGGGCGTCTCGGTGGTGAACGCGCTGTCCCATGAACTGCGCCTGACCATCCGTCGCCACAACAAGGT
CTGGGAACAGGTCTACCACCACGGCGTTCCGCAGTTCCCACTGCGCGAAGTGGGCGAGACCGATGGCTCCGGCACCG
AAGTTCACTTCAAGCCGTCCCCGGAGACCTTCAGCAACATCCACTTCAGTTGGGACATCCTGGCCAAGCGCATCCGC
GAGCTGTCCTTCCTCAACTCCGGCGTCGGCATCCTGCTGCGCGACGAGCGTACCGGCAAGGAGGAGCTGTTCAAGTA
CGAAGGCGGTCTGAAGGCCTTCGTCGAGTACCTGAACACCAACAAGACCGCGGTGAACGAGGTATTCCACTTCAACG
TCCAGCGTGAAGAGGACGGCGTGGGTGTGGAAGTCGCCTTGCAGTGGAACGACAGCTTCAACGAGAACCTGCTCTGC
TTCACCAACAACATCCCGCAGCGTGACGGCGGCACCCACCTGGCCGGTTTCCGTTCGGCGCTGACGCGTAACCTGAA
CAACTACATCGAGGCCGAAGGCCTGGCGAAGAAGTTCAAGATCGCCACCACCGGCGACGATGCCCGCGAAGGCCTCA
CCGCGATCATCTCGGTGAAGGTACCGGACCCGAAGTTCAGCTC
>AU29014
TTGCACGGTGTGGGCGTCTCGGTGGTGAACGCGCTGTCCCATGAACTACGCCTGACCATCCGTCGCCACAACAAGGT
CTGGGAACAGGTCTACCACCACGGCGTTCCGCAGTTCCCACTGCGCGAAGTGGGCGAGACCGATGGCTCCGGCACCG
AAGTTCACTTCAAGCCGTCCCCGGAGACCTTCAGCAACATCCACTTCAGTTGGGACATCCTGGCCAAGCGCATCCGC
GAGCTGTCCTTCCTCAACTCCGGCGTCGGCATCCTGCTGCGCGACGAGCGTACCGGCAAGGAGGAGCTGTTCAAGTA
CGAAGGCGGTCTGAAGGCCTTCGTCGAGTACCTGAACACCAACAAGACCGCGGTGAACGAGGTATTCCACTTCAACG
TCCAGCGTGAAGAGGACGGCGTGGGTGTGGAAGTCGCCTTGCAGTGGAACGACAGCTTCAACGAGAACCTGCTCTGC
TTCACCAACAACATCCCGCAGCGTGACGGCGGCACCCACCTGGCCGGTTTCCGTTCGGCGCTGACGCGTAACCTGAA
CAACTACATCGAGGCCGAAGGCCTGGCGAAGAAGTTCAAGATCGCCACCACCGGCGACGATGCCCGCGAAGGCCTCA
CCGCGATCATCTCGGTGAAGGTACCGGACCCGAAGTTCAGCTC
>AU29142
TTGCACGGTGTGGGCGTCTCGGTGGTGAACGCGCTGTCCCATGAACTACGCCTGACCATCCGTCGCCACAACAAGGT
CTGGGAACAGGTCTACCACCACGGCGTTCCGCAGTTCCCACTGCGCGAAGTGGGCGAGACCGATGGCTCCGGCACCG
AAGTTCACTTCAAGCCGTCCCCGGAGACCTTCAGCAACATCCACTTCAGTTGGGACATCCTGGCCAAGCGCATCCGC
GAGCTGTCCTTCCTCAACTCCGGCGTCGGCATCCTGCTGCGCGACGAGCGTACCGGCAAGGAGGAGCTGTTCAAGTA
CGAAGGCGGTCTGAAGGCCTTCGTCGAGTACCTGAACACCAACAAGACCGCGGTGAACGAGGTATTCCACTTCAACG
TCCAGCGTGAAGAGGACGGCGTGGGTGTGGAAGTCGCCTTGCAGTGGAACGACAGCTTCAACGAGAACCTGCTCTGC
TTCACCAACAACATCCCGCAGCGTGACGGCGGCACCCACCTGGCCGGTTTCCGTTCGGCGCTGACGCGTAACCTGAA
CAACTACATCGAGGCCGAAGGCCTGGCGAAGAAGTTCAAGATCGCCACCACCGGCGACGATGCCCGCGAAGGCCTCA
CCGCGATCATCTCGGTGAAGGTACCGGACCCGAAGTTCAGCTC

>AU16821
TTGCACGGTGTGGGCGTCTCGGTGGTGAACGCGCTGTCCCATGAACTACGCCTGACCATCCGTCGCCACAACAAGGT
CTGGGAACAGGTCTACCACCACGGCGTTCCGCAGTTCCCACTGCGCGAAGTGGGCGAGACCGATGGCTCCGGCACCG
AAGTTCACTTCAAGCCGTCCCCGGAGACCTTCAGCAACATCCACTTCAGTTGGGACATCCTGGCCAAGCGCATCCGC
GAGCTGTCCTTCCTCAACTCCGGCGTCGGCATCCTGCTGCGCGACGAGCGTACCGGCAAGGAGGAGCTGTTCAAGTA
CGAAGGCGGTCTGAAGGCCTTCGTCGAGTACCTGAACACCAACAAGACCGCGGTGAACGAGGTATTCCACTTCAACG
TCCAGCGTGAAGAGGACGGCGTGGGTGTGGAAGTCGCCTTGCAGTGGAACGACAGCTTCAACGAGAACCTGCTCTGC
TTCACCAACAACATCCCGCAGCGTGACGGCGGCACCCACCTGGCCGGTTTCCGTTCGGCGCTGACGCGTAACCTGAA
CAACTACATCGAGGCCGAAGGCCTGGCGAAGAAGTTCAAGATCGCCACCACCGGCGACGATGCCCGCGAAGGCCTCA
CCGCGATCATCTCGGTGAAGGTACCGGACCCGAAGTTCAGCTC
>AU20339
TTGCACGGTGTGGGCGTCTCGGTGGTGAACGCGCTGTCCCATGAACTACGCCTGACCATCCGTCGCCACAACAAGGT
CTGGGAACAGGTCTACCACCACGGCGTTCCGCAGTTCCCACTGCGCGAAGTGGGCGAGACCGATGGCTCCGGCACCG
AAGTTCACTTCAAGCCGTCCCCGGAGACCTTCAGCAACATCCACTTCAGTTGGGACATCCTGGCCAAGCGCATCCGC
GAGCTGTCCTTCCTCAACTCCGGCGTCGGCATCCTGCTGCGCGACGAGCGTACCGGCAAGGAGGAGCTGTTCAAGTA
CGAAGGCGGTCTGAAGGCCTTCGTCGAGTACCTGAACACCAACAAGACCGCGGTGAACGAGGTATTCCACTTCAACG
TCCAGCGTGAAGAGGACGGCGTGGGTGTGGAAGTCGCCTTGCAGTGGAACGACAGCTTCAACGAGAACCTGCTCTGC
TTCACCAACAACATCCCGCAGCGTGACGGCGGTACCCACCTGGCCGGTTTCCGTTCGGCGCTGACGCGTAACCTGAA
CAACTACATCGAGGCCGAAGGCCTGGCGAAGAAGTTCAAGATCGCCACCACCGGCGACGATGCCCGCGAAGGCCTCA
CCGCGATCATCTCGGTGAAGGTACCGGACCCGAAGTTCAGCTC
>AU23316
TTGCACGGTGTGGGCGTCTCGGTGGTGAACGCGCTGTCCCATGAGCTGCGCCTGACCATCCGTCGCCACAACAAGGT
CTGGGAACAGGTCTACCACCACGGCGTTCCGCAGTTCCCACTGCGCGAAGTGGGCGAGACCGATGGCTCCGGTACCG
AAGTTCACTTCAAGCCGTCCCCGGAGACCTTCAGCAACATCCACTTCAGTTGGGACATCCTGGCCAAGCGCATCCGC
GAGCTGTCCTTCCTCAACTCCGGCGTCGGCATCCTGCTGCGCGACGAGCGTACCGGCAAGGAGGAGCTGTTCAAGTA
CGAAGGCGGTCTGAAGGCCTTCGTCGAGTACCTGAACACCAACAAGACCGCGGTGAACGAGGTATTCCACTTCAACG
TCCAGCGTGAAGAGGACGGCGTGGGTGTGGAAGTCGCCTTGCAGTGGAACGACAGCTTCAACGAGAACCTGCTCTGC
TTCACCAACAACATCCCGCAGCGTGATGGCGGCACCCACTTGGCCGGTTTCCGTTCGGCGCTGACGCGTAACCTGAA
CAACTACATCGAGGCCGAAGGCCTGGCGAAGAAATTCAAGATCGCCACCACCGGCGACGATGCCCGCGAAGGCCTCA
CCGCGATCATCTCGGTGAAGGTACCGGACCCGAAGTTCAGCTC
>AU30307
TTGCACGGTGTGGGCGTCTCGGTGGTGAACGCGCTGTCCCATGAGCTGCGCCTGACCATCCGTCGCCACAACAAGGT
CTGGGAACAGGTCTACCACCACGGCGTTCCGCAGTTCCCACTGCGCGAAGTGGGCGAGACCGATGGCTCCGGTACCG
AAGTTCACTTCAAGCCGTCCCCGGAGACCTTCAGCAACATCCACTTCAGTTGGGACATCCTGGCCAAGCGCATCCGC
GAACTGTCCTTCCTCAACTCCGGCGTCGGCATCCTGCTGCGCGACGAGCGTACCGGCAAGGAGGAGCTGTTCAAGTA
CGAAGGCGGTCTGAAGGCCTTCGTCGAGTACCTGAACACCAACAAGACCGCGGTGAACGAGGTATTCCACTTCAACG
TCCAGCGTGAAGAGGACGGCGTGGGTGTGGAAGTCGCCTTGCAGTGGAACGACAGCTTCAACGAGAACCTGCTCTGC
TTCACCAACAACATCCCGCAGCGTGATGGCGGCACCCACCTGGCCGGTTTCCGTTCGGCGCTGACGCGTAACCTGAA
CAACTACATCGAGGCCGAAGGCCTGGCGAAGAAATTCAAGATCGCCACCACCGGCGACGATGCCCGCGAAGGCCTCA
CCGCGATCATCTCGGTGAAGGTACCGGACCCGAAGTTCAGCTC
>AU19092
TTGCACGGTGTGGGCGTCTCGGTGGTGAACGCGCTGTCCCATGAACTGCGCCTGACCATCCGTCGCCACAACAAGGT
CTGGGAACAGGTCTACCACCACGGCGTTCCGCAGTTCCCACTGCGCGAAGTGGGCGAGACCGATGGCTCCGGCACCG
AAGTTCACTTCAAGCCGTCCCCGGAGACCTTCAGCAACATCCACTTCAGTTGGGACATCCTGGCCAAGCGCATCCGC
GAGCTGTCCTTCCTCAACTCCGGCGTCGGCATCCTGCTGCGCGACGAGCGTACCGGCAAGGAGGAGCTGTTCAAGTA
CGAAGGCGGTCTGAAGGCCTTCGTCGAGTACCTGAACACCAACAAGACCGCGGTGAACGAGGTATTCCACTTCAACG
TCCAGCGTGAAGAGGACGGCGTGGGTGTGGAAGTCGCTTTGCAGTGGAACGACAGCTTCAACGAGAACCTGCTCTGC
TTCACCAACAACATCCCGCAGCGTGATGGCGGCACCCACCTCGCCGGTTTCCGTTCGGCGCTGACGCGTAACCTGAA
CAACTACATCGAGGCCGAAGGCCTAGCGAAGAAATTCAAGATCGCCACCACCGGCGACGATGCCCGCGAAGGCCTCA
CCGCGATCATCTCGGTGAAGGTACCGGACCCGAAGTTCAGCTC
>AU27145
TTGCACGGTGTGGGCGTCTCGGTGGTGAACGCGCTGTCCCATGAACTGCGCCTGACCATCCGTCGCCACAACAAGGT
CTGGGAACAGGTCTACCACCACGGCGTTCCGCAGTTCCCACTGCGCGAAGTGGGCGAGACCGATGGCTCCGGCACCG
AAGTTCACTTCAAGCCGTCCCCGGAGACCTTCAGCAACATCCACTTCAGTTGGGACATCCTGGCCAAGCGCATCCGC
GAGCTGTCCTTCCTCAACTCCGGCGTCGGCATCCTGCTGCGCGACGAGCGTACCGGCAAGGAGGAGCTGTTCAAGTA
CGAAGGCGGTCTGAAGGCCTTCGTCGAGTACCTGAACACCAACAAGACCGCGGTGAACGAGGTATTCCACTTCAACG
TCCAGCGTGAAGAGGACGGCGTGGGTGTGGAAGTCGCCTTGCAGTGGAACGACAGCTTCAACGAGAACCTGCTCTGC

TTCACCAACAACATCCCGCAGCGTGATGGCGGCACCCACCTGGCCGGTTTCCGTTCGGCGCTGACGCGTAACCTGAA
CAACTACATCGAGGCCGAAGGCCTGGCGAAGAAATTCAAGATCGCCACCACCGGCGACGATGCCCGCGAAGGCCTCA
CCGCGATCATCTCGGTGAAGGTACCGGACCCGAAGTTCAGCTC
>AU26901
TTGCACGGTGTGGGCGTCTCGGTGGTGAACGCGCTGTCCCATGAACTGCGCCTGACCATCCGTCGCCACAACAAGGT
CTGGGAGCAGGTCTACCACCACGGCGTTCCGCAGTTCCCACTGCGCGAAGTGGGCGAGACCGATGGCTCCGGTACCG
AAGTTCACTTCAAGCCGTCCCCGGAGACCTTCAGCAACATCCACTTCAGTTGGGACATCCTGGCCAAGCGCATCCGC
GAGCTGTCCTTCCTCAACTCCGGCGTCGGCATCCTGCTGCGCGACGAGCGTACCGGCAAGGAGGAGCTGTTCAAGTA
CGAAGGCGGTCTGAAGGCCTTCGTCGAATACCTGAACACCAACAAGACCGCGGTGAACGAGGTATTCCACTTCAACG
TCCAGCGTGAAGAGGACGGCGTGGGTGTGGAAGTCGCCTTGCAGTGGAACGACAGCTTCAACGAGAACCTGCTCTGC
TTCACCAACAACATCCCGCAGCGTGACGGCGGTACCCACCTGGCCGGTTTCCGTTCGGCGCTGACGCGTAACCTGAA
CAACTACATCGAGGCCGAAGGCCTGGCGAAGAAATTCAAGATCGCCACCACCGGCGACGATGCCCGCGAAGGCCTCA
CCGCGATCATCTCGGTGAAGGTGCCGGACCCGAAGTTCAGCTC
>AU19425
TTGCACGGTGTGGGCGTCTCGGTGGTGAACGCGCTTTCCCATGAGCTGCGCCTGACCATCCGTCGCCACAACAAGGT
CTGGGAACAGGTCTACCACCACGGCGTTCCGCAGTTCCCACTGCGCGAAGTGGGCGAGACTGATGGCTCCGGTACCG
AAGTTCACTTCAAGCCGTCCCCGGAGACCTTCAGCAACATCCACTTCAGTTGGGACATCCTGGCCAAGCGCATCCGC
GAACTGTCCTTCCTCAACTCCGGCGTCGGCATCCTGCTGCGCGACGAGCGTACCGGCAAGGAGGAGCTGTTCAAGTA
CGAAGGCGGTCTGAAGGCCTTCGTCGAGTACCTGAACACCAACAAGACCGCGGTGAACGAGGTATTCCACTTCAACG
TCCAGCGTGAAGAGGACGGCGTGGGTGTGGAAGTCGCCTTGCAGTGGAACGACAGCTTCAACGAGAACCTGCTCTGC
TTCACCAACAACATCCCGCAGCGTGACGGCGGCACCCATCTGGCCGGTTTCCGTTCGGCGCTGACGCGTAACCTGAA
CAACTACATCGAGGCCGAAGGCCTGGCGAAGAAGTTCAAGATCGCCACCACCGGCGACGATGCCCGCGAAGGCCTCA
CCGCGATCATCTCGGTGAAGGTACCGGACCCGAAGTTCAGCTC
>AU15152
TTGCACGGTGTGGGCGTCTCGGTGGTGAACGCGCTGTCCCATGAACTACGCCTGACCATCCGTCGCCACAACAAGGT
CTGGGAACAGGTCTACCACCACGGCGTTCCGCAGTTCCCACTGCGCGAAGTGGGCGAGACCGATGGCTCCGGCACCG
AAGTTCACTTCAAGCCGTCCCCGGAGACCTTCAGCAACATCCACTTCAGTTGGGACATCCTGGCCAAGCGCATCCGC
GAGCTGTCCTTCCTCAACTCCGGCGTCGGCATCCTGCTGCGCGACGAGCGTACCGGCAAGGAGGAGCTGTTCAAGTA
CGAAGGCGGTCTGAAGGCCTTCGTCGAGTACCTGAACACCAACAAGACCGCGGTGAACGAGGTATTCCACTTCAACG
TCCAGCGTGAAGAGGACGGCGTGGGTGTGGAAGTCGCCTTGCAGTGGAACGACAGCTTCAACGAGAACCTGCTCTGC
TTCACCAACAACATCCCGCAGCGTGACGGCGGCACCCACCTGGCCGGTTTCCGTTCGGCGCTGACGCGTAACCTGAA
CAACTACATCGAGGCCGAAGGCCTGGCGAAGAAGTTCAAGATCGCCACCACCGGCGACGATGCCCGCGAAGGCCTCA
CCGCGATCATCTCGGTGAAGGTACCGGACCCGAAGTTCAGCTC
>AU12175
TTGCACGGTGTGGGCGTCTCGGTGGTGAACGCGCTGTCCCATGAACTGCGCCTGACCATCCGTCGCCACAACAAGGT
CTGGGAACAGGTCTACCACCACGGCGTTCCGCAGTTCCCACTGCGCGAAGTGGGCGAGACCGATGGCTCCGGCACCG
AAGTTCACTTCAAGCCGTCCCCGGAGACCTTCAGCAACATCCACTTCAGTTGGGACATCCTGGCCAAGCGCATCCGC
GAGCTGTCCTTCCTCAACTCCGGCGTCGGCATCCTGCTGCGCGACGAGCGTACCGGCAAGGAGGAGCTGTTCAAGTA
CGAAGGCGGTCTGAAGGCCTTCGTCGAGTACCTGAACACCAACAAGACCGCGGTGAACGAGGTATTCCACTTCAACG
TCCAGCGTGAAGAGGACGGCGTGGGTGTGGAAGTCGCCTTGCAGTGGAACGACAGCTTCAACGAGAACCTGCTCTGC
TTCACCAACAACATCCCGCAGCGTGATGGCGGCACCCACCTGGCCGGTTTCCGTTCGGCGCTGACGCGTAACCTGAA
CAACTACATCGAGGCCGAAGGCCTGGCGAAGAAATTCAAGATCGCCACCACCGGCGACGATGCCCGCGAAGGCCTCA
CCGCGATCATCTCGGTGAAGGTACCGGACCCGAAGTTCAGCTC
>AU17108
TTGCACGGTGTGGGCGTCTCGGTGGTGAACGCGCTGTCCCATGAACTGCGCCTGACCATCCGTCGCCACAACAAGGT
CTGGGAACAGGTCTACCACCACGGCGTTCCGCAGTTCCCACTGCGCGAAGTGGGCGAGACCGATGGCTCCGGCACCG
AAGTTCACTTCAAGCCGTCCCCGGAGACCTTCAGCAACATCCACTTCAGTTGGGACATCCTGGCCAAGCGCATCCGC
GAGCTGTCCTTCCTCAACTCCGGCGTCGGCATCCTGCTGCGCGACGAGCGTACCGGCAAGGAGGAGCTGTTCAAGTA
CGAAGGCGGTCTGAAGGCCTTCGTCGAGTACCTGAACACCAACAAGACCGCGGTGAACGAGGTATTCCACTTCAACG
TCCAGCGTGAAGAGGACGGCGTGGGTGTGGAAGTCGCCTTGCAGTGGAACGACAGCTTCAACGAGAACCTGCTCTGC
TTCACCAACAACATCCCGCAGCGTGATGGCGGCACCCACCTGGCCGGTTTCCGTTCGGCGCTGACGCGTAACCTGAA
CAACTACATCGAGGCCGAAGGCCTGGCGAAGAAATTCAAGATCGCCACCACCGGCGACGATGCCCGCGAAGGCCTCA
CCGCGATCATCTCGGTGAAGGTACCGGACCCGAAGTTCAGCTC
>AU9276
TTGCACGGTGTGGGCGTCTCGGTGGTGAACGCGCTGTCCCATGAACTACGCCTGACCATCCGTCGCCACAACAAGGT
CTGGGAACAGGTCTACCACCACGGCGTTCCGCAGTTCCCACTGCGCGAAGTGGGCGAGACCGATGGCTCCGGCACCG
AAGTTCACTTCAAGCCGTCCCCGGAGACCTTCAGCAACATCCACTTCAGTTGGGACATCCTGGCCAAGCGCATCCGC

GAGCTGTCCTTCCTCAACTCCGGCGTCGGCATCCTGCTGCGCGACGAGCGTACCGGCAAGGAGGAGCTGTTCAAGTA
CGAAGGCGGTCTGAAGGCCTTCGTCGAGTACCTGAACACCAACAAGACCGCGGTGAACGAGGTATTCCACTTCAACG
TCCAGCGTGAAGAGGACGGCGTGGGTGTGGAAGTCGCCTTGCAGTGGAACGACAGCTTCAACGAGAACCTGCTCTGC
TTCACCAACAACATCCCGCAGCGTGACGGCGGCACCCACCTGGCCGGTTTCCGTTCGGCGCTGACGCGTAACCTGAA
CAACTACATCGAGGCCGAAGGCCTGGCGAAGAAGTTCAAGATCGCCACCACCGGCGACGATGCCCGCGAAGGCCTCA
CCGCGATCATCTCGGTGAAGGTACCGGACCCGAAGTTCAGCTC
>AU18234
TTGCACGGTGTGGGCGTCTCGGTGGTGAACGCGCTGTCCCATGAACTACGCCTGACCATCCGTCGCCACAACAAGGT
CTGGGAACAGGTCTACCACCACGGCGTTCCGCAGTTCCCACTGCGCGAAGTGGGCGAGACCGATGGCTCCGGCACCG
AAGTTCACTTCAAGCCGTCCCCGGAGACCTTCAGCAACATCCACTTCAGTTGGGACATCCTGGCCAAGCGCATCCGC
GAGCTGTCCTTCCTCAACTCCGGCGTCGGCATCCTGCTGCGCGACGAGCGTACCGGCAAGGAGGAGCTGTTCAAGTA
CGAAGGCGGTCTGAAGGCCTTCGTCGAGTACCTGAACACCAACAAGACCGCGGTGAACGAGGTATTCCACTTCAACG
TCCAGCGTGAAGAGGACGGCGTGGGTGTGGAAGTCGCCTTGCAGTGGAACGACAGCTTCAACGAGAACCTGCTCTGC
TTCACCAACAACATCCCGCAGCGTGACGGCGGCACCCACCTGGCCGGTTTCCGTTCGGCGCTGACGCGTAACCTGAA
CAACTACATCGAGGCCGAAGGCCTGGCGAAGAAGTTCAAGATCGCCACCACCGGCGACGATGCCCGCGAAGGCCTCA
CCGCGATCATCTCGGTGAAGGTACCGGACCCGAAGTTCAGCTC
>AU17766
TTGCACGGTGTGGGCGTCTCGGTGGTGAACGCGCTGTCCCATGAACTACGCCTGACCATCCGTCGCCACAACAAGGT
CTGGGAACAGGTCTACCACCACGGCGTTCCGCAGTTCCCACTGCGCGAAGTGGGCGAGACCGATGGCTCCGGCACCG
AAGTTCACTTCAAGCCGTCCCCGGAGACCTTCAGCAACATCCACTTCAGTTGGGACATCCTGGCCAAGCGCATCCGC
GAGCTGTCCTTCCTCAACTCCGGCGTCGGCATCCTGCTGCGCGACGAGCGTACCGGCAAGGAGGAGCTGTTCAAGTA
CGAAGGCGGTCTGAAGGCCTTCGTCGAGTACCTGAACACCAACAAGACCGCGGTGAACGAGGTATTCCACTTCAACG
TCCAGCGTGAAGAGGACGGCGTGGGTGTGGAAGTCGCCTTGCAGTGGAACGACAGCTTCAACGAGAACCTGCTCTGC
TTCACCAACAACATCCCGCAGCGTGACGGCGGCACCCACCTGGCCGGTTTCCGTTCGGCGCTGACGCGTAACCTGAA
CAACTACATCGAGGCCGAAGGCCTGGCGAAGAAGTTCAAGATCGCCACCACCGGCGACGATGCCCGCGAAGGCCTCA
CCGCGATCATCTCGGTGAAGGTACCGGACCCGAAGTTCAGCTC
>AU18005
TTGCACGGTGTGGGCGTCTCGGTGGTGAACGCGCTGTCCCATGAACTACGCCTGACCATCCGTCGCCACAACAAGGT
CTGGGAACAGGTCTACCACCACGGCGTTCCGCAGTTCCCACTGCGCGAAGTGGGCGAGACCGATGGCTCCGGCACCG
AAGTTCACTTCAAGCCGTCCCCGGAGACCTTCAGCAACATCCACTTCAGTTGGGACATCCTGGCCAAGCGCATCCGC
GAGCTGTCCTTCCTCAACTCCGGCGTCGGCATCCTGCTGCGCGACGAGCGTACCGGCAAGGAGGAGCTGTTCAAGTA
CGAAGGCGGTCTGAAGGCCTTCGTCGAGTACCTGAACACCAACAAGACCGCGGTGAACGAGGTATTCCACTTCAACG
TCCAGCGTGAAGAGGACGGCGTGGGTGTGGAAGTCGCCTTGCAGTGGAACGACAGCTTCAACGAGAACCTGCTCTGC
TTCACCAACAACATCCCGCAGCGTGACGGCGGCACCCACCTGGCCGGTTTCCGTTCGGCGCTGACGCGTAACCTGAA
CAACTACATCGAGGCCGAAGGCCTGGCGAAGAAGTTCAAGATCGCCACCACCGGCGACGATGCCCGCGAAGGCCTCA
CCGCGATCATCTCGGTGAAGGTACCGGACCCGAAGTTCAGCTC
>AU17787
TTGCACGGTGTGGGCGTCTCGGTGGTGAACGCGCTGTCCCATGAACTACGCCTGACCATCCGTCGCCACAACAAGGT
CTGGGAACAGGTCTACCACCACGGCGTTCCGCAGTTCCCACTGCGCGAAGTGGGCGAGACCGATGGCTCCGGCACTG
AAGTTCACTTCAAGCCGTCCCCGGAGACCTTCAGCAACATCCACTTCAGTTGGGACATCCTGGCCAAGCGCATCCGC
GAGCTGTCCTTCCTCAACTCCGGCGTCGGCATCCTGCTGCGCGACGAGCGTACCGGCAAGGAGGAGCTGTTCAAGTA
CGAAGGCGGTCTGAAGGCCTTCGTCGAGTACCTGAACACCAACAAGACCGCGGTGAACGAGGTATTCCACTTCAACG
TCCAGCGTGAAGAGGACGGCGTGGGTGTGGAAGTCGCCTTGCAGTGGAACGACAGCTTCAACGAGAACCTGCTCTGC
TTCACCAACAACATCCCGCAGCGTGACGGCGGCACCCACCTGGCCGGTTTCCGTTCGGCGCTGACGCGTAACCTGAA
CAACTACATCGAGGCCGAAGGCCTGGCGAAGAAGTTCAAGATCGCCACCACCGGCGACGATGCCCGCGAAGGCCTCA
CCGCGATCATCTCGGTGAAGGTACCGGACCCGAAGTTCAGCTC
>AU15031
TTGCACGGTGTGGGCGTCTCGGTGGTGAACGCGCTGTCCCATGAACTGCGCCTGACCATCCGTCGCCACAACAAGGT
CTGGGAACAGGTCTACCACCACGGCGTTCCGCAGTTCCCACTGCGCGAAGTGGGCGAGACCGATGGCTCCGGCACCG
AAGTTCACTTCAAGCCGTCCCCGGAGACCTTCAGCAACATCCACTTCAGTTGGGACATCCTGGCCAAGCGCATCCGC
GAGCTGTCCTTCCTCAACTCCGGCGTCGGCATCCTGCTGCGCGACGAGCGTACCGGCAAGGAGGAGCTGTTCAAGTA
CGAAGGCGGTCTGAAGGCCTTCGTCGAGTACCTGAACACCAACAAGACCGCGGTGAACGAGGTATTCCACTTCAACG
TCCAGCGTGAAGAGGACGGCGTGGGTGTGGAAGTCGCCTTGCAGTGGAACGACAGCTTCAACGAGAACCTGCTCTGC
TTCACCAACAACATCCCGCAGCGTGACGGCGGCACCCACCTGGCCGGTTTCCGTTCGGCGCTGACGCGTAACCTGAA
CAACTACATCGAGGCCGAAGGCCTGGCGAAGAAGTTCAAGATCGCCACCACCGGCGACGATGCCCGCGAAGGCCTCA
CCGCGATCATCTCGGTGAAGGTACCGGACCCGAAGTTCAGCTC

>AU12176
TTGCACGGTGTGGGCGTCTCGGTGGTGAACGCGCTGTCCCATGAACTGCGCCTGACCATCCGTCGCCACAACAAGGT
CTGGGAACAGGTCTACCACCACGGCGTTCCGCAGTTCCCACTGCGCGAAGTGGGCGAGACCGATGGCTCCGGCACCG
AAGTTCACTTCAAGCCGTCCCCGGAGACCTTCAGCAACATCCACTTCAGTTGGGACATCCTGGCCAAGCGCATCCGC
GAGCTGTCCTTCCTCAACTCCGGCGTCGGCATCCTGCTGCGCGACGAGCGTACCGGCAAGGAGGAGCTGTTCAAGTA
CGAAGGCGGTCTGAAGGCCTTCGTCGAGTACCTGAACACCAACAAGACCGCGGTGAACGAGGTATTCCACTTCAACG
TCCAGCGTGAAGAGGACGGCGTGGGTGTGGAAGTCGCCTTGCAGTGGAACGACAGCTTCAACGAGAACCTGCTCTGC
TTCACCAACAACATCCCGCAGCGTGACGGCGGCACCCACCTGGCCGGTTTCCGTTCGGCGCTGACGCGTAACCTGAA
CAACTACATCGAGGCCGAAGGCCTGGCGAAGAAGTTCAAGATCGCCACCACCGGCGACGATGCCCGCGAAGGCCTCA
CCGCGATCATCTCGGTGAAGGTACCGGACCCGAAGTTCAGCTC
>AU11650
TTGCACGGTGTGGGCGTCTCGGTGGTGAACGCGCTGTCCCATGAACTGCGCCTGACCATCCGTCGCCACAACAAGGT
CTGGGAACAGGTCTACCACCACGGCGTTCCGCAGTTCCCACTGCGCGAAGTGGGCGAGACCGATGGCTCCGGCACCG
AAGTTCACTTCAAGCCGTCCCCGGAGACCTTCAGCAACATCCACTTCAGTTGGGACATCCTGGCCAAGCGCATCCGC
GAGCTGTCCTTCCTCAACTCCGGCGTCGGCATCCTGCTGCGCGACGAGCGTACCGGCAAGGAGGAGCTGTTCAAGTA
CGAAGGCGGTCTGAAGGCCTTCGTCGAGTACCTGAACACCAACAAGACCGCGGTGAACGAGGTATTCCACTTCAACG
TCCAGCGTGAAGAGGACGGCGTGGGTGTGGAAGTCGCCTTGCAGTGGAACGACAGCTTCAACGAGAACCTGCTCTGC
TTCACCAACAACATCCCGCAGCGTGACGGCGGCACCCACCTGGCCGGTTTCCGTTCGGCGCTGACGCGTAACCTGAA
CAACTACATCGAGGCCGAAGGCCTGGCGAAGAAGTTCAAGATCGCCACCACCGGCGACGATGCCCGCGAAGGCCTCA
CCGCGATCATCTCGGTGAAGGTACCGGACCCGAAGTTCAGCTC
>AU15873
TTGCACGGTGTGGGCGTCTCGGTGGTGAACGCGCTGTCCCATGAACTGCGCCTGACCATCCGTCGCCACAACAAGGT
CTGGGAACAGGTCTACCACCACGGCGTTCCGCAGTTCCCACTGCGCGAAGTGGGCGAGACCGATGGCTCCGGCACCG
AAGTTCACTTCAAGCCGTCCCCGGAGACCTTCAGCAACATCCACTTCAGTTGGGACATCCTGGCCAAGCGCATCCGC
GAGCTGTCCTTCCTCAACTCCGGAGTCGGCATCCTGCTGCGCGACGAGCGTACCGGCAAGGAGGAGCTGTTCAAGTA
CGAAGGCGGTCTGAAGGCCTTCGTCGAGTACCTGAACACCAACAAGACCGCGGTGAACGAGGTATTCCACTTCAACG
TCCAGCGTGAAGAGGACGGCGTGGGTGTGGAAGTCGCCTTGCAGTGGAACGACAGCTTCAACGAGAACCTGCTCTGC
TTCACCAACAACATCCCGCAGCGTGACGGCGGCACCCACCTGGCCGGTTTCCGTTCGGCGCTGACGCGTAACCTGAA
CAACTACATCGAGGCCGAAGGCCTGGCGAAGAAGTTCAAGATCGCCACCACCGGCGACGATGCCCGCGAAGGCCTCA
CCGCGATCATCTCGGTGAAGGTACCGGACCCGAAGTTCAGCTC
>AU8215
TTGCACGGTGTGGGCGTCTCGGTGGTGAACGCGCTGTCCCATGAACTGCGCCTGACCATCCGTCGCCACAACAAGGT
CTGGGAACAGGTCTACCACCACGGCGTTCCGCAGTTCCCACTGCGCGAAGTGGGCGAGACCGATGGCTCCGGCACCG
AAGTTCACTTCAAGCCGTCCCCGGAGACCTTCAGCAACATCCACTTCAGTTGGGACATCCTGGCCAAGCGCATCCGC
GAGCTGTCCTTCCTCAACTCCGGAGTCGGCATCCTGCTGCGCGACGAGCGTACCGGCAAGGAGGAGCTGTTCAAGTA
CGAAGGCGGTCTGAAGGCCTTCGTCGAGTACCTGAACACCAACAAGACCGCGGTGAACGAGGTATTCCACTTCAACG
TCCAGCGTGAAGAGGACGGCGTGGGTGTGGAAGTCGCCTTGCAGTGGAACGACAGCTTCAACGAGAACCTGCTCTGC
TTCACCAACAACATCCCGCAGCGTGACGGCGGCACCCACCTGGCCGGTTTCCGTTCGGCGCTGACGCGTAACCTGAA
CAACTACATCGAGGCCGAAGGCCTGGCGAAGAAGTTCAAGATCGCCACCACCGGCGACGATGCCCGCGAAGGCCTCA
CCGCGATCATCTCGGTGAAGGTACCGGACCCGAAGTTCAGCTC
>AU18422
TTGCACGGTGTGGGCGTCTCGGTGGTGAACGCGCTGTCCCATGAACTACGCCTGACCATCCGTCGCCACAACAAGGT
CTGGGAACAGGTCTACCACCACGGCGTTCCGCAGTTCCCACTGCGCGAAGTGGGCGAGACCGATGGCTCCGGCACCG
AAGTTCACTTCAAGCCGTCCCCGGAGACCTTCAGCAACATCCACTTCAGTTGGGACATCCTGGCCAAGCGCATCCGC
GAGCTGTCCTTCCTCAACTCCGGCGTCGGCATCCTGCTGCGCGACGAGCGTACCGGCAAGGAGGAGCTGTTCAAGTA
CGAAGGCGGTCTGAAGGCCTTCGTCGAGTACCTGAACACCAACAAGACCGCGGTGAACGAGGTATTCCACTTCAACG
TCCAGCGTGAAGAGGACGGCGTGGGTGTGGAAGTCGCCTTGCAGTGGAACGACAGCTTCAACGAGAACCTGCTCTGC
TTCACCAACAACATCCCGCAGCGTGACGGCGGTACCCACCTGGCCGGTTTCCGTTCGGCGCTGACGCGTAACCTGAA
CAACTACATCGAGGCCGAAGGCCTGGCGAAGAAGTTCAAGATCGCCACCACCGGCGACGATGCCCGCGAAGGCCTCA
CCGCGATCATCTCGGTGAAGGTACCGGACCCGAAGTTCAGCTC
>AU16000
TTGCACGGTGTGGGCGTCTCGGTGGTGAACGCGCTGTCCCATGAACTACGCCTGACCATCCGTCGCCACAACAAGGT
CTGGGAACAGGTCTACCACCACGGCGTTCCGCAGTTCCCACTGCGCGAAGTGGGCGAGACCGATGGCTCCGGCACCG
AAGTTCACTTCAAGCCGTCCCCGGAGACCTTCAGCAACATCCACTTCAGTTGGGACATCCTGGCCAAGCGCATCCGC
GAGCTGTCCTTCCTCAACTCCGGCGTCGGCATCCTGCTGCGCGATGAGCGTACCGGCAAGGAGGAGCTGTTCAAGTA
CGAAGGCGGTCTGAAGGCCTTCGTCGAGTACCTGAACACCAACAAGACCGCGGTGAACGAGGTATTCCACTTCAACG
TCCAGCGTGAAGAGGACGGCGTGGGTGTGGAAGTCGCCTTGCAGTGGAACGACAGCTTCAACGAGAACCTGCTCTGC

TTCACCAACAACATCCCGCAGCGTGACGGCGGTACCCACCTGGCCGGTTTCCGTTCGGCGCTGACGCGTAACCTGAA
CAACTACATCGAGGCCGAAGGCCTGGCGAAGAAGTTCAAGATCGCCACCACCGGCGACGATGCCCGCGAAGGCCTCA
CCGCGATCATCTCGGTGAAGGTACCGGACCCGAAGTTCAGCTC
>AU14282
TTGCACGGTGTGGGCGTCTCGGTGGTGAACGCGCTGTCCCATGAGCTGCGCCTGACCATCCGTCGCCACAACAAGGT
CTGGGAACAGGTCTACCACCACGGCGTTCCGCAGTTCCCACTGCGCGAAGTGGGCGAGACCGATGGCTCCGGTACCG
AAGTTCACTTCAAGCCGTCCCCGGAGACCTTCAGCAACATCCACTTCAGTTGGGACATCCTGGCCAAGCGCATCCGC
GAACTGTCCTTCCTCAACTCCGGCGTCGGCATCCTGCTGCGCGACGAGCGTACCGGCAAGGAGGAGCTGTTCAAGTA
CGAAGGCGGTCTGAAGGCCTTCGTCGAGTACCTGAACACCAACAAGACCGCGGTGAACGAGGTATTCCACTTCAACG
TCCAGCGTGAAGAGGACGGCGTGGGTGTGGAAGTCGCCTTGCAGTGGAACGACAGCTTCAACGAGAACCTGCTCTGC
TTCACCAACAACATCCCGCAGCGTGATGGCGGCACCCACCTGGCCGGTTTCCGTTCGGCGCTGACGCGTAACCTGAA
CAACTACATCGAGGCCGAAGGCCTGGCGAAGAAATTCAAGATCGCCACCACCGGCGACGATGCCCGCGAAGGCCTCA
CCGCGATCATCTCGGTGAAGGTACCGGACCCGAAGTTCAGCTC
>AU10014
TTGCACGGTGTGGGCGTCTCGGTGGTGAACGCGCTTTCCCATGAGCTGCGCCTGACCATCCGTCGCCACAACAAGGT
CTGGGAACAGGTCTACCACCACGGCGTTCCGCAGTTCCCACTGCGCGAAGTGGGCGAGACTGATGGCTCCGGTACCG
AAGTTCACTTCAAGCCGTCCCCGGAGACCTTCAGCAACATCCACTTCAGTTGGGACATCCTGGCCAAGCGCATCCGC
GAACTGTCCTTCCTCAACTCCGGCGTCGGCATCCTGCTGCGCGACGAGCGTACCGGCAAGGAGGAGCTGTTCAAGTA
CGAAGGCGGTCTGAAGGCCTTCGTCGAGTACCTGAACACCAACAAGACCGCGGTGAACGAGGTATTCCACTTCAACG
TCCAGCGTGAAGAGGACGGCGTGGGTGTGGAAGTCGCCTTGCAGTGGAACGACAGCTTCAACGAGAACCTGCTCTGC
TTCACCAACAACATCCCGCAGCGTGACGGCGGCACCCATCTGGCCGGTTTCCGTTCGGCGCTGACGCGTAACCTGAA
CAACTACATCGAGGCCGAAGGCCTGGCGAAGAAGTTCAAGATCGCCACCACCGGCGACGATGCCCGCGAAGGCCTCA
CCGCGATCATCTCGGTGAAGGTACCGGACCCGAAGTTCAGCTC
>AU18081
TTGCACGGTGTGGGCGTCTCGGTGGTGAACGCGCTGTCCCATGAACTACGCCTGACCATCCGTCGCCACAACAAGGT
CTGGGAACAGGTCTACCACCACGGCGTTCCGCAGTTCCCACTGCGCGAAGTGGGCGAGACCGATGGCTCCGGCACCG
AAGTTCACTTCAAGCCGTCCCCGGAGACCTTCAGCAACATCCACTTCAGTTGGGACATCCTGGCCAAGCGCATCCGC
GAGCTGTCCTTCCTCAACTCCGGCGTCGGCATCCTGCTGCGCGACGAGCGTACCGGCAAGGAGGAGCTGTTCAAGTA
CGAAGGCGGTCTGAAGGCCTTCGTCGAGTACCTGAACACCAACAAGACCGCGGTGAACGAGGTATTCCACTTCAACG
TCCAGCGTGAAGAGGACGGCGTGGGTGTGGAAGTCGCCTTGCAGTGGAACGACAGCTTCAACGAGAACCTGCTCTGC
TTCACCAACAACATCCCGCAGCGTGACGGCGGCACCCACCTGGCCGGTTTCCGTTCGGCGCTGACGCGTAACCTGAA
CAACTACATCGAGGCCGAAGGCCTGGCGAAGAAATTCAAGATCGCCACCACCGGCGACGATGCCCGCGAAGGCCTCA
CCGCGATCATCTCGGTGAAGGTACCGGACCCGAAGTTCAGCTC
>AU19324
TTGCACGGTGTGGGCGTCTCGGTGGTGAACGCGCTGTCCCATGAGCTGCGCCTGACCATCCGTCGCCACAACAAGGT
CTGGGAACAGGTCTACCACCACGGCGTTCCGCAGTTCCCACTGCGCGAAGTGGGCGAGACCGATGGCTCCGGCACCG
AAGTTCACTTCAAGCCGTCCCCGGAGACCTTCAGCAACATCCACTTCAGTTGGGACATCCTGGCCAAGCGCATCCGC
GAGCTGTCCTTCCTCAACTCCGGCGTCGGCATCCTGCTGCGCGACGAGCGTACCGGCAAGGAGGAGCTGTTCAAGTA
CGAAGGCGGTCTGAAGGCCTTCGTCGAGTACCTGAACACCAACAAGACCGCGGTGAACGAGGTATTCCACTTCAACG
TCCAGCGTGAAGAGGACGGCGTGGGTGTGGAAGTCGCCTTGCAGTGGAACGACAGCTTCAACGAGAACCTGCTCTGC
TTCACCAACAACATCCCGCAGCGTGATGGCGGCACCCACCTCGCCGGTTTCCGTTCGGCGCTGACGCGTAACCTGAA
CAACTACATCGAGGCCGAAGGCCTGGCGAAGAAATTCAAGATCGCCACCACCGGCGACGATGCCCGCGAAGGCCTCA
CCGCGATCATCTCGGTGAAGGTACCGGACCCGAAGTTCAGCTC
>AU8660
TTGCACGGTGTGGGCGTCTCGGTGGTGAACGCGCTGTCCCATGAACTACGCCTGACCATCCGTCGCCACAACAAGGT
CTGGGAACAGGTCTACCACCACGGCGTTCCGCAGTTCCCACTGCGCGAAGTGGGCGAGACCGATGGCTCCGGCACCG
AAGTTCACTTCAAGCCGTCCCCGGAGACCTTCAGCAACATCCACTTCAGTTGGGACATCCTGGCCAAGCGCATCCGC
GAGCTGTCCTTCCTCAACTCCGGCGTCGGCATCCTGCTGCGCGACGAGCGTACCGGCAAGGAGGAGCTGTTCAAGTA
CGAAGGCGGTCTGAAGGCCTTTGTCGAGTACCTGAACACCAACAAGACCGCGGTGAACGAGGTATTCCACTTCAACG
TCCAGCGTGAAGAGGACGGCGTGGGTGTGGAAGTCGCCTTGCAGTGGAACGACAGCTTCAACGAGAACCTGCTCTGC
TTCACCAACAACATCCCGCAGCGTGACGGCGGCACCCACCTTGCCGGTTTCCGTTCGGCGCTGACGCGTAACCTGAA
CAACTACATCGAGGCCGAAGGCCTGGCGAAGAAGTTCAAGATCGCCACCACCGGCGACGATGCCCGCGAAGGCCTCA
CCGCGATCATCTCGGTGAAGGTACCGGACCCGAAGTTCAGCTC
>PAO1 NC002
TTGCACGGTGTGGGCGTCTCGGTGGTGAACGCGCTGTCCCATGAACTGCGCCTGACCATCCGTCGCCACAACAAGGT
CTGGGAACAGGTCTACCACCACGGCGTTCCGCAGTTCCCTCTGCGCGAAGTGGGCGAGACTGATGGCTCCGGCACCG
AAGTTCACTTCAAGCCGTCCCCGGAGACCTTCAGCAACATCCACTTCAGTTGGGACATCCTGGCCAAGCGCATCCGC

GAGCTGTCCTTCCTCAACTCCGGAGTCGGCATCCTGCTGCGCGACGAGCGTACCGGCAAGGAGGAGCTGTTCAAGTA
CGAAGGCGGTCTGAAGGCCTTCGTCGAGTACCTGAACACCAACAAGACCGCGGTGAACGAGGTATTCCACTTCAACG
TCCAGCGTGAAGAGGACGGCGTGGGTGTGGAAGTCGCCTTGCAGTGGAACGACAGCTTCAACGAGAACCTGCTCTGC
TTCACCAACAACATCCCGCAGCGTGACGGCGGCACCCACCTGGCCGGTTTCCGTTCGGCGCTGACGCGTAACCTGAA
CAACTACATCGAGGCCGAAGGCCTGGCGAAGAAGTTCAAGATCGCCACCACCGGCGACGATGCCCGCGAAGGCCTCA
CCGCGATCATCTCGGTGAAGGTACCGGACCCGAAGTTCAGCTC

>node62_contains the qbs locus
GCCCGCTCGCCACAGAAAGCCCAGATGCCACGGTGATGCCCGCCTGGTCCAGGGCTGTGCGGGTCGCTTCCACCAG
GTCCAGGGCGCCCGGTGTATCGCTGTGCAAGCAGATGGAATCGAATTCGATAAACAGGTCTTCGCCTTCGACGGTG
CGCACCAAGCCGGTCTGGCAGGCACGCAGCACCCGCGCTGCCACCGTCGCGGGGTCCAGGGCGCGTACGTTGCGGG
TAAACACAATGGAACCAGACAGATCGTACTCACGGTCGGCATAAAACTCGCGAACCACCGGCTGCCCCAGCTCCTT
GGCAATGCGCCAGATCACCGAACCGGGCATGCAATACAGCAGCAGTTCCGGCTCCAGGCGCTGCAGGTTTTCTACC
AGCAGCCGTGCGGCCTCTTCGTCCCGGGCCAGGTGCATGTACAGCGCGCCATGGGGCTTGATGTGCTGCAAGGTCA
CGCCCTGCACCCGCGCCAATTCGCGCAGGGCACCCAGTTGGTAGAGCATGTCGTCTACCAGTTCCTGGGCCGGGGC
GTTGATATGGCGGCGGCCAAACCCCACCAGGTCGCGAAAGCCCGGGTGCGCGCCGATGGCTACGCCCAAGGCCTTG
GCGCGCTCGATGGTGCGGCGCATGGTGCCGGGGTCACCGGCATGGAAGCCGGTGGCCACGTTGGCGGAGCTGATAA
ACGCCATCAGTTCGTGATCCACACCGTCGCCGATGGTCCAGGGGCCGAAGCTTTCGCCCATGTCCGAGTTGAAATC
CACTGTTTGCATCGGGGCCTGCTCCGCAAGAGTTCGTGCCATCGAAAATAGGCCGGCCCTTGATCCCTTGGGAAGA
TCTATTATCAGATAGGGTATCTTCTGAAAATCAGATACCACGACGAGCAATCTGTATGTCCTTGACGTTGCGCCAG
GTTCGTTACTTTGTCGCCACCGCCGAAATCGGCCAGATTTCCCAGGCGGCGATCCACCTGAACATCTCCCAGTCGG
CGGTGACCACCGCGATCAAGGAGCTGGAAGCCATGCTCGGTGCCCAGCTGTTTGTGCGCTCGGCCCAGGGCATGAA
CCTGACCGATGCCGGGCGGCATTTTCTCAACCGCGCCTATGTGATTGTGCGCAGTGTCGACGACGCGCTGAACAGT
CCGCTGCCAGACTACCGCGCCAGTGGCGTGCTGCGGGTTGCGGCCAGTTACACGGTGTTGGGTTACTTCCTGCCGC
ACCACCTGCAACGCATGGAACACTGGCATCCGGACGTGACCATCGAGGTCTTCGAGCAGGAGCGCCAGGCGATCGA
ACAAGGGCTGCTGGATGGCCAGTTCGATATGGCCGTGGTACTCACCGCCAACCTGACCCACCCGGATATCGTCTCG
CAGATCCTGTTCAATTCCGAACGCCGCCTGTGGCTGCCCAGCCACCACCCGCTGTGCGAACGCCGTGCGGTGAGCC
TGGCCGATGTGGCGCGCGAGCCCTACATCCTGCTGACCGTGGATGAGGCAGAACAAAGTGCCATGCGCTACTGGGA
ACAGGCCGGGCAAGCGCCCAAGGTGCGGCTGCGCACCAGTTCGGTAGAAGCGGTGCGCAGTATGGTGGCCAATGGC
AGCGGCGTGGCGATTCTGTCCGACCTGGTGCACCGCCCCTGGTCTTTGGAAGGCAAGCGCATCGAGACCTTGAGCG
TGACCGACAAGGTCACGCCCATGAGCGTCGGCCTGGCCTGGCACCGCGAACGCGCGTTCACCCCGGCGATGCAAGC
ATTGCGCGACTACTTCCACGACGCCTTCCTCGCGCCCCAGCAATTATCCACACGCCGTTAAAGCAGGCCCTTCAAG
GTGGCCGCCAACCAGTCCATCAGCACCCGCACCCGTAGCGGCAAGTGCCGCTGGCCGGCATACAACATCGACACAT
CCAGCGCGGGCGCGGGGTAGTCGGGAAGCACCGCCACCAACTCGCCCTTTTCGAGCAATTCGCGGATGCCCAGCCC
CGGCACCTGGATGATCCCGAAACCGGCGAGGCACGCCGACTGGTACGCGTCTGTGCTGCTGACCGTGACGCGCCCA
GCCATGGCAATGCGCTGCACCTTGTTGCCCTGCGCATACTCAAAACCCGTTGAACGCGCACCCAGCGGCCGCACGT
AGTGCACCAACTGATGTGCGGCCAGGTCGGCCAGGGTCTTGGGTTCGCCATAGCGTTGCAGGTAGCCGGGGCTTAC
GCAGTTGATCATCGGCATGCTGCATAACAGCCGTGCGACCACCGACTGGTCCGGCTGTACGCCCACGCGCAGCACA
CAATCAAAGCCTTCGGCCAGGAGGTCGACCTGACGGTCAGAGCTGCTGATTTCCAACTCGATCAAGGGATGCCGAT
CCATAAACTCCGGCAGGCGCGGCACGATCACCTTGCGCGCCATCACATTGGGCATGTCGATACGGATCCGCCCGGT
CAATTGCGCCTCATCCTGGCGAAACAGCCCTTCCAGCTCCTCCATATCGGCCAGCAGGTCCTTGCTGCGCTCATAC
AGCACCCGCCCGTCCTGGGTGGCCTGCACCTTACGCGTGGTGCGCTGCAACAGACGCGTGCCCAACAGGTCTTCCA
GGGCGCGCACCTGTTCGGATACGGTCGAACGCGGCAGCCCCAGGCTATCGCCGGCCTGGGTGAAGCTCGACAGCTC
GGTGACGCGGACAAAGGTGCGCAACAACTCCAGCTTGTTCATGGTGGTGACCCGTGATTGTTCGTCTGATCCGACC
AGTGATTCCGGATTAGCCATGTTTATCACGTATTGCCCGGACAAATACACTCAGTCCCAGCGTTACCCACCCGCTC
CGAGGACTGCACCATGACCCGTAAAATCGCACTGATCACCGGCGCCAGCCGTGGCTTGGGCAAAAGCACCGCCCTG
CACCTGGCCGCCCAAGGCATCGACATTATCGGCACCTACTACAGCAAGGCCGAAGAGGCACAGGCAGTCGTGGCGC
AGATTGAACAACTCGGTGGCCGCGCCGCCATGTTGCAATTGGATGTGAGCAAAAGCGCCACATTCGACGGCTTCGT
CGGCGAAGTTGGCCGCACGCTGAAGGAAGTTTTCGCACAGCAACAGTTCGATTTCCTGATCAACAACGCCGGTATC
GGCACCCACGCCAGCTTTGTCGAGACCACCGAAGAACAGTTCGACCAAATGGTCGCAATCCACCTCAAGGGCCCGT
TTTTCCTGACCCAGAAGCTGCTCGCGCGGATCGTCGATGGCGGGCGCATCATCAATATTTCCAGCGGCCTGGCGCG
TTTCAGCCTGCCGGGCTATGCCGCCTACGCCTCAATGAAAGGCGCGGTGGAAGTACTGACCCGCTACCAGGCCAAG
GAGTTGGGTGCACGAGGGATCAGCGTCAACACGCTCGCCCCTGGCGCCATTGAAACCGACTTCGGCGGCGGCGCGG
TGCGCGATAACGCCGAGATCAATGCCTTTGTCGCCAGCAACACGGCCCTGGGTCGGGTCGGCCTGCCGGACGACAT
TGGCGGGGCGATCTCCACGCTGCTGGCCGATGGCAGCAAGTGGATCAATGGGCAACGCATCGAGGCGTCGGGTGGG
ATGTTCGTCTGACGGCCCGCGTCATCGTTAACGCCCTTCGCGAGCGAGCTCGCTCCTGCACAGGAATTTTTCCCGC

AGCACGTCGTAGACCCAGTGGTACACATAGGTGTAAGGCAGGAAGAACAACAGCACGCCGATATCCAGGATAAACG
CCTGCATCAGGCTGATATTCAGCCACGCGGCAATCAGCGGCACGGCAAACAGAATCAGCCCGCCCTCGAACAGCAG
GGCGTGCACGATCCGGGTCTTGACGCTGTTGGCCAGTTGCAGGCGCACCTTGAGGCGGTCGAACACGCTGTTGAAA
ATCATATTCCACGTCAGCGCCAACAAACTGATGGCCAGGGTCACCGCGCCCATTTCCAGGGCCGGCCGACCGGTGA
TCCAGACGAGCAACGGGGTACAGATCAGCAAGGCCAGGCCTTCGAAGCCAAGGGCCTGGAAAACACGTTCGGTGAC
GGACTTGGGGGTGTTCATAAGTGTGGCTCTGGGAATGTCTTGGGTTGCCATTATCGATCCCTAACTCGATACTTAA
TAATCAATAACCATCGATCAAGGCGATAGATGATGGCTTCGCATGAAGTGTTGCAAGCGTTTGTGCAAGCGGCGAC
CCAAGGCTCGTTTTCGGCGGCGGCGCGCAAACTGGGCAAGAGCCAATCAACGGTCAGCGCCGCAGTGGCCAGCCTG
GAGATTGATCTGGACGTGGTGCTGTTCGACCGCAGCAGCCGCAAACCCACCCTGACTCCGGCCGGGCATGTGCTGC
TGCAACGGGCCGAACACGTGCTGGAGGCCAGCAGCCGCCTGGAGCTTGCCGCCAGCCAGTTGTCCCAGGGGCTGGA
GCCCAAGCTCAGTATTGCCATGTCCGATACTTATCAGTCGGGACGCTTCGAAACCCTGCTCAGCGCCCTGGAACAG
CGCTATCCAGACCTGGAGCTGGAGTGGCTGATTGCCGAATGCGAAGACCTCATCGCCCTGGTGCAAAGCGGGCGGG
CGCAGCTCGCGTTTATCGAGGCGCAGGAGGTCTACCCGCCAGACCTGACCCATGCCACCGTCGATGAGGGCGCGCA
GCTTGCGCTGTTTGTTTCCCCGCGGCATGCACTGGCCAGCCTCGATCCCATCAATCAGCAAAGCCTGCAACAACAT
CGCGAGCTGCGCCTGGCAAGCATCATCAACCCCAATGAAAACCGGCAGACCGGCCGAGTGTGGTCCGCCCCCAGCT
TTTTGATGCTGATGGAAATGGCCATCCTCGGTTTTGGCTGGGCAGCCTTGCCGCGCTGGTTGGTGGAGCGCTTTGC
TAATGGCCAACTGGTGGAGCTCAAGGCTCGCGGCTGGCCACGCTCGGTGGCCGTGGATGCCTTATGGTCACGGCAA
CACCCGCCAGGCACTGCGGGGAGCTGGCTGCTGGAAAAAATGCTCGAATAGCCTCTGTGGGGGCCGGCTTGCCGGC
GCCTACAAGGAGGTTTCGGTGCAGGTGGCTAGGCGACGTTCGATGAAGCGTTTTTCCGGGGTTTGATGGGTCAGGG
CCAGGGCTTTTTCGTAGGCCTGGCGCGCCTCTTTCATACGCCCCAATTGCCGGCAGAACTCCCCACGCGCCGAATG
CGCCAGGTGGTAGTCCAGCAACTCGCCACGCGCCAGGATTCCCTCCACTTGCTGCAACCCCGCCAAGGGCCCATCG
CGCATGGCCAGGGCAGCAGCGCGGTTGAGTTCGATCACCGGCGACGGCAGCATCCTCAGCAACACGTCATACAAAC
CAATAATCTGTGGCCAATCCGTCTGCGCCGCGCTCGGCGCTTCGGCGTGCACTGCCGCAATCGCCGCTTGCAGGCA
GTAAGGGCCAAAGCGCCGGGTGGCGAGGGCCTGCTGCACCAACGCGCAACCTTGGGCAATCAACCCTGCATCCCAT
AGCGTGCGGTCCTGTTCATCCAGCAGCACCAGTTCAGCGACAGGTGATAACCGGGCCGGGCGCCGGGACTCGTGGA
GCAGCATCAAGGCCAGCAGGCCCATGACTTCCGGTTCCGGCAGCAACTCCAACAGCAGATGGCCCAGGCGTATGGC
CTCGCGGGTCAAGTCATCACGGGTCAGGTCGGCGCCCATGGACGCCGAATAGCCCTCGTTGAACACCAGGTAAATC
ACCCGCAGTACGCTGTCCAGGCGTTCGGGCAACTCCGCCAGGGCAGGCACTTGATAGGGAATCCTCGCTTCGCGGA
TCTTGCCCTTGGCGCGCACGATGCGTTGGGCGATGGTGGCCGGCGTGACCAGGAAAGCCCGGGCAATTTCCTCGGT
GGTGAGGTCACAAATCTCACGCAGGGTCAGCGGCACCTGGGCATCCGCCGCCAGCGCCGGGTGGCAGCACGTAAAG
ATCAGGCGCAGGCGGTCGTCTTCCACGTCTTCGGTACTCCAGTCAGCCGCTTGCAGCGCATCGGCCTGCTCCTGCA
GCAACGGGGTGAAGCGGGCCTGGCGACGCAGACGGTCGATGGCCTTGAAGCGCCCGGTCGAGACCAGCCACGCCCG
CGGGTTGTCAGGCACCCCCTCCTGCTGCCAGCGCTCGACCGCGACGAAGAACGCCTCATGCAGGGCTTCTTCGGCG
AGGTCGAAATCCCCCAGCAGGCGAATCAAGGTCGCCAGGATGCGCCGGGACTCGCTGCGGTAGATCGCCTCGACCT
GGTCTTTGACGGTCAATCGCGCAGGCTCTGGGTCACCAGGCTCACCAACCGATCCAGGCTCTCGCCCCAGCCCTGA
TGAAAGCCCATGGCTTCATGGGCCTGCTTGTCGGCTTCGCTCCAGTGCATGGCGCGGGCGGTGTAGCGGGTCTTGC
CCTCGACCTCCTCGAAGGTCACTTCGGCGCTCATGAACGGCTTGCCCGATGGAATCCAACCCGGCAGGAATGCATC
GGTAAACACCAGCCGGCGCGGCGCGTCGATCTCCAGGAATACGCCCTGGGTCGGATACTCGCTGCCATCCGGCGCG
CGCATCAGGGTGCGAAACAGACCACCGACCCACAGGTTCATTTCGCACTCCGGCGTGGTCATGCCGTGGGGGCCCC
ACCATTGCTGCAACAAGGCCGGCTCGGTCCAGGCACGAAACACCCGGCTCGGTGGCGCGTCGATCACGCGGCTGAT
GGACAGTTCGAATGCAGCAGGTTGAGTGTTCATTGGGTAAACCTCCTGAGTCTTATGATTGTTGGGTTCAGAGATT
CAATTCACGCACGGGGCGCACTTCCACACTGCCGACCCGCGCCGCCGGGATACCACCGGCTACCTGCAGCGCCTCA
TTCAAGTCCTTGGCCTCAATCAGGTAGAAGCCGGCAAGCTGCTCCTTGGTTTCGGCGAACGGCCCATCGGTGATCG
ACAGCTTGCCGTTGCGCATGCGTACGGTGGTGGCCGTGCTCACCGGCTCCAGGGCCTGCGCCGCGAGCATGCGGCC
ACTGGCGTGGATCGCTTCGGCGTAGGCGTGGCATTCCGGGTCCTTGGGGCTGTCGGGCAGGCTGTGCAGCAAGGCT
TCGGCGCTGTAGACCAGGCACAGGTATTTCATGGCGTTCTCCGTTTGCGGACTACTGACTATAGTTGGCGGCGATC
AAGGTTTCAGGTCGAACAGACCGGCGCCGGTTTCCATATCGAACGGTGCAGACCAGTGCTCATGCACCACCTGCCA
TTGCCCGTTGGTCCGCTGATAACCGGCGGTGACGCGCATCCAGCAGGTCTTGAGCACGCCGTCTGGCCCCGAACCA
CCACAGTGCGCGAGCCAATGGGCAAAGGCGCTGTTTTCGGCGGCGACGATATGCAGCTCATGGAACTCAAAGACAC
CGGGGCCCGGGCAAGACTCCATGCAGGCCTGCCAATGGGCGCGGTAGGCTGCCTTGCCTTTGAACTGCAGGGCATT
GACAGCATCGAACGCGACGATATCGTCGGCGTAAAAGCTGACAATCTGCTGGATATCCTTAGCGATGACTGCTTGT
TTCCATTGCTCGATCAGGCTGTTGATTGCGTTGTTCATGGCGATGCTCCTCGGGGTGCAAATCACGGAAGACACCT
TTGGTCGAATGCCCCGCCGGCAAATCGACAAGCCTGCGAAAAATAATTTCGCCGAGGGTAAAGCCGCTAGAATTCG
CGACTTCCCTGTAGGACTTCGGATACCTGCCCGATGGAAACCCGCCTCGCGCCCTATGAGACGCTGACCGCTGTCC
AGCAACAGCAACTCGCCACTCTGGAAGTCCACCCCGAGCAACTGCCCTATAGCGGCGACATCTACTGCGCGCTGAA
CAGCCTGCAGGTCAACCCCAGCCCCGGCATCAGGGGCTTCGCCCTGCTGGCCGACGACATTCCCGTAGCCTTCCTG
CTACTCAAGCGCCCGCCCTGCCTGCCCCATTGGGCCAGCGAACACAGCGCCACCCTGCACGCCCTGCAAGTCGATC

GCCACCAACAAGGCCGGGGCTTGGGCAAAGCCTGCCTGCAAGCCCTGCCCGCCGCCGCCCGCCAGGCCTGGCCGCA
GATCACCGCCCTGGAGTTGTCGGTGGACAGCGACAATGTGGCGGCGATGAGCTTGTACTTGCAGCAAGGCTGGGTA
GACCGGGGCGAGGCGTATAAGGGGCGGGTTGGCTATGAGCGGCGGTTGTCGCTACTGTTCTGACGACGCACAAGGA
GACTCCCATGCTAACCAGCCTCGAACAGCTCGAAACCCTTTACGGCAAGCCCCACGAGCGCGCCCTGCACAAGGAA
ATCGGATTTCTCAATGAGGATTACCAGGCGATGGTGAAAGCCTCGCCGCTGGTGATTGTCAGTTCTTGCGGGCCTG
ATGGCCTCGACGGCTCGCCTCGGGGCGATGTGCCGGGGTTTGTGCGCATCATCGATGAGCGCACCCTGGCGATTCC
GGATCGGCCGGGCAATAACCGGCTCGATACCTTGCGCAACCTGCTGGTGGACCCGCGCATCGCCTTGCTGTTTATC
ATCCCGGGGATCGGTGAGACGTTACGGGTCAATGGGCGGGCGCAGATCTCCATCGAGCCTCAGTTGCTGGAAAGCT
TTGCGGTGAATGGCAAGCCAGCGCGGTCGGTGATTGTGGTGCAGGTGGACGCTGCGTATTTCCACTGCTCCAAAGC
CTTTGTGCGCTCCGACTGCTGGAACCCGGACAAGCATCTGCAGCGCACGGCGCTGCCATCAGCGGGCGCCATTCAC
AAGCGTTTGAATGATGGGCAGTTCGATGCCGAGGCCTATGACCGGGAGATGCCGGAGCGGGTAAAGGCCACGTTGT
ATTGAGGCCCCAGCGGCGATGGCGCCCTTTGGTTCGCCATCGCCGGCAAGCCGGCTCCTACAGGTTATGCAAACTT
TTTGACAGGCAGCCAGATTTCCACGGTGCCGGTGCCCTTATCCACATCGAAGTCGGCGCTGTAGCGCTCGAAATCC
GCGCCCGCCACTGTGTAACCCGAGGTCGGCAGCCACTCGTACATGACTCGCTCATAGGTCTGCGACAGCGCATACA
CCGGCCCGTAATGCGCAAAGACCGCATAATGATTGGCCGGCACTTCGATGGATTCGAAGTTGCTTGGCACATCGCC
CTTGGCCGGCACTTCGACCCCGGCGATGTAATCGAATTCTTCTTGCGTGGCGTTATAGCAAACGCCGTAGGTCACG
CCGCCGATGCGGTTCTTGATCTCTTTGATGCAGGTGTCGAACAACTCCCACAACTTCGGGATGTCCCCCAGCGTGG
CCTTGCCGTAATGACCGCCGACCCCGGCGATCACCATGGCCTTGCCCTCTTCCATGCGGGGTTCGAGGGGTCGCGT
TGCCTGTTGGTTCATCTGCACAATCTCCATGGGTGAGTGACCAAAACCATACCGAGTATAGGGGCATACGCACTTC
ACACTCCAGATGGAAAAATTTCCTACGCCATCTGGACAACCGGCTGTCATCGCCCGTATTGTCTGCCCCGCAGGCC
ACCGCAGTGGCCGACGTCGCTCGGACGGTTCCGGGCGCTTACGTACTCCGAGGCAACAATGGCTGACCAAGGTTCG
CCGCGCCGCTTTGCGCGCATAGATCGACTCCCCCCTTATGTTTTCAATATCACTGCCGAGCTGAAGATGGCTGCGC
GTCGGCGCGGCGAAGACATCATCGACTTGAGCATGGGTAACCCCGACGGCCCGACTCCACCGCATATCGTGGAGAA
ACTGGTGACCGTCGCCCAGCGTGAAGACACCCACGGCTACTCCACCTCCAAAGGCATCCCGCGCCTGCGCCGCGCT
ATTTCGCGCTGGTACAAGGATCGCTACGAAGTGGATATCGACCCGGAAACCGAAGCCATCGTCACCATCGGTTCCA
AGGAAGGCCTGGCGCACTTGATGCTGGCCACCCTGGACCAGGGCGACACGGTCCTGGTGCCCAACCCCAGCTACCC
GATCCACATCTACGGTGCCGTGATTGCCGGCGCCCAGGTGCGCTCGGTGCCGCTGATTCCCGGCGTGGATTTTTTT
GCCGAGCTGGAACGGGCGATTCGCGGTTCGATTCCCAAGCCCAAGATGATGATCCTCGGCTTCCCGTCCAACCCCA
CGGCGCAGTGCGTGGAGCTGGATTTCTTCGAGCGGGTGATCGCCCTGGCCAAACAGTACGACGTGCTGGTGATCCA
CGACCTGGCCTACGCCGATATCGTCTACGACGGCTGGAAAGCCCCGTCGATCATGCAGGTGCCGGGCGCCAAGGAC
ATTGCGGTGGAGTTTTTCACCCTCTCCAAGAGCTACAACATGGCCGGCTGGCGCATTGGCTTTATGGTTGGCAACG
CTGAACTGGTCAACGCCCTGGCGCGCATCAAGAGCTACCACGACTACGGCACCTTCACCCCGCTGCAAGTGGCGGC
CATTGCCGCGCTGGAAGGTGACCAGCAATGTGTGAAAGACATTGCCGAGCAGTACCGCCAACGCCGCAACGTGCTG
GTCAAGGGTCTGCATGAGCTGGGCTGGATGGTGGAGAACCCGAAGGCGTCGATGTACGTCTGGGCGAAGATTCCCG
AGGCGTATGCCGCCATGGGCTCCCTGGAATTTGCCAAGAAGTTGCTGCTGGAAGCCAAGGTGTGTGTCTCGCCGGG
GATCGGCTTTGGCGAGTATGGCGATGACCATGTGCGCTTTGCGCTGATCGAGAACCAGGACCGCATTCGCCAGGCG
GTGCGCGGGATCCGGGCCATGTTCCGGGCTGATGGGCTGGTCACGAAAGCCTGATAGCTGCCCGATAACGAATGTG
GGAGCGGGCTTGCCCGCGATTGCTGTGTGTCAGGCAATACATCTTGCACTGATACACCGCAATCGCGGGCAAGCCC
GCTCCCACATTTTGCTTGGGTTACACCACCAACGACAGCAACATGATGAAGCCCAGTGCCACGATGGACAGAATGG
TCTCCATCGCCGTCCAGGTCTTGAACGTCTCGGCCACGGTCATGTTGAAGTACTGCTTCACCAACCAGAACCCTGC
ATCGTTGACGTGCGACAGAATCAACGACCCCGCCCCCGTCGCCAGCACCAGCAACTCCAGGTTGACCCCCGGCACC
ATCCCCACCACCGGCACCACGATACCCGCGCCAGTAATGGTGGCAACGGTCGCCGAACCCGTGGCAATGCGAATCA
CCGCCGCCACCAGCCAGGCCAGCAGGATCGGATTGATCTGCGCCGTCACCGCCATATGGCCGATCACGTCACCCAC
GCCGCTGGTCACCAGCATCTGCTTGAAACCACCGCCGGCACCGATGATCAGGATGATCGCGGCGGTCGGCGCAAGG
CTGGCGTCGAGCAGCTTGAGGATCTGCTTGGAGTGGATGCCCTGGCGATGGCCAAAGGTGTAAAGCGACAGCAGCA
ACGCCAGGAGCAAGGCACTGATCGGGTGGCCGATCATGTCCATCCAGTTGCGCACCACATTGCCTTCGGCAAAGGC
GATGTCAGCAAAGGTTTTGAGCAGCATCAGGAACACCGGCAGCAGCACGGTGATCAGGGTGATGCTGAAGCTCGGC
AGGTTTTTCGAGTCGGTCTCACGGGCCAGTTGATCCACCAGCTCCTGGGACGGGTTGCCCGGGATGTGCTTGGCAA
TGAACGTACCGAAGATCGGCCCGGCAATGATGGCGGTCGGCAAGGCAACGATCAGGCCGTAGAGAATGGTCTTGCC
AATGTCTGCGCCAAACACGCCGATGGCCAGCAACGGCCCCGGGTGCGGCGGCACCAGGCCATGGACCGCCGACAGG
CCGGCGAGCAGCGGGATGCCGATCTTGATCAGCGATACACCGGTACGCCGCGCGACGATGAACACCAGCGGGATCA
GCAGCACAAAGCCGATCTCGAAGAACAGCGGGATGCCCACCAGGAAGGCGGCGAACATCATCGCCCATTGCACCTT
GTCCTTGCCGAAAGCACGGATCAGGGTTTGCGCGATCTGATCGGCACCGCCGGATTCGGCCATCATCTTGCCGAGC
ATCGTACCCAGCGCCAGGATAATCCCGACAAACCCGAGCACACCGCCAAAACCGTCCTGGAAGGCCTTGATGATCT
TGTCCACGGGCATGCCCGAGGTCAGGCCAAGGAAGCCTGCGGCGATGATCAGGGCAATAAAGGGGTGAACCTTGAA
CCGGGTGATCAACACGATCAGCCCGATGATAGTGACCACTGCATCAAGCAGCAGGTACGACTCGTGGGACATTCCA
AACATGGGGGTCTCTCCTGTTTGTTGTTGTTATTAAAGCGGGTGTTGAGTTTCGTGCCGCCAGATAGCGCTATCTT

TCGGCCAAAAAATCATCGGGAAGGTTCAAAGCCGTGGGTCAGCCACCAGGCGTGGGTCTGCGCGGCCAGGTCCTCG
ATGCTGTCTTCACTGGCATTGAGGGCCAGGGTCAGCGGCTCGCCGATAGGCGATTCGAGGGTGGCGAACTGGCTGT
CGATCAGGCTGGCCGGCATGAAATGGCCGGGGCGATGGGACACGCGGTCGGCGGCCACCTCGCGGGTCAGTTCCAG
GAAGACAAAGCCCAGGCCCGGCGCGGCTTCGCGCAGGTGGTCGCGGTATTTTTTCTTCAGCGCCGAACAGGTCAGC
ACTGGATGCTCGCCAGCCTTGAGCGCACGGCGCAGCTCATCGCAGAGGATATCCAGCCAGCCGGCACGGTCGTCGT
CGTTGAGAGGATGGCCTGCGCTCATCTTTTCGATATTGGCAGCCGGGTGAAAGCTGTCGCCTTCAATGGCGGTGGC
GCCGTTCAACAGGCACAGGGCCTCGCTGACACTGGACTTACCACAGCCGGAAACACCCATGATGACCAGGGCGGTA
ACAGGTTGACTCATGAAACACCTCAGGACGCAGATAGCGCTACCTTTGCACGCCCTAAGGCTAGTGCAAAAACAGG
CTTTTCCCGCGCCTAATTGTCATTTTTATGGAGTGACGCGTGCATCCGCTCCAACCGTTTGAGCAAGGATCAGGCA
ACCCTTTGCGCAAGTATTTGCAGGCCTTTGGAGACAGCGCTACCTTAGTGCCTCGATTTTTGTTTGGCAAGCCGCC
TGATGACCTCCAAGAACGATAAAAATACCCGCACGACGGGTCGCCCTACCCTCAACGAAGTTGCCCGCCTGGCGGG
CGTCAGTCCGATCACGGCCTCCCGGGCCTTGCGCGGCATCAGCACCGTGGCCCCCGACCTGGTGGAAAAGGTGCAA
AAAGCCGCCGCCGAGCTGAGCTACGTGGTCAACCCGGCCGCCCGCGCCCTGGCCTCTGCACAGAGCCAGTCGGTGG
TAGTGCTGGTGCCTTCGCTGTCCAACCTGCTGTTTATCGAAACCCTGGAGGCCATTCACCAGGTGTTGCGGCCCAA
GGGCTTCGAAGTGCTGATCGGCAATACCCACTATTCCCGGGACGAAGAAGAAAACCTGCTGCGCAACTACATGGCC
TATCAGCCGCGGGGCTTGCTGCTGACCGGTTTTGATCGCACCGAAAGCGCCCGGCGCATGGTGGAGTCGAGCAATG
TACCTTGCGTGTACATGATGGACCTGGACCCCACGGCCGGCCTCAACTGCGTGGGTTTCTCGCAGATCAGTGCCGG
CGAAACCGCCGCCGCCCACCTGATCTCCCGGGGTCGCAAGCGCCTGGCCTATGTTGGCGCACAGCTGGACCAGCGC
ACGCTGCTGCGCGGCGAAGGCTTCCGCCAAGGCCTGCAGAAGGCCGGTTTGTACGACCCGGCATTGGAACTGCTGA
CCCCGCGCCCCTCGTCGGTGGGACTGGGTGGTGAACTGTTCCTGCAACTGCTGGCCAGCCATCCGGACGTGGACGC
GATTTTTTTCGGCAACGATGACCTGGCCCACGGCGCCTTGCTCGAAGCCTTGCGCCACGGGATCAAGGTACCGCAG
CAAGTGGCGGTGCTGGGCTTCAACGACCTGCCCGCCTCCAGCTTCATGGTGCCGCGTCTGAGCAGCATCAGCACCC
CGCGCGAAGCCATTGGCCGACGCGCGGCAGAGCATTTGCTGACAGTGATGGCAGGCAACAAGATCGCCCGGCCGGT
GGTGGATATGGGGTTTGAATTGAAGGTGCGCGAGAGCACCTGAACCAATGTGGGAGCGGGCTTGCCCGCGATAGCG
GTGTATCAGTGCCAGATGTATTGCCTGACACACCGTTATCGCGGGCAAGCCCGCTCCCACATTTTTGAGCATGCCC
ACTTAACTCTATCGCTGTCCTCCCGCGCCCCTGCTAAATTGCCCAACTCACCTTTTGCCAGGGAAGCACCATGGGA
CACGCGCTGAAAATCTTGGGTCGCACTTCGTCCATCAACGTACGAAAAGTCCTGTGGACCTGCCAGGAACTGGCTA
TCCCCTACCAGCGTGAAGATTGGGGCATCGGCTTCACCCCTACCCAGTCACCCGAATTCCTTGCCTTGAACCCTAA
CGCCCAGGTACCGGTCATCCTCGATGACAATGGCGTGCTATGGGAATCCAACACCATTTGCCGTTACCTGGTGGGC
TTGCACCAACGCCACGACCTGCTGCCCGCCGCCCCTGCGCCACGGGCCAGGGTCGAGCAATGGATGGATTGGCAAG
CCACCGAGCTCAACCCCTCCTGGGGCTACGCCTTCCATGCCCTGGTGCGCAACAACCCGGACTTCCAGGACCCGCA
GCGCGTTGCCGCCGGCGTGCGCGGCTGGAACGACAAGATGGGACTGCTGGAGCAACAATTGATCAAAACCGGCGCC
TATGTGGCCGGGGATAACTTCACCTTGGCCGATATCCTGATCGGCCTGTCAGTGCACCGCTGGCGGATGACGCCCA
TGGAGCGCCCTGCCTACCCGGCTGTGGACGCGTACTACGCCCTACTCAGCCAGCGCCCAGGTTTCCAGGCTTTTGC
CCTCGACGGTCACAACTAAGGACTACACGATGAAAGGACTCAACGTACTGCTCACCGGTGCCTGCGGCAGAATCGG
CAAGACCTTCTTCGAGGCCTCGAAAGATCGTTACCGCTTCACCCTCACCGACCGCGTCACACCAGGCTTCGCCCTC
GACGGCCATCGCTTCGTGCAGGCCGACTTGAGCGACAAAACCGCACTGCCAGCGCTGCTCGACGGCATCGATGTGA
TCGTGCACCTGTCGGGCATTCCCCACGCCAGCGCGACCTTTGACGAACTGCTACCCAACAATATCCTCGCCACCAC
CTATCTGTTCGAAGCGGCCGTGGCCGCCCACGTCAAGCGCCTGGTCTTCGCCAGCAGCGCCCAGACCATCGAAGGC
TATCCGGTGGACCGGCAAATCACCCCCGGCATGCCGGTGCTGCCGGCCAACCTGTATGGCGTGAGCAAATGCTACG
GCGAAGCGCTGTGCGGGTATTACGCGGCCAAAACCCCACTCTCCACTATCGCTCTACGCATTGGGGCCTTTGAATT
TGCCGAAACCCACGACCTGAACAACGCTCGCGACCTCAGCGCCTGGCTCAGCCCTCGGGATGCCGTCCAGTTGTTG
CAGCGTTCGGTGGAGGCGGAAGGGGTCAAGCACCTGATCGCCCATGGCATTTCCAACAACCGCTTCAAGCGCCTGG
ATTTGAGCGAGACCACGCGGGTCCTGGGTTATCGACCGGTAGATGATGCATTCCAGCTATTCGAGATTCCCATCAC
CTACTGAAGCCTGTGGCCGCCGGCGGCAAACATTCGCCGTTGGCGGCCCCCCTCATGACTATGCAGCGCCCTCAAA
GCCAATGAATCCGGGGGTGTTGGCTCTCGGCACGCTAATTGCTAAATCCTCTGCATCGTTCAAACCCACAGGTGCC
CGATCATGTTGGTCAATACCCAAGTCTCGTCCGTGATCCAACAGTCCAAACGCCCCGACCTGGACCCCGCCAACGC
CGCCGCCAGTGCGCTGTACAGTGGCGTGATGCAGAACGTGCAGAATGCTGCTGCCGCGACCCAGGTGCAGGCCACG
CAAAAGGCCACTGACGCCACCGCAAGCAACGTGGATGCCGCTTTTGCCAAGACCCGCCTGCAACTGCAAGCGACCC
CGCCGACCAGTGCCACGCCGACCACCGCGACCAGCACCCAGGCCCGCGCAGAATTCACCGACTATATGACCAAGAC
CCCGGCCGAACGCATCCGCGATCAGATCCTGCTGGAAAAGGGCCTGACCGAAGACGATGTAAAAGCGATGCCCGTG
GAACAACAAGACGCTATTGCCCAGGAAGTGGCTGATCGCCTAAAGATGCAGGTAGCGGAGCAGTTCGCCGAGAAGA
CTGCTGATCCGCAGATCAAGGCCGTGAAAGACGCCCTCGCCGCAATCTGACTCAGCACAACGCCCTTTGTAGGAGC
GAGCTTGCTCGCGAAAAACCTGAGACCGCCACGGGGAACCAGATACCCCGCGTTATCGTTGAAGTTTTTCGCGAGC
AAGCTCGCTCCTACACAGAGGCACTATTGCAGTTGCAGGGTGGAGTTGAACTGGCCGATGGCGTCCACCACATGCC
GCGAGCCTTCCTGGATCTCCAGGATCACCTGCCCTGCTTCATTGGCCAACTCCACCCCCAGCCCGGTACGACTCAG
GCTCGACTGCATGCTCGACACCGCGCTCAGGGACAAGTCGTGGTTCTTGCGCACCACATCGACAATCTCGATTGTC

GCCTGGCTGGTACGCGCCGCCAGGCTGCGCACCTCGTCCGCCACCACCGCAAAACCCCGCCCGTGCTCACCGGCCC
GTGCCGCTTCAATGGCCGCGTTAAGCGCCAACAGGTTGGTTTGATCGGCAATCCCGCGAATGGTCTGCACGATGGT
GCCGATAATGTCCGACTGCTTGCTTACCGCGTCGATGCTGACTGCCGCTTGATTGAGGTCGCGGGAAATTTCTTCG
ATGATCTGCACCGTCTGCTGCACCACTTGCGACCCCTTGCGGGCACAGGCGTCATTCTGCACCGAGGTGGCATGGG
CCGAATCGGCAGCGGTGCGCAAGGTAGTGACTTGGTCGGTGATGTCACTGGCGAACTTCACCACTTTGTACAGACG
CCCGTTGGCATCGAAGATTGGGTTGTAGGAGGCTTCCAGAAATAGCGTCTGACCCTGTTTGTTGCGCCGCTCAAAA
CGATGGGAGTGGAATTCGCCCCGGTTCAGTGACGCCCAGAATGCTTTGTAGGTCGGCGATTCCACCTCGGCGCGAT
GACAGAACATGCTGTGGTGCTGGCCGACAATTTCACCGAGAGAGTACTGCACCGTCTTCAAGAAGTTCTGGTTGGC
ATTGAGGATCTGCCCCTGGGGCGTGAACTCGATGACCGCCATGGAGCGGCCTATGGCATCGATCAGGCTTTGGTTT
TCGTGCTCGCGGTGAACCCGTTCGGAAATGTCCGCAGCCACCTTGATCACGCTCTGCACTTGTTTATCCGGACCGT
AGACCGGCATGTAACTGGCCTCCAGCCACACTTCCTGGCCAGTTTTGGTCAAGCGCATGAACGTGCCGCTGATGGG
TTCGCCCTGGCCCAGGTCGCGCCACAATTTGGCGTAGTCATCGCTGCGGTAATAGGACTCTTCACAGAAGATTCGA
TGGTGTTTACCGCGTACTTCCTCGGCGCTGTAACCCATGACCTTGCAGAAGTTTTCGTTGGCATCCAGTACCACGC
CGTCGCGATCAAACTCGATCATCGCCATGGAACGGCTGATGGCCGCCAACTTGGCTTGGGCCTGGGTCAGCGCGCA
GGAAAAGCGCTCGATTTCCAACAGGTCGGATTTGTGGTGGAGGTTAAACATGATCGGATCACCTTCAGCGCTGTCT
TTGAATGACGGTTAGTTCTTGGATTTGACCCAGTACAACTTTCCACAAAACAGGCAAACGCAGCTGTCCATAAGGC
AGGACAAGTGTCAGGCGATAGATAAATGATGGCTAATCGGAGAGTCCATGGGACAACGCTCGTTTCAAGGCATCCT
TTTCTTGATGACCCCAGCCGGGGCCCGCTTTAACCGACACACGTAAATCCATTTAACCTGCAGCTCCTTTTTCCGG
CGCTTTGACATACGCACTTATCTATTCACAGTTTGTATAGTGCTCATCAACCACTATGCAAACAGCCACCCGAAAC
GCTTCATATGGCAAGCGACATAAGTGGTTATGGGTGAGCATAGACAGGGCTTGGCGCTATGCAAGGCCATTAATCA
GCCAGCATTTATAGACAGGTGTTGGAGGGGATGTGCAGGGGCAGGATGTATCGATGCCGCTGTAGGAGCGAGCTTG
CTCGCGAAAAGCTCGAGAGCGCCGTGTTTTTCCTGCGAACACGCGTTTTCGTTAACGCTCTTCGCGAGCAAGCTCG
CCCCTACAGTGAAGTACTCAGTGACCGAACAGACCTGTCTCGGTCTTCTTGGCTTTTTTGTCCGCACGTTTTTCAT
CAGCGGTCTTTGCCGGTTTTTTCTTCGCGGCTTTCTTTGAATCCATACCTTTGGCCATGCTGCATGCTCCACTAAC
AGAGGAAGTAACAAGGGGTATAACACCTATTGTCCACCAGAAGGCGCTTTGCCCGACTTATAATCCCCGCCCCTGG
ATCACACCTGTCATACCGTCATGCCCGAGTTGCACATTGATCGACTGGCAGAGCCCCTGTGGCCGCTGCTTAACAA
GTTTTATCGCAGCCACAACTCATCGATGAAAGCCCTCAAGGGCGGCCAATTGTGGGTGGCGCGTAATAGCGAGATT
GTGGCCGGTCTGTGCCTGAGCCCCGTGGTCGGCGGCCAATGGCTGACCGGGCTGTTTGTCGATCCGGCATTACGTG
GTCAAGGGCTGGCGACGCGGCTGATTGCGCAAGCGATCGCGCCTGTCGAAGGCTCGGTATGGCTGCTGTGCCACCC
GGACCTGGAAGGGTTTTATGCAGCCCAGGGCTTCAGCCAGCAGACGGTGTTGCCACAGTCCCTGGCCGAGCGCCTG
GTGCGCTATAAGCGCAACAAGCCGATGATCGCCATGGGCGTGTGCCGCCAGGCCTAGGCGTCGGCGGCAGGATCCA
GTTCAGGGAACATCACCTCGATAAACCCGAACTTGCTGAAATCAGTGATGCGCGAAGGGTACAGGCGACCGATCAG
GTGGTCGCATTCGTGCTGCACCACCCGCGCATGGAAACCATCGGCAATCCGCACAATCGGCTCGCCCTTGGGGTCA
AACCCTTCGTAGCGAATCTGCTGGTAGCGATCCACTGCACCACGCAGGCCTGGCACCGACAGGCAGCCTTCATAGC
CCTCTTCCAACACCGGGCTCAGCGGTGTAATCAGCGGGTTGATCAGGATGGTCTGGGGCACCGCCGGGGCATCCGG
GTAGCGCTCGCTGCTTTCGAAGCCGAAGATCACCAGTTGCAGATCGACACCAATCTGCGGCGCGGCCAGGCCGACG
CCGCCCACATGCTCCATGGTCTGGAACATGTCATCGATCAGTTGCCACAGCTCGGGGCTGTCGAACATTTCCGGTG
GTACCGGTGGCGCGATACGCAGCAGGCGTTCGTCGCCCATTTTCAGGATTTCACGAATCATCGGTCAGGTTTCATC
AGTGGTGGGCTTGGGAATCGAGTGGTCCCGGCCGAGGCCCGAGACATGCTGTTTTTCATCGGGGTGGTGTTCGCCA
AAATCCTTCTCCCCCGGATCCTTGCCTTCGGCGGACATATGTTCGATCACCGCATTCATTTCCGCACCGAGCAACA
ATACGGCGGCGGAAATGTAGAAATACAACAACAGCACGATGATCGCACCGATGCTGCCATACATGGCGTTGTAGTC
GGCGAAGGTCTTGACGTAATAGCCAAAACCCAATGAAGCCACGATCCAGACGACCACCGCCAGCACTGAGCCTGGC
GTGATAAAGCGAAATTTCTGCTTCACATCCGGCATCACGTAGTACATCAACGCCACGGCGAACATCAGCAGGATCA
CGATCAAGGGCCACCGCAGGATGGTCCACAGGGTAACGACAAATTCCTGCATGCCGATCTGCCCGGCCAGCCACTC
CATCACTTGCGGCCCCAGCACCATCAGCGCGGCAGCGGCCAGCAACATGCCGGCGATGCCCACGGTGTAGAGAATC
GACAGCGGGAAGCGCTTCCAGATCGGCCGGCCTTCCACCACGTCGTAGGCGGCATTCATCGCGCTCATCATCAGGC
GCACGCCGGCCGAGGCGGTCCACAGCGCGATGACGATACCCACCGACAGCAGCCCGCCCTTGGATTGCTGCAATTG
GTCGATCACCGGGTTGACCTGCTCCAGGGCCTGGGGCGGCAGCACCAGTTCCGATTGCAGGCGCAGCCAACTGAAG
AAATCCGGCAGGTGCAGGAAACCGATCAGGGCAATCAGGAACAGCAGGAAGGGGAACAGCGAAAACAGCATCTGGT
AGGCCAGTGCCGAGGCGTAGGTGGGCATCTCGTCATCGATGAATTCTGTGACGGTGCGCATCAATACGCGGTGCAG
CGGCAGATCTTTTAATACCGGAAAAATCATAGCGTCTCCTTTCGCCGCAAAAAGGTTGAGTTCGTGGGCGACTCAG
GGGCCGTTTTCTACATCAAGGTAGCCTATTTGGCGACCTTGAAACAATTTCGAAACTTTAAACATCGGGCGTGACA
TAAAAACGGCCATCCGTGGATGGCCGTTGTACTGCGTGTTGCAAATGCTGGAATCAAGCCTTGTCGACGGTATTCT
TTACCGCATCCTTGACCTTGCCGACCGCTTGCTGGGCTTCGCCCTTCTTCTCTTGAATCTTGCCTTCGGTCTCAAG
TTTGGTGTTGTCAGTGGCTTTGCCGACACCTTGCTTGATGTTGCCGATGGCTTCGTTCGCTAAACCTTTTGCCTTA
TCCGCTGTGCTGCCCATGGTATTTCTCCGTAAGAACAATCAAGGGTTTTGGTCATTACGTAAGGGTTGACCCTGGG
CCTCTGCGCAGAGTTTCAATTATTTGCGTGAGGCATTTCATCACCCCATACAGGTTTGGCTTTATGTTTTGCCGCC

AACCCCCGAGAATGCCCGGCAGATTCAGGCTCAATCGCTGAATAACAGATCCCGTAGGAAGGTTATGAAACTCAAT
AAATCACAGGCCATCGCCCGCAGAAACCTGGAACTGGGCGGTGCCGTGCTCGGCGTCAACAACTGCCACTTCACCG
ACCTGGACCGCAAGCGCAACATCTGGTGGTTCGACCTACCGGTGGCCCGCATTGCCATTGGGCAGTACGAGTGGAT
TCACTTGTTGATGCACAACGCCGAGACCGACCAGTTGCTGCACTTGAAAGTACCGACCGCGTTCCTGCGTGAAAAG
CTTGAAGGGTTGGTGGTGCGCAATGCGGGCAAGCGCAAGCCGGAGATTACCCTGGAGCTGAGCGCGGACAAGGACT
CGTTCCTCAAGGATGTACGCCCGGCAGGCGCTGGCGTGAGCTTCGCGCAGTTCGCCCTGTAAGCAGGCGCTCAAAA
ATGTGGGAGCTGGCTTGCCTGCTCCCACATTTGGATTGCAGTCCAATCAGCGCCCAATAAAAAGCCCCGTATTCAC
GGGGCTTTTTGTTTACTTCTTCAGGCCCAACTTCTTCAGCTCTTCATCACGCAACTCACGACGCAGGATCTTGCCC
ACGTTGGTAGTCGGCAGCGCTTCGCGGAATTCAACAGCCTTCGGCACTTTGTAGCCGGTGACGTTGGCGCGCATAT
GCTCCATCACCTGCTCCTTGGTCAGCGTCGCGCCCGGCTTGACCACGATGAACAGCTTGATGTGCTCGCCCGACTT
CTCGTCCGGCACACCAATGGCAGCGCACTGCAGCACGCCTGGCAAGCTTGCCAGCACGTCTTCCAGCTCGTTGGGA
TAGACGTTGAAACCGGAGACCAGAATCATGTCCTTCTTGCGATCAACGATACGCATGTAGCCGTCTGGCTGGATGA
TCGCGATATCACCGGTCTTCAACCAGCCTTGGCTGTCGAGGACTTCGGCGGTGGCGTCTTCACGCTGCCAGTAACC
TTTCATCACCTGCGGGCCCTTGACGCACAGTTCGCCGATTTCGCCCAGGGGCAACTCGACGCCCTCGTCCGAGATG
ACCTTGCACAGCGTCGAAGGCACCGGAATGCCGATGGTGCCGATCTGGATATTCTGGATCGGGTTGACCGTGGCCA
CCGGGCTGGTTTCGGTCATGCCGTAGCCTTCACAGATGTTGCAGCCGGTCACGGCTTTCCAACGCTCGGCCGCCGC
CAGTTGCAGGGCCATGCCGCCCGACAGGGTGACCTTGAGCGCCGAGAAATCCAGCTTGCGAAAGCCTTCGTTGTTG
CACAAGGCAACGAACAGGGTGTTCAGGCCGACAAAGCCGCTGAACTTCCACTTCGACAACTCCTTGACCATCGCCG
GCAGGTCGCGCGGGTTGCTGATCAGGATGTTGTGGTTGCCGATCAGCATCATCGCCATGCAATGAAAGGTGAAGGC
ATAGATGTGGTAAAGCGGCAGCGGTGTGATCAGGATCTCGCAACCTTCATTGAGGTTGGAGCCCATCAGCGCCTTG
CACTGCAGCATGTTGGCCACCAGGTTGCGATGGGTGAGCATCGCGCCCTTGGCTACGCCGGTGGTGCCACCGGTGT
ATTGCAGCACGGCCACGTCTTCGCTGGCGGGACTGACTTCGTTGACCGGCTGGCCATGGCCCTTGGCCAGGACGTC
GTTGAACTTGATCGCCTTGGGCAAGTGATACGCCGGGACCATTTTCTTCACGTACTTGATGACGCTGTTGATCAGC
AGCCGCTTGAGCGGTGGCAGCAGGTCAGCCACTTCAGTGACGATCACATGCTTGACGCCAGTCTTGGGTACGACTT
TTTCCGCCAGGTGCGCCATATTGGCCAGGCAGATAAGGGCCTTGGCCCCGGAATCGTTGAACTGGTGTTCCATTTC
CCGCGCGGTGTACAGCGGGTTGGTGTTGACCACGACCAGGCCGGCACGGATGGCGCCAAACACGGCCACCGGGTAT
TGCAGGAGGTTGGGCAATTGCACGGCGATTCGATCACCGGGCTGCAAGTCGGTATGCTGTTGCAGGTACGCGGCAA
AGGCACCGGACAGTTCGTACAACTCACCGTAGGTGATGGTCTTGCCCAGGTTGCTGAAAGCCGGTTTGTCGGCGAA
GCGCTGGCAGGACTGTTTCAGTACCGCCTGAATATTTTGATACTCGTCTGGATTGATGTCTGCAGCAATCCCGGCG
GGGTACTTATCCTTCCAAAAGTCTTCGATCATGGAAGCCCACTCCTCAGCGACGCGAATTCATTCACCGCATTTGA
TGCGATTATTATTTGTGTGTGTTTTTTTAGGTGATTCTGGCTGTTTCAAGCAGGCCGAGAGGTCACAAAGCGCGCC
GAGAGTAGCAGCTTTGCAAAGGGCCGCCTAGAGCCAAAAGAGGGCCCTACAGTCATAAACATGACTCAAGAATATG
CAGTAGTCATTTTTAGAGCAAAGATTCTATAACCCCTGAAACAGCTCTATTTTGCGGACTTACACCGTAAATGCAG
GAGCTGGCTTGCCAGCTCCCACATTTGAATCGCATTTCAATCCAGGTCAGGCGATGTCGCGCAGCTCCCGGCGCAG
GATCTTGCCTACCGGCGTCATCGGCAACGAGTCCCGCAACACAATGTGCTTGGGCACTTTGTAACCGGTGAAGTTG
GCCTTGCAGTAGGTCTTCAATTCCTCAAGGCTGACGCCCTGGGCCCGCGCCACCACAAACAGCTTCACGGCCTCGC
CGGTGCGTTCGTCGGGCACGCCGATCACCGCGCAGTTGGCGACGTTCGGGTGGGCCATCACTACGTCTTCGATCTC
GTTGGGGTACACATTGAAGCCCGAGACGATGATCAGGTCCTTCTTGCGATCGACAATGCGCACAAACCCGTCCGGG
TCGATCACCGCAATATCGCCCGTCTTGAGCCAGCCCTCGGCATCCAGGCTCTCGGCGGTGGCCGCCGGTTGCTGCC
AGTAGCCCTTCATGACCTGCGGGCCCTTGATGCACAACTCGCCACGCTCGCCCAGCGGCAACTCCACGCCGGCGTC
ATCGATGACTTTCATCGCCGTGCCTGGCACGGGAATACCCACGGTGCCCAGACGCGATTGATCACCATAGGGGTTG
GTGCTGGCCACCGGCGACGTCTCGGTCAGGCCGTAGCCTTCGCCGATGGAACAGCCGGTGATCTGCTTCCAGCGTT
CGGCCGTGGCCTTGACCAGGGCCGTGCCGCCGGAGTTGGTGATCTTCAGGTGGGAGAAGTCCAGGGTCTTGAAGTC
CGGGTGTTCCATCAGCGCGACAAACAGGGTGTTGAGCCCCAGCAGCCCGGTAAACCGCCACTTCTTCAACTCCTTG
ATAAAGCCGCCGATGTCCCGGGGATTGGTGATCAGCACGTTGTGGTTGCCGGTGACCATCATGCACATGCAGTTCG
CGGTGAAGGCATAGATATGGTAGAGCGGCAACGGCGCGACCATCACCTCCTGCCCTTCCTTGACCAGCGGATGCCC
GTCTTCGCGCACCTGTGACATGCACGCCCGCACCTGTTGCATGTTCGCCACCAGGTTGCCGTGGGTCAGCATCGCG
CCCTTGGCCAGGCCGGTGGTGCCACCGGTGTATTGCAGCACCGCGATGTCATCCAGGGTGACCGGATGGCGGGTCA
CGGCCAGGCCTGCGCCCATGTGCAAGGCGCGCTTGAACGACATCGCCCGGGGCAGGCTGTAGGCCGGCACCATCTT
CTTCACCTTGTCGACTACAGTGTTGATCAGCCAACCCTTGGCGGTGGGCATGAAATCGCCCATTTTGGCTTCGATC
AGGTATTCGATCTCGGTGTCGGCCGCCACTTCCTGCACCCTGGAACCGAACAGGTTGAGGTAGACCAGGGCACGAA
TGCCTGCGTCCTTGAACTGATGACGCATTTCCCGCGGCGTGTACAGCGGGTTGGTATTGACCACCACCAGCCCGGC
GCGCAAGGCGCCGAAGACGGCAATCGGATAATGCAGGACGTTGGGCATCTGCACCGCAATACGGTCGCCCGGTTTC
AGGTCTGTGTGGTGTTGCAGGTAGCCGGCGAACGCCGCGCTCTGGCGCTCCAGGTCGGCGTAGGTCAGGGTGATCC
CCATGTTGCTGAATGCCGGACGGTCGGCAAAGGCCTTGCAGGAACGCTCGAAGACTTCGATTACTGACTTGTACGC
CGACAGGTCTAACTCATTGGGCACACCGGCCGCGCGTTTGTCATTCCAGAAATCAGGTTGCATTATTCTTGTCCTC
TTACCCGAGTGTGCCCGACCGCTTCCGGCTGTTTACGGGAAGCGGAGCTTCGGGGACGTTAGCAGTTATGGCCAAA

CAGGCAAATACACAAAAACGTGTCATTGATTGTATGAATCTTGCCCCTGAGGCCAAGGGTGATCAGACGCCCGCCC
ACAGATGAGCTATACACTGCAACGACCCTGAGCAAAGGAAGCGCCATGAACCACACCACTTTCTGGCTGACCGCGA
ATGACCGCAGCCGCCTGCACGTCAACCAGTGGCTCCCGGACGAAACGCCCACGGCGCTGGTGATGCTGGCCCATGG
CATGGCCGAGCATAGCGGCCGTTATGCGCGCCTGGCCCAGGCCTTGTGTGGCGCCGGCTACGGTGTGTATGCCCCG
GACCTGCGCGGGCACGGCCGCACCGCCGATGAAGGTACCCTGGGCCTGTACGCCGAGCGGGATGGCTGGAACAAGG
TGGTGGGTGACCTGGCCGCCCTCAACCAGCATATCGGCCAACAGGCCCCCGGTGTACCGATCATCCTGTTGGGCCA
CAGCATGGGCAGCTATATCGCCCAGGCCTATCTGCTGCACCACAGCGCCAGCCTGCACGGGGCGATTCTCAGTGGC
TCGAATTTCCAGCCGGTGGCCCTGTACCGCGCCGCCCAACTGATCGCCCGCGCCGAACGCTTGCGCCAGGGCCTGC
GGGGGCGCAGCGCGCTGATCGAGTTCCTGTCGTTCGGCTCGTTCAACAAGGCCTTCAAACCCAACCGAACGGCATT
CGACTGGTTGAGCCGCGATCCGGACGAAGTGGACAAGTACATCAATGATCCCTTGTGCGGCTTTCGCTGCACCAAC
CAGCTGTGGATCGACTTGCTCGGTGGCTTGCAGCAAATCAGCAAAGCGTCCAATCTCAAACAGATTGATCCGGGCC
TGCCGATCCTGGTGATGGGTGGCGAATGTGATCCGGTCAGTGAAGGCAAGCGTCTCACCAGCCTGGCCAACGCGCT
GCGCGATGCGGGCTGCCAGAACCTGCAATTGAATATTTACCCGCAGGCCCGGCACGAACTGTTCAATGAAACCAAC
CGCGATGCGGTCACCGCCGATGTGCTGGCGTGGATGGCCCAGGCCGTGAACCATCGCCGGCCGGCACGCTGCGAAT
GAACAGAAAAATATTTTCGTTTAAAATCAACGAATTAAGACTATCGATTAGCCACAGGATGCACGATTAGATGACC
CAGGTTACCAACACGCCTTACGAAGCCCTCGAAGTCGGCCAGAAAGCCACCTACAGCAAAACCGTGGAAGAGCGCG
ATATCCAGCTGTTCGCGGCGATGTCCGGCGACCATAACCCGGTGCACCTGGATGCGGAGTTTGCCAAGGCGACCAT
GTTCAAGGAGCGTATCGCCCACGGCATGTTCAGCGGCGCATTGATCAGCGCGGCAGTCGCCTGTGAGTTGCCTGGG
CCGGGCACCATCTATATCGGCCAGCAGATGAGCTTCCAGAAACCGGTGAAAATCGGCGACACCCTGACCGTACGTC
TGGAAATTCTCGAGAAGCTACCCAAGTTCCGCGTGCGGATTGCCACCCAGGTGTTCAACCAGCGTGATGAGTTGGT
AGTGGATGGCGAAGCGGAGATCCTCGCACCGCGCAAGCAACAGGTCGTGACCTTGACCCAATTGCCAGCGATCAGC
ATCGGCTGAAAATCCCTGGTCCTGTGAAAGCAAAATAATGTAGGAGCGGGCTTGCCCGCGATGGCGGTGAAGCAGT
CGATATCTAAATTGACTGATCCACCGCCATCGCAGGCAAGCCAGCTCCCACAGTTGTTTTGGGGCGTACTCAAACA
TCCTGCCCGGCACAAACCCCTTGTGGGAGCGGGCTTGCCCGCGATGGCGATGTGGTAGTCGACATCTAAATTGACC
GATCCACCGCCATCGCAGGCAAGCCAGCTCCCACAGTTGTTACTGCGAGGCTACTCAGGACTGGGCGCGGGCCTGG
TTACGCAGGGCTTTGACCTGGTCATGGTTGCGCTGTGCGCCGTGGTACTGACGCTCAACCAGGTCACGAATACCGA
CCAGGTTGTGCTTGTTGATTTTCTCGATGGCTTCTTTGTAAGCCTTCAACGCATGGTCTTCACCACGCTCGGCTTC
GTTGAGGACTGCTTCTTCATCCTTGCCAGTCACCAGCGACTTCACGTCAACCCAGCCACGATGCAGGGCGCCAGCA
ACGCTGCCGGAATCTTCCGGGTCACCACCCAAGGCACGCACGGCTGTCTGCAGTTCGGCAGCCGCCGTGGCGCAGT
CAGTTGAACGCTTGGTGAACAGCGCCTTGAGCTCTGGGTGCTTGATGTCTTCTGCGCAGGTCTTGAAGCCTTCCTG
GCCGTCTTTACTGGTTTCGATCAGGTCGTTGAGTACGGAGATCGATTCTTTGTTGATGTCAGTCATTTTTCAATTC
CTTCACAGGTTTAGAAACGTACTGGGGTAGTTGCAGCGCCCGTGCCAGGTCTTGAATTAAAAATTAAACCTTAATT
TTCAACGAGTTATAAATAAACTCCAAATCTGTATCCGCTTTATTTGCATGATCTGTCATTTGGCCTTCATGCAGAA
TGCCTGTATTTTCCAGGGCATCCTCCTGATTGCCTGAAGCACCTATGAACCCCGAAAAACTCGAACTGCTGATCAC
CCGCCAAATGCCGTTCGGCAAGTACAAGGGCCGGATCATTGCCGACCTGCCCGGTCCTTACCTGAACTGGTTTGCC
CGCGAGGGTTTCCCCCACGGCGAGTTAGGCGGCCTGCTGGCGTTGATGCAGGAAATCGATCACAACGGCCTGTCCG
AACTGCTCGAACCGCTGCGCGCCAAACACGGTAAACCTGCCCCTCGCCACTAATCGAGCCCGACCATGCCCTACAC
CCGCGACGAGGCCTACTGGCAGGCCATTGCCCAGCGCTATGAGCTGGAACCCGGCCCCATCAACCTGGAAAACGGC
TACTTCGGCCGTATGAGCCGGGCGGTGCTGGCGCAATATCAGGAGCATGTGGCCTTTATCAATCGCAGCAACTCGG
TGCATGTGCGCCAACGTTTCGAGCAAGGAGACAATGTCGAGATTCGCCGGCAACTGGCCGAATTAATCAATGTCGA
TCCAGAAGCCATCGCCTTCACCCGCAATGCCACCGAAGCCCTGCAATCGTTGATCCGCAACTACAACCGCCTGCAA
CCGGGCGACCAGGTGCTGATCAGTGACCTGGAATACGACACGGTCAAGGGCGCCATGCGTTGGCTCGCCGGCTACC
GGGGTGTGGAGGTGATAGAAGTGTCCCACGCCCACCCGGCCAGTTTCGACAGCCTGGTGCAGACCTATCGCGATAC
CTTCAGCCGCTACCCGCGCCTGAAGCTGATGGCGCTGACCTATGTCACCCACCGTACGGGCCTGGTTATGCCGGTA
GAGGCCATCGCCCGGGCTGCACGGGAATCTGGCGTCGAAATCATCCTCGATGGCGCCCATGCGTTGGGCCAGATCG
ACTTCGACCTGGCCCAACTGGGCATCTCGTTCGCGGGTTTTAACCTGCACAAGTGGATCGGTGCGCCGCTGACCCT
GGGCTTTTTGTACATAGCCCCTGAGCGCCTGGCCCAGATTGATCCGGACATGGCCGAGTTCCATTACCCGATCACC
GATGTGCGTGCCCGTACGCCCTACAGCACACCGAATTTTCCGGCGCTGATGACCTTACCGCTCGTGCTTGAAGAAC
ATCGAGCCTTGGGCGGCGCGCATGCCAAGGGTGCGCGGGTGAAGTACCTGCGTGACCTGTGGGTGAGCCAGGTGCG
GCCATTGCCGGGGATCGAGGTGCTGACCTCGGATGATCCACGGCTGTATTGCGCGATTACGGCGTTCAAGTTCATC
GGGCGCGACCAGCAGGTGATGGCGGATCGATTGCTCGATGAGTACAACCTGTTTACCACCCTGCGCAGCGGGGCGG
CATTTGGCAGTTGTATCCGGGTGACGCCGGGGTTGGTGACCTCGGCGGCGGACATCGGCGTGCTGGTCAACGCGAT
TACCGAATTGAACACCGATTAAAATGTGGGAGCGGGCTTGCTCGCGAATGCGCGGTGACAGTCACTGAATGTGTTG
AATGACCCACCGCATTCGCGAGCAAGCCCGCTCCCACAGTTCGATTGCCGTGATTTCAAGGTTTTCTGTAGGCAAA
AAAAAGCGGCACACCGACCAAGTGCACCGCAAACATGCCGTGAAGCACAGCAACAACGATTCGGTAAATCAGATCA
ATCGAGCAGCGCCAGCGCCTCGGCGGTGACTTCCTGGATACGCCCCCAGTCGCCGTCCTTGATCCACTGTGGATCA
AGCATCCAGCTACCGCCCACGCACATCACGTTTTTCAACGCCATGTAGTTTTTGATGTTGGCCGGGCCAACGCCGC

CAGTCGGGCAGAATTTCACTTCGCCAAACGGCCCACCCAAGGCCTTGATTGCTGCCACGCCGCCGCTGACTTCCGC
GGGGAACAGCTTGAAGCGGCGATAACCCAGGCCATAGCCTTCCATGATGCCGGAGGCATTGCTGATGCCAGGCAGC
AGCGGGATCGGGCTGTCGACACTGGCTTCGAGCAGGTCGCGGGTAATGCCGGGGGTGACGATGAACTGCGAACCCG
CCGCCTCGGCTGCCGCCAGCATGGTGCGATCGAGCACCGTGCCCGCACCGGTGACCAGCTCCGGACGCTGCTCACG
CAGGACCTGGATAGCCTTGAGGCCGAACTGCGAACGCAGGGTCACTTCCAGCGCGGTCAAGCCACCCGCCGCCAGG
GCATCAGCCAGGGGCAGAATGTCCTGCTCGCGGGCAATGGTGATCACCGGCAGGATCCGCGCCTTGGCGCAGAGGC
TGTCGATCAGGGCAACTTTATCCGCCATGGACACGGTCGGGTGAGGGCTTTTCATAGCGGCTGATCCTTGGCTCAT
GGGCACCAGTAAATCTCTAGAGTAGGTTGCAAAAACGCGCGAATCGGCATGCCGGCAACATCGTCGCCAGCCAGCG
CTTCGCTCAGGGTGTGGAGTTTCGAATCGCCGGCAATCGACAACACGGTGTAACGGGCACTGGCCAGCAACGCACG
GCTCATGGTCAGGCGCTGATGGGGCACGCTCGGCGCCAGCATCGGCCAGCAACGACGAGTGCCATCCGGCTTCAAG
GCTTCGGCCAGGTTCGGGCTGTTGGGGAACAGGGAGGCGGTATGCCCGTCATCGCCCATGCCCAGCACCAGCACAT
CAATTGCCGGCAGTTCAGCCAGCAGGCGATCGGCCTGTTCGGCAGCACCGTCCAGGTTCACCGCAGCACTGTAGAG
GCTCAAGAACCGGGCCTTGGCCGCCGGGCCTTGCAGCAGATGTTTTTCAACAGGCCGGCGTTGCTGTCGGCATGC
TCGACCGGCACCCAGCGCTCGTCGGCCAGGGTGATCGTCACCTTGGACCAGTCCAGGCCCTGCTTGGCCAGGTTCT
GGAAAAACGCCACCGGGCTGCGGCCACCGGACACCACCAGCGTCGCCTCGCCCCGGGCACTGATGGCCGCGCGCAG
TTGTTCGGCCACGTCATTGGCCAAGCCTTCGGCCAGCAGTACCGGCGTGCGGTACTCGTGGGGTGTTACGCCTTGC
GGCAGTTTCAAATCAGATATCGCCATACCACGACCTCCCATCCCGCGTGATCAGTGCAATGGAGCTCATCGGCCCC
CAGGAACCCGCCGCGTACGGCTTGGGTGCATCACCGGACTTCTTCCACCCGGCGATCAACTGGTCACACCACTTCC
ACGCGGCTTCGATTTCATCTTTACGGACAAACAGGTTCTGATTGCCGCGCATCACTTCCAGCAACAACCGTTCGTA
GGCATCGGGGATCCGCGTACTACGGTAGGTGTCGGAAAAATTCAGTTGCAGTGGCCCGCTGCGCAGTTGCATGCCT
TTATCCAGGCCCTGCTCCTTGGTCATCACACGCAAGGAAATACCTTCGTCCGGTTGCAGGCGGATGATCAGCTTGT
TGCTGATCTGCAGGCGCTGCTCGGGGGCGAAGATATAGTGGGACGGTTCCTTGAAATGGATGACGATCTGCGACAG
TTTTTGCGGCATGCGCTTACCGGTGCGCAGGTAGAACGGCACCCCGGCCCAACGCCAGTTGCGGATATCGGCACGC
AGGGCAACGAAGGTTTCGGTGTCGCTCTGGGTGTTGGAATTCTCTTCTTCCAGGTAGCCCGGTACCGGTTTGCCGG
CGCTGTAGCCGGCGATGTACTGGCCGCGCACTACCTGGGTGGTCAGGCCTTCGGGGCTGATCGGCGCCAGGGCCTT
GAGCACCTTGACCTTCTCATCGCGGATGCTATCGGCCGACAGGTCGGCCGGCGGGTCCATGGCAATCAGGCAGAGC
AATTGCAGCAGGTGGTTCTGGATCATGTCCCGCAGTTGCCCGGCCTTGTCGAAGTAGCCCCAGCGGCCTTCGATAC
CCACCTGCTCGGCCACGGTGATTTCCACGTGGGAGATGTAGTGCTGGTTCCACTGGGTTTCAAACAGGCTATTGGC
AAAACGCAGGGCGATCAGGTTCTGGACCGTCTCTTTGCCCAGGTAGTGGTCTATGCGGTAGGTACGGTTTTCCGGG
AAAAACTGCGCCACGGCGTCGTTGACCTTGCGCGAGGATTCCAGGTCCGAACCGATGGGCTTTTCCAGGACAACGC
GGGTATTTTCCGCCAGGCCAACCTTGGACAGGTTCTCGCAAATCGCGCCATACACCGCGGCCGGGGTGGCGAAGTA
GGCGATCAGGCGTTGTTCGGTGCCGGCCTTGGCGGCCAGGGCCACGTAGTCATCGGCGTTCATGAAGTCGACGTGC
ACGTAGGCCAGACGCGCCAGGAAGCGCGCGGCCACCGCTTCATCCAGCTCTTGGCCGACGTACTTGCGCAGTTCGT
GCTCGATGTGGTCCAGGTGCTGCTGCTCGGAACCGGCCTCGCGGGCCAGGGCCAGGATGCGAGTGTCGGCGTGCAA
GAGCCCGGCGCCATCGAGTTGATAGAGGGCAGGAAATAACTTGCGCAGCGCCAGATCACCCAAGGCGCCAAACAGG
GCAAAGGTGCAGGGTTCCACGGTTATCGAAGGCATGATGTTTGTTCTTTTATCAAGTTAAGCTACAAATACCTTTT
TTCAAGGCATCACTCAAGGAAAAATGTAGTAATAACCACAACATTTTCCCAAAAAACGCATTGCGAGTGGTGGTGT
TCAACTACCCTCAGTAGGATAGGCCACCGCCAAGGGCCGCTCGTCGACCTTTTACTGGCCCCTACTTGCATCGTCG
CCCGAAGGAAAGACTGAATGGACCGCGTGCGAAATCTTCTGGAACAGATCCGGAACCGCCTCGAAGAATTGAACAA
GGCCGAGAAAAAAGTCGCCGAGGTCATCCTGCTCAACCCACAGCAGGCGACCCGCTTCTCTATCGCGGCCCTCGCC
CAAGCCGCCTCGGTCAGTGAACCGACGGTCAACCGTTTCTGCCGCTCGTTCGGCGTCAGTGGTTACCCTGAACTGA
AATTGCAGCTGGCGCAAAGCCTGGCCAGTGGCGCGGCCTATGTCAGCCGCGCGGTGGAGGCCGATGATAACCCCGA
GGCCTACACGCAGAAGATTTTTGGCAGCGCCATTGCCTCACTGGACAGCGCCTGCCAGGCCCTGGACCCCAACCTG
ATCAGCAAGGCCGTGGACCTGCTGATCCAGGCGCGGCAGATCCACTTCTTTGGCCTGGGGGCTTCGGCGCCGGTGG
CCATGGATGCCCTGCACAAATTCTTCCGTTTCAACCTGTCGGTGACCGCCCATGCGGACGTGCTGATGCAGCGGAT
GATTGCCTCAGTGGCGCATACCGGCGAGCTATTTGTGATTATTTCCTACACCGGCCGTACCCGTGAGTTGGTGGAA
GTGGCGCGTATTGCGCGGGAAAACGGTGCTTCGGTGCTGGGCGTAACTGCCGAGAACTCGCCACTGGCCAAGGCCA
GTACGGTGAGCCTGAACATCCCGTTGCCGGAAGACACCGACATCTATATGCCAATGACCTCGCGGATCATTCAGTT
GACGGTGTTGGATGTACTGGCTACGGGCATGACCTTGCGTCGCGGGGTGGATTTCCAGCCGCATTTGCGCAAGATC
AAAGAGAGTTTGAACGACAGCCGGTATCCGGTTGGGGATGAGTTCAACTAAAGCTGGAACCTCCTCAAGCAGGGCC
GGCCCTATCGCGGGCAAGCCCGCTCCCACATTTTTGATTGGTGAACACATTCGAATGTGGGAGCGGGGTTGCCCGC
AAAGACGCCTTCAGCAACACCGCTAAACCCCAGCCCGGGCCTGCAGACTCAAATGCGCCCTCTCCCCCGGCGCCAG
GCAGGCCCCGGCCATGGCCGACTCGACACACACAAAGCCCGACGCTTCATTAAAGCTCACCCCCAGCAATGGCCGG
CTGCCCGGATGCCAGACCACCGTGTCTGCACTGTCACCCGTATCAATGCACAACTCGCGCTGCCACGCATGGTCCT
TGAGCTGCAATTCGCCTTCATGCTGGAACACGCGCTGGCAGCCGCCATCGACGCGCAGCTCACCTTCCTGCTGGCA
GGCCTGGCGGCTCAACTGGTCGTAACCTTGCGCACCCTCTAGCCCAGACAGCGCTATCTCACCAACGTGACCAATA
CGCCAATAAGCGTGCAAAGCGTGGCTCAATTGGCACGGCAGTTCGTCCTGGTGCTCGGTGCTCAAGCGCAATTGCA

GGCTTTCACCCAAGTGCGCATGCAGGTCCACCTGCCAATCGCACAACTGCAATTGCCAGTGCAGGCGCACGCCGTC
CTCGCCGGTGCTGCTGTCCAACAGCTTCCAGTCGATCAGGCGCGCCCAGCCATGGGAAGGCCAGGCGTTTTCGCTG
GGATGACGGCCATACCACGGCCAGCACACGGGCACGCCGCCACGAATCGCCCCAACTTGCGGCCACTTTGCCGCGC
ACCACAGCCATGGCTTCTGGCCCGTAGGCTGGAAGTGCAGCAATTGCGCGCCCTGGCGGCTGAACACCGCCTGGCA
GAGCGGGTGATCGATCACCAGCACGTCACGCATCTGGAAGCGCTCCCAGGCAAACACCGGTTGCTCGCGCAGGGAT
TTGAAAAAGCGTTGCAGCGGTTGCTCATGCATGTGCCACGGTCCTGATAATCATGTCGCGCAAATGCGCCCATACC
TGTAGGAGCGAGCTTGCTCGCGAAGATCGTTAACGCTGACGCGGGCATTTTGGATGAACGCGGCGCTCTCAAGTTT
TTCGCGAGCAAGCTCGCTCCTGCAGGGCTGTTCGCCCTTCGCAAAAAAAAGCGGATAGCCATGGCTACCCGCAAAT
GCGCACAGAGAGAAGGAGCTTATCGCAACAGCGTTAGAACACCGACTGAATTTTCAGGCCGGCCACCAGCGCGTTG
TCGACTTTATCCACGCCGCCCGGTTGGACAACGTATTGCAGGTTGGGACGCACGGTCAGCCAGTTGGTGACGTGGA
AGCCGTAGTTGATTTCGAAGTTGTACTCGGTTTCGCGCAGCGGCGAGAACCGCGCATTGTCGTAGTCACTCACACC
GTTGGAAATGTTGCGCAGCTCGGCGTTTTTCTTCACGTCGTCGTTGACGTGCAGACGTGCGGCGCCAATCCCTACG
TCATCTTTTGGACGCGCGTCGAATGGGCCTTTGTACACCAGCATGATCGACTGGTAGTTATCGACGGTGTTGGTTT
CTTTGTCGTGGAACGTGGCGTTGGCAGCGATATTCAGACCACGAGAGGCGTCGCCGTTGTGGGTGGTGAGTTGCTG
TTGCGCAACGAACCAATAGCCTTTCTTGCTGCTGCGGGTGCGGTAGTCAGCGCCAGTGGTGGCGGCATCGTTACCG
TTGACGTCCTCACGCACATCAGGAGCATCGGCGGCGCTCTTGTAGTAACCCACGCGGTATTCGCCCGGCAGGTTAT
TGACCTTCGGCGACCAGACCAACTCCACCGGAATCACGGTGCCCTTGGTGCCACTGCCGCTGAGTTTGAAGCCGTT
GCCGTGCTCCAGTTGCGACGGGTTCTGGTTGTACGCACCGATCTGCGCATACAGCTCAGGGGTGATGTGGTACTTC
ACGCGGATCGCGGCCTGGCTGACCGGCCAGTTGTACCAGGTGTTGACGTAGTTACCCACTTGGGAGCCGCAGAACG
ACAGGTTCTGGAAGTCGCATGGGAAGGTGTTGAAGTCTTCGCCTTCACCGAAGTAACCGAGTTTTACATCCAGCTT
GTTGTCGAACATCTGGTGCTGAATCCAGAACTGGGTCAGGCGCACCATATGGCCACGGCCGTACACTTCTTGAGAT
GAACTCAAGGTGCCTGCACGCGGGTCGCCAATACGGTCATTGGAAATGTTACGACCATTACGGCTGGTCATCTGGA
TCTTGGCCTGGGTGTTGTCCCAGCCCCACAGCTTTTGCAGGTCCAGCGCTACGCCGAGGCCGAACTGGTCAGCGTA
ACGACCGGTCTTGTCATCGTTGTAGCCGCCGCGCGCGTTGTAGCCCGCTTCCCCAACGTAGTCGGCCTTGATGTCG
ATACCCTGCTCGATCAGCTTGGTTCGCTCGCCGCCCCAATCGCCGGTCATCCATTTGGAGTCGGCGCTGAAGGCTT
CGTCTGCCATCGCATTGGCGGACAAAACCAGGGCTGCTGCCGCTGACAGTTGGCAGATCAGCCGGGTGTTGTTGTG
TTGCTTTTTCATCCCTACATCCTCGTCTTTATTGTTATTAACTGTTTTTATCTAACGCGGTTTACATCTATTTTTA
TCTGAAGTAGGAGCGAGCTTGCTCGCGAAGATCGTTAACGCTGACGCGGGCATCCTGGATGAACGCGGCGCTCTCA
CGTTTTTCGCGAGCGAGCTCGCTCCTACAGTTATTGGTCTTCAGCGGCCCTTGAATTGGGTGACGTTGTCGGCATG
CGACTGTGCCGGCTGCGCAGCAGCGGTGCCCAGGCGTTCACCGGTCTTGGCGTCGAACAGCAGCACCTTGGCCGGA
TCGAACTGCAAGGTCAGGCTCTCGCCCACTTGCGGTGCCACGTCCGGCGCCAGGCGGCAGCAGACCTTGGTGTCAT
TGAGCTGGACAAACACCAGGGTGTCCGGGCCGGTGGGCTCAGTGACCTGGACCTCGGCACGAATGCTCGGCAAACC
ATTGCCATCACCGGCAGCCAGCACAATCTGTTCCGGACGCAGGCCAAGAATCACATCACGCTCTTCAAGACCGGCG
TCGCTCATGCCCAGCGGCAGCTCGCAACGCGCCTGGCCACTGTCGAGCAGGGCCAGCAGACGGCCTTCCTTGCGCT
GTAAACGCAGGGGGATGAAGTTCATCGGCGGCGAGCCAATGAAGCTGGCAACAAACAGGTTGGCCGGATCGTTGTA
GATCTCTTTGGGCGTGCCGAACTGTTGGATGATGCCGTCCTTCATCACCGCCACTTTGTCGCCCAGGGTCATGGCT
TCGATCTGGTCGTGGGTCACGTAGACCGTGGTGGTTTTCAGGCGCTGGTGCATCAGCTTCATTTCGGTGCGCATCT
CGACCCGCAGCTTGGCGTCGAGGTTGGACAGCGGTTCGTCGAACAGGTAGATCTTCGGCCGCCGCGCCAGCGCCCG
GCCCATGGCCACCCGCTGCTGCTGGCCGCCAGAGAGCTGGCCCGGCTTGCGATTGAGCAAGTGTTCGATCTGCAGC
AGCTTGGCCACCCGCGCCACTTCCTCATCGATGGCCGGCTGGCTCATCTTGCGGATTTTCAGGCCGAACTCGATGT
TCTCGCGCACGCTCATGGTCGGGTACAGCGCGTAGGACTGGAACACCATGGCGATGTCCCGATCCTTGGGGCTCAT
GCCGCTGACGTCCTGGTCACCGATCATGATCGCGCCGCCGCTGATGTTTTCCAGGCCGGCGATGCAGTTCATCAAG
GTGGATTTGCCGCAGCCCGAGGGCCCGACGAGGATCAGGAATTCGCCGTCCTTGATCGACAGTTCGATATTTTTCA
GGGTGTCCGGCAGGCCAGCACCATAGGTCTTGTTTACATTGCGAAGTTCGAGCGTAGCCATGATTACCCCTTGACC
GCGCCGGCCGTGAGACCGCGCACGAAATACTTGCCTGCGACCACATAGACCAGCAGGGTCGGCAGCCCGGCGATCA
TCGCCGCCGCCATATCAACGTTATATTCCTTGGCCCCGGTGCTGGTGTTGACCAGGTTGTTCAGCGCCACTGTGAT
GGGCTGCGAATCACCGCTGGAGAACACCACGCCAAAGAGGAAGTCGTTCCAGATCTGGGTGAACTGCCAGATCAGG
CAGACCATGATGATCGGTGTGGACATCGGCAGAATGATCCGACGGAAAATGGTGAAGAACCCTGCACCATCCAGGC
GTGCTGCCTTGACCAGCGCATCCGGGATGCTCACGTAGTAGTTACGGAAGAACAGCGTGGTAAACGCCAGGCCGTA
GACCACATGCACAAACACCAGGCCGGTGGTGGTACTGGCCAGGCCCATCTTGCCGAGGGTGAACGAGGCTGGCAGC
AAGACCGTCTGGAACGGCAGGAAGCAGCCGAACAGCAACAACCCGAAGAACAACTGCGAGCCGCGAAAGCGCCACA
TCGCCAGCACATAGCCGTTCAATGCACCGATGGTGGTGGAGATCAGCACCGCCGGGACGGTGATCAGGAACGAGTT
CCAGAAGTAACCGTTCACCGTGGCCCAGGCCTTGACCCAGCCGATCCCGGTGATCACCGTCGGCCAGCTCAGCAGG
TTGCCGGTGCTGATATCTTCCGGGGTCTTGAAGCTGGTGAGCAGCATGACCACCAGCGGCACCAGGTACAGCACCA
CGGCCAGGATCAGCACCGCATGGATCGCGACACGGCTGAAGCTGATGGAAGGTTTGGCGAGACTAGTCATGGCGCT
TGGTCCTCAGCTCGGAGTACAGGTAAGGCACGATGATCGCAAGGATCGCCCCAAGCATCAGGATCGCACTGGCCGA
GCCCATGCCCATCTGGCCACGGCTGAAGGTGAAGGAGTACATGAACATCGCCGGCAGGTCGGATGAGTAGCCTGGG

CCACCGGCCGTCATCGCGGCCACCAGGTCGAAGCTCTTGATCGCGATGTGCGCGAGGATCATCACCGCACTGAAAA
ACACCGGGCGCAGGCTGGGCAGCACCACGCTCCAGTAGATGCGCGGCAGGCTCGCACCGTCGATCTGTGCGGCACG
GATGATCGATTGGTCAACGCCACGCAGGCCGGCGAGGAACATCGCCATGATAAAGCCCGAGGCTTGCCATACCGCC
GCGATCACCAGGCAATACACCACGCGGTCCGGGTCGATCAGCCAGTCCAGGCGAAAGCCTTCCCAGCCCCAGTCGC
GCAGCAGTTTGTCCAGGCCCATGCCGGGGTTGAGCAGCCATTTCCAGGCGGTACCGGTGACGATCATCGAGAGCGC
CATCGGGTACAGGTAAATAGTGCGAATGAAGCCTTCGCGGCGGATACGCTGGTCAAGGAATACCGCCAGCAGCACA
CCGATCACCAGGGTGATGCCGATAAACATGCCGCCAAACAGCGCCAGGTTTTTGCTCGCCACCCACCAGCGATCGT
TGTCGAACAACCGCGCGTATTGCGCCAGGCCTACCCACTTGTAGGTCGGCAGGAAGGTGGAGTTGGTGAACGACAG
TACGAACGTCCACAGGATGTAGCCATAGAAGCCCACCAGGACGATGAACATGCTCGGGGCCAGCACCAGTTTAGGT
AGCCAGCGCTGCAATGCATCGAACGGCGAGGCCTTGCTGAACACAGCAACAGAACTCATGGGGAAATCCAGGGCAG
GGAGTAAGGGACTAGTGGATAACCGTGTGGGGGCCGGCTTCAGGTGGGAGCTGGCTGGCCTGCGATGGCATCACCT
GGGTGTCATAGATACACCCAGGTGCCTGCATCGCGGGCAAGCCCGCTCCCACACTCAAGCCCAGCCTTCCAAGGGT
TACTTGGCAGACTTGATCGCCGCACCCAGCTTCTTGGCGGTGTCGGCCGGGTCGGCTTTCGGGTCGTTGATGAAGT
TGGTCACCACATCAAAGAACGCGCCCTGTACCGCCAGGGTGGTCGCCATGTTGTGCGCCATGCTTGGTTGCAGGCC
GCCAGACTTGGCATCGACCAGGAAGTCCTTGGCCGCAGTCTGGGCGCAGCTGTCAAAGCCGTACTTGGCCATATCG
CCGAGCATGTCGTTGCGCACCGGGATCGAGCCCTTGTTGATGCTGAAGACCTTCTGGAAGTTCTCGCCCAGCACCA
CCTTGGCGATGTCCTGCTGCCCGGCCGAGGTGCCGGCATCTTTCTGCTTGAACACTGCCAGGGAGTCGATGTTGTA
GGTGAAGGCCTTGTCGGTGCCCGGGAAGGCTACGCACTCGTAGTCCTTGCCGGCGACTTTCTTGGCGGCAGTCCAC
TCGCTCTTGGCCCAGTCACCCATGATCTGCATGCCGGCCTTGCCGTTGATGACCTTGGCGGCTTCCAGGTTCCAGT
CCTGGCCCTTGCCGTCGGCGTCCATATAGGTCGCGACTTTCTTCAGCTCCGTCAGCGACTTGACCATATCCGGGCC
GGTCAGGGCTGCGTTGTCGAGGTCGACCAGGGCTTTTTTGTAGCCATCAGCCCCCATGACCGACAACACCACGGCT
TCGAACACCGTGCTGTCCTGCCAAGGCTGACCACCGTGGGCGAGCGGGATGAAGCCTGCGGCCTTGAGCTTGTCGC
CGGCGGCGTAGAATTCTTCGAGGGTAGTAGGGTTTTTGGTGATGCCGGCTTTCTTGAAGACTTCCGGGTTGATCCA
CAGCCAGTTGACGCGGTGGATATTCACCGGCACGGCCACGTAATCGCCTTCGTACTTCACGGTATCGGAGACTTTT
TTGTCGAGCAGGCCATCCCACTTCTCGGCCTTGGCCACGTCCTTGAGGACGTCGGTATCGAGCAGCCCGGTGGAGG
CCCACTCCTGGATGTCCGGGCCCTTGATCTGGGCAACGCCTGGCGGGTTGCCGGCGACCGCGCGGCTTTTCAGCAC
GGTCATGGCAGTGGCGCCGCCACCACCTGCAACAGCCCCGTCTTTCCAGGTAAAGCCGTCTTTCTCAACTTGGGCC
TTCAGGACATCCACCGCCGCCTTCTCACCACCCGAGGTCCACCAATGCACAACTTCAACCGTCCCTTTCGAGTCGG
CGGCAAATGCACTGAGGGGAAACAACGAGGCAATGGAAATAGCAACGGCGAGGCGATTAATCGCGTTCATCTGAGT
ACCTTTTTCTTGTTGTTATGCATGCAAGTCTGGAGCTTGCGCTGCATAGGATTCTAAACAGGGATTTTTCCCAGGC
ACGTAACAAAGGGACGGCCAAATGTCACCACTTGGTGACATAAGCACCCACCCCCAACGCACTGGCCATGCTTGGG
GATAAAGGCAGACCCGGCAGCAATACCGCTTGCCAGGCATGGTACAGATCCGGCTTGCCGCCCCAGATCTTGCTGG
CGGGCTGGTTGAGCGGGTCGAGTTCATGATGCCAACTGCCCTGGACCCGGTCGATCAGGTGAGTTTCACAAAACTC
CCAGAAACGCCGGTACCAGGTTTCGTAATGCCACTCACCCGTGCGCTTGAGCAGGGCCTGGGCGGCTGCACTGGCT
TCGGCGTGGGTCCAATGCAGGCGCTCACGCACCACCGGGCGAAAGTGCCAGTCCAAGGTGTAGACCAGGCCCGGGG
CACCGTCCACCGACCAGCCATACTCGCAGGCACTGGCGAACAAACCCTTGGCGTCCTCCAGCAACCACTGCGGCGT
GACGAGCCCTGCCTGCTGGCGTGCGGCTTCGAGGTGCAGCACCAGGCGCGACCATTCGAAGCCATGGCCAGGGGTA
ATGCCGTAGGGACGAAAGCCGTCGGCCGGGTTGTCTTCGTTGTAACGATGCAGGGGCTGCCAATAGCGGTCGAAAT
GTTCGATCACCATAAATTCATTGGCGCTGGCGTGCTGATGGATCACCCGCTCGACAATGCGCAACGCCCGCCCCAG
CCATCGGCTGTCGCCGGTGACATCAGCCAGGGCAAGGAACGCTTCGGTGGCGTGCATATTGCTGTTGGCCCCGCGA
TAGGCTTCCTCGCCGCTCCAATCCTGGGCGAAGGATTCAAGCATCGCGCCCTCCTCCTCGCACCAGAAATGCTCGT
CGATGCATTGGACGGCGTCCGCCAGCAAGGCTTGCGCCCCAGGGGCACCCGCCACTACCGCGGAGCTGGCGGCCAG
GGCGACAAAAGCGTGCAGGTAGGCGGCCTTGCCTGTATTGCCATCACGCGCGCCGGCGACGGCGAACCAGCCGCTA
TGTTCGGCATCGCGCAACGGGCCACTGAGGCTGGCCACCCCGTGCTCTACCAACTGTGCATAGCCTGGCAGCCCTT
GGGCGTGAGCCATGGCAAAGCTGTGGGTCATGCGGGCGGTGTTCATGGTTTCGGCGCAGGCATCGGCGGGCAGGCG
GCCCTGGACGTCGAGGTTGCCGAAGCCTTCAGGCAGCCTGGAAGCCTTGGCAAACGCCAGCAGGCGCTGCCCCTCG
GCGGCAAGCCAGGCGTGATGGGCAGGTGCGTTCAGCCAACTGCTGGCAGGCAGTGGTTGGATGGTCATGGGTGGCC
CTTTTTGTTTTTGTGGGATGACCGCAGTCTAAACAAGGGCCGAGAAGGGACATGTAACGAAAGGGAAGACAAATGT
CACTGAACGGTGACACTTGCCGGGGATGGCGGCCCTGGAATCAGCGCAAGGCTACTGGCACCATCGCCGCATAGGT
ATATACACAACTTTCAGAAACTGACGCCCCCCGTGGCGAGCGGGCTTGCCCCGCGCTGGGCTGCGAAGCAGCCCTA
AACCCTGCCATCGCGGTTTATCTGAAGAAACGTGGAGGGCTTTTTGGGCTGCTTCGCAGCCCAGCGCGGGGCAAGC
CCGCTCGCCACAGGAGAGTTCGTCAGTTTCTGAAAGTTGTGTAGATACCTATGCCCATCGCCGGCAAGCCGGCTCC
TACAGCAATTGGCTCATTGGCCCAGGGGCGTAGCCCTGGCCGGTGCGCTGACTTCCCTCTGCAGGTTCTGCATCTG
GGTCTGCAAGGTCGTGATGTTGCGGGTGGTCTGGATGCGGAACACGTCGAACTCTTTATTCGACGGCCCTTCCGTG
GCGGCGCGGTTGTCCAGTTCGCTCTTGATTACCAACAGGTCCTGTTCCAGGCGTGCAATGGCCGTGGCCGGGTTGC
CCTGCTTCTTCAACGCTGCCACATCCGTGCTCAGTTCCTTGACTTCGGCATTCAACTTGGCGGCTTCGGCCTGCGC
AGCCTTGAGGGCCGTCAGTTCTGTCGCCAGGGTTTGCACCTGGGCCTGCATCTGGGTATTGGCAGTGGTCAGTTCC

GTGGTGCTGGCACTCATCTGCGCCAAGCGCTGGTCCAGCTCGGTGGCCTGGCCTGCCACACCCAACTGCTGCTTGC
CCTGCTCCAGCAACTGGCTTTCCAACTGTTTGATCTGCAGCTTGAGCGCTTCACTGCCGGTATTGACGCTGGCCTC
ACTGGCCACCACCTTGCCGGAAATGTCCTGCAAACGGCCGGCAGCTTCTTCGCTGATGCGCGCAAAACTTTCCTGG
GTCGCCACCAGTTGCTGCCCCATCAGGGAAATCTGCTGGAAGCTCCACCACGCCAGGCCCACAAAGGCGAACAGCA
GCGCCCCGACCAAGGCCCACAACGGCCCGGTGCTGGCGCTCTTGACCTTGACCACCGGCGCCGGACGCGAGCGCAT
GGACGTGGCGGCGGTCGGCACAAAATCATCGTCATCGCCAACGTCGGCCCGCAGGCTCGGCACATGGTCGAAGTCG
TCTTTAATATCGTTACGCATGAATCAACCTTGAAATAGCCGCATAAAAAAACTGAGCGGCGGGAGTATAAACCCCT
GGCCCGTCGCCTTGGTCGACCGGCAACCGAAAAATCGGTTCCCGGTGTTGCCAACGCTAGTTGGAGCCCGGTTCCT
GGACTTTCCACCAATGACAGAACTCATCAAGGGCCGTCCACAGGCTGACCTTGGGCTGGTAGTCGAGATAGTGCTG
CGCACGGCTGATATCGAGGCTGAAATCCTTGTTCATCACTTGCATCCCCAGGCGTGACAGGGAAGGTTCCGGACGC
CCCGGCCACATCGCGCAAAAGGCCTCGTTGAGCGCCGCGATGCTATAAGACAAGCCGTAGGAGCGGTAACGAGTGA
CCTGGGGCATGTCCATCTGGCGCATCACATAGTTGACCACGTCCCACACCGGCACCGGCGCGCCATTACTGATGTT
ATAGGCCTTGCCCAGCGCCGAGCCGGTGGCCAGCAGGCTGCTGAGCAGGGCCTCGTTGAGGTTGTGCACACTGGTG
AAGTCGACCTTGTTCAGGCCATCGCCAACGATGGCGAGACGGTTCTTGCGCTGCATTTTCAACAGGCGCGGGAAGA
TGCTCATGTCGCCAGCCCCGGTGACAAAACGCGGGCGCAGGGCCAGCACTTCCAGGCCGAATTCCTGGGCACCAAA
GACTTTTTGCTCGGCCAGGTACTTGGTCGCCGCATAGGGATGCTTGAAGCGCTTGGGCACTTGCTCTTCGGTCAGT
CCCAGGTGATCGCGGCCATCGAAATAGATCGATGGCGACGACAGGTGCACCAGGCGGCCGACCCGTTGTTTAAGGC
AGGCTTCGACCACGTTTTCGGTGACCTGCACATTGCCCTGATAGAAGTCCTGGTAACGTCCCCACAGCCCTACGGC
GCCGGCGCAATGAACCACGGCCTCAACGTCGCGGCACAGGTTGCGCGCCAAATCCGGATCATTCAGATCACCCTGG
ATAAACTCGGCGCCGCGCCGCACCAGGTGCTCGACACCCTCGGCGCGCCGCCCGTTGACCCGCACGTCCAGGCCCT
GCTCCAGGGCGAAACGCGCAAAGCGCCCGCCAATGAAGCCGCTTGCGCCGGTGACCAGAATTTTCATGTGTTACCC
CGTGTTCTTTCATTTTTCATCGCAGTTGCGTTGACGCCGACGGTCACTCCAACGGCACCAGCAATTGGCCGGTAGC
CCGTGCCAGATGCTCGGTCAACAAACCGAGCAATTGCCCGCCACTGCGCCAATGATGCCAGTACAGCGGCACATCG
ATGGGTTTATCTGGCAGTAATTCCACCAGCACGCCCCGTTGCAGCTGATCACGCACCTGCAACTCCGGCACCAAGC
CCCAGCCCAGGCCGGCCTCGGTCAGGCGGATAAAACCTTCGGAGGACGGGCATAAATGGTGTTCAAAACCACCGTC
CACGCCCAGCGACGCCAGGTAGCGATGTTGCAGGAAGTCATCCGGACCAAATACCAGCGCTGGCGTACGCGCCAGT
TGATCGGCCCGTACTCCGCCGGGGAAGTGCCGGGCAATAAACGCCGGGCTCGCCAATGCCCGATAACGCATGGCCC
CCAGCAACAGGCTGCGGGCACCGGCCACCGGGCGTTCGCTGGCGCAGACGCAAGCCGCCACCTCGCCGGCGCGCAT
ACGCTTGAGGCCGACGGTCTGGTCTTCCACCACCAGGTCGAGCAGCAAGTGCTGCTCGGCGCAAAAATCCCCGACG
GCCTGCGCCCACCACGTCGCCAGGCTGTCGGCGTTGATGGCGATGCGCAGCCGCTCGGGCATGCCCTCTTCGTCCA
ACGCCGGCACCTGGCTTTGCAGGTCGCGCTCCAGCAAGCGCACCTGCTGCACATGGTTGAGCAGGCGGCGGCCGAT
TTCCGTGGGGGTCGGCGGGGTCGCGCGTACCAGCACCGGCTGGCCGATGCGGGCTTCGAGCAATTTGATGCGCTGG
GAAATGGCCGATTGCGACACACCAAGCACCTGGGCCCCGCGCTCGAAACCGGCCTGTTCCACCACTGCCGCCAGGG
CGGAGAGCAATTTATAGTCGAACATCAGTTTTCCTAATGAGCGATCAGCAATATTTGTTTTTCTTATACAACCTCA
ACGCCGACAATCGCCAGCATTGATTAGCAAGCAGAGTAAATATTATGTGGCAGAGTTATGTGAACGGCCTGCTGGT
CGCCTTGGGGCTGATCATGGCCATCGGCACGCAAAACGCGTTTGTTCTGGCCCAGAGCCTGCGTCGCGAACATCAC
TTGCCCGTGGCGGCCTTGTGCGTGGTGTGCGATGCGTTGCTGGTGGCTGCCGGCGTATTCGGCCTGGCGACAGTGC
TGGCGCATAACCCGCTGTTGCTGGCGATTGCGCGCTGGGGCGGCGCGGCGTTCCTGCTATGGTATGGCGCCCTGGC
CTTGCGCCGCGCCTGTTCACGTCAAAGCCTGGAGCAGGGTGAAGGGCAAAAAGTGCGCTCGCTGCGCGCGGTAATG
CTCAGTGCATTGGCCGTGACCCTGCTCAACCCCCACGTCTATCTGGACACGGTGTTGCTGATCGGCTCCCTCGGCG
CCCAGCAGACCGAACCCGGCGCTTATGTCGCAGGTGCTGCCAGTGCGTCCTTGTTGTGGTTTTCCGCCCTGGCCCT
CGGCGCGGCGTGGCTGGCCCCGTGGCTGGCACGCCCGGCGACCTGGCGCCTGCTGGACCTGCTGGTGGCGGTGATG
ATGTTCAGCGTGGCCTATCAATTGATCAGCAACTGAGGAATATTCCAAAACGCTCTGGAACCTCTATTCCACACAG
TTGTTGCGTGGTTTTGCCGCACCCCCGGTGCTATGATCCAGCCCCTGCGCCGCAAAGAGTACAAACTCCCCGGCGC
TTGTTTGGCCGCCCGTGATCGGCCTTGCGCTCACCGCAACTGACCTGATTAGGAGAATTATCATGGCTTTCGAATT
GCCGCCGCTGCCGTACGCACACGATGCCCTGCAGCCGCACATCTCCAAGGAAACCCTGGAGTACCACCACGACAAG
CACCACAACACCTACGTCGTGAACCTGAACAACCTGGTGCCAGGCACCGAGTTCGAAGGCAAGACCCTGGAAGAGA
TCGTCAAATCCTCTTCGGGTGGCATCTTCAACAACGCCGCGCAGGTCTGGAACCACACTTTCTACTGGAACTGCCT
GGCACCAAACGCTGGCGGTCAACCAACTGGCGCATTGGCTGAAGCCATCAACGCTGCGTTCGGTTCGTTCGACAAG
TTCAAGGAAGAGTTCACCAAGACTTCCGTCGGCACCTTCGGTTCCGGTTGGGGCTGGCTGGTGAAAAAGGCTGACG
GTTCCCTGGCCCTGGCCAGCACCATCGGCGCCGGCAACCCGCTGACCAGCGGCGACACCCCGCTGCTGACCTGCGA
CGTCTGGGAACACGCCTACTACATCGACTACCGCAACGTGCGTCCAAAGTATGTGGAAGCGTTCTGGAACCTGGTC
AACTGGAAGTTCGTTGCCGAGCAGTTCGAAGGCAAGACCTTCACTGCGTAAGTGACCCTTGCAGTACAAAGAGCCC
GGCAATTGCCGGGCTTTTTCATGCGCTCGATCTGGAACCCGATGTGTCGCTTCTGCCACCTAGTGGATGAGGAATA
TCCCTCTCATCCCTGTCGGAGATTTCACAATGTCTATGTCAGCACTCGCAGCGCTTCCAGTGGTTGGTGAACTGGC
CACAGCAGCTGCCGATATCACCAAATCGATCGCTCCCCTGATCCAACCCTTCGCCGACGTCGCCGCAAATACCTTG
GGCGACACTCTGAAAAACACCAGCGATGGGCAAAACACGTCCATAGATTTCGACGTAGCGAAAAAAGAACAGAGCG

CAACAATCACGTTCAGTTGAGAGATCAACCTGCCCATCTGACCGTCCAAACCCGCCTCTTCGTTGAACAGCGCCCA
CCCCGCTACCGCAAGGCGGATGCAAATCCGCCTTGCGCCATCGATGCCCGCCTCACCAGGAAACGACCCCGCGCCG
CTTCAAAGCTGCAACTGCTTCAATGTCCCTTTGACTGCCACCCCGGGATTGCCAATACTCATGGCATATTGAGGCT
ACCCGAATGGAACAAGGAATGACCCTTTGAAGCTGGAACTCAAAAACAGCTTGTCGGTGAAGTTGCTCCGGGTGGT
GCTGTTGTCGGCACTGATTGTGGGCGTGGCACTGAGCGTGGCGCAGATCGTCTTCGATGCCTATAAGACGCGCCAG
GCCGTGGCAGGCGATGCCCAGCGCATCCTCGACATGTTTCGCGACCCTTCCACCCAGGCCGTCTACAGCCTGGACC
GTGAGATGGGCATGCAAGTCATCGAGGGCCTGTTCCAGGATGATGCAGTGCGCATGGCCTCCATTGGCCACCCCAA
CGAGCCCATGCTTGCCGAAAAGAGCCGGGAACTGCAGCACTCCCCAAGCCGCTGGCTGACGGACGTGATTCTCGGC
CAGGAACGCACCTTCACGACGCCCCTGGTGGGGCGCGGGCCCTACAGCGAATATTACGGCGACCTGAGCATCACCC
TCGACACGGCCACCTATGGCCAGGGCTTTATCGTCAATTCGGTGATCATTTTTATCTCCGGGGTGCTGCGCGCCCT
GGCCATGGGCCTGGTGTTGTACCTGGTCTATCACTGGCTGCTGACCAAGCCCCTGTCGCGGATCATCAGCCACCTC
AGTTCGATCAACCCCGACCGGCCCAGCGAGCACAAGATCCCCCAGCTCAAAGGCCACGAGAAAAATGAACTGGGGC
TGTGGATCAACACCGCCAATCAATTGCTGGCCTCTATCGAACGCAACACTCACCTGCGTCATGAAGCCGAAAACAG
CCTGCTGCGCATGGCCCAGTACGACTTCCTCACCGGGCTGCCCAACCGCCAGAAGCTGCAAGAGCAACTGGACAAG
ATCCTCATCGATGCCGGCCGCCGTCAACGCCGGGTCGCGGTGTTGTGCGTGGGGCTGGATGACTTCAAGAGCATCA
ACGAGCAGTACACCTACCAGGCCGGCGACCAACTGCTGCTAGCCCTGGCCGACCGCCTGCGCGCCCACAGTGGCCG
GCTCGGCGCCCTCGCCCGCCTGGGTGGCGACCAATTCGCCCTGGTCCAGGCCGATATCGAGCAGCCCTACGAAGCC
GCCGAGCTGGCGCAAAGCATCCTCGATGACCTGGAAGCCGAGTTCGCCCTCGACCACGAACAGATCCGCCTGCGCG
CCACCATCGGTATCACCTTGTTTCCCGAAGACGGCGATAGCACCGAGAAGCTGCTGCAAAAAGCCGAGCAGACCAT
GACCCTGGCCAAGACCCGCTCGCGCAATCGCTACCAGTTCTATATCGCCAGCGTCGACAGCGAAATGCGCCGGCGC
CGCGAGCTGGAGAAAGACCTGCGCGAAGCCCTGGGCCGCGACCAGTTTCATTTGGTGTACCAACCGCAGATCAGCT
ATGTGGATCATCGCGTGGTCGGCGTCGAGGCGCTGATTCGCTGGCAACATCCGGAACACGGCCTGGTACCTCCCGA
CCTGTTTATCCCGTTGGCGGAACAGAACGGCACCATCATCCCCATTGGCGAGTGGGTACTGGACCAGGCCTGCCGG
CAATTGCGCGAATGGCACGACCAGGGTTTTACCGAGCTGCGCATGGCGGTCAACCTGTCCACGGTGCAACTGCACC
ACGCCGAACTGCCACGGGTGGTCAACAACCTGTTGCAGATCTATCGCCTGCCGCCGCGCAGCCTGGAGCTGGAAGT
GACCGAGACCGGCCTGATGGAAGACATCAGCACCGCCGCCCAGCACTTGCTGAGCCTGCGCCGCTCCGGCGCGCTG
ATTGCCATCGACGACTTCGGTACCGGGTACTCGTCGCTGAGCTACCTCAAAAGCCTGCCCCTGGACAAAATCAAGA
TCGACAAGAGCTTTGTCCAGGACCTGCTGGACGACGATGACGATGCCACCATCGTGCGGGCGATCATCCAACTGGG
CAAAAGCCTGGGCATGCAGGTCATTGCAGAAGGCGTGGAGACGCCCGAGCAAGAGGCCTACATCATCAGCGAAGGC
TGCCACGAGGGTCAGGGCTATCACTACAGCAAACCCCTGCCGGCCCGTGAACTGGCGGCCTATCTCAAACAGGCCG
AGCGCAACAACGCGGCGATCTTATAGCGCAATGCTGGCCACCCAGGGCAAAGCCTTGGGCGGCCAGGGCGCGAATG
TGAAACTTTTACTGCGTTAACTCTTTACAGAAAATGCAAATCTTTCGCATTATGTCGCAGTTTTGCGCGCTGCATT
GCGCCCCGATCAACTACCGAAGCAGGATGTTCGCCATGATTCGTATGCCCCTGGCCACCGCCAGTCTGCTGGCCAT
CGCTATTTCTCTCGCCGGCTGCGGCGAGGGTAAAGACAAGGCCGCCGCTCCTCAGGCGCCAACGCCAGCTGCCAGC
ACTACCGCTCCAGCGGCTGCCGCTACCGCCGGCCAGGTTGACGAGGCTGCCGCCAAGGCGGTCGTCGCGCATTACG
CAGACATCGTGTTCGCGGTGTACAGCGACTCCGAGGCCACTGCAAAAACCCTGCAGACCGCCATCGATACCTTCCT
CGCCAACCCCAACGACGAGACCCTGAAGGCCGCTCGCACCGCCTGGATCGCTGCACGCGTACCGTATCTGCAGAGC
GAAGTGTTCCGCTTTGGCAACACCATCATTGATGACTGGGAAGGTCAGGTGAACGCCTGGCCGCTGGACGAAGGCC
TGATCGACTATGTCGACAAGTCCTACGAACACGCCCTCGGCAACCCGGGCGCCACCGCCAATATCATCGCCAACAC
CGAGATCCAGGTCGGCGAAGACAAGATCGACGTCAAGGAAATCACCCCGGAGAAACTCGCCAGCCTCAACGAGCTG
GGCGGCTCCGAAGCCAACGTCGCCACCGGCTACCACGCCATCGAATTCCTGCTGTGGGGCCAGGACCTGAACGGCA
CCGGCCCTGGCGCCGGCAACCGTCCAGCGTCGGACTACCTGCAAGGCGAAGGCGCCACCGGCGGTCATAACGAGCG
TCGCCGCACTTACCTGCGCGCCGTAACCCAACTGCTGGTCAGCGACCTCGAAGAAATGGTCGGCAACTGGAAACCC
AACGTCGAAGACAACTACCGCGCCACCCTGGAAGCCGAACCTGGCACCGACGGCCTGCGCAAGATGCTGTTCGGCA
TGGGCAGCCTGTCCCTGGGTGAACTGGCGGGCGAGCGGATGAAAGTGTCCCTGGAAGCCAACTCGCCGGAAGACGA
GCACGACTGCTTCAGCGACAACACACACTACTCGCACTTCTACGACGCCAAGGGCATCCGCAACGTCTACCTGGGC
GAGTACACCCGCACCGACGGCACCAAACTGACCGGCGCCAGCCTGTCGTCCCTGGTGGCCAAGGTTGATCCGGCTG
CCGATGAAGCCCTCAAGGCTGACCTCGCTGCCACCGAAGCCAAGATCCAGGTCATTGTCGACCACGCCAACAAGGG
TGAGCACTACGACCAACTGATCGCTGCCGGCAACACCGCAGGCAACCAGATCGTGCGCGATGCTATCGCATCCCTG
GTCAAGCAGACCGGCTCCATCGAGCAAGCGGCTGGCAAGCTGGGCATCGGTGACCTGAACCCGGATACCGCTGATC
ACGAGTTCTGATCCGCGCGGTTTGAATGAGGCGGCCTTCGGGTCGCCTTTTTTGTATTACCCCCACAAAACCCATG
TGGGAGCTGGCTTGCCTGCGATAGCGGCGGGTCAGTCAAGCAGTCGAACCTGACGCACCGCCATCGCAGGCAAGCC
AGCTCCCACATCAGATCCGTACCCCCCGGTTTACAAGCCCTGCACCCTAGGTAGAATGGCGCCTCAAACTGTCACC
CGAGCGCAATTCATGGCAATGCCGACCCTGCGAATCATTGGTTTCATCATCGGCATCTTCCTGATTACCCTCGCGG
TCGCCATGGTAGTGCCCATGGCCACCCTGATGTTTTTCGACCGCACCAGCGACCTGCCGTCCTTTCTCTGGGCAAG
CATGATCACCTTTATCGCAGGCCTGGCCCTGGTGATTCCCGGTCGCCCGGAACAAGTCCAACTGCGCCCCAGGGAC
ATGTACCTGCTGACCGTGAGCAGTTGGGTGGTGGTGTGTATCTTTGCCGCGCTGCCGTTCTTGCTGACCCAGCACA

TCAGCTATACCGACTCGTTCTTTGAAAGCATGTCCGGCATCACCGCCACCGGCGCCACCGTGCTCAGTGGGCTGGA
CACCATGTCACCGGGCATCCTGATGTGGCGCTCGATGCTGCACTGGCTCGGCGGCATCGGTTTTATCGGCATGGCG
GTGGCGATCCTGCCGCTGCTGCGCATCGGTGGCATGCGCCTGTTCCAGACCGAATCCTCGGACCGCTCCGAGAAGG
TCATGCCCCGCTCGCATATGGTGGCGCGCCTGATCGTGGCGGCCTATGTCGGCATTACCATCCTCGGCAGCCTGGG
CTTCTGGTGGGCCGGCATGGGCCTGTTCGACGCGATCAACCACGCGATGTCGGCCATTTCCACCGGCGGTTTCTCC
ACCTCCGACGAGTCATTGGCGCACTGGAAACAGCCCGCCGTGCATTGGGTCGCGATAGTCGTGATGATCATGGGCA
GCCTACCGTTTGCCTTGTATGTCGCCACCTTGCGTGGCAACCGCAAGGCGCTGATCAAGGACCAGCAGGTACAGGG
CTTGCTGGGCATGCTGTTGGTGACCTGGATCGTGCTCGGCACCTGGTACTGGTGGACCACCAACCTGCACTGGCTG
GATGCGCTGCGCCATGTGGCGCTGAACGTCACGTCCGTAGTGACCACCACCGGTTTTGCCCTGGGTGACTACAGCC
TGTGGGGCAATTTCTCGCTGATGCTGTTCTTTTACCTGGGTTTTGTTGGCGGTTGCTCGGGCTCCACCGCTGGCGG
AATCAAGATTTTCCGCTTCCAGGTTGCCTATATCCTGCTCAAGGCCAACTTGAACCAGCTGATTCACCCCAGGGCG
GTGATCAAGCAGAAATACAACGGCCATCGCCTCGACGAAGAAATCGTCCGGTCGATCCTGACGTTCTCGTTCTTCT
TCGCCATCACCATCTGCATGATCGCCCTGGCCCTGTCGTTGCTGGGCGTGGACTGGATGACCGCCCTGACCGGCGC
CGCCAGCACCGTTTCCGGCGTGGGCCCGGGGCTGGGCGAAACCATCGGCCCGGCCGGCAACTTCGCCACCCTGCCA
GATGCCGCCAAGTGGATCCTGTCCCTGGGCATGCTGCTCGGCCGGCTGGAGATCATTACGGTGTTTGTACTGTGTA
TGCCGGCGTTCTGGCGACACTGACACTCGCCGAAGTCTCCAATAACCGCGCCCGGTATTCGCCGGGCGTGGCATCG
AACCAGCGGCGAAACGCGCGGAAGAAGTTGCTCGGGTCGGCAAACCCCAGCAGGTAGGCGATTTCCAGCAAGGTCA
TGCTCGGCTGCGCCAGGTACTGCTCGGCCAATTCGCGGCGGGTGTCGTCGAGCAAGGTCTGGAAGCTGGTGCCCTC
CTCCTGCAAGCGCCGCTGCAGCGTACGCTGGGACAGGTGCAACGTCTGCGCGACCATCTCACGCTTGGGCTCGCCC
TGGGGCAGCAACCGGCAGAGCACCTGGCGGGCCTTGTGGGTCACGCGGCTCTCGGAAAACCGCGCCAGGTATTCCC
CGGCAAACCGGTCGTGCAGCAGCGCCATCGCTTCATTGGCGGTAGGCAACGGTGCTTCCATATCCGCCCGCTCGAA
GATCAGCGCGTCATACGGCGCGTTGAACACCAGCGGCGCATGAAAGGCCTGCCTATAGGGTTCCAGGTCGGCTGGC
TGGGCGCCTTGCACCAACACTTTGCGCGGTTGCAACGTGCGCCCGGTCAGCCAGCCACACAACGCCAACGCGCAGG
CAAGCGATGCCTCGGCGCTTTGCCGGGTGGGCGGCAGATGATCGCCGTGTACCGTAAGGATCAACGCATAGCCTTC
GGGCAACAGGCGGAAGCTCAAGTCAGCGCTTTCGGCGATGATCCGCTGGTAGCGCACCAGCCGGGTAAAGCCCTCG
GCCAGGGTACTGCTGGACATCAAGGCATAGCCGGCCACATGGAATGACGCCGGGCGCACTACCTTGCCCATATTCA
GGCCAATGGCCGGGTTGCCCGAAACCTCCACCGCACGCTGCCAGAGCCGGGTCATGGAGTCCTGGGGAAAGCGCGC
GTCGGGGTCATCCAGGGCGCTGTAATCCAGGCCCAGCTGTTTGAACAGCGCGCGGCAATCCAGGCCCTCGAGCTCC
AGCGCCTTGACTATCCCCATCGCCCAGCTTGCAGAAGTCGTTCGTTCGTTCATGGCGTTATCTTGCACGCGTTCTA
TGACGGGACGGCTGAAATCGCAGCCAGGAACCCAAGGATACTAAAGTGGCGTCTATTGTCACTGGCCCTTGCCAGT
GATGACTCTAGACTCAAATAAGCTCCCCGCCGAACATCCGTCGGGCACAACAATAACCAGAGGGCCGGCCAACGTG
GAAAATGTCAGACAATTCAATAGCTTCGCCGAGTTCTATCCGTATTACCTGAGTGAACACGGCAACAGCACCTGTC
GGCGCCTGCACTTTATCGGTACCAGCCTGGTGATTTTTATTCTCGCGTTCGCCATCGGCAAGGGTGCCTGGTGGCT
ACTGTGGGTACTGCCAGTGGCCGGCTACAGCTTTGCCTGGGTCGGGCACTTTTTCTTTGAAAAGAACCGCCCGGCG
ACATTCCAGCATCCGTTCTACAGCCTGCTGGGAGATTTCGTGATGTACCGCGACATGATCCTGGGCAAGGTGCCGT
TCTAAACCATCCAAAACAATAAGAGACACGCCGATGAGCCAGCAGGCCCGCTTCACCCATATGCAAGACGGCACCC
AGGAAGACTGGTCCATCATCGCCGCCGACTTCAGTGCCTATGCCCGCCAGTTGCCCGGGCGAATCCTCGCGCACCT
GCGGCTGCTGGAGGGTGACTTCGGTGGCTTCCCGGTGGACCGCCTGACCCACTCCCTGCAGACCGCCAGCCGCGCC
TGGCACGATGGCCGCGACGAAGAATATGTGGTCTGTGCGCTGCTCCATGACATCGGCGACACCCTGGGTTCCTACA
ACCACCCGGACATTGCCGCCGCCATCCTCAAACCGTTCGTCAGCCCGCAAAACCTGTGGATGGTGGAAAAGCACGG
GATTTTCCAGGGCTACTATTTCTTTCATCACCTGGGCATGGATCGCCACCTGCGCGAGCAATTTGGTGAACACCCG
CAGTATGAGCAAACCATTGAATTCTGCGCGCTGTACGACGCGGCCGCCTTCGATCCGGCCTATGCCACCCTGCCCC
TGAGCTTTTTCGAACCCATGCTGGCACGGGTCTTCGCCCAGCCAAAAAACTCCATCTACAAGGCCGCCATGGAGCG
CCAGGCCAGCGTGTGACCTCTTGTCACTTGCGTCTTGGGGCGAAAGGTTCGTGCATTTGGCGAGATAAAAGTCTTT
TTGTCATTTGCAGTGCTGTATCCTGCCCAGGCTTTGTAAAAAGCGTGGCACATCTGCAATCCCACAACAGACCTTG
CGAGGAGCGACGACGATGCACGAGATTCCCAATCTCCCCTTCCCAAGCCTGCACGAGACCGAGCAGCCAACCCCAC
AACAGGCCGGCAGCGATAAACAAGCGCCAGCACAAGCAGCACCGGTCGACAGCGATAGCGACGACTGACCGCAGTA
CCCAGACCGTGTGGAAAGCGTGACATTGCGCGCTTTCCACACCGCCTGAATCAAGAGACACGCAACATGCCTGACA
CTCCCAAGCATTCCCCCGATGACGCGATACTCGGCGCCGCCCTGCAAATGGTCGATGTGTGGAGCCGCCTCAGCCC
GGAAAAACAAGCGCTGCTGCTCAAGCGCTTTGGCACCCAGGAAAACGCCCTGGCCGCGCTCGTGACCACGCGCATG
CTCAGCCCCGCGCCACTGTAGGCGCCGGCTCGCCAGCGATGGCGGGCGTTCAGCCGCCAAAGGTTTTATGCCCTGT
GCGGCGCAAAGCACCGGTATCATAAGCAGCCTATCTTTTACTGCCGCGACAGTGGACCCCTCCCATGCCAGATACC
CAGCGCCCCATGGCGGTCACGCTGCAAGTCGTTTCCATCGTGCTGTTCACCTTTATCGGCTATCTGAATATAGGCA
TACCCCTGGCCGTATTGCCGGGCTATGTCCACAGCGACCTGGGTTTTGGCGCGGTGATCGCGGGCCTGGTGATCAG
CGTGCAGTACCTGGCCACCCTGCTGAGCCGGCCCTATGCCGGGCGCATCATCGATAACCGCGGCAGCAAGCGCGCG
GTGATGTACGGCCTGGCCGGTTGTGGCTTGAGTGGGGTGTTCATGCTGGTGTCCGCGGCATTGACCCATCTGCCGG
CCTTGAGCCTGACCAGCCTGCTGATTGGCCGGCTGGTCCTGGGCAGTGCCGAAAGCCTGGTGGGATCGGGCTCCAT

TGGCTGGGGGATTGGCCGGGTCGGTGCGCTGAATACCGCCAAGGTCATCTCCTGGAACGGTATTGCCAGCTACGGT
GCCCTGGCGATTGGCGCACCGCTGGGGGTGCTGCTGGTCAGCCGGTTCGGGCTGTGGAGCATGGGCGCCAGCATCA
TGGTGCTGGCGGTGCTGGGCCTGCTGCTGGCCTGGCCGAAAGTCGCGGCGCCCATCGTAGTCGGCGAACGCCTGCC
CTTCATGCATGTGCTGGGCCGCGTACTGCCCCACGGCTGCGGGCTGGCCCTGGGCTCCATCGGCTTTGGCACCATC
GCCACGTTTATCACCCTGTATTACGCGACCCAGAACTGGGATAACGCGGTGCTGTGCCTGAGCCTGTTCGGGGCCA
GCTTTATCGGCGCGCGGTTGCTGTTCGGTAACCTGATCAACCGGATCGGCGGGTTTCGCGTGGCGATCGCCTGCCT
GTCGGTGGAAACCCTTGGCCTGCTGCTGTTGTGGCTGGCGCCGAATGCCGAACTGGCGTTGGCGGGCGCGGCCCTG
AGTGGCTTTGGCTTTTCCCTGGTGTTTCCGGCGCTGGGCGTCGAGGCGGTCAACCTGGTGCCTGCCTCCAGCCGTG
GCGCGGCGGTGGGGGCCTATTCGCTGTTTATCGACTTGTCTTTGGGCATCACCGGGCCATTGGCCGGCGCTATTGC
GGCGGGTTTCGGCTTTGCTTCGATCTTTCTGTTTGCCGCACTGGCGGCCTTCGCCGGGCTGCTGTTGAGCCTGTAC
CTGTATCGCCAGGCCCCCAAATACCGCGACGAACGCAACGCCGGCTAGAAATCCACCTTGCCGCGCCCGGCCTTGA
TGCTGCCGCGCTTGGTCTTGGATTCCAGGCGACGCTTTTTCGAGCCCAGGGTGGGTTTGGTCGGACGGCGTTTTTT
CTCGACCTTGGTCGCGCTGAGGATCAACTCCACCAGGCGCTCCAGGGCATCGGCGCGGTTCTGTTCCTGGGTCCGG
TATTGCTGGGCCTTGAGGATCAATACGCCTTCGCTGGTGATGCGGCTGTCACGCAGTGCCAGCAGGCGCTCCTTGT
AGAACGGCGGCAACGATGAAGCGCCGATATCGAAGCGCAAGTGCACCGCGCTGGAAACCTTATTGACGTTCTGCCC
CCCTGCGCCCTGGGCGCGGATGGCCGTCAACTCGATTTCAGCATCGGGCAGGTGCACGTTACTGGAGATTACCAAC
ATGGAAAGCGGGTCCGTTGACACCTGCAGGAGCCGGTTTACCGGCTCCTGCAGGGTAGGCTAGTTGTTCAGCGAGG
ATACCGCACTCAGGTTTTCCCGAGCCTCGCGCTTGCTCTTGAAGAAATAGCAGACCCACATGAACGCCAGCCAGAT
CGGAATCGCATACACCGACTTCTGAATGCCCGGTGTCATCAGCATGATCGCCAGGATAAACACCACGAACGCCAGG
CACACAAAGTTGCCATAGGGGTACCACAGCGCCTTGAACAGCGGCACCTGGCCGGTGCGGTTCATGTGCTGGCGGA
ACTTGAAGTGCGAGAAGCTGATCATCGCCCAGTTGATCACCAGCGTGGCCACCACCAGGGACATCAGCAGTTCCAG
GGCGTGCTGCGGGATCCAGTAGTTCATCAACACCGCCACCAGAGTGACTGCCGCCGAGGCCAGGATCGAACGCACG
GGCACGCCGCGCTTGTCGATCTTCGCCAACGCCGCCGGGGCGTCGCCTTGTTCGGCCATGCCCAGCAGCATGCGGC
TGTTGCAGTAGGTGCCGCTGTTGTACACCGACAGCGCCGCCGTCAACACCACAAAGTTGAGGATATGCGCGGCAGT
GTTGCTGCCCAGCATCGAGAACACCTGCACGAACGGGCTGCCGCTATAGGCATCGCCGGACGCGTTCAAGGTCACC
AGCAGGCTGTCCCATGGCGTCAGCGACAACAGCACCACCAGGGCGCCGATGTAGAAAATCAGGATGCGGTAGATCA
CCTGATTGATCGCCTTGGGGATCACGGTCCGTGGCTGGTCCGCTTCAGCGGCGGTAAAGCCGAGCATTTCCAGGCC
GCCAAACGAGAACATGATGATCGCCATGGCCATCACCAACCCACCGACGCCATGGGGGAAGAACCCGCCGTGTTCC
CACAGGTTGCTCACCGAAGCCTGTGGCCCGCCGGTGCCGCTGACCAGCAGATAGCTGCCCAGGGCGATCATGCCGA
CAATCGCCACCACCTTGATAATCGCAAACCAGAACTCGGCCTCGCCAAACACTTTGACGTTGGCCAGGTTGATCAC
GTTGATCAGCACGAAAAATGCCGCCGCCGAGACCCAGGTCGGAATCTCCGGCCACCAGTAGTGCACGTACTTGCCG
ACGGCTGTCAGCTCCGACATGCCCACCAGGATATAAAGGATCCAGCAGTTCCAGCCCGACAAAAAGCCCGCGAAAC
CGCCCCAGTACTTGTGGGCAAAATGGCTGAAGGAGCCGGCTACTGGCTCTTCGACAATCATTTCGCCCAGTTGGCG
CATGATCATGAAGGCGATAAAGCCGCAGATGGCATAGCCGAGGATCATCGACGGGCCGGCTGACTTCAGCACCCCC
GCCGAGCCCAGGAACAGGCCGGTACCGATCGCGCCACCGAGGGCGATCAATTGGATATGACGATTTTTCAGGCCGC
GCTTCAGTTCACCTGAAGGCGAGTTGTTTGCACTCATGAAAAGGGTCTCACGCAAGGTTTGATGATGTTGATTACA
CGATGCTGCTTTGAAACCGACTCAAGCAGCGCCGATCAAACCCCAGCGCAGGCACCAGGAGTTCAGCGTCTTTCTT
ACGGTCATGCGTCACCTGTTTGTTTTTATCTGTGACGAAATCGAACCCGTCCGGCTTATGGCCAGGCGGAGTTAAC
AAGGCAGCCAGCCTTGAAGGCCTTTTGCATGGGTATGCAAGGGGGGTCACAGTAAAACGCGGCGCATTGTACACCG
CTCGATTGGTTAGGGAGCCTTGCCGGGGAGATTGTTGACCGATCGGCAAGTTTTCTTTACAGATAAAAAAACACGA
GACCAAGACAGGCGTAGGCTCAGGCCATGCGTAAAATAATTGTTACAGCGCGGAAGACATTCATGACGACCGGGCT
TGCCATCATCTGTAGGAACGAGCCTACTCGCGGCAAACGCCCGAGCACCGCGTTCATCCTGGATAAGCGTGACAGC
TCTGGTTTTTTCGCGAGCAAGCTCGCTCCTGCACAAAATCCGACGCAAAAAAAACGCCAACCCTCAGGGTTGGCGT
TTTCTCTTACCGCTCGCAGCTTAAAACTTGCCGCAGCCCTTAAGGCTTCTTGCGACCAAAGCCCGGACGCTGCCCG
GAACCTGCCGGTGCACCACGGCGCTTGCCCGATGGCTCGTCAGCCACCAGTTTGAGCCCAGGGCGCTTTTTCGGCG
CTGGCTTGGCTGGGCGCTTGGCGCCTTCGGCTGGACGATCGGTCTGCGGCGCACCGCGGCCTGCATCGCCTCGGTT
GCTGCGGCCACCTGTTGGCGCTTCGCCACGACCGGCCGGACGTGGAGTACGCGGCGCACGCTCGCCATCCTGACGG
GCTGGAGCCGATGGGCGACGATCACCCTCGATCTGCGGCTGACGGGAAATACGACCGCCAGTGGCCGGTGCATCCA
GGGCCGGGCGCAAAGTGCGGGCAACGCGCTCGGTACGAGCCACTGGGCGCGACGATTTACGCTGCATACGCTCCAG
CTTGTCTTTGCTCTTGGCGTTCATCTGCGGCATGGCCACCGGGGTCAGGCCCACTTCAGCACTGAGGATGTCGACT
TCGTACTGGCTCATTTCGCGCCAGCGACCCATCGGCAGGTCAGAGTTGAGGAACACCGGGCCAAAACGCACGCGCT
TCAGGCGGCTGACCACCAGGCCCTGGGATTCCCACAGGCGACGTACTTCACGGTTACGCCCTTCCATCACCACGCA
GTGGTACCAGTGGTTGAAACCTTCGCCACCTGGAGCCTGCTTGATGTCGGTGAACTTGGCCGGGCCGTCTTCCAGC
ACGACGCCAGCCTTGAGGCGCTCGATCATCTCGTCATCGACTTCACCACGTACACGCACCGCATATTCACGGTCCA
TCTCGTAGGACGGGTGCATCAGGCGGTTGGCCAGTTCACCGTCGGTGGTGAACATCAGCAAGCCGGTGGTGTTGAT
GTCCAGGCGACCGATGTTGATCCAACGGCCTTCTTTGGGCTTGGGCATCTTGTCGAACACGGTGGGACGGCCTTCC
GGGTCGTCGCGGGTGCAGATCTCGCCGTCGGGCTTGTTGTACATGATCACGCGGCGCACCGATTCGGCGGCCTCTT

CACGCTTGATGACCTTGCCATCAATGGTGATCGCGTCATGCAGGTCGACGCGCTGGCCAAGGGTGGCCTCGACGCC
GTTGACCTTGATGCGCTTCTGGGTGATCCAGGCTTCCACGTCGCGACGCGAGCCGACGCCGATACGGGCGAGAACT
TTTTGCAGTTTCTCGCCTGCTGGGCCGATTTCCTGGCTGTCGTTCTGGTCTTGGTCTTTCATCTTAAGCACCTCCC
GGTGTGTCGATTCAGGCGTGGCCTGAAGGTATTGAAACTGGGTTCTTGGGCGAACGCATCGCCGAAGGGTCGCGAA
TCATACGCTCATGGCGCGCATTGCGCATCAGAGACTAGTCGATATGGGCCGGCTCATTTACTTTTCCGCCGACCGG
TGGCACCCAGGGCGAGCAGCCGTAACTCGGCTTCGGCCAGCACCGCGCGCTTGTCGACCTTGTCGAGTTTCTTCCA
GGCCCGGATCTCACGCTTGCTGCGCCCGCAGCCGAGGCAGATATCGTCGCTGAATTTGCAGATGCTGATGCAGGGG
TCTTTGGTGGAGCTCATCACTACCTCACTGCACAGGGCTTATCTGTGGGAGCTGTCAGTCTTCGAATTCGCGGCGT
TCGTTTTCGATGGCTTCGGCCAGTGCTCGGGCTTCGGCTTCTTCTTCGCTCAACTCGGGCTGTTCAAGGGCGGCGA
CGGCGGCCAGCAATTTTTCGCGAGCGGCTGCCACACCCAAGATGTCTTCCAGAGGTTCAGGTTCGACCGCTGGCGT
CGTTTCGACGTTCACCGGCGTTTCGGGCTCGGCCGCCCCATCGCGCAGCAAGTCATCAAAATCGGTCTTGATCCCC
TGCTCCATATCGTCCAGCTCCAACAGCAGCGTATGGAAACTGGTCTCGTCCTTGGGCTCTTCCGGCTCGGCGCTGG
CATCAGCCAGCTCCTGCAAGCCAGCCGGCACTGGCGCATCGTCGAAATCGAGCACCGGTTCGGCCTCGATCTCCCG
CAATTCGGCCAATGGCGGCAGGTCGTCGAGGTTCTTCAGGTTGAAGTGATCGAGAAACACCTTGGTGGTGGCAAAC
ATCGCCGGTTTGCCCGGCACGTCGCGGTAACCGACGATACGGATCCACTCGCGCTCCAGCAGCGTCTTGACGATAT
GACTGTTGACCGCCACGCCGCGTACGTCTTCGATCTCGCCACGGGTGATGGGCTGGCGATAGGCGATCAGCGCCAT
GGTTTCCAGCATCGCCCGGGAGTAACGCTGCGGGCGCTCTTCCCACAGGCGCCCGACCCACGGCGAAAACTTTTCG
CGAATTTGCAGGCGGTAGCCCGAGGCCACTTCCCGCAGCTCAAAGGCCCGGCCTTCACAGGATTTGCGCAGGATCT
CCAAGGCCTTCTTGAATACCGGCGGCTCCGGGCGCTCAGCCTCTTCAAACAGTTCGAACAGGCGCTCCAGTGATTG
CGGCTTGCCCGAGGCCAAGAGGAAGGCTTCCAACAGCGGGGCCAGTTCGCGGGGTTCAGTCAGATTCATCGATTCA
GCTCGTTATTCGGCTCGCGCCCGCACGTGGATAGCCGCAAAAGGCTCATTCTGTACCAGCTCGACCAAGGACTCCT
TGACCAATTCGAGGATCGCCATAAAAGTCACCACCACCCCCAGGCGCCCTTCTTCAGCGGTGAACAGCTCGACAAA
CGGCACAAAACCACCGCCCTTGAGGCGCTCCAGCACATCACTCATGCGTTCGCGAGTGGACAAGGCTTCACGGCTG
ACCTGGTGGCTTTCAAACATATCGCCACGGCGCAGCACCTCGGCCATGGACATCAGCAATTCTTCCAGGCTCACAT
CTGGCAACAATTTGCGCGCCCGGGCTTCCGGCGCATCCAGCTTGGGCACCACCACGTCACGGCCCACGCGGCTCAA
GCCGTCAATGCCCTCGGCGGCTGCCTTGAAGCGCTCGTACTCCTGCAGGCGGCGGATCAGTTCGGCGCGCGGGTCG
TCCTCTTCGGCCTCGATCGTCTCCGAGCGCGGCAGCAGCATGCGCGACTTGATCTCGGCCAGCATCGCGGCCATCA
CCAGGTATTCGGCGGCCAGTTCCAGGCGCACCGACTGCATCAACTCGACATAGCCCATGTACTGGCGGGTGATTTC
TGCCACCGGGATATCAAGGATATTGATGTTCTGTTTGCGGATCAGGTACAGCAGCAAGTCCAGCGGCCCCTCGAAG
GCCTCAAGGAAGACTTCCAGGGCATCCGGCGGGATGTACAAGTCCAGGGGCATTTCCATGACCGCCTGGCCGTAGA
CCATGGCAAATGGCAGCTCCTGCTGGGCGCCGGCCTGGCTGTCGACGGGTTCCACGGCCGACATTCAGGCCTCGAC
CATGAACGGCGTGGGATCGCCGCAACCGACGCGCACCACTTCAGGCTCGCCGTCGGCCAGGTTGATCACAGTAGAC
GCCTTGTTGCCGCCGTAGCCGCCGTCGATGATCAGGTCTACGTGTTTTTCCAGGAGTTGGCGCATTTCGTAAGGGT
CGTACAGCGGCTCGGACTCTCCGGGCATGATCAGCGACACGCTCATCAGTGGCTCACCCAGCTCGGCCAGTAACGC
CAGGGCGATGGGATGCTCCGGCACCCGCAGGCCGATGGTGCGCTTCTTCGGGTGCAGCAACAGGCGCGGCACCTCG
CGGGTGGCATTGAGGATGAACGTGTAGGGCCCTGGCGTATGGGCCTTGAGCAGGCGGAAAGTGCCCGTATCAACCT
TGGCGAACAGACCCAGTTGCGACAAGTCGCTACAGATCAACGCGAAGTTGTGGTTCTTATCCAGGTCGCGCAGGCG
TCTTACGCGCTCCACCGCATTCTTGTCGCCGATCTGACAACCAATGGCGTAGGAGGAGTCCGTGGGGTAGATCACC
ACGCCACCGGCACGGATGATCTCCACGGCCTGCTTGATCAGCCGCGCTTGCGGGTTTTCCGGATGAATCTGGAAAA
ATTGACTCACGTTTTCTACCTGTTCAGACGGTGGCGGTAATGGGGTCATGCTTGAATCGCCCCCACAGGAGCGGCA
AGTCTTCCGGGACCGGGCGGTATTCGCCGATCTCCGACCAGCCGCCTGGGCCATGGAAATCACTGCCGGCGCTGAC
CAGCAGCCCAAATTCACGGGCAAGAATCGCCAGGCTGCCAACCTGTTCGGCGGGTTGATGCCCATTGACCACTTCG
ATGGCGTGGCCACCTGCTTGAATATAGTCGCCTATCAGCTTGCGGCGTTTACTGCGGGTGAAATCATAGTGCCAGG
GATGCGCCAGGCTGACCCAGGCCCCCGAAGCGCGCAGGGTGGCCACGGTGTCTTCGAGGGTCGGCCAGTGCAACTT
CACGTCCCCCAGCTTGCCGGCGCCCAGCCATTTGCGGAACGCTTCGGCGCGATCCTTGACGTAACCTGCGCGCACC
ATCCAGTCGGCAAAATGCGGACGGGCCGGCGCGTTGCCACTGTCGCCCAGCTCCTGCTGGATGGCACGGGCGCCCT
CCAGGGCGTTGGGCATGCCTTTGAGCGCCAGTTTGCGGCTTATTTCTTCGGACCGCAGCCAGCGGCCATCGTGCAA
ACTGGCAATGGCCGCCACCAATGGCGGGGCGTTGACGTCGAAACCGTAGCCCAGTACATGAATGGTCGCCCCGCCC
CAGGTGCAGGACAGTTCGACGCCATTGACCAGATACATCCCCAGTGCCGTGGCGGCGCTGCGCGCCTCGTCCAGGC
CTTCGAGGGTGTCGTGATCGGTCAGCGACAGGACTCGCACGCCTTTTTCAAACGCACGCGCAACCAGTACCGCGGG
CGCCAGGGCGCCGTCGGAGGCCGTGCTGTGGCAGTGCAAATCAACATTCACGGGAGTGTGTAACCTCAAGTCAGCT
GGCGCTATCGCAACCAAGGATGTTTGTTATTATGCCGCCACATCCAGCTTCTGGCTCTTACTGTGAAACAATTCAT
CGACTTCATCCCGCTGTTGCTGTTTTTCATCGTCTTCAAAACCGACCCACGGGTCTTGGATATCGGCGGCCATGAG
CTGTCGTTCGGCGGCATCTACAGCGCCACCGCGGTGCTGATCATCAGTTCCCTGGTGGTCTACGGCGCGATCTTCA
TCTCCCAGCGCAAGCTGGAAAAAAGCCAATGGCTGACCCTGATCGCCTGCCTGGTCTTTGGCGGCCTGACCCTGGC
CTTCCACAGCGAAACCTTCCTTAAATGGAAAGCCCCGGTGGTCAACTGGCTGTTCGCCCTGGCCTTTATCGGCAGC
CACTTCATCGGCGATCGCCTGTTGATCAAGCGGATCATGGGCCATGCCCTGAGCCTGCCGGAGCCGATCTGGACCC

GCCTGAACATCGCCTGGATCCTGTTTTTCCTGTTCTGCGGCGCGGCCAACCTGTTCGTGGCCTTTACCTTCCAGAG
CTACTGGGTCGACTTCAAGGTGTTCGGCAGCCTGGGCATGACCGTAGTATTCCTGGTTGCCCAGGGCATCTACCTG
TCGCGCCACTTGCACGATACCGACTCCACCACGCCAAAAACCGAGGACTGACATGCTCTACGCAATCATTGCCACC
GACGTCGCCAACTCCCTGGAAAAGCGCCTGGCTGTGCGCCCGGCCCACGTGGAACGCCTCAAGGCGCTGCAAGCCG
AAGGACGCATCGTGCTCGCCGGCCCACACCCGGCAGTCGACAGCAATGACCCGGGTGAAGCCGGCTTCACCGGCAG
CCTGATCGTTGCCGAGTTCGCGTCCCTGGCCGATGCCCAAGCCTGGGCCAAGGCCGACCCGTACGTAGCGGCCGGT
GTGTATGCCGATGTCGTGATCAAACCCTTCAAGCAAGTCCTGCCTTGATCCGGGTGGCGCTGAACCTATTGGGTTC
GGCAAACGCCTGATCGCTCATTATTCTCGGTAACCTGCCGACAACGTTCTGAATATTCGTGTGGAATCAGGAGTTC
CGATGCGCTTAAGTCAGTTGTGTCTGTTGGCAGTGTTAACGATCGGGGCCACGGCCCAGGCTGAAGAAACCTCGAA
CACCGGCAGTTCTACGCCCCTGTCCTTGAGTGCCGGCAGCCAGATCACCGAGTTGCAGCAACGCTTGAAAGAAAGC
GAACGCCTGCGCGAAGAACTGAGCAAACAATTGCAAACTGCCGATGCCGCCCGCGAGAGTGCGCAATTGAGCCGCT
TGCGCCAGGAGAACCAGCGCCTGGCCCAGCAACTCAAGGACGCACAAGGCAGCACCCTGCCCCGCTGGCTCACCGA
CCAGCAGCAATGGTTCGTGATTGGCGCAGGAGTTGCGCTGGTTGCCCTGCTGTGCGGTATCTTCGCCAGTGGCGGG
CATCGGCGCCGTCGACAATGGCTAAATTGAGTGAGTCATGAGCGAGCTGTTACTGATAGATGATGACCAGGAGCTC
TGTGAGCTGCTGACCAGTTGGTTGAGCCAGGAAGGTTTCCAGGTGCGCGCTTGCCATGACGGCGTGAGCGCCCGCA
AGGCCTTGGCCGACGCCGCGCCGGCGGCCGTGGTGCTGGACGTGATGCTGCCCGACGGCAGCGGCCTGGAGCTGCT
CAAGCAATTGCGCGCCGACCACCCGGAACTGCCGGTGCTGATGCTCTCGGCACGGGGCGAGCCCCTGGACCGGATC
CTCGGCCTGGAGCTGGGCGCCGATGACTACCTGGCCAAGCCCTGCGACCCCCGCGAACTGACCGCCCGCCTACGGG
CGGTGTTGCGCCGCAGCCATCCGACGGCTGTGTCGACCCAACTGGAACTGGGCGACCTGTGCTTCAGCCCGGTGCG
CGGCGTGGTGATCATCGACCATCAGGAACTGACCCTCACCCTGTCCGAAAGCCGCCTGCTGGAAGCCTTGCTGCGC
CAGCCGGGCGAGCCGCTGGACAAGCAGGAACTGGCGCAGATTGCCCTGGGCCGCAAGCTGACCCTGTATGACCGCA
GCCTGGACATGCACGTCAGCAACCTGCGCAAGAAAATCGGCCCGCATGCCGACGGCCGTCCGCGGATCGTCGCCCT
GCGCAGCCGTGGCTACTACTACACCCCCTGAAATCCCCTGTAGGAGCCGGCTTGCCGGCTCTTACCTGTTTTGTAA
GCCCCCACCCAACCCGTCTTTACCCAAGCTTTACCCTCCCCTGACCGCCGCTGACCTTGATCCTCGTAATCTACTC
ACATCCGGACTCACCGGAACCGAGACAAGGAGAAACACCATGCGCAAGACCCTTATCGCTCTGATGTTCGCTGCTG
CCCTGCCTACCGTTGCCATGGCCATGCCAGAAGGCCCAGGCCCGGTGGGCGGTCCAGAAGGCCATATGATGGGCGG
CCCGGGCCACGGCGGTGAACACGGCATGCGCGGCAAAGGCGGCCCTTTCAGCCAACTGGACCTGACCCGCGAACAG
CGCCAGCAGATCGGCAAGTTGATGGGCGAGCAACGCGAAGGTCGTCAGCAACTGGTCAAAAAGTACCTGGACAAAC
TCCCGGCCGCTGAGCAGAAAGCCATGAACGACGAGATGGCCGCCGCCAAGCAGAAAACCCAAGCCGATATCCGCGC
CCTGCTCAAGCCTGACCAGCAGAAGAAGTTCGACGAAATGGCGAAGAAACGCGCCGAGCGTCAGGCCGAGTGGCAG
CAGTTCCAGGCCTGGAAAGCGCAACAACCGCAAAAAGCGCAATAATGCGTTAGCAATCTCCCAGCCCAGTGGCCCG
CGCCACTGGGCTTTTCCTGTTTGAGGGTTTCCTGTGCGTTCACTCTTCTGGCGCATCCTTGCCAGTTTTTGGCTGG
CCATCGCCCTGGTTGCCGGGCTGTCGATCCTGCTTGGGCATATGCTCAACCAGGATGCCTGGATTCTCAGCCGCCA
CCCTGGCCTCAGTAACCTGGCCGAGGAATGGACCCAGCGCTACGAAACCCAAGGCGAGGATGCGGCCCAGGACTTG
TTGCAACAGCGCAAGCGCCAATACCACGTCGATGTGCAAGTGCTCAACGAAAGCGGCGACCCGGTGGTACGTGGCA
CCTTCCCGCGCCGTGCCGCCGCCTTCGAAGCGCGACAGCATGAGAGCCACGAGGGCCGGCTGCCCTGGCGTCGCCT
GACGGCCGAATACACCAGCGAGAAGACCGGCGATACCTACCTGCTGATCTACCGCATCGCCCTGCCGGAGCTGGAT
GCCTGGCACCGCAGCAGCCTGCTCTGGCCATTGAGCGCCCTGGCGATTGCGCTGGTGGTGCTGACCCTGTTCAGCC
TGCTGGTGACCCTGTCGATCACGCGGCCTTTGAGCCGCTTGCGCGGCGCGGTGCATGATCTGGGGCAGACCACGTA
CCAGCAAAACAGCCTGGCTCGCCTGGCCGACCGGCGCGATGAGTTTGGCGTGCTGGCCACCGACTTCAACCGCATG
GGCGCGCGCCTGCAAAGCCTGATCGGCAGCCAGCGGCAACTGCTGCGCGACGTCTCCCATGAGCTGCGCTCGCCCC
TGGCCCGCCTGCGGATCGCACTGGCCCTGGCGGAACGGGCCACCCCCGAGGCACGGGAAAAGCTCTGGCCGCGCCT
GACCCTGGAATGTGACCGCCTCGAAGCCCTGATCAGCGAGATCCTGGTACTGGCGCGAGTCGACGCCGACAACGCC
AGCGCCGAAGACATCGACCTCAATAGGCTGCTCGAGACCCTGCAAAAGGACGCCCAGCTCGGCGCCCCGGACCAGG
AAGTCCGGCTTCAGGCCGACCCGGCGTTAAACCTCAAGGGCTGGCCCCATATGATCGAACGGGCGCTGGACAACCT
GTTGCGCAATGCCCAGCGCTTCAACCCGCCCACCCAGCCGATTGAAATGCGCGCCCAGCGTGAGGGCGAACGCATT
GTGATCAGCGTGCGCGATCACGGCCCGGGCGTAGATGCCGAGTGCCTGCAAAAACTGGGCGAGCCGTTTTACCGTG
CCCCCGGGCAAACCGCCCAAGGTCATGGCCTGGGCCTGGCCATCGCCCGGCGCGCCGCCGAGCGTCACGGCGGCAG
CCTGAGCCTGGCCAACCACCCCGAGGGCGGTTTTATCGCCACCCTGGATTTGCCCTTGCAACCGGGGATTGCAGGA
CACCCCTGAAGATCTTCTATGATGGCCCTTCTGTTAAGGAGGGCCCCTGATGACTGATCTGCTCACATCGATCCAA
CTTGCACTCGATCTGCCGGCCGCACCGCTGGCGACCACAGAGGCCGGTGCCCTGCCGTCGGCCTTCGCCGTCACCG
AGTTGGCCTGCGCCAGCATGGGTGCGGCGGGTCAGGCAGTGGCCCGCCTGCTTGAACAACAGACCGCGCGCTTGCC
CGCCGTCAGCGTCGACCGGCGCCTGGCCTCGTTCTGGTTCTCCAGCTCCCTGCGCCCGCTGGGCTGGAGCGTTCCG
CCATTATGGGACCCGATTGCCGGCGACTATGCCTGCGCCGATGGCTGGATTCGCCTGCACACCAACGCCCCACACC
ATCGTGCCGCCGCCGAGCGAGTACTCGGACAGGCGGCGCAGCGGACGGCAATGGCCCTCAAGGTCGCCCGGTGGAA
CGCTGGCGAGCTTGAACAAGCGATAGTCGACGAAGCCGGCTGTGCGGCGCAGATGCGCACCTGGCAGGAGTGGCAG
GCCCATCCACAAGGGTTGGCGGTCAATCAGGAACCCCTGATCGCATGGGAGCGCTTCGACGCGCGCGGCAAAAGCC

GCTGGCTCGGCAGCGTTGCGCGCCCGCTGGCGGGGGTCAAGGTCCTGGACCTGACCCGCGTCCTGGCCGGCCCCGT
GGCCAGTCGTTTTCTTGCAGGCCTGGGTGCCGACGTATTGCGCATCGACGCACCCGACTGGAACGAACCCGGGGTT
GTCCCGGAAATGACCCTGGGTAAGCGCTGCGCGCGCCTTGATCTTAAACACGCCAACGACCGGCAACTATTCGAAA
ACCTGCTCAGGGATGCCGACATTCTGCTTCACGGTTATCGGGCAGATGCGCTGGAACACCTGGGTTACAACGCAAC
CGCACTGCAGCAGATTGCCCCGGGCCTGATCGACGTCAGCCTTAACGCCTACGGCTGGAGCGGCCCGTGGCGCAAT
CGCCGGGGCTTCGACAGCCTGGTGCAGATGAGCAGCGGCATCGCCCACGCCGGTATGCAGAGCAAACAGTTGGATC
AACCCGTGCCGCTGCCAGTGCAGGCTCTGGACCATGCTACCGGCTACCTGATGGCCGCCAGTGCGATACAGGCCTT
GGCCCAGCGCTTGAACAGCGGGCACGCAGGTTCGGCACGGCTGTCGCTGGCGCGTACGGCAAAATTGTTGGTCGAA
GCGGCACACGGCAAGCAGCCCGCATTGCGTGAAGAACAACCCGCTGATCAGGGGCTGCTGGTGGAACAGACGGCCT
GGGGGCCGGCACATCGTTTGCTGGCCCCGGTCACTATTACCGGGACACCGCTGCAATGGGTCTTGCCGGCCGGGGA
ATTGGGCGCGCATCGGGCACAGTGGTGACCTTGGGATTGTCATCGCGGGCAAGCCCACTCCCACAGTTGATTGTGT
ACAACTCAGTCAATGTGGGCTTGCCCGCGATGAAGCCCGCACAATCGCTCATAGGCTCAACACACCACTGTACAAC
CCATACGCCGCCAGGCTCGCCCCAGCGATCACACAGCTGAAAATGCCTTTTTCCAACAAGGTAAACAGCGGCTGGC
CCTGCTCGCGCTTGGCCTGGGCAAACAGGATGACGCCCGGCGCATACAGCAGCGCTGACAGCAGCAGGTATTTCAA
GCCACCGGCATACAGCAGCCAGACCGCATAGCCCAGGGCAATCACGCCCACCAGCAGGTCCTTGAGCCGCTCGCCC
TGGGCGCCCTCATAGGTTTCCCCGCGCCCGCACAGCAGCACGGCATAGGCCGCCGACCATAAATACGGCACCAGAA
TCATCGACGAGGCCAGGTAGATCAGCGTGGTGTAAGTGCTGGCGGAAAACAGCGTGATCACCAGGAACAGCTGGAT
CATCACATTGGTCAGCCACAACGCATTGACCGGCACACGGTTGGCATTTTCCTTTTTCAGGAAGGCCGGCATGGTC
TTGTCATGGGCGGTGGCGTAGAGAATCTCGGCGCACAGCAAGGCCCATGACAGCAACGCCCCCAGCAAAGACACCG
CCAGGCCGATGCTGATCAACATCGCGCCCCAGGGGCCCACGATATGTTCCAGCACGCCCGCCAGTGACGGGTTCTG
CAAGTTGGCTAACTCGGGCTGGCTCATGATCCCCAGGGACAGCACGTTCACCAGTACCAGCAGGGCCAGCACCCCG
ACAAACCCGATCACCGTGGCGCGCCCCACGTCGGAGCGTTTTTCGGCGCGCGCCGAGTACACACTGGCGCCTTCGA
TGCCGATAAACACAAACACGGTGACCAGCATCATGTTGCGCACCTGGTCCATCACCCCACCGAATTGCGGGTTGCC
CAGGCCCCAGATATCCCGGGTAAAAATGTCTGCCTTGAAGGCCACGGCGGCGATCACGATGAACATCAGCAACGGC
ACGATCTTGGCCACAGTCGTGACCTGATTGATAAACGCCGCCTCCTTGATGCCGCGCATCACCAGAAAATGCACGG
CCCACAGCAGCAACGACGCGCAACCGATCGCCACGGGCGTGTTGCCCTGGCCGAACACCGGGAAAAAATAGCCCAG
GGTGCTGAACAGCAACACGAAGTAGCCGACATTGCCCATCCAGGCGCTGATCCAGTAGCCCCAGGCCGACGAGAAG
CCCATGTAGTCACCAAAACCGGCCTTCGCGTAGGCATACACACCCGAGTCCAGGTTGGGCTTGCGGTTGGCCAGGG
TCTGGAACACAAAGGCCAGGGTCAACATGCCAATGGCGGTGATGCCCCAGCCGATCAGGATCGCCCCCACCTCTGC
CCGCGCCGCCATGTTCTGCGGCAACGAGAAGATCCCGCCGCCAATCATCGAGCCCACCACCAACGCCACCAGGGCG
CTGAGGCGCAGCTTTTGTGCCGGTTGCGACATAGAAACTCCTGCAGATAAAACCGTCATTGAGCTAACAACTGTGC
ACTCGGCTAATTTATCGAAAACATGCAACGTTTATTATCTATAGCGAATAACACTGCTCGCCTCATAACTTAATGA
CATTTCAGTTAGTCGAGTTTAGGCAAATTTAGAATTTAAATACCCCTTGTCTTTTATCCTTGAGAGGTATTTGGCA
AAGCCTTTTGAAAAAGTTTGCACATCTGCGAAAACGGGCTAGCTTCAAACATCCACGCCTTACGCATATCGCATTA
ATCCAAACGGATAAAAGCTCTGGGACGCGCTTATCCGAGTAATTATACGCCCGCAGCATTTATTCATAAGTCTTTA
ATTCACAATGGAATGTGCCAATGAAGTGATCTGAGTCAGCTGTTTGAATAGCGCACAGTTTTATTCTGTGGTCTCT
CTTCTCCTGCATTGGAGTCACGCAATGTCTGAATCTCCCGGAAAACTTCGATTAGGCGCCCTTGTTGCTCTAGTTG
TGGGCTCAATGATTGGCGGTGGGATCTTCTCGTTGCCGCAAAACATGGCCGCCAGCGCCGATGTTGGTGCGGTATT
GATTGGTTGGGTTATCACCGCCGTAGGTATGTTGACCCTGGCCTTTGTGTTCCAGACCCTGGCCAACCGCAAACCC
GACCTGGACGGCGGTGTATACGCCTACGCCAAGGCCGGTTTTGGCGACTACATGGGTTTCTCGTCGGCCTGGGGCT
ACTGGATCAGTGCCTGGCTGGGCAACGTCGGTTACTTCGTGCTGCTGTTCAGCACCCTCGGTTACTTCTTCCCCAT
TTTCGGCGAAGGCAATACCCCGGCGGCAGTGATTGGCGCCTCGGTGTTGTTGTGGGCCGTGCATTTCCTGGTGCTG
CGCGGCATCAAGGAAGCAGCGTTCATCAACCTGGTCACTACGGTCGCCAAGGTCGTGCCGCTGGTGCTGTTCGTAT
TGATCGCACTGTTCGCGTTCAAGCTGGACATCTTCACCGCGGATATCTGGGGCGTGAAAAACCCCGACCTGGGCAG
TGTGATGAACCAGGTGCGCAACATGATGCTGGTTACCGTCTGGGTATTTATCGGTATCGAAGGCGCGAGCATCTTC
TCGTCCCGTGCCGAAAAGCGCTCGGATGTGGGCAAGGCCACCGTCATCGGCTTTATCACCGTGCTGCTGTTCCTGA
TGCTGGTGAACGTGCTGTCCCTGGGGATCATGACTCAACCGGAACTGGCCAAGCTGCAGAACCCGTCGATGGCGGC
CGTGCTCGAGCATGTGGTCGGCCACTGGGGCGCGGTGCTGATCAGCGTCGGCTTGATCATCTCCCTGCTCGGCGCG
TTGCTGTCGTGGGTGCTGCTGTGCGCAGAGATCATGTTCGCCGCTGCCAAAGACCACACCATGCCGGAGTTCCTGC
GTAAGGAAAACGCCAACCATGTGCCGGTCAACGCCCTGTGGCTGACCAACGCCATGGTGCAGATATTCCTGGTAAT
CACCCTGTTCTCCGCCAGTACCTACCTGTCGCTGATCTACCTCGCCACCTCGATGATCCTGGTGCCTTACCTGTGG
TCGGCGGCCTACGCACTGTTGCTGGCGGTACGCGGCGAGACCTACGAGGCGGCCCTGGCCGAGCGCAAGAAAGACC
TGTTCATCGGCGCCATCGCCCTGATCTACGCGGTCTGGCTGTTGTATGCCGGCGGCACCAAATACCTGCTGTTGTC
CGCCCTGCTCTATGCCCCTGGCGCGATCCTGTTCGCCAAGGCCAAGCGTGAGCTGGGCAAACCGATTTTCACCAAC
GTCGAGAAGCTGATTTTCGCCGCAGTGGTCATTGGCGCCCTGGTGGCGGCCTATGGGCTCTACGACGGCTTCCTGA
CCCTGTAACACCTGAATCTTTTGTTCACTGGAGGATCTGTAATGACCACGGAAAAAGTTAAGTACGGCGTACATTC
CGAAGCCGGCAAACTGCGCAAAGTCATGGTGTGTTCCCCAGGTCTGGCCCATCAGCGGCTGACCCCCAACAATTGC

GACGAACTGCTCTTCGATGACGTGCTCTGGGTGGCCCAGGCCAAGCGCGACCATTTCGACTTCGTCACCAAGATGC
GCGAGCGCGATATCGATGTGCTGGAAATGCACAACCTGCTGACCGAGATTGTCGCCATCCCCGAAGCGCTGGACTG
GATCCTGGAGCGCAAGATCACCGCCAACACCGTGGGCCTGGGCCTGGTCGATGAAGTCGGCTCGTGGCTGCGCAGC
CTGGAGCCACGCAAGATCGCCGAGTTCCTGATCGGTGGCGTGTCGGCCGATGACCTGCCCAGCAGCTTCGGTGGCA
AGACCATCGAGATGTTCCGCGACTTCCTCGGCCACGCCAGCTTCATCCTGCCGCCGCTGCCCAACACTCAGTTCAC
CCGTGACACCACGTGCTGGATCTACGGTGGCGTGACACTCAACCCCATGTACTGGCCGGCGCGACGTCAGGAAACC
CTGCTGACCACCGCCATCTACAAGTTCCACCCCGAGTTCACCAACGCCGACTTCCAGATCTGGTACGGCGACCCTG
ACCAGGAGCATGGCGCTGCCACCCTGGAAGGCGGCGACGTGATGCCGATTGGCAACGGCGTGGTGTTGATCGGCAT
GGGCGAGCGTTCGTCCCACCAGGCTATCGGCCAACTGGCGCGCAACCTGTTCAAGAACAAGGCCGTGGAAAAGGTC
ATCGTTGCCGGCCTGCCGAAATCCCGCGCGGCGATGCACCTGGACACCGTGTTCAGCTTCTGCGACCGCGACCTGG
TGACCATCTTCCCGGAAGTGGTGAACCAGATCGTGCCCTTCACCCTGCGTCCAGATGAAAGCAAGCCCCACGGCAT
TGATATCCAACGGGAGAAAACCAACTTCCTCGATACCGTGGCCGCCGCCCTCGGCCTCAAGGCCCTGCGCGTAGTG
GAGACCGGCGGCAACAGCTTCGCCGCCGAACGCGAACAGTGGGACGACGGCAACAACGTGGTGGCCGTGGAGCCTG
GCGTGGTGATCGGCTACGACCGCAACACCTACACCAACACTCTGCTGCGCAAAGCCGGTGTGGAAGTCATCACCAT
CAGCGCCGGCGAACTGGGGCGCGGCCGTGGCGGCGGCCACTGCATGACCTGCCCGATCATCCGCGACCCTATCGAC
TACTAAACGACCATCTCCCCGGCGGCGTCCACCCGATGCCGCACGGGCCGATTACCGAATCCAAGGAGAATCATCA
TGGCGTTCAACATCCACAACCGTAACCTGCTGAGCCTGGAACACCACACCCCGCGCGAACTGCGCTACCTGCTGGA
CCTGTCCCGCGACCTCAAGCGCGCCAAGTACACCGGCACCGAGCAGCAGCACCTCAAAGGCAACAACATCGCGCTG
ATCTTCGAGAAAACCTCGACCCGCACCCGATGTGCCTTCGAAGTGGCGGCCTATGACCAGGGCGCCAACGTCACCT
ACATCGACCCGAACTCGTCGCAGATCGGCCACAAAGAAAGCATGAAAGACACCGCCCGCGTACTTGGGCGCATGTA
CGACGCCATCGAATACCGTGGCTTCAAGCAGGAAATCGTCGAAGAACTGGCGAAATTCGCCGGCGTGCCGGTATTC
AACGGCCTGACCGACGAGTACCACCCAACCCAGATGATCGCCGACGTGCTGACCATGCGTGAACACGCCGACAAGC
CGATCCACGACATCAGCTACGCCTACCTGGGCGACGCCCGCAACAACATGGGCAACTCGCTGCTGCTGGTCGGCGC
CAAGCTGGGCATGGACGTGCGCATCTGCGCGCCAAAAGCCCTGTGGCCCCATGACGATCTGGTCGGTCGCTGCAAA
AAATACGCAGAAGAAAGCGGTGCACGCATCACCCTCACCGAAGACCCGAAAGCGGCGGTCAAGGGTGTGGACTTCA
TCCACACCGACGTCTGGGTGTCCATGGGCGAGCCAGTAGAGGCTTGGGCCGAACGTATCGAGCAACTGCTGCCGTA
CCAGGTCAATGCCCAACTGATGAAAGCCACCGGCAACCCACGCACCAAGTTCATGCACTGCCTGCCGGCGTTCCAT
AACAGCGATACCAAGATCGGCAAGCAGATTGCCGAGCAGTATCCGCACCTGGCCAACGGCATCGAAGTGACCGACG
ACGTGTTCGAGTCCCCGGCCTGCATCGCCTTCGAGCAAGCGGAAAACCGCATGCATACCATCAAGGCGATCCTGGT
TTCGACCCTAGCGGACCTGTAACCCGGCACCGCTTCCACTGATGCAACACGGTCAATGTGGGAGCGAGCTTTATGT
GGGAGCTGGCTTGCCTGCGATGGCATCACCTCAATATGTCTGGCAGACCGAGGTGACTGCATCGCGGGCAAGCCCG
GCTCCTACATAAAGCCTGTTCCCACACTTGGCCCAGTGTTCACAAGGCAGAATTCTAGAAGGATTGCATTATGCGT
ATCGTCGTTGCACTGGGCGGTAACGCCCTGCTCCGCCGTGGTGAACCCATGACTGCGGACAACCAACGCGCCAATA
TCCGGGTCGCCACCGAACAGATTGCCAAGATTCATCCCGGCAATGAGCTGGTGATCGCCCACGGCAATGGGCCGCA
AGTCGGCCTGCTGTCGCTGCAAGCGGCCGCCTACACCCAGGTTTCCCCCTACCCGCTGGACGTGCTGGGCGCCGAA
ACCGAAGGCATGATCGGCTACATCATCGAACAGGAACTGGGCAACCTGCTGGACTTTGAAGTGCCTTTCGCCACCC
TGTTGACCCAGGTCGAAGTGGACGCCAAGGACCCGGCCTTCCAGAACCCGACCAAGCCGATCGGCCCTGTCTACTC
CAAGGCCGAAGCCGAAAAACTGGCCGCCGAAAAAGGCTGGGCAATTGCCCCCGACGGCGATAAATACCGTCGGGTA
GTGGCCAGCCCACGGCCCAAACGCATCTTTGAAATCCGCCCGATCAAGTGGCTCCTGGAAAAGAGCAGCATCGTGA
TCTGCGCCGGCGGCGGCGGTATCCCGACCATGTACGACGAGAACGGCAAGCTCAAGGGCATCGAAGCTGTGATCGA
TAAAGACCTGTGTTCCTCGCTGCTGGCCCAGCAACTGGAAGCCGACCTGCTGGTGATCGCCACCGACGTCAATGCG
GCCTTTATCGACTTCGGTAAACCGACGCAAAAAGCCATCGGCCAGGCGCACCCTGACGACATGGAAAAACTCGGCT
TCGCCGCCGGCTCCATGGGGCCCAAGGTCCAGGCCGCCTGCGAGTTCGCCCGCAACACTGGCAAAACCGCAGTGAT
CGGTTCACTCTCGGACATCGAAGCCATTGTCCAGGGCACCGCCGGCACCCGTATCAGCACGGCAAAACCTGGCATC
ACCTACTTATAAATACACCCCGGGCGGTTGTTGCAGCCGCCCTTCTCCAATGCCTTGTTAGGGAGAGACGCCTATG
GCCATTTTTGAACCCGGTCACTTGCATGTTGAACGCCATGCATTGAATGCCCAGGATCACAGCTACGACCTGTGCA
TCGACTACGAAGTCAGCCAGGACCCAAAGGAAGGCAAGGGAATGCTCTTCAAGATGCACGGTTCGGTGCAGGGCAA
AGACCTCAAGGAAGAATTCTTCCTGCCCAAGGACCAGGCCTTCGACTTTGCCCGCCACGCCATGAACATCGCGCAG
AAGTACGGCATGCCGAAAACTGCGGTGCTCAACGGTGGCATGCATAAACAGTACGACCTGATGTTCGAGGATGTGC
GCCACCAACTGGATGTAAAACCGGGCGACCCGATCAGGCCAGAGCATCTGGAATAACCTCGGTTCCACTGCCATAA
CCAGTCAAATGTGGGAGCTGGCTTGCCTGCGATAGGCATGAGTATCTACGCAACTCTTTGATGGTTCTTGGGGATG
TGTACATATCCGTTGCTGCGGTCACGGCGGCTATGGGTTCCGCCCTGACGGCGGGTCACTTTCGAAAAGCGCGAAA
GTAACCAAAGCGCTCTTGCCCCCACCACTCGGTGCCTCGCCTAGGCTCGGCATGCCCGCAGTCAGGCATTGCTCCG
TGGGCCCGCCGCGNNNNNNNNNNGCGCATAGTCGCGACATGAAAAAGCCCTGGGGTCGCACCCAGGGCTTTTTTTA
TGGGGGCCGGTCAAGGCGCATCAACAGTGGGCGGTTTGCCATTTCTTGAAGTACATCACCTCAAGCACCTGGCCCG
GAAGGGTTCGGATCCCATCTCCAATGCTGTAGCCTTGTGCCCCAATTGCCATCAGCGTTGTTACCGGTCGACTGAT
CGGGATGAATTCACCACAGGGTTTTACTCGAAAATCGATAGATTGATATCCGAGTAGACTCCTGCGGTCACAGCGT

ATGCATTCCAAAAAAATAGTAGATAGGACCGAGGCCACCCCGTGAACCCAGACATGCTAGAAGCCGGCCCAGTGGG
CGCCAAGGTACTTTCAGTCTTCGACTTCGACGGCACCCTGACCCGCCACGACAGTTTTGTGCCGTTCCTCAAGTTT
GCTTTCGGCAAGCAGGAATTCAACCGCAGGATGCTCAAGCTCGCACTGCCTGGCGTGCGCTTCCTGTTGCGCCAGA
TGAGCCGCGATGAGCTCAAGGCCCAGTTGATTCGCACCTTTATGACCGGTGTGGACAAGGCTTGGGTCCAGCAGAA
AGCCGAGGAGTACTGCAAGGCCTCCTGGAACAAGCTGATGCGTCCTGCCGGCCTGCAGTCGGTGGCCGACGAGATT
GGCTCCGGCGCCGAGGTGACGCTGTGCTCGGCGTCGCCGGCAATCGTGTTGCAGCCTTTTGCTGATCGGCTGGGGA
TCAAGCTGATCGGCACCGAGCTTGAGGTGGTCGATGGGGTGTTGAGTGGGCGCCTTACGGGCAACAACTGCCGCTG
TGAGAACAAGGTGCTGCGCCTTGAGGCGATTTATGGCGACTTGGCTGAGTATCGGCTCAGGGCCTGGGGTGACACG
CGCGGCGATCGCGAGTTGCTGGCAGCGGCGCAGGATGCCCATTGGCGGCATTTTCATTCGGCCAAGCGCCGTGGCA
AGCCGAAGCTGAAAGCCCGGTAATCGCAGGCGCTCTCTTGTAGGGGTAGGCACGCCCCTACACAGTGGAGTGCCGA
TGGATGTTTGAAGTACCGCCGCGATCCCGCTATTAATAGCGCACCTTCCTACGGATCCGCCTGCGATGAAGTTCGA
CACAGCCTATTGCCTGAGCCTGGATGAAAAGCTGTCGATCTATGACGTGCGCGACCTGAATTTCGACGAAACCGCC
GCCTTCGATTCCGACAAGGACAGCTTCCTGTGCCCCAACGATGACTGCCGTGCGGCGTTTGCGGCGGGCAATGTAC
TGGGCACGTTCAATGGCAAGAACGTCAACTACCTGCGCACCCCGCATTTCAAGAACCTGCCCAGCACCCGGCATAT
CGAGGGCTGCCGCTATGCCAGTCACAAAGCCACGGCAGGAGAAACCGAGGATGGGCGCGAGGAGAACTTCCCATCG
GAGTTTGTCCTGACTCGGCGTCAGTACGAACGCAAGGTAGCTATCCAAGGCGCCACAGACGTTATCCCGCAGGCTC
CGGCGAAAGAGCCCTCGAGAATCTCCAATACTTCCCACAGTGCCAGCGAAACCACCCCGGACAAGACCAGCGTCTT
TGCACACCCGGTCGAATGCTACGTGTCCAACATCGATAATAAGGACAAGCTCAAGGGCATGCCGCTGAAGATCGCT
GAGCAGACCGCGACCTATTGGGCGTTTTTCAAGAAAATCGAATACCTGCAGGACAACAAAGGCTTGATCTACTGGG
GCCGGATCAAGGCGATCAAGGACTACACCAGCAGCTTTCGCATCGACTTCGAAAAGAAGGTGTGGCTCGACAAGAA
GCCCTACTCCGTCAACGTGTACCTGAACAAGAAGCTGATCGAGAACTACCGCAAGCGCAAGGCGTTCCTGGAGCAG
ATCAAGGCCGCCGTAGACAGTGAGCGCCCCTTGTATTGCTTCTTTTATGGCGTGACGCCGGAGTTGAAACAGGTGC
CGAGCAAGAAAAACCCCGAGCAGACATTCGGGGTGTTCAGTGCCAATATCCAGAACCTGGACCACCTGCTTATCCG
CGAGGCACCGGGGCTGGAGGAGAAATGACCTGAAGCGCCCGCCAATCAAGGGCCGGCCACGGGGGAAGTGAACCAG
TTGATGAACGGTCAAAACAACTGTACAAAAACACAGTATAGTTTGTCCTCCCCCGTCGGCATCCTGGAGAAACCCC
ATGTCCTCTCTGGCAGTGAGCACCACCGTAGATCAGCAAATCGTCCTTCATCAGTTCACCCCACGGCACAGTGTGC
AAGCACGCGCTTTGCTGGGCTGGAGCCGGGAAGACCTGGCCCGGCACGCCGGGGTTGGGGTGGACGCCATACAACG
CTTTGAAAGCCATCATGAGGTGGACGACGCCATCCGCATCGCCCTGGCGTTTCGCCTGGAGCACGAAGGCCTGGTG
TTTTTCCCGGGTTTTGCGCCGGGGTGGGGCATGAGTGGACGCCGCTCCCAAGCGTCACAGGCTGGTGCCGAACCGG
GCCGCCGCAGCCTTATTGCGCGGCTGCTGGGCGCGACATCGTCGAACACGCCGCATCCCAACAGCGCATAGGCTCA
CTGGCCACGCAGCAGGTTCACCTGCCGGGTATGTCGGCACGTGCCTGCATCGCCTGTTGATAAACCCGTATCAAGG
CCGACAGTGATTCCCGTGCCCAGCGAGCACCGTAGAACCAGAGCACGACCTTGGTGCCATGCAGATCGTCGTGGAA
TATCTGGCACACGCCTGGGTATCCGGTTTTGCAGAAGCCATGGAAAGCCGGGCTTTGGCCATCGGACACCAGGGCG
TCGAGGCGATGCTCGCTCACCCGCTCATCGATCTCGTTGCTGGACGCCAAGGTGAAATGTGCAGTGGAATCCGGTA
GCAGCGCCAGCACTGGCACCACTTCAGCACCGGCGGCCCGTAACACCTCCAAAGCCTGGAGAAATACGCTGGAGTG
CTCCACCATTTCACGTGCATTGTTGCGCAGGTATTCGTCGGCATAACCGATACGCCAATCGGCCAGGGCAGGAGCT
GATTTCGCGGCATTGTTTTCCATGACGGGCGGGACTTCCACCATAACCGGGCCCACGGGATCGACGCCACTCAGGG
CCAAGAGTGATAACGACGGATCACGCACGGTCTTCATCATCGGCCCCGGAATATCGAACCTGGGGCCCTGGACAAC
AGGCGTTGCCGCAGGCTGGAACTCAATGCCACCCCGAGGCGGCGCCTTATTGTCCGTCGTTCCAGCCGAGGGGTTC
AGTGCACAAGGTGCTGTGTCCAGGATGTTGAGCATCGACGGTACTCCCCCAGAACAGGAAGCACCTGGCACCAAGA
TCTTCCTACATTTTTCCTCCATCAAGCCCACTCCCAGGAAATGACTTTCCCGAGATCATCGCATCCTTTTCCTGTC
GGTTCTCAAGGCGGAAACACCTTGAAAGCCCTTTCACCGACAGCGCATTGCAAACCCCGTCAACTCACCAGCGGTT
GCGCCGCCTGGGCCTCGACATCCAGCCACCAGGCATGGCCGCGCTCAGGTGCAGCCAGCATGAAAGCCTGCCCCAT
GGCTGGCGTGGTAATCGCAACATTACGCTCCCACGCCAGGGCCATGATGCGGTCAAACGGCTCATGCCAGGCATGG
AATGCCAGATCAAAGGTGCCGTTGTGGATCGGCAGCAACCAACGCCCCTTCAGATCGATATGGGCCTGCAAGGTTT
GTTCAGGCTGCATATGCACATGGGGCCAGTCGACGTTGTAGGCACCCGTCTCCATCAGCGTCAGGTCAAATGGCCC
GTACTGCTCGCCAATACGCTTGAAGCCATCGAAATAACCGGTGTCGCCACTGAAAAAGATGCGCCGGGTACCCTCG
ATCATTACCCACGATGCCCAAAGTGTCTGGTTGCCATCAAACAGACCACGTCCGGAAAAGTGCTGGGCTGGGGTGG
CGACAAAACGGATGCCCTCCACTTCAGTGGCCTGCCACCAATCCAGCTGCCGCACCTTGTCTTGCGCAACGCCCCA
TTTGATCAAGGTGTCGCCGACGCCCAGTGGTGCGAGAAAATAGCGGGTCTTGGCGGCCAGCTGCACGACGGCCTGG
CGGTCCAGATGGTCGTAATGGTTGTGCGACAGGATCACCGCCTCAATCGGCGGCAGATCCTCAAGGCTGATGGGCG
GCTGGTGAAAGCGCTTGGGCCCCGCCCAACTGAAAGGCGAAGCACGCTCGGCGAACACCGGATCGGTCAGCCAGAA
CTTGCCGCGCATTTTCAGCAGTACGGTGGAGTGCCCCAGGCGGTACACACTGTGATCCGGGGCACTGAGCAGTTGC
TCGCGCGTCAGGCGCTGCACCGGAATCTCCCCCACTGGCCGCGTACTGCGAGGCTTGTGAAAAAGCATTTTCCAGA
AAATCTTCAGGGTCTTGCCAAAGCCAGCTCGGTTGACCGGCGCATGATTGTTGAAGTGACCTTGCTGCTGCTCGGA
TGGTTTGAGCGCTGCCGAGGGGGTGTCGAGGCGGTTAGAAATCGTGGCCATTACAGAGTGACTCCGGTACGTTCTG
CTGATGCAGACCAACAACTACACTGCACAGTGTAGTTTCAAGATTGCAACAAAACCCGGACCAAGTAAACTACCGA

GTGTAATTTTCTTCCCAGAGCCGAAAAGCCCCATGACTGCACCTCAACGCCTCACCGACCGCAAACGCGAAGCCAT
CGTGGCAGCGGCCATCGCCGAGTTTCGCGACAACGGCTTCGAGGTAACTAGCATGGACAAAATCGCCGCCACCGCC
GGCGTATCCAAGCGTACGGTGTACAACCACTTCCCCAGCAAAGAGGAGTTGTTCACCGAGATCCTGCATAAGCTGT
GGGCCAGCAGCGTGGCGCAACTGGATGTCAATTACTGCAGCCAACAACCCCTGCGCGACCAATTGCGCGTATTGCT
GCAAGCAAAGATGAAAATGATGTCGGACGCCAACTTCCTCGATCTGGCCCGGGTGGCGATTGCCGCGACCATCCAT
TCCCCGGAACGCGCACAGGACATCGTCAATCGCCTGAGCGAGCGCGAAGAGGGGTTCACTTTGTGGGTACGTGCCG
CCCAGGAAGACGGGCGACTCAAACCCGCCGACCCGTGTTTTGCCGCCCATCAGGTGCAAAGCCTGCTCAAGGCGTT
CGCCTTCTGGCCGCAAATCACCCTCGGCCAACCTACCCTGGACGCCGCCACCCAGGCCAACGTGATTGAATCGGCG
ATGGATCTGTTCCTGGCCGGCTACGAGATTGCTACCTGCCCCCGATAAGCCCCCGTGTCGCAATACGACGCAGCAG
GTCGTTTTTGCCCTAATCCCTGTGGATCTCCACGCAAATGGCTTGGAAGGACACTGATTATTCCTGCGTAAATCAA
TGACAATATTCGCCTCTCATCTGGCGTAAATGGCCTTTGGTAAACGTACGCCGTCGCCCCTTAAAAAAAGAAAAAC
CTGACGCAGGGATCTATGGAAAACCTTCGAGGCAAAGGGCTGTCGTTTGCCCGGCGCATTTATCGGCCGCGCATTA
TCGGGCTGGGCATTGGCAGCCTGTGCGTGCTGGCTGCCCTGTACCCGCTGGCCATGCCCGGATGGATCTGGGCGTT
GTGGCTGCTCAACGCATTTATCTGGCCGCACCTGGCGTACCAGTTATCGAGCCATTCGGCATTTCCCTACCAGGCC
GAGCGCCGCAACCTGCTGTATGACTCACTGTCCGGTGGGTTCTGGGCCGCAGCTACGCAATTCACACCACTGACCG
CAGTGACCATCCTCGCGATGATGACCATGCACAACGTCGCGGCGGGCGGCAAGCGGTTGATGGTCCAGGGCATGCT
CGCGCAACTGGTGGGCGTGGCACTCGCCTGGCTGATATTCGGCCCTTCATTCAACCCCAACGTCGGCCTGATCCAG
GTGTATGCGTGCCTGCCGATGTTGATCCTCTATCCCGTCGCCATCGGCATGGCCAGTTATCGTTTGGCCATCAAGC
TGTCGGAACACAAGCGTGCCCTGAGCGCCTTGAGCCGTACCGACAGCCTCACCGGCCTGCTCAATCACGGCTCCTG
GAAAGACCTGCTGCAACTGAAGTTCCACAAGTGCCAGCAGCAACAATCCCAGGCCACCATTGCGCTGATCGATATC
GACCACTTCAAGCAGATCAACGACGCCTATGGCCATATCGTTGGTGACCGAGTGCTGCGACAACTGAGCAAGGAAT
TGCAGCGCAACGTGCGGGAAAATGACCTGGCGGGCCGCTACGGTGGCGACGAGTTCTGCGTGATCCTCCCGGACGT
TGCGCTGGAACAGGCCAGCTATGTGATGGAGCGTCTGCGGGAAATGTTCAGCCGCTACCGCGACCCGCAGGTCCCG
GAGCTGCGGGTCAGCCTGAGCATTGGCCTGGCGGCCTACCAGGTGCAATTTACCCAGGCCCATATGTGGCTCAACG
CAGCGGACAAGGCGCTGTACGCCGCCAAGGGCACCGGGCGCAATCGGGTGACGGTGGCTGAATCCCTGGTGGCCCG
TTCCGCCTGAATAGAACTTCCCTCGGGGGCCAAGACTCAGAGCTCTGATCTGTACTGTCTGCCGGTAGAAGGGAAG
CCGGCAATGAGCACGTCTGCCGTGGGCCTGGGGCTACACGGTGAGCAAGCGCAACCATGGATACATTCTCGCACTG
TGCATCACTGAGCCCGCGACCATGCTATTTACTGCCCATAAAAAAACCATCACTACCCTGCAAGAAACGATCAGCC
AACAAGCCGGCCTGCTCGATGCTCTCGAGCGCTCCATGGCCGTGATCGAGTTCGATCTACAAGGCAGCGTTTTGCG
CGCCAATGACAACTTCCTCAAGACCATGGGCTACCGCGCGGATCAAGTCGTCGGCCAACCCCATCGCCTGTTTTGC
ACCCAGGCGTTTGCCCGCAGCGCCGATTACGGCCAATTATGGACGCAACTGCGCAACGGCCAGTTCCAGTCGGGCA
CCTTCGAGCGCGTCGCCGCCGATGGCCACTCGGTGTGGCTGGAAGCCAGCTACAACCCGGTGCGCGACCAGGCCGG
CCGAGTGGTGAAAGTCATCAAATATGCCCTGGACGTCAGCGCCAGGATGCAGGCCGAAAGCGAAGCCAATGCCAAG
CTCGAGGCGATTGACCGGGCCATGGCGGTGATCGAGTTCAACCTCGACGGCACCATCATTACCGCCAACCAGAACT
TCCTCCAGCGCCTGGGTTACAGCCTGTCGCAGATCCAGGGCAAACATCATCGCCTGTTCTGCAAACCGGAGCTGGC
CAACAGCCAGGAATACAGCGATTTCTGGCGCCGGCTGAACCAGGGTGAGTTATTCAGCGGCCAGTTCGAGCGCATC
ACTCACACCGGCCAGACGCTGTGGCTGGAAGCCAACTACAACCCGGTCTACGACGCCAGCGGCCGCTTGTGCAAGA
TTGTGAAGTTCGCGTCCGACGTCACCGCCATGGTGGAAAAACACGCCGAAGATGCCCAGAGCGCCACCCAGGCCTA
TCACATCTCCCTGCAGACCCGGGAGATCGCCGAAAAAGGCGCCGAGGTGATTCAGCAGACCGCCAGCGGCATCCAT
GACATTGCCGCCGACATAGAAAGCTCATCCAGACTGATCGCCAAGTTGGGCGAGCGCTCGCAGCAGATCACCGCCA
TCGTCAACACCATTCGCGCGATTGCCGACCAGACCAACCTGCTGGCCTTGAATGCCGCCATTGAAGCCGCACGCGC
AGGTGACCAGGGCCGCGGGTTTGCGGTGGTCGCCGATGAAGTCCGGCAACTGGCCGCCCGCACCAGCGGCTCCACG
GCAGAGATTTCGAGCATGATCGAGATGATCCAGAGCGAAACCCGCCAGGCCATCGACAGCATGGACAGCACCCGCG
ACCGGGCTGCCCAAGGCGTGGACCTGGCCAACCAGGCCGGCACGGTGATCCTGCAGATTCGCGAAGGCACCAGTGA
TGCCGTGCGGGCAGTGAGTATGTTTGCCAATGACCGGGTCGGGTTGTAGAGACTGGCTTGCCGGCACCTACGAGGG
GTGGCGCAAGTGCTGGCCCAGTTCATCGAACAACGTCACCACTGATCGCAGCGCCCGGCAGTCCGGGCGTGTGAGC
AACCACAGGGCCGTATCATGCCCCGCGAGTGCTGGCCCCAGTGGCTGCAACCCATGGCCTTTCCCCAACAGAAAAT
CCGGCAGCGCCGCCACGCCCAGGCCGGCGCGTACCAACTCGGTCGCGGACAACATGCTGTTGCAGCGGTAACTGGG
CCTGACGCCGGGCAAGTGCTGGCGACGCCAAGCCACGGTGGGGTGGTCGGGCAGGAAGTCATCCGGCGCGATCCAG
GCCAGGCTGGCCAAGTCCTGAATGTCGTGCTGCTGCGCATAGCCGGGGCTGGCGCAGACGCGATAAGACACCGTCC
CCAGGCACCGCCCCACCAAATGCTCCGCAGGAGTGCGGGTCAGGCGCAAGGCGATGTCGGCATCGCGCCGACTGAG
GTTGGCAAAGTCGTTGGAAGTGCTCAGTTCCAGAGTCAGCGCCGGATAGCCCGGCATGAACCGGGCCAGGGCCGGC
AGCAACAGGCCCTGCAACACCGAATCGGTGCAGGTCAGGCGCACTGTGCCGCTGATCACCTCGCCGCCCTGCTCCA
CCCCAACCCGCGCAGCTTCCAACGCCTGCTCGGCACGTTCGGCCTGCTGGGCGAGTGTGGTGGCCAGGCTGGTCGG
CAGGTAACCGGCACGGCTCTTTTCGAACAGCGCCTGGCCCAGTGACGACTCCAGGCGCCGCACAGCCCGAAACACC
GTGGACACATCCACCCGCAGCAAGGCCGCCGCCCGGGCCAGGGTGCCACCACGCACCAGGGCAAGGACCAGCGCCA
GGTCGGGATAGTCGAGTTGATAGTGCGTGGCTGCATTGAGCATGTGGAGTAACGCCAATATTGATTGCGTGAGCGC

CAATCTATAGTGCGCTCCAGGACTCGACAAGCCACTGGACGCCAGCCATGAACCCCACTGCCCTGCACCTCGCCCT
GATTGGCGACTACGACCCGCAAGTGACCGCACACCAAGCCATCCCGATTGCGTTGCAACAGGCCAGCGCACAGCTG
GGCCTGACGATCCATGCTCAGTGGCTGGCCACTGACACCCTCCTCGACGCCCAATCCCTGCAACCCTTCGACGGAT
TCTGGTGCGTACCTGCCAGCCCCTATCGCGATATCGACGGTGCCTTGCGGGCGATCCGTTTTGCCCGCGAACAGCG
GCGTCCTTTCCTCGGCACATGCGGCGGTTTTCAACATGCCGTGCTGGAATATGCGCGCAATGTGCTGGGCTGGGCC
GATGCCGAACATGGCGAACTGGCCCCCGATGCGCCCCGTGCGGTGATCGCCCCCTTGGCATGCGCCCTGGTCGAGA
CCATCGCCCCGTTACGCCTAATGGCATTCACACGGATTGCCGAAGCTTACGGCACTCTTGAAATTCAAGAGGCTTA
TCGTTGCCGCTATGGCGTCAATCACGACTTCGAACAGGCATTGCTGGAGGACAATCTGATTGGCAGCGCCCATGAC
TCCACAGGCGAGGTGCGGGCGATTGAACTGCTGGACCATCCGTTTTTTGTCGCCACGCTGTTCCAGCCAGAACGCG
CGGCACTCAAGGGAGCCAACGTGCCGCTGGTCACGGCATGGCTCAGGGCCTGCCAGGAGCGCTCGGCATGATTGCC
CAGACACCCGCCCCGCCCTATTACGCCGTGATCTTCACCTCCCTGCGCACCGACACCGATCCAGGCTACGCACAGG
CCGCCGAACGCATGCTCGAACTGGCCCGCCAGCAACCGGGGTTTCTCGGTGTCGAGTCGGCCAGGGGCGAAGAGGG
GTTGGGAATTACCGTGTCCTACTGGGCCAGCGAGGCCGCCATCCTGGCCTGGAAACAACACACCGAGCACCGCGAA
GTGCGCGAGCAAGGTAGGGCCTCCTGGTACTCAGCCTTTCACACACGGGTATGCAAGGTGGAGCGGGCTTACGCCT
TCAACGTCCTGTAGTTGCAGGGTTGAACTGGCGATCGGTGTCCCCCTGTGGGCGCTGGCTTGGCGGGGCGTTCTTG
CTGGCGCCATCGCTGCAAGCCAGCGCCTACAAGGGGTGAGCGTGATCAACTGACCAGGCTGCGCACCGCGCTGATC
TGCGGGATTTCAACCCGGCGCATGTACACCCGCAGCGGTTCGGTGATGTTGATCCGGTCGTCGATGTTCTGGTCAA
GCAGCAGTTGGATGTGCTCGCGGGACAGGGTCATGGCCTGGTCGGCCGCGGGCTGCCAGACGAACTCGCTGGTGGG
GATGATGCCGTCGTCGGCGACGTCCATGCCGAACGAGTCTTCGCTGAAACGTACGATGTACTGGCCGGTCTTGCGG
TTCAGGCCGACAAAGCCATTGAGTTGGTCGGCGGCCTGGCAGATGAGTTCTGAAGTGATGCGCATGCTAAACCTCA
CTGAAAAATCCCACAGGGGAAGTGAACGATGGTTTACACGCGAGGCAGAAAAGTAAGGCAATACAGGTTTCCCTCT
GGCGGGGAAGCTCGGACCAAGAGTACTGCATAGCACGGCACAAAAGCGCAGAAAAATAGCGCATGCGCACGTTAAT
GTCGGTTTGGTGATAGCGCGATTTGCATGCAATTGTTAAAAAGGAACCCTTCTTGAAGCTACCTCCACGAAAGGAC
CTCGCACATGCCTGCTACGTTTACCAAGAGTGCCCTGCTGCTCGCTCTGATGATGGGCCTTGGCCACGCACAGGCT
GCAAGCCCCATCAGCCCGGTTGAACTGGCGACCAAGGACGGCATTCCCCACCCTGCCGTGATCGCCCACCGAGGTG
CATCCTTCGATGCCCCGGAGTCCACGGCCGCCGCCTACAAGCTTGCCCGCGACCTGGGTGCCGACTACCTGGAACT
GGATCTGCAACGCAGCAAGGACGGCGTCCTGTTCGCCTTGCATGACAACAATCTGCAACGTACCACCGACGTGGCC
ACCAAGTTCCCTGAGCGCAAAGACAGCCCGGCCAACGAGTTCACCTGGAAAGAACTGCAAACCCTGGATGCCGGCA
GTTGGTTCAACGCCGCCTACCCGGATCGTGCGCGCCCGGGCTTTGTCGGTTTGAAAATCCTCAGCCTGGACGAAAT
CATCAAGATCGCCGAAGGCAACCCCCAGCACAAACCCGGCCTGTACATCGAAACCAAGGAGCCCAAGCAGTTTCCG
GGGATCGAAGCCGATCTGAAAGACAAGCTGCTGGACAAGGGTTGGCTCAGTTCCGTGGGCTCCAAGCTGGGCAAGA
GCAACACGGGCGTCGGCCAGGGCAAGGGCCGCGTGGTGCTGCAGACCTTCGAGACCGCCAGCCTCAAGGAACTGCA
GAAAGAAATGCCCAACACCCCGAAAATCCTGCTGCTGTGGGTGGGTGAAGGCAGCATCGAGCCCAAGTCCAAGGTG
ACCTTCGCCGAATCCGGCGAGCCGACCAAGGCGGCCTACTATGCCAAGCAGGAACCCAAGGACGCTGCCGAATTCG
AGAAGTGGGTCGATGAAGCCAAGAGCCTCGGGGCGATTGGCACCGGCCCGTCCGCACAGCTGACGAACCTCGGCGA
CCAGAGCTATTCGGACCTGGTCAAGCCCGAGATGAACCAGCTGACCCACGACAAAGGCCTGCTGGTGCACGTCTAT
ACCGTCGATGAACCGGTGGATTTCGAGAAGGTCATGAGCGCCGGGGTCGATGGCATCTTTACCAACCGCGCCGCCG
AACTGCTGAAGTTCTACAAGCGCTGGCCGTCGTCCAGCGTGCAGGACCTGCTGCAGGACAACGGTTACTGAGTGAA
CGAGTGGTCAGGGCAACTGTGGCTGGGCCGTGACTACGGCCTGATCCTCGGTGAGACGGGGCGCACTGCGCTCCAC
GCTCATTACGCCCATCAATTTATCCTGTCTTCGGGCAGCCCCGTCACCGTCAGCCTTGATGGGCACCTGCAAACGG
CCTATCGCCTGTTCATCCCCTCTCGCCAGCCCCATGCCATCGTCCACGCCCCCGGCACTGTGTTGATGGCTTATGT
GGAACCCGGGGCATTCGACCTCGATTCGTTGCAAAAAACCCTGGAGACGGCCGAGTTTTCCCCCGAACGCCTTGAG
CAGTCTTTGCGCCAGCTGCCACGTCGCCAGGCTGACGATGAGCGGGTGCAGCGCGCACTGCACACCCTTGATCAAC
AGTTGAACGGCAAGGTCTCGGCGCAAGCGTTGGCAGAAGCGGCGCACCTGTCCTTGAGCCAGTTGGAGCGCCTGTT
CGTCAGCCAACTCGGCGTGCCGGTGCGGCGCCTGGTGATGTGGCGACGCTTGCGCCTGGCCCTGGACCTGGCCCTG
AACGGCCACACGTTGACACAAGCTGCCCATGGCGCGGGGTTTGCCGACTCGGCGCATTTTTCCCGGACCATGAAGC
AACTGTTTGGCGTGACCGCCGGGGCCTCGTTACGCCATCTGCGGGTACACCTCCTCCCCCTGTAGGAGCGAGCTTG
CTCGCGAAGAACTCGAGAGCACCGCATCAAACCAGACAGCTCACGCCTACATTTGGAGAGCGCTTGGGACAGGCGG
TTCTTTACTCAACTGGGTCGCCGTAAAGGGGTCACGCAGTTGCCCAAGATAATCCACCGGGAAATCCGGACGGCTA
CCAATCCCCAAGTCGCCCTCGCCCATTCGATAGTCCTCGGTGTAAAACGCAGTCCCCAACAGCCAGTCCCACAGGT
TGAAAAAAAACCGAAATTCACATCCCCCGCGCGCCCGTACTTCATGTGGTGAAAGCGATGCACCGGCGCCCAGGCA
AACACCCAGCGCAGCGGCCCCAGGCGCATATCGACATTGGAGTGCTGCAACAGCAACTGCACCGCAATGGCCAGCG
CCAGCAACATCGCTACCTCCACTGGAATGCCCAGCAGAATCAGCGGCAGCAAACCCGCCGTAGCCTCAAGCATCTG
GTGCAAGGGGTGCTTCATCAAGCCATTGAACCCATAGAGCCGCTGCACGCTGTGGTGCACCGCGTGCAAACGCCAG
AGTGCGGCGATGCGGTGACTGGCGTAGTGCATCAGCGTGATGCCCGCATCGGCAATCACGATGGCCAGCAGCAATT
GCGCCCACAGTGACCAGGCGCGCGGCCAGGCGCCCTCGATCGCCAGCCAGGCCGTGAGCAGCGGCAATGCCAGCAA
CCCCAGGGCATTCAGGCTTTCATTGACCAATGCATGGAGGACATCCCGGCGTCGGTCGCCCAGCGGGCGGTTCCAC

TGAGGCTCGTAGGGCAAGCACAGCTCGGCAATGAACGACACGCCGATGGCCAGCGCAAACACCGCCAGCAACCACT
GCGACGCGCCAACCGTGACCAACCAAAGCCCCCCACCTATAAACCCCACCCAGAACACGGGTGCATATAGCCAAGC
CAGTATCTGTTTCATGTGTGCCTCCTGAAGTTGAGACACCAGCATGAAATTTCCCGGCGCGGGGCGATTGAACAAA
CAACGCAAACGTCCTACACAATTAAGGGTGAATTAAGTTGCGCGGGTTAATCTGATCCTACTTAAACAGCTCACCC
CAAAGGACACCACCATGAAAAACCTGACCGCTCTGTTCGCCGCTGCTGCCCTGACCCTGACTGCTGGTTTGGCCCA
GGCCGATGTTCGCCCGGACCAGATTCCAAGCCTGCTGCAATCGGGTGCCGTGATGCCATTTGAAAAACTGAATGCC
GCCGCCCTGGCCCAACACGCCGGCGCGACCATCAACGACACTGAACTGGACAACGAAGCCGGTACCCTGGTCTACG
AAGTGGACCTGACCGACACCACTGGCAAGAAATGGGACGTGAAGCTCGACGCCAAGACCGGCAAAGTACTGGAAAA
CAAGCAAGACACCTGAGTTACCCCAGCACTTCTGGTAACCGGTCCAGGACAAGCCCCCCCCGTGGCGAGGGGGCTC
GTCCCCCGCTGGGCTGCGCAGCAGCCCCAAAACCTGCACCCTCGGTTTACATGACACTCATCGGTGGTCTTATCGG
GGCTGCTGCGCAGCCCAGCGGGGGTGTAGAACCGTAGACATCCTTTACATCTGAAACCGGGGACATCGNNNNNNNN
NNCTGGCGGTACTGGTATCACCGTCGCTGTCGTTCAATTGATAAGTGAAGGTTTCAGTGCCGCTGCCGCCACCATT
CAGAGCCATGAACTGTGGGTCGGTCTTATCGAGGGTGTACACATAGCTGCCGTCGGCGAAGAGCTGCAAGGTGCCA
TAAGTACCAGTGAACGTACCCGCAGTTACCGGGCCGGTGGCGACCCGATCCGCGCCTTGCACGTCATTGCCCAGCA
CGCTGCCAGTCAGGGTCAACAGGCTTTCAGACGCCACTTGCGCATTGCTGTCATCGGCAGCGGTCGGTACGTCGTC
CACCACACTGATATGCAGCGTGCTGGTGGCCGTGCTGTTGTTGGTATCACTGGCGATCACGCTGAAGCTTTCGGTC
AGTTCGTTCACCCCCGCCGCCGTTGGATGCGCCTCGGTGCCGGTCAAGGTGTAGCTGTAGGTGATTACTCCGGTGG
CCGGGTCAAAACCGGTGATGGTCAGGGTATTGCCCAGGCCGGTGGCGATCGATTGCGGGAAGCCGGCGGCAACACC
ACCGGACACTACGCTGATACCGCCCACGGACAGGTTCTGCAAGCCGTCCGCTGCCGACACGGTCAGGCTGCCGGTG
CGGGTCAGCGCGCCAGCGTCGGGGCTGCTGCCGTCCACCAGGTTCTTTTCATAAAGCGTCAGGTCGGCACCATTGA
TGCTGATGCCACCGATCACCACCGGATCGTCGTTGTTATGCACTTGCAGCACCAGGTTGGCGGTGCTGCTGTCGCC
ATCGGCGTCGGTCAGGGTGTAGGTGAAGGTTTCCGTGCCGTTGCCACCGCCCTGCAGCGTGACAAAGTCAGGGTCG
GCGGTGTTCAGCGTGTAGGTGTAGCTGCCATCGGCAGCCAGGACCAGGGTGCCATATTGGCCGGTGAAGGTACCGC
CGACGATGGGGCCGGTGGCGATACGGTCCGCTCCCTGGGTGTCGTTGTCCAATACGTTGCCGCTGAGGGTCAACAG
GCTTTCCGACGCACTGCCCTCGTGGCTGTCGTCCAGCGCCTTGGGCACGTCATCGATCACGGTGATATCCAGGGAA
CCGGTGGCGGTGTCGTTGTCGCCATCGGTGGCAACCACCGCAAATTGTTCGCCCAGGCTGTTGGTGCCGCCACCTG
ACGGATGGGCCTCATTGCCGGTCAAGGTATAGCTGTAGCTGATCACCCCGGTGGCCGCGTTGTAGCCGGTGATGCT
CAGGGTGTTGCCCAGCCCGGTGGTGATGGTTTGCGGGAAGCTGCTGACCACCCCGCCGCTGACCACGCTGATGCCG
CCCACGGTCAGGCTTTGCAGGCCATCGGGCGCCGAGACGTTGAAGGTGCCGCTTTGCACCAGCGCCGCCGGATTAC
TGGCCGAGCCTTGGGCAAGATTGGCCTCGTCGAGCGTCAACTCACCGCCCTCGACCTCCAGCCCGCCCAGGCTCAC
CGGGTTGTCGGGAGGCACCACCACCGGCTGGTCGTTGCCGGTATCCGGCTGGCCGGCGACCTGGAACAACGGAAAC
TCGGGAATCGCGCCAAAGCCCGCCGTCGGAAAGCCGATGACCGGCTCCACGCGCCCGGCGACTTCGGTCAGCAGCA
CAAAGGAGTGCCCACCGCCCAACTCACCCGCCGCTGTGCTGCCGGTCTGGCCAGCAGCCGTGGCTTCACCGGTCTG
GGTCGGGTCGGCACCGGCAGCAATGGCTTTTTGCAGTTGCTGCACATCGGTCAACTGCGCCTGGCTCGGCGCAAGG
TCTTCAGACCCATGAACCTGCGGCGCCTGGCCGGCCAGCAGTTGCGGGGAGAGGCCCAGGCTGCTGTCGCGCCCCA
GGGTCAGCTCATGGCCGTTGGTCAGTTGCACCGCCACCGCACCGGCCGCGCCGGTCTGCAGCTGCTCGCCGGCAAA
CAGTCGGTCGCCTTCGATCAGGCTCCGGCGGCTGCCATCTCCCGCCACGGCAAAGACTTCCCCAACCACCTTGCTG
ACAACACCGATTAACTTGGCCATGTGCTTATCTCCCTGTCCGACCCCAGTAGTCGGCACCTCGGTTACGTGCACAA
ATGCAGTGGATCCAGTGGCCCGGACCTTCGCCCGGAAGTGGCACAAAACTGATTCAAATCAGCCGCCAAACCGCGC
TTCGACCCGCTTGCAGAGAGCCTTCCCAGGGACCGAGTGACAAAAAACCGTCACCCTTAAATTCCCTTTTTTCCGC
TACCCCACGATCCGCTTGCACTTGCGATTGAACTTTCCAAGGCCCTGAAAATGCGCCGAAGCGCACATAACTCAAG
GCTTGTGCTTTGAACAATGTCTTGCTTCACGCAGAACGCATAAGTTTTTTTGGACATAAACGTTATGAGAAATTCT
TCTTAGGTCCTCCTGCAAATGTTCTTAGCTCTGTTGTTACAAGCCTGAGACGGTTTGCCCCCTAAATTCGTCAAAT
AAATGGCGACAGACCAGCAGCACTGATACACAGGAGATGTTCCCATGCGCGTCTTTACCCCCCTCTGCAGCGCCAT
TCTGATCGCCATGGCATGCACCAACACCCAAGCGATGTCGCTGACCCAAGCGATCCAAAGCACGCTGGACACTCAC
CCGGAACTCAATGCCAGCCGTAACAGCCGGCTGTCGGCCGACGAAGATGTGAAAGTCGCCAAGGGTGGCTTCTATC
CCAGCCTCGACCTGAATGCCGCCTATGGCCGCGGCTACAGCGACAACAGCACCACCCGCGCCCGCGGCGGCAACCA
CCACACCGAGACCGACAATTACACCCAGTCGGAACTGCGCCTGCGCCAGATGCTGTTCGACGGTTTCAACACCTCC
AACGAAGTGCAACGCACCGGCGCCGTGGTCAACTCACGTGCCTATTACACCCGCGGCACTGCCGAAAGCCTGGCCC
TGCGCACCGTCGAGGTGTACCTGGAAGTGCTCAAGCGCCGCGACCTGCTGACCTTGGCCAAGAACAACCTGCAGGC
GCACCTGCGGGTCACCGATCAGATCGGCCTGCGCACCGAGCGTGGCATCGGCAGTACCGCCGACCGCGACCAGTCC
ACCGCCCGTCGGGCCCTGGCGGACAACAACTACCAGACCGCCATGGTCGACCTGCAAGACGCCGAATCGAATTTTT
ATAGTGTGGTAGGGCGCATGCCGGACGAACTGGAAAGCCCAGCCTCGATCAAGGGTCAATTGCCCGCCGACCAACT
CGGTGCGCGGCGCAGCATGCTGGAGAACAACCCCTATCTGAAATCCGCCCAGTCCGATGTGATGGCCGCCGAGAGT
CAGTACGAAGTAGCCAAGTCGCCGTTCTACCCACGCTTCGACGCCGAGGCTGCGGTGGGCGCCAATAACAATGTGC
AGGGCGACCTGGGCCACGACAATGAATGGCGGGTGGGCGTGGTGATGAACTACAACCTGTTCCGTGGCGGCAGCGA
CAAGGCCCGACTGGCGTCGGACTCGCACAAGATCAACCAAGCCATGGACATCCGCAACAACGCCCTGCGCATGCTC

AACGAGAACCTCAACCTGGCCTGGCACGCCATGGACAACGCACGCATCCAGACCCCCACCGCCCGCGAATACGCCG
AAACCACCCAACGGGTGCGTGCGGCCTATCAGGATCAGTTCGGCCTGGGCCAGCGCACCCTGCTGGACCTGCTGGA
CAGTGAAAACGAGCTGTACAACGCCAACCGGCGCTATACCGAAGTGCGCTACACCGAAGAGTTCTCCATGTACCGC
GTGCTGGCCAACATGGGCGAGCTGCTGAACAAGGAGCGCGTGGTGGTGCCGGCCGAAGCCATTGCCCAGTCGGAAG
TCAGAAGCGAAGCCCACTTGCCTGACCTGAAGTAGCCAACGACGTGGAGCAGACCGCCGTGAGCCAGATGGAACCG
CTGAACCCGGGCGTCGACCCGCGCCAGAGCTTTGATGACCCGTTGCTCGACGGCCTGCTGATCCTGTGCAAGCTGC
ATGGCTGTACCGTCAGCCGCGCCAGCCTGAGCGCCGGCCTGCCCTTGGCGCAACAACGCCTGAGCCTGGACTTGCT
GCCACGGGCAGCGGCCCGGGCCAGCTTGCAGGCGCGGGTATTGCGCCGGGACTTGAAGGCAATATCGGCGCTCAAC
CTGCCGGTGCTGCTGCTGCTCAACGAAGGCCGCTGCGCGGTGCTGCGGCGTTGGAGCGAGGATGGCCAGGCGCTGA
TCCTGCCCAGCGAAGCCGAAGGCGGCGAACAGTGGGTCAGCCGCGAGGAGCTCGACGCGGCCTACAGCGGCCAGGC
CCTGTTCGCCCGGCCACGGCACGAGATCGAAGACCTGCGCGCGCCATTGGTGCCGCGGGTCGAGGCGTGGTTTCGC
GACACCTTGAAGCTGTCGCGCTGGCTGTACAGCGATGCGATTCTCGCCAGCTTGCTGATCAACCTGCTGGGCCTGA
TGGTGCCGCTGTTCGTCATGCAGACCTACGACCGTGTGGTGCCGAACCAGGCCACGTCGACCTTGTGGGTCCTGGT
GATCGGCCTGCTGATCGGCACGGGTTTCGAGCTGGTGCTGCGGGTGGTGCGCGCTCACTTGCTGGACACCGCCGGC
AAGAAGACCGACGTGATTCTTTCGGCCACCCTGTTCGAGCGCATCACCGGCATGTCGATGAAGGCGCGCCCGGCCA
CCATCGGCGGTTTCGCCCAGAGCATCCATGACTTCCAGGGCCTGCGCGAGTTTCTCACCGCCGTGACCCTCACCAG
CCTGATCGACCTGCCCTTCGCCGTGCTGATGCTGGTGGTGATCGGCCTGCTCGGCGGTTGGCTGGTGATCATTCCT
TTGTGTGCGTTCCCGCTGACCATCGGCTTTGCGATGCTGATCCAGGCTCGCCTGCGCGACACCGTGCAAAAAAGCC
TGAGCCTGGGCGCCGAACGTCAGGCGCTGTTGATCGAGACCCTCGGCGGCCTGGAAACCCTCAAGGCCTGCAGCGC
CGAAAGCGAGCGCCAGCACCAGTGGGAAAGCACCCATGGCGCCCTCACCCGCCTCGATAGCCACGCGCGCAACCTC
TCGGCGCTGGCCACCAATGGCACGTTGTTCATCCAGCAGTTTTCCGGGATGGCAACGATTGTCGCCGGGGTCTACA
GCATCATCGCCGGCAGCCTCAGTGTCGGTGCGCTGGTGGCCACCTACATGCTCGGCAGCCGGGTACTGGCGCCGCT
GGGGCAGATCGCCGGGTTGATCACCCGCTATCAGCAGGCGCAATTGACCATGCGCAGCACCGACGCGTTGATGGCC
CTGCCCCAGGAACGCGATCCGCAACAGCGCCCCCTGGAACGCACGCAACTGACGGGCGCCCTGGAAGTCAGCCAGG
TGACCTTCCACTACGCCGGACAAAACGCCCCGGCCCTGAGCCAGGTCAGTTTCCAGCTCAAGGCGGGCGAGCGCGT
GGGGATTATCGGGCGCAGCGGCTCGGGCAAAAGCACCCTGGGCCGGCTGGTGATGGGTTTTTACACCCCAGAAGAG
GGCCAATTGCTGCTCGACGGTCTCGACCTGCGGCAACTGGACGTGGCCGACCTGCGCCAGCAAGTGGGCTATGTGG
CCCATGATCTGCCGCTGCTGGCCGGTAGCCTGCGCGACAACCTGACCCTGGGCGCGCGCTATATCAGCGATGCGCG
GATGCTCGAAGTGGCCGAGCTGACCGGCGTCAGCGAGCTGGCCCGTCAACACCCCCAAGGCTTCGACCGGCCGGTG
GGCGAGCGCGGGCAACTGCTGTCCGGCGGCCAACGCCAGGCGGTGTTGCTGGCCCGCGCGCTGCTGCTCGACCCGC
CGATCCTGTTGCTGGACGAACCCACCAGCGCCATGGACAACAGCAGCGAAGAAACCCTGCGCCAGCGCCTGGAGCT
ATGGGTCAAGGGCAAGACGTTGCTCCTGGTCACGCACCGCACCTCGATGCTCAGCCTGGTCGACCGTTTGATCGTG
CTGGACAACGGCCGCATCGTGGCCGACGGCCCCAAAGAAAGTGTGATCGATGCATTGCGCAAGGGCCGTGTCGGCT
CGGCCGCCGTCTAGGAGCCCCACCATGTCAGCCACTCCCTCATCACCGGGCTACTTCAAGAGTTTTGGCAAAACCG
CCGACAGCGAATTCATGCCCGAACTGGCGGGCGCTGCACTGCAGGATTCGCCGCGCCGTTCACGGATTATCGTGTG
GCTCACCGCCGGCCTGCTGATCTGCGCCCTGCTGTGGGCCAACTTTGCCGTGCTCGATGAAGTGACCATGGGCGAA
GGCAAGGCCATCCCGTCGAGCAAAGTGCAGGTGATCCAGAACCTGGAGGGTGGCATCGTCACCCAGATTTACGTGC
GCGAAGGACAGATGGTGGACAAGGGCGACAAGTTGCTGCAACTGGACGACACGCGGTTTCGCTCGAACAAGGGCGA
AAGCGAAGTCGACCGTTATGCCCTCACCGCCCAGGTCGAACGCCTGTCTGCCGAGGCCGAGGGGCGGCCGTTCAAA
TTGTCCGATGAAGTGATCGCCAAGGCCCCGCAAGTGGCCGAGGACGAACGCTCGTTGTTCGAACAACGCCAGCGGC
GCTTGAGCAGCGAACAGCGGACCTTGAGCGAACAACTGCGGCAGAAAACCCAGGAGCTGGCGGAATTCCGCTCCAA
GCAGGGCCAATTCAGCTCCAGCCTGGCGCTGCTGCAGCAAGAGATGAACATGTCGGCGCCGCTGGTGGGCACCGGC
GCGGTCTCGCCAGTGGAAATCCTGCGCCTCAAACGCAGCGCCGTGGAGATCCGTGGCTCATTGAACGCCACCACCC
TGGCGATCCCCCGGGCGGAGTCGGCGATCAATGAGATCAAGAGCAAGATCGACGAATCGGTGCAGACCTTCCGCTC
GGAAGCGGCCAAGGAGCTCAACGAAAAGCGCACTGATCTATCGAAAATCACCGCCACCAGCATCGCCATTGATGAC
CGTGTCAGCCGCACCACCGTGGTCTCGCCGGTCCATGGGGTAATCAAGCAACTGAAGGTCAACACTATCGGCGGCG
TGGTCCAACCGGGCAGCGACATGGTGGAAATCGTACCGCTGGAAGACAACCTGCTGATCGAGGCCAAAGTGCGGCC
CCAGGACGTAGCGTTCTTGCATCCGGGGCAGAAGGCGATGGTCAAGTTCAGTGCCTACGACTACACCATCTACGGC
GGGCTCGCGGCAAAACTGGAACTGATCGGCGCCGATACGATTACCGACGACAAGGGCAACAGTTTCTACCTGATCC
AGGTGCGCACCGATAAGAACCATTTGGGCGGGGAGAAAAAACCGCTGCTGATCATCCCGGGGATGGTGGCAACGGT
GGACATTATTACCGGGGAGAAAAGCGTGCTGGATTACTTGCTCAAGCCGGTGTTGAAGGCGAGGACGGAGGCGATG
CGCGAGCGTTGAGGCGCGACCCGGCGCGCCTCAACGTTAATCTGTCAGCGTCACACGACACTGCTGTGTCAGCTTT
GCCCTGTTGGCGCTGGGCGGTATGCAAGGCTCAGGCAATTTGATATGCCAACTGCTCTCGAAACAGAGCGGGGTTG
AGTGTCCAGGTTTCCATATCAACCATCTCCCCCTCATTCATACCAGCGCACAGGCGATCCAGAATCCGCTCAAGCG
TGCCGTCGTCTCGCCAATCCCGATAGCGTTGATACACCGTCGACCATGCTCCGAACCGCTCCGGTAGATCACGCCA
GGCTGCGCCGGAGCACACGATCCAAAGCACTCCGTTGAGCATCAGGCGATCGTCATGACGCGGTCTACCCGCTTTT
TGCGTTGTCGACATCAGGTCCACGATTGAATTCCACGCGCAATTGGGAAGTTCATAGCGCCTAACCATTTGAGACT

CCTTCTACCTAGGTTAATGACTTGGCTACCAGGGAAATGCCATTTGAATATCGTCCTGAACCTTTCGCCGGACTTT
TACCGAAAGCCCGATCAATCGAACCGACCTACCTTCACGACGGGTATCCCAGAGCAGTCGACAGAGAGACTGAACG
GTGTGCGCGCACAGGCTGTTGGCCGACGCCTCGGTGGTTGAGATCGAAAAATCGGAAAACTTGAGTTTTACTAAAT
TTTTGGTGATCAGGTAGTCATCTCCCATGTCTGCAATACGCTCCCTCAAGCCTTGCGACAATCGCTCCAACTCCTC
CAGGCAATCGCACAACGCATCGCAATCAAAGGGCAGTGTTCTTTCAATGCCGACAGACTTGATATCACTGCCCCGC
TCATCCGCCTCTTCGATTCCCTGGCACCGCTCCCATACGTAAAACCCATGCACGCCGAGATAACGCATCAATGCCG
GAATTTTGTCCTCGGTAATGTCTTTGCACTGGCGGCAACCCAGGTTTTCCAGCACCGCGTAGGTCTTGGGGCCGAC
GCCAGGAATTTTACGAACATCCAGCTCGTCGAGAAATCCACGCACCTGACTCGGTGTAATGACGAACATTCCATTG
GGCTTTTGCATTTCCGAAGCAATCTTGGCCAAGTATTTCAACGGCGCCACTCCAGCAGAGGCGGTCAGGCCGAGTT
CTTGCTGGACCGTGGCTCGAATGTGCGCAGCTATATGGGTGGCCGACCCGTTGAAGCATGTTTGCCCCGTCACTTC
CAGAGCCGCTTCGTCGAGTGACTGAAACTCTACTTTGTCGGTATATCGCCGGAAGATCTCGTCGAGCTTTCGCGTG
ATCGCTTCATACGCCTCGAAGCGCGTTGGCAGCAGTACCAAATCCGGGCACAAACGATGCGCCGTGGCGGTGGCCA
TGCCGCTTTTGACGCCAAACTTTCTTGCGATGTAATTCGCTGTACTGATCACTCCACGCTCTGCAGCCGTCCCCCC
TACGGCGACGGGGCAATGCATTAAATGGGGCGCGTCGAGAAGCTCGACAGAGACAAAAAAAGAGTCCATGTCGATA
TAAATGAACTTTCGCTCGCTCGCACCTGCTGTAGTACCCACCATCGATTGTCCTGTGCGCATGGATATCAAACAGT
TACGCCATCAAGTACATGGACCTGAAGGGCCAAAGTACCGTCATCATGTTGGCCGTAGTGCACCCCTTGGATATGA
GGCGCAAGGTCGGGATAGAGATTGGCCAAGGCTTGCACGGCCAGGAGGTACCCAACCAACGCCGAATTCCAGCACT
CCCCGGGAGCGATGCACATAACCCCAGGCGGATAAGGAATGACCCCTTCAGCCGCCACACAACCAACGGCATCTGC
GATGTCGATCAGACTGGAGCGCCCACGTACGAAGGCTTGGTTTGCCACATACGGCGTCAACACGGCCTGCGCCTGC
GCCGCCGAGCTGAATATGGAGGATTGGAGCTGTTGGATCTGATGATGCTGGAACAGCTGGTTGATGACGCAGCACA
ACTCGCGCAAGGAAATGTTTGCGTACCCCTGCCCACTGCTCGCAAGCGACGGCAACACGTCGCTTACCCGATCACC
TTGCGCCAGCGCTCGCTCCAGTCGCTCCAGCGCGTGCATCAGCCGGGTCAACTGCGTTGCGTCGGAAGAAGGCGAC
ACCAGCAGGGTGAAGTTGTAGAAATCAGATTTTTCCGGCGTGAAGTTCATCTCTCTGAGGTAGCAAGTGACGATGC
TTGCAGGGATCGAAACGTACCCGCAGGCAACCGCGTCCTCATTCGCACGCGTCGTGAACATGACCTTACAGGGGTC
CAGGTAATGCAGGCCGCGTTCGATTGGATTTGCGTCGCGACTGACAGGGCAAAAGTCCTGCGTATAGACCTGCGAA
GCAAGATCGCCGGGTGACGTCCGGTTGCCGTAGTACGGCTTGATAACCGTGCAGTGCTGATCGACCTGGCGCCTGA
ACTCATTGGCCATTGAGACCGCGCCACTCCATAGCCGTTGACCGTGGCCATCAGCATGAATAGCCGCATTGACCTC
CAAGGACATGAACAACGGATAAAACGGACTGGTAGAAGAGTGCAACATGAAAGCGCTATTGAACACGCGACTGGAG
CAATAGCGCTGCTGGAACTGGATATGTTTATCCTTTTTATGGATCTGCGAGGTTTGTGAAAATCCGGACATTTGCT
TGTGGACAGACTGCGTCACAATGATACCCGGGGCATCCGGCAGTAACGGGATGTGCAATGGCGATCGGTGGGCAAG
CAGATCGATAAACGGTTCGTAGCCTAACCATGCGGAATCAAAGAGCACGTAATCGCACAGGTGACCGATTCGTTCC
AACAGTGCACCAGCATCCACGACAACACCATCGCAAGTGGCGTGTTGCACGATTGCCAGGCGAAACGGCCTTGCCT
GCCTCGCCTTCGCCTCATCTATTTTGCTCGCCCGGGCTCTGAGCTGATCCTCTTGCAAGGCTCCTTTTCGGTAGCC
GCCCAGTACGCCGCAGTCATCGCGAAAATTATCGAGATACACCGCACGCGCGCCACTTTGAACCAGCGCCCCCAAA
TACACCGATTTGTGATTGTTCCGATCCATCAGTACAAGGTCGCCCTCGGTAAGCAAGGCACTGGTGACAATCTTGT
TGGCGGTCGAGGTGCCATTGAGGACAAAGTAGGTTTCGTCGGCATTAAATACCTTGGCAGCGAGTTCCTCAGCCTG
CTTGATCGGCCCTTCATGACTGAGCACATCACCCAGCTCGGGCGCAGCGTGGGGCACATCCACACTGAACACCTCT
GCCCCAACCAACTGTTTGAAACGCACTCCGGCAGGGTGCATATCCAGACATTGCCCTCCTTGATGCCCTGGGCAGG
CAAACGTAGGTCGTTTCTTTGAGACAAACTGCGCGATTGCCGAACTGAACGCTGGTAGAGCCCTGGATTCGAAGCT
CCTGGAGGCATCAATGATGCGATTGGCTGCGTGCTCCGATAATGGTGCCTGAAGCAAAGTCGGCTGCAGTTTGGCT
AGTGCGGGAGAGCACTGCTCTTGCCCGTCGTGATGTATCACAAACAACGGAATCTGCCATTTTTCCACGCAGTCGA
TTACGCGAGCTATCGATGCATCTTCCACGCTCAAAACCAGCGCGCCAGCCCCCAGTAAATCATGGCTCCCTGCGAA
AGCTGCAACCACCACCCCAGGCCGCTCCTCAAGATGGACGGTTGCGCTACAAACGATCCTTATCTGCATCATCTCT
TCTCAGGCTCCAACCCAAGGGTGCAATACACAGCATGAACGTCATGCCAATAAAAGTAGCCGGCCCCACGATGAGA
AACATAAGGCACGCGATAAGCCCGGACATAGCATCGCCAATGACCGCCGTAAAGTAATACAACGAAAAGTTCTTAC
CCACTTCCTGTGGATTCGACAAACATTGAATATTGGTAATAATTGCAATATCGACGAACGCACCGATAAACCCCAC
GCACAGCAACAGCAAAACAAACAAATACGTATTGAATTGCAAGGCGATAGCCGTGGACAAAAAAAGCACCCCATAC
AACAACCAATAGACAATGACTGAACGCGCCGTGGTCGTCGCATTGAATTTCACATACAACATACCGCCCATCACCG
TACCCAAGGCAAGCAGGGAATAAATGTAACCAACAGCCGCTTCCGACTGAAAATGCTCGATCACGGCAGCCGGCAA
TATAAAACGTATCACTGAAGTGGCAAACATCGCACACAACGTAGTGCAACAGATGACCGTAAAGAGTGGCTTGTTG
AATTTTCGCAGACTGCCAACGCTGCGTGTCGCTTCTTTGAAAATAGCCAGTATATTGAAAGACTCTTGTGTAGTTC
TTGCAGACGACAGCAACATCAACATACAAGCGGCCGACACCAACAAACTTACCGCAAAAAATGCAAACGCATAGCT
TGGCGCAAGAACAATTGCAATAAGAGAAAATATAAGCGGGCCAATGATCGAAGAGATATCTTCAATGACCTGTATT
GCACTATTGACAGAAGGCAAGTCATCGTTATGCACAATATCGGGAAAGTAAGCACGAAATGTCGGTGTATACAAAC
AGTCAAGTGCCGTCATGAGCATGGAGGCAACGATGACCATCGCCACCGTCGGTTGCGAGTACTCGAACATCACGAA
CATCGCAACTGCCAAGGCGCACTTCATTAATTCAACAACTACCAGCGTAGACTTTTTTTCAACGTTATCCGCCAGC
CAGCCGCCTACGGGAGAAAACAATGCACTGGGTAAGTAACGAAAAAAGTAGACCAAGCCCATCAGTAGAATATCGG

CATCGGTCAACGTTACAACGGCTACAGCAAACGCCGTCTCGAAAGCAAACTCACCGCATTTCGAGAAAAAAAAAAGC
GATCGAGAGCCGTTGGGTTCCATGATTGAGTGTAAGTGCGCGCATATCCACTGCCTACCAAGTGGTTCTAACCTGC
AGAATAAATCAACTCATCCCGAGTGATATCTCTTCCGTCGGCACTAAGCTTCTCAAACACTATTTTTTTCAAGATA
TTTTGTTCACTTTCGGGAATTAAGTAAAACATAATGTTGCTGTACCACAACCAGCCCAGCTTGGTCAGTTCAAAGC
TTGTCGGGGTTTCAGTGACAAGGTCCGCTTCCACCAACTCCTGCAGACGCGCCAGGAGTCCTTGGGGTAGTTGGCT
CATCGCTACCCTGGATTTGTCGATTTCTCCATGATAGGGAAGACGCAGCACAACCGGCTTGACCAGATCCAACACC
GGGGCATGCTGGCTGACCTGACAGACATAATCACCAGCGCTCATCTTGGCGATATATTGTTCGCGCAACGAGGTGT
TGGTAATCACGTTTTCCGCTACAGAGGAGATAGCGCCCACGCCAAAACCCAGCAGGTCGTGGTCGGCATAGCCGTA
AACGTGCTCATGGTAGATAAATGAGTAATCTGGGGTAATCAACGTCGGTTGCGCACTGGTGCGCCGCACGTAGCCA
TGCCCGTTGTAAGGCAGGTAGCCCTTCTGTCGCATGTGCTCATCAATCATCAGTTTCATGTTCAATTTACGCATTG
CCGGGAAAACTTCAGCAGTACGCTCTTTGATTTGCTTGTGGAGCTTTACCGAGGTGACGACGTTGTTGATGGGGTA
AATGTCTATGTTACTAAGACCCAACTCGACGGCTTTGTCGATATCTGCCAGCGTCTGGGCTTCATTGCTTCCGTTC
ATACCGTACATGATGTCGCACAGGACATGCTCGAACGTGGGCACCAACAGATCGGCTGCGCGCTCGATTTCGACAA
TATCCTCATTGAGATTGAACAGTTTGCGCCAGGTAGGATCAACCGTCTGCAAACCAAAGCGCGCATGCGTAACGCC
AATGTCAGCCAGCGCTACATTGCGTTCACGCGTGACACTGGTGATGTTGAACTCGAAAGAGAATTCTTCAACCGTC
GACATGTCAAAATATCGATGCAAGGCCTCACCGATATCATTGATATTCTGTGGTGAGAGCAGCGAAGGCGTACCGC
CTCCGAAGAATATCGCCCTGACCGGAACTGCTTTAAAGTCCATTAGCTGTGATTTATATTCGATTTCCTTTATCAA
CGCCTTAGTATAACGATCAATATCCTCTGCATTTTTGTACAAGCCCCGAGTGAACGGACAAAAAGAGCAGATAGCC
TCGCAAAATGGTACATGAAAATACAATGCCCGCGACTTCGCCTTGGCATTGGCGCCATTCAGCAAGTTTGACAACT
GCGTTTCGCTGGGGCGAAAGACATTCATGGACTGCGAAGGAAAAAAGAAATTGTACAGTGGAAACTGATAATCGAA
CTTAATAAATCCATTATTGACAAGTTTCATTGTCCACCTCTTTCGCAACTACCACATTGCTGCCACGAAGCAACGT
ACGGCTTTTGACTTTTACATCACCACCAAAAAAAAACGCGAATCCCTTCATTGATCGACAAGTTATTCGAGCCGCAC
GTATAGACGTCGATGCTAATGTAATGACTTTCTGGATAGGTGTGATAGGAAAGGTGAGATTCGGAAAGTAGATAGA
GCCCTGTCACCCCCTCGCCGCCGACATTGAATTTGTGGGTCAAATGGTCAAGTACAGTAAAGCGTGAACGTTGCAA
AGTACTGAGGAAAAAATCATGCAGTGCCGGCGCATCTTTTAACAAGCGCGCATCTGCACCTGTGATATCCCAAATC
GTATGATAACCAAAGTCGTCCATACGAACATTGCTCCTATCCTTGGGCATGAAATATTTCAAATAAAGAGAAGGGG
AGATTAACTCCCCTTCCCCCTTTTGCATTTACCAGAGGCAAACACCTTTGGTAGTAACGCCAGTCACTTCAGTTTG
AAACAGATCGATTGCAGATTTCATGATACGTTCCTTGTTCAAGATTAAGTGTTGCCCACCGTTATAGGGCAACTTA
AAACTAGCAGGAAACTTTTCCTAAAAAAGCTGGGGAAATGTAAAAATTTCTTTCAGGCAAGCCAAATCTTATACTT
TTAAAAATATTTCCAAACATGTCATCTCGGCAACCTCCCAACTACAGCCGCTTACTAAAACTCAATCTAGCCCCAA
ATCCTCCACTAGTGCTGGCACTTGCCGCCTCTGGGAGTAGCCGTTCAAGGGGGAAGCCCCCTCCTGCAAGGGGTCT
CGAGTGGACTTTTCGTACAGGAGCACACCTAAAATCTTTCAAAAAAAAGTAGCCGTTCTACTCATTATTTCGACAC
ATCTCACTTGCTTTACCCAGATAACTGACCTGAAGTCAGCGTCCAATGCCTCTGCTTCAACACGGATCAGGCTCAA
AATGCGCTAAATGCAGCGCCCCTCAAGACCTCGACTGGAGCAACCCACCCCACTGCTCCTGACTCCGTTAGACAGA
ACTCGTATCCCCGTGATTCCTACGGTAGATATGTTCGCCCATCGCCGCAATCTGGCCCTCTATCAGGGCTTCAAAA
GGCCGCAATAAGGGCTCGAAAGAAGTCGGCGCCTCCAGCACCGTCAACGCCTGCTCGATCGCCTCCACCGTCGACA
ACGCCCCCGGCCCCGGTGCCTTGCGCAACCGATAACGGGACACGGCGCCCGCCGTCAGCGTCACCCGTGGCAACGC
CGCCAGCAGCGGGTTGAGGTGCAGCAATTTACGCGCCTTGCGCCAGGTGCCGTCGGGCACCACCAGCAGCAGCGGT
TCGTCGGATGGGCTGTAGGCCTGCAGCGGCTGCGCGTCCTCGCCGGGAAACAGCAACCGCGCCTGATACCCAGGGG
GGTTGAGCAAGGTCTGCAAGCCTTCGAATACTTCACCCACCACCAGTTGCGCATTCACCAGGCCCAACGCGGCCAG
ACGTGCGGTGTTGAGTGCATGGTTGACCTCGCTGGGATGTTGCAACAGCAACACTCGGGTGCGGCTGTCCAGGCTC
GGGATCAGCGCGCACAGGCAGTGGGTGGTCGGCCGCAGGCAGCGGGGGCATTGGGGTCTGGACATGTTCAGGCCTG
ATTGAGCTGTGCTTTGAGTAAGTCGCGGAAGGTCTGGATCAACGGTTCGCGGCTGCGCCCACGGCGCATGATCAGC
GAAAACGGCGCCTGGTAGCCAAAAGTCGCGGGCAGCAGCACCCGCAAGTCGCCCTTGTCAGCCCAGGCCTGGGCGT
AGTGTTCGGGCAAGTAGCCGATGTAGGCACCGGACAGCACCAGGATCAACTGCGCTTCCATACTTTCTACCGTTGC
GGCGCTGTGTTTGAAGCCGTGGCGCGCCAGTTCCGCCTGGCTCCAATACCCGCGCCCGACCATGCGTTGCTGGGTA
ATCACCTGTTCGGGGATGCGCCGCTCGCTGAACAGTGGATGGCGCGAACTGCAGTAAAGCCAGTGTTGCTCGCGGT
ACAACGGCATGTAGATCAAGCCGCTCATGCGGTTGGAGAAGGCGCCGATGGCCAGGTCCAGGCGATTGTCCTGGAC
CCCGAGTTGCAGCTCGTAGGGGCTCATGACCGACAAATGCAAATGCACTGCCGGATGCTCAAGGCTGTAGGCGCCG
ATGACTTCGGCGAATGGCAGGGCCTTGTCGCTGACGGTGGAGTCGAGCACGCCCAGTTTGAGCGTGCCACGCAGCT
CGCCTTTGAGCGCCGCCGCGTACTGCTCGAAGCCCTCCAACTCGGCCAGCAGGCGCAAGGTTTCCTGATGAAACAG
CTCACCCTTGCTGGTCAGGCTGAAACCGCCCCGCCCACGATGGCACAACACCAGGCCCAGGGCGCCTTCCAACTGG
CTCATATAGGTACTGATGGCGGACGTCGATAGGTTGAGTTCGTGCTGGGCACTGGCAAACCCTTGGTGGCGCACCA
CGCTGACGAAGATGCGCAACAGTTTCAGGTCGGGCAAGGCGCTGGCCATGGAACCTCCAGGCAAAAAATCGGCTGA
CAGGCCCATTCCCTGTAGGAGCGAGCTTGCTCGCGAAAAACCTGAAAGCGCCGCGTACGTTCTGAATGCCCGCGTG
ATCGTTAACGTTTTTCGCGAGCAAGCTCGCTCCTACAGGGAACAGGTTTTGCACACCGGTGGAGTCTACCCCAGGC
ATTAGTTCAGAAAAATCTGAACTAAGTATTTTCCCGCAGCGATTCTTCCCGTGTCCGACATTTCGCAGAATCGGCC

TCTGATAACCACAACAACGATGAGGCACTCCCGTGGACAAGATCTTTCACCAACCACTGGGCGGCAACGAAATGCC
GCGCTTTGCCGGCATCGCCACCATGATGCGCCTGCCCCACCTGCAAACCGCAGCCGGCCTGGATGCGGCCTTTGTC
GGTGTGCCCCTGGATATTGGCACCTCGCTGCGCGCCGGGACCCGCTTCGGGCCCCGCGAAATCCGCGCCGAGTCGG
TGATGATCCGCCCCTACAACATGGCCACCGGCGCCGCACCGTTCGACTCGCTCTCAGTGGCCGACATCGGCGATGT
GGCGATCAACACCTTCAACCTGCTGGACGCCGTGCGCATCATCGAAGAATCCTACGATGAGATCCTCAAGCACGAC
GTGATCCCGCTGACCCTGGGCGGCGACCACACCATCACCCTGCCGATCCTACGGGCGATCCACAAGAAGCACGGCA
AGGTCGGGCTGGTGCATATCGATGCCCACGCCGACGTCAATGACCATATGTTCGGCGAGAAAATCGCCCACGGCAC
CACCTTCCGCCGTGCCGTCGAAGAGGGCCTGCTCGATTGCGACCGCGTGGTGCAAATCGGCCTGCGCGCCCAGGGC
TATACCGCCGAAGACTTCAACTGGAGCCGTAAACAGGGGTTCCGCGTGGTCCAGGCCGAAGAGTGCTGGCACCAGT
CCCTCGCGCCGTTGATGGCCGAAGTCCGGGAAAAGGTCGGCGGCGGCCCGGTGTACCTGAGTTTCGACATCGACGG
TATCGATCCGGCCTGGGCACCCGGTACCGGCACCCCGGAAATTGGCGGGCTGACCACCATCCAGGCGATCGAGATT
ATCCGTGGCTGCCAGGGCCTGGAGCTGATTGGTTGTGACCTGGTGGAAGTTTCACCGCCCTACGACACCACCGGCA
ACACCTCGTTGCTGGGCGCCAACCTGCTGTATGAAATGCTCTGTGTGCTGCCTGGCGTCACGCACCGCTGATGTAG
CGCGGGGCCATGTGCCCCGCGCCTGAATACCGATAAAAATAATAATTGGAGATTCGCCACCATGGCTGTGGATCTA
TTCGTCGTACTTATCTACGCCGCCGGCATGCTCGTGCTCGGCTATTTGGGCATGCGCCGGGCCAAGACCCATGAAG
ACTACCTGGTCGCCGGGCGCAACCTGGGGCCGACCCTGTACATGGGCACCATGGCGGCAACGGTACTCGGTGGCGC
GTCCACCGTGGGCAGCGTGCGCCTGGGTTATGTCCACGGTATTTCCGGCTTCTGGCTCTGCGCAGCGCTGGGCGCG
GGGATCATCGCGCTGAACCTGTTCCTCGCCAAGCCCTTGCTCAAGCTGAAAATTTTCACCGTGACCCAGGTCCTGG
AGCAGCGCTACAACCCCACGGCCCGCCAGGCCAGCGGGTTGATCATGCTGGCCTACGCGCTGATGCTGGCAGTGAC
CTCGATCCTGGGGATCGGCACCGTATTGCAAGTGATGTTTGACCTGCCCTTCTGGGCCGCCATCCTGCTGGGCGGT
GGCGTGGTGCTGGTGTATTCGACCATTGGTGGCATGTGGTCATTGACCTTGACCGATATCGTGCAGTTCGTGATCA
AGACCGTCGGCCTGATGTTTATCCTGCTGCCGATCTGCCTGTACCGCGTGGGCGGCTGGGATGAGCTGGTGGCCAA
GTTGCCAGCGGCCAGTTTCAGCTTCACCAGCATCGGCTGGGACACCATCATCACCTACTTCATGATCTACTTTTTC
GGCATCCTGATCGGCCAGGACATCTGGCAACGGGTCTTTACCGCCCGGGACGAAAAGGTCGCCAAGTATGCAGGCA
CTTTCGCCGGGTTCTACTGCATCCTCTACGGCCTGGCCTGTGCCCTGATCGGCATGGCCGCCCATGTGCTGATCCC
GGACCTGGACAACGTCAACAACGCCTTCGCCGCCATCGTCAAACTGTCGCTGCCCGATGGCCTGCGTGGCCTGGTG
ATCGCCGCGGCCCTGGCGGCCATGATGTCCACGGCCAGCGCCGGGCTGCTCGCCGCATCCACCGTGCTCACCGAAG
ACCTGCTGCCACGCCTGCGCGGCGGCAAACAGTCGAGCCTGAACATCAACCGCCTGTTCACCCTGCTGACCGGCAT
CGCCGTGCTGGCTATCGCCTTGGTGGTAACCGATGTGATCAGTGCCTTGACCCTGGCCTATAACCTGCTGGTCGGC
GGCATGTTGATCCCGTTGATCGGGGCGATCTACTGGAAACGCGCGACCACTTCGGGCGCAATCAGCGCCATGGCAC
TGGGGTTTGCCACGTCGCTGGTGTTCATGATCAAGGATGGTCTGGAGGCAAACACACCGATCTACTACAGCTTGGG
CGTGAGTGTGGTGAGTTTTGTGCTGGTGAGCATGCTGTCGCGCCGGCCACAGGTGGCGGTGGCCGACGCGGCCTGA
GTAAAGCTTGCTTCAGCGGATGCGACGTCGGTTGTCGCATCCGTTTTTTTATTTAAAACACCAAACAAAAAGTGGG
AGCGGGCTTGCTCGCGATTACGGTAGGTCAGTCAACCGTTCAGTCACTGATTTACCGTAATCGCGAGCAAGCCCGC
TCCCACATTGAATCTGCGGTGTTGCGTGGTCCTACAACCGCGCTCAAACCCTGTGTTTGAGCGCTTTTCCGGGCTT
ACCTGCGACGCGGGCGGCGCTGTTCGTCATCGGTGCGGATAGGCACCGGTTGCAGCGGTGGCTCGATCAAGCCGAG
TGCGACGCCGAGGTCGTGCAGCCAGTTTTGCAGTTTTTCTTTCATGGTGCCCCCTTTGAGGGTAGTGCCGAGCAGC
TGGAATTCTGTGGCTGCTTCCTACAGATAGTAGTACTAAGTCCCACGCAATGCTTGCCTGAAACCGCAACGAACCA
TTACTGAATTGATACAAATCCTGACGATGCGGTCAGGCAATCGCTCGCGCCTCTTGTTCAGGCCGCGTCTTGCCCT
CTTGCTGCAGGTCAATCCACGCGTTGAGATTAGCCCCGAGCATGCCCTTGCGCCACATCAGCCAGGTGGTGGCGTG
GGCAAACGGCTCGGCCAGGGGGTGGATCGAGACGCTGTGCTTGCCGGGCAGGCTGTCGAGCATCGATTCGGACATC
AACGCCACCCCGGAACCGGCGATCACACACGCCAGCATGCCTTGATACGACTCGATCTCGATGGCCCGCCCCATGG
CTACGCGGTCGTGGGAAAACCACGCTTCCAGGCGCGCCCGATACGAACAACTGCGTCGGAAGGTGAACACCGAGCG
CCCGGCCACATCCTGCGGGCCGCGTACCGGCGGGTGGTCCGCCTCGCAGATCAGCAGCAAGCGTTCCTCGCACAAC
GGCACACCGTCCAGGGTCGCCAAGGTCAATGGGCCATCCACCAGCGCCGCATCCAGACGCCCGGTGATCAGGCCTT
CAAGCAGCTCACCGCTGGGCGCCGACTGCACTTGCAGGTTCACCATCGGGTAAGCCTTGTGATATTGCGCCAGCAG
CTTCGGCAAATGGATCGCGGCGGTGCTGTACATGCTGCCCAGCACAAAGTCCCCGGCTGGTTGCCCGCCCTGTACG
GCGCCGTGCGCTTCGTCGTGCAGCGCCAGAAGCCGCGTGCTGTAGTCGAGCAGCACCTTGCCAGCCGGCGACAGTT
GCAGGCGCTGGCGCTCGCGCACGAACAACTCGACACCCAGTTGCTCTTCCATTTGCTTGAGCCGGGTCGAGAGGTT
CGACGGCACCCGGTGCAGGCGCTCTGCCGCACGGGTGATAGAGCCCTCTTCGGCCACAGCCTGGAAAATACGCAGT
TGGCTGAACTCCACGACCATTCTCCTTAACTGAACAAGTTGCTCAGTATTATTCATTTTTACTGAACAACAATCCA
CCTTAACCTGGGTCATCGCTTCTTGCAGGAATTTGCCCCATGTCCCCCCTGATTCGCTTACTCGCCAGTTTTATCG
CCCTGATGATGGCCATGGGCATTGGCCGCTTTGCCCTCACCCCGCAAATGCCACACTTGCTCAGCGAAGGGCAAAT
CGACCTGACCGAGGCCGGGCTGATCGCCGCAGCCAACTACCTGGGCTATTTCGTGGGGGCCGTGGACTCGATGTTC
GCGCGCAGTCATCACCACATACGCGGGCGGCTGCTCGGCGGGTTGTGGTTGTGTGTGCTGCTGACCCTGGCGTCGT
TCTGGGCCAGCGGCTTCTGGTCGCACCTGATGTTGCGCTTTGGCACGGGCGTGGCCAGCGCCTGGGTGCTGGTGAT
GATTACCGGCCTGAGCCAACCCCTGGCGATTGCCGCCGGGCGCCCGCGATTAGGAGCATTGGTATTTGCCGGGCCT

GGGCTGGGGATACTGCTCACGGGGCTGTTGGCATTGGGCTCGAACCTGCTGGGCGAGGATTCGGCGACGTTGTGGC
TGGTGTATGGCGTGGTCGCCCTGGGCATGTTGCTGGTGATCCTGCCGTTGCTGCCCAAGCCTGACAGAGGACAGCC
GCAGAGCAGCAGCGCCACTACCGGGAGCAATGGCAGCATCGCGCATCTGGGCTGGGTTTACCTGTTGTTTGGCTTG
GGCTACATCATCCCCGCCACGTTCCTGTCACAAATGGCCAGCGCGCAGTTCAAGGGCGCCTGGCAGGCGGACCTGT
TCTGGCCGTGCTTTGGCCTCGCGGCAGCCCTGGGTGTATTGCTGGTCAGCCTGCGCAAGCCCAACCCCGACACTAC
ACGCTATTGGTTGATGGCCACCCTATGGCTGCAAGCGGCCGGCGTCTTCGCTTGTTTGCTGGGCAATGGTGTGGGT
TTGGCATTGGGCGTAGTGCTGTGCGGTCTGCCGTTCTTGGCGTGCATGCAACTGGTGATGCAGCGCCTGCGGGAAA
TCGCACCCCACGGTGCGCAACGCAGCACGGGGCTGCTGACGGCATGTTTTGCCCTGGGCCAGTTAAGTGGCCCACT
GCTGGCATCGTTGAGTAGCCACCTTAGCGGCGGCTTGCAGCCGGCGCTGATGGTCGCGGGCAGCGGCCTGATACTG
GCCGGTGCACTGCTGCTACGACCGGCCCCGGCACCGATTGATTGCCGGGCGCGGCTCAGCCCCTGACGCGGTGCGC
CGCCCCGTACCCCAGCAAGCAGCACGCAGCCGTGAGGAAGGTGCCCCACGCCATGTCCATCAATGCCAGGCCCGCC
GACCAGCCTTGCAAGGTCGCCCAGTTGCTCAGGTCATAGGTGCCATAGGCCACCAACCCGAGCAAGGCGCCCAGGC
GGGCAGCCCGTTGCCAGCCACCACTGGCCAGCGCCGGCAGCACCACAAAAGCCACACAGCCGAATACATAAAGGAA
GTAGAACAGTGCCGCCGGCAGCAATCGCGGTTGTTCAAGCATCAGCGGCCCCAGCAGGGCCTTGTAGGTCGACCCC
ATCAACACGCCAAGCCAGAGGCCATCGAGCACTAAAAAGGCCAATAACGTACCGAGGTAAGCAAACACGACTTTTT
TGCCCATGACGACACCCTACTGTAGGAGCCGGCATGCCGGCGATAGCGACTCCTACACAAGAGCAGGTTGCAGTCA
GCTTAGTTCAATGCGATCAGCGTGAATCACGATCTGGCCCTGCTTGTACAGCGCACCAATGGCCTTCTTGAAGTTG
CCCTTGCTCACGCCAAACAAGTTGCTGATCACGGTCGGGTCGCTCTTGTCGCTGACCGGCAGGCTGCCGTTGTTTT
CACGCAACTTGGCGAGGATCTTCGAGTTCAGGCTGGACGCCGCTTGCTCGCCGACCGGTTGCAGGCTCAGGCTGAT
ATTGCCATCGGCGCGGATTTCCTTGATGAACCCCTTCTCTTCCTTGCCCGGACGCAGGAACTTGAACACTTCGTTC
TTGTGGATCAGGCCCCAGTGCTTGTTGTTGATGATCGCCTTGAACCCCATATCAGTGGCTTCGGCAACCAGCAAGT
CAACTTCCTGGCCCACGGTGTAGTTGGCCGGGGTCTTGTCCAGGTAACGGTCCAGGCGCGCAGTGGCGGTGATGCG
CTTGGTGTGCTTGTCGAGGTAGACATGCACCACGCAGTATTCACCGGCGGTCAGTTGGCGCTTTTCCTCGGAGTAA
GGCAGCAGCAGATCCTTGGGCAGGCCCCAGTCGAGGAATACCCCGATGCTGTTGACTTCCACCACTTTCAGGCTGG
CAAACTCACCCACCTGCACTTTTGGTTTTTCAGTGGTGGCGATAAGTTTGTCATCGCTGTCCAGATAAATAAAAAC
GTTAAGCCAGTCTTCATCTTCACTGGGAATATCTTTGGGAATATAACGATTAGGCAAGAGGATTTCGCCCTCTTGC
TCACCATCCAGATACAAACCAAAGTTAGTGTGTTTAACCACTTGCAAACTGTTGTAGCGCCCGACTAAAGCCATTT
CCAATACCCTCATTGCGTGGGCGGCATTCTACCCGACTTGAAGGCAGCGGTCCCGTGTCCAGGCGCGCAACCCTGA
AAAATCGCGGGTTTGCCAGGGGATTTCAGGCAATCGCTCCGCTCGTCAGGTCGGGCTACGTGCTGGCATCGATGGC
CGGCTGGTTTTTCCCCCTCGGGTTTCTATTTAAAACAGCGAGTTAGGGCCCTCAACCATGGATCAACTTAGGTTTT
TATAATAACGCAGCTTATTCCTGGGAGATATTTACCAAGCAATTGTCAAGTGTTTCATGTACGATGCCTGGCCAAG
TTAATTTTCTACAGGTTAGTGGCCGCCATGCGCGTAAAAGCATCCAACAGCAAAGCAAAGCCAGCTCCCGCCGTTG
AAACCAGCGAATCGATCAACAACCAAATCGCCGCGTTCCTCAAGTCCGGTGGCGAAATCCAGCAGATCGCCAAGGG
CGTGAGCGGCCAGACTTTCGGCCCGTCCAAGCAGATCACCCTGGGTAAGAAGTAAGCGCGAAGCCGCCTACGCCTC
CCACCTGGTCCCTAAGCGTCTTGCCTATCAAGGCGCTAGGACTAGAGCCCGCACCCTCCCCCCGACACACTTTCAA
CCCCGCCAATCGACGAACGGGCCTCAGCCCCTGACTTTCGCCGGTATGCTTGCACACGTCTAGATCGGGCGTTTGC
CCGATTCCTTCTGTTACTGCTCCTCGCTTTTCATGGAGTGACGCATGCTCAAGCCGTGCATTTTCCTGGTCAGCGC
CCTGCTCGCCAGCCCCCTCGCCGACGCGCAGGTTTTCCAGCGTGAACTGGGCGACTTCGACTTGAAACTGGGGACT
ACGCCCAGCCGCAGCATGGCCCAGGGCCTGGTCAAACCCACCTCTCCGGGCAGCAACTCGTTCCACGGCGGCCTCG
ACCTGAGCCACGCCAGCGGTCTGTATTTCGGCCAGTTTTCGCCGAGCATGGGCCTGTCACCCGCCAGTAACCTGGA
AGTCGATTCCTATTTGGGCTTCAAGCGCCCGTTCGACCAGACCCTGGGCTATGAACTGGGGATGATCCACTACAGC
TACCCCAAGTTGAGCCCGCTCGACAGCCAGGAGTTCTACGGCGGCCTGAACTTGCTGGGCAATCGCTTTGGCGTGT
CATTGGGCAACGATCCAGATCGTCGCGACAGCACCCTGTTTGCCGACCTGGGCGGACCCAGCCGTTTGGCATTGG
CGTGAGCATGAAGTACACCACTCATCAGTTAGGCGCTCCGGCATCGGTGGAGGGCGGCTCGATCCGTAGTTTCAGC
GACTGGTCGGTGAAGTTCTCCCGGCCGTGGATGGGCGTCGACCTGGACCTGATCTACAGCGACTCCAGCCTCAGCG
GCAGCGACTGCTCGGCCTACTCCGGACACAATTCGCAATGTGATGGCCTGTTGACCCTCAAGGCCGAGCGATCGTT
TTATTGATGGGCTGAACTGTCACGCCCCCGGCGAGTTCAGATGAACATCCCTGGACTGCGCAAGGACTCGCCCATG
CTGTATCGGTTAAGAATCCTGGTGGCCTTGCTGGCCCTGAGCGTGCTGCTTGCCGGCTGCAACCGCGTGGGCCTGG
CCTATCGCAACCTCGACGTGATCATCCCCTGGACCCTCAACGACTACCTGGACATGAACGCCGGGCAGAAAAGCTG
GTTCAACGACACCCTCAAGGACCACCTGGCCTGGCACTGCACCACCCAATTGCCCGGCTACCTCGACTGGCTCGAC
CGCCTGCAACAGATGGTCGACAACCATCAGGTCAGTGATGCGGCCCTGCAAACCCGCACCCTCGAAGCCAAGCAGG
CCATCGCCGACGTCGCCCGGCAGATCACTCCATCCGCCATCCAATTGCTACGCGGGCTGGACGAGCAACAGGTACG
GGAGATGGATCAAGCGCTGGCCAAGGACCTGCGTAAACGCCAGGACGAATACCTCAAGCCTCCCCTGGCGCAACAG
ATCAAGGACCGTAGCGAGCGCATGAGCAAACGCCTGGACGCCTGGCTCGGCCCGTTGAGCGCCAGCCAGCAAGAGC
GTGTGCTGGCCTGGTCCACCGCCCTGGGTGCGCAGAACCAGCAATGGATCGGCAACCGCGCCCATTGGCAGGCGCA
GTTCATCGCGGCCGTCAACCAACGGGCTAGCAGCGACTTCCCGCGCAAGATGGAGCAACTGCTGGTGGACCGTGAA
AGCCTGTGGACCGCAGATTATCGCCAGGCCTATGCCCAGACCGAAGCGGCGGCCCGCAGCCTGCTGGTGGATTTGA

TGGCCGAAAGCACGGTGCAACAACGACAGAAGCTGGTGCAGAAAATCGATAAAGTGCGCAGCGACTTCCAGTCCCT
CAAGTGCCTCAAGGGCAGCCCAGCCTAGCCCGACGCCATTGGGCCCAGCGTCCCCAGCCAAGCGACCAGCCCCAGG
ATCAGCAGCACCGCACCGAACTCCACGCTCAGGCTACGCCGCAGGGCGCGACAGGCCAGCGTGTACTCGCCAACCT
CCACCGCCCAGGTCGCCAGCCACAGACTGGCGCTAGGCCACATTGCACCGGTCAAGGCAACCAGGGCGGCCACCAG
CAATACGCCGATACGCAGCATCCAGGTCAAGCCAAACTCGGTCTGCCCCAGCATCATCTGCAGATGCGCCCCAAGT
GCCTGCCACTCTTGCTCACCGCTCATGGCCCGGGTCATGACCAGCAGCGAAGCCATCGAGAGCAACATGCCAAGAA
GCGCGGTCCCTGCCAGCCAGCCTTTGAAATTCAACACCGCGCCGGATAGCCGCTCCGATGCCTTCAGCCCATACAG
ACCAAACAAGCCCAAGCCGAACAGCAGCATCAGGTCCAGGTACAGCGCAAACCGCAAGGCAATGCCCACAGACTCA
CTCATGGCCGGACGCTGAACACCACATCGCCGGTAATGGGGTGGGTGTCTGACGACACCGCACGCCAGTCCACCCG
GTAGGTCCCGGCCGGCAGCGGCGCCGCAGGCTTGATCAGCATCACCTTGGGGTCGCTGCTGCCCGACACGCTGGCC
TTGATGGGCATGGGCGAATGGGCCATGCCGGGCATCGCCGTCATGATCAGCTTGGCGCCCGAAAACTGGGTCAGCA
GGTTTTCCGAAAAGTGCAGCTCAATGGTCTCGGGGGCCGGCCCGCTTGCCCCGGCCGCAGGCAGTGAAGACAGCAA
CTTCGGATGGGCCCAGGAAGGCCCGCTGAGCAATAATCCGGAACACAGCGCAACCGCCACCCACGTGGTTTTAAAC
ACAGACATGCAAGGCTCCTGGCCGCTCATGGCGACAGGTTATAGGTAGAGTTTTTTAATCAGCATTCATCAAAACC
ACAGGCGCACACCCAGTACCCAGCGCACCTCGCTGCGGTCCTCGCCCTCGTCCCTGGCGTACTCGGCCGTCTGGCC
GTAGGCACGATTCCAGGTCACCCCGACGTAAGGCGCAAACTCGCGACGGATTTCGTAACGCAGCCGCACCCCAACC
TCGGTTTGCGCAAGGCCTGAGCCAATCCCGCGCCCCGGATCGTTTTTCCCGTAAAAGTTGGCCTCGGCGGTAGGCT
GCAGCACCAGCCGATTGGTCAGCAAAATATCGTAGTCCCCTTCGAGGCGCGCCGCGGTCTGCCCGCCTTCACCGAC
AAACAGCGTGGCCTCGGCCTCGAAGTTGTACAGGGCCATGCCTTGCAGGCCGAACGCCGCCCAACTTTGGCTGTCA
CCGGGCTTGAAGTCCTGGCGCATACCCGCCACCACATCCCACCACGGGCTGATGGCGTGGCCCCATAACGCCTGGA
CCTCAGCCTTTTCAGTCTTGCCCTTGGCGCGCTCACCTTCGGTGCGCAGCCACAAGCGGTCGATATCACCGCCCAT
CCAGCCCTTGGCATCCCAGCCCACGGCGCGGCCATCGTCAGAGCCTTGCCATTCCAGTTGGTCGATCACAAACAAC
GAGTTGATGCCACTGTCATGCACCACATGCCCGCCCGGGGCGTTGTAGACCGCGGCGCGATCGGCGTCGGTCAGCG
GTGGAATCGGTGTACGACTATGACTGCTGGCCGCAGGTGTTGCTGGCGTCATGCCCTGCATCTGGCTGTGGTCCAT
GGGTTCGGCGCTGGCCAACGCTACGGGGGCAAATGTCGCGATCAGTAGCGCAGCGGGGCGAAATAGATGGCTCATG
CTCGGTTCCTCATTCTTGCACCCGGACTTCACGGAACATGCCCATTTCCATGTGGTACAGCAGATGGCAGTGATAG
GCCCAGCGCCCCAGGGCATCGGCGGTCACCCGGTAACTGCGGCGCGATCCTGGCGGCATGTCGATGGTGTGTTTGC
GCACCATGAACTGGCCGTTTTCGTCCTCCAGGTCACTCCACATGCCATGCAGGTGAATCGGGTGCGTCATCATGGT
GTCGTTGACCAGCACCACCCGCACCCGCTCGCCATAGGTCAACCTGATCGGCTCGGCATCGGAGAACTTCACCCCG
TCGAAGGACCAGGCGAACTTCTCCATATGCCCGGTCAGGTGCAACTCGATGGTACGACTGGGCTCGCGGCCGTCGG
GGTCTTCAAAGGTGCTGCGCAAATCGGCATAGGTCAGGACCTTGCGCCCGTTGTTGCGCAAGCCGATGCCAGGGTC
GTCGAGCTTGGGTGAGGTGCTCATCGCCTGCATGTCTACCAACGGGTTGTTGGTTTCGCTGGCCGGATGGCTTTGC
ATTGGCATCGCCATCCCCGCCATGGCGCTGTGGTCCATGCCCTCCATCGCCCCACCGTCCATGCCGGCCATCTGAC
TGTGATCCATGGTCGGCATATCTGCCATGGCCCCGTGATCCATTGCACCGTGGTCCATACCGCCCATGCCCATGTC
GTCCATGGTCAACCAGGGGCGAGCGTCCAGCGCCGGGACCGGCGCCAGCAAGCCCGGCTCACGGCTGAGGGTGCCG
CGTGCGTAGCCGGTGCGGTCCATGGATTGGGCGAACAGGGTGTAGGCCGCCTCGCTCGGTTCGACGAGCACGTCGT
AGGTCTCGGCCACGGCGATGCGCAACTCGTCCACGCTGACCGGTTTCACCGGCTGGCCGTCGGCTGCCACAACCGT
CATCTTCAGGCCCGGAATACGCAGATCGAAGTAGGTCATGGCCGAGCCGTTGATCAAGCGCAGGCGGATCTTCTCT
CCGGCACGGAACAGCCCGGTCCAGTTTTGCGCAGGTGGTTGGCCGTTCATCAAGAAGGTGTAGGTCGCGCCGCTGA
CGTCCGCCAAGTCCGTGGGATTCATCTTCATTTGCGCCCACATCAGACGGTCGGCAGCAGTGGCCGACCAGCCCTT
GTCGGCCACATCCTTGATGAAGTCGCCCACGGTGGGTTTATTGAAGTTGTAGTAATCGGACTGTTTTTTCAGGGTC
TTCATCAGGCTGACGGGATCTTCGTCGGTCCAGTCCGTCAGCATCACCACATAGTCGCGCTGGTAAGTGAAGGGTT
CCGGCTCCCGCGGATCGATCACCAGCGGGCCATAGACCCCTTGTTGCTCCTGGAGCCCGGAGTGGCTGTGGTACCA
GTAGGTGCCGTGCTGGTTGACCTTGAACTGATAGACATACACCCCGCCCGGCTCGATCCCCTTGAAACTCAGGCCC
GGCACGCCGTCCATATTGGCCGGCAGGATAATCCCGTGCCAGTGGATCGACGTCGGCTCTGCCAGGCGGTTACGCA
CACGCAAGGTCACGGTATCGCCTTCGCGCCAGCGCAGCAGCGGCCCCGGCAAACTGCCGTTGATGGTCAAGGCGGT
GCGCACGTTGCCGGTGAGGTTGACCGGGGTCTGGCCAATGGACAGTTCAAAATCTCTGCCTGCCAGCACGCCCGGT
TGTCCGGGACTGGCCAGGGCCCAGACCGGTGGCCGCCACAGGCCTGCGCCGCCCAGCAGGCCGGCCGCAGCCAAGC
CTTTGACGAAGGTTCGTCGAGAAGTGTTGGAGTGCATGCCGATTCCATTCCCGTCAATTGAATGACCCAAGAATAG
GAATCGGCGCCTGTCAGTTGGCTTAGCGCCAGATTACCGATTTGACAGTTTCAGCCGGCTCAGGCGATTTGCGCCT
TGGAGGCCACGACCTCGAACGTCGCCGGATCCAGCGCATCGGCCTGCTCGTCCAAAACCTGGCGCGGGTGATCGTT
ACCCGGGATGGAACTGTCGATCAGCGCCAGCAGGCGGGCCCCCAGCGGCGTCAAAATGAAGTTCTCGCCATTGCCG
CCCTGATCCTCTGGACGGGACTCGATAAAGCCGCGCTTGAACAACAGCGCCTCATAGTCGGCAGCGGTCTTTTTCA
GCGCATCGAGGTCGCCGTTGAAGTCGCCGGCCGTGGCCTTCTCGGCGGCTTCCTGCTCGGCATACTTGCGCGGGGC
AAAGCTGCCCTCGCCATTTTGCACTTCATGTAACAGACGCTCGATCAGATCCCAATTGTAAGTACTCATCCTGAAT
CCCTCCTGCAGGCACACCGACTGGCGGCCTTCAAAGGTTGTGACCGGCGTGTGCGGGCGCCGTTCAGCCGGATGGA
CGAGCGTGATTGCACTGAACTTTTCGCGGGCCGTAGCCCTCAACCGAGTATCACCGCCAAGGAGGTCCACCCATGA

AAACCCTGATGCAACTGGCCGTCGCCGCCACCCTGTTGAGCGCCCTGCCCGCCTGGGCCTGTACCCCGGAAGAGGC
CACGGAAAAACGTGAGCAACTGGCCAAGGAAGTCAGCACCCTGACCCAGCAGAACCCGACCAAGGCCAAGGAAATC
AACGATGAACTGCAGCAAATGGACCTGGATACCGAAAGCGCGAAGTTCCCCGACAAGTGCCAGTTGATCGAGGCGC
GGCTCAAGGAATTGAAGGAAGCGGCGGCCAAGGCAAAAAACTGAAGGCATAAAAAAACCGGACATTGTCCGGTTTT
TTTTGTGCGCCGCCGTATTATTCGGCAGCCGGTGCTTCTGGCTTGCGGCGCTTGAGCGGTGCCATGCCGTCCTTGC
TGACCAACGACAGGTTGTCGGTCTTCGGCCGGTTGGCGATCTTGCGCTTGGTCGGCGACTTGGCGCCGGCTTTCTT
CTTGTCGCCCTTGGCGTCGACCTTTTTCTTCTTCACGCCCACGGCCTTGCCCGAGGCCTTGACCTTCTTCGGCCCG
GTGTAGGTGCCTTTGACTTCCTTGATGGTGCGCCGCTCAAACGACTGCTTGAGGTAGCGCTCGATGCTCGACATCA
GGTTCCAGTCGCCATGGCAGATCAGCGAAATGGCCAGGCCATCGTTGCCGGCACGCCCGGTACGGCCGATACGGTG
CACGTACTCGTCGCCGCTGCGCGGCATATCGAAGTTGATCACCATGTCCAGGCCTTCGACGTCCAGGCCGCGCGCG
GCGACGTCGGTGGCTACCAGGATCTTCACGCCGCCCTGCTTGAGGCGGTCGATGGCCAGCTTGCGGTCCTTCTGGT
CCTTCTCACCGTGCAAGACAAACGCCTTGTACTCCTGAGCCACCAGGCGCCCGTAGATGCGGTCGGCCATGGCCCG
GGTGTTGGTGAACACAATGGCCTTCTGATACGTCTCGTTGGCCAACAGCCAGTTGAGGATCTGCTCTTTGTGCACA
TTGTGGTCAGCAGTGATGATCTGCTGACGGGTGGTCGAGTTCAGATCGCTGACGTTGTTGACCTGCAGGTGCTCAG
GGTTGTTCAGGACCTTGGCGACCATCTCGCGCAGGGTCGAACCGCCAGTGGTGGCGGAGAACAGCATGGTCTGCTG
GCGGTTGACGCACTCTTCGACCAGGCGCTGCACGTCGTCGGCAAAACCCATGTCGAGCATGCGGTCGGCTTCGTCG
AGCACCAGTACTTCGACTTCCTTGAGGTCCAGGTTGCCGGCGTTCAGTTGCTCGATCATCCGCCCAGGGGTGCCGA
TCAGGATATCCGGGACCTTGCGCAGCATGGCGGCCTGCACCTTGAAGTCTTCACCGCCGGTGATCAGGCCGGACTT
GATGAAGGTGAACTGCGAAAAGCGCTCCACTTCCTTCAAGGTCTGCTGGGCCAGTTCACGGGTAGGCAACAGGATC
AGGGTCTTGATGCTGACGCGGATCTTGGCCGGGCCGATCAGACGATTGAGGATCGGCAGGACGAAAGCGGCGGTCT
TGCCACTCCCGGTTTGAGCCGTCACCCGCAGGTCACGCCCTTGGAGCGCGAGCGGGATGGCCGCTGCTTGCACAGG
CGTAGGCTCGACAAATTTAAGCTCGGCCACGGCTTTGAGCAGGCGTTCGTGCAGGGCGAATTGGGAAAACACGGGT
GCTACCTCGAAGAAATACAAAAAATCAGCTGCATAGGGTAACGGTTTCGGGCGCTCAGGCCGAGTTTCTTTACGCG
AAGTGGGCCAATCAGATGGTTTTTTGTCAGGTGATTTGTCTGCCTGCACAACTTTGCAGCACTTAAATGCTCTAAT
CGCCCCGTCATGTCTTACAGAAGAATCGCTTTAAACCATGGATATCAAACAGCTCTGGCTCAACGTCCAAGACCTC
TGGGGTGCCCTGGACCAACATCCGCTGCTGCATTCAAGCATCGCCTTGCTGGTGCTGCTGGTCGTGGCCCTGATTG
TCGGACGGGTGGCTCGCTACCTCATCCTGCACACCGTCAAACTGCTCGGGCGCCAGCCGGCGCTGCACTGGCTCAA
TGACCTGCGGCACAACAAAGTCTTCCATCGCCTGGCACAGATGACGCCGTCCCTGGTGATCCAGTTCGGCCTGCAC
CTGGTGCCGGAACTGAGCAAAAACGCCGCGTTGTTCATCGGCAACGTGGCGCTGGCGTTCACCATCCTGTTCCAGG
TCCTGGCCATGAGCGCCCTGCTCAATGCCCTGCTGGATATCTACGCCCGCACCGAACACGCCCGCACGCGCTCGAT
CAAGGGCTATGTGCAATTGGCAAAAATGGTGTTGTTCGTGTTTGGCGCGATCATCATCGTCGCCACGCTGATCGAC
CGCTCGCCGCTGTTGCTGTTGTCCGGCCTGGGTGCGATGTCGGCGGTGATCCTGTTGGTGTACAAGGACACGCTGC
TGTCGTTTGTCGCCAGCGTGCAATTGACCAGCAACGACATGCTGCGGGTCGGCGACTGGATCGAAATGCCCCAGGT
CGGCGCCGATGGCGATGTGGTGGATATCACCTTGCATACGGTCAAGGTGCAGAACTTCGACAAGACCATCGTCTCG
ATCCCCACCTGGCGCCTGATGTCCGAATCGTTCAAGAACTGGCGCGGCATGCAGGCCTCGGGCGGGCGGCGGATCA
AACGCAGCCTGTTTATCGACGCGGCCGGCGTGCGTTTCCTGCGTGACGATGAAGAAGTGCGCATGACCCAGGTCCA
CCTGCTGACCGACTACATCAGCCGCAAGCAGGCGGAACTCAAAGCCTGGAACGAAGCCCAGGGCCACAGCGCGCAA
CTCTCGGCCAACCGCCGGCGGATGACCAACCTGGGGACGTTCCGTGCCTACGCCCTGGCGTACCTCAAGAGCCATC
CCGATATCCAGCCGAACATGACCTGCATGGTGCGCCAGATGCAGACCACCTCCCAGGGCGTGCCCCTGGAAATCTA
CTGCTTTACCCGCACCACCGCGTGGGCGGATTACGAGCGCATCCAGGGAGATATTTTTGACTACCTGTTGGCAGTG
CTGCCGGAGTTTGGCTTGAGCCTGTACCAGCAGCCAAGTGGCAATGACCTGCGGGCCGGGGTGTTGCCGGCGGTGC
TGGGTGCCAGCCATTTGCCAGCCCCAGAAAAAGCTGCGCTATAAGCTGAACCCTTGTGGGGGGGGGTTGCTCGCGA
ATGCGGCATGTCAGTCAGCATATCTGGCTCTGACCCACCGCATTCGCGAGCAAGCCCGCTCCCACAACACTCATCA
CCGGTTGGCAAGCCCTAAAGCCGGGCGCACACCCAACCGGCTGATCCACACCGCCGCCACGATCACCGCCCCGCCC
AGGAACAACCGCCCCAACGGTTCATGCTGATTCCAGATCAGCAGGTTCAGCAGCAACCCCACCGGCACATGCAGGT
TGTTCATCACGGCCAGGGTGCCGCCGTTGACCAGGCAGGCGCCCTTGTTCCACCAGTACAAGCCCAACGCGGTGCT
GACCAGCCCCAGAAACACCAATACCCCCCACTGCAATGGCGCTTCGGGCAGGAAGTTGGCTTTGCCGAACAACAGG
AACGCCGGCAACACCACGGCCAATGCTCCCAGGTAGAAATAGCCAAAGCGCCGGTAGTGCGGCAAGTCGCTTGGAT
GGCGCGCCACCAGGCGCCGGTACAGCACCTGCCCGGCGGCGTAGGTGAAGTTGGCCAGTTGCAACAACAGGAAGCC
CATGAAGAAGTCCGGGCTGATCTGGTCAAAGCGGATCACCGCCGCGCCCGCCACCGCCACCAGCGCAGCAATCAAC
GCCCACGGGTTGAAGCGCCGGTTCAGTGCGTCTTCGATCAACGTCACATGCAACGGCGTGAGGATGGTGAACAACA
ACACCTCGGGCACCGTCAGCACGCGAAAGCTCAAGTACAAGCACACATATGTCACACCAAACTGCAACGCGCCGAT
CAGCAGCATGCCGCGCATGAACGCAGGCTCCACCGAACGCCAGCGGGTCAGCGGGATAAACACCAGCCCGGCCAGC
AGCACGCGTACCAGCACGGCGAAATAGCTGTCGACATGACCGGCCAGGTATTCGCCGATCAAGCTGAAGGAAAACG
CCTGGATCAGCGTGACAATCAGTAAATAGCCCATGTGCGCCTCGTATTTGAATGGGCGCGACATTAGCGTTTTTCA
CGGCGGGGCGAAACTCGGGGCAAAAAAAACCCGACCTCGCTAAAGCGGCAGTCGGGCAGGAGCACTCAGGAGCAAC
AAGTGCAAAGGGAGGTTCGATACGCGAACTTGAAGGGTTGCGTGGGGTTATAAAAACACCACGCGATTATCTGGCG

ATTAACGCTTCAGGCAACCTGTGCCAGCAGGGCCTTGGCGTGGTTGACGCCCTTTTCCTGGAAGTCGCCACCCAGG
TTCACCCCTTCGGCATGAATGAAGGTGACGTCATGGATACCGATAAAGCCCATGACCTGGCGCAGGTACGGTTCCT
GGTGATCGCTGCTCGCGCCGGTATGGATACCGCCACGGGCGGTCAACACGACAGCGCGCTTGCCGGTCAACAGGCC
CTGTGGCCCGGTGGCGGTGTACTTGAACGTCACCCCAGCCCGCAACACATGGTCAAGCCAGGCCTTGAGGGTGCTG
GGGATGGCAAAGTTGTACATCGGCGCGGCCATTACCAGCACATCGGCGGCCAGCAGTTCGTCGGTCAGTTGGTTGG
AACGGTCCAGGGAAGCCTGCTCATCTGCGTTGCGCTGCTCGGCAGGCTTCATCCAGCCGCCCAACAGGTTGGCGTC
CAGGTGCGGCACCGGGTTGATGGCCAGGTCACGCACGGTGATCTGATCGGCCGGGTGGGCCGCCTGCCATTGGCTG
ATGAACTGTTGGGTCAACTGGCGGGAGATGGAATCCTGCTGGCGGGCGCTGCTTTCGATGATCAGAACATTGGACA
TGGCTTTGTAGGCTCCATCGGTAATTGCTGTAAGTCGATGGAGTGAAGGTTAAACAGAGCCCGTTCGATAAAAAAG
CGTAAAAAACTGCTATAACCCATCTATAAAGTTGTTTATAAGCGGAGCATAGCGCTGGCCGCGCCCCGCTTGTGCC
TTTATTTTGGCTCGCAGGTCAGTTTGATGCGCATCTTGATAATCGCGCGGCTGAACTTGACCGTGGTGTTGGTGCT
TTTGCCGGCAGGAACCGTGACATTGCGTACCCGCGCAGGCTCCGGACCATTGGTAAATGCGACCCTGCACGCAGCG
TCGTTGCTGCCGAAGTTACTGAGTTGGATGGAGCTGATCTCACTGTCCACATCGGACGCCATGTAATCGATGCTCA
CCCCCTTGATCGATTTTGACACGTCGATCGGATAGGCAAACGCGCTCAGCGGCAACAGTGCCAGCACTACACAACA
GAATTTTCTCATTCGGCAGTCTCCAATAAGGACCGCCAGCTTAGGACAAGAGGAGCTCATCTTGAAAGCGCCCCGC
GTGACCCTTGATCAATGGCGCACGTTGCAAGCCGTGGTCGACCATGGTGGCTTCGCCCAGGCGGCCGAAGTACTGC
ACCGCTCGCAATCCTCGGTGAGCTACACCGTCGCCCGCATGCAAGACCAGTTGGGCGTGCCCCTGCTGCGCATCGA
CGGGCGCAAGGCCGTGCTCACCGAGGCCGGCGAAGTGCTGCTGCGCCGCTCCCGGCAACTGGTGAAAAACGCCAGC
CAGCTCGAAGACCTGGCCCATCATATGGAGCAAGGTTGGGAAGCCGAAGTACGCCTGGTGGTCGATGCCGCCTACC
CCAACGCCCGCCTGGTGCGCGCCCTCACCGCCTTTATGCCGCAAAGCCGTGGCTGCCGCGTGCGCCTGCGTGAAGA
AGTGCTGTCGGGTGTCGAGGAACTGCTGATTGAAGGCGTCGCCGACCTGGCGATCAGCGGCTTCAGCATCCCTGGC
TACCTGGGCACGGAAATGAGCGATGTGGAATTTGTCGCCGTGGCCCACCCCGAACACCCCCTGCACCGGCTTAACC
GCGAGTTGAGCTTCCAGGACCTGGAAAGCCAGATGCAAGTGGTGATCCGCGACTCCGGCCGCCAGCAACCACGGGA
CGTCGGCTGGCTCGGCGCCGAACAGCGCTGGACCGTGGGCAGCCTGGCCACCGCCGCCACCTTCGTCGGCAGCGGC
CTGGGGTTTGCCTGGCTGCCCCGGCACCTGATCGAGCGCGAGCTTAAAGAAGGCGTGCTCAAGCAACTGCCCCTGG
AGCAAGGCGGCAGCCGCCACCCGACGTTCTACCTGTACTCCAACAAGGACAAGCCCCTGGGGCCCGCGACGCAGAT
TCTCGTCGAACTGCTGCGCACCTTCGACACCGCACCGCTGGACGCCCCCTTTGCCGCCCCCCGGCAAGCCTGAAAC
GGAGTTCACCCATGGCCTATTTCGAACATGAAGGTTGCACCCTGCATTACGAGGAATATGGCCACGGCGAGCCGCT
GATCCTGATCCACGGCCTGGGCTCCAGTTGCCAGGATTGGGAACTGCAAATACCGGTATTGTCCCGGCATTACCGC
CTGGTGGTGCTGGACGTGCGCGGTCACGGCCGCTCCGACAAGCCTCGGGAGCGCTACAGCATCGCTGGTTTTACCG
CCGACCTGGTGGCCTTGATCGAACACCTGCAACTGCCCGCCGCCCATGTGGTGGGCCTGTCGATGGGCGCGATGAT
CGCCTTTCAACTGGCGGTCGACCAACCGCAGTTGCTCAAGAGCCTGTGTATCGTCAACAGCGCACCCGAGGTCAAG
GTGCGCAGCGCCGATGACTATTGGCAATGGGCCAAGCGCTGGACCCTGGCACGTGTGCTGAGCTTGAAGACCATCG
GCAAGGCCCTCGGTGACAGGCTGTTTCCCAAACCCGAACAGGCCGACCTGCGGCGCAAGATGGCCGAGCGCTGGGC
AAGAAACGACAAACGTGCTTACCTCGAGAGCTTCGACGCGATTGTGGGCTGGGGCGTGCAGGAACGACTTTCGAAA
ATCACCTGTCCAACCCTGGTCATCAGCGCCGACCATGACTACACCCCGGTGGCGCAGAAACAAATCTATGTAAAAC
TGCTGCCCGATGCGCGGTTGGTGGTGATCGAGGATTCGCGCCACGCCACACCGCTGGACCAACCCGAAACCTTCAA
TACAACCCTGCTCGATTTTCTGAAGACAGTCGAAAGCACTTCCCAGGATCACTGACCCATGCTGAAAAAAATCGCC
GTTTTTGCCGGTTCTGTTCTGTTTGCTGCCAACCTGATGGCGGCCACGCCGGCCAAGGCACCACACGTATTGATCA
CCACCACCAATGGCGAGATCGAAATCGAGTTGGACCCGGTCAAGGCGCCCATCAGTACCAAGAACTTCCTGTCCTA
TGTCGACAAAGGCTTCTACACCAACACCATTTTTCATCGGGTGATCCCGGGGTTTATGGTGCAAGGCGGCGGGTTC
ACCCAGCAAATGTCGCAAAAGCCAACTGAAACGCCGATCAAGAACGAAGCCAGCAACGGCCTGCATAACGTGCGTG
GCACCTTGTCCATGGCGCGTACCAGCAACCCGGATTCGGCCACCAGCCAGTTCTTCATCAACGTAGCCGACAACGC
CTTCCTCGACCCGGGCCGTGATGCTGGCTACGCGGTATTTGCCAAAGTGGTCAAGGGTATGGACGTAGTCGATATC
ATCGTCAACTCGCAGACCACCACCAAGCAAGGCATGCAAAACGTGCCTATCGATCCTGTCCTGATCAAGTCGGCCA
AGCGTATCGACTAAGGTCCACAGGGCGTTTCGCGCGCCGCCCACAAGGCGGCGCGTGTATTTGAAAAGGAGAGCCC
GCCCGGCGGGCGTCAGACCTAATGCTCTATCGTCGCTTTGAACAACTGATCGATATCTTCCGCGACGCTCCCAGCG
CGGCACCTCCCGATAAAGTCCTGCCCTTCTACCTCTATTACCTGCGCCAGGTGTGGCCGTGCTTTGCCGCGCTATT
GGTGGTCGGCCTGATTGGCGCGCTGATCGAAGTGGCGCTGTTCAGCTACCTGAGCCGCATCATCGACCTGGCCCAG
GGCACACCGCCGGCCAATTTCTTCCAGGTCCACGCCACCGAGTTGATCTGGATGGCCGTGGTCGCCCTGCTGCTAC
GCCCGATCTTCGGCGCCCTGCACGACCTGCTGGTACACCAGACCATCAGCCCCGGCATGACCAGCCTGATCCGCTG
GCAGAACCACAGCTATGTGCTCAAGCAGAGCCTGAACTTTTTCCAGAACGATTTTGCCGGGCGCATTGCCCAGCGC
ATCATGCAGACCGGCAACTCGCTGCGCGACTCAGCCGTCGCCGCTGTCGATGCCATCTGGCACGTGGCGATCTACG
CCATCAGTTCCCTGGTGCTGTTTGCCGAGGCCGACTGGCGCCTGATGATCCCGCTGGTCACCTGGATCATCGCCTA
CAGCCTGGCGCTGCGCTACTTCGTGCCACGGGTCAAGGAACGCTCGGTGATCTCCTCCGAGGCGCGCTCCAAATTG
ATGGGGCGCATCGTCGATGGCTACACCAACATCACCACCTTGAAGCTGTTCGCCCACACGCAGTCCGAGCAGGAGT
ACGCCAAGGAAGCAATCATCGAGCAGACCGAAAAAACCCAGCTGGCCAGCCGCGTGGTGACCAGCATGGACGTGGT

GATCACCACCATGAACGGCCTGCTGATCGTCACCACCACCGGCCTGGCCCTGTGGCTGTGGACCCAGTCGCTGATC
TCGGTGGGCGCCATTGCCCTGGCCACCGGCCTGGTGATCCGCATCGTCAATATGTCGGGTTGGATCATGTGGGTGG
TCAACGGCATTTTCGAGAACATCGGCATGGTCCAGGACGGCCTGAAAACCATCGCCCAGCCACTGGCGGTGATCGA
CCGCGAGAACGCCCCGCGCCTGCGCGTGCCCCACGGCGAAGTGCGCTTCGAGCAAGTGGATTTCCACTACGGCAAG
AGCAGCGGCATCATCGGGGGCCTGAACCTGGTGATCAAACCTGGGGAAAAGATTGGTCTGATCGGTCCTTCCGGTG
CCGGCAAGTCGACCCTGGTCAACCTGCTGCTGCGCCTCTACGACCTGCAGGGCGGACGCATCCTGATCGACGACCA
GAACATCGCCGACGTGGCCCAGGAAAGCCTGCGCGAACGCATCGGCATGATTACCCAGGACACCTCGCTGCTGCAC
CGTTCGATCCGCGACAACCTGCTGTATGGCAAACCCGACGCCACCGACCAGGAACTTTGGGAGGCCATCCGCAAGG
CGCGTGCCGATGAATTTATCCCGCTGCTCTCAGACGCCGAGGGCCGTGTCGGCCTGGACGCCCATGTGGGTGAGCG
CGGGGTGAAACTCTCCGGCGGGCAACGCCAACGCATCGCCATCGCCCGGGTCCTGCTCAAGGACGCGCCGATCCTG
ATCATGGACGAAGCCACGTCGGCCCTGGACTCGGAAGTGGAAGCGGCCATCCAGGAAAGCCTGGAAACCCTGATGC
AAGGCAAGACGGTGATTGCCATCGCTCACCGCCTGTCCACGATTGCTCGAATGGACAGGTTGGTCGTCCTGGAAAA
AGGCCAGATCGCCGAGAGCGGCAGCCACGCCCAACTACTGGCACAGGGCGGCCTGTACTCGCGATTGTGGCAACAC
CAGACCGGCGGATTCGTCGGTATCGACTGATTCTGGACTACTAACGGCGCTATCAATTCCGAACAATTCTGACAGT
TTTTTCTAACTCGCTGGCCGTGTATCACAATCCTGCTGTACTGCAATCAGGGCCCAGGGAGGGCCCTGACATCAGA
CTGCAGGGAAGCCTCACCGACCAGTTGATTTAGAAGGACGCGTTCATGTCTCTGTTCAAACGTTCCGTTACAGAAG
GGTTAGGTACGTTTTGGCTGGTGTTGGGTGGTTGCGGGAGTGCGGTTCTGGCCGCAGCGTTCCCCGATGTCGGAAT
TGGTCTACTGGGGGTATCCCTGGCATTTGGACTGACGGTGCTGACCATGGCGTTCGCCATTGGCCATATCAGCGGC
TGTCACCTCAACCCGGCAGTTACCGTGGGCCTGGTGGTCGGCGGCCGGTTTCCGGTCAAGGAGTTGCCAGCGTATA
TCGTCGCGCAGGTCATAGGTGGCGTCGTCGCGGCGGCCCTGTTGTATTTCATTGCCAGCGGCAAGCCGGGCTTTGA
ACTGGCTGCGGGGCTGGCCTCCAATGGTTACGGCGAACATTCGCCGGGCGGGTACTCGATGGCGGCGGGGTTTGTC
ACCGAACTGGTGATGACGGCGATGTTCCTGCTGATCATCCTCGGCGCCACTGACCGTCGTGCACCGGCGGGCCTGG
CCCCCATCGCCATTGGCCTGGGGCTGACGCTGATCCACCTGATCTCCATCCCGGTCACCAACACCTCGGTCAACCC
GGCCCGCAGCACCGGCCCCGCGCTGATTGTCGGCGGCTGGGCGATCCAGCAGTTGTGGCTGTTCTGGCTGGCGCCG
ATCCTTGGCGCGGTGATCGGTGGCATCACCTACCGTTGGCTGGGCAAGGAAGAAAACGCCTGATCCACCCTGTAGG
AGCGAGCTTGCTGGCGAAAAACCTGAGGGCGCCGCGTTTATCCAGAATGAATGCGCTGTACTTGAGTTCTTCGCGA
GCAAGCTCGCTCCTACAGGGGAACAGGTCGAGTTCCCTGAGCGTTATGCCTGCCGATACGGCAACGCCGACCGCGC
CTCTTCGGCATACGCCAGAATCCCTACCCGCTCGCGCTCCAGGAAGTCTGCCACGGCGGTTTTCAAGCCCGGATGA
CGCAAGTAATGCCAGGAGCGGGTAATCACCGGCTCAAACCCCCGGATCAACTTGTGCTCGCCCTGGGCACCGGCAT
CGAAACGTTGCAGTCCATTGGCGATCGCGTAATCCATGCCTTGATAGAAACAGGTTTCGAAATGTAGCCGGTCGAA
TTCACCCAAGCAGCCCCAGTAGCGACCGTACAGGCTATCGCCACCCACCAGGCTGAAAGCCATGGCCACCGGCCGC
GCCCCTTGCTTGGCCAGCACCACCCGAATGGCCTGCGGCATGCGCTCGGCCAACAGGCTGAAAAACGCCCGCGTCA
GGTACGGCGACTGGCGGCGCACGGCATAGGTGTTGGCGTAACAGGCGTAGACAAAATCCCACTGGGCCTCGCTCAA
CTCATGGCCTTGCAACCATTCGAACTCAATCCCCTGCCCCGCCACTTGCTCGCGCTCCTTGCGCATCTGCTTGCGC
TTGCGCGAACTCAGGGCGTCGAGGAAGTCCTGGAAATCGCGATAACCACGGTTCTGCCAGTGGAACTGGCAACCCA
GACGCGCAAGCCAACCGGGTTGCTGGCCCAGTGCCGCATCGGCCATGGCATCGGTGAAGTTGATATGGGCGCTGGA
AAGCCCTTCGATCTCCAGGTAGCCAGGCAAGCTCTGCAGCAGCTCCAGGCCGTCTGCGCCATTGGCCGCCAGCAGG
CGCGGCCCGCTGACCGGGCTGAAGGGCACGGCTGTCAGCAACTTGGGGTAGTAATCGATGCCGGCACGCGCGCAGG
CGTCCGCCCAACCATGGTCAAACACGTATTCGCCATAGGAGTGCCATTTGCGGTAGCTGGGCAGCGCCGCGACCAG
CCGCTCATCCTCGTAGTGCAGCAAGTGCTCGGGCTGCCAGCCAGAATGCCGACCAAGGCTGGCACTGTCCTCCAGC
GCACTGAGGAACGCATGGCGCAGAAACGGCTGATCCTCGGGGACCAACGCGTTCCACTCATCAGGCGATATTTCGG
ACAGGCTATTCAAACGTTTCAACGGCATGCCATTCCCCGACGGTAAACGCTCACTCACGATATTCAGGCCATTCTG
ACAGAACCCACTGCCTGCGCGGGCAGCAAAAAAAAACCCCGCCAAATGGCGGGGTGAAAAAGAGGATCGCGCTTTA
ACGGATGAAGAAAGAGCGCGTTGGAGCGGTATTACAGTTGCTTAGAACACGTACTGCACACGACCGACGATGGAGT
CACCGCTGTCATCGCCATTGGCGTTGGTGATTTTGTCGGTGCTGACCTTGCTGTAGTTCAGCGACAGCTTGACCGC
CTCGTTGGCGTACCAGTTCACACCCAGGGTGTTCACGTTGGCCTTGGCGTCGCCGGTTTCGCGGGTAGCGCTGCTG
ACGACGATGTTCTTGTCGTCAACCTTGATGCTGTCGTAGCGGTAGAACACTTCCCAGGCGCCGAATTGCTTGTTCT
CGGGCTTGATCGCGTCGAACTTGCCGCCGTCGAGCTTGTAGGCACGGGACTCGCCGGTCAGGGTGTAGGCCAGTTG
GCCGTAGAAACCGCTGGTTTTCACGTCCTGGTAGGCATTGCTGTCAGCTTTCATCTTGCGGCTCAGGGCTTCAGCC
TGCACCGAGAATGGGCCGGTTGCGAAGGCAAACTCACCACCGAACACGGTGTCGGTGTCATAGGCGCCCACTGCGG
TCAGGTTGCTGGCGTTCAGGCCCAGACCACCGCCGAAAGTAGCGCGGTTGCCGTTGGAGCCGGCATCGTTGCCACC
GGTGGTCGCTACGCCACGAGCGCCGAGACGAGGACGAATCCGCGAGTCGAAGGCTGCATCGTTCAGATCACGTGCC
GCCACGTTCAAGCCGAAGTGCAGGACGTCGCCGCCTTTGTTCATCGGCGCCAACACGATACGGCCGTTGAATTGCT
TCACGCCCTTGCCGTCTTCGTCGTTGATGTCCTTGGAGGCCAGGCTGGCGGAGCCGTAGAACATGTCCGCCGCAGT
GCCGCTGACCTGGATGCCCATGCCGTTTTCGTGGGTGTTGACCCAGTCCGCAACCTCGTAAGCGGAGTTACGCTCC
ATGGCGGTGATCCACTTGGAGCTGGTGGCTTTTTCCAGGCCAAAATCCGGGTCAAAACGACCGACGCGGATGGTTA
CTGGCTTGAAGCCCACATAGGACATGGACGCTTCGTCGAAGTAGCCGTCGTCGGAGTTACCCGAGTTGTGGGAGAA

GTCGTAGTTGAGCTGGTATTTGAAATCCTTGTAGACGGTGCCACCCAGCTCCAGGAAGGCGCGACGGAAGTAAGCG
GCGTCGCCCGAGTCACCGTTCTTGGTGTAGAAGCCGTCAAATGAGCCGTAATCGGCTTGCAGGCGACCACCCAGCT
TGAAGCTGAAGTTCTGGTCGGTAGTAGCAACCTCAAGGCCGCCCTTGGTTTTGACAACGATATCGGCGCCGTCAGT
AGTGACAGTACCAGCGAAAGCCTGGGCGGTAACGGCCAGGGCCAGGGCGCTGGCCGCGAAACCGGCGAAGTGCTTA
CGGATCATCGAAGAATTCCCCTAATTGGTGGTCTTTTGCGTTGGAAAACACACGGGTTCTGCCCCGCTGGTGTTGG
GAGGGGATCTTGGCGAGGGGATATTTCAGGCAAGTTGCTGCAAGATAAATATTTTATTACAGGGGAACTTTATTAC
AACCACTGGAAATAAGCGCAAAGCTAGAGCCGGCGCGGGCTTGAGCAAAAAAAGTACGAGAAATGCCAGGTGAAAA
AAAGGTGAAAAAAACTTACGAACCTGCAATGTTTTTCAGCGCAGGGCTTTTCGCCGACACAAAAAAGCCCACGACG
GCGACGTCGCGGGCGTTGGGTCCAGCAGAAGGCGGTCAGGAATTGCGCTTGAGTTCCGAGGCCAGGCGTTCGGCCA
ACCCTTGTGCATCCGGCACCTCGGGTTCGGGCAGGATGCGCAAGGTGGCTTGCATCAGCCGCATCTGGCGAATGAA
GCGCCGGCAATTGGCACAGAACATCAGGTGATGGCGCATCAGCAGGCGCTCGCGAAAGGTCAGTTGCCCATCGAGA
TAATCACTGGAGCGTGCCACTTGTTCTTTGCAGGTCAACATTCGCCGGTTTCCTCGAAATGCTCCACCGTGGCGAA
GACTTTAAGCCGCGCCCGATGCAGCAGTACACGCACATTGGAGAGCGAGACTTCCAGAAGATTACAAATTTCTTCC
AACTCCAGGCCCTGGCGTTCGCGCAGCAGCAGCACGCTGCTTTGCAGCTCGGACAGACTGAGCAGCGTGTGCTCCA
GACATTCACGCAGCTCGTTTTCGGTGAGCAGCGCCTCGGGGGTGTCCTGATGCCAGGCAAAGGGCGCGATCAGCCA
ATGCTCGTCGCCGGCGGCAAAGCGCTCATCACCAATGGTCCCGTGGGGCGACGGCAAGTCGTCGAGCAAAACTTCC
CGGCGGTTCTGTTTGTAACGCCCCTTGGCCGAGTTGGCGGTGATGGTCAGCAGCCAGGTCTTGAGGCTGGAGCGCC
CTTCGAACTTGGCGAGGCTGCGTACCACCGAAAGCCAGGCGTCCTGGACCACTTCGTCAGCGTGGCGCTGGCCAAC
GATGGCGTAGGCCACCGCACGCATGGCGCTCTGGTACTGGGTGACTAACTCCTTATAGGCCTGCTGCTCGCCCTTG
AGCAGGCGCTCAAGCAAGTGCGCGTCGTCGGCTGCTGCCATTCAGAATCTCACTGTGTTGAGGGGAGCCGTTGTGG
CGAGGGGGCTTGTCCCCCGCTGGGCTGCGCAGCAGCCCCCATAAAACCCACTGAGTAGTGTCAGGCAGACCGAGGG
GGTGGTTTTGGGGCTGCTGCGCAGCCCAGCGGGGGACAAGCCCCCTCGCCACAACAAGCCTCCACCGATAGGCCTC
TTGGCCACTCAGCGCTTGCGCAGGATCACGCTGCCAATCGAATACCCGGCGCCAAACGAGCTGAGTACCGCCAGCG
CGCCCTTGGGCAGGTCGTCCTGGTAGGTGTGGAACGCAATCACCGAACCCGCCGAACTGGTGTTGGCGTAGGTATC
GAGGATCACCGGCGCCTCTTCGATCTCGGCTTCACGGCCCAGCAGTTTCTTGACGATCAGGTGGTTCATGCTCAGG
TTGGCCTGGTGCAGCCAGAAGCGCTTCACGTCGGTCACGCTCAACTGGTTTTCAACCAGGTGCTGGCCGATCAACT
CGGCAACCATCGGGCAGACATCACGGAAGACTTTGCGGCCTTCCTGCACGAAAAGTTTATCTGGCGCACCAATGCC
CTCTTCAGCAGCGCGGTTGAGAAAGCCGAAGTTGTTGCGGATGGTATTGGAGAACTTGGTCAGCAACTTGGTGCTG
ACAACGTCGAACTGGTGCTCGGAAGTGGCCAGGTCGGCACGCTCCAGAATCACCGCGGTGGCCGCATCGCCAAAGA
TGAAGTGGCTGTCGCGGTCACGGAAGTTCAGGTGGCCGGTGCAGATCTCCGGGTTGACCATCAGGATCGCCCGGGC
CTGGCCCAGTTGGATGCTGTTGCAGGCGTTCTGGATGCCGAAGGTCGCCGAAGAACAGGCCACGTTCATGTCAAAA
CCGAACCCCTGGATGCCCAGGGCTTCCTGGACTTCGATGGCGATGGCCGGATAGGCGCGTTGCAGGTTGGAGCAGG
CAACGATCACCCCGTCGATATCGGCCGCAGTCTTGCCGGCGCGCTGCAGGGCTTGCTCGGCGGCGCCCACGGCCAT
CTGGCAGAGGATCGACCACTCGTCGTTGGAGCGCTCTGGCAGGCGCGGGGTCATGCGCTTGGGGTCCAGAATGCCT
TCCTTGTCCATGACAAACCGGCTCTTGATACCCGAAGCCTTTTCGATAAAGGCGGCGCTGGATTCAGTCAACGCCT
GAACGTCACCCTGCTCGATAGCCACGGCGTTGTCGGCATTGAACTGTTGAACGTAAGCATTGAAAGACTGCACCAG
CTCTTCGTTGGAGATGCTGTTGGCCGGGGTGTACAGGCCTGTGCCGCTGATGACGACGTTATGCACGGTCGTTCCT
CTAAATCTGTTCAGGCAGAAGGGATTGGTACCTTCGTACCAAACCGTAAGTCGCTCTAGTCCGCCGCGCGGACATC
AAACGGGGAAACGCTTTGTCCCGGCGCGTCGAGGCTGATACAGCCAATCTAGACGATCCTGGCATTTATAACGGCG
AAGTTTGCCATAAACCGGGGGGTTTGGCGCCTGTCACACGCGCACCTGCCCCCGGGATTGCAGGTCAAGGCTCCAC
CTGGCTCCACTGTTTGCTCAAACGCTTGTCGGAAATCGGTACCTTGGTGCCCAACTGCTGGGCAAACAGCGACACC
CGGTATTCCTCCAGCCACCAGCGGTAAAGCTCCAACTGCGGATCGCGCTTGCCTTCCTGGGTGTGCTTGTTCAGCC
GCGTCTGGTACTGGCTCCACAGGCCGCTCAACTCGCCGCTCCAGACCCGATCCTTCTGCACCTGGCTGGGCAGTTT
TTCCAGGCGCAGTTCGATGGCCTTGAGATAACGCGGCAGTTCCTTGAACCACTGCGCCGGGGTTTCGCGCACAAAG
CCTGGGTACACCAGGTTGCTCAGTTGTTGCTTGATATCGTTGAGTGCCACGGCCTGGGCCAGGTCGATCTTGCCCT
TGAAGCGCTTTTGCAGGCCGTGCCACAGCTTCAATACTTCCAGGGTCAGGCGCGCCAGGCGTTCGGCATGTTCGGT
CCAACTGCCGCGTTTGCGCTCGGCCAGGGCCGCCAAACCAGCGCCATCCCTCGGCAGGCTGGCCTCGCCTTCCAGC
ACGCAGCTGTCGAGGCTGGCCAGCAGAATGTCTTCCACCAGGGCATCGATACGTCCCAGCTCGCGGTACATCAGCC
CCAGCTCGGTAAGACCGGGCAACTTGCCGCGCAGGAATTTTGCCGGCTCGGCCAGTTGCTGCATCAACAGACGCTG
TAAGGCACGGCGATGCTGGAACTCGGCTTCGGCAGCAGTGGAGAAGCGCCCTTCCTTGACCGTCCCGTTTTCCTCC
ACCAACGCCGGATACACCGTCATCGACAGCCCGGCAATCTTTTGCTGGGTGGTCTCGGCCACGGCGGCGAAGACCT
TGGCCTCCACCGGCAACTGGCTTTTGGCAGTCTGCGGCACCGCCAGGGCGGCCTGGCTGGCTTCGACAAAACGTGC
TGTCAGCGCGGCCAGGTCGCGGCCCTCGCCGAGGAACTTGCCCTGGGCGTCCACCACTTCCAGGTTCATTTTCAGA
TGGTTTTCCACCTGCTGCGCGGCTTCGCTCCAGGCTTCATCGCTGACCCGCGCCCCCGTCATGCGCAGCAGCTCGC
GCCCCAGCGCCTGAGGCAGCGAGCCCTGGGCAAATTCGATACGTTGCAGCGCAGCCTTGACGAAGTCCGGCACCGG
CACGAAGTTCTTGCGCAGGGCCTTGGGCAGGTTGCGCACCAGGGCAATGCACTTGGCCTCGATCAGCCCGGGCACC
AGCCACTCCAGGCGCTCGGCAGGCAAGGCCGGCAACAATGGGGCGGGCACGCGCACGGTCACGCCGTCGCGCGGGT

GATTGGGTTCAAAGTGATAACTCAAGGCCAGGGCCAGGTCACCCAGGTGCAGCGTGTCCGGATAATGGGCAGCGGT
GACTTCACTGGCCTCGCGCGCCAGCACGTCTTCTTCGCGCATGATCAGCAGTTGCGGGTCTTTCTGGCTGGTGATC
TTGTACCAACTGTCGAAGGTCGCCGTCTGGTGGATCTCCGCCGGCAGGCGCGCATCGTAGAAGGCGTACAGGGTTT
CTTCGTCGGCCAGGATATCGCGGCGCCGGGCCTTGGCTTCCAGTTCATCCAATTGCTCCAGCAACTGCTGGTTGGC
GGCCAGGCACTTGGCCCGGGACTGGATCTCGCCACGCACCAGGCCTTCACGGATAAACAGCTCGCGCGACACCACC
GGGTCCACCGGCCCGTAATGCACCGGCCGGCGTCCCACCACAATCAGGCCGAACAGGGTGATCTGCTCGAAGGCCA
CGACCTGGCCGCGCTTCTTCTCCCAATGGGGCTCGAAGTGGTTTTTCTTGATCAAGTGCCCGGCCAGGGGCTCGAT
CCAATCGGCATCGATCTTGGCCACCATGCGCGCATACAGCTTGGTGGTTTCCACCAGCTCGGCGGTCATCAACCAT
TGCGGGCGCTTCTTGCCGATCCCCGACGACGGGTGAATCCAGAAGCGCCGCTGACGGGCACCCAGATAGTCGCCCT
CTTCGGTCTTCTGGCCGATCTGGCTCAGCAGGCCGGACAACACCGCCTTGTGCAGTTTCGGGAAATCCGCCGGCTC
CTTGTTGACCGTCAACTGCATGTCACGGCAGATCAGGCTCAACTGGCGGTGGGAATCGCGCCACTCGCGCAGGCGC
AGGTAGTTGAGGAAATTCTTGCGACACCAGTTGCGCAACGGGCTGGCGGTCAATTCCTGGCGCTGCTCTTCAAAGC
CGCGCCACAGGTTGACCAGACCGGCGAAGTCCGAATCGGGATCCTTCCATTGCGCGTGGGCCTGGTCGGCCGCTTG
CTGGCGCTCCGGCGGGCGCTCGCGCGGGTCCTGGATCGACATGGCACTGGCGACGATCAGCACTTCCTGCAAACTG
CCGAGCTTGGCCGCTTCCAGCAACATGCGGCCCATGCGCGGGTCCACCGGCAGGCGCGCCAGTTGCCGGCCCAGGG
GCGTCAGTTGGCTGTTGCGGTCCACCGCCGAGAGTTCCTGCAGCAGGTTGAAGCCATCGCTGATGGCCTTGCCATC
CGGTGGCTCGATAAACGGGAAGTCGGTGATTTCGCCCAGGCGCAGGTGCAGCATCTGCAGGATCACTGCCGCCAGG
TTGGTGCGCAGGATTTCCGGATCGGTAAATTCCGGGCGCCCGATAAAATCTTCTTCGCTGTACAGGCGCACGCAAA
TGCCCGGTTCAACCCGGCCGCAACGCCCCTTACGCTGGTTGGCGCTGGCCTGGGAAACCGCCTCGATCGGCAGGCG
CTGGACCTTGGCGCGGTAGCTGTAGCGGCTGATGCGGGCGGTGCCGCTGTCGATCACGTAGCGGATGCCCGGCACG
GTCAGCGAGGTTTCCGCGACGTTGGTCGCCAGCACCACGCGCCGCCCCGGATGGGACTGAAAAATGCGCTGCTGTT
CGGCCGGCGACAGGCGCGCGTACAACGGCAGGATTTCCGTGTGCTTGAGCTGGGCCTTGCGCAGCATGTCGGCGGC
ATCGCGAATCTCGCGCTCACCGGGCAGGAACACCAGCACATCGCCCGGGCTCTTGCGCTCGCTGCGTTCAAAGGCG
GCAATTTCATCGAGGGTGGCGAGGATCGCCTGGTCCACGGTCAAGTCGTCTTCGACGCGGTTGCCCTCTTCGTCCT
GCTCCAGGGTCAGTGGGCGGTACCAGGTTTCCACCGGGAACGTACGCCCCGAGACCTCGACAATCGGCGCATCGTC
GAAGTGCTTGGAGAAGCGCTCCAGGTCGATGGTGGCCGACGTGATGATGACTTTGAGGTCCGGGCGACGTGGCAGC
AGGGTCTTCAGATAGCCGAGCAGGAAGTCGATGTTCAGGCTGCGCTCATGGGCTTCGTCGACGATGATCGTGTCGT
AGCGCTCGAGGTAGCGGTCGTTCTGGGTCTCGGCCAGCAGGATGCCGTCGGTCATCAGCTTGATCAGGGTGTTGGA
GTCGCTCTGGTCCTCGAACCGCACCTGATAGCCGACCAGCGCGCCCAAAGGCGTCGCCAGTTCTTCGGCAACCCGA
CTCGCCACACTGCGCGCCGCAATACGCCGAGGCTGGGTATGGCCGATCAAGCCGTACTGGCCGCGACCGATTTCCA
GGCAGATTTTCGGCAGCTGCGTGGTTTTACCCGAGCCGGTCTCGCCCGCGATGATCAACACTTGGTGTTTTTGCAG
CGCCGCCTTGATTTCATCGCGCTTGGCGGCGATGGGCAGGCTGTCGTCGTAACGAACCTGCGGCACGCTGGCCGCG
CGCGCCGTGACCTGGGCACAGGACGCCTGCATGCGTGCCACCCACTGCGCCAGCTTCTCCTCATCGGGCTTCTTGC
GCAGCTCAAGCAACTGCCGGCGCAAGCGGTGGCGGTCGGCGAGCATGGCGTGATCGAGGTTCTTCAGGAGTTGGTC
GATAGCGGGCGCTTGGTCAGTCATCAGGTACTTTGCAGGTCGTCTATTTATGCACGGCCGGCAATTGTGGCAGAAA
AGCGCCCGCAACGTTAAAGGCCGCGTAAATCACGTTTGGGAGCTGTCGAGCCCCACCCCAGCGAGGCTGCGATGAC
GGCGGCACTGCCTGCATCAATGTTGCCTGCCCCACCGCTATCGCGGGCAAGCCCGCTCCTACACGGATTAGTGCCC
TTATTCGTTATCCAGGCCCTTGCGCCGGTACGGAAACACATCAATCACTTTCCCCGCCCGAATCGCCTCTTGCAGG
CCTTTCCAGTAGTCGGCGTTGTACAGCTCACCGTGCAACTCATCGAACAAGCGCCGTTGCCCGGCATCCGCAAACA
GGAACGGCGGGAACTCCTCGGGGAATACGTCCAGCGGGCCGATGGAATACCACGGCTCGGAGGCCATTTCATCTTC
CGGGGTTCGGGGTGCCGGGATATGGCGGAAGTTGGCTTCGGTGAGGAAGCAGATCTCGTCATAGTCGTAGAACACC
ACGCGACCGTGGCGGGTGACGCCAAAGTTTTTCAGCAGCATGTCGCCGGGAAAGATATTCGCCGCCGCCAGCTGTT
TGATTGCCAGGCCGTAATCTTCGAGGGCTTCGCGCACCTGGGCCTCATTGGCGTTTTCCAGGTAGAGGTTGAGCGG
GGTCATGCGCCGTTCGGTCCAGCAGTGACGAATCAGCACCGTGTCACCTTCCACCTCGACCGTGCCCGCAGCCACT
TCCAGCAGTTCGGCCAGGCATTGGGGCTCGAATTTGCTCAACGGGAAACGGAAGTCGGCGAACTCCTGGGTATCAG
CCATGCGCCCGACCCGGTCGACACTTTTGACCAGGCGGTACTTCTCGATAACCGTCGCACGGTTGACGTTCTTCGA
CGGCGAGAAACGGTCCTTGATGATTTTGAACACCGTGTTGAACCCCGGCAGGGTGAACACGCTCATGACCATCCCC
CGCACGCCCGGGGCCATGATGAACTGGTCGTCGGTGCTGGCCAGGTGGTTGATCAGCGCCCGGTAGAACTCTGACT
TGCCGTGTTTGTAGAAGCCGATGGAGGTGTACAGCTCGGCGATGTGCTTGCCCGGCAGGATGCGTTTGAGAAAACC
GATGAATTCCGCCGGTACCGGCACATCCACCATGAAATAGGAGCGGGTGAACGAGAAGATGATCGACACATCGGCT
TCGTCAGTGATCAGCGCGTCGATGCGGATGCCCTGCCCTTCGCGATGCAGCAGCGGGATCACCAGCGGCCACTGTT
CGTCGCGGGTATAGAGACGACCCACCAGGTATGCGCCTTTGTTGCGATAGAGCACCGAAGAAAACAGCTCGACGTT
GAGGTCCGGGTCCTTGCAGACCCAATCCGGCAGGCTCTCGCGCAGTTGCGCTTCGAGGCGCTGCAGGTCGGCGGCC
AGGTCGGCGTAGGGCTCGCTGAAGCGGTAGTCGCTGAAAATCTGCTCGAGCATGCCCGCCAGTTGGCCCTGGGGCT
TGTAGGTGCGGGTTTGCGCAGCGCGGGCGCGACGCAGGCTCGGGCGAGTGGTGTGGATGAACATGCAGCCGTCGCT
GATCAGGTCGTGGCTGAACAGCCCGCAAAAGATCGAGTTGTACCAGGTTTCGGACAGCTCATCGTCAAACCGCAGG
TCGATCAGGCTGATATAGGCGCTCTTGACCAGTGGCCAGGACTTGATATCCAGCAACGCGCCTGCGTCAAAGGTTG

CCCGCAGGCGCGTGGTGACTTCACCGACCTTTTCTTCGTACAGGTTGATCCGCGCCGCCGAGGCGGTTTGCGCCTC
CTGCCACTGGGCCTTCTCAAAGCGCTCGCGGGCGCCGTCGGTGATCTGGCGAAAATGCTCGCGGTAATCGTCGAAG
CCATCAAGGATCATCTGTGCGATGGACTGCGCGGTGTCGATGGCAGGCGTTTGCTGCGGCATGTCGGAGACCTCTG
CGGGCGTTCGGAAGCCCTGAGATTAGCCAGAGTCCGCAAGCAGGAGAAGTACAATTTTCGCCGGGCGATGCCACGC
GGATCGGTGCGCGACAAACAAATGTTTACGATTTTTTAATTCGACCATCCGGTCAATAAAACACCCCAAAGTCACG
TCGCGTCGATGAATCGATTCACCTGAAGGCCGCAAAAGCCGGGCGCTGGCACCAAAAGCAGGGAACAACTCAGGCC
GGCGTGGGCTCTGCGGCCTTATACAAACGTGGATCCAATGTAAGGGAAACCCTGATTACCTCAGTGATGGCACTTT
GCTATATAGCCACCCCCTTCCCTGATAACAAGGTCAAAAGACCGGCTCGGGAACGGCTTTAAATCGCCCAAGGAGC
ATTGCAATGTCATTGAGGAATATGGGGATCGGCCTGCGCGCCAGTCTGAGTTTTGCGGTATTGGCCGGCCTGCTGG
TGCTGGTCGGGTTGTTTGGCCTGGGGCAGATGGCCAAGCTGCGCGAAAGTGCCGCCATCATTGAAACGGCTTGGAT
GCCGAGCATCGAGAACATCCACGACAGCGCCGCCAACGTCGCGGCGATCCGCCTGGAGGCGCTGCGCTTGTTGACC
ACCGATGAGTCGGCGGTGCGCGAGCGCAGCAAAGGTTTGATCGCCAAGGAGCGCGCGCAGTTGCAGGAGCGCCTGG
ACAATCACCAGGCCCTGTTGAGCAATGATCAGGAGCGTCAGCTACTGGACCAGCTCAAAACCGCCGCGGCGGGTTA
TATGACCATCGTCGACCAGTTGATCCAACGGGTAGACCAGGATGAGCAGATGCAGGCCCTGACCTTGCTCACCGAC
AAGTTGACGCCCCAGGGCGTGATCCTCGGCCGGAGCCTGGACGCGTTGATCGACTTCAACCAGCAAGGCGCGCAGG
ATGCGGCGCAATCGGCGGCGCAGGTGTATCAGAGTGCACAGTGGATCGTCGGCGTCATCATCGCAGTCGCCCTGAT
CGCGACGTTGCTACTGGCCTGGCTGTTGACCCGCAGTATCACCACGCCCCTGGCGCAGGCGTTGCGCGCGGCACGT
ACGATTGCCTCCGGCGACCTGAGCCAGCCGATTCACGTGCAGGGCAAGGACGAACCGGCACAGTTGCTGGGCGCCC
TGGCCACCATGCAGGAGCAATTGCAGGCGACCATTCGCGGTATCAGCGAATCCGCCCAGCAATTGGCCTCGGCCGC
CGAAGAGATGAGCTCGGTGATGGAGCAAAGCACTCGCGGCCTGCAAGCCCAGAACGATGAAATCGAGCAGGCCGCC
ACCGCCGTCACGCAAATGAGCGCGGCAGTGGATGAGGTAGCGGGCAATGCGGTCTCCAGCGCCGAAGCCTCCCAGG
CGTCCGATGAAGACAGCAAGCACGGGCACTATCAGATCAGCGAAACCATCAGCTCGATCCAGAACCTGGTCAACGA
GGTGCTCGACGCCTCGGGCAAGGCCGAGGGCCTGGCAGTGCAGGCGCAAGACATCAGCAAGGTGCTGGAAGTGATA
CGCGGGATTGCCGGGCAGACCAACCTGCTTGCGCTCAACGCCGCCATTGAAGCCGCCCGCGCTGGCGAGGCCGGGC
GGGGCTTTGCGGTGGTGGCCGACGAGGTACGTTCCCTGGCACAACGGACCCAGGACTCCACCGAAGAAATCGAGCA
GATGATCACCGGTATCCAGCAAGGCACGCAGGACACGGTCGGCGCCCTCACCAGCAGCGCCGAGCACGCCAGCCAG
ACGTTGCAACGGGCCAACAGCGCAGGCAGCGCCCTGGAAAAAATCACCGCCGCCATTTCGCAGATCAACCAGCGCA
ACCTGGTGATCGCCAGTGCTGCCGAGCAACAGGCGTCGGTGGCCCGGGAGGTGGATCGGAGCCTGGTGAACATCCG
CGACCTGTCGACCCAGACCGCCGCCGGGGCGACCCAGACCTCGGCCGCCAGCCAGGAGCTGTCGCGCCTGGCGGTG
GACCTCAATGGTTTGGTGACGCGCTTTATTGTGTAGTGGATACGTAAAAGATTGTGTCAGACAGGTCCCTGGGCAA
CACTCTCGCCCTGATTAAAAAGGAGCTGTCCGTGAGACCTGTCGACACCCTGTACCTGCTGGTACTGGCCGCCATC
TGGGGCGCGAGCTTTCTGTTCATGCGGATTATCGCCCCCGAGATCGGCACGATTCCTACAGCATTCTTCCGGGTGT
CAATTGCCGCAGCCGGTTTGCTGGTGATCCTGGCAATCCTGCGGGTGAGCTGGGATTTTCGCGGTAAATTCAAAAC
CGTGCTGTTGCTTGGGGTGATCAACTCCGGGATCCCTGCGACCATGTATTCGGTGGCGGCGCAGGTCTTGCCCGCA
GGTTATTCGGCGATTTTCAACGCGACGACGCCGTTGATGGGCGTATTGATTGGCGGGCTGTTTTTCCATGAACGGC
TGACGCTGTCGAAGATTACCGGAGTAGGCCTGGGTTTGTTCGGCGTGGGCATCCTCACGCGGGCCGGGCCGGTGGC
CTTTGACCTGGAACTGCTGATGGGCGCCCTCGCCTGCCTGCTGGCAACCACCTGCTACGGCTTCGCCGGCTTCCTG
GCGCGCCGCTGGCTGGATCAACAGGGTGGCCTGGACAGCCGTCTTTCGGCCTTGGGCAGCATGCTCGGTGCCACAT
TGTTTCTATTGCCTTGGTTTGGCTGGAGCGCCATCAGCCATCCGCCCGCCAGTTGGGGTGGCTGGAATGTGTGGTT
GTCGCTGCTCGGTTTGGGGCTGTTGTGTACGGCGTTTGCCTACATCCTGTACTTCCGCCTGCTGACATCCATCGGG
CCGGTGAAGTCGATGACCGTTACCTTCATGATTCCGCCGTTCGGCGTGTTGTGGGGCGCATTGCTGCTGGATGAAC
CGTTGTCCATGGCCCATCTGTATGGCGGGATGTTGATCGCGGGGGCGCTGTGGCTGGTGCTACGCCCAGGGAAACT
GATCAAGACCGTGTAGGAGCCGGCTTGCCGGCGCCTACACGGGGTATCAGGCTTTACGGAAAACGAACACCAAGCC
GACGATGATCAACCCCATCCCCAGCACACTCAGCAGCGCCAGCCGATTGCCAAAGATCAGGTAGTCCATCACCGCA
GTCACCGCTGGCACCAGGTAGAACAGGCTGGTGACATTCACCAGGTTGCCCCGGGCGATCAGGCGGTACAGCAGCA
GCGTCGCCAACAGTGACACCACCAGCCCCATCCACAACACCGGCAGGTAAAAGCGGCTGCTGTGCTCGAAATGAAA
AGGCTGGAACGGCACGAACACTGCGCACAACAACAAGCCCGCCAGGTACTGCACTGGCAAGGTGCCCAATGGGTTC
TCGGTAATGCGCTTCTGCATGATCGAGCCCAGGGTCATGCTCGCCAGCGCCAGCAAGCCAAACAGCATGCCGGCCA
GGGACATGCCGGCCAGACCGATGCCCTGGTACACCACCATGATCAGCCCCACCAACCCCAGCGCCAGGCCGAATAT
CCGACGCCATGAACGCTGGCGCTCCATCAACACCACGGTGAGGATCGGTTGCACGCCCATGATGGTTGCCATCACA
CCAGGGGTGATCTTCAAGTCCAGGGCCAGCAGATAGAAAATCTGATAGGCCCCCAGCAGCACCAGCCCGGTGGCCA
CCGCATACCACAGCGGTTTGCCCGGGCGCGGCAACCTGAGCTTGAGGATCGGTACCAGCACCACCAGGCCCGCCAG
GGCAATGGCAAAGCGGATCAACAGGAAGGCAAAGGGTGATGCATGAGCCAGGCCCAGCTTGGAGAAAATCGCCCCG
CTGCTCCACAGCAGGACAAACAGGCTCGTCGAGGCCGCCGCGGCCACGGATTGTTTCGAAAGAACAGACATGATGA
ACACCTGTAGTCAGGCAAAAAGCCAACTCAGCCAAGGCTGAAATTCAGTAGGTTCGGTACAGGCAGGCAACGGGCA
ACAGTGATCTGCTGATCAGCCCGAAATGCCAACGCCCGGCGGTGATACGACTGCGTACACCGGCGTGCTACTGACA
GGTGGAGGGTAATGACTGATCTGCGCAGGCTGCTGGCTCCCACACGGCACGACCGCAGCGTTGACCGCTGAATCGA

CTACCGCTATGCGTGGGGAAGCAGGCATGGGCTGATCTTTTTTGAAGGGATTAAAGGTGCTGTGAAACACCTGGCG
CCGACTATAACCAGCCGGTGACACGCTTTGCAATGGCTTTGCCAAAAGGCCAGTAAGGACAAGTTGAAAAACCACC
TCTAGCCTTGGGAAAAACCAACTCACTCCTCTTCAGGAGGCTTTCGACTCATGACCGCCAATGCCAAAAACACGCG
ACTCCTTGCGATACCAGCTCTACCGATCCAAAGCTTTACGCCTTGCGAATGCATCGACTCAACAGTGGTACAAAAA
CCTGAAACACACCGACGCCCAAGGCGCAGAGATCGACTTGACCCATGTCCGTTTTGAATTGATCGACAGCCATGCA
TTGGCTGCCTATCAGGACTGGGCAGAAGACTCTCACTTCTCATGGGACGAGGTTGCGACATGGAAAGGCCGTGAAC
CGATGGCGTTCGATTTGTCCCTCTGGTACGACGAGGTGCTCTGTGGGCTGTGCTTTGCCAATCCCAATCAAAGCCG
ATTGCGGATCAAGGTCATCCGCCTGGAGGGAAGACCTGGAAAATCCCACCCTTTGAAAAGCCGCATAGCATCACTG
ACGATGATTGCCATCGACCATTACGCGAGAATCATTGGCAGCCAATGGATAGAAATTCAGGAGCCCGCACCCGGGG
CTATTCCCCTCTATCAAAACCTGGGCTTTGACTTCGATTCACGGGGCAGGCTTGTGATAGCGGTAGAGAACGAATA
GCATCGCAAACATGTTCAATTAATGATCAATCGTGGAGGCTGCATGGGAGAACTTATTAAAAAACCCGTCGCCCGA
AAAGCGTCGTCCAAGGCCGGCCAGGAACCTAAAAGCACTGTGCTGACCGCCAATCAGCGCCGTTTTCTCAGCGCGG
CTATGGCCGGCAACGTTGAAGAACCAAGCCTTTTGGCGGGTCACCCGGAAAAATAAAAGTCATAAAAAAACCGCTC
ATACGAGCGGTTTTTTTGAACCCATCAGCCAAACAACCAATACACCAACGCCCCCACCACCACCGCCGCCAATACC
GGCCGCATGACGCGATAGGCTTTGGGGTGCTTGCGCTTCCACTGCTTGACCACACCACTGAAGCGATCGCTGAAGC
TCTTGCTCCAGGCATAGGCCTGGTTGATCCCGCCCACGCGCTCGTCGTCCAGGTTCTGTGGCGCAGTCGCGCGGCC
CAACTGGGCACTGACCCAGCGGTTGATGCGGGTCATCAATCGGTTGCTCAGGGGGCGTTCGATATCGCAGAACAGA
ATCACCCGGGTCTTCTCGGTTTCGTTCTTCACCCAGTGCACGTAGGTTTCGTCGAACATCACATCTTCGCCATCGC
GCCAGGCGTAGATCTGGCCATCGACGAAAATGCGGCAGTCGTCGGAGTTGGGCGTCGACAACCCCAAGTGATAGCG
CAGGGACCCGGCAAACGGGTCGCGATGGGGATTGAGGTGGCTGCCGCCCGGCAACAGGGCAAACATGGCGCCCTTG
ACGTTGGGGATGCTGCTGACCAGGGCCACGGTTTTTGGGCACAGCAACTCGGCCGACGGCAGTGGTTTGTCGTACC
ACTTGAGGTAGAAGCGCTTCCAACCCTTCTTGAAGAACGAGCCAAAACCGGCATCGTTGTTCTTCTCGGCGGCGCG
GATATAGCCTTCGTCAAACAGGTGCATGGCCTCGTCGCGGATCACTTCCCAGTTGTCCTTGAGCACATCCAGTTCC
GGGAACTTGCTGCGGTCCAGGTACGGCTTGGACGGCACAGCCGAAAAGATGTACATCAAGGCGTTGTACGGGGCGA
ACAGTGCCGAATGGTTGACGAACTGGCGCAGCACCGGCAAACGCGCCTTGCCGCGCAAATGCACGTAAAGCGTACT
GCCGATAAACAACAGCAGCACCGACAGCTTGGCGGCCAAAGAAAAGGTCATGCAGCAACTCCTGGAAATAGCCTCC
CGGCAGCGGGAAACCCGACATAGCAGCCGGCCATGATAAACACTCCAGGCCGGAGGAAAAACCCGCGTTATCCAAC
AATACGTTTTAAGGGGTGCGCGGCGAGTAAACAATTATGCATAAAGGGCATCAAACCGGGCTGATTTGGATCAACC
CGATTTCTGTAGGAGCGAGCGTGTCTGCAAGGGGCATTCAGGGTCTTCGTGGGCAAGCTCGCTCCTACAGCGTCAG
GCCTGGTTTTCCTGGTCGGTGAACAGATCGCTGAACAGCATGCTCGACAGGTAACGCTCGCCGGAGTCCGGCAGGA
TTACCACAATGGTCTTGCCCTGCATTTCTGGTTTCTCCGCCAGGCGTACCGCGACGGCCATCGCCGCACCACAGGA
AATCCCGCACAGGATGCCTTCTTCCTGCATCAACCGCAGCGCCATGGCCTTGGACTCATCGTCACTGACCAACTCC
ACCCGGTCGACCATCGACAGGTCGAGGTTCTTCGGCACAAACCCTGCGCCGATCCCCTGGATCTTGTGGGGGCTGG
GCTTGATTTCCTCACCGGCCAACGCCTGGGTAATCACCGGCGAGCTGATGGGCTCCACGGCCACCGAGAGGATCGG
TTTGCCCGCTGTATTCTTGATGTAACGCGACACACCGGTGATGGTCCCACCCGTGCCGACACCCGCTACCAGCACA
TCCACGGCGCCATCGGTGTCGTTCCAGATTTCCGGGCCGGTGGTTTTTTCATGGATGGCCGGGTTGGCCGGGTTGT
CGAACTGGGACGGCATGAAGTAGGTCGCCGGGTCGCTGGCAAGGATTTCGCCAGCTTTCTCGATCGCGCCCTTCAT
GCCCTTGGCCGGCTCGGTCAGCACCAGTTCAGCGCCCAGGGCCTTGAGCACCTTGCGCCGCTCGATGCTCATGGAG
GCAGGCATGGTCAGCAGCAACTTGTAGCCACGGGCAGCGGCGACAAAGGCCAGGCCAATCCCGGTGTTACCCGAAG
TCGGCTCGACGATGGTCATGCCAGGCTTGAGTTTGCCGCTGCTTTCCGCGTCCCAGATCATGTTCGCGCCGATCCG
GCACTTCACCGAATACCCAGGGTTACGCCCTTCGATCTTGGCCAGGATGGTCACGCCACGGGGCGCGATGCGGTTG
ATCTGAACCAGGGGCGTGTTACCGATGGAGTGCGCGTTGTCTGCAAAAATGCGGCTCATGGCGGGGTCCTTTAGGC
GGTTTCAAGAAGGCAACAAGGGTATGCCTCGTACTCCAAGGCGTCCAGTCGAGGAACGTTGGCGCTGCCTGCGTAG
TCAACCGCCTATACACAGGGAGGAAAAACCTATGAAGCGTCGCTACAGTTGGCCGATATGGACGGTCGCCGGGCTG
GTGGTCGTGCTGGTGGCACTGGATATCGCCCTGCCCTACCTGGTGCGCAACTACCTCAACGAAAAACTCGCCGATA
TGGGCGACTACCGTGGCCAAGTGACTGATGTCGACCTGGCGCTATGGCGCGGCGCGTACCGGATCAACGGCCTGCA
GATCGTCAAGGTCGATGGCAAGGTGCCCGTACCGTTCGTCAAGGCCCCGGTGATCGATCTGTCGGTCAGCTGGCAT
TCCTTATGGTACGACCATGCGGTTGTAGCCGAAGTGCAATTCGTCGAGCCAGAACTCAACTTCGTCGACGGCGGCC
CCAACAAACAGGCGTCCCAGACCGGTCGCGGCACCGACTGGCGGGCACAACTGGGCAAGTTGCTGCCCATCACCCT
CAACGAAGTGCGCATCGACGACGGCAAGATCGCCTTTCACAACTTCAATTCAAAGCCGCCGGTCAACATCGGCGCC
ACCCAGGTCAACGCCAGCCTCTATAACCTGACCAATGTGGTGGACGTCGAAGGCAAGCGCGATGCGCGCTTTGACG
GCAAGGCCATGTTGCTGGGGCATGCACCACTGGAAGCCAGCGCGACTTTCGACCCGCTGAGCAACTTTGAAGACTT
CGAGTTCCGCTTCCGCGCCCGTGATATCCAGCTCAAGCGCATGAACGACTTCGCCTCGGCCTATGGCAAGTTCGAC
TTCAAAGCCGGCACCGGCGACGTGGTCATCGAGGCCCAGGCCGAAAAAGCCCAACTGACCGGTTATATCAAGCCAC
TGTTGCGGGACGTGGAAGTCTTCGACTGGCAGCAGGATGTGGAGAACAAGGACAAGGGGATTTTCCGCTCGATCTG
GGAAGCCGTGGTCGGCGCCAGCGAAACCGTCCTGAAGAACCAGAGCAAAAACCAGTTCGCCACCCGAGTCGAGCTC
AGTGGCAGCGTGCACCAGCAAAATATCAGCGCATTCCAGGCGTTTTTGCAGATTTTGCGCAATGGTTTCGTCCAGG

CGTTCAATGCCCGCTACGAACAACCCAAGCCCAGCGCGGATTGACCGTGGAGTGACTGGTCATTCAGAGACTGAAT
AACCCGTCTGCGTTCACAGTCACCGGGGCTGCGCGCTATAGTCGCAGGCATGCTGACTACCGGCGTCATGTTCGAG
GATTGAGAAATGAAGTTCGAAGGCACCCAGGCCTACGTTGCCACCGATGACCTGAAACTGGCGGTCAACGCCGCCA
TCACATTGGAGCGCCCGCTGCTGGTCAAGGGCGAGCCGGGCACCGGTAAGACCATGCTCGCCGAACAACTGGCCGA
ATCGTTCGGCGCACGCTTGATTACCTGGCACATCAAGTCCACCACCAAGGCGCACCAGGGCCTGTATGAATACGAT
GCGGTCAGCCGCCTGCGCGACTCGCAGCTGGGCGTGGACAAGGTCCATGACGTGCGCAACTACCTCAAGAAAGGCA
AGCTGTGGGAGGCTTTCGAGTCCGAAGAGCGGGTGATCCTGCTGATCGACGAGATCGACAAGGCCGACATCGAGTT
CCCCAACGACCTGTTGCAAGAACTCGACAAGATGGAGTTCTACGTCTACGAAATCGACGAGACCATCAAGGCCAAG
AAGCGCCCGATCATCATCATTACCTCCAACAACGAGAAAGAGCTGCCGGATGCGTTCCTGCGTCGCTGCTTCTTCC
ACTACATCGCCTTCCCCGACCGCACCACCCTGCAGAAAATCGTCGACGTGCACTACCCGGACATCAAGAAAGACCT
GGTCAGCGAAGCGCTGGACGTGTTCTTCGACGTGCGCAAGGTGCCGGGCCTGAAGAAAAAACCTTCCACCTCCGAA
CTGGTGGACTGGCTCAAGCTGCTGATGGCCGACAACATCGGCGAAGCGGTGTTGCGCGAGCGTGATCCGACCAAGG
CCATCCCGCCACTGGCCGGCGCCCTGGTGAAAAACGAGCAGGATGTGCAACTGCTTGAGCGCCTGGCGTTCATGAG
CCGTCGCGGCACTCGATAAGGGCTTTTGCCATGCTGCTCAACCTGTTCAATGAAATGCGTGCAGCCAAGGTGCCGG
TCTCGGTGCGTGAGCTGTTGGACTTGATCAACGCCCTGAAACAACGGGTGACCTTCGCCGACATGGACGAGTTCTA
CTACTTGTCCCGGGCGATCCTGGTGAAAGACGAGCGCCATTTCGACAAGTTCGACCGTGCCTTCGGCGCCTACTTC
AACGGCCTGGAAAAACTCGACGATCACTTGCAGGCGCTGATTCCCGAAGATTGGCTGCGCAAGGAGTTCGAGCGTT
CGCTGAGCGATGAAGAGCGCGCGCAGATCCAATCTTTGGGCGGCCTGGACAAGCTGATCGAAGAGTTCAAGAAACG
CCTGGAAGAACAAAAGGAACGCCACGCCGGCGGCAACAAATGGATCGGCACCGGCGGCACCAGCCCGTTCGGTTCC
GGCGGCTTCAACCCCGAGGGCATTCGCGTGGGCGATGCCGGCAAGCGCCAGGGCAAGGCGGTCAAGGTCTGGGATC
AACGCGAGTACAAGAACCTCGATGATTCGGTGGAGCTGGGCACGCGCAATATCAAGGTGGCGCTGCGCCGCCTGCG
CAAATTTGCACGCCAGGGTGCGGCCGAAGAGCTGGATATCGACGGCACCATCGACCACACCGCCCGCGACGCTGGC
CTGCTGAATATCCAGATGCGCCCGGAACGGCGCAACACGGTCAAATTGCTGTTGCTGTTCGACATCGGCGGCTCGA
TGGATGCCCACGTGAAGATCTGCGAGGAGCTGTTTTCGGCCTGCAAGACCGAGTTCAAGCACCTGGAGTACTTCTA
CTTCCACAACTTCATTTATGAATCAGTGTGGAAGAACAACATGCGCCGCACCTCCGAACGCACCTCGACCCAGGAC
CTGCTGCACAAGTACGGCGCTGACTACAAGGTGATCTTTATCGGCGATGCGGCCATGGCGCCTTACGAAATCACCC
AGGCCGGTGGCAGCGTGGAGCACTGGAACGAGGAGCCGGGGTATGTGTGGATGCAGCGGTTCATGGAGAAGTACAA
GAAGCTGATCTGGATTAACCCGTACCCGAAAGATACCTGGGGCTATACCTCGTCGACCAATATCGTGCGGGATTTG
ATTGAGGACCAGATGTATCCGCTGACCTTGCGGGGATTGGAAGAAGGGATGCGCTTTCTCTCTAAATAGGTGGTGA
ATTCACTGACGCTATCGCAGGCAAGCCAGCTCCCACATGGAATGCATTCACATACTTTACTGTGTGAACGCAGTTT
AAATGTGGGAGCTGGCTTGCCTGCGATGCCTTTAGCGGTTCATAAATCGCTGCAAATAGCTCAGATGCTCCCGGTG
CGCCGTCTCCTGCATCACCGGTGCCAACCGCACCTTCTCCCCCGGCAGGCACTGGGCCAGGCGCGCCAATGACAAC
GGCGTCAACGCACCCAATCGCGGGTAGCCGCCAATGGTCTGCCGGTCATTGAGCAACACAATCGGCTGCCCATCCG
GCGGTACCTGGATTGCGCCCAAGGGGATGCCTTCGGAGATCAGCGACGCGCCCTGATACGCCAGCGGCGTGCCCAG
CAGGCGCATGCCCATGCGGTCGGCGCGGCTGTCCAGGGCCCATTCGGTGTTAAACGCATCGAACAGGCTCTGGCCG
CTGAACTGGCCGATCTGCGCACCGAGAATCACGTCCAGGGGCGCCGCTGGGTGTAATTGTTCCACAGGCAACTCAC
GCAAATTGCTGCTCGTGCCGCAATAGGCAAGCGTAGCGCCTTCAGCCAGGGCCCGGCCAAACCCGTCCAAACCGCC
CAAGGCCTCGCGGCCAACACTGGCACAACTGCCCAGCACCTTGGGCGCATCAAAACCTCCCGGCGCCGCCAGGTAA
GCCCGTGCGCCACGGTGTGGTTGGGTGAAGCGCAAACGCTGGCCTTTTTGCAGAATGAAACTACGCCCTGGGCTGA
CGGCACGCTCGTCGATGTACGCCCCCAGGTCAGCACCGGCCAGTGCCAGCACGCAATAGCCTTGGGCCTGGACGGT
AAAACCGCCCAAGGTGATCTCCACCACCGGCGCATCCAGTGGATTACCCAGCAGCCAATTGGCCCAGGACATCGAC
ACCCAATCCAGCGCGCCACCCTGGGTCACGCCCAGATGCCGCACACCAAAACGCCCGGCGTCCTGCAACAGGCACA
GCGCGGTGCTGGCCTCGATCAACAAGCGGCTCATGCCTGGGCCTCCAAGGGCGTGTCATCGCCACCCAGGTTGATA
AATTCAGCGCGGCCGATGGCTTCGAAACGCACACTGTCGCCTGGCTGCATGAGGCTGTAGCCATCGCGCTCGCGGT
CGAACAGTTTGGCCGGGGTGCGGCCGATCAGGTTCCAGCCCCCTGGGGACTCCACTGGGTAGGCTGCGGTCTGCCG
CTCGGCGATGCCGACGCTACCCGGCGCCACGCGTTTGCGCGGGGTGTTCAGGCGTGGCGCGGCGAGCTGTTCTTCG
ACCAGGCCCATAAAAGCAAAACCTGGGGCAAAACCGAGGGCGAAAACCTGATAGTGATGCTGGCTGTGGCGCCGAA
TCACCTCGTCGACCGCAAGCCCGCTGCGCTGGGCGAGCAAGGTCAGTTCAGGGCCGACGCTCAGGTCGTACCACAC
CGGCAACACGTGACAGTGCCCGCTGCCCTGTGCCTGTGGCTGCAGGTCGGTAAGCGCCTGATCGATCAGCTCCCGT
GCCTGTGCCGGACTCAGCGCCAGCAGGTCGTAATGCACCATCAGCGTCGTGTAGGAAGGCACCAGGTCCACCAACA
CCGAGCCAAAGCCCTCGCGCAGGCGGCGCGTGGCGGCGAGCATCCAGGGCATGTTGTCTTCGGCTATTGCATCAAA
CAGGCGCACCATCAGGCAGTCGATGGCCACTACTTCAATCCGTGGCTTCATGGTGGCGTCAACGCGTGGCGAATGC
GCTGCACCGCCGCCACCGAGCTGGCGTTATCGCCATGCACGCAAAGGGTGTCGGCCTGCAACAGCAAGGGGCTGCC
ATCGCTGGCAACCAGGGTTTCGCCACGGGCAATGGTCAGGGCCTGCTGGAGGATGACCTGCGCATCATGATGAACG
GCCCCGGGCAACTGGCGCGAGACCAGGTGCCCGGCGTTGTCATAGGCGCGGTCGGCAAAGGCTTCGAACCACAGGG
TCACGCCATACTCATCACCCAGGGCCTGGGCGGCGCTGTTGTCGCGGGTAGCCATGAGCATCAACGGCAGCCTCGC
GTCGTACGCCGCCACGGCCTGGATCACCGCGCGCAGTTGCGCAGGCTTGGCCATCATGTCGTTGTACATCGCGCCA

TGGGGCTTGACGTAGCTGACCCGGCCACCCTGGGCCCGGCAGATACCGTCGAGGGCACCGATCTGGTAATGCAGCA
GGTCCTGGAGTTCCTCGGCCCCATACGCCATGGAGCGGCGGCCAAAGCCTGCCAGGTCCTGATAGGCCGGGTGCGC
ACCGATCTGCACGCCATGCTGCAATGCCAGGGCGACGGTCTTGCGCATGATGCTCGGGTCGCCGGCGTGGAAGCCG
CAGGCAATATTGGCGCAATCAATGAATGGCATGACCTCGGCATCCAGACCGATGGTCCAGGCGCCAAAGCTTTCGC
CGATGTCGCAATTCAGTAGCAGGCGGCTCACGGTGAAGACTCCTGTAGGCTTTATTTTTTTGTAGTGTGGGATCTT
TTACCAAGATCCCGCAACGATGTTGTCCTGAGCCCCGACGACGCTTATCGTGAGACAGCCCGATTTTTGGCGCGCA
GCCCCTGCTGCGTCCAACTAATAAAAACCGATCCATGCATAAGAAAAAGTTGATCCCATGAATTTGAAGTTCCTCG
AAACCTTCGTCTGGGTAGCCCGGCTCAAGAGCTTCCGGCTGACGGCAGAGAAGCTGTTCACCACCCAGGCCTCGAT
CTCCAGCCGCATTGCGGTACTGGAAAGCGAGCTGGGGGTGAAACTGTTCCTGCGTGACTCACGAGGGGTGAGCCTG
ACCCCGGATGGGATTAAGGTGCTCGATTATGCCGAGCAGATGATGGTCACCATGCAGGGGCTCAAGCAGTCGCTGG
AAACCACCAGCAGCAAGGTCGGACGCATCCGCATCGGCGCGATGGACACGGTGATCCATACCTGGCTCAGCCCGCT
GGTGACCGAACTGATGGACCACTTCCCCCTGGTGGAAATCGAGCTGGTCGCCGATACCGCGCTCAACCTCAGCGAT
CAGCTGCAAAAAGGCTTTCTCGATCTGATCCTGCAAACCGACCTGCTACGCCAGGAATCGGTGCGCAGCCTGGAAC
TGGCCAGCCACCCCATGGGCTGGATCGTCGCCAGCCATAGCATCTACAACCGTGACTACGCCTCCCTGGCCGAGTT
GGCCCAAGAGCGGATCATCACCTATTCGAAAAACTCCCACCCGCACCAGGACGTGCTGAGCCTGATGCAGGCCCAT
GGCGTGGCGGCGCCGCGTATGAACTGTGTGAATTCGGTGTCGGCCATCACCCGCTTGCTGCGCGACGGCTTCGGTA
TCGGCGCGCTGCCGCCGGTGCTGGTCAGCGAAGAGTTGGCGCGGGGCGAACTGGTCATGCTGCCCATGACGCAACG
CTTACCAAACCTGCAGGTGGTGGTGTCGTGGCGGGTGGGCGTGGAGTTGGTGGAGGAGATTGTTGCGTTGTGCCAG
AGCGTCGTGGCGCGGTATGCCGAGGAAGTGGGTGAAGAACGCATGGTGCTCAGTCACCCACCCTCACTGAAGAAAC
CCGATTGAAACGGCAGGGGCTGGATTGGGTGGGAGCTGGCTTGCCTGCGATAGCCTCACCGCGGTGCAACTGTTGT
ACCGAGGCACATTTTTGCCGCAACGCGCAAACCTAAGCAGATAGAGCCCTTGTGGGGTGCAGGCCAGTGCCTACAC
AAACCCCGTTCCACATTTGGATTTACAAACCCTTGAGGTCCCGCTCTTCAATCGGTCGGCTCTGGCGCAGACGCTT
GCCACCCAGTACCACCCAGTCGATCAGGCGGAACAGGCATTCCAGGCCGAACGACAACAGCATCGCCCCGCCCAGG
CCCCAGCCCATGGCTTCGGGGGTCAGGAGGATCTGGTAGCTGTAGCCATTCCAGGTCTCCTTGCGGATATCCGGGT
CGGCGGCCACCGCCACCTGCAAGGCGCGGATGTACCACGGCCCCTGCATGGCCTGGAACTGCTTGTCCAACGCCAC
CTGGCGATCGAGCAAGGTGCCAAGGCTGTTGGCATCGCTGCGGAACACCGGATCCTCACTGGCGCGGTAATGCGCC
ACCAATGCCTGCAAATCGCCATTGAAGAACTGCTGCGCAGTGCTTTCAAACCCGCGCAGGCCGGTCTGGGCTTCGA
TCAAATGGGCTTCGACGCGCTTGGCGTAGTCGTTGATAAACCCCGGGACTTGCACCCCGACCAACAAGCCGATGGC
AAACAACACCAGGCGCAGATAGCTGAGCAACATAATCGACGTCCTTATGCGGTTGTACCCTGGCGCACGCATTCAC
CGCGCCGCCACAGGCTCCACTGGCCCGGTTCGTAGCGGGTCCAGGTCTCGTTTTCGGTCAAGGGTTCGGTGGCAAT
CACCGTTACTACATCGTTTGGCGTGGTTTCGGCCTGAAAATCGACGATCACGTCGACATCTTTCAGGCGCGCAGGG
CCAAATGGTGCGCGACGGGTGATCTGCGCCAGTTTGGTCGAGCAGTAGCAGAACAGCCAGTCACCATCACTGAGCA
GGCAGTTGAACACGCCTTTGCTGCGGTATTCGGCGCAGGCGGCGATCAGGTCCGGCAGCACTTGCTCGATATCCAC
CGGCTCGGGAAACGCCTCACGAATACGGTTGAGCAGGTCACAGAACGCCGCTTCGCTGTCGGTATCGCCTACCGGC
CGGTAGAACGTGGCCTGGGGCGTGAAGTCGGCCAACTGGCCGTTATGCGCAAAACACCAGTTGCGCCCCCACAATT
CGCGCACGAACGGATGGGTGTTGGACAGGCTGACCTTGCCGACGTTGGCCTGGCGGATATGGCCAATCACCACTTC
GCTCTTGATCGGATAACGCTGCACCAGCAGCGCCACCTCAGACTCGCTGCTGGCCGCCGGGTCCTGGAACAGCCGC
AGGCCACGGCCCTCGTAGAAGGCAATGCCCCAGCCGTCGCGGTGCGGCCCGGTACGCCCACCGCGCTGCATCAGCC
CGGTGAAGCTGAACACGATATCGGTCGGTACGTTGGCACTCATGCCCAATAATTCACACATGCCAGGACTCTCGAT
GCAGGGCTTACAACGTTAAAGACGCGGCTCGACCCGCATGCCACTGACAGGGGCCGGCGGCCGGCCATAGGGTTCG
TCGTCTTCAGGCTCGGCCGCGACCGCTGCCGCAGCGGCGGCCTTCTGCTCACGCCGGGCCTTGGCGGCACGCTCGA
TGGGCCAGCGGATCAGCACAAAGACCAGGTACAGGCCAAAGGCAATCATCGCGTACATGAGCAAATCGGAAACCGC
TCGCCAGGCGTTATTGCCCACCTTGAGCAGCAGATCCAGGGCGGTGATGGCCACGGCCGGGGCAAACTGCTCCTTG
ACCGGGTCGACAATGGTCGGGCTTAACAGCAGCACCGCCATCAGCAACCGCAGCGGTTCGCGCAGCCAGCGCCAGA
TCCAGCGGGTCAGGCGAAACCACACCAGCAGGCAGCCCAAAGCGGCGAAGGCGTAGAGGCCCCAAGCGGTCAGATA
GTCGTTCTCGGTCATGGTGTCCATGGCAAGGTAGGCAAACAGGCCCGTATGATAACGGCTTTTCCGTGCCACGGCT
GCAATGCCGTCGGCCCACTGCCAGACAGAGAGTGATCCATGCCTCCATCGACCCACATCACTGCCGCCCCGATTGC
CCGCAGCGCCCCAGGCCAGGACCCATATGCCTGGTTGCAGGAACGCGACAGCAGCGAGGTGCTCGACTACCTCAAG
GCCGAGAATGCCTGGCAAGAGGCACAGCTCGATGATCAAAAAGAGCTGCGCGAAACGCTGTTTCAAGAGATCAAGG
GGCGCATCCTCGAGACCGATCTGTCACTGCCCTCGCCCTGGGGGCCCTACCTGTACTACACGCGCACCACCGCCGG
TGACGAATACCCACGCCACTATCGCTGCCCGCGCCCGGCCGACGACAGCCAGCAGATCGATGAGAGCGCCGAAGAG
TTGCTGCTGGACCCGAATGTGCTGGCCAATGGCGGTTTTTTCTCCCTCGGTGCCTTCAGCATCAGCCCGGACCATA
AGCGCCTGGCCTACAGCCTGGACACCAATGGCGAAGAGATCTACACCCTGTACGTGAAGGAATTGTCTTCAGGCAA
GGTCAGCGAACTGACCTTCGAAAATTGCGACGGCAGCATGACCTGGGCCAACGACAGCCTGACGCTGTTCTTCGGC
GAACTGGACGACACCCATCGCCCACACAAACTGTATCGCTATCGCCTGGACGGTACCGCTGCCGAACAGGTGTTCC
ACGAGCCCGACGGCCGCTTCTTCCTGCATTGCTACCGCTCCAGTTCCGAGCGCCAACTGCTCCTGGCCCTGGGCAG
CAAGACCACCAGTGAAATCTGGGCCCTGGACGCCGAGCAGCCGCAACAGGCATTTGCCTGCCTGGCGCCACGGGTT

GAAGGCCATGAATACGATGTCGACCACGGCAAGCTGGACGACCAGTGGACCTGGTTTATCCGCAGCAACCGCGACG
GCATCAACTTTGCGCTGTTCCAGGCGGCGGACAACGGCAGCGTGCCCAGCGAAGAGCAGTGGCACAACCTGATTGC
CCACGATGACGCGGTGATGCTCGATGGCGTGACGCTCAATGCCCGCGCCATGACCCTGAGCCTGCGCATTGGCGGC
CTGCCGGTGATCGACGTGCACCCACAAGGTCTGCCGAGCTATCGCGTGGAACTACCAGACGCGGCCTACAGCCTGT
ATGTGCAAAACAGCCTGGAGTTCGACAGCGACAAGATCCGCCTGCGTTACGAAGCGCTGAACCGCCCGGCGCAGGT
TCGCCAGTTGGAACTGGCCAGCGGCGCACAGCAGGTGCTCAAGGAAACCCCGGTGCTGGGTGAGTTCAACGCTGAC
GACTACGTCAGCCAGCGACTGTGGGCGACGTCTGCCGATGGCACCCAGGTGCCGATCAGCCTGGTGGTCAAGCGCG
ACCAGGTGGGCCAGCCAACGCCGCTGTACCTGTATGGCTACGGTGCCTACGGTTCCAGCCTCGATCCGTGGTTCTC
CCATGCACGCCTGAGCCTGCTGGACCGGGGCGTGGCGTTTGCCATCGCCCACGTGCGCGGCGGCGGCGAACTGGGC
GAAGCCTGGTACCGCGCCGGCAAGCAGGAGCACAAGCAAAACACCTTCAGCGACTTTATTGCCTGTGCCGAACACT
TGATCGCCCAAGGCCTGACCACTTCCAAGCAACTGGCCATCAGCGGCGGCAGCGCCGGCGGGCTGTTGATCGGTGC
CGTGCTCAACCAGCGCCCGGAACTGTTCCAGGCAGCGATCGCCGAAGTGCCCTTTGTCGATGTGCTCAACACCATG
CTCGACCCGGACCTGCCACTGACCGTCACTGAATATGACGAGTGGGGCAACCCACAGGAGCCCGAGGTGTACGAGC
GGATCAAGGCCTATGCGCCGTACGAAAACGTCAGCGCCCAGGCGTACCCGGCGATGCTGGTGATCGCCGGCTATAA
CGACAGCCGTGTGCAGTACTGGGAAGCCGCCAAATGGGTGGCCAAGTTGCGCGCCACCAAGACCGACGACAACCTG
CTGCTGCTCAAGACCGAACTGGGCGCCGGGCATGGCGGCATGAGCGGGCGCTACCAGGGGCTGCGTGACGTAGCCC
TGGAATACGTCTTTGTGTTCAAGGCCCTGGGGTTGGTTTAAGAACCCGCTGCCGGCGCCTTCGGCTTGCTGTCGCT
TTGCTGCAGGCCGGGCAAGGTCTGGTCCTTGGGCGGCTTGGGCATTTCAATCGGCGGCAACAACGGCGCCCCGGCC
CCTTGTGGCTTGGGTGCCATCGGCGGGGTGACTTGCGGATAGGGGGTCGGCGTTGCAGTACCCGGTGCACCCGTGG
TGGTCGCCGGTGTGTTCGGCACGGGTTGCAACTGCTGGGCATGTGCGGCACCCAATGTGAGCACACTCCCTACAAT
GACCGCTAGAATGCTGCACTTCATCGATGGCTCCATGGCCTGACTGTTAACCAGTCCCAAGGCTACTCCCAACCGC
GCAAGAATGCCCTTCCCAATGAGATTTCCATGAAACGTTTCGTGCTGCTCGACACCACCCCCATCCCCGATAACGG
CGGCGCCCTGTGCCTGTTCGAGTACGGCGAGGATTTTGTGATCAAGATCCAGGGTGGTGACGGCGGGCAATTGATG
AACACGCGCATGCACGGTTCCGAAGACGCCCTGGCAGAAATCCCCTGTCGCAAAGTTGCCGGGCGCCCCGGCTCGC
GGGTATTGATTGGCGGCCTGGGCATGGGGTTCACCCTGGCCTCGGCGCTCAAGCACCTGGGCAAGACGGCGCAAGT
GGTCGTGGCAGAATTGGTGCCGGGCGTAGTGGAATGGAATCGCGGCCCTCTTGGAGAAAAATCCGGCAACCCGTTG
CAGGATCCGCGCACCGTGATCCGCCTGGAAGACGTGGCCAAGGTCCTGCAAGCCGAGCCCCAGGGCTTTGATGCGA
TCATGCTCGACGTCGACAACGGCCCCGAAGGCCTGACGCAAAAAGCCAACAGCTGGCTGTACTCCGCCGGTGGCCT
CAGCGCCTGCGCCAAGGCCCTACGCCCCAAGGGCGTGCTGGCCGTGTGGTCGGCCAGCGCCGACAAGCAGTTCAGC
GACAAACTGCGCAAGGCCGGCTTCAAGGCCGAGGAAGTGCAAGTCTTCGCCCATGGCAACAAGGGCACCCGGCATA
CCATCTGGATTGCCGAGAAGCTCAAGGGCTGAGGCAAATTGCCAGGCGAGCAGCTAGAATCAACGTCATCCGTGAT
CTATCCAATAAATGAACAGGAGCCATGATGAGCTCGACCAACCCGCCCTCCCACACCGCCAAGCTGGACCGCATCC
TCGCCGATGCCCAGCGCGACCGGGAAATGGGCTACCGCGACAAAGCCCTGAAAATGTACCCCCACGTGTGTGGCCG
TTGCGCCCGTGAATTCGCTGGCAAGCGCCTCAGCGAACTGACCGTGCACCACCGCAACCACAACCATGACGACAAC
CCTCAGGACGGTTCCAACTGGGAGTTGTTGTGCTTGTACTGCCACGACAACGAACACTCCCGCTATACCGACCAAC
AGTATTTCGGCGAAGGCTCCACCAGCAGCCCGACCATCGCCAAGGCTACGCACAACCCGTTTGCGGCGTTGGCGGG
GTTGATGAAAAAGGACGACTGACGCTCGGCGCTGAACGCAACGACGCTATCGCGGGCAAGCCCGGCTCCCACAGGT
GAATGCATTTCAAATGTGGGAGCCGGGCTTGCCCGCGATGACAATCCCCCAGACCCGCCATTTCTATACCGACCGC
TGTTCGAGCAATCCCCGTATAATCGCGGTTTTTCTCGAAGGCACCCTCTCCCGTGGGCAATAAACGCTACAGCTGC
ATCGGTCTGTACAACCCCAAATCCCCCGAAAACGTTGGCTCGGTCATGCGTGCCGCCGGCTGCTATGGGGTGGCTT
CGGTGTTCTACACCGGTAAACGTTATGAGCGGGCCCGGGACTTTATTACCGACACCAAGAAGGTCCACCACGATAT
CCCGCTGATCGGCATCGACGACCTGAAGAAAATCCTGCCCCTGGGCTGCATCCCGGTCGCCGTGGAACTGGTAGAG
GGCGCTCGGCCGCTGCCGGAATACACCCATCCTGATCGGGCGCTGTATATCTTCGGCCCCGAAGACGGCTCCCTCG
ACAAAGAGATCCGCGACTGGTGCGAAGACGTGATCTACATCCCGACCACCGGCTGCATGAACCTGGCAGCCACCGT
CAATGTGGTGCTCTACGACCGCTTGGCCAAGGGCAATAACACCCGTTCGGGACCCAAGTACTGACTTTTTGGGAAC
ATCCTTGAACTGCCGACAGTCAGTTGCATATCAATGGCCCTTTGTTGGAGACAGACCATGAGCGACAGCAGAACCC
TCGAACCTGTGATTGAATCGACGCCGTTCCAGACCCAGCCGACCCAGAACGTCCAGGGCTGGGAGCGCATTGGCTC
CCTGGCCGGTGGCGTGGTGATGATGGGCAAGGGCATTCGCCGGGGCGGTTTTTTCGGCCTGATCCAGGTGGCGATT
GGCGGCGTGGCATTGGCGCGCGGATCAGCGGCCATAGCTCGGCCAAGTCGCTGCTGGAAAAAAGCCGTCAGGATA
TCAATAGTGTGCGGACCAAGATCGAGCGGGCCGGCGAGGAATTGAAGAACCTCAAGACCCGGGCTGAAGTGGCTGC
CGAAAAAACCACCGGCTGACATTAATCAAAATGTAGGAGCTGGCCAGCGATAGCGTCACCTCGGTACATCTGATGG
ACCGAGCCGCTTGCATCGCTGGCAAGCCAGCTCCTACACGGGCCCGGTCAGTGCAGCAGCTTGCTGTCGAGCACCA
CGGACGATTCGCCGATCACGCTTTCAGCCAACTGCACAAACTCCTTGGTACTGATACTGTCCAGGCGCATCAGCGC
TTGGGTCAGGTCATCCAGTGAGCGCTTGTTGTGGGTTTTCAGGCGAATTTCGCGATCCAGTTCTTGCAAAACCACC
ACCGCCCGCGCCACCGTGGCACCGCTGACGTGTTCGCCACGCAAGGTCGTCACGCCCTTGCCATCCTTGCTCAGGC
GCGCCTGGATCGCCACATAACGCTCATCGCTGATACCACCCGCACGCCGCAGCAGCTCGATGGCGTAGTACTCCGA
AAGCCCCTCACTGATCCAGTCACTGCCCTGGTGGTCGTTGAACCGTCCGATGGCTTGCACCACTTCGCGAATCAGC

GGGCTGCTGCCGTTCTCGCTGACCAGCGGCGTGCGGCTATTGAGGTATACCGAATCGCGTGCGGCAAACGCGCCAC
GGCGCATTGGGTCGCTGGCGCCCACTACCAGCAGCTTGGCCGGGTGCCTGGGAAACGCCGCTTGCACCTGAGGCCA
GACGAACGTCAGCAACGTCAGCACATCCATACGCCGCATGGCCTGGCCCTTGGGCGAGGCCACGGTGACTTCGGTT
TCGCCCAGGCGCACACGGCGGCTGCCCAGGCTGCCGGCGAGCATCCAGCCAGTGGGCCGGTCGAACAGACGGGACA
CATTGTCGATACGGAATTTGTGCTTGCCGATCCGCGGCCAGGGCGTCTCGACGCTTTTCCACCCCGCCGGCAGCTC
GAAGGTCAGCCGCGACACCAGTTCCACGCCATCCTGCTGGTCCAGACGGGCCGCCGGCACCAGGTCCTCGCCGCGA
AACAGCGCCCAACTGGGGGTCATGCGTGATTCGTAGATACCGGCCTTGCGCGCATGGCTCAAGCGCACGCGGTAGG
TCAGGCTGGCCTTGTCGGCGCCCGGCTGCCACAGGCCACGACTGGCCCCCGCGTCAGGCCTGACCTGCCACTGGCC
GTCGGCCTTGAAATCGCTGTAGTCACCGTCGCGCCCCAAGTCGAAGTCCAGGCTGCGCACCGCCGAGCCCTGGGAC
AGGCTTAAACGGACTTCGGCCTGATCGCTCTGGGGCAAGAGTTTGACGTGATAGTCGAGGTCGACCTTTTTCGCCG
ACCACGCCGACGCGCTCAACCCCAACAACAGCAGGCTCACTGCCAGCTTGGCTTGGACCGTCATACGCTCTCCTTT
TCAGCCTGCGCGGAAAATCAGGTAATCCTCCCAATCATCCTCGGGCACGCTGCCTTCGCTGAGCATGCGTCCGGAC
TGGGAAATGCGTTCGTGGTGCACGGCATCGCGATCACCACAGACCAGGTGGTGCCAGAGCGGCAGGTCCTTGCGCT
CACTCACCAGGCGATAGCCGCAGGTCGGCGGCAGCCATTTGAACTGATCGGCTTGGCCCGGCGTGAGCTGGATGCA
ATCGGGGACCGAGTCGCGGCGGTTAGGGTAGTCGGTGCACTGGCAGGTTTTCAGGTCCAGCAGTTTGCAGGCGATA
CGCGTGTAGTAGACGCTGTTGTCGTCTTCATCTTCGAGCTTTTGCAGGCAGCACAGGCCACAGCCGTCGCACAGCG
ACTCCCATTCCTCCTGGTCGAGGTGCTCAAGGGTTTTGCGTATCCAGAAAGGTTCGACTTTGGCGGCCATGGCTCT
ACATCAGTTTCGGTATGTTAAAAGGCCGCCAGTCTAGTGCCCAAGCCCCCTGGGGCCAAGCGCTTACGACTACCGG
TAGGGCAAGACTTGTCAGGTACGCGGTGGCGCAGTAGCTTTGAAGGCCGGTTTTTCCATCAGGATGCGCTTATGAG
CACTGAAGCACGCGTTGCCGATTATCCGATCCACCCGCAGTTCACCGAACGTTGGTCGCCGCGTGCGTTTACCGGG
GAAAGCATCCCGGAGGAAACCTTGCTGAGCTTCTTCGAGGCCGCGCGCTGGGCGCCTTCGGCCTACAACTCGCAGC
CTTGGCGCTTTCTCTATGCCCGTCGCGATACGCCGGACTGGCAGCGCTTCCTTGGCCTGCTGAACGAATTCAACCG
TGGCTGGGCGCAACATGCCTCGGCGCTGGTGATCGTGGTCTCGAAAACCGATTTTGCGGTGCCGGGCGCCAGCGAA
GAAACCCCTGCCCTGTGGCACACCTTTGATACCGGCTCGGCCTGGGGCCACCTGGCGCTGCAAGCCAGCATCAGCG
GCTGGCACACCCATGGCATGGCCGGTTTCGATCAGGAACTGACCCGCAAGGAACTGAAAATCCCCGAGGGGTATGC
GCTGCATGCGGCGGTCGCCGTGGGCAAGCTGGGAGACAAGTCGACCCTGGCGCAATACCTCCAAGCCCGTGAAGCT
CCAAGCCCGCGCCGGCCATTGAGCGAGTTGGTGGCTGCCGGGGACTTCAACCTCTAAGACTGCCCCCCGCCGCTGA
CACGCGGCGGGGTGTCCAGGCCCCCCATCAAGAAGGTTTAGCGACAAGGCCACTGTAGGCCACGCGCAGCGCCTCT
TTATGCCTGTTATATGTGTCCATGACCACCGGCACCATAAACTCCAAGCCCTGGGAATCCATATCCACCGGGTAAA
CTTTCTGGTCTTTGGCAGAGTACATAAAGTTCTTTAATTGCAGGTCACTGTGATAGATATCCTTGGCTTCCATTTG
CGCCAATACATCATCCAGCAATGAACGCGCCTCGGGTGGCAGGCCATTGCGCTCGAACTTGCTCAGATCCACACCT
TCGATCCGCCCCATTTTTATATATTTACGCCCATCCTCGATGATTACCCTGGCAAAACCCTCGCCGTAATATTTAT
TCAGCGAGACCACTTCCGTATTGATGTACTTATCCGATGGCTTGATTGATGTAGGCCCCAGATCCTTGTAGACACT
TTTGCCATCAAGGCTCGCGTAGATAATGCCCTCGCCACCTTGCCCGATTACAGCGCCCAGATCGGCTGCCACCGGC
ATCGGAGCAGCGCCCAACAGATTCAGGCGCTGCCATTGATCACCTTGTACATGCTGGACCAATAGCGGCGTCTGCC
GGCCACGGTGATAGATCCGTGCCTGCACCACGCCCTCTTCGGTACGGGTGACTTGCCTAACCTCATACACAGCCGT
CTCACCGGCACTGTCGGTATGGCGGATATGGGACAAGTGGCCGTCAGCCGCAACATAGACGCCCTGGCTGTTGCGC
GACAGCCCGGCTATTTTCGAGGGCGCAACGCTGTACTCGACGAGCATCCTGTTGCCCAAGTCGCTGCCCGGCGCGA
CACGTCCCATGGCAAGCGCGCCGTCCACGGCCCTGGTCTGGGCCACAAAGTCCTGCAGAGGCCCACCATAGGCACG
TAGCCTGTCGGCATCAAAGGCATACCATTTGCCGTTATGCAGGACGGCCCCTGCTTCGACACTGCGCCCTGCCACC
TTGAACCTGCCGGTGGCCGCTACGCCATAGTCGTTGCTGACAGCCTTCAACAGGTCATAGCTGCCGGAAGTGCCCC
TGAGCCTATTGACCCCGCCGACACCTTTGGCGAGCCCTTTGCCGACCAGCCTGGCACCGCCGACGCTCAAGTCACC
CAACCCGCCAAGCGGGTTCAAGGCGCCAATCGTGGCCGCACCAATGATCTTCGCTCCCTGCAAGGCCTTGGCCGTC
CCGGATATGGCTGCGCTGCCTAACTTGCCAAGCTTTCCGGCGACGCCCGCGCCGGCAGTCAGGAAGCCAAAGATAT
CCAGCGCTAGGTCAAAGGCGCCCTCGCCATAGTTGCCCTGTTGGAAATTGACAATCGCCGAACGCAGCGGAACCAG
GTTCAATAAAAACGTTTCAACAACCTTGGCCCTGCCCCGCTGTGCGTCCAGCGTGGTGTGCCCTCTCGCGTGTTCC
TTTATCGCGGGATCATCAAGATCAAGGTGTTCGACAAATGCCTGCGCAATGTATTGAGTGCGTGCCGTCGCAAAAC
TATCAAGTAACGGGTTACCCACGGGTGTTGTTTGTTCTTGTTGCTGGGCTTGGCTGGCGGCAGGTGTGAACGCCTT
GGTCTCGTAGACCAGGTTGGCATTTCTGGAACGACGCTCCTCGGCTTGCCAGGGCTCAGTTGATCGAATAGTGCCT
TGGGCAAAGTTTATTTCGTACGCCGCGGTCTTTCCCTCGCGTTCGGTTTTTACCAGCAGCTCCACCCCCTTGGGAT
GATGGGTTTTATCGGAGAACCCCAGCCCGAGCGTATGGGATGAACGTTGATAGAAGGCGATCTTTCCATATTCAAA
GTTCTTTCGGTCCTCCAGGGGAAGCTGCGCGATCAAGTGTTTGACGGCTGTGGTGACCGCCGCCTTTTTACTCTGG
ATGACATCATTGAACTGCTGGTTGAACGACTCGGTTGTGCCAAACCGGTAGTTGGCATTCAACTCGGCCAGGGGGA
TGCGGCTGTCCGCCGAATAGAACAAGGCTGGAGAAGGCAGGTCCATCATGGCGATATCCAGCAACGAATGGAGGCC
GACCAATCGGGTGGCCGGCGTTCCCGGCGCTCCTCGCGGGGAACTTGTGGTGCTGATCAACCTTTCTTCAAAAAGC
CCACCGAGATCGCCAAACCGTTGTTTGAGTTCGGCCAGCGCCAGTGCCTTTCGACCAGGGAGGGCCTGGTCCAATG
ACCGGCTGGCACTGATCATTTGCTGCTGGCGGGTATTGAAGGTGTTCTGGAGGGTGTCCAGTTCTTGGTTGGTGTA

GCGTTCGTCCGCCTTGCGCACCAGCAGGCCATTGACCACCCCCCAGTCCACCAGCGCAGCCTGCTGTGCACGCTGG
GTGATGTCCGGATCAGCCAGCCTTGCGCGCTCGGCCTGCTGCATGACCTGCACAAAGGTCATGTTCGGTACTTTGC
CCGGCGACTGGGCCTCGATGGTCGCCGCCGCAATCGCCAGGCTGACCCAGGCCGAACTGCCATAGGTCACGCTGGC
GGGGATGTCTTTGATAAGAAATTCGGGAGCCGCTTTGGCCAGCAACAGATAGGCGCCCACCCCGGCCATTTCCGGG
CTGGTCTTGCCTCCAGCGCTGAGGTGCTCGCTCAACCCCGCTACCACCGTTGAAGCGGGTTGGCCCCAGTGCTTCT
CGCTGGCCAGGTCGAAACCCGCCACCTTGTTGCGCTGGGGAGCGCCGATGGGCTCGGGATCCATCTGTACAGCCAT
GGCGGCCAGCAAATAGTCGCCGGCACTGCGGTCAGTGGCTATCCCGCTCATTTGTTGCTGCAAGTACTTCCCCAGC
CGCTGCCCCTCGCTGGAACTGACCAGCGACTCCAGAGCCTTGGCGGGGTTGCCCAGCACAGGCGCGGCGACTGGCT
GGCCAGCGTGCAGGAAACCCAACACCCCCTTGCCTTGCCTGCCTTGTGGCTGCTCACCCAGATAACTCTGGGTCAA
CACGCCAAGTTTGTGCTGCTCAGTTGGGCTCATGGGCACCGGCCAGGACAACCCACCGCCAAAATCGCCAAGAGGG
TGCTCCAGGGCTTTTTCATCCAGCGCCTGGGCCAGGCTGGTGAGATGAAAATGACTGGAGGGCAGGAATAAACCCA
GATGCTTGATAAAGGCTGCCAAGGTGATGCTGCGACCGGTCTGCAGCGCATGGGACGACTCGGGGTCGATGCCCAT
CGGCGTACTCTCCAGCGCCGCCAGCACTGCCTGAGGCGCAGCGTCTACGCCCAATCGGGTGGCGACCTCGTGGAGC
TTGCGCGCCAGGAGCTTGAGGTTTGCCTGGTCTGCCAGCGCGGCCCGCAGACCCGCCGGCTGCTTGGTTCGGGCCG
GGATGACCTGGGCGCCTGCATCAACAGTGGTCGGATCAGGTTGCGGCTGCAGCGGGCGGGAAGCGTCAACGGCGCG
ATCAGCCGCAGGCATGAGAAGCGGGGATAGTGGCGGGGGAACAGCGCGCAAAACGAGCATGGAGAGGCCTCCCTTC
TTAAGGTCAGAGAGCCTCCCACTTTTGCCGCCAGCGATAAAATCAGATGGGTGCCACGCACCGTGTGGGGTTTTTC
AATATCCCCGCTCGAAACTCACCTCGCCCCGCAGCGAATCCTTGGCCTGGTACGCCCGCAGGTTTTCCACAAACAG
CGCCACCATCCTGCGCGGGCAGGTCGGCGCGGAGCTGTGGCCCGTCAGCAGCAAACCCCAGGCGGTCCAGAACGGA
TGGCGCTGGGGCAGTGGCTCCTGGCGGCAAACGTCGATCACCGCACCGGCCAGGTGACCTTCCTTCAAGGCCTCGA
CCAGGTCGGCATCCACCACCGCCACGCCACGCCCGACGTTGATAAACAACCCGGTCGGTTTGAACTGCTTGAACAG
CGCTGCGTCGTACAGGTCGTGGGTGTTGGGCGTGTTGGGCAGCAGGTTGACCACATAGTCCACCTCGCCCACCAGG
CGGCCCAGTTGATCCGGCCCGACCACCTCGATAAACGGTGCCTGCTCCCGGGCTTCGCTGGCGATGCCGTACAACT
CCACGCCAAAGGGCAGCAGGAACTGCGCCACGCTCTGGCCGATATCGCCGGTGCCGACGATCAGCACCTTGCGCCC
CGCCAGGCTCTGGCCCATGCGGTTGTCCCACTTGCGCTCGACCTGGCTCACCAGGCGCGCCAGCACTTCACGCTCG
TGACCGAGCATATAGGTGAGCACGTACTCGGCCATCACCTGGCCAAAAATGCCGACGGCACGGGTCAGCCGATAAT
CCCGAGGCAGGCCATCGGCCAGCAGCGGCGTGATGCCGGCCCAGGTCGATTGCAGCCACTGCGGGTGGTGGCCCTG
GCGCAGCAGGGTTGCCAGCAGGTCTGGCTGGCCCAGCCACACCGGGCAATCGGCAGCCAGGCTCGACAATTGGGCC
GAGTCGCCACTGGTCAGGACTTCAATATCCGGCGCCGCTTCGCGCAGCAATCGGGCGTACAGCGGGTGGTCCTGTT
CTGCTATCAGAACGCGCATGGTTCAAACCTTTCAAAAACAGTGCAGGCGGCCACCGCCAGCAAACTGACTGCAGGG
CTCGGGGCTCGCCAGGTCATTCGATTCAAACGTGTCCCGAACGGGACACTGGTCGCTCACATCGGGTCGTTACGGC
GCAGCAACTCTTCGGGAAGGTGTTCGATGTACTCGTCATCCGGCGGTGGCATTTGCAGGTGGTAGCCCTGCTTTTC
GAGGTTGGCCAGGACCGCCACGATATCCTCCTGCTGCAACTTGCGTTCCGGCGTCAGTACCAGGTTGAACGCGTGT
ACCGGCTTGCCGAACACCGTCAGCAGGCCTTCGGGCACGCGCTCGAGGGCATCGCTCTTGAGCACGTAGAGGTACA
TGCCGGCACGTTTCGGGCTGCGGTAGATGGAGCAAATATGTTTCAAGGCTGTTCTCCGGCAGTGGCCAGGCTGTCC
AGCAGCGCCTGGCCCATCAACTCGCGGCGCCAGCCACGCAGCGAGTCAGGCAATTGGTAAGGCCCATCGGGGTAGC
CGCTCTTGACCAGCGCCTCAAGGGTTTTCTTGCGCAGCATCAGTTCCGGGGCCATGTCCAGGCGTTCGGCGAAGGT
CTGGCCCAGGGCGCGCAGTTGCTTGATCAGCACCGCGGCATCCACCGGCAACGGCTCGGGCACGGCCGGTGGCCAT
TGGTCCATCGGCACACTGGCGGCACGCTTGATCAGGCCCAGCAGGAACTCGCCGTCCTGGCGCACGGTGCGCGGGT
GCATGTCTTCGATTTTGCCCAGGGCAGCGAGGTTATCCGGCTGCGACTTGGCCAGGGGCCATAGTGAGTGTTCGCG
GATGATGCGGTTGCGTGGCAGGTCGCGGGCACGGGCCTGCTGCTCGCGCCAGGCGCACAGTTCGCGCAACACGGCG
AGCTGGGCCCGGGACAGTTTCCAAGCCAGCTTGGCGTCGCGGTAGACCTCGTAGGGGTCGATTTCCCGGCGCAGGT
TGGCCACCAGTTCGGCGCCATCTTCCAGGACCCAGGCGTACTTATCGTCGGACAACCGCGGACGCAGGAGCGTATA
GACCTGCGCCAGATGCACCGCGTCTTCGGCGGCGTAACTGACCTGGGTCTCGGACAGTGGCCGCTGCAGCCAGTCC
GAACGCGTCTCGCCCTTGGGCAACTCGATATCCAGCACCGCTTGCACCAGGCGCGAGTAGCCCATGGAGAAGCCGA
GGTTCAGGTAGGCCGCCGCCAATTGCGTGTCGAACAATGGCACCGGCAGGCTGCCGGTCAGGCGCAGCAATACTTC
CAGGTCTTCGCTGCAGGCGTGCACCACCTTGATCACCGCTGGATTTTCCAGCAACGCGGCCAAGGGCTGCCAGTTA
TCGATGGTCAGGGGATCGATCAGATAAGCGCGTACACCATCCCCGATCTGGATCAACCCGGCGATGGGATAGAAGG
TGTCGACCCGCATGAATTCGGTGTCGAGGGCGACGAATGGCAACTGCTGCCATTCGGCGCAATGCTGGCCGAGGCT
ATCGTTGTCGCAGATCCAGTGAATATCGATGGCCACACGGCTCTCCCTTGAAGAATGGCGCGCAGTATATATCGCC
TGAGGGTCTTTCGAGCACCCGCAGTGTGCCAGCCATCATGAAAACTCTGACAGGAGACGAAGAATAGTCCGACGTG
TGCGGCTTATCCTCTTTTACGCAGCACATAAACCCGCCACAAGGCCGAGCCGGGCATGAACTCTTGCTGGTTCAGC
ACCGTGAAGCCCGCCTGGCGGAACTCCGCTTCCACCTCGCTGCGATGGGCCAGTAAAGGTTGCCCCGCGCCTGCCG
GGTGGCGTTGACGATGGCGGCTGTCGCTGTCTACCGAGACAATCACCGTGTCGCGGCTGACCCGGTGAAATTCGCG
CAACAACGCCAGCCGCGCGTCAGCCGTGGGGACGTGGCGAAACAATTCCAGGCAAAAAATGCAGTCCACCGCGTTG
GCCGACAGGCCGATGGAAAAGGCCGAGCCCTGGAACGTCTTGATCCGCCGCAGAAATGCCGCGCCATGGTGGCTCT
GTGCATGATCGAGCATGACTTGGGAGGCGTCCGAGGCCAGGATCACGCGGTTGCTGTGCTCAGCCAGCACCGGCCA

GAAACGCCCCGCCCCGCAGGCCAGGTCCAGCACCAGGCCGGGCTCCCCCGCCACCTTCAGGGCCTGGCGCACCAGG
CGCTCATCGCGCCATAACCACAAGCGCCGCAGCAAACCAGGCGGTCGCGTGTCACCACAGACCCGAGCGTGGTCAC
GATCACAGCGCGCGGCGAATTCAGCCTCGATGGACGATAGGGGGGGTGGTGACATCACGGGACTCATGTAGATGAA
CCGGTCTCTACGGACACTGGAACTTGAGCTTAACCACCCCTGCGTGAAAAAAAGGTCGAAAGGCCATCGTCACGCC
TGGTGCAAGTACCAACGCCAGTCCTGCTCACCCACTTCGCCCATGAACTGCCGATACTCGGCCCGCTTGACCGCCA
GGTACACGCCGAGGAACTCCTGGCCGAACGCCTCACGCGCCCAGGCCGAACCTTCCAATGCGCGCAAGGTGGTCAG
CCAATCGGTGGGCAGCAACTCCTTGGCCTGGGCATAACCATTGCCCGTCACCGGCGCGCCCGGATCGCGCTGTTCG
CGAATGCCTCGGTGAATCCCCGCGAGAATGGCTGCCGCGGCCAGGTAAGGGTTGGCATCGGCGCCGCAGATGCGGT
GTTCGATATGCCGCGAATACGCCGGCCCGCCTGGCACACGCAGGCTCACCGTGCGGTTGTCGACGCCCCAGGTGGC
GGCCAGCGGCGCGTAGCTGTTGCTCTGGAAGCGGCGGTAGGAGTTGGCGTTGGGACAAAACAGCAGCAAGCAATCG
AGCAAGGTACTGAGCATCCCGCCGACCGCCTGGCGCAACAATGGCGTGCCGTCGGGCGCCTCGCTGGCAAACAGGT
TGTTGCCGTCGGCATCCGCCAGGCTGACATGCATGTGCATGCCTGTGCCGGCCAGGTCGTCGAACGGCTTGGCCAT
GAAACAGGCAGTCATCCCGTGTTTGTGGGCCACGCCCTTGACCAGGCGCTTGTAACGCACCGCCTCGTCCATCGCC
TGCAACGCATCGCTGCGATGCTCGAGGGTGATCTCCACCTGGCCTGGGGCGTATTCGGAAATTGCCGTGCGCGCGG
GGATGCCTTGCAGTTTGCAGGCACTGTACAGGTCGGCCAGAAACGGCTCGATCTGCTCCAGTTCGCGCAGGCCATA
GACCTGGGTCGAGCGCGGGCGAGCGCCATCCACATCCCGCGCCGGTTGCGGGCGGCCATTGCTGTCGGGTTTCTGG
TCGAGCAGGTAGAACTCCAGTTCCGCCGCCATCACCGGGTAATAACCGTCGGCCTTGAGCCCATCGATGACCTTGG
CCAGCAAGTGCCGGGGATCGGCAACCGTCGCCGGCAGGCCTTCCTCGGGGTGCATGCTGACTTGCACCGCTGCCGT
GGGAATCAAGCGCCACGGCATGCGTTGCAAACTGCCGCTGATCGGGTAGGCACGGCAATCGATATCGCCCACCTCC
CACACCAGGCCGGAGTTTTCTACGTCATCGCCATTGAGGGTCAGGCCCAGGATGGTGCTGGGCAGCGGCCGACCGC
TTGCATAGACCGCCAGCAGTTCATCACGGTGCAGCAACTTGCCCCGGGGCACGCCGTTGCTGTCGAGGATGAACAG
TTCGAACATCTCGATATCGGGGTTGTGGTCAAGAAAGGACTGGGCTTCGTGCAGGTTGGCAAAGACGGTACTCATG
GCAGCTCTCTTACGTTTGCGTCAGGCAGGCGCGCACGACGGCACGCCCTGGATGGGGGCAATCAGCGTAAGGGCGT
GGTGTCTGGCGGGCGGGCGTGGCGGCAGTGCCACAGCGCCCAAAAGGCCAGTTCAGAGGGCAGTGCATCAGCACCC
AACCGACATACCGGGCAGCAGGGCGCGATGGCGCCAGGCGAGGCCTCGGGGTGAGCGTCGGGGGAGCCGTCCATGC
AGTGATCACAGTGAAGAGATGAACACCAGCGTCACACGGTGCAATAAGTCGTTAAATTCACCCTTGATGGAGTTTG
GATGCGACCTCACTAAACATTCAGAAGCGACTTACATCGTTTGCTGAATATTCATGCATAGCTCACAGCCGTCTAC
GCTGTTGGGACATTCACAAGAACGAGGGACGGGTCAATGAAGGGAATGCTCCGCGCCACCTGGCTGCTGTTGCTGT
TGTGTGCCAGCCAGGCCCACGCCGCTACCGCCCAGGAACAGGACGCCCAGGCCGCCAAGGCGCTGCTGGAGAAGGC
TCTGGCCTACTACCACGACAACGGTGACAAGGCCTTCGCCGCCTTCAGTCGCCAGGGCGAGTTTGTCGACAAGGAC
CGTTATGTGTTCGTGGTCGATACCAAGGGCGTGCTGCTGGCCAGTGGCGGCCCGTCATCGGCGTTGATCGGGCGCG
ACGTGTCCGAAGTGCTCGGCCCGGACCTGCGCCAGTCCTTCAAGGAGGCGTTGAAAATCCCGGAAGGCAATGGCAT
CCAGCAAGCCGAATACCGCTGGCAGAATTGGAACGATGGCAAGGTTGAACGCAAGCGTGTGTTCTACCAGCGCATC
GGCCAGCGCATCCTCGCCGTCGGCTATTACCTGCCCCGCGCCACGCCCGAACAGGCCAAGGCCCTGCGGGACAAGG
CGGTCAAGGACCTGGAGAAAAACGAAGCGGGCACCCTGAAGGCAATCAACGCACTGCAAGGTGGCTTCCTGCAGGA
CGACCTGTATGTGTTCGTGGTCGACCTGGATACCGGGCGCTACGTGGCCCATGGCACCAACCTGCGTCTGATTAAC
ACCGACTTTGCCAAGGTCAAGGACCCGGACGGCAAGCCCGTGGGTGAGCCGATCCTGGCACTGATGAAGGAGCAGG
GTCAGGGCGAATATGAGTACCGCTGGAAAAACCCGGTGACCGGCAAGGTGGAAGACAAGCATGCCTACCTGCGCAA
GAGTGGGCATTTTCTGGTGGCAGTGGGTTACTACAGCCCATAAAGCGCCCGCGCTAAAGAATCCCCGTGGCGAGGG
GCTCATCCCCTCGCCACGGGCGGGGTTCGCGCTTAATTGAGCGTCCTGGGTCGAAGCGTTTATCGCGCCTTGTCCT
CACGCCCACGCAACAGGTGGTTGGGCATGGCAATCGCCGCCGCCAGCCCCAGCAGCGACACTGCCGCACTGATCAT
CAATAAATGCCGGAACGTACTCAGCAACTCCCCACGCAAGGCATCCTGCGCCGGGCCGGGCGCGGCATTGAGGCCA
TCGAGCAGGACGTTGCCGGAACTGCCTTCAGCCATCATCGAGCCACCGGCCAGATGGGCAATACTCGAATCCTGCA
ACAACGCCAGCAGCAAGGCCGACATGCATGCCACGCCGACCGCGCCACCCAGGGAGCGGAACAGGTTGGTGGTGCT
GGTGGCGACGCCAATGTCGTGTTGGGCCACCGAGTTTTGCGTGCCCACCAGGGATGTCGGGAACTGCATGCCTGAG
GCAATGCCACTGAGCACCATGAACAGGCTGCTCAAGACCAGGGACTGGGGTGCGCTGAAGGCCATGCCGAAGATCG
CAATCGGCGTGAGCACTGCCCCGGCCAGGATCATGGGTTTGTAGCGCCCCGTCACCGAGGTCATGCGCCCGGCGAA
GAACGCGCCCATGGGCAAGCCCATCGCCAGCGGCAGCAGGTGCAGCGCAGCGCTGTCGGCGCCGGCGCCCGTGACG
CTCTGGTAGCGCAGGGGCATCAGTACGATCAGCGAGATGGCCTGGAAACTGGTGAAGAAAATCGTGCACCAGCACA
GCACCGCGCTGCGGTTGGCGAACAGGTGCATGGGCAACAACGGCTCCCGCGCCCGGCGCTCGTGCCAGACAAACAG
CGTCAACGCCACCAGGGCACATGCCAGCAGGCCCAGCACTTGATCATCGCGCCAATGATGGCCCTGGCCGATTTCG
GTGATGCCCAGCAACAACGCCGTCAGGCCAACGATCATCAGCAGCGTGCCAAGGTAATCGATGATCGGCTTGCGCT
GTGGCACCGGCAGCCCCACCAGGGTGCGATGGGCCACCCACCAGGCGCCCGCGCCCAATGGCAGGTTGATCAAAAA
CACCCAGCGCCACGACAGGTACTCGGTCATATAGCCGCCCAGCACCGGCCCGGCCACGCTGGCGACGGCGTACATG
CTGCTGAAGTAACCCTGGTAACGCCCGCGCTCGCGGGGTGGGATGATGTCGCCGATGATCGCCTGGCTCACGGAAA
TCATCCCGCCCGCGCCAATGCCCTGGAGGATACGCGCCAGCACCAGTTGTTCCATGCTTTGCGCCATGCCACAAAA
CAGCGAGGCGACGGTGAACAGGCCCATGCCGATCAGCATCATGGGCCGGCGCCCGTACAGGTCGCCGAGCTTGCCG

TAGATCGGCACCGCCACCGTCATCGCCACCATGTAGCCAGAGATGACCCAGGCCAGCAAGTTGACGTCGTTGAACT
GGGCGGAAATGGCCGGCATCGACACCGCCACGATGGTCTGGTCCAGGGCGCCGAGGAAAATCGCCAACATCAGCGC
GGCGAGGACACTGCGTACGGAAGGTGTGGGTTGAGTCACGGCAGAACCTGCAGGCAAGGCCACGGAAAAAGAGGCC
CGCGGCAATGCCCGTCATGTTACTCGATAGCCAGCTATTGGATAGCTTCGTAGGGAAGTCCGACGTAATTTTCTGC
AATGGTTTTACGCCCGGCCTCAGACTCGACAAAGTACTCCAGCTCGCTCTGGGCGATGCGTTGGCTGAAGCCATCC
GCCTCGGGAAACTGGTGCAGCATCGAGGTCATCCACCACGAAAACCGCTCGGCCTTCCAGATACGGCGCAGGCAGA
TCTGTGAGTATTTTTCCAGCAGCTCCACGCGCCCTTCCCGGTACACCTTGCACAGGATCGTAAACAGGGTGCTGAC
ATCACTGGCCGCCAGGTTCAGGCCCTTGGCCCCGGTGGGGGGCACGATGTGGGCGGCGTCTCCCAGCAGGAACAGG
CGCCCGTACTGCATGGGCTCCACCACGAAACTGCGCAGCGGGGCGATGCTCTTTTCGATCGACGGCCCGGTGACCA
ATTGCGCCGCCAGATCCGTGGGTAAGCGCGCCTTGAGTTCATCCCAGAAGCGCTGGTCGGACCACGCGTCAATCGA
CTCCGCGGCCGGCACTTGCAGGTAATAGCGGCTGCGGCTGGACGAACGCATGCTGCACAGGGCAAAGCCGCGCGGG
TGCTTGGCGTATACCAATTCGTCGTGGACCGGCGGGGTGTCGGCAAGAATGCCCAGCCAGCCGAAGGGGTAAACCC
GCTCGAAGGTCTTGAGCACGTCGCTGGGGATCGACTGACGCGCCACGCCGTGGAAGCCATCGCAACCGGCTATGTA
GTCACAGTCCACGCGTACCTGCTGGCCTTGATGCTCGAAGGTCAGCCAGGGCCGCTCGCCCTTGAGGTCGTGGGGT
TGTACGTTGCGTGCTTCATAGAAAGTGATGGCGCCGGCCTGCTCGCGGGCGGCCATCAGGTCGCGGGTGACTTCGG
TCTGGCCGTAGATCATCACGGTCTTGCCGCCGGTCAGGGATTTCAGGTCGATGTGTTGCAAACGCCCATTCAGAGC
CAGCTCGAAGCCTTCATGCACCAGGCCTTCGGCGTCCATCCGCTGGCCGACGCCCGCCTGGCGCAACAGCTCGACC
ATGCCCTGCTCCAGCACCCCGGCGCGAATCCGCCCCAGAACATAGTCCGGGGTCTGCCGCTCAAGAATCAGGGTTT
CAATCCCGGCGTTGTGCAGCAACTGGCCGAGCAGCAGCCCCGAAGGACCGGCGCCGATGATGGCGATTTGGGTTTT
TAGCGTTTTCATTATTTTTATGACCCGCCTGCTTCCCCCGAACGTAACCGGTGAAAGCGTTGTCAGTGGTTTTGGT
ACTGGCATTTTTCCCTTGAGTGACCGCCACAAGAAGGTGAAAACTGAGCCAAAGCCTGCGCTTTTCGCCAATCGGG
GCGATTACCGAACCACTGTCCTGAGTATCGGATAAAAACATGACCAAAAATGCCAGCCCCGCGATTCCAGTGTTCA
AACTCTACGGGGAAAGCCAACAGTGGCCGACGCCAGACTTACTGCACTGTGAAACCATCTCCCGGCGCAGCCGTGA
GTACCAGTGGGAGATCCAGCCCCACCGGCATGCGGACCTGTGCCAATTGCTCTACGTGTACAAGGGCCAGGCGCAA
CTGGAGATCGAGGGCCAGCGCACGACGCTGACGCAGTCGACCCTGCAGGTGCTGCCGCCGCTGTGTGTGCATGGGT
TCCGGTTTTCCGAGGATGTCGAAGGATTTGTGGTGACACTGGCCGCGCCACTGGTGGCGCACCTGCAAGGCCAATT
GGGTTCGGCGGTGGACGGCTTGAGCGCCCTGGGCAGCTACCCGGCGGGCCAGGACAGTGACTATCTCAATAGCCTG
TTTACGCGGTTGCAGAACGAATACAGCGACGACTTCCCGGCCCGGGACATGATGATGCACGCCCTGGTCAGCGTGC
TGCTGGTGTGGATCAGCCGCCAGGCCATCCAGCGCCGCCATCCACGGGCCCCACGGGGCCGTGAGTACTTCCGGCG
GTTCACCCAGTTGGTGGAGCAGCACTACCGTGAACACCCGAAAATCGAAGACCTGGCGCACAAGCTGGGGATCTCT
GTGTCGCACCTCAATGGCACCTGCCGCGAATTGGGCGGGCAGCCGGCCTTGCAGATCATGCATGACCGTCAGTTGC
TGGAGGCCAAGCGCCTGCTGACCTACACCAGCATGACCATCAACGAAATGTCTGAGGTTTTGGGGTTTTCAGATCC
GACCAATTTTTCACGCCTGTTCCGACGACGCGTGGGGTTTTCACCCAAGGCATTTCGCGAACAGCTGAACGCGGCG
CCTAACGATGGCGTGTCAGCGCAGAGCGCTCAACGTCGCTGAGTAGGTGCATTGCCCATGGTTGCAACTCGCGGCC
ATGCCCGGTTGCTGGCGGGCCTGTTCGGCGCGGTAGGCGGCGGTGCCGTACAGGGCTGTGGCGAAGGCGCACAGGG
CAAAGATCATCAGGTACTTTCTGGTTTTGATCGTCATGGCGGTGACCTCCGGACAGATCAAGAAGGTGCTGTCTTA
AGCATAGGTCAGCCAGGGCGAGTTGCCAGTATTGACGGGTATTCAGGCGCCTTATCGGCCCTACAGCCCCGCATCC
CATTGCGGCTCGGGCGGGAAGCGCAATACCAGGAAATCCAGAAGGCTGCGCAAGGCCTGCGGCATGTGCTTGCGCG
AGGCGTAGACCGCGTACATGTTCATCCGCCGGGGCTCGGCATGGGGCAGCAGGCGGATCAGTTGCCCGCTGCGGAT
GTAATCGCCGGCCTGATAGGTCGGCAACATCGCCACCCCCGCGCCGGCCAGGGTCAGCCGTAGCAAGGTGCTGGCC
TCATTGGCGCTGATATTGCCGTGCACCGGCACCGACACCGGTTCGCCATCCTCCTCGAAATGCCACAGGCTTTTGC
CGAAGTAAGAATGGGTCAGGCAGTTATGCCCCGCCAACTCCTCTACCCGCTGGGGTTGCGGGTGCTCCTGCAGATA
CGCCGGCGAAGCGCAGATCACCGAGCGGCATACCGTCAGTTGACGGGCGATCAGGTTGGGATCCAGGTCATTGCTG
GTGCGGATCGCCAGGTCAATGCGCTCATCCACCAGGTTCACCGTGCGATCGAGCATCTGCAGGTCAATGCTGACCA
ACGGGTAACGCTTGACGTAATCGGCCATCGCCTCGGCCAACTGCGCCTGGCCAAACGAGGTGCTGACACTCAGGCG
CACCAGGCCACGGGGGGCGTCGTCCGGTTCGCTGACGGCGGCCTGCATGTCGCAGGACAATTCCAGCATCTGCCGG
CATCGTGGCAGGGTTTCGCTGCCGGCGGCGGTCAGGCTGAGCTTGCGGGTGGTGCGGTGCATCAAGCGTGCGCCGA
CCCAGTCCTCCAACTCCGCCAGATAACGCGACACGACAGGGCGCGACAGGTCCAGGTGATCGGCGGCGGCAGATTG
GCTGCCCAGGTCGACCACGGTGACAAACACGCGCATTGCTTGAAGACGATCCATGATTTGCCCGATTTCAGAAACA
AACTATGTTCCAGCATCGCATTTTTTGCTGCGTTCAGTGCAACTAAGCTCTGTCCATCGCCTGCCAAGGCACCCCT
GACCTGGACCTGAACATGCTCACCACCCTCAAGCGCTTTGTATTGGCTACTACCGTTCTCGGCTTTGCCGCCCACG
CCGTCGCCGCCGACCTCACCCTCGATGTCTACAACCCGGGCGAATCGGCGATCTTCCCTGTCAGTTCGGTGCTGGT
CAGCGGCGAGAAAGACGCGATCCTGGTGGACGCGCAGTTTGGCAAAGGCCAGGCCGAACAACTGGTGCAGAAAATC
CGCGCCAGCGGCAAACAACTGACCACCATCTATATCAGCCATGGCGACCCAGACTACTACTTCGGCCTCGACACCC
TGGCCAGCGCCTTCCCCCAGGCCAAGATCCTCGCCCCGCAGCCCGTGGTCGACCATATCAAGGCCACCGTTGCCGG
AAAGATCGAATACTGGGGTCCGAAAATGGGCGACGACAAGCCGGCCAAAACCATTATTCCCCAAGTCCTCGAAGGC
CATAGCCTGACGCTTGAAGGGCAAGCACTGGAAGTGATCGGCCTGGATGGCCCGCAGCCGGATCGCAGCTTTGTCT

GGATCCCATCGATCAAGGCCGTGGTCGGCGGCGTGGTGGTCTCGCAAAACATCCACCTGTGGATGGCCGACACCCA
GGGCGCGAAATCCCACGCCGATTGGCTGGGCACCCTGCAACGTATCGAAAAACTCAAACCGGTCACCGTGGTACCC
GGTCACTACCTGGGCACACCTTCGGCCAAATCTGTCGCCTTCACTGCCGACTACATCAAGGCGTTCGACGTAGAGA
CCGCCAAGGCCAAGGACTCCACCGCACTGATCGCGGCGATGAAAAAACGCTACCCGCACCTGGCCGATGAAAGCTC
CCTGGAACTGAGCGCCAAAGTCGCCAAGGGCGAAATGAAGTGGTGAATTGATCCAACCCTTACCCACCCTGGAGAA
CGTCATGAGCAAGATTGCAATCATTGGTGCCACCGGCCGTGCCGGTAGCCAACTGCTGGAAGAAGCCCTGCGTCGC
GGCCACACGGTCACTGCCATTGCCCGTAACACTGGCGCTATCGCGGCGCGTCCCGGCCTGACCGTCAAACAGGTCG
ACGCCCTGGACGCGGCAGCTCTGCAACAGGCCATCAGCGGCAACGACGTGGTGATCAGCGCCGCGCACTTCGCCAC
CCTGCCCGCTGCCGCAGTGATCGGCCCGGTCAAACAGGCCGGCGTAAAGCGTTTGCTGGTAGTGGGCGGTGCGGGT
TCGCTGCTGTTGCCGGGCGGCGGGCGGGTCATCGACAGCGAAGGTTTCCCTGCCGAATACAAGGCCGAGGCCAGTG
CGGGTGCGGTGTTCCTCGACACCTTGCGCCAGGAAAAGACGTTGGACTGGACCTTTCTTTCGCCCTCGGCAGAGTT
TGTCGCGACTGAGCGCACCGGCACGTTCCGCCTGGGCCAGGACGACCTGCTGGTGAGCAGCGAAGGCCGCAGTTGG
ATCAGCTTTGCCGACTTCGCCATTGCGTTGATCGATGAAGTGGAAACACCCAAGCATTCGCGCCAGCGTTTCACCG
TAGGCTACTAAACACACACCCTGTGGCGAGCGGGCTTGCCCCACCACTCGGTGCCTCGCCTAGGCTCGGCATGCCC
GCAGTCAGGCATTGCTCCGTGGGCCCGCNNNNNNNNNNGTTCTGGCATATCGCCACTGTTCACCCCGACAAATTTG
CACAGTTATTTCTGAGCCAGAACACTAGAATAGACGCCTGATCATTTTAGACTTAGAGCCCTATATGTCCGACCCG
GTTGATACCCCTCAGGTGTCCGACCTACCTCTGGAGGACCTGGTTGCCTGTCATGAGTGCGACTTGCTGATGCGCA
AGCCAGAACTTGCGCACGGTGAAAAGGCCCTGTGCCCGCGCTGTGGTTATGAGATGTACGCCCACCGCTACAATGT
CGTCGAGCGCAGCCTGGCCTTGGTGATCGCGGCGCTGTTGTTGTACATCCCGGCGAACTTTCTACCCATCATGCAG
CTCAATCTACTCGGGCAGTCTTCTGAAGACACGGTCTGGAGCGGCGTAGTCGCCCTGTTTAATACCGGGATGCAAG
GCGTGGCCGTGGTGGTGTTCCTGTGCAGCATGGGCATTCCATTGCTTAAGTTGCTGTGCCAACTGGCGGTCTTGCT
CAGCATTCGTTGGAACGTAGGCCGCAACTACGGCCTGTTGCTGTACCGCATTTATCACCATCTGCGCGATTGGGGG
ATGCTTGAGGTCTACCTGATGGGCGTGCTGGTGGCGATCGTAAAGTTGGCCGACATGGCCGCCCTCAGCGTAGGCC
TGGGACTGGCCTGTTTTGTCAGCCTGTTGATGGTCCAGGTGCTGCTGGAGGTAGTCATGTCACCTCAACAGATCTG
GCAAGCGTTGTCCGGGGAGGACGAACATGCGGGCGATTGATGCTGGCATTCTGATCTGTACTGAATGTCACGAATT
GAACAGGCAAGACCCGGACACAGACGAGCAAACCTGCACCCGTTGCGGCGCGCTGGTCCACGCTCGCCGTCCGAAC
AGCCTGATGCGCACCTGGGCATTGCTGATTACTGCGGCAATTCTGTACATCCCGGCTAACCTGTTGCCTATCATGA
CGGTCAACTCCCTGGGGCAGGGCGCGCCGAGCACGATCATGGCCGGCGTGATCGAACTGGTTCAGCACGGTATGTT
CCCGATTGCCGCCGTGGTGTTTATTGCCAGTATCCTGGTACCAACCTTCAAGCTGGTAGGCATCGCGCTGCTGCTG
TTCTCGGTGCAGCGTCACCAGCCGCTTTCTGCCCGACAACGCATTATCATGTACCGCTTTATCGAATTCATCGGCC
GCTGGTCCATGCTGGATATTTTCGTGATCGCTATTCTGGTTGCGGTCGTAAACTTTGGGCGACTTGCCAGTATCGA
GGCCAATCTCGGTGCCGTGGCATTCGCCAGTGTGGTGATTTTGACGATGCTTGCTGCAGTAACTTTCGATCCCCGA
CTGATTTGGGATAACACGGAGTCGGACGACGACCATGACTGATTTGCCAAAGGCTAAAACCCGCCCCGCTTCGAAC
TGGTCGGCCATATGGGTACTGCCCTTGATCGCCCTGGTGATCGGCGGCTGGCTCGGATGGCGTGCCTACAGCCAGA
CGGGCATCGAGATCCAGGTTCGCTTTGAGAGCGGCGAAGGCATCCAGGTCAACAAGACAGAAGTGGTCTACAAAGG
CATGCCTGTGGGCAAGGTCAAGACCCTGGCCCTGGATGATGAGGGCAGTAATCGCGGGGTGATTGCCACCATCGAG
ATGAACAAGGATGTCGAGCAGTACCTCAAGGCCAATACGCGCTTCTGGCTGGTCAAGCCAAGCGTCAGCCTGGCTG
GCATTACAGGCCTGGAAACCCTGGTCTCGGGCAACTATATCGCTGCCAGCCCTGGCGACGGCGAGCCTACGCGCAA
GTTCAAGGCGCTCTCCGAAGAGCCACCTTTATCCGACGCCAAGCCCGGCCTGCACCTGACCGTCAAGGCCGAGCGG
CTCGGCTCGCTCGACCGTGGCAGCCCGGTGTTCTACAAACAGATCCAGGTCGGCCAGGTCAAAAGCTACTTGCTGT
CCGCCGATCAGAACACCGTCGAGGTGAAGATCTACATCGAGCCGACCTACGCCAGCCTGGTGCGCAAACATACGCG
CTTCTGGAATGCCAGCGGCATCAGCATCGACGCCAACCTGTCCGGGGTCAAAGTGCGCAGCGAGTCCCTGGCCAGC
ATTGTCTCCGGTGGTATCGCCTTCGCCACGCCAGAGAATCGCAAGGACAGCCCGCCCACCGATCCGAGCCTGCCGT
TCCGCCTCTACGAAGACTTCGATGCCGCCGCCGCGGGTATCCGCGTCAAGGTCAAGCTCAGTGACTTCGAGGGCCT
GCAGGCAGGTCGCACACCTGTGATGTACAAAGGTATCCAGGTCGGCAGCCTCAAGGCTCTGAAGGTGGACTCTGAT
CTGTCCAGCGCCAGCGCCGACCTGACCCTCGACCCATTGGCCGAGGACTACCTGGTGGAAGGCACCCAGTTCTGGG
TGGTCAAGCCGTCCATTTCCCTGGCGGGCATCACTGGCCTGGAAGCGTTGGTCAAGGGTAACTACATTGCCATCCG
TCCCGGCGACAAAGGCGCCAGCCCGCAACGCGAGTTTGTCGCGCGGCCTAAGGCGCCACCGCTGGACCTGCGCTCC
CCAGGCTTGCACCTGGTTCTGCTCACCGAGAGCCTCGGCTCCCTGGAGGTCGGCAGCCCGATCCTCTACAAGCAGG
TCAAGGTCGGTTCGGTACAGAGCTATCAGTTCTCGCGCAAGCGCAAGCAACTGGTGATCGGCGTGCATATCGAGAA
GGAATACGAAGGCCTGGTCAACGGCTCGACGCGTTTCTGGAACGCCAGTGGCGTGACCCTCACCGGTGGACTCACC
GGCGGCATCCAGGTCAAAAGCGAATCCCTGCAAAGCCTGATGGCCGGTGGTATCGCCTTTGAAACCCCTGAGCCGA
ATGTGCCGCTGAGAAATCGCATTCCACGTTTCCGCCTGTTTGCCGATCATGAAGCCTCCAGCCAACGCGGCACCCT
GATCACCATCAAGGTGGACCGCGCCGATGGCCTGCGCACCGACACGCCGATCCGCTTCAAAGGCCTGGACGTGGGC
AAAATTGAAAGCGTCGACCTGAGCGCCGACATGCAGTCGGTGATGCTCAGCGCACGCATCACCGAAGTGCCTGAGC
GGATCGCCCGCGCCGGCAGCCAGTTCTGGGTGGTCAAGCCCGAGTTGGGCCTGATGAAAACCGCCAACCTGGAAAC
CCTGGTCACAGGCCAGTACATCGAAGTTCTGCCACCGGTGAAAAGCACCGGCCCGCAAAAGAACTTCGTGGCCCTG

GCCCAGCCGCCAGAGACCAATGTGCAGGAGGCCGGCTTGAGCCTGGTGCTCAGCGCCGCCCGTCGTGGTTCGTTGA
AAGTTGGCGTACCGGTCACCTACCGTGAAGTCACCGTGGGCAAGGTCACTGGATACGAGCTGGGCAAGACCGCTGA
TCGCGTGCTGATCCATGTGCTGATCGAGCCCAAGTACGCACCGTTGGTTCGCAGTGGCAGCCGTTTCTGGAACAGC
AGTGGCTTTGGTCTGGACTTCGGTCTGTTCAAAGGCGCGACGGTACGCACCGAGTCCTTGGAAACCCTGATTCAGG
GCGGGATCGCCTTTGCCACGCCAGACGGCGAGCGCATGGGCAACCCGGCGCTGCCTGAGCAGACGTTCCCGTTGTT
CGACAAGTTTGAAGACGAGTGGCTGATCTGGGCGCCGAAGATCCCCCTGGGGAAATAAATCCAGGAGCTACAAAAA
AGGCCGCGATCCAAAAGATCGCGGCCTTTTTATTGCTTCGCAGGAGCCGGCAAGCCAGTTCCTACAAGGTGTGCAG
CATTCGGTCAGACCTCATCCAACTCCGGCTCGTCCGCCTGCACATTCACCGTTGCCTTCACCACATCATGGCGACG
GATGTACTTCCAGTCCGCCTCATCGATGTAGATCCCGTTCGGCCCGCTGCCGCCTTCCAGGTCGATCGCCACCTGG
GCGGAAACCTGCGGCTTCACACTGGCCAGGATCGGCACGAAGCCCAGTTGCAGGCTGGTTTCCAGCAGGGCTGCCT
GGTTCTTCTCGTCGATATCCGCTGCCTCATCAAGGTAGTACGGCAGGCGCACACGACCGGCCTGGTCGCGGTCCAT
CAAGTGCAGCAACAAGTACATGTTGGTCAGCGCCTTGATGGTCATGGTGGTGCCGTTGGACGCGGCACCATCGATG
TCGGTGTGAATCACCGGCTGGCCATTGACCTTGGTGATCTCGAACGCCAACTCGAACAGATCCTTGAGCCCCAACT
GGTTATGGTTGGCGGCGACCAGGCGCGCCAGGTATTCCTTGGCCTCTTCGTTCTTGTTGTCCTGCTCGGCGCTTTG
GCTGAGGTCGAACACCGACAGGGTCTCGCCTTCCTCATACTGACCAGCGCTGTGGATGATCTGGTCGATATGCTTG
AGCGCTTCCTTGTTTGGCGCGAGCACAATGCGGAAGCTTTGCAGGTTCGACACCTGGCGCTTGTTGATCTCGCGGT
TGAACAGCGCCAACTGGTGCTCAAGGCTGTCGTAGTCGCTGCGGATATTACGCAGGGTCCGCGCGATATCCGTCAC
CGCCGCACGGCGTGCCTTGCCCAGGGTCAGGGCTTCGTCGGTACGGTGTGCGTACGCGTTGATCAACAGGTGCAGG
CGACGCTCCATATCGTCTTCGCTGTCGAACTTGGCCACGCCCTTGAGGCGCACCTGGGCATACAACGCCTCGATCT
GGCCATCGGCGCGCAGCAAACCCTGCCAGCTGTCCTGATAGTCGTTGAGCAGTGGCAGCAGGTTGTCCATGGAGTC
GTCGACCGGGTCCATGAACGGCGTGCCGAACGGCAAGTCAGCCGGCAACAACTGGCGACGGCGCAGCGCGTCATCC
AAAGTGCGTTGCTTGGCTTCCATATCGCCGATTTGCCGGCCGACCAGCTGCAACTTGGCCGACAGCTGCTGGACGC
GCTCGGTAAACGCATCGCTGGAGCGTTTCAACTCGTCCTGCGCGGCTTCCATTTGCGCCAGGTGCTCCAGTTTTTC
GCCTTCCTCGGCGCTCAGGGTCTGGCTGCGACGGAAGTCTTCCAGGGCTTTCTGCGCATCCAGTACTTGTTGGTAC
AGCGCTTCAGTCTGGGTCTTGCTCGCCGCCCGGTCAGACGCTACCGCCTGCTGGGTTTTGAGTTGCTTGAGTTCTT
TTTCCAGGCGTTCTTTCTGGTCGCGCAATGCCGCACGATCCGCCAAGGCTTGCAGGGCAGGTGGCTCGATATGCGA
GATGTCGATGGACAGCCCCGGCACTTCAAAGCGCTCGCCGGTGAAGCCATCAAGGATCAGCTCCAGGGATTTTACC
CACTGGCCGTCTTCATCCAGGGTGATGCCATGTTCGCCCAGCGGCAGGCTGAACAGCGAACTGTTGAACAGGCGCA
TCAGACGCTCGACGTCTTGCTGTGAGAATTCTTCCCGCAGTTTGGCGTAGCTGTTGTTGTCGGCGTGATCGAGTTG
CTGCTTGACCGATTTCAGGCGTTTTTCCAGGTCGCGCAGGCGCTCATCCAGATCCTCGGCGCTGAACTGTCGCGAC
TGCGCCAGGGCGCCGGCCAGTTCGTCGTGCGCATCCTTGGCCGCCAGCAGTTGCTGCTCCAGGACCTTGACGTCAT
CCACCAGCGCAAAGCGATGCTTGAGCACCGACAACTCGCCCAACCAGCGCTGGATGCCGCTGATTTCCCGCTCCAG
GCGCATCAGTTCCTGGGTGCCACCGCGCTGATCGTTCTGCAGCGCATCCTGCTCGTTGCGGTAGTGTTCGGCCTGG
ATTGTCAGCTCTTCCTTGCGCGCACTGGCGTAGTCCGACCAGGTGCCCAGCAACGAATCGAGCAACGGCGACAAAC
GGTGAAGCTTGCCACGCAGGACGTCGCGCTGTTTCACACCATTGGCCAACGCTTCGACCAAGGGGCCGGCGGCGAC
CAGGGAGTTGTAGTCCTGCTCCATGCGCCGCACATCGCGGAAGGCTTCTTCGCACGCGGCGATGTAATCCACACTG
CCAGAGCGCAGGCTGTGCTCGAACGCATCGAGGAACAGTTGCTTGAGCTTGGCCGCAGTGATTTCGCGCATGTGCA
GCAGGTTGATAAACAGTGCGCGGAAGGTCTTCAGGCTTTGCTCGCTGGTGGAGCGCAGCGGAATCAGGGTCAGGTC
CAGCGGGATCGAAGTATGGCCACCCACCAGCAAACGTCGCAGTTCATCGGGCTTGAGCTCATAGGCTTTCAGGCCT
TCGCGCTCAAGGTTGGTGAACAGCTCTTTCTGGCGCAGGCAGGTGTCGTTCTTCTGATAGTGGGCCAGGTCCAGCT
TGCCGGCGTAGGCAAAGAACTGGTGACCGAAACCGCCGCCCGGGCCGCGGCCGACCACGCCGATCACGTGGGGGCC
ATGGGGCAGGGACACTTCGACGAGGATGTAGCTGGTGTCGGTGGCAAAGTAGAAGCGTCGCGACTGTTCCAGGGTG
TACTTGCCGAAGCTCATGTCCGACATGCGCGCCAGGATCGGGAACTGCAAGGCGTTGATCGAAGCCGATTTACCGA
GGTTGTTCGCGCCATACACCGACAGCGGCTCTTCCAGCGGGAACAGGCCCAGGCTGTAGCCGGCGGTATTCAATAG
GGCGAAGCGGCGAATGCCGTAGCGTTCCTTGCTCATGCATCGGTCTCCTGTTCTTCGGCAATGGCGCGGGCCAGGG
CGTCTTCCTCGCTTTGTGTTTCAAACTCGCTGAGGTCCAGCGGGTCATCGGTCTGCAGCAGTTTTTCATCGCTGTC
TTCGTCGATGATCACCGGCGCCGGCAACGGCAGCACGCTGTGTACACTGGCCGCCAGGTCGCGGTCGGCCTGGACC
GACAGGCACACGTCGAGGAAACGATGCATTGGCGGCAGGAACCGGTAGACGCCGTTATCTTCACTGGCAAAGCCGA
GCTGGGTCATGCGGCGCATGATTTTTTCTTCCAGCTCTTCCTGGGTCTGCACTTCGGCCTGGATAAACAGGTCGCG
GTATTTCTCCAGCAAGGAGGGCAACTCATCGCGGCCCAGGCTGCCACCATCCAGCACGGCAATCGGGTCGCGGCCC
TGATCGGCCAGGTGCTCGACGATGATGAAGGTGAACAGTGCCAGGCGCTGTGCGGTCTTGTTCACCTGCGCGGCGG
CTACGGCGGTGTCCGGCACGAAGTAGTAGAAACCACGGGTATCGCACACCAGCTCAAAGCCCAGGGCCTTGAACAG
CGTGCGGTACTGGTCCTGGAAGTTCGACAATTGCGCGTACAACTCCGGGTCGCGGCGGCTGACGTGGTAACCCTTG
AACAACTCGCGAAAGATCGGCGCCAGTTGGGACAGTTCGGATAGATCAAGATGCATGAGGTGTGCTCGCAGAGTGC
TCGGCCGCGTCGCTGGCCGGGAGCAGGGCGAATGAGCGCAGGCTGACCTGGTGCTCGTGAGTATGGTAATCCTGGC
GTTCCAGGCGCTCGCGCTTGAAACGCTTCTCCCGCGACAGGCGCGAGAACCAGTACAGCAATTCGTCGGTGGCACC
GTCCGGTTCCTGCTCCAGCAGCCAGGTCATCAGGTCCGGCATCGGCAGGGCGTCTTCGCAACGCTCCAGCATCTCC

TTGACCGTACGCGGCGCACGCTGGGCTTCGCCCTTATGGGATTTGTGCGCCTTGGGGAAGCGCGCCGGTTTAGGTT
CGAAGTTCGCCAGGGCATACACGTAGGCCTCGACCTGACTGGCACTGCCCAGGAAGGTACTTTGCGGCCGGGTAAA
CATCGGCATCGCCGCTTGCGGCACCGCGTCCAGGCCCTTGCGCCGGATCGCCGACAGGGCCAGCGCTGCGCCACGG
GTCACGGCGTTGTGCCGGCGTGCTTCTTCACGCAGCGGCAGCAGCAGTTCGCGGGCGTGACGCAAGGTCAACTGGG
CGCTGGTCTGCATTTCGAGGATGCGCGCGTGGGTGCGCAGCAACATGTCGTCATCCACCAGGTGGCCCAGGCGCTG
CTGCTCGGTGAGCATGCGCAGCAGCACGTTCTCCACCTTGCGTACGCCTTGCTCGAAGGCACCGTCGGCGTTCACC
AGTTGAATCATCGGTTCGACGTACTCATCCCAGGTCGCCAGGACCTCGGCATAGCGCTGGCGCAACGGGATCTGTC
GGTCGCTGGTCTTGGCGCGGTCGGCCACGGCAGCGAGGGCCTGTTCGTCGTTGGCGAGTTTTTTCAGCACGTCACG
CACGCGCATGTCCAACAACCGCAGTTGGCGGGCCAGGTCATGGCCGTCGCGGATGTCGAAGGCGTCCTGGATATAG
CCGGCCAGGCGCTCGAGATGGCGCAGGTAGGCTTCGATTTCCAGGCACAGGCCTAGCCGGTGCTCCTTGCGAAGAT
AGGCCAGGAAGTCATGGATCTGCGCATTGAGCTCGAAACGGTTCGGGCTTTTGGCGACAGGCACCAGGATATCCAG
GCGAATCCACACGTCCAGCAGGCTGGTGATGTCCTGTGGCGTGCTGTCCAGTTGTTGGGCGGCCAGTTGCGCGCGC
AGTTCGCCAAGGCTCAGGGTGCCTTGGTCGAAGTGTTCGCACAGTGGCTCAAGCAGGGCCCAGTGTTCGGCGAGGG
CGCGCAAGACGCGCTTGGGTTCGATCATCGGAATGGCCGGCTGGTTGGCGATTAAAAGCGGCGATTGTACTGCATC
AGGCGCGGGATTTATCCACAGCCGGTCAGGGCGCCTTCGCGATGGAGGGTCAGGAATGCAGAGAAAAGCATCAACG
GTGGGCGATCGTTGCCGAAGGGCGGTAGAATCTGCGCTCTTTAGTTATCCACAAGTGGCCGAGCCTTGCTTATCGA
GTCCCGACGTCGCGCTTACTTGACCGCCATGCAGGTGGTCAACTGGCTGCCGCGCACCGAATTGCCCTTTGCCGCC
CCGTCGCGGCCCGAACTGCTGCAAGCGCTGGAACCGTTCGAGCCGTTCGCTGCCTCGGGCGAAGAAGCGGCGGCGC
CGGTTGCGGTAGTCAAGCCCGCGGCTGAAGCGTCCGTGCGGCCATCGGAGCGGGTCAAGATCGAAGTGCCGCGCCC
ATCAATGGCGCCCAAGGTCGCCGCAGTGGCCGAAGATGCCCCGGCGCCGGTCGCCAAGGCTCCGGTCGTACCGCCA
CCGCGTTTTGCCTTGCAACTGCTGCGGGCCGGGCGCTGTTTGTTGTTGGTGGAGTTGCCGACCGGCGAACGTTTCC
AGGCCCGCGATCCTGCCTATCTGCTGCTCAAGGACATGCTGCGCGCCGCCGGCCTGCCCGACAGCCCGCAGATTGT
CGGCGAGCCCGTGCGCTGGCCGCTGCTGGTGCGCGGCAATATGGACCAGGGGCCGGACGCCGCGCGCGACTTCGTG
CAAGGCTTTGTTTCGGCCCGCCTGGAAGATGAACCCTGCGTGTGCCTGTGGCTGATCGGCCTGCCCGCCGTGCGGT
TTGCCGGTGAGGCGAATGCCGAGGCCTGGTACCGCGAGCTGCAGGTCGACGGCCTGGGTTCGGTGTGGGCCTTGCC
GGGCCTGGAATTATTAATGGAAGAGCCACAGCGTAAGGCTGATGTCTGGCAAGCCATGCGCCGGCTGATGGCGCGC
TGGAAAACAACCGATGAGTGAGGCTTTATCCTTTCGCCCGATGACCGAGGCTGACCTCGACGCGGTGCTGAAAATC
GAATATGCGGCGTTCAGCCACCCCTGGACCCGTGGCATCTTTCTCGACGGGCTGGGCAAGTACCAGATCTGGCTGA
TGTTCGAAGGCGAGCAGCAGGTGGGCCACGGTGTAGTGCAGATCATCCTCGATGAAGCGCATTTGCTGAACATCAC
CGTCAAACCGGAAAACCAGGGCCGCGGCCTGGGCCTGGCGTTGCTGGAACACCTTATGTCCCGTGCCTATGCCGCC
AGCGCCCGGGAATGCTTCCTCGAAGTGCGCGACAGCAATACCGGCGCATTTCGCCTGTATGAACGCTATGGGTTCA
ACGAAATCGGCCGTCGCCGCGATTACTACCCTGCCGTCGGTGGTCGCGAAGACGCCGTCGTCATGGCCTGCACCCT
GGTCGACTAAACACCCCTTGTAGGAGCTGGCTTGCCAGCGATAGCGGTATTGAATTCACCATCGCTATCGCAGCAT
AGGTATCTACACAACTATCTTCTTGGTGTGTGTACATATCCGTTGCTGCGATCACGGCCACTTAAGGTTCCGCTCT
TACAGCGGGTCACTTTCGAAGAGCGCGAAAGTAACCAAAGCGCTCTTGCNNNNNNNNNNGGTTTTGGCCACTGGCT
TGGCGACCGATTTTGCAGGCGCTTTTTTAGCAGCAGGCTTGGCCGCAACTTTAGCCGAAGCCTTGGCCGCAACTGC
CTTGGCCGGTGTACGAGTGCTCAACACTTTGGCCACTGCTTCTTTCACACGACCAACGCCCTGGGCCAGTTTCAGG
CTCTCTTGGGCATCACGCTTGAGTTGAAGGATGTAGGTGCGGGTTTCGGATTGACGATCCTTGAGGGCATCGAGCA
AGTCCTCAAGTTCTTTGACAGCGCCCTTGGCTTTGGTTTGCGCCTTGGCTTTGCCGGCAGTCGCGGCGTCTTGCAG
TTTGGTGCGGGATTTATGCAGTTTTTCTTGAGCCTTACCGCGTTGCTTTTCCAGCTTGGCGAGCAATTTCTCTGCA
TCAGCCAAGGCTTGGGAGCAAGCACTTTCCAAATGTTCGAGCAGGCTGCCCGAGAGTTGTTGGAGCAAGTGCAACG
GGGTATTAACAGGCTTCTGTTTGGCCGACATGGTTTACCTCCTGGCTGACGTGGGTGCGGCTCATACTAGCCCTCT
GCTCTTACCGCCGCTAGGGCATGTTGACAGTATCGAATGCGTTGCGTTGCACCGCACAAAAATTCTTATCGATATA
ACGAAAAACCACTCCATTCTTTACCCCTCCACACTGGCATAATCCACCGCACTTTCGGCTGGAGAATACCCATGTC
GCGTTACCTTTTTTTCATCCTGGGTTTGTCGTTTTCAGTGGCCAACGCGAGTGAGCCAGCAGCGTCCAAAGACAGC
CACGACCTGGCCTACAGCCTGGGCGCCAGCCTTGGCGAACGCCTGCGCCAGGAAGTCCCCGACCTGCAGATCCAGG
CCTTGCTCGACGGCCTCAAGCAAGCCTATCAAGGCAAGCCCCTGGCCCTGGATGACGCACGCATCGCTCAGATCCT
CGCCCAACACGAAGCGCAAGCCAGCAGCGAGGCACAAGTGCCGCAAAGCGAAAAGGCTCTCGCCGCCGAACAACAA
TTTCTGAGCACGGAAAAAGCCAGGAACGGTGCGCGCGAATTGGCAGACGGTATCTTGTTCACGGAGTTGGCCCCTG
GAAGCGGGAAAAAGCCAGGCGCCAATGATCGGGTGCAGGTGAAGTACGTGGGGCGATTGCCGGATGGGACGGTTTT
CGACCAGAGCACGCAGCCCCAATGGTTTCGCCTGGACAGCGTGATCAGCGGCTGGCAAAGCGCGCTGCAACAGATG
CCGGTAGGCGCAAGATGGCGCCTGGTGATTCCTTCTGCACAGGCCTATGGCGCAGACGGTGCGGGCCAGTTGATCG
CACCTTACACACCGCTGGTTTTCGAGATTGAGCTGCTCGCAATCGGTGCCTGAGCCCAACGAAAAACGGTGCGCCA
TGCGCACCGTTTTTTGTCTTGCCGCTGAGGCTCAAGCCTGGGTTGCAGGCTCTTCCTTGTGGGCGTTGTGCAGCAC
TTCGATCAAGCAGTCTTCCAGTTCGAAACGCTCATGGAGCAGGCCACCCAGCTCTTTGAATTTCGCGGCAACGCAC
TCACCCTTGTCGCAAAGGTCGTTAAACGCCAGGAGCTTTTCGGTGATGACGTCGATACGCGGGTAAATGGTCTTGG
CCAAATCCAGGCCACGCTGGTCATCGAAGGCCTCGGCCTCCTTGGTCAGTTGCTCATAGACCCCGAAGTGCCCTGC

CGAGACGTAGTCCACCAAGACACCACAGAACTCCTGCAGAGGCTTGCGGTTCTCAGCCAGCGCTTCAGGCTTCTCA
CCCAGAGCATCAAAGGCCCGAACCAATTCGTGACGTGCCTTCAACCAGCTGTCGATCAGCTTATGCACACCACCCC
AGCGTTCCTGAGCATTCTGACAACTTTCCAACATGATGATCTCTTCCCTTATGGGCGGTGCCGCTTTCACCCGCGC
GATGCGTCGAGGATAAAGCGGCAAGCGGACAACGCCGCATCGAACAAACTGTTTCAATAACGCGTGCGGGCCAGAT
TATGCCCCCACGACTATGGCTTCAAGGTACGCAGACCAGAAAGTTCATACAAGTGTTTAATCCCATCCTACGAACC
AACCCAACCAGTCAGTGCTTCACAAACCGTAGATTCTGACCGGGTAACAGTCGGGAAAACTGCGTCACCCCCAGTA
GGAGCATTGCCACGAAGAACAGCAGGCTCCACTCCGGGATGCTCAGGTCAAACAACGTCCAGTTGATTTCCACACA
GTCGACAGTGCCCTTGAACGCCAACTGCAGGGCTTGCCCCAGCGACAGGTTTTCGATCATGTATTCCAGGCTTGGC
CAGCAGTTGGGAACCTGGTCAGGCGGGATGTTCTGCAATAGCACCTGGCGCACCGCTGTCACTGCGCCGAGCAACG
CACAGCCCATGCTCGCCGCCCAGTACAGGTAGATGCCCACGCGCCTGGGGCCGTGGATCGCCGCTATGAAGTTGAT
CAGGGTGAACACCGCCAGCAGGCTGCGCTGGATCAGGCATAGGAAACAAGGCCGCAAAAGAGCCCCGTATTCCAGG
TAAAACGACGCGCCCAACGTCAGCACACCGGCCATGAACGCCAGGAAGAACAAGGAGCGTGAAGGGGCCAAAGACA
TTGTTTATCCGTAACGCAAGAGATAGGTCGTTACGGTAGAGGAAAGACCTTACCCCTTTCAATACGCGCTGGAGCA
GACACTTCCGCGAGATCGCAGGGATATCCCGTCAGAGGGCAGGTAACTTTACGTGGTGCCCTGTGGGAAACCACCT
CAAGCCCCTGAATCCGCAAAACCGAGGGATTTTGAAGGACTGGTTGCCAGATGCTTCTGAGCCAGCCCTTCAAAAC
ATCCTACGTCAGACCCGTGCAGGCACCGGTAGCGGTGAAGCCAGCAGGCGGCTGTCCAACAGCCCCAGGCCCTCCT
GGAACAGTTGATTACTGCGCTCGGTATCACCCAGCTGCGCGAGCAATCGCGCCAACTCGGCACAGGCCTCGGGGTT
GCGTTGAACCTGCAGGCTGCTTTCCAGATAGTCCCGCGCCTTGCCCCACAAACTGTTTTGCAGGCAGAGCCGACCC
AGCGTCAGCAACAAACTGGCATCGCCCGGATGGTCCTTGAGCCAGCCTTCGGCGAACTTCAACTGCCGTGCCGGAT
CGCCACCGCGCAGCAGGCCATACAACCGGATCAGGTGACTGTCGTAGCCGCGCTTGAGCGCAACACGCAAAGCCTC
TTCGGCCTTGGCATCGGCGCCCAGTTGGCGCAACTGTTCGGCATAGGCCAGTACCAGTTGGGGCTCCTGGCGCTGG
GCCGAGGTCAATTGCTGCCAGGCCCGCTCAAGGGACTGCAGGCCGGCCTCACCATGCTCTTCACGCTGGACGGCCA
GGCTCAGGTTTTCACCCCAGGCCCGGCGCTCCAGATCCGCCAGCTCGGTGGCTGGCAGCACTTTATCCTTGCGCAG
CTCCGGCAAGAGCCGGATCACCGAGGACCAGTCACCACGCTGCTGGTGCAGGCGCTGGAGCTGGCGCAATACCTGG
GCGTTGTGCGGATGGCGCTCATGCATGGCCTGCAGCGTCACGAGGGCTGCGTCCGTGTCGCCACGGTCCATCTGCA
ACTGTGCGTGGCTCAAGGCAATCGCCAGCTCGGCCTGGGGCTGACGCTCCAGGGCACGCTCCAACAGGCTGTCGGA
CTCTTCATAGCGCCCCTGCTCGTTGGCCGCCCGAGCCGCGCCGAGGTAGTAGAGCAATGGCTGGCGTTCGGCTTCC
GCCGCACGGTGCAGGTGCCGCTCGGCACTGGCCCAGCGGCCTTCCGCCAAGTCCATCTGCCCCTGCTCGATAGCCA
CCTGCACCCGCCGGCTGCGATTGCGCCGCGACCAGGGATTGACCACGCCACCCGAAGTCGTCACCAGGCCCAGCAG
CACGCGCAGCAGATAGATCGCCAGGCCGACGACAAACACTGCCACCAGGGTCGACCACAGGCCGGACTCATAATGC
AGCACATGGGGATAGCTGATCAGCACATAACCGGTGTGTTTCGAGATGCCCACCGCCAGCGCCAGAGCGAGGGCAA
TGGCCAGCACCAGGACCACATAGAAACGCTTCATCGGCTTTACTCCTGGGTCGCCGGCTTACCGGCGGAAGCCTTG
GCTTCGTCGGCAGACAGATGGCGGCGTTCAAGGTAAGCCTGCACCGCAGACAAACTCGCGGCGAGGTCCGGGGTCA
CTACCGACACGGCCTTGGGCTCAAGCTCGGCAATACGCGCCAGCATGGCCTGGCTTTGCGGGTTGTCCTGGTTGAA
GTTGCCCTGCAATACGCTGCGCGCCTCGCCCAGCGCCCGAGCGTACACCGCCGGTTCGCCATTGAGTGCGGCCCAT
TGCGCCTGCTCCAGCGCCAGGCTCAGGGCCAGGCGCACCTGGTTCAGGCCTTGGCCTGCCAGCAATGGGCGGATGT
TGTCATCGGGGTTGAAATCGATACGGAAGTAGCGGGAGATCTGCTCCCACCATTGGCTCCAGCGACTGTCAGTGTC
GGTGCTCGGACGGCCCTGGGGGCTCTCTTGCAACTGATATTCCGGGGACACGGCGGCCAGTTGCACCACCTGGTCA
CGCAGCGCCGCCAACTGCAGGTACAACCCGGTGCGGTCGGGCTGCTCAACGCTGCGCAAGGCCGCCAGGCTCTTGG
CCAACTGCTCGCGGGCGGCGTAGGAACCCGGATCGCTCTGCTCACGCAGGATCTCATCGGCGCCCTGCACCAGCGC
CTGGGCACTGTTGATGTCCTGCAACGCTGACAAGCGCAGGCTGGCCAGGCGCAGCAGATGCTCGGCCTCGGCCAGG
CGCCAGTCCTTGCGGCTGGCCCCCAGGACGGTTTCCAGACGCTGGTTCAGTCGCTGCTGGTCGCCCTGCAATTGCG
CCACCAGGCGCCGGCGTTCTTCCAGTTCATCAGCACCCGGCAATTGCGCCAGGCGTGCGGCCAATTGCTGCTGCGC
CTGCTGGATGCCCTGGGATTGTTCATCCAGGGTGCGCACCTGCACCAACTGTTGCTGGCTGCTCGTTTGCAGGGCC
CGGACCTGCCAGAGCCCCCAACCGCCTGCCGCAACACCCGCGGCGCCAAGCAGCAAAGCAAAGACCGCCAGGCCGT
TGCCACGGCGAGGCGTAGTGACCGGGGTCTCAACAGGCGCATCAAGCGCGGGCTGAGCTTCATCTTTAGGCAAGGC
TGTTTCGCTCACGTGTCCATCCTTTGCGTTATTAGAGAGTGGGAACGGGCTGGCTCCGTAACGCCACTAGCAAAGC
CGCGGCACTGGCACCGCGGCAATCCACAACTTTTTTCGCGCCCGCAGCCCGGGCCATTTCGGCAACTCGTGGGCTG
GGCACGAACAACGGCAACTGTGCCACGCGTGGCCAATCGGCACCGGCGAGGCCATGCAGATGCAAAAAACCCTGCC
CACTGCTGACCACCAGGCCGTTCAAGCGTTCCACATCAATGCGCTGGGTGAGCACATCCGGGTCGTAGGTCGGCAA
AAACCGACGATACAACTCCAGATAATCGACACTAGCACCTTGGCCGCGTAAACGCTCAGCCAGCAACTCTCGCCCC
CCCTGCCCACGTAGGATCAATACGCGCGCACCGTCAACAGCGACAGCCTCGCGCAACCTGGGCAATTCAAGCAAGG
CCTCGCTGTCGTCCCCAACCTCGGGGTAGCTCACATCCAGGCCATGGTCAGCCAGAACCCGGGCCGTTGCCGCGCC
GACGCTGAACCACGGCAGGGTCGGCACCGCTGGGGCGAACTGAGCGAGCAACTGCAGCGCCCGGCGCGCGGCCGGC
TTGCTCACCACGATCACCGCGCAATAGCAGTTCAGGCGCTGAAAAACCGCCTGGTGCTCGGCGGTGATGGGCAATG
GTTGGGTTTCCAGCAACGCCAGGCTGCTGCTGAAGATACCGGCTTCGGACAACGAGGCCGCCAGGGCTGCCGATTC
CTCGGCAGGCCGCGTCAGCAGCACACGCCAACCGGTCACTGCGGACCGGCCTCGCCATAGACTTTTTGCAGAATGG

CGCCGGCGCCTTTTGCCAGGAGTTCTTCGGCAACCTGCACGCCCAGGCTCGCAGCCTCGGTTTGTGGCCCGCGGGT
TTCAGCCGTGAGCAGCAAACCGCCGTCGGGATCACCCACCAGGCCGCGCAGCCAGAGGTTGTCACCCTCAAGTACT
GCGTAGCAGGCAATCGGCACCTGGCAGCCACCGTTGAGGTGCTTGTTCAGGGCACGCTCGGCGGTGACCCGCACTT
CGGTTGGCTGGTGATCGAGGGGCTTGAGCAGCGCCTGGATTTCCAGGTCTGCGGTGCGGCATTCGATACCGACGGC
ACCCTGGCCTCCAGCCGGCAGGCTGTCTTCGACGCTGATGGCAGAGGTGATGCGCTCTTCAAAGCCCAGGCGAATC
AGGCCAGCGGCCGCCAGGATAATCGCGTCGTACTCGCCGGCATCCAGCTTGGCCAGGCGGGTATTGACGTTGCCCC
GCAGGAAGCGGATCTGCAAGTCCGGGCGACGGGTCAGCAACTGGGCCTGGCGACGCAGGCTGGAGGTGCCGACGAT
GCTGCCCTGCGGCAGTTCATCAAGGGAGGCGTAGGTATTGGACACGAAGGCATCGCGCGGGTCTTCACGCTCGCAG
ATGCAATACAGGCCCAGACCTTGGGGAAAGTCCATCGGCACGTCTTTCATCGAGTGCACGGCGATGTCGGCTTCAT
TCTCCAGCAGAGCGGTTTCCAGCTCCTTGACGAACAAGCCCTTGCCACCGATTTTCGACAGCGGCGAGTCGAGCAG
CTTGTCGCCACGACTGACCATGGGCACCAGGGACACCGTGAGCCCCGGATGGGCTTGCTCAAGGCGGGCTTTGACG
TATTCGGCCTGCCACAAGGCCAGGGCGCTTTTACGGGTGGCGATGCGGATTTCGCGAGAGGACATGGATCAATCCG
TACTGAATAGATACGGCAGATAATAACAGCTCAGGCAGAACCGCCTTGATTTGAATCAGCAAGTGCGGGGCCTCCC
TGGCCGCTGCGCACCGGGATTCGGGGCATGCAGGTCGCTACAGCCCTCGGGCTAAAGCTGCTGCATCATCTTGCGT
ACGCCTGCGACATGGCGCCGGCTGACGATCAACGCGTCGCCGTTGAGCCCTTTGAGGAACAACTGAAAATGCCCCA
GGGGCGTGCGTTGCAGGCGTTCGATACGCTCGCGAGCCACCAGCGCGTTGCGGTGGATACGCACGAAGCGCTCGCC
AAATTCGTCTTCCAGGGCCTTGAGCGGCTCATCGAGCAGCACTTCACCGTGCTCGTGGCGCAAGGTCACGTACTTG
TGATCGGCAATGAAATAAATCACCTGGTCCACGGGAATCAGCTCGATCCCCTTGCGGGTCCTCGCGCTGATATGGC
TGCGGGGGCCATTGCCACTTTGCGCCGCAGGCTGGGTCAGGGCAGCAAGCTGCACGCGATTGGGCCGCTCGGCCTT
CTTCAACGCCTTGAGCAGCGCTTCGGCAGATACCGGCTTGGCCAGGAAGCTGACGCCGCTTGCCTCCAGGGTCTCG
GAGGAGAACTCCTCGTCGGTCGCGCATAACACCAGCGCCGGCGGCGACTCGCGCTCGCTCAAGCGGGCGGCGACCT
GCAACCCATCAAGGCCAGGCATACGGATATCAAGGAGCACCACGTCCGGCTTGAGGCTTTCGATCAAGGCCAATGC
CTCGTCGCCACTGGACGCGCTCGGTTCAAGGGCAGTGTATCCCTCGAGCTCACTGACCATACGGCTCAGTCGCTCG
CGGGCTTGGGGTTCGTCATCAACGATCAGGACATTCATATTGCGCTGGATTCCTGCGTGAGTCTCGCACAAGGATA
GCGTAGACAGGTGCGGTGACTTCCGTCACGGCGATCCACGCTAAGACTAGCGTGAGCGGCAAAAAGTGCCCCGAGA
GCGACACCAATATTTACCCGGACCTGTTCAATACCGCCCAAAACCCGATACCGCAGGGCATCGTCATAGGGTTTGC
TGATACTCAATATGAACACCCCCTCTACTTTAAGGTTAGCCTGGAGTTCGACCTCATCGCCTGGCATTGGTGCACT
GCACCCCTGTCAGTGCAACTGTAGACGCTCACGAAGACGACATTGCTCAATCGTCAAATATCGTTTCAATAAACAT
CCTTTGTTTACCTCAGGATCCGGTCGGGTGAAACCCCCGCTTTCACTCCCACCTCCAGTCTCATCCCGGACGCGGC
CAACGGCAAGGCACTTTGCCATCCATCAAACAAATAAAAATGCCGACGCGGCGTATCAACGCCTCCACGGGCAACC
CTGTTATTATCGGCGGCACACTTTCATGCCTCTTTTAAGCAGATCACGAGCGAATCCATGAGCACCGACAAGACCA
ATCAGTCCTGGGGCGGCCGCTTCAGTGAGCCCGTCGACGCCTTCGTCGCGCGCTTCACCGCCTCCGTCAATTTCGA
CCAGCGCCTCTATCGCCACGACATCATGGGCTCGATCGCCCACGCCACCATGCTGGCCAAGGTCGGCGTGCTGACC
GATGCCGAGCGCGACAGCATCATCGATGGCCTGACCACCATCCGTGGCGAAATCGAGGCCGGCACCTTCGACTGGC
GCGTGGACCTTGAAGATGTGCACATGAATATCGAGGCGCGCCTGACCGACCGCATCGGCGTGACCGGCAAGAAACT
GCACACCGGGCGTAGCCGCAACGACCAGGTCGCTACCGATATCCGCCTGTGGCTGCGTGATGAAATCGACCTGATC
CTGGGCGAAATCACCCGCCTGCAAAAAGGCTTGCTGGAGCAGGCCGAGCGTGAATCGGACACCATCATGCCCGGCT
TCACCCACCTGCAGACCGCGCAGCCCGTGACCTTCGGCCACCATCTGTTGGCCTGGTTCGAAATGCTCAGCCGCGA
CTACGAACGCCTGGTCGACTGCCGCAAGCGCGCCAACCGCATGCCCCTGGGCAGCGCTGCGCTGGCCGGCACCACC
TACCCGATCGACCGCGAATACACTGCGCAACTGCTGGACTTTGACGCCGTGGGCGGCAACTCCCTGGACGGCGTGT
CGGATCGTGACTTCGCCATCGAATTCTGTGCAGCGGCCAGCATTGCGATGATGCACCTGTCGCGCTTCTCCGAAGA
ACTGGTGTTGTGGACCAGCGCGCAGTTCCAGTTCATCGACCTGCCGGATCGCTTCTGCACCGGCAGTTCGATCATG
CCGCAAAAGAAAAACCCCGACGTGCCGGAACTGGTGCGCGGTAAAAGTGGCCGTGTATTCGGTGCGCTGATGGGCC
TGCTGACCCTGATGAAAGGCCAGCCCCTGGCCTACAACAAGGACAACCAGGAAGACAAGGAACCGTTGTTCGACGC
CGCCGACACCCTGCGCGACTCGCTGCGCGCATTTGCCGACATGATCCCGGCGATCAAGCCCAAGCACGCGATCATG
CGTGAAGCGGCCCTGCGCGGTTTCTCCACCGCCACTGACCTTGCGGACTACCTGGTGCGCCGTGGCCTGCCGTTCC
GTGACTGCCATGAAATCGTCGGGCATGCGGTGAAATACGGCGTGGATACCGGCAAGGACCTGGCGGAAATGAGCCT
GGAAGAACTGCGCCAGTTCAGTGACCAGATCGAACAGGATGTGTTTGCGGTGCTGACCCTGGAAGGGTCGGTCAAT
GCCCGTAACCACATCGGCGGCACCGCACCGGCCCAGGTCAAGGCGGCAGTCGCACGCGGCCAGGCGCTGCTCGCCA
GCCGCTGAAAGTATCGCAGGCAAGCTAGCTGCCACCTTTGACCGGGGCCTTCAATTCAACTCGGTCAACTGTGGAA
GCACGCTTGTGTGGGAGCTGGCTTGCCTGCGATAGCATCACCTTAGTGTCACTGACACACCGAGGTGCCTGCATCG
CGGGCAAGCCCAGCTCCCACACAAGCCCGCCCCCCCATTTACTACTTCTTCGCCGCAATCATCGCCATGAAGGCTG
GCATCGCCGCCTCCTTGTCCGCCGCCACTCGCTGGGCGTTGGGCATGTCTTGCAGCCTCTTCAGCAAGGCCTTGGC
CTTCGGAAGCTCAGCCAGCAAATCCAGGCCAAACAGCTTCCCACCGACGGCACACGCCAAGTCCACGCTGTACAGG
AAGTACAAGTCGGCCAGCGTAAAACTCTCCCCCGCGACATACGGCGCAAACTTGCCGTGTCGCCCCAACGCCGCAA
TTCCCAACAGCAACTCGGCCCTGGACTTGTCCTTGATCGCCTCTGGCACCGACATGCCAAAGAACGCCTCGGCGTA
ACACGCGCGGGCTGGCAGTTCGATGTACAGCTCGATTTCCCGGCACAACGCCAACACCTGGGCGCGCTGGAATGCA

TCGCCTGGCAGCAGGGCCGGGCCCGACTGTGTCTGTTCGAGGTATTCGAGAATCACGCTGGTTTCGTTGATGAAGC
CTTTCTCGACCTTCAGCACCGGCACCTTGCCGCGCGGGCTCACCGCCAGGGCTTCAGCGGTTTGCCCGGCATAAAA
CGGCACTTCTTCAAAGGGCAGGCCCTTTTCCAGGAGCGCCAGCTTGACCATGTTGTAGTAGTTACTGACCGAAAAT
CCGTAGAGCTTGAGCATCACAAAGCCTCCAGGCCGTGCAGGGGTTGGCTGGGTAGGTTATAGATCGTGACGCACGG
CCTGACCAGCAGCATCACCTGGCTGAATGGGGGTAGACTGGCGCCCTTTCCTGACAGGAGGCTGCCATGAGCGAGC
CAACCGATATCGATAACGACGAAGAAGAGTTCGCCGAGTCCACCTTGACCCAGGCGATCGAAAACCAGATCGAAAG
CGACAACCCGCCGGCAGCCAAGGCTACGTTCAACAAGCTGACCCTGGTCGGTTACGAGCGTGAAGACATCCTGAAC
CTGATGGCCCACGTGCTGGCCGTGGAAATCGACGCGATGCTGGAAACAGACCGCCCGTTCGATACCGATTGGTATG
AAACGGCGCTGCGCGCACTGCCTGAGCTGCCGCCGGAAAAACAGTAGGTCCCTGGAACCGCCATCGCCGGCAAGCC
GGCTCCTACAGGAGTGCGGCTTGCCTCCCCTGCGCCCATATCGCACTGGACAGTTCGCTCAAGTGCGTTCACCTTA
GGCTCTGCTAGCGTCTGACAATTCCTAGAACGTCTGGAGTCCTTATGTCGTATACCCCTGAGTTGGTTGCCGAACT
GGAAATCCTTGTACTTTTCCCCCTGGATAGCACCCATGAAGGTCTGAAAGTCCATCAGACTGCTGCTCCCACTGCC
ATTGCTGCTGCAAAGCGCCTGTTCGACAAAAAACTGATCGATCAGCCAGATGGCGGCTACCTGACCAGCCTGGGCC
GGGATGCTGCAGAGCAAGCGCAAACGTTGCTGACGATTCTGACCACCGCGACCACCAAAGAAGCTGCCTGACACTC
CGTATCGCCCTCGGGATCCTCTGTTCACGGATTCCGCGGGCATTCCTGTGTGTGCGATAAAATTCTGACGTCAAAA
ACAAATTCCCCTTAAACCTCCTACGATTCTGTCTGTAAACTCCTACACGGCTGCCCACGCCGGGCCCTGCGAGCCG
CTGAGTTCGACATGACCAGTACCCATGAAATCCGCCCTGACTTGGATGAAGGCATTGACCGCAAGGTACTGGCCCA
ACTGCGCTCGCGCTTCCTGACCCTCAATGCCGGCCGCATGGCCCGGGCCGTCGAAGGCCTGACGCCACGCCAGCAA
AGCGTGTTGGCGCTGCTGCCGCTGTTTTTCCACGTCAATCACCCGCTGCTGCCGGGCTATGTCTCGGGCAGCACCC
CGGCCGGGCTGGCGAATTTTGAACCCGAGGCCCAGGTCCTGGCCGAAGCCCAGCGCCTGACCCGCTCGTTTTCCTA
CAAACCACGCCACGGCAACCCGCCTACCCCGATCCACGGCCTGTTCCTGATGGGTAGCCTCGGCACGCTGGCCCAG
GCCGACCAAAGCGATATGGACGTCTGGGTATGCCACGCCGACGACCTGAACGAGGCCGAGCTGGGTGAGTTGCGCA
AAAAATGCCAACTGCTGGAGGCCTGGGCCGTAAGCATGGGCGCCGAAGCGCATTTTTTCCTGGTCGAGCCGAACCG
CTTTGCACAAGGTGGCCGCGATACCCAGCTCAGTTCGGATGACTGTGGCACCAGCCAGCACTATCTGTTGCTCGAC
GAGTTCTACCGCACCGCCATCTGGCTGGCCGGGCGCACGCCGATCTGGTGGCTGGTGCCGGTCTACGAAGAGGCTC
GGTACGCCGAATTTACCCACACGCTGATTTCCAAACGCTTTATCCGCGCCGACGAAACCCTCGACCTCGGGCACTT
GGCCCATATCCCGCCGGGAGAATTTATCGGCGCCGGCCTGTGGCAGTTATTCAAGGGTATCGAGTCCCCGTACAAG
TCCGTACTCAAGCTGTTGCTCACCGAGGTGTATGCCAGCGAACACCCCAAGGTGCAGTGCCTGAGCCTGCGCTTCA
AACGCGCGGTGTTCGCCAACCAGCTGGATCTGGATGAGCTGGACCCCTATGTCGTGGTCTACCGCCGCATCGAAGA
ATATCTGCGCGGGCGCAACGAGCCCGAGCGACTGGAACTGGTGCGGCGCAGCCTGTACCTGAAGGTCAACCGCAAG
CTCACCACCGGCCAGCGCACCATCGGTTGGCAGCGCGCACTGCTGGAACGCCTGGCCAATGAATGGGGCTGGGACC
AGCGGCAACTGGCCCTGCTTGATAGCCGCAGCCAGTGGAAAGTGCGCCAGGTAGCATCCGAGCGCCGGGCCCTGGT
CAATGAACTCAATTACAGTTATCGCTTCCTGACCCAATTCGCACGCACCGAACAGACCGTGAGCCTGATCAACAAG
CGTGACCTCAATGTACTGGGCCGGCGCCTGTACGCGGCATTTGAACGCAAGGCCGGCAAAGTCGAATTCATCAACC
CCGGGATTGCCCCGGACCTGGCCGAAGACACGCTGACCCTGGTGCATTCGCCCAACCGCAAGGAGCCGGGTCAGTT
TCACTGGGGTTTGTACAACGGCAGCCTGACCGCCCTGGAATGGGAGCACTTTGCACCGATCAAACGCAGCCGTGAC
CTGCTGGAGATGCTCACCTGGTGCCATCGCAACGGCGTGATCGACAGCAGCACCCGCCTGGCCCTGCACCCGGGCA
GCAGTGACTTGAGCGAATTCGAGCTGTTCAACCTGCTGGGCAGCCTGCAGCAGACCATCGCCCTGCCGTTGGCCAC
GGTGGATGAGGAGCATTTGCTGCGCACGGCGATACCCGATGAAGTGCTATTGCTGATCAACGTTGGGGTCGACCCG
CTCAAGCACCATCGCGACCTGAATATCCTGATGACCACCGAGCGTACCGACTCCTTGAGTTATGCCGGCGTGCGGG
AAAACCTGGTCCTGACCCTGGACCAGGTGACGCTCAACAGCTGGAACGAGGTGATGGTCAGCCGCTACGACGGCCC
CCATGCCCTGCTCGACTGCCTGCGCGACTACCTCAACCAACTGCCGCGCAACCAACTGCCACGGCTGCGGGTCCGG
TGTTTTTGCCACAACCGTGCGCAATTCATCGCGCAACGTGTAGAAGAGATTTTCGACACCGCGCAGAACCTGCAAC
TGAGCCAGCTCAACCACCGCTACCTGATCCAGGTACAACAGCACTATCACGTCATGGAGCTGGCGCCGGGGCAAGC
CAATCATGTGTCGCTGCCCAGCCAGCAGGCGCTGGTGGACTATCTCAGCGAAGAATTGGCCAGCTACAGCCCGCTG
CACCTGGATGCCATGGCCCTGGAAGACCACGACCTGGCAGCGTTGTTGCCCATGGGCCTGCCGGAATGTGTGCAGG
TGTTCTACCGCATCAACGAGGGCTTTGCCGAGCTGTACGTACTCGACGAGTTCAATGCCCTTTGGCAGCAGCGCCT
GCCGTTTCATGATGAACAGAGCTTGTTGGCGCCTTTGCAACGCTTCCTGCAATCGATCATCTATCGCCGCGACGCG
CTGTCACCGCTGGACCCGCAGCAGCCATCAGGGGCCTTGCAGGTGCTGTATTACCAACTATTGTCATCGGCAGGCG
GCCGCGCCCGTCGAGTAGAGCCACGGCCTGCGCCACAGACTCCGGCCAACAAACCGTTCTATGACGTGCAGGCGAT
TATCGGCAAGGGTTCGCCAGGCCAGGTGGGGATTACCCTGTATTGCAATCAGCGGGAGTTTTCCGAACTGGAATTT
GGCGACCAACTGTTTGCAGTGGTCGCCCAGGAGATCGTCGGGCAGCGTCGGGAAACCGAGCGCTATCGTTGCTACA
TCACCGACCTGGACCTGTCCGGGCTACTCGGTGATGTTCAAAGCCCAAGCAATCTGTACCTGCGCTACAAGGCCGA
GTTGGAGCAGGCGCTCAACCAGGCGTTGAACCAGATTTAGAGCGTGAAGGCGCCGCCGTCTTTAGGCTGGCCTTCG
ACACTCAGCAACTCAAGCTTGAGGGTCTTGCCGCCCGGAGCCGGCCAATCGATGTGCTGGCCGACTTGCAGGCCCA
GCAACGCACTGCCCACAGGAGCCAGGATCGAAATCTTGCCCTCGTCGGCATTCGCGTCCTTGGGGTAGACCAGGGT
CAGGTGATAGTCCTTGCCGCTGCTCTGCTCGCGGCAATGGACACTGGAGTTCATGGTCACGACACCGGCAGGCACT

TCATCGTGGCCAACCAAGTCTTCGGCACGGTCAAGTTCGGCTTGCAACGCCTCGACGCCAGGAAATTCGTCGCCCA
GACGGTCGATCAATTGCTCAAGACGTTGCACATCGAGACGGGTGAGGATGATGGAAGGTGCAGTCATGATTGCGGC
AGACTCCTTTTTTTCTGCACAAAAAAGCAAAACCCCGCCAGTAAACGGCGGGGTTCTCACGGACCTCGATGAGTTG
AGGTGTACCCGGACACTACCACAGCACAACAAATAAACAAGATGGGCCGGCCTGAAGGTCAGGCGCCAGCCTTGCG
TTGCTCGGCCTGGGCGACGATCACGCGGCGCCGCTGATCGTCGGCGGAGCGCCATTCCCGGATGTCTTCCACGTGC
CGGAAACAGCCAAGGCAGACTTTTTGCTCATCCAGCCGACACAAACTGATACAAGGTGACGGCACAGCCGGGCTGA
CATTGCTGAACAGCGGCTTGGGCGGGCGGGCGGAAGCAGGCTGGGTCACGATCAGATCTCGTCGAAATCCAGCTCG
ACGCCGGCTTGTTCCAGGGTGATGCGTGCAAGCATTTCACTCAACAGCTCGTCGCTGGTGTCGTTCGACCAGCGGT
TTTCTTCTTCGTCATAGTCGAAGTGAAAACCTCCGGAGCGCGCCGCCAACCACAACTGACGCAGCGGCTCTTGGCG
GCTGAAGATCAATTGGCTGCCGTTCTCGAACTTGACGGTCAACACGCCTGCAGAATTTTCCAGATCCACGTCCAGG
CCACTCTCGTCGAAAATATCCTCCAGCGCTTGCTGGGTCGCATCGACCAGGTCGTGAAAACGGGCTTCGGTCAAAC
TCATGGTGGGAACCTCAAAAAGTGTCTACTCACGCTCAAGCGCCGCACGATACGGAGGCGCCATCATGATTGCAAA
GGATACCGATTTCATTCCCGTACGCCGCGTGAAATATCTCAGAGTGATGTAGGTCACTTCACGACATACACGGCAA
AGCCCCGCCGGACGGGTGTCCCGGCGCATAGGCAAGCTGCCGGGTGGTCGGTATACTCGGGCGCAATTAATGCATA
TTCAAGGATTTAGCCATGAAGCGCCTGATCTCTTCCCTTGCTGCGCTCGTCGCGGTCGCTTGCCTCGTCAGTGCCT
GTGGTCAAAAAGGCCCGTTGTACCTGCCTGATGACAGCAAAGACCCCAATGAACAGGCGCAAACGTCGCAATCCAA
AGCGCACAAGCACGACAACAACTAAGGGAACATCATGGACGCTTTTAACTACCGGGACGGCGAGCTGTTCGCGGAG
GGCGTGGCGCTGTCCGCGATTGCCGAACGCTTCGGCACCCCGACCTATGTCTACTCCCGTGCCCACATCGAAGCGC
AATACCGCTCCTTCACCGACGCCCTCGATGGCGTACCGCACCTGGTGTGCTACGCGGTAAAGGCCAACTCCAACCT
GGGTGTACTCAATGTCCTGGCGCGTCTGGGCGCTGGTTTCGACATCGTGTCCCGTGGCGAACTGGAGCGTGTCCTG
GCGGCTGGTGGCCAGGCTGACAAAATCGTGTTCTCCGGTGTCGGCAAGAGCCGTGAAGACATGCGCCGCGCCCTGG
AAGTGGGCGTGCATTGCTTCAACATCGAATCCACCGACGAGTTGGAGCGCCTGCAAGTCGTAGCCGCCGAGATGGG
CGTGCGTGCGCCGATCTCCCTGCGCGTCAACCCGGATGTGGATGCCGGCACCCACCCGTACATTTCCACCGGGCTC
AAAGAGAACAAGTTCGGCATCGCCATTGCCGACGCCGAAGACGTGTACATCCGTGCCGCCCAGTTGCCGAACCTGG
AAGTGCTGGGTGTGGACTGCCATATCGGCTCGCAACTGACCAGCCTGCCACCGTTCCTCGATGCCCTCGACCGCCT
GCTGGCGTTGATCGATCGCCTCGGCGAGTGCGGCATCTACCTGCACCACATCGACCTCGGTGGTGGCGTGGGTGTG
CGTTATCGCGATGAAGAGCCGCCGCTGATTGCCGACTACATCCAGGCCGTACGCGAGCGCACCGAAGGCCGCGACC
TGACGCTGATGTTCGAGCCGGGCCGCTACATCGTAGCCAATGCGGGCGTACTGCTGACCCAGGTCGAGTACCTCAA
GCACACCGAATACAAGGATTTCGCCATCGTCGATGCGGCGATGAACGACTTGATCCGCCCGGCGCTGTACCAGGCC
TGGATGAACGTCACCGCCGTGACGCCGCGTAACAGCGAAGCGCGCGCCTATGACATCGTCGGCCCGATCTGCGAAA
CCGGCGACTTCCTGGCCAAGGACCGTGAACTGGCCCTGGCAGAAGGCGATCTGCTGGCCGTGCACTCGGCCGGTGC
CTATGGGTTTGTCATGAGTTCCAACTACAACACCCGCGGGCGTACCGCCGAAGTGTTGGTGGACGGTGATCAAGCG
TTTGAAGTGCGTCGCCGCGAGACGGTAGCCGAGTTGTTTGCTGGCGAAAGCCTGCTGCCGGAGTAAGCCATGCTGC
TGCGTTTTACCAAGATGCACGGCCTGGGCAATGACTTCATGGTCCTTGACCTGGTCAGCCAGCACGCGCACATCCT
GCCCAAGCACGCCAAGCTGTGGGGCGACCGGCATACCGGCATCGGCTTCGATCAACTGTTGATCGTCGAGGCGCCG
AGCAACCCGGAAGTGGACTTCCGTTACCGGATCTTCAACTCCGACGGTTCCGAAGTGGAGCAATGCGGCAACGGCG
CGCGCTGCTTCGCCAGGTTTGTGCTGGACAAGCGCCTGACCGCCAAGCGGCAGATTCGCGTCGAGACCAAAAGCGG
CATTATCGAGCTGGATATCCGCAGCGACGGCCAGATCAGCGTCGACATGGGCGCCCCGCGCCTGGTGCCGGCCGAT
ATTCCGTTCGAGGCCGATGCCCAGGCGTTGAGTTATCCGCTGGAGGTCGACGGTACTGTGGTCGAGGTCGCCGCCG
TGTCCATGGGCAACCCCCATGCGGTGCTGCGGGTCAACGACATCAACAATGCGCCGGTGCATGAGTTGGGGCCGAA
GATCGAACATCACCCGCGCTTTCCGGCACGGGTCAACGTCGGCTTCCTGCAGGTCATCGACCGCTCCCGTGCGCAA
TTGCGCGTCTGGGAGCGCGGCGCCGGGGAAACCCAGGCTTGCGGCACCGGTGCTTGCGCCGCCGCCGTGGCCGCGA
TCAGCCAGGGCTGGATGGATTCGCCACTGTTGATCGACCTGCCCGGCGGACGCCTGTCCATCGAATGGGCAGGCCC
AGGCCAACCGGTGAAGATGACCGGCCCGGCATCCCGTGTATACGAAGGACAGGTCCGTCTATGAGAGCGACTCGCC
AATGACCGATAAGCCCCAGGCACCCGCCAAGCAGCCCGAAGGATCCCCTTGCGAAAGCCTGGAGGCAGCGGCCGTC
GCCGCTTACCTGGAGGCTAACCCGGACTTCTTCGTCGAACACGACGAGCTGCTCCCGGCCCTGCGCATCCCTCACC
AGCGCGGCGACACCGTGTCGTTGGTAGAACGGCAGATGAAAATCCTGCGCGAGCGCAACATCGAAATGCGCCATCG
GCTTTCGCAGTTGATGGACGTGGCCCGTGACAACGACCGCCTGTTCGACAAAACCCGCCGCCTGATCCTGGCGCTG
CTGGATGCTGGCAGCCTGGAAGACCTGACCATTGCCGTCGAAGACAGCTTGCGCCAGGACTTCCAGGTGCCGTTTG
TCAGCCTGATCCTGTTCAGTGACAACCCGATGCCGGTCGGGCGCTGGGTCAAGGGCAGCGAGGCGCAAACCGCCAT
TGGTGGCCTGCTGTCAGAAGGCAAGACCATCAGCGGCACTCTGCGCGAGCATGAGCTGGACTTCCTGTTTGGCGAA
GACCAGCGCGTGCAGATCGGCTCGACTGCCGTGGTCGCTCTCAGCCATCAGGGCTTGCACGGCGTCCTCGCCATCG
CGAGCCGTGATCCGCAGCACTACAAAAGCACCGTCGGTACCTTGTTCTTGACCTACATTGCCGAAGTGCTGAGCCG
CAGCCTGCCGCGCTTTACCACCGCCCTGCGCGCGGTGCGCTAGCCATGGAACGACAACTGGACGCCTACTGCGCTC
ACCTGCGCAGCGAGCGCCAGGTGTCGCCCCATACGCTGGAGGCTTATCGGCGGGACTTGAACAAGGTCCTGGCCTT
CTGCGAGAAACAACAGATCGGTAGCTGGAAGGCCCTCGACATCCAGGGTCTGCGCAGCCTGATTGCCCGCCTGCAC
CAGCAAGGCCAATCCTCCCGCAGCCTGGCGCGCCTGCTGTCGGCGGTGCGCGGCCTCTATCACTACCTTAACCGCG

AAGGCCTGTGCGACCACGACCCGGCCAACGGCCTGGCGCCGCCCAAGGGTGAACGTCGCCTGCCCAAGACCCTGGA
TGCCGACCGCACCCTGCAATTGCTCGATGGCGCGGTCGAGGATGACTTCCTGGCGCACCGCGACCAGGCGATCCTG
GAGCTGTTCTACTCTTCGGGCCTGCGCCTGTCGGAGCTGACCAGCCTCAATCTCGATCAGTTGGACCTGGCCGACG
GTCTGGTACAAGTGCACGGCAAAGGCAGCAAGACCCGCGTGCTGCCCATCGGCAAAAAAGCCCGCGACGCCCTGCT
GATATGGCTGCCCCTGCGGGCATTGAGCAATCCGCCGGACGATGCGGTGTTTGTCAGCCAACAAGGCCGACGCCTC
GGGCCACGGGCCATTCAGTTACGGGTCAAGGCCGCCGGCGAGCGGGAACTGGGGCAGAACCTGCATCCGCACATGC
TGCGGCACTCCTTTGCCAGCCACCTGCTGGAATCGTCACAAGACTTGCGCGCCGTACAGGAACTGCTCGGCCACTC
GGATATCAAGACCACCCAGATCTACACCCACCTGGACTTCCAGCATCTGGCGACGGTGTACGACAGCGCCCACCCA
AGGGCCAAACGCATTAAAGGCGGCGACTCATGAGCATCAAGCTAATCACCTTCGACCTGGACGATACCTTGTGGGA
TAACGTCCCCGTCATCATCAGCGCCGAAGCATCGATGCGCCAATGGCTGGCAACCCATGCGCCCAAGGTCGGCGAC
CTGCCCCTGGAGCATTTCGCCAGCCTGCGCCAGCAGGTGCTGCAGCGTCATCCCGAGCTCAAGCACCGGATCAGCA
TCCTGCGCCACCGGGTGCTGATGCATGCCTTTGAAGAGGCCGGTTATGCACAACCGCAAGCCACGGAGATGGCCGA
TGTGTGTTTTGAAGCTTTCATTCACGCGCGGCACCAACTCACGGTGTTCCCGGAAGCCGAGCCGATGCTTCAAGCC
CTGCGCCAACACTTCCTGCTGGGGGTGATCACCAATGGCAATGCCGACGTACAACGCGTGGGCCTGGCGGATTACT
TCCACTTTGCCCTGAGGGCCGAAGATATCGGGATCCCCAAGCCAGATGCGCGATTGTTTCAGGAAGCGTTGCAGCG
TGGCGGGGTGGAGGCCAGTGCGGCAGTGCACATCGGCGATCATCCAGGTGATGACATTGCCGGCGCGCAGCAGGCG
GGGTTGCGAGCGGTGTGGTTCAACCCGACTGGCAAGGCCTGGACAGGCGATAAGTTGCCGGATGCGCAGATTCGTA
GCCTGACCGAGCTGCCCTCACTGCTTCACAGCTGGAAATAACTGTAGGAGCCGGCTTGCCGGCGAAAAACCCGAGA
GCACCGCGACCCTGCGTTATCGTTGACGATCTTCGCCGGCAAGCCGGCTCCTACAGAAGTCATCACCGCAGGAAAT
AGAGGCGCCCATGAAAAAGCCCGCAGCGACGGCGGGCTTTTTCAGCAAGCAAGTCACCGACCTCAGATAGGACGGC
TGCCGTACTTGTTATCCGGCTTCTTCGGTGGATCTGCCACCACGTTAGCCTCGACTTCCTGAACTTTGCCACCTTT
GGCGAGGAACTCTTCCATCGCACGGGCCAGCGCATCGCGCTCTTTGTTCTTGGCCTCGACGCTCGGCAGTTCGTCT
ACCGAAACGGCAGCCTTGGCCTTGCCTTTGGCGGCCGGAACAGGTGCGTCATCGCCACCGTCGTCGTCAGCAACAT
CTTCCGCTGCCGCTTCAAGACCTTCTTCGGTGTCGTCTTCGTCACCTACTTCCAGTTCGTCGTTTTCCAGATCATC
GTCGCTCATGTTCTACCTCATGACTTGCGAAAAGCAGATTAGTTATAGCCCAGCTTCACCATCTGTCGAAGGCTGC
CGGAAAAAAATCAACGTCCACCGGATAACCAGTGGCTTATGCCCCGTCACCCTGCAAGGTACCGAGGACTTTACGA
GCACCGCCCTGATCACGCTGCTCGCCCAGATAAACGCGTTGCCATGTGCCCATTGCCAAGTGGCCGGCCTTGACCG
GCAAACTCAGTTGGCGACCTGATAGGTTGCCCTTGAAAGGACCCGGGGCTTGGCCCACAGGGTAATCAGGGTCTGC
CGCCGTATACAGTTCTGCGCCCATCGGCGCGCATTCTAGCGCGCTCTGGGAAAAAACAAAGTGCCGAATACGCCTA
CATGCCTGTAAGACATTCGCACAGTGAAAACTGCCTTGTGGATCCGATCACAAACTCGCCACAAAAAATCTCACAA
GCCTTCACCACAGGCAAATACCAGACAAAAAAATGCCCGGCAAGCCGGGCAAGTTTTTTCATAGGTTCTTACAAGT
TGTAGCCGCGTTCGTTGTGTTCCGCCAGGTCCAGGCCAACGGCCTCTTCCTCTTCAGTCACACGCAGGCCGATGGT
CAAGTCCAGCACCTTGAGAATCACAAAAGTCACGATACCGGTGTAGATCACGGTAAAGCCGACGCCCTTGGCCTGG
ATCCAGACCTGTGCGGCAATATCGGTCACTGTGCCGAAGCCACCCAGGATCGGCGCCGCGAATACGCCGGTGAGAA
TTGCCCCGACAATCCCGCCCACGCCGTGTACACCGAAGGCATCCAGGGAGTCATCGTAGCCCAGCTTGCGTTTGAG
GCTGGTGGCGCAGAAGAAGCAGATCACACCCGAAGCCAGGCCGATCACCAGGGCACCCATCGGGCCCACGGTACCG
GCGGCCGGCGTAACGGCCACCAGGCCGGCAACCACACCTGAGGCGATGCCCAGGGCGCTTGGTTTGCCGTGGGTGA
TCCACTCGGCGAACATCCAGCCCAGTGCCGCAGCAGCGGTGGCGATCTGGGTCACCAGCATCGCCATGCCGGCCGT
GCCGTTGGCCGCTGCAGCGGAGCCGGCGTTGAAGCCAAACCAGCCGATCCACAGCATTGCCGCACCGATCAGGGTG
TAGCCCAGGTTATGCGGGGCCATCGGCGTAGTCGGGTAGCCTTTACGCTTGCCGAGCACGATGCACGCCACCAGGC
CAGCCACACCGGCGTTGATGTGCACCACGGTGCCGCCAGCGAAGTCCAGCACGCCCCAGTCCCACATCAGGCCGCC
GTTGCCGCTCCAGACCATGTGCGCGATTGGCGCATAGACCAGGGTGAACCAGATGGCCATGAACAGCAGCATGGCG
GAGAACTTCATGCGCTCGGCGAATGCACCGACGATCAGCGCCGGGGTGATGATCGCAAAGGTCATCTGGAAGGTGA
TGAACACCGCTTCCGGGAACAGCGCCGCAGGGCCAGTAATGCTGGACGGTGTGACACCCGCCAGGAACGCCTTGCC
CATCCCGCCGAAGAAGGAGTTGAAGTTGACGACGCCCTGCTCCATGCCGGTGGTGTCGAACGCAATGCTGTAGCCG
TAGACGACCCACAGGATGCTGATCAGACCGGTAATGGCGAAGCACTGCATCATCACGGAAAGAATGTTTTTGGAGC
GGACCATGCCGCCGTAGAACAGCGCGAGGCCGGGGATGGTCATGAACAGCACCAATGCGGTGGCTGTCAGCATCCA
GGCGGTGTCGCCGGAGTTGAGGACTGGAGCCGCCACTTCGTCTGCCGCCATGGCCAGGCTGGGCATTACGATGGAC
AACAGGGCTCCTAGCCCTGCGAATTTACGCAGAGTCATATTGTTTTCTCCTGGGGCGTTGGGGTTTGGCGGCTTAG
ATTGCGTCGGTATCGGTTTCGCCGGTACGGATGCGAATAGCCTGTTCCAGATTGACCACGAAGATCTTGCCGTCAC
CGATCTTTCCGGTGTTGGCCGCCTTGGTTATCGCCTCGATAACCCGATCAAGATCCTTGTCGTCAATGGCGACATC
AATCTTCACCTTCGGCAGGAAATCGACTACATATTCCGCGCCGCGATACAGCTCGGTATGACCCTTCTGCCGACCG
AAGCCTTTGACCTCAGTGACGGTAATGCCCTGCACGCCGATCTCGGACAGTGACTCGCGTACATCGTCCAACTTGA
ACGGCTTGATGATGGCAGTGACTAGCTTCATGAAAACTCTCTCCCGAATTGGTGGACTTGCCCCAGGAAAACAAAC
CCGTCTCAAGTCTAAGCGCAGTGCCTGGCTTTGTAACGCATCGTCGCCTTGGCAATTGCCTTTGCGACGCCAGCTA
ACCGTTGGTGACGAAACAAGTACCCTGATCCGTCAGCGCACTGCATTCGTCACAGCGACTGCATCAGTGCATGGGT
CATGATCGTCTAAGCAGAAACCTTGCCAGCTCCGTAAAAATCACTGAAATCAATCCGTTGCCCGCCTACATCCGCC

CACAGGCCTGTGCTGCGGCCTGGATACGCACAAAAACGGTGCATACCCATTCGGGCCGATGCGCGAAAAGCGTGCG
TAGATCACCTTGAAAAGACTGTAGGCGCTGCGTGATACACTGCCGGCCAACTGTTTTCCGGAATGTTTCCCATGCT
CGCGCCCAAAGACCTCCTCGACGCCCTGAGCGGCCATGCCTCTCGCCTGTTCAGCGGCGAAACCCCGCTGCCTCGC
AACGAAATCGAAAGCCAGTTCAAGGCGCTGCTGCAAAGCGGGTTCAGCAAACTGGACCTGGTGAGCCGCGAGGAAT
TCGACAGCCAGATGGTGGTCCTGGCCCGCACCCGCGCGCGTCTGGAGAGCCTGGAGGCGAAGGTCGCGGAGTTGGA
AGCCAAGTTGACGCCACCCGCTGCTGAATAAACGCGGTCAGTGTGGGAGCTGGCTTGCCTGCGATAGCATCGCCTC
AGTCTTTCTGATACACCGAGGCGTGTGCATCGCAGGCAAGCCAGCTCCCACATAAAGCGCTGCGAACGACTCCGGA
TACGTTCTGTAAAACCTCCCCCGCCTCGCTCTTCCCCACCTCGTCTACCCTTGAAAAACCCGCAGGAAGCGGCCTC
TCTTTCAAGGAACGAGCATGTCCCTCGCCATCGTCCACAGCCGCGCCCAGATCGGCGTCGAAGCCCCTGCCGTCAC
GGTCGAAGTGCATATGGCCAATGGCTTGCCATCGTTGACCCTGGTGGGCCTGCCCGAGGCTGCTGTGAAGGAAAGC
AAGGACCGCGTACGCAGCGCCATTCTCAACAGCGCGCTGCAATACCCCGCACGCCGCATTACGCTCAACCTGGCCC
CCGCCGACCTGCCCAAGGACGGTGGGCGGTTTGACTTGGCGATTGCCCTGGGGATTCTCGCGGCCAGTGTGCAGGT
GCCAGCGTTGATGCTCGATGATGTGGAGTGTCTGGGCGAACTGGCCCTGTCCGGCGAGGTGCGAGCGGTGAAAGGC
GTGTTGCCGGCCGCCCTGGCGGCACGCAAGGCCGGGCGCACGGTGATCGTGCCTCGGGCCAATGCCGAGGAAGCCT
GCCTGGCGTCCGGGCTGACGGTGATTGCGGTGGATCATCTGCTGCAGGTGGTGGCGCACCTTAATGGGCAGGTGCC
GATTGAACCGTTCAAGTCCGACGGTTTGCTGTACCTGAACAAGCCCTACCCCGACCTGAGTGAAGTGCAAGGCCAA
CTGGCGGCCAAGCGAGCGCTGTTGATTGCCGCAGCAGGCGCCCACAACCTGTTGTTCAGCGGCCCGCCGGGCACCG
GCAAGACCCTTCTCGCCAGCCGCCTCCCCGGCTTGCTGCCGCCGCTGAGTGAACAGGAAGCCCTGGAAGTGGCGGC
GATCCAGTCGGTGGTCAGCCTCGCTCCGTTGAGCCACTGGCCGCACCGGCCATTCCGTCAGCCGCATCATTCCGCG
TCCGGGCCGGCGCTGGTGGGTGGCGGCTCGAAACCCCAGCCAGGGGAAATCACCCTGGCCCATCATGGGGTGCTGT
TTCTCGATGAGTTGCCAGAGTTTGATCGCAAAGTGCTTGAGGTGCTGCGCGAGCCGCTGGAGTCCGGGCATATCGT
GATTTCCCGCGCCCGCGACCGTGTGAGTTTCCCGGCGCGGTTTCAACTGGTAGCGGCGATGAACCCCTGCCCTTGC
GGCTATATGGGCGAGCCCAGCGGGCGCTGTCGTTGCACGCCGGAGCAGGTCCAGCGCTACCGCAACAAACTGTCCG
GGCCGCTGCTGGACCGCATCGACCTGCACCTGACCGTCGCCCGGGAAAGCACCGCGCTGAACCCTGCCAAACAAAC
CGGCAATGACACGGCCAGCGCTTCTGCCCTCGTCGCCGACGCCAGGGATCGCCAGCAGCAACGCCAGGGTTGTGCA
AATGCTTTCCTCGACCTGACGGGGTTGCGCCGCCACTGCGAGTTATCGAAAGTCGACGAAGGCTGGCTGGAGACGG
CCTGCGAGCGCTTGACCCTGTCGTTGCGCGCGGCCCACCGGCTGCTCAAGGTCGCACGCACCCTGGCGGATCTGGA
GCAGGTGGAAAGCATCATGCGGCATCACTTGGCGGAGGCGTTGCAATACCGGCCAGCAACCTTGGGCTAGAGATCC
CGGGGTAAGTCCAGCAACCACCCCGCCACTTCCAGCACCTCACCCAGTGCCGATTCAGCGGACTCGGTGGAGGGGT
TAAGCATCAGGTCATCGGCATACCCCATCGCCAAGGCAAAGATCTGGCGTGCGGCGCGTGTGGATGAGTCGCAACG
GAACACCTGCGCGGCCACACCCCGCTCGATCACCTCCGCCAGCATGGCTTGCCACTGGGCGTTGATCGACAGATAG
GCCTGGGCCATTTGCGGGTCATACAGCGCCTCGTCCCAGGCGTCCAGCCACAGTGCCCAGTCGGCATCGGCGCTGC
TTGGCAGGCAGTCACGCAGGAACCCCAGCAACGCTTCGAGAGGCGGCGCCAGCGCCAGGGGGGCACGCACTTCGTC
CAACTGGTCGGTGGCGAACCGCACGAAGGCTTCGCGGCGCAGTTCTTTCCAGTCGCTGAAGTAGTGATACACGTGG
CTGCGGGACAAACCGGCATGCTCCGCCAGATCGCGGGTGGAGACATCGGCAAAACCCTTGCTGCGAAACAGCTCCA
GGGCAGCGGTGATGATCTGGTCTTTACGGTCGATACGCGACATGGGCAATTCCTTACGGGCACCGGCAGATAAGCG
GCGCTTGCTTTTTGAGCGATCGCTCAAGGATACTTGAGCAGTCGCTCAATAGTAGCGCCTATCACTCCTTTGGTGC
ACCCATGCCATCCGTAACGCCGCACATCCTGATCGAAGAAAGCAAGAAAATCGGCCAGCAATCCGCCAGCCAAACC
CTCAAGGCAATCGCCAGGGCTTACCCCTGGAAGCTGTTTGGCACGTTGTCGTTGGTCGCGCTGGAGAACGCCTTGC
TGTTGGCCTACCCGCTATTTGCGGGTTTCGCTGTGGACTCGATCCTGCGCGGCGATGCCGGCAGCGCGTTGATTTA
TGCAGCCGTGGTCATGGCTTTCTGGGTAGTCGGTGCCGCACGCCGGGCCCTGGATACCCGTACCTTCACCCGGATC
TACGCCGACCTTGCCGTGCCGGTGATTCTCAACCAGCGCCTGCAAAACCACAGCACGTCAAAGGCTGCCGCGCGGG
TAGTGCTGGCACGGGATTTTGTTGATTTTTTCGAAAAACACGTGCCGGTGATTGCTACGGCCCTGGTATCCATCGT
TGGCGCAGCCGTGATGCTCCTGGTGATTGAACCCTGGGTCGGCCTGGCCTGCTTTGCCGCGCTGGTGCTGTGCATC
ACCCTGCTGCCGCGCTTTGCCCGCCGTAACGAGCAATTGCACAAACGCTTGAATAACCGCCTGGAGAAAGAAATTG
GCCTGGTGGAAAAGGTCAGCGCCCAGACCCTGCGCCGCCATTACCAACTGCTGTCACGCCTGCGTATCTGGCTATC
GGACCGCGAGGCCGCGGCGTTCCTGTTTATCGGCACGCTGGCGGCGCTGCTGTTTGTGGTCGCCATCAGCCAGCTG
GCCCTGTCGCCTGCAGTCAAGGCCGGCCACGTGTATGCGGTGATGACGTACTTGTGGACCTTCGTCAGCAGCCTCG
ACGAAGCACCAGGGATGGTCGATCAACTGGCGCGGCTCAAGGACATCGGCAAGCGCGTGGACCCAGGGCTCGGCGA
TCGAACCGAGGCTTGAGAGCCGAGTCAGCCGTCATCGCGGGCAAGCCCGGCTCCCACACTGAACCGGTTTCTTCAA
TTGAACACGGTCAACTGTGGGAGCCGGGCTTGCCCGCGATGAGGCCCTGACAGTCAATCAACGAAACCGATCAACC
TCATGCCGCAGCCCCGCCGCCAGCGTCTCTAACTCCCTGGCGGTAATCGCCAGGTTCGAGACCACTTCACGTTGTT
CACTGTTGGCCAGCGCAATGCTCTGCAGGTTGCTGCTGAGCAAGGTCGCCGTGCTGCTTTGCTCCTGGGTGGCGGT
GGTGATCGCGGCGAATTGCTGCCCCGCCGAGCGGCTTTGTTCGTCAATGCGCGCCAGGGCCGAGGCAACGTCGGCA
TTGCGCGACAAGCCTTCCTGCATCAGCACATTGCCCTGCTCCATGGTGCTGATGGCATTGCCGGTTTCCTGCTGGA
TGCTCTGGATCATCCCGGAGATCTCGTCGGTGGCCTGGCGGGTGCGTGAGGCCAGGTTGCGCACCTCATCGGCAAC
CACGGCAAACCCACGCCCCTGCTCCCCGGCCCGTGCCGCCTCGATGGCCGCGTTGAGGGCCAACAGGTTGGTCTGG

TCGGCAATCGAGGTGATCACGCCGACGATGCCACCGATTTCCTGGGAACGCTGGCCCAGGGTGTTGATCACCGTGG
CCGTGCTGTTGAGTGCCGTAGCGATATGCTCCAGGGACGCCGACGCCTCCTGCATCGAGTTGCGGCCAATGCGGGT
CTGCTGGGCATTTTCCTGGGCCAGGCGTTCGGTATTGCCCATGTTGTCGGCAATGTTCAACGAGGTGGCACTGAAC
TCTTCCACCGCACCGGCCATGCTGGTGATCTCGCCGGACTGCTGCTCCATGCCTTCATAGGCCCCACCTGACAAGC
CCGACAGCGCCTGAGCGCGGCTGTTGACCTCCTCGGCCGCCTCACGGATATGCGAGACCATGGTCGACAGGGCCTC
GCCCATCTGGTTGAAACTGCGCGCCAATTGACCGATTTCGTCATGGCTGGACACATTCAACCGCGCACTCAAATCA
CCGGCGCCCAGGGCTTCGGCCTGGCGCACCAGGTCGCTCAATGGAGCCAGCTTACTGCGCAACAGCCACACCGTGG
CGCCTACTGCCAGCAACATCGCCAGCACACTGCCGATCACCAAGCGGATGCCGACTTCCCAGGTCACTGCGCGAAT
TTCAGCCTTGGGCATGCTCGCTACCACCGCCCAGGGGCCGCCTTCGAACGGCACCGAGACGCTGTAGAAATCTTCG
TGCTTGTCGCTCCAGAAACGGCCTGCGCCCGGTTTCTTCGCCAGGTCGAGCATCACCGCAATGGCCTCGTCCGGCG
CCTGCACCCCAGCCGGCGCCACCAGCCAATGCTTCTGTTCATCCAGCAGCGCGAGCGAGCCGGTCTGGCCGATGCG
AAAACGTTTGAGGTTTTCAAACTGGGCGTTCTGGGCGTCGGTGTAATCAAAACCGATGAACAGCACGGCGATGACC
TTGCCACTGCTGTCACGCACCGGGCTGTATTGAGTCATGTAGGAACGGTCGAACAGCACCGCACGGCCGATGTAGG
TCTGGCCGCCGGCCACGCGCTGGTAGGCCGCGCCCTGACGGTCGAGCACGGTGCCGATGGCGCGGTTGCCATCCTG
TTTGGTCAGGGAGGTACTGATGCGGATGAAGTCTTCGCCGCTGCGCACAAACACCGTGGCCACGCCGCCGGACATC
TGCTTGAACTCATCGACCTCGTCGAAGTTGTTGTTGAGCACCGCATCGCCCAGGTAAAGACTCGGGGTCGCTACCC
CGGCCACATTCACCGTCTGCTCGGCATGCACACTCAGGCCTGCGCCAAAGCGCTTTTCAAACAGCCCGCTCAAACG
CTGAGTGCTCTCGCGCAAGGTGCTGTGGAAGGTATTGAGTTGGTCGGCGAGCAAACGCGCTTCGCTGGCCAGGTGT
TCTTCACGGGTCTGCAGGTTGGCCGAATCAAGGGAGCGCAGGGCAAACACGGTGCTGCCGCTGATCACGACAGCCA
ATATGACCGCCAAGGCGATGCCCAGCTGCGAAGCTATGCGGGCGCGGGGTTGAGACATGACTGCTCCTGGCCGAGG
CCCGGATCTTCCTGATCTCATAGCGCTAATCGGCGAATTTCTTGAATTGGGGGCACGCTGAGCCGCAACTAATGGC
AGCTGCGACACACCTACTTCGGCGGCCGGGGCCAATACTTGAGCAAATCACAGGGGTATCACTCCAACGCCTTGTT
GGCGGGCGCTGCATCGATTCAGTCGAGCTGTTCCACTGCCGGCAGTGCCATGGCCCGCACTTCACTTTGCAGAAAG
TCCGACAGCCTGCGCAGGCGCTCACCGCCGGGGCGGGTCTTGGGCCAGACCAGGTAGTAGCTCTCGCCGCTGGCCA
CCGCAGTAGGCCAAGGCAGGCTCAAACGGCCCTGGGCCACATCCTCGGCGACCATCAGCAAGTCGCCCATGGACAC
GCCGTAGCCCCGGGCGGCAGCAATCATGCCCAGCTCCAGGGTATCGAACACCTGCCCGCCCTTGATGGCGACGCGA
GAGGCCAGCCCCGTGCGTTGCAACCAGCGGCGCCAATCACGCCGGTCCGGCGTGGGGTGCAGCAGCTCGGCGCCGG
CCAGGCGTTCCACATCCCAGGGGCCATCGTTAAGCAAGTTCGGAGCGCCCACCGGGATCAGCAATTCAGGAAACAG
GTAACTGGCTTCCCAGTCCGCAGGAAAGTGCCCATCGCTCAGCAGGACCGCGCAGTCGAACGGCTCCTGGTTGAAA
TCCACCTCATCGATGCTCATCCACGCGCTGGTCAACTGCACCTCATTGCCCGGCTGCAGATGCCGGAACCGACTCA
ACCGCGCCAGCAACCAACGCATGGTCAGGGTCGACGGGGCCTTCATGCGCAAGATGTCGTCCTCGGCATTCAAGGT
ATGGCAAGCGCGTTCCAGGGCGGCAAAACCCTCGCGCACGCCGGGCAACAGCAGGCGCGCCGACTCGGTGAGCTGC
AAGGTGCGCCCGCTGCGCTGGAACAGACGGCAGGCGAAGTGCTCCTCCAGAGTGCGGATATGCCGGCTGACCGCGC
TTTGGGTGATCGACAACTCCTCGGCGGCACGGGTGAACGAATTGTGCCGGGACGCCGCTTCAAACGCGCGCAGGGC
ATACAGCGGAGGAAGACGACGAGACATGAAGAAAACTCCGACGGCTTGATCCGCAAACCCTACCAGAATCGTCCAG
GCATGAGTTTTAATCATGTGACTGATCGCATTTATCCCTTTGTGCAAAGCGCTGAGAGCGCCGAGAATCAACCGCT
CTACCCTTTATACAAATATTGAGCGTGATGATCATGCAGCATCCGGCGCGTACCGAACTCTGGGCCATTCTGCGGC
TGTCAGGGCCGTTGATTGCCTCACAGTTGGCGCACATGCTGATGGTGCTCACCGACACCTTGATGATGGCCCGCCT
GAGCCCCGAAGCCCTGGCCGGCGGCGGGCTGGGGGCGGCGAGCTACTCGTTTGTGTCGATCTTTTGCATCGGCGTG
ATAGCCGCCGTGGGCACCCTGGTCGCTATCCGCCAGGGCGCCGGCGATATCGAAGGCGCCACCCGCCTGACCCAGG
CGGGACTCTGGCTGGCCTGGCTGATGGCCCTGGTGGCGGGCCTGCTGCTGTGGAACCTCAAGCCCGTACTGCTGAT
GTTCGGCCAGACCGAAACCAACGTGCAGTCGGCTGGCCAGTTCCTGATGATCCTGCCGTTCGCCCTGCCCGGCTAC
CTGACGTTCATGGCCCTGCGCGGCTTCACCAGCGCCATCGGCAAAGCCACGCCGGTGATGGTGATCAGCCTGTGCG
GCACGGTGATCAACTACCTGCTCAACCATGCGTTGATCGAAGGCATGTTCGGCCTGCCCAAGCTGGGCCTGATGGG
CATTGGCCTGGTCACCGCGATTGTCGCCAACTGCATGGCCCTGGCGCTGTTGTGGTACATCAAATACAACCGCGCC
TACGCGGCCTACCCATTGGGCAAGGGCCTGTTGCGGCCCAACCTGCACTACCTGCGCGAGCTGTGGCGCCTGGGCC
TGCCGATTGGCGGCACCTATGCAGTGGAAGTCGGCTTGTTCGCCTTCGCGGCGCTGTGCATGGGGACCATGGGCAG
TACGCAGTTGGCAGCGCATCAGATTGCCCTGCAAATCGTCTCGGTGGCGTTCATGGTGCCGGCGGGCATGTCTTAT
GCGGTCACCATGCGCATCGGTTTGCATTACGGCGGTGGGCACCTGCTGGGCGCGAGGCTGGCCGGGCGGGTCGGGA
TCGCCTTCGGCGCAACGGTGATGCTGGGGTTTGCCATGGTGTTCTGGTTGTTTTCAGACCCGCTGATCGGCCTGTT
CCTCGACCATGACGACCCGGCCTTCGCCCAGGTGATCAGCCTGGCGGTCAGCCTGTTGGCGGTGGCGGCGTGGTTC
GAGTTGTTCGACGGCGTGCAAACCATTGCCATGGGTTGTATCCGCGGGCTCAAGGATGCCAAGACCACTTTCCTCG
TCGGCCTGGGCTGCTACTGGTTGATTGGCGCGCCAGCGGCCTGGTTGATGGCGTTTACCCTGGATTGGGGCCCGAC
CGGCGTGTGGTGGGGACTGGCGCTGGGCCTGGCCTGTGCGGCGATTACCCTGACGTGGGCGTTTGAAAGAAAGATG
AGGCGAATGATTGCCCAGGAACCGCAAATCGACATGGCCCCACAGCCGAACTGAAGCATGTACTCGGTCAATGTGG
GAGCTGGCTTGCCTGCTCCCACATTTTTGATTGGGTTTACAAAGTCAGATCACTTCACCCAACAACGCCTGTTGGC
TGCTGCCAAATGTCAGGTATTCCACCAACTCCGGCAACGGCAATGGCTTGCTGATCAGGTAACCCTGGGCCTGGTC

GCAGCCAAACAGACGCAATAACGCCAGTTGCTCCGGCGTCTCCACACCTTCGGCCACCACTTCCAGGTTGAGGTTA
TGGGCCAGGTTGATCATGGCGTGCACCAGCTTGCGGTTTTCCTCGCGCTCTTCCATGCCGCCGACAAAGCTCTTGT
CGATCTTGAGCAGGGCAATCGGCAGGCTGTTGAGGTGTACGAAGGATGAAAACCCGGTACCAAAGTCATCCAGGGA
AAAGCGTACGCCCAGGCGGCCCAAGGCATCCATGGTCTGCTTGACCAGGTCGCTGCGGCGCATCACGGCGGTTTCC
GTCAGTTCGAATTCCAGCCACTGCGCCTCCACCCCACGCTCGGCAATCAGCCGGCTCAAGGTCGACAGCAATTGGC
TGTCCTGGAATTGGCGGAACGACAGGTTGACCGCCATGTGCAGCGGCGGCAGGCCGCGTTCGCGCAGGTCCTGCAT
GTCGCGCAGGGCACGGGAAATCACCCAGTAGCCCAACGGCACAATCAGGCCGCTCTGCTCGGCCAACGGCACGAAC
TCGCTTGGCGGCAACAGGCCGCGCTCGCCATGGCGCCAGCGCACCAGGGCTTCGAGGCCGACGATATGCCCATCGT
CCAGGTCCAGACGCGGCTGGTAATGCAGCTCCAGCTCATCACGGCGCAGCGCCCGGCGCAGTTCGCTTTCCAGGTC
GGCAAGGCTGCGGGCGTTGCGGTTGATTCGTTCATTGAAGATATGAAAGGTGCAGCCCTGGGTGCTCTTGGCCTGT
TGCATGGCGATATGCGCGTGCCACATCAACGGGTCGGCGCCGGCCTTGGCGCGGGCATGGGCCACACCGAGGCTGC
AGCCGATCAGCAGGCTCTCGCCATCGACCCAGTAAGGTTCGGCCATGGCCTCGGTGATGCGTTCGGCCATCCACTC
GGCGCGTTGCGGCGCGCGGCGGGTGTCGATCAACAAGGCGAATTCGTCACTGCCCAGGCGCGCCAGTTGGTCGCCG
GCTTCGAGCTGGCTCTTGAGCCGCGAGACCACTTGCAGGATCAGCCGGTCGCCCGCCTGGTGGCCGAGGGCATCGT
TGGCATGGCGGAAGTTGTCCAGGTCCAGATGGCCGAGTGCCAGGCCACGCCCGTCGTTTTCTGCCAGGCGCGCGGC
CAGCAGGGTCTGGAACCCCTGGCGGTTGGCGATGCCGGTCAGCGGATCCTGTTCGGCCAGGCGTTGCAGAGTGTTT
TCCAGCAAGCCGCGCTCGCGCACATGGCGCAGGCAGCGGCGAACCGTATCGACATCAAACGCACCGAGGATCAGCC
AGTCGCTGGCGCCCAACGGCGCAACCAGCGGCTCCTGCTCCAGCAGCAAGACGGTCGGCAGGCTGCAACGGCCTGG
GCCGGGTTGCAGGCTGGGGGTGGTGAGCAAGACGGCGGTGCGGTCGTCGTCGAACAGGCTGCTCACCGACTCCCAA
TTTGGCGCACTGATCAGCACTGCCCCGTCGCCCATCGGCGCCAGGCACTCGCGCAACAACGCTGCCCACGTAGGCG
TATCGGCCAATAGCAGCAAACGCAAGGGTTCGACAGGCGTAGACAAGCTAGCTCCCTAGACTGTGCAAGATTTCGT
TGGCGGCGGGCATTATGACGTGCAGCCTGATAATCACCAATGATATTGGTTATCAAATACGCGCAGTGCGGCTCTA
GATCGAACGATAGACCCAAAAACCCCGCACATCCTGCGGCAAAGTAACAAAAGCGGCAAATTTAGATCGAGTGGTA
CGTCACACTGTCGTTCAGAGGCAGCAGAATCTTGCCAGCCTGTTAAAATGCCCGCCCTTTTGAACAATGACTCCCA
AAATTCCGTATGTCCCGACTCAATCCCCGGCAGCAAGAAGCCGTGAACTACGTCGGCGGCCCTCTATTGGTGCTCG
CCGGTGCTGGCTCCGGCAAGACCAGCGTGATCACCCGCAAGATCGCGCACTTGATCCAGAACTGTGGCATCCGTGC
CCAGTACATCGTCGCCATGACCTTTACCAACAAGGCCGCGCGCGAGATGAAAGAGCGCGTCGGCACCCTGCTCAAG
GGCGGCGAAGGCCGTGGCCTCACCGTGTGTACCTTCCACAACCTGGGGCTGAACATCATCCGCAAGGAGCATGCGC
GGCTGGGCTACAAGCCGGGCTTCTCGATCTTTGACGAGACCGACGTCAAGGCCCTGATGACCGACATCATGCAGAA
GGAATACGCGGGCGACGACGGCGTCGATGAGATCAAGAACATGATCGGCGCCTGGAAAAACGACCTGATCCTGCCC
CCCGAAGCCCTAGAAAACGCACGCAACCCCAAGGAACAGACCGCCGCCATCGTCTACACCCACTACCAGCGCACGC
TCAAGGCGTTCAATGCGGTGGACTTCGACGACCTGATCCTGCAGCCGGTCAAGCTGTTCCAGGAACACGCCGACAT
CCTCGAAAAGTGGCAGAACAAGGTGCGCTACCTGCTGGTGGATGAATACCAGGACACCAACGCCAGCCAGTATTTG
CTGGTGAAGTTGCTGATCGGTACACGCAACCAGTTCACCGTGGTCGGCGACGATGACCAGTCGATCTACGCCTGGC
GCGGCGCGCGCCCGGAAAACCTGATGCTGCTCAAGGTCGACTACCCGTCGCTGAAAGTGGTGATGCTGGAGCAGAA
CTATCGCTCCACCAGCCGCATCCTGCGTTGCGCCAACGTGCTGATCTCCAACAACCCCCACGAGTTTGAAAAGCAG
CTGTGGAGCGAGATGGGCCATGGCGACGAAATCCGCGTGATCCGCTGCCGCAACGAAGATGCCGAAGCCGAGCGCG
TGGCCGTCGAGATCCTCAGCCTGCACCTGCGCACCGACCGGCCCTACAGCGATTTTGCGATCCTGTATCGCGGTAA
CTACCAGGCCAAGCTGATCGAGCTGAAGCTGCAGCACCACCAGGTGCCGTATCGCCTGTCAGGCGGCAACAGCTTT
TTCGGACGCCAGGAAGTAAAGGATCTGATGGCCTACTTCCGGTTGATCGTGAACCCGGATGACGACAACGCCTTCC
TGCGCGTGATCAACGTGCCGCGCCGGGAGATCGGCTCCACCACCCTGGAAAAACTCGGCAACTACGCCACCGAACG
CAAGATCTCGATGTACGCCGCCACCGACGAAATCGGCCTGGGCGAACACCTGGATACGCGCTTCACCGATCGCCTG
TCGCGCTTCAAGCGCTTTATGGACAAGGTGCGCGAGCAGTGCGCCGGCGAAGACCCGATCAGCGCCCTGCGCAGCA
TGGTCATGGATATCGACTATGAAAACTGGCTGCGCACCAACAGCTCCAGCGACAAGGCCGCGGATTACCGCATGGG
CAACGTCTGGTTCCTGATCGAAGCGCTGAAGAACACCCTGGAAAAAGACGAAGACGGTGAAATGACCGTCGAGGAC
GCCATCGGCAAGCTGGTCCTGCGCGACATGCTCGAGCGCCAGCAGGAAGAAGAAGATGGCGCCGAAGGGGTGCAAA
TGATGACCTTGCATGCATCCAAGGGCCTGGAATTCCCCTATGTGTTCATCATGGGCATGGAAGAGGAAATCCTCCC
ACACCGCTCCAGTATCGAAGCCGACACCATTGAAGAAGAACGGCGCCTGGCCTACGTGGGCATTACCCGCGCGCGT
CAGACCCTGGCCTTCACCTTTGCGGCCAAGCGCAAGCAATACGGCGAGATCATCGACTGCGCCCCGAGCCGCTTCC
TCGATGAGCTGCCACCGGACGATTTGGCCTGGGAAGGCAACGACGACACCCCGACTGAAGTCAAGGCGGTACGCGG
CAACACTGCCCTGGCGGATATACGCGCGATGCTAAAGCGCTAGAATCGACTACTTTTTAATCTACTTTCGGCGCCC
CTTGCGCCACCAGAGGAAGCTTTTGTGGAAGCACTGCACAAGAAAATTCGCGAAGAAGGCATCGTGCTTTCCGACC
AGGTACTCAAGGTCGACGCCTTTCTGAACCATCAGATCGACCCGGCGCTGATGAAGCTGATCGGCGACGAATTCGC
CGCGCTGTTCAAGGATTCGGGGATCACCAAGATCGTCACCATCGAAGCCTCGGGCATCGCGCCGGCGATCATGACC
GGCCTGAACCTGGGCGTGCCGGTGATTTTCGCCCGCAAGCAACAGTCCCTGACCCTGACGGAAAACCTGCTGTCGG
CGACGGTGTATTCCTTCACCAAGAAAGTCGAAAGCACCGTGGCGATTTCCCCGCGTCACCTGACCAGCAGCGACCG
CGTGCTGGTGATCGATGACTTCCTGGCCAACGGCAAGGCGTCCCAGGCGCTGATTTCGATCATCAAACAGGCCGGC

GCCACCGTGGCAGGCCTGGGGATTGTGATCGAGAAGTCGTTCCAGGGCGGCCGTGCAGAGCTGGATGCCCAGGGCT
ACCGTGTTGAGTCGCTGGCGCGGGTGAAGTCCCTGGCCGGTGGGGTTGTGACCTTCATCGACTGAAGGTGAGCGAG
CACAGTTAAAAATGTGGGAGCTGGCTTGCCTGCGATAGCGCAGTGTCAGGCGACAGAGATGTCGACTGTCAGGCCC
TCATCGCAGGCAAGCCAGCTCCCACATTTGCCTTGTGTTACCTATCAGTGTGCGGTGGCCTGTAGGCCAGCAAGCA
ATAGGCGCTGATACAGATCCTCTTTAAACCCCTCAGGCTTATCCAGGCGCATGCGCACCAGATGCTCGGGAAACGC
CGCAGGTTCCGGTGCATCCAGCGCCGCCTTGCCCAACTCCAGAATCTCCGTCAGCTTGAATTTGCTCTTGAGCCAG
TTCAGCGCGCGCAACAAATCCCGCTCTTGATCAGTGAAATCACTGCCCAGCGGATACTCCGGAAACAGCCTTGAAT
GCCGCGCCTGCACCGCCTGCAAACGCTCGGGAGTGTTGTCGGCAAAGCGTGGGTCGAGCTGGAAGTCCTTGGGCAA
CTTGCCGGCTTTTTGCGCCTGTTCGATCAAGTCGGCCTGGAACCGTGAGTCGGTGATATTGAGCAACGCCTCGATC
ACCTTGGCGTCGGTCTGGCCGCGCAGGTCAGCGATACCGTATTCGGTGATCACGATATCGCGCAGGTGCCGTGGAA
TCGTGCAATGGCCGTACTCCCAGACAATATTGGAGCTGACCTCGCCAGCCGATTCGCGCCAACTGCGCAGGATCAG
GATCGAGCGCGCACCCTCCAGCGCATGGCCTTGGGCGACAAAGTTGTATTGCCCACCCACACCGCTGAGCACGCGG
CCATCTTCCAGTTGGTCGGCAACGCCTGCACCCAGTAGCGTCACCATGATCGCGCTGTTGATAAACCGCGCATCCA
CGCGCTGTAAACGCTTGAGTTCTTCCTGCCCGTACAGCTCGTTGATATAGCTGATGCGGGTCATGTTGAATTCCAG
ACGCTTGGCGTGGGTCATTTCCTGCAAGCGCTGGTAAAAACTGCGGGGGCCGAGGAAAAACCCACCGTGGATCGAT
ATGCCATCGGGCTGCGCCGCATCGTCCAGCAGGCCTGCGTTGGCCTGCTCCTGCGTCGCCACATCCGGGTACACCT
TGCGCCGCACAATCCCGGCATCGGCCAGCACCAGCAGGCCGTTGACGAACATTTCGCTGCAACCATAAAGGCCCCG
GGCAAACGGTTCGACCCCACCCTCACGGCTGATCAGCGGCGCCCATTGGTACACATCCAGGTCGGTGAGCAACTGC
CGATAGCTCTCGTTATCGGCCTGACGCGCCAGCAACGCGGCGGTCAAGGCGTCGCCCATCGAACCGATACCAATCT
GCAAGGTGCCGCCATCGCGCACCAGGGTGCTGGCGTGCAAACCGATGAAGTGGTCCTGCAAACCCACTGGCATGTT
GGGCGTAGAGAACAGCGTGGTGCGGTCTTTTTCGTCGATCAGGAAGTCGAACTGATCCATGCCCAGTTCGGCATCA
CCGGGCATGTAGGGCAAATCTTCATGCACCTGGCCGACCACCAGGATGGTCTCGCCTGCCGCGCGGCGCTTGGCGA
TCATGGGCAACAGGTCGAGGGTGATATCCGGGTTGCAACTCAGGCTCAGGCGATCCGGGTGCTCACTGCTGCTGGC
CACCAATTGCGCAACCAGGTTCAGGCCGGCGGCGTTGATATCGCGAGCGGCATGGCTGTAGTTGCTGCTGACGTAG
TCCTGCTGGGCCTGGGCGCTGTGCAACAGGCTGCCGGGCTGCATGAAGAACTGCTGCACGTGGATATTGGCCGGCA
GGCTGTCCTTGCGCAGGGCGGCAAGGTAATCCAGCTCGGGATAATCACCGAACACCCGCTCGACAAACGGCTCCAG
AAAGCGCTTCTGCAAACCATCACCCAGCGGCGGGCGGCCCAGGCTCAACGCGGTATAGATCGTCAGCGACCGCTCG
GGCAGCTTGGCGATACGCCGGTACAAGGCATTGGCAAACAGATTCGGCTTACCCAGACCCAGCGGCATACCCATGT
GGATATGCGCCGGTAGGCGCTCCAGTACGTCGTCGACCGCTTGCTCGATTGAACACAACTGCACCATCCGACCCTC
CTGACGTTCCGTGATTAGGGGTTGGACCGAGCTTGCCGGGTCTTTGCTGCAATGAACAGCCTTGGCCGGCAAATTG
CGGGCACAAAAAAGCCGCTGGTCAGCGGCTTTTTGGCGAAGATCGGTTTATTTCAAACCAGACATCTTCTCGATTG
CAGCCTTGAGGTCGTCATCCGAGCAATCGGAGCAGGTGCCTTTAGGCGGCATGGCGTTGATGCCGGTGATGGCCTT
GGCCAGGATGCCGTCGAGGCCGCCCTGGTGATCGGCGCGCTCTTTCCAGGCTGCGGTGTCACCGATTTTCGGTGCG
CCCAACAGGCCAGTGCCGTGGCAAGCATTACAGTGTTTGGCGACGATCTCGTCCGGCGTTTTTGCGCCGCCGCCGC
CCGCTGTCACAGCGACTTCCATGCCCTTGCATTCCTCACCTTGAATACAGACCTTCCCCACGGGTTCAAGGCGCTT
GGCAATATCATCATTGGTCGCAGCTTGAGCGCTGGCAGCCCAAAGGGCCAGTACGGTTGCTGGTACAGCCAGCATT
TTCATAATTAGGTTCACGCGTTCACCCTCATGGTGGCTATTCACGCCTGCGGCCACGGTTTCGCAGGCGGCGAAAG
TATAACGGTTAGGCCGCCTCACTGAAACAACCCTATTATCCAAGGGGAGATTGAGCATGGCGAAATACAACTACGG
GCACCTCTGCAAGGTAGGCAGGGCGCCTCTTCTGTTAAAAGTTCGCGGGTGTGGCTGCGCTGATTAGTCGCGCCGG
CACATCGAACGGATTGCGAAAACGGTGCGGCTTGGTGCTTTCAAAGTAGTAGCTATCGCCGGCTTCGAGCACAAAA
GTTTCGACGCCCACGACCAATTCCAGACGACCTTCCAGCAGGATCCCGGTTTCCTCGCCGTCATGGGTCAGCATTT
CCTCACCCGTATCGGCGCCCGGCGGGTAGATTTCGTTGAGAAACGCAATCGCCCGGCTCGGATGTGCCCGGCCCAC
CAGCTTCATGGTGACCGCGCCATCGGAGATGTCGATCAGCTCATTGGCTTTATAGACGATCTGCGTCGGTTTTTCC
TGGAGGATCTCCTCGGAAAAGAACTCGACCATGGACATGGGGATGCCGCCCAGCACCTTGCGCAAGGAGCTGATGG
AGGGGCTGACACTGTTTTTCTCGATCATCGAAATGGTGCTGTTGGTGACGCCCGCACGCTTGGCGAGTTCACGCTG
GGAAAGACCTTTCAGTTTACGGATGGATTGCAGTCGTTCGCCGACGTCCAATGCAGGAGCCTCCTAGGATTCAGGC
TTTGTTGTAATTGAGCGTTATCATGGCGACAGCGTTCAGTATTTACAACACTTTGGTCTGAATCCCGCAGGGCTTC
ATCTGCAGCTGGCGGCTAAGCGCCGGAATAGAGCCGTGGCACACGGCGCAGGTTGCAGAAAATCTGATAAGGGATG
GTCTCGGCGGCGGTCGCCACATCGCTGGCGAGGATGTTTTTGCCCCACAACTCGACGGTCGAACCCAGACCCGCCT
GGGGTACATCGGTCAGGTCGATGCACAGCATGTCCATCGACACCCGCCCCAGCAACTGGCTGCGCTGGCCGGCGAC
CAGTACCGGCGTACCGGTCGGCGCATGCCGTGGATAACCGTCGGCATACCCCATGGCAACCACACCGATACGCATC
GGCTTGGGCGTCACGAAGCGCGCGCCATAACCCACGGGCTCGCCCGCAGGCAGCTCGCGTACGCAGATGACTTTCG
ACTCCAGGGTCATTACCGGCTGCAAACGCGCGGCAACCGCCTGGTCTTCGCCAAACGGCGTCGCACCGTAAAGCAT
GATGCCGGGCCGTACCCAGTCGCTGGAAACGCTCGGCCAGCCCATCACCGACGGCGAGTTGCGCAGGCTGACCTGG
GCCGCCAAGCCCTGGCGCGCGGCCTCGAAGACCGTGACCTGTTCATCGCTGCTCGCGCAGTCCAGCTCATCGGCAC
GGGCGAAGTGGCTCATCAACACGATCTTGGCCACTTTGCCGCTGGCCAGCAGGCGCTGATAGGCGTCGTGATAATC
CTTGGGATGCAAGCCAACCCGGTGCATGCCCGAATCCAGCTTCAACCATACCGTCAGCGGCTTGCTCAGGCGCGCC

TGTTCAATCGCTTCCAGCTGCCACAGCGAATGCACCACGCACCAGAAGTCATGCTCGATGATCAGCGGCAGTTCGT
CGGCCTCGAAGAACCCTTCGAGCAACAATACCGGCCCACCAATCCCGGCAGCGCGCAGTTCCAGCGCTTCCTCGAT
ACAGGCCACGGCAAAACCGTCGGCCTGGTCTTCCAGGGCCTGGGCCACGCGCACGGCTCCATGGCCGTAGGCATCG
GCCTTGATCACGGCAAGGGCCTTGGCCCCGGTCAGTTCGCGGGCCAATTGGTAGTTGTGGCGCAGGGCTTGTAGGT
CGATCAGGGCACGGGCAGGACGCATGGCGGCAGGCTTCTAGGCGGCGGTAGGAAGAAAAAACCGGTACCGACCGAC
AGCGCCGGCACCGGAAGAGGGATCGTTTAAGGCAGAGCGGCCACGACAGACAGCTCGACCAGGATTTCGGGTTCAC
ACAGTTTCGATTCGACCGTGGCACGGGCGGGTGCGACACCTTTTGGCAGCCACTTGTCCCATACCGCGTTCATGCC
GGCAAAGTCGGCGTCGATGTCTTTGAGGTAGATCGTCACCGACAGCAACCTGGTTTTGTCGGTACCGGCCAGATCC
AGCAAGCGCTCGATGTTGGCCAAGGTCTCGCGGGTCTGCTGTTCAATCCCGGCACTCATGTCGTCGCCGACTTGCC
CTGCCAGATACACGGTACCGCTGTGGACAACGATCTGGCTCATGCGCTCATTGGTGAGCTGGCGCTGGATTGACAT
GTTTTGTGGACTCCTGAGGGTTGCCGTAACGGGAAATATCGAGGCCTGCGGCGCTGATCTGCGGGGTTTTCTTGGC
CATCAGGTCCGCCAGCAAGCGGCCAGAGCCACAGGCCATGGTCCAGCCGAGGGTGCCGTGGCCGGTATTCAAGAAC
AGATTCTTGAACGGGGTAGCGCCGACAATTGGCGTACCGTCCGGGGTGGTCGGGCGCAGGCCGGTCCAGAACGTGG
CTTCGCTCAGATCACCGCCCTGAGGATAAAGGTCGTTGACGATCATCTCCAGGGTTTCGCGCCGGCGCGGGTTCAG
CGACAGGTCAAAACCGGCTATTTCAGCCATGCCGCCAACCCGGATGCGGTTGTCGAAACGGGTGATCGCAACCTTG
TAGGTTTCGTCGAGAATGGTCGAAGTAGGGGCCATCGCCGGGTTGGTGATCGGCACGGTCAGGGAGTAACCCTTGA
GCGGATACACCGGTGCCTTGATCCCCAGCGGCTTGAGCAACTGCGGCGAGTAGCTGCCCAGGGCCAGCACGTAGCG
ATCGGCGGTTTCCAGCTTGCCGTCGATCCACACACCGTTGATACGATCACCGGCGTGGTCCAGGCGCTGAATGTCC
TGCTCGAAACGGAACTCCACGCCCAACTGCTTGCACATATCGGCCAGGCGGGTGGTGAACATCTGGCAGTCGCCGG
TCTGGTCATTGGGCAGGCGCAAGGCACCGGCCAGGATATCCGTGACGCTGGCCAGGGCCGGCTCGACGCGGGCGAT
GCCGGCGCGGTCGAGCAGCTCGAACGGTACGCCGGACTCTTTCAACACGGCAATATCCTTGGCCGCACCATCGAGT
TGCGCCTGGGTACGGAACAATTGCGTAGTACCCAGGCTACGGCCTTCGTAGGCAATGCCGGTTTCGGCGCGCAGTT
CGTCGAGGCAGTCGCGGCTGTACTCGGACAGACGCACCATGCGCTCCTTGTTCACCGCATAGCGGTTGGCCGTGCA
GTTGCGCAGCATCTGCGCCATCCACAGGTATTGGTCGATATCGGCCGTGGCCTTGATCGCCAGCGGCGCATGGCGC
TGCAGCAGCCACTTGATGGCCTTGAGCGGCACACCCGGCGCAGCCCATGGCGACGCATAGCCGGGCGAAACCTGGC
CGGCGTTGGCGAAGCTGGTCTCCATTGCAACGGCCGGCTGACGGTCGACCACGACCACCTCAAAACCGGCACGCGC
CAAATAGTAGGCACTGGCCACACCAATGACACCGCTACCCAAGACCAAAACGCGCATTTTTATATCCTCATCACGG
CTTGACCGCTGACGTTTGTTGTTCAGCACAAAGATGTGGGCAGTATAAAAAGCAATGACCAGTGCATTTCACTATA
TAAGTGCCTATATTTGGCGACAATTCTCGGCAAAAACCCTTTTCACGGAGGAGCATCCCCTATGCGGACCAATACC
CAGACCAAGCGGGAGCTGGACAAGATCGACCGCAACATCCTGCGCATCCTGCAGGCGGACGGGCGGATATCGTTTA
CCGAGCTGGGGGAGAAGGTAGGACTGTCGACCACGCCCTGCACCGAACGGGTCCGGCGCCTGGAGCGCGAGGGGAT
CATCATGGGCTACAACGCCCGCCTCAATCCGCAGCACTTGAAGGGTAGCCTGCTGGTGTTTGTCGAGATCAGCCTC
GACTACAAATCCGGCGATACCTTCGAAGAATTCCGACGTGCCGTGCTGAAACTGCCCCATGTGCTGGAGTGCCACC
TGGTCTCGGGGGATTTCGACTATCTAGTGAAGGCGCGGATTTCCGAAATGGCGTCGTACCGCAAACTGCTGGGCGA
CATCCTGCTCAAGCTGCCCCATGTGCGCGAGTCCAAGAGCTATATCGTGATGGAAGAGGTGAAGGAGAGCCTGAAC
CTGCCGATCCCGGATTGATCCTGTGGGAGCTGGCTTGCCTGCGATAGCGATCAACCCGCCGCCATTTTCATGGCCT
GGCACACCGTCATCGCAGGCAAGCCAGCTCCCACCTTTTGACCGAGTACATCTGTCAGACCAGCACCTGCCGCGTC
GTCGCCATGTACTCGTGGATCTGCTTCTCGACCCGAGGGTGAATCAGTTCCACCGGCCCACGGTTATTGGGGCATG
GCAGGCTGGGCGTAGTACCAAACAACCTACAGATCAATGGGCGCTCGTCGTACACCGTGCAGCCGTTGGGCCCCAG
GTGTACACAGTTCAGCTCATCCATGGCCGCTTCCTGCTCGGCCGCGGTCTTGCGCGGCAGGCGCGACATTTCCTCG
GTGGATGTAGTCACCGGCCCACAGCAATCATGGCAACCCGGCACACACTCGAACGAGGGAATCTGCCGGCGCAGCG
CGTTGACTTTCTGGCTGTTGCAACTCATCGAAACCCATACCGAACGGCGAATAGGCGTGGATTTTGCCCTAAAAGC
CCCGAGTGAGACAGCTTCACCCGACCGCTGTATCCTGCGTCAAATTTTCCAAACACGGATGCTCCCCATGACCGCC
AGCGCCCGGCACACCGCTTCCTACTACGCCGCCAGCAGCCTGCCGCAACCCGATTACCCGGTGCTGGCGGGTGAAG
TCGTCGCCGATGTGTGCGTGGTGGGCGGTGGGTTTTCCGGGCTGAACACCGCGCTGGAACTGGCTGAACGGGGCTT
CAGCGTGGTGCTGCTGGAGGCCCGCAAGATTGCCTGGGGCGCCAGCGGGCGCAATGGCGGGCAGTTGATTCGTGGC
GTCGGCCACGGCCTTGATCAGTTTGCCAATGTGGTCGGCACCGAAGGCGTGCGGCAGATGAAGTTGATGGGCCTGG
AAGCCGTGGAAATCGTGCGCCAGCGGGTCGAGCGCTTCCAGATTGAATGCGACCTGACCTGGGGTTACTGCGACCT
GGCCAACAAGCCCCGCGACCTGCAGGGCCTGGCCGCCGATGCCGAGGAGTTGCACGACCTGGGTTATCGCCATGAA
CTGCGCCTGCTGCAAGCCGGGGAGATGGGTAGCGTGATCGGTTCCGACCGTTACGTAGGCGGCATGATCGACATGG
GTTCCGGCCACCTGCACCCGCTGAACCTGGCCCTCGGCGAAGCCGCCGCCGCGCAGCAACTGGGAGTCAGGCTGTT
CGAGCACTCCGCAGCGGTGCGCATCGACTACGGCCCTGAAGTCAAAGTCCATACCGCCCACGGCAGTGTGCGCGCC
AAGACTCTGGTGCTGGGCTGCAATGCCTACCTCAACGGCCTCAACCCCCACCTGAGCGGTAAGGTGCTGCCCGCCG
GCAGCTACATCATCGCCACCGAACCCTTGAGCCCGACCCAGGCCGCCGAACTGCTGCCGCAAAACATGGCCGTGTG
TGATCAGCGGGTCACGGTGGATTACTTCCGGCTGTCAGCCGACCGCCGCCTGCTGTTTGGCGGCGCCTGCCATTAT
TCCGGGCGCGACCCTCAAGACATCGCGGCTTATATGCGGCCCAAGATGCTCCAGGTCTTCCCGCAACTGGCCGAGG
TGAAAATCGACTACCAATGGGGCGGCATGATCGGCATCGGTGCCAACCGCCTGCCGCAGATTGGCCGCTTGGCCGA

CCAACCCAACGTGTACTTTGCCCAGGCGTATGCCGGCCATGGCCTGAACGCCACCCACCTGGCGGGCAAGCTGCTG
GCCGAAGCCATTAGCGGCCAGCACCGTGGGCGCTTCGATCTGTTTGCCCAGGTGCCCCACGTCACCGTCCCTGGCG
GTAAGCACTTGCGCTCGCCGTTGCTGGCCCTGGGCATGCTCTGGCACCGCTTGAAAGAACTGCTCTGACTCACCCG
CGCCAGAACGGTTTCAAGCCTTCCTGGCGCGCTTGCTCAGCGCTTAAGCCGATATCGCGCAGTTGCTCCGGGGTCA
GGTCCAGCAGGGCCTGGCGCGTGTGGCGACGGCGCCAGAACAGTTCCCAACGGCTCGGCTGACCAGGCGCCAGCCC
AGTCAGCCCACGCTCCTGCCCTGCTTCCAGTTCCTGACTGTGTAGCGCCAGCCGCACATCGCTCAAGCCACTCATT
GTCGTGCCCCTCATTTGCCTGTTGCCATGAGTGACTAGAATGGATGGCGCGGCAAAACCATTACAGATTCAACCAA
CCTTTATTAAATCCATACAGATACTGCCATGCAGGGGCTGAATCCTGTATTTTCCCTTCATCTGTACTGGTCCACC
GGGAGCGACCGCCATGACCCTCTACGTCAACCTCGCCGAATTGCTGGGTACCCGCATCGAGCAAGGCTTCTATCGC
CCCGGCGATCGCCTGCCCTCGGTGCGTGCCCTGAGCGTGGAACATGGCGTCAGCCTGAGCACCGTGCAACAGGCTT
ACCGGTTACTGGAAGACAACGGCCTGGCGACTCCAAAGCCAAAATCCGGTTACTTCGTGCCCGTCGGGCGCGAGCT
GCCGGCGCTGCCCGCCGTGGGTCGGCCGGCCCAGCGCCCGGTGGAGATCTCCCAGTGGGACCAGGTACTGGAACTG
GTGCGCGCGGTGCCGCGCAAAGACGTCATACAGATGGGCCGCGGTATGCCAGATGTAATGTCACCGACCATCAAGC
CCTTGCTGCGCAGCCTGGCCCGGGTCAGTCGCCGCCAGGACCTGCCGGGCCTGTACTACGACAACATCTACGGCTG
TATGGAATTGCGCGAGCAAATAGCCCGTCTGTCATTGGATTCCGGCTGCCAGCTCGACGCCCAGGACATCGTGATC
ACCACTGGCTGCCACGAAGCCTTATCCTCCAGCATCCGCGCGATTTGCGAACCCGGCGATATCGTCGCCGTGGACT
CGCCAAGCTTCCATGGCGCCATGCAGACCCTCAAGGGCCTGGGCATGAAAGCCCTGGAAATCCCCACCGACCCGCT
CACCGGCATCAGCCTCGAAGCCCTGGAACTGGCCCTGGAGCAATGGCCGATCAAGGTTATCCAGCTGACGCCCAAC
TGCAACAACCCCCTGGGCTACATCATGCCCGAGGCGCGCAAGCGGGCGCTGCTGACCCTGGCCCAGCGCTTTGACG
TGGCGATCATTGAGGACGATGTGTATGGCGAACTGGCCTACAGCTACCCGCGACCGCGCACCATCAAGTCCTTCGA
CGAAGATGGCCGCGTCCTACTCTGCAGCTCGTTCTCCAAGACCCTGGCCCCCGGCCTGCGGATCGGCTGGGTAGCG
CCCGGACGCTATCTGGAGCGGGTGCTGCACATGAAGTACATCAGCACCGGCTCCACCGCCACCCAACCACAGATCG
CCATTGCCGAATTCCTCAAGAACGGGCACTTCGAACCGCATTTGCGGCGCATGCGTACCCAATACCAGCGCAATCG
CGACCTGATGCTCGATTGGGTCAGCCGCTATTTCCCCGCTGGCACCCGCGCCAGCCGGCCCCAGGGCAGCTTTATG
CTGTGGGTGGAACTGCCCGAAGGCTTCGACACCCTCAGGCTCAACCGGGCCCTGGTCGAGCAGGGGGTGCAAATTG
CCGTAGGCAGTATCTTTTCCGCTTCGGGCAAATACCGTAACTGCCTGCGGATGAACTACGCTGCCAAGCCAACCCC
GCAGATTGAAGAGGCGGTGCGCAAGGTCGGGGCAACGGCGAGCAAGATGCTCGCCGAAGCCGAACAGCGGATGGAC
TGACCTTTTGCCAGCAATCACCGTCATATGCCCACCACCGTCCTGATTCGGAACCTGTCTCCTTGATGAGCCAACG
GCTATTAGCGTTCTTTTTGCTGGGGTTCCTGGGCCTGGGCGGTTGCGCCACCCTGGACGTGCCCCGGGTGCCCAGC
GAGGCACTGCCGGCGACGCAGTCCGCCTTTGGGCGCTCGATCCAGGCCCAGGCCGCGCCCTATCAGGGCCGCTCCG
GGTTTCGCCTGCTGCCCAACAGCAGCGAAGCGTTCATGGCCCGCGCCGAATTGATCCGCAATGCCCAGACCAGCCT
CGACTTGCAGTACTACATCGTCCATGACGGCATCAGCACGCGCATGCTGGTGGACGAGCTGCTGACGGCGGCCGAC
CGTGGCGTGCGGGTGCGCATCCTCCTCGATGACACCACCAGCGACGGCCTGGACCAGATCATCGCCACACTGGCTG
CACATCCGCAGATCCAGATCCGCCTGTTCAACCCCTTGCACCTGGGGCGCAGCACCGGCGTGACACGGGCCATGGG
CCGGCTGTTCAACCTGTCGCTGCAACACCGGCGCATGCACAACAAGCTATGGCTGGCCGACAATAGCGTGGCGATT
GTCGGCGGGCGCAACCTGGGGGATGAGTATTTCGATGCCGAGCCCAACCTGAACTTCACCGATATCGACATGCTCA
GTGTGGGACCCGTGGCCGAGCAACTGGGCCACAGTTTTGATCAGTACTGGAACAGTGCGCTGAGCAAACCTATCGC
CGACTTTATCTCCAGCGCCCCGTCCTCCCGCGACCTGGCCACGGCGCGCGGGCTTCTGGAGAAGTCCCTGGCCGAA
TCGCGCCAGCAAAACCATGCCCTGTATAACCGCCTGCGGACCTACCAGACCCAGCCACGCATGGATATCTGGCGCC
GGGAACTGATCTGGGCCTGGAACCAGGCACTGTGGGACGCGCCGAGCAAGGTCCTGGCCAAGGCCGATCCCGACCC
ACAACTGCTGCTCACGACCCAACTGGGGCCAGAGCTGGAAGGCGTGCACAGTGAGCTGATGATGATTTCGGCCTAC
TTCGTGCCCGGCCAGCCGGGCCTGGTGTACCTCACCGGCCGCGCGGACGCAGGCGTCGACGTGCGCCTGCTGACCA
ACGCCCTGGAAGCCACCGACGTGCCGGCGGTGCATGGCGGCTATGCGCCCTATCGCAAGGCGCTGCTGGAACACGG
GGTGAAGTTGTACGAACTGCGCCGCCAACCGGGTGACGGTGGCGGCAGTGGCCCGCACCTGCTGGGCAGCCGCTCG
CTGCACGGTTCGGACTCGAGCCTGCACAGCAAGGCGATGATTTTTGATCGGCAGAAGTCGTTTATCGGCTCGTTCA
ATTTCGACCCGCGTTCGGTGCTGTGGAACACCGAGGTCGGCGTGCTGGTGGACAGCCCCGAACTGGCCGAGCATGT
TCGTAACCTGGCCCTGCAAGGGATGGCGCCGGCGTTGAGTTATGAGGCAAAACTGCAAGATGGCCAGGTGGTATGG
GTCACGGAAGACAACGGCCAGATGCACACCCTGAGCCGCGAGCCGGGGAGCTGGTGGCGCCGGTTCAATGCCTGGT
TTGCCACCACCGTGGGTCTGGAGCGCATGCTCTAAGAACCACGAATATCCATTGTGGGAGCTGGCTTGCCTGCGAT
AGCGGTGGGTCAGTTGGCCCATTTGCTACTGATACACCGCTATCGCAGGCAAGCCAGCTCCCACAGTTTTGATCGC
ATTTCCAGGTCAGGCTGGTTGCTCGAGGCCAAACGCGCCTTGGCGCAACAACAGAATCACCAATCCCAAGGCACCC
GCGGCCATCAACAACGGCAACGCATGCCCGCTGATCCACTGGCTGCCAGCCCCCGCCACCAGCGGCCCGATCAGGC
AACCAATCCCCCACAACTGGGCAATATGGGCATTGGCTCGCACCAGCGCATCGTCACGGTAGCGCTCGCCGATCAG
AATCAACGACAAGGTGAACAAGCCCCCCGCACTGGCACCGAACAGGACCCAGATCGGCCAGATCAGCCAGGTATCC
AGCAACGGCGCAATGGCCAGGCTCGACAGCAGCAACAGCACCGCACAGCCGAGAAACAGGGTGCGCCTGGGCAGGT
AGTCGGCCAGCGCACCAATCGGCAATTGCAGCAGGGCATCGCCTACTACCACGGTGCTGACCATCGCCAACGCCAC
TTCCGCAGTGAAGCCCTGTTGCAGGCAATACACCGGCAACAGCGTCAGAATCATCGCCTCGAACGCGGCAAACAAT

GCCACCGCCCAGGCGATCGCCGGCAAGCCCCGGCAAAATCCCCATAAGTCGCCAAAGGTCACGCTGAAGGCCTCTG
CCGTCGGCGCCCCGCTACGCCCCAGCAGCAGGAAGGGCGCAACGATCAACAGACCGACGCCAACCCAGAAGCCATA
GTCATGATCGGTGCCCAGTACGCCGAGCAACAACGGCCCCGACAGCTGGCTCAAGGCGTAGGTACAGCCATACAGC
GCCACCAGCCGGCCACGCCACTGCTCCACCACCAGTTGGTTGATCCAGCTTTCGCCGAGGATAAACACAATGGTCA
GGATCACCCCTATCATCAACCGCAGCACCAGCCACACCGGATAGCTGGGCAATACCGCCAGCAACCCGATGGAAAC
CGCCCCGGCCCACAGGCACAGGCGCATCAGATTTGCCGTGCCCAGCCAGGAAGCCAGGCGGCTCGACACCTTGGCG
CCCAACAACACACCGAACGCTGGCATCGCCGCCATGATGCCGATAGCAAAGTTGCCGTAGCCCCAGCTTTCCAAGC
GCAGAGACACCAGCGGCATGCTGACGCCCAGGGCAAGGCCGACGCTGAGTACTGAAGCCAGGACGGCAAAGTAAGT
CGCCCAACGCATTGCCACGCTCCTGTGGATAACTCATTTGTGGACGGTAAAACGAACTGTAGGAGCGAGCTTGTGT
GGGAGCCGGGCTTGCCCGCGATGCTGGCACCTCGGTACATCAGTTGCACCGAGGTGATGCTGTCGCAGGCAAGCCA
GCTCCCATACAAGCTCGCTCCTACAGGGGATCAGCGGGGTTGCTTACAGCTTGATCCAGGTTGCCTTCAGTTCGGT
GTACTTGTCGAACGCGTGCAGCGACTTGTCGCGACCGTTGCCCGACTGCTTGAACCCGCCGAACGGCGCGGTCATG
TCGCCGCCATCGTACTGGTTGACCCACACGCTACCGGCGCGCAGCGCTTTGGCGGTCAGGTGCGCCTTGGAGATAT
CCGAGGTCCATACCGCAGCGGCCAAGCCATAAGGCGTGTCGTTGGCGATGGCTACGGCTTCTTCAGCGCTGTCGAA
GGTGATCACCGACAGGACCGGGCCAAAGATCTCTTCCTGGGCAATCTTCATGGCGTTGGTCACACCGTCGAAAATC
GTCGGCTCGACGTAAGTGCCGCCGGTTTCCTGCAGGGTGCGCTTGCCACCGGCCACCAGTTTGGCGCCATCGGCGT
GCCCGGCTTCGATGTACGACAGCACGGTGTTCATCTGCTGGGTATCCACCAGCGCACCGACGTTGGTGGCCGGGTC
CAGCGGGTTGCCCGGCTTCCAGCCCTTGAGGGCCTCGATCACCATCGGCAGGAATTTGTCCTTGATCGAACGCTCC
ACCAGCAGGCGCGAGCCGGCGGTGCAGACTTCGCCCTGGTTGAAGGCGATGGCGCCAGCGGCGGATTCGGCAGCGG
CTTGCAGGTCCGGGGCATCGGCAAACACGATGTTCGGGCTCTTGCCGCCGGCTTCCAGCCAGACGCGCTTCATGTT
CGACTCGCCGGAGTAGATCATCAGTTGCTTGGCGATCTTGGTAGAACCGGTGAACACCAGAGTGTCGACGTCCATA
TGCAGCGCCAACGCCTTGCCCACGGTGTGGCCGTAGCCCGGCAGCACGTTGAGCACGCCTTTGGGGATACCGGCTT
CAACGGCCAGGGCGGCGATGCGGATGGCGGTCAGCGGCGACTTTTCCGACGGCTTGAGGACGACCGAGTTACCGGT
GGACAGCGCTGGCCCCAGTTTCCAGCAGGCCATCATCAGCGGGAAGTTCCACGGCACGATGGCCGCCACTACGCCG
ACGGGCTCGCGGGTCACCAGGCCCAGTTGGTCATGGGGGGTGGCGGCGACTTCGTCGTAAATCTTGTCGATAGCCT
CGCCGCTCCAGCTCAGGGCTTGCGCCGCACCTGGAATGTCGATGCCCAGGGAATCACCGATTGGCTTGCCCATGTC
CAAGGTTTCAAGCAGGGCCAGCTCTTCGGCATTGGCCTTGAGCAGCGCGGCGAAACGGATCATGGTGGCTTTACGC
TTGGCCGGGGCCAGGCGTGACCAGGCACCCGAGTTGAAGGTGGCGCGGGCGTTTTCCACGGCGCGCTGGGCGTCGG
CGACGTCACAGCTGGCAACCGTGGTCAGCAGGCGGCCGTCAACCGGGCTGAGACATTCGAACGTGTCGCCGGAAAC
GGCGGCGGTGTATTCGCCATTGATATACGCGCGGCCTTCGATCTTCAGGTCCTTGGCGCGTTGTTCCCAGTCGGCA
CGGGTCAGGGTGGTCATGCGAGTGTCCTCCTCTTATTAAATATAAGGGCCCGGCGTAATCACCAGTTCCCTTCCAA
ATGCTTGCCCGGCCAGCCAGCTATTCGGTCCGAGGCACCCGCCACCCTAAACCAGCCGCCGGGGATGTTTCAATAT
ATTTGACATAACGGCCGCAAACGCCCTTGCGATGTTCATTTTAATAAACATAGACTTTGGCTCTCCAACCCCAGGC
CTAGACGGGATACGTGCATGAACATTCAGGATGTCGTCGATTTCAGCCAGGCGAACACCGCCGCCGAACGCTACCG
CCCCGCCACCGAAAAAATCCTCAAGGGCGACCCCGAGCAGACGCTCTTCAACCACTACAACAGCCCGTGCGGCCAG
ATGAACGCCGGGGTGTGGGAGGGTGAAGTCGGGCAATGGACGGTGAACTACACCGAACACGAATATTGCGAGATCG
TGCAGGGGGTTTCGGTACTTCGCGATGGCGACGGCAACGCCAAGACCTTGCGCGCCGGTGATCGCTTCGTCATCCC
GGCCGGCTTCAGGGGCACCTGGGAAGTGCTGGAGCCGTGCCGCAAGATCTACGTGGTGTTCGAAAAAAAGGCCTGA
TCGGTCGATCGCGACTTTTCAAAGACGAAAAAAAACCCGCATCGTGAGATGCGGGTTTTTTTCACAAGAAAGAAAA
CCAATTACTTGATTTTGCCTTCTTTGTAGATCACGTGCTTGCGAACGCGCGGGTCGAACATTTTCTTTTCGAGCTT
GTCCGGGGTAGTACGCTTGTTCTTGTCGGTAGTGTAGAAGTGACCAGTACCGGCGCTAGAGATCATTCGAATCAAT
TCACGCATGATATAGCTCCTTAGATTTTGCCGTCGCGGCGCAGTTCAGCGAGCACGACAGTGATGCCACGCTTGTC
GATGATGCGCATGCCTTTGGCAGATACGCGCAGACGGACAAAACGTTTCTCTTCTTCAACCCAGAAGCGGTGATGC
TGCAGGTTTGGCAGGAAACGACGACGGGTTTTGTTGTTTGCGTGGGAAATGTTATTCCCAGTCACCGGACCCTTAC
CGGTAACTTGACAGACTCTCGACATGCCTCAGCCCTCTAAAACCACATGCCCAACCCGGCATGGGTTGGCCGCTTA
ATCTCTCAGTCATTTGGCGCCAGGCGCCGCGTTTCTTTAAGGGTCTTACCGGCTACACCTACAGTGAAGGAACCGG
GCCCCTAGAAAAGAGCGCTGCTTTATACCAGAAAGACCCCAGTGCAACAACAGCCGGTGTGTTTTTCCCGCTGTAA
ATCTCGCCCGAACAGGCCTCAAGCGCTGCCAGGGTGGGGCGCCGTGCTGCAGAACCGCTCGTCGCACCGAATGATT
TGTCGCCGCTGCTTGCAGGCCAAGGCCCTGGGCAAAATGACCTGAAGCGCCATCAAAACAGTATTTGCCGTAAATG
CACAGGCAAAAATGGGCTAGTCATTTATGAATGAGCCCACTACGGTAGGACTTTTCCAGACTGCACTCGCAGATGG
GCCTCCGATCTGCAAAGGAAACCGACCATGCGTCTCGCTGCCCTACCCTTATTGCTCGCCCCGCTCTTCCTCGCCC
CACTGGCCCAGGCAGCCTCTACGCTGAGCGTCTGCACCGAGGCCAGCCCCGAAGGGTTTGACGTGGTGCAATACAA
CTCGTTGACCACCACCAACGCCTCGGCGGATGTGCTGATGAACCGCCTGGTGGACTTCGATGCCGCGAGCGGCAAG
CTGGTACCGAGCCTGGCCGACAGCTGGGAAGTCTCGCCGGACGGCCTGACCTATACCTTTAAATTGCACCCGGACG
TCAAATTCCACCGCACTGCGTATTTCAACCCAAGCCGCAGCCTCACGGCTGAAGATGTGCGTTTCAGCTTCGAGCG
CATGCTCGACCCGGCCAACCCCTGGCACAAGATCGCCCAGAGCGGCTTCCCCCACGCCCAGTCGCTGCAACTGCCA
GCGCTGATCAAGAAGATCGACGCCCTTGACCCGCTGACCGTGCGTTTTACCCTGGATCACGCCGATTCCACCTTCC

TCGCCGCGCTGAGCATGGGCTTTGCCTCGATCTACCCCGCCGAATACGCCGACAAGCTGCTCAAGGCCGGTACCCC
GGAAAAGCTCAATAGCCAACCCATCGGCACCGGGCCGTTCGTGTTCGGGCGCTTCCAGAAAGATGCGGCCATCCGC
TACAAGGCCAACCCGGATTATTTTGCCGGCAAGCCGGCTGTGGATAACTTGATCTTTGCTATCACCACGGACGCCA
ATGTGCGCCTGCAGAAACTGCGCCGGGACGAATGTCAGATCGCCTTGTCACCCAAGCCCCTGGATATCGGTGAAGC
GCAAAAGGATCCTGCACTCAAGGTGGAAAAAACTGCTGCCTTCATGACGGCTTTCGTCGCCATCAATAGCCAGCAC
CCGCCACTGGACAAACCCGAAGTGCGCCAGGCAATCAACCTGGCCTTCGACAAGGCCAGCTACCTCAAGGCCGTGT
TTGAAGGCACCGCCGAAGCTGCCAATGGGCCTTACCCGCCCAATACCTGGAGCTTTGCCAAGGAGTTGCCGGGTTA
TCCACAGGACATTGCCAAGGCCAAGGGGCTGCTGGACCGCGCCGGGCTCAAGGACGGTTTCAAGACCACTATCTGG
ACCCGGCCATCCGGCAGCCTGCTCAATCCCAACCCCAGCCTGGGCGCCCAGCTGTTGCAGGCAGACCTGGCCAAGA
TCGGCATCCAGGCAGAGATCCGCGTGATCGAATGGGGCGAGCTGATCCGCCGCGCCAAGGCCGGCGAGCACGATCT
GTTGTTCATGGGCTGGGCCGGCGACAACGGCGACCCGGATAACTTCCTGACCCCGCAGTTTTCCTGCGCGGCGGTC
AAATCCAGCACCAACTTCGCCCGTTACTGCGACCCGGCGCTGGACAAGCTGATCAGTGCCGGCAAGACCACCAACG
AGCAAGGCGTGCGCAGCAAGCTCTACCAACAGGCGCAGGCGCAGATCCAGCAGCAGGCGCTGTGGTTGCCACTGGC
CCATCCGACGGCCTTTGCCCTAACACGCAAGAATGTCGAGGGGTATCAGGTCAGCCCGTTTGGGCGCCAGGACTTC
TCCAAAGTCAGCGTCACCCCCTGACCCTTCGTGTGTAGGAGCCGGCTTGCCGGCTCCTACAAGGGGTTGCGTTATA
TCCAGCCATATTCGGCCATCGACAGCGGATCACCGTCGCCGACGATAATGTGGTCGAGTACCCGCACATCCACCAG
ATCCAGGGCTTTTTGCAGCACTTTGGTCAATTTTCGATCAGCGATGCTCGGCTCGGCATTCCCAGAGGGGTGGTTG
TGGCACAGGATCAACGCCGCAGCGTTGTAGTCCAGGGCGCGCTTGACCACTTGTCGTGGGTAAACCGTGGCGTTGT
CGATGGAGCCCTGGAACAACGCTTCGAAGCCCAGCACCCGATGCCTTGAATCGAGAAACAGGCACCCAAAGATCTC
ATGGGGTTGATGGCGCAGCAGTGCCTTGAGGTAATCACGCACCGCCACCGGGCTTTCCAACACCGACTCATTACGC
ACATGCTCGGCCAGATGGCGCCGGCCCATTTCCAGCACCGCCTGCAGTTGGGCGAATTTAGCCGGCCCCAGCCCCA
GATGCTGGATGAATAAGGCCTGCCGGGCCTCCAGCAATGCGCGCAGGCTGCCAAATTGCTCCAGCAGATGACGCGC
CAGATCCACTGCACTTTTACCCGAGACGCCGGTGCGCAGGAATATCGCCAGCAACTCGGCATCCGAAAGACTCGCC
GCGCCCAACTCCAAAAGCCTCTCCCGCGGACGCTCCGCTGCAGGCCAATTGCGAATACTCATGCCACCTCCCTGTG
CATGTGCGCCGCTGTTGCATGGCGGACGCTGTGTTATCTTAGCCCCTCTTTTTTGCGCGCGATTTCTCCTGGGGAG
GGGGGATCGCAACGCCATCACTGAACTGGAAAGGCAAACCAATGCAGCGTCTGTATCGGAAACGCATCGTTCTCGG
CGTCGGCGGCGGCATTGCCGCCTACAAGAGCGCAGAGCTGGTTCGCAGGCTCCTGGACCAAGGCGCCGAAGTGCGC
GTGGTCATGACCCGTGGCGGCAGTGAGTTCATTACCCCGCTGACCATGCAGGCGCTGTCCGGCCACCCGGTTCACC
TGGACCTGCTGGACCCGGCGGCCGAAGCTGCCATGGGCCATATCGAGCTGGCCAAATGGGCCGACCTGGTGCTGAT
CGCACCCGCCACCGCCGACCTGATTGCGCGCCTCGCCCAAGGCATTGCCGACGACTTGCTGACCACGCTGGTACTG
GCCACCGACGCCACTGTCGCCATCGCCCCGGCGATGAACCAGGCCATGTGGCGCGACCCCGCCACCCAGGCCAACA
CCCAATTGCTGCAAAGCCGTGGCCTCAAGGTCTTCGGCCCGGCCTCCGGCAGCCAGGCCTGTGGCGACGTCGGCCT
GGGCCGCATGCTCGAAGCCACCGACCTGGCGCTGTGCGCCGCCGAGTGTTTCCAGCACCTGGCCCTGACCGGCAAG
CACGTGCTGATCACCGCCGGCCCGACCCAGGAAAACATCGACCCGGTGCGCTACATCACCAACCATAGCTCAGGGA
AAATGGGCTTTGCCCTGGCTGAAGCGGCGGTTGAGGCAGGCGCACGCGTGACCCTGATCACCGGCCCGGTGCACCT
GCCGACCCCGGATCGGGTCACCCGCATCGACGTAGTCAGCGCCCGTGACATGCTTGCGGCCTGTGAGGCGGCGATT
CCCTGCGATGTGTTTATCGCCTCGGCAGCGGTCGCAGACTACCGACCGGAAGTAGTTGCGCCGCAAAAACTCAAGA
AAGACCCTACAAGCGGCGACGGCCTGCTCCTGCAAATGGTCCGCAACCCGGATATTTTGGCCACCATCGCCACCCG
CGCGGATCGTCCGTTCAGCGTCGGTTTCGCCGCCGAAACCGAAAACCTGCTGGACTACGCCGCACGCAAACTGAAA
GACAAAAACCTCGACCTGATCGTTGCCAATGATGTCGCCAACCCGAGCATCGGTTTCAACAGCGAGGAAAACGCCT
GCAGCGTGATCGACCGCGAACTGCACGCGACCCTTTTCGCCCAGACCAGCAAGGGCAAGATTGCCCGCCAACTGAT
CTCTTTTATCGCCCAACGGCTGAACCAGGTTTAATTTCCATGCACGCTTTGCAAGCCAAGATCCTCGACCCCCGCA
TCGGCAGCGAATTCCCACTGCCGGCCTACGCCACACCGGGCTCCGCCGGCCTGGACCTGCGCGCCATGCTCAAGCA
GGACACCGTGCTTGAGCCGGGCCAGACCCTGCTGATCCCCACCGGCCTGTCGATCTACGTCGGCGATCCCGGCCTG
GCCGCGCTGATCCTGCCGCGCTCGGGCCTGGGCCACAAACACGGGATCGTCCTGGGCAATCTCGTTGGCCTGATCG
ACTCCGATTACCAGGGCGAATTGATGGTCTCGTGCTGGAACCGTGGCCAGACCGCCTTCAATATCGCCGTTGGCGA
ACGTATCGCCCAACTGGTGCTGGTACCGGTGGTGCAGGCGCATTTCGAACTGGTGACCGAGTTCGACGAAACCCAG
CGTGGCGCAGGCGGTTTCGGGCATTCCGGCAGCCACTGACTACGCTCAGGCATGCCGGGCTCCGCGCCCGGCATGC
CCAGATGGCATTTTCGCACCACGAACTCTAGGCCAAAAACGCCGTCTTACCCTTCAGTTTGAGCCTGCCGGTCGCC
ATATTCAGGCCTGTCCGCCCCGTATCGATGGAGTTTCCCCAGTGATGAGCAACGCAGCCCCAGTCGCACCGACGTT
TCCCGACAGTATTTTCCGCGCCTACGACATCCGTGGCGTCGTCCCGAAAACCCTGACCGCCGAAACCGCCTACTGG
ATCGGCCGCGCCATCGGCTCCCAAAGCCTGGCCCAGGGCGAACCCAACGTCTCGGTAGGCCGTGATGGCCGCCTGT
CCGGCCCCGAGCTGGTGGAACGCCTGATCCAGGGCCTGGCCGACAGCGGCTGCCATGTCAGCGACGTCGGCCTGGT
GCCGACGCCGGCGCTGTACTACGCCGCCAACGTGCTGGCCGGCAAGTCGGGCGTCATGCTCACCGGCAGCCACAAC
CCGTCGGACTACAACGGCTTCAAGATCGTGATCGCTGGCGACACCCTGGCCAACGAACAGATCCAGGCCCTGCACA
CGCGCCTGAAAACCAATGACCTGACCAGCGGCAAAGGCAGCATCACCAAGGTCGATATCCTCCAGCGCTATTCCGA
CGAAATCACCCGGGACGTCAAGCTTGAGCGTCGCCTGAAGGTCGTGGTGGACTGCGGCAACGGCGCGGCCGGCGTG

ATCGCCCCGCAATTGCTCGAAGCCCTGAACTGCGAAGTGATCCCGCTGTTCTGCGACGTCGACGGCCACTTCCCCA
ACCATCACCCGGACCCGGGCAAGCCTGAAAACCTGGTGGACCTGATCGCCAAGGTCGAAGAAGTGGGCGCCGATGT
CGGCCTGGCCTTCGATGGCGATGGCGACCGCGTGGGCGTGGTGACCAACACCGGCAGCATCGTGTTCCCCGATCGC
CTGCTGATGCTGTTCGCCCGCGACGTGGTGGCGCGCAACCCAGGCGCCGAGATTATTTTCGACGTGAAGTGCACCC
GTCGCCTCACACCGCTGATCAAGGAATACGGTGGCCGCCCACTGATGTGGAAGACCGGTCACTCGCTGATCAAAAA
GAAAATGAAGGAAACCGGCGCCCTGCTGGCGGGCGAAATGAGCGGCCATGTGTTCTTCAAGGAGCGCTGGTTCGGT
TTCGACGACGGCATCTACAGTGCCGCACGTCTGCTGGAGATCCTCAGCAAGGAGAAATCCAGCGCCGAGGATCTGT
TCGAGACCTTCCCGAACGATATTTCTACGCCGGAAATCAATATCCATGTGACCGAAGAGAGCAAATTCAGCATCAT
TGACGCACTGCACGATGCGCAATGGGGTGAAGGCGCCAACCTGACCACCATTGATGGTGTGCGAGTCGATTATGCC
GAAGGCTGGGGCCTGGTTCGCGCGTCCAACACCACACCGGTGCTGGTCCTGCGTTTCGAGGCGGATACCGAGGCCG
AGTTGCAGCGCATCAAGGACGTGTTCCACGCCCAGTTGAAACGTGTTGCCCCTGATCTCCAACTACCGTTCTGATT
ATTTGCCCCGGAGCCCTGAATGACCCTCGAACGCGAAGCCGCCGCCAACACCGCCAAGGTCCTTTCCGAAGCGCTG
CCTTACATTCGACGCTACGTCGGCAAGACGCTGGTGATCAAGTACGGCGGCAATGCCATGGAAAGCGACGAGCTGA
AAACCGGCTTTGCCCGCGACATCGTGCTGATGAAAGCCGTCGGGATCAACCCGGTGGTGGTCCACGGCGGCGGCCC
GCAGATCGGTGACCTGCTCAAGCGCTTGTCCATCGAGAGTCACTTCATCGACGGTATGCGCGTCACCGATGCGCAG
ACCATGGACGTGGTGGAGATGGTCCTTGGCGGCCAGGTTAACAAAGACATCGTCAACCTGATCAACCGTCATGGCG
GCAGCGCCATCGGCCTGACCGGCAAGGACGCCGAGCTGATCCGGGCGAAGAAACTCACCGTGACCCGCAAGACCCC
GGAGATGACCCAGCCGGAAATCATCGACATTGGCCAGGTAGGCGAAGTGATCGGCATCAACACCGACTTGCTGAAC
CTGCTGGTCAAGGGTGACTTCATTCCGGTGATCGCGCCGATCGGCGTGGGTGCCAATGGTGAGTCCTACAACATCA
ACGCCGACCTGGTCGCCGGTAAAGTAGCCGAGGCCCTGAAGGCCGAGAAGCTGATGCTGCTGACCAACATTGCCGG
CCTGATGGACAAGGAAGGCAAGGTCCTGACCGGCCTGACCACCCAGCAGGTCGACGATTTGATCGCCGACGGCACC
ATCTACGGCGGCATGCTGCCGAAGATCCGCTGCGCACTGGAAGCCGTGCAAGGTGGCGTAGGCAGCTCGCTGATCA
TCGATGGCCGGGTGCCGAACGCGGTGTTGCTGGAAATCTTCACTGATACCGGCATGGGCACGCTGATCAGCAATCG
CAAGCGTCCCTGACCGCTGAAACAAAAAGCCCCCGTTCAACCTGGTTGAACGGGGGCTTTTTTATGTGCGCCGACC
TGCAGCCTTGTGGAGATCCCCTGTAGGAGCGGGCTTGCCCGCGATGAGGGCTTGTCAGTCAACATCAGTGTTGAAT
GTACTACCGCTATCGCGAGCAAGCTCGCTCCTACACGAGCTCATCAGCTACAAGGGGTTGTGTCAGACGCCAAACT
GCTCGCGGTACGCCCGTACAGCCGGCAGGTGTTGCTTGAGCTGTGGGTCGTCTTCGAGGAACTGCAATACCTGGTT
CAGCGAGACAATGCTCACCACCGGGATTGCGAAGTCACGCTCCACTTCCTGGATCGCCGACAACTCACCGTTGCCA
CGCTCCTGGCGGTTCAGCGCGATCAACACGCCAGCGGCCTTGGCGCCATCCTGGGATGCAATGATCTGCATCACTT
CGCGGATCGCGGTGCCGGCGGTGATCACGTCGTCGATGATCAGCACATCACCGGTCAACGGAGCGCCGACCAGGCT
GCCGCCTTCGCCGTGGGCCTTGGCTTCCTTGCGGTTGAAGCACCATGGCAGATCCCGGTCGTGATGTTCAGCCAGG
GCCACCGCGGTGGCCGCAGCCAACGGAATGCCTTTGTAGGCCGGGCCAAACAGCACGTCGAAGGAAATGCCGCTTT
CAACGATGGCGGCAGCGTAGAAACGCCCCAGCTGCGCCAGGGCAGAACCCGAGTTGAACAGGCCGGCATTGAAGAA
GTATGGGCTGGTGCGCCCGGACTTGAGGGTGAACTCACCGAAGCGCAAAACCCCGCGATCGATGGCAAAACGAATG
AAATCGCGTTGATACGCCTGCATGAAAAAAGCCTCAGATACCACGGATTTAGCTAAATAGGTAGACGGCGTGTATC
ATACACGCACGCGATTTTTGGGGCCATTTATGCGGATCATCAGTGTGAACGTTAATGGTATTCAGGCTGCAGTCGA
GCGTGGTTTGCTCAGTTGGCTGCAAGCCCAGAATGCCGACGTCATCTGCCTGCAGGACACGCGCGCCTCCGCCTTT
GAACTGGACGACCCAGCCTTCCAACTGGATGGCTACTTCCTTTATGCCTGTGATGCCGAAGTCCCTGCCCAAGGTG
GCGTGGCTCTGTATTCACGGTTGCAACCCAAGGCGGTCATCAGCGGCCTCGGCTTCGAGACAGCCGATCGCTACGG
GCGCTACCTGCAAGCCGATTTCGACAAAGTCAGTATTGCTACCTTGCTGCTCCCTTCGGGGATGAACGGCGATGAA
GACTTGAACCAGAAGTTCAAGCTAATGGACGATTTCGCCCGTTATCTGGATAAACAGCGACGCAAACGCCGCGAGT
ACATTTATTGTGGCTCGCTGTACGTGGCGCAACAGAAGCTGGATATCAAGAACTGGCGCGACAGCCAGCAATCCCC
GGGCTTCCTGGCGCCGGAACGGGCCTGGATGGACGAGATTGTCGGCAACATGGGCTATGTAGATGCCCTGCGTGAA
GTCAGCCGTGAAGGTGACCAGTACAGCTGGTGGCCGGACAACGAACAGGCCGAGATGCTCAACCTGGGCTGGCGTT
TTGACTACCAACTGCTGACCCCCGGCCTGCGCCGGTTTGTCCGCAGCGCACGCCTGCCGCGTCAACCGCGCTTCTC
GCAACACGCGCCGTTGATCGTGGATTACGACTGGACCTTGACCATCTGAGGTCATGTTCCAGGTACAAAAAAACCG
ACATCGCTGTCGGTTTTTTTGTGGGCGATCATTATTTGATCAATCGCCATGTGCACGGGTAACGGTAGGCAATGCC
TTTATTGGCCTTGATGCCCCCAATGATGGTCAGCACCAACGCGGCAATCACCAGCACCGCCATCATCAGGAAACCA
ATGATCACAAAACCCAGGACAAAGCAGACCAGCCAGGCAATGGCCACGGTCAGCTGGAAGTTCAGCGCTTCCTTGC
CCTGATCATCGATCAGCGGATCCATGTCCTTCTTCACCTGCCACAGGATCAAAGGCCCCAGCACACTGCCGAAGGG
GAACACCAGGCCAAGGAACGCCGCGAAGTGACAGAGCATGGCCCACTGGCGCACTTCCTTGGTCTGGGCTGGCACA
GGAAGCTGGTTGTCACTCATGGCGTTGCTCCTTGAGGCGGGTTTCAGTCAGCCAGTGCGGCGGTCTGTAGTTCGAA
GATTTCGCTCATGCCTTTCTGGGCCAGGGCCAGCATGGCGTTCAGGTCTGCTGGCTGGAACGGCGCGCCTTCGGCG
GTGCCCTGCACTTCGATGAACCCACCGGTGCTGGTCATGACCACGTTCAGGTCGGTCTCGGCCGCCGAATCTTCCA
GGTAGTCCAGGTCCAGCACAGGCTCGCCCTGGTACATGCCCACCGATACAGCTGCAATCATCTGCTTGAGCGGGTC
GCCGCCTTTCAGGCCGCCGCGCTTCTTGATCACTTTCAGCGCATCGACCAGCGCGACCATGGCGCCAGTGATGGAC
GCGGTGCGGGTGCCGCCGTCGGCCTGGATCACATCGCAGTCGACGTACAGGGTGACGTCGCCCAGCTTGGACATGT

CCAGCGCAGCGCGCAGGGAACGGCCGATCAGGCGCTGGATCTCCAGGGTGCGACCGCCCTGCTTGCCACGGCTGGC
TTCACGCTGGTTACGCTCGCCGGTGGCGCGCGGCAACATGCCGTATTCGGCGGTCAACCAACCCTGGCCCTGGCCC
TTGAGGAAACGCGGCACGCCGTTTTCAACGCTGACGGTGCAGATGACCTTGGTATCGCCGAACTCGACCAGTACAG
ATCCCTCGGCGTGTTTGGTGTAGTTGCGGGTGATGCGGATCGAGCGGAGCTGATCGGCAGCGCGACCACTTGGACG
TTTCATAGGGGATACCTGTACTGAGGACGAAAAACTGCCGAGCATTATAGAGCCGCAGGCCGCTTCGCGGCACTGC
TAATAAATTCGGTTACCAAAGGGCGGCCCTGGGCACCCTTTGTCACAGCCAGCATTTGGGGGCGCTTGCCCCGTTG
CGCTACAATCCTGCGCCTTCGCAGCCAGTCGGCTTCAATCTACCTACCAGCCCGCCCGTTTGCGGGACTGTATCGC
GAGGTACCTCCATGGTGCATAGCATGACCGCCTTCGCCCGCGTCGAAAAAGCCGGCGCCCAAGGCACCCTGAGCTG
GGAGTTGCGCTCGGTCAACAGCCGCTACCTGGAGCCGCACCTGCGCCTGCCCGAGTCCTTTCGCGACCTTGAAGGC
GTCGTGCGCGAAGCCCTGCGCGCGGGCATCTCACGCGGCAAGCTGGAATGCACCCTGCGCTTTACCGAGGAAACCA
CCGGCAAGCCACTGCAGGTTGATCGCGAGCGCGCTGCACAGTTGGTCGCTGCCGCTGAAACGATTGCCAGCCTGAT
CAAGCAGCCGGCGGCACTGAACCCCCTGGAAGTCCTGGCCTGGCCCGGCGTACTGGTGGCTGACGCCACTGACCCG
CAGGCACTGAACGCCGAGGCCCTGGCCCTGTTCAACCAGGGCCTCAAGGAGCTAAAGGCCGGCCGTGAGCGCGAAG
GCGCCGAACTGGCCCGCCTGATCAATGAGCGCCTGACTGCCATCGAAGAAGACGTCGTGACCCTGCGCGAGCTGGT
GCCACAAATGCTGGCCACCCAGCGCCAGAAAGTCCTCGACCGCTTCACCGACATGAAGGCCGACCTCGATCCGGTA
CGCCTGGAACAGGAAATGGTCCTGCTCGCACAAAAAAGCGACGTCGCCGAAGAACTCGACCGCCTGAGCACCCACA
TCCTGGAGGTGCGTCGCGTACTCAAGTCGGCCGGTGCCGCTGGTCGGCGCCTGGACTTCCTGATGCAGGAACTCAA
CCGCGAAGCCAATACACTGGGCTCCAAAGCCTTCGACCCGCGCAGCACCCAGGCTGCGGTCAACCTCAAAGTGTTG
ATCGAGCAAATGCGCGAACAAGTACAGAATATTGAGTAAGGCAACTCCCATGACCCACAGCACCGGCACCCTGTAC
ATCATTTCCGCCCCCTCGGGCGCGGGCAAAAGCAGCCTGGTCAAGGCCCTGACCGACACCAACCCGGAGATCCGCG
TATCGGTCTCCCACACCACCCGCGCCATGCGCCCCGGTGAGGTGAACGGTGTGAACTATCACTTCGTCGAGCGCAG
CGAGTTCGTGAAGATGATCGAGCACGGTGACTTCCTGGAGCGCGCCGAAGTGTTCGGCAACCTCTATGGCACCTCC
CAGAGCCACCTGCAGCAGACCCTGGACGAAGGCCACGACCTGATCCTGGAAATCGACTGGCAAGGCGCCGAACAGG
TCCGCCAATTGATGCCCAAGGCGCGTTCGATCTTTATCCTGCCGCCTTCACTGGAAGCCTTGCACCAGCGCCTGAA
CAATCGCGGCCAGGACAGCGAAGAGGTCATCGCCGGGCGCATGCGCGAAGCTGTCAGCGAAATGAGCCACTATGTG
GACTACGACTACCTGATCATCAACGACGATTTTGCCCACGCCCTGGACGACCTGAAGGCGATTTTCCGCGCCAATC
AGCTCCAGCAAAAACGCCAGCAGCAGCGTTTTGGCAAATTGTTGGCCGAACTGCTCGGTTGAATGGCTTTTCCCAA
AACCGCTGCAAGGGCCTTACATTGGCACTTGTAGCGTGTTTGCCGGGGCCTGCGCAAAATCAGCGCTTCCCTAAAC
GCTGGTGATTTTTTAAACTGTTGAGTCCGCTCGCCCAACCGGGCAGCGCGCATCTTGCATTCGCTCCGAGGAATAC
CATGGCCCGCGTAACCGTTGAAGACTGCCTAGAACACGTGGATAACCGCTTTGAGCTGGTCATGCTCTCTACCAAG
CGTGCCCGTCAATTGGCCACTGGCGGCAAAGAGCCCCTGGTCCAGTGGGAAAACGACAAGCCTACCGTTGTAGCCC
TGCGTGAAATCGCTGAAGGCCTGATGAGCTACGAGTTCATCGCCAACGCCGAAATCGTTGAAGACGAACCGCTGTT
TGCAGCGTTCGAGGACGAGTCCAACGAGGCCGTCTAAGCCTATGCCTGGTCGACGTAGCACGGCGCGGGGTCACAG
CCTTCGGCAGGAGTTAACACTTTGCCGAGTATAGACGCCCTCGCCGATCGCTTATCGACCTACCTCGGCACCGACC
AGGTCAACCTGGTCCGCCGAGCGTACTTCTACGCCGAACAAGCCCACGACGGCCAGCGCCGCCGTAGCGGCGAGGC
GTACGTCACCCATCCTCTTGCGGTGGCCAATATTCTTGCCGACATGCACATGGACCATCAAAGCCTGATGGCCGCG
ATGCTGCATGACGTGATCGAAGACACCGGTATTGCCAAGGAAGCGCTCAGCGCACAATTTGGCGAAACCGTGGCCG
ACCTGGTCGACGGGGTCAGCAAGCTGACCCAGATGAACTTCGAGACCAAGGCCGAGGCCCAGGCGGAAAACTTCCA
GAAGATGGCCATGGCCATGGCCCGGGATATCCGGGTAATCCTGGTCAAGCTGGCCGACCGGCTGCACAACATGCGC
ACGCTGGAAGTGCTATCCGGTGAAAAACGCCGGCGCATCGCCAAGGAAACCCTGGAAATCTACGCGCCCATCGCCA
ACCGGCTGGGCATGCATGCCATTCGTATCGAGTTCGAAGACCTCGGCTTCAAGGCCATGCACCCGATGCGTTCGGC
GCGCATCTACCAGGCGGTCAAGCGCGCCCGGGGCAATCGCAAGGAAATCGTCAACAAGATCGAAGAGTCCCTGAGC
CATTGCCTGGCAATCGACGAGATTGAAGGCGAAGTCAGCGGGCGGCAAAAGCATATCTACGGCATCTACAAGAAGA
TGCGCGGCAAGCGCCGGGCCTTCAACGAGATCATGGACGTGTATGCGTTCCGGATCATCGTCGACAAGGTCGATAC
CTGTTACCGCGTGTTGGGCGCTGTACATAATTTGTACAAACCCCTTCCGGGGCGCTTCAAGGACTACATCGCCATC
CCCAAGGCAAACGGCTATCAGTCGCTGCACACCACGCTGTTTGGCATGCATGGGGTGCCGATCGAGATCCAGATCC
GCACCCGGGAAATGGAAGAGATGGCCAATAACGGCATCGCCGCCCATTGGCTGTACAAATCCACCGGCGACGAGCA
GCCCAAAGGCACCCATGCCCGCGCCCGCCAGTGGGTCAAGGGCGTGCTGGAAATGCAGCAACGTGCCGGCAACTCG
CTGGAATTCATCGAAAGCGTGAAGATCGACCTGTTCCCGGACGAGGTCTACGTGTTCACGCCCAAAGGCCGGATCA
TGGAGCTGCCCAAGGGCTCCACGGCGGTCGACTTTGCCTACGCGGTACACACCGACGTCGGCAACAGCTGCATCGC
GTGCCGGATCAATCGTCGCCTGGCCCCGCTGTCGGAACCGTTGCAAAGCGGCTCCACCGTGGAGATCGTCAGCGCG
CCGGGGGCCAGGCCGAATCCGGCGTGGCTCAACTTCGTGGTCACCGGCAAGGCGCGCACCCACATCCGCCACGCGC
TGAAGCTGCAACGCCGGTCCGAGTCCATCAGCCTGGGTGAACGCCTGCTGAACAAGGTACTCAACGGCTTTGACAG
CTCCCTGGAGAAAATCCCCGCCGACCGCGTGCAGGCGATCCTCCACGAGTACCGCCAGGAAACCATCGAAGACCTG
CTCGAAGATATCGGCCTGGGCAACCGCATGGCCTATGTCGTCGCCCGTCGCCTGTTGGGCGAAGGCGAGCAGTTGC
CAAGCCCCGAAGGCCCGCTGGCCATTCGCGGCACCGAAGGCCTGGTCCTCAGCTACGCCAAGTGCTGCACGCCGAT
CCCGGGCGACCCGATTGTCGGGCACCTGTCCGCCGGCAAAGGCATGGTGGTGCACCTGGATAACTGCCGCAATATC

AGCGAAATCCGCCACAACCCGGAAAAATGCATCCAGCTGTCATGGGCCAAGGATGTGACCGGCGAATTCAACGTCG
AGCTGCGAGTGGAGCTGGAGCACCAGCGCGGGCTGATCGCCTTGCTGGCCAGCAGTGTCAACGCGGCCGACGGCAA
TATCGAAAAAATCAGCATGGACGAACGCGATGGTCGCATCAGCGTGGTCCAACTGGTGGTCAGCGTGCACGACCGT
GTGCACCTGGCCCGTGTGATCAAGAAACTGCGTGCCCTGACCGGGGTTATCCGCATCACTCGCATGCGCGCATAAC
GCCTCCCCTATAGCCCGGACATTACAAGGAGTCATTCATGACCAAGACTGTTATCACCAGCGACAAGGCACCTGCC
GCCATCGGTACTTACTCCCAAGCGATCAAGGCGGGCAACACCGTCTACATGTCCGGCCAGATCCCACTGGACCCAA
AGACCATGGAGCTGGTTGAAGGCTTCGAAGCCCAGACCGTACAAGTCTTCGAAAACCTCAAGTCGGTAGCCGAAGC
AGCGGGCGGTTCGTTCAAGGACATCGTCAAGTTGAACATCTTCCTCACCGACCTGAGCCACTTCGCCAAGGTCAAC
GAGATCATGGGCAAGTACTTCGAACAACCCTACCCAGCCCGCGCCGCCATTGGCGTTGCCGCCCTGCCAAAGGGTT
CGCAGGTTGAGATGGACGCGATTCTGGTCATCGAGTAATACATTCGGCGCAACCCCAGGGGTTGCGCCGACTTCGT
TTTAAAAGGATTTCGTAATGCGCAAAGCGCTCGTTGCCTCCACGCTGCTCACCCTGTTCCTTGGCGGCTGCGCCAG
CAATCCTGCCGCCCGGGATGTGAGCGGCACCTGGATCAACCAGGTCGCCATCGATGCGGCGTCCAAGGGCGGCCCT
TTGCGTGAAGCCCTGCAGGCCTATGGTCCGAACCTCGAATGGGAGGTCAATACCCGGGCCAACCAAGCGCGCTACT
TCAACGGTTTTGAACGCCCTGAAGGCACGTTGGAGGGCGAGAAGGCCGGCACCTGGAATGTCGACTTCTACGGTAG
CTCAACCACCGAGCTCAAGCGTGATGGCAAGCAGTTGCTGCAAGTGGCCAACGACAATGAGCCCGAACAGCTCTTT
GACCGACCCAAGGACCCTGCTCCCGAAGGCGCGCCCCTGGGTGCCAACTTCGAACGGGCGCTGTATTCGGCCTATA
TGGGCGGCAGCTGGAAAATCACCAGCGGCAATGGCGTCGGCGCGACAGTGCAGTTCCAGGCCAACGGCCAAGTCAC
TGGCCTGCCCGGCGTAGACCAATACTCACTGTGCCTGGCCGGCGATTGCGCCTCCATGAGCGGTGGCTACGACAGC
ATCTGGCTGCAACAAAGCGGCCAGGGCAACCCGTGGATCTTCAGCCGCAATGACAAGCAACTGGAGATTTTCCAGG
CGATCAATACGTCCCAGGCCGACGAAGTACCTTCGTTCACCCCGGGGCCACGCCAGTGGTTGCTGGATAAACAGTA
AAGTCACTGCAGGCACCTCGGTGTGTCAGTGACACCAAGGTGATGCTATCGCAGCGATGCGGCGACCCGACGAGCC
AGCTCCCACTCAAGCCGGTAGTGCTCAGCCTTTGAGGATGGCCGCGTAGCCTTCGCGGTAACTCGGATAGGTGGGC
GACCAGCCCAGTGCCTTAGCCCGCGCATTACTGCACTGCTTGCTGCCCGCGCGGCGCACGCTGGCGTCTTCGGCCC
ATTCGGTCACCCCAAGGTAGCCACGCAGCCAGTCCACGACTTCGGCCAGCGGTGCGGGCGCGTTGTCGACACCGAT
ATAGACCTTCTCCAACGCCCCGCCCTGCTCTACATGCAGCAGCAGATAAGCCAGCAGGCCTGCCGCGTCATCGACA
TGTATGCGGTTGCCATATAAAGGCGGCTCGACCGCCACGCGATAGCCTTGGCGCACCTGGCTCAACAACCACTCAC
GCCCAGGCCCGTAGATCCCGGTCAAGCGCACAGTCGTAGCCGGGATACCGCTGTCGAACGCCACTTGCTCGGCTTC
CAACATCACCTGCCCGGAATAGCCGACGGCCTGGGTGGGCGAGGTTTCATCGACCCATTCACCGTTTTGCTGGCCG
TAGACACTGCTGCTGGAGACGAAAACAAGGTGCTTGGGCTCCTGGCCATAGTCGCCCAGCCACTCCAGCACATGCT
GCAGGCCCTGGACATAAGCCTTGCGGTAGCCCGCCTCGTCGTGATCGGTTGCAGCGGCGCAGTACACCAGGTAATC
CACGCCGCCGATCGGCCACGTGTCGGGACACTCTTTATTGAACAGGTCGCCGGCGATGCCGATCACCCCTTCCGGC
AAGCGCGCGACATTGCGCCGCAGGCCATGAACCTCCCATCCCGCGGCCAGCAATTGACTCGCCAGCCGACTACCTA
CATCACCACAACCGGCAATCACCACAGAAGGCGCAGACATCACAAAACTCCGTTCTCAAAGGTCTAGATTAGCCCT
CACAGGTGACCAGCGGCCAGAAATAGCACAAATAAAGTTACTGTATTACTTCTGTTAACAAGAATTACTTGCAATA
ATAACCGCCCATTTGTCCTCGGCCGTCCTCTGGCCTGGCAGGACAACTACTTATTTTCAACTCTCAGGTCCGGCCA
GCATGACACGCAATAACCTCCCCGCTTCGCCAACCAAGCCTCACAGCCCATCCCGCGCCTGGCGCGCGATTGCTGC
GGTGCTGTTCAGCGCCCTGCTGGCACCGACCGCCGCGTTTGCAGATGCCACGGCGCCGGCAACCCCGCCTGCCGCT
ACGGCACCAGCCCCAGCTGCGGCCGCCGCACCTGAGCAAAATGCCGCCGTACCAGGTGCCCCGGCTGCCGCACCTG
CCGCCACCGACCCGGTCGCCGCCGAGGGTGTGACCCCGGAAGATGAAACCGGCGTGGTCCTGGAAGAAGACAACAC
CCTGGGCATGGCACACGACCTGTCGCCGTGGGGCATGTACCAGAACGCTGACATCATCGTGAAGATCGTGATGATC
GGCCTGGCCATCGCCTCGATCATCACCTGGACCATCTGGATCGCCAAAGGCTTCGAACTGCTGGGCGCCAAACGCC
GCCTGCGCAATGAAATCGTCAACCTGAAAAAAGCCACCACCCTTAAAGAAGCCAGCGAAAGCGCGGCCATCAAGGG
CACCCTGGCGCACCTGCTGGTGCACGACGCACTGGAAGAAATGCGCCTGTCGGCCAACACCCGCGAAAAAGAAGGC
ATCAAGGAACGCGTGAGCTTCCGCCTCGAGCGCCTGGTAGCGGCCTGCGGTCGCAACATGAGCAACGGCACCGGCG
TGCTGGCAACCATCGGTTCCACCGCGCCGTTCGTCGGTCTGTTCGGTACCGTGTGGGGCATCATGAACAGCTTCAT
CGGCATCGCCAAGACCCAGACCACCAACCTCGCCGTTGTCGCCCCCGGCATCGCCGAAGCCCTGCTGGCAACTGCG
CTGGGCCTGGTCGCCGCGATTCCTGCGGTGGTGATCTACAACGTGTTCGCCCGTTCGATCGCCGGCTACAAGGCCC
AGGTGTCGGACGCTTCGGCAGAAGTCCTGTTGCTGGTCAGCCGTGACCTCGATCACCTGCCTACCGAGCGCAGCTC
GCAACCGCACATGGTGAAAGTGGGGTAATCGGCCATGGGCCTGCATTTGAATCAAGGTGACGACGAGCTCGTCGAG
AACCACGAAATCAACGTCACGCCGTTTATCGACGTGATGCTGGTGCTGCTGATCATCTTCATGGTGGCAGCACCCT
TGGCTACGGTGGACATCAAGGTCGACCTCCCCGCCTCCAGCGCGAAACCCGCGCCCCGGCCGGAGAAACCGGTGTT
CCTCAGTGTCAAGGCGGACCAGCGCCTGTTCCTGGGCGAAGAAGAAGTCAAATCCGAAACCCTCGGCGCGGTGCTC
GACGCCCGTACCCAGGGCAAGAAAGACACGACGATCTTCTTCCAGGCCGACAAAGGCGTGGACTACGGCGACCTGA
TGAGCGTGATGGATGCCCTGCGGGCAGCCGGCTACCTCAAGGTCGGTCTGGTCGGACTTGAGACGGCAGCCAAAAA
ATGATCACGACGCGCCACAAACTGACGCGTTATGGCACCAGCCTCGCCGTCGTGCTGGGCGTCCATGCCGTCGCGA
TCATCCTCGCGTACCAGTGGTCGGCGCCACATATGGTGCAGTTGCCACCGGCAGCCATGGTCATCGACCTAGCGCC
GCTGCCGGCACCACCGCCTCCGGCCCCGCCGAAGGTGGTGACGCCTCCACAACCGCCAGCACCGGTGGAAGAGCTG

CCATTGCCGAAACTGGCAGAGGCCCCCAAGCCGACGATTTCCGTGCCCAAGCCGGTCAAACCCAAGCCCAAGCCAC
AGCCGCCCAAGCCTGAGAAGAAGATCGAGCCGCCCAAGGAGAAACCTTCCGAGGATCCGCCAAGCGAAAGCCCGGC
GAATAATGCGCCTTCGGAAAAGTCCGCGCAGCCGCAACCCGGCCCTTCGCCGGCGCAGATCGCGGCCAAGGCAACG
TGGGAAAGTACGCTGCTCGGTCACTTGGCGAAGTACAAGAAGTACCCGCCAGGCGCCCAGGCCCGTGGCAAGGAAG
GCTTGAACCGCTTGCGGTTCGTGGTGGATGCCGAAGGCAAGGTGCTGTCCTATGAGCTGGTGGGCCGCTCCGGCAA
CGCCGATCTGGACCGCGCTACCCTGGAGATGATCCGTCGTGCCCAGCCGCTGCCCAAGCCACCGGCCGATATGTTG
AAAAACGGCAGCATCGAAATCGTTGCGCCGTTTGTCTACAACATCGAGAAGCGCCGCCGCTGACCCAGTGGGAACG
GGCTTGTCGGATCGCGGCGGTGCGGCGATCCGACAAGCCCCCTCTCACATTCAGGACCCGCTTGAGCGTTAAGCAA
AATCCCCCGCACAAACCGCTCAATCCCCACTCTAGTTCTGATAACGTGCGCCTATCGATTGCAGCCGGTATGCTTG
GCCCGCAACCTCATGGACGCCCGCTATGACTCTTACAGAATTACGCTACATCGTGACCCTCGCCCAAGAGCAACAC
TTCGGCCATGCCGCCGAACGTTGCCATGTCAGCCAGCCGACGCTGTCGGTGGGCGTGAAAAAGCTTGAAGACGAAC
TCGGTGTGCTGATTTTCGAGCGCAGCAAAAGCGCCGTGCGCCTCACCCCGGTCGGCGAAGGCATTGTCGCCCAGGC
CCAGAAGGTGCTGGAGCAGGCCCAGAGCATTCGTGAACTGGCCCAGGCCGGCAAGAACCAACTGACGGCCCCGCTC
AAAGTCGGCGCGATCTACACCGTCGGCCCATACCTGTTCCCGCACCTGATCCCGCAACTGCACCGCGTCGCGCCGC
AGATGCCGCTGTATATCGAAGAAAACTTCACCCACGTACTGCGCGACAAACTGCGCAACGGTGAGTTGGACGCGAT
CATCATCGCCCTGCCGTTCAATGAAGCCGACGTGCTGACATTGCCGCTCTACGACGAGCCGTTCTATGTGCTGATG
CCCGCCTCTCACCCGTGGACGAAGAAAGACACGATCGACGCTGGCCTGCTCAACGACAAGAGCCTGCTGCTGCTCG
GTGAGGGTCACTGCTTCCGCGACCAGGTCCTGGAAGCCTGCCCGACCCTGACCAAGGGCAACGACGGCGCCAAGCA
CACCACGGTCGAATCCAGCTCCCTGGAGACTATTCGCCATATGGTGGCGTCCGGCCTGGGTATTTCGATCCTGCCG
TTGTCGGCCGTGGACAGCCATCACTACGCCGCCGGCGTGATTGAAGTGCGTCCGCTCACCCCGCCCGTGCCGTTCC
GTACCGTGGCGATTGCCTGGCGTGCCAGTTTCCCGCGCCCGAAAGCGATTGATATCCTCGCCGACTCGATCCGCCT
GTGTTCGGTGGCCAAGCCGCCTGCTGCGAGCTAAGCAAGCGTATGACAGAGCTGTCGCAGGTGTCGGTGACGGCAC
TCAAGGGTGTCGGTGACGCCATGGCCGAGAAACTGGCCAAGGTTGGCCTGGAAAACCTCCAGGACGTGCTGTTTCA
CCTGCCCTTGCGCTATCAGGACCGCACCCGCGTAGTGCCCATCGGCCATCTGCGCCCTGGGCAGGATGCGGTGGTC
GAAGGCACCGTCAGCGGTGCAGATGTGGTGATGGGCAAGCGCCGCAGCCTGGTGGTGCGCCTGCAGGACGGCACCG
GTGGCCTGAGCCTGCGCTTCTACCATTTCAGCAACGCGCAAAAGGAAGGCCTCAAGCGTGGCACTCGTGTGCGTTG
CTACGGCGAAGCCCGGCCGGGCGCATCGGGCCTGGAAATCTACCATCCGGAATATCGCGCCATTACCGGCGACGAA
CCGCCGCCGGTAGACACCACCCTCACCCCCATCTACCCGCTCACCGAAGGCCTGACGCAACAGCGCCTGCGCCAGC
TATGCATGCAAACCCTGACCCTGCTCGGCCCGCAAAGCCTGCCCGACTGGCTGCCGCAAGAGCTGGCCCGCGATTA
TCAGTTGGCGCCCCTGGCTGACGCGATCCGCTACCTGCATCATCCACCGGCCGACGCGGACGTGGATGAGTTGGCC
CTGGGTCATCACTGGGCCCAACATCGCCTGGCCTTCGAAGAGCTGCTGACCCATCAACTGTCCCAGCAACGCCTGC
GCGAAAGCATGCGTTCCCTGCGCGCGCCAGCGATGCCCAAGGCCACGCGCCTGCCCGCGCAATACCTGGCCAACCT
CGGTTTTGCGCCGACCGGTGCGCAGCAGCGCGTAGGCAATGAAATCGCCTACGACCTGAGTCAGAAAGAACCCATG
CTGCGCCTGATCCAGGGCGATGTCGGGGCCGGCAAGACCGTGGTCGCCGCCCTGGCTGCGCTGCAGGCCCTGGAGG
CTGGTTACCAGGTGGCGCTGATGGCGCCGACCGAGATCCTCGCCGAACAGCACTTCATCACCTTCAAGCGCTGGCT
CGACCCCCTGGGCCTGGAAGTGGCGTGGCTGGCGGGCAAGCTCAAAGGCAAGAACCGCACGGCCGCCCTGGAGCAG
ATCGCCGCCGGCGCGCCGATGGTGGTCGGCACCCACGCGCTGTTCCAGGATGAGGTTAAATTCAAGAACCTGGCCC
TGGTGATCATCGACGAGCAGCACCGTTTCGGCGTGCAGCAACGCCTGGCCCTGCGTGAAAAAGGCGTGGGCGGGCG
AATGAACCCGCACCAGTTGATCATGACCGCCACCCCGATCCCACGCACGCTGGCCATGAGTGCCTACGCCGACCTC
GACACCTCGATCCTCGACGAACTGCCGCCGGGCCGAACGCCTGTCAACACGGTGTTGGTCACCGACACCCGCCGCG
TCGAAGTGATCGAACGCGTGCGCGGCGCCTGCGCCGAAGGGCGCCAGGCCTATTGGGTGTGCACGCTGATCGAAGA
ATCCGAAGAGCTGACCTGCCAGGCTGCCGAAACCACCTACGAAGACCTCACCAGCGCCTTGGGCGAGCTCAAGGTC
GGGCTGATCCATGGGCGCATGAAACCTGCGGAAAAAGCCGCGGTGATGGCCGAGTTCAAGGCCGGCAACCTGCAAC
TGCTGGTGGCCACCACCGTGATTGAAGTCGGCGTGGATGTACCCAACGCCAGCCTGATGATCATCGAAAACCCCGA
ACGCCTGGGCCTGGCGCAACTGCACCAACTGCGCGGCCGTGTCGGCCGGGGCAGTGCCGCCAGCCACTGCGTGCTG
CTCTACCATCCACCGCTGTCGCAGATTGGCCGCCAGCGCCTGGGGATCATGCGCGAGACCAACGACGGCTTCGTGA
TCGCCGAAAAGGACCTGGAACTGCGCGGCCCCGGCGAGATGCTCGGCACCCGCCAGACCGGGCTGCTGCAGTTCAA
GGTGGCCGACCTGATGCGCGACGCCGACCTGCTGCCCGCCGTGCGCGACGCCGCCCAGGCCCTGCTGGAACGCTGG
CCGCACCACGTCAGCCCGCTGCTGGATCGCTGGCTGCGCCACGGCCAGCAATACGGTCAGGTCTGACCCAGCCCCC
CTGTAGGAGCGAGCTTGCTCGCGAAGAACACCCAGGCGCCACGGATATGGCGGTGCCCCGCGTCACCGTCAAGGTT
CTTCGCGAGCAAGCTCGCTCCTACAGGAATGGGGACCACCATCACAAACCACAGTTAGACCCCCCTCAGCCGGACC
AAGCTGGTTATACTCCAAGCAAATGTAGGAAATTGGATCCGGCCATGTCAGAAGTAGCCCACGCCACAGCCCCACC
GACCGCACCCTCGGTCATTCGGGACCTGCTCGCAAAACTCGCCGTCAGCTACAAGGAAGTCATCGAACATCCAGGC
CTGAACCCCGCTCGCAAGGTTCAAGCGGTGCTGCTCGACGATGCCGTGGGCGCCCTGATGGTGCTGTTCCCCCAGA
GCCAACTGCTGGATCTCAATCGCCTTACCGAACTGACCGGCCGCAACCTCACCGCCGTGGCACCGGAACGCCTGGA
GCGCATGCTCGGCAAACACAGCCTGAGCCTGCTGCCTGGCCTGCCACCGCTGACCAGTTCGCCGTGCCTGTACGAA
GAAAGCCTGCTACGCGAGCCGACCTTACTGGTGCATTCGGGCGAAGCCACGGTGCTGCTGGAAATCGCCAGCGATG

ACTTCAAGCGCATGCTGAGCAAGGCCAGTGCGGCCAACTTCGGCGAGCCCTTGAGCAGCATCCGGCCCAACTTCGA
CCGCCCCAACGACGACCGCGACGAGATCTCCCAAGCGATGCAGGCGTTTACCGCCCGGCGTATCCAGCAACGCCTG
GAAGCCACCATCGAGATCCCGCCGCTGGCCGACACCGCGCAGAAGATCATCAAGCTGCGGGTCGATCCCAACGCGA
CCATCGATGACATCACTGGCGTCGTGGAAACCGACCCGGCCCTGGCCGCGCAAGTAGTGAGCTGGGCGGCATCGCC
GTACTACGCCTCGCCGGGCAAGATCCGCTCGGTAGAGGACGCCATTGTCCGCGTGCTGGGCTTCGACCTGGTGATC
AACCTCGCCCTGGGCCTGGCGCTGGGCAAGACCCTGAGCCTGCCCAAGGACCATCCGCAACAGGCCACGCCTTACT
GGCACCAGTCGATCTACACCGCGGCGGTGATCGAAGGCCTGACCCGCGCCATGCCCCGCGCCCAGCGCCCGGAAGC
CGGCCTGACCTACCTGGCGGGCCTGCTGCACAACTTTGGCTATCTGTTGCTGGCCCACGTGTTCCCGCCGCACTTC
TCACTGATCTGCCGGCACCTGGAGGTCAACCCGCACCTGTGCCACAGCTATGTCGAGCAACACTTGCTGGGAATCA
GCCGCGAGCAGATCGGCGCCTGGCTGATGCGTTACTGGGACATGCCGCAAGAACTGTCCACGGCCTTGCGCTTCCA
GCACGACCCGAGCTACGACGGCCAGTACGCCGAATACCCCAACCTGGTGTGCCTGGCCGTGCGCTTGCTGCGCGGG
CGTGGGATTGGCTCGGGGCCCGAAGCCGAGATTCCGGACGAGCTGCTGGAGCGCCTGGGCCTGGACCGCAGCAAGG
CAGAGGATGTGGTGAGCAAGGTGCTGGATGCTGAGGTGTTGCTGCGGGAGTTGGCGTCGCAGTTCAGCCAGGGCTG
AAGCGGGCGTCTGTCAAACCGCTATCGCAGGCAAGCCAGCTCCCACCGTTGACCGAGTTCCAACGTTGGAATGCGA
TCAAAATGTGGGAGCTGGCTTGCCTGCGATGAATCCAACTCGGTCTCTCAGGCCTTCTTTTTCTTCGGCTTCAAGT
ACTTGGTCAACCCCTGGAACCACATCACCAGCGCCGGGTTGCCCTTGATCTGGATCGACTTGTCCTGAATCCCCGT
CATGAACGCCAGTTGCTTGTTCTTCGCCTGCATGGTGGCGAAGCCATAGGCGGCGTCTTTGAAGGCAATGGCGAAC
GCAGGCTCCGGGTGGACACCCGAGCGGCTAGTGATGCGCTGGTCTTTGACGATGAAGTGACGGGCAACCTTGCCGT
CGAGGGTCTGCAGCTGGAACGCCAGGTCCTTGTCACCCAACTGCTGCTGGAACGCGGGGTTGTTGCGGCTGGCTTT
GGCCATCATCAGGCCCAGCACCCACAGGAGAAAACGAAATTTCATGCACACGGCCTCAGTGTAATTATTGGGTGGC
CGCAGCAGTTTAACGATTTGTGCGCGGAACGCTACCGATCTCCCGCGTTAGCAGGAGATCGGCTGGCGTTTACCGC
TTATAGCAACAACTTGGCTTAAACGTTAGGGCAAGGTACCGGCGCCCAGTCGGCCAGGTTCGGATCGCGGCAAGTG
CCTTCGATCTGTGCTTTACCGGCGCTGGCCACCTGCTTGCTTTCAGTCTGCTTGACCTTGGCGGTCAGGATGCTTT
TCTCGGTGCTCACCGCTTCGGTCGGCACCTTTGGCAGTGGCGGACGCTTGGTCACGACTTTGCCGGTGGATTTGCC
TTTCTTCGCCGGGACCACGGTCTTGGTGGTATCGGCGGTGGCCACGGTCACCACATCATTACGCTGCACACCCACC
TGTTGCAGGTCTTTCTCGTAGGCCTGGGTGTAGTTGTTCAGGTCTTCGCTGGCCTTGCCATTGACCGCGACGATCA
GGTCATTGGACTCCTTGAGGCCGGAGACGATTTCCGCCAGGCGCTTGCGGCCTTCGGTGTCGTTGACGGTCTTGGC
CTTGCGGTCGGCCACAAGCTTGGTGAACGCGCTCTGGTAGCACTGCTGGGACGCCTTGGCGTACGCGGTGCTGCGG
TCGATGTCGGAGGCGCTTTTGTTGAAGTCAGCGGCGTAGGACGCGATGCGCTGGTTGTCGTCGCTGATCTGCTTCT
GGCGCTCGGTGTAGTAACCCGCCGCGCCACCTGCCAGGGCACCGCCCGCTGCACCAATGGCGGCGTTGCGGCCGCG
CTTGTCCGAATCCCCGGTCAAGGCGCCCAGCAGCGCACCACCCGCCGCGCCGAGGGCGGCGCCGGTGACGACCGAC
TTGGTCATGTCCGAATCGGTGGCACGCAAGTGCTGCACAGGCTCGTAGCAATTGGGGTAGTACTCGACCTTGGTGC
TCGACGCGACCTTGGAGGCCGGCGACGTGGCGCAACCAGTCAACACGGTGCTGAAACCAGCCGCGATCAGCAGCAA
GTAACGCTTGGAAACCGCCTTACGGGAAAAAAGCATAGGTGTGTTCTCTTTTAAGTTTGACTTGCCCAGCACGGCC
CATGCCGCCTGAGTCCCTTCCAAGCTCGCCAGCCTGCGAGCGATCAAGACCGCTGACCGCTCGCTGCGAGCAATTC
CTTCAAAATCGTTGCCGGGTCGGCTCGCTGTTGTTTGCGATAGTTACCGACATGGCGAACGAACAACGTCGCGCGC
GTCATCAGGCTCTTGCCCAATACCGGCTTGCGCTCCAACTCTTGCTTGACCCTTTGAAACTGCGCCTGGATCACCG
CATCGGTGTAGCGCGTGCCGGTGGCCAGGTTGTCGATATAGATCGCCACCGCACCGTCCAGCGCGCTGCGGCCATT
GGCGATGGCCGTGGCGAACAGCTGCGCGCTGGCCTCGTCCTTGGCGTCCAGGGACTGCTGGCGACTTTTCTGCAGA
TTGATCAGGCGGATGTTGTAGTTATTGATGGTCTCGGCAACCAGGGCGGCAGACTGGATCAGGTTGCCGGCCGCTT
CCTCACGTTGCTGGGCAATCAGCGACACATTGCGCTTGAGCTCTTCGTTGACCAGCCCCAACTCGGCCTGCAGCCG
CGGGTCGACGCTGGCGGCGATGATGCTGTCCAGGCGCTTGGTCGGGTCCTGGGCGTCGTCGATGGCATCGGTCAGC
GCGGCCAGGCGCCCCTCTTTCTGCCACTGCGCAAACAGTTCGTTGTAGCGTGAACGCGGTGCCGACAGCGCTCCGA
TCGCTACGCGAAAACCGGTGGTTTCGTTGCTTTGCTCGGTGCCATCGGCGCCGAACAGCGGGTACTCGCGGCGCAT
GCCGGTGAACAGCGTGCCCTCGCCTTCCAGGTAGTTGCCACCCTTGACGATAAAGCCGCCATACGTGCCCTGGCGG
CGGCCGGCATGGACCAGTTGGAACGACTCCTGGACCATTTCGGCGGCGTTGCCGATCACGTCGAACATGCCGATAG
GGTTCGGCAACCTGGTGCCGATGGGCATCAGCCGCGCGGCCTGGCCGGTGCCGCCGGCGACCTGGTTGAACACCGC
CCAATCGCCCAGCGGGCCGTCGCTGTCGCTGCCCTCGATGCGCCGTGGGAACAGCCGGCCTTCGAGGTCCTGGCGG
CTCACCGCCTGGCCGCCGCGTGCGGCAAACTCCCATTCCACTTCGGTGGGCAGCCGCACAAAGCCCAGGCCACCAT
CCTCTGCCGAAGATCCGCGACCACTGACCGGCAACAACTCGCGGTGATATTTCATCAACCAGGCGCTGTACACCGC
CGCAAAGCGCTCGGCTTCAAACCGCGACAGCTTGACCTTGGGCAGGCGCCCGGCCATGCCCTCAGGCACCTCGCAC
GCCGGGGCTGGCTCGCCGCTGGCCAACGACTGGGCCTGGGCCATGACCTGGGCATACTGGCGCGCGGTCACTTCGT
ACTTGCCGATAAAGTAGAGCATCGGCTTGAGCGGGGTCTTGGCGTCGGTCTTGGGCATCAGCGGCGCGATGACCTT
GTTCCAGCCCTTGGGCAAATCCTTGAGGGTGAATTGGCCGTTGATGAAGTCGCGGCGATAGCCGGAGATAAACGAC
TGCTTGTAGCCCGCCTCGCCTTCGCTGAACGGGTAGCCGAGGCTGACTTCACGGTCGTCCAGGGTGCCCTGGGCCA
AGACATAGGCGTAGCGAAACACCATATTGCCCTCGCACGGCAGCGGCAGGCTGACGTCATCGGCCAAAGGCTTGGG
GTTGTCCAGCTTGTCGCCGGCCTCATCGGCCCACGCCCCAGCAGCCAGGCTCAGCGCCACGGCGGCGCCCAACAAC

TTATACATCTCTGATTCCCTCGCAAGCCTGGATACGCGCCACACGCCACCCACCACACGCGGCCGCCACGGCGCTG
ACGCCGAGCACCGCGACCAGCGCCAGGGCGTAATGCCGCACCAGCAGATGGCTGGCGTATTCGCCCGGCACCTGCA
CGAATAATCGATTCAAACCGGCCTCGGCCAAGCCATACAGGCCGGCACTGAGCAGCGCGGCAAACCCTGCGCCGTA
GAGCGCCTGCAACGTCACGAACAGCAACAGCGCGCCCGTGGAAAACCCCAGCAGGCGCAACACCGACAACTCCCGA
CGCTTGCGCACCACCGCCGCCAGCGCCCCGGCGAAGATCGCCGCAAATGCTCCCGCCAAGGCCAGGCCGGCGATGA
TCCAGAACACGATGGACAAGTTGCGGCTCAACGACTGCACCTGGGCGATGCTCAGCGCCTGGGTCGAGACCAACAG
GTTCTGCGCGGCGAAATGCACCCGCAGCGGCTCTACATCACTGAGGTTGCGCGCATACAAACGAAACCCCGGATAC
ACGCGCTGCGCGCCCTCGCCTGCCGCGTCACCGGGCCAACCCAGCGCCAGTACCGCCCGGCCATCACGGTAATCTT
CCGCCGCTTCCAGCAGGCTCAAACCGGCAAACAATCCATCCCGGGCGAATGCCTCCAGGGGCAATACCGCCAGCAC
CTGCAGCCGCGTGCCCTGGGCCTCGACACGCCCGGCCACTTGCCGCGCAAAGCGGGTTTGCAACCAATCCCCCGGC
CGCGCCGCGAGCTTCTCGGCGGCGGTGTGGCTCAGCACGATCTGGTCCAGGCCTGCGGGCACTGGCAGCCCGCTCA
GCAACGGATCACCCGGCGCGGTCGGCAACATTTCCAGGGTCAGCGCGCCTACCTGGGCCGTGGCCGCGATCTGCCG
TGTGCGTGGGATGGCGAACGCCACATCGCTGCGCTGAGCAAGCTGCTCGACAAAAGCGTGGCTGAATCGACCACCG
CCCAATGGAATGATTTCCCGGGTCGCCGGGTCATTCTCCAGACGCTCGGTCAGGCTGCTGACCAGGCCGAACTTGA
GGCCGAACAACACCAGCAGCGGCGCGATCACGGCCACCAGCGCCAGCACCGAACAGGCCGACAACCAGCCATCGTC
GCGGTAATCCTGCCAAGCGAGGGACGCTACCAAAGGAATGCGCATCAGCACGCCTCCCCAAGGGTTGCGGTGACGC
CACCGTCGCTATCGCGCTGGCAACTGATGCGCCGCACCTGCAAGCCACTGGCGCGGGCCAGGGGCTCGTCATGGGT
GGCGATCACGCAGGCGGCACGGTGCTCGCGGGCCTGGGCCAGCAGCGCCTGCATGACACGCTCGGCATTAAGGGGG
TCAAGGGACGCGGTTGGCTCATCCGCCAACAGCAGTTGCGGGGCATGGGCCAGGGCACGGGCGCAACTCACGCGCT
GACGCTGGCCCACCGATAACGCGCCAGGCTTCTTGGCCAACTGGTCACTGATCTGCAACTGCGCCGCCAGGCGCGC
CACGCTGCCATCGTCCTTGAGCCCCAACAGTTGCCGGGGCAGGGCAATATTGCTGCGCACGTCGAGAAAACCCAGC
AGGCCGCCGGTCTGCAGCACATAACCGAGGTGCCGGCTGCGCAACCCGGCCAACGTGGATTGCTGATTGGCCCGCC
ATAGACCCTCAATATCCAGTTGGTTGAATTCAAACCGGCCCACCTGATCCGGCGCCAATACCAACGCCAACAGATC
CAGCAGGGTGCTTTTGCCGCAGCCACTGGGCCCGACAATCGCCAATTGCTCCCCCGCGCACAGGTGCAACCCTGGA
ATCACCAGGCTATAGCGCTGGCTACCGACGCCCCGGCTCTTGTGCACCGCGCTCAGGTTCAGCATCAAGGCAGCGT
CGACAGCGGCACGCGGTACAACGCATCGCCCGGCTCGGCATCACCGAAACGGACCCAGTTGGCCACGTCGTTATGG
AAGGTCTCAAAGAGGCGGATTTTCGAATCCAGCTCATCGATAAAGTCTTCCTGTTCGGCCACGCTCAACGACAGCC
ACAAGTCCTGGGTCATGTTCAGCGACTTGCTGCGGTACGGCAGGCCTTCCAGGTATTCGCCGAGGATGCCGCCATC
GGCGAGGTTGCCGCCCTTGCGCAAGGCCTGCGGGTCGCGGCTCATGTAGGCCGAGGCGCTGGCGATTTCCTGGAAG
AAATCCTTGGGCGAGGTCTGGGTCTTGCGCGCCGCATCGACGATCAGTTTGAGCGACTGCTGCAAGTCGTTGAGCT
GCAATTTGGTGAGCATCACGCAGACCTGGAACGCCGGCAGCGCGGGGTTGGTCAGGTCGCGGTCGGCGGTCCAGGC
GCTGACCAGCTGCGGCGCCTGACTCGCGCCTTTGTGGCCGAGGAAGTCCATGTGCATGGCATAGCCGACGGCCGCG
GATTTGTCCGCCAGGCTCGGCGCGTTGCTGAGCAACGGCACCGGCTGCGGGGTGTTGCTGCGCACTTGGTGCACCA
GGTTGGCGAACACCGTGCCGATCTCATCGACGCGCTCGCCCAGCTTGCGCACATCACCGCCCGGCACCGCTGTGTA
GAGGTCGCCGATCTGCGGGTTGGCGTCGGCGGTCAGGGTGCGGTACTGGGACTCGGCGCCGCCATGGGTTTTCTTG
CCGGCGTCGGTGCGCAGGTGCAAGGCATAGATCTTGATCTGCTTGCCCAGCGCGGCCTGGCGCACTTCGGCTTCGT
TCATCTGGGTGGCGGCGAACGGGTCGTTCTTGCGCAAGGCGCCGGCATCGGTCACCAGCAGGATCAGGCGCCCGCC
ATAGCCGGACCAGTCCATACCGTTCACCGCCTGCATCACCCCGGCGAACGCGTCTTCGTTGAAGGCGTGACTGGAA
ACGGTCGAGGCCTTGACCTGCCGCGCCCTATCGAGAAAACGCTGGGGGTCGCGGCCCTGATCGAGGGTGATCAGGG
TCTTGGCCACGTATTCCAGGCCCGGGGTTTTCTTCACGCTGCTGCGAAAGCCGACCATGCCGAAACTGACGCTGTC
CAACTCGCCACGCTCGGCGATGCGGGTCTGCAACTCGTGGACGACGTCGCGCACTTGGTCGATGTAGGGCTGCATT
GACACGGTGGTGTCCACCACCAGCACCACAGCGGTGCGGAACGCATCGGCATTCGCACTGATCACCGGGGTCGCCG
GCTTGGCGGCGGCACTGCCGGGGTCGATGGACGCCACATTGAGTAACTGCACCGGCTGGCCATTCTCGTCGAAGCT
CTCTTTGGCATCGAAGATCGGCAACAGGTAGAACTGATTCTGCGGCACGGCGCTGGCGGCGGGCTCCAGGGCCAGG
ACCTGCTGATTGTCGCCGGGGTTGTTCTGCGCCTTGAGCAACAGGTTCTTCGCCGCGACTGGATCCGCCAGGAGTT
TTTCCACCTCGCCGGCCTGGCGCAGGAACATCACCGGCGCACGCCCCGAACGCTCGGTGAACTTGAGTACCAGGCT
CTGCTTCCAGTCGCTGACGTGCACGGCCGGCAACCAGCCGTCGCTGCGCCCATCGGTGGCGGCGCCCACACGCAGC
CACGGGCTGCCGTCGATGTCCTTGCGCTGGTACACATAGAGCACGGAAAACGCCGGCAAAGCCTTGCCCGGTGCGC
CGCCCGCCGCGCTGGAGAGCTTGGCGCCGGGCTTGCTCAGGACCCGCTGGAACAGGGTTTTCTTGCCGGCCATCAG
CAACGGGCGCTGGCCGCCGTCGATATCGGCTGCCACCGGTGTGACGGCAGCAGGCGTCACCGGCCCTGGCGCAGCA
GGCGGCGGCAGCGGGGCGTCGTCACTCAGCCACCCGTAACTGGCACCACCGATGGCCAGCGCAACCGCCACCGCCA
CGGCGGCCAATGCCAGCACCGGGCCGCGCCGCTGCTCGGACACCGCACGTGGCGGCGTGGGAGCCATCACCGGCTG
GCGCACCGCTTGCGGCTGCGCAGTGGCGCTGGGCACTTCGATGGACACCGGGGTTAACCCGGCCAAGTCGTTGACC
GGGCGAATCAAGGTCGCCTCCAGCGCGTCTTTCGGTGCTTCCAGCGGCAGTCGATCCAGCGCCAGCAGCAGCGCGG
TGGCATCCGGGAAACGTTGCGCCGGGTCCTTGGCCAGGAGCTTTTTCAGCACCTCTTGATAACGACCATGGTGCAG
CGGCAACTCCGGCAAGGGCTCGGTCAGGTGTGCCAGGGCCGTGGAGAGTGCATCGGTGCCACGGTAAGGCAACTGG
CCGACGAGGATTTCATAGAGCACCACCCCGAGGGCGTAGAGGTCGGCACGGCCGTCGATTTCCTGGCCGCGCGCCT

GCTCCGGGCTCATGTAGCTGGGGGTGCCCACGGCAAACCCGGCCTGGGTGAACTGCGTGCGGTCATCCAGGGACTT
GGCGATACCAAAGTCCGACAGCACCGCCGTGCCATCGGCGCGAAACAGGATATTCGCCGGCTTGACGTCGCGATGC
ACCAGGCCCTGGGCATGGGCGTAACCCAGTGCCGAGGCGATCTGGCGGATATACACCAGCCCCTGTTCCGGCGTCA
GGCCGGCGGCGATGCGCTCCTTGAGCGTGCCGTTGGGCAGATATTCCATGGCCATGTAATACAGCTCGCCAACATT
GCCGATGTCGTGGATCGTCACCGTATGCGGGTGCGACAGCCGCGCCAGGGTCTTGCCCTCGCGCAGGAAGCGTTCG
CAAAAGCTCGGGTCGGCGGCCAGCGCGGCGGCCATGACCTTCAACGCCACCTTGCGCTCCAGCGATCGCTGGGTCG
CCAGGTATACGCTGGCCATGGCGCCTTCGCCGATTTCGCCGTCGATGTCGTAGCCAGGGATGATCAGGGTCATGGC
GACACCTTGACGACGAGGGTGGTGATGTTGTCCGGCGCACCGCGGTTAAGCCCCAGGTGCACCAGGCTGCGGGCAA
TTTCGTCGGGCTCGGCATGGCTCAGTACATCGCGGATCTCGTGGTCTTCGACGGTCTTGTTCAGGCCATCGCTGCA
CAACAGGTAACTGTCGCCGGGGGCGATCAACAGGTCGACCACCGACAACTCCAGTCGCGCCTCCACGCCAATGGCG
CGGGTGACGATATTGGCCCGTGGATGCACCCGCGCATCGGCCTCGCTGAGCAGGCCGCTGTCCTGCAGGTCCTGCA
CGTAGCTGTGGTCGCGGGAGATGCGCTCCAACTCGCCATCGCGCAGGCGATACAAGCGGCTGTCACCGGCCCACAG
GCACACGCCGCGCAGACCACGTGCGGCCAGCAGCACCACGGTGCTGCCCATCATGGTCACGCCGCGATTGGCGGTT
TCTTCGCGCACGTCCGCGTTGACCCGGTTCAACTCGGTGCGCAGCGCCGCGGTGTATTCGTCCAGTGACCGGCCCA
CCGGCAGGCTGCGCAGGCTGTCGACGATCAAGCTGCTGACGTAATCGCCTGCGGCATGCCCGCCCATGCCATCGGC
CACCACCCACAGGCGGTTCTGCGGCAAGTCCAGGCACGCGTCCTCATTGACCTGGCGCACCATGCCCACATGACTT
TTGCTCGCGGATTTGTACGTCGACCCCATCTACACCACACCTTCTTGTCCGAGCAGAAATTGCGCAAAATCGCCGG
CGGCAGGCAAACCCTGGCACCGCAATAAACCGGGGGAAATGCGTTGCGAGCCACGCCCCCACCACAGGCTGGCGCC
TTCGCAGGCCTGTTCGGCAAGGGCCGTCATGCGAGCATGGGGCTCGGTGGCGGCTACCCGCTGCAGGCCGGCGAAG
CGGCTGTCCAACGCGCGCGCCTCGCTGCCCGGCAAACCCAGGCGCTCCAGGCCATCGCTGAACCCTTCGAACGTGG
CGCCGGGGTCCAGGGTGCTGAGCAACAACTCCTCGGCCTGCTCGAACCAGGCATCCGGGCCGCCCACCAGGGACGC
TGGATTGGCGTCGTGATCCAACAACGCGACCACCGCCAGCGGGAAATACCGCCCGACCCGATCAATGCTCGGCATC
ACCACCCCCGCCGCCGCATCCGGCCCGCACACACCCGGCGCCAGCACAAAACGCCACAGCGGGCTGACCAGGTACA
CGTTGAGCCAATCGCCGCCCAGGCTGTGTTGACTGGCCAGCAAACCGGCGGCCAGCCAACTGTCCCAAGGCCCGAT
AAAACTCTGGGGCAAGGCGCGGCTGACAAAGTCCCCGCGACTGGCCAACTTGCCGTAGAACCCCAAGGTGGTCATA
ACCGCTCCGGCAGGCTGAACCCGCTCAGCACACGGCTCTTGAACGGGTTGAAGGCGCTGTTGGCCCGCAACTCATA
AGCAATGCTGGCGCCATCGACCCGCAGACGCAGGTTGAAGCGATCCGGCGAATTGCCCGCCGAAAGCTCCGATTGC
TCCAGCAGGCGGAACCACGCCCACGGGCCATCCAGGGTCACGCCCGAACGTCCGCTGGCGGCTGGCGGCATGATCG
AGATCCGCACCCCGCCGCTGCTGCCGGGGTTCGGCCACTGCATCGCCACCGGCCGGCTCGGGCCGTGGTCGTAGCT
CAGTTGCTGGCCGTCGAGGTCGAGCAGGAACTGGGTGATGGTCGCGTCCATTGCCACCGGCTTGAGCTCGAAACGC
ACGATGGGCTGCGTGCCCCCGGCACGGAAGAACGCATCACGAATGGTCGCCGCACGCTGGAAGGTTTGCAGCACAC
CCGGCGCGATCCCGAGTTTCTGCGCCGCCCCCGGCTGCCAGCGCCAGGTCTGCGCGGAGGTGTCGACGTAGGGCTG
CAGGTACTTGCGGAAGTAGTTGTCCATCACCCCGCCCACCCCGAAGAACTGACCGAAGTCGTCCAGGGTGGCATCG
CGGGCACTGCCAGGGGACATCGGGTAACGGCCGGCCAGGGACTGGCGGTAAACGTTGACCACTTCGCTGACCCAGG
CCGCGTTCAGTTGATTGCGCACCCCACCCATCATGCTGTTGGTGGTGGAGTTGACCACCGACTTGACCATGCCCTG
TACCAGCGGCGGCTGGCGCTCGGCATTCAGGCTGACCCGCGTGGCGGCGGCAGCGGCCTGGTTCTTGGCTTCGCCG
AGCAAGGCATCGCCACTGGCACCGACCATGGCGCTGACCTGCACATACAGCGCATTCATGTCCGCCAACAGGCCGT
CGATGGCCGCCGGCTCGCCTTCGTTCTTGGCGACGATGCTGTTGAGTTCGGCAAAGTGCGCGGTCACCGGGTCTTC
GCTGAGGGCTGGCGCGCTGGCCGCCGGTTGCGCCTCGCCGAGCAGGCTGCCGAGGCGCTCCTTGAGCTTGTCGACA
CCCCCTTCCACTGGCACGCCCTTGGCAGCCAGTTGGCGCTCTTCAGCCTGCAGATCGGTTTCCTTGGCCACCGCCA
CCAGCAGCTTTTTCAGGGGCGAGGTCGGGCCGGAAATCACCCGCAATACGTCGGCCGCCTGGGCCACGCTGGTGAT
GGGCACAAAGTCGATATCGGCCAGCAAGGCGTCCCACTGGCGCTGATAGTCCTGGAAATACAGGCGCCGCACATCG
GCGGCAAGGCTGGCGACGTTCTGTTGATCCGCCGGTGCATGCCCCAATACCCATTGCTCTTCAGCCAGGGTGCCGG
CCTGGTTCAGGCTGGTCAGCAAAAAACCCTGGCGATAGCCCTTGGCAGTAAAGAACCCACTCAATGGCTCGCCCAG
CGGCTTGCCACTTTTGCGGCTGAATACCAGTGCGGCATCGCGGCCGGCAGCTTCGTTGATGCGAAAATCCGTCACG
CCTTCGGGCAGCTTGTGGCGCTTGACCCGGTCGTATACACGCTGGGCCACCGGCAGTTGTTGCAACTGCCGACGCA
AGTCATCGATCAGCCGTGGGTCGAGTCGCGCATTCGGTGGGTGACGATCGAACAACGCCTGCAAGTGCCCGGTCAG
TGCCTGGCGCTGGTCGGCGGGCAGGTCGCGTGGCAGGTTGCGGTCCCAGTCCAGGGCGATCCAGGCCTTGATGAAG
TCCGGGTCGTAATGCTCGCTGTCAGCCAGCATCAGGTAGGCCTTCAAGCCTTCGTAGAGGAAGTCGGAACTGCCGC
CGCTGTGCAGTTGCTCTTCGATGCGCGTTACCAGCCGCGGGGCGAACACCGCGATCAGCAGCTTGCGATAGACACT
GGCTGATTCGGCTTCGAGCATGTCGCCCTGATACAGGCCCAGGCCTTCGGACCAGCTTGGCGAATCGCCCGCCAGG
TTCTTCACCGCGTTGAGCAGCGGCAACACGGCCAGCACCTCGCGCTGTGCCGGGCTCAGGGTCTGCACGGCCTGGC
CCAGTGGCGCGACCTTGTGGTCGACCTGGGCGATATACGCCTGGTTCGCGCGATAACTCACCCACCACAGCGTACT
GACCACCAACACCACCGCCACGGTGGCGGCCAGGACGCCACGGGCCAGCCATTTGCGACGGCGCTCGACCTTCGGG
TTGACCCCCACCAGACCACGCTCGGCAAAGGCCACGGCGCTGAACAGCTTTTCGATGAAATAACTGCGCCCGGTGC
CGCTCTGGCGCGCCAGGTGCTGGCGGTCGAGGTTCATGCTCTGGGCCATGGCGCCGATCAGGCGATCGATCGGGCT
GCCTTCCTGGGTGCCGCTGGTGAAATAGACGCCGCGCAGCAGCACGCGCTCTTCAAACGCATTGGGCTTGAATACG

CCTTCGAGGAAGCCTTGCAGGCAATCCTTCAGGGCACCGAACTGCTGCGGGAAGCCGTAGATCAGGTCGCGCCGCG
CCGGGTCGCGCTCCTGTTGCAGGCGCTCCACCAAGCGCTCGTTGAGGCGCTGCTCCAGGCCGGCGAATTCGCTTTG
CAAATGGGCCAACGGGCTGTCGCTGTGCTTGCCGTCGTCCAGGGCAAAGGTCATGCCCCACACCTGGGCGCGGTCT
TCCTTGCTCAGGTTGTCGAAAAACTCCATGAAGCCCGGCACCAGGTCGAGCTTGGTCAGCATCAGGTAGATCGGGA
AGCGCACGCCCAACTGGGTGTACAACTCCTGGATGCGCAGGCGGATCGCTGCTGCATGGGCGGCGCGCTCGGCGTC
GCTGCCTAGCAGCAGGTCCGAAAGGCTGATGGCGATAAAGGCGCCGTCGATCGGGCGACGGGCGCGCTGCTTTTTC
AGCAGGTCGAGAAAGCCCAGCCAGGCCGCTTTATCCACCGTGGAGTTGCTGTCCTGGGTGGTGTAGCGCCCGGCGG
TGTCCAGCAGCACGGCTTGATCGGTAAACCACCAATCGCAATTGCGCGTACCGCCCACGCCCCGCACCGCGCCGGC
ACCCAACTGGGCGGCCAGGGGGAAATGCAGCCCGGAGTTGACCAGCGCGGTGGTCTTGCCCGAGCCCGGTGGGCCG
ATGATCACGTACCACGGCAACTCGTAGAGGTTGCGCCGCTCGTCACCGCCCAACTTGGCCTTTTTCAGCAACGCCA
GGGCTTCGTCCATGCGCTGGCGCAGGGTGGCCAGTTCTTCTGCAGTGGCAACGCTGTTGGGGTCGACCGGGGTTTC
GGCGGCCAGGCTGCGCATCACTTCGCCAGCCTGGCGCCGGGCCTGGATGATGCGAAACACGCGGTAGGCGATCCAC
ACCGCGAAGACCAGGATGATCAGTGCCCAGCGCCGGCCTTCGGGGACCAGCACGTCGAGCAACGGCCCGACAAACC
AGATGATCAGGCCCAGGGCAATCAGGCCCAGCACAGGGATCACCCAGCGAATCATGAAACTGAAAAACGCCTTCAC
TCGACGCCCTCCGCCAATACGGTGATTTCAACCCGACGATTGCGGGCACGGCCTTCGGCTGTGGCGTTGGTCGCCA
CCGGCTCGGTATCGCTGCGCCCCTCGGCGCTGAAACGCTCGCCCTGGCCGGTCTTGGCCGCGAGAATCTCCAGTAC
CGACTTGGCCCGCGCTTCGGACAAGGCCCAGTTGGACGGGAAACGCAGGGTGGCAATCGGGCGGTTATCGCTGTGC
CCGGTCACGCGCACCTGGCCCTTGACCTTGCGGATGGCATCGGCGATGCGCAGCATCAGGGGCTGGTAGTCATCGA
CAATGCTCGAGCTAGCGGAAGCGAACAGCTCATCGCCACGGATCGTCACCACGGAGCGGTCAACGGCGTCTTCCAC
TGCGACCCGGCCTGCCTTGATGTCTTCCACCAGAAAGCCCGCCAGGCGCGGGCGCTCGATGATTTTCGGCTGCGCC
ACCGGGCGGTCGATGGCCTGCACCGGGATTTCCCCCAGGGCATGGATGTTCTTGAACACCGGCTCGGCATCCGACG
CCAGCTTCAGGCGCAAGCCAAACAACAGCGCCAGCAACAACGCCAGGCCGATGGCGACTGCAATCCACGGCGGCAT
GAATTGCGCCAGGCGATCACGGGCCACGGTCACGCCGCGCCAATGGGGCGACAACTCGCGCTCATGCTCGCCACGG
GCACTGCGAATCGCCGCCGCCGTGCGTTCGCGCAACGCTTCCAACTGGCTGCGCCCGTCGTTCATCACCCGGTAGC
GGCCTTCGAAACCCAGGCACATGCACAGGTACAACAGCTCCAGCAGATACAGGCGCTCGCGCGGGCTTTGCAGGCA
GTGATCCAACAACTGAAAGACCTTTTCGCCGCCCCAGGCTTCGTTGTGCACGGTAATCAACAAGCTCTGCTTGCCC
CAATCACTGGCGCTGCCCCAGGGGGTGCTCAGCACCGCTTCATCGAGGGCGGTGCACAGCGCGTAGCGCGCCAGCA
GTACCTCGTTACGGGCCACGCCGGCCGCCTCGGCGCGTTCTTCGAACTGGCGCAGGTAGGCCAGCAACTGCGCACG
CAGGCTCGATGGCGCGGGATGGGCGATGGTGTTGCGCAGGCGTGTCAGCAACGCCAGCAACGGGCCGGCGGCGCTT
TCCAGGGGGTTGAGGCCCTGGCTTTTGCCGGTGAGGATCGGCGCCGCCGGCATCGACAAAGGCGCCGGTTCTGCGC
GTGCAGGCTCCGGCGCACGGCCACCCGGGCGCGGCATGAACTGGGTGCGGTCGTCATCATTGGGATGCATCGCGGA
TTATCCTCGGATCGCCCAGAAGGCCAGGTTCAAGCCCGGGAACTGCCCGGCGATATGGAAGGCAAAGCCGCCGGAG
TGGATCAACTGCTGCCAGTGTTCGCTGCCGCGGTCCAGTTCGTAATAGGTGGAGCCCGCGTGATACGGGATCTGGC
GTGGCGCCACCGGCAGCGGCAGCAGGCCGATGCCCGGCAGTTGCAGGTTGACCAGGTCGCGGATGTGTTCCACCGA
GCCGACCTTGCTCTGCTGGCCGAAGCGCGCACGCAGGGTTTCGCCCGGCACATCGGCACGGACCACGAGGATGAAG
CTGGCGCTGTCGATCAGGGTTTTATCCGCCAGCATTGCCACGTGCACGCCGTAGGCTTTCTCGACAATCGGGATAG
GCGTGGCCTTGCTGTCGATCAGCATCGACAGCGCCTCGCGCAGGGCCTGCATCACCGGGGCGAAGCTCAGGGCCAG
GTCGTCATGCTGATAGCGCGGGTATTCCTCAGGGCGCCGCCCTGAGGTGGAGAACGTCGAGAACTCGCCGGCCAGG
CTTACCAGCTCACTGAAGAAACGCTCCGGGTGCAGCGGGCTCAACTGGCTCAGGTGCTGGATCAGCGGCTGGGCGC
GGTTGACCAGTTGCAACAGCATGAAGTCGGCAATCTCTGAAGCGCCACCGGCGCCCGACGCCACCACGCGCCCAGC
CAGGGCTTCGCCACGCTGGTGCAGCAGGCCCAGCAGTTCACTGCGAAACGCCGTCAGCGGTTTGCTCGCCGCCACA
TCGAGCACCGGCGGGATGTAGCTGTCATCCAGCACCAGGGCGCGGTCGGCGCGCTTTTCCTTGATGCGCACCAGGC
CGATGGCGGCGTAGTCGCTGATGCCATCGGCGCCGGTGAGCAAGCGCAAGGCACGGGAACCGACGGCCACCGGTGC
GCGGTTTTCGAAGGGTGCGTTGTCATCGCGCACCTCGCGCACCTGGCTCAGGTAACGCGCGGCGCCCAGGGGCTCG
CCTTCATCTACCGTGTCGCGGGCGCCGGCACGCTTGAGGGGCAAGGCCAGGTACACCAGGCCGTCACGCAAGTTGT
CGTCGATATTCAGCGGGCTCGGCGCCAGGTCATCCTGGGGAATATTGAACGGCGTGCCATCGGGCAACAGGCCACG
GGCCGAGACAATCGCCAGCTTGCCCTGGGCCAGCAGGCCCTGGTCGATCAGCAGTTCGCAAAACCCCCAGGCGCCG
GCCGACAGCGGACGGCTGCGGGCATCGATCAGGTTTTCCAGGTAACGGTCATGCTGCTGGAAGTGCTGCGTTCCGA
TGAACATGCCTTCCGACCAGACCACGCGATTGTTCCAGGACATGGGGGCTCCGATTGCTTCTTATTGGGCAGGACT
GGATGGGGCAACGCTGAGGTGCGCACGTACGGCGCGCACATCAAGGCTGATCTGGTATTCGCTGGCCTGGCGGGGC
GGCACGCTGAGTACCGTGCGCCATTGCGCACGGTCCAGTTCGCGATAGCCCACCAGCAGGCCGATCTGCCGAGTGG
CCGGGTCGAGGTCGCGCTGCAGGCTCAATTGCTGGCCGGGCTGGATCACCACTTCATCCTGGTCCAGCAGGTCCAG
GCCGAGGGTCGGCTGGGCGCGATCCGCCAGGGCGAAGTAATCGGCACGACTGAAGGTCGCGGCGTTTTTCAGTTCG
AAAATCCGTACCCGCACCGGCGCTGGCTGGCCCGTGGTGCCGGGGTTGAGGCCGCTGATGGCATGGAAATGCAGCT
CGACGCTGGCAGTGTCGATTTGTGCCTCTGGCGCGGGCGGCGCAGCATCCTTGGCACAGGCCGTCAGCAGCAACAG
GCCGGCGGCCGCGAGTAAAAACCTGGAAATCATCCTGCGTCCTCAATGACGTATGTGGCTGTCCGTTGTTCCAGTT
TCAGCGCCGCTGGCGTGCGCTGTGTGCTTCATAGGCCCGGCTGAATTCACGGCCGAACAGGTCCTGGAAATCCTCC

TGGGCCTCACGGGAAATACTGTTGTAGAGCTCGGTGAACTGTTGCCAGTACTGGGCCTGGCGCGAACCGTTGAACA
GGCTCGACAGCCCGCCAGGCTTGCCCATGCGCTCTTCCAGTTGAGCGGGTTCGAAGCGTGTCAGCAGATGTTTGAT
GGCCGCTTCGACACCGGCCATCACCGCCAGTTGGTGGGCCCGCAGGTCGTCGAAACTGTCGCGCACGGCCAGGTCC
GGCGCCATAAACGCTTGGTTGCCGTGGCGAAGCAGCAGCAGTAACGCTTCGTCGGCATTCGGGGCGAATTTCAACG
GATTGTTTTCCACCGGCTGGATCGTCGTCTGCTGCATGCGGAACTCGCCCTTGAGGCTGCTGCGCGCACGTAATAC
GTCGATCAGGCCTTCGACCATCAGCCGGTAGCTGCGGCCGATACTTTCCATCTGCGCCTCGGCCTGGGCCTTGTCC
AGGCGCAGGTGATCAAGACCGGCACCGCGCAGGAAGGCTTGCAGCAAATCGGGCTGGGTGGCGTCTGCCAGCGGCA
GCGGGCGCTCGACCACAGGAGGTTCAGGCGGCGCGATGGGCGGTGGCGTGCTGAGCGGCGGCGCGGGTGTTTCGCC
AAGCAGGTCCCAGTCTTCCGGAATCACCGAGCCCGGGGTCGCCGGCTTCTCGGGCACAGGCACGTTAACAGCGACG
GGCGGGCGAAAATCATGCTGCTCGGCCGGCACATGGTCAGGCTGGCTGGGCGGCGGCACCGCCGTTGGTGTGAGGA
AATCGAACAGGTCCGGCAGCGTATCCATCGACGACGCGCCCTGGAACTGCGGCGCAATCACCGGGGTGCTTGGCAG
CGGCGCGGCCACCCCCGCACCCTGGCGGCCCATGAGCGCTTCGAAACTGTTGGAGGCATCGGCGGCGAAAGGCTGG
GAGCCTGTCACGTTCACCCCGCAATCAATCCGTGCCTGGATCTCGTAATCGCCGATCCGGATCAACTCACCATCTT
GCAATGGCTCGCTGTTCCCCCGGCGCAAACGAATACCGGCATTAACCAATTCCACACCATTAGTACTGTTATCGGT
TAGATAGTAACGGCCATCTTTGTATTGAATAACGCAATGTTGCGCGGAGACCAAACGCTCTGGATCAGGCAATATC
CAGTCGTTTTCACTAGAGCGGCCAATAGCCATCACTCCATTATCCATGGACTTTTCCGGACATTGACCTGGGGTGA
TCTTGTGATAACTAGTGATAGTCAAACACAGTGACATCTTGCCTCCTTGCTGAACCTCACGCGCGGTCGGCCAAGG
GAAATACTTTCCCAAGCGCGTCTCGACAAATCCTTTAAACAACCTTAGAAGCCTGCTTTTACCAGCATGATCGCTG
ATCTTAACCGGCAAATGTGACAAAAATCCTCAGGCAGCGCCTACTTTGACCCACGGGTTGCATAGGATGTTAAACA
CCGCACATCTGTCACACAGGGCGAATAGCTTACCTTGACAACGCATAACTACTACACCAAAAATGCACAACTTCTT
GAAGATAAGTAGTGGCACATTGAAATCAATGTGCTGGCCTGACCAAGAAAACATGCCGATAAATTGCGCAACTAAA
CCGCGCAACTAACGCATGCACTGCCCGAGACTAAAGGGCCGATATGGAGATCGAATTTAGTGGATGTGCCGTTGCT
GCTCACCGCTGTTTCCGCGACTTTGCCGTGTGGCGAAGACCTGGAATATGACGCGGACTTCCTGCACTTGGAACGT
GCTGCCCAGGGTCAACCCGAGCGCAGCATGGGCGACGCTATTTTGCCCGCCGAGCCCCCAGACTGGCGCAGCATCC
AGCAACAGAGCCTGGACTTGCTGCAGCGCAGCAAAGACCTGCGCATCACCCATTTTTTGCTGCAAAGCTCCCTCGC
CCTGCAAGGCGTCGGCGGGCTGGCCAGCGCCCTCACCCTGATCAGCGAACTGCTCCAGCAGTACTGGGCCGACCTG
CATCCGCGCCTGGATGCCGATGACGACAACGATCCCACCGTACGCATCAATGCCCTCGCCGGATTGACCTGCGACA
CGAACATCCGCCTGCTGCGCGAAAGCGTGCTTACGCGCTCGCGAGCGTTCGGCGCCGTGAGCCTGCGTGGTGCCCT
GAATGCCAGCGGCCTGCAAAGTTTTCCCGGCGAGAGCCTCGGTGCCGAGCAACTGGCCGCCGCCTTCCTCGACAGC
GACCCGGAGCCGTTGCAAGCCACCCACGCCGCCCTGGTCGAGGCCCGTGCGGCCTGTGAAAACATCGAAAAATACG
TCAGCGAGCAAGTCGGTTCCGCCCAGGGCGTGGACCTCGGCGCGCTGAAACATCCGCTCAAGCAGGCCCTGCAGAT
TTTCAGCCAGTTCACTGCACAGAGCGACGACAGCCGTGAACCCGAGGTGGTCAGTGACGACAGCCACGCCTCGGTC
GAACACGCTAGCGCCCCTGCAGCCCCCCGCAATGCTGGCGAGATCGCCAGCCGCGACGAGGTGCTGCGCGACCTGG
ACCGGATCCTTGCGTACTACACCCGGCACGAGCCTTCCAGCCCGCTGCCGGTACTGTTGAACCGGGCGAAAAACCT
GGTGCACGCCGATTTTGCGGCCATCGTGCGCAATCTGATTCCCGACGGCATGTCCCAATTTGAAAACCTGCGTGGC
CCGGACGGCGAGTAAGTCGCCAGGCTTTGCAGTAACAACACCGTCGCTCAAGCGACCAGGAGCAGCAACGTGGCGA
AGCAAAGTTCTCAGAAATTCATCGCGCGCAACCGTGCGCCTCGAGTGCAGATCGAGTACGACGTCGAGCTTTACGG
CGCCGAGAAAAAGGTCCAACTGCCCTTCGTGATGGGTGTGATGGCCGACCTCGCCGGCAAGCCCGCCGAGCCTCTG
GCGCCGGTCGCGGACCGCAAGTTCCTTGAAGTGGACGTCGACAACTTCGACTCGCGGCTCAAGGCCATGCAGCCGC
GGGTGGCATTCCACGTACCCAACGAGCTGACAGGCGAAGGCAACCTGAGCCTGGATATCACCTTCGAAAGCATGGA
CGACTTCAGCCCCGCCGCCGTGGCGCGCAAGGTCGACTCGCTGAACCAGTTGCTGGAAGCCCGTACCCAACTGGCC
AACCTGCTGACCTACATGGACGGCAAGACCGGCGCAGAAGAAATCATCATGAAAGCGATCAAGGACCCGGCATTGC
TGCAGGCCCTTGCGAGCGCGCCCAAACCTGCTGGGGACCAGTAATCATGACCGACACCGCCCGCGAAGGCGCCCAG
AACCTGGGTACCACCCAAGAAGCCAGCGAGTTTGCAAACCTGCTGCTGCAAGAATTCAAGCCCAAGACCGAGCGTG
CCCGCGAAGCCGTCGAGACCGCCGTGCGCACCTTGGCCGAGCAGGCCCTGGCGCAGACGGACCTGGTATCCAACGA
CGCGATCAAGTCGATCGAATCGATCATCGCCGCCATCGACGCCAAGCTCACCGCCCAGGTCAACCAGGTTATCCAT
CACCCGGATTTCCAGCAGCTGGAAAGCGCCTGGCGCGGCCTGCACTACCTGGTCAACAACACCGAGAGCGATGAGC
AGCTGAAGATCCGCGTGCTCAATATCTCCAAGACTGACCTGCACAAGACCCTGAAGAAATTCAAGGGCACCGCGTG
GGACCAGAGCCCGATCTTCAAGAAGATGTACGAGGAAGAATACGGCCAGTTCGGTGGCGAACCCTACGGTTGCCTG
GTGGGCGATTACTACTTCGACCAGTCGCCTCCTGACGTGGAGTTGCTGGGCGAGCTGTCGAAAGTCTGCGCCGCCA
TGCACTCGCCGTTCATCGCTGCCGCATCGCCGACCGTGATGGGCATGGGCTCCTGGCAGGAACTGTCGAACCCACG
TGACCTGACCAAGATCTTCACCACCCCGGAATACGCTGGCTGGCGTTCGCTGCGCGAATCGGAAGACTCGCGCTAC
ATCGGCCTGACCATGCCGCGCTTCCTCGCGCGCCTGCCGTATGGTGCCAAGACCGACCCGGTGGAAGCCTTCGCCT
TCGAAGAAAACACCGACGGCGCCGACAGCTCCAAGTACACCTGGGCCAACGCCGCGTACGCGATGGCGGTGAACAT
CAACCGCTCGTTCAAGCATTTCGGCTGGTGCTCGCGCATCCGTGGCGTGGAGTCCGGCGGTGAAGTGGAAAACTTG
CCGGCTCACACCTTCCCCACCGATGACGGTGGCGTGGACATGAAGTGCCCGACCGAAATCGCCATCAGCGACCGCC
GTGAAGCGGAGCTGGCGAAGAACGGTTTCATGCCGCTGCTGCACAAGAAAAACACCGACTTCGCCGCGTTCATCGG

CGCCCAGTCGTTGCAGAAACCAGCCGAATACGACGACCCGGACGCCACCGCCAACGCCAACCTGGCTGCACGCCTG
CCGTACCTGTTCGCCACCTGCCGTTTCGCCCACTACTTGAAGTGCATCGTGCGCGACAAGATCGGTTCCTTCAAAG
AGAAGGACGAGATGCAACGCTGGTTGCAGGACTGGATCCTCAACTACGTCGACGGGGATCCTGCGCATTCCACCGA
AACCACCAAGGCCCAGCACCCATTGGCTGCCGCCGAAGTGATCGTGGAAGACGTCGAGGGCAACCCGGGGTACTAC
AACTCCAAGTTCTACCTGCGCCCGCACTACCAGCTCGAAGGGCTGACCGTGTCGCTGCGCCTGGTATCGAAACTGC
CTTCGGCAAAAAGCGCGTAAAAAATCGGGACTGGCTCGCTCCCTGTCGGAGCGAGCTTGCTCGCGAAAAACTTGAA
GGCGCTGCGTTCATTCAGGATGCCCGCGTCACCGTTGACGACCTTCGCGAGCAAGCTCGCTCCTACAGGGGTCGGC
GGCGTAAACAAATATCGTGGCTCAGACCACACAGGGAGAAAACATGGCTGTTGATATCTTCATCAAGATCGGCGAC
ATCAAGGGCGAGTCCATGGACAAGGCCCACAAGGACGAAATCGACGTGCTGAACTGGAGCTGGGGCATGGCCCAGT
CCGGCAACATGCACGTTGGCAGTGGCGGCGGCGCGGGCAAGGTGAATATCCAGGACCTGTCGTTGACCAAGTACGT
CGACAAGGCTTCGCCTAACCTGATGATGCATTGCGCCAGCGGCAAGCACATCGACAAGGTCAAGCTGACCGTGCGC
AAGGCCGGCGGCGAAAGCCAGGTCGAGTACATGATCATCAACCTTGAAGAAGTGCTGATCACGTCCCTGAGCACCG
GCGGCTCGGGCAGCGATGATCGCCTGACCGAAAACGTCACCCTGAACTTTGCCCAGGTGATGGTCGACTACCAGCC
GCAGAAAGCCGACGGCACCAAGGACGGCGGCCCGATCAAGTTCGGCTGGAATATCCGTTCCAACACCAAGCGTTGA
TACGAAGACCCCTGGCGCAACGCGCCGGGGGTTTTGGTGCTTGCTCGAATTTCGACCTTCCTCTTTTCGGTATCTG
AGTCATGGCCAAACCTTCGTTTATCAATTTGTGGGCGAACTACCCCACAACATCAACACCCTGTGACGGTCCTTGG
GACAACCAATGCGCCATCCGTATGAGCATCACGCTTAACGCCGAAAAAACCATCAAGGTAAACAAATCGACGTATA
GCGAACCCAAATGCGCGCATGACCACGCCAGAGGCGCGGAGTCGCTGGCTAACTGGCTGTGGCGTCATCATCTCGG
TCGCCCAACCATTCTTACGGGCAGCGCCGAGGACCGCCGTACGCTTTACCAAAAAACCGGAATTATTTTCTTCAAA
GATTGTTTTGCCCGGATGGGCCAATCTCTGGACGCTCGTACCGGCGACCATATTGACCTATGGAATCGAGGTTCAG
TAACAGGTGATTTCGCCGACCAGGCGCACCGGTCCCGGCAGGTCTGGTTTTGGGAGTTGAAATGATCAAACGCACC
CTGCAATGCGTACTCTCAGTGAGCATGCTGTTGAGCCTGAATGCCTGTGCGGAGCAAGCTGAGCAAATCGACGCCA
CCCAATCAAACCCCGAGGTGGTGGTTGGAGGTCAACGACTGACCTTGGAGAATCATGCACAACGCTGTGCATTGCG
AAAGCCCGACCAGAGCCTGTTGACCCTGGATATGCCTTGGCCTTGCCATCTCAGCGTCGACCGCAAGGACCAGCCA
AGGGTCGAGACCTTTAACAATGCACAGATCATCATTGTGCAGCACTTTGCACCAGAACCTGCACCGAGCCAGGAGT
GTCGCTCTCAGTATCAAGCCATCAGACATATTGAAGGTCGCTTGGAGGCGTCTATGGTTGCCAACGGAGGTCGTTG
TCTGCTTGGGGCTATGGACCAGAAAAACTTCGTGGCACTCTTTACGTGGTAACCGAAATCGCCACCCGCGACCGCC
TGCAACCGTCCCTGCTGGACCGGTTGACCGACGACGAGCCGAGCAACCCCAAGGAAAGCGCCGACAAGCGCGTGCT
GTCCCTGACCCAGCTCAAAGCCTCGGTGCTGCGGGACCTGGCGTGGCTGCTCAATACCACCTCGCTGCTCAATGCC
GATGCCACGCTGCACACCCCGGCCGGGACGTCAGTGATCAATTACGGCCTGCCGGCGCTGGCGGGCAACAGTGCGT
CGAGCGTCGATATCAATGCTCTGGAAGCCCTGATCTACCAGGCAATTGCCACCTTTGAGCCGCGCATCCTGCGCCA
CACCCTGCGGGTCAAGGCGCGTGTGGCCCATGGCGAGATGAACCACAACGCCTTGAGCTTCGAGATCGAAGGCGAC
CTGTGGGCCCAGCCGGTGCCCTTGCGTCTGCTGCTGCAAACCGACCTGGACCTGGAGTCTGGCCATGTACGCGTGG
TCAACGCCGACCAGCGGAGGCGCCCATGAACCCGCGCCTGCTGGAGCTGTACAACCAGGAGCTGCATCACGTGCGC
GAGAGCGCCGCGGAGTTCGCCAAGGAATACCCGAAGATCGCCAGTCGGCTGACCCTGTCCGGCATGGACTGCGCCG
ACCCGTACGTCGAGCGCCTGCTCGAAGGCTTCGCGTATCTCACCGCCCGGGTGCAGCTCAAACTCGACGCCGAGTA
CCCGACCTTCACCCATAACCTGCTGGAAATCGCCTACCCGCACTACCTGGCGCCGACCCCATCGATGACCGTGGTG
CAGTTGCAGGCCGACCCCGATGAAGGCTCCCTGACCAGCGGATTTCCATTGCCCCGCGATACCGTACTGCGCGCAG
CCCTGGGCCGCGAAACCCAGACCTGCTGCGAGTACCGCACCGCCCACCCGGTGACGCTGTGGCCGTTGCAGGTCAG
CCAGGCCGATTACTTTGGCAACCCGTCCGCCGTGCTCGGGCGCCTGGCCGCCAGTGAGCCCAAGGCCAAGGCCGGT
TTGCGCCTGACCCTGCGCACCGGCGCCGAGCTGCCATTCAACAGCCTGGCCCTGGACAATCTACCGCTGTACCTGA
GCGGCGCCGATGAGCAGCCGTTCCGCCTCTACGAGCAATTGCTGGGTAATGTGTGCGCGGTGTTTGCGCGCCAACC
CGGCGGTGACTGGGTGGAGCGCCTGCCACAGGACGCGCTGCGTTCGCGCGGGTTCGACGACGCCGATGCGGCGATG
CCCGTGGTATCGCGAGCCTTCCAGGGTTACCGCTTGCTGCAGGAATACTTCGCCCTGCCCCATCGCTACCTGTTCG
TCGAATTCGCCGAACTGAGCCGCGCCGTACAACGCTGCGATGGCCAGGAACTGGAGCTGATCGTGCTGTTCGACCG
CCACGATCCCAGCCTGGAAGGCAGTGTTGGCGCTGCGCAGTTCGTGCCCTTCTGCACCCCGGCGATCAACCTGTTC
CCCAAACGGGTGGACCGGATTCACTTGTCCGAGCGGGTCAACGAACACCATGTGATTGCCGACCGCACCCGGCCCA
TGGACTTCGAGATCCACTCCCTCAGCGCCATCACCGGCCACGGCACCGGGCCGGAGCAACCCTTTTTGCCGTTCTA
CGCCGTGCGTGATCCGTCGCGCTACGGCCGCGACCAGAGTTACTACAGCGTGCGCCCGCGAGCCGCGGGTGCTGTCC
AGCGACCAGCGGCGCAATGGCCCGCGCTCCACGTATGTGGGCAGCGAGACCTTTGTCAGCCTGGTGGACAGCCAGC
AAGCGCCCTACCGCCACGACCTGCGCCAATTGGGCGTGACCGCGTTGTGCACCAACCGCGATCTGCCACTGTTCAT
GAGCGTGGGCAACGGCAAGACCGACTTCACCCTGGCCGACAGTGCCCCGGTGCTGGCGGTGCGCTGCGTCGCCGGG
CCCAGCCGCCCGCGCGCCAGCCACGCCCATGACGCCAAGGCCTGGCGCCTGATCAGCCAGTTGTCGCTCAACTACC
TGTCCCTGAGCGAAGGCCAGGGTGCCGCGGCGTTGCGTGAACTGCTGCGCCTGTATGGCGACAGCAACGACGCCGC
CCTGCAATTGCAGATCGAGGGCCTGCGCGAGGTCAGCAGCAAAGCGGTGACACGGCGCCTGCCGATGCCCGGCCCG
ATCGTGTTTGGCCGTGGCCTGGAAATCACCCTGGAATTTGATGAAAACGCGTTCCGCGGCACCGGTGTATTCCTGC
TCGGTGCAGTGTTGGAACGATTCCTGGCGCGTTACGTGTCGATCAACAGTTTTACCGAGACGGTGATTCGTACCAC

CGAACGCGGCGAGATCATGCGATGGAAAGCCAAGCCCGGACGGCGTCCGACCCTGTGAGTACCCTGGATGCGATGC
ATCAGGAGCCCTGGGAATATGACTTCTTCCAGGCGCTGCGCCGTATCGAGTGCGAATCTCCCCAACTGCCGCGCCT
GGGGCATTCCCTGCGCCTGGCCGATGACCCGTTGCGCCTCGGGCAACAGGCTGACTGCACCTTCGCCCCGGCCACC
CTGGCGTCGGTGAACCCCGGCGGCGACGGCACGCCAGCGCGCCTGGAGCAGTTCTTTTTCGGCCTCGGCGGCCCCA
ATGGGCCGTTGCCGCTGCACATCACCGAATACGTGCGCGAACGCCAGCGCAACAACGCCGACAGCACCAGCAAGCG
CTTCCTGGATGTATTCCACCACCGCCTGTTGACCCTGTTTTATCGGGCCTGGGCCGAAGCGCGGCCGACCGTCAGC
CACGACCGCCCCGACGATGATTACTGGTCCGCACGCCTGGCGGCGCTCAGCGGCCGCGGTATGCCCAGCCTCCTCG
ACCAGGGCCTGATCCCCGATACCGCAAAACTGCACTACAGCGGCCACCTCTCGGCGCAAACCCGCTACCCGGACGG
TTTGAAGGCAATTCTCAGCGAGTATTTCGGCTTGCCGGTGGAGATTGAAGAGTACGTCGGCCAATGGCTGGAGCTG
CCCGAGCGCAGCCGGGTCAGCGTGAGTGCCAATCAATTGGGCGTGGATTTTTGCCTGGGCAGCCATGTGTGGGACC
GCCAGCACAAATTCCGTATCCGCCTCGGGCCCCTGACGCTCGACGACTACATGGGCATGTTGCCCGGCAGCCAGCC
GTTCAATGAGCTGGTGGCCTGGGTCACCGAATACCTGGGCCATGAACTGGACTGGGACCTGAACCTGATTCTTCAA
CAACCGCAAGTGCCGCCGTTGCAACTCAACGGGCAGTTCCGGTTGGGTTTCAACACCTGGCTCGGCCAACCCGGGC
AAGACGCCAACGACCTAATCCTGGCCCGGCATTACGCCGATCAAGCCACCACCTCAAGGAATCCAGAGCATGGGTG
AAATCAGTCGCGCCGCGCTGTTCGGCAAACTCAACAGCGTGGCCTACAAGGCCATCGAAGCCGCCACGGTGTTCTG
CAAATTGCGCGGTAACCCCTACGTGGAACTGGCCCACTGGTTTCACCAGTTGCTGCAACTGCAGGACTCTGACCTG
CATCGCATCATCCGCCAATTCAACATCGAGCCGGCACGGCTGGCCCGAGACCTCACCGAAGCCCTGGACCGCCTGC
CCCGTGGCTCGACGTCGATCACCGACCTGTCGTCCCATGTGGAAGAAGCCGTGGAACGCGGCTGGGTATACGGCAG
CCTGATGTTTGGCGAAAGCCAGGTGCGCACCGGCTACCTGGTGCTGGGCATTTTGAAGACGCCGAGCCTGCGGCAT
GCACTGCTGGGCTTGTCGTCCGAGTTCAACAAGATCAAGGCCGAGGCCCTGAGCGAGCGCTTTGATGAATATGTCG
GTGATTCGCCGGAAAACGCCCTGAGCGCCAGCGATGGTTTCAATGCCGGTGCCGTGCCGGGCGAAGCCAGCGGCGC
CATGGCCCCCAGTGCCATGGGCAAGCAGGAAGCCCTCAAGCGTTTCACCGTGGACCTGACCGAACAGGCGCGTAGC
GGCAAGCTCGACCCGATCGTGGGGCGTGACGAAGAGATCCGCCAACTGGTGGACATCCTGATGCGTCGGCGCCAGA
ACAACCCGATCCTCACCGGTGAAGCCGGGGTGGGCAAGACCGCCGTGGTCGAAGGCTTTGCCCTGCGCATCGTTGC
CGGCGACGTGCCGCCAGCGCTCAAGGACGTGGAGCTGCGCAGCCTGGATGTGGGCCTGCTGCAAGCCGGCGCCAGC
ATGAAAGGCGAATTCGAACAGCGCCTGCGCCAGGTCATCGAAGACGTCCAGGCGTCGCCAAAACCGATCATCCTGT
TTATCGACGAAGCCCACACCCTGGTAGGTGCCGGGGGCGCCGCCGGCACCGGTGATGCGGCCAACCTGCTCAAGCC
GGCCCTGGCCCGTGGCACCTTGCGCACCGTGGCCGCCACCACCTGGGCCGAGTACAAGAAGCATATCGAGAAAGAC
CCGGCGCTGACCCGCCGCTTCCAGGTGGTGCAAGTGGCCGAGCCGTCGGAGGACAAGGCGCTGCTGATGATGCGCG
GCGTGGCCTCGACCATGGAAAAGCACCACCAGGTGCAGATCCTCGATGAAGCCCTGGAAGCCTCGGTCAAACTGTC
CCACCGCTATATCCCGGCGCGCCAGTTGCCGGATAAATCCGTGAGCCTGCTGGACACCGCTTGCGCCCGCGTCGCC
ATCAGCCTGCACGCGGTGCCGGCAGAAGTGGATGACAGCCGTCGCCGCATCGAAGCCCTGGAAACCGAGCTGCAAA
TCATCGCCCGCGAGCATGCCATCGGCATCGCCATTGGCGCACGCCAGAGCAACAGCGAAGCCTTGTTGAGTGCCGA
ACGCGAGCGCCTGGCTAGCCTGGAAAGCCGCTGGGCCGAAGAAAAAACCCTGGTGGACGAGTTGCTCGCTACCCGC
GCGACCCTGCGCGAGAAAGCCGGCGTTGTCGACAGCGGCAACGACGAGTTGCGCGCAAAACTGGTGGACCTGCAAC
AGCGCCTCAGTGCCCTGCAGGGTGAAACCCCGTTGATTCTGCCGACCGTGGATTACCAGGCCGTAGCCTCGGTGGT
CGCCGACTGGACCGGAATCCCGGTAGGCCGCATGGCCCGCAACGAACTGGAGACGGTGCTCAACCTCGACCAGCAC
CTGAAAAAACGCATCATCGGCCAGGACCACGCCTTGCAGATGATCGCCAAGCGCATCCAGACCTCCCGCGCCGGCC
TCGACAACCCAAGCAAGCCGATTGGCGTGTTCATGCTGGCCGGCACCTCGGGCGTGGGCAAGACCGAAACCGCCCT
GGCCCTGGCCGAAGCCATGTACGGCGGCGAGCAGAACGTGATCACCATCAACATGAGCGAGTTCCAGGAAGCCCAT
ACCGTGTCCACCCTCAAGGGCGCGCCACCGGGCTATATCGGCTATGGCGAAGGCGGCGTGCTGACCGAAGCCGTGC
GGCGCAAACCCTACAGCGTGGTGCTGCTGGATGAGGTGGAAAAAGCCCACCCGGACGTGCATGAGATTTTCTTCCA
GGTGTTCGACAAGGGCGTGATGGAGGACGGCGAAGGCCGGGTGATCGACTTCAAGAACACCTTGATCCTGCTCACC
ACCAACGCCGGCACCGAGCTGATTTCCCACGTCTGCAAAGACCCGGCAAACGTGCCCGACCCGGAAGACATCGCCA
AGGCCCTGCGCCAGCCGCTGCTGGAGATCTTCCCACCCGCCCTGCTCGGGCGCCTGGTGACGATTCCGTATTACCC
GCTCAGCGATGTGATGCTCAAGGCGATTACCCGCCTGCAACTGGACCGCATCAAGAAGCGCGTGGAGAACACCCAC
AAAGTCGCGTTCGATTACGACGACACGGTGATCGACCTGATTGTCTCGCGCTGCACCGAAACCGAAAGTGGCGGGC
GGATGATCGACACCATCCTCACCAACAGCCTGCTGCCGGACATGAGCCGCGAGTTCCTCACGCGCATGCTCGAAGG
CAAGGCGCTGGCGGGGGTGCGGATCGGCAGCCGGGATAACGAATTGCACTACGACTTCAGCGACGCCGAATAACCT
GACGAAGTGCTATCCAAATGTGGGAGCGGGCTTGCTCGCGAATGCGGTGAGTCAGTCACCGGATGTATCAACTGAT
ACACCGCATTCGCGAGCAAGCCCGCTCCCACATTGGATTCGGTTTCTTCAGTTATTAAGACACTTACGGGACAACC
CATGCTTTTCAACCAAGCCTCACGCCTGGCCAAGATCACCAGCCCCCTGGGGCCGGATGTGCTGCTGCTCAATGAA
ATGGGCGGCGGCGAAGAGCTGGGGCGGCTGTTCAGCTACGAGCTGCAACTGAACTCGTTGGACGCCAACATCGACC
TCAACCAGTTGCTGGGCAAGCCGATGAGCGTCGGTCTGCAACTGGCGGACGGTGGCGAGCGGTATTTCCATGGGAT
CGTCGCGCGCTGCAGCCAGAACATCGACCAAGGCCAGTTCGCCAGTTACCAGGTGACGCTGCGCCCGTGGCTGTGG
CTGCTGAGCCGTACCTCCGACTGCCGGATTTTCCAGAACCTGAGCATCCCGCAGATCATCAAGCAGGTGTTTCGCG
ACCTCGGGTTTTCCGACTTCGAAGACGCCCTGAGCCGGCCGTATCGCGAGTGGGAATACTGCGTGCAGTACCGCGA

GACCAGCTTCGACTTCGTCAGCCGCCTGATGGAACAGGAAGGCATCTACTACTTCTTCCGTCACGAGCAGGATCGC
CATGTGCTGGTGCTGGCCGATGCCTATGGCGCCCACACCACGGTGCCCGGCTACACCTCGGTGCCCTACTACCCCA
AGGACGAGCAGCAGCGCGAACGCGACCATATGCACAACTGGCACCTGGCGCAGCAAGTCCAGCCGGGTTCCCTTGA
GCTCAACGACTACGACTTCCAGCGCCCCAGCGCCAGCATCGACGTGCGCTCGGCCATGCCGCGCCCGCACACCGCC
GGTGATTATCCGCTGTACGACTACCCCGGCACCTACGTCAAAAGCGAAGACGGCGAACACTACGCACGTACCCGCA
TCGAAGCCCTGCAAACCCTGCACGAACAAGTGGAGTTCAGCGGCAATGCACGGGGCCTGGGCTCTGGTCATCTGTT
CAGTCTCACCGGCTTCAGCCGCCGCGACCAGAACCGCGAATACCTGATTCTTGCCGCGCGCTACTACATCACCCAA
GAACGCCTGGAATCCGGCGGCGGCGCGGCGTCGGCGCAGTTCGACAGCAGCCTGACCTGCATCGACGCACAACAGA
GCTTCCGCCCACTGGCCAACACCCATCGCCCTGTCGTCAAAGGCCCGCAGACCGCGCTGGTGGTCGGGCCCAAGGG
CGAGGAAATCTGGACCGACCAGTATGGTCGGGTGAAGGTGCATTTCTATTGGGACCGCCACGACCAATCCAACGAA
AACAGCTCGTGCTGGATTCGCGTGTCGCAATCCTGGGCCGGGAAAAACTGGGGCTCGATGCAGATCCCACGCATCG
GCCAGGAAGTGATTGTCAGCTTCCTCGAAGGCGACCCGGACCGCCCGATCATTACCGGCCGGGTCTACAACGCCGA
ACAGACGGTGCCCTACGACCTGCCGGAAAACGCCACCCAAAGCGGGATGAAAAGCCGCTCCAGCAAGGGCGGCAGC
CCGGCGAACTTCAATGAAATCCGCATGGAGGACAAGAAGGGCCTGGAGCAGTTGTACATCCATGCCGAGCGCAATC
AGGACATTGTGGTGGAGGTGGATGAAAGCCACTCGGTGGGGCATGACCGCAACAAGAGCATCGGGCACAACGAGAC
GGTGACCATTGGCAACAACCGCCTGCGCATCGTCAAGCAGGAAGACGTGCTCTCGGTGGGCCAGAAGAAGACCGAC
AGCATCAGCCAGAGTTATGTCATCGAGGTCGGCGAAAACCTGCGCCTGGTGTGTGGGCAAAGCATCCTCGAGCTGA
ACGCCAGCGGGCAAATCAACCTCACTGGCGTGCAAATCAGCTTCTATGCCAGCGGCGACGCCGAGTTCAACACCGG
CGGCGTGCTGCACCTCAACAACGGCGGCGGGCCTGGCGCCACACCGGATGGCCAGGGCATAAAAACCAGCATCGAC
GCCAACATCAAAGCCGCGTTCCCCACGCCCAAAAGCTCCTGACGAGACCTGTTTGCCATGACGTATCGCATCAACG
AATTCCAGTTCCAACTGCCCGCCGGCGAGCTGCAGGACGCAACGATCAATATCCTCAAGTTCCCGGAGCTGGGGAC
TTCACTGATCGTCAGCCGCAGCTTGCTGGCGCAAGGCGAAACCTTGCACAGCAACTTCGACGACCAGCTCAAGCGC
CTGGAAAAACAGGTGCAGGACTTGCGCTGCCTGCCCAGCGTGCCCGTGCGCCTGGGTGCTGCCCAGGAGGTTGAAG
GGATCGAGTTGCGCAGCCAGTTCAGCAAGGGCAATGACAAGGTGTTCCAGTATCAACTCGCCCTGGTGTTGCCGGG
TACGCGCAAGATGCTCGCCTTGAGCTATGTGAAAGCCGACAAGCTCGGCGATGCCGAAGCGGCGCACTGGGCGACT
ATCAAAAATTCGCTGTCGTTCGACGTGCCTGCGTGATGGGCATCTTGCATGTCTGACGCGTTATGGGCCGCACGCC
TGGGCGATGCGATGGAACACACCTCGATGATGGCCGACATTCTCGGCGGCGTGCTGGAGGTGGCGGCGAACATCGC
GATTANNNNNNNNNNCAAGAGCACCCAGACGGTCAAGATCACCGGTACTGAAGGCGGTAACTTCGAGAAGCTGGTG
GTTGCAGGCGACGGTGCAACAACGCAGATCAACGACACCATCGACGATGTGAAGGTTGTGCTGACCGCCACCACCA
ACGTCGCCGAAGGTGGCCAGATCGTCTACACCGCCAGCCTCGTGGATAAGAACGGCGTGGCCGTGACCAACGTGGG
TTCGGACCTGGTGATCAAGTTGGACAACGACTTGAACATCACCATCGGCAATGGCAAGACCAGCGGTACTGCCAAC
TTCACCGCGCCGAATGACTTCTACCTGGGCGCCAAGGACATTTCCGCCAAGATCACCAATGTGGTCAGCGGTGGCG
ACAAGTATGAAAACCTGATCCCGGTCGGCACGCCAGTAATCACCAAAGTGACGGACGTGACGAGCAACACCGTGAT
CAGTATTGCGGGCGATACCTCGGTGACCGAAGGCCAGACCGCTCACTACACGTTGAACCTGACCCAGCCGGCGCAA
ACCGAAGTCACGGTGACCCTGTCGTACAAAGGCGTGGCCCAGGACGGTTCAGACTTCAACGGCGTGTACACCGTGA
AGATTCCGGCCGGCCAAAGCAGCGCGACGTTCGATATCAAGACCATCGACGACAAGCTGACCGAGCCTACGGAGAA
GTTCGAGATCAGTATCTCGGGCACTTCCGGCGGTAACTTCGAGAACCTGGCTATCAGCACCACCAACGGCAAAATC
GAAACGTCGATCATCGATAACGATGCGCCACCGGTTATCGACCTGGATGCCAACAATTCCAGTGGCGCCACTGGCA
ACGACTACAGGACTACGTTCACCGAAGGCGGCAGTGGCGTGTCGATTGCTGATACCGACATCAAGATCACCGACCC
GGACAGCACCCAGCTGACTGGCGCCACGGTAGTCCTGACCAATACTCAGCCGAGCGACACGCTGGACTACAGCAAG
GTCACCGGTATGAACGTGACCTCGGTCACGGACCCTGTCACCGGCAAGATCACCCTGACCCTGACCGGTACGGCGT
CGCTGGCTGACTACATGCAGCAGATCAAGAACATCACGTTCGGCAACAGCAGCCACGACCCGAGCACCACGCCACG
GACCATCACCGTGACGGTGACCGACGGCGGCAATTACTCGAACGTGGCAACCACCACGGTCAACGTAGTGGCGGTC
AACGACGCACCGGTTGCCACCGGCGGCGCCGTCACCGGCACTGAAGACACGTCGCTGAAGCTGACCTGGGCCAACT
TTGGGGTCAGCGATGTGGATTCGCCACAGGCCAGCCTCGGGGTGAAAATCACCGAGCTGCCAGTAGCCGGCAAGCT
GCAGTTCCTGGCAGCGGATGGCACTACCTGGACCAACGTGACAGCCGGCCAGACCTTCACCAAGGCGCAGATCGAC
GGCGGCCAGTTGCGCTTCACGCCAAATGCCAACGAATCCGGAGCCGACGGCTACGGCGGCACAGGTGTGGGCAACA
AGCAGGCTGATTATGCACAGTTCAAGTTCCAGCCAACCGATGGCAAGGACCTGGGCACCAGCGCCACTGTAAAAGT
CGACATCACTCCGGTTGCAGACGCGCCGACCTTGAGCGTTGCCGATAACAACATCGCCTCGGTCGGCCTGACCAAG
CAGAGCTGGAACAGCATTGCGAACCTGGGCACCAACGGCAATGGTGCCTCGGCGACCGTGTTGAAAAATGCCATCG
ACAACGCCGGCACGCCAACCAGCACGACGGTTTCGACCACCGTCCTACCTACTGCAGATGTGCCTGGCGGTGCAGG
CTCGAAGACCTCCGGCCTGATCTACCTGGAAGCCGGCAAGTCGTACACCTTCAGCGGCTACGGCGATGACAGCATC
CTGGTCAATATAGGCGGCAAAGATGTGGCTAGCGGTACCTGGGGCTCGAACTCCGGCCAGTTCAACGGTACGTTCA
CCCCAACCAGCAACGGCTACTACAGCATTGATATCTACCATGCCAACCAATCAGGTCCGGGCAGCTTTGATCTCAA
GCTGTCGGTCAACGGTGGCACCGCGACCAACCTGAGCAACACCACGATGCCGATCTACACCGGCATCAACGACCTG
ATCAGCGCCGGCGCGCAAGTGTCGGATCTGCACGGCACAAACGGCCAGGGTTACTACGACGCTTACAAGCTCAACG
AAGGGCTGGAAAACGGCACGGTCAAACTGACCAAAGTCACCACCGGCCTGACCGATACCGATGGTTCGGAAACCCT

GAGCGTGAAGATTGGCAGCATCCCGGTGGGCGCAGTGCTCAGCGACGGTGCCGGGCATACCTTCACGGCGACCCAG
GGCCATACCGAAGTGGACGTCACCGGTTGGAACCTCAACAACCTGAGCCTCAAGCCACTGCCTTACACCAGCGGCC
AGTACAACCTGACCGTGACGTCGACCTCCACCGAAAGCCTCGGCGGCTCGGCCACAACCGTGGCCAATCTGCCGGT
CAAGGTGTACCCGGCAACCTATAGCGGGAGCACGGCGACTTCGGGCGATGACAACGTCACCGGTACCAGTGGCAAC
GACATCATCGTGGCGGACGTTTCGGGCCTGAACGTAGTTCAGGGCAAGAACTACAACATTGCGTTCATGATCGATA
CGTCGGGCAGTATGGGTTCGGACTCGGTGGCGGCGGCGAAAGCCTCGCTGAACACCGTGTTCAACTCACTGAAAAA
CAGTATCGGCTCCAGCACCTCCGGGGTCGTGAACATCTTCCTCGCGGATTTCAGTGACCAGGTCAATCGCACGGTT
ACAGTCAACCTGAAAGACCCAGGTGCACTGGAGGCGTTGCAGAAAGTCCTGGATAAGATGGTGTCGACCGGCGGCA
CCAACTACGAAGACGTGTTCAAGGCTGCATCCAACTTCTTCCAAAGTGCCCAGGCGACTGGCAACAAGGATGCAAT
CAACACCACGTACTTCATCACCGATGGTGAGCCGACGTTCTACCAGCGTTCCGAGCAGACCAACCCGACCTTGTAC
GGCAACGTCAAGCTCGATGACGTCGTTAACATGAACAACTACACCATAGGTACCGCCACTCGGATCAATATCGATT
CGAATAACTACCTCGATATCAACTCTGCCGGTAAGGTGACCCTCTTTTCCTACGGCTGGGCCTACAACAAGGGCAT
CATCCACGCCCAAGGGGATGGCACCTATGAGATATCGGTTCTTGACGGTTATGGTAACTACACCGATACAGCCACC
GCTACCAACTCCCAGAACGCGTTCGCATTGCTCAAAGGCCTGTCAACGGGTGGCGTAGAAGCCATCGGTCTTAAAA
GCGGGGTCGGCCTGGAGCAGTTGGCACCGTACGACAGCGACAACAAGCCACAAAGCAACATCGATCCGAAGGACCT
GGCCAACGCCATCCTCGGTCACAGCGAGGCTACCCTGCCGGGTGCCGACCGTGTCGATGGCGGTGACGGCAACGAC
ATCCTGTTCGGCGACCTGGTGAGCTTCACGGGTGTTACCGGCGAAGGCTACAACGCCATCCAGTCCTACGTGGCAG
GGAAAAACGGCGTGGCGGTGTCTGCAGTCACCGGCCAGGATGTGCATAAGTACATCACCGAGCACTACAACGAGTT
CAACACCTCTGGTGCCAAGGACGGCAATGACACCCTGCTGGGTGGCTCGGGTGATGACATCCTGTTCGGCCAGGGC
GGCAACGACTACCTGGACGGTGGCAAGGGCAACGACATCCTGTTGGGCGGTACCGGCAACGACACGCTGATCGGCG
GCCAGGGCAATGACATCCTGATCGGCGGCACCGGTGCCGATACCTTCGTCTGGAGATCCGGTGACGTCGGCAACGA
TGTGATCAAGGACTTCAAGGCACTGGAAGGCGACCGCATTGACCTGCGTGACCTGCTCAAGGGTGAAACCGACGGC
ACCATCGACAACTTCCTGAAGTTGACCACGGTGGATAACGTGACAACGCTGCAGATCAGCTCCGAAGGCAAGCTCA
ACGCCGCTGGCGGCCTGGCTAATGCGGATGTGACGATCAAGCTGGAAGGTAACAACTGGTCCGGCCAGACGATCAA
TTCGTTGATCAGTGGTGCTGACCCGACCATCAAGGTCGATCACACCTAAGCTGAACCACTGAACCGGCCGCCTCTT
GAGGCGGCCGATTCATTTGCGGCCACCGTTCGCTGGCATGGGGTCGCGATTGCACGGCATACTCACGCCCAATCGC
GCGCCGTTGGCCTATGCTCTGACAGGCTGTCGTCAGTTCTTCTCATGAGGGATGTTCAATGTTCTACGTGCAACGT
GATGCACAGGGTGAGCTGATTCGCGCCGAAGCGGTTGCCTTCGCCGAATCGACGGGCACCCTGCCCGCGGATCATC
ATGAGATCCAGGCCTGGTTTGCCAATGAAGAAGCGGAGCTGAGCCTCAAGCAGCTCAAGCACAGTGACTCGGAGAT
GATCCGGGTGCTGGAAGACTTGATTCAGGTGCTGATCCAGAAAGGCGTAATCAACCTGACGGACCTGCCGGTCGCC
GCGCAAGCCAAGCTCAAGGACCGCAGCAACGCCCGGGAATCACTGGGTGGCCTGAGCCACCTGATCAATGATGATG
AGACCGGCCTGATCTGAGGCCGGCCACGGCCTCAGCGCCAGGGCGCCGGCTCACCAAACAGCTGGCCCTGCACGCC
AAAAATCCCCATCTCGCGAATTACCTGCAACTCACCCTCGGTTTCGACCCGCTCGGCAATCAGCGGCAAGTCGATG
CTGTGGGCTGCGCGCTGGATGGCTTCGATGAACAGGCGCTTGTCGCTTTCCTGGTCGATGTCGCGGATGTAGCTGC
CATCGATCTTCAAGTAGGCCAGGCCCAGGCGCGCCAGGTTGCCGATCATGCTGAAGCGCCCGCCAAAGCGTTGCAG
GCTGAGGGAAAAGTTCAGCTCCCGCAGGCGGTGGGTCAACTGCTCCAGCACCGCCTGTTCGGGCAGTTGCTCTTCA
CCGATTTCCAGGGTCAGGCGCGGGCCCAGGTTGGCATGTTGGCGTAGCAGGTCGAAGATTCGGTTCAGGGCTTGCG
GGTCTGCCAAGGTCGCCGCAGACAGGTTCAGGGCCAGCGAGTGCTCGTGGCTGGCCATTTGTTTGAGTACCAGCTC
CAACATCACCCGATCCAGGCGCGCCGACCAGCCAAACCGCTCAAGCCAAGGCAGGAAACGGCCGGCCGGGATCGTG
TGGCCATGACCGTCGACCAAGCGCGAGAGCACTTTGTAATGCAGGACCAGTTGCGGATCCTGGCTGGCCACCACCG
GTTGGAAGTACAACTCGAAGCGGTGTTGGGTCAGCGCCTGGTCCAGCAGTGTGTGCCAGGCATGATGGTCGTCGCC
GACGCTGGCCGCTGCGCTGTGGTCCAGGCACACCCAACTGACATCGCCCTGGGTTTCGGCCTGGGCCAGCGCCTGG
TCGGCCAGGGTCAACAGGGCTTGTGGCGAATCACCATGATTGAACGGCGCCAGGCCGATACAGGCAACCGGCGAAA
CGTCACTGGCGCCGGTGGCTTGCAGGCTTTGCAGGGTGCTTTCCAGGTTCTGCGCCAGCTGCAACGCCTCTTCACG
CATCAGCCCCGGCGCCAGCACGGCAAACTCGCCACCGCGGATACGGGTGACCAGGTTGTGGGTTTCCGGATACGGC
TCGCACTGGCGCAGCAACTGTTCACCGACCGCCTGCAGCAGTTGGTCGGTGCGTTGGCCGCCGAGGCGCTGGTTGA
GGCCGGCCAGGTCCTTGACCCGCAACACCAGCAGGTAGCCTGAGCTGGTTTCTTCGGGGTTGCTGACCCGCGCATT
GAGTTGCATCTCGAAGTAACGGCGGTTGGCCAGGCCGGTGAGGTTGTCTTGATAGGACTCCACCCGCAGTTTTTCG
CTACGCTCGGCCTGTTCCTGGAACAGGGCCTTGAGCTTTTCCACCATCTGGTTCATCGCCTGCACCACGCGGCGCA
GTTCCGGGGTGCGCGGCAGATCCGGCAGGCTCAGGAATTCGCGGCGGGCAATGGCGTGGGACTGTTTGACCATGTA
GTCCAAAGGCTTGAGTTGCCGGCGCAGCAACAGCGCGCCCAGCACCGCACTCACCGCGCCGCACAGCAATAACCAG
CCGAGGCTGCCCAGGGCGCTCTGCCACAGCTTGGCCAGGGCGAACATCGGATGGCTGACCACCTCGACCCGCGCCG
CCTGCTCCCAGCCACGGCTGACCAGGGCGTCACCACCGGCCGGTTCCAGGCCGATCAGCTTGACGAACCAGTTGGG
CACGCCGTTGTTGTCGGGGATGCCACTGCGTTCGACGATGACCTGGTCGTTGGTCACATCGATCACCCGGATGCTG
GCGTAATAACCACTATCGAAAATCGAGCTGACCAGCAGCTCGACCATCGCCGGGTCGTCGATATTGGGTGTCAGGG
ACAGCGCCAGGGCGGTCGCCGCGTCCTGCGCGTGGGAGCGCAGTTGGTTCACGTACTGGGTACGCGAGCTTTCCAG
GCTGACCATGAAGCTGCCGCTGAAGGCGACCACCAGGAACAGACAGATAGCGATCAACAGCTGTTTGAACAGTGAC

ATCTGAACAGTTACTCCTAGTTGGCCGGCTCGGCTGGGAAGCCTTCCGCACGCATTTTTTTCAACACATCCTGCCA
GCGCGACAGGCGTTTGGTGTCGCCGACCTTTTTGTTGCCCTTGGCGCCCGGCAGGTACAAGCCCTCGGCGTTGAAG
GAGTAGACCGGCAGCAAGTCGGTCCGTTGGCTGGCAGGCTGGATTTCATCGATCAGGCTGTCGAGTACCAGGGGCA
TTGCCTCTGGCGTCGAATAATAGGTCAAGACCATATGGGCGCGATTCTGCCGCAATGCCTTGACGTAGGTAATGCG
CAATTTGTCGCTGGAGACGCCCAGGTGACGCAGGCTGAAATACTTGGCGATGGCATAGTCTTCACAATCACCGGCG
CCTTTCCACAGGGCTTCGATGGGCGTTTCCCAATAATCGACCTGGTTCCACAAGTCGATATCTTCGACATAGCGCA
CGCGCTTGTTGAAGAACAGGTTGACCACCTTGAGCTGCTCCGCCTCGCTGCCTTGTTTTTGCGTGGCCAGCAGTTG
CTGCCAGTCATCAATACGCTGCTGACCCGCACCCAACGGCCCGTAGAGCGCGGTGGCGCGCCGGCTGATCTGGGAG
AAATCCCAATCGGCATGCAGCCCGCCAGGCAATAAGCCAGCCAGCAGCAGCGCTGAAAACAGCCAGCACAGAAACC
GCGAGGGAGTAAAACGTGCCGCCAATGGTTTCAGTCCGTAGAAAGAGACGCAGAATCAATCGATGGTGAAGGGTCG
GTCGGCAAAAAACAATGGCACTGTGCAATCACTCAGTCCCCATTACGCTAAAGCAACCGCGCCTTGTAAGACGCCG
GCAAGCCATAATGTGCGATGAAAAACCGCTCTGTTGAGTCGCTTACAAAGGGAAACCTTGCCTGTTTGACAAGCTT
GCATGCAATCTCTAGTGTTCTTTGGATCCAATTAGCCAAAACCCATTCCAACAAGAAAGGAGGATAGCGTAGTCGT
GACGCACAAGCCGAACCCTTTGAGCAGCATCAAGATCAGTGGGCCCATTCCTGCTCACCTCGCTCGCTCCGTTATT
GAAGAAACACTGCGCAACGCCATACTGGATGGCCGTCTGCCCTGCGGCACCGCCATGCGCCAGCAAGAGCTGGCCA
GCCTGTTCGGGGTCAGCCGGATGCCGGTGCGCGAAGCCCTGCGCCAACTGGAGGCACAGTCGCTGTTGCATGTTGT
GACCCATAAAGGTGCCGTGGTCGCCCCGCTGATTGAAGATAACTCGGCGGAGACTTACGCCCTGCGTATTCTCCTG
GAATCGGAGGCGCTGCGCTTATCGATCCCGTTGCTCAGGCAAAGCGATATCGAGCAGGCCGACGCCTTGATCGACG
CCCTTGAGCGCGAAACCGACTACAGCGAAATCGGCCGGCTTAACCGCCTGTTCCATATGGCGCTGTATGGCAAGGC
CCCCAACCAGCGGCTATTGAACCTGGTGGAGCATGGGCTGAACGAGGAGGAACGCTTCCTGCGGTTCAACCTTGAA
GCCATGGGCCTGGGCGAAACCTCCCAGGAAGACCACCGTGAACTGCTGAACCTGGTTGCCCAGAAAAAGGTCGAGC
AGAGCGTGCTTACCCTGCGTAATCACCTGATGCGCGGGATGGAAGTGATCACTCACTACCTGAACAGCCTTGAACA
GGGCGATAAAGAGCAGCGCTGATTGCTCCCGACTTTGGCCGCCCAGTGCGCGACCTGGATCGCCAGGCGCCGCACA
CGTCCAAAGCCGGCCTCTGCTGCTGTCGCTCTTTGGACCGTTGCGGTAACAAACTCGATATTGACCGCCATGACAC
TTCCGGCATTGCAAATGCAATAGCGGAATACGTGCCAGTCATTCAGCTCAATAATTTAAACTTGCAAACAACAAGG
AAAGTTCCCACATCGAATATAAAGTTCGACTACGAAACACCCCTTCCTGCAAAAAAACCTTATATAACTTAGTAAG
TATTTGAACACTTAACCAAGTGATTACTCTTTAATTGGACTTGGCGCTGCAGTCGAATGCGCAGCCATGGGTTTGG
GCATGGCTGTGGACGGCGTTCAACCCAATAAGACTAAGTAGCTTTGTCACTATTTGTTGCTTGCCGGGCATCCAAG
CCAGCCGGGACATCTCCGTGACTGCGCGACTAAAACCAACCCCAACACACTTGGTTCTAAACAACCCTTTTAATCT
GAGTCGGCATAACGACTTATAGGCATTAACCATGTTTCAACTTCAAGAACTACTCAATCATAAAAAGCTGCAACTA
CAAGAACATCCCATTTTCCTGGAAATCAATTCTTTCGCACAGCTGCAACATTTCATGCAAAACCATGTTTTCGCCG
TCTGGGATTTCATGACGCTGACCAAACGCCTGCAACAGGAACTGACCTGCATGCGCCTGCCCTGGCTGCCCCCAAC
AGACCCGCAGGCCGCGCGACTGATCAACGAAATTGTGCTGGATGAGGAATCCGACCGCCAGCTGGGCGACGGGTAC
GCCAGTCACTTCGAACTGTATCTGGAGGCCATGCGCGAGGTAGGCGCCAGTACCGCCACCATCGAACGCTTCATCG
CGTTGCAGCGCGCGGGTGCAAGCGTCGATAGCGCCCTGGCACAGATCACGATTGATCCTGCGGTTGAACGTTTCGT
CCGGAACACCCTGGACGTTGCCTTGAACGCCCCGACCCATTGTGTAGCCGCTACCTTCCTGCATGGCCGCGAAAGT
GTCATCCCGACGATGTTCAAACGCCTTCTGGAAGGCTGCAATATCGCCCATCGGCAAGCCCCCAGCTTGTGCTACT
ACCTCCAGCGGCATATCGAGCTGGACGCCCAGGATCATCAGCCGGCTGCAGAGCAACTGCTGCGGCGCCTGGTCCA
GGCAGACCCGCTGCACGAACAACAGGCCTGGCATGCCGCTCTGAGCGCCGTGGAGCATCGCATTGCCTTGTGGGAC
GGCCTGCGCCAACACGCAGCAAGCCAGGAGGTGAGCCTGTGAACGCACACTACCGCCCGTTTGCCAGCGACTGGGA
GCATCAGGCGACCGTCCGTACTCGCCCGCGACGCGTACTGGAAGAAGACGACAAGTTTATCTATCCGCTGTGTCGC
CAACCGTTGGTGCTCAGTGCGGGGTTTGTTGAGCACTGCCCACAGTGGCGCGACTTTGTCCTGGTGCAGACGTTCT
ACAAATTCATCAATGACGTGGTGATCTTTGAAACAGAGATTGTCGATAAAACCGCACGCAACATCGCCAAGAACCG
CTTTTCAATCCCCTTCCCTGCGGCCTGCCGCTACGACGCCATGACGGTGGTGGTCGATGAGGACTACCACGCGCTC
GTCGCCCTGGACTTCATGCAGCAAACCCTGGAGCAAACCGGTATCGCGCCACTGGAGCTGCCCGCGGCCATCGAAT
TGAGCCGCGCCATACCCATCGCACAGGCACAGGCGCCGACACATCTACACGATGCCGTAGAACTGATTTGCGTGGC
GATTGCCGAGAATACGGTGACCCATGACGTGGCGGCGTTTGCCAAGGATGACAGCGTCAAACAATCGGTCAGGGGG
TTGATGGCCGACCACCTGGCTGACGAGGGTCGCCATGCCATCTTCTGGGCCAGCCTGGTCAGGCACTACTGGCAAA
CGGCGAGCGATGAAGACCGCTACGGTATCGCCCAGGTCCTGCCGGTGTTCCTGCAACACTATTTGACCAATGACCT
GCAAAGGGACTTCGATCTGCTCTTGATCGAGCATCTGGATATCAGCGCCGATGCCCGTCAAGTCCTGCAGGCGCAG
GTAAGTGCCCTGACCTTTCCCATCACGCCTCAGCACCCGCTGATCAGCAACATCATGGGTTTGCTGCGCCGCAGCG
GGCTGCTGCAAACACCTTGTGTCGCCCAAGCGCTAAGCGCCTATCTGCCCGTACCCGGGAGCCCATCATGAGACGC
CTCGGGATTCGGTTGCTCGGCACCAGTGCGGCCTTGGATGAGCTGAGGCAGGCGCTGGCACCCTATGGCCATGAAC
TCGCCGCACAGGCGCCGGCGATTGACCTGCTGATTGAAGACGGCAGCCAGCCAGTCCCGCAGGCCTTGGCCGGCAT
GGCCGTGATGAGCCTGCGCCTGTCCATCGGCGCCATGAGTGAGTGGGGGCTGCCGGTCCTGCAAATACGTTGCTAT
GACCGCCTCCAGCAATTACTGGCCGTGCTGGACATTGCTCCGCCCTCCTGCGGCAACGGCCAACAGTTGCGACGCC
AGGCGATCGCACGCCTAATCGAGTGGGTCGCGCTGCAGGTCAGCGGCTTTTCGCGCAACCCTGGGCACTTTGCGCA

GCGTGCGAGCGCTTCCACGTTTCCAGAGCAAAGCCTCGAAGGTCTGGACGGGCTGGCCTACCTGCATCGTTTTAAC
CGAACCGATGATCCGGGGCTGATGCAAAAAGCCCGGACACCACTGATCAAGCAACTGGAGCATGGCTTACGGACCT
TTGCCGACAGGCCGGCCCTGGAGATCGCCGGCCTCACCCTGAGTTATCGCCAATTGTTGCGACACAGCCTGGCCAT
CCAGCAGGTTTTGCAGCCGCTGCTCGGGCCTGGGCAGGCGCCCGTGGTCGGCATTTGCCTGGACAAGTCGGCTGGG
TTGTACGCCAGCATTCTGGCGACCCTCGGTTGCGGCGCGGTGTATCTGCCGCTGGAACCGGGCCACCCGCTGCCGC
GTCAACAAGCCATGCTGGAGAATGCCGGGGCCGTCGTGCTACTCGATGATGGCCAGCATCCCTTGCGCAGGCATTT
CATGGCTGTGGATGTCAGCCGCATCGAAAGCCAGCACATGGACGACAGCGCGCCCTTGGCACTTACCTCTCCCACG
GCTGATAGCGCCAGCATGGTGCTTTACACCTCGGGCACCAGCGGCCAACCCAAGGGGGTTTTGCTCAGCCAGCAGA
ACCTGGCCCATTTCTCTGCGTGGTACAGCCAGTATGTTGAGCTCACTGAGCACAGTCGCGTCCTGCAGTTTTCGTC
CCTGAGCTTTGACTCGTCACTGGTGGACCTGTTTGCGGCCCTATGCCAAGGCGCGACCCTGATCGTGCCCAGTGAA
GAACAGCGTCGCGACCCTCACCAATTGGTTGAGTTGATCCAGCAACAACGCGTGTCACACGCCTTCCTGCCGCCAG
CCCTGTTGAGCGTGCTGCCACTGGATCGCCCGCTGGGCCTGGAGCACCTGCTGACCGGCGGTGACATCTGCGAGCC
CCATGTCATCGCGCAGATGGCCGGGCAATGCGTCCTGCATAACCTGTATGGCCCTACAGAGGCGACGGTGCTGGTC
AGTCGCCGTCGAATGCAGGCTGATGACAGCAATCTGAACCTCGGCACGCCCATCGCCAACAGCCAGGTGCTGATCC
TCGACGAGCAGCGTCGGCCAGTCGAAGATCAGGTGGTGGGCGAACTGTACATTGTCGGCCCCGGGGTGAGCCTTGG
TTACGTGAACCAGCCACAACAGACCGCCGAGTGCTTTGTGCGCCTTGTGCTGCCTGGCGGCCAGGCCTTGCGCGCC
TACCGCACGGGCGACATGGCGAAATGGACGGCAAAGGGCATCGAACTGGTCGGGCGCCGGGACCTGCAGGTGAAGA
TCCGTGGTTTTCGGGTCGAGCCCCAGGAGATTGAGCAGTGCTTGCGTGCCAGCCAGCTTTTTCGCCAGGTTGCGGT
GGTGATTGACCGTGATCGACGAATCCTCGCGTTCGTGGCGCACGCAGATGGCAGTGACTCAGGTACGGCCCTGGCC
GCGTTAAAACAACATGCCGGGCAACGGCTGCCGGACTATATGCAGCCAACCGTGTGTACAGAGTTGCCGAGCCTGC
CTTATGGCAGCAATGGCAAGGTCGATCGCCAGGCCTTGCTGGCGCTGGCGGTTCAAACTGTCTCGCAGCCTCCTCA
GCGCCGCCCCCAAACACCGGTGGAAGGGCAATTGCTGGACCTGTGGAGTGAGCTGCTGAGATTACCGGCCGACGAG
ATATCGACTGACGACAGTTTTTTTCATCTCGGCGGCCACTCCATCCTGCTATCAACCATGCTCCTGCGCATTCGTG
AGTTGTACGGGCGCAGCCTGCCTCTCAATCGGTTTATCGAGGCGCCCACGGTGCAGACGCTCGCGGCGCTGATGGG
TGACAGTGCACCAGGTGCCGCTCCCACCGAACGGGCGATCAGCGATGCGCTGCGCACGCTGGATCTGGCGCTGTTA
CCAGCAGAGCGTGCCGGTGATCGTCACAAGGCTATCGTGACCGGCGCCAATAGTTTCCTGGGTATACATATCGTCG
AGGCCTTGCTGATGGCCGGAGCGACCGAAGTCGCCTGCCTGGTGCGGGAACACCCAGGGCAATCGGCAACTGCACG
CTTTGCCGAAGCCTTGCGTGAGTACCGCCTGGAACACCTGGACCTGAGCCGGGTGCGTGTATACACCACCGACCTG
CGCCAACCGCGCATGGGGCTAGAGGCGCCGGTCTATGAGTACCTCGCCCGCCATTACGGGGTGCTGGTACACAGCG
CTGCCCACCTCAATCACGTCATGGACTACGCGTCACTGGCCAAGGACAACGTGGAACCGGTGCTTGAATGCCTGCG
CCTGTGCGAAACCCACTGTAAGAAAGTGCTCAACTTCGTGTCCACGTTATCTGCGTCAAGCAGCGTCGGGGCCGAT
GGGCAGGTGCTTGAAGCGCCTGCGGCAAGTACGCCACCGCTCTACATCAAGAACGGCTACAACCTGTCCAAATGGG
TCGCTGAACGTCTGTTGGGGCGTGCCGCCGTACAAGGTGCCTGGGTCAATATCTACCGCCCCGGGAACATCAGTTT
CAATAGCCGCAATGGCGTCTGCCAACCCCACAAGAACCGCCTGTTGCTGATGCTCAAGGGATCGCTGCAGTTGGGC
CGGGTGCCGCGCCTGGGATTGAACTTCGACCTGATGCCGGTGGATTTCCTGGCACGTTTCATCGCCTTCCACAGCG
GCAATTATGTTGCCCAGCGCAGCGTCTTCAACCTGCACAACCCGCAACCCTTGAAGTGGGAAACCTATGTCGAGTC
GTTTCGCCAGGCAGGGCATGCGTTCGAGTTGGTGAGCGTAGCGGATTGGCAACACCATCTGCGCGAAGTGGATCGC
GAGAATGCCCTTTTCGGTGTACTGGGGTTCTACCTCAATGGGCTGGGAGAAGACACGGGCGACATCTCCATGATCC
GCCATGAGAACGCACGCCATGGTGTTGAACAGATGGGCGCGCAGTACCCCGAAAAAGAACCTGCGCTGTTACGCAG
AGGGTGCCAGTACCTCAATGCCATTGGCTTTCTGTGAGCCTCGCCATCAGGAAAAACTTATGAGACAGCTGCAACC
CGATACGTTGATTAAAAACCCCCAGGGCAAGCCTCTGGTGTCATCTGTGGTGATTGCCTGCAATGCTTCCAAGCTG
TGGAAGGTTGTGGGGCGCTTTGAGGGTTTTGACGCGTTCATTCCTGCCCTGGCACGCATCACGATGACGGGCAGCG
GCGTGGGATCGCTACGCAAGAAATACTTCCACGATGGCAATATCGCCGTGGAACAGCTCAATAGCCGCGATGAGCT
GGCGATGCATATGACCTGGACGACAATCTATAACACCTTGGGCGTGGCCCGACTGTGGGCGGCAATGAGTGTTGAG
TCGCTAGGGGAGAACCTGGCTCGGGCGACCTGGACCATTATCGCCGAGCCTGTCACTGCCGGTGATAACGAGGGCT
TCGAACAGTTCATCCAGGATTTCGCCGACCGCGCACTGAATAACGTTTGCCATTTGCTTGGTTAAAAAAATGGCGC
AGTGCACTGCTGCACTACGCCATGTTTATCGTGAGGTGATCAGATCTTGAAACTGTCGACCAACTGCTTCAAGCGA
TTGGCTTGTTGTGACAGCGCGTCGCAGTCTTTCAAGGTCTCGTTGAGGTTGGCCACGCCTTGCTGGTTAAGCAGGT
TGATCTGGTTGATATCGACATTGAGCGTCTCCACCACGGCGGTCTGTTCCTCAGTGGCTGCCGCCACCGACTGGTT
CATGCCATCGATCTCGACGATCCGCTGAGTCACACTGATCAAGCGTTCACCCGCCTGGTTGGCGACCTCGACGCTT
TCTTCACTGGAGACCTGGCTGGCGTTCATCGTGGTGACCGCTTCGCGAGAGCCGATCTGCAATGAGGTGATCATCT
TGTGGATCTCCTCCGCCGACTCCTGGGTACGGTGAGCCAGGTTGCGTACTTCGTCGGCCACCACGGCAAACCCACG
GCCGGCTTCACCGGCACGGGCAGCTTCAATGGCAGCGTTGAGCGCCAGCAGGTTGGTTTGCTGCGAGATGCCTTTG
ATCACATCGAGAATATGCCCGATGTTGTCGGTGCTGGCATTCAGGGTTTCAATCTGCGTACAGGACAGGCTGATCT
TCTGCGACAACTCGGACATCGCCAGGATGGTTTTCTCTACGACCTGGCGACCGTCATCGGCTTGCTCGCTGGCGCC
GCTGGCATGTTGCGAGGCGTCAGCGGCGTTACGGGCGATTTCCTGAGTGGCGGCACCCAGTTCGTTAATCGCAGCG
GCAACGCTGTTGGTGCGGGCGCTCTGCTCGTCGGAGCCGATAATCGAGGCGTTGGACGATGCCATCACGCGCTGGG

ACAGATCGTGCACATGCCTTGTCGCGGAAGACACTTCGCTGATCGAGGCGTGGATACGCTCGACAAACTGGTTGAA
GGCGCCACCCAGCTCGCCGAACTCGTCCTTGCTTTCTACGACCAGGCGACGAGTCAGGTCGCCCTCGCCCTGGGCA
ATGTCTTGCATCGCACGGCCCATGGTTGTCAGTGGGCGCATCAGTACCTGAATCAACAGGCTCAGCAATACCGCAA
TCGCGGTGACGGCAATGAACATCGCGATCAAGGCGGAGGTGCGGAACTTGCTCAACGGGGCGTAGGCTTTGTCTTT
GTCGATCGACAGGCCTATGTACCAATCTACGCCCGGCAAGCCGCTGATCGGGGTGAAGGACAGGATACGGTCCTGA
CCATTGAGGATCACATCCTGGTTAACCTTCTCAATACGGACGCTGCTGCCTGGGTAGATGTCCTTGAGGTTTTTCA
TCACCTGTTCCTGGTCAGGGCTGACGATCACCTGCCCGTCGCCACTAACCAGGAATGCATGACCAATACCGCCGAA
GTCGACCGAGTTGATGATCTTCACCAGGGTTTGCAGGCTCAAATCCCCCCCTACGACACCAAGCAGCTCACCATTC
TTCTTGACCGGCATCGCGATGGTGACGATCAAGCCACCGACAGCGGCCATATAAGGCGGCGTCAGCATGGGTTTGT
CAGCCATGACCGCTTGCTTGTACCAGGGGCGCTGACGTGGATCGTAGCCATCTGGCATTTTCGCGTCAGGGCGCTG
GGTGAAGACGCCGTTGGTTGAACCGACATACGTGAACTGGAAGTTCGAGGTGAGTGCCGGTTGATCAACCAAGCCA
GGAAGGTCGGCATTGCTACCTTGGTGAGCAACGTTCTGCGCAAGATTTTCCAGGACTAGCACCCGACCACCGAGCC
AGTTCTGCACGCTGCTGGCGGTCAGGTCACCCGACTGCTGGATGGATGATTCCAGGTTTTGCCTGATGGTATTGCG
CTGCAGGTAGTCGTTGTACAACGTGAACAGCGCGAAGGCCAGAACCACGACGCCTGACGCTGCCAACAGAATTTTA
TGACTGAACTTGAGATTCATTTCATCGACTTCTTTTTGCCAAAAGGGGGTGAGCGTGCCGAATGCAACATTCCATG
TACGGTATAGGCGAACTCTACGACCGCTCTATTTCGGTGCACGCATGGCTCATCAGGCTTTCGGCCAAGACTGGAT
AAATCTTAGGAATTTGTGGGATAGATCTTCTTTTTAAGTAGGAAACCGACAGGCGGTCTGGATAGGGCGCTCAGCC
CTAAAACGACAAAACCCCCGCCTGCGTTAGCAGACGGGGGCTTCGGAATTTGATCTTGACGATGACCTACTCTCAC
ATGGGGAAACCCCACACTACCATCGGCGATGCATCGTTTCACTGCTNNNNNNNNNNCAAGCCATCGAAAAAGACGT
CAGGGGAACCTTCGATTATAGGGTGGGTGCCATGCCCCGACATTGGGCATGCCGTAGGGTCCGTAACGCGGGCGGC
GGGTTTTCCACTCATATGGCTTTTCCAACAGGAGACAATGTGCGCCTGTTGATAACCGTTTTTTTCGCCTCCCTCT
ATCATCTAACTCAGTTTGAGAAGTCGGAAATTTCCCAACTTCAGACCGAGCTATCCTGTGGAAGATGTATTGATGA
ACTAACCAGCCGGTATATCCCTCCCAGCCCTCAGCAGCAGATTCAACTCATCCATTACTTGCGCCCAATCCGCATC
TTCCAGAATTTCTTCACGCAAAAACGCCTGTTGGCTATCCGTCCAGAAAAACGCATCTTCAAGTTTCAACTCCGCC
TTGAGCGGCGAATGCACGGCAACAAAACGCTCAATGCTGAGGGCGTCATCGGGCAACCCAAGCTGTTTGAACAATG
ACGGCAGACTGTGAACAGGCGATTCCATGACAACTCCAAAACCAGGTAATTGCCCACAGTCTAGACCGATAAAACT
CAGCGAATGGCTGCCACCGGTGCAACATCTTGCAATGTTCACGCTATCATCTTCACCTTTGCCCAGCGGCATTCGC
GGACTCCCGGAATAAGGACAATTCATGCGCATCGGTCTTCTGTCATCCCTGGCCCTGGCACTGTGTGCCGGCGTTT
CCCTGCAGGCCCAGGCCAGTGGCGACGACTCCTGCTACCCCGACTGGCGCGTCTCCCAGGATTCGCTGACCGCCTG
TAGCAACATGCCTTTTCTGAGCCCCGGCAATGACAGCCGGACCAACCTGCGCCTATTGCTCGCGGCGAAAAAGAAC
GCGCCCCTGACCCCCAATGCACTGGGCGAAGATGACCTGGCCCAGGGCTTTGGCCCGGTCCCGTTCCCGGTGTATC
GCCTGGTGCCCCGCCCTCCCGAGGGGCAGGCGCCTGAGGAACAAGGCGCGGAGCTTGCGCAGCTGCTGGCCTCGCT
GGGCATCCAGCGCGAGGCCACGGCAGCGGCAGGCGATCAGTTTTTGTGGGGCGAAGGCAGCCGCTGTCGCAGCAAC
AATGTGCAGAGCGCCACCGAATTTGTACGCCAAGTGATCAAGGCCGATGTGCCGACGAATGAGCGGGTGCTGCTGG
CCAATGCACGCCTGAAATTGCTTGGCACCTGCGACTGGCAGGGCGAAGTGCTGCTCGAACCCCAGTTGATTGCATC
CACCCAGGGCCGTGAACTGACCACCTACCTGCTTGCCGCTGCCGACTTTTACAGCGGGCGCTTTGTGTATGCCGAG
CGTGGGTTTGCCGCCGTCACCGTGACCACGTTGCCATGGCTGAAGGAAACGGCGGTGTACATGATCGCCCGCGCGT
CGCTCAACCATGCCCAGGAAAACGCCTTCGATGAATACGGCCTGCCCCAGCGCCAGGCTGTGGATAAGTTGGCCCT
GGATGAAGCCGAGCACAGTTTTCTCAGCTACCTGAAGACTTATCCACAGGGCGACTACGCGGCCTCCGCTCGCGGC
CTGCTGCGTCGGGTCCATTGGCTGGCCGGCGATGCGGCCAAGCTGGCCGACGACTACGCCTGGCAACTGACCGAGG
CCAGCGATGCCGAGCGCAATGGGTCTATCGATGAACTGGTGGAAGAAACCGACAACAAGCTGCTGACCAGCTACGC
CGAGACCATCCGCAACCCGCTGTTGCTGGCGATCAACGATCTGATGTGGATGCGCGCCAACAACCCGCCCAAGCTG
ACCCGCGCCACCCTCGACGCCCAGAAGGCTGCATTTGCCAACGAGCCGGCGTTGTACGACTACCTGCAGGCGGCGT
TTGCCCTGTATGTCGAGCACCAGCCGGACACGGCGCTGAAACATCTGCCACCAGCGCTGCCAGCGAGCCTGGATTA
CTTGGCATTCAGCCAGCAAACCCTGCGCGGCCTGGCCCTGGAAGCCAAGAACGACTGGAAAGGCGCAGAAACGCTG
TGGCTGCAACTGCTGCCACTGGCCAAGCAACCGTTGCAGCGCGAGCAACTGGAATTGGCCCTGGCGATGAACTACG
AACGCAGCAGCCAATTGGCCAAGGTGTTCGCCGCCGACTCGCCGATCCAGACTGCGCAAGTGCGCTATATCCTGCT
ACGCAGCGTCGCCGGCCCCGAGTTGCTGCGCCAGCAGATCAGCCAGGCCAGCGACCCGCTTGAGCGCCGTACCGCG
CAATTTGTATTGCTTTACAAAGACCTGTTGCGCGGCCAGTTCGCGACTTTTGCCGAGGATTTGCAGCAACTGCCGG
CCTCGCCCGCCGCAGATAAAATCGGCACTAGCCTGGGCTATGTGTACGACGGTGGCCAGGCGCTGACCTTGTTCCG
CTGGAGCGGCGACAAGGCGCCGTCCGGCTACACCTGCCCGACCATCGCGCAAACCGCCAGCACCTTGCAAAACGAC
GCGAAAAACCCACACGCCCTCAACTGCCTGGGTGAGTTCATCCTGCGCAACGGCCTGGACGGCATGCCCCTGGAAG
AGGCGCGCTCGGCCGGCAGCCTGGGCAGTACCGCATCGGGTTTCAGCGGTGAAACCTTCTCGCGGCTCGATGGCTA
CAAACAGGTGATCGCCAACCCCAAGGCGCCCCGGGACGACAAGGCTTATGCGCTATTCCGGGCGATCAACTGCTAT
GCACCTTCAGGCTATAACAGCTGCGGTGGCGAAGACGTCGACAAAGCGGTGCGCAAAGGCTGGTTCCGCCAGTTGA
AAACCGGGTTTGCCGACACGCAGTGGGGCAAGTCGCTGCAGTACTACTGGTGAAAAACCTGTGGCTAGGCTTGCTG
CTGTTGGCGAGCCCGGCCTTCGCCACCGTCGACGCCCACGACTATGACGCTTTCTGGTTGTGGAGCGGCGTCGCCC

CACAGCCGGTGCTCAAGCAGGCCAAGACCCTGTACATCCTCCAGGGCCAGATCAACTCGACCCGCCGCCAACCCAG
CCTTGGCGTGCAGTTCATCGCCCAGGGCATGAGCGTGCCGCGCATCCGCCAGGGTGAGGTGTGGGTGGTCTACCGC
GCCCACACCCTGCGTTGGCCAGAGCCGGTCTACACGAAATTGCTCGGGCAGGTGCAACGTTGGCGTGCAGCGGGTA
ACCCGGTGGTGGGCATCCAGATCGACTTCGATGCCCGCACCCAATACCTGCACGAATACGCCGACTTCCTCAAGGA
CCTGCGCCAGCGCCTGCCAAGGGATTTGCGCCTGAGCATCACCGGCCTGATGGACTGGAGCAGCAACGCCGACCCG
GCCGCCATCGCCCAGCTCAACGGCGTAGTGGATGAAGTGGTGGTGCAGACCTACCAGGGGCGCCATAGCATCCCGG
ACTACGCGGCGTACCTGCCACGGATGAACCGGCTGGGGCTGCCGTTCAAGATCGGCTTGATCCAGGGCGGGGATTG
GCAGGAGCCGGAGTATTTGCAGGAGAGTGAGTGGTTTCGCGGGTATGTGGTGTTTTTGCAGAATCCACCAGATCCT
TGACACCTGCGCAAAATCCATGCAATAGATTGTCCACAGTCCATGGACAGTGGACGGAGAGGACTGGATCAAAATG
AAAAGTCAAGACATCGTAATCCTCTTTAAGTTGATCAGCCTAAACCTCCAAGCCGAAGGAGAGCGGGTCAAAAAGC
GCAATTTCACTGTGAATCTTGATCTTGAAGAGGCCATGGATCTGGTGCCGATTGATTCCCAGGATTATCTGGATCG
CTTCCTTGACCTGCGCATGCAGGGGAATATCGAAAAAATCGACCTCTCACAGGGCCAGCATTGGGAGGGATGGGAA
GATATTGATGAGGATTCGATCGCACCGAATATGGAAAGCTATTCCGTACGAGCGCTCGCAGCTTCGCTGGCGTTAA
GTAAAAGTGAGGTTTCCAACTCACTGAATCGCTGCCGTGACATTGGCCTTATTCATGCAGACCGCCTGAGCGGCCA
GCCGATTGTCAGGGCCGTGGCTCTTCTGGACTTTGTAAAATATGGTGTGCGTTATGTGTTCCCTGCCAAGCCAGGT
CCTATCGTGCGAGGCATCCCCACAGCATTTGCCGCTCCGATTATGGCCGGCAAAGTCATGACTGGCGGCGACCTCA
TTCCCGTATGGCCAGACGCTTACGGAAAGAACAAAGGTCAGGAAATCACCCCCTTGTATAAAACGGTTCCTGGAGC
CGTGAAAAAAGACGAACTGCTTTATCATTTTCTAGCTCTCGTAGACGCCATTCGTATTGGAGGCCCGCGAGAAACC
AAGGTCGCAGATGCCATGCTGCGAGAGTGGATCCTGTGAACTCAACCAAAGCAATGATGCTGCTGATGCTTGAAGA
GGTCGCCCAAGCGCTGGGCGAAGAGCTGAGGAGCCAGGTGGCCTTTGTCGGGGGATGTACAACCGTCCTGCTGATC
ACCGATGAATACACTCAAGAATCAATTCGCGGCACCGACGATGTGGACCTGGTCATTCAACTGAGCAGCACTGGCG
AGTGGTATCGACTGGAAGAAAGACTCAAATCCCAAGGTTTCAAGAACACCGGCCAGGACACTGTCACCTGTCGCTT
GCGATTAGGTGCCCTGAAGGTGGACTTCATGCCGACTGACGAAAAGGTACTGGGCTTCTCCAATGCCTGGTTCGTG
GGAGGTCTGGAGAATGCCATCAATCACCAACTGCCAAATGGCACTTGGATCAAGGTGTTTGCGGCACCCTGGTTTC
TTGCGGCGAAGCTGCAGGCGTATCAGGGTCGGGGTCATGGCGATGTGCTGATGTCCAACGATATTGAAGACATCGT
TGCCCTGCTGGATGGCCGCGAAGAACTGCTTGAGGAAACAAAACCGGCGCCTGCTGCATTGCGGCGCTTCGTCAGC
GAACAACTCGCCGCATTGCTGAGCTTCACAGCCTTCCATGATGTCATCCAGAGCACGGCCCAAAACGCCGAGCGCG
AATCGCTGATTCACGAGCGCATCAATAAGCTGATTGCAGATGGGACATACGCTTGAATCTACATTCGAGCGCCACC
CGACAAGGAACCGGAAGAACAGAAAACCGCGATGTCACAGGCTCTGCGTACAACAGCGGCACGTACCTCTACGTGA
TAGCAGACGGCACCTCCAGGCCCGACAGTAACAAGTTGGCAACAGCTCTGACTTGCGGCCTTCTCAAGTCTTTTTC
CAACGCAAGGCCATCAAGCTTATCTTGCCTCGACACAGCCCTTGAATTAACGCTGTCCTCACTCAGCGCAATACGC
TCAACACTCTGCCCTGAATACCCCTTTGCATCAGCCAGTTACCTCGTTTTACTGGTGCTTGGCGATACTGCTATTT
CAATACATGCAGGGGACTGTTGCCTGGGATTGATTGATAAAGAAAAAAACCTGAAGTGGCTGACAGCGCCCCACTG
CGGCCCCAACTGGAAGGGGAACCTGAGCCACGCCTTCATCGCCAACAGCCCGGCCCGCAAAACGCTGCTCAACTGC
ATGAGCCACCGCAGGCCACATGAGCCACAGGTTCAATCCTTGAGAGTTCTGCCTGACACCCGATGGATTTTGGCTA
CCGACGGTTTTTGGGCCGAACTCTCTCCCACACACCAGTTCAAGGCGATAGAAAACTCCAGTCTTGAAGGCTACCC
AGGTGAGGATGATTGCACCTTTATGCTGCTACCTTAGAGGCGGCGCATCCTTCAAGTGCTGAATGACACGCCGCTA
TCGCCGGCAATCCGGCTCCTACAGGGTTGTGCCTTACCAGTCGTAGGTCAGGCTCGAGACCACCGTGCGGCCTTCG
CCGTAGTAGCAGTCCAGGTCACTGGTGCACTGCGACACATAGGTTTTATTGGCAAGGTTCTGCACATTCATCGCCA
GCTTCACGCCTTTGAGCTTGAGGGGCGAGGCGCCCAGGTCGTAGCTCAGGCTGGCATCGTAGACGGTGTAGGACGG
CACCGTGAAGGCGCCGGCAAAATAGTCACCCATGCTGCTGCGCGAGTAGCGGGCACCCAGGCCTGCGCCGAGGCCG
GCCAGGGGGCTGTCGCCGATCACCGTGTAGTTGACCCACAACGCCGCCGTCAGGGGTGAGATGCCCGCCGGGTGGC
GGCCTTCGCGACCTTCGTCGTCCTTGGTGTACTTGATGTCGTTGCGCGCGGCCGAAGCGACCACATCCCAAGCGTC
GCTCAGCACCGCCTTGGCTTCGAACTCGACGCCGCGCGAACGCATGGCCCCGCTCTGGCTGCTGACGTAGCTGAGC
GGATTGGCCGTCAGGATATTTTCCTGGTCCAGTTGGTAGACCGAGACCTGCACAAAACTTTCCTGGCCCGGCGGCT
GGTACTTCACGCCGACCTCATACTGGTTGCCGGTGGACGGGTCGAAGGGCTTCTTGTTGGCATCGAGGCCGGACAG
CGGCAGGAAGGATTCCGAGTAGCTGATAAACGGCGCCAGGCCGTTATCGAACAGGTATACCAGGCCCGCGCGCCCC
GTGAATGCCTGGTCCTTGCTGCTGGTGCGGTCGCCGCTCAGCGGCACCTTGTTGACCACGTTGGCCCAGTCATAAC
GCCCGCCCAGCACCAGCGCCCACTTGTCCCACTTGATCTGGTCCTGCACATACAGGCCGGTCTGGGTGATGGTGTT
GTCCCAGCGGTACGGCTGGCCAAAATTCAGCGGCTGGCCGTAGACCGGCGTGAACAGGTCGATGATCGGCGGATTG
CGGTCGTAGAGGCCGAGGAACTTGGAATTGAAATGGTAGTAGTCCAGGCCCACGATCAGGGTGTGGGAGAACTCGC
CAGTGTTGAACTCGGCCTGGGCGATGTTATCCACGCCGATGACTTTGTTGTTCTGCCGCCAATCCACACCAAAGCG
CTGCAGGTAGCGTTGATCCATGGCGCCGGTGGCCGGGTTGGCGACAAATCGATAGCCGTGCAACGGCGCCACGTAG
CGGTCGTCCACTTCGGCATAGCGCGCGTTTTGCTTGAGGGTCCAGGTGTCGTTCAAACGATGGGACAACTCGTAGC
CGAAGGCGACCTGTTCGCGGTTGTATTTGTTGACGCCTGGCTCACCGATAAACAGATCACGGTCGATCTTGCCGTT
CGGGTTTTTCCAGATCGTCCCGGAGGCCGGCAGCCCCTGGGCTTCGGGCACGCCCCTTGTCTTTCTGATATTGGGCG
AAGAGGGTCAGGCTGGTGTCGTCATTGGGTCGCCACGTCAGGCTGGGCGCGATGAATTGGCGCTTGTTTTCGAAGT

AGTCGATCTCGCCCTGTTCGTCCTTGAGTCGTCCAGTAAGGCGATAGAAGAATTGGCCTTGGTCATCCAACGGGCC
ACTGAGGTCGAGGGCCGCGCTCTTGTGCTCGTAGGTGCCGGCTTCGAGCACCACCTGGCGCACCGGCGTCTCGCTG
GGGCGCTTGCTGACCATATTGACGATGCCGCCCGGCTGGTTGTGCCCGTACAGCACCGATGCCGGGCCCTTGAGCA
CTTCGATGCGCTCCAGGGAATAGGGCTCGATCTGCAACGCACCGCCGGTACTGCCACCGCCGTACGGCAAGTGCAG
GCCGTCGAGGTACAGGGGCGTTGGCGAGAAACCACGGGAGGTCGGCTCATCGAACAGCTTTACCCGGTCGGAAAAA
CCACCGGCGCTCATGCCTGGGGTGTAGAGCAACGCCTGGGTCACGCTTTGCGCACCGCGCGCCTTGATTTCGGCGG
CGGTGATGACGTTGATGGTCTGGGGGATTTCCACCAGTGCCGCATCGGTCTTGCTGCCCGTGGCGCTACGCGTGGC
GACGATGCCGTCGACCGGGCCCCAGGCGTTCTCCTGGGCCTGCTGGGCGCTGATCCGCGTCGCGCCCAATTGCACC
GAGCCGTCCCCCGAAATCGCCTGCACCGACCAGCCACCCGCGGATTGCATCGCCACCAGGCCGCTGCCCGCCAACA
ACTGCTCCAATCCAGGCCCGGTGGCATAGCGACCTTGCAAGCCAGGGCTGCGTTTGCCAGAAGTCAGTGCCGCGTC
CACCGAGAGCAAAATCCCCGAGGCGCTGGCGAAACGGTTCAACGCCTGGTCCAGGCTGCCGGCGGGGATGTTGTAT
TGGCGCAAGGCTTCGCCGGCGCTCTCCTGGGCCTGAACCATGGTCGGGACGACCGGTACAGTCGCCAGCACACTGA
TAAACAGGCCACGGCGCAGGGCGCGGGCCAGAGGTTGGATTGGGTGGTGCGGCATTGCAGGACTCCCCCGTGAGAA
CGCTTCTTGATTGGCTTTCAAGAGGTATCCCGCACGGGATTGGAAAACCGACAAAAGAAAATCAGATTTTTTTCAA
AGACCCTGTAGGAGCCGGCTTGCCGGCGATGGCGGTGTGTCAGGCCCTGGGTTTGATGGTGACCCAGTAACGGGTC
ACCGCCTGTGCCTGCACCGGTAGCGAGCGCTCCAGCGCTGCGAGGATGGCGTCGGTGTCATTCAGGGGAAATACGC
CCGTCAGCAACAGGCCCGACACCGATTCATCGCAACGCAAAACCCCTGGCCGATAGCGGCTCAGTTCAGCGACAAA
GCGCCCTAGCGGCTGGCGCTCGGCGATCAGGCGATTCTGGGTCCAACTGCTGGCGTTGGCATCCAGCGCGGTCACC
GCCGAGGTCTGGGTTGCGCTGAACCACAGGCTGTCCCCCGCGTTCATCAGCACTGGCACCCCCTGCACCGGGCGCA
CCTGCACGCGCCCTTCATACAGATCAACGCGGCTACCGTCGTCCAGTTCGCGCACGGCAAACCGGGTGCCCAGGGC
CTGGATATCGCCGGCGGCGGTCTCGACGATCAACGGGCGGGATGGGTCGTGGCCGCTGGTCAAAAGGATCTCGCCA
CGAATCAGGCGGATGCGCCGCTCAGTGGCACTGAAGCGCAGGTCGATGGCGCTGTCGCTGTTGAGGTCCAGGTGGG
TGCCGTCGCTCAAGGTCAGGTGGCGAATCTCGCCGGTAGCGGTGCGATGCTCGGCAAAGGCCGCCTGCCAGGGCTG
ACTGCCCTGGACCCAATAACCGCTGCCACCGGCCACCAGCAGCAACCCGAGCAGTTTCAACGCGGCCCGACGCTGG
GGGTCTGGCGCGTCGCGCAACACCGCACGGGCGCTGTCGGCGGGCGCGCCGCCCAAGGTCTGCTGTAACTGCTGCA
AACGCTGCCAGGCGCGGCGATGCTCCGGGTCGGCAGCCTGCCAATGGGCAAAGGCTTGGCGCTGGGCCCCGTCGAA
TTCGCCGCCCCATTGCAGCATTAACCACTCGCTGGCCTGCTCGACCACAGCCGGGGCAATCGGCGCGTCATGGCGG
GCTCTCATTCTTCGTAGGCCACCTGGTAGCAGGCGATGATCGCGCGGGTCATGTACTTCTGCACCGAGCTGATCGT
CACCCCCAGGCGTTCGGCGATCTGCGCGTAGGTCAGGCCTTCGAACTGCGACAACAGGAACGCGCGGCGCACGTTC
TCGGGCATGCGGTCGAGCATGGCGTCGATCTGCATCAGGGTTTCGAGGATCAGGGCGCGGGTCTCCAGTGACGGCG
ACTGCGGCTCGGGCAAGTGCGCGATGCTTTCCAGATAGGCGCGCTCAATGCGCAGGCGCCGCCATTGGTCGATCAC
CAGGTTGCGCGCGATCTGCGTCAGGTAGCTGCGGCTTTCCTTGGCCCCGGGAAAACGCCCGGACACCAACAGGCGC
AGGAACGTGTCCTGGGCCACATCGGCGGCGTGCTCACGGTCGCCCAGGCGCTTGCGCAGCCAGCCTTGCAGCCAGC
CATGATGGTCGCGGTACAGGAGATGAAGCTGTTGCTGGCCGTCAGTCGCGGTGTCCATGAATCGCTCGGATACATT
ATTGAGAGCAATTATCATTACAGCTTGTGTCGCGACTGGCAAAATCTTTGTTTTCAGAGCCCCTGGATTGTGCCCC
GCTCACCACAACAAGCCCATTACCAAACCAACAGGATCTAGCTGGACAACGGGTACACCGACTCCCAGCCTCCACC
CAGTGCCTTGTACAGGCCGACCATGGCCAGGGACACGCCGGTGGAGCTTTCCACCCATTGTTCCTGGGTCGCCAGC
AAAGCGCTCTGCACCGTGAGCACGTTGACGAAATCCACCACCCCTTCGACGTACTGGTGCTGCGCGGTGTCCAGGG
CGATCTGGTTCTGGCGCACCGCTTCGGCGAGGCTGTCGCGGCGCAGTTGGCTGGCGTTGTAGCGGGTCAACTGGTC
GTCGATCTCATGCCAGGCGCGCAGCACGGTCTGCTGGTAAGCCAGAGCCGCTTCCTGCTGCTGGGCCTCGCGCAGG
TCGAGCATGCCTTGCAGGCGGCCACCGTTGAACAGCGGCAGGCTCAGCGACGGGCCGAAGGCAAAGGTGCGTGAAC
CCCAGGAGCCCAGATCCGACAATTGCATGGCCTGGGAACCGAGGCTGCCCGACAGGGTGATACGCGGGTAGAAGTC
ACCCTTGGCCACGCCAATGCTGGCGGTCGCCGCATGCAGGCGTGCTTCGGCCTGGCGGATGTCCGGGCGGCGCTGT
GCCAGTTGCGACGGCAGGCCAATCGCAACCTGGCGCTGGGTCTGGGGCACTGCGCCGTCCTGCGACAGTTGCGCAT
GCAAGGCTTGCGGCGCCTGGCCCATCAACAGGCTCAGGGCGTTGATCAACTGGTCCTGGCGCTGCTGCAAATCCGG
CAGTCGCGCTTCGATGGCGGCCACTTGCGCAGCGGCTTCAGCGACATCCAGGTGGGTCGCCACGCCATCGGCCAAA
CGCAGCTGCGAGAGTTTCAGGCTACGGCGCGCCACGTCGAGGTTTTGCTCGGTGACCGCACGGGTGCTTTGCACCC
CGCGCAGTTGGATGTAGTCCTGGGCGGTCTCGGCCAGCACCGACAGGAGTACGCTGCGGCGGTCGTTTTCGGCCAC
TTGCAGGGTGGCGTCGGCAGCTTCGGTTTCGCGCCTTACCCGGCCCCAGAAATCCAGCTCCCAGGAAGCGGCAAAA
CCGGCGTCCCACAGGTTGAAGGCCGAACGGCCGTTGTTGCCGGACGGGTCGCTCAGGCCATTGGCGCTGTTGCGTT
TGCGGCCGTAGTTGCCATCGGCGCTGACCTCTGGATAGCGCCCGGCGGTCGTCACCTGGCGCACCGCACGGCTCTG
TTGCAGGCGGCTGCTGGCCAGTTTCAAGTCGAGGTTGTCGCTCAGTGCCCGGCGCACCAGGGCCGACAGTTGCCGG
TCGTGGAACACGTCCCACCACTGTTCTTCCAACGGGTCGCTGACCGCGCGGCTGGCGGCCTGGCGGCCCTGGGGCT
CGGCCCATTGCGTCGCCGACTGGGTGTCGGGCCGCTTAAAGTCCGGGCCGACGGTGCAAGCGCCCAGGCTGATCAT
GCTCAGAGTAAGCAGCGCGAGTTTTCTCATTGCTGCGCCACCTCACGTTGCTGCGCACTGGGCTGGGTGTTGACGC
TGGCCTCCACCGACATACCGACCCGCAGGCGATGAATATGCGCCTGCCCTGGCTCCAGGAGGATTTTCACCGGAAT
ACGCTGCACCACCTTGGTGAAGTTGCCGGTGGCGTTGTCTGGCTTGACCGAGGCGAAGGTCACGCCCGTGGCCGGC

GCCAGGCTTTCCAGGTGGCCCTGGAGCACTTCACCGTCGAGGCTGTCGACCCGCACTTCAACCGTTTGGCCGGCAT
GCATGTGGGACAGTTGGTTTTCCTGGAAATTGGCCACCACATACGCGTCGGCCAGCGGCACCACGGCGAGGATCTT
GCTGCCGGGTGTGACAAAGGCCCCGACCCGCACCGCACGCTCGCCGACCATGCCGTCCACCGGCGCGACGATGCGT
GTGTACGACAGCTGATAGCTGGCCATTTCCAGCGCGGCCTGGGCCCGCTTCAAACCGCCTTCAGCTGCATCGCGCT
GGGCCGTGAGGATTTCCACCTGCTTGCGTTCGGCGGCCAGTACCGCTGTGGCGTTGGCCAGGCGCGCATTGGCCTG
GTCGATGCGGGTCTTGGCTTGCTGGGCGTTCTGCACAGTGCCGGCGCCGACACCGGCAAGGTGGTTGTAGCGGCTC
AATTCGTGTTCGGCAAAGGCCACTTCGGCGCGATCGGCGGCCACGGTGGCCTGGGCCTGGGCGATCACCGAGCTTT
GCCGCTCAAGGGTGGCTACTGCGTTTTTCAGTTGGGCCTGGGCCATCAGGGTCTCGGCATCGGCTGCCTGGGCGGC
GGCGCGCAAGTCGCGGTCATCGATCAGCGCCAGCAACTGCCCGGCCTTGACCTGTTGGTTGTCCTCTACCAGCACC
TCTTTGATAAACCCGGCCACCCGTGGCGCCACCAGGGTGTAGTCGGCAGCGACGAAGGCGTCGTTGGTGGTCTGGC
GCTTGCTGCCAAACACGCCCGGCGCCAGCAGGTACACCAGCACGCCCACGGCGCACACGGCGATAACAGTCACGGC
AATCTGGTCTTTACGTTTCATGGGAATCCTTTGTTTCAGTCAACAATCAGGCGGGTGCGCGCGGCGGGAAAATCCG
CGTCGGCATCCAGAAAATCAGCAGGATCAACGCCACCGCGACACCGGCCATGACGTAATAGAGATCCGAAGACGTC
AGCACCACGGCCTGCTGGTGCAGCCGATGGGCCAGGCCCGTGGCATCGCCGTCGGCCAGGGGTGAGTTACCCAGGG
CGTCCACCAGCATGGTCGAGTGGAAATGCAGGCGGTGGGTGGTCAGTACCTCGATCACGCCGGTGGCGACCACGGC
GGCCAGGCCCTTGACCGTGTTGAACCAGGCCGAGGCAAACGGCCCGTCGGTGGGCACGATGCTGCCGGTAGACAGC
ATCAACAGCGGCAACACCGCCATCGGCTGCCCGAAAATCTGCAGCAGTTGCAGCACGTAGAACTCGTCCCGGATCC
ACGCCGAGGTCAGTTGCGCGCCACCGATACACGAGAGGGTCAGCATGCCCAGGCCAATCCCCAGCACCCAGCGGCA
ATCCACCCAGCGCAGGTTGCACAGGGCCGCTACCAGCGGCAGGGCGATCAACTGCGGCAGGGCCATGACCAGCATT
ACCGGCGCGGTCTGCAGCGGCCGATAGCCCTGCACCTGCGCCAGGTAACTGGACGGCAGGATGATCACCGCCTGCA
GCACCACCAGCACCCCGGCCAGCACGATCAAGGCAAACGACAGGTTGCGCAGGCCGAGCATCTGCAACTTGAAGAA
CGGCATCGGGTGTGACCATTCATTGATCATGAACAGCACCAGCAACAGCACCCCGCCCACCAGCAGCGTGGTGATC
AGCGGCGACTCGAACCAGTCCAGACGATTGCCCTGCAAGATGCCGATTACCAGCATGCAGATCGCCGGGAACCCCA
GCAGCAGGCCGCGCCAGTTGAATTGCTTGAGGCGCTCCAGGCGCAACGGATCCTGGGGCAGGCCGTAGGCCACGGC
GGCCATCGCCAGCAGGCACGGCGCGACGATCTGCCAGAACGCCCACTGCCAACCGACATATTCGGTCCACAGCGCC
GCCAGCGGTGTGCCGAGGCTGGGGCCGAAGGTAGCGGTCAGGGCGTAGCCGGCCAGGCCGTAGAGTTTGACGTTGG
CCGGCAAAAAGCGCAGGGCCACGGTCATCAGCATCGGCGGTAGAGCGCCACCGGCCAGGCCTTGCAAGGTACGCAG
CAGCAGCAGGCTTTCGTAGTTCGGTGCCAAGGGGCACAGCACCCCCAGCAGGGTAAACAGGCCGATGGCCCACAGG
GTGAAGCGGCGCAGCGAAAACGTCACCGAACACCACGGCGCAAAGGCCATGGCCGAGACGGAGGTGGCCGTGTAGG
CGGCAACCAGCCAGGTGCCTTCGTCAAACCCGATGTACAGCGCACCGCGGATATCGGCGAGGGCGACCTTGGTCAC
CATTTCGTTCAGGCCCGAAACCAGAACCGCCAGCAGCACGCCGACCAGGCCGATGATGATCCGCGGGCCAAACACA
GGGGGGCTGACGGCGGCGGGTTTGGCTGCAGCCAAGGGAGCGGGGGCAGCGAGGGAAGTCATGAAGGTACAACTCG
GAAATAGAATACCCGAGCAGTTTAATAAGGCTTAGACATGACGAAAACTGACTTATGGGAAGGTTATAACTGCGCC
ACACGCAATGATCGAATGTGGGAGCTGGCTTGCCTGCTCCCACACAAGCTAGGCCAGCTCCACACATTTGGATCGA
AGTTGTCAGGTGGGCTGGGCTTCGCGCCAGGTGGCCGCGCAGCTTTCGCGCATGGTCTCACGCAGCCATTTGTGCG
CCGGGTCTTTGTCGAAACGCGGGTGCCAGGCCTGGGTCAGCACCAGGGTCGGCAGGGAGATCGGCAAGGTGAATGC
GCGCAGCGGCAGTTTCAGGCGATGGGCGCTGTACAGCGCTTCCTTGGGCACCGGCAGGATCAGGTCGGAGTCGGGC
AGCATGAACATCGCGCCGTGAAACCCCGGCGCGATCATCGCCACCCGGCGTTCCAGGCCCTGGGCATTGAGGGCGG
TGTCGATCGGCCCACGGGCAATACCGCGCCGGGAAATGCTGATATGGGAGTAGCTGGCAAAGCGTGCGGCGGTGAT
TTCTTCGTCGAACAGCGGGTGGCCTTCGCGGGCCAGGCCGACGAAGGTTGTGGAGAACAGGTTCTGCACCTTTACT
TCCGGGGTGATCGGCATGGTGTTGCCGACGCGCAAGTCCAGGCGCCCTTCGCGCAGGGCTTCGTCGTCGCTGTCGC
CTTCGGGGACGAACCGCAGCTCGCAATGGGGTGCCATCCGCTCCATGGTGTCGAACAACCGCCCGCCATACACGCC
GACAAAAAAGTCATTGGCGCGCACGCTGAAGCGCCGGCGCAAGGTGCTCAGGTCGACTTCATCCGCCGAACGAAAC
AACAACGCCGCCTGCTCCACCACATTGCGTACCTGGCCTTGCAACTCCAGGGCCTTGGGCGTAGGCACCAGGCCGC
GCCCGGCGCGCACCAGGATCGGATCGCCCACCGCTTCGCGGATGCGCGTCAGGGTTCGGCTCATGGCGGCCGGGCT
GAGGTTCATCCGCCGCGCGGCGCCCACCACGCTGCCCTCGTCGAGCAAAGCGTCGAGGGCCACCAACAGGTTCATA
TCCGGTAATTGCATGCCAAGCACTCATGATGGGTTTGGAGTACAGCGCACGCAATCGTAGCAGGCTTTACCGGTAC
TGCCCGGTGGCGAGGGGCTCCAGGCCCCCGCACGGGTTTATCGCTGCATGCCCCAGCGCTTCACGGTGACGCGCTC
CAGGGTGTCGAACACCAGGTTCTCTACGAGCAGGCCGATAAGGATCACCACCGCCAGCCCCGCGAAGACCTTGTCG
GTGTACAGCTCGTTGCGGTTCTGGAAGATGTACCAGCCCAGGCCACCCTTGCCGCTGGTCGCGCCGAACACCAGTT
CAGCGGCGATCAGCGTACGCCAGGCAAACGCCCAGCCAATCTTCAGCCCGGCGAGGATCGACGGCAACGCCGCGGG
AATCAGGATAAACAGCACAAAGCGCATGCCCTTGAGGCCGTAGTTGCGACCGGCCATGCGCAGGGTTTCCGACACG
CCCATGAATCCCGAGTAAGTGTTGAGTGCCAAGGCCCACAGCACCGAGTGCACCAACACAAAAATCAGGCTGTTCT
GCCCCAGGCCAAACCACAGCAACGCCAATGGCAGCAGGGCAATCGCCGGCAGCGGGTTGAACATCGAGGTCAGGGT
GCTCAGCAGGTCACGGCCCATCTGGGTCGAAACCGCCAACGTGGTCAGGGCGAATGCCAGGACAATCCCGATCAGG
TAGCCCTTGAGCAGCACCACCAGCGAGATACTGACCTTACCCAACAGCTCGCCACTGGCAATGCCGTCGTAGAAGG
CGTGGAAGGTCTGCAGGAAACTGGGCAGCAGCAGGTCGTTGTTCTGATAGCGCGCCGCTGCTTCCCAGAGGAGCGC

CAGCACGATCAGGATCAAGCCTTTGCGCAACCAGCCTTGTTGCCACAGGCGCTGGCGCAGGGGTAAGGCACGCTTC
ACGGGCACGCCGGGCAGCGGTTGCAGGGTGATTTCATATTCCTGGCGCATGGGCACAGTCCTTCAATAAGCGATGC
GGATATCGGCGAAATCCAACTCGGTTTCTGCCTCGGGCGTTTCATCGAACAACAGGCGATGAATACGCCGCGCCGA
CGCCTGGAACTCCACACCGCCAAGGCTGTGCAGGTCATATTGATGGCTGTGGATTTCGGCGCGCACACGCCCTGGA
TGGGGCGACAGCAGCAGGATGCGGTTACCCACCACCAAGGCCTCTTCGATGGAGTGGGTGACGAACAGCAAGGTAA
AACGCACCTCCTCCCACAGCAGCAGCAACTCCTCTTGCATCTTGCGCCGGGTCAGGGCGTCGAGTGCGGCGAACGG
TTCGTCCATCAGCAGGATTTTCGGCTGCATGGCCAATGCCCGGGCAATCGCAACGCGAGCCTTCATGCCACCGGAG
AGGGTGTGGGGATAGGCGTCGGCAAAGGCGCTCAAGCCGACCTTGTCGAGGTAGTACAACGCCCGTTCTTCGGCCT
CGCGACGCTTGAGGGTCTTGGACGCCAGCAGCGGGAACATCACGTTCTGCTTGACGGTTTTCCACGGCGGCAACTG
GTCGAACTCCTGGAACACCACGATGCGATCCGGGCCCGGCTGCTCGACCTTCTGCCCTACCAGGCGAATTTCGCCT
TCGCAGGGCTTGATAAAACCGGCTACCGCCTTGAGCAGCGTGGACTTGCCGCAGCCCGAAGGCCCCAGCAGTACAA
AACGGTCGGCCGGATCGATCTCGAAACTGACCTGGTGGGTGGCCCGCACTACCCGTTCGGGCGTGCGGTATTCAAG
GCTGACGTGATCCACCGAGAGCAGCGCCTCAGTGGTGTTCAGGTTGCTGGCCGTGTGGCCTTGCAAGGGTGCGTTC
ATCTCAGCTCCCTTGCAGCGGCTTGGCATCCTGGAAGAAGTAATCCTTCCACGACTCGGGTTTGTTCTTGATCGCG
CCCACGCGGTAGAGGAACTCGGCCAGCGGGTAGGTGTTTTTCGGGGTGACGCTGAATTCGAATTGCGGGTTGTCGA
TAATTTTCAGCAGCGCGGCACGGTCGATCTTGGCCTTGGTCACGCGGATGTAGGTGTCGGCAGCGGCGCCCTTGTC
GTTCTGCGCAAACTGCGCGGCCTCGGTCAGTGCATCGATAAAGGCCTTGTAGGTTTTCGGGTTGTCGTTGCGAAAC
TTCTCGGTGGCGAACAGCACGGTCGGTGAGTTCGGCCCCAACAGGTCATAGGTGTTGAGCACCACATGCACGTTCG
GGTTGGCCAGGGCCTGGTCCTGGAACGGCGGGTTGGAGAAGTGCCCGGTCAGCTCGGTACCGCCGGCGATCAGCGC
AGCCGTGGCGTCCGGGTGCGGCACGGCGATGGTGTATTTATCCAGGCGATTGAATTCCTTGTCGCCCCATTGCTTG
GCCGCCGCGTATTGCAGGAACCGCGACTGCACGGAAACCCCCACGGCCGGTACCGCGATGCGATCTTTTTCGGTGA
AGTCGGCGATGGTCTTGACCTTGGGGTTGTTGCTGACCAGGTAGTAAGGGAAGTTGCCCAGGGAAGCCACGGCCTT
GACGTTCTGCTTGCCATGGGTGCGGTCCCAGATGGTCAGCAGCGGGCCGACACCGGCGCCGGCAATGTCGATGGAG
CCAGACAGCAGCGCGTCATTGACCGCCGCACCTCCGGACAATTGGGTCCAGTCGACCTTGATGTCGATACCTTCCT
GCTTGCCGTATTTTTCGATCAGGTTCTGGTCGCGCACCACATTGAGCAGCAAGTAAACGATGCCGAACTGCTCGGC
AATGCGAATCTCGCCTTCGGCCTGGGCGACGGCGGGAGCAACCAAACTGCCGGCCAACAGGCTGACGCCAAGGCCA
ACGGTCGCGGCCAATCGTGCAAAGGGGGTTTTCCTGGACATGGTCATGCTCCGAATCAGAAGGGGGCGTCGCCCTG
GATGGTGGTGCGAAACAGTTTGCGACGCAGGTGGCTGGGGCAACCGGCGGCCAGGTGGATCAGCGAGCGGTTGTCC
CAGAACACCAGGTCGTGGGGCTGCCAGCTGTGGCGGTAGATGTTGTGCGGGTGGATGCTGTGGGCGAAGAGTTGGG
CCAGCAGGTCGCGGCTTTCGTCTTCGGGCAGGCCGACGATACGGGTGGTGAAGCCTTCGCTGACGAACAGCGCCTG
GCGCCCGTTTTCCGGGTGAGTACGCACTACCGGGTGCACCACTTCGGCGACCTGGGCCAGTTGTTCGGGGGTCAGG
GTCGGGCGCCAGTTGCCTTCGAATTTGGTTTCGCTGTAGCGCGCGGTGTACGAATGCGCAGCGCTGCGACCTTCCA
CGGCCTTGCGCAGATGCTCGGGCAACTGGTCCCAGGCTTTGTGCATGTCGGCAAACAGCGTATCGCCGCCTTCGGA
CGGCAGTTCCTGAGCGTGGAGCATCGAGCCCAGGCTCGGCAGTTCCTTGTATGACAGGTCCGAGTGCCAGAATTTG
CCCGCGTCGCCCAGGCCGATGGACTGGCCGTTTTCGATGATGTTGGAAACGATGAGGATTTCCGGGTGGTTGGCCA
GCAGGAACTGTTTGAGCACATGGATCTGCAACACGCCGAAGCGGCGGCTGAAGGCGATCTGTTGCTCGGGGGTGAT
GCGCTGGTCGCGGAACACCACCACATGGTGGTCCAGGTGCGCACGGTGGATGCGGGCGAAGTCCTGGTCGTTGACC
GGGCGCGACAGATCCAGGCCAATGATCTCGGCGCCTACGCTGCCGGGGAATGGGCGGATGTCGAAGGTTTGGGCGG
TGGATAAGGCGGCTGTGGCGGTGGACATGTGATCACTCCCTGGCAGTCAGGAGAGTGACTTTATAGATATAAGAAT
TGGAATTTAAATACCGTTAGAGAATAACGATATGGCCTTTTTGCGGCAGGGCCTGGGGGATGGGGTGGGGTTGAGG
GCCCCATCGCAGGCAAGCCAGCTCCCACATTGAACGGCGTACGTGGTCCAAAATGTGGGAGCTGGCTTGCCTGCGA
TGAGGCCAGGCCAGGCAATCGAGATCAACGCTCCTGCAGCGACTCGGCCCGGGCCTTGATGATGGGCTTGAGCAGG
TAGCTGAGGATGGTCTTCTTGCCGGTGATGATGTCGACCGAGGCGACCATGCCGGGGATGATGATCAGTGGCTTCT
CATCGGTGCCCAGGTGGCTGCGGTCGGTGCGCAAGGTGATCATGTAGTAGGTGGTTTTCTTGTCTTCATCGGTGAT
GGTGTCGGCACCGATGCGCTCCAGCTTGGCCTTGAGGCCGCCGTAGATGGTGTAGTCGTAGGCGGTGAACTTGACC
ACGGCTTCTTGCCCCGGGTGCAGGAAGGCGATGTCTTGCGGGCGGATCTTGGCTTCTACCAGCAAGGTGTCATCCA
GCGGCACGATTTCAACCATGTCGCTGCCCGGCTGGATCACGCCGCCAATGGTATTGACCAACAGTTGCTTGACGAT
CCCGCGCACCGGCGAGGTGACCAGGGTCCGGCTCACGCGGTCTTCGAGAGCTTTGCCGGTGGCCTGGGCCTTGTTC
AGGTCAGTACGTGCCTCGTTGAGCTCGGTCAGCGCTTCACTGCGGTATTTACCACGCGTTTCGTCGATCTTGCGTT
GTACTTCCTTGATCGCCGAGTCGGCACGGGGGATGGCCAGGGTGGTAGCGTCCAGTTGCCCACGGGTTTCCATTTC
TGCGCGCTTGAGGCGCAGCACTTCCACCGGCGACACCGCACCCTGGGCGACCAGGGGTTCGGACATGTTGATTTCC
TGGCGCTGCAACGACAGTTGGTTACGGTACTGGCCTTGCTTGGAGGTGTATTCGCGCATCTCCTGCTGGCGCTGGG
TCAGTTGTTCCTGCAAACCACCGATTTCATCCTTGAACTGCTGGCGCCGGCTTTCATACAGCGACTGCTCGTTGAT
GGCCTGGGTGGGCGCAGCGGCACGCACGTCTTCAGGAATGTTCAGCGGCCGGTCTTCGACCTGGGCGTTCAAGCGC
TCGATGCGCAGTAACAGCGACAGGCGCTGGGCCTCGGTTTCACCAGCGTTGGAGACAAAGCGCGTGTCATCCAGGC
GGATCAGTGGCGCGCCAGCCTCGACGATCTGCCCTTCCTTGACGTACAGCTCGGCGACGATGCCGCCCTCAAGGTT
CTGGATCTTCTGCAACTTGGAGGATGGAATAGCCTTGCCATCACCGCGCGTCACTTCGTCGACCTTGGCAAAACCG

GCCCACAGCAGCATGCACAGGAAAAACCCGATGATCCCCCAGATGGTCAACCGCACCACACGCGGGGCATCTTCGA
TCAGGGCCTTCTTGACCTCTGGCAGTGGCTGGCCATCCAGCGAATCGGAACCAATGAAATAGCGACGGACCGCGTC
CTTGAATTTACGCAACACTGATCTGCCCCTTCTTCAACGCTTCCATCACTACGGCTTTCGGGCCATCGGCAAGGAT
TTGCCCACGGTCGATGACCAACAAACGGTCCACTAGGGACAACAGCGACGCACGGTGCGTCACCAGCACCACGGTC
TTGTTTTCCACAACGGCTTGCAGGCGCTGTTTCAAGCGTTCTTCACCGGTGTTGTCCATGGCGCTGGTTGGTTCGT
CCAGCAGCAGGATAGGCGGGTTGAGCAACAGGGCGCGGGCCAGGGCGACATTTTGTCGCTGGCCGCCAGAGAGGTT
CTGGCCGCGCTCGCCCACTTGCAGCTCGTAGCCTTGTGGATGCAAGCGGGCAAATTCGTGGACGCCCGCCAGCTCA
GCGGCTTGCAGTACCACTTCGTCTTCAACGTAACGGGCGCCGGAGACCAGGTTGTCGCGCAGGGTGCCGGCCAGCA
GTTGGATGTCCTGGGGCACATAGCCGACGTTGTGGCGCAGTTCGCTGACGTCGATCTGGCGAATGTCCACGCCGTC
CACCAGCAAGGCGCCGGAGTCGGGTTGGTAGAGGCCCACGATCAGTTTGGCCAGGGAGCTTTTACCCGAGCCGCTG
CGCCCGATGATGCCGATCTTCTCGCCGGGCTTGATCACCAGGTTGATGTTCTTGAGTGCCATGTTCTGCTGGTTGG
GGTACGTGAAATTCAGGCCCCGGCACTCGATGGCGCCCTGCAGGGTACGGCGGCTCAAAGGGCGTTCTTCGAAGTT
GCGCTCTTGGGGCAGCTCCATCATCTGGTCGGTGGAGACCATGGTGACCTTGGCTTGCTGGTAGCGGGTCATCAGG
CCCGACAGCGACGCCAGCGGGCTGAGGGCGCGGCCACTGAGCATGTAGCAGGCAATCAGGCCGCCCATGCTGAGAT
TGCCGTCGATGATCTGGTAGACCCCGAAGACAATCATGATCACACCCGCCAATTGCTGGATCAGCAGGGTGATGTT
CATCGACAGCCCAGAGAGCATTTTCACCCGCAATTCAAGGCGGCTGAGGGTGCCAATGGTTTGCTCCCACTGGTAT
TGGCGCTCGCTTTCAGCGTTATTGACCTTGACCGCATCCAGCCCGGCCAGGGTTTCGATCAGGCTCGACTGGCGCT
CGGAGGCCAGGGCCATGGTGCGCTCCATGGTGGCGACCAGCGGCTTTTGCAGCAGGTAGCCGATGCCCAGGGCAAT
CGGGAAGGCCAGCATAGGGATCCATACCAGATGCCCGCCGAGGATCGCGATCACCATGAATATCAGCAGGGTAAAC
GGCAAGTCGATCAGGCTGGTGAGCGTCAGGGAGGCGAGGAAATCACGCAGGCTCTGAAACTCGTGGATGTTCTGGG
CGAAGCTGCCGACCCGTGCCGGACGGTACTTCATCGACATGCCGACGATCCGCTCGAACAACGTGGCCGAGATGAT
CAGGTCGGTTTTCTTGCCGGCCAGGTCCAGGCACAGGCTGCGCAGGCTCTTGAGGACCAGGTCGAAGACGTAGGCG
ATGAAAATACCCGTCGCCAGCACCCACAGGGTGGCGGTGGCCTGGTTGGGCACGACGCGGTCGTAGACGTTCATCA
CGAACAGCGGCGCTGCCATGGCAATCAGGTTGATCAGGAAACTGGCGGCGATGGCATCGGCGTAGAGCCAGCGCGA
ACGCTTGAGGGTGTCACGAAACCAGGAGCGGGCCCGGGGGATCAGGGTGCCGTGGCTGACATCGAATTTGTGTTGG
GGCTGGGCAAAGAACACTTTACCGGTGTAGTCATCGGCCAACGCCTGGCGCTTGACGATGGTTTCACCGCCATCGC
TTTCGCTGAGCAGCACCTGCGCCTCGTCCTCACCCTGCCAGCCGAGCAGCACGGCACTACGGCCATCCTTGAGCAA
CAGCAGCGCCGGCATGGCAATCGGGGGGATCGAATCGAGCTTGCGTTGCAGCAGCCGCCCTTGCAAACCGGCGCGG
GCCGCGGCCCGGGCCAGCAAGTCGGCGGTCAGTTGCTGTGCCGGCAATGGCAGACCGGTGGTCAGCATCGCCGCAC
TGGCGGGCTTCTGGTGCAGGGCGCAGAGAGCCAACAACCCGTCGAGTAACGGGTCGTCGTGCAATGTGCGTGGATC
ATGACTGAGATGGACTCGACTGACTTCTGATTCCACGCGGGCTCTCTCAACTAACAGATGGGACTATAAGGAATAG
CTCAATTCATCCCAGGCAGTTGGACTTTCGGCTTCACGTCGTTCTGGACGACGGAGGCCATCGGTGCGACCACTCC
CTGGCTCTTGAGCAACTCGCCCATGGTCGCCTTGATTCGGTACTGAGTAAATAACTGAACGTTTTTTATCTCTTCC
AAACGACGCGAAGCCGTGAACAGCTCGTTTTCACTGTCGAGCAAGTCAAGCAGAGTTCGTTCGCCCAGGCTGAATT
GTTTCTGGTAGGCGCCGCGCACCTTGCTGCTGTGGTCGACGTATTGCTGGGCAATCGGCACCTGGGCATTGGCGTT
GTTCATGGCGTTCCAGGCCAGGCCCAGTTCTTCGTTCAACTGGCGCAAGGCGTTGTTGCGGATATCCAGGGCCTGG
TTGGACTGGTAGGACTTGGACTCCAGCTCAGCCTTGTTGCTGCCACCGGCGTACAGGTTGAAGCGCATGCGCAGCA
TGGCTTCCCAGCCGTTGTTGTGGCTGGCGTCGCCGTCGATGTTGTTGTCGGCGTTACGGCCCAACTCGGCGTCGAA
ACGTGGGTAGAAGGAAGACTTGGCGGCTTCGTATTGTTTTTCGGCAGCGGCGATGTCGGATTCGGCCGAACGCAGG
ATCGGGCTGTTATCGAGCATCTGGCGACGCGCTTCATTCAGGTCTGCAGGCAGCAGCGCCATGAAGCTGGGCGGAC
GCTCCAACTGATCGGCTTCTTGGCCGACGGCACTGAGGTAGTTGGTCTGGGCGTCGGCCAGGTTGGTCTGCTCGGT
GATCACGTTGTTACGGGCTTGGGCCAAACGGGCTTCAGCCTGGTCCAGGTCAGCCCCGCTGCCGACACCGCGCTGG
GTGCGCAGTTTGATCTGGTCGTAGATGCGCTCGTGGTTACGCAGGTTGTCTTCGGCCAGACGCACGAACTCACGAC
GGGTCAGGACGTCCAGGTAAACCTGGGCTACCGTCAATGCAGTGCGCTCGGAAGTGCCAAGCAACGAAAAAGCGCG
GGAGTTAACGGTGGCTTGTTGACGCCCTACTTCACTGGAGGTGGCAAAACCGTCAAAAACCATTTGCTGCAGGCGC
AGGCTCGATTCGCCGCGATTGAGGGTGTCCCAATGGCCGCCACGGGCGCGAGTGGATGAACTGTCGGTGCCTTCAC
GGCCATAACCCGCGTTCAAGTCGACACGGGGCAGGTAACCACCCTTGGCCGCACGCAACTGATAGTCAGCCGCGAT
ACGAGCGTTGACACCTGCCTGAATCTCAGGGTGCACATCCAGCGCTTGCTGCATGGCCTGGGGCAGGGTTTGGGCT
TGTACGAAACTGGCGGCGAGAACGAACGGTATTGCCTTAAGCAGGTGCAAACGCATTTTAAGAATCCCCAGGACTT
CTTGTCTCAAGTCACAGCAACTTGGTTGTGCTGCGTTAGAAAATGGCTACCGTAATGACCGTACGTCGGAAAGTTC
AAAAGCGGAACTGGTTCACACATGTAGGACGGTTCGCCGCATGAATATCAATGTGACATTAGCGAGGCGATTGTTT
AGGATGGCGCCAAGAAGGTCAATAGTTTGGCATAAAGTAAATCGCCAAAAAATATAGCCAATTATTTGACGTCACT
CGTTTTATAAACTACGCACCTCCCAGGCTGATGAATCCCATCGGGTCTCAGCCTTAGGAACACCCACAGAATGGAT
CGCCGGAGAAATCTTCAATGAGCAGTGTTGTTGCAATCGTCAAAAGCATTGTTGGCCAAGTTTTCGTCATTTCTCC
GGAAGGCGCCCGTCGGGTGCTGGTTGAAGGTGATCGCCTGTTTGCCGGCGATCAGATAGACACCGGCATGTCGGGC
TCCGTGAGCCTGGAGCTGGCCGATGGCCGTACGCTGGACCTGGGGCGTGATACCCAGTGGAGCGCCAACGCGCCCG
ACTCCAGCACTGACCTGGCCCAAGCCACTGCACAGGCCGCACCTTCGGTTGCCGAACTGCAGCAGGCCATTGCCGC

CGGCGCTGACCCGACCACCGACCTGGAAGCCACCGCCGCAGGCGCGACAGCTTCCGGCACGGGCGCTGCCGGTGGC
GGCCACAGCTTTGTGATGCTTGATGAGACCGCAGGCTCGGTCGATCCGACCATTGGGTTTCCGACTGCTGGCCTGG
GTTTCGCAACCAATGCGTTGACCAACGAACTCGGCGGGCAGCCGACTGACCCACTCACGGCCGCACCGTTGCCCTC
GACCCTGACCCTGGGCGCCACACCGACCATCACCGAAGCCGGCGGCGTGCTGACCTACACCGCCACAGTGACCCAG
CCGTCCACCAGCAACCTGACGATCACCCTGTCCAACGGCGCGGTGATCACCATCCCGGCCGGCCAAGTGACCGGCT
CCGTCAATGTGCCTTTGGCGCCCAACGACAGCCCGTACATCGATCCAGGCCAGATCAGCGTCACCGTCACCGGCAC
CACCGGCGGTAACAACCTGATCTTGACCGTCGACCCGACACCGGCAGTCACCCAGATCACCGACACCATCGACACC
ACCACTGTGACCCTGACAGCAGGCGAAACGGTGACCGAAGGCGGCCAGATCACTTACACCGCGACCCTGACCAACC
CGGCACAGACCCCTGTCACCATCACCTTGAGCAACGGCTCGGTGATCACCATTGAGGCAGGCAAGTCCACCGGTAC
GGTGGTGGTCGATACGCCGGCCAACGACGTCTACAACAATGGCAGCACGGTCAGCACCACCATTACCGGGGCAACC
GGTGGCAACTTCGAAAAGCTGGAGCCCGACACCACCCCAGCTGTCACCACGATCACCGACTCGGTGGATGACACTG
GCCTGACCCTCACCGCCACCAATACCGTGACCGAAGGCGGCCAGATCACCTACACCGCGACCCTGACCAACCCGGC
GCAAACCCCGGTTACCGTGACGTTGTCCAACGGTTCGGTGATCACCATCAAGGCCGGCGAATCCGTCGGCACCGTG
GTGGTCGATACCCCGGCGAACGACGTCTACGTCAACGGCAGCTCCGTCACTACGACTATCACCGGGACCACCGGTG
GCAACTTTGAAAACCTGGTGCCCAACACGACGCCAGCCGTCACGACCATCACCGATTCGGTCGACACCACCACCGT
GACACTGACAGCGCCAGGCGATGTCAACGAAGGCGGCCAGATCACCTACACCGCCACGCTTTCCAATAAAGCAGAC
ACCGACGTTACGCTGAAGCTGGATAACGGTTCGACCATCATCATCAAGGCGGGTGAAACCGTTGGCTCCGTCACCG
TCGATGCTCCGGGCGATGATGTGTTTGTCGACAAAAGCACGCAAACCGTGAAGATCGTCGAGACTGATGGCGGTAA
CTTCGAAAAACTGGAAGTGGCCGGTGACGGCGCTACCACCACCGTGAACGACACCATCGACAAGGTCGATGTGGTG
CTGACGGCGACCACCACCGTGGGCGAAGGCGGCCAGATCGTTTACACCGCATCGCTGGTGGACAAGGCGGGTAACC
CGGTCACCAACACCACCAATCCGTTGACCGTGACCCTGGGCAATGGCCAGACCATTACCATTGGCGTCGGCCAATC
CGCGGGCACTGCCAGCACTGTTGCGCCTAATGACGTGTATGAGGGCAACCAGACCGTCACCACCGCGATCACCAAT
GTGACCGGCGGCGCGCACTTCGAGAACCTGGTACCTGGCACCACGCCAGTCAACACCACCGTGACCGACACACCCG
GCACCACTGACACCACCACCGTCACCCTGACCGCACCGGGCGAGGCCGACGAGGGTGGCAACATCACCTACACCGC
GACCCTGAGCAACAAAGCCGGCAGCGACCTGACCCTTACCTTGAGCAATGGTGACGTCATCACTATCGCCAAGGGC
GAGACCACAGGCCAAGTGACCTCCAAGGCACCGACCGACGACGTATTCAAAGATGCGGGTCCGATCAACGTCACGA
TCAATCCGGACTTTGTGGGTGGTGGTTTCGAGAAGCTTGATATCGCTCCGGGTGGGGCGACGACGCAGATCAACGA
CACCATCGACAAGGTCGATGTGGTGCTGACCGCGACCAAAACCGTGGGTGAAGGTGGTGAGATCGTTTACACCGCA
ACCTTGGTGGATAAGAACGGCGCACCGGTGACTAATATCACCAGCCCAGTGACTGTGACTCTGGATAACAACCAGG
TCATCACCATTGGCGTGAATCAGTCGAGCGGGACTGTCTCGGTTGTGGCTCCGGATGATGTTTACAAAGGTGATCA
GACTGTTACGACCGGCATCGAAAAAGTCACCGGTGGCGAGCACTTCGAGAACCTGGTTCCAGGTACCGATAAAGTC
ACGACCGTCGTAACCGACACACCAGGCACCGATAACACCACTACCGTTACGTTGACGGCTCCGTCGGAAGTGAGTG
AAGGCGGCACGATCACTTACACCGCCACCTTGAGCAACAAGGCTGACACCGACGTCACCCTGACGCTCGATAACAA
ACAAACCATCACCATCAAGGCCGGCGAAACCGTTGGTACTGTGATTGTTGATGCCCCTGGCGATGACGTGTTCATC
GACAAGAGCACCCAGACCGTCCAGATCACCGGCACCGCGGGTGGCAACTTCGAGAAGCTTGTGGTTGCTGGCGACG
GCGCGACTACGCAGATCAACGACACCATCGATAAAGTCGATGTGGTCCTGACTGCCACCAAAACTGTCGGCGAAGG
CGGCGAGATCGTTTACACCGCGACTTTGGTTGATAAAGACGNNNNNNNNNNTTATGCGGGGCTTTTGTGTTTCTGG
CGTTCGATAATTGGACTGGGTTTTAAACTAACAGTTTCCGCATCGTTTACGACGCTTTCCGCAACTACTGTCAGAT
GCTCTATCAACGTGTCGGTTTCACGATCTCGCCAACGCGCCTATAAACCGTCTCGGTTATCCGCTTATCCGTGTGC
CCAAGCAGGCGACTGGCATCGCCAAGGTCCAATATCTCACTGGCTGCTTTTGGTCGGATGTCGCGGAACTGGAACT
GCCGGATGCTCGCCGCAAGCACGCCGTCTCCCGCCTCGTTAGCAATGGCAATTGCCTTGTTCCTGGCATCGTCAAA
GCGTAGGCGTAGCATCGGCTTGGTCACCTGCCGGCCATCTTCAGTGATTATTAGGTATGGAGTCCGTACTCCACGC
GAGCGCCGCTGCTCTATCAGTCGCTCGACCAGTGTGCCCAGGTCATTAAGGTTTCCGGCAGCGGTAAGGCGGATCC
GGAGCTTCTTGGCCGTCTTGCCTTGCGACACCTGCAGGAATTCATTTACCGCATCAGCCTCGCGCATCGAAAGCAC
ATCTGCGGGGCGCTGTCCGGTCAGGTAGGCCAAGTCCATAGCATCGCGAAGCTCCGAGGCTGCGACTGCGTAAACG
GCGCTCCAGATCTCCTCAGTTGCGTAGAAGTCTCGAGGCGCCTCCTTGTTCTTGCGCACGCCGGCGGCAGGGTTGT
TCTCGGTGAGCCCCCACTCCCGCGCAATGTTGTAGATGTGCGACAACAGTGATATTTCCCGATTGGCTCTTACCTT
GGCGCTCCGGCTATCCCTGTACTGCGCGATGATCTGTGGTGTAACCGCATCAATAGGGGCGTCGCTGAACGCTTTG
CGCAGTTGCTTCAGGCTCAGGAGATTATCGCTCTGCGTCTTGGGTGCCTTGCCTGGGATGATCTCGCGCTCGTAAC
GGTCAAACACCTGGCCCAGCAGGGCTCCTTTCTTCGGTACCGGCTTGCAGTCGAGCTTTGCCCATTCAGCCTTTGC
AATTTCCAGGTCACCCCCGAGCGGAATCTCCACCCGCTTCCCGTCTTCATTCCTCCCGTCGTAGTAATACCCAACC
CACTCTTTTCCGCCTTTCAGCGTGCGTACACGCCGAATCATTCGCGGCGGCAGGTCCCTGTTTGCCGCCTTTTTTG
CTCGCATGTCTTTATCCTACGTTTGAAAGATTCAGTGACCAGGCTTCGGCCGCGACGTTTTCTGCTGATGGCTTCA
CTCCGGCCAACTTCATCCGGGCGTAAACGCGGCCCACCACTGGTCGGCGCGAGCGAGTCAGTACGTATTTCCAGCC
GTTTTTGTTGAGCCACTCGATCTGCCTGGACGGGATCATGTAGCCCGTTATTGCCGCGATCTCTTCCTCGGCAAGC
GTTTCGCTTTGCATTTCCATGGTGTGCACCTCTATGCCCGGGGACTGTCATGCGGGCCAGTAGGCCGCGCTGTCTT
GATGATGTGGATGATGAAACCGAAGGTGAGTAGCATCCAGGCGACGGTGCCGGCGAATGCTGCGATCAGGTGTTGG

TCGGTACCGGTGGAGAGCAGCTCCGGCACCTGCCAGAAAAACCAGAAGATGGACCCCGCCACGTACAACACGATGG
CCGTGATGAGCAGGGTGAGTTTCGTTGCGTACATGGGGTGTCCTCGCCACGCTGGGCAGCAGAAGTTGATAGCAAA
TTGCCTCGATGGAAATGGGGACGGATAAGGGCTACAAACACGGCTCAATTTGGTTTCTGTGTCAGGGAGGGCGCGT
GCCACACAATCTTGATGTTCCAGTCGTTCACGCCTATCGGGGGCATACGTTGTTTCTCAAGTTCGAATGGAGGCGA
CCAAACGATGACGTACCCGCATTTGCGAGAATCATCGAGCCGGCAGCCGTTGAAGGATTGGGCGAAGTTGCCGCCG
AGTTGACGGGTCCATGGCCTGACTATCCGTCGGCACTGGGTGATGCGATGGCAGCTGCGGAGCGATGGATCGACAG
CCAGTTACCGTGAGTTCGCTCACCGGCAGGCATGTAGGGGGATTGGGGTTAGGCTTGTTTGATGCGAAGGAAACGT
CGGCACTGGCATTGCGTATCGTCGCAATGGAGCTCGCCAACCCATTCCGGGAATTGATCGCCTTCGCAGCGCTCAA
TGCGGGAGTGGTTGAGCCCGCTTCGCTTCACATCTTTCACAAAGCGCTGCAGGTCTTTGGCGTTGTCATCGCGATC
CGGGCTATCCCATCGACTCATTACCAAGAGGCCGCAATCTGTCTTTCCGTGGTCCATCCAGCCGAGACTCTTCGGG
GAATCGCTCATGGCGTCACCCGCTTGAACTCGACCACCCAGACCCACGGGTTGGCGTCCCAGTTGCCGCCGACGGA
TTGCCAGAGATCGCGGAAGGCGGGCACCGGGTACTTGTGGCAGGCGCCACCTTCGTCGCTCGCGCACCACTCGCGT
AGCGGGCCTCGGTGAACGCCCTCGGCAGCCGCCTGCTCGTAGGTGATGTCCTGCAACCGCTCGACGCGCACGTCGG
TGATCTCCAGCAGGATGCGGCAGGCAGCGCGAGGCATGTGGATGCTGGGCTTGTACTTCAGGCCGAAGTCTTTCCT
TGCCTCGTCGCTGTGTGACCCAGGGCGGCAGTCGGCAGCGTAGGCGTAGCGCTGGAGCGGGCCGTCCGGGTCTGGG
CGATGCTCAACACCGGTGCCGCGCAGATCGATGAAAGTCTCGCGCACCCACAGCCGGTCGCCTGGCTTGCCGTAGG
GGCACTTGCACCCAGACTCGCCATCACGCCACCAGGCGCCAAATATCTCTGGCCCTGCCTCTTGGTAGCCGCGACG
ATTGGTTACTGCCACGTTGTACGACTCAACCGTGATGCTGGCATCCGCGTCGGGCTGGCACTTCATTGCCCGGCGC
GTGACCGTCTTCCGGCCTTCCAGGATGGCGCGCACTATCGGCGCCGAGAACAGAATGGGGCGTTCCTTCGCTTGAG
ACATGCGGATTCCTTGCTGCTATAGCGGCTGACTTTGAAGGGAGTAGGGGTTACAGGTTTTTGCACTAACTAAAAA
AACTAGTTACCCGCATATTTACAGAGGTATTCTCACCGGATAATTTTGGTCGTTTGGAACGTCGTAGATTGAGTAA
TCATTTTTCAGGGACGGTAGCCCGTTACGCGGTGACATTGATTTGGGCCCTTATATTTCTTAGCTCAAGCAGCTGG
GTTCTTGTCGGCTCTGTTGCCTACTGGGTAAAAAATGGGTGGTTGCCGCCAGATAGTGCGGGATGGGCCCAGGCAA
TCGGTGGGTTGTTAGCGGTGATCGTGGCAATATCTGTGCCTGCTTATCAGGGCTACCAACAGCAGAAGCAACTTCA
AGATAAAGATTTGAAGGTTCGATCTGACGGGTTGCAAGCCACAAGGGCTCTCATGGATCATTTGCTGGGCGTTCAG
AAGCGATTGCGTAAGGGGCTTATGGATTTTCAACTTCGTAGAGGCTCATTTACCCCACTCGACGGCGCCAGGGCTT
CTGCTCACGATGCCAAGCAGGCTGCTGCAATGCTTAGAGAACTTTCAGTGGTGGCGCTGAGTGTTGAAATGGTTCA
TTTCGTCGTGGGGATGCGAGAAGTTGCCAGTTATGGAGAGTTTTCAGCTGCAATAATGGACAGCACCCATTCGGTT
CTCACGCCTGGCATGCTCCAACAGTTAGATGCAAACATAATTTCTTTGCAGAAATGGATTACTGAGCTGGATGATT
TGGAACAGTATTAATTATCCATCGATTTTCTTTTTCTAGAATTACTCAATTACTTTCGGTATATAAGTTAGCGTGC
CGTCAAGGATCGCTTCCTTGATGGCGTTGAACTCCCAGGCGTAATACTGGGATTCGACGTAGACCCGCATGCCTTT
GCCATAGTCGTGCTTCTTGCGCCGGATGAATGCCTCGGCGGCGTCTTTGGTGAAGTGCGCGTTGATGTACTCCCAG
TTTTCGCACCAGCCAGTGACCGCATGTCCTTCCAGCTCGCCGAGGACGTACCACTGATCCGACTCGTCCGCCTTCA
TGAACTGGCATCCTGACCAAGCCTGCATAGCCTTGTTCAAGGAGGCCCGCTCATCTTCGTCTTGTTCGTCCCAGTA
ATCCTTTGGCGAGAACCACTCACCCTCATTGCAGTGATTGATAAGCACGCGTTTGTCGCTGTAGTCGGTGTCTATC
CCGTAGACGATTCGTCTGGCCTGGACGATGAAGGTCGCTTCAGAGGTGCAGTGATCATGCACGCCAGCACCAACGC
AGTCATGGCGAAGGCGGGTCACGAAGTCGGCCCAGGTAGCAGCATCCAGGTCGCAGCCGGTAGCAAGGCTCAGCGC
GGGCTTGGGGGTTTGGTTTTCTGTAGGCATCGGGAATCCTTGCCGGGCCATGCCCGGGCGGTGGAGTTTGATAGGG
GATGAGCTAAAGTTCTGCTACAAGCCAAACGGAGGTGGTGATGGATAATTACGTATGTGTTATCTGCGGCGGAAAG
GGCAAGGAGCGGTATTCAGTAGGCGAGTTCAGGGATGTCGAATGCCCGGGCTGTGGGAAGTACCTAATTTCATCAA
CGGTGCTGGCCGAGATGAGGGAACGAGGCAATCGATTCAACGTGCCAGCTACTAGGGCGTGCATCAATGGCTTCAT
TGCAGCTGGACTGCCTGCAGTAATTTCCAGGGCTGAAATCGCCTACTACCAGCTCTTTTCTAGTTGACCGCCAGCT
GTTGCAGCAGGCGCCGGCCAATCCAGCGAACCACGGTTACGGCCTTGCTGTTACCGATTGCCTTGTAGCGGGGACC
GTCTGGGCACAAACCCAAAACCCTTCCTCGCCAATGGATGCGCGTGTAGTCGTCGGGGAAGCCCTGAAGGCGTTCG
CACTCCCTCGGCGTGAGGCGGCGCACTGAGCTGGTAAAGGACACTACGTTGGGGCCCAGGGCTGAATCAGTGTTGT
CGACCTGCTTCCCGTAGTTGCAGGTCAGCGTCTGGGCCACTTCTCGACATGCAACAATCGGCTGGCCGCGCCCGGT
ACCGTCCTCGCTGCCGTCGAAACCCTCAGCCTTCAGGGTGTGTGTGACATCACCGGTGATACACACGGCCACCTGG
CCGCCGGCGTTGGCGTGGCTGCCTGAGTGATTCATTGCGCGCAGCGTAGGTGCGATTACTCCAGCATCTGCGCCAT
GATCCTTGCAGGAGAACGCGAGTACTGCGTTTTCCTGCCCGTTGTTCCGGCCCAGGGCAAAGGCAAGCCTTTCGCT
GACACCAGGGTCTTGCGTGCCATGCACCACCAGCAAGCCTGATTCGGCATCCTGTTGGGTTGCGCTGCCGGCCGCC
TTACCGCCGGCCTGAAGCGTGCCGGCCACGACCAGCAGGCTTTCCCGACTTTCATGGTCGCCGTACTGGTTTGTTG
TGAGCGGTGCGGTCACAACGAACGTTTCGGTTTCAGCATCCATCCTTGTACCGCCAGGGTGCGCGCTGAGAGCCGT
AGCTACTTCGGTCTGTTCGCAGTTGGCGCCGCCGCCGAATGCGGTTGGCTGGAGATGACCTGCTACTGCGTGATCG
ACATCGCTGCCGCCGTCAGTGCTTCTAAGAGTGCCGGCGGGAGAGTCCGCTTTCTCGCTTCGGCGCGGCGCAGTAT
CCCGGCGCACGCCTTCGCGCTCAAAAAGTACCTCGGCGGGATCGAACCCGTCTCGAGCACTTGCGACAACGAACAC
ACGACGGCGTCGTTGGGCCAGGCCGAAATATTGGGCGTCCAGGACCCGCCACGCGATTGTTCTTTTGGGTCCATAC
ACACAACCAGCGTCCTGCCATTTCTTCCTTGGAGGCTGCAGCTCGCAGTCCTCCCCAGCAAGCGCGCCAAGAAAGC

ATCCGAAGGCGTTCCCTTTGTCGCTGAGGACGCCGGGGACGTTCTCCCAGACGATAACGCTGGCGGGCTTTCGCTG
GCCTGCTCGAACATAGTCAACTGCATCTGCAAGCTCCACGTATTTGATGGTGAGGGCGCCGCGCGGGTCGGTGAGG
CCTTCCCGCATACCTGCGACCGAGAAGGCCTGGCATGGGGTGCCGCCGACCAAGACGTCCGGCGCCGGGATCTTGC
CGGCCAGCACCAGAGCGGCCAGCTTGGTCATGTCGCCGTGGTTCGGCACGTCCGGGTAGTGGTGGGCCAGGACCGC
CGAGGGGAATGGCTCAATCTCGGCGAACCAGGCGGCGCGCATGCCCAGCGGGTGCCACGCTTGTGTCGCGGCTTCG
ATGCCGCTGCACACGCTTCCGTAAGTGATGGCCATGAGGCATTCTCTGTTCAATTTTTGCAGACTCAAAAGGAATT
ACTTATGGATGAGTCAACCCTGACCAGCGTTGCCATTAACATTGGCTGTGCGTTAATCGGTTTTTTTGCCAACAGC
CTCCGGCTCAAGGTCGTAGATGTTTGGGATGCGCGGAAGATCTACAAATGGCTGGTTGCGGAATCGGTAAAGCCGG
ATGCGGTATCCTTTAGAACGACCAAGGCGATCAGTAGAGGCGTATGCATGAGCCCTGAGCGCGTTTCCGACTTGTG
CGGCAGGTCAAAAAACATTCATCTGTCTACCGGAAAAGAGGATGGGCTATGGACTATCACGCGCCATTCGTCCCCG
CGTGGATTTTTTGATATGTAGCCACATTGATGCCGGCTATGCGGCCTGGTCTTGTTGCTTCGGCGGCCACTCCGAA
AACCCCACGCGGGGCGCCTTTGTTTTGGGGTTGATGATCGGCTTGCCCTTGGCGTCCACCAGCACAGCCTTGGCTC
TGATCTGCATGTCGCGGCATCTCAGCGTCTTGCGGGCCAACTCAATGAACTGCTCGGCGTACTGCGGGGCATCGAA
GAGCGGTGAGAGCTGGCGGACCGTACCCCCCCCATAATCTTCTCGGTACGCTTGGCGACCAGCTCCAGCCACTCGG
CTTCCGGTATCGGCTCGACGCCGCCAGGAGCTTTGGCGTTCTTCCTGGTGCCAGCCGTCTTCTTGCGGGCCTCTGT
CTTGGCGACATCGAGGGTCATTCCAAACACTGCAAAGGTGCTCATGGGTTATCTCCAGGTGTGCGCCTGCCTCGCC
GGCTGGCGTGATTCGTTGATAGAAGTGATAATCTTTTCGAGTGAGTTGTCGGGGAGAGTGGCAATGGCCAGTAAAA
AGCTTGAAGTGTTCCGCGAGTGGTTCACGCCGCGTAAGCGGCTATGGACAGGGGTAGGCTTGTTTGCAATTGCTAT
CGCCTTACCTATTGTCAGTCCGGGAACGACCGCCGCTTGGCTCATAGGGCCTGCGACTGTCTTTTTCTTGGGTAGC
TTTATTCCCGATACGAACAAAAAGCGTTAGGGCCAAGGCGGCTGGCGTGATTCGTTGAGATGGGGTATTACGGGTG
ACCGGCATGGAGCCGGATAAAGGAGAAAGAGTTGGTCAACCTAGAACTAAAAAAAGCAGACTTCGCATATCGAGGT
ATGAACGTTGAAGTTACCTATTTCCTGAGCTCTTCGGGCGCCGTTCTTCGGTTTGACGTGTCCGCCGCACTGTCTC
AGAAAGAGGAAAAAATTGGAGCATCGATCCAGGGCTGGGAGACAGAGAGCGATGCCATGACCGCTGCGAGAACTCT
GGCGGCAGAGGCCATCGACAAGCACTTGCTGGGAAAATAGCTGGGGTTGGCAGCGCCGGAGGGTCAGGCCCGGCGA
ACCTTGAAGCCGAAGATGCACTCGATGTCGTGGTACTCGCAGCTCTCGTAGGCCTTGTACTTGGCCTGTGAAGGCG
TGCTGGCGAACATATCAACAATTGTTCGGTTGGTGATGTCCCACCAGTCCCAGCCAGCGACCAAAACCTGGTAGCG
CTTCAGTGGCAGCTTCTCTGCCATCTCGCCGTACTGCATCTCCCAAGTTGGGTGGTAGTTTCGAATGCGCTTCTTC
GGGTCGCTGTCGAGGATGACTCCGATGTAGTGGCCACGGTCGGCCATGATCACGCCAGGCTCACCGTTGGCGATGA
CGCGGCGGCCGACTTCGGCGGGCACCTGATAGTGCTGCCGAACGTATGCGCAGTTGTGGCTCATGGATTATCTCCA
GTCAGGCGCCGCCCTCCGGCTTCCGGTGGTGGCAATTTGGTTTTGGTTGGGGTATGACAGATGTCTGGCACTGGGT
CGGATCAAGGAGTGAACATGCGAAAGCAGGTCGGTTATCTGGATAAAATGGGTGATGAGCAGTTCTTTTATTTGGA
GTTCGATGAAGAGACTCATCAGTTCAGCTACGTGATCGAATACATAACCCGTAAGGGCGATCACAGCGAGCGAACC
GTGCCTCTTCATGAGGCCAAAGAGGAACGGGGCTTTCACAAGGCCGTTCAGCTGATTAAAGATCGTTTGTTTGCCG
AGGATGCATGACCTTGTGCGGTGAGCGGGCTGGACTATTCGTCGCCTTCTTCTTCGGCGTTCATTTGGAGTGATTC
GGCAAAGCCAGCCTGCCGTAATTTACGCGCCACGTTTTGAGTTATGTCGATTCCGTGGCGCTTAATCTCGAACATT
GATGCGGACCTCTCGGCGCCCAGTGAGTGTGCATGCACGATCAGTCGCCAGATCGTCGCGCGGTCCTTCTTTTCGC
CCAGGTCTGCGGTGAGTGCTGCAAGGCGGTCGCGCATCCCCTGACGGAAGTAATGGCGAATGATGTCGGAGGTGCC
TTTGACCTTCGGCGGCGCCGGCGGCAGATCCTCGGCCCGGCCATTCAGCACCAGCAGTTGCACCGCCTCGCTGACT
TCCTCGACCTTATGCCAGAGCATCAACTCGTCGAGCATCTGCCGGGTGCCGTGCGGTACCGTGTGCCGCAACTCCT
GCTCGCCCAGTTCCTGCCGCTTCTCGGCAAGCTTTGCCGTGCGCTCCTTCTGTTCGGCTGCCATGGCCTGCCTCTT
CTATTCCGCTGGCCGGCAGTGCGAGCCAGGTTTGATGTTTGCGTTGCTGGGTGCAGGCTATTCGGCGCATGTAGCG
ACCTTCTGCTGATTCCACGCGCCGACCGCTTCGAAGATCCGCGCGGCGTGCGCCTCTTCCAGTGATAAAGCCTCGG
GAATGGCAATCCAACCTGAAGCCACCATCTGGCTTTGATTCGCCTCGTCGCGCAGCTTCTTGTAGCAGTGCTCGAT
CACGTCTTCCAGGTGGTCGGAGAGATAAACGCCATCGGGCGCCACCTCCACCGATTTGCTGTAGCGGTCGCCGCGG
GCGTCGATGCAAAGCGCGCTGAGGTAGATCGTCCACCGGTGGGGAATGCTGCAGACGGCCTGGCCAATCTTCCCCG
GCGCAATGTTCTTCAGCGACTTGTAATTGATCATGCCCTGGCGGCCGCAGGGGTCGATGTTCACGACGGCGACGCG
GTTGGATGCCAGCAACGACCGGCACGACCGGGCAATGCGGGCCTGCAGGTTATGCGCCTTGCGTTTGCTCACAATG
CCTCCGCGAGTTTGCGCAGCGCCTTACGTTCTGCCGCTGTGATAGGCGGCTTGCGGCGCTTGAGGATGGTTTCGGG
ATCGATCTTGGTGGAGCGCTTCGGCGGTGGAGGGTTGATCGCCGGGCTTTCGCCCAGGTAGATCGTTCCGCCGGCT
GCCAGGAACTGCGCCGTGCGCACCGATATTGATTCTGCGTGCTGACGCTGCTGCTTAACCAGGCTGAGGTGGTTGC
TAATCATGCTGCCGCCTTGGCCAGCGTCACCCCGGCCATGCTGAAGCTTGATCCCTGCTCTGCGACCATCGTGTCG
AGCGCTTCCCAGTTGACCAGGAGGACGGTGATTGGCGCCTGACCATATGCCACGGCTTTCACCAGAACCTCGAAGT
CCGTCACGTTGGCCTGCAGCGCTACCTGCTCGACCGCATGGCTCGTTACTGACGTTGTGGCCTGGGCTACGGGGGC
CGCCGTCTGGACAGGAGCGGTGCGGACGGGCTCTGATGTCGAAACTTTCTCCACGACCGGATCTGACTTGAAGGCT
GCCAGGCGCTGGGCCTCTTGTTCGTCGGCGATTCGCTTCGCGTCGGCCTTATCCTGTTCCGCCTTCTGGTGTTCCG
AGATACGGAATTTGATCAGCGTCACCAGGTCATCATTGGCCTTGGTCACCAGTTGCTGCACGTCGCTGAACAGGAA
GGCGTAATCAACGGCGAGCTCCGCCAGGCTGGTCAGATTCAAGCGAATACTTTCCGCTGCCTGACTTGCATCGATC

TTCGCCCGGGCCAGCTCGGTATCAACTGCGTCCTGGAGGCTGGCAATTGTGCGTTTGTTCTTCATGGCGCCGGCGA
AGTCCGAAACGACAGGAGGCAGCGTGACTTTGCCAAGGGTCTTGTTGATTGCGGCAATGTGATCCGCCAGTGCCAG
CTCGGCTTTTTGCTTGATGTTGGTCTTCACCAACAGCTCTTGAGCCTTCACCAGCTTGTCGACCTTCAGGCGAGTC
TCGCGAGCATGGGCACTGATGCGATCCAACGACGAAAACAGCTCGTCGATGCTTTGGGTCTGCGACAGAGCCTGTT
TCTTGGCGACAGCGACAGCCTCCTCGACATCGCCACACCACTTGACCGCCTTCTTGGCGTCGGCGAAGTCTTGGTC
GGTGGAGAGCGTGGTTTTCACCGAGTCGATGACGGCCAGTGCCGAATCTTCAAACACCTTCAGGTTGCTGGCGGTG
ACCATGCCGGTCAGCTCGATGCGCAGGGCTGGCAGTTCATCAGGTGCCTTGCCGACTACGATTGAAGGAGCGTCGG
CCATTTCGAAGTTGGACAGGTCTGCCTCGAACTGTTTCCAGCCTTCGATCAACTGGGCCGCGCGCCCGGCGACGGG
CCGGTATTCCATGTGCACGAAGTTCTCCGGGGTGCCGTCCGAGCAAACAAAGATCACTCGTTCAGCGCCGCTTACC
AGCAGTTGCTGCTCAAGCTGCCAGTAGTAGTGCGGAGCCAGGTCGCCGGCTTTCAACTGGGCCACGACTGACTCAT
TCCACAGTTTGTGCTCGAACAGTGTCTCGCCGAGCATCGTGGCGCCATCCATGGAGGCCAGCAGGTTGCCCTCGGT
CGCAACGATCGGATACAGCTCTTCGCCGATCAACGCCTCAGTCAGTGGGCGGGCCAGGGCTTCAGTAGCGTGGCCT
TTGTCGAAGATGAACTGCTGGGAATGCGTGACGTCGGGCGTGATCCCGGTCTTCTTAATCGCCAGCAGATCGGTCC
GCGTTTGATACTTCGATGCGCCCATCATGGCGGGAGCTTCAGAGGCTGTGAAATGCTGCGCGCGCAGAGCGTGCCA
CTCGGCAGAGCCTTGAACTACGTTATGAATTTTCATGTCGCGTCTCCTTCTTGTGATGGGGCGATGATTGCGAGAA
TGGTTTTTTTCTGGTGTTCGGTCAGGGTTGCTTTCAGTCCAAGCATCACGACGATGTGCTCCGCTGTCTTTTGCCC
GCCCAGAATCATGGCAACCCACTTAGGGAAGTTTTCAGCGAACTTTTCAGCTGGATACGGTGGAAGGGCCTCAGGC
TCTGGTGGCGAATCCTTGGGAAGGGGGGCTCCCGAGTTCCCGTCATCGTCGTCATCTGTTGTGGTGACGTTGAAAA
TCATCTGGGTCAGGTATCGCCGGCCATAGCTGATAGTTGAGCCGTTCGCCTGCACTCCGGTCTTGTTTGTCTTTCC
GCCTGATCCAGCAGCGTCAATTGGAAGGTCGAGCTGATATAGCTTCGTATGGCCGCCTGCGTGCATGCATTCACAG
GTGACGCGAAGATGGCCTGGCAGGCGAGAGTCAGCAGTTCCGAAGGAAAGCGAAAAGCCTTCCTCCGTGAAAATCG
GGCTGATCTCTCGGTCGATATCTTCAAGTCTGGCGTAGGTGCTGGCCGTGTGCTTGTTGAGAGCATTACGTGTCAC
CGGCTTGATTCGTTTCTGGGCGCTGACCATCGCAATACTGAATGCAACTGACGCCTGTCGATCGACGTGACGCTCG
TGCATTTGCATCAGCCGCTCCATGTTTCCGACATCGACATTTGGATTGGTGGCGACCTGGATGATCACGGACATGA
TGCTGGCGGCATCACTGCTTTGAGCTGAAATATCAGTCGACACACTTTGCGAGCGACGGGGATCGATAGTGGTAAC
CACACTCGACTCAGCATCTTCCACAATAGGGCTGGGCTTTCTCTCTGCGGACATGGGGATTCCTTGCCGCGATGTT
CGCAGCGTTCAAAATTATTGGGTGCTTGGGGGATATTGAAGGGCGGGCCGGACGTAGGTTTAGCTCTGCTGTTGAA
CTCTATAGCGGAGCACCTGAAGCACTCGACCGCCATAACCAGGCTCTGCGTACTGCTCAACTGGCGCACCGAAGAA
GCCGCGGCGTTCGGCCAGGCTGTAGGCCTCGCGAAGGTTATGAGCGCTGATATCTTCGAGCTGCTCGTCGACCAGT
GATTTAACCGGTGCGGTAGTCATGCCTTCTCCTTACGCCGCCGGCTTACATCCAGGAGGCGTGCGCTATACCAATG
AAATTCTTCTGCGTTGATGGCGCCCGAGGTGAAGTGACGCACGATCAAACCCTCGGCCAAGATCTCGTCGAGGGCG
GGCTTATCTGGATCTTTAAGCGCAAACAGCGCTTTATCGATCTCAACGTAGGGGCTCACAGTTCGTTGTCCTCGGC
CTGGGCGATCAGAGCGTCATCAGCCAGAGGTTGAAGGAGGCGTTCGGCAATCTCGCCGAGCTTCCCAAGTGGGTGG
TCGCTTCGGCCGAGTAGCTCCAGTGCTTCCGACTTCACCCTTGAGCCCGCCATCCCAGCGATCAGCAAGTAGCCCA
GGGCTGGAGCGTCGACGCCGCAGTCGGCCAGAAGGTTGTTGGCGTACTCATCGACAGCCAGTGCGAACTGGTAGGC
CGTGACGCCTTGCTGCGGGTGCAGGTGGCGCTTGAACACCACGCTGCCACCTCTGGCCAGCTCTTCGGCTGCGTTG
TATAGCCATTCGCTGCGAGCCAATTGCTGAGAGGTTTCGCTCACCGGAGGCGGCAGTTGTTCGTCGTGCTCAAACT
GAGCCTTGCGTAGTGCTGACATGGTCGCCTCCAAAGTGGCGGGTTGTTCACCTGTATTCGTCAACACTCATGCCTT
CCGCTGGTTGCCGATGGGCGCGGGGTAAGTGCTGACGGGTAGAGGTGGGGAGGGGTATAAGTAGGGAAGTTCGAAA
TAAGGAGTTTTCTATGTCCCTGGTGGATCTTGATTTGATCTCGAAGGCCTTGCCTCAAGCTTGGAAGTCCACCGTT
CTAGGACGGGTAGGCGGCTCCAATATCAAAGTTCTGCGCATGGATGAAATGTCCGTTGAAGCAGAGGTGCATGACT
ACACTGAGGGATTGTTAGTCATCTCCGGGCAGTTATTGCTGGGAGTCGATGGTGAATCCGTGCGAGTGGGAGAAGG
ACAAATCTATCTGGCTGAAGCTGGTGTTCCGCATGCAGTTTTGTCTGGGAGCTACGGGACACTTGTCATCATTGAT
GTTTGAAAGGCCGCAGGTGCCGGAACTGGCCCGCACGCAGCTGACCAGACAGTTAACGACAGGCTGTCCTGGTGGC
CTCTGTTAAGCGGTCAACCTAGGACATTCCGTCCGGCGCACCATCCGAACCTGAGCCATTCGCCTCTCCGGTGCCC
TGCGATCACGACGCATTGAGTCGTCACTGATCATTGAGTGCATGGCAAGCAGCGCCGCCAGTACAAAGCAAACCGG
CGTGATGATCTGGCGCTTCATGGCCTCAGCCACTAACGCCGTACGCTTCGTCACGCCCAGCTTGAACATCGCATTG
GTCAGGCGCTTCTTGACGGTGCCTGCCTCAATCCCTGCTTCGCGGGCGATCTCTTTCGAGGTGAAGCCCTGGGCAA
TCCAAAGCAGGAACTGAAGTTCTCGCGGCGCCAGGCCACGTCCGAGGTGACCCTTCCATGAACCGTTGATGATTGT
TGCTTCCATCGTCGTGACTCCCGGGTTGGTTTCCCGTTAGGCCCTGTTGCCAAGGCCTATCGGTGAAACCCCCGGC
CTCGCTACTGGCGACAGGCCGGGGTTGTTGCGTCAGCGGTGATGTGCGCCGGTTGCCCGCTGCTGATTGCAGGGCT
GGCTATCTGTGGGTGGGCTTCGAGCTTCCTACTCACGGCGTCAAACAGCATCTGTTCGCCGTGGATCACAGGTCCT
TACAACATGTACGCTACAGCTCTGAATGCCCTGGTTGAGTGGGGCAGGGTGCATGAGGTCCGGCGTTCCCAGCCGA
GGCTATCGGGACCGCTAATTCTGTTCAGTTGAATCTCCCTTCTTCCGCTGGGATTCGCGGGGCGCATTGCTTGCCA
GGTCATTCGCACGGTTCGAGCGTTTCGCTCTCGATCAGCCGTACAGGGTTCTCCTGTCGTGGGCGGGCTATCTGAC
CCGTCTGATCGCCGGTCGCCGGTAGAGGCAATGCGGTCTGTTGGTATTTCTGTATTGCGCTGACTGTTAAAGAGCG
GCGCAGCTCTCGCTGCTGGCCGGCAATGTTCGTTGCTGGCTTGAGATAAACATTACTAGCGATAATATTATCAGTC

AAGACCGCGGGTAATAAAAATATTCACAGGTAATAAAAATCCCGCTCACTGGCGGGCTAATTTATGAATCGCAGTA
CTCACGCCAGCCGATTCTGACGGCACCGTCATCCAAATGATCGATCCTGATACCGGCTGTGTCGCCGATATCCTGG
ATGACCTGGCGCCAGGCTTCAGGGCTTTCATCGTCGCGCCTGGATACCTCGACCAATTGAATCCGCTGTACCCGGG
GAGAGGCGATCAAGCCTTGCAGGCGGCGGCCAACAAGCTCGTAGGAATTTCGTGGTTTCGATGCGGGGTAGGGTGC
CTGGCTCATGCTTCGCTCCTTGCGAAAACTGTATAAATAAACAGTATTCCTGCTGGCATGCATTGGCAATAGGACG
GCAACAGATTTTATGCATAAATGCATAAGCCATTTGCTAAGGAAATCCGGAGCTAGGAAAAGCTGGGCATGTATTG
CTGCACGCGAGGAATTGCCTGAGCTTTCTCAAAGCAAAAAGGTGGAAGGCGATCTAGCCCGATCGGCGAGCGCAAT
CTAGATCCAAATCGCAGGCAAAGAAAAGCCCGCGATGGGGATTAGCGGGCTTAAAGGGATTTTCTTTAGGAGCTGG
GGTAACCATATGCGTTCAGCTGTGAAAGGTTTGTGAAATGTCGGACGTCAAACTCTGCGAGATTAATTAATCATCA
GCGCCCAGTTGGTCAGCTTTCAATGGAGGGACACGCAACTCTGGCAGAGCAACGGCTTCCGGCCATCGAAGCTCTT
GCCGTGGCGTGTCAGTGCTAGTCGATCTGTTCACCTTCGCATGGACGTAAGCCACTTCTCGGCTCACGACATGGGC
CACATAGCTCACGCTGACGCACACACTCATGAGTGCGGTGATTTTGACGAGCTTGGTTGCGATGGGTTTCATGCAG
CCCCCTCCATACGGATGCGATCACCGATCCTAACTGGTAAGGACAGAAACAAGAAGCCCGGCGCTGGGCCGGGCTT
CGATGTAATGCGTCAGAAACTAACGATGTTTGTTCTTGTGTTTATGCTTGTTGTTGCCTTTGTGCTTCTTACCATC
CGATCGATGATTGCTCTCATCGTCCGCCAGGTTGTTTCCTAGAGCACCACCCGCAGCGCCGCCGAGCCCTGCACCG
ATAGTCGAGCCAGTTGTGCCGCCCAGGCGATTACCAATGAGTGAGCCACCAGCCGAGCCGATACCGCCACCTAATG
CAGCTTCAGCTCTATTGCCTTTTTGAGCACCAACAGCACTACCGGCAGCACCACCTACGCCTGCGCCTACCGCTGC
GCCTGTCGAGCCTCCGAGCTGTTGGCCTACGATGTTGCCTAGCGCACCACCAACGCCGCCTCCAAGAGCGGCTGTG
CCATCTCCGGCTGCGATCGCTCCTTGCGAAATGAGGACACCCAAAGCTAGGGCGGGTAGTGTCATTTTCATTTTGT
GAACCTCTATGGGTTGTCAGCAGGCGCTCACGGTTTAAAGCACGCCTAGCATTAGGACTAGGGTGAAAGTTTGAAG
TTCTCTACGGGGGCTGCCTACGGGCGAATGGTTGTAACAAGGTATGCCGTAATGGATCAGGCTGCTGAAGGCGGGC
TTAAGCGGTTTCACTAGGAATTGAGATGACCAGAGGAGCCGAGCGTGAAAAGGCAGAAACAAGAAGCCCGGCGCTG
GGCCTGATTATCTGTTTACTCAGGCTAGTCAGTATTCGTGGGAGTGCATGGCGTTCGCTCCACTCGATGACGTGGA
AAGCGTAGCAGCGGGCAAAAAAAGGCCCGCTTGTGTGCGGACCTAAAGGGAATTCTTCAAAGGAGTAGGGGGAGTT
TTGCCCAGGCCCTGTAAATGCCAGGTGAAAAGGATGTCGCAGAAACGAAAGGTTTGGTGACGCGGTCGGTATCAGA
ACGAAGTTGCGATCGCCCGGGCGAGTTGGGTGTCTGACATCAGCGGCGGCGGGCTGGTCGAGCGGTAGTACCTGGA
GGCTTGCTCAAACTGGGCGCCGCGAATTTCGCCGTCCGAACCTATGAACGCCAGGGCGTCAGTCTTGGCTGACTTG
AAAATTTTAGGCGGCTCGGTCGTGAGAGATGTCGTCGCCCCAATTAAAATGGTTGGCGCGGAGATTGTGAGAAATA
TCGCAGCGGCGATAGGGTTGGCGCCATCACCTGACACGGCCTGCGTGCTGACCGATGCTAGCAGGGCAATCCCCAG
AGCCTTCCATGAATTCATTCTTCGATGCTTCCATTGCTATCAGAGGGCGACACCGTAGCAGAACTGGGCGATCGCC
AGATACAAGAAGCCCGGCGCTGGGCCGGGCCTACTATGGCGTCTTAATGATCAGTGGATCAGCCGCGCCACTCCAA
AGGCGACCGCAGAAAGCGCGGCCGCAGTGCCTACAAACCACTTAAGAAGCCTGGTTTCAAGCGATTGAATGTCTGT
TCTGAAATTGGCTATGGCTTGGTTCAGCCCGCCAGTCTCTTCCAGAAGGTCGGCCTTGGTAACCATATGCTTTTCA
ATCGAATCAATTTTTGTCTCGACGCGCGCCAACCGCTCCTTTATTTCAGGGAGTGACTTCTCAATAGACTCAATTC
TCTTTTCCATAAAATCACCGCCACCAGTACCGCCGCCTCCGCCGCCGCTCCCGCCATGCCTGTCTGGATGGGGAAG
AAATTTTTCGGCATTTTTGAAGCGCTCTTCAAGGTAGTGAATATTGTCACTCATTTTTTTGCGCTCCAGCATTCAA
GAGTATACGACCTTTGGTATCCCCGTACGTTTTCAGCAACTCAATGACACCCTTAAGCTCCTCAAACGCGGACTCG
CCCAGGGCTTGTATTTGCTCGTCATCAGAGTGGTTCCTATGGACAACCGCAAGGTCTAACGCGGCGCTCGTTAGCT
TAACGATTGCATCCTCCAATGGGCTGAAAAGCTCGAACAGTTCGCCATGGGTGACATTCTTACTGGCGATTTCGGG
TGGTAGCTGTTTCATCGTTACTCCGTTTGAGTCAGGAATGCCTCTTGGCGCCGCTATCCAAAGTTCGAGAATTTAC
GCATCAATGCATCCCTCCGCGCCAAACCATGCGCTTACTCGGGCTTGCTGCGATTTATCTGTCCCGCCTTCACCTC
ATCCGCCCGCGCCATAGAAGTGGTTCAGCGCTATCAGCTCAACCACCGCCACGATGGTGCAGAGCACAACGAAGCC
AGGGCTGAAGACTCGCTTTCGACCTGATGAACCCCAACAACTAGGACCCGCGTCGGAAATAAAAGGAAGCACCATG
AGCAACGCCAACCAAGCCCAGACCCAAAACTTGCTCCAGAAGCTCTGCTCTCGCCATGCAGTCATTTTGTCTGTTT
CGAAGCCCGGCTTATCCGGCCGGCCTTCACCTCATCTGCGTAGCCCACAAGCCTGTCAGCTTCCTCATGGAGCGTG
CCGACCAAGCCTATTAGTGCTATGGCATCGGCGTCGCTGAGCTTTTCCGCCAGCTTACCCAGGCTTACGCAGGACT
GCTCAAGGTTTGAAGCGATAGCTTTGAGGTCGCGGCGCAGATGCTGGTTGGGCCTATTGAGAGGCATGATCAATTA
TCTCGTTCAGCTCGGCCAGTCAAAATCCAATTTACTAGTCGTCCGGTGACCTTGTCATACAGGGCGGACAAGAAGA
GTCCAATGATCACAAGGTAGCTACCGTAAAAGACCGCAGGGTACTCCCTGATGCGATATTTATTCCAAGCGATCAC
ATCTAACCAGCGATCGAAAAAACGCGATGAGCTCAAATAGCGCCCACCCATATCGTAAAGACCAACTGACAGGGTA
ATCAGGCCAACGAGCAAAAGAACAATACCTATGCGCTTTGTTACAGTATTCGTGAACTGTTCCATGCTGACCTCCT
GTCAGCTCGCTATACCAAGAGGCATAGTCAAGACACTTCGTTGATCAGTAAAACTTCTGCAGCGCCTGCACAACCA
CGCCCACGATCCGGCAATTCTCGTCTACCGCCTCAATTGGGTAGCTCGGGTTCAGTGGCTTCAGGAACAGCCGGCC
GCCATCGCTGACCAGCTTCTTGAACGTCGCCTCATTGCTATCCGGCAGCTTGGCCACCACCAGCTTACCTGGTGCA
ACCTCAGCCTCTGTGTCCACCAGGATCAGCGTGCCCTCGGTGATGCTCTGGCCGGCGGGCGCCGTCATTGAGTCGC
CTTTCACCTTCAACCAGAACGCCATCCCCTTTGAGTCGTACTCCGAGAACTCATAGCTATCAGAAAAGCCGGCCGG
GTAGGGCTCAACGGCCTCCGCCCAGGCGCCGGCGGCAACCCAGCTGATTACTGGGTAGCGGAATGATTTTGTCGGC

TGTTGTGCATCGCGGACATTGGAGTCAGACGAACTGCTGATCCCCTTTATTTCCGCCGCCAGGCGCTCACTGAAAT
CAGAAACTGGAACTTGTAAATAGCGGGCGAAGACTGCTGCTGCCTCGATATTTAATGCGTTTCTGCCGGTTAGGTA
GTGGCTCGCAGCGCTTTGGCTGTTTGCCTTCAATCCTTCCATTGCGATTTTTTCTTGGCTGATGCCAAGGGCCTTC
TTCTTTGCCTTGTAAATAGCGTTCAGCGCCGCGCACTCCGCCGCCTCTACTTCTGTGAGGGGGCGCTTTTTGCCTG
GCTTTTTGTCAGTTGTTTTCATCGAGCAACTGTATGACTTCAGGTATTAACATTCTAAGACCGCCGATATTGACTT
AATAAAGACCGCAAGTAATACTTGCGTGTGCATCGCGCAGGAGAAAACGTATGCAGCGCACACCACTTAAAGAGTT
TGTGACAAGGGTCGGGCAGATCAAGGCTGCCGAAGAGCTCGGCATGACCCAAGGCGGAATCAGCAAAGCACTGCGA
GCTGGTCGCGAGGTTTATGTGATCGAGCAAGGGAATGGGAAATACAAGGCTGAAGAGATCAAGCCATTTCCAGGCC
AGGTTCAGCGACTCGCGAGCTAGTGAGATCCGTATTCGCCAATCCATGTTGCCAGTATCGGCTCAGACGGCCTGCG
AAGTAAGAAACCTGAAGTCGCTGGAGTTTTATCCAGTGGGTTTTAAGAGAGGCGAAGCGGGGAGCAGGGTGGGGAG
GTTGGTGCTTCGGCGCTGAATTACACGCACAAAAAAGCCGACGGTCGAGGTCGGCTAATTCGATAACACTTTGTGA
GGCCGATTATATGCAAATCCAACCAAATATCAATAGCGCTACAGATCTCGCGCCACGTTTTCCGCAATCTGAAAAC
GTGGCGCGTATTAGCTCACATTCAGACCCCTTGGCGGCCTGACATGCAATACACCGTCACGATTAACCAGGTGAAG
GCGCTGGAGTGGGGGCTGAATGCTCAGCAGGCCCTACTGTTCGCTTTCGTCTACGGCTGTCCGAGCTGGACCAAGC
CAATCAAGACCGATGACGGGATCTTCTTCGCGCTGAGCAAGGCCAAGATCACTGAGGAGCTGCCGCTACTCACTGA
TAAGCCCGACACCGCTTACCGCATGCTGAAGGCCCTGGAAGAAGCCGGTTTGATTGAGCTTTCCAGTACTTCGAAC
ATCACGCTTTTCAGGCTGACAGAGAAGGCCATCGAGTGGAACCAGAAGCTCGACGGGTCGGAAAAATATCCGACCC
CGCCAAATAACAAGGGTCGGAAAAATATCCGATCTACCTCGGATAAATCTCCGAGCAAGGTCGGAAAAAAATCCGG
GCAAGGGTCGGAAAAATCTCCGACAAATCAGGATACCAATCATCAGGATACAGATCAGGACACCAGTCAGTCCTTG
CAGGACGCCACCGGCAAGCCGGCTCAATCCCGCGGCTTGGTTCTCGTGGTTGATCGTATCGACGCCCCACGGGTTG
AGATCCCTGCCGACATGCCGGGCCCCAAAGACCAGACCTGCAAAACGTTCAAGGTCTGGGCGAACTACGCAATGGC
CTACCGCAAACGCTACCTCGGCGCATGGCCTGTTTGGAACGCCAAGGTTGGTGGTCAGCTCGGCCAATTGGTCGAC
CGACTCGGCGCCGATGTCGCTCACCACGTCGCCGCGCATTACCTGAAAACCAACGATGCCGCCGTTTTGCGCAAGT
GCCACAGCCTCAACGAGCTGCTGGCCAACGCCGAGAGCTACCACACCCAGTGGGTGACCGGGCAGCGCGTCAACGG
CACTACCGCCCGCCAGATGGAACGAACCGAGGCCAACCTGTCCGCAGCGGAGCAGGCCGCTCAGATGGTTCTGGCC
AAACGCCAAGCAGGTGACCGCAATGAATACCTCTGAAATGAATGACCAGCAGGTTGCCGGGCTGGCCGCCGCCATC
TGCGCCACAGCCGAGGCCATGGGCCAGGAAATGAACCCAGGCACTGCCGCGATGATGGCCGAAGACCTCTGCGCGT
ACCCGGTACCCGTCGTCAAAGCCGCCTTGAAGGCCTGCCGTTTCGAGGTTAAGGGCAAGCTGGCTATGGCTGACAT
CCTGCAACGCGTCCAGTCCTCTGACGGGCGCCCGGGCAAGGACGAGGCCTGGGCAATCGCCATGACCACCAACGAT
GAATTCGAAACTGTGGTGCTGACCGATGAGATCCAACTGGCCCTGGCTGCCGCGAAACCCATCTTGGATGGTGGCG
ACAAGATCGGTGCTCGCATGGCGTTCATCGACGCCTACCAGCGGTTCGTGAGTCAGGCCCGTGAGGATGCGAAGCC
GGTCAACTGGCATGTGTCCGTGGGTTTCGACGCCAACCGTCGAATCCAGGCCGTAACCAAGGCCATGGAGCTGAAG
CGTATTCCCCGCGAGCACGGCCAAAAGTACCTGGCAGACCTGAGCGTCGAGCCGGTCACCGAAGACGGTCGCGCGA
TCGCTGGCTTGCTCACCGGTACCGTCACCCGGCCGGAACCCGCTATTCGCGCAAAGCTTGAGATCGTTAAGAGCTC
GATGCTTGAAATGCGCAAGGCCAGCGCAGAGCGAAAGGATGAGATACGGATTGCAGCGGCCAACGAGTTGGCGGAT
CGCCGGGCGCTGCTGATCAAACAGGTCCAGGAACTGGAAGAGAAGAGGGCGGCGCAATGACCAAGCCAGCTAAGCC
TCGCCCAATGCCCGTGTATCTGGTGCTGCGCCGCCTGATTGATCCTGCCACCGGCAGGGAGGTGGCCGCGTTCGTG
CCGTCCTCCGACGCTGACCGATCGATCCTTCGTGAGCGTGATTTCCGGATCAATACCAAGATCCGCGCCGAACTCA
AGCAGCCACGCAATCCAAGGTTCAACGGTTTGGTCCACGGCCTGGGTCGGGTACTGAGCCAGAACATCGATCGGTT
CTCTGGCAAGCAGTCCCACGACGCAATTAAGGCCCTGCAACTGGAGTCGGGCGTGTACTGCGACGAGGAGCAGTTC
GACATCCCTGGCCTGGGCCAACTCACCCGTAAGACACCCCGCAGCCTTTCCTACGATTCGATGGGGGAGGAGACAT
TCCAAGATTTTTGGCGCCAGTGCTGCGCGTACCTGGTGCTGCATGACTGGCCGACGCTCACGGAAGAGCGCCTGAC
CGAAATGGCAGAGTTCGAAGCATTCAAGGAGGCCGCATGAAGCGCACCCCATTACAACGCAAAACCCCGCTCACGT
CCGGTGGGCCACGCCGCAAACGCTGCCCAGAGTGCCGAGTGATGTTCACGCCTGCCCGCGGCTCGCAGGCGGTGTG
CGGCGAGATCGAGTGCGCTATCGCTTACGGCAGGTCGGAGAAGGGGCAGGCGAGCGCCAAGAAGGCCCTAGCTGAT
GTTGGTCGCCGGGACATCAAGGTGCGCAAGGAGAAGCTGAAGAGCAGGGCGGACCACCTGCGCGAAGCTCAGGCTG
CGGTGAATGAGTACGTGCGCCTGCGTGACGCGCACCTGCCCTGCATTAGCTGTGACTCCACTCCGAACGACAACGA
CCTCATGACCGGCAGCCGGTGGGACGCTGGGCATTACCGATCCGTGGGTGCCTGCCCAGAGCTGCGCTTCGAGCCG
CTGAACATCCACCGCCAGTGTGTGAAGTGCAATCGCAACCTATCCGGCAACGCTGTGGAGTACCGCATCCGCCTGG
TGCAGCGCATCGGCGCCGAGAAGGTCGCCTGGCTGGAGGGGCTGCACCCGGCCTGCAAGTACACCGTGGATGAGAT
CAAGGCCATCAAGGCCGAATATCGAGCGAAGACCAGAAAGCTAAAGGAGAAGGCCGCATGACCTATCGCAACGTTG
TTTCAGCAGTAGTTCGAGCTCTCGCGGCCGAGACCATCACTTCCGCCGGCGGCTGCGACTTTGAGCCCAAGGTGCA
GTGTGCGAAGCAGAAGGGGGAGATCGTTGGCAAGGAGGCTGCGTTTCTTCAGGATTGCTGGGTGTTTGGGCGGCTG
CACAAAGCTTTGACCCCTGCGCATTGGCGCGCCCTGGTGGCAAAGTACTCTACCCACGAAGAGCGCAAGCACGGCG
CCATACTGGAACTGTTGAATTCGGTGAAGACGCCCGCGCCGAAACGTTTTCGTGAATGTGCTGTGTTGACTTGGGC
CATTCCTCAGGTTGTCGGGGCCGAGGGTAAGCGTTCCGCGGCCGTCCTACCTGCAGCCTGGTATGACATCACCAAT
TGGGGCAACGACGGTAAGCCGGAGTCGACACGGTACCGGTGGCGCTCATGTATTCGTAGGGCACTGGACGACCAGG

TCAACGAGGCGTTGACTGCCGTCCAGGAGCTGCTCGACGCAGAAGGTTTAATTGACACCGTTGCTGCGTAGAGCAG
GTTTAGAAATCGCAAGCCCGTGTCTCGCCGTTGCCAATATTCGGTTCTCTTGCCGTGCCAAAATTAAGGGTGAAAC
CCTCCCTTTTCCAACCACTGAGTCAGTAGCGCTGCGCTCAGTGGTTTAGTGATCAAGTAACCCTGGGCTTCTGTGC
AGCCCCATCGGCTAATCAGAGAAAGGGTCTTTTCCGTTTCAACGCCCTCTGCTACCACCCGATATCCCAATCCTTT
GGCAAGTTCAATTAAAGTTCTAACTAGGCGTTTGTCTTTTTCATTTGCGTGGAGGTTGCTGATCAGTGATTGGTCG
ATTTTAACCGTGTTAACTGGGAGTTGTCTCAGGTACGACCAGTTACTATATCCGGTACCGAAGTCATCTACTGAAA
CCTCAATTCCCAATCCTTGAGCTCGCTCTAGCTGGGCTATAACTGTATTGGGGTCAGTCATAAGCATGCTCTCGGT
AAATTCGAGCTCTAAAGCTTTTGGATCGAGTTCGCCTTTTTCTATGAGGGCAACAATTGCGTCGACAAATTTTGAG
TTTTCAAGATCGCTAACAGTCACATTCATAGCGATGCGCAGTTTTATTCCTTGGTTCTTCCATTGGTTAGCTTGAG
CTGTAACGGCTTCAAGTACCCATATAGTTAAACTATGCATCAATGCTGTTTTTTCGGCCAGGGGTATGAACTCGGC
CGGACTTATGGGGCCAAGAGTGGGGTGGTTCCAGCGTATTAATGCCTCGACGCTTTCACATTCAAAGCTTGGTAGT
TTTATTTTTGGGTGAAACACTAAATTTAGTTGGTCTGTCGATCGGACGGCATCTAACAGTGAGCTTAGCACTGCGA
ATACCCGTTTCTGCGCAGCATCTAGTTTGGGCTGATACATTGTCCAGCCGATATTGCGGACTCTAGCGTCATCGGC
AGCGCCAACTACTAAACGAAGCCAGTCTTTCTCTTCTGAGTCGATGATAGGCAACACTCCGATGCCTGTCTGCATT
AGTATTGGAATTCCTTGACAGTCAACTGGGTCGTTAAAATCTGATAGGATTTTCAAACAAATAGCTTCGATGGGCT
CGTCTGCACTCAGGAGGAAACCAAATCGTGTGGGGCTAATTTTATATAAGGTGCAATTTTTGGGCAGCACTGATTG
AAGTCGAGCCTTTACGTTAAGCATTAAATCTTGCGAGAAGCTATATCCTAGGGCCTTCACGACATCGTTTAGAAAC
TTTGGAGAAATTACATCTACTGCGTAAAGTTTGTGTCGTTTGCCGCTAGAACTCACTTGTCGGATGTCTTCCTCCA
ACCGAAGTCGATTGAATAGACCTGTAGGTTGATCAATGTAGTTGCGGGAACGTAGCCCCATGATTCGCATTATCAC
GAGTTGCGAGAAGTAAACGAGCATTACTGCATCTCGCTCACTCATTGGATCTCTAGGTTTAGTATCGATTATACAA
AGGCTACCTAATGAAAAACCATCCTTTGTAAGGAGCGGGGCGCTAGCGTAATAGTTGATATATGGTGGTCCGGTCA
CCATAGGGTTATCTTTGAAGCGCTCATCCTTGCGTGCGTCTAAGATTTCCAATGGTTCTTTGTCATGCAGCGAATG
AGCGCAGAAAGAAACATCTCGCGGAGTTGAATTATCTTCAATACCAATCCGCGCCTTGAACCATTGCTCATGCTCA
TCCACGATTGAAATCAGCGCGATCGGTGCATTGAAGTACTCCGAAGTCATCGCGATCATTTTTTCGAATACTTCAT
CTTGTTGATCGTCGCGCGAGCAAAATTCTGTCACGCGCTTCAGGCGCATCGTTTCGTAATCTGGGATTGAGTCGTT
AGCGTCCATGGTCTCCCTCGCGTGGAACGTCTAAATACTCGAAAGGTATCAGATGCCCTAGCCTGCTACGACTGCA
TTTATCTATGGAAGCTATAGCATTAAGTGTTTCGAGCCCACTGGTGCTGTGCGAAGTGATGCACCCGGTATTTTGC
GCTACGTAGCCCAAATTCCACCTCAGGGTTTTGAAGGCCTAATTCGTTAGGTTGACGAAAAAACATATCTTTTTGC
AAAGCGTGGTTGCACCGAATGAGAAAGTGAGAGAGTATTTACCCATCCTGTCGATCTTGCGCGTTGAGGTTGCACA
GTAAAGCCCGGCATGAAGTCGGGCTTTTTCATGAGAGGCACAGGACGATGCGAACTCTCTTACTTCTCGGAATGTT
GCTCTCGCCATTGGCGTTCGCCGACCTAACCGAGCCCTCGCATGACTGCAACCAGCCGGACGTGCCGTACGAGTTT
CAAGACCAGTACGAGCGTGACCAATTTCAGGCTGATGTTGAGGAATACGAAACGTGCATAACTGACTTCGTAGAGG
AGCAGCAGGACGCAATTCGTAAGCATAAGTCGGCGGCCGATGACGCCATTGAAGAGTGGAACTCGTTCGCTCGATC
GACATAATCGCTACGTATTTTTCTAAGCCTCGCCAAGTGCGGGGCTTTTTATTGCTCGCCGAAAAGACAATGCAAA
AGGAATTTGCAAATGTTGAAAGAATTCATGTGCGGTAACTGCAAAAGACTTATCGCCCGTACGGGTGCCCGCCACT
GAGCGGGCTTTTTTATTCCCAACTCCCTGACGGGGAGGAACCGAGATGTCCAACATGCCAGACAAACCAGACACCT
GGCTCATCGTGCTTGCATGGCTGAGTCAGCATTCGCCGACGCTTTATGCCGCAGGCCTATCTGCCTTGATGGCTGG
TATCAGAATCATCTATGGCGGCGGTACCCGGCGCCAGGCTCTGCTCGAGGCGGCGATCTGCACCTTGATCACCATT
GGCCTGATTCCGGTCCTTGAGTACTTCGGACTGCCGCAGAACCTTGCGACGGCTGCCGGAGTGTTTATCGGGTTCC
TGGGCGTGAAGAAGCTGGCCGACTTTGCTGATCGGGTCGCCGAATTCAAGTTTCCCAGGAGCGGTGCAGGGCAGTA
ATCCGCGCCACGTTTTCGAATGCACCAAATCGTGGCGCGGATAGCATGTTCTTCTATGGGAAAGTGATATCAGCTC
TGATCATGGGAAGGTTGCTTGCATCGTAGGAGCAATGATACGAGCGCCCATCATCGGTGTTCTTTTTCGGGGCATC
TTCACATTTGAATGAAACAGTGATGCCGTATTTTGTAGCTGTGAACCCTTTGACTGCTTCTTCAGGGGTAAGCACC
GCAACGAGCTCACGATTGAAACTCAGTGTGATATCGGGACCACCGAAGACTTCATATTTCTGCGATCGGATACAAA
GGCTGGCCCCGACCAATTTGCCATTCAGCGTATTGGTGCCACTCGTACACGCTTTCGGTGAGATGAAGTTGCCAAC
GCTCATTTTATTAGTGGGAATTTCTGTCTTCACTGCCGGCGGTGTGTACGGCCCGCTCTGGCAGGCTGTGATAAGG
CTAGCCGCAAATATCAGAACGGCAGCTTGAATCATTGATTTCATAAAGGGCAGCATCCTTTGGCTGAGGTGTCGTG
AGCAAGTCCAATCCACGGTTCTTGAGCTTGCGAACAGGGTTGCCGTGGCTTATGTAGGGACACCGCCGTGTTTTAC
TTCACGTATTTTACGGTTGCACTCACTAGCAACTGATCGTTGACCGTGACTTTGCAAAAACGCTTCGTAACTGTTT
TGTTATCAGTCGCGAGCGTAGGCTCGCATTGCAAAAACACTTTTTTACCGTTCCAGTCCTGAGCTATCCCAGCAGT
GGCTGCGCTAGCGTACTCTTTCATGAATTTCCGATTCCATGGTCCGAAATAGACTTCAGGTTCGCCGCCTCCGAAG
AAACCAGATTCCTGCGTGGCGCAGAGCGTACCCTCCATCCTTTCTCCGTCGATGATATTGGCATCGGTGTAGCAGT
TCATCAGGCCGTTCCAGACATTCATATCTACTGGGCCCCTGTTAACCCAGGTAGGAGGGGTAACGCATCCTGAGAG
AGTTGCGGCGGCAAGCGCCAACGCGATAAGAGAAGTTGACTTAATACGGGGCATTGAACTGAGCTTCCTTACTTTT
GAATTTGCATCATCAATACCGGCAGCCAGCCACGATTTCAAGTATTGCAGTGAGTTCGTGGTAGCCAAGCAACCCG
AATGGGAGGCAATCGAACGCGCCTACCGGGCTGGTTCGCTTTCCATCAAATCCACAGCCAGGTGTAATGGCATCTG
TGGCAACTATGATGAAGTACACTCTGCAGAATTTGACCGTAGGATTGCGATCATGAGCTGGCAAATCAGAGAGGCT

GTCCCCAGCGACTTTAGATCGTTGGTGTGCGTGGATTCTGTAGCGGAGAAAGACGATGGCCGTCGGGCGCAAATAG
CAAGTGCAATAAGCAAGGGCCACTGCTGGGTTGCTTGTGACGAAGACGATCCCGCTGTCCCGGTCGGTTACGGATG
CTTGGATAGAAGTTTTTTCGGTGAGTGGTTTGTTCCGCTCGTAATCGTCTCGGATGCACACAGAAGATGCGGAATA
GGTAGGAAGATCATTGCTCACTTAGAGCGTTGTTCCTCTGCCAAAAAGATCTTCACTTCAACCAACAACTCCAATA
CACCCATGCGGCAATTGCTCGCGCAGCTTGGATACCAATCTAGCGGCGTAGTGGAGAATCTTGATCCTGGTGATCC
GGAGTTAATTTTCATGAAGGTTCTTGACGAGTAGAAGCCCCTGTATTCCTCTGATTCATCTGCACTGATAGTGGCT
TGCCATTCATGTTCATATCTCGGGGAGGACTCATGTGCAGGCCGTACCCTCCGACGTCTTTGCTTGAACTGTCCGA
CTTCGGCGTCCGCCTCACTCCAGCTCCCGAAGTGTGGGAGTGGCTCCAAGCCGAGATCCTTGCCGACACCGGCAGC
ATTCACAACGAAGACCACGCCCATCTACTGGATGCAGACATCCGGATCATGTGGGCGTCGTCGAGCTTCGAGAAGC
AGGGCCGCACAGTCCTGGACCAGGCCGAGCAGGTAGCATTCCGCGCTGGCGGCTGGCAGAAAGCCCGGATGGAGCA
ACAGATGCGTGATTGGTTCGGCGATGTGCCGGCCTTCATCATCACTCTGGCTGCTGACTACTGCGCCCAGTGCAGC
GACCTTGAGTTCTGCGCCCTGATCGAACACGAGCTGTATCACTTGGCTCACGCGACCGACAAGTACGGTCAACCAG
CATTCACCCAAGATGGCGCACCGAAGATCAAGCTGCAGGGCCACGACGTGGAAGAGTTCGTCGGTGTGGTCCGCCG
CTACGGTGCAAGCCCTGACGTTCAAGCGTTGGTAGATGCTGCAAACAGTCCTGCTGAGGTGGGGAAATTGAACATT
GCGAGGGCCTGCGGAACCTGTCTGCTGAGATCGGCCTGATTTTTGACAGGCTCTAGACGGATGAGAATTTATGGCA
GCCCTGAAAAATGAGGTGAAGAGCTTCATCGTTCAGGCGCTGGCGTGCTTTGACACCCCCTCCCAGGTGGTGGAAG
CCGTCAAGAACGAATACGGGCTTGTGGTGAGCCGCCAGCAGGTGGAGACGCACGACCCAACCAAGTCTGCAGGGAA
GGGGCTGGCGGTGAAGTGGGTGACCCTGTTTCACGATACTCGCAAGCGATTCCGCGAAGAGACCGCAGAGATACCC
ATCGCCAACCGCGCCTACCGGCTTCGTGGCCTGGGGCGAATGGCTGAAAAGGCCGAGAGCATGCGCAACCTGGCGC
TGACCGCTCAGTTGTACGAGCAGGCCGCCAAGGAGGTGGGCGACGTCTACGTGAATCGTCGCCTCGAACCTGAGAA
ACCACTGGGCTCCCAAGCGGACCAGCAGCACGCCGTTGCTGAGTACACCTTGGAGCCTGATGAGAATGTCCCCGCT
ACCCCGTACCTTTGACCCGCCGGTGAAGCTGACGCCCAAGCAGGCGAACATTTACTGCTGGGGCTTCCAGCCTGAG
GCCCGCTTCCGCGATGCGGTGTGTGGCCGCCGGTTCGGCAAGACGTTCCTGGGCAAGGCCGAAATGCGCCGTGCTG
CCCGCCTGGCTGCTGAGTGGGGCGTGAGTGTTGAGGACGAGATCTGGTATGGCGCGCCGACGTTCAAACAGGCCAA
GCGCGTGTTCTGGAGGCGCCTGAAGCAAGCGATCCCCGAAGCATGGCGTGCACACCGCCCGAACGAGACCGAATGC
TCGATCACCCTTAAGTCCGGCCACGTCATGCGCGTGGTGGGGCTCGACAACTACGATAACCTGCGGGGCTCTGGCC
TGTTCTTCGTCCTGGTGGATGAATGGGCAGACTGTCCGTGGGCGGCGTGGGAAGAAGTGCTGCGGCCGATGCTATC
GACTTGTCAGTACCAGATTCCAGACGTTGGTATGCGAAAGGGAGGCCATGCGCTGCGCATTGGCACTCCGAAGGGC
TTCAACCACTGCTACGACACTTATCTGGACGGTAAACCAGGCGGCGAGCCGGACCACAAGAGCTGGCAGTACACCT
CGTTACAAGGCGGCAACGTTCCTCCTGAAGAGCTTGAGGCGGCACGCCGGAAGATGGACCCGCGCACCTTCCGGCA
AGAGTACGAAGCCGGCTTTGAGAACTACGCGGGCGTCGTCTACTACACGTTCAATCGAGATGAGTGCCGAACCAGC
GAGCGAATCAAGCCGGGCGAGGCGTTGCACATCGGCATGGACTTCAACGTCATGAAGATGGCGGCCGTTGTCTATG
TCGTGCGTAACGATCTGCCGATGGCCCTGGATGAGTTTCATGGTGTTCGTGACACGCCTGAGATGATCGAGAAGAT
CCAGTCACGGTTCCCGGGTCACTCGGTGGCGGTCTATCCCGACGCCAGTGGGCAGAACACCAGCAGCAAAAACGCG
AGCGAGTCAGACTTGTCACTGCTGAAGAAAGCAAAATTCACGGTGATCGTCGACTCCACAAACCCTGGTGTGAAAG
ACCGCGTGAACTCGGTAAACGCCATGTTCCTGAACGGCTACGGCGAGCGACGACTGAAAGTCAACATCGACCAGTG
CCCTCAGCTCACCTTGTGCCTTGAGCGACAGACCTACACCGACAAGGGCGAGCCTGACAAAGACCCGAAGAAGGGC
CATGACCACATGAACGACGCGGCGGGCTACTTCATCGCCAAGCGTTACCCGATCAAAGCAATCGTCACCTCCATCA
AAATGGGATACGCCCGATGAGCAACGACGTCTCCTTCAAGCGGGCGGAATACATAGCTGTGCTGGATCGCTGGGCG
ACCGTTCGCGACGTTTGTGCCGGCCAGCACCGGGTTGTCGACCGACTGCCGTACATCAACTCACACGACAAGTCGC
CTGAGAACGTAGACCGGAACAAGGCCTATCGCGAACGTGCGGTATTCAAGAACGCCACTGGGCACACCCGTAACGG
GCTGCTCGGCCTGGCGTTCCACAAAGACCCAACGTTGACGGTGTCGAAGAAGCTGGAATACCTGCAGGACAACGCC
AACGGTTCGGGCGTGAGCATCTACCAGCACTCGCAGGGCACGCTTGAAAAGGTGCTTGAGGCTGGTCGCCACGGCC
TGTACGTCGACTATCACCAGGACAATGGTGTCGGCGGGCACTCGGTGATCCTTTCCTACTGCGCCGAGGACATCAT
CAACTGGCGCACGGGCATGGTGAACGGGCACAGTGTGCTGACCCTGGTGGTGCTGCACGAGTCTCCCGAGATTCCG
GAGGGTTTCGGGTTCAAGACAGTTGAGCAGTACCGGGAGCTGGCACTGGAAGATGACGGTTTCGTTTGTCGCGTTT
GGCGCCGGTCTGGGCCAAAAGGTGGCGGGCCACTGGCGGTCACCGAGGAATTTAGGCCGGAAGGAGTCACGGGGCG
TCTCAAGGAGATCCCGTTCACCTTCGTCGGCGCACAGAACAACGATCCAAGCATCGACGAGTCGCCGCTCTACGAC
ATCGCGATGATCAACCTAGGCCATTACCGGAACAGTGCTGACTACGAAGATAGCGTTTTCTGGTGTGGGCAGGCTC
AGCCCTATATCTCTGGGCTCGATGAGCAATGGCGTGACCACATGGAGAAAAACGGCATTTACGTTGGTTCCCGGGC
GCCAATGCTGCTTCCTGCCGGCGGTGCCTTCGCCTATGCGCAGCCGCTACCGAACACCCTGGTCAAAGAGGCCATG
GCCGACAAGAACCAGATGATGATCGAGCTGGGCGCCCGGATGGTGGTTGCGTCACTTGCTACCAAGACCGCTACGG
AGTCCCGCGGCGATCAGTCAGCCTCAACATCGGTGCTGGCCGGGTGCGTGGCGAACGTGAGCGAGGCCTACACCCA
GGCAATCATGTGGTGCTGCGCCTACATGGGCATCACCGACAAGAAGGTCGCCTACCAGGTCAATCAAGAGTTCGTC
GAACTGACGGCTGATCCGCAGATGATCACGGCCTTGGTGGGCTTGTGGCAGAACGGAGGCTTTGCGAAAGCGGATC
TTCGAGCATATCTGCGCAAGCTTGGGCTGATTGCGCCAGAGCGCACCGACCAACAGATCGATGGCGAGCTGGAAGA
GCAGGGCGACGGCCTGGGACTGGACGACGAGGGCAAAGTAGATGGCGGTGAACCAGGCGATACTTGATGCGACCAT

CCGGCACGCAGTGTTCCTCGAAAAGCTCAAGGCGGGGGAGGTGGGCAAGTTCGCTCCCTTCCTCAAGGAGATTGAC
CGGTCTATCCGTGATCGGCTGACCCAGTCGGACCTGACCGAATACAACGTGAAGCGCCTGGAAGCGTTGCTGAAAG
AGGTCGACAGCCTGTTACTGGGAATTTTCGACCGCTACAGCGCGCAATTGAACCTTGACCTGGTGGACATCGCCAA
CTACGAGGCTGAGTTCGAAGCGACGAGCTTGGCCCGATCGGCACCGGTTGGCGTGTCGCTGGATGTGGTCGCCCCG
ACAGCCGCTGCAATCCGCACCGCAGTGCTGACGAACCCGCTCAGCGTGCGCGGTACCGGTGGCGGGAAGCTGCTGA
GGGCTTTCATCAAGGGCTGGACCAGCGCCGAACATGAGCGCGTCACCGGCACCATCCGCCAGGGCTTCTTCGAAGG
ACAGACGAATTTCCAGATCATCCGCAACATTCGCGGTACCAAGGCGGCGGGGTACAAAGACGGCATCCTGGCGACC
ACCAACCGTAACGCCAGCAGCGTCGTGCACACCGCCATTCAGCATGTGTCATCCCAGGCAAGGATGGAGGTGGCCA
AGGCCAATAAGGACATCGTGTCCGAAGTCGAGATGGTCGCTACGCTCGACAGCAAGACCAGCCAGCTATGTCGCTC
GATGGACAAACGCCGGTTTCCAGTCGATTCAGGCCCACGGCCGCCGTTTCACCCGAACTGCCGCACCACCTTCATC
CTGCTGACCAAACTTAGCGAGATGTTCGCCAAGGGCGCTACCCGAGCTTCAGTGGGTGCCAATGGCGGCCAGCAGG
TCAGTGCAAGCCTGGATTACTACCACTGGCTCCAGCAGCAGCCAGCTTCGTTCCAGGACGTTGCTATCGGCCCAGT
CCGGGCCAAGCTTTTCCGGGAGGGTGGGCTGACCGTGGAGCGTTTCGCCGAGATGCAGCTTGATCGCAACTTCGCC
CCGTTGACTCTGGTGCAGATGCGCGCTCTAGAACCGTTATCATTTGAAAGGGCTGGTCTGCTAATCTGATTGTTTT
AGCGCTATATCAAGGAAGGTTTAGGATGCTGGGTAAACTCCAAGGACAAGAGCAAGGAGACCTTCTCAATGATTTT
GTGCGAGCTTTTGGGTTAGCAGGATTTCATAGTTTTGGTGATCGGCTGTGGAATCAACGCAATGCTATTTTCTGCA
TTAAAGTGTTTGCCCATTCCCACCAGTATAAGGATGATATTTTGCTGGTGCTGGCAAGTATTCGGAATAATAGATG
GTTAAGATTGCACGTACGCGAACAAGCCCACACTTTTATTTTTGCCCTCTCTGGTAGCAAAGAGGAAGTTGAGGCG
CGACGCAGATTAATTGCGATCACAGACCTTATATGCGAGTTCGCTCAAGATGATGAAACACTTGAGACATTAGAGA
TGTATTTAGAATAAACGATTATGAATATAACCCGCTTCGGCGGGTTTTTTTATGCCTGCAAAACGGGCGAAACATA
CCCAAGGGGTGCATCAACGTGGCAGAAGAAAACGAAATTGACCTGGAAAACCCGGCAATCAAGGCCGCTATCGCGA
CTGCCGTTGAAGCATCCGTTTCTGGTCTGAAAACTAAAAACTCGGAACTGCTGGGTAAGCTGAAGGAAACCTCCGG
CAAGCTGACCCAGTTCGAAACCCAGTTTGAAGGCATCGACATCGACGCCGTCAAAGGCCTGCTCAGTCGTGCCGGC
CAGGACGAAGAAACCAAGCTGCTGACCGAGGGCAAGGTGGACGAGGTCTTCAACCGCCGCACTGAGCGTCTTCGCG
GCGACTACGACAAGCAGTTGAAGGCCATCAGCGAGCGCGCCGAGAAGGCCGAATCCTTCGCTGCCAAGTTCCAGGG
CAAAGTCCTGGGCGACTCGGTGCGCGGCGCAGCACTGAAAGCCGGCGCTCTACCTGAAGCAACCGACGACATCATC
CTGCGCGCCAAAGGCGTGTTCACCCTTAACGAAGATGGCGATGCGGTCGCCGTTGATGAATCCGGCCAGGTCATCC
TCGGGAAAGACGGCAAGACCCCTCTGACTCCGCTCGAATGGGCGGAATCTCTGCGCGAAAGCGCACCTCACCTGTG
GCCAAGGGCTTCAGGGACATTTGCCCCGGGCGGGGGTGGCGGCAAGGCTGCATTCAAGCGCTCCGAAATGACCTCC
GAGCAGAAGCGCGACTTCCAGCGCAAGCACGGCCAAACCGCATATCTCGCATTGCCCAAGTAAGGGGATTAACCCA
TGGCTACAACCGTTAACAGCGACCTGATCATCTATAACGATGAGGCGCAAACCGCATACCTGGAGCGAGTCCAGGA
CAACCTCGACGTGTTCAACGCATCGTCCAACGGCGCGATGGTGCTAGACAACGAACTGATCGAGGGCGACTTCCGC
AAGCGCGCCCTCTACAAGCTGAACGGCTCCCTGGAACACCGTGACGTCAACTCCGAAGGCAAGGTAACTGCCAAGA
AGATCAGCGCCGGCGAAGCTGTCGGCGTCAAGGCTCCCTGGAAGTACGGCCCATACCAGACCACCGAAGAGGCGTT
CAAGCGCCGCGGTCGTCCGGTCGAGGAGTTCTCCCAGATCGTTGGTGCCGACGTTGCTGACGCGACCCTGGAGGGC
TTCATCCAGTACGCAACGGCTGGTCTGCGTGCCGCTATTGGCTCCAACGCTGACATGGTGGTCTCGGCCAACATCG
AAACCGACGGCAAGAAAACCCTGACTCGCGGCATGCGCAAGTTCGGCGACAAGTTCGGCCGGATCGCCTTGTGGGT
CATGCACTCCAGCGCTTACTTCGACATCGTCGACGAAGCCATCACCAACAAGATCTACGAAGAAGCGGGCGTCGTG
ATCTACGGCGGCCTGCCTGGCACCCTGGGCAAGCCTGTACTGGTAACCGACACCGCGCCGGCGGATGTCATCTTTG
GCCTGTTGCCGAATGCCGTAACAATCACCGAGTCGCAGGCCCCGGGCTTCCGTTCGTACGAAGTGAACGACGAAGA
GAACCTGAGCATTGGCTACCGTGCGGAGGGCACCGTGAACATCGATGTGCTGGGCTACAGCTGGAAGGCCACCACT
GGCGGTTCCAACCCAACCCTGGCCGCTGTCGGCTCCGCTGCCAACTGGGTCAAGCACGCGGGTAGCAACAAGGTCA
CCGCCGGCGTGATGATCAAGCTCACTGCGACGCCTCCTGCCTCTGGCGGCTAAGTCTCAAACTCAACGCGCGGTCA
GCGATGGCCGCCTTGGAGAAATACATGGAATTGACTTACAGCAACCAACTCTCCGGCTTCGACCCGGAGAAGCGTT
ACCGCAACCCGGAACACTTCGACAAGCCAGAAGCGGGAGTAACCAGTGTGCTGGTAGTCGGCCATTGGCCGAGCGT
TGTTGATGCGTACGAAGCGGCCGGCATTGACGTGTCGGTGAAGGAGGCCAAGCAGGTAAAGATTGTTGGCGCCGCC
AATCAGGGCGAACTGGAAAAACTCATCGCAGCTTTACGGGCTGAACATGGATCTATCCAGGTCCTGGTTGACGGCC
TGGAAGCTGGCGAGATTCACCGTCCCGGGTCGGGCGAGCTGGCGGTGCGTTTGTATGAAGTACTGGGCACCATCCA
TGCCTCAGTTGGTGAGCTGACCGCCGAGCGTGACGGCCTGCTTCTTACTGTCGACGAGCTGCGCGATGATGTTGAA
GCTCTGAAGAGAGCCGCCATCACACCGCCGGCTGGAGAGGTCGATGAAATCGCCGCGCTGAAAGCAAAGCTCGATG
AGGCCAAGGTTCCGTACCGGGCCAATGCTTCGAAAGAATCCCTGGAAAAGCTCGTCGCTGAGCTGACCAAGGAGTA
ATACTGCTGGCTGTCGGTTACCTGGCGGCCACTCTTCAAACCATTCCAGCGAGTTAACGCATGACACTGATCATCG
AGGATGGCACCGGCAAGCCTGACGCCGAAAGTTACGCGAGCGCCGAGGATCTGGCCCGGTATGCCGTGAAATTCGG
CACGGTCATTCCTGCAGGTGTTCCTGAGCAGGAAGCGTTGCTGCGACGGGCCGCACTGGCGATGGATGGCATGACC
TGGAAAGGGCGCAAGACGAATAGCGAGCAGGGCTTGTCCTGGCCGCGCCGGGAGGTGCGGTTGGATTATGAGATCA
AGCCAAACAACTACCTACCGGCGCGGATCCAGTACGGGCAGATGGCCCTGGCTGCTGAGATCCATCAGGACGATAT
CGACCCGGTGGAGAAGCGCAAGGGCGCGGTTCTGCTTGATCGTGTTGAAGGGGCGGTGACCCGGCAGTACGCGGCG

ATCCCCAACACCAGCAATCGGTTGTTGCCGGCAGCGCCGGATCGACCGAGTGCCACTCAGTTCGCTGACTACTTAC
AGAAGCGTGGATTGTTTTCGGTGCGGGTCTAAATCAAATATCCAACATTTTCAGGTTTGTATAGTGGACAAAAACA
CTGAATAAATCTTCGTTGCTGCTAGCAGATACGAGCTCAGTAAAAGTAGAAAATTCATTTGAGTGGAACGGGACAT
GGTGTTGAGCATGCTCCGCGATCAGCTCGTCGGTAAATTCTTCTGCGCGCGTTCCAGCTAAGACGGTGCGTATTGC
CTTTTTGAAATCCTTTCTCGCTGAGCATTTGTCGATGCTAGTGAAGTCTGCCAATGCATTGTTTAGACGGCCAGCG
GCGTCGATCAAAAACAATGCTTTTTCGCCGTTAGTCATCGCAGGTCCTTATAGGGTAACTAAATGGCATTCTACGA
AGAAATGGCCGTGATGGCTCTGGAGATGATCACAGAGTTCGGCCAGCCCGTGACTATCAGCAAGACGCAGCCAGGC
GAATACGACCCTGATATCGGAGGCGAAGCTCCTGGCGTAACCGTCGAACAGGTCGCCCAGGGCATCCTGCTCGACT
TCACCGGCCAAGAATTCCAGAACAATAGCCTCATCAAGCAGGGCGACAAGAAGCTCAAGATCGCCGCGCAGGGTTT
GGCCTGGGTGCCCGGCCTGCTCGACAAGGTAATCGCCCAAGGCCGCATCTGGTCAATTGTCCCTCCGCTGAAAGAG
GTCAACCCCGCCGGCACGCCGATCCTGTATGAATTGCAGGTACGGTCATGACAAACAAATACGCGAGCATGAACGG
CAGCTTCGCCGAGAACATTCGCGACTTCGCTGAGCGCGCCCAGGCTGGTATTGACGCAACTATCCGAGAGATCGTT
ATCGAGATCGGCAGCAGCGTTATCCGCATGTCACCGGTGGGCAATCCAGAGATTTGGGCTGCGAACATCGTTCACC
GACAGGCGAACAAGCGGGCCGCCGATGACTACGACTTCAAGGTCGCGGTCCGCAATACGATCATCAACCTCAACGA
GTCGAATTTCACGAAGGCCGGCAAGCTGCGGCGCGGCGTGAAATATGCCAAACCCCTGACAAAGACCGAGCGCGAC
CAGAACTTCAATGTGAACGGCTTGGTCGCCGGCAGGGACTACGTCGGTGGGCGGTTCCGCGGGAACTGGCAGTTTT
CCATCGGCACACCCGCAGAAGGCACGCTTGATCAGGTCGACCCGGCCGGTGGCGTGACGCTGGCCAAGCTTAAGCT
GCAGGTGGAGTCGCTCACGGCAGGACAAACTGCCTATATCGTGAACAACTTGCCCTACGCAATCCCGCTTGAGTAC
GGGCACTCGACCCAAGCGCCCGGCGGCATGGTCCGAATCACTCTGGCCCGCTTCCAGCAGATCGTCGACGAAGCCA
CAAGGAACAACCAGGTATGAGCCACGCCATCATTGCGTCCATTTACGAGGCCAAGCTGATTGCCTGGAGCAAGGCG
CGCACGGAGCCGATCAAGGTGGTGTTCGAGAACGTTCAGTACGACCCGGCGGACGCCGAGACCTATCTGCGGGCGT
TCATGCTCCCAGGCGACACGGCAAGCAGCACGCTTGCCGGCGACCACCGCGCTTTCATCGGCGTCTACCAGGTCAG
CATTGTGGCCCCGGCCAATACTGGCAAAACCAAGACAAATCCGCTTGTAGCCGAGCTGGCAGTGCTGTTCCCGCTT
TATGCTCGAGACACAAAGGCAGGCGTCACCGTCATCACGATGTCGCCGGTTGATCTTGGGCCTGGCATTCCTGACC
CGCTCACATACACCGTGCCGGTGTCGTTCGAGTATCGAGCTGACATCGCCATCTGAATACGCCCGTTGGGCAAACC
CCGAAACCCGCCTCTGTGCGGGTTTTGTCATTTCTGATAAGAGGAAACACCCATGGCCGGCATCCAAATGCCCAAC
GGCGCCACCCTTGAGATTGCGTCGACTTACGGCGTTGCAATCCCATTCACTGCGCTGACCAATGCCAATCCGGCAA
TCGCAACTGCTGCAGCCCATGGCTTGGCCGAGGGCGATGTAGTCGCCGTCAGTTCCGGCTGGACTCGCCTCGACGG
CCGCGGTGTCCGTGTGGGGGTAATTGCCAGCGGCACCTTCGCCCTGGCTGGCGTGAACACCACCAACGCTCAGCAG
TACCCCGCGGGCTCCGGTATCGGCTCTGTCCGCGAGGTGACGGCCTTCACTGAGATCTCGCAGGTCACTGAAATGA
ACTCGGCCGGTGGCGATCAGCAGTTCCTTACGTTTGGTTTCGTGGCCGACGACAACGACCGCCAGATGCCGACCAC
CAAGAATCCGATCACGCTGACTTACACCGTTGCTGACGATCCGTCCAAGCCCTATGTGGCTGTCTGCGAGGCGGCG
GACGATGACAAGCAGCCTCGCTTGCTCCGCCTCAACCTCCCAGGTGGTAGCAGCATCCTCTACAACGGCTACGTGT
CGATCACTGCGACGCCGACCATGTCGCGCAACAACCTGATGACCCGTGTTATCAGTGTTGCCCTGACTGGTCTTCC
TACCCGCTACGCGGCCACGGTGTAACCCATGGCCAAGTTCAAGCTGATCCAAAAGCCAACCTTCAAGGCTCCGGTG
ATGATCCAGCGCGCGGGCTACAACGCTGAAAAGGTTGAGTTCGAATTCAAGTACCTGGACCGAACCGCGCTAGCCG
AGCTGTATACCGGCTGGAACGAGCGGCACGACGAGCTGGGAAAGCAGGTCGGGGACATGGACCTCAAAGCTTTCAC
TGCCGCCCAGATCGCCCTGCAAGCCGACCAGCTATTGGATGTGGTGGTGGGCTGGGATATTGAGGAGGAGTTCACG
CCTGAAAACGTGCGCATCCTCGTCAACTCGATCAACTCCGCCCCGAAGGCGGTGCTGAATGCTTACGCAGAGGCAT
TCAGCGAGGCCCGCCTGGGAAACTCCTAAGCGCTTCCCGCGCCCTGTATGAGCCTGGGCCATCAGATGCAGACCTG
ATGGCCTTCGGCTTGTCCCGCCAAGACATTCCCGACAAGGAAGTTGGGATCTGGCCCGACAACTGGGACGTCTTCA
AAGTCTTTGAGGCCATGAGTACCCAGTGGCGTACAGGCTCGTGCGGCGCAACAGGCATGGACTACAGCGTTCTCCC
GGGTGTGATTCGGATGTGCGGCGTGCCGATCGGCCAGCGACAAACCATTTTCAGCGACTTCCGCCGTATGGAGGCT
GAAGCCCTGGAGGTGATGGCGGAACAGAGGGAGAGCGCCAGGGGCGGGTAAAGCGTCGGTGTACTGCTATAGAAGA
CTAGCAGCCTAGGGATGAGCCCCCCCATGCGGGGCTTTCGTGTTGCTCACTCGTTGATGATAAAGTTCCGCGATAA
CTCAACGAGGGAACGACATGAAATTATTCGTAGGTGCTCTGGCGTTAACGTTGCTGGCTGGTTGTGCTACATCGCC
AGTACCTTCTGACAAGGCGCGTTCAGCACCATCTGAAAGGGTGAGCGGTTACCAGAAGCCAGTACCTGGTGGCAGC
TCGCTGATTGTGACGAGAGACACTGGGTTTCAAGGTGGCGGATGTTTTGCAACGATTTTCCTAAATGGTGCACCCG
TGGCGAAGCTGGACACTGGCGAGAAGGCTGTCTTCCAGGTGCCTTCAGGCGAGTGGTTGCTAGGTGCCGCGCTGGA
CGGTTCAGCGCTTTGCGCAGCTAACCCCGAACGAATGGAAACGTCAGTAGTTTTGAAGCAGGGCCAGCAAAAGAAA
TTCAGGGTTTTTATTCCTGCGGGTGGTGGGTCGATAGCCGTACAGCCGAGCAGCTTCTAAGTATTTAAGGAGAAGT
TGATATGTCACAGGTGGTCGTTTTGGGCTGGCTTATTGTTGTCGGACTCACAGCCGTGGCGGCGAACGCGAAGAAC
AGATCTGCGTTCGAAGGCTTTTTGGTGGGGATTTTCTTTCCTGTTCTCGGACTGGTCATTTACCTAATAATCAAAC
CAAACCCACAAAAAACTTCAACTATTAAAGAAGGTCTTCTCGTCAACTCTAGCGGCGAGAGAGCCTGCCCTAGCTG
CTCAGAACTTGTAAAAATGCAGGCGTCTAAGTGCAAGCACTGCGGAAGTGGTATCACGCCAATTACTGACGCTGAA
GCAAGCTCTGCGCACGAGGCTGTGTACGGCCCCATGAGAAGCCACCGAACGATTCTAGTTTTGTTGCTAGGAATAG
CTGTTGCGGTTTTCGGCTACTTTAGCCAGCACTAAATATATTTCCATAAGCCCGCCTAGAGCGGGTTTTTTTACGC

CTGGAGAAAGCATGGCTACCAACTTCACGTCCTTGGGCATTGCCGTAAACTCATCGGAGGCGGTGAACGCTGCCGG
CGACTTGGACAAGTTGGTCGACTCGGCAGAAGGCGCGGAGAAGGCGATTGTCGATCTGGGAAAAGCCGGCGATGGC
CTGGCCAGCACCGGAAATAAGATCACTCAAGCTGAAAGTGAGGTGGCACAGGGCGTCGACAAGTCTACTGCCGCGA
TAGATCGCAGGTCCGGTGCAAGTCGCAAAGCCGCCGACAGTGCGGTAGCTGAGATCAATGTCATCAGCCAGCTAGA
CAAGGCGATGAGCGGCAATATCACCAGCATGGAGTCGCTGATCCAGGCTGAGGGATTGCTCGAGCGTGCCAAGAAA
GGTGGACTGGTAACGATAGAGGACCAGGTCAAGTATCAGGATCAGTTGGGGAAGGCCCACGACAGGCTTGAAAAGG
CTGAAGCCAAAGAACTGGCGCAGAAGCAGCGCCTGATAGAGGTTGAGGGCCGACGGATCGAAGCGCTGAAGCGCAC
CGTGAATGGAATGGACCCCTTAACCTCCAAGCTGGCGAAGCTTGAAGAGCGTGAGCGAGCACTCAACGAGCTTCAT
AAGCTAGGCGAGACTGACGCCAACCGCTACAACGAAGCTTTGGCAAAAATCGGCAAGGATCGTGCAGGGCTGACAG
CTACGGAAACCGCGTTCGACAAGTTGAAGCTTGGCACTCGCCAGGCACAAGAGAACGTGATGCAGCTCGCCAACGC
CCTGCAATCTGGCGATCTGGGGAGTGGTGCGCGGGCCATTGCGCAATTGGGTGCCGGTGCCGGCGAATCAGCTCGA
AGTCTGGCGGGGATGATAATTCCAGCCGGCCTACTGGTGACCGTTCTTGGGTCGCTTGGATACGCCTATTTCGATG
CAATGAAGCAGGCTCGCGAGTTCAATGCTGCTATCAATGGCGGCACCAACGGCGCCGGTCAGACAATCGCCAGCCT
GAAGGATATGGCCGAAGGTGCCGGGCGCATCACTGGAAATCTATCTGGCGCGCGCGAAGCGGTTGTGTCGCTTGCG
TCGGGGGCCGCCACAAGCGGCACGCAGATGCGCAATCTGGCTGAGGCTGCGACAGCGATCAGCGAGATCACGGGGC
AGGGTGCCGGAGAGCTCGCAAAGTCATTCGCCACTGCTGGGGATACAGCTACGGAGGCTGCGGGCAAAATCAGCAG
CCAGTATGGGCTGCTGACCCTCGAGCAGTACCAGGTGATCAAGGGGATTGATGACCAGGGCGATCATCAGCGCGCG
CTTGATGTCCTGAGTGGCAATTTGAATGAGGCGGCGTTAGAGCGACTCAAGGCCTATCGCGGGTCTCTATCCGATA
TTGAGCGAGACTGGGACGACGTCGGGGAAGCTACGAAACGCGCTTACTCATATATTAGGTCGGAAGCTTTCCCAGA
CGTCGCAAAGCAGATCGAGATTACCCAGCGCGTGCTGGATACCCGTAAGGGTGGCGGATTCGCCGGCGCAGTCTCC
AGCGGGCTGAGCTCGCTCAATACTGCGTTAGGCTTGGGTACCGGCGAGCATGACGACTCTACTGAGGCGCTGGAGA
AGAAGCTCGCGAACCTGAAAGCCAGGCAAGCAGCCAACGCCAATCTGGCAATCATCACTGGCGAGAACACCGACGC
AAACCAGAAAGCTATTAAGATCCAAAAAGAATTGGATGCGCAGCTCGATGAGATTAACCCTCTAAATAAGCGCACA
GCAGGGCTGGACAAGCTCAACGATAAATTCAGAACGCTTTACGAGAATGCTGAGAGGATCGGCCAAAAGTCTAAGC
TTCTGGAGGGTGTCGATTTTGACGGTAAAAAGTTCTCAGGGGGGGCTTACGATACCTTGGTTAAAGGGCTTAACGA
TAAAAACAAAGGCCCGAAGGCCGCCTCAGCTCAAGTCGATCTGACCAGTTTCAATAACGCTAAAAACAACCTGTCC
GACATCGTCACGGACTACCGAAACGCCCAAAAGGAACTGGAGGCCCAGCAGAAGGCCGGCGTGGTGTCGCTGTCGG
ACTACGCAAAGCAGCGATCGGCCCTGATCAATCAGGAAAAGGACGATGTGACGGCCGCGTACCAGGCTGAGATCGA
TGCCCTGGAAGCGGCCAAGGCCAAGAAAGACACAACGGCGGCGCAGAGCATCCAGCTTGACCAGAAGATCGCCGAT
GCAAGGCAAGGGATGGTCAAGGCTCAAAAGGATGCCGATAGTCAGCTGACGGTCATCGCTACCAACGAGAAGGGCC
GCCTGGAGCAACTGACGGCCGCCTCGGAGGCCTACGTCAATCAGCTGGAGCGCCAGCGCGCCGCACTGGAAGCGGC
CGGTTCCCGTGCAGCAAACGGGCTGGGTCTGGGCGACCGGCAAGCAGCACTGCAGGCGAGCCTCGACGCAACCACT
GATAAGTTCAACGACGAGCGCGCCAAACTTCTGGATCGCCGTAAGACAGCGCCTGATAAGTACAGCCAGGAAGACT
ACATGCGCGACCTGGCCAGCCTGGAAGATGCGGAAAGGAAGTACCGCGACACAGTGGTCGACAACTACGACAAGAT
GTCGGAGGCCCAAGGCGACTGGCGCAGCGGCGCTTCTTCTGCCTTCCAGAACTACCTGCAATCAGCCAACGACGTT
GCCGGGCAAACCAAAAGCCTGTTCACCAATGCCTTTTCCAGCATGGAGGACGCGGTCGCTGACTTTGCCATTACCG
GCAAACTTTCGTTTTCCGACTTTACCAAGTCGATCATCGCTGACATGGCGCGTATCGCTACTCGTCAGGCTGCGTC
CGGTTTGCTGTCGGGGCTGGCGGGCACTGCGATCGGTGCCTGGTTCGGTGGCGGTGCAGCGGCGGGTGCAGGCGCT
GGCAGTTTCGGCTCCAGCATCGGCAGCGCTCTAGTAGAGGGTAGGGCGTCAGGTGGTCCTGTCGATCCGAACACCC
TGTATGAGGTAAACGAAAGGGGGCCTGAGCTGTTCAGTCAGGGCGGCCGCTCTTATCTCATGACGGGCGCACAGGG
CGGCAGCGTCACTCCGCTGATGACTGGCGGCAACTCAATCATGGCTGCAGCTGGCAGCGGAGGGGGTGGGGGAGGC
GGCAACACCTACAACTTTCCCGTGTCGGTATCCGTGCAAACGGCTGGCGACGGCGGTAATGCAACTCAGGAAGACA
CCTCGCAGGCCGGAAGGAATATCCAGCAGGTCGCCAAGACCGAAGCCGAGGCTGCCATCGCACGCGGTGTTCAGCC
TGGCGGGGCTATCTGGCGAGCTATCAACGGGAGGTAACGATGGCGATTGAGACATTCACCTGGGCCACCCAGAACG
GGGAGGCACCCGATATTACCTATCGGGTGCGCACCTCGCAGTTTGGGGACGGCTACAAGCAAGAGGTCGGGGATGG
GATCAACAACAAAGTCGATGCCTACCCAATCACCCACACCGGCGGCCAAGCAAGAGCGCTGGAAATTATGGCGTTC
TTCGATCGTCACAAGGGTGCCAAAGCCTTCCTCTGGACAACCCCGCTTGGCCAGCTTGGCCTTTTTACCTGCAAGA
ACCCGACCCCCACTCCCATGGGCGGGGGCGTATTCAAAGTGACGGCGACGTTCGAGCGCGCCTTCCACCCGTAAAG
GTCAATCCATGTCGCTGATCAATGCTATCCAGACCCTTGAGCCTGGGAACGAAATCATGCTGTTTGAACTGGATGG
CAGCGATTACGGCGCCGATGTGCTGCGCTTCCACGGCCATGCGATACCGCATACGCCTGCCGAACTGCTGGCCGCC
GGCAGCAACGCTGACCAGCTGCCGGCCAAGTCGATCTGGTGGAAGGGCGAAGAGTATGGCGCCTGGCCAATGCAGT
ACGAGGGCAGCGAGGCGAACGGCGACGGCACCGCAGTACGGCCAAAGCTGTCGGTGGGCAACGTCAATGGACGAAT
CACCGCTCTATGCCTGGCCTTCGAGGATCTGCTCGAGTTCAAGGTGACAGTTCGCAACACGCTGGCCGAGTTCCTG
GACGCGGTGAACTTCGAAGACGGCAACCCCGCGGCCGATCCCACCCAGGAATCGATCGAAGTCTGGTATGTCGACC
AGAAGACCAACGAGGACGGCGAGACGGTCAGCTGGGACTTGGCCAGCCCTGGTGATGTCGGCGGCGAAACAATCGG
CAGACAGATGACAACCCTGTGCCACTGGGCGCTAACCGGCGGGTACAGGGGGCCCGACTGTGGCTACACAGGGCCG
TACCGGGACAAGGATGGGAATCTGACAGATAACCCTGAGAATGACATACCTGACGGAACGCTGGGCCGCTGCTGCA

TTCCCCGCTTCGGCGAGGGAAACCCTTACCCATTCGGTGGATTTCCCGCTGTGTCACTCATTGCCCGGAGCTGAGA
AATGCGCAAACACATTATCGCCGCCATCCAGGTGCATGCGGCGGCCGAGTACCCGAAAGAGTGCTGCGGGCTTTTG
ATTTCCGTGGGCCGGGCACAGAAGTATTTCCCGTGCCGGAACATCGCCACGGAGCCGAACGAAGAGTTCCGGCTTG
ATCCAGAGGACTACGCCGCGGCGGAAGACCAGGGCCAGGTGATCGGCATCGTCCATTCTCATCCGGACGCCACCAG
CCGCCCGTCATCGCGCGACCTGGCCATGTGCGAGGCCACGGCCTTGCCCTGGCACATATTGAGCTGGCCCGAGGGC
GACCTGCGCACTGTCACCCCGAGCGGCAACACGCCGCTGCTAAAGCGCCCATTCGTGCATGGTGCCTGGGACTGCT
GGCAGGTCTGCGCCGATTGGTACCGACGTGAGTGGAGCCTTGAGTTCGAAGCCTTCCAGCGAACAGAGGGCTGGTG
GGAGAGTGCAGATAGCACCAGTCTGTACGAGGCGAATTATACCGCTGCCGGTTTCGAGCAAGTCGACAAACCACAG
CGCGGCGACATGATCGTGATGGAGGTCGGGCGCACGGCGCATCCGAACCACGCGGGAATCTACCTGGGAACTGACC
CGGCGCTGCCCGATGAAGAATCCGGCGTCTTTGGTCCCGGGCCCTTCGTCCTGCACCACCTCTATGGCCGGCCATC
AGAGTTGATTGTCTACGGCGGGCCATGGCTTCAGCGCACACGCTTAATTCTTCGACACAAGGAGGCACGATGAGCG
CCATCGTTTATTCGCCAATGACCACCGTCAAACTGTCCGGCTCGCTGGCTCAGAAGTTCGGCAGGCTGCACCGGCG
TCAGGTTGGCCCGGGTGATACGTGGGAAGTTTTCCGGGCGCTTAAGGCCACCATTGAGGGATTCGAGGCCGAGATT
CGCCGCCTTGACCGCCTGGGTCTTCGCTTCGCCATCTTCCGCAACCGTAAAAACGTCGGGCCGGATGAGTTCGGCA
TGGGCGGGGCCAGGGAGATAAGGATTGTCCCAGTCGTAGAAGGTCGCAAGCGGGGCGGCATTTTGCAGATTGTGCT
GGGCGTCGTGCTGATTGCGGCCAGCTACTTCGGCGCGCCGACGGCGCCCGCCGGTATCGCGTTGCTCGCCGGGGGG
GTGATTCAGATGCTCAGCCCGCAGGCATCAGGCCTTAAGCAGAGCGCAGCGCCTGAAAACATGCCCAGCTACGCAT
TCGGCAGCGCGAAAAACACCACGGCCAGTGGCAACCCCGTCCCCATCTGCATCGGTGAGCGCCGGTGGGGCGGCGC
AATCATTTCGGCCTCTATCTACGCCGAAGACAAGACATAACCTCGACACGTCGAGCAAGCCGCCCAAGAGGCGGTT
TTTTATTGCCTGGAGGAAAGCATGGGCGCAGCACAGAATCTGGATATTCACGGTGCCAAAGGTGGCGAGAGCAAGC
CAAAGGCGCCGGTAGAGGCACCCGATAGTCTGCGCTCCACCAACGTGGCCAAGATCCTCATCGCAGTGGGCGAGGG
TGAGTTCGATGGTGTGCCCACCGCACGCGACATTTTTCTCGATAACACCCCGATCCAGGACGCCAGCGGAAACTAT
AACTTCAGCAATGTGAAGTGGGACTGGCGACCAGGCTCGGTTGAGCAGACTTACATCCCAGGTATTCCGTCAGTCG
ACAACGAGACCTCCCTGAATATCGAGCTGCGCAGTGGCACGCCGTGGGTGCAGTCGTTTACTAACCTTCAGCTCTC
TGCGGCGCGTATTCGCCTGGCCACACCGCGACTGGCGAGCCAGGATGGCGCAAACAACATCAACGGCTATCGCGTC
GAGTATGCGGTCGACGTGGCAACTGATGGCGGCGCCTACCAGCAGGTTCTGTTAGGTGCCATGGACGGTAAGACGA
CCACGCGCTACGAGCGCTCATTACGCGTCGACCTGCCGCCGGCTGCCAGCGGCTGGCTGATTCGTGTTCGCCGAAT
CACCCCCAACTCGCAGAGCACTGACAAAGTTGCCGACAGCCTGTTCATCGCCGGCTACACCCAGGTGATCGACGCA
AAGCTGCGCTACCCGAACACCGCGCTATTGTTCGTCGAGTTCGACGCCGAGCAGTTCACCAACATCCCGGCCGTCA
CCGTGAAATGCAAAGCGCGCCGTTGGCAGGTTCCGAGCAACTATGACCCGGTGGCGCGGACATATACCGGTGCCTG
GGATGGCACAATGAAAGAGGCGTGGACCAATAACCCGGCTTGGGTCACCTACGGCATCTGCACCCAGGATCGTTTC
GGACTGGGTCGCCGAATCAAGCCGTGGATGGTGGACAAGTGGGAGCTATACCGCATCTCACAATATTGCGATCAGT
TGGTGTCCGATGGTGCCGGCGGCGTTGAGCCGCGCTTCTTGTGTGACATGAACCTGCAGGGAAAGGCCGATGCATG
GTCATTGCTGCGGGACATCGCCGGCATCTATCGCGGCATGACGTACTGGGCTCAGGGCCAACTGGTGATGCAGGCT
GACATGCCTCGGGCACAGGACATGGACTACGTCTTCACCCGGTCGAATGTCATCGACGGCAAGATCTCCTATGGCA
GCGCATCGGCGAAGACTCGCTTCACGCGCTGCCTTGTCAGCTACGATAACCCGCTGAACAACTACGATACCGATGT
CACAGTCTATTCCGATTTGCCGCTGCAGCGCCGCCTGGGCGACAAGCCGACGGAGATCAGTGCCATCGGCTGCACT
CGGGCATCTGAGGCCCAGCGCCGTGCCAAGTGGCTGGTGCTGAGCAACAACCAGGACCGCACCATCAGCTTCAGGA
CCGGCATGGAAGGCCGTATTCCGCTGCCTGGGTTCATTATCCCCGTGGCCGACTCGCTGTTGGCGGGCCGGGAGAT
AGGCGGGCGCATTGCAGCCGCGGCGGGGAAGGTCGTCACCTTGGACCGCGATACCCTGGTCAAGGCCGGTGACCGG
CTGGTGATTAACCTTCCCGGCGGGCGGGCGGAAGGGCGCACAGTGGAAAGCGTGAACGGCCGCAAGGTAACTGTGA
CTGTCGCCTACAGCGAGGCGCCGGCCGCGCAGCTTCAGTGGGCAATCGACGCTGATGATCTGGCTATTCCTCTGTA
TAGGGTTATGAGGACCGCCCGGACCCCAGAGGGCGACTACGACATCAGTGCGCTGCAGTTCGAGCCGAGCAAGTTT
CCAAGTATCGACACTGGCGCACGCCTTGAAGAACGCCCAATCAGTGTTGTGCCGATCACAGTGGTACCGGCACCTG
CGAGCGTCGACATCACGTCGAACCTCGCGATCGACCAAGGCCTGGCAATCAGCACCATGAACATCTCCTGGCCATC
CGTACCTGGTGCGGTCGCTTATGACGTGGAGTGGCGCAAGGACAGCGGCAACTGGATCAAGCTGCAGCGCACTGGC
TCGACGAGCGTGGACGTTACTGGCATCTATTCGGGCGCCTATCTGGCCCGGGTACGGTCTGTGAGTGCCTTCGAGA
TCTCTTCGATTTGGAAGAGCTCAAACCTGACCAACCTGAAGGGTAAAGAGGGTCTGCCGCCGGCGGTAGCGTTCCT
GACCACCTCCAGCTTGGTCTACGGCATCGGCATCCAGTGGGGCTTTCCACCAGGTGCAGAGGACACCCAGCGCACG
GAACTCTGGTATAGCGAGGCGCCCGATTTGGAGACTGCTGAGAAGCTGAGCGACTTCAGCTACCCGCAGGCCAAGC
ATGAGATGCACAGCCTGCTGGCGGGGGCGAGCCTGTTTTTCTGGGCGCGCCTGGTGGATCGTACCGGCAACGTCGG
GCCGTTCTATCCGATCCCAGGTGCGGTAAATGGCCAGGCCAGTTCGGACCAGAGCGAGTACGAGAAGTACTTCGCG
GACAAGATCGGCAAGGGGGCTCTGTACCAGGAGTTGCGCGAAGAGATCGAGCTTATTTCGGGAGATGGTCCTGGCT
CGATGAATGCGCGTTTGCAAGAGCTGGACGCCCAGCTACAGGCGCAAATTGATGCGATTGCGGATATTGCAGACTC
GGCGGCTTACGATCCTGAAAAGCCTTACACGGCAGGTCAAAGCGTGCTGTTCACTGACGGCATTCTGTATCAGGCC
AAGGGTAGTGTTCCCGCTGGCGCAGCACCGCCAGATGCAAACTACTGGCTCAACGTGGGGCAGGCTGTAACGACTG
CCAACGGCGTCGCCGCGCGTGTCACTACGTCAGAAACCAAGATCACCAATATCGAGGGGGTGAACACCGCTCAATC

CACGGCAATCACTGGACTGCAAACCAGCCTCACCACCACCAACGGCAACGTCACTGCGGCGCAAAACGCTGCAAAC
GCGGCCAATACACTGGCAGGCGGTAAGGGCAAGGTGCTGGTGCAGGCGGCAGCTCCAGGGGCTGCTGATCAGCTTG
TGCAGAATCTTTGGATCGATATCACTGGAGGTGCAAACACGCCGAAACGCTGGACGGGTTCCGCCTGGGCGGCTGT
TAGCGACAAGGTGGCCACGGATGCAGCGACGGCAGCTGCCAATGCATTGTCGGTCGCGCAAACCAAGGCAGATGCA
TCGACCGTCACAGCTCTGGGCAACACCGTGACGCAGCAAGGCAACACCATCACGGCGCAGGGTACGGCGATCACCG
GCATCCGGGCTTCTATCGGCCAGCAACTGGATAACCTGCTTATTCGAGGTAACTTCGAGGATGGCCTGGCAGATCC
TTGGACCACATCTACTGGGTCCCCAGCAACAGCGGTCGTAGCCACTGCATCCAGTTCGACGGCATTCAGCAAGGCG
ATCGCCTTCTATGCCAACAGCTTTTGCGGGATCAACTTCAACATCCTGACTGTGGCGGGTGAGCAGTTCGATGTGA
CGGCGGAGATTTCTGGGGATGAGATGACCCCGGGGCAGACCGGCAACTTCCAGATGCAGTTCTACAACAGCGCCAA
TGTGAACTTGGGCTATGTGACGGCCTTTTCATTCGCGGCCACCACTGGCGCGGGTTTCAAAAAGTTCTCGGGCCGT
ATCACTGCGCCGGCGAATGCGGTTTCTGCCAGGTTCGTCACCCGAATCATGGTCGCGAACAGTACGGGGCGGGCTC
TGTGGTGCAACATCGCGGCTCGCCGAGTTACGGCAGCAGACGCTGCAAATGCGTCCGCTACTTCAAGCTTGGCGGC
CATGGTTAGCGATATCGATGGCAAGCTGGTTGCAACATCTGAAAAGGTTGATGGGGTTTACGCTCAGATCAATCCA
GCCTATGCAGGCGACACCGCGACCAGTGCAGGCGACAACACTGTCAGCGCGGGCGTGTGGTCCGTGTGGTCAGCGA
TTGCCGAGGGTGATTCAGCCCAAAGTAAACGAACTGACTTGGTTGAGGCAAAGGTCAACGGTAACGCGGTTGCCAT
TGCTGCTGAGACCACTGCGCGAGTTAATGCTGAAGGGGTAATGGGTCAGCAAATTGCGACCGTAGAAACCAATCTC
AGCGGAGCCAACCTATCGATCGCACAGAACGCCCAGGCTATTCAGACGGTCGGCGACAAAGTTTCGGCTACCTGGT
CGGTGCGCCTGCAGTACACCTCGGCTACAGGTGAATATAAATATGCTGGTGTGGGGCTCGGCCTGGAAGACGGCCC
TGGCGGCTTGCAGTCCAAGTTCATTATTGATGCGGATTTGTTCGCCCTTGGGCAGGGAGGCACGATTCCTTTTGCC
GTAACGGACGGCCAGACGTTTATTAAAGCCGCGTTTATCCAGGATGGCACGATCACCAATGCAAAAATCGGGGCTT
ACATCCAGTCCGATGACTATGTTGCGGGTTCCGTTGGATGGCGACTGAGCAAGACCGGGGTTTTTGAAATAAACGG
ATCTGTTCCAGGGCAAGGGCGCATGACCATGACCAACAGATCTTTGCGCGTATACGACGCCGCTGGACAAAAAAGG
GTGCAGCTAGGAGACCTATCGGAATGAGTTTCGGTATGCGGATATGGGGGGCTGACGGAGCCCTCCAGCTGGATGA
AACATCCTTTACTGTCAGGGTTATATATTCGGCGCTTATACCCAAAACGGCCGGGCGCTTTATCGACATAGCTATT
GCGGGCGTAACGCCCGCAACAGACTCCTGCGTCTGTATCCCTATCGTGCCCTACAGTACGAACGGGCAAAGCATCG
ACGCGATAGCATTTATCCCCTACGTCTATGAGGGCTATGTCCGGGTCTTCTTTGGAAGTCCGGCGGCCACTACTGG
GCCGACCGGGTTAACCACTCAAAGACTGATAGTAATGAGGAATAGATAGTGTCGTTCGGACTGACATTCACAAACA
ATGACAACGTCGTGACTCTGGACTCTGAATTTTCAAGACTTGTAATTCTCGATAGCGGAACGTGGGCGGGCAGCGG
CGGTATAGGCATTGCCTTTTCAAGTGTAATTACAACTGACGAGCCCCCGCTTGTGTTTGTTAGGCCAGACCAGGAC
GACTACTTCGCCGCTTGCCTCGTCCAAGGGAGCGCTGGAAACTGGCACGCCTTTAGGTTCAACACACTGAAAGGAA
GTCACTCCAGCGGAAAGTGGTTTATCGCCGCATTCAAATCCGCGCCAATAGCTGATTACGGCCTGCGGCTGTGGGA
TGCGACCTCGAAGATTATTTTTGATAGCGGAACTCCGTGTGCACAATTTACGCGCACCATATCCGCCTGGAACTAT
CTCGGGGCAGAGCAAACTCAGCCGGGGGTTTTCAGGTACAGCTGGTCCGCTTTTTCACCGATAAACACTGGCGACT
ACATGATGCTAAACAATATTGCCATGGACATGGCGGGCACGGTGTCGAGGCAGGGCGTAGTCCAAGCGGTATGGGA
TTACGGCGGGAACAGGTTAGTTATCCAGGCCACTGGGGTGGACCTGCCGACCGCACAGTATCTCTCGGTAGTCTTT
GCGAAGCCGATTTCTTAAATTAGATAAAGAGAACCTACTTATGGCCAGACAGGCAATTAACCTCGGCGCATTGCCG
AACGGTGTAGGGGGCGATACTCCGCGCTCTGCGAACACTAAGATCAACGATATGACGCAAGAGCTTTATGATGCGC
TGGGCGCTACTACGGGTGGAAGTAATGCCAACGGCACATTTATTAGGTTCGCCGATGGCACGATGATTTGTCATGC
CGCTCTTGTTTCGATTCAAGCGACGGCCAATGTAAGGAAGGATACTAATTTGAATTTTCCTGCCGCGTTTTTGGCG
ACGCCGACTTTGGTTCCAACCCTTCAATATCCCACGGTAGTCGATAACAACAATGTCGCGGTTAAGCGCTTTACCG
GAACAGCGACTGGCCCGAGCGCGGCTTTAATTCAACTGACTACTGCGGAAAATAATAACTATATGGTCGGCTACAT
TGCAGTTGGGAGGTGGAAGTAATGATCATCAAGTTGTCACCGTTTTCCCCCATCGACCCCGCCCAGAAAATAACCC
TCGAAAGACGCGGTGAAGCGTTAGTAATTAATGGTGAGTGTTTCGACTTTGGGCCCGTTCCAGAAGGCGCCACGCT
GCCAATGGAGGCGATCCTCAGCGAGTGGATTGCTCAGCCTGTCAGCCGTGTCGACGGGAGGCTGATCATCTGCATT
CGACTACCTGTTGGCCCTGATGCATCTGCGTCTGCGCTTTACCCCGCCGACATCTTCAATCCACCCAGCGGCAATG
TGAGGCTTCCCCAGTGAGCAATATCGATTACAGCATGATGAAGACTGCCGAACAGCTGCAGCAAGAGCGTGAGCGC
GAAGCGCTGGAGGCCGCCCTGGCTTCGCGGCGAGCAGCGTATCTCTCCGAGTCGGATCCGCTGCGCCTGGAAGCTG
ACTACGATGCCTTGAGTCAGGGGCTGGAGCCCGACTACACCGAGTGGCTGGCATCCGTAGCGGCGATCAAGCAACG
CTTCCCGCTGCCAGCCGCCGCCGAACCTTTGAGCGACTGAGCTTTTGTCGAACACCGCCACCCGCGATCGAGCGGG
TTTATTTTTGCCAAAAATACCTGGAGCACACCGTGCCTTACATCGTCATCAATTCCAGCAACGCCTTCGATCCCCT
CAACCTTATGGAATTCGCCACGGCGGATGAAGCGGATTCAAAGGCTCGCGAGCTGCTGGCCTCGCAGCCCCAGGCC
GTTGTCCGTACAGCGCAGCTGCTGAACACCTACAGCGCCAAGGTAACTGTGAAGGTTGAGGCCGTGCCTGAGATCG
TCCCAGCCGCCGACGAGTAGCTACGTACCGATCGGCCTGACACCCGCCCTGTGCGGGTTTATTTTTGCCTGGAGAA
AAGCATGACCACTTCCGATAAAGACCGGGACATCCTGGCGCGCACGCTCTGGGGTGAAGCCCGCGGTGAAGGTTTG
GCCGGCCAGATCGCCGTGGCCTGGACCATCCGCAACCGCGTGTTCGACGGCAAGGCCAAGTCCTGGTGGGGCGAGG
GCTACGCCGAGGTGTGCCTGAAGCCGTGGCAGTTCAGCTGCTGGAACCAGAACGACCCGAACTACGTCTACCTCAG
CGGTGCGAAGCCGATACCGGCCGCGCAGCTCGCCCAGGCCCAGCGTGCTGCTGACCAGGTGATGACCGGCGCTGTA

CCGGATCCGACTGGCGGGGCCACTCACTACTATGCAACCACGATGCCCAAGGCCCCGGCCTGGGCGGCGAAGGCCA
AGCAAACGCTGCGCCTCGGGCACCACGTCTTCTTCAAGGATGTGCCGTGATGACGCCCGTACAGAAGCTGGCCGGG
TTGGTGGTGTTGATCCTGGCGCTGATGGCCAGCGCCGCCGGCGTCACCTGGCAGGTTCAGGAGTGGCGCACGGGCA
AGACGCTTGCCGAGCAGGCCAGCCTGCACCAGGACGAGCTGGACAGGATCAGCGCGGTGGCCATCAAGCAAGTGCG
CGACGAGCAGGACAGGCGCCTGGGCCTGGAGAAGACGCTGGCCACCTCCGATCAACAACACACCCGAGAACTTTCC
GATGCTAAACGCAACCAGACCCGCCTTCTTGACCGCCTTGCTACTGCTGATGTGCGGCTGTCAGTCCTTCTCGACG
CCACGGATTCAGCCAGTGGCTGCGGAGTGTCTGCCACCTCCGGCGCCGTCGGCGTGGTTCATGGAGCCCGTCGAGC
CCAACTTGACCCAGCGCATGCTCAACGAATTATCGCCATCACCGATGCCGGCGACCAAGGATTGATCGCTCTGCGG
GCGTGCCAGGCGTATGTCAGGGCCGTCGCGCCCTGACCTACATTCCCGGTCGCAAAAATTAACATGACACCAATAC
ACACCCACTTTGCAAAGGATTGCAAAAATGACAAACCCAATCGTTCCATGGATGGGCGGCAAACGTCGTCTGGCTG
ATCGCCTTATTCCACTGTTCCCACCGCACGAATGCTACGTTGAAGTGTTTGCTGGTGGTGCTGCGCTATATTTCAT
GCGCCCTCAGGCCGCGCCAGTTGAAGTCCTCAATGACATCAACGGCGACCTGGTGACGCTGTACCGCGTCGTGCAG
AACCACTTGGAAGAGTTCGTGCGCCAGTTCAAATGGGCGCTCAGTTCTCGGCAGGTGTTCGAGTGGCAGAAGATGA
CCCGCCCTGAAACCCTTACCGACATTCAGCGTGCTGCTCGATTTTTCTACCTTCAGCACCATGCCTTCGCCGGCAA
GGTGACGGGGCAGACGTTTGGTACTGCGACCACTGGTCCGGCAATCAACCTGCTTCGGATCGAGGAAAACCTTTCT
GCAGCGTGGCAGCGTCTTTCCGGAACCTATGTTGAAAACCTCGGCTGGCTTGAATGTGCCGAACGTTACGACCGGC
CTCACACCTTCCATTACATGGACCCGCCTTACTGGCAGACCGCTGGTTACGGTGTGGACTTTCCGTTTGAGAACTA
TGAACGGATGGCTGATTTCATGCGGCGCTGCAAAGGAAAGGTGATGGTCAGCATCAATGACCATCCGGACATACGG
CGGGTATTTGAGGGGTTTCACTTTGAAACTGTAGATATTCGATATTCCACGACGAACCAGCGGCAGGGGAAGGCTG
AGGTCAGCGGGGAGTTGGTGATAATGAATTGGAAACCCTCTGACCTCGGCGGGCTTTTCTAGGCCAGTCGCTTTAT
CAGTCCCAGGCCTTTGTTCCGTACGTTGCCCACGGCCGTATCGACTTTGAACCATTCGAAAGCCTCGGCCGGCTCG
CCCTGGTGCAACACCATCTGCTCGGCACGCTCCTTGGGCGTGGCTGGGTCCAGCCATTCCCTGGCCAGCTCTGGCG
TTAGCACCACGGGCCGCCGGTCGTGAATGTCCACCATGCCGCCGGCGCTGTCGGCGGTGATGATCACAAAGCCGTC
ATGCTCGCCTTGGCCTTCATCTGCGTCGGGTAGCTGGCCTATGGCCGCGCACAGCACAGGCGCGCCATCCCGCCGG
CGGATCAGGTAGGGCTGTTTCTTTGGCCCGCCTTCATCAATCCACTCAAACCAGTTGTCCACGGGCGTGATTGCTC
GGTGTGGCCAGATCGCCCGGAAGAATGGGCCGTAGGCCACTTTCTCGACTCGAGCGTTTATCGCCGCGGCACGGTC
CTTGGCCCAGTGCGGCCGCCAACCCCACTGCACCAAGTCTGCGTGCAAGGTTTCATCTTGCAGGTGGAGCAGGGCG
ACTTGGGTTGTCGGTGCCACGTTGTAGCGCTCAAGTGGCGCGTCACCCACGGAATTCACCAGGGCGTTCGGCATGC
TCAGTGCTGCAACGAAGTCATGAATTCCGCTGTACTGTGAAAGTCTTCCGCACATCGGCAAGCCCTCGGTAGAAAT
TTCAGCTTAGATCACGCGGCAAAATTTCTAAGGGTTTGCTTCCCCAGGCCCCATTTGTCGCTCGCGCAAGCCCCCA
TTCAAGTGAGTTGAGTTTGCTTCTGACAAAACCCAGGTCTGTCTTGCAGCTAGCAAGGGCGCAATTGGAGGTCTTG
AGCTCTGCCCGAAGTTTGTCGCGTTCGGTAGATAACTCAGCGTGCATTCGCACCAGGCCATAGATGTCCTCTCGGG
CTTTGCGAAGCTGCAGGTGCAGTTCCTGGATCTCGTTTTCCAGGAGCAGGGCGTGTTGCTTGTGCATTTCTAGAGG
CGTAGGGCATCCAAGCCACTCGCAGGTGTCTTCATCGATTTTCATGGTGGGGTAATCCGAAGCTGTATGTTTATAC
AGTAATCGAGATTCGTGCGGACTGAGGTTGCGAGGCGACGAGCTGCAGGTGTTCCCGACGATCAGTTAGGGAGCAT
TGAGAACGGCCAGGGTCAGCTTGATGAACTCTTCGTTCCGGTCGATGGCGTCCAACGCGCCGCGAACGTTGTCGGC
GATTTCGGTGCCGCCTTGCTGCTCAACCCAGTTCGTAAGCTCCATGATGGCCGCCTCAAGGGCGAGTTGGTTTTCG
TTGATTTTGGACAGCAGGGAAGGGAGCAGGTCTGAGTTCGGCATCGTTGTTCCTCCTTGGAGTGAACAGCGTAGCA
GTTGGTGTGCGCCACGCATGTTCTAGGGGGGGCTCCTTTCGACCGATTGTGTTGAAAAAGTCGGCTTGCCCAAAAC
GCTCGAATACTGATGGGTGAAATGACCTCTTTTGCGCGCTGCTACGTGAATTCAAAGCTTTGAACCGTCAGCCCAT
AACTCAGATTTCAATCTCTGGCGCGTTCTTTTCTGCCGTAGAGTTCGAAGCCGACTTTTTCAACAGAATTGACCCA
TAGCGGATAGTGGAGAAACTTAGGTGTGCGAGTATTAAGTAAGCGTCCGGAAACTCACTCTCCGGACGGTGAGTAG
CAGTGGGAAGTTGCTAGCTGGCCCTGTTTTTGTTGCTGGCGCTTGCTGCACCGTATGCCTACACTCCAAAAACCTA
TAATACCAGTGCAGAACGGCGTACAAGCTCGTGCAAGGGCGTTCACAAGGATATGTGGAGTTGGATAGTGAGCAAT
TACCTACCAGCAGGAGCATTTTGAAAACAGAGCCGCGCTTGGGAGCGTTGCTTGCACTGGTAAGCTTGTTGGTGTT
GGGCATGCCATGCGTGGAAGCACGTACGCTGTCCCAGATTCGGGAAAGCGGCGTCCTGCGCATATGCGTGGCTGGC
TCAAGCGCTTCTTTCTACCGGACTAACGCGGAGGCGTTCGCGCGGTCACTCGGTTTACGCCCACAGGTAACGGAAC
TGCCGAGTTTCGATGCGCAGTTTCACGACGATCGGGGCGAGACCGTACGCGAGGCAAGCTACGACCCGAAGCTACT
CGCCGAAGGCCGCTGTGATCTTTATCCAAATGACTTGCAGATCGTCCCTTGGCGCGAAACGAAGATGCTGCTCGTG
CCCTATTACCGGGTACGCAACCTGGTCATCGCCCATCGCTCCAACCAGTCCCTAGGCAATGAGGTGTCTGATCTGC
TCGGTCTGACGGCGGCAGTGCAGAAGGGCACGGGCTACGAGCAGTGGATCGATGAAGCCAATCGCGGTCCTCTCTC
CAGTAAGCCGGTCAGAATGGTCTATGCGACAACCGAACAGGCGGTGAAACTGGTAGCCGATGCGAAGGTGGATTTC
ACGGTGTTGGGTAGTGAAAGCGCGCTGCGCTGGGCCCGTGAAGATCCCGAACGTCTCTCCATTGCGTTTCCGGTCA
GCGAAGCGACCAGCGTTGGGTGGGGTGTTGCGCGCAACGCCCCGGATCTTGCGCGAGCACTGGAACAGTTCTTCCA
GTCCAGCAATCGTATTGATTCGCCGCTCGATGCGAGCTGGCGGCGGTACTATCGCGTATCGCTCATGGAATACCGG
CTGTTCGAGGAGTCCCTCAACGAGGACAGCCTTGATATCAAAACCGTAATGAGCTGGGCGATCCCGAGCTTGGTCG
CGGTCCTGCTGTTGCTCGGCGCCATGCTGGCCTGGAACCGACGCCTCAACCGAGAAGTGGACGAGCGCAAGCGCGT

TGCCGCAGAGCTAGCCGAGCGCGAGGCTTTCACTCGCGCGCTCATGGATACCAGCCCCGCCAGCCTGGTGCTTGCA
GATCGGAACGGGGCCGTCAGGGAAGTAAGTCAACGCTTTTCACAGGCGACCGGTTATGTCAGCGAGGACCTGCTAG
GGCGCGACGCCACGAGCCTGTATGTCGACCCTGAGGAGCGCGAACGCTTCCTCGAACTGCTGACACGAAACGGCAA
GGTCGAAAACTTCGAGACCCGCCTGTTGCACAAAAATGGATCAATTCTCTGGGTTCTGGTTGGTGCATCATTCGTG
ACAATCCACGGTGAGACCCTGGTCGCCAGCTGGGTGCGCGATGTCACGGATCATCATGCGGCAGCAGCGGCGCTTA
GCGAAGAACGCGCGCGGCTGCAGGCAATCCTCGATACGAGTCCAATCAACATAGCCTTTTCTACCGAGGGCGTGAT
CCGCTTCGCCAACCCCCGCTTCTGTGAAACGTTCGGCGTAGGCGTCGGTGACGCTGCTTCGCAGATTTACGCGAAT
GAGCATGATCGCGAAGCCATCGTCGAACGCCTCACGGACGACGGCATTGCAAAGGACCAGGAAGTACCGATGCGTG
ACCGGCAGGGGCGCGAACGCACCATACTTGTCACTTACCTGCCAATCCAGATCCAGGGGGAGCAAGGGCTGCTCGG
CTGGCTGCAGGATATCACCGAGCGCAAGGCAGCAGAGCACGCGCAGCAGCGGGCGAAGGAGATTGCTGAAGAGGCG
ACGCGGGCAAAAAGCGACTTCCTTGCAAACATGAGTCACGAAATCCGCACGCCCATGAATGCGATCATCGGCATGT
CTTACCTTGCGCTGAAGACTTCGCTGGACCAGCGGCAGCGCGATTATGTCTCGAAGATCCACAACGCGGGCACTTC
GCTGCTTGGCATTATCAACGACATCCTCGACTTCTCCAAGATCGAGGCCGGCAAGCTTAGCGTCGAGGCAGTGCCA
TTTGAGATAGACGCAGTGCTCGACAACGTGTCCTCGCTGATTGCGCAAAAGGCCTACGACAAGGGCATTGAGCTGC
TGTTCGACCGGGCGTCGGACGTTCCCGAGACGCTGCTTGGAGATCCGCTGCGTCTGGGCCAGGTCATCCTGAACCT
GGTAGGCAACGCAGTGAAATTCACCGAACAGGGGCAGGTTACGGTCGTGGTTCGCAACCTGGACCGCACCGGCGAC
AAAGTTCAACTGCAGGTTCGTATCCGCGATACCGGTATCGGCATGACTCCCGAGCAAACCGGGCGACTCTTCGAGG
CCTTTGCCCAAGCCGATAGCTCCACCACGCGACAATTCGGCGGTACCGGCCTGGGCCTTGCCATCTCGAAGCGCAT
CGTCGAGCTGATGGGCGGAACGATCGCGGTTGACTCAGAACCGGGCAACGGCAGCACGTTCTCTTTCAGCGTCTGG
CTTGGTCTTGACCAGCACATGCGTACGCAGCGCCAGATTGTCCCTGCGGAGCTCGCCGGAGCGCGCGTCCTGGTCG
TCGATGACAATGCTGACGCGCGCCAGATCCTTTCCGACATGCTTCGCGTAGCGGGGCTTTCGCCGGTCGCGGTGGC
GTCAGGCGAGGCAGCTCTGGAGAGCCTTCGCGGTGCTGCGGCCGACGACCCGTTCCGGGTGCTATTGGTGGATTGG
CAAATGCCAGAGATGGACGGCATCGAGACGACGCGCCGGGCGCTTGGCCTGCAGCCTGCACTGCACGCTGTCATGG
TCAGTGCATTTGGGCATGACGAAATGCACGCCGCTGCGCAAGCGGCCGGCATTCGCGCGTTCCTGGTCAAGCCGGT
CAACCAGTCCTCGCTGGTAGAGGTCCTGGTCAACTTATTCGCGCCGCAAACGGGCGTGGTGGCGCTAACCGTACCC
ATTGCGCAGGCGAAGGTGCTCGCCGGAGTGCGCCTGCTCGTCGCGGAGGACAACGAAATCAACCAACAGATTGCCA
GGGAGCTTCTCGAGGCTGCCGGTGCCCAGGTGGAAGTTGCCGGCAACGGCTGCGAGGCGCTGTCGATGTTGGCTGC
AGCGCAATACGACGCGGTGCTGATGGACGTACAGATGCCGCAGATGGATGGTCTCGAGACGACCCGGCGTATTCGC
GTCGGGCCTCACTGCGCACAGATCCCGGTGATTGCTATGACCGCACATGCCAGGATCGAGGACCGGGAGCGCTGCA
TCGAGGCGGGCATGGTCGACCATGTGACCAAGCCCGTGGATCCGCAAGCACTCATTGCCACGGTTCTGCGCTGGGT
CGTACCTCGGTCGGCGGCAGTGGAGCCGTCCAGCGCCGCATCGGGCGAGGAGCGCCTACCGGACATTCCAGGGCTA
GATAGCGCTGACGGACTGAGGCGAGTGGCGGGCAATCGCCAGCTATACATCAAGCTGCTGGGTCAATTTGCCGACC
GCAGGGCTTTGGAAGGGCAAGCGCTGGGGAACGCGCTACGCGCGGGTGATCGGGCGACGGCCGAGCGAATGGCCCA
CACCGTGAAAGGCGTTGCAGGCAACCTCGGCTTCAGCGGCCTGCAAGCCATGGCCGGTAAGTTGGAAACCGCGATC
AGGGCGCACGCTGAGTCGGACGCATTGGTGAATGAATTGATCGAGGCGCTCTCGCTGGCCGTGCATGCAATCCGCC
AAGCGCTGGGTAGCGGCCAGGCAGCACCGGCGGTGGTATCGAGCGTCGACTGGGCACAGCATGCCATTGCGCTCAT
CCACCTACTGGAGGCCAATGACGGAGCAGCGCCGGATTATATGGAACAGTATGCGGATTCGTTGCGCGGAGCACTC
GGCCAGGACAGTTTCGAGCAGTTGATGAATGAGGTGAACAACTTTGACTTCGAGGCCGCACGGCTAACACTCAAAC
GTGCATCGACGGCCCGCGATACCACGTGCTGGAGGGAGCATCATGAGTGAAACGCAAAACTACTCACTTCGCCCGA
TCATCCTCATTGTCGATGATACGCCGGAGAACATTACGCTGATCAATGGCCTGCTGAAGGACATCTACCGCATGCG
GGTGGCCATCAGCGGTGAGCGCGCGCTGAAGGCAGCGGTGCAAGAGCCTCGCCCGGACCTGATTCTGCTCGACATC
ATGATGCCTGATCTGTCGGGGTACGAGGTAGCAGAGCAGCTCAAGCGCGATCCGCGCACCGCTCATATCCCGATCA
TCTTCATCACCGCCATGGCGACAATGGAAGACGAGATCCTGGGTTTGCAGATGGGCGCCGTGGACTACATCACCAA
GCCGATCAACCCACCCATCGTGCTGGCACGGGTGGAAACCCAATTGAAGATCAAGGCGGCCGCCGACTTTTTGCGT
GATCAGAAGGCTTACCTTGAGCAGGAAGTCCAGCGCCGCACCGCCGAGGTCATCGCCATCCAGGATGTGACCATTC
AGGCCATGACCTCGCTGGCCGAAACCCGCGACAATGAGACGGGTAACCACATTCGACGAACTCAGCACTACGTCAG
ACTACTGGCCGAGATGCTTCGCGAACATCCGAGGTTCCAGCAGTTTCTCAATGACGAAAGTATCCGCCTGCTATTT
AAGTCAGCTCCGTTGCATGACATCGGCAAGATCGGCATACCCGATGACATACTGCTCAAGCCGGGGCGCCTCACGC
CGGAAGAGTTCGAGACGATGAAGACCCATACGATACTGGGACGCGATGCCATCCGGCATGCCGAAGAGCAGTTGGG
CATCTCGGTGGGTTTTTTGCGCCTTGCCAAGGAGATCGCCTACAGCCACCACGAGAAGTGGGATGGTACCGGTTAT
CCAGACGGGCTGAGTGCCGATGACATCCCCATCAGCGCTAGGCTCATGGCGGTGGCGGATGTGTATGACGCGCTGA
TTAGCCGACGCGTATACAAGAAAGGCATGTCACACGATCAGGCGCTGGATATAATCCGAGAGGGATGTGCTACGCA
TTTCGATCCAGATATCTGCGAGGTTTTCCTGGCCAACCATGAGCAATTCCGGGCTATCGCGGTGCGTTTTGCTGAT
CGCGGTCAGGATGGCTAATCAAAGCGTCGCCGTGAAATCAATGTCAAGGGCTTTTGGTCATTTTTTTGAGGTTTTG
AAATGCCCCTTAGAGGCAATGCGAACTCCATCGATTATTTGGCATCATCTGCTTGAACGTCCGTTATGGGTCGATT
GTGTTGAAAAAGTCGACCCGGCCTGGTGACCCATGCATTGAGCGGTGAAAGCGCCTTTTTTGCACGCGGCTACGTC
AAATCTGAGTCCTGAACCTTCTGCATTCAACACAGATTTCAATCTCAGGCGCGCACTTTTCTGCCGTGGAAACCAA

AGCCNNNNNNNNNNNGGCGGTTCCCGAGTTGAGCGCTAGCCTGTAGATGGCTCATGCAGGCGCGATCAGTTGCGCGC
CTGCATGAGCAATGGCGATCAGGCGCCCAGGCTTTCGATGTCCCTGGCCAACATCACCAGTTGATGCGCCAGGGAG
CTTTGCAGGGTGTCGGTAGGCAGCTGGAACTTCGGCGCGCCACAGGTCAGGGCCATGATGCTGCCGTCTGCCAGGC
GCAGCGGAACGCCTGCGGCGTTGACGTTGCGGTCCCACTCGCCGAGGGACAGGCAAAAACCGAACTCTTCATACTC
GCGGAACGACTCGTTCAACGAGATCTCCAGGGCCGGCCATTCGTCACCGGCTTTCTTGGCTATTTCGCCGATCAGA
TGCGCCCGCTCGTTTTCCGGCGTGGCGGCTAGGTAGGCGCGACCTGCGGCAGTGGTGCTCAACGGCAGGCGCACAC
CGGCTTCCATACGCAGGCTGGCGACGTCGGGTGGGCGGCAGCTTTCCACGTAGATCATCGACAGGCGATCGCGGCA
GGTGAGGCCAACGGTGGTGTTGGTACGCCGTGCGAACTCGTCCATCAGCGGCTTGCCCAGCTGGCGTACCCGCAGG
TTGTTGACGTAGGCGTAACCCAGGGCCAGCACCCCCGAGTCCAGTTGGTACTTTTCCACGTCCTGGTTGTAGCTCA
GGTAGCCAAGCTGCGTCAGGGTGTAGGTCAGGCGCGAAACCGTGGGCTTGGGCAGGCCGGTAATCCGCGCAATGTC
CTGGTTGCCACGGGTGACGTTGCCGTGGGTGAACGCGCGCAGCACTTCGAGACCGCGACTCAGGGCTTCGACGAAT
TTCCGGTCCTTGGGTTCGTTCTCGGGCGTTTCGATTTCTTCAGTCATGGAGGCTCGTGTGGGCAGCGGACAGGCAG
TGGAAGAGGTTAGTCGATAATGCCCGGCAGTGTGGGCAGTAACCGTCGCGATTTCAATGCAACGACTTATATAGTC
GCGCAAAGTGGCAGCATAGCGGAATCCTGTTCCGCTAGTGGAACCATTGCACGACCCACGACAGGGCCAGCAACAC
CGGAATGGCGATCAGCGCGAAACGGCCATTCCAGGTGAGGACAATCGTCCACGCCGAGGTGCCAGCCGTGCGCGCC
AGGATCAGCACCGCAGGCCCAAAGGGCGAGAGCAGCATCGCCAGGGAAAACGCGCAGACCAGGCTGACGGCAGGCC
CCAGTGGGTTGACGCCGGCCACCTGCAATTGGGCCAGCAAGCCGGCACAAATGCTCAGGGTGATAATCGGCGCAAT
GCCAACCAGGGCGAGCCCGACCACACCCAGCAGCGCCATGCATTGCACCCAGAAAACCTGAGAGGAGCCACCGAGC
AACGGCACCACCTGCTCCAGGGAAACCAGTTGATTGAACAGCGCACCGAACAGCGCCGAACAGCCAAATATAAAAA
TCTCATTACGCATGCCCGCCAGATGCTCGGCCAATTCGTCGTAGGCCTTGCGCGGGCTTTCGAGGAACAGCCAGCC
GCTGACCAGTAGCGGCACCACCATCAGCGATGCCTGGGTGATGTTCAGCCAACTCAGGCTGGCCAGCGCCACAATC
GCACCAATGCCCACGGCGGTGCCGAGCGCCAGCGGGTTGAGAGACGCGGCGGGGATTTCGGGTGATTCGTCGACCA
GGGTCTGCAAGTGGCGGTTTTCCGTGCGTGCGCCAATGAGCATGAGCAGCACCGTGGCGATCACCCCGTAAGGCAG
CAGGTCGGCCCAGGCCAGGCTCGGTACTTCACGGGTGATGATCGCCACGACAATACTGGTCGGCGCAACCAACGGC
ACCAGGGCAAAACCGCGAATGGCAGCGGTCAGCACGCCGCGCTGGCCATCACGCCGGCCCGGATGATCCGGCGCAA
ACGGCTGCAGGTAGCTGTGCAGGGTCGAGCACATCAGGTTGAGCATGCCGAAACTGAGGATTGACCCCAGCCCCAA
TGCGGCCAGCAGATAGCGCGGATAGAGCAGCGAACGTGGTCCACGCAACAGAAAACGATGCACCTCCCGCAGTGCC
GGCAGGCGCTTGACCAGGCAGCCCATCAGCCCCAATCCACCAATGAACGCTGCGTAATAGGCGCCAGCGCTGCTCA
TGCGCTGGACGGCGGGCAGATCCAGCGGGGCCTTGACCAGCCAGATCGCGAGCAACACTAGCGTCGCCAGGCCCAG
GCGCCGAGGGTAGGGCATGAGCGACTGCCAGTGGCGGATAAAAAACAATCCGAACAGCAGCGCCGCGATCAGGCTG
AACGCACTGGTACGCGTGCCCAGGGCAACCAGTTCGCAGAGCAGGGCCAGTGGCAGCAGGCGTGTCAGCTTCACGC
GGGCAGGCGGCGCAGGCGGCTACGCTTGAACACACCTTCGGCGCTGGCCAACAGGTGGTCCTCGGCATCGAGCACA
TCACACGCGGCCATGTAGATTTTCGGCCCGCCGCCCCGGACCCGGGCGATGACCCGAACCCGACTGCCTGCGGGGG
CGGGAGCCATGTAGTTGATGTTCAGTGACAGGGTCAGGGACAAGCGCCACAGGTCCGGGTCGATGTTCCAGGTGCC
GCACATGCCCAGCGCCGCGTCGGCCATGGCGGCAATCACACCGCCGTGCAGGTTGCCGGCCTGGTTGAGATGCTCG
GGGCGCACCTGCAGCGCGATGACGGTTTGCGCCTGGCCGTATTCAACCAGATCAAGGCCCAGGTACGCGGCGAATC
CACTGAACAGGCCTTGCAGGTCGGCGGGTGGTGAGACGGTCATGGTGTGTTCTCTTTTTGTTATGGGGTGAACGGC
GGCACTTGCTATGCACGGTATGTTCCGCAAAGAGCCTTTTTTAGATTGAAGTTGAGCCTGACAATAATTAGTATTC
TAGTCAATACGGAATTAGGTTCTGCACAGCGAAACATAAAATCGGAGAAACCATGTTTTCACGTATCGGTGTGATT
GGCAGCGGCGCCATGGGCCGTGGCATTGTTCAAGTGTTTGCAACCTCAGGCTGTGAGGTGCTGCTGTATGACGTGC
GCCCTGAAGCGGTCGAACAGGCGCTGAGCTTTAACAAGGACCTGTTGGAGCGCCAGGTGGCCAAGGGCAAGCTCAG
CCCCGAGCAGTTGGCGCAGACGCTGGCGCGCATGCAGCCTGCTGCGAGCCTGGAAGCCTTGGCCGGTTGCGATCTG
TTGATCGAAGCCATCGTCGAGAACCTGGAGGCCAAGCAAGGGCTGTTTCGCCAGTTGGAAGCCATCGTTGCGCCGA
GCGCGGTATTGGCTACCAACACGTCGTCGCTGTCCGTCACGCAGATCGCCAGCGGATGCCAACACCCCGAGCGGGT
TGCCGGTTTTCACTTCTTCAACCCCGTACCGCTGATGAAGATCGTCGAGGTAGTGCGCGGTGAACGTACCCAGGTG
CAGGTGGTGGAGCGCCTGTGTGCGCTGGCCGAGGCGGCTGGTCACTTTGCGGCTCAGACCCCAGATACGCCGGGCT
TTCTGGTCAATCATGCCGGTCGTGCCTATGGCACTGAAGCGTTGCGTATCCTTGGCGAGAACATTGCCGATTTCGA
GCAGATCGACCGCATCTTGCGCGATGGCCCGGGGTTTCGCATGGGGCCGTTCGAGCTTTTTGACCTCACCGGCCTG
GACATCTCCCATGGCGTGATGGAGTCGATCTACGCGCAGTTTTATGGTGATCCGCGTTATACCCCGTCTTACCTGG
CGGCCCAGCGGGTGGCCGCAGGTTTGCTGGGGCGCAAGAGTGATGGGCAGGGCTTTTACCGCTATGTCGATGGTCA
GCAACTGCGTTCGCCGCAGCCCCAACGCGCGCATGTGTCGGTTGACCGCAGCTTCTGGCTCGACAGCCAGGATGCG
CAGGTGAGGGGTGAAGTGGCGGCTTTGCTCGGCGCCGGTGGGGCGATGCTCGAAGAGGCCGCTCAACCCTCGGCCA
ATGCGATCTGCCTGATCACCCCGCTGGGGGAAGATGCCAGCAGCGTGATCGCGCGCCAGGGGTTGCCCGCCGAGCG
CACCCTGGCCCTGGACACCTTCTGCGACTTCTCCAAACGCCTGGTGCTGATGCGCCAGCCTGCGCTGGACCCGGCA
TTCGAAGCCCAGTCGGTTCAGGCCTTGGGCAGCAACGGGGTACCGGTGGAAGTGATCAATGACTCACCCGGTTTCA
TCATCCAGCGGGTAGTGGCAAGCGTGGCCAACCTGGGAGCGGAAATCGTCCAGCGCGGCATCGCCACCCCGGCCAC
CCTCGACCGCGCGGTGATGCTGGCGCTGGGTTATCCGCGCGGCCCACTGGCCTTCGCCGAGTTCTATGGCGCCGAC

AAAATCCTCGCGGTGCTGCGTGGCATTCAAGGCTGCTATGAGGAGCCGCGCTACCGTCCCAGCCCCTGGCTGCGTC
GCCGGGTCCAGTTGGGCCTGAGCCTGCAAACTCCCGATCAACCCTCTGCCGTGCAGGAGCAATAACCATGTCTGCT
TATATCTATGATGGCCTGCGTACACCTTTTGGCCGCAGCGCCGGCGCCTTGGCCAGCGTGCGCCCCGATGACTTGC
TGGGTGAGGTCATCCGCGCCCTGGTGGCGCGTAGCCCGTTCGCTGCCGAGGACTACGAAGACGTCGTGGCGGGCTG
TGCCTGCCAGGCCGGTGAAGATGCGCGCAACGTCGCCCGCAATGCGGCATTGCTCGCGGGTTTGCCATTGACCACC
GGTGGCTTGACGGTCAACCGCTTGTGCGGCTCGGGGCTGGCGGCGGTGCTGGATGTGAATCGGATGATCCGGTGCG
ATGAAGGCCAGCTGTTTATCGCCGGCGGCACCGAGAGCATGAGCCGTGCACCGTTTGTTGTCGGCAAGAGCGAGGC
GGCCTTCTCCCGGGCGTTCCAGGTTTTTGACAGCAGCATCGGTGCGCGTTTTCCCAACCCGCGGATCGAGGCCGAA
TTCGGCGCCGACAGCATGCCGCAGACCGCCGATAACATTGCGCACGATCTGGGTATCAGCCGTGGCGACAGTGATG
CGTTCGCGGCGCGCAGCCAGGCGTTGTATGCCAAGGCGCTGGCCGAGGGTTTTTACGAGGAGGAACTGCTGCAGGT
CCTGGTGCCCCAGGGGCGCAAGTTGCCGCCCAAGGCGGTGGTCGCCGATGAGCACCCACGGCCGAGCACCGACGAG
CCAGCGCTCGCCCGTCTGGGCGCGCTGTTCGAGGGCGGCGTGGTCACGGCGGGTAATGCCTCGGGGATCAACGATG
GCGCGGCGGCGCTGATTATGGGCAACCGCGCTATCGGTGAGCGTCACGGGCTCAAGCCACGAGCACGGATCGTGGC
CGGTGCGGTCGCGGGGGTCGCACCACGGGTGATGGGCCTGGGGCCGGTACCGGCGATTCGCAAAGTCTTGGCGCGT
GCCGGGCTGAGCCTGGCGGATATGGACGTGATTGAAATCAACGAAGCCTTCGCCACCCAGGTACTGGGGTGCGCCA
AGGAGTTGGGCCTGGCCTTCGACGACCCACGCTTGAATGCCCAGGGCGGTGCCATCGCCATCGGCCACCCGCTGGG
CGCCTCCGGTGCCCGCTTGGCCCTGACCGCCTTGCGCAGCCTGGAGCGCCAACAAGGGCGCTACGCATTGGTGAGC
CTATGCATCGGGATTGGCCAGGGCATTGCCTGCGTGATCGAACGGTTTGACTGAAACCACCGCATGCAAATGCGGA
CTTGTTGTGGCGAGCGGGCTTGTCGGAACGCCGCTTGCCACAGGGTGAGTCGTCAGTTTTTGAAAGTTGTGTAGCC
CAGGCAATCCAGCTCCCCCATGTGATGGGTGGCCAAAGACTCTGCGCCTGGCACAGATCCCCTGTGGGAGCGGGCT
TGCCCGCGAAGGCGGCGGGTCAGAAAAATAGATTCTGACTGACCCACCGCTTTCGCGAGCAAGCTCCCACACAGAT
GCAGCGTCGCACGCTGGTTCTGCGTCAGACCACCTCGAACAACCCGGCAGCGCCTTGCCCGCCGCCGATGCACATG
GTCACCACCACATATCGGGCGCCGCGCCGTTTGCCTTCGATCAGGGCATGGCCGGTCAGGCGTGAGCCGGTGACGC
CATAAGGGTGGCCGATGGAGATGGAGCCGCCGTTGACATTCATCTTCTCCAAGGGAATGCCAAGCTTTTCCCGGCA
GTAGATCACCTGCGAAGCGAAGGCTTCGTTGAGCTCCCACAGGTCGATATCGTCGATCTTCAGCCCGTTGCGCTTG
AGCAGGCGCGGGATGGCATAGACCGGGCCAATGCCCATTTCGTCCGGCTCGCAGCCGGCCACGGCAAAACCCCGGA
ATATCCCCAGCGGCTCGAGGTTATTGCGTTCGGCGTAGAGGCTGTCCATGACCAGGCACACCGATGCCCCATCGGA
CAATTGGCTGGCATTGCCCGCCGTGATGAATTGCCCGGCTCCGCGCACCGGCTCCAACAGGGCCAGGTTTTGCAGG
GTGGTGGAGGGGCGGTTGCATTCGTCTTGGGTGAGGGTGACGGGCTGCTCGCTGACCTCGCCGCTGTGTTTGTCCT
TGACCAGCATGTGCGTGGTGAAAGGAACAATCTCGGCATCAAACAAACCGGCGGCCTGGGCCGCGGCGGTGCGCTG
CTGGCTGAGCAGGGCGTATTCGTCCTGGGCCTCGCGGCTGATGTTGTAGCGATGGGCAACGATGTCGGCGGTTTCA
ATCATCGACAGGTAAAGCTCGGGTTTGTGCTCCAGCAGCCATGGGTTCTGACTGCGATAGGTATTGAGCTTGTCGT
TTTGCAGCAGGCTGATGGATTCCACGCCGCCGGCGATCATCGCCGGGACTTTTTCCGACACGATACGTTGTGCCGC
GAGGGCGATGGCTTGCAGCCCCGAGCTGCAAAAGCGGTTGATCGACAAACCCGCGGTGGTGGTAGGCAACCCGCCA
CGGATGGCCGCCAGGCGTGCCATGTTCTTGCCCTGGGCGCCTTCCTGGAACGTCGCGCCCGAGGATCACATCCTCAA
TCAGCCCTGGATCAATCCCGGCACGGGCCACGGCCTGTTGGATCACATGCCCGGCCAGGTCGATGCTGTGGGTGTT
GTTCAGGGCGCCCCGGTAGGATTTGCCCAGGGCGGTACGCGCGGTGGAGACAATAACGGCGTCAGACATCAATCAG
CTTCCTGTGCCAGTAACGGAGGGGGTGTGGGCGGCCCATTCTTCATCCTGCAATTGGCGCCAGAGCAGTTTGCCGG
CACCGGATTGCGGCAGGTGCTCGCGAAACTCAATGGTGACGGGGACTTTGTAGGCCGACATGTTGGCCTTGCACCA
CTCGATGATGTCGCTGGCCGTGGTGTCCTGTTGTCCGGGGTGCAGGGTCACCAGGGCTTTGACCGCTTCCCCTCGG
CGTGCGTCGGGGGAGGAGATGACGCAGCACTGCTTGATCGCCGGATGGCGGTACATCAGGCTTTCGACTTCCGAAG
GCCAGACCTTGTAGCCGGAGGCGTTGATCATGCGTTTGATGCGGTCGACCATGAAGAAGTAGCCGTCTTCATCGTA
GTAGCCGAGGTCGCCGGTGCGTAGGAAGCGTTTACCTTCCAGTTCGATAAAGGCTTTTTCGGTGTCCTGCGGGAGT
CGCCAGTAGCCTTTGAACACCTGCGGGCCCCGGCAGACAATTTCGCCCACCTCGTGGGGCGCCAGCTCCAGGCCGG
TGGTGCTGTCGATCACGCGCGAGTCGACGTCGAACGCCGGAATGCCCAGGCATTGGGATTTGCTGCGCGCCTTGGG
GTTCATATGGGTCGCGGCCATGGTTTCGGTCATGCCGTAACCTTCGATGTAGTCCAGGCCCAGCAACTGCTTGAGC
TTGTCCTTGACCGGGCCGGGCATCGCCGCGCCGCCGCCGCCAATGTAGTTGAGGCTCGACAGGTCATAGCGGTCGA
GCCCGTCCTGGGTCAGGAAATCCACGGCCATGGTGACGATATTGGACCAGTTGCTGACGCGATATCGCTGCATCAA
CTGTGCGGCGACTTCACGGTTCCAGCGGGTCATGACCACCACACAGGCGCCACTGAAAATCGGCCCGTTCATCGAC
GATTGCATACCGGCGACATGGAACAGCGGCAGGGTGGTCAGCACCACGTCTTCGCAATTGCGGTTGGTCCAGGCTT
GGCGGTGCACCGTTGTCGCCATCACCGACCCATGGGTATGCAGGCAGCCCTTGGGTACGCCGGTTGTGCCTGAGCT
ATAAGGCATCACGCAGTGATCGTCGGCGTCACTCAGGTGGGGGCCGGGGCGTTGCCCGCAATCGAGGGCGGCCTGC
CAGCGTGTCACACCCGGCAACGTTGCCGTTTCAACCGGGGCCAGCAACTCGGGCGGCAGGGGCAGGTCGGTGGGTG
TACGCAGGTACTCGGAGTAGGCCGTGGTCAATACGTGGCGCAGCTTGAAGCGGCCGAGCAGCGGCGTGACAAACGG
CGCGAGATCCTGGGCGCAGACGACGATCTCGCCGTCAGTATCGTGCAGGTAGTGTTCCAGCTCGCTGGCGCGGCTC
ATGGGGTTGAGCGGTACCACCATGGCATCGGCGCGCAGCGCGGCGTAGTAGGTGATGATGAACTGCGGCGAGTTCT
GCATGAACAACAGGACTCGGTCGCCGCGCTTGACCCCCTGGGATTGGAAATACCCGGCCAGCCGCTCGACCTCATC

GAGCAACTGGGCGTAGGTGATGAGGGTGTCGTAGTAGATGATCGCGGTCTTTTGCGGATAACGCCGGGCAGAGATT
TCCAGGTTACAGAACAGGCTGGTATCCGGCAGTGTCAGGTGGTGGGGCGCACCCTCCGGCCAATGGGGCAGGTGAC
GGTTGAACATTCTAAAACCCTATTGTTATGAGTCTGGGTTAATCGATATGCGGGCGGTATGGCGGTGCCATCTCGC
TGGAGATTTGCACGCAATACGGCTGTATGTCAAATATATGGAATCGTGTTTCGCTATGCGGAACTATTGATCGGTT
ATTCGTAAGCCGTTGAAGTCTGCGGGGCGTTTGTCAAAGAATGCGGCAATCCCTTCGGCAGCTTCATCGTCCCCCA
TGGACACCACCATATGGTTGGCCTCCAGCTCCAACTGTTGTTCCAGGGTCGTGCGGCTGGCCTGGCGGCACAGGGT
CTTGATGCGTGCCATTGCACGCTGGGGGCCAGCGGCAATCCGGGTTGCCAGGGTGATGGCTGTCGGGAGCGCGTCA
CCGGGTTCGGTCAGCGCATTGACCGCGCCCAAGGCATGCAAGCGTTCACCGCTGATACGCTCACCTGTCAGGCACA
GTTGCGTCAGTACCTGGCGCGAAACAAACTCGGCGAGGAACGCCGTGGCACCACCATCGGGCGTGAGGCCAACCTT
GACGTAGGCCACCGAAAACGCCGCGTTACGCGCCACGACCAGCATGTCGCAAGCCAACGCAATCGATAGGCCGGCG
CCGGCGGCGGCGCCTTCGACGGCGGCAATCACCGGCTTGCTGCAATTTTGGAGCGCGCGGATCAGGTCATGCAGGC
CTTCAAGATTGACCCGGCGCTCCGCTGGCGTCATGGCCCGACGCAATGCCAGCCGATGCAGATCGCCGCCGGCGCA
GAAATGGCCGCCAACCCCGGTCAGCACCACGGCGCCAATCGACGGGTCTGCCTCGGCCTGGGCCAGGGCCTGGGGC
AATGCTGCATAGAACTCGGGCGTGAGTGCATTACGTGTGGCGGGGTTGTTATTGGACAGCACGAGGACAGCACCTT
CACGCCCCACAAGAATCGGTTCACCCATGGTTTCACCTGTTGTGCTCAGTGGCTGGTTATCCCAAGGATTATCGGA
AGGCAAAGCTTCATTGACAATCACAGCCCTGATTGACAGGCTGTTCAAAATTCTTTGACAGTGATCCCCATGGACC
TCAAGTCGCTTACCTTGCTGGTCGAAATCATCGATGCAGGCAACCTCAGCGAGGCCGCACGGCGACTTAAAGTCAG
CCGGGCCAATATCAGCTACCACCTCAGCCGCCTGGAGCAGTCGATCGGTTTGCAACTGGTGCGGCGCACGACGCGC
CGCATTGAGCCGACCGAGATCGGCCTGCGCCTGTATGAGCACGGGCGGGCCATTCGCAATGAACTGCTGGCGGCGC
AAGAGTCGGTCTCATCGCTGGGCCAGAGCTTGCAGGGCAGGGTTCGGGTGAGTGTGCCCAGCGGTTACGGGCAGTG
GGTGATGGCCGATTGGCTGCTGGACTTCAAGCGGCTGTACCCGGGGATCGTGCTGGATGTGATGTTCGAAAATCGC
GTCGAAGACCTGATGCGCGATGAAGTGGATATCGCAGTACGGGTCATGTCCGAGCCGCCGCAAAACCTGGTGGCTC
GGGACATGGGCGTGTTGCGCTACGTGGCTTGCGTCTCGGCGGCCTATGCCCGGGAACATGGCGTGCCCGGACAACT
GAGCGACTTGCGCACCGCACCGCTGATTACCGCGGCGGTGGTCGGCCGACAATTGCGCGTTGCTGCGTACCTGGAC
GATGAGCGCCATGAGGTACTGCTGGAGCCCACCCTGATTTCAGAGAACTTTCTGTTCCTGCGCCAAGCCGTATTGG
CCGGGCTGGGTGTAGGGCTGGTGCCGGACTACCTGGTGCAGGACGACGTACGCCAGGGCACGCTGGTGACCGCACT
GGACGCCTGGCGTCTGAGCATCTTCGGCACCAACATGTATATGCTCTACATGCCCGACCACCACCACACTCGCGCG
ACATCGACGTTCATCGACTTCATGCTCGCCCGCTCCAGAACCACGGAGATAGACTGCGCCCCATGTCCATCCAACA
CGCACTCCTGACCTCACTCCTGGAAAAACCCTCGACGGGGTATGAGCTGGCCAACCGGTTTGATCGCTCCATTGGT
TACTTCTGGCAGGCCACCCATCAGCAGATCTATCGTGAGCTGGGGCGCATGGTCACAGCCGGCTGGCTCATGGCGC
AGGACTGTCCCGAGGCCGACAAGCGGCGCAAAAAAATCTACCAGGTGCTACCTGCGGGGCGCGAAGAGTTGCAGCG
TTGGGCGAGCGAGCCGCAAGAGTCGGGGGACCAGAGCCAGGCACTGCTGATCAAGCTACGTGCCGAGGCGGTCATC
GGGCCGTTGGGCCTGCGTGAGGAAGTGCTGCGCCTGTTCAAGCAACACCAGGCCATGCTTGAGACATATCGGCAGA
TTGAGCAGCGCGACTTTTCGTCTGCGTCGTTGACCCACGCACAGCGCCTGCAATACGTGGTGCTGCGGCGCGGAAT
CATGCTCGAAGAAAGCTGGCTGGCCTGGGCGGACGAGTTATTGCCATTGATTGAGGCGTGAGGCCAGGCAGGGTTC
GACTCCGACCCACCGCCAATCCCTGTAGGAGCTGTCGAGCCCTTGATCTCCACCCAGACTGAAATCCCCGCCCCAG
CGCCAATCCCTGTGGGAGCTGTCGAGCTTCAGCNNNNNNNNNNTGAGCGCTTTGGTTACTTTCGCGCTCTTCGAAA
GTGACCCGCCGTAAGGGCGGAACCCTAAGTGGCCGTGACCGCAACAACGGATATGTACACATACCAAGAAGATACT
TGTGTAGATACTCACAGCTATCGCCGGCAAGGCCGCACATTACTTGCTTGTGCTCACTGAGGATCAGATCCGCACA
TTGCCCCGTGGTCCGGCGATGGCCCAGATAATCAGGCCCAATACCGGCAACAGCAGGATCAACAGGATCCACAACA
CTTTGGCGCCGGTGCTGGCGCCGCTTTTGAACACGTTGATGATCGCCCAGATATCCAAAGCCAGGATGATCAAGCC
AATCAGGCTGTTAAATGTGGAACCCATGGTGTCGCTCCTAAGTGAGGGCTTGCCTGCTTAGGATAGCCAGCCCTGG
CGAGGGTTCCGTTTTATTTCATACGTGCAAGGCGATACGCAGGGCTTCCAGGGACGGCTCACCCTTGATCCCGACT
GCAGCACACAACTCCAGCACCCGTGGTACATCGTTGCCGTACACCAACACCGCCTGGATCTCGTCATCGAGCAACT
GGCTGAAGTTCATCAGGGTGTAGCCGCCGTTTTCCGGGTTCATGGCAGCAAGTTGGATCTGGATGCGATTGAGCGC
CGTGAGCGCCTCGGTCTTGGCCAATTGCTTGGGCTTGAGGTTGAACGCCACGTCGGGACCAAACGAGGCGACGATC
TGGGCGAACAGGTCCTGGTAGGTATCGGCCTGGAACAGCACCGTTTCCGGCAGGCTACCGACCACAATCCACTCAC
CCAGGGGGATGGGGAAGCTGTCGTCGTAGTTGATATCGGGGTTGGCCGCCAGGAACCCGTCGGCATCCGCGTAGGC
TTGGGCAGCCTCGTCGGCGATCTGCGCGATCTCGGCCTCGCCCAGGCAACCGGAACTGATTTTAGTGATGAGTTCG
ACGAGCGCGGTGTTCATGAGCGGATCCTGTGGCGAGGCTGATTTTGAGGGCGCAAAGCCTACACCAGTTTTTGCAA
CTGCGCCGTCGTATCCACTGCCCCCATGGTCTGGGCCGCCACCAGCGCAGTCACGCCCTGGGCATCGGTGGCCTTC
GGGTTGGCCCCGACGCTGAGGAGGTAATCGACTATCTCGGTGCGGTTGAACATCGCGGCCATCATCAGCGCCGTGC
GGCCATCGGCAGATGCGCCTTCAACCGGCGCGCCACCTTCGATCAACGCCTTGACCACCGCGAGGTTGCCTTTGAA
CGCAGCACCGGCAATGGGCAACTGATTTTTGTCATTGGCCATCTGCGGGTCGGCCTTGAACTCCAGCAGCACCTTC
ACCGCCTCGGCATGCCCGTGATAGGCGGACAACATCAACAAGGTATCGCCATTGTGATTGCGCAGATTGACCGGCA
AGCCCTTGGCCAGCAGCGCGGCCAGCATGGCGGCATCGCCCCGGCGGGCCACGTCGAAGACCTGCTCGGCAAATTC
GGCGGCTTCATCTTCGGTCATTTGTTTGGCTTGATCTGACATCTGCGGCTCCCTGTGGGGTGTGGCGTAAGGCCGT

CAGTTTCCCGAGCAGCGCCACGCCTGTCACCCCTTTTTTGCCGAAAGCCGCAATAGTCAGGCTCTATCACCCACCG
TCAGTAGCGATATTTTCCGCCCTGAATATCCACCAGCACCTGCTCGGTCACAGGCACATAGGCATCCGTGCCTGGC
AGCCAGGCATAGAGCGGATCATCACCGACCCTGGCCGGGTCGAACGCCTCTTCCTTGAGGCGCACCTTCTGGTACT
TGAAGGTGCCTGTGGTTTCCATCTTGACCTTGATCCGCAGGAACAGCGGCACCGCATAGGCCGGCATCTGGCTCAG
GGCGAACTGCAACAGCGCCCGCATATCCAGGGTTGCCAGGGACTCGGACGGGGTAATCGCGGCCATCCCGGCGCGG
CCGTTGGTATTGGCGATTTCCACACCGTAGGCCACCGCCTCGGTGATCTGCGGGTACTGCATAAAGATGTTTTCCA
CCTCGGTGGTGGAAACGTTCTCGCCCTTCCAGCGATAGGTGTCGCCCAGGCGGTCGACGAACTGCGCATGGCCAAA
GCCGATGCTGCGCAGCAGATCGCCGGTGTTGAAATAGCGATCGCCCTTTTCGAAGACATCGCTCAAGACCACCTTG
CGGTTCTTTTCCGGGTCGGTGTAGCCATCGAACGGCGCCTTGTCGTCGATCCTGGCCAGCAGCAGGCCCTGCCCGC
CCTTCTGCACTTCACGCATGAAGCCATTGCTGCCACGGATCGGTTCGCAGGTGTCATGGGCGTAGTCCACCAGCGC
CCAGTGCATCAGGGAAAAGCCGATGGTGTTGTCGAAATTCAGCACGTTGGTGAAACCGATATTGCCGTCGCTGGCC
GCATACAGCTCGCAGATATGCTCGACGCCAAAGCGCTCCTTGAACGGCCCCCACACCCCGGGGCGCAAGCCGTTGC
CGATCATCTTCACCACGCGGTTGTCGCGGTCGCGCGGGCTTGGCGGCTGGTCGATCAGGTAGCGGCACAACTCACC
GACATATCCCAGGGTGGTTGCGTTGAATTTGCGCACATCGTCCCAGAACTGGCTGGCGCTGAACTTGCGGCGGATG
GCAAACCCCGACGCGCCGGCAATCGCCGAACCCCAGCACACGCAGAGCCCGGTGGCGTGGTACAGCGGCAGGGTGC
AATACACGACGTCCTCCGGCCCCATGTCCAGGGCGATGGTGCCGAAGCTCGCCGAGGATTTCATCCAACGGCCATG
CTTGAAGATGCCAGCCTTGGGCAAACCGGTGGTGCCGGAGGTGTAGATATAGAAGCATGGGTCGTTGAAGTAGATC
TGCTGGCTGCTTGCGGGGTTATCCGGCGAGCAGTCCGCACTGGCAGCCATCAGGTCGACATAGCCCGCGGGCACCG
GCGCCGCCGGTTGTTCGGCAACGTACCAGGTGCGCTGAGGATCAATCGCCACCTGCCCGCGCACGGCGTCGTAGGC
TTCGACCAGCTCCGCGCCGACCACAATCGCCACCGGATCTACCAGGGTCAGGCTGTGGACCAGCACTGCCTGGGTT
TGCGCGGTGTTGAGCATGGCGCAGATGCCGCCGACCTTGGCCACCGCCAGCACGGTCAGCAGCAGTTCGGGACGGT
TTTCAATAAACAGCGCGACCACATCCCCCTTGCCAATGCCCTGGGCTTGCAGGTGATGGGCGATGCGGTTGGCATG
CTGGTTGGCCTGGCGGTAACTGAGCACCTGGTCGCCGTACAGCAGCGCCGCGCCCTCGGGGTTGCGTTGGGTGGCC
TGTTCGAAGGTCCAGCCCAACCCGCAGGGTTGATCGGGGTCAGTGATGTTGGCGACGCGGATACCCCGTACCAGGC
GTGGCAGGGCCCGGACAATGGTCGGCACCTTGCGCAACATCATGCCGAGGGTAATCATCTCGCTGTTCGGATTGCT
CATGTACAGAAGTCCTTTTTCTTATTATTCGTAGGGTCTGCAGGCCGTCATGGGACGTAGGACCGTTCCTGAATGC
CTTTCAGGAAAATACGTCAGCCATTCCTTGGCTGTACACGCAGGATTTGCAATGTTTTGTATCCCACAGCGCGGTG
CCGCCCAGCACCGTTCGTCACCGGCCAATAGCGCAAAATGCAGGATGCACGATGGCCATTCATGCATATTGCACGA
AACCGAAAAATTCAAATTTTTATAAGATACTGATTTGTAAGGATTTTTTATAAAAAGCAATACTGGCACAATCACT
GCACCTATCACTCCATGCCGCACAACACGAGCCAATGGAGCCGATAGACATGAGCCTGATCCAAGACAAATTCGCT
TCTGTATTCTCCAACTACGACGTCACCACCCAAGCGCGTCCTGACGGTGGCATCCTGCTGACCCTGCGCAACACCG
AGGGCAAGCAGATCAAACGCTCGATTTCGTACCAGCAGCTGCACACTGCCGACCAACTGACGTGGGTCATCAGCGC
CATCCGTCGTGACCTGGCAGAACAGGCCAGCGAGCTGCCGCAGATTTCGATGCTGCAAAGCCAGCACCGCTTTGCC
CTGCCGACCTACCACTCGGCCTGAGTACCGGTTTGTTTTCCAGGGCCGTGAAGCCGTCAGGCTTCACGGCCTTTTT
TTATCTGCACAAACCGCGCTTGATCGCCTCGCTCACCGCCTGGACCCGGTTGTTGACGCGCAGCTTGCAGTAGATA
TTGCGCAGGTGAAATTTTACCGTGGCTTGTGAGACGCAACGGATCCGACTGATTTCCCATACGGTCTTGCCCTCCG
TGGCCCAATGCAATATTTCCAGTTCCTTCGCAGTCAGCGGCTTGTTCGGCAACGCCAGTTGTGCGCCATCTGCCCT
GTGGTCGGACGCCTGTTCACAGGCCTCGTTTGCCGCAGGCGCCACGGATGGGATCGACTCATCTGGGCTGCTCATG
CATACCTCTCCGTCTCTTATTGAATTGCACTGCCAATATTCGGACTCCTCCTATTGACAGATAAATACACAGCGAC
GGGAGAGTTACATAAAGCCTGGAATTAACACATCGACCCACAGACAAATAGCTATAACTTACAAACTATAGAGCCC
ATTCCCACAAACTTATCCAACTTCGGCGTATTGAATAAGTTTGTGAAAAGCGAACTTGGATCAATCATCCAATCCG
GGGAACGCTTGGGCGATATCACTGCCGGTTACTTGCGCCACGGCCCCGGCGGCCGTCTGGAACAGGCTGATGGCAA
CCACACGCGGTTCTTCCCCCAAGTCAAACCAGCGCGGTATGCCGGCCGGGATGCGCAGCAGGTCGTGTTTTTCGCA
GAGTACCGTGAAGATGTAATCGCCGATGTGTAGGCCGAGCAGCCCTCGCCCGGCGGCGAACAGGCGGATTTCATCG
GCATCGTGGCGATATTCATCGCGCAAACCCGCAGCAACGGCGTCCTTTTGCGGATGCTCGCGGCTCACACTCAATA
CCTCTACGTGCGCGTAGCCCTGGGCGGTCATCCAGCTGTCGATGGGCCCCTGGTACGCCGCCGTCACCTCGGCCGC
GCTGGCACCGGCCTCGACGCGGGTGTTCGCCTGCCAGCGCTCGAAGCCCACGCCGTGCTCGGCCAGGGTCGAGGCA
ATGTCGTCAGCGTGGGTCAGGACCTTGTTCGGGGTGTCCGGGGTGGCAACGTGATAAACGAAGAGAATGCTCATAA
AAGGGGTCCTCGGGCGGTAAGAGTCTGCAGATGATGGGCCGGGCTCACGGCCGTTCCTCGGGCAAGCGCAGCAGCA
TGAGCAACGCGGCGGCCAGGGCCGCGAGGCTGGCGATACTGAAGGTCAGGGTCGGCCCCAGTTGGTTCCAGCTATA
ACCGGAATACAAGGCGCCCAGCGCGCCGCCGGTGCCGGCCAGCGCCGCATACAGGGCCTGGCCCTGGCCTTGCTGG
CGTGCACCGAAGCTGCGCTGCACAAACTGGATGGCCGCCGCATGAAAGCTGCCAAAGGTCGCGGCATGCAGCAATT
GGGCAAATAGCAGCACCCACAGGAACTCGGCGAACGAACCCAGCAACAACCAGCGCAACGCCGCCAGCAGGAAACT
CGCCGCCAGCACCCGGCGCACCGAGAAGCGCGTGAGAATCCGGCTCATGGCCATGAACATCAACACTTCTGCCACC
ACGCCCAAGGCCCACAACAAACCGATCACACCACGGCTGTAACCGAGGCTTTCCAGGTGCAGAGTAAGAAAGGTGT
AGTACGGGCCGTGGCTCATTTGCATCAAGGCCACGCAGGCATAGAACGCCAGCACACCAGGGCTGCGCAATTGCCG
CAGGAAGCCGTCGCCGGCCCCGCGCTCCCCGGTGGACGTCGGCTGGGCATTGGGCACCCACCAACTGGCACCGATG

ATGCCGGCCATGATCAACACGATTGCCACCGGGTAGATGTCCAGGCTCAGGGCTTCAAACAGCCGCCCCAGGGCAA
CGACGGCGAGGATAAAACCCACCGAGCCCCAGAGGCGGACCTGGCTGTAGCGTGAGGTCTGGCCGCGTAGATGCGC
CAGGGTGATGACCTCGAACTGCGGCAGCACCGCATGCCAGAAGAACGCATGCAGGGCCATCACCAGCGCCAGCCAG
GCGTAGCTCTGGCTGACGAAGATCAGCGAAAAACTCAGCAACGTGCACACCGCGCCAAACCGCACGATGGCCAGGC
GCCGACCGGTGTAGTCCCCCAGCCAGCCCCAGATATTGGGTGCCACGCAGCGCATCAGCATCGGGATCGCTACCAG
CTCGCCAATGCGCGCGCTGGAGAACCCCAGATGATGGAAATACAGCGCCAGGAATGGCGCCGTCGCCCCCAGCAGG
GCGAAATAGAACAGATAGAAGCTGGAAAGTCGCCAGTACGGGAGTGCTGTCACGGTCAGACCGTCACAGCTGGCCC
AGCACCGGGGTACTTACACACACATCGGCATTCTGCCCACGGTGGCGCAGCAGGTGGTCCATCAGCACAATGGCCA
TCATTGCCTCGGCAATCGGCGTGGCGCGGATGCCCACGCAAGGGTCATGGCGGCCCTTGGTGATCACGTCCACCGG
GTTGCCATGCACATCGATGGAACGCCCCGGCGTGGTAATGCTCGATGTCGGCTTGAGTGCCAGGTGCGCCACGATC
GGCTGGCCGGAGGAGATGCCCCCAAGAATGCCGCCAGCGTTGTTGCTGACAAAGCCTTGCGGGGTCAGCTCATCGC
GATGCTCGGTGCCACGCTGGGCAACGCAGGCGAAACCGGCGCCGATTTCCACACCCTTGACCGCGTTGATGCTCAT
CAGCGCATGGGCCAGTTCAGCGTCCAGGCGGTCGAAGATCGGCTCGCCCAGGCCCGGCATCACCCCTTCGGCGACC
ACGGTGATCTTGGCGCCTACCGAATCCTGGTCACGGCGCAACTGGTCCATATAGGCTTCCAGTTCCGGCACCTTGT
CCGGGTCGGGGCTGAAGAAGGCGTTGTCTTGCACGCTGTCCCAGGTCTTGAACGGGATTTCGATCGGGCCCAACTG
GCTCATGTAGCCACGAACCACAATGCCCTGGGTCGCCAGGTACTTCTTGGCGATGGCCCCGGCAGCTACGCGCATC
GCGGTTTCCCGCGCCGAACTGCGGCCACCGCCACGGTAGTCGCGCTCGCCGTATTTGTGGTGATAGGTGTAGTCGG
CGTGGGCCGGGCGGAACAGATCCTTGATCGCCGAATAGTCCTTGGACTTCTGGTCGGTGTTGCGGATCAACAGGCC
GATGGCGCACCCGGTGGTGCGGCCTTCGAACACGCCGGAGAGGATTTCGACTTCGTCGGGCTCCTGGCGCTGGGTG
GTGTGGCGGCTGGTGCCGGGCTTGCGGCGGTCGAGGTCGCGCTGCAGGTCGTCCAGGGACAGCTCAAGGCCGGGCG
GGCAGCCGTCGACAATGGCGACCAACGCCGGACCATGGCTTTCGCCTGCGGTGGTGACAGTGAACAGCTTGCCGTA
GGTATTGCCGGACATGCAGAGCGCTCCGTGAAATCAGTTGAATCAACTCAACCGTGATAACTAAGCGCGCCAGTAT
ACGCAGGCTCACCCAGTAGTTCATCCTCGAAACCTTCCCGGTAGACGTTGGTCCAACCGGCACCTTCCTCATGATG
GCGCGATGATGCTGCGAGTTTTACTGTTTACCCTCACCCTGATCTGTACCGCCGCCCACGCCGCCGCCCCTGTGGT
GCTGCAACGCCCCATCAGCCTGGACACCGGCAGTGGCGAGTTGTTTGGCTTGTTGCTGCTGCCGCAATCCGCCAAG
CCCGTGCCCGTGGTGCTGATCATTGCGGGCTCCGGCCCCACCGATCGCAACGGCAACAGCGCCGAAGGGGCACGCA
ACGACAGCCTCAAACGCCTGGCCTGGGTCCTGGCCCGCCACAACATTGCCAGCGTGCGCTACGACAAGCGCGGCGT
GGCCGCCAGCCTCAAGGCCACGCCCGATGAGCGCAACCTGACGCTGGATGCCTATGTGGCCGACGCGGTGGCCTGG
GGCAAATCCCTCAAGGCCGATTCGCGTTTCGGGCCCCTGATAGTGCTGGGCCACAGTGAAGGCGCCTTGGTCGCAG
CCCTGGCCGCCCCTGAGCTCGACCCCGCCGGGGTGGTTTCCCTGTCCGGCAGTGCCCGCCCGGTGGACCAGGTGAT
CCGCCAGCAACTGGCCGATCACCTGCCACCGGCCCTGCTGCTGCGCAGTAACCAGATCCTCGACCGACTCAAGGCC
GGCCAGGTCGATGCCGATGTGCCCGACCCACTGCAGGGTATTTTCCGCCCCAGCGTGCAGCCTTATCTGATCAGTC
TGTTTCGCGCAGATCCTGCGGCGGCATTCGCTCGCCTGAACATGCCGGCCCTGATTGTCCAGGGCACCACCGACCT
GCAAGTGGGAGTGGCCGATGCCGAAAAGCTCAAGCAGGCCAAACCCGACGCCGAGCTGGCGGTGATTCCCGGCATG
AACCACGTCATGCGCATCGTACCCAACGATGTGAAACAACAGTTGAGCTCCTACAACAACCCACAATTGCCCCTCG
CCGACGCGTTGGGCAGGCGCCTGGTGAGCTTTATCGACGGACTTCGCCCCCGATAACCCACTATTTACCCTCCAGT
CCTGGAGAAAACGGCCGATAAGCACAGGGTCGACAGCAAAAAGACTGCCGACTCGCTGGGCTTGGACAGGATCGCG
CCGAATGACGAACACCGAAGCAGTACCGGAATCTCCCGCCGAGAGCACCGCCGAAACCGAAGCGGCGGCTGCGGCG
GCTCTGCCGTGGGCGGAGGTTGAGGCCGAACACTTCAAGATGCTGCGCCTGGCGCCCTTGGCCACTGACCGCGCGA
CCGGCGCCCGGCCATTGCGCTTTGTGCAATTTGGCTACGCCGAACGCAACAACAAGGACCACAGCCTGCTGCGCAT
GGTCATCCAATTGCCCGCCCAACGGGTGCGGCGCGAACAGAATCACCTGGATGTGTGGGTCGACCACGCCACCCAC
CGCGTGCATTTTGGCCCGGGCAACAGCCTGGAAATCGAGCCGGCCAACCGTGGCCTCGGTCGTTTCCTGGTGGCCC
AGGGCGCCGCGTGGGCGAAGAAGAAGTGGTCGCACTATCGCGTCGACGGTGTGGACCTGGCAAACAAAGACGCGCT
GAACGAAGCCACCCGCCTGCGCCGCGATCACTTTTTGCGGATCCAGGGTTTTGATGTGGCCTATGCCGATGTGCAG
CACCTCAAAGGGCACGTGCAGCCGGGCAAGGTCAGCGAAGTGCTGGACAGCTGGAACACCGAGAAGGTGCAGTTCG
TGGAAATCCTGGAGGCGGCGCAGATGCTGCAACAGGCCGAGCAGAACCTGGCGGAACAGGAAGTGAAACTGCGCCA
CGAAGAAGAAAAGGTCATCAAGTTCAAGCGCGAAGACAGCGGCTTGCGCTTCACCATCACCTGCCTGGTGGCGTTT
ACCGTGTTCCAGGCGGGGCTGTTGATCTGGATTGCGACGCACCGCTGACAACACGCTTTAACTGATCAAATCAAAT
CAAATGTGGGAGCTGGCTTGCCTGCGATGGCATCAACTCGGTGTATCAGCTACACCGAGTTGATGCCATCGCAGGC
AAGCCAGCTCCTACATGGTCCGGGCTAGACCCTGGCGGCAAACACCGCCTGATGCGCCCGGCACTGCTCCGCCGTG
AGCATGAACACGCCATGACCACCGTTCTGGAATTCCAGCCAGGCAAAATCCACTTCCGGGTACAGCGCCTCAACGT
GCACCTGGCTGTTGCCCACCTCGACAATCAACAAGCCCTTCTCGGTCAGATGATCGGCCGCTTCGGCCAACATCCG
GCGCACCAGGTTCAAGCCGTCGTCACCGCAGGCCAGGCCCAGTTCGGGTTCGTGCTGGTATTCATCGGGCATGTCG
GCAAAGTCTTCGGCATCGACATACGGCGGGTTGGACACAATCAGGTCAAAGCGCTGGCCGGGCAAACCGTCGAAAC
CATCACCCTGCACGGTGTACACGCGCTCGTCGGCACCATGGCGCTCGATGTTCTGGTTAGCCACTTCCAGCGCTTC
GAAGGACAAGTCCCCCAGCACCACCTCGGCCTCTTGGAACTCGTAGGCGCAGGCAATACCGATGCAACCGGAGCCG
GTGCACAGGTCGAGGATGCGCGCCGGCGGCTGGGCCAGCCAGGGCTCGAAGCGGTTTTCGATCAGTTCGCCAATCG

GTGAGCGCGGAATCAACACGCGCTCGTCGACGATAAACGACATGCCGCAGAACCAGGCTTCTTTCAACAGGTAGGC
GGTGGGCACGCGCTCGTGGATACGGCGATGCAGCAGGCGCTGGACGTGGGAAATCTCTTCTTCTTCGAGGTTGCAA
TCCAGGTAGCTGTCGGCAATTTCCCAAGGCAGGTGCAGCGCACCCAACACCAATTGCCGGGCTTCATCCCAGGCAT
TGTCGGTACCATGGCCAAAAAACAGGTCTTCCCCATGGAAACGGCTGACAGCCCAACGGATATGGTCGCGCAAGGT
GCGCAGGCGGGAAGTGATCACGGGGCAAGCTCCAGGAAAAAACGACTGGCAATTCTAACAGCCTTAAGCTGTACGG
ACGATGTATGAAAAACCTTTGCAAAACGTCAGATCCAACCTGATTCAAGGTTTTTCTGTTACACAGCAGCCCCTCT
TGACAGGGCTGTAGGCCAACAACGGCGCACCTTACGTTAGTAGCGATTCACAGAACCGCTCAGCCAGAGGACAATG
TCGCAAAAGCCCCACTCACAGGAGCCCCAGAATGTCCGTTCCAAAGACGATGTTTCAACTCAGCGGTCGCGGTTAC
GCACCGGCCAATTTGAGCCACGCGACCCTTGTGATCATCGATGCCCAGAAAGAATACTTGAGCGGCCCGCTCGCCC
TGTCGGGCATGGACGCCGCAGTAGAAAACATCAAGCAACTGAGCGCTGCCGCACGCAAGGCCGGGCGGCCCATCGT
GCATGTGCGTCACCTGGGCACCGTTGGTGGCCTGTTCGACCCTCAGGGCGAGCGCGGCGAGTTTATTCCCGGCCTG
GAGCCACAAGACGACGAAACCATCATCGGCAAGCTGCTGCCCAGCGCTTTCCATGGCACGCTCCTGGAAAAAACCC
TGCAGGAGCTCGGCTCCCTGGACTTGATCGTCTGCGGTTTCATGAGCCACTCCAGTGTCAGCACCACCGTGCGTGC
GGCAAAGAACCTGGGCTTTCGCTGCACCCTGGTCGAAGACGCCTGCGCGACCCGCGACCTGCCTTACAAAGGCGGC
ATCCTCAGCGCCGAGCATGTACAGCAGACAGAAATGGCCATCATGGCCGACAACTTCGCCACCCTGGCCCTGACCC
AAGATCTGATCTGATCTGGTCGCCCTGTCGATGAGCGGCGCGGCCACCGCAAAGGCCCCGCTCATCCGCTAAAGCC
TGTCATTTACCGCAATTGCCTCCCAGCCCGGAACAAGTTGTTTAACTTCCGGTCGAAGGGCCGATACCCTGGAGGA
AAGGTCGGAATGAAGATATCCGATGGTTTTGACGCTCGTCGCTTGCGCCCCAAGGGCCCGAGCAACTGGCGTCTGC
GCCTGGGCGTGGCATTTGCCGCGCTGCTGGCGTTGTGTGGCTTGCTGCTTTCGTTGGCCGGTGCTGCCGGTCTGTT
CGGTCATTCGCCCGCGCTGGGCGAACTCAATGCCAGCCCGGGTGGCGCGGCGGTGCTGTTGGTCAGCGGGCTGTTG
ATCCTGTGGCTGGGCGTCTGGCTGTGGCGCCGTTGCCGGCGCCGTATGCGCCAGCCACTGTCGCTGAACATCGCCT
CGCACCTGATGAAAAAGCACGATTGAGGGAACACTGATAGCGTTTTGTCCCGCATCAACCGCCTCGATTTGGGTAA
ACTGCCCGCCCCGCGCGGAGGCTGACATGCAAGACGACGATTTTTCCCTATTCAAGAACGAGCTGCGCGGCGTCAA
GCCGATCAAGCACGATCGCGCCGATACCGGCAAACCCAAGACCGACCGCGCGCAGATCGCCAAGCTGCGCCAGTCG
GCCACGGTGCGCACGGATGCCACCACGGTGGACGGCCTATCGGACCAGTTCGTGATTGACGTCGGCCCCGAGGACG
AGTTGATGTGGTCCCGCGATGGGGTCCAGGAAAGCCAGGTGCGCAAGCTCAAGGCCGGGCAGATCCCCTTCGAAGG
CAGCCTCGACCTGCACGGCATGACGGTGGAAAAGGCCAGGGAAACCCTCTGGGCGTTCCTTGCCGAAGCCACCAAG
TTCGAGATCCGCTGCGTACGCGTGACCCACGGCAAGGCCGTGCGCCTGGACGGCAAGCGCCCGATGATCAAGAGTC
ACGTCAATACCTGGCTGCGCCAACATGCCCAAGTGCTCGGGTTCACCTCCTGCCAACCGCGCCATGGTGGCGCCGG
TGCGGTGTATGTGATGCTCAAGCGCACGATGCTTGAAGGGCGTGACGAGTGAATGACCTGGGAAAGGTTCATCTGT
GGCGAGGGGGCTCGTCCCCCGCTGTGCTGCGAAGCAGCGCCAATCTATAAGGCCCACTGCGCTTTATCAGATACAC
CGAGGCGCCAGGTTTTGGGGCCGCTTCGCAGCCCACCGGGGGTGTAGCCCCCTCGCCACGAATGCAAACCCCGCAT
TCCAGGGAATTACACCTCTCGGCATACCTACGCTACTATGTGCGCCAATCACTGGCGACGCTGCACGACCGCTCGC
TGGCGTACCGCTTCACATTGGTACAGGCATACACCGTACCGTTCTGAAACCCATACGGAAACAGTCCTGTGACATT
GGAACAAAACTACACCGCAATCCTCGGCCAAATCGGCGAGGACGTTTCCCGCGAGGGCCTGCTCGACACGCCAAAA
CGTGCCGCCAAGGCCATGCAGTACCTTTGCCGCGGCTATGAGCAGACACTGGAAGAGGTCACCAACGGCGCCCTGT
TCAGCTCCGACAACAGCGAAATGGTGCTGGTCAAGGACATCGAGTTGTACTCGCTGTGCGAACACCACCTGCTGCC
GTTCATCGGCAAGGCCCATGTCGCCTATATCCCCAGCGGCAAGGTCCTGGGCCTGTCGAAGGTCGCGCGGATCGTC
GATATGTATGCCCGCCGCCTGCAGATCCAGGAAAACCTCAGCCGCCAGATCGCCGAAGCGGTCCAGCAGGTGACCG
GTGCCCTGGGCGTTGCCGTGGTGATCGAGGCCAAGCACATGTGCATGATGATGCGCGGTGTGGAGAAGCAGAATTC
GTCGATGATCACCTCGGTGATGCTGGGTGAGTTCCGCGAAAACGCGGCAACCCGCAGCGAGTTTCTCAGCCTGATC
AAGTAACAGGCTGCGTAAGAAAAAACCGGCATTCATCGCCGGTTTTTTTCGCCTGTGGAAATCAGGTAAGCTGCG
GCCCCTTCTGTCATGGGCAAGAGGTTTCAGCCATGTTCGTCAAAGCACTTCGAGTGGGCCTCGGCCACGTCATCAT
CGCGGGCGACTTCCTTACCCGCCCACGCAAAAAACAGCGCCCTGCCGAGCAACAGGCACAGGTCAATGAAGCGGCC
AAGGGCCTGACCCTCTACCAATTCCACGCCTGCCCGTTCTGCGTGAAGACCCGCCGCACCCTGCACCGCCTGAATG
TACCGGTGGCGCTCAAGGACGCGAAGAACAACGAGCAGGACCGCCAGACCTTGCTTACCCAAGGCGGCAAGATCAA
GGTGCCGTGCCTGCGTATCGAAGAAAACGGCCAGACCACCTGGATGTATGACTCCAAAGTGATCATCGACTACCTG
GACAAACGCTTCGCCGCGATCTGACAGACAGGCATGGCCCCTTGTGGGAGCTGGCTTGCCTGCGATGGCGGCGTGT
CCGTTGATGGATAGTTGACTGACACACCGCCATCGCGGGCAAGCCCGCTCCTACAGTGATGCCGTAAATCAGTCGA
GCATCGGCACGTGCCGGGGATGGCTGGCCACGCGTGCAAGCCAGGCTTGCACGGCCGGATAGGGGCTCAGGTCAAA
ACCACCCTCATGGGCCACATGGGTGTAGGCATACAGCGCAATATCGGCAATCGAGTATTGATCGCCCACCAGGTAG
GGCGTGGCCTGCAGCTGTTTCTCCATGACCCTCAAGGCCTTGTAGCCGCCTTTATGGGTAGTCTTGTACTCCTCCA
GCCGATCGTCCGGCAGCCCCAGGTAAAACTGGATAAACCGCGCCACCGCGATGTACGGCTCATGGCTGTATTGTTC
GAAAAACTGCCACTGCAACACCTGGGTGCGCAGGCGCGGTTCGGTGGGCAGGAACTCGCTGCCGTCGGCGAGGAAG
TTGAGGATCGCGTTGGACTCCCACAGGCAGGTGCCGTCTTCGAGCTCCAGTACCGGGATCTTGCCGTTGGGGTTCT
TCGCCAGGAACTCGGCGGTCTGGGTGTCGCCGTTCAAGATGTCGACATTGACCCACTCATACGCGATGCCAAGCAG
GTTGAGCATCAACTTGACCTTGTAGCAGTTGCCCGAACGGTAATCGCCATAAACCTTGTACATGAAGCTCCCCTTA

TGCCGCTTGCGCGCGCTGTGCTTGACGGATCACCGCCGCGAGACGCTTGAGACCTTCGTCCAGGCGTGCCGGGTCG
ATGTGGCTGAAATTGAGCCGCAGGTGCCCGTGGTGCTGGTCCGGCTCGGAAAAGAACGGTTCACCGGGCATGAACG
CCACGTCCTGGTCGAGCGCTGGTGCCAACAAGGTGCGGGTATCCAGAGGCTGTTTCAAGGTCAGCCAGAAGAATAA
TCCACCCTGGGGCACTTGCCAGTCGGCCAGTTCATCGAAGTGGCGCGTCAATGCTGCCTGGAACCCATCGCGACGC
TCACGGTAAAAACTGCGCAGTTGCACCAGGTGTTGCTGGTAGCGCTCGGTGCCGATCCACTGCATGGCCTGCCATT
GGCCGACGCGGTTGGTGTGCAGGTCCGCCGACTGCTTGAGCTTGAGCAGGTGCGGGAACAGGTCGGGGCTGGCGAT
CAGGTAGCCGACCCGCAGGCCCGGGAGCAGGGTTTTCGAGACGGTGCCGGTGTAGATCCAACTGGCCTTGCGCAAG
CGGCTGACAATCGGTCGGGCGCTGCCGCCATCAAAGGTCAGCTCGCGGTACGGCTCGTCTTCGATCAGGGTCACGC
CGAATTCATCGAGCAGCGCCGCCACGGCATCGCGCTTGGCTTCGCTATAGCGCACCGCCGACGGGTTCTGGAAAGT
AGGGATCAGGTAGATGAACGCCGGGCGATGGCGTTCCAGATTGCTGCGCAGCGCCGTCAGGTCCGGGCCGTCGGCT
TCCAGCTCCACGGTCAGGCAATCGGCGCCGAACAGTTGAAAGATCTGCAGGGCGGCCAGGTAGGTCGGGCCTTCCA
GCAGGATTTGCGTGCCTTGGTCGATGTACAACTTGGCCGCCAGGTCCAGGGTTTGCTGCGAACCACTGACCACCAG
GACCTGGCTGGCCTCGCAAGCCACCCCCAGCGACCGCGCCTCGGCGGCCAGCAACTCGCGCAATTGCGGCTCGCCC
TCGCTCATGCCGTATTGGCCCATGGCCGGCGGCATGCCTTGCCAATCCAGCGTCGGCAACATGGCTTCGGCGGGCA
GGCCACCGGCAAATGACATCACCTGCGGGCGCTGGGCCGCCGCGAGGATCTCACGGATCAAGGAGCTTTTAAGGCG
CGAAACACGTTCAGAGAAAGCCATAGGGGTCACCGGTAGCAAAGCCTGGGGAAAATACGTCAAACTGGTTGACCGA
AATTACGACGCCCAAGGCAGATACGTCAATATGCTTGACCTTAAAAACCAGACTTCGCAGCAAGCCGCCATGGAAG
CGTTTTTCTTCGGCTACCAGGCCTTTACCGCCAAGGCCGACGCAATGCTGGAGCGTCGGGGCTTGAGCCGGGTGCA
TCAGCGCATCGTGTTTTTTATCGCCCGCTACCCCGCCTTGAGCGTCAAGCAACTGCTGGAGCTGTTGGGGGTGAGC
AAGCAGGCGCTGAACATGCCGCTGCGCCAATTGCAGGAAATGCAGCTGGTCGACAGCGTTGCCTCGGACGCTGACA
AACGTAAGCGCCTGTTGCAGTTGACCGAGGAAGGCCAGCGCTTCGAACAGTCCTTGCGCCGCGAACAAGTCAAGCT
GCTGCAGCACGCCTTCGCCGAGGCCGGTGAAGAGGCGGTGATGGGTTGGCTGGCAGTGAACAAGGCCCTGACCCTG
CCTTGAAATTGGCCTTTCATCTTTTTTGCACACATAATCGCAAACATTATTTGCTTTCTTTGTATACAAAAGCATA
ATTCGCTTCGTGCGAGTTCCTGACCTGATGGTCAACAAAGCCCGCTGGTACCTCAAAGCACCGCTGCCCACCCCAG
TGTCCGGTGCTGGAAATAACAATAAAACTCTTGAGGAGTACTTGCTGTGGAAAGCCATAAATCCGACGCCCCGACC
CTGGACCTCAGTCCGCCCCACCTTTCGACCGCAAAGCAGGGCTGGCTGGAACGCCTGTTCAAACTCAGCTTGCATG
GCACCACAGTGAAGACCGAACTGATCGCCGGCCTCACGACGTTTATCACCATGGCCTACATCATCTTCGTCAACCC
CAACATCATGGCCGACGCGGGCATCGACCACGGCGCCGCCTTTGTCGCGACGTGCATCGCCGCAGCCCTCGGCTGC
CTGCTGATGGGCCTGTATGCCAACTGGCCTGTAGGCCTGGCGCCGGGCATGGGTTTGAATGCGTTCTTTACCTACA
CCGTGGTCGGCACCATGGGCTACACCTGGGAGACCGCGCTGGGTGCGGTGTTTATCTCCGGCGTGCTGTTCATGAT
CCTGACGCTGTCGCGCATCCGCGAATGGCTGCTCAACAGCATTCCGGTAAGCCTGCGCCACGCCATGGGCGCCGGG
GTGGGTCTATTCCTGGGGGTGATCGGCCTGAAAACCGCCGGCATCATCGTTGCCAGCCCCGCCACCCTGATCAAGC
TCGGCTCCCTGCATGAACCGGCCCCGCTGTTGGCGGCCGTGTGCTTCCTGCTGATCGCGATCCTCAGTTACCACCG
GGTATTCGGCGCAATCTTGATCAGCATCATTGCGGTCACCCTGGCCGGCTGGGGCCTGGGCCTGGTGCAGTACCAG
GGCATCGTCGCCGCCCCGCCGAGCCTGGCGCCGACCTGGATGGCGATGGACGTGATGGGCGTGTTCAACGTCAGCA
TGATCAGTGTGGTATTCGCCTTCCTGTTTGTGCACATGTTCGACACCGCCGGCACCCTGATGGGCGTTGCCCAGCG
GGCGGGCCTGGTCAATGCCGACGGCAAGATCGAGAACCTGTCGCGGGCCTTGAAAGCCGACAGTGCTTCCAGCGTG
TTTGGCGCAGTGGTCGGTGTACCGCCCGTGACCAGTTATGTGGAAAGCGCCGCGGGCGTGGCGGCCGGTGGGCGCA
CCGGGCTTACGGCTGTGACCGTGGGCGTGCTGTTTGTAGCGGCGATGTTTTTCGCCCCGCTGGCAGGCATGATTCC
TGCGTATGCCACGGCCGGTGCGCTGATTTATGTGGCGATGCTGATGATGAGCGGCATGGCCCATATCGAATGGGAC
GACGCCACCGACAGCATTCCGGCGATTGTCACGGCAATCATGATGCCCCTGACCTTCTCGGTCGCCGACGGCATCG
CGCTGGGCTTTATCACTTACGTGGCCCTCAAGGCCGGCACGGGCAAGTACAAGCAGATTTCCATCAGTCTGTGGGT
GCTGTGCGCGATTTTCATCGCCAAGTTTATCTTCCTGTAACTCGGAAGCCGGCGAAAACCGCCTCACCCTTGGGTG
GGGCTTTTGCATGTATGGAGGAATGTAATGAGCGTGGAAACCTGGCTGCTGTTCAGCGGCGCTGCTTTGATCGTGA
TCCTGATTCCGGGGCCGCTGTCGCTGCTGATGATCAGCAACAGCCTGAACTACGGCCTGCGCCGTTCGTACCCGGC
GTTTCTTGGCGGGGTGTTTGCCTCGATCTGCCTGTTGAGCGCCTCGGCCCTGGGCCTGGGGGCCTTGTTGCTGGCG
TCGGAGCAGTTGTTCAGCGCCTTGAAGATCGTCGGTGCGCTGTATCTGTTCTACCTCGCGTGGCAGAGCTGGCAGC
AATCGCGCCTGCCGTCCCAGGGCGCCGTGGTGCCGGACGCAGCCCCCGTGCCGCGTTTTCGTGCACTGTTTGGCCG
AGCTTTTGTGCTGGGCGCCAGCAACCCCAAGGACATTCTGTTTTTCGCCGCCTTCCTGCCGCAGTTTCTCAGCAGC
CAGCAGCCGTTCCTGCCGCAACTGCTGATCATGATCGCCACCTGGACCGTGCTTGACCTGCTATGCAAGCTGGCCT
ACGGCCTGGGTGCCCACGGCGCCGCGCGCTACCTGCGCACCGGCAAGGGCCAGAGCTGGTTCAACCGCTTCAGTGC
CGGGCTGTTTGGCAGCGCCGGGGCGGCATCGCTGCTCAGGGGCTAGTGGCTTCCTGACGCCGTGTAGGAGCGAGCT
TGTCTCCGGGCGGCGCTCCGACGAAGAACTTGAGAGCGCCGCGTGTAGCCAGACAACACGCGTTATCGTTGACGTT
TTTCGCGAGCAAGCTCGCTCCTACAGAGCGGGCAAAAAAAAAGCCCGCAGTGTGAGCGGGCGAAACCAACGAAGAG
CATTTGGGGGTGTGAAGCGAGGCGACGACTCAGCTACCGCGGTAGGTGGAGTAGCTGTAGGGCGAAATCAGCAGCG
GCACATGGTAGTGATCCTGTTCGGCATTGATGCCAAAACGCAGCACTACCACATCGAGAAACGCCGGTTCCGGCAG
TTGCACACCACGGGCGCGATAGTAATCCCCGGCACTGAACTGCAACTGGTAGACCCCACTGCGGTAGTCGTCACCT

TGCAGCAGCGGTGCGTCGCAACGGCCGTCGCTGTTGGTCAGGGCGCTGGCGACCAGCTCCAGTTGCGCGCCCTCGA
CGCGGTACAGTTCAACCTTGATGGCGCTGCCTGGGCAGCCGTGTGCGGCGTCCAGTACGTGTGTGGTCAGTCGTCC
CATGGATTGGAGCTCCCGTGTCATCGATTAATAAGAGGCCGCACCTTTGTGGGGCATTGAAAAAGCCGGCGATGGC
TGATTAAGACACTTTTCAAAAAAATTGTACACAATAAATTCAACAACCCCCCTCTTCCCCAATGGGCGCTGGCTTA
CCGGCGATGACGGTGTATCTGCGAGCAGTGCGGTTACACGAAGATCGTTATCGCCGGCAAGCCCGCTCCTACACTA
AAGCGGTGCCGCAGGTTTTTGATATTTAGTTGACCAGTCGGGCAAGTTTCTTGCAAGGATAGGCTGGCACGACCAC
TCGGAAAAAATGCAGAAATACAGGCTTACAAAGTGACCATTAAGTTGTATACAATCAGCCCATCGCTGTGACGCAA
CGGCCGCCACACCATCGATCACGAACAAGAAGGAAGACTGCAGTGAGCGCTGACTACCCACGCGACCTGATCGGTT
ACGGCAGTAACCCACCGCACCCTCACTGGCCGGGCAAGGCGCGTATCGCCCTGTCGTTCGTACTCAATTACGAAGA
AGGCGGTGAGCGCAATATCCTGCACGGCGACAAAGAGTCGGAAGCCTTCCTCTCGGAAATGGTCTCGGCCCAGCCG
CTGCAAGGCGCGCGCAACATGAGCATGGAGTCGCTGTACGAATACGGCAGCCGTGCCGGCGTGTGGCGCATCCTCA
AGCTGTTCAAGGAATTCGACATTCCGCTGACCATCTTCGCCGTGGCCATGGCCGCCCAGCGTCACCCCGATGTTAT
CCGTGCGATGGTCGCCGCCGGTCACGAGATTTGCAGCCACGGCTACCGCTGGATCGACTACCAGTACATGGATGAG
GCCCAGGAGCGCGAGCACATGCTCGAAGCCATCCGCATCCTTACCGAGCTGACCGGCGAGCGCCCACTGGGCTGGT
ACACCGGGCGCACCGGCCCGAATACGCGGCGGCTGGTGATGGAAGAAGGTGGCTTCCTGTATGACTGCGACACCTA
CGACGACGACCTGCCCTACTGGGAACCGAACAACCCGACCGGCAAGGCGCACCTGGTGATCCCCTACACCCTGGAC
ACCAATGACATGCGCTTCACCCAGGTCCAGGGTTTCAACAAAGGCGACGACTTTTTCGAGTACCTCAAGGACGCGT
TCGATGTGCTGTATGCCGAAGGTGCCGAGGCGCCGAAGATGCTGTCCATCGGCCTGCATTGCCGCCTGATCGGCCG
CCCGGCGCGCCTGGCCGCACTCAAGCGCTTTATCGAATACGCCAAGGGCCATGACCAGGTGTGGTTCACCCGCCGC
GTCGACATTGCCCGCCACTGGCATGACGTACACCCTTTCCAGGGAGCCGCCAAATGACTGCTTTCCAAACCTTGAA
GCCATCGGCCCTCAGCCGCGACGAATTTGTCGCAGCCTTCGCCGATATCTATGAACACTCGCCATGGGTGGCCGAA
AAGGCCTTCGACCTGGGCCAGGACGCCTCGATCGATGAGATCGAAACCCTGCACCAGCGCATGAGCGATATCCTAT
TGAGCGCCGATCACACCAGGCAACTGGCCCTGATCAACGCTCACCCGGACCTGGCCGGCAAAGCTGCCGTCCAGGG
CCAACTGACCGAAGCCAGTACCCATGAACAAGCTGGCGCCGGTATCCACCAATGCTCGAGCGACGAGTTTTCGCGC
TTCACCGAGCTGAACGACGCCTACAAGGCCAAGTTCAAGTTTCCCTTCATCATGGCGGTAAAAGGCAGCAACCGGC
ATCAGATCCTCGCCGCGTTTGAAACGCGCATCCATAACCCGGTCGAGACCGAGTTCAAGTGCGCACTGGCAGAGAT
CAACAAGATCGCGTTGTTCCGTTTATTGACCCTCTAAACGACCATCCCCAGCCACTCTATCTAAGGCAGACAAGAA
GAATGAAAGCGCAACTCCTACCTTTCGAGAAGTTAGTCAACCTGGCCGACGCCCGCCTGGGCACCAAGATCATCTC
TGTGACTGATGACTGGTTCGCCGACGCCAACCGACTGTTCCAGCCGACCCCGGCCGTATGGAAGGAGGGCGTGTTC
GATGATAACGGCAAGTGGATGGACGGCTGGGAGTCACGCCGCAAGCGCTTCGAAGGCTTCGACAGCGCGGTGATCC
GCCTGGGCGTACCGGGCTCGATCAAGGGCGTGGACATCGACACTTCATTCTTCACCGGCAACTTCCCGCCATCGGC
GTCCCTGGAAGGTTGCTTCCTGGCGTCGGGCGAGCCCGATGACAACACCCAGTGGGTCGAAGTGCTGTCTGCCGTA
GAGTTGCAGGGCAACAGCCATCATTTCCACGAAATCAACAACGACCAGGCATTCAGCCACCTGCGCTTCAACATCT
ATCCCGATGGTGGCGTAGCGCGTCTGCGGGTGTACGGCATTCCGTTCCGCGACTGGTCGGCCGTGGGCGACAACGA
ACAAGTGGACCTGGCCTCCTCCCTCAATGGCGGCCGTGCACTGGCCTGCTCCGACGAACACTTCGGGCGCATGGGC
AACATCCTCAACCCGGGCCGTGGCATCAACATGGGCGATGGCTGGGAAACCGCGCGTCGTCGAACACCTGGCAATG
ACTGGGTGATCGTCGCGCTGGGGCATGCCGGCGAGATCGAGAAAGTGGTGGTCGACACCCTGCACTTCAAGGGCAA
CTACCCGGACACATGCTCGATCCAGGCCGCGTTCGTCAAGGGCGGCACTGACAGCCAGATCGAGACCCAATCGCTG
TTCTGGCGCGAACTGCTGCCGGCACAGAAACTGGAAATGCACGCCGAACACACCTTCGCCGAGCAGATCAAGGCAC
TGGGCCCGATCACCCACATCCGCCTCAACGTATTCCCGGATGGTGGTGTAAGCCGCCTGCGGGTTTTCGGCAAGGT
TTCGAAGTAAGCGGTGATGCCATTCGCGAGCAAGCCCGCTCCCACAGGGGAATGCATTCCAAATGTGGGAGCGGGC
TTGCTCGCGAAGGCGCCAGAACAAACAACACAGAATCCGGATAAGAAGACAGCCATGCGCACACTGATGATCGAAC
CCTTGACCAAAGAAGCCTTCGCCCCTTTCGGAGACGTTATCGAAACCGATGGCAGCGATCACTTCATGATCAACAA
CGGCTCGACCATGCGCTTTCATAAACTGGCGACGGTAGAAACCGCCACGCCAGAAGACCACGCCATCATCAGCATC
TTCCGCGCCGATGCGCAGGACATGCCGCTGACCGTGTGCATGCTGGAAAGACACCCGCTGGGCAGCCAGGCCTTTA
TCCCGCTGCTCGGCAACCCCTTTCTGATCGTGGTCGCGCCGGTTGGCGATGAACCTGTATCAGGCTTGGTCCGCGC
CTTCGTCACCAACGGCAGGCAGGGCATTAATTACCATCGCGGCGTCTGGCACCACCCGGTGCTGACGATCGAAAAG
CGGGATGACTTCCTGGTGGTTGATCGCAGTGGCACAGGCAATAACTGCGATGAGCATTTTTTCAAAGAGGATGAGC
GGTTGATCCTTGCCCCCCACCAATAAGAGAAGGTCCGATCACCCGACAACAAGGGTGACGGGCGAGAGGTAAAGAC
TGTGGAAGCACATCTGTTGGAATGGCTGAACCTAAGCGTGCGCTGGGTTCATATGATCACTGGCGTGGCCTGGATC
GGTGCGTCTTTCTATTTCGTATGGCTGGAAAACAACCTGAACCGCGTCAACCCCAAAAGCGGCCTGGCTGGCGATT
TATGGGCGATCCACGGTGGCGGTATCTACCACCTGGAAAAATACAAACTGGCCCCACCGACCATGCCGGACAACCT
GCACTGGTTCAAATGGGAAGCCTATTTCACCTGGATGTCGGGCGTCGCGCTGCTGTGCGTGGTGTTCTACCTCAAC
CCGACGCTGTACCTGCTCGCCCCCGGCAGCAGCCTCAGCGGTACCGAAGGCGTGTTGCTGGGCATAGGCTCACTGT
TCGCTGGCTGGTTCATCTACTCCTTTCTGTGCGACTCGGCCCTGGGCAAACGCCCTGCCCTGCTCGGCTTTATCCT
GTTCGTCCTGTTGATCGGCGCGGCCTACGGCTTCAGCAAGGTGTTCAGCGGCCGCGGTGCGTACCTGCATGTGGGT
GCGATCATCGGCACCATCATGGTCGGCAACGTGTTCCGTATCATCATGCCGGCCCAGCGTGCGCTGGTGGCAGCGA

TTGCGGAGAACCGCACCCCAGACCCGGCACTGCCGGCCAAGGGCCTGCTGCGCTCGCGTCACAACAACTACTTCAC
CCTGCCGGTGCTGTTCATCATGATCAGCAACCACTTCCCGAGCACCTACGGCAGCCAGTACAACTGGTTGATCCTG
GCCGGGATCGCGGTGGCGGCGGTGTTGGTACGGCACTACTTCAACACCCGTCATGACAGCCAGAAATACGCGTGGA
CCCTACCGGTCGGCGCCCTGGCGATGATCTGCCTGGCCTATGTCACCGGTCCTAAACCCGTGGCGACCACACCGGA
CGTGGCCAAGGCGCCAGCAGCGATCGAGTACCAGCCATTGCCGGAAACCGCGCTGGGTGGCGGCCTCAAGCCAGCA
GCTCCCGCCGCGCCAGCACCAGCGGCACAAGCGCCTGCGGCCCAGGCCTCGACGGATTTTGGCAAGGTGCACAGCG
TGATTGAAGAGCGTTGCACCGTGTGCCATTCGGCCAAGCCCACCAGCCCGCTGTTCAGCACCGCGCCGGCCGGGGT
GATGTTCGATACCCCGGCGCAGATCCAGCAGCAGGCGGCGCGCATTCAAGCGCAGGCGGTGGCCAGCCAGATCATG
CCATTGGGCAACATTACCCAGATGACCCAGCAGGAGCGGGAGTTGATTGGCAGTTGGATCAATCAGGGGGCCCGCA
CCAATTAACTCTTTGGTGGCTTGACGGCCGCCATCGCCGGCAAGCCGGCTCCTACAGGTGAATGCACGCCTTGTAG
GAGCCGGCTTGCCGGCGATCAACCGCACAGCGGTTGCCAAACAACACAAAAATAAAAAAGATTCGAGGTGTTGCAT
GTCCCAGTTAGAAACGCAGATTCCTGCCGCGCCCGCCATGGTGCGGCTTCCACTTTTGCAACTGATCCTGGTAGGC
CTGCAACATGTACTGCTGATGTACGGCGGCGCGGTGGCCGTACCGCTGATCATCGGACAAGCCGCGGGCTTGAGCC
GTGAAGAAATCGCCTTTTTGATCAACGCCGACCTGTTGGTGGCGGGGATCGCCACCATGGTCCAGTCGTTTGGCAT
CGGCCCGCTGGGTATCCGCATGCCGGTGATGATGGGCGCCAGTTTCGCCGCCGTCGGCAGCATGGTCGCCATGGCC
GGCATGCCAGGTATCGGCCTGCAAGGGATTTTTGGCGCGACCATCGCCGCCGGGTTCTTTGGCATGGTCATCGCCC
CATTCATGTCCAAGGTGGTGCGCTTCTTCCCACCTCTGGTGACCGGTACCGTGATTACGGCTATCGGTCTTTCGTT
GTTTCCTGTCGCGGTGAACTGGGCCGGTGGTGGCGCTACCGCTGTGCAATTCGGCTCACCGATCTACCTGGCCATC
GCCGGCCTGGTGCTCGCCACCATCTTGCTGATCAACCGTTTTATGCGTGGCTTCTGGGTCAATATCTCGGTACTGA
TCGGCATGGCACTGGGCTACGCCTTGTGTGGATTGCTCGGTATGGTCGACCTCGGCGGCCTGGAGCAGGCGCCGTG
GCTGCAGGTGGTCACACCGCTGCACTTCGGTATGCCCACGTTCGAGTTGGCGCCGATTCTGTCGATGTGCCTGGTG
GTGGTGATTATCTTTGTCGAGTCCACCGGCATGTTCCTCGCGCTGGGTAAGATCACCGGCCAGGAAGTCACCCCGA
AAATGCTGCGGCGCGGCCTGCTGTGCGATGCCGGGGCGTCGTTTTTCGCCGGGTTCTTCAACACCTTCACCCACTC
CTCTTTCGCCCAGAACATCGGCCTGGTACAGATGACCGGTGTGCGCTGCCGTTCGGTGACGATCATGGCGGGAGCC
TTTCTGATTGTCCTCAGCCTGCTGCCCAAGGCCGCATTCCTGGTGGCATCGATCCCTCCTGCGGTGCTGGGCGGTG
CGGCTATCGCAATGTTTGGCATGGTTGCGGCGACCGGGATCAAGATCCTGCAGGAAGCCGACATCGCCGACCGTCG
CAACCAGTTGCTGGTAGCGGTCAGTATCGGCATGGGCCTGATCCCCGTGGTGCGCCCGGAGTTCTTCGCCCAGTTG
CCCTTGTGGATGAGCCCGATTACCCACAGTGGCATCGCCATGGCCACGCTCAGTGCGTTGTCGCTGAACGTGCTGT
TTAACATTCTGGGCGGCTCAGAACGCCCCGCAGTCGCCCATACGCATTGATTCCTTCGTTACTACAAAAATAACAA
CGAACAGGGAACACCAACATGCACACCACCAACTTGGGGCTGGCCACACGCCGTGCCCCATTCTCGCCCCCTGCGC
TCTTTTACAGCGCACTTGAGCACAGGCTCTGGAGCCAGTATTCTCCGCGCCCGCTCGAATCGACTCAAAAAAAAAC
AGCGAATGTAAAAGCTGACTTAAAGGCCAACTTATCCCGGCCTTATGTCTGCTGCCAAAGTGCGCAAAAGTCCTCT
GAACCCGACTGCATCCCATGCAGCCGGCGGGGATTTTTTGGCTTTTCAAACACTTTGGCGCAGCTCTTGCTAAAAG
CATTAAAGCTGACCGAACAGACGATTTTTGCAGCGAACCAAAAGGCGCCACACCAGAGCGCCTGCGTAGAAGAAAA
ACCACAAAACTTTAGCTCGGGAGCAACCGAATGAAACGTACCGTGAACAGCCTGATATTGGCCGGATCCCTGTTGG
CCGGGGGCCAAGCAATAGCGGGTGACCTGTTGCAGTGGCAGAACAACAGCCTGACTTACCTGTGGGGCAAGAACTT
CAAAGTCAACCCAGAGATCCAGCAAACCGTTACCTTCGAACATGCCGATGCCTGGAAGTACGGCGACAACTTCTTC
TTCCTCGACCGCATCTTTTACAACGGTAAGGAAGACGGCAACGTCGGCCCCAATACTTACTACGGCGAGTTCAGCC
CGCGGCTGTCGTTTGGCAAGATCTTCGATAAAGACTTGTCCTTCGGCCCGATCAAAGATGTATTGCTGGCGTTCAC
CTACGAGTTCGGCGAAGGCGACAACGAGTCGTACTTGGTCGGCCCGGCGTTCGACTTGAACATCCCTGGCTTTGAC
TACTTCCAGTTGAACTTCTATCAGCGCCAGACCGAAGGCAATCGCCCGGGTGACGGCGTATGGCAGATCACCCCGG
TCTGGTCCTACACCATTCCAGTGGGCAACTCCGATGTGCTGATCGACGGTTTCATGGACTGGGTGGTGGATAACGA
CAAGAACGCCCGTGGCACTTACCACGCCAACCTGCACTTCAACCCACAGGTCAAATATGACCTGGGCAAGGCCATG
AGCTGGGGCGCCAAGCAGTTGTATGTGGGTTTTGAATACGACTACTGGAAGAATAAGTACGGGATCCAGGACAGCG
GCGCGTTCGAGACCAACCAGGACACCGCGAGCCTGTTGGTCAAGTACCACTTCTAAAGCCAACCCCGGCCTCCCCT
GTGGGAGCGGGCAAGCCCTCTCCCACACAAGCCCGCGCCCCGATTCAGTTCGGGGTCAAGCCCAGGAACCGCTGCA
ACTCTTCGCGCTTGGCCAGCGCATCGCGCCGGCCCAACTCGATCAACTCACTGCAATACCCCGCCTCGAACAGCAG
ATAGCTGAGCACCCCCGCCCCGCTGGTTTTGGTCGCCCCTGGCCCACGCAAAAACAGACGCAACGCCGCCGGCAGT
TCCTGGCGATGGCGTGCCGCGATTTCATCGATGGGTTGGCTGGGCGCAATCACCAGTACTTCAACCGGTGCAAGAC
CGCGCGCAGCGCTGTTGGCCGGTTGCAGATGGCTGAACTGGTTGAGGCGCTCCAGTAACTCGATGTCACTCTCCAG
GCTGTCAATGAACGTGCTGTTGAGCATATGCCCACCGATCTGCGCCAGGGTCGGCTCCTGGCCGGTGTAGGTGCGC
TGCTGCGGCACCGAGGGGTCCGGGGAACGCGGGTTGCCACTGACACCCACCACCAGTACCCGGCTGGCCCCCAGGT
GCAACGCCGGGCTGATCGGCGCTGATTGGCGGACGGCGCCATCGCCAAAATATTCCTGATCGAGTTTGACCGGGGC
AAACAGCAACGGAATCGCCGAACTGGCCAGCAGGTGCTCCACGGTCAGTTCCGTGGGCAAACCAATGCGCCGGTGG
CGCAACCAGGCGTCGATGGTGCCGCCGCCCTGATAGAACGTCACCGCCTGCCCCGACTCATAGCCGAACGCCGTCA
CGGCGACGGCGTGCAGGTGTTTGTGGCGGATTGCTTCATTGATGCCTTCGAAATGCAGTTTTTCCTGCAGCAGTTC
CCGTAGTGGAGAACTGTCGAGCAGTGCCACCGGCACTGGTGAGCCCAGGCCCAGCAAGCTGCGGGTCAGGAAGCGG

CTGGCCTGGTGAATCACCCCCGGCCAGTCGCTGCGCAGCACCAAGTGGCTGCGAAAGCCCTGCCAGAATGCGGTGA
GGCGCGCGATTGCTGCGCGAAAGTCCATGGCGCCGCTCGCCAGGCTCACCGCGTTGATCGCCCCGGCCGAGGTGCC
AACAATCACCGGGAAAGGGTTGGCAGCCCCTGGTGGCAACAGTTCGGCAATCGCCGCCAATACACCCACCTGATAC
GCCGCACGCGCCCCGCCGCCGGAAAGAATCAAGCCTGTGACCGGTTCAGTTGGGCGCATTGCATAACTCCATGGGC
GGGGACAATCCGTTTATTCAGCGGCGCTTTTCGTACAGCTTGGGTTCGCCCGGTGGCCGGCTCTTGAAGCGGCGAT
GAGCCCACAGGTATTGCTCGGGGCACTCGCGCACCGCGGTTTCCACCCATTGGTTGATACGCAGGCAATCGACTTC
ATCGGATTCGCCGGGGAAGTCGCTCAAAGGCGGATGAATCACCATGCGGTAGCCACTGCCATCGGCCAGGCGCTCC
TGGGTAAACGGCACCACCAACGCCTTGCCCAGGCGCGCGAACTTGCTGGTGGCGGTGACTGTGGCGGCCTGGATGC
CGAACAACGGCACAAAAATGCTCTGCTTGGCGCCATAGTCCTGGTCGGGCGCGTACCAGATCGCCCGGCCGGCGCG
CAGCAGCTTGAGCATGCCACGCACGTCTTCGCGCTCTACCGCCAGGGAGTCGAGGTTGTGGCGCTCGCGGCCACGA
CGCTGAATAAAGTCGAACAACGGGTTGCCGTGCTCACGGTACATGCCATCGATGGTGTGCTTCTGCCCCAGCAAGG
CCGCACCGATCTCCAGGGTGGTGAAATGCAGGGCCATGAGGATCACGCCCTTGCCTTCGCGTTGGGCCTGCTTGAG
GTGCTCCAGCCCTTCGACATGGGCCAGGCGCGCCAGGCGCTGTTTGGACCACCACCAACTCATGGCCATCTCAAAG
AAGGCGATACCGGTGGATGCAAAGTTTTCCTTGAGCAAATGTTTACGCTCCTGGGCGGACATTTGCGGGAAGCACA
GTTCCAGGTTTCGCGCGGCAATGCGCCGGCGCTCACCGGCCACCCGGTACATAAACGCACCCAGCACACGACCAAT
TCCCAGCAGCACGCGGTATGGCAACTGGGTAATCAACCACAGCACACCGAGCCCCAGCCATAGCAGCCAGAAACGC
GGGTGAAAAAATACAGCTCGAAAACGCGGGCGATCCATTACAGATTCCGGTAAAGACAGGGCCGCGCATTCTACAA
CGGTTCGACCCGGCTTGCGGCTAGCGGTTGTTCTCGTTATAAGTCTCGACACTTTTCGTGACAAGCCGCTTTGCCG
ACCATGAGCCAAACCGAATCGCTAGACCAAGATCCCGTCTTCCAGCTCAAAGGCAGCATGCTCGCCATTACTGTGC
TGGAACTGGCCCGTAACGACCTTGATGCCCTGGACCGCCAGCTCGCCGCCAAGGTAGCCCTGGCCCCGAATTTCTT
CAACAACGCGCCGCTGGTGCTGGCCCTGGATAAATTGCCGGCCGGCCAGGGCGCGGTCGACCTGCCCGGGTTGATG
CGTGTTTGCCGCCAGCATGGCCTGCGCACCCTGGCCATTCGCGCCAGCCGTATCGAAGACATCGCGGCCGCCATCG
CCATTGAACTGCCAGTATTGCCACCGTCCGGCGCCCGCGAGCGTGTGCTCGACCCGGTGGAAGGTGAAGTGAAGAA
AAAACCGGAAAAACCACCAGAGCCGACGATCAAGCCTACAAGGATCATCACCTCTCCCGTACGCGGCGGGCAGCAG
ATTTACGCCCAGGGCGGCGACCTGGTGGTGGTCGCTCCGGTCAGTCCCGGCGCGGAACTTCTCGCCGATGGCAACA
TCCATGTATACGGCCCAATGCGTGGCCGGGCACTGGCTGGAGTCAAGGGCGATACCAAGGCCCGGATTTTCTGCCA
GCAATTGAGCGCTGAGCTGGTGTCCATCGCCGGCCAGTACAAGGTTTCGGAAGATTTGCGCCGTGATCCGCTGTGG
GGGGCCGGGGTACAAGTCAGCCTGTCGGGCGATGTGTTGAACATCATCCGTCTTTAACGGATACTGCCGCATTTTC
CAAGCATCTCTGGACTCTGATAGCACAGCGAAACCGGCAAGACTTGCGTAGGAATAATTGAAAGTTGGCGTTCTCC
CTACGGGAGGCACATCTTTTTCCTACGAAGACTGTCCGCCTGCAGCGAGTTTCAAGAGATGTTTTTCAGGGACTGA
GAGGTCCTTTTTCCTTAGGGGTGAAACACCTTGGCCAAGATTCTCGTGGTTACATCCGGCAAGGGTGGTGTGGGTA
AGACCACCACCAGCGCCGCTATCGGTACCGGCCTCGCTCTGCGCGGGCACAAGACAGTCATCGTCGACTTCGACGT
CGGTTTGCGTAACCTCGACCTGATCATGGGCTGCGAACGCCGCGTGGTGTACGACTTCGTCAACGTGGTCAACGGC
GAAGCCAACCTGCAACAGGCCCTGATCAAGGACAAGCGCCTTGAGAACCTGTATGTACTGGCCGCCAGCCAGACCC
GTGACAAAGACGCGCTGACCAAAGAAGGCGTAGGCAAAGTTCTGGCCGAGCTGAAAGAAACCTTCGAATACGTGGT
CTGCGATTCCCCGGCGGGTATCGAGACCGGCGCTCACCTGGCGATGTACTTCGCCGATGAAGCCATCGTCGTGACC
AACCCGGAAGTGTCCTCGGTACGTGACTCGGACCGCATGCTCGGCCTGCTGGCCAGCAAGTCCAAGCGCGCCGAAG
ACAACCAGGACCCGATCAAGGAGCACCTGCTGCTCACCCGCTACAACCCTGAGCGCGTAAGCAAGGGCGAGATGCT
GGGCGTAGAAGACGTTAAAGATATCCTGGCAGTTACGCTGCTGGGTGTGATCCCAGAATCCCAAGCGGTACTCAAG
GCGTCCAACGAGGGCGTACCCGTTATTCTCGACGACCAGAGCGACGCCGGCCAGGCGTACAGCGATGCTGTCGATC
GCCTGCTGGGCAAGACCGTGGAACATCGCTTCCTCGATGTAAAGAAGAAGGGATTCTTCGAGCGTATCTTTGGAGG
CAACTAAATCATGAAATTTCTCGACTTCTTTCGCGCCAACAAAAAGCCAAGCACCGCGTCGGTAGCGAAAGAGCGT
CTACAGATCATCGTGGCGCACGAACGCGGCCAACGCAGCACCCCGGACTACCTGCCAGCCCTGCAGAAGGAGCTGG
TTGAGGTGATCCGCAAGTACGTCAATATCGGCAACGATGACGTGCATGTCGCCCTGGAAAACGACGGCAGCTGCTC
GATTCTGGAACTCAATATCACCCTGCCTGATCGTTGATCGAACAGGCGGTCGCCACGGCGGCTGCGCTACCCGCCC
CTACAGGGGTGGTTGGCGCAGTCGCCGTTGGCGTTTGTTACGAGACTGTTTAATGCCGCTGTCCAATATTCATATC
CTTCACCAGGATGACGCTGTCCTGGTGGTGAACAAGCCGACCCTGCTGCTCTCGGTGCCTGGTCGCGCCGACGACA
ACAAGGACTGCCTGATCACCCGCCTGCAAGAAAACGGTTATCCCGAGGCCCGCATCGTCCATCGCCTGGACTGGGA
AACCTCGGGGATCATCCTGCTGGCCCGGGATGCCGACACCCACCGCGAACTGTCCCGCCAGTTTCACGACCGTGAA
ACCGAAAAAGCCTACACCGCTTTGTGCTGGGGCCAGCCGGATCTGGACAGCGGCAGCATCGACCTACCCCTGCGCT
ACGACCCACCGACCAAGCCCCGCCATGTGGTGGACCATGAGTTCGGCAAGCACGCCCTGACCTTCTGGAAAGTCCT
GGAGCGTTGCGGGGATTGGTGCCGGGTTGAGTTGACGCCGATTACCGGGCGTTCACACCAGTTGCGGGTCCACATG
CTGTCCATCGGCCACCCGCTACTGGGCGATGGCTTGTATGCCCACGAACAGGCCCTGGCCGCCTGGCCGCGCCTGT
GCCTGCACGCCAGCATGCTGAGCTTTACTCACCCGCAAAGCGGCGAGCTCCTGCGCTTCGAGTGCCCAGCGCCGTT
CTGAATGGCTAGGCACAATAATTGTAGGAGCGAGCTTGCTCGCGAAAACCCCGAGAGCGCCGTGTATATCCAGGAT
GCCCGCATTATCGTTGGCGTTTTTCGCGAGCAAGCTCGCTCCTACAATGAATCGGTTGGGCCAATACGGTAAACTC
GCGCCATTGCTGTCTGGAGCTACTTATGCGCGAAGCGTTGAATCAAGGCCTGATCGACTTCCTCAAGGCCTCTCCC

ACCCCCTTTCATGCCACTGCGGCCCTGGCCCAACGCCTGGAAGCTGCCGGTTTCCAGCGCCTCGACGAGCGCGAGA
CCTGGACTACCGAGGCCAACGGTCGCTACTACGTGACCCGCAACGACTCCTCGATCATCGCGTTCAAGATGGGCCG
CCAGTCGCCCCTGCACGACGGCATCCGCCTGGTCGGCGCCCATACCGACAGCCCATGCCTGCGAGTCAAGCCACAA
CCTGAGCTGCAACGCCAGGGCTTCTGGCAACTGGGCGTGGAAGTCTACGGCGGCGCGCTGCTGGCGCCGTGGTTCG
ACCGTGACCTGTCCCTGGCCGGCCGCGTGACGTTCCGCCGTGACGGCAAGGTCGAAAGCCAGTTGATCGACTTCAA
GCTGCCGATCGCCATCATTCCCAACCTGGCCATCCACCTGAACCGTGAAGCCAACCAGGGCTGGGCGATCAATGCC
CAGACCGAGCTGCCGCCAATCCTCGCGCAGTTTGCCGGTGATGAGCGCGTGGACTTCCGTGCCGTGCTCACCGACC
AACTGGCCCGCGAACATGGGTTGAACGCTGATGTGGTGCTCGACTACGAGCTGAGTTTCTACGATACCCAAGGCGC
GGCGGTGATCGGCCTGCATGGCGACTTTATCGCCGGCGCCCGCCTGGACAACCTGCTGTCGTGCTACGCCGGCCTG
CAAGCGCTGCTCACCAGCGAGACCGACGAAACCTGCGTGCTGGTGTGTACCGACCACGAAGAAGTCGGTTCCTGCT
CAGCCTGCGGTGCCGACGGCCCGATGCTGGAACAGACCCTGCGGCGCCTGCTGCCTGAGGGTGATGAGTTCGTACG
TGCGATTCAGAAATCGTTGCTGGTGTCTGCCGACAATGCCCACGGCATCCACCCCAACTACGCCGACAAGCACGAC
GCCAACCACGGCCCGAAACTCAATGCCGGCCCGGTGATCAAGGTCAACAGCAACCAGCGCTACGCCACCAACAGCG
AAACCGCCGGGTTCTTCCGCCACCTGTGCATGGCCGAAGAAGTGCCGGTGCAGAGTTTCGTGGTGCGCAGCGACAT
GGGCTGCGGCTCGACCATCGGCCCGATCACCGCCAGCCACCTGGGTGTGCGCACCGTGGACATCGGCCTGCCGACA
TTCGCCATGCACTCGATCCGCGAGCTGTGCGGCAGCCACGACCTGGCGCACCTGGTGAAGGTGTTGAGTGCGTTCT
ACGCAAGCCGCGACCTGCCCTGAAGACTCCTTTGTGGCGAGGGGGCTTGTCCCCCACTGGGCTGCGCAGCAGCCCC
ATTCAGCCTCACTGAGATCTATGAGATACAACGAGGTGGCAGGTTTTGGGGCTGCTGCGCAGCCCAGTGGGGGACA
AGCCCCCTCGCCACGACAGCGCCTGGCCACATAGCCTTCGCCCTATCTGACACACATCAACCCCACTTGCGCCAAT
CCAGCCTAGACTGGTAATCATCTTTCTACGACAAGGCCGTCCCCACCATGATCCCGATGTCCGCCTTCCACGCCAT
GCTCATCCCCATCCTCACGGGCATGATCCTGCTGGCCGTTGGCTTCAACTTCCGCGACAAAAACCTCGGTGTGTTT
GGCATGTGGGTCGGCATGCTGCTGATCCTCGGCACCATCGTTTACCGGATCCTCGCAAAACTGGCCGAATAACAAA
TGCACTCGCAATGGGCATAGCGCCCTCGTACACTCGGTCGATCTGTCGTTTCCAAGGTTGACCGCCTCGTGCTTGC
CCGTCTGTTTGCCCTGCCCTACTGCCTGCTCCTTTGCCTGCTGATCCTATTGCCTATCGCCCCTGCCCAGGCGGTC
GGCCTGCCAGGCATTCTGGGCAGCAGCACCAAAGCCCAACCACAAGCCGACGTGCCGCTGGGCCAGTCTTTGGACG
AGGTGATCAAGACCCTGGAAAACGACCAGCAGCGCACCAAGCTGCTGGCCGACCTGAAGAAGCTGCGCGAAGCCAC
GAAAAAAGCTCAACCGGCAGCCGAGCAAGGGGTGCTGGGCTTGATCGGCGGCACCCTGGCGAGCCTGGAGCAACAA
CTGTCCGGCAGCGACAGCCCGCTCAATCGCTGGTCCGTCGAGTTCGACCAGGCCAAGGCCGAGTTTTCCGCCTTGA
TGCTGCCAGCCAGCGAATGGCTGCCAATCATCTTTGGCTTTGCCATGGTGCTGATGCTCTGGAGCCTGCTGGCGGC
CGCGCTGATCTGGCTCAGCCATCGCGTGCGCCTGCGTTTTGGCCTCAGTGAAGAACTGCCCCAACACCCCAAGACC
TGGGACATGCTGCGTTTTGCCCTGCGCAAACTCGGCCCCTGGCTGATTGCCCTGGTGATCACCGTCTACTTGAGCT
ACGCCCTGCCCTCGTCCCTGGGCAAGTCCCTGGCAATGGTCCTGGCGTACGCGCTGGTGGTCGGCACCTGTTTCTC
GGCGATCTGCGTCATTGCCTTTTCCGTGCTCGACGGCCCGCACCGCCACCGAGCCCTTTATATCCTGCGCCACCAG
GCGTTTCGCCCGCTGTGGCTGATCGGCAGCTTCGCCGCCTTTGGCGAGGCACTGAGCGACCCGCGCCTGACCGAAG
CCCTGGGCACCCACCTGGCACACAGCGCGGCGACCGTCGCCAATGTGCTGGCCGCACTGTCTACCGGCGTGTTTAT
CCTGCGTTTCCGCCGACCCATCGCGCACCTGATCCGCAACCAGCCACTGTCCCGGCGCCTGACCCGTCGCGCCTTG
AGCGACACCATCGAGATCATCGGCACCTTCTGGTACCTGCCGGCGCTGCTGCTGGTGGGCATTTCGCTGTTTGCCA
CGTTCGTGTCCGCAGGCGATACCAGCACCGCCTTGCGCCAGTCATTGTTCTGCACGGTGTTGCTGGTGCTGTGCAT
GGTGATCAACGGCCTGGTGCGCCGCCATGCCCTCAAGCCTCAACGTGGGCATAAGCGCCATGCGCTGTATTCCGAA
CGCCTGAAAAGTTTCGTCTACACCCTGGCCCACCTGGCGGTGTGGCTGGTGTTCATCGAGCTCGGCCTGCGGGTCT
GGGGCTGGTCGCTGATCCGCTTTACCGAGGGCGATGGGCATGAAATCAGCGTCAAATTGTTCGGCCTCGCGGGTAC
CCTGCTGTTCGCCTGGCTGATCTGGATCCTCAGCGACACCGCGATCCACCACGCCCTCACCCGCTCGCGCAAAGGC
CTGGCGAATGCGCGGGCGCAGACCATGATGCCGTTGATCCGCAACGTGCTGTTCGTGACGATCTTTATCATCGCCG
CCATCGTCGCCCTGGCGAACATGGGCATGAACGTCACGCCACTGCTGGCCGGTGCCGGTGTGATCGGCCTGGCCAT
CGGTTTTGGCGCGCAGTCACTGGTAGCGGACTTGATCACCGGCCTGTTCATCATCATCGAAGACTCCCTGGCGATC
GATGACTACGTGGACGTCGGCGGCCACCTCGGCACCGTCGAAGGCCTGACCATCCGCACCGTGCGCCTGCGCGATA
TCGATGGCATCGTGCATACCATCCCGTTCAGCGAGATCAAGAGCATCAAGAACTACTCGCGGGAATTCGGCTATGC
GATTTTCCGCGTGGCGGTGCCTGCCAGCATGGACATCGACACCGCGATCAAACTGATGCGCGACGTCGGCCAGAAG
ATGCGCGCCGATCCGCTGCAGCGCCGCAATATCTGGTCGCCCCTGGAGTTCCAGGGGGTGGAGAGTTTCGAGTCGG
GCAACGCCATCCTGCGCGCGCGCTTCAAGACCGCGCCGATCAAGCAGTGGGAAGTGTCGCGGGCGTTCAACCTGTC
GCTCAAGCGCCACCTGGATGAGGCCGGCATGGACCTGGCAATGCCGCGCTTGAGTGTGCAGGTGGTGACGGCGGCG
AGTGGGCCGCAGGACGCTCAGGTCACATCGACCCCGACGTGAATCGCATCATGGCGCCAGAACTCCAGGTCGCAAT
CGATCAGGCGCTGGTGCTGATCGTAATTGACCCGGGCGATCCGCAGCCCGGGGCTGCCTGCCGAGACCTTCAGGGC
GGCTGCCGCTTCCACCTGCAATGAAGTCGGCACAATTTCAAAGCGCACGCGCCCGTAATGCAAGTCGTACTGGCGG
GCGTACAACTCGGTCATCGACTCGTTCAGGTCACACGCAAGGATCCCCGGGAAATACTGCGGGTTGAGGTAGTGCT
CGACATACAGCACCAACCGCCCATCAATACGTCGCCGGCGGCAGATCTGGATCACGCTCGATAGCGCCGGCAGTTG
CAGCCACGCACAAACAGCGGCCGAGGCCGGTTGCAAGCGAGCCGATATGACTTCAGTGCAAGGCACCCGCCCCTGG

GCGCCGACCATGGCGTGGAAGTGGCTGCGCTGCATCAGGTTATAGGCCAGCCGGGGCGGCGAAACGAACCAGCCAC
GCCGCTCCTCGCGGTAGATCTGGCCCTGGGCTTCCAGTTGCAGCAACGCCTCGCGCACGGTGATGCGGGTGGTACC
AAACAACTCACTGAGTTTGCGCTCGGCCGGCAATTTGCTCGCCGGGCTCAATAAGCCGTGGCTGATCTGCTCTTGC
AGCGCCAGGCCTATGGCTGTCACCGCTTTGGTTGCCTCATCACGCATCAACGTTACCTATCTGGACTAGGCCAGCA
CTGTTTGGGTGCGCCAAATGCCCCTGGAATGGGCGACGGCTAGTGCGTGCAAGCCTAGGCATCACACATGACCGAC
AGATGACAGCGCCGTGCGCCGCCGTCTCACATGCCCTGCAATACATCGGCCAAGTCCAGGTATTACGGGGTCATGG
CCGTGGTCTACGCTTACCCGGCAGGCGCCTGCCCAGGCGCTTTAAAAACGACATCGACATGAAACTGTCATCCAGC
GCGCCTAAATTGGCTCGTGTATTGCTGACCTAGACCAAACCAACCGCGCCATCAAAAAATCGCAGCGTTGAAAACG
CCCAAAGGAGCTTCGGATGAAACAGCTTTTCCTGGCATCACTGTTAGGCTCGACCATTGCCATGTGCACCGCCGCC
ATGGCCGCTGACACCGATTTGAAAACCCTGGAAGCTGCCGCCAAGGCAGAAGGCGCTGTGAACAGCGTCGGCATGC
CCGATGACTGGGCCAACTGGAGAGGCACCTGGGAAGACCTTGCGGCCAAGTACGGCCTCAAGCACATCGACACCGA
CATGAGCTCGGCCCAGGAAATCGCCAAGTTCGATGCGGAAAAAGACAATGCCAGCGCCGATATCGGCGACGTCGGT
GCAGCCTTTGGGCCGATTGCGGCCACCAAGGGCGTCACGCAACCCTACAAGCCCAGCACCTGGGAACAGATCCCCG
ATTGGGCCAAAGATAAAGACGGTCACTGGGCACTGGCCTACACCGGCACCATCGCGTTTATCATCAACAAAAATCT
GCTGCACGGCTCCGAAGCCCCGACCAAGTGGGCGGACCTGAAGAACGGCAAATACAAGGTCTCGATTGGTGACGTG
AGCACCGCCGCCCAAGCCGCCAACGGCGTGTTGGCAGCCGCTCTGGCCAACGGTGGCGACGAGAAGAACATCCAGC
CGGCGCTGCTGATGTTCGCCGACATTGCCAAGCAGGGGCGTATTTCCCTGGCCAACCCGACCATTGCCACCATGGA
AAAAGGCGAAGTGGAAGTGGGCGTGGTCTGGGACTTCAACGGGCTGAGCTACAAGGCGAAGATGGCCAACCCGGAT
GACTACGTGGTGCTGATCCCATCGGATGGCTCGGTGATTTCCGGCTATACCACCATCATCAACAAATACGCCAAGC
ACCCGAATGCGGCCAAGCTGACCCGCGAATACATCTTCAGTGATGCCGGGCAGATCAACCTGGCCAAGGGCAATGC
GCGGCCGATCCGTGCCGAACACCTGACCCTGCCCGCCGAAGTGCAGGCCAAGCTGCTGCCTAACGAACAATACAAA
AACGTGACGCCCATCAAGGACCCGGCCGCCTGGGAGGCCACCTCCAAGACCTTGCCGCAGAAGTGGCAGGAAGAAG
TCATCATCAATATGCAGTAAGCCCTGGGTGGGAGCTGGCTTGCCGGCGATAGCGGTATGACATTCAGCACTCGTAT
CGGCTAATGCACCGTTATCGCAGGCAAGCCAGCTCCCACAGTTGATCCGGTTCCAGAAGAACCACGCAATCAAGCC
CAATTTGTCGCGGAGCCCCTGCCCCTATGAAGCACAACGTCATCCTTGTGGTGCTCGACGGCCTCAATTACGAGGT
CGCCCGGCATGCCATGGGGCATCTCCAGGCCTACGTCGGTGCCGGGCGTGCGGCGCTGTACAAGCTGGAATGCGAA
TTGCCCGCCCTGTCCCGCCCGCTGTACGAATGCATCCTCACCGGCGTACCGCCGATCGACAGCGGCATCGTGCACA
ACCACGTCTCGCGCCTGTCCAACCAGCGCAGTATCTTCCACTACGCCAGAGCCGCCGGCCTGACTACCGCAGCGGC
GGCTTACCACTGGGTCAGCGAGCTCTACAACCGCTCGCCGTTCATCGCGGCACGGGACCGGCACACCGACGACGAA
GCCCTGGCGATCCAGCACGGCCACTTCTACTGGAGCGATCATTACCCGGACGCCCACTTGTTTGCCGACGCCGAAA
GCCTGCGCCTCAAGCACGCGCCGAACTTTTTGCTGGTGCACCCGATGAATATCGACGACGCCGGCCACAAGCACGG
CCTCGACACCGCGCAATACCGCAACAGCGCCCGCACAGCCGACATTATCCTGGCCGACTACCTGCAAGGCTGGCTC
GATGCCGGCTACCAGGTGCTGGTAACGGCCGACCACGGCATGAACAACGACCGCTCCCACAACGGCCTGTTGCCAG
AGGAGCGTGAAGTCCCGCTGTTTGTCTTCGGTGATGCCTTCAGTCTCGACCCCAAGGCCCAGCCCAAGCAAACCGA
TATCTGCGGCACCGTCTGCGCCCTGCTTGGCGTTGCCCACGAAAAACCTGTCTGCCAGGAGCTATTGAAGTGAGTT
CAGCGATCCGTGGCAAATGGCTGGCCGCCTTGTGCCTGGTACCCTTCGCCCTGTTTTTTATCGTATTCCAGATCGC
GCCGCTGTTCTGGGTGATGGTCAACAGCCTGCAATCGGAGGAGTTCGGCTGGGGCCTGGCCAACTTCAGCAAGATC
TTCAACTCCAAGTTCTACCTACAAGCGATCAAGCACAGCCTGGAGATCAGTTTCTACTCCAGCATCTTCGGCATCA
TCATCGCGTTGCTGGGCAGCTACTCGCTGCGCAAGGTGGATTCGCCGCTGCGCAACTTCGTCACCGCCTTCGCCAA
CATGACCAGCAACTTCTCCGGCGTGCCCCTGGCCTTTGCCTTCATCATCCTGCTGGGGTTCAACGGCAGCATCACC
ATCATGCTCAAGCAGGCCGGGATCATTCAGGACTTCAACCTGTACTCCAAGACCGGCCTGATCATCCTGTACACCT
ACTTCCAGATCCCTTTGGGCGTGCTGCTGCTCTACCCGGCCTTCGACGCCCTGCGTGAAGACTGGCGTGAGTCCGC
AGCGCTCCTGGGAGCCAATGGCTGGCAGTACTGGCGGCATATCGGCCTGCCGGTGCTGACGCCGGCGCTGCTGGGT
ACTTTCGTGATCCTGGTGGCCAACGCCCTGGGCGCCTACGCCACGGTGTACGCGTTGACCACCGGTAACTTCAACG
TGCTGCCGATCCGTATCGCGGCGATGGTTTCCGGTGACATTTCCCTGGACCCGAACATGGCCAGCGCCCTGGCCGT
GATCCTGGTGGCGCTGATGACCGTGGTCACCGTGGTCCATCAACTGCTGCTCAAGAGGAGCTACCATGTCTCGCGC
TGAAGCCAGCCCCGCTGCCCTCTATCATCGCGTGGTGGTGTACCTGCTGTTTGCGATCCTGGTGCTGCCGCTGATT
GGTACCCTGGTGTACTCCCTCGCCAGCAGTTGGTCGGCCACCATCCTGCCCGCCGGCTTTACCTTCAAGTGGTATG
TGCAGCTGTGGAGCGACCCGCGCTTTTTACATGCCTTTGGCCAGTCGCTGCTGGTATGCGTGGGTGCGCTGATCCT
GTCGGTGGTGCTGATCCTGCCGCTGCTGTTCGTGGTGCATTACCACTTCCCCCGGCTCGATGCGCTGATGAACATC
CTGATCCTTTTGCCCTTCGCCGTGCCGCCAGTGGTGTCGTCGGTGGGGCTGTTGCAGCTGTATGGCTCGGGGCCGC
TGGCCATGGTCGGTACGCCGTGGATCCTGATCGGCTGCTACTTCACCGTGGCGCTGCCGTTCATGTACCGCGCGAT
CACCAACAACCTGCAAGCGATCAACCTGCGTGACCTGATGGACGCCGCCCAACTGCTCGGCGCCAGCACCTGGCAG
GCCGCGATCCTGGTGGTGCTGCCCAACCTGCGCAAGGGTTTGATGGTGGCGTTGCTGCTGTCGTTCTCGTTCCTGT
TCGGCGAGTTTGTGTTCGCCAATATCCTCGTCGGCACGCGCTACGAGACCTTGCAGGTTTACCTCAACAACATGCG
CAACAGCAGCGGCCATTTCACCAGCGCCGTGGTCATTTCCTATTTCCTTTTCGTGCTGCTGCTCACTTGGGCCGCC
AACCTCTTGAACAAGGACAAAAGCCAATGAGCTTCGTCAGCGTCCAGCACCTGCAAAAAAGCTACGCCGCCACCCC

GGTGTTCAGTGATATCAACTGCGAGATCGCCAAGGGCGAATTCGTCACCCTCCTCGGCCCGTCCGGTTGCGGCAAG
TCCACGCTGCTGCGCTGCATCGCCGGCCTGACGCCGGTGGACAGCGGGCAGATCCTGCTGGACGGCCAGGACATTG
TTCCGTTGAGCCCGCAGAAGCGCCATATCGGCATGGTGTTCCAGAGCTACGCACTGTTCCCCAATATGACCGTGGA
ACAGAACGTCGCCTTCGGCCTGCGCATGCAGAAAGTCAACGCCGATGACAGCCACAAGCGGGTCCAGGAAGTGTTG
CAACTGGTCGAGCTCAAGGACCTGGCCGGGCGCTACCCGCACCAGATGTCCGGCGGCCAGTGCCAGCGCGTCGCCC
TTGCCCGCTCACTGGTCACGCGGCCGCGCCTGCTGCTGCTTGACGAGCCGCTGTCGGCCCTGGACGCACGGATTCG
CAAGCACCTGCGCGAACAGATCCGCCAGATCCAGCGCGAGCTGGGGCTGACCACCATCTTCGTGACCCACGACCAG
GAAGAAGCCCTGGTCATGTCCGACCGCATCTTCCTGATGAACCAGGGCAAGATCGTGCAGAGCGGCGACGCCGAGA
CCCTCTACACCGCTCCGGTGGATGCCTTTGCCGCCGGTTTTATCGGCAACTACAACCTGTTGGATGCCGAGCAGGC
CAGCAAGCTGTTGCAGCGCCCGGTCAACGGGCGCATCGCCATCCGCCCTGAATCCATCGAGCTCAACCGCAGCGGC
GAACTGGATGCCCTGGTGCGCAGCCATAGCCTGCTGGGCAATGTGATTCGCTATCGTGTCGAGGCCCGGGGCGTGG
AATTGGTGGTGGACGTACTCAATCGCTCGGCCCAGGATCTGCATGCGGATGGCCAGCGCCTGGCACTTTCTATCGA
TCCCGCTGCGCTGTGTGAGGTAGCCTGAGGATGCAGCGTTTATTGAGGAAGCATGACTGATGGCTTTGGTAATTTT
TGATCTGGACGACACCCTGATCCACGGCGATTGCGCCACCTTGTGGAGCGAACAGATGGGCCGCCTGGGCTGGGTG
GACCGTGAATCGTTCATGCGCAAGAACCATGAACTGATGTCCGCCTACAGCCGCGGCGAGTTGGCCATGGAAGAGT
TCATGGACTTCAGCCTGGAACCGATGATCGGGCGCACCCCGGAAGAAGTCGCACATCTGGTAGAGCCATGGGTGGA
AGATATTATCGAGCCGCTGATCTACAGCGACGCCACCAAGACCATCGCCCGCCACCGCCAGAACGGTGACCGGATC
CTGGTGATCTCGGCCTCGGGCACGCACTTGGTCACGCCGATAGCGGCGCGTATTGGCATCGATGAGGTGCTGGGGA
TTGAACTGGACGTGGCCCATGGGGTGTACAGCGGCAAGACCGTCGGTGTACTGACCTACCGGGAAGGCAAGATCAC
CCGCTTGATGCAATGGCTGGAGCAGGAAGGTGAAAGCCTGGAAGGAGCGTATTTCTATTCGGATTCGCGCAATGAC
TTGCCGCTGTTGAGCAAGGTCGATTACCCACAGGTGGTGAACCCGGATCCTGTGCTACGGGCCCACGCTGAACAGG
CGGGCTGGCCGATTCACCAGTGGACCTGACAGCGCGTTTCCTGACTTGAAACCCAATCAAAATGTGGGAGCGGGCT
TGTCGGATCGCTCCCACACTTTTGACCCGGTTTCAATTCGAGATTGGCATTTAAGCCAGGCTTTCGTCGATCACCA
GCACCAACTTCCCGGAAATCTGGTTGGTCGCCAGCTCCGCAAACGCCGCCTCGGCATCCGTGATCGCAAAGGTCTT
GGCCAACTGCGGGCTCAAGCGCCCTTCACCAAACAATGGCCACACCTGCTGGCCCAGGTCGCTCAGCAGATCAGCC
TTGAACTGATCATCGCGGCTGCGCAAGGTCGAGCCCAGCAACTGCACGCGCTTGGCCAGCACCTGCGCCAGGTCCA
ACTGGGCCTCGCGCCCGCCCATCAAGCCGATCAATACCCAACGACCGTCGCGGGCCAGGAGCTTGAGGTTGATGGC
TGCATAATTGCCGCCCACCGGGTCGAGAATCACGTCGAACGGGCCAAAATCCCCCAGGCTCTCGATGCCGTCAGTA
CGTACCACACCGCCTTGGGCGCCGAGCTCTTCGCAGTAGGCCAGGCGATCCGCCGAGCCGACGCTGACCCAGCACG
GGCTGCCAAAGGCCTTGCACAGCTGGATCGCCGCCGAACCGACGCCACTGGCGCCCGCGTGCAACAGCACTTTTTC
ACCCGGCTTGAGGGCCGCCAGTTGGAACAGGTTCAGCCAGGCAGTGCTGTACACCTCAGGCAGAGCGGCCGCTTCA
GCCAGGGACAGGCCCTCGGGCACCGGCAGTACGTGGCGCGCGTCCACCACGACTTCCTGCGCCATGCCACCGCCGG
CCAGCAGCGCACAAACCCGATCCCCTACTTGCCAGGAAGACCCGGCCCCCACCTCACTGATAACCCCGGAGCACTC
CAGGCCCAGCACCTCGCTGGCACCTGGGGGTGGCGGATAGAGCCCCGCACGCTGTAATAAATCCGCCCGATTCAAG
CCGGCTGCTGCCACCCGAATGCGAACTTGCCCTACATCGCACGTAGGACTTGGGGTCTCAACCCACTCCACATGAC
CTTCAACGCCTTGCAATGCTTTCACAGTGCCTCCATAGTGAGTCTGGACTGAGCCCGTTGCTGTAGCGCCGGGCTT
TTTGCATTATGCGACCGGCCCAAATGGAACCGGCGACTTCAAAGACGGCCTAATATGCGTTATCAATTGCCCCCGC
GTCGAATCAGCATGAAGCATTTGTTCCCCAGCACCGCCCTCGCTCTTTTCATTGGTCTCGGCCTGTTGCCGCTGTC
GACCAATACGTTCGCAGCCAACAGCTGGGACAAACTTCAGCCTGATCGCGATGAGGTGATCGCCAGCCTTAACGTC
GTCGAGTTGCTCAAGCGCCACCATTACAGCAAGCCGCCGTTGGACGACGCGCGCTCGGTGATCATCTATGACAGCT
ACCTCAAGCTGCTTGATCCCTCGCGCAGCTATTTCCTGGCCAGTGATGTTGCCGAGTTCGACAAGTGGAAGACCCA
GTTCGACGACTTCCTCAAGAGCGGCGACCTGCAGCCTGGCTTCACCATCTACAAGCGTTACCTGGACCGTGTCAAA
GCGCGTCTGGACTTCGCCCTGGGTGAGCTGAACAAAGGCGTCGACAAGCTCGACTTCACCCAGAAGGAAACCCTGC
TGGTGGATCGCAAGGAGGCCCCGTGGCTGACCAGCACCGCCGCCCTGGATGACCTGTGGCGCAAACGCGTCAAGGA
CGAAGTCCTGCGCCTGAAGATCGCCGGCAAAGAGCCCAAGGCGATCCAGGAACTGCTGACCAAGCGCTACAAGAAC
CAATTGAGCCGCCTTGAGCAGACCCGCGCCGAAGATATCTTCCAGGCCTATATCAACACCTTCGCGATGTCCTACG
ATCCGCACACCAATTATCTGTCGCCAGATAACGCGGAAAATTTTGATATCAACATGAGTCTGTCCCTCGAAGGCAT
CGGTGCCGTGCTGCAAAGCGACAACGATCAGGTCAAGATCGTGCGCCTGGTGCCCGCTGGCCCTGCGGACAAGACC
AAGCAGGTCGCCCCGGCCGACAAGATCATTGGCGTGGCCCAGGCCGACAAAGAGATGGTCGACGTGGTCGGCTGGC
GCCTGGACGAAGTGGTCAAGCTGATCCGTGGGCCCAAAGGCAGCGTGGTGCGCCTGGAAGTGATTCCGCACACCAA
TGCGCCGAATGACCAGACCAGCAAAGTGGTGTCGATCACCCGCGAAGCGGTGAAGCTCGAAGACCAGGCCGTGCAG
AAGAAAGTCCTCAACCTCAAGCAGGATGGCAAGGACTACAAGCTGGGCGTGATTGAGATTCCGGCGTTCTACCTGG
ACTTCAAGGCCTTCCGTGCCGGTGATCCGGACTACAAGAGCACCACCCGTGACGTGAAGAAAATCCTCACCGAGCT
GACCAAAGAGAAGGTCGACGGTGTGGTCATCGACCTGCGCAACAACGGTGGCGGCTCGTTGCAGGAAGCCACCGAG
CTGACCAGCCTGTTTATCGACAAGGGCCCTACCGTGTTGGTGCGCAACGCTGACGGCCGTGTGGATGTGCTGGAAG
ACGAAAACCCCGGCGCCTTCTACAAAGGCCCGATGGCGTTGCTGGTCAACCGCCTCTCGGCTTCGGCCTCGGAGAT
TTTCGCCGGTGCGATGCAGGACTACCACCGCGCAATGATCATCGGTGGCCAGACCTTCGGCAAAGGTACGGTGCAG

ACCATCCAGCCGCTCAACCATGGCGAGCTGAAGCTGACCCTGGCCAAGTTCTACCGGGTTTCCGGGCAGAGCACCC
AGCACCAGGGCGTACTGCCGGACATCGATTTCCCGTCGCTCATCGACACCAAGGAAATCGGCGAAAGCGCCCTGCC
CGAAGCCATGCCATGGGACACCATCCGCCCAGCCATCAAGCCCGCATCGGACCCGTTCAAGCCATACCTGGCCCAG
CTCAAGGCCGAACATGACCTGCGCTCCGCCAAGGATGCCGAGTTCGTGTTTATCCGCGACAAGCTGGCCCTGGCCA
AGAAGCTGATGGCCGAGAAGACCGTCAGCCTCAATGAAGTGGACCGCCGCGCGCAGCACACCGATATCGAGAACCA
GCAACTTGCCCTGGAAAACATCCGCCGCAAGGCCAAGGGTGAAGCCCCGCTCAAGGAGCTGAAGAAAGAAGACGAA
GACGCGCTGCCGACTGAACCTGAAAAGACCAAGCCGGAAGATGATGCCTACCTGAGCGAAACCGGGCGGATTCTGC
TGGACTACCTGAAAATCAGTAAGACGGTGGCCAAGCAGTAAATGATGGCAATTTAATGTTGCCGCTCCACTGAGCG
TCATCAAACAGACATCATTCTGTCGTGAAATGAAGGACCGGGCGCCCCAGGCACCCGGTCCTTTTTTTTCGACCGA
GATCATCATGACCATGACCGAACAGCTGAATGCCCTGGGCTCCATCCTGGCTCAGGGCAGTTTGCACAGTCTGTTC
CAACCCATCATCAGTCTCTGCGAACGCCGCATCCTCGGCTACGAAGCCCTGAGCCGCGGCCCCTCCAACAGCCCGC
TGCACTCGCCCCTCGCGCTGTTTAGCGTGGCGCGCCAGGCCGGACGCCTGAATGAGCTGGAGATGGCCTGCAGGCA
AAGCGCCTGCAAGCGCTTCAGCGAGCAGAAGCTGCCGGGCAAGCTGTTTCTCAACGTCTCACCCGAGTCGCTGCTG
GAAACCACCCATCAACCGGGGCGCACCTTGCAGATGCTGCGCGAGTACGGGATCGCCCCCAGCCAGATCGTGATCG
AACTCACGGAGCAAGCACCGACCGACGATTTCCAGCTGCTGCAGACCGCCCTGCACCACTATCGCGACATGGGCTT
TTCAATTGCCCTGGATGACTTGGGCGCCGGTTACTCCAGCTTGCGTTTGTGGTCCGAGTTGCGCCCGGACTACGTG
AAGATCGACCGGCATTTTATCGACGGCATTCACCAGGATGCGCTCAAGCGCGAGTTTGTCGGCTCCATCCTGCAGA
TCGCCAGGGCTTCCCGTGCGCAGGTAATTGCTGAAGGCATCGAATTGCCAGAAGAATTGGCGGTGCTCACGGAGAT
GGGCGTGGATCTGATCCAGGGCTACCTGCTGTGCCGTCCCCAGGAGCACCCGCCGACCGAAGCCCGGTTGATGCTG
GCCAGGCCCGACAGCACCAGCGTCTCGCTCAACGAGGACGCCAGTGACCTCAGCGCCCTGCTCAACGAACAACCGG
CCGTGGCCCAGGACATCGCCACAGCAAAAGTGCTGGAAGCGTTCCGCAGCCAGGCCAACCTCAACTCCCTGGCGGT
CCTCGATGGGCGCGGCCAGCCCATCGGCATCGTGCATCGGCATTCGTTGTCCGACGCCCTGCTCAAGCCATTTGCC
ACTGATCTGTTTGCGCGCAAGCCCATCAGCCGCTTGATGAGCGACGACTTCCTGGCCGTGGAGCTGAGCCAGTCGT
TGCAACAGGTCAGCCGCCTGCTGACCAGCCGCGCGCGGCAGCGCATCGAAGAAGACTTCATCATCACCCAGAACGG
CAACTACCTGGGATTGGGCCGGGTGATCGACGTGCTCAAGCTGATTACCGAACTGAAAATCCAGCAGGCGCGCTAC
GCCAACCCACTGACCCTGCTACCGGGCAACGTGCCGATCCAGCAGTGCCTGACGCGGCTGTTGCAGCAGCAGCGCG
AATCGGTGATTTGCTATGTGGATATCGACAGCTTCAAGCCGTTCAACGATATCTACGGCTATGGCCGTGGCGATGA
AGTGTTGCTGTGCCTGGCGCAATGCCTGAACGACCGGGTGGACCCCAGTCGCGATTTTGTCGGGCATATTGGCGGC
GATGATTTTTTACTGGTGCTGGGCGCCCAGGATTGGCGCAAGCGTCTTAACCAGTTGGTGGATGATTTCCAGACCC
AGTGCCGGCGTTTCTACCGCAGTGAGCATTTGGAGGCGGGCTGTTTTATCGCCCTCAATCGCCAGGGGGCGCGACA
GGAGTTTGCCTTACTGTCGCTGTCTATCGGCGTGGTGCATCTGCATACGCTGAGTTGTGGACACTTGGATGCCAGC
CAGTTGGCGGAACTGGCCTCACAGGCCAAGCATCACGCCAAGAACGTGGCCGGCTACAGCATCCACGTAATCGACA
GCCGCGAACTCCTGTAGGAGCCGGCTTGCCGGCGCTTACAGTTGCTCCAGGCGCATCTGCGCCAGCGGATGTCCGG
CCTTGGCGGCGAGGGTCCACCAGCGCGCGGCTTCGACTGGGTCCGGGGCCTTGCCCAGGCTGCCAGCCAGGCTCAG
CACGCCGACTTGATAGGCAGCCTTGCCATCTCCAGCCTGGCCGGCCAAGCGCAGCAAGCGCACGCCCTCTTCCCGT
GCGCCAAGACCCTTGCCGCGAAAGGTCAGGATGTGCCCATAGAAACTTTGGGCCTCGACATTACCCAGGTTGGCCA
TGCGTGCAAACTGCCCCTCCAGCCAGTGCCAGCCACGGGGCTGACGCACGAACCATGACCAATGAAACAACCTGCG
CGCCAGCCAGTAGCTGGCATACGCTTTGAGCTTCCAGAACACTCAAGCCTCCGCTGATTCAGGATATTCGTACTCG
AACACACGGACCACTTCCGAGGCGTGCCATGACGCCGCGGCGACGCCATCGGATGGCCCGCAGAAACGCCCAAGGC
GTTCGACACATTCAAAAAAACCGGTGCGCGGCAAGCGACTCGCGCCCTGGCTGATCACCAGGGAACTGCGCAGGGG
TTGCTCGGCCTTGGCATCAAGGGCGGCCAAGTGCTCAAGTGCAGCGGTCAGGGTTTGCATCGCCGGCGTCGGAAAC
TGCAAACGCTCAAGCAATGCGCGATAGGTCAAAAGATGGCGTTGGCGACGGGCCTGATCAAGTTCGTTGAGCAATC
CATCCCAATGCTGACGGCTGATGCGCACACTCACACTGCATCCCTCCAACCCGCAATCCCCAACTCCCAGGCCAGG
CTGCGACGGATCGCGGCATCCGGCTGGCGCTCACCACTTTCAATCAAACCCAGGTAGGACGGGCTGATACCCACAG
TACGGGCCAATGTCTCGATCGCCAGCCCCTTGGCTTCGCGCAGGCTGCGTAATTGCTCCAGGGGGCGTAGATCCTG
CTCGGTAGCGATGGGTGGGATCTGTGCAGAAGGCGTGGCTGGTTGCTGCAGGCCGGCGGCTGTCAACAGCGCCTGG
TACTGAGCCCATGGCAGAACCGCGTATTCGGGTTCGCCATCCCGTGAAATGATTTGTATATCCATGACTGCCCCGT
AGGATCACAACACTTACGAAGTAAGTACTTTCCTTAGGAGTGTAATCCTAACAGCCACAAAGAGCGCAGGGGGATA
ATCCGCGTCTTCAGTTTTTTTTCGGGTTTTCCTGCTCCAGAAGCTGTGGCGTGGCGGGCAAGCGCTCAACCACCAC
CAACTTCTCCGGGCGCTGGCGTTCACGCCAGGCGCGGAAAGCACTCAGTTCACCGTCCAGGGACTTCATGACCCAG
GCCAGAACCGCGATGTCATCGAGCATGCCGAACATCGGCAGGAAATCCGGGATCGCATCAATCGGGCTGAGGAAAT
ACATCAAGCCCGCCACTACCGATATCAGCGCCTTGGGGCTGATGGCCCGATACTCGCCGCGCCAGTAGGCCAGGCA
CAACGCCTGGAGCAGCTTCAGGTCGTCCTTGAGCTTGCCCAGGCGGCTGCCCTGGCTTGAGCCTTTGGCAGCGACC
GCGAACAACAGGGTCGGCAGGCGTCCACGCCCAATCAGGCGTGCGGCCAACGGTAGGAAACGGGTGAAATTCCAGG
GTGCTTTCATCGTCACTCCAGTTCCAGGCGAGAGAAAAGGTTATCCACACAACTTGTGGATAACCTTGTGAACAGA
GCCCATTTTCGCGGCTGAAAGCCACGTAATACAAGGCCTACAGTCAGATCGGGCGTTTTTTGCGCACATAAAAAAA
TCCCAAGATTTCATTGACTTGGGGTGCATCTGCGGCTACCCGTGCCGAGGTATAGCATCAGACTGCTCTAAATCGC

AGGTGTTCGTTCGGATTGCAAGGGATCCGGTTTTCAGCGAATTCACCCTGTGGAGAGCAGGCTTGCCACAATCAGC
CCGCTCACCACAGATAAATAGAACCCGCAGAAACAACAACGCCCCGTATAAACGGGGCGTTGGGTGTTCAACTGCT
GCTTACTTGGCAGCAGGTGCTTCAGGAGCGTCAGCCTTGGCCGGGTCCTTGATTGCCAGCAGCTCCAGGTCAAAGA
CCAGTACCGAGTTGGCTGGAATCGCCGGGCTCGGGCTCTGGGCGCCGTAGGCCAGGTCGGACGGGATGTACAACTC
GACCTTCTCGCCTACGTGCATCAGTTGCAGGCCTTCGACCCAACCCGGGATCACGCCGCTGACCGGCAGGTCAATC
GGGCTACCGCGATCCACGGAGCTGTCAAAGGTGGTGCCGTTGGTCAGCTTGCCGGTGTAGTGCACAGTCACTACGT
CGGTAGGCTTGGGTTGCGCGCCTTCGGCCTTCTTCACGATCTTGTATTGCAGACCGGAAGCGGTGGTGACGACACC
GTCTTTCTTGGCGTTGTCTTCGAGGTATTTCTTGCCGGCGGCTGCCGATTCTTCGCTCATCTTGGTCATGCGTTCT
TCAGCACGCTTTTGCAGTGCGGAAAACGCTTCTACCAGTTCTTCGTCCTTCAGCTTCTGTTCTTTCTTGCCGACGG
CATCTTCGATGCCTTGGGCAACGGCCTTGGAGTCCAGGTCATCCATACCTTCCTGAGCCAGGCTTTTGCCCATGTT
CAGGCCGATACCGTAGGAAGCTTTCTGCGCCGGGGTTTTCAGCTCTACGCTGGTTTGCGAATCACAACCCGCAAGT
ACCAGGCTAACCAGGGCCACCGCCGCCGCCAACCGATGCTGTTTCATGCTATTTCCTTGTTCATGCGCCAAAAGGG
CATTCGAATAAAGTCGCGAGCTTATCAGGCGGCCACGACCAATGGCTACCGGCATGTGAGCAGGAAACTTCTGATA
AGTTCACGTGTTTAAAAGCATTTTCATCTGCCACAAAGCCCGGCGAAGGACAGCCGATGTACCGAAAACCGCCTGC
GTAGACGGATCATGGCTACTTGCCGCCTACCGCGCTCAGTGGCATAAGGACACAACCACTCGACAAGGATAGTTAT
CTTGCGCATTTTTTCCCGTGCTTTACGCCTGATCCTGTCACTTCTGGGACTGGGGCTGGCCGTCGTACTCTATTAC
GTGGCCAACCCGAAGCTACCGGATTACGTGCCGGTACAGCAGGTGCACTACCAGGACCAATGGAGCGCCGCCGACC
GCCAGGTCTACTACTTCACCCCCCAGGGCACCCAGGTCAAGGGCCTGCGCTACAACTGGTTCTCTGCGCTGGAGCT
GCCGTTTTCCGAGCAGCGATTTGCCGAGCCGGCCAACCTGGCGCGCTTTGGTTTCCTGGTAGACCCGCGGCAACAG
GCCACACCCCAGAACCCCGGCAACCTGCCCGTAGGTTTCGCCCGGCACAAGAATGCCGGCAGCAACGATGAGTTTC
TCGATATAACCTGCGCCGCCTGCCACACCGGCGAGTTGCGCTTCAATGGCCAGGCCCTGCGCATCGACGGCGGCTC
GGCGCAACACGTGCTGCCCTCCAGTGTCCCGACCCTGCGCGGTGGCAGCTTTGGCCAGGCCCTGGTGGCCAGCCTC
GCGTCCACTTATTACAACCCGTTGAAGTTCAGGCGCTTCGCCCACCGCGTACTGGGCGATCAATACGACGCCCAAT
ACGATCAACTGCGCCAGGACTTCAAGAAGTCCCTGGACAACTTCCTCAAGGTGGCCTGGAACGATACCCACCGTGG
CCTCTACCCCACCGAAGAAGGCCCGGGGCGCACCGATGCGTTCGGCCGCATCGCCAACGCCAGCTTCGGCGATGCC
ATTTCGCCGGAAAACTACCGTATCGCCAACGCGCCGGTGGACTACCCACAGTTGTGGGACATGTGGACCTTCGACT
GGGTGCAATGGAACGGCTCGGCCCAACAACCCATGGCGCGCAATATCGGCGAGGCACTGGGCGTAGGCGCTACCCT
GGCGTTCTTCGACAGTGCCGGCCAGCCTCTGCAGGGCGATGCGCGCTACCCTTCCAGCGTACGCGTGCGTGACCTG
AACCTGATCGAAGAAACCCTGCAACGGCTAAAGCCCCCGACCTGGCCTGAAGCGCTGTTCGGCACTATCGACAAAC
CCCTGGCCGCCCGTGGCCGCGCATTGTTCACGGAAAACTGCGCGGCCTGCCATGTGCCCAGCGTGAGTGAAGTCAA
CGGGCGCCCGGTACAGCAGTTGAAGATGCTGGCGGTGGACTACATCGGCACCGACCCTGGCACCGCCAGCAACATT
GCCGACCAACGCTACGACCTCAGTGCCCTGCAATGGGACCCGGCCGAATTGGCGCAGTTAAACGTCGAACTGCACC
CCGCGCCGACCGAACCGCTGGACCTGCGTAGCCTGTCCGTGGCCAAAGGCCTGGCCTATGTCACTGCATTTGTCGA
AGAACATGCTTACCGCGCCGCCGGTATCACCCCGGCCGAACGCCCCCGCCTGGACGGTTTCGGCCTGCCCATCGGC
GTGCGCGAGCTGCGCGCCTACAAGGCCCGCCCGCTGGCCGGTGTGTGGGCCACGCCGCCGTTCCTGCACAACGGCT
CGGTGCCGACGCTCTACCAATTGCTATCACCCCAGTACGAGCGCAGCCGCACCTTCTATAAAGGCACCTTCGACTA
CAACCCACGTCATCTGGGCTATCGCACCGAGGCTTTTAAAAACGCATTCCTGTTCGATACGAGCATCACCGGCAAC
CATAACAGCGGCCACGAGTTCCGCGCGGGCAAGCGTGGCAACGGGGTGATTGGCCGGGGCCTGGAGCCTGAGGAGC
GCTGGGCGTTGCTGGAATACCTGAAAGTACTGGGCGGCCCGCTGGAGCAGCAGCTGCCATGACTTCCCCATTTCGA
TACAAGGACCGTTCCATGCTCGCTCGACTGTGGCTGCGCCTCGGCGCGTTTCTCGGCAAGACGCTGTTATGGCTGG
TAGGCCTGGGCCTGCTCGGCTGGTTGCTGTCGACCCTGTGGTTTGCCTGGCACCACAGCGGCCCGGTGCCTACCGA
GGAGCAGATACCGCCGGGCGAAGCGGCCATGACCCAAGGCATCATCCAGACGGCGGTGCGCATCGTCGACCAGCAT
CGCGAAGGCACCCGTTACCTGCGCGATGCCCATGCCAAGGCCCACGGTTGCGTGAAGGCCGAAGTCCGCGTGCTGC
CGGACCTTGAGCCGGCGCTGCGCCAGGGCGTGTTCGCCGAACCGGGCAAGACCTGGCAGGCAATGATGCGCCTGTC
CAACGGCAACGCTTATCCACAGTTCGACAGCATCCGCGATGCTCGCGGCATGGCGATCAAGTTGCTGGACGTGCCG
GGCAAGCAATTGCTGGCCGATCAGCAGGCGCGCACCGAGCAGGACTTTGTCATGTTCAGCCACCCGAATTTCTTCG
TCAGCGACGTGGCCGAATACGCGCAGAACGTCGGGGCCCAGGCCGATGGCAAAAAAGCCATGGCGTTTTTCCCAAG
CCTCGACCCACGCAGTTGGCAGGTGCGGCATCTGTTTATCGCCCTGGCCACACTTGCACCCGCACCGGCCAGCCCG
ACCGAGGCGACGTACTTTTCGGTATCGCCCTACAAGTTCGGCAGCGCAAATGCCAAATTCCGCGTCGCCCCAGACC
CGCAAAGCTGCCCGGAGTACAGCCTGCCCAAGCAAAACCAGGACTTGCCGAACTTCCTGCGCAGCGCCTTGAGCCA
GCAACTGTCCACGGACCGCATCGACGCGTGTTTTGTGCTGCAGATCCAGCGCCAGAACCCGCAGAAATACATGCCC
ATCGAAGACACCAGCATTGAATGGAAAGAAAGCGACGCGCCCTACGAGAGCGTGGCAACAATCCGCATCCCGGCGC
AGGACTTTGATACTCCGGCCCTGAACCTGGCCTGTGACAACCAGTCGTTCAACCCATGGTTTGGCGTGGAGGAACA
CCGGCCGATTGGGGGGATCAATCGGCTGCGCAAGGCGGTGTACGAGGCGGTGAGTGATTATCGGCATAGCCGCAAT
ACCCCCTCTGATCGTTGACCTGTAGGAGCGGGCTTGCCCGCGATGTTCGTTAACGATAACGCTGGTTGCCTGACAC
CCTACGGCGTTTTATCGGACACCATCGCTGGCACGCTGGCGCCTCTTCGCGTAGGCGCAGGTCAACGTGTATGGGA
GGATTTGTCCCTGGCCACAGAATCCCAGGCACAAAAAAGCCCGCTCAATGAGCGGGCTTTGCGTGGTGACCTGGCG

TTCAGCATTCCAGGTTACCGAATATGGCGCAGCGGACGGGACTCGAACCCGCGACCCCCGGCGTGACAGGCCGGTA
TNNNNNNNNNNGACAGCACCGCCCATCCACCCCTGCATGACAGCACCTACCCGACCTTCGTCGACACTGACTAACC
CCGAGCATCATGCGAGACTGTCGCCCCGGGCCTGGAATGCCTGGGTGTTCTTATGTCCGGAGTCCTCATGACGCTG
TCCAGCGGGCTGATCGCCGCCGTTGCCCTGGCCTATATGGCCATTATGTTTGCCATCGCCTTCTACGGTGACCGCC
GCCACGCGCCGCTGCCGCCACGGGTGCGTGCGTGGGTGTATAGCCTGTCGCTGGCGGTGTACTGCACCAGTTGGAC
CTTCTTCGGCGCCGTAGGCCAGGCCGCCGAACAATTGTGGGCTTTTTTACCGATCTACCTGGGACCCGTACTGCTG
CTGGTACTCGCGCCCTGGGTCCTGCAGAAAATGGTGCTGATCAGCAAGCAGGAAAACATCACCTCCATCGCCGACT
TCATCGCCGCGCGCTATGGCAAATCCCAGTCACTGGCGGTGGTGGTGGCGCTGATCTGCCTGGTAGGTGTGCTGCC
TTATATTGCCCTGCAACTTAAAGGCATCGTGCTGGGGGTCAACCTGCTGATTGGCGCCGGCGCCGATGCCACCGGC
ACACGGGCCCAGGACACCGCCCTGATTGTGTCCCTGGTGCTGGCGCTGTTCACCATTGTGTTCGGCACACGCAACC
TCGACGCCACGGAGCACCACCGTGGCATGGTCCTGGCGATTGCCTTTGAATCCCTGGTCAAGCTGTTCGCGTTTCT
TGCCGTCGGCGCGTTTGTCACCTACGGCCTGTACGACGGTTTCAACGACCTGTTCAGCCAGGCAATGCTCGCGCCG
AGGCTGGAGGAGTACTGGAAAGAGACGGTGAACTGGCCATCCATGGTGGTGCAGACCGGGGTGGCGATGATGGCAA
TTATCTGCCTGCCCCGGCAGTTCCACGTCACCGTGGTGGAGAATATCGAGCCCCAGGATCTGCGCCTGGCCAAGTG
GGTGTTCCCGGCCTACCTGGTGCTGGCCGCGCTGTTTGTGGTGCCGATTGCCCTCGGCGGCAAGATGCTGCTGCCA
AGTTCGGTGCTACCTGACTCCTACGTGATCAGCCTACCCCTGGCCGAAGCCCATCCGGCCCTGGCGCTGCTGGCAT
TTATCGGCGGTGCTTCGGCGGCGACCGGCATGGTCATCGTCGCGAGCGTGGCGCTGTCGACCATGGTCTCCAACGA
TATGCTGTTGCCCTGGCTGCTGCGTCGCACCAACGCCGAGCGCCCGTTCGAAGTCTTCCGCCACTGGATGCTCTCG
GTACGCCGGGTCAGCATCGTGATTATTCTGCTGCTGGCCTATGTGAGTTATCGCCTGCTCGGCTCCACCGCGAGCC
TGGCGACCATCGGCCAGATTGCCTTTGCCGCCGTGACCCAACTGGCGCCTGCAATGCTCGGCGCGCTGTACTGGAA
GCAGGCCAACCGTCGCGGCGTGTTTGCCGGCCTGGCGGCGGGCACGTTCCTGTGGTTCTACACCCTGGTGCTGCCA
ATTGCCGCCCATAGCCTGGGTTGGTCGCTGAATATCTTCCCTGGCCTCTCCTGGCTGCATGGCAACCCGCTGGGGC
TGCCGATCACGCCGCTGACCCAAGGGGTTGTGCTGTCCCTTGCCGGTAACTTCACTCTGTTTGCCTGGGTCTCGGT
CCTCTCGCGCACTCGGGTATCAGAGCATTGGCAGGCCGGGCGGTTTATCGGCCAGGAAACCAGCCAGCGCGCCAGC
GCCCGCTCCATGTTGTCGGTGCAGATCAATGATCTGTTGAGCCTGGCGGCGCGCTTTGTCGGCGAGGAACGGGCGC
AACAGAGCTTCATCCGCTTCGCCTACCGCCAAGGCAAGGGCTTTAACCCCAACCAGAATGCCGACAACGACTGGAT
CGCCCATACCGAGCGGCTGCTGGCAGGCGTACTGGGTGCCTCATCTACGCGGGCGGTGGTGAAAGCAGCGATTGAA
GGCCGCGAAATGCAGCTGGAGGACGTAGTCCGTATCGCTGACGAAGCATCCGAAGTGCTGCAGTTCAACCGGGCAC
TGTTGCAAGGTGCAATCGAGAACATCACCCAGGGCATCAGCGTGGTGGATCAGTCCCTCAAGCTGGTGGCCTGGAA
CCGCCGCTACCTGGAACTGTTCAACTACCCCGACGGACTGATCAGTGTCGGGCGGCCGATTGCCGACATTATTCGC
TACAACGCCGAGCGCGGCCTGTGCGGCCCCGGTGAGGCCGAAGTCCACGTGGCGCGGCGCCTGCACTGGATGCGCC
AGGGCCGCGCGCATACCTCGGAACGGCTGTTTCCCAACGGACGCGTGATCGAGCTGATCGGCAACCCGATGCCGGG
CGGCGGGTTTGTCATGAGTTTCACCGACATCACCGCCTTCCGCGAGGCCGAACAGGCCCTGACCGAGGCCAATGAG
GGCCTGGAACAGCGCGTCACCGAGCGCACCCATGAGCTGTCGCAACTCAACGTCGCGCTGACCGACGCCAAAGGCG
TGGCCGAATCGGCCAACCAATCGAAAACCCGCTTCCTCGCTGCGGTCAGCCATGACCTGATGCAGCCGCTCAACGC
TGCGCGGCTGTTCTCGGCCGCCCTCTCCCACCAGAGCGAAGGATTGTCCGGCGAGGCTCGGCAGTTGGTGCACCAC
TTGGACAGCTCGCTGCGCTCGGCCGAAGACCTGATCAGCGATTTGCTGGATATCTCGCGCCTGGAAAACGGCAAGA
TCAATCCCCAGCGCCAGCCGTTTGCCTTGAGCGAACTGTTCGATGCCCTGGGTGCAGAGTTCAAGGTGCTGGCCCA
GGAACAAGGCTTGCGCTTCCGCCTGCGCGGCAGCCGCTTGCGCATCGACAGTGATATCAAGCTGCTGCGGCGCATC
CTGCAGAATTTCCTGACCAACGCCTTCCGTTATGCCCACGGCCCAGTGCTGCTGGGTGTAAGACGGCGTCGCGGTG
AGTTGCGTCTGGAGGTGTGGGACCGTGGACCGGGGATTCCACTGGATAAGCAGAAAGTGATCTTCGAAGAGTTCAA
GCGCCTGGACAGCCACCAGACCCGTGCCGAGAAGGGCCTGGGGCTGGGCCTGGCGATTGCCGACGGGTTGTGCCGG
GTACTCGGCCACCGCTTGCGCGTGCGCTCATGGCCGGGCAAAGGCAGTGTGTTCAGCGTCAGCGTGCCACTGGCCA
AGGCGCAAACCAGCTTGCAGCTACCGGTCACACCGGACAAGGGCCTGCCACTGAGCGGCGCCCAGGTGCTGTGCGT
GGACAACGAAGACAGCATCCTGATTGGCATGCGCAGCCTGTTGAGTCGTTGGGGCTGCCAGGTCTGGACCGCACGT
GACCAGGCGCAATGCGCGGCGTTGCTGGCCGAGGGCATGCGCCCGCAATTGGCACTGGTGGATTACCACCTGGACC
ACGGCGAGACGGGCACCGAGTTGATGGGCTGGCTGCGCGCGCAACTGGCAGAGCCGATTCCAGGGGTGGTAATCAG
CGCCGATGGCCGGCCAGAGATGGTGGCCGAGGTGCATGCAGCGGGTTTGGATTACCTGGCCAAGCCAGTCAAGCCA
GCGGCGTTGCGGGCGCTGCTGAACCGGCATTTGCCGCTGTAGCTTGTGGGTGCTTTAAACCCAAGCCGATAGGGCT
GCTGTGGCGAGCGGGCTTGCCCCGCGTTGGGTGGCGCAGCCGCCCCAAAACCTGCCGGCTCGGTATGTCTTAAACA
ACCGCGATGCCTGGGGTGGGGCTGCTGCGCAGCCCAGCGCGGGGCAAGCCCGCTCGCCACAGGGAGAGTCATCAGT
TTCGGAGCGTTGTGTAGATACCCGATGCCACATTTGGGCTCTGCGTTTAGTCAGGTAACTGTGCGACGCCATCTTC
ATCGGTCATCGCTTTCTCCAACAGGTCGGACGGCAAGCCTTTGCTCGCGCGCGCGCCCAGCAGCTTCAACTGCTCG
ACCCGGCTGACCAGATTGCCGCGACCATCTGTGAGCTTATTGCGCGCTGCACTGTAAGCCTTATCCAGTTGCTGTA
ATCGATTGCCCACTTCGTCCAGATCCTGGATAAACAGCACGAACTTGTCGTACAGCCAGCCGGCGCGCTCGGCGAT
TTCCCGGGCGTTCTGGCTTTGCCGCTCCTGCTTCCACAGGCTGTCGATCACGCGCAGGGTCGCCAACAAGGTGGTG
GGGCTGACAATCACGATATGGCGGTCGAAGGCTTCCTGGAACAGGTTGGGCTCGGCCTGCAACGCGGCGGAAAACG

CTGCTTCGATGGGCACGAACAACAACACGAAATCCAGGCTATGCAGGCCTTCCAGACGCTTGTAGTCCTTGCCGGC
CAAGCCTTTGACATGATTGCGCAACGACAGCACGTGCTGCTTGAGAGCGACCTGGCCGATCACCTCGTCATCGGCC
GCCACGTACTGCTGGTAGGCCGTCAGGCTGACCTTGGAGTCCACCACCACCTGCTTGTCGCCGGGCAACATGATCA
ACACGTCCGGCTGGAAGCGCTCGCCATCCGGGCCCTTGAGGCTGACTTGGGTCTGGTACTCGCGGCCCTTCTCCAG
GCCGGCATGTTCCAGCACCCGCTCCAGAATCAACTCACCCCAGTTACCCTGGGTTTTCTGCCCCTTGAGGGCGCGG
GTGAGGTTGGTGGCTTCGTCCGACAGGCGCAGGTTCAATTGCTGCAGGCGCTCCAGCTCCTTGGCCAGGGAAAAAC
GCTCGCGCGCCTCGTTCTGATAGCTTTCTTCCACACGCTTTTCGAACGATTGGATGCGCTCTTTCAACGGATCGAG
CAATTGCCCCAGGCGCTGCTGGCTGGTCTCGGCGAACCGCTGTTCGCGCTCATCGAAGATTTTCCCGGCCAGTTCG
GCGAACTGCGCACGCAACTCGTCCCGCGAGCCTTGCAGGTCATTGAGGCGCTGTTGATGGCTGTCTTGCTGCTCCC
GCAGTTCGGCACGCAGGGAGGCCGACAAGGCGTCCAGGCGGCGCAGTTCGCCTTCCTTGGCGCTGCGCTCGATGTT
CCAGGCATGAGCGGCATCACGCGCATTGTCGCGGTCGATCTGCAGCAACTCGACTTCCCGGCGCACAGCGGCCAGA
TCTGCCTGCTTGGCGGCATTGGCCTGGCTCAGATCGCTGATCTCGTCACGGCTGGCGTCCAGTTGCGCGGCCAGGC
CTTCCTGGGCCATCTGCGCCGTCGCCAGGCGTTCCTCCAGCAGTTCCCAACCGGCGCTGCGTGCATTCAGGCGGCG
CTGGATCTGCCAGCACAGGACCGACAGAGGCAACGCCGCAGCGGCGAGACCCAGTGCAACACTGGTCCAGTCAAAA
GCCATAGCCATTCCTGCCATTACGGTAAAAGAGGAAGGTTAACCAAGCGGGCGCTTCGTGGACAGCTCAGTCTTTG
ATCTGGCCCAGTTCACGCTGGGCGCCCACGTCGCCGGCACGGGCGGCCTGGCGCAGAAGATCGTGGCCGATGCGGC
GGTCTCGGGCATTGCCGCACTCACGACACATCAACAGGCCCAGGCGGCTCTGTGCCACAACCACCCCTTCACGGGC
CGGCTGCTTGAGCAAACGCCCGGCAAAATGCTTGATATTGGGGTGATCGCCAATGCGTGGGCTATCCAGCAGCCAC
AAGGCGACTTTCAAGGAGAAGCGCTTGGGTGCGGTAACACTTGGAGGGTTGTCGGTAACAGAAGATGATACTGAGC
GAAACTTCATAAAGCACTGTAGGGCAGAACGGAAGGCGCGCCACTCTACTCCTTTTTTCGTACGGGTAAAGCTGAA
AAAAACCTGCACGCCCGTTCTAGAGCAAGCGCTTGGGACAATCCACAGAAGCTGTGGATAACTCAGTGGACAACCG
TGCTTTAACTCGCGCAAAGGCCCATGGAACGGGGGCCGCGCTTAAACTGACGATTTTTTCACCAATAAAAAAAAGC
CAGATTTTTCATTGACTTAAATTTTCGATGCAGGCATTCACGGGCCAATCCGGCGCAGCACCTTCCCGGTGACACA
CTGCGCAACCAATGTGCACAACTACCTGCCTGACGGTTATATCCCGCACTTTTGTGGCCCGAGGGCACAACAAAAC
GTTACGCGCGACACACAAAGGCCACTTTTCAAACGTGCCTGCGCTCGCCACACTGTTCGCCCTACAGCAAGGAGGT
GCGCCACTTCATGAAAGGAATCCTGGTGTTCGCGTTATTGCTGTTCGCCCGTTTGTGTGATTTCCCTGGGCACCAAT
GCCGTGCTGCCATCTCCCGACGGCGCGCTGTTTGCCATCGCCATCCTGTTGTCGGCCGCTTTCACGGCGGTCAGCT
TGTGTATCGAGTTGGGTGACGGACGGGTCAACGACCTGCGCGATGTGGTGAAAGTCATCGAAACCGGCCAATCCCT
GCGCTTGACCCTCGCCGCCTATGCACTGGGCTGTTCATTGGCCGCAGCGTCCACGGTGCTGATTTATCGTGGGCTG
ATGGGCGGCTGATTGAAGTATTTTTGCTAACACACTTGCTTTAGCTGTTTCAAGCCATTAGTATCCGCAGCGTTAG
TACCAAGCTGAAAGTCAATTCTGGTCGAACTAATCCCCGCGACGTAGCTTCCCTAAAGAGAAGTTCTGTCAATCTT
GATGGGATCGACCACCTCGATGGTTTCCAGGTATCACTTGCGACGACACTGCCTTTGAACAATGCAAAGGGTGTCG
CGAAGTATCCATACTCTGACCGAACCAACCTGCAAATTGATCAGGATCTTCACCCTGGGCCCAGAACCTTTGCCCC
TGTTGTGTTGCCTGCCCTCCTAAGTACCTACCTGCCAGCCCAAGCGCGCCAAACTATTTAGCGCTTCAAACTGGCT
GCTTTGTTCCAGTCGGGTTCTTCGCATGATCAATGGTGATCAGCGTCACTGGAACGTTTTAACGTTGCACGGTTCT
TATCCGTGTCATTTGTAGGAACACCAATAATATGTCCACTCAAATCCATACTCAGGATGCCATTCGCACCCTAACC
AATGCTTTTGCTCCAATGAACTGCCTGATCATGGCCGCTCGCAAAGGCTGCTTCAGCTTCACCCTGGTCAACGAAC
ACGGCATCGCTCGTCATAGCGAACGCCTGTACCCCGATCAATACTCCAGCGCAGAACCGCTGCAGGCCGTGATCGA
GCGTACCCGTCAGGCACTGGTTGCCTGATTGACCCGACGCTGAACACCGAAAACCCTGCCCGCAAGGCAGGGTTTT
TTATTGCCCGCGATTCGGCTTTTTTAGCGGCTCCCCTTAGGCCAAACAGATATAACGCTTATAACCCGTAGCCGGA
AATATTTTAAAAACAGTCCTTTACGTCGTGAATCTGACACTACACTTCAACTCAGGCGGCATGTCCCGCCTCCGGC
GAGCCTGAGATCCGATTCACCGCTGCCAAGCCCCCCCTTCTCAGGCCTCGTCGACCACTTTCACGGTTTCGAGGGT
TTTATGGGCATTGCCGCCGGCGAGTTGTGTCGTTATGTGATCCGACCCACGTTGATTTACCTGGGCCGCCACAGCC
AGACCGCAGAAACCCTGCTGCTGGGGGTTGCCGCCAGCCAATCGGAACTGGGCTCTGCCTTGCACGACAGGCGCGG
CCACGGCCTGTATCGCATTACCGAACCGCGCCACAGGGCCCTGTGGGACGACTACCTGGCCCTCGACCCAGAGCGC
GCCAGCCTGGTGCGTGGCCTCGCCAGCCAACATGCTTTCCTCAGCGGGCCGCAACTGGAGCTGACGGTCAACCTGC
GCTACGCCACCGCCATTGCCTGGCTGCTGGTGGAGCAACATCGCCCCGAACTCCCCCAGCCCGACGACTTGCTCGC
CATGGCCCGTATCTGGAAAGAAATTTTTCACCCCCAGGGCCGGCTGCGCGATTACACCCAGGCCTGGCAAACCTGT
GTAAACCCAATGAATCAGGTCGCCTGTTGATCGGGCACTTTGCAAGATTCGGCAAAAATCGGCAATTTTGGTCGGA
TTGTCCTACAAAACCGCTCTATCTCCTACATTACAGCCTATAGCGCTGCGTTAATTTTATTGGTACTTTCCGCAGC
GGTGATCACCTCGGAGTTCTAATAATGAAAAAAGTAATGCTCAAGACCACACTTAGCCTCGCCGTTGCCATGGCCT
CTTCCCAACTGTTCGCCAGCGGCTTTGCCCTCAACGAACAAAGCGTCAGCGGTATGGGTACCGGTTTCGCAGGCCG
TTCTTCTTCTGCCGATGATGCAAGCACTGTTTTTGGTAACCCTGCCGGTATGGCTCGCCTTGAAGGCCAGCAAGTC
ACCGGCGGTGTTGCAGCGATTGATGCGTCCACTGATATCAAAGATGTCAGCGGTCGCTCCCGCGGCACCAACAAGG
GCGACATGGTCCCCTTCACGGCGGTACCGTTCGGCTTCTACACCAACAAACTCAATGACCAGTGGGCAGTCGGCTT
TGGTGTGTACGCACCGTTCGGCCTGATTACCGACTACGAAAGCGGCTATCAGGGCCGTGGCTTTGGCAGCAAAAGT
GAAGTGCAAGTCATCACTTTCCAGCCGACCGTCAGCTATGCCTTCAACGATCGGGTTTCCGTCGGTTTTGGCCCAA

CCTTCAACCGCATTGCCGGCACCCTGGAATCCGAGCTGACCCTCAACCCGGCCGTGCCAGATACCAAGATCAAGAT
CAAGGGTGACGATACTGCCATCGGCTTCAACGCCGGCGTATTGGTCCAGGCTACCGATACCACCCGCGTCGGCCTG
ACCTACCACTCGAAGGTCAAGTACAAGCTCGAAGGCCACACCGAAGTCAACGCGCCTACTGCGACCTCCAGCGCCC
TGGCAACCGGCCGTTATGACGCGTCGCTGAAAATCGACACGCCTGAATCCTGGGATCTGTCGGTTACCCAAGACAT
GAACGACGCCTGGAAGCTGTATGCCGGTGCTACCTGGACTCGCTGGAGCCGTCTGAAGGACATCACCGTCAACAAC
AAAGGCGTGACAGCAGCCGGTGGCGGCCTCGCCCCAGGTCTTGTGGGCACCATCAAGGAAGACCAGAACTGGCACG
ACACCTGGGCTTATGCCGTGGGTACCTCGTACCAGTTGACCAAGCAGGTGGTGCTGCGTACCGGTTTGACCTTCGA
CCAGTCGCCAACCAACAACACTGATCGTTCGCCACGTATCCCTACCGGCGACCGGACCATCTTCAGTGTTGGCCTG
GGCTATGCCGTCATGGATAACATGACCATCGACCTGGCCTACTCCTACCTCAAGGAAGAAGAAGTCAGCGTCAACC
GCTCCAACGCTCTGGCAAGCTACAACGCCAAGTACAAAAACAGCGCCAACGGTTTTGGCCTGGGCATGACGTACAA
GTTCTGATCCAACGCTGCATAAAAAAGCCCCGCACTCCTGCAAAGGAGGCGGGGCTTTTTAGTGGCCGATAATCAG
GGCTTGGAAGCCAGCGCCTGCTCCACGGCCTCGATCAGTTCCGGACTGTCGGGTTTGGTCAGGCTGGAGAAGCTGG
CGATCACCTTGCCCTGGCGGTCCACCACGTACTTGTAGAAATTCCACTTCGGCGCGTTGCTCTGCTCGGCCAGGAC
CTTGAACAGGTGTGCCGCATCCGGGCCCCTGACCTTCTGTGGCTCGGTCATGGTGAAGGTCACGCCGTAGTTCACA
TAGCAGACCTTGGCCGTCTCTTCGCCCGTCTTGGCTTCCTGCTTGAAGTCATCGGAAGGCACGCCAATCACTTCCA
GGCCGTCGCCCTTGTAGCGCTGATACAACGCCTCCAGGCCTTTGAACTGCGGGGCAAACCCACAAAAGCTGGCGGT
ATTGACGATCACCAGGGGCTTACCTGCAAAGCGCTGGCACAGGTCGACGGACTCCTTGGCGCGTAACTTGGGCAAT
TGACCTTCGAGCAACGACGGACAATCGGCGGCCATGGCGACACCGCCAAAAGCCACCATCAGGGCAGGTACTGCAA
GCCAGCGCTTCAACATGTCGAGGGTCCTTGGACAAAGTCAGGCAAGCAACTTAACAGATCGCCATGCCCAGTTGCA
TCAACGCCAGCCCGCTACGCTGCCAGCCCCACCAGGCCAGCAGCAACAGCGTCAGCAACCCGGCTGCGCCAAACCA
GTATCCGATCGAATTCATGCTACCCCCATCTGCGCCTGCAGCCTTGCCACCGGTCGCTCACGCACCGGCCAGTTCA
GGGCGGCCGCGACCAGGCTCAAGATAATCGCCACCTGCCAGATCAAATCGTAGTTGCCCGTTCGATCATAGACCAC
GCCCCCCAGCCAACCGCCCAGGAACGAACCTAGCTGGTGAAACAGGAACACAATCCCACCCAGCATCGACAGGTTT
CTGACCCCGAACAAGGTCGCCACCGTACCGTTGGTCAGCGGCACAGTCGACAGCCACAAAAAGCCCATGGCCATGC
CGAACAGGTAGGCGCTCATCTGTGTCACCGGCGCCCACAGGAACAGCACGATCACCACCGCCCGCAGCAGGTACAA
CGCCGTGAGCAAGCGTGGCTTGGACATCCGCCCACCGAGCCAGCCCGCCGTGTAGGTGCCGAAGATATTGAACAGG
CCGATCAGCGCCAGCACCGTAGTGCCGACGGTCGCTGGCAGATGCTGATCCACCAGGTAGGCCGGCAAGTGCACGC
CGATAAACACCACCTGGAAACCGCAGACAAAAAAACCAAAGGCCAGCAACCAGAACCCGGAATGGGAACAGGCTTC
CTGCAGGGCCTCGCGCAGGGTTTGCTGGCCGGCCATGACCGGTAGCGGGCGGTCCTTGATCATCGTCACCAACGGC
ACAATCAGCGCCACCAGCAGGCCCAGCACCAGCAAGGCCGCGGACCAGCCCAGCCAGCCGATCAGGCCCAAGGTGC
CCGGCAACATGGCGAACTGGCCGAAAGAGCCGGCAGCACTGGCGATGCCCATGGCCATACTGCGTTTTTCCGGGGC
CACGGCACGCCCTACCACGCCGAGGATCACCGAGAACGAGGTACCGGACAGGCCGATACCAATCAGCAAGCCGGCA
CTCAGGGACAGCGACCACGCCGAATCAGACAAGCCCATGAACACCAGGCCCAAGGCATAGAGCACGCCACCGATCA
TCACCACTTTGGCCGCACCAAAGCGGTCGGCCAGGGCCCCGGTAAACGGCTGCGCCAGGCCCCAGATCAGGTTTTG
CAAAGCGATGGCGAAGGCAAAGGTTTCACGGCCCCAGCCGAACTCGGCGCTCATGGGCGCCAGGAACAGGCCAAAG
CCATGCCGTACGCCCAGGGAAAGAGCCAGGATCAGCGCACTCCCGACAAGAATCCAGCCACTGGTACGCCACATCG
AGGTCATTATTATTCTCCGCCTGCGGGTATATACCCGCTTGTATTCGAACTAGCCCGCGTTTAAGCGAGTTCGTCC
AGCAAGGCCAACAGGGTTTCGCGTTTTTGCACACCCAATTTATCGATCAATTTCTGCTGCGCCGCTTCCCAGGCCG
GCAAAGCGGCCGTCAGGCGCTGCTTGCCTTCCTCCGTGAGCACTACCAGGCGATTGCGCAGGTCATCGCCCTCGAC
CAACCGTACCAGGCCTTCACCTTCCAGCACCCGCAGGTTACGCCCCAGGGTGCTGCGGTCCAGGCCCATGGCATCG
GCCAGGCTGGAAATGCTCGGCTGGTCCAGGCGCTGCAGATTACACAGCAAGGAATACTGGGCGACGTTGATCCCGA
AGCCATCGAGGGCGCCGTCGTAGTGCCTGCTGACGCCACGGGCGGCACGACGCAGGTTGGTGCATAAACATTGGGA
GGCAAGCATGGAACGTGTATATACCCGCGATTAAGGAAATGCAAGAAAGTGTTACAGAGCCAGGCCGATCAACACT
GCCACTTCGAGCAGCTCCAGCAAGGCGCCGGCCGTGTCACCGGTGGTGCCGCCCAGTCGATTGATCATCACTTGGC
GCAGCCCCATAAAGCAGATGGCAGCGAGCAGGACGGCGATCCCACCGCTGAAACCACCGATCAACACACAGGCCAG
ACCGGTAAGGATCAGTACTTGTTGCCCGACAATCCGCGGCAAATGGTCGGCCAGCGCCTGGCCCAGCCCCCCGGCA
CGCACATAGCGAGTCGTGAGGAACAACGCCAGCAGCGAACCGCGACCGATCAATGGGGCGAGGATCAACGCAGCGC
CGTTGTGTTGCTCGATTAGCGCTACCAGCGCGGTGAATTTGAGCAGCAGCACCAAGCCCAGGGTGACCACGGCAAT
CGGGCCGCTGCGCGGGTCCTTCATGATGGTCAGGGTGCGCTCGCGGTCGCCAAAGCCCCCCAGCCACGCATCGGCA
CTGTCCGCCAGGCCATCCAGGTGCAGGCCGCCACTGAGCAGCACCCACACCGTCAACAGCAGCGCCGCGTGCAACA
GCAACGGCGCGCCCATCAACAGCGTGTTCAAGCCCCACAGCAGCAGCCCGAACAGCAGGCCCACCAACGGATAAAA
CAGCAGCGAACGCCCCAGTTCCTGCGGTTGCGGCATGCCTGGCAGGCGGATCGGCAGGCTGCTGAGAAACTGCAAG
GCAATCCAGAATGGCAACATCAGGCGCCCTCGCTCAACACGCCATCGGCCTCGACCCGCAGGCTGAACAAGCCGCC
GTGCCCCACCTCGACATTCAACAACTGCTCACGGGGCAAGCCACGGGCCTGGGCCAGCAATAAACGCATGACACCG
CCATGGCTGATCAGCAGCACCCGTTGCCCGGCGTACAGCTGGTGCAGGCGCAGGACCGCACCGTGTACGCGCCGGG
AAAACCCTTCGACAGGCTCGCCATCCGGCGGCGTAAAGCTATACGGGTCAGCCCAGAACAGCCCCAGTGCCTCGGC
GTCGGTTTCCATCAACTTCGCCGCGCTCTGCCCTTCCCAGGCACCGAAATGCAGCTCCTGCAGATCCGGCTCCAAG

CTCACCGGCAAATCCAGCCGTGCGCCCAACTCTTCAGCAAAACGCGCGCAACGCTGCAGCGGCGAACTGACCAGGC
GATCCCACGGCCCGCGCGCCAGCACCGCCTCGCGCATCTGCGTCCAGCCCTTGGCAGTCAGCGCATCATCCAGGCT
GCCGCGCAAGCCGCCGCCCAGTTCGGTTTCGCCATGGCGCAGCAGGTCCAGGTGCAAGGTCATGCCGGGCGATCCG
CCACTGCCGCCTCAGCAAACGTCGCCATCTGCCCGTGCAACGCACAAGCCAGGCGCAACAGTGGCACCGCCAGCGC
CGCGCCGCTGCCCTCGCCCAGCCGCAGGCCCAGCTCCAGCAGCGGCTCGGCTTGCAGGCTTTGCAGCACGTGGCGA
TGGCCCGGCTCGGCGCCCCGGTGGCCGAACAACAGCCATGGCCGGCACGACGGATTCAGACGCGTGGCGACCAGCG
CCGCGACGCTGCAGATAAACCCGTCGACCAATACCACCACGCCCGCTTGGGCGCAGGCCAGATAAGCCCCCACCAA
CCCGGCAATCTCCAGGCCACCGAGGTTGAACAGGGTCTGCAACGGGTCACCGCGCTGCCCCGCATGCAATGCCAGC
GCGCGCTCGATCACCGCCACTTTGTGGCTCACGCCCTGGGCATTCAGGCCGGTGCCGGGGCCGGTCAGGTCGCTGA
CCTGGCAATCGAGCAGCGCACAGGCCAGCGCGCTGGCGGCAGTGGTGTTGCCGATGCCCATTTCGCCGCCAATAAA
CAGCTGCGTACCGGCGTTGATCGCGCGCTGCACGCTGTCACGCCCCGCCGCCAAAGCCAGCCGTCCCTGGGCCTCG
GTCATGGCCGCGCCCAGCACGAAGTTGGCCGTGCCTGGGGCGATATTCAGGTGGCGCACGCCCGGCAGGTCCAGCG
ATGGCGTGACAGTGCCCAGGTCCAACACTTCCAGCCGTGCATCCAGTTGGCGCGCCAACACGCTGATCGCCGCGCC
ACCACTGACGAAGTTATGCAGCATCTGCGCGGTGACTTCCTGGGGATAGGCCGAGACACCTTCCGCGACCACGCCG
TGATCACCGGCAAAGATGCTGATCCACACGTGATCGACCGTCGGCTTGACCCGGCCCTGCAAACCGGCCAATTGCA
CCGCCAGCGCTTCCAGTTGGCCCAGGGATCCGGCGGGTTTGGTCAGTTGTTGCTGGCGGGCCAGCGCTTGTTCATA
GGCCGCAGCGTCCAGCGCCTTGCAGGGGTCCAGCCACCAGGATTGGCTCATAACGCAGTACCTTTCAAAGTCAGGG
GCAGGCCCGCAACGGTCAGCACGACACGCTGACAACGCTCGGCCAGGGCTTGATGCAGCCAACCGGCTTCATCCAC
GTAGCGGCGAGTCAATTCGCCCAGCGGCACGACACCCAGGCCGGTCTCGTTGCTGACAAAAATGATTTCCCCCGGC
AGCGACGCCAGGCAGTCCAGCAGTTGTTCGCGCTCCACGCCCAGGCGCTGCGGGTCGTCGAGCATCAATAGGTTGG
TGAGCCACAAGGTCAGGCAATCCACCAGCAGGCACTGGCCAGGGGCGGCGTGTTCGCGCAGGACCCGCGCCAGTTC
CAGCGGCTCTTCGATCAGGCCCCAATGGCTTGGGCGACGTTGGCGATGCAGGGTCACGCGCGCATTCATTTCACCG
TCCAGGGGTTGGCTGGTGGCGATATACGTGACAGGCATGGCGCTGTCGCTGGCGAGTTTTTCCGCCAGGCGGCTCT
TGCCGGAACGGGCGCCGCCGAGGATCAGTTGCAACATGGTCAAACCCTCAAATGCCACATAACTGGCGCAGCAACC
GGGTGTCCAGATGCTGCTCCACCAGGTCCGCCAGGCGCTCGATATCGCGCTCGCGCAGCCCGTGGTAATCCACCTC
CTGCACATCCTGCAAACCGGCCCAGCGCAACAAGGCGCTGCACGCAGCGGGCGTTTCGAACAGGCCGTGCAGATAG
GTGCCGAGGATCTGCCCGTCATCGCTACAGGCGCCGTCACTGCGCCCGTCATCCAGGCGTACCGCCGGCTGCTCCA
GGGCCGTGCCACGGGTCACGCCCGCATGAATCTCATAACCGCTGACCGGCGCCTCTTCCAGCACCAGCCGGCCACG
CACATTGCGCAGTTGTTTTTCGGCTTCCAGGGTGGTGCTGAACGCCAGCAGGCCCAGCCCCTCACTGGAGCCCGCA
GAACCTTCCAGGCCGAGCGGATCGTGGACCTGCTCACCCAGCATCTGCAGGCCGCCGCAGATCCCCAGGACCTTGC
CGCCGTAGCGCAAGTGCCGGGCGATCGCACGGTCCCAGCCATTGGCGCGCAGGTAGGCCAAATCGCTGCGCACGCT
TTTCGAGCCGGGCAGGATGATCAGGTCGGCGGCAGGGATCGCCTGCCCCGGCCCGACAAACTGCAAATCCACCTGG
GGATGCAGGCGCAAAGGGTCAAAGTCGGTGTGATTGCTGATGCGCGGCAATACCGGCACCACCACTTTGAGCACCT
GCGCCGCCTTGTCGATCTGGCGCTGGTCGATGCCATCCTCGGCTTCCAGGTGCAGGTCCATCACATAGGGCAGCAC
GCCGACCACCGGTTTGCCGGTGCGCGCTTCAAGCCAATCGAGGCCCGGTTGCAGCAGCGCAATATCGCCGCGAAAG
CGGTTGATGATAAAACCCTTGATCCGCGCCTGTTCCGATGGCGATAGCAACTCCAACGTGCCGACCAGGTGGGCGA
ATACCCCGCCGCGATTGATATCGGCAATCAGCAGTACCGGGCAGTCCACCGCTTCGGCGAACCCCATGTTGGCGAT
GTCGTTGGCCCGCAGATTGATCTCGGCCGGGGAGCCCGCGCCTTCGACCATCACCACTGGGTAGGCCGCACTCAAA
CGCGCATGGGAAGCCAGTACCGCCTGCATGGCGATGGCCTTGTAGTCGTGATAGGCCACGGCATTCATGCTGGTCA
CGGCATGGCCATGGATGATGACCTGGGAGCCGGTGTCGCTGTTGGGCTTGAGCAGCACCGGGTTCATATCGGTGTG
CGGCGCCAGGTTGGCAGCCTGGGCCTGCACGGCCTGGGCACGGCCGATCTCGCCACCTTCTGCCGTGACGGCGCTG
TTGAGCGCCATGTTCTGCGGCTTGAAGGGCACCACCGCCACGCCCTGGCGCACGACCCAGCGGCACAGTGCGGTCA
CCAGGGTGCTTTTGCCCGCATCGGAGGTGGTGCCTTGCACCATCAGGGTGGTCATGAGGCGTGTTTCCAGGCAGTC
AGGGCTTGTTCAAGGCGCAGCCAATCGGCGTCGGTCCCGGGCAGGCCAAAACGCAGGCTGCTGTCGTGGACAAACA
GGCGCAGCAAGATGCCGCGCTGGGCCATGAACTCATGCAACTGCTCGGCACGATCGGCGATCAGCCACTGGAACAG
CGCACAACCGCCTTGCGGGCGCAGGCCATACTGGTCCAGCAGATTGAACAGACGCTGGCTGGCTTCCTCGCAGCGC
TGGCGCTGCTGGGTGTGGCCGGCGCTGTCACGCAGGCAGACCTGGCCCAACTCCCGGGTCGGCCCGCTCACCGCCC
ATGGCCCGACCTGTTCGGCCAGCAGCTTGAGCAGCTTGCGCTCGGTCAGGACAAAGCCCAGGCGCACCCCGGCCAG
GCCGAAAAACTTGCCGAAGGAGCGCAACACAATCAACCCCACTTGGTTAGCCTGGCTTGCCAGGCTCAAGTGCGGG
GTGTTGTCCATAAAGGCTTCATCCACCACCAGCCAGCCACCGCGCTGGGCCAGCCGCGCATGCCAATCCAGCAGGC
GCTGGGGGGTAAGGCTCAGGCCGGTTGGGTTATTGGGATTGACCACCACCAACACGTCGAGGCTGTCGAGGAAGAA
GTCGACTTCCTGCTCCAGCACCTCACGCACGATGTAACCGGCGCGGCGCCAGGCTTCGGCATGCTCGGCATAGCAC
GGCGACAATACGCCGACCTTGCCCGCTCGGCGCAGGCGCGGCAGCAGTTGGATCGCCGCCTGGGAGCCCGCCACCG
GCAACACCTGGGCGGCGCCGTAGTAGTCGCAGGCGGCCTGTTCCAGGCCGTCATCGGTCTCTGGCAAGCGCGCCCA
GGCCCGTAACGGGATGGGTGGGATTGGCCAGGGCCAGGGCGCGAGGCCACTGGACAGGTCCAACCAGTCCTCCTGG
GCGATGCCGTACTGCATCGCGGCTTTGCGCAGCCGGCCACCATGTTCAAGCATAGAATTGTGCCCCCGCGCACAGG
ATCAATACCCACAACCACACGCCGCGCTGCACCAGTTGCCAGCCACGCTCGATGGAATCGGCATCGGCTGGAGGCC

CTTCACCCAGGACTGGACGCTGGTGCAATTCACCGTGATAAATCGCTGCACCGCCCAACTCGACGCCCAGGGCGCC
CGCACCGGCGGCCATCACCGGGCCGGCATTCGGGCTGTCCCAGGTCGGGCCCTGGCGGCGCCAGCATGTGAGCGCC
AGGCGCGTCTTGCCGAGCACAGCGTAGGTCAACGCGACCAGGCGCGCGGGAATGTAATTGAGCAGATCGTCAATTT
TTGCCGCCGCCCAGCCGAAGCGTTCAAAGCGTTCGTTGCGATAGCCCCACATGGCATCCAGGGTATTACTCAGGCG
GTACAACACCACGCCCGGCACCCCGGCCACGACAAACCAGAACAGCGCGGCGAACACCGCGTCGCTGCCGTTTTCC
AGCACCGACTCGGTGGCGGCACGCGCCACTTCAGTGCTGTCCAGTTCGCTGGTCTGGCGACTGACCAGGTAACTGA
CGCGCGTACGCGCCTCGTCCAGGTCCCCACTGCGCAGGGCCTGGGCCACCGGCAACACGTGCTCCCCCAGGCTGCG
CATGCCCAGGGCCAGGTACAACGCGAGGATCTCGATCACCCAGCCGAAGTAAGGCGCCCAGGACAATGCCGTGGCC
AGCAAGGTCAGCGGTATCACGGTGATAAACCACGCGGTCACGCCATGGCTGCGCCAGCCGCGACCTGCGGAGTTGA
AACGCTGCTCGATGCGCCCGGCAAAATTGCCGAACGCCACCAGCGGATGCCAGCGCCTGGGCTCGCCCAGCAGCGC
ATCCAGCGCCACTGCCGCGACACTCAGCAAGGCCACACTCATTGACTCACTCCCCAATAATTCTCATACAGCATGT
CACTCAACGGACGCGGTTCGGTCCAGCCTTCGAGTTGCAACATCGGTGCCGGGTAGAACTCCGTCACCGGGCCCAG
GCACAGCACGGCCAGCGGCTTGGCGCCAGGCGGCAGGCCGAGCAGGTCGGCCAGGTCCCGGGGTTCGAACAACGAC
ACCCAGCCCATTCCCAGGCCTTCGGCGCGGGAGGCCAGCCACAGATTCTGGATCGCGCAGGACAGCGAGGCCAGGT
CCATTTCCGGCAACGTGCGCCGGCCGAAGATATGCCGCTCGCGATCATCCATCAACGCTGCCACCAACACTTCGGC
GCAGTCGTTGATGCCTTCGACCTTGAGCTTCATAAAGTCGTCCGAGCGCTCGCCCAAGGCGGCCGCGGTGCGGATG
CGCTCGGTTTCCACCAACTGCTGGATCTGCCCACGCAGGTTGCGGTCGCTGATGCGGATAAAGCGCCAGGGCTGCA
TCAGGCCGACACTGGGGGCCTGGTGCGCGGCGTGCAGCAGGCGCTGGAGCAGTTCGGGAGCCACGGTGCCGCCGCT
GAAGTGGCGCATGTCGCGGCGTTCGGCGATGGCGCGGTAGATGGCCTGGCGCTCGGCTGCGGGGAAGGCGTTGTCG
GTCATGGCCCTATCGCGGGCAAGCCCGCGCCTACAGTTGCAATGCAATCCCCTGTGGGAGCTGGCTTGCCGGCGAT
AGCGGCGTCCCGCTCGGGCGCAAACAGCGCAGCCACCGCAGCCGGGTTCGACGGGAAATAAAAGTGCACATAGGAG
GCCGTCATCCGCCCCTGGCGATACACCGCCTCGGCACCGCGCCCGCCATTGGGGCTCAGGCCACGGGCAATCGGCT
GCCATTGCGTGCTGGTCAGGGAGTGATGATAGGTATGGCCGCGCAGCACGCCTTCCGGCAATTCCACCGTTTGCAG
GGCCAGGGCCGCCAGGCGTTTCTGCATGACCGCATCGCCTTGCAACAGCCCCAGCAGTTCGGCGCGAGTGCCCTCG
ACATCGGTCAATGAGTCGAGCAAATAGAGCATGCCGCCACATTCGGCCAACAACGGCTTACCGGCAGCGTGATGGC
CACGGATCGCCTGCAGCATCGCGGTGTTTTGCGACAACGCCTGATGATGCAGCTCGGGGTAACCACCGGGCAGATA
GAGGCTATCGGCATCGGGCAACAGTTGGTCATGGATCGGCGAGAAGAAGCGCAACTCGGCGCCCATCGCCCGCAGC
AAGTCGAGGCTCGCGCCATAGGTGAACGCGAAGGCTTCATCACGGGCCACGGCAATCCGCACCCCGGCCAGCAGTG
GCTCGGCCTCGATCAGCTCGGGTGCGGCGAACTCCACCGGCGGTGGCAGCGCTACCTCGCAACTGCCAGCCAGGGC
TTCGGCGGCCGCATCCAGACGCAGGTCCAGGTCGTTCAACTCGCTGGCCTGCACCAGGCCCAGGTGGCGGCTGGGC
AACTCGATCCCGGTTTCCCGGGACAGTGCACCGTACCAACGCAGGCCTTCGGTCAGGCTGCCTTCAAGCAATTGCG
CATGGCGCACGGTCCCGACGCGGTTGGCCAAGACCCCGGCGAACGGCAGGTCCGCCTGGTAACGCGCCAGCCCCAG
CGCCAGGGCGCCAAAGGTCTGGGCCATGGCGGTGCCATCGATCACCGCCAGCACTGGCACACCAAAGTGGCGCGCC
AGGTCGGCGCTGGAGGGGGTGCCGTCAAACAGCCCCATCACGCCCTCGATCAGGATCAGGTCCGCCTCCCCCGCGG
CTTCCCACAACAAACGGCGACTTTCCTGCTCGCCCACCATCCACATGTCCAATTGATAGACCGGCGCGCCACTGGC
GCGCTCATGGATCATCGGGTCGAGAAAGTCCGGGCCGCATTTGAATACCCGGACCTTGCGCCCCAGGTTGCGGTGC
AACCGGGCCAGGGCGGCGGTGACAGTGGTTTTGCCCTGGCCTGACGCCGGTGCGGCGATCAGTACGGCTGGGCAAT
GGCGCGACTGATTCAAAGTTCGATGCCTTTCTGTGCCTTGATACCGGCCTGAAACGCGTGCTTGAGCATGCCCATT
TCGGTCACGGTGTCGGCCATTTCAATCAGTTCTGGCTTGGCGCCACGACCGGTGACCAGCACGTGCTGCATCGGTG
GCCGGGCTTGCAGGTCGCTCAACACCTGGTCCAGGTCGAGGTAGCCGTGCTTGAGCGCGATGTTCAGCTCATCCAG
CACCACCAGGCCGATGGAAGGGTCCTGCAACATTTCCCGGGAGACTTGCCAGGCCGCTTCGGCGGCGGCGATATCG
CGCTGGCGGTCCTGGGTTTCCCAGGTGAAGCCCTCGCCCATCACATGAAACCGCACCTGCTCGGGGAAACGGCGGA
AGAACAGTTCTTCACCGGTGCTGTTACGGCCCTTGATGAACTGCACTACGCCGCATTGCATGCCGTGCCCCATGGC
GCGGGCCAGCATGCCAAACGCCGAACTGCTCTTGCCTTTGCCATTGCCGGTCAATACCAGCAGCAAACCGCATTCA
TTCGGTGAATTGGCAATGCGCTCATCAATCACCGCTTTTTTGCGCAGCATGCGCGCCAGGTGGCGTTCGTCACGAT
CAGGGGAATCAGTCATGGCAGCTCTCCGTTGGGGCTGGACAAAAACGGCGGGCAGGAAAAGACAATTTCTATGAAG
CCAAGCATCGCCCACCGTGATGCTGTTGGATGAATCAGGCCGGTCTCCGGGCTCATGAGTGGCGCCCTGTTGACCA
GGGCGGGCCGACGATGCGCCTTCCCATATCGCACGCGATACAGTGGCAAAAGCAGCGTCTTGACTCATTTACCGTT
GCGGGGGCAGCGCCGGGATCGTGGCCGCACTGTAGAAAAGTGCGCGCCCTCACCGGCTTCCCTGTTTCACTCGATC
GACGCGTACGCCACAGAGCACCTGAAACAAGCGGCGAAGGTTAGTGGGTTGGGGGTACAGCGTCAATTAAAGCCGG
CCACGCACTTGAACCATCGCCACGGGCCGTACCTCTATGACTACAGGCATTGAGGAGAAAAACATGCACAAGACCC
GACTCGCCGTACTGATCATGGCCGCAGGCGCACTCGCCGCCTGCGGGGAAAGCTCAACGTTGCAGGTGTCCGACGG
CACCGGGCCTTCGCCCAAGCTGCCGGAACCTAACAAAACCCTGATCCCCACCGTCAACATCGCCCCGGCCGTTGGC
TGGCCCGATGGCGCCAAGCCCCAGGCCGCCGCGGGTACCCAGGTCGGCGCCTTCGCCGAAGGCCTGGACCACCCGC
GCTGGCTGTATGTATTGCCCAATGGCGATGTGCTGGTGGCCGAGACCAACGCGCCGCCCAAGCCGGATGACTCCAA
GGGCATTCGCGGCTGGGTCATGGAAAAGGTCATGGGCCGCGCCGGCGCGGGTGTGCCCAGCCCTAACCGCATCACC
CTGCTGCGCGATGCCGACCATGATGGCGTGGCAGAAACCCGCACGGTCTTCCTGGAAAACCTCAACTCGCCCTTTG

GCATGACCCTGGTGGGCAACGATCTGTATGTGGCTGATTCGGACAAGCTGCTGCGCTTCCCTTATCAACCGGGGGA
AACCGCGATCAAGGCGCCAGGCACCAAGGTCATCGACCTGCCGGGCGGCCCGCTGAACCACCACTGGACGAAAAAC
GTGGTGGCCAGCAAGGATGGCAGCAAGTTGTATGTGAGCGTGGGCTCTAACAGCAACGTCGGCGAAAACGGCCTGG
AAGCCGAACAGGGCCGCGCTGCGATCTGGGAAGTCGATAGGGCCAGCGGCAAACAGCGGATTTTTGCCTCCGGCCT
GCGCAACCCCAACGGCATGGCCTGGGAGCCGCAAAGCGGCAAGCTGTGGACGGCGGTCAACGAGCGCGATGAAATC
GGCAGCGACCTGGTGCCGGACTACATCACCTCGGTCAAGGATGGCGGCTTCTATGGCTGGCCGTTCAGTTACTACG
GCCAACATGTGGATGTACGGGTCACGCCGCAGGACCCGGACCTGGTGGCCAAGGCCATCGCGCCGGACTACGCCGT
CGGGCCACACACCGCGTCCCTGGGCCTGACCTTCGCAGAAGGCAACACGCTACCGGCACAATTTAGCCATGGCGCG
TTTATCGGCCAGCATGGCTCGTGGAATCGCAAGCCGCACAGTGGCTACAAGGTGATCTTTGTGCCGTTCGAAGGCG
GCAAGCCGAAGGGGCAACCGGTGGATGTGCTGACCGGGTTTCTCAACAGTGATGAGAAAGCCATGGGGCGGCCGGT
GGGGGTGGTGATCGACCAGCAAGGCGGGCTGTTGGTGGCGGATGATGTGGGGAATAAGGTGTGGCGGGTGTCGGCT
GTTAAATAAATAATTTGCCTCCCCAAAAAACAACTGTGGGAGCGGGCTTGCCCGCGATGGCGGTGGATCAGCCAGG
AATCTATCCACTGATACACCGCCATCGCGAGCAAGCCCGCTCCCACATTTTTTATCGGTGCAGGTCTTAAGGGTTA
CGCGCCAGGTGCTCGGCCGGCAACACGCGCTTGGCGCTCAGGTAGGCATTCTGCCAATAGGCCTTGGACAAGGTAT
CAAGCTTCACCGTACCGCCAGTTTGTGGCGCATGGACGAAGCGCCCTTCCCCTACATAGATGCCGGCATGGCTGAC
CTGCGAACCACCGCCCGTGGCAAAAAACAGCAGGTCGCCGGTTTGCAGCGCATCTTCACGCACGTCCTGGGCACGC
ATCACGATCAACTCGCGAGTGGTGCGCGGCAGCGAGATGCCAGCAGCATCGCGGTAGACAAAACCAATCAGGCCGC
TGCAATCAAACCCCGAGTCCGGCGTGTTGCCGCCCCAGCGATAAGGCGTGCCGACCAGGCCCAAGGCGCGAAACAG
CACATCTTCGGCAACCGGCGAAAAGTTCTGGGTGGTGTAGTTGAATACCGGCTTGGGCTTGACGGCCACAGGCGCA
GGTGGAGGACGGCTGGCGCAGGCACTGAGCAGCGCGGCGCAGACAAGAAGAATTAGGCGGGCCGAGGTCGACATGG
GCAGAACAATCCTGATCTGGATGCGGCTTTCGCTGCCGAACGCTGAAAACTAGACCGCGCAAGCAAGCTTGCGCGG
TCAGGTTCGCAACAATATCTCAGGATTCTAGCGGTTACGCGTCAAACTTCAAGTATCACTTTAAGTTTACTTGCGA
GCGGTAACGGTAGTCGGGGCCATCGCCAGTGCGCGCTTGGCTTCGATGAAGGTTTTGCTCCAGTAGCTGTCGCCCA
GCTTATCGACCCGCACCCCACCGCTGCGGCGGCTGCTGGAGTGGATGAACTGGTCATCACCCAGGTAGATACCGGC
GTGGCTGACACGACCACGGCCATTGGTACTGAAGAAAAGCAGATCGCCAGGCTTGAGGTTGTTCCGTGCGACCAAC
GGTGCATCCACGTTGATCATTTCGCGGGTGGAGCGCGGCAGGTTCATGCCAGCTTCTTCACGAAACAGATAACCGA
TAAAACCGCTGCAGTCGAAACCGGCTTCGGAAGTACCGCCGAAACGGTAGCGGGTACCGATCAGGGACATGCCGCG
TTCGAGGATGCTGTCGGCCAGCAGTGGCAACTGATAAGGCTTGCTGTCGGCGAACTGGGCCAGTTCTTTTTCGGTC
GCCACCTCTTCTTCATAAACAGAAGAAGACTGTGTTGCGACGAATTTGGCTTGATGTTGTTGCTGTGGCTTTTGCT
GCTCTACCACCTGCTGGGGGTGGGTGGCGCAACCAAACAACAGGGTAACGAGTGCGAGAGGCACGAGGGGTGCGAA
GCGATTTAGCATGGGCACGACCGTGGCTGATATGTAAAGAAGTCGAGACTATGCCTTCTATGACATCGATTTGCAA
ATTCAATCGTGATCTATGTGACTTCTTGTTTGACCTATGTCATCTAAGCCCTTAAACCCCATTTAGAGCCTTTTGC
GTGGCCAGAGCGGCCCAAACGCAGGTTTTCTGGCGCCTGGAATTTGCCAGAAGCCCCGGTTTCAAAGGGCTGGCTA
CCAGCCGAGGGTCTCTTTGAGAAAGGGGATGGTGAGCTTGCGCTGGGCCTGCAGGGAGGCCTGGTCGAGCTGTTCG
AGCAGGTCGAACAACGCGCTCATGCTGCGGGTGCCACGGGTAAGGATGAAATGCCCGACTTCATCGGTCAGGTGCA
GGCCACGGCGCGATGCGCGTAGTTGCAAGGCACGCAGCTTGTCTTCATCGGACAACGGACGCATCTGGAAGATCAG
CGCCAGGGTCAGCCGCGACTTCAGGTCCGCCAGCTTGACCGGCAGCTCCCGAGGCGAGGTTGACGCGGCAATCAAC
AGGCGCCGACCGCTGTCGCGCAGGCGGTTGAACAGATGGAACAGCGCTTCTTCCCAGTCCGCCCTGCCGGCGACCG
CTTGCAGGTCATCCAGGCACACCAGTTCGTATTGCTCGAGGTTGTCGAGGATGCCGATGCCACGGTCCAGCAGTTC
GGCCAACGGCAGGTACACCGCCGGTTCACCCAACTGCTCAAAGCGCAGGCAGGCTGCCTGCAACAGGTGGGTACGC
CCCACGCCGTCCTTACCCCAGAGGTAGATCAGGCTTTCCGTCCACCCGGCGTCGGCTTCGCAGAGCCGCTCGACAT
AGCCGAGTGCAGCGGCATTGGCGCCTGGGTAGTAATTGATGAAGGTAGCGTCGTCACGCAGACGCACACCTAGGGG
CAGCTGAATCGGTTTCATGCTGACTGAACGGCTCCAATCGAACCGTTAGTGGCCTCTGTGAAAAGTTTGCAAAGTT
TATACCCGTGACGCCGGGCGCACAATGCAACAGACCACAAGCAAAATCAAAGGTTTGCGTTAACTCATTGGATTTT
TTGGAGATTGTTTGACGCGATGGCTAAATGCCAAGCCCTGAAGACAACAAACCCGGCCTTGCGCCGGGTTTGTGAT
CAGCCTTACAACTGAGGGTCATCCGCGCCCGTGTACACGTCCGAATCCTTGTACACATCATGCACATGGCGCACCA
GCACCATGATCACCGCCGCCACCGGCAGCGCCAGCAGGATGCCGGTAAAGCCGAACAGCTCGCCGCCCGCCAGGAT
CGCAAAGATCACGGCCACCGGGTGCAGGCCGATACGGTCGCCCACCAGCAACGGTGTCAACACCATGCCTTCCAGC
GCCTGACCGACCATGAACACCGCGACAATCCCCAGCATCGGGTACAAGTCGCCGCCAAACTGGAACAGGCCGGCCA
CCAGCGCCGCGCCAATCCCGATGACAAAGCCCATGTACGGCACAATCGCCGCCAGGCCGGCAATCATGCCGATCAA
CAGGCCGAGTTCCAGGCCAATCGCCATTAGCCCGCCAGCATAGATAATCCCCAACGCCACCATCACCAACAACTGC
CCACGAACGAAGGCACCCAGCACCTCGTGGCACTCCCCGGCCAGGGACACGATGCGCTCCTCACGATCACGGGGCA
GCAGGCTGCGGATCTTGGCGATCATGATGTCCCAGTCGCGCAGCAGGTAGAACGCCACCACCGGGATCAGCACCAG
GTTGGTCAGCCAGCCGATCAAGGCCAGGCTGGAGGCCGTGGCCTGGCTGAGAATGACCCCGACGATATCGGTGGTC
TGGCCCATGTGCTCGCTGATCGCGGCCTTGACCTTGTCGAACTTCCAGAAGCCATCGGCCAGGCCAAGCTTGGACT
GCGCCCAGGGCATGGCTGTGTGTTGCAGCCAGTCGAGCATCTGCGGTGCCAGCTCATATAGGCGGACCAGCTGCTT
GGCCAGCATCGGCACCAGCACCAGCAACAGCGCCATGACGATCAGGGTAAACAGCGCAAATACCGCCACCACCCCC

CAGGTTCGCGACAAGCCGGCCTTTTCCAGGCGATCCACCACCGGATCGAACAGATAGGCCAACAGCAACGCGACCA
GGAACGGCGTCAGGATCGAATGCAGCAAGAACACAAAGACGCACAGCAGGACAATCCCGCCAAGCCACACCCAACG
ACGCGTATCCGCCATAAACCACTCCATCTGTCATTTGCTTCTATATAAGGAAGAACATTTACCAACGAAAACGCAG
TTGCGGCTCAGGCTCGGCTGGCGGCAGCGGCGCAGGTGCAACCCCCTCGGCCACGGGCGGCTGTACAGGCTGCGGC
GCAAGTGCCTCACCGGCGGGCACTTCCTGCAACTTGGCCAGGCTCAACTGGCTGCGCAGTTGCTCGCTGCTGCCAT
TGACCCGATAGACGATCCGATCGCCATCCACCCGCTGCAACTGCGCGCCAAAAGGTTCCAGCAGATGACCGAGGGC
GGCGTAGCGTTCCAGGGTCATGCCTTGCACTTGCAGCAACTGCTCGCTGGACACCCCGGCTTTTACCGCAAAACGG
GGAGCAAGACGCTCACTGACCGCCAGCAACACGGCATCAGCCAAGGCAGCCGTGTCAGCGCCCTGTACGTTGCCCT
GCTCGGCTTTGTCACCCAGCCACAGGCGCCATTTTGCTTGCCATTGGCCCGCCTCTTCACGGGCGTGCACCGCCAG
CAGGGCATCGGCGCCATAACGTTCGGAGGCCGAACGCAGGGGCGCGGGATCTGCACTTTCAAGATTAGGCGCGGTG
GCAACGATCTGTTCATCGAGATCACCCAGTGGCAGGCGCAACGGCAAGCCGCGATGCTGGGCTGCACGACGCAGTG
GCTCGGCCGCCGACTGGCCATCCCCCACCAGGCTGCTGCCTTCAGTGGAATCGTTCAGCCACCAGCCAAGGATCGA
CGGCCGATTGTTGCCCCACAACGCCAGGCCGGCATCGCGCAGGGCGCGGTCGGTGCTGACCGGGTCGAAATCCACT
TGCAGGCTTTCCGGGGGGCCCGCGTCGTAGCCGTACTGGCTGATGATCTGTTGCGGATCCTTGCGGATCGCTGCCA
GGCCAGGACCCTCGGCGGCCTTGGCGTCGCCGGTCAGGCGGATCACCAGGGTTTGCAGGGCTCGCTGGGTGGCTTG
TTCACGCTCTTGCGGCGCCTGGCTGCTGACAGGCTCCAGCACTTGATAGAGGCCATTGAGGGTTTCGGCATGACTC
GCCAGGCTGACCAACGACAAACAGCCGACAAAAAAGTATCTACTCAGACCCATGAAAAAAGATTCCCGAACGACAA
AAAAGCGGCTGGAACAGACCGCGTGAAGAAGGTTAAGCAAGGCTGTGACCACAGCGACCGGCAAAACATTCACAAG
GCCCCGGTAAGTTTTCGTACTGTCATAACGATAGCGGGTTAAAGGCTATACCTTAATACAGGCGATCGCGGCGCCG
ATTAAGGTTTTTTTAGCACCATATGCCTCTGCCCGTCGGCCCGAGGATGGCCGCTGCCCCTCAAGCCTGATAAAAT
CGCGCGCCTTCGCAGACCGTCAACGGCTGGGCGTCCTGAAGTGTTCAACTTCCGTACCCCCACCGCATCGGTCGTT
ACCCCTGAATCCCCCCCTAAAGGCCTGGATCATGAGCAAGCAACCCTCCCTGAGCTACAAGGACGCCGGTGTAGAC
ATCGACGCCGGTGAAGCATTGGTCGAACGCATCAAGAGCGTCGCCAAGCGCACTGCGCGCCCCGAAGTCATGGGCG
GCCTGGGCGGTTTCGGCGCCCTCTGCGAGATCCCGGCCGGCTACAAGCAGCCTGTGCTGGTCTCCGGCACCGACGG
CGTGGGCACCAAGCTGCGCCTGGCGCTGAACCTGAACAAGCACGACACCATCGGCATCGACCTGGTAGCCATGTGC
GTCAACGACCTGGTGGTGTGCGGCGCCGAGCCGCTGTTCTTCCTCGACTACTACGCCACCGGCAAGCTGAACGTGG
ACACCGCTGCACAAGTGGTCACCGGTATCGGCGCTGGCTGTGAGCTGTCGGGTTGCTCCCTGGTCGGCGGCGAAAC
CGCTGAAATGCCGGGCATGTACGAAGGCGAAGACTACGACCTGGCCGGCTTCTGCGTCGGCGTTGTAGAAAAAGCC
GAGATCATCGACGGTTCCAAGGTGGCCGCTGGCGACGCCCTGCTGGCCCTGCCATCGTCCGGCCCGCACTCCAACG
GCTACTCGCTGATCCGCAAGATCATCGAAGTGTCCGGTGCCGACATCGAAAACACCCAACTCGACGGCAAGCCCTT
GACCGACCTGCTGATGGCCCCGACCCGCATCTACGTCAAGCCATTGCTCAAGCTGATCAAGGACACTGGCGCCGTC
AAGGCCATGGCCCACATCACCGGTGGCGGCCTGCTGGACAACATCCCGCGCGTGCTGCCAAAAGGCGCCCAGGCCA
TCGTCGACGTGGCCAGCTGGCAGCGCCCGGCGGTCTTCGACTGGCTGCAAGAGCAAGGCAACGTCAATGAAACCGA
GATGCACCGCGTGCTGAACTGCGGCGTGGGCATGGTCATCTGCGTGGCTCAAGAACACGTGGAAACTGCGCTGAAC
GTACTGCGTGAAGCCGGCGAGCAGCCATGGGTGATCGGCCAGATCGCCACCGCTGCCGAAGGTGCTGCCCAGGTTG
AACTGAAGAACCTCAAGGCTCACTGATGCCAGCAACCTGTGATGTCGTGGTGCTGCTTTCCGGCACCGGCAGTAAC
TTGCAGGCCCTGATCGATAGCACGCGCGCCGGCGACAGCCCGGTGCGCATCGCTGCGGTGATTTCCAACCGCAGCG
ACGCCTACGGCCTGCAACGCGCCCAGGACGCGGGTATCGATACCCGCTCCCTGGATCACAAGGCGTTCGACGGTCG
CGAGGCCTTCGATGCAGCCTTGATCGAACTGATCGACGCCTTCAATCCCAAGCTCGTGGTCCTCGCCGGTTTCATG
CGCATTCTCAGCGCTGACTTCGTGCGCCACTACCAGGGGCGCCTGCTGAATATCCACCCTTCCCTGCTGCCCAAAT
ACAAAGGGTTACATACCCACCAACGGGCCCTTGAGGCCGGAGATACAGAGCACGGCTGCAGCGTGCACTTTGTCAC
CGAGGAACTCGATGGCGGGCCTCTGGTCGTACAGGCAGTAGTTCCGGTAGAGTCTGCTGACTCGGCGCAGAGCCTT
GCGCAACGGGTTCACACCCAGGAACACAGGATTTACCCGCTGGCGGTTCGCTGGTTTGCCGAAGGACGCTTGGTGC
TCGGTGAACAAGGTGCATTGTTGGACGGCCAGTTACTTGCGGCCAGCGGCCACTTGATTCGTACCTAGGAGATTTT
ATGCGTCGTGCCCTGCTCTTCGCTTTTGCTCTGCTTGCCCTGCCTACCGTGCACGCGGCAGATCTACAGCCTTTCT
CCGCCAGCTACACCGCCGACTGGAAGCAGTTGCCCATGAGTGGCCAGGCCTCCCGCAGCCTGGAAAGCACCGGTAA
CGGCGTCTGGAAGCTCAGCTTCAAGGCCTCGATGATGATCGCCAGCCTGACGGAAGAAAGCACCCTGACCCTGGAC
AAGGACACCCTGCTGCCCCAGTCCTACCACTTTGAACGTGGTGGCCTGGGCAAAGCCAAGAAGGCCGACCTGGACT
TCGACTGGACCGCCAAGATGGTCACCGGTACCGATCGCGGCGACGCGGTCAAGATCCCACTGAACCGCGGTATGGT
CGATAAATCCACCTACCAGTTGGCCCTGCAGCATGACGTAGCGGCCGGCAAGAAAACCATGAGTTACCAGGTAGTG
GATGACGGCGAAGTCGAGACCTATGACTTCCGCGTACTGGGCACCGAGAAAGTCGACACCAAGGCCGGCAAGATCG
ATGCCATCAAGGTTGAGCGCGTGCGCGATCCGACGCAAAGCAAGCGCACCACGGTGATGTGGTTTGCCAAGGATTG
GGACTACCTGCTGGTTCGCCTGCAACAAGTCGAAACCGACGGTAAGGAATACAACATCATGCTGTTGGACGGTACG
GTCAATGGCAAGGCTGTGAAAGGCAGCTGATCGTTGATTGATTGAAAGAGCCCCGCTAACTGCGGGGCTTTTTTTC
GCCCGCATTTTTTCATGGATGGCTGATACCGCTATCGCCGGCAAGCCGGCTCCTACAGGTCTAAACAGCCTAACCA
ACATGAAACTTTTGTCATAAACCGCCCCCTCCTGCGACGCAATGTCGCGGTTTACGGGGCCTGGAGCATTGTTGCG
GGTACTGCAATGAATTGTTGCACGCAAAGTCAGTTTACTTAGCAAGCTAACAAAATATATAACAAAGGCCTGCGAC

GCCTTGCCGCAGATCAACCAGAGACTGGAGCAAGCAGATGACAGTAAAAGTAACTGAACGCGACGATGCCCATATG
TCCCACGAAGCCCTGGCCCACGGCATTCATATCTGGGATGTGCATCAACAAGACCTGCTGGTAGGCATGTTTCACA
ACGAGTGCGACGCCCACAATTATAAAAGCGAGCTCGAAACCCTCGAAATGAAACGCCAGGCATCCAGCTCCTGACT
CACTGGAATGCTATGGGCCGCTCCGTGGCTATGACCCCGGAGGTGGAAGTGATTGTGGCGAGCGGGCTTGTTGTGG
CGAGCGGGCTTGCCCCGCGCTGGACTGCGAAGCAGGCCCAATAAGGCCACCGCGATCTTTAAGTTACAACGGTGAG
CTTACGTTTTGGGGCCGCTTCGCAGCCCAGCGCGGGCAAGCCCGCTCGCCACAACAAGCCCGCCTGCCACAATAGC
CCCGCTTACAACCGCAAGGTTGTTACCACATCAGATCATCAGGGATCTGGTACGCGGCGTATGGATCATCCTCATC
CGGCACCTGGGTCTCGGTGAGGATGTTGAGCTGGACGATGCGCTCAGGCGCGCGCTCCTGGATTTTCAGGGCGGCC
TCCCGTGGAATCACTTCGTAACTACCGCCATGGTGGACAATCGCCAACGAGCCGTTGCTCAGCTTGTTGCGCATCA
AGGTGTTGACCGACAGGCGCTTGACCTTCTTGTCGTCAACAAAGTTGTAGTAGTCCTCGGTGGTCAGCTTGGGCAG
GCGCGAGGTCTCGATCAATTGCTTGACCTGCGCGGCACGGGCCTTGGCCTCGACCTTTTCCTGCTGCTGACGGTTG
AGCTCCTGGTCGCGCTTGACCTTCTCGGCCTGCGCTTCCAGGGCCAGGCGCTGCTGGGTGTCATCGGCCTGGGCCT
GGCCTTTGTGAACCAGGCGCTGCTGCTTCTGCTTGTCTTTGCCGACCTGCTTGGCCTGCTTCTGATTGACCAGGCC
TGCTTTGAGCAACTGGTCGCGAAGGGAAATGCTCATGGTGCTTACTCACTTAGGCAACTGCTCAACCGCAGTTGGA
CACATTCTTTTCCTGGCGTTTGGCTTCACCCCACAGGGCGTCCAACTCTTCGAGGGTGCAATCTTCCATGGGACGG
TGGGTGTCGCGCAATGCCTGTTCGATAAATCGGAATCGCCTTTCGAATTTGGCATTGGCACCGCGCAGGGCGGTTT
CCGGGTCCACCTTGAGGTGCCGGGCCAGGTTGACCGCCGCAAACAGCAGGTCACCGACTTCATCGGCAATCGCTGC
CGGGTCGTTGTCGGCCATGGCTTCGAGCACCTCATCGAGCTCTTCACGCACGTTATCCACCACCGGTAACGCCGCT
GGCCAGTCAAAGCCTACCTGGCTCGCGCGTTTTTGCAATTTGGCCGCACGGGACAGTGACGGCAAGGCGCTGGGCA
CGTCATCGAGCAGGGACAGTTGCTCGGGGGCGTCAGACTTTTCTGCGCGCTCCTGCGCCTTGATGGCTTCCCAGCG
CTCCTTGACCTGTTCTTCACTTAACTGAGGGATATCCAGCGGCGCATACAGGTCTCCGGTGGGGAACACATGGGGA
TGCCGGCGAATCAGCTTGCGGGTGATGCTGTCGACCACCCCGGCGAACTCGAAGCGCCCTTCTTCCCGGGCCAATT
GGCTGTAATACACCACCTGGAACAACAGGTCGCCCAACTCGCCTTGCAGATGATCAAAATCCCCGCGCTCGATGGC
GTCGGCGACTTCGTAGGCTTCTTCCAGGGTATGGGGCACGATGCTGGCGTAGGTTTGCTTGATGTCCCACGGGCAA
CCATATTGCGGGTCGCGCAGGCGGTTCATCAGGTGCAGCAGGTCTTCAAGTGAATACATCGGTCAATCTCTGTCCG
CTGAAATGGTCATGCCACCGATCAATGTGGGAGCCGGGCTTGCCCGCGATGCAAGCGCCTCGGTGTGCCTGGCAGA
CCGAGTCGATGCGATCGCAGGCAAGCCAGCTCCCACACAAGCCGGCTCCAACAAGGGATCAGGTCATGGCGTGCGG
TTACGCCGCGTCTCGATGATGTTCGGCAACTGGGAGATACGCCCCAGCAACCGCCCCAACGCGTCCAGCCCCGGAA
TCTCGATGGTCAGGGACATCAGTGCAGTGTTGTCCTCTTTGTTCGAGCGGGTATTGACTGCCAGTACGTTGATCCG
CTCATTGAGCAGCACTTGCGAAACGTCCCGCAGCAGACCCGAGCGGTCGTAGGCACGGATGATGATGTCCACCGGG
TAGGTGAGCACCGGCACCGGCCCCCAACTGACCTGGATGATCCGCTCCGGCTCACGCCCGCCCAACTGCAGCACCG
AGGCGCAGTCCTGGCGGTGAATGCTCACCCCACGGCCCTGGGTGATGTAGCCGACGATGGCATCGCCCGGCAACGG
CTGGCAGCAGCCAGCCATCTGGGTCATCAGGTTGCCTACGCCCTGGATCTGGATATCGCCGCGCTTGCCCGGCTTG
TAGCCGGTGGCCTTGCGTGGGATCAGTTCCAGTTGCTCGCTGCCACGCTCCGGTTCCACCAGTTGCTGGGCCAGGT
TGACCAACTGCGCCAGGCGCAGGTCGCCGGCACCGAGGGCAGCGAACATGTCTTCGGCAATCTTCATGTTGGCCTT
TTCGGCCAACTTGTCGAAATCCACCTGGGGCAGGCCCAGGCGCCCCAGCTCGCGCTCGAGCAAGGTCTTGCCGGCG
GCGACGTTCTGGTCCCGCGCCTGCAGCTTGAACCAGTGGACAATCTTCGCCCGCGCACGCGACGTGGTGATGTAGC
CCAGGTTCGGGTTCAGCCAGTCGCGGCTCGGCGTACCGTGCTTGCTGGTGATGATCTCGACCTGCTCACCGGTCTG
CAGGCTGTAGTTGAGCGGCACGATCCGCCCGTTGATCTTGGCGCCACGGCAGTTGTGGCCGATCTCGGTGTGCACG
CGGTAGGCAAAGTCCAGCGGCGTCGCACCCTTGGGCAAGTCGATGGCGTGGCCGTCGGGGGTGAAGATGTAGACCC
GGTCGGGCTCGATATCCACCCGCAGTTGCTCGGCCAGGCCGCCGATATCGCCCAGTTCTTCGTGCCATTCCAGTAC
TTGGCGCAGCCAGGAGATTTTCTCTTCGTACTGGTTGGAGCCCGACTTGACGTCGGTACCCTTGTAGCGCCAGTGC
GCACACACGCCCAACTCTGCCTCTTCGTGCATGGCGTGGGTACGGATCTGCACTTCCAGCACCTTGCCCTCGGGGC
CGATCACCGCCGTGTGCAGCGAGCGGTAGCCGTTCTCCTTGGGGTTGGCGATGTAGTCGTCGAACTCTTTGGGAAT
GTGCCGCCACAGCGTATGAACGATACCCAGCGCGGTGTAGCAGTCGCGCATTTCCGGCACCAGCACACGCACGGCG
CGCACGTCGTAGATCTGGCTGAAGGCCAGGCCCTTGCGCTGCATTTTGCGCCAGATCGAATAGATGTGCTTTGCCC
GGCCGCTGATATCGGCTTCGACACCGGTAGCCTGCAATTCCGAGCGCAACTGGCCCATCACATCGCTGATGAAACG
CTCACGATCCAGCCGCCGCTCGTGCAGCAGGGTCGCGATCTGTTTGTATTGATCGGGCTCCAGGTAGCGGAAGGAC
AAGTCCTCCAGCTCCCACTTGATATGGCCGATGCCCAGGCGGTGAGCCAGGGGCGCGTAAATATCGAAGACCTCAC
GGGCGACGCGATTGCGCTTTTCGTCATCGGCGGTTTTCACCGCACGGATCGCGCAGGTGCGCTCGGCCAGCTTGAT
CAGCGCGACGCGTACGTCGTCGACCATCGCCACCAGCATCTTGCGCAGGTTTTCCACCTGGCCCTGGGTGCCGAGC
ACCATCGACTGGCGCGGGCTGAGGCTGGCGCTGATCGCAGCCATACGCAACACGCCATCGATCAGCTTGGCCACCA
CCGCGCCGAAACGCTGGCTGACCAGCGGCAACGCAATATGCCCTTCGCGCACACCGCGATAGAGCACCGCGGCGAT
CAGCGAATCCTGGTCCAGCTTGAGGTCGGCGAGGATCTCGGCGATTTCAAGCCCGGTGCGAAAACTGGATGTTCCT
TCGGCCCAGAGGTTCTTGGCCGCATTGTCTTGCTGTTCTGCCTCGCGAGCGAACTCGCAGGCGGCTTTCAAGGCTT
CACGGTCCAGTGCCGGATCGACACTGATGGCATGATCCAGCCATGCCTCGAGATTGATACTGCCGTCGGTGTTGAT
CGGCTGGTGTGCTCTCACCTGTACCATCTTGCTTACCTTCCCTACGACGCGCCTGTAGCGCGTCAAAATCGCTGAC

CCTTGTAACGCGAACTCCCTGGCAGGTCACAGGCCTTGGAGAACGCAGGCCAGTCGGACTAAGCGAGCATCCTAGC
TCGCTTCAAATAACGCCATCGCCTCGACATGCGCAGTTTGCGGAAACATATCGAGGATCCCGGCACGTTTTAGCCG
GTAGCCTTGCTTGACCAACTCGACAGTGTCCCGCGCCAGCGTGGCTGGATTGCAGGACACATACACCAGGCGATCA
GCCCCCAGAGTGGCGAGCTTGCGCACAACCTCCAGGGCACCATCGCGGGGTGGGTCCAAGAGTACCGCAGAAAAGC
CTGCTTTGGCCCACTGCGCATCACTCAAAGGCTGGGACAAATCGGCCTGAAAAAACTGCACATTATGCAAATTGTT
GCTTGCGGCATTCAGCGCCGCGCGGTCCACCATGGCCTGCACGCCCTCTACCGCCACCACTTCACGCACTTGGCGC
GCCAGAGGCAGGGCAAAGTTGCCAAGGCCGCAGAACAGGTCCAGCACCCGTTCGTCGGAACGTGGTGCCAGCCAAT
CCAGCGCCTGGGCAACCATCGCTTCGTTGACCCCGGCGTTGACCTGCACAAAATCCCCAGGCCGGTAAGCCAGGTC
CAAGTCCCACTGCTGCAGACGATAGCCCAGGCCCGCCCCGGCCGCGAACGGCTGCGGCTCGCCCTCTCCATGCAGC
CACAACTGGGCATCATGGAAGGCACAGAATTCTTGCAGCACCACCAGGTCGGCATCCGACAGCGGCGCCATATGCC
GCAGCAACACGGCAATCGACGAGCCACTGAACAACTCCACATGCCCCAGGGCCTGAGGTTTGCTCAGGCGCCGCAA
CATGTTGGGCAGGCGCTGCATGATCGGTTGCAAGGCCTGTACCAGCACAGGGCAATCATCGATGGCCACGATGTCC
TGGCTGGCCACGGCGCGAAAGCCAACGTCCAGGCGCTTGGCCTTGACGTCATAGCGCACGGCCACGCGGGCCCGGC
GGCGATAGCCGAACTCGGGCCCGCTCAACGGCGCGGCCCATTCCTGGGGCTCGACCCCGGCAACACGGGACAATTG
CTCGGCGAGCATGCGCTGTTTCAGGGCAAGCTGTTCGTCATGGGGCAAGTGTTGCAGGCTGCAACCGCCACAACGG
CCGGCATGGGCGCACGGCGCCGGGCGACGCAGTTCGCTGGCCTTGAACACCCGTTCGGTGCGGGCCTCGACCACTT
TGCCATGGGCACCCAGCACCCGCGCCTCGACCTCTTCGCCGGCCAGTGCACCCACTACAAACCAGGTGCGGCCTTC
GAAAAACACGATCCCCCGGCCATCGTTGGCCAGGCGTTCGATGGTCAGGCGCTGTTTTTACCGACCGGGATTTGT
GGCGCCCGATTACCGCCCGTTGGCTGGAAACGCAGGCCTCGCTCTTGCTTGGCCATCAGTTGGGCGCGTCGAAAAT
GCCGGTCGACAGGTAACGGTCGCCTCGGTCACAGATGATCGCGACGATCACCGCGTTTTCCAGTTCTGCAGACAAG
CGCAACATGCCGGCCACAGCGCCACCCGAGGACACACCGCAGAAGATACCTTCTTCACGGGCCAGGCGACGGGTAG
TGTCTTCGGCTTCACGCTGGGCCATGTCGATGATGCGATCGACGCGGGCGGCGTTGTAGATCTTTGGCAGATACTC
CTCGGGCCAGCGGCGGATACCGGGGATCGCCGCGCCTTCCATCGGTTGCAGGCCAACGATCTGGATCGCCGGGTTC
TGCTCCTTGAGGTAGCGCGAGTTACCCATGATGGTACCAGTGGTGCCCATGGAGCTGACGAAATGGGTGATGGTGC
CCTGGGTCTGGCGCCAGATTTCCGGGCCGGTGCTGGCGTAGTGCGCTTCCGGGTTGTCACCGTTGGCGAACTGGTC
CAGGACCTGGCCACGGCCTTCGGCGGCCATGCGCTCGGCAAGATCGCGCGCGCCTTCCATGCCTTCTTCCGGGGTG
ACCAGCAACAGCTCGGCACCATAGGCGGTCATCGCCGCCTTGCGCTCGGCGCTGCCGTTGTCGGGCATGATCAGGA
TCATCTTGTAGCCCTTGATCGCCGCGGCCATGGCCAGGGCAATCCCGGTATTGCCGGAAGTGGCTTCGATCAGGGT
GTCGCCGGGCTTGATCTGCCCACGCAACTCGGCGCGGGTGATCATCGACAACGCCGGGCGGTCCTTGACCGAGCCT
GCCGGGTTATTACCCTCGAGCTTGAGCAGCAAGGTATTGCTGGTGTTACCCGCCATGCGCTGCAAGCGGACCAGGG
GGGTGTTGCCGACGCAATCGGCGATGGTTGGGTACTGCAAGGTCATGGCGTATTCGCAATCCAGACTGCGGGGGCG
CCTATCATACCGGCAAACGCTGGCGGGCCATATCACGCAAAGTGTGGTGCTTATAACTCAACGAACTAAAGCCCAG
GGCTGTCGTGGTGAGGGACTGTTGTGGCGAGGGGGCTTGTCCCCCGCTGGGCTGCGCAGCAGCCCCAAAACATGCC
ACTTCGGTGTATCTGATAAAGCGCAGTGGATCTTAGGAATTGGGGCTGCTGCGCAGCCCAGCGGGGGACAAGCCCC
CTCTCCACAAAAGCCCCTCTGCCAGAACAGCTCCTTGGCCATAGGGGCTACACGGTATCCACCTTCAATGAATGAT
CGTTGAGCATCCCGTTAATGATGGTCGCCGTGTCTGCCCCTCCCGCAATCAGGCCACCCGCCCCCGGTGTCACGCC
GTATTGGGTCGCCAGGTTGACGCCAGCCAGGTCGATGGTCTGGGTAGCCGTGGCCCCTGCAACAGAGCTGATGTCG
ATGGTGGTCGTGCCGCTCACCACCTTGAAGTGCAGGTAATCATCCAGGGACGCCGTCGTCGCATTCTCCCCTTGCA
GCAATTGCGACAGGTCCAGCTTGTCGGTGCCCACGGTAAAGTCGGTGATGACGTCGTGCCCGCTACCGCCGGTGAG
GTATTGGAAGGTGTCGGCACCCGCGCCGCCGGTCAGGGTGTTGTTGCCCGGACCACCGATCAGGATGTCATCGCCG
CCCTCGCCCTTGAGGGTGTCATTGCCCAGCCCGCCCGAGATCAGGTTGTTGCTGCCGTTGCCGGTGAGGAAGTCGT
TGAAGTTGGAACCGATCAGGTTCTCCAGGGCCGTCAATGTATCGGTCCCGGCGCCCCCCGTGATTTGCCCTCCCAC
CGTGCCAAGGTTGACGGTCACGCCCGACGTGGCATTGGCGTAACTCACGGTATCGATGCCCGCACCGCCATCGAGC
AGGTCATTGCCGATGCCACTGAACAGCAGGTCATTGCCATTGCCGCCGTGCATCTCGTTGTTGCCATTGCCAGCAC
TCAGTACATCATTGCCATCGCCGCCATTGAGGATGTTGTTACCGGGCCCGGCCATGAGGATGTCATCACCGTTGGT
ACCGGTGAGGGTGGTGCCGGCCTGGTAGGTGATGTCTACGGCGGCGCTGTCGCTGCCGCCGTGGTTATCGCTGACC
GAGTAGTTACCGTGGTAGTCCGGCGTGTAGTCCTGGGCGCCGGCATAGTTGATCTTCATATCTAACTGGTAGCTTT
CTGCGCCATTGACGTTGCCACCTGCTGGATTGGTGGTGTTGGCAATGTGAATCTGATAGCTACCGTCCGTTAACGC
AGTCAGCGTCGCGCCATCCGCGAGAGGGAGTTCTCCGCCACCGTTGAGCGAATAGGTCATGACGATGTTACCGACC
GGCCGGTTGTGATCCAGGTTGAGGGTCTCCCCCGCCCTGAGTGTGACCGTCACGCGATCGTCGGCCGTAGCGCTGC
CGACACTGCCGAGACTGCCGGTGATCACAAGGGCTGCGGTCAGGGCCCCGATATCGGCAAACGAGGTACGTACATC
CCCCAGGGTTGTACCGGTAAGCGTTCTGGTCCCCGTCCCGGTAAACGCCGCGTTCTTGGCCACCCAGCCGGTTGGA
AAACTGGTGGGGATAGCCGTCAGGTTGTCATGGTTGGCATCGCTGTCATTGGCCAGCAGCAACTGCGCAGGCACCA
CAATGTTGCCCGACAGGATATTGGTGATCACATGGTCATCACCGGCCACCGGTGCCACGTTGGGGTTGACCTTGAC
CACCAGGCTGGAGGTCGCCAGGTCGCCGTCGTTATCGCTGACGGTCACGCCAAAGGTTTCGGTGACCAGCGAGCCC
GTCAATTTCGGCGGGTTGTAGCGGAACTCGCCGTTGTCCATGTCGACGATCAGCGTGCCGCCCTGGTTGGTGGTGA
TGGTAATACTGTTGCTGACGGTGTCGAACGTGCCGTGCCCGCCCGGCAGGTTGGCGCTCATCGAGCCCCCCGCCGC

CGGGTTGTAGGTGTAGGTGATGCCATCCACAGTCAGGGATTTGACGAAGCCACCGTCGGCACCAAATGTGCCATCC
GTCAGCAGGCTCCCCGTGACTGGCGCGCCCTGCACGGTGCCGGCCAGCACCGCATCCAGGCCCGCCAGGTTGGCCA
CCACTACCGCATTGGTATTGGTGTGGGTGCTGCCGTCGTAGGCCACCGGGTCCAGGTAAATGCTGTTGACGCCCGC
GCCCAGGCCAATGGCATAGGCCTTGATGCCGTTGGCATCGAGGAAGGCCCGCCATACGCCTTCTTCTGTGGCATCG
ATACCGTCGCCGTACTCCGGGTTGACCACGTTGCCAGGGTTGTTCGGGTTATTGGTGTTGGACGTGGTGGGGTTAC
CGTCCGAGAAGAAGTACGCCACGTTCTGCGCACCTTCCAGCTTGCCGGCGGTGCCGTAGGCGGTCTGCGCAGTGCC
CAGGGCCGAGTCGTAGTTGGTGCCATTGAACGCGGTGAGGCCGTTGACCAGGGTCTTGGCCGTGGCGACGCTGACC
CATTGGCTGCTCAACTGTTGCGAGCTGTCGTTGAAGGTCACGATCTGCACCTTCACGTCGCCCATATCGTCATAGC
GGTTAAGCAACTGGGTGATGGACGCCTTGGCCAGGTCGAGCCGCGTACCGCCGGCCCCTGACGCGTCATTCATGCT
GCCGGAAATGTCGATGATCAGCAGCAGGTTGGAATCCACCTCCGTCGCCGTGACCTCACGCGCGCTGGACACCACT
ATTGGCATGTCGTCGACAATACTCACCACGATGCTGCTGGTGGTGGAGTTGCCCAGTTGGTCGGTGACCTTGTAGG
TGAAGGTCTCGGTCAGCACATTCGGCCCGTCATTGCTGTTGGGCAAGGTCTTCGGTGCCGACGTCAGGGTGTAGGT
GTAGGTGCCATTGGCATTAAGCAGGATCTGCCCATAGCTGCCCGTCGCACTGCCGACCAGGCTGAAACTCAACGGG
CCAGTGCCGCCGGTGGTCGAGCCCACCAGGGTGCCAGTGGCCGTTTCGCTGGTGGCGGTCGGGTCGCTGCCGATCA
CAGTGCCGGGCGCCAGGTCGAGGCCATCGAGTTTCAGGTCCAGGGCTTTTTCGTAGACAGTGACATCGCCATCGAC
GCTAGCCACCAGGCCACTGTCGGCCACATTGATGGTCAAGGTCGTGGTGCTCAAGTCACCGTCTGCATCACGCACG
GTGTAGACAAAGGTGTCCACCGCTCCCGGTGGGTTCACCGAGTTGGGGTTGCTGTGATACACGGCATTGCCGTTCG
CATCCAGGGTCAGGTAGCCGAAGCTGCCGGTGATATTCCCGCCCAGGCCACCACTGGCCGGCGTACCGACATTACT
CCCTGCCCGCACCCCGACCACCGCACCCGCCAGGGTGGGGCTGTCGGCACCGCCGACATCATTGCCCAACACATTG
ACGCTGACCACACCGCCCTCTGCTACCGAGCGCACATCGGGATTGGCGATGGGCAGGTCATCGATGATGTTGATAT
CAATCGTGCCCGTCGCCGTATTGGTGTCGTAATCGCTGGCCGTGACCGGAATTTGCTCGGACAGGCTGTTGGCACC
GCTGGCATTGGGGTGCGTTTCGTTGTCGAGCAGGGTGTAGCGGTAGCTGACCACCCCGGTGTTGGGGTCGTAGCCA
GTGACAACAAAGCTGTTGCCCAGCAGGGTACCTTCCGACAGCGGGAAGGTGGCAACCACCCCGTTGGTCACCACAT
TCAGGCCACCGACCGTGAGGTTTTTCAAACCGTCCAGGGCGGTCACGGTGAAGGTACCGGTCTGAGTCAACAAGGG
CGTGTTCGGGTTGGAACCATCGACCAGGTTTTTTTCAAAGACGGTCAACTCCCCGCCAGTCAGATCCAGGCCGGTG
AGCACTACCGGGTCGTTGTTGTTATGCACCTGGAGCACCAACTTGGCGGTGCTGGTATCGCCGTCACTGTCGTTCA
ACTGGTAGGTGAAAGTCTCGGTACCGTTCCCCCCGCCGTGCAGGGCGATAAATTGGGGGTCGGTCTTGTCGAGGGT
GTAGGTGTAGCTGCCGTCGGCAAACAGTTGCAGGGTCCCGTAGGTGCCAACAATGGTGTTTGGCGTGACCGGGCCG
GTGGCGATGCGGTCGGCGCCTTGTACGTCATTGCCCAGCACGCTGCCGGTCAGGGTCAGCAGGCTTTCAGAGGCCA
CACCGCCGGGGTTGTCGTCGAAGGCTTTTGGCACATCGTCGACCACATTGACGTCCAACGTGCCCGTAGCGCNNNN
NNNNNNGAGCCCCCTCGCCACAGGGGCGGTGTTCAATTAGTTGCCGCTAAACACTGCCGGCCGGCGCTGGGCAAAG
GCTGCCAGGCCTTCGCCAAAGTCGGCGCTTTCGCAGGCCTGGCGGCGCAATTCGGCGATCTGTTCCATGGCCTGCA
CCGGCATCGGTTGCAGGTCTTCGAGGATGCGCAGTTGTTCCTTGACTGCCGCCACGGCCAGCGGCGCTTTGCTGGC
GATGCCTTGCGCCAGTTCCAGAGCGGTGGCTTCCAGCATATCGCTGTCGACCAGGCGGTTGACCAGGCCAAAGCGT
TCGGCGCGTTGCACATCAAGCTTGTTTGCCGTGAAGAACAGTTCCTTGAGCACATGAATCGGCAAGTTGTGGAAAA
AGCGCAGCAGACCGCTGGTGGTGTACGGCAAGCCAATGTTTACGGGGGTCATGGCGAAGCTCGCGCTGTGATCTGC
CACGACCATGTCGCAACTCATCACCAGGTCCACTGCGCCGCCCCATACCGAGCCGGACACCAGGGTAATCACCACC
CCGGGATAGGCGCGGATGCTGCGCAATACGCGCTCGAGCGGTTTGCCGTAGGCAATCGGGTCGCGGTCGTGTTGCA
GTTCACGGATGTCGTGGCCGGCACTCCACACCGACTGGCCGACTTCGCTGCCCAGGATCACCACCGGGATGCGTTG
CGCCGCCAGGGCCGCGAGGCTCTCGTCAAGCGCCTGCAGCAACTGTGCGCTGAGTGCATTGCGGTGGGTGGGATTG
CTGAAGACCAGCCGCGCGATGCGTGGGTCGACCTGCTGGACGGTCACGGTGGCGCAAGACGTTGGATTCATGGTGC
GGTTCCTGTTGATTGGGCACAGTCTTGGAACACTACTTGCGCGGCGAGCAGTGCGTTGATCGATGCGGCGTCGAAA
CCCGCTTCACGCAGTACTTCGACGGTGTGTTGCCCCAGCAGTGGCGGCGGTCGGCTGGCGGCGCCGGCGGTGGCGC
CAAACACCGGGGTCACGACCTGGCGCAGTGCGCCGAGTGTGGGGTGCGTGAGGGTCTCGGTCAGTTGGCTGTGCAA
TACCTGGGGGTCGGCGAAAACCTCATCGAGGGTATTGATGGGGCCAGCGGGTAAACCGGCGTCGACGAACATGGCC
GTCCATTCGCGCTTGCTGCGGGTTTTCAAGCGGCTCTCCAGAATGCCGCGCAATTCGTCACGCCGTTCGACACGCG
CCTCGTTGGTGGCAAAGCGCGGATCGTCCGGCAGCCCAGGTAACTCCAGTAGGACACACAGGCGCCCCCACATGGC
TGAAGTAATCGGTGCCAGATTCAGCGGGCCATCCTGGGTCTGGAATACCCCGTAAGGCGCAATCACTGAATGGGCG
TTGCCGGTGCGCCGTGGCACATCCCCCAGGCTGAGATAGCGTTGGCCGTGCACACTCAACAACCCCACCAGGCTGG
CCAGCAGCGAGGTGCTGACATGTTGGCCACAGCCGGTGCGCTCACGCTCCAGCAGCGCGGCCAGCACCGCCGTCAC
CAGCCACATGCCCGAGGTCAGATCACCGATGGCGGTGCCCGTGCGCGTCGGGTCGCCATCGACGAAACCGGTCAGG
CTCATCAGGCCCGAGTAGCCCTGGGCGATCTGGTCGAAGCCCGGCCAACTGCTCATCGGCCCGTCGGTGCCAAAGG
CATTGATGCTGCCCATCACCAGTCGTGGGTTGCGGGCGCTGAGCACTTCATAGTCCAGGCCCATGCTGTGCAGCGT
GCCGGGTTTGAAGTTCTCGATGACGACGTCGGCGCCATCGATCAAGCGCTGGACGGTCGCCAGCCCCTCGGGGTTG
CGCAGGTCAATGCACATGCCGCGCTTGTTGCGGTTGCAAGATAAATAGTAGGTGCTCACGCCCCGGTCGAACGGGC
CCCAGGTGCGACTCATGTCGCCCGTCGGGCCGGGCTCGATCTTGATCACATCGGCCCCCAGGTCTGCCAGGACCAT
GGTGCAGAACGGTCCCGACAGCGCGCGACTCAGGTCGACGATTTTCACGCCTTGCAAAGCTTGCATAGGGTTCTCC

AGTGAAGAACGCTTGGTGGGTTGTGGTCCGAGTCTAGGCGCCGTATCTCATAAACTAAAAATATGATATTCCTCTA
CTCTCATCGGAAAATCATATGGCTCAAGAGGTACCCATGGACCTGCGCCAATTGCGCTACTTCGTCAAAGTCATCG
AGTGCGGCAACATTACCCGCGCCAGTGAAGCGCTGCATATTGCGCAGCCGGCAATCAGTCAGCAGATGCGCAATCT
GGAACTGGACATGGGTATGCCGTTGCTCGAACGCAGCGTGCACGGCGTAGTCGCCACGGCGGCCGGGCAGACCCTT
TACCGGCATGCCATCGAGCTGCTGCGCCAGGCCGATGGCACCCGGGAACTGCTGCGCCAGGACGCCGACTTCCCGC
AGGGTAAAGTCTCGGTCGGCATGCCATCGAGTACCGCACGCATGCTGGCCATTCCCTTGGCGCGCGCGATTGGCCA
GCGATATCCCGGCATCAAGCTGGAGTTGATCGATGCCCCCAGCGCCGAACTGGGGCGCCTGATCACCGTGGGGCGG
GTGGCACTGGCGGTGAACGTTGATGTGGTCGAAACCCGAGGCATGGCGTTCCAGCGGCTGTTGACCGAAACCCTGT
ACCTGGTGGCGTGGCCGGCGTTTGAATTGCCCGATGAACCGGTCTCCATCGAGGCCTTGGCAAACATGCCGCTGGT
GCTGCCTTGTGCGCCCAACACCATTCGCAATCGGGTCGAGTTTGCCCTGCAGGAGGCCGGCCTCAAATGCGAGGTT
GAGTTTGAAGCCAACTCCACCGACCTGTTGTTCTCAGCCGTCAAGGCGCAGTTGGGTGTCACGATCCTGCCGTGGG
CAGCGGCCCACGCCGAGTTGGTGCAACACCGGCTCAAGCTGGCCAGGATCGATCATCGACTGTTCAAGCGCGATCT
TTCACTGTGCTGGCATGACACGGTGGTCGAGAGCAACGCGGTACAGAAGGTCAAGGCAACCATCATTGAGCTGTTC
GAGGCGTTCGGGCGCCAGCCGGGGTGGGCGGACGGGCAGTGACAAGAGGCTGGGGCTCGACGCGGGCGAATAAACG
GTACCATTGCCGCGCCTGGCCCACTGATCGGTTCAAATGAAGGAACAGAAGACATGATCGAAACCCGCCTGCTGCG
CCAGTTCATTGCCGTGGCTGAAGAGCTGCACTTTCATAAGGCGGCGATCCGCCTGCACATGGCTCAACCGCCGCTG
AGCCAGGCGATCAACCGCCTGGAGGGCAAGCTGGGGTTCAGTCTGTTCAACCGCAGCAAACGCGGGGTAAAACTGA
CCCCGGCCGGTGACGCGTTCCTGCACGCCGCCTATAGCACCCTCAAGGAACTGGAGCTGGGCATCGAACACGCTCG
CCAGGTGTCCGAAGGGATCGCGGGCAAGCTGACAGTGACCGCCGTGTCGATCACCTACTACGAGTCGCTGCTCAAT
AGCCTGCGCCGGTTCCGCGAACACTTTCCCAAGGTGCAGTTGATCATCAAGGAGATGCCCTCCTCCAACCAGGCCA
AGGCGATTGTGGCGGGAGAAGCGGACATTGCCTTCATGCGCAAATTGCCGATTTCGGCGCAAAACGTCGAGTCGCG
CCTATTGCTGGATGAAGAGATCGTGATGGCGCTGCCTGCCCACCACCCCAAGGCGGATGCTGGCCAGATCGATTTG
CGGCACTTTGCTGGCGAAGACTTCGTGTTCACCCCCCAAGCCCTGGGCACTGGCTATCACAGCCAACTTATCGCCC
TGTGTGAAGCCGCCGGCTTTTACCCCAGGGTGGTACAGGAGGCCGCGCAGATCCATACCTTGATCGGCCTGGTGGC
CTGTGGCTTCGGGGTCGCGCTGGTGCCTGAATCGATTGCCCACTCAATCATGCGCGACAAGGTCGTGTTTCGGCGG
ATCGGCCCCGTGGGCGCGACCCCCAACCCGACCATCGGCCTGTATATGAGTTGGAACACCGACAACCCATCACCGT
TGCTGGACAGTTTTATCGCCATGCTCGATTTCAATCCTGCCTCGGTGCCATCGACCAGGGGTTAGTCGTCGTCAAA
GGTATCAGGCAGAGACTTTTATAGTATTTCCCAGCGGGCGAGCGGCGCCCCATCATCCCCTAAAGCCTCGCTCGCC
CTCCTCCCGTCACTGAGTTCTTATGCCTGCCGTCATCTACATTTTTTCACTGTGTACCTTCACATTCGGCTTGTCC
GAATTCGTCGTCGCCGGCCTGGTATCGGCAATCTCCCTCGACCTGAGCGAGTCCATCGCGCAAGTGGGTTCGGCCA
TTGCAGCCTACGCCCTGGGTGCGGCCATCGGCGCCCCGGTCATCACCGCCATGCTGGCCACTTGGCGCGACAAAAC
CGTGTTGCTCCTGACCCTCGCCGTGCTGGCCATCGGCAGCGTAGTCATCAGCCTGGCGGCAGATATCGTGACCCTG
CATGGCATACGCTTCGTTATCGGCCTGGCGCACGGTGTATTCATGGCCGTGGCCTCCAACGCCGCGGTCAAGCTGG
TTGACCCGCTACGGGCCGGGCGTGCGCTATCACTGGTGTGGATTGGCCTGACCCTGTCGATCGCCTTTGGCGTGCC
GATCGGTACCTTTTTCGGCAGCTACTGGTCATGGCGCATGGTATTCCTGGCAATCGGCGGCCTGGGCTTGATCAGC
ACCCTGGGCCTGATTCTGCTGATGCGCCAGCCACCCGCCGACGCACTGCACAAAGACCACGGCCTGAAACAAAGCC
TCGCCGCCCTGCTGCAGCGCGAACTGTTGATGGCTGCCAGCATCGCAATGCTGGTGAGCGTGGCGACTTTCTCATT
TTTTACCTTTGTGTCGCCGTTCCTGCTGGAGATCACCCGCGTTCAACCACAGGTGCTGAGTTTTGCGATGCTGGTG
TTCGGTGTCTGCTCCATCCTTGGCAACGTGCTGGGCGGTTACCTCGCGGATGCATTCGACGCCGACCGATGCCTGC
TCGGCGGGTTGAGCGCATTGATGTTCAACCTCTTGGGTTTGTATGTGTGGGGCGACTCGATCATCGCCACCTTGAT
GCTGGTGGGGCTGTTGGGGGTGGTGTTCTTTGCCATTGTGACCCTATCGACCCTGCGCTTGCTGCGCCTGGCACAC
CAGCATATTCCCCAATCCAGTGCAGTGGCTTCAGGGCTCAATATTGCCGCGTTCAACCTCGGCACTGCGCTGGGTG
GCATCGGGGCAGGCTGGGTGATCAGCCAGTTCGGGCTGGCCTATATACCCATTGCGGGTGCCGGCGCGGCGTTAGT
AGCCATGGGCGTGCTTGGGCTGCAGATGAACCATGTACGATTGAACAACCCCTGCCACCCAACGGTTTTGTAGGAA
CTGGCTTACCAGCGAAAGACTCAAGCGCGTCGTGTTCCTTCCGGTTGCCCGCGTTATCGTTGACGATTTTCGCCGG
CAAGCCGGCTCCTACAAGATGCTGCATTGATTGCATTGATGATGACTATCAAACCGACCGGCCTGTTTAATTGCCA
ACGCTGATTCCTATAATCGCCTCCACATTCTCCCCCGGCCTGGCTTCAGGCTGCATTCCCCCCGGATCTCAACACC
ATGCCAAGCGCCAGCCAGGTCCCTCGCAGCCTTCCCGAACATAATCAACAGAGTGTCAAACAGCAGTGGCTGGCGA
TTCTCTCGGTGGCCGTGGGTGCCTTTGCCCTGGTGACCAGTGAGTTCCTGCCGGTAGGCGTGCTCAATGATGTCGC
CAGTGACCTGGGTATCACTGCCGGTCACGCCGGGCTGATGGTGACCCTGCCGGGCATCATGGCCGCCCTCGCCGCG
CCTTTGCTGTCGGTCAGCATTGGCACCATGGACCGGCGCTATCTATTGATCGGCCTGACGCTGATCATGATCATCG
CCAACTCGGTAGTGGCTTTTGCCAGCGACTTTGGCCTGCTGCTGTTGGGCCGCGTGCTGCTGGGTATCAGCATCGG
GGGCTTCTGGGCGACCGCCATCGCTCTGAGCGGCCGGCTGGCGCCCAAGGGCGTGGGTGTCGCCCAGGCGACTTCG
ATCATCATGGTCGGCGTGACCCTCGCCACCGTCCTGGGCGTGCCCGTAGGCACCTGGCTGAGTGGCCTGATGGGCT
GGCGCATGACCTTCCTGGTCACCGCGCTGATGGGCGTCCCGGTGCTCCTGGCGCAGATATTCCTGCTGCCGCGACT
CAACCCCGAGAAAGCCATCCACATCCGTGACCTGCCCGCCTTGTTTATCAACCCCCAAGCGCGGGTCGGGTTGATC
GCCGTATTGCTGATCGGCCTGGCGCACTTTGCCGCGTACACCTATGTCGCGCCGTTCTTCAAGCACAGTGCCGGCT

TCGACGGGCCAACGATTGGCTCGTTGCTTTTGCTCTTCGGCGTGGCTGGGGTGGCGGGAATATTTTTGCCGGCTTC
GCCGCCAACCGCAGCGTACGTCACACCCTGTTGCTGGTGGCGCTGATGATCGGCACCAGCACCGCCCTGTTCCCCC
ACTTCGCCACCGGCATGACCGGCGCAGCCATGTTGATTGGGCTCTGGGGTTTTGCCTTCGGCGCCTTCCCGGCCTG
CGCCAGCATCTGGATGTTTGTCGTGGCGCCCAAGGATGTCGAACGCGGCATGCCGCTCTTCGTCGCGCTGTTCCAG
GTGATCATTGCCCTGGGCTCATTCTTCGGCGGGCGGATCGTCGACCAGTTGGGCAGCTCGGTGCTCCTGAGCCTTG
CCACGGTGCTGGTGGGCTTTGGTTTTGTGACCGTGCTGGTGCTGGGGCGCAATGTCAGCAACAGCCTCGCGGCCCA
GCCCTGCTAGTGGGCTAAGTGTGGCTCTCCCATCAGCCGGGGGAGCCTAGTCGATTTTACGAGACAGCCTGACTTT
CGCTTTACCGATGGGGAACTCTTGCAGTCCTGCCCTCAATGCCAGGGAAACCCTGGTGTTTTGCGCGTCAACCTTC
ACTAGCAGGTCGATATCTTTTTGCGTGCCCAGCTTCTGGAAAAGATCAAAGATTTCCTTCTCATCGAAAGCGTCGA
TCCACACGCCATACCGGCTGGTACCGTTGCGCCAATAGAAGCCACAGAACTTGGGAATGGCCCTTTGTTCATAGCG
ATTATCCAGTAGAGCCTTTGCGAATATATTTTCTTGCTCGCCGTTGTAGGCGCTGAATGCAAAATCCAGCCATTCG
CCCTCTTGCAGGACAACCACAGGTCTCCAGCTATATTTTTCCCGGTAGACGCTATAGGTTTGCGGCGCAGGATAGC
CTTGCGCTTGGATTTTTTGCCGGATTTGCGGGCTGAAGGTCTTATAGGACGGATCATTGAGCATGCGGTCTGCATA
GCCGTCTTTAAACATGTACTGGGCGTTGCTGTAGGCGCTCTCCTTGGTCATCGTCGTGGCCTGCGCCTGAAAGACC
CCGACCTCCTTCTGGTTTCCCGCCCCGCTCAGCCAGAGCACCACCCGACCCTGCGGCGCCAACCCAACGACAAAAG
TGTTGTAGGTGTCTTTTTTACGGGTGTCCTGGTCGATAAAGCCCTCATCCAGGAGGCGCTTTATCTGGTCTTTGTC
CAAGGCCCAGTTACCGGTATAGAACGTTCGCTCCACCAGCGAGTACCACGTAAATTCCAGCTGATAAGGCACACGC
TTTTTTTCGATCGCGGTGGTCATCGTTCCACCGTGGTTGCCCCAACCGGTATTCTGCGTGCCCCAGATCGCGTCAA
AACCGTAAGTAAAACCCTCAGCGATGATGGCGCCCCGGTAAATCTCCATAGGGTATTCCTGGGGTGCGGATACCGT
TCCCGACCAGCTGTAAGTTTCAGCCACGTCGTTACTCTTGCAAGCCGTCAGGGAAAAACAGGACAAGACAGCAACC
ATCACTGTCATCAGGCTTTTTATTTTCACGCACCCCTACCTCGTCATTGCCCATCGCCAATCGCTGCAGTTCCTTC
TCCCGGAATCAAGACCATGCTCTGGGGGGAAGACAATGTAATGCCCATTTCCTGAAGGCGCGCCAGCAGGATAAAC
AGCAAATCGCTGCGGGTACCGGACACCGAACGCGGGCTGTTGACGTAGCCGCTGACACCAATGATCAGGCCGTCCC
TGGCCAAGTCCTTGAAGGTGACCGAAGGTGCGGGGGTGGCGAGAATCGCCTCATGCTCGGTAAACGACTGCATCAA
CAGTTCGCGAACTTTCAATACATCGGTGTCCAGCGGCAATGTGAGGGTGATACCCACCACGCCGAGCGCGTTGCCC
ATGGTGACGTTGCGCACGTTCTGCGAGATGAACTGTGAGTTGGGCACGATCACCGTCGAGCGGTCGGACATCTGGA
TTTCGGTGGCCCTGACGTTGATCCGCCGAATATCGCCCTCGACCCCACCCAGGCTGACCCAATCGCCGACTTTCAC
CGGCCGTTCGGTCAGCAGGATCAGCCCGGAGATGAAGTTCTGCACAATGGCCTGCAGGCCGAAGCCGATCCCCACC
GACAAGGCACTGACGACCCAGGTCAGGCTGGTCAGGTTGATGCGCAAGGTCGACATCACCAGCAGACTCAACAACA
CAAAGCCCAGGTACCCCACCAAGGTGACCAGGGACGCACGCATGCCGGCGTCCATGTTGGTTTCCGGCAGCAGACG
CTCGCTCAGCCAGCGCTTCAAGGTACGCACGGCGAACAAGCCGATGACAAACATCAGCAGCGCAACGAGGATGTCC
TGGGGAATGATATGCAGGTTGCCCAGCGTCTTGCTGGACTCGCCCAACTGGCTGAAACCCTGGAGCAACTCGCTGG
GGCTGGAGCCCGCCGGCAAAAAGGCCGCCATCACTGCGGTGAGCAGCAACAACGTACGGCCGACGCCAGCCAGCAC
CGTACAAGCCTGGGCCTGGTGCCGTGGCGACAGCCCCAATGCGTTGGCCAGTGCCAGGCCGCTCGGCTGTTTGGGC
GACAGCAAGGTTTCACAAACGTCGCCGTAAAAGGCGATCAGCAGGTAGGCCGTCGCCGCCACGACGCTGACCCACA
GCAGCTTGACGGTCAGGAAGTACGCCAGGGACAGGTAGCCGGTAAACAACGCCAGGACAATCACCGCCACCCATAT
CGACGCGACAAAAGGCAGCATGCCGGCGAGCCCGCCCGGGCGCTCCAGCGCAAGCTTTTTGCGCACGTGGCGCACG
CGCAGCAACGCGGCGATAAAGATCAGCGCCGTGGTCAGCGCCGTCAGGCCATTGAGCGCCACGGTCAACGCCAGGC
TGGCGCCCGTGACATTGTTGATCCGCTCTTCGGTGGCCACCAGCATCAAGGCCACCGCCAGAATCGCCGGGAAAGG
CCCAAGCGCACTGGCAATCTCATCGGCAATGGCCGGCAACCGCCAGGACGGGCGCGGCAGCATCAGCAAGGCGCGT
CCAAGGCCCGCAATAAACGCACAGAAGGTGGCCAGGGTGACCAGATGATCGGAAAGCCGGGCCAGGTTCTCGCTCA
AGACGGCATTGCTTTCCAAGCCCCAGCGCATCAGCGAGACGGCGCCCATGATCGTCAGGATCGACGATAACCCCAC
CGCCACCGCCAACGTGCTGCGCCGCAAGCGACCCTCCGGCAGCCAGCGGATCATCGCCCAGGTCAGCAGATGCTCA
AGCAGTCGCCTGCCGAAACTCCAGATCAGCACTGCCGCCAACAACGTGCCGAAGAACGCCCAACGGCGCTCGGGGC
TGACCGCACTTTTCAAGGCGACAATGCCTTCGGCCTTTAGATTGTCCAGGCGTTGCAGGTCTTCCGGGGTCGGGCG
GATCAGGGTCGACCAGAACGTGGGGCTGAAGGGAGTATCGGTACGTGAAGTGACCTGGGAATTAAACAGATTGCGC
CGCAGGTTGACGATTTGCGCAGCGAGCTCGCGGCTCGTCTGGCTCAGTTGTTTGGCCTGCTGCTCATCGTCCAGCA
GGCTGTTCTTTTTGCGCGTAAGGGCATTGCGCTGGTCGGTAAGGCTCTGCGCTTCCCCAGGTTGGGCCTCGCCCAA
TACCTTGAGTTGATCGTCGAGGCGCTCCATCTCGGTAGCGCGTACCGTGAGCAACAGGGCAGCCTGTTGCTGTACC
TGCAGTGCACCCTGTCGAAACTCCGCCAGCTTCTCGTCACTGGTGCTGATCGGCACGCTCTGGCGAATCAGCTCAA
GCTGCTGGCTAAGCTGCTTGAGGTCGGCGTCTTCGGGCAACAGCGGCAAACTGTCGACGACATCGACTGGCGCCGG
CGCTGCGGCCGCATCTGCCCATCCCGGCAGGCTGATAGCCAAAACCATGGCCAGCAAGGCCTGGCAGATGACGCCC
CTATACATTCGCCGCATCACTTCACCCTTTTGTGTCTTTTTTCCATTGGCATTATGGACCGCCAGGGCAGAAAGGA
AATTCCGCCCTGACGGCAAAAGAGTAGAGGCGGCGTTTTCTTCTCAGTCAACCAGCGTGGCCTTGGGCGCCAATGC
CCACGGCGCCATCAAGCGTTGCGGGGTCTGGCAGGTCTTGCGCTTAAACACGAAGTTACGGCACTTCTCGGCGAAC
TGTTGGCGGTTTTCCACCATGTCCAAGCGCATCTGAGCCAGCTCGTTTTCGCGGCAGCGTTCAATCTGCTCAAGGT
CCAACTGGGCTTTGCGCGCTCGATGCAGGGCGTACTTTTGATCGGTCAACGCAGCACTGGCCTGTTGCAGCAACCA

GGCGCCAAATGCCCGCGGGCAGTGTGGACCGATATCCTGGATCATCCCCCACTGCCGGGCCTGGAAGGTACTGATC
GGCAGGCATTCTTCGATAAGTTTATGGGCGATTTCACTGCCGACCGCGCGCGGCAGGCTGTAGGTCCAGTATTCAG
AGCCATACAAACCCATGGTTTTGTAATGCGGATTGAGTACCACACCTGCCCGGGCGAATACGATGTCGGCCGCCAG
CGCCAGCATCACGCCGCCGGCTCCGGCACCCCCCGTCAGGCCACTGACCACCAGTTGCCGGGCCGTCAGCAGCTCC
TGGCAGACATCGTCGATAGCCTGGATATTGGCCCAGGCCTCCAGCCCCGGCACCTCGGCCGCCTGAATCACGTTGA
GGTGCACACCATTGGAAAAGCTCCCGCGCCCGCCCCGGATCAGCAGCACCTGGGTGTCCCGGGCCTTGGCCCAACG
CAGCGCAGCGACCAGCCGCTGGCACTGTTCGGTGCTCATGGCACCGTTGTAGAACTCGAAGGTCAGTTCGCCAACA
TGACCCGACTCTTGATACCGAATCGGTTGATACGCCATCTCGTCGAACCCTTGGCTGGCAATCGAACCATCCAGCA
CCGGTACACCCGCCAACTGCCCAGCAAGTACGTGACGGGCCGGCAACTTGAAGGTTTCCTCGCCGGGCTGGGCCTT
GCGCTTGAGTGAGCCGATCCACAGGCTGTGATCGCCGGTCGCGACCAATACCGCGTCATCCTGCACCGCGAGGATT
TCCCCCGGCGTACCGCGGCGCGAATCCAGGTGCGCGTCATACAGGTAATACTGCCCGCCGGCGAGACTCGCCAACA
CACCGGGCTGGCCGTCGGCCGCATCGATATTGCGTTTGATGAACCGGGCGCAATCGTACCAACCGAAGGTGCGATC
AGCCTGCTTCATGTTCGGCTGCAAACGTCCGATAACGTCAGGTTGCGTGTAGTCCAGTGGCGTCGGTACGAAACCG
CTGGCAAACTTACCCACCACGTCACGGATGCAATAGAGCGCAGCGTCACTCACCGGACCGTTGTACAACTCGGATT
TACGCACATCGGCGGGCATGTCGAACTCACAGGTCGACCAGACTGGGCCGGCGTCCATCTCCTCCACCGCCTGCAA
CGCGGTGACGCCCCAGCGCCTGACCTGCTGAGTGATGGCCCAGTCCAGGGCGCTGGCGCCACGATCGCCCACGATC
CCCGGATGAATGATCACCACCGGGCGGTCCGCGTTGCTCCAGAGCTGCTGCGGTACGCGGTCCTTGAGAAACGGGC
AGATCACCAGATCCGCCGCCGACTCCTCGATCTGCCGGCATACCGTGGCTTCATCGGTGAACAACACCACACTGGG
CTCGTGCCCCGACTGACGTAAATCCAGCCAGGCCCGTTGGGTCAGACCGTTGAACGCCGACGACAACAAAATAATC
TTCAATGACTGCATGACTGACTCTTCCTTGAGAATGCATGGCCGGTGGCTCTCCATGAGCCATCCTTGGCCTTTTA
TGGAAACGCCAGCATAGAGGGTGTGTCAGGTGGGGTAGATGATCGAGATCAATCTTGTGCCAGCAAGTGTGCCTGG
TGAAGCGCGGGGGCGTTATCCATCATGCGCAGCGCCTCAGCGTTGCGTGCCGCTGTCGCACCGCCCCTCCGACGAT
GGATGCCAGTTTGGTGGGCTCTGCGCTCAATCGCAGCACTATCCAGTCGACCTTGAAAGATCGCTCCTACGTCTTT
ACCAAGCGCTTCCGAAGGCCCCGTCCGAAAAACCCGACAGTCACGAAAAATGACGAGCCCTTCTTTACAATAAACG
TTATGTATTCAGTTGTTCTGGGCAAGAAGCCCACATATCCATTCATCTCATGAAGAATCAGGATCATGCTAAATAC
TTCGTTTTTACCCACGCTGCGTGCCGCCCTCGGTACATCCCTGCGGCCCCGACAGCTCGACCGAGAAGAAGAGCGA
TTGATCCGCTACTACCGTTTATTGTCAGAGCAGGATCGTGAAGCAGTGCGCTGCCTGTTGTTTGCAGTCAAGGAAA
CCAGCCGGTCCGAATGCATTAAGCAGTCGCAAGTGATATCAGCCGATTCAGCGACCAAATAAAGTGGAAGGCTCTA
GGTCCGATCGGACTATTAAGACCCACGACCAGAAACACAATGTTTTTGCGCACCTCTTTGGCAGTACGCTCCAGTT
GCTGATGGGCCCGGGCATTGACCCACTGAAAAGTGCGCCCACAAGAACTGGATAGGAGTGTTTCAGGGAAGAGCGC
AGCAAAAGCCAAAGAAAGACTTTTCTTGGGACGAAAAAAATGGACCTCTTTTCAGAGATCCATTTTTTAATGTTTG
GAGCGGGAAACGAGACTCGAACTCGCGACCCCGACCTTGGCAAGGTCGTGCTCTACCAACTGAGCTATNNNNNNNN
NNCGGCAGCACGCCCTGCAACTCGCCATCGGTCAGGGTCAGCAGTTCTTCGCCAGTGACCATGGACACCGGGCAGC
CATGGGTCGCGGTTTTATCGGCGCTGGCGCTGGCCGCGCCGTTGGGGTTTTTCGCGGAGGAGGGCGCGTCGTCCAC
CGCCTCATGTTTGATCAAGGCCGTCTTGGGCGCGGATTTTTCCCGTACCAGTGGTACCGGATTGGCGACTTCAATC
GCGCCGGCCTTGAGTGGCACGCTGGGCACCGTTTTGATCTGTGTTGCGGGGCTGCCTAGCAATAACGGCTTGAGCG
CCTCGGCATGGGGCCCCAACCCGGTTTGTGCAAACGGTCGGGCCAGTTGCTCTAGCCACTGGCGGACGCGCCCCGA
CTTGACCTTGCCCAGCACCTGAGCGCCCAGGCGCACTGTCACGCCCATGCCGGCGGTGACCCACACCAGCAACAGG
TTGATCAGCACTTCGGCGCTGATTTGCCCCAGCAGTTCGTTCAAGTACGGCGGCGGCAGCATGCGCAGCCAACTGA
CCATCGCCGACAGGTAGATGAACAGCAGCGGCTCGTCGCTGAGCACCAGCAGGCCATTGGCGATGGCCTCCTGGCT
CAGTTCCAGCAGGGCATCCAGTTCGGCCTGGGTGAGGTATTGCAGGAGTTTCTCGCTGTTGGGCCCAAGGTCCGCC
AGCCATTCATACAATTGCTCGATGTTGTCCCAGAGCTCATGCAGTGCGTCGCCAAATCCACGGGCATAGGCGTGGT
CCAGCAGGCGGTAGCGCTGCACAAAGTTGGCTTCGGAGTAGTCCTGCCACAGTGGCTCAAATGTGCTGGTCCAGTC
GTTGCGCAACTGGCTTTCCAGCGGTGCAATGAGCGACTGATAAGAGGCATACAACGCCTTGACGTGGTCCGCGCAA
ACATTGGGGTAGAAGGTGATCCGATACGACTGGTCGCGGGTACAGTCGGTGACTTCAAGAATGCCGCTGGGGCCGA
TGGTTTTGTAAATGGGTTCGCCCAGCGGGCCACCGTCCTTGGGGACGGTTTGCAGCATCACCGGGGTGTTGCCGAT
GGGCACAAAGCGTGTGCTTTGGAACAGGTGCACCAGGGTCAACGAGCCACTGGCCTTGCACATCGCGACCGTGCTG
CTGGGGGCTTTGGACGAAATGGGCGCCACCAGCGCCACCTCATCGCCCACCTTGAAGACCTGCTCGACCTCCAGCG
CCGAGAAGCTCCAGAAACTCTCGGCCCACTGCTCATAGGTATTGATGCATTGCCGGAAGTCGCTGAGGACCGCTTC
GACGTCCCGTGTTTTGAAGTCCATGGCCGCCAGGACCATAAAGCCAATGGCGGCATTCAAGAGGTGGTCCCCAGGA
CGGGGGCATCAATTGGAAAGTACATCGGCGGTCCCTTGCGCTGTGTGATCAGGCGAGGGACTTTGCAGAGGTTTTC
AGCGGGTATAAGTAGAGTTTATCTGGCCGCAGGGTGGGATATTTCGGTGGATGGCGTTGGCCAAGCGTTGACCTCG
TTGTAGGAGTGGGCTTGCCCGCGATGGCGGCGTGTCAGTCACTGTAGATATTGACTGATCCACCGCTATCGCGGGC
AAGCCCGCTCCTACAGGGGGATGGTGGTTTTTAGGGGCGGGTTTGGGGGATTGCTGCCTGCTGCTTGTCCCGGCCC
AACACCAAGCTGCACAACGCTGGCAAGAACAGCAGCGTCAACACCGTCCCCACCAAAACCCCACCAATCAGCACAT
AGGCCAGCGACGACCAGAATACTGACAAGGTCAGCGGAATAAACGCCAACGCCGCGGCCAGTGCGGTCAATATCAC
CGGCCGTGCGCGGCGTACGGTGGCTTCGACAATCGCTTCGCTGGTCGCCATGCCGTGTTCCTGGTTCTGGCGGATC

TGATCGGTGAAGATCAGCGTGTTACGCATCAGGATCCCGCCAATCCCGATCAGCCCCAGGATCGCATTGAAGCCGA
ACGGCTGGTTGAACAGCAGCAGGGTCGGCACCGCGCCGATCAAGCCCAGCGGCGCGGTGGCGAAGACCATGAACAT
CACGCCGAACGAGCGCACCTGGAACATGATCACCGTCAGGGTCAGCAGGATCATGATGGGGAATAGCGCCGCGAGG
GCCACGTTGGCCTTGGCGCTTTCTTCGACGGGGCCGCCGATGTCCAGGTGATAGCCGGTCGGCAATCGTGCGATCA
GCGGTTGCAGGTCTTTGTAGACGGCCATTTCCACATCGGGCGGCTGCACGCCGTCGACGATATCGGCGCGTACTTC
GACGGTGCTCGCGCGGTTGCGGCGCTTGAGGATCGGTTCTTCCATCACCGCTTGAAAATGCCCTACCTGAGCCAGG
GGCACCGATACCCCGGCGCTGTTGGTCAGGGTCATATTGGCGAGGTTGCCGAGGTCCTCGCGCTGGCTGCCCTGGG
CACGGGCTACCACCGATACCGTACGGTTGCCTTCACGCACTTCGGTGATGGGGTTGCCGCTGAGCAGGGCATTGAG
TTGGGATTTCACCTCGTCGGGGGTGAAGCCCAGCAGGCGCAGGCGATCCTGGTCCAGCACCAGGCGGTAACCGCTG
CTGCGCTCGCCCCAATCGAGGAACGCATCCTTGGTCAGGCGGTTGGCGGCGACCACCTGGCGCACTTCCTCAGACA
GGCTGCGCAGCACATTCACGTCCGGGCCAGACACGCGAAACACCACCGGGAAGGGTACCGGCGGGCCGAACAGCAA
TTGCGTCACCCGTACCCGCGCGGCGGGGAATTCACCGTCGGCGATACGTTCGCGCATGCGCAGTTTCAAGGCGTCC
CGGGCATGGGCATCGGCGGTTTGCACGATCAGTTTGGCGAACGCCGGGTCGGGCAATTCGGGGTTGAGCGACAGGA
AAAAGCGTGGCGCGCCGCCGCCCACGTAGGTGTCGACCATGCGGGTCTGCGGTTCCTGCAGCAGGGCCTTTTCCAG
TTGCGCCGCCACCGCCTCGGTGCTTTTGAAGGCACTGCCCGGCGGCATATACACCTCCAGGATCAGTTCGGAGCGG
TCGGAGTTAGGGAAGAACTGCTTCTTCACCACGGCCATGCCCAAGCCGCACAGCACAAACGCCGACACGACCAGGC
CGGTCACCAGCCAGCGTCGGCGCACGCAGGTTTGCACCAGGTCGCGCAGTTTCTGGTACCAGCGGCCGGCATAGAT
TGCGTCGTGGCCGCCGGGCACCGGCTGGATGTGCGGCAGCAGCTTGACCCCCAGGTACGGGGTAAATACCACCGCC
ACCAACCAGGAGGCGATCAGGGCAAAGCCAACGATCCAGAAAATATTGCCGGCATACTCGCCCGCACCCGAACGGG
CAAAACCCACCGGCAGAAAGCCGATGATCGTCACCAGGGTGCCGGTCAGCATGGGGGCGGCGGTGGAACTCCAGGC
AAAGGTGGCGGCGTGGATGCGGTCGAAACCTTCTTCGAGCTTCACCACCATCATTTCAATGGCGATGATCGCGTCG
TCCACCAAAAGCCCCAGGGAAATAATCAGCGCGCCGAGGGTGATGCGGTCAAATTCGCGGCCGGTCAGCAGCATGA
TCACAAACACGATGGACAGGGTCAGCGGCACCGCGGCCGCCACCACCAAACCGACGCGAAAGCCCAGGGCCAACAG
GCTGATGAGCATCACCACCGCCAGGGCGACGAAGAATTTGAGCATGAATTCATTGACTGCCAGGCTGATGTTTTTC
GCCTGGTCGGAGACTTTCGCGAAGTTGACGCCCAGGGGCAGGTCTGCCTGGATCCTGGCTTCCTCGGCCTTGAGGC
TCTTGTCCAGGTCCAAGCCGTTCCAGTGTTTTTCCATGATGATGCCGAGCATCAACGCGGGGTCGCCCTGGTGGCG
GATGCGGTAGCTGGGCGGGTCTTCATAGCCACGGCTGACGGTGGCGACGTCAGCGATGCGCAGCACTTTGCCGTCC
ACCGGCAGCGGCACGTTTTCGATCAGCGCCAGGCTGTCGAAGGCGCCGTCGATACGGATATAGGCGCGGGCACCGG
CGGTTTCGACAAACCCCGACGGGGCCACGGAGTTTTGCGCAGCGAGGGCGGCGAAGATCTGTTCGGGTTTGATCCC
CAGGGTGGCCAGGCGCTCGTAGGAGAACTCGACAAACACCCGCTGCGCTTGTTCGCCGAGGATATTGACCTTCTTC
ACCCCCGGTAGCCTGAGGAAGTCCTGGCGCATGGCTTCGGCCATCTGCACCTGTTGGCGATGGGGCAGGTGTTCGG
CTTCCAGGGCGTACAGGGCGAAGTACACATCGGAATATTCATCGTTGAAGAAGGGCCCGATCACGCCCTTGGGCAG
CCTGGCCGCTTCGTCGCTGAGTTTTTTGCGGGTCTGGTAGAACAGGTCCTGGATTTCATGGGGGCGCGTGGACTCG
AGAAAGGTCATGCGCATCGACACAAACCCCGGTTGGGCGATGGTTTCCACACGATCGTAGTAGTCCAGTTCCTGCA
GGCGCTTTTCCAGGCGGTCGGCCACTTGCTCCTGCATCTCCTGGGCTGTGGCGCCGGGCCAGGCGGCGGTGATGGT
CATGACCTTGACCGTGAACAACGGGTCTTCGGCGCGCCCCAGTTTGCCAAAGGAAAAAATCCCGGCGGCGAGTATC
GCGAGAATCAGGAACAGCGTGACGGCGCGGTGTTTGACCGCCAGTGCGGAGAGGTTCAGGCCATGCATGCTCAAGG
TTCCTGCCGGCGATTGAGGGTCAGTGCCTGGGCGGGCAACAGGCGCACGCTGTCACCCTGGTGCAGCAGATGTGCG
CCGAGGGCGACGATCAGTTGGCCGGGGCTGACGCCGCCAGAAAGCAAGGCTTCTTCCTGGCCCAGGCTGGCCACGG
TGACCGGGGCGAAGCTGACCTTGTCATCTGCTCCAATCAGCCACACCCCGGTGCCGCTGCCGGCATCCTGCAGGGC
GCCGATCGGCACGCGGGTCTGTGGCGGCTGGCCGTTGCCTTGCAGGCGCAAGGTGATGGTGGAACCGAGGGCGAAA
CGCTCACCCGCACCGTGCAACACATAGCGCGCGCGATAGGTGCGGGTGACGGGATCGGCACTGGCGGACAGTTCAC
GCAAGGTCGCGTTGACGGCTTGGTCCGGTGCACCGAAGGGCAAGGCCAGGGCTTTTTGCGAGGCGCGGTCGCGCTG
GTTTTCCGGCAGGTTGATGACCGCTTCCCGCGCTCCGTCGTGGGCCAGGCGGGCGACGATCTGCCCTTCGGCCACC
ACCTGCCCGCGCTCTACCCGCACATCGGTGATCACTCCGTCGCCATCGGCCCTGAGCACCGAGTAGGTACGACGGT
TTTCGATCTGGCTGGCATCCGACTGGGCGGCGGCAAGCTCCGCTTCGGCGACCCGCAGGTTAGTCGCGGACTGGTC
GAAAATCTGCCGTGACACCGCGCCGGTACTGGCCAGGCGCTGGTAGCGGTTTGCGTCATCGCGGCGCTGGCGTAAC
TGGGCCTGGGCGGCGTTGACGCGGTTTTTCGCCGAACGCAGCGCCAGTTCGAAGTCGCCGATATCCAGCACCAACA
AGGTATCGCCCCGGGACACATGCTGGCCGGGATCGACCTTGCGTTCGATCACCTTGCCGCTGACCCGGAAGCCCAG
GTCACTTTCAGTACGGGCCGCCACCACGCCGGTATAGGCGTTCTGCTGGCTGGCGGCGGCTTCGACCTTGGCCGCC
AGCACCGGGCGCGGCGAGGCGGCGTTGGCGCTGGGGTCGGCCTGGCTGTTGCAGCCGCTGAGCAGGAGCAACAGCA
TCAGGGGCGGGAAAAGGCTGTGAAGGGGAGGGCGTAGGCTCATTTCAGGCTCCTGGCGGCGCTGGGTCGGTAAAAT
TACAAGCGTAATATTTTGCCGAATATTACGACCGAAATATAATTTAAGCCAGCCCTTGTTCAGGTTTTGTAGGCGT
CGGCTCGCCGGTGATGGTCGTTCAGGATGAGGTCAGATACCTGGCGCTCTGGGGTGTTCTTGCTGGCGTCATCGCT
GGCAAGCCAGCTCCTACAAAAGCAAGCGGTCAGGGACGTGGGTGGAGTTGTCACACTTTGTCCAAGTTATTGGGGT
GTAGATGATTACGATTCTCTTTTGAGAATAATCCTGAAGGTCTACGCCCATGCTTCCTGCTTTTCGTCTTGCGCCG
CTGGCCCTGCTGGTGGCCGGCAGTCTCAACGCTCACGCCAACGAGCCTGCGTCACTGGAACTCAATGAAGTGGTGG

TCACCGCTTCCGGCTTTGAACAGAACGTCGAAGACGCACCCGCTTCGGTCACCGTGATCGACGGCGAGGCCTTGCG
CCGCAAGTCGTACCGCGACCTGGGCGATGCCGTGCGGGATGTGGAGGGCGTTACGGTCAATGGCGGGGCCAATGAA
ACCGACATCTCGATCCGTGGCATGCCCGCCGACTACACGCTGATCATGGTGGATGGCAAACGCCAGAGCGCCCGTG
AATCGCGGGTCAATGGCAACAGTGGTTATGAGCAGAGTTTTGTACCGCCAGCCGCCGCCATCGAGCGCATTGAAGT
GGTGCGCGGGCCAATGTCGTCGCTGTACGGTTCCGACGCCATCGGCGGCGTGATCAACGTGATTACGCGCAAGGTC
TCGCCACAATGGGGCGGTTCGATTGGCTATGACTACTCGGCCCGCCAGCACAGCGACCAGGGCAATGCGCGCCAGA
CCCAGTTCTACCTCAGCGGCCCGCTCAAGGAAGACCTGCTGGGCCTGCAGGTGTGGGGCCGTTACCTCGATCGCCA
GGCCGATGATGATATCGAGCAGACCAATGGCTTCAGCAAGGCGGATCACCGCGATCTCACCGCACGACTGGCCTTC
ACCCCAAGCATCGACCACGACATCCTCCTCGAAGCCGGGGCCACGCGCCTGAAAAACGGTGATGGGATCAGCGCCA
ACTGGGCCACCCGCGAGCAGGAGAACAACCGCGATCACTGGTCGCTGTCGCACCAGGGTCGCTGGGGCTGGGCCAC
CTCGGACATCGCCTTTTCCCAGGAAACCTCCACCCGTGAAGGCCGTGCCACGCCAGCCCAAACCGATATTTTCGGA
CGCAAGCCCGAGGTCAAGAACAGCGTGTTCGACGCCAAGTTGGTAGTGCCCACCACCCATAACGTCAGCACCGTGG
GCATGCAGTGGAACGAAAGCCAACTGACCGACTGGAACCAGGGCCTGGGCGATCGCGTGGATTACGAGTTTTCGGT
GGTGCAAAAAGCCCTGTTTGCCGAGAACGAATGGTCGGTCACCGACAGCTTCGCCCTGACCACCGGCCTGCGCCTG
GATCATCACGAAGAGTACGGTGCCCACGTCAGCCCACGGCTCTACGGAGTGTGGCGCGCCACCGAGCAGTGGACAC
TCAAGGGTGGTATCGCCCGTGGCTTCAAGGCCCCGGAGATCCGTGCAGTCGTGGCGGACTACGCCTACCTGCGGCG
CAACCGTTTTGTGATGTTCGGCAACCCCGACCTCAAGCCAGAGACCAGCACCAACTATGAAGTCTCGGCGCTGTGG
AGCAACCGTGATGATCTGTCGGCGGGCGTGACCCTGTTCTACAACGACTTCCAGGACAAGCTGTCCACCGTGACCA
CCGACCAGCGCTGGAATGGCTACACCATCATGGAGCGGGTCAACGTCGACAAGGCAATCATCCAGGGCGTGGAGCT
CAACGGCCAATGGGATATCAACCCCAGCGTGCTGCTCAAGGCCAACTACACCTACACCGATTCCGAACAGAAAACC
GGCGCCAACGCCGGCGCGCCCCTGGCCCTGACCCCCAAGCAAAAGGCCAATGTGCGCACCGAATGGAGCATCAACG
ACCGCGCCCAGGCCTGGGCGTCGCTGAGCTACTACGGCGAGGAAACCGGCAACACCCTCACCAGCGAAAAACCGGC
GCCGGGCTACGCCACGGCCGACCTGGGCGGCTCGTTCGATGTGACCGACTCGCTGGTACTGAATGCCTCACTCAAC
AACCTGGCCAACAAGCGCCTGGATGACGAGACCTACGGCACGGTGAACTACGGCCGCACCTTGTGGATGGGCGCAA
CGCTCAACTTCTAGGTCAGTTGCAGACGGCCTTTGTGATGGCTAGAGGTCTTGTTGTGGCTATGTGACTTGCTGTG
GCGAGGGGGCTTGTCCCCGCTGGGCTGCGTAGCAGCCCCAATAAGCCTCACCCCGGTTTATCTGATAGACCGAGAT
AGCAGGTTTTGGGGCTGCTACGCAGCCCAGCGGGGGACAAACCCCCTCGCCACATCAAGCCCTACGGCCACATCAA
TCTAAGCCGCCTGGGGCAGGCGCACCACCGCGCACAGCCCGGCCGGTGGGCGGTTTTCCAGGAGCAGTTCGCCGCC
ATGGGCCTGGATGCACGAGCGTGCAATCGCCAGGCCCAACCCCAGGCCACCGTCGCTGGCACCCAACTGCACAAAC
GGCTCGAAGACCTTGTTCAGCCAGTTCTGGGGCAGGCCGCTGCCATGATCGAGAATCTCCACGCACAGCACACCGC
TGTCCTCGCGCTTGAGGCTGATACAGGCATCGCCACCGTGTTTGACGGCGTTGTCGATCAGGTTGGTCAGGGCACG
CTTGAGGGCCAGCGGGCGGCAATCGAGCAGCAGCGGTGGGGCGCCTTGCCAGGTGACCGGTTGCTGCATGCGCCGG
TAGCGTTTGGCCAAGTCATCCAGCAAAGCGCTCAGCGAGGTGCGGCGGGTGGCTTCCTGCACGGCATCGTCGCGCA
CGAAGTTCAGGGTTTCGCGCACCATGGCGCTCAGTTCGCCGAGGCTGTCGAGCATGTCGTCGCGTTCAGGGCCAGG
TTCCAGCAACTCCACCTGCAAGCGCAGTTCGGTGATCGGGGTGTTGAGGTCGTGACTGACCGCCGCCAACATCCGC
GTGCGCCCTTCGACATGCCGCGCCAGGTTGGCCTGCATGGTATTGAAGGCGGTGGTCAGGTCGCGGGCTTCCTGGG
GGCCGCTCAAGGGCAGCGGCGCGATCCATTCGCCCCGGCTGACCCGTTCGGTGGCCTGGGCCAGGGTCTTGACCGG
GCGCACCACCCGCGCGATGAAAAACATCACGATCAGCAGCACTGGCACCGTGGTCACCGGCAGGCTGTAGGCCAGG
ACGCGGCCCCATTCATAAGCCCCCGCGGGATGCTGCAGCGCGTTCAGGTACTGGCCGTCCGGCAGGGCGATGCTGC
TGCGCAAACGCAACGGCGCCCAGCCGGCGGGGCTGAACACGTGGGTGCGGGCATTGGCGCCGCTCACCCGCTCCAA
CTGCATGCCGATGCTCACGTCTGGCGGCAGTTGCAGTTGCTGGCGCAATTCGTTGGCCAGGCGGCGCTCTTCGGGG
CGCAGTTCAAACGGCTCGACTTCGGCGGTGGGGGCGACCCAGAAACGGGCGCCTTCGTTGCTCATGGCGGCCATCA
AGTGCGCGGGTTCTTCGCCGCGCAGGTCGACGGCCGCGTGGTGCGCGATCACCAGGCGTTCCAGGCTGAAACTGCG
CGATAGCGGGTGGATCAGGCTGCCGGTGCGTTGCAGGAACAGCATCGCGATGGCGTTGGAGGCCAGCAGCGCCAAC
AGCAGCAACAGGCTCAATTGCTGCACCAGCGTGCGCGGCCATAGGCGGCGCAAACGGTTCAAGAGACGCACGGGGT
CACATCAGCCGCCAGCAGGTAGCCGCCACCCCAGATGGTGCGCAACAGGTCCACGGCTTCGGTGTCGTGGGCCAGT
TTGCGGCGCAGGCGGCTGACCTGGCGGTCGATGGCGCGGTCGCTGACATCGCTGTCGGCGGCGGCGGTCAGGTCGA
TCAGGCGCTCGCGGGGCAACAGGGTATTGGGGTGGTTGAGGAACACTTGCAGCAACCGACTTTCGGCGGTGCTCAG
TTGGACGGGGGGGCAGCCTTCGCGGGTCAGCACTGCGCTGATGGTGCTGAAGTTCCAGCCGGCGAAGCCATAGGTG
CGGCTGGTCGGTGCCAGGGGGGCATGGGTGCGGCCCTGGCGGCGCAGCACGCTGTTGAGGCGTGCCACCAGTTCCC
GGGGTTCGAAGGGCTTGGTGATGTAGTCATCGGCACCCAGGTCCAGGCCCCGGATGCGGTCGGCGGCGGCATCGCG
GGCGGTCAACAGGATCACCGGGGTGTTGCTGCGCCGATGCAATTGGTTGCACAACTCGAAACCGTCGCCATCGGGC
AGCAGCACATCCAGCACGATCACGTCGAAGGCCTGGGTCTTGAGCAATTGCCACATGCCCGCTGCATCTTCAGCGG
TGCGCACTTCGAAGCTGTGGCGACGCAAATAGATGGCCAGCGGCTCGCGGATCTTGCGGTGGTCGTCCACCACCAG
GACGCGCGGCGCTGCCGGCGCAGGAGCAAATGGCATGGTGGCCCTCGGGGAGTTGAGCGGGAGAGCGGGGATATTA
CTACGAGTCATTATCATTCACTCGCTTTATGCGCCACAGCTCCAGAGTCGTCCCATGTATGGAACGATGCGAGCCA
AATCCGCGATCTACCGCCTTATTGGTATTGGATTTAAGGTGTATGCACATTGGAGGACCATCATGAAAAACATCAT

CGGTATCTACACCAGCCCACGTGGCCATTGGGTTGGCGACGGTTTCCCGGTTCGCACGCTGTTTTCCTACGACACC
ATGGGCAAGCACATCAGCCCGTTCCTGCTGCTGGACCACGCCGGCCCGGCCGATTTCACCCCCACCACCCAGCGAC
GTGGCGTGGGCCAGCATCCCCATCGCGGCTTTGAAACCGTGACGATCGTTTACGAAGGTGAAGTCGAGCACCGCGA
TTCAACCGGTGCAGGCGGCACCATCGGCCCCGGCGACGTGCAGTGGATGACGGCAGCCAAGGGCATCCTGCATGAG
GAGTTCCATTCCCAAGCCTTCGCCAAACGAGGTGGGGCCCTGGAAATGGTGCAGCTGTGGGTCAACCTGCCGGCCA
AGGACAAAATGAGCGACGCCGGCTACCAGACCATCCTCGACGGCGATATCCCCGCGTTGCCCCTGGCCAACGGCGC
CGGCAGCCTGCGCCTGATCGCCGGTGAGTTCGACGGCCACACAGGCCCGGCGCGCACCTTTACGCCGATCGATGTG
TGGGATCTGCGCCTCAATGGCGGCAAGGCCGTGACCTTTGACCTGCACGCCGGGCGCAATACCGCGCTGGTGGTGC
TCAAAGGCACCGTGCAAGTCAACGGCGGTGAACTGGCACGCGAAGGGCAACTGGTGCTGTTCGAGCGTGATGGCGA
GCAGATCAGCCTGGAGTCCAATGCTGACGCCATGGTGCTGTTGCTCAGTGGTGAACCCATCGACGAGCCCATCGTC
GGTCACGGCCCGTTCGTGATGAACACCGAGCAAGAGATCCACCAGGCCTTTGCCGACTTCCAGTCCGGCCAATTCG
GCCAACTGCACGGCGCCGCCAGCTGATTGGCGGCGCCCTATACCCGCCAACTGCATTTCGTGCGATCTTCAGGGCA
TTCGCCCTGGAGTCGCCTGGATGCTGTCTTTTATCTCCGATCACCCGATGCTGTGCGCCCTGGCGCTGATCATTAT
CGATATCTTCGTCTGGCGTATGTTTTCGGCCAGCCCCGGCAACTGGAAACTGATCGCACGCCTGGGGATTTTCGCT
GTGTTCAGCGCCGTGCTGTTCAACGACGGCATGAACCCCATGCAACTGGCACCCTATGCCGACAACACCCCGCTGC
ACCTGGCCGCCACGGCATTGCAGATCGGCTGGTGGTTGTTCGCCGCGCGCACCCTGACGGTCTTGCTCGGGGCCTT
GATGATGCAACGGGTCGGCCATACCGGGCGGTTGCTGCAAGACCTGATGGGCGCAGTGATCTTCCTGATCGCGATC
ATCGCGGCCATGGCCTACGTGCTCGACCTGCCAGTCAAGGGCGTGCTGGCGACGTCCGGCGCGGTGGCGATCATCG
TCGGCCTGGCCTTGCAAAGCACCTTGAGCGACGTGTTCTCCGGGATCGTGCTCAACACCACCAAGCCCTACCAGCT
GGATGACTGGATCTCCATCGACGGCACCGAAGGCCGGGTGACGGATATCGACTGGCGCGCCACGCGCCTGCAAACC
TCCCAGGGCAGCATGGCGGTGATCCCCAATTCGTTGGCGGCCAAGGCCAAGATCATCAACTTCTCGCGCCCGGCCG
ATATGTTCGGCCTGGCGGTGAGCCTGCAAGTCAGCCCCCATGCGCGCCCACAAACAGTGCTCGAAGCCTTGGAGCG
TGCCATGCAGGGCTGCCGGCAATTGCTGGCCAAGCCGGCGCCAAGCGTGGCGTTCAAGACCTCCACGGGCGGCGGC
GTGGAATATGAAATCAGCGGTTTTGTGCCTGCCATGGGCCTTAAACGCGAGGTGCGCAATCAGCTTTATGACCTGG
CCTACCGGCATTTGCAGGCGGCTGGCGTCAGCCTGCTGTCGAGCGCCGAAAGCCCCACCTCGCCGACCACCTCGCC
GGCGCGGGCGCTGCTGGAAAGCTCCAACATTTTCTCGACCCTGCGCCAGGAAGAGAAGGAAACGTTCAGCCAGAAC
ATGACCCTGCACACCTTCCGCGCCGGCGAGATGATCCTGCCGACGGGGGAGGTCAGCGATCATCTGTTTATCATCG
AGTCCGGGGTGGTTTCGGTGATGCTGGCCAAGGCCGGGCACACGTTCGAAGGCGGGCGCATGGGCCCCGGTGAAGT
GATCGGCGAAGCCGGGATCCTCGGTGAGCAAGGCGTGCCGGCGGACTTTGTGGCGAAGACCTACTGCACCCTCTAC
CGCATCGAAAAGGAATACCTCAAACCGTGCCTGGATGCGCGCCACGATATCAGCGAAGCGATGCAAAACCTCCTGG
ATTTCCGCCTGCATGCCGCCCAGCACCTGACCCAGGATGCGCCCATCGTGCCGGTGAAAAAGGGCTTCCTGCATTG
GCTGCGCAGCCGGGCCTGACTTGCCTGGCATTGGCTACCTGTTCTTCGCCCGGATTGGCTATTTGTGCAGGGCAGT
GGGGTGATTAAGCTGGGCACCAATCCCCACTGTCCTGGAGTAACACCATGTCTGCCCGTATCGATTTCTACACCGC
TTCCCCTGACGCGATGAAAGCCATGCTTGCCCTGGAAGCGGCCGTCGGCAAATTGTCCATCGAACTGCCCCTGCTG
GAGCTGGTGCGCCTGCGCGTATCGCAGATCAACGGCTGCGCCTTTTGCCTGGACATGCACACCGCCGATGCACGCA
AGGGCGGCGAGACCGAGCGCCGCCTGTACACCGTCTCGGCCTGGCGTGAAACACCATTCTTCACCCCGCGCGAACG
TGCCGCCCTGGCCTGGGCCGAAAGCCTCACGCTGATCAGCCAGACCCACGCCTCGGACGAAGATTACGCGTTGCTC
AATGACCAATTCAGCCCGGCCGAACAGGTTGACCTGAGCGTGGCCATCGCCACCATCAACAGCTGGAACCGGCTGG
CGGTGGGCTTTCGCAAGATGCCTAAGTAACGCCATATAGCGGGACTTGCCATTGTGGCGAGCGGGCTTGCCCCGCG
CTGGGCTGCGAAGCAGCCCCAAAACCAGCGGTTACGGAGTATCTGATACACCGCATTCGGCTTACTGGGGCTGCTT
CGCAGCCCAGCGCGGCGATGCGGCGTTCCGACAAGCCCGCTCGCCACGACAGCCCGTTACTTGGGGCGGGGTCAGA
AGTTGACGGAGGCGCTCAAGCGCGCCGTCAATGGCGCGCCCAGGAACAGATAGTCATCGCCCATGTATTCGCCCGC
GTCACGCCAGTAGCGCTTGTCGAACAGGTTGTCGACGCTCAGGCGCAACACCGTTTCATACCCCTCGACCTTGGTG
GTGTAGCGGCTGCCAATGTTGACCAGCGCATAATCCCCCACCTCTACATTGCCGGTGCGGCTGGCGTTTTTGCTGG
CGCTGTATTGCACGCCGCCGAGTACCGCCAGGCCGTTGACCCAAGGCAGGGCGTAGTCGGCGTATACGCTGGCGCG
CAGCTTGGGCACGTTGATCGTCTGGTGACCTTCATACTGCGGCGTGCCGCTGCCGTTCACCCGTGCGCGAATCGCT
GCCACGCTGGTGGCGATCTGCAGACGCTCGGTGGCCCAGCCATTGGCCGACAGCTCCAGGCCGGTGTTTTTCTGCT
GGCCCTGCTGCACGTAGGTGAAATCACCGGCGCTATCAGGTTTGGCGTACTGGTAGGCCTGGCGTGTCTGGAACAC
GGCGGCGGCGAAGCTGATGCGGCGCCAGTCGTATTTCACCCCGGCTTCGATCTGCCGGGAGACGGTCGGCGCGAGG
ATTTCCCCATCATTGCTCGCGTACCACGGTGCCTCGCCGCCCAGCGACAAGCCTTTGCTGTAGCTGGTGTACAGCG
ACAGATTGGCCATCGGCTTGTAGATCAGCGCAGCGTTGGGCAGGAACTCGTACTGCTGGGTGTGACGCTCTTGCAC
GCCATTTTGATCGAAAGACTTTTCATCCAGGCGCACTTCACGGCCACCGAGCACGGTTTGCCATTGCTCGTTGAAA
CTGATGCGGTCGGTGACGAACAGCCCGTACTGGCGGCTGTCGAGGCGCCGGTGACTGTCCTTCAGGGGCTCGTCGG
TGGGCTTGAAGGTTTCCGGGTCCTGATCGATATTGCCGCTGCCGATGTACTCGTTGACTGACTTGCGCTTGTCGAT
CACCCGGCGGAACGCGCTGGTGCCGAAGGTCAGTTCATGGCCTATCCCCGCAGTGTCGAACAGCCCGGTCATTGCC
GCCTGGATTTCATCATTGCGTCGGGTGTCGTCGGGGCTGCGATAGTCAGCAATATCGTAGTTGCCTTCCGGGCTGA
AGTAGTTCGGCACTTTGACGCCAGCGCAACTCGGGGAGCCGTAGCAACCCCACGCAAACGAACTGTAGTCATCAAT

CACCACCTTGCTGCGGGCCGCGCTGACGCTACCTTTCCACTGGTCGCTGAAGCGGTACTCGAACTTGCCGTTAAGG
TTCAGCGAGTCGATGCCCACCTGTTTGGAGCCGCTCTGATGGCCGAGCAGTTTTTTCGGCGAGGCGTCATGGGGCA
CTTCGGTGCCGCCAAGCAGTTGGTAGCCGGGCACCGAGCGCTGCTGCTTGTCCTGGTATTCGGCATCCAGTTGCAG
CACGGCGTCGGGGCTGATGTTCCAGTCGAAGGCCAGGGACACAAAATCCCGCTGGCCATTGGCGTGTTCGACATAA
GAGTTGAGGTCTTCATGGGCGACGTTGGCGCGCAAGCCAAATTGCTGCTCGCTGCCAAACCAGCCGCCGACATCGG
TAGCGATGTAGCCGCTGCCGCGATCATCGGTGGACACCGTGACCGAACGCACGTCTTCCGGGCGCTTGGTCACGTA
GTTGATCACGCCGCTGGGTTCGGCAATCCCGCTTTGCAGGCCCGCCAGGCCTTTGAGCAATTCCACCCGCTGTTTG
TTTTCCAGGGCGACGTTCTGCTCGCCGGTGACGGTGCGGCCGTTGATCTTGTAGCTGCTGGCGGCATTCAGGGAAA
AACCCCGGACCACGAAGTTTTCGTAGTAGCCGATCGGCGCGTAGCTTTCGCCCACCGAGGCGTCGTTGCGCAGCAC
TTCACTGAGCAGGCGTGCCTGCTGGTCCTTGATCAACGCGGCATTGATCACGGTGATCGAGGCCGGGGTGTCGAGC
AGCGGCGCTTCATCGAAGCCGCCGACCGACGCGCTTTGTGCGCGATAACCGGACTCATCCTGGCCCTGAATGTTCA
GCGCTGGCAGTTCAAGCTCAGCCGCCAGGCTGTTGCCGATACCGCCGCTGAGCAGCAGGCCGAGGGCCAAATGTGA
AGTGACTTCGGGGCGAAAACGCAAAACCATGGGGGCAAGGCCTTAAAGCGCGGGGCGGGGGCGCATAAGCTAGGCA
TAACTGCGCGCCTTTACAAGTACTGGCCGTGGCGCGTCGGCTTGAGACGTTGCGTCTCAAGCCCCACGCCAGTTCA
TCCGGGCCTCGACAGCGCGACCCCGCACCTGCGATAGACAAGGCTTCGCTTATCGTTGCAGGAGATCGTCATGGGT
ATCGCTGGAAAAGTTGCACTGGTGACCGGCGCCGGGCAGGGCATTGGCCGGGCCATCGCCCTGCGCCTGGCGCGGG
ACGGGGCGGATATCGCCCTGGTCGACATCAATAGCGCCAAGCTCGAAGCCGTGGCAGCCGAGGTCGTGGCACTTGG
ACGCAAAGCCTCGGTGTTTACCGCCGACGTGTCCAAGCGTGAACAGGTCGGCGCGGCGGTCGACCATGCGCACCAG
ACCCTCGGTGGCTTCGACATCATCGTCAACAACGCTGGAGTGGCGCAGATCGATTCGCTGCTGGATGTGACCCCGG
AACAGGTTGAACGCACCCTGGGCATCAACGTGCAGGGCGTGTTGTGGGGCATCCAGGCCGCCGGCAATAAGTTCAA
GGCGCTCAAACAGAAGGGCAAGATCATCAATGCCTGCTCGATCGCCGGGCATGAAGGCTTTGCACTGCTGGGCGTG
TACTCGGCGACCAAGTTTGCCGTGCGCGCCCTGACCCAGGCGGCGGCCAAGGAACTGGCCAGCGCCGGGGTGACGG
TCAATGCCTATTGCCCGGGTGTGGTGGGCACCGATATGTGGGTCGAGATCGACAAGCGCATGGCCGAGATTACCGG
TGCGCCAGTGGGAGAAACGTACAAGAAGTACGTGGATGGCATCGCGCTGGGCCGTGCAGAAACGCCGGAGGACGTG
GCAGGCCTGGTGGCATACCTGGCCGGCCCGGATTCGGACTACATGACCGGCCAGGCACCGTTGATCGACGGCGGCC
TTGTATACCGCTAAACCCAAGCCCGACCCGGTAAAAATGTGGGAGCGGGCTTGCCCGCGATAGCGGCGGGTCAGTC
AAAGATGCATTTACTGTCACTCCGCCATCGCGGGCAAGCCCGCTCCCACATTTGGATTGCAGGGTTTCAGGGGTGT
TTACCGGGCATATCGATGCCCAGTTGCTTGACCCGGCGGTACAGGGTCGCGCGGGAAATCCCCAGCGCCTGCGCCG
CCGGCAACGGCTTCCAGCGATGGCGCACCAGCGCATCGAGCAGCACCTGCCGCTCTGGGCTCGCACTGTGCTCGAC
CTCGGCCTCCACCCGCTCGCCGCGCACTTCGACCGGCAAATCCGCCAGTTGGATCGTCCCGCCTTCGCACACCGCA
CAGGCATACGCCAGCACATGGTGCAGTTGCCGCACGTTGCCTGGCCATGCATAGCCCAGCAGTGCTTCAAGCGCCG
CCTGGCCGATTCCCACCGTGATCCCGCTGTTGCGTGCTTCCTGCTCCAACAACCGGTTGATCAAGCCCAGTTTGTC
GGTGCGCTCGCGCAGCGGCGGCAGGGTGACGCGGGCGCAGCCCAGGCGAAAGTACAGGTCTTCACGAAAACGCCCG
TCATTGACCAGGGTCGCGAGGTCGCGGTGGCTGGCGCAGATCACCTGGATATCCACCGCGCGGGTTTTCGCCGCGC
CCAGCGGGGCTACTTCACCTTCGGCCATCACCCGCAGCAAGCGCGTCTGCAGGGCCAGGGGCATATCGCCGATTTC
ATCGAGGAACAACGTGCCGCCGTGGGCCTGTTGCAACAGCCCGGTCATGCCCTTGCTGGAGGCCCCGGTAAAGGCT
CCGGCGACGTAGCCGAACAGCTCGCTTTCAATCAGGTTTTCCGGGATCGCCGCACAGTTCACCGCCACGAATGGCC
CGTCGCGGCGCGCACTGCGGGCGTGCAGTTGGCGGGCGAAGACTTCCTTGCCGGCGCCGGTTTCGCCTTGCACCAG
CACCGGCAGGTTGCGGTCCTTGACCCGCACGGCCAGGCGCAGGTGTTCTTCCACCCGTGGGTCGACCTGGGCATGG
GGCAGCGCCCGCGCAGGTTGGCGCCGCGGGGTGTTGACGCGCACATGCAGCACGCCAGTTTCTCCCAGCCACTGCA
GTTGCTGGACCGATTGATCGGTCACCGCGCGCAACGCATCGAGGTCGAACACCTCGCCGATATGTACCGGCACCTG
GCCAAAACGCCGGCGCAACGCCTGGCGTGCCTTGCTGTTCAAGGCTTGCAGGCGACCGTCCTGGTCCCAGGCCAGC
AGCAGGTCGGGTTGGCTGTCCACATAACCCGGTGTGCTGTGGGCCTGGAGCACCCAGTGGCGGGCGGCGCTGTGCA
TGAAGAAGGCGTTTTCGATGGCCTGGGCGCTCTGCGCCACCATCTGGCGCACCAGGTGCTGGCTGCGTCGGTCATC
CGGCGATTGCAGCGCCGAAGCGTCCATTACCCCCAGCAGGTTGCCCTGGGGGTCGAAGATCGGCGCGGCGGAGCAG
GTCAGGTTGATAAAGGCGGCGCGGAAGTGATCGCGCTTGTGCACGGTCACCGCCGCTTGGCTGGTCAACACCGTGG
CCACGCCGCAGGTGCCTTCTTCGGCTTCCGACCAGCAGGTGCCCAGGTACAGGCCGGCCTTGCGGCAGTCGCTGCG
GATCGCGGCATCGACCCGGTGGTCGATGGTCTGGCCCTGGGCGTCGGTGAGCATCACGCAGTAGTCGGCATGGCGC
ACGCGGTGGTGCAGTTGGCTGACTTGCTCGCTGGCAATACGCATGAACAGCTCGGCGCGCTCGCGGCATTCCTTGA
GCAACGGTTCGGTGAGGATCCGTGGGCCCTGCAGCGAGCCGGGGTCCAGGTGGTGTTGCTCCATGGAACGGCGCCA
TGAATCGAAAATCAGCGACGGCACTGGCGCCTGGGGCAGGCGCTCGGCATTGCGGACCACGCGACTGACACAGTCG
ACATGCTCCCTGGAGTTCGCGGCGAGCATAAGGCCTCCGGTTCTCGATCTTTGTTGTTATGCCCGCCATTAAGCGC
CCAGGGGCGCAAGGGGACAAGGGCGGCCTGACCAACTGCCCGCCGTGAGACGCAACGTCTCATCCTGCCTGAGACG
CAGCCTGCACGCTGCTATGCGTCGTCTCATTGTGCGGTGGCATGCCGCAGCGGCACAACCGCACGGCACTGCTCTG
GATCAAGGCTTTGCCAGGTTTTGCCGCGCCTTGGCACAGGCTGTGCTGAACAGGTTGCAGCTGGCCGCAGCCAGCC
TTTTGTGTGTCGAGACAACCTGGAGAACAACAAGATGTCCACTCACCTGTCCACTGATCAACTGCTGCATGCCTAT
ACCGTGATGCGCACCATCCGTGATTTTGAAGAACGCCTGCACGTGGAATTCGCCACCGGCGAGATCCCCGGCTTCG

TGCACCTGTATGCCGGCCAGGAGGCTTGCGCCGCCGGGGTCATGGCCCATCTCAACGATGAAGATTGCATCGCCTC
CAACCACCGTGGCCACGGCCACTGCATCGCCAAGGGCGTGGATGTGTTCGGCATGATGGCCGAGATCTACGGCAAG
AAAACCGGGGTCTGTGGCGGCAAGGGTGGCTCCATGCACATTGCCGACCAGGAGAAGGGCATGCTCGGCGCCAACG
GCATTGTCGGTGCCGGCGCACCGTTGGCGGCCGGCGCGGCGTTGGCGTCCAAGCTCAAGGGCAGCCAGGGCGTGGC
CGTGGCGTTTTTTGGCGACGGCGGTTCCAACGAGGGCGCGGTATTCGAGGCGATGAACCTGGCCGCGATCATGAAG
TTGCCGTGCCTGTTTGTCGCCGAGAACAACGGCTACGCGGAAGCGACCGGTTCCGGCTGGTCGGTGGCGTGCAAGG
ACATTGCCGAGCGCGCCGTGGGTTTTGGCATGCCGGGGGTGATCGTCGATGGCAATGACTTCTTTGCCGTCCACGC
AGCGTTGGGCGTGGCGGTGCAGCGGGCGCGCCAGGGCGAGGGGCCGACCCTGGTCGAGGTCAAGCTGCACCGCTTC
TACGGCCACTTTGAAGGCGACGCGCAAACCTATCGCGGCCGCGATGAAGTGAAAAACCTGCGCGAAACCCAGGACT
GCCTGGCGCTGTTTCGCCAGCGCTGCAGCGCCGAAGGCTGGCTGGACGCGGCGCAGTTCGAGCGTATCGACGGGGA
AGTCGCGCAGTTGATCGACGACGCCGTGCGCCTGGCCAAGTCCGATCCCAAGCCCCAGGCCGCCGACCTGCTCAGC
GATGTCTACGTCGCCTACCGCTAATAACAACAAGACCCGGAGAGAATTTTATGGCTCGCAAAATCAGTTATCAGCA
GGCAATCAACGAAGCCCTGGCCCAGGAAATGCGCCGCGACAAGAGCGTTTTTATCATCGGCCAGGACGTGTCCGGC
GGCACCGGCGCCCCGGGTGAGCAGGATGCCTGGGGCGGTGTGCTGGGGGTGACCAAGGGCCTGTACCCGGAGTTTC
CCGAGCGCGTGCTGGATGCGCCGCTGTCTGAAGTCGGTTACGTGGGCATGGCCGTTGGCGCCGCCACCCGGGGCAT
GCGCCCGGTGTGCGAGTTGATGTTCGTCGACTTTATCGGCTGCTGCCTTGACCAGTTGCTCAACCAGGCCGCCAAG
TTCCGCTACATGTTCGGCGGCAAGACCACCACGCCCCTGGTTGTGCGCGCCATGTACGGCGCCGGCCTGCGTGCCG
CGGCCCAGCATTCGCAGATGCTCACCTCGATGTGGACGCACATCCCTGGCCTGAAGGTGGTGTGCCCGGCCACGCC
CTATGACGCCAAGGGCATGCTGATCCAGGCGATCCGCGACAACGACCCGGTGATCTTCCTCGAACACAAGATGCTC
TACAGCCTGCAAGGCGAAGTGCCGGAGGAGCTGTACACCGTGCCATTTGGCGAGGCCAACTTTGTACGCGAAGGCA
ACGACGTGACCCTGGTGACCTACGGGCGCATGCTGCATATCGCCCTGGAAGCCGCGGCCAACCTGGCGCGCCAGGG
TATCGACTGCGAGGTGCTGGACCTGCGCACCACCAGCCCACTGGATGAAGACAGCATCCTTGAAAGCGTGGAGAAA
ACCGGGCGCCTGGTGGTGATCGACGAGTCCAACCCGCGCTGCTCCATCGCCACGGACATCAGCGCCCTGGTGGCCC
AGCGTGGTTTTTCGTTTCTGCGCGCGCCGATCGAGATGGTCACCGCGCCGCATACCCCGGTGCCGTTTTCCGATGC
CCTGGAAGACCTGTACATCCCCAATGCGGCGAAGATCGAGGCCGCTGTGCGCAAGATCGCTGACAAGAGGACTGCG
GCATGATCCATACCTTGACCATGCCCAAGTGGGGCTTGTCGATGACCGAAGGCCGGGTCGATGTCTGGCTCAAGCA
GCCGGGCGACCGGGTGGAGAAGGGCGAGGAAGTGCTCGACGTGGAGACCGACAAAATCTCCAGCAGCGTCGAGGCG
CCGTTCAGTGGCGTGCTGCGCCGGGTGCTGGCGCAGAGCGATGAGACATTGGCGGTCGGCGCGTTGCTGGGCATTG
TGGTAGAAGGCGAAGCCACGGAGGCAGAGATCGATGCGGTGATCGACAGCTTCAATGCCGGCTTTGTCTCCAGCAG
CGCCGAGGCGCAGGCGTCGGGGCCGAGCGCGCAGAAGGTCGAGCTGGATGGGCGCCTGCTGCGTTATCTCGACCTC
GGCGAGGGCGGCACGCCGCTGGTGCTGATCCATGGTTTTGGTGGCGACTTGAACAACTGGCTGTTCAACCATCCGG
CCCTGGCCGCCGAGCGCCGGGTGATTGCCCTGGACCTGCCGGGGCATGGCGAATCGGGCAAGCACTTGCACAATGG
CGATGCCGAGGAATTGAGCCAGGCGGTGCTGGCGTTGCTCGATCACCTCAACCTCGAACGCGTGCACCTGGCTGGG
CACTCCATGGGCGGGCTGGTTTCGCTGACGGTGGCGAGCCAGGCGCCGGAGCGCGTAGCGTCCGTGGTGCTGATCG
CCAGCGCCGGCCTGGGCGCGGATATCAACGGCGATTACCTGCAGGGATTCACCGAGGCCAATAACCGCAACGCGCT
CAAGCCGCAGTTGGTGCAACTGTTCAGTGACCCGGCATTGGTCACGCGGCAGATGCTTGAAGACATGCTCAAGTTC
AAACGGTTGGAAGGAGTGGATCAGGCGCTGCGCCAACTCAATGCCAAGCTGTTCGATGGTGGGCGGCAGATGCTGG
ATTTGCGCGCGGTGGTAGGGCAGCAGCCGAGCATGGTGATCTGGGGCAGTGACGATGCCATCATCCCGGTGCGGCA
TGCCCAGGGGTTGCAGGCCCAGGTGGAGGTCTTGCCGGGGCAGGGGCATATGGTGCAGTTGGAGGCGGCGGAGCAA
GTGAATCAGTTGCTGAGTCTCTTCCTCAAATCTCAAGCCTGATAGCAATCAAATGTGTGAGCGGGCTTGCTCGCGA
ATACGGTGGGTCAGTCAGACATTCATTGCCTGACTCACCGCTTTCGCGAGCAAGCCCGCTCCCACAGGGTTGACCG
TTTATCAACCATGACGCTTGAGGGTTTCGCTGGGCAGTTCCTTGAACAGCGCCCGGTAGCTATTGGAAAACCGCCC
CAGGTGCCAGAACGACCAATTCATCGCCACCTCGGCCACCGTGGTGGTGGCTGAACTCAACAACTCGCGCCGCGCG
CCGTTCAATCGGCGCAGGCGCAGCCATTGTGCCGGGCTCATCCCGGTGTAGGTCTTGAAGCCCTGCTGCAACTGCC
GCAGCGGCACACCGGCAATCTGCGCCAGTTGCAGCAGGTTGACCGTTTCATCCGGCGCATCTGCCGCCCACTCGCC
AATGCGTGCCATCAACTGGCGTTCTGCGCCGCGTCGCTGCAATGAGTGGCGGTCCAGGCAGACACAGGCGTTGTCG
AGAATAAACAGGCAATCGTCCAGCAGTTGCCGGGTGAGGGCGTCGCGGCTGATCGGGTCGACCGCACAGGCCAGGC
GCGTCAGGGTGCCACTGAGCCAGCGACTGAACAGCGCGTTTTGCTGGCAGGTCAACGGGGCCATGAACAAACTTTC
GAGGCTGGCCACATCCAGGCCATGGCGCTGCACAAATTGCGGGCCGAACACCACCGCCACTTCACGGTAGTTTTCC
GGGGTGATCCAGGTATTGCGGCTTTCGCCATTGAGCAGATACAGCGCGTTGTCGCTGCCGTCGAAACAGAAGGCGA
GTGAGCCCGGGGGCGCGTTGAAGTGCTGCTCGACACGGGTATTCATGCACTCTTCGTACACCTGCACACCCTGCAG
GTCGAGGTAGCGCACCTGCCCGGCAAAATGCCCTGGGGACATCTGTTGGTACTGCTGCACCCAACCGGGTGTTGCA
CTGCATTGAGCGGCCACATCACCGGTGGTGAAGGCCTGTACGCGCAAAGCTGTTGCCTGTGTCATGGGTGATCCGT
GCGCACTCTATTGGTGCGTTGTGGTTCGTGCAAAGTGGATAGATGCCCTTCGAAGGCTGGCCCAAGATAGACCTCA
ATGCGCCGGGAGTACAACTGGGCGCCGCATCCAACCCATGCCGAGGTCTTTATGAATGCCCCTTTCGATCAGCTGT
CCACCTGGCTGAAAGAACACAAGATTACCGAAGTCGAATGCGTGATCAGCGACCTGACGGGCATTGCCCGCGGCAA
GATCGCGCCCACCAACAAGTTCCTGCATGAGCGCGGCATGCGCCTGCCGGAAAGTGTGTTGCTGCAAACGGTAACC

GGGGACTTTGTCGACGACGATATCTATTACGACCTGCTGGACCCGGCCGATATCGACATGATCTGCCGCCCGGTGG
CCAACGCCACCTACGTGGTGCCGTGGGCCATCGAACCCACCGCCATCGTGATCCACGACACCTTCGATAAACAGGG
CAACCCCATCGAACTGTCGCCGCGCAACGTGCTGAAAAAGGTCCTGCAGCTGTATACGGACCAGGGCTGGCAGCCC
ATTGTCGCGCCGGAAATGGAATTTTACCTGACCCAGCGCTGCGAAGACCCGGACCTGCCGCTGAAAACCCCGGTGG
GCCGTTCCGGGCGTGCCGAGACCGGGCGCCAGTCGTTCTCCATCGACGCGGCCAACGAGTTCGACCCGCTGTTTGA
AGATGTCTATGACTGGTGCGAAGCCCAGGGCCTGGACCTCGATACGCTGATCCACGAAGACGGCCCGGCGCAGATG
GAAATCAACTTCCGCCACGGCGACGCCCTCGACCTGGCCGACCAGATCACCGTGTTCAAGCGCACCCTGCGCGAAG
CGGCGCTCAAGCACAATGTGGCCGCGACCTTTATGGCCAAGCCTGTGGCCGACGAGCCGGGCAGCGCCATGCACCT
GCACCAGAGCGTGGTGGATATCGCCACCGGCCAACCGGTGTTTGTCGACGCTTGCGGGCAGATGAGCCAGCTGTTC
TTGCAGCACATTGGCGGGCTGCAGAAATACATCCCGATGCTGCTGCCGATGTTCGCGCCCAACGTCAACTCGTTCC
GCCGCTTCCTGCCCGACACCTCGGCGCCGGTCAACGTGGAGTGGGGCGAAGAAAACCGCACCGTCGGCCTGCGCGT
GCCGACCTCCAGCCCCGACGCGATGCGCGTCGAAAACCGCCTGCCGGGGGCCGATGCCAACCCCTACCTGGCCATC
GCCGCCAGCCTGCTGTGCGGCTACCTGGGAATGATCGAAAAGATTCAGCCAAGCGCGCCGGTGGAAGGCCGCGCTT
ATGAACGCCGCAACCTGCGCCTGCCAATCACCATCGAAGATGCCCTGGCGCGCATGGAAGCCTGCGACACGGTCAA
GCAGTACCTGGGGGACAAGTTTGTGCGGGGGTACGTCGCGGTGAAACGTGCCGAGCATGAGAATTTCAAACGGGTG
ATCAGCTCCTGGGAGCGTGAGTTCCTGCTGCTGAGTGTCTGACTCCACCACCAACCATAAAAAAGGTGTCGATATG
CGTCTCGTCAAACAGCTTCTCCCGTTGGCCCTGGCCGCAGTGTTCAGCAACGCCAGCCACGCCGCACCGACGGTCA
GTGTCTACAACTGGACCGATTACATCGGCGAGACCACCCTGGCCGACTTCCAGGCCAAGACCGGGATCAAGGTGGT
CTACGACGTGTTCGACTCCAATGAAACCCTGGAGGGCAAGCTGCTGGCGGGGCGTACCGGGTATGACGTGGTGGTG
CCGTCCAATCACTTTTTGGGGCGCCAGGTAAAGGCCGGGGCGTTCCTCAAGCTGGACCGCGCGCAATTGCCCGACT
ACCCGAACCTGGACCCCAAGCTACTCAAGCTGCTGGAGCAGAACGACCCCGGCAATGCGCATGCGGTGCCGTACCT
GTGGGGCACCAATGGCATCGGCTACAACGTCGACAAGGTCAAGCAGGTGCTGGGCATCGACCACATCGACTCTTGG
GCAGTGATGTTCGAGCCCGAGAACCTGAGGAAGCTGCAGCAATGCGGCGTGGCGTTCCTGGATTCGGCGGATGAAG
CGTTCCCGGCGATCCTCAACTACCTGGGCATGGACCCGCGCAGCGAGAAGGTCGGCGACTATGAAAAGGCCGAGGC
CAAGTTGCTGACCCTGCGCCCGTACATCACCTATTTCCATTCATCCAAGTACATCTCGGACCTGGCCAATGGTGAT
ATCTGCGTGGCCTTCGGCTACTCCGGTGATGTGTTCCAGGCGGCCAACCGCGCCAAGGAAGCCAAGAACGGGGTGA
ACATCGCCTATTCGATCCCCAAGGAGGGCAGCAACCTGTGGTTCGACCTGCTGGCGATTCCCGCAGACGCCACCAA
CCCGAAAGAGGCCCACGCGTTTATCAACTACCTGCTGGACCCCGAGGTCATTGCCAAGGTCAGCACCTATGTCGGC
TACGCCAATCCCAACCTGGCGGCGAAGGCCTTCATGGCACCGGAACTGGTGAATAATCCCGAGGTGTACCCGCCCC
AGGAGGTGCTCGACAAACTGTACATTTCCACCACTCCCAGCCCGGCCATCATGCGTGTGATGACCCGCGCCTGGAG
CAAAGTGAAGTCCAACCGATGAACCAGTCCCAAGACCACGCCCGCTCCTATTACCTGGCTTCGGCCAATGCCATGC
CCCAACGCCCAGCGCTGGCCGAGGACTTGAGTGCCGATGTGTGTGTGATCGGCGGCGGCTTTACCGGCGTCAACAC
CGCCATCGAACTGGCCCAGCGCGGCCTTTCGGTGATCCTCCTGGAAGCCCGGCGTATTGGTTGGGGCGCCAGTGGG
CGCAATGGTGGGCAGTTGATCCGGGGGATCGGCCATGATGTCTCGGGCTTTGCCCGGCATGTGGGCGAGGAAGGCG
TGCGATACCTGCAACGGGCCGGGATCGATTCGGTGGCGTTGGTGGGTGAGCGTATCCGTGAGCATGGAATCGACTG
CGACCTGCGCTGGGGCTTTTGTGAGTTGGCCAATACGCCGGCGCAATTTGCCGCGTTCAAGGGCGAGCAGCAAAGC
CTGGCTGCTCTGGGTTATAAGCCTGAAACCCGGCTGGTGGCGGCGCAGGATATGGCGCAGGTGGTCGGCTCTGAGC
GGTACGCCGGTGGTTTGGTGGACATGGGCTCGGGCCACTTGCATCCGCTCAACCTGGTGTTGGGCGAGGCCCGCGT
GGCCGAGGCGCTGGGCGTGCGGATTTTCGAGCAGAGCCCAGCGCTGCAACTGAGCCATGGCGACACCGTGCAGGTG
CGCTGCGCCAGCGGCACTGTGCGGGCTGCCAGCCTGGTATTGGCGTGCAATGCACACCTCGAGGAACTGGAGCCAC
GCCTGAGCGGCAAGGTGCTGCCGGCCGGCAGCTATATCATCGCTACCGAGCCGTTGCCGGCCGCGCTTGCCGATGA
ACTGATCCCGCAGAACCTGGCGCTGTGCGACCAGAAAGTCGGCCTGGACTATTACCGGCTCTCGGCGGACCGTCGG
TTGCTGTTCGGAGGTGCCTGTCATTACTCAGGGCGCGACCCGGTGGATATCGCCGCTTATATGCGCCCGAAAATGC
TCAAGGTGTTTCCACAGTTGGCCACTACCGCCATCGAGTTCCAATGGGGCGGCAAGATTGGTATTACCGCCAATCG
CTTTCCCCAGGTCGGTCGGTTGCAACAGTACCCCAACGTGTTTTACGCCCAGGGCTATTCGGGGCATGGGCTTAAT
GTCACCCACTGGTGCGCGCGGCTGCTGGCCGAAGCGATCCATACCGGCCACAGCCAGGGCCTGGAGACCTTCAGCC
GGGTGCCACATATGACGTTCCCGGGCGGCAAGGCCTTGCGCTCGCCTTTACTGGCGCTGGGCATGTTGTGGTACCG
GCTGCGGGAAATGCTGGGCTAGGGCTTGTGAGCGGGAGGGCGGTTTAAAACACAGCATGATCGGATCCAATACGCT
ATAAACCCTCGGTCGCTGTGTAAATCCGAGGCCCCGCTTTTCATGCAGAACCCTTTGCAACGCCCGCTGTGGCAGG
TCTACCTGTTCTTCCTTGCGCCGATGGTGCTGTCCAACTTCCTGCAGAGTTTTTCCGGCACCCTCAACGGTATCTA
TGTTGGGCAGATGCTCGGCACCCAGGCGCTGGCGGCGGTGTCGGGGATGTTTCCCATCGTGTTCTTTTTTATCGCC
CTGGTGATCGGCCTCGGGGCGGGCGCTTCGGTGCTGATCGGCCAGGCCTGGGGCGCGGGCGAAACGGCGTTGGTCA
AGGTCATCACCGGCGCGACCCTGACCCTGGGCGCGTTGATCGGGCTGATCGCTGCTGTGCTCGGCAGCCTGTTCGC
GCGTCCGGCGATGCAAGCTTTGGGCACGCCGTTGGATGTACTGGACGACGCGGTCGGCTATGCCCAGGTGATGATG
CTGATCATGCCGTTGTTGCTGGTGTTCATTCTCTACACCCAGCTGCTGCGGGGCGTGAGCGATACGGTGTCACCGC
TGCTGGCGCTGATCGTTTCGACGGTGGTTGGCCTGTTGCTGACCCCGGCCCTGATCCGCGGCTGGCTCGGGCTGCC
GCCCATGGGGATCCAGAGCGCGGCCTATGCCGGCCTGGCGGCGAATGCCGTGGCCATGTTGTTTCTGGTGCTGCGC

CTGCGCCACAAAAACCATGTGATGGCGCCGGACCGCCTATTGCTGGCGGCCTTGCGCCTGGACCGGGCGATCCTCG
GCAAGGTCCTGCGCATCGGCCTGCCCACGGGCTTGCAGATGGTGGTGCTGTCGTTGTCGGAGCTGGTGATCCTGGC
ACTGGTCAATGGCCACGGCTCCCAGGCCACAGCGGCGTATGGCGCGGTCACGCAGATCGTCAACTACGTGCAATTT
CCGGCGCTGTCGATTGCCATCACCGCCTCGATCCTCGGCGCCCAGGCCATCGGCGCCGGGCGCCTGGAACGCATCG
GGCCGATCCTGCGCACGGGGCTTGCGATCAACCTGTGCCTGACCGGTGGCCTGATCCTGCTCGGCTACGCCTTGTC
CCACTGGCTCCTGGGCCTGTTCATCACCGATGACCAGGCGCGGGTCCAGGCCGAACACCTGCTGCACATCATGCTC
TGGAGCATTCTGGTGTTTGGCTTCCAGGCGGTGATAGGCGGGATCATGCGCGCCAGCGGCGTGGTCCTGATGCCGG
TGGCGATCTCGATCTTCTGTGTGTTGGGCGTGGAGCTGCCGGTGGCGTATCTGCTCAATGCACGCTTCGGCCTGGA
AGGGGTGTGGATGGCGTTCCCGGTGACCTATTTGGTGATGCTTGGTTTGCAGACCGCGTATTACCGGTTGGTGTGG
CGGCATAAGCAGATTAAGCGGTTGGTCTAGAAGCTGGATTGGCGACGGCAAGCAATTGCTCTGCTTAATAGGAAGC
ATTACTATTCACGCCCCGCCTATCCAGTGCACCCCCGATGACTCCCCAGCCGCCCCGCAGACCAGGCTTTTTCGCG
TACTACGAAGAGTTGGTCGGGACCTGGACGCGTCGCCTGCGCAATCGCCAACAGGCCGAGGATCTGGCCCATGACA
CGTTTGTGCGGGTCTTGGAGTCCAAGTCGGCGCTGGTCGAGCAGCCGCGTGCCTATTTGCACCAGACGGCGCGCAA
TATTGCGGTGGATGCCTATCGCCGTGCAGATCGGCACGACGCGGTGGCGTTGCAGGCCTTTGAACCGAGTGCGTCG
CACAGTGGCGACCCGGAGCATTTCATGCACGCCGTGCAGTTGGCGGACTCCATCGAGCGGGCCCTGGCTGAATTGC
CCCTCAATTGCCGCAAGATTTTCATCTGGCAAAAGATCGAGGGCCTGACCCAGCAGGAAATCGCCGAGCGCCTGGG
GCTTTCTAAAAACATGGTGGAAAAGTATATGATCCGCACCCTGCGGCATCTGCGTGACCGCCTGGACGCGCTGGCC
CCATGACCAATGCCCGCTTTGAAACAGGATCTTCCATGATGGATACCCGTGAGTGTGCCTGTGGCAGCGCAACGGT
GCGTGACGAGGCGGCGCAGTGGTTCGTGCGTTTGCAGGACCCACCCTTGAGCGTTGAGGACCAGCATCTTTTTGAC
GCCTGGCGTGGGGAGCATCCTGCGCACCAGTATGAATTCGAGGTGCTGCAAGGGCTGTGGAGCGTCGCCGACCTGC
TGCCCAGGCACCGTTTGCAGGCTCTGGTCGAGACGCCTGTACCGCGCAAGCGCCGTGCGCTGGTGCGCTATGCCAT
GGCAGCGAGCGTGCTGGCGGTGGCCGTGGGCCTGGGGTTGTTCAGCGGCCTGGACCATCCGGCGGTGTACGACGCA
CAGTTCAGCACGGTGTTGGGTGAGCGTCGCCAGGTGGCGTTGCCCGACGGCTCGCTGATGGACCTCAATAGCCGCA
GCGTGGTGGCGGTGCACTACGAAAAGGGCCAGCGTGGGATTGAGCTCAAGCAAGGCGAAGCGCTGTTCAGCGTCGA
GCACGACAGCAGCCGACCGTTCGTGGTGCAGGCTGGGGCAGGGCAGGTGACGGTCACCGGTACCCGGTTTGATGTG
CGCCGCGACGATGACCAGACCCGCGTGGTGGTCGAGGCCGGAACGGTCAAGGTGCAAGGGCATGCCGCGCAGCCGG
TGGTCACACTGACGGCGGGCCTGGGTACATCGATCGACAATCAGGGCGCGGTCGCGGCGGCCCACCCGGTCAACGC
CCAGGCCCTGCTGGCTTGGCAGACCGGCAAGCTGGTGTTCAACGACGCCAGCCTGGCCGAGGTGGCGCGGGAAGTC
TCGCGCTACCGCGAAAAACCCTTGCGGGTCAACACTGCGGCCAGCGAGTTGCGCCTGACCAGCGTGTTCAAGTCCA
ACGACACCGATGCCTTGCTCAAGGCGTTGCCGCACATCCTGCCGGTGGCCCTGCGCACGTTGCCTGACGGTAGCCA
GGAAATAATTTCACGCTAGGTTCAGGTTTTTTTCGAGTTCTTCGTCTTCTTGTCCAACTGCAACTGGTTTGCATTA
ACCGACGCGCTCTCTTGGCATCACTGGACTAGGTACGACGTGAAAACACTCGCTGCGAACAACAATAAAACCTCCC
GTTGGGCCCCCCTTGCCCTGGCCTTGGCAGTCACTGCCGCCATGCCTGCGGCCTACGCCGACGAGGCCATTCATAT
CAAGGCCCAGCCCCTGGGCGCGGCGCTGAGCCAATTGGGCCAGCAAACGTCGCTGCAAGTGTTTTTCAGCCCGCAG
ATGGTGGCCGGCAAACACGCCCAGGCCGTCGACGGCAACCTGTCGCCGGAACAGGCCCTGCGCCAGCTGCTGCAAG
GCAGTGGCCTGGACTATCAGATCGACTCGGGCTCGGTGACGTTGCATCCAGCCTCCACGGGCACCGGTGAAGCCGG
TTCACCCCTGGAATTGGGCGCCACCGCGATCAAGGTCGTGGGCGATTGGCTGGGCGACGCCAATGCCGAAGTGGTG
CAGAACCATCCCGGCGCGCGTACCGTAATCCGCCGCGAAGCTATGGTGGAGCAGGGCGCAATGAACGTCGGCGACG
TGCTGCGCCGGGTTCCCGGGGTGCAGGTGCAGGAAGCCAACGGCACCGGCGGCAGCGATATTTCCCTCAACGTCGG
CGTACGCGGCCTGACGTCCCGCCTGTCGCCGCGTTCCACCGTGCTGATTGACGGGATTCCCGCGGCGTTCGCCCCC
TACGGCCAGCCGCAGTTGTCGATGGCGCCGATTTCCGCCGGCAACCTGGAGAGCATCGATGTAGTACGCGGCGCCG
GTTCCGTGCGTTATGGACCGCAGAACGTTGGCGGTGTGATCAACTTCGTGACCCGCGCGATCCCCGAGACCTTTTC
CGGCGAAGTCGGCACCACCCTGCAAACCTCTTCCCATGGCGGCTGGAAGCATGTCGACAATGCCTTTATCGGGGGG
ACCGCAGACAACGGCATCGGCATGGCGCTCTTGTATTCCGGGGTCAATGGCAACGGTTATCGCGACAGCAACAACG
GTAACGACATCGACGATGTGATCCTCAAGACCCACTGGGCACCGACCGATCAGGATGACTTCACACTCAACTTCCA
TTACTACGACGCCCGGGCCGATATGCCGGGAGGCCTGACCCAGCAGCAGTTCGACGCCAATCCTTATCAGTCGGTG
CGCGACTGGGACAACTTCAGCGGCCGGCGCAAGGATGTGTCGTTCAAGTACATCCGCCAGGTTGACGACCGTACCC
AGGCGGAAGTGCTGACGTACTACTCCGACAGCTTTCGTGGCAGCAACATCGCCAGCCGTGACCAGCAGACCCTCGG
TTCGTTTCCTCGCACCTACTACACCTTTGGCATCGAGCCACGGGTGTCCCATGTGTTTGACCTGGGCCCGACCAGC
CAGGAAGTCAGCGTCGGCTACCGCTACCTGAAAGAAGGCATGCACGAGCAGGCCAGCACTCTGAGACTGGTCAACA
ACGTCCCCACCCCGGTCGGTCAAAATGACGGCCACGTTTATCAGGACCGTACCGGTGGCACCGAGGCCAACGCGTT
CTACATTGACGACAAAATCGACGTCGGCAAATGGACGATCACCCCAGGTATCCGCTTTGAGAGCATCCGCACCGAA
TGGCACGACCGCCCGGTAGTGGGCTTGAACGGCACGCGTACCCAGGAGAAGCACCGCGAGATCAAGAACAACGAAC
CGCTGCCGGCCCTGAGCGTGATGTACCACGTCTCCGATGCCTGGAAACTGTTTGCCAACTACGAAACGTCCTTCGG
CAGCCTGCAGTATTTCCAATTGGGCCAGGGCGGTAACGGCGACCAGACAGCCAATGGCCTGCAACCGGAAAAGGCC
AAGACCTACGAGGTCGGCACCCGCTACAACGATGAGGTCTGGGGTGGCGAAGTCACGCTGTTCTATATCGACTTCT
CCGATGAGCTGCAGTACGTCAGCAATGACGTGGGTTGGACCAACCTCGGCGCCACCAAACACACGGGTATCGAAAC

CTCGGCCCACTACGATCTGTCCAATCTCGACCCACGCCTGGAGGGCTTGAGCGCCAACGCCGGCTTCACCTACACC
AGGGCGACTGCCGAGGGCGATGTGGCGTTCAAGGGCCGTGACCTGCCGTTGTATTCGCGCCAGGTGGCGACCCTGG
GGCTGCGATACGCGGTCAATCACTGGACCCACAACCTGGACGCCTACGCTCAGTCCGGCCAGCGCGCACCCGGCAC
CACCAGCACCTATATCACCCAGCCGACCGCTGACGGCCAGTACGGTGATATTCCGGGATATGTGTCGGTCAACGTG
CGCAGTGGCTATGACTTTGGCGCCCAACTGTCGAACCTGAAACTGGGCGTGGGGGTAAAAAACCTGTTCGACCAGC
AGCACTACACCCGTTCCAGCGACAACAACGCCGGGATTTACCTGGGTGAGCCGCGTACGTTCTTTGTGCAGGCGAG
CGTGGGCTTCTGATCAGGTGACGGCTGCGTCGCCGCGCCTCGAACGAGCCGCGGCGATGTGCCTGCTGATCCAAGA
CGGCTGCTGACGCACTTCAGCGTTTTTCCTTGTACCGCCGCGTATAAACCTGACGATTCTTCTGTGCAGCTTGAAG
AGGTGAGGGTTGCATTTCCTACCTTTTTTATGCGGCTTTCATCGCTTTCAGGAACCGAACTCCGTAGGCCTGCCAC
ACACCATCAACCCACCGTGCACGGGTGCCTTGTGATAACTGCGTTGTCGAGGTGCTTGGGTGCGTGAGGTGGACCG
TGTCAACAAGTCGTACTGGAGAACATTTATGTCGGTACCTGCGTTCAATCAGCCAGTTGTCAATCTACCCACTGAC
GACACACGTCAAGGGGCCCCTCAAGGGGCCAGCGCCAAAGAGGCAAAGGCTCCGGTTATCGGGTCGCCTTCCAGTG
GCCCGAGTTTCAGCGCGCCTGTGGCTAAGTCGGGCCCGGTGTTCGCCGGGGCACCACCGTTGGCGCCTGCCGCTGC
GGTGGCCCGCGACCAGGGGGCCAGCCAGGCTTCCACATGGCAGAACGACTCATCTCGGGGAGTTTTCGGCCGTTTG
AAAGGGTTTTTCTTCCCCGCCAGGCCGCCCCATTGCGGGCACATGCCGCACAGGCCTCAGCCGGGCCATGGGCATC
CACCGCACATGTCCCATCCGGGTCACGGGCATCTGCCGCACAGGCCCCATCCGGGCCACGGGCATCCGCCGCAAAG
GCCTCATCCGATCCATGGAGGCCCGCCGCCAAGGCCGTTTCCCCAGCAGCCCGATCCGCACTATTCAAAGCGCAGC
CATGAGCAGTTGGCCCAACAGCTGCTGGATAAATTCCAGGCATTCACGGACCCGAGAAACCCGGGCTACGTGACGC
GGCAAAGTTTGAGCAACATGGCGGCAAGACCGTTGACGGGCAATCCGGGCATGGATCAGAACATCAGGCTGGCCAG
GGAAATCCTGAAGCGGCCGGAGCTCATGGGCGCCCTGGACCTCGACGGGCGCACGGGCGTGCAGGACAACCGGTTC
AGCTGGCAGAACATCAACGATGTTGTACGTTCCAGCAATCCGTTGAAGTTCCAGGATGACAAGCAACTGGCCAGTG
CGATGCTGGAGAACTTCAACAAGTTGAAGGGCGGGTTCTGGAGCCAGACCATCAAGGTCGCTGATCTGCGTGCCCT
GGCCCAGCGTCCTCTGACAGGCAACCCGCAGAGGGATCAACTCATCCTCCTGGCGAAAGAAGTCGTCAGCCGGTCG
AGCCTCTTGCAACAAATGGACAACATCGCAGGCCATCACTACGACGGCCGGATTAGCCGACAAACGCTACGTCAAC
TGATGTCCTAGTCAACCATCGGTACTGCGCAGGTGTTCCAACAGCGGGAAAGGTCGCAGGCTCTGCGCGGCTTTTA
CCGGTGCGCCTGTGCCGTATCAATCGGCAGGAACTGTTTTGGGCTGTTTGCCACATAACGTGTATTCGACGGCCTT
TATGACGCCGACGGTTTCACTCAAAGAGAGGGTGTTGACATGACTTCAACGTCACTTGGAACTCAAACTCCCGGTC
TGCCGCAATCCCTTCTTGCACCCACCCGTGACAATGGCGAGCAGGGTCGACGCTTCGGTGCCCAGGCTCAGCACAA
GCCTGTCGAAGAGGGGGCTGAAGCCCTGCGAGAAACGTTCTTTCCCAGGCAGTACACATTCAAGGCACCGGCGGCG
GATGATTTCGCCAAAGGCCTGCTTCCCCATTGTGCCCCCCATACCTCTGCTGCCCCGATGCCGGAAATGGCGGGCA
CGACAACCGGATTGATCAGTGCATTGAAGGTCTTGCTGGGTGTTTCCAGATAGGTCGCGTTGGGGTGTCGAAAGAC
GGACTGAGGCTGCCCCAGGTCCTGCCAGTTGTTTACATGCAGTCATCGAAATTGATCGGCCGCCCCGGAACCATTG
TGGATGCCAGGCCACATACTTTGCGTTTGTTGGAGCGCCACTATTGCAGTGACAAGGGTGGTAGCCGGTCTGAGTA
GGTGTCTAGAAAAGGTGGATATGAAACATGGCAATTGACGGTGGGGTACCTCTCCTGCGGCCCGGTAGTACGAGCC
GCCATGATGCGATACCGGATGACGCACAACAAAAAGCTCATGCTGCTTCAAGCGAAACCACACCCCGTGGCTGGCA
GGATGATAATCAGCCGTTGCCCAGTACGATTCGTTCTTCGGGTTTCCGGCGTCCCCTGCGCTGGGCCACGCCCGAC
GAGATCACGCTGCATCATCAGGAGCTGAGAAACACGCCGTTGGGGCGCGCAACCGATGTAAACCCCTTTACCCAAC
TGCCTGATAGCCAGCTAGTGACGGCGCTGGGTGAGCATTTTTCCGTGCTGGAAAGATTTCATGAAAAATGAAGGAT
TGAAACTGTCGGCGCTGCGGCAAATCGCCAGTGAAAAAATGACCGACACACCCGCACGCAACAACGCCATCCTGCT
GGCCAGGGCACTTGTCCAGCGCCCGCGGTTGATCGACGCGATTGTCGATGAGCGAGGGGTCATCACGCGGCAAAGC
CTGTCAAAGGCCGTGCAGGCAGTGTTCGGCAACAGCGACCCCAATGCCTTCAGCTCGGACCCTTTTCACGCCAAGA
CCAATGTCGAGCTGGTGCAGGCATTCCGGGATGCATTTGATGAACTTCGGGACAGGTCCCGGGACCGCATGAACTT
TTTTGAGCAGGTCGGCTTCGTCCAGATCGAGAGGCTTGTCTCAATCAGCAAGGACCCGGACGAAACGGACACCCAA
GGCACGGTTATCCGGGACCCCGCCACGGGTCTGCCGAAAAAGATGTACAGCGAACAGCTGGTGTACATGTCAAAAA
ACCTGGTGGACCGTCCCAGGCTATTGAACTCACTGGCGCGCATTCATTCTGGCTGGCGACGCATTTATGGCAGTCG
CTACCAGAAGGAGTGGTTGAGCAGCAAAGACCTCGACGGCTGGCTGGAAAACAACAAGAGCCAGTAGCCCCCCGGA
CTCATGACACCCGCAGGATTTTCCCGCTGATCGCCACGCCCAGCAGGAGCACGGCGATCAACACAAAGGCGACGCT
CAAGCTGCTGCCATGGGCGATAAAGCCGATCAGCGCCGGGCCCGCAAGAATGCCCGCATAACCCAGGGTGGTGATG
GCAGGCACCGCAATGTGCTCCGGCATGACCGTTTGCTTACCGACAGCGGTGTACAGCACCGGCACGATATTCGAAC
AGCCGGCGCCAACCAGCGCATAGCCCAGCAGTGCGGTTTCCCACGCCGGGAGCAGCGTGGCCAGGAGCATGCCGGC
GGTGGCCAGCAAGCCACCGATCACGATCACCCGTTTGGCCCCCAGGCGACGAACAATTGCATCGCCGGTCAGGCGC
CCGATGGTCATGGTCAAGGCAAACGCCGCATAGCCCAGGCCCGCATAGGCCTCGTCGAGGCCGCGTTCGTCACTGA
GGAACACAGCGCTCCAGTCGAGCACGGCACCTTCAGCCAGGAACACGATAAAACACAGGCAACCGATAAACAGCAC
CACGCCGTGGGGGATGGCGAAGGCGGGGCCGGAACTTTCACTGCCGTAGGGCAGCAAATGGGGGGCCGCCTTGAGC
AGTGCGGCCAGCGTAATCACGATCACCACCAGGGTCGCCGACAGCGGTGACAGGCCCAGGCCGAGCAAGGCGCTGA
CGCCCGCCGCCCCCACGATGCCGCCCAGGCTGAACAGCCCATGGAAGCCCGACATCATCGTCTTGCCACTGGCCCG
TTCTACGATCACGGCTTGCAGGTTGACGGTGGAGTCCACCGTGCCAAGGCCGGCACCAAACAGAAACAACCCCGCC

ATCAGCAACGGTATCGAGCTGACGGTAGCCAGCATCGGCAAAGCCACGCAGAGGAGAATGGTCCCGGCGCTGAGCA
CGCGTCGGCAACCAAAGCGTGACGCCAGCGCGCCCGCTACCGGCATGGCGATGATGGACCCCACCCCCAGGCACAG
CAGCAACAGGCCCAGCGTACCTTCGTTGAGTTGGGCACGGGCCTTGGCGTAGGGTACCAGCGGTGCCCAGGCCGCG
ATGCCAAAGCCGGCAATGAAAAACGCGATGCGGGTCGACATTTGTTCCAGACGTCCTGGAATCACAGGCGCTGGAG
TGGGGATGGCAGTCATGAAGGTCCTTGATTTTGCGTTCAGCGAACGATGTCCGGCGCGACATCCTTGCACATCAGG
CCGTATCGAGCGAGCCAGGTTCCCGTGGCCCCTGCGAACTTTTTTTTTACAACATGGAACACTTTGCCGGTGAGCG
CCACATAGAGGCTACCGGGCTCGCTTGAGGTGGCCTGGATGGTGCGTAATCCCTGGGAGTGGCGGTGAACCCCAGG
CTGCTTGCAGAGGATTACCACCTTCCAGGCCACTTTGGCGGGTGGTATCCGGGGCCGATATTCCCCACAATCACCC
GGCGCTTTTCCCATGCCGAGGTGGATGACCCCATGACCCTGTTCTACGACGCCCGCGGCAATATCTACGGTGTGGC
CAGCCCGGCCCTGTTACGCAGCCGGGGCATTGCCGTGCCCGATAGCGCCGCCCTGGCCGCCAGCACTCGCCAGGCG
TGGGCGGCGTCGGCGATAGCGTCCGAATGCGGCTGGGGCACGTCGCCGCGTCCAGCCCAGGCCAAGGCCTATCGCT
GCGATGGTTTGCTGGTCGGGCCGTTTCAGGCATTGCCACCGTATGACCTGCTGATCATCAACACCGATGGCTCCCT
GGCCGAGCGCAGCGGCAATGGCTTGACGATTTTTGCCCAGGCCCTGACGGACGCGGGGCTGATGACTGGGGCCTGT
GAGCTGCGGGTGCACCATGACAAACCGGATGCCCCCTCAACGGTGGCGACGTGGGTTGAGCCGGCGGTCCACCAGC
AGGTTGCGGGGTTCTGGTTGACGTTGGGCCAGCCAGCATTCGGGCCCCAGGCAGTGGGGGCCGTGGGGTTGCCGAG
CTACGAGCCCGCCCATGTGAGTGCGCTGGCTGCGATCAACCCGCAATGGGCACGCAGTGAGTTTGTGCGTATCGGC
AACCCCCATTGTGTGACCTTGGTCGAGCACGCATCGGCCTTGCCGGACAATCGGCAAATGCATCAGCCGGGGTTGT
TCGCGCCCTTGAAAGCGATTGCCTTTGCCCCGCCGGCAGGTGATGGCCGGCCTTGTGCTGCCGGGATCAACCTGCA
GTGGGCCGCGCGCGATTCTGCCAATCGGGTGATCGCCCGGGTATTTGAGCGCGGCGAAGGCCCCACGGCATCCTCC
GGGACCAGTGCCAGCGCGGTGGCCTGTGCAGCATGGCGCGCGGGGTGGGTCGAGGCGGGGGAGGTTGCGGTGGTGA
TGCCGGGTGGCACGGCGCCGGTACGTTTGCATGTTCAGGCCGGGACGTTGCTCAGTGTCAGTCTGTTCGGGGCCGC
AACGCTTCAAGGCTGAGGACGCAAGTATTCGGTAGCTTAAATAAGTGTCAGTTGTTTCCTTATTGTTTAGTTATCG
CCTGATGTATTACGTAAGTGATTTCTTGTTATTAATGCATGATTTGTTTTGCGGATTTCTCATTTTGTAAAAGAGA
CTTCCACCTTGTTCGTACGAAACTTCTTTTTTATTTCAAAGTGCCGAATGTTCGCGGATCTCGCTAATCTTTAGA
GCGTTCAGGCACTTTCTTTGGCCGCGCGATTGAGCTTGGCGGGGGAGAGTATTTCAATCAGATAGCGGACGGCGGG
TAACAACGGATGGTTAATGCGCTTGTTTCGTCACGTTTCAAATTTATCCTGCAAAGTGTGGAAAATGACTTTCAAG
TCCTGGCATTCAAAGGCCGCGAAGCCCTTGACCAGCCCTACTTCATCGAGGTGCAGTTGGTCAGCGAAAACCCTGG
CCTTGATCTGGAAACATTGCTGCACCAGCCGGCCTACCTGGGCTTCGGCGAGGAGGGCGCAGGCCTGCATGGGCAG
GTGTATGCAGTCGGCCGTGATGACCCCGGGGCCCGGCTCACCCGTTATCGTCTGACCCTGGCACCGCGCCTGGCGT
GGCTGGAACACCGCCGTGACCTGTGCATCTTTCAACAACGCAGCGTGCCGCAGATCATTGCGGCCGTACTCGAACG
CCACGGTATCCTGGCCGACGCGTATGCCTTCGAATTGGGTCCGGTGGTCTATCCGCCACGTACATTCTGTGTGCAA
TACGCAGAAACCGACCTGCACTTTATCCAGCGACTGTGTGAAGAAGAGGGAATTCACTACCACTTTCGCCACAGCC
CGCACGCCCATGTGCTGGTATTTGGTGATGACCAGACGGTGTTTCGCCGCTTGCCTGTGCAGCGTTATCGCCCGGC
CAGCACCGAGGCGCGGGGCATCCAGCGCTTCGATGTGCGCCTGGCCACCCGCAGCCGCCAGGTCGTGCGTCGTGAT
CATGACTTCGAGCACCCTGCGCGGCGCCTGCAAGCGCAAGCCGGGGCGCCATCGGTGCTACCGCTTGAGGACTATC
ACTACCCCGCAGGTTTTACGGGGCAGGCACGCGGCGTGCAACTGGCGCGTCGCAGCCTGGAGCGCCATCAGCGTGA
CCATCACCGGGCCTTGGGCAAGAGTGATCAACCGGCGTTATGCAGTGGCCATTTCCTGCCGTTGAGCGATCACCCG
CAGCCCTCCTGCAATGACTTGTGGCTACTGACTTCAGTGCGTCACGAGGGGTATCAACCACAGGTGCTGGAAGAGG
CGATGCCGGGCAGTGAAGTGTTCCAGGGATATCGCAACCGCTTCAACGCCACACCCTGGCGCGCGGTCCACCGCCC
GGCGCTCAAGCACCCCAAACCGACGATCACTGGTAGCCAGACGGCCACCGTGACGGGGCCGGTGGGCGAGGAGGTG
CACTGTGATGCCTATGGTCGGGTGAAGGTGCGGTTTCACTGGGATCGGTTGGACAAGACGAGTGATAAAAGCAGTT
GCTGGGTGCGAGTGGCCTCCGGGTGGGCTGGTGACGGCTTTGGCGCGATCATGACGCCCCGGGTCGGCATGGAGGT
GCTGGTGACGTTCCTGGACGGCGATCCTGATCGGCCTTTGATCAATGGCTGCCTGCCCAATGCGCGGCATCTGCCC
CCTTATCCGTTACCGCAACACAAGACCCGCAGTGTATTGCGCAGCCGCAGTTCCCCCGGTGGCGCGGGCGCCAACG
AACTGCACCTGGAGGACCACCTGGGCAAGGAGCTCATTTACCTGCGGGCCCAACGGGACCTGCAGCAACAGGTGGG
GCATGACTGCCACCTGGACGTCGGGGGCGAGCGGCGTGAAACCATTCGCGGCCTGAGCACGGCGTACCTGGAAAGC
GAGGAACACCGCGACGTTCGCGGCAATCGCAAAGTCCTGATCGGGGCCGGCGATCACCTGCAGGTAAAGGGCGACA
GCCAGGCCTACATCGGCCAGGCCCTGGTGATCGAGGCGGGGCAACAGATCCATCTCAAGGCGGGCGCCAGCCTGGT
GATCGACGCGGGTGCGCAGTTGAGTTTCAAGGCGGGAGGCGAACACCTGCTGATCCAGGCCGGCGGAATTTTCAGC
AGTCGCCCCATCACCCAGGGTGGCTTGCCAGGGGTCGCACGCTCGGCCAACCCCGTGGGCCCGGGCGATAACCCAT
CATCGCCTCCGCGCGCCGCGGGTGTTCCCTCTATTTCAGTTATCCAGGGCGAAATCATGCACAGGGCGAGGCAACT
GGATGCGGATTTTTGCCCAGTGTGCGAGCGCTGCCGCGAAGGTATGTGTGACATTCGCGGGAGGGTCGCGTGATGT
CGCCCGGCCAATGGATGGCCCAACAGCAGCAGGCAGGGCGCCGCTTGTGTCTGGTCCTCGAGGGAAGCAGCGATGC
GCGCTTGCCGTTGATAGCGTCCCGCAACCTGGCGCAATGTCTTAGCGTGTACGGTGGCAGCGCAGTGGCGGCGCTG
GCGGCTGCAGGGCCGGTGATCCTGCTGGTGGAGCAACTCGGTGAACCCGCGCTGGTGAGCTTGCTGGAGCACCCGC
AGGCGAACTGGGGCTGGCTGGGCAGCTTGCCCGACACAGACTTGGGTGGCGTTATCGAGCATTGGCACGCGCGGAT
GCTGGTGGGGCCGCCGGGCAAGCAGGCCCTGTACCGGTTCCACGACAACCGCACCTTGGCGCGAGCGCTTGCCTGC

TTGCCGGTAGCGCAACGGCCAGCCTATCTCGGGCCGCTGATCAGTGTGTGCTACTGGTATGAAGACCAATGGTGCA
GTGCCAGCAACCCTGCGCCCGGCCATTACCCGGTACCTGATCCCGCCCCATGGCTCAGCATTCCCAACCCCCAGGC
CTCGGCCATTCTGCACGCCAATATTCTGCGCTACCTGCTGGCTGAGCACAGTGAAGATCTCGCGGCATTGGCTGAA
TTCCAGGACCCGAGGGTATGGCTGGCCCAGGTACTGGAACAGGCCCGCACCTGGCAATGGCGCGGGGCGCAACAAC
TGGAGTTCTTGGTGGTACGCCGGTTGGAGGAAGCGACGGGGGCCAGTGTGATCCGTTGGCAGCCGTTGGCGGGTGA
GGCGCCGGGAGATCACTTTGCACGGGTGTTGGCGCAGTGGCACACCTTGAGGGAAAAACATGAGTAGGCGGGCGTG
CGGTGCGTTACTGGTCGTGGGCATGGCGTGGTTGGCGGGATGCGCAAGTGTGCCCGCAGAGCGTTTTACCTTGCAG
GTAGACCTGCCGGCCAATTTTCGATTCAAAAGCGCGGCCAACTACAGCCCCGCCGCAGGCGAAAGCTGCACCTTGC
CAGTACGCCGAGGCAAGAGGCCAGAGCGCAAGGTCTTCAGCGTTGCGTATAACACCGCAGCCAGCCGCGTGACCCA
GGACCTACCGCTGACTGAAACGGTAGAAGGTTGCCCGTTGGTGTTGCGCAGTGTGGAGTTTGATTTTTACGCACGG
TGGGGAACGCGGGACACGGATATCGGGGGGGATATTGCGGCGATCTACATTCAGGACCGGGCAGGAGGGGAGACGC
CTGGGTTGGAGACGTTGGAGTTGAAAGGGCGATGTCAGTGGTTGTTTCGTACCCTCGGAACACAGCATGCCATCAG
GAAAATTCTTAAATGCAATTCACTGGACAACGAAGGGCAACTACAGAAGACACGGGCAGGTGGCGTAGTACTACAC
GATCAACTCCCCGGAAATACACTTAACTTGGTGATGGAAGTTTCGGACGAAGAGAGGCCTTATATGGGGAGTACCT
GGATCCGTTTCCCTCACGGGTGGAAGCGTTGTCTGGGGAAAAACATGGACGATCAATATGGTTTTTGCGCGAAAGA
TGCTCCCCCTTTCAGGTCTTTCAAGATGCCTGACGGGCGTGAATGCAATGTTTACCCAACCTGCACTGAATAAGGA
TATTCAGCTATGGCTATCGAAGAATGGAAGGCACCGTTTTTCAGCGAAAAAATGCCGGCTTGCCCGTTACGTGGAT
GTTGGCTGAGTTTTTCCCTGGTTGATGAAACCGGCAATGGGAAAGCCTATGGAGGGCTTACCTATTCGGTCCTGGA
TAGTAACGGACAGCGGCACATTGGGCGCTTGGACGGTGATGGCTATGTGAAGCTGGCCAACCAATACAGAGGCCCA
TTGGTTCTGACTTTCGAGGCGGTTCATGAGTCATCTGGAGCTGATTATCACGGAGTATTGCAAGCGCGAGATCACT
ACCCCTTACCTATCACCGAGCTTCAATTTCGAGCTGAACATACTCGATTTGTGCGCAATGACGGCCTGCGGGTCGA
GCAAAATCCGGCCCAGGAGCAAGCGAATCGTTTCTACCAAGTCGAAGTCCGCGACCTCGTCCGCCATGCCGCCCAT
TTGCCGCCTCCGGCGCCCCGGCATTTCGCCCCCCGACGCCATGCCCTGAAAATGATGGCCGACCTTGGATTCGGCC
CACCGCAGCCTGCATTGTCGGGTGTGGTGCTATTCCCCAACCAGCACAGCGTACTGGAGGTCCGGCCGTTGCGGGC
GTTGCGCCCGATGCTGTCCACCGAGGATGGGTTCTGCGCCCTGAACCTGTACCAACTGGCATTGATGGCGACCTTG
AGCTACTGCCCATTTGGCCAGGAGCCGGCCGCCCAACCACAAGATGAGGTCCGTTTTCCCGTGGACCCAAGCATTG
GCCATCTGTTTGCCGAAAAACTCTCGGGGTATGAGCAAGCCTGGAGGGTCGACGCTGGGCAAGTACAACGGTTTTA
CCCGCTGTATGAAGAGGTCGCCTATTCCCGGCGTTTCGAGATCCTGCCCTTCGATCCCCAGTTGTATCCGCAAAAC
AGCCCGAAGCTCGCGGGCGAGCAGGAGCATCCGGCGAACCTGCATTTTTTCGATGATGAAGAGCTGGGTACTGATA
CCCAGGCCTTTATCAGTCATCACGATGAGATCGTGCTGATTTCTGTACGGGGTACGGCCAGTGCCGCTGATGGCCT
GCGTGATGCAAACGCCCACCAGGTGCCGTTCGCGGAGGGCATCGGCAAGGCCCATGAAGGTTTTTATCAGGCCTAT
CGAGCCATGCGCGACTTCGTGCTGCGCTACCTCGATCAGTTCTACAGCGGCCAACGTATCGTGATCTGCGGCCATA
GCCTGGGAGGGGCAATCGCCCTGCTGCTGGCAGAGGGACTGCGCCGGGTCAAAGATGCCGACTACAACGTGCTCCT
CTACACCTACGGTGCGCCGCGTGCCGCTGACGCGGAATTTATCAGAGGCGCGGCAGACCTGGTCCATCACCGTATC
GTCAACCACAATGACCCGGTTCCCAGCGTGCCGGCACCCTGGATGAACACCACGGCCAAACTCTGGGTTCCCGGCG
CGGTGACGATGTTCAGCGCCCCCGCGCCCGGAGGCCTGCTGTTTGCGGCGGGCCTGGTACGCGTCGGTGGCAATCC
TTACTGGCACCACGGCGAGCAACAACACTTCATGCCGATCCTGCTCCCGGACGGAACGCATTCATCGGTGCTGTGG
AAGCCCGGGTGTGAGTCGCTGGAGGAGGCGGGATGCAATCGCGCCTTGTACCTGAGCGGCGATATGCCCGAGCGCG
ATAACCTGCTCAAGCAGCTGTTCCAGGCCAGCCAGCATTTTATGGTCAGCAGCTACGTGCCGGCAGCCTGGGCCAC
CTTGCGTCGTTGGCAACAGACCCTGGATAACCGCGGCGCCCTCGTGACGTCGCGGGAGTACGCACTGATCGACGAC
GCCTTGGGCACCATGGGCCGCCAATTGCAGGACAAACAGTTGGAACTGCACCGTCGCCGACCAGCGAACGGGCGTA
GCCATGAGCACTACGACGGCTTGGCTGCTGAAATAGACCGCTTGCAAATCAGTCGCCAGCGCCTGGAAAGTTTGCG
CTGGCGCCGCCTGCAAGCCCGTGATGTCTATGGCAGCCATGCTCAGGGTGCAAACTTGCCAGCCAGCCTCAAACGC
TGGTTCAGCCACCGGGAAAACCGTGAGCTATCGCAAGTGGCAAGCATCCCGCCGGCCTTGCATGACGAGCATGGCA
AAGGCCAGCCGTTGGACCTCGACTCGATCGTTTAGCCCAGGGTTTTACAAAAAAGCCACCTGCGCCAGCTGGCGCT
TCATCAACACCTTGCCGTTGCGGATCGAATACAGCGCCAGGCCCTGGCTGCGAATGACCTCGTAGTCGCTGTCGGC
CGAGAGAATCAGCAGGTTGGCCGGCCGCCCCGGTTCCAGGCCGTAGCGATCGCCCAGGGCCATGGCTTTGGCGCTG
TTGTCGGTGACAAGGTCCAGGGCCCTTTGCAGGTTGCTGTAGCCGAGCATATGGCAGATATGCAGCCCGGCTTCGA
GCACCCGCAGAATATTGCCGTTGCCCAGTGGGTACCAGGGATCGACGATGGAGTCCTGGCCGAAGCACACGTTCAT
GCCGGCCTCCAACAGTTCGTTGACCCGGGTTACGCCCCGGCGTTTCGGGAAGTTGTCGAAGCGGCCCTGCAAGTGA
ATGCTTTCGGTGGGGCAGGAAACAAAGCTGATGCCAGAGTGCCCCAACAGCCGGAACAGTTTTGCGCAGTAGGCAT
TGTCGTAGGAGCCCATGGCCGTGGTGTGGCTGGCGGTGACCCGGCTGCCCATGTCGCGACTGCGGGCCTCTTCGGC
CAGCACTTCGAGAAAGCGCGAGTGCGGGTCGTCGGTTTCATCGCAATGCACGTCGACCAGGCAGCCCGTGCGTTCG
GCCAGGTCCATGAGGAACTTGACCGAGCTGACCCCTTGGTCGCGGGTGTATTCGAAATGGGGAATGCCGCCCACCA
CATCGGCGCCGAGGCGAATGGCTTCTTCCATCAATTCACGGCCATTGCGGTAGGACTCGATGCCTTCCTGGGGAAA
TGCCACGATCTGCAGGTCGATCAGGTGTGAGCTCTCCTGGCGCACTTCGAGCATGGCCTTGAGCGCCGTCAACTGC
GGGTCGGTGACGTCCACGTGGGTGCGTACATGCTGGATACCATGGGCCGCCAGGGCCTGGATGGTTTGTTTGGCGC

GGGTCTTGGTGTCTTCATGGGTGATGGTGGCCTTGCGTTCGCCCCAGCACTCGATCCCTTCAAACAGCGTCCCGCT
CATATTCCAGCGCGGCTCGCCGGCAGTGAGGGTGGCATCAAGGTGGATATGCGGTTCGACAAAAGGTGGTACCACC
AGGTTACCCGCAGCATCCAGGTCGTCCGGGCCCATGGGCGGCGTGGCAGTTTGAGGCGTAATACTGGCGATCCGGC
CATGTTCCAGGTGCAGATCATGCAGGCCTTCACGGTTGCGCAGGCGGGCGTTGATGATATGCATGGGCAAGTTCCT
CTTGGGCAAAAAGCAATCAGGCAGACAGCCGCCAGATCATCAGCGCGACGCCGATGTCCAGACGGTCATTGGGATT
GTCCAGCGACAGCCCGCACAGGGCCTCGATACGTTGGACGCGGTGGGTCAGGGTGTTGCGGTGGACCGCCAACCGC
TGGGACGCAGCCACAAGGTTACCATTGTCCAGGAACCAGGCTTGCAGGGTCGGCATCAGGCTGGGGCCGTGGCGCT
GGTCATGGCGCAGCAGCGGCCCCAGGCGTTCATGGAGGAACTGCTCGAGCAGCCCGCGATCGCGTACCGCGCTCAG
TAGCTTCAGCAGCCCTAGCTCATCGTAGACACAGACGCCGGCATGCTCGCCAAAGCGCCGGGCAACCACCAGCGCC
TGGCGAGCTTCGTCCAGGCCCTGGGCGAGGCCGGCGGGCGGATGTTTTGCCGCGCTCAAGCCGATGCATAGCTTCA
GCGGGGCCAGGCGCAGGTTCAACGGCCTCAGCCAATTTGCCAGGCGTTGCCGGTGATCTTCGGTGGCCGGCAGCAG
CAAGGTCCATTGCCCAGCCCTGCCAAGTACGGGCAGGGGTGAGGGATGCTCGGCGGCGAACTGGCGCAATCGGCGG
GTAATGCCTTCGTGTTGGCGAGCGAGTTGGGCTTCGGCCTCCCCAGCCACGGTTTGCGCAAATAACGCGTCGCCAC
CCTCCAGTTGTAACTGCGCCACTTGCCAGTGTCCGGCCAATGGCATGCCCAGTTGGCTGGTGCGATGCAACAGCAG
GTCGAGGGATTGGTAGTCCCCCGCCAACAGGCGTTCCAGCACCTCATGCCGTGAACGCCCCAACTGCTCGGACTCG
ATCAGCGCCGAGCCGATGGCCTGGGTCACCAGGACCATTTTCAGCGAGTACGGTTGTTCGATCAGCGGCATCCCGG
CGGCGTCGGCGGCTTGCAGCAACCCAGGGGCAATCCCCTGGATGTAATCCGCACCGGTCAGGATCACCAGGCCGGC
GACACGCCGCGCGCAGGCTTCATCCAGCAGGCGCAGGAGATTGGCCTCATCACGCGGGTGGTTGATACCGGTGATG
AACACCAGTTCGCCGCCCAGCACCCAGTCTGCAATGCCGCTGTTCTCGGCCACATAAGGCCAGCGCACGCCATTGT
CCAGGCCTGCGCTACCGGCTCGCAGGGTCATGGACTCCAGCCCGGGCAGGGCCAGCACATCCGCCACGGTCAGGCT
CATGCCTGGGCGTCAGCCAAGGGCACCCGCACCCGCAGGACCGCGGTGAGCAAGATGTAGGTGGCGGCCGCGGCGA
TAATTCCCACCAGCGGCGCGACCCATGGCGAACTGAACGCCAGCACCGTACCCACCCCATAGGCAATCAGCCCCGG
CCAATTGAACGCGGGCAAACGCACCTCCGCCAGCCGAGGGTACTGACCGCGATAGCGATAGAAGAAATCGGCCATG
ATCACCCCGCCAATCGGTGGAATCACGGTGCCCAGCAAAATCAGATAGGGCACCAGCATGTCGTACATCCCCAGCA
AGGCGAGCAGGGTGCCGATCACGGCGCCGACCAGGGTCACAGTCTTGCGGCGTTTGGTACGCAGCAGGTTGCAGCC
GGCGACGGCGAAGTTGTAGATGGTGTTGTCCTGGGTGCTCCAGATATTGAGCAGCAACATGGCCATTGCAGCCATG
GCGAAACCTTGCAGCAGCAACACCTCCACCACATCCGGCTGTTGATAGACGATAGCGCCGTAGGCCCCAATCAGCA
CCATCAGGCCGTTGCCGATAAAAAAGCCGATCAGGCTGGCCAGGACTGCCACCTTGGCCGAGCGGGAAAAGCGCGT
CCAGTTGGTAGCCTGGGTTGCGCCACTGACAAAGGTGCCGAATACCAGAGTAATGGCCGTGGACAAATCCAACTCA
CCGCTGGGTACCACGGCAAGCAAGCCTTCCAGCCCCCCGATGTGCACCGTGGCCACCCACATCGACAGCAACAGCA
GCAGGCCCATGGCCGGGACTGCGATGTAAGAGAGGATTTCCAGGCCGCGATAGCCCACGTAGGCCGTGGCGCAGAA
CACCAGGCCGAACAGCACCATCAGTGCCATCACGCTGCCCTGGCCCAGCTCGAAATACTTACCCAGTACCACGGCC
GCCGTGGCGGTGCCCCAGGCGTACCAGCCAATCTGGGTGAAACCCAGGATCAGGTCACTCAACTTGCTACCCACTT
CGCCAAAGCAGAACCGCCCCATCAGCACCGAGTTCAAGCCGCTCTTGAAGGCGATGTAACCCAGGCCTGCGGCGTA
GATACCCAACAGCAGGTTACCCAGGGCCACGACACCGAGCATCTGGGTAAAACTGAAGGCCACCCCCAGTTTGCCG
CCGGCGAACATGGTCGCGGTAAAAAAGGTGAAGCCCAGCAACACCATGGCCGTGGAGCCAAGGCCCTTGCGTGCAT
GCATGGGTACTTCGCTGAGAGGGTAATCGTTGCCCGGTTCGTGCTGCGTCATGTGCCGTTCCCCTGTGAGTGACCC
AGGGCCCGTTGCAGCCGGCGTGCCAACTGCCGGGCAGTGACGGCTGCACGGTGGACACTGTGCAGGTTGCACACTA
TTCAGGGGGCGGGCGGGGCAGATAGAGGGGATTGCTTGTTGGTTATGCCCAGTCAGGCGCGATAGCGGTGCAGCGG
TGATACAAATTGGATCACCGTGCCGGGGTGTCAGTCGAACAGGGTCTGGGTGATTTCGCCGCGGTGAAAGGCGAAG
GCTGCTTCAAGATCACTCTCGATGGTGCGGCCGCGTGACAGGGGCGGCAGTTCGTCGAGGCGGAAGAAGCCGACGT
CGGTGGTTTCAAAGCCCGCCGTGGGGGCGGCCTGGTCGGTGCGCTCGCAGAGAAAGTACAACTTATAGAAGTCACG
CACGTCGGGCCGGTACAGGCCCTTGGCTTTGTGGGTCACGCTGTACAGCGTGCGTGCGGTGACGCTTAAGCCAGCT
TCTTCGCGAAGCTCCTTGACGATGTTTTCTGCAGCCGACAATCCGATATCGGCATAACCCCCCGGTAATGCCCAAC
AGCCATCCGTTGCTTCGCGTACCAGCAGGATCCGCTCGTCTTCAATCAGCGCGCCACGCACATCGATCATCGGTGT
TGAATAGCGCTGCGCGAAGTCCGAGACCAGGCCGGTAATGCGCTCCAGAGGCACTTGTCCCAACTGCGCAAGCATA
CTGTGGGCAATATGGGCGATCTCTTCCAGGCGTTCGCGCTCGAAGTCATCAGTGCAAAAGTGCAGCCCGGTGGATG
CCAATGCCTGCAGGCGCTTGGCCTGGGCCAGCCAGGTGGATTCCATGGTGTGGTTCCTTGAGTTGCGGCGAAGTCT
GGTTTTAATCGCTGTGATCCTCGAACGCAAACCCTTACGCCGAGGGTGCCTCAAAAATCGCCTCGATCGGCCGTTC
AAACAGCCTGGCTATTTTGAAGGCCAACGGCAGGCTGGGGTCGTAGCGGCCCGTTTCGATGGCGTTGACGGTCTGG
CGCGACACTTCAAGTTGTGCCGCCAGATCCGCCTGGGACCAGCCTCGGTCGGTACGCAATTGGCGCATCAGGTTGT
TCATCGATAACGCCTTGCGCCGATCACCGTGGCTACTGCCCAGATACCGCCCATGACGGGCCATACAAAAAACATC
GACAGGCGGGGCAGGCCGGCGGTTTCCAGGAAGCCATAGCTGAAGGTGATCAGCGCCGTGCAGATAAATGCAATGC
CCAGGGCCTCCAATTGAATCTTGCGGCCCATCTCATCCATTCGCCCCAATTGGCGGATCGCCGCCAAGGCCATGGC
GACGAGGGGAATGACCGGGACCAGGGCGAGGGAGATTTTCCCAGCGCCCTCGGGGGCGTCGGCCAGAAAGTGGGTA
CTGACAAGCACGGCAACCACATAGGCGGCCAGGGCGATGAAGAATTCCACAAAATAGCGTTTGATTGGCATGCGCG
CGTTCCGTAAGTAAAGGGAGCTTTACACTAGGAGGGCAAATGTCGAGTGTCAAGCTCGCTTTACATTTTGCAGGCT

CGATCCTGCAATCAATTCCAGGATTTACTCACCGCGATAAATACAGCCACTGGTGCAGGTCTCGTGGATACGAATG
GCGCTGAGTTCCGGCAGCAAGGGCTTCAACTCGTTCCAGATCCACTTGGCCAGCACTTCGCTGGTCGGGTTCTCCA
GGCCGGGGATGTCGTTGAGGTAGTTGTGGTCGAGGCGCTCATAGAGCGGCTTGAAAATCGCCTTGATCTCCGAAAA
GTCACGGATCCAGCCGGTATGCGGGTCAAGGTCGCCGCTCAGATGGATGGCCACCTTGAAGGAGTGACCATGCAGG
CGCCCGCACTTATGGCCTTCGGGGACGTGAGGCAAGCGGTGGGCGGACTCGAATGTGAACTCTTTGAAAATTTCCA
CGGTATGACCTGTAAGGCAGGAGTTTCTAGGGCGGTGTGCGGTTGTCAGTGTCGGGTGGGCGCTTGATGGGCCCAG
GCGACCGACTTGGCGCGCATTCTAACCTGTTTTAAGGGCCGACCTGGTGGCGTGTATCAAGCGTGGTTATTCCTGG
GACCACAACGTTGTACTGACCTTGACCCCGTCCCCACACGGCAGTGAGTACTGCGACCCTCGCCGCTGCAACTCTG
CGAGTGCACGGGCACACTGCACCTGGCCTTGGGCCGCCGCGTTATCAATGCTGATGTTGCCGGTGGTTTCAGACAC
AAGGTCGCCGTTTTTCTCGACCCGGATCAATGGCCGCTCGGGCTTGATTTCAAACCGGCTTTTCGTCGGCTCGATG
TTCAGTGCCAGGTCGGCAGGGGCGGCGGGCAGTTGATCTTCGGTAACACCATACCGCTTGAGCAGCGAGAGATTGG
GCAAGTCTTGTGCGCTTGTTGGGCCCACTGCCAGGAGCAGGAACAAACCAAGGCCATACATTATTCGGCGCTCAGA
ACCCATGATTGTATCTTTCCATGGTTGATTCTTGATGGGCTGGATTGAAGAACAACTTGTTGTTATCGCAACCGCG
TTTAGGGCATGGAGTGTCCAACCAGAAAGAAGAGCCGTAGCGGTTGCCGAACTGCGTGCCTGGTTCATAAACCTTG
AACGAGGACGGGTTGAAACTACCGGCGCTGCAGCCGCTGGTAATCAGGGCAACAGTAAAGAGGCCCAGGAGCTTTA
TGGGGTTCATAGGGGGTAATCCGTTTTAAGTTGATCAGTGTTCTACAGCGCTGCCCAGGAGCCTGGCAGCATGGTC
CAAGGCCTAGGCCTCTTCACTCTCGAATTCCAGCAAGTCGCCGGGCTGGCAATCCAGAGCGATACATAGCTTGTTC
AGGGTTTCGAGTTTAAAACCTTTGATTTTTCCGTTTTTCAATAGCGATAGATTGGCTTCCGTAATCCCAATGGCTT
CTGCAAGCTGATTGGAGCGGATCTTTTTCTTCGCCATCATGACGTCCAAACGAATAATGATCGTCATGGTTTATAT
GATGCTTTTGTTTTCATCGTTAATAGCCTTGGACTCAGCCACTATTAATGAGAACGCACACAGAAATATGCCTTGG
ATGATTTCATAGATCGCGGTGGATGAAATACCAATGTCGATGCTGATGGCGCGCTCTCCGGAAGGCATGTCTATCG
CCAGGGCCAAACCCTGGAGGGTGTAAATCACAGGTGCCAACAGTGGGCTGATAATAAAAAGAAGTCCCATCCACCA
CAGCATCTTTATATTTGATTCGATCCAGATCTGTCCTTTGGAAAGACGCATGAAAAAAATGCCGGTCAGTAACAGC
GCAACCACCATCAGGTCGGCAGGCAGCATATCCAATACCAGAAGCAGGCCGGTTGCCAGGGACGATGGCTGATAGT
CCGGGGCCAACAGCAAAGGCATCTGGCTGCCATAGGTGCTCAGGTGACTGGCCAGGATATTCGAATAGTCGAAGAT
CCAGAGCAATGGGGTACAGCCGAACTCGACAAGTGCCACGGCCCACGCCAGCAACGCGGCAAAGGCTCCGATGGAG
CGCAGGCGGCTGTTGGTGGAGACGGAATCCAGATTGTTGTCCATGTGTCGTTCCAAACGCAGTTGATGGGGCGAAT
AATCCATGAATAATTATCGAATTACAATAATTTTTTCCAAAAAAATTAATGGCGCCCTCTGCATCCTTAAACGTTC
GGGGGTAGCAATAAAAGCGAGTCACAGGGCAATCAGGCGCTCACCCAATCGGTCGTTATCGGCCAGTTCTAGGAAC
TCATCACCCAGCCTCTGGCTTTCACTCATTGCGGTTTGCCAGTACTGATTGCGTCGGGCGTCATCCCCCAAAAAGC
GTTTGAAGTCGCTGCGATCTGGCAATTTGCCGTAGGGCAGGCGAGCCAGGTACTCCCGTGAGGGCGCCAATAGCAA
CACATCCTGCAAACCCTGGGGGTTGCTGCGCCGCCACGGCAGGCCCTTATCGAACCAGCCGGGGATGACCCGGTCG
GTGAAGTGCGGGTACAGCACGATGTCATCGCCGTGGTAGGGCAGGTCGAGGTGGTAGTCCAGCAGGCCGCCATCGC
GATAGGTGCCGGCACCGGCGCCCGGCAGGTCGCGCACGCCTTGCATGACCATGGGGATCGAGCCGGACGCCAGCAG
TGCCTGGCGCAGGTTGCCCAGTTCCAGGTCGAGGAAGCGCGAAGGGAAATCCTTGAGCGGGTGCAATGGCGGCGCC
TGGCGCGGGTCGTGGATGATCAGTCGTTCAAAATGCCGGGACAGCCGCGCACGGCCACGCAGGTTGTCGGCGATTA
CCGAGGACAGGCCCAGGCCCAGGCGGCCACGGTGATCGTCGGCCAGCAGGCCATGGCTTTTGACGACCACGATATT
CAGGCGATAGTGCGGGTTATCCAGCAGCGTCGCATCGCGCCCGGCCAACAGGTCATCGAGCATGCGCTGGCAGCTC
TGGCTGACTTCGGCCATGGTCACGCCCTTGGCAAAGCGTTGCTCGTTGTACAAGCGACCAAGGTCGAGCAGGCCTT
GTACAGGGTCTGGCAGGCAGGCGCTGGCAAAGCGCCAGGAACCGATGGAGGCACCGATCAGCGAGCGCTCGCGGGG
CGCGCGCGCCAGCCAGTCACCGAACAGCGCCAGGTCCAGGCCCTGTATGCCCAAGGCCTTGGGGCCCCCGGCTGCG
CCGGGCAAAATTCCTACGTCGGCAGGCTGCAGGCCTTGTTCGCGAATGCGTGCAAAGGCGCGCTGGCCAGCCTTGA
GCGTCAGGGCTGGAAACTTGATGTGGATGGCGGTCATACCGATCTCTGTGCAGGCGAGCAGGGCAGTATAGGAAAT
CTTCCCGGGGGCCTGGCAAGGTGTTGTGGGACAATTGCCATGGTGGCAATTCAGTTTCAGTTAAGTTGCGATCCGT
ACTGTAGGGAGCGTAGGCAAAACATCCTGAAATGGAGAATCATCATGAAATACCTCACAGCCGTTATCGCCGCCAG
TATCATCGCCCTCACCACCGGCCTGGCCCAGGCCCGGGACCTGGGACCGGATGAAGCGCTGAAACTGCGTGACGCT
GGTACCATCCTGTCGTTCGAGAAGCTCAACGCTGCCGCCCTGGCCAAACATCCCGGTGGCACTGTGCATGAGACCG
AGTTGGAGCAGGAGTACGGCAAATACATTTACCAGGTTGACCTGCGCGATGCCCAGGGCGTCGAGTGGGACGTGGA
ATTGGACGCTGTCACCGGGCAAGTTCTCAAGGATCATCAGGATACGTAATGAAGGTAAATCTACGCACCGGCAGTC
GACTGGCGCTGGTGCTCCTGGCCTTTTGCTCGGTGGTGGCTGCCCGAGACCTCAATCAGGACGAAGCCCTGGCGCT
GCGCCAGCAAGGGGTGATCCTGTCCCTTGAGCAACTGCTGCAGCAGGCCATGGCGCGGCATCCCGGGTCCCGGTTG
CTGGAGGCCGAGCTTGAGAGCAAGCACGACCGCTACGTCTATGAAGTAGAGCTGGTGACCACCGATGGCGTGGTTC
GGGAAATTAAACTGGACGCGACCACGGGCGCGCTGCTTAAAGACGAGGAAGATGACTGATGCGCCTGCTTCTGGTG
GAAGACCATGTGCCCCTGGCCGATGAATTGCTGGCCGGGCTCAAAGCCCAGGGGTACGCCGTGGATTGGCTGGCGG
ACGGTCGTGACGCGGTGTATCAGGGGCGCAGCGAGCCGTATGACCTGATCATCCTCGATCTGGGCTTGCCCGGGCT
GCCGGGGCTTGAGGTGTTGAGCCAATGGCGCGCCGCCGCCCTGGCGACCCCGGTGTTGATCCTCACCGCCCGTGAC
TCCTGGGCCGAACGCATCGAAGGGCTCAAGGCCGGAGCCGACGATTACCTGAGCAAACCGTTTCACCCCGAAGAGT

TGTACCTGCGCATCCAGGCGTTGTTGCGCCGCTCCCATGGCCAGGCCAACCAGCCGACGCTGCAGGCCGCCGGCCT
GCATCTGGATGAAGGCCGCCAGTGTGTGATGCGCGACGGTGCCGAGGTGCAACTGACCGCTGCCGAGTTCCGTCTG
CTGCGCTACTTCATGCTGCATCCCGAGCAGATCCTGTCCAAAAGCCACCTGGCCGAGCACTTGTACGATGGTGAAA
CCGAGCGCGACTCCAACGTCCTCGAAGTGCACGTCAATCATCTGCGCCGCAAGCTGGGGCGTAGCGTGATTGAAAC
CCGGCGCGGCCAGGGTTATCGCTTTGGCGCCAGCCCCACGTGAGGTCGATCCAGCGGCGTTTGAGCCTGGGGCTGA
TCAGCGTGATGGTGATCGTCGGCCTGGTGCTGGCGCAAACCAGCCTGTGGCTGTTTGAAATGGGTTTGCAGCGCTA
CCTGGAGTCGGGCCTGCGCAACGACAGTGAAAACCTGCTGGTGGCCCTGGTGCGTGGCCCCGGTGGCGTGCAACTG
GATGAACAGCGCTTGTCGCCGGCCTATCAGCGGCCGTTCTCCGGGCATTATTTCGTATCGACTTCAGCGACAACC
ACTGGCGCTCCCGCTCCCTGTGGGACCAGGAACTGCCGCAGCTGCCCCAGGCCGGCCTGCAGGGCAATCTGCAGTT
GGGCCCGGAAGGCCAGCAATTGCTGATACTGCGCTCGGACTACAAACGGTTTGGCCAGGCGATTTCCATCAGCGTG
GCCCAGGACTACACACCGGTGCGCGAGAGTTTTCGCCTGATGCGCCAGGTCGGCCTGGTCCTGGGGATGGCGGCGT
TGCTGCTGATCCTGCTGCTGCAACGGGTCACTGTGCGTCGGGCCCTGCGCCCGCTGGAGACCGCCCGCGAGCAGAT
CGCGCAGTTGCAGCAAGGCCAGCGCTCGCAACTGGACACCCAGGTGCCGGTGGAACTGGAGCCGCTGGTGGCACAG
ATCAATCACCTGCTGGCCCATACCGAAGACAGCCTCAAACGCTCGCGCAACGCCCTGGGCAACCTCGGCCACGCCT
TGAAGACGCCCCTGGCAGTGCTGTTGAGTGTGGCGTCCAGCGAGCAGCTCAAGGACCAGCCGCAATTGGCCAAGTT
GTTGCGCGAGCAGTTGCAGCAGGTGCAGCAACGGCTCAACCGCGAACTCAACCGCGCGCGCCTGGCCGGTGAAACC
CTGCCCGGCGCACTGTTCGACTGCGACAGCGAGCTGCCAGGCCTGCTGGCGACCCTGAACATGATCCACGGCGAGC
ACCTGGACCTGAGCTACCACGTACGCCCCGGCCTGCAGTTGCCTTGGGACCGTGAAGACCTGCTGGAGCTGCTGGG
CAACCTGCTGGACAACGCCTGCAAATGGGCGGATGCCGAGGTGCGCGTGAGCATTGACGAGGCGCCCCATAGTTTT
CAGCTGCGGGTCGAGGATGACGGGCCGGGGATTCCCGAGGCTCAGCGTAGCCAGGTGTTCAGCCGAGGCACTCGAC
TGGATGAGCAGACCGATGGCCATGGCCTGGGGTTAGGGATCGTGCGGGATATCGTTGAGGTGTGGGGTGGCACTTT
GCAGTTGCATGAGAGTGAGTTCGGCGGCCTCAAGGTGCTGATCGAGTTACCCCGACGCCAGGGCTGATGGCACTTT
GTTTTCGATTTGTAGGGATTTGCAACAAGGCTTTTCCTTGTAACGCCTGGCCGCAACCATTTCGCTTATAACAGCC
ACCTGGCTGACCATGCCGAGCGTTTTTTTCGCTCAAGGCATGGTGTCTGTCAGAGCACTTGCCAGCCGGGCACTCG
CTTGCTCGGTACCTAATAAGAAAGATAAAAGGTGACTCCCATGCGAATGACCAAGCGTTTCAGTGTGTTTGCTGCT
GCCGCAGCCTTTGCCACAAGTACTGCAGCACTCGCCGCCCCCACATTTATCAACGTGCTGACGGGGGGTACCAGCG
GCGTGTATTACCCGATCGGCGTGGGCCTGTCGCAGCTCTACAGCAATGGCATCGAAGGGTCGAAGACCTCGGTGCA
GGCCACCAAAGCGTCGGTAGAAAACCTCAACCTGCTACAAGCCGGTCGCGGCGAGCTGGCCTTTGCCCTGGGTGAC
TCGGTAGCCGATGCGTGGAATGGAGTCGAAGAGGCCGGCTTCAAAGCCCCGTTGCAAAAACTGCGGGCGATTGCCG
GCACCTATCCGAACTACATCCAGATCGTTGCCAGCCAGGAGTCCGGCATCACCACCCTGGCCGACCTGAAAAACAA
GCGCATCTCCGTCGGCGCGCCGAAATCCGGCACCGAGCTTAACGCCCGCGCGATCTTCAAGGCGGCTGGCCTGAGC
TATGAAGACCTGGGCAAGGTCGAGTTCCTGCCTTACGCCGAGTCGGTCGAATTGATCAAGAACCGCCAGTTGGACG
CCACCTTGCAGTCTTCGGGCCTGGGCATGGCGGCGATCCGTGACCTGGCCGCAACGATGCCGGTCACCTTCGTTGC
CATTCCCCCAGAGGTCATCGCCAAGATCGACAACGCGGCCTACCAGGCCGGCACGATCCCGGCAGGCACCTACGAC
GGTCAGGCCAGCGATGTACCCACAGTGGCCATCACCAACCTCCTGGTCAGCCATGACGGTGTGCCCGATGACGTGG
CCTACCAGATGACCAAACTCATGTTCGACAACCTCGATCGCCTGGGCAATGCCCACTCGGCCGCCAAGGATATCCA
GTTGGAAGGCGCGGCCAAGGGCCTGCCGATCCCGCTGCATCCGGGCGCCGAGCGCTTCTATACGGAGGCAGGCGTC
CTGTAGGCGGCAGGTGGGTGCTGTGTGCAGCACCCACCGCTGCGCGCACTTGCCTGGGTTGCGAATAAAACCTTCG
TACATCGCGCATCGGGTTTTGTTGGTCTATCGACGTTGTAACGTGGGAGTACCCGCATGAGTGAGCAAGACCAAAG
CCTCGCCGCCAATCCCAGGGATTGGCCCCGAGCACTGTTTTATGTCGCGCTGTTGTTTTCCGTGTTCCAAATCATC
ACCGCAGCCTTCCATCCGGTCTCCAGCCAGATTCTGCGGGCCGTGCACGTGGGTTTTTTGCTACTGGTGGTATTTC
TGTGCTTTCCGGCGCGGGGCAAGGCCCAGCCCTGGCAACCGCTGGCCTGGCTATTGGGGCTGGCCGGGATGGCCAC
GGCGTTCTACCAGTGGTATTTCGAAGCCGCGCTGATTCAACGCTCCGGCGACCTGAGCGGCAGCGACATGGTCGTC
GGCCTGGTGCTGATCGTGCTGGTGTTCGAAGCAGCGCGGCGGGTGATGGGGATTGCCTTGCCGCTGATCTGTGCGC
TGTTCCTGGCTTACGGCCTGTTTGGCCAGTACCTGCCGGGCGAGCTGATGCACCGCGGCTATGCCATCGACCAGAT
CGTCAACCAACTGGCCTTTGGCACCGAGGGGCTGTACGGCACGCCGACCTATGTCTCGGCCACGTACATCTTCCTG
TTTATCCTGTTCGGCGCGTTCCTCGAACAAGCCGGGATGATCAAACTGTTCACCGACTTCGCCATGGGCCTGTTCG
GCCATAAGCTCGGTGGCCCGGCGAAGGTGTCGGTGGTGTCGTCGGCGCTGATGGGCACCATCACCGGTTCGGGGGT
GGCGAATGTGGTGACCACCGGCCAATTCACCATCCCGTTGATGAAGCGCTTTGGCTACAAGTCGGCGTTTGCCGGC
GGCGTGGAAGCGACCTCGTCGATGGGCAGCCAGATCATGCCGCCGATCATGGGCGCGGTGGCTTTTATCATGGCCG
AGACGATCAACGTGCCGTTCTTCGAAGTGGCCAAGGCCGCGTTGATTCCGGCCATGTTGTACTTTGGCTCGGTGTT
CTGGATGGTTCACCTGGAAGCCAAGCGTTCCAACCTGTGCGGCTTGCCCAAGAGCGAATGCCCGAACCCCTGGGCT
GCGGTGAAGCAGCGCTGGTATCTGCTGATCCCGTTATTCGTGCTGATCTACCTGCTGTTCTCCGGACGCACCCCGT
TGTTCTCCGGCATGGTCGGCCTGGCACTGACCTCCATCGTCATCCTGGGCTCGGCGATCATACTGCGGGTGTCGTC
CACGGCCCTGCGTTTGGTTTTCTGGATCGTCCTGGGCGTGCTGTGTGCGGGCTTCTTCCGGCTCGGTATCGGCATG
GTGTTTGGAGTCGTGGCGCTGTTGGTGGCGGTGTGCTGGTTCATCAAGGGCGGCCGCGACACCCTGACAATCTGCC
TGCATGCCCTGGTTGAAGGCGCACGCCATGCCGTGCCGGTGGGCATCGCCTGCGCGCTGGTGGGGGTGATCATCGG

GGTGGTGTCGCTGACCGGGGTGGCCTCGACCTTTGCCGGCTACATCCTCGCTATCGGCCAGGACAATCTGTTCCTT
TCGCTGGTACTCACCATGCTTACCTGCCTAGTGCTGGGCATGGGCATACCGACCATTCCCAACTACATCATCACCA
GCTCGATTGCCGCGCCGGCCTTGTTGGACCTGGGCGTGCCGCTGATTGTGTCGCATATGTTCGTGTTCTATTTCGG
CATCATGGCCGACCTCACGCCGCCGGTAGCGCTGGCCTGTTTCGCGGCGGCACCGATTGCCCGGGCAAGCGGCCTG
AAAATCAGCCTGTGGGCGATCCGCATCGCCGTGGCCGGGTTTGTCGTGCCGTTCATGGCGGTGTACGAGCCGGCAT
TGATGCTGCAGGGCGACAGCCTGCTGGCGACCCTGTATGTGGTGGCCAAGGCGCTGCTGGCCATCGGTATCTGGGG
CGCGGTGTTCACTGGCTACCTGCTGACCAGGCTCAGTGGGTGGGAGCGGGTGGTGGGCTTTGCCGCCGGCGCCAGC
CTGATTCTGGCAACGCCGTTAAGCGATGAAATCGGCTTCGTCCTGGCGGCGCTGTTGATTGCGCAGCACTGTTGGC
GTGCCCATCGCGCCCAGGTGGCTGCGGTGTGATCGGTTTGTGCCTGGGCTTGGCGGGTACGGTGTGGGCGCAGGTC
CCCACGCCGGCCTTCACCCTGGCCTGGACGCATACGATCGAGAAAGTGCGCTGGGAAGAGGATTACCGCGTGACCC
CGGAGGGGTTGGTGCTGGGGGAGGCACGGGTCAAGGGCTCGGGGGCCGGTATGGAGATTCCGGACGGCGCCCAACT
GCGCGAGGGCAGCTGGCATTACCAGCGTCAGTTGCCCCCGTTGCAACCCCTGCGCTTGGGGCGAACCCCTGAGGCC
GGGGATTATCAACTGTGTTTCAACCAGCACTGCCACTGGATAAGTACCTGGCTTGGCCCGCCACAGGCGAGCCAGC
CAGCATTGGAACTCTGGAGTTGTGAACTGGCGACGTACGGCGCCCAACCCAGTGCATTTCAAATTGCCATCAAACC
CGGAACTGATCCATCAAGCCTTGTTGCTGATTGGCCAGGCTATTGAGTGTCTGGCTGACCCGGGCCGACTCATTGG
CCTGACCCGACAGCGACTCGGTCACGTCGCGAATCGTCGCCACGTTGCTGTTGATCTCCTCGGCCACCGCGCTTTG
TTGCTCGGCGGCGCTGGCGATCTGCAGGTTCATGTCGCTGATCACCGTCACCGCATCGCCGATCTGGCGCAATGCC
GTTACTGCCTGGCCGACCTGTTCCACACTGCCTTGGGCCTGGCGATGGCTGTTGCTCATGGAACTCACCACTTCCT
GGGTGCCGTTTTGCAACTGCTCGATCACCTGGCGGGTTTCTTCCACCGACTCCTGGGTGCGCCGTGCGAGGTTGCG
CACTTCATCGGCGACCACCGCAAAACCACGCCCGGCCTCACCGGCACGGGCAGCTTCGATGGCGGCGTTGAGGGCC
AGCAGGTTGGTCTGTTCGGCAATCGCGCGGATCACTTCCAGCACAGAGCCGATCTGCTCGCTATTGGCCGCCAGGC
CTTCGACCTGGGTCATCGCCGTACTCATATCGGCGGCGAGGTGGCCGATGCTGGTGGTGGTGCGGTCGATCACGGT
CAGGCCTTCGCGGGTGGCCTGATCGGCATCGCGGGCGGCGTGTGCGGCCTGGGCAGCGCTGCGGGCGACGTCCTGG
GCGGTGGCGCTCATCTCGTGGGAGGCGGTGGCCACCTGGTCGACCTGGCGGTATTGCTGGTCCATGCCGGCACTGG
TCTGGCTGGCAATCGCCGCCGATTGGTCGGCGGTGCTGCGCGCGTCCTGCACCGAGCGTTTGACCTCGGCAATGAT
CGGTTGCAGCTTGTCAAGGAAACGGTTGAACCAACTGGCCAGTTCACCGAGTTCGTCGCGCTTGTCGTAGGCCAGG
CGCCTGGTCAGGTCGCCTTCGCCGCTGGCGATATCCTCCAGCATGCGGGCCACGCCGAGGATTGGCCGGGTGACGC
TACGGGCCATCAGCCACACCAACAGCAAACCGACAATCGCGGCGAGCAAGCCCAGGCCCAGTTCCAGCAAGGTGCC
TTTGGTGTTGTCGGCATCCAGTTCGGCCTTGAGGGCTTGGGCGGGGCCGATCAGGACTTGTTCCGGCACGTCCAGC
AACACGCCCCAGGACTTGCCATCGGGAATTGGCTGGAACGGCGCCAGCACCTTGAGTTGCTGGCCGGTGCGCAGGC
TGTAGGTTTGGGTACTGTTGGCCATGGCACTGATCAGTTGTGCGCCGCTGGTGGTGTCGACGCTGTCCAGGCGCTG
GCTGAGCTTGCTTGCATCCGGGCTGTAGCCGGCGAGCAAACCTACCGGGCTGATGATGCTGACCTTGGTCTGGCCG
TCATACAGCTTGCGGCTGGCATTCTGGCTGACCGCCTGCAGGCTGTTGAGGTTGATGTCCACCGACAGCGAGGCGA
TGACTTTGCCATTGACCGTGAGGGGGAACACGATGCTGGTGAGCAACACGTTTTGCCCGTCGATCACATAGAAGTA
CGGTTCGATCACGCACGGTTTGAGGGTAGTGCGCGGGCAGGTGAACCAGGTGTTGGCCTTTTCGCCGCTGGGGCCG
ACGCTGGTGTCGGACATGTCGCTTTCCGGCAGTGCCATCGAGGTCAGCTTGCCGGGTGTCGGCTGCGACCAGTACA
GGGCGAAGCGGCCCTTCTCGTTACTGCCCAGCTCGTCCTGGCCGGTAAACAACTCATCCTTGCCATCCAGGGCATT
GGCTTCGAACACCAGGGAAAGGCCCAGCAGGTCCGGATTGGCCTGCAGCGCGGCCTTGACTTGGCGCGTCAGGTCT
TCACGCAAGTCAAAGGCATCAAGGAAGCGCTTCTCGGCCTGTTCCCGCAAAAACAGCACCTGGCGCGAAAAACCAT
GGCCGTACTGGTAAGCGTCCATAAACTGTTGGCGGATGCCCAGCGCCTGGACTTCCCCCTGGGCTTCGATGCGCGA
TTGAGCCGCGACATTGAGCATCTGCGTGCTGGAGGTCTGGACCCTATGTGAGCTCTGATCCATCCGATACAGCGAA
AGACCCACTAGCAGGGTCACGATCCCCAACAGACAAAGGCCGGCGAGGAGGGTGATTTTCCATTGAATGGACAGCT
GCCTGAGCGACATGGAGATCTTTTAACCTTCGTTACGACAGAGATGATCCGTTGTATCGGCCGCCCGGTTTTTTTC
TTTATTCATACGATCAATTGAATTCTGATTACGACGTCGCGCTTTGACATGGCGTGTCGGCTGCTGCAAAGTGCGC
GCCCTCTAATAAAACGCCTTTTCTGCGTGTGCCGTGGTCGCCATTTGGCCAGGGTGCCGGCGCGTGTTGTTTTTAT
GTTTCTGTCTTGAGGTAACCATGATTAACGCAGTAATCGCCGCGGTCGGCACCATGCTCGTGCTGAGCCTGTCCCG
CGTGCATGTGGTCATCGCGATCATCGTCGGCGCCCTGGTCGGCGGCTTGACCGGTGGTCTTGGCATCGACGCGACA
CTCAGCGCATTCAACAGCGGCCTGGGCGGCGGCGCCACGGTGGCGCTGTCCTACGCATTGCTCGGTGCTTTCGCGG
TGGCGATCGCCAAGTCCGGCCTGGCCCACGCCCTGGCGGACAAGGCGCTGCTGCTGGTGGATCGCCAGGAAGCGAC
GGGCGGCAGTCATGTCAAATGGCTGCTGATCTGCCTGCTGGGCGCGGTGGCGATTGCCTCGCAGAACATCCTGCCG
ATCCATATCGCCTTTATCCCGCTGCTGGTGCCGCCTCTTCTGTATGTGCTGACCAAGTTGCAGATTGACCGGCGCC
TGATCGCCTGTGTCATGACCTTTGGCCTGATCACCCCGTACATGTTCCTGCCGGTGGGCTTCGGCAACATCTTCCT
CAACCAGATCCTGCTGGCCAACGTCGCCAAGAGTGGGGTGGATATCAGCCAGGTCAATGTGACCCACGCCATGGGC
ATCCCGGCGCTGGGCATGGTGTTCGGCCTGTTGGTGGCGGTGTTTATCAGCTACCGCAAGAAGCGCGTCTACGACC
TGGAGAAAATCGAGCGGGTCGAGCAAGTGGCGGTGCAATACAACCCGCTGACCCTGCTGGTAGCCGGGCTGGCCAT
TGCCTCGGCGTTTATCATTCAACTGTGGCTGGACTCAATGATCATTGGTGCCCTGGCGGGTTTCCTGATCTTCTCG
GTGTCGGGCATCGTGCGCTGGCGCGACACCGATGACCTGTTCACCGAAGGCATGAAGATGATGGCGATGATCGGCT

TCATCATGATCGCGTCCTCCGGGTTTGCCCAAGTGCTCAAGGCCACCGGCCAGGTGCAGACGCTAGTGCAGGCCTC
TGCGGCGTTTATCGGCCATAGCCGTGGCGTGGGCGCGCTGTTGATGTTGCTGGTGGGGTTGCTGGTGACCATGGGG
ATTGGCTCGTCGTTTTCCACGGTACCGATCCTGGCGGCGATTTTCGTGCCGCTGTGTGTGCAACTGGGCTTCAGCC
CCCTGGCCATTGTCTGCATCGTCGGCACCGCTGGCGCCCTGGGCGATGCCGGTTCGCCAGCGTCGGACTCCACCCT
GGGACCGACCTCGGGTTTGAACATCGACGGCCAGCACCACCATATCTGGGACACCGTGGTCCCGACCTTCCTGCAC
TACAACCTGCCGCTGCTGGTGTTCGGCTGGTTTGCCGCGATGACCCTCTGACCTCTCCTGTAGGAGCGAGCTTGCT
CGCGAAGGATTCAAGGGCACCGCGTCCATCCAGGATGCCCGTGTTATTGCCGACGACTTTCGCGAGCAAGCTCGCT
CCTACAGTTTTTTCTCTCAGGGCCGTTAACCCCTTAACGTTGCCCTATAAGAGAGAAGATCCATGCGTCTGAGTCT
GAAGGCCAAAGTCCTGTCCCTCGCCGTACTGCCGGTGTTGCTGTTTGCCGTGGTCATCAGCCTGACCACGGTATGG
ATCCTCAAGGAACAGGCGCGCAGCGAGGTGGCTGAAACCCGCGAGCGGCTGCTCAACAATGCCAAGGCCACCCTGC
AGAGTTACGTGGAAGTGGCGATGAGCACCATCAAGCCGCTGTACGACGCCGCCGCTCCTGGCGATACGCAGGCGCG
GGCGCAGGTGGTCAAGCTGTTGTCCAACACCACCTACGGCAAGGACGGATATTTCTTTGGCTACGACTCCGAGACC
ATCCGCCTGTTCAAGGGCAACAGCCCCGACGGTGTGGGCAAAAGCTTCAAGGACAACCGTGACCCCAACGGTGTCT
ACGTCAACCGCGACCTGGTGAAGGTCGGCAAGGACGGCACCCACTACCTGCAATACAGCTCGACCCAGCCGGGGCA
AACCGAACTGGTGCCCAAGCTTGGTTACACCGAATACTTGCCCAAGTGGGACATGGTGATCGGTACGTCGGTCAAC
CTCGACGGTATCGACGCCCTGGTCAGTGAGGTGGAAGCCAAGGTCAACGAACGTATGGAAGGGGTGTTGTTGAGCA
TCGTCGGTATCGCCGTGGTCCTTCTGTTGCTGATTGCGGCAGTGGGCATGGTGCTGGCCAATACCATCCTGCGCCC
CTTGCACCTGATGAAGGCCAACCTCGACGACATCGCCGCCGGCGAGGGTGATTTGACCCGCCGCCTGGCAATCACC
AGCCAGGACGAGTTGGGTGAACTGGCGGGTTCGTTCAACCGCTTTGTCGACAAAATCCATGGCTTGGTGCGCCAGA
TCACCGACATGACTTCGCAACTGACCGGCCTGGTCAGCCAGGTATCCGAGCAGGCCCACCGTTCGGAACAGGCGAT
GGAGCGCCAGCGCCAGGAGACTGACCAGGTGGCTACGGCGATCAACGAAATGTCGTCTGCCGCCCATGAAGTGGCC
CGCAGTGCCCAGGGCGCCGCGGTGGCGGCGCAGCAGACCGACGCCGAAGGCCAGGCCGCCAAACGCGTGGTGGATG
GCAGCATCGCGCAGATTCATGCACTGGTGAATGACATCCGCAGCAGCGGCGTGTCCCTGGACAGCCTGCAACAGGA
CGTATCGTCGATTGTCAGCGTGCTCGGGGTGATCCGCTCGATTGCCGAGCAGACCAACCTGCTGGCCCTCAACGCC
GCCATCGAAGCGGCCCGCGCCGGTGAAGCCGGACGTGGCTTTGCGGTGGTGGCCGATGAAGTGCGCGCACTGGCCA
GCCGCACCCAACAAAGCACCCAGGAAATCCAGGGCATGATCGACCGCCTACAAAAGGGCACCGAGGCGGCTGTGGA
ATCCATGCGCCGCTCCAGCGATGCCGGCGACGGCACCTCGGCCCAGGCCAATGAGGCCGGGGCGTCCCTCGATACC
ATGGCGCAGTTGATCGGCACCATCAACACGATGAACGCGCAGATCGCCAGCGCCGCGGAAGAGCAAACCGCCGTGG
CGGAAGAGATCAACCGCAGCGTGCATCAGATTGCGGTGGCGGTGGACAGTGTGGCGGACGAAACCCAGCAGGGCGC
GCAGACTTCACGCAGCCTGGCGGATCTGGGGCAGCGATTGGGGCAGTTGGTGGGGCAGTTCAGGATCTGACCTGGC
AATTGCAGTGCTTGTTCTGGCCTCATCGCGAGGCCCGCTCCCACATTTGACCCTGTTCACAGACCAAGTGTGGGGG
CGGGCCTCGCGATGAGGCCCTCACAGATACCTACACATCCCGCATCAACAACCCAAACCCGATATCCATCTCCAAC
GGCACCGGCACATACACCGTATGCCCATCCCCGGGCGCCACCTCGATCGCCTCGCCCTTGGCGTTATGCATCCTCC
CCAAGTCAAAATGGAAATTCCCCGCCGGGGTCATCAACTCCAGGTGGTTGCCCAGCGCAAAGCGGTTCTTCACCTT
GACCTCGGCCAGCTCCCCGCGCCGCTCGCCGGTCAACTCGCCGACAAACTGCTGGCGCTCGGAAACCGAGCTACCG
TTCTGGTAGTTCTGGTACTCGTCATGCACATGGCGGCGCAGGAAGCCTTCGGTGTAGCCGCGCTGGGCCAGGGATT
CGAGGTTGGTCATCAAACCACGGTCGAACGCGCGCCCGGCCACCGCGTCATCAATGGCCTGGCGGTACACCTGGGT
GGTGCGCGCCACATAGAAGTGGGATTTGGTGCGGCCTTCGATCTTCAGCGAGTGCACGCCCATCTGCGTCAGGCGC
TCGACGTGCTGTACCGCCCGCAGGTCCTTGGCGTTCATGATGTAGGTGCCGTGCTCGTCCTCGAAGGCCGGCATCT
GCTCATCGGGGCGGTTGGCCTCCTGGAGCAGGAACACCTGGTCGGTGGGCGCGCCAAGGCCCAGGGTCGGTTCGGG
CTGATACTGCTGGACGATATCGCCCACGCTGTTTTCGACGGCGGGGCTGGCCTGGTACTTCCAGCGGCAGGCGTTG
GTGCAGGTGCCCTGGTTGGCATCGCGCTTGTTCATATACCCCGACAACAGGCAGCGCCCGGAATAGGCCATGCACA
GTGCGCCATGCACAAACACTTCCAACTCCATGGCCGGCACTTGCTGGCGGATTTCGGCGATTTCTTCCAGGGACAG
TTCCCGCGACAGGATGATCCGGCAGATGCCTTGCTGTTGCCAGAACTCGACGCTGGCCCAGTTCACCGTATTGGCC
TGTACTGACAGGTGGATCGGCATCTGCGGGAAGTGCCGGCGCACCAGCATGATCAAGCCGGGGTCAGACATGATCA
GCGCGTCCGGGCCCATGGCGATCACCGGTTCCAGGTCCTTGAGAAAGGTCTTCAGCTTGGCGTTGTGCGGCGCGAT
GTTGACCACGACATAAAAGCGCTTGCCCTGGGCCTGGGCCTCGGCGATACCCAGGGCCAGGTTGGCGTGGTCGAAC
TCGTTGTTGCGCACCCGTAAGCTGTAGCGCGGCTGGCCGGCGTAGACCGCATCGGCGCCGTAGGCAAAGGCGTAGC
GCATGTTTTTCAGGGTGCCAGCGGGGGCGAGCAATTCGGGAGCGAGGACAGGGGGCATGGGGATTCAGGTCGCAAA
AGGGCGGCAGGGTAGCCGAGCCGGGCCGGGCGCTTATTGATCTGGGTCTACGCTGTAGAAATAAAGATGGCACTCG
CCGGATGCGCGGTGGACTAAGCAACAGGGCGCGCTGGGCGCCCGGCGTTTTTCTGGTACGGATCGGACATGAACCA
AACGAATCTGCAATTCAAAACCCTGCTGTTGCTGCTGGTCGTAGTGACTGTCGGCTTCTTCTGGATCCTGTTGCCG
TTCTATGGCGCGGTGTTCTGGGCGATCATCCTCGGGATCATTTTTGCGCCCATGCAGCGGCGCCTGCAGCTCAAGT
TTGGCTGGAACCGCAACCTGACGTCGCTGTGTACCTTGATGGTGTGCCTGGTGATCGCGATTTTACCGGTGATCAT
CACCAGCGCCTTGCTGGTGCAGGAGGGGGCGACGCTGTACAAGAACGTCGAGAGCGGCAAGCTGGATGTGGCGGGC
TACATCGAACAATTCAAGAACTTCCTGCCGCCGTACTTCCAGCACCTGCTGGACCGCTTCGGCATGGGCAACCTGG
AGGGCCTGCGCGAGAAAATCGTCAAGGGCGCGATGCAGGGCAGCCAGTTCTTCGCCAGCCAGGCGTTTAGCTTCGG

CCAGGGCACCTTTGATTTCCTGGTGAGCTTTTTCATCATGCTGTACTTGCTGTTCTTCCTGCTGCGCGACGGTCCC
GAACTGGTGCGCAAAGTGCGCACGGCGGTGCCGCTGGCCGAGCCGCAGAAGCGTCGCCTGCAACTGAAATTCAACC
GCGTGGTGCGCGCGACCGTCAAAGGCAATGTGCTGGTGGCGGTGACTCAGGGCGCATTGGGTGGCCTGATCTTCTG
GTTCCTGGACATTCCCAGCGCGTTGCTCTGGGCGGTATTGATGGCGTTCCTGTCGTTGCTGCCTGCGGTGGGGGCG
GGGATTGTGTGGGGGCCGGTGGCTGCGTACTTCCTGCTCAGCGGTTCAATCTGGCAAGGAGTGGTGCTGGCCTTGT
TCGGGGTATTTGTGATTGGCCTGGTGGACAATGTGCTGCGCCCGATCCTGGTGGGCAAGGACACCAAGATGCCGGA
TTACCTGATCCTGATCTCGACCCTGGGCGGGCTGTCGGTGTTCGGCCTCAATGGCTTTGTCATCGGCCCGTTGATC
GCCGCGCTGTTTATCTCCAGCTGGGCGCTGTTTGTCGAGACCAAACCCCGGGTGCGGCTGCCATTGCCCTAGGCTG
AGAAGCAGTGGGCCATCTTGCGGGACAGGGCGTGGGCGTCGGGCAACGATGCCAGGGGCCCGCTGATGGCTTCACC
GTCGCGTACCAGGTACCAACAGGCGAGCAAACCCAGTTTGCGCAGGGGGCCGGGGACGGCACTGCCAACGACGGAC
ATGATCTGGATAGTGGGCATATGTACCTCCATTGCTGATGGAGTTCACCTTACGGCGGCCGAGCGGCGAGGGAAAA
TCACCCTGCTCGATAGTCAACATCACTGCCATCGAGGCTGCGCCGACAGGGCTCAGTCCACCACCTGGTCCAGCAT
GTGCAGCACTTCTTGTTCATTGAGCAGGCCCTTGTGGACCAGGTTTTCTGCCAGCAACGAGAGAAACTTGGCGCTA
CGGTGGCCTTCAAGGTGCTTGAGTTCAGTCAAGGCGCTGTAGACCTTGCTGGAAGTGCAGAGGCCGGCGGTGCGGT
GCGGGTTTTGTGTGGGCATGCTGGGGCGTCCTTGTTGTTATGACCTGTTATGGGGGACCGTTTCGATAGTGAGAGT
GCCGCGTGACACAAACATGACAGCGCGCCCCCCGGTTGGGCAAGTACACTTTGGCCTTAATAATGTCGGATTTAAC
GGTCGGCGATGTCTCCATAACGACCCCGGCGAGATTTATTCAGACCCTGCACCCACAAAAAAGCCCCGCCTGGAAT
GCGGGGCTTTGTATACCGGGCTTACCAGCCGCGCCCGTAATAGCCTCGGGGCGGGCCGTAGTAGCCGCGTGGGGGC
CCGTAATACACCGGCGCCGGGCGGTAATAAACCGGCTGCTGCACGTACACCGGTGGTGGCTGGTAATAGACCGGTG
GCGGGGCGGCATACACCGGCTGCGGCTGGTAGTACACAGGTTGTTGCTGCACATACACCGGCCGATCATGGCTGGC
GATCACTGAGCCCACCACGGCTGCGCCGACAACTGCACCCAAAACGGCCGGGCCACCCCAACCACCACCATGGGCG
GAGGCTTGGCCGGCTACAGCAAGTGCACCGATCAGCAAGGCGATCCTGGGGATTTTACGCATCATGGTCTTTCCTC
GGTTGGCCCCTGCGCTTGTGGTCTGAGACTCAAGGATTGTCGCGGGGATACTTTTAAGACAACGTATTTCAGCAAA
ACAGCACTGCCGCTAGGTAAAGGTTGTGTAAGGTCTGTACCGATTTGCTTACTTGAAGAGGAGTTACCGATGCTGA
TGAACCCCAACAAGGACACCCAGTTGTGCATGTCGTTGTCGGCCCGCCCGGGTAATTTCGGCCTGCGCTTTCATAA
CCATTTGTATGAGCAGTTGGGGCTGAATTTTTACTACAAGGCTTTCAGCAGCCAGGACTTGAACGGCGCTGTAGCC
GGGATCCGCGCCTTGGGCATTCGTGGCTGTGGGGTGTCGATGCCGTTCAAGGAAGCCTGTATCGAACTGGTGGATG
AACTCGACGACTCGGCCCGCGCCATCGCCTCAATCAACACCATCGTCAATACGGCCGGCCACCTCAAGGCCTACAA
CACTGATTACATCGCCATCGAGCAACTGCTGCAAAAGCACGCGGTGCCCAAGGACTCGACCTTTGCACTGCACGGC
AGTGGCGGCATGGCCAAGGCCGTGGCCAGTGCCTTGCGTGACGGTGGCTACGCCAATGGCCTGATCGTTGCCCGTA
ACGAAGTGGCTGGCCGGGCCCTGGCGCGCAATCTGGGGTATGAGTGGCAGGCGACATTGGGTAACGCGCGGCCGCA
GATGCTGGTCAACGTAACGCCCATCGGCATGACCGGCGGCGCCCAGGCCGAGGACCTGGCATTCGAGGCCGACGCC
ATTGATGCGGCAGAGACTGTATTCGATGTGGTGGCGATCCCCGCCGAAACGCCGGTGATCGTGCGTGGCCGGGCCC
AGGGCAAGCGGGTGATTACCGGCCTGGAAGTGATCGCGATCCAGGCACTGGAGCAGTTCGTGCTGTACACCGGCAT
ACGCCCGACGGATGAGCAGTTCCAGAAAGCCGTAGCGTTTGCCCGACAGGGCTAAGGCAGGCCTTGTAGGAGCCGG
CAAGCCGGCGATGGCGGCAAGGGCCTTATCGCCGGCAAGCCGGCTCCTACAAAGGGGCGGTTATTCGAGGCGTGAG
AGGCGCTCTTCCAGCGCCGCAATCCGCGCCTCCAGCTCCTCGATGCGCTCCAGAGAAACCCCGCTGCCAGCGCTGC
TGCGCTCCACCGGGCTGCCACGGGCGGCCAGGATCGCCTCGATATCCGCCGGGTCACCCAGGGAGTGGGTGTAGCG
GTCCTCACGCTGGCCGGCCTGCTTGGGGACCAGCACGGCAAGGTCGCGGGCGATCAGGCGTTCGAGTTGGTGTACC
ACCTGCTCGGCATCTTCAAAGTCATGCATGCGCCCGCTGCGGGTCAGCAATTCATTGACCGTCTGCGGGCCACGCA
GGAACAACAGGCCAGTAAGAATCACCTGCGCCGGCACCAGTTCCAGGGCCTTGTCCACCCGGTGTTCCCAGCGGTC
GGCACGGCTGCCCATCACCAGTTTGGTAAACCCCTGGCCTTCCAGGGCCCGCAGGCTCTGGCCAACCTGGCCCTGG
CTGAGGTTCATCACAGGTTCCCGGCTGGTTTTCTGGTTGCAGGCCAGGACCAGGGCATTGAGGGTCAGCGGGTAGG
TTTCCGGGTTGGTGGCCTGCTTTTCGATCAGGCAGCCCAGAACACGGATTTCCGTGCTGTTGAGGCGCGGCTCGGA
GGAGGTGGTGTCGTGCTCGGCGGTCATCGCGCTTTCCCTATGCAGTGAACCCACTAGCCTAATCCCGAAAAAATAA
AAGACAAGCAGCGCTCATGTCTATAATCGCGGCTGTTAAAACCCTGCCACCACTGTCGAGACTGTCATGACTATTT
CCCTGTACGCCGCTTCCCTTCCGGTCTTCAAGCAAATGCTCAACGCTTTGAGCGATGTGCTGAACAAGGCCGAGGC
CCACGCCACCGCCAAGAACATCGACCCAAACGCCTTGCTGCAAGCGCGCCTGTTCCCGGACATGTTCCCGCTGGTG
CGCCAGGTGCAGATCGCCGTGGATTTTGCCAAGGGCGTTTCCGCGCGTCTGGCCGAGATTGAAGTGCCGAAATACG
AAGACAACGAAGTCACCTTCGCTGACCTGCAAGCGCTGATCGCCAAGGTTCTGGGGTTCATCGACACCATCACCCC
GGCGCAGATCGATGGCAAAGAAGGCATCGAGATCGTCACCCGTCCGGGCACCCCTAAAGAGAAGCGCTTCAGCGGC
CAGTCCTACCTGTTGACCTACGGCCTGCCGCAGTTCTTCTTCCACGTCACCACCGCCTACGCCTTGCTGCGTCACA
ACGGTGTGGAAGTGGGCAAGCGCGACTATATGGGCGCGTTCTAATTACTGAGTAACACCGCAAAACAAATGTGGGA
GCTGACTTGTGTGGGAGCTGGCTTGCCTGCGATGGCCATGAGTATCTACACAACTCTCTAATACTTCTGGGCTCTG
GGTTTTGGGCTGATGTAGGGGGCATATCCATTGCTGCGGTAACGGCGGCCATGGNNNNNNNNNNCTCGCAGAGATA
ACGGACATGTCCCTACGCACACTGCTCACCGCCCTACTGCTATCCGCCAGTTTCACGGCGATGGCAGCCACTGAAG
TCCTGCCCTTGAGCAACCGCACCAGCGCCGACCTGCTGCCGGTCGCACAGAATTTCATTGGCAAGGATGGCACGGT

CAGCGCCTATGGCAATCAACTGATCGTCAATGCCGAGCCGGACAAGATCCAGGGCCTGCGCGCCCTGCTCGCACAA
CTGGACACACCGTCCAAGCGCTTGCTGATCACCGTCGACACCAACGAAAACAATCAGCAGAGCAACGGCCAGGTCA
TCACCTACAGCACCGAAAGCCGAGAGGGTGGAATCCAGCAGATCCAGGCCAGCGAAGGCGTGCCCGCGCTGATCCA
GGTCGGTCAGAGCGTGCCACTGACCACCACCCAGTCCGATACCTATGGCCGCCCGCAAAACCAGACTCAGTACCGC
AACGTCACCCAAGGTTTCTACGTCACCGCCAGCGTCACCGGCGAGACCGTACACCTGAGCATCAGCACCAATCGTG
ACCGTATGAGCCAGGAGCGTCCCGATGTAGTGAATGTGCAAAGTACCGACACAACTGTCAGCGGGCGCCTGGGCGA
ATGGATCACCCTGGCCGGTATCAATCGCCAGACCCAGGCCGACAAACAGGGCGCGACCCGCACCTACTCGACCCAG
GGCCGGGATGACCTGACACTGCGGGTCAAAGTGGACACCTTGAACTGAAGCACCAAAAACTGACTGATTAGTCGTA
TTAGACCAAAGATGTAGTGCTATAAAAAAAGCACTACAAAACATTTGACGATCCAAAAAAGCATGGGCATGATGGC
CTCGCTCCCGCTAATCAGGGGCCCTGGCAAGGGCCTTCGGATCGTCGCTCTAAGCTACCCACCTGAGCCGATTCGT
GTCTGTACCGCCCACAAGGTGTGTTTGACGAGGTTGCGACTGGAACGAAGTTGTCCCGAGGGACGGAAGCTAACCA
GGTAACCCGGCAACACACTGATGGATCGTACCAAGGCCCACGACGCCCGAAGACTGTTCTCGGTTCGCCTCTACCT
GCTCACTTTCTCCCTTGAGCCTATCGTTCATCCCGTCGCCTTCCCCGCCAAGCCCACTTTGACCGCCTAAGCTTCT
GGTCAGCGAGCAGCCAATCCCACGCACTGAATACGTGGCTGGCAAATGGAATTTTCAACCCAGTAGTTTTTCCACA
AAAGACGCGACGAGGTTTTTCCCCATGGCACTGACACGCGAACAGCAAATTGCAGCCCTTGAAAAAGACTGGGCTG
AAAACCCACGCTGGAAAGGCGTGACTCGCGCTTACTCCGCTGCTGACGTCGTCCGCCTGCGCGGCTCGGTTCAACC
TGAGCACACCTTCGCAAAACTGGGCGCCGAGAAGCTGTGGAAGCTGGTTACCCAGGGCGCCAAGCCGTCCTTCCGT
CCCGAGAAAGATTTCGTCAACTGCATGGGCGCCCTGACCGGCGGCCAGGCTGTGCAACAAGTCAAGGCCGGCATCC
AGGCGATCTACCTGTCCGGCTGGCAAGTGGCCGCGGACAACAACTCCGCCGAGTCCATGTACCCTGACCAATCGCT
GTACCCGGTGGATTCCGTGCCAACCGTGGTCAAGCGCATCAACAACTCGTTCCGCCGCGCCGACCAGATCCAATGG
AAAGCCGGTAAAGGTCCGGGCGACGAAGGCTACATCGACTACTTCGCGCCAATCGTGGCTGACGCCGAAGCCGGTT
TCGGTGGCGTACTGAACGCCTACGAGCTGATGAAGAGCATGATCGAGGCAGGCGCCGCCGGCGTGCACTTCGAAGA
CCAACTGGCTTCCGTGAAAAAATGCGGCCACATGGGCGGCAAGGTACTGGTTCCGACCCAGGAAGCCGTACAGAAG
CTGACCGCTGCTCGCCTGGCTGCTGACGTTGCCGGCACCCCGACCATCATCCTGGCACGTACCGACGCTAACGCGG
CAGACCTGCTGACGTCGGACTGCGACCCGTACGACCAGCCGTTCGTGACTGGCGAGCGCACCCAGGAAGGCTTCTA
CAAGGTTCGTGCAGGCCTGGATCAAGCAATTGCCCGCGGCCTGGCCTACGCGCCGTATGCCGACCTGATCTGGTGC
GAAACCGCCAAGCCAGACCTGGACGAAGCCCGTCGCTTCGCCGAGGCGATCAAGAAGGAATACCCGGACCAACTGC
TGTCCTACAACTGCTCGCCTTCCTTCAACTGGAAGAAAAACCTGGACGACGCGACCATCGCCAAGTTCCAGCGCGA
ACTGTCCGCCATGGGCTACAAGCACCAGTTCATCACCCTGGCCGGCATTCACAACATGTGGCACAGCATGTTCAAC
CTGGCGCACGACTACGCCCGCAACGACATGACTGCCTACGTGAAGCTGCAGGAGCAGGAATTCGCTGACGCCGCCA
AAGGCTACACCTTCGTGGCGCACCAGCAGGAAGTGGGCACTGGCTACTTCGACGACATGACCACCGTGATCCAGGG
CGGCACCTCGTCCGTAACCGCGCTGACCGGTTCGACCGAAGAAGAACAGTTTCACTAAGTAGCTGAGTACACGGCC
ATTGCGGGCCCGATAGAAAGCTAACCGCAGTGCCGCACGATCTGACGCCCCGACTGGTTCGGGGCGTTTTTTTTGC
CCCGAGAAAACCCTCCCACCCCTGCGAAGATCAAAATGTGCGGGCTTGCTCGCGAAAGCGGTGCATCAGCCAGCAG
TTTTTTGACTGACACACCGCTTTCGCGAGCAAGCCCGCTCCCACAATAAAAATGCGGCCAGCCTGACGTTTTTGCG
CAAAACCTTGATCCAGAGCGGTATTCGCCCAGGAAAATTCAGTTAAAAGTTGTGCGCTGACAACTTGCGCCTAACG
GGCAAGTAACAACAACTTTTCCCGCGACGCACTCAACACCCGCTTAAAACTCCCGCAAACAATATTCATTATCATT
TAGAGCATTAAAACTTCGTTACACCCTAAACAAAACACAATTGATTAAAATCAAGCTAATGCCCGCAGCGTCTGGG
CTACAGCCCCATAAAGGTGCACTATGTCCTTAATTCAGCGAATAAATTAGCTACAGGAATTTTACTTGCTAGGTGT
TTAGCCATAAAATCACCGCGATTGATTGCGCTGCGACATATCGTCACTGCGTCGTTACTTTTTCGAGCTCAGAGAC
CTTTGCTCTCTGTTAAGGATTTCCAGCATGACCGAAGCGACAGGACTCATGGCCCACAACTGGGGCTTTGCCATTT
TCCTCCTCGGTGTTGTCGGCCTTTGCGCCTTCATGCTCGGTGTTTCCAGCCTCCTCGGGTCAAAAGCCTGGGGCCG
CAGCAAAAACGAACCGTTCGAGTCCGGCATGCTACCTACAGGTGGCGCCCGCTTGCGGCTCTCAGCCAAATTCTAT
CTGGTCGCGATGCTGTTCGTGATCTTCGATATCGAAGCCCTCTTTCTCTTTGCCTGGTCTGTGTCCGTCCGCGAAA
GCGGCTGGACCGGATTCGTCGAAGCTCTCGTTTTCATAGCAATTCTGTTGGCAGGTCTTGTCTACCTGTTCCGAGT
GGGCGCCCTTGACTGGGCTCCGGAAGCTCGTCGCAAGCGGCAAGCGAAGCTGAAACAATGAGGCTTTGGCGATGCA
ATACAATCTCACCAGGATCGACCCGGATGCTCCTAACGATCAGTACCCCATCGGCGAACGGGAAACCGTTTCCGAT
CCGTTAGAAGATCAAGTCCACAAAAACATCTACATGGGCAAGCTGGAAGACGTGCTGAGTGGCGCGGTCAACTGGG
GGCGCAAGAATTCCCTGTGGCCGTACAACTTCGGCCTTTCGTGCTGCTACGTGGAAATGACCACCGCCTTCACGGC
GCCCCATGACATCGCGCGCTTTGGCGCCGAAGTTATCCGGGCATCGCCGCGCCAGGCGGATTTCATGGTTATCGCC
GGTACCTGCTTTATCAAGATGGCGCCGATCATCCAGCGTCTCTACGAGCAAATGCTCGAGCCAAAGTGGGTTATCT
CCATGGGTTCGTGCGCCAACTCCGGTGGCATGTACGACATCTACTCCGTGGTTCAGGGGGTGGACAAGTTCCTGCC
CGTGGACGTCTACGTGCCTGGCTGCCCGCCCCGTCCTGAAGCATTCCTGCAAGGCTTGATGTTGCTGCAGGAATCG
ATTGGCAAGGAGCGTCGCCCACTTTCCTGGGTCGTCGGAGATCAAGGCGTGTACCGCGCCGAGATGCCGTCACAAA
AGGAACAGCGCCGCGAACAGCGAATCGCAGTCACCAACCTGCGCAGCCCCGACGAAGTCTGATCCAGCACCGCTTC
TTTTATAGAACGAAAACCTGGCTTCATTCTTTACGTTGACCGAAAGCGATAAAAAAACCATGACTACAGGCAGTGCT
CTGTACATCCCGCCTTATAAGGCAGACGACCAGGATGTGGTCGTCGAACTCAACAACCGTTTTGGCCCTGACGCCT

TCACCGCCCAGGCCACACGCACCGGTATGCCAGTGCTGTGGGTGGCGCGCGCCAAACTCGTCGAAGTCCTGACCTT
CCTGCGCAACCTGCCCAAGCCGTACGTCATGCTCTATGACCTGCATGGCGTGGACGAGCGCCTGCGCACCAAGCGC
CAGGGGCTGCCGAGCGGCGCGGACTTCACCGTGTTCTATCACCTGATGTCGCTGGAACGTAACAGCGACGTGATGA
TCAAGGTCGCACTAAACGAAAGCGACTTGAGCATCCCGACCGTGACCGGTATCTGGCCGAACGCCAGCTGGTACGA
GCGTGAAGTCTGGGACATGTTCGGTATCGACTTCCCGGGCCACCCGCACCTGACCCGCATCATGATGCCGCCGACC
TGGGAAGGTCACCCGCTGCGCAAGGACTTCCCGGCCCGCGCCACTGAATTCGATCCGTTCAGCCTCAACCTCGCCA
AGCAGCAGCTTGAAGAGGAAGCCGCACGTTTCCGCCCGGAAGACTGGGGCATGAAGCGCTCCGGCACCAACGAGGA
CTACATGTTCCTCAACCTGGGCCCGAACCACCCTTCGGCCCACGGCGCATTCCGTATCATCCTGCAGCTGGACGGT
GAAGAAATCGTCGACTGCGTGCCCGACATCGGCTACCACCACCGTGGTGCCGAGAAGATGGCCGAGCGCCAGTCGT
GGCACAGCTTCATCCCGTACACCGACCGTATCGACTACCTCGGCGGCGTGATGAACAACCTGCCGTACGTGCTCTC
GGTCGAGAAGCTGGCCGGCATCAAGGTCCCGGACCGGGTCGACACCATCCGCATCATGATGGCCGAGTTCTTCCGG
ATCACCAGCCACCTGCTGTTCCTGGGTACCTATATCCAGGACGTTGGCGCCATGACCCCGGTGTTCTTCACCTTCA
CCGACCGTCAGCGTGCCTACAAGGTCATCGAAGCCATCACGGGCTTCCGCCTGCACCCGGCCTGGTACCGCATCGG
CGGTGTGGCCCACGACCTGCCGAATGGCTGGGAGCGCCTGGTCAAGGAATTCATCGACTGGATGCCCAAGCGTCTG
GACGAGTACCAGAAAGCCGCCCTGGACAACAGCATCCTCAAGGGTCGGACCATCGGCGTCGCGCAGTACAACACCA
AGGAAGCCCTGGAATGGGGCGTCACCGGTGCGGGCTTGCGTTCGACCGGTTGCGATTTCGACCTGCGCAAGGCGCG
CCCGTACTCCGGCTACGAGAACTTCGAGTTCGAAGTCCCGCTGGCGGCCAACGGCGATGCCTACGACCGCTGCATC
GTGCGCGTCGAAGAAATGCGCCAGAGCCTGAAGATCATCGAGCAGTGCATGCGCAACATGCCGGCAGGCCCGTACA
AGGCGGATCACCCGCTGACCACGCCGCCGCCTAAAGAGCGCACCCTGCAACACATCGAAACCCTGATCACGCACTT
CCTGCAAGTCTCGTGGGGCCCGGTGATGCCGGCCAACGAGTCCTTCCAGATGATCGAAGCGACCAAGGGTATCAAC
AGTTATTACCTGACGAGCGATGGCGGCACCATGAGCTACCGCACCCGGATTCGTACCCCAAGCTTCGCGCACTTGC
AGCAGATCCCTTCGGTGATCAAAGGCGAGATGGTCGCGGACTTGATTGCGTACCTGGGTAGTATCGATTTCGTTAT
GGCCGACGTGGACCGCTAAGCATGAACAGCACGCTTATCCAGACAGACCGTTTCACCTTGAGTGAAACCGAGCGCT
CGGCCATCGAGCACGAGCTGCATCACTACGAAGATCCGCGCGCAGCGTCGATCGAAGCCTTGAAGATCGTCCAGAA
GGAACGTGGCTGGGTGCCCGACGGCGCCCTCTACGCCATCGGCGAGATCCTCGGCATCCCGGCCAGCGACGTTGAA
GGCGTGGCCACGTTCTACAGCCAGATCTTCCGCCAGCCGGTTGGCCGCCACATCATTCGCGTGTGCGACAGCATGG
TCTGCTACATCGGCGGCCACGAATCAGTGGTTGACGCGATCCAGAGCAAGCTGGGCATCGGCCTCGGCCAAACCAC
TGCAGACGGGCGTTTCACCCTGCTGCCGGTGTGCTGCCTGGGTAACTGTGACAAGGCGCCGGCGTTGATGATCGAC
GACGACACATTCGGCGACGTCCAGGCCTCTGGCGTCACCCAATTGCTCGAGGGCTACCCATGACCCTGACTTCCTT
TGGCCCGGCCAACCTGATCAAGCGTTCGGCCGAAACCCACCCGCTGACCTGGCGCCTGCGTGACGACGGCGAGCCT
GTATGGCTTGACGAGTATCAGGCCAAGAACGGCTATGCCGCAGCACGCAAGGCTTTTGCCGACATGGCCCAGGACG
ATATCGTCCAGACCGTGAAGGACGCCGGCCTTAAAGGTCGCGGCGGTGCTGGCTTCCCCACGGGTGTGAAGTGGGG
CCTGATGCCCAAAGACGAATCCATCAACATCCGCTACTTGCTGTGCAACGCGGATGAAATGGAGCCCAACACCTGG
AAGGACCGCATGCTGATGGAGCAACTGCCCCATCTGCTGATCGAAGGCATGCTGATCAGCGCCCGTGCGCTGAAAA
CCTACCGTGGCTACATCTTCCTGCGTGGCGAATACACCACTGCCGCCAAGCACTTGAACCGTGCCGTGGAAGAAGC
CAAGGCCGCAGGCCTGTTGGGCAAGAACATCCTCGGTTCGGGGTTCGACTTCGAACTGTTCGTGCACACCGGCGCC
GGGCGTTATATCTGCGGTGAAGAAACCGCCCTGATCAACTCCCTCGAAGGCCGCCGCGCCAACCCGCGCTCCAAGC
CGCCCTTCCCCGCTGCTGTTGGCGTGTGGGGCAAGCCGACGTGCGTTAACAACGTCGAGACGCTGTGCAACGTGCC
GGCGATCATCGCCGACGGCGTGGACTGGTATAAATCCCTGGCCCGCGAAGGCAGCGAAGACATGGGCACCAAGCTC
ATGGGCTTCTCCGGCAAGGTCAAGAACCCGGGCCTGTGGGAACTGCCATTCGGCGTGACCGCCCGCGAGCTGTTCG
AGGACTACGCCGGCGGCATGCGCGACGGCTACACCCTGAAAGCCTGGCAGCCAGGCGGCGCCGGTACCGGCTTCCT
GCTCCCTGAGCACCTCGACGCACAAATGTATGCCGGCGGCATCGGCAAGGTGGGCACCCGGATGGGTACCGGCCTG
GCGATGGCGGTGGACAACACCGTGAACATGGTCTCGCTGCTGCGCAACATGGAGCAGTTCTTCGCCCGTGAATCCT
GTGGTTTCTGCACTCCGTGCCGCGATGGCCTGCCATGGAGCGTCAAGCTCCTGATGGCCATCGAGAAAGGCGAAGG
CCAGCCGGGGGATATCGAGACCCTGCTGGGCCTGGTCGGTTTCCTCGGCCCTGGCAAGACCTTCTGTGCTCACGCA
CCGGGCGCCGTGGAGCCACTGGGCAGCGCAATCAAGTACTTCCGCTCGGAGTTCGAAGCCGGCATCGCGCCCAAAA
GCGCCGCCGTCCCGCCTCTGGCAAAGCCGATCGTAGTCGGCGCGTAACGCTTCAATAAGCGAAGGGTCCGTGCCCT
TCGCTTTGTCATGTGCTGACGCCTTGATGGCTGTGTTGATGCACATGAATAACAAGATTCCATTAGCCACGCCCGC
TGACAACGGGCCAACGAAGAACTTTGAACCATGGCCACTATCCACGTAGACGGCAAAGCGCTCGAAGTCGATGGGG
CAGACAACCTGTTACAGGCGTGTCTGTCACTAGGCCTCGACATCCCTTATTTCTGCTGGCACCCCGCGCTCGGTAG
CGTCGGTGCCTGTCGCCAGTGTGCGGTCAAGCAGTACACCGACGAAAACGACACCCGTGGTCGTATCGTCATGTCC
TGCATGACCCCAGCCACCGACAACACCTGGATCTCCATCGAAGATGAAGAATCCAAGGCGTTCCGCGCCAGCGTTG
TCGAATGGCTGATGACCAACCACCCCCACGACTGCCCGGTCTGTGAGGAAGGCGGTCACTGCCACCTGCAAGACAT
GACGGTGATGACCGGCCACAACGAGCGCCGTTACCGCTTCACCAAGCGTACCCACCAGAACCAGGAACTGGGCCCG
TTCATTTCCCATGAAATGAACCGCTGCATCGCCTGCTACCGCTGCGTACGCTTCTATAAAGACTACGCCGGCGGCA
CCGACCTGGGCGTCTTTGGCGCCCACGACAACGTGTACTTCGGTCGCGTTGAAGACGGCGTGCTCGAAAGCGAGTT
CTCCGGCAACCTCACCGAGGTCTGCCCGACCGGTGTGTTCACCGACAAGACTCACTCCGAGCGCTACAACCGCAAG

TGGGACATGCAGTTCGCCCCGAGCATCTGCCATGGCTGCTCCAGCGGTTGCAACATCTCCCCGGGCGAGCGCTACG
GTGAACTGCGTCGCATCGAAAACCGCTTCAACGGTTCGGTCAACCAGTACTTCCTGTGCGACCGTGGCCGTTTCGG
CTATGGCTACGTCAACCGCGAAGACCGTCCGCGCCAGCCGTTGCTGGCCGATGGCGCCAAGCTGAGCCTGGACGAC
GCACTGGATAAAGCCGCCGACCTGCTGCGCGGCCGTAACATCGTCGGTATCGGTTCGCCACGCGCCAGCCTCGAAA
GCAACTACGCGCTGCGCGAGCTGGTCGGTGCCGAGCACTTCTACAGTGGTATCGAAGCCGCTGAGCTGGAGCGCAT
CCGCCTGGTCCTCCAGGTGCTGAACGACAGCCCGCTGCCAGTGCCAAACATGCGCGACATCGAAGACCACGACGCA
ATCTTCGTACTCGGTGAAGACCTGACCCAGACCGCTGCGCGTATCGCGCTGTCCCTGCGCCAGTCGGTCAAAGGCA
AGGCCGAAGAAATGGCCGACGCCATGCGCGTGCAGCCTTGGCTCGACGCTGCGGTGAAAAACATCGGCCAGCACGC
GCTGAACCCGCTGTTTATCGCCAGCATCGCTGAAACCAAGCTCGACGATATCGCCGAAGAGTGCGTGCACGCAGCA
CCCGACGACCTGGCACGCATCGGTTTCGCCGTGGCCCACGCCCTCGACGCCAGCGCCCCTGCCGTCGAAGGCCTGG
ACGCAGAAGCCGTGGCCCTGGCCCAGCGCATCGCCGACGCCCTGCTGGCGGCCAAGCGCCCATTGATCATTGCCGG
TACCTCCCTGGGTTCCAAGGCACTGATCGAAGCCGCCGCCAACATCGCCAAGGCCCTGAAGCTGCGGGACAAGAAC
GGCTCCATCAGCCTGGTTGTACCGGAAGCCAACAGCCTTGGCCTGGCCATGCTCGGCGGCGAGTCCGTGGACGCAG
CCCTGCAAGCGGTGATCGACGGCAATGCCGACGCCATCGTGGTGCTGGAAAACGACCTGTACACCCGCACCGATGC
CGCCAAGGTTGACGCTGCACTGGACGCTGCCAAAGTCCTGATCGTCGCCGATCACCAGAAGACCGCTACCAGCGAC
CGTGCCCACCTGGTGCTGCCAGCCGCTACCTTCGCTGAAGGCGACGGTACCCTGGTCAGCCAGGAAGGCCGCGCCC
AGCGCTTCTTCCAGGTGTTCGATCCGAAGTACATGGACGCCAGCATCCTGGTTCACGAAGGCTGGCGCTGGCTGCA
TGCCCTGCGCGCAACCCTGCTGAACCAGCCGATCGACTGGACCCAACTCGACCACGTCACCGCTGCCGTCGCCTCC
AGCGCACCGCAACTGGCGCGTATCGTCGATGCCGCACCGTCCGCCGCGTTCCGCATCAAGGGCATGAAACTGGCCC
GTGAGCCGCTGCGTTACTCCGGACGTACCGCCATGCGCGCTGACATCAGCGTGCACGAACCGCGTACCCCGCAAGA
CAACGACACCGCGTTTGCCTTTTCCATGGAAGGTTACTCGGGTTCGGTCGAGCCACGTCAGCAGGTGCCTTTCGCC
TGGTCGCCGGGCTGGAACTCGCCGCAGGCCTGGAACAAGTTCCAGGACGAAGTCGGTGGTCATATCCGCGCTGGCG
ACCCGGGCACCCGCCTGATCGAAAGCACCGGCGACTCGCTGAACTGGTTCGCGGCCGTACCGCGTCCGTTCAACCC
GGCCCAGGGCACCTGGCAGGTTGTGCCGTTTTTCCACCTGTTCGGCAGCGAAGAGAACTCTTCCAAGGCCGCGCCG
GTGCAAGAGCGCATCCCGGCTGCCTACGTGGCGCTGGCCAAGTCCGAAGCCGACCGCCTGGGCGTCAACGACGGTG
CCCTGCTCAGCTTGAACGTAGGCGGCCAGACCCTGCGCCTGCCGCTGCGCATCAACGATGAGTTGGGTGCTGGCCT
GGTTGGCCTGCCAAAAGGTATCGCTGGCATTCCGCCTGCGATCTTCGGCCACACCGTTGACGGTCTGCAGGAGGCA
GCGCAATGACTTGGTTCACTCCTGAAGTGATCGACGTGATCATCTCGGTGGTCAAGGCCATCGTGATCCTGCTGGC
CGTGGTCGTCGCGGGCGCCCTGCTCAGCTTCGTCGAACGTCGCTTGCTGGGCTGGTGGCAGGACCGTTACGGTCCG
AACCGCGTTGGCCCGTTCGGCATGTTCCAGATCGCTGCCGACATGCTGAAAATGTTCTTCAAGGAAGACTGGACCC
CGCCGTTTGCCGACAAGGTGATCTTCACCCTGGCACCGGTCGTGGCCATGAGCGCCTTGCTGATCGCCTTCGCGAT
CATCCCGATCACCCCGACCTGGGGCGTGGCGGACCTGAACATCGGCTTGCTGTTCTTCTTCGCCATGGCCGGTCTG
TCGGTCTACGCGGTGCTGTTCGCCGGTTGGGCCAGTAACAACAAGTTTGCCCTGCTGGGCAGCTTGCGGGCCTCGG
CGCAGACTGTGTCCTACGAAGTGTTCATGGGCCTGGCGCTGATGGGCATCGTGGTGCAGGTTGGCTCGTTCAACAT
GCGCGACATCGTTGAGTACCAGGCGCAGAACCTATGGTTCATCATTCCGCAGTTCTTTGGCTTCTGTACCTTCTTC
ATCGCTGGCGTGGCCGTGACTCACCGTCACCCCTTCGACCAGCCGGAAGCAGAACAGGAACTGGCCGACGGTTACC
ACATTGAATACGCCGGCATGAAATGGGGCATGTTCTTCGTCGGTGAGTACATCGGCATCATCTTGATCTCGGCCCT
GCTGGTCACGCTGTTCTTCGGCGGCTGGCACGGTCCGTTCGGCATCCTGCCGCAGTTGGCGTTCTTCTGGTTCTTC
CTCAAGACCGCGTTCTTCATCATGTTGTTTATCCTGCTGCGCGCTTCCATTCCGCGTCCACGATACGACCAGGTGA
TGGATTTCAGCTGGCGCTTCTGCCTGCCGCTGACCCTGATCAATTTGCTGGTGACTGCTGCCGTTGTGTTGTTGAA
CACGCCAGCGGGCGCGGTTCAGTGAGGATTTGACCCATGTTCAAATATATTGGCGACATCGTTAAGGGTACCGGTA
CCCAATTGCGAAGCCTGGTGATGGTTTTCGGCCATGGCTTTCGCAAACGCGACACCCTGCAATACCCGGAAGAAGC
GGTGTACCTGCCGCCGCGCTATCGCGGCCGCATCGTACTGACCCGCGACCCCGACGGCGAAGAGCGTTGCGTAGCC
TGCAACCTGTGCGCCGTGGCGTGCCCGGTGGGCTGTATCTCCCTGCAGAAAGCTGAAACCGAAGACGGTCGCTGGT
ACCCGGACTTCTTCCGCATCAACTTCTCGCGCTGCATTTTCTGCGGCCTGTGCGAGGAAGCTTGCCCGACCACCGC
GATCCAGCTGACACCGGATTTCGAGATGGCCGAGTTCAAACGTCAGGACCTGGTGTACGAGAAAGAAGATCTGCTG
ATCTCCGGTCCCGGTAAAAACCCTGATTACAACTTCTATCGTGTTGCAGGTATGGCCGTTGCCGGTAAGCCCAAGG
GCGCCGCACAAAACGAAGCCGAGCCGATCAACGTGAAGAGCTTGCTGCCTTAAGGAAGAAAGATGGAATTCGCTTT
CTATTTCGCATCGGGTATTGCAGTGGTGTCCACACTTCGCGTGATCACCAACACCAACCCCGTGCACGCCCTGCTC
TACCTGATCATTTCGCTGATCGCCGTGGCCATGACCTTTTTCAGCCTCGGCGCACCGTTTGCCGGTGTCCTGGAAG
TGATCGCCTACGCTGGCGCCATCATGGTGCTGTTCGTGTTTGTGGTGATGATGCTCAACCTGGGGCCGGCTTCGGT
CGCCCAGGAGCGCGTCTGGCTCAAGCCCGGCATCTGGCTTGGCCCGGTGATCCTGGCAGCGCTGCTGCTGGCTGAA
CTGCTGTATGTGCTGTTCGCTCACCAGAGCGGCCAGGCCATCGGCCACACCACCGTAGACGCCAAGGCCGTGGGCA
TCAGCCTGTTCGGCCCGTACCTGCTGGTGGTCGAACTGGCTTCGATGCTGCTGCTCGCTGCAGCCATCACTGCCTT
CCACTTGGGCCGCAACGAAGCCAAGGAGCAATGACGATGCCTGCTATCCCTTTGGAGCATGGTCTGGCGGTCGCCG
GCATCCTGTTCTGCCTTGGCCTGGTCGGCCTGATGGTTCGCCGTAACATCCTGTTCGTGTTGATGAGCCTGGAAAT
CATGATGAACGCTGCAGCACTGGCCTTCATCGTGGCAGGTGCGCGTTGGGGCCAGCCGGATGGACAAGTCATGTTC

ATCCTGGTGATCAGCCTGGCAGCCGCCGAGGCCAGTATTGGCCTGGCGATCCTGCTGCAACTGTATCGTCGCTTCC
ACACGCTTGATATCGACGCTGCCAGTGAGATGCGCGGATGAACATGATCTTTCTGACTTTCGTATTCCCCCTGATC
GGTTTCCTGCTGCTGTCGTTCTCCCGTGGACGCTGGTCGGAAAACCTCTCGGCCCTGGTCGGCGTGGGTTCCATTG
GCTTGTCGGCGATTGTCGCCGCCTACGTCATCTGGCAATTCAACGTTGCACCACCCGAAGCCGGTCACTACACCCT
GGTGCTGTGGCAATGGATGGCGGTCGAGGGCTTCAAGCCTGACTTCGCCCTGTACGTCGACGGCCTGTCGATCACC
ATGCTCGGCGTGGTGGTAGGCGTGGGTTTCCTGATCCACCTGTTCGCGTCCTGGTACATGCGCGGTGAGGCCGGTT
ACTCGCGCTTCTTCGCCTACACCAACCTGTTTATCGCCAGCATGCTGTTCCTGGTGCTGGGCGATAACCTGTTGTT
CCTGTACTTCGGCTGGGAAGGCGTGGGCCTGTGCTCGTACCTGTTGATCGGTTTCTACTACAGCAACCGCAACAAC
GGTAATGCAGCACTCAAAGCCTTTGTCGTGACCCGGATCGGCGACGTGTTCATGGCCATCGGCCTGTTCATCCTGT
TCCAACAGGTGGGCACGCTGAATATCCAGGAACTGCTGGTGCTGGCACCACAGAAATTCCAGGTCGGCGACTTCTG
GATCACCCTGGCGACCCTGATGCTGCTGGGTGGCGCTGTCGGTAAATCGGCACAACTGCCACTGCAAACCTGGCTG
GCAGACGCCATGGCCGGTCCTACCCCGGTGTCGGCACTGATCCACGCCGCCACCATGGTGACCGCCGGTGTCTACC
TGATCGCCCGTACCCACGGCCTGTTCACCCTGGCGCCGGAAATCCTGCACCTGGTGGGCCTGGTAGGTGGCGTGAC
CCTGGTACTGGCCGGCTTCGCCGCGCTGGTGCAGACCGACATCAAGCGCATCCTCGCCTACTCGACCATGAGCCAG
ATCGGCTACATGTTCCTGGCCCTGGGCGTCGGCGCCTGGGACGGCGCGATCTTCCACCTGATGACCCACGCCTTCT
TCAAGGCCCTGCTGTTCCTGGCTTCCGGTGCGGTGATCGTTGCCTGCCACCACGAGCAGAACATCTTCAAGATGGG
TGGCCTGTGGAAGAAACTGCCGTTGGCCTACGCCAGCTTCATCGTCGGTGGTGCCGCACTCGCAGCCCTGCCACTG
GTGACCGCAGGTTTCTACTCCAAAGACGAAATCCTCTGGGAAGCCTTTGCCAGCGGTAACCAAGGCCTGCTCTACG
CCGGTCTGGTGGGTGCGTTCATGACCTCGCTGTACACCTTCCGCCTGATCTTCATCACCTTCCACGGTGAAGCCAA
GACTGAAGCCCACGCAGGCCACGGCATCTCCCACTGGCTGCCGCTGTCGGTGCTGATCATCCTGTCGACCTTCGTC
GGCGCCATGATCACCCCGCCACTGGCTGGCGTACTGCCACAAAGCGTTGGACATGCTGGCGGTGACGCCAAGCACA
GCCTGGAAATCGCCTCGGGCGCCATCGCCATCGCCGGTATCCTGCTGGCGGCCATGCTGTTCCTCGGCAAACGTCG
CTTCGTCACTGCCGTGGCCAACAGTGGCATTGGCCGCTTCCTTTCGGCCTGGTGGTTCGCCGCCTGGGGCTTCGAC
TGGATCTACGACAAACTGTTCGTCAAGCCTTACCTTGCGATCAGCCATGTACTGCGCAAAGACCCGCTCGACCAGA
CCATTGGTTTGATCCCGCGTGCTGCCAAGGCCGGTCACACCGCCCTGAGCCGCACCGAGACCGGCCAATTGCGTTG
GTATGCGGCTTCCATGGCGGCTGGTGCCGTGCTGGTGATCGGCGCCATCGTAGTGGTAGCGGTCTGATATGAACTT
TGCGAACTTGCGAAAGGAAACGAGCCCGTCATGATTCTGCCCTGGCTAATCCTGATCCCCTTTATCGGCGGCCTGC
TCTGCTGGATGGGTGAACGCTTCGGCGCCACCCTGCCCCGCTGGATTGCGTTGCTGACCATGTCCCTGGAACTCGC
ACTCGGCCTCTGGCTGTGGGCCCATGGCGACTATTCATTTGCTCCGGCACCGGGTGTCGATCCAACCTTCGCGCTT
GAGTTCAAGCACGTCTGGATCGAACGCTTCGGCATCAACGTGCACCTGGCCCTCGACGGCCTGTCGCTGTTGATGA
TCCTGCTGACCGGCCTGCTGGGTATCCTCTCGGTACTCTGCTCCTGGAAAGAAATCGAGCGTCACGTGGGCTTCTT
CCACCTGAACCTGATGTGGATCCTGGGCGGCGTGGTCGGCGTATTCCTCGCCCTCGACCTGTTCATGTTCTTCTTC
TTCTGGGAAATGATGCTGGTGCCGATGTACTTCCTCATCGCGCTCTGGGGTCACAGTTCTTCGGACGGCAAGAAAA
CCCGGATCTACGCGGCGACCAAGTTCTTCATCTTCACCCAGGCTTCCGGCCTGATCATGTTGGTGGCGATCCTGGG
CCTGGTACTGGTCAACTTCAACAACACCGGCGTGATTACCTTCAACTACGCCGACCTGTTGAAAACCAAGATGTCG
ATGACCACCGAGTACATCCTCATGCTCGGGTTCTTCATCGCCTTCGCGGTGAAGCTGCCGGTGGTGCCGTTCCACT
CCTGGCTGCCTGACGCTCACGCCCAGGCGCCAACCGCAGGTTCCGTGGACCTGGCCGGTATCCTGTTGAAGACGGC
GGCCTACGGCCTGCTGCGTTTCGCCCTGCCGCTGTTCCCGAATGCTTCGGCCGAGTTTGCGCCGATCGCCATGACC
CTGGGTCTGATCGGGATCTTCTACGGTGCATTCCTGGCCTTCGCGCAAACCGACATCAAGCGTCTGATTGCCTTCT
CGTCCGTTTCCCACATGGGTTTCGTACTGATCGGCATCTACTCCGGCAGCCAGCTGGCGTTGCAAGGCGCAGTGAT
CCAGATGTTGGCCCACGGTGTTTCAGCCGCCGCACTGTTTATCCTCAGTGGCCAGTTGTACGAGCGCCTGCACACC
CGTGACATGCGTGAGATGGGCGGCATCTGGTCGCGTATCGCGTACCTGCCGGCGATCAGCCTGTTCTTTGCCGCCG
CGTCCCTGGGCTTGCCGGGTACGGGTAACTTTGTCGGTGAGTTCCTGATCCTGATGGGCAGCTTCGTCAGTGCGCC
ATGGATCACCGCGATTGCCACTTCCGGCCTGGTGTTCGGTTCGGTCTATTCGCTGATCATGATCCACCGCGCCTAC
TTCGGCCCGACCAAGTCGGACGCCGTCCTCAAAGGGATGGATGCTCGCGAACTGATCATGGTGCTCGGCCTTGCGG
TACTGCTGGTGTACATCGGCGTGTATCCGCAACCGTTCCTCGACACCTCTGCCGCCACGATGCATGGCGTGCAGCA
GTGGTTGAGCACCGCCTTCACTCAACTCGCTTCGGCCCGGTAAGAGCGCTATGGAATTCACGATCCAACACTTTAT
CGCGCTTGCGCCGCTGCTGATTACCAGCCTCACCATTGTGGTGGTGATGCTGGCAATCGCCTGGCGCCGCAATCAC
TCGCAGACGTTCCTGCTGTCCACCGCAGGCCTGAACCTGGCCTTGCTGTCGATTATCCCGGCACTCAAGGTCGCCC
CGCTGGCGGTCACCCCGCTGGTCATGATCGATGACTTCGCGCTGCTGTACACCGCGTTGATCCTCGTTGCGACACT
GGCCTGCGTCACCCTCGCCCACGCTTACCTCGGCGAAGGCGGCACCGGCTATCCGGGCAACCGCGAAGAGCTGTAC
CTGCTGATCCTGCTGGCTGCGGCCGGCGGTATCGTGCTGGTCAGCGCGCAGCACCTGGCCGGTTTGTTCATCGGCC
TGGAACTGCTGTCGATCCCGACCTACGGCCTGGTGGCGTACGCCTTCTTCAACAAGCGCTCCCTGGAAGCCGGCAT
CAAGTACATGGTGCTGTCGGCCGCCGGTTCCGCGTTCCTGTTGTTCGGTATGGCCCTGCTCTACGCCGAAGCCGGC
AGCCTGAGCTTCAGCGGTATCGGCCACGCCCTGGCCACCACCAGCATGCCTGCGCCCATCGCCCAACTGGGCCTGG
CCATGATGCTGATCGGCCTGGCGTTCAAGCTGTCGCTGGTACCGTTCCACCTATGGACCCCAGACGTGTACGAAGG
CGCCCCGGCGCCAGTGGCCGCGTTCCTGGCAACCGCCTCTAAGGTTGCAGTGTTTGCGGTGATGGTGCGTCTGTTC

CAGATCTCCCCTGCCGCCAACACCGGCGTGCTGAGCGACGTGCTGACCGTGATCGCCATTGCGTCGATCCTGTTCG
GTAACCTGCTGGCCCTGACCCAGAACAACCTCAAGCGTCTGCTGGGTTACTCGTCCATCGCCCACTTCGGCTACCT
GCTGATCGCCCTGGTGGCGAGCAAAGGCCTCGCCGTGGAAGCCATCGGCGTGTACCTGGTCACCTACGTGATCACC
AGCCTCGGCGCGTTTGGCGTGATTACCCTGATGTCCTCGCCTTACAAAGGCCGTGACGCCGACGCCCTGTACGAAT
ACCGCGGCCTGTTCTGGCGCCGTCCGTACCTGACCGCCGTGCTGACCGTGATGATGCTGTCCCTGGCCGGCATCCC
GCTGACCGCAGGTTTTATCGGCAAGTTCTACATCGTCGCGACCGGTGTCGAAGCCCACGAGTGGTGGCTGGTTGCC
TCCCTGGTGCTGGGCAGTGCCATCGGCGTGTTCTACTACCTGCGCGTGATGGTCACCCTGTACCTGATCGAGCCAA
ACCTGCGCCGTGTGGATGCCGAGTTGCACTGGGAACAGAAAGCAGGCGGCGTGATGCTGCTGGCTATCGCCCTGCT
GGCATTCTTCCTGGGCGTGTACCCACAACCGTTGCTCACCCTGGTGCAGCATGCGGTGATGGCGGGTTGATCGCTT
AAGCTCAAGCAGTAAACAGAACGGCACCTTAGGGTGCCGTTTTGCGTTTCTGGGAAAGATTCAAGCTCCCTGCCCA
GCTCAAGGAATATTCCTTACTGAAATGCCTCCGTGTCTGGCATATACCTGCCAGAGCCGCTCGTATGGTGTATGCG
CTTTAGGAAGAATCCCACGTCAGGCTCACTTGACCCCTGGCAGATGGGCCACCAAACTATACGCAGGCTACGTACA
GAACATGGATGCTCTATTTATCGAATTGCCGCCCTTTGAGCGGTACCGCAAGGACTACCTGACTGACGAACTGTTT
CACGGCTTTCAACAAGAGCTAATGAAGAACCCCGAAGCGGGAGCCGTGATGGAAGGCACCGGTGGGTTACGCAAGC
TGCGCTTCGTCGATGAACGACGAAACAAAGGCAAACGGGGTGGATTACGCGTCATCTACTACTGGTGGTCAGGCGG
CACTCAATTTTGGTTGTTCACGCTGTACGGCAAGCACGAGCAAAGCGACTTGAGCCCACAACATAAAAAAGCCCTC
AAGCAACTGCTGGACAGGGAAATCAACGCGAGAGGATTCACATGAAACGTGACATTTATTCCGAACTGGTCGAAGG
CTTCGATGCCTTGGCCAACGAGCGCCAAGGCAAGGTCACCCTACGTACTCATACCGTGCAGCAAAACAAACTGGCC
CCTCTTACCGCTGAAGACGTGATTGCAGTGCGACAACAACTCAATCTTTCCCGTGCTGTATTTGCCATGTACCTAC
GCACCAATACACGCACCCTGGAAAACTGGGAGCAAGGTCGCGCACGCCCCAATGCCCAGGCAGCCACGTTGATTCG
TCTGGTATCAAAATTTCCGGATACGGTGGAGCGCCTGGCGTTGCTTAGCTGATCGTAGGTGAAGCGATCTTTCCCA
CATCAGCAAAAACGGCACCTCACGGTGCCGTTTTGCTATCCGCCCTACCTCACTTGCGCGCCGCCTCCCACGACTT
GAGCAAATCGGCATAGTTCACGGTTTCACCCTTGGGTTTCTCGTTCGCCAGTTTCGGCTTCGGTGCGCCCGGCTGG
TCGAACCAGTACTGGGCATCGCGCTCGGGGTTCATTTTCGGTCCGCAGATCGGCTGCACCTTGGAGCGCTCCAGGC
GCGTCATGATCGCGTCCTGGTCCTTGGCCAGGCCATCCAGCGCCTGTTGCGGGGTTTTCTCGCCGCTGGCGGCTTC
CGAAATGTGGCTCCACCACAGTTGGGCCAGGCGCGGATAATCCGGCACGTTGGTGCCCGTCGGTGTCCACTGCACC
CGCGCCGGGCTGCGGTAGAACTCCACCAGGCCGCCGAGTTTGGGGGCCAGGTCGGTCATGGCCTGGGAGTTGATGT
CGGATTCACGGATGGGCGTCAGGCCGACGATGGTTTTTTTCAGGGAAACGGTTTTCGATGTCACGAACTGCGCGTA
GAGCCAAGCCGCGAGTTTTTGTTTCTCAGGCGTGGACTTGAGGAACGTCCAGGAACCCACGTCCTGATACCCCAGC
TTCATCCCCTCCTCCCAATACGGCCCGCGTGGCGACGGTGCCATGCGCCACTTCGGCGTGCCGTCGGCGTTCATCA
CCGGCAAGCCGGGTTTGGTCATGTCAGCGGTAAAGGCGGTATACCAGAAGATCTGCTGCGCGATGTTGCCCTGGGA
CGGTACCGGCCCGGACTCGGAGAAGGTCATGCCTGCCGCTTCCGGTGGCGCATAGGCACGCATCCAGTCGACATAC
TTCTGCGTGGCAAATACCGCAGCCGGGCCGTTGGTGTCGCCGCCACGGGTCACGCTAGACCCCACCGGGTGGCAAT
CCTCGACCCGGATCCCCCACTCGTCCACCGGCAAGCCGTTGGGCAGGCCCTTGTCGCCACCACCGGCCATGGAGAA
CCAGGCATCGGTAAAGCGCCAACCCAGGGAAGGATCTTTCTTGCCGTAGTCCATATGGCCATACACGCGCTTGCCG
TCGATTTCCTTGACGTCTTCACTGAAGAACTTGGCGATGTCCTCATAGGCCGACCAGTTCACTGGCACGCCTAGTT
CATAGCCGTACTTTTCCTTGAACTTGGCCTTGAGGTCGGCGCGCTCGAACCAGTCGGCACGGAACCAGTAGAGGTT
AGCGAACTGCTGGTCGGGCAACTGATAGATCTTGCCGTCCGGCGCGGTGGTAAACGAGATGCCGATAAAGTCCTTG
AGGTCCAGGGTCGGCGAGGTGAAGTCCTTGCCTTCGTTGGCCATCAGGTCGGTGATGGACTCGGTCTTGCCATAGC
GAAAGTGCGTGCCAATCAGGTCGGAATCGTTGACCCAACCGTCATAGATATTCTTGTCCGACTGCATCTGGGTTTG
CAGTTTCTCCACCACGTCGCCTTCCTGCAGCAGGTCGTGGGTCAGCTTGATCCCGGTGATTTCACTGAAGGCCTTG
GCCAGCACCTTGGATTCGTACTCGTGGGTGGTGAGGGTTTCCGACACCACATTGATCTTCATCCCACGAAACGGCT
CGGCGGCCTTGATAAACCACTTCAGTTCTTCAAGCTGCTGGGCCTCCGTCAGGGTGGACGGTTTGAACTCGCTGCC
GATCCACTTCTTCGCGGCATCTTCGTACGCATCGGCCCAGGCCGCAGCACTCAAACCGCTGAGGGCCAAGACGGCG
GCCAATGAAATGCTATGTCGCAGCTTATTGTTTTTATCGAACATAGAGACCTCCTGGTTGATTTCAGACGGGTCCG
ACAGAAGCCCCGCCCCGTGTAGGAGCGAGCTTGCTCGCGAAAAACCCCAAGACACCGCGTTAATCCAGACAACCTG
CGTTATCGTTGACGACTTTCGCGAGCAAGCTCGCTCCTACAGGGGTATGGGGACCACCTCAGCCCCAACGCATCAC
AGACAACAGCCACACCACAGACAGCGCGGACGCCACCCAGATGCTCCAGCCGGTAACGCCGATCACCAGCAGATGC
AGGTAGGCGCTCCCGAGAAGACCGATAAACAAGCGATCACCACGTGTCGTGCTGATCGGTAACAAACCGCGCCGGG
GAATGCTCGGCGAGCGCAGTTCCCACGTGGTCATGCCTGCCAGCAATAAGGCAATTGCCGCAAAAAACAGCGCAGT
CGGGGTGGTCCAGGCCATCCATTCCATCATCGTTTCCTCAGACCCGGCCCAGGGCAAAGCCCTTGGCCACATGGTT
GCGAACAAACCAGATCACCAGCATGCCCGGCAGGATGGTCAGCACTCCGGCAGCCGCCAGCACGCCCCAGTCAATG
CCCGACGCGGACACGGTGCGGGTCATTACGGCGGCAATCGGCTTGGCATTGACGGACGTCAGGGTGCGCGCCAGCA
GCAGTTCGACCCAGGAAAACATAAAGCAGAAAAACGCCGTGACACCGATCCCCGAGCCAATCAGCGGGATAAATAT
CTTCACGAAAAACTTCGGAAAGCTATAACCATCAATGTAGGCCGTTTCGTCGATCTCCTTGGGCACCCCTGACATA
AAGCCTTCCAGGATCCACACCGCCAGCGGTACGTTGAACAGGCAGTGGGCCAGGGCCACGGCGATATGGGTATCGA
ACAGGCCAATGGACGAATACAGCTGGAAAAACGGCAACAGGAACACCGCCGGCGGCGCCATGCGATTGGTCAGCAG

CCAGAAGAACAGGTGCTTGTCGCCAAGGAAGCGATAGCGCGAAAACGCGTAGGCCGCCGGTAGCGCCACGGTCAGG
GAAATAACCGTGTTCAGGCTCACGTAGTACAGCGAGTTGAGATACCCGGTGTACCAACTGGGGTCGGTGAAGATCA
CCTTGTAGTTGGCCAAGGTGAAATCCTGCGGAAACAGCGTCAGGCCGCCGAGGATTTCGGTGTTGCTCTTGAAGGA
CATGTTCAGCAGCCAGTAGATCGGCACCAGCAGGAACAGGATGTAGATCAGCAGCGGAATGACCTTGCGCTTGCTC
ATGTTGGCGGCCTCAGCGGTTGGCGTCGGAGTGGGTCATGGCGGTGTAGAACAGCCAGGACACCAACAGGATGATC
AGGAAATACACCAGGGAAAACGCGGCGGCCGGGCCCAGGTCGAATTGACCGACAGCCATCTGGGTCAGGGTCTGAC
TGAGAAAGGTCGTCGCATTTCCCGGCCCACCGCCGGTCAGTACAAAAGGCTCGGTATAGATCATGAAACTGTCCAT
AAAACGCAGCATCACCGCGATCAGCAGCACGCTCTTCATCTTCGGCAGTTGGATATGTCGGAATACGGCCCAGGCC
GATGCCCGATCAATCCGCGCCGCCTGGTAGTACACATCCGGTATAGCCCGCAGCCCCGAGTAACAGAGCAGCGCCA
CCAGGGATGTCCAGTGCCATACATCCATCACCAGTACTGTGACCCAGGCATCCATGGTGTTGGCGGCGTAGTTGTA
GTTGATGCCCATGGCATTCAGGCTCGAGCCCAGCAGGCCGATATCGGCACGGCCAAAGATCTGCCAGATGGTGCCG
ACGACGTTCCACGGGATCAGCAGCGGGATGGCCATCACAATCAGCACCAGGGACGACCAGCGGCCTTTGGTGGGCA
TGGTCAGGGCAATGGCAATGCCCAGGGGGATCTCGATCAGCAACACACATGCCGAATAGATGAACTGGCGCAGCAG
CGAGTCATGCAGGCGTGGGTCCAGCAGCACTTGTTTGTACCAGTCGGCCCCGACGAAATAGCGGCTGGACTGATCG
AAAATGTCCTGCACCGAGTAGTTGACCACGGTCATCATCGGGATCACCGCACTGAACGCCACCAGCAGGAACACCG
GCAACACCAGCCACCAGGCCTTGTTGTTCTGCACCTTGTTCATGGCCGCGCCTCCAGCAGGTAATCGTCGGCATAC
ACCATCAGCCATTGCGCCGGAAAACTGATCGAGGCCCGGCCCTCGGGCACCGGTTTGTCTTCGGCCAGACGCACTT
TCAACGGCGCGCCGTCGAGGTTGAGGGTCAAGATCTTGTAGGTGCCCAGGTCTTCGACATGTATGACGTCGGCTTG
CAACGCGTCGGTATTGGGCTCATCCCAGACATGGATAAACTCGGGACGAATACCGACCTGGAGTTTTTTGTAGTCC
TTTTCCGCGAGTTGCCGCTGCTGCACCTCGGACAACACCAGATGTGTTCCGCCAAACCGTACCCCACCTGCATCGG
CCTGCACCTCAATCAGATTCATCCCCGGGCTACCGATGAAATAGCCGACAAAGGTATGACTGGGACGCTCGAACAA
CTCCCGCGGTGTGCCGAACTGCACAATCTGCCCGCCGTACATCACCGCAATCTTGTCGGCAAAGGTCGAAGCCTCC
AACTGGTCGTGGGTGACGTAGACCATGGTGATGTTGAACTGCTCGTGGATCTGCTTGAGCTTGCGCCGCAGTTTCC
ACTTCAGGTGCGGATCTATCACCGTCAGCGGCTCGTCGAACAGAATCGCGGAGACGTCATCGCGCACCAGGCCACG
GCCCATTGAGACCTTTTGCTTTTCATCAGCACTGAGGTTGCGGGCTTTTTTGCCCAGCAGGTTCTGCAGGTCGAGC
ACTTCGGCAATTTCCTGCACCTTGCTGTGCACTTTCGCCTCGGCCATCCCCTGGTTACGCAGGGGGAATGCCAGGT
TATCGAACACCGTCATGGTGTCGTAGACCACCGGGAACTGGAACACCTGAGCGATATTGCGCTTTTCCGGGGTAAG
GTCATTCACCACCTTGCTGTCGAACAGCACCTGGCCTTCCGAAGGGCTGAGCAAGCCGGAGATGATGTTGAGCAAG
GTTGACTTGCCACAGCCCGAAGGACCGAGCAAGGCGTAGGCGCCGCCCTGCTCCCAGATGTGGTTCATTTCGCGAA
TGGCGTAATCTTCAGGCCCTGCAGGTGTAGGGCTGTAGCTATGCGCCAGGTTCTGCAAATGGATCTCGGCCATCAG
GCAACCCTCGCGACACGACGCCCGGGGGCTTGGACCAACCGGCCCTGGCCATCGAACACAAACAGTTTATGGGTCG
GAATATAGATCCGGATCGGCGCATCCACGTCGTACTCGTGGACTCCCGGCAAATGCAGCACCAGCAAGAAATGCTC
GCTGCGCACATGCAGGAAGGTTTCCGAGCCGCTGATCTCCGCCACTTCCACGGTTACCGCCAGTTCCAGGTCATCG
TCGTTGCTGGGCACCAGGCTGATATGGCTGGGGCGCACGCCGAACCGGAACTCGCCCTCACCAATCGGGCGCAGGT
CCACGTTCAACGGAAAGTGCACATCGTTGGCGAAACTCACTTCATTACCGCTGATACGCCCCGGCATCAGGTTGAT
GGGCGGTTCGGAAAACAGCTCGGCCGCCAATACCGTCTGCGGTTGGTGATAGACCTCGGCGGCCTTGCCGCTCTGG
ATCACCCGGCCTTCGTGGAGGATGGTGGTGGTGCCGCCCAGCGCCAAGGCCTCGTTGGGCTCCGTGGTGGCATAGA
TGGCAATGGTGTTGCGAGCCTTGAACAGCTCGCGCATTTCCTGACGCAGCTCTTCACGCAGTTTGTAGTCCAGGTT
CACCAGAGGCTCGTCGAACAGAATCAATTCGGCGTCCTTCACCAGCGCCCGGGCCATGGCCGTGCGTTGCTGCTGG
CCACCGGACAGCTCCAGGGGATGACGTTGCAGGAACTTGTCGATGCGCAGCATCTTCGCGGTTTCCAGTACCTTGC
TGCGGATCAGTTCGTTGGACACCCCGGCCTGGCGCAAAGGCGAGGCGATGTTCTCGAACACGGTCATGGTCGGGTA
GTTGATGAACTGCTGGTACACCATTGAAACATTGCGCAAGCGCACCGGCTTGTGGGTGACATCGACGCCGTTCATC
AGGATGCGCCCGTTGTCAGGCTTGTCCAGGCCGGCCATCAGGCGCATCAGGCTGGTTTTGCCGGACAGCGTACGGC
CCAGCAACACATTGAAGGAGCCGGCTTCAAAGCGCAGGTTGGCGTCGTCGATCCAGGTCTGGCCTTCGACAACGCG
GGTCACATGTTCCAGGGTCAATGACATGACACGCCCTTTTTCTTATTGGAATTGTTCTTCCAAGGTCAGAGCGAGT
TTCGTGCCAATAACAAAAAGTATTTATTTAATAGGGACTTAGGCGTAACTACTGCTCAGTGCTGAACAGAAATGAA
CATTCATAACTGAACAATTGAACAACCTCCGGGTTGACAATGAACAGTTCTGAACAACACTGAATAAGCTCAGCAA
AACCGTTCAACCTGTGGGAGCAGGCTTGCCTGCTACCACAAGGTGAGCGGTGCACGGTTCATAACAACAATAAAAA
GAAGGTCACCGCCATGGCCGCACCTGCCCCGCTTTTGCCCCACGACACCATCATCCAGGACTCCTGGCGCCGCTGC
CGTGCCTTTGGCCTGGATCACCAGAGCGCGCCCAGCTTCGACCAACTGCCCGCCGAGGGCATCAGCCAGCTGTTGG
AGAGCCAGCACGCACTGGTCCAGACCACCCACCAGGAAGTGCTGCCCTACTACGAGAACATCCTCAGCAATTCCAA
CTGCCTGATCATGCTTGCCGACAACCAGGGCCAGGTGCTGACCTCCTGGGGCACCCAGCGCTTTATCGAACCCAAG
CTCGCCCGTGGTTTCAGCGCAGGGGCCAGTTGGCGGGAGCGCTCTACCGGCACCAACGCCATCGGTACCGCACTGG
CCTGCGCCCAGGCGGTGCACATCGAGCATGACGAACACTTCCTCAAGGCCAACCGTTTCATGACCGGTTCAGCGGC
GCCGATCTTTGACGCCAACCGCGAAATCATCGCGGTGCTGGATGTGTCCAGCGACAGCTACCTGCCGCCCTCCCAC
ACCCTGGGCATGGTCAAGATGATGAGCCAGACCGTGGAAAACCGGCTGATCCTCAACCTGTTTGGCGGCGAGCACT
TCCAACTGACCTTCAACACCGGTCTGAACAACCTCGACAGCCAATGGGCCGGCCTGCTGATCTTTGATGAAAGCGG

CCAGGTGCTGTCAGCCAACCGCCGCGCCGACAACCTGCTGGGCATCAGCCTGTCGCGGGTGATGATCGACAGCCTG
TTCAAGGTCTCGCTGCTGGAGCTGCTCAACCAACCCGAAGGCCTGCCCTTCGCCCTGCAAGCTGCGGGGCGCAACC
GTTTCCAGTGCCTGCTCAAACGCCCCAAGCAGGCGCCGGTGCAGGCCAGGGTCTTCAGCGAGCCGGCCGCCGTGCC
CATCGGTCTCAAGACCCTGCACTTTGGCGACAGCCGCGTGGAAAAAGCCGTGCGCCAGGCCGAACGCCTGCTGGAA
AAAGACATCCCGTTGCTGATCCATGGAGAAACCGGCGTGGGCAAGGAAGTCTTTGTCAAAGCCCTGCACCAGGCCA
GCTCCCGCAGCCAGCAGGCTTTTATCGCGGTCAACTGTGCGGCAATTCCGGCCGAGTTAGTGGAATCGGAGCTGTT
TGGCTACGAAAAAGGCGCGTTCACCGGCGCCAATCAAAAGGGCAGCATCGGCCTGATCCGCAAGGCTGACAAAGGC
ACGCTGTTTCTCGATGAGATCGGAGACATGCCCCTCCCAACCCAGGCCCGGCTGCTGCGGGTATTGCAGGAGCGCT
GCGTGCAACCGGTGGGCAGCAGCGAATTGTTCCCGGTGGACCTGCGCATCATCTCGGCCACCAACCGTTCGCTGCG
CGAGCAGGTGCAGGTCGGGCGGTTCCGCGAGGATTTGTACTACCGCATCGGCGGCCTGACCCTGGAACTGCCCCCC
CTGCGCGAACGCAGTGACAAGCAGGCGCTGTTCAAGCAACTGTGGCAACAGCACCGCGAACCCACGCAGTGGGCCG
GGCTCAGTACTGAAGTGCTGGCGTTATTCGAGCGACACCCTTGGCCAGGCAACTTGCGCCAGGTCAGCAGCGTGAT
GCAGGTGGCGCTGGCCATGGCCGAAGACCAGCCGATCCGCCCGGAGCATCTGCCCGATGATTTTTTTGTCGACCTG
GGCCAAGCCTCGCCCAGCGCACCGCCGGTTATGGGCGATCTGGACGATAGCCAAAACCTTCAGCAGCGACTGGCAG
CGGTCGGCGGCAATATCTCCCACTTGGCGCGCGAGTTGGGGGTCAGCCGCAATACCTTGTACAAGCGGTTACGCGC
CGCTACGTAACGGTAACCGCCCCTACGGGCAGCCAGCCTCAGATTTATCCGACAGTGTTTTAGGGTTGCTGCTCGG
AAGATGCCCCAATAATCTTCGGCAGTCGCTGCAAATTCAGCGACCGGGTTTCGCAGCCCGGTTAAACTCTAGACGC
ACAGCGTCCACCACAAGATAACGGGCGCTTTTTTATGCCTGATGTCATGGCGGCTGTGCGCGGGATACCTTCGGGT
ATGCCGGGTTCCTAGAGTCCCGGTCTGCGAACCTGCGTACAGCTGCCACCCAGGTATCGTTTCGCAGCGATGGGTA
GCAGTTCCATAACTCTGGGAGCCTTGCCATGATCAAACACAGCCCAAACCCGCCGTTAGACTCAACCTCGCTTCAG
GCAGCTGCTCAACGAGCGATTGACTACTATCTCAACCCTCCTGCCGAAACCAAGCGGCCTACTGAAAACCACCGCC
TTTTCACCGTGCGCGAAGGGTTAAATGCAGAGGTATTGTTGATCAACGCCTCGGAAGACCTTGCATCGGCTCAAGT
ATTGGCAACCCATTTGGCTTTTGAAGTAGACGGCACTGCACGGCGTGTGGCGCTAGGAATTTGTCGGATGCTGGAG
GGAGTTGAGCTATTGGTGAACAGCGCCGTCGTTCAATGTGGAACTGAGCAAGAAAGAACAAGGCCCACTCACAATC
CAGATTAGTCTGCGATAGCCCGTCCGCCATCGCTGGCAAGCCAGCTCCTACAGTTGAGCAGTGTTGATCGGCAAAA
CCTGCACAAATAAAAACGCCCCGAACCAGTCGGGGCGTTTTTATTCAGCTTGAAGCAATCAGCCGTCAGTCAGCCA
AGCGCCAGGTCGTCCCGCCCTTCCCGTCTTCCAACACCACGCCCATGGCGGTGAGTTGGTCGCGGATGCGGTCGGA
TTCTGCCCAGTCCTTGTTGGCACGGGCATTCAAGCGGGCCTGGATCAGTGCGTCGACTTCGGCTGCGTCCACACGC
CCCTCGGCGCCGGCTTGCAGGAAGTCGTCGGCTTCCATTTGCAGCACGCCCAGCACGCTGGCCAGCTCTTTCAAAC
GGGCCGCTAAACCAGCCGCTGCATCCAGATCGCTCTCGCGCAGGCGGTTGATCTCGCGGACCATTTCGAACAGCAC
GGCGCAGGCTTCGGGGGTGCCGAAGTCGTCGTTCATCACGGTGGTGAAGCGTTCAACAAATGCTTCGCCACCGGCC
GGCGCAACAACTGGCAGGCCTTTCAACGCATGGTAGAAACGTTCCAGGGCGCCCTTGGCGTCCTTGAGGTTGTCTT
CCGAGTAGTTGATGGCGCTGCGGTAGTGGCTGGACACCAGCAGGTAACGCACAACTTCCGGGTGGTACTTTTCCAG
CACGTCGCGGATGGTGAAGAAGTTGTTCAAGGACTTGGACATCTTCTCGCCGTTGATGCGAATCATGCCGCAATGC
ATCCACGCGTTGGCGTAGGTCTTGCCGGTGGCCGCTTCGCTTTGGGCGATTTCGTTTTCGTGGTGCGGGAACTCAA
GGTCGCTGCCGCCGCCATGAATGTCGAAGGTCTCGCCCAGGCAGCAGGTGGACATCACCGAGCATTCGATGTGCCA
GCCCGGACGCCCGGCGCCCCACGGCGACTCCCAGCTCGGCTCGCCCGGCTTGGTGCCTTTCCACAGCACGAAGTCC
AGCGGATCCTGCTTGGCTTCGTCGACTTCGATGCGTGCACCGATGCGCAGGTCTTCGATTTTTTTGCGCGACAGCT
TGCCGTAGCCCATGAACTTGGCGACGCGGTAGTACACGTCGCCATTGCCCGGGGCGTAGGCGTAGCCCTTGTCGAT
CAAGGTCTGGATCATCGCGTGCATGCCAGGGATGTGGTCCGTGGCACGCGGCTCCATGTCCGGCTTGAGGATGTTG
AGGCGCGCCTCGTCCTCGTGCATGGCAGCAATCATGCGCTCGGTCAGCGCGTCGAACGACTCGCCGTTTTCATTGG
CGCGATTGATGATCTTGTCGTCGATGTCGGTGATGTTGCGCACGTACGTCAAGTCGTAGCCGCTAAAACGCAACCA
GCGGGTCACCAGGTCGAAGGCGACCATGCTGCGGCCATGGCCGATGTGGCAGTAGTCGTACACGGTCATGCCGCAC
ACGTACATGCGCACCTTGTTGCCATCCAGCGGCTTGAAGACTTCTTTGGTCTTGCTGAGCGTGTTGTAGATCGTTA
GCACGATGTTTCCCTTAAGACTTGATCACTGGCCCCACGAATCACGCAAGGTCACGGTACGGTTGAATACCGGAGC
GCCTGGTTTCGAGTCCTTGATATCTGCGCAGAAGTAGCCTTCGCGCTCGAACTGGAAACGGTCTTCCGGCTGTGCG
TCGCCAAGCGATGGCTCGGCACGACAACCCGTAAGTACTTGCAGGGAGTCAGGGTTGATGTTGTCCAGGAAGCTGG
CGCTGTCTTCGGCCTTCTCAGGGTTGGCCGAACGGAACAGACGATCGTACAGGCGCACTTCGCACTCGACGCTGGC
GGCTGCCGGCACCCAGTGCACCACGCCCTTGACCTTACGGCCTTCAGGGTTCTTGCCCAGGGTGTCGGGATCGTAC
GAGCAACGCAGTTCGACGATGTTGCCATCGGCGTCCTTGATCGCCTCAAGGGCACGGATCACGTAGCTGCCGCGCA
GGCGCACTTCGCCGTTGGGCTCCAGGCGCTTGTAGCCTTTTGGCGGCTCTTCCATGAAGTCATCGCGGTCGATGTA
GATTTCACGGGCAAACGGCAGCTTGCGCACGCCCAGTTCTTCTTTTTGCGGATGACGCGGCAGTTCGAGGTTCTCG
ACCTGGCCTTCCGGGTAGTTGGTGATCACGACTTTCAACGGACGCAGCACACACATGGCGCGCGGGGCATTGTGGT
CCAGGTCCTGGCGGATGCTGAATTCCAGCATGCCGAAGTCGACCACGCCGTCGGAGCGGTTGGTGCCGACCATCTC
GCAGAAGTTGCGGATCGACGCCGGGGTGTAGCCGCGACGGCGGAAGCCCGACAGGGTCGACATGCGTGGGTCATCC
CAACCAAACACGTGCTTTTCATCGACCAGTTGCTTGAGCTTGCGCTTGCTGGTGATGGTGTAGTTGAGGTTCAGGC
GGCTGAATTCGTACTGACGCGGATGCGCCGGTACTGGCAGGCTGTCGAGGAACCATTCGTACAGTGGACGGTGGCT

TTCGAACTCCAGGGTGCAAATGGAGTGGGTGATGCCTTCGATGGCGTCCGACTGACCGTGGGTGAAGTCGTAGTTG
GGGTAGATGCACCACTTGTCACCGGTCTGGTGGTGATGGGCGTGGCGGATGCGGTACATGATCGGGTCGCGCAGGT
TCATGTTCGGCGAGGCCATGTCGATCTTGGCGCGCAGTACGCGGGCGCCGTCCGGGAACTCGCCGTCGCGCATGCG
GGTGAACCAGTCCAGGTTCTCTTCTACCGAACGGTCGCGGAACGGGCTGTTCTTGCCCGGCTCGGTCAGGGTGCCA
CGGTATTGCTTGGCCTGTTCCGGGCTCAGATCGTCAACGTAGGCCTTGCCGGCCTTGATCAGTTCCACGGCCCAGT
CGAACAACTGGTCGAAATACTTGGAGGCATAGCGCACTTCACCGGACCATTCGAAGCCCAGCCACTTGATGTCGCT
TTCGATGGCGTCGATGTATTCCTGGTCTTCCTTGGCCGGGTTGGTGTCGTCGAAACGCAGGTGCGTGACGCCACCG
AACTCCTGGGCCAGGCCGAAGTTCACACAGATCGACTTGGCGTGGCCGATGTGCAGGTAACCGTTGGGCTCAGGCG
GGAAACGGGTGACGATCTGCGTGTGCTTGCCCGAATCCAGGTCCGCCTGGATGATCGGGCGCAGGAAATTGACCGG
GACGGCAGGTCCGGCCTTGGAATTCGAGGTAGGGTCGACAGTGGGCTTGCTCATAGGATCCTTGAACAGACAGGTA
CGTGGCCGGGTCAGGCCAGACAAACAAAGGGCTTATCATAGCCGATGCTGTCAAGCACCTGACAGGGCATGCTCAA
AAACTGCTGCATTTATTGCATGGGCGGTAAAAAACCACCTCGAAATTCCTGCAGGGCACGCTAAACTGCGCGCCTT
GGCAATGTCTTGCCAGACAGGTGCTGGCGTGTGACGCGCCAGAACCCACGAATTCCTTGAAAGAGTAGTGAACATG
ACTCAAGTCAAACTGAGCACCAACCACGGTGACATCGTCATCGAGCTGAACGCCGAGAAAGCGCCGATCACCGTCG
CCAACTTCATCGAGTACGTGAACGCCGGCCACTACGAAAACACTGTTTTCCACCGTGTCATCGGCAACTTCATGGT
CCAGGGCGGCGGTTTCGAGCCTGGCATGAAAGAAAAGAAAGACAAGCGCCCAAGCATTCAGAACGAAGCGGACAAC
GGTCTTTCCAACGACAAGTACACCGTCGCCATGGCCCGTACCATGGAGCCGCATTCGGCCTCCGCGCAGTTCTTTA
TCAACGTGGCCGACAACGGCTTCCTGAACCACAGCGCCAAGACCGTACAGGGTTGGGGCTACGCGGTATTCGGTAA
AGTCACCGAAGGCACCGACGTCGTGGACAAGATCAAGGGTGTCTCCACCACCATGAAGTCCGGCCACCAGGACGTA
CCAGCAGAAGACGTGATCATCGAGAAAGCCGAGATCATTGCGTGATATTGCTGATTTCAGATTTGCATCTGGAAGA
GGAGCGCCCGGATATTACCCGGGCGTTTCTGGATTTACTCCACGGCCGCGCCCGTGACGCGCAAGCGTTGTACATC
CTGGGGGACTTTTTCGAAGCCTGGATTGGCGACGATGGCATGACCCCGTTCCAGCGTTCGATTTGCACCGCTCTAC
GTGAGTTGAGCGACAGTGGCACCCAAGTATTCCTGATGCATGGCAACCGGGATTTCCTGATCGGCAAGGCGTTCTG
CAAAGCAGCAGGCGCGACCTTGCTCAAGGACCCCAGCGTCGTGCAGATGTATGGCGAGCCCGTGCTGTTGATGCAC
GGCGACAGCCTGTGTACCCGCGACCTCGGCTATATGAAGCTGCGGCGCATCCTGCGTAACCCCATTGTGTTGTTTA
TCCTGCGCCACCTGCCCTTGCGCACTCGCCATAAACTGGCGCGCAAGCTGCGCAGTGAAAGCCGTGCGCAGACGCG
CATGAAGGCCAACGATATTGTCGATGTGACGCCCGAGGAAGTGCCACGGGTGATGCAGCAGTTCGGCGTGCGCACC
CTGGTCCACGGCCACACCCACCGCCCGGCCATTCACAAATTGCAGATTGGCGAGCAGGCGGCCAAGCGCATTGTGC
TGGGGGATTGGGACAAGCAAGGCTGGGCGTTGCAGGTAGATGAGCAAGGGTTTCAATTGGCGGCGTTTGACTTCGT
CAACCCGCAGTTGGCGTTGCCTGGCGCCTGACTTACAAACAACACGAATCAAATGTGGGAGCGGGCTTGCTCGCGA
ATGCGCTGTGTCAGTCGACACCTTAGGTGACTGACACAGCGCATTCGCGAGCAAGCCCGCTCCCACATTTTTGACC
GCGTGCGCTGCAAGATCAGTGACCCGACGCCGCCGGCCCTGCCTTCGCGGTAAACGGCGGCTTGGCCAGCCACACC
AGCAGGATCAAGCCCATGAACATCCACCCCAGCAGCGTGAAATAGTCCACGGTGGACATCATGTATGCCTGGCTGG
TGAGCACCTGGTCCAGTTGCGCATAGGCCTTGTGGCCCGCACCGCCCAGGGTGTTCAGCGCCTCGCGGGTGGCCGA
GTCGTACACACTCATGCTTTCGCTCATGTAGGCATGATGCTGGTCAGCCCGGCGAATCCAGATCCAGGTAGTCAAC
GATGCCGCAAAGCTGCCGCCCAGGGTTCGCAGGAAGGTCGCCAGGCCCGCACCGTCGGCGATCTGGTGCGGCGGCA
GGTCGGACATCAGGATGCTCAAGGTCGGCATAAAGAACAGCGCCACACCGATGCCCATGAACAGTTGCACCAGGGC
GATGTGCTGGAAGTCCACTTCATTGGTGAAACCGGCGCGCATAAAGCAGCTCAGGCCAATCGCCAGGAACGCCAGG
CCGGCCAGCAGCCGCAAATCGAACTTGTGTGCGTACTTGCCGACAAAGGGCGACATGAGCACCGGCAGGATGCCGA
TCGGCGCCACGGCCAAACCGGCCCAAGTGGCGGTGTAGCCCATCTGCGTCTGCAGCCATTGCGGCAGGATCAGGTT
GATCCCGAAGAACCCGGCATAGCCCAGCACCAGCACAATGGTGCCGATGCGGAAATTGCGGTGGGCAAACAGGCGA
AGGTTGACCACCGGATGTTTGTCGGTCATTTCCCAGATCACGAACACCGCCAGGGCGATCACCGAGATGGCCGCGC
CGATGATGATGAAATTGGATTCGAACCAGTCCAGGTCGTTGCCCTTGTCGAGGATGATCTGCAAGGCACCGACCCC
GACGATCAGGCTCAACAGGCCGACATAATCCATCGGCTGATGGCTGGTGACCACCGGCCGCTTCTTGAGCTGCGAA
CGTACCACCATCACCGCAAAAATCCCGATGGGCACGTTGATGAAGAAGATCCACGGCCAGCTGTAGCTGTCGGTGA
TCCAGCCGCCAAGAATGGGACCTGCAATCGGCGCGACCACCGTGACCATCGCCAGCAGTGCCAGGGCCATGCCGCG
CCTGGCGGGTGGATAGACGGCGATCAGCAAGGTCTGGGTCATCGGGTACAGCGGCCCCGCCACCAGGCCTTGCAGC
ACCCGGAAGCCGATCAGCTCGGGCATCGAGGTGGAGATACCACACAGGAACGAGGCCAGCACAAACAGAAGCGTGG
CCCACAAAAACAGCTTCACCTCGCCAAAGCGCCGGCTCAACCAACCGGTCAAAGGCAGCGCAATGGCATTGCTCAC
GGCAAATGAGGTGATGACCCAGGTGCCCTGCTCCGAACTCACCCCCAGGTTGCCAGAGATGGTCGGCAGCGCCACG
TTGGCGATGGTGGTGTCGAGCACCTGCATAAAGGTCGCCAGGGACAGGCCAATGGTGGCCATCAGCAAGCTGGGCG
GCGTGAAGGAGGCGTTATTGCTCATCAGCGTTGTGCTGCCTTGGGCGCGGCCACGCTGTTGTCATGGATCAACTGG
GTGATCATCGCGTCGGCCTCGGCCAACTGGCGGTCGTACACATTGGTGCTGAACGAGGCCTTTTGCGGCGGTTGCT
GCGCCAGCACCGGGCCGCTCTGGTCGTGCAGGTTGACTTCCACCTGGGTCGACAACCCCACCCGCAACGGGTGCTG
GGCCAACTCCTGGGCATTGATATGGATGCGCACCGGCACCCGTTGCACGATCTTGATCCAGTTACCCGTGGCGTTC
TGTGCCGGCAGCAGGGCAAACGCACTGCCGGTGCCGGTGCCGAGGCTGTCGACGGTGCCGCTGTATTTCACATCAC
TGCCGTAGATATCGGCCTCGATGTCCACCGGCTGGCCAATGCGCATCTGGCGCAGTTGGGTTTCCTTGAAGTTGGC

GTCGATCCACAGTTGGTCCAGGGGAATCACCGCCATCAACGCCGTACCCGGCTCGACCCGCTGCCCCAGCTGTACG
GTGCGCTTGGCCACATAGCCGGTGACCGGGGCGATCAAGGTGCTGCGCGCATTGGCCAGGTAGGCCTGGCGCAACT
GCGCGGCAGCGGCCTGCACATCCGGGTGGGACGATATCACCGTGTCATCCACCAGCGCGGTGGAGGTATTGAGTTG
CTGTTGCAGGTTGGCCAGTGAGTTGCTCGCCGCGCTCAGGGCATCACGGGCATGGGACAGTTCTTCCAGGGAAATC
GCGCCACCCTGGGCGAGGATCCTGCGGCGGTTGAAGTTGTCCTGGGCAGTTTGCACTTGGGCCTTTTGCGCCTGAA
CCTGGGCTTTCATGCCATCCACATTGCTGTACAGGCCGCGCACCTGGCGTACGGTGCGCGCCAGGTTGGCCTGGGC
ACTTTGCAAGCCGACGGCCGCGTCGTTGGGGTCGAAGTTGATCAGCACCTGACCTTCGTGGACCAGGTCGCCATCA
TCGGCGCCAATGCTGACCACGGTGCCGGTGACCAGCGGGGTGATTTCCACCACGTTGCCGTTCACGTAGGCGTCGT
CGGTGCTTTCGCTCCAACGGCCATATAGCTCGTGCCAGCCCCAGACGCCGACGACGCAGAGCACGACGATCAGCGC
CAGGCCCAGCAACATGATCTTGCGTTTGCGCGGGTTGCTGTCGTCGGGTTGGGAGGGTGCGGTGTTGTTTGCTTGG
GCAGTAGCCATGACAAATACCTTGCGTTATTCAGCGCGCGTGACTGGCGTGTTCATTGAGTCCCGACCCGTTGCAG
GGTGATGGGGTCACCCGCAGCGATCAGGATTTTCTTGAGGATGTATTCCAGGGTCTGCAATTCCCCCGGCTCCAGC
ACGCCAGCCAGCTGGTTCAGGGCCTGGGCGCCGATCTGCGGCAACATGTCCGCCAGGCGCTGGCCGTCGGCGGTCA
ACACAAGCTGCACCTGGCGCCGGTCCTGCTCGGAACGCTTGCGTGCCAGCAGGTGTTTTTGCTCCAGGCGGTCGAG
CATGCGGGTCATCGAGCCACTGTCCAGGGACAGATTGCGGCACAGCTCCGCCGGGGTATCGACGCCAAACTGGGCC
ATGATAATCAACACCTTGAACTGCGCGGCGGTGATGCCGTGGGGTTCCATATGGGTGTCGATGATGCGGTCTTTGA
GAATCGCGGCGCGCCCCAGCAACAGGCCCAGATGGCAGTTGTGGAAGTTTTCCGGGGTGAAGTGTTTCATCGAAAG
TCACCTTATTACTGCCTAGGCAGTGAATATGTGACCAAATATTACTGCCTAGGCAGCGAATGTCAAATAAATAGTT
AGGTTGCTTGGTAATCAGGCGATGACTGGAGCCATGGGAAGGTTCTGAATCCACGACAAAACAAATGTGGGAGCTG
GCTTGCCTGCGATTGCGGTGTGTCAGCCACTAGAAATAGTGACTGAACCACTGCTATCGCAGGCAAGCCAGCTCCC
ACAATTTGATTGACGTTTAAGCGTCAGAAATCCCGTTTGTAGAAGATATCCAGGGAGCTTGCCACGCCGCTGGCCA
CTTCCAGATACACCCGCTTGCTCAGCAGGTAGCGCAAGGCGATGGTGCTGGCCGGTTCAAACACCCCCACGCCGTA
ACGCAAGCTGAGCTTTTCGGTGATCTTGCCGCTGGCCACCACCGCCGTATTGTTGCCGCTGCCCTGGGTGTCCAGT
TCGAAGTCCTGGATTCCCAGGTTCTTGGCAATGTCCGACGTCACCCCGGCACTGCCCATCAACCCCAGGCCCAGCG
CCGCTTGCGCCAGCATGTTGTTGTCTTCACCGGTGGTGCTCAACGGACGTCCCAGCACCAGGTAGGACAGCGCCTG
CTCCTGGCTCATGGCCGGTTCCGAGAAGATTTGCGTGGTGGGTTGCTCGGCACTGCCGCTCAGGCGGATACCCGCG
ATCACGTCGTCGGTCTTGCGGATGGCTTCGATATCCAGATACGGCTGGTCGAGCGGCCCGGCGAACAGCAGGCGCG
CACGGCGCACATCGAGTCTCTGGCCGTAGGCACGGTAACGGCCGTCGTTGAGCCAGAGTTCGCCACGGGTGTCCAT
GTTATCGCCGATATGCACCCGGCCCTGGACCTTGGCCGTGAGGCCGAAGCCCGCGAAGTTGAGCTGATCCTCGCCC
ACCACCACGTTGATATCCATGGCCATGGCCATCGGCGGCTTGCCTTCTTCGGTCTGGCTGCCAACGATCACCGTGT
CATCCGAGACCTTGACCGTCGAAGGCGGCAATTCGCGCACGGTGATATCACCGCGCGGGATGTGGACATTACCGGC
GATCGCCAACTTGTCGTCCTTGAGGCTGATCTTCAAGTCGGGCGCCACTTCCAGCTTGGCGTAGGGTTCGACCGTA
ACCGGTAATTGCGAACCCTGCAGGCTGAGGTCAACGATCAGGGCCCGGCCCCAGTCGATCTGGCCCTTGAGGCTGC
CCTGCCCGGCCTTGCCGCTGCGCCAGCCGCCGTTGATCTGTACGCTTTCACCGGCAATCAGCGCCTGCACGTTCAG
GCCCTCAAGGCTGATCGGCAGTTCGGGCCCGGAAATCTCGCCCGCGATCAGGTTGATATTGCCGTTGACCTGCGGC
GCCAGCAGCCCGCCGGAAATACGCCCGCTACCGTTGAGTTTGCCGCTGAGTTTTTCCACCATCGGCACGAACGGTC
GCGCTACCGCCAGGTCCAGGCCGGTGAGGCTGAAGTTGCCGGAAATCGGCTTGTTTTTCGCCAAGGGGTTGAGCTG
TGCCTGCAACAGCAACTCACCGAGTTTGCCGCCGCGGAAATTCAGCTGGGTATCGATGCGCGTAGGGTTGAGCGTG
GTTTCCAGCTTGAGGGTGTCGTAGGGGAAATCCAGCCACTGGTCCTTGTCTTTGACCCGCAGTGTGCCGCCACTGG
CATCCACCGCGATCAGGCCTTTGGGGCCACTGGCCGGCAGGTCGAGCTGGATATCGGCGTTGAGCTTGCCCTGCCA
GGCAAAATCCTTGGGCAGCCATTGCGCCAGGCTGTCGATGGGGAATTGCTTGAGGTGGTAGCGCAGCTTGGGATCG
GGCATCAGCCGCTGGTCTTCGCCACACAGGCTGGCGGGCCCGGAGACCCAGCAATGGGCAGCGAAGGTCAGCTTGC
CGTCGGCCATACGCTCGATTTTTGCCGGGGCCTGCAGTTTCCAGTCCTGGCCACCGGCCTGTACATCACCACTGGC
CAGGCGCCCGCGCCAGTTGCCTTTGTCGAGCGTGCCGTCCAGGGCCAGGGCCAGCTTGACCAGGGGGCCCATCAGG
TCCAGTTGGACTTTCTGGCTTTTGATATCGCCCTGGGCGCTGGCGGTCAGGGTGCCGAGCTGGGTGTCACCGGCCT
GGATGCCGCTGCCCTTGAGGTCGATCTTCGCCCGTTGCGCGCTATCAAGTGTCGCGTCCAGGCTGAGGGCTTGCAG
GCGATTGTCTTCAAACACCAGTTGCTGGCCCTTGAGGTCGAGCTTGCCCTGGGGGGCCTTTAAAGTGCCGGCCACA
TCGAGCCGCCCATTGACCTGCCCACGCAACTGCGGCCAGAGCTGGGCCAGGCGTGCGAGCTTGATGTCGATCTGCC
CGGCCAGGCGCTGTTGCAGGCTGCCACTGCCATTGATGCGGTTGTCACCCAGGCGGATATCCAGGTTGGCCAGGGT
CCATTGCTCACCCGCGCCTTCGGCCTTGGCCGCCAGCACCGCGGGCTGGCCGCGCAAACGGCCCTTCAGATCAAGG
TCAGCAGTGAGCTTGAGTTGTTCGTTTTTCATTTCACCCTGGCTGCGCAGCGGCCCGGCGAGCGTGCCCGGCAGTT
CCGCGACCCAGTACGCCGGGTTGATCGCCGACAGGTCAAGCGCCGTATCCCAGGCGATGCCATCGGCAAATTGCAG
GTTCAGGTGGCCTTCGGCCTTGCCTTGGCCGGCAACCAGTTTCAGTTCTGGCAGGAACACTTGGGTCAGGTCGCCA
CTGAATGGCGTAACCAGGCTGAACTTGCCGGCCGGCCCATCGAGGTCGGCCTTGAGATTGCCCAGGTAGTTGCCGT
CCTTGTAGGAGACTTCGCCGTTGAAGGTACGCAACACCACTTGCGGCTCGTCAATCAGCGGGTACAGGCGGTGCCA
GGGGAAATCCAGCCAGTCGATTTTTGCATCTGCGCTGAAGCCCTGCTGCCAATCCAGTTGGGCACTGAGCTTGAGG
CTCTGCTTATCCCCGGCATTCAGGTCAAGGCCGGCGATCTGTGCGCCCTTGGCGTCGACCCGGCCTTGCAGCAACA

GGTCAACCGGGCCCTTTTCTGCGGGTAATACGGCCTTGCCCAACAGTTGATAGCCCTTGCCCAGGTCGCCCTTGGC
CGTCAGGTCCAGTTGGTTGAGCTGCAGGGTGTCCGGCAAGTCGGCGGCGGGCTTGAAGCCATCGGCGGTGATCTGC
AGTTGCGCCGGCAGGTTCTCGACCAGAGGTTGCAGTTCGCCCTTGAGCTTGGCGGGCAGGTAGCCCGTGCTGTCGG
CGTCGAGCTTGAGGGTCTTGAGCAAATCACCCGTGACGTTCAGCGCCACGGCCCATGGCGCGCCACCCGGAGCGTA
GGGCAGGCTCAGGTTACCGGTCGCCGTCAGCGGCCAGTCACCGGTGGGTTGCAGCATACCGCTGAGGTCCAGCACT
AGGCCGTCGCGCTGCAGGTGCACCGCGTCGATCTGCATTCCTTCGGCGGTCCAATGGGCCGCCAGTTGCAGGCCCT
GGAGTTCTTCGCTGCCGTTGAACAGCAGGCTGCCTACACGCACGTCGCCCAGTTCGATGGCCAGGGGCAGGTTCAA
GTCTGGCAGGGTAATCGGGCCGCTGCTTTCTTCGGTGCTGGGTGGAAACTGCAGGCTCACCTGCTCCACGTCCAGT
TGCCGGATGCACAAGGTCATGCGCAGCAGGCACGCCAGCGACCAGTCGAATTTCGGCGCATTGAGTTCCACGCGGC
TGCTGTCCTGCTGCCACAGCAGATGGTCGGCGCTCCATTGCCCGCCCAGGTGGCCCTGGAAGTTGTCCACGCTCAA
CCCCGGAACCTGGCCGAGTGCCCAGCGACTGCCGACTTGCGTACCCAGTACGCTCCATACGGCCAGGGGCACCAAG
GCGACAAGCGCCACCACACTCAGACCCGCTATTTTCAAACCACGCTTCACAGCTCAGGCCCCATGGAAAAGTGCAA
ACGAACGCCGCCCGGATCTTCCAGGGCATGGGCCAGGTCAAGACGAATCGGACCGACTGGCGACACCCAGCGCACG
CCGATGCCGACCCCGGTTTTCAGGCTCGGCAACTCAAGGCTGTTAAAGGAATTGCCCTGGTCGACGAAGGTCGCGA
TCCGCCATTTTTCGGCGATGGAATATTGATACTCGGCGCTCAAGGCCACCATATAGCGGCCACCGATACGGTCGCC
TTTGTTGTTTTCCGGGGACAGGGTCTGGTACTCGTAGCCACGCACGCTCTGGTCGCCACCGGCAAAAAAGCGCAGG
GACGGCGGCACCGACTTGTAGCCGTTGGTGGCGCTGCCGCCAAACTGTGCACGGCCGAGGAAGCGGTGCTTGTCCC
ACACCGTGGTCAAGCCCTTGATCATCGCGGTCCCGTACAGCAGGTTGGTGTCCGAGCCCAGCCCCTCTTTGGCCAC
CTTGGTATCGAACTGCAGGCGATAGCCGTTATGGGGGTCGATGCGGTTGTCGCTGCGCAGGTAGGAATAGCTGATT
CCCGGCATCAGCAAATTGCTCAAGCCGGAGTCATCCCCCAGGCGATATTCTTCGCGCTGCCATTTGAGCGAGATCA
CCCGTGTCCAGCCGCTGGGCAGCTTGCTGTGCCATTCCGGGCCGAGGGTGAGCAGTTTGCTGAGGGTGTCGGTGTT
GGCCAGTTCTTCGTTCTGGTAGCCACCAGCAAAGCGCAGCTTGTCGGTCAGTGGCGGGTCGAGGGGGATGTCGTAC
CACAGGCCGACGTTCTGCCGGGGCGCCGACAGTTCCGCCTCCCAGCCATAGCTATGCCCCTGGGGGTTGACCCAGT
GCCGCGTCCAGTTGGCCTTGCCGCGCGGGCCGACGTCGGTGGAAAAACCCAGGCCCAGGCCCATGGTCCGGGGTTT
GCGGGTTTCCAGCTGGACTGCCACCGGGATCACATCATTGGCCGCTGCGGCCGGGGCCGCGTCCACCCGCACGCCC
TCGAAATAACCGCTCGACTGCAGGGCCTGGTTCAGCTCGGCAATCAGCTCGGAATCGTAAGGGGTGCCACTCTGGA
ACGGCACCATGCGTTGCAACAGTTCTTCATCAAAAGGGGTATCGCCGGCAAAGTTGACCTTGCCGAGGGTGTAGCG
CGGGCCGCTGTCGTAGACCAGCTCGATATCGGCCACGCCCGCCTGGGGATCGACAGACAGTTTCTGCTGGGTAAAA
CGGCCACTGAAATAGCCGAAGCGCGAGGCCTGGTTCTGGATCAGGCGCTTGGCGTCTTCGTAATGACCATGATTGA
GTACGGCGCCGGACTTGAGGTCTTGGCTATCGGGCACGCGAAAGGACTTTAACTCGGCCGCCGGGCCGTCCACCCG
AATCGTGACATTACGCAGGTGCACAGGCTCGCCAGGGTCGATGCTGAGGATCAGGCGCGGGTTCTTGCCGGCCTTT
ACCTCACTGGCAATCTGCGGCTGATAAAAGCCCAGTGCCTGGGCGGCCTTGCGCGCCTGCTCCTCGGCACCGCGAC
TGAAGCGCAGCAAGGCCTCCTCATCACGCTCGCCAACCCCGCCGATATAGCCTTCGATATTGGCCTTCAGCGCATC
GTTTGAAGGTTTGACCCGCACGTCCAACTCGCTTTGCGCCAAGGCGCCGCAGCTTGTGAACAGCAGAATCAAGCCG
CTGGTAAATCTATTGGAAAACTTCATAGGCGCGGATGCTACACGAGCTGGGGAGCACCTTAGAACCGGGAATTGTC
TGAATAATTCTGCGTTAAGGCGTTGCAGGTTGTAACACTTGGGGGTTTGGGTGAAAAAACACATGCTCCAGGATAG
GTCCTACAGCCACCTCGCCCACTTCGTCATAGCCCTGGCGTTTATAGAACTCCAGGTAGAGCGGATTGCCGGTGTC
CAGCACCACCCCTTGTGAATGCGGGTCTTCGGCGCACCAGTTATGCACGGCTTCGAGCAGTTGCTCGCCGTAATGC
TTGCCCTGGAATTGCGGGTGAATCCCCAACAGCGGCAACACGTGTACCGACTCGGACGGCAGGCACGCCAACACCG
CCTGATGGTATTCGAGGTAACGCCGGGTACACCTCAAGCCAGTGCTCAACCACATACGTAATTGCCAGGCCCAACT
TTCAGTGATACCCAGGCGCCGTTGCGGAGGGGCAATCAGGGCGATGCCGATCAGGCGGTCATTGACCAGCAGGCCG
ATCGCCGGCAGTTCCTGGAGAAAATGCTGCTTGACCAGTTCGCGCACCGTAGCCCTGACCCGCTGCTCATACCCGG
AGCGTTCGGCTTCAAACAGGTAGGCGAAGGTCGGCTCATGGCGATAGGCGTGGTACAGCAACGAGCGCGCTTCGCG
GGAATAGCCGCTATCGAGCATGTGGACCTGGGCAATGGCGGTGCTAGTGTCAGGCATCAGTTGTATCTCCCCTGGG
CGCAACTCAAAAGGCCGCGCTCTTATTGTTACGAGCCCGGGGCAACTGAGTCGTTCCATGCCATTGGCTCCTTGAG
AGACATTAGCAGTGCATCTGTCCTACCGCCACGCTGGCCCCCGTCCGACTTGTCGGCTAGCATCGCCCTTTTGCCA
GACTGGACTGCCGACCATGAAAATCGTCTCCTTCAATATCAACGGGCTGCGTGCCCGGCCTCATCAGCTGGCGGCC
CTGATCGACAAACATCAACCGGACGTGATCGGCCTGCAGGAAACCAAGGTCCACGACGACCAGTTCCCGCTGGCTG
AAGTCCAAGCCCTGGGGTACCACGTGTACTACCACGGGCAGAAAGGCCACTACGGTGTCGCCCTGCTCTCGCGCCA
AGAGCCGCTGGCCCTGCACAAGGGTTTTGCCAGCGACGATGAGGATGCCCAGCGCCGTTTTATCTGGGGCAGCTTT
GCCGATGAACACGGCAACCCGATCACCATCATGAACGGCTATTTCCCACAGGGCGAAAGCCGCGATCACCCCACCA
AGTTCCCGGCCAAGCAGCGCTTCTACGAAGACCTGCAACAGTTGCTGGAAACCCAGTTCAGCAATGACCAGGCGTT
GGTGGTGATGGGTGATGTGAACATTTCCCCTGAGGATTGCGATATCGGCATCGGTGCCGACAACGCCAAGCGCTGG
CTGAAGACCGGCAAATGCAGCTTCCTGCCGGAAGAACGCGAATGGATGGCGCGCCTGAAAAACTGGGGCCTGACCG
ACAGCTTCCGGCACCTGAACCCAGATGTGGCGGATCGCTTCAGTTGGTTCGATTACCGCAGCCGCGGCTTTGAAGA
TGAACCCAAGCGTGGCCTGCGCATCGACCTGATCATGACCTCCAATGGCCTGCTGCCACGGGTCAAGGATGCGGGG
GTGGACTACGAACTGCGCGGCCTGGAAAAACCGTCGGACCATGCGCCGATCTGGCTGGAACTGAGCTGATTTTTGC

GGCAACACCATCCAAAAATGTGGGAGCGGGCTTGCTCGCGAAATCGGTGGATCAGTCGCTGGATGTGTTGAATGAC
ACACCGTATTCGCGAGCAAGCCCGCTCCCACACAAGCCAGCTCCCACATTGGGTTCAGTTGGGCATCAGTCATATT
CTGGCAACCTTTCTGACTTAATCTGCCCGCACGTCCTTTGGCTTGAGAAGGTGGCGGCATGATGCTGCGCGTTCTG
TACCTGCTGGTTCTGTGTAGCACCTTCCCTCTTTCGGTCGTTGCAACGCCCCTGCCCTTTCCCGAACAGGGTCCGG
CCCTGCGCCTGCAGGGCTCCAACACCATTGGCGCGGCGCTGGGCCCGGCGCTGGTCAAGGGTTTGATGGTGCAACA
GGGCCTGAAGGCCGTGCACAGTGAACCGACGAACCACCCCAATGAGCAGCGCCTCGTCGGCACGAGCCCCCAAGGC
AAACGGGTCCTGGTAGAAATCGCCGCCCATGGTTCCAGCACCGGCTTTGCCGCGCTGAAAAATGCCGAAGCCGACC
TGGCCGCCGCGTCGCGCCCGATCAAGGACCGCGAGCTGGTGGAGCTTGAACCCCTGGGCGACCTGAAAAGCCCCGG
CGCCGAACAGGTAATTGCCATCGACGGCCTGGCGATCATCCTGCATCCACGCAACCCCTTGCAGACGTTGACGACC
GAACAACTGGCGAGGATTTTCAGTGGCGAAACCAGCACCTGGGAAGCCTTGGGCGGCACCGGCGGGAAGATCCGGC
TGTATGCCCGCGATGACCAATCCGGAACCTACGACACCTTCAAGGAGTTGGTGCTCAACCCTCGCGGTAAAACCCT
CGACAGCGCGGCCAAACGCTTTGAGTCCAGCGAGCAACTGTCCGATGCGGTGAGCCGCGACCCGCAGGGCATCGGC
TTCATCGGCCTGCCCTACGTGCGCCAGGCCAAGGCCGCAGCCATCAGTGATGGTGACTCCCAAGCCATGTTGCCGG
TGAACAGCCTGATCGCCACCGAGGACTACCCCCTGTCGCGCCGTTTGTTTTTCTACCTGCCGCCCGGCAACAGCAA
TGCCTGGGCGCAGGCGCTGGTGGACTTTGCCCAGAGCGCCCAGGGCCAGGCCATCGTTGCCGCCAACGGGTTTGTC
GCCCAACAGGTCCAGGCCATCAGCGTGCAGCCAAGGCCACCGATGCCGGACGCCTACCAGGCCATCGCTCGCGAGG
CACAACGCCTGAGCGTGAATTTTCGCTTCGAAGAAGGCAGTGCGAGCCTGGACAACAAGGCACGCCAGGACCTGGC
ACGGGTGGTGGCTTATATAAGCAGCCACGACAAACGGGACAAGCGGGTTACGCTGGTGGGTTTTGGTGATGCCAAG
GATGACCCGCAGCGCGCCGACCTGTTGTCCAAGTTGCGGGCGATGGCGGTGCGTCGCGAGTTGGTCAAGAGTGGCG
TGGTGTTGCGCGACATCCGCGGCTTTGGCGCCGAGATGCCGGTGGCAGCGAATACCGTGGATGAGGGGCGGATCAA
GAATCGCCGGGTGGAAGTCTGGGTTTACTGACGCCTGAAGATTGGGGTTGACTGGCCCACCGCTATCGCGGGCAAG
CCCGCTCCTACACTGAATGTGGGAACGGGCTTGCCCGCGATAAGGCCGGCGTGGTGTTACTGACCGCTGCGCATCA
GCTCTTTAGGCACATATTTGCCAATCTCGAACTTGCCGATCGCCGCCCGATGCACTTCGTCCGGGCCGTCGGCCAG
GCGCAGGGTGCGCTGCATGGCGTACATATAGGCCAGCGGGAAGTCGTTGGACACCCCGGCACCACCATGGATCTGG
ATCGCCCGGTCAATTACCTTGAGTGCCACATTCGGTGCCACGACCTTGATCTGGGCGATTTCACTTTTCGCCACTT
TGTTGCCGACCGTGTCCATCATGTACGCCGCTTTCAAGGTCAACAGGCGTGCCATGTCGATTTCCATCCGCGAGTC
GGCGATTTTGTCGATATTGCCACCCAGGCGCGCCAGCGGCTTGCCAAAGGCGGTGCGGCTGACAGAACGCTTGCAC
ATCAATTCCAGGGCGCGTTCAGCCATGCCAATGGAGCGCATGCAGTGGTGAATACGTCCTGGGCCAAGGCGCCCCT
GGGCAATTTCAAAGCCGCGGCCTTCACCCAGCAGCACGTTTTCGTACGGCACGCGCACGTTGTCGAACAGTACTTC
GGCATGCCCATGGGGCGCGTCGTCGTAGCCGAACACCGGCAGCGGCCGGACGATTTTCACCCCGGGCGCATCCACC
GGTACCAGGATCATCGAGTGTTGCTGGTGACGCGGCGCATCCGGGTTGCTCAGGCCCATGAAGATGAGGATTTTGC
AACGCGGGTCGCACGCGCCGGAGGTCCACCACTTTTTACCGTTGATGACCCATTCGTCACCCTGGCGTTCGGCGCG
GGCGGCCATATTGGTGGCGTCGGAGGACGCCACATCCGGTTCGGTCATGGCAAACGCAGAGCGGATCTCACCGCGC
AGCAGCGGCTCCAGCCAGCGCTGTTTCTGCTCTTCGTTGGCGTAGCGCACCAGCACTTCCATATTGCCGGTGTCGG
GTGCCGAGCAATTGAACGGCTCCGGGCCGAGCAAGGAGCGGCCCATGATTTCTGCGAGCGGCGCGTATTCCAGGTT
GGTCAGGCCCGCGCCCAGCTCGGACTCCGGCAGGAACAGGTTCCACAAGCCTTCGGCCTTAGCCTTGGCTTTGAGC
TCTTCCATGATGGCGGTGGGCTGCCAGCGATCACCTTCGCTGACCTGGCGCTCAAACACCGGTTCGGCTGGGTAAA
CGTAAGCATCCATAAACGCGGTGACGCGTTCACGCAGTTCCTGAACCTTGGGTGAATAGGCAAAATCCATGAGCAG
CTCCCTTCTTTGAGAGGTTGTTTAGGTCATGCAATCGATGCTAGAACAGCGCTGATAATTTACCTAGCCTATTCTC
GGCGTGTATTAACATTCATCACCGATATATGATCGGCGTATGAAAACCTAACAAGAAGAGCGCAACGAAATGAATC
TGAGCAAGGTCGACCTCAATCTTTTCATCGTGTTTGACGCGATCTACACCGAAGCCAACCTGACCCGCGCCGGGCA
GATCGTCGGCATTACCCAGCCGGCGGTATCAAACGCCCTGGCGCGCCTGCGCGAGACCTTCAACGACCCGCTGTTC
GTGCGGACCGCCCAGGGCATGGTGCCGACCCCCATGGCCCAGAACATCATTGGCCCGGTGCGCAATGCCCTGTCGC
TGTTGCGGGTGTCGGTGCAAGAGAGCCGGATCTTCAACCCCTTGCAGGCGGCCAAGACCTACCGCATCAGCATGAC
CGACCTCACTGAAGCGGTGATCCTGCCGTCGCTGTTCCAGCGCCTGCGCCGCCTGGCGCCCACGGTGATCATCGAA
AGTTTCCTGTCCAAGCGCCGTGAAACCACCAAGGAACTGGCCGCCGGCCGCCTGGATTTTGCGGTGGACGCGCCCC
TGAACACCGACCCGCAAGTGCGCCACGTCAAGCTCATGGAAGACCGCTACGTGTGTGCCATGCGCAAGGGCCACCC
GATGGCGGGCAAAGAAAAATTCAGCCTGGATGACTACCTCTCGCTGACCCATATTCATATTTCCAGCCGACGCAAT
GGCCTGGGCCACGTCGACCTGGCCCTGGGCAAGATGGGGATCCAGCGCAAGATCGCCCTGCGTTCGCAGCATTACC
TGATGGCCTCCCAGGTATTGCAGCAGACCGATATGGTGATGACCGTGCCCGAGCGCTTTGCAAGGCGTAACGAGCT
GCACTCGTTCAACCTGCCGGTCAATGATGTGCCGCCGGTGGAAACCCACCTGTACTGGCATGAAAGCACCGACCAG
GACCCGGCCAACCGCTGGATGCGCGAACAGATGATCGAACTGTGCCAGCAGGTCACGGCGCGTGAGAAGAAGCTGG
ACCAGCAATCGACCTGACCTTGCCGGCTCCTGCAATGGGCTTGATTCGCTTGACGTTTACGTCAAGCAGCCATTAG
CGTAGCGCCGAGACCCTCCTTGAGCGCCACCATGAGCACGACCTACAGCATCTCCGACCTGGCCCGCGAGTTGGAC
ATCACCACCCGGGCCATTCGCTTCTATGAAGAACAAGGCCTTCTGAGCCCCGAACGCCGTGGCCAGGAGCGTATCT
ATTCGCCTCGGGACAAAGTCAGCCTGAAGCTGATCCTGCGTGGCAAGCGCATCGGTTTTTCCCTGGCCGAGTGTCG
CGAGCTGATCGAGCTCTACGACCCCACCAGCGGCAATCAAAAGCAATTGCACAGCATGCTGGCGAAGATCAGCGAA

CGCCGTGAGCAGTTGGAACAACAGTTACTGGACATCGAACAGATGAAGCTGGAACTGGACACCGCTGAAGAACGTT
GCACCCAGGCCCTGGAACAGACGATCCAGAGCCAGGTCGGCCAACCCCAATAAAAAGGTAGATCCCATGTCTCTCC
CCTCCCATGTGCGCCTGGTCGAAGTCGGCCCGCGCGACGGCCTGCAAAACGAAGCACAGCCCATCAGCGTGGCCGA
CAAGGTGCAACTGGTCGACGCCCTCAGTGCGGCGGGCCTGGGTTACATCGAAGTCGGCAGTTTTGTCTCGCCCAAG
TGGGTCCCGCAAATGGCCGGCTCAGCCGAGGTGTTTGCGCAGATCCAGCGCAAGCCGGGCGTGATCTACGGCGCGC
TGGCGCCGAACCTGCGCGGGTTCGAGGACGCGCTGGCGGCCGGGGTCAAGGAGGTCGCGGTGTTTGCCGCAGCGTC
GGAGGCGTTTTCCCAGCGCAATATCAATTGCTCCATCAGCGAGAGCCTGGAGCGCTTTGCGCCGATTATGGCGGCG
GCGCAGCAACAGGGGGTGAGCGTGCGCGGCTATGTGTCCTGCGTGCTGGGTTGCCCTTACGAGGGCCAGGTGGCGC
CGGAACAAGTCGCCGCCGTGGCGCGAGAGTTGTATGCCATGGGCTGTTACGAGGTGTCTCTGGGCGACACGATCGG
CACCGGCACCGCCGGCGCCACGCGCCGTATGTTTGAGGTGGTGGGCAAGGACGTGCCACGGGCAAAGCTTGCCGGG
CACTTCCATGACACCTACGGGCAGGCCATCGCCAACCTGTATGCCAGCCTGCTGGAAGGTATCAACGTGTTCGACA
GCTCTATCGCCGGCCTCGGCGGCTGCCCCTACGCCAAGGGCGCCAGCGGTAACGTGGCCACCGAGGACGTGCTGTA
CCTGCTCAACGGCCTGGGCATCGAGACCGGTATCGACATGGACAAGCTCCTAGTGGCGGGCCAGCAGATCTGTTCG
GTACTCGGCCGCCCCACCGGCTCACGCGTGGCCAGGGCCCGTAGCGCCAGTTGAGTAGCGTGCCCGTGGCGATGTG
TTACCTCGCGGCTACACATCGGGTAACACGGGAACATATTTGTCGCATTCTCTGAAGTAAATTTCCTACTAAAAA
ATTAAATCATTGATTTTAAAGGCTTTTTAAAAGTTGGCACGGCTTCTGCTATCTCTATCGCATAACAAGAATAAAA
AGCACCAAACCTAATAAAAATAAGACGTAACGACTCTGACATAACAAAAACAACACGGCAGAGACGCAGCTAACAG
ATTTTTTTGGAGAAGATGTGCTTCGCAGGGTACGGCGCAAGCCGCCACTCGCAACCGGGCAGAGAACAATAAATCT
ACCTCAAGGTAGCTACCCACTGGTTGGATCGCGTGCGAAGAAGCAGATCAGCGCTCAAAAAAATACGTTTGCTCTT
GACCCCGGATGGGGGTCGCCAAAAACAGCGGTAAAGGGCCACGGTTGCCAAAAACAACAATAGACCGCCCCTCAAT
AATAAAAAAAGAGCACGTGACAACAAATTAAAGGGGAGCTTCGGCTCCCCTTTGTGCTGCCTGGGTGAAAAGTTAT
TGTGGGAGCTGGCTTGCCTGCGATAGCGGTGTGTCAGCTACTCAATTGTTAACTGATAAACCGCAATCGCAGGCAA
GCCAGCTCCCACAATTTAGATCGGGGTGATCAGGGCTTCAGCGTTCGCAGTTCCTCAACTGTAATCTCGCGCATAC
GAAACTTCTGGATCTTGCCGGTCACTGTCATCGGAAACTCTTCGACAAACCGGAAATGCTTCGGCGTCTTGAAGTG
CGCAATGCGCCCCTTGCACCAGGTTTGCAGCTCCAGTTCGTTGGCAACCTGCCCCGGGTGAAACTTGATCCAGGCC
ACGATCTCTTCGCCGTAGCGCTCATCGGGAATGCCGATGATCTGCACATCGGCCACCGCCGGGTGGGTGAAGAAGA
ACTCCTCCAGCTCCCGCGGGTACACGTTCTCGCCACCGCGGATGATCATGTCCTTGTTGCGCCCGGCGATGCACAC
GTAACCCTGCTCATCCATGCTCGCCAGGTCGCCCGTATGCATCCAGCCCGCCTCGTCGATGGCTTCGCGGGTGCCC
TCGGGGTTGTTCCAGTAGCCGAGCATCACGCTGTAGCCACGGGTGCACAGCTCGCCGATCTGCCCGCGCGGCACGG
TGTTGCCGGCTTCGTCGATGATCTTGCTTTCCAACTGGGGTTGCGTGCGCCCGACCGTGGTCACGCGCCGCTCCAG
GTCGTCATCGGCCCCGGTCTGCAGCGACACCGGGCTGGTCTCGGTCATGCCGTAGGCGATTTGTACCTCACTCATA
TGCAGGTCGCTGATCACCCGGCGCATCACCTCGATCGGGCAGGTGGAGCCGGCCATGATCCCGGTGCGCAGGGTCG
ACAGGTCGAACGTGCCGCGTTGCGGATGATCGAGCATCGCGATAAACATCGTGGGTACGCCGTAAAGGCCGGTGGC
GCGTTCTTCGGCCACGGCGGCCAGGGTCAGCAGCGGGTCGAAGCCGTCATTGGGATAGATCATGGTCGTGCCATGG
GTGATGCAGCCCAGGTTGCCCATGACCATCCCGAAGCAATGGTAGAGCGGTACGGGGATCACCAGGCGGTCCTGGG
CGCTCAGGCCCAGGCTTTCGCCGACCATGTAGCCGTTATTGAGGATGTTGTGGTGGCTGAGGGTGGCGCCCTTGGG
GAAGCCCGTGGTGCCGGAGGTGTACTGGATGTTGACGGGTTGATCGAAGTGCAGGCTGGCCTCGCGGTAATGGAGC
TGTTGCGGGGGGATGCCCGTACCCAGGGCCGACAATTGCGACCAGGGCAGGAACCCCGAGGGCGGTCGCGCATCCA
GGCTGATCACCCCGCGCAGCTCGGGTAACAGCTCGCAGCGCAATTGGCCAATGCCCTGCTCCGCCAGTTCCGGTAC
CAGCGCCTGCAGCATCCCGTGGTAGTCGGAGGATTTGAACGAACCGGCACAGATCAACCACTGGCAGCCCGATTGC
TTGAGCACGTATTCCAACTCGCTACTGCGGTAGGCGGGGTTGATGTTGACCAGGATCACCCCGAGCTTGGCACTGG
CCACCTGGCTGATGCACCACTGCGCGCAGTTCGGCGCCCAGATGCCAAGACGATCCCCGGCTTGCAACCCCAGGGC
CAGCAGAGCGCGGGCGTGCAGGTCCACAGCTTCGGCCAATTGCTGCCAGGTGTAGCGCAACTGCTGATGGCGCACC
ACCAGGGCCTCGCCCTGCGGGTACTGCGCCACCGTGCGGTCGAATGCCTGGCCAATGGTCATGGCCAGCAGGGCCT
TGTCCTGTGGGCCACGGCTGTAGCTCTGATTCGGTTGATCCATGACGACCCCTATTGTCTTTTTTGTAGGTGGACG
GTGCGCCGGTCAGGCGCGAGCTTGACGTTAACGTAAGCTACGATTGACAGTCATGCAACGCAAGTTTACGTTAACG
TAAAGGTGATCGCCCAATGGCGTCGCCAGCCCCACAACAATAATGTTTATAGGTGCCCTCCCATGAGTTACCCGAC
CCTGAACTTCGCCCTCGGTGAAACCATCGACATGCTGCGCGACCAGGTGCAGGCCTTCGTGGCCAAGGAGCTGGCG
CCCCGGGCAGCACAAATCGATATCGACAACCTGTTCCCCGTGGATATGTGGCGCAAGTTCGGTGACATGGGCCTGT
TGGGCATCACCGTGCCGGAAGAATACGGCGGTGCCGGCCTGGGTTACCTGGCCCACGTGGTGGCCATGGAAGAAAT
CAGCCGTGGCTCGGCCTCGGTGGCGTTGTCCTACGGCGCGCACTCGAACCTGTGCGTCAACCAGATCAACCGCAAC
GGCAGCCACGAGCAAAAGCTCAAGTACCTGCCCAAGCTGATCAGCGGCGAGCACATTGGCGCCCTGGCCATGAGCG
AGCCCAATGCCGGTTCTGATGTGGTCTCGATGAAGCTGCGCGCCGATAAGCGCGGCAGCGTGTATGTGCTCAACGG
CAGCAAGACCTGGATCACCAACGGTCCCGATGCCCACACCTACGTGATCTATGCCAAGACCGACCTGGAAAAAGGC
GCCCATGGCATCACCGCGTTTATCGTCGAGCGCGACTCGAAAGGCTTCAGCCGCAGCAACAAGTTCGACAAGCTGG
GCATGCGCGGCTCCAACACCTGCGAGCTGTTTTTCGATGACGTCGAGGTGCCTGAAGAAAACATCCTCGGCGCGCT
CAATGGCGGCGTCAAAGTGCTGATGAGTGGCCTGGACTACGAGCGCGTGGTGCTCTCCGGCGGACCGACCGGGATC

ATGCAGGCCTGCATGGACCTGATCGTGCCGTATATCCACGACCGCAAGCAGTTCGGCCAGAGCATCGGCGAGTTCC
AACTGATCCAGGGCAAGGTCGCCGACATGTACACCCAGCTCAACGCCAGCCGCGCCTACCTGTATGCCGTCGCCCA
AGCCTGCGAACGCGGCGAAACCACACGCAAGGACGCGGCCGGGGTGATTCTGTACAGCGCCGAACGCGCCACGCAG
ATGGCCCTCGATGCAATCCAGATTCTCGGCGGCAATGGCTATATCAATGAATTCCCGGCCGGCCGCCTGCTGCGCG
ATGCCAAGCTGTATGAAATCGGCGCGGGCACCAGTGAGATCCGGCGCATGTTGATTGGCCGCGAACTGTTCAACGA
AACCCGCTGAGGGAGTGCACCATGGCCACCCTGCACACCCAACTCAACCCACGCTCGGCAGAATTCGCCACCAACA
GCGCGGCAATGCTCAAACAGGTCGACGCCCTGCACACCCTGCTCGCCCAGGTCCAGCAAGGCGGCGGCAGCAAGGC
CCAGGAGCGCCACACGTCCCGGGGCAAGCTGTTGCCGCGCGAACGCATCAACCGCCTGCTCGACCCGGGCTCGGCA
TTCCTTGAGCTCAGCCAACTGGCGGCGTATCAGGTCTACGGTGAAGAGGTGCCGGCCGCTGGCGTGATCGCCGGGA
TCGGCCGGGTCGAAGGCGTGGAATGCATGATCGTCGCCAACGACGCCACGGTAAAAGGTGGCTCCTACTACCCGCT
GACCGTGAAAAAACACCTGCGCGCGCAAACCATTGCCGAGCAGAACCGCCTGCCCTGCATCTACCTGGTGGATTCC
GGCGGCGCCAACCTGCCGCGCCAGGATGAAGTGTTTCCCGACCGCGAGCACTTTGGGCGGATCTTCTTCAACCAGG
CCAACATGAGTGCCCAAGGCATCCCGCAGATTGCCGTGGTCATGGGCTCCTGCACCGCCGGTGGTGCCTATGTGCC
AGCGATGGCCGACGAAGCGATCATGGTGCGCCAGCAGGCGACCATCTTCCTCGCCGGCCCGCCGCTGGTGAAGGCC
GCCACGGGCGAAGTGGTCAGCGCCGAAGACCTTGGCGGTGCCGATGTGCACTGCAAGGTCTCCGGGGTGGCCGACC
ATTACGCCGACAGCGACGAACACGCCCTGGCCCTGGCCCGACGTAGCATCGCCAACCTCAACTGGCGCAAACAGGG
GCAGTTGCAGCAGCGCACGCCAGTCGCGCCGCTGTATGCCAGCGACGAGTTGTATGGGGTTATCCCGGCCGACGCC
AAGCAACCGTTCGATGTGCGCGAAGTGATTGCGCGGGTGGTGGACGGCTCGGTCTTCGATGAATTCAAGGCGCTGT
TCGGCACCACGCTGGTCTGCGGTTTTGCCCATTTGCATGGCTATCCCATCGCGATCCTCGCCAACAACGGCATCCT
CTTCGCCGAAGCTGCGCAAAAAGGCGCGCATTTTATCGAACTGGCCTGCCAGCGCGGGATTCCCCTGCTGTTCTTG
CAGAACATCACCGGCTTTATGGTCGGCCAGAAGTACGAAGCCGGTGGCATCGCCAAGCACGGCGCCAAGCTGGTGA
CGGCGGTGGCCTGCGCCAAGGTGCCGAAATTCACGGTGATCATCGGTGGCAGCTTTGGTGCCGGTAACTACGGGAT
GTGCGGGCGCGCCTATGACCCGCGCTTTTTGTGGATGTGGCCCAATGCCCGTATTGGCGTAATGGGCGCCGAACAG
GCAGCCGGCGTGCTGGTGCAGGTCAAGCGCGAGCAGGCCGGACGCAGTGGCCAGGGGTTCAGCGCCGAGGACGAAG
CGCAGATCAAGCAGCCGATCCTCGACCAGTACGAGACCCAGGGCCACCCCTATTACTCCAGCGCCCGCCTGTGGGA
TGACGGCGTCATCGACCCTTTGCAAACCCGTGACGTGCTGGCCCTGGCCTTGTCCGCTGCGCTGAATGCCCCCATC
GAGCCGAGCCGCTTCGGCGTGTTCCGCATGTAATTGGAGCTGCACCCATGAGCGATTTCAACACCGTTGAACTGGT
GAACGACCCCCGTGGCTTTGCCACGCTGTGGTTGAGCCGGCAAGAGAAGAACAACGCGTTCAACGCCGAAATGATC
CGCGAGCTGATCATTGCCCTGGATCAGGTGCAAGCCGACGCAACCCTGCGCTTTTTGCTGATACGCGGGCGCGGCA
AGCACTTCAGCGCCGGCGCGGACCTGGCCTGGATGCAACAGTCGGCCGAACTGGATTACCACACCAACCTTGACGA
CGCCCGCGAGCTGGCAGAGTTGATGTACAACCTGGCCAAGCTGAAAATCCCGACCCTGGCCGTGGTCCAGGGCGCG
GCGTATGGCGGCGCCCTGGGCCTGATCAGTTGCTGCGACATGGCCATTGGTGCCGATGATGCGCAGTTCTGCCTGT
CGGAAGTACGCATCGGCCTCGCACCGGCGGTCATCAGCCCGTTTGTGGTGCAGGCGATTGGCGAGCGCGCGGCCCG
GCGTTATGCCCTCACCGCAGAACGCTTCGACGGCCAGCAGGCACAGGCGATCGGGCTATTGGCGCAAAGCTATCCC
TTCGATGAGCTGAATCAGCAGGTCGAGCAGTGGACCGCCAACCTGCTGCTCAACAGCCCGGCCGCCATGCGCGCCA
GCAAGGACTTGCTGCGTGAAGTCGGCAACGGCGCGCTCACCCCAGCGTTGCGCCGTTACTGCGAAAACGCCATCTC
ACGCATCCGCGTCAGCGCCGAGGGCCAGGAAGGCTTGCGCGCGTTTTTGCAAAAGCGTGCACCCAGCTGGCAGTCC
CAGGAGCCTCGTTCATGAGTTCACCGTTGATTACCTGCGTGCTGGTGGCCAACCGTGGCGAAATCGCCTGCCGCGT
CATGCGCACGGCCAAGGCGATGGGCATGACCACCGTTGCCGTGCATAGCGCCACGGACCGCAATGCGCGCCACAGC
CGCGAAGCTGATATCCGCGTGGACCTGGGCGGCAGCAAGGCCGCCGACAGCTACCTGCAGATCGACAAATTGATTG
CCGCCGCCAAAGCCAGCGGCGCCCAGGCGATTCATCCTGGCTATGGCTTTCTTTCCGAAAACCCGGGCTTTGCGCG
GGCAATTGAAGAGGCGGGGCTGATTTTTCTCGGCCCGCCGGCTTCGGCCATTGATGCCATGGGCAGCAAGTCCGCC
GCCAAGGCGCTGATGGAAACCGCCGGTGTGCCGCTGGTGCCGGGTTACCACGGTGCCGGGCAGGACCTGGAGACCT
TCCGCCAGGCCGCCGAGCGCATTGGCTACCCGGTGCTGCTCAAGGCCACCGCTGGCGGCGGCGGCAAGGGCATGAA
AGTGGTCGAGGACGAAAGCCAGCTGGGCGAAGCCCTGGCCTCGGCCCAGCGTGAAGCGCTGTCGTCGTTTGGCAAT
GGGCAGATGCTGGTGGAGAAGTACCTGCTCAAGCCACGCCACGTGGAGATCCAGGTGTTTGCCGACCAGCATGGCA
ATTGCCTGTACCTCAATGAGCGCGACTGCTCGATCCAGCGTCGCCACCAAAAGGTCGTCGAGGAAGCACCCGCCCC
CGGCCTCAGCCCTGCCCTGCGCCAGGCCATGGGGGAAGCGGCGGTACGTGCGGCCCAGGCGATTGGCTATGTGGGC
GCCGGCACCGTGGAGTTCCTGCTGGATGCGCGCGGTGAGTTCTTCTTCATGGAGATGAACACCCGCTTGCAAGTGG
AACACCCGGTGACCGAGGCCATCACCGGCCTGGACCTGGTGGCCTGGCAGATTCGCGTGGCTCAAGGCGAGCCGCT
GCCAATGACCCAGGAGCAGGTGCCGTTGCGCGGCCACGCCATCGAAGTACGGCTGTATGCCGAAGACCCGACCCAT
GACTTTTTACCGGCCACCGGGCATCTGGCGCTGTACCGCGAATCGGCCCCAGGCCCGGGACGCCGGGTAGACAGCG
GCGTGGAGCAAGGTGACGACGTGTCGCCCTTCTACGACCCGATGCTCGGCAAGCTGATCGCTTGGGGCGAGGACCG
CGAACAGGCGCGCCTGCGCCTTTTGAGCATGCTGGACGAGTTTGCGGTCGGCGGCTTGAAGACCAACCTGGGTTTT
CTACGGCGCATCATCGGCCATCCGGCGTTCGCCGCAGCGCAGTTGGATACCGGGTTTATCCCGCGCTATCAGGAAC
AACTGCTGCCTGTTGCAAGCGCGTTGAGCGATGAGTTCTGGCAGGCGGCTGGCCACGCCTTCATACAAAGCCAACT
GCCTCGACTGCGGGACGACGATCCCGCCTCACCGTGGGCTGCGACACAGGGGTTTCGTGCAGGCCTGCCTGCCGAG

ACATCACTGCACCTGAGCTGCGACGGCCAGGACCGCCTGGTGACGCTGCAAACCACCACTGCGCCGCAACTGCGGG
ACGAACACCTGCTGATCGAAGACGCCGGTATACGCCGCTCGCACCTGGCGGTGCGCCAGGGGCAGACGCTGTTCCT
GCGCTGGCAAGGTGAGATGCGCAGTGTGACGCTGTTTGATCCGATCGCCGCGGTCGATGCCGGGCAGGCGCATCAG
GGCGGCCTGACTGCGCCAATGAACGGCAGTATCGTGCGGGTGCTGGTGGAGGTCGGCCAGACGGTTGAAACCGGTA
CGCAATTGGTGGTGCTCGAGGCGATGAAGATGGAGCACAGCATCCGCGCGCCCCATGCAGGTGTGGTCAAGGCGCT
GTATTGCCAGGAAGGCGAAATGGTCGGCGAAGGCAGCGCCTTGGTGGAGCTGGAGGCCCAGCCCTAGAACTTCACC
GTGGCTTGTACCACCACGCCGATGATCCGGCAGTCATCGGTGAGCAACGCCTTTGGGTAGGTCGGGTTGAGTGGCA
CCAGGTAACGCTGGCCGCTTTCTTCCAGCAGCTTGCGAAACGTCGCCTGACTGCTGCCTGCCCATTGGGCCACCAC
CAGTTTGCCAGGCTCGGCGTCGAGGGCCGGATCCACCAGGATCATCATCCCATCGGCGATACTCGGCCCGCTCGGC
GCGGTCATTGCATCGCCGCTCACCTGGAGCCAGAACGCCTCGCCGTCGGCGTCGTAGTCGCTTGCCTGAAAACGCC
CGGGTCCGTAGGCCAGACGCTGTTCGTGGACTTGGCAGGGCTGTTTCCAGTCTTTTACCGGGTAGCGAAAGTACGG
GTTGTATTGAACGACCGGCGCGACTTTATCGTCTGCCGGGGCGTGGCCCTTCTCGCGAATCACCAGCGCCGCTTCG
AGATACCCCAGGCCCAGTTCTTCGAGCACCCGGTTCATGTCGGCGAGGCTCGGTACACGGCGCTTGCCCAGCCAGT
GGCCAATGCCGCCCTGGGACATGCCCATGCGTGCAGCCAGTTGATTCTGCGTGACCTTGCGGGCCTTCATGTTGGC
TTTGACCAACGCTATCCATTTATCCATGGGGCAGACAATACCGGGTGTATTCGCGGCGACAATAAACAGTTTGTAG
TAATCAATCAAATGACATAAATACAGATCGTATTATTATCAGTCGTACCTGTTCACCTTCCCACCCAGAGCACCCA
CCCCATGACCACAACCCCGGATCACTTGGACGGTACACCTGATACCGACGAAATAAAGTTCTTGCAGGGCAGCGAC
GCAGCTCGACGCGCACTGGATTACTACTTGAAAGAAGATACAGACCCTGCATCGGACGATATGGCCTGGTTCGAGG
CCAGCCCCACCGTCAGCAGTGAAGAAGCACTGATCCACGCGTCGGACTTGCTGCGCAGCGCCGCCGCCATCGCGTA
TGAGCTGGCGAACAACAAGCTGGGAGGCACCCGGGACCTGGCGTTTTCGGTGGTGTATTTAATTGATATGGCCAAG
GCCATGCTGGAGCGTCCACTGCAACTGGCAGGCGGGAAAACCTCGTAACCAGGTAGGCGTGCAGGAACAGAAGGAA
AAATATTTCTATCTGGCAGATAAAAACATTTGACTTGCAAATGATAATGATTATTATTGCAAGCAGCTGGTCGCGA
GATCAGTCGATAGACCAAGAGACCTTAGGTCGGACTCTTGGAATATCTCCTCATCAGGCTAATCACGGTTTTTGAC
CCGGCTCTTTGGCCGGGTCTTTTTTTTGCCAGTTATTCTGGCGTGGCTTCAGGCTAATGAAGACTGTGTGTTGCTT
GATGGCGCGCATGGTAGCAAAAGACCATCGCCGAAAGAAAGCCGAGAAAGCGCTCTGGTCCGGTTCTTTCCAAATT
AGCACTTGAGAATCAATCTCATAGACTCTAAGCTGCTGCTGCGTCACGGACGACGCCCCCTCCTTTTTCGCAACAA
TTTGCGTCCCAAACTTTACCCCGCCCCTGTGTAAACTGCCTGTCACATCTCTCATATTCAGGCAACCAGACTATGA
CCGTGGCCTTGACCTCCCTTAAAATCAGCAGCGATTTCGACAGCGGCAATATCCAGGTGCTCGACACCCGCGATGC
CCATCAACCGCTGCTGGCGATCAAACCCGATACCCGCAGCCAGCACTTCCAATGGTTCCACTTCAAGGCCGAAGGT
ATGCACATCGGTCATACCCATACCTTTCGCCTGAGCAACGCCGGCCAGTCCTCCTACAAACATGCCTGGAGCGGCT
ACAACGCAGTGGCATCCTACGACCATATCAACTGGTTCCGCGTGCCAACACGCTTTGATGGCGAAACCTTGCACAT
CGAATTCAAGACCGAGCAAAAACACGCCTGGCTCGCCTACTTCGAACCCTACAGCCGCGAGCGTCACGCCTGGTTG
ATCGACCGAGCGCTGAACCGCGCAGGCACCCAATTGCTGGCAACGGGCAAAAGCATCGAGGGCCGCGATATCCCCC
TGCTGCGCCGTGGCAAGGGCGGTGCGGGGCAACGCAAAGTGTGGATCATCGCCCAGCAGCACCCCGGCGAGCACAT
GGCTGAATGGTTTATGGAAGGCATCATCGAACGCCTGCAACAGGACGGTGATGATGAGCTGAAAAAACTGCTGGCC
GCCGCCGACCTCTACCTGGTGCCGAACATGAACCCGGATGGCGCGTTCCACGGCCATCTGCGCACCAACGCCATGG
GCCAGGATCTCAATCGGGCCTGGCAAAACGCAAGCCAGGAGCAGAGCCCGGAAGTGCTGTTCGTCCAACAGCAAAT
GGAGAAATACGGCGTCGACCTGTTCCTGGATATCCACGGCGACGAGGAAATCCCCTACGTGTTCACCGCAGGTTGT
GAAGGCAACCCCGGCTATACGCCACGTATCGAGGCCCTGGAAAAACACTTTCGCAGCCACTTGAGCGAACTGACCC
GCGACTTCCAGACCACCCATGGCTACACCCGCGACTTGCCAGGCCAGGCCAACATGACCCTGGCGTGCAACAGTGT
GGGCGAGCGGTTCGATTGCCTGTCCCTGACCCTGGAAATGCCGTTCAAGGACAACGACGATGCACCCGACCCGCAT
ACCGGTTGGTCCGGCAAACGCTCGCTGCAACTGGGCAAGGACGTGCTAAGCAGCGTGGCGGATATCGTCGACGTAC
TGCGCTGACCCTGGCCAACCCGGGGGGCATTCAGCCCCCGGATTGCGCCGCCACCCGCAGACATTCCTGCGGCCCG
AGCACCCGGCCATCCGCCGCACGCACTTGCAAGGGCTGCAGGGCCTGCCCGTGCTCTCGGTCCAGCATCAACGAAT
GCGCCTCGCCCTCAGCGTAAAAAAAGCCCTCGCCCCACTGCCGTAACCCAATGATCACGGCAAACAACCCCCTGCC
CTTTTCGGTCAACACATACTCCTGATAAGCACTGCCATCGGAGGCTGGGACGATCTCGAAAATCCCATGGGCCACC
AGATTGCGTAGGCGCGCCGAGAGTATGTTCTTGGCCATCCCCAGGCTGCGCTGGAACTCGCCAAAACGGCGGATGC
CATCGAAGGCATCACGAATGATCAACAGCGCCCACCCATCGCCAATGGCCTCCAGTGAGCGCGCGACCGGACATTC
GGCTGTTTCAAAGCGGGTGAGTTTGACCATGGATGATGCCTGCCTGACAAAAAATGTAGTTGCAATATAAAACCGG
AATACTTACCGTACAACTGGTTTCATTTCAAAACTATAACGAGCATCCAACATGCCAAAATCCCCCTTGAGCGGCG
CGGTCGTGCTGCTGTTCGCCATTGCCTGCGGGTTATCGGTGGCGAATGTGTATTACGCCCAACCCCTGCTCGATGC
CATGGCCAGCACGTTCGCCATGGACCCGGCCACCGTCGGCATCGTCATCAGCCTGACCCAGGTCGGTTATGGCATC
GGCCTGTTATTGCTGGTGCCACTGGGAGACCTGCTCAATCGCCGCCACTTGATTGTCAGCCAGTTGTTGCTCTCGA
CCTGTGCGGTTCTGCTGGTCGCGCTGTCCAGCAGCGGCCCGTGGTTCCTGGCGGGCCTGCTGCTGACGGGCCTGCT
CGCCGTGGTGGCCCAGGTGTTAGTGGCCTATGCCGCCCACCTGGCACAGCCCGAGCAACGGGGGCACGTGGTCGGC
CTGGTGACCAGTGGCATCGTGGTCGGCCTGCTGCTGGCGCGCACGGTATCCGGGATCATGGCGGACCTGGCCGGCT
GGCGTTCGGTGTACCTGTTGTCGGCCGGCCTGACGCTGCTGATGGCACTGTTGCTGTGGCGTGTGTTGCCCGCCGC

CGAACAGCCTCGTACGCAGGATTCCTACGGACAATTGCTACGCTCGGTGTTCAGCCTGTTGCGCGAGGAAAAAGTC
CTGCGGGATCGTGCAGTGCTGGCGTTGCTGACGTTTGCCGCCGGCACCGTGCTCTGGACGCCTTTGGTGTTGCCGC
TGAGCACCCCGCCATTGTCGTTGTCCCACACCCAGATCGGCCTATTCGGCCTTGCTGGTGCCGCCGGCGCGCTGGG
TGCGGCCCATGCCGGGCGCCTGGCCGACCGAGGCCGCGCCCAGTGGACCAGCGGTGCAGCCTTGATGCTGATGCTG
CTGTCGTGGCTGGCCATCGCCTTCACCCAGTCCTCGTTATGGGCGTTATTGCTCGGTGTGATTACCTTCGACCTCG
GCCTGCAAGCCGTGCATGTCACCAGCCAAAGCCTGATCTACAGCGTGCGCCCCGATGCCCAGAGCAGGCTGGTGGC
CGCCTACATGCTGTTCTACTCGGCAGGCAGCGCCCTGGGCTCGGTCATGGCCACGATGATGTACGCCTGGGCCGGT
TGGCTCGGCGTGTGCGCCCTGGGCGCTGCCATCAACCTGCTGGCCCTGGTTTACTGGCGGCTTACCCTCGGTGCTC
AGTCCTTGCCCAGCATGCTTTGCAACACGCCATCACGACGCACCCAGCCATGAAACAACGCCGCCGCCAAATGCAG
CAGCACCGTCAGAAACAGTAAATAGGCGAGGTACCCGTGAGCCTTGCGCAACACCGCAAACAACTGGGCATTGGCC
GGCACCCACGCCGGCAACTGCACGGTGCTGGAGAGCATCACCGGGTCGCCCGCCGCCGAAATCATCGCCCAACCCA
GCAGCGGCAACACCAGCATCAAGCCATACAGCAGCAGATGAGACGCCTTGGCCGCCAGCACCTGCCACAGTGGCAA
ATCAGAAGGCAACGGCGGCTGACGCGTAGAAAAACGCACCCCCAGGCGCACGATCACCAACGCCAGAATCGCAATA
CCCAAAGGCTTGTGCAGGTGCAGCAGCCACTCATGCCGTTGAGAAACCGACGCCACCATGCCGGCACCGATAAACA
GCATGGCGATGACCATCAATGCCATCAGCCAGTGCAGCAAGCGCGCCAGCGGGGTAAAAAACCGGGATGGGGCATT
CATGGCTTCGACTCCTGAACCGCGTTGTGCAGTTGGCTGACTTCACCGGTACGCCGCAGATACGAACTGGCATAGG
CGGCAGAACGCGCCGCCAGCAGCGGATCGTTGGACGCTTCAATGCCACTCGGCAGGATCAGCGGATCAAAATTGAT
ATCACGACAATCGCCGCTCAGTTGCGGCTGGCTGCTGTCGAGCACCAGCGTGCCGGCATTGAGCACCGTATGCTCG
CCAGCCCACACTTTGCTGGCGTCATCCACGGGGTCAGCGGGGTTGGCCAGGGTGATCAGCAATTGCCAGCGCACGG
GGCCGGCGAGCAAGCGTTGTTCCAGGTCCTGTTGCAAGAAATCCGCCCCCGCAGGCGCCGCAGCCTCCTTTGTGTC
CTGGCTGATTGGCACCACGCCCCAGCGCACGGCCTGGCGCTGGCCATTGGCGTTGATCAGGTAAAACCCATTGATG
CCGTTGTAGGACTCGGTGGCATAACTGGCCGAAGGCTTGGCGGTCTTGACCCATGCCAGAAACGGCGCAGCCTCGG
GATGGCTGGCAAAAAATGCCGGCATGCTGGCCGGGTTCGGCTTGCCGGTCACCGGGTCAGGAGCGCCCGCCTTGAG
CATCTGGTAGAACGCCTCGGGCGTGCCCACCGGGAACACCGGCATGCTGTTCATCCCGGTGCGCCACTGCTGGCCG
TTGGCCTGACTGAACTGCAAGGCCAGGCTACGGATCGGCACGCTGCTGTCGGGGGCATACGGGTTGCCGCTGGGCA
AGGCGAAACGCCCGATCACCGGAGTGCGTGCCTCGTGGAACACTTGCGCACTGGAATACGCACGCGCCGCAGGACC
GCTCTCAAAATAGCCCGCGACACACACGCCCTTGGAATGGTTGCGTCGGTAGCCGGGGTGAACGCCGTTGTTGGTT
TCCAGCGCATTGACCAGAGTCTTGGGCCGCAAACGTTGTGGGTCGAGTGTGCCATTGACGTAGGCAAAGGCTCCCG
CCACTGCGCCGACAACCACACCGATAGCGGCCAGGCGCAGCAGCAGGCTTGCACGCCCCAACGGCCGACGTGACGG
TGATGAGTGATCTACCATGAAATGCTCCGAGGCCCACGGCCACAGGGGAAGGTGCAATAAGACGCTGCTGACGCAG
GCTTATTCCCCTGAAGCTGTAGGTATTTTCAGAACAAGTGGCAAGTAGTCCTATTTCTCGGACTCATCCCACCCCT
GTTGGCGGGGCGGCGTGCCAAACCGTTGCTCAGGAATCCCAGATCGCGCCGTAGGCCGGTATGCCCCGGGCTTCCA
TCTCTTCAACCACCCGGCGGGTCAAGGCCTGGTTGGCAATGCAGATCACCGCTTCGGCCCCTGCGGCTTGCACGGC
AGCGTAGGCCAACTGCACCAGATCCGGCTTGCCCCGCACATCGGTGTCCCAGATCACCGCATCGGGTTGTGCCCGC
ACAATTTCCGCGACTAATTGCGCGCCATAGGTCTTGGCGGGGCTGCGGGTGGACCACACCAGTTGGATCGGTACCT
GCGCCGCCAGCAAATGCGGCATCACCGGGCCGATGCCACTCCCTGTGGCGATGTACACCACCGACTTGAACAAGGT
CTCGACGTTGGCCACGCCCGCGGTAGTAATCCCCTTGACCCACACATGCGATGGCAAGCGGTCGATATAGCGCCCC
GTCCAATCCCCGGCCCGGGAAATAATCAGGCGAAAACCCGGCTGGCCCGGAGCTGGGATATTGGCAAACGAATGCC
ACTCCAGCAGCGGCTGGAGACTGATTGCAGTGGACGAGCCGGCAAACGGCATGGTGTGGCACAGGCGGGTCACTAT
TGCATGGTCGGACGGTCGGTGATGACTGACCGCCACCCTGCGCAGGCGCAACCAAGGCAGGGCAATACTCAGGCTC
AGCAGGAGCAACACCCAGAACTCCATGGCATGCAGCAGCCTGCCCTGGGGTACCGCCATCAGCGTCTGCGCCCAGA
ACAGCAACAAGGCTGCCCACCCTGCAAACCGATGCACGCGCTCGAAGCCGTTATGGAAGCGACCGCGAATCCACGG
CTGCGCCATCACCACCATCAGCACCAACATCCCGAACAATGCCCCACTGACCAGCAACCACGCCGCCGAAACGCTG
TGGGGCTGTTGCAGGTAACGCCATCCCTGGCTGCCGACCAGCAACGCGAACCACAACGTCGCCGCCACCGCGCCAC
CGCTGTGCAGCCCCCCCAAGTGATAGACCTTGGCCGCATGGCGACGGATGCACAGCGGCCACGTCACGGGGATCCG
CGTCGCCAGCCAGAACAACCCGTTGATCACGTACTGCTGGCGCACCAGAATCGCCAGGGCCAGGTTGAACATCACC
AGTTTCGACACCACCTGCACATCCACAACAGGCAGCTGCAGGCTGGCCAGCGCAAACCAACCGTTGGCCACCAGCA
CCAGCGCTACCAGCCGGTTGTAATGAGAAAACAGCGGCGCACCAGACCAACGTCGCCACCCACCAGGCCGTGCAGG
CAATGGTATTTCCATCAACCTGTCAATCATGGGCGGCCACCTGCTTGCAGGGCGAGTTGCTGCCCCTCGACGCTGT
CGCGCGCCCATTGCAGCAGCAAGGTCTTGTCGATCTTGCCGCGCGCCGTCAGCGGCAGATTCTCCACAGCAAAGAT
CAACGCGGGAACGCAGTAGTAGGCCAACCGCTGCGCGCAGTACTGCCTGGCAGCCGGAACATTCGCACTGGCCGGC
GTCACAAACGCCACAAGGTGCTGACTGTCGAACTTCAGCGTCACCGCCCGGCTACACGCCCCACACGCCTCCAAAG
TGCTTGAAACCGCATCCAACTCCACGCGAAAACCACGCACCTTGACCTGATCGTCCACTCGCCCCAGATGTTCCAG
GGCCCCGCTGTCGGTCCAGCGTCCCAGGTCCCGTGTGCGAAACATCATGCGGCCCGCCCCCAGGAACGGATCTGGG
CAATAACGTTGCGCGGTCAATAGCGGGTTGTTCAGATAACCCGCCGTCACCCCGTCGCCCCCGGCCCACATCTCAC
CGACCGCCCCTGGCGCGCAGGCCCGCCCGTGTTCATCCAGCACATACACGGTGTTGTTGGGCGTAGGCGTGCCGAT
GGTCAAGGGTGCCCCCGGCGTGTGCCGATGCAGGGTGTTGACGATGGTGGTTTCGGTAGGCCCGCAGCCGTTGTAG

AACACACAATGGGCCGCCCATCGGTCCGCCAGTGCCTGTGGGCACGGCTCACCCGCCACGGCTACCGTCTCGAGGT
CGGGGCACTGTTGGGGGTCCAGGGTGCCGAGGATTGACGGCGTTGCGATCAGCACCTGGCAAAGGCGCGCCGTCTG
CCCGATATCCTGGGTGCGGATATGCAATGTTGCGCCGTGGGCCAGGCAGCCGAGAATTTCCCAGGCGGCCATGTCG
AATGCAATGTTGAGAATCTGCCCGACGATCATGCCCGGCTTCATGCCCAGGTTTCCCGGCGCCGTCAGCAGCACGT
TGCACACATTGCGATGGGTCACCTGCACCCCGTTGGGCCGACCCGTGGTACCAGAGGTAAACAATACAAAACACAA
GTCGCCGGGGCGGGGCACAACGACGTTCTGGTCTGCGTCCTCTGCCGGCCACGGCGCCTGCGCGAGAAACTCATCC
AGGCACACGCACGGCTGATCCGCTGCCAGCGGCAGGCGATCCTTGAACCCCGACAATGTCAGCACCACCTGGCTCG
ACAACGACTCAAGGATCATCGCCAGCTGGGCCGGTGGGGTAATTCGGGCGTCCTGGGGCACATACGCAGCACCGAC
TTTCAGCACCGCGAGCATCCCCACCAGCATGGGGATCGAGCGTTCGACAAACAGTGCCACACTGTCGCCCGGCACA
ACCCCCTGTTCGATCAGGTACGCCGCCAGCCGATTGGCTTGGCGATTCAACTGCCGATAGCTGATGCTCTGGTCCA
GCCAGCGGGCGGCTATCGCCTCGGGCGCTGCCGCCGCGTGCGCTTCAAAGGCCTGGTGTATAGAGGTGTACGGCAG
TGGGCTGACCGCACCGCATCCCCAGGCCTGGAGGGTATCGCGTTGCCGGGCCGGCAAATGGCCTAGCGCCCTGGTA
CTGACAAACCCCCCACTCGTTGCTGTTGCCGACATCATAGGCTTACTCCTCATCTGGCTCCTGCCCCACAGTGACC
GGGCAGTCAGGGCGCTTGATTGAGGAACTGACACGACATTACAGACTTGCGAGAGGCTACAAACGATAGGGAACGA
GGCCATTCACTGAATTTATTTTTCCTACGAATGGAATATCCATCGCTGGCTAGCGTCTCCCTGAGCACAGCCCATT
GCCACACACGCGCCGCAGGCCATCCATGCATGAACTCGACGAACAGTTGCGTGAGCTACTCCCCAGGCTGCGGCGC
TTCGCCGTCTCCCTGACACGCAACGCCAGCAGCGCTGACGACCTGGTGCAGGCCAGCCTTGAGCGGGCCATCGTCC
ATTGGCGCGACAAACGCGTAGACGGTGACTTGCGGGCCTGGCTGTTCTCGATCCTCTACCGGCAATTCCTCGATGC
ACACCGCCGCTCCCGGCGCTACGCGCGAATGCTGGAGTTCTTTACCGGCCGCGATGACGCGCAGCCGTCCGCCGAA
CGCACAGTGATCGCCCAGACGACCCTGCAAGCCTTTGACCAATTGACGAGCGAGCAACGCGCCCTGCTGCTGTGGG
TTTCGGTGGAAGGCTTGAGCTACAAGGAAGTCGCCCACATTCTTGAGGTGCCCATCGGCACCGTGATGTCGCGCCT
GTCCCGCGCGCGCCAGGCCTTGCGCCAACTCAGCGACGGTGACATCACCAGCCCTTCCCTGCGGAGACTCAAATGA
TCAGCCTGCCCCCCAGCGAGCGCGACCTGCACGCTTACGTCGATCACCAGCTTACGGAGCCTGATCGACGCCAACT
GGACATCTGGCTCGCCGCGCACCCCGAGGTCGCGGCCCAGGTCCACGCCTGGCAGCAGGATGCCCAGCACCTGCGC
GCGGCGCTCGGTGCCCAGCCGGCAGCGAACCCTGGGCTGGACCCGGCGCTGATTCGCCAGCGCCTCAAGCGCCACG
CGCGCCGTCGATTCGCCAGCGCGGCCGTGCTGTTGCTCGCGGTCGGGCTCGGTGGCCTGGGGGGCTGGCAAGCGCG
GCAGGCCACGCTTATCACCACGGCACTGCCGATGACCGATGCCCTGCAGGCCTATCGCCTGTTCGCCCAAGACCAG
ATCATGCCCGCCGACTATCAGGTCCACGACAACGGCGATATGCAAGCCTGGCTCGACCGCTACTTCGCCCAGGCCC
ATCGCCTGCCAGACCTGAGCCCGGCCGGCTTCAACCCCGTCAGCGGGCGCCTGCTCACCACGGACCAGGGCGCAGC
GGCGATGGTGCTCTACGAGAACCCACAAGGCCGGCGCATCAGTTTCTATATTCGCCCACCGGGCCCGCAGAACACC
TTGCTGCCACGGGGCAGCCGCAGTGCCGACGGGCTCCAGGCCCAATACTGGTCCAGCCCCGGCTACAACTACGCGA
TGGTCAGCCCAGAGGACGAACCGACCACCCGCTTGCTCAAGCAAACCACACGGTTCTAGGACGCCAACGCCATGCC
CTTTCACGATAACCGCCCCCGTCGCCTGACCTTGCTCAAACGGTTGCTGGGCAAAAAGACCGCCACCGATGGCGCG
CCAGCCATTCACCGGTTCTTCGCGGACAAGGCCCGGCAGCAGGGCTACACCCTCAGCCCCAGCCAGTTGCGGGTGA
TCGATGCCATGGCCCGGCAAACCGCACAATTGTTAGCCAACCGCCCGGCCCGCAGCCTGTACCTGCATGGCGCCGT
CGGCCGCGGCAAAAGTTGGTTACTCGACGGTTTTTCCAGGCCTTGGCGCTGCCCGGCAAACAGCGCGTGCATTTC
CATGACTTTTTCGCCCGCCTGCACCGGGGCATGTTTGCCCACCGCCAGCAGGCCGATGCCCTGGGCGCGACCTTGG
ACCAGCTACTAAACGGCTGCCGGGTGCTGTGTTTCGACGAGTTCCATGTGCATGACATCGGCGATGCGATGTTGAT
CACCCGGCTGTTCAAGGCCCTGTTCCAACGCGGCATCCTGGTGCTGGTCACCTCCAACTACCCACCACAAGGCTTG
CTGCCCAACCCGCTGTACCACCAGCGCTTCAAGCCGGCGATCGACCTGATCAGCGCGCGCATGGACGTCATGGAAG
TCAGCGCGCCCCAGGACTACCGCGGCTTGGCGCAAAACCACCGGCAGCAGCGTTTTACCCACGGCCAATACGTGTG
GCCCGGCACCCCGGCCCAGCGCCGTGAACTGGGGCTGCCCGCAACCGATGGCCCGCCCCTGGCGCTGACGGTCGGC
TCGCGGCAACTGCGGGTGCGCTGGCAACAGGCACGCAGCCTGGCCGTGGCCTTCGAGGACCTGTGTGAACAGCCCA
CCGCCGTAATGGACTACCTGCTGCTGTGCGAGGACTTCGACCACTGGATCATCGACGGCCTGCCCCGGTTGGACGA
CTGCCCGATTGCCGTGCAACAGCGGTTTATCAACCTGATCGACGTGCTGTACGACCAGGACAAGCACCTCACACTG
CTGGGCCAACAGCCACTGGATACGGCCCTGCAAGGTCAGGCGATTGACCTGGCCCGCACCCGCAGCCGCCTGGGCC
AGCTGCAACAAGTCAGCTCGCCGGCGCTCAAGTCGCTATCATGAGCGCCTTTGCCCTTGTCGAGTGACCGAAGCTG
TTCATGGATACCCTTGCCCAACTCAAGGCTGGCCAACTGGCTGGCATCAAGCGCCTGGACCTGTCCTGCGGCCTGA
CGCAGTTCCCCCGGGAAATCTTCGACCTGGCCGACTCCCTGGAAATCCTCAACCTGAGCGGCAACGCCCTGAGCAG
CCTGCCGGACGACTTGCACCGCCTGCCCCACCTGCGTGTGCTGTTTTGCTCGGACAACGCCTTCACCGAACTGCCA
GCGTGCCTGGGCCAATGCCAGCAATTGAGCATGGTCGGATTCAAGGCCAACCAGATCGAGCATGTGCCCGCCGCCG
CGCTGCCGTCGCAGTTGCGCTGGTTGATCCTCACCGATAACGGCATCAGCCACTTGCCGGACGCACTCGGTGAGCG
GCCCCTGCTACAGAAGCTCATGCTGGCGGGCAACCGCCTGGAGCAACTGCCGGCGAGCTTGGCCCAGTGCCACAAC
CTCGAGCTACTGCGCATCGCCTCCAACCGCTTGACGCAATTGCCGCAATGGCTGCTGACGCTGCCGCGCCTGACCT
GGCTGGCCTATGCCGGCAACCCGGTGGAAAGCGCGGTGGACCTGGTAAATGATGAGGCGACGCCGAATATTCCCTG
GGCTGAACTGCAACTGGGCGAAATATTGGGCGAAGGCGCGTCAGGGGTGATTCGCAAGGCGCTCAGGGGAACATCG
GGTGCACCTGTGGCCGTCAAGCTCTATAAGGGCACCATCACCAGCGACGGTTCGCCGCTGCACGAAATGCAGGCCT

GCATTGCCGCCGGGCTGCATCCCAACCTGATCAAGGTGCAAGGCCGCGTGATCGGCCACCCCACTGAACAGGCCGC
CCTGGTAATGGACCTGATCGACCCGAGCTACCGCAACCTCGCCGATCTGCCCAGCCTGGCGTCCTGCACCCGGGAC
GTCTACGACCCTGCCACCCGTTTCAGCCTGGAAGTGGCATTGCGCATGGCGCGCGGCATCGCCTCCGTAGCGGCGC
ATCTGCACCGGCACGGCATTACCCATGGCGACCTGTATGGCCACAATATCCTGTGCAATGCCGAGGGTGATTGCTT
GCTCGGGGACTTTGGCGCGGCATCGTTCAGCGCCACGGCAGACACCTTGGAAACCAGGGCGTTGCAGCGCATTGAA
GTGCGCGCGTTCGGGGTATTGCTGGGGGAGTTGCTGGAGCGCATTAACGAAGGCTTGAGCGATGAACAGCGCCAAG
TGCTGGAGGCGCTGCGTGACAGTTGCTGCCAGCCGCAGGTGCTCACGCGTCCGGCGTTCCAAGAGATCGAGACACT
GCTGTCCTCCCTCTGAAACGCCATGCACATGTGGGAGCGGGCTTGCCCGCGATAGCGGTGTAACTGACCCACCGCT
ATCGCGGGCAAGCCCGCTCCCACACTAGCCCCCGTCCATGATTAAAGGCCGGTGTCAGCCCGCCAGGCCGACGAAC
ATATCTTGCACATCGTCATGGTTGTCGAGGCCTTCGAGGAACGCTTCGACTTCCGCCATCTGCTCGTCGCTCAAGC
CGCTGACCGGGTTTTTCGAGATGTAGCCCAACTTGGCCGACAACACGGTAAACCCCTGCTCCGGCAAAGCTTTCTG
TACCGCATCCAGGTCCGTGGTCTCGGTAATGAACAGGGTCGTGCCCTCTTCTTCACCTTCTTCAAAGTCCTGGGCG
CCGGCTTCGATGGCGGCCATTTCCGGGTCGGCATCCGGGGTATCCGGCGAGGCCTCGATCAACCCCACATGGTTGA
AGTCCCAGGCCACCGAGCCCGAAGCCCCCAGTTGCCCCTTGCGAAATGCCACGCGGATTTCCGCCACGGTGCGGTT
GATGTTGTCGGTCACGCATTCGACGATCAACGGTACCTGGTGCGGGGCGAACCCTTCGTAGGTCACTCGATGGTAC
TGCACGGTCTCGCCCAGCAGGCCCGCGCCTTTCTTGATCGCGCGTTCCAGGGTTTCCTTGGGCATCGAGGCCTTTT
TGGCCTGTTCGACCACCAGACGCAGGTGGGCGTTGGTCGCGGTGTCCGCACCATTACGGGCGGCAATGGTGATCTC
TTTCACCAGCTTGCCGAAGATCTTGCCCTTGGCATTGGATGCCGCTTCTTTATGTTTAACCTTCCACTGTGCGCCC
ATGACTCACTCTCTTGATATCCGTGGCGCCGAGACGTCTACTGGCCGGCGCTTTGGCCGCAGAGTTTATAGGGCAA
GACCCCTTCGTTCCACCAAAAAACCGGCCAGCATTGCAGGTGCCTCAGGTGCGCAGGGGTTTGATCGCCACTGAGC
GATCCGGCACAAAGTCGCCCACTTGCGCCAAAACCGCAGGCCGGGCGTTCCAATAGGGCATCAGCACCAACGCCGA
AAACAACGCGGCGCCGGCGAACACGATAAACACCGTCACCGTATCGAAGTAGCCCGGCAACAGGCCACCGAGAATC
GCCCCCACCGAACCGCAACCGTTGACGAAGCCCGCCGCCGTCGCACCTGCCTTGGCCGTGCCGAAGTCGATCGCCG
CCGTACTGCTGATCATCGAGTCCGGCCCGTACAGGGTCAGGCCCATGACAAACAGCAGGGCCATCACCAACAGCAC
GCTACCGGTATGCAGTGCCCCCATGAACAGCGCCAGCGACACCGTCAGCGCCAACAGGCTGAGGACACAGGCCGGC
ATGCGCCGGGCGCCAAACAGTTTGTCGGACGCCAGGCCAATCATGATCGGCCCCAACAGGCCCGCCAGTTCGAACG
CCGTGGGCACAATAGCGGCACCGACCTTGCCCACCGAGGGCATCTGCTCGTAGACGATGACCGGGCCCCACAACAG
GATCGCGTAGCGCGCCGGTTTCAACAGGAAGTAGGCCAGGCCCAAGGTCAGCACCGTGCGGTTGCGCAAGATCTCG
CGCAGCGGCTCAAGGATGCTGATTTTCTTGGCCGCCGCCTGCTCGTCGGCGCTCATTGCCGGCTCAGGCTCCACTT
CCGGCAGGCCGACGTCCTGGGGCGTATTGCGCTGGAAAATGAAGAACAGCACCGCCACCAACGCCACCACCGCGGC
ACTGGAGATAAATGCCGCGTGCCAGCTGCCGATCAGGGTATAGGCCCACCAACCGGCAAACGGCGATGCCACCAAC
CCGCCAAACGCATAGCACGAACTCCACAAGCCCAACACCCGCCCGCGCTGTTGCGCCGGAAAGAAACTGCCAAGGT
TCTTGCACAGCCCCGACCAGCCAGTGGACTGCGCCAGGCCCTGGATCAACATGCACGTGACGAAGATCGGCAACGT
GGCGAACGTGCCCATGACCAAGGCGGCGGCCGCAGAAATCACCAACCCTCCCAACACCACCACCCTGGGGCCGAAG
CGGTCGGCGAGCATGCCCCAGGTGAACTGGCCAACGGCATACGCCGCCAGGTACAGGCCATCCAGGTTGGCCATCA
TCATCTTGTCCAGCGGGAAACTCGGGTCGTCGACGATCCCCAACTTGGCCACGGAAAACGCTTTGCGGGTGAAATA
GAAGGCGGCATAGGCCAGCCAGGTGATGGCGAAAATCTGCACGCGCCAACGCTTGATACTACCCATAGGGATATTC
ATGGTGGTTCTGACCTCAGGTGTGAGTGTGCCGGCAGAATCGAAAAGCAATTGCCTGTGTTTTTATTGTTTTGCAC
TGCAGTACCGGGTCGCTGGGCGCCGGTACTGGTCAGATGAGTCCTGCAAGGTTGCACGCAACGACCCATGATCGCC
GAAGTTGTAGGACTCAACCTACGGCGACTGGGGGCTATCAAAGCAATGGCTGACTAATAGGTAAAATCGATTTATC
GTATTTCAAATATAAGCCCAGCTTGTAAGAGGTCGTGCCATGTCGGTTTCCCACGCGCAACTCAAAGCCTTCCATG
CGGTAGCGGTCCACGGCAGCTTTACCCTGGCCGGCGAGCGGCTGTTCCTGACCCAGCCGGCCATTTCCGACCAGGT
GCGCAAGCTCGAAGAGCGCTTCGGCGTGCTGTTGTTTCATCGCAACAAGCGCTCCGTGCGCCTGACCGACCTGGGC
GAACGCCTGCTGAGTATCACCCAGCGTCTGTTTGTGGTCGCCGCCGAGGCCGAGGAGCTGCTGCAAGACTCCAAGG
CCTTGCAGACCGGCAGCCTGACCCTGGCCGTGGATGCGCCGGTGCATGTACTGCCGCAGATCGCGCAGTTTTGCGA
ACGTTATCCGGGGATCACGGTAAAGATCGAGACAGGTAACACCGATGATGCGCTGTATCGCCTGCACAACTATCAG
GCTGACCTGGCGCTGGTGGGGCGCGACGTCGACGATGAACGCCTGCTCTCAATCCCGCTGCGCGATGACCCGATGC
TGGCCTTTGTTGCACGCAACCACCCGTGGGCCGGGCGGGAATTCATCGACCTGGCGGACCTGGACGACACGCCCCT
GGTTCTGCGGGAAACCGGCTCAGTGACCCGTCAGACCCTGGAACGGGAGATGCACCAGGCCGGGTTGCGCATTCGC
CCGGCGATCCAGGTGGAAGGCCGCGAGGCCGCCCGTGAAGCGGTGGTGGTAGGGATTGGCGTGGGCGTAGTCTCGG
CCTCGGAATTCGGTGCAGACTCCCGGGTGTATGCCATGCCAATCCGCGACTGCACCCAGCGCATGAAGGAAACACT
GGTGTGCCTCAAAGAACAAAGCTCGCGACGGGTGGTGGCGACGTTTCTGGAGATTGTCCGCGAGAGCCTGGATACC
TGATCACCGACCTGTGTGGCGAGGGGGCTCGTCCCCCGCTGGGCTGCGCAGCAGCCCCAAACCCTGGTGCCGCGGT
CTATCTGATAAAACTCGGCGGTCTTACTGGGGCTGCTGCGCAGCCCAGCGGGGGTGTAGAACCGTAGACATCCTTT
ACATCTGAAACCGGGGACATCNNNNNNNNNNTATCGCGGGCAAGCCCGCTCCCACACAAAACCACCTTTTAGCGCT
GTACCGCCTTGGGCCTTAGGCCAACAGGGAGTACACCACCGCTGTGATCGCCACCAGACCTACCGCCGTAACAAAC
ACATTGGACGCCTGCCCACGGTATTTGGCCATGGCTGGTACCTTGCGAATCGCGTACATCGGCATCAGGAACAGGA

TCGAGGCGATGATCGGCCCACCCAGGGCCTCAATCATTCCCAGGATGCTTGGGTTCAGAGTGGCCACGATCCAGCA
CACCACCAGCATGAAGCCCGCAGTCATGCGGTCCAGGGCCTTGGCACCCGGGCGGCGGCCGCTCTTGACGATCAGG
CCCTTGAGGCCTTCGCTGGCGCCGATGTAGTGGCCCAGGAACGACTTGGCAATTGCCACGAACGCAATCAATGGCG
CCGCAAAGGCGATGGTCGGGTTATCGAAGTGGTTGGCCAGGTAGGACAGGATCGACAGGTTCTGCGCCTTGGCTTC
GGCCAGTTGGCCCGGCGACAAGGTCAGCACGCAGCTGAATACGAAGAACATCACCATCGCCACCATCAACAGATGG
GCGCGGGACAGGATCTGCGAACTGCGCTCTTCGGCGTGGGCGCCGTACTGGCGTTTCTGATCAACGGCGAAGGCCG
AGATGATCGGCGAGTGGTTGAACGAGAACACCATCACCGGAATCGCCAGCCACATCGTATGCAGCAGCGCCGACGG
TGCCGGCACAGTGCTGGCGGTGCTGAGGATACCGCCGTTCCAGTGCGGCACCAGGTACACCGCCAGAAACAGCAAG
GCGACAATAAACGGATACACCATCAGGCTCATGGCCTTGACGATCACCTGTTCGCCGCAACGCACCACCCCCAGCA
GGCCCAGGATCAGCACAAACGACAACAGCGCCCGTGGCGGCGGTTCAATGTGCAGTTGGTGCGACATGAAGCTGCC
AACCGTATTGGTCAACGCCACGCTGTAGATCAGCAGGATCGGGAAGATCGCGAAGAAGTACAGCAAGGTGATCAAC
GCACCGGCCTTGATGCCGAAGTGTTCTTCAACCACCTCGGTGATATCCGAGCCTGCACGGCCCGACAATACAAAGC
GCGTCAGGCCACGGTGCGCGTAATAAGTCATCGGGAACGCCAGCAGCGCGAGGATCAGCAACGGCCAGAAGCCGCC
CAGGCCAGCGTTGATGGGTAGAAACAAAGTACCGGCGCCAATCGCCGTGCCGAACAGGCCAAGCATCCAGGTCGTG
TCCTGGCGGCTCCAGCCCGTGAGGCTTACAGGTGTCGTTGCGTAGCGTTCATCGACGCTATTGGCCTGATCATTCA
TCCGCTCGGTTCTCCGCTTTCATACGATCGCGACGAGTCAGAAAAACCTGACAGGCAGCGCCACGACCGTAACAGG
GGCGCGATTGTCCGGGATTCGCCTGCATAAGCAAAGACTTAGCTGACGAATGCAGAGCCTGCAAAAAAATCGCGCC
GTCAACTTCAGCCTCTGAGGCGGCCCCTCCAGCACCCATATGCAGCCCGCCTGAAGACTGCTACCGATGCGTATTT
GTATGTCTTGGTTTGTTCTTTCGGGAACGCGATGCACCAGCACCACCACACAACGCTGCACGCTATAAAAGGCGTG
TCACGTTCACCACCTTCCCTCGTACCTGGAGCACTCAAAATGACCTCAATTGCATCTAACAATCAGCAATCCTTCT
TCAATACCGGCCTACTGGCGGACACCATCAGCCAATTCGTCGCATCACACCAACCCGACCAACAGCCCCAGCCCGG
GCAACCACAACATAATCCCAACACCTTCAGCTTCAATGGTGTCGAGTTTCAGGCGCAAAAACCACGCACCACCCTG
ATTCCCTTCCCAAGAATCGGCTGAGTCTCATCAAGTGACGTCGCGGCGCTTCAGCCCGCGACGCACTTTCTGCCTT
AAACATAATTCATATTAAAAGCTAAATTTTAGGAAACAACAACTTAACTCTAGGAAGCTTCCCACGCACAAATTC
AAAACAAACAAAACATCAACTTTTTAACATCGCTGAATTTGTTACAAACATATGCTCCAGCATTGATAGATGCTCT
CAGCAGACCTATATTCTTCCACTGTTGCTTCCAAGCAGTGCATCGCCGGTAACGCCCTCAACCCCATACAGGAATA
ATTCAATGAAAAAATATAACTGTATATTTACCGGGCTTTTTCTTATGACCCTGTTCACCCCACTCGCCAGCCAAGC
GCAACCGACAAACGCCGATATCCGCGCATTCGCTTCATCCCCTCAATGGCTGACCACCAACGTTTATATAGAGGGC
GCCCCCCACGTTGACGTCAAGGACAAGTACCCAGGCGTAGTAGGCATATCCATGTGGGACCCCGAGCGCAATCGCT
ACGAGTTCTTCTACGCCGACACCAGGCTGTCAAAATACGATAATGGCGGTGGCGGTTACTTCGTTGTTACGGGCGA
CAAAAAGACTCACATCCTGATCCCTGACAAAGGCCCGACCAGAACAGTGGTGAGGCGACTTGAAACCCTCAATCAC
AAAGAATTTACCTATTCGCGGGAGGTGCCGCACGACATGGTCGAAACCAACGAGAAAGTCCGCATCTACGTGGTTC
ACGCACCTTACAAAGGCCCGATAAAAACCACCACGTCTCACTGAATATTCTTCATCTATTGAATACACTTTCCAAC
AACTTACGAAAACAACAAAAAGACCCAATCTATGAAGACTCAACTCAAGTCACTCTTAATATCCATGGTAGGGTTT
TTTGCTCTACTTATCGGCTGTGCCAATGCACAACCCACAGCCACCCAAGCCGCAAGTGCACCTGCCGCGACAAAAG
ACAATGCAATCATGCTGACGGTCTTTCTAAAACATGATCAATCCCGACCATTAAGTGAACTAAGAGCGCAACTTTC
CAAGCAGGAGTTCTATAAAGTCTTTCCACCGGAGGGGGTAGAGGTCGTCAGTTGGAACATCACCATGGGTATCGGA
CAAGTCATCGTCCTGCGCCTGCCCGCCTCTCGTCTGTCGGCGGTCAACCTCGCATTGGAAAACACCGCATGGGGCC
CCTACCGGACCGAGTTTTTCCCTACCTATGATTTCAAGGACATCGCCCAGGCGGAACGGAACAAGGCGAGGCAAGC
CCAGTAGCACCGGAATCGATGCACAGCGTCTTCAGGCGCTGTCATCCCCATCTAAAGAGCTCCCATCAAATAAAGA
TCTTCCACCCGCTGGCATTTCGTACTTAAAAAATGCCGCATAATCCCGGTCTCAAACCCCACAACAAGAGCTGCCC
CATGTCCGCCACTGTTCTGGTCCTGGTTGAAACCATCAACGAATACCTGCACATCATCGAGAGCAATGACTTTCAT
GTGATCCTCGCGCCGACGCCCGCCGAACGCGCCCAGGCCATCAAGGCCCATGGCACGCAGATCAAGGCCGTGCTGA
CCCGTGGCCCTTTGGGGTTGCATGCCGAGGAAATCGCCGCCCTGCCGCTGCTGGAAATCATTTGCGTGATCGGCGC
TGGCTATGAGCAGGTCGACCTGCAGGCTGCGAGCAACCGGGGCATCGTCGTCACCAACGGTGCCGGCGTGAACGCC
CCTTCGGTGGCGGACCACGCCATGGCGCTGCTGCTGTCACTGGTACGTGACATCCCCCGCGCCGACGCAGCGGTAC
GGCGCAGTGAATGGCCGAAGGTCATGCGCCCGTCCCTTTCGGGCAAGCAACTGGGGATCCTGGGCCTGGGCGCGGT
CGGCATGGCGATCGCCCAGCGCGCTGCGCTGGGTTTTGACATGCAGATCAGTTACCACAGCCGCCAGCCCCGCAGC
GATGTGGACTACACCTACTGCGCCACACCGGTAGAACTGGCGCGCAACTCGGACTTCCTGGTGATTGCCACGCCCG
GCGGGCCGGGCACCCGTCACTTGATCGACCGTCAGGCGCTGGACGCCCTCGGCCCCACGGTTTTTGGTGAACAT
TGCCCGCGGCAGCGTGATTGTCACCGCCGACCTCATCAACGCCCTGGAACAACGACGCATCGGCGGCGCGGCGCTG
GATGTGTTCGATGACGAGCCCAACGTGCCGGACGGCCTCAAGCGCCTCAACAACACAGTCCTCACCCCGCATGTGG
CCGGGCTGTCGCCGGAAGCGGCCCATGACACCGTGCAACGGGTGGCGGACAATCTGGTGGAGTACTTCGCCGGGCG
CCCGGTACTCACGCCAGTCGCCTTGCCGGCGCCCGAAAAGTGACCGATCAGGCACGCTGATTATCATCCAACACGC
TAACCTATTAAGCCGTGCCAGCCTTGTGGCGGGTCGGCTGTGCGCATTAGATTAGCCAATAGTCCAAGGCCTCAAG
AATAAGCAGAAGGGATAAGCATGGCGCTCAATGACCAATCCACCCAGATTCGCCCTGGCGAAGAACTGGATGCCAG
CCTCATCGACCCCTACCTCAAGGCCCATATCCCAGGCCTGTCCGGCAGCGCAAAAGTCAGCCAGTTTCCAGGTGGC

GCCTCCAACCTGACCTACCTGCTTGAGTACCCTGACCAGGAGTTCGTCCTGCGTCGCCCGCCCTTTGGCCACAAGG
CCAAGTCCGCCCATGACATGGGCCGCGAATTCCGCATCCTGAACCAACTCAAGGACGGTTTCCCCTACTGCCCCAA
GGCCTACCTGCACTGCACCGACGAATCGGTGATCGGCGCCGAGTTCTATGTGATGGAGCGGGTCAAGGGCATCATC
CTGCGCTCGGACCTGCCGCCGGAGCTGGGTCTGGACGCCGGGCGCACCGAAGCCCTGTGCAAAAGCTTCATCGACA
AGTTCGTCGAACTGCACCAAGTCGACTACCACGCCTGCGGCCTGGCCGACCTGGGCAAACCCGAAGGTTACGTGGC
ACGCCAGATCAGCGGGTGGAGCGATCGCTACGAAAAAGCCCTGACCCCCGACGCCCCGAAATGGGAAGCCGTGCGC
GCCTGGCTCAACGACAAGATGCCGGCTGATCATCCGACCTCCAGCATCGTGCACAACGACTATCGCTTCGACAACG
TGATCCTCGACCCCAACAACCCGATGCAGATCATCGGCGTGCTGGACTGGGAACTGACCACCCTCGGTGATCCGCT
GATGGACCTGGGCAACAGCCTCGCCTATTGGATCGAAGCCGCCGACCCTGCGCCCGTGCAACTGATGCGCCGCCAG
CCGAGCAACGCGCCCGGCATGCTCACCCGCCGCCAGTTTGTCGATTATTACGCCGAGCGCGCCGGCATCCAGATCG
ACAATTTCGACTTCTACTACACCTACGGCCTGTTTCGCCTGGCCGGCATCGTGCAGCAGATCTACTACCGCTTCTT
CCATGGCCAGACCCAGGACAAACGCTTTGCGCAGTTCATCCAGATGAACAAGCTGCTGGAGCAGATGAGCCTGAAC
GTCATCCACAAATCCACGCTTTGACCGACAAGGAACCACCATGTCCAAGACCAACCTGTTCGACCTCGACGGCAAG
ATTGCTTTTGTCTCCGGCGCCAGCCGTGGCATCGGTGAGGCCATTGCCAAGTTGCTGGCCCAGCAAGGCGCCCATG
TGATTGTGTCGAGCCGCAAGATCGATGGCTGCCAACACGTGGCCGACGCGATCATCGCCGCCGGTGGCAAGGCTAC
GGCCATTGCCTGCCACATCGGTGAGATGGAACAGATCACCCAGGTGTTTGCCGGCATCCGCGAGCAATTCGGGCGC
TTGGACATCCTGGTCAACAATGCCGCGACCAACCCGCAGTTCTGCAACGTGCTGGATACCGACCTGGGCGCCTTCC
AGAAAACCGTCGACGTGAACATCCGTGGTTATTTCTTCATGTCGGTGGAAGCCGGCAAGCTGATGCGTGAAAACGG
GGGTGGCAGCATCATCAACGTGGCCTCGATCAACGGTATTTCCCCCGGGCATTTCCAGGGTATCTACTCGGTGACC
AAGGCCGCCGTGATCAATATGACCAAGGTCTTCGCCAAGGAATGTGCGCAGTTCGGTATTCGCTGCAACGCCCTGC
TGCCGGGCCTGACCGACACCAAATTTGCCTCGGCACTGGTCAAGAACGAATCGATCCTCAACATGGCCCTGGCACA
GATCCCACTCAAGCGCGTGGCCGACCCAAGCGAAATGGCCGGCGCCGTGCTCTACCTGGCCAGCGACGCCTCGACC
TACACCACCGGTGTGTCGCTGAACGTCGATGGCGGCTTCCTGTCCTGACACCGGCGGCTTGTGCGGCGAGGTGGAG
CTTATGTGGCGAGGGGGCTTATCCCTCGCTGGGCTGCGCAGCAGCCCTAAACCCAGGCGCTGCGGTCTGCCTGATA
AAAAGCGGCGGTCTTATAGGGGCTGCTGCGCAGCCCAGCGGGGGAAAAGCCCCCTCGCCACAGCAAGCCCCCTTCT
CCATAGCCGCCTTTGCCACAACAGCGCTCTCCCCCCACCACCTCCCCTCTAACGCACCGGGCTCATATCAATCTTG
AACACATACTGAGTGCCATTGGGTGCCGTGGCGCCGACTAGCTTGAATAGCTGTTGATTGCCCAGCGCGTGCCTGA
CGGCATCCCCAGCCGGTGAGTTCGCAGCCTCGGAATGGGCCAATACCGCGCTTACCCAGCGGTCTTCCATCTGCCA
GTACCCCAGCCCCTTCGCCATGCTGACACTGGACAAACGGTTAGTGTTACTCGGTGGAATATGTTTGATTTCCAGC
AGATAAATCGCATCCGCTCGCCCTTGTAATACCAGGTCAAAACCGTTCTGCCCCCGGCCATAGGTGCCTCCTGGAA
TAATCCGGTAGCCATCCTCGACCAACATCTGACGATACATGCGCTCATTGGCCTCACCACGCTGCCTGGTGGCGTG
GATGTCAAGCGCACTCCATTGCTCGAACAACCTTTGGTGTTCATCACTTCGGCGCCGCCTGGGGTAATGAAGCTGG
GCGTTGCTGCTGACGCTTTGAACGTTGAAGCTGCCGCCCTCACGCACGCTGACGGTTGCCGTACCCTGGAGCGGAT
CGAACTGCGCGATGCCCGCCGGCAAGGCGCGACCATCCGCCTGGGCACTGATCGCCTGGCGCAAACGGTCATAGGG
ATAACCATGGCGCCAGATATGCTCGTCAATCATGCGATCCCGATCCCGTACCGGCGCCTGGTGTATAGCCGTCGTC
TCGGAACCGATCAGTTGCCGGGCATATTGCTGCATATAGACATAGGTGTTCTTGTCCATGGTCGACATGACTCCCT
GGCTCATGTCGCTGCTGACGCCGGTGTAGCCCAACACCCTGCGCGCCTGCGAAATAATATCGCCGATATCGCGCAA
TTGAAGCTGCGGCACCCAGGAACTCAAATGGCCGCCCGTATCCAGGTAAATCGCCTCGACCCCATTGCCGAGCAGC
CGCCATTTCTGGCGCACGTTATCGAGCTCGATACGGCTGGTAACCCCCCCGACATGCAGATAGGTCAGGGGCTCGC
GCCCTGAAAGCCGGATGATCCCGTCCTCGGCAAACGAGCCACTGGGGCGCAAGTTGCCGTTAGGCCCGCGCTGCTG
GATATGGCTTTGCTTGATCCTGAAGGCCTTCAAGTTGAACTGCGCATTCACGTAGTAACGCGTGCCCTGGACCACA
AAGTCGTTCATTCCCGCCATCGGAAACAGCGGCACATTGCTCGCCTGGCTGACGTCATACCGCTGCAGGTCCCACA
CCGGGTCCAAGCGCCCTCTGGCCGGGACCGCTGTCGGCAACGAGACCGGGGCCTGCGCCACAGCGTTGCGCCTGGC
CCACAACGAACTGCCCGCAACCCGCTGCACCGACGCGCCCGTGGCGGCCAATTGATGAGGCGCGCGTTCGCGCCAC
TCACCGGTGTGCGGGTCACGCACCACCAGTACCGTCTCGCCGGTTTCCAAGTCCAGATAAGTGCGACCGTTGTGGG
TATACACGCCTTCGGCGCTCGGCGCCGGCAGCAGCGACCCAGTGACCAGCACGGCCATCGGCGGCATGGGTCCCGG
CACGTCCAGATCGCTGATTTGCGGCACTGTAAGCGACGCATCGTGGCCGCGTACCAGCGGGCGGCTCGCCTGACGC
TCCGTAAAAGGGCTACGGCCCTGAGGACCAAAATCCCTGACCAGGCCGCCAGCGCTCCCTGCCTCGACGCGAGGGG
CAGGCCTGGAGGGCCCGGGCCGGGGTGAATGAGGTGTCTGTTGCATAGAAATCCCTTTCCAGTATTAGTTGAGTGA
CACGCTTATGCGCATCAGGTCTGCCGAAGCCACGCTACGCGTGGGGTCACCGTGGGCGACCCACCGATCGAGCAGC
GCGTCGTAATCAATCGCGCTGTCAGTGCCATCCGCCAACTGCACACCGAGCACCCGCAAGCGGCGCGCCATCAACC
TTGAGTGCTGACGCATGCGGCGCAGTTGCTGCCAGACCTCGGCTATGGTCAGGTAACGCGAGGCATCCAGTTGCAC
CAGGCAAACGCTGCCCAGATCGAGGCGTATCTCGTATCGATCACCTTCAAGGGTCTGCACGTACAGTTGCTCCAGC
GGCACCTGCGGGTCTGTGACGATGGGCAGGATCAGCGTCACGCTGCCCACCTTGAGCCGGTGCTCGGCCAGAATGA
TCTGCTTGTGGTCGATGGGCTCATCCAGGCCGCTGGTGTTGATCTGCCAGGTGGAGGCATCCGAGGTCTTGGCCAG
GGCTCCTGGGGTAGACAGGGCAATGGTGGCGCCGCTGCTGGGGTGCACCTCATACACCGACCCGCGCCCCACCAGC
GCATAGGTCTGACGATGGGGCCCAGCCGGGATCCGCAGGGTCTGCGGCAGCGCCTGCAGCCACACGCCCTTGGCAC

TCAGCGGCCGGAAAAACCCATGGCTGCCAACCCGCGCCAACAGTGCCGAACGGCCGTCAAAGACCCTGAAGCCTGG
CCGCTCACGGTCTTGCTGGTAGGATTGCTGCAACGGTTGAACCCGATAAGACATGCCATCGTTGAAGACCAGCACA
CACTCGCAGTCGGGCTTCAGGCTGGCCGCCCGGATATTGGCGGCGACATCGGCGCGCACCGCACCGCGGTCGCTGG
CGAACTCGGCCAGGACCAGGCGGCCGCCGTCCGCGAAAGTCAGGGTCAAGGTCACATCACTGTAAGTCAGCGTGTC
GACCGGGCCTTGGCTGCGCAGGGTCACGCGATACCCGGCCACCACATCGATCAGTTCCTCGGTGCGGTAGTCCAGG
TACAGGGTACAGGCCGTGGCGCCGCCCGCGATTCGAACCCGCACACGTTTTTCGGCCACGCCACGCTCGATAAAGC
AGCGGCTCGACAGGCCGAATGGCAGCACCCATTCGCCGCCCGCCAACACGCGCTGAAACGCCGGCACAGGGCTTAT
CGAGCGGGCAGCCACCTTGATCGGCTGGTCGCCCTGCGCGCTCAGCAACGGCGGGAGAACCGGCTCCAACGTGTAA
CCGTCCGCCGTCAGTAAACGCAGATTATGACCGGCCAGGCGCCGCTCGCCGGATTCATGCAGGTAGAGGTTTTCAA
TCCTGACCAAGCGCTCCGGCAACTCGCCGTCGAGGCCGCGCAGGGAGCGGATCACCAACGCACATTCCTGGATCAC
CCAGTCAGCAATGCTGGACAGCTCCCACCCCAGCTCAATCAGCGTGTCTTCCTGCCAGTGCTGCTCGTGCAGCGTG
ACGGTGCCCCGGTTGCTCGCAATGACGTAGCGGTCGCTACCCGTTCCACCGTCGACCCAGGCATCGGTACCGTTGA
CGAAGATCGTGTCATTGCCGGCCCCGGCCTCGATCCGGTCGTTGTTGCCTTCGGCAATCAGGATATTGGCCTGCGC
CGTACCCTTGACGTGACTGGCTACGCCCGCAGGCGTCGCAACGTTCTCGATCGAGTCCAGGGTTATCTGGTTCGAC
AGCCCGCTGCGGCCGGCATCCTTGCGCCGTGTGACGCTGCCGGCCTGCAGATCGATGGCAAGCCCCTCAATGCCCT
CGACGTAAGGGGAGCGGTAGCCAAAATGCAAGGCATCACTGCCGCTGCCACCGTGCAGCTCACTCGCCGGGCCCGG
GGTGGCAACGGCCTCAAACGTATCCGCTGGCAGCTCAAATATGAACTGGTCATCGCGGATGCCACCCGTGAGTCGT
TTGTGCCCCGCCCCGAACTGAAAGATATTGCGCATCTCCAGCAGGCCCTGGACGTTGTCGTTACCATCGCCCAGGC
GCCAGAGGATCGCAGCGTGGGTAAACGGTTTGCCCAGCACCACCCCGGGCAGTTGGCTGACGCCGGCATGGGAGGC
ATCGACCACATCATCACCATCATGGATGGCCGGCCCAGTGGTATCGCGGTAGGGCAGCTCTCCCGCCGCTTCGTCC
CAACGGTGCTTCCACTCCCGCACGGGGATCATGCGCACTTCGAACCGCCCATTGACCACCGCGTGTACGGTGCTCT
TGAGCGCACCGCGCAGCCACTTTTCGGCCACGCCGGTAAGGTAGGTTGCGTGCTGTTTTTCCATCTTCGCCACCAG
GTAGCGGTCGCGCACATCCTTGTCCAGCTCCCGGCCTGCAAACGACAGCCAGCCGGCCCTCAAGCGCTCGGACACG
GTCAGTTCGATGTAATCGTCAATGTCATCCACCTGACGCACTGCGCTGTAGATCTGCGCCCCGAGAATCAGCACCA
CGGCCGCGGCAATCCCCAACGGTCCGGCATAGCTGAAACCCGCCAGGGCGGCGATCCCCAGCACCACGCCCAGGGC
TGCACTGGCCAGGCTGAAGCCGGCATTGACGTAGTGGTCCAACGCCTCTTTGCCCTGGGCCTTCGCCGCCTCATTG
AGTGACTGCACTGCACTGAAGATGTCGAACGGCAGGGTCAACACTTGGGCAAACAGGCCCGCGCCGCGCCGCAACA
ACTGGCCTACGCTGCTCGCACAGAACCCCTTGAACACACGCCCGCCGTTCTCCAGCATGCGCTTGCCCAGACGCTC
CAGGGCCAACTCCAGGGCGCTGGACGTCAGCTCGGCGAGCAATTCGCCACCGGCGGCCAGGGTCTGGCGAATATCA
CCGGTCTTGATGGCCTGGGCCAAGGCGTTGATCGCGCTGTAATAGCCATAGACCTGCAAGCCAGTCCCCATCGCTT
CCAGGGTTCGGCTTCGGGGAGTGTCGACCCAGGCGGGCAAGCCTTCACGTTGCAGGGCCTGGTTGAGGTTGGTGTT
CTGCACGGCGCGCAGCAAGTCATCGAGCCGGGTCATCGTGTCACCGCTGTCCAGCCTCGACGCCCCCTCAAGCAGC
GGCTTGGCCGGGGTTGAGCGTAATGCGGCGAACTCGAACAGCAGGCTGGCGCTGCTGTCACTGACGGTGGCGTCCG
TGCCCAGGAATGCCTTGAGCTGCGGCGCCGCAAAGCGGATCGAATCGAGCAGCGTCTGGTCGGCCTCGCGCCCAGC
CAGGTGGTGCACGTTGATGGGCTTGCCGTTTATCTGCGCGCCCATTTGAGCCAGGACGGTACGGCGAACCGGTAAA
ACACCGACCAGCAGATCCCCCAGCCGGGCATCCAGATCGGCCAGCGCCAAAGGTGCGTCCACTGCGGGTGTATCAG
GTTGAGTCATGAGTCGTTCCCTATGCCATGACGTGGGTCACATGGTTTGCCGGCAACAAGTGCCAGGCATAGGGCG
ATGGTGCTACGGGCCACAAGCGCCGCAGCGGTAGATCTTTATCCGGGTGGGCTTATTCTGGCGGCAACTGGCCAAA
GGCGACGGCCAGGCGGTTCCAGCCGCTGATGGTCGTAACCGCCATGGTCAGGTCCACCAGGGCCTGCTCGCCGAAC
TCGGCACGGGCGGCGGCGAACAGCGCATCCGGCACGCCTGCACACGCCAGCAACGCCGTGGCCTCGCTCCACGCCA
GGGCAAGTTGTTCGCGGCGAGTGAACAGCGGCGAATCACGCCATACGCACAACGCCAAAAGACGCCGCGTGCTTTC
GCCCCGCGCCAACGCTGCCATGGCATGCATGTCACTGCAAAACCCACAGTGGTTCAGTTGCGAGACGCGGATCCTT
ATCAGGTCAAGCAAAGGCCTGTCGATGGACAGCTCGAACAACGTCGCCTCGAGCACCATCATGCCTTTAAGCGCTT
GGGGCGAAGCCGTGCAGTAATCCAGACGCGGTTGCATAGGGGGCGGCCTCGATGGGCAAAGACGATGGCCGCCAGC
TTAAGACCGTGCCGGCCCGTGCCCTATAGCCAAAGTGGCGCTTTATGCCTAGACCGCCTTGGCCGTAGGCGCGCCC
CACGGTTGCGGGTAATCTGGGCGCAGTCTGACCGCCTCGGGATGCTCCATGGAACTGCACGTCGTCATCAATGGCC
GCAAGGATCTGGCCGAACAACTGTATCGGCAATTGCTCGCGGCCATCCAGTCCGGGCGCCTGGCCTCGGGTGCGCA
GCTGCCGCCCAGCCGCCTGCTGGCCGAGCAGTTGGGGGTGTCGCGCAAGACCGTCTCCGACACCTATGCGCTGCTG
ACCTATGAAGGGCTGCTGGTGGGGAAGATTGGCCGTGGCACCTTCGTCAACAGTCAGGCAAACCGCCCTGCCCGGC
GCCAGGACGCTGCCGACCTGGCCTGCGCCGCGCACTTGGCCAAATGGCAGGCGCTGCCCTCGCCCATGCGCCATCC
GACCCGCGACGGCACCTTGCGGTGCGAGTTCATCGGCGGCGCCACCACCCGCAGCCTGTTCCCCCAGGACGAATGG
CGGCGCTGCACCCTCGACGCCTTGCGCCGCACTGCCCAGAACAGCGGGTTCTATAGCCAACCCGAAGGCCTGCCGG
CCCTGCGTAGCGCGATCGCCGGGCACATCGCATTTTCCCGGGGGGTCAACTGCCGCGACACCGACATTGTGGTGTG
CAATGGCGCCCAGCAAGCCTTGGACCTGATCGCCCGAGTGGTACTGGAACCGGGCAGTCTGGTGGCCATGGAAGAC
CCCGGCTACACCCCCGCCCGCTTGATGTTCGAGGCCGTAGGGGCAACGGTGGCCAGCGTGCCGGTGGATGCCGAAG
GTATTCAGGTCACGCAGATTCCCCAGGGCACACGGCTGATCTACGTGACGCCTTCACACCAGTTCCCCCTTGGCAT
GCCCATGAGCCAGGCACGTCGCGAGGCGCTGCTGGCACGCGCGCTGGAATTGGGCGCGATCATCATCGAAGACGAC

TACGACAGTGATTTCCGCTACGAAGGCCGACCCACCCACTCCCTGCAAAGCATGGACGAGCGCGGCATTGTGGCCT
ACGTCGGGACCTTCTCCAAAACCCTGCTGCCGGACCTGCGCCTGGGTTATGCAGTACTGCCGCCGGCGATCCATGG
CGCGGTGGTCAAGGCCAAGCAACTGACCGACCAGCACACCTCGACACTGCCGCAATGGGCACTCGCCAAGTTCATC
AGCGAAGGCTACCTGCTCAAGCACATTCGGCGCTGTCACACCGTCTACGCTACCCGCCGCGAGCGCATCCTCAGCC
GCCTGGCCGGCGACTTGTCACCCTGGTTCGAAGCCGTGCCCAGCGTGGCCGGGTTCCACCTGGCAGCGCTGTGCAA
GGGGGCAGTAAACATTCCCTTGCTGATCGAACTGGCGCGCCAAGTGGAAGTCGGGCTGTACCCACTGGACGTGTTT
TTTCATGAAGCACCGGTGCGGTGCGGCCTGATTATCGGCTTTGGGGCCATCGAGACTCTGGATATCGACCCATCGC
TGGACAAGGTGCGCGATATCCTCCAGCAGATTGGCTAAGGGGTTTTCCAGAGGATTGGCTATAGGTGCGCCACGGT
GCCGGGCGTAGGGTGATGGGGTCTGCAACTCTGGAGAACTGCACCATGAAAATACTGCTCGGCACTACCCTCCTGC
TGGCTTCGCTAAGCGCCTTTGCCCATGAGCCGGTCTACAACCAGGAATCGATCAAGGTCCTGCAAGAGCATGCCTT
GAGCAACGTGCCTGGCAAAAAAACCATCATGCTCACCGTGGACTACGCACCGGGCCAGGCCACCGTCCCCCACAGC
CACACCGGCACGGCGGTGGCTTATGTACTGGAGGGTGAAATCACCTCGCGGGTCAACGATGAACCGGCCATTACCT
ACAAGGCCGGGCAATCGTTCTACGAACCGGCCGGCTCGCGCCACTTCGAATCCAGCAATGCCAGCCAGACCCAGCC
GGCCAGGTTGCTGGTGGTGATGGTTCTGGATGACAAGGCTGATGTGCTGACGCCCCTGCCAGCGCAGAACTGATTG
CTAGACGCTGGCTTGTAGAGCCCCTGTGGCAAGCCCGCTCGCCACAGGGGCTTGCTTGAACTGGCGCCGCAGTTCT
TCGATCGGCTGCCGGCAGGCGCAGCCTTCCAGGTGGTCATTGACCATTCCCATGGCCTGCATGAACGCGTACATCG
TCGTCGGCCCCACGAATGTCCAGCCGCGTTTCTTCAAGGCCTTGGACAAGCGCACCGAAGCCGGCGAGGTGGGATT
GCCCGTCCAGTAAGCCAGGTCCACCACCGCTGGCCGCTCATCGGCAGTCGGTTCAAAGGCCCATAGCCAGGCCGCC
AGCGAACCGCTTTCTGCCACCAGTTCACAGGCGCGGCGTGCGTTGTTGATGGCTGAGAGAATCTTGGCGCGGTTGC
GCACGATGCCCGGATCGGCCATCAAGCGCTCGACATCGCGCTCGGTGTATTGCGCCACCTGGCGAAAATCAAAACC
CTCGAACGCCCGGCGAAAGTGCTCGCGTTTGCGCAGGATGGTGATCCACGCCAAGCCTGCCTGGAAACCTTCCAGG
CAGATCTTCTCGTACAACGCAATGTCATCGGCAACCGGCACGCCCCACTCGTGATCGTGATAATCGGGGTATTGGG
CGGCGGCCGTGCGCCATGTACAACGGGTCAGCCCCGTTTCATCCGTCGTCAGTCCTGGCTTATCCATTTATCACCT
CAACGGCCAATCGCCGCATGATAAGCGAAAAAAATTCCACGCCGCCAACCGCTCAAGCATCACGCTTACCGAGCAA
CTGGTCAGACCGGACATCCTAAACACCCCACTTCCCCCTTGTAGTTTGCAAAAAACCGGGCGTAGACTGGCCACGC
ACTGGACTTACCGGTCAGACCACAACAATTAAGCCCTGGACCCACCAGGGCACCGAATAGAGATCCTTCCCATGCT
CAGATGGTGCTCGCGTTCGATTTTCCTGCAAGTCGTCATAGGCCTGATGCTCGGCATCGCTTGTGGCCTGGCCCTT
CCCGAATTCTCCTCACAACTCAAACCCCTGGGTGACGGCTTTATCAAGCTGATCAAGATGCTGATCGGCCTGATCG
TGTTCTGTGTGGTGGTCAGCGGCATCTCCGGGGCCGGCGACCTGAAGAAGGTCGGGCGCATCGGCCTCAAGTCGGT
GATCTACTTTGAAATCCTCACCACTATCGCTCTGGTGCTTGGCCTGGTGCTGGCCTTCAGTACCGGGATCGGCAGC
GGGGCCAATATCCACCTGGAGCAGTTGTCATCTGCCGGCCTCAATGAATTGGCGGATCGCGGCCAGCATATCAAGG
GCACCAGCCAGTTCCTGATGGACCTGATCCCCAACTCGGTGATCGGCGCCTTTGCCGACAACAACGTGTTGCAGGT
GCTGTTGTTCTCGGTGCTGTTTGGCAGCGCGCTGAACCTGGTGGGTGAAGCGGCCTCGGGCATTTCGCGACTGATC
AATGAGTTGAGCCATGTGATCTTCCGCATCATGGGCATGATCGTGCGCCTGGCGCCGATTGGTGTGTTCGGTGCCA
TCGCCTTTACCACCAGCACTTACGGCCTGGACTCGCTGCAACACCTGGGCAGCCTGGTGGGTTTGTTCTACCTCAC
GTGCATGGCCTTCGTCGCCATAATCCTCGGCGCCGTGATGCGGTTTTCGGGCCTGCGGATGTTGCCGCTGCTCAAA
TACCTGCGCGAAGAACTGCTGATCGTGATGGGCACCGCCTCGTCCGACGCCGTGCTGCCACAGATCATGCGTAAAC
TGGAGCACCTGGGCATTGGCAGTTCCACCGTCGGCCTGGTGATCCCCACCGGTTACTCGTTCAACCTCGATGGTTT
TTCGATCTACCTGACCCTGGCCATCGTGTTTATCGCCAACGCCACCGGTACGCCGTTGTCGATGACCGACCTGCTG
ACCATCCTGCTGGTATCGCTGATTACCTCCAAGGGCGCCCACGGCATACCGGGCTCGGCCCTGGTGATTCTGGCGG
CAACCCTCACTGCCATCCCCGCCATCCCGGTGGTGGGCCTGGTGCTGGTGTTGGCGGTGGACTGGTTCATGGGGAT
CGGCCGGGCGCTGACCAACCTGATCGGCAACTGCGTAGCCACCGTCGCCATTGCCCGCTGGGAAAAGGACATTGAT
ATCCAACGGGCCAACAAGGTGCTCGATGGGCAGCAAGGCTATGCGTTCCAGGCCAAGAAACCGGTGCTGCCGGTTC
ATCAGGAGTTCTGAACCTTCCCCACACACTGCAGGAGCGAGCTTGTTCGCGAAAAACTTCAAGGCACCGCGCGCAC
CCAGTCTGCCCGCGTTATCGTTGACGGTCTTCGCGAGCAAGCTCGCTCCTACCGTTGAAGGGTTATTTAAATTCAA
GGAGCCCACGACGTGATCAGCACCTCAACCGTCGTCAATTCAGTCGTAGAAAAACTGCGCGCTGCCCTGGCCCGCG
GCCAGTGGCGGCGCGGGGAGATGCTGCCCGGCCAGCGCGAATTGGCCGAACAACTGGGCATCAGCCGCCCTAGCCT
GCGCGAGGCAGTGATCGTGCTGGAAACCCTCGGCCTGGTACGCTCGATGCCCGGCAAAGGCGTGGTCGTACTGGAA
ACCAGTGTCAACGACCCCCAGGCCCATGACGCGGTGGCCGATGCCAGCCTCGCCGATATCCTGCAACTGCGCTACA
CCCTGGAGCCGTTTATCGTCGGCCTGGTGGCCCAGTCCATCACCAGCAAGGAAATCGGGCAACTGCGCCTGACCTT
GATGGACATGCGCGAAGCCCTGGAAGCCAACGACAGTGAAGCGGGGATGAACGCCTACATTGCCTTCCACGAAGAG
CTGTTCGCCCTGACCTCCAACCCCATCTTCCAGAACGTGGTGCAACAGACCAGCAACGCCCTCAAGCAAAGCGCCC
AGGTGTTGCGCAACTCCCCCGAGCACCTGGCCGAGCGCCTGCAGGAAAACGAAGCCGTGGTGCGCGCCATCCGCAA
CAAGAACAGCGCCCTGGCCAGCGCCGAAATGCGCCGCCACATCCTCAACGAGGGCCAGCGCATGGGCACCGCCCTG
AACATTCCCGACGATCATCTCGGCAGCTGACTTTGGAGACTGGCCATGAGCGCTCACGCCTTGCAACGCAACATCG
TTGTCACCGCCCCGCCCGCCATGCGCCTGGTGCCCGGCAAGCGGCCGTCGGTGGAAGACCTCTACCCGCGCCTGTT
CGATGCCATCCTGGAACAGCGCATCGTACCCACCAGCCGCTTTACCGAAGAAGGCCTGGCCCAGGAATTTGGTGTC

AGCCGCAGCGTACTGCGCCGGGTCCTGGCACGCCTGGCCGAACAGCAAGTGATCATCCTGCGCCCCAACCTGCGCG
CCCAGGTGGCGGCGCCGGATGCCGAGCAGGTGAAACATATTCTTGAAGCGCGGCGCTTGATGGAAGTGACCGTGGT
GCAACTGGCGTGCGCGCAGGCCAACCCAGGGCAAGTGCGGCGCCTACGCGAACTGGTGGCCCGCCAGCGGGCATGC
GTGGAACAAGGCCAGCAAGGCCCGGCCATCCGCCTGTGTGGGGAGTTTCACCTGTTGCTGGCGGCCATTGCGGGGA
ACCGGCCATTGGCGCAGTTCCTTAATAGCCTGGTGCCATTGACCTCGCTGGTCATTGCCCGGCAAGGGGCGTTCTC
CGGCGCCTGGCGTGAGCAGGCAGAGATTGTCGAAGCGGTGGAGCGCAAGGATGCGGGCACGGCCGCAGGATTGATG
GGCCGGTATCTGGATCACCTGGAGTCGAAGCTTTCGTAAGCCCGTGGCGATCAACATGGGGGAACTGGCCTGCGAT
AGCGGTGGGTCAGTTGATACATCTCTGCCTGAACGGACGCCATCGCGGGCAAGCCCGCTCCCACANNNNNNNNNNG
CTGTGTGGTGGCGCGTATAATACTGATTTCTAAGGATTATTCAACACCTTATTTAAATAAATCAGAAATAACGCG
TTGGGTCAGTAATTCCGGCCGCTTGGAAGCCTTCTGCACGTAGTCGGCAGCTGTCACATTTCCTACATGCACGACC
GTCATTGTCTGCCTGGTAGCAGGAAACAGTCAGCGAGTAGTCGACACCAAGCCTTACACCCGCCTTGACGATATCC
GCCTTGCTCAGGTTTTGCAGTGGCGCCTGGATACGGAAGCCGTGCCCTTCTACGCCCGCCTTGGTCGCCAGGTTGG
CCATGCGCTCGAACGACTCGACGAACTCTGGACGGCAGTCCGGGTAGCCGGAGTAGTCGACGGCGTTGACGCCGAT
AAAGATGTCGCGGGCATTGAGTACCTCTGCCCAGCCCAGGGCCAGCGACAGGAACACGGTGTTGCGTGCCGGCACA
TAGGTGACCGGGATGCCCTCAGTGGGCGCCTCAGGCACGTCGATGGTGCTGTCGGTCAAGGCCGAGCCGCCAATGC
CGTTGAGGTTCAGGCCAATCACCTTGTGCTCCACCACGCCGAGGTCGCGGGCGACACGGGCGGCGGCGTGCAACTC
GGCATGGGAGCGCTGGCCATAGTCGAAGCTCATGGTATAGCAGCTGTAACCCTCGGCACGTGCCATCGCCACCACG
GTGGCGGAGTCGAGGCCGCCAGACAGCAGGATTACGGCACGTTTTTGCTCAGTGGTTTGTTCAGTCATTTCAGCGT
CCTGGCTCGTCGTTCCAAAGATATTTATGCAGCTGCAATTGCAGGCGCACCGGCAGATTGTCCGCGACCACCCAGT
CAGCCAGGTCGCGAGCGTTCAAGTCATGGTGGCTTGGAGAAAACAGCACCTCGCCGGCGCGTCGGTCCAAACCGTA
CTGGATCAGCTTGGAATTGGCCCAGTCATAGTCATCCCGCGAACAGATGACGAACTTCACTTGGTCGTTGGCAGTG
AGCAACTCGATATTTTCATAGCGATTGCGATGCGCTTCCTTGGAGTCCGGCGTCTTCAGGTCAACCACTCGGCTGA
CGCGAGGATCGACTGCGGAAATATCCAGGGCACCGCTGGTTTCAAGGGACACCTCGTAACCGGCGTCACACAGCTG
CTTGAGCAAAGGAATGGCATTGGGCTGGGCCAACGGCTCGCCGCCTGTAACACAGACGTAGCGCGGCTTGTAGCCT
GCCACCTGCTCCAGGATATCGTCGAGGGTACGAATAGTGCCGCCGCTGAACGCGTAGGCACTGTCACAGTACTGGC
AGCGCAACGGGCAACCGGTCAGGCGCACAAATACTGTGGGCAGGCCGGCAGTGCGCGTTTCACCCTGTAACGAGTA
AAAAACTTCGGTGATGCGTAATGTGTCTTGCATAGTCGCCACGGGCGTAACAGCTAAACAGGCTGTCCGCCTCCGT
CAGGCACTTCATGGGATCCCGGCAAAGCGTGAACCCCAAAAAGCGTATTTCATAAAAGGGCGTGGATTCTAACGAA
AAAACCCGCGACAGGCGCGGGTTTCTTCCAAGCGGATCAGCAATGCTTACAAACGCTGCAAATCGCGCTGGGCCAA
CTGGGCGGCAGATGTGCCCGGGTATTGGGCCACTACTTGCTGCAGAATGCCTTTGACCTTGTCGGTATGACCCAGG
CGGCGTTCTACATCAGCCAGTTTGTACAGCGAATCAGGCACCTTGGCGTGCTTGGGGTAAAGCTGGCTGACCTTGG
CAAACGCCTGGCCTGCACCCTGCAAATCACCCTTGGCAAGGTTGACCTCACCCAACCAGTACTGGGCATTGCCCGC
ATACTGGCTGTTTGGATAACGACCCAGGAAAGCGGTGAACGCCTTGCTGGCCTTGTCGAAATCCTTGGCCTTGATC
AGGTCGAAGGCTGCATCGTAATACAGCTTTTCCTTCGCCGGGTCAGCCGGTTCGCTGCTGGCGGCAGGGGCCTGTG
TGGCGCCTGCGGCTACACCACCGGCGGCGCTTGCGGCGCCACCGGCAGAAGAATTATCAGGAGTAGCGGCTGGTGT
AACGCCGGTTCCTATACGTCGGTCAAGATCCTGGTAACGCTCCAGGCCTTCTTGCTTGAGCTGACGCACCTCATTC
TGCAGAACTTCAATGGTGCCTTGTTGCTGCGCCAATTGATCCTGCATGCGTTGCAGTTGGTTGAACAGTTCGCCCT
GTGCCGAAGGAGCGGCCGTGGCCGCTCCCCCCGCATAGGCGCCGTTCGTGCCATAACCTGCAGGTGGATAGCTGCT
CCCACTATTGTTATAGCCAGAGTTGCTATCTTCCACGGGAACCGCAGCCCACACCGCAAGCGGTGCGAGGCTGAGA
GCCAATACGGTTACAACACGACGGCACGTTCGCATGACGAATTACTTACGCAGTTCGACGCGACGGTTTTGAGCCC
AGGATTGCTCGTCGTTGCCGGTAGCAACTGGACGCTCTTCACCGTAGGAAACCAGTTCCAGCTGAGCTGGGGAAAC
GCCTTGCAGTACCAGGTAGCGCTGAACGGCTTTCGCACGACGCTCGCCCAGTGCCATGTTGTACTCACGAGTACCA
CGTTCGTCGGTGTTGCCTTCCAGTACAACGCGAGCGCCGTTACCTTTCAGGTCTTTCGCGTGAACGTCCAGAGCGC
GCATGGCTTCTGGCTTCAGGTCCGAACTGTCGTATTCGAAGTAGAAAGTGGTGATCGCGCGCAGAGCAGCTTCTTC
GCTCAGGGAGCCGTCAACTGCACCAGTGTTGGCGCCGTAACCAGCGTTTGGATCAACAGCTGCGCCTTCACCGGCA
TTGTCGCCGCCTTTAGACGAGCAACCAACGGCTACGGACAGAGCCAGAGCCAGAGCAGCAAACTTACCAAACTTCA
GCATTTCCATCGTGAAACTCCTAATGAACCCCAGTGTGTTAAGTACTTCTTTTTGTAGCGCCGCGTCAGTTCAGGT
AAGGGGACCAGGACGGTTCTCTGACTTCGCCTTGTGCGGTAGGAAGCGGGAGCCTTACGCGTCCATTAATGGACAC
GAGCATCAAGACTCCCCGGCCCTGCTGGCGGGTGGCGTAGATTACCATGGTGCCGTTGGGCGCAACAGTAGGCGAC
TCATCAAGAGTGCTATCTGTGAGGATTTTTACGCTTCCGCGCTGCAAATCCTGGGCCGCCACCTTGAAATTAGTGA
AGCCATCCTGGCGATGGATCATTACCAGGGTCTTTTCATCGGCCGAAAGCTTCGGATTGGCGTTGTAGTTGCCGAT
AAAGGTCACACGTTCTGCACCACCACCGCCAACACTGCTCTTATAGATCTGCGGTTTGCCGCCACGGTCTGATGTG
AAGTAGATGGTCGAACCATCCTTGCCCCAGAACGGTTCAGTGTTGATGCCAGGGCCGGAAGTGACACGGGTGATCT
GGCGCGAACCGAGGTTCATCACGTAGATATCCGGGTTACCGTCCTTGGACAGTACGAATGCCAGGCGATTGCCATC
CGGCGACCAGGCTGGTGCACCGTTCAAACCTTCGAAGTTGGTGATCTGCTCACGGCGGCCGGTGTCGATGTGCTGG
ACGAAGATGCGCGGACGCTTCTGTTCAAACGACACATAGGCGATACGCTTGCCATCCGGCGCAAAACGCGGCGACA
GGATCGGCTCGCGCGATTGCAGCAGGGTCACTGCCCGGGCACCGTCGTAGTCCGAACGCTGCAGGGTGTAGCGCGT

GTTGTTCACCGAGAAACGCTCAGCCGTCACGTACAACATGCGGGTAGAGAACGCACCTTTGATACCCACCAGTTTT
TCGAACGACTGGTCGGCGATGTAGTGCGCCATGTCCCGCAGTTGGTCGACGCTGCCCGACACGCTACCGGTCAGCA
CTTGCTGCTCGGTGGCCACGTTGAACAGAGCGTATTGCACCTGCAGGCGACCGCCCGCTGGCACGATACTGCCTAC
CATAAGGTACTGGGCGCCCAGGGCTTTCCAGTCACGGTAGATCACTTCGCTGCCTTGGGTCGGCTGGCTGATCATG
TTCTGCTTCGGAATAGGCGCGTAGTAACCCGAGTTGCGCAGGTCATTACCAATGATTTCCGCCATATCGTCCGGCA
GTACGCTACCGCCCTGCCAACCAAACGGCACTACTGCAATCGGGGTCGCCCGATCGCTACCGCTGGTTACCAGGAT
GTTCTTTTCATCCGCCGCCGCTATCCCTGCCATACAGCAAATAACGACAAGCATTCCTCGAAGAAGGTTTCTCACA
AGGCTAGATCCTCAGGTGTGAATGTCATCTTGAATGAACGATAGGGAGCGAAGTCGCTTGGTTTCATTCCCTGCAT
CTCGGTCAACCGGCCAATGTTCTTGACCGCTGCAACCGCTGAACTGTCGAACGAACCGTCGCCACTGGACTTGGCC
ACGCTGACCGAAGTCACCGTACCGTCCGGCAACATGCCGATCTGCAGCACCACTGTCATGCCTTTGCGTGCCGAAG
GTGGACGTGTCCAGCCCTCTGCTGCCCGCGCCCGAATCAGGTCGTCGAAACTGCCCGCGACTTCATCACCACGTTC
ATCGGCCAAGGCTTGCTGACGTTGCGGCGTGTCGGAAAGCAAATCAGCCAAGGCCTGGGCCTTTTTATCTTCGGCG
GATTTACGCGCTGCTTCCTGGGCCTTTTTCTTCGAGGCATCGGCGGCAGCTTTCTTCTTCGCGTCATCGGCGATTT
TCTTCTTCGCCTCGTCTGCTTCAGCTTTTTTCTTGGCGTCTTCGGCGGCTTTCTTCTTCGCGTCTTCGACTATCTT
CTTCTTGGCTTCTTCAGCGGCCTTTTTCTTGGCCTCTTCTTCAGCCGCTTTCTTGGCTTCTTCTTCAGATTTCTTC
TTGGCTATATCAGCCAATTGTTTCTCTTCGGCTTTTTTAGCTTCGGCAGCTTTCTCGGCTTTCTTGGCTTCATCAG
CCTTTTTCGCCTCGTCGGCCTTCTTGGCTTCGTCAGCCTTTTTCGATTCTTCGGCCTTTTGAGCCGCCTCGTCTTT
CTTTTGTTCCGCAGCAGCCTTCACCGCTTCCTGCTCGACCTTCTTCTGTTCCATCTGTTCGACTTCAGTCTGGCGC
GCAGCCGACTTCTGGGCCTCACCCGCAATCTTCTGATTGGTCTGGGTGGTGGCCTGACTCTTCGATTTCAGCTGAT
ACAGGGTCGCCTGCACGATCGGCTTGGCTGGCGGCAGCTCCGGCGTCATGGCGAAGCTGACAAACAGCATGCCAAA
CACCAGAACGTGCAGGGCAATTGCCCAGACGCTAGGCCAGAAGAAGCTTTCCGAGGCGGACGGCTCTCGCTGTTGC
TGCATCAGGGCGCCTCGGTAATCAAGCCAACATTACCGACCCCGGCCTTCTGCAGCCCGCCCATGGCGCCCATGAC
GGAGCCGTAGTCGACCGACTTGTCACCGCGGATGAACACCTGGGTGTGCTTGCCGCCTTCATTGCCGGCGCGGATG
ATCTTGGTCACCGCGTCGGTCATTTGCGGCAAGGTCATGGCCTTGTCCTGTTGCTTCTGGGTGTCGACTTCGCTGC
CAAGGTTCCAGTAATAGGTCTTGTCAGCCTTGATCGAGATGGTCAGGACCTGGGTGTTGTTGTCCTGCGGCAAGGC
TTCGCTGGAAACCTTGGGCAGATCAACCTTCACACCCTGATTGAGCATCGGCGCGGTCACCATGAAGATCACCAGC
AGCACCAGCATCACGTCGATGTAAGGCACTACGTTCATCTCGGCGACCGGCTTGCGCTTTTTGCGAGCTCGAGCGA
TTAAAGCCATTGGGAATTACCTGCTTATTCTTCGCTGGTGTGCACTTTACGGTGCAGGATCGCCTGGAATTCATCG
GCGAAGGTGTAGTAACGGCCAATCAGGGTTTCGCTGGTGGCGGCGAAACGGTTGTAGGCAATTACTGCGGGGATTG
CAGCGAACAGGCCGATGGCAGTGGCGATCAGCGCTTCGGCGATACCCGGGGCCACGGTGGCCAGGGTCGCTTGCTG
GGCCGTTGCCAGGCCACGGAAGGAGTTCATGATGCCCCATACGGTACCAAACAGGCCGATATACGGGCTGACCGAC
CCGACGGTAGCGAGGAACGGCAGGCTCTGTTCGAGCTTTTCTTCCTCACGGGAGATCGCGACGCGCATCGCACGGG
CCACGCCTTCCATGACCGCTTCCGGGTCAACGCCGGACTGCTGACGCAGGCGGGAGAATTCCTTGAAACCGGCGCG
GAAGATCTGTTCTACGCCCGAATCCGGGTCTGGGTTGCTGCCTGCCTGGCGATACAGCTTGGACAGGTCGATACCC
GACCAGAAGCGCTCTTCGAAGCTCTCCAGGGCACGTCGACCGGCACGCAGCAGGTTGCTGCGCTGAAAAATCATGA
CCCATGAGGTAACCGATGCGGCTACCAGGGTCAGCATCACCAGTTGAACCACGACACTGGCATTGCTGACCAGGCT
CCACATGGAGGAATGGTCGACGACGGTAGGTTCCACGCTAAATCTCCTGCTCTGATTGTTTACCGGCGCCGCTCAC
GCCGGCAAAGGCCGCACGTAAAGCTTCGGGAATGGCCCGGGGTTTCAAACTATTGGTGCGCACACACGCCACCAGG
AACTGCCCCTCACAGAGCAGCGTTGCATCCGTTGCCCGCCTGACCTGCTGTTTAAAGCGCAGGCTGACACGGTTCA
ATTCGATTACTTCGGCGCTGACCAGCAACTCATCGTCCAGTCGCGCCGGCGCGTGGTAACGGGCCTCGCTGGAATG
CACGACAAATAACAGGTCCTCCCCTGCAAGCTGGGATTGGGCAAAGCCCAGCTCCCGCAGCCGCTCGGTTCGAGCC
CGTTCCATGAATTTCAGGTAGTTGACGTAATACACGATGCCGCCCGCATCGGTGTCCTCGTAATAAACGCGACAGC
GATGTGCGAACGACTGATCCCCGTTTTGCGCGCGCATACTCTAGTGCTTACTCCTCAGGTTGCCAATCCGGCCAGG
CAACTGTTTTTCATTCTCTGCAACATTTCTAACGAAAGAATGACGACGCCAGCCACTAGGACAGCACAAACGTCGA
ATAAATCGACTCGCCATGCCTTTTTAATCGTCCACTGCATCGAGGAATTCGTCTACCACAGGCATCTCCCCCAATC
GTGACGGAATGTTTAGCCCGAAGTGCAAATACGCATGGCGCGTGACCACACGGCCCCTCGGTGTACGCATGATGTA
ACCCTGTTGAATCAAGTAAGGCTCCAAAACATCCTCGATGGTATGGCGCTCCTCACTGATGGCAGCGGCCAGGCTG
TCCACGCCCACCGGCCCACCATCGAATTTTTCGATCATGGTCAACAGCAGGCGCCGGTCCTGATGATCGAAACCAT
GTTCATCGACGTCCAGCAGGTTCAGCGCTAGGTCGGCCACGGCCTTGGTGATATGCCCCTTGGCTCGCACTTCGGC
AAAGTCCCGTACCCGACGCAGCAAACGGTTGGCGATCCGTGGCGTGCCACGGGCGCGCCGGGCAATCTCAAAGGCA
CCTTCCGGATCGAGCGGCAAGCCGAGAATCCCCGCCGAACGACTGACAATCGTCGCAAGGTCAGCAGTGCTATAGA
ACTCTAGACGTTGAACAATGCCGAAACGGTCTCGCAACGGGTTGGTCAGCATACCCGCACGGGTGGTGGCGCCAAC
CAGGGTGAACGGCGGCAGATCCAGCTTGATCGAGCGCGCAGCCGGCCCTTCGCCAATCATGATATCCAGCTGGAAG
TCTTCCATAGCCGGGTACAGCACCTCTTCGACGATGGGCGATAACCGGTGGATTTCGTCGATGAACAGCACGTCAT
GGGGCTCAAGGTTGGTCAACAGCGCGGCCAGGTCGCCCGGGCGCTCCAACACCGGGCCGGAGGTGCTCTTGATCGA
CACCCCCATTTCCTGGGCGATGATATTGGCCAGGGTGGTCTTGCCCAACCCCGGCGGGCCGAAGATCAACGTGTGG
TCCAGGGACTCGGCACGCCCGCGCGCCGCCTGGATGAACAACTCCATCTGCTCGCGCACGGTGGGTTGGCCGATGT

AGTCGGCAAGGCTCAGGGGGCGAATGGCCCGATCCTGGACCTCTTCACGGTCGCGAGGGCCGGTGGCTGCAATCAG
ACGATCAGCTTCAATCACTTAAATCATTCCCTTCAGGGCTCGGCGGATCATGTCTTCGGCGCTCAGGTTCTTGTCC
TTGATGGCTGACACTGCCTTGCTGGCTTCCTGGGGTTTGTAGCCCAGGGAAATCAGCGCATTGACGGCGTCGCTTT
CGGCACTGGCAACCTGGGCAGCCGGCAGATCCGGCTGGTTGGGTACCAGGGCAAACATGCTTGGCACCACTTCCCA
GGCCTTGAAGCGGTCCTTGAGCTCTACCAGCAGGCGTTCGGCGGTTTTCTTGCCGACCCCCGGCACCTTGGTCAGT
GCCGAGGTGTCCTGGGCCGAAACGGCACGCACCAACTCGTCCACTTCCAGGCTCGACATCAATGCCAGGGCCAATT
TCGGGCCGACACCATTGAGGCGGATCAGCTCGCGGAAGAAGTCCCGGTCGCGCTTGCCAATGAAACCATAGAGTAG
TTGCGCGTCTTCGCGCACCACCAGATGGGTGTGCAAGGTTATCGGTTCACCGACCGACGGTAGGCGATACAGGGTG
GTCATGGGCACTTCCAGCTCATACCCCAACCCGTTTACATCCAGAATCAGGTGCGGCGGCTGTTTCTCAGCCAGGG
TGCCGCGCAAGCGTCCAATCACGGTTCAGATCCTTAAAGCTTGAGGTCAGATCGAGGCCCGACCATAACGATTGCG
CTGATGCTATCAGAGACGCAGGCGCCCGCCACGACTGCGTGCCGTACCAAGGCCGTGGGGCAGCAGGCTGGAACGG
GTGTGGGCATGGCAAATGGCGATGGCCAGCGCGTCAGAGGCATCGATCTGTGGTTTTGACGTGAGCTTGAGCATGT
GCATGACCATCATCTGCACCTGCTCTTTATTGGCCGCACCGGTGCCGACCACGGCCTGCTTGACCTGGGTCGCGGT
GTATTCGGCGATTTCCATACCCTCCTCCGCCCCGGCAACAATGGCTGCGCCACGGGCCTGGCCCAGCTTCAGGGCC
GAGTCGGCGTTCTTGGCCATGAACACCTTTTCAATGCCCATGGTCACCGGCCCGTAGGTCTGGATCACTTCGCGCA
CGCCGCGATAGACAATCTGCAAGCGTTCGGCCAGCTCGCCAGCGCCGGTACGGATACACCCCGAGGCGACGTAGAT
GCAGCCACGCGGCGTCTGCTGCACCACGCCAAAGCCGGTGATGCGCGAACCGGGGTCGATACCTAGGATTAAAGTC
ATAACGCCTGCGGGTTTATGGGTACATAAGATCCAATGTGGGAGCGGGCTTGCCCGCGATAGCGGTGTTTCAGTCA
GAAACTCTGTATCTGATAAACCGCTATCGCAGGCAAGCCAGCCCCCACATTAGATTTTCATCGCGCTTAAGTTAGC
TGTTCGGCGACGGATTCCGGGATGTCGGCATTGGAATACACGTTCTGCACATCATCCAGGTCTTCAAGCATATCCA
GCATCTTGAGCACCTTTTGCGCGCCATCCAGGTCCAGTTCGGCGCTGGTGGTCGGCAACATGACGATTTCCGCGTC
GGTGCCCTTGAAGCCAGCAGCTTCCAAGGCGTTACGCACCGCGTAGAAGCCGGCAAACGAGGTAAACACGTCGATG
GAGCCGTCTTCGTTGGTCACCACGTCGTCGGCATCCGCCTCCATCGCGGCTTCCATCAGCGCATCTTCATCCGTCC
CCGGCGCGAAGCTAATCTGCCCCTTGCGCTCGAACAGATAGGCCACCGAACCATCCGTACCCAGGTTGCCGCCACA
CTTGCTGAACGCATGGCGTACGGCAGCTGCAGTGCGGTTGCGGTTGTCGGTCATGCACTCGACCATTACCGCGACA
CCGCCCGGGCCGTACCCTTCATAGGTCAGTTCGACCATATCGTCGGTATCCGCCGCACCCGCACCACGGGCCACGG
CGCGGTCGATGATGTCGCGACTCATGTTGGCGCCCAGGGCCTTGTCCAACGCCAGGCGCAGGCGTGGGTTGGAGCC
TGGGTCACCGCCGCCCTGGCGGGCTGCGACGGTCAGTTCGCGAATCCACTTGGTGAAGATCTTGCCTTTCTTGGCA
TCCTGACGCTCTTTGCGGTGCTTGATGTTCGCCCACTTGGAATGGCCAGCCATAACACAACTCCGAAATTCTCTAG
AAACCTTTCAATCCCACCCCAGGCAGGATTTCCCTCTTTTAAACGCAAAGGCGCATCCGAAGATGCGCCTTTTTGA
CTGCGTACAACCTTATTGCGCTTTCACGCCGCAGCCTTACTCAGCCTTCGGCGCCTCGCGCAGACGGATGTGCAGC
TCGCGCAGTGCCTTGGCATCCACCACACCTGGCGCCTGGGTCATGACGTCCGCAGCACTCTGGGTTTTCGGGAAGG
CAATCACTTCACGGATCGACTGGGCACCGGTCATCAGCATCACCAGGCGGTCAAGGCCGAAGGCCAGGCCACCGTG
GGGTGGAGCACCGTATTTCAGGGCGTCGAGCAGGAAGCCGAACTTCTCTTCCTGTTCCGCTTCGTTGATACCCAGC
AGGCGGAAGACCGACTGTTGCATTTCTTTGCGGTGGATACGGATCGAACCGCCACCCAGCTCAGTGCCGTTGAGCA
CCATGTCGTAGGCACGGGACAGAGCGCCAGCCGGGTTGGCTTCCAGCTCTTGCGGCGTGCACTTGGGCGCGGTGAA
CGGGTGGTGCAGGGCGCTGAAGCTGCCGTCGTCGTTCTCTTCGAACATCGGGAAGTCGACGACCCACATCGGGGCC
CACTTGCAGGTCAGCAGGTCGAGGTCGTGACCCAGCTTGATCCGCAGCGCGCCCAGGGCTTCGCTGACGATCTTGG
CCTTGTCGGCGCCGAAGAACACGATATCGCCATCAACGGCACCGACGCGATCGAGGATCACATTGAGCTTGTCTTC
CGGGATGTTCTTGACGATTGGCGACTGCAGGCCTTCAACACCCTTGGCACGCTCGTTGACCTTGATGTAAGCCAGG
CCCTTGGCGCCGTAGATGCCGACGAACTTGGTGTAGTCGTCGATCTGCTTGCGCGGCATGCTCGCGCCGCCAGGCA
CACGCAGTGCGGCGATGCGGCATTTCGGGTCGTTGGCCGGACCGCTGAACACCTTGAAGTCGACTTCCTTGAGCTG
ATCGGCAACGTCGACCAGTTCCAGCGGGTTACGCAGGTCTGGCTTGTCGGAACCGTAGCGGCGCATGGCCTCTTCG
AAGGTCATGTGCGGGAACTCGCCGAATTCCAGGTCCAGCACTTCCTTGAACAGGTTACGGATCATTTCTTCGGTCA
GGCCCATGATCTCTTTTTCATCGAGGAAGCTGGTCTCGATGTCGATCTGGGTGAATTCAGGCTGGCGATCGGCGCG
CAGGTCTTCGTCACGGAAGCACTTGGCGATCTGGTAGTAACGGTCGAAACCGGCAACCATCAGCAGTTGCTTGAAC
AGCTGCGGCGATTGCGGCAGGGCGAAGAAGCTACCGGCGTGGGTACGGCTCGGCACCAGGTAGTCACGTGCGCCTT
CTGGCGTAGCGCGAGTCAGGATTGGCGTCTCGACGTCCAGGAAGCCATTCTCGTCGAGGAAGCGACGGATGCTGGT
AGTCATGCGCGAACGCAGGCGCAGCTTCTCGGCCATTTCCGGGCGACGCAGGTCGAGGAAGCGATAACGCAGGCGG
GTTTCTTCGCCAACGTCAGAGAACTCGTTGAGTGGGAACGGCGGGGTTTCCGACTCGTTCAGCACTTCCAGTTCGT
AGCCCAGGACTTCGATCATGCCCGACGCCATGTTGGCGTTGGTGGCACCGGCCGGACGCAGGCGAACCTTGCCGGT
GATTTTCACGACGTACTCGCTGCGCACGCGGTCGGCGGCGGCGAAGCTCTCGGCGCGATCCGGGTCGAATACCACC
TGGGCCAGACCGTCACGATCACGGATATCGAGGAAAATCACCCCGCCATGGTCGCGGCGACGGTGAACCCATCCGC
AAAGGGTGATTTCCTGACCTTCCAGGGTCTCGTTCAGTTGGCCGCAATAGTGGCTGCGCATCATGGTAGTGGTTTC
ACTTCTCGTAATTCGAAATTCGGTGGAGTTCCTGCTCGGGCACCGCACCTGTTGAGTCAGGCCACGGATAATGCAG
GAGCTCGCGCGTAGAGTTCAACTCAGTCTGCTTTGTCGCCGCCCGCCAGATTCTTCTTGGCGCCGGTCTTGAAGTC
GGTCTCGTACCAGCCGCTACCGCTCAAGCGAAAGCCCGGCATGGACAACATCTTTTTCAGCTCGGGCGCCCGGCAA

GCGGGGCAATCGACCAACGGCGCGTCGCTGATCTTCTGAATGGCTTCCAATTGGTGACCACAGGAAGCGCATTGGT
AATCGTACATGGGCATGGAGTATCTCGGCGATCACGTCACTGCTGCGCCACGCCCCGTACCAGCCGGGTGCGCAGC
AAAGGGCGAGATTATATCCGTTAAATCGAGGCTGTGCAGCCGTAGCAACGTGCAGCGCACTATATAGAGAACCGCC
CAGCCCCTGCACTACCGCGCAACCCGACTACATGAGGCCGGCCTGGCTTGTGGCCGATGCCTGCCCTTTGAGCGCA
TGAACCACACACACCACCCGGACCAGCCCGCTGAAATTCTTGACTCCGCCATAACGTAAATGCACTTCGCGATCCA
CGTACGACAGCAGCGCACTGATCGAGCAGGTATTGATCCTGGCAATCTCAGCAAGAATATTCCAATAGACTTCTTC
AAGCCGCAGGCATGTCGAGAAACCATTTAATCGGACAGACCGCGACAATGGCCGCGCCAGCGCCATATCAAACCCT
TTGACAAAGGGATCAATCTTGATCTTTTGAAAACCGGAAGTTTCCATCACACCATTACCCATACCACGCGACATAC
ACTTGACACTCCCTTGCCAAACGGCTGTTGAATGAACTGCTCTTAGTTGCCGTTGAGCCTATAAAACATCAAGCAG
CCACGAGCAGCCAGAAGACGGGGAGTCGGTGTGCGTAGGACAAGCCAACGTGAGTATCCGGAAGTGATCAGGCCAA
GCCAGTGGCGCCTGCCAGGCACCACTGGCCCGTAGGGTTTATTTGCCTTCGAGCAGCGAGCGCAGCATCCAGGCGG
TTTTCTCGTGGATCTGCATGCGCTGGGTCAGCAAGTCCGCGGTCGGCTCGTCGCTGACCTTATCAAGCAACGGGAA
GATCCCGCGCGCCGTCCGGGTAACGGCTTCTTGCCCATCGACAAGTTGCTTGATCATGTTTTCAGCGCTGGGCACG
CCTTCTTCTTCCTTGATCGAGGATAAACGGGCATAGATGGAATAAGCGCCCGGCGCAGGAAAGCCCAGGGCACGAA
TACGCTCGGCAATGGAGTCAACTGCCAGCGCCAGCTCGTTGTATTGCTCTTCAAACATCAAGTGCAATGTACGGAA
TTGCGGCCCCGTGACGTTCCAATGAAAGTTATGGGTTTTCAGATAAAGTACATAGGTATCTGAAAGCAGCCGTGAA
AGTCCGTCAACAATGGATTTACGATCTTCTTCACTGATACCGATATCGATTGCCATGTCTATTCCCTTCAATTGAT
GAAAATTCATTGATCAGGTGCTTACCCACTCTAGCAAGAGTACCCGCCTTGCGCAGCCCTGACGCCGCAGCAGGCC
TGCGGCAAATCGCCGTGTTACAGCGCGCGTAACCACGAGCGGCAGGGCGTGGCGTGGCAAAAAGCAGCCTGACCTG
TCTAGGACAAGGGTTTGCCGCACAAATGCCATGATCTTCGCAACGCCTTGAAACGCTTGATTTGAGAAGGCACAGG
CTTTGCTGTTAAATAGGCAGCGTGTCGCCACCGCTATTTCCTGCGGCAAGCGGCATAGGCTGATAACCGCGCACGT
GCCCAACGCCTCCCATTGTTCAGAAGCGAACCGTGCCAGTCAGCTCTTCCTTGTGATCCGTCTTAACGTGAGTTAA
TCAAAATGTTGAAAATCGTCCACCTGCTAACGGGCGCTGCAGCTTTGCTGCTGTCCTTTATCCCGAGCCTGCAACC
AGAAAGCCTGCCGTACCTGCAACAACACGACGCGCTGTACCTGGCTCTGTTCGGCCTTCTCAACCTGACGCTGGCG
CCAGTGATTCCCTACTGGAACAAAGGCACACGCCATCAACTGCAAAACCTGGTCAGCGCATTGCTGGTACTGACTG
TCGTCGTACAAACCCTCACCCTCCTGGCCCCCATGCCTGAAGTCGGCGGCCATCCAGCCATCCTGCTCAGCCTGGC
CATCGCTGTGGTCGCTATCGTTCTTCACCTGGCCATCAGCTTCTACCGTTCGTCTGCTCCGGCCACTTCGCAAAAC
TACGACATGACCAACCGGGATACCGGGACCGTCAAGTGGTTCAACACCTCCAAAGGCTTCGGCTTTATTTCCCGGG
ACTCGGGCGACGATATCTTCGTCCACTTCCGGGCTATTCGCGGTGAAGGCCATCGCGTTCTGGTAGAAGGCCAGCG
CGTGGAGTTTTCCGTCATGAACCGTGACAAAGGCCTGCAAGCTGAAGACGTGATCGCCGCCCTGCCCCGTCGCTGA
TTCAGACCAGGCCCGAAAAAAAACCGCGATGCAGTTGAAACTGCATCGCGGTTTTTTATGGCCGCAAGTTTTCCCC
AGCCCTCAATAATGAGGCGGCGGCGCCTCCTCCTCGGAGGATTCGAACTGCCCGCCCATTTCTTCCTGACGCTTGA
GCAAGGCAGCCATCTGCAATTGCAGGCGCTCTACCGCACGCTGCTGGGCAACCAGGATGTCATTCAATGTCTCGAT
GGTGTCGTCCTGGAAAGCCAGGCGGCTCTCAAGGTCCATCACGCGGTCTTGCAGGTCCATGGTTCAATCCTGGGCA
AAAGTGAATTCGGGCGGCATCAACTCCCGCAGGCGGGCTCGAATCAGCGCTACCTGGTCGTCGGAATAAGGTACCG
CCGGGTGCCTGCCCCAGACCGGTGCGGGCCAGGCCGCATCGTCGCGCTTGCGCACAATCACGTGCACATGCAGCTG
GTTGACCACGTTACCGAGCGCACCGATGTTCATCTTATCGGCGCCGAATCCGTCATTGAGCAGTTGCGCCAACGTG
GTGGTTTCGTGCCACAACCGGGACTGGTCGCCAGCATCGAGCTGGAACACTTCGCTGATACCGGCAATGCGCGGCA
CCAGGATAAGCCAGAGGTAGTTAGAGTCGTTGGACAGCAGCAGCCGGCACAGGGGGAATTCCCCGATGGGCAAGGT
GTCGTTTTGCAGACGTGGATCCAAGGCGAACACCGCAGGCACTCCGTTCGGCTAGTCAGTTTCAGGCGCCCAGCAT
ACCTGCGAATGACGGCTGCTTCACGGGGCAATCGCCCCACACCCCATGGGCAATCTGCCGACCGCAAGCCAACGCA
CCAAAATGAGTCTTTTATGCCCCGCATCGGGCAGGGCCTATCCGCCGATTACTCACTGCGCAGGGTGAACCGGTAA
CATTTCTGACGGAAACGTATTTTCACCCGTTCGAAATCCCGCACAACCAACGCCCCGAATTCCAAAAAAAACCACG
CAAAAGCCCCGCATCCAGGCGGTTTTCACAGACTCACCACATTTTTCACGAAAAAATGACATCTGCCTGGCAGTTT
GAGCACGCTTGTTGCATTCACTCCCATATCGTCGACAACGGCACCTGCCAGGAAGGCGGTGTTTCACGATAAAAAG
AACAGTGAGTCAATACACAAACAGGAAGTTTTAAAACTTTGAACAGGGTACTTTGCACCTCTGTCCAGGGTATTAA
AAACAGTATTGAAGTTGTCAGTCTGGCTACAAACAGACTGTAGATTTGCGACATGGATCTAGCAATTTACGACAGC
CTCGTAAAGAAGCTGAAAGGACAGATGTGCAAGTATCGCCAATTATTGCCAGCGTGCTATAAGTTTGCGCTGTCAC
AAAAAAGAAAGAGCCGCCCAGACAAAAATACAGGTGGGACGGCAGTACTCTTCCTAAAACCAAAGGAGCAAATCAC
GATGCGCGTGATGAAGTGGAGCATGATCGCCCTCGCTGTTTCAGCAGGCACCTCACAGTTCGCAATGGCGTCCGCC
CAAGATGATGCCAAGGGCTTTGTCGAAGACAGTACGTTCAGCATCAATACCCGCACCCTGTACTTCAGCCGAGATA
ACCGCAACAACAAGTCGGGTACGAGCTACACCCGGGAAACCGGCCTGGGCTTCAACGGTTTGTACCAGTCGGGTTT
CACCCAGGGCACCATCGGTGTGGGTGTTGACGTTATCGGCCTGCTGGGCGTGAAACTGGACAGCGGCAGAGGCCGT
AATGGCACCGGCCTGTTCCCGGAAGGTTCGGATGGCCGCTCAGAGGATGAATACTCCAAGGGCGGCGGCGCCATCA
AGTTCCGCCTCTCCAATACCGTGCTGAAAGTCGGCGACCAGTACACCACCGCACCGGTATTCGCATCCGACGACAG
TCGCTTGCTGCCAGAGCTGCCACAAGGTATCTCGATCGTCAGTAACGAGATCAAGGACCTCAAGCTGGAAGCCGGC
CACTTCACTTCCAGCGTTGCCCAGGCGCAAACCTTCAAGGACAGTATCCTCGACTTCCCTGAAACCTCCCGAGGCA

TCAAGCAAGCCAATTTCGTGGGCGGCACCTACGGTTGGACGCCTGAGTTCACCACCAGCCTCTACTACTCGAAAGT
CGAAGACTACTGGCGCAAATACTACGCCAACGTCAACTGGACCCACGCCCTTAGCGATGACCAGTCGGTGGCCGTA
GACTTCAACATCTACGACACCAAGAGCGACGGAGCCGGCCTGCAACGCGCCTTCAAAGACGACGTTACCAAACTCG
ACAACCGCGCGTTCAGCTTGCAGGGTGCCTACACCATCGGTGCACACACCTTCACCCTGGCTGCACAGAAGGTTAC
CGGTGACGGCGATTACGGCTACGGCATCGATGGCGGCGGTACAGTGTTCCTGGCCAACTCCGTGGCCCGTTCCGAC
TTCAACGCCGAAGGCGAGAAATCCTTCAAGGCCCGTTATGACCTCGACATGGCCACCTTCGGCGTACCTGGCCTGA
GCTTCCTGGCTGCGTACGTCACTGGTAGCGGCGCCAACACCGCCCTGACCTCGAACGGCAAAGAGTGGGAACGCGA
CCTCGAAGCCAAGTACGTGATCCAGAGCGGCCCGGCCAAAGACCTGAGCCTGCGTATTCGCCAAGCGACTTATCGT
TCGTCTGACGGTGTTTACTACGGTTCTTCGTCCATCGACGAACTGCGTTTGATTGCGCAATACCCGCTGAACATCT
TGTAATCAAAGATGCCCGAGGGCTTCGGCTCTTAATGAAAAAGCCGCTCACTTTTAACCAGTGAGCGGCTTTTTTA
TTACACTTTTATTTCTGCGACCTTGATAACTAGCAAGCTAACTATCAAGTTTATTGCCGAGACGAAACCTGACTCT
TCAGCCAAGTTAAATCCCTGTACCCGTTTATTTGGCAGGCGCGTCTTGCATCACGCGAATAACACGCTGCGGGAAT
GGGATATCAATTCCAGCCGCTTTCAAACGGTCACGCGCCAGTTCATTGAACATGAACATCACATCCCAATAATCCG
CCGTCTTGGTCCATACGCGCAGGGAAACAGTGATTGAACTGTCGCCCAGGGTCGAAACCACAGCGACCGGCTCTGG
GTCCGCCAGAACCCGTGGGTCCTTGGCCAGTTCCAGCAGTACTGCGCGGGCTTTCTGCAGGTCGGCTTCGTAGTCG
ACACCCACATCAAACACCACCTTGCGGGTTGGCTGGCGGTTGGTGTTGGTGATGATGCCGTTGGACAAGATGCCGT
TAGGCACGATGACCGTCTTGTTGTCGCCGGTACGGATCACCGTGTGGAAGATCTGGATGCTATCGACGGTACCCGC
CGTGCCCTGGGCTTCGATCCAGTCACCGATACGGAACGGACGGAACAGCAGGATCAGCACGCCACCGGCAAAGTTG
GCCAGGCTGCCTTGCAACGCCAGGCCAATGGCCAGGGTCGCGGCACCAATCGCGGCAACAAACGAAGTGGTTTCGA
CGCCAATCATTGAGGCCACGCTGACGATCAGCATCACCTTGAGCACGATGTTCGCAAGGCTGGTCACGAAGCCCTG
CAACGCCAGGTCGGCATTGCGCAGGGCCAGCAGGCGCCCAACCTTGTGGGTCAGCTTGTTGATCAGCCACCAGCCA
ATGGCCAGGGTCAATACCGCCAGCAGCACCCGGCTGCCGTATTCCATGATCATAGGGACCCAGGCTTGGGAAGCCT
TGATCAAATGATCCACTTCAGCATTCAAGTCCATCTAGATTCTCCTGTTTACCACTGCATATATAGGTCGCCACAG
CAATTGAGCCCCGAAGGGCTCAATCGTTTCTGCAGGTGCTGGGGCGTGGGACGTCGAAAAGCCCCGCGGGTTCCCC
AAAACCGGGTCAGTCGCGGAAGTTATTGAACTGCAGCGGCATATCGAAGGTCTTGGCGCGCAGGGCGGCAATCGCG
TCCTGCAAGTCGTCGCGCTTCTTGCCGGTGATACGCACCTGCTCGCCCTGGATCGCGGCCTGGACCTTGAGTTTGG
CATCCTTGACATGAGCGACGATTTTCTTCGCCAGCTCCTTGTCGATACCTTCCTTGAGCACCGCTTCCTGCTTCAT
CAACTTGCCCGACGCATAGGCCTCCTTGACTTCAAGGCACTGCACATCGATCTTGCGCTTGACCAGGGCCAGCTTG
AGGATCTCGATCATCGCTTCCAGTTGGAAGTCCGCTTCAGCGGTCAGGTTGACCGTCAGTTCCTTTTCCTTGAACT
CGAAGCTGCCTTTGCCTTTCAAGTCATAACGACGGTCCAGCTCCTTGACGGCGTTCTCGACGGCGTTGGTGACTTC
GTGCTTGTCCAGTTCAGATACCACGTCGAACGACGGCATGTAGTTTCTCCAATAAAAAAGGGGCGCGGCTCAGTAG
AGATGGAGCGCGCCTGGCTTGCGGGTAAAATGCCGGGGCATTATAACGATTCTTTACCCTGAGCTGCTTGGCTCAC
CCAAAGGCCGACGGAGCAAAATTGATGTCCACCACCTGGCATGTCCTCGGCGCCGGCAGTCTCGGTACGTTATGGG
CCGCACGCCTGGCCCGCGCAGGCTTGCCTGTCACACTGATCCTGCGCGACCAGGCACGCTTGGCCCGCTATCGGAC
AACGCCCGGGCTGACGTTGATCGAGCAGGGGCATGCCCAGTCGTACCCGGTTAGTGCACAAACATCCGACAGCCCC
GAGCCGATTGATCGCCTGCTGGTCGCGTGTAAAGCCTACGACGCACCTGGCGCCGTCGCACAGCTAGCCCGACGCC
TGACACCCAATGCCGAGGTGATCCTGCTGCAGAATGGCCTGGGCAGCCAGGATGCGGTTGCTACGCAACTGCCCCA
GGCCCGGTGTATCTTCGCCTCCAGCACCGAGGGTGCGTTTCGCCAGAGCGATGGCAGTGTGGTTTTCGCCGGCCAT
GGCTTTACCTGGCTCGGTGACACTACCCATCCCACGGCGCCGCTGTGGCTGGACGACTTGGAGGCCGCCGGCATTC
CCCACCAGTGGAGCACCGATATCCTCACGCGCCTCTGGCGAAAACTGGCACTCAACTGTGCGATCAATCCACTGAC
CGTGCTCTATCAATGCCGCAATGGCGGGCTACAGCAGCACCAATGCGAAGTAGCGACCCTGTGTACCGAACTCGCC
GAACTGCTGGAGTACTGCGGCCAGCCAGCGGCGGCGCAGGACCTGCAGACCGAAGTGGCGCGGGTGATCCAGGCGA
CGGCAGCGAATTTTTCGTCGATGTACCAGGATGTGGCTGGCGGCCGACGCACTGAAATCCGCTACCTGCTCGGCCA
TGCCTGCCAGGCGGCCTTGCGCCATCGCCTGAGCCTGCCGCACCTGCAACAACTGCATGTGCGCCTGGCCGGTTAC
CTGGACGCACGCGGATTGCCCAGCGACTGAAGCACGCGCTACCCTGCCCACTTGATCCTTGCCTGCGAACAACCTG
ATGCCACTGCGCCAGCGTCTCGAAAATCTCCCCGTTGGGCAGAAACTGCTGGCGGCCCTGCTGGTACTGCTGACCA
CCGTGCTGCTGGTGGCCAACCTGACCTTTATCAGCGCCGCCTACTGGATCTCCCAGGAAAGCATGGCCCCCCAGGC
CCTGCAGGCAATTGGCCGACTGGTTTCCAATCCGGCCCTCGCCGCGCAAGCCCTGGACTCTCCCGCCAAGGCCGAT
GCCCTGCTCAACGAGCTGACCAGTTACTCGCCACTGCGCGCGGCGGCCCTCTACGACGGCGAAGGCAGCCGCCTGG
CGCAGTTGCAACACGGTGACCGCCTGCAATTGCCGGCGCACTATCGTGACATCGAAGCCTGGCGGGTCACCGAGTT
TCGCAGCAATCAAATCATCACCCTGCCCCGCCCCGGCCTGCCCTCGGGGCACCTGCTGCTGGTGGCCAGCAGCGAA
CTGCCGGTGGCCTTCTACACCGGCACCCTGACCGCCAGCCTCGGCATCCTGGTTTTCAGCATCCTGCTGTGGATGG
TGATCGCCCGGCAGATCAAGCGCCTGATTACCCGGCCCATCCACGAGTTGGAAGAGTTGTCGCGCCAGGTCACCCG
CGAGGAGAACTACGCGTTGCGGGCCGGTCGCGGCAACCATGACGAAATCGGCAGCCTGGCCGAAGCGTTCAATACC
ATGCTGTCACGCATCGAAGCCCGCGAGCAGCAACTCAAGCGCGCGCGGGACGACTCCCAGGCCGCCTATGACCAGG
CCCAGGGCCTGGCCGAGGAAACGCGCCATACCAACCGCAAGCTGGAACTGGAAGTCCAGGTGCGCAGCAAAATCGA
GAAAAAACTCACCGGGTTCCAGAATTATCTCAACAGCATCATCGACTCCATGCCCTCTGCCTTGATCGCCCTGGAC

GAGCAGCTTTACGTTACCCAATGGAACCAGGAAGCCAGCGCCCTTTCCGGCACACGCCTGGACCAAGCGCTGAACC
AGCCAATCTTCCTCGCCTTCGAACCGCTCAAGCCCTACCTGCCCCAACTCAAGGCCACGGTGGAGCAACATACGGT
GGAGCGCATCGAGCGCGTCACCTGGATCAAGGACGACGAGCCCAAGCACTACGCCCTGACCTTTTACCCGCTGATG
GGCGGTGCCGGGCGCGGGGTGGTGATCCGCATCGATGACATCACCCAGCGCCTGTCCCTGGAAGAAATGATGGTGC
AGTCAGAGAAGATGCTGTCCGTCGGCGGGCTGGCCGCCGGCATGGCCCACGAAATCAACAACCCCCTGGGGGCGAT
CCTGCACAACGTGCAGAACATCCGCCGACGCCTATCGACGGAGCTGCCGAAAAACCTGGAGCACGCCGAGCAGGTG
GGCGTGGAACTGGAAACGGTGAACCGTTACCTGCAAAGCCGCGAGGTGCCGCAATTGCTCGACGGTATCCAGCAAG
CCGGGGCCCGCGCGGCGAAAATCGTCACCCATATGCTCAGTTTCAGCCGCCGCAGCAACCGCCAGATGGCGCCTTG
CGACCTGCCAGCCCTGATCGATCAGGCCGTGGAGATCGCCGGTAATGACTTCGACCTGGCGATCGGCTTCGACTTC
AAGGGCCAGGCGATCATTCGCCAGTTCGACCCAGCGCTGGGCCCGGTGCCCGGCACCGCCAACGAGCTGGAGCAGG
TACTGCTCAACCTGCTGAAAAACGCCGCCCAGGCCATCCACCTGCGCGAAGACGACAGCGAGCCAGGGCGCATCAT
CCTGCGCACCCGGCTCAACCCGCCCTGGGCCGAGATCCAGGTGGAAGACAATGGCATCGGCATGAGTGAGAGCGTA
CGTAAACGCACGTTCGAGCCGTTCTTCACCACCAAGGAAATCGGCCAGGGCACGGGGCTGGGCTTGTCGGTCTCGT
ACTTCATCATCACCAACAATCACAAGGGCCAGATGGAGGTTCACTCCACCCTGGGCCAGGGCACCTGCTTTACCTT
GCGCCTGCCGTTGGCCGGCAGCCAGCTTGCCCCCCTCGAACTGACTCAACTGGAGCACTGACCATGGGCTTTCGCC
TGTCGAAAATTTACACCCGTACCGGCGACAAAGGCGAAACCGGCCTGGGTGACGGTCGCCGCGTGCCCAAGGATCA
CCCCCGGGTGGAAGCCATCGGCGAAGTGGACAGCCTCAACAGCCAATTGGGCCTGCTGTTGGCCGGGCTCGAGGAG
CAAAGCGGCCAGCATCCTGCGTTGAAGGATGTGGTCGAGGTCCTGACACCTTGCCAGCACCGACTGTTTGACCTGG
GTGGCGAGTTGGCAATGCCGGTCTACAAGGCACTGAATGCAGGCGAAGTGGACCGCTTGGAAGCCGCGATCGATCG
CTGGAATGAAGAGGTAGGGCCTTTGGAAAACTTCATCCTGCCCGGCGGTTCGGCACTGATCGCCCAGGCCCACGTA
TGCCGCAGCCTGGCCCGCAGCGCCGAGCGCCGCTGCCAGCAGTTGAATGCACTGGAGCCGTTGGAAGGCGTGGGAT
TGGCGTATATCAATCGGCTATCGGATTTACTGTTTGTCGCAGCGCGAGTAATTGCCAGGCGCCAGGGGGTTGCGGA
GATTTTGTGGCAGGCGGCAGCTAAGCCGCACTGAGGTTTTGTAGAGCTTTATCGGGCCTCATCGCAGGCAAGCCAG
CTCCCACACTTGAGCGGTGAATACATTCAAATGTGGGAGCTGGCTTGCCTGCGATGGGGCCATCACTGACGACTCA
AACCTCAGGCCAGAACGCCCGAATCCCCGCCACACCCTGCGCTCCGTTCTGCCAGGCCTTCTCCCGCTCGTCAGGC
CCAACCCCACCCAACAGAAACACAGGCTTGCTGAAGCCCTTGATCAACTGCGCGGCCTCTTCCCAACCCAATGGTT
GAGCATCAGGATGAGTTTGGGTCGGCTGCACCGGCGACAAAGTGACAAAGTCCACGTCCATCAATTCGGCCAATGC
CAGCTCCTCGGCATTGTGGCAAGACGCTGCCAACCAGCGATCCTTGGGCAATGGCCGGCCTTTGCTGGCGTATTTG
CGCAGTTGCGCCGAAGTCATGTGCCAGCCCGCCGCCGGGAAATCACCGAGCCATTCAAACGGCCCCTTGAGCATCA
ACTGTGCCTTGCCGGCACACAACCCCACGGCATCCACCGCCAGGTCGCGGTACTTGGGGTCATAGCCATTGGGTGC
GCGCAATTGGATCAGCTTGATGCCACCGGCAATGGCTTTCTGAATACCGCGCAACAACACTGGGGTTTCCAGTTCA
CCTGGGGTGATCAGGTAGTCAGCGGGCAAGCGCGCGGCAGCGACGATTGGCGCGTTGGCAGCTGGAAACTCGTAGT
TCTGCAAGTCCCGTGGCGCCACCCATTCGAGGGGTTGCCCTTCTGCGCCATGGGGCTCGCCGGTAAACGCCGAGAC
CTCCCAGACATCCAGCAACACCTGCTTGTCCGGGTAATCATGCTGCACCTGGATCAACGGCCGCGCCGTGGTGACC
TGGATACCCAGTTCCTCCTGCAGCTCTCGGCCCAGTGCGGTGAAAACCGATTCATCGGCCTCCACCTTGCCTCCGG
GAAATTCCCAGAGGCCGCCCTGGTGCTGGGTATCTGCACGACGCGCCAGCAAAATCCGGCCATCGATACCGCGAAT
AACTGCGGCTGCTACATGAACTCGTTTCACGGTATTACGTCCTCTAAGCCGGCGTCTGCCGGGGCATTGCCAATGC
CCCGGGCAGGTATCAAGTGCGGTATTCGGCGTTGATTTTCACGTATTCGTGGGACAGGTCGGTGGTCCAGATGGTT
TCGCTGCACTCACCGCGTCCCAGTTCGATGCGGATGGTGATTTCTTCCTGCTGCATCACCGCCGAGCCCTGGGCTT
CGGTGTAGGTTTCGGCACGGGCGCCACGGCTGGCGATGCACACATCACCGAGGAACACGTCGATCTTGCTCACGTC
CAGGTTCGGCACGCCGGCACGACCCACCGCTGCGAGGATCCGGCCCCAGTTCGGGTCGGAGGCAAACAGCGCGGTC
TTGATCAGCGGCGAGTGGGCCACGGTGTAGCCGACATCCAGGCACTCCTGGTGATTGCCGCCGCCATTGACTTCAA
CGGTGACGAACTTGGTCGCGCCTTCGCCATCACGCACGATGGCCTGGGCCACTTCCATGCACACTTCAAATACCGC
CTGCTTCAACGCCGCGAACAGCGGGCCGTTGGAGGAGGTGATTTCCGGCAGGTTGGCTTTACCAGTGGCAATCAAC
ATGCAGCAGTCGTTGGTCGAAGTGTCGCCGTCGATGGTGATGCGGTTGAACGACTTGTTGGCGCCATCGAGCATCA
GGTTCTGCAGCACATCGCGGGAGACTTTGGCGTCAGTGGCGATGTAGCCGAGCATGGTCGCCATGTTCGGGCGGAT
CATGCCCGCACCTTTGCTGATGCCGGTCACGGTGACAGTCACGCCTTCATGCATGAACTGGCGGCTGGCGCCCTTG
GGCAGAGTGTCGGTGGTCATGATCCCGGTGGCCGCGGCCGCCCAGTTATCCACCGACAGGTCGTCCAGCGCGGCCT
GCAGGGCGCCTTCGATTTTTTCCACGGGCAACGGCTCGCCGATCACCCCGGTGGAGTACGGCAGCACCTGGCTGGC
GTCCACCCCGGTCAACTCCGCCAGCTTGGCGCAAGTGCGCGCAGCCGCAGCCAGGCCAGGCTCGCCGGTACCGGCG
TTGGCATTGCCGGTGTTGGTCAGCAAGTAGCGCACCGGGCCTTCGACACGTTGCTTGGCGAGGATCACGGGGGCTG
CGCAAAACGCGTTCAGGGTGAACACGCCAGCGATGGTCGAGCCTTCGGCACAGCGCATCACTACCACGTCCTTGCG
CCCGGGGCGCTTGATACCGGCCGAAGCGATACCGAGTTCAAAACCGGCAACCGGGTGCAACGTGGGCAAAGGACCA
AGACCAACAGCCATGAATGCGCTCCTTAACGATGTGATGTCTGCACCGTCGTCGTCGACGGTGAATAAATGGCAAA
ACGCCGCGACGGCTGATGCCGGTCGCGGCGCGGGGTATTGCAGCGTCAGGCAAAAATCTTAGTTGATTTCGCCATG
GCAGTGTTTGAACTTCTTGCCCGAACCGCACCAGCATGGCTCGTTGCGACCCAGCTTCTGCTCGTTGCGCACAGGA
GCCTGGGCCAGGGCCACATCGACCTCTTCACCCAACACTTCCGGCGACTCAAGGCCCGGCGCTTCGTCATGCTGGA

ACTGCATGCGCGCGGCCAGGGCTTCGGCTTCCTGGCGCAGGCGGGCTTCTTCTTCGATCGGGTCTTCGCGACGCAC
CTGAACGTGGGACAGCACACGAATCGAATCGCGCTTGATCGAATCCAGCAACTCGGAGAACAGCGTGAACGACTCG
CGCTTGTACTCCTGCTTCGGGTTCTTCTGGGCGTAGCCACGCAGGTGGATACCGTGGCGCAGGTGGTCCATGGTCG
ACAGGTGGTCTTTCCACAGGTCGTCCAGCACACGCAGAACGATTTGCTTCTCGAAGGTGCGCAGTGCTTCGGCACT
GGCTTGCTCTTCTTTCTCGTTGTACGCGGCCAGCAGTTCGGCCATCAGTTTTTCGCGCAGGGTTTCTTCGTACAGG
TGATCGTCTTCGTCAAGCCATTGCTGGACCGGCAGGTCGACACCGAAGTCGCTCTTCAACGCAGCTTCCAGGCCGG
CAACATCCCACTGCTCAGGCAGCGACTGTGGCGGGATATGGGCACTGACGGTGGCGTTGAGCACGTCCTGGCGGAA
GTCGGCAATGGTTTCGCCAATGTTGTCGGCGGCCAGCAACGTGTTACGCATGTGATAAATCACTTTACGCTGTTCG
TTGTTGACGTCATCGAACTCCAGCAGTTGCTTACGAATATCGAAGTTGCGGCCTTCGACCTTGCGCTGGGCCTTTT
CGATGGCGTTGGTCACCATGCGATGCTCGATCGCTTCGCCAGACTGCATGCCCAGGGCCTTCATGAAGTTCTTCAC
CCGGTCAGAGGCGAAGATGCGCATCAGGCTGTCTTCCAGGGACAGGTAGAAACGGCTGGAACCGGCGTCGCCCTGG
CGACCGGCACGGCCACGCAGTTGGTTGTCGATACGGCGCGATTCGTGACGCTCGGACGCGATCACCTGCAAACCAC
CGGATTCCAGCACGGCCTGATGGCGCTTCTGCCAGTCGGCCTTGATCTGGGCGATCTGCTCAGGCGTCGGGTTGTC
CAGGGATGCCACTTCCACTTCCCAGTTACCGCCCAACAGGATGTCGGTACCACGACCGGCCATGTTGGTAGCGATG
GTCAGTGCGCCTGGGCGACCGGCCTGGGCAATGATCTCGGCTTCTTTTTCGTGGAACTTGGCGTTGAGGACCTTGT
GCTCGATACCTTCCTTGTTGAGCAGGTTCGACATGTGCTCGGAGGTTTCGATGGTTGCAGTACCCACCAGGACCGG
ACGGCCTTGGGCCATGCATTCCTTGATGTCGGCAACGATTGCAGCGTATTTCTCGTCGGCGGTCAGGAACACCAGG
TCGTTGTAGTCTTTACGCGCCAGCGGCTTGTTCGGCGGGATGACCATCACCGACAGACCGTAGATCTGGTGGAATT
CGAACGCTTCGGTGTCAGCAGTACCGGTCATGCCGGACAGCTTGTTGTACAGGCGGAAGTAGTTCTGGAAGGTGGT
CGAGGCCAGCGTCTGGCTCTCGGCCTGGATGTTGAGCACTTCCTTGGCTTCGATGGCCTGGTGCAGGCCTTCGGAC
AAACGACGACCGGGCATGGTACGGCCGGTGTGTTCGTCGACCAGCACGACCTGGCCGTCCTGGACAATGTATTCGA
CGTTGCGATGGAACAGCTTGTGGGCGCGCAGGCCGGCATACACGTGGGTCAACAGGCCCAGGTTGTGTGCCGAGTA
CAGGCTTTCGCCTTCAGCCAGCAGGCCGATCTGGGTCAGCATCTCTTCGACGAACTGGTGACCGGCTTCGTTAAGC
TCGACCTGGCGGGTCTTCTCGTCGATGGAGTAATGACCTTCTTTGGTCACGACGCCTTCCACTTCCTCGATATGTT
GCTCAAGGCGCGGGATCAGCTTGTTGATTTCGGTGTACAGGCGCGAGCTGTCCTCGGCCTGGCCGGAGATGATCAG
CGGAGTACGGGCTTCGTCGATGAGAATGGAGTCGACTTCGTCGATCACGGCAAAGTTGAGTTCACGCTGGAATTTT
TCTTCCATGCTGAACGCCATGTTGTCGCGCAGGTAGTCGAAACCGAATTCGTTGTTGGTGCCGTAGGTGATGTCGG
CGGCGTAGGCGGCACGCTTCTCTTCCGGCGGCTGGAACGGCGTTACCACGCCGACGGTCAGGCCGAGGAATTCATA
CAGCGGGCGCATCCAGTTGGCGTCCCGGCGAGCCAGATAGTCGTTCACCGTCACAACGTGCACGCCCTTGCCGGAC
AGTGCGTTGAGGTAAACGCCCAGGGTTGCCACCAGGGTCTTGCCTTCACCGGTGCGCATTTCGGCAATCATGCCTT
CATGCAAGGTCATGCCGCCGATCAACTGGACGTCGAAGTGGCGCATACCCATTACACGCTTACCGGCTTCGCGGGC
GACCGCAAAGGCTTCGGGCAGCAGTTTGTCGAGGGTTTCACCTTTGGCTATGCGGGCCTTGAACTCTTCGGTCTTG
GCGCGCAATTGCTCGTCCGACAAAGCAACCATCTGCTCTTCGAAGGCATTGACCAGCTGCACCGTCTTGAGCATGC
GTTTGACTTCGCGCTCATTCTTGCTTCCAAAAAGTTTCTTTAACAAAGGCGCAAACATATCGGCAGGTTCTTCCAC
ACATTAGGGATGAAGGGGCGCCCCGTAAGTCGCCCGAGCAGCCCTCACGGCCGCATGCGAACGAGCATTCTACCCG
GAAACGAGGGTGAGGAAAGTGGCGTTGTTCCACGATGCAGGCACAGCGTTCCGGGAGGGCTTGTTTAAAATAAGGG
CTTTTTGCTGACTTTCAACCCATTGGAGCGAGAAGTTACTTATTGATTTAATGGATAAACTGCTGACAAAGCAATG
ACCACAGTGCCGCACACGCTTTCTGCTACCATGGCGGCTCTGTTACCTAAGGTGTCTAGACCATGGCATTTCGCCC
TCTTACGGCCCGAGCTCCCGCCGTGCTGCTGCGCGAAGCCAAGCCTTTAAAAGCCATCTTCGGCCATGCCCAGCGC
CTTGGCCACCTGCAACGCCTGCTGGAAAGCCAACTGCAACCGGCGGCCCGCGAGCACTGCCATGTAGCATCGTGGC
GTGACGGTACGCTGCTGCTGATTGTCACAGATGGGCATTGGGCCACCCGCCTGCGCTATCAGCAAAAACGCTTGCA
GCGCCAGCTCCAGGCGTTTGAGGTCTTTGAGAGCCTGCTGCGCATCCAGTTCAAGGTCCAGCCTCCGACCGTGCAG
CAAGGTGCGGTGGGGCATACCATGGATTTGTCGGTGAATGCCGCCGAAACCATCCAGGCGACAGCGGACGGCATTA
CCGATCCGAAGCTGCGCGCGGCGCTGGAGCGGTTGGCCGCTCATGCCAAGCCCAAAACCTGAGAGCGGCACAACCT
AATGTAGGAGCGAGCTTGCCCGCTCCTACAGTTGATTCCATCTGCCGTAAGAACTACTTACGCTTGCTACCGCCCA
GCAAAGAACCCAACAGCCCGCGCACCAATTGGCGGCCCATCTGATTGGCAGCCTGCTGCATCGCCGACTTCAACGC
CTTGCCCGCTGTAGTTCCCAGGAACGCCCCGGCCTTGTCAGTAAAACTCGGCTCTTCTGCGGCAGGCGCAGCACCC
GCCTCCGGCCCCAGTTCCTTGCGCGCCATCAGCACTTCATAGGCCGACTCACGATCGACCGGCTTGTCGTAGCGCC
CCAAGAGCGACGAACTGCCGATCAAGGCCGTGCGCTCAGCGGCGGTCAATGGCCCGATCCGCGATTGCGGCGGCGC
TACCAGCACCCGCTGGACCACCTCGGGCGTGCCTTTTTCCTGCAGCGTGCCCACCAGCGCCTCACCGGTGCCCAGC
TCGGTCAACACCGCCAGTGCATCGAATGCCGGGTTCGGCCGAAAACCATCCGCCACCGCCCGCAGGGATTTCTGCT
CCTTGGTGGTGAATGCCCGCAGGCCATGCTGGATACGCAAACCCAATTGGGCCAGCACGTTGTCCGGCAGATCGCC
CGGCGACTGGGTGACAAAATACACACCCACCCCTTTCGAACGAATCAGCCGCACCACTTGCTCCAGACGGTCCTGC
AAAGCCTTGGGCGTATCGGCAAACAGCAGATGCGCCTCGTCGAAAAACAGTGCCAGCAACGGCTTGTCGGCATCCC
CGCGTTCCGGCAACTGCTCGAACAACTCCGCCAACAACCACAACAGGAACGTCGCATAGACCTTGGGCGACTCGTG
CACCAGGCGGCTGGCGTCCAGCAGATGAATGCGCCCTCGCCCATCGCTGGCCGGTTGCAGAATATCTTCCAGCTGC
AGCGCCGGCTCACCGAACAACGCTTCGGCGCCCTGCTGTTCGAGCACGGCCAAGCGCCGCAGCAAGGCCTGGCTGG

AGCCGGTGGTCATCAGGGCGGCGTCATCGCCCAGCAGTTCGGGGTGGAACTTGAGGTGGTTGAGCAGAGCCTTGAG
GTCCTTGAGGTCCAGCAGCAGCAAGCCTTCGCGATCCGCCACTTTAAAGGCTGCATACAACGTCGACTGCTGGCTG
TCGGTCAGTTCCAATAGACTGCCCAGCAGCAATGGGCCCATTTCGCTCAAGGTTGTACGAAGCGGATGACCGGACT
GACCATGGATATCCCACAGAGTTACCGGATAAGCCTTAGGCGCGTAATTCAGGAACGGCATACCGGCAATACGCTC
GGCCACCTTGCCCTGGGGGTTGCCTGCGGCGCCGAGGCCACACAGGTCACCCTTGATATCGGCGGCGAACACTGCG
ACGCCAGCATCACTGAACGCCTCCGCCAGCCGCTGCAGCGTCACAGTCTTGCCCGTGCCGGTGGCACCAGCGATCA
AGCCATGGCGATTAGCCAGGCGCATGGCCTGGGCGATGGGCTGACCTTCGAGATCTGCGCCAATAAGAAGTTGCGA
GGAGTCAGACATTTTGTCACCCATGGTTAATCTTTAGCGCTGCACGGTCGATACAGACAAAGCGAGAGACCCAAAA
AGTCTAAAAAAGTTAGACCGGATAATTCCTGCAGGAGCAGATGGAAATATCACACTTTATTTAATGCTGCCATTTG
CGCGTCTGCATAAAAGACGCGCCTCGGACACTAAGACCTTAGCGGACACGACGAGCCATGAATAAAAATCTGCGCT
TCAGCCACAAAATCCTGCTTGCAGCCGCCCTTATCGTCATTGCCGCATTTGCGTTGTTCACGTTGTACAACGACTA
CCTGCAACGAAACGCAATTCGCGACGACCTCGACAGCTACCTGCATGAAATGGGCAGCGTGACTGCCAGCAATACC
CAGAGTTGGCTGGCCGGGCGCATCGTCCTGGTGGAAAACGCCGCCCAGAACATCGCCATCAACTCCGAGCCGGCCG
TGGTTGCCAGCCTGCTGGAGCAGAAAGCCCTGACGTCATCGTTCATGGCCACCTACCTGGGCGACAGCAAAGGCGG
CTTCACCATTCGCCCCGATGCGAAGATGCCCGACGGCTTCGATCCCCGCGTGCGCCCCTGGTACAAAGGTGCCCAG
ACCAGCAACGGCTCGACCCTGACCGAACCTTATATCGATGCGGCCACCGGCCAGTTGATCATTTCCATCGCCACCC
CCAGCAGCAAGGCTGGGCAGAGTGTCGGTGTGGTCGGCGGCGACCTGAGCCTGCAAACCCTGGTGGACAACATCGG
CGCGCTGAGCTTCGGCGGCATGGGCTACGCGTTCCTGGTCAGTGCCGACGGCAAGATCCTGGTACACCCGGATAAA
AGCCTGGTGATGAAAACCCTGGGCGAGGCGTACCCGAAACAGGCCATCAAGATCAGTGCCGACTTCAGCGAAATCG
AGGTTGATGGCAAAACCCGCATTGTCACCTTCGCCCCGATCAAGGGTTTGCCGTCGGTGAACTGGTATATCGGTCT
GTCGGTAGATAAAGACAAATCCTTCGCCATGCTCAGCGAATTCCGCACCTCGGCCATCATTGCCACCGTGATAGCC
GTAGCGATCATCATCGCGCTGCTAGGCATGCTGATCCGCGTGCTGATGCAGCCGCTGTACGTAATGACCCGCGCCA
TGGAAGACATCGCCGATGGCGAAGGCGACCTGACGCGCCGCCTGACTATCCAGAACCATGACGAATTCGGCATCCT
CGGCAAAGCCTTCAACCGCTTTGTGGAGCGGATTCATACCTCGATTCGCGAAGTGTCTTCCGCCACCGGCCAGGTC
AATGAAGTGGCACTGCGCGTGGTCAGCGCCTCCAACTCGTCGATGGTCAACTCCGACGAACAGGCCAACCGCACCA
ACAGCGTCGCGGCAGCAATCAACCAGTTGGGCGCCGCCGCCCAGGAAATCGCCCGTAACGCCGCCCAGGCCTCGCA
CCAGGCCAGCGATGCACGGCACCTGGCCGAAGATGGCCAGCAAGTGGTGGAGCGCAATATCAAGGCCATGACACAG
CTGTCCGAAATGATCAGCGCCTCAAGCAGCAATATCGAGGCGCTCAACAGTAAGACGGTGAATATCGGGCAGATCC
TCGAAGTGATCACCAGTATTTCCCAGCAAACCAACCTGCTGGCGCTCAACGCCGCCATTGAGGCTGCGCGCGCCGG
GGAAGCCGGCCGTGGGTTTGCGGTGGTGGCAGACGAGGTGCGCAACCTGGCCCATCGCACGCAAGAATCGGCGCAA
CAGGTGCAAAAGATGATCGAGGAGCTGCAAGTCGGCGCTCGGGACTCGGTCAGCACCATGAGCGAAAGCCAACGCC
ATAGCCTCGACAGCGTAGAAATCGCCAACCTGGCAGGTGAACGCCTGAGCAGCGTGACCCAGCGTATCGGTGAAAT
CGACGGCATGAACCAATCGGTGGCTACCGCGACCGAGGAGCAGACCTCGGTGGTTGAATCGATCAATATCGATATC
ACCGAAATCAATACGCTCAACCAGGAAGGCGTGGAAAACCTGCAGTCCACCCTGCGCGCCTGCTCCGACCTTGAGC
AGCAAGCGGCACGCCTGAAACACCTGGTGGGGAGTTTCCGTATCTAAACCGGAGTACAGCGCAAATCAACTGTAGG
AACCGGGGCTAGACCTCTCCTACATTCAACCTGCGTTGTTTCTGTCAGGGCCGGGCTTGTCGTGCGGCTCTGACTC
TTCCTGCAACTGCTCCCACAACCGGGCGGCGCCGGGAAAATCCGTGCCGTCCTCGGCATCCAAGTCATCCGGGTCA
TAGCGGCTCAAACACCCTTCGCCCAAGGTAGCGGGCGCCTTGGACGTGGCTTTATCCAGGGGATCACTCATCGTTG
GATCCTCATCGCTGACACAAATAAAAAGGGCCTGGGTGATTTAACGCCCAGGCCCTTTTTGCTTCAACTAAAAACA
GTCGGTCAGAACACCACGGTCTTGTTGCCGTGTACCAGCACGCGGTCTTCCAGGTGATAGCGCAGGCCGCGGGCCA
GGACCATCTTCTCGACGTCGCGGCCGAAGCGCACCATGTCTTCAATACTGTCGCTGTGGCTGACGCGCACCACGTC
CTGTTCGATGATAGGACCGGCGTCCAGCTCCTCGGTGACATAGTGGCACGTTGCGCCGATCAGCTTCACGCCGCGC
AGGGAGGCTTGGTGATACGGCTTGGCGCCGACAAACGACGGCAGGAAGCTGTGATGAATGTTGATCACCTTGCCTG
CATATTCGCGGCACAGTTCCGGCGGCAGGATCTGCATGTAGCGCGCCAGTACCACGACGTCCGCTTGATGCTGCTC
GACCAGGCGCGAGACTTCGGTGAAGGCCGGCTGTTTGTTCTGCGAGTCGACCGGCACGTGGTAGTAAGGAATACCG
TGCCACTCAACCATGCTGCGCAGGTCGTCGTGGTTGGAAATCACGCAGGCGATTTCACAGTCCAGCTCATCGCTGT
GCCAGCGGTGCAGCAAGTCCGCCAGGCAATGGGACTCGCGGCTGGCCATCAGCACCACACGTTTTTTCTGCTCGGT
GTCGGTAATGCGCCATGTCATCGAAAACTCTTCGGCGATGGGCGCAAACGCCTCGCGAAATGCCTCAAGACCAAAG
GGCAGTGTGTCGGCACGAATTTCGTGGCGCATGAAAAACCAACCACTGAGATTGTCCGAGTGATGACTCGCTTCGG
TGATCCAGCCGTTGTGGGAGGCCAGAAAGTTACTGACTTTAGCGACGATACCAACCCGATCCGGGCAGGCAATCAC
CAGACGAAAAGTGCGCATTAGGGGAAAACTCCAGAACTTCGCAAAGGCCGCCATTCTAGCGATACCGTAGAAAAAC
TGCAGTATTCCTCAACGCTTATTCGAGCGATTGCTGGCGGTATCCCTAAACAGCGTACGGTTTTTACCCCAGCACT
ATGTCAACACAAGCATCTGTTACCGCAATAAGCCAAGATCTAATGAGAATATCTTTAGCCGGTCGAGCGCACTAAA
TAATGCACTCAGCGTCTCTGACTACAAACGTTCAAAGTAAATTACATAAAACAGAGCTTAAATGTTTACTTGGGGA
AACTGTCTGACTATTATTAGGCCACTATCCCTGTCCCTCAGCGCCCCGCATAAGGTAGTCCACATGTCCCTGATCA
ACGAATACCGTGCCACCGAAGAAGCTATCAAAGAGCTGCAAGCCCGTTTGAAGAATCTGTCCCAAGACGACAAACT
GCAAACCGAGCTGGAATTCGAAGGCAAATTGCGCACCCTGATGGGTGAATACTCCAAATCCCTGCGTGACATCATC

GCGCTGCTGGATCCGGAATCGAAAGTTAAAGCACCTCGCGGCGCAGTAAAAGTTACCGGCACCAAGCGCGCCCGTA
AGGTCAAGCAATACAAAAACCCGCACAACGGTGAAGTGATTGAAACCAAAGGTGGCAATCACAAGACTCTGAAAGA
GTGGAAAGCCAAGTGGGGCGGTGACGTGGTTGAAGGCTGGGCCACCCTGCTGGGCTAAGGCTCGCTGGGTCGTCAC
CTGATTCGCGACACAATAAAAAACGCCAGCTTGCTGGCGTTTTTTATTGCCCGCTGTTTATCAAGCGCTGATTACA
GGCTCAGGCGCTGACGTAAAGCATCAGCATATTGCTGCCATTCATCCAGTACCTGACGCTGAAACGTTGTAGCACT
AACGTCCAATTCAAGCCTGGCCTTATTAAAACTCTCCAGGGTATTGGGTGCACCGTAATCCGCGTCTGACAAGCGT
TGCTGACAGAATAGTTTCCAGCGTTGTTGCTCTTCGTCATCCAAGGTGTACGCAAAGTTACGGGCACGATAGCGAA
ACAATAATTCCGGCAAACGATGATCATCAAAAGGCCACTGCTGGCGTGCTAGTTGCGCAGGTTCGGCAGCCCGGAC
TTGTTCGCATAAACGCCGATCACGATCGCCAATAAAACCATCGTAGAGCTGTTGTTCCGGATCGGCGGTTGCGGCG
AAACCTTCTTCGGCATAAATCGCCGGCAATTTATCCCGCCAAAGTTCCTGTGCGTCAGTTAGCCGCAGCGCCCGGG
CCTGATATTCACCCATGTCCAGATTCAAACGCTGCTGGTCTTGCGCCCGCAACACACTCAGCGGTGCGACCACCGG
GCAGCGGTTGATGTGGACTAATTTGAGCGGCACCGGCAACTCGCCTTCAGCCAACTCATCGCGCCGGGTGTACAGA
CGCTGGCGCAGTGTTTCGGCATCCAGGTCCAGCAATCCCTGTGGGTCGAGCCCCAAGTCACAGACAATCAACGCGT
TGCGATTGCGCGGATGCCACGCCAACGGCAGGACCACCCCCAGGTAATGGCGCTCGGCGGAAAAACGCCCGGAAAT
ATGCACCATGGGCTGCAACAGGCGGATTTGATCCATGACCCGCTGCTTGCTGCGCAGTTGAAACAGCCAATCGTAC
AACTTCGGTTGTTTTTCCCGGACCAGGCGCGCCAATGCGATGGTGGCACGCACATCGGCCAATGCCTCGTGGGCCT
GGCCATGGTCGATCCCATTGGCCGCCGTCAGGCGCTCCAGCTTGAGGGTCACGCGCCCGTCCTGCTCGGGCCAGAC
GATGCCATCGGGCCGCAGGGCATAGGCCGTGCGCAGCACGTCGATCAAATCCCAACGGCTGTTTCCGCCCTGCCAT
TCCCGCGCATAGGGGTCGAAAAAATTGCGATACAGACTGTAGCGGGTCATTTCATCGTCGAAGCGCAGGGTGTTAT
AGCCCGCGCCGCAGGTGCCGGGGGCCGCCAGCTCGGCATGTACCCGCGTCATGAAGTCGGCTTCGGCCAGGCCTTT
TTCCGCCAGTTGGCCGGGGGTAATGCCCGTGATCATGCACGCCGCGGGGTGCGGCAGGATATCGTCGCTGGGCTGG
CAATAGAGGTTGACCGGCGGCCCTATCTCATTGAGGTCAAGGTCGGTGCGAATCCCGGCGACCTGCAGCGGGCGGT
CGCTGCGTGGGTTGATGCCAGTGGTTTCATAGTCGTACCAGAAGATCGAGGTCACGGGCTATTCCTGTGCTGAAGA
CCGACAAAGTCTAGGCGCTGGACGCCGCCCGGGGCCAGCGCAACGTGCACTGGATAAACATTCAGTGAAACCTTGG
CGCATTTTCAGGTGTTACTTCCATCGACGACACTGCTAGCATCGGCCTCTCAATTTGCCGGCCGCCCCAGAAGGTC
GCCCATGCCCAGCCACACCGCACTGCCAGGGAACGCAATGCCCACCGCCCCATTGGACACTCGCTACCAGATCGAG
ACCCCGGAAGGTATAGACCTGCCCTTGCGCCCCGCCGGGCTGATGCCCAGGGCACTGGCATTCGGTTTCGACCTGG
GACTGCGCGGCCTGATCCTCGGCATCCTGTTCATCGCCCTGGCGTTTCTCGGCAACCTGGGCATCGGCCTCGGCTC
GATCCTGCTGTTTTTCGTCAGTTGGTGGTACATGGTGCTCTTCGAAGTGCTCAACCAAGGCTGTTCACCGGGCAAG
CAGGTCATGGGGCTGCGCGTGGTGCAGGATGACGGCACGCCCATTGGCTGGTCCGCGTCCTTGATCCGCAACCTGC
TGCGTTTTGTCGATATGTTGCCGTTCGGCTACTTCCTCGGTGCCATAAGTTGCCTGCAACATCCCCACTTCAAGCG
CCTGGGTGATATCGCAGCCGGTACCCTGGTTGTTTACCGCGAACAGCCCCTGGCTCGCCCCCAGTTGCCCCAGGCC
CCGGCCTTGCGCCTGCCCTTTGCCCTTGAGTTGAACGAGCAGCGCGCAATCCTGGGTTTTGCCGAACGCCAGGGCG
AACTGTCCGCCGAGCGGACCCATGAGCTGGCGGCGATTCTGGCCACGCCCTTGCAGGTTTCCCCGGCACGCGCCGT
GGAACAGCTCAATGGCGTGGCCCGCGGCCTGCTGGGGCCGACATGAAACAAAGCCTGTTCGAAACCCGTCACCAGC
CGCACTGGCAAGCCTTTGCCGCGCAACTGGCACTGCTTGAACAAGGCAAGGCCAAGGCCCACGACCTGACGCGCTT
CCCCCATGATTACCGACGCCTGTGCCAACACTTGGCGCTGGCGCAGGAGCGCGGCTACAGCAGCTACCTGGTAGAC
CCGCTGCAACAACTGGCTCTGCGCGGCCATCAACAGCTGTACCGCCATCGCAGCCAGCTCGGCGCCAAGACCTTGA
GCTTCCTGCTGGCCGACTTTCCCCGGACGGTGCGCGAGCAATGGCGCTTCGTGCTGGTGGCCAGCCTGTTGTTCTT
TGGCAGCCTGGTCGGCATTGGCCTGCTGGTCTATCTGTTCCCCGACTTGATCTACACCATCGTCAGTACCGAGCAA
GTGACCGAGATGCAAGGCATGTACGACCCCGACGCCAGTCGCCTGGGGCGCGCGGCCGAGCGGGCATCGAGTGAGG
ACTGGATGATGTTCGGCTACTACGTCATGCACAACATCGGCATTGCATTCCAGACCTTCGCCGGCGGCTTGCTGTT
CGGCCTGGGCAGCGTGTTTTTCCTGTTTTTCAATGGCCTGATGATCGGGGCCGTTGCCGGGCACCTGACACAAATC
GGCTACGGGCAGACCTTCTGGTCCTTTGTCATCGGCCATGGTGCCTTCGAACTGACAGCCATTGCCCTGGCCGGCG
CGGCAGGCCTGCAGCTGGGCTGGGCGTTGATCGCTCCGGGAGCGTTGCCCCGGGGTGAGTCATTGCGGCTGGCAGC
CCGCAAGAGCGTGCAAATGATCTGCGGCGTGATGGTATTGCTGCTTATCGCAGCTTTTATAGAAGCCTATTGGTCC
TCCACCACCGCCGTGCAGCCCTGGGGCAAATACCTGGTGGGCGCTTCACTCTGGGCGCTGGTGATCGCCTATCTGG
TGTTCGCCGGCAGGAGTCGTCATGCGCCTGAGTGACGCCAGCGTGGTGATTCGTCCTCGCTCCTCGTGGGAAGCCA
TGGACCTGGGGGTATTACTGGCCCGTAAGCACCGCGTGCTGTTGATGAGCAGTTGGGCCCTGGTGAGCCTGCCAAT
ATTTATCCTGATCAGCGTAGTGCTGTGGGATTACCCGTCTGTAGCCGTGCTGGTGTTCTGGTGGTTGAAACCGGCC
TTTGAGCGCTTGCCGCTGTACATCCTGTCCAAGGCGCTGTTTGGCCAGACGCCCACGCTCAAACAGGCGCTGCGCC
AATGGCCGCACCTGCTCAAGGGGCAACTGCTGGCCAGCCTGACCTGGAGGCGCTTTAGCCTCAGTCGCAGCTTTAC
CATGCCCGTGGTGCAACTCGAAGGCCTCGCAGGCCTGGCCCGCCAACAACGGTTGGGGATCCTGCAACAGCGCAAC
GCTGGCGCGGCGCGCTGGCTGACACTGATCGGCATTCACCTGGAAACGGCGCTATGGGTGGGCTTGATGGCGCTGT
TCTACCTGTTCCTGCCACAACAGGTAGAACTGGACTGGGACTGGCAACAGTTGGCGCTGGCCAGCAGCCAGGATTG
GCTATGGCTGGAGCACCTGAGCAATGCCTTCTACGCCCTGATCCTGGTGTTCTGGGAGCCCATCTACGTGGCGTGT
GGATTCAGCCTCTACCTCAACCGGCGTACCGTGCTCGAAGCCTGGGACCTGGAGTTGGTGTTCCGCCGCCTGCGCC

AGCGCCTGGAAGCAGCAGTGGCCGTCCTGCTGCTGGCGGTCGGCCTGCTCGTGATCGCGCCAACGCCCCAGGCCAT
GGCCAATCCACCGGTCGACTCGAAACCATTGAGCACGCCGCAAGCCAGCCAATCGATCAAGCACCTGCTGGAACAA
CCCCCGTTCAAGAATCCGGAAACCGTTACCCGCTACCGTTTCGGCGAGGACCAGGGCGCCAACAAAACCAAGCCCG
GCGCCGATGCCAAGTTGCCTGCCTGGCTTGAATCCTTTCTGAACAACCTCAACAGCGACACATTCAAGTCCCTGGC
CCTGGTGATGGAGGTACTGCTCTGGGGCCTGCTGATCGCCGGGATTACCCTGCTCGCCTGGCGTTATCGCGACTGG
CTGCAGGCCTTTGTCAGCCGTCGCCGCGCCCCGCGGCCCAAGGCGGAGCAGCCCGAGCCGTCCCAGCTGTTCGGCT
TGGACATGGGCGTGGCAACCCTGCCTGCAGACATTGCCGGTGCCGCCGAACAACTGTGGGCCACCAAGCCCCGCGA
GGCCCTTGGCCTGCTGTATCGCGGCCTGCTCAGCCGGCTGCTGCATGACTTCAACCTGCCACTGAAAAACGCCGAC
ACCGAAGGCCAGGTCCTGGAACGTGTGCGCCACTTGCAGCAGCCGCAATTGCTGGCATTCAGCGACGACCTGACCC
GGCACTGGCAAAACCTCGCCTACGGCCATCGCGTGCCGCCTGCCCCGGCCCAGCAACAGCTCTGCGCAGATTGGCG
CAGCTTGTTCGACAGCGAGGCCGCGCAATGAACCGGCCGCTGATCTGGTTCGGGCTGATCCTGGCTTGCCTGCTGA
CAGCCGGGGGCTTCTACGCCTGGCACAAGGCGATTCCCTACAACGAAGTGGTCGACCGCGGGCCGTCACCCGAAGC
CCAGGCCAACCCCTACCTGGCTGCAGAGCACTTCCTGGGCCAGCAAGGCCTGGCCGTCGAACACGCCCTCAGCCTG
GAGCGCCTGGCCACCCTGCCCGCCAAGGGCACAAGCCTGCTGTTGCTCAGCGAGCGCAGCAACATGACCCCGCACC
AGGTCGACCAACTGCTGGAGTGGACTCAGGCCGGCGGCCATCTGCTGTTGGTGGCCGAAGCCATATGGGATGAAGA
AACCCAGGACAGCGGCGACCTGCTGCTTGATCGCCTGCAGTTGCGCCAGGCCTTCAGCGATGACTTCGATCAGCCG
CCCAGCGCCCGCAAGGGCAAGAAACCTGACCTGACACGCCTGTATGTCGATAACGAAACCGCACCGGCGTTTTTCA
GTTTCGACACGGACTACACCCTGATCGACCCCAGGCACCTGGCCCAGTTTTCGGCCAACAGCGCCAAGTCCAGTCA
CCTGATGCAACTCAACCTCGGGCATGGCCGCGTGACCGTGATCACCGACAGCGAGCTGTGGAAGACCCCGGCCATC
GGCTTGCACGACAACGCCTGGCTGCTCTGGTACCTGACCCAGGGCAGCGATGTAACCCTACTGTCCAACACCGACG
TCGAAAACCTGTTCAGCCTGTTGCTGCGGTATTTCCCCCAGGCCCTGGTGGCACTTGCCGCCCTGATTCTGCTGGG
GTTGTGGCGTGCCGGCATACGCCTGGGGCCGATCCGGGCGCCCGCCCCGAAAGCCCGCCGCCAATTACAGGAACAC
CTGAATGCCAGTGCCGATTTCCTGCTGCGCCGCAGCGGGCAAAACAGCCTGTTGCAAGCCTTGCAGCGTGACATCC
TGCGCACCGCGCGTCGCCGCCACCCCGGATTCGAGCAACTGGATACCGACGGCCAACGGCAGGTACTTGAACGCCT
GACCCGCCAACCGTCTTCAATTATCAGCCAGGCCCTCGGCCCTCTCCCGGCAAAGCGGCTCTCCAGCGCCGACTTC
AGCCGCCAGGTGGCTTGCCTGCAAACCCTCAGGAATGCCCTATGAGCGACTCTCCAGTGGATGTATCCACCCCCCT
CGACACCGGGCGAGCCACCCAACTGGCCCAGGCTTTGCGCACCGAGCTGCGCAAAGCGGTGATTGGCCAGGATGCG
GTGATCGATGACGTACTGACTGCGCTGATCGCCGGCGGCCATGTGCTGCTCGAAGGGGTCCCGGGCCTGGGCAAGA
CCTTGCTGGTACGCGCCCTGGCCCGCTGCTTTGACGGCGACTTCGCGCGCATCCAGTTCACCCCGGACCTGATGCC
CAGTGATGTTACAGGGCATGCCGTGTACGACTTGCACACCGAGCAGTTCAAGCTGCGCAAGGGCCCCTTGTTCACC
CACCTGCTGCTGGCTGACGAGATCAATCGGGCCCCAGCGAAAACCCAGGCCGCCTTGCTCGAAGCCATGCAGGAAC
GCCAGGTCACTCTGGAGGGTGAAGCCCTGCCCATCGGCCAACCCTTCATGGTGCTGGCCACCCAGAACCCCATCGA
ACAGGAAGGCACTTACCCGCTACCGGAGGCTGAACTCGACCGTTTCATGCTCAAGGTACGCATGGACTACCCTGAC
GCCCAGCAAGAGTTGGAGATGGTGCGTGAAGTAACCCGCTCCTCCCGCGCCGACATGCTCGACGTACAGCCACTGC
GCACCGTGCTCCAGGCCGAAGATGTGCTGATGCTGCAGCAGGTCGCCAGTGAATTGGCCCTGGATGAACAGGTGCT
TGACTACGCCGTGCGCCTGGCACGCACCACCCGCACTTGGCCCGGCCTGGCCCTTGGCGCCGGTCCCCGCGCCTCC
ATAGCCCTGGTGCGAGGTGCCCGCGCCCGGGCGTTGCTGCGTGGTGGCGAGTTCGTGATCCCGGACGACATAAAGA
GCTGCGCGCTGGCGGTGTTGCGCCATCGGGTACGCATCGCCCCGGAGCTGGATATCGAAGGGCTGGAAGTCGATCA
GGTACTTGCCCAGTTGCTCGACCAAGTGCCGGCGCCGCGCCAGTGACTTGCCCATGAGACCCACTCGCCTGTTGCT
GGCCTGGCTCGCGGTACTGTTGGGTCTGGGCATCGCCCTCGGAACAGCCATGGCCCTGCAACTCAAGGTGCCAGCC
GCCCTGCACTCCGTGGCATGGGGCCTGCTCCTGGCGCTGCTTTTGCTCGCATTGCTCGATGCTGTCCGCCTCAAAC
GCCGCCCCTCGCCACGCCTGCGGCGGCAGATGCCGGGCAGCCTGGCGCTGGGGCGCTGGAGCGAAGTGCGGATTAC
CCTGGAGCATGACTATGCACACCCCCTGGCCCTGGAGGTGTTTGATCATGTGCCGGATGGGTTGAGCGTGCAAAAC
CTGCCGCAGTCCATCGCACTGCGTCCCAGCGAACACAGCGAGCTGGGCTATCGCCTGCGCCCCTTGAGCCGTGGGC
ACTTCAGCTTTGAGCGTTGTGAGGTGCACCTCCCCAGCCCTCTAGGATTATGGACCGCAAAACGCCTACTGCCACT
GCAGGATGACACGCGGGTCTATCCGGATTTTGCCCGCCTGTACGGTGCGCAATTGTTGGCGGTAGACAACTGGCTC
AGCCAACTGGGTGTACGTCAGCGCCAGCGGCGCGGCCTGGGCCTGGAGTTTCACCAACTGCGCGAATTTCGCGAGG
GTGACAGCCTGCGCCAGATCGACTGGAAAGCCACGGCACGGCAACGCACGCCGATCGCCCGGGAATACCAGGATGA
GCGCGACCAGCAGATTGTCTTCATGCTCGATTGCGGCCGGCGTATGCGCAGCCAGGACGGCGAACTGTCGCATTTC
GACCACGCCCTCAACGCCTGTCTGCTGCTCAGCTACGTGGCCTTGCGCCAGGGCGATGCGGTGGGCCTGTGTACCT
TCGCCTGTGACCAGCCGCGCTATCTGGCACCGGTCAAGGGCAGTGGCCAACTGAACCAGTTGCTCAACGCGGTCTA
CGACCTGGACAGCACCCGGCGCGCGGCCGATTACCAGGCCGCAGCCAATCAGTTACTGGCTCGTCAGAAACGTCGA
GCGCTGGTGATCGTCGTGACCAACCTGCGTGATGAGGACGACGAGGATCTGCTGACTGCGGTCAAGCGCATCAGCC
GCCAGCATCGGGTATTGGTGGCAAGCCTGCGCGAAGAAGTACTGGATCATCTGCGCCAGGCCCCTGTGCAAACCCT
GCCGGAAGCGCTGGCCTACTGCGCCAGCGTCGACTATCTAAACGCCCGGGAAGAACTCCATGACCGTCTGAAGGCA
CAGGGCTTCGCCGTACTGGATGCTCACCCGTCGGATTTGGGTGCGGATCTGGTAACGCGTTATCTGGGCTGGAAAA
AAGGCGGCGTGCTATGAAGGTGCACCTGGTACAGGTATCCGTTCAACGATCAGCACGGGGCCGACAACGTTCGTCG

CGCTAGGCCGAGGCCTGCTGGGGAACAAAACTGAAGTACTGCGACAGTGCCAGGATCAATTGCCCGTACTCGGCAG
GTGCCTGTAACAGCATGAAACCCGAATCATAATGCTGAGGGTTGATGTCTTCACGGCTCCACAGGCACCTGGCAGT
GAAGTCAATTGCATGCACATCTCCCTCAAGCCCGGGGATCTTCAAGCACAGCTCGAAATCCACGTTGATCATCATC
GGTAGCTGACTGGCCAACATCAGGCCTTCGCTGGAAATATTCCCCAGATAACCGATGGGTTTGTCCGTGAAGCGGT
TGAACACCTGCAAGCAACAAGGCAACTGGTGCCGCTCAATCCGTCGATTGGTAAACATGATGCCATCCAATGGCCA
TGATTTTGGCCGTTCAGTGGTTCGGACCGGAGCGTTATCAACAACACCCCACTCTCCATGGTTCTCGGTGGGACAG
CAACCTCCGCCCTGGCACTTTACCGCCGCCGAATCCGAGAATTGCATCTGTTTGGATTGAAAGTTGTACAAACAGA
CATCCAAAGGATAGCCCAACTCCGGCAAGAGACCAGCTATGAAATGATAGACACCATCACAATGCAGCTGGCCTTG
CTCGTGCGGGGCTTGCACCTGGGTAATGCCCCAGTTGTTGCAGCGTATCCAGGCGTGCACGCGCGCGGAACGCGTA
TTCGCTCGACGGGTACTGGCTGATGATGAACTGATACGTCTGCACCGCGTCCACAAACAACTTCTGCCGCTCCAGG
CACTGCCCACGCAGCATCGAGACCTCCGGCTGCACGTAGCGCCGTGAGCGGCTCTCGCGGTCCACCTGGGACAATT
CCAGGGTGACCCGTTCGCAGTCGCCTACGTTGTAGGCACGGTAGGCATTGTTCAAATGATGGTCCATCGACCAACG
GGTGCAGCCGACAACACTGACGGCCAGGGCAGCAATGATCAAAACTCGCATGGGGTGTTCTCCTGTCTTGAGCAGT
GTATCGACCCTTGGGCGAAAATCTTCAGGGTTGTTCGTCCCGCTCCTTAGAGTGAAAACAAGCGAATCGATAATAA
GTAGTGCAAACGAACAATGACTACAGCCAAAGAGCATAGTAGCCTTCCCCAGCGCTAGAACTCAGGAGTCCGTGCA
TGTCCGTCCGTCGTACCAAAATCGTCGCCACCCTTGGCCCGGCCAGTAACTCGCCGGAAGTCCTCGAACAGCTGAT
TCTGGCTGGCCTGGACGTTGCCCGTCTGAACTTCTCCCACGGCACCCCGGACGAGCACAAGGCTCGCGCCAAGCTG
GTACGTGACCTCGCCGCCAAGCATGGCCGCTTCGTCGCGCTGCTGGGTGACCTGCAAGGTCCGAAGATCCGGATCG
CCAAATTCGCCAACAAGCGCATCGAGCTGAAGATTGGTGACAAATTCACCTTCTCCACCAGCCATCCGCTGACCGA
AGGCAACCAGCAGGTTGTGGGTATCGACTACCCGGACCTGGTGAAAGACTGCGGCGTCGGCGACGAACTGCTGTTG
GACGATGGCCGAGTGGTCATGCGCGTCGACACCGCCACCGTCACCGAGCTGAACTGCACCGTGATCATCGGCGGCC
CGCTGTCCGACCACAAAGGCATCAACCGTCGTGGTGGCGGCCTGACTGCACCGGCCCTGACCGAGAAAGACAAGGC
TGACATCAAGCTCGCCGCCGAAATGGAAGTGGACTACCTCGCCGTTTCCTTCCCACGCGACGCTGCCGACATGGAA
TACGCCCGCCAACTGCGCGACGAAGCCGGCGGTACTGCCTGGCTGGTCGCGAAGATCGAGCGCGCTGAAGCCGTGG
CCGACGACGAAACCCTCGACGGCCTGATCAAGGCGTCCGACGCAGTGATGGTTGCCCGTGGCGACCTCGGCGTAGA
AATCGGTGACGCGGAACTGATCGGCATCCAGAAGAAAATCATCCTGCACGCACGCCGCCACAATAAAGCGGTGATC
GTTGCGACCCAGATGATGGAGTCGATGATCCAGAACCCGATGCCAACCCGTGCCGAAGTGTCCGACGTGGCCAACG
CCGTGCTCGACTACACCGACGCCGTAATGCTCTCGGCGGAAAGTGCCGCCGGCCCGTACCCGCTGGAAGCGGTCCA
GGCCATGGCGCGGATCTGCGTCGGCGCTGAAAAGCACCCGACCAGCAAGACCTCCAGCCACCGCATCGGCAAGGTG
TTCGAAAGCTGCGACCAGAGCATCGCCCTGGCCGCCATGTACACCGCCAACCACTTCCCGGGTGTGAAGGCGATCA
TCGCCCTGACCGAAAGTGGCTACACCCCGTTGATCATGTCGCGCATCCGCTCCTCGATCCCGATCTACGCGTTCTC
CCCGCACCGCGAAACCCAGGCCCGCGCGGCCATGTTCCGTGGCGTGTACACCGTACCGTTCGACCCGGCGTCGTTG
CCGCCTGAGCAAGTCAGCCAGGCTGCCATCGACGAACTGCTCAAGCGTGGCGTCGTGGAGAAAGGCGATTGGGTGA
TCCTGACCAAGGGTGACAGCTACCACACCATCGGCGGCACCAACGGCATGAAGATCCTGCACGTTGGCGACCCAAT
GGTCTGAGTGACCTGCTGAAAGCAAAGGCCCTGTCATGTGAGTGACAGGGCCTTTTGCATTTCTAATCAGGGTGAG
GGGGTGGTTGCACTGGCGCTTCGATTTCAGCGCCGGGCAATAAACCCCGACAACGCCGCAATCGCCTCCGGCGACC
TCAACCGCTGGGTAAACAGCGCGCCCTCCTCCTCAATCACCTGGCGCAACTGCTCGCGATCAACGCTCTTCATCAA
CTGCTTGCTGATTTGCACTGCACCCGGCGCCAGAGTCTCGAAGCGTTCGGCCACCTCCCGCGCCTTGGCCAAGGCT
GCCTCACCACTGTCCAGCGCTTGCGTGGCGATGCCCCACTGTGCCGCTTGCTCGCCGCTAAACCCTTCGCCCAGCA
GCAACAATTCGGCAGCCTTTGCATGCCCAAGCAAACGCGGCAGGATCAGACTGGAGCCGAACTCCGGGCACAGTCC
CAGGTTGACAAACGGCATGCGCAACCGGGCATCACGGCTGACATACACCAGGTCGCAATGCAGCAACAGCGTGGTA
CCAATGCCCACTGCAGCGCCCGCCACACCGGCAATCACCGGTTTGCGGCAATTGAGCAGGCTCTTCATAAAGAGGA
ACGGTGGGCTGTCCAGGTCCGTGGGCGGTTGCTGCAGGAAGTCGCCGATATCATTGCCGGCGGTAAAACACTCGCT
GCTGCCCTGGATCAGCACCGCATTGACCGTGTTGTCGGCATCGGCCTGCTCCAACGCCGCGGCCAACTGGTGGTAC
ATCGCGCGGGTCAGGGCGTTTTTCTTGTCGGGGCGATTGAACTGCAGAATCAGCAGCCCACGGTCACGTTGCAGCA
CAATGGCATCGGTCATGGTGAGTCTCGCAGATAGGCGCTCAACCACGGGGCAGGAACACATCCGCCAGCAGTTGAT
TACGCGGCAAGCCCGCCAGGAACAGGCGCTTGGAAAAGGCCTCGACACTGCCGGGATGCCCGCAGAGTAAGGCCAG
GGTTTGCCGGGAAACAAGCCGCAGTTGCGCCAAAGCACCGGCCAACTGCGCCGCTGTCCACAGCTCCACGATGAGG
TTTTCATGGCTGGCGGCCAGTTGGGCCAGGGGCTCGGCCAGATAATGCGCGTGCGTATCATGGGCCAGGTGAATCA
CGCGGATGACGCCCTGGTGATCCTGGCGCAAGGCCTCACGCAACACGCCCCACAACGGGCCCAGGCCAGTGCCGGA
TGCCAGCAGCCACAGCGGCCGGGATTGCCAGTCCGGGTCATAGTGCAACGCCCCACCGCGCAATTCGCCCAGGCGC
AGTTGATCACCGGCCTGTAACTGCCGGGCAAGATCGCTGAACTCGCCGGGCAGGCGGCAATCGAGATGGAACTCCA
GGAACGGATCTTCCCCGGGCAAGCTGGCCAGGGAATAAGGCCGCGCCACCGTACCCGCCCATAGCACCAGGTGCTG
CCCGGCTCGGTAACGCAGGCCTCGCTCCGGTTGCAGGCGCAGGCGCAGCACCGTCGGGTGCAGCCAGTCCGCGCTC
AGTACCGTGGCCGCCGTGCCGTCGCGCAATGGGTCAAAGGCCTCAACCTGCACATCACCTGTAACCTGGCACTGGC
AGGCCAGGCGCCAGCCATCCTGGCGCTGGGCCGGGCTCAGGGCGTCGGGTTGCTTGTCCTCGACCTCACCCCGGCA
ACGCACCAGGCAGGCATGGCAACTCCCCGCCCGGCAGCTGTAGGGCACAGCCACACCGGCCTGGTTCAAGGCATCC

AGCAGATTGCTGCCGGTGGAGACCGACCAGTGGCGTGCGCCGACGTGCAGTTCAGGCATAAAACTCTTCAGCTGTA
GGAGCGAGCTTGCTCGCGAATGTCGCTAACGATAACGCGGGCTGCCTGACGAAACCCACCAGATTCACGTTTTTCG
CGAGCAAGCTCGCTCCTACAGCTAAATCGAGCCGGCCATTCATGACCATCGTCGGGTGTATCAGCCACCGCGCCGG
GTTATACTGCCGCGCCTTTTTAGCGTCGCGCCAGCACTACCCGGCGTGCCTTGGAAAAGGTGGCTCCAGCGACCGA
TACACCCATCTGAAGCCACCTTTATTGAATGTTCCCTTATAGAGGAGCGCGACTCATGACCGTGATCAAGCAAGAC
GACCTGATTCAGAGCGTTGCCGACGCCCTGCAATTCATTTCCTACTACCACCCCGTTGACTTCATTCAGGCAATGC
ATGAGGCCTACCTGCGCGAAGAATCGCCGGCCGCCCGTGACTCCATGGCCCAGATCCTGATCAACTCGCGCATGTG
TGCCACCGGCCACCGCCCGATCTGCCAGGACACCGGGATCGTTACCGTGTTCGTACGCGTCGGCATGGATGTACGT
TGGGATGGCGCCACCATGGGCCTGGACGACATGATCAACGAAGGCGTGCGTCGCGCCTACAACCTGCCGGAAAACG
TCCTGCGGGCCTCGATCCTCGCCGATCCGGCAGGTAGCAGGAAGAACACCAAGGACAACACCCCAGCGGTCATCCA
CTACTCCATCGTTCCGGGTAACACCGTGGAAGTGGACGTGGCCGCCAAGGGTGGTGGTTCCGAGAACAAGTCGAAA
ATGGCCATGCTCAACCCGTCCGACTCGATCGTCGACTGGGTCCTCAAGACCGTGCCGACCATGGGTGCCGGCTGGT
GCCCACCGGGCATGCTCGGCATTGGTATCGGCGGCACCGCCGAGAAAGCCGCGGTGATGGCCAAGGAAGTGTTGAT
GGAATCCATCGACATCCACGAGCTGAAAAAGCGCGGCCCTTCCAACCGTATCGAAGAAATGCGCCTGGAGCTGTTC
GAGAAGGTCAACCAACTGGGGATCGGCGCCCAGGGCCTGGGTGGCCTGACCACCGTGCTCGACGTGAAGATCATGG
ATTACCCGACCCACGCCGCCTCGTTGCCGGTGTGCATGATCCCCAACTGCGCCGCCACCCGTCACGCACACTTCGT
GCTCGACGGTTCCGGCCCGGCCTCGCTGGAAGCGCCACCGCTGGACGCCTACCCGGAAATCGTCTGGGAAGCCGGC
CCGTCGGCCCGCCGCGTCAACCTCGACACCCTGACCCCGGAAGACGTGCAGAGCTGGAAACCGGGCGAAACCGTGC
TGCTCAACGGCAAGATGCTCACCGGGCGCGACGCGGCGCACAAGCGCATGGTCGAGATGCTGAACAAGGGTGAAAC
CTTGCCGGTAGACCTCAAGGGTCGCTTCATCTACTACGTCGGCCCGGTCGATCCGGTGCGCGAAGAAGTGGTTGGC
CCGGCTGGCCCGACCACCGCCACGCGGATGGACAAGTTCACCCGCCAGATCCTCGAGCAGACTGGCCTGTTGGGCA
TGATCGGCAAATCCGAGCGCGGCCCGACCGCCATCGAAGCAATCAAGGACCACAAGGCCGTGTACCTGATGGCCGT
GGGTGGCGCGGCTTACCTGGTGGCGCAAGCCATCAAGAAATCGCGTGTGGTGGCCTTCGCCGAACTGGGCATGGAA
GCGATCTACGAGTTCGACGTCAAAGACATGCCGGTCACAGTGGCTGTCGACAGCAACGGCGAGTCGGTCCACATCA
CTGGCCCTGCCATCTGGCAGAAAAAGATCAGTGAGAGCCTGGCGGTAGAAGTGCAGTAAGCACCCTGCGCTGAAAA
AAGCCACGCTACCTGAATCGGTAGCGTGGCTTTTTTACACCTGTTTCTGTGCCATTGGGTTCGGTTCTACTGCTGG
TCGTAGGGACTATTCACACTTAACGACGCGCTCTGGCCCGAATAATAAGAGGTATAGACCCAACCATCGCGGGATG
TTTTCTGGTGATACACCACCCCATCTTTGGTGAGGGTCACGGAAAGCTGGGCAGGATTTCTCCGGTAAACGCCGA
GGCAACACGCATACCCTTGTAATAGTACTCTGCAGTTCCACCCATTTGGACCGGCCCGTAGGTTTCCGAGACAGTG
CCCTTGTTATGCGCCAGCCCAATCGGCAACACCCGGGAGGTGACATCCCAGGCGGCCACCTTCATTTCCAGCCCGG
CATTCACATCGGCACTCGCCTCGGAGCAATCCACCGTCCAAGTCAGCCCGGCTTGCTCCATGACCCGCGCAGTATG
ACCGACCGGCTGCAGGATCATGCCGTGATCGGCAGCATCGGGTAACCAATCCAGCGTCACCTCCTTGCCGATAATT
TCGGTATCAGCCTCAGGCACCACCAGGATCGTGTGCGTTGCCCCCCTACACAAAAGCAAGGGCTCGCCGAAGGATT
TTCCACGGAACGCATCCAGTCTGACCTCTACCAGATCCCCCAGGTCGTCTGAAAGCAGTTTTCCCTGCAGGTACCA
ATCCGGCAGGCTGGGGCTCATCAATTTCAACCCGAAATGCCCTTCGTCACCCAACAACGAGGTGACGGACCAGGAC
ACACTCTGCCCCTCAACGCTGCGTGGCTCTCCCAAGGGTGGAGAAAACACCAGGCCCAAGGCTTGTGCGCCGGCAA
AGTCTTCCAACGTGACGGTCGAGCCCAACAGCGGACTGTCCTCGTTGACTTCCAGCTCCAATTCATACCGCCTGCC
ACGCCGCAGTTGCAGGCCGGCAAAGGCCAGATCAACAGTCTTGTGGTTTAGATAAAGTACGAAGTGCTGATCCCAT
GGGTTGTCCTCAAGCGCAGTAACGGTGAACCCGACCTGGGTGACAACGCCTTCGTTTTCGCCAGTCATATCAGCGA
TAACCTGATGCACACCTTCAGTCTGTGGCCGGTAGAGGTATTCAGCCCATCCGAATCTATCGGTGGTGGTGATCGA
CCATAAAGCCTCCTCCCCATCAACGACCCAACGCACTTGGAGCCCGGCCTGACCTTCTTCGGTTTCAGCGTTCAGC
ACCTGAAAACGTAGCAATGCTCCCTCATCTTTTCTGACAACGGGTTGAGCCGACACCGGGCTGATCTTATCAATCA
GATACCGCGCATCTACCACCTCGAACTCGCGTACAGCCGACCATTCGGATGTGTCCCCATCTACAGCCTGTTGCAC
TTGAACCCCGTGCCGCCCTAACGCCCAATCAGCCGAAGCACTCCATCGCCAGGACCTGACACCCGGGATCGTCGCC
AGCACGTTACTCAGATCATTGACATGGCGTAGCAACAATTGCCCCCCCGTCACCCCGGTGCCGGTGAATTGTGGTT
TGCGCGATACGCGGCTGTTGTGTGTAGGCGTCCTGATTTCCGGCGTTTGTGGCCGAGGCATTACGTGAAAGGTATG
CGGCTCGCTCCAGGGCGATTGCGGCCCAGTTCCTTTCTGCTGTTTGGCCTGAAGCGCCACGGGGCCTGGTTTGAAC
GGTTCTTCGATGACGAATTCGAGCGTCCCGTCCTCTTCGGTCACTTGTCCGTGCAGGACCAGCTCACCCTCACCCC
GTAGGCTTACCAGCGCCCCAGGCAGGCCTTTTATGCGGATAAGCGGACGGGACGACGACTGCGACCCCGCTAGCGG
CAGTTCGAAATACGGTGCGTCGGGTACCGGTTCTCGAATCACGATAAAGACACAGGGCTCGCTCACTACAGACGCA
ACGCCTTCCACCACCTGGTTCGCAGTCACCTTGTTTGCGTCGTACGGCCAGTCCTGATCAGGTGAGAACACTGCCT
CCTTATCCCCGATCACGAACGTTCCGGAAACCTGATGCCCGTTTTCATCAACCAGATTCAAGGTCGCGCCTGCCGA
ATACACCCCCGTGATCAACAGCGTTTGCCGCAGGTTGTTTGTCTCAGCCAGCGGGACAATCGCCAACCGGGGTGGC
CTGACTCGGAATGTCCGAAGGGCCTCCTCAGAGACCAAATCCTGTAAATACACCCGCACCACAATTTCATGGACTC
CCGGGCCGTGAGGTAGTGCTTCATGCGCTGTCCAGTGCCCGTCAGGCAAGGGCACGGCGTGCGCCACTTCGACGCC
GGTGAGCGGATCGCTTACCACGACAGACGCACCTATGCCCGGCCAGCCACGACTGCCGGAAAGAATGGCTCCGATA
TCAATGACCTGATCATGTTGGGGATACTCGATGATTGGCGCTTGGATCCGTACGTCGAAGCGACACATCTCACTGG

GCTCGGACATGACGTCATTGACTGTCTGAACGGCATAAACCTGATTGACCTTCGCCCAGTTTTTAGACGCAATGAA
CTGCCGAATCGAACCCTCTTCGGAAAACACGCCAGGTACGGATTGATCGTTCTCATCAAACATCGCCAGCAGCACG
TTGCCGCTACCTACATGACGAACCGTAAGGGCCTGGGCTGGCATGGCCGGGTTGGTTGGAGGAAGAATTTCAACGC
GATCAGGACGCAGATAGAACCCACGCACCGGCCCTGGGCGGGACTCAATACCTTGTCGCACCTGCATGAGCTGCTT
GTGTTTGAACCCGGGTGCCGACCAAGTGTGAATGCCATACCACTGACCGCCCTGCACCGGCCAAGATGCAATGCGC
TCGCCATCGGTGTCGAGCAGTACCACCGTAGCGTCCGATACGCCCGTTCCGCTCAACTCAAATTTCAGCGGGTAGT
ACTCGCCCTCCGCCGGGGCAATGAGTTGCGGGACGGGCGGGCGAGCAATGAAATCGACCGGCGTACTGGGGTTGGA
AACGATATCCCCCAATTGCTGGGTCACGGTGATCCGATTCTCATGAGGCCAGGGCTGTGCAGGCACAAACTGGCGA
GAAGACCCGCTCCCTGTCATGTGCCCGGTCACTGCATGCCCCTTGTTGTAGACACGCACGGCCACCGCTTCGGAAT
AGAGCCCCGTAATCTCGAGCATTTGATCCGATTCGGCAGGATGGGCGGGTTTTCCAACTAGGGGAACAGCCAATGA
AATATAAAAATGACGAACCGGCCCCGGAGCGGATTCAACGCCATTGACGACTTGCACAACACGCTTTTGCTTGAAC
CCCGGTATCCAGTGATAGATTCCTGTCCAGAGGGTACCGATGGCCCGCCAGCTCCCCACCACGCTGCCGTCCAGGT
TCAACACCTTGACCAGAGCGTTCGGATGACACCGCCCTGTCATTTCGAAATCCTGCTCGTAGTAGCCTCCCTCTTG
GGGCGTATCGAATTGAACTGTAAAGGGTCTGGCCGAGAACACGCACATCTCGCTGGGCAAGGACTCTGTGTCATTG
ACCACCTGTGTCGCCCAGACCTTGTTGGTTCCCGGCGCCCAGTCCAGGTCAGGCACAAAGGTGTAGGTCTCACCGT
AGCGGGTAAAGATGCCAGGAACATGATGTCCATCGAGAAACACCTTCACATTCTCGACCGCCGGGTGAACACCGAC
CAGTGTCAGCGCCTGCGTGGCGTCTGCCGGGTTCGGCGGCTGAATGATCGTCAAAGGCAGCGGTTGCACACAAATC
ACTCGGGCCTCACTGGCGTCGGAGGCAACCGAATTAACGACCTGGAACACCTTTACCGTATGTTTACCGAATGACC
AATCCTGGGCCGGCGTAAACACACGCTCACTGCCGGTTCCCGTAAAAATACCCGGAATTGGCGTGTCATCGTCCCT
AAGCATCTGCAAGGTCGCGGCACTCGGATACACATGACTGATCTGCAGCGTCTGGGCTGGCGCTGCCGGGTTGGCC
ACCAGCGCAATCGAGGGCGGGTCAGGCTTGATAAAAAACGCACGGGCCGGGCTTCTCGCAGAGTCGACATCATTGA
CCACCTGCATGACCTGCACCCGTGCCTCTCCGGGCACCTGCCAGCGGTGCCGGGCTATCCAGCCATTCCCATCCAC
CTGCGCCTTGACCAGGATATTACCGTCGATGGCGAGTACATAAACAGTCGCCCCCACGCGACAAGTGCCGCTCAAA
TGCACCTCGAACGGCTGGTGCGACTTATCCACTGGCTCATTGATCACCGGCGCCAGCGGGCTGACGAAAAAGGTCA
CGTCATCGCTTCGATTAGACGTCACGCCTGCCACCTGCTGGGTCACCCATACCGATTGCTCTCCCAGCGTCCAGTC
TTGAGCGGGCGTAAACAGATAGCGAGAGCCACTGATGACATTACCCGTCACCTGACCTGCGACGTTATAAACCTTC
AGCGTAGCGCCAGCCAGGACCCCGCTGATTTGCAACGACTGCCGGGGACTAGGTATAACGGGTTCCAGCAGCACCG
GTTTGGGCGGTCGACTGTAAAAGCGACGCTCCAGGAGGGTGGAGAGCAAGCCTCGCCATTGGACATACACACGAAG
CGTATGCTCACCCGGCTGCAGCGATAACGCCGTTGAAGTCCAATTCCCATCGGCATCAGGGATGGCCGAGCCCACT
TCGTGGTCAATATTGCCGGCGTACAGCACGACCCTGGATCCTGGCCCAACATAGCCTCGCTGCCCGGAAAACGAAT
CGCCTATATCGACAACCCCGTACTGTGCTGGATAAAGCACCGTCGGTGCTGCAAACACCACATTGAAGGTCACACT
GTCGCTGGGATCCGATACAAAACCGTCGACGGTCTGCGTCGCGCTCACCGATTGAGCGCCAATCGGCCAGGCAAGT
GTGGGCGTGAACTGACGCGTGCTCCCTGTCCCCGAGAAGGTGCCGGCAACCACGGTGGCGCCCCGGGAAACCGTCA
CATGGCCCGCCTTGGCATGCACTGCGCTGATCGTCAGCACCTGCGAGGGCAGCGCCGAATGGGGTGGTTGACTGAG
TGTTGGCTTGCTCGGCCGGACGGTGAACTTTATGTCCCGCGCCCACCAGGACGCGTGCTGAGGGTTCTTACCCACC
TGTCTTGCCGTAATTTCGTAGGCCTGCACGGCCCACGGGCTGTTTGCAGTGACTGACCATGTGCCCTGCGCTTGTA
CCGTCGCGCTCAACTGAACCCCGCCTCCACCACCGGTATGAAAAACCTCGATGGTTGCCCCCGAAAGACCTCTGCC
ACTCACCATGGGCCGACCATCGGCTAAAACCGTCACCTCAGTGATCACGGGCACCAGCAGCACGGTGAAGCTCACA
GGTGCCGATACTGAAGTCATCCCGTTCACTGTTTGCCTTGCTTCGAGAATCAGGGGATCAGCCGACCAGAGGTCCC
TCGTCAGTAGCACGCTCCAACTACTCTGCTGCACGACCGTCGTAGCAAACGGCACATTGAAGCCCCGCTGATAAAT
GCCGATCAGCGCGCCGCTCACACCGCTCCCGCTCAATATGGGCCGGGCAACCGTAACCAGGCCAGGATTGTTGAGG
CCTGGCGGGGCGGGTGCCACGGTAACTATTTTTTCATCCGACCAGTCGGAATAAGCGCCGGTCAGCGGCTCGCGCT
GTCGCGCGACGATACGGTACGAACCTGCGGCCCATGCACGGGTTGCCGTCGCCTCCCATGTGACCCCCGATTGCAC
GGTCGTCAGTTGGACACCACCACCCCCACCGACAAAAAAGATTTCGATAGTCGCGCCCACCAGGGCGCCTCCCCCT
GCGACGGACGGTACGCCGCCGACACTCACGCTCACACCTGTGATCATAGGCTTGAGCAACAATACGAAACTGACCC
AGTTGGAAGAACCAGACGTTTCGCCGCCCAGCGTTTGCCGCGCGAACACCTCATGAACTCCCTGGCCTATCCAAGG
ACCAATCGTAATACTCCACGTCCCATCCCGCACTTGACCGGTCCCTAAAGGCAGCCTGATTCCGGCTTGGAACCGC
GGCAATAAATCGACCTGCGCGCCCGTTACCCCGGTACCGCGAAGGGTGGGATAAGGCGTCCACTGCGTGCCTGGGC
GGGTTATGACCGGAGGCGTCAACACTTGCAATACCGGCGTTACATAAGCCCAGGGGCTATAGGTTGTCGCTTGGAA
CTCAACCCTTACCGAAATAATGGAAACTGCCTCCGCTGGAACTTCCACGGTGGCCCTCACCTCAGAACGCCAATGC
GCCAATAAAAAATAAGAGGTGAAAGAGTTAGCGACTCTGCGCATATGCTGCTTGCCAGCGCTATCCCTATAAAAAA
GCTCCACATTCCATTTATGTATCGGAGTGCTCAATGCCCACCCACTGGCCCTAATGACAAAACGCCGTCCCACACT
TGCGTTGGCTCTGGGCTCGCTGATATCAGGGGTCGCCCAAAAAAACAGCGGGCCTATCCCTCTCTTACCGTCAGGC
TGACGTGCTTGCACAGTGGTGTTGGCGCGATTGTCGCTCATGGCAGGGTACTCCGGTCGCTAACGCCTTCATCGAT
GTCGCCGGTATATACCGATGCGCGCCCCTTCAGCACCTGTGAAATCTACCAGTTACGACGAATGGCTAAACTCGCG
ACCTGTCAAGTTTCACAGGTTACAAGCGATTTTTTCTCCACTACCTTGGACCTCGACGTCTCAGTTCCCTTCTCCC
GTTCGGATGAGAACCCATGGCCAGCACACGCAACGTTCTGCTCAAGCACCTAGTCGCTGCTGACGCCCAGCCGGTC

AAAGGCCAAATGACGTTCGAGGCGGCCCTCAAAGTAATGGACGTGACGTCGGTCTTCGATATCGTTCGATTGTCCC
GACCGGACTTCATCCGACAGTTGGCCCTGCACAGCAACGCCAATGGTGCGCAAGCCTACGACAACGCCATGGCCTA
CGCCTCTGTGCTCGCGCGCCTATACCGCGAACACCGGACGTCGTCGGGCGCATCCCACCCGCTGGCACAGCGCGAC
GGCGTCAGGGCACTGGTGCCCTATGGTCCGACCTACGCCAATCTATTCAAGGAAAACTGGAGTGAGTTCTGCAAAG
TCGGAGCTCTGGCCGCTAACAACTCACCCGGCGCCTATCTGAGCGCACTTCGCGTGTTCATCAAGCAACTGGAAAA
CACCAGCAGCGATACCTCTCGCCTTTTGCTGGACCGGCGTAGGCCAGACCTCAAGGAGCTGTTGATCACCCACGAG
AGCACCTTCACCCCACGCCCAATGCTGGAGATCGTCAACCAGGTGCTGGGCGCTAATCTGCGTCAATATCTGCAAC
GCGTACCGGCAGACCAAAACAAGACGGTGCACACGGTACTGACTGAACGCCAGTACCCCTTCGAGCTGCCCTACAA
CTACTACCACCAGCAATGCCTACTGGGCCTAGCGGGAAACAAACCTCGGCTGGGTGAGCTCAGTTACCAGATCAGC
GACCGGCTGCCGATTGAACAGAACGCCTTCAACCATTACGGCGCCGTGCAACATACGTCGATGCGGGCCCAGCAAT
TATTAAATGGGCTGAGTCCACAACAGCAGGCACTGTTGATCGCAGCTTCGCCATTCAGTCATTTTTACCTGAACCG
CACTCACCTCGTCGCAGGATGGAAAAGTCCGGCCAACTGTCATCTAAGCCCTCATGCTCCTTTCCCGATGGCTTAT
CTACTGCCGACGGGACAGCCGGATGTCGGCGTAATCACACCGGCATTGAACGTCCCTGTGTTAGCCGCCAGCGGTT
CGAATAATGTCCCTCTGACCTTTCGAAAGCCCGGACAGACCTCAACACATACCGCGACGATTCAATTTGCCAACGC
CACCCCTGATAACACCACGCTTTACCGATTCAACTCGCTACAACAGGCGTCACAGGCGAGCCTGTGCGTTAACTTC
AAGGCCGTTGGCACGCTCCCCGTACCCACTGCAACAGGCTATACCGCCACATTCGATACACTCATGGCGACGGGTA
CGATAGCGGCCCCTGTCAATACCGCCAGGCGCCGCTTCACATTGAGCCTGGATGAGCAATACCAGGCGAATGCCCA
AGAAAAAGCTTATTTCCTAAGCCTCTACGGCATTGAAATCTCAGGCGCCAATGCCGGCACGCTCACCGACCTCAAC
ATCTTCATGCAACATACGCAGCTACACGCCGAGCAAGTGGAGATGCTGTTGTCGCGGCGCACCTACGCGGTACGCC
TGTCGCCCAACTGCCTCAGCAAAAACCTTCAACACCTTGGCCTGCGCGGCGACAGGGACTGCCCTTTTCCCCATGC
AAACCACTATGGGGCTTGCTACGTCAACGGCCAGGGTACGGATGGATCCGAGCCGGGCAGCACAGACCTCTCACGT
AGGCGCAATCAGTTCGACAACGCCATGGATCTCGTACAGGAGGAAGTCGGTGACGGTAAGACCTGGCATCTGACCA
AGACCTCCCTGGACCGTTTTGACCGCCTGCAACGCATGATTCGCCTGCAGCGCTGGACCGGCATCGCCTTCTGCAA
ACTCGACACATTGGTCGTCAGTGCCATGCGTGCCGAAGGTAAAGAAAACCTGGATTTGCAACTCAACGACAATACC
CTGCGGGCACTCGGCGTCTACCGTCACCTGAATCAACGCCACGGCATCGACCCTCAGGAGTTTGCCGCGTTCATGC
ATTACCTCACCCCCTACACGACGGTCAGGGATGAGCTGGCGTTGTTTGACCAGGTGTTCAATGCGGTCCAGCTCTT
CGACACGCCGCTGGTGCTGGACCATGGCGTATTCACCGTCACCGACACGGCATACGCCGCGCGTAAAACCGTGCTG
CAACTGTGCGCGGGCCTGGGCTTGAAGCCGACCGATGATGAGTTCCTGCTGATCGCCGCCCACACGCGTAGCCTGT
TGGGCAGTCTCAAGCGCGACCTGGCGACCGTGTCCTCGCTGTATCGCCAGGCACGTATCGCCAGGCTCTTTGGCTG
TACGGTTGCCCAGTTACTGACGCTGGCCGGCCTACTTGGCGGCCAACCGTTCAGAACCGCGCTGGCCAGCGGTGTG
CTGCGCGCCCCCGCCAACGTACACACGCCGGATATTCTGGACGTTTTGATGCAGTTGCAGTGGGCCTTCGACTGGC
TCGCCGACAGCCATCAAACCATCGCCCAATTGCAGCAGCGCCTGGGAGCTCCCGTACCGTTGGTACACATTCACGG
CGCTACACAGGACACCGCGCCAACCCCGAGCGCCGAGCCCGAACCTGTAACGGATGATATGCGTGCCCGCCTGAAC
ACGCTGCAGGCCGATACCGTTCGCAGCGCGGTCACCGCTGCCGAGGTCAAGGCATTGAACCTGCCCGCGTACAACA
CTGACAATGCGCCAATGGACTGGCTTGCCTTACTGAACGGCGCACACATACTGGACGCCGCAGGCCTACTGCCAGG
GCTTGACCGTGAGTTGACCCTGGTGGACGAACCCCAGGTTTGGTTAAGCCGTTCACTTGACACACTGCTGGCCGCG
CAAAAACTCAGCGATGCCGCCAAGCAGTCCAGCAAGCAGCAACTCCTCGCCCTGCTGCTGAACGCCCATGACCGGC
AAACCCAACTACTGGCGGATCTTTTCCAAGAAGTCGCCCAACTGCCGGCAGAACGTGCCGTGGCCGTGATCAACTG
GGCGAAAACGTCGGTGTATGCCGTGCTGCTCGATGCCCAAGACAACGATAACGCGCTGATAGAGCAGGTCCAACGC
ATTACCCGGCACGCTGAAATCGTTGTGCAACTGCGCTTGAGCAACCGCGCGTTACGCCTGTTTGTCTGCAATCCCC
AGTGGCTGGGCGACTACTCACTCACCAACAGCGAGCCCAGCCTGGCGGATCTGTACCTGTTCGAGCGCTTGAGTCA
TTGGTTGTATGCACAGGACAAACCCGAAGACACCCTGCTCAGCTACTTCAGCCTCGCCAACCCCGTGCTGAAGCGC
CCGACAAAAAAAATCCGTCAACAACTCGCCACCGACGCCAATCTGGAGCTGGCTCGGCTACTGGGTTGGTCGACAG
AAGAGGTCGCAGTACTCACCGCGCGCCTGCCCGATGCCATTGCCAAGTCGATGGCCCAGGTGGATTGGGTGCGCCG
CTGTCAGGCAACCTGCAAAGCCAGTGGGCTATCGGCCAAAGCGCTGCTGCAAGCCACCGCGTTGCAGGTCGACAGC
AGCCTGGATGACTGGAAAATGCTGGGCGACGCCGTGACGGCCGCCAATACCCAAATGCAAGCGTGAGGTGGACGA
TGGCGACTTCCTTCGACGGCACACTCAACGAATCCCTGCGCGATGCCATGCTCGCGATGTATTTGCACGAAGCCGT
GCCGAAGGATGCCTACATCATCGAACAAAACCTGGTCGATCGTATCAAGACGCCCAGCGACCTCTACGAATACTGG
TTGCTGGATGTATTGGTCAGTCAAAACGTGCCCACCAGCCCGGTCGCATGCGCAATTGCCAGTCTGCAACAACTGG
TCAACAGCATGATGTTGAACATGGAACCCGGTTATCGAGACAACAGCCTGAGCACCGAGCAGGTCGACACCTGGCA
GACGGTGCTCAATCACTATCCGATCTGGGCAGGGTTGCAGCAATTGCAGGATTTTCCGGACATCTACCTGGACCCG
ACGCTGCGTAAAACCAAGACCGATAGCTTCAAGCAGTTTGAAGCCGACATCACTCAGGCCAAAATTCAGCCTGACA
CCATCATTTCAGCCCTCATGGCCTACCTCGCGCGGTTCGAGGAAATTGCCAACCTGAACATCTGCAATGGCTATAT
CGACGGTGACGATTTCGCCAACAGCACCTACTACTTCATCGCCCGATCGCCGGCCGAAAATGCTTATTACTGGCGA
TCACTGGATATGCGTCAACGTCCGGCAAAGGCCGCGGCGTCACGGGCCGGTGAGGCTCCCTTCAAGTATGACAAGC
CGTCGCCCCATGCCTGGACAGATTGGCAGAGGGCCAATGTACCGATCTCCGATAAAGTCCTTGAACATACGATTCG
CCCCTGCTGGTTCAACAATCGGCTGTTCGTGGTGTGGGCCCAGGTGGAGTACCCGGACACAGAATTTTTCAGTCCA

CAGGCGCGGGAAGGCACAGCTCAGGAAACCAGCAAGCCCTACCCGCTGTTCCGCCTGTACGCCAGCTACAAGAAAT
ACGACGACAGTTGGAGCACCCCCCGTGTGTATGCCGAACACACCTGCAAGACACCTGGCCTGATCATCCAGACGCC
CGAAGAGATTGCTGCAAACACCCATACGATCGCGGTCTACGACCACTCGACCTCTCCTGAGTCACTAGTACTGATG
GTGTACAGCGGCTACCGACAAAACGCCGACCCTAGCGGAGACAAAGATCAATACGACTTTTTAAGGTCGGTGCGCG
TCGACAAAAATTACAAAGTCACGCCATTGTTTCCCGCCGCTGGCAGCGTTCTGCCGCCTTTTTCCGAGGAAGCTCT
GGAACAAGACTCGAACAGAGATCACGTGCTGTTGATCGGCCATACCTTCGCCAGTAATCAAAAAAATCAAGGCCGC
TTTCAATACTGGCTCCCTTCCAGCGTCCCGGTTTTTGGCTCCCTCGTGCACGGCAATCCACCTGTCGATTCGACAC
TCTGGAATTTTGAGCAACTGCAATCGAATGTCAAAACGCTCGAGAAGGACGTTGATATTGCCTATGACCGGAAAAA
CTCAAACATCGAGTTGAACGTGCGCCTTCAAGGCAGCTTCGAGCATGTAAGAGAGGAGAATTTCATCCTTTTCGAA
GACGATCAAAACACGCGCCCGGTTCTGGAAATAAAACTGACCTTCAACGAAACCAGCTTGCACAAGGGGTCGTATA
CGCTACTACCCGGTTCTGAATTGATACCTCTCGACAGTGATTTTTTTGACCGCCAAGGTGGGATCTACCTAATTTA
CGTTTACGCCTCTCGAATCGTGCACGCCATAATTCTCGCAAATGACGGGAAAGATAGGTTTGAGTTGCCCCGTACA
GCTGCGGGGCAAACAGCCTCCCTGGCGGGTAAACGTTTGGCAAAAAAAGGCCTTGAGCTGGCGAAAAAAAACCAGG
CGTCATTCGATGTAGCGCGCCTAAAACCCGATGAGTCCTATCTGGCCGTCAGCAAAAACAGGCATCACACAAACGC
GGGGCCGTTTTTTTACCAGTGTTTTGTCGGCCATCCGCTGGATGTCCAAACCAGCACACTCCCTACGACCTTTGCG
CAAATACGCCCGCTGTACGCGAGCGGCATCGAACCTGCCTTGCGCAAAGATTCCTGGCTTAGCTACAGCTTTTCCA
TCGACCAAGTCACCCATCAGGCCGGCGGCTGGCCAGTGATTTGGCCCGAGAAACAAGAAGACATCAAAATTCCGTT
GATCTATGGAGTATTGATCTATGAAGAACGTGCAGCGTTTCCCCCCATGCATCTGGTGGGTGGTGCATTAAAAACT
GCGGCCATTACCTGGGGCGCCGAACTCAATACCGAGCTTCAGATCGCTCCCAGCATCAACAGCCTGACGGACCCGA
CCCAAGGTGACAGTCCGTCCCTGGGCAGGGCGCAGTTCATTGACTTCACCGGCTCCGCCATTGCGTTCAGCGATGG
CGAGTTGAGCCACAAAAAGCCCCTGCGGCGTGACCCCATCCGCATGAACACGACCTTCGTGCGCCATCTGATCGGC
TTGGCCGAATCCAGTATGGAGTCCCTGCTCAACTGGAATACCCAGCATTTGCCCGAACCGCCGATCGGGGGCAGCC
TCGGTGCCGAACCCATGGATTTTTCCGGCGGGTATTCCCTGTACTTCCTGGAGTTGTTCCTGTACTTGCCATGGCT
GGTGGCCCACCGCTTGAATCAGGAACAACAGTACGACGACGCTAAACGCTGGCTGGCCTTTTTGTTCAACCCCGCG
CGCCAAAGCAATGAGCAGGGACACCCGGGATACTGGCAGTCCGTACCACTTGAAGAGGCGGTATGGCCGACCCAGC
CAGATCCCAGCCAGGCGGTGCTTTATCCCTACGACCCACACCGGATTGCCCTGAGTTTCCCGGTGCATCTGCGCAA
GGCGTTGTATGGGCTCTACATCGATATCGAATGCAACCAGGCCGATGCCGCTTACCACGAGCTCACACCCGACGGG
TTGGCGCAAGCCAAGCTGCGCTACACACATCTGCTCGATATGCTGGGACCACGCCCAGATGTAAGACAGGCAGACG
ACTGGGTGCCGGTGACGCTGGATCAACTGAGTACCAGCATCAATCCACACCTGCGCACGTTCGAGCAGCAGTTGAT
CAATGCTCAGCAATCGCTGCACAAGCATCCGCCGGTACATGTCGGCCCCGCCGCGGCCTCGCCTCAGGCACCACTG
CTGTGTCTGCGAGCGTTTACGGCGACGCCCGAATTATCCGCCCTCGACAACCCTTACCTGCGTCGTCCATTCAACC
CCGAACTTATCCGGCGCTGGGAGCGGATAGAAAGTCGCCTGTACAACCTGCGGCATAATCTGAACATAGACGGCAC
CCCCTTGAATCTGCCGCTGTTTGCCAGCCCGCTCGACCCTCGCACGCTCCTCAGCGCCCAGCAACAAGGGCTCATC
GGCCCAGGGTTGAATCGCTCGCTGACGCCACAGATTCCCCATTACCGCTTCAGCGTCATGTTCGCCCTTGCCCAAA
ATGCAGTCGACGGCGTGATTCAATTCGGCACGACCCTGCTCTCGTTGATCGAGCGCAAGGAGCAGGCGCAATACCT
TGAGTTGCAACAGCGACAGGCGTGGAATCTGGCACGCGTTGCCGTTGATATCCAACTGCAACTGGAAAAAGTAGAT
GAGGCCAACCGAGAGGCTATGCGAGCCTCCCTGCACATCGTCCAAGGACGCGTCAGCTATTACGACAAGTTGATCA
ACGATGATGTAAGTGTGGGGGAAAGGCTCGTCGGCGGCCTGCTGCTCAATGCCCGGGTGGCAGAGACCGGCCTGCC
AGCGCTGGGTGCTGAGGCTGAATTCTCCAAGCTACCACCGAATATTTTTGGTTTTGCCGTTGGTGGCCAACGCATG
GAAGGCGTCTCGCAAGTGCCCATGTATCTCTTGCAAGGGCTTTCGAGTGCCTTGCACGGCCAGGCCGAGGTCCTTG
CTCAACATGAACAATTCCGGCGCCGTACCCAGGAGTGGAACCTGGCGCGCGACCAGGCAAAACTGGAGTCGAGGCA
TATCGAGATGCAACTCAAGGTCCTCGAAGAACAACACCGCGCGACACACTTACAACTGCTCCAGGCACAAACCGCG
CTCAACCAGGCCCAAGCCACCCATGCGTTGCTGCTGGGCAACCAACGGTTCAGCCAGTCACAAACCTATGACTGGC
TCAACAGCAAATTCAGCGGTTTCTATTACAGCGCCTTCAACGCAGCGCAATCCATCTGCCAGGCTGCCGAAGCCTG
CTGGCAATACGAAACCTGCCAGTTCGACCAGACCTTTATCCTTCCTGGCGCGTGGAACAACAGCTACCGCGGCCTG
GGTGCAGGTGAGGCGCTGAAAATGGGTTTGCAACAGATGTACCGCGAATACGTGCAGCACAACGTGCGGGATCTGG
AAATCCGTAAAACCCTGTCGCTCAAAGACCTCAAGGCCAGGGACCCGGACTCCACGCTCAACAAAAGCTGGGAAGA
GGTCAAAGCAGCACTGCTCAACACCGGCAGTTGCGAATTCGAAGTGACGCAAAAAATGCTCGACGATGACTACGAT
GGGCAAAACCATTACCTGCGCCGCATAAAAACCATCAGCGCGTCCTTCCCTGTCGTGCTCGGGCCTTATGAAGACA
TCAGGGCAATTCTGACGCAGTCCTACAGCAAGGTTGAACTGTTACCTTCCGTGGGCACAAACATCAGGGAAAACCT
GAAGGCCAACCAACAGATCGCGCTTTCCCATGGGGTTAACGATGACGGTCAGTTCCAACTCAACTTTCAAGATGAG
CGCTACCTGCCGTTCGAATACACCGGAGCCGTTTCCCGTTGGCGTTTGACCTTTCCAAACGCGCCGGAACAAAGAG
GGGCCCTGAACTCGCTGACCGACATCATCTTTCACCTCAGCTATACGGCAAGATCCGGCGGGAGTATGCAATGACG
GGGCAAACACCGCTGCAGATTGAATCGCCCGCCCTACCCAAAATGGGCGGCAGCGTCCAGAGCATCGGCAAAGGCT
GGGGGGCCATCGGCACCGGGGGTGAGGCCGGCCTGAGCGTGGCATTGCCGATTTCCCAGGGACGAGGGTTTGCTCC
GACACTGGAGTTAGGCTACAGCAACCAGGCCGGCAATAGCATGTTCGGCATGGGATGGACCGTGAACGTCAATGCC
ATCACCCTGCGTACCACCCAAGGGGTGCCGCACTATGACGGTACCGACCAGGTGGTCGGGCCCGATGGCGATGTAT

GGATGCCTGAGCGTGCCGATGACGGCAGCCTGATTGCCAGCACCATCTCGACCTACAACGGCGTCTACACCGGCGC
GCCACACCAGGTGGTGCGCCACTGGCCCAGGGTAGAAGGCGAACATGCGCTGATCGAACATTGGTCCTGCGCCGAG
GATCCGTCTGGGTTCTGGCTGGTGCACAGTGCAGACGGCGGCCTGCACCTTTACGGCAAGAACGCCGAGTCACGCC
GCTTTGCCCCCCTAGCCCCGGAACATGTCGGTGCCTGGTTGATCTGCGAGAGCCTCAACCCCTTCGGCGAACATAT
CGTCTATGAATACAAACACGAACAGGAACCCCCTGCCCCGCCGGACATGCGCGATTATCGCGCCCAGCGTTACCTC
AAGCGTGTCCACTATGGCAACGCAACAGCCTACGAGCATCTATACGGCTGGAACGAGGACAGTTGGAAGCAGCAAC
ACTGGCACTTTCACTTGGTATTCGATTACGGCGAGCACAGCACCGACCCGCAGGCCGCCCCCACCTTTGACGAACA
GCGTGCCTGGCCCGCGCGCGCCGACCCCATCTGGAACCAAGCCTATGGCTTTGAAATAGGTACCCGGCGCCTGTGC
CGGAACATACTGATGTTCCACGCATTTGCCGGTGAACTGGGGCCGGTGCCGATGCTCGTGCAGCAACTGATGCTGG
AACATCGACAGAGCCCGTGGGGCTACAGCCTGCTGGCCGCCACCCATGCGCTGGCATACGACAGCCTCGGACAGGC
GGAGCGCCGGCCACCGACCGAGTATGACTACACTCCCTTCACCCTGTCCCCCAGCGCCCCAGGCTGGTCCCGGTTC
GACGCGATGCCGGGGCTCGACGACGGCTATTCTTACCAACTGGTGGACTTGTACGGCGAAGGGCTGCCAGGAGTGC
TGTCTTGCCAAGACAAGGCTTGGTATTACCGCGAACCTCTACGTGACGCCACCGGGGGCGACCAGCTGCGTTATGG
CGAATGGGCGCGCCTGGAACATATCCCCGTCGCCAATACCGGCAAAGCAGTCCATCAATCACTGGCCGACCTGACC
GGCGACGCCAGGCTCGACTGGATCGTGTCGGCGCCCGGCATGCATGGCTACTTCACGCTGGACCCCGATCGCGAAT
GGTCACACTTCGTGCCCTTCAACGCGTTTCCCAAGGAATTTTTCATCCTGCCGCGCAGATGGCCGACCTGATCGG
TAACGGCCTGAGCGACATGGTGCTGATCGGTAGCCGCAGCGTGCGCCTGTATGCCAACCGACGTGACGCGGGATTC
GCGCCGGGGCTCGATGTACCTCATCCGCAGGACCAGTACGAGGGCGATGAGCTACCGCTTGCAGGTACCAGTCCCA
CGCAGTTGGTGGCATTCGCCGATGTGCTGGGAAGTGGCTCCGTGCAGTTGGTGCGCATACGCCACGATGAGGTCAA
ATGCTGGCCGAACCTGGGGCACGGGCGTTTCGGCAAAGGGTTTGTGCTATGTACGCTGCCCTTCAACTCTACCCAG
TTCAATGCTGAACAGATACGGCTGACGGATCTTGACGGTTCCGGCGCGGCCGATCTGATTTGCCTGACACCCGATG
CCGTCAAAGTATTCATGAACCACGCGGGCAGCGGCTATCTCAGCACCCCGGTGGAACTGCCTTGGCCCGACGGGGT
GCGCTATGACCGTTTCTGTCAGGTCAGCACCGTGGACCTGCAAGGCATAGGATGCGCCAGCCTGGTGTTGACCGTA
CCCCATATGACACCGCGCCATTGGCGTTACGACTTTGTAGACTCCAAGCCCTATTTGCTGAGTACGACCTGCAACA
ACATGGGCGCCCGCACCCATGTGACCTACCGCAGCTCAGCCCAGGAATGGCTGGATGAAAAGCAGGAGAAACAGAG
CGATGCCCTGTCCGCGCCGGTCGCCCACTTGCCGCTGACCCTCAATCTGGTTAAGCACATCGCACTGATCGACGAA
ATCAGCGGCACCCGTCTGACACAAAGTTACGCTTACCGAGCTGGCCATTACGACAGCGTAGAGCGCGAGTTTCGTG
GCTTTGGCCTGTTGTTGCAAACCGATACCGAAGCGACGCCATCACAGCGTGTTCAAGCAGGCTTCAGCGCGCCGCT
GCTGAGCAAGACCTGGTTCCACACGGGCATCGAGATAGACCCGCCCTGCGACGGCAGTAGCGTCCATGACCCGCAG
GCCGTGCCCCTCAACCCAACGCTGGTGTGCACCGGCCATCCCGACGACAAAGCCACGCGATTACCCTGGGCACCGG
ACGAGCAGATGGCCCGCGAAATCGCGCGCAGCTTGAGTGGCCGGGTATTACGCAGCGAGCTGTTCAACGCTGACGA
CCCGCGCGCGCCCTATGCAGTCATTGAACACCGTGCGTGTGTTGGCATACTTCGGCCCAAGGGCCCGCACCAACCG
TATGCGGTCGTCCAGCCCGATAATCTGGAAACCATCAGTTACCAATATGAACCACACATCCCCGATGATCCGCTCT
GCCAGCACCTGCTCAATCTTGCGTGGGACGAATACGGCAACGTCACACATGCCGTCACCGTGTACTACGCCCGGCG
CAAGACCGCCAACGACGAGCCGCCCTTCAGCGACGAACATCAACAGCAATGGTGGCGTGACGCTCATGATGACGCA
CAGCAAAAGTGGTACCTGACCCAGGAGAAGGCTCAGTACATTCACCACTTGGGCGAGGCGCAAGCCAACCTCCAGG
CCTGGCGCCTGAACCTGCCTTACCTGGCGCGCAGCAACGCGCTGGTGCTGGAAAAATCGACCCTGAGTGCAGCCGA
CATCAACCATGAGAACCTGCGGGTCCGAACTTTGGACAACAGCGAATGGGCCAGGCAAGCGGTACTCACCAGCATG
TCCCGTCAGTTCTACATGGACACGCTCAGCAACCAGCCGTTGGCCGAGGGCAAGGCTTCATTTCAGGCCCTGGTCG
CCCACCGTGAAAGCGCAGAACTGGACGCCAACGCTTTGGGGGCTTATGACCGACTGCTCGAAGAACGCGCCAACCC
GCCGTTCAACCTCAAACAGCTACTGGAATCACCGCAAGTCGGTTACCACATCATGCAACCTTGCCTTCCCACTGTG
CCGAACCGGCCCTTTCAGCACAGCGACAATGCGAAGGATTATTTGTGGTGCATCCATCAGGGGTTCCCCGAGTACG
AGGGGCCAAGCGGCTTCCATGCCCTGCGCAGCTACCGTCAAACCCGCAGCCATGGCGTCACCACGTTGACCCGTGA
CCGTTACAACTGCTTGATCACTGCCGTACAGCTGCCTGATGGCTGTACCACGCGTATCCTCGACATCGACTACCGG
ACGTTGCTGCCCGGCACCCTCGAAGATGCCAATCGCAACCTTCAACAAGCACGCTATACCGGTTTTGGTGAACCCT
TCGTCACCACGGTCCACGGCACTGAAAAGGGCGTACCCAGCGGGTTTCATCCCTTGGACAGCGATACCCTGACAGG
CGATCGTGACCCGCGTGTCGCACTCGAAAACAAGCAGAGCGCCTTGGGTAAATTCGCCAGCGCCGGTTTCTGGGAC
CTGTTCAGTTGGATGGGATGTATAGACCGTGCGGTGTTGCCTTTGCCCGCATGGATGAACTGGGCCACGCAAGAAG
GTTTTATCTTGCCCAGCGGACACTTTTATGACCGTGCCCGGCGCCATCTGGGCACCCTGAAAAACCCCGATGCTAA
TGAACGACTTCTCAAAGAACTGATTGACGCGGCGCCCCGTGAGCCGGTGTACAGGGTCACGCTGATAGCCGACCAG
TTTGCGGATCAAGCAGCACCTGTGCAGATACGCGCGAGCATTACCTGCCTGGACGGCTTTGGCCGAACCTTGCAAA
CCAAGCAGGAAGTCGAGCCCGGCATGGCCTGGTGCGTCGGTGACGACGGCGCACTGTTGCTCAACCCCGATGGCAG
CCCGAAGGAAGCGCTGGCAGCCCGGCGCTGGCGGATCAGCCAACCGGTCGAATACAACAACAAGGGGCTGGCGGTG
CGTGAGTACCGTCCCTATTTCGCGGATAAATGGCGCTACATCAATGACCGCTCAATGCGCCAACACGCCCTGCATG
ACCAGCAGTTTTACGATGGCCTTGGTCGCCCGACCCACACGGTGCTGGCCAGAACAATGCTCCAGGGCGACCCGGC
CCAGGAACAGCCATTGCGCCGCGAGACCTGGTACTGGCTGTGGAACACAGTAGCGTTCGACGAAAATGACTTGTTT
GTTCCACCTGCCGCCGCGCGACGGCATCAAGTGCCTTGAGAGCATGACACCATGAACCTCTGCACACCCACCCTCA

ACGTGATCGACAACCGCAGCCTCAATGTCAGGCAAGTACACTATTGTCAGTTGCCCAACGCCGCTCAAGCCCAGAC
GCGCGTGCACCGCCGACACTACGACGCCGCCGGACACCTCTTGAGCGAACACGACCCGCGCCTGAGCGCCGCAACC
CCTAATCAGACCCACCTGCACAGCCTGAGCGGCGCGCGCCTGCTCAGCCAGAATGTCGACGCTGGCTGGCGCATTG
AACTGCGTGGGCAAGCCGCACAAGTGGTACGCCAATGGGACCAACGCGGCGGTCAATGGCAGACCGACTACGACCC
ACTGCTTCGCCCCATCGCTCGGCACGAATGGTCTGGCCAGCTACCTCGTCGCACCTTGGAGCACTTGGTCTATGGC
GATAATTCGCAAACGTCAGCCCAGCACAATCAGTGCGGTCGACTGATTCAGCATTTTGATACTGCCGGCTCAGTGG
CCATGCCCGACTACGGGTTGTTATCGGCGCCCCTGACTCAAGTGCGTTGCTTTGTGCAAGGCCTGGAACCGGTGGA
CTGGCCGCTCAGCCAACTGGAACCGAACGACCACACCACGCATTGGCAGTACAACGCGCTCAATGAAGTGCTAATG
CAAACCGACGCTAAAGGTCATCGCCAGCTATACGCCCTGGATATTGCAGGCCAACTCAAGAGCGCGTCTTTGCAGC
TCAGGCATGCAGCCGTGGCGCAGACTGTCATCGACACCTTGCATTACAACGCGGCAGGCCAACCACTGGGGCACAG
GGCTGGAAACGGGGTGTCGAGTCATCTGGCGTTCAACCCCAGCGATGGACGGCTGGTGCATATCAAGGCGGTGAAA
AGCGCTCAGGTGTTCCAGGACATGAGCTATGAATACGACAGCGTCGGCAACATCGTCTCCTTGAACGACCATGTTC
AGCCCACACGATACTTTTCCAATCAGCGTGTCGACGGTTCGCGGCAGTTCACCTACGACAGCCTCAATCAGTTGAT
CACGGCCACCGGTGCTGAAACTGTCGGCGCCACGACCCGGCCACAATTGCCCGACCTGATCACCCCAATCGATCTC
ACCCGACGCAGCCGCTACACCCAGCACTACACCTATGACGACGGCGGCAACCTGACCAAGCGGGAGCACATCAGCC
CGATTCCCGGCCAGAGCCACACCTGTGTGATGGACATTGCTCCCCAAAGCAATCGCGCAGTGAGCTGGACACGCAG
CGACTCGACAGCCCTTACGATGGATTACGACGTCAATGGCAATCTGCAAGCATTGCAACCCGGCGCACAACCCTTG
CAGTGGAACCTGCTGGACCAATTGCAACGCGTGACGATGTTGCACCGAGAAGAAGCCGAAGACGATTTTGAACACT
ATCTCTACGCCAGTGACGGTCAGCGTATGTACAAATGGCGAAGCACTCGCGCCGCTTCGGTCACCCATATTCACAA
GCAGTACTACCTGCCGGGGCTTGAAGTTCGCACTCAAGACGGACGCGAATCGCTGCATGTCATCAATCTGCAGGCG
GGCATTGGCCAGGTGTGCTGCCTGCACTGGCCTGAAGGGGCACCCGACGGTATTGAGCAAGACGCCCTGCGTTACA
GCCTGAGGGATCATCAGGACTCCAGCCTGATCGAGCTGGACGGTGAAGCTCAATTGATCAGCCAGGAAGCGTATTA
CCCCTTTGGCGGCACAGCGTGGTGGGCCGCTCGTTCACAAGTGCATGCCAGCTACAAGACACTGCGCTACAGCGGC
AAGGAGCGTGACAGCAGTGGCCTCTATTACTATGGCGCGCGTTATTACGCCCCCTGGTTATCACGCTGGATCAATG
CAGACCCCAGCGGAACCGCAGATGGTCTGAACCTGTACTGCATGGCTCATAACAACCCGATCAACAAGGTCGACGG
CCGGGGGGAAAACGCCGAAATTGCCTACCTGATCGCAGGCACTGCCACCCTGGTCGCGATTGGCGCGGCGGCCCTG
CTTAGCAAAAAAGCGTCACCGTCGCTGGGTGCTGAAACGGTGCTGCCACCGCTTGATTTTGAACTGCATCCGACCG
AGCGGGAAAACCTGCAGGCGTTCAATAAACAATCTCCCACTGGCCAGTCCGTAGAGGTCAGAAAGCTGGGGGATGG
ATCTGTATGGGCCTATAGACCCAAGAACCTCACCCACGCCACAAACCTGTCGAATGCCAGTGACATCAACTTACAA
AAGCAAATCAGCAAGCCGGGTTACCCCGCCATCAAACTGCGGGAGGCCCCACCACCACCGGCGAAGAAGGCCGCTG
CACCCACCCCCTACACAGCATTTGAAGTCGCTACGACAACCAAAGCCAGTTCAAAAAAAGCCGAACGCATAGCGGC
TGTCGAAGAGGTTGTGCAAGCCAGCGTGCAGTCGACTTCCACCGGGCCGGTTGGCGCCGAGGTCGACGAAACTCAA
TTTAGACTCAGCCGCCACTTTCAAAAATGGTCCCCAGAGCAACAAGCAAAAATAACAGGTGTGCTGGACGAAATCA
GAATCTCTAGGTTTGCAGCCAACAATCACAGATACAGCCACGACAGAGAAGTGGATCAAAACATCCCAGTCGAGAT
GGGCAAGCCAAAGGTCATGCGCGATATCCACACCGTGGACGTCACCTTCTTTGATGGCGCGGCAGGCGGGCGAGGC
GATTGGCGATTGGTCATGTACCTGATCGATGGGGTGTACTACCCGCAACGCATGGCTAGCCATAGGGATATCGTCA
ACCGGGCCAGGAGATAGACATGCCTTGAGTGACCAAACCTGCTGCAGGCCACTGCTCACATGCTATGGTGCGATCC
CCGTTTCCATCGTTAGCCATAGCATGATGCTGACTTCCCGCCCGTTGCGCTTGACGCTGTACACTTTGCTGATCAT
CGCTGGCGCCATTGGCGCAGCCACACTGGCCATCCGCCATGCCGAACGCCAGGCTCTGGTGGATGATGCTGCCCGC
GCCAATCAGCAATTGGCCTTGTATGCCAATTCCTTGCACACCCTGATCGAACGTTACCGCGCCCTGCCCGCCGTGC
TGGCCCTGGACTCGGAAATGATCCGCGCCCTGAAAAGCCCGCTGGATCCGGCGACCCAGGAGGTGCTCAACCACAA
ACTGGAGCGCATCAACGGCGCTGCGCAGTCTTCGACCCTGGAATTGATGGACCGTACCGGCCTGGCCGTGGCTGCC
AGTAACTGGCGCTTGCCCAGCAGTTATGTCGGCCACAACTATGCGTTTCGTCCCTACTTCAGCCAGACCCGCAGCC
AGGGCACCGGGCGTTTTTATGCGGTGGGGGTGACCACCGGGATTCCGGGGTATTTCCTCTCCAGCGCCGTGGTGGA
TGAGCAGGAACAATTCCTCGGTGCCATGGTGGTCAAGCTGGAATTTCCGGAGCTGGAGCGCGAATGGGCCCAGGGC
GACGACCTGCTGCTGGTCAGCGATGCCCGGGGCATCGTGTTTATCGCCAATAAACCCGGATGGCGTTATCGCAACC
TGCGCCCGCTGTCGGCCAACGATCTGGCCGAACTCAAGGCCACGCGTCAATACGACAAGAAACAACTGCAGCCCCT
GGACACCCAAACCTTGCAGCGCTTCGACGAAAACAGTCACCTGGTCCGCGTGGCCGGCCCTGAAGGCAACGCCAAC
TACATCTGGGAATCCCTGCCGCTCAAAGCCGAAGGCTGGACCCTGCACCTGCTGCGCAAACCCCAGGTGGCCTTTG
AAGATCAGCGTAACGCCGGCCTCGCCGCCGCCGGCCTGTGGTTGGCGCTGGTGTTCCTGCTGTTGTTCCTGACCCA
GCGTTGGCGCCTGGCCCGCTTGCGCCAGCGCAGCCGCGAAGAACTGGAGCGCCTGGTGCAAGAGCGTACCCAGGCC
CTGCGCACCGCCCAGGATGGCCTGGTGCAATCGGCCAAGCTGGCGGCGCTGGGGCAGATGTCTGCGGCCCTGGCCC
ATGAAATCAACCAGCCGCTGACCACCCAGCGCATGCAACTGGCGACCTTGCGCCTGCTGCTTGATCACGGGCGCAT
CGACGACGCGTACCAGGCGCTGACGCCATTGGACGACATGCTCACCCGCATGGCCGCCCTGACCGGCCATCTGAAA
ACCTTCGCCCGCAAAAGCCCCAGCGGCCTGCGCGAACGCCTGGACCTGGCGACCGTGGTCGACCAGTCTCTGCACT
TGCTCGACGCCCGCCTGCGCGACGAAGCCATCGGCACTGTGCTTGACCTGACCCGCCCGGCCTGGGTACGCGGCGA
CGCCATTCGTCTGGAACAGGTGCTGATCAACCTGCTGCGCAATGCCCTGGACGCCATGGCCAACAAACCGCGCAAG

CGCCTGGAAATCCGCCTGCACGCCGATCAACAACTGTGGCACCTGAGCGTCAGCGACAGCGGCGGCGGCATCGCCG
AGGAGCACCTGAACAACGTGTTCGACCCGTTCTTCACCACCAAGCCGGTGGGTGACGGGCTCGGTCTCGGGTTGGC
GGTGTCCTACGCTATCGTGCATGAACTGGGGGGGCGCCTGAGTGTTGCCAATCAGGGCGACGGCGCCGTATTCACC
CTGACCCTGCCCATCGCACTGGAGGCGCCAGACCTATGCTGAACGCGGTGATCGTGGTTGATGACGAAGCCAGCAT
CCGCACGGCCGTGGAACAGTGGCTGAACCTCTCCGGGTTCGAGGTGCAGTTGTTCAGCCGCGCCGAGGACTGCCTG
GCGCAACTGCCCAGGGATTTCCCCGGGGTGATTCTCAGTGACGTGCGCATGCCGGGCCTCAGCGGCCTGGAGCTGC
TGGCCGAGGTGCAACGACGCGATGCCGACTTGCCGGTGATCCTGCTGACGGGCCACGGTGACGTGCCAATGGCCGT
CGAGGCGATGCGCGATGGCGCCTACGACTTCCTGGAAAAACCCTTCAGCCCCGACGCCCTGCTCGCCAGCCTGCGC
CGGGCCCTGGACAAGCGCGGGCTGATCCTGGAAAACCGTCGCCTGCACCAGCAGGCCGATCATCGGGCCAAGCTGG
AGTCGAGCCTGCTGGGGGTGTCCCGCAGCCTGCAGACACTGCGCCGCCAGGTCCTGGATTTGGCGGCCCTGCCGGT
CAACGTGCTGATCCGTGGCGAGACCGGCAGCGGCAAGGAACTGGTCGCCCGCTGCCTGCACGACTTCGGGCCCCGG
GCGAAGAAGCCGTTTGTCGCGCTCAACTGTGCGGCAATTCCCGAGCAACTGTTTGAAGCCGAACTGTTCGGCCATG
AAAGCGGCGCCTTCACTGGCGCCCAGGGCAAGCGTATCGGCAAGCTGGAGTACGCCGACGGCGGCACGTTGTTTCT
CGACGAGATCGAAAGCATGCCCCTGGCCCAGCAGGTGAAACTGCTGCGGGTGCTGCAAGAGCAGAAGCTCGAGCGC
CTGGGCTCCAACCAGAGCATCAAGGTCGACCTGCGCATCATTGCCGCGACCAAGCCCGACTTGCTGGACGAAGCCC
GCGCCGGGCGGTTCCGTGAGGACCTGGCCTACCGCCTGACCGTCGCACAATTGCGCCTGCCGCCGCTGCGCGAACG
CCGCGAAGATATTCCGCTGCTGTTCGAACACTTTGCCCAGAGCGCCGCCGACCGCCTGGGCCGCGCGGCCGCCCCC
CTCAGCGGGCCGCAGCTGGGGCGACTGCTCAGCCATGACTGGCCCGGCAACGTGCGCGAGTTGGCCAACGCTGCCG
AACGCCAGGTACTGGGCCTCGGTGAGCCGGAGCCGGAGGGTATCGAGGCCGGGCAGTCCCTGGCAGCGCAGCAGGA
AGCGTTCGAGGCGCACTGCTTGAAAGCCGCGCTGACCCGGCACAAGGGCGATATCAAGGCGGTGCTGGCCGAGCTG
CAACTGCCACGGCGTACGTTCAATGAAAAGATGCAGCGCCATGGGCTGACCCGGGAAATGTTTCTGTCGATGGACT
GAGGGGGGCTGCTGGAGGCCTCATCGCCGGCAAGCCAGCTCCTACCGAATGTATTCGCAAATCAAAATGTAGGAGC
TGGCTTGCCAGCGATAAGCACAGCCGCAATAAGCGGATTTCCGCTCATCACCCAGAAATCATCAGCGACTTTCCGC
TCAAAAAATCCCCCCCAACCCTTCTAAACCGGGCCTTCACCTGCTTGGCACAGCTCCTGCTATAGCTTGCGCCAGG
CTGCGCTGACGCGCGCTCCACAAAAACAATGACATGAAGGATCCTTCAATGGATAACTCCAACTCCCTGCCTCTGG
GGTCGGCCGCCGTGCCGGCAAAAGAACGCACTACCTCCAGCCGGATCAAGTCGATCTTCAGCGGATCGGTCGGCAA
CATGGTCGAGTGGTACGACTGGTACGTCTACGCCGCCTTCTCGCTGTACTTCGCAAAAACCTTCTTCCCGAAAGGC
GACACCACTGCTCAACTGCTCAATACCGCCGCGATCTTCGCCGTAGGCTTCCTGATGCGCCCGATCGGCGGCTGGT
TGATGGGGCTGTACGCTGACAAAGTCGGGCGTAAAAAGGCCTTGATGGCCTCGGTCTACCTGATGTGCTTCGGCTC
ATTGCTGATTGCCCTGAGCCCGGGCTATGAAATCATCGGTATCGGCGCGCCGATCCTGCTGGTATTTGCCCGTTTG
CTGCAGGGCCTGTCGGTGGGCGGCGAATACGGCACCTCCGCCACTTACCTCAGCGAGATGGCGACCAAGGAACGTC
GCGGTTTCTACTCCAGCTTCCAATACGTGACCCTGATCTCTGGCCAGCTCATCGCATTGGCGGTGCTGATCGTGCT
GCAACAGCTCCTCACCACCGAAGAACTGTATGCCTGGGGCTGGCGTATCCCGTTCGCCATTGGTGCCCTGTGCGCG
GTGGTGGCCCTGTACCTGCGACGCGGCATGGAAGAAACCGAATCGTTCACCAAGAAGGAAAAAGCCAAGGAAAGCG
CCATGCGCACCTTGATGCGCCACCCCAAGGAACTGATGACCGTGGTCGGCCTGACCATGGGCGGCACCCTGGCCTT
CTACACCTACACCACCTACATGCAGAAGTACCTGGTGAACACCGTCGGCATGAGCATCTCCGACTCCACCACCATT
TCTGCGGCCACGCTGTTCCTGTTCATGTGCTTGCAACCGATTGTCGGCGGCCTGTCGGACAAGGTCGGTCGCCGAC
CGATCCTGATTGCCTTCGGTATCCTCGGGACGCTGTTCACCGTGCCGATCCTCACCACCCTGCACACCGTCCAGAC
CTGGTGGGGTGCATTCTTCCTGATCATGGCGGCGCTGATCATCGTCAGCGGCTACACCTCGATCAACGCGGTGGTG
AAGGCCGAGCTGTTCCCTACCGAAATCCGTGCACTGGGTGTAGGCCTGCCTTACGCACTGACCGTGTCGATCTTCG
GCGGCACCGCCGAATACATCGCGCTGTGGTTCAAGAGCATAGGCATGGAAACCGGCTACTACTGGTACGTGACCGC
GTGTATTGCCGTGTCGTTGCTGGTCTACATCACCATGAAAGACACCCGCAAGCATTCGCGGATCACCACCGACTGA
CCAGCAAGATGCAGTAAACCCTGTGGGAGCGGGCTTGCCCGCGATAGCGGTGAATCAGTCAACGGATGTATCGACT
GACACTCAGTTATCGCGGGCAAGCCCGCTCCCACATTTGGTTTCGCAGTTCACACAATTGCGTGCACTATCGTGTC
CTTTCCGGAACCACCAGCAGGAACCCTGTCTATGCCCGACGATATCCATTTCTACGAACCCGCCAACGGCCACCGC
CTGCCCCATGACCCGTTCAACGCCATCGTCGGCCCGCGGCCAATTGGCTGGATTTCCTCCCAGGACAACGAAGGCC
GCCTGAACCTGGCGCCCTACAGTTTTTTCAACGCGTTCAACTACATTCCGCCGATCATTGGTTTTTCCAGCGTCGG
GCGCAAAGACAGCCTGAACAACATCGAGCAGACCGGCGAATTTGTCTGGAACCTGGCCACGCGCCCACTGGCCGAG
CAGATGAACCAGAGCTGCGCCGCCGTGTCACCCGAGGTCAACGAGTTTGAACTGGCCGGGCTGACGCCGGTGGCGT
CCAAGGTGATTGCCGTGCCCCGAGTGGGCGAAAGCCCGGTGTCGTTCGAGTGCAAGGTTACGCAGATCATTCAGCT
GCAGCGGGCTGACAAGGAACTGGTGCCCAGTTGGCTGGTGCTTGGCGAAGTGGTCGCGGTGCATATCGCCAAATGG
CTGCTCAAGGATGGGGTATACGACACGGCGGCTGCCGAGCCGATTCTGCGAGGTGGTGGGCCGGCGGATTACTTCC
AACTGGGCCCGGAAGCGCTGTTCAAGATGTACCGCCCGGGTGCCGGCAAACCCTGATTGGATCGAATGCAATTAAA
AATGTGGGAGCGGGCTTGCCCGCGATAGCGGTGTATCAGTCAACTCATGTATCGACTGACACTCAGTCATCGCGGG
CAAGCCCGCTCCCACACAGAACCCCACAGCTGGGTCGGTGGCGCTCTTTAGTTTGCTGCGGTCGCCCAGGCCAACT
GACCCTCTTCATCGATATCGATCAAGCGCTCCAATTGCTGGGCCGCTGCATCGTCAGCGTCATCAGCAGTCTTGAA
CAATTGGCCATCGAGGATCTTGTGAAAGCGCGGGGCGCTCATGCCATCCAGTGCCTTGACGGCGATGGCGGCGCTA

TAGCCCCCCTGGTCTTCACGCACTAGGGCGGAAACAGCTTCGTGGTGGGCAAACTCTTTACGTGCCATGTTGCAGG
TCCTGGCCAATGGAAAGTCGGCCATTCTAAACCCTAACCGGCCAATTGCAGGTACTCGCCGTAGGCATTGGCCGCG
GATGCGTCGGTGAAGGTCTGGAAGTCCATGCTGCGCACGACCATGTCGTTCAACAGCTCGGTAAACAGCATCAAGG
CCGGCGAGTTGAAGTAGGCACTCATCGCCTGTTCACTGCTCCAGAAACCTGAGACCAACCATACATCGGGGTCAAC
CTGAGAGTGCTGCAACGAGAACTGCAGGCAACCCTGAGCCTGGCGGCCCGGTTCGATCAAACTGCTCAAGCGCGCA
CCCAGTTCGGTGCTGCACCCGCTGCGGGCGCGGATAAAGGCCATATGGCTCGCGGGAATGGGGGTGGACATGTTCG
ACTCTCCCGTTGAGAAGTGATGCTCGGCAAGCACTGTGGGGGCGTGCTGCCACAGGATCAAAGATACGGGCGCCTG
CCCCTGGCCGGTTAGTCGATTCCTGCCGGCTTATTGCACAATCCTGCCAGAAGCCTGGAAAGACGGTTTGGCGCCC
GCCTTTATTCCACGCATAGGCGCCTTGTTTCAGGGAGATGACAGGCCATGGGCTTGCCTGATTCAAGACGCGCCGC
AGGATCAGGCAAGGATTGTGCAGAATCGACCTAACACGACTGGAACAGGCACCGCTAAGCTGTGCCCATCAAAGAA
GCATGCAGAGGACGCCCCATGTCGTCGCCCGAACAGCAGTCCATTCCCCTCGATGCCGAGATGGAAAAACAGCGCG
CCGAACTGGCCGGTATCGTGCACAGGCATACCTGGGAGGACGGTTCCTACGGCACGGCGATCACTTCGTTGTACCT
GAACCGCCACAACACGCCGCGCGACTTCATGCCGGTGCTGGTGGAACCCGCCCTGTGCATCCTCGCCAACGGCAGC
AAGGAGGTGCGCCTGGCCGACGAAATCTTTGCCTACGACCCTCTCAACTACCTGGTGTTCTCGGTCGCAATGCCGG
TGGCCGGGCGGGTCATCGACGCCACGCCCGAAGACCCCAACCTGTCGGTGCGGATCAATATCGACCCGGCGCAACT
GACCGCCTTGATTGCCGAAGCCGGCCCGATGGGCGTGCCTACACGCCCGACATCGCGCGGGATGTATGTCGACCGG
ATCGACACCCAGTTGCTTGATGCCGTACTGCGCCTGGCACGCCTGCTGGATACGCCCAAAGACATCGCGATGTTGG
CGCCGTTGATCAATCGCGAAATTCTCTACCGTCTGCTGCGTGGGCCACAGGGCTATCGCCTGTACGAAATTGCCGT
GGCCAATAGCCAAAGCCATCGGGTCAGCCGTGCGATCACCTGGTTGAACGGTAACTACGAACAACCGCTGCGCATC
GACGACCTGGCCAAGGAAGTGAATCTGAGCGTGTCGACCCTGCACCATCGCTTCAAGGCCATCACCGCCATGAGCC
CGCTGCAGTATCAGAAACAGCTACGCCTGCAGGAAGCACGACGGCTGATGATCGCCGAAGGCCTGGAGGCCTCGGC
GGCGGGGTATCGGGTGGGGTATGAAAGCCCGTCGCAGTTCAGCCGGGAATACAGCCGTTTGTTTGGCGCGCCGCCG
TTACGCGACTTGGCCAGGCTACGCCAGAGCATCTGACCCAGCGCTCCTACAGGGTGACGCGGTGTTGTTTAGTCCA
GGGTCCGGGGCAGTTGCAAGGTCACCCGTAGGCCACCTTCGCGCAAATTCTGCAGGCTGACTTCGCCGCCATGGCT
GTGGGCGATATTGCGTGCAATCCCCAACCCCAAGCCATAGCCCTGCTGCTGACCGGCCAGGCGAAAGTGCGGTTCG
AAGACCTGTTCCAAACGCTGTTCCGGCACACCCGGCCCCTCGTCATCCACATGCAGGATGAACTGCGCACCATCGT
CCTCGATACGCAGGTGAGCGTTCTGCCCGTACTTCAAAGCATTGTCGATCAGGTTGCCAATGCAGCGCTTGAGCGC
CAGCGGCTTGCCCGGGTAGGCCGTCAGCGCCCGACCGTGCTGCGTCACCCGCCCATTGCCATGGGGCGCCAGGTAC
GGCTCCACCAGACAGTCGAGCACATGGTTGAGGTCCACCGGCTCGATGTTCTCGTGGATATCGGTGTCCTTGACGC
ACTGCAACGCGCCCTTGACCAGCAGTTCCAGCTCATCCAGGTCACGGCCGAACTTGGCTTGCAGCTTTTCATCTTC
CAGCAGCTCCACGCGCAGCCGCAGGCGTGTGATCGGCGTGCGCAGGTCGTGGGAAATCGCGCTGAACAACTGGCTG
CGTTCGGTCAGGTAGCGGCTGATCCGCTCGCGCATGGCATTGAACGCACGCCCCACTTCCACCACTTCGCTACCGC
CACCTTCGGCCACCGGTTCCACGTCGGCGCCCAGGGACAGGTCCCGCGCCGCCCGCGCCAGACGCTTGAGCGGCCG
GCTCTGCCAATGCACCAGCAAACCGATAAACAGCAGCAGGAAGCCACTGGTCAGCACGATAAACCACACCTGTTGG
GAGGGCAGGCCCTGTTCTTCGAGGCTGGTGTAGGGTTCGGGCAGCAACGAGGCGATATACAGCCACTCGCCGGCGC
CCAACTCGATCTGCGTCACCAGCACCGGCGGGTTGACCGGTTCCAGGGTCAATGCGTAATGCGCCCAGGAACGGGG
CAACTCATCGAGCTTGAGCCCGGCATTGAAAATGCGCAGGTCTTCGGCACTGACAAACTCCACCGAAAAATGCACG
TCACTGCCCAGGGTCTGGCGCAGTACTTCATCCACGGCACCGAGCACCGCCTGTTTGCGCGGGGTTTGCGGCAGGA
TCTGCATATCCAGGGGCTTGTCGTTGAGCGTTACGACAAAGCGCGTACCGCCCATGCTGCGCAACTGATCAAGCAC
CAGCGGCCGATAAGCCACCGGCAGTGAGCGGAAATAGCTGACGCTGGCAGTCATCGAGTGGGCCAGGCTGCGGGCG
CTGGTGACCAGGCCTTCGAGCTGGGTAGCGCGCAATTGCGACACCCAGATCACACTGGATAAAGCCTGGGCAAACA
GCACTGCCAACAGGGTCAGCAGCAGCATGCGCCCCAGCAGTGAGCGCGGCACCGGAATACGCCGGCGCTCAGTGGG
CATTGCTGGCAACCACACTGGCCGCCAGTTGGTAACCGCTGCCACGCACGGTGCGGATCAGCCGTGGAGGTTTCTC
GGTATCGCGTAGGCGTTGTCGCAGGCGGCTGACCGCCATATCCACAATCCGATCAAGGGGCATCAGGTCGCGGCCG
CGGGTGGCGTTGCCAATGGTGTCGCGATCGAGGATTTGCTGCGGATGATCAAGGAACAGTTTGAGCAGGGCAAAGT
CGGCGCCGGACAGGATGACTTCCTCACCATCGAGGTGAAACAGCCGATGGCTGATCATATCCAGCCGCCACTCATC
GAACACCAGCACATCGCCGCCGGTACGTTCCTGGCCAAACTGGCAGCGGCGCAGCAGGGCCTTGATGCGGGCCTGC
AACTCTCGCGGGCTGAAAGGTTTGCCGATGTAATCGTCGGCGCCCAGTTCCAGGCCGATGACCCGGTCGGCTTCGT
CGGAACTGGCGGTGAGCATGATGATCGGCACCTGGGCCTGGCGCGGGTGCTGGCGTACCCAGCGGCAGAGGCTGAA
GCCGTCTTCGTCGGGCAGCATCACGTCGAGGATCACCAAGTCGCACGGAGCCTCGTCCATGGCCTGGCGAAACCCC
GCGCCATTGGGCACGCCCCGCACCTGGAAGCCGGCGCGGCTGAGGTAGGTTTGCAGCAACTCGCGGATTTCCTCGT
CGTCATCGACGAGGAGAATGGATTTACTGATTACGCTCACGGGGGCCTCCTTGTTGTTATGGGCCAAGCCTTGAAG
CATGGGTTGTTCTCAGGGGCCTCATCGCGGGCAAGCCCGCTCCCACCTTTGGAATGCATTGCCCGCGATGAGGCCA
TCACTGCCTACGCAAATGCCTGTTCGAGCGCCACACCCGCGCCGGTCAACCCCGAATACGGCGCCGTCACCAACCA
CACCGGGATCCCCTTGAAGTAGTCACTCATGCAGCCTTTGTCAGCAAAGCTCTTGGCAAAGCCACTGTTGATAAAA
AACTCGGCAAACCGCGGGATCACCCCACCCACGATATACACACCGCCCCGGGCACCCGTGGTCAGCACGTTATTGC
CGGCGACGCGGCCCAGCCAGATGCTGAACTGGTCGAGGACCTCCAGCGCAACCGGATCGCCAGCCAGCCCCGCCGC

TGTAATCGCCTCTGGGGTTTCCAATACCGCCGGATGACCGTCCACCGCACAGATCGCCCGGTACAAACGTGGCAAT
CCGCCGCCGCTCAGGGCCGTTTCGGCGCTGACATGCCCGATCTCGTTGTAGATGTGCTGCCACAACTGGGTTTCCC
GGGGGCTGCTCAAGGGCAGGTCGACATGCCCGCCCTCCCCCGGCAACGCTGCCCAACGGCCGGCGCCCAGGTCGAG
CAAGGTGCCCACGCCCAAGCCCGTTCCAGGGCCAATCACCACCGCCGGGCGCCATGGCTCCGGGGTGCCGGCGCAC
ACCACGCGGTATTCGTCGGGTTGCAAGCGGGTCATGCCCAGGGCCATGGCCGAGAAGTCATTGACCAGCAGTAGTT
CGTCCACTTGCAGGGCCTTGCAGAACGCGGTCTTGCTCAGGCGCCAGTGGTTGTTGGTGAACTTGAACTCATCACC
GCTTACCGGCCCGGCGACCGACAGGCATACCGCGCCTATGGAGCCCGGCTCCAAGCCTTCCTCGGCGAGGTAGACC
TTGATCGCTTCCTCGGGGCTGGCGTGATCCGCCGTGGCCAACACTCGGATCGAATGCAGTGCCTGGTCCCGCCACA
ACGCAAACCGGGCGTTGGTACCGCCAATATCACCTACCAGCGCAAGCTTCACTTAAGTGTCTCCAGGGCAGAGGTA
AAGGCGCTGGCGCCCTGCTCTGCGGAGCTTGCGGCCAGACGCATAAATGCAAACAGCTCGCGACCGGCCCCCACGT
TATTGCCCAACAGGCCCGTGGCAGGCGCGCGCGCTGCAAATTCTTCGGCGTCCACCTTAAGCTCCAGGGTGCCTTT
GACGCCATCGACGCGAATGATATCGCCATCCCGGACCCGCGCCAGCGCCCCACCGCGCTGCGCTTCGGGGCTGACG
TGAATTGCGGCGGGAATCTTACCCGAGGCGCCGGACATGCGCCCGTCTGTCACCAGTGCCACCTTGAAGCCACGGT
CCTGGAGCACGCCGAGGAACGGCGTCATCTTGTGCAATTCCGGCATGCCATTGGAACGCGGGCCCTGGAAGCGCAT
CACGGCGACGAAATCCTTCTCCAACTGGCCGGCCTTGAACGCATCGGCCAGGTCCTGCTGGTCCTGGAACACCACG
GCCGGCGCTTCGACGATTTGATGTTCGAGGGCCACCGCCGAGACTTTCATTACCCCACGGCCGAGGTTGCCTTGCA
TCACACGCAAGCCGCCCTCCGGGGAGAAGGCACGGGCGACGGGGCGCAGGATGGTTTCGTCGAGGCTTTCGATCGG
GCCTTCGCGCCAGATCAGTTCGCCGTCCACCAGGAAGGGTTCGCGGGTGTAGCGGCTCAGGCCATGGCCGGCGACG
GTGTTGACGTCTTCGTGGAGCAGCCCGGCTTCCAGCAGCTCGCGGATCAGGAACGACATGCCGCCCGCCGCCTGGA
AGTGGTTGATGTCAGCCTTGCCGTTCGGATAGACGTGGGACAGGGTCGGCACCACTTCCGAGAGGTCGGCCATGTC
CTGCCAGGTGAGGATGATCCCCGCCGCCATGGCGATGGCTGGCATATGCAGGGTGTGGTTGGTCGAGCCGCCGGTG
GCGTTGAGGGCGACGATGGAGTTGACCAGGGATTTTTCATCGACGATCTCGCCAATCGGCGTGAAGTTGCCATTGG
CCTTGGTCAAGCGCGTGACCTGGTGCGCGGCTTCACGGGTCAGCGCATCACGCAGCGGCGTGTACGGGTTGACGAA
CGAGGCGCCCGGCAGGTGCAGGCCCATGACTTCCATCAGCAACTGGTTGGTGTTGGCGGTGCCGTAGAAGGTGCAG
GTGCCGGGGCTGTGGTAGGACTTCATCTCCGACTCCAGCAGCTCTTCACGGCTGGCCTTGCCTTCGGCGTAGCGCT
GGCGCACGTCGGCCTTCTGCTTGTTGGAGATCCCCGACGGCATCGGCCCGCCGGGCACAAAGATCATCGGCAGGTG
GCCATAACGCAGGGCGCCCATCATCAGGCCCGGGACGATCTTGTCGCAGATCCCCAGCATCAGCGCGGCGTCGAAC
ATGTTGTGGGACAGCGCTACCGCCGTGGACATGGCAATCACTTCGCGGCTCAGCAGGCTCAGCTCCATACCGGGCT
CGCCCTGGGTCACGCCGTCGCACATGGCCGGGGTGCCACCGGCGAACTGGCCGACCGAGCCAACCTCGCGCAGGGC
TTTCTTGATCTGTTCCGGGAAATGTTCGTAGGGCTGGTGCGCCGAGAGCATGTCGTTATATGACGAAACAATTGCC
ACGTTGGCAGCGTTCATCATTCGCAGGCTGTTTTTGTCTTCGGTGCCGCATCCGGCCACGCCGTGGGCAAAGTTGG
CGCATTGCAGCTTGCCGCGCATCGGCCCGTCGCTGGCCGCGCCGCGAATCAGCGCAAGGTACGCCTCACGGGTTGC
CCGGCTGCGGGCGATAAGGCGTTCGGTGACCTCAAGTACGCGGGGATGCATGTGTAGAACTCCAGGCTAACGGATG
TGGCGACCTGAGTGTCTATGCTGGTCAAAAGCCCGCCGCGAAGAGGTTGGCAGGGAGTTACTTGACCAATCGGACC
AGTTGATTCAGGTCACTCGTTGTAGATTGAACAAAATATTGCCACTAAAAAGGCTTGTTTTCTATTTTTATGCGAA
TAATCTTGTAATTCTTACAACAAATCGACGACAGGCGCTTTCAAATGACTCTTCGAATTGCAATCAATGGTTTTGG
CCGCATTGGCCGCAACGTCCTGCGCGCACTTTATACCCAAGGCTACCGCCAGGATTTGCAGATCGTCGCCATCAAC
GATCTGGGCGACAGTTCGATCAATGCCCATCTGCTCAAATACGACACCGTCCATGGCACTTTCGATGCAGAGGTCG
CTCACGATCAGGAAAGCCTGACCGTCAATGGCGACCGGATTGCCGTCAGTGCCATTCGCAACCCCGCCGACCTGCC
GTGGGCTGCACACAAGATCGACGTGGTGTTCGAATGCACCGGCCTGTTCACCGATCGTGACAAGGCTGCCGCCCAT
ATTAGCGCCGGTGCGCGCAAGGTGATCATCTCTGCCCCGGCCAAGGGCGCGGATGCGACCGTGGTCTACGGGGTGA
ACCATGACATTTTGCGTCAATCGCACCAGATCATCTCCAATGCGTCCTGCACCACCAACTGCCTGGCGCCTGTCGC
CCAGGTGCTGCACCGCGAGCTGGGCATTGAAAGCGGTCTGATGACCACCATCCACGCCTACACCAATGACCAGAAC
CTGACCGACGTCTACCACACCGACCCGTACCGCGCCCGTTCGGCCACGCAGAACATGATCCCAAGCAAGACCGGCG
CCGCCGAAGCGGTGGGCCTGGTACTCCCGGAACTGGCAGGCAAGCTGACCGGCATGGCGGTGCGAGTGCCGGTGAT
CAACGTGTCGCTGGTGGACCTGACCGTGCAATTGAAAAAGAAGCCACGGCCGATGAGGTCAACGCGCTGCTCAAG
GAAGCCAGCCAACACTCGAAAATCCTCGGTTACAACACCTTGCCGCTGGTTTCCAGTGACTTCAACCATAACCCGC
TGTCGTCGATCTTCGACGCCAACCACACCAAGTCCAGCGGCAAGCTGCTCAAGGTATTGGCCTGGTACGACAACGA
ATGGGGCTTCTCCAACCGCATGCTGGATAACTGCCTGGCGCTGTGTAACGCTGAATAACCCCACTGAAAACCTGAT
CGACCCGATCACTGTAGGAGCGAGCTTGCTCGCGAGAAACGCAAAGGCGCCACGCTCATTCAGGATGCCAGCGTTA
TCGTTGACGATCTTCGCGAGCAAGCTCGCTCCTACAGGGTTGAGCAGATTGCAGCAAAGTGATACTTGCCATTTGC
GTTGATGATAAGCATTATCATTAACTGCCTATCGGTCTGGTACCCCCGTGAGCCTATCTCGCTTCAATCAAGTCTT
CCTCAGCCAACGGCTGGTGCTGCTGCGAACCCTGCAGCGGATGGTGAACAACCCCAGCACCGCCGAGGACCTGTTG
CAGGAAACCTACCTGCGCGTGACGCGGGCCCTCAGCGAACGACCGATTGATCACCTCGAACCTTTCGTGTTCCAGA
CCGCGCGCAACCTGGCTCTGGATCATCTGCGCTCGCGCAGGATCCAGTCCCGCACCTTGCAGGAAGATGTCCCGAT
GGACGTTCTGCAAAGCGTCGCCTCCCCAGTCACCACCCCTGAAGATGCCGCCCACGCCGAACAATTGCTGGAGCAC
CTGAGCGTCAGCCTCGGGCAGTTGAGCGCCCGTCAGCAGCAGATCTTTATCCTCAGCCGCCTGCACGGCTGCAGCT

ATCAAGAGATCGCCGACCAGTTGCAAGTGTCCTTGAGCACCGTGCAAAAGGAACTGAAGTTGATCATGGCCATCTG
CATAGGTGTGGCCGAACGACTCGATCACCCTTAAGCTTTGTCGGGACAAGCCGACAGAAATGTCGCAAAAATACGC
AAGACACTGAATAAAACGTGGCGAAGACCCGAGGAACACCGTGACGGACCCGAACAAACTGCATCCCCATGAGCTG
GCTCATGAGGTGTTGCAACATGCGGCTATGGACCAAGCCCTCGACTGGCTGATCGCGTTGCAGTGCCCGCAGCCTG
GGCAGCAAGCCGAGTTCCAGGCCTGGCTGGATGCCGACCCGAGTCACGCCCAAGCCTTCGCCAAGGCCCAGGCCGC
CTGGGGCGGTGCGCCCGTGCACAGCGCCGCCGTGGCCCTCAATGCACCACGCAAGCCCAGCGCCTGGCGGCGGATC
AAGCCGCACTGGAAGCCCTTGGCCACGGCGGCGGTGTTGCTGCTCGGGCTGTTCAGCTTCAGCAACCTGCCGGTAC
GCCTGCAAGCCGACCATTTGACCGTGGTCGGCGAGCGCCAGCGCTTGCAACTGGATGACGGCTCCAAGGTACTGCT
CAATACCAATTCGGCGTTTTCCAGCACCATCGACGAGCATCAGCGCATTGCCCGCCTGTATCAGGGCGAGGCGTTT
TTCGAAGTCGCCGCCAACCGCGGCCTGCCCCTGCAAATCGACGCCGGCCCGGTGCGCGCCAGCGTCAGCGATACGG
CATTTGCCGTGCGCTACCTCAACGGTGAGGCGCAGGTCCAGGTGCAACGCGGGGATGTCGACCTGAGCACCACTTT
CGGCAACAACCGGGTGCGCCTGAGTGCCGGCGAGAGCATCCGCGTCGGCCCCAAGGGCTTCGGCCAGCCAGCCAAG
CTCGATGCCACCAAGGAACTGGCGTGGGTCCAGGGCCGGCTGATCTTTGAAAACTGCCCGATGAGCGAGGTGCTGG
CTGAGCTGCGGCGCTATTACCCGGGCTGGATCGTCAATACCAACGAGCAACTGGCCAGCGTCGCCGTGACCGGCAA
TTACCGCCTGGACCAGCCGCTGGACGTGGTGCGCTCCCTGGCCCACATCACCTCCGCCAAGCTCTCGGAGTTCCCG
GCGCTAGTAATTCTCAACTGAGAATAATTATTTTTACTCGATAGTCCCTGCTGGTACGTCTCGTCTTAGCCAATGC
AACTGATTCCTATTTGATTCAGGCTCGCAACTATAAGACTCGTCCCTCGGAGCGCTCTCGATGTCCTCTCGTTTCA
ACCGCCGGTCCTCTTCGCCCACGCTGTGCCTGCTGACCGCCGCGATCCTGCTGGCCGGCAGCCCGGTGCTCACGGC
CAATGCGGCCGAACCCGCTGCGCGCAGCCACGCCAATTACACTTTCAGCATCGAGCAGCAACCCCTGGTATCGGCA
CTCAACGCCTTTACCGCCGTCACCGGCTGGCAGATCGGCCTGCCCGCCGAACTGGGCCAAGGGGTTTCCTCCCCCG
GCGCGCGCGGTTCGCTGACCCCGGAAAAGGCCCTGGACCGGCTGTTGGTGGGGACCAACCTGAACTACCGCAAACT
GGGCAATAACAACATCGTCCTGGAAAAGCGCACGGCGGGGAGTGCCATCACCCTGCAACAAATGACCATCAGCGCC
ACCCGTACCGAACAGGCGGTCGATAGCGTGCCCAGCACCGTCAGCGTGCATGAGCGCGAGGAACTGGACCGCCAGA
ACGTCAACACCATCCGCGAACTGGTGCGCTACGAGCCCGGTGTCTCGGTGGGCGGTGCCGGCACCCGCTCCGGTAA
TTCGGGCTACAACATCCGTGGTATTGACGGTGACCGCATCCTCACCCAGGTGGACGGCGTAGAAGTCCCGGACAAC
TTCTTCAACGGCCCGTACGCCAAGACCCGTCGCAACTATGTCGACCCGGAAATCGTCAAGCGCGTGGAAATCATCC
GCGGCCCGGCCTCGGCCCTGTACGGCAGCAGCGCCATCGGCGGCGCCGTCAGCTACTTCACCCTCGACCCGGACGA
CATCATCAAGCCCGGCCAGGACGTTGGCGCGCGCCTGAAGACCGGCTATAGCTCGGCCGACGAAAGCTGGCTGACC
TCCGGCACCGTCGCCGGGCGTGTCCAGGACTTCGATGGTTTGCTGCACCTGAGCCAGCGTAATGGCCATGAGATGG
AATCCTACAACGGCAACAACGCCACCGGCCTGGCCCGTACCGGCGCCAACCCGGAAGACGTACGCACCACCAACGC
GCTGGCCAAGCTGGGCTGGAACTATGGCGAAGACAACCGCCTGGGCCTGACCTACGAGAAGTACAAGGACGACCGC
GACATCAACCAGAAAAGCGCGGTGGGTGGGCTTTTCCTTCAAGGTCAAGGCATGAACTTCTATCGTGACCGTCGCG
GTAACGACACCATCACCCGCGAGCGTTTTGGCCTGGAAAACAAGTTTGCACTCGACGCCCCGTTTGCCGATCACAT
CAAGACCAGCCTCAACTACCAGATCGCCAAGACCGACCAGTCCACCGCCGAGATCTATCAGGTGGGCCGTCGCGTG
CTGCGTACCCGCGACACTCTCTACGAAGAAAAACAGTGGGTGTTCGACGCCCAACTGGACAAAGCCTTCGCCATCG
GCGACACCGGTCATGTCGTGACCTATGGCACCACGATCAAGCAACAGAAAGTCACCGGTTCGCGCGAAGGCGCGGC
GACCTGCCTGGCTGTCGGTGCCGGCTGTACGGCCATCGGTGCCCCAAGCCCATCGCCCAGCGACAGCGTGAAAAAA
GCCAGCGACTTCCCTGACCCAACCATCAACAGCTACGCGCTGTTCGCCCAGGATCAGATCAGCTGGAACGACTGGA
CCTTCCTGCCCAGCCTGCGCTACGACTACATCCAGCTCAAACCCAAGCTGACCGAAGAGTTCCTCAACACCACCGA
CCCGGACCGTGAGTTTCCCCACGACGACTCGAATAAAACCTGGCACCGCTTCTCGCCGAAACTCGGCGTGACCTAC
GCGTTGACCGAGCAATACACCTGGTTCGGCCAGTACGCCGAAGGTTTCCGCACACCCTCGGCCAAAGCGTTGTACG
GCCGCTTTGAAAACCTGGATGTAGGCTATGTCGTCGAGCCCAACTCCAACCTCAAGCCGGAAACCAGCAAGAGCTA
TGAAACCGGGATTCGTGGCAATTTCGACGCCGGTAATTTCGACGTCGCGGTGTTCTACAACAAGTACCGCGACTTC
ATCGACGAAGACAACGCCGCGCTGGGCCCGTCTGGCACCATCTTCCAGCCAGGCAACATCAAGCGCGCCACCATCA
AGGGCATGGAAGTCAAAGGCCGCTTGAACCTGGACGCCTTCGGCGCACCGCAAGGCCTGTACACCAAAGGCGCGAT
TGCGTACGCCCACGGTCGCAATGACGAAACCGGCCAGCCGCTCAACAGCGTCAACCCGCTCAAGGGTGTGTTTGGC
CTGGGCTACGACCAGGATAACTACGGTGCGCTGGTCAGTTGGACCCTGGTCAAGCAACAGAATCGAGTGGACAGCA
CCACCTTCTTTGCACCGGACGGCATCACCGCCAACGGCCCGTTCAAGACTCCGGGCTTTGGCATCGTCGACCTGAC
GGGTTTCTACAAAGTCACCAACGATCTGACCGTCAACGGCGGCCTGTACAACCTGACCGACAAGAAATACTGGAAC
TGGGATGACGTGCGCAGCTACGACGGTGTGGGTGAAGCCGGCGTGACCTCGCCGGCCAGCCTGGACCGGCTGACCC
AGCCCGGTCGCAACTTCGCGATCAACCTGATCTGGGACATCTGATCCCTGCAACCCCACCCCGCCGGATTTATCGC
CAGCGGGGTGAGGTTTTTTTACTGTGACGCGTCTTCTTGTTCGTCTAGTAATAACAGCCTGGCAATAAGGCGCTTT
TCCTTCTCAAGGACATCCCATGACTGCTTCCACCACCGCAGAACGTGCAAGCCTGCGCTCCCAGCGCCTGAACCAG
ATCACCAACGAGCCACACACCAAGCTCGACGCACTGGTCAAGGCCCACGCCCCGTTCGAAACCCAGGCCAACTTCG
CCCGTTTTGTCGTCGCCCAGTACCTGTTCCAGTCTGAACTGGTGGCGCTGTACAACGATCCCGAGCTGATCAAGCT
GTTCCCGGACCTGGCCGAGCGCTGCCGCGCCGAAGCCGCCAAACTGGACCTGGCCGACCTGGAAACCGAAGTACCC
GCCCCTGTGGCCGGTGCCGTGCGGAACCCGAGCAAGGCCCAAGCCCTGGGCTGGCTGTTCGTGTCCGAAGGTTCCA

AGCTTGGCGCTGCGTTCCTGATCAAGCGCGCCGTCGGCCTGGGCCTGAGCGAAACCTTCGGTGCCCGCCACCTGGG
CGAACCAGCCGGTGGCCGTGCCGAAGGCTGGAAGAGCTTCACCCGTACCCTCGACGGCCTGGAACTGAGCGCCGAA
GAAGAAGCGGCAGCGGAAAAAGGTGCGGTGGATGCATTCGTACGCTTCACCGTGTTGCTGGAACAGGCGTACGCTA
GCGCCCCAGAACTGGCCTGATCCCCTGCTGGTGTAGGCGCTGGCTTGTGTGGGAGCTGGCTTGCCTGCGATAGCAT
CGCCTCGGTTCAACTGATACACCGAGGCGGCTGCATCGCAGGCAAGCCAGCTCCCATAGAAGCCAGCTCCCACACA
GGCCAGTTCCTACATATTGATCGCACTGCACTTTCAGATTTTTCGCACCCCATACAAATCGTACCTATGACCGGCA
CAACCCAATCCTCCTCCAAACTCGCCCGAATCCTGTTCGGCCTGCTGGCCTACGTCAGCCTGGGGATCGGCTTGAT
TGCGATTGTCGTGCCGGGCCTGCCCACCACCGAATTCATCCTGCTGGCTGCCTGGGCCGCCACCAAGAGTTCGCCA
CGCCTGAGCGCCTGGCTGGAAAACCACCGCCTGTTCGGGCCGATCCTGTTCAACTGGCGCAACGGCAAGATCATTG
CGCGCCGGGCCAAGGTCAGCGCCACCGTGAGCATGCTGCTGTGTGCCGGCCTGATGTTGGTGATGCTCGATCACGG
CTGGCCGATCTACCTGGCCATCGCCGGGATGAGCCTGGGCAACCTGTGGATCTGGTCACGCCCCGAACGTCTCGCG
CAAACCGCGACCTGAGCGCTACCGCTGATCGCCTGCCCCTCATGAATTACCCGCCGGTGCCGATGTTGCGGGCATA
GCGCCGAATGGACTTGGCCGTTGCCCTGCTTGCACACCAACAAGTCCAATCGCGAGTAAGCCCATGTTCGACACCC
TCTCCATCCGCTTGAAAATCGTGCTGCTGTCTGGCCTCTGCCTGTTGGGGGTGATTGCATTGATCGTCAGCCTGAA
CATCTACCAGACCAACCAGAACGACCAACTGGTCAGCGCCTCCAGCTCGCGCATGCTCACCACCAGCGTGGAGAAC
CTGCTCCAGTCCAAGGCCGCCGAGCAAGCCGTGCAGTTACAGAAAGCCTTCGGCGAAAGCCTGTTGGTGGTCACCG
CCCTCGCCGACCAGGTCAAGGACTTGCGCAACATGGCCGCCAAGCGCTCGCTGGAAGCCGGGGCCGTGCGCGAGGA
GCTTAACCAGCGGATCAAGACCGCCTTCGAGCGCAATACCAAGGTGCTGGGGATCTGGTTGTCGTTTGAACCCAAC
GGCCTGGATGGCAGGGACAGCGAATTTGTCGACGACAAACTGCGCGCTTCCAACGAAGTCGGGCGCTTTTCCAGCT
ACTGGAGCCGCGCCGGCGGTGAAGGCCTGAACACGGTGATGGTGGAAGCGGACCTGACCAAGACCACCCTCAACCT
CAGCGGTACGCCCTACAACATCTGGTACACCTGCCCACGGGATACCCGCAACGTTTGCCTGCTGGACCCGTACGCC
GACGAAGTCGCCGGCAAGGCGATGCTGATGACCACTATCTCGCTGCCGCTGCTGGCAGATGGCAAGGTGATTGGTG
TGATCGGCGTCGATATTGCGCTGGACGCCCTGCAAGCGGCGGCCAATACCGCGCAAAAAGAGCTGTTCGACGGCGC
CGGCGTCATGGAAGTCCTCTCCAGCAGCGGCCTGATCGCCAGTTACAGCGGTGAGCCGGTCAAGGTCGGCAAAAGC
ATCGTTGATGTGCTGGGTGGCGAAGGCAAAGAGATCGTGCAACTGCTGGCCAGTGACACGCGCAAGGTGCGCGAGC
AAGACGACACGATCCGCGCGGTGTACCCAGTCAAGCCGATTGCCGAGGCCAAGCCCTGGGGCGTGGTGATCAAGCT
GCCCAAGGACGTGCTGCTGGCCGATTCGGTCAAGTTGCAAGGCGTGCTCGACGACGCCCAGGCCCGAGGCACACTG
CAAGCGCTGCTGGTGGGCGCCGCAGCCTGCCTGCTGGGGCTGTTGCTGATATGGCTGACGGCCACAGGCGTGACTC
GCCCGATCAACAGCGTGGCAGCGATGCTCAAGGACATTGCCAGCGGCGACGGCGACCTGACCCAACGCCTGGCCTA
CACGAAAAAAGATGAACTGGGGGAGCTGGTCAGTTGGTTCAACCGCTTCCTCGACAAACTGCAACCGACCATTGCC
CAGATCAAACAGAGCATCACCGAAGCCCGAGGCACTGCCGATCAGTCCTCGGAAATCGCGCGCCAGACCAGTGAAG
GCATGCAGGTGCAGTTCCGCGAGATCGACCAGGTGGCAACCGCCTCCAACGAGATGAGCGCCACCGCCCATGATGT
GGCCAACAGCGCCTCTAACGCTGCCAGTGCAGCTCGCGGCGCCGATCAGTCAGCCCGCGAGGGCCTGGCGATCATC
GAACGCAGCACCCGGGATATCACCACCCTTGCCGAGGAAGTCAGCAAGGCCGTGACCGAAGTTGAAGCCCTGGCGG
TCAACAGCGAGCAGATCGGTTCGGTGCTGGAAGTGATCCGCAGCATTGCCGAGCAGACCAACCTGCTGGCCCTCAA
TGCCGCGATTGAAGCCGCGCGCGCCGGGGAAAGCGGGCGGGGTTTTGCGGTGGTCGCAGACGAAGTGCGCAACCTG
GCGCGACGCACCCAGGATTCGGTGGAGGAAATCCGTCTGGTGATCGAACGCATCCAGAGCGCGACCCGTGGGGTGG
TGACCACCATGCATTCGAGCCAGAGCCAGGCGCAGAGCAATGCCGGGCAGATCCAACAAGCCGTACAGGCCCTGGG
CAAGATCAGTGATGCGGTGACGGTGATCAGCGACATGAACCTGCAAATCGCCAGCGCCGCCGAAGAACAGAGCGCC
GTGGCCGAAGAGGTCAACCGCAACGTCTCGGCGATCCGTACCGTGACCGAGACCCTGACCGGCCAGGCGACAGAAT
CAGCGGCGATCAGCAGCCAGTTGAATGCATTGGCCAACCAGCAGATGAAACTGATGGATCAGTTCCGGGTGTAGAC
CCAAATACCCATGGGGGAGCTGGCTTATGTGGGAGCTGGCTTGCCTGCGATAGCATCACCTCTGTTTCACTGAAGC
ACCGAGGCATCTGTATCGCAGGCAAGCCAGCTCCCACACAGTGTGAGGTCAGAGCCCGGTCCAGGCACTGACAAAC
GCGGTGAGGTCTTCCTTTGCCGCCGTACGCGGTGGGTTCTGCGCGGTACCCAGATACAGGAAGCCAATCACCTCCT
CACCCGCGCCCAGCCCCAACCCCTTGGCCACATGCGCCGAATACGACAACTCCCCGGTGCGCCACACTGCGCCAAT
CCCCTGGGCATAGGCCGCCAGCAGAATACCGTGGGCCGCACAGGCCGCCGCCAGCAGTTGCTCGGACTTGGGCACT
TTGAAGTGTTCTTGCAGACGGGCAATCACCACCACCACCAACGGTGCACGCAACGGGCCGTTCTGTGCCTTGTCGA
TCACCGCTTGCAGTGCCGCGGGGTCTTGCAGGCGGGCCGCTTCGGCCAGCAGGGTGCCCATCTGCTCGCGGGCCGG
GCCTTCCACGGTGAGAAAACGCCAAGGCCGCAACTGGCCGTGATCCGGCGCACGCATGGCTGCCGCGAACAGCACT
TCGCGCTGTTCGGCAGTAGGCGCCGGCTCCAGCAGGCGCGGCACGGAAACACGGTTGAGCAAAGCGTCGAGAGCCT
GCATTGGCCACCTCCAGGAAAAATGTGCGGCCATTCTAGCGGGAATGCATCAGATTGGAGCAAACGATAATTGCTC
TTATTCAATCCGCCCCGCCCTGTTAGACTTTGCCCCCTGAATTTCATCCACCGTCGCCCGTCTTCCAGGATTATCG
ATGCCCCGTTTGCTTCTTCGCCTGTCTGCACTCTTGGTGGCCGTGAGCCTGAGTGCCTGCGATGACGCCGCGCCGC
CGACGTTTACCCAGGCCGAGCCGGGTGAGGCGCGTTCGGGAGGCAGCACGACGGTAAGGAAGACAGACCAGAATGC
CTTTTCGCTGCCTTCAGCGAACCTGGCGCCGACCCGTCGCCTGGACTTCAGCGTCGGCAACAGCTTTTTCCGCAAT
CCGTGGGTGATCGCCCCGGCCACTACCACCGCCCGCGATGGGCTGGGGCCGCTGTTCAACACCAATGCCTGCCAGA
ACTGCCACATCAAGGACGGGCGCGGCCATCCGCCCTTGCCCGAGGCCAGCAATGCGGTGTCGATGTTGGTGCGTCT

GTCGATCCCGGATTCGCCGCCCTATGCCAAGGTCATCGAGCAACTGGGTATCGTCCCCGAGCCGGTGTACGGCGGG
CAGTTGCAGGACATGGCCGTGCCCGGCGTAGTCCCGGAAGGCAAGGTGCGGGTCGACTACACGCCGGTCAACGTCA
CATTCAAGGACGGCACAGTGGTCGAGTTGCGCAAGCCGAGCCTGCAGATTACCCAGCTCGGCTACGGGGCGATGCA
CCCCGATACGCGGTTTTCCGCTCGCGTGGCTCCGCCGATGATCGGCCTGGGTTTGCTCGAAGCCATCAGCGACGCA
GACATCCTGCAAAACGCCAGCAAACGCCCGGACAAAAACGGCGTGTTCGGGCGCCCGAACTGGGTCTGGGATGATG
CCCGACAAAAAACCGTGCTCGGCCGTTTCGGCTGGAAAGCCGGGCAGCCGAACCTCAATCAACAAAATGTTCACGC
GTTTTCGGGTGATATGGGCCTGACGACCAGCCTGCGCCCCTTCGATGACTGCACCGACGCACAGGTTGCCTGTAAG
CAGGCGCCCAACGGCAACGGCGAAAATGGCGAGCAGGAAGTCAGCGATAACATCCTGCGCCTGGTGCTGTTCTATA
CCCGCAACCTGGCCGTACCAGCGCGCCGTGATGTCAACGAACAACAAGTGCTGGCCGGCAAGAACCTGTTCTTCCA
GGCTGGCTGCCAGTCGTGCCATACGCCGAAATTCACCACCGCAGCCAACGCCGCTGAACCTGAGCTGGCCAACCAG
GTGATCCGCCCCTACACCGACCTGTTGCTGCACGATATGGGCGAGGGCCTGGCCGATAACCGTACCGAATTCAAGG
CCGGTGGCCGCGACTGGCGCACGCCGCCGTTGTGGGGCATCGGCCTGACGCAAAACGTCAGTGGCCACACCCAGTT
CCTGCATGACGGCCGCGCCCGCAACCTGCTTGAAGCCGTGCTGTGGCACGGCGGTGAAGCCCAGGCGGCGCAGCAG
CATGTGTTGTCATTTAGCGCCGAGCAGCGCGCCGCGTTGCTCGCGTTCCTGAATTCTCTATAAGCTTTGCCCCAAT
TAATGAAGGGAGCTCGACATGTTCCGTCCCAAGTTGTTGTTCACCAGCCTTGCCGCCCTCGCCCTGGGCGCTTGCT
CGCCACAGGATCCGCAAGCGGTTACTTCGGCCGCCATCGCCAAGCAAGTGATCCTGCCGACCTACAGCCGTTGGGT
CGAAGCTGACCGGCAATTGGCCGTCAGCGCCCTGGCGTATTGCCAAGGCAAGGAATCCCTGGAGACCGCCCGCGCC
GACTTCCTCCACGCGCAAAAAGCCTGGGCCGAGTTGCAACCGCTGCTGATCGGCCCACTGGCCGAGGGCAATCGTG
CCTGGCAGGTGCAGTTCTGGCCCGACAAGAAAAACCTCGTCGGCCGCCAGGTCGAGCAACTGGCCACCGCCCAGCC
GCAGATCGATGCCGCCGCCCTGGCCAAGTCCAGCGTCGTGGTGCAAGGCCTGTCGGCCTACGAATACATCCTGTAT
GACGCCAAGACCGACATCGCCAACGACGCCCAGAAAGCCCGCTACTGCCCGCTGCTGATCGCCATCGGCGAGCGTC
AGAAACAACTCGCCGAGGAAATCCTCGCCAGTTGGAACAGCACCGACGGCATGCTCGCGCAGATGACCAAGTTCCC
CAACCAGCGCTACGCCGATTCCCACGAAGCCATCGCCGAACTGCTGCGCGTGCAGGTCACGGCCCTCGACACCCTG
AAGAAAAAGCTCGGCACGCCCATGGGTCGCCTGTCCAAGGGCATCCCCCAGCCGTTCCAGGCCGATGCGTGGCGCA
GCCAGTCGTCCCTGCAAGGCTTGCGAGCCAGCCTCGCGGCGGCCCAGACCGTATGGGTCGGGGTCGACAACAAAGG
CCTGCGCGGTCTGCTGCCCGCCGACCAGAAGCCGTTGGCCGAGAAGGTCGATGCGGCCTACGCTGCTTCCCTGAAA
CTGTTCGACAGCAACCAGCGCTCGCTCAACGACCTGCTGGCCGACGACGCCGGGCGCCAGCAACTCAACGATATCT
ACGACAGCCTCAACGTCGTACACCGCTTGCACGAAGGCGAATTGGCCAAGGCGCTGGGCATCCAACTGGGCTTCAA
CGCCAACGACGGTGACTGATGATGCTCAGGCGACAGGCTTTGGCCATCGGCAGCGTGCTGCTCAGTGCAATTACTC
TGGGCGGCTGGACGCTGTTCAAGCGCAAGGGCGACAGCCCACTGCTGCTGTCGGCCCGGGATGATGGCGACGGCAA
GCACTACGCCGTGGGCTATCGCCTGGATGGCACCCAGGTGTTCGCCACCCAGGTCGGCCAGCGCTGCCACGACATC
ATCAACCACCCGACCCTGCCCATCGCCCTGTTCGTGGCCCGCCGCCCAGGCACCGAGAGCTACCTGGTGGACCTGC
GCGACGGCACGCTCCTGCAAACCGTGACATCCCAGGCCAACCGGCACTTCTACGGCCATGCGGTGATCCACAAGAG
CGGCGACTGGCTGTACGCCACCGAAAACGACACCACTGACCCGGGTCGCGGCCTGTTGGGCGTGTACAAGTTCGAA
GGTGAGCGCCTGGTGCACACGGGTGAGATTTCAACCCACGGCATCGGCCCGCACCAGGTGTCGTGGATGCCCGACG
GTGAAACCCTGATCGTGGCCAATGGCGGTATTCGCACCGAGGCCGAGAGCCGGGTCGAGATGAACCTCTACGCCAT
GGAGCCCAGCCTGGTGCTGATGCAGCGCGACGGTACGCTGTTGAGCAAGGAAACCCTCGGTCAGCAGATGAACAGC
GTGCGCCATATGGGGATCGCCAGCGATGGCACGATCCTCACCGGGCAACAGTTCATGGGCCCGTCCCAGGAGCGTT
CCGAGCTGTTGGCGATCAAGCGTCCGGGCCAGCCGTTCGTGGCGTTCCCGGTGGCCGACGAGCAATTGCAGGCCAT
GGGGCACTACACCGCCAGCGTAGCCGTGCACAGTGAGTTGCGCCTGGTGGCACTGACCGCACCACGGGGCAATCGC
CTTTTTATCTGGGACATGGACAGCGGCGAACTGCGCCTGGACGGGCCGTTGCCCGACTGCGCCGGCGTGGGTGCAG
TGGCGGATGGGTTTGTCGTGACTTCAGGCCAGGGCCGCTGCCGCTTCTACGATTGCCGGCAGCCACAACTGGTGGC
CAAGCCCCTGGAATTGCCGGCAGGGCTCTGGGACAACCATCTGCATCTGATCTGATCGCAGGCGCCGGCTTGCCGG
CGCCTACAGGGGGGCTGACGGCAAAAAATGCCAGTTGGAATCCCCCAGGCACTCGGAGTAATGTGCCAAATCGTTC
GTCTTCGTCTGCCTCAGTTTTTTCCAAGGAACCGGAATATGCTGCGTCGTCGCATGCTGATCATGTTGGCAGTTGC
CCTGCTAATCGTCCTGCTGTTGGCGGGCTATAAGGCCTTTTCCATCTATCAGCAGATCCAGGTCTTTTCCGCGCCC
AAACCACCGATCAGCGTGGCGGTGGCCACGGCGGTCGAACAGCCCTGGCAACAGCGCCTGCCGGCGGTGGGTACAC
TCAAGGCGTTGCAAGGGGTGAACCTCGCCCTGGAGATTGCCGGCACCGTCAAGAACGTGCAGTTCGAGTCCGGGCA
AAAGGTCAAGGCCGGGCAACCGCTGGTGCAACTGGACAGCGCAGTGGAAGACGCCCTGCTGCAAACCGCCCAGGCC
GACCTCGGCCTGGCCCAGCTCGACTACGGGCGCGGCAGCCAACTGGTGGGCAGCCAGGCGATCTCCAAGGGTGAGT
TCGACCGACTCTCGGCGCAACTGCAAAAAAACAAGGCCACGGTCAACCAGCTCAAGGCGTCCCTGGCCAAGAAACA
CATCGTCGCGCCATTCAGCGGCACCATCGGCATTCGCCAGGTGGACGTCGGCGACTATCTGGCCAGCGGCACGGTG
ATCGCCACCTTGCAGGACCTGAGCAGCCTCTACGTGGACTTCTATGTGCCCGAGCAATCGGTGCCCAAGCTGGCGG
TCGGCCAGGAGGTACAGGTGCATGTCTCGGCCTACCCGACACTGACGTTCCCCGGCACCCTCAGCGCCATCAACCC
CAAAGTCGAAAGCAGCACCCGTAACGTACTGGTGCGGGCAACCCTGGCCAACCCGGATAACAAACTGCTGCCGGGC
ATGTTCAGCAGCCTGCAAGTGCTGTTGCCCGATCCGCAGGCACAGGTGGTGATACCGGAAAGTGCGATCACCTACA
CCCTCTACGGCAACTCGGCCTATGTGGTGGCGCAGAAGAAGAGCGAAGACGGCAGCCTCGAAAAGGATGCCAACGG

TCAACCGATATTGATCGCCGAACGGCGCTTTATCGAGACCGGCGAACGACGCGATGGCCTGGTGCTGGTGACCAAG
GGCCTGAGCAGTGGCGAGCAAGTGGTCAGCGCCGGCCAACTCAAGCTCGACACCGGCGCGCATATTGCTATCACCG
CAGACAAAAACCTGCCGGCCACTCCTGGCCAACCTCCGCGCAACGACGGTTCAAGGAACTGAGCCATGGCGTTTAC
CGACACGTTTATCCGCCGTCCGGTGCTGGCCATGGTGGTCAGCCTGCTGATCGTGCTGTTGGGTTTCCAGGCCTAC
AGCAAACTACCGTTGCGCCAATACCCGTCCATGGAAAATGCGCTGATCACGGTGACCACCGCTTACCCGGGGGCCA
ATGCCGAGACCATCCAGGGCTACATCACACAGCCGTTGCAGCAAAGCCTGGCGAGCGCCGAAGGCATCGACTACAT
GACCTCGGTCAGTCGCCAGAACTTCTCGGTGATTTCCATCTATGCGCGGATCGGCGCCAACAGCGACCGCCTGTTC
ACCGAACTGTTGGCCAAGGCCAACGAGGTGAAGAACAAGCTGCCGCAAGACGCTGAAGATCCGGTATTGAGCAAAG
AGGCCGCAGATGCGTCGGCCCTGATGTACATCAGCTTCTCCAGCCAACAACTGAGCAACCCGCAGATCACCGACTA
CCTGTCGCGGGTGATCCAGCCCAAGTTGGCGACCCTGCCTGGCATGGCCGAAGCCGAGATCCTCGGTAACCAGGTA
TTTGCCATGCGCCTGTGGCTGGACCCGGTCAAGCTCGCGGGGTTCGGCCTGAGCGCCGGCGACGTCACCAATGCCG
TGCGCAACTACAACTTTCTCTCTGCCGCCGGTGAAGTGAAAGGCGAGTATGTGGTCACCAGCATCAATGCCAACAC
CGACCTCAAGACCGCCGAAGCCTTCGCTGCCATCACCGTGAAGACCGATGGCGACAGCCGGGTGCTGCTCGGCGAC
GTGGCCCGGGTGGAAATGGGCGCGGAAAACTACAACGCCATCAGCTCCTTTGGCGGCACGCCCTCGGTGTATATCG
GCATCAAGGCCACGCCCAGCGCCAACCCGCTGGATGTGATCAAAGAGGTGCGCAAGATCATGCCGGAGCTGGAATC
CCAGCTGCCGCCCAACCTCAAGGCAGAAATTGCCTACGACGCCACGCTGTTTATCCAGGCGTCCATCAACGAGGTG
GTCAAGACCCTGTTTGAAGCGGTGCTGATCGTGATCGTCGTGGTGTTCCTGTTTCTCGGGGCGCTGCGCTCGGTGG
TGATCCCGGTGGTTACCATTCCGCTGTCGATGATTGGCGTGCTGTTTTTCATGCAACTGATGGGCTACTCCATCAA
CCTGCTGACGCTGCTGGCTATGGTCCTGGCCATCGGCCTGGTAGTGGACGATGCGATTGTGGTGGTGGAGAACATC
CACCGCCACATCGAAGACGGCAAGAAACCACTGGACGCCGCGCTGGAAGGCGCGCGGGAGATTGCGGTGCCGGTGG
TGTCGATGACCATCACCCTGGCGGCGGTGTATGCGCCCATCGGCTTCCTGGAGGGGCTGACCGGGGCCCTGTTCAA
GGAATTCGCCCTGACCCTGGCCGGAGCGGTGGTGATTTCCGGTATCGTCGCCCTGACCCTGTCGCCGATGATGTGC
GCCCTGCTGTTGCGCCACGACGAAAACCCCAGCGGCCTGGCCCATCGCCTGGACCGGATCTTCGAGGGCCTCAAGC
GCCGCTACCAGAGCCTGTTGCACGGCACCTTGAACACCCGGCCGGTGGTGATCGTGTTTGCCCTGATCGTGCTGTG
CCTGATCCCGGTGTTCCTCAAGTTCACCCAGTCGCAACTGGCCCCGGACGAAGACCAGGGCATTATCTTCATGATG
GCCAGCGCGCCGCAGCCGACCAACCTGGACTACCTGAACACCTACACCGACGAGTTCATCGCGATCTTCAAGGACT
TCCCGGAGTATTACTCCTCGTTCCAGATCAATGGCTTCAATGGCGTGCAATCGGGTATCGGCGGCTTCCTGCTCAA
GCCGTGGAACGAGCGCGAGCGCACCCAGATGCAAATCCTGCCGGAGGTGCAAAAGCGCCTGGAGCAGATTCCGGGC
TTGCAGGTATTTGGCTTCAACCTGCCATCGCTGCCGGGCACTGGCGAGGGCCTGCCCTTTGGTTTTGTGATCAACA
CGCCCAATGACTATGAGTCATTGCTGCAAGTGGCCGATCGTGTGAAAAAACGCGCCATGGAGTCGGGCAAGTTCGC
CTTCGTGGACATCGACCTGGCGTTCGACAAACCCGAGGTGGTGGTGGATATCGACCGAGCCAAGGCGGCGCAGATG
GGCGTCTCGATGCAGGACCTGGGCAGCACCCTGGCGACTTTGCTGGCGGAAGCGGAAATCAACCGGTTCACCATTG
ATGGCCGCAGCTACAAGGTCATCGCCCAGGTCGAGCGGGCCTACCGGGACAACCCCGAATGGCTGAACAATTACTA
CGTGAAAAACACCCAGGGCGAACTGCTGCCGCTGTCGACCCTGATCAGCGTCAGCGACCGCGCACGGCCGCGCCAA
CTGAACCAGTTCCAGCAACTCAACTCGGCGCTGATCTCGGGTTTCCCGATTGTCAGCATGGGCGAAGCTATCGACA
CCGTGCGCCAGATCGCCCTCGAGGAAACCCCGGCCGGCTACGCCTTCGACTACAGCGGCGCCTCGCGCCAGTTCAT
CCAGGAAGGCAGCGCACTGTGGGTCACCTTTGGCCTGGCACTGGCGATCATCTTCCTGGTGCTGGCGGCGCAGTTC
GAGAGCTTCCGTGACCCGCTGGTGATACTGGTGACAGTGCCGCTATCGATCTGTGGGGCGTTGATCCCGTTGTTCC
TTGGTTGGTCGAGCATGAACATCTACACCCAGGTGGGGTTGGTGACGTTGATCGGGCTGATCAGCAAGCACGGCAT
TCTGATCGTCGAGTTTGCCAACCAGTTGCGCAAGGACAAGGGGCTGACAGCCCGCGAAGCAGTGGAAGAAGCGGCA
GCGATTCGCCTGCGCCCGGTATTGATGACCACGGCAGCGATGGTATTTGGCATGGTGCCGTTGATCTTTGCCACGG
GAGCGGGGGCGGTCAGTCGGTTTGATATCGGGATGGTGATTGCCACGGGGATGTCGGTGGGCACCTTGTTTACTTT
GTTTGTATTGCCGTGTGTGTATACGTTGCTGGCCAAGCGTGATCCCTTGGCGGATTGAGTACCTATCCATTTGTGC
GGCAACGGCACTGACTTGTGGGGGGGGGCTAGTCCCTCCCCACAAGTACGCACAAAACAAAAGGCCTCGCTATGCG
AGGCCTTTTATCGGGGGGTCAATCTCTTCAACTCGCAGGCCTCATCCCTTGGGACATCCCGAACATAAACAGCAAA
AGCTGATGATCAGGCTGCGTAACCCCCGTGGTTTTGGCGATCCGTGGTAGCGGGCATTGCCCGCCACTGTGCACCG
CGCTGAGGACTTCTGTGCGGGGCTGCTCCCAGGCAGCCAGGGCCAGGGAGGCGATGCCCAAGGCCCCCAACAGAAA
CAAACCTCGTGCTATTTCTAGCTTCATTAGGTTAAACCCTTGATAGCGCTGCCAAACGCCGCTTCGTAAAAGTAGC
TGAGTTTTTGCCAGTCAGCGGCATTCCACGACGAATGGCGACGCAATTGCTGCATGTCATGGGCTGCGGCCCGTCG
GGCGGTCAAACGCTGGCGGCACTTTTCCAGGTCCAGCAGCGCCACTTCGACCGTCGCGCCCTCTCCCGCACCGCTG
ACCCGCACAAAGATATGCTTGCTGTACAAGCAGCCATGCTGCCAACGCCCTTTGTGCATGCGCGCCAGGGTTTGCG
CCAGTTCATCAAGCAGCCGCTCATGCACCAGTTCCCCGCATTGCTTGCGGCCACCGGCGGCGTACCAGTTGTCGAT
TTCCTCGAAACCATCCAATGACGCCGTCACCAACAAGGCCTTCCATTGATGTTCAGGGTCACGTCGGGCGCCACAG
AACACCAGCTCCGGAACGCCGACATCGAGCAAACGCAGGCCCCTGAGCGCATCACGCTCACGCAGCACCGTAGGCC
GCCCGAAGGGGTGCAGCCAGCTGCGATAGGTATGGCCGGTCTGACGCTTGCTGTAGAGCAGCCGCCCATTGGTCCC
GATCACCCGCTGCACGCCGCTTTCGCCACCTCGACGACGATTGGGTGCCTCGACCCACTCGCCCTGCTGCCGCCAG
AAATATTCGAACCGCTCTTCGGGAGCTACGTGACTGCCTGCTACGTACTCAACCACCATCCTGCTACCTCTTGCGC

AATACATACACTCGCCACATGGCATACAGCGGTATGAAGTCCAAATACTCCTGGACGCGAAAACCAGCTTGCTCAA
ACTCAGCCTCAACAGTAGCAGCCGGTAACACAAATCGATTTCGGTAACTCTCCTGCTCACTTTTGTTGCGGCGCTG
CACTTCCAGGCGCTTGCGTTTCCAGGCCTTGAAGTTGCCATCCACCCACAATGAAATGATCACGCTGTCGCGGGTA
ACTCGTTGAAATTCCTTCAATATCGCCATTCGGTGGGCCGGCTCGCCGATGTGGTGCAACAGGCGCATGCAAAAAA
TGCTGTCCACGGCGCTGTCTGGCAAATCGATATCAAAGGCAGATGTCTGCAAGGGTCGTACCTGCTTGACCACATC
CGCCGGTTGCGCCCCCATGGCGATGTTCAGCATGGACTCGGAATTGTCCGCACCGATAACGATCCGGTTGGGCTTT
TCTGTGAGTAACGGCCAGAAACGCCCGGCGCCGCACGGCAAGTCCAATACCAGGCCAGGCTCACCGGCCATCGCCA
GCGCACCACGGGCCAATTGTTCGTCGCGTTTGTGGGACAGCCGGCGCGCCAACGTGCCCTGGTGTTTGAGCAAATA
TTGCTGAGCGTGGCGGTCGTCGTACTTTGCGGAAAATTCAAGCTTGATCGGAGTAGCCATCAACGAGTCTCCTGGA
CTGATGCCTACGACTTTAAGCAGCCGCACGTCATCCACAGGTCAACCCTTTGTGAAAAAAACGTCCCGTCTAACCC
TAAAGAATTACAAAGTTTTACAGGGAAAAAAGCATGGCAGGAAGCCATCTTCGTCGAACAGCAGGGAATCTGCCCC
AACGCAAGGGATCAGGGTTTGACGTGGCTCAATTCAACATGGAAACGGCAGCCGCTAGGCTCCATGGTGCTGAGGC
TGACCACCCAGCCCTGGTTATCGCAGATCCGCTGCACCAGCGACAAACCCAGGCCCAGGCCATCGCCGCGTTTTTC
ATTGCCGCGCACAAAGGGCTCGAACATCGCTTCGCGCTTGTCCTCGGGGATGCCCACGCCGGTGTCTTCCACCACA
AACCCCGTGGGTTCCAGGGTCAGGCGGATAAAGCCCTGCTCCGTGTAATGCAGGGCGTTGCGCAACAGGTTCCCCA
TCACCGCATGCAAAAAGGTGGCGTTGTAGCGGATATCGAGGGGGTTGCCCGGCTGGTAGAACAGCTCGATGCCCTT
GCGCTCGATGGGTTCACGCCAAATCCCCAGCAAGTCCTCGGCGACCTGGGCCAGGGTGACTTGGGGTGACATGGTG
GTGTCGTCATGCTGGGCGCGGGCGAGCATCAGGAAGGTCTGCACCAGTTCGCGCATTTCCTCGCAGGCCCGGGCGA
TGCGCTCGACCTGTTTGCGCCCCCGCGAATCGATGGCCGGGTTTTCCAGCAGCAGTTCGCAGGAGCTGGCCAGCAC
CATCAGTGGCGTCCGCAGTTCATGGCTGACATCACTGGTAAACAGGCGCTCACGGGTCAGCGCCTGGCGTAGGCGG
CCCAGGGTGGCGTCGAAGGCCACTGCCAGCTCGCCCACTTCGTCGGCGGCATAGTCCGGGGCCAACGGCGGGGCCA
GGCCCAGCAGTTGGTCACGATGACGCACCTGGCGCGCCAGGCGCACCACTGGGGCCATTACCTTGCGGGCCAGGAC
CCAGCCGAGGAATACCGCCAGCGCCAGGCTGAGGACAAACCCCACCAGCACCACCGCAAACAGCACACGCTCACGC
TCTTCGAAATCGCTCTGGTCTTGCAGTAGGACATAACGCCGGCCGTCGACGATCTCAACCATGGCATGGTAGGACA
GCGCTTCGCGAAACACTTCGTGAAAGCCCGCATCCAGATGCCGCAAGTCCTTGGGCAGCTCAAAATCGCCGCGCCC
GCCGCTGAAGTAGAACAACTGGTCCGGCTCGGGCCGGTGGCGCCAGTCTTCGACACTGTCCATCAGCAGCAAGCGT
TGCAAGTCGCCGCCCAGGCCCGCCGAAATCAGTTTTTCTTCCACCAGGTGCACCGTGGCGACGATGCCCATGGCAA
AGGCCCCCGCCACCAGCGCGCTCATCAGCGCAAAGGCGATGATGATCCGCTGGGCAAGGCTTTGCTTAAACTCCAT
CACGGCCCTCGGCCAAGCGATAACCCACGCCATGCACGGTTTGCAGCAATGGTTTGGCAAATGGCTTGTCGATCAC
CTGGCGCAGTTGGTGGACATGGCTGCGCAGGCTGTCGCTGTCCGGGCAGTCATCGCCCCACAGGGCTTCTTCCAAC
ACTTCGCGACGCAGGACGTGAGGGCTTTTCTGCATCAGCACGGCGAGCAATTTCAGGCCGACCGGGTTGAGCTTGA
GCAAACGCCCTTCGCGGGTCACTTCCAGGGTATCGAGGTCGTAGGACAGATCGCCGACTTGCAGCTCACGCCGCCC
GCCGCCCTGGGCCCGGCGCAGCACCGCTTCGATGCGTGCGGCCAACTCCGATAGGGCAAACGGCTTGAGCAGATAG
TCGTCGGCACCCGACTTGAAACCTTGCAGGCGGTCGTCCAATTGATCGCGGGCGGTGAGCATGATCACCGGCGTAT
CGCGGCGCGCATCTTCACGCAGGCGTTTGCACAAGGTGTAGCCATCAATGCCGGGCAGCATGATATCGAGCACGAT
CAGGTCGTAATGCTCGGTGGCGGCCAGATGCAGGCCCGACAAACCGTCCTGCGCACAGTCCACGGTATAACCCTTG
AGCCCCAGGTAATCCGCCAGGTTGGCCAGAATATCGCGGTTGTCTTCAACCAATAAAATTCGCATGGGCAGTGTCT
CCGTACGCGGTAACGGCCGTGTTGGCTCGCGCAGCTTAAGGCCAAGTGTGCTCCACGACTAGGGCGCCGTAGCGAG
TCTATAAGTTTTTTCAACCTATTGATGGGCTTCACGAGTTTTTCACTATCACTTCACCCGCCGCGCACAGAAAGAC
CATCACACTGATGCAGGGTTACTACACCCAGGCAAAAACCAGGCATTAAAAAACCCCGCCTGCATCGCTGCAGGCG
GGGTTTCAGTACAGGGGTAAGGCTGGCTTACATCATGCCGCCCATGCCGCCCATACCACCCATGCCGCCCATGTCT
GGCATGCCGCCGCTACCTTCAGCCTTCGGCTTTTCAGCAATGCCGACTTCGGTAGTCAGCAGCAGACCAGCGATCG
AAGCTGCAGCTTGCAGAGCCGAACGGGTGACTTTGGTTGGGTCCAGAATACCCATTTCAACCATGTCGCCGTATTC
GCTGGTTGCAGCGTTGTAACCGTAGTTACCTTTACCGTTCTTCACTTCGTTGACCACAACACTTGGCTCGTCGCCG
CTGTTGGAAGCGATCTGGCGCAGTGGGGATTCAACGGCGCGACGCAGAACAGCGATACCGACGTTCTGGTCAGCGT
TATCGCCTTTGAGGTCAACCAAGGCTTCCAGAGCACGGATCAGCGCAACGCCACCGCCAGGTACCACGCCTTCTTC
AACGGCTGCACGGGTCGCGTGCAGGGCGTCTTCAACGCGGGCTTTCTTCTCTTTCATTTCAACTTCGGAACCAGCG
CCAACCTTGATCACTGCAACGCCGCCGGACAGCTTGGCCAGACGCTCTTGCAGTTTTTCACGGTCGTAGTCCGAGG
AGGTTTCAGCAACCTGGGCACGGATCTGGGTGATACGCGCCTGGATGTCCTGCTCAACGCCAGCACCGTCAACGAT
GATGGTGTTTTCCTTGGAGATGGTCACGCGCTTGGCGCTACCCAGGTTTTCCAGGGTGGCGCTTTCCAGGCTCAGG
CCGATCTCTTCGGAGATTACGGTACCGCCAGTCAGGACTGCGATGTCCTGCAGCATGGCCTTGCGACGGTCGCCGA
AGCCTGGAGCCTTGACGGCTGCGACTTTAACGATGCCACGCATGTTGTTCACAACCAGAGTCGCCAGGGCTTCGCC
TTCAACGTCTTCGGAAACGATCAGCAGTGGGCGGCCGGCTTTGGCAACGGCTTCCAGCACTGGCAGCATTTCGCGG
ATGTTGGAGATCTTTTTGTCGACCAGCAGGATCAGCGGGCTTTCCAGCTCGGCAACCATGGTTTCTGGCTTGTTGA
CGAAGTACGGGGACAGGTAGCCACGGTCGAACTGCATGCCTTCTACAACCGACAGTTCGTTTTCCAGGCCAGTGCC
TTCTTCAACGGTGATCACGCCTTCTTTACCGACTTTTTCCATGGCTTCGGCAATGATGTCGCCGATGGAGCTGTCG
GAGTTGGCGGAGATGGTGCCTACCTGAGCGATAGCCTTGGTGTCAGCGCATGGCTTGGACAGGTTTTTCAGCTCGG

CAACAACGGCGATGGTCGCCTTGTCGATACCGCGCTTGAGGTCCATCGGGTTCATGCCGGCAGCGACGGCTTTCAG
GCCTTCGTTGACGATCGACTGAGCCAGAACGGTGGCGGTGGTAGTACCGTCGCCTGCGTCATCGTTGGCACGGGAG
GCAACGTCTTTGACCAGCTGCGCGCCCATGTTTTCGAAACGGTCTTCCAGTTCGATTTCTTTTGCTACGGAAACGC
CGTCCTTGGTGATGGTCGGAGCGCCGAAGCTCTTCTCGATGATCACGTTACGGCCTTTCGGGCCCAGGGTCGCTTT
TACTGCGTCAGCCAGGATGTTGACACCGGTGAGCATTTTCTTACGGGCGGAATCGCCGAATTTAACTTCTTTAGCA
GCCATGATCGATATTCCTTAAATACTTTGTAGTAACGGGAAAATGAGCGGGGAATCAGTCTTCCAGAACAGCGAGA
ATCTCGTTCTCAGCCATAACCAGCAGGTCTTCGCCGTCAACTTTCACAGTGTTGCTGCCGGAGTAAGGACCGAATA
CAACCTTGTCACCGACTTTAACGGCCAGCGCACGCACGTCACCATTTTCCAGAGTCTTGCCTGGGCCTGCAGCGAC
GATCACACCGTGGTTGGCTTTTTCAGCAGCCGAACCTGGCAGAACGATACCGCCAGCGGTTTTCTTTTCTTCTTCG
CTGCGACGGATAACGACGCGGTCGTGCAGAGGACGAAGCTTGCTCATTGTCGATCTCTCCTAATTGTAAGTTTCAT
CGGCCGGTATCAGTACCGGCTGGTGGTATTCCGGCGGGGCCGGTCGCGGTTCGCCAGGCGAAACGCGGAAGTCTGT
CTGGTGTCGCCACCAGAAACCTTGCGGTGACCGTTACATAAGGGCGTATATGCTTATTACAAGGGCCACAGGTGAA
AATTTTTGTGTCAGCAGGGCACCCGAAGGCACCCTGCCTGACATGGCGTTACTTGGAGTCGCGGTGCTCGAACTCG
CCTTCGATCACATCACCTTCACGCCCCAGCGGCTGGCGAGGTGCCGGGCCGCCACGTGGCTGCAGGTCGTCGGCAA
AGGCGCGCTGGCGCACCGCCGCTTCTTCGGCACGCTGGCGCAACTTGCCTGCCAGCAACCGACGAGTGAATGGCAG
CAGCAGAATCAAACCCAGCACATCGCTGACAAAACCCGGCACGATCAGCAGGCCACCGGCCAAGGCCATCATCAGG
CCTTCAAGCATGGTCTGGGCCGGCAGTTCGCCGCGATTCAGGCTTTCACGGGCACGCAGTGCGGTGGCCAGGCCGG
CGATGCGCAGCACGAACACGCCGAGCATCGAGCCGAGAATGATCAGCAGCAGCGCCGGGAAAAACCCGATAGCCCC
GCTGACCTTGACGAATACGAACAGCTCCAGCACCGGAAACAGCAGAAAGAGCAATAAAAAAGGGCGCATCAAATGG
TTCCTCAACGCAAGAATGCCTTGCTACTAGACCTTAGATGACGTCGCCCTTTCGTGAATTCAAGCTTGGGTGGTGG
ATTTTTTCGGCCATTGCTCGGCGTGAGCCAGGAAAACCAAGGCTTCGCGCACTTGTGTCGGCGTATTGCAAGTAGC
CGCAAACGGCAACCAGTGCAAGCACTGGCCGATGCGCAGGTGCATGCCTTCACTATCGATACCCGCCAACTGCGCG
GGCTCGGAGGTCGGCAGGCCGGCCAACTCGACATAGTGGGCAATGGCTTTGGTGTGATCGCTGTTCATGTGCTCGA
TCATGCTCAACTCGGCCTTGCCGGCAAACGGGTTGGCCAGGGTCAGTTGGTCGACCCAATGGATCGCGCCGAAACC
GCCGATATAGCGATGACGCACCGGCTTGAGCACCCAGAAATCAAAGTCATGGGCTTTATGGTAGTTAGCCGAGTCG
GGGAAATACCGGTAGTAACGCTCGGCGGCGGCGGCGATGGCCACCTCGTCCTCGAGTTTTTCGGCTTCAGCCAGGT
AAGTCAGGCGGCCCACGGCCTGCACATCATCAGCCTCACGCTCGCCTACCAGCAACGAGCATTTCGGATCTTTTTG
CAGGTTATGGGTGTGCTGGGCGATACGGCTGATCAGGATCAACGGCCGGCCCTCTGCGTCCAGGCAATAAGGCACT
ACCGAACCAAAGGGAAAACCGGGCATGGCCTTGGAGTGCGTGGCGAGCGCCCCACGGTATTCCTTGAGCAGTAGTT
CTCGGGCATTCTTGGCAACGTGGACGCTCAACTTATGACTCCTTCGATAAGATCCGTCACAAAACGGACTTGGACA
ACCGCTTAAATCTGCTCACAGGACATGCGAATGCATCTCAACGACAAAGTAATCATTATCACCGGCGGTTGCCAGG
GTTTGGGCCGCTCCATGGCCGAGTATTTTGCAAGCAAAGGTGCCCACCTGGCGCTGGTGGACCTCAATCAGGAAAA
GCTCGATGCCGCAGTGGCTGCATGCCAGTCCCATGGCGTCACCGCACGCAGCTATCTATGCAACGTGGCGGATGAA
GAACAGGTGACCCATACCGTGGCACGGATCGCCGATGACTTTGGCGCGATCCATGGTTTGATCAACAACGCCGGGA
TCCTGCGCGATGGCTTGCTGCTCAAGGTCAAGGACGGCGAGATGACCAAGATGAGCCTGGCCCAGTGGCAGGCGGT
GATCGACGTCAATCTGACCGGCGTGTTCCTCTGCACCCGTGAAGTGGCGGCGAAGATGGTGGAGCTGAAAAACCAG
GGCGCCATTATCAACATCTCGTCCATCTCCCGCGCGGGCAATGTGGGCCAGACCAACTATTCGGCGGCCAAGGCCG
GCGTGGCCGCAGCAACGGTGACCTGGGCCAGGGAGCTGGCGCGTTATGGCATACGTGTGGCGGGGATTGCGCCGGG
CTTTATCGAGACCGAAATGACCCTGGGCATGAAGCCCGAAGCATTGGAGAAGATGACGTCGGGGATTCCGCTCAAG
CGCATGGGCCGGCCGCAAGAGATCGCCCACTCGGCGGCCTATATCTTCGAGAACGACTACTACACCGGGCGCATCC
TGGAGCTGGATGGCGGGTTGCGCCTGTAACACCACTGAAAAACAAATCAGGGGGGGCGCTACGCGCCCCAACGCGG
GCGATGCGGCGTTCCGACAAGCCGGCTCGCCACAAAAGCACGATCACACCCAGGCAGGCTTACCAGGTCACGCCAA
ACCCTGCGGTGTAGCGGGTCTTGCTCAGGTCGCTGTCCGAACCGCCGCTGATGATGTCGCGCTCGGCCTTGAGGTT
GAGCGAGGCCCATTCGGTGACTTTGTAGCGCAGGCCCATTTCGGCATCGAGCGCGTAGTCGGCGGGGCCGGCCAGG
GGCTTGCCCACTTCACCGTTGGTGAAGAACTCCACGGTCTTGCCGATCAGATAGCGGTTGTAGTCCCACTTCATCG
CCAGGGAATAGAAGTTGTCCTTGCCGCCATCCTGGTACTCGTAGTCAGTACGGTTGAGCAATGACCCCAGGGAGAA
AGCCCCCAGTTCATCATCCCAGAACTGATAACCCGGACCGGTACCGACGGTGCGCTGGCGACGCAGGTCTTCGACC
TTGTCGTGCTTGTAGGTCAGGCGCCCCTGCCAGAACCACTGGTCGGTGATAAAGCGGTCCAGCGCGTACTCGGCGC
TCCAGTTATCGGTGGTGACCACATCGTTCTGGAATTCGCGGTTGTATTCGCCTTCTGCAATATGGCGCCACTTGCC
GTGACGGGCAGTGGTCTTGAAGTCGATGTCATAGTCGTCGGTGTCTTTTTCCGCACGCTTGTAGTCCAGCGCCACG
TCGACATTGCCCTTCCACACCAGGTCCTCGATCACAGGCTTGGGCTTGATGATCTGCTGGATACTGGCCAACTCCA
CCGTCTTCGGTGTTTCGCCGTTGGCCAGGACCACCTTGCCGTCCTCGGCCGGATGCAGGGACTTGGCCTTCTCGCC
GATATAGGCATCCTGCTTGACCAGCAACTCCTGGTCGCTTTCCAGGGTTTTCACCTGTTTCCAGTCCACCGGGATC
GCGCCCGCGTATTCGGTCTGGATCAGCAGCTTGCCGCCATCGAAAACCTTGATCTTGCCGGTCAGGCGATCACCGT
TTTTCAACCAGACCGTATCGGCCAGCACAGGGGTAGAAGCAGTAAAGACAGCGAGGCACAGCAGGGTTCTGGACAA
CATAAGCGGTTACGGGGCTCAAGTTTGGCGAAAAAGGAGGCATTATCGGCGTAGCTGAAGTCTTAGCAAGGACTGA
CTCAAGGTTTTGTATTGAGTTCAATTCTCAACGGCATCTTTACAGACGTTTCTTACATATCCACGACTATTTTCAA

CTGAGCCGCCAGAACAGTGAACGAGCCCGCTGACACCCCGCAAAACCCGGCCGAAATGCGCCGAACTGCGCTGTAC
CTGACCTTGGCCCAAGTGCCCGAAGGCTGCGTGGTCAGCTACGGCGAACTGGCCCACCTGGCTGGCCTGGGGCGGG
CCGCACGCTTCGTGGGCCGTACGTTGAGCCAACTTCCCGAGGGTTCGAAGTTGCCCTGGCACCGGGTGGTCGCCGC
CGGCGGTCGGATAAGTCTGCCGGTGGGCAGCGTCTCGGGCGATGAACAACGTGCGCGTTTACGCAGTGAAGGTGTC
AGTATCCTGAACAATCGCGTTGATATTCAGCGTCATGGCTGGCGCCCGGTAGAGCACAGCGGTTAGAGTGCGCGCT
TTGTTTCCGTAAATCTGAGGCAGACTCCAGCCCATGCCCCGTAAAACCTGGCGCGCCGCGCTCGCTGCCTATGCCA
GCCCCTCGACATTAGTGCTGTTACTGCTCGGCTTTGCCGCCGGCCTGCCTTACATGTTGGTGTTCTCGACGCTCTC
GGTCTGGTTGCGCGAAGCCGGTGTGGCACGCGAGACCATCGGCTATGCGAGCCTGATCGGCCTGGCGTATGCGTTC
AAGTGGGTCTGGTCACCGCTGCTGGACCAATGGCGCCTGCCGCTGTTGGGTAAGCTGGGACGTCGTCGTTCCTGGC
TGGTGCTGTCCCAATCCCTGGTGATTCTCGGCTTGATCGGCATGGGCTTCTGCGACCCGCAGAAACACCTGTCCTG
GCTGATCGCCATTGCCGTTATCGTCGCATTTGCCTCCGCAACCCAAGACATCGCGGTGGACGCCTATCGCCTGGAA
ATCGCCGACGACAGCCGCCAGGCCGCCCTGGCCGCCAGCTATATGTCCGGCTATCGCATCGCCGCCCTGCTGGCCA
CGGCGGGCGCGCTGTTCTTTGCCGAAGGCTTTGGCTCCACGGGGTTCAACTACAAGCATTCGGCGTGGACCGGCAC
CTACGTGCTGTTCGGCGTGCTGATGATCCCGGCCCTGCTCACCAGCTTTTTCATGCGTGAACCCGATGTGCCCCTG
CGCACCCAGTTGCAGGCCGGGCGCTATACGTTTGCCCACCAATTGATGTCGGTATTTGTGCTGATCATCTTGTTGG
TGTCGGTACCGGCGATGTTCACCCAGTTGTACAACACCGACTTCGCCAGCGTGCTGTTCGAAGGCGTGAGCCTGCT
CGACCTGTTGCTGGAAGACCGCGCCTTCCTGCGCGCCATCCTCTATATCACCCTCACCGCCCTGTGCCTCTCGGCC
ATGGGCCGCCGGGGTCTGGCGCCTGTGCTGACGCCGGTCAACGACTTTATCCTGCGCTACCGCTGGCAGGCCTTGC
TGCTGTTGGGGCTGATCGCCACCTACCGGATGTCGGACACGGTGATGGGCGTGATGGCCAACGTGTTCTATATCGA
CCAGGGGTTCACCAAGGACCAGATCGCCAGCGTCAGCAAGATCTTCGGCCTGATCATGACCCTGGTCGGCGCCGGC
ATGGGCGGCCTGTTGATCGTGCGGTTCGGCATCCTGCCGATCCTGTTTATCGGCGGCATCACCTCGGCCGGCACCA
ACCTGCTGTTCCTGATGCTCGCCGACATGGGCGCCGACCTGCAGATGCTGATCTTCACCATCTCCCTGGACAACTT
CAGCTCAGGCCTGGCGACCTCGGCATTCGTCGCCTATCTGTCGAGCCTGACCAACCTGAAGTTCTCCGCCACCCAG
TACGCCCTGCTCAGCTCGATCATGTTGCTGTTGCCACGGCTGATCGGCGGCTACTCGGGGGTCATGGTGGAAAAGT
TCGGCTATCACAACTTCTTCCTGATCACCGCACTGCTCGGCGTGCCAACGCTGCTGCTGATTGCCTTGCACTGGTA
CCAGGAAAGCCGGCGGATCCGCTTGAATCCCCCCGAAGAAGGCTGACACCTGCAGGGCCGGGCTTGTGTGGGAACG
GGCTTGCCCGCGATAGCATCACTGCGGTTTGCCTGACACACCGAGGCGCCTGCATCGCGGGCAAGCCCGCTCCCAC
ATTGACGCCGGCGCCCCACAGAAATCCCGTCACCACCTGATGCCTGTACTCTGGCGGCGACCGCCCGTACAATCCC
AGGTCACTTCAAGTCCAAGCAACCGACAACGGCCTACCATGCGCACCAGTCAATATTTGCTCGCCACACAGAAAGA
AACGCCTTCCGACGCGGTCGTGATCAGCCACCAGCTGATGCTGCGCGCCGGCATGATCCGCAAACTGGCCTCCGGC
CTGTACACCTGGCTGCCCATGGGCTTGAAGGTGATGCGCAAGGTCGAAGCGATCGTTCGCGAAGAAATGAACGCCG
CCGGCTCTCTGGAAGTATTGATGCCGAGCACTCAGCCGGCTGAACTGTGGCAGGAATCCGGGCGCTGGGAAGAGTA
CGGTCCGGAATTGCTGCGCTTCAAGGATCGTCATGGCCGCGACTTCTGCGCAGGCCCGACCCATGAAGAAGTGATC
ACCGACCTGATGCGCAACGAGTTGAGCAGCTACAAACAACTGCCCCTCAACCTGTATCAGATCCAGACCAAGTTCC
GTGACGAAATCCGCCCACGCTTCGGCTTGATGCGTGGCCGCGAATTCATCATGAAGGACGCCTATTCGTTCCACGC
CGACCAGCCTTCGCTGCAAGTCACCTATGACCGCATGCACCAGGCCTATTGCAACGTGTTCACCCGCCTGGGCCTG
AAGTTCCGCCCAGTGGAGGCAGATAACGGCTCGATCGGCGGCGCGGGCTCCCACGAGTTCCACGTACTGGCAGAAT
CGGGCGAAGACGACATCGTCTTCAGCGACGGCTCCGACTACGCAGCCAACATCGAGAAAGCCGAAGCCGTGCCACG
GGAAACGTCCCGTCCGGCGCCGGCTGAAGAGCTGCGCCTGGTCGATACCCCAGACACCAAGACCATCGCGGCCCTG
GTCGAGAAATTCAATCTGCCGATTGAAAAGACCATCAAGACCCTGATCGTGCATGCCGAAGAAGAAGGCAAGCTGA
TCGCCCTGGTTATCCGTGGCGACCACGAACTCAACGAAATCAAGGCGGCCCAGCAACCTGGCGTGGCCAGCCCGCT
GGTCATGGCCTCGGACGCCGAACTGCGTGACGCCATTGGCGCCGGCGCTGGTTCCCTGGGCCCGCTGAACCTGCCA
CTGCCGATCATCATCGACCGTTCGGTCGAGCTGATGAGCGACTTCGGCATCGGTGCCAATATCGACGACAAGCACT
ACTTTGGCGTCAACTGGGAACGTGACCTGCCGGTGCCTACCGTGGCCGACCTGCGCAACGTGGTTGCCGGTGATCC
AAGCCCGGATGGCAAGGGCACCCTGGAAATCAAGCGCGGTATCGAAGTGGGGCACATCTTCCAGCTGGGCAACAAG
TACAGCAAGGCGATGAAGTGCGAAGTACTGGGCGAAAACGGCAAGCCGGTAACCCTGGAAATGGGTTGCTACGGCA
TTGGTGTTTCCCGCGTGGTGGCGGCTGCCATCGAGCAGAGCCACGACGACAAAGGCATCATCTGGAGCGACACTTT
GGCGCCGTTCCAGATCGCCCTGGTACCCCTGCGCTACGAAACCGAGCAAGTTCGCGAAGCCACCGACAAGCTGTAT
GCCGAACTGACGGCCGCTGGCTTCGATGTACTGCTGGACGATCGCGACAAGAAGACCAGCCCGGGCATCAAGTTTG
CCGACATGGAACTGATTGGCATCCCTCACCGGATCGTGGTCAGTGACCGCGGCCTGGCCGATGGCAATCTGGAATA
CAAGAGCCGGACCGAAGCCCAAGCCCAACCGTTGCCGGTGGCTGACGTGCTGCCCTTCCTTCAGGCGCGTATTCGT
CGCTGAAAACCAGATCAAGAGAAGTCATGTTCAAGCGAAACACCAAAGCCCTGGGGGGCGCCGCCTTGTGCGGCGC
CCTGCTGGTCAGCGGCTGTGCCAATCAGATGTCGCAGCGTAGCGAGCACGAGGAACGGATCGAGCGCAAATTGCTC
GACCACAGCCTGCAGATTGATGTGGGCGAACCCAAAGTACTCGAGTTGCCGCAACGCCGCGTGCGCATTCATGAGC
AGCGGACTTTCGAAGTCACTGAATTTGAAGTCACGCGTCACTATGATCGCTACACGCCCTACCAGCCCTGGCGCGA
ACTCTACGAGATCCCGCTGGGTGCCGTGGCAGTGGTGGCGGGCGCCGGGGCCAATGTGGCGAACATCTTTGCCCTG
GGCAACCTGCCAGACAGCATGACCCATGACTGGATCAGCTACGGCGTGGCCGGGCTCAACCCGTTCATGAACGTGC

AGTCCCATGGCCGGGCGCAACAGAACCTGGCGGGCATCGATGAAGTCCAGCGTGACCGACGCCTGGAATACACCAG
CCTGCCGTGGAGCGAGCGACCGGTGCAGGTAAAAGCCGGCAAGCAGACCCACGAGATGACCACCGACCGCAACGGC
GTCCTGCGCCTGAACCTGTTGGACAGCCCGTTTGCCGAGCAGGACCTGAGCAAGGTCCGCACCTTGCGGATCAGCA
TTGAAGATGCCCAGGACGATGTGCATTCGGACTCATCCCTGGCCATCAGCACCACCCTGCGTGGCAAGCTGCTGGA
AGCACACGCCTTGATCTACGACGACCTGGAGGATGACGAAGTCAACCAATGGGTCCATCGGGTCAAGCGCTTGTCG
GAACTGGGCCTGGAAGAAGAAGCCAGCGAACTGGAACAGAGCCTGATTGAACTGACACGCAATGATCCTGAATTGC
AGCAGGAATTTCTCAAGGCCCTGACCAAGGACGGCGGCCGACTGGTGGCCGACCCCGGCACCCGCTGACCCCCTGT
AGGAGCCGGCTTGCCGGCTCCTACAGGGACAGCTCTAGCTGTTCATTTCCTACGCTCAAATCGAGCAGCCGCACAC
CAATCCCCAGCAAGCGCACCGGTTTTGCGCCGCGATTGAACGCCTGGGTCAGCAACTGCCGATAGCTCTCCAGATC
CCGCCCCGCCCCCGCCTGCTCCAGGGTGGTTTGGGTAAAGTCATGGAACTTCACTTTGACAAAGGGTTTGCCCGGC
CGATAACTGCTGTCGATACGCTGTATGCGCCCGGCCAGGGTTTCCATCAGTTCCGGTAGCTTTTCCAGGCAACTGG
GCAGGTCCGGCAGGTCGACATCGTAGGTGTTCTCCACGCTGATCGATTGCCGGCGACTGTCGTTATGCACCGCGCG
ATCATCAATCCCACGCGCCAAACTCCATAACCGCTCACCAAAGCTGCCGAATTCGCGCACCAGCGCCAGCTTGTTC
CACTCGCGCAATTGCAGGCAATCCTCGATCCCCAGGCGTGCCAGCTTGTCGGCGGTGACCTTGCCGACGCCATGCA
ACTTGCTCACCGGCAGCTGCGATACGAAGTCTTCCACCTGGTCCGGGGTGATCACAAACAGCCCATTGGGCTTTTT
CCAGTCGCTGGCGATCTTGGCCAGAAACTTGTTGGGAGCCACTCCCGCAGAAACCGTGATGTGCAACTGATTGGAA
ACGCGCCGACGGATATCCTGGGCGATGCGCGTGGCGCTGCCGCCAAAGTGCGCGCAATCGGAAACATCCAGGTAAG
CCTCGTCCAGGGACAACGGCTCGATCATGTCGGTGTAGTCACGGAAAATCGTGTGGATTTCCTTGGACGCTTCTTT
ATAGGCGTCCATGCGCGGCTTGACGATGGTCAGGTCAGGGCAGAGCTTCAACGCATGGCGCGACGACATCGCCGAA
CGCACGCCGTAGGCCCGGGCCTCGTAGTTGCAGGTAGCGATCACCCCGCGCCGATCAGCCGAGCCCCCTACCGCCA
GCGGCTTTTGCGCCAGGCTCGGGTTATCCCGCATTTCGATGGCAGCGTAGAAACAATCACAGTCGACATGGATGAT
TTTGCGCTGCGTCATATAAAGGGAGGCGTGATCGGGACATAAAGTAGACGGATCGCCAGTATCGCATTCACACCTG
TATATAGCACCAGTACTTTGAATCATCTGACAAAGCAGTCGGAAAAAAACCTTGATGAATTCATTTTCTCAATCGAA
AGGCTCCCCTCAATAGAGCTGGAAGCCACGGCCAGACTGGCCCCAAACCCTTCCAGCGGCCCTCTTGGAGAGCTAA
GCGATTGAAGCACAAGCGCTTTTCTTTAAATCAGCGGTTGACACACCGTCATTCCTCTGTAGAATGCCGACACACA
GACGCGGGATGGAGCAGTCTGGTAGCTCGTCGGGCTCATAACCCGAAGGTCGTCGGTTCAAATCCGGCTCCCGCAA
CCAAACATCAAAAAAGGCTACTCGAAAGAGTGGCCTTTTTTGTATCCGTAGAAAAAGTTCTTTTGAAACAATGGCA
TGGCATCTTTCATGAAACCGCGCGCGGGTTTCGGAGTCCTTCCATTTGCAGGCTATGCTCAAGTCTCGAGCACATC
CTTGCGCGACTAATGGACGCTGTCGCCGGCCCCGGTGATCCGCGTTACTATTTGTAATTATTTTGTCCATAGGGAT
TGGTAACTTGGCTGGATACCCCCATCCTGTCGCGCACAATCCAAGAGGTGATTGATGCGCGCCAATTCGTCTGAAC
CACAAGACAACATCACAGCTACACAACCGATCGCGCCCACCCGCCTGCGCTGGCTGGATCTGTTGAGCAAGTATCG
GCAACCCATTGGGTTGGCGGTCACGTTGCTGCTATTTGCAATCGCTTTGATCGCCTGCCGCCACTTGTTGCTAGAG
CTGGATCTGTACGCCCTCCACGATTCGATCCTGGAAGTACCGAAGCCGTCCCTGTTGGGCGCCTTCGCCGCTGCGA
TCGCCGGGTTCATCATTCTATTGGGCTACGAATTTTCCGGGGCGCGCTATGCCGGAGTAAAACTTCCTGCTAGAAC
CCTGGCCTACGGCGGCTTTACTGCTTTCGCCATCGGTAATGCGATTGGCCTGTCGATGCTGTCTGGCGGCTCAGTG
CGCTACCGTTTATATGCACGGCATGGCATTGGGGCTTCGGAAGTGGCCCATATGACCGTATTTGCCAGCCTGGCCC
TGGGCTGTGCCCTGCCGCCGCTCGCCGCACTGGCGACCTTGAGCAACCTGCCCGCCGCGTCCAGTGCCTTGCATTT
GCCTGCAGCACTGCTCGGCGCAATCGCAGGCGCGGTACTGCTGCTCTCTGCCGTGCTGTGTATCGGGATCTATCGC
CGCCGCTTGCCAGAACAACCCTACCCCGACAACCTGCTGGTCAAGGCCGGCCGCCGCACCTTGCGCCTGCCTGGGC
GCCGCCTGACCTTCCTGCAATTGATCATCACCGCCCTGGATGTCGCTGCCGCCGCGACCGTTCTCTATCTGTTGCT
ACCAGAAGCCCCGCCGTTCGGCCCGTTCCTGCTGGTGTACCTGCTGGCGCTGGCGGCGGGTGTCCTCAGCCATGTA
CCGGGCGGCGTCGGCGTGTTCGAAGCGATTTTGCTGGCCGCGTTTGCCGACAAGCTCGGCGCCGCGCCGCTGGCCG
CCGCCCTGCTGTTGTATCGCATGATCTACGTTGTGCTGCCGTTACTGATCGCGTGCATCTTCCTGCTGATCAACGA
GGCCCAACGGCTGTTCCAGACCCAGCAGAGCCTGCGGGTCGCCTCAGGCCTCGCGGCCCCGGTACTGGCCGTGCTG
GTTTTTTTGTCCGGGGTGGTGCTGCTGTTCTCCGGCGCCACCCCGGAAATCGACTCACGCCTGGAAAACATCGGCT
TCCTGATTCCCCACCGCCTGATTGACGCCTCGCACTTTGGTGCCAGCCTGATCGGCGTGCTGTGCCTGCTACTGGC
CCAAGGCCTGCGTCGACGCCTGTCGGCAGCCTGGATGCTGACCATGGTGCTGTTGCTGGTGGGCGCCCTGCTCTCG
CTGCTTAAAGGCTTCGACTGGGAAGAAGCCAGCCTGATGACCACCACCGCAGTGCTGCTGGCGATCTTCCGGCGCT
CGTTCTACCGTGCCAGTCGCCTGACCGAGCTGCCCTTTTCGCCGCTGTACCTGGTGGCCAGCGTCTGCGTGCTGGG
TGCTTCGATCTGGCTGCTGCTGTTTGCTTACCAGGATGTGCCTTATAGCCATCAACTGTGGTGGCAGTTCACCCTC
GACGCCAACGCCCCGCGCGGCCTGCGCTCGCTGCTGGGCGCCGCCATCTTGCTGGTGATTGTGTCCCTGACCTGGC
TGTTGCGCACCGCCCGCCCTGTGATCCACCTGCCAACCGCCGATGAGCTGGAGCGCGCCACCAAGATCCTGATGGC
CTCGTCGCAACCCGACGGCGGCCTGGCACTGACCGGCGACAAGGCCCTGCTATTTCACCCCAACGACGAAGCGTTC
CTGATGTACGCCCGGCGCGGCCGCAGCCTGGTGGCGCTGTACGACCCGATCGGCCCGACCCAGCCCCGGGCCGAGA
TGATCTGGCAGTTCCGCGACCTCTGCGACATTCACCACGCCCGCCCGGTGTTCTACCAGGTGCGCGCGGAGAACCT
GCCCTTCTATATGGACATTGGCCTGACTGCGATCAAGCTGGGCGAAGAGGCCCGGGTCGATCTCAAGCGCTTTGAC
CTGGAAGCCAAGGGCAAGGAGATGAAGGATTTGCGCTACACCTGGAACCGCGGGACCCGGGACGGCCTGTCCCTGG

AAGTCTTCGAGGCGGGCCAGGCGCCGATGGACGCACTGAAAGTCATCTCCGATGCCTGGTTGACCGGTAAGAACGT
GCGCGAAAAAGGCTTCTCCCTGGGCCGTTTCAGCGACGAATACCTCAAGCACTTTCGCATTGCGGTGATTCGCTTC
GAAGGCCAGCCGGTGGCGTTCGCCAACCTGCTGGAGACCTACAGCCATGATCTGGCCAGCCTCGACCTGATGCGCG
CGCATCCCGAAGCGCCAAAGCTGACCATGGAGTTCATGATGATCGGCTTGATCCTGCACTATAAGAGCCACAACTA
TGCACGCTTCAGCCTCGGCATGGTGCCGTTGTCGGGCTTGCAGCCGCGCCGTGGCGCACCGCTGACCCAGCGCCTG
GGCTCGATGGTGTTCCGCCGTGGCGAGCAACTGTATAACTTCCAAGGTTTGCGCCGCTTCAAAGACAAGTTCCAGC
CTGACTGGGAACCTCGTTACATGGCCGTGCCCGCAGGACTTGATCCGCTGGTGGCACTGGCCGATACCGCCGCCCT
GATCGCGGGCGGCTTGACTGGATTGGTGAAACGCTGATGATTCGACGCTCCTGGCGGTATGTATTGGCAAGCCTGG
TGGTGCTGGCCCTGATCCTCGGTGGCGGCTACTGGTACTGGAATCGCCCCGCCCCTCAACCGACCCTGGAACAACT
GCCCCAGGCCGACGGCTCGACCATGACCCGTGTGACCCCTGGCACCAGCGCCAAGGCCCGTGTAGCGGTAGCCGTG
ATGGCCGACGCCAGCCTGACCGACAGCCAGCTGATTGCCCTGAGCCAGGGCGGCAGCGCGCAGATCGTGCAAGTGA
TCCTGCCCAAGGACGACTGCAAGCTGCAAGAACAAGCCCTGCAAGCGGCCCTCGGCCAGCTCAAGGGCCCGGCCAC
CCTGGTCAGCGGCATCGGCCCTGGCGCCGCGCTGGCCTGGCGCTGGCTGGCAACGCAGAACGACGACAAGGCCAAC
GCCATCTCCGTAGGCTTCGCCCTGGTCCAGGAAAGTTGCAAGGACCCACTGCCCAAGACTGCCGCCCACGGCAACT
GGCTGGTGGCCTGGAACGATAACCCTGACGACGAAAGCGCCAGCTTCGTACGCGACACGCCGCGTGCCACCACCAG
CATCAGCGACTACGACATCAACTACCCGCAGGTATTGAACAACGAGCTGCGCAAGCAACTGGTGGGTTCGGACAAT
GGCGGCCTGGCGATCCCGGTGGTTGAAGTGCCGGCCGGCCAGGCCAAGGACACCGTGACCCTGTTCCTTTCCGGCG
ACGGCGGCTGGCGCGACCTGGACCGCGACGTGGCCGGCGAGATGGCGAAGATCGGTTACCCGGTGGTCGGTATCGA
CACCCTGCGCTACTACTGGCAGCACAAAAGCCCGGAGCAGAGCGCCAAGGACCTGAGCGAGCTGATGCAACACTAC
CGGCAAAAAATGGGGCACCAAGCGTTTTGTGCTGACCGGCTATTCGTTCGGCGCCGATGTGCTGCCAGCCATCTACA
ACCGCCTGCCGGAAGCCGAGCAGCAACGGGTAGACGCTATTATCCTGCTGGCCTTCGCCCGCACCGGTAGCTTTGA
GATCGAAGTGGAAGGCTGGCTGGGCAATGCCGGCAAAGAGGCAGCCACCGGCCCGGAAATGGCCAAGCTGCCAGCA
GCCAAAGTGGTGTGCATCTACGGCGAAGAAGAGGTCGACGAGAGTGGCTGCACCGACAAGACCGCCGTGGGTGAAG
CCATGAAACTACCGGGTGGCCATCACTTCGACGAGAACTACCCGGCACTGGCCAAGCGCCTGGTGGATATCATCGA
GAAGCGCCAGGCCAAGGCCGAGTAAACGCCCATCCTGTAGGAGCCAGTTTCCTCAGGCACTAAAAAGCCCCCGCTG
CCGCACGGCAACGGGGGCTTTTTGTTGGGCTACATTTCTACCTGGGTGCCCAGCTCAATCACCCGGTTCACCGGCA
GCTTGAAGAAGCGCAGGTTGCCGTTGGCGTTCTTCAGCATGAAGGCGAACAACCCTTCCCGCCAGCGCGCCATGCC
CTCAAGCTTGGAGGCGATGACCGTCTCGCGGCTGAGGAAGTACGTGGTGCGCATGGGGCTAAAGTCCAGGTCATCC
AAGTGGCACAGCTTCAGGGCTTCGGGCACGTCCGGCTCATCGGTAAAGCCGAAGTGCAGGATCACCCGGAAGAAGC
CGTCACCGTAGGAGTCCACCTCGAAGCGGCGCTGGGCCGGGACGCGCGGGATGTCTTCATAGACCACCGTCAACAG
CACCACTTGCTCGTGCAGCACCTGGTTGTGCAGCAGGTTATGCAGCAGGGCGTGGGGCACCGCGTCCGGGCGGGCG
GTGAGGAACACCGCCGTGCCCTGTACGCGATGGGGCGGTTGCACGCGAATGCTGCTGATAAAGATCGGCAGCGGCA
GCCCGCCCTCGTCCAGGCGCTCCACCAGCAATTGCTTACCGCGCTTCCAGGTGGTCATCAACACAAACAGCACGAT
CCCCGCCAGCACCGGGAACGCGCCGCCCTGGACGACTTTCGGCACGTTGGCCGCGAAGAACAGGCCATCCACCAGC
AAAAAGCCCAGCAAAACAGGCACTGCCAATACCGGCGGCCACTTCCACAACAGCAGCATCACGGCGGATACCAGGA
TGGTGGTCATCAGCATGGTGCCGGTCACAGCCACCCCGTATGCCGAGGCCAGGGCGCCCGAGGACTCGAAGCCCAG
CACCAGCAGGATTACACCGACCATCAGCGACCAGTTCACCGCGCCAATATAGATCTGGCCCTGTTCGGCGCTGGAG
GTGTGCTGGATATGCATGCGCGGGATGTAACCCAACTGGATCGCCTGGCGCGTCAACGAGAACGCACCGGAAATCA
CCGCCTGGGATGCAATCACCGTCGCCAGGGTGGACAACCCCACCAGTGGCAGCAATGCCCAGCTTGGCGCCAGCAG
GTAGAACGGGTTGCGCGCGGCTTCGGGGTCGCCCAGCAACATCGCGCCCTGGCCAAAATAGTTGAGCACCAGCGCC
GGCAGCACCAGGATGAACCAGGCGCGGGCAATGGGTTTGCGGCCGAAGTGGCCCATGTCGGCATACAGCGCCTCGG
CACCGGTCAAGGCCAGCACCACCGCGCCTAGGATCGCCACGCCGATACCCGGATGCACCATGAAGAAACGCACGCC
CCACATCGGGTTCATCGCGTTGAGCACTTCGGGGTGCTGGACAATGCCATAGATGCCCAGGCCGCCCAGCACCAGG
AACCAGGTGACCATCACCGGCCCGAACAGCTTGCCGATGCGGTCTGTCCCGTGTTTCTGGATCAGGAACAACCCCA
CCAGCACCACCAGCGCAACGGGCACCACCCAGTGCTCCAGGCCATCGAACGCCAGTTCCAGGCCCTCGATGGCCGA
CAATACCGAAATCGCCGGGGTGATCATGCTGTCGCCATAGAACAGCGCCGCGCCGCATAGGCCGCACACCACCAGG
AAACTGCGCAACCGCGGGTGATTGCCGGCCGCCCGTCGGGCCAGTGCGGTAAGGGCCATGATGCCGCCCTCACCCT
GGTTGTCGGCGCGCAGTACGAACAACATGTACTTGATCGACACCACCCAGATCAGCGACCAGAAGATCAGGGCCAG
GATCCCCAGCACCCCATCATGATTGACCTGGACCCCATAACCACCGGAAAACACCTCTTTAAGGGTGTAGAGAGGG
CTGGTACCGATATCACCGTAGACCACCCCGACAGCTGCCACCAACATGCCAATTGGCTTTGCGCTGGAATGCCCGG
CACCTGCCGCCTGACTATTTGCCTGACCCATCAACCACTCCTGCCCTTTTGACCCGAGGTCTTTTATAAACAGCAC
AGCTGTACAGCTAAGCCTAAGCTGTCCACAGCATGCGCTGTTTTACTTCTAGTAACAGATGATTTGTTGACCATCG
GCTTTACACAAGTGCAACGGCGCGAAGCATAGCGCAGCACTCGTCGTATTTCCCTGCATAAAGCTGGTCAAGCGCG
TTTCCCGCCGCTAGAATTGCGCACTTTTTGATCAGAGGCACATCAAGTGCCCGTCCGCTGCCCTGTCGTGCCCGAC
TGGCGGCGTCATTCAATACCGAGGTTAGACATGTCCACCACCATCGCAAAAGCCAACCCCAAGGTTGGCTTTGTTT
CCCTGGGTTGCCCGAAGGCGCTCGTCGACTCCGAGCGCATCCTCACCCAACTGCGCATGGAAGGTTACGATGTCGT
GTCCACCTACCAGGACGCCGATGTGGTGGTGGTCAACACCTGTGGCTTCATTGACTCGGCCAAGGCAGAGTCTTTG

GAAGTGATCGGCGAAGCCATCAAGGAAAACGGCAAGGTGATCGTCACCGGCTGCATGGGCGTGGAAGAAGGCAATA
TCCGCAACGTGCACCCAAGCGTGCTGGCCGTGACCGGCCCGCAGCAGTACGAGCAGGTGGTCAACGCTGTGCATGA
GGTGGTGCCGCCGCGCCAGGATCACAACCCGTTGATCGACCTGGTGCCGCCGCAAGGCATCAAGCTGACGCCACGT
CATTACGCTTACCTGAAGATTTCCGAAGGCTGCAACCACAGCTGCAGCTTCTGCATCATCCCGTCGATGCGCGGCA
AGCTGGTCAGCCGCCCGGTGGGTGATGTGCTGGACGAAGCCCAGCGCCTGGTCAAGTCTGGCGTCAAAGAGCTGCT
GGTGATCTCCCAGGACACCAGCGCCTACGGCGTCGACGTGAAGTACCGCACCGGCTTCTGGAATGGCGCGCCGGTG
AAAACCCGCATGACCGAACTCTGCGAAGCCTTGAGCAGCCTGGGTGTGTGGGTACGCCTGCACTACGTGTACCCGT
ACCCACACGTCGACGAATTGATCCCGCTGATGGCCGCCGGCAAGATCCTGCCGTATCTGGACATCCCGTTCCAACA
CGCCAGCCCGAAAGTGCTCAAGGCCATGAAACGCCCGGCGTTTGAAGACAAGACCCTGGCGCGGATCAAGAACTGG
CGCCAGATCTGCCCTGAGCTGATCATCCGTTCGACCTTTATCGTCGGCTTCCCCGGCGAAACCGAAGAAGACTTCC
AGTACCTGCTGGATTGGCTGACCGAAGCCCAGCTCGATCGCGTCGGCTGCTTCCAGTACTCGCCAGTGGAAGGTGC
GCCAGCCAACCTGCTGGACCTGGCCGTGGTGCCGGATGACGTCAAGCAGGACCGTTGGGAGCGTTTCATGACCCAT
CAGCAGGCCATCAGCTCGGCACGCCTGCAACTGCGTATCGGCAAGGAAATCGAAGTACTGATCGACGAAGTGGACG
AGCAAGGCGCGGTAGGCCGTTGCTTCTTCGACGCTCCGGAAATCGACGGTAACGTATTCATCGACGATGCCAGCGG
TCTCAAGCCAGGCGACAAGGTGTGGTGCACAGTCACCGACGCCGACGAGTACGACCTCTGGGCGCAAAAACGCGAC
TAAGCTGTAAAAAATTGAAAAAGCCCTGCTTCTGGACAAGATGCGGGGCTTTTTTAGGTCTATCGTTTGCCCCATC
ACCGCCAACAAGCAGCAAGGAGCAGCGGGCATGGGCCAGCACTCGGTTATTCACACGCCTAAACCCAGCGACTATC
AGGAGCTGACCCGGATCTGGGAGGCATCGGTGCGGGCCACCCATGACTTCCTGCCGCAAAGCTACATCGAGCGCCT
GCGACACCTGGTGCTGACGCGTTACCTCGATGCGGTGATGCTGATCTGTACCAAGGACGCGCGCCAACGGATCACC
GGATTTGCCGGTGTCGCCGCAGGCAAGGTCGAGATGCTGTTCATCGACCCGCAGTACCGGGGCCAGGGCCTGGGGC
GGCAATTGCTGCACTATGCCGTTGCGTCGATGAATGCCGATGAACTGGACGTCAACGAACAGAACCCACAGGCCCT
GGGCTTCTATATCAAGCAAGGTTTTGAAGTAATCGGGCGCACCGAGCATGACGGCATGGGCCAGCCCTATCCCTTG
CTGCACATGCGTTTGCGCCAGGCCCAGCAAACACGCAGCGGCTGACACAACCCAAATCAAATGCAGGAGCTGGCTT
GCCTGCGATGCGGTCAACTCGGTGCATCAGGTAGGCCGAGGTGATGCCATCGCAGACAAGCCAGCTCCCACAAAAA
GCGCGAGCACTGAAATGGGGCCGGGATTAACCGGCGCCAGGCAGGTACAATAGCGGCCCCCTTTTGTTACGGCCCT
TGTCATGACTGACCCCATACGCCTCTCCAAACGCCTTATCGAACTGGTCGGTTGCTCCCGTCGGGAGGCCGAGCTG
TTCATCGAAGGCGGCTGGGTCTCGGTGGATGGCGAAGTGATCGACGAGCCGCAGTTCAAGGTCACGACACAAAAAG
TCGAACTTGATCCCGAGGCCAAGGCCACGGCTCCGGAGCCGGTGACTATCCTGTTTCATGCCCCGGCCGGCGTGGA
CGTCGACAGCGCGATGCAATCCCTGAGCGCCGAGACGCTGTCGGAAGAACACCGCTTCAGCAAGCGCCCGCTCAAA
GGCCACTTCCTGCGCCTGACCGCCAGCGCCGATCTGCAAGCCAAGGCCAGCGGCCTGCTGGTGTTTACCCAGGACT
GGAAGATCCTGCGCAAGCTGACGGCCGACGCCGCCAAGATCGAGCAGGAGTATGTGGTCGAGGTCGCAGGCGACGT
GGCAGAGCACGGCCTGAACCGCCTCGCCCACGGCCTGATGTACAAAGGCAAGGAATTGCCGGCGGTCAAAGCCAGC
TGGCAGAACGAAAACCGCCTGCGCTTTGCCCTGAAAAACCCGCAGCCCGGCATCATCGCCCTGTTCTGCGAAGCCA
TTGGCCTGAAGGTTGTGGCCATTCGCCGCATCCGCATTGGCGGCGTGTCCATCGGCAAGGTGCCGGTCGGCCAGTG
GCGCTACATGTCCGGCAAAGAAAAGTTCTAAGCCACCCTATCTATCGACATCGCCCGCCCGGGCGGTGTCTACAGT
CGAATACCAGGATTGCCCACCATGATTCACAATGACGTACTGCGCAGCGTGCGCTACATGCTCGACATCAGCGACA
ACAAGATGGTCGAGATCATCAAGATCGGCGGCATGGAAGTGTCCAAGGAAGACCTGCTGACCTACCTCAAGAAAGA
CGAGGAAGAAGGCTTCGTGTTCTGCCCCGACGAGGTCATGGCGCATTTCCTCGATGGCCTGGTGATCTTCAAGCGC
GGCAAGGACGAAAGCCGTCCACCACAGCCGATCGAAACCCCGGTGACCAACAACATCATCCTCAAGAAGCTGCGCG
TGGCCTTCGAACTCAAGGAAGACGACATGCACGCCATCCTCAAGGCCGCCGAGTTCCCGGTGTCCAAGCCAGAGCT
GAGCGCGCTGTTCCGCAAGTTCGGCCACACCAACTATCGTCCGTGTGGCGACCAGTTGCTGCGTAACTTCCTCAAG
GGCCTGACCCTGCGGGTTCGCGCGTAAGCCATGAATTACAACGTCTCGCCCGTGGGCTTCGTGCGCTCCTGCTTCA
AGGAGAAGTTCGCCATCCCGCGCCAGCCACAACTGGCGCCCGCCGCACGGGGCGTGTTGGAGTTGGTGGCGCCGTT
CGACCAGGGTGAGGCGGTGCAAGGCCTGGAGCAGGTCAGCCATGTGTGGCTGCTGTTCCTGTTTCATCAGGCCCTG
GAAGACAAGCCGCGCTTGAAAGTGCGCCCACCGCGCCTGGGCGGCAACACATCCATGGGTGTATTTGCCACCCGCG
CAACCCATCGCCCCAATGGGATCGGCCAGTCGGTGGTGAAACTGGACAAGGTGGAGCCGGGCCGGCTGTGGGTTTC
CGGGATCGATCTGCTCGATGGCACGCCGGTATTGGATATCAAGCCGTATGTGCCGTATGCCGACATCATCGACACG
GCCACCAACAGCATTGCCAGTGCCGCACCGGCGCTGATCCCCGTGCAATGGCTGAAGACCGCCCTGCAACAGGCTC
ACACCCACGCTCAGCGCCTTGGCGAGCCGTTGGTGGAGCTGATTGAACAATGCCTGGCGCAGGATCCACGCCCGGC
CTATCAGACGCCCGGGCCAGAGCGCGAATACGGCGTGCGGTTCTGGGATGTGGATGTGCGTTGGCACTATCCCGAG
GCGGGGATGATTTGTGTGCTTGAAGTGGTTGCCGCGCAGTAAATCGCAGCAATGAAAAAGCCCGCACTGCCTTCAC
AGGCAATGCGGGCTTTTCTTTGCAAACGCCTTCACCTGTAGGAGCGAGCTTGCTCGCGAAGAACCCAAGGGCAGCG
CGTCAACTCGACCGCGTTAACGTTGACGACTTTCGCGAGCAAGCTCGCTCCTACAGATGTGCCGGGGTCACTTCTC
GACGAAGGCGCGTTCGATCAGGTAGTCACCCGGCTCACGCATCCGTGGCGAAACCTTCAGGCCGAAGCTGTTCAGC
ACTTCGCTGGTCTCATCCAACATGCTTGGGCTGCCGCACAGCATGGCGCGGTCGTCTTCAGGGTTGATCGGCGGCA
GGCCGATATCGCTGAACAGCTTGCCGCTGCGCATCAGGTCGGTCAGGCGGCCTTCGTTTTCGAAGGGTTCGCGGGT
CACGGTTGGGTAGTAGATCAACTTTTCACGCAGCGCCTCGCCGAAGAATTCGTTCTGCGGCAGGTGTTCGGTGATG

AATTCGCGATAGGCGACTTCGTTGACGTAACGCACACCGTGGCACAGGATCACTTTTTCAAAGCGCTCGTAGGTTT
CCGGGTCCTGGATCACGCTCATGAAGGGGGCGAGACCAGTACCGGTGCTGAGCAGGTACAGGTGCTTGCCCGGCTT
CAAATCGTCCAGCACCAGGGTGCCAGTCGGTTTTTTGCTGATGATGATCTCGTCGCCTTCCTTCAAATGCTGCAAT
TGGGAAGTCAGCGGGCCATCCGGAACCTTGATGCTGAAGAACTCCAGATGCTCTTCCCAGTTCGGGCTGGCGATGG
AGTAAGCGCGCATGAGCGGGCGGCCGTTAGGCTGTTGCAGGCCGATCATCACGAACTGACCGTTCTCAAAGCGCAA
GCCCGGGTCGCGGGTGCACTTGAAGCTGAACAGAGTGTCGTTCCAGTGATGAACACTGAGGACACGCTCGTGGTTC
ATGTTGCTCATGTACGGGGAACTCCTGGAAATGGGTCTGCGCCAAAATGATAGGTGCGCAATTGCACAGTATTCTA
ATGGCGGCGACAATATCTGTTAACTGGATTATTAAGATAAGGGTTATCGGTTATATCGATATGCGATTTACTCTCC
GTCAACTGCAAGTCTTCGTCGCCGTCGCCCAGCAGGAAAGTGTGTCACGCGCTGCTGGCCTTCTGGCCTTATCTCA
ATCCGCCGCCAGCACCTCGATCACCGAGCTGGAGCGCCAATCCAGCTGTCAATTATTCGACCGCGCCGGTAAACGC
CTGAGCCTCAACGCCCTCGGCCATCAACTGCTACCCCAGGCGGTGGCGCTGCTGGACCAGGCCAAGGAGATCGAAG
ACCTGCTCAACGGCAAGTCTGGCTTCGGTTCCCTGGCGGTCGGCGCCACCCTGACCATCGGCAATTACCTGGCCAC
CCTGCTGATCGGCAGTTTCATGCAGCAGCATCCCGAGAGCCAGGTGAAGCTGCATGTACAGAACACTGCGCATATC
GTGCATCAGGTTGCGCATTACGAAATTGATCTGGGTCTAATCGAAGGCGACTGCAGCCACCCGGACATCGAGGTGC
AAACCTGGGTCGAGGATGAGCTGGTGGTGTTCTGCGCGCCGCAGCATCCCCTGGCCCAGCGCGGTCAGGCGACCAT
GGAGCAACTGACCCATGAAGCCTGGATCCTGCGGGAACAGGGCTCCGGGACACGCCTGACCTTTGATCAAGCCATG
CGCCATCATCGCAGCGCGCTGAACATCCGCCTGGAGCTGGAGCACACCGAGGCGATCAAGCGGGCCGTGGAGTCAG
GATTGGGGATTGGCTGCATTTCACGCCTGGCGTTACGGGATGCGTTCCGCCGTGGCAGCCTGGTGCCAGTGGAGAC
GCCGGACCTGGACCTGGCCCGGCAGTTCTACTTTATCTGGCATAAACAGAAGTACCAGACCTCGGCCATGCGCGAG
TTCCTCGAACTGTGCCGCGCCTTCACCGCTGGGGTGCAGCGCAGCGACGAGATCGTACTGCCGAATATCGCCTAGA
TCAGGATTACGGCCCACACCACGGTGATCATGGTCAGCGCCACGAATTGCGCGGCGCTGCCCATGTCCTTGGCGTT
TTTCGACAGGGGGTGGCGATCCAGGGAAATCCGGTCGATTGCCGCTTCCACGGCCGAGTTGAGCAACTCGACAATC
AACGCCAGCAGGCACACGGCGATCAGCAGTGCGCGCTCTACGCGGCTGACCGTGAGGAAGAAGCTCAACGGAATCA
GGATGACATTGAGCAAGACCAACTGGCGAAAGGCCGCCTCGCCGGTGAAAGCCGCACGCAGGCCATCGAGGGAGTA
ACCCCCGGCGTTGAAGATACGTTTGATACCGGTTTGACCCTTGAAAGGCGACATAGGTAGGCAACTGAGCAAAAAA
GATGGGAGGAAGCTAGAGCACGCCTGGTCAAAAAAGCGTGAATCGAGCCACCCTACGAGTGGGAAATTGACTCAAG
TTGTTGCAACAGCAGCGCCGCCTGGGTCCGGGTGCGCACACCCAGCTTGCGGAAAATCGCGGTAACGTGCGCCTTG
ATGGTCGCTTCGGATACGCTCAATTCATAGGCGATCTGCTTGTTCAGCAAGCCTTCGCAGACCATGGTCAACACAC
GGAACTGCTGGGGCGTAAGGCTGGCGAGCCCTTCCTGGGCAGCCTTGGCTTCGGCGGACACGTTGATTTCTTCGAA
GGCCTGGGGCGGCCACGAGACGTCACCGTCCAACACCGTACGCACCGCTGTTTGAATCTGCTCCATGGAACTGGAT
TTGGGGATAAAACCACTGGCCCCGAACTCCCGGGAACGCACTACGATATCGGCCTCTTCCTGGGCCGACACCATCA
CCACCGGAATCTGCGGATATTGCCCGCGTAACAGCACCAAGCCGGAAAACCCGTAGGCGCCGGGCATATTCAGGTC
CAGCAGCACCAGATCCCAATCGGTCTTGTCAGTCAGGCGGGCTTCCAGTTCCGCGATGCTGCCCACTTCCACCAGG
CGTACATCCGGGCCCAGGCCCAGTGTGACCGCCTGGTGCAGCGCGCTGCGGAAAAGTGGGTGGTCATCGGCAATCA
GGATTTCGTATGTGGCCATTTATTAAATGATCCTGTTTTTTATGGGAGCACCGATGGGTTCGGTGCTCACCAACGT
ACGAGGTGCCAGCCAGCGGCTGTCACGAACAGTTACGCTTGATGGCATTTTAGCCGGCTCAGGCGTTGCAGTGCCA
TCATGCAAAGGTCATCTGGCCCCAATGCCTACAGATAAGGGGCAAGACGCGTGTGGACGCTGTCCGTCGGGTCCAG
GGCCAACTGGAACTTGGCCGCCAGGTAATGGGTACTGAACACATCCAGGTAGGCATCCAGCACTTCACTGGCCTGC
TGGTCCTGGGCCAGTTCCAGGCACAAGGCAGCCACTTCGGCCGTGCAGAAATGGTCATCGCGTTTGGAACGGCGCA
ATTTGTAGCGCGATAGCTGCTCCGGCTCCAGGCTCAGTACCGGCAGGTGTTCCAGATACGGGCTCTTGCGGAACAT
CTTGCGCGCTTCGCTCCAGGTGCCGTCCAGCAGGATGAACAACGGGCGCTTGCCATCCGCCACCTTGACCTGGTTG
ACCACCCGCTGCGGCGCCACAAACTCGCCGGGAAAGACGATGTAGGGCTGCCATTGCGGGTCGGCCAGCAGCGCCA
GCAGTTCAGGTTCGACGGCCGTGCGCGCCCAGGGGAAGGCGCTGGTGTCGTCGATGACATCAGCGATCAGCCAACC
GGTATTGCTGGGTTTCATCGGTTCCACGTCGTGCATCAACAGGCACATGGCCGACTTGGCCGCGACCTTGGGGCGC
CAGGCGCACAGGCAGTATTCGGGGATGACCCGGCACCTGGGGCAACGTTCGGCACGCGAGCCACGGGCAACAAAGG
GGCTCACGGCACGGGCCAGGCGCTGGGTACGCAGACGGAACACTGCGTGGCTCATAGCGGGTGCCGTTGTGAAGCG
ATGAAAATCAACACGGACAAAACTCGGGGCAGGCAAAAGTGCCGGCAGTTTACCAGAGCCGCCCAGCCAATACGCC
TGGCCGTCGCGGCTGCCGGTCACCTATAATCCCGCGCCACTGAACGCACAGCGCAGTGACGGGTCTATGGACCAGT
AACCGAATCAGGAGAGTTTCATGCTGCGTCTTATGTTGCGTCTTGCCGCCCCCACCGTCGCCCTGGCGCTGGTATT
GCCCTTGGGCGCCCAGGCAGCCTCCCTGGTGGAGCTGCAAATGAACAAGAAACTGCAAAGCGTCGCGGCAGAAAGC
AACAAAGACCTGCCCAGGGAAATTGATGAACAAACCCTGGAAGTGGCCTACACCGTTGAAGGCATGCATCTGATTG
ACCACCTGAGCGTGCTGCCAGAACGGGCCGAACAAATGCGCGCCAACCCCAAGGCTGTGTATTTCCAGCTGGGGCA
AAGCGTTTGCCTGAACAAAGGCTATCGCGAGTTGATGGCCAAGGGCGCCGTGATGCGCTACGAAATCACCGAGAAC
AAGACCAATCGCCCGGTGGCGTCGGTCAAGTTCGTTGAAGCTGATTGCCCGGCCCCAGCCAAGAAGAAAAAGTAAG
TACCTTCCCCAAGAGCCATGTGGCGAGGGGGCCTGTCCTCTCACCACAACGCCTCCTGCAAAACCTTCGTCAATCC
CCATTAAGTTATTGCCAGACAAGACTTTAGGGGCTCATGATCGAGTGATCCGGTAACATTTCCAGGGTAAAAAAGG
TTGCCCTCGAGTGGCTTGCAGTGCCAAGGTTTTTAATCACAAGGAACATACAGAGCCTGTTTCGTCGCCGTCAGCA

GCCTGCAATAAGACGCCTGGGCTCTGTAACGAAGGAGAAGCTGAATGCCTGATCAAACGAATGACCTCCTGACCCG
TCATTTTCCAGAGAACGGGGCCGACCTCACGCAGCAGGTCGAAGCGCAACTCAACCTGATTGCCCCCAGCAGCCCG
AATATTCCGCTTTATCGCGACATGATCCTCACCGTGCTACGCATGGCCCAGGACGACCGTGACCGTTGGAACGCAA
AGATCACCTTGAAAGCCATTAATGAACTGGACCATGCATTTCGCGTGCTCGAACAGTTCAAGGGCCGCCGCAAGGT
CACTGTCTTCGGTTCGGCCCGTACCCCGGTTGAAAGCCCGCTCTATGCCCTGGCCCGGGAAGTGGGCGCAGCCCTG
GCCCGCTCAGACCTGATGGTCATTACCGGTGGTGGCGGCGGCATCATGGCCGCGGCCCACGAAGGCGCTGGCCTGG
CCCACAGCCTGGGGTTCAATATCACCCTGCCCTTTGAACAGCATGCCAACCCGACCATTGATGGCACGGAGAACCT
GCTGTCGTTCCATTTCTTCTTTACCCGCAAACTGTTTTTCGTCAAGGAAGCCGACGGCTTGGTGCTGTGCCCAGGG
GGCTTCGGCACCCTGGATGAAGCGCTGGAAGTACTGACGCTGATCCAGACGGGCAAGAGCCCACTGGTTCCCGTGG
TGCTGCTAGACGAGCCAGGCGGTGGATTCTGGAAAGGCGCCCTGGATTTTATTCGCAGCCAGTTGGAGGCCAACCG
CTACATCCTGCCTACGGATCTAAAGCTGGTGCGCCTGGTACACAGCGCGGAAGAGGCAGTGAATGAAATCAACCAG
TTCTACGCCAACTTCCACTCCACCCGCTGGCTCAAGCGCCAGTTTGTGGTACGCATGAACCACCCGCTCAGCGAGC
GCGCATTGGCGCATATGCAGACCGAATTCGCTGACCTGCGGTTGAGTGATGACTTCCAGCAACTGGCCTATGGGGG
TGAAGAACACGATGAAGCACGCTTCAGCCACCTGACGCGTCTGGTGTTCAATTTCACCGGCCGCAACCAGGGGCGG
TTGCGGGAGTTGGTGGATTACATCAACTTGCCGGAAAACTGGGCGCAGGCCCAGGGTAAAACACAGCAACGGCTGA
CGCCCGAGCCAGCGTGATGCCGTAAAACGCAAAAAAGGCCCGCTATTTTCATAGCGGGCCTTTTTGTATAAACGCA
AAATCTTTGTAGGAGCTGGCTTGCCAGCGATAGCGGTCTATCTGTCATTGTACTGATACCTGGCCCGCCGCTATCG
CCGGCAAGCCGGCTGCTACAAAGGGTTCACGCTTCAGTCGTCCAGATCACGACCGCTCAACAAGCGGCTGATCATC
TCCATCGAAAATCCGCGATAGCTCAAAAAACGCCCTTGCTTGGCACGCTCACGGGAATCAATCGGCAGATGGCCGG
CAAACTTGCGGCGCCAGGTGTCTTCCAGTTGTGCTTGCCAACTGATGCCACACTCACGCAGCGCCAGATCGATATC
GGCGCGTTGCAAGCCTCGCTGGCTCAGCTCTTCGCGAATCCGCGCAGGCCCGTAGCCCGAGCGTGCCCGGTAGGAA
ACAAAACTTTCGAGATAACGGGCTTCCGAAAGCAGCCCCTCTTCCGTCAAGCGGTCGAGGGCTGTTTCGATCATTT
CGGGAGGGGCGCCACGCTGACGCAGTTTACGCGTCAGCTCGACACGACCATGCTCGCGACGTGCGAGCAGGTCCAT
TGCCGTACGCCGTACCGCAACCAGCGTATCGAGTACAACGGTCATCGTTGGGCTCAGATATCAGCGTCGGCTTCTG
CCATGTCGTCAACAGGTTCGCGGTTGGCGGCGGCCTTGACGTCTGGCGCTGGGGTCAGCAGCTTGTCGCGGATCTG
CTTCTCAAGGGTCGCCGCAATATCGGGGTTGTCCTGCAGGAACTTGGCCGAGTTGGCCTTGCCCTGACCGATCTTG
CTGCCGTTGTAGGCATACCAGGCACCGGACTTCTCGACAAAACCGTGCAGCACGCCCAGGTCGATCATCTCGCCGT
TCAGGTAAATACCCTTGCCGTAGAGAATCTGGAACTCAGCCTGACGGAATGGCGGAGCCACCTTGTTCTTCACAAC
CTTGACGCGGGTTTCGCTACCGACAACCTCGTCACCTTCCTTCACCGCGCCGGTACGACGGATGTCCAGGCGGACC
GAAGCGTAGAACTTCAGCGCATTACCACCGGTAGTGGTTTCCGGGCTGCCGAACATCACGCCGATTTTCATCCGGA
TCTGGTTGATGAAGATCACCAGGCAGTTGGCGTTCTTGATGTTACCGGTGATTTTACGCAGGGCCTGGGACATCAG
ACGGGCTTGCAGGCCGACGTGCATGTCGCCCATTTCGCCTTCGATCTCGGCCTTCGGCACCAGGGCCGCCACGGAG
TCGACCACGATCACGTCGATGGCGTTGGAGCGCACCAGCATGTCAGTGATTTCCAGCGCTTGTTCGCCGGTGTCCG
GCTGCGAAACCAGCAGGTCGTCAACATTGACGCCCAGCTTGCCAGCGTATTCAGGATCCAGGGCGTGCTCGGCATC
AACGAACGCACAGGTGGCGCCCATTTTTTGTGCCTGGGCAATCACCGACAGGGTCAGGGTGGTTTTACCAGAAGAC
TCCGGACCGTAGATTTCAACGATACGGCCTTTTGGCAAGCCGCCAATGCCGAGCGCGATGTCCAGACCCAGAGAGC
CAGTGGAAATAGCCGGGATCGCCTGACGGTCGTGATCGCCCATACGCATTACGGCACCCTTGCCGAATTGACGTTC
GATCTGACCCAGGGCCGCAGCCAAGGCTTTCTTCTTGTTGTCGTCCATTAAAGTCCTCACGTAATCAATAAGGCCT
GACGGCCAACACCTGTATAAGTAGACAGTATTGTTCCATAAAGATCGGGGATCGCCTACCCCTGATTTTCTATTTC
TGCTGCAGCTCGTCGCAACAGCCCCTCTAATGCGGCCTTTACCGTTTGTCGGCGGACCTCGTCGCGGTTGCCAGGG
AAGTGCTGGCACTCGGCCGTAACCTCGTCACCCACACCCCAGGCCAGCCAGACGGTGCCTACCGGTTTGTCCGGCG
AGCCGCCATCGGGGCCCGCCACACCGCTGACCGCCACGGCAAACCGCGCCAGGCTTTTTTCCTGGGCGCCACGGGC
CATCGCCTCGACCACTTCACGGCTCACCGCGCCGACCTTGGGGAACAACCCTTGCGGCACATTCAACTGGCGGGTC
TTCTGCCGATTGGAGTAGGTCACGTAGCCGGCCTCGAACCAGGCCGAACTTCCGGGGATCCGCGTGATGGCTTCAG
CGATACCGCCACCGGTACAGGATTCAGCAGTGGTGACGTGGGCATTGAGCACCTGCAAACGCCGGCCAAGTTCGGC
GGCGAGTTGGGTTATTTCCTTCACGGTCGTCTCCAGGAAAGTGCGGGGGTTTGCCTACCCTACAGGAGCCCATCGG
CCACGCAAGCTGCAGACAGCGTCAACAGATTACCGGGCGATAGCCTGCACATAGGCCTGGCAGGCCCGCAGCGCGA
TCAATCCGTTATCACCGGCATCGGTGATGCCGATAATTCGTTGAGCATGCGCCGGGTCAAGTCGGCCTCGCGGGGT
TCCATGAACCACGCCGCGGGTGGCGGCGGTGGCTCGCATTGCGGGGCTGTCGACGGGGTCGCTGGCGTCGAGAAGG
ACTGACAGGCGCACATCAGCAGTGGCCAACCGATCACGCAGATAAACCTGATTACGCTGTGCATCGCGCAACTCCC
GTGTGTGCTGTTGGTCGCTGGCGCGCAGTTGTTGCTCCAGAGCCTGGCGCTTGTGTTGTTCGGCCTGGTGCTGAAC
CAGTGCCGCCTGGCTTTGCTGGCTGAGCACCTGTGAATGCCGCGCGGCCTGGCGTTCAAAGGCCAGGCCATGGCGC
CAGGCCTGGACCTGCCAACTGGCCGCCGCCGGGATGAGCATCAGCAGAGCAATAACCAGCATGCGCCCGCTCAGTT
GCATAGCACCGCCCTCGCCTTCGCCCACAGTTGCTGGCGATGCTCCAACCCATTGAGCCCGCCATTGATCCGGCGG
GTGATGGTGGTGAATTGGTCCTGGTCAGCCAGTTCATTCAAGCCATGGCTGTGCCAGAACCAGGCGGCGGATTCGG
CCGCCCATTGCGGTTGCTCCAGCAACTCAGGAAAGTGCAGCAGGCGCTCATCGCCAAACAGCGCCCGGCTACAGGC
CAGGTAATTGCTTCGCCCGGTAATCTGGATCAGCCCACGGCCGCGGTACTTCTGGCCGTCACCGTCGGCTTCGGGG

CTATTACCCAAGCGTGCGGCCAAGGTGCCAGTGTCGTATTTGCTCAGGTAGCGCTCGCCACCCAGTTCACGCACGT
AGCGCAACTCACCGGACTCATGGCCGACCTGGGCGATAAAGGCTGCCATGCGTTGGGGGGCAGTGATGTCGCGGTG
AAGCATGGCGGCGTTCAGCGCCGAAATAAAAACGCCCGCTTGGGCGCGGGCATTGGGCAGGATCTGTTGCAATTGC
GTCAGTGTCATCATCCTTGATCTCCACGGCCTAAGAGGCCAATCCACGGGCCATGATCGAACTGCGATAGCCCGTC
GCCGTGTCACCTGCATGGGTCACCTTGTCAATCGACCAGCGCCCCTGCATATACCCCGGCCAGGTCTCATCCAGCA
GCAACAGCCCTTCGGCGGACAGCAACGGGTTGCCAGGGCAATCGATGGCGATGGTCAGCCCTTCGCGACCCACCCG
GCGCAACTCGCCCTCGGCCACGGAGCGCGCCTCGGTTTCGCTCTGGCAACGTTGGCGCAAGGTCTTGAACGGCGCG
ATCCCCACCTCGACCACACGCTGGCGAGAAGCCGCGGCATCCCACCAATTGACACGGCAGCCCTGGTATTTGGCCC
GGGCACTTTCATCCACCCGCGCGCTGATAAATGCCTGCTCGCCAGGACGGTTGTCATGGGTCACCGACAGCCGCAC
ATCCGGCAACTGCTGGCCCGACAACGACTTGACCTGACCGCTTTCGGCCAGCACATACAACTCGTTGATCGGTTTG
GTCACTGCTTTGTAGCGCCTGGCCAGGCGGGTGATGAAGCCCATGTCAGTCTCATTCGACTGATCGATATGGTCGA
TCACAACCCCGTCCAATGCCGGCGCCACCCGGGGTGAAAACCCGTGCCGCGTGGCCAGTTGGCGAAACAACGCGCC
CAGGGTCGTGGGACCATGGCTGGCGGAGCGGCGCTGACGGTAACCGCTTGGGTCCATCACGCTGAACGGCGCCGCC
GTGGCCACGATCGCCAGGCGCATGGGAAACAGGTAGGGAGTGCGCTGGGTGATCACAAACTCGCCTTTTTCCACCA
GCCCCGACTCGCGGTACCCCACGCGCAAGCCGATCTTGGCGCCCAGGCTCGGCAGGCCCTCCAGGCCCTCGATATT
GATGGTCAGCTCCAGACGGTCGGATTCGATGCCCGCCGCGTCGGTGTGTTTCCACTGCATCAGCCGTGGGTTGATC
AACGCCGCGTTGGCGCCGTAGATCTCCACGACCGGGGTAAATCCCATAGCCATGCCGCCTCCTTAATCCCAGGCTG
AAACGGGTGTTGCGACACCCGGTTGCGCATCCATCTCGGGCACGATCACCCATACGCCCGCCGGCAACACCGGGCC
CTGCTCGGCAAGCCCGGGGTTCAAGCGCCAGAGGGTTTCTTCGGCCGCGTCATCGCAACGCCCCAGCTCGCGGTAA
AGCAGCAGGTTGACCGAATCACCGGCAATACTTCGCACTCTACGCATTGACGAACTCCTCCAGCACCAGCGACCAG
GTGACCACCATGGCCGTTCCGTCATCGATCACATTGGACTGGTTTTCCGTGAGCGCGGTTATGCGCCACAGCCCCC
AGTTGCGGCCGATACCATCGACCAGCGGCAGCGGCGCCCGGGCATCCTGCAAGGCACGCAATTGATCGAGGCGCTG
CATGCCAACGGCCGCCATGGCGGTACCGGCGAAGGTCAGTTTTTCCAGCTTCTGGCCGTTCTGTCGCGACTGGGGC
TTGCTGGCAATAATCTCCAGGTCGCCCCAGCCGCCATCACTGCTGCGCTGGAGCGTGGAATAGGCGAACCCTCGGG
CCAGCCCGAAAATAAAGTCGCCCAGCACCATCTGCTGTCGCATTAATCACCTCCATCGGTCAGTGCCGCATTGCGC
CGAACCGCCAAAGAGTCGGTGAGCATCGGCACGCATTGGTTTTGCAGGGATTGCAGAACCCGGTCGACCACCTGCT
GAGCGTCTGCGGGGTTGACGCCGGTGATCTGGATGCTCGGTGCCAGGGTGACCTGGACGTTGTCCGAGCGAGCGCT
GTTGAGGTTTTTGCTCAAGGCCTCGGGTGATGGCAGGCGGTCACTGGGGCCGAACAATTTTTCACCGAGCCAACTA
CCGCCCTCGCCGCCCAACAGGCCACCGATAACGCCGCCCACCGCAGTGCCCACGCCAGGGAATACCAGGGTGCCAA
GGGCCGCTCCGGCAGAAGCGCCGGCCCATGCGGCACCGGCGGTACCGAGGCCGCTGCCGACGGCTCGCATGTTGCC
GCTGCGCACGCCCTGGACGACGTTGATGGCGGCATCGGCGGAGCGCAATGGCGTCAACCTGCGCACGCCGATGGAC
CCCAGCCGGCTCAGGGTTGGGACCAGGCGGGAGGCTGGGGCCTTGCGCAGGCCGAGGGCTGATGCACTCTTTAATA
GCAACGCCTTGCCTGCGCTCAACCCACGGGTAGAAACCCTTCGCCGGCTGACGCTTACCGCAGTCACCCGCGCCAG
GAACCCCGTTTTCAGGCCGTCAAACATCTTGCCTGCGCGCTCGCCCAGGCTCCTCATGATTGCCATGGGCCCAGTG
GCGCTCCTGCCGGTCCCTGAGGTTTTTCTCGGCATCGGTGGGCTGGTGCGGCCAGTGCCGTTTTGCCTCACCGTTT
TTCGTGACCCTTTGGGCCCGTAACGAGCCCGCCCGGTCAAGGGTTCGCTGGCGCCTGGGCAGCAGCAGTCCTTGCT
ATCGTCCCGGAACAATTTCCCGACGTAGGGCAACTTGCCCAGCGTTGCATCGACTACCTTGCCCGATACCCGGCTC
TTGACCGTATCAAGCAAGCTTTCACCCACCCATTTGGAACTCTTGCTCAACCAGGACTCAGTCGCCGAGGCCTTGT
CTTCGGGCGCAGCTTGCGGCGCACTGGTCATGGCTTCAACGACCGTGCCGCCCAGCGGTCCACCCGTGATAAACAG
GGTGCCATTGAGGGTTTCCAGGCTCTCGCGCAGGCGCACCTGTTCCTCGGTCAACGCGTGGATATGCAGGCTGGCG
TTGGCCAGGGCCAGGCTCAGCTCAGACGGCGGCTCGGTTGCGCCGCCCACACTGGAGGCGCCAACCAGACTTTCGT
CCTGTGCTTCAAGCAGGCCCGGCAGTTGCAAGGCTCCACTTTCGGCCAGCCCGGGAAAGGTCATCCAGCGTTTGTC
TTCGTCGGCGAGCTTGAGCGTATATCGAGGGTCTTGCATCTCGCTCTACTCCTATTTGACGCCAAGGCGATTAATC
GCGATGTCGTAGCGGCGCAATGCTTTTGCGGCATCCCATTCGAGAATTTCCGCTTCGTTTACCGAGTAAACCAGCG
GGACGACGTCGAGGATTACTTCGATGTCGCGCTGCGAAAGAAGTCCGCCGGTTTGTTTAAAAAATCGTCGATGCGC
TCCTGCAGTTGTGTCCAGTCGGGCACGGCCAGGCCGTCCAGATCGGGGATCATCAGGCCGGTGCAGTGCGAGGTAA
TGAACTCGGCGCGCTCTTTGTTCGTGGCGAGTTTTTTCATCACCTTGGTGGCACGCAGGGCGGGCATTTCCAGGGT
GACGCTGGTCAACACGCGGCCGGCCACTTCCAGCGGCAGCAGCAGTTGCACCTGCTCGCCAGGCTCGGTGGAGTCG
CCCAGAAAGAACGAGGTCGGGCGCGTCGACATGTCGTGTACGTGCTGGGCAATGCTCACGTAGTCCGGGCGCTTAA
GTTGGTCGAGTTCCTTTTCCGAGAGGCCGGTGGCCAGTTTGGCCAGTTCGAAGAACTGGTCGTCCTCGTCCTCGCC
GGCGCGGGCCAACGCTTGTTTTTGCGCGGCGTAGAACAAGGGTTTGAGTTGGATCTGCTCGATGCTCGCACCGCTG
TCGGCGGTGATCGGCGCCAGCAGGATATGCAGGGGTGGCTTCCAGGCCATGGGCGAAATTCCTTGAGTAAAAATGT
TGGACCCGTCGTGGGCACGGTCAATGGAGGAGCTGGCTTGCCTGCGATACAGGCAGCGCGGTCTTTCAGTTGAACA
GAGGTGATGCAATCGCAGGCAAGCCAGCTCCCACACTGACCGTGACTATTTCAGATGGGTGAAAAGGCTTACGGCA
TCAGCACCGCGCGCCGGGCATCACCGAGGATGTCGACGCCGTTGAGCATGAACTTCTGGGTGCGTACGTCGATGTC
GATCACCGGCACGCCGTTGTCCAGGCGCTGGTAGGTGCGGCAGGACAGATCCAGTGTAGTGGTGGCCTTGTCGCCC
ATCTTCAGCTTGGCCTCGTCCAGGGATTTGAGCTTGCCGCCGACGGTGTGGTAGGTGAAATAGGTCTTGCCGTCCT

GGTCCTGGCCCGCTTCGCGCACGTTGAGCAGGATGTCGTCGCCCAAGCGCACACCCAGGGCCAGCATGATTTCAGG
ACCGGCGCCTTGCAGCACCAATTTGGCGTTCAGCACCTTGGCGCTCTTGGCCATTTCTTCGGCGATAAAGCGCCCG
CCGGACATGGGCTCCATTTCGAATTCGATCTTCGGCGGGGTGAACTCTTCGACGGTCGCCGACAGCGGCAGGCCCT
GGAGGGTGGCCGCAATGGCCTGTCTGACTCGGTTGGTAAACATTAGAGAACGTCCTCCAGGAATTGCTCGATGATT
TCATCGCGGGCATTGAGTTGATAAACCATGTGTTCGTTCGGCGCGTAGCGGCCGTAGTCGATCACGATGAACCAGG
TGCCGTTCTTGTACTTCTCGACACTGTTGAGTTCCGGGTGCAGGTACACGCTGCCGCCCGGGATGGTCTCGTCGGC
CACCAGGGTTTGCAGCCAGTCGTTGATGCGCTTGACCTCCTGGTCCATGAACGACTTGGTCAGGTTGTGCGCCATG
GCTTTCTGCCCGGCCTTGACCAGCTTGCGGCTGATGGCGTCTTCCAGGCCCACATAGCTGATGAACTTGCCGGTGA
TGGAGCGGTTACCCAGCAGCGAGAAGCCGCCGAGGATGGTGCGCGCGTAATAGCTCACGCCGTAGCGGTTGAGCAG
GTCGCCCTCGGTGGAGGTGTCGAGGATGTTGTACTCGACCACCCGCGACACGTCTTCGGCGAAGGTCACCTGGTTG
CCTGGGCTCTCCCACTGCTTGACCTTGGCCAAGGCGGCGATGGCCAGGGACGACGGCGCGAGGAACACGTTTTTCT
TCGCCGCCTTGGAGTACACCGACGGCATGTTGTGTACCAGCAGGCAGCGGTCGAAACCCAGGTCGGCGCCACCCAG
CTCGCCGCTGTAGGTCACTTGATCGGCGACGCTGGCGTCTTTGCCGTCCAGCACCACGCGGGCCTTGATGCGCTTG
CCGAAAGCGGCAAACTCACCGGCCACGGCCTTGGTCCCGGTAAAGCCTGGCGCGCCAATGATCGTCAGGTCTTCCG
GGACGCTGCCAAGCGCCGCGAGGCCCAGTTTGCGACCGGTCACCGGCTCATTGCCGCCGATCACGTTATTAAGCGT
ATCGGCGGGCGTGCTGCCCTCTTCGACGATCACCACATAGACCGGCACCTTGACCACTTTGAGGATCTGGTACACC
GCATGAAACAGCGTGCCCGACTCGGCGCCGGTAGGGTCCAGCAGTGCCTGGGCGGTGAAGCTGTTGATGCGAAACG
GGGCGTTTTTCGGGATCGACAGGTGCGCATTGGGCGCAGTACCGACCAGGCCGATCACGTTGTCACCCAGGCCACC
CATGGCCTCGGGGGATTCGCTGGCATTGACGGTGATGCCGTTATGCTCGAAGTTCAAAACCTCAGCCATGATTATT
CAGCCTTCTTGGTGACGGCCTTCTTGGCCGGTGGGGTGTCTTGGGCGGCAGCCAGCACGCTGGTCAGTTCCAGGCG
GCCGGCAGTGCGCAGAGCGGATGCTTCGACGTCCAGCAGGTCCAATTCCTGGCCGACGGTGGACCAGTGGCCACCT
CCGGTGGGGAATGGGATGAGGACGGTGTAGGTTTGGCGGTTGGCCATGGGGGGAATTCTCCGGGTGCAAAAAGCGC
AAAGCCCCTGCGGGAGGGGCTTTGGGCGGGCGAAAAAAAACCGCTTTCGCGGTGGGATTTACTTTTCTTCTGGCAA
TGGGAATCGGGTTTTAATCTCGGCAACTTTGTCACGCCAAACTCTTTCAGAATCGGGCTTCTGATCGTATTGCCAC
TCCAGATAGAGCGGGTCAGCTTCTGCCACATAAGCCTCCCGCCTCGCTTGCTGGGCAAACACCACGCGCTGCGTCA
CCAATGTCTCGTTAATCAGGTCTACCGGGATACCCGCGCCAACAAGATACTCACGAGAGCATTCAGTGTATTTACG
CGAAGCGAACTCCAAACTTTTAACAATCTCCATATCACTCAATCCTTAATATTCGTTAGAGCATTAGACATATCCT
GAGAGATCAACTCCGCCCAAGTAGACGCATCTTGCCGAACCATTTCTATTCCATACAAAGCAAATGGGACCGCAAC
ATTATGGTAACTCCCCATCAAAAACTGATACCCCGTAGAGACCGGCAAAGTACTCACGGACACTTTATTCAACAAG
ACATTACGCATAAACAAGTCAGCGACACCAATTGACCCCGACGTATGCTGATACATAAACGCACCGTTATTCAACT
CTATCGAACAATGCTGAAGCATAACAAAGCTTTTTGAATTATGGGTTTTGATAAATGATGTACGCCAAACATCATG
CCCTTTACCTTGGAACACCGCCGACAGTTTAGCCGTTTTTAGTTTGCAACCATAAAACACTAACACTGAATTGTAA
CCTAGATGAAAACCACCCGCCTGAGCCCTGCCATCCTCGCCTACTACAGGCTCAGCCAGCAATTCTACATAGGAGT
TCGAATCATAAGCTACGAATTCACTAGGTTGAATAGAGATCGTCTTATTGTCACAACTCTGATCTTTGGGCAGTGA
ATAAGCAGAACCAGGTTTAAGAATTATAGAGTTCCTTCCTCCCGAAATCATTCTGACAAAAGCTCCAGCAATAGTC
TTTACTGCTGTTGCCCAACTGAGGCCATCGTTCGTGTCGCTACCGGAAACGCCATCCACATAAAGCGCGATATCCA
TGGCCCGCCGTACAGTCTGAGGAACAACAGTCACGGCTTGCTGCACCTTTAAATCGATATCACCAATTTTAGCTGA
GATCATTTTTGTCAAATCATTCGCAGCGTCGACTACTGCAACCAGCTTCTGTTCTGCACTCACAATAAAAGTCCTT
TTAACTGATCGATAAAATCCATATCGCTTCCACTACAAAGCGTTAAAACAATACAACCAACTCTAAAACATAGAAG
CACTAGCCTGCTTCTAAAGCCATAACACGAAACAAGATAGACACTTCTCTATTCATATTGTCGATCGTGGCAGCAG
AGAGTACCGCCAATTCTTGCGCCAATAACAAGTTGAGGTTTTCACTGCCCACCACAATCGTCACGCTATCCACCGG
CAACGGCGAAATATCCAACGTAAACTTCTGCAACACCCGCGCAGCCGCCGCCTTATACGTCAGTAACTTCCCCGCC
ACCGAGTACACGGCCAACAACGTGCCACTGGCGAGATAAAACCCAAACTCGCCAATCTCATATTCGCCATCGCCAT
CAAACAGCGCGGCCATGCGCAGTTGGCGCTGGCCCAGGTCTTCGTAATCCACAATCGCCACGCGCTGGCGCTCGTT
GCGCAGGGCGGTTTCGTTGCCGGTGGGGTTGTAGCGGCCGGTGCCAGCGGCGATGTGGGTGATTTCGCCTTTCAAG
CCTTGGTTCTTTGCCTGCAGCACTTCATCCAAACCCTTGGAGGTGAAGCGCACCAGGCGCGTAGTGTCATCGGTCA
TGGCTGCGCCCTGAGGTCGTAGTCGTTAATGGTGTAGTGCTGGGCGATGCCCGCACTGTTAAGCCTTGAACTCAAT
ACAAGTTCCGGCAGCGCGCCGCACAACGACAACTCGCCGTCGTTCAGCGCCCGGTGGGCGGCGCCGGTGATCGGCA
GCCCACCGAACACTTCATGCACCAGGGTGATGGTGGCCAGGTCGCGCTCGCTCTTGGCCGCGTTGATGCGGCGGAT
CAGGCGGTTGTGGTCGCCACTGGACCAACTGCGGCCGATGATCGCTTGCACGTCGAAGGTGTAGGGCTGGGCCAGC
GGACGCTGTTGGTACCAGGCCAGGATGTTGGGGGTAAATCCCAGGGATTCGACCGCGTGGCTCAACGCCTTGGGTG
TGCCGGCCTGGCGCTGGATCTGCCAGGACAGGGCGACCGTGAGGCGTTTTTCGGTTTCGTCGGCTGCGGCGTTCCA
TTCACTCACGCCACGGTCGGCGGCCAGGTAAGGCAGGAAGGCCTGCGGCGTTTGCAGCGGATTCATCAGCGTCGGA
AACGGCGGCTCGACCCGGTCGAGCAGCTTGCCCAAGCCCAGGTCCAACGCCTTCTCCAGCGGTGAACTGTTGGCCG
GCAATAAACTGGATGTGTGCTCACTCATAGCGTCAGCACCTGCACCTCGACACCCGTGCAATACGGAGCCTGGAAG
GCTGTGGTGACGATCGGTGCCAACGGTTCGAGAATCTCCAGTTGCGCAGCCCCGGCGCTGTGAATGGTGTAGTCGA
TCCAACTCGGGTCCACGCGCCCTTCAAGGCGGTGGCAGGATTCGGCATAACGTTGCAGCAACTGCTGGGCCGCCAC

TTGCGTCAGCCCCGAGTCCGGGCCGGCGTTGATCCGGGCGACCACGCGGATCTTGTAGGGCACGATCTGCGCGCCC
TGCACCGTCACCCAATCGGTTTCCGGGCGCACATCGGGCCGGGCGAAATGGCGGCGCACACCTTCAAGCAAGTCTG
TCGATGGCGTGCCATTACCCGAGCGGGAAAGCACTGTGACCATTACCTCGCCAGGTGCCGTGCGCCGGGCGTTGCC
ATCCTTGACCTGGGCGGCATAACCATCCGGGTCGAAGGTATAACTGACCGTCACCACGCCTGCAGCGGCACTCTGT
ACTTTCACCACCGGCCGCTCGCCAAGGGTGAAGACCTCACGGCGATACTGCATGCGCGAACCCGCAGCCGGCGCGT
GGGGCGCCAGGTAGTAGCGCAGCCGCGCATCGTCATCGCTTTCCAGGGTCGGCGGCACCGGCGGGAACGCCGCCGG
GTCGCCCGGGTCGAGTACCTGGCGTTCCAGGCCCATATCGGCCAGGCGCGCATCCAGGTTGCTGCCGGTGGCCCAC
CACGCCAGCATCTGCTGGATGCGGGCGTTGTACTTGCGCTCATGGGTTTGCAGGCGCACGCAAAAGGCTTCCAGGG
CCAGGGTCAACAACTCGCTTTCGTTGTCCAGGCTGACCTTGAGCTTGGCCGCGCTTTGCGGGGCGCGGGCGGCCAC
ATAGTCGACGACAAACGTCTTGAACTGCGCCAGCAACGGTTCGAATTCATCGACGGCGATGAGGTCGGGCGGCGCC
AGTTGGTTCTGCCCAGGGATCAACATGCTCATGTCACCACCTCGAAGGATTGCTTGCGGTTTTTCCAGGTGCCGGC
AAACCGCAACAGCAGGCCTGCGCCCTGGCGATTGGCGACGATGACTTGCGGCACAAAATCGCCGATGCCGTTCTGC
GGATTGTAAAAGGCCTGGGCGGCATGGCTTTGGGCGAGGATCAGCAGATCGTCGCCCAGGTTCTGCCCCAGCAGCT
GCGGGATCAGCGAACCGTAAAGCGGGCGTTTCTGCCGTGTGCCCAAAGGCGTGGTCAGGGCCCGGGTGGCGCGCTG
GACGAATTGCGGCCAGTCGTCCACCACGGCCCCGGTGTTTCTCTCAATTCCGATCATGGCAAATCCTTATGCCGTG
CTGATAACTCGGCCCTGGTGATCCACCACCGGGCCACTCAAGTGCACGCCGGATGCATCCACCATCATGCCGACGG
CTCCGACCTGCAGACTGATGGTTGCGGCGGTCATGACTAAACGCGCGGCGCCCAAACTCATTTCCACTTGCTCGCG
AGAGCCGCTGAATGCCGCTGGTCCATTGTTCCAATCCAATACATGTGCTGCGGCGTCGTAGGCGCTTTGCGTGCCG
TCCTCATAACGCCGGCTCGTCAGGCTGGCCACACTGGACAGCGGCGGAAAGCGGTCACTGTTCAGGCCAAACAACG
CCACCGACTGGCCCCCGCCCTCGCCGCCGCCGTAGTTGAGCAGCAGGCATTGCTCACCCACCGTGGGGATCCGTGT
TTCCGTCTGCGCCCCGGCGCTGGGGTTGAAAAAGCGAATCGCCGGGGTCAGCAATTCACCATGGCTGACCTTGCAG
GTATTGCTGGCCGCGTCGACCTCCTGGCACACGCCGATCCGGCAAAAGCTCTCGGCCCGCCGATAGAGGTCTTCAA
GCTGGGTTTCCATCTGCGCTAGCCGCTCGACGATGGGTGCCAGTTGCAGGCGTAACAGTGCATCGAACATGGGCTA
CTCCTGCAATGGCCGGTATTGATCGGGGTCGTCGATATTCGACACCTCCCAGGTGCAGGCAAACAGCGGCTTGCCG
ATGGGGTCGTCAAGCAGCGGCGGGCCGAGGTACAGGGTTTGGTTGAAGGACACGGTCCAACTGTCGTAATGGGTCT
CGGCACCCTCCAGGGTCGCCGGCAGCGCGACGATATTCATGGGCACATCGCATTGGGCCGCCGGCAATTGCCAACG
GTTATCCAGCACCAGGTCCATCAACTGACTCGCCAGGTCACAGGCCTCAAAGGGCTGCGCGCCCTGGGCGGCCATG
ATCTTGAGTGAAATCGACAGCACATGGGCCTTGCGCCCTTCAAGGGAGCGTACGCCCGGGCCATTACGCTCGAGCG
TGATCGCCACACCGGTTTCATCCGCCGCCAGAACGCCCGGCGGGCTGCCTACCTGTAGCTTGGGGAACGCCGCACG
CAGCGCCTGGCCGATGGCAATCGGCAGCTGTGAAGGTTTTTCAATGAGGGTCATGGCGTGGCTTCCTTGCAACAAT
CATTTTGAATCCGGGGGCTGGTTGATCCCGATGCGCTTGGCGGCCCAGCGCTCATAAAGGCCGATGGCGACATCGG
CACCGGCCATGGCGGTCAAGCACCCCAAGGCACCGGCCGTCCAGATCGACATGCCAGCGGCGTACAGCAACATCAA
CGCTGAAACCCCGCACACCATGCATGCCCCGGAACGCAGGGCCAGGCGGCGGACCAGCGACCAACCGCGGGCGCCC
TCCTTGTCGGCGCGCCACATCTCCCCGGATACGCCGCCGATCAGGGCCAGAATAATCACCAGCCAGATAGGCATTT
CTGCCAACGCTTGCTGCTCGTTTGTCATGTCACGCCTCCTGGAATGCGTAAAACCGGCAGATGGCCGGTACGTTGA
TAAATAGATGGATGGGGTTGTCGGTAGGCATTCCAAAAAGCCCGGTGGCCCGGGCTTTTCAGTAATACGGTCCGTG
CGATCTTTCGGCGCTACTGGCGCGGTACGGATCTTTCCTCGATGTTTGTCCGACCACGATCCCTGTCTGCCGGATA
ACTGCTTCTGGTGCTTTACGCTGCACACCCGGGTCAGTTGCCAACCCTCTGAACCGTCAAGGCCGGTTCATCGCTG
CCTGTTGTAAAACTGTGAAACTAAAGAGCGTCGGCATCCTTGCCGGTGTTGCCTGGCGTCCTTGCCATCACTCGAT
GGCATCCATGCCAGTGTTGCGTGCCTTCCTTGTCTTCCTTGGCAGCATCCTTGCCGCCTCCACCAGGCCTTGTTGG
CTGGCTTGAGATGGAGAATATGCATGTATGCATATACAGTCAATGCATAAATGCATTTTATTTTGCAAATGAAATG
CACAAACGCATTAACTCCTTAAGCAGCGGGCACTTGGAGGTTTTCCACAGGCAAAAAAAAGCCCGCTCTCAGGCGG
GCTGTTTCTTACCGATAAGGTTTAGCGGGCGTACATGCCCCACCAGAAAACGTGGCCGAGAATGCTGATCTGCTCC
TCCTGGATATCCTGGAAGCTGTAGTCCTCATCCGGATGCTCATCGCGATTGAAGCTGCGCAGGCGGATCCCCGAAG
GTAGACGATAGAGCTGTTTGACCCGCAATTGGCCGTTGTGGTTGATGGCATACAAATCGCCATCCACGATATCGCC
AATCCCGCTCTTGCCGGCGTTCACCCCCACCGTCGCCCCATCGCGCAACACCGGCAGCATGCTGTTGCCGCGCACC
GTCACACACTTGGCCTGGTCGAACTGCACACCGTTATGCCGCAGGCTGCGCTTGCCGAACCGCAAGCTGGCCTTCT
CGCTTTCCTCGATGACGAATCTTCCTGATCCAGCAGCCAATTCAACCTCGCGCAAAAAGGGGATCGACACCTCGTC
ATCATTAACGGGCGTGTCGTCGTCCCACAGGCTTATGTCCTTGAGCTCCGAATGCATCGGGTCACGCTCTTCCTGC
CACGCCGACCCCGCGCGCCCGCGCAACTGGTCAGTGCTCACCCGAAAATAATCGGCGATACGCGAGATGTGCTTGT
CCGACGGATCAACGATCTTGCCGCTGAGAATCCGGGACAGAGTGGATTGAGGCACGCCGGTACGCCGGTGAAGCTC
CGTGGGGGAGATCCCGTCGCGATCGAGCAGCTCTCTTAAGACGATTGAAACGTTGCGTTTTTGCATAAACCGGATA
GTGACGGGAGTTTTGGGGGTTGGCAAATGCTAATTTGCATATTTTGTGCATGAAGCATGCGGTTGTACCTAGATGT
TTGTGAACTGCGAAGCACTGACCTCGCATGTTAACCTTGCCGCCATCGCAAAAAATGCAGAGCTTGTTTGATGGAA
AACGGTGCAGATCCAAGTGATCAACTGATGACGCCGAACGAGAGAAAAACCTTAGACGACGCCTTGAGCTTTGCTC
GTTCAAACAGGAAATCCATCGCCAAACGCCTGACCGATAAGAGTATTTATCTACCAGAGGAAGCTCCGGTCTCCGT
CTATATGGCGGGCTCACCTGGTGCCGGAAAAACCGAGGCTTCAATTGCCTTGGTAAATCTGTTTGCCGACAGCAAG

ATCCTGCGAATTGATCCTGATGAACTGCGTGGCGAATTCGCCGCCTATACAGGAGGAAACTCATGGTTATTTCAAC
GCGGCGTTTCGATTCTAGTTGAAAAAATCGTGGATCTTGCGATGGAGCAACGGCAGTCATTTCTGCTCGACGGCAC
CTTCTCAAATATTGAGGTAGCCAGGAAGAAACGTAGAACGCTCCTTGAGAAAAGGAAGATTCGTCCAGATATTGTA
TGTCTACCAAGATCCAATGCTTGCGTGGGGCTTCGTCAAAGCCCGCGAGGCAGCTGAAGGAAGAAAAATTCGCAAA
GAGCACTTCATCGAGCAGTACTTCGCTGCACGTGACGTGGTCAACGCACTTAAGCTAGAGTACGGAAGCGATATTC
ATGTGGATTTGCTGCTCAAGCATATCGACAACTCGGGACGGCTCTACAAAGCCGGCGTCGAAAAAATTGACTACCA
TATTCCCGAACGACACTCGCGGGCTGATCTAAAAGCCAGGCTCGAACTACCACCATCGGGGGCGCAATGATGATTT
CGATCAAGCTGGGCTTGTCCAGAGGAGCGAAAAACCCCTTTGCTGACTTTATTCGTAACGCAAAATCGGATCAAAA
AAAGCGCGTCTACAGCCAGGTGCTAATCGAAGCCACCAAGCAACAGAATCTTGTCATGATGCAGGCAGAAACAAAG
CGGACCTGACGTCTATCCCGCCTATGGACAAAAGCCCGACCTAATGTCGGGCTTTTTATTGGCCACCCTTCAAGTA
AATGCGTCACCTCGTTTTACGTTTTACCTAGCCGCTTGCAACCTGCGAACGGCCGAGCTCGCATGTTAACCTTGCC
GCCATCGCAAAAAATGCTGGGCCAAGCGCCCCCTTTGCCCCATCACTTTCAACGAATTTGCCTACTATCCAATGAG
TAAAAACACGTCCGATCTGTCCTCCCACACGCCGATGATGCAGCAGTACTGGCGCCTGAAAAACCAGCACCCTGAT
CAGTTGATGTTCTATCGCATGGGCGACTTCTACGAGATCTTCTATGAAGATGCGAAGAAGGCCGCCAAGTTGCTGG
ACATCACCCTGACCGCGCGCGGGCAGTCGGCTGGGCAGGCGATTCCGATGTGTGGGATTCCCTATCACTCGTTGGA
AGGTTACCTGGCCAAGCTGGTCAAGCTCGGTGAGTCGGTGGTGATCTGCGAGCAGATCGGCGACCCGGCCACCAGC
AAGGGGCCGGTGGAGCGTCAGGTGGTGCGCATCATTACACCGGGGACGGTGAGTGATGAGGCCCTGCTGGATGAGC
GGCGTGACAACCTGATCGCGGCGGTGCTGGGGGATGAGCGGCTGTTCGGCCTGGCGGTGCTGGACATCACCAGTGG
CAATTTCACGGTCCTGGAAATCAAGGGCTGGGAAAACCTGCTGGCGGAACTGGAGCGGATCAATCCGGTGGAATTG
ATGATCCCGGATGACTGGCCCAAGGATTTGCCGGCAGAAAAACGCCGTGGGGCCAAGCGTCGTGCGCCGTGGGATT
TTGAGCGCGATTCGGCGCTGAAAAGTCTGTGCCAGCAATTTTCCGTACAAGATTTGAAGGGTTTTGGTTGCGAGAC
CCTGACCCTGGCCATCGGCGCCGCGGGTTGCCTGCTCAATTACGCCAAGGAAACCCAGCGCACCGCCCTGCCTCAT
TTGCGCAGCCTGCGCCATGAGCGCCTGGACGACACGGTAGTGCTCGATGGGGCCAGCCGACGCAATCTGGAGTTGG
ACACCAACCTGGCAGGTGGGCGCGACAACACCCTGCAGTCGGTGGTGGACCGTTGCCAGACCGCCATGGGCAGCCG
CTTGCTGACCCGTTGGCTGAACCGCCCGCTGCGCGACCTGACCGTGCTGCAGGCACGCCAGTCCTCTATTACCTGC
CTGCTGGACGGCTACCGCTTTGAAAAGCTGCAACCGCAGCTCAAGGAAATCGGCGATATCGAGCGGATTCTCGCGC
GCATCGGCCTGCGTAATGCCCGCCCGCGCGACCTGGCGCGCCTGCGTGACGCGCTGGGCGTACTGCCCGAGCTGCA
ACAGGCTATGACCGAGCTGGAAGCGCCGCACCTGCAGCAACTTGCCGTCACGGCCAGCACCTACCCGGAACTGGCT
GCGCTGCTGGAAAAAGCCATTATCGACAACCCACCGGCGATCATCCGCGACGGCGGCGTGCTCAAGACCGGCTACG
ACAGCGAGCTGGACGAGCTGCAAGCCTTGAGCGAGAACGCCGGGCAGTTCCTGATCGACCTGGAAGCCCGCGAAAA
GGCGCGTACCGGCCTGGCTAACCTGAAAGTCGGCTATAACCGCGTGCACGGCTACTTTATTGAGTTGCCGAGCAAG
CAGGCCGAACAGGCGCCTATCGATTACCAGCGCCGCCAGACACTCAAGGGTGCCGAACGCTTCATTACCCCCGAAC
TGAAGGAGTTCGAAGACAAGGCGCTGTCGGCCAAGAGCCGCGCCCTGGCCCGGGAGAAGATGCTCTATGAGGCCTT
GCTCGAAGACCTGATCGGCAAGCTGGCCCCCTTGCAGGACACCGCCGCAGCCCTGGCCGAGCTGGATGTGTTGAGC
AACCTTGCCGAACGCGCCTTGAACCTTGACCTGAACTGCCCGCGGTTTGTCAGCGAGCCGTGCATGCGCATTGTCC
AGGGTCGCCACCCGGTAGTGGAACAAGTGCTGACCACCCCGTTCGTGGCCAACGACCTGTCTCTGGATGACGATAC
GCGCATGCTGGTGATCACCGGTCCGAACATGGGCGGTAAATCCACCTACATGCGCCAGACCGCATTGATCGTGTTG
CTGGCCCATATCGGTAGCTTCGTGCCGGCGGCCAGTTGCGAGTTGTCCCTGGTGGACCGCATCTTTACCCGGATCG
GCTCCAGCGATGACCTGGCCGGTGGCCGATCGACCTTTATGGTGGAAATGAGCGAGACCGCCAATATCCTGCACAA
CGCCAGCGACCGTAGCCTGGTGCTGATGGACGAAGTGGGACGCGGCACCAGTACCTTCGACGGCCTGTCCCTGGCC
TGGGCTGCCGCCGAGCGCCTGGCGCATCTGCGGGCCTATACGCTGTTTGCCACCCACTACTTCGAACTGACCGTGC
TGCCGGAAAGCGAGCCACTGGTGGCCAACGTGCATCTCAATGCCACCGAGCACAACGAACGCATCGTGTTCCTGCA
CCATGTGCTGCCAGGGCCGGCCAGCCAGAGTTACGGCCTGGCCGTGGCCCAGTTGGCCGGTGTACCGAACGACGTG
ATTTTGCGCGCCCGCGAGCACCTCAGCCGCCTGGAAACCACGGCCCTGCCCCATGAGAATGTGGTCGTCAGCCCAG
CCAAGGCCGCCAGCAAACCGGCGGCACCGCACCAGAGCGATATGTTCGCCAGCCTGCCCCATCCGGTGCTGGATGA
GTTAGCAAAGCTTGACCTGGATGACTTGACACCGCGTAAAGCGCTCGAAATGTTATATGCACTGAAGACTCGGATA
TAACGCAGACGCTTGCAAGCTGGTAGACTCTCGCGCGGTTTGGGATGCTGCGGGCTTTTAGCCTGGCCTGCAGACT
ATCGCTCCCGAACCTCGCGAGCCCTGCCACAAGGGGTTTCGCTGCCGCCGCCTGAGGAGAGAATTAGAAATGACCT
TCGTCGTCACCGACAACTGCATCAAGTGCAAGTACACCGACTGCGTAGAAGTGTGTCCGGTGGACTGCTTTTACGA
AGGCCCGAACTTCTTGGTAATCCACCCGGACGAGTGCATTGACTGCGCCCTGTGTGAGCCAGAATGCCCGGCCGTT
GCGATTTTCTCCGAGGATGAAGTCCCGGAAGAGATGCAGGAATTTATTCAGTTGAACGTTGAGCTGGCGGAAATCT
GGCCAAACATCACTGAGAAGAAAGACTCGCTGCCAGACGCAGCCGAGTGGGATGGCGTAAAAGGCAAGATCAAAGA
CCTCGAACGCTAACTGCGTCGCGCCCCTCCAAAAGGCCCCTTGCGGGCCTTTTTGCGTTTCTGCGTGGCGGCCTTT
CACTTTTCTGCAGGCAAAAAAAAGGGGCGGTTTGACCCGCCCACATTTTTTCCCTAGTCCCTGTGTTCCTTTTCAT
CGTCCTGATGAATCGCATCCTGCGAGGTTCCTTGAGTCATCGTTCCTTGATGACCGTGTCAATCCGTGGACACAGG
GCTGATATTAGAGACTTCCCGAACAAGTTCAACGGGATCCATTCTCAAGCACAACACGCAATCGCCCTCCTCATAA
AAAAATTAAATGTTATTTTTCAATAAGATATAAATGTATCCGGGTAATTTGAGACGTCCCGGCACGGTTAAAAAAC

CAAACACTTACGAAAAAGTAAGCGAATGCTTACACGGGCCATTAGGGAAATGCGTACCCAGCCTCCCAAAAGGTTT
ACGCCAGACATTAAAAAGCCCCGACAAGGTCGAGGCTTTTTTACCTTGAGGTCGATCAGTCGTCGCTGACAGTGAT
GGTCGGCATTGCCTGGGAACCGGCTTCCTGCAAGACGATCCGCGCGCCCACGTGACGGGCAAGCTCCTGGTAGACC
ATGGCGATCTGCCCGTCGGGCTCGGCGACCACCGTCGGTTTGCCACCATCGGCCTGCTCACGAATGCCCATCGACA
GCGGCAACGACGCCAGCAGCTCAACGCCATATTGGGTGGCGAGTTTCTCGCCGCCGCCTTCGCCGAACAGATGCTC
GGCATGACCGCAGTTGGAGCAGATGTGCACCGCCATGTTTTCCACCACACCCAGCACCGGGATGTTGACCTTGCGG
AACATCTCCACGCCTTTACGCGCGTCGAGCAGCGCCAGGTCCTGGGGCGTGGTCACGATCACGGCACCCGCCACCG
GCACCTTTTGCGCCAGGGTCAGTTGGATATCACCGGTGCCTGGCGGCATGTCGATCACCAGGTAGTCGAGGTTGCC
CCAATCGGTCTGCATCACCAGCTGCATCAAGGCCCCGGAGACCATCGGCCCACGCCAGACCATAGGCGTGTTGTCA
TCGGTGAGGAACGCCATCGACATCACTTCCACGCCATGGGCCTGGATCGGCACGAACCACTTCTGGTCCTTGATCT
TCGGCCGCGAACCTTCGGCAATGCCGAACATCACGCCTTGGCTGGGGCCATAGATATCGGCGTCGAGAATCCCCAC
CCTGGCCCCTTCACGGGCCAGGGCCAGGGCCAGGTTGGCCGCGGTGGTCGACTTGCCCACGCCGCCCTTGCCGGAT
GCCACGGCCACCACGTTCTTGACGTTGGCCAGGCCAGGGATCTGGGCCTGGGCCTTGTGCGCGGCGATCACGCACT
GGATGTCGACCTTGGCCGAACGCACACCGTCCAGGCCTTCAATGGCCATTTGCAGCATCTGTGCCCAGCCACTCTT
GAACAAACCTGCGGCGTAGCCCAGTTCAAGCTGGACCGTGACCTGATCGCCCTGCACATCAATGGCCCGGACACAG
CCGGCGCTGACCGGGTCCTGGTTAAGATAGGGATCGGTGTACTGGCGAAGAACGGCTTCCACCGCTGCGCGATTGA
CGGCGCTCATGGGCTACTCCCGGAAAAGACAGACTAAAACAGGCGGCTATCCTAACCGTTCCAGCGCTTGAGGGGC
ATGCTTTCGCGACATTGGAAAACCCTGAAACAGCCTCACGGGGTGAAATAAATTTCCCGGCGCTTTATAGTGGCCG
ACCTCCGTTTCATCAAGTAGCCGAGCCCCATGTCCGAGCCACGCAAGATCCTCGTCACCAGCGCCCTGCCCTATGC
CAATGGTTCCATCCATCTTGGCCATATGCTTGAGTACATCCAGACCGATATGTGGGTGCGCTTCCAGAAGCATCGC
GGCAATCAATGCATTTATGTCTGCGCGGACGACGCCCACGGTTCGGCCATCATGTTGCGCGCCGAAAAGGAAGGCA
TCACTCCGGAACAACTGATCGCCAACGTCCAGGCTGAACATAGCGCCGACTTTGCCGAGTTCCTGGTGGATTTCGA
CAACTTCCACTCGACCCACGCCGAAGAAAACCGCGAGCTGTCGAGCCAGATCTACCTGAAACTGCGTGACGCTGGG
CACATTGCCACGCGCTCGATCACCCAGTATTTCGACCCGGAAAAGAAAATGTTCCTGGCCGACCGCTTCATCAAGG
GCACCTGCCCCAAGTGCGGCACTGAAGATCAGTACGGCGACAACTGCGAAAAATGCGGTGCCACCTACGCACCGAC
CGACCTGAAGGATCCGAAGTCGGCGATCTCGGGCGCCACCCCGGTACTCAAGGATTCCCAGCACTTCTTCTTCAAG
CTCCCGGACTTCCAGGAAATGCTGCAAACCTGGACCCGCAGCGGCACCCTGCAGGACGCGGTGGCGAACAAGATCG
CCGAATGGCTGGATGCCGGCCTGCAACAGTGGGACATCTCCCGCGATGCGCCGTACTTCGGCTTCGAGATCCCCGA
TGAGCCGGGCAAATACTTTTATGTATGGCTGGATGCGCCGATCGGCTACATGGCCAGCTTCAAGAACCTCTGCGAC
CGCACGCCTGAGCTGGACTTCGACGCGTTCTGGGGCAAGGACTCCACCGCCGAGCTGTACCATTTCATCGGCAAGG
ATATCGTCAACTTCCACGCCCTGTTCTGGCCAGCAATGCTCGAAGGTTCGGGCTACCGCAAGCCAACCGGCATCGC
CGTACACGGTTACCTGACGGTCAATGGCCAGAAGATGTCCAAGTCCCGTGGCACCTTTATCAAGGCGCGCACCTAT
CTGGACCACCTGTCGCCGGAATACCTGCGCTACTACTACGCCTCCAAGCTGGGCCGTGGCGTCGACGACCTGGACC
TGAACCTGGAAGACTTCGTGCAGAAGGTCAATTCGGACCTGGTGGGCAAGGTGGTCAACATCGCCAGCCGTTGCGC
CGGTTTTATCCACAAGGGCAATGCTGGCGTGATGGTGCCGGTCAACGCCGCGCCGGAGCTGACCGACGCCTTCCTG
GCCGCCGCGCCAAGCATCGCCGATGCCTATGAAGCCCGCGACTTTGCCCGTGCCATGCGCGAGATCATGGCCTGG
CCGACCGTGCCAACGCCTGGATCGCCGACAAGGCGCCGTGGTCATTGAACAAGCAGGAAGGCAAGCAGGATGAAGT
CCAGGCCATCTGCGCCACCGGCATCAACCTGTTCCGCCAGTTGGTGATCTTCCTCAAGCCGGTGCTGCCGTTGCTG
GCCGCTGACGCCGAGGCGTTCCTCAACGTTGCACCGTTGACCTGGAATGACCACACCAGCCTGCTCAACAACCATC
AGCTCAACGAATTCAAGCCGCTGATGACCCGTATCGACCCGGTCAAGGTACAGGCCATGACGGACGCCTCCAAGGA
AGACCTGACCGCCAGCCAGACCGATACCGGCCAGGCAGCCCCAGCGGGCAATGGCGAGCTGGCCAAGGATCCGCTG
TCGCCGGAAATCGACTTCGACGCGTTTGCCGCGGTGGACCTGCGGGTTGCACTGATCATCAAGGCCGAAGCCGTGG
AAGGCGCCGACAAGCTGTTGCGCCTGACTCTGGATATCGGTGATGAGCAGCGCAATGTGTTCTCCGGGATCAAGAG
TGCCTATCCGGATCCGTCCAAGCTCGATGGTCGGCTGACCATGATGATTGCCAACCTCAAGCCACGGAAAATGAAG
TTCGGTATTTCCGAAGGCATGGTGATGGCGGCAGGTCCCGGCGGCGAGGAAATCTACCTGCTCAGCCCGGACAGCG
GCGCCAAGCCTGGCCAGCGCATCAAGTAACCCGAAGTGCCCTGTCGCGGTGATTGGCCGCGACAGGGCGGTTTGCG
CTAGTACCCTCTTCCTGCTTGCCGGATAATCAGGCCCTGTTTGCTTAACCGGCTCGACCATGACCGAACTGCTTGT
CGCCCTTCTCAGCGCCGCCCTGATCAGCCATTACCTGCTGCGTCTGACGCTGCCCGGTGATCCACAGCTGGAGCGG
CGGCGGGTGCATGCCCTGGGGTTGGCGACCACGTTGTTGGTCGCATTCAGCGGCCTATCGGCCTGCCTGCTGGATA
AATTTGTATTACAACCCTTGGCGCTGGGCGCGTTATGGCTGTTTGCTTATGTACCCGTGGTGATTCTATTGAGTCA
GCCGTTGCTGAATGCGCTTGCCCGCCGCGTGCCCGCACTGCCCTTCGATGGGTTATGGGTGCCGCTGCTGGCTAGC
ACCGGCGTGCTCAGCGTGGCCCTCCTGGGCCCGGGCAACGCAGGCGAGCTGATCTATAGCCTGGGCATCGGCCCCG
CGTTCTGGCTGGTCCTCAGCCTGTTCAACGACCTGCGCCAGCGAATAGACGAAAACGATATCCCCAGGCCCTTCAA
AGGCCTGCCCGTGGACCTGCTGAGTGCCGGGCTGCTGGCGGTGGCGTTCCTGGGCTTAAACGCAATGATCAAACCA
TGAGCCTGATTGAACGTATCGATGCCCTGCTGCCCCAGACCCAATGCGGCAAATGCGGGCACCCGGGTTGCAGACC
CTACGCCGAGGGTATCGCCCAGGGCGAAGCCATCAACAAGTGCCCCCCGGGCGGCACCGAGACCATTGCCGAGCTG
GCGCAGTTGTTGAGCTTGCCCCCCTTGGAGCTGGACCGCGCCCGTGGCGAAGCACCCGCGCAGGTTGCCTTCATCC

GCGAGGCCGAGTGCATCGGCTGCACCAAGTGCATCCAGGCCTGCCCGGTGGATGCGATTGTCGGCGCGGCCAAGCT
GATGCACACCGTTATCGTGGATGAATGCACCGGCTGTGACCTGTGTGTCGCGCCTTGCCCGGTGGATTGCATCGAC
ATGCTGCCACTGCCCCAGGCCACGGTACTGCCGATTGTCGCGGGCTATGCCACTACGGCCGAGGAGCGCCAGGCGC
GGGCGACCAAGCGTGATCGCGCCCGGCGTCGCTTCGAGCAGCGCAATGCACGGCTGCAACGCGAGGAAGCGCACAA
GCTGGCCGAACGCCTGGCCCGCAACCAACGCCCGGCCCCCAGCGAAGCGATCAATAGCAATCCGCTACAGGCCGCC
ATCGAACGCGTGCGTGCGCAAAAAGCCGCAGACATCGACGCAGCGCTTAAAAAAGCCAAGATCAACCTGGCCATGA
GCCGGGCCCAACTGCACAAATCCTTGAAAGCCTTTGGCCATCCGCCGACCTTTGAGCAGCAGTCGCAATTGATCAT
CCTGCAGCAGCAATTTGAGTGCGCCGAACAGGCATTGATCGCGCTTCAAGCAAACGCCCCTGCGGCGACGCCTCCC
AAGGCCATTAAAGATCCTGGGCTCAAGCAGGCAAAAATCCAGCTGGCCATGCGCCGCGCCGCGCTGAAAAAAGCCC
AGGACACCCTTGCCGATGCCGAGCAACTGGCCACGCTGGCTGCAGCCCTGACCGCCGCCGAACAGGCCTTGCATGC
CGCCGAAGAAACCTCCGAGCAGCCTCGGCCTGACCTGCAACGTGTCGAGAAGCGGCCCATCGACAGCCAGCTTCGC
CAGTTGAAGACGGCGCTGGCCTATGCCCGCGCCGACCTGAGCAAACTCGAGCGCCAACCTGCCAGCAGTACAACCG
AGTTGACGAACGCCAGGCAGCGCCTGGATGACGCCCAGCGCTTGGTGGATGAACATGTCGGCGCCTGAGGTTATCG
AGCGCCAGCAACAGGCCATGCAGCGCCTGTTAATGGCGGCAACGCCTGGCTTGCTGGTGCTGTTCTGGCTATATGG
CTGGGGCGTGTTGATCAATCTGTTGCTGGCCGGCACCTGCGCATGGGCAGTCGAAGCCGGGGTGCGCCGGTTGCGC
GGTCATGGCTCCAACGACCTGAGCGGCCTGGTCAGCGCGACGCTGCTGGCCCTGGCGCTGCCCCCTTATTGCCCGT
GGTGGTTATGCCTGGGCGCCGTCAGCAGTGGATTGCTGTTGGGCAAACACCTGTATGGCGGCAAACAGAACCCCTT
CAACCCGGCCATGCTCGGTTATGCGCTGATGCTGCTGGCTTTCCCCCAACCGATGACCCATTGGCCGGCGTCCCAC
GGCCTCGACCTGCTGGCCGGCCTGCAGCAGGTGTTCGGCCTGCACGGCGAGACCGCCGATGCCTGGGCCCGAGCCA
CGGCGCTGGACGCTTTGCGCATCAACAACAGCCTGACCATCGATGAGCTGTTTGCCCGCCACCCGGCCTTCGGCCA
AATCGCCGGTAAAGGCAGCGAGTGGGTGAACCTGGCGTTTCTCGCGGGCGGCCTCTTTTTGCTGCAGCAACGGGTC
TTCAGTTGGCATGCGCCGGTGGGCATGCTTGCCAGCCTGTTTGTTATCAGCTTGCTGTGCTGGAACGGCTCCGGGT
CCGACTCCCACGGCTCGCCGCTGTTTCATCTGTTGACCGGGGCCACCATGCTCGGGGCCTTCTTTATCATCACCGA
GCCCGTGTCGGGTGCGAAAAGTGCGCGGGCGCGCCTGCTGTTTGGTGTAGGCGTGGGAGTGCTGACCTATCTGATC
CGCACCTGGGGCAGTTACCCGGATGGCCTGGCGTTTGCGGTGCTGATGATGAACCTGGCGGTGCCGGCACTTGAGC
GCTGGACGCAGCCGCAGGCGGTGAAGGCATGAAGCGCACGTCTCAAGCCGTGATCGTGCTGCTGATCGCCATCGGC
GCGGCGGGCCTGACGATAGGCCTGCAACAGCTGACCGCCAAGCCGATTGCCGCGCAGCTGCGTGAAATGCACAGTC
GGGCGCTGTTGGATGTGCTGCCCGTCGGCAGCTATGACAATCAACCGCTGGAGCAGCCTTTAGGCATCACCTCGGG
AATGCTGGACAACAGCCAACTGCTGGGTGGTTACCGCGCGACCTTGGCCGGCACCCCCAGTGCGGTGGTGCTGCGC
TCGCAGGTCGATGGATACGGTGGGTGCATAGAGTTGCTGATTGCCATCGACCACAACGCCAAGCTGCTCGGGGTAA
AGACCCTGGAGCACAGAGAAACGCCGAGCCTGGGCGGGCACATTGGCGAGCCTGGTAATGCGTGGCTGGCCTCATT
CATGGGCATGTCGCGGGAAAACCCACAGGTTTGGGCGCTGAAGAAAGACAGCGGCCAGTTTGACCAGATGGCCGGT
GCGACTATTACCTCCCGCGCGGTGATCAACGCGATCCATGATGCCTTGCGTTACTTTGACGAGCATCGTCAGGCGC
TGCTGGAGACCACCGGCCATGACTAAGCCATTCGCCCTCGGCAGCCTGTTACTGCTGCCTGCGCTGCTGGGTGTCA
GCGCTACGCTGGCGAGCGCTGTGGCCTTCTGGGCTCTGTGGGTAACGATCTTGACGTTGCATGGCTGCAGCTGCGG
CTGGGTACGCAACCACCTCAGTGGCAACTGGCGGCTGGCTGCCAATGTGTTGCTGGCCACCACCTGGGTCAGTTGC
GCGCATTTAGTGGCGCAAGCCCTGGCCTTAGGCCCGTCTACCCCCGTGGGGGCATACCTGGCGCTGATCGGTATGC
AATGCGTACTGCTTGAGCATGAGGGTTTGTTCGCCACCGACCGGCGCAGGGCGCGGCTCCAACTGTTCGCCGTAGC
CGCCGGGTTGCTGCTCGCTCTCGCCCTATTGCGTTTTTTACTCGGCAACAGCATCGCCATCCTTGCCCCTACAGGG
TTTATCCTGCTCGGGTTACTGCTTGCCGGGTGGCAAGCCTGGACCCAACGCCGCACACCTCTTTGAAGGAACAGCT
CGCCCCATGAATGCCGCAAAACGCCTGGAGATCTTTCGCCGGTTTCACGAGGACAACCCGGAGCCCAAGACCGAAC
TGGCCTACTCGTCGCCGTTCGAGTTGCTGATTGCCGTGATCCTGTCTGCGCAATCGACGGACGTGGGCGTTAACAA
GGCCACGGCCAAGCTGTTCCCGGTCGCCAACACCCCGGCCGCGATCCACGCCCTGGGGGTTGAAGGGTTGTCGCAG
TACATCAAGACCATTGGCCTTTACAACAGCAAGGCGAAAAACGTCATTGAAACCTGCCGGTTGCTGGTCGAGTTGC
ATGGTGGAGAAGTGCCAGAAACCCGGGAAGCACTCGAGGCATTGCCCGGCGTAGGCCGCAAGACCGCCAATGTGGT
GCTCAATACTGCATTTCGCCAATTGACCATGGCGGTGGACACCCACATTTTCCGCGTCAGCAACCGCACCGGAATC
GCTCCGGGAAAGAATGTGGTGGAAGTGGAGAAGCAACTGATGAAGTTTGTACCCAAACCTTACCTGCTCGACTCAC
ACCATTGGTTGATCCTGCACGGTCGTTATGTGTGCCAGGCCCGCAAGCCGCGCTGTGGCAGCTGTCGCATCGAAGA
TTTGTGTGAATACAAGGAAAAGACGTCGGACGATTGAGCAATCATTGCTTTTATTGATCAGTCGATTGAAAAAATC
TTTTTTACCCGGTGCACGATTGTCGATATAAGGAGCCCCAACGGCAGTCTTAGCCTGGAGTTAACCTTATGAGCAC
TGGCAAAGAACAACTGGATGTAGAAGATGACTTTGTGGCCACCGACGCCGACGAGGCAGCCGAGGCACCTGTAGAA
GTTGCCAAGACCAATTTGAGCAAACGCCGCACCATCGACAACCTTCTGGAAGAGCGACGCTTGCAAAAACAGTTGG
CCGAATACGATTTCGATCTCTAGCCAAGCGATCACACAGAAGCCTCCGCCATGGAGGCTTTTCCTGCCTGTTGCTC
CCTGCTTCTTAAACCAGGCCGTTACGCTGAGCGAGCTCGATCAGGTCCACCAGGGACCGGGCATTGAGCTTGAGCA
GCAAGCGAGTCTTGTACGTGCTCACCGTCTTGTTGCTGAGGAACATCCCATCAGCGATCTCCTTGTTGGTCTTTCC
CTGGGCCAATTGCTGCAACACCATCATCTCCCGGCCTGAGAGCCGCTCGACCATATCGGCTTCACTGGTGTTGCCC
TGTGTGGAGCGCACCGAGTTGAGTGCCTGGTTGGGGAAGTAGCTGTAGCCAGACAGCACCGCCTTGATCGCGCTCA

GCAACTCGGTGAGGTCCTGCTGCTTGCACACGTAGCCCGCTGCGCCGGCCTGCATGCAGCGCATGGAAAAGTGGCC
GGGCGCCTGTGACGTCAGCACCAGTACCTTGAACGGTTGCACCTGTTTTGTGGAAGACAGGCGACAAATAACTTCC
AGGCCGTCAAGTTTGGGGATTCCAATATCCAGGATGACAATATCCGGCATATGCTCCCGTGCTAGTTGCAACGCGT
CGACTCCGTTATCGGTCTCTGCAACGACCTCATAACCATGACGTTCCATTAGCATGCGTACAGCAAGACGAATGAC
GGGATGATCATCCACGATCAGCACTTTATTCATGGGCAAGTCCAATTTCGCTGTTCGAATTATTCAGAACCAGCAC
AATAGCCTAGTCGTTTCGTCCTTGGCATAGCACTCCCCCCCAAGTAACGACAAGCAGAGACCCAACCCACAAAGTT
ATGAGAATAGGACTACAAAAAAACGCCTATATGGATGTGTCGGTTTTTGTTCAGCCTTTCAGAACTTTCCGCGGCC
AAACATATCTTGAGCAAAAAAAGCCCCCGGTGCCCCGGAAAAGTAAACGGGACGCACGCCAATCACAGCAAATACC
TATTGCACGGCCCGCTTGTGCGGGCGCCGCAGCCGCCTAAGGCATTTCAAAAAAGTTCCCTTGTGCAATAGCTACT
GACAATACCTGGAATTATCTGACAGATATTTCCACAAACTAAATAAAACACCCCGTCCGCCCTACTAAACAACCGA
ATGGATTTAAACACAATTAACACCACGCACCGGGCGGACCAACTTTCAGATACAAAATCCAAGCGCAAAAACACTA
AGAAACACCGCAAGCCTCACTCTCGAGTGACAACATTAAACATCAATAAACACTCACCCTAATTCCAGGCAAAAAA
AATGGCCCCTCTTACCGAGGGACCATTTTTATGAGGCTTCAGATACTTCAGAACAACTTGCGGCCTTTGTTCGCCG
CAATGCGCATGCGCAGTGCATTGAGCTTGATGAAACCCGCAGCGTCAGCCTGATTGTAAGCACCGCCATCTTCTTC
AAACGTGGCGATGTTGGCATCGAACAGCGACTCATCGGACTTGCGACCGGTAACGATCACGTTGCCCTTGTACAGT
TTCAGGCGCACAACACCGTTCACATGGACCTGGGAAGCGTCGATCATCTGTTGCAGCATCAGGCGCTCAGGGCTCC
ACCAGTAACCGGTGTAGATCAGGCTGGCGTACTTGGGCATCAGCTCATCTTTTAGGTGTGCCACTTCGCGGTCCAG
GGTGATGGACTCGATGGCGCGGTGAGCGCGCAGCATGATGGTGCCACCTGGGGTTTCGTAGCAGCCACGGGACTTC
ATGCCCACGTAACGGTTCTCTACGATATCCAGACGACCAATACCGTGCTCGCCACCGATACGGTTCAGGGTCGCCA
GTACAGTGGCCGGGGTCATTTCGACGCCATCCAGCGCGACGATATCGCCGTTGCGATAGGTCAGTTCAAGGTACTG
CGGCTTATCGGGAGCGTTCTCCGGGGAGACGGTCCACTTCCACATGTCTTCTTCGTGCTCGGTCCAGGTGTCTTCC
AGCACGCCGCCTTCATAAGAGATATGCAGCAGGTTGGCGTCCATCGAGTACGGGGACTTCTTCTTACCGTGGCGCT
CGATCGGGATGGCGTGCTTTTCAGCGTAATCCATCAGTTTTTCGCGGGACAGCAGGTCCCACTCACGCCAAGGGGC
AATCACTTTTACGCCGGGTTTCAAGGCATAGGCGCCCAGTTCGAAACGCACCTGGTCGTTGCCCTTGCCGGTGGCG
CCATGGGAAATGGCGTCAGCGCCAGTTTCGTTGGCGATTTCGATCAGGCGCTTAGCGATCAGCGGACGTGCGATGG
AAGTACCCAGCAGGTACTCGCCTTCGTAGACGGTGTTGGCGCGAAACATCGGGAAAACGAAATCGCGGACGAATTC
TTCGCGCAGGTCGTCAATGTAGATCTCTTTGACGCCCATGGCTTGCGCCTTGGCTCGTGCAGGTTCGACCTCTTCG
CCCTGACCCAGGTCAGCGGTAAAGGTCACCACTTCACAGTTATAAGTATCCTGCAGCCACTTGAGGATCACCGAAG
TGTCCAGGCCGCCGGAATACGCGAGAACGACCTTGTTTACGTCGGCCATGCCATCACTCCACGGGGTTTTACGGAA
AGGCGTCGATTCTACCGATCAAAACCGTGAAATTACAGGGGCGCGACAGCAAATGAAGATAAAGCGACAGATTATG
TCGGGCGAGCGACGAATGGCCGCACCTCAAAACGCCTTAGCCGTGGGCATCAGATGCCCAGCGTCATCGTTGATGA
CCACCGCTGGCAGGCAGCTCCCACAGAGGAATATCAGGAGGTCGCTGCCGCATTCGCCGGGGCCGTGGCCTGGGGG
GCCGGTTTTTCGGGGGCCGGTACGCGATCGAGGTGGATATTGACCCGCCGGTTCTTCGCCCGGTTGGCCGGATTGG
TATTGGGTACCAACGGATAACGCTCACCATGGAAGCGCAGGACAATCTGCGACTCGGGGATGCCGTTGGCCTTGAA
GAAGTCCATCACCGCCAAGCCCCGGCGCCGGGACAGGTCGCGATTGGTCAGGCGGTTGCCGCTGTTGTCCGAGTGG
CCGTCCAGTTCGATATGGTTGACCGTAGGGTCGGCCTTGATGAATTCGAGCATCACCATCAGCTTGGCCTTGGCTG
CCGGGTCCAGGTCGATACCCCCGCCCGGAAAACCTACTTCCGCCTGCTTGACCTGCTCGAAGTTCATCGGCAGCAA
CTTCGCGGTACACAACTGGTAGTCGCTATAGGCCTTATTGAATCGCACCGGCAGCAGGCGGATTTCCGAATAACCG
CCGTCCCGGGAATAGTGGCGCACGGTGGGGCTGCGGCCATCCAGCAAGCCGTTGAACAGGCGTCCGGCCTGGGCCT
GGGAGCTGTTGAACAGCACATCGCCGCTGCCCGCACGCACGGCACCGAGGTTGATATCACCGCGCCCCGGCTGCCA
CGGCGCGGCCGCTGCCAATAGCGTGGCAGATCCAACGCCCAGCGCGCCGTTATAGGCTTTCAGACGAAATGTCGCC
TGCTCGCCGGCCCTGCGCACGAACTCCCCCGAACCAAAATCGGCGATCGGCTGGCTCAAGCGGCACTCGAACTTGT
CGCCTTCCACCTTCCACTCGATATTCTCCAGGCGCGTCTGGAATGTCAGGGCCATCGCGGGCAGGCTGGCGAACAC
GCTGAGCAGGGCTAGATATTGCTGGCGCACGGGCGGCTCCACTGGTTTCTACAACACATCAAGGCGTCATACATCG
TTAAGGCATACCCAGGGATATCGGTCGCACCCTGCAAAACTTGATAGCGAGTGCCTGCAAGAGTCTTTTCCGGTAG
CATTCCCACCAGATTGACCCGCCTGGAATCCCCAATGTCCGACCGCCTGACCCTGCTGCGTCCCGACGACTGGCAT
ATTCATCTTCGCGATGGTGCTGCGTTGCCCCAAACCGTGGCCGATGTTGCGCGCACGTTTGGCCGCGCCATCATCA
TGCCTAACCTGGTACCTCCGGTGCGTAATGCCGCTGAAGCCGACGCCTATCGCCAGCGCATCCTCGCTGCACGACC
GGCCGGTAGCCGCTTCGAACCGTTGATGGTGCTTTACCTTACCGACCGCACCCAGCCCGCCGAAATCCGCGAGGCC
AAGGCCAGCGGTTTCGTGCACGCCGCCAAGCTGTACCCGGCCGGCGCCACGACCAACTCCGACTCCGGCGTAACCA
GTATCGACAAGATCCTGCCCGCCATCGAAGCCATGGCCGAAGTAGGCATGCCGCTGCTGATCCACGGTGAAGTCAC
CCGTGGCGATGTGGATGTCTTCGATCGCGAGAAGATCTTCATCGACGAGCACATGCGCCCGCGTGGTCGAGCTGTTC
CCGACCCTCAAGGTCGTGTTCGAGCACATCACCACCGCAGACGCCGTGCAGTTCGTCACCGAGGCTTCGGCCAACG
TCGGCGCAACCATCACCGCGCACCACCTGCTGTACAACCGCAACCACATGCTGGTGGGCGGGATTCGGCCGCACTT
CTATTGCCTGCCGATCCTCAAGCGCAACACCCATCAGGTGGCATTGCTCGATGCGGCTACCAGCGGCAGCGCGAAG
TTCTTCCTCGGCACCGACTCGGCGCCCCACGCCCAGCACGCCAAGGAAGCCGCCTGCGGTTGCGCCGGTTGCTACA
CCGCCTACGCGGCGATCGAGCTGTACGCCGAGGCCTTCGAATCGCGCAATGCCCTGGACAAACTCGAGGCCTTCGC

CAGCCTCAATGGCCCGCGTTTCTATGGCCTGCCGGCGAATACCGACCGTATTACCCTGGTCCGTGAAGACTGGACC
GCCCCCACCAGCCTGCCATTTGGCGAGCTGACCGTTATCCCGCTGCGCGCCGGTGAAACACTGCGCTGGCGCCTGC
TGGAGGAACACCCGTGAGTGAAGACCATTACGACGACGAACAGGAACACGGTGGCGGCGGTTCGCGTCACCCGATG
GCCGAGCGTTTTCGCGGCTATCTGCCGGTCGTTATCGACGTAGAAACCGGCGGTTTCAATTGCGCCACCGATGCAC
TGCTGGAAATTGCGGCAACCACCATCGGCATGGACGAACAGGGCTTTGTGTATCCAGAACACACCCATTTCTTCCG
CGTCGAGCCGTTTGAAGGCGCCAATATCGAAGCAGCAGCCCTGGAGTTCACCGGGATCAAGCTCGACCATCCGTTG
CGCATGGCCGTCAGCGAAGAGGCGGCATTGACCGACATCTTCCGTGGCGTGCGCAAGGCCTTGAAGGCCAACGGCT
GCAAACGCGCGATCCTGGTCGGCCACAACAGCAGTTTCGACCTGGGCTTCCTCAACGCCGCCGTGGCGCGCCTGGA
CATGAAACGCAACCCGTTTCACCCGTTCTCCAGCTTTGACACCGCTACCCTGGCTGGCCTGGCTTATGGCCAGACC
GTATTGGCCAAGGCATGCCAGGCAGCCGGCATCGACTTCGACGGACGTGAAGCCCACTCGGCTCGCTACGACACCG
AGAAGACCGCCGACCTGTTCTGCGGCATCGTCAACCGCTGGAAACAGATGGGCGGCTGGGAAGACTTCAGCGACTG
ATTGCGTCGCCAAATCCCACTCATAAAAAAACCGGCCTTCTCAGGCCGGTTTTTTTACCGCAACCCTGGCTTACA
GCTTGCCAGCGTTCTCGGTCAGGTAAGCGGCAACGCCTTCTGGCGAAGCGTTCATGCCCTTGTCGCCTTTTTTCCA
GTTGGCTGGGCAGACTTCGCCGTGCTCTTCGTGGAATTGCAGAGCGTCGACCAGGCGGATCAGCTCTTCCATGTTA
CGGCCCAGCGGCAGGTCGTTGATGATCTGCGAGCGCACAACGCCCTTGTCGTCGATCAGGAACGCGCCACGGAAAG
CCACGCCGCCTTCGGACTCAACGTCGTAGGCCTTGGCGATGTCGTGCTTCATGTCGGCAGCCATGGTGTATTTGAC
TTTGCCGATACCGCCATCATTGATGGCGGTGTTGCGCCAGGCGTTGTGGGTGAAATGGGAGTCGATGGAAACGGCA
ACCACTTCAACGTTACGCGCCTTGAAATCGTCCATGCGGTGATCCAGGGCGATCAGCTCGGACGGGCAGACGAAGG
TGAAGTCCAGCGGGTAGAAGAACACCAGGCCGTATTTGCCTTTGATGGCTTCAGACAGCTTGAAGCTGTCAACGAT
TTCGCCATTGCCGAGGACGGCTGGTACGTCGAAATCCGGGGCTTGTTTGCCGACGAGTACGCTCATTGGTTATCTC
CTGATGGTAATTGAAGTAAATGGACCGCCCTTGAGTCTGCCGCAAATTGACGACAGGCCTGTGACGCGATCACGCT
TCGTAAAGACCGACCATCATACCTGAAAAAAGCCGTTCGTCAGTGAGGGCCCCACGGGGGGCAACGGTTTGAGAAA
GCACTTTGACAATCATTCTCGTTAACATTAAGATCCATCGCACTTAAGCCTTAAACCGCGATGGTTCTCCCTTATG
TATGTCTGCCTCTGCACTGGCGTCACCGACGGACAAATCCGCGAAGCGATCTATGAAGGTTGCTGCAGCTACAAGG
AAGTGCGTGAAACCACCGGCGTTGCCAGTCAATGCGGCAAATGTGCCTGCCTTGCCAAGCAAGTCGTACGGGAAAC
CCTGACAAAACTGCAGGTAGCCCAGGCCGCGATCCCCTACTCGGCAGAATTTACACACGCCTGAAACAGCCTATTT
TAAAGAACCGGACTTAGTGTCCGGTTTTTTTATGCCTGTAATTCAAATAGTTAGGCCCAAGACGCGGAACACAAAC
ATTCTTATTCCGATTAATTTTCATTTATTATTCAATAACTTAGGTTTGACACTAGTAATCGTGCAGCTCAAACTCC
GCCCTATATACAGCGAACACAGGGCAGGACCCCAATCATGAAAGGCGACATCTCAGTCATCCAGCAACTCAACAAA
ATCCTTGCCAATGAACTGGTCGCGATCAATCAGTACTTCCTGCATGCACGCATGTATGACGACTGGGGCCTGGAAA
AACTCGGCAAGCGTGAGTACAAGGAATCGATCAAGGCTATGAAGGACGCAGACGCGCTGATCAAGCGCATCCTGTT
CCTTGAAGGCCTGCCGAACGTACAGGACCTGGGCAAGCTGAACATCGGCGAGCACACCCTGGAAATGCTCAACAGC
GACCTGGGTTTCGAGCGCAAGAGCCATGCCGACCTCAAGGCCGCCATCGCCCACTGCGAGACCAAGGGCGACTTCG
GCAGCCGTGAGCTGCTGGAAGATATCCTGGAAGACCAGGAAGAACATATCGACTGGCTGGAAACCCAAGTTGGCCT
GATCGACAAAGTGACCCTGGAGAACTACCTGCAATCGCAGATGGGTGAGTAATCTCTAATTATTGTAGGAGCGAGC
TTGCTCGCGAAAAGCGTGAAGGCGCCGCGTTCATTCAGAATGCTCGCGCTATCGTTAACGTCCTTCGCGAGCAAGC
TCGCTCCTACAGGTACAAAAAAGCCCCGCTCTCGAATGAGAAGCGGGGCTTTTTATTGTGCGCCAATTACGCTTCG
GTCTTGGCAGCCGCTTTTTCAGCAGCCTCCTTGATCAGCGCCTGCAACGAACCATCAGCCGCCATTTCGGTGATGA
TATCGCTACCGCCGACCAGCTCACCGGCCACCCACAGTTGTGGGAACGTTGGCCAATTGGCGTACTTCGGCAGGTT
GGCGCGGATTTCCGGGTTTTGCAGGATATCCACGTAAGCGAACTTTTCACCACACTGCATGACAGCCTGGGAAGCC
TTCGCGGAGAAGCCACACTGCGGGGCATTCGGGGCGCCCTTCATGTAAAGCAGAATGGTGTTGTTGGCAATCTGCT
CTTTAATCGTTTCGATGATATCCATGGAACACCTCGGCTGGAACTTTGCGACTCACAGGTCGGCACGGTGGCGCAT
TGTAACGCAAAATCCCAGCGTGGTGCTCGGGCTCCCCGACAGACTCACTGCGTCACGCCGCCGCGACCTGCACCGG
CACGCCATTCAATGCCGCATTGCCCGACAACTCATCCAACTGCCGTTCATCCGTCAGGTCATTGGCACTCGCCCCA
GGCTGGGCACTGGCGATGTTCATGTGTACACCCGGGCGGTCATGGCCCCAACCGTGGGGCAGGCTGACCACGCCGG
GCATCATTTCCAGGCTGGCCAACACCTGTACTTCGATCATGCCGATCCGCGAACTGACCCGCACCCGCTGCCCGTC
ACTCAGTTGGCGACGGGCAAGGTCATCGGGGTGCATCAGCAATTGATGGCGGGGTTTGCCCTTCACCAGCCGGTGA
TAATTATGCATCCACGAATTATTGCTGCGTACATGCCGGCGGCCGATCAACAGTAACTCGTCGACCTTGGGCAGCG
GCAACGCCGCAAACCGCGCCAGGTCCGCCAGGATCACCGGCGGCGCCGCCTGGACCCGGCCATTGGCAGTTTTCAA
GCGCCCCGCCAGATTGGCCTTCAGGGGCCCCAGGTCCAGACCATGGGGATGATCGGCCAGCATGGCCACCGACAGC
TTGTGCGGGGAGGCGTCACCATAGGCCCCCGCCCGCAAGCCAAAATCAATCATCTGCGCCGGCGCCATCGTGGGCT
TGAGCGGGCTGCCGGTACGGGCCGCAAACGCCTGGGCCAAGCCGACGAAAATCTCCCAATCGTGCAACGCCCCCTC
GGGCTTGGGCAGAATCGCCCGGTTGAAGCGGGTGACGTTACGCACCGCAAACATATTGAACGTGGTGTCGTAATGA
TCGTTTTCCAGGGCCGAAGTCGATGGCAGGATCAGGTCGGCATAACGGGTGGTTTCATTGATATACAGGTCGACGC
TGACCATAAACTCCAGGCCATCCAGCGCCTGCTCCAGTTGCCGACCGTTCGGCGTGGACAACACCGGATTGCCAGC
CACTGTGACCAGCGCGCGGATCTGCCCTTCGCCCGCAGTAAGCATTTCTTCAGCCAGGGCCGACACGGGCAACTCG
CCGCTGTATTCGGGACGCCCGGACACCCGACTCTGCCACCGATTGAAGTGCCCGCCGGATGTGGCCGCCACCAGGT

CCACCGCAGGCTCGGTGCACACGGCGCCACCTACCCGGTCCAGATTGCCCGTGACCAGGTTGATCAACTGCACCAG
CCAGTGGCACAAGGTGCCAAATGCCTGGGTCGACACCCCCATGCGCCCGTAGCAGACGGCCTTGTCTGCGGCCGCA
AAATCCCGCGCCAACTGGCGGATCTGCTCGGCCGGCACCGCGCATTGAGCGCTCATGGCTTCGGCGGTAAAGCCCG
CGATGGCGCGGCGCACCTCGTCCAGTCCGTCCACCGGCAAGTGGCTATCACGGGTCAGGTGTTCGGCGAACAATGT
GTTGAGCAGTCCGAACAGCAACGCCGCGTCCCCACCGGGGCGCACAAACAGATGCTGGTCGGCCATGGCCGCCGTC
TCACTGCGCCGGGGATCGACCACCACCACTTTGCCGCCCCGCGCCTGAATCGCCTTCAGGCGCTTTTCCACGTCCG
GCACAGTCATGATGCTGCCATTGGAAGCCAGTGGGTTGCCGCCCAGGATCAGCATGAAGTCGGTATGGTCGATATC
CGGGATGGGCAGCAACAGCCCGTGCCCATACATCAAGTGGCTTGTGAGGTGATGGGGCAGTTGGTCCACCGAGGTC
GCGGAAAAGCGATTGCGCGTCTTCAACTGCCCGAGGAAATAGTTGCTGTGGGTCATCAAGCCATAGTTGTGCACGC
TGGGATTGCCCTGGTACACCGCCACCGCATTCTGCCCATGCCGCGCCTGGATGCCCGCCAGCCGCTCGGCCACCAG
GGCGAAAGCCTCGTCCCAGGGGATCGGCTGCCATTGGCTGCCCACCCGCAGCATCGGCTGGTGCAGGCGATCGGGA
TCGTTCTGGATATCTTGCAGGGCCACGGCCTTGGGGCAGATATGGCCACGACTGAAGGTGTCCTGGGCGTCACCCT
TGATCGAGGTGATGGCAATACTGCCTGCGTCCGAGGTGGTGGTTTCTAGGGTCAGGCCGCAGATCGCTTCGCACAG
GTGGCAGGCACGGTGATGGAGAGTCTTGGTCATGGCCAGTCTCTTTATTAGGGCTTTGGCCCGACTATGGGCCGCG
CCCTCCAGCGACGCCAGCAACGTTCGCCCTGTGAATCAACGGGCATCAGGCCAGGCCATGGGTAAGCATGAGTAAA
TGTTTCAAAAACCTCCCGCACCGCCCGGGAAACTGACGCGACCTGGCCCGAGGCCGCCATCACACATTGCAAAAGG
TCGCGGCGCCAGTGTACAAATGACCCGCTGCTGTCCGTAAACAGGCCTCAAAAGTGCAATTCCCGCCCCTTTGACC
CCCTCTAAAAAACCGTAGCCTATTGGTAAGACGCGACATTTAGTGTCAATATCGCGCCTCCCCCTATTTCGTCGCC
CAGTGCGGCTTTCGCCGCAGGTCTCGCCCGTTGTCACAATAAACAAGGCTTTGAGTATCTGCGGTCTGTTGCAAAA
AGGTAGTTAATGATGAGCGCAAGGCACTTTCTCTCCCTGATGGATTGCACGCCCGAAGAGCTGGTCAGCGTGATCC
GTCGAGGCATTGAGCTTAAAGACCTGCGTAATCGCGGCGTACTGTTCGAGCCTTTGAAAAACCGCGTACTCGGGAT
GATTTTCGAGAAATCGTCCACCCGTACCCGCCTGTCCTTCGAAGCCGGCATGATCCAGCTCGGCGGCCAAGCCATT
TTCCTGTCGCCCCGGGACACCCAACTGGGCCGGGGCGAGCCGATCAGCGATTGCGCCATTGTCATGTCGCGCATGC
TCGATGCGGTGATGATCCGTACCTTCGCCCATAGCACCCTCACCGAATTTGCCGCCAACTCGCGGGTGCCGGTGAT
CAACGGGCTGTCCGATGACCTGCATCCGTGCCAGTTGCTGGCCGATATGCAGACGTTCCTCGAACATCGCGGCTCG
ATCCAGGGCAAGACCGTAGCCTGGGTCGGCGATGGCAACAATATGTGCAACAGCTATATAGAAGCGGCGATCCAGT
TCGACTTCCATCTGCGCATTGCCTGCCCTGAAGGCTACGAGCCCAGCGCCGAATTCCTGGCCAAGGCCGACGCCCG
CGTTCAAATCGTGCGCGACCCGAAAGACGCGGTGGTCGGCGCGCACCTGGTGAGCACCGACGTCTGGACCTCCATG
GGCCAGGAAGACGAGACCGCCAAGCGCTTGGCCTTGTTCGCACCTTATCAAGTCACCCGTGAATTGCTCGACCTTG
CCGCGCCGGATGTGTTGTTCATGCACTGCCTACCGGCCCATCGCGGCGAAGAAATCAGCCTCGACCTGTTAGACGA
CCAGCGCTCCGTCGCCTGGGACCAGGCAGAAAACCGCCTGCACGCCCAGAAGGCCCTGCTGGAGTTCCTGGTGCCA
CCGGCGTATCACCACGCATGAGCCAGCCACTTCTGCTGAACCTGCGCAATCTGGCATGCGGCTATCAGGACCAACG
GGTGGTGCAGAACCTCAATCTGCACCTCAATGCCGGGGATATCGGTTGCCTGCTGGGCTCTTCCGGCTGCGGCAAG
ACCACCACCCTGCGCGCGATTGCCGGGTTTGAGCCGGTGCACGAAGGCGAAATAAACCTGGCCGGCCAAGTGATCT
CCAGCGCCGGTTTTACCCTGGCACCGGAGAAACGCCGCATTGGCATGGTGTTCCAGGACTACGCACTGTTTCCACA
CCTCTGCGTGGCCGACAACATCGGCTTCGGCATTCGCAAACACCCGCAAAAAGAGCGCGTGGTGGCTGAATTGCTG
GAGTTGGTCAACCTGAAGAACCTGGGCAAGCGCTTCCCTCACGAACTTTCCGGCGGCCAGCAACAGCGTGTGGCCC
TGGCCCGTGCGCTGGCACCCGAGCCGCAGTTGCTGCTGCTGGACGAGCCCTTCTCCAACCTTGATGGTGAGCTGCG
GCGCAAGCTCAGCCATGAAGTACGGGACATCCTCAAGGCCCGTGGTACCAGTGCGATCCTGGTCACCCATGACCAG
GAAGAAGCCTTTGCCGTGAGCGACCACGTGGGTGTGTTCAAGGAAGGCCGGCTGGAACAATGGGATACGCCCTACA
ACCTCTATCACGAACCGCTGACGCCTTATGTCGCAAGTTTTATTGGCCAGGGTTACTTCATCCGCGGGCAATTGAG
TTCGCCGGAATCGGTCAGCACCGAGCTGGGCGAATTGCGCGGCAATCGTGCCTATACCTGGCCGACTGGCAGCGCG
GTGGATGTGTTACTGCGCCCGGATGACGTCGTTTATGCACCCGACAGCACGCTGAAAGCGCAGATCGTCGGCAAGA
CGTTCCTCGGTGCCTCGACCCTCTACCGCTTGCAGTTGCCGACCGGGGCCCAGCTGGAATCGATTTTCCCCAGCCA
TGCCGACCATCAGGTGGGTGCGCAGGTGGGAGTCCGCGTGGCGGCCGAGCATCTGGTGCTGTTCCAGGCCTCGGGG
AGCACGGCGGCGCAGATTGCGCAGGTGGAGTCAGGTGTGCGGCGCTACAGCACTACCAGTTGAAGAAACCCGATCC
AAAATGTGACAGCGCGCTAGTGTGGGAACCGGGCTTGCTCGCGATGCGGGCGCCTGGGTCTGTCAGTTGCATCAAG
GTGATGCTATCGCAGGCAAGCCAGCTCCCACCGAGGCTGGCTCGCACATTCGATCCCGCTGTTCTTGACGTTACGC
CCGGCCAATCGGCGCAAATGTCGCCTGGGTGTGCTCTGCCAACACCGCCGCCGCCAACTCCACCTCAAGTCCTCTC
CTGCCTGCGCTGACAAATATTGTCGCAAAAGGCTGCGCGCTCACATCGATGAACGTGCGCAAGCGCTTCTTCTGCC
CCAACGGGCTGATCCCCCCCAACAAATAACCGGTAGAGCGCTGCGCCGCTGCCGGGTCGGCCATCTCGACTTTCTT
CACGCCCGCAGCATGCGCCAGGGCCTTCAAATCCAGACTTCCGACGACCGGCACCACCGCCACCAATAACTCAGCT
TTTTCGCTGCTGGCGAGCAAGGTCTTGAACACCCGCGCAGGATCCAGGCTCAATTTTTCCGCGGCCTCCAGGCCAT
AAGAGGCGGCCTTGGGGTCATGTTCGTAACTGTGGATGCGATGTTCGGCGCGAACTTTTTTCAACAAGTCCAATGC
GGGTGTCATTGGGGGCTCCAGGCTTGTGGGAAACGTGGAAAAGCAGACCGCCGATTCTAGGACAAACCCAGGCAAA
ACGCTCTAGTGCAGGGCACCGTGCGACCAATCCGCGCAGCGCCTGGGCACGCTTGCCGCCCGTTGCGCCAACGTAG
TAGCGCGATCATTCATCACACTAATAGTTTGAAAATGACCGACCGTTCACTTTCGACCTTTGACAGCGGTGTTTCT

TGTCTATATTTTTTCGTTTCCGAATACTGTAGGAATACACCCACAGTATCCGAGCAGTAGCCGTGGCCGGGATGGG
GATTTTGGTGACGGTGAAAATCGCGCGAGGCCCGTTGAGGCACAGCGCCAGACAAGAAAAAAAACGAGGTTTTCAA
TGACAACTGCTCTTCAACAGCCTTCACTTTCAAGCCAATGCATGGCCGAGTTCCTCGGCACCGCGCTGCTGATCTT
CTTCGGCACCGGCTGCGTCGCTGCGCTCAAGGTCGCGGGTGCCAGCTTCGGCTTGTGGGAGATCAGCATCATCTGG
GGGATCGGCGTGAGCATGGCGATCTACCTGAGTGCCGGCATTTCCGGGGCTCATCTCAATCCAGCCGTCAGCATCG
CGCTGTGCATCTTTGCCGATTTCGACAAGCGCAAACTGCCCTTCTATATCATCGCCCAGGTTGCCGGCGCCTTCTG
TTCGGCTGCGTTGGTCTACACGCTCTACAGCAACCTGTTTTTCGATTACGAACAAACTCACCACATGGTTCGTGGC
TCGGCGGCCAGCCTGGAACTGGCTTCGGTCTTCTCCACCTACCCTCACGCCCTGCTCAATACCGCCCAGGCATTCC
TGGTGGAGATGGTTATCACCGCCATCCTGATGGGCGTGATCATGGCCCTGACCGACGACAATAACGGCTTGCCCCG
TGGCCCCCTGGCGCCACTGCTGATCGGCTTGCTGATCGCGGTGATCGGCAGTGCCATGGGCCCGCTGACCGGGTTT
GCGATGAACCCGGCCCGGGATTTCGGCCCCAAGCTGATGACGTTTTTCGCCGGTTGGGGTGAAATGGCCTTCACCG
GCGGGCGCGATATTCCTTATTTCCTGATTCCGATCCTTGCACCGATTGTCGGTGCCTGCCTCGGCGCTGCGGCCTA
TCGCGGGTTGATTGCCCGCCATCTGCCCAACGCTGCACCTGCTACAACTGATGCAGCCGACACCGCCGCCAGCGGT
AACACCCGAACTTCCTGAAAGCGGTAGCCTGAGCCCTTCTGCCCTTTGCGGCCCAGGCTACTTACCCACTCCCTTT
TTCCGTCCAAGGCAATCGACATGACCGACACACAGAATAAGAACTACATCATTGCCCTCGATCAGGGCACCACCAG
TTCCCGGGCGATCATCTTTGATCGTGACGCCAACGTGGTGTGCACCGCCCAACGTGAATTCGTCCAGCATTACCCG
CAAGCCGGTTGGGTCGAACATGACCCGATGGAAATCTTCGCCACCCAAAGCGCCGTGATGGTCGAGGCCCTGGCAC
AGGCCGGCCTGCACCACGATCAGGTGGCCGCCATCGGCATCACCAACCAGCGTGAAACCACGGTGGTCTGGGACAA
GATCACCGGCCGCCCGATCTACAACGCCATCGTCTGGCAATGCCGCCGCAGCACCGAGATCTGTCAGCAACTCAAG
CGTGACGGCCATGAGCAATACATCAGCGACACCACCGGCCTGGTCACCGACCCGTACTTCTCTGGCACCAAGCTCA
AGTGGATCCTCGATAACGTCGAAGGCAGCCGCGAACGCGCGCGCAATGGCGAACTGCTGTTCGGCACCATCGACAG
CTGGCTGATCTGGAAATTTACCGGCGGCAAGACCCACGTCACCGACTACACCAACGCCTCGCGCACCATGCTCTTC
AATATCCACACCCTGGAGTGGGACGCGAAGATGCTCGAGGTGCTGGACATCCCGCGCGAGATGCTGCCGGAGGTTA
AATCCTCTTCGGAAATCTACGGCCGCACCAAAAGCGGGATTGCCATTGGCGGGATTGCCGGCGACCAGCAAGCGGC
GCTGTTCGGCCAGATGTGCGTCGAGCCCGGCCAGGCGAAAAACACCTACGGCACCGGCTGCTTCCTGCTGATGAAC
ACCGGCGACAAGGCGGTCAAATCCAAGCACGGCATGCTCACCACCATCGCTTGCGGCCCACGTGGCGAAGTGGCCT
ACGCCCTCGAAGGCGCAGTGTTCAACGGCGGCTCCACCGTGCAATGGCTGCGCGACGAGCTGAAGATCATCAACGA
TGCCCACGACACCGAGTACTTCGCCAACAAGGTCAAGGACAGCAACGGCGTGTACCTGGTGCCAGCGTTCACCGGC
CTCGGCGCGCCGTACTGGGACCCCTATGCCCGTGGCGCACTGTTCGGCCTGACCCGCGGCGTACGTGTCGATCACA
TCATTCGTGCGGCCCTGGAGTCGATTGCCTACCAGACCCGCGATGTGCTCGACGCCATGCAGCAGGACTCTGGTGA
ACGCCTCAAATCCCTGCGGGTGGACGGCGGCGCCGTGGCCAACAACTTCTTGATGCAGTTCCAGGCCGACATCCTC
GGCACCCAGGTCGAGCGCCCTCAAATGCGCGAGACCACCGCATTGGGCGCGGCCTACCTGGCCGGCCTGGCCTGTG
GCTTCTGGGGCAGCCTGGATGAGCTGCGTGGCAAGGCGGTGATCGAGCGCGAATTCGAACCGCAACTGGACGAACC
GGCCAAAGAAAAACTCTACGCCGGCTGGAAAAAAGCCGTCAGCCGCACCCGCGACTGGGAACCCCACGAAGGCGCT
GAATAAGCCACGCGCCGGACCCACTTAGGGTTGCAACTGGCAGGGAGCAGATTCCTGCGTCATCATGGGCCACTTT
TTTGTACGGCAGCCCAAAGGACGCCCCATGAATCTGCCTCCCCGTCAGCAGCAAATCCTCGAACTGGTCCGCGAAC
GCGGCTATGTCAGCATCGAGGAAATGGCGCAGCTGTTCGTTGTTACCCCGCAAACCATCCGCCGCGACATCAATCA
ACTGGCCGAAGCCAACCTGCTGCGCCGCTACCATGGCGGCGCCGCCTATGACTCCAGCGTCGAAAACACCGAGTAC
GCCATGCGTGCCGACCAGATGCGCGATGAAAAACAGCGTATCGGCGAAGCTATTGCCGCACAGATCCCTGATCACG
CCTCGCTATTCATCAATATCGGCACCACCACCGAGTCCATCGCCCGGGCGCTGCTCAACCACAGCCACCTGAAGAT
CATCACCAACAACCTCAACGTCGCCATGATGCTCAGCGCCAAGGACGACTTCGACGTGCTCCTCACCGGCGGCAAT
GTGCGCCGTGACGGCGGCGTGGTGGGCCAGGCCAGCGTGGACTTTATCAACCAGTTCAAGGTCGACTTCGCCCTGG
TGGGGATCAGCGGTATCGACGAGGACGGCAGCCTGCTGGACTTCGATTACCAGGAAGTGCGGGTTTCCCAGGCGAT
CATTGCCAATGCACGCAAGGTGATCCTGGCGGCGGACTCCAGCAAATTCGGGCGCAACGCCATGATTCGCCTGGGC
CCGATCAGCTTGATTGATTGCCTGGTTACCGATCAGCAGCCGGTGCCGGCGCTGGTGCAGTTGCTCAACCAGCATA
AGATTCGCCTGGAAGTCGTATAAGCCTCCGACTGAATCTCTGGTGCCTGTACTGCCGTCATCGCGGGCAAGCCCGC
TCCCACATTGGAATGTGATCAACCTGTGGGAGCGGGCTTGCCCGCGATAGCGACCTACAAGGCCACAACTCTCTTG
GGCCAACACCAGGCTCAATGTTCACAATTTTTCCTTTTCCGGCCCTTCGATGAGTTTTTTCAATCGAAGGCGACTG
GCTGCGCCCGCGTTTATGAGCTACCATTTTCGCAAATGAACATTAATGTTCGAATTCAAATATAAATAAAGATCGC
GAGGCCAGCCGATGACCCCCTCCACCTTGCCTGCTCCACCCCTTGCCGAAGTCTATGATGTTGCCGTTATCGGTGG
CGGGATCAATGGCGTGGGCATCGCGGCAGATGCAGCCGGTCGCGGCCTGTCGGTATTCCTTTGCGAAAAGGACGAC
CTGGCCAGCCACACCTCCTCCGCCAGCAGCAAGCTGATCCACGGCGGCCTGCGCTACCTTGAACATTACGAATTCC
GCCTGGTGCGCGAAGCCCTGGCCGAACGCGAAGTGCTGCTGGCCAAGGCCCCGCACATCGTCAAGCAGATGCGCTT
TGTGCTGCCCCATCGTCCACATCTGCGCCCGGCGTGGATGATCCGCGCTGGCCTGTTTCTGTATGACCACCTCGGC
AAGCGCGAAAAACTCGCTGGCTCCAAAAGCCTGAAGTTCGGTGCCGACAGCCCGCTGAAAAGCGAAATCACCAAGG
GTTTCGAATACTCCGACTGCTGGGTCGATGACGCACGCCTGGTGGTGCTCAACGCCATGGCCGCCCGGGAAAAAGG
GGCACACATCCACACCCAGACCCGTTGCGTCAGCGCCCGTCGCAGCAAGGGCCTGTGGCACCTGCACCTGGAGCGC

GCCGATGGCAGCCTGTTCTCGATCCGCGCCAAGGCGCTGGTGAACGCGGCCGGCCCGTGGGTTGCCAAGTTCATCA
AGGATGACCTGAAGCTGGATTCGCCCTACGGCATCCGCCTGATCCAGGGCAGCCACCTGATCGTGCCGAAACTGTA
CGAAGGTGCCCACGCGCACATCCTGCAGAACGAAGACGGGCGCATCGTCTTCACCATTCCGTACCTCAATCACCTG
ACCATCATCGGTACCACCGACCGTGAGTACACCGGCGATCCAGCGAAAGTGGCGATTACCGAAGGTGAAACGGACT
ACATGCTCAAGGTGGTCAACGCCCACTTCAAGCAGCAGTTGAGCCGCGACGATATCGTGCACACCTACTCCGGCGT
GCGTCCGCTGTGCAACGACGAGTCGGACAACCCGTCGGCCATTACCCGCGACTACACCCTGGCGTTGTCGGGCACA
GGTGAAGAAGCGCCGATCCTGTCGGTGTTTGGCGGCAAGCTGACCACCTACCGTAAGCTTGCGGAGTCGGCACTGG
CGCAACTGGCCCCCTACTTCCCCCATATCAAGCCAAGCTGGACCGCCAAGGCCAGCCTGCCCGGCGGCGAAGAAAT
GACCACGCCAGAAGCCCTGGCCACGGACATTCGCAGCAAGTACGAATGGATCCCCAGCGAAATCGCCCGCCGTTGG
TCGACCACCTATGGCAGTCGCACCTGGCGCCTGCTCGAAGGCGTGCAATCCTTGGCCGACCTCGGCGAGCATCTGG
GTGGCGGGCTGTACACCCGGGAAGTGGACTACCTGTGCGCCGAGGAATGGGCTACCCAGGCCTACGACATTCTATG
GCGTCGCACCAAGCTCGGGCTGTTCACCACCCCCGAGGAGCAGGAAAACCTGCAGCGCTACCTGATCAAGCTGGAG
CAGAACCGCAGCAAGATCGAAGCAGCATGACTACCCAGCCCCTGCACCGAAAGGTCCAGGGGCTTTTTTTGGCTTG
CGCCTCAAGCCGGCCCCTACAAATTCGGTTTTCCGAACGCGTTACTGCCGGCGATTCGGTTTGCCGGACTTAAACA
CCGGATAAACATCGTCACATAAAGTTAATCACTATATAAATCATATAGTTATATCTTTATTAGCAGTCTGGCACGA
CTCATGCTCTACACTTGGGACGTGTTTGCCTGAGATGCTTCAGGAGCCGTCACAGGCATTCGCTGTATCGAAAGAG
CCGTCCAGGCTTCATAAAAAAAAACAAATGTCGAGGAAGTATTGATGCGTATCGTTCCCCATATTTTGGGCGCAGCC
ATCGCTGCCGCTCTGATTAGCACTCCAGTCTTCGCCGCCGAACTGACCGGCACGCTGAAGAAAATCAAAGAGTCGG
GCACCATCACACTGGGCCACCGCGACGCCTCCATCCCGTTCTCCTACATCGCGGACGGCTCTGGCAAGCCAGTGGG
CTACTCCCACGACATTCAACTGGCCATCGTGAAAGCTGTCGAGAAAGACCTCGGCATGAAGGAAGGCGAGCTGAAG
GTCAAATACAACCTGGTCACCTCCCAAACCCGCATCCCGCTGGTGCAGAACGGCACCGTAGACGTTGAGTGTGGTT
CCACCACCAACAACGTCGAGCGTCAGCAACAAGTCGACTTCTCCGTTGGCATCTTCGAAATTGGTACCCGTCTGCT
GTCCAAAAAGGACTCGACCTACAAGGATTTCGCTGACCTCAAGGGCAAGAACGTCGTAACCACCGCCGGTACCACC
TCCGAGCGCATCCTCAAGTCCATGAACGCTGACAAGCAGATGGGCATGAACGTCATCTCCGCCAAGGACCACGGTG
AATCCTTCCAGATGCTGGAAAGCGGCCGTGCCGTTGCCTTCATGATGGACGACGCACTGCTGGCCGGTGAAATGGC
CAAGGCCAAGAAGCCAACCGACTGGGCCGTGACCGGCACGCCACAATCCTTCGAAATCTACGGCTGCATGGTTCGC
AAAGGCGATGCCCCGTTCAAGAAGGCTGTGGATGACGCCATCGTCGCTACCTACAAGTCCGGCGAAATCAACAACA
TCTACACCAAGTGGTTCAGCTCGCCGATCCCACCAAAAGGCCTGAACCTGATGTTCCCGATGAGCGACGAACTCAA
GGCCCTGATCGCGAACCCGACCGATAAGGCGGCCGACGACAAAACGGCTGAAAAAAAGTCCTGATTCCGAACTAAC
CTTATCCCCTGAGGGAGCCAACCCTCCCTCAGGTGTCTGTTACTACCTGCTGGCATTATTTGGAACACTCGACCTG
GCGGTTTCCGAGCCGATCTTGTGTGCCTGACGTTCACCGTCAGGCGGGAATGGATTTTCCCCAAGCGGGTGCTTGT
ACATCGATCGATTTCGGGGGAGACCCTAATGAATTACAACTGGGACTGGGGCGTATTCTTCAAGTCCACCGGCGTG
GGCAGCGAGACCTATCTCGACTGGTTCATCTCCGGTTTGGGCTGGACCATCGCCATCGCCATCGTGGCCTGGATCG
TTGCCTTGCTGCTGGGCTCGGTACTGGGCGTAATGCGCACGATGCCTAATCGGCTCGTGGCCGGCATCGCCACCTG
CTACGTGGAACTCTTTCGTAACGTGCCACTGCTGGTACAGCTGTTCATCTGGTACTTCCTGGTACCCGACCTGCTG
CCGCAGAACCTGCAGGACTGGTACAAACAAGACTTGAACCCGACCACCTCGGCCTACCTGAGCGTTGTCGTGTGCC
TGGGCCTGTTCACCGCCGCCCGCGTATGTGAACAAGTGCGTACCGGTATCCAGGCCCTGCCCCACGGTCAGGAAGC
CGCCGCGCGCGCCATGGGTTTCAGCCTGTCGCAGATCTACTGGAACGTGCTGTTGCCCCAGGCCTACCGGATCATC
ATTCCGCCGCTCACCTCGGAATTCCTCAACGTCTTCAAGAACTCCTCCGTGGCGTCCTTGATCGGCCTGATGGAGT
TGCTGGCGCAAACCAAGCAGACCGCCGAGTTCTCGGCCAACCTGTTTGAAGCCTTCACCCTGGCCACGCTGATCTA
CTTCACCCTGAACATGAGCCTGATGTTGCTGATGCGCGTGGTAGAGAAGAAAGTCGCAGTGCCCGGCCTGATTTCC
GTGGGGGGTAAATAATGGAATTCGATTTCAGCGGCATTATCCCGGCCTTCCCTGGCCTGTGGAACGGCATGGTCAT
GACCTTGAAGCTGATGGTCATGGGCGTGGTCGGCGGCATCGTCCTCGGGACCATCCTGGCGTTGATGCGCCTGTCC
TCCAGCAAGCTGCTGTCCCGCGTGGCCGGCGCCTACGTGAACTACTTCCGCTCGATCCCGCTGCTGCTGGTGATCA
CCTGGTTCTACCTGGCGGTGCCGTTCGTGTTGCGCTGGATCACCGGCGAAGACACGCCAATCGGCGCGTTCACCTC
CTGCGTCGTGGCATTCATGATGTTCGAGGCCGCGTACTTCTGCGAAATCGTGCGGGCCGGCGTGCAGTCGATCCCC
AAGGGCCAGATGGCTGCAGCACAAGCGATGGGCATGACCTACGGCCAGACCATGCGCCTGATCATCCTGCCCCAGG
CGTTCCGCAAGATGACCCCGCTGCTGTTGCAGCAGAGCATCATCCTGTTCCAGGACACCTCGCTGGTCTACACCGT
GGGCCTGGTGGACTTCCTCAACTCCGCTCGCTCCAACGGCGACATTATCGGCCGCTCCAATGAGTTCCTGATCTTT
GCCGGTGTCGTCTACTTCATCATCAGCTTTGCCGCCTCGCTGCTGGTCAAGCGTCTGCAAAAAAGGTTTGCCGTAT
GATCTCTATCAAGAACATCAACAAGTGGTATGGCGACTTCCAGGTGCTGACCGATTGCAGCACTGATGTCAAAAAA
GGCGAAGTGATCGTGGTGTGCGGGCCGTCCGGCTCGGGCAAATCCACCCTGATCAAGTGCGTCAACGCCCTGGAAC
CGTTCCAGAAAGGTGACGTCGTGGTCGACGGCACCTCGATTGCCGACCCGAAGACCAACCTGCCGAAACTGCGCTC
GCGCGTGGGCATGGTGTTCCAGCACTTCGAACTGTTCCCGCACATGACCATCACCGAAAACCTGACGGTCGCGCAG
ATCAAGGTGCTGGGCCGCAGCAAGGAAGAAGCCACCAAGAAAGGCCTGCAATTGCTGGAGCGCGTGGGTCTGTCGG
CGCACAAGGACAAGCACCCGGGCCAACTGTCCGGCGGCCAGCAGCAGCGCGTGGCGATTGCCCGTGCACTGGCCAT
GGACCCGATCGTCATGTTGTTCGACGAACCGACTTCGGCTCTGGACCCGGAGATGGTCAACGAAGTACTGGACGTG

ATGGTGCAACTGGCCCAGGAAGGCATGACCATGATGTGCGTGACCCACGAAATGGGCTTCGCCCGTAAAGTGGCCG
ACCGTGTGATCTTCATGGACGCCGGCAAGATCATCGAGGACTGCCCTAAAGAAGAGTTCTTCGGCGATATCAGCGC
CCGCTCCGAACGTGCGCAGCACTTCCTCGAGAAAATCCTGCAGCACTAAGCACGACACAGCGTCCACTGTAGAACC
CGGGCCCTGTGGGAGCGGGCTTGCCCGCGATTACGGTGTATCAGTCAACCTATCTGTGGCTGACCTACCGTCATCG
CGGGCAAGCCCGCTCCCACACCGGTCCAGGCAAGCGCTGGTTGACCCAAGGCATCTGTGATGAAATGCGACCCCAC
CCTCTATCGCGCCGCACCGCCATCACTTGCCGTGAAGCCCCGTCTGATCCGCCAATTGTTCCTGCCGCCTCTGATC
ATCCTGTTGATGATCGGCCTGGGTTTTATCGGCTTCTGGACCAGCGAGTACTACGGCATCCGGACCCTGGGCGAGA
ACGGTGAGCGCCAGTTGGAGCTGCATGCGCGCACGGTCGAAAGTGAGATCAGCAAATACACCTACCTGCCCAGCCT
GCTGGAGCTGGAGTCCAGTGTCTCCAAGCTGCTGGCCGAGCCAAGCCCGGAACACCGGCAAACCGTCAATGAATAC
CTCGAAGGCCTGAACCGGCGCAGCCGCAGCCGGGCGATTTATGTGATGGACACCACCGGCCGCGTGATGGCCACCA
GTAACTGGCGCGATATCGACAGTTACCTGGGTGAAGACCTGTCGTTCCGCGCCTATTTCCAGAATGCCGTGCGTGG
CCAGCCCGGCCGTTTCTATGGCATCGGCAGCACCAATGGCGAACCCGGCTACTACCTGGCCCATGGCCTGGAAGAA
CGCGGCAAGATCATTGGCGTCGCGGTGGTCAAGGTGCGTCTCGAAGCGTTGGAAGAACGCTGGCAGCGCGCGCGCC
TGGAAGCCTTCGTCAGCGATGAGAACGGCATCATCATCCTCTCCAGCGACCCGGCGCGCCGGCTCAAGGCCGTGCG
CCCGCTGAGCGACGAGACCAAGGAGCGCCTGGCCCGCAGCCTGCAGTATTACTGGGCGACCCTCAACGAACTGCAA
CCCCTGGCCCGCGAGCACCTCGACGCCGGCACCGAGAAGCTGACCTTCCCCGCCAACAGTGAAGTGGTGGCCGACG
ACCAGGAAGTCACCTACCTGGCCCAGACCCGGCCACTGAACGACACGCCGTGGAATTTCACCCTGCTCACCCCGCT
CAACGACCTGCGCCAGGCCGCGATCAACCAGGGCATCCTCGTGGCGGTGGCCTTTGCCCTGGTGGCGTTCCTGCTG
ATTGCCTGGAATGAGCGGCGCAAGGTGATCGCCACACGCCTGGCCGCCCGCGAGGCCCTGCAGGAAGCCAACAACC
AACTGGAGCGGCGGATTGCCGAGCGTACCACCGACCTGCGCGCCAGCAACGAACGGCTCAAGGCGCAGATCCGCGA
GCGACGCCAGGCCGAAGAAACCCTGCGCCGGGCCCAGGACGAATTGGTCCAGGCCGGAAAACTGGCGGCCATCGGC
CAGATGTCGACCAGCATCGCCCATGAACTGAACCAGCCCCTGGCAGCCTTGCGCACCTTGTCCGGCAACACCGTGC
GCTTCCTGGAGCGCGGTGCCCTGGATACCGCCAGCACCAACCTCAAGACCATCAATGAATTGATCGACCGCATGGG
CCGCATCACCGCCAGCCTGCGCTCGTTTGCCCGGCGTGGTGACGACCAGGGTGAAGCCAGCCTGGCCAAGGCCGTG
GACGCGGCGTTCCAGGTCCTGGGCAGCCGCCTCGACAACCTGCCGCTGACCGTACACCGCGGTTTCAGCCAGGCCA
TGTTGCAAATCGACCAGACGCGCCTGGAGCAGATCCTGGTCAACCTGATCGGCAACGCCCTGGACGCGATGCACGC
CCAACCCGCCCCCGAGCTGTGGCTGGAAAGTGATATCCATGAAGGCAAATACCGCCTGCGGGTGCGGGACAACGGG
CATGGCATCGACCCAGAGACCCGCAAGCACCTGTTCGAACCCTTTTTCACCACCAAACCCGGCGAGCAAGGTTTAG
GCCTGGGCCTGACCCTGTCGGCCAGCCTCGCGGCGGCCACCGGCGGCAACCTGGCCGTCGAGCACCCGGCCGATGG
TGGTACGGCCTTTGTCCTGAGCCTGCCCCTGGCAGGCCATCAACCCGGCGAATCGATATGAACAGCCAACCCAATA
CCGAACTGACCGTGCTGATCGTCGAAGACGATCCCCATGTGTTGCTCGGCTGCCAGCAAGCCCTGGCCCTGGAAGA
TATTCCCAGCGTCGGCGTGGCCAGTGCCGAAGAGGCCCTCAAGCGCGTCGGCGAGAACTTCGCCGGCATTGTGATC
AGCGATATCCGCCTGCCCGGCATCGACGGCCTGGAGCTGTTGACCCGCCTCAAGGCCCTGGATAAAAGCCTGCCGG
TGGTGCTGATCACCGGCCACGGCGACATCTCCATGGCCGTGGGCGCGATGCGCAACGGCGCCTATGACTTCATGGA
AAAACCTTTTTCCCCGGAACGCCTGGTGGACGTGGTGCGCCGCGCCCTCGAACAGCGCGGCCTGGCCCGGGAAGTC
TGGGCGTTGCGCCGGCAGCTGGCCGAGCGCGACTCCCTGGAAGGCCGGATCATCGGGCGCTCGCCGGCAATGCAGA
ACCTGCGGGAATTGATCGCCAATGTCGCCGACACCTCAGCCAACGTGCTGATCGAAGGCGAGACCGGCACCGGCAA
GGAACTGGTCGCCCGCTGCCTGCACGACTTCAGCCGCCGCCACGCCCACCAGTTCGTGGCCCTGAACTGCGGCGGC
CTGCCGGAAAACCTGTTTGAAAGCGAGATTTTCGGTCACGAAGCCAACGCCTTCACCGGCGCGGGCAAGCGGCGGA
TCGGCAAGATCGAACATGCTCACGAAGGCACGCTGTTCCTCGACGAAGTGGAAAGCATGCCGATGAACCTGCAGAT
CAAACTGCTGCGGGTCTTGCAGGAGCGCACCCTGGAACGCCTGGGCTCGAACCAGAGCGTGGCGGTGGATTGCCGG
GTGATCGCCGCGACCAAGTCAGACCTTGACGAATTGAGCCGCGCCAACCAGTTCCGCAGCGACTTGTACTACCGCC
TGAACGTAGTGACCCTGGAACTGCCGCCCCTGCGCGAGCGTCGCGAAGATATCCTGCAACTGTTCGAACACTTCCT
GCAGCAGTCATCCCTGCGCTTCGACCGCATCGCCCCGGAGCTGGACAACCAGACCCTGTCCAGCCTGATGAGCCAC
GACTGGCCCGGCAACGTGCGTGAACTGCGCAACGTCGCCGAACGCTTTGCCCTGGGCCTGCCGGCCTTCAAGAAGA
GCGGTAACAGCCCCGACAACCATGGCCTGGCCTTTACCGAGGCGGTGGAAGCCTTTGAGCGCAACCTGCTCAGCGA
CGCCCTGCAGCGCAGCGGCGGCAACCTGACCCAGGCCAGCCTGGAGCTGGGCATGGCCAAGACCACCCTGTTCGAC
AAGGTCAAGAAATACGGCCTGAACCACTAAGGAGTACTGCGTGGATTTAGTTTTCAAGGCCGCCCTCGGTGCGGCG
GTGGTATTACTCCTGGCAGTGCTGGCCAAGACCAGGAACTACTACATCGCCGGCCTGGTGCCGCTGTTTCCGACCT
TTGCCCTGATCGCCCACTACATCGTCGGCAAGGGCCGCTCGGTGGACGACTTGAAAACCACGATTGTGTTCGGCAT
GTGGTCGATCATTCCGTACTTTGTGTACTTGGCGACCTTGTATGTGATGGTCGACCGCATGCGCCTGGAGGCCTCG
CTGGCAGTCGCCGCAGTAGCCTGGCTGATGGCCGCGACGGTACTGGTCAGCGTGTGGGTACGCATCCACGCCTGAG
CCTGCGCTGATCCCCTGTGGGAGCTGTCGAGCCCCGGCGAGGCTGCGATAGGATCAACTCGATACACCTGTAAAAC
CGAGTTGCCTGCATCGCAGCCTCGCCGGGGCTCGACAGCTCCCACATTTGATCTGTGTAACTCCCAGGGCTCAGCG
CACGATCTTGTCGATCTGGATCCCGAGCTTTTTCAAGCGGTACCAGAAGCTGCGCTCGGAAATCCCGATCCTTGAC
GCCGCAGCCGCCTGCACGCCATTGCTCTCCTGCAACGCCGCCAGGATGTACGCCTTCTCTACTTCTGCCAGCGCGG
CATCCAGGTCCCGAGGCACGCCCGGGCCTTCGCTGAGAATCGTCGTCACGTCACCCGCCGTCGGCTGGGACGCGAA

CAGATAGGCCGGCAAGTCGATATCTTCAATCACCGGGCTCGCCGCGACGATGGTCGCGCGCTCCACGCAGTTTTGC
AGCTCGCGGATATTGCCCGGCCAGCTGTAGGCCGCCATGGCCTGCAAGGCGTCTGCGCTGAAGCCGGTGATGCGTT
TACCGGCATTGGCGCTCAGGCTGCGGGCAAAGTGGCGGGCCAGCGGGGCGATGTCTTCGACACGTTCGCGCAGGGC
GGGCAGCGGGATTGGGAACACGTTGAGACGGTAGTAGAGGTCCTCGCGAAACTCCTTGTTCGCCACGGCTTCCAGC
AGGTTCTTGTTGGTGGCGGCGATCACCCGCACATCCACCTTGCGCTCGCGGGGGTCGCCCACCGGCTCGATCACCC
GCTCCTGCAAGGCGCGCAGGATCTTGGCCTGCAACGCCAGGGGCATATCGCCCACCTCGTCGAGAAACAGCGTGCC
CTTGTCGGCCTGCATGAAACGCCCTACCCGATCGGCCACAGCGCCGGTAAAGGCGCCCTTGCGATGGCCGAACATT
TCGCTTTCCAGCAAGCCTTCGGGGATCGCAGCGCAATTGACCGCGACGAAGGGTTTATCGGCGCGGTTGCCATGCT
TGTGAATGGCGCGGGCGACCATTTCCTTGCCGGTGCCGCTTTCGCCGGTCAACAGGATGGTGGCGTTGCTTTCGCG
CACCGAATCCACCGCTTGCAGCACACGGCGGAACGCCGGGCTGTCGCCCACCAGGCTGTCGAACTGCGCATGCTCG
TCGAGCTCGGCGCGCAGCCGCGCGTTGTCGCGCATGATGTCGCGAAACTGCAAGGCCTTGGCCACGGTGATGTCCA
GCTCGTCGATATCGAAGGGCTTGGCAATGTAGTCGTAGGCGCCGTTGCGCATCGACTGCACGGCATTTTTCACAGT
GCTGTAGGCCGTCATCACGATCACCGGCAACTGTGGGTAGCGTACCTTGATCTCGGCCAGCAACTGCGGGCCGTCC
ATGCCGGGCATACGCCAATCGCTGATCACCAGGTCGATATCTTCCTGCTCCAGCACCTTGAGGGCATGCAGGCCAT
TGCCGGCGATAAACACCTGGATATCGTTCTGGCTCAGGGCCGAGGCCAGCAAATCGCAGAGTTTGGGCTCGTCGTC
GACCACCAATATGTTATGCGTCATGACTGTCCTCGTCGTCGCCGTCATCTTCACCGCTGGCCGGAATGTACAGGCT
GAAGGTTGCGCCGGCATCCTTCTCGCTGGTGCATTCGATGCGGCCATCGTGACTTTCCATGATCGAATAGACTTTC
GCCAGGCCAAGGCCAGTGCCCGAGGCCTTGGTGGTGACGAAGGGGGTAAAGATCCGCTCGATCATCTCTGCCGGGA
TGCCCTGCCCGCTGTCGCTGACGCTGATCACTGTGGACTGGTCCGCGCGGCTGATGCTTAACGTCAGGCGCCCGCC
CTGGGGCATGGCATCGATGGCGTTGAGAATCAGGTTCAGGCACGCCTGCTTGAATTGCTTGGCGTCCACATAGAGG
GTCGCGCCAGGCGCCTGGTCGTCGAGATGGGCGTCGATGGCGTGGCTCGCCAGCTCCGGCGCGCAGAACCCCAGGA
TGTCTTCCACCAGTGGCCGCGCCAGCTGCTGGCTGCGCAGGGGCTCGCTGGGTTTGGCGAAATCGAGAAAGTCGGT
GATCAGGTCATTGATCCGGCTGACTTCGCTGACCACATATTCCAGGTGGCGCTTGTCGGTCTCCGGCAGGTCCGTG
CGGCGGTGCAGCAGTTGGGTCGCGGTCTTGATGATGCCCAATGGGTTGCGAATTTCGTGGGCCAGGCCCATCGCCA
CTTCGCCCAGGGCATGCAGGCGGTCGCGCCGACGCAGTTGGGCCTCGAGGTGATGCAATTCGCCCAGGCGCTCGGT
CATGTGGTTGAAGGTGCTGCTCAGTTGCGCCAACTCGTCGCCGCCGGTGACGGCCACGCGGTGCTGGTAATTGCCG
CTGATCACCGCACTCACGCCCTGGGACAACTCACGCAGCGGCCGCGTCAGGTGCCTGGAAACCAACACACCGGCGC
CCAGGGACAAGGCGGAACTGAGCAGGAAGATCAGCACGAACAGATTGCTCTGGTTCACCAGCCCCACCAGGCTGGT
GTGGCGCAGCAGGCCGCTGAAGATCACACCCTGCAACTCGCCGGTGTCATTGAAGATCGGCCAGTACAGGCCACTG
TAGTTGCTGGTGAATTGCTCGCTAGGCTGCTTGGTGCTGCGCAGCAGGGTTTCCACGGATTTCGGCACGCGGGTCG
GGTGATCTTCAAAACGCTGGGTCGAGAAGATCTCCGAGAAGCCGTCGGTGTTGGCCAGGTACAGGCGCAAGTCCAG
GGAGTGGACCTCGGCGACACTGGTCAGGAAGCTGCTGTCCAGGTACGTGCCCACCAACAGTTCGTAATCGACACCG
TCCTGAGTGGTTTCGAACGAGGAAATCACCGCCCCGGTGGCCACGCCGCCAACCTGCACGGTTTGCAATACGGCAT
TGGGGGCGAGGCTGATTTGTTTCACCACATCATCGCCCACGGTGCTGAACATCACTTTGCCGTCGCTGCTGCGAAT
CAGCGTTACCACGTCAATATCAGTGGCGCTGGCAATGTCCGCGATCAAACGGTCATGCCGCATCGCTTCATGGGTG
GAGGGCGGACGCACATAACGCAGGAAGATCTGCGCCACGCGGGCGTTGTCGTGCAGGATGTCGCTGACTTCGTCCT
TGACGATCCGGGTCGACTCTTGCAGCCAGATGCGCACGTTACTGTCGAAAATCTGCGACAGGGTGGTGGCCGCCAG
CTCCGCGGCGATCATCGTCGGGATCACCGTCACCAACCAGAACGCCAACACCAGCTTGCGCTGCAGGCTCCAGCGG
GAAATGGCAAACGGGCGGGCTTTCTGGCGGGTTTTGGCAATCATCGGGTTTCGCTTATTGCAGCAGCCATGGCAGG
GTCGGATCGGTCCGGGCGAATAAACACTTTTAGCACTTTGAGCAGGGTGTCGAGCAAGCGGTTGCGGTGCTGGAAA
AACAGGGTATTGCCGGCGAACCCGCAGCCCAGGCGTAGCTTACTGGCATACCCCGCCTGCCCGCACTCATACAACG
CAATATTGTGCTCGATGCAGTAGTCGACGTTGGTCAGCCAACTGCGAAAGTACAGGTTATGTTCGCGGCTGTGGGC
CAGGTCATGGGCAAAGAACTTGTCGATCAGCCGATGCTGGTCGAGCAGCACCAGGTTGAACGCCACCAACTGCCCA
TCCACCCAGTAAAGTACACAGCGCGCCCGTTGTTCAAGCTGGGCGAGCACCTGGCTGAAATAACCGGCGGGCAGGC
GTTCGAATTGCAGGTCGGCGCGGGCCAGGGTCGCTTCGTAGAGACGCATGATATCTGGCAATACGTCATCGACATT
GTCCCGCCACTCCACCTGTGGCCCCGGCGCGCGTAGCTTGCGCCGCAAGTCCTTGCGCGTGGATTTGCCCAGGGAG
CCCAGGTAGGCATCCACCGAACCGTAGGGCAGCGGCAGCAGCCCGGTCGGCAGGCTCGGCATGCTGTGCAGGCCGG
CGGCCTTGCAGCTGTCGGCCCAGTCCTGATCCTGGGTCGGAGCATCCTTGACCGCCAGCAAACCGATGCCGAATGC
ATCGGCATCCCGCCGTGCGGCGGCCAGCAGTTGTTGCAACAAGGCACCACGGCGCGCCGCCGGCACATGGCTGGCG
ACACCGGCATGGCATTGCTCGGCCACCGGCGAACCGATGGCGTACAGGCCCAGGCACAATGCGCCCGGCCACCAGC
GTTCCAGGCGTTGGGTTAAGCGCTTGCCAATACCCGAGACGGTGGTGTCGAGGCGGTAATGGGTGATGAACGCCGG
GGCCACCGCCAGCAGCCGCCCCTCCTCATAAAGGGCCAGGTAGCGCCATTGGAAGTCGTCGATAGCGGCGTTTTCC
ACGGCGACGTAGTAGTCCCAATCCTCCAGGGCCTGGGGAAAGCAGTCGTTCCAGGCACTGCGGTCGATGGCTTGGA
TACTCGGGAAGGTTTGCGCGGTTATCACATCTGATCCCTGTCAATACTGTGGCAAGCGGACAACCCGGCGCCTACA
GGGAGAACCTTGTAGGCGCTGGCTTGCCAGCGAAAAACTCGAGGCCGCCGCGTTGAACCAGGCGCTACCTGTCATC
GTTGGCGACCATCGCGGGCAAGCCCGCTCCTACAGGGGGCGGTGTCAGGCCCGGTGATATTCGTCCACAGGGGTGG
TGTGCTGCTCAATGAAGTCGGCACTCAACTCGATCACCCGCCGGTATTGCAGGTCGACGGTGATCATGTGGTAACA

GTTGTCCAACAGGATCTTGGTCACCGGGCCGCCGAGGTGGCGTTCCACGTAGTCGGCGTTCCAGCGGCTGGTGATG
TCGTCCTCGATGGAGTGCAGGACCAGCGCCGGGGTCTTGATCGACGGCATGCGTTTCTTCACCACGGCATTCATCC
AGTGCAACTCACGCACGGTGACGCCTTCCATGGTCAACAGCCCGGCCTCACTGCTCTCGCCCTCTTTCATCTGCCG
CTCGACGATCGCCCGCAAGCGTTCGTTCTTGATGCCGTAGGGCGGCTTCTCGGTAAAGCGGAACAGGCGCACGCCG
AAGGGAATGCGGATCAGCAACGGCGTGATGAACGCCATTTTGTTGATGCTCCAACCGTCGTACTTGAGGGTCGTGG
AATACATCAGCAACCCCGCGACCTGGCCCGGAAATTCCGAAGCCAGGTACATCGACATCACCGCGCCCATGGACAA
GCCACCGACGAACACCTGTGAATGACGCTGCTGCACCCCGACAAAGGTCTTGCGCACGCCCTCGTACCAGTCGCGC
CAGCCGGTGGCCTGCAGGTCCTCGTTGCCCCCGCAGTGCCCGGCCAGGGTTGGCACGTACACCGTGCAATTACCCG
CCTTGACCAGGCCCTGGGCAACCCGACGCAATTCGGTCGGGGTGCCGGTCAGGCCGTGGATCAACAGGACCCCGAC
CGGACCGTTGCCGAGAACGAAGCCGGCGTTGCCTTCGCCGAGATCGATATCGGCCAGACTCACCGCTTCATCGCTC
GGGTCAGCAGTTGCTCCAGCAGCTTCAAGCCCAGGTCGATTTCCGGGTAGCTGATTTCCAGGGACGGCGCCAGGGT
GATCACGTTCTTGTAGTAACCGCCCACATCGAGGATCAGCCCGAGTTTCTGGCCGTCTACCAGCATGTCGCCTTTC
ATGCCTTCCTCAACCATGTAGTCCAGGGTCGCCTTGTCCGGGGTGAAACCATCGGGGCCGCAGATTTCGCAGCGCA
GGGCCAGGCCCAGGCCATCCACATCGCCGATGATCGGGAAGCGCTTTTGCAGGTCCTGCAGGCCTTCAAGGAAGTA
TTTGCCCTTGGCCATGACCATCGCGCCGTAGTCGACTTCGCTGGTCATCTTGAACATTTCCAGGCCCACCGCCGTG
CCCAGCGGGTTGGAGGCGAAGGTGGAGTGGGTGGAACCCGGCGGGAACAGTTTCGGGTTGATCAGTTCTTCCTTGG
CCCAGATGCCGCCCAATGGGTTGAGGCCGTTGGTCAGGGCTTTACCGAACACGATCACGTCCGGTTGCACATCGAA
GTGCTCGATCGACCACAACTTGCCAGTGCGCCAGAAGCCCATCTGGATTTCGTCGACCACCATCAGGATGCCGTGC
TGGTCGAGCACTTGCTTGAGCTCGCTGTAGAAGTTCATCGGCGGGATCACGTAGCCGCCGGTGCCCTGGATCGGCT
CGACGTAAAACGCGGCGTATTCGCTCTGGCCGACTTTCGGGTCCCAGACACCGTTGTATTCGGTCTCGAACAGGCG
CGCGAATTGCTGCACGCAGTGGCTACCGTATTCTTCCTTGGTCATGCCCTTTGGCCCACGGAAGTGGTACGGGAAC
GGGATGAAGTTGGCGCGCTCGCCAAAGTGGCCGTAGCGGCGACGGTAGCGGTAGCTGGAGGTGATGGACGACGCGC
CGAGGGTACGCCCGTGGTAGCCGCCTTCAAAGGCGAACATCAGGCTCTTGCCGTTGCAGGCGTTACGCACCACCTT
CAGGGAGTCCTCGATGGACTGCGAACCGCCGACGTTGAAATGCACGCGTCCATCGAGGCCGAATTTTTTCTTGGCA
TCCACCGCGATCATTTCCGACAGCTCGATCTTGCCCTTGTGCAGGTACTGGCTGGCGATTTGCGGCAGGGTGTCGA
TCTGCTGTTTCAGCGCATTGTTCAGGCGCGGGTTGGCGTAGCCGAAGTTGACCGCCGAGTACCACATTTGCAGGTC
GAGGTAGGCCTGGTCTTCGGTGTCCCAGACGTAGGAGCCTTCGCAGCGGCTGAAGATACGCGGTGGGTCGATGTAG
TGGACGGTGTCGCCGTAGGAGCAGTATTTGGCTTCTTTATCCAGCAGAACCTGGTCTTCGGCGGTAGCGATACGGA
TATCAGACATGATGAAAGAGTTCCTGAGTGTCAGAAGTAAAGTGAGTGGCGTTGGCGGCCATGCCCTGGGGCAGTC
GGGCCAGTAATGCAGGCAGTTCGGCAAAGCTGTCGAAGCGCAGATGGGGGATGGCGTGGGCCTGGCAGTACTGCGC
CAGGCTGCCTTTGGCGAAGACGAAATCGGCGGTAGTCGATACGCACATATCGGACTTGCCGTCGCCGATCACCAAC
ACCCGCTTGTTGCGCGGGGTGGACTTGCATTTGCAGTTGCCCGAGGCCGCGCGGCAGGCGTCGCTGGCATGGGGGA
AGTCGATGCGCCAGCTGTTCTGGTCGACCTGGCGCAGGCGGTTGGCGATGATCGGCAGCAAGGTCACGTAGTTGCG
CGAGAGGATCCGCGCGATGCCCTGCTCGATGCCGTCACTGACCACCTCAAGGGAGGCACCGAGGCCAAGGACATGG
TCGACGAAGTCCGGGAAATCCGGATCGATCTCGACCGTGTCGAAGTACGCCAGCAGCTCGGCCGGCGTCGCCTTGA
TCAGCGCCAGTTGGCGGCTCAGGCATTCGCGCGAACCGATATGGCCGTCCAGCCATTGCTGCTCGATGGTTTCCCA
TTCTCTGCCTGCGAAGCGTTGGAGAACGTTGTCGATGACATCGGTGGGTGTGATGGTCCCATCGAAGTCACACACG
ATATGCCAGTCATTCATCTGCGTTTACCTTGGATGCTAGCGCGCTGATGCGCCGTTGACAGGGATAGAGCAGGGAC
TGTGCCAAGTGGGCAAAACCCAGCAGGGCTGGGGTTCGCGGGCATGCGCGGGTGATAAAGGACGCGGCCAAGCTAC
AAATTTTGTAGGTCGCTACAAACAACATGAGTGCTTGCACAAGCCCCCGCGCCTGCGCGTTCATGCGCGCATGTTT
TGCAAGAGGAGCAAGCAATATGTTCAGGGTGACTTCGCTGGTGTGCGCCGGCGTCCTGGGGCTATGGATGGACTCG
GCAGCGGCGGCCGGCATCAGCGGCGAGGTGGGCCTGGGCCTGAGCTATCAGCCACGGGAACCCAGCGCCAGCCGCT
ATCAGACGGTACCGGTGCCGTACTTCGACCTGGATTGGGGAGATGTCAGCCTCGATACCGATGATGGCCTGACCTG
GAGCGTGCTCAACAGCAATGGCTTGAGTGCCGGGCCGTACCTCAACTACCTGCCGGGACGCACGGCCAACGGGCCG
CTACAGGGCCTGCGAGACGTGTCGGACATGGCTGAGGTCGGTGGGTTTATCCAGTACGCACCGGCCGACTTCTGGC
GGGTCTATGCGCAGCTGGGGCGCAGTGTTGGCGGCGCTCATGACCAGAGCGGGATATTGGGCAAGCTCGGCGGCGA
ACTGGGTTACCCACTGGGCGGCGGGGTCATTGGCAGTACGGGGCTGGTGGCGCACTTTGCCGACGATAACCAGACC
CAGACGTTTTTCGGGGTCGATGAGCATGAGGCGCAAGCCTCGGGAATTCGCCCGTACAACGCCAGCGGCGGGTTCC
AGAACCTGACATTGACCCAGAGCCTGCAGATCCCGCTGGCGCCCAAGTGGTCGCTGCTGGCCAGTGCCAGTTGGGT
CCACCTGACAGGTTCGGCGGCCGACAGCAGCATCGTGCGCCAGACCGGCGAGGTGAACCAGGGGCAGGTGCAGACG
GCGATCAGCTACACCTTCGATTGATTGTCCAAAGCCCAAGCCCCATCAAAATGTGCAAGCTGGCTGTTGTGGGAGC
TGGCTTGTGTGGCAGTTGGCTTGTGTGGCAGTTGGCTTGTGTGGCAGTTGGCTTGCCTGCGATAGCATCACCGCGG
TTTAACTGATGCACCGAGGCGCTTGCATCGCAGGCAAGCCAGCTCCCACAAAAGCCAGCTCCCACACAAACCCGCT
CCCACATTTATCCAGCACTCCAGCCTGCGCTCAGTTTTCTTCGCCCTCGGCCAGCAGCGCGATCCCGCCAATGATC
ACCACCACGCCCACCCAGTGCAGGAAGCTGATGTGCTCGCCCAGGCCCAGCCATGAGCCGAGCAACACCACCACAA
ACACCAGCGAACTCAGGGGGAAAGCCAGGGACAGGCTGCTGCGCCGCAGGATCAGCATCCACACAAAAAATGCGCC
GATATAACAGGCAATGGCCGCCAGGATCCCCGGGTTGACCGCTACCGCCGCCAACCATTGCCAGTTGAAATCCATC

TGCCCCAGCTGATCGCCGGCCACCTTGGTAAACAACTGGCCGCCGCTTTCGGTCAAGATCAACAACGCCCACAACA
CCAGCGTCCCCAGGCGACCGTGCAGCCAGCCAACCGGACGGATAGCGATATTTTCCGAATTCATGCTTGAGTCCCC
GCAACAGCAACCAGCATCACGCCGGCCGTAATCACCAGGGTACCCAGCCAGCGACGGCGGCTGACGGTTTCCCCCA
GTACGACTTTGCCCACCAGCACCACCAGGCAATACGCCAGCGCCGCCAGCGGAAACAACAGGCTCAGCGGCGCACG
GGACAAGGCCTCCAGCCACACGAAAAATTCAATCACATAGGCGCCGATGCCCGCCCACAGCAACGGTGCATTGAGC
ACCTGGCCCCAAAATGCACCCAGGCGAAAACCGCCATTGATTTCGGGCAAGCGGTCCACGCCCAGCTTGAAACACA
ACTGGCCAATCACATCGAGCACGATGGAAAAGACCAATAACACCACCACGGTCAGGGTCACGGAAACAGCTCCTCG
AACAGGTTGATCAACGCCTCGTTGGTCGCCGCGTTGCGCAGCAAATCGTCCGCGCTCCAGCGCTCGGCCGGCGGTT
GGTCAGACTTGGCTTCCCAGCGTTTGAACGCGTTGCTGAATGCAGGCTGGGCAAACTCGCCGCACACATCGATACC
GATGATCCGCTTGCTCGCCGCCAACGCCCGCAGGGCTTGCAGCAGATGCGTCAGGCGCATGCCGCCCTGGTCCCAG
TTGGTGGCGGCGTCTTCGCTGGCCAGTACGTCTTTGTCCAGGGTGATCCACACGGCCTCGGTGGGCAGGCTGGCGA
TCATTTGCTCAAGGAATGCGCCCCAGTCGAGCTCAGCCAGGTTGCGCCAGTGCAGGTGGTTTTCCTGTTGTGCGTG
GCCGGCGCCATCCCCTACCCGCCCCCAGACTTTCGACGGCGCGTGCTGCCAGGGAAACAACTGCAAGTGCCCGCGC
TTGAGGGCGCCCAGGTTGCCGCCGCGCAGTTGCGGGTTATGCAGGTCATCGCTGCACGGGCCGAGGGTGACAATAC
GTTTGATCGCCGGCATCTTCAGTGCCTGGTTGACCCACGAACCGCAGTGCCGGCGCGGCGCCAGGCGCACCCAGTC
GGGGTGGTTGTCGAAGTGGATCAGGCTGATGGGCTCCTGCACCTCGGCGAGAAAAGCCGGGGTCAGGTGGTGATAG
TCGCCAGAACCGACAAAGAAGATTTCGGGTCGCGCCATGGTCGGCCGGGGCCGTTGGGCCAGGCGCTGGGCAAAGC
GCTTCCAGGTTTTTTCGGTGGACCACAGGCGCAGTTTCGGGCCCAGGTCGAGCAGGTCCAGGCGCGTGGCGTGACC
GCTGGCCAGACGCCGGGCAATGGGTGCCTGGGCTGTGAGGCTGTGGTCGAGATCGAGGATATTCAAGGTAATGTTT
CACCCCTTGGCAGCGCCCAACCGCACGCCGGCTGGGCTTTAAGGGGGTAATGCAAGGGATGGGCCAGGGGGGGACA
ACAATGATCCAATGTGGGAGCGGGCTTGCTCGCGAAAGCGGTGGGTCAGTTAAGAGATACTTAGCTGATATACCGC
TTTCGCGAGCAAGCCCGCTCCCACATTTTGCTTTGCGCTCGACTTTAGGCCTTGGACTCCTGGATGATCTGGCGGA
TCGCCCCGACAAACGCCTCAGCCGGCTGCCCGCCGCTGACCGCGTACTGGTCGTTGAACACAATGGTCGGCACCGA
GGTCACGCCACGGGAGACCCACAGTTGTTCCTGCTCGCGCACTTGTGCGGCGTACTCGTCCGAGGCCAAGATCTCG
GCTGCGCGCTTGAGGTCCAGCCCGACGCTTTCGGCAATGATCGCCAAGGTAGCGTGGTCGGAAGGGTCCTGGCCAT
CGCTGAAATAGGCCTTGAACAGCGCTTCCTTGAGGTTGTACTGCAAGCCCTCGAGCCCGGCCCAGTGCAGCAGGCG
ATGGGCGTCGAAGGTGTTGTAGATACGGCTCTGGCCATCGGTACGAAACGCAAAGCCCAGCGCGGCGCCCATGTCG
CGGATGCGGGCACGGGTGGCCTGGGATTCTTCGGCGGTGGAGCCGTATTTCTCGGTGATGTGCTCAACGATGTTCT
GCCCTTCGGCGGGCATGTTCGGGTTCAGTTCGAAGGGCTGGAAGTGGATCTCGGCCTGCACTTCGGCGCCCAGTTG
GTCCAGGGCTTCGGTCAGGCCGCGCAGGCCGATGATGCACCAGGGGCAGGACACGTCGCTGACAAAATCGATTTTC
AGGGAAGTACTCATTCAGGCAACCTCGCAGGCATTACACGCCATAAATGTCGCACCATACACCCCTAGGCGCTGCT
CATAAAACCTTGGCCGGCAGCGCGCGATCAACCTGCGCCCAATGCGCCGCCTCTTCGCGGTGGGCTTGCAGGTAGG
GCAACACCGCCGCCAGCAACGGCGCCTTGAATGCCTCCTGGAAACGATGAGCCAAGCCCGGGATCAGCCGCAACTG
GCTGCCCTGGATATGCGCCGCCAGGTGCACGCCATGCATGACCGGCAGCAACGGGTCGGCCGTGCCGTGGACCACC
AGGGTCGGCACGCGCAATTGATTGAGCAATGGCACCCGGCTCGGTTCGGCAAGGATTGCCATGATCTGGCGCTTCA
CACCCTCGGGGTTGAATGCCCGGTCGTAGGACTGCGCCGCCTGCTGCAGCAAGGCCTGGCGATCATCCTTGACCTC
AGGGCTGCCCAGGGCCGCGAGCAAATCGGCTTGCTGCTCCAGGGCCACTTCGCGGTTGGGCGCGCTGCGCCGTGAC
AGCAGTTGGACCAGCGCGGCATTCGGCGCGGGCAGGCCTTCGGCACCGGAGCTGGTCATGATCAGGGTCAGGCTTT
CCACGCGCTGCGGGGCCATGGCCGCCAAGTGCTGGGCGATCATGCCGCCCATGCTCGCACCCAGCACATGGAACTG
GCGCACCTGCAGCGCATCCATCAACCCCAGCGCGTCGTCGGCCATGTCAGTCAAGGTGTAGGGCGCAGCCACCGGC
AGGCCCAGTTTGTAGCGCAGCACTTCAAAGGTCAGGTTGGCGCTGGCGGGCGCCTGGCGCCAGGTGGACAGGCCAA
CGTCGCGGTTGTCGTAGCGGATCACCCGAAAACCCTGTTGGCACAGGGCGACTACCACTTCGTCCGGCCAGTGGAT
CAACTGCCCGCCCAGACCCATCACCAGCAACAAGGCCGGATCGGAGGCACGACCAATGCTCTGGTACGCCAGGCTC
ACCTGGTCCAGGTCGACCCGCTCAGTCGGGACATTGACATCACATCGAGAAGCCGCAAAAGACGGCAGGCCGAACA
GTAAGGCGGCCAGTAAAAACAACACGCGCATGAAAAACACCGAAACGCAGAACCCCAGTAGAGCGCGAGTCTGATG
AAGTTTGTTCAAGCGCGCTGCCACAGTTACGTGACAGTTTGATGAAGAGCGCCCAGCGGTCATGGTCGACAGATTT
GGCAACATGAGACAAACTCACGCCATTGGAACTTGTCACAAATTATCTTCATGGAGAGCCCGTGCTCGAAATCCGT
CATCTCAAGACCCTGCACGCCTTGCGCGAAGCCGACAGCCTGGTGGAAGCCGCCGAACGCCTGCACCTGACCCAAT
CGGCGTTGTCCCACCAGTTCAAGGAGCTGGAGGAACGCATGGGCATGCCGCTGTTTGTGCGCAAGACCAAGCCCGT
GCGCTTCACCAGCGCTGGCTTGCGCCTGCTGCAACTGGCGGATGCCACCTTGCCGCTGCTGCGCGGGGCCGAACGG
GATATTGCGCGCTTGGCCGGTGGCACTGCCGGGCGCCTGCACATGGCGATCGAGTGCCACAGCTGCTTCCAGTGGC
TGATGCCGACCATCGACCAGTTCCGCGATGCCTGGCCGGAAGTAGAACTGGACCTGGCCTCCGGCTTTGCCTTTGC
CCCACTGCCGGCCCTGGCCCGTGGCGACCTGGACCTGGTGGTGACCTCCGACCCACTGGAGCTGGCGGGCATCACC
TATGTGCCGCTGTTCACCTACGAAGCAATGCTCGCGGTGGCCAACCAGCACGCGCTGGCGAACAAGCCGTACATCG
TGCCCGAGGATTTGCTCAGCGAAACCCTGATCACCTACCCGGTGGAACGCGACCGCCTGGATATCTTCACCCGGTT
CCTGGAACCGGCCGATGTCGAGCCCGCACAAGTCCGCACTTCGGAGCTGACGGTGATGATGATGCAGTTGGTGGCC
AGCGGTCGCGGCGTGTGCGGCATGCCCCACTGGGCGCTGCATGAGTACAGCTCGCGGGGTTATGTGAAGGCCAAGC

GGCTGGGGGAGAAAGGTTTGTTCGCCACGCTGTACGCCGGGATCCGTGCGGATATGCTGGATGCGCCGTACATGCG
CGACTTTTTGCTGACCGCCAAGGACACGTCGTTTTCAACCCTCGATGGGGTCAGCGCGGTCCGTTGATTGTTCACG
CGCTGCGCCGTTGCTCGAGCATCAGGCGCACCGCCAGGGCTGCCAGCACGAACCCCATGAAATAACGCTGGACCGC
CAGCCACGTGGGGTTGTGGATAAACCACGCGGCGATGCTGGCAGCGCACAGCGAGATCAGCAAGTTGACCACAAAG
CTGACGCTGATCTGGGTAAAGCCCAGCATCAGGCTCTGGGTGAAGATCGAGCCGTGCTCCGGGGTGATGAACTGCG
GGAATACCGACAGGTAGAACACGGCGATTTTCGGGTTCAGTGCGCTGGTCAGAAAGCCCATGGTCACCAGCTTGCG
TGGCGAATCAGCAGGCAATTGCTGGGCTTGGAACGGTGAGCGGGCACCCGGTTTCACCGCTTGCCAGGCCAGCCAC
AGCAGGTAGAGCGCACCGGCCCATTTCAGTACTTCATAAGCCAGCGGCACAGTGAGGAACACCGCCGTCAAACCGG
CGGCAGCGGCGAACAGGTGCACGAAAAAACCCGCGACCACCCCCAGCAACGACGTCACCCCGGCCTTGCGGCCCTG
GCAGATCGAGCGGGAAATCAGGTAGATCATGTTCGGCCCCGGGGTCAGCACCAGCAGCAGCGAGGCGGCGGCAAAA
ATCAGCAAGTCATTGAGGGGGATCACGGCTCAGTCCTTTGTGCCCGACAGCTCAGGCAATAGTGGTCAGCGATGCG
CGATAGAAAGGCAGGATCAGGTCACGTGTCAAGGGCGCCAACTGCAGGCCACCGTCGCCGGCCGGATCGATCCAGC
GCACTTCTTCGATCTCGGCGGCGGGTGTCACCGGTACGTCGATACGCACTTCAAACAACTCGGCCTCCACGGTAAA
CCCCGGCTCGTTGGCCGCCGGTGCCGAAAAATGCCCAAGGTACTGCGCCGCTGCCGGGTCGATCTGCAGGCCAAGC
TCTTCGTGCAGCTCACGCGCCAAGGCCTGCGCCGGTTGCTCGCCGGCATCGATCTTGCCGCCCGGTTGCATAAAGG
CCTGGGTGCCACGCTTGCGCACCAGCAGGGTCTGGCCGTCGCTGCCGATCAACAGGGCGGCGGCAATACGGATGGT
CTTGGACATGAAATAAACGCCTTGGAATAAAGGCGCGAAGGATCACATGACTGTGCTCCGTGCTGCAATGCCACAG
CCACAGCTGGCTTACTCTTCGGGGTCCTTGATGAACACGCTGTACTTGGCACCTTCCATGGCTTCAAACGCGATCA
GCTTGTCTACCAGCGGCAGGTTCAGCACCTTGCCGGCATTGAGCAGCAGCATGCCGTTGTCGGCATTGAGGTTGCG
CGCCAGGACCATGCCTTCGGCCAACTCGCGAGTGCCCAGCACCCTGACCGTCGGGTCGCCCAGGGTCACGTCGCTG
AGGAAGGCCGCGCAGGCCTGGACGAAGTCTTCCACCATGTCCGGGTCATACAGGCGCCCGGCGTATTTGCGGATAT
ACAGCAGCGCCTCGTCGCTGTTCATCTGCCGCTCAAGAATCAAGCCGCGCTGCAGTTCGATAAAATCCACCGCCAG
CTTCAGCAAGCGCGAGCCGAAAGGAATGGCCTCGCCCTTGAGGTGATCAGGGAAACCACTGCCGTCCCAGCGCTCC
TGGTGATGGCGGATCAGCCGCGCGGCGTCCTTCATCGGTTCCAGGGTCATCAACAACGATTCGCTCTGTGTGGCGT
ACTCGCGAAAACGCGCGCGGTCGGTGCTGTGCAGCAAGTCCGAGGGCGCGACCATCATGCTGTCGCTCCAGCTCAA
CTTGCCGATGTTGTACAGCGCCGCCGCCATGGTCAGGTCGCGGCTGCTGGCATCGTCCAGGCCATGGAGCACGCAC
CAGGTGCGCACCAGCTCGATGATCTGGCGGTTGGTCTGCTTACTGCGCGGCAGGCGCAGGTTGGCCAGCAGCGAGA
ACACCTCGGTGCCGGTGGCGTAGCTGTGCTTGAGTTCTTCGTAGGCCAGGTCGAGCATGTCGGCGGTCTGCTGCAA
TTCACTGGTGCGCGAGACCACGCGCTTTTCCAGGGATGCATTGAGCTGCTTGAGCTCCTCGTTCTGCTGGCGGATC
AGTTGTTCCAGGCGCAGGCGCTCACGCTCGGAATGCTGGTGGGCCAAGGACTGACGCAGCGCCAGCAGCAACTCTT
CGTCCTTCCAGGGTTTGCTCAGGTAGCGGTAGATCTGCCCTTCGTTGATGGCCTTGATGATCAGGGTCAGGTCGGT
TTCGCCGGTCAACAGCAGGCGCACGGTATGGGGGTAGTCGTGATGGATCTGTGCCAGCAACGTGGCCCCATCCATA
TCCGGCAGGCGTGCAGCACTCATCACCAGGTCCACAGGCTGGCGCGCCAGCGTGGCTAACGCTTCGACCGCCGTGG
TGGCGCTGCACAGTTCAAAGGGTTCCAGGCGCAGTAACTGCGCGAGGCGGTCATGCACGTCCGGGTGAGTGTCGAT
CAACAGCACCACGGGACGATCGATTTCAGAAAAGGTAACTGACTCTCCCACGGGACACCTCGGATCTAGTCGCGTC
GGTGTGCGCTTCCTACGGTCAAGGTAACAGCCTCCTGGCTATTACCTAGCAGTTTAGTCAGTTTTTAGCCGGTAG
TGGCCTAATGATGCTATTTACCACTACCGCGCCTGAAGCCTCGAACCGGTCAGGCCTCGGCGGCTTTGCTGGAGGA
ACCAAACGAGGCAAAGCGCTTGTTGAAGCCGGCAATCCGCCCTTCGACCTGGGTCTTGCGCTGTTGCCCGGTGTAG
ATCGGGTGGGAAGCGCTGGACACGTCGAGAGCCATGTAAGGATAAGTATTGCCATCGCTGTGTTGGTGGGTGCGAT
CCGTGTCGACCGTGGAGCCGATCAGGAAGAACACGTCGGCGGCAGTGTCATGGAACAGTACGGTGCGATAGGCGGG
ATGGATACCAGCTTTCATAGGGCCTCCGGGGGCATCTGGGCAAGATTAATACGTTATACAGTAACGCAAATAATGC
ACCCAAACGAGAACGTATTGCAATACCGATAGAAGCCCAGGTTCTATGCGGTTGCCCTGGCAACGAACTCGCCCGT
AAGCTGGTGCTCCGTGAGAGCACAGGCATCCATAGCCTGGAGATCCTTGTAGGAACTGGCTTGCTATCGAAAACGC
CCTCGCCGGCAAGCCGGTTCCTGCAGGTCATAAAGAGTCCGAACCCAAGGAAACCGTATGAGCCTGTCGTTGCTTA
GCCGTTATGCATTCTTTGCCGTGTGTGTCATTTTCACCCTCGCCAGCCTGCCCTTTATCCAACACGAATGGCTGTG
GCCGTTTACCCTGGTCACCGGTGTCCTCAGCCTGATCGGCGTGTTCGACCTACTGCAAAGCCCGCACGCCGTGCGC
CGCAACTACCCGATCCTGGGCAATATCCGCTACCTGGTGGAAGGCATCCGCCCGGAAATCCGCCAATACCTGCTCG
AATCCGACAGCGACGCCCTGCCCTTCTCCCGATCCCAGCGCTCGCTGGTGTATTCGCGGGCCAAAAACGAAACCGC
TGACAAACCCTTCGGCACCCTGATCGATGTGTACCAATCCGGTTTCGAGTTTATCGGCCACTCCATGCGCCCGGCG
CCGTTGAGCGATCCCAGCGCGTTCCGCGTGATCGTCGGCGGCCCGCAATGCACACAGCCGTATTCGGCGTCGGTGT
TCAATATCTCGGCAATGAGCTTCGGCTCCCTCAGCGCCAACGCCATCCGCGCGCTCAACCAAGGGGCCAAGCTCGG
CAACTTCGCCCACGACACCGGCGAAGGCAGCATCAGCCCCTATCACCGCGAACACGGCGGCGACCTGACTTGGGAG
CTGGGCAGCGGCTATTTTGGCTGCCGCACCAGCGACGGCCGCTTCGACCCGGAGCGCTTCGCCGTGCAGGCGCAAA
ACCCCCAAGTGCGGATGATCGAAATCAAGATGAGCCAGGGCGCCAAGCCCGGCCACGGCGGGATCCTGCCCAAGCA
CAAGGTGACCAAGGAAATCGCCGAGACCCGCGGCATCCTGATGGGCGAAGACTGCATCTCGCCGTCACGCCACAGC
GCGTTTTCCACGCCGATCGAGCTGATGCAGTTCATCGCTCAATTACGTGAACTGTCCGGCGGCAAACCGGTGGGTT
TCAAATTTTGCCTGGGCCACCCGTGGGAATTCATGGGGATTGCCAAGGCCATGCTGGAAACCGGAATCCTGCCGGA

CTTTATCGTGGTCGACGGCAAGGAAGGCGGCACCGGCGCCGCCCCCGTGGAGTTCACCGACCATATCGGCGTGCCC
CTGCGCGAAGGCCTGCTGTTTGTGCACAACACCCTGGTAGGCCTGAACCTGCGGGACAAGATCAAGCTCGGCGCCA
GCGGCAAGATCGTCAGTGCCTTCGACATCGCCAGCGTCCTGGCCATCGGTGCCGACTGGGCCAACTCGGCCCGGGG
CTTTATGTTCGCCATTGGTTGCATCCAGTCGCAAAGCTGCCACACCAACAAATGCCCCACCGGCGTCGCCACGCAA
GACCCGCTGCGCCAGCGGGCGCTGGTGGTCCCGGACAAGGCCCAGCGCGTATTCAACTTCCACCGCAACACCCTCA
AGGCCCTGGCCGAAATGCTCGCGGCAGCAGGGCTGGATCATCCGTCGCAACTGTCGGCCAAGCACCTGGTGCGGCG
CATGTCGGCCACCGAGATCAAGTTGTTCTCACAGTTGCACGTGTTCCTCAAGCCCGGTGAATTGCTCACTGGCGAG
GTGAACGGCGAGTTCTATTCGCGGATGTGGCAGATGGCGCGGGCAGACAGTTTTGAGCCCCATGAGGTGGAAGCCG
CGTAAACAGTCGGCCACGACAACCCTGCGCAGGTCAAGGCCCTGCGCAAGGGATACTGGCCGGCAGTCATGACACG
CGGGGGATCCTCGATGCCTAGCGGGCGCGCATGAGACATTCAGTAATACCCAACCTTTGCAATTGCTTACAAATAC
CGCCAACCTGCGCCCGCCCGACGCACGGCAGGACCATTCGCGTCACTATTCAACCCATTGTTCAAGAGCCCGCAGC
CATGCTTAAAGGACGCGACCCGCGCATCGATTTTTTTCGGGGCCTGGCGTTGATCTTCATTTTCTGGGATCACGTC
CCCCAAAACCCACTGGGCCAGATCACCCTACGCAACTTCGGTTTCAGCGATGCCGCCGAGGTCTTTGTGTTTCTCG
CCGGCTACGCCTGCGTGCTGGCCTACGGCAAAATCCTGCAGGGCGAAGGTTATTGGATGGCCAGCCTGAAAATCCT
GCGCCGCGCCTGGGTGCTGTACGTGGTGCATATCTTTTTGCTGGCGATGCTGATGGGCATCGTGTTCTTCGCCAAC
AGCAAGGTAGAAACCCGCGACCTGGTCCAGGAAATGGGCCTGACGCACTTCATCACCAACCCCCAACAAGCCCTCA
CCGATGAGTTGCTGTTGCGCTTCAAGCCCAACCTGATGGACCCGTTGCCCCTGTACATCGTGTTGTTGCTGGGCCT
GCCGCTGGTGCTGCCGCTGTTGCTGCGCAAGCCGTTGGCAGTGGTGGCGGCGTCGATGACGGTGTACCTGTTGGCA
CCACGCCTGGGCTGGAACCTGGCGGCGATTGCCGACGGCGTCTGGTATTTCAACCCGGTGACCTGGCAGTTTCTGT
TTATTCTCGGCGGCGCGGCGGCGGTGCATGCCAGCCAACCGCGCGCACCCGATACGCGGCCGCTGCACCGGCAACC
AGTGTTTATCGCCGCGGCCACCTACACCCTGCTGGCCGCGGTGATCACCCTGTCCTGGCGCTGGCCCGAGATCCAC
GATGCGGTGATGCCCGCCGTCATCAGCGACCTGTTGTACCCCATCAGCAAGACCGATCTGTCGCCGGCGCGCCTGT
TGCACTTCCTTGCCCTGGCCTATGTCACCGCCAAGCTGCTGCCGGGTCGGGCGTGGACGCAAAACTGGTTGGCCCG
GCAAAGCTGTCGCATGGGCCGCTATTCACTGGAAGTGTTTTGCCTGGGGGTGTTGCTCGCGCCGTTGGCGGACATG
CTCAACGCCCAGGTCAACGACGCCTGGCCGATGCAGATTTTCAGTGCCCTGCTGGGCCTGATGCTGATGGCGGCGC
TGGCGGCCTGGCTGGAGTTCAACAAGCACCTGGATGCGTCGCGACAACGCCAGTGGGTGGCGGGTAGATAGTTAGC
CCCAGGTGACGCACCGGCGATGATTACATCGGCCTTCCACTTAATTTGTGAAAGGCCAGTCCCTTATGCCGACCTC
TGAGTTACTCCACGGTTTATCCGGCAACCTGCAATCGAGCACCTCCTGGGAACAACAGGAGGCCATTGAGCAGATC
CGGCAAAACCTCACGCAAGCCTTGAGCGGCCTCACGCCCGATGAACAAAAGCGCTATGTGCAACTGCAGCGCGAGT
CCCTGACGGCCCTGTCTGCAGTCGAAACCGAAAAGGACCGCCTGGTCCAGGCCTTCAAGACCGAAGGCCTGGCCCA
ACTGCGCAGCCGGATTGGCGGGCGCGATCCCGAAGCCTTCCGCTTCGATACCACCTACCGGGAAAAAATCGAAAAG
CCTTTCCCCTGGGAGCCGAAACGCGACGAACGCACTCGCTTTCGCCGCCGCTCCTACGCAGAAGACTGGGACTACA
TCGACCACGTTAAAAGCATGACGTTATGGGAGGCGGCCTGCCTCAACTTTGGCTTTACCTACGGCAGGATTACCGA
GTCTGGCTACAGCCTGGTCCAAGCCAGCAAGGTGATTGGCCCTGGCGATGACCGCAGCCTCTCGGCGCAGGAGTTT
ATCGACGTGGCACGCGAACTGGATCTGGGTGGCCAGCTGAAAAACCGGATCCACGCCACCTTGGGCGATAACGGCC
TGCTTCGTCGCCTGATGGGCACTTCGGCGCGCACGCTCCTGCAGTTCGACGTGCTGGACGCCTGGCGCAACCGGGC
CCAAAGCGGTCTTACCCGTGAAATGTACGACAAGCTCAATGCGGCGATTGAGAACACCGGTGCTCAACCGGAGATT
GAAACACTAGGCCTGACCTCAGGCGTAACCTTGGTGGTGGCCGTTCCCTTCGTCCCCTGGGAAAACATCATTCCAG
TGCCCCTGCTGCTGATTCGTGTCGCAGCCCTGGGTGTGCTGTCGTACTTTCCATTCCGCCCAGGGGGCGCGCTGCG
CTATCACCCCGACGCACGCGCCGCCGAACAGGCGTTCCGCCAGCAACTGCACGATAGCCATCAACAACATGATCTG
GGCTGGTTTTCCCGGCAGTTACCCCTGGTAGGCATGTCCGTCTTCAACAAACTGTTGAGCCATAAGACGCGTCCGC
AAAACCTGGGTTGGCTGGAGGGGGCCCTGTATGACGGGTTTCACAAAGCCTTCCCGCGCAGAACCCTTGACGACAT
CCGCTTCACCGCCGACCCCAAGTCAGGGCCACCAGTAAGCCTGGTGCAGGCCCTGACTTACCGACAGATCCAACGC
TGCAAGACTGACCTGGATACACTTGCCGTTGATCGCGCCGAGGCCGACTGGGAAGCCCTCAAGAATGCGGCGCAGG
AGATCACGGGCGAAGTTCTCGGTTTACTCCTGATGCCTATGCCCGGTGGCCTCACCGGCATGATGCGCATCACCCA
ACTGCTGATGCTCGGGAGCCTGACCTACAGCGTGATCCAAGGCCTGGATCAAGCGGTGAAAGGCGAAGCCTTAGCG
TTTGCCAGCAGCCTGACAGATGTGGCCGACCTGATCGTCAGCGGCCGGCTGACCGCAACCGCCGGCCGCTTGCAGC
GCCGGCGTATGCAAAAACTGATGCAGAACATCGGCAACCCGCGCAAAACCAGCAACCAGCGCCTGTGGAAACCAGA
CGCACAGCCCTATGCCCATGACCGCCAGGCGCTGCTCGACGGCCGCAGGCCCGATGCACTGGGGATCTACTACGTC
GATGGCGAGCAATACGTCAAGCTGAACCAGAATGAGCAATTTCTGGTGGCCAAGGTCAAGCACGACGACCAGCAGA
TGCGCTACGTGCTCAAGCACCAACGCAACGAGGGCTACGCACCGCCGATTTTTTTTAATCCAGCAAAACAGGCGTG
GGAGGTCGACCTGCACAACGCCCACGCGCTCTCGGACATCGAGTTGCTGCAACGCATGCTGCCCAACGGCGCCTCC
GTGGCACCGGCGACAGATCTGCAAACCATGCTGCGCAGCACCGCCACCACCCGTACTGCGCTGGACAAGGTCTGGC
GCTCGGAGCCGGCGCCGTTGAACCTGACCGAAGGGGTACGGCGCGTGCAGGCTGATCAATTGATCCAACTGCTGAT
CAACCGTTTCAACGAGCCCGGCTACCTCCCCCCCTACGGCGACAGCGCGGTGTTTTGCCTGCTGACACAACTGCCG
CAGTGGCCCGCCAACGCCACCTTGAGCATCCGCGACCCCCAGCACACAGTGATCGAAAGCTTCAGCAAAACCGAGG
TCGTGCCCCTCGACGCACACACCATCAACATTGTCCGCGGCGATGATGGCCGCTATAGCGCCCTGGGCGCTGCGCA

CCCGCCATCCAATACCAACGAGCCGCTGTTGAGCCTGATCATCGTGCTGCAACCTGGCACCTCGCGCCTGGGCATC
GAAGGCCACGCCGCTCAGACTACCGCGCAACGCATCACGCGGCTGCGCCAGCAGGTCACGACCCTGGTCCGCGACG
AGCGGCTGAGTGTGTTCAGCGCGCTGGTGAATTACGCCGGTTACGAAAAAAGCGAGCTGCTTGCCCCGGCGAATGT
ACGCCGTTTTCTTCCCTTTCAAGCTCCGCCGCCTTTGGTGACCGTCACCCCCTTACTGAAAAAGCTGCGTGACCTC
AACCGCCCCTTGTCACCGGCCAATCTGGAACGCCTGTTGCAGGAGCACCCTCTGACGCCACGCCAGCAACAGGCTT
ACTTGCAGCATGGTTCGCTGCCGGCTGCCTTTCATGAGCGGTTGGACAACCACCGTACCGCGCTGCGCATCGATGC
GGCCATCGACGGCTTGTACCACCCCCGCGCCTACAGTAACGATATTGACCAATGGGCCAGGGAGTTCGCCTCGGCG
CTGGTGCGCAACACCCTCAAGCGCCCGTTCGTCATCACCGAAGTGGTCACCGGCGATACCGCCAAACCTTACGTTT
CAAGCGGGCCACAGGATCCCACCGTGGAGTTGCGCCATTACGGCAACGGGGTCTATGAGGCATACGACATGCTTAA
CGGCGGTACGATTCCAGTGCCCGCCACCGTAGACAGTTTCTACCTGGCCATCGGTTCGGTGTTGCAGCCCCACGAA
CGTCTGTTGCTGGGCATGACCAGCGCCACCGACACCCTGGGCCTGCGCACCAAACTGGGCAATTACATGAGCAGCC
AACGCAGCCCGGCGGGGTATGTCAGCCTGGCCAACGGTTCGCTGATGCAATACGAACAGACCCTGAAGCTGCCCAC
CGACCTGGCGCCGAACGCCCAGGGTATTTTTCAATGGGATGCTCAACACTTCCTGCCGCTCTACGGCTCGCTGTAC
CGGGTCAGTTTTGACAAGAGCCTGTACAAATGGCGCCTGGTCCACCCCGAGAAAATCGGGGTCGAAACCCCCGTGC
TGTCTCACAACGGCCAAGGTGCCTGGCGCCTGGCCAGCGAGAACCCGATGATCTGGAGCGACCACGAGTTGCTCTA
CCGCCTGGGCAATCACCCGTACGCCTTCACCCAGGCCGAGTCGGTCAATATCCTGGCCCTCACCGACACCCCGCCC
AGGCTCCTGCGCCACATCCACCATGCAGGGCTTACCGCTCCCCCATTGCTGGCCGATACCTGCAAGCGCCTGAAGA
TCGAACAGGAAATCCTGCAGTTCATCGAAGCACTGGGCAACACCCCTACCTCGCGTATCGCCCGCCCGGACTTGCA
ACTGCTGGTGCTCTCCAGCCTGCCAAGCTGGCCCGACAGTCACGCCATTCGTGTAATCGACAGCGACAACCGGGTC
ATCCAGCAATACCCAGATCGCAACCCGCCAAACGACAAGGCATTGCTGGTCAAAAAGAGTGACTATGAAAACGCCA
GGCTGCTGGACAACGTCATCGCCAACGATGAGTTGACCCAGGCCTTGCTCGGTGAACTGCCAGAATCCAGCAATGA
CCGCTTGTTCAAGCTGGTGAAACAGATCGTCGAGTTCACCGAAAAGGAAAAATCGCAGTTACTCGACTCGATCTAC
CAACGTAGCGAAAACGATGGGCCTGAATCGGTCCGGCAATTCAAAGCCCACCATCCACACTTGCCCACCAGTAGTG
TCCAGGCGATCCTCGGACAAGCCACCCCCCGGGAACTCAAGCAACTGCAACAAAAGAAAGCCCCCGGTCTGCGCCT
TGCCGAGCAGGCGCGCCTGTCGGCCGATGAGGTGCGCCTGAACCGGGCCTATGAAGGTCTGTACAGCGAAGCGTGC
AGCAACGCCGACAGCGACCGGATCACCGCACATTTGCTCAAGGATCTGCCCACCTGGCCGGCGGAACTGCGCATCG
ACATCCACCAGGCAAACCTGCAGGGGCACCTGCTGCAAAGCGCAGGTCCGCGCACCGCCAGCCAACATCGACAGAT
CGCCCGGACCCAAGGTGGTTATCAGGCTTACGACGCAGCGGGCAAGCCTGTGGGGCAACCGTCGAACCGCTTGCTG
GAGGTGATTGTGTCTACGTTGTCCGAGAGTGAAAAAGTCGCGTTGGAGGTGACCGACGACGCGGGCATTGCCGCGC
TGCGCCAACAGATTCTCACCCTGGCGCTCAACCAACGGGTAGCGATCAAAAGCCTGCTGGACCTCAAACACCTGCA
ACCCTGGATGGTGCCGCCGATGGGGGGTGAGCGCACACTCCTGGTGTACCCGTTCTGGAGCCGGTTCTGGCCGTTC
AGCGGTAATCGATCGCCCGACCTGGTCACCAAGGTCCAGGAACTTTACCCCCGGATGGACAACGATCAAGCCCGGC
AACTGATCCAATCGATGAACCTGAGCGAGCCGGCGGCGCTGATTGAAATCGAACGGCGGCGTACCGAATACCAAGC
GATGGAAACGGCACTGTCACGCTGGGCCGACACACCCGCGAGCGAGGACGAACACCTGCGCGACCCACTGGGTATG
AGCCTGGGCAACCGCCGCTACATTGCCCAGCAGTTGTGTTCAGCGTGGCGCCGGGAAAGTCGTTCGCTCTATCTGG
CGGGGATATTGGACGCCCAATTCGTCGAGCTGCAATTGGACGACGGCAACCTGCCCCCCGCCGATTTCATCAGCGG
CACCCAGGGGTTTGCGCACATCGACTTCCTGAAAATCGCGGGTAATCACTTGCCTCAAACGGCCAGTGACTTCCTC
AGCAAGTTCGCCGGGTTGCGAGGGCTGCATCTGGATTGCATGTTGACCGAGTTACCCAGCGCCATCACTGACATGA
CTCAGCTCACACACTTGAGCCTCAGTGGCAACGACATCCGACTCACCGAGGCTTCGGCCCAGCGCCTGGCCTCCAT
GGTCAACCTGCGCGAGTTGAATATGGACAACAACCCCCACCTTGGCGTGACCCCGGATGTGAGCGCCATGAGCCAG
CTGCACCGTCTGCAGTTGGTGAATACCGGGTTGACCCAATGGCCCATCGGAGCGGAGACGCTCGCCCGCCTGGAGG
AACTGCACCTGCAGGAGAACGCCATCAGCGAGATCCCTGAAGCGGTATTCACCCAGGAACGCATCGGCTTGCCCCG
CCGCAATATCCTGCTGCACGACAACCCCCTCTCGCCGGCCACCCTGGAGCGTATCGAAGAGTACCGACGCAACACG
GGCGTGCGGCTGGGCGGTGTGCTTGGCTCCAGGCATCAACCGCCAGCGACGGACGGCGTGTCCCAATGGTTGAGCG
GTGTACCCGCTGCCGATGTTCCGGCCCGCAGGGCGTTATGGGAGCAACTCAAGGCCCATGAAGGCGCCAGCCCCGA
CGATGCCTTCCGGGTGCTCACGGACCTGACTCAAACCTATGCGTACATCGCAGCGGGCGCGTCGCGCAACGCGCTG
ACCCAAAGGGTGTGGACCTTGCTGCAGGCCATGGGGGAGTCCACCGAATTGCGCAACAACGTGTACCTCAACACCT
ATGGTTCAGGTGACTGTGGCGACAGTGTGTTGTTGGCCTTTACCAACATGGAGCTTCAACACCAGATTCACCTGGC
CAAGTTGCAAAGCCGCAGCTATGCCAGTGACCGCGCACTGATGGACTTGTCCGCCGGGCGGTTCTACCTCAACCAA
CTGGACCATATCAGCGACAAGTTTATTCACGATCGGGAAGTGGCCGGCCTGGAAGTCGACCCGGCCGAGGTCACCA
TTTTCATGCGTGCCGAATTGGCCAAGGAGTTCAAACTGCCTTTCTATCCACTGGAACTGCTGTACACCGTCGAAGA
CTACGTGACGAATGAAGTCATCAGTGGCGCACGGGAAAAACTGCGCAGGCTCGGCCAGAGCCCTGCCATGCAGGAA
TGGTTGCTTATGGAAGGATTCTGGATCGAATACCTGGCCAGGAGCCACCCGGAGCCTTTTGCTTCCGTCAAGGACA
CCATCCGCTACAAAGTCGGCCTGCTGGAGCAGGAAGTACAGGACAAGTACTCGGACGAGTACCTGGAGCGCCGTCA
GTCACTGGTCGACCTGGAGCAGGCAGAGCAGAACCGCCTGGTGCGACAACTGACGGTCGCCACCCAGGCGGCATTG
CAACGCACGTAAGACATGCCCCGGCGCGTAGGGCCGGGGCATGTTGCGTCAAACCGAGCGAGGCGAGCCCTGGCTG
ACCTGGGTCGGTTGCAGTTTGAACGTGTAGAACAACACGGTCAGCAACACCAGGAAGGCCGGGCCGACATACAACG

CAATGCGGGTATCGGGGAAGTACGCCATCAGGCCCACCACCAGCACCAGGAAGGCCAGCGCCAGGTACGAACTGAC
CGGGTACAACCACATGCGGTACTTCAACGCCTTGTTCTCGGCGGGGCTCAGGCTCTTACGGAACTTGAGCTGCGCC
AGGAGGATCATCACCCAGGTCCAGATCGCGCCGAAGGTGGCGATGGACGTCACCCACACGAACACTTTTTCAGGCA
CCAGGTAGTTGAGCAGTACGCCCAGCAGCAAGGCGAAGATCGACAGCAGTAGCGCACGACGCGGTACGCCATTGCT
GGAGGTGGTGCCGAATGTCGCTGGGGCCTGGCCGTTCTGCGCCAGGCTGTAGAGCATGCGCCCGGTGCTGAAGATC
CCACCGTTGCAGGACGACAGCGCCGCGGTAATCACCACGAAGTTGATGATACCGGCCGCCGTCTTGATGCCCAGAC
GCTCAAAGGTCATGACAAACGGGCTGCCCTGGGTACCGATTTCGTTCCACGGGTAGATCGACAGAATCACGAACAA
CGCACCGACGTAGAACAGCAGAATCCGCCAGAACACCGAGCCGATGGCGTTCGGGATAGTCTTCTGCGGGTTCTTC
GCTTCGCCGGCGGTCAGGCCGATCATCTCCACGCCGAGGTAGGCGAACATCACCATCTGCAACGACATCAACACGC
CCTGCACACCATTGGGCAGGAAGCCGCCGTGGGCCCACAGGTTGGAAATGCCCAGCGCCACACCGTCGTTGCCGAA
GCCAAAGGCGATGATACCGACGCCGCCGATGACCATGGCAATGATGGTGACGATCTTGATCAGGGCGAACCAGAAC
TCGAACTCACCGAACGCCTTCACGGCGATCAGGTTGATCGTGCCCATGCTCACCAGGGCCGCCAGGGCCCAGATCC
AGCGGGGCGTATCGGGGAACCAGACGCCCATGTACACGGCGACGGCGGTGATTTCGGCCACACAGGTCACCAGCCA
CAGGAACCAATAGTTCCAGCCAGTGAGAAAGCCCGCCAACGGGCCGAGGTAATCCTGGGCATAACGGCTGAACGAA
CCGGCGACGGGGTTATGCACCGCCATTTCGCCGAGGGCACGCATGATCACCAGGATCGCCAGGCCGCCGATGATGT
ACGACAGCATGATTGCCGGGCCGGCCATTTCGATGGCCTTGGCCGAACCCAGGAACAGACCGACACCGATACAGGC
GCCAAGCGCCATCAGGCGAATATGCCGTTCGCCCAGTTCACGTTTGAGCGGGCCGCCGGAAGCGGTCTCGCCGTGG
GGCAGGTGATTGCCAACGGGCATAGGGGTACAACCTCATCTTGTTATTGGATAGGAACCACCCAGTGTCCATCCTC
ACGGCGGGTCGCCATAAGGATGTCGTACCAGGCTCGCCTTTTGGACAAGCCCGTCTGGCCCGGTTAAACCGGCCAG
CTTGGCGGCGGTGCAGTATAAAAAGCCGCAAGTAGGGCTTTTCACTCAATAAGCAGCAAAGTTCAGCGATAAGTCC
CGGAAAACCCCGGCTCGACAGAGGAGCATTCCCTGAGACCTGTAGGAATCGCCTTCAATAAACGCCGGCGAGTATT
GCACAGGGCGGGGGCGCCGTCATGCCCCTACCTTCGGCGGATATACCTGCCTTAAAGGCTCTAAACCAGCTATTCC
AGGCCAGCGCCCATCATGGCGAACGGGCTATCCGCCCAGTGACTCCAGGGGCATTGAGCAAGCAGGTTTTGGGGCG
GCTTCGCCACCCAACGGGGGACAAGCCCCCTCTCCACATTTACCGCGCCAGCCCCCCGACTGCGGCCCGAGCACCC
GTTGTGGCAAGCGGGCTTGTTGTGGCGAGCGGGCTTGCCCCGCGCTGGGCTGCGCAGCAGCCCCAACAAACCCACC
CCAGTTACCCCAGACACACCGAGCAAACAGGCTTTGGGGCGGCTTCGCCACCCAACGCGGGACAAGCCCCCTCTCC
ACAGCTGACCTCCTACGCAGCAGCCCCCACTCCCCACAGGGCACAGTTTTTTTGCCAATACCCCCGCGCTAGTAAC
GCGCATCCACCGGCTTGCCGCCGTTCGGCCAGTCCTTGACCTTATCCGGGAAGTCCGGGCGTGGTTTTTGCGAGAA
GTAGGCGGCAACGTCCACCGCTTCCTGGTCCGACAAGCCGCCCTGGCCCAACGGGAATTTTTCGTGGAAGCCGATC
GGCATGTTGCGTTTGACGAACGCGGCCGCGGTGTAAGTGCGGGCCATGCCGGCACCGATGTTGAACGACTGATCGC
CCCACAACGGTGGGTACACCAAAGTGCCGTCCGCTCGGACCAGGCCTTCGCCGTCATTGCCGTGGCATACCGCGCA
TTGCTTGGCGTAGACCTGTTCGCCATTGGCAAGGTCGGGCTTGATGGCCGGGTCGATCTTGCCGACACCCCGGCCT
GGCACCTTGTCCTCGGGCTTGGTGTTGTTTTTCATCCAGTCGAAGTACGCCACCATCGCCTGCATGTCCGCCGATT
GTGGCGGCACCGGTTTGCCATTCATCGAGCGGCGGAAGCAACCGTTGATGCGTTCTTCCAGGCTCACCACCTTGCC
AGCACGCGGGGCGTAGCTGGGGAAGAAGGCCGACACGCCCACAAACGGCGAACCATCCGCCACGGTGCCGGCATTG
AGATGGCAACTGGTGCAGTTCAGCGAGTTGCCGACATTGTCTGGCAGCAGTTCCTTGGTTTGCAGGTGCAAGCGCA
TGCCGCGTATCACTTGGTCGGCATTCGAAGCTGCCAGCAGATTGGCCAGGCGCGGGGTTTCGAAGGCCGATGAGTC
GGTGCTGACCGGGTTGAGCTGGTCACGCATTTTCTTCACCTGGGCGGTACTGATCGGCGTGCCTTCGTTACCCCAG
CGCGTGCGCACAAAGGTGAGAATTTCCGCCACTTCGTTGTTCCCCAGGCGCGCAAAGCCCGGCATGGTGTAGACCC
GTGGATGGGCTGCGGTCTCGGCGGTTTCCCAACCGGTAAGGGTAATGTGCAGCAACGAGGTCGGGTTGCTGGCGAC
CACGCCGGCGTTGCCGGCCAATGCCGGGAACAGCCCTTTCACACCGGCGCCGTCGCTGCGGTGGCAATCGCTGCAG
AACTGCATGTAACCCAGGCCGCCACGGCTGGTGAACAAATCCTTGGGCAGTTCGGCAGGACCTTTGGCGACCGCAG
GCATCGGCAGGTCGTCCTTGCCCGGTGGCAGGGATTTCAAATACGTCGCGACGGCCGTCAGGTCGGCGTCCGTGAA
ATGCTGGGTACTGTGACGGATCACGTCCACCATGTTGCCCGACACTGTGGCAAAGCGGTTCTGCCCGGTCTTGAGC
AGTTGCACGGTGTCTTCCACCGTCCACAGGTTGCGCAGACTCAAGGCGCGCCAATGCTCGACGGTTTCCCCGCCCA
GGTAGTGCTCGCCGCCGCTGCCGGTGTCGCTCATGGCTTTTTCCTGGAAGGCGATACCCCGTGGGGTATGGCACGA
GCCGCAGTGGCCGAGGCCCTGGACCAGATAGGCGCCGCGGTTGAGCACTGCATCCCGGGCCGGGTCGGGCTGGAAC
GGTTGTTTGTCGAGGAACATGAAGTTCCACAACGCCAGGCCCCAGCGCTGGTTGAACGGGAACCCCATGTCTGCTT
CGAGGTTGGCCTGGCTCACCGGCTTGACCTCATGCATCAGGTAGGCGAACAAGGCGCGCATGTCCTCTTCGCTCAT
CTTCGCGTAGGACGGATACGGCATGGCTGGGTACAGGTTCATACCAGCCGGTGTCACACCTTCACGCATCACGCGG
TCGAACTGTTCAAAGCTGTAGTTACCGATGCCGGTCTCGCGGTCCGGGGTGATGTTGCTGGAATAGATGGTGCCCA
TGGGCGTGCTCAGTTCCAGGCCGCCGGCCATCTGTGGACCTTGCTTGGCGGTGTGGCAGGCGATGCAGTCACCGAG
TTGGGCCACGTATTTGCCTTGGGCGATCAGCTCGGTATCGGGTTTGGCAGGTTCGTTGGCGTGCAGGGGAAGCGCA
AGACACAGCGTCGCCCCCATCAAGGCCAAACGTGACAGAGAAAGGGCCATCGCATTATTCCTTTAATAACCTGGCC
TGGGGGTTGGCCCCTGGGTACAGGTTTATCTTCGTTTTTATGAGGTCATCAGGCGGACAACCGGGGCTTTGTAAGC
CCCGGTTTGTTGTAGCGGATTTTCGAGGGTGGCCCGTTGATATGGATCAGGGCCGGGGGAGGTTCAAGCGTTTTGC
AGGTATTGCGCAGCGTCGAGTTCGATCATCACCGGTTGGCGGCTGGTGGCCAGGGTTTGCAACAACGTGTGCAAGG

CCTGTGGCGTGTCCGCCGTGTGGTGCTCGACATGGCAGCTGCGGGCCATCGCGGCAAAGTCCGGGGTCATGATATC
CACGCCCAGCGGGGTGATTTCCCGGGCTTGCATATAGTCGCGAATCTCGCCGTAGCACTGGTTGTTCCACACCACC
AGGATCACGCCGATACCTGCCTCCCGGGCTGCGATCAGTTCGTTGCTGGCAAACTGCAGGCCGCCATCGCCCACCA
GCGCCACCACCGGTCGCTCGGGCTTGGCCAGCTTGGCACCGATAGCCGCCGGCAACCCGTAGCCGAGAGTGCCGTA
GCCAGTGCCAGCATTGAACCAACTGTTGGCCGCCGGCGCCTGGTAACCCACTGCGCCCTGATACACCGGCTGGGTC
GAATCGCCGACGATCAATGGGTCGACCAGGCTGTCGCGCAGCAGGTCCAGCAAGCCTTGCAGCCCCTGTTGCTTGG
CATTCCATGTCTCGCGCTCCAGACGGTTGACCCGCTGCACGCTGTCCTGTGCCCAACGCCCGCTCGGGGCAGGTGG
GCGGACCTCATCCAGCAGCACCTGCAACCCTTCGCGGGCATCGCCCACCAGCCCGACATGGGCGCGTTGCACGCCC
ATGACTTGCATCGGGTCGATATCCAGACGGATCAGTGGTGCCTTGAATTTCAGTGGGCCAAGGCCGAAGAAGTCGT
AGTCGGTTTCCCCAAGCTCCGTGCCCACGGCGAGGATCACATCGGCTTCGTCAAACAGCGAGCGGCCATGTTTGGA
TGACTGCACGCCGTCCAGCAATAAGGGGTGGTTCATGGGCAGCAAGCCACGGGCATTAGTGGTCAGGGCCACCGGC
GCTTGCAGGCGCTCGGCCAGTTGCTGCAACACCTCGGCGCAATCACGGGCACCGCCGCCGGCGAGAATCAGCGGCC
GGCGCGCGTCGTTGAGCAAATTCAACGCCGCACGCAACGAGGCCGGCGCCGGGGCCGGTGCGCCTGGCAGGCTGCG
GGGCATCAGGTTGAGGTCGATGGCCGGCATGTCCAGCACATCCAGGGGGATTTCCAGGTGCACAGGGCGCGGGCGC
GCACATTTGAACACGGCGAAAGCGCGGGCCAGCAGCTCGGGCAATTGCTCAGGCGATTGCAACGTGTGGCTGAACG
CACACACCCCCGAGACCATCGCGCGCTGATCCGGCAACTCGTGCAGATGCCCATGGCCCAGGCGCAGATGTTCGCG
GCGGTTGGTGGTGGAAATCACCAGCATCGGCACCGAGTCGGCATAAGCCTGGCCCATGGCGGTCAGAATATTGGTC
ATGCCCGGGCCGGTAATGATGAAACAGACGCCCGGCTTGCCACTGGCGCGGGCGTAGCCGTCGGCCATAAAACCGG
CACCCTGTTCATGGCGCGGGCTGACATGGCGCAGGCGGCTACCGTGCAGGCCGCGATAGAGCTCGACGGTATGCAC
GCCGGGGATACCGAACACGGTATCTACCCCATAACCTTCCAGCAGGCGAACCAGGGACTGGGCGCAGGTGGTCATT
GCTCACTTCCTTCTTATTGTTCTTTGTCGATTGGCCAGACCTTAGGGTGCGACCCGAGGGCGGACAAGCAAAAAAT
TTACCGGCCATTGGCAAAAAAAAATTCACGGATTGCCTGGCGTCAAAGCCCGCCGCCCAACTGCGACTTCACCCACT
CGCCAAACGCCTGCACCACCGGTCGTTCGACTTTCTGGTGATCGGCCACCAGGTAATAGCCACGGTTGGACTCGAT
CTCGGTCTCGAACGGTCGCACCAGCAAGCCCCTGGCCAGGGCTTCACCGCTGATCAGGTTGTCGCCCATGGCGATA
CCCTGACTGGCGATGCACGCCGACTGCACAAAGTGCGCATCGGCAAACACGATGCCGCGCTGCACATCGACATTCG
GCGCCCCGGCGGCGGCCAGCCAGACGCGCCAGTCGGAGTAGTCGATCATATGCAGCAAAGGTGCGCCGGCCAGGTG
CTCCGGGTTCTTCAGGCCGCCCATGGTGTTGACCAGGCGCGGGCTGCACACCGGGAAGTAGCGCAGCGAAACGATC
TTCTGCACATGCTGGTTGGGCCAGTCGCCCATGCCGTAGGCGATAAACAGGTCGGCGCCGTGGTCGCTGGTGTCGT
CTGGGGTACGCGGTGAAATCAGGTGCAGTTCGACCTGTGGATAAAGGGCCTGGAAATCAGCGATATGCGTGCACAG
CCAATAGGTGGCAAAGCCCGCCGGGCTGCTGATACACAGGCGCCCGCTGACCTGCTGGCCATCAAAGCGCTGGCCA
GCCTCGAGGATATTGGCCAGCAGCCCATGGATATCCCGGGCATAACGCTCGCCTTGCCAGGTGATGCGGATATTGC
GCCCGGTGCGCTCGGTCAGGGCAAAGCCCAGGGTTTGTTCCAGGCTCTTGATCTGGTGGCTGATGGCGCTGGGGGT
GAGGTTCAGCTCGGTAGCGGCCTCGGACACACTGCCCAGCCGGGCCACCGCGTCCAGCGCCCGCAAGGCGGTCATG
GAGGGGTAACGCATGGATGTTCACCTGCTCAAAGATACCCGCACCATAGCCGTAAACGGCCGTCAGACATAGCCAC
CGGCAAAGCGGACAGTTGAATTAAATTTCAAGATCGACGGAAAAATGCTCGATTGACCTCCAATGGCCAGTGGCTA
TTCTCGACCTTCAGCACGGCACGCCCAAACTTGCCGGCGTTGGTCGTCAACGATGACATGGGCAGGCTGGATAAAC
GTGGTGTTCTTGCGTTTTTCGCTGGCAAGCCAGCTCCCACAGGGGGCGGTGTCCAGGTTGATCAATTTACAAAAAT
AAAGAGGGAAAACCCTTGAACCCAGTCACCCCAGTGGTCGCCATTGATGCTCTGCACAAGAGCTACGCCGCGCACC
ATGTTCTCAAAGGCATTTCCCTGCGCGCCAACGAAGGCGATGTGGTGTCGCTGATCGGTTCCAGCGGTTCGGGCAA
AAGCACCCTGCTGCGTTGCATCAACCTGCTGGAAATCCCTTGCAGCGGCAGCCTGCGTATCAACGGCGAAGAAGTG
CGCCTGAAAAAGGACCGCGCTGGCAACCCGCTGATCGCCGACCCGGATCAAGTCGCTCGCCTGCGCACGCAACTGG
GCATGGTGTTCCAGAGCTTCAACCTGTGGCCCCACCGCACCGTGCTGGAAAATGTCATCGAAGCCCCGGTATATGT
GCTGGGTGAAGACCGCAAGGACGCCATCGCCCACGCCGAGCACCTGCTGGAAAAAGTCGGCCTGGCCAGCAAGCGC
GATACCTTCCCCGCCTTCCTTTCCGGTGGCCAGCAACAACGCGTGGCCATCGCCCGCGCCTTGGCCATGCGCCCGC
GCGTATTGCTGATGGATGAACCTACCTCGGCCCTCGACCCGGAGTTGGTCGGCGAAGTGCTGAAGGTGATCCAGGG
CATCGCCGAAGAAGGCCGCACCATGATCCTCGTCACCCACGAAATGGCGTTTGCCCGCGACGTATCGAGCAAAGTC
ATGTTTTTGCACCAAGGGTTGGTTGAAGAAGAAGGCACGCCACAGCAAGTCTTCCAGAACCCCACCAGCGAGCGTT
GCCGCCAGTTCGTCATGGCCCAGGACAATCGCGTTTGAACTGCCGCTTCCATCCTTCAAAACAATAACTTCGAAAG
GGTCAAGCCATGCACAACACTCTGAAATCCCTCGCCGCCGGCTGCGTGCTCGCCGTGCTTTCCCACAGTGCCGTTG
CCGCCGACAAAATTGTCTTTGGCATCGCCCTGGAACCGTACCCACCGTTCTCCGAAAAAGCCGGCAATGGCGCCTG
GAGCGGTTTCGAACCGGACCTGATCAAGGCCCTGTGCGAACGCCTGCAAGCCCAATGCCCGCTCAAGGAAGTGTCC
TGGGACGGCCTGATTCCGGCGCTGCAATCGAGCCAGATCGACGTGATCCTCAACTCCCTGAGCATCACCGAGGAAC
GCGAAAAGGTCATCGCCTTCACCGCCCCTTACTACCAGACCCCGGCCATGTGGGTCGCCGACAAGAGCCTGGAGCT
GGTCACCACCCCCGAAGGCCTCAAGGGCAAGCTGATCGGCGTGCAGGGCTCGACGTCCAACGCCACGTATCTCAAG
GCTTACTACGCCAAGGGTTCGACCCTGCGCTACTACAACACCCAGGACGACATGACCGCCGACCTGCAAAGCGGAC
GGATCGACGTCATGCTGGCCGACGCCCTGACCATCGAGCCGATGCTCAAGTCTGATGCTGGCAGCGGCCTGGCCGA
CAAGGGCCTGGCGCCCAAGGATCCATTGTTCGGTTCGGGCATCGGCGCCGCCGTGCGCAAGGGTGATGACAGCCTG

CGCGAGCAGCTCAACACCGCGCTGGCGGCGCTGAAGGCTGACGGCACCTACGACAAAATTCGCAGCCGCTATTTCA
GCGTCGATATTTCCGCGCAATAACGTCCAACGGAACGCGCCATGATTTCTTTGTCCGAACTCTCCCAGCTGTTCGT
CGCCGACGGCTGGCTCAACGCCCTGCTCCAGGGGGCGGTGGTGACCTTGCAGATTTCCGCCGGGGCCTTTGTGCTG
GGCCTGGGCATCGGCCTGCTGGTGGCCATGATCAAGCTCAAGGGCCCACGCTGGATGGTGCGCCTGGCCAACCTGT
ACACCACGCTGTACCGCGCGGTGCCGGAGTTGCTGCTGATTTTGCTGCTGTATTACGCCGGCACCGACCTGCTGAA
CATGGCGATGGCCGCCATGGGCCGCGACAGCGTGACGGTCAATGGCTTTATCGCCGCCGTGCTGGTGCTGGGCATT
GTCCAGGGCGCCTATTCGGCGGAGATCATTCGTGGCGCGATCCAGGCCATTCCCGTCGGCCAATTGGAAGCAGCGC
GGGCCTTTGGTATCGAGCGCTGGTTGCTGGTACGCCGGGTGTTGCTGCCCAGCATGCTGCCGTTTGCCATGGCCGG
CCTGTCGAACCTGTGGCTGGTGCTGGTCAAGGACAGCGCGCTGATCAGCATCGTCGGCTACAGCGAGCTGCTCTCG
GTGGGCAAGCAGGCGGCCGGTTCCACCAAACATTACCTGGTGTTCTACCTGGCGGTGGCAGCGGTGTATTTCATCA
TCACCCTGATTTCCAACGGCGCCTTCCGGGTGTTCGAGCGACGGATCAACCGCTGGATGCCGCAGCACTAAGGAGC
GCAGTGGATGGATTTTTCATGGATCAACAGTTTTGGCAACGAGCTGCTGCATGGCCTGGGCATCACCCTGAAATTG
CTGGCGTTGTCCGGGGTGTTCGGGTTCTTGCTGGCGGTGCTGGTGGCGTTGGGGCGCTTGTCGTCCAACCCGCTGA
TCAGTGCGCCGCTGCGCGCCTACACCGCGGTATTCCGGGGCACGCCGCTGCTGGTGCAGATCTACATCCTGTACTA
CGGCGTAGGCAGCGTGTTTGCCAGCTACCCGCTGATTCGCGGCAGCTTCCTCTGGCCGTACTTGCGTGAAGGTTTC
TGGTACGTGGCCCTGGCGCTGATCCTGTGTGTCGGCGCCTATGTCGGCGAAGTGTTGCGCGGCGCCCTGCGTGCGG
TGCCACGGGGCGAGTTGGAAGCGGCGCGGGCCTTTGGCATGAAGCGCCTGATGGTGCTGCGCCGGGTCTGGCTGCC
GCGGGCGCTGGAGCTGGTGAAGCCGACATTGGTGGGCGAGACCGTGCTGCTGCTCAAGGCCACCGCGCTGGCTTCC
ACCGTGGCCGTCACCGACTTGCTCGGCGCCGCCAACCTGGTGCGTGCGCAGACGCTGCGGGTGTACGAACCCCTGC
TGGCCGTGGCGGTGATCTACATTGTCCTGGCGTTCGTGATCGAACACGTCTTCTCGCGCTGGGGCAAAGTCCCGCA
GCGCCAGGCTTGAATCAACTTTAGGAGAACAAGGATGCGTTATTCACCGTTTGTCGAGCGCATTAGTGGCGAGTCC
GTCAGCGCCTGGGATATTCACTACGCGGCCGTCGAAGCCCGTGGCCGTGGCGAAGATGTGATTGTGTTGAGCGTGG
GCGACCCGGACTTCGCCACCGCCAGCGCCATTTGCGAAACCGCCGTCGATGCCCTGCGCGCCGGCGACACTCACTA
CACCCACGTCCTCGGCCGGCCGGCCCTGCGCGAGGCGATTGCCGCCAAGCAAAGCCGCCTGCAAGGGATTGCCGTA
CACGCTGATAACGTGGCCCTGGTGGCCGGTGCGCAAAACGGCCTGTTCGCCACCTCGCTGTGCCTGTTCAGCCAGG
GCGATGAAGTGCTGGTTCCAGAACCGATGTACCTGACTTATGAAGCGTGCATCCACGCTTCCGGTGCGCGCATCGC
AACCATCGAGCAACCGGCGGCCAACGGTTTCCGCCTGACCCGCGCCGCCCTGGAAAAAGCCATTAGCGCCAAGACC
CGGGGCATTGCCCTGGCCACGCCCTGCAACCCCACCGGCAACGTCTACAGCCGGGAAGAGCTGGAACTGGTGGCCG
AAGTCGCCCGTGAGCATGACCTGTGGGTGATTTCCGATGAAGTCTACGGCCAGATCACTTACGACCAGCCGCACTT
GAGCATCGCCTCGCTGCCGGGCATGGCTGAGCGCACCGTGGTGCTCAACAGCCTGTCGAAAACCCACGCCATGACC
GGCTGGCGCGTGGGTTGGGTGGTAGCGCCGACTGCGTTGGTGGGCCACCTGGATAACCTGCTGCTGTGCATGCTCT
ATGGCTTGCCGGGGTTTATCCAGGAAGCGGCGATCAAAGCCCTGGAACTGGATGACGAAGTGGTCAGCGATGCGCG
CACGGTGTATCGCCGCCGGCGTGACCTGGTGGTAGCGGGCCTCGGCGGCATTGCCGACCTCGATTGCCGCGTGCCC
CAGGCCGGGATGTTCATGCTGGTGGACGTGCGCCGCACGGGGCTGTCGAGCATGGACTTTGCCTGGCAGCTGTTCC
GCGCCACCGGCGTTTCGGTGCTCGATGCCCAGGCGTTTGGCGCCAGTGCCGAGGGCTTTGTGCGCATCTCCTTTAC
CGTCGCCGACGACACCCTCAAGGACGCCTGCCAGCGCATTGCCGGGTTCGTCGAGGGCCTGAGAGCCAACCGCTGA
TTATGTGAACACTGGGATTGATGTGGGAGCTGGCTTGCCTGTTCCCACATTTGATCTTTGCTCGTCTTGAATATTG
AGGTTTCCCCCGTGACCGCTTCCGACCAATTCACTTCCACCCACTCCGTTGTCGGCGAGCACGCTGACCTGGTGCT
GCACCATGGCCGTTTCTACACCCTCGACCCGAGCCAGCCGTGGGCCGACGCGGTGGCGATTCGCGGGGGACGTTTT
ATCGCGGTGGGCACGCTCGCCGAGCTGCAACACCTGATCGGCCCGCAGACCCGCCAGCACGATCTGCTGGGCGCAT
TCTGCATGCCCGGCCTGCACGACATGCACACTCACCCCGACCTCGCCCTGGCCCCGCGCTACAGCGATGACCTGGA
CGTGGGCATCGAAGACCCCACCCCCGAACAACTGGCGGCGGCGATCCACGCCTATGCCGACGCCCACCCCGGCGAT
GGCTGGATCTACGGCCAATACTGGGTGCGCTACACCTTCCGCGAAGCCGGGCTGACCCCAGGTCGCGAGTGGCTCG
ACAGCATCATGCCGGACCGCCCGGTGGCACTGCTGGACCGCATGTGGGGCACCATGATGGTCAACTCCTGCGCCTT
GAAACTGGCCGGCATCGACCGCCACACCGCCGACCCGCGCAACGGCTATATGGAGCGCGACGAACTCAGCGGCGAG
CCCAATGGCCTGATGATCGACGGTGCCTACGCGCTGATCCACGCGGCCATGCCGCCCACACCGGTGGCGGTGCTGC
GCCGGGCCTATCGCGACGGCGTGCACTTCCAGAGCTCACGCGGCGTTACCGCGACCAAGTACGTGCACGTCTGCGA
GCGGCGCCTGGACGCCCTCAAGGAACTGGACGACGCCGGTGAGTTGAGCGTGCGCGTGGAAGCGGCGATCAGTTGG
CAGGACGATATTTTCCCGGTGCGCCGGCGCTGGGAACTGCTCGGTGGCGAGCGTCACTACTACCGCAGCGCGCGGC
TCAATGCCAACGCGGTGAAGTTCCACTTCGACGGCACCGTGGAGCCCAAGTCTTCGTACCTGATGACACCCTGGGC
CGCCGACTCCACCTGGCGCGGCAAGCTCAACCTGACCCCGGAACACATCACCGACATGGTGGTGGACATGGACCGT
CGCGGCCTGCGGGTGATCGCCCACTGCACTGGCGACGGCGCCTCCGATGTATTCCTCGATGCGGTGGCCGAGGCGC
GGCGGGTCAATGGCGACAGCGGCATCCGTCACCAGTGCGCCCACAGCACCCTGCTGCATCCGGGCAACCTCAAGCG
CTTCAAGGCGCTGAACGTGATCGCCGAGTTCTCCCCGGCGGCCTGGTACCCGACGCCGTTTGCCAGTGGCGCCCGC
TCCGGTTATGGCGCCGAACGCCTCAAGCGCATCTACGACTTCAAAGGTGTGCTGGCCGAAGGTGGCCTGGCGGTGA
TGGGCACTGACTGGCCGGTGGCCTCGATTGACCCGTGGCTGGCCCTGGAAACCATGGTCACCCGGCAGAACCCGTG
GAACCAGGAACCGGACTGCTTCGGCGAGCCGATCACCCTGGAACAGGCGCTGCAAGTGGCGACCCTCAACGGCGCC

CACGCCATGGGCCTGGAGCATTCCACCGGCAGCCTGGAAGTGGGCAAGTGCGCCGACCTGATCGTGATCGACCGCG
ACCTGTTCGCCCAGCCGGCGCGCAACTACATCCACCAGACCCAGGTGCTGCTGACCTTTGTCGATGGCCGTCCGGT
CTACGACCGTCTCAACGCGTTTGAAGGCACAGCCTTGCAAGCTGTGTGGCAGGGCCTGCCGCCGGTGATCGAATGA
ATAACGACAGCATTGCCGTCACGGTGGTCTCGGGCTTTCTCGGCGCCGGCAAGACCACCCTGCTCAACCGCATCGC
ACAGCAGCCGCAACATGGCCGGATGGCGGTGATCGTGAATGACTTTGGCGAGCTGAATATCGATGCGGCGATCATC
GCCGAAGTCACCGATGCGGTGTTCAGCCTGCAGAACGGCTGCATCTGCTGCACCGTGCAAGAGGACCTGCTGGCGC
AACTGGTGAGCCTGAGCCAACTGCGCCCACGCCTGGATCGCATCGTGATCGAGTGCAGTGGCGTGTCCGACCCGCA
GCGCATTGTGCAGACCCTGGGTTATCCACAGCTCAAGGCGCACTTGCATCTGGATACGGTGATCACCCTGGTGGAT
GCCAGCGGTTATGCCGCCCTGGAAGGTGAGTTCGCCCGTTTGTCCCGCGCTCAGGTGGCCTGTGCCGACCTGGTGC
TGCTGAACAAGGCCGATCTGGTCAGCGCCGCCGAACTGCAAAGCCTGCGCGCAACTATCGGTGCGCGCACACGGGT
GATCAGCACCGTGCAGGCGCAGATCCCCGACGCCCTGCTGCTGGGCGAACGCAGCCCGCGCAACGGCTTTACTCCC
GTGCCGGCGCCGCACCATGAACTGTTTGAAAGCTGGACCTGGCAAGGCACCCAGGCCCTGCCGGCCAAGGCCCTGC
GCGACTGGCTGGGGCAACTGCCCAAGGACGTGTTCCGCCTTAAAGGCCTGGTGCATTTGCAGGGCAACGACCAGCC
CTTCTGGCTGCAACATGTGGGCAACCGCAGCCAGTTCACCCTGGCCAGCGGCGAGCATGCGCGAAATTCGGCACAA
CTGGTGTTTATCGCGCGGCGCGGCAGTGACCTGCGGGAGCGGTTGGAGGCGCAATTGCAGGAGATACAGGGCCTGT
AGACGCGGATGACGATGAAGGCAGAGTGCCGCCTTGACCTTGCTACCCTGGCCCTTGAAAATGCCCGACGCTTTTT
CGTCTCCCCGCTTACAGAAGTAGTGAAATTTCAATGTCTGATTCCCGTACCGAAGAATCCGTAGTCACCTTGCAAT
CCAAAGCTGAATACGAAAACGCCATCAATCTTTCACAGCATGTGCCAGCGGCCAAGAACATCAGTGAAATGGTGCT
GGACGCGTTCCATACCTCCAAGGAAAGCGACCAGATCCGCGAGTTGCGCGTGGCCATCCGCCAGGCCCACGACGCC
TTTGACGATGACAAGGCCTACGACCTGATGGGCCAGCTCAAGCAGCTCAAGGACGCCGAAGCCGCCGACAACGCCG
CCCTCGAAGACCTGAGCAGCAAGTTCTCGATCAGCCGCATCCTTTCCAGCTTCAAGGACGACCCAGAGTTCCAGGA
ACTGGTCTACGGCCTGGCGCTGAAGGTGCTGAACCAGACCCACCAGGCCATCAGCAACCCAAGCGCCGGCAAAGGC
AAGGCCGCCCGGGTGAAGAAGGAAGTGGAAGTGTTTGTGATCAGCAAGGATGGCATCAGCGTCACCCTGCCATTGC
GCACACCGCGCTCCAAGCTCAATGTCGACCGCGAGGCGCTGGAATTCCTCGGTTTCAGCTTTGTGGGTGAAGGCGA
CGAGGCCGAGCTTGAGAGCGAGACCTTTGTGGATAACTCCGGTGCCGAACAGCCTTTGACGCGCAAGAGCATCGTC
ACCGCGCTGCAGCAGCAGACGGCGTTTGACGGTTACAGCATCGCCCAGCAATAAGCCCTACCCCCCAAAAAATGTG
GGAGCGGGCTTGCCCGCGATAGCGTTGGGTCAGCCAACACATGTATCGACTGATTCATTGCTATCGCGGGCAAGTC
GAATCGTCGCACCGCCGCTCCCACAGGGTTCTGCGGTATTTTCAGAGCTCAATAAAAAGCCCCGCTCTTCTCTCGA
AGGCGGGGCTTTTCATTGCATCCCAACTCAGTGGGCGTAAGTCAGCAGCAACTCTTTCGGCACCTGGAAATCCAGG
GACATCATCACGCTCAAGGCGGTGATGGTGAAGATCGAGAAGACGAACAACTTGCGCGCCCAGACGGTGTCATCCA
CCGCCTTGTAGCCGGTCCAGGCCATGTACAACCAGTACATGCCCATGGCCGCGGCGACGGCGAGGTAGCTCATGCC
GGCGTAGCCACTGAAGGTCAACATCAAGGTCGCCACGAGGAAGGCCAGGATGTAGAGCAGGATGTGTTTCTTCGCC
ACCTGGATGCCGCGCTTGACCGGCAATACCGGAATCGAAGCCGCCAGGTAGTCGTTGAAACGGAAGATCGCAATGG
CGTAGGAGTGCGGCATCTGCCACAGGCTGAACATCACCAGTAGCACCAGCGCGGCCATGTCGAAGCTATTGGTCAC
AGCCACGTAACCAATCACCGGCGGCATCGCCCCCGACAGACTGCCCACCAGCGTGCCGTGAACCGACTTGCGCTTG
AGGTACAGGCTGTAGAGGCCGACGTAGATGACAAAACCGATCACGGCAAACAACGCCGCCAGCGGATTGGCCACCG
TGTACAGCAACGCCACGCCGACAACACCGAGGACGGTCGCAAAGATCAGTGCCAGTTTCAGGGAGATAAGGCCCTG
GACCAGCACGCGATTCTTGGTGCGCTCCATCTTGATATCGATGTCACGGTCGATGCAGTTGTTGAACACGCAACCG
GAGGCCACAACAAGGGAAGTGCCGATCATTGCCGCCAGGAAGATGGCCAGATCGACATGCCCCTTGGAGGCCAGGA
AGAAACCGCCTGCCACAGAAAGCACGTTACCGAAAATGATCCCCGGTTTGGTGATTTGGATAAAGTGCTTAAGCGA
CATCGGGTCTTACCTCACTTCGCCATCATGTAGGTGTGGATGCTGAACATGATCCACAACGACAGGCCAACCAGCA
GCAGGATCACAATGGCCGTGAACACAAACGCAATCACGTTGTTGCGCTGGGCCTGGGAGCGATCCAGGTGCAGGAA
GTAATACAGGTGGACAACCACCTGGATCACCGCGAACAACAGCACGATAGCCAATGTGGTGGCCTTCGGCAGGGTC
GGGTACATCACTAGGCCGAACGGGATGATCGTCAGGATCACCGACAGGATGAAGCCGATAGCGTAAGACTTTACGC
TGCCGTGGCTCGCATCATGGCTGTCATGGGAGTGTGCATTAGCCATTACAGAGTCCCCATCAAGTAGACAACGGTG
AAGACGCAGATCCAGACCACGTCCAGGAAGTGCCAGAACAGGCTCAGGCAGCTCAGGCGCGTCTTGTTGGTCGACG
TCAGGCCATGCTTGTTGACCTGGTACATCATGATCGCCATCCAGATCAGACCAGCGGTTACGTGCAGACCGTGGGT
ACCGACCAGGGTGAAGAACGCCGACAGGAAGCCGGAACGGCTAGGGCCGTAGCCTTCGGAGATCAGCAGGTGGAAC
TCGTTGATCTCCATGGCGATAAAGCCAAGGCCGAGCAGGAAGGTCAGGCCCAGCCACTTGAGCACGCCTGCCTTGT
TGCCACGGAAGAACGCCAGCATGGCGAAGCCGTAGGTGATCGAACTGAACAGCAGCAAGGCGGTTTCGCCCAGTAC
GTAGTTCAGTTCGAAGATGTCGTGGCCCGACGGGCCACCCGCTACGTTGTTTACCAGTACCGCGTATACCGCGAAG
ATCGATGCAAACAGGATGCAGTCGGTCATCAGGTAGAGCCAGAAACCGTATACGGTCATCTCGCCCGCGTCGTGGT
GGTCATCGTGCCCATGGTCATCGACATGGGCGTGTCCAGCATTGGTCACTAAGTTCGACATGGTTTAAGCCTGTTC
CAACGAGGTTTCAACACGGTTGGCCGGAATCTTCTTCTCGGCTACCAGGCGAGCGTGCTGCTCGGCTTCGATGCGC
TCGATCACGTCGACCGGCACCATGTAGCCTTGATCATCACGAGCCGCGTGGATCACGAAATAGATCACGGTGCCAG
CCAGGCTTGCGATAGCCAGCCACCAGATGTGCCAGATCATCGCGAAACCGAATACCGTCAACAGCGCGCCCATCAC
CACACCAGTGGCGGTGTTGTTCGGCATGTGGATCGGCGAGTAGCGCACAGGACGCTGGTACGCCGTACCGTTTTCC

TTGGCTTCGGTGAATGGGTCGATGGTCTCGGCCTTAGGCAGCACGGCGAAGTTGTAGAACGGTGGTGGCGACGAGG
TCGACCATTCCAGGGTGTGTGCATTCCATGGATCGCCGGAGTCGCAGGCGTTTTGCTTGCGGTCACGGATACTCAC
GTACAGCTGGATCAACTGGCAGGCGATGCCGACAGCGATCATCACCGCACCGAACATGGCGACGTACAGGTACGGC
ACCCACTCAGGGTTGGTAGTGGCGTTCAGACGACGGGTCATGCCCATGAAGCCCAGTGCATAGAGCGGCATGAACG
CGACGAAGAAGCCGGTGATCCAGAACCAGAATGCAGCCTTGCCCCAGCCTTCGTGCAGCTTGAAGCCGAACGCTTT
CGGGAAGTAGAAGCTGAAACCAGCGATGTAGCCGAATACCGCGCCGCCGATGATCACGTTGTGGAAGTGAGCGATC
ACGAACAGGCTGTTGTGCAGTACGAAGTCAGCACCCGGGATGGCCAGCAGTACGCCAGTCATGCCGCCGATGGCGA
AGGTCACCATGAAGCCCAGGGTCCACAGAACCTGGCTGGTGAAGCGCAGGCGGCCGTGGTAGATGGTGAACAGCCA
GTTGAATAGCTTCACCCCCGTCGGGATCGAAATCAGCATCGTCGCCAGGCCGAAGAAGGCGTTGACGCTGGCCCCC
GAACCCATGGTGAAGAAGTGGTGCAGCCAAACCATGAAACCCAGCACCGAGATCGCGCCGGAGGCGTAGACCATCG
AGTGGTGACCAAACAGGCGCTTGCCGGTAAAGGTCGAGATCACTTCGGAGAAGATACCGAACGCTGGCAGGATCAG
GATGTACACCTCAGGGTGACCCCATGCCCAGAACAGGTTCACGTACATCATTGGATTGCCACCAAGTTCATTGGTG
AAAATGTGGAAATCCATGTAACGGTCAAGCGTCAGCAGTGCCAGGGTAGCGGTCAGGATCGGGAACGAAGCCACGA
TCAGGACGTTGGCCCAGGTGCAGGTCCAGGTGAAGATCGGCATGTCCATCAGTTTCATGCCTGGGGTACGCATTTT
CAGCACGGTGGCCAGGAAGTTGACCCCCGTTAGCGTCGTACCCAACCCGGATAACTGTAGCGCCCAGATGTAGTAA
TCCATCCCCACGCCCGGACTGTATTGCAGCCCCGACAACGGCGGATAGGCAACCCAACCGGTCTTGGCGAATTCGC
CGACGCCCAGGGACAGGTTGATCAGCACGACGCCGGAAACCAGCAGCCAGAAGCTCAGGGAGTTGAGGAACGGGAA
CGCAACGTCACGCGCACCGATCTGCAGCGGCACTGCCAGGTTCATCAGGCCGGTGAAGAAAGGCATCGCCATGAAG
ATGATCATGATCACACCGTGGGCGGTGAAGATCTGGTCATAGTGTTCAGGCGGCAGGTAGCCAGGCGAACCTTCGG
TGGCCATGGCCAACTGGGTCCGCATCATGATGGCGTCGGCAAAACCGCGCAGCAGCATGACCATGGCGACGATGAT
GTACATCACGCCGATTTTCTTGTGGTCGACCGACGTCAGCCACTCAGTCCACAGGTAGGTCCACTTCTTGAAGTAG
GTGATTGCCGCAAACAGCGCCAGACCACCCAGCGCGATCATGGCGATGGTCACCATCACGATCGGCTCGTGGAATG
GGACCGCTTCCCAACTTAATTTACCAAACATCGTTTACTCCTCTGCCCCGGCAGCTGAATGCGAATTTGCGTCCGT
CCCTTCCACAGCAGCCACTTCTTTCTTCTCGTGCTTGACCGGCTTGCCTGGCTTCATCCCTTCATACTTATCGATG
ATTTTCTGAAACAGGTCCGGCGTGTACGAGGAGTACAGCTCTACAGGGTTGTTCTGGCTTGGCTTGGCAAGGGCTT
CGTATTCAGCTTTTTCAAGCTGTTTTGGTGAGCTCTTGGCTTCTTGTACCCAGGCGTTGAAGTCTTCCTGAGTGGT
GGCGATTGCTTTGAATTTCATGCCGGTAAAGCCCGCGCCACTGTAGTTGGCGGAGATACCGTCGAGCTCGGCGTTC
TGGTTGGCAATCAGGTGCAGCTTGGTCTGCATGCCCGCCATCGCGTAGATCTGGCCACCCAGGCCCGGGATGAAGA
ACGAGTTCATCACCGCATCCGAGGTCACTTTGAAGTTGATCGGGGTGTTGGCCGGGAACACGATCTTGTTGACGGT
GGCGATCCCTTGCTCGGGATAGATGAACAGCCACTTCCAGTCCAGCGCGACCACTTCGATAGTGATTGGCGGCACG
TCGGATTCCAGCGGACGGTAAGGGTCCAGCTCGTGGGTCGACTTATAGGTGATGTAACCCAGGGCGATGATGATGA
GTACAGGTACCAGCCACACCGCGATCTCGATCTTGGTGGAGTGCGACCACTTCGGCGCGTAGGTGGCCTTGGTGTT
GGACGCGCGGTATTTCCAGGCGAACGCGAAGGTCATCACGATCACCGGCACCACGACCAACAACATCAGCAGCGTA
GCGGTGATGATCAGGTTACGTTGCTCCACGCCGACCTGACCCACAGGGTCGAGCAGCGTCCAGTTGCAGCCTCCCA
GCAATAACATGCCGAAAAGCGGCACTATGCCTAGTAATCGGGTGTACCTGTTTTTACTCATCTCACGACCTCTAAA
GCAGCTTGCGCAATGCAGTTGGGTTTTGATCGCCAACACTTCACCCTGCCAAGGGTTGGCATTTCTCTTCGATTGA
ATAAGGGCCTGCCCGGCGCTTCATGCGCTGATCGACACGTCCAGGTCTGGCGGTGAATTCTTATTCGATTTCGTTG
GTCAAAGGCCTTGTTACAGACCAATTCCATTTGGTGCGGATAGTTGAAAGGCTGCCGGCACCTGGGGTCGCATGAA
GCAATGTTTTCGCCTCTCGACCGCCCTATTTGCTCAGGGTTGAGCAGCACCGGACACTCAGTGCGGGCGATTGTAG
TTAGCTAGCGATGTATAAACCATGACTTATAAAGAAATAATTTTTATCGATTCCAGCAATAATCCTGCGCCATTGT
TGCAAAGGTTCGGGATCGTATCGCGTTTGATTCTCAACAAAAACCACAAAAAATGCCGTGTTATTGCCCATTGTGT
CGCACCCAAGGGGTCCTTCCCGGGTCGCCAAACCCCAGGCAAGCCCTCTAAAAACAAGGCATTCGTAGATCACCGC
AAGTTCTGCAACGCACGCCGTTCGCGGCTCGGCTCCAAGCAGAAAGTGACAGGTAAAAGCAACTGCCCGAGGGTGG
ATTAACCGGCAAACCCAGGGGTAGGAAACCTCCTGCGGCACCTCCACTGTGACAACGTGTCGCACTTCGCGCACCG
CCAATCTTGTCCAGCGTCACGGTCCGTTCACAAAATGCCCACACACAAAAACGCCCCGGCCTGCATCAGCAGGACC
GGGGCGTTTTGTAGGACGATTTAAGCGCTGTGCTTGCGATTGCGGTAAATGCCCAGCGGCACCAGGATCACCGTCA
ACACGAACGCCACCAGCGCCCACTGGGCCAGGGACAAGCCCAGTACCGGCGGATACGGCGTGCTGCAGAAACCATC
GACCTGGAAGCCCAGGGGGAAGATCTTCGCCAGGGGCAGGTCATCGACAATCGGCTGCAGTACATCGACACCGCAA
CTGATGGCCGGATAGAACTGGGTATACACATGGTGCCCGGCGGCAGCGACACCGCCCAGGGCGCTGAGCACCACCA
GTACTTCAAAGATCGTGACACTGCGCTGGGTACGCATTGCCGCGCCAATAAAGGAAAAGAGCGCAATCAGCAGCAA
GGCATAGCGCTGCAGGATGCACAGTGGGCATGGCGCTTCACCCAGCACTATCTGCATATAAAGCGCGCCACCGATC
AGCGCCAGGCAGATGATCCCCAGCAACACCAAAAAGCGCCGCTCACGCCCCAACCGCATTTCGTCAATCATCCCCG
TTTCCCTTTGCCTAATGATGGGTGTGCCAATGGCCGCAAGTTTACACACAGGAAGTGAAAATTTAGCAGTAGGCGT
CGGCATCTTAGGCGCAGGCGGGTTAACGACACATTAATGTGGGGGCTTTCGGACGCCATCGCAGGCAAGCCAGCTT
CCACAATTTGATCTGTGAATACATTCAAATGTGGGAGCTGGCTTGCCTGCGATGAAGCCAACGCGGTTTAAAGAGC
GGCCGCTTATTCCAGCGCCGCCGCCGGCCCGAAGAACTCATACCGGCTCTGCTTTTCAGGCACACCCAGGGCCTTG
AGGTGACGCTTGATTGCACTCATAAACCCTTTGGGCCCAAGGAAGTAGGCATCCACATCACGCTCCTCAGGCAACC

ACGCCGCCAGTTGCTCCTGGGTCAACATCCCCAGCTTGTCCGCCGCCGGGCTGATGCCGTCGTCTTCGGCGTAGCA
GTAGAAACGCTGCAGTTGCGGGTGACGCGCGGCCAGGGCATCGACCCAGTCGCGAAACGCATGCACGCCACCATTG
CGCGCGCAGTGGATGAAGTGCACCGGGCGCTCGGTCGCCAACGCCGCTTCCAGCATCGGCAGGGTTGGCGTGATGC
CCACACCGCCGCTGATCAGCACCAGGGGTTTGTCGCTGTCCACCAGCGTGAACTCCCCCGATGGCGGGAACAGGTC
GATGCTCGCCCCCACGTGCATCTGGTCATGCAGGTAATTGGACACGCGCCCGCCCGCTTCGCGCTTGACGCTGATG
CGGTATAGGCCCGCGTCGGTACGCGCCGACAATGAATAGTTGCGGCGCACTTCTTCGCCGTCGAGGATCAGCTTCA
GGCCGATGTACTGGCCGGGCTCGGCGGCGAGGATCGGGCCGTTGTCCACCGGGGCAAAGTACAAGGAGATGATTTC
ACTGCTCTCCTCCACGCGCTTGACCAGCAAAAACGGGCGCGCCCCGCGCCAGCCGCCAACGGCCTGGGCTTTTTCG
TCGTAGATGGCACTCTCTGCGCCGATCAGGATATCGGCCAGTTGCCCGTAGGCTGCGCCCCATGCACTCATCACTT
CAGGGGTGGCGATTTCATCACCCAGCACCTCGGAAATCGCCCGCAGCAGGCAGTTGCCGACAATCGGGTAATGCTC
CGGCAGGATTTGCAGGGCCACATGCTTGTTGATGATCTTGGCCACGAGGTCGCCCAACTGATCCAACTGGTCGATG
TGCCGGGCGTACATCAGCACACCGTTGGCCAAGGCGCGAGGCTGGTCGCCGCTGGCCTGGTGGGCCTGGTTGAACA
GCGGGCGCACTTCGGGGTACTCGGACAACATCATGCGGTAGAAGTGGGTGATCAGGGCTTCACCACCGCTTTCGAG
CAGCGGCACAGTAGATTTGACGATGGCACGGTCTTGAACGCTAAGCATGGGGAGACTCCTGAGCCTTTGGGCTTCT
GAATGATTACCTTGGGATACTCAGTATTCGTGCCAACAAATAATCCTTTAATTATCAAGGGTTTAAATTAACAGTA
GTCAATATGACTTCCCACAACTTATAGTCATTAGGACTACAAGGAGTCATTATGACTGCACACTCCCTGCTCACCA
CCCTGCTGCCACTGGTTGCCGACCTTTCCCGCGAACTGCCGGAAGGCGAGCGCTACCGACGCCTGCTGCAAGCCAT
GCGCACCCTGCTGCCCTGCGATGCCGCCGCACTGCTGCACCTGGACGGCGAATGGCTGGTGCCCCTGGCGGTGGAC
GGCTTGAGCCCTGACACCCTGGGGCGACGCTTCAAGATCAGCGAACATCCGCGCTTTGCCGTGCTGCTGAGCAGCC
CCGGCCCAACCCGCTTTGATAGCGACAGCGAATTGCCCGACCCCTACGACGGCCTGGTCGATGGCCTGCACGGGCA
TTTGGAAGTCCACGACTGCATGGGCTGCCCGCTGTTTGTCGACGACCATCCCTGGGGCCTGCTGACCCTGGATGCC
CTCGACACCGAACGCTTCGAACGTGTGGAACTGGACGCCTTGCAGGCGTTTGCCAGCCTCGCCGCCGCCACGGTCA
ATGTCGCCGAACGCATGGAACACCTGGCCCTGCGCGCCGAGGACGAACACCAGCGTGCAGAAATCTATCGCCAGGC
CAGCGGCCAGCAGCACAAGGAAATGATCGGCCAGAGCAAGACCCACAAACGCCTGGTGGAAGAAATCAAGCTGGTG
GGCGGCAGTGACTTGACCGTGTTGATCACCGGCGAGACCGGGGTCGGCAAGGAGCTGGTGGCCCAGGCCATTCACG
CAGCCTCGCCCCGTTCAGAGAAACCGCTGATCAGCCTCAACTGCGCAGCACTGCCAGAGACCCTGGTGGAAAGTGA
GCTGTTTGGCCACGTTCGCGGCGCGTTCACCGGGGCGCTGAACGAACGGCGGGGCAAGTTCGAGCTGGCCAATGGC
GGCACCTTGTTCCTTGATGAAGTGGGTGAGTTGTCGCTGGCGGTCCAGGCCAAGCTGTTGCGGGTGTTGCAAAGCG
GGCAGTTGCAGCGACTGGGTTCGGATAAGGAGCATCAAGTGGACGTGCGCCTGATCGCCGCCACCAACCGTGACTT
GGCCGAAGAAGTGCGCAACGGCCGTTACCGCGCCGACTTCTATCACCGCTTGAGCGTGTACCCGCTGCAAGTTCCG
GCCTTGCGTGAACGCGGGCGTGACGTGTTGCTGCTGGCCGGTTTCTTCCTGGAACAGAACCGCTCGCGCATGGGCC
TGGGCAGCCTGCGCCTGACCAGCGATGCCCAGGCGGCGTTGCTGGCTTATGGCTGGCCCGGGAACGTGCGAGAGCT
GGAACACTTGATTGGCCGCAGCGCGCTGAAAGCCTTGGGCAACCACCGGGAGCGGCCGAAGATCCTCAGCCTCAGC
GCCAAGGACCTGGACTTGCCGAGTGAAGCGGCGCCACTGATTGAGGAACAGGCCGAGCTAGCCGTGGTGACCGGTG
ACTTGCGCCAGGCCACCGAGCACTACCAAAGGCAAGTCATCAGCGCCTGCCTGGAACGCCATCAGCACAACTGGGC
CAGCGCCGCCCGCGAACTGGGGCTGGACCGCGCCAACCTGGGGCGCATGGCCAAGCGCCTGGGCCTGAAGTAACAC
CGATCAAACTGTGGGAGCTGGCTTGCCTGCTCCCACATCAGATTTGCTTCGATTCCCAAAGCGGACTCACAGGCGT
AATCGCTCGGGGCTTACGAAACACCAAGACATTCCCCAGCATCACCAGCACCAACCCGCCCAGCGCCGGGGCGGTC
CACTGGTAGCCTTCGAGCAGCGCCGAGACGTTCAGCGCCACCACCGGGAACAACACCGTGCAATAGGCCGCCCGCT
CCGGGCCCATGCGGCCGACCAGGGTCAGGTAGGCGGTAAAGCCGATCACCGAGCCGGGGATCACCAGGTACAGCAA
CGAGCCGATGTAGCGAGTGTTCCATTCCATTTCAAAAGGAATGCCCTGGATCACGCAGTACAACGCCAGCATCGAT
GCGCCGTAAGCCATGCCCCAGGCGTTGGTGGTCAACGGCTTGAGCCCGGCTTTCTGTTGCAGGCTCGACAGCATGT
TGCCCGCCGAGAAACACATCGTCCCCAGCAACGCCAGGCCCAAGCCCAGCAGCGTCTCGGGGCTGGCGGTATGCCC
GACCAGTTCCGGCCAGAACAACAACCCCAGGCCGAGCAACCCGAGACCGCCGCCCAGCAACACGTTACGCGCCACT
TTCTGGCCGAAGAACACTCGGGCATTCAAGGCATTCCATAGCGTCGCGGTGGAGAACACCACGGCCACCAGGCCAC
TGGGGATCCATTGGCTGGCGGTAAGGAAGCACATGAAGTTCACGCAAAACAGGCACAAGCCCTGGGCCAGGCAGAT
CAGGTGCCCACGGCGGTTCATCACCTGCAATTTGTGACTGAGCAGCAGCAACACAAACAGCACCAGTGCCGCCAGG
CCGAAGCGATAGACGATGGATGCCGCAATCTCCACCACACCCAGTTGCCATTTGAGGGCGATCCAGGTGGTGCCCC
AGATCAGCACGGTCAGTAAGTACAGGGATAGGTTCATGGCGCACTCCGGTGGTTGAGCCACAGTTTCACCCCGACT
GGCTGCCAGGCACTTGCAGATTCTTGCGCTTTTGTCGGGCCGAGGGATCACAGCGCCAGCCGCCGGGAGTAGGATG
CAAGGCGTCGGAGAGAACCCCATGTCTGCACTGGAAAACCTACAAGTCTTCAAAGCCCTGAACAGCTCGCCCAACG
CTCGCCTTGAGCTGAGTGCCGAGCTGGGTGACGGCTTGTCTGCGGCTTTGTGGAGCAACCACCACGACGCCCAGGA
CTACGAGGCCCCCAGCCATCACACCCTGTCTTGCTACGTGGGCGGTGGCACTGAGACATTTCGCCGTGGCCAGCCC
GGCACCAAGGGCGGCCCCGACAAATTGTGCATCCTGCCCGCCGAGCATCAGTCGGCCTGGGTGATCAACGGCGAAA
TCCGCCTGGCCCATGTGTATTTCAGCCCGGAGCACTTTGCCCTGGGTTGCGTCACCCTGCTGGACCGCGAACCCCG
GGCGCTGCAACTGCGCGAAAGCACGTTCCTGGAAGACCCGCAGCAGGCCGGGCGTTTTCATCGGTTGATCGGCCTG
AACTGGCAGGAGCCCGCCGAACGCCTGCTGACCAGTAGCCTGGCCCATGAAATGCTCAGCCACACCCTGCTCAGCC

AGGTCGGCCTGCGCGAAGGCTTGCGACTCAAGGGTGGATTGGCCGCACACCAACGCCGGCAGTTGGTGGAATACAT
CGAACAGCAACTGGCCGAGCCGCTCAGCCTGGGGCAATTGGCGGGGTTGTGTGCGTTGTCGGAGTACCACTTTGCG
CGGATGTTTCGCACCAGTTTCGGCCTGCCGCCTCACCAATATGTGCTGGCCCGCCGCCTGGTGCGGGCCCAGGCCA
TGTTGCGCGGCGGATCACTGCCATTGGGGGAGATTGCCCTGGCGTGCGGGTTTTCCAGTGCCAGCCACTTTACCAA
CCGGTTTCGCCAGGCGATGGGCGCGACACCAGGTGAATACCGGCAGGCGTTCCAACCCCGCCCCCTGTAGGAGCGA
GCTTGCTCGCGAAAAACCTGAGGCCAATGCGGGGCGTCAGGTTTCCCGCGTTAGCGTTAACGACCTTCGCGAGCAA
GCTCGCTCCTACAAGGGGCTTTGGGCCGGGGCCGTTGCNNNNNNNNNNGTAAAGGGTTTATCTGACTGCCACGCCA
CTGCCTTTATCCGGCGGGAAGGCGCAGCCAGCGCCAGCCACCAGGCCGGCAAGCCCCAAGCCAGGAGACCTGCCTC
GCAACCGATTTTCCATTTCAACCGGGCGGGGTGATCCGGTGACGAATCCAGCGCCTGCGCGTCGCCGCAGGGCCAG
TCGTCCCGTATGCCCGCCCCAAAGGGCATCCAATGAAAACACTGGCCAAACTCCCCGTTACTATCGTCACCGGCTT
TCTCGGCTCGGGCAAAACCACCTTGCTGCGTCACATGCTGGATAACGCCCAGGGCCGCCGTATCGCCGTGATCGTC
AACGAGTTCGGTGAGCTGGGTATCGACGGCGAGATCCTCAAGCAGTGTTCCATCGGTTGCACCGAAGAAGAGGCCA
ACGGCCGTGTGTATGAGCTGGCTAACGGCTGCCTGTGCTGCACCGTCCAGGAAGAGTTCTTCCCGGTGATGCGTGA
ACTGGTCGCGCGTCGTGGCGATCTGGACCATATCCTCATCGAAACATCCGGCCTGGCCCTGCCAAAGCCGCTGGTG
CAGGCTTTTCAGTGGCCGGAAATTCGCAGCGCCTGCACGGTTGACGCGGTGATTACCGTGGTCGACAGCCCAGCTG
TGGCGGCTGGCACCTTCGCTGCCTTCCCTGATCAGGTCGACGCCCAGCGCAAACTGGATCCCAACCTGGACCACGA
ATCGCCGCTGCACGAACTGTTTGCCGATCAACTGGCCAGCGCCGACCTGGTGATCCTCAACAAAACCGACCTGATC
AGCGCCGAAGACCTGGCCCGCGTGCGCCTGGAAGTCGCCGAAGAGCTGCCTCCAGCAGTGAAAATCGTCGAAGCCA
GCAGTGGTCGCGTGCCCCTGGACGTGTTGATCGGCTTGGGTGCCGGCTCCGAGGAGCATATCGACGGGCGCCACAG
CCATCACGATCATCACCACGAAGGTGAAGACGATCACGATCACGATGCTTTTGATTCCATCTCCATCGACCTGCCA
CAGGCTGACGAAGCACGGCTGCTCGATGCCTTGACCCAATTGGTGGTGCAGCACGGCATCCTGCGTGTCAAAGGCT
TCGCAGCGATTCCAAACAAGCCGATGCGGTTGCTGATCCAGGGCGTGGGCACGCGTTTCGACAAGCACTTCGACCG
TGCCTGGAGCGCTGACGAAGCGCGCATCACGCGCCTGGTGCTGATCGGCCAGGATCTGGATGCTGCTGGCCTCGAA
GCGCAACTGCGCGCCGCCCTCAGCGTCTAACCATGCACCTGCTCAGGACCCAGCCCGGCGGTTTCGTCTCTGATGA
CAATATCGCCGACCTTGGACAGACTCCCGCCGAGTTGGTGATCCTGTGCAGCGGTGATTCCAGCCTGGCGCTGCTG
GCCGAAGCGGCCCAGCAACTGCCCGACGACTATCCGAGCCTGCGCCTCGCCAACCCGATGCAGGTGCAGAACCACG
CGTCGGTGGACCTGTACGTGGACGAGGTGCTGCGCCACGCCAAGGTCATCCTGATATCCCTGCATGGTGGCATTGG
CTATTGGCGCTACGGTATTCAGCGCCTGGTGGAGTTGGCCGAGCAGGGCGTGCAGTTGATCCTGGTGCCGGGGGAT
GATCGTCCCGATCCCGAACTCAGCGGCCTGAGCTCCGTGGATGCGCCGGCGCGTGACCGTCTGTGGCACTTTTTGC
GCCAGGGTGGACTGGGGAATGCACTGGATTTCTATCGCTGCCTGGCCAGCAGCTATCTGGAGCGCGATTACGCCTG
GGACGAGCCGCAAACCCTGCCGCGCACCGCGATTTATCATCCGCATAAGGCCAATGCGCGCCTGAATGACTGGCAG
GCAGACTGGAACGCTGCATGGCCGGTGGCGGCGGTGCTGTTCTACCGCTCGCACTTGCAGGCGGCCAATACCGCTT
TTATCGATGTGTTCTGCCAACGCTTGCAGGCTGCGGGCCTCAACCCGTTGCCAATGGCGGTGGCCAGCTTGAAAGA
GCCGGGCTGCCTGGCGGTGGTCGAGGATCTGCTTGATGAGGTGCAGGCGGCGGTGATTCTCAACACCACCGGCTTT
GCCCAATCCAGCCCCGAAGCGCCCCATCTGCGCCCGTTTCGCCGCAATATCCCGGTGATACAAGCCATCTGCGCCC
AGGACAACCAACCCGGTTGGGAAGCCAGCGAGCAAGGCCTTGGCCCACGCGACCTGGCCATGCACATCGCCTTGCC
GGAGCTGGACGGGCGCATCATCAGCCGCCCGATCAGCTTCAAGGATCTGGCCTGGCGCAGCGAGCGCAGTCAGTCG
GACGTAGTGTGCTATCGCGCGGCGCCCGAGCGCATGGATTTTGTGGCCGAACTGGCGCGGCGCTGGGTCGAACTGG
CGCGGGTGCCGAATGCAGATAAACGCATCGCCCTGATTCTGGCCAATTACCCGACCCGCGACGGGCGCATCGGCAA
CGGCGTTGGCCTGGATACCCCGGCCGCCGCCTTGAACATCCTGCGGGCCCTGCAAACCGAAGGTTATCCGCTGCCA
TCCACCTTGCCGGACAGCGGCACGGCCTTGATCCATGAGCTGCTCGGCGGCGTGACCAACGACCTCGACAGTCTCG
ACCTGCGACCCTGCCACCAAAGCCTGGGCCTGGACGATTACCAGGCGATGTTCAATCGCTTGCCCGAGGCCAACCG
CCAGGCTGTGCTGGAGCGTTGGGGTACGCCCCATAACGATCCGATGTGTCGCGACGGGCGCATGATGATCGCCGGC
CTGCGCCTGGGCCTGACCTTTGTCGGCATCCAGCCGGCGCGGGGTTACCAGGTGGATGCCAGTGCGGTGTACCACG
ACCCGGACCTGGTGCCGCCCCATGCTTACCTGGCGTTCTATTTCTGGCTGCGCCACACCTACGGCGCCCATGGCGT
GATCCACGTCGGCAAGCATGGCAACCTGGAATGGCTGCCGGGCAAAGGCGTCGGGCTGTCCGAGCACTGCTGGCCG
GATGCGCTGCTGGGGCCGTTGCCCAATATCTACCCGTTTATCGTCAACGACCCGGGCGAGGGGGCCCAGGCCAAGC
GGCGCACCCAGGCCGTGATCATCGACCATTTGATGCCGCCGCTGACCCGCGCCGAGACCTATGGCCCGCTGCGCAA
CCTGGAGCTGTTGGCAGACGAGTATTACGAAGCGCAATTGCTCGACCCAAGACGCGCCCTGGAGCTGCAAAAGGAC
ATCCTCAAGCTGGTGCGCGAAACCCGTATCGACCAAGAGCTTGAGCTGGATAACGATGCCGACGCTGCGGTCTGGT
TGCCGCGCCTGGATACCTACCTGTGCGATTTGAAGGAATCGCAGATCCGCGACGGCCTGCATATCTTTGGTGAATC
ACCCGAAGGTCGGCTGCGTATCGACACCTTGCTGGCCCTGCTGCGCATCCCGCGTGGCGACGGGCGCGGCCCGCAA
TCGAGCCTGCTGCGCGTGTTGGCCAAGGCCTTCGAACTGGGCTTTGACCCACTGGACTGTGCCTTGGCAGAGCCCT
GGACCGGGCGCCGCCCCGAGGTGTTGCAGAAAATCGACGGCCAGTTGTGGCGCACCGCCGGTGACACCCGCGAGCG
CCTGGAGTTGTACGCGGCGCGGCTGATCGAACAGGCGCTGGAAGGGCCGCTCGAACAGCTTGAGGAGCCGGGCTGG
GAAGACGTGAAATCGGTGATCGAAAGCCTGCGCATCGTCGTCGCGCCGCGCCTGGATGCCTGCGGCCCGGCGGAAA
TGCGCGGCTTGCTGGATGCCCTGGGCGGTCGTTTCGTACCGGCCGGCCCCAGCGGTGCGCCGAGCCGGGGGCGCCT

TGATGTACTGCCGACCGGGCGGAACTTTTTCACCGTCGATGTGCGCAATCTGCCGACCACCACCGCCTGGCGGATC
GGTTTCCAGTCCGCGAACCTGATGCTCGAGCGGCACCTGCAGGACCACGGCGACCATTTGCGCCAACTCGGGCTTT
CTGTGTGGGGCACCGCAACCATGCGTACGGGTGGTGACGATATCGCCCAGGCCATGGCGCTGATGGGCGTGCGTCC
GGTGTGGGCCACGGGCAGCCAGCGTGTCGATGACTTTGAAATCCTGCCTATCAGCTTGCTCGACCGCCCGCGAGTG
GACGTGACCCTGCGGGTGTCCGGTTTCTTCCGGGATGCGTTCGCCAACCTGATCCGGCTGTTCGATGCCGCGGTGC
AGGCGGTTGCGGCGCTGGATGAGCCGGACGATATGAACCCGCTGGCAGCCAAAGTGCGCAGCGAGCGTGAGGCGCT
GCTCAAGTCTGGGCTGGATGAGGAGGCCGCGGCCAGGCAGGCCGGCTGGCGCATTTTCGGCGCCAAGCCCGGCGCG
TATGGTGCGGGTGTGCAGGGCGCAATTGACGGCCGCCTGTGGCAAAGCCGTGAAGACCTGGCCGAGGTCTACCTCA
ATTGGGGCGGCTACGCCTATGGCGGTGCCGATGAAGGCACTGGCGCTCGCGAACAGTTCGCCCAGCGCCTGAGCCA
GGTTCAGGCAGTGCTGCAGAACCAGGATAACCGCGAGCACGACCTGCTCGACTCCAATGACTACTATCAATTCCAG
GGTGGCATGCTCGCCGCCGTAGAAACCCTCAGTGGGGCCAAGGCCGCGAGTTACCATGGCGACCATAGCCAGCCGG
ACCTGCCGAAAATCCGCACGCTGAAGGAAGAGCTGAACCGGGTGATCCGTTCCCGCGCGGCCAATCCCAAGTGGAT
CGAGGGGGTCAAGCGCCACGGCTATAAAGGTGCGTTCGAGATGGCGGCAACGGTGGACAACCTGTTCGCCTTCGAC
GCAACCACCGCGCTGATCGACGATCATCAGTATGCATTGCTGGCCGATGCCTACCTGCTCGACCCAGATACCCGGG
CGTTTGTGCAACAGCACAACCCCGATGCCCTGCGTGACATGACCGAACGCATGCTCGAAGCCCAGCAGCGCGGCAT
GTGGCAGGAGCCTGGGGCGTACCGCGAGGCATTGGAAAACCTGTTGCTGGACATAGAAGAAGACACCTGATGCTGA
TCGTGCTTGCCGTGAAGCTCAGTGCCAAAAGAGCGCACGCCTCACTGTAGGCGCGAGCTTGCTCGCGAAAAACCCA
AGGGCAACGCGCTCATCAAGGCTCGCGTCAACGTTGACTGTTTTCGCGAGCAAGCTCGCTCCTACAGGGGCGCTAC
AGAATTCATTGAGATATCAGTATGACTGACACCCCACATTTTCCATTGTCGGCTGTAGTCGGCGCCGACGACCTGA
AGCTTGCGCTGTGCCTGGCCGCCATCGATCCGAAGATCGGCGGCGTACTGATCGAAGGCCCGCGCGGCATGGCCAA
GTCCACCCTGGCCCGCGGCTTGGCCGATCTGTTGGCCAGCGGGCAGTTTGTCACCTTGCCCTTGGGCGCCACTGAA
GAGCGCCTGGTGGGTACCCTGGACCTTGACGCTGCGTTGGCCGAAGGCCGTGCGCAGTTCTCTCCGGGCGTGCTGG
CTAAGGCTGACGGCGGCGTGTTGTATGTGGATGAAGTCAACCTGCTGCCCGATCACCTGGTGGACCTGCTGCTCGA
CGTCGCCGCCAGCGGCACCAACCTGATCGAGCGCGACGGCATTTCCCACCGGCACTCGGCGCGTTTTGTGCTGATC
GGTACGATGAACCCGGAAGAGGGCGAGTTGCGCCCGCAACTGCTGGATCGTTTTGGTTTCAATGTGGCGCTGAGCG
GGCAGACACTGCCGGCCGAGCGTGGGCAGATCATTCGCCGCCGCCTGGATTTCGACAGCGACCCTGCGGCCTTCTG
TGCGCAGTGGGCCGATCAGCAAGGGGCGCTGCGCGAACGCTGTACCCAGGCTCGTGCGCGCCTGGCCAGCATCGCC
CTGGATGACCAGGCCCTGGCGCAGATTAGCGAGCGCTGTTTTGCCGCCGGGGTCGATGGCCTGCGTGCCGACCTGG
TGTGGTTGCGCGGGGCGCGGGCCCATGCCGCGTGGCGTGGTGCCGTGGCCATCGCTGAAGAAGATATAGAGGCCGT
GGCGGAATTTGCCCTGCGTCACCGTCGCCAGGAACACTCTTCGCCTGCGAGCACGCCCGAGCAGGGCCAGGCGCCC
CAGCCTTCAGATAACGCGCCAGGCCAAGGTCAGTGGGGCGATATGCCCGCGCCTGCGTTGCCCATGGGCGCTCGCC
GAGAGGTGCCGGCCTGGCCAAAAAAGCCCTAGGCATCCGCCCCCGACCTGACACGGGGGTGGATGCCCGCCCCAAG
GCTGGGCGATTGAGTAGCGGGCGGCAGGGTAAGGCGCGCAGCGCGCAGCAGGGCCTGGTCAACTGGCCGGGCACCT
TGCTGGGGGGGCGGCCGCAATCTCGTGAAGACCTGCGCTACCACCTGCGCAGCCGCTCGGCCCATGAGTTGTGGCT
GGTGATTGTCGATGCCTCGGCGTCCACGCGGCGCCATCAGGCGTTGAGCGATGCCAAGGGCTTACTGGCGCAGGTG
TTTGACGATGCCTATCGGCAACGGGCGCGGTTGGCATTGCTGACGGCCAGCGGGCAGGTGCCGAGCTGGCAGGTCC
AAGGCTTGAAGGCCGCGAAGGGCTTGGGCCGTTGGTTGGACGGGCTGGGCGCGGGCGGCGGTACGCCGTTGCTGGC
GGCATTGACCGAGGCCGGGCATTGGCTGGTAGCACGGCGCAAGCGGTATCCGGCTGAACAGCAGCGATTACTGGTG
ATTACCGATGGCAGGTTGAAAGATATCGGCCAGTTGCCCGGGTTGGAGTGCCCCGGGCTGTTGGTGGATATTGAAC
GCGGCCCGATCAGGTTGGGTCGGGCCCGTGAGCTGGCCGCCGGGTTGCAGGTGGATTATCGGCATATTGATGGTTT
GTAGCGCTGCGGCTAGCGATTTCGCGGCGGTGCGGCGTTCCGATAAGCTGACGACCTCCCCTGTGGCAAGCCCGCT
CGCCACAGGGGAGGTCGTCAGTGTCTGAAAATTGTGTAGATACCGATGCTGCCATGAGGCCCTCAAATCCACCATC
GGAATATCAGCCCGCCCCCGGAACATTCGGCCAGAGATCCGCCACCAGAAACAGCCGCTCCGCTTCCTCCCAATCC
CCGCCATTCTGAATCATCCGCACCAACAACTGCGCTGGCGCCATCGGGTCAAGGTCGGCAACCCAGCGTTGCATCT
GCTCGGCAGTCCACACGTCCTCTGCCGGGTAATGCGCCGGCGCCAGCCAGGCGTGGCGGGGCAGGGGCTGCCAGCG
GCCCGCTGGGCTGTTGCGGATAAAGTCCGACCAGTCACGCTGATGCAACCAGCGCCCCCGTAAGTGCTGGGGATGG
GTGCCAAGGGGTGGCCCAGCCTCGCCGGGCCAGGGGTACAGCAAGTAGCCTCCCAGCCACAGATGGGCATCGAACT
GTTGGATATCCAGCGCCGCCAGAACCTCCCGGCTTTCGGCTCTGGCCGAGATTGGCAACTGATGGTCGGCCAGGTG
CGCCAGCTTGCGGTCGAGGCGGTCGTGACAACCTGGGCCGAGCCATTGCGCGGGATCACGACCGTCACCGTCTTGC
GGGCCGAGGTAGAGTTTGATCGCCAGTTCCAGGTGATGCACGCCGTCACGGTCGCGCAGCAGCATATCCAACTCAC
CCAAGGTATGCCCGGCCTGGCGGATCGGCAGGTTGGCCGCCAGCAACTCCACGCCGGGGGCGTGCTGCACGGCAAA
CTGCCACAGACGCTCGTAATACAGGCCCAGGCGCCGGGTGCGGGCCAGGCTCAGCCAATGCTGCAGGGCGCTGCTG
TCCTGGTCCAGCTGCCTGAGCCAGTGCTCCAGAGCCGCAGGCCGCTGCACCCACTCGCTGCCGGCCAGTGGGTGGC
GTTGCGGCCAGGGGGGCTGTTCCAGCATCGGCGGGGCGAGGATCACCCACGCCAGGTCCCGCACCTCGGGATGGCG
TAGTTGACGGGGCAGGTCCTGCAAGTCTGGGAACGGATTCATGGTCCGAGCATAGCTGTTTCCTACTGCCGGGGTG
ATCGTGCCGGGTGTTGAGAACCGTTGTCGTCTGGGCCCTGCGGCGACAAAGGATTTTGTCTAATGCGGCCTTTCGC
CCATAATCGTTGTTTTTCGCCACACCCAAACCCTGCAGGAGCCCCATGGAGCAATTTCGCAATATCGGCATTATCG

GTCGCCTGGGCAGTACCCAGGTTCTGGACACCGTCCGCCGGCTGAAAAAGTTTCTGCTTGAGCGCCACCTGCATGT
GATCCTCGAAGACACCATCGCCGAGATTCTGCCGGGCCACGGCCTGCAGACCTCGTCGCGCAAAATGCTGGGTGAA
GTCTGCGACATGGTGATCGTGGTCGGCGGCGACGGCAGCCTGCTGGGCGCCGCTCGCGCCCTGGCGCGGCACAACG
TGCCGGTGCTGGGGATCAACCGCGGCAGCCTGGGGTTCCTTACCGATATCCGCCCCGATGACCTGGAAGTCGAAGT
GGCCAAGGTGCTGGACGGTCATTACCTGGTAGAAAACCGCTTCCTGCTGCAAGCCGAAGTGCGTCGCCACGGCGAA
GCCATCGGCCAGGGCGATGCCCTCAATGACGTGGTGCTGCACCCTGGCAAATCGACGCGGATGATCGAGTTCGAGT
TGTACATCGACGGCCAGTTCGTCTGCAGTCAGAAAGCCGACGGCCTGATCGTCGCCACGCCCACCGGTTCCACGGC
CTACGCGCTGTCGGCGGGCGGGCCGATCATGCATCCCAAGCTCGATGCCATTGTGATCGTGCCGATGTACCCCCAT
ATGTTGTCGAGCCGGCCGATTGTGGTCGATGGCAACAGTGAGCTGAAAATCGTGGTTTCCAAAGACATGCAGATCT
ACCCGCAAGTCTCCTGTGACGGGCAGAACCACTTCACCTGCGCCCCCGGTGACACCATCACCGTGAGCAAAAAGGC
GCAGAAGCTGCGATTGATCCACCCGCTGGATCACAACTACTACGAAGTGTGCCGCACCAAGCTGGGCTGGGGCAGC
CGCTTGGGGGGTGGAGGCGACTGATGCTCGATCCCGCGCGTAGCTACGACCTGATTGGTGACGTGCACGGTTGCGC
TCATACCCTTGAGCACCTGCTCGACCAGATGGGTTACCACAAGCAGGGCGGCACCTGGCGCCATCCTTCACGCATG
GCGGTGTTCCTCGGCGATATCATCGACCGTGGGCCACGGATCCGCGAGGCATTGCATATCGTCCATGACATGACCG
AAGCCGGCCAGGCGCTGTGCATCATGGGCAACCATGAGTTCAACGCCCTGGGCTGGAGCACCCTGGCGCCTCCCGG
CAGTGGCAAGCAGTACGTGCGCGAACATTCGCCACGCCACGCGCGGCTGATCCACGAGACCCTGACCCAGTTTGAA
CACCATCCTGGCGACTGGCATGACTTCCTGGCCTGGTTCTACGACATGCCGTTGTTTGTCGACGCCGGGCGCTTTC
GCGTGGTGCACGCCTGCTGGGATTCGGGGTTGATCGAGCCGCTGCGCAGGCAATTCCCCGACGGCTGCATTGACGA
GCACTTCCTGCAGGCGGCGGCGGTGCCTGGCAGTTTTGCCTGCAACGCGTTCGACCGCCTGCTGCGCGGCACCGAT
ATGCGCCTGCCGGACGGCCTGACGCTGACCGGTGGCGACGGCCTGACCCGTTCGTTCTTCCGCACCAAGTTCTGGG
AGGACGACCCGAAGACCTACGGCGATATTGTTTTCCAGCCTGACGCCTTGCCTGACCCGGTGGCGCGTACACCGTT
GTCCTCGACTGAAAAAAACTCCCTGCTGCGTTATGGCGTCGATGAGCCGCTGTTGTTCGTGGGGCATTACTGGCGC
AGCGGCAAACCGGCGCCGATCCGCTCGAACCTGGCCTGCCTGGACTACAGCGCGGTGCTCTACGGCAAGCTGGTGG
CCTATCGACTGGATCAAGAAACCCGCCTCGACCCGAACAAATTTGTCTGGGTCGATGTCGAGCGCCCTGAGGTGAT
TTCATGAGTACCGTTGCGGTGTTGCGCTTGCCCCTGAGCGTGGACCTGGGTGGCTTCGTCAAACTGCTGCAGCGCA
TGCAAGTGCCCCATCGCGTCAGCGAAGAGGCCGGCGAGCAGGTGCTCTGGGTGCCGGACGCGATCAGCGAGGACGT
ACGCAGCCTGTATCAACGCTTTCCTGCTGGCGACCCGGACCAGCAATTGGACCTTCCCCCGGAGCAGGTGGGCAAG
CGTCCCGGTTTCCTCCTGCAATTGCGCCACAGCCCACTGACCGCCACGGTGCTGCTGCTGAGCCTGATCGTCGGCG
CTGTCACGATGCTGGGTGACAATCTGCAAGCAATGAGCTGGTTGACGTTCCTCAATTTCCACGTGGCCGGCGAATA
CCTGTACTTCACCCCCCTGGCCGACAGCCTGGCGGCGGGGCAGTGGTGGCGGTTGGTAACGCCGATGCTGATCCAC
TTCGGCATCCTCCACCTGGCGATGAACGGCATGTGGTACTGGGAGCTGGGGCGGCGGATCGAGTCGCGCCAGGGCA
GTATCAACCTGCTGGGCCTGACGTTGCTGTTCAGCCTGGCCTCCAACTACGCGCAATACACCTTTGGCGGCCCCGG
CCTGTTTGGCGGTTTGTCCGGCGTGCTGTACGGCTTGTTGGGCCATTGCTGGATCTATCAGTTGCTCGCGCCCAAC
CCGGCGTATCGCCTGCCCCGTGGTGTGCTGGTGATGATGCTGGTGTGGCTGGTGCTGTGTTTGTCGGGCCTGGTGT
CGATGATCGGTTTCGGCGAAATCGCCAATGCGGCCCATGTCGGCGGCCTGGTGGTGGGTTGCATCACCGGTTTGCT
CGGCGGGTTGTACAGCCGCCATAAACGTAACGCTTGAATCTCTGCCCATTCTTTAAGGAAGAGTCTTATGTCCTCT
TTTGCTGAAATGATCGAAAACATCACTCCGGATATCTACCAGAGCCTCAAGCTCGCGGTAGAAATCGGCAAATGGT
CCGACGGCCGCAAGCTCACCGCCGAACAGCGTGAACTGTCCCTGCAGGCGATGATTGCCTGGGAGCTGCAAAACCT
GCCGGAAGACCAGCGCACCGGCTATATGGGCCCGCAGGAATGCCAGTCCAAATCGACCCCGGTGGAAAACATCCTG
TTCAAGTCGGATGCCATCCATTGATTGAGATTGGCCGTGGTGCAGTCAGCAAGATGTCGGCGCAACTGGGTTCGCC
GACCGTTCAATACGCTTTTCGCCTGGGTGACACCGAGGTGCCGGTCAACCCGTTGATTGGCACTCATATACGCCTG
GAATACCTCGGTGCGATCCACTGCACCCATTGCGGACGCAAGACCAAGACCAGTTTCAGCCAGGGCTATTGCTACC
CATGCATGACCAAGCTGGCCCAGTGCGACCTGTGCATCATGAGCCCTGAGCGCTGCCATTTCGATGCCGGCACTTG
CCGCGATCCGGCCTGGGGCGAAAAATTCTGCATGACCGACCATGTGGTGTATCTGGCGAATTCGTCGGGGATCAAG
GTCGGCATCACCCGCGCCACCCAGTTGCCCACACGCTGGCTGGACCAGGGGGCAAGCCAGGCGCTGCCGATCATGC
GCGTGGCTACACGCCAGCAATCAGGTTTTGTCGAGGATGTACTGCGCAGCCAGGTCGCCGACAAGACCAACTGGCG
CGCCTTGCTCAAGGGCGACGCGACCACGGTGGACCTCAAGCAGGTGCGCGACGAGCTGTTCGTCTCCTGCGCTGAC
GGCCTGCTGCAATTGCAGGAACGCTTTGGCCTGCAGGCTATCCAACCGGTGACTGATATCGAGCCCCTCGAAATCC
GCTATCCGGTGGAGCAATATCCGACCAAGATCGTCAGCTTCAACCTGGACAAGAACCCGATTGCCGAAGGCACGCT
GCTGGGGGTCAAGGGCCAATACCTGATCTTCGATACCGGCGTTATCAATATTCGTAAATACACGGCTTACCAGCTC
GCCGTGCATCAGTAGAAGGATTTCACGCATGCGCACCGAACAACCGAAGATGATCTACCTGAAGGACTATCAGGCG
CCCGAGTACCTGATCGACGAGACCCACCTGACCTTCGAGTTGTTCGAGGACCATAGCCTGGTCCACGCGCAGCTGG
TGATGCGCCGTAATCCCGAGCGCGGTGCCGGCCTGCCGCCACTGGTGTTGGACGGCCAGCAACTGGAGCTGTTGAG
CGTGAACCTGGGCGACCGGGAACTGAGCGCTGCCGACTACCAGTTGAGCGACAGCCACCTGACATTGCAGCCGCTC
AGCGACAGCTTCACTGTCGACACCAGCGTGCGGATCCACCCTGAAACCAACACCGCCCTGGAAGGCCTGTACAAGT
CCAGCGGCATGTTCTGCACCCAATGCGAGGCCGAGGGTTTTCGCAAGATCACCTACTACCTGGATCGCCCGGACGT
GATGAGCACGTTCACCACCACCGTGATCGCCGAGCAGCACAGCTATCCGGTGCTGCTGTCCAACGGCAACCCGATT

GCCAGCGGCCCCGGCGAAGACGGCCGGCACTGGGCAACCTGGGAAGACCCGTTCAAGAAACCGGCCTACCTGTTTG
CCCTGGTGGCCGGTGACCTGTGGTGTGTCGAAGACACCTTCACCACCATGAGCCAGCGCACCGTCGCGCTGCGCAT
CTATGTCGAGCCAGAAAACATCGACAAGTGCCAGCACGCCATGAACAGCCTGAAGAAGTCCATGCGCTGGGATGAA
GAGGTCTACGGTCGCGAATACGACCTGGATATCTTCATGATCGTGGCCGTGAACGACTTCAATATGGGCGCCATGG
AAAACAAGGGACTGAATATCTTCAACTCCAGTGCGGTGCTGGCCCGCGCCGAGACCGCGACCGACGCCGCGCATCA
GCGGGTCGAAGCAATCGTCGCCCACGAGTATTTCCACAACTGGTCGGGCAACCGCGTGACCTGCCGCGACTGGTTC
CAGCTGTCGCTTAAGGAAGGCTTCACGGTATTCCGCGACTCGGGCTTCTCTGCCGACATGAATTCGGCCACCGTCA
AGCGGATCCAGGATGTGGCCTACCTGCGCACTCACCAATTCGCTGAAGATGCAGGGCCAATGGCCCACGCCGTGCG
CCCGGACAGCTTTATCGAGATTTCCAACTTCTACACCCTGACCGTGTACGAAAAGGGTTCGGAAGTGGTCGGCATG
ATCCACACCTTGCTCGGCGCCGAAGGGTTCCGCAAAGGCAGCGACCTGTACTTTGAGCGCCACGACGGCCAGGCCG
TGACCTGCGACGACTTTATCAAGGCCATGGAAGACGCCAACGGCACCGACCTGACCCAGTTCAAGCGCTGGTACAG
CCAGGCTGGTACGCCACGCTTGGCGGTGAGCGAGTCCTACGACGCCGCAGCCAAGACCTACAGCCTGACCGTGCGC
CAGAGCTGCCCGCAAACCCCGGACAACGTGGAAAAGCTGCCATTCGTGATTCCCGTGGCTCTGGGCCTGCTGGACG
CCGATGGGGCTGATTTGCCGCTGCGCCTGGCCGGTGAAGCCGCCGCGCAGGGCACCTCGCGGGTGTTGTCGGTGAC
TGAGGCCGAGCAGACGTTCACCTTTGTCGATATCGCCGCACAGCCACTGCCGTCGCTGCTGCGTGGTTTCTCCGCG
CCGGTGAAGTTGAGCTTCCCCTACAGCCGTGACCAGTTGATGTTCCTGATGCAACACGACAGCGACGGTTTCAATC
GCTGGGAGGCGGGCCAGCAACTGGCAGTGCAGGTATTGCAGGACCTGATCGGCCAACACCAACAGGGCCAGGCCCT
GGTGCTGGATCAGCGGCTGGTCAATGCACTGCGCAGCGTACTGAGCGACGACAGCCTGGACCAGGCCATGGTCGCG
GAAATGTTGTCGTTGCCCGGTGAAGCCTACCTCACCGAAATCAGCGAAGTGGCAGACGTCGACGCGATCCACGCCG
CCCGCGAATTTGCGCGCAAGCAGTTGGCCGACCAACTGTTCGAAGGCCTGTGGCTGCGCTACCAGGCCAACCGCGA
TCTTTCGCGCAACACGCCGTACGTGGCGTCGGCCGAGCACTTTGCCCGCCGCGCCCTGCAGAACATCGCGCTGTCG
TACCTGATGCTCAGCGGCAAGCCCGAAGTGCTGGCCGCGACCCTGGAACAGTTCGACGCATGCGACAACATGACCG
AACGCCTGACTGCATTGGCGGTACTGGTCAACTCGCCGTTCGAGGCCGAGAAGGCCAAGGCCCTGCAAGTGTTTGC
CGAGAACTTCAAGGACAACCCACTGGTCATGGACCAATGGTTCAGTGTCCAGGCCGCCAGCGTATTGCCGGACGGG
CTTGCGCGGGTCAAGGCCTTGATGCAGCACCCGGCGTTCAATATCAAGAACCCGAACAAGGTACGCGCGCTGGTTG
GCGCCTTTGCCGGGCAAAACCTGATCAACTTCCACGCGGCCGATGGTTCGGGCTATCGCTTCCTGGCGGACCTGGT
CATCGAGCTGAATGCCTTCAACCCGCAGATCGCCTCGCGTCAGTTGGCGCCGCTGACCCGCTGGCGTAAATACGAC
AGCGCCCGCCAAGCGCTGATGAAGGCTGAACTGGAGCGCATCCGCGGTTCGGGCGAGCTGTCCAGCGATGTGTTCG
AGGTGGTCAGCAAAAGCCTTGCATAGGGTTTTCTGTAGTGAAGGTGCTTGTCTGTGGCGAGGGGGCTTGTCCCCCG
CTGGGCTGCAAAGCAGCCCCAAAACCTGCTGCTTCGGTGTGTCAGATAGAGCTCAGGAGCCTACTGGGGCTGCTTC
GCAGCCCAACGGGGGACAAGCCCCCTCGCCACAGATGAGCCATTCTGGCTATGGATCGGTATGATTTTTGATTCAC
CCTTGGCCGCGTACCAGCAGGCCCTGCATGAGGGTTTTGTCGCTGACGATGCCCAGCGCCATGCCGTGCAAATCTT
GCAGGATTGCCATGAGGCCTTGCATCAGGGCACCCATGGTCCCGTGACCGGGGTTTATCTCTGGGGCCCGGTGGGC
CGTGGCAAAACCTGGCTGATGGATCAGTTCTACCAGAGCCTGCGGGTGCCTGCGCGGCGCCAGCATTTTCATCACT
TCATGGGCTGGGTTCACCAGCGCTCGTTCCAGTTGACCGGCACGCCCGACCCTTTGCAGGCCCTGGCCCGTGAATT
GGCCGGGGAGGTGCGGGTGCTGTGTTTTGACGAACTGTTCGTCAATGACATCGGCGATGCGATCATCCTCGGGCGT
TTGTTCCAGGTGATGTTCGACGAAGGGGTGGTGATGGTCTGCACCTCCAATCAACCGCCAGAGCAGCTCTACGCTG
ACGGTTTCAACCGGGACCGCTTCATGCCGGCCATCGAGGCTATCAAGGCCCATATGCAGGTGGTGGCGGTAGCGGG
CAGTGAAGATCACCGCCTGCATCCCGGCACCGCTGAGCAGCGTTACTGGGTCGATGAGCCACAGGCACTGGCACAG
GTATTTGAGCGATTGAGCGCGGGACAAGCCTGCAGCGATGCGCCGGTGGTGGTGGGCTCGCGCAGCATTGCCACGG
TGAAATCCTGTGCAACGGTGCTGTGGTGCCGTTATGCCGACTTGTGCGAGCAACCGTTGGCGGCCATGGATTTCAT
GCAACTGTGCGATCGTTACAGCGCCATATTGCTCGGCGAGGTCCCCTGCTTGAGTGCTCAGCAGCGTCCCGGACGG
ATTGCCCGCGGCACCGAGGACGGCGCCCAGCGTGTGGAGGCGGGGGATCGTGAGTTGCCGCAATTGTCTGTGCACG
ATGACGGCGTGCGACGCTTTATTGCCCTGGTCGATGAGTGCTACGACCGCAAGGTGCCGTTATACATCGAAGCCAA
AGTGCCCTTGGTGTCGCTTTACACAGAGGGTTACCTGGAATTTGCGTTCCGACGAACGTTCAGTCGCTTACAAGAA
ATGCAGCTACAGCGCTTCGGCCGATAATGGCCTTGAGCCACTTTTGTTCGACAGGAGTCGTGACAGCGCTTTCCTG
CACAAACCTGTAGTTAATCTCCTTCATCTATGGCCGACAGCCCGCTTACAATCGAGGTTTTGCGGGGAGGTGATCC
CTGCCTAACTGTTATCGAGGAAGGAAATGGAAGCCGCAGAAATACTGGTGTTGCAAGCCAGCTACAGCAATCCTGT
ACAGGCCGAGGCCATTGGCCTGCTGCTCAATCACTACGCGCAAGACCCGATGGGCGGAGGCAAAGCCCTGTCCGCC
GATCTGCTGGAGCAACTGCCGGCTGAATTGGCCAAGCGTGCCCACGCATTCAGCGTGCTGGCCTTTGTGGGTGGCG
AGCCGGCCGGGCTTGTGAACTGCTTTGAGGGGTTTTCGACCTTCGCCTGTCGCCCGCTGGTCAACGTGCATGACGT
GGTCGTCATGGAGCAGTTCCGTGGCCTGGGCCTGAGCCAGAAAATGCTGCAAAAGGTCGAGGAAATCGCCCGTCAA
CGCGGCTGCTGCAAGATCACCCTCGAAGTGCTGGAAGGCAATCCGGTGGCTCAGGCGTCGTACCGCAAGTTTGGGT
TTGATGACTCGGTGTTCGACCCCGCCCATGGCCGCATGCTGTTCTGGAGCAAGGTGCTTTAAGGCCGCGTCGGTGG
TAAGGGGCTTATCCCGTCGGGTTGCGAATGCCCCTCACCACAGACAGCCTTGATGAACGTCTCCATCAGAGCGTAA
GTGGCGCGTCAGCTCTTGGGTTGTTGACTATCCCGAGCCTGCTCGGCAGAGCCGCCTTCAGCGCCCTTGGCATCGG
TCGCGGCAGTCTGCTCAGCATCGCTGTGGATTTGTTTCTGGGTGAAGCTGAAGTTGTCGAAGTGCTCCTTCACGCG

TTCCGCGCCACCTTCGGCGAAGGCTCCGGCCGAGGTCAAGCCCAGTACGCCGGCGAGCATCAAGGTTGAAAGTTTC
ATTGCATGTATTCCCATTAAGAGTGATCGGTATCTGCACCGTCCCTGATCAGATTTATGGGAATAGTCTGTAGGAA
GCTTATTAAATGAGAATTAACGTCAAGAACGTCCAAAGTTAATCACCCGGTTAACTTTGGACGGGGCGGGGGCGTT
TACTTCAGCACCACATTCCTGGGGTCGGCGCTGACCTTGGGCTTGATCAGGCTGAAGTCGATCAAGGCCTTCTGCG
AACGCTGGTAGGGATCGCCAATCAGCAATGGCCGCGGGGTGAAGCTATCGCTGACCAAGCTGCGGCTGCGGTCCAG
TTCGTCGAAGCTCAAGCCGGCAAGGTCGGCCCAGGTATGAATCAACTGCGAGCTGCTGTACGGCCGTTGTAAATCG
CCTCGCATATTCCAGTCGTGGCTGGCGCGCCATTTCGGCGAGGCCCAGGTCATGAAAGGAATGGTGTACATCGGCG
CCGTCGGCTTGCCCTCGTTGCGGCCCAGGGTGTCGTGGCCCGCCGAGTCGAACACATCCTCGCCGTGGTCGGAGAG
GTACAGCAGGAAACCGTTGGGATCGGTCTTGGCGTAGTCCTTGATCAGGCTCGACACCACGAAGTCGTTGTAGCGC
ACCGCGTTGTCGTAGCTGTTGTAAGTCGGCAACTGGTCGTCGCGCACGCCATCCGGGACGCCCTGGCGGTCGGTAA
AGGTGTCGAAGCTCGGCGGGTAGCGGTACTGATAGCTCATGTGGGTACCCAGCAGGTGCACCACGATCAGCTTGCG
CTCGGCCTTGTCGGCGAGGGCCTTGGAGAACGGTTCCAGCACGTCGCCATCGTACTGGCGGGCGTTCTGGTTGCGG
TTGTTATTGAGGTATACCTGTTCGTCGGCCTGTTCCGAGAACGTGGTGAGCATGGTGTTGCGCTTGGTCATGGTCT
GCTGGTTGGTGATCCAGAAGGTCTTGTAGCCGGCCTGTTTCATCATGCTGACGATCGATGGGGTCTTGAGGTACAG
GTCCGGGTGGTCTTCGTCGGCAAAGGTCAACACCTGTTGCAGCGCCTCGATGGTGTAGGGGCGCGGGGTGATGACG
TTGTCGAACACGTCCAGTTGGTCACGCAGCTTGTCCAGTTCCGGGGTGGTCTTGCGTGGGTAGCCGTAGAGGCTCA
TGCGCTGGCGGTTGGTGGACTCGCCGATCACCAGCACCAGGGTCGATGGCTGGCCGGCCATGCTGTCTTTGAAGTT
GCTCAGGGGCGGGATCTTGCTGACGTTGTCGAGCATGCCCTGCATGTTGTTCAACTGCTGGGTATAGCGACGATAG
GCGACGATCATCTGCCACGGCACCGCAGGTTCGACACGGGTTTCGAAACCATCGATGGCTCGTTCCAGGGTGTCGT
TGGTGGCGATCTGCTTGATCAACGGATAACCGACAATCGCCAGCACAATGGCGGTGGCCGCGACCATCGCCCGTGC
CTTGGGCAGATAGACCGGGCGCACGCGGGTCCAGAGGAAGATGGCCAAGGCCGTGTGGGCAATAAAGGCCAGCACG
ATCCACCAGGCAAAATATTGGGTTGCGTACTCGCCCGCTTCAGAGATGTTCGACTCGAACATGATGAAGATGACGC
TCTGGGAAAATTCCTGTTGGTAAATAAAGAAATAGCCCAGGCTGGCCATGGAGCAGGCCCACAACACCACGCCGAT
CAGCGCCGCCAGCAGCCTGGCGCGACGCGGGAACAGCAGCATCGGCGCCAACCACAGTGCACTCATGAAGAACGCC
TGGCGGAAACCACTGAAGCCGGAAGTACCGGTCAATTGGATCAGCAGCTGGGTAATGCCCGAGAAATACCAGAAAA
ACAGGAACATCCAGCCAAGGCCGGCCCAATCAAACCCTGTCGCAGACTTTGTGCTGCGTTTGAACATCGCCATGCA
GCGCTCCAACCCGATAATTGCCACCGCCCATGGGTGAGGCGGCGTTCTGGTGGAGGATTATCGGGAAGGGCGGGTG
AAAACTTCGTGAGTTTGATCCGAGATGGCGACCCCTGTAGGAACCGGCTTGCCGGCAAGTCGGCCCCTACAAAAGG
TCAGCAAGTGATGGCTTGATTCGTGAGCTGGGCCTGCAATTGCGCACGCAGGCGGATCACTTCCTGCTGCAACTGT
TCACGTTCGTCTTTCAACTGGCGCAACTCGTCGCGGCGGACGGTGACGTAAAGGGTTTGTGGCGGGCGTGTGATCT
GTGCAGTGCGCATTGCTTACCTCGCAAATAGCCGGTGTATCGCGGTGCCAGGGGGCTTGAGTCGAGGCCCTCGGCA
GGAGTCAGGGGCGATTCTGATTTCTTTTTGGCTCAAGGGAACTTTTTTATTTTTCGGGGTCGTTGTGACTTTTCA
GCCGGTGATGGTGAAACGATGGCCTGTTTTTGTGATGAAACTAGTGCGATTGGCGCCGGACTAATTCGTCATTGCG
CTATAAACTGTGTCACACATTTTTTATTTGTAGTTGGGAGCATCAATACATGCAACTGGGGATTATCGGACTAGGC
CGCATGGGCGGGAATATTGCGCGCCGCTTGATGCTCAATGGGCATACCACCGTGGTCTACGACCGCAATGAAGCAT
TCGTCAAAGGCTTGAGTGAAGAGGGCGCCACTGGCGTTGCCGATCTCGCCGCCCTGGTCGCGGGCCTGCAAAAACC
GCGGGCTGTCTGGGTCATGCTGCCGGCGGGCGAACCCACCGAAACCACGATCAACGACCTGAGCCAACTGATGGAA
CCCGGCGATGTGATCATCGACGGCGGCAACACTTTCTATAAGGATGACGTGCGTCGCGGCAAGGCTCTGGCGGAAA
AAGGCCTGCACTACATCGACGTCGGCACTTCCGGTGGCGTCTGGGGCCTGGAGCGTGGCTACTGCATGATGATTGG
CGGCGACGCCGAGACCGTCAAGCACTTGGACCCGATCTTCGCCACCCTGGCGCCAGGCCTGGGCGATATCCCGCGG
ACCAAGGACCGTACCGCCACCGATGACCGTGCCGAACGTGGTTACATCCATGCTGGCCCTGCCGGTTCCGGGCACT
TTGTGAAGATGATCCACAACGGCATCGAGTACGGGATGATGCAGGCCTTTGCCGAAGGGTTTGACATCCTCAAGAC
CAAGAACTCGGAAAACCTGCCGCCAGAACAGCGCTTCGATCTGAACGTGGCCGACATCGCCGAAGTCTGGCGTCGC
GGCAGCGTCGTATCTTCCTGGCTGCTGGACCTGACCGCCGATGCCCTGGCGACCGACCCCAAGCTCGACGGTTACT
CCGGTTCCGTGGCCGACAGCGGCGAAGGGCGCTGGACCATCGAAGCGGCCATGGAGCAATCGGTGCCGGTGCCGGT
GCTGTCCAACTCGCTGTTCGCGCGGTTCCGCTCGCGCCAGCAAAGCACTTACGGTGACAAGCTGCTGTCGGCTATG
CGCTTCGGCTTCGGCGGCCATGTGGAGACTTCCAAAAAATGACGGCCAACGGCAAGAAACCCAAGGCCGAACCCGC
TCCACCCACTACCTTGTTCCTGTTTGGCGCCCATGGCGACCTGGTCAAGCGCCTGCTCATGCCGGCGCTGTACAAC
CTCAGTCGCGATGGTCTGCTGGGTGAGGGCCTGCGGATTGTCGGGGTGGATCACAACGCTATCAGCGATGTCGACT
TCGCCAAGAAACTCGAAGACTTCATCCGCACCGAAGCCGCGAGCAAGGTCAAGGGCAATGCCGAACATGCACTGGA
CCCACAGTTGTGGGCACAGTTGGCCAAGGGCATCAGCTACGTGCAGGGCGATTTCCTCGATGATGGCACCTATGCC
GACATCGCCAGCAAAATCGCTGACAGCGGCACTGGCAACGCGGTGTTCTACCTGGCCACCGCGCCGCGCTTCTTCA
GTGAAGTGGTGCAGCGCCTGGGCCGGGCCAACCTGCTGGAGGAGAGCCCCGAGGCTTTCCGCCGCGTGGTGATCGA
AAAGCCGTTCGGCTCCGACCTGGCCACCGCCGAGGCGTTGAACGCCTGCCTGCTCAAGGTCATGAGCGAAAAGCAG
ATCTACCGCATCGACCATTACCTGGGCAAGGAAACGGTACAGAACATCTTGATCAGCCGTTTCTCCAACGTGCTGT
TCGAAGCGTTCTGGAACAACCATTACATTGACCACGTGCAGATCACCGCTGCGGAAACCGTCGGGGTCGAAACCCG
GGGCAGTTTCTTCGAAAACACGGGCACCCTGCGCGATATGGTGCCCAACCACCTGTTCCAACTGCTGGCGATGATT

GCCATGGAACCGCCGGCGGCGTTTGGCGCCGACGCGGTACGTGGCGAGAAGGCCAAGGTGATTGGCGCTGTGCGCC
CCTGGTCGCTGGAAGATGCGCGGGCCAACTCGGTGCGCGGCCAATACACTGCCGGCGAGATCGGTGGCAAGGCGCT
GCCGGGCTATCGGCAGGAGGCCAATGTGGCCCCGGACAGCAGCACCGAGACCTTTGTGGCACTGAAGGTGATGATC
GATAACTGGCGTTGGGTCGGCGTGCCGTTCTACCTGCGCACCGGCAAGCGCATGAGCGTGCGCGACACCGAGATTG
TCATCTGCTTCAAGCCGGCGCCGTATGCGCAGTTCCGCGACACCGAAGTCGACGAACTCAAGCCGACGTACCTGAA
GATCCAGATCCAGCCCAATGAAGGCATGTGGTTCGACCTGCTGGCGAAAAAGCCCGGGCCGACCCTGGACATGGCC
AACATCCAACTGGGCTTTGCCTACAAGGACTTCTTCGAGATGCAGCCGTCCACCGGCTACGAAACCCTGATCTACG
ACTGCCTGACCGGTGACCAGACGCTGTTCCAGCGCGCGGACAACATCGAAAACGGCTGGCGTGCGGTACAGCCGTT
TCTCGATGCGTGGAAAGAAGACGACGGGATCCAGGCCTACAAGGCTGGCGAAGATGGCCCGGCGGCGGCAGATGCG
TTGCTGGCCCGTGATGGCCGCGCTTGGCATAGCCTCGGATGAGTGCAGACGCGATTCAACCCATCCGTTTTTTGCT
GAGTGATATGGACGGCACTTTGTTGTTGCCCGATCACAGCCTGAGCCAACGCACCATCGAAGCCGTAAGGGCGCTG
CGCGAGGCAGGGGTGTTTTTCAGCCTCGCCACCGGGCGGCCGCCCAGGGCCATGTTGCAGCAGATCGAAGCGCTGG
GCGTCGACGTGCCCACCGCGGGGTTCAACGGTGGCACCCTGGTCAACCCGGATGGCAGCATCCTGGTTGCCCACCA
TTTGCCGGCTGAGGCGGCGCTGATCACCCTGGCGCTGCTGTGCGAGCAGCCAGAGGTGGAAGTCTGGGTGTTTGCC
GAAGGCGACTGGCTGCTGCGCGATCCCACCGGCCCCATGGTGCCTCGGGAGCAGCAGGGCCTGGGATATGCGCCGG
TGGTGGTGGAGAGTTTCGAGCCGGTCCTGGGGCGGATCGACAAGATCGTCGCCGCCAGCGCCAACGCCCAGTTGTT
GATTGACCTGGAGGTGCAGTTGCAGCCCAAGGTCGCAGGCCTGGCCCAGGTATCGCGTTCGCAACCGGTGTACCTC
GATATCACGGCCATGCAGGCCAACAAGGGTGATGCCCTGAAAACCCTGGCCGCTCATCTTGGCGTGGCGCTGGAGC
AGACAGCGGCCATCGGCGACGGCGGCAATGACCCGGCGATGTTTCAGGTGGCCGGGTTATCGATTGCCATGGGCCA
GGCCGAAGAGGCCGTCAAGCGCCAGGCCGATGTCGTCACCGGCAGCAATCTCGAGGACGGCGCAGCCCAGGCTATT
GAACGGTTTATCCTTCACAGCCAATAGCTGACCGCGTACCAGCCCAGCACGCCCATCACCACGGTGTACGGTAGCG
CCATCCACACCATCCGCCCGTAGGACAGGCGAATCAGCGGCGCAATCGCAGAGGTCAGCAAAAACAGGAAAGCTGC
CTGGCCGTTGGGCGTCGCTACGCTGGGCAGGTTGGTGCCGGTATTGATCGCAATGGCCAGGGTCTCGAAATGCTCG
CGGCTCATATGGCCGGACACAAACGCCTGCTTGACCTCGGTGATGTAGATCGTCGCCACAAACACGTTATCGCTGA
TGGCGGATAACAGGCCGTTGGCAATAAACAGCATGCCCGGCTGTTGTTCGGCGGGCAGTGCCAGCACCCATTGGAT
CAATGGGGTGAACAGCTGTTGATCATGAATCACTGCGACCACGGCGAAAAACACCACCAGCAACGCGGTAAAGGGC
AGGGCGTCCTTGAAGGCATTGCCGATGCGGTGTTCTTCGGTAATCCCGGTGAAGGCAGTGATCAGTACGATCACCA
GCAAACCGACCAGGCCCACTTCGGCGATATGAAACGCCAGGCCGGCGATCAGGATCAGCGCTGCCAGGCCTTGAAC
AATCAGCGCCGCACGTTGGCGCGGGGTGCGTTCGGCGTTGTCTTCACGGGCGTAGTTGGCCAGCACTTGGCGCACG
TTGTCCGGCAGCAGGGTGCCGTAGCCAAACCAGCGCAGCTTTTCCAGCAGCACGCAGGTCATCAGGCCGGCGACCA
GCACCGGCAGCGATACCGGGGCAATTCGCTGGAAGAACTCAGTGAAATTCCAGCCCATCTCATGGCCGATCAACAG
GTTCTGTGGTTCTCCTACCAGCGTGCACACGCCACCCAGGGCGGTGCCGACGGCGCCGTGCATCAACAGGCTGCGC
AGGAAAGCGCGAAATTGCTCAAGGTCGGCGTGGTGCAAGGTGGGCAGGTGGCGGTCGTCGCTGTATTCGCTGTCCT
GGCGTGGATCATTGCCTGAGGCTACGCGGTGATACACCGAATAGAAGCCCACGGCCGCACTGATAATGACCGCAGT
GACCGTCAAGGCATCAAGAAAGGCTGAAAGAAAGGCCGACAAAAAGCAGAACATCAGCCCCAGCAGCGCTTTTGAG
CGCACGCCCAGCAGCAGGCGCGAAAACAGGAACAGCAGCAAATCCTTCATAAAGTAGATGCCGGCCACCATAAACA
TCAGCAGCAGGATCACCGGGAAGTTATGCAGCAGCTCGTCGTACAGTGCCTGGGGCGTGGTCATGCGCAGCAACAG
TGCCTCGACCATCAACAGCCCACCGGGCATCAACGGATAGCAGTGCAGCGCCATGGCCAGGGTGAAAATGAACTCG
GCCACCAGCAACCAGCCAGCCGCAATCGGGCCAAAAGCCCAGAGCACCAGTGGGTTGATGATGAGGAAAGCGAGGA
TGCTGGCCTTGTACCAACGGGGCGAGTGCCCCAGGAAGTTATGGGCGAAGGCCTGGGCCATTGAACCGGACATGGG
GTGCTCCTGGGGGCGAGAAGCGCGCAAAGTGCCCGATGCTCGCAGGAAGATCAAGCGGTCATGGCATGGCAGCCAT
ATAAAGAAGGGAGATACTTAAATGTCAGGCACGCAAAACCCGTGGCGGGAGTCGAACTCACGCGCCACGGGTTTAA
TAGTCTTAACAAGTTGCAGCCAATATAAGAAGCGACGGCATGTCGCAGTGGCTGCCTTAACTTACTTGGGCATTGC
CCAGGACTGTAGTTGATAACCTTCGCCATTGAGTTCGGCGCGGGCTTGTAGCAGCAGGGTTTCCAATTGTTTTGGA
TCGGAGTAAGTGCTGCACGGAATCTGTTTGCGCCCGATGCGCTTCTCGGTGCGGTCGATCACGGTCAGGCTCAGTT
CGCCAGTGCCGTCTTGTGGGGCCCAGGCCACGCAGTGAAAGGGTTGGAAAGCACGGTCTGCGATCAAAAGAGCTTC
GTTGATACGGAGCGGGGCGTTCATGGGGTCTTCTCTCTAAGTGCCCAATCAGAAGATGTGCCGTCGGTTGGGCTTA
CCGTTCGGCTAGTACCTGTACTGATGCACGATGACGGGGTACGGGTCACATGTTAGAACAAGAAAATTCAACTTTT
CGTGATTAACGTCATGTAAGGCGCGATTTTGAAAGGTGAATGAAAGATTGAAGTAGCGCTTGCCGAGGGCCTGATC
GCTAAACGCTGTATCGGTGATGGTTTTATCGCGAAACGATACAAGGCTAGCGTCAAGATCTTGCTAGGGTTATGAG
CAGCCTGCCCAAACTATTGTTTCTAAATAACTTTAATGATTTTGACGCCAAGGAATAGTCATGGTTGTTACACCAG
CCCTGCGTCGCTTGCTCGTGGTCGATCCCTGTGACGACTGCCACCAGTTATTGCCTGGTTTGCGGGCGGTTGGCTG
GGATGTAGACAGTTGCGCCCTGGATGCGTTGGGGGATCGTACCTGTGATGTGGGGTTGTTGCGGCTCCAGCCCATG
CACTTGGAGCGACCCGAGGCGGTCAAGGAGCTGATCAGCCGCAGCGGTACCGAATGGATCGCCGTGCTCAGCCCAG
AAATGCTGCGCCTGCAGAACGTCGGCGACTTTGTCTGCGAATGGTTTTTTGACTTCCACACCTTGCCCTTCGATGT
ATCACGGGTCCAAGTGACCCTGGGCCGCGCCTTTGGCATGGCACGCCTGCGGGGTAAAGGCGCCGCTCATGTGAAT
GAACTGGAACATGAATTGTTGGGCGACAGCCGGCCAATCCGGGAGCTGCGCAAACTATTGGCCAAGCTGGCCCCCA

CCGAATCGCCGATTCTGATCCGTGGCGACAGCGGCACCGGCAAGGAACTGGTGGCCAAGACCCTGCACCGCCAATC
CCAGCGCCACGCCAAGCCGTTTGTGGCGATCAATTGTGGGGCGATCCCAGAGCACTTGATCCAATCCGAACTGTTT
GGCCATGAAAAGGGCGCATTCACCGGCGCCCATCAACGCAAGATCGGGCGTATCGAAGCGGCCAACGGCGGCACCC
TGTTCCTCGATGAAATCGGCGACTTGCCCATGGAGTTGCAGGCCAACCTGTTGCGCTTTCTCCAGGAAAAACACAT
TGAGCGCGTAGGGGGTAGCCAACCGATCCCGGTAGATGTGCGGGTGCTGGCCGCGACTCACGTCGACCTTGAAGCC
GCCATCGAGAGGGGGACTTTTCGCGAGGACTTGTACTACCGGCTCAATGTCCTGCAAGTGATCACCGCGCCGTTAC
GTGAGCGGCATGGCGATGTCGGCATGCTGGCCAACCATTTCTCCAGGTTCTACAGCCAGGAAACCGGGCGCCGTCC
ACGCAGCTTCAGCGAAGACGCCCTGATCGCCATGGGCAAGCATGGTTGGCCGGGCAATGTGCGTGAGTTGGCCAAC
CGAGTGCGGCGTGGCCTGGTACTGGCCGAAGGGCGGCAAATCGAAGCAGCCGACCTGGGCTTGCAAAGCCAGCAGG
CAATCGCGCTGCCCATGGCTACGCTTGAGGACTACAAGCACCGCGCTGAACGCCAGGCGCTGTGTGATGTGTTGAA
CCGGCACAGTGACAACCTGAGTGTCGCCGCCCGTGTGCTGGGGGTTTCCCGGCCGACGTTCTACCGGTTGCTGCAC
AAACATCAGATCCGCTAGGGCGGGTAAAAAAGCCCCGTCGCGACCCTTATCGCGACGGGGCTTTTCCTTTTCTCCG
TTGTAGGAGCGGCGGTTTTAGAAGTAGTACGGGAATTTCAGGCTGAAGGTGAAGTCCGGTGCGTCATCGGTCATAC
CAATAGCCAGGTTGGGCACGATGGTCAGGTTCTCACTGGCGGCGATGGTCATGCCGACGTTGAAATAGCCGGCGTT
GGCGTCACTTGAGACAATCGACTGCCAGTCCCCGCCATCAGGCTTGAGCTTGCTCTTGCGCTGGATCAGGTCGGAC
ACCGAGAACGACATACTCATGCGCTCGTTCAAGGCAAATGCCACACCCACGCCGAACTGGAAGCTGTCGCCCAGGC
GGACCTTACCCCCGACCTTCTGGTTGACGTCGCTGCTGATGTCATCAAATGAGTCTTCGAGGTTGTGGGTATAGGA
CACCGAACCAAACAGCACGGCCGGGTCGAAGGTCTTGACCAGGGAAATCCCCGGCGTGATCGACCACACACCGTTG
CCGGTGGGCAGGTTCTCCGGCACGTACAGGTTGTCGTTGGCGGTCGAACGCACCAACTTGATCCCGAACGGCTCCT
TGCCGGTTGGCGCCTTGACCCGCACCGATACCACGGCATCGGGCATGGTTGGCGATTCGTCGAGGAACTTGTAGGC
GATCCCGAAGTTGACGTCGCCGATGGTCGGGTCCCGGCTCACGGACTCTTCGGAAGTGGCCTGCGGATCGCCGCCA
CTGGCGCCACCCGATTGATAGGTGGACTCGCGGTACACCACCGGGACGTTGACGTCGAACTGCCAACGGTTATCGA
GGTTGTAGCGGCCGGTCAAGTCCAGGGTCCAGTTATCGGCCTTGATCCGGTCGAGGTTGATATTGCCGAGAAAGAT
CGAATCCAGTGCGAGGAAACCATTGAGGGTCAACTGGCGGGCGTCGTAACGCGCGTAAGTAATGCCGGTTTCAAAA
CTGAACTTGCCGTTGCCGAAGAAGCCGCTGGCTTCGTTATACAAGTTACTGACACTTTGCGCGGGCGCCGAATCAT
CCTTGAGTGATTGCCCGTAGGAGCTGCCACCCGTGGCCCCCCCCGTACCGCCGCTGGCGGCCACGGTGCTGCCGCT
TTGCTTGAAGTCTGCCGGCGACTTGGCCAGGCGCTTGGGTGCCGGTACAGCAGGCTGGTCTTCCACCTGGCGTACC
CGTTGTTCGAGGACTGCCAGGGCTTTTTGTTGGACTTCGTAGCGCTGCTTGAGCTCCAGCAGTTCTTGTTTGAGGG
TTTCGATATCCGCGTCTGGCGCTGCGTACAACATACTCGCGGGCAAGAGCGTACTTAAACAGACAACGGCACGTAG
TGATAACGATCGGTGCATGAAGTAAGCCGTCCTTTTCTAATGCGTAAGTGTCGAGGGTCAGCGTAGTTCAATAACC
GAGTGTGCGTAGGCCCTTGAGTTGATCCAGATTGCAGTTCAGAGCCCCGGCGCTCAGGCCATTGCTGCCAAGCGCG
ACATTGAGCTGGGTCATGTTGTTGACGAAGTTGCTGTTGCCTTGCAACACCGTGCCTTGCAGCACGTTGCCGCCGC
CGATCTGCTGCAGCGCGTTGCCCTGGTTGTTGTGGGCCGCGATGGCCATCTGCACGCCGCCATTGTTGGCCGACAC
ACTGACCCGTCCGGCTGCACTGTCTGTTGTAAAGGAGCCGCCCGCCATCAACGCCTGCCCTGATTGCGCCACGTTG
GCCGGTGACAGGCCGTTTTCACTGACATTGATCCCGACGTTGTTGTACGCCGTGTTGCCATCGCCGGCGGCACGTA
CGGTCTGGGTCACACCCTGGCCGCTGCCCAGCCCGGCGCCACCGACCACGGTGCCGGTGCCCGGGTCGCGTTGGGT
GCCATTACCCACCGACCCGCTGGATTGCACATAGAATTGCGGAGTGATCGTGGCTGAGTCGATATGCATGTTGGCG
CTCACCGTACTGGTGTCGCCCGCAGCATTGGTCCAGGTGGTGCTCATGACAATGCCGAAACTGATAATGCGCCCCG
GCATCACGAAGCGGCCGCGCAGTTCAGCCAGTTCCGCGTCGTTCATTTCAATGGGCTTGAACGCCGAGGACGCATA
AGCAGGCATCGAGGCTGCCAGGCACAGGGCCGTGAGCCAACGGGAAGTGTTCATCATCTGCTCCTGGGGCGTCCTT
CCCCTTATGGCGCTTCTAGAAGAAGTCGCTCTGGATGAAACCGAAGTCCATCAGCTCGGCGTCGCCCACGGGCCGG
AACTCGTTCATTTTGGTCTTGGCGGTCAACGGCGTAGGGGGATCGAGCAGGGCGTTGGTCTTGTCATAGCCTTCAC
CCAGTACGGCGAACACGATGCCATTCCAGCCTGCGACGAAGTCATCCCGCGAATAGCGCTTGTTGCCCAGGACCGG
ATCGCCGATATAGACCCAGTCTTTTTCGGCCCGCTGCATCACCACGAAGTGCTTGTAGCCACGGATCTCCAACAGC
ACCACTACCGGAATCTTTACCGTCAGCAGAGTGTCGGGGCCGATCTTGTAGCCCCTGGCCCGCATGCCGATGCTTT
CCAGGTAGCGCTTCATGTCCAGCATGGAAAAGCCCTGGGTGCGCACCAGATCCTGGTCAGCGTTGATCAGCATGCC
CTTGATGATGTGTGCTTCATCGACGTCCAGCCAATAGGCCTGGCGCAGGATGGTGGCGAGTGCCGCGGCACCGCAG
CTGAAATCGGTTTTCTGTTCCACCAGGTTGGCGAACCGGCGTTCCCGGATGCTCTCGACCTTCTTGAAGATCACTG
CACCGCCGGGCATGGCGGAAATCGCCATTTGCCCTGCCCAGGACGGGCCGGTGAGTAAAAACAAGAGGCAGAGGGT
CGCGAGGCGCATGATCATGACCTGGTGGACACTGGAAATAAAAAGGGCCTTGTTGCCAAGGCCCCGTTGCATCAGA
ACGCCAGTTTGTTGCTGGTGCCTCCTGCGCAGACGGTGCAGCCGCTGCCCAGGGTGAGGGTGCTGCTTTGTTGGTT
GCCTGAACCTGCCGCCACGTTCACGCCGATGTTGCCGGACGCGTTGTTGGCGGAGTTGAGCAGGCCAGCGTTGTTG
ACCACGTACTGAGGTTGATAGCTGTGTTTGGAGGACGCTTCGTTGAGGAAGTGGTTATCCAGGGACACTTGTTCGG
TGTTCGAGGCAGACGCGACGTTTTTGCCGTCAGCTGTCACGATTGCCAGGTTGTTTTTCTGCTGGTTGCCTTGACC
GGCTGCCACGTTCACGCCCACGTTACCGGAGCTGTTGTTGGCCGAGTTGACCAGCGAAGCGTTGTTCTGCGTGCCT
TTGTTAATGAACTTGTTGTCTTTGCTTTCTTGATAGATATCAGCTGCGGCGAAGACACTGGCCGCATCATCTGCGC
TACTGGTGATGGCAGCACCATTGTCTTGCTGGTTGGCATCACCGGCTGCGATGTTGGCACCCAGGTTACCTTTGCT

GCTGCTCAACGAGCCGTTGGCCCCTGCATTATTGAGGGTCTTGGTATTGTCGAACTTGTTGTCCTTGCTCACTTGC
GTGTCAGTCACAACGGCAGCCACGGTGCCGGTTGGAACTGGGGTTGGTTTCCATTTTTCGGCAGGACTTGCCTGTG
CAGCAACAGCCATGAGCGCAGCCAGAGCGAAAACCAGTGGTTTGAGAGCCATTGTAGGTTTCATGGTGTATCTCCT
TGCTTCTATTAGTTGGTTAAGTGTTGGTACGGTCTAACAAACACCTATCAAGCTGTTACTTGATAGTCACACCGAG
GGTGTTAGCCATCCGGTTCCCCACCCCTGCACTCTGATTCACCTGTACCACTCCACGGCTGCCGGTGAAGGCCTGG
TCGCTGGTAACGACCTGGCGGCTGCCAGTGGTAGAGGGGGATCGTGAGTCTGGTAACAGCGTCATGTTCTGTTGCG
ACAGGACGCTGTCATCGATGCTCTGCGGACCTGTGCTGATCCGCACCGCATTGATCATCTGGTTATTGGCCCCGGC
CGACTGGTTGACACCCAGGATGCCGTTGCCATTGCTGAATGAGTTGCCCTGGATCGCCGCGCTGGCGTTAAGGGCG
GGATCAACCTTGCCGTCGAGTTTCTGGACAAGCGCGTCGCTGGCCTGGCCACCGAGGCTGATCGCGCGGCTGTTGA
TCTGCTGTTGTTGATTGCCGGCGGCCTGGTTGACTGACAGCACACCGGTGTATTGCTTGCCGGCGTTGTCGATCAG
CGCCTGGTGGCTGCTATCGGCCATGGCCGCCGAACAGCCGATTACGGTGAGGAACAGCCACGAACGGTTCATTGCT
GGACCCCCATCATCGCGCCGATGTTGTTCAGCGGCGCCAGGCCGCGCTCGATCGAGCCGCTGATCTGCCCGCCCAG
CGTGTTGCCGCTGGCATGTCCGGCCGCCGCACCGGCGCCCAGGCCAGCGGAGTTGCTGCCCAGGGTATTGCTCAAG
CCGGGAAGGTCGCCGTTAGGCAGGATGGCGCGGGTGATGCTCGAGCCGCTGCTGACCCCCGCAAAATCGTTGTCGC
TGAGTTCGCCGCCGGTAGCGCGCAGGATCTGTTGGCTGGGGTTGGCGTTGGCGGTGGATGGGTACGGGTCTTTGCC
AAAGGCGGGCCGGCCGACCATAAACCCTTGCACATTGCGGCCCGTCACGATGATGCCGTCCTCGGCGCAGGCGGGC
AGGCTCACGCTGGCGCTGAGTACACAGCTGATCAGCAGCAGGCCTATGGAGCCTTGATTGAGAGTTGCCACGGCAT
CATTCCTTGTTTTGTGACGCTGACCCTAAACAGCGCTGTAAGGAATAGAGCAGAAGCTGTGCCGCTTTTTATATGT
TCCAGCTTTTCAATGGCTTGGGATTTATGGCGGGGTGGAGGTGGCGTGGGGTGTATCAGGCATGAAACAGGATGCC
GGCGGATGACCAGCGCCTTGCAGGGGGAAGGGGGTGTTTCAGCAAAGCAACACTATCGATGACGAATCGATGAATC
CGCAGCCACAGGGCGGTTGCGCGGCGTTGAGTGTTTCAAAAGTGGACACAGCGGCGCGAGCAACACCCTGGGCAGA
GCGTGCTGCTCAGATCTTCGGTGCTTTGCGCGGTATGGAGTTGAGCACGGTGTTGCCATCCTGGTTCTGGGTCAGA
TAGACCGGCAGCACCTTGGGCAGGGAGGACACCAGATGGGTGACTTCATGGATATCGTAGATCCCGCCAATACGGA
TATTGCCGGTACTGGTATCGGCCAGCATCACCGGTTTGTTGAGGTAGCGGTTGATCAGGGGCAAGGCATCCGCCAG
TGCCAGGTTGTCGAGGATCAACTTGCCCGTGCGCCAGGCCAGTGCCGGATCATGGGGAGAGAACGCCTGGAGCTGT
GGCTGCGCGTCACCGCGGTTGTAACTGGCTTGCATCCCGGGCGTCAGGTTCAGCCCGCTGTGGCTGGCATCGCTGG
TGATCTTCACCGAGCCTTCGAGCAGCATGACCCGCACCTGATCGTCATACTTCCAGACGTTGAACTGGGTGCCTGT
CACACGGATCTGCCCGGCGCCGGCGCGCACCACAAACGGGTGTTGGCGGTCATGGCTGATATTGAAGAACGCTTCG
CCCTTTTTCAGGGTGGCCCGGCGTTCGTTTTTATAGTTGCTGTAGACCAGTTCGGTACCCAGGTTCAGCTCCACCA
CACTGCCATCGGCCAGCGTCACTTGGCGCAGGCCGTTTTCGGCATCGTAATGTTCATAGGAACTGGGCAGCCAGCC
CGCTTCCCAGCCAGTGAAGGCGGCCAGGGGCAGGGCCACCAACGAGATGGCCGCAGCTACGGCGTAGTTGCGCCAG
CGGCTGGGCGGCGGCGCCAGTGCAACGGGCGGGGAAGGTTGGCCTGTGCGTGGCAGATGGTCGGCCACATCCCAGA
TTTCCAGCATGGCGGCATATTCGAAGGCATGCAGGGGATGCGCATCGTGCCACTGTTCGAAAGCCTGCCGCTCGGC
CTCTGTGCAATCAAAAGCGTGCAGGCGCATGCACCAATGCGCGGCGGCATCGGTGACGGCATCATATTCGGCTTCT
GAAAAAGGCTGTTGGGTCATTGGTGCTTCCTGATTTCGTACATTCTAACCTTCCCGGTAGGGTGCCGAGAACAGCC
ATCATGGCGAATGAGCATCAATGTGGAACTAATTGGGCCCGCAAACACCCTAAAAATCTGGATCTGTAAGTCATTC
TCTATAACGGAGTAAGAAAATGCTGAAGAAAACCCTCACCGCCATGCTTGCCACAGCCACCTTGCTCGGCGCTGGC
GCCGCCCTGGCCGATAAGCCAGGTGCAGGCTGGATCACCATCGAAAAAGCCATCGAGACTGCCAAGACCAAGGCCG
GCTACATCGAGGTCTACAGTATCGAAGCCGATAACGACGGTTATTGGGAAGGCAAGGGGCGCAAAGCTGACGGCAG
CGTCTATGAGTTCCGTGTCGATGGCGCCTCGGGCAATGTGACACGCGACCAGAAAGACTGATCGCAAACCGATCAG
TGCGCGGACGCCTTTCGATCGAAAGGCACCGGGTCCAATTGACGTCCCAGGTTACTGATCAGGAACGCCACCGAGT
CGGCATTATCCAGGATAGTGTCGACTCGTTTGCCGAGATTGGTCATGAATATCCCCCTCGCATTACTCAGGTCCAC
ACCGCCCGTAGTACGCAGGATCTGATCCCTGATGTATTCGGTTTCAGGTGTCGAGCCGAAGTCTCTCCAGACGTTG
CCCGCGACGTCCACGTACTTGAACAATTGGCTGACGGGCGTCCTGCGCGAATACCCCGTGGTTTGCCGATTTTTCT
GGTAGGCGGCCAACTCGCGCCCGGCCTGGGTCAGTGTCTCGATCAGATCGGAAAACGGCATCACCGAGTCCAGGTA
CGCTGCATCGGCCGTCGTGGCCACCAGATGGCTCAACTCGTGAATCAACAGCGTGGCCCGCGCATGGACCTCCCGG
TCGAAGTAGTCGGTCAGACGATTGTCGTAGTCGCTGAAGGGCGGTGCAAAAAAGTGCTCAGTCAAAAAGATCCGCC
GGTCCGGATCTTTGTCGATGGTGAAGCCCCAGTTGTTCTGTGGGTCGATTCGATGGGACCCCACCACGAACCGCTT
CGAATCGAGTGCATACAGGCTGGGGTCCAGCAGGCCGGCGAGAATCTGGTCGACGACGCGATGCAGCTTTTGCACC
AGGGCCGGGGCAATCTCTTGAGGCCCCTGGTCCGGGTTGATATCGATCCCGAAGAAGCTGTTGACGAAGCGATGTA
TACGGGTCACCGGCGCGATGCCTGGCGCCAGTAACGCCAGGTTCAGCTTGCAGTTCTGCACATAAAACGTTGCCAG
GTCCATGGCCTCCACAATCATCCTGGCCCGCTGGGGATACAACCGGCGAATCGCCTTCATGCCGATCGCTTCGATA
TTCATGGTGGTACGGGCTGAGGCGCTGACATCTGCCCGGTCCTGCAGGCGGCCCAAGGCCTGGCCATAGCGCGCGA
TCTTTTGCCGGGTGTCGAGGCGCCATTGGCCCGCGCTGTCGCGATAGACAAACGGGCCATGGTTAGCGGCCGTCTT
GATCCGCCAGAACGTACCGAGGCTCTCTACCCGGTAGACCTTGCCCGCCAGCGCAATGTACGGCACGCCTTTATGC
TGGTAAGTCCCGTTGAATGTATTGAGCGTCATACCCGTCAATCCCACATCGTGCACTTCGCTGGGCGACAGCAGGG
TGCGCTGTGGGGCGGTGATGTCCAGTGTCGACCAATTGGCCGAGGGTTGCGTCGGCGCGACGGGGGGTTGCTGCGC

CGGGTCGAAGATCAAAGCCTTGGCGTCCTGCAAGGCCTGGCCGAGCGCGGCCATTTGTCCCAGCCCGTTGATGAAC
GTCTGCAAGGCCAGGCCCCAGTGCTGGTCCTGCAAGGCCTCTGCGGACTCAGTGAACAGTGAAATGCTTTGCCAGA
TGGCCCAAGGCAGTGCCAGCTTGCCGGGCAGCAAGCCGAGCATCCGGCCCGCAGTGCCATCGAGCACCTGGGTGAG
GGTTTGCCACTCGGCCTCGGCCCCTTGGATCAATTGGGCACCCAGCAGATTGAGGAGTAACTGGCGGTTATCGTCG
AACAGGCGGCCCAGCAGGTTGCCCTGGATCGGATTGAATCCCAGGGTGATCTCCGAGGTAGCGCCTTGGGAACTGG
CCAACAGGTTTCGATAGGTGGCGTCCTGGGGCGCAGCCAGCCGCCTGAGCACCCAGTCTTGCAGGGCGCCTGGAGT
ATTCAGGGCTTGCACGAACTGCGCCTCGCTTTCGAACTCGCGCAAGCTCAAGGTTGGGTGATAGGGCGCGTACAAC
ACCTGCGGCCCCCCGCGCCCGGCGCTGATGAAGTACAGCCCCAGGGCCGGGGCCAGCGAAGCGCCGGCGGTGGCGA
GCAATTCCAGGGGCCTGACCTGGGCCGTGGTGCCTGGCACCAGTGCACGGGCCACCGCATCCGGCATGTCGAATGC
CTGTTGCAGCAGTTCGAAACCCACATCTGACAGGCGCTCTTGCAACTTGAGGGCATGGGCGTGTTGCAACAGTTGC
CAGGGCAACTGCTGGCTGAAATCCTGCAAGCGCTGTGCGACACCGGCTGCGCCCGCCGACAGTTTTTGATTCAGGT
ACGTGTAATAGTCGCTCTGGATATTCAATTGCGTCAGCATCTGACGCACCACGGTTTGCGTCAGCCCGGTGGCCAG
GGACACCACGTCAAATTCGGGCGCTTGCGCACTGGTGGGGCCCATGGCGAAATCCAGCAGGCTTTGGCGCCGTCCT
GCCAGCGCCAGCCTGGGCACAATCTCGACGCCCGTGGCATAGGTTGAGTAATCGACGAGCATGTCGTTCAGGCGGT
TCTCCACATGTTGACGCAACGAATCAAGCTCGTAGAGATAGTCCCGGCCGTGCGAGGTGTGGATGCGATATTGCTC
CAATATCTCGGCCTGGCGCCGTTGCTCCCTGGGCGGCGCTGCGCCCAGCCGGTTGTGCAGTGAGTGCCGCAGCTCT
GCCAGGCGTGCGATGTCCATCGCGCGCTGCAGGTTATTGGCGGGGACTTCGCTGCGCAGTTGTCCCAGAGCCTGGG
CCAGCGACGAGGCTTGGGCCTTGAGCGTCAGGGTGTGTTGGCGTTGGGCAAGATAATGCTCGATCCAGGATTGCTG
GCATTGCTGCAATGCGGGCTTGGTCATCAATTGCAACGGCCCCAGCTCATAACGTCGATGGGGGATGTGCTGCAAG
GCCGAGAGGTTTTCCAGCAGGCCCAGGCGCTGGGCCGGGCTGCGTAGCCGTTGAGCCAGGACCTCGCGCAAGTCGC
TGATCGAAGCGAAGGCTTCCACGCCTGCCGCCGGCGTCCACAGCAGCGCATGCCCGGAGTGGTAATGATCGAGGCC
CCCGCGTTCAGTGAGCACAAAACAGTTGGTCAGTCGCAGCAGGCTGGTCTGGCCAGCGGGCAACACTGACACTGTG
AATGCTTGTGGGGTGAAGCCTTTCAACACAGGATCCTGTCCGCGCACTGTGTTGTCCGGATCCAGCATCGTTTGCA
GGATCTGGATATCGCGCTCATCAAGGGTCTTGTCCAGCCCTCTGAGCCGTGCCTCTTCAAGAATCCCCACGGACAT
GCTGTGCGCCAGCTTGGGGGTCAAGGCCTCCAGGCCCGCACGGGGGATTTGCGCGGCGGGCTGGCGGCTCAGACCG
GCTAGCCACTGGGTGACCATACTATTGAGGCGGACAATGTCCAGGTCCGGTCTTTTCGTGGCCTGGCCGCGAGTCC
GTGGTCCATATACCCCGTATTGCGCAGTGTCAGGGATGGCCTTTGCCTGGTGCAGCAGGCGGGCGACACAGTGCAC
CAACAGGCTCTGGCTGGACTGTGGCGTGCGCCGCTCCTGGGCGTCGGCATTGCCAGCATAGCGATTGATAAAAAGG
TCTTGTGGGTTGAGCGCGGCCACCTCAGCGGCCGGGAATGTGGCATGCAACTGCTCGCTGATGACGCGCTCCAGGC
TGGGAACCTGCGCTATCAGGGTGTCCAGCGCATGGCTGACGCTGTGCAATCGCCGGTGGGTCAGGCGGGCCTTTTC
CGCCGGCACCGACGGCGCGCAAGGATGGGTGCTCCAGCGTCCCTCGGCGTCCAGTGCCCGTAACCTGTGGTCGAGC
ATCGCGCGGATATCCAGGCCGTAATCCAGCATGGCGTAGGCGTTGACCGTACCCTGGTTGCGTCGGTACAGGTCCA
GGCAGTATTCGAGGTTCTGTTGCTGTTTGCCGATGACCGTTTCCAACAAGGTGCTGAATACCGGCCCAGTGAACGG
CACCGAGCTGAACTGCGGGTTTTCAAACCCGACAAACCGTGCCCGCTCTTCCAGTGACAAAAACCCGAAGAAATCA
TCCTCATGCCCCTGCGCAACGGCCATTGCCTTGAGCGTGGCCTGCAAGTCGTGCTCGTCCTTGAGCACTTGCCAAC
CTCTGGACTGGGTGTAGAGGTAGGCGTCGGGATGAGTGACCACCAGCGTGCTGGCCAGCTCGACATAGTAAGAGGT
GTGCTCCCACAGGCGGACTTTTTCAGTCAACGGCCCGTGCTCGCGTTCGACGGGCTGGTAGAGCGCGTTCAGTTGC
TGCCACTGTCCGGGAGTGAAGATCTCCTGCTGACGCTTGAGCAGTAAGTCGACCCTGGATTTATCGCTCATGGCCT
GGGCAAAGTATTCGCGCCTGGACCACCCGGCGTAAACGTCGGTGTTCCAGTAAGTGAGCAGTGAGTTGTTCAGATA
CAGGCGCAGTTGATCGGACGCTTGCTGGATGCCATCGTCCCAGCGGCTGAGGTCGCGCGCCTGGGGGCTGGTGCCT
GAGGGCGACAATATCTCGGGGTTGGTGTAGACCCGGCGCAGCCCCACCGGCCAGGCATGCTGGCGGTGGAACAGCA
GTAGGGCATCACGCAACAACAGGGCATTTACCCATTTTTCCTGGTCGGTGGATGATGAAACCCGTGCGTAAAAAAA
TACATGGGTCTTGCGCTGGTCCACGCTCGGGAAGGGCTTGCGCAAGGTGCTGGACAACAACTGGTCCAGCAGGGTT
GCCACCAATGGCAGCTTGCTCAATTCATTGAGCAGGGTCTGCACGGTGCGGTTCTGGCCACGTTCGATACAGGCTC
GCTGTTCATCAAATACGTTGCCCGTGATCAACTGTCGGGTGAGGCTGGCGCCGGGCTTGAAGTCCAGGCCTATGCG
GTCGTCCAGGGCCAGGAAGTGCAGGAGTTCGCCCCGTTGTGCATCGTCCAGCAGGCGTTTTTCCAGGGTATCGAGC
AGTTGCCTGGCATCACTGAACTTCTCCAGCCCGGTTTGCGGGCCGTAGAAAAACACAGGGGTGTAGGCGTCTTCGG
AACTCATCATGAAGGCGCCGGCCAATTCGACCGGCTCGTGCCCCGGCAAGTTGATCAACAACCGTTCGGCAATCAT
TGGCGGGGTTTGCGCCTTGCGTGCATCGGCATCGACAAAAAACAGATTGCGCAGGAATTCCCGATCCTTTTCGGTA
ATGCCGCGTTGTCGCTCACGCTCGTTCAAACGCTTGATCACCCGTAACGCTTCGGGGAAAAAATAGGGTAGAGGCT
GGTCGTCCATGATCGTTTGTGCACTGGCTTGAGGAAGGGCAAAGCCTACGAGTACCGATGGGCGCAGCGGCGCTAA
ATAAATAGGCCGCGAGCCCGCACAGGTGTTGACAGGCGGGTTGCCGGGCGTTGTTTAATCAGGGCGTTGAGTCCAG
CAGGCTGTAGCCCCCTCCAATAACAATTCCGGAGCTTCAGATGTCAGCCACGCCCGTTACTGCAAACACATCCATG
GCCTTCAGCGAACTGGTGGCCCTGCTTACACGCATCTTCATTCGTCATGGCACCTCGCCCGATGTGGCCGCTGTCC
TGGCCGATAACTGCGCCCGTGCCGAACGGGACGGCGCGCACAGCCATGGGGTCTTTCGTATACCTGGCTACGTCTC
GACGCTGAACAGCGGCTGGGTCAATGGTATGGCCGTGCCGCAGGTCGAAGATGTCGCGTCAGGCTTTGTCCGGGTC
GATGCCGACAATGGTTTTGCGCAGCCGGCCCTGGCAGCAGCGCGCCCGCTGTTAGTGCAAAAGGCCCGCAGCGCCG

GGATCGCGGTATTGGCCATTCGCAACTCCCATCACTTTGCGGCGCTCTGGCCCGATGTAGAGCCTTTCGCCGAGGA
AGGGCTGGTGGCGCTGAGTGTGGTCAACAGCATGACCTGTGTGGTGCCTCACGGCGCAGATCGCCCCTTGTTCGGC
ACCAATCCCATCGCGTTCGCCGCGCCCCGTGCCGATGGGCCGCCGATTGTGTTTGACCTGGCCACCAGCGCCATTG
CCCATGGCGACGTGCAGATCGCCGCGCGCAAGGGCGAGCGCGTGCCACCTGGCACCGGTGTCGACAGCCTGGGCCA
GCCGACCCAGGACCCTCGGGCCATCCTTGAAGGGGGCGCGTTGCTGCCGTTCGGTGGGCACAAGGGCTCGGCCTTG
TCGATGATGGTTGAACTGTTGGCGGCCGCATTGACGGGGGGCAATTTTTCCTTTGAGTTCGACTGGTCCAACCATC
CCGGCGCCAAGACGCCCTGGACCGGCCAGTTGCTGATCGTCATCGACCCGAGCAAAAACGCTGGCCAGGGCTTTGC
CGAACGCAGCCAGGAACTGGTGCGCCAGATGCATGCAGCAGGGTTACGACGTTTGCCGGGCGACCGCCGTCATCGG
GCGCGGGCCAGGTCTGAGGAGGAGGGGATTATCATCGACGGTGAAGAGTTGGAGCGCTTGCGGGCGTTGGCCCAGG
CCTGAACACCGGCGCGGACGACGCCGCGCCAGTGATGACCGTTATCAGTGGCGACGGCCCAGCAACAGGCCGACGA
CCAGGCCGAAACCGGCCGAGATTGCCACCGTCTGCCACGGGTGGCCACCGATGTAGGTTTCGGTAGCATCGACCAC
CGGCTTGCTGCGCTCACGCACGTTGGACACCGAGTCCAGGGCCTGCTTGAGCTTGATCGCGACCTGCTCGCGCAGG
GTTTCACCTTCTTCTCCCACCAGCGACGCGCTGCTTTTGAGCAGTTTGTCCGATTCTTCGATCAGGGCCTGGAGTT
CGCTGAACGCCTGGTCCTTGATTTGCTCTTCGACTGCTTGGGCGGCGGTTTTACGGGCCATTGAGTAGCTCCTTGC
GGGGTAAGTGGGCAGTGAACAATGGAGTCTTGCAGTAGGCTAAAAGTTGCAGTTTTTTTCGCTGACTCTCAGCACG
GGATAAAATCCGAATTCGGACCTTTCGACCTACAGTGTAAGATGTCACCTATTTGTGCAGCAGGTAATTCCACATG
AGCTTCAATCTGGCTAACAAGCCCCTTGCCGAGCGCGCTGCGCTTGAAGATGAAAAGTCGCGCCTGTACGACTTGT
GGCAAAGCAACCTGGGCAAGGCCAAAGGCGAGGGCGCCCGACTGTTTGGCGAGCGCTCCAAACGCAAGGGCAAATG
GGCTGAATGGGTCCGCTCGGAATTGGACGGCATGTCGCCACCGGAGTTTGCCAACATGGTGCGCAGTGAAGTCAAC
CGACTGATGGCGGCCAAGTAGGGGCCGGCGATTGCGGTAGTTCAGTCACTACATGTGCTGGCTGGTATACCGCTAT
CGCGGGCAAGCCCGCTCCTACAGGGGTTGTTTCATCCATTCTTCGCGGGTGATTTCCCAAATTTCCTTGGGGAATA
CCCCCGCCACGAAGCGTCCTTCTTCCGTGCGGATCAAGCGCATCCGGGTTTTTTCCGAAATCCTGCGCGACCCCAG
GTTCGGCGCGGCCTTGGGCACCCGCAGCAGTGGGCGTTCGAGCACCTTGAACCAATACTCGGTCACCGCCGCACTG
GCTTCGCTCATTAGCCCCTGACCTTGCCAATGCGGGGCCAGCCAGAAGCCCCGGTTGTTATCCAGCTCATCCATCA
GGCTCACATTGCCGATCAACTGCCCGGGCTCTTCGCGCAGGCGGATCGACCAGTGCCACTCGCGCCCCTCGACCAT
GGCCGGCAAGGCTATTTGCTCCAGGAAGCTGCGGGCGCCATCGGCCGGGTAGGGCCAGGGCACCAAGGCATTGAGG
TAACGCACCACCTCCCAGTGGGGAAACTGTTGCTGGATACCCTCGGCGTCGTCCAGGGTCAGCGGTTGCAGGATCA
GGCGTTCGGTGTGCAGGGTGGGCAAGGTGTCCATGTTGTTTACCACGTCACTGTGGTGGGCAGGTCCAGCGTCCCG
CGATCAACAAAACGCACGGTGCCAAACAAGCCACCGGCCAGCTTGCCGCGCAGGACATAGGGCAGGTTGTTCAAGC
TCTGGGTCTGGCTCAGGCCAAGGGTCTGGCGCAGCACGGAAAATGCCGAAACGCTGACCGGGACTGTCAGCACCGT
CTCAGAAAAGCGCGGGATCGATCCCTGTTGATCGCTGACCCCGGCCGCCAGTGGCCGTCCATTGACCTCCAGGTCC
AGGGCCACGCCGTTGTAGTCGATCGCTGTTTCGTTGGGGTTCTGCACCCGCAGCTTGACCGCAAACCGCACTTCCA
GGTCCTGGCTCTGCAACGGTTCGATGCCCACCACATTGATATGCAGCGCGTCACGGTCGGGGAACAGGGCGCAGGC
GCTCAGGCTGAGCAATACCAGGGATAGGGTCAGGCCGATCAATCTGCGCATAAGGGTTCCTATTTCGTCGGGGCCG
GGTCCTGCATCACTTTGAGGGTCGAGGCTTCCGGATCAAATTCATCTTCCTGCAACTCTATGAACTCTTCAGGCAG
GAAGATGTTCAGCAGGATCGCACAGAATGCACCGACGGTAATCGGCGACTCGAAGATGTTATGCAGGGCCTTGGGC
AACTCGCGCAGCACTTCTGGCACCGCCGCCACACCCAGGCCCATCCCCAGGGAAATGGCCACGATCAGCACATTGC
GTCGATGCAGCCCGGCCTCGGCGAGGATCTTGATCCCGGCCACGGCCACGGTACCGAACATGATCAGGGTCGCGCC
ACCCAGCACCGGCTTGGGCATCAATTGCAAGACCGCGCCAATCATCGGGAACAGACCGAGCAGCACCAGCAGGCCG
GCAATGAAATACGCCACATAGCGGCTGGCCACGCCGGTCAACTGGATCACGCCGTTGTTCTGGGCGAAGGTCACCA
TCGGCAGGCTATTGAAGGTGGCGGCCATCACCGAGTTCAGGCCGTCAGCCAGCAGGCCAGACTTGATGCGCTTGAT
GTACAGCGGGCCTTTGACCGGTTGCTGGGAAATCATCGAGTTAGCTGTCAGATCTCCGGCCGCCTCCAGCGGGGAA
ATCAGGAAGATCACGGCTACCGGGATAAACGCCACCCAATCAAACGAAAATCCGTACTTGAACGGCACCGGCACGC
TGACCAACGGCACCTGGGGCAATGCCGCCAGGTCGACCCGGCCCAGCCACCAGGCCACGACAAACCCCAGGGTGAG
GCCGATGACAATCGAGCCCAGACGCAGGAATGGGTTATTGAAGCGGTTGAGCACCACGATGGTCACCAACACCAGG
GCGGCCAACCCCATATTGCTGGCGGCACCCAGGTCGGCGGCGCCGTAGCCGCCGGCCATGTCAGTGACGGCGACCT
TGATCAACGACAGGCCCATCAAGGTGATGATGGTGCCGGTAACCACCGGGGTGATCAGTTTGCGCAATTTTCCGAT
GAACTGGCTGAGCACCACTTCGATAAACGCTGCAAAGAAGCAGATGCCAAAGATCGTCGACAGGATCTCATCGGTG
CCGCCGCCCCGGGCCTTGACCATGAACCCGGCACTGAGAATCACGCTGATAAACGAAAAGCTGGTGCCTTGCAGGC
ACAACAAGCCCGAGCCCACCGGACCAAAGCGCCGCGCCTGGACAAACGTGCCCAGGCCCGAGACGAACAGGGCCAT
GCTGACCAGGTAGGGAACTTCGCTTTCCAGGCCCAGCACGCCGCCGACGATCAGGGTCGGGGTAATGATCCCAACA
AAGCTGGCGAGCACGTGCTGCAGGGCGGCAAAAATCGCCGCGGTGAAGTGCGGACGGTCTTCCAGACCATAGATCA
GGTCCGATTTATAGCGGGGGCGGGGGGCTTGAGCGTCAGACAAAATACGTGCTCGGGTCAGAAAATGGACGCGCAG
GATGCCAGAAAGCGCCCGCTGTCAAAAGTGTGAAAATATATTTCTCGGATCCGTGCAAAAAGCCTCAAGCCTGCCG
ATATCCCTTATAGGCCCACATAACCCTTACAACAGTGGCGCCGAACAGTCACCTGTGTGCGTTGACGCACAGTGAC
CGTGTCGCGGCAAGGACGCTCGCAGCCATCCCTTCTTGAGAGCTTTCCTATGTCCCTCCGTAATTTGAACATCGCA
CCTCGGGCGTTTCTCGGTTTTGCGTTTATTGCGTTGCTTGTGATTGTTCTAGGGGTGTTTGCCGTCAACCGCATGA

CGCTGATTCGCCAGGCCGCGTTGGACATGGGCGCCAACCAACTGCCGAGTGTCGGCTACCTGGCCAATATGACCGA
GAACGTGTTGCGCCTGCGCATTCTGTCCTTCCGTATCTTGGTCAATCGTGAGCCGGCAGCCCTGCAAGATGCCCAA
ACGCGCATCGGGGTGCTGGTCGACAAGGCCCGCCAAGCCCAGGCAGGTTATGCCGCGATGCCGGCCGGCGCCGAAG
AAGCCGCGCTGTACAAGACCTTTGCGGCCACGCTCGACAACTACCTCAAAGCCCAGGCCGAAATGCTTGCGCTGTC
CCAGCAGAACAAAGTCGAGGAGATGCGCGCCCTGATCAACAGCCGGATCAAGGACGGCACCGATCGGATGGGTGAA
CAACTGAACCAGTTGATTGCGATCAATGCCGCCGATGCCAAACAGGCCGGCGAAGACGCCGGGCGCAGCTATAGCG
ATGCGATCAGCGGGATCATCGTGGTGGCGGTGGTCGCGGCGCTGATGACTGTATTGCTTGCCTGGCTGCTGACTCG
CAGCATCGTTACGCCCCTGCGCAAGGCCGTGGCGGCCGCCGAGACCATCTCCAGTGGCGACCTGAGCAAAGCCATC
GAGGACGACGGCCAGGACGAGCCGGCCCGCCTGATCGGTGCCCTGGCGACCATGCAGAACAACCTGCGCCAGACCA
TCCAGCACATCGCCGGCTCAGCTACGCAATTGGCCTCGGCCGCCGAAGAGCTGAGCGCAGTTACCGAGGAAGCCTC
CAAGGGCTTGCAGCAGCAGAACAATGAAATCGACCAGGCCGCCACGGCGGTCAATGAAATGACCGCCGCCGTAGAG
GAAGTGGCGCGCAACGCGGTGTCGACCTCCGAGGCCTCGACCCAGTCGAACCAGGCCGCGCGGGAAGGGCGCGACC
GTGTGGTGGAAACCGTGGGCGCGATCCAGACCATGACCCAGGACGTGCAGAACACCTCGGTGTTGATCGAAGGCCT
GGCGACCCAGGGCCGTGATATTGGCAAGGTACTGGACGTGATTCGCGCGATTGCCGAGCAAACCAACCTGCTGGCC
CTCAACGCGGCCATCGAGGCCGCACGCGCCGGTGAAGCCGGGCGTGGGTTTGCCGTAGTGGCCGATGAAGTGCGCG
CCCTGGCCCATCGCACCCAGCAGTCGACTCAAGAGATCGAGAAGATGGTGGCCGGGATCCAGAACGGTACGGGTGA
AGCGGTGCAATCCATGCAGCAGAGCAACCAGCGTACCCAAAGCACTTTGGAAATGGCCCGCGCGGCGGGTGTGGCG
CTGGAGCAGATCACCCAGTCCATCAGCCTGATCAACGAGCGCAACCTGGTGATCGCCAGCGCCTCTGAAGAGCAGG
CCCAGGTATCACGGGAAGTGGACCGCAACCTGGTGAACATCCGCGACCTGGCCACCCAATCGGCGGCCGGTGCCAA
CCAGACCAGCGCCGCCAGCCACGAACTGTCGCGCCTGGCGGTGGACTTGAACGGTATGGTGGCGCGTTTCGTGATC
TAACGACTGGTGGGGAAAGTGGGGGGCTGCGGCTGCTGCCCCGGAACCAGCCACCTTGGTGTACCTGGCAGAACAG
GGTGAGGCTGTTGGGGCGGCTTTGCCGCCCAGCGCGGGGCAAGCCCGCTCGCCACAGACAAGTCCTCTCACCACAG
ACGAGTCCTCTCACCAATCAGGCCAGGCTCATTGCTTCCAGGCGCCGGTGTTCACCAGGTTTTTCGGGCGTTCGCC
GGCCAGTGCTGCCAGCAGGTTGTCCACCGCACATTTGGCCATGGCTTCGCGGGTTTCGTGGGTCGCAGAGCCGATA
TGGGGGGTCGCCACCACGTTGCTCAAACGCAGCAACGGCGAGTCGTGATTCAACGGCTCACGCTCGAACACATCCA
GACCCGCAGCGCGAATCGTCCGCTGTTGCAGGGCTTCGACCAGTGCCGCTTCGTCCACCACTTTGCCCCGGGAGAT
ATTGATGAAAATCGTCTCCGGCCCCATAAGCGCGAACTCCTTGGCGCCAATCAACCCCTGAGTCTCGGCAGTCAAC
GGCAATGTCAGGCAAACAAAATCGGCTTGCTGCAGCAGATCCGGCAAGCTGTGGTATTGCGCGTCAAACCGCTGAT
CTACCGCAGGCTTGGGCGAGTGGCTGTGGTAGATCACCGGCATGCCAAAGCCAAAGTGCCCACGCTGTGCCAGCGC
TTCGCCAATACGCCCCATGCCAATGATGCCCAGGGTCTTGCCATGCACATCGCTGCCAAAGTGCAGGGGGCCGATG
TTCTGGTTCCAGTTGCCGGCGCGCACCATATTCGCCAACTCCACCACGCGTCGGGCGGTGGCCAGGATCAGCGCAA
AACCGGTGTCGGCGGTGGTTTCGGTGAGCACATCAGGGGTGTTGCTCAGCAGGATGCCGCGGTGGGTCAGGTAGTC
GATGTCGTAGTTATCAACGCCCACCGAAACACTGGCCACCGCCTCCAGCTGCGGTGCCAGGTCCAGCAGGTTCGCA
TCCAGGCGCAGGCTGGCGCCCAGCAAGCCGTGGGCGCTGGGCAAGGCATCACGCAATTGTGCCAGGCCGGTGGGGC
CGAGGTTTTCAATCAGCGTGACCTGGGCCGCTTCGTGCAGGCGGGCCATCAGCGGCGCGGAGAGTTTCTTGTACAA
CACGACATGCTTTTTCATCAGGTTCTTACCTTCAAATCCAGGGAATGCAACGGTGCTGTCGGGGAGGGTTCACGTG
CACCGGTCTTGAGGAAAATCGTCAGCACCACCGACAGCAGCAACGCACCGCTCATCAGCAGGTACGACGCGCCCGG
CGAGCCGGTGCTGCTGTTGAGGTAGCCCACCAGGTACGAGCCGCCAAACGAGCCCAGCGCGCCCATGCTGTTGATC
AAGGCCATGGCCCCACCGGCGACGTTGGCCGGGAGGATTTCCGGGACGATGGCAAAAAACGGCCCATAGGGCGCAT
ACATGCACGCGCCGGCGATCACCAGCAGGGTGTAGGACCACCAGAAGTGTTCGGCCCCCAGCAGGTAGGAACCGTA
GAACGCGATGGAGGCGATCAGCAGTGGCGGCCACACAAAGCGTTTACGTTTTTGCAGCTTGTCCGAGCCCCAGGAC
ACCAGCAGCATGGCAATCACCGCGGCGAGGTAAGGCAGCGCCGATAACCAGCCGGCTTCGACCATATCCATCTGCA
GGCCCGCCTTGAGGATCGAAGGCAGCCACAGCACAAAGCCGTAGACGCCGATGCTCCAGCAAAAGAATTGCAACGC
CAGGATGATGACCTTGGGCGAGCGGAAGGCTTCGGCATAGTTTTTCACCGCCTTGAGACCCACCTGCTCGGCGGCC
AGGGCGGTTTCCAGGTCGTGTTTCTCGCCGGCACTCAGCCATTTGGCATCCGCTGGGCGCTCATCGGCCAGGCGCC
ACCAGATAAAGGCCCAGAACACCGCCGGCAAGCCTTCGACGATAAACATCCAGCGCCAGCTGTAATGCTGAACCAG
GTAACCCGACACGACCGACATCCACAACATGGTCACCGGGTTGCCAAGGATCAGGAAGGTATTGGCGCGCGAGCGT
TCGGCACGGGTGAACCAATGGCACAGGTACACCAGCATCGCCGGCATCACTGCGGCCTCGACCACCCCGAGCAAAA
AGCGGATGGCGATCAGCATATAGGCGTTGGACACCACCCCGGTCAGCGTGGCCAGGCCGCCCCAGAGGATCAGGCT
GACGAAGATCAGCTTCTTCACGCTGCGCCGCTGGGCGTAGATCGCGCCCGGCACCTGGAAGAAAAAGTAGCCAAGG
AAAAACAGTGCGCCGAGCAGTGAAGACATGCCCGGGGTAATCATCAAGTCTTCGGCCATGCCGGAGGCGGCGGCAA
AGCCGTAGTTGGCGCGGTCCAGGTACGCCAGGCTGTAGGTAATGAAGACGATGGGCATGATGTACCACCAACGGCG
GGTGGCGAGTTTCAGGTTATTCATGGGGTTGCTCCTGAGCTTGTTGTAGTTGTGGCAACAGGTAACGGTGTATTCG
GGTCAGCCGGCTTTTTGCAGGGCGGGCAGGTCGGCGCGCAGGGGCAGGCCTTCCATATCGCCACGGCTCTGAACCG
CGCGGCTGCCGATCCAGTTGGCGCGCTGCACTGCCTGGGCAAAGCTGAGGTTTTCAAGGAGCGCGCTGATCATGCC
CACGGCAAAGCCATCACCGGCACCCACGGTATCCACGACTTTGGTCACCGGCACGGCGGCGATAAACCCCTGGTCC
AGATGGCTACGGTAGTAGGCGCCGTGGGCGCCGAGCTTGATCGCCACGGCTTCGGCACCCTGGTCGAGGTAGAAGG

CGGCGATGTCCGCAGGGTCTTCGAAACCGGTCAGCAGGCGACCTTCGCTCAACCCCGGCAGCACCCAATGGGCCAG
GCGGGCCAGAGCGTTGATTTCGCGGATCATTTTTTGCTCGCTGGGCCATAGCGAGGGGCGCAGATTGGGGTCGAAG
GACACACTGCGGCCTGCGGCGCGCATCTGGCTCATCAACTGGCGCGACAGCTCGGCGGCGGTGTCCGACAACGCCG
GCACAATGCCGGTGGCGTGCAGGTGCCGCGCTTGCAGCAGCGCAGGCGTAATCACGGCACTGGACAGGTGGCTGGC
CGCCGAACCGCGGCGAAAGTACTCGACCTGCGGATCGGCGCCGGTCTCTTCCCGAGACTTGAGCTGAAAACCGGTG
GGGTGCGCGGGGTCGACCGTCACATGTCGGCAGTCCAGGCCTTCGCGCTCGAGGGTCTGCACCACGAAACGCCCCA
ATGAGTCATCACCGACCCGACTCAACCAGGCCACATTGAAACCCAAGCGCGACAGGCCAATTGCGACATTGCTGTC
GGCGCCGGCGATCCGTTTGTGAAACTGGCCGACCTGGGCCAGGTCGCCGGTTTGCTCGGCGACGAACATCGCCATG
GTCTCGCCAAACGACAGGATATCGATCTCAGACATGGGCATTCTCCACACGCGGTTGGCCAAGGACGCTAAGGGTG
GCCACTTGCTGGGCAGTGACCTCCAGCAGGTCGTCGCCTTGCAGTGGGAATTCCACTGCCCGGCTGATTCCTTGAG
GCATCAGCCCCAGCAATTGTTCCCACAGATGCAGGTCGGGGGCGCCCGGCGGCAGGGCCACCAGCTTGCCGTCGGC
GCGACGTGCCACCGCCTTGCAGTGCAGGTAATCGACATACCGCCCCAACAGGCGCGCGGCGGTCTGGGGGGACTGG
TCCTGCCAGTGCCAGTTGCCGATATCGAAAGTCATCTTCACCGGGACGCTCAAGCGTTCGGCTTGATCGAAGAAGC
GTTGCATCGGCTCGATGCGCCCACCGTGCAGGGTCTGGTCGTTCTCCACCAGCAAGCGTACCGGGTGGCGGCGGAG
CAGGGCGCTGAGGCTGTGCAGGTCGTTGGTGTCGGTGAAGTAGCCGAGCGAAACCTTCAACCAGCGCGCACCGAAG
GCCTGGGCCCGGTCGAGGGTGGCGGACAACTGTGCATTGGCTTGCGCGCGGCCGGCCACCCACAGCTCCAGGGGCG
AGGAAAACACGCACTCCAGGCCCTGTTCGGCGGCGGCTTGGGCCAGCTCGCCGGGGTGTTCGCTGGTGAGCAGTTC
TTCGCGCCATTCAATGCGCGTGGCGCCGGCAGCGGCCAGCACCTCGATGAAACTCAATTGGCCGCGCTGGCGAACC
AGGTCGGCGCCGTAGCTGGAGAGGCTGATGGAAACGGGATATCTGTGCATTGTTATTAATCTCTGAAACCGGTTTC
ATTTGTAAATAAAAAAACACCCTTCAGGAGCCGGCTTGCCGGCGATAAGGCCCTTACGCCGGGCACCTCACCGGCA
AGCAGCTCCTACACGGGTTGGGTGGAGCCTCGAAGAATGAGTTGCGGAGCAAAATCCAGGGCACGGGGCGGCGAGG
TATCGCCGCGCAAACGCTTGAGCAAACAGTCGAACGCGCTGGCGCCAATCGCTTCGGTAGGTTGGGCCAGGGCGGT
GATGCCGCTGCCCACCAGCGGGTACCAGTCCAGGTCATCCAAGGCAATCAGCCCGACGTCCTCGAACAACCGGCAA
CCCAATTGGCGAACGGCTTTGGTGCAGGCAAGTGCGGCGACGCCGTTGGCACAAAACAGGGCTTTGGGGCCGGTGT
CGCAGGCGCCAAGGAATGCCTGCAAACGGCTGGTCAAATCATCGCCAATTTCCAGCACGGCGGCATGCAGGCTGTT
GCGGCGACTGATCTGGGCATGGAAGCTTTCCATGCGCTCAACCCGCGAACTGGTGCCATCAGCGGCTTCGCTGACC
AGCAGGATATCCCGGTAGCCCTGTTGTTCCAGGTGCTCCAGGGCCATGCGCACGGCCAGCGGGTTGTCCAGGCCCA
CCAGATCGCTGTGCAAGGCCTCGACCTTGCGATCGACCAGCACCAGCGGCATTTCCCGCTGCAACTCCAGCAGTTG
CTCAAGGTGATGGCCCAGGGTGTTCACGATCAAACCTTCGATGTTGTAAGCCCGCAGGGCCTCCAGGTGCTGGCGC
TCCTGCTCGTCATCGCGGTCGGTATTGCACACCACCAGGCTGTAGCCGTGTTGCCGGCAGGCGGTTTCCACACCAT
GCATCACGGCAATGGAATAGGGGTTGCGGATATCGGCCACCAGCATGCCGATCAGGCGCGTGCGCCCACGCTTGAG
GCCGCGGGCCATTTGGTTGGGGCGGTAGCCCAACTCCTCGATGGCCTGCTCGATACGCAGGGCGATCGTGTCGGAG
AGCAGGGCGCGGTCATCGCCGATAAAACGCGACACGCTGGCCTTGGACACACCGGCACGGTTGGCTACATCAAGCA
TGGTCACGCGGCTGCGCTGGGCGGCAGAGAATGAAGTCACGGTAGCGGGCCTTTCTTATTTTGGATAACGGTACAT
GAATCAATTTGAAACCGGTTTCAGTGGACGCCATGTTCGACGCCTCGTCAAGGAATTTTCTGAAGGCACATGGCTT
TGAAAACACTTGAATCCGACGAATGGAATGCGGCACCTGTTAGTTTTGACAGTTACGGACAATGCAAATCAGGCGT
TTAATTTCTCCAGCAGTCGATCCTTAAGTCACTGGCACCTTCATGTTTCATCGTTGAGGTAATCGTCATGTCCGGC
ACCTCCAAGCCAACCACTCAAGCTGTGGTCACTCAACCAGAGCAAGGTGTGTTGAATCTTTTTCCGATGACCATTC
CTGGTGTGGACCCTCTGGACCCAAATGGGCTGATCCCCGCAGCATTGATGCTCTCACCGCTTCAAGTGCAAGTTCC
CATGTGGACACCGCCCCCTGAGCCGCTGGATACCCCCCATATCCTCATGATTTATTGGGAGCGCGCAGGCACTGTG
GTTTACAGCGACTCGATCATTGTTCAGTCGCCGCCGCCACCGGTACCTGACATGTACACCCTGTTCATTCCTTTGC
TGGTGCTACGGGCACAGTCCGGGCCCGTCGAACTGTATTATTCAGTCACCGATTCGTTTGGTGGGCGCAGCGAACT
CAACCCTAAAAGAACACTGACGGTCGACATGGATGCGCCGCAATTATTAAACCCCGCCGACCAACTGACGTTTGTT
GTGCCTCCTGCACCCGCAGTTGATGAGGCGTACTTGGCCAGTCATCCACAGGTGGCGCTGCACGTTCCGCCCTATA
GCGGGCGCAATGACGGGGACACGATTCATTTCTACCTGTCCAATTTAGCCAACCCACCGGTTGCGGGTGAAGATGG
TGTTTATGAACTGGTGTCCAGCGCTGATCCGTTGATTGCCACCCTGGATGCAGCCGCTTTTCGTACGCTTAGCAAT
GGTGACGCTTACGTCTTTTTCAGGGTTTTTGACAAAGCGGGCAACTTCAGCGACCGCAGTGCCGGCCTGCCATTTC
AGCTGGCGCTGACTGCAATGCCGGGGCAGTTGCCCCTTGCGCAAATCGTGCCTCCTCGTTATGACGATTTACTGAT
CAATCGCGAAGACGCCAGGGCCAGCGTATTTGTCCGCATAGGTTCCTACACCGACTGGGCTGCGGGGGATGAAGTC
ATTGTGTATTGGAAAGGCCGTCCTACTGTCAGGCATCCGGTCAACGGTTTTCCCACCGATGTCGAGGTTCCATGGG
CTGTTATGCGTGGCCCGCTGACCGATCCGTTGGTGCCCGAAGCGGTACCGGTACGCTATGAGATTATTCGAGGTGG
CCTGCCGCCATTTCCTTCGTTCAATATTCTGGTGAATGTGAACCTCACGGTCGCTGGACAGGACCACGCGAATGCA
CCGGCACTGTTCAATCCGGATTTGCCCGTGCCGCAAGTCTGGGGGCTTGTTTCCAACACCCAGAACGTCGTCAACC
ACAATGACAATCCCGCCGGAGCCCGTGCCCGCGTCTTGCTTTATGAAAACCCGCTGCCTGGAGAAATTCTGCGGTT
TTATTGGAATGGCACCGGGCCGGTTGCCAGTTACACCGTGCAGTTGGGAGATGTAGCAGGGCAACTGGTGTTTTCC
ACGGTTATTCCCTGGGCGGCGATGGCGGGTTTTATTCACCCAGCATTGCCGGTGTATTACACCACCAGTAATGGCG
TGAATGATCAGCAATCGGACAATGCGCTGGTAAACGTAAATACGGGTGCATTGATTTCTTTCCCGGCTCCACTTCT

AAACCATACTTTAGTGGGGCCCGCTGGCTACTTGGGTTGTTGTTCAAAGCCCGAAATATTCCTGGGGGTAGAGTGG
TCATTGGCGCCGGATGATCGCTTTGAACTTAATGATGTGGTGACTTTTTTTTGGGAAGGGTTCCTGGTTAACAATT
GGGAACCGCCGGTTATTGAAGAATCAAGGTATGACGATAGCAAAACATTCAGTACTGACGCAGATTTGATGAATGG
ATTGCACTTCCGGGTCGCGCCCTATGAGGAAAAAGTCGTGCCGATGCGTAATTACGGCTCGGCTCGGGCCTGGTAT
CAGGTGAGGCGTCGCGGGGCCCTCATAGGTGAGTCGCTGCCTAGAAGGATTCGTGTGGATCTGCTGTCCGCTGGCT
CAGGTCAGTATTGTAAGTTAGGCGATGTCATTGTGTGTTCAAACGACGGCGTGGCAACCATTATCGGAAACAAGAG
CAACTAAGTTTTGATGTTGGCTTGATCGCAATATTGTCGTTGTTTTTAAAGTGTGCAGTTACTCCAGTGCGCAAGA
GGCTAGCCCGTTTGCAACTTGCATTGGGTGCTGCTGTACACCCTGAAAAAGCTGTAATACTCAATCAGCGTTGGAG
AAGAAAATGGACAGGAAAACGTTAGAAACCATCCAAAGATTCCAAACTCGTTGCGCGACAAATAACACACTTCTTA
GCTTGCCCGCGGGGACCTATGAAGGTCAGGTTCCGGGGTTTCCCGGGTTGATTGATCGCAACACCGCACTTAAAGA
GACAATGGAGTTTTTAATTCCTCGTTGGGATGATTTCAGCACCGATCCAAGTTTTCCAGACACCGTCAGAGTCTGG
ATGTGGCCATTGGATGACCCTCAGCCGTCCGAACCCTTTACGACATTTACCGTATCTTCACCTGCAGCGGGCGGGG
TGCCGGTCAACATTGCATTGGCTCGGCGTCCACCTGGCGCGCACCTTATCAGGTATGAACTGTTCGTCGACAATAT
CGGAAATTCCACCCAGTCAGATTCGCAATTGCTGATCGTGGACCTCGCGCCTCCCTATTACAGCCATGTGGGGCCG
ATTCCCGCTCCCTTACCTCCAGCAGGCCTGCCGACCCCCGCATCATTGGCTTACTTCCAGGGTTTACCCAATCAAG
CGGCTATGTTTCGCGTTCCTGACTATGTGGCGAACGGGCGCGCACCTGGAGACTATTTATTAGCGTATTACAACAA
TTCGGATACGCCTTATCTGCCTACGGCAGCCAGTACTGATCCGAAATGGGTTCTGCCTGCCGATTTGAGCTTCCCC
TTGCCCTTGTCGGTCGTCCAGGGTAGCCCGGACGGGCTACGTAGTTTGCGCTATGAGTTGTACGACGCGGCAGGCA
ACCCTGCGAGGTTGTCATCCCAATTTGTATTTGACGTAGGGTTGTTTCCTGCGCCCAGTAATTTCATCGCTCCAAC
CATTGATCTGGCGGTTCCCGGTGACCTGCTGATTGATCGCGGTGATGTCGCACAGCTCAACGGCGCCATCATCCGC
ATACCTGCCTATAGTAATTTTCTGCGCGGCGACGAAGGGGACATGATCAGTGTGATCCTGACCACATCCCTGGGTA
CGGTGACCCTGCCCGACGTGCCGTTGGGCAGTAACCCCTTCCCGCTGCAGGTACATGTTGCATTCCCTACGCTGGC
TCTGCTTTATGGCGCCACTGAAGGGTTACTGTCGATGACGGCGTCCTACGCGGTAATGCGTCGTTCGGTCAGCTAC
CCTTCGACGCTGACCGCAACAACTGACCTTGACCTTTTCGTAGTTGGGCCAGCAAACCCTAACGAGCCGGATCCGG
TCAACCCAGATCTGAACCCCGTAGTGGTCAGGGGTGAGGATGCAACGGGCGTCGAGGGATCGCCCAATGAGTTGCT
ACCCGAACACGCAAACCGGCCAGCCAATGCCTATATCACACTCTGGGACGAACCGCCGACTCCGGATGCCAGCGCA
TTTACCATCTACCTCTACTACGAAGGCGAACTGGTGGATTCGCTGTTTGTCGCAAACGGTATCGCCAATCAGGTCG
TCCAGTTGCAGATTCCCTGGAACGTCATTTCTAACCATAACAACGGTACCAAGCGGGTGCATTACACCATCGGTGC
CGCAGGCAGTTCGAATCGACAACAGGCCCCCATCACACAGGTGGAGGTTACAGCCAACATCATATTCCTGGCGCCT
CCGATCGTTCGAAACCTTGTCGGCGGCGGCATGGGGACTATCAACTGCAGCACCTTCAGGCCAGTCGGGCCGCCCC
CCGGTAACATCGTAGTGTTTGTTCCGCCCTCGGAGCATTTCTCGCTGAATATGATCGTTACCGTGCATTGGCGGGG
TTACCGGGATGATGCGGGCACCATTGAAGTTCCCGCCGTCGCAGGCAGCAAAGCTTCGGTGCCCCTGACGCAGGCG
ATGATCAACCTTGGGTTCGAGATCGAGTTGGAGGACTACTTCACTCTCTTCAAGCCGATTCAGCCGACCATGGATG
ACCGGCTTGCCGGTTCAGCCAGGATTCACTACAGCATTGTTCTGCCCTCGGGCACGGTCAACTCCTCTGACGCTAC
ACCGCGAGTGCGTGGGCATCAGATCGGCGGTGCAACTACGTTTTTTTGCGATGGAACGGTCGCGCCGGCGTCTTGA
TCTTTTGAGCTGTTGCCAAAGGGGAGCATTGCTCCCCTTTGGCATTTTGATGTGGATGATCGGCGGACGCCAGGAA
TCAGCTGGCGACAATTAAAGGCCTTGAGCCACGACCGCAAGCACACTTAAGGATAATCCTACATAAAGCGGTAAGT
ACTCTGGATACCGTTAGCACCGCTCGCTTGGCAGGGTCCCACACAGTTTGGAACCGGCCAGTTTCTTGCGAATTGG
AACGCCAACCCAGAGGTCCGCTAAATGCCATCCGTATTCAATTTCAAACCATCACCGTTGAGCCTCGCATTGAAGG
CCAACACTGTCACTTTTCTTATGCTTCTCGGCCATGCAGCCGAAGCCCGTGTCGTCACGGGGCCCGACGAGTATGT
GCGCCCAGGTCCGGCGCCGGATCGCTTTGAGCTGCGCACAGGTTCTGGCCTGACCGTCCAAGGTACCGAAACCCTG
GAAATCCGTGCCCAATCCGCAACCCTGGTGGTCACGCCAGGCAGCTTGACCAAGGACATCGTCGCTTTTGATTCCC
AGATCCGTGTAGACGGTTCGGATGTCATTGCTCGCAGCACGGCAGATGCCGCTATTCGCGCCACAGGCAGTAATGT
ACTGGTCAGCAACAGCAATCTCATCCACAACAATGGAACCGGCCTGATTGCCGGCCGCTCACTGGGCGGCACTGAT
GGTTCCAGCGTCAAGGTCATCAACAGCACGATTACCGGCACTAACCGTGGGGCCAGCGCCAGTGCCTACAGTGTGC
TGGACTTCAGCGGTTCCCATATCGAGGCGACGAACGACAATGGCATGGGCCTGTCGCTGATGAGCGGGACAGCGTT
TGCGACCGATAGCCGAATTGTAGGTGGCGCCAACGGCGTGCAAATGGCCGTCGACGGATCGTCCGGAATCATGGAC
AGTGCGTTGCTGCTGGACGGTTCCAGCGTGGTAGGTACGAACGGTGCTGCCATCCTGACAAGAGCGGGTACCAACG
CTGATATTCAGGTGCTGAACGGTTCGTCCCTGACTGGCGGCAACGGCAACATCCTGGAAGTCACTGGTGGCTCCAC
CGCCAACATGAACGTTGACCGCAGCGCCCTGGTGGGCGACGTGGTTGTCGAGGATGGCAGCAGCGCCAAGGTGCAG
CTCAACAACGGCTCCTGGCTGACCGGCCAGTTGAAAAATGTCGCCGAGTTGAGCGTGAACGATGCCTCCCACTGGG
TCCTGGTGGGCGACAGCCAGGTCGACGCCTTGAAGATGGGCGGCGGCGCCGTGCACTTCGGCGGGGAAGATGAGTT
CTACCAATTGAACGTCAAGAGCCTGGAAGGCGAGGGCACGTTCATCATGCACACCGACTTTGCCACCCACCAGACC
GACTTCCTCAACGTCGAGGGCAAGGCTGAGGGGCAGCACAGCCTGATGCTGGCCGCTACCGGCTCGGAGATGGCCC
GTGGCGAGCCGATCAAGGTTGTGCACACCGAAGGCGGCGATGCGCACTTTTCCCTGGTGGGTGACACCGTTGACGT
CGGCGCCTATGCCTATGGCTTGAAGCAGGAGGGCACCGACTGGTTGCTCGACCCGACCCGTCGCGGCACCAGCACC
AGCGCCCACTCGGTACTCGCCCTGTTCAACAGCGCGCCGACCGTGTTGTACGGCGAGATGAGCATCCTGCGTACGC

GCATGGGCGAATTACGCTTCAGCGAAGGCGCTGGCAACGGCCTGTGGATGCGCAGCTACGGCAATAAATTCGACGT
GGCAAGCAACAAGAACGGCGCGGGCTACAAGCAGGTCCAGAAGGGCTTTACCATCGGTGCCGACGCGCCGTTGCCC
GGTGGTGATGGTCAGTGGATTGCCGGCGTGATGGGCGGCCACAGTACCTCGGACCTGAGCCTGACCCGAGGCAGCA
GTGGCGAGGTCAAGAGCTACTACGTGGGTGGTTACGCCACCTGGCTGGACGCTGAAAGTGGTCTGTACTTCGATGG
TGTGGCCAAGCTTAACCGCTTGCACACTGAGAACGATGTGACCATGAGTGATGGTAAAAAAGCCAAGGGCAACTAC
ACGCAAAACGCCGTGGGCGCGTCGGCCGAGTTCGGTCGCCACATCAAGCTGGATAACGATTTCTTCGTTGAACCGT
ATGGTCAGTTGTCGGCCACGATCACCCAGGCGCAGAGCTATGAGCTGTCCAATGGCCTGAAGGCCAAAGGTGATCG
CTCCAGCAGCGTGGTCGGCAAAGTGGGTGTGACTGCGGGCAAGAACATCCAGCTGGAAAGTGGCGGTGTGTTGCAG
CCTTACCTGCGTACTGCGGTGGCCCATGAGTTTGATCAGAGCAACAAGGTGTTTATCAACGATCAAAGCTTCAACA
ATGACCTGTCGGGTTCGCGGGTTGAATTGGCCGCCGGTGTTGCCATGTCGGTGTCGCAGAACGTCAAGGTGCACGC
GGACTTTGAAACCAGCACAGGCAAGAAGATCGACCAGCCTTGGGGTGTCAACTTCGGTATTCGTTACGACTTCTAA
GACCCCCACCCACAAAAAAAGGAAGCTTCGGCTTCCTTTTTTTGTGGACGTTGTTAGGGGTCAGCCGAACAGTCCG
GCGGCAATATTGATCGAGAAGCCCAGGATCGCCGTGTTGAACAGGAACCCGATCAACGATTGCCCCAGTACCACCT
TGCGCATGGCGCGGGTGGCGACGCCCACGTCCGAGGTCTGGACGGCGACGCCGATGGTGAAAGAAAAGTACAGGAA
GTCCCAGTAGTTGGGTGTCAGCGGCCCCTCGGCAAAACGCAGCGCTGGCTCCTTGCCCTCCCAGGTGTAATAGAGG
CGGGCGTAATGCACGCTGAAAATCACCCCGATCAGCAACCACGAGCCAATCACCGTCACCCCGGTAAAGCCGTAGT
GCAGCAGGCGTTCGGCAGTGCCCAGGTCTTTACTGCCGGCCAGGCCAAAGGCGATGGTCGCCAGGCTGGCAATCGC
GGCAATACACACCATGAACAGCACCAGGCCGGCGTTCTCGTCCTCGATTTCAGCGATACGCTTGACGTCTTCGGCC
TTGGCCTTGATGGTCAGGCGCAGCATCAATATCAGGTAGGTCCAGACCCCGGCGTTCCAGCCGATCAGGATTTTGG
TGAGGATCGAATCGAAGGGCGCGAGGATGCCGATTGCCAGGCCCAGGACGGCGGCGGCGGACAGGCGAGGGTGGGT
TCGGGCGAGAAAGGGCATGGTGCTCACAAATGCAGGTTTGGTGAGCAACCATAGCCTATTCCTGTAGGAGCCGGCT
TGCCGGCGAAAATCATCAACGATAACGCGGTTATCCTGATGCCCCGGAGCGCTCTCTGGTTTTTCGCCGGCAAGCC
GGCTCCTACAGGGTGGCGCTATCAATCTTTGCGTTTGCGCACCAGTTTCATCACCACCACGAAGAACACCGGCACA
AACACGACCGCCAATGTCGCGGTGATCATCCCGCCGATCACCCCGGTACCGATCGCCTGCTGGCTCGCCGAACTGG
CACCGGTGGCAATGGCCAACGGCACCACGCCGAGGATAAACGCCAGCGAGGTCATGAGGATCGGCCGCAGACGCAA
GCGTGCAGCCTTCAACGTTGCCTCGATCAGGTCTTCACCCTGGTCATACAGGTCCTTGGCAAACTCGATGATCAGA
ATCGCGTTTTTCGCCGACAGCCCGATAATCGTGATCAAGCCGACCTTGAAGAACACATCGTTAGGCATGCCGCGCA
GCGATACCGCCATGACCGCGCCCAATACCCCCAACGGCACCACCAACAACACCGAGGTGGGGATCGACCAGCTTTC
ATACAGCGCCGCCAGGCACAGGAACACCACCAGCAGCGACAGCATCAGCAGCAGCGGCGCCTGCGAGCCCGACAAC
CGTTCCTGCAGCGACAAGCCGGTCCATTCCTGGCCCAGGCCAGCCGGCAGTTGGCTGACCAGGCGCTGGATTTCTT
CCATGGCCTCGCCGGTGCTGTGGCCCGGTGAGGCCTCACCGGAAATGGCGATGGCCGGGTAGCCGTTGTAACGGGT
CAATTGCGCCGGGCCCTGGACCCAACGAGCTTCGACAAACGCCGACAACGGCACCATCTTCCCGGCCTCGTTGCGC
ACGTGGATCTTCATCAGGTCTTCGACCTGGCTGCGTTGATCACCTTCGGCCTGTACCACCACCCGCTGCATGCGGC
CCTGGTTGGGGAAGTCATTGATATAGGCCGAGCCAATGGCCGAGGAGAGGATATTGCCCACATCGGCAAACGACAC
GCCCAAGGCATTCGCGCGTTTGCGGTCTACCTCAAGCTGCACCTGCGGTGCTTCTGCCAGGGCGCTTTCACGCACG
TTAGCCAGGATCGGACTTTTTTCCGCCGCCGCCAGCAACTCGGTACGCGCCGCCATCAACGCCGCATGCCCCACAC
CGCCACGGTCCTGCAAGCGGAACTCAAAGCCGCTGGAGGTGCCCAGGCCGTCCACCGGCGGCGGCAGCACGGCATA
GGCCATCGCATCCTTGAGCTCGGTGAAGGCCATGTTGGCGCGGTCGGCAATCGAGGCCGCCGAATCGTCGCTGCTG
CGCTCGGACCAATCCTTGAGCGTGGTAAACGCCAGCGCCGCGTTCTGCCCCGAGCCGGAAAAGCTGAAGCCCATGA
TCATCGTCGTATCACCGACGCCTGGCTCGGTGGCGTTATGCGCCTCGATCTGCTCGGCCACCTGCACCGTGCGGTT
CTTGCTCGCGCCCGGTGGCAGCTGGATATCGGTGATGGTGTAGCCCTGGTCTTCCACCGGCAGGAACGAGGCGGGC
AGGCGGCTGAACAAATAGCCCAGGCCCACCAGCAATACCAGGTAGATCAGCAAATAGCGGCCGCTGCGCTTGAGGG
CATAGGCCACCCAGCCTTCGTAACGGTCGGTCAGTTGCTCGAAGCGGCGGTTGAACCAGCCGAAGAACCCGCCCTT
GGTGTGATGCTCGCCCTTGGCAATTGGCTTGAGCAACGTGGCGCACAGGGCCGGGGTCAAGGACAGGGCGAGAAAC
GCCGAGAACAGGATAGAAGTGGCCATCGACAACGAGAACTGCTGGTAGATCACCCCCACCGAGCCGGCCATGAAGG
CCATCGGCAGGAATACCGCCACCAGCACCAGGGTAATCCCGATGATCGCCCCAGTGATCTGGCCCATGGCCTTGCG
CGTGGCTTCCTTGGGCGACAGGCCCTCGGTCACCATGATCCGCTCGACGTTCTCCACCACCACGATTGCATCGTCC
ACCAGGATGCCGATGGCCAACACCATGCCAAACATGGTCAGCACGTTGATCGAGAACCCCAGCAACAGCATGGTGG
CGAAGGTGCCCATCAGGGCAATCGGCACCACCAGCGTCGGGATCAGGGTGTAGCGGATGTTCTGCAGGAACAGGAA
CATCACGGCAAACACCAGCGCCATCGCCTCGAGCAGGGTGTAGATCACTTTGGTGATCGAGACCTTGACGAACGGC
GAGGTGTCGTAAGGAATCTTGTACTCCACATTGGCCGGGAAATAGCGCGACAGCTCATCCATCTTCGCCCGCACCA
GGGTCGCCGTGCTCAAGGCATTGGCGCCCGGCGACAGCTGCACGCTGACGGCGGTAGATGGCTTGCCGTTCAAGCG
CGTGGAGAACTGGTATTCCTGGCTGCCGATTTCGACGCGCGCCACATCGCCAATGCGCACGGTGGAGCCGTCCGGG
TTGGCCTTGAGCACAATGTCGGCAAACTCGGCCGGTGTGGAGAGTTGGCCCTTGACCAGGATCGAAGCGGTGATTT
CCTGGGTGTTGGTACCCGGCAAATCACCAATGCTGCCCGCCGAAACCTGGGCGTTCTGCGCGGTGATCGCGGCATT
CACGTCGGCCGGGGTCAGGTTGAAACCGATGAGTTTCTGCGGATCGATCCAGATCCGCATCGCCCGTTCGGCGCCA
TACAACTGGGCCTTGCCCACGCCGTCCAGGCGCTTGAGCTCGTTCATCACGTTGCGCGCCAGGTAATCGCTGAGCG

CCACGTCGTCGAGCTTGCCGTCGCTGGAGGTCAGGGTCACCAGCAGCAGGAAACCGGCGGACACTTTCTCCACCTG
CAGGCCTTGCTGGGTTACGGCTTGCGGCAGGCGCGGCTCCACGGCCTTGAGGCGGTTCTGCACATCGACCTGGGCC
ATTTCCGGGTTGGTGCCTGGCTGGAAGGTCGCGGTGATAGTGGCCGAGCCGAGGCTGCTCTGGGATTCGAAATACA
ATAAGTGGTCGGCACCGTTGAGCTCCTGCTCGATCAGGCTGACCACGCTTTCGTCCAGGGTCTGGGCCGAAGCACC
CGGGTACACCGCGTAGATTTCCACCTTCGGTGGTGCGACGTTGGGGTACTGCGCCACCGGCAACTGCGGGATGGCG
AGGGCGCCCGCCAGCAGGATAAACAGGGCGACCACCCAGGCGAAGATCGGGCGGTCAATAAAGAACTGCGGCATTG
TCTCTGATCCCTTTGATTACTGGCCGGCTGCCTGGGCAACAGGCTCGGGGCTGGCATCCACTTCGACTTTTTCACC
GGGGCGCGCGTGTTGCAGGCCTTCGACGATGATGCGGTCACCGGCCTTCAGGCCGCTGTTGACCACCCAGCGATCG
TTGACCGCCGCGCCCAACTCCACCGGTTGCTGGCTGACGTTCTGTTCGGCATCCACCAGCAACACCATGGGGATAC
CGGCGCTGTCGCGGGTGATTGCGCGCTGCGGCACGCTCAGGCCTTGCTGGTCGACCGCCTGCTCCAGGCGCACACG
GATAAAGCTGCCGGGCAACAGGTCGAGGTCCGGGTTGGGGAACTCGCTGCGCAGGATGATCTGCCCGGTACCGGGG
TCCACGGAAATCTCCGCGAACAGCAACTTGCCCGGCAGCGGGTACAGGCTCCCGTCATCCTGGATCAGCGTGGCCT
TGGCCTGGTCCTGGCCCACCTGCTTCAAATGCCCGGCGCGAAAGGCCCGGCGCAGGTCATTGAGCTCGCGGGTGGA
CTGGGTGAGGTCCGCGTGGATCGGGTCCAATTGCTGGATCAATGCCATGGGCGTGGCTTCGTTCTGGCCCACCAGG
GCGCCTTCGGTCACCAGCGCGCGGCCGATACGCCCGGAGATTGGCGCCGTCACCGTGGCATAGCCCAGGTTGAGCT
TGGCGCGCTGCACCGCGGCCTGGTTGGCCGCGACATCGGCGGCAGTCTGCCGGGCGGCGGCGCGGGCGTTGTCGTA
GTCCTGGGCGCTGATGGCATTGCCTTCGACCAACTGGCTATAGCGCTGCTCTTGCAGGCGCGCCTGGAAGGCATTG
GCCTCGGCCTTGCGCAGGCTCGCCTGGGCGCTGTCGAGGTCAGCCTTGAACGGCGCCGGGTCGATACGGAACAGCA
CGTCGCCCTGTTTCACGTCATGGCCTTCGTTGAACACCCGCTGCAAGACCACGCCTGCGACCCGTGCGCGCACCTC
GGCAATGCGCGGCGCGGCAATCCGCCCGCTCAGTTCACTGCTGATGGACAGGGGCTTGGCTTCAAGGGTTTCAATC
CGTACCTTGGCCAGGGGCATTTGCGGGGCTTCGTCCGCAGAATTGCCACAGGCCCCCAGGGCCAACGTCAGTGCCA
GCAGGCAAAACGGCGCAAACAGATTCTTTGACATGCTCTTATCCCAATATTGACTGGCGCATCCTACGGGCGCCCC
CGACCCGGTGCTGTGAAGCTATGTAGGCGGTGTGTGAAGAAATGTAAGGTACGGCGCACGCCGGGGCACGTGCGTA
TATCCTTCACGGCTGTGGATCCTAACAAGCTCTTCTGATCAATGACGATCTAATGTGGGAGCGGGCTTGCCCGCGA
TAGTGGTGTGTCGGTAGATACATCCGCGACTGATGCACCGCCATCGCGGGCAAGCCCGCTCCCACATTGGATTTGT
GTACGACAAAAAAACGTATTAATTGGAAACACCATGCCCAACATCCTCCTGGTCGAAGACGACGCCGCGCTGTCCG
AGCTGATTGCCAGTTACCTTGAACGCAATGGCTATCACGTCAATGTGCTCAGCCGTGGCGACCATGTGCGCGAACG
GGCCAGGCTCAATCCACCGGACCTGGTCATCCTCGACCTGATGCTGCCTGGCCTTGATGGCCTGCAAGTCTGCCGC
CTGCTGCGCGCCGACTCGGCGGGCCTGCCGATCCTGATGCTGACCGCCCGCGATGACAGCCATGACCAGGTGCTGG
GCCTGGAAATGGGCGCCGATGACTACGTGACTAAACCGTGCGAGCCACGGGTGTTGCTGGCCCGGGTGCGCACCTT
GCTGCGCCGCAGTAGCCTCAGCGAGCCACAGGCGGCCAATGACCAGATCCTGATGGGCAACCTGTGCATCGACCTG
TCGGAGCGCTCCGTGACCTGGCGCGAGCAGGTGGTGGAGCTGTCCAGCGGCGAATACAACCTGCTGGTGGTACTGG
CCCGGCATGCAGGTGAAGTACTCAGCCGCGACCAGATCCTGCAGCGCCTGCGTGGCATCGAGTTCAACGGCACCGA
CCGCTCGGTGGACGTGGCCATTTCCAAACTGCGCCGCAAGTTCGACGACCATGCCGGCGAGGCCCGCAAGATCAAG
ACGGTGTGGGGCAAGGGCTACCTGTTCAGCCGTTCCGAGTGGGAATGCTGACACCATGTTCCGCGTCCTGATCCGT
CTCTACCTGCTGACCATCGTGACCTACAGCGCGGCGATCTACCTGATCCCCATGGGCATCATCGAGGTCTTCCACA
ACCGCTACATGAACTACAACATCGAGCAGTCCCGGGGCTTGCAAAGCCTGATCGTGCGCCAGTACCACAGCCGGCC
GGTGGAGCGCTGGTCCGAGGTCACCGAGCAGTTGTCGCGGGACTTCGCGCCATTGAAGGTGCAATTGATGCATCGC
CAGGACGCCCGCTATACACCAGATGAGGAGCGGTTGCTGGAGCAGGGCAAACCGGTGATCCGCCTGGGGGAGTGGG
GCTGGATGGAAGAAATCAGCTCGCCGCTCAATGAGCAGTTGGCGGTCAAACTGACCATCCCGCCCGACCCGCTGGA
CATGAACCTGCTGTACTGGAGCATGAACGTGCTGATCGGCGCGGCGTTGCTGGGCGGGTTGTTGATATGGCTGCGC
CCGCATTGGCGCGACCTGGAACGCCTCAAGACCACCGCCGCGCAACTGGGCCGCGGCCATCTGGAAGAGCGCACGC
ACATCCCCCCCAGCTCCAACATCGGCAGCCTGGCGGCGGTGTTCGACACCATGGCCAACGACATCGAACACCTGCT
CAATCAGCAGCGCGACTTGCTCAATGCCGTCTCCCACGAACTGCGTACCCCGTTGACCCGGCTGGACTTCGGCCTG
GCCCTGGCACTGTCCGAAGACCAGCCGGCCGCCAGCCGCGAACGCCTGCAAGGCCTGGTGGCGCACATCCGCGAAC
TGGATGAGCTGGTGCTGGAACTGCTGTCCTACAGCCGCCTGCAAAACCCGGCGCAACTGCCCGAGCGGGTGGACGT
GGTGTTGGATGAGTTTATCGACAGCATCCTGGGCAGCATCGACGAGGAACTGGAGAACCCCGAGATCGTGATTGAT
GTGGTGCTGGACTGCGCCATGGAGCGTTTTGCCCTCGACCCGCGCCTTACCGCCCGTGCGCTGCAAAACCTGCTGC
GCAATGCCATGCGTTATTGCGAACGGCGGATCCAGATTGGCGTCAAGGTCTGCGCCAAGGGCTGCGAAATCTGGGT
GGATGACGATGGCATTGGCATCCCCGAAGAACAGCGCGCGCGGATTTTCGAGCCTTTCTACCGCCTGGACCGAAGC
CGCGACCGCGCCACCGGAGGTTTCGGCCTGGGCCTGGCGATCAGCCGCCGGGCCGTCGAGGCGCAGGGCGGCACAC
TCACCGCACAAGCCTCGCCCTTGGGCGGCGCGCGCCTACGGCTGTGGCTGCCTACCTGAGCAGTGTGGCCAGAGGG
TTTGTTGTGGCTAAGGTCGCTTGTTGTGGAGAGGGGTTTGTTGTGGCGAGCGGGCTTGCCCCGCGTTGGGTGGCGA
AGCCGCCCCAATAGCCTCACCACGTTCTGCCAGGTATACCGAGGTAGTTGGTTTTGGGGCTGCTACGCAGCCCACC
GGGACAAGCCCCCTCGCCACGAAAGCCCGCTCAAAGCTTCTACTCCAGTCCTGCTTTTGGCACATGCACTGCCTTA
CCGCCCCTGATGCACCCTTGCTATAGTTCGGCCCTTATCAGCCGTCAGCCAAGGATCACTGCCATGTCCGAATACC
AAGCCTTTGTCGTCGAACTCCACGGCAACGTCGCCCATGTGCAGATCAATCGCCCAGAAAAAATCAATGCCATGAA

TGCGGCGTTCTGGAGCGAGATCATCGAGATTTTCCAATGGATCGACGACACCGACGCCGTGCGCGCGGTGGTGTTG
AGTGGTGCCGGCAAACATTTTTCCTCGGGCATCGACCTGATGATGCTGGCCTCGGTGGCCAATGAGTTTGGCAAGG
ATGTGGGGCGCAATGCGCGCTTGCTGCGGCGCAAGATCCTGCAACTGCAGGCCTCGTTCAATGCCGTCGACAACTG
CCGCAAACCGGTGCTGGCGGCGATCCAGGGCTACTGCATCGGCGGCGCAATCGACCTGATCAGCGCCTGTGACATG
CGCTACGCCGCTGACGACGCGCAGTTCTCGATCAAGGAAATCGACATCGGCATGGCCGCCGACGTCGGCACCCTGC
AACGCTTGCCGCGCATCATCGGCGACGGCATGTTGCGGGAGTTGGCCTACACCGGTCGCACCTTTGGCGCCGAAGA
GGCGCGCAGCATCGGCCTGGTCAACCGCACCTTTGCCGATCACCAAAGCCTGCTGGCCGGAGTGATGGAGCTGGCT
GCGCAAATCGCCGCCAAATCACCGATTGCCGTGACCGGCACCAAAGCCATGATCAGCTACATGCGTGACCACAGCA
TCAACGATGGCCTGGAATACGTTGCCACCTGGAACGCTGCTATGTTGCAATCCAACGACCTGCGCGTGGCCATTGC
GGCCCATATGAGTAAGCAGAAACCCGAATTCGTGGATTGACTGAACATGACCCCAGGCTGGATTACCACAACGCTG
CTGGACAACGACGCCCCTGGCGGCTGGGCTGTGGCCCGTAGTCGCGAAGGTTTTTTACTCGATGACAACGGGCCGC
TGTTCCCCAGGGAGTGGCTCAAGCGCCAGGACCTGTCGGTGTTGGCCGAGCATGGTATTGGCCACCTGGACGGTGA
GCCTGTGTATCTGCTGGAACTGAACAGCCCTGGCGATGTGCCGGGTTGCCGCTGGCAAGGTTTGCGGGCCTTTATG
CTGCAGGGCGATCACCGTTTGTACAAAGTCCTGGGCTATGCCGCACAAATCGGTACCTGGGCCCGCGAGCACCGGT
TCTGCGGCAGTTGTGGCCAGGCGATGGTGCATGTACCCCGGGAGCGCGCGATGTTCTGCCAGACCTGCGACCTGCG
CAGTTACCCGCGCATCTCACCGAGCATGATTGTGCTGATCACCCGTGGCGATGAGATTCTGCTGGCCCGTTCACCG
CGTTTCGTCACCGGGGTCTACAGCACCCTGGCCGGGTTTGCCGAGCCGGGGGAGTCTGCCGAAGACTGCCTGATCC
GCGAAGTGCGCGAGGAAGTGCAGGTCGAGGTGCAGAACATTCAGTACGTGGGCAGCCAGTGCTGGCCGTTTCCCCA
CTCCATGATGCTGGGCTTCCACGCCGAGTATGCCGGGGGCGAGATCGTGCCCCAGGCCGACGAGATCGAGGATGCG
CAGTGGTTCAACATCCACCAGTTGCCACCGCTGCCGGCGTCGCGCTCGATTGCCCGCTACCTGATCGACCTGTATG
TGGCGCGCCGCTTAGGCCACGCTGAACCAGTGCTGCCAGGCTAGGCGCACAGTCAGGCCGAGGACCACGGTGATAA
ACACCGGGCGAATGAATTTGGCGCCACCGCTGATGGCACTGCGGGCGCCAAAGAACGCGCCCACCATCACCGACAG
GCCCATGCTCAGGCCGACAATCCAGTCTACCGAGCCATTGAAGATAAACACCGACAGCGCCGCAGCGTTACTGACG
AAGTTCATGCTGCGCGCCACGCCGCTGGCCTTGACCAGGTCGACGGGGTACATCAGCAGGGTGCTGACGGTCCAGA
ACGCGCCGGTGCCCGGGCCGGCCACGCCATCATAGAAGCCCAGGCCAAAACCCTGGGTCGACTGCCATTTCTTCTT
GATCGGCGCATCTGCGTCCAGCGGCGCCTTGGGTGTGCCGCCAAACAGCAGATAGATGCCACAGGCGAAGACAATC
ACCGGCAGCATCTTGTTCAGCCATTCGGCGGGCAGGTAATGGGCGACGACGGCCCCGACCAGCGCGCCCACCAGCG
TGCCGACGATGGCATGCACCCACTGGCGCGGGTGGAACAGCTTGCGCCGGTAGAAGGTAAAACTGGCCGTGGCCGA
ACCGAAGGTCGAACTCAGTTTGTTGGTGCCCAACACCAGATGGGGCGGCATGCCGGCAGTGAGCAAGGCCGGGGTG
GTGAGCAAACCGCCGCCGCCGGCAATGGCATCGATAAAACCGGCGATAAAGGCAACCACTGCGAGAATGGCCAGGG
TGGTGAGGTCAACGCTGAGTTCAAAAGGCATGGAGGCGACTTATTTTCGGCGAGGCGCAGAAACAGCTGCGCGAAA
GTCCGCTAGCTTACCTACAACCGATTGTTACGGCGACTGTTGTGTCAGCAACAGTGATTCAACAAACCATCCGCCC
GGGCAACATTCGCAGGTCCAGGTTCGGAGCGGTCGCAGGCGCCTGCCACGGGGCTTTGTCCACGCTGGCACGGTTG
CTGCTCCAGACCCTGCACCATCCCTGTGTGCGAGGTAACGAACGATGACTCAGCAAGCCGTGAAATTCGCCTATTG
GGTGCCCAATGTCAGTGGTGGCCTGGTGGTGAGCAAGGTCGAACAACGCACCGACTGGGGCATCGACTACAACCGC
AAATTGGCCCAACTGGCGGAGGAGGCGGGGTTTGAGTACGCCTTGACGCAAATCCGCTTCACCGCAGGCTACGGTG
CCGAGTACCAGCACGAGTCCGTAGCCTTCAGTCACGCGTTGCTCGCCGCCACCAGCAAACTCAAGGTCATCGCGGC
GATCCTGCCGGGGCCCTGGCAGCCGGCACTGGCGGCCAAGCAACTGGCGACCATCGACCAACTGACCAATGGCCGG
GTGGCGGTGAATATCGTCAGCGGCTGGTTCAAGGGCGAGTTCCAAGCCATTGGCGAGCACTGGCTGGAGCACGACG
AGCGCTATCGCCGCTCTGAAGAGTTCATCCGCGCCTTGAAAGGCATCTGGACCCAGGACGATTTCACCTTCAAGGG
CGATTTCTACCGCTTCAACAATTACAGCCTCAAGCCCAAGCCACTGGGCCAACCGCACCCGGAGATTTTCCAGGGC
GGCAGCTCCCGCGCGGCCCGGGATATGGCGGCGCGGGTGTCGGATTGGTATTTCACCAATGGCAACACACCAGAGG
GGATCAAAGCCCAGGTCGACGATATCCGCGCCAAGGCGGCGGCCAATAACCATTCGGTCAAAGTCGGGGTCAATGC
GTTTGTGATCGCCCGCGACACCGAGCAAGAAGCCCAGGCAGTATTGGCCGAGATCATCGACAAGGCCGACCCGGAA
GCGGTCAACGCCTTTGGTGACGCCGCCAAACAAGCAGGCAAGGCATCGCCCGAAGGCGAGGGCAACTGGGCCAAGT
CCAGCTTCGCCGATCTGGTGCAGTACAACGACGGCTTCAAGACCAACCTGATCGGCACGCCCCGTCAGATTGCCGA
GCGTATCGTCGCGCTCAAGGCCGTGGGCGTGGACCTGGTGCTGGCGGGCTTTCTGCACTTCCAGGAGGAGGTGGAG
TATTTCGGCCAGCGCGTATTGCCCCTGGTGCGCGAGTTGGAAGCCAAGGCCGCCGCGACCGGCAGCGCCAGGCCGG
AGGCGTTACCTGCATAGCACCGCCGCCGCCCGTAGCGGGCAATTACCTGGGCGGCCATCCGCTGGTGCGCTGCCTA
GGTACTTGCCTGCAGGGTCAGCACTTCAAAACCATCAGCGGTGACGGCCACCGTGTGTTCCCACTGGGCTGAAAGG
CTGTTGTCGCGAGTCACCACGGTCCAGCCATCCTTGAGGCTGCGCACCTTGGCGCTGCCCTGGTTGAGCATCGGTT
CGATGGTGAACACCATGCCTTCGCGCAGGGTCAAGCCGGTGCCCGGTCGGCCGAAGTGCAGGATTTGCGGTTCTTC
GTGCATCTCCCGGCCGATTCCGTGGCCGCAATACTCGCGCACCACGCTGTAGCCATTGGCTTGGGCGTGGCTCTGG
ATCGCATGACCGATATCCCCCAGGCGGGCGCCGGGCCTGACTTGGCGGATACCGGCCCACATCGCCTCGAAGGTCT
GTTCCACCAGGCGCCGGGCCTTGGGTGCGACGGTGCCGATCATGTACATCTTGCTGGAGTCGGCAATAAACCCGCC
TTTTTCCAGGGTGATGTCGATATTGACGATATCGCCGTCCTTCAACACATCCTTGGCACTGGGCATGCCATGGCAC
ACCACTTCGTTGATCGAGGTGTTGATGCTGAACGGATAGTCGTACTGCCCAAGGCTGGCGGGGCGTGCTTGCAGGT

CATTGCGGATAAACGCCTCGACCGCCGCATCCAGTTCCAGGGTGCTCTGGCCGGCGCCGACTCGGCTGTCGAGCAT
GCTGAATACCTGGCCCAGCAGGCGCCCGGCTTCGCGCATCACGGCAATCTGTGCAGGGGTTTTGATCATCAGCTGC
GCCCCCGGATCAGGTCGGGTTTGTCGAGCAACAGCTTGTTGATCAGGTCGTTGTAGGCCAGGTGCGGGTTGAGCTC
GGCCAACAGGCCGATCTTGATCCAGAACTCTGCCTGGGCGTTGATGGAGCGGTCCATGGTGGCGCTGGCCAGGCGG
ACTTGTTCGTGCAAATGATCGGTAATCTTGACGATGCCCATGAGCTTCACTGTGTAGGGTGGATATACAAAGCGTA
TATGTTTCGTATATCGCAAGTAAACCCCGCAGTTGACTTGTAATAGGCTGCCGGTTAGTTTCGTTGCATGACATTT
CAAACCTATCGTTCCCAGCCAAGCATTATTACCGCCATTCCTCATATGGCGGGATAGCGTTCGGCTGTACCCAAAC
CCGCCCTAGAGGCGGGTTTTGTTTTTCCGTCTCCAGGGCTCTGTCAAACGCCAGGAGACACCCATGAGTAGCCCAC
TGCCGCAGTCCACGCTCGACGATGGCTTGCTTGAGCATTACGTGAAGAAAATCCTCGCCGCGCCGGTCTACGACCT
GGCGGTGCGCACGCCGTTGCAGCCGGCCCCGGCGCTATCGGCGTTGCTCGGCAATCAGATCCTGCTCAAGCGCGAA
GACCTGCAGCCGACGTTTTCCTTCAAGATCCGCGGCGCCTACAACAAACTGGTGCAGCTTACCGATGGGCAAAAAA
ACTGCGGGGTGGTCACTGCATCTGCCGGCAACCATGCCCAGGGCGTGGCCCTGGCGGCGCGTGAACTGGGGATCAA
GGCGACGATTGTGATGCCCAGTACCACGCCCGAATTGAAAGTGCTGGGCGTGCGCTCACGGGGCGCCGACGCGGTG
CTGCACGGCGCCAGTTTTCCGTTCGCCCTGGCCCATGCGTTGCAGTTAGCCGAACAGTCAGGGGGCACTTTTGTGT
CGCCCTTTGATGATCCAGACGTCATCGCCGGACAGGGCACCGTCGCCATGGAAATCCTGCGCCAGCAACAAGGCGC
ACTGGACGCGATCTTCGTACCGGTGGGCGGTGGCGGGTTGATCGCTGGCATCGCGGCATACATCAAGTATCTGCGC
CCCGAGGTGCGGGTCATTGGCGTGGAGCCCGAAGGCTCCAGTTGCCTGCTGGCGGCCTTGCGTGCCGGCGAGCGGG
TGATATTGCCCAGCGTGGACAGCTTTGCCGACGGCACCGCCGTGGCCCAGATCGGCGCCTACGGCTTTGAAATCTG
CCGCCAGCATGTCGATGAAGTCATCACCGTCAGCAACGATGAACTGTGCAGCGCGATCAAGCTGATCTACGACGAT
ACCCGCTCCATCACCGAGCCTTCAGGCGCCCTGGCGGTTGCCGGAATCCGCCGCTATGTGCAACGCACCGGGGCCC
AGGGGCAGACGCTGGTGGCGATCAACTCCGGGGCCAATATCAACTTCGATAGTTTGCGCCACGTGGCAGAGCGGGT
GGCGGCACAAGCCTGACGCCCCTTGTGGCGAGCGGGCTTGCCCCGCGCTGGGCGGCAAAGCCGCCCCAATAGCCTC
ACCACGTTCCGTCAGATACACCAAGGTGGCTGTATTTGGGGCTGCTTCGCAGCCCAGCGCGGGGCAAGCCCGCTCG
CCACAGAAGGTCGATCTGCGCGTTACTGCTCGCTAGCCTTCTCGACCGTGGTCGATGCCGACCAGATGCGGTAACG
CACTTCGACATCTTTTGGAACATATACAACGATGGGCAACTTGCTGTTGTAACGCAGCACAAAACCGTCGCCGACC
ACCGGCACAAAGTCCTCGCGGGTCTTGCCTTCGGGGCAGGCCATCATCGTGCTCATGGGGCCGCTGACCTTCTCCA
GGCGGTAAAACGGATAGCCCCAGCCTTCGAGGTTTTTCTCTTCCAGCACACCGCCCAGGCGCTGCTGGTTGCAGTC
CACCAGCAGGGTTTTGCCGGCGAGGATTTCGACCTTGAAGTCCTGTTCCTGGGACTTTGCCGGCAAGTGGATGACC
TGGCGAACATGCCCGGCGTCGGCCTTGGGGTAGGGCGCGATATCTTCAAGTTTGGCGGCATTGGCCTGGGCGGCCA
GCACGCTCATGGCCAGTACCGAAAGGAGGGTGAGAGCAGAAGAGGTCATGGGACTTCCTTATCTTAGGGGGCAATA
AAACAGCGGCTGCCACGATGCACGCCAACGTCCGGCCGTGCAAACCAGCAGCAACTTATATAAGGGTTGCTGGTGC
TTATGAGGAAAGGATCCTACGCCTACAGACACAAGGAGTAGGCTTTGCAGTTAAAGCGCCTGCCAGGGTTGGCAAG
CGTGAAGTCAACCGTCCTGACGCGGCGGTCAGACTGTCGGTATCGATAAAAATTCATTGGCGGTCTCAAAAAAGGA
GAGGTTCAGCCATGCTTTCTGAATTGGAATTACGCGGCCTTATTGAAGGCAGCTTCCTGCCCAGGCGTTGTGAATG
CATCAAGGCTCAGGATGCGTCGTTGACCATCAAAATCTACGATGACGACGGTACGGATGTCACCTTCACCGGCATC
CAGGCCCACGAACTCAACAGCAGCCGAGCGATCTGCAACTTGATCACCGAGTTGCGTGAAGACCTCAAGCATGCCC
ATGCCCCGGCTCCGCGCAGAGCGGGGGCGAGACTGTACTGATCAGCGTACGGCAGGATTGCGGGCCTGGATAAACC
CCAGGCCCAGCGCCAACTCTGTGCCCACGGCCAGCAAAATCCCTGGGCCGGCATGGCCATTGAGCCATTCGCCGAT
CCCCAGCCCCGCCAACCCCAGGCAACCGACGATAAACCCTGAAACCAGTGCGCTGCGCGTGGGGGATGGGGGCGTG
TGGCGCAACCGATAAAACATGACGCCGATCGCCAGGAACAGCATGGCGTTGCGTCGAGCGACGAGCCCACTGGCTT
CGGAAAATTCCACGTCCCACAGCCACAGCAGCCAGTCGGGCCGCAAACCCCAGGCAATTGCCAGGGCCAGGCACAG
TACGGCGGTAAAAGTGGCAAGCGGGGGGAAGCTGATGCGCATGGCGAGTCCTTTCGGCCGTTTCAGAAGGGCCCAA
GAGTACGCTGTGCAGCAAGCCTCAGACAAATCCGATACGGCGCCGCCCGCTTGCGCTGCTACTGTCCGGCCACATC
TGTCGCCAGTGGCTGAGGTAGGGCGTGCAACAAAAAATCGCAGCAGAGTTTCTCGGCACTTTCTGGCTGACCTTCG
GCGGCTGTGGCAGCGCCATTCTGTCGGCCGCATTTCCCGGCCTGGGCATTGGCTTTGCCGGGGTAGCCCTGGCGTT
TGGCCTGAGTGTGCTGACCATGTCCTACGCGGTGGGCAGCATCAGTGGCGCGCATTTCAACCCGGCCATTACAGTC
GGCCTATGGGTGGGAGGACGAATGCCGGGGCTGGATGTGTTGCCCTATATCATCGCCCAGGTCGCCGGGGCGACCG
TCGCGGCGGCGATTCTCGCGCTGATCGCCAGCGGCCGGCCAGGCTTTGAACTCGGTGAGTTCGCAGCCAATGGCTA
CGGCGCATTGAGTCCGGGCCAGTACAGCCTGTTGGCCGCACTGGTGGTAGAGAGCGTGGGAACGTTCTTCCTGGTG
TTTATCGTCATGCGGGTCACCACACCCGGCGCCGCGCCAGGGTTTGCGCCAATTGCCGTGGGCCTGACGCTGACCC
TGATCCACCTGGTATCGATTCCCGTCACCAACACCTCGGTCAACCCGGCGCGCAGTACCGGGACGGCGCTGTTTGC
CGGCACCGATTACCTGATGCAGCTCTGGTTGTTCTGGCTGGCCCCGCTGGCTGGCGGCATCGTCGGCGCCTGGGTG
GCCCGTGGCGTGCGAGTGACGGCGAAGTGAGACCGCATGGGGTTTATTCGTTTAATTGGTGGCGATAGGGCAGGTC
TGGCCCGGTCTTGCCACACGTCAGCGCGGCGGCACGGATGGCGAACTTGAGCATGCCGTCAATCTGCTCGCGGCCC
AGGCCCTGCACACCTTCCACCGAATCCAGCTGCTGCTCGGTGAGCCAGGCGATCAACGCCGCCTGGAAGGTGTCGC
CAGCGCCCACCGTATCGGCCATCACCACCTTGACCGCCGGGGCCGACCAGTTGCCGTGCTGGCGGCTGAACACGCT
TGCGCCATCGCCGCCACGGGTCAGGAAAATCAACTGGCAGCGGTGCTGTAGCCAACCCTGCAACACGCTTTCCGGC

GCCTGGCCGGGGTAGAGTAGGTGCAAGTCTTCATCGCTGACCTTGATCAGGTCGGCATAGTGCACCAGTTCAGCCA
CGCGCCGGCGCCACAGTTCGATATCCGGCTCGGGGTTCAAACGCACATTCGGATCAAGGCTGATCAGGCGCTTGCC
GCTTTCCCGGCGGATCAGGGCCAACAGGCTGTCAGCGACCGGCTGCACCACCAGGGAAAACGAACCGAAATGCAGA
CCGCGTACGCGCCCATCGAGCACCGGCAGATGTTCGATCTGCAACTGCCGATCGGCACAGCCTGCGCCGCGAAAGC
TGTACTGCGGTGAACCGTCGGCCCCCAGGGCCACCATCGCCAGGGTGGTCGGCGCGGCAAACTCCACCAAATAATC
GGCGTGCACGCCTTCATCCTTGAGGACCTGGCGCAGGCGCTGGCCCAGGTAATCGCTGGACAGCCCGGCGAACAAC
CCGGCTTCGATCCCCAGGCGGCGCAGGCCCACGGCGACGTTAAACGGCGAACCGCCGGCAATTGCCTTGAAATTGA
CTTGGGACGCCTGCGCGCTGGCATCCTCCTGGCTGAAAAAATCAAACAGCGCTTCGCCACACACCAGATACATAGT
TATTCGCTCTTAAAGGGTTGCCACATGCTGTTGATAACGTTCATAGGCCTGCTGATAAGCCCTGACGCTGGCGGCG
ACAGGCTGGGTGCGGCTGGCCGGGTCGACGCTGACGCAGCGTGCACACAGCGCCTGCAGGCTTTCGCCAGACTGGC
ACCACGCGGCCTGGATCGCCGCGCCCAGGGCCGCCGCTTCGCTTTGTTCGGTACACACCACTTCGGTGTCCATGAT
ATCGGCGACCATCTGCCGCCACACCGGGCTTTTCGAGCCGCCGCCGGTCAAGCGGATACTGTGGCTTTGCAGGCCG
GTCTGGCGCAACAGGTCCAAGCCTTGGCGCAAGCCGAAGGTGGTGCCTTCGACCACTGCGCGGCATAGATTGCCAC
GGGTCAGGTTGGTCATGGTCAGGCCATGCAGGCTGCCGGTGGCGTGGGGCAGGGCGGGCACGCGCTCGCCATTGAG
GAACGGCAGCATGCTGACGCCCTCGGCGCCAATCGGGGCCTGGGCCACCAGGGCGTTGAACGCTACCAGGTCCAGC
TCGAACAATTCGCGGATGACCCCGGTGGCATTGGTCAGGTTCATGGTGCAGATCAGCGGCAGCCAGCCACCGCTGG
AAGAACAGAACGTCGCCACCGCAGCTTCGGCGCTGACCGTCGGTTGATCGGCAAACGCATACACGGTGCCCGACGA
ACCAAGGCTCATGGTAAACACCCCTGGCACGATATTGCCGGTGCCAATCGCCCCCATCATATTGTCGCCGCCACCA
CTGGACACCATCGCCCCGGGGTTGATCCCCAGGCGTTCGGCGATGGCCGGCAGAATGTTGCCCACGGGCTGGTTGG
CCTCGATCAACACGGGCAAAGCCGCCTCCAGGCGCCCGCTGGCATCGATGTGCCGCAGCAACGCAAGGTCCCATTG
GCGGGTACGCACATTGAAATAACCGGTGCCCGAGGCGTCGCCATACTCGGCGCAGGCGCGGCCAGTGAGCCAATAG
TTGAGGTAATCGTGGGGCAGCAGCACATGGGCGATGCGTGCGAAAACGTCCGGGTGTTGCTCCAGGGTCCATAGCA
GCTTGGACACGGTATAGCCCGGGGCAATCGCGACGCCCAACCGCTCCAGGGAGCCGCTTTCACCGCCCAGGTGCTC
GAGTAAACGGGCGTTTTCGGCGCTGGTTTCGGTGTCACACCACAGCTTGGCCGGGCGCAGTACCTGGCCGTGCTCG
TCGAGCAGCACCAGGCCGTGTTGCTGGCCGGAAACCCCGATGCCGAGGATGTCCTGGCCGTCCACGCCGGCTTGTT
GCAGGGCGCGGTGAGTGGCTTCGGTAAAGGCGTCCAGCCACTCCTGAGTGTGCTGCTCGCGCCGGCCATTGGCGCC
GCTGATCAGTGTGTGGGCAGCCGCGCCCAGGCCCAGCACCTTGCCGCTGGCAGTGTCGAGGACGATGGCCTTGGTG
CCTTGGGTACCGCAGTCGATGCCGAGGAACAGAGATTGCCGGGTCATAGTTATCGCTCAATGGAACGCGGTCAAAA
TGTGGGAGCGGGCTTGCTCGCGAAAGCGGCGTATCAGCCAAACATTTATCAACTGACCCACTGCTTTCGCGAGCAA
GCCCGCTCCCACATTATTTTGCTCAAGTTGTGTCAGTGAAGGTTGGCGAGCAGGTGCTTCAGGGTCTCTGCGACAC
CTTTGTCACGCAAACTGGCAAAACACCGCTCAAACGCTGCCACAAACTCAGGTGAGTTGGGAATCGCCGGGCCAAA
AATCTCTTCCACACCCAACAGCCGCTGACTGACGCAAGCGTCATCCACCACCAGTGCCCGGCAGAAGTCCGCGCGC
GGGTCGGGGATGCGGTAGCGCACGCCGTTCTCGTCCACACCTCGCAGGTACAAGGCCCAGGCCGCCACCACCAGCG
CTGCTCGCTCGGTGTCGCGACCCTCGGCAATCAGGCGGTTGATGGTCGGTACCGTGAACTTGGGAAACTTCGACGA
ACCGTCGGAACAGACCCGCTCCAGTTGATCGGCAATCGCCTGGTTGGAGAAGCGCTCCACCAAGGTCTGCTTGTAC
TGGTCCAGATCGATGCCCGGCACCGGCGCCAGGTTGGGGGTCACGTCCAGGTCCATATAGGCGCGCATATACGCGA
CAAACAGCGGGTCGTTCATGGTTTCGTGGACAAACCGGTAACCCTTGAGGAACCCCAGGTAGGTCAGGGCCAGGTG
GCTGCCGTTGAGCAGGCCGATCTTCATTTCTTCATAGGGCGTGACGTCGTCGGTGAACTGCACACCCACCTGCTCC
CAGGCTGGCCGGCCGTTGACAAACTTGTCTTCCAGTACCCATTGCACAAAAGGTTCGCACACCACCGGCCAGGCAT
CGTCGATGCCGTGTTCATCATGCAGTTGCAGGCGGTGGGCGGTACTGGTCATGGGCGTGATGCGGTCGACCATGGC
GTTGGGGAAACTCACGTTAGCCTGGATCCAGTCATGCAGTTCGGCATCGCGCAACGCGGCGAACGCCAGCAACGCC
TTGCGGGTCACGGCGCCGTTGTGCGGCAGGTTATCGCAGGACATCACGGTAAAGGCCCGAATGCCTGCAGCACGGC
GCTGGCTCAGGGCCGCGCAGATAAAACCGAATACGGTCTGCGGCGCCGCAGGGTGGGCCAGGTCATGCTGGATCTG
CGGCAAGTGGGCCATGAACTCACCGTTGCTGTCGTCGATGCAGTAGCCGCCCTCGGTGATGGTCAGCGACACGATG
CGGATTTCAGGGCTGGCCAGTTTGTCGATCAGGGCCTGGGCGCCGTCCTGCGCCAGGAGCATATCGCTGATGGCGC
CGATCACCCGCACTTGGGTGTCGTCGGTGTCGCCCAGCTCATACAAGGTGAACAGATAGTCCTGGCCGGCCAGGTC
ATCCCGCGCCTTGCGGTCTTCGGCGCGCAGGCCGATGCCGCAAATGCTCCAGTCCAGGCCGACACCGGTGTTCATC
AGGGCGTCGGTGTAATACGCCTGGTGTGCGCGGTGGAAACCGCCGACGCCGATATGGGCGATGCCCTGGCGTGTGT
CGGCAATGGCATAGGCGGGCAGGCGCACTTCGGGCGCCAACTGGGTGAGATTGTGTTGATTGAGTTTCATCAAAAT
GCTCGCGAAATCAGGCGGCAGCGCGCAGTGGACGGGCCACCGCAACGCCGTCGGCGTCGAATAGATGGCAGTGCGC
CGGGTCCAGGTGCAATTGCAGCGTCTCGCCATACTGGCTGGCCATATCGCCACGGATCCGCATGGTCAGCGGCTCG
CCACTGGCGGTGAGCACATGGCAGAACGTGTCGCTGCCCAGGCGCTCGCCCACGTCGGCGGTAACGGTGAGGGTGG
TATCACCCGGCGCGGCCAGTTCCAGGTGCTCGGGGCGGATGCCCAGGGTCACGGCGCTGCCCACGCGCAAGCTGGC
GCTGCTCAATGGCAGGCTGATCAGGGTGCCAGCGTCCAGTTGCACATCGCAGCCCTGGCCTTCGACCCGCGTAACC
TGGCCCTTGAGAAAGCCCATTTTCGGCGTCCCCAGAAAACCCGCGACAAACAGGTTGGCCGGCTGGTGATACAGCT
CCAGGGGGGAGCCGACCTGTTCGATGCGCCCACTGTTAAGCACCACCACCTTGTCGGCCAGGGTCATGGCCTCGAC
CTGATCGTGGGTCACGTAGATCATGGTGGCTTGCAGTTCTTTGTGCAGGCGCGCCAGTTCCAGGCGCATTTGCACG

CGCAGGGCGGCGTCGAGGTTGGACAGCGGCTCGTCGAACAGGAAGATCTTCGGGTTGCGCACAATCGCCCGGCCGA
TGGCCACCCGCTGACGCTGGCCGCCAGACAACTGCTTGGGCTTGCGCTCCAGCAGCGGGCCCAGCTCCAGGATGCG
TGCAGCCTCACTGACTTTGCTGTCGACGATCTTCTTGTCTACGCCCGCCAGGTCCAGGGCAAACGACATGTTCTTG
CGCACGCTCATATGCGGGTACAGGGCATAGGTCTGGAACACCATGGCCAGGTCGCGCTTGGCCGGGGTCACTTCGG
TGATGTCGCGGCCATCGAGTTCGATGGTGCCTTCGGTGACTTCCTCAAGGCCGGCAATCAGGCGCAGCAGGGTGGA
CTTGCCACAGCCCGAAGGCCCGACAAAGACCACGAATTCCTTGTCGTTCACTTCCAGGTCGATGCCCTTGATGATG
GAGAAGCCTTCGAAGCCTTTTTGCAGATTCTTGATTTTCAGGTTGGCCATGATGGGCCTCCATTTTTTATAAGGGT
CGTTATTTCACGGCGCCAAAGGACAGGCCGCGCACCAGTTGCTTCTGGCTGATCCAACCGAAGATCAGGATCGGCG
CGCAGGCCAGGGTCGAGACGGCGGACAATTTGGCCCAGAACAAACCTTCGGGGCTGGAGTAGGAGGCGATCAACGC
GGTCAGCGGCGCGGCATTCGACGAGGTCAGGTTCAGCGACCAGAACGCTTCGTTCCAGCACAGGATCAGCGACAGC
AATACCGTGGAAGCCAGGCCGCCCTTGGCGATGGGCAGCAGCACCCGCACCATTTCCTGCCACAGGGTGGCGCCAT
CCAGGCGCGCGGCTTCGAGGATGTCCTTGGGAATGTCCTTGAAGTAGGTGTAAACCATCCACACCCACAATCGGCAG
GTTGATCAGGGTGTAGATGATGATCAGCGCGATGCGCGTATCCAGCAGGCCCATGCTCTTGGCCAGCAGGTAGATG
GGCATCAGCACACCCACGGGTGGCAGCATCTTGGTCGACAGCATCCACAGCAGCGTGCCCTTGGTGCGCTGGGTTT
CGTAGAACGCCATGGAGTAGGCCGCCGGCACCGCAATCAGCAGGCACAAGGCGGTGGCGCTGAAGGAAATCACCAC
TGAGTTCCAGGCGAAGCTTACGTAATTACTGCGCTCGTTGATGTGCAGGTAGTTCTCAAGGGTTGGCGTGAAAATG
AACTGCGGCGGCGTGGCAAACGCGTCGATTTCGGTCTTGAAACTGGTGAGTACCATCCAGAAGATCGGGAAGAAAA
TCACGATCGCGATGGCCCAGGCCAGGGTGCCGAGCAACAAGCTTTGCAGGCGGCGGGATTGTTGAAGCGTCATGGC
GCGGCCCTCAAGGCTTGTCAGTCAGGTTTTTGCCGAGCATCCGTACCAGGATGATCGCTGCGATGTTGGCAATGAC
CACGGCAATCAAGCCGCCGGCCGAAGCCATGCCCACGTCGAACTGCACCAGCGCCTGGTTGTAGATCAGGTAGGCG
AGGTTGGTCGAGGCGTAGCCGGGGCCGCCGTTGGTGGTGGTGAAGATTTCGGCGAACACCGAGAGCAGGAAAATCG
TCTCGATCATCACCACCACGGCGATGGGCCGGGCCAGGTGTGGCAGGGTCAGGTGCCAGAAGATCGCAATCGGACC
GGCACCGTCCAGGCGCGCCGCTTCTTTCTGTTCCTGGTCCAGGGACTGCATGGCGGTCATCAGGATCAGGATGGCG
AAGGGCAGCCATTGCCAGGACACAATGATGATGATCGACAGCAGCGGGTAGTGCGCCAGCCAATCCACCGGCTGCG
CGCCAAACAGCTTCCACACCGCCGCCAGCACGCCGGACACCGGGTGGAAAATCAGGTTCTTCCAGATCAGCGCACC
CACCGTGGGCATGATGAAGAACGGCGAGATCAACAACACCCGCACCAACCCACGACCGAAAAACTCGCTGGCCTCC
AGCAAGGCACTGATCAACACGCCCAGCACCACGCTGATCAACAGCACGCTGCCCACCAGCAACAGGGTGTTGGTGG
CGCCGGGCAGGAAGCCGGAATCGGTGATGAAGTAGGTGAAGTTCTCCAGGCCCACGAATTGGTTTTCGCCGGGGTA
AAGCAGGTTGTAGCGGATCAGCGAAAAGTACAGGGTCATGCCCAGGGGCACGATCATCCACAGCAGCAACAGGGCC
ACCGAGGGGCTGACGAGGAACCAGCCGGGGTTGAGCAGGCGGCGTTTAGACGTATTCATGGCAATCGAAACCCATT
AGAGGCAGAAAGAACCGGCCTTGGAATGCAGTCAATGTGGGAGCTGGCTTGCCAGCGATAGCGGACGATCAGTCAA
CCGATTCATCCACTGACAGGCCGCCATCGCAGGCAAGCCAGCACCTACATTTACAGGGGTGGACCTGGGTTATTTG
GGATAGCCGGCGCGCTTCATTTCCCGCTCGGTGGTGGTTTGCGCGGCAGCCAGGGCCTGGTCCACCGTCTGCTGGC
CGGTCAGGGCGCCGGAGAAGAACTTGCCCACCTGGGTGCCAATCGCCTGGAACTCAGGAATGGTCACCAACTGGAT
ACCGATATACGGCACCGGCTTGAGAGTCGGTTTGGTAGGGTCCGCAACCTTCAACGACTCCAGCGTCACCTTGGCA
AAAGGCGCGGCCTGCATATATTCCTCGCTGTAGGTCGAGGCGCGGGTACCTGGCGGCACGTTGGCGATACCATCGG
TCTTGGCCACCAGCGCGCCGTACTCCTTGGAGGTGGCCCAGGCACTGAAGACCTTGGCTGCGTCCTTGGCCTTGGA
GCTGGTCGGGATCGCCAGGGACCAGGAATACAGCCACGAGGTGCCCTTGTCGGTGACCTGCTGAGGGGCAAAGGTG
AAGCCGACGTGATCGGCGACCTTGCTCTGGGTCTTGTCGGTAACAAACGAGCCGGCGACGCTGGCATCCACCCAGA
TCGCGCACTTGCCGCTGTTGAACAGCGCGAGGTTTTCATTGAAACCATTGCTGGACGCACCCGGTGGGCCGGATTT
CTTCATGTTGTCGACGTAGAAGTTCAGTGCATCCTTCCATTCGGGCCCGTTGAATTGCGGTTGCCACTTTTCATCG
AACCAGCGTGCGCCATAACCGTTGGCCAGGGTGCCGATCAGCGCCATGTTCTCGCCCCAGCCGGCCTTGCCGCGCA
GGCACAGGCCGTATTGTTCCTTGCTCTTGTCGGTGAGTTTTTCGGCAAACTCGCCAATCTGTACCCAGGTTGGATG
CTCAGGCATGGTCAACCCGGCCTGCTTGAACAGGTCGGTGCGGTAGTAAGTGATGGAGCTTTCTGCATAGAAGGGC
AGGGCGTACAGCGTGCCCTTGACGGAAAGGCCGTCGCGCACTGAAGGGAACACATCGTCCAGGTCGTAGGACGCCG
GCAGATCCTTCATCGGTTCCAGCCAGCCCTTGGCGCCCCAGAGTGCGGCTTCGTACATGCCGATGGTCAGCACATC
GAACTGCCCGCCCTGGGTGGCGATATCGGTGGTCAGGCGTTGACGCAGGACGTTTTCTTCCAGTACCACCCAGTTC
AACTTGATCTGCGGATGCTCGGCCTCGAAGGTTTTCGACAGCTTTTGCATGCGGATCATGTCGCTGTTGTTGACGG
TGGCAATGGTCAGGGTCTGTGCGCCCAGGCTGACGGCGCTGAGGGTCATGCAGGTACAGGCAAGCAGTGCTTTTGC
AGGGAAGTTCATCGCGCACTCCGTTTCTGCGCCCAGGGGCTGCAGAAGGACAGTTATTGTTGTTGTGTCTTCCATG
GCTGCAGGAAGAGTGTGCGTTGATTACAGCCTTCTAAAGGCGCGGTGACAAATCCTTGAGCGCACTTGGACTGATA
CTTTTTTGCACTCTATCGCGCAGCGATAGAGATACTTGATGGGGACTTGGAAATGAAGCAGCCGTTACAGTTGCCT
GCTGCAGCTGCGGTGTTAGGCGAATTACTCGGCGAAGTCGAACACGTTTTTGATCATGGGTTGAGTGAAAGTGCCC
TTACCGGTCCCCTCGATCAGCTTGTAAGTACCATCGGGATTGATATCCACACCCAGTGAGCTGGCGATGATGGTCT
CCACGCCCGGCTTCTTAAGTATATCTAACAGCGGTTTTCTACCTTGGACCTCCTGCCCCAATACGACCCCCATGCC
GACGGGTGATTGAGAGTCCACACCGCCTACGAAGATGACCTTGCCGCCTTTGGCTAATGACTTCTCCAACTCATCC
ACTGTCTGGGCGGCGTTTGCGTCGAACAGGCCAAACTCCAGGTTGCTGCCGGTCAGGTGCATTAATGTGCGTTTTT

GATAAACCCCATCTTTTAGATCTGTCAGCACGGCAAGGGCGGAGCAGTCTGTCAGACTGCGGGTCGATAGTACCGA
GGTTGAGTTTTCAACGGTGTGAGAGTAGCCCATCAGCACTTCTATCGGTTTCTGCGGTGCCGTTTTGGTTTTCGGA
CTTCCAAAGGTACGAGGCTGATAATCGACCCGGGTTCTATAAAACACAGGCTTTTCATCGCTGCCTATATTAATTT
CAATTTCCGTGCCGTTATCTTTGTTGATGTTGAAGTCGAGGTCACGCCCCGCAAGCTTGCGGTTCAGTTCTTTTTC
GGCCAAGAGATGAATATCACTGTCACTGAGGGCTGGATATTGTTGGCGATAGCGTGTCGATGCATCTTCGTAGATC
AAGAACGGGTCTTCACCGTCAACACCTTTGTAATCTACGATAATTTCCCTGCGGAAGCCTTTGACGCGCACGCCCG
TGAGCGTCTGAAGCTGCGCGGCAAGTGATTGATCACCGCCACTGCTGGAATAGCAGGATAACAGCCGTACCTCGCT
GTAGTTTCGAATGTCTATGCCGCGGGCCAGCAATTCCTGGTTGATATCTTCAGCTCCGTAACTTTTGTTATCTTCT
CCTAAAATGCGCGATGCCCCGTCCCCACTGGGTGCCTCGGCGTGGCCCATGATGTTAAGACGCTGCTCGCCTTTGT
AAGTATCCACGAAGATTTGAATATCACCAGACAGCGGTTTGATATCCGCCGGCCCTCCCAAGACAATGCGGTTTTT
TGCCGGTGCTGCGGTACTTGCCACTTCATCGATTAACAAGCTTGTGCTATGTGGCGCTCGAGTCGAACCGCCGACG
GCACGCTCACTCGATGAGCGCACTTTGAAGTGAATCAATAAAGACGCAAGGTTGACCAGCAGATCAGTCAGGGCCG
CCTCTTTGTTTTTCACGTCATCAGCGCTCAGGACGTGTACGTCGTTGAGGATACCGTCAGCTTGTAGCAGCACACC
CAGTGTGGCACCGGGACCACGCATCGCTGGCAGTAGAGCATTGAGCAATAGAAACCCGCCCTCTTTCAGGGAAGCC
CAGCGGCTTTCTGCATCGGATGTCGATTGTTGTTTAGCCAGTGTTATCAAGTTCTGAGCGTTAGCCGTGTACAGGT
ATTCAATCAATTTACCATCGTGCAGCGCTTGCTGCAGACGATCATCCACCGCGAGGGGAATGGTTTCATTCAGGCT
CGTAGATCCAAGGTTGTAACCGAACAGCACTACGTTGGGGTGTGTGAAACCGTTGCCGCGGTAAAGCTGTCGCGTG
CTTTCGTCAGGTAGCCACGCTAGGGTGTCGGTTTGCAGTTTGCCTCTCTTTTGTATGGCTTCCAGCAACGCCTGGC
GAGAAGGGAACTGCATAAGTGGTGCATCGGCAATCAACGGCCGATATAAGATATGGGGGCCGGTGGCACTGTCCTT
GGGTTCGATCAGGTAGGCACCCTCGACTACATCGATTTTCCCATTGGCTGTGTTATCAAAAGCCAGCGGACGGATC
ACGATTTCCTTGCCGTCAACCATCCTTGGGGCGGGCCCCGGCTTGAGTATTTCCTGGATGAAACGAAAACCTGTAG
TGGTAACCCCGGCTGTTCCCTTTATGCTCAACTCCAGTGCCTTCATCTGCAGTTCGATCGGCACCTGCTGGGCGAA
AAGCGCTTGACGCTCTGCGGTTTTGCCGGGCTCGCCGAGCAACTTTTGTTTCAGCAAAGCAGGATAGGTTTTGCCG
ATATCAAGATCGTTGATCATTTGTTTGAGCACCCCCTCTTTTTCCAGCTGGGGAACCCTGACTTTTATGTCGACTC
CATTTTCCTTGAGCCCTGTCTTGGCAAATACTTCTATCTGGCCATTCGGCAGCCCCGACAAGTTCTTCAGCATCAT
GTCGGTCAGGCTCATTGACTGGCGATCGATAATGCCTGCGGTCATCGGTGCGCCATAAGGCACTTTGAAGACCACC
TCCAGTTGATTGGCGGCATACCGGGTTAACTTGTTCTTTTTAGGCAACCTGTCGAACTTCTCCTCTGCGAAGGTGC
GGATGTCCTTGATATCGCTGTTGTACATTTGTCCCTTGTTCAACAGCAGGAAGTTAGCCAACTCAAGGCTGAGTTC
ATGGAACACAAAACGTTGCGCGTCGCCGGCTTTACTCATCCAATCGGGCAGCTTGGTTTTAGAATTGTCGTCATAT
AGCGGTGGGGCGGCGGACAAGCTGTTCCCTTGATGCTGTTTGATAAGAACATCGGCCTGCGCATCGAAGGTATTGC
CGTTTTGAATTGTCAGGTTTCGTCTTGCGTACGGTAGGTCGTCCCAGGCGTTATAAGCGGTGATTTTTCCGCTGGG
TTCCACGAGCAGGAGTATCTCGCGGTGGGGTTGCGACAAGTAGCGATGAATCAGTATGTTCGGTATGGGGCCAGGG
GCGGCATAGACGTCGGCGTCAGAGGTTGTGGGGTCAAGGGGGAGGGCGCGCGTCGAACCGTTCGGACGCATCGTGA
CCATGGCCACGGTTTTACGTTGTTCATCATCCAGGCCAGGTTGCTTCAGGCCAGCTTGCTGCAGGTTGCTACGCAA
TATGCTGCTAACCAAGTCACGGTGGCTGCCGGGAAAGGCGGTGCCGGTAGCAGTTGGGGTACTGAAGGCCGGTTTA
TTCCAATAGGCTTGTGAATCGACGTTTAACGCCTCGTTGAGTTGGGTTGGCAGTGCGCGTAGTGCCGCGCTGAGTG
CCGGTAAATCCAGGCTCAGCGGGATGCCAGCATCGTTGCCTGTATGCTCCAGCACGTTCGGGCGGGTATCAAACGC
TCGATGGCCATTGGTGAAGTCGGGTGGAGTGCCGCCAGCCAGGTAGTCCATTACCAGTGTCATCAGTTGCGTTTTC
TTGAACTGCCCTGGATGTTGCGGGTCGGGTGTGGCAATGGCGATCTGGTTGGCGTCGACATCCAGTCCAGGAGCGA
TCGCTTTTATCCTGTCGCTGACCAATCGGGAGGTCACCGAATGCAACGTAGGCTCACTGCTAAACTGGCTGCGTGC
GAGATCGGCCATAGTATTGATGGCCGGGGCTGGTTTGTCAGTGGGTAAGACGGGGTTGGCGAGCCGCTGGATAGCG
GCATCTTGCCGTCGTCGAACCGCAACGGAGCGTTTATCAGTTGGCGGATCGGCTTTGCTCAACGCGTGAAAGCCGT
GGTGCGTGAGTGATGAGGTATGTTTGAGCGCGCCTTCCAATGTGCCATCGCTGGCCGGTTCGCCATAGAACCTACC
GATCCAGTCGAGTGTCAGTTGGGTGTTTTTACCACGCGGCTGCGTTGAGTTGCGCAGGGTTTGCCCAAGTGTTGAG
TGTTCAGGTATTTCAGACACCGACAAAGTCGCGCGGGGTGCATTAACCAGGCGCCCCATTTCCGGCCTGAGGCTGA
GTACGGCTCTCCCGTACAACCGTGCTAACGCCAAGTCGGCGTCCTCAACTGACGACTTTGGATTGGGTTCGGCGAG
TGAAAACCGTTGTGGATCGGCTGCAATTTTTGCAGATAACGCCTCTATTGCCTTTTCTTGCTGCTGGCGGACCGGC
AAATGTGCGGGACGGTTGAACGCAGGCGCTGTGGAGTCCGGTTCGCGCAGAGCGTCGAACGAGCCATGCTGGTTCT
GCAGGGCGATTAGATAAAGCTTATCGGCTTGCGTACCGTTGGTGCGTGCGGCATAGAAATCGCCTATCACCTCTGC
GGGCGCACTCGTTGCGCCGGTGTATTCAACGTCCAGCTTGAGTGTCGGGGTAAGCGCCCGAGCGGCCGCTATCACT
GCTGCGGTAGCCTTCCACCAGCCAGGCGTGTATTTCGAAAAAGAGGCAGGTTTTCCGTCTGCAATACAATCAAGCG
TCCATCCATCACGAGTGTTCAGTCGCAGGGTGCCTGGCTCGATCTTGTGTACCCTGGCGAAAGTCGCAAATGGCTC
GCTTTGAATGGCTTGGGTAAGCTGACCCCAGGCCATACCGAATGTGGATAAGGGTGGAATATTCGACACAAATTTG
CCAGCTGGCTTTGCCTCCCCGTCAGTGGGGCTAGCGAGGATTTGTTCCATTAATGCAGCATTGTATTGGTAAGCGA
GCGTCGCGTCGCCAGACCTGATGAGTTCGGTGGCAAGTTTTTTGTTCTCTTGGGTTATTGCCTGATTCGTACGTTT
GACACGTATTAACCCAGGAGCGGTATTTTCAAACGCAGAGTCGGTGGTATTTGCAGTAGGGGAAGTTGCGGGCATA
CGCAATGATTCGACTTTCATGAGTAGTAACTACCATTGAAGGGAAATAATGATAAGGGTGAATCAATCAAAGTTTG

AAGCAGGCGTTTTGATCAGTCAGTGAAGTCGAGTTTCTCTCTAGTCATATCCGTACTTGCGCCTGCATTAGTCCCT
CTCTCGGCACTATCCAACGTTGGTTTATTACTCAGTTAGAGGACGCAGGAGAAGCCTGGACATCCGTGTGCCCCTT
ATAAAAGACTTGGATAGTTTGGGGCGTCATTGAATCGGTATTGCCGGGCGGTGGGTTTAGTTTGTTCGTGGGGAGA
AGGTGGGAAATATATGACGGATTAATGCTGATCATGAGGCTGCCTCCTACCAGGTTATTTCATAAATACCCCGTCG
GTTAGGCGAGCGAGGTACTTAGATACGTGGTCAAGGTTTCGCTGAACGTTCCTGTATCGGAGAATGAAATGCCTGC
GTGATCTGTAAGAAATCTTTTGGGGCGTGTATTTTGGCCAGGTTCTGCTCTGTGAGCCGCTGGACCGCCAGGCGCC
GATAATGGGAAGGTGTCATGCCCTTGAGCTGCTGAAAGCGCCGGTTGAAGTTGGAGATATTGTTGAACCCCGATTC
AAAGCACACATCGGTGACGGCCTTGTCACCATCGGCCAACAGTTCGCAGGATTTACTGATGCGCAGCCGATTGACG
AACTCGATGAAGGTGCGCCCGGTCGCCTGCTTGAACACCCTGGAAAAGTACGTCGGCTTCATCCCCAGATGGTCGG
CTACTTCTTCCAGGGGCAACTCTCGGCCGTAGTTGGCAAAGATATAGTCCACCGCGCGGTTGGTGCGGTCGATGCT
GTGCTCATCGGCCAGTTGCGGGGTGGTCACCCCGGACAGTAACTGGTAATCCTCGCAGGCACTCAACACCTCCATC
AGGATCAAAAAGTGCCCGAGCCGCGCCACGCCGCGTGCCTCTTCGATCTGCTGCATCAGCGTCATGGCCTGGGCGA
TGGTGCGCTTGCAGCGAAACTCGATGCCGTACTTGGCCCGTTCCAGCAACGGCGCCAGGGCCTTGAGCTCACTGAA
GACCGGGACGGCGCTATCAAACAACTCGTCGGTAAAATTCACCAGCATGTCGCGCTTGGGCACCACCTCATCCTCG
GCCACCTGGCTGATCCAGTTATGGGGCAGGTTGGGGCCGGTGAGGAACAGGCTTTCGGGATAGAAATTGCCGATGT
AGTCGCCGATAAACACTTTGCCGGAGCTGGCAATAATCAGGTGCAGCTCGTATTCCTTATGGAAATGCCAGCGCAC
CAGCGGGCAGGGGAAACCATGCTGGCGATAGATGATGGACAGGCCATTGTGGTCGTCCATCAATTCATAGGAGGGG
TCGGTGATTCGCGCGGCTCGAGTCATGCTGGCGTCGCTTTTATTGTGGTTGCGCAACCGATAATGCCCCGTTGCGC
AGCACCTCGCCAGCATTTAGAGCGTGCGGTTTTTTGCCTCGATCCACTGCGCCATGTACTGGGTACTTTTATGCAG
ATGGTGGCGCAACATGCTGCCGGTGAAGTTGTTGCGTCGATGTTGCGCCAGTTCGCCACGGCAAAGTTGCAGGCGC
TCGACCAGCGCCGGGCCATGCAGTTGCGGGGCATAGCGTCGGGCCAGCAGTTCGCGGCCAGCGGCTTGGGCCGCGG
TCCAGGCTTGCGGGTCTGAGTACAGTGCCACGGCAGCGGCGGCCAGGGCGCTGGCGCTGTGGGCGATCGCTCCGGG
CCAAGGCTGCCCGTCGGCCATGCCTTCGGCACCGATGGGGGTGGTGATGCTGGGTGTACCGCAGAGCATGCCGTCC
GCCAGCTTGCCCTTGATCCCGGCGCCGAAGCGCAAGGGGGCCAGGCAGATGCGTGCGGCTGACATGACTTGCAGTG
CGTCTTCGGCCCAGTTCATCACATGAAAGCCCTGGGCCGGGTTATGCAGCGCGGTGGCCTTGGGCGGAGTGTAGGC
GCCGTAGATATGCAACTGGGCACCCGGCAGTTGCTGGCGGATCAGGGGCCAGAGGCTGTTCTTCATCCACAGCACG
GCATCCCAGTTGGGGGCGTGGCGGAAGTTACCGATACTCAGGAAGTGCGCACGGTCTTCAAACGGCACAAATGCTT
CGCTGGGCTGCTCGATCATCAACGGGCACCAGTGGAGCAGGGCGCGGGGCACCTTGAAATGCTCGGTGAGCAGTTG
GATCTCGACGTCGGAAATCATCAGGCTGATATCACAGCGGTAGATCGCTGCGATTTCGCGCTTGGCCAGGTCGGTT
GGCGCCATCAACTGGAATTCCTCATCCAGGGCGGGAGCGAACAGCGGGCTGAAATCATCTGCGTCAGGCTGGCTCT
TGAGATGGTCCTTGAGGCGCTGATGGCGGGCATCGCGCAGGCTCTGCAAATCCGAGGTTTCCAGCACGCGCAAGGC
GTCCGGGCAGTGCTTCTCGACCCGCCAGCCGAACTGTTCCTCCATCATGAACCGGTCGAACAGCACGATATCCGGG
GCGAGTTCCCGAATAAATTCGTCGAAACTGCTGTTATTGAGCTCGATGGCGCATTCGTGGATGCCCAGGGCGGCAA
GGTCGGCCTTGTGCTCGCCGATGGCCGCCGGGCTGCTGAAGGTTATTTCCCAGCCTTGAGCAAGAAAACTCTCGAG
AATCTGCATCATATGCCCGCCCGCAGCAGAGGAACGGGGCTCCGGCCAGACATAACCAATGACCAGGACGCGGGTG
GCAGGCTGATTGTTCAACGATCAATTCCTTCAAGAGCAGGGCAAAAGCGGCGCAATTAAACCACAGCCGCCCGGCC
CGGGCGCAGTCATGACATTTTGTGTTCGGCCATAGGTGTCAATCGCACTTTGTGGTTAACTTCTGCCTCTCGAATT
CGTTTCATCTAACCCAGCATAAGGATTCCGTTCATGGCTCAAGTCACCCTCAAAGGCAACCCGGTCCAGGTTGAAG
GCGAGTTGCCGCAAGTTGGCACCCAGGCCCTCGACTTTTCCCTGACCGCAGGCGATCTGTCGGACGTCACCCTGGC
GACTTTCGCCGGCAAGCGCAAAGTACTGAACATCTTCCCAAGCGTTGACACCCCGACCTGCGCGACTTCGGTGCGC
AAGTTCAACGCCCAGGCCAACGACCTGAACAACACCGTTGTACTGTGCATCTCCACCGACCTGCCATTCGCCCAGG
CTCGCTTCTGCGGTGCCGAAGGCCTGGAAAACGTGAAGAACCTGTCGGACTTCCGCAGCGCCGACTTTGCCGTTGA
CTACGGTGTATCCATTGCCGATGGCGCCCTCAAGGGCCTGACCGCCCGCGCCGTGGTGGTTCTGGATGAAAACGAC
AAAGTGCTGCACAGCGAATTGGTTGGCGAGATCGCCGAAGAACCAAACTACGAAGCGGCCCTGGCTGTACTGAAGT
AATTGTTCCAGCCCGTTACTGTCGCTTTGCAGTAACACGTATGATGCATGCTTGCGGCCTGGCCTAGTCCAGGCCG
TTTTCATTTGTGCTTCAGCGGTTTAGCCCAAATTGCTGATGTTTCACCTCGAAAAGTAAATGTTTGTAAGCCCGAA
GGTAAATAGCCGGTAAAGGCGCTTTTCTAAACGCGCCCCAGTGCTTATCTTTCAGCCTCCCAAAGAAGAAGCCCTC
GCATCCAATGGTTGATCAATCCATGCAATCGTCTCCCCGTAGTTCCCGCCGCTGGCTGTTTGGCTTGCTTGTCCTG
TTGGTGATTGCCGGCCTGTGCTGGAAGTTCTGGCCGGCAGGCTCCGCCCAGAAAGACGCCCCGGCAGCCAGGACCA
GCAAGACGGGGATGATGCGTCCCGGCTTCGGCGGCTCCGGCGCTGCAGTGCCCGTACGTGTTGCACCTGCGGTCAC
CGGGGACTTCCCGGTGTACTACAAGGCGCTTGGCACTGTGACGGCGCTGAACACCATCAACGTGCGCAGCCGGGTT
GGGGGAGAGCTGGTGAAGATCGCCTTTGAAGAAGGGCAGATGGTCAAGGCCGGCGACCTGCTGGCGCAAATCGACC
CGCGCAGCTACCAGAACGCCTTGCTCCAGGCCCAGGGCACGCTTCTGCAAAACCAGGCCCAACTGAAAAACGCCCA
GGTTGACCTTGAGCGTTATCGCGGCCTGTATGAACAAGACAGTATCGCCAAGCAGACCCTGGACACCGCCGCCGCG
CTGGTCCTGCAATACCAGGGTACGGTCAAGACCAACCAGGGTGCGGTGGATGACGCCAAGCTCAACCTTGAATTCA
CCCAGATCCGCGCGCCGATCTCCGGCCGTGTCGGCCTGCGCCAACTGGACGTCGGCAACCTGGTGGCGGCCAACGA
CACCACCGCCCTGGCAATCATCACCCAGACCCAACCGATCAGCGTGGCCTTCACCTTGCCGGAGAACAGCCTGCAA

ACCGTGCTGGCCCGCTACCGTACCGGCAACAAGCTGCCGGTGCAGGCCTGGGACCGTGGCGATCTGCAGCTACAGG
CCACCGGCGTGTTGCAGAGCCTGGACAACCAGATCGATGTCGCCACCGGCACCCTGAAATTCAAGGCCCGTTTCGA
TAACCAGGACCAGGTGCTGTTCCCCAATCAGTTCGTCAACGTGCGCCTGTTGGCCGACACCCTGAAAAACGTGGTG
TTGGTGCCATCGGCGGCGATCCAGTTCGGCACCAACGGCACCTTCGTCTATGCCCTGGACGCTGACAAGAAGGTCA
AGATCCGCACCCTGGTCATTGGTGATACCGATGGCCAGAACACCGTGGTCAAGGAAGGCCTGGCTGCCGGCGATCG
CGTGGTACTCGAAGGCACCGATCGCTTGAAGGACGGCACCGACGTCGAAGTGGTCAACGACAGCAACCAGGTGCCA
ACCACCCCCACTGAACATTTGCAGGGCAAGCCCGCGGCACAAGGCGAAAGCAGCCCGGCGGCGGATGCCGGCAAGG
CGCAAAAGGCCGGCGCATGAACCTGTCACGGCTGTTCATCCTCAGGCCCGTCGCCACCACCCTGAGCATGCTGGCC
ATTGTCCTGGCTGGCCTGATCGCCTATCGCTTGATGCCGGTGTCAGCCTTGCCCCAGGTGGATTACCCGACCATCC
GCGTCATGACCCTGTACCCCGGCGCCAGCCCGGATGTGATGACCAGCGCGGTCACTGCGCCCCTGGAGCGCCAGTT
CGGGCAAATGCCCGGCCTGACGCAGATGGCCTCGACCAGTTCCGGTGGGGCCTCGGTGCTGACCCTGCGCTTCAAC
CTCGACGTCAACATGGACGTCGCCGAGCAACAGGTGCAGGCGGCGATCAACGCGGCCAGCAACCTGCTGCCTTCGG
ACCTGCCGGCGCCCCCGGTGTACAACAAGGTCAACCCGGCCGATACCCCGGTGCTGACCCTGGCCATTACTTCCAA
GACCATGTTGTTGCCCAAGCTCAATGACCTGGTCGATACGCGCATGGCGCAAAAAATCGCGCAGATCAGCGGTGTG
GGCATGGTCAGCATCGCCGGCGGCCAGCGCCAGGCCGTGCGGATCAAGGTCAACCCCGAGGCCCTGGCCGCCAACG
GCTTGAACCTGGCGGACGTGCGCAGCCTGATCGCGGCCTCCAACGTCAACCAGCCCAAGGGTAACTTCGACGGCCC
GACCCGGGTGTCGATGCTCGACGCCAACGACCAGTTGGTATCGCCCGAGCAGTACGCCGAACTGATCCTGGCCTAT
AGCAACGGTGCGCCGCTGCGCCTCAAGGACGTCGCGCAGATCGTCGATGGCGCCGAGAACGAACGCCTTGCCGCCT
GGGCCAATGAAAACCAGGCGGTGCTGCTCAATATCCAGCGCCAGCCGGGCGCCAACGTGATCGAGGTGGTGGACCG
GATCAAGGCGCTGCTGCCCAGCATCACCGACAACCTGCCCGCGGGCCTCGACGTGACGGTGCTCACCGACCGCACC
CAGACCATTCGCGCCTCGGTCACCGATGTGCAGCATGAATTGCTGATCGCCATTGCCCTGGTGGTAATGGTGACGT
TCCTGTTCCTGCGTCGCTTCAGCGCCACCATCATCCCGTCGATTGCCGTGCCGTTGTCCCTGATCGGCACCTTTGG
CGTGATGTACCTGGCCGGTTTCTCCATCAACAACCTGACCCTGATGGCCCTGACCATCGCCACCGGGTTCGTGGTG
GACGATGCCATCGTAATGCTGGAGAACATTTCCCGCTACATCGAGGAAGGCGAGACACCGATGGCCGCGGCGCTCA
AGGGCGCCAAGCAGATCGGCTTCACCCTGGTTTCCCTGACCCTGTCGCTGATTGCGGTATTGATCCCGCTGCTGTT
CATGGCCGATGTGGTCGGGCGGCTGTTCCGCGAATTTGCCATCACCCTGGCGGTGGCGATCCTGATTTCCCTGGTG
GTGTCCTTGACCCTGACGCCGATGATGTGCGCGCGCCTGCTCAAGCGCGAACCCAAGGAAGAAGAACAGGGCCGCT
TCTACAAGGCCAGCGGCGCCTGGATCGACTGGCTGGTGGCTGCCTACGGGCGCAAGCTGCAATGGGTGCTCAAGCA
CCAACCGCTGACCCTGCTGGTGGCCATCGCCACCCTGGGCCTGACGGTGGTGCTGTACCTGGTGGTGCCCAAGGGC
TTCTTCCCGGTGCAGGACACCGGGGTGATCCAGGGCATTTCCGAGGCGCCGCAGTCGATTTCCTTCGCTGCCATGA
GCCAGCGCCAGCAAGAACTGGCGAAGATCATCCTCGAAGATCCGGCGGTGGAAAGCCTGTCGTCCTACATTGGGGT
AGATGGCGATAACGCCACCCTCAACAGTGGCCGCCTGCTGATCAACCTCAAGCCCCATAAAGAGCGGGACTTGAGC
GCCGTCGAGGTGATTGCGCGCCTGCAGCCGACGCTGGACAAGCTGGTGGGCATCCGCCTGTTCATGCAGCCGGTGC
AGGACCTGACCATCGAAGACCGCGTCAGTCGTACCCAGTACCAGTTCAGCATGTCCTCCCCGGACGCCGACCTGTT
GGCACTGTGGAGCGGCAAGCTGGTACAGGCCCTGAGCCAGTATCCGGAACTCACCGATGTCGCCAGCGACCTGCAG
GACAAGGGCTTGCAGGTGTACATGGTGATCGACCGCGATGCCGCCTCGCGCCTGGGCGTGTCGGTGGCCAATATCA
CCGACGCCCTGTATGACGCCTTTGGCCAACGGCAGATTTCCACGATCTACACCCAGGCCAGCCAGTACCGCGTGGT
GCTGCAGGCCCAGTCCGGCGAAACCCTGGGGCCGGATGCGCTGAACCAGATCCATGTGAAAACCACCGACGGCGGC
CAGGTTCGCCTGTCGAGCCTGGCCCGCATCGAGCAGCGCCAGGCGCAGTTGGCGATTACCCATATCGGCCAGTTCC
CGGCGGTGATGATGTCGTTCAACCTGGCACCCGGCGTGGCCCTGGGCAAAGGTGTGGAGCTGATCAACCAGGTGCA
GAAGGACATTGGCATGCCGGTCGGCGTGCAGACCCAGTTCCAGGGCGCCGCCCAGGCGTTCGAGGCGTCGCTGTCG
AGCACCTTGCTGCTGATCCTGGCGGCGGTGGTCACCATGTACATCGTGCTCGGCGTGTTGTACGAGAGCTACATCC
ACCCGATCACCATTCTCTCGACCCTGCCATCGGCAGCGGTGGGGGCCCTGTTGGCGCTGCTGCTCACGGGCAACGA
CCTGGGGATGATCGCGATCATCGGCATCATCTTGCTGATCGGCATCGTGAAGAAGAACGCGATCATGATGATCGAC
TTCGCCCTCGACGCCGAACGCAACCAGGGCCTGGACCCGCAGACCGCGATCTACCAGGCGGCGCTGTTGCGCTTCC
GGCCAATCCTGATGACCACCCTGGCGGCGCTGTTCGGTGCGGTGCCCCTGATGCTGGCCACCGGCTCCGGCGCCGA
ACTGCGTCAGCCCCTGGGCCTGGTGATGGTCGGCGGCTTGCTGTTGAGCCAGGTCCTTACGCTGTTCACTACCCCG
GTGATCTACCTGTACTTCGACCGCCTGGGCCGTCGCTGGCGCAAGACCCCCGAAGCGCTGGAGCCGGTTGAGCCAT
GAACCTGTCCGGACCTTTTATTCGCCGGCCGGTAGCCACCATGCTGCTGAGCCTGGCGATCATGTTGCTGGGCGGT
GTCAGTTTCAGCCTGTTACCGGTGTCGCCGCTGCCCCAGATCGACTTCCCGGTGATTGTGGTCTCCGCCAGCCTGC
CTGGCGCCAGCCCGGAAGTGATGGCGTCTACCGTGGCTACGCCCCTGGAGCGCTCCTTCGGCGCGATTGCCGGGGT
CACCACCTTGAGCAGTTCGTCGAGCCAGGGCTCGACCCGGGTGATCCTGGCCTTTGACTCCGACCGCGATATCAAC
GGCGCGGCGCGGGAAGTGCAGGCGGCGATCAACGCCTCGCGCAACCTGTTGCCCAGCGGCATGCGCAGCATGCCCA
CCTACCGCAAGATCAACCCGTCCCAGGCGCCGATCATGGTGTTGTCGCTGACCTCGGACGTGTTGCCCAAGGGCCA
GTTGTATGACCTGGCCTCGACCATCCTCTCCCAAAGCCTGTCCCAGGTACCGGGTGTGGGTGAAGTGCAGATCGGC
GGCAGTTCCCTGCCGGCGGTGCGCATCGAGCTCGAACCCAAGGCTCTCGACCAGTACGGCGTGTCCCTGGATGAAG
TGCGCGACACCATTGCCAATGCCAACGTGCGCCGGCCCAAGGGCTCGGTGGAAGACGGCGAGCGTAACTGGCAGAT

CCAGGCCAACGACCAGTTGGAAAAGGCCAAGGACTATGAGCCGCTGCTGATCCGCTACCAGGACGGCGCGGCGTTG
CGCCTGAGCGATGTGGCGAAGATCAGCGATGGCGTCGAGGACCGCTACAACAGTGGTTTCTTCAACAATGATTCGG
CGGTGTTGCTGGTGATCAACCGCCAGTCGGGCGCCAACATCATCGAGACCGTCAAGCAGATCAAGGCGCAGTTGCC
GGCGCTGCAGGCGGTGCTGCCGTCCAGCGTCAAGCTCAACCTGGCCATGGACCGCTCGCCGGTGATCACCGCCACC
CTGCATGAGGCGGAAATGACCCTGCTGATTGCGGTGGCCCTGGTGATCCTGGTGGTGTACCTGTTCCTCGGCAACT
TCCGCGCCTCGCTGATCCCGACCCTGGCGGTGCCGGTGTCGCTGGTGGGCACCTTTGCGATCATGTACCTGTACGG
GTTTTCCCTGAACAACCTGTCGCTGATGGCCCTGATCCTGGCCACTGGCCTGGTGGTGGACGATGCCATCGTGGTG
CTGGAGAACATCTCGCGGCATATCGACGACGGTATCGCGCCGATGAAGGCGGCGTATCTTGGGGCCAAGGAGGTCG
GTTTTACCCTGCTGTCGATGAACGTGTCGCTGGTGGCGGTGTTCCTGTCGATCCTGTTTATGGGCGGGATTGTCAC
CAGCCTGTTCCGTGAATTCTCCATCACCCTGGCGGCGGCGATCATCGTTTCGCTGGTGGTCTCCCTGACCCTGACG
CCGATGCTCTGCGCGCGCTGGCTCAAGCCCCATGTCAAAGGGCAGCAGACCGGCTTGCAGCGCTGGAGCCAGAAGG
TCAACGACCATATGGTGGCGGGTTATGCCCGCAGCCTGGACTGGGTGCTGCGGCACAAACGCCTGACCCTGCTCAG
CCTGCTGGTGACCATTGGCGTGAATATCGCGCTGTATGTGGTGGTCCCCAAAACCTTTATGCCGCAACAGGACACC
GGGCAATTGATCGGTTTTGTGCGCGGCGATGACGGCCTGTCATTTAGCGTCATGCAGCCGAAGATGGAGATTTTCC
GCAAGGCTGTGTTGCAAGACCCGGCAGTGGAGAGCGTGGCCGGCTTTATCGGTGGCAGCAACGGCACCAACAACGC
GGTGATGCTGGTGCGGCTCAAGCCGATCAAGGAACGCAAGCTGTCGGCCCAGGCAGTGATCGAGCGCCTGCGTAAA
GAAGTGCCGCTGGTACCGGGTGGTCGCTTGATGCTGATGGCCGACCAGGACCTGCAATTTGGCGGCGGCCGCGACC
AGACGTCCTCGCAGTACTCCTACATCCTGCAAAGTGGCGACCTGGCCGCCTTGCGCCTGTGGTACCCGAAAGTGGT
CGCCGCGCTGCGCGAGCTGCCGGAACTGACCGCGATCGACGCCCGCGAAGGCCGTGGTGCGCAACAGGTGACGTTG
ATTGTCGACCGCGATCAGGCCAAGCGCCTGGGCGTCGACATGAACATGGTCACCTCGGTGCTGAACAACGCCTACA
GCCAGCGGCAGATTTCCACCATCTATGACAGCCTCAACCAGTACCGGGTGGTGATGGAGGTCAATCCAAAATACGC
CCAGGACCCGATCACCCTCAATCAGGTGCAAGTGATTACCGCCGATGGCGCGCGAATCCCGCTGTCGACCATCGCC
CACTACGAAAACAGCCTGGCCGACGACCGTGTCAGCCACGAAGGGCAGTTCGCTTCCGAAGACATCGCCTTCGACA
TGGCCCCCGGGGTCACGGTGGAGCAGGGCACGGCCGCTATCGAGCGGGCGATTGCCAAGGTCGGCTTGCCCGAAGA
CGTCATCGCCAAGATGGCGGGCACCGCCGATGCCTTTGCCGCCACTCAGAAAAGCCAGCCGTTCATGATTCTCGGC
GCGTTGGTGGCGGTGTATCTGGTGCTGGGGATTTTGTATGAGAGCTACATCCACCCGCTGACCATCCTTTCGACCT
TGCCGTCGGCCGGGGTTGGTGCGCTGTTGTCGATCTACGTGCTGGGCGGCGAGTTCAGCCTGATCTCGTTGCTGGG
CCTGTTCCTGCTGATCGGCGTGGTGAAGAAAAACGCGATCCTGATGATCGACCTGGCACTGCAACTGGAGCGCAAG
GAAGGCATGCAGCCCCTGGAATCGATTCGCAGCGCCTGCCTGCTGCGCTTGCGACCGATCCTGATGACCACCCTGG
CGGCAATCCTCGGTGCCTTGCCTTTGCTGCTGAGCACCGCCGAAGGCGCGGAAATGCGCCAGCCCCTGGGCCTGAC
GATTATCGGCGGGCTGGTGTTCAGCCAGATCCTGACCCTTTACACCACCCCGGTGGTTTACCTCTATCTCGACCGC
CTGCGCCACCGTTTCAACGGTTGGCGCGGGGTGCGTACCGACGCTGCCCTGGACACTCCGTTATGACCGCAACGCA
CCTCCCTGTAGGCGCTGGCTTGCCAGCGAAAAACGCCAACCATGACGCGGGGCACCTGACTCACCGCCGGGGCCTC
AGGTTTTTCGCGAGCAAGCTCGCGCCTACCGTGTTTTGCACCGTATTGCTCAGCGCTTGCGCCATCGGCCCGGATT
ACCAGCGCCCGCAAGTGGCTGAGCCGGCGCAATACAAAGAAGCCCAGGGCTGGCGCCAGGCGGCGCCCAGCGATGC
CTTGGCCCGTGGCGCCTGGTGGGAGTTGTATGGCGACCAGCAACTCAACAACCTGGTGGACAAGCTCAACAACGCC
AACCAGACCGTGGCCCAGGCCGAAGCCCGCTACCGTCAGGCCCAGGCCACTGCGCGCAGTTCCCGTGGCGCCTTTT
TCCCCACGGTTGACCTGAGCGCCGGTAAAACCCGCGCCAGCCAGGGCACCGGTAGCAGCAATGCAAGCCTGAGCAG
CTCCAGCAGCGGTATCCGCGACACCCTCAACACCCAGTTGGGTGTCAGTTGGGAAGCGGATATCTGGGGCAAGTTG
CGCCGTGGCCTGGAAGCCAGCAACGCCAGCGAGCAAGCCAGCGCCGCAGACCTGGCCGCCATGCGCTTGAGCCAGC
AGTCGGAGTTGGTGCAGAACTACCTGCAACTGCGGGTCATGGACGAACAGACCCGCTTGCTGCAAAGCACCGTCGA
GACCTACCAGCGCTCGCTGCAAATGACCGAAAACCAGTATCGCGCCGGTGTCTCCGGCAAAGACGCCGTGGCCCAG
GCGCAGACCCAGCTCAAAAGCACCCAGGCCAGCATGATCGACCTGATCTGGCAGCGTGCGCAGCTGGAAAACGCCA
TCGCCGTACTGATCGGCGAAGCCCCGGCCACCTTCAACCTGGCGGTGAGCAAGGATATTCCGGCCTTGCCGCAGAT
CCCTGCCAACCTGCCATCACAATTGCTGGAGCGCCGCCCAGACATCGCCTCGGCCGAACGGGCGGTGATCGCGGCC
AATGCCAATATTGGCGTGGCGAAAACCGCCTACTACCCCGACCTGACCCTGAGCCTGGCCGGTGGTTATTCCAGCA
GCACCTACGCCGACTGGATCAGCCTGCCCAACCGTTTCTGGTCGGTGGGGCCGAAACTGGCGATGACCCTGTTCGA
CGGCGGGCAACGCTCGGCGGAAGTTGACCGCGCCGAAGCCTCCTACGACGAAACCGTGGCCAAGTACCGCCAGACC
GTGCTCGACGGTTTCCGCGAGGTGGAAAACTACATGGTCCAGCTCAAGGTCCTGGAAGACGAGGCGGGTGTGAGCA
ACGAAGCCCTGGCATCTGCCCGCGAATCCCTGCGCCTGACCCAGAATCAGTACAAGGCGGGCCTGATCGCCTACCT
GGACGTGGTTAACGTGCAGGCCACGGCATTGAGCAACGAACGCACGGTGTTGACCTTGCTGCAAACCCGGCTGGTC
GCCAGTGTGCAACTGATCGCCGCATTGGGCGGCGGATGGGACGGTCAGATGCAGCCACGCGAAGAGTAATCGGTAT
CTCTGACAGCAGTTGGTGAGGCCATTGGGGCGGCTTCGCCACCCAGCGCGGGCGATGCGGCGTTCCGACAAGCCCG
CTCGCCACAGGGAAGGCTGTTCGTCAAAGGGCGACTTCAGGTGATGGCCTTGTGACAATATTTCACCTATTCAAGA
ATTATTCTCGCTACAATCCCCGGCCTTTCCTGGCATGGATGCCGCTCGCACTGTCAGCCTTAGAGACGCCCCATGC
TTACCGGCAGCTATTCCCCCGCACTGGTCCTGATCTCCCTGTTTGTGGCGATCCTTGCTTCCTATACCGCCCTTGA
CTTGACCGGCCGCATTGCCACGGCGAGGGGGCGGGCGGTGTACCTGTGGATGGCCGGCGGGGCGCTGGCCATGGGG

ATTGGCGTGTGGTCGATGCATTTTATCGGCATGTTGGCCTTCCGCTTGCCAATCGCGCTGGGCTACGACATCGGCA
TTACCACGTTGTCGCTGGTGATCGCGATTTTCTCCTGCGGGTTCGCCCTGTGGCTGGTCAACCAACCGCGCCTGCC
TATTTGGCAACTGGGTTTTGGTGCCCTGGTCATGGGGGCCGGTATCAGTAGCATGCACTACACCGGCATGGCCGCC
ATGCGCATGACCCCGGGGATCGACTACGACCCCATCTTGTTCGCTGCCTCACTGCTGATCGCCGTCGGCGCTTCGG
CCGCCGCCCTGTGGATTGCCTTCAACCTGCGGCGCAATACCCCCTATGTACGCCTGGCGCGGGGTGGGGCAGCGGT
GGTGATGGGCGTGGCCATCGTCGGCATGCATTACACCGGCATGGCCGCCGCCCGTTTTGCCGATGGCAGCTTTTGC
GGGGCGTCGGTGAATGGCCTGAGCGGCAGCGGCCTGGATAACCTGGTGCTGGTGACCACCCTGGCGGTGCTGGCCA
TTGCGCTGTTGACCTCGTTGCTTGACGCGCGCCTGGAAGCCCGCACCGCCGTGCTGGCCGATTCCCTGTCCCTGGC
CAACCAGGAACTCACCCACTTGGCCTTGCACGACACACTCACCGGCTTGCCAAACCGTACGCTGTTGGCCGATCGC
ATTCAGCAGGCTATGCAGTTGGTGGCGGAGCAGGGCGGCTGTTTTGCCCTGATGTTTATCGACCTGGACGGCTTCA
AACCGGTCAACGATGCCTTTGGCCATCATATGGGTGACCAATTGCTACGCGAAGTGGGCCTGCGCCTGCGTGAAGA
TCTGCGCAGCCAGGACACCCTGGCCCGGATCGGTGGCGATGAGTTCGTGTTGCTGGTGCAATTGAGCCAGCCCGAC
GATGCCCTGCGCCTGGCCGAGCGCCAAGTCGGCCTGATCAACCAGTCGTTCCGGGTCGCCGAGCATGACTTGAACA
TCTCCGCCAGCGTCGGTATCGCCCTGTTCCCCGGCAACGGAGCAACGCCCCAGGAACTGTTGATGAATGCCGATGC
GGCGATGTATCACGCCAAGAGCATGGGCAAGAACGGCTACAGCTTTTTTGATGTATCGATGAACACCAACGCGCGC
CGGCAACTGCAACTGTTGCAGGATTTGCGCAATGCGGTGGACCAACAGCAGTTGCGCCTGTACTACCAACCCAAGT
TCGACGCCCTCAGCGGCCGCGCTGTCGGCGCCGAGGCGCTATTGCGCTGGGAGCATCCACAACATGGCTTGTTGCT
GCCGGACAAGTTTATCGAGCTGGCGGAAAAGACCGGGGTGATCATTGCCATCGGCGACTGGGTGCTCAACGAAGCG
TGCCGGCAAATGCAGGTGTGGTTTGCCCAGGGGTATCGGGACTGGCGCATCGCGGTCAACCTGTCGGCCCTGCAGT
TCTGCCATGCCGGGCTGGTCACCAGTGTGGCCAACGCCCTGCAGCGTCACCAGTTGCCGGCCAACAGCCTGACCCT
GGAAATCACCGAAACCACCGCCATGAGCGATGCCGATGCCAGCATGACGGTGTTGCAGGAACTCTCGGAAATGGGC
GTTGACCTGTCAATCGACGACTTTGGCACCGGTTATTCCAGCCTGATGTACCTCAAGCGCCTGCCGGCCAACGAAC
TGAAGATCGACCGCGGTTTTGTACGGGACCTGGAGCATGACAGCGACGATGCCGCCATTGTCTCGGCGATTGTCGC
CCTGGGCCAGGCCCTCGGCCTGCGCATCGTCGCCGAAGGGGTGGAGACCGACATGCAGCAGGATTTCCTCACGCGT
CTGGGCTGCGACTCGTTGCAAGGCTATCACCTGGGCCACCCGCTGCCGCCGGATACATTTATGAGCGCCATCGTGG
CCAAGGAACAACTGACCCGGCCGCCGTTTACCCAACCCTGAGATCAATTGACCCCGGTCCATGCAAAACCTGGCAG
GCACAGGTATTCTTGGATCCAACAGCCTGGTTGAATGGGGAGTCAGTACACATGGACAAAGTCGTCATCATTACCG
GTGGCAGTCGTGGGATCGGGGCCGCCACGGCCTTGCTGGCGGCCCGGCAGGGCTATCGCATCTGCATCAATTTCCA
GTCTGACGAAGAAGCCGCCCTGCGTGTGCTGGAGCAGGTCCGCGCACTCGGCGCCCAGGCCATCGCGGTGCGCGCC
GACGTGAGCATCGAAGACGAAGTGATCAGCCTGTTCAACCGTGTCGACACCGAATTGGGTCGCGTCACCGCGCTGG
TCAACAACGCCGGCACCGTCGGCCACAAGTCGCGGGTCGACGAAATGTCCGAGTTCCGCATCCTGAAAATCCTCAA
GACCAACGTGCTGGGCCCGATTCTGTGCGCCAAGCATGCATTGCTGCGCATGTCGCCCAAGCACGGCGGGCAGGGC
GGTAGCATCGTCAATGTGTCCTCGGTGGCTGCACGCCTGGGCTCGCCCGGTGAGTACGTAGACTACGCAGCGTCCA
AGGGCGCGCTGGACACCTTTACCATTGGCTTGTCCAAGGAAGTGGCGGGCGAGGGGATCCGGGTCAACGCGGTGCG
CCCGGGTTATATCTTCACTGAATTCCACGCCTTGAGCGGCGACCCGGACCGGGTCAGCAAGCTTGAGCCGGGCATT
CCCATGGCCCGTGGCGGGCGCCCTGATGAAGTGGCGGAAGCGATTGTCTGGTTGTTATCGGACAAGGCCTCTTACA
CCACCGGGACATTTCTGGATCTTGGCGGCGGGCGTTGATTCTGCCTGGGTGCTGAGAGCCCCCTCACAAAGGGGCG
GGGCTTCCTGGCTTTTCGTGGCGAGCGGGCTTGTCGGAACGCCGCATCGCCCGCGCTGGGTGGCGAAGCCGCCTCA
ATCGCCTCACCACGTTCTGCACGAACCCGTGTCAGAAAGTGCCTAAAGCCTGATACCCCCGACGCGCAAACTGGTC
CAGGCGGTCTGCCTCCGAGTCATGCAGGATGGTGACGCCCAAGTCGTAAAACCCGTCGATCTTGCCGTTACGTTCC
ACGCTGCCCTTGAGCTTGTCTTCGTCCAGGTTATCAATGAAGCGCAGCACTTTATCCAGGTTGTAGATCGCAGTGG
CGCGGGCCTGGGGGGCGTTGTTGGCCGGCGTCCAGTCCCCCACGTGGCGCTTGAGGTCCGGCTGGAGCTGAAAGCG
ATCATTGCCTGCAAGGAAGTCCCTGAGCACCGGGCTCTGTTCGATAATCCTTTCTGCCGAACGATGGTCACCTGTC
GGGCGCCCGGTTGGCCCGTTCAACTGGCGAACTTCGCGCTGTTCGGCCACCCGCATGTAGTTGGGTGGGAGCGGCA
GCGGAAGATGGAAGAAGGGCGGCAGTACACCTCGCATCAAACGTTTCCTGAGCAATGGAGAAAATGGACCCTCCTA
CGTGGCACGTTTCAGTGTTTGGTTCCCTTTCAAGCGCAGCCCCTTAAAACGAACGCACAATGCGCCCCAACGTTTC
CATGGCCTTTTCCGCCGCCTCATCCCAGGGGCTGCCGTAGTTCAAGCGAATGCAGTTGCGAAAACGCTGGGTCGGC
GAAAAAATCGGCCCCGGCGCGATGCTGATACCTTGGGCCAGGGCCATCTGGAACAGTTTCAACGAGTCGGTCTGTT
CTGGCAGTTCCAGCCACAGGAAATAGCCGCCGGCCGGTTGGCTGACCCGGGTTTGCGCCGGAAAGTAACGGCCGAT
GGCGGCGAGCATGGCACTTTGCTGCTCTTCCAGGGCATAGCGCAATTTACGCAGGTGCCGGTCGTAGCCGCCGTGT
TGCAGGTAATCGGCAATCGCCGCCTGGGCGGGCATCGAGGCACAAAGCGAGGTCATCAGCTTCAGGCGTTCGATCT
TTTGCGCATAACGGCCGGCGGCCACCCAGCCTACGCGATAGCCAGGGGCCAGGCTCTTGGCGAAGGAACCGCAGTG
CATCACCAGGCCTTCGGTGTCGAAGGCCTTGGCGGGCTTGGGCGCCTGCTGGCCGTAATACAACTCGGCGTACACG
TCGTCCTCGATCAGCGGCACCTGATGCCCGCGCAACAACTCAACCAACGCCTGCTTCTTGGCCTCGGGCATGGTTG
CCCCCATGGGGTTCTGGAAACTGGTCATGCACCAGCAGGCCTTGATCGGGTAGCGCTCCAGGGTCTGGGCCAGCAC
TTGGAGGTCAATGCCATCGCGCGGGTGCACGGGGATTTCCACGGCCTTGAGTTTCAAGCGCTCGAGCACTTGCAGG
CAGGCATAAAACGCCGGGGCCTCGATGGCCACCAGGTCTCCCGGCTCGGTCACCGCCTGCAGGCACAGGTTCAGCG

CTTCGAGGGCGCCGTTGGTGATCAGCAACTCCTCCATGGGCAGCATCAAGCCGCCGACCATGTAGCGCAGGGCAAT
TTGCCGGCGCAGTTGCGGGTTGCCCGGCGACATGTCGGTGACCACCATGCGCGGATCCATCTCGCGGCTGGCGCTG
GCCAGGGAGCGGGCCAGGCGTTGCAGGGGGAACAGCATCGGGCTGGGGAAGGCCGAGCCAAACGGTACGGTCTGCG
GGTCCTTGATCGAATCGAGTACCGAGAACACCAGCTCGCTGACGTCCACCTCAGTGGACTCGTGCACATGGGCGCT
GACCGCAGGCTCGGAAAACGGGCTGGGGGCATGGGTATTGACGAAATACCCGGAGCGCGGGCGCGCACGGATCAGG
CCACGGCGCTCCAGCAGGTAATAGGCCTGGAACACCGTGGATGGGCTGACGCCATACGTTTGGCTGGCATAGCGCA
CCGACGGCACACGCTGGCCGGGGCCGAGGACGCCGGAGCGGATCAGTTCAGCGATGTCGTCGGCAAATTTCTCGTA
GCGTTTCATCGTGATCCTAGGTCTACACAGGGCAAAGTGTGGGAGCGGGCTTGCCCGCGATTACGGTGTGTCAGTA
GCAGATATTTCGACTGACGCACCGCTATCGCGGGCAAGCCCGCTCCTACAAGGGAGGTTGCGTGTGTCAGCGGTTC
AGCGGCGCCACAAAACGGCTGTCGGCGACACTATAAACACTTGGCTCATCCTTATCCGTTACCTTGAAGCGGATGG
TTTGCGAGCTACTGGTCGGTTTGTCACGGGTCAACGCTACAGAAACCGGCAAGTCGGTGATTTCCCCCGGCGCCAG
GCTGACCTCGGTCTTGCCCTGCAGCTCGAAGCCCTCGCCATCCAGCAGCTCCACGCGGTAATCCTGGCGCTGTTGG
GTCTTGTTGATGATCTTCAGGCTGTAGATGTTCTCGATCAGCCCCTGGCTGTTTTCCCGGAACATCCCACGGTCCT
TGGTCACGTCCAGCGACACCATTGGCCGCTGCACCAGCGCCATCACCAACGCAGCGATCATCACCAGCAGCACCGC
ACTGTAGCCGATCAATCGCGGTCGCAGCAGGTGGGTCTTGCCGCCCTGCATCTGATGCTCGGAGGTGTAGCTCACC
AAGCCCCGCGCATAGCCCATCTTGTCCATGATCGAATCACAGGCGTCGATGCAGGCTGCGCAGCCGATGCATTCCA
TCTGCAGGCCGTCGCGGATATCGATGCCGGTGGGGCAAACCTGTACGCACAAGTGGCAGTCGATGCAGTCGCCCAG
GCCGACGTCGGCCGGGTTCACATCACGTTTGCGCGGGCCACGGCTTTCGCCACGGGCGGCGTCGTAGGAAATGGTC
AGGGTGTCCTTGTCGAACATCACGCTCTGGAACCGCGCATACGGGCACATATGCATGCACACCGCTTCACGCAGCC
AGCCGGCATTGATGTAGGTGGCGCCGGTAAAGAACAGCACCCAGAACAGGCTGACGCCGCCCATCTGCCAGGTCAG
CAGTTCTTCGGCCAGCGGCCGGATCGGGGTGAAGTAGCCGACAAAGGTCAAACCGGTCAGCACGCTGATCGCCAGC
CACAGCGTGTGCTTGGCCGCACGGCGCGCCAGCTTGTTCACGCCCCAGGGCGCAGCCTGCAGCTTGATCCGCTGGT
TGCGCTCGCCTTCGGTGACTTTCTCGCACCACATGAACAACCAGGTAAACGAGCTTTGCGGGCAGGTGTAGCCACA
CCAGATGCGGCCGGCAAAGACGGTGATCGCAAACAGCCCGAAGGCACAGATGATCAACAACGCCGACAACAGGATG
AAGTCCTGGGGCCAGAAGGTCGCGCCAAAGATATGAAACTTGCTTTCCGCCAGGTCCCACAACACTGCCTGGCGTG
AGTCCCAGTTCAGCCACACGGTGCCAAAAAACGCCAGGAACAAAAAGCCTGCGCCACTGATGCGCAAGGTGCGGAA
CAGGCCGGTGAAACTGCGGGTATGGATCAGGTTGTCGCTGGATTTGGCCTTCATCTTCTTTGGATGTGCAGGCTCG
AAATTTTCTACTAATCGGACGGGGATTCTATCGCTCATGGTCTTTCGCTCATCAGCCTCCATCAGGCGGATGAACT
ATGGGCCGCGATCTGTTTGCATAACAGACTCAGGTAAATCGATAAAAAGCGGATCAGATGGGTTTTGGCGCCCGCC
CTGCGACAATGTGCAGCACCCCGCAAGCCGTGGGGCCGGTGCCTGGATCAACAGTGCCCACGGCATGCTGATCCAG
ATCAATCAAATCACCGAATCACCGTCAGTGGCCTTGAGGTGCTTACGCCCATCGATGGCGCCGGCCACGGTCAAGG
CGTCGGCTTGCGCCTCAGTGATGTAAATCCGCTGGCCATCGATCTCCACCGCCGACATGGCTTTATCGCTGTCGGT
AATCACCGTTACCTCCAACACGCTGCAACGCTGCTCTTCGCCGCTGTCGTCAATCTTGAAATAGCAGCTCTGGTTT
TCGATACGTACGGGCATGGTGTTCTCCTGGTTGCACAGTCCCGTTGGTTAGGTGTCGCGCCCTTGCCAATGGTTCG
GCGCAACTGACTGGCGGTCGTTGCTGTCCATCTTCTATCCCCCCCATGAAAAGGACAGGACCATGGACGCCTGGTG
GCAAGAAGTCTGGCAAACCCTGCAAAGCGAATTCGCCGATATCGGCGATGCGCGGCAAATGACGCAAATCACCGTA
CGCCTGCTGATTGCAGCGATCCTCGGTGGCATCCTCGGCTTTGAACGTGAACAAAAGGGCAAGGCCGCGGGTGTGC
GCACCCATATGCTGGTGGCGATGGGCGCAGCGTTGTTCGTGCTGGTCCCGCAGATAGGCGGTTCACAGGCCGATGC
CATGAGCCGGGTGATACAGGGGGTGATCGCCGGGATCGGTTTTTTGGGCGCGGGCACCATCCTCAAGGGCAAGGAG
GAGGAGGCCGGGCAGCACGTCAAGGGGCTGACCACTGCGGCCGGCCTGTGGATGACCGCCGCCATCGGGGTGGCGG
CGGGGCTGGGCAAGGAGTCCACTGCGGTGCTGAGCACCGTGCTCGCCCTTGCCGTGTTCAGCGTGATGCCAAGGAT
TGTGAAGCTGTTGGACAAGCCTTAGCAGCGGCTCCTACACGATAACCGGCGGCATCGTCGTCGGCGGTTCTTCCTG
TGCCGGTGGCACGCTTTCCGGAGGTTCCTGCTCGGGAATCGGCTCCGGTTCGGTCGGCGGCAGGGTGGGTTTGTCG
ATATTCGGGTCTGGGGTTTCAGCAGGAATGGGGATGTTCATCGCGGTGGCCTCGTTTGTGCTTTGCTCAACCGGTG
GACAACCGTCATGGCCGTTTGATTCCCGCCCGGTGGCGGTACATCCACCTGAACTTTTCCAACGCGCCCGGGCTCG
GACCTTATGTGGCCGGCTCAGGGAGAGCCCGCTTGCAGCACTGACAATCAAGCACAAGAGCGTCCATGGGGCGTAA
GGGGAGATGCTCGATGACTGCTGAAAGACCCGCACTACCACTGTCCCAGGCACTGTTGCTGCCCAGGATCGCCATT
GAAGACACCACACCTGTGATCGACGCCGGGGCCTTTGCCGTCAAGGCCGTGCAAGGCCAGCGCATCCAGGTGGCGA
GCAATGTCTTTGCCGATGGCCATGACCAGTTGGCCGTCTTGATCCGTTGGCAGGCCTTGGGTGAAGACAGCTGGCA
CAGCGTGCCCATGAGTGACCAGGGCAACAATGCCTGGCAGGGCGCGTTCAGCGTGACCGAGCAAGGCCCCCACCAG
TATTGCGTCGAAGCGTGGATCGATCATTTCGCCAGCTTCTGCTACGAATTGGGCAAGAAACACACGGCCGGGGTTC
CTGTCAGCCTGGAGCTGCAGGAAGGTCGCAACCAGGTGCTGCAAGCTGCCGAACGCAGCGAAGGCGAATTGCAGGA
GCGCTTGCAGCAGTTGCACCAGCAATTGTCCGGGCTGCTGGAAACCGAGCAGGTCGCGCTGTTTCTGCACGAAGAC
AGTGCACAGCTGATGGCCCAGGCTGATTTGCGCCCCTACCTGAGTATCAGCCCGCTGTACCCACTGGATGTGGAGC
GCCCACAGGCGCTGTTTGCCAGTTGGTACGAGCTGTTCCCGCGCTCGATCACCGACGATCCGGCGCGCCACGGCAC
CTTCAATGACGTGCATTCGCGCCTGGCGATGATCCACGACATGGGCTTTGACGTCCTGTATTTCCCACCGATTCAC
CCCATTGGCCGTAGCCATCGCAAGGGCAAGAACAACTCACTGAGCGCTGGCCCCGATGATCCCGGCAGCCCCTATG

CGATCGGCAGCGAGGAGGGCGGCCACGATGCAATCCATTCGCAACTGGGCACCCGCGAGGATTTCCGGCGCCTGGT
AGCGGCCGCCGCCGAGCATGGCCTGGAAATCGCCCTGGACTTTGCCATCCAGTGCTCCCAGGACCACCCGTGGCTC
AAGCAACACCCCGGCTGGTTCAACTGGCGTCCGGACGGCACGATCAAATACGCGGAAAACCCGCCGAAAAAATACC
AGGACATCGTCAACGTCGACTTCTATGCCGCCGATGCGATCCCCAGCCTGTGGCTGGAGCTGCGCGACATTGTGCT
GGGCTGGGTCGAGGAGGGGGTCAAGACCTTCCGCGTCGACAACCCGCACACCAAGCCGCTGCCATTTTGGCAATGG
CTGATCAGTGACGTACGGGCCCAGCACCCGGAGGTGATCTTCCTCGCCGAAGCCTTCACCACCCCGGCGATGATGG
CGCGCCTGGGCAAGGTCGGTTATTCCCAGAGCTACACTTATTTCACTTGGCGCAACACCAAGGCCGAACTCAGCGA
GTACTTCACCCAACTGAACCAGTCGCCCTGGCGTGAATGCTACCGGCCGAACTTTTTCGTCAATACCCCGGACATC
AACCCAGGCTTCTTGCATGAGTCCGGCCGCCCGGGCTTTTTGATCCGCGCCGCGCTGGCCACCATGGGCTCGGGCC
TGTGGGGCATGTACTCGGGCTTTGAACTGTGTGAAAGCGCGCCAGTGCCGGGCAAGGAAGAGTACCTGGACTCGGA
AAAGTACGAGATCCGCCCCCGGGACTTCAGCGCCCCCGGCAACATCATTGCCGAGATCGCCCAGCTCAACCGCATC
CGCCGGCAGAACCCGGCGCTGCAGACACACCTGGGCCTGACGCTCTACAACGCCTTCAACGACAACATCCTGTACT
TCGGCAAACGCAGCGAAGATGGCAGCAACTTCATCCTGATCGCCGTCAGCCTCGACCCGTTCAACGCCCAGGAGGC
CCATTTCGAGTTGCCGCTGTGGGAGATGGGCTTGTCGGACGACGCCCAGGTCCAGGGCGAAGACCTGATGAATGGC
CATCGCTGGACCTGGTACGGCAAGACCCAATGGATGCGCATCGAGCCTCACATGCCGTTTGGCATCTGGCGCATAA
CCCATTCTTAAGTCGAACACGGTCTTCAGAACCCTGAGGATCCAATGTGGGAGCTGGCTTGCCTGCGATAGCGGTG
GGTCAGTGAAAAGCATCTTTGCTGATCCATCGCTATCGCAGGCAAGCCAGCTCCCACATTAACCACAGTATTTATT
CAGGAGTTTCCAATGGCGAAGAAACCCAAGGCTGCCACCTTTATCAAAGACCCGCTCTGGTACAAGGACGCGGTGA
TTTACCAGGTTCACGTCAAATCCTATTTCGACTCCAACAACGACGGCATCGGTGACTTCCCCGGGCTGATCGCCAA
ACTCGACTACATCGCCGACCTGGGCGTGAATACCATCTGGCTGCTGCCGTTCTATCCCTCGCCACGGCGTGATGAC
GGCTATGACATCGCCGAATACCGGGGTGTACACAGCGACTACGGGACCATGGCCGACGCCAAGCGCTTTATCGCCG
AGGCGCACAAGCGTGGCCTGCGGGTGATCACGGAGCTGGTGATCAACCACACGTCCGACCAGCACCCCTGGTTCCA
GCGGGCACGCAAGGCCAAGCCCGGTTCCAGTGCGCGGGACTTCTACGTGTGGTCCGATGATGACCAGAAATACGAT
GGCACCCGCATCATCTTCCTCGACACCGAAAAGTCCAACTGGACCTGGGACCCGGTGGCCGGCCAGTACTTCTGGC
ACCGTTTCTATTCCCACCAGCCGGACCTCAACTTCGATAACCCGCAAGTCATGAAAGCCGTGCTGTCGGTGATGCG
CTACTGGCTGGACATGGGCATCGACGGCCTGCGCCTGGACGCGATTCCTTACCTGATCGAGCGCGACGGCACCAAC
AACGAAAACCTCCCCGAAACCCACGATGTGCTCAAGCAGATCCGCGCCGAGATCGATGCCAATTACCCCGACCGCA
TGCTGCTGGCCGAAGCCAACCAGTGGCCGGAGGATACCCAGCTGTACTTTGGCGACCAGAAAGGCGATGACGGCGA
TGAGTGCCACATGGCCTTCCATTTTCCGTTGATGCCGCGCATGTATATGGCCCTGGCCCAGGAAGATCGCTTCCCG
ATCACCGACATCCTGCGCCAGACCCCGGAAATCCCCGCCAACTGCCAATGGGCGATTTTCCTGCGCAACCATGATG
AGCTGACCCTGGAAATGGTCACCGACAAGGAGCGCGATTACCTGTGGAACTACTACGCCGCCGACCGCCGCGCGCG
TATCAACCTGGGTATTCGCCGGCGCCTGGCGCCACTGATGGAACGCGACCGCCGGCGCATCGAGCTGCTCAACAGC
CTGCTGTTGTCCATGCCCGGCACGCCGACCCTGTACTACGGGGATGAAATCGGCATGGGCGACAACATCTACCTGG
GTGACCGCGACGGCGTGCGTACGCCGATGCAGTGGTCCATCGACCGCAATGGCGGGTTTTCCCGCGCCGATCCGGC
CAGCCTGGTGCTGCCGCCGATCATGGACCCGCTCTACGGTTACCAGTCGGTCAACGTCGAAACCCAGGCCCAGGAC
CCACACTCACTGCTGAACTGGACCCGGCGCATGCTGGCGATCCGCAAGCAGTCCAAGGCATTTGGCCGTGGCAGCC
TGAAAATGCTTTCGCCCAGCAATCGCCGGATCCTGGCCTACACCCGCGAATACACCGGCGGCGACGGCAAGCACGA
AATCATCCTGTGCGTGGCCAACGTGTCGCGCACCGCCCAGGCGGCGGAGCTGGACCTGTCGGCCTTCGCCGGCATG
GTTCCGGTGGAAATGCTCGGTGGCAATGCATTCCCGCCCATTGGCCAGCTGAATTTCCTGCTGACCCTGGCGCCCT
ATGGCTTCTACTGGTTCGTACTGGCCACCGAAAACCAAATGCCCAGCTGGCATGTGGAACCGGCCCAAAGCATGCC
GGACTTCACCACCCTGGTCCTGAAAAAACGCCTCGAAGAGTTGCTCGAAGCGCCATCGCGCACCACCCTGGAGCAA
ACCTCCTTACCCACCTGGCTGCCCAAGCGGCGCTGGTTTGCCAATAAGGACGCGGCCATCGACAGTGTGCACATTG
CCTATGGCGTACGGTTTGGCGATGCGCAGCACCCGGTGCTGCTCAGCGAGATAGAAGTGACCAGCGGCGGGCAGAC
CAGCCGTTACCAATTGCCCTTCGGCCTGCTAGGCGAAGACCAGTTCACCAGCGCCTTGCCGCAACAACTGGCCCTG
GCGCGGGTCCGCCGAGTACGCCAGGTGGGCTTGATCACCGATGCCTTCAGCCTCGACAGCTATATCCGCGCGGTCA
TCGAGGGCCTGCGTGCGCAGACCGTGCTCAGCAGCAGCGACGGCGAGATCCGTTTCGAGCCGACCCCGCAATTGGC
CAACCTGCCGGTGGGGGACGAGTTGCAAGTGCGCTACCTGGCGGCAGAACAATCCAACAGTTCCGTGGTGGTGGGC
GAAAGCCTGGTGCTGAAGTTGATTCGCAAAGTCAGCGCCGGGGTCCACCCGGAACTGGAAATGGGCGCCTACCTGA
CCGCCGCCGGCTACGCGCATATCTCGCCCTTGCTAGGTTCGGTGATCCGTCGCGATGCCGAGGGCCAGGACAATCT
GTTGATGATCGCCCAGGGCTATCTCAGCAACCAGGGCGATGCCTGGAGCTGGACCCAGAACAACCTGGAGCGGGCG
ATCCGCGACGAACTGGCCCAAGCCATTTCCGAACAGGAACAACACTACAACGCCCTCGGTGAACTGGCCGATTTCG
CCGGGCTGCTTGGGCAGCGGCTCGGGGAGATGCACAACGTCCTGGCCGCGCCTACGAGCAATCCGGACTTCCAGCC
TGAAGTCACCTCGGCCAAGGACTGCCAGGGGTGGGCCAAGCAAGTGGGGGCCCAGGTCGAGCGCGCCCTGCAGCTG
CTCAAACAGCATCAAAGTGAATTGAACCCTGACGATCAGGCGCTGGTCACTCAACTGCTGGCGCAGAAAAAGCCCA
TCGCCAGCCACGTGCAGGAGTTGGCAAAAGCCACCCTGGGCGGTTTGCGCATCCGTGTGCATGGGGATTTGCACTT
GGGCCAGGTGCTGGTGGTCAAGGGCGATGCCTACCTGATCGACTTCGAAGGCGAGCCCGCACGGCCGCTGCACGAG
CGCCGGGGCAAGCACAGCCCGTACAAGGACGTCAGCGGCGTGTTGCGTTCGTTCGACTATGCCGCCGCAATGGCCC

TGAATGTGCAAGCACAAGGCCTGGATCACTCGGCTGATGCTGATGCGGCACGCCAGCGGGTCGCCGATCGTTACCT
GAGTGAAGCGCGACAGGCATTTATCCAGGCGTATCAACAGGCTACGTCTACACTGGCGCATGACTGGCAGGATGCC
AAAGGCGCGCAGGCGGCACTGGCGTTGTTCAGCCTGGAGAAGGCTGCGTACGAAGTGGCCTACGAAGCGCAGAACC
GCCCGACCTGGCTGGCGGTGCCATTGCGTGGGTTGCATGGCTTGTTGGCCGGCCACATTTAGGGGGTTTGCTGTGT
CTGACCTTATTCAAAACCACAAGCAGTGGGAGAAATCATGAGTTTTACACACAAAGAACCGCTGCAATCGACGTTC
AAGATCATGCCACCCCCCAAGGATATCGAAGCCCTGGTGCGAGCCGAGCACCCGGATCCGTTCGCGATCCTCGGGC
CCCATGAGGACGAGGCAGGCGGCCAGGTCATCCGCGCGTTTTTGCCCGAGGCCCTGAGCGTCGAGGTCCTGGCCCG
TGGCAGTCACGAGGTACTCGGCAGCCTCGACGCCACGCCGGTCCCAGGCTTATTCATTGGGCATTTCGCCACACGC
CAGGGCTACCTGCTGAAGATCCAGTGGGCGGGCGGCGAGCAGATCACCGAAGACCCCTATAGCTTCGGGCAGTTGC
TCGGGGAAATGGACCTGTACCTGTTTGCCGAAGGCAATCACCGCGACCTGAGCAGTTGCCTCGGCGCCCAGGTGAT
GAGTGTTGACGGCATCCAGGGCGTGCGCTTTGCGGTGTGGGCGCCGAATGCGCGGCGAGTCTCGGTGGTGGGTGAT
TTCAATATCTGGGACGGGCGCCGCCACCCGATGCGCCTGCGTCATCCGGCTGGGGTCTGGGAGATCTTTATCCCTC
GCCTGCAACCGGGGGCGGCCTATAAATACGAAATCCTCGGCGCCACCGGGATCCTGCCGCTCAAGGCCGATCCCAT
GGCCCTGGCTACGCAACTGCCGCCCGACACCGCGTCGAAAGTCGCCGCACCGTTGCAGGTGGAGTGGGCAGATGAG
GCGTGGATGCAAGCCCGGGGCGAACGCCAGGCGGCCAGCGCGCCGCTGTCGATCTACGAACTGCACGTCGGCTCCT
GGCAATGCGAACTCGACGAGTTGGGCGAAGTGGCGCGCCAATACGGTTGGCGCGAGCTGGGTGAGCGGCTGATTCC
CTATGTGCAGCAACTGGGTTTTACCCATATCGAGTTGATGCCGATCATGGAGCACCCTTTCGGCGGCTCCTGGGGC
TATCAGCCGCTGTCGCAATTTGCGCCCAGCGCGCGTTTCGGTTCGCCCGATGACTTTGGCGCGTTCGTCAACGCCT
GCCATCAGGCGGATATCGGCATTATCCTCGACTGGGTGCCCGCGCATTTCCCCACCGATACCCACGGCCTGGCGCA
GTTCGACGGCACCGCGCTGTACGAATATGGCAACCCGCTCGAAGGCTTCCACCAGGACTGGGACACCCTGATCTAC
AACCTGGGGCGCACCGAGGTCCACGGCTTCATGCTGGCCTCGGCGTTGCACTGGCTCAAGCACTTCCATATCGATG
GGCTGCGGGTGGATGCGGTGGCGTCGATGCTGTATCGCGATTATTCGCGCAAGGCCGGCGAGTGGGTGCCCAATCG
CCACGGCGGGCGCGAGAACCTGGAAGCCATCGACTTCCTGCGCCATCTGAACGACGTGGTGGCGCTGGAAGCCCCC
GGCGCCCTGGTGATCGCCGAGGAGTCCACGGCCTGGCCGGGCGTCAGCCAGCCGACCCAGCAGGGCGGCCTGGGCT
TCAACTACAAGTGGAACATGGGCTGGATGCACGATTCGCTGCATTACATCCAGCAAGACCCGGTGTACCGCGCCCA
CCATCACAACGAACTGAGTTTCGGCCTGGTGTATGCCTGGTCCGAGCGCTTTGTGCTGCCGATTTCCCATGATGAA
GTGGTGCATGGCAAACACTCGCTGATCGACAAAATGCCCGGCGATCGCTGGCAGAAATTCGCCAACCTGCGCGCCT
ACCTGAGCTTTATGTGGATGCATCCGGGCAAAAAACTGCTGTTCATGGGCTGTGAGTTCGGCCAATGGCGCGAATG
GAATCATGACCAGCAACTGGACTGGTACCTGCTGCAGTACCCGGAACACCGCGGCGTGCAGAAACTGGTGGGCGAC
CTCAATTGCCTGTACCGCGAAGAGCCGGCGCTGCATGAGCAGGACGATGCGCCGCAGGGCTTCCAGTGGTTGATTG
GTGACGATGCCATCAACAGCGTGTATGCGTGGCTGCGCTGGGGTAAAAGCGGCAAGCCGGTGTTGGTCGTTGCCAA
CTTCACCCCGGTGCCCCGAGAGGCGTACAAGGTCGGCGTGCCCTTGGCCGGCCGCTGGAAAGAGCTGATCAACAGC
GATGCCGATACCTATTCCGGCTCCAACTACGGTAATGGCGGCGAAGCCTTTACCCAGGACGAGCCGCTCCATGGGC
AGGCCGTATCACTGTCGTTGAATTTGCCGCCCTTGGCCGTGCTGATTCTGCGCCAGGAGTAATAACCGTAGGAGCC
GGCCTGCCGGCGATGGACGATAACGCTGATGTTGGGTACCAGACACCCCGCAGCGTTTATCGGCCGCCATCGCCGC
CAAGCCGTCGCCTGAAAACAACCCGCGCCGTTGCCCTGACGAGCGCCGTTACCAATTCCTCTGGAACAATTTCTTA
CGTGTGATCCACACAAGGCTGTCAGCGATCAGGCGTAGGATCTCCGTTCACCTCGTTTCGTCGCTGTCCCTGTGTA
TCACTGCCAGAGACGCAAACGCCATGACCCCAGCAAGCTTGAATACCCGACCCGCCACTCTTTCGCCTCAGGCGGC
TCCGCCGGTTGATGCCCCACCTGTGGCCCAGGGCAAATCCAGCCTGGCCGCGGTGCCAACAGCGGAGCCACCTGCC
AGTTCGCAACAGGCCGAGGTGCGAGGAAAACTGGGGGATCTGGACGAAATGCTGAAGACTTTGAGGGAGCTTGCAG
CCTCGAAAAACGCCGATACCCCCGTGAACAAAAGCGTGTTCCTGAACAAAGACTCACCGTTGATACCGCCGAACGG
TTCCACCGAGCCGGGTACCAACCGGCTGGAGAACATGCTCAAGTTTTATGGCATCGTGCCATCTGATCCAAAGACA
CCGGAGCAACTGCAAGCAGCCATTTATGCCGTGGCAGATGCCCGCAGCGAAATCGCCCTGGGTCTGGATAATACTT
CCAGTAATGCCGATGGTTTGTTGCACAAGACTAACGAGCCGGCTATCTCTGCATGGGTCACCGAGCAGGAGGCCAA
GCACAAGCGGCCTTTGCTCAGTCTGTTGACTGAGGGGATCTCGCCACAGGCCCTTGAAAAAGCCAAGGACACACCT
GCAGCGGTACTGGAACTGATACTTAAGTCACCCAAAGCCAATGAGCTGATTCAACAGTTGCTCAAGACCCTGGGCG
GGGCTGAATTTCCACTGGATGGCGTCATTACCCGTGAGGTCAAGATCAAATTGTTGCTCAAGGCCCTGACCTTGAG
CCTGGACCCGGTGCAAGGCCAAAAGCCGGGGCATATCGCCGGCTTTGACCTAGCGTCGAGTGAGCGTTGGAACCAA
AGCTACCCAGCCATCAAGTCCGAGTTCAGGCAACACTTGGTCAGCACAAGGCAGTTATCCAGCGACCAGGCCTATC
TGGCGGCCTATATCCTGAAACCCCAATTCCCGGCCGACTTTGCCGTACGTGATACACCGGCAGAACTGCCTTACCA
GGGATCGGCGGCGTGGGTGAACTTCAAGCACGGCGTCAGCCTGGCCGAAGCGATAAAACCGGGCTCTTCGCGGCAC
ATGAGTTTTCAGGAGCTGGTTGACCTGCCAGCCAAGTTCAGCGAGCAAGCGACGACGGACGAGCAGTGGGCGGCTA
TCGCGTCGACCCGTATCCCGGCGACGTTGGATTGGGCCGTGGCACAAGGGCTGCTCCCACAAAAACAGGACTACTC
CCAGGAGGAGATCAATACAGCAGTCGCCGCATTGGATGCCCACGTCGATAAGGTGGTGTCGGCAGCCCAGCAATTG
TCAGCGGATGTGCCGATGCGCAAGGATTATTTCGAGCGTATGGCGCGGGGTACTTCACGCACCGAAGTGCTGAGCC
TGTTCAGCCCTTTGTTTCTTCTGTACAGGCATCTCAATGGCGAGAAAATCACGCCGGCCGAGGTCAGCTATAAATA
CCCTGTCTATGATCAAGCCACGTTCAAAACGGCGTTCGACAAGTACCTTAAGGATACAAAGTCAGCCTATGCAAAA

CTGATCACCGACCAGTTGTCCCAACTCCCGCTAAAAGACCGCAGGGCCATTGAACAGGGAGATGTCACGGTTTACG
CACTTAGGACTAAACCCGACTCAGCCAAGGAAACTGATGACGATAAAAAGGCAGCAATCACACGCCCGGCATTTAT
TCTCAAGGCGGTACATGAGGGGCAGACAACCTACTACGAGATCAATCCTGTCAAAGGTATCGCTCGGCGTCGTGAC
GACTTGAAACCGATTCTGGAGTCCTACAACCCGGCCGATAAAAGCTATGGCAAAGACTTTATCTCGATCGAACACT
CCGCTGACGGTGTGCCTTCTGAAGAATTGGTTGTGCGCGACGCTCGTGCAGGTGGCAGGGCTGAGTTCCAGAAAAA
GTGGGCGAGCTATAAGTCAGGCAAAGGCCCCAAACCTGCTAGTTTTTCTATCGTTGTCCCTCAACAACTCGCCCAC
TTTCCATCATCTGCCGCCCCCTCTACACGAGCCGTGCCACAAACCCTCACTTCCGAACGGTCTGAGGCTATCGCAA
AGACTGCTACTGATGATCTGTTCTACGTCGATACAGACAGGCTGAAGGAGTGGGCCGAGTCCGATCCTGAGCGAGA
AAGTGCCGCACAGAAAAAAGATGAGGCGTTCTGGGAGCGCGTCAAAGGTACCGCGCAGATGGTGGTTCCGCTCTGG
AGCGGCATTGAAAGTCTCGTCAAGGGTGACACCAAACAGGGTGTGTTGGATCTGGTTAACGATGGGCTGTCAGCGT
TAGCGGGCCCTATGGGCAAGTTTGCCGCGGGCTCGGTGCGTTTGGTTTCGCGTGCAGGCAAGATCGGAGTGCGCGC
GCTACTGCCTAAGTTTGGCTCGTTGGTAAAGAAGTTTGCGGTTTCTGTGGTGCGCAATCTCAACCCCCTTGACCCT
GTGATGCCTGCACTGAGTAGCAGTGGCAACAGGCTGCTGAAGTTCGGCGGCGCGCACCTGCAAAATGTTCAAGCTG
GTATTGCACAGTTCAAAAAATCTGCCGTGCTTAAACTGCCCGGCCGAGGGCGTTACGACCTCTCTCCAACAATTGA
CCTGACCCCCAGGCCCGTTGCCCAAGGACAGACCTTGCGGGTGGCCGAAGGTATTTCCAGTGAGCAGGCACGGGTG
GTGAAACGCAATAAATACAGCGATGTGATGGTCGGCGATGACGTCTATCGCTATAACCCGGGCAAACCCGAAAGCC
TGGTCAAACTAGGCGGGCCTGAGGATATAGGGCCGCTTGAAGGGTTTGTGATGACCTGTGGCGCCGGAGGGCGCCG
TCACAAACGGGATCTCGACGACCTGTGTTACACCAAACAGATCGAACCTGGCGGCACCCCAGTGTTCCAGGACGCC
CAGGCCCTGGAGCATCGCCGCCTGATTCCGGGGGTAGGGCAGGCGAAAGGTCCGCGCACGGTCATTCACGAGCATC
GTCGTTACCGCGTCAATGAAGCGGGAAACCATGAACTACTACCAATGGCCAGTTCACAGCCTGTGACGTACAAGGG
CCGTACCACGGGGACCGTAGTCAATGAACCGGACTTTGGTTATGACGACTTTGGTGTGCCTCGGGCGGTCAACCAG
CAAACGGTTGTAGTCAAGATGGATGCGATCAGCGATCTGAGTAATGACCAGCGTGTCGTCAGGGGCCTGAAGGTCA
ATCATGACGGCCGGCAGTACGTGGTGGTCGAGGCCGATACCGGTGTGCACTACTACGCTGAGCTGAACGGCAATGG
CCCACTGGAATTTCACCGCATGACCCGCAAGGATCCACTGGACGTTGAATTTATCAAACTGTACGACCAGCATAAG
GATATCTATGGCTTTGCCGCCCAGGGCCTGCCGGACAACCCGTTGGTGGTGCTGCCGACTATGGATAGCCTGGTCA
AGAAGATCGTCGCTGACGAACCCATGACGCCTAAGGAAATCAACCATCTTGCCGGCGTGTTGCAAGGACTGCCCCC
CGAAAAACAGCGTGAAGTATTGATGGGGGTGTACGCCGCGGGGAGCAACCCCGGCCATGTGGTGGTCGCGGCAAAG
CCGGTGCGACTGGCGCCGATCAAGAAGCCGGCTGACTTTGTCCAACTGCCGGCAGAGCAGCAGAACCGCTTGTATG
CAGAAGGGGCGAGAAAAGCCGTCGACGAGCAATTCCAGGCCACTGGCATCCGGTCAGCCAATCAACAGGTTCCAGG
TCTTGCCGGTGAGGTCGCACGTAGCAACACCGCCACGGAAATGGTGGGCTGGCTTTACACACGAACAGGCGCCCCC
AACTATAGCGAAATAGTCCTGAAAACCGGTGCCGGCAATTGCGACCAGATGGCCAAGGTGGCCGTTGATACCATCA
ATACCAGTGGCGGCCATGCGCGTATCGCGCAAGTCAAGGGGCATACTTTCGCTATCATCGGCGGCCCGCCAGGGCA
GCCTCGCAGCAAAGGGTTTGTGGGGCCGGAGTGGGATGACGCCTGGGTCGTTGATCCCTGGGCCGGTATCACCTGC
CGAGCTGCGGATTACCCCGCGTTGTTCAAGGCGCGAATGCAAGAGTGGAGCCAGTCGGGCCGGCGCATCCTGATCA
GCGATGGGGCGACGCCACCCCGTTCAGTGTGGAGTGACCCCATGGAACCACGCTGGATAGCGGCGACCGTCGATGG
CGAGGCTCAGGTGTTCCAATAGTCCCGGGCGCATCACCCGCGACAGAAAACACCCACGCCCCTAGGGTTTTCAGAA
CTCCACCCTGACGCCCACAGTGCCTTTGCGCCCATCCAGCGCATTGCTATCGAGGTTGCGTTCATAACCCAGATCG
CCATACAGGCTTACGCTGCTGGACATCTGCAGGGTTGCACCGGCACTCAGGTCCAGGGTGGTGGATTTTTGCTCGG
TGTCGATATCCGTGACCCCGTTGAAACGCACCCGATTGGTGCCGGCCGAGGCATGCCAGACATTGGCACGCAGATA
CGGCTGGACCGGCATCGACGCCACGGTATAGCTGCCCCGCAGCCTGGCGCCGAGCCGCGTGGTGATGGCGGTATCG
GCGGAAAACGAAACGTCGGAAACCCCGTCATTCTGGCGATCGAGCCGGGTTTTATTGACGATCAACTGCAATTGCG
GCTCCAGCACCCAGTTTGGCGCGACGGGCAGCGGTACGCCGACTTCCACCGAGCCCAGCACATCGTGGCCTTTGGT
CTTGAGCTTGAGCCCGCGATCAGACTCGCTGCTGCCATTGAGGCGGGTGCCCATCAGCACGGCATCGACGTACCAG
CCATACGGGTCGATCAAGGTCCAGTACAGGCCCAGGCTGTCGCCACGCAAGGTGGTGTTGCCGGCATCCAGATGCT
GCCAGCCGCGATTGAAGCCGTCGACATTGCCCCGCAGGCGGCTATGACCGACGAAGAAGCCCACACGTTGGGTCTG
CCCGTTGGCGGTGGTCCAGGCATGCACGTCGCTGCCCACCTGGAAACCGGTGACCGAACTGTCCAACCGCGGGCTG
ACCGTGCCGGCATAGGCCTGGCGGCTGCTGTTGCCGTACACCCGGCCCCAGCCCGCCGGGAATGAGCCGCTGTTGG
CCTGGCGTGCCTGGTCGCCCATGCGCTCATGGTAGGTGCCGAGCATGGCCCGCACCGTCTGCTGCGCCGCGGGGAA
CAGCGCCGAATACACGGGTACTTCGGGGCGATAGATCGGGATCGGCGCTTCCCCGGCCACCGGCGCAGGCAAGTCC
ACCGGTGTGCCTTCGGCGGGAATCGGCACCGGCAGCGGTTCCACGGGGCCCTCCGGGGCGGGCAGCGGCGGCGCCA
CCACGGCCGAGCGCAGGTACCACTGCTGCTGGGAGTCTGCGCTGACACCGCCCTTGAACAGGAAGTACTCGAACGC
CCCGGCCGACACCGAGCCCCCCGCCAGGCTGAAGGCCTGGTCACTGCCGGTCGCGCCGTTGAGCGCTTGGACCACC
TGAATGCCATCGCTCAGGGTTTCGGCGCCAGCCCCCCCAAGGTTGCTGACGTTGAGGGTCGTGCTGCCCTGAAGCG
TGCCCTGGTTCACCACCAGTCTGTCACTGGCCGAGCCATCACCGCCCAATACACTTTGCAGCGCCATCTGGCCATT
GTTGCCGGTGTAGTTGCCATTGACCGTCAGGCTGTCGGTGGCGCTGCTGCTGGCACTGGTCATATCAATCAGGCCG
CTGTTATGGAGGGTGGCCAACTGGCCGGCAGTGAGGGGGCTGATGCTGCCCTGGCTGACCAACAGCACGCTGCTGC
CGTCGACATTCAAGGTGCCAGTGCCGGTGCCTGTATCGCCGAGTTTGAAATCCCCAGCCAGGCTAAAACGCGAGCC

ATTGTCCAGGTTGAGCGTTTCCCAGTTGGCGTAGCGCTCGGCGTTGGTCGCCTGGGTGTTGTCAAGCGTCAGGGTA
TCGTTGCCCAGGCCGCCATCGATCAAGGGGGTGGCGGCGAGGGTGGCTTGCGTCAGGTTCTGCAGGAGTGCGGTGT
CGTTATCCGGGCCCATATTGACCGCAGAGTTGATCGTGCCGGCGCCGATCCAGTTGAACTGATCGTTGCCAAAGCT
CATGAGCACCTGGCCGTTGACCACGCCGCCGGTCAGGGTAAACAAATCATTGCCGCCGCTGACACTGACGTTACCG
CCGATATACCCCCCGGAAATGCTGATGGTGTCCAAGCCAAACCCGGTCACCAGGTTGCCGTTGATCCTCCCGCCCA
GCAGGGTGTAGAAGTTGTTGTCGAGCTTCATGTCGACCCGCCCGATCGTGCCCGCCGATTGGGTGGCAACATCGCC
ATCCTCGAACGCCCCGACGATCTCGCCGCCACTCATGTTGAACGTATCCCGCCCATCGCCCTGGGCCAAACTGGCG
ACACGACCGCCGCTTTGCGCGTAGGCATCGATGCCATCACCCTGGTTAATCGCGCCAACGCTGCCGCCGCTGATTT
GGATGCTATCGGCGCCGTCGCCCTGGGTCACCGCCCCCACGACAGCATCCAGCTCCACACGGAAAATATTCGCCCC
ATTGCCCATGTTCAGCGCGCCCATGCTGCCCGATTGCGCTTGCACCAGATCGTGGCCGTCGCCAAATGTCACGGCG
CTGGTGATTGCGCCATTGCCACCGGTAGGCAGGTTGAGGGTGTTATTGCCGTTGAGGTCGGTAAAGCCGGGCGTGG
TGCCGCTGTCGCAGGTGGTGGTGTCGTCGCCGGCCGTGACCACCGGTGTACAAGCCGCGTAGGCCGGTAGCGAGAG
GGTGGTCATCAGGGTCGCGCCGGCCGTCCAGGCGCTCAGGTGCAGCAGAGAATATCGCCGTGTGCTCATGATCCAT
CCTCACTCGATGGCAACTGACATCCCGTCGCCTGCATGGGCAGGCGCGGTACCCACCATGGCAACGAGAAGCACGA
TAACGATAAGGGACGGCTGGTTCCGTTTCGTAAAACATCGCCAATGCAAGGGGTTGGCATGGCTTTCTCGACTGCA
CCTGAACCGGTGCAGTGCTTGTTCCACGTTAGCAACAGCCGCAAAGGGCCGCCACTGTCAGAATTAACAGGTACAG
ACCAGTTACAAGAACACAATCTCGTAGGGTTCGCGCATCCGTTCCAGCAGTACCGGTGGCAGCATCGGCGCCAGGC
CGATCAGCGGGTCGCCAAGCAGTTGACTGGCGTAGGCGTGGGCGGCGTGGCGTTTGCGCGCCACACTCCAGGTGTC
GAGGCGCACCTTGCGCGCGCACTGCCAGGGCATGCGCCCGGCTTCACGGGCGGGGCGATGCCAGGCCCAGATCGGC
AATTCAAGAAGGCGCGCGCCAGTCAGCGCACAGGCAATGGCCGTGGCGCGGCCGACGGCGTCGTGGTCGGCATTGC
CATCATTGCGCCAGGTGGTGAACACCACATCACCGGGTTGCAGGTAGCGGGCGATAAAGGGGCTCAATAGCTGTTC
GCGAGCGGCCAGGTCGTTGTCGCAAAAACCGCCGCGAATCCACTTCAGGCTATGCAGGGGCAAGCCGAGGCGGCGC
AGGGCCTCGACACTTTCCTGGGGGCGAAACACGCTCAGGCGCTGCGCCGACCATTGGTTGGAACCCGGATGACTGG
CGCTACCGTCGGTGATCGAGATCAGCTTCAAGGGGTGGTCAAGGGCGCTGAGCAGTTGCAACAAACCACCGCAGGC
CAGCACTTCGTCACCCGGGTGCGGGGCAACTATCACCACCCGCGAGCCGGCAGGCACCAGGAGGGAAGGGCTGATG
GTGGGGATTTGTGCCAGTTGCGGTGCGCTGTTCCAGATTTGCGCCGGCGTATGGCGACCGTCGTTGAGAGTGGCGG
GTTTGATCAATGTCACATCCTTGTTCTGATGATGCAGGTCGTGCCCCGTGGACGGGTCGACTCTCCTTTTTTATCG
CGCAGGCGCCAAGGCCTGTGCGGCACACGGGCAATCCGAATCCTGGATTGCCAAGCTTGAGTATTGTTCATCACCC
GGATTTCGCCGGGCAAAGTTGCAACTTTTTCAGGAAGGCTGCATCAGGCCCTTCAAATAATCGCCAAAACCGCCCT
GTGCGCGGCAGTCCAGCCGCGCACTGGTGATGACCTGGGGCGCATGGCTCCAGGCAATGGTTGCGCCGCATCGCTC
CAGTTGGCGCACCAGTTGCACATCTTCATGACAGGCCAGGGGTTCGAAGCCGCCGGCACGTACATAGGCACCGGCG
CTGACGCCCAGGTTGGCCCCATGGATGTGCCGATGCCCGTCACAAGCCCGATAGGCCTGGTTGTAGCGGATCTGCG
CGGCCTCATCGAAACCTTCGCTCCAGGCATCCACCGTGACCGTGCCGCATACCGCGTCCACGCCCAGGGCCAGTTG
CGCCACCAGCCAATCACTCGCCACGCGGCTGTCGGCGTCGGTGCAGGAAATCCAGCGCGCGCCCTGGTTCAACAGG
TGCCGTGCGCCTATTCCACGCACCTGGCCGACGTTGCGGGCCTCTACGTTCAGGCAGTACACCCCGAATGCCTGGG
CAATCTCGCTGCTGCGGTCGGTGCAGCTGTCGAGGACCACCAGGATCTGCACCTCTTCACCGAGCAAGCCCGGATG
GCTGGCGGCGATGATCGCAGCCTTCAAGCACTCGGGCAGCAGCGCCTCTTCGTTGTGCACCGGGATCAGAATGCCG
ATCATCGCAGCCCCTCAAGGTGGGCGACCGAGGCGCTTTCGCGAGACCACAGGTCGAGTAGGAAATCACTGTCAGC
CACTTGCGCCACGCGCGGCATGCCCAGGCGCTCGTGCAATAACTGGTGGACCTGTTCCGCCGTCTGCGGGCAGCCC
TTGATAGGCGGGCGCCAGTGGCAGGCCAGCACCTGACCGTCGTGGGTCAACGCGGCCAGGGCCCGCTCGATCAACT
CGCACAGGTCATCATGGTCGAGGTAATAGCACCACTCGCTGAGCACGATCAGTTCGAACCGGCCCTGCGGCCACTG
TGCCGGCAAGCGGCTCTGGCACACCTGGGCGTGGGCAAAACCCTGCAGGCGCGCCTGGGCCAAGGCCACCGCCTTG
GCCGCGGTGTCGCAACACACCAGCTGGTCACAGCGCGGCGCCAGTTCGGCGCTTAATTCGCCATTGGCGCAGCCGG
GCTCGAAAATCGAGGTGTAGCGCGGTTTGCTCAGCAACCCCAGGGTCAAGGCCCGTTTGCGCTGCTCGTACCAGCG
CTGGCGAAAGGCCCAAGGGTCGTCATTGTCGACAAACAGTTGATCGAAATAGGCGGTGGCAACACTCATACAAACA
CCACTTCAAAAGGTTGCAGTAGCCGCTCCAGCACATGGGGCGGCAATACGGGCGGCAAGTCGATCTGCGGATCACC
CTCCAACTGGCTGGTGAACGCGTGCAGGGCGTGGCGCTTGCGCGCTACGGCAGCGGGCGTCAGGGCGATCTTGTGC
GCACGGTGCCAGGGCACCCGCGGGTCCTCTGGGGTTGCCCAGTGCCAGGTCCATACCGGCAACTCGATCAACTGCG
CGCCTACGGCCCGGGCCGCCTGGGCACTGGCTCGGCCTACGGCTTCGTGGTCGCAATGGCCGTCTTCGCGCCAGGT
GGTGAACACCACGTCGTGGGGGTGCAAGTGTTGCTGGATAAATTCAACCAGTGCCGGTTCCCGCGCCTGTACCTGG
CTGTCGGAAAAACCACCACGCAGCCAGCTCAGCCGTTGCGGCGACCAGCCCAGGCGGTGCAGCGCCTCGGCCGACT
CCTGGGGGCGCACCTCGCGTAAACGCTGGCGCGGCCAGCGGGCCGAGCCGGGGTGGCTGGCGCAACCATCGGTCAC
CGAGACCAGCAGCAACTCGCGGTCCAGTGCCGCCAAGCCTTGCAACAGGCCGCCACAGCCCAGTACTTCATCGTCT
GGGTGGGGCGCGATAATCACCGCGCGACAGCCAGCAGGCACCAGTTGCGCGATGCTGATCAGGGGCAGGCCGGCGA
CCCGAGTGGAGCCTTGCCAGGCGCGCAGCGAGGTGCCTTGGCCGACAATCGGGTTGGCGTTCACAGCTGCCACGTC
CCTGGTGATTGCTGCGACAGCTGCTGGCCGAGGGCCGCCAGGTCACGCTCGGCGTGGCTTTGGCGCAAGTACACCG
GCAAGTCGGCGATCAGCCGGGCAAAGTGCGGATCCTGGCAATACGGCCCGGCGCCCATGGCCCGGCCAACGTGATG

GATGACCTCGTTGGCCGTCGCCTCAATGACTGCTCGCGCTCGGCGGGCGAGCAGTTGGGCATTGGCCTGAGGTTGC
TCATCGATCTGCCGTGCAGCGCTGCGCAACACCATGGCGCCAGCATGCAACGCCATGTCCACCGCGCCCAGGTGGG
CCAGGGCATGGGGTTCTTCGCGGCGGCCACAGTGTTCGCGCAGGGCCTCGGCCAAACGTTGCGCCGCGCCGTACCA
ACAGGCGGCGATACCAACCCCGCCTTGCCAGAAACCCGGGCGTTGCAGGTAATCCCCTGGCCCGCCGACCGCTACT
GCAACGGCGGTGTCGAAAACCACCTCGACACTTGCACAGGCTTGCATGCCCACGGCTCGCCAGCCGTCGCCGGTCA
CTTCGACACCCGGCTGAGCCATCGCCACCGCGACCAGCTGTTGGCGTTGCTGCTCATCCCAGGCAGTCAGTAATCC
ATGACTGACCACCGCAGCGCCGGAGCACCAGGCCTTGCGGCCCTCCAGCAGCAGCTGGCTGCCGGCGCGTCTGACC
TGCACCCGGGCCGCCGGCGGTTCGGCGGCCCACATGCCCCAAGTGCTGCCCGCAGGCGGGGCCTGGCTGCCGAGTT
CGGCCATGATTGCCAGCGCGTCAGTGTGCCCTTCGTACAACTTGCACAGGCTCAAATCGTGGCCGGCCACCTGGGC
CAACTGGCTGAAACGCACCAGTGTCTGCCCGCCACCGGGGCGCGGCAACTGGTCCAGGCCCGCGTCCACCAGGGCC
TTGAGGGTCAGGCCAAGGCCCTCGATTTCAGCCAGTGGGTGGCGGTTGAGTTGTAGAAAATCGTGCAATGCCATTT
CACACCTCTTCCTCATGCTGCAGTTCGAACAGTAGCAGCGAGCGCCCGGTCACGGCATATTCGTGGTCAAAGTCAA
AACGCTCCTGACCGCGTACGGCCGGCTGGTTGGTGTCGACCATGCAGGTCCAGAAGCCGCCATCCGGCACCTGGGG
CAAACGGAAATTCACCAGGTCATGGTGCGCGTTCACCACCAGCAGCAGCGTAGCATCGGCCCCTGGCCGGCGAATC
CCGGTTTCCTGGGCGCGTCCGTCCATCAACATCCCCAGGCAACGGCCCTGGCTGTCTTCCCATTGTTCGATGGACA
TTTCGTTGCCATCGGGGGCCAGCCAGGTCACGTCCTTGACCCCGATGTCTTCGTTGTAGTCGCCCACCAGGAAGCG
CCCGCGACGCAGGATGGGGTAGGCCAGGCGCAGCTTGATCAGGCGCTTGACGAACTTGAGCAAGGACTTGCCATCT
TCATCCAGCTCCCAGTTGACCCAGCCGATTTCACTGTCCTGGCAATAGGCGTTGTTGTTGCCGTGCTGGGTACGTG
CGAACTCGTCCCCGGCCACCAGCATCGGCGTGCCCTGGGCCAGCAATAGGGTGGCGAAGAAGTTACGCATCTGGCG
CAGGCGCAGCGCGTTGATCTCGGGGTCTTCGGTGGGGCCTTCGACGCCGTGGTTCCACGACAGGTTGTTATTGCTG
CCGTCCTGATTGTTCTCGTCGTTGGCTTCGTTGTGCTTGTCGTTGTAGGACACCAGGTCGTGGAGGGTGAAGCCAT
CGTGGGCGGTGATGAAGTTCACCGAACTGTAGGGGCGGCGCCCGCGCTGGTTGAACATTTCGCCGGAAGCGGTCAT
GCGCCCGGCAAAGTCGGCCAGTTGCCCATCATCGCCTTTCCAGAACGCGCGCACGGTGTCGCGGAAACGGTCATTC
CATTCCACCCAACCTGGCGGGAAGTTGCCCACCTGATAACCACCGGGGCCCACATCCCAGGGTTCGGCGATCATTT
TCACCTGGCGCAGCACCGGGTCCTGCCGGCAGGCGACCAGGAAGCTGTGGCGTTCGTCGAAACCGTCGTGGTAGCG
CCCGAGAATGGTCGCCAGGTCGAAGCGGAAACCATCCACATGCATTTCCGTGGCCCAGTAGCGCAGGGAATCGGTG
ACCATTTGCAGCACGCAGGGGTGGCTCAGGTCCAGGGTGTTGCCGGTCCCGGAATCGTTGATGTAGAAGCGCTTGT
CGTCGGGCATCAGGCGGTAGTAGGAGGCATTGTCGATGCCGCGCATGGACAGCGTCGGGCCCTGCTCGTTGCCTTC
GGCGGTGTGGTTGTAGACCACATCGAGGATCACCTCCAGCTTGGCTTCGTGCAGGTGCGCGACCATTTCCTTGAAT
TCGGCGATCTTGCCACTGGCCAGGTAGCGCGGGTCGGGGGCAAAGAAGGCGATGCTGTTGTAGCCCCAATAGTTGG
TCATGCCTTTTTGCAGCAGGTGCTGGTCATTGACGAAGGCATGGATTGGCAGCAGCTCTACGGATGACACGCCCAG
TTGGCGAATGTGCTTGAGCACGTCATCGACCATCAGCCCGGCGCAGGTGCCGCGCACCGCCTCAGGGACCGAAGGA
TGGCGCATGCTGATGCCGCGCAAGTGGGTCTCGTAGATGATCGTGCGATCCCAGGGCGTGCGCACCGGCTGGTCGT
TGCCCCAGGTGTGGGCCGGGTCGATGACTTTGCATTTGGGCACGAAGGGCGCGCTGTCGCGCTCATCAAAGCTCAG
GTCGGCATCCGGGTGGCCGATGGTGTAGCCAAACAGGGCTTCGGACCACTTGAGCTCGCCCACCAGTTGCTTGGCA
TAGGGGTCGATCAACAATTTGTGATGGTTGAAACGATGGCCATTGGCAGGATCGTAGGCGCCGTAGACGCGGTAGC
CATAGATCAGCCCGGGGTGGGCGTCGGGCAAGTAGCCGTGGAACGTCTCGTCGGTGTATTCCGGCAGTTCGATGCG
CTCCAGTTCTACTTCGCCGGTATCGTCGAACAGGCACAGCTCGACCTTGGTGGCATTGGCCGAGAACAGCGCGAAA
TTGACCCCCAGACCGTCCCAGGTCGCGCCGAGGGGGAAGGGCAAGCCTTCACGAATCCGCGATGGCTCGGTATCGG
GTGTCTTGCTCATAGTGTTGCTCCTGCAAAGTTTTTCCGGGCCGAACGGGGCCGTGCTGACATCAGGTCAGATCAA
TCGTTGAGCTGTTTTGTAGGAGCCGGCGAGCCGGCTCCTACAGGAAGGTGGGGTCAGCCGACGGGCTTGCGTGGGG
CTCGGGGCTTTTTGGCGGCGGCGGGTTTTTCCGCAGGCGGTTTTGCCTTGGCCGCCGGTTTGGCCTTGGGTTTGCT
GGGTGTCAGGGCCTCGGCTTCGGCCAGTTTGCGGGCCATTTCCCAATGGCGGGCTTCCTGGCCTTCGGGCTTACCT
TCGGACTCCCAGATCTGATACGCGAATTCACGGACGCGCTTGTCTTCGGTGCTCATCGCAATGCTCCTCACAGAAA
ATTTCAGCTTTCTTGATCGTCAGAATGGATAAAGACATTCACCGGGACATCCCCCAGGGCAGCGCTGATCAGTAGC
TCCTTGTTGGGTGTGACTGCGCCTGTTTGCAAAAGTCCCTTCCAATTTTGCATCGTGGCGTCGAACGGTAATTTGA
TCCGGGTATCGCCCCAAACTTGCGCATCTACCCGGGGTTGGGCGCTGTTTTCAAGCAGGGCGTGGGTCCAGCGCGG
TACCACCACCAACAGCCATTGGCCTTGATACTCGCGACTGAAAGCCACCACCCGCTGGGCATGCTTGCCCAGCACT
TGCAGCGGTTCATAGCGGCCCCTGGCAAACAGCAACGGGTGGGCCTTGCGCAGGTTCAACACCTGGGCAATCAACG
CCTGCTTCAGGTGCCCGTCACGCCAGCGCTCAAGCAAGGCGCCCGGTGCTGCCGCCAGTGCCCGTTGCCGGGCGCT
GAAATCCACCGCCCGCCGGTTGTCCGGATCCACCAGGCTCAAGTCCCAGAATTCGTCGCCCTGGTACAGGTCCGGC
ACGCCGGGCACGGTGTTGCGCAGCAAGGATTGCGCCAGCCCGTTCAGGGCCCCGGCGGGGGCGATGGCCTCGGCGG
CAGCGTGGATCGCCGTGCGCAAGGGCAGGCCGACCGGGTCGAGCAGCAGGCGTGTGAGGAACGCTTCGACACCCTG
CTCGTAGAGGTCGTTGGGCGCACTCCAACTGCTGTGCAATTTGGCTTCGCGCAGGGCTTTTTGTTGCCATTGCCAG
AGGCGTTGCTGGTAGTCGTCGATGTCGTGCAGGTCCAGGGGCCAACTGCCCAGCAGCACCTGGTAGAGGATCAGCT
CATCGCCGGCCGAGGGAATGCCCGGCTCATCGCGCAACGGCATGGCCAGCCGGCGCCAGTGCTGCACCTGCTCGAC
ATACCACGGCGCACATTCACTGAGTACCGCCAGGCGCGCGCGGCTGTCTTCGCCGCGCTTGTGGTCATGGGTGGCG

GTGGCCAGCAGGTTATCGGGGAAAGTCTGCAGGCGTTGCTGGTTGGCGGCATGAAAGTCAGCCAGGGGCGCGCTGA
ACTGTTCGGTGCAAAAGCCTACGTCATTGCGCGAGAGCAATACCGCCGAGCGGTAGAAGGCGGTGTCTTCCACGGC
CTTGGCGGCTGCGGGCGAGGTCAGTTGCTGGAAACGCACGCAGGCGTGCTTGAGGATCTTGCGCGGTTGCCCCACG
GGACGCTCGCGCCAGGTTTGGCCACCGAGCCACTGTTGCAGGTAGTCGAGCACTGGCCAGTCGGCTTCGCTCAAGG
TCCCTCGGGCGCCGGCCAGGGCCTGTTGGAACACGCTCTCGTCAGCGGCGCTGCGGCCACGGGCGCTGATATAGGT
GCGATACACCGGGAAGTGCACGACCAGCTCCTGCAATGCACGGCGGATCGCGCCCAGGGTCAAGTCGCGGCTCATC
ACGTCGGCGCGGGCTACTTGCAGCAGGGCCTGGGCCACGCTTTCAAAATCCCCGGCCAGGGAGCCGTTGAGGATCT
GTTGCCGGGCCAGCCAGGCTTCCTCGATAAACGCCGAAGGCCGTTCAGTGGCCTCGCGCCAGAGCCGGGCCAAGGC
TTCGAACCCCTGGGGGTCGTGCTGCACCAGCGACACCTGGTTCATGAATTCGTAGCCGGTGGTGCCATCTACCCGC
CAGTCTTCACGCAGGGTTTCGCCGGCGCCGAGGATCTTCTCGACAAAGATCGGCAAGTGGCGGTTTGGCGACAGTC
CATCCACGCGCCGCCGCAACTTGCGGCAGTAACCCCGTGGATCGGCCAGGCCGTCGATATGGTCAATGCGCAGGCC
GTCCACCAGGCCCTCGCTGATCAGTTGGAAGATCTTGCTATGGGTGGCCTCGAACACCGTGGTGCGCTCCACCCGC
AGGCCGCCGAGCTCGTTGACGTCGAAAAAGCGCCGCCAGTTGATGTCATCCGCTGCCGTGCGCCAACTGGCCAAGC
GATAGGCTTGCTGTTCGAGCAACTGATGCAGGCGGGCAAAACCTTGAGGTTGGCGCGCATCAAACATTTCCAGGGC
GTGTTCGATGGCCGCCAGGGTCAGTGGGGCCTGCGCGCGCTGCGCCAGTGCCTGTTTCAGGCTGGCGGCTTGAGGC
CAGGCATCGGCCTGATACGCCAGGGCGCTGAAGCGGTCGGCCAGGTCTTTGAGGGGAGGATGGGCGCGCAGGATAT
CGCCGTAGTCACTGGGGCAGATCGGGAAGCGGTGTTCGTAGTGTTCGACAAAAAAACTGCCCTGGGCCGGGTCAAA
ACGCAGGGGCAGCGTACCGCTTTGCAAGGCTTCGCCGTAGTCGCTACCGAGGAAAGGCATTAACAGTTGGCCCTTG
AGCAGCGGGTCCGGCGAGTGCCACTGGATATCGAAGAATTCGCTGTAGGGGCTCAGGCGCCCCCATTCCAGCAGGT
CCAGCCACCAGGGGTTGTCGCTGCCGCCGACGGCCATGTGGTTGGACACGATATCCAGAATCAGGCCCATGTCGTG
CGCGCGCAAGGCCGCCACCAGGCGACGTAGCGCCGGTTCGCCGCCCAGCTCGGGGTTGACCGTGGTTGGGTCGACG
ACATCGTAGCCATGCATCGAGCCGGCGCGGGCCTTGAGCAGGGGCGAGGCGTACAGGTGGCTGATGCCCAGGCTGG
CGAAATAGGGCACCAGCGGCACCGCATCATCGAGGGTAAAGCCCTTATGGAATTGCAGGCGTTGGGTGGCCCGTAG
CAAGACGGCGTTCATCGATCACGCTCGAAGGCCTGATTGCGCGCCACCGCCAGCAACTCCAGGCGGCGGGCGGCGT
TCTCATTGTCGAGCAATTGCGCAGCGTCCCCCGGCAGGCGCCGGCGCCAATTGGGATGGGTGTCGATGGTGCCGGG
CAGGTTGGCTTGTTCTTCGACGCCCAGGGCGTCTTCCAGGGGCAGTATGACCAGCGGTGCGCGGGTGTGGCCGAGG
TAACGCACGCTGGCATCGAGTATATGGTCGGTTTCGTTGCGCCACTCATCGCGAAAATTCTGCGGGTCTTGCGCCA
GGGCCCGGCGCAGAGCCTCGCGCTCACCCTGGCGGTGCTCGCTCCACTGCTCAACGGTGGGCGCATCGATCAGCCC
CAGTTGCACATTCCAGTCGATATCGCGGCTGTGCCACCAGCCGTTGAGTGTCGGCAGGTCATGGGTGCTGGTGGTG
GCCAGGGCGTTGTCCGGCCAGTCGAGGATCGGCGTGAAGCACCCGCCATGGTCCTGTTCAAACAACAGCACGCGCA
TGCCAAGCATGGCGCGGGCGCTGAGTTTTTCCCGCAGGCCATCGGGCACCGTGCCCAGGTCTTCGCCCAGCACAAT
CGCCTGGTGCCGGCTGGATTCCAGGGCCAGCAGGCGCAGCAGGTCATCCACCGGGTAATACAGGTACGCGCCTTGT
TTGGGCGAGGCGTCCTTGGGGATGACCCACAGCCGTTGCAGGCCCATCACATGGTCGATACGCAAACCGCCGGCAT
GGGCGAAGTTGGCCCGCAGCATTTCGATGAACGCCCGGAAACCATTGCGTTTGAGGCCTTCGGGGGAGAACGCGGA
GATGCCCCAGCCTTGGCCAGCACGGTTGAGGATATCCGGTGGCGCGCCGACGGTCAGGTCGGCCAGCAGTTCGTCC
TGACGGCTCCAGGCCTGGCTGCCGCCGCCATCGGCGCCCACGGCCAGGTCGGCAATCAGGCCGATGCCCATGCCAC
TGCCGCGTGCGGCTTGTTGTGCGCGCTCGAGGCAGCGGGCGATCAACCATTGGTTGAAGGCATAAAAACCAATGGC
TTCGGCCTGTTCGGCGGCAAATTGTGCCAGCGCGGCGCTGCCCGGGTCGCGCCAGTCCTCGGGCCACTGGCGCCAG
TCGAGGCTTTCACCGTTGGCGGCGCGCGCGGCTTGCACGGCTTCAAAGCGGCAGTGGTTTTCCAGGGCCTCGCCAC
CGGCCTGACGGAAACTCATGAAGTCAGCCTGCTGCGGGTGTTCACCCTGGCGGAAATCCTCGTACAGTGCCCGCAG
CAAGCGTTGCTTGGCCTTGGCGGCGGCGGGCCAGTCGATCAGGCTTTGTTGTTCCAGCTCTTGCAGTTCATCGGCC
AGCCCGGTGGACTCGATGGCGCTGCGCAGTGCGCGCTCGCCGAGGATGCAACCGGGCGAGGCATACAAGCTATTGA
GGAACAGGCGGCTGGACGGTGAATAAGGGCTGAAATGCTCGGTATCGGCACTGAACATTGCGTGCATCGGGCTGAT
CGCCAGGGCGTCGACGCCCCGTTCCGCGGCAGCGCGAGCCAGTTGTTCCAGGGCCTGGGTATCACCGAACCCACCA
TCGCCTGCGCGGCGCAAGGCATATAGCTGGGCACTCAGGCCCCAGGCGCGGGCGGGCTGGCTATCCACTGCCTCGG
CAACGCTGAAACACTGATGGGGGGCAACGGCCAGGGTGAAGGACTGGTCGGCGATCTGCACTCGGTGGTAGCCCAC
GGGCAGGACGCCGGGCAGGCTGGCGTCGGCATCCAGCGCCAGGCCTTGGGTGCTGCCGTCCTCCAGGTGAACCTGG
CATGCCGTACCAGGGGCAAAGTAGGCGGCCAGGTCAAGGCCGGTGCCCACGTCCACGGTCAACAGCGGCGCCAGGT
GCCTGTCCTGCTGCACGCGCTCCAGTTCTTGCAGGCTGGCGGCGATGGCCGCGTCGGTGTCGGCGGGGTGGCCGAG
GCCTTGGAGCACGGCGCGCAACGCGTCGGCGCTGACATGCTGCGGGCGGCCGTTGGCGTCGATCCAGTCCACCGCC
AGGCCCGCACGGCTGGCGAGTATTTCCAATTGCGCTTCGCTCAAGAGTGCTCTCCAACAGATGATGAGGTGTCACC
CGGCGTCAGGCTGACGCGGGCAGAGAAGGGCGTAAGTGACTTAAGGTCGCTGCTGGTGCTGAACAGTTCGCGGAGG
CCGGCCGGGTGATGCAGGGTGGCCGGGCTCAGGTTGAGATCGATCTGCAATAGGCTGCCGTTGCCCAGGCGCCAGC
GTGCAGACACCGCGCCCTGGCCCAGGATTTGCGCGCCGAGGGCAGTGCTGCCGGGCAGGTGGGGCACGATGTGCTG
TTGGCGCAGGCCCAGCAGTTGGCGGTACAGCGCGGCCGGCGCCTGGGTAAATTGCGGAATAGAGCGCTCAAAGGTC
TGCAGCGCGTTGGGGTCGGGAATCTGCGCGCGACGTGCGGGGTCACTGAAGGCGGCGAAGTCAGCAAATTCATTGC
GCCGGCCTTCGCGCACGGCTTCGGCCAGTTCGCCGTGATGGTCGGTGAAGAACAGAAACGGCTGTTCGGCGTTGAC

TTCATCACCCATGAATAGCAGGGGGATCATCGGCGACATCAATAACAGCGTGGTCGCCGCCTGCAACGCCTGGGGC
GAACACAGTTGGTGCAGGCGCTCACCGAGGGCGCGGTTGCCGATCTGGTCGTGATTCTGCAAAAACAGCACAAAGG
CGCTGGGCGGCAAATGAGCGCTGGGCTCGCCGCGCCGAGTACCGTGGCGGGTGGTCTCGCCCTGATACACAAAGCC
TTCGCCCAGGCAGCGGGCGAGTTTGGCGGTGGGATCATCGGCAAAATCGGCGTAGTAGGCGTCGGTCTCGCCGGTC
AACAGCACATGCAGGGCGTTATGCCCGTCATCGTTCCATTGCGCATCAAAGCCCTGCTGTAACAGGTCGGCCTGAT
TAAATTCGTTTTCCAGTACCAGCCACACGTGGCGCCCGGGCTCTACCTGCTCACGCACCCGTTGCGCCAGCTCCTT
GAGGAACGCCGGCTCGTTGATGGCATGCACCGCGTCCAGGCGCAGGCCGTCGAAGCGGTATTCGTTGAGCCACATC
AGGGCGTTATCGATAAAGAAGTCGCGCACTTCGCGGCGGCGAAAGTCGATCCCGGCGCCCCAGGGGGTGTGGCTGT
CTTCGCGGAAAAAGCCTTTGGCATACTGGCCTAGATAATTGCCATCGGGGCCAAAGTGGTTGTAGACCACATCGAG
AATCACCGCCAGGCCATGCTCGTGGGCCGTGTCGATCAGGTGTTGCAACTGCTCGGGGGTGCCCAGGGATGCCTGC
GGTGCGTAGGGCAAGACCCCGTCGTAACCCCAGTTGCGTTCGCCGGGGCACTGGGCCAAGGGCATCAGCTCGATGG
CGGTAACGCCCAACTCTGCCAGGCGTGGCAGGTGCTGCTCGACTGCGGCGTAACCACCCAGTATCCCCACATGCAG
CTCGTAGATCACCGCTTCATGCCAGGGCCGGCCTTGCCAGGTGGCGTGGCGCCAAGGGTAGGCCAGGGGATCGACC
ACCACGCTGTCGCCATGCACGTCGGCATGTTGCAGGCGTGAGGCAGGGTCCGGCACCTTCTGCTCACCATCAATGG
TGTAGTGGTAGCGGGTGCCGGCTCCACAGCGTACATGGGCGACAAACCAGCCCTCTGCCTGGGGCAGCATGGGCAC
TGACTGACCATTGTGCAGCTCGACGCTGACATCAGACGCGTCAGGCGCCCACAGGGCGAAGCGGGTATGTTGCGCA
TCCTGCATGATCGCGCCGTGGGGCCATGTTTCCAGAGTCCGTAACGGCATCTATCCCAACCTCTTTATGCTTTCCC
TGATTTTCCCAGCGCTTTCGCTACCAGTTGTTCGTAAAGTTCGGCGTAGGGTTCCACCGCCTGGCACCAGTTGAAC
GGCGCGGCCATGGCCCTGCAGCGCATGGCGTTGAGCAGGCCGGGGAAGGCAAACACCTTGAAGGCGCGCGCGAGGG
CGGCTTCGTAGCTCTGCACGGTGGATTCGTCGAACAAAAAGCCGGTCACGCCGTCTTCGATGGTGTCTGCCAGGCC
GCCGGTATTACGCGCCACCGGCAACGAGCCGAAGCGCTGGGCGTACATCTGGCTCAGGCCGCAGGGCTCGTAGCGT
GAGGGCATCAACAAAAAGTCGCTGCCGGCAAACATGCGGCGGGCATCGGTTTCGTTGAAGCCGATCCGCACCCCGA
TGCGCCCGGGGAACCGCAGGGCCAGGGCGCGCATGGCCTGTTCTTCTTCCGGCTCGCCACGGCCGATAATCGCGAT
CTGCCCGCCGTTTTCCACGATAAAGCCGGCGACCGCTTCCGTCAGGTCCAGGCCTTTCTGGTACACCAGGCGCGAC
ACTACGGCGAACAACGGGCCGGTGGACTCGTCCAGGCCGAACAACTTGCGCACGTGGCTGGCGTTGACCGCCTTGC
CTTGCCAATCGCCGATATTGAACGGGTGATCCAGGTGCGGGTCGGTGGACGACTCCCAGCTTTCATCGATGCCGTT
GGGAATACCACTGAGCAGGCCTTGCTGGGTCTTGCTGGCAAGGAAGCCATCCAGGCCGCAACCGAACTCGGGGGTG
GTGATCTCCCGGGCGTAGGTGGCGCTGACCGTGGTGATATGGCTGGAATACGCCATGCCGGCCTTGAGGAAGGACA
TCTTGCCGTAGAACTCCATGCCCTCCTGTTGCAAGGCATGTTCGGGAATCCCCAGCTCCGGGCACGAGGCCAGGCT
CACCACACCCTGGTAAGCCAGGTTATGAATGGTGAACAGGGTGGGGGTGCGTGACCCACGCCAGTGCATATAGGCT
GGCGCCAGGCCAGCCGGCCAGTCATGGGCGTGCACCAGGTCCGGGCACCAGTGGATTTGCGCGAGGTTGGCGGCAA
TATCGGCGGCTGCCAGGCCCAGGCGGGCGAAGCGGATATGGTTGTCAGGCCAGTCGCGACCGTTGTTGGCGCCATA
GGGCGTGCCTTCACGCTCGTACAGTTCGGGGCAGATCAGTACATAGATGACCAGGCCATCCTTGAGGTCCATGCGC
CCGATCTTGCAGGGCGGCAGCGCGGCATGGCCGCCCAGTTCGCCGATGATATGGATCGGGTTGTCGCTGTCGAGCA
CCTGGCGGTAGCCGGGAATCAACACCCGTACATCGTGCAGGTGCGCCATGGCACGGGGCAAGGCGGCGGACACATC
TCCCAGGCCACCGGTTTTCACCAGGTCGGCCAGTTCCGAGGTCACAAACAGGACTTTCTTGCGATTGGGGTTTTGA
GACGAGACCGGCCGCACTGCGCCGGGAAGGTCAACCAAGGCGGTTGGCCCATCGACCGGCTGACTGAAACGCTGTC
CCTGAATTCCCGAAGCGGCACTGATCATAATTATTCTCTCCCATGTGTTGGTCAGCTGAGGGCCTGGCGCCCTCAG
CGAAAGTCAACCGCGTCCTGCGGTCACGCGCACTAGCACTGCACAGACTGGCAAGGCTTATGCCAATTGCACCGAA
CTGATAAATAGATTGGATGGGCGGACGTCCGGCTGGCGCCGGTAGCGTGCGTCCTACCTTAAACCTGACCTACGGC
AGAGTTGGAAAGTTTCGATTTATTGCGGGGGCTTTTGTTCTGATCGTGACCCATCGGTCATGAGTCTAGGACAGAG
CCCAGAGCCTGCAAGGGTTGTAGGAAAATTTTGTAGGACAATTATATTTTTCAGCCAGGCGCTCCCCTTGTAGAAG
CTGGCTTGGCGGCGATGGCGGCCTTGAGAAATGCCGCGTTGCCCAGGGCTTCATCGCCGGCAAGCCGGCTCCTACG
GGGCGGGTGCCTCTTCGTGGGGCATCGTGGCTGGGCTGCTCGATAGGAGTGCAGTCGGCTACTTAAAGTGATTTGT
TAAGAGTTGATATCTATATAAATCAATGCTTTATAAGAAATATGTGAAGTAAATAATCAGATAAGGCTGCGCAAGG
TCTGCCCACTGGCCCACGCCTGGATGTTTTCGATCATCTGCCCATAGAACTGCCGGTAGTTCTGCTCGCTGACATA
ACCCACATGGGGCGTCGCCAGAACATTGGGCAGATGGCGAAACGGGTGATCGACCGGCAACGGCTCCTGGCCGAAC
ACATCCAGGGCCGCGCCCGCCAATCGCCCATGGCGCAGCGCATCGACCAACGCCGCCTCATCGACGATCGGCCCCC
GTGCGGTGTTGACCAGTCGCGCACCGGGTTTCATCCAGCCCAGGGCCTGGGCGTCCACCAGGCCACGGCTGCGTTC
GCTGAGTACCAAGTGAATCGACAGTACATCGGCCTGCTCGAACAGTTCGCGCTTGCTGACCCGGGTCACACCCACT
GCGGCGGCTCGCTCTGCGGTGAGGTTTTCACTCCACGCGATCACAGACATACCAAACACCTGGCCAAACTGCGCCA
CCCGCTGGCCGATGCTGCCCAACCCGAGGATGCCCAGGGTCTTGCCATGCAGATCGACGCCCAGCCCTTGCTGCCA
CTGGCCGGCGCGCAGGGAGTTCGCTTCGGCCAGCAGATTGCGGGTGGATGCCATGATTAATGCCCAGGTCAGCTCG
GGCGCCGCGTGTTTGTAGCTGTCGGTACCGCACACCTGGATGCCCAGGGCCTTGGCGGCGACAAGGTCGATGGCGG
CATTGCGCATGCCGCCGGTAACCAGCAATTTAAGGGCCGGCAGCCCCTGCAACAGCGCCTTGTCGAAGGTTGTGCG
CTCACGCATCAGGCAAATCACCTCAAAACCCTGCAAACGCTCGATCAGGGTCGCGGTGTCGGCGGGATAGTCATGC
AGGAAGTGCACCTTGCCGATCGCTTGCAGGGCGGACCAATCCACTACGTCACGTGCCACATTTTGCCAATCGTCAA

TGACTGCGATCTGTACTGACATACACACCTGCCTCGGGTTAAGTGGAATTAGAGCGCCTTGAGTCCTTGCAACAGC
GCCTGGTGAAAGCGCGCGGGTTCTTCCATCTGCGGGGCATGGCCCAGGCCAGGGAACTCCACCAGGGTCGAATGGG
GGATGAGCTTGGCCACCTGCTTGCCCAGTTCGTTGTAGCGGCCGATCCTGGCCTTGACCTCGGGCGGTGCGTTATC
ACTCCCGATGGCGGTGATATCAGACGTACCAATCAGCAGCAGGGTGGGCATCTGCAGGTCCTTGAACTCGTAGTAC
ACCGGCTGGGTGAAGATCATGTCGTAAATCAACGCCGAGTTCCACGCCACCCGTTTGTGGCCAGGGCCCTGGTTCA
AGCCGACGAGCATGTCGACCCAGCGCTCGTATTCCGGCTTCCAGCGCCCGTCGTAGTAGGTCTTGCGCTCATAATC
ACGCACGCCGTCGGCATTCAGTTTCAACTCGCGCTCATACCATTGGTCGACGCTGCGATAGGGCACGCCCAGGGCT
TTCCAGTCCTCCAGGCCGATCGGGTTGACCAGCGCCAGTTGCTCGGTTTGCGTGGGGTAGAGCAGGGCATAGCGGG
TAGCCAGCATGCCACCGGTGGAATGGCCGACAAGAGTGGCCGTGGAGATGCCGAGCTTTTGCAGCAACTGATGAGT
GTTGATTGCCAACTGCTGGAAGCTGTACTGATAGTGCTCGGGCTTGCTGGAGGTGCAGAAGCCGATCTGGTCCGGC
GCGATCACCCGGTAGCCGGCTTCGCTCAGGGCCTTGATCGACGCCTCCCAGGTCGCGCCGCAGAAGTTCTTGCCGT
GCATCAACACCACATTGCGGCCATTGGCAGGGCTTTTGGCGGGCACGTCCATATAACCCATTTGCACGGATTGGCC
CTGGGACTGGAAGTCGAAATGTTCGACCGGATAGGGGTACTGGAAACCTTGCAACTGCGGGCCATAGCTTGGGCTT
TGCGTGGCTGCCAAGGCAGGCAGGGCGCAAGCGGCCAGCAGACCGGTAAGGACAAACGAGCGTGTCAATGGCATGG
GAAAGCTCCAGGGGCGATGCTCCCGGATGCTGTGGTGGGTGGATTAAGCGGGGATTAACCGGTTACATCCAGTGCA
GCGTCAGCATTGCCACCACGGCATAACGAGCCGCCTTGGCCAGGCTGACGATTAACAAAAAACGCCACAGCGGCTC
CTTCATCACCCCGGCCACCAGGGTCAGTGGATCGCCAATAATCGGCAGCCAACTGAGCAGCAGTGACCAATGGCCC
CAACGTGAATAGTGGCGGCGGGACTTTTCCAACTGCGCGGCGCTCACTGGAAACCAGCGGCGATCCTTGAACAGTT
CGACCGAGCGCCCCAGCAACCAGTTGACCCACGAGCCCAGGACGTTACCCAGGGTCGCGACCCCCAGCAGTAACCC
TACCCAATAGCGGTCATTGAGCAGCAGCGCTACCAGCACCGCTTCCGATTGCAGCGGCAACAGGGTGGCCGCTCCA
AAAGCGCTGAGAAACAGCCCCGCATAGCCTGCAAGCATTGCTCAGTGAGCCGGGTATTCGGCTACCACCACATCTT
TGCCGGCACGGGTCAGGCCAATGACTTGATAGGCGTCGCTCATCCCATCCATTTCCATGCCTGGCGAGCCCATGGG
CATGCCCGGTGCGGCGATGCCCAGCAGGTCATCACGTTTGCGCAGGGCCAGTACCTGATCGGCGGGTACGTGGCCT
TCGACGAATTTGCCGTCGATCACGGCGGTATGGCAAGACGCCAGGCGGGGTGCTACGCCCAGTCGCTGTTTGACCG
AGCTCATGTCGGCTTCAACATGGTCGTTGACCTTGAAGCCGTTGCTTTCGAGGTGGCTGATCCATTTTTTGCAGCA
GCCGCAATTGGCATCACGGTGCACGTCAATCGGGATCAGTTCGGCGGCCTGGGCCAGGGTGGTCATGAACAGGGCG
GACAGTAAAGTCAGGCGCAGGGTGGTTTTCATAGACAGGTCCCATGGGTTACGGAGTGCGCAAAGAGGGCGCATTG
TCCGCGCTTTTGCGCATCAGGTGAGGTGGTTATGTTTCGAAATTATACGAGCACGCCACAACCCCGAAGCTTATCG
TCCGCAGCTTTCAGCAAACTGAGAGGAAGATGACAAATTTGTCAGTTCAGGATGATTCCAGGCGCTGCTTGAGCTG
GTCCATGGCCTGATTCGACAGCTCTACCATGCGCGCTTCCAAGGTGTTGAGCTTCAGTTCGGTCTCGTAGCTGAGC
ACGCGAGTGAACAAGGTGCCGCTGCCGCGAGGTTGTAGATAGTAGTGAATGCAGCCATCTACCGCGAGCGAGGTAA
AAACCGTCTTGAACTCCCGCGGCGGCTCGGCAATCTGCACCCGGTAGCTCATGGGCACACGCACCCCGAGCAAGTC
GATCATCTCGGTAAACCGCTGGCCGGCAGGCAGCGAGCCGCTGGTGCCGGTGTCGGCGCTCAGGGATGTGGGGTGC
CATTCGTGCCAGCGGTCCGGTTGGGTGACGTAGTCGTAGACGGCTTCGATGGGGGCTTGGATATAGCGTTCCTGGC
TAATCTGCCTGACGTGGGCCTGGTGCTCGACAGCGTTCATGACACACCTCCTGTGTGGTGAGACGGAGCATTAGGA
GCTGTCAGAATAGTCGTACTTGGCGACAGTGCGCGCAAAGATGTGTCGCCGATTGAAGCCTGTGTGAGTAAGCCGA
AATTAAAAATGCCCCGTTTAGACTGTTATATCTGACAGGTTCTTTTTTTTGAGTTTCGCTCTCTAATGATCAGGCT
CCTCCCATTAACTTAGCAATCATGAAATGGTGAAGAAAATGCCTAGTGACGCGCGCATAGAAATTAAGAACGGTGA
CGTTAAATCGACTGGAAGGATGGAGGGTGACATTTCAATCAGTGATTCTGATTCTAAGAGCAGCTACTACTTCCGG
TCGAATAGCGTTAATTACGTCAACAGTCGTTCAGCCCTCCAAGTAATGGGCATACAGGGGGAATCAGAGAAAAGTA
CAATTATCGCAGCAGATTTAACGCCGGGAACGCCTTCGGGCTCCTATAAATTTGGAGGGAGTGAAATCACCAGCCT
GAGCTATTCCCCGCCGCGTGGAGAACTTTTATGGAAGGTGACAGATGGCGGGATCATTATCACATTTGATGAAGAT
GGGAAACGTGCTCACGGAGCCTTGCAAATTATCGCCGAGCGTGGAACACAAACTCTAGAAGCGTTTGTGAGTTTCG
ATGTCAGAAATTAATCAAAGGCCTACTGAGTGATGAATGTTCCCGATCAAGAGAACAATCATCACTTTTTATCAGA
TCTGATAGTTCTTCAATTCCCGGGCAATCACCAGCCGTTGGATCTCACTGGTTCCCTCATAGATCTGGGTGATCCG
CGCATCGCGGTAGTAGCGTTCCACCGGGTAGTCTTCCAGGTAGCCATAGCCTCCATGGATCTGCATGGCCGAAGAA
CAGACCTTCTCGGCCATTTCCGAAGCGAACAGCTTGGCCTGGGACGCTTCGGACAGGCACGGCTTGCCGGCACTGC
GCAAACGGGCGGCATGGAGGATCAGCAGGCGCGCGGCATTGAGGCGGGTCTGCATGTCGGCCAGCAGGTTGGCCAC
GCTCTGGTGTTCAATAATGGCCTTGTCAAACTGCACCCGGTCACGGGCGTAGGCCAACGCGGCTTCAAAGGCCGCG
CGAGCGATGCCCAAGGCCTGGGCGGCGATGCCGATGCGCCCACCTTCCAGGTTGGAGAGGGCAATGGCCAGGCCCT
TGCCGCGTTCACCCAGCAGGTTAGCTTCGGGGATGCTGCACTGGTTGAGGGTCACCGCGCAGGTGTCGGAGGCGCG
GATGCCCATCTTGTGTTCCGTGCGGTCCACGGTAAAGCCGGGCGTATCAGTGGGCACCAGGAACGCCGAAATACCT
TTCTTGCCCAACTCCGGGTCGGTGACCGCAAACACGATTGCCAACTTGGCGCGCTTGCCGTTGCTGACAAACTGTT
TGGCGCCGTTGATCACCCACTGGCCATCGCGCAGTTCGGCGCGGGTGCGCAAGTTGTGGGCTTCGGAACCGGCCTG
GGGTTCGGTCAGGCAGAAGCAACCGATGGCCTGGCCACTGGCGAGTTCGGCCAGCCAGGTCTCTTTCTGTGCGGGT
GTGCCGTAGTTGAGGATAGGGCCGCAACCCACTGAATTATGGATACTCATCAGCGCGCCTACCGCACCGTCACCCG
CAGAAATCTCCTCCACGGCCAAGGCGTAGGCCACGTAGTCGACGTAGGTGCCACCCCATTCTTCCGGCACCACCAT

ACCCAGCAGGCCCAGTTCACCCATCTTGGCCACCAGGGCGTCATCGATCCAGCCGGCTTTCTCCCAGGCCTGGGCA
TGGGGGGCGATTTCACCACGGGCAAAGTCGCGCGCCATGTCGCGAATCATTACCTGTTCTTCTGTAAATTCAATAT
CTTGCATGATTTATCTCCCGCTCTTTGCGAAGTCTTGGAAGTAGCTGTCGACGTGGCTCTGGTCCAGTGCCGCTAA
CGTTGGCGGGTTCCAGCGCGGGGATTTGTCCTTGTCGATCAACAGCGCACGCACGCCTTCGATCAGGTCGCCACGG
GCAAACCACTGGCGGTCCAGGTGCAGTTCCAGAGCAAAGCAGTCCTCCAGCGCCAGGTGCCGGCCACGTCGCAGCA
TCTGCAGGGTGACGGCCATGGCCAAGGGCGAGCGAGTCTGCATCAACTGGGCGGTAGTCACGGCCCATTCATGGCT
GTCGGCGACGGTCACTTGCTGCAATTGCTCGACAATGCTCGGCACGTCCGGCTGGGCAAAGAAGTGATCGATGGCC
GGGCGCAGGGCCGCCAGCGGCGCATCCGGCAATTGTTGCACCGCAAGCTTGGCCAGCAGGCCTTGCAGATCCTTGA
GCGGTGAGTCTTGCCACTGCAGGTGGTCGAGTTTCTGATCCAGTTCGGCCAACCTGGCGCTGTCCAGGTACCAGTC
GGCGAGCCCGCAATACAGGGCGTCGGCGGCGCGGATCTGCACCCCGGTCACGCCCAGGTAAATCCCCAGCTCACCG
GGAATGCGCGGCAGGAAATAGCTGCCGCCTACATCCGGGAAATAACCGATGGCCACTTCCGGCATGGCCAGGCGAC
TGCGCTCGGTCACCACCCGCAGGTCGGCCCCCTGGACCAGGCCCATCCCACCGCCCAGGACAAAGCCGTCCATCAG
GGCTAGGACCGGTTTGCGGTAGTGATGAATGGCCAGGTCGAGGGCGTATTCTTCGACGAAGAAGGCTTCATGCAGC
GTGTCGCCGCTCTTGAAACTGTCGTACAGCGAGCGGATATCGCCCCCGGCGCAGAAGGCCTTTTCACCGGCGCCGC
GCAGAACCACCGCGTGCACCTGGGGATCAGCGGCCCAGGCGTGCAAATGCGCCGACAGGCTGCGGACCATGTCCAG
GGTGATGGCGTTGAGGCCGGCGGGGCGGTTGAGGGTCAGGTGGCCGATATGGTTGCGTACCTCGGCCAGGACGCTG
TCCTGGGGGGACTCGGTGCTGCATGTCGCCTGGGACTGAGCCTGAGCAGTCATCTGTAACTCCCTGCTTTTATTGT
TCTTTATCAAAATGTTCGCGGACGAACCATCACCGGATCGTACCAGTGCAAATTTGCCCAGTACAACCCGGAATCC
TGCAGGTCATCTTTGCATTTATGCAGGGGGGTGTTCGATAAAAAACCAGGGCAGGCGTGCTTAGACCCGACTGGCC
AGTACTTGGCCAATGCTGCGGCGGCGAGCGTGGTCTTCGGCGATATGGATCAGCTCTTCGAGCTCAGTGGGGGTGA
CGTCGAAAAACTGCTCCATTTGCGCCAGCGCTTGCTCCAGGTCCACAGCGGTGATTTTTGGATCTACCTGCGGCGC
CGCAGACGGCACCATGACCGGCGCTTCGGCTGGGCCCTTGGGGTAGCGAGTGCGCGTCAGGTTGTTATAGGCCAGG
GCCGAGAGCAACAACGTGGCGGCGCCCAACATGACCGGCGCGACTTCCTGCCAGCCCAGGGCGATGGTGGCCGGGT
CCGCCAGCACCAGGGTCATCGCCAGGCCCCCCGAAGGCGGGTGCAGGCAGCGTAGCCAGCACATCAGGATCAAGGC
CATGCCCGCCGCGAGGCAGGCGCTGCCCAGGGTTCGCCCCAATACGCGGGCCACCAGCAAAGCCACCACGGTGGCG
CACAGGTAGCTGCCGATGATCGACCACGGCTGGGCGAGGGCACCCGATGACACGGCGAATAACAGCACGGCGGAGG
CGCCCAGCGGGCCCAACAGGTGCATGGCCACTTCCAGGCCATACACCTGGGAGCAGAGCCATACGGTCAATAGAGT
GCCCAGGGCCATGCCGATGGCGGCACGGCTCCATTCGGCGGGGCGGGTGTTGATGGCAGCGGGTAACCAGCGAGCG
AGCATTGCGGGGCAGTCCAATCTTGAAAGGCGATCACAAAACAATGCCGCTCAGCTACACCTGCAGGTTTGTGCAG
GACTGAGTGACATCGGGCAAAAAAAGGGCTTTGCGGTTTGCCGCAAAGCCCTTCGAAAGTTCCAACATTTGGGGGA
GGAACCGGCGCAGTGTGCCGTTCAAACAAAACTGTCGCCAATGCATATTAATGCAGCTTAAGTGCAGTAATTTTGC
ATCAAAACCGCTCATACCCCGGGCTCACCTTGCCCAGTACGCGGTCGTGGTTGGCGCTGCTGTTCAGCGACTGCTG
CACGCTCTCCCGCAACCGTGGGTCGCTGGCCAAGGGGCCCTTGAGGGCGACAAGGGCGTCGCGCAACCCCATCAGC
CGAGCCAGGGTGGGCAGGGGGATATAGCCGACGTGTACCGGCTCGATAGGCAATGACTCGTCAGGCACGGCAGCGG
TCCTCTTAGGGGTGTTTTTGAACGCAGGCGGTAACTAAATAGCGCAATGTGCAGCGAGTGTAACGCTGGGGTCATA
GGGCCGGTAGAGGCGCCGCTACCGGCATTTACGGATACAAACGTAGGAGCTTTCTTGATAAAAACCCACCACTCAG
TCATCCGTACCCAGGTGCAGCAACACATAGGTGTTTTTATCGAACTGCCCTGTCAGAACCGGCGTCATGCCATGCC
AGCGTGGCTCGCCCAGACGCATGAAATCCGCCTTGTCCATCACCAGCCATGCAGGCACCGGCACTGCTGCCAGGTC
GCCAGGGGCTTGGGTGAACAATGGCACCCGGTCACATTCCACATTGACCATGAATTTGATGGCCTTGGCGTCTTTA
CCCAGGCCATGCAATACCAGGGGCGCCGGGTGCTGCCGGATCAGGGCATGAGCCTCGCGGGCAAAGGTGCGGGTGT
CATACAGGCGCCGTTCCAGGGGTTCGACCACCAGGATATATACGCTCCACACCGCCAGCACCGCGCAAAACGCCGG
CCCGACCGGGCGCCAGTGCCACTTGAGGAGCATCCCGGCGACAACGACCTGCAAGCCAATCAGCAGGCCAACCACG
CCCTGGAAATGCTCCAACTGCGCACCAAAGCGATGGCGCGCCACCACTAGCGCCCCCATCAACAGCCCCGGGCTCA
CTGCCCATAACGCCAGCATCAAGCCTCGCAGCGCCTTGAACAGACGGCCATCTGCCCGATGGAACGGGTACGCGGC
AATGATCGCCACCATGGGCATCATGGGCAACAGATAGCGGGCTTTCTTCGCCTCGGGGATCGACAGGCCGAGCATC
ACGATCAAGCCCGCCGCCGTACAGCCCAGCAGCAATTGCCAACCGCTGTCAGCCTTACGCCGGCCATTGAGCAGCA
CCGCAAGCAGCGCCAGCAGGGCCAGCGGGTATGCCAGGGCGTAGTTGCCCAGGGAGCTGGTGAAGTAATACAACAC
GCTGCTTGAACCTTCGCGGCCATCCATGCGCCCGAGGAACTGCATGCGGATCACATCCTGCATAAAGGCCTCGCCG
CCACTGACCTTGGCCAGCAACAACAGCAACCCTACGCAGGCCACCAGCAACGCCAGGGCCAGCAGGCCGAAGCTGA
ACAACGGGCGCCACTGGCGATTGAGCAGGTAAAAGCTGCACAACACGCCGGCAGGGATCACCAGGCCGATGGGCCC
GCGAATCGCAAAGCCGAGCAATAACAGGAGCAATAGCCACGGCAGGCGCCGGGGGCTGGCGAAGTGATCGTGGGCG
TAGCCCAGGTAAAACACTGCCAGCGTCACCGCCGCCAACATCTGGTCCAGGGACACCGAGCGGGTTTCGCTGATAA
ACGTACTGCTCAGCAACAGCAACGCAACACTCAGCAACGCCCAGCGCCGCGAGTAGGGCGCGGTCAGGCGGTAGAC
CAGGGCGACGATCACTGCCGACGCAATGGCCGTCGGCAGCCAGGCACTGAGGCTGGTGACGCGCCCCAGGGGCAGC
GACAACAGCCAGGTCAACAAGGTTGAGGTCGACAGGTAGTCGGCATACGGCTGGCCATAGGTCGAGGGGAAAAATC
CCGGCCCATGGCGCAGCATTTCCTGGGCGAACAGCACAAAGCGCGAGTCGAAACCGATAATTGCATGGTCCCAGTT
GCCGGCGACAAACAGCAGCAGCGCCAGCAGCCCGATACCCCAGGACTGGCGGCGCAAGGTTGCGCCGAGCATGTTG

TCCACGCCTCAGGCCTCCAGCAACCGTGGCAGCCACTGTTGCTGGGGGATCGGCAGGTCGCAGGATTGCCCGCGGC
CCATGGGGAAGTAATGGAAGCCCTTGCGCGCCAGGCGCTCGGCGTCATACAGGTTGCGCCCGTCAAAGATCACCGG
CGCCTTGAGGCGTGCGTGCAGCAGGTCGAAGTCCGGCGCCTTGAATTGCTGCCACTCGGTGCAGATGATCAAGGCG
TCGGCCCCGCCCAGGGTCGATTCCGGGGTGCCCATCAGCATCAGGCGCGGGTCATCGCCGTACAGCCTCTGGGTTT
CCTGCATGGCCTCGGGGTCGAAGGCCCGCACGTTGGCGCCGGCGGCCCATAGCGACTCCATCAGCACCCGGCTCGG
TGCGTCGCGCATGTCGTCGGTGTTGGGCTTGAACGCCAGGCCCCACAAGGCGAAGGTCTTGCCGCGCAGGTCGCCT
TTGTAGAACGCGCGGATCCGTTCGAACAGTTTGCTTTTCTGCCGCTGGTTGATGGCTTCCACCGCTTCGAGCAAAT
CACTCGAGCAATTGGCTTGGCGGGCGCTGTGGATCAGCGCGCGCATGTCCTTGGGGAAGCACGAGCCACCGTAGCC
GCAGCCAGGGTAGATAAAGTGGTAGCCGATCCGCGAATCGGCACCGATGCCCAGGCGCACCGACTCGATATCGGCG
CCCAGGTGCTCGGCCAGTTCGGCGATCTGGTTGATAAAGCTGATTTTCGTGGCCAGCATGCAGTTGGCGGCGTATT
TGGTCAGCTCGGCGCTACGCACATCCATGAAGATGATGCGGTCATGATTGCGGTTGAACGGCGCGTACAAGTCGCG
CATTACCTCGCGCACCTCTTCGCGCTCGCAGCCGATGATGATCCGGTCGGGGCGGCGGCAGTCGGCCACGGCAGAG
CCTTCCTTGAGGAATTCCGGGTTGGAGACAATATCGAACACCAGGCTGCGCCCGGCCAACGCCTTGTCGATGTGCG
CGCGCAAGGTGTCGCCGGTACCCACCGGGACCGTGGACTTCTCCACCAGGATCACCGGTTCGACGCGATGACGGGC
CACCGCATCGCCCACCGACAGTACGTACTTCAAATCGGCCGAACCGTCCTCGTCCGAAGGTGTCCCGACCGCGATA
AACAGCACTTCGCCATGCTCGACCGCGAGTTTTTCCTGGCTGGTAAAGGTCAACCGGCCGTTTTCCAGGTTTTCCC
GCACCAGGCTGGCAAGCCCCGGCTCGAAAATGCTCACATGGCCCTGTTGCAGCAGGCGCACCTTGTTCTCGTCAAC
GTCCATGCAGATCACATCGTGACCGACCTCGGCCAATACGGTGGCCTGTACCAGGCCAACGTAACCACTACCAAAT
ACGCTGATTTTCATGGGGCATTCCTGGGGCTTGTGTTGCCGATACGACGGGAGTTGATTGTCAAGACGCCGAGGAT
GACCAGTGCCACCCCCAAGGTTCGCGACAGGGAAAAGGGTTCGTTGAAGCCCGGCAGGCTGGCTGCCAACAGGTAC
ACCAGCACGTAGCTGATACTCAGCAACGAATAGGCGCGCCCGAGGGGCAAGTACTGCAGTGCGCCGAGCCAGCAGA
GCATCGACAGCGCATAGGCAACAATCGCCATGATCACTACGCCCAACGCCAGCGGATCCACCGTGGCGTTACCGAG
TGCAGTGAGCCAGTCGGCGGGCAAGGGCAGGCGCGTCATGCTCCAGCGCATGCCCAACTGGGCAGCGCTCACCAGC
GCGACGCTGGATAACGCCAAGGTAAAACCACGTAACCGGCTCATACCTGGCCCCCCAGCAACAGCACACCCGCCAT
CACCAGGGCCACGCCAAACCAGTGGCGGCGATCCACCGGCTCATGGAACACAAAGCGCGCCACCAGGGTAATCAAG
ACAAAGTTCAGGCTGAGCATCGGGTAGGCAATCCCCACCTCCAGGCGTTGCAGTACCAGCAGCCACACCAGCAGGC
CCAGGCCCAGACACAACAGGGCCAGCCACAGCCAGGGCGAGCGCAGTTTCCAGGCCCAGCCCACGGGCTGGTTGCG
CCAGCCTTCGACGGCATACTTTTGGGCGATCTGGCCCATGCAGGTCAGCAGGCACGCGGCCAGAAGCAGCAGCAGG
GTGATCAAGGCGCCACCTGTGGGAATACCAGGATCACCATGTTGCCTTGCTCGTAGCGCTTGCCATCTTTGGGCAG
CAACTCGACCTCGCGTACTTCACTCTCGCCCTTGACGCGCATCACCACGCCCACCGAGCCCTGTTTGCGGGCCTCG
GTCATCCATTGCTGGACTTGCGTGGTGTCGACCCGGCGATTGGCCGCATCGGCGTAGGCAAGGCCATATTTGACTT
CGCCCAGGGTGTCGTACAGCGTCACGTCCGGGCGCTTGAGCCGCCACGCCAGGGACGAGGCGGCGCCCAGGTCGTT
GCTCAGCAAGCGTTGGGTGCCTGCCAGCTCTTCGACGTGGTCAATGATGAACTGGTCCGGGGTCTTGTTATAAACC
ACCGAGTGCGGCAACGCCGCCGGCACCAGGGCCACCAGCAACCAGCTGCCCAGGGCAGGCGCCGCCCACAGGCGCA
ACGGGCGTGCCACCTGTAGCAGGTTGGCGATGATCCAGCCCAACAGCACGATAAACACCAGCACCAGGCTCAGCAG
TTCCTGCTGGTGGGCAAAAATCGGCTTCTTCAACTGGAAATACACGATGGCCAGCAGGCCCAGTACGCCAAGGATC
AGGTTGAGCCCGCCGTTGATTTGCAACACTCGGCCACGGGCCTGGGCCAGTTTGTCCGCCAGGGTGTTGCCCATCA
GCAATGCCAAAGGCAGCAGGCACGGCAGGATGTAAGCCGGCAGCTTGCCCTTGCTCAGGCTGAAAAACGCCAGGGG
CATCAGCAGCCACAACAATAGGAAGCCGGTCTTGTGCGTACGCTTTTGTTGCCACGCCTGCTTCAGGGTGGAGGGC
AACAGCGCCAGCCACGGTAGGCTGAACGCGGCCAGCAACGGCAAGTAGTACCACCAGGCCTCTTCGTGCTGGGCAT
CTTCACCGGCAAAACGCTGGATGTGTTCGTGCCAGAAAAAGAAGTTCCAGTAGTCCGGTTCCTGGGCATGCACCAT
CAGCGCCCAGGGCAGGCTGACCACAATTGCCACCACCACCGCGACCACGCCATAGGTCAGCAACTCACGAAAGCGT
TTTTGCCAGACCATGTAGGGCAGGGCGACCAGCACCGGCAGCAGCCACGCCAAAAAGCCCTTGGTCATGAAGCCCA
TGGCGCAGGCCAGTCCCAGCAATGCCCAGGCGCTCAGGCGGGCGCGCCGGCCGTGGCTGTCGATACACAGCCACAG
CGCCACGCCGCTGAGGTTGACCCAGAAGCTGAACTGCGGGTCGAGGTTGGCGTAGCCACCGGACATCGCTACGCTG
ACAAAACTCATGTACAGCACTGCGCTGGCCAGGCTCTTGCGTGGATCGTTCCACAGGCGCCGGGCCATGAAGTACA
CCAACAGGATGCTCAAGCCCGTGGCCAGCGCCGAGGCCACACGCACACCGAACAGGTTCTGCCCGAACACGGCCTG
GCCCAGGGCGATCATCCAGTAACCGGCCGCCGGTTTTTCGAAGTAACGCAGGCCCAAAAAGTGCGGGGACGCCCAT
TTGCCCGTCAGCAGCATTTCCTGGCTGATCTGCGCGTAGCGGCTTTCATCCGGAATCCACAGGCCATGGGTGGCCA
TCGGCAACAGGTAACACAGGCCGAAGACCAATAGGAGCAGGGGCAGTGCCCAACGTCGGATCATGCCTTTTGCACT
CCCAGCCAGCCTTCGCGGCCGCCGAGTACGCCGCGTGTCAGATGCCCCTGGGGCAGGCTCGCCGGATCGGCCGGCA
ACAAGTTGCCCAAGGGCTGGAATTGAATGCCGCGCTGCCGTGCCTGGGCAAGCAACTGGCGAAATTCATTGGCCAT
CAGAATCCCTTCTACTTCTGCGTGGATCGTATAGACGTTGAGTGTCGATTCGGTGAATCGATCAAGAATGAAGCCG
TTGAAGTCCTTGGCCGCAACCTGTGGACCGACCACTTCGTCGAAGGTCGGCAAGTCCACCGGGATCTGTGGGGTGC
CGGCACTGCCGTCGGCGAGCGTCGGGCGAAACACGCTGGTGCCGCGACAATCGCTGTTGTAGCGCAGGCCAAAGCC
TTGCTTGGCCTGCACCACACGCTCGTCGGCGCGCCAGCCGGCGGCGGCGGAACAGTCCACGGGCTGACCAAGAATG
TCGCTCAGGGTGTCGAGCCCACGGCGCATCTGTTCCACCAGTTGCGCGTTGCTCCAACGGCCGGCATTGGCCTGCC

AGCCATGGTGATCCCAGGCGTGCAGGCCCACTTCGTGCCCGGCGGCCCTGGCCTGGCGCATCAAGTGCCCCAGGTC
GCGGCCAATCGGCTTGCCCGGCCAGGCGGTGCCGGCCAGCAGGATGTCCCAGCCGTACAGCCCGGCCGCGTTGGAG
CGCAGCATCTTCCACAGGAACTGCGGGCGGATCAGGCGCCACAGATGCCGGCCCATATTGTCCGGGCCGACACTGA
AGAAAAACGTCGCCTTGATGCCGGCCTCATCCAGCAACTCCAGCAACCTTGGCACCCCATCGCGGGTGCCTCGGTA
GGTGTCGACGTCAATGCGCAGACCTGCCTTCATTATTTGCCTGCTTCGCTGGACTGGGCGATTTCCAGCATCGCTT
CACGCAGGAAGAAATCCAGGGTGTTGCCGATGGTTTCGCTCATCTCCACGCTCGGCTCCCAGTTCAGCAGGCGCTT
GGCGTTGGCGATGCTTGGCTTGCGGTGCGCCACGTCCTGATACCCGGCGCCGTAGAACGCCTTGCTTTCCACTTCG
CGAAAACCCGCGAACGGTGGGAAGTTGCCACGCAATGGGTGAGCCTCGAACTGGCGCAGCAGCTCTTCGCCCAACT
GGCGGATGCTGGCTTCGTTGTCCGGGTTGCCGATGTTGATGATCTGGCCGTTGCACGCGTCGTTATCGTTGTCGAT
GATCCGCGCCAGGGCCTCGATGCCGTCGGCGATATCGGTGAAGCAGCGTTTTTGCTCGCCACCGTCAAACAGGCGG
ATTGGCGTACCTTCCACCAGGTTGAGGATCAACTGGGTGATCGCCCGGGAGCTGCCGATACGTGCCGAGTCGAGGC
GATCCAGGCGTGGGCCCATCCAGTTGAACGGGCGGAACAAGGTGAAATTCAGGCCCTTGGCGCCATAGGCCCAGAT
CACCCGGTCCAGCAGTTGCTTGGATACCGAGTAGATCCAGCGCTGCTTGTTGATCGGCCCCACCACCAGGTTGGAG
GTGTCTTCGTCAAAATTCTGGTCCTGGCACATGCCATAGACTTCGGAGGTCGATGGGAAGATCACGCGCTTGTTGT
ACTTGACGCAGTAGCGCACCAGCTTGAGGTTTTCTTCGAAATCCAGCTCGAACACGCGCAATGGGTTGCGGGTGTA
TTCGATTGGCGTGGCGATGGCCACCAGCGGCAGGACCACGTCGCACTTCTTGATGTGGTACTCGATCCACTCGGAG
TGAATGCTGATATCGCCTTCCACATAATGGAAGTTGGGGTGGCTGCGCAGGCGTTCGATAGCGTCAGAGCCAATAT
CCAGGCCGTAGACTTCGTAGCGATCATCGCGCAACAGCCGCTCGGACAGGTGGTTGCCGATAAAACCGTTGACACC
CAGGATCAGCACACGGGTCCGCCGTGGCTTGCGGCCCGACTCGGCGCCACGCAGCACCGAGCCATCCACCAGCCCC
AGCTCATCGGCCAGGGCCGGGCCTGTGAGGTACAGGCCGTGGTCGTTGCGCTGGCCGAAGTTGATCACCAGGGAGT
CTTCACCGCAGGCAATGCGCAGCGGGTTGACGCTGATCACGCGGCCGGGTGCCTGGCCTTCGTTGCCCTTGACCAC
CTCGGCTTGCCACACGATCAGCTTGTGCTCGCCCACGGCACAGAAGGCGCCGGGGTAGGGCTGGGTCACGGCGCGT
ACCAGGTTGTACAGCGCCTCAGCAGGCTTGCTCCACACCAGCTTGCCGTCTGCCGCGGTGCGGCGGCCGAAGCAGG
TAGCCTGGCTTTCGTCCTGGGCAGTTTCGCTCAACGTGCCCTTGGCCAGTTGCGGCAGCGCATCGCGCAGCAGTTG
GGTGGCGGCCTCGCGCAACTTGGCGTGGAGCGTCAGGCCGGTGTCGCTGCGCTCGATCATGACCTTTTGCTGGGCG
AGGATCGCGCCGGCATCGGCACGCTTGACCATGCGGTGCAGGGTCACGCCGGTTTCGGTTTCACCGTTGACCAACA
CCCAATTGGCCGGCGCGCGGCCACGGTACTTGGGCAGCAGGGAACCGTGCAGGTTGAATGCGCCCTGGCGCGCGGT
GGCCAGCAGTGCCTCGCCCAGCAGGTTGCGGTAGTAGAACGAGAAGATGAAATCCGGGTTCAGCTTGGCGACACGC
TCGACCCACAGGGGGGTGGTTGGCGTCTTCCGGCGCGTGCACCGGGATGCCGTGGCGTGCGCACAACTGGGCGACCG
AGCCGTAGAAGGTGTTTTCCTTGGGGTCGTCGGCATGGGTGAACACTGCAGCAATGTCGTAACCCGCAGCGAGCAG
GGCTTCAATGCCTGCACAGCCAATGTCGTGGTAAGCGAAGACAACAGCTTTTGAACTCATGACGGAACCTGATTGG
AAGGTGAAGTGGAAGGCTGGGAAGAGGGCAGGCCATCGACGATGACCACCGGCGCGGGCGCTGCTGGCTCGTTGCG
CAGCACCTTTTCAATAAAGAAGCGTGGGCGCGCGCGCACATCGCTGTACATGCGCCCCAGGTACTCGCCCAACAGG
CCCATGCCGATGAACTGGCCGCCGGTAAACACAAACAGCACGGCGAACAGCACGAACAGGCCATCACCGGCCCAAT
CGGCACCAAAGGCCAGGCGCATGACGATCAGGGCGAACGCAAACAGCATGCCCAGGGCCGCCAGGCTGAAGCCGAC
GATGGACAGCAGGCGCAGGGGCGTGGTGGTCATGCAGGTCAGCAAGTCGAACATCAGGCTGATCAGGCGCATGGCG
CTGTATTTGGATTCGCCGTGTTCGCGCTCGGCGTGGTGCACCGGGATCTCGGTGGTGTGGCGCGCAAAACCGTTGG
CCAGGATCGGGATAAAGGTGCTGCGCTCACGGCAGGCGAGCATGGCGTCGACAATGGTGCGCCGGTAAGCGCGCAG
CATGCAGCCGTAATCGCTCATGGCCACGCCGGTGGAGCGTTGCACGGCCAGGTTGATCAGGCGCGAAGGCCAGCGG
CGGAAGGCCGAGTCCTGGCGGTTGTTGCGCACGGTGGCAACCACGTCGTAGCCCAACGCGGCTTGCTCGACCAGGC
GAGGGATCTCTTCGGGCGGGTTTTGCAGGTCGGCGTCGAGGGTAATCACCACCTCGCCCCTGCATTGCTCGAAACC
GGCCATGATTGCCGCATGCTGGCCGTAGTTGCGGTTCAGGATCACGGCCACCACATGGCTGCCTGGTTCGCCTGCG
GCGTCTTCCAGCAATTGCGCGGAAGTGTCGCGGCTGCCGTCGTCCACCAGGATGATTTCGTATTCGTAGGCCAACT
GCTCACACGCAGCACGGGTACGGCGCAACAGTTCGGGCAGGCTCTGTTCTTCGTTGTAGACCGGGATAACGATCGA
CACGCAATGAATAGGGTAGGGTTTCAAAGATTCATGACCTCGGTTTTAGAGCAACTTCAAGCGTAGAGATGACAAC
AAACAGCGGATGAAAGTGCGGGCAACTGAATAAAAAACAGCCGTGTGCATAGCCATCCTGAGTAAAGCCCATAACG
ACCAAAGGTAAAGCATTATCAAAGGGTTAGTCAGCAAAACGCGGTTTGCCCCAAGTACTGCGGCCAAGATTAAGCG
CAAATGTGTCCAGCGAATATGAAAGGCGGATGAAAAACTCGTTTATTTGTTGGGCAGACAGCGAGTGTTCAGCTAG
ACAATAAAGAACCCAGACAAACGATAACTGTTTGCGCTGTAGTCCCAGTCTGTAAGTGCTTTAGTGCGTATGTACG
GTGAATTAACCGGGTTTGCACTCAGTCCCGCCTGACGAATCTTCTCTGCATCCCCCTGCAAGTCCCGGTAAAGTAA
GCGATCTCTTGCCTGGGTAGTCGGATGAATAGCGTGCAGTTTTTACGCGGCCAGTTACGGCCGGTCTTGATGGTGA
TGGCGCTCGGGGCGGCCCATGCAGCTGTGGCGGCCGAGGAAATGAAGCTGACGCCGCTCAACCAGGTTCCGCTCGG
GATCGAGGTCCGGGGCAAGCAGGTGGCCGCGTATAGCTGGGATGATCGCCAGGGCCGTAACCTGCTGGTGTTGGCC
GAGCAGGAAGCCGGCGAGCGTGATGATGACGGGACGCAGTCGGCGTCAATCTATGCCGCCCAGTACCTGCTGGAGG
GGGATCGCCCCAAGCGCCTGTGGATGCTGCATGACGATGTGCAGCGCTGCGAATTCGACGCCAGCCTGCACTTCGA
TAACGCCGCGACCCGCGTCACCGACCTGCTGGATGACGGCCTGAGCCAAGTGACCGTGGGCTACTCGCGTACATGC
ACCAGCGATGTCAGCCCCCGGGAGTTCAAGCTGATCATGCGCATCGGCAAGTCGCAAAAGTACGGGCTGCGTGGTG

TCGACCGCTACGGAGCCAGTTGGTGGGACGAAGAAGCCGGTGCGCTGCGCGGTATGCCGCTGCCCACCGATTGCAG
CGTCGCCGGGCAGCAGGCGTTGGTCAGTCAGTACAAGGAGCAGGGCACCGACCTGCCATTGCCAGGCTGCTACAAC
GACGAGCATGACTTCGCCAAGGCGCCCGCCACGCTGTTGGCTTTCATGCGCCAGCAGTGGTTTGCGCTGATGCAAA
AACAGGACGTGGACTGGGGCCAGCAGCAAAGCCCGGCACCCGCAGAGGATGAGCCCTAGTTTGGTATGCGGCGCCG
ACCTGCGTTAGACTCGGCGCTCGCCAAACCACTAAATACCGAGATGAAACATAGGCTTAGTCTCGATATTTAATCT
CAAGGCTGATCTATTTTGACCGAGATTTCCCGCAATCCCCTGACCCTGTATCTCGCGCGCCTGGCGCCCTCTAGCC
AACTGACCATGCGCTATGTGCTGCAAGATGCGGCCGACCGCCTGGGTTTCGAAGAGATCAACCTCGAAGATATCGA
CTGGCACCTGCTGCAACCGGAGCACGTGATGGCTTTGGTCGCCGTGCTGCGTGAAGACGGGTATGCGCCCAACACA
TCGTCGCTGTATGTCAACGCGGTGCGCGGGGTGATGAACGAAGCCTGGCGCATCAGCCTGATCAGCCAGGAACACT
TGCTGAAGATGCGCTCGGTCAAAGCCACCGCCGGCACGCGCTTGAGCCAGGGGCGTAACCTGCGTCGCACCTTGAT
CCGCGAATTGATGGACGTCTGCGCCGCCGACCCAAGGCCGCAAGGGCTGCGGGATGCGGCAGTCATCGGGATCCTG
TATGGCTCGGGGATGCGCAAGTCAGAATCGGTCAACCTCGACCTGGCCCAGGTGAACTTTGCAGAGCGCAGCCTGC
GGGTGATGGGCAAGGGCAATAAAGAGTTGATCAAGTACGCGCCGGCCTGGGCGTTTGCCAAGTTGCAGGCCTGGCT
GGAGTTTCGCCGCGAGCACTTGAAGGAAGGGGAACAGGACGATAATTTCCTGTTCAACCGTATCCGCCGGGGCAGT
CATATCACCCGCGAGCGCATTACCAAACATGCCATTTACTACATCGCCCGCCAACGCGGCGAACAGGTGGGTGTGA
AAATCATGCCCCATGATTTCCGGCGCTCGTTTATTACTCGGGTGATCGAGGAGCATGACTTGTCGATTGCACAGAA
ACTGGCGCACCACACCAATATCCAGACCACCGCCAGCTACGATGTGCGTGATGACAACGAGCGGCGGCGCGCGATT
GACCGGTTTGACCTTTGACACCCCTTGTGGCGAGCGGGCTTGCCCCGCGCTGGGTGGCGAAGCCGCCCCAATAGCT
TCACCCAGTTCTGCCACAGCGTTCCCATTTAACCATGAGCCAAAAGCTGCTTTTTAAAGACCTTGAACGCTGTGAC
ACACCAACGCCTGGCGCTCGTAGTTCAGCGCCTTGTTTTGTGCACGCAGCGCATACGGCGCAGTACATTGCTGGTC
ACCCCACTTGATCGTCAGGCTGCCTTGGTCCTCTTCGACCAGTACCAAGGCATTGCCGCTGGGATCGGCGATGGCC
AGCTGCTTGCCGCTGGCGTCTTCCAGTGAGGCGCCGAACGCCATGCGCTGGTGCTGCGCATCAAACAGCTCAAACA
GCACGCGCCGCCCGGTTTTGCCGCTGTAGCGGGCCAATACCACCGCACCGCGGCGCGGTACTAACTGCTGCGTGGC
CTGGGTGATTTCAACATCGCCGCCCAGGTCGCGGGTGTCGAGGCTGATCCAGTTCACCCGATAAGGCTGTGCCGAG
GGCACCACGGCATAGCCGTTGCGCCCGGTTTCAACCCCGCTGTAGCTGCTGACTTTAGCCCCGCTGATACCCGGCA
CCTGAACCAAGGCAAAGGTTTCGCTGACGGTCTGCCCCAGGTTGATGCCACCGGCGTGCGCCACCACTGAGCCGGC
AACGTTAAGGTTTTGTGCGTCGTACCCACGGCCCTGGCTATACCCCAGGCTGATATCGGCCATGGATGTGCGGCTA
TTGATGTTGGCCGATCCCGATTGGCCACCCGTTCGGCTATGCCCGGCCTGGACCGAATAGAACACATCGCTGGTAT
CGGCCACATAACCGTTGATACCCGCCTGGGTGCTGGTATCGCCTTGCTGGGTGCTGGTGGTGACGAATGCACGCGG
CGCCCGGGGCTGGCTGCCCAAGGGGAACGAAAAGGACAGGTTGAGCTGCGTGTCGGAGTCCGGTGCGCCCCAACTG
GCAATCTGTTTGGTGCGTGTCACCCCCAGGTTGTAGCTCAGGTCGCCCCAGTTGTTGTTGTACCCCGCCGAAAAGC
TCTGGGAGCCGCCACGGTTCCAATAGCGCTGGTCGGTGGCGTTCAAGTAAAGGCTGCCGAACTCGCGGTTGCGCCC
GATGCTTTGGTTGATCGTCAGGTCGGTGCGGGTTTTCGAGTTGCCCGTACGCTTGACGGCACCTTCACTGGTGTCC
TCGACGTGGTCGGTCAGGGTGCGGTAGCCCTCGGTCGAGTAACGATAGGCGGCCAGGGTGAAGTTGGTGTCGGTGC
CGGCAAAGGTCTTGGCATACAACGCACGCACGCTGTTGCCCTGGGTCGTTTGCCCTTGGGCGGCGCTTGAGGAGTG
AGTGACGTCCATCGACACCGCGCCCAGCGACGTGTTTTTGCCAACCCCCAGGGAGAGTGCCTTGTAGTCTTCGCTC
ACTTGCAGGCCGACAATCGCGCTGAGGTTACTGGTGAGACCGTAGGCAAGGGTGCTGCTGACAAATTGCGGGCTCG
CCAAGCCGTCAGCATTGCTGCTGAATTTACCGGCTGAAACGCTGTATTTGACCTGGCCTTCACGCACCATGATCGG
CAGGCTGGAGAACGCCTGGGTGGTCACGCGCCGACGGCCGTCGGCCTCGATGATGGTGATTTCCAGGTCGCCGTTC
GAGCCGCTGGGGTAGATATCGTTGATCTCGAAAGGCCCGGGCGCGACGTTGGCGGTGTAGAGGATGTAGTCGTTCT
GGCGGATTTCTACCGTGGCGTTGGTCTGTGCCACGCCACGAATCACCGGTGCATAACCGCGCTCGCTGTCGGCGCG
CATGCCTTCGTCGGACGCCAGTTTCACGCCGCGGTAACGCACGCTGTCGAACAGGTCGGTATCGGAAAAAATCTCC
CCGGCACTGAACTGGCCCTTGAGCGTCGTCACGTCATGCTGCACATAGCTGCGGTTGCTGGTGAAGTTGCTCGGCC
GCCCCGTGCCGTTGCTCAGGTTCGATTCGTTACGCAAACGCCATGCGCCCAGGTTGATGCCGTTGCGCAGCCCCAA
GTTGTTGTTGATGCGGGTTTCATAGTCGCCAGCGGTGCGGCTGCTATTGAGTTGATAGTTGATAAACGCTGCGGGC
ACGCCATCGTCCCATAACTGGGGGTCGACATAACCGCGCATGCCCCGTTGCATCGCAACCTGTGGAATGCTCGCCG
CCAGGCGTAGGCGACTGGCGTCATAGCGCAGGGTGGCCTGGTCGATCAGCGTCGGTAGGTCGTAGCAATCCTGGGG
TTGGCTGGCGTCGAGCTTGCCTTGGGCTTGCAAGAGGGTCAGGTCGATCCCCAGTTGCTTGAGCAAGTCCAGGGTC
AGGCACGGATCGACCCGGCCGGTCTGCGGGTTGCGCTTGAAGTCGATGTCGCGACGGCCCACCAGGACCTCATTGC
TATACAGGTCGACTCGGTAGTTACCGGGCAGCACGCTGTTGGCCGACAGCAGTTGCTGCAGGTCTACCGACGATTG
AGCGCCCTGCAAAAAGGTGGTGTTGAACACTTCCAGGGACGGCTCATCGGCAACGGCCCAAACGGGCGAGCCGGCA
AGAATGGCCAATGACAGCGTACTCAACTTCAGAGATGTGCACGTTGTAGACACGCGTGAAGTGCGCTCGCTTGATA
TGTAATGATTGGGCCTGCTGCCTAAAATAAAAAACATGCAAAGAACCTATATCATCCCTGTTTATCGGACAGGCGA
GGCACGCGAGTACGCACTCGAATCGAACCTGGAGGGTTTAAGGGAAACGCTTATTGCCGGGTGCGCGCCTCAGTGG
CCTTGGCCGTCAGGCGCGTGGAAAGTTGTGCGGTGTATTTATCTTGTGCGCCATAGTCGTTAATGCTGGAAAAAGA
CAACTGCACTGTTTCGTAACCACCGAGTTGTTTAACGCTGAACTGTTTTTCGGCGCCCGGGGCAATCATTGTCGAA
TCGGCAGCCAGTTCCGTTTGTTGCCCGGCCTGAAGTTTTATATCGGCCATGGACACGTGATACAGCGTCGGGTTTT

TTACGCTCAGCCACGACTCGGCGCCACGCTTTTGCAGTTGCCATTCCAGTTGCGCCGGCGCCGACAGGGCGCTACC
TGGCAAACCGGCTGGCCGGAAAAATATCTTGATGCGCTGGCGTACCGCCAGTTGCAACGTGTTGTCGGAGGCGGCG
GCCTGCGGGATCTCTTGCACATTGAGCCAGATCACGGATTCACGATCCGCCGGCATACCGCTGCCCTGGTAAAGAA
TGCGCAGCAATTGCTGCTCCTTGGCAAATATCCGTGCCAGTGGCGGGGTCACGGCGAACGGTGCAGCACTGGCTTG
CGCATCGCTGGTATCGACCCAGGATTGAATCAATACATCCTGGTTACCGTTGCGCACGGTGATATTGGCTTCTTTG
TGCTCGCCATCGAAGACGATACGCGTGGCACTCAATGAAATGCTGGCGCCAGCCTGGGTCGCCATCAGCAGGGTCA
ACAACCCCAGGCCTGCGGCAATGGAACGAGGCAACATGATGTCTACCGTTTATGGGTCGGATAAATGGCGAGGCCC
TGGGGCCTCGCGCGTGGCATTTACAGCCTGGGGGGTTATTCGTATTGCAGGATGAACGGCAGGGTGGCGTCACCAC
GACCCGCGGTGGAGGCGCCGGCTGCACCGGTGGTGACGTAGGCTGCGGCAAAGCTCAAGGTTGCATCGCCACCTTC
GGTGCCTTGGCCGTGCATGCTGCTTTCGATGCGGGCGCTGGCCGGGGAGCTGAGGTCGATCAGTGCGCCTTTGCTG
TCGAGCAGGGCGATGCCGACGTTGCTTGCGCTGCCGTTGCCCGGGGTCAGGGCGAGGACTTTCTTGCCGGTGACCA
GGCCCGAGCCGCCGCTGTTGGCGTCAAAGATCATGGCCACTTTGGTGCCCAGGTTGCAGTTCACGTTGAGGTTGAA
GTCTTTGGCTGTCACACGGCCCGAGGCCGGGTTTTCAGCGGTGCCCATGTCTTTGATCGATACCGAGCCCATGTCC
ACCGAGATCACGCGATCGGAGTTGGCGCCGTCGACCGAGCACGCGTCGTTGTTGATCACACCGGTGAAAGTGATTT
TGCCGCTGCCACCCAGGGTCGGGGTCGGCGCAACGGGATCTTCGGCAAATGCACTGCTGCTGGCGGCAATAACTGA
CACAGCGATAAGGGCCAGGGAAAACTTCTTCATGATGATGTCCGTTAGTGTTGGTTGTAGAGAGGGTTGAAACTGC
CAACGAACAATAGTGATTTTAGGCCTTAGAGAGAATAAGACGATTCTTATAAAGCACTGCAAGGCAAGTTAGTTAA
GGAGAAGATTAGTTGATAAGGCACGGATTAGTTGAAGATATTTTGCGCCAGGCAATAAGCCAACAAGTCTTGGTCA
CTACTGACTTCAAGTTTGCGCATGGCCGAAATCTTTTGCGCACTGATAGTCTTTGAACTTCTATTCTGGCTGCGCG
CTATATCACTGACACTTTTGCCTGAAATAAACAAACGCAGTATCTCGAATTCCTTCGGCGACAGGGTCGAGAAACG
CGCATCGATAGCCGTCATCGACTCGACCACCGAAGTCTTGGCGGGCTCCAGGCTGCGATACAGCGCCTGCTGCACA
ATAGACTTGAGGGCTACCTGGATTTCGGTGTGCAACTGGCTTTTCTGGATGACGCCCACCACGCCCATCTCTTGCA
GACGGGTCAGGATCAGATGATTGGAGATCATGGTCAGGATCAGAATCTGCACCTGGGGGAAATGCCGCTTAAGGTA
TTCCACCAGTTTCAAACCATCGCCGTAGGGTGAATCGCCGGGCATGTTGTAATCGGTAATCACGATGTCGATAGGC
TGCTGTTCAAGCAGCCCGATGAGGCCTGCCGAACAGATCGCTTCGCCCACTACTTGAAAGCGCGCGTCTCGCTCGA
CGAGTTCTCGCACGCCCAAAAGCACGATGGGGTGATCATCCGCAATAACCACAGTGAAGGTTTTCATAAGGGTTGC
CCGTAATGCTGGTTCAGTGAGTGAGGAGGGCTCATGGCTGCACGCTGAGCAAGGCTGAAAGCCTACTGCTGACCTG
GTTTATGCGCATTTCCAGCGCGGGATTGAGCCGGCCGCTGGCCAACTGGCTTTCCAGTTCGACACACGCCTGGGCC
AGGTCCAGCGCCTGCACCGCGCCCAAGGCCCCGGCCACCGAGTGCAAGGTTTCGGCCAGGCGCCGGCAATCGCGTT
GCTCCAGTGCGGCGCTGATGCGCTGCAGATCGTGCTGTACGCTGCTGACAAACAGTGGGCGCATTTTTTCTGACAT
CTGCACCGTGTCGATCACCGCCGGCAAGACGCGTACCGTCGGCCGGCACAGCTTGATCAGTTGCGCGCGCAGGGTT
TGCAGGCTCAAGGGCTTGACCAGCCACGCATTCATCCCGACTGCCAGGCAACGCATGCCTTCTTCGCGCATGGCAT
TGGCGGTGACGCCGATGATCGGTAATTGCGGGTCATGTTCCCTGAGGGTGCGGGCCAGTTCGTAGCCATTCATCAA
GGGCATGTTGACGTCGGTCAGCACCAGGTCGAAGGCCTGGGGTTGCCACAGGTGCATGGCCTGCTCGCCATTGTCT
GCCAAGGTAACCGTACAGCCCAGCGCTTCCAGTTGCTCCTGGATGATGGCCTGGTTTACCGGGTTGTCCTCGGCCA
CCAGAATGCGCAGTTGCAACTGCCCGGCGCTCTGTTGTTCGGCGTGCTGTACGGCCACCTGCTTACCCTGCTGCGC
CAGCGAGACCACCGACGCAATGGCACGCACATCATGCATATCCACTTCCCAGCCCTGGGCCGTATACAGCGGTGGG
CTGTGTGCACCCGGCACACACAAGACGCGCGGCCCGGCCCAGGACAATCGCGGCCCGGCGGGCAACATGTCGACCA
GTACCGCCTGCGCATTCTGTGAGGCCAGGGGCAGCGTTGCCACCGAGGCCTGGATACCCAGGCGCCCGAGCCAGTC
GCACAGCGCTTCGGCCAACTCGCGCACCGGCGCCTGTACATAGACCGGCGTCGCGCTCGGCTCAAGAAGGGGAAGT
CCGGGCAACTCGCCACGGCCTATCGGCAAGCTCATTTTCAAGGTGAAACTGCTACCCAGGCCGGGCTCGCTGACGA
CCTTGATCTGGCCATCCATCATTTCGGCGAGCCAGCGGCATATCGGCAGGCCCAGCCCGGCACCGCCTTGGCCAGC
GGTGTCTGGTGCCTGGTAGAACAAGTCGAACAGCTTGGCTTGCTGCGCCTGGCTGATGCCCACGCCAGAGTCGGTC
ACTTGCCACTCCAGGTCCACCTTATCGGCCCCCCGGCGAAGCGCCCGGGCCCGAATCACCACGCGTCCTATGTCAG
TAAATTTGAGCGCATTGCTCACCAGGTTGTTGAGGATCTGGCGGATCCGCATCGGGTCGCCGACCACACAGTCGGG
CAACTGCGCATCAATGCAGCCATACAGTTGCAGGCCCTTGCGCTGGGCGAAGGCGGCGTAGGTGTGCAAGGTGTCT
TCGAGCATGTCCAGGGGGCAAAACTCGATGGACTCGATTGCCATCTGCCCGGATTCGATTTTCGATACGTCGAGCA
CATCACTGATCAGTTGGAACAACGTGGCAGATGAGCGTTGGATGGTGTGCAGGTAGGTTTTCTGGCGCGGGTCCAG
GGTCGTCAACCCGAGCAATTCCAGGGTGCCGAGCACGCCGTACAAAGGTGTGCGGATTTCATGGCTCATGGTTGCC
AGGAACAGCGTCTTGGCTTCATTGGCCGCATCGGCGGCGCGCCGGGCATCTTCCAGGGCCAGGGCGTCTTCAATAT
GCCGGGTCACGTCGTTGAACGCATAAAGCCGCACATCCTGGCCTTGATAACGGGTCGACACGAAACCGACCTGCAG
GTGCCGGCCTTCGATTTCCAGGTGGGTTTCGCCACTGTCGGCGCTGTCCTGGAAGCGGCTGAGGGCCGCCACCAAC
CTGGCGGTGCCCGGCCATTGTTGGGCGCGCTGGTTCTCGATCAGCACCTGGCCGTCCGCGCGGCCCACCACACACA
GGCCGGTAGGCGCGGTGTCGATAATCACCCGACTGAATGCCACGCTTTCGGCAATCCGCTCATGGGCCTGGCGCGC
CGGACGGATCACTTGCTTGCGGTACCAGCGGCTGCCGGCCCAGACAATCGCCAGTACGGCACACAGAAACAGCACC
AGGCTGGTCAGTGGCCACAGGGCATAGCGGAAAAAATGCTTGTAACTGATCCCGTAGATTGCAGTCCACTGCGACT
GGGCGTCGTGGGTGATTTTGAACTCCAGCCCATCGGCGCGAATATTCAGCCCTTCACCCAAGGCAGAGGCGGGGAC

CTGGGGCCCCGTCAGCGTCTGCCCGGCGGGGGAGATCAGGGTGAATCGATCGTAGATGGACGACTCCATGGTGCGT
TCCACATCGTTGACGTGTGCCATATCCAGCAGCGTGGCAACCATCACCTGGTTAGTGCCTTGACCCGCGCGCAAAA
CCTGAGGGTTCAGGGGGATGCCGATATACGCCAACAACTGCGGAGCCCCGGAGGCCTTCCCGGTTGGGGTATAGGT
TTCCCAGTAAAGTCTGTCGTCTACCCGGGGCAGTTGCTCGAGCCTTTGCTGCACCGCGTCGGCCTGTTCGGCCGTG
GGGCCGTTGAACGGGGTTTTGCCGCCACGCTGGTAGCCGACCGCAGGCACCGCAATGTGGTAATTGACCAGGCGGT
TGAGGATAAAGACGTGGGGCGATGCGTAGGGCGACGATGACCAGTACGCGCTGTAGTAACTGGCCAGATGCACCCC
CAGGGCAAAGATCTTCTGCTGCTCGCCAGGCGTGACCCGTTGCGCCTCGAACTGTGCACTGAAGGGCACCGAAAAG
GCGAACTCACGACCTTCATAGCGGGTGTTGTGCATGTCCAGGCGATGGCTCTGGAGGCTGGCCTGGGTGTTCTTGT
CGCTGATCAAGTCCCGTTGGATCGCCGGCTGCGCCAGGGTCCGGAGGAAGGTTTCCTGTTCCTGGATATTTTCCAT
CAGCCGGGCAAAATGAAACCCCACCGCAGCGTGCTGCTCATCGATGATCCGCTGCAACGCCCAGTAACTGGTGCCT
GCCAGCAACAACGCGACGCCGAGCACGCCCATCAGCCCTTTGTTCAAGCGCAGTGAGCTGCGGGTGAATGCTTCCA
GGATCGCGTTGTTGTGCTTCATTGATGGTTTTCGCAGGCGGCTGAGAAATCGGCGCTGGATGGAGTTTACTCCTTA
TACAGGTTAGACAAATCCGAGCCGGGGGCTAATGAGATGTCGCTGGGCCATCATTGTTCAACAGTTGCTGAAATTG
TTCTGAGGAGACCGCGTGGGAGATCAGAAAACCCTGCACCTGGGTGCAGTCGATTTTGCGTAGCAACGCCAGTTGC
TGGGGCGTCTCCACACCTTCGGCCACCACCGTCAGCCCGAGCTTGCGGCCCAGTGCGATGATACTCGCCAGGGCCG
AGGTCATGCCTTCGTTGTCATGGCAGCCCTGTACCAGCGCCTGGTCGATCTTGAGCTCGGTAAACGGGGTCGAGAC
CAGGTTCAGGTAAGAGCTGTAGCCCTTGCCGAAATCGTCCTGGGACAGGCCGAAACCCTTGATGCGCAGGCGGCAG
GCGCCGGCGTAGAAGTTGCTGATGTCCTGGGGCACCGAGCATTCCATCAGCTCGAAGCAGATCATCCCCGGGATGC
CTTGGTGGTCGAGGACGAACGCCAGCAGGCGGTCTGCCAAGTCATGGCTATTGAGCAGGTGGGTCGGCAAATTGAT
CGACACTGGAATCTCATACCCGCGCTGGCGCCAGCGTTCCTGGGCCAGGATCGCCTGTTTGAGCACCAGCCATAGC
AGGCGTTCTTCCAGGTCGAAGGTCTTGATCGCCGGCAGAAACGCGGCGGGCAACATCACCCCTTGCTCGGGGTTCA
TCCACCGCACCAGCGCTTCAGCGGCGACAATCCGGCCGTTGGCCAAGGATTTTTTCGGTTGGAACCAGGCCTGGAT
CTGCCCGTTGCCCATGGCCTGGAGGAGTGTGTGCCGGTCAATCGCCGGCGGCGTGGCAGGCGCGGGTGAGGGGCTG
CGGTCCTTGTGGTTGAGCTGGCTGACCAGGCTGCGCAAGGCGTCGGCTGCGACCGGCTTGGAAATCAGGCCGATGA
CTTTCACATCGAGATTTTTCGCCACCAGACTGGCCGCCATCAACATACGCCGGGAGGCGGCACTCATGATGGCCAG
GGCAGGCTTGCAACGCAGGGTCGCCAGGCTCTGGATAAATTGCACACCGTCCATGCCCGGCATCAGCAGATCGGTC
AGCACCAGGTCGAAATCACGCTGGCGCAAGCGCTCCAGTGCTTCGTTTCCATCCTGGGCGGACTCCAGCATGAAGT
CGCCCAATTCATTGAGCAGGTTCTGCAGGTACAGGTGCTGCAGGGGATGGTCCTCGACGATCAAAATACTATAGGG
CTTCATGGGGGATCCGTGTGTAGGGCTCCAGGTAGTTGAAGAGGGCCCGCAACGCAAAGGGTTTGACCAGGCAATG
GTTCATTCCGGCGGCCAGGCACAGGTCTGCCTCGCCGCGCATGGCGTTGGCGGTTGCGCCGATGATGGGCAGCGCG
CAACCTTGGCTGCGCAACTCCCTGGCTAATTCATAGCCGTTGACTCTGGGCATATTCACATCCGTCAGTACCACAT
CGAAGTGTGAGGCCTTGCACAACTGCAGCGCTTCATGACCATCGCAGGCCAACTCCACGGTGCACCCAAGTTCCTC
AAGCTGGTCACGCAAAATCAGTTGATTGATAACGTTGTCCTCAGCCACCAGCACATGCAGGTCGAGCTTGCGCAAC
TCACGCTTTTGCACCTGCTCGTCGACCCGGGCGACCCACAATCCCTGGGCCTGGCTGACCGCCTGGTGAATGGCGC
TCAACTGGTTGAGGTTGGCATGCCACACGCCGGTCTCGGCATGCTGGCCATTGCTGCTGTTGCCACTGACCAGGAT
CACCGGCCCCGGCCAGTCGGGCACCAGGGGCTGTTCGATAGTGCCGGGATGCAGCTCCAGCAGCAGTTGCCCGTCG
GGGTGGGTGGGCTGGCCGGTCTGGGCCCGGGCGCCCCAGCGACGCAGCCAACCGCTGATCGATTGAGCGAGTTCGG
GAATGGGCGAAACCACGTAGACAGTCTCGGCGAGCAAGCTGCTCATGGGCGCGCAGGGCGCCACTTCGAGCGGTAG
GGTGAGGCTGAAACTGCTGCCCAGGCCCAGTTCGCTGACCATGCGGATATGCCCATTCATCAATTGCGTCAGGCGC
TGGCAAATCGGCAAGCCGAGCCCGGTACCGGCGACGACGTTGGTGTTGCCTTCGCTTTGATAGAACGGCTCGAAAA
TCATCGCCTGGTCTTCCTGGGCAATGCCTTTGCCGGTGTCGGACACTTGCCACAACACACTGGAGCGCTCGTCGTC
GCGGTCAAGCAACTTGACGCGCAGCACCACGCGCCCGTAGTCGGTGAACTTCACCGCGTTGCTCAACAGGTTGTTG
AGGATCTGCCGCAGGCGGGTAACATCGCCCATCAGCCGCTCGGGTAACTTGGGGTCAAAGCAGGCGTACAGCTGCA
ACCCCTTGCCCATTGCGGCCGCCGCGTAGCCCTGGATGATTTCATTGACCAGGTCCAGGGGCGAGAACTCGCTCAG
TTCCAGTGCCAATTGCCCGGCCTCGATCTTCGATACATCCAGCACATCGCAGATCAGTTGCAGCAAGGTGGCCGAA
GACCCCTCTATGGCATGCAGATAGTCTTTTTGCTGGGCATCGAGCTGGGTGCGGGCCAACAGTTCCAGGGTGCCCA
GCACGCCGTACAGCGGTGTACGGATTTCGTGACTCATGGTCGCCAGGAACAGGGTTTTCGCGGCATTCGCAGCGTC
GGCCGATTGCCGGGCTTCTTCCAGGGCCGCTTCCACCTGCTTGCGCGCGCTGATGTCGCTGAATGCGCAAAACAGC
ACATCCTCGCCTCTATAGCGGGTGGGTGCACTGCTGAGGTAAAGGTGGCGGCCGTCGAGGGTCTCGAAGTAGTCAC
AGTGCCCGGAGGGCTCACCATCGAATGCCTGGCGGATCCAGCCGGCGCAGAGGGTTTCACGCTCCAGGCCGGTGCC
CAGCCACTGCTGGGCCAGGGTGTTTTCCAGTACCACCTGGCCGTCGGTGCGCCGCAGCACACACAGGGCCACCGGC
GCGGTCTGGATCACATCGCGCCCGAAGGCTTCGCTTTCAATCAGCGCCTGGATGCCATTGATTGACGGGATGATGA
AGCGTTGATTGACCCGGCGCATCACCAGCCAGATCAGCGTGACGCTGAACAGGCAAAACACCAGCGACCCGAGCAG
CTCGCGCCACAACCCCCGCACCACATCGCGCAGGTTGATCGTGTACATCAGTTGCCAGTCCGACGATTTCAGTTGC
TTGCGAATCACCAGGTGATCCGGCACCAGGCCATCGCCGACAAACCCGAAAAAATTCTCGCCCTTGGGCTTGAGCA
GGGACTGGTCCAGCCTTGGGTCATGGCTATTGGTAAATACCAGCATGCCTTGGGAGTTGAGCATCATGAACTCGCC
CGCGCTCTGGTCATTGAGGGTTGTGGAGACTTCCTGGCTTTCCATCTCCAGGCCCAGCCAGCCGGAGTCGGGATCG

CGTTCATCCAGCAAAATGAACAGGTAGAGGTGGGAGTGCCCCTCGGTTTGGTCGCTGAGCCAGAGTTCGCCGGGGT
CCAGTGGGTTGTGTTGCTTCAACGCCCGGAGGCGGTTGAGCATGCACTTGGAAAACGGCAGTACCTGCGGCGTCGA
GTCATAGAGCCGGGTCAGTTGCGGATCGGGGCCGGCGGTCACGTAGAGCAGGTTGACCTGCTTGGTCTTCAGGTAA
CTGCGCATGCGCGAAGTCAGCCAGATGCTCCACTGATGGCCTGGGGCATTGCCCAGTTGCAGGCGCTGCTCTTCGC
TGGAGGCCGGGTAAGCCTGGGCCTTGGCCTGGGTGGATTTGCGTACCGCTGACAGGCTCAGGCTTTCGAGCAGTGC
TTCACGGGTGGTGAAGAAGGTATGAGCCTCGGCAATGGCGCTACTCATATAGCCACGCCGCTGGGAGATGTCATTG
TTGAAGGTGGACAGCAGGAACGTGTAGACACCGCTCAAGATGCCGACCAATAACACGGCGGCGAAAAGACGAAGCA
GCCTTCTGGCTGCTTCGGGGCGGGAAAGGACGGGATTGATCTGCAGCAGATAACTTTTAAATCGCATCGGCGGATT
TTAATCGGCCACCCAGCGCCTGGGCATAAGACGATCCTTAAAAGCAGCCACGTATCTATTTGTAGAACATGTCGAC
CACGACCGCCGCATCGAACGCGCCGGGAACGGGCAGGGTGCGCCACTTCAGGTCCGCCCTGAAGTCCTGGCGGGTG
GTCTGGTTGGCCCCCTTCATCTGGGCCAGGGGGAACCACTCATTGAAGGGCAGGTTCTGGTTCGTATAGTCCCAGG
ACAACGCGATGCCCACGGAACTGTTGTTGGTGGGCACCAACAAGCCATTGACCACGTTCCCGCCGCCCGGGGTGGT
GGCGAAGCGCGCGCTCACCGTATACGGGGTATCGCAGGCTTTGCGCAGGCTCAAGCTGAAGGCTGCGGTGCTGGCC
ACTTCACCGATCTGCGCCTTGCGCGAGGGCTTGGGGAAGCTCACCACATTGGGCGTGATCGTCAGGTCGGGCGTGC
AGGGCACGAAGCGTATGTCGTTCACGCCGGTGACGATGTAGTTGAGGTTGCTGTTGGGGCGGTTATTGAGACCGGT
AATACCGTCCAGTTGAAACACCCGGTATTGCGACAGGGTACTGGCCTGGCCGTTTTGTGGGGTGGTACCAAACTTC
TCGATAAATACGCTGAAGTTGAGGGTGAACCGGGCCTTGTCCCAACCCACGCAACTTGACAGGGTGCAACCGCGGT
CGGAGTAGAACCCAGTACCGTACTTGCCGCTGCTTTGGGTGATGGGCACCGAGTTATAGCGAATCCCGGCGCGAAT
ACCCTGGCCGATGCTGACGCTTGCGGGGTTCAAGTAGAAATACACTTCCTCGCGCCCATGCAAGTAGTCGTCTGCG
CAGATCACATTGACCGAGCGAGGGCCGGACTCCCAGATAATCGTGCCATCTGGCGCATCGGCCGCCACGGCCAATG
CGGTGCCCAGGGGAGCGGTGTCGGCGATCGAGCCGCTACCGTCCACCCGGCAGGCCAGCGCGTAGCTGGTAGCGGG
GAGGGCGGCGCAGGCAAACAGGGTGGCCACGAGCAGGTATTTAAGAGGTGACATAGGTCGGTATTCCCCGCGATCC
ATCACTTGAGGCCCGCGCGATGGCGGGCCTGCAAGCCGATATCGGTGATTTCGGTAAAGTTGATTTGGCTCCCGGC
GCTGATGGGAGCCTTGGTGCTGAAGACCTTGCGTTCCCCGGGCTTGAGCAATACGTAGTCCTCCAACGCCTGAACC
TGGCCGCCGGAATCGCTCTGCACATCCACCAGGGACAGATGAAACGCACTGGGATTGGTGACGGTTACCGATTGCG
CATCGGTGCGGCTCCACACCAATTGCTGCACGGCCTCGGACGAACTTCCTGCAAGCCCTGGCGGGCGATAGAACAG
CTTGAGACGCTGGCGCACGGCAAATTGCACACTGTCGGCGGTCTCCGGTTTCAGCGGGATTTCCATGACGTTGAGC
AGGAACATCGACTCCACGTCGCCGGGCAAGCCCTCGCCGGCGTACAGAATGCGCAGCAGGTGATGCTGTTGAGCAT
CCAGGCGAACCAGCGGCTGGACCACGGCGAAGGGCACCGCCTGGGTTGCATCATCGTTGTCCCGAGTGACCCAGCT
TTGCACCACCAATTCGCTGGGGGACTGATTGACGATATTGATGCTGGCTTCTTTGAATTGGCCAAAGTAGATCAGG
CGCGTGCCTTCAATCTTGACCGCCGCCTGCGCGCAGGGCGCCAGGGCCAGCAGCGCTGCAACCAGCGCGCTGATCC
AGAGCAAGGGACGGCACAATGGCCAGTGGTTGAGTGGTGACATGAGGGCCCGTAGCTTTCCTGGGTATGTTCGCAG
TGGCTTCGGGGTGCATGGTAGGAGGTGGGGGCCGGGGATTCTCTCAGGCGATTCTTAAAAGCCCCAATCAGGTTCA
TGGGCCAGGTTAACGAACCGCTGTCAGCCTGGGCGGCCTTGAGTCGAACGCGCCTGCCAATCGCGCAGCCATTGCA
AACCTTGGGATGTGTTGCCCCGTGGCCGGTATTCGCAACCCACCCAGCCGGTGTAGCCCAACTGGTCAATCAGCTC
GAACAAATAGGGGTAGTTCAACTCGTTGTGCAGGTCCGGTTCGTGGCGATCCGGCACGCCCGCAATCTGGATATGG
CCGATACCGGCGAAATCCCGGCGCAGCGTCGTCGCCACATCGCCTTCGACGATCTGGCAATGGTAGATATCGAATT
GGACTTTCAGGTTGGCGTGGCCGACCGCTTTGCAGATGGCGTGCGCCTGATGCTGGTGGTTGAGGAAAAAGCCCGG
GATGTCCCGGGTATTGATCGGTTCCAGCAGTACGGTGACGCCGATTTTGGCGGCCTGGGTCGCTGCGTAGTCGAGG
TTCTCCAGGTAGGTCTGCTGGTGCCTGGCGCGATCGGCCTCGTCTGCCAGCAGCCCGGCCATTACGTGAATGCGGG
CGTTGCCCAGGACGGCGGCATATTCCAGGGCCTGATCGAAACCTGCGCGAAACTCCGATTCGCGGCCGGGCAAGGA
TGCGGTGCCTTTTTCCCCTGCTGCGATATCGCCGGGCGGCGCGTTGAACAGGGCCTGGACCAAGCCATTGTCGTCA
AGCCGCTGCTTGAGTTGCTGCGCGGGATAAGCGTAGGGGAACATGTATTCCACGGCCCGGAAACCATCACTAGCGG
CGGCGGCAAAACGGTCGAGAAAATCATGTTCGGGGTAAAGCATACTGAGGTTGGCAGCGAAACGAGGCATGAAGAC
TCCTGCGGTGAAAAATCAATCGTATGGCCTGGGTTACCACTGAACCCCAAAGACCTCGTGCAACTCATCCAATGCC
GACTGAGGCAGGGGAGTCGGTCGTGGCTGGTTCATCAACCACAGGCGTGCGGTTTCTTCCAGCTCTTCCAGGGCGT
AGCTGGCCTTGGACACTGAGCTTTCCCAGATCACCGGCCCCAGGCGTTCAAGCATTACACCGCGCACCAGGTTAGC
CAACTGCGCCACGCGCTCGGCGACCTTGGGGGAGCCGGGACGCTCATAGGCGATCTGCGGAATGCGCCCCACTTTC
ATCACTTGATAAGGCGTCAACGGCGGCAGGATCATATCCTCCTGCCAGACGCCGGCCAGGGTCAGCGCCACCAGGT
GAGTGGAGTGGGTGTGGACCACGCCGCCAACCCCGGGGTTGCGATCGTAGACTTGACGGTGCAGCGCCAGCGTCTT
GGAAGGCTTGTCACCGCTGACCCAGTCCCCGGCCAGGCTGACCTTGGCTATCGCCGCCGGGTCGAGGCGCCCCAGG
CAGGCGTCCGTGGGGGTGATCAGCCAGCCGTCGTCCAACCGCGCACTGATGTTGCCGGCGCTGCCCACCGTGTAGC
CACGGGTATACAACGAAGCGCCAACCTCACAGATCTCTTCACGCAGGGTGTTTTCGTTGCTCATGCCGGAACTCCT
GCCAGTTGCTTTAGAGCCTTGTCGAAGAAGTCCACACCACCGAAATTGCCCGACTTGAGTGCCAATGCCAGCGGTT
CCCCGGGCGTGCTGACGCAGGCGGGTACTCCAGGGTCGATTTGCGCGCCAATCTGCAATAGCTGGATATTCAGCGC
CTGGACCACGGCGCCCGAGGTTTCCCCGCCGGCGACCACAAAGCGCCGTACGCCGGCCTCGCGCAGACCGCTGGCG
ATCCGCCCCATGGCTTGCTCGACCAAATGCCCAGCACGCTCGGCGCCCAACTCGCGCTGGACCGCCTTGACCTCAT

CGGCCGTGTTGGTGGCGTAGATCAGCACAGTTTGCGCATGGTCGGCAGCGAAGGCCAGCGCGTCCTCAACCACCGG
CTTTCCCGCCGCCAACGCCAGCGGGTCGACCCGCATCGCCGGACGACCTGCCTGCAGCCAGTTCGCGATCTGGCCC
AGGGTGGCCTGGGAAGCGCTGCCCGCAAGTACCACCGCCGCGCCTTGGACCACGGGCACGTGCGCGGCGTCCAGGT
CGCGCAGCTTGCCGGCACGCCGGAAGTTGCCCGGCAGGCCCAGCGCCAACCCGGAACCGCCGGTGAGCAGCGGCAA
GTCCGCACAGGCTTCGCCCAGTAGATAGAGATCGGTATCACACAGGGCGTCGGCGACAGCCATGCTGACACCGTCG
GCGCGCAGTTGAGCAATGCGCGCGCGTATGCTCTCCACGCCGGCGGACACGCTGTCGTGGCGCAACAGGCCAACCT
TGCCTTGGGTCTGCGCTTGCAGCACGCGAACCAGGTTTGCGTCTGTCATGGGTGTCAATGGGTGATGCTGCATGCC
CGACTCGCTGAGCAATTGGTCCTGAACGAACAAATGCCCACGAAACACCGTGCGGCCGTTTTCCGGGAAGGCGGGG
CAGGCAAGGGTGAAATCGCTGCCAAGGGCCTGCAGCAAGGCTTCGCTGACCTGGCCGATATTGCCGGCCGCCGTTG
AGTCGAAGGTTGAACAGTATTTGAAGAATATCTGTTCACAGCCACGTTCACGCAGCCAGTGCAGCGCTTGGAGTGA
TTCCTCGACGGCATCGGCGGCCGGCACCGTGCGCGATTTCAGGGCGATGACAATCGCATCGGCATCGAGTTGGTCG
GCAACATCGGCAGCGGGAATGCCGATGCTTTGCACCGTGCGCATGCCGCCACGTACCAGCATGTTGGCAAGGTCGG
TGGCACCGGTGAAGTCGTCGGCGATGCAACCCAGCAGCGGCCGGGCAGGGGCATTCAACATGTCGAACTCCTCAGC
GGGTTTAACCGGGCTGGTATTCATGGATGAACTGCTCGATCACATCGGTGAAACGCTCATCCGCGGTGAAGCCCAG
GTCCCGGGAGTAGGTGGCACAAAACGCGCCCGGCCAGGAACCGACGATGCGCTCGATCGCCTGATTGGGTTCCAGG
CGAATGCGTTCGGCCACTTGCGCGCCGGCTGTCTGGCGCAGGGCTTCGATCATGCGCTCTACCGTGATCGACAGGC
CCGGCATGTTGATCACTCGGCCCTCCGTCAACTGCTCGCTACCCAGTTCGTGGCCATGGATCAGGTTGCGAATGGC
CTGTGCAGGCGACATCAGCCAGAGCCGGGTGTCCAGTGGTACCGGGCAGACGCTGGTCTGGCCGCTGAGCGGTTCG
CGGATGATACCGCTGGCGAAGCTGGAGGCTGCAAGGTTTGGCTTGCCCGGGCGCACGACGATGGTCGGCATGCGCA
GGCTGCGACCGTCGACGAATGCACGCCGGCTGTAATCGGCGAGTAGCAGGTCGTTCATGGCTTTTTGTGTACCGTA
GGAGCTTTGTGGCGCCCATACCTGGTTATCTGGCACCTGTTCCGGCAACTGCCCGCCGAACACCGCGACGGAGCTG
GTCATCACCCATTTTGGGCATGTGCCAAGCTGGCGCACGCGCTCCAACATGTGCTGGGTCGCACTGAAGTTGATGC
GCATGCCCAGTTCAAAATCGGCTTCGGCCTGGCTGGAAACCACCGCAGCCAGATGGAAAATGCTGTCGGTATTGGC
ATCGATCAAGCGCTCCAGCACATGGGCGTTGGCGATATCGCCACACACCACTTTTACCCGGGGGTCATTGATCCCC
GTCAGTTCGACCATGTCGAACGCGACAATCTGCTCGATGGGCCGGGACTGGCCCGTGCGGTCGGTGAGCGAACCAC
GCAGCAGCAGCGCTTCGATCAGGCGGCGGCCAAGGAATCCGGCTGCACCGGTCACGAGAATATTCATGAGTTACTC
CAAACGTCAGCGGTTGACGAGTTTTCCGGGAATTCTGAAGACCAGCAGGGCGCCGAGGAACAGTGCGCTTGCCATC
ACATACATGCCGATTGTGGTGCTGTGGGTCAGGTCCTTGAGGAAGCCCATCAGGTAGGGCGAAACGAAGCCCGCCA
GGTTGCCCCAGGAGTTGATCAGGGCAATCCCGGCAGCCGCCGCCGTACCGCCAAGGAACGCAGTCGGCAGGCTCCA
GAACAGCGGCAGGGTGCTCATGGCGCCCATCGAACCCAGGGTCAGGCCGATCATCGACACCGTGAAGTTGTCGCTC
CAGGTCGCAGAGATGAACAGGCCGATGCCCCCGAGCACGGCGGTCAACGCCAGGTGCCAGCGCCGTTCGCGCAACC
GGTCGGCACTGCGCGAAACCAGGATCATGGTCAGTGCAGCGGCGGCGTAAGGGATGGCGGTGAGCATGCCCACCTC
GAACACATCGCTGACACCGGCCTGGCGGATAAGGGTCGGCAACCAGAAACCGACGGTGTAGAAACCGGCGATCATG
CAGAAGTAGATGGCCGTCAACAGCCAGATACGCGGCTGCAGGAACACTTCTTTGAGGCTGTGTTTCTGGTGGCCGA
CGGCTTCTGCGTCAATGCGCGCCTGCAGCAGGGTTTTTTCTTCACGGGTGAGCCAGCCAGCGCTGGAGATACGGTC
GCTGAGGCAGAAAACCACCAGGAAGCCGACAGCAATCGAGGGTAGCCCTTCGAGGAAAAACAACCATTGCCAGCCC
GCATATCCATGAACCCCATTGAAGGCGCTCATGATCCAGCCGGACAACGGCGCGCCTATCAGCCCGGACAGCGGCA
CGGCGGTGGCGAACAGCGCGTACATCTTGCCGCGGCGGTGGGTCGGGAACCAGTAGGACAGATAGAGGATGACCCC
CGGATAGAACCCGGCCTCGGCGATCCCGAGCAGGAAACGCATGATGTAGAAGGATGTCGGCGTCGTCACCAGGGCC
ATGGCCGAGGAGATGATGCCCCAGGTGATCATGATGCGTGCGATCCACAACCGCGCGCCGACGCGATGCAGGATCA
GGTTGCTGGGCACCTCGACGAGGAAGTAGGCAATGAAGAAAATCCCTGCGCCGAGGCCATAGACGGCTTCGGAAAA
TTGCAGATCGTCTGACATCTGCAACTTGGCGAAACCGACGTTTACCCGATCCAGGTAGGCGACCAGGTAGCACAGC
AACAACAGCGGCATGATCCGCCACGCCACTTTGCGATACGCCAGGTTCTCGAAGCGATCTGCGGGTACGACGGCAC
TGGTTTGAATCACTGATTCAGTCATGTGTTTCCCCCATCCTTATTTTTATAGTGGGTGGCGTTGTTTGTGTCGGAT
TACATGATGGGGCGTTTTTTATCATAATACGTCATACGAATTCAACGACTAAATCCAGAGAGGACGGCGGGTCTAA
AATGCAGTACCTTTCACAGATTTGAGCCAGAGTCGTCTATGCCCACTCCTGACAAATCCTCCTGTAGTGCCGATAC
GCTGGGTACCCTGAAGGACTCCGCCAAGGGCACACTGTCCGATCAGGTGACGGCGGCCCTCAAGGCCTATATCGCC
AGCGGCGAAGCGCTGCCAGGCCATCGCCTGCCCACCGAACCGGTACTTTCGCAGCGTTTTGGCGTCAGCCGCACGG
TCATCCGCGAGGCCATTTCGCGCCTGAAGTCGGCCGGCCTGGTCGAGGTCCGCCAGGGCAGCGGTACGGTGGTCAG
CGAAGGGGCACACATCAAGGCATTCACGATTGACCTGGATGTCGGCGGGTCGATCGAGGCTGTATTGAGAGTCACC
GAACTGCGCCGCGGCATCGAAGGCGAGGCGGCGGCATTGGCGGCCCAGCGACACTCACCGGCGCAACTGGAAGCCA
TCCACCATGCGCTCAAGGCCATTGATGCAGCGGAGCAGGGGAGCCGCGATGGGGTTGAGGAAGACTTGGCGTTTCA
TCACTCTATTTCCAAGGCGACCGGCAATCCGTTGTACCCGTCATTGCACGAGTTCATCGCGCAATTCATCAAAGAG
GCGATCCGCATCACACGCTCCAACGAAGAGCGGCGCCGAGACTTGGCCAAGACCGTCCGGGCCGAGCACTTTGCCG
TGTACGCGGCCATTGCCGCGCGTGATCCAGAAGGCGCAAGGAAAGCGGCGCTGGAGCATATCAATAATGCGGTAGA
GCGACTTAAAAGTGCGGATCCGTCGTTTTGGCAGAGTCCGGGGGCGAGGAAGGTTTAGTAGTTGAGCTTGGGAATG
TGAGTGGGGCATTATAACTAAACTAAATAGTTATTAATTTCATTTATAAGCTATTGAGATATAAAGAGTAGGGCAC

ACCCCACATTACCAGCTCTGCGATTGTTGAATATGGAAATAACCACTATCGTCAAAAGCGCTAAAAAACTAGTAGT
TCCTACAGTAGCCACAACCCTGGAATGCCCGGAACATCGGGTTTCCAGCACCTGGTGGTGCCTATGAGGAGAACGA
ACATGAATAAGATCTACGACGATGAACTGAAGGCTATTTCGACCAGGCTCGCTGCCATGACGGCTGTTGAGCGGCA
ACGCGCGTTGGACTTCTTGAATGGCAAGGCTGAACTGCGTCCCGATATGGGGAATAGTGCAAGCGTGGCTGGTGTT
ACAACGACTCCCGTTAAACCCATTGAGTGACAAGGTGCCGCCATCCGGCGTTGAGCCACACGAAGCATTCACGCCA
ATATCCGGCAACTTGGCCAATATTGCGTACGCAGAGTAAACCTCCGGTGATCTATAAGATTCGGGCGGGGTTTCTT
TGCGTACGGAACACCTGGGGCGATTTGGTTGGATCGGTATTGCGGCGGCCTCTGGGAATAATGAGCGTCCTGCAGC
ACGTGTCCAATCGGTGGCCATCGCCAAACATGAGGTGTGGGTTGCAGCGCATCAGCACGAGGACTTGACCAATCCT
ACGGCAACATTCATAGGGTAAATAGGGGAGGAGGGATGCATTTATCGCCTGACACCTTGTTGATTTAGCCGCTAAA
TTTTAGGTCGATTCCTCCGGCGTTCGATCTGCTTTTCACCGTGAAAAATAGAGTAGGTAATCGGTTCCGACTCGGT
TGAACTATAACTAAACTAAATGGATATTTAGTTTAGTTATATTTATTCCTTATATTACAAGGGCAAAGCTAGCTCA
TCCTTCGCGCGAGTGTGGGCACATGCACGATACTTAACATTTGAGCATCATAAAGGGACGGTCTATATGCGGGGAA
TGCTTACGTCAGGTCTATGGGTGGCATGTTTCGTGACCAGTGCCACGGCGCTGGCGGATGAACTGCCGCGTAGCGA
GCAGATAAAAGCGTTCCGCGCTGCTGGATTTAACCAAGAGGCGTCGGAGTGCGAAATCGAAACAACAGGTAGCTAC
ACACCGGCAACCATGGAGTTGGTTGAAGACCTGAACGGTGACGGCCGGCCGGATGCCTTGATTACCGAAGGCAGCA
CTGACTGTTATGGGCAAGCAGGTACTGGATTTTATCTAGTCAGCCAGCAACCTGACGGTCACTGGAAACTCATGCT
CAAAGAAAACGGCGTCGCTGAGTTTCTCGCTAGCAAAGGCAGTAATGGTTGGCCTGATATCGAAGTCGGCGGCCCG
GGCTTTTGTTTCCCAGTACTGCGTTACGACGGGCAACGTTATCGATTTCACCACAGCGTTGGTGAACGGTGTGCTG
ACTCGCCGTAAACGTCCGGTGGCCCAAGGGTCAAACGAAGGCAGGTCAACGAGGGCGACGGTGGCTGCTTTTGAAT
CTGGGGTGCGGTTGGTTCTATGTCGCTTAGTGAAATGCACCGAACAGAGCCCCTGAAGGACAGCCCACTTGGTTCG
CATAATGTATATTATGTTAAATTATGTATGTACAAAGCTCCTAGCGCTTTCCCTCCCATCCACTTCCTCCATTCGT
CATCAGAACCCTATCGATCTCGGCTACCACCCGCATCGTCGCGTCTTCGATCGTTCCCTCATTGCGGCATACGATC
CAGCCGTGTTCGGTCATCACGCGCTCGAATGCCTCGGTACACGCAACATGTTCTGCCAGCGTATGGAGCACAGTAC
TGTTCAACCCACGCCGTCTTGCCGCGGCCCTTGCCCATGAAACTTCTGGGTTGGTGACAACCCCCACGTGCAGGTC
TGGCACTTGCACCTGTCGATCGATGTTCAACTGCAGAATCTCGGCAAACGGGACTATCCGGCGAAACGCCGCATCG
GATGGGTACCAGCGATCCATCAAGACGATGCTGTCTGCGGGCTGCTTAGGCAAAATCTGTTGGGAAATCCAGGCTC
GACTATCAGCAAGGCGCTCACAAACCGCCCACTCCAAATCCCGGCTGGGATTTCTGACGAGTCTGTTGACGAGGTT
CATTGTGTCACCCCTGTAGGGATCGCTTTTCTTCTCGCACAGCCGGATCACCTTTTTGCCCGCGCCCCTCAGCACG
GTCGTCACGGCTTCCAACAGTGTGGTTTTGCCGGTGCCCTTGGGTCCATCGAGAGAAACAAACAGCGCACGTTTCA
TTCTTCGTACAACACGTTATAAACAAGCACTCTAACCTGATTGCCCGGCACCGCGCCAACCCCCGCATTTTGCCTG
CGCCGGCCTTAAACCAGGTGTGGCTGTTTCAATGGCCGGCTAAACTGCCCGCCAGATTATTTTTGGTACAAGAGAA
CTCCCATGACCCAGCAAGACCTCAGCTATACCCCCGACCTCGACCCGGACTCCATTTCCTCGGATGTCATCGGCTT
CAACGGCATCCTGGTCTCCACGCAAATCCCGGTGCGCGCCGATGGCAGCCTGGAACTGGGCGATATCACCGCGCAA
AGCGAATGCACCCTGCAAGCCCTCAAGGTCGCGCTTGAACGTGCCGGCAGCAGCATGGACCGGGTCATGCACTTGA
CCATTTACCTCACCGACATGGCCGACCGCGCCGCATTCAACGAGGTGTACAAGCGCGTCTTCGCTAAACCCTGGCC
AGTACGCGCGGCGGTTGGCGTGGCCGCATTGGCGGTCGACGGCATGCGCGTCGAAGTCACAGCGATGGCAGCCAAA
GGCTGAGCCCTGCGCGCCATTGGGCATCCAGCGGGTGCCCACCCGGCGATTCACGGCTACAATGCGCACCTCAACC
GTGACAAGCCTGACTAAAAAAACTATGTCCTTGCCCAAGCATCATCTGGAATTGCTCAGCCCTGCCCGCGATGTCG
CCATCGCGCGCGAGGCTATCCTGCACGGCGCCGACGCCGTGTACATCGGCGGCCCAAGCTTCGGTGCGCGCCACAA
CGCCTGCAACGACGTGAGCGAGATCGCCCAACTGGTGGAATTCGCCCGTCGTTATCACGCCCGGGTGTTCACCACC
ATCAACACCATCTTGCATGACGATGAACTGGAGGCGGCCCGCAAGCTGATCCACCAGTTGCACGATGCAGGGGTTG
ATGCACTGATCGTCCAGGACCTGGGCGTGATGGAGCTGGATATCCCGCCCATCGAACTGCATGCCAGCACCCAGAC
CGACATCCGCACCCTGGCCCGGGCCAAGTTCCTCGATCAGGCCGGATTCTCCCAACTGGTGCTGGCCCGCGAGCTG
AACCTGCAGGAAATCCGCGCGATTGCCGATGAAACCGATGCCGCCATCGAGTTCTTCATCCACGGCGCGTTGTGCG
TGGCGTTCTCCGGGCAGTGCAATATTTCCCATGCGCAAAATGGCCGCAGCGCCAACCGTGGCGATTGCTCCCAGGC
CTGCCGCCTGCCCTACACCCTCAAGGACGACCAGGGTCGTGTTGTCGCCTTTGAAAAACACCTGTTGTCCATGAAA
GACAACAACCAGAGCGCCAACCTGCGCGCCCTGGTAGATGCCGGCGTGCGCTCGTTCAAGATCGAAGGCCGCTACA
AGGACATGGGCTATGTGAAGAACATCACCGCCTATTACCGCCAGCGCCTGGACGAGATCCTTGAGGACCGCCCCGA
CCTGGCTCGCGCCTCCAGCGGCCGCACCGCGCATTTCTTCCTGCCGGACCCGGAAAAGACCTTCCACCGTGGCAGT
ACCGACTACTTCGTCAGTGATCGCAAGATCGATATCGGTGCCTTCGACACCCCAACCTTTACCGGCCTGCCAGTCG
GCGTGGTGGAAAAAGCCGGCAAGCGCGACTTGCAGGTCATCACCCATGAGCCGCTGTCCAACGGTGACGGCTTAAA
CGTGCTGATCAAGCGCGAAGTCGTGGGTTTCCGCGCCAATATCGCCGAGCCCAAGGGTGAGTTCGACGAAGACGGC
GAGAAGCGCTACCGCTACCGCGTCGAGCCCAACGAGATGCCGGCGGGCCTGCATCAGTTGCGGCCCAATCACCCGC
TCAACCGCAACCTGGACCACAACTGGCAGCAAGCGCTGCTCAAGACCTCCGCCGAGCGCCGGATTGGTGTGTCCTG
GCAAGCCTGCCTGCGCGAAGACCAACTGCAACTGACCGCCACCAGTGAAGAGGGGGTGAGTGCCAGCACCACCCTG
CCCGGCCCGTTCGGCGTGGCCAACAAACCGGAACAGGCGCTGGATACCTTGCGCGACCTGCTCGGCCAACTCGGTA
CCACCCAGTATCACGCCACGGCCATCGAACTGGATGCGCCGCAGGCGTATTTCATCCCCAACTCACAGCTCAAGGC

CCTGCGCCGAGATGTGATCGAAGCGCTGACAGTGGCCCGTGTCGAGGCCCATCCCCGTGGAGGCCGCAAAGCTGAA
ACCACGCCACCACCGGTGTACCCGGAGTCGCACCTGTCGTTCCTGGCCAACGTCTACAACCAGAAGGCTCGCGATT
TCTATCATCGCCATGGCGTGAAATTGATCGACGCGGCCTACGAGGCCCACGAAGAAACCGGTGAAGTGCCGGTGAT
GATCACCAAGCATTGCCTGCGCTTCTCCTTCAACCTGTGCCCCAAGCAGGCCAAGGGCGTGACCGGCGTACGCACC
AAGGTTGCACCGATGCAGCTGATTCACGGCGATGAGGTGTTGACCCTGAAGTTCGACTGCAAGCCTTGCGAAATGC
ACGTGGTGGGCAAGATCAAAGGCCATATCCTCGACTTGCCGTTGCCGGGCAGCGGCGTGCAGCAGCAGGTCGTGGG
GCATATCAGCCCGGCCGACCTGCTCAAGACCATCCCCCGCGCGCCACACTGACCCACGCCCTTGTAGGATCCCGCT
TGCCAGCGATAGCGCCCTCCAGATCGCCATCGCCGGCAAGCCGGCTCCTACAAGGGGCGCCTGGATGTCGGCAGCT
CGTGTTCAGCACGCCACCACCAGCCTCGCTGCAACCCCGCCGCCGCCAGCAATATCGCCGCAACCCCCAACCACTG
TGCCCAGCCCAAGCGATGCCCGTAGGCCATCCAGTCAACCAGGATCGCCGCAACCGGGTAGATGAACGACAGCGCC
CCGGTCAAGGCCGTCGGCAGTTTTTGAATCGCGCCATACAGCAGCACGTACATCAGGCCGGTATGCACCACGCCCA
GCGTCAACAGCGCGGCCCAGGCATCGACGGGCGCCGATAGGCTTTGCCACGAGACCAGCGGCACCAGCAACACCGC
GCCGGTAGTCACTTGAATCAGTGCCAGTAGGTGCGGCGGCACGGCCTTCAGACGTTTGATGATCAGCGCAGCGACG
GCATACAAAAACGCCGCGCCAATCGCCTGCACCACGCCCAACAGGTAATGATCACCACTGCCCTGCTGCTCGCCAT
GGGCACTGACGATCGCCAGCATGCCCACGAAAGCCACACTCAACCAGGCGAGCTTCTGTGCGGTGATCTTTTCGCC
AAGAAACAACGCCGCGAGCATCACCAGCATGAACGGCTGCACGTTATAGACCGCGGTGCTGATCGCAATGGAGGCC
TTGGCGTAAGACGCAAACAGCAACAGCCAATTACCGACAATGGCCACGCCGCTCAACATCGCCCAGCCGAGTGTCG
AGTAATTCAACCATCCGCCCCGCAAATACCCCAGCCGCCAGCACACCAGCAGCAGAGTCAGGCCGCCGATGACGCA
ACGCCAGAACACCACTTCGATGACCGGCACCCCAGACACCAGCACAAACCAGCCAATGGTGCCGGAAATCAGCATG
GCGCCGACCATTTCCCATGAGCCGCGACGTATGGATATGTCCATGATTTAGCTCCTCGAATGAGGCTAAATTATGG
CAACCTGCGCCAATCAGGCTCCAGCGCCTGCAGAAGGTAAAAGGTGGTCCTTACCTATTCTTATTAGGCAAAACAG
CAAATCTGCTTAAGGAGCCCAGGATGATTGATGTTATCGACCAACAACTGATCGCCGCCCTGATGGACGACTCGCG
CTGCTCGCTCAAGACCCTGGCGGGCATTACCGGCCTGTCCTCGCCCAGCGTGGGCGAACGTTTGCGACGCCTGGAG
GAGCGCGGAGTCCTCAAGGGCTACACCGTGGATATCGACCCCAAGCACTTTGGCTATCTGTTGCAAGCCATCGTGC
GCATCCGCCCCCTGCCCGGCCAGCTACAAGAGGTGGAACGGCAGATCCAGGCGATCCCGGAGTTTACCGAGTGCGA
CAAGGTCACTGGCGATGACTGCTTTATCGCCCGACTGCACGTACGCACCATGGACCAGTTGGATACCTTGCTCGAC
CGTCTCAACGCTTACGCCGAAACCAACACCGCCATCGTCAAGAAAACCCCGGTCAAGCGCCGGCTGCCGCCCTTGG
CGGGCCAGTGATGCTCTGTTGATGGCTATCTCGCCCAGGCTGATGCCCTACGCTAAGCGTCAGCATCGCCTTCTTC
AGCCAAATCATCCTGCTCGATCAGCGGCACCTGCGCATCATCCATCAACGATCCCGGGTCTTCATTGCCGGGATCA
TTGATCGGCTCCTCTTCGGCTGGGGTATGCATCGCCCTAGCCGCCCGGGGTATCAATGTCAGCGTCCGTTTCTGGC
TTGGGCTCGCGCTCGGGCTTGAAGTCCGGGCTGTACTCGTTGTCCTTGGCGACCTCATCAGTCGCGGGTTGTGGGT
CCTTGGGGGCCGTACCGGGAGCGGGTATTTTATGCGTGTTGTCATCGATGGCCGGATCATTCCTGCCAATGACTTG
CGGGTCTTGACTGTGATTCGCGCTCATGGGGACCTCCTTCCAAGCTGATGTACAGTTAAGGCGCCACGGAGATGGG
TGAAGTTCAGTTCAAACGAACCGCGCTGATCAATGGTCACGACCCCGGATTATCCACCTGGATATAAAACGCAAAC
GCCGCCAGCAGCATCCAGAACACCGTGAACAACCACGGTGTACGCACCGAAAACAGTAATGACTTGCGATAGCCCT
TGTGCGACGACTCGGTCTCGCAGAACAGCGGCGACTCATCGTAGGCCATGCCCATCACCGGTTCGCTGCGCAACAA
CTCGCTCTGCTTGAAGTGCCAGTGGTCAATGATGTCGTAGGCTGCACGTATGCCGGGCCAGGCGTTGAGCGAGCTG
AACACGCCGAGCATCGACAGCAGCAGCGGCACCACCAGGGTGAACAGCTTGCCCCACTCGGGGTTGAGGTTGCCCA
TGCAGGACACAAAGGCAATCACCAGGAACGACTGCGCGGTGAGGTAAGCATCGGTGCGGTTGGCAAGGATGGTGGT
TTCGTATTGGATTTCCCGGCGATAGAAATCCAGCCGGTCCTTGGGCGAGCCAAAGATCTTGGCATTGTGTTCGGTA
TGGTCAGTCAGCGCCGATTCGGGGCTATGGGGGGAAATGATCCGGGGCACGATCGGTGATCCTTACGGTGCGTAAT
CCCGTTAGAAGAACCGGTGATGCCCGAAGTTCAGGTCAATTACCTGCACCGTTATGGAGCATGGCGTATGCCTGGG
GCGCACTGCACTTGCGCAGGCCGCTCCGATAACGGGCTTACACACTGTGTAATCCCTATAAACGCAGTTTCGGCAC
AGCCATTGCTTTACTCCTTAAATCCCCTGCCTGCCCAGGTGAACACAGGAGCAAACGTTACCCCCCCGACTCAGAG
CCGACTCCAAAAGAAAGTAAGGACAAGGTCCGTTGGCGCCAAAAGCCACGGGCTCATAACAATGCGTTTTCTAAAG
CGGCAGTGCCTGCAACGTTTCACCCGGCACTGACAAGGTAATTGGAATGGCTCGATCTTTCTTTGATGAAATGAAT
GACGCAAATGGCGTGTGCCGTACGCACTATCAGGATTTTTCCCGCTGGCTGGCCAACACACCGCCGGAACTGCTGG
CCCAACGCCGTCGCGAGGCCGACCTGCTGTTCCACCGCGCCGGGATCACCTTCACCCTGTACGGCGACGAGCAAGA
CACCGAACGCCTGATTCCCTTCGATATCATCCCGCGCAGCATCCCGGCCAGTGAGTGGAGCGTGATCGAGCGCGGC
TGTATCCAGCGGGTCAATGCGCTGAACATGTTCCTCGCCGATATCTACCACGACCAGCGCATTATCAAGGCCGGGA
TCATTCCCGCCGAGCAGGTGCTGGGCAATGAAGGCTATCAAAAAGCCATGGTCGGCCTGGACCTGCATCGCGATAT
CTATTCGCACATCTCCGGTGTCGACCTGGTGCGTGATGGCGACGGCACCTACTACGTGCTCGAAGACAATTTGCGC
ACACCCAGCGGTGTGAGCTATATGCTCGAAGACCGCAAAATGATGATGCGCCTGTTCCCTGAAGTGTTTGCCAAGC
AGCGCATCGCGCCAGTGGATCACTACCCCAACCTGCTGCTCAAGACCCTGAAAAGCGCCAGTCGCCTGGACAACCC
CAATGTCGTGGTGCTGACCCCGGGGCGCTTTAACAGTGCGTTCTTCGAGCACGCGTTCCTGGCCCGAGAAATGGGG
GTGGAGCTGGTAGAAGGCGCCGACCTGTTCGTGCACGACCTCAAGGTGTTCATGCGCACCACCGACGGCCCCAAAG
CAGTGGATGTGATCTACCGGCGCATCGACGATGCCTTCCTTGACCCGCTGGCCTTCAACCCGGAATCCATGCTCGG

CGTTCCCGGCCTGGTAGCGGCCTACTGCGCTGGCAACGTGGTGCTGGCCAATGCCATCGGCACCGGCGTGGCCGAC
GACAAGTCTATCTACCCCTACGTGCCGGAGATGATCCGCTTCTATCTGGATGAAGAACCGGTGCTGCAGAACGTAC
CGACCTTCCAGTGCCGCAATCCCGCTGAGCTGTCCCACGTACTGGCGCACTTGCCCGAACTGGTGGTCAAGGAAAC
CCAGGGCTCCGGCGGCTACGGCATGCTGGTGGGCCCGGCCGCCTCGGCCGCCGAGATCGAGGACTTCCGCCAACGC
ATCAAGGCCCGGCCCCACGCCTATATCGCCCAGCCGACCTTGAGCCTGTCCACCTGCCCGACTTTTGTCGAAAACG
GCATCGCGCCACGGCATATCGACCTGCGGCCCTTTGTGCTGTCGGGCAAGGAGACCCGCCTGGTGCCGGGTGGGCT
GACCCGGGTGGCGCTGCGCGAAGGCTCATTGATCGTCAACTCGTCGCAAGGCGGCGGAACCAAGGACACCTGGGTG
GTGGAGGGCTGAGTATGCTGAGTAGAACCGCCGCAGATCTGTACTGGATGTCGCGCTACCTGGAACGCGCGGAAAA
CCTGGCGCGCATGCTGGAGGTCAGCTATTCGCTGTCGTTGATGCCCCAGGCCGGACGCAGTGATGGCCTGGACGAA
CTGGCGATGTCGCTGCTCAGCAGCGGCACCCTGGACAGCTATCTGGAACGCCATCAACAGCTGGACGCCGAGCGCA
TGCTGCACTTCTTCGCCCTCGACGAAGAAAACCCGGCCAGTATCTACAACTGCCTGCGCGCTGCCCGGGGCAATGC
CCATGCGGTGCGTGGGCGGATCACCGCCGACATGTGGGAAAACCTCAACGCCACCTGGCTGGAAATGCGCAGCATC
GCCGCCGGCGGCCTGGCACGCCATGGCATCAGCCATTTCTGCGACTGGGTCAAACAGCGCTCGCACCTGTTCCGTG
GCGCCACCTCCGGCACCATCATGCGCAACGACGCCTATCGTTTTATCCGCCTGGGCACCTTTGTCGAACGCGCCGA
CAACACCTTGCGCCTGCTGGATGCGCGCTACGAGATGTTTGGCGAAGAATCCGAGGAAGTCAGCGACCTGTCCGCC
CGCGGCTACTACCAGTGGAGCGCCCTGCTCCGTGCCTTGTCGTCATTCGAGGCCTACACCGAGCTGTACCCCAATG
CGCTGAACGCGCGTTCGGTGTCGGAGTTGCTGCTGCTACGCAGTGATGTACCGCGCTCGTTGCATGCGTGTATCGA
GGAGTTGAGCCATATCCTCGCCGACCTGCCAGGTAGCTACGGCCGCACGGCCCAGCGCCTGGCTGCCGAATTCGAG
GCGCGGCTGCGTTATACCGGCATTGACGAGATCTTGGAGGAAGGCCTGCACAGCCGGCTGACCGAATTCATCGACA
CCGTGCGCGAGCTGGCGCGGGCGATCCACAGTTCCTACCTGGAAGTGGTGTAAGCGTCGCATCACCCTGTAGGAGC
GGGCTTGCCCGCGATAACGGTGGAACAGTCGGAAATTGTTCATCTGACCCACCGCTATCGCGGGCAAGCCCGCTCC
CACAGGTCTGGTGTTTGCTCCAGACATATAACTGTCAGTTTGGATCATTGATCCCACCTCACTGCTGCTCTTATGG
TTGCTCAACACCCATAAGAACAGGTACAGACCATGTCCGAAGCTCTGCAATTATCCCCTGGGGCCGCCCATACCCT
GAAGCGCTGGCACACCATGCTGGCCACTGCCGACCTCAAGGCCCTGCCCGAACTGCTGGCACCTGACGCGGTGTTT
CGCTCGCCGATGGCCCACACCCCCTACCCCGGCGCCCCCGTCGTATCGATGATCCTCAACACCGTGTTTACTGTGT
TTGAGGACTTCCAATACCACCGAGAACTGGCCAGCGCCGACGGCCACAGTGTGGTGCTGGAGTTCAGCGCGCGAGT
GGGCGACAAACAGCTCAAGGGCATCGACCTGATCCGCTTCAACGAAGAGGGGCAAATCATCGAATTCGAAGTGATG
GTCCGCCCCTTGAGCGGTCTCCAGGCCCTCGGTGAGGAAATGGGCCGACGCCTGGCACCGTTCCTGGCCAAGGCCA
AGGGCTAACGCTGCAGGAACTCCAGCATCAGCGTGTTGACCTGCGCGCCCTGCTCCACCTGGATCCAGTGCCCGCA
GTTGGCCAGCACCTGCTGCTCCAACTGCGGCACCACCTCGGGCATGCGTTTGAGCGTATGCGCCTCAAACACCCCC
ACCGGGTCGCGGTCGCCGATCAAAAACAGCGTCGGTTGCAACACCTGTCGGCCCGCCAGGAACTCGGTGCGCTGCC
AATTGCGCTCGAAGTTGCGGTACCAGTTCAGCGGCCCGCGAAAGCCATTGGCAAAGGTCTGCACATAGATGTCCAG
GTCGTGCTGGCTGCACCAGGCCGGCAAGGCGCTTGGCAGCGCGACACCGTCCAGCAAGGTGGCCGTGGCTGGCTTT
TGCTGCAGGAATACATCCTGGTCCTGCATGAACAGACGCAAGGTGCGTTCGATATCGGCTTCCAGTTCCTGCTCCG
CCACGCCGGGCTGCTGGAAGTACAGGATGTAGTTGAACCGTTCGGCATACAGCTCACGCATGATCTCGATCACCGG
CCTGCGGGAACGACCGGCGAACGGCACCGACATCGTGACCAGCGCGTTGATACGCGTAGGCTCCAGCAAGGCCAGG
TGCCACGCCACCACCGCGCCCCAGTCATGGCCGACCATCGCTACCCGTTGATGGCCGAACAGGTCCATGGCCCTCT
GGATATCCGCGCATAGGGTCAACAGGTCGTAGTCGGCAATCTCGGCGGGCGCGCTACTGCGCCCATAGCCCCGCAT
TTCAGGCACAAATACGCGATACCCGGCGGCTGCCAGCACGGGGATCTGTTGGCGCCAGGAGTGCCAGCATTCGGGA
AAACCATGGAGCAGCCACACGGGCGGGCCGCTCTCGGGGCCGGCGATATGCAGGCTCAATTGAATGCCATTGACCT
GGATCGTCTGTTGTTCGATAGCACTCATCACGCAGCTCCATGGGGGGACTGCTGCAGACTGCCCCACCGGCGCCCC
CCGCGCCAGCATCATTGACGGGGCAAATACCTCAGGTCACCGACCACCAGCGCGGTAGCAGCGGCCGCACCTTGGG
TTCGGCAAAACGGTCATCGATCAGCATCACCACCCCCTGGTCCTGCTGGGTGCGAATCACCCGGCCTGCCGCCTGC
ACCACCTTCTGGATCCCGGGATACAGGTAGGTGTAGTCATAACCGGCACCGAACATCGCGCCCATGCGCAGTTTCA
TCTGCTCGTTGACCGGGTTGAGTTGCGCCAGGCCCAGGGTGGCGATAAAGGCCCCGATCAAGCGTGCGCCGGGCAA
ATCGATACCCTCGCCGAACGCGCCGCCTAACACGGCAAACCCTACGCCCCGGCTGTGCTGGGTGAACTGGTCGAGG
AAGGCCTGGCGCTCGGCCTCGGCCATGCCCCGTGATTGATGCCAGGTAGGAATCGACGGATACCGCTCGGCCAGCA
ACTGCGCCACTTGCTGCTGGTAATCAAAGCTGCTGAAAAACGCCAGGTAGTTGCCCGGCGTACGCTCAAACTGCTC
GGCGATCAACGCCACGATGGGCGCCAGGGACGCCTGGCGATGTACAAAGCGCGTGGAGATGCGGTCGATGATGTGC
ACCTGCAACTGCGCGGCATCGAAGGGCGATTCCACGTCCACCCACGCGGTATCCGCCGGCAGGCCCAGCAGGTCGG
CATAGTAATGCCGGGGGCTCAGGGTCGCGGAAAACAGCACGCTGCTGCGGGCCGCCGTCAAACGTGGGCGGATAAA
TTCGGCCGGCACCACGTTCCGCAGGCTCAAGCGCGAGAAGCGACGCTTGGCCTGGGGGTCGCGCTGAGCGATATCA
AAAATAAACTGCTCGTTGAACAGCTCCGCCACCTTGGCGAACTGCAACGCCTCGAAATAAAAGCCTTGCAGCTCGC
CCGTGAGCGCCTGCGGGTGATCATTGAAATACTCGCCCATGGCGCTGGTACACAGGGCCAGGGCCTGCAACAGCTT
GTCAGGCTTGCTGGCATAAGCCTGATACACCGTGACCTGCTCCTTGTGCAGCGCATTCCATTCACGGTTGAGCCGC
TGCAACGGCTTTTTCAACGGCTCGGGGGCGGTATCGCGCAAGGTCTTGAGGGCAAATTGATCGAGGTCGGCGCTGT
ACATCGCGCGCCCCCGCTCCACCAGGTTATGGGCTTCGTCCACCAGCACCGCGACCCGCCATTGGTTGAGCTGGGC

CAGGCCAAACAGCATTGCGCCAAAATCGAAGTAATAGTTGTAGTCGGCGACCACCATGTCGCACCAGCGCGCCATC
TCCTGGCTCAGGTAATACGGGCATACACCGTGGGCGAGCGCGACCTCGCGCAGATTGCGTTGGTCCAGCAACCGGA
CCTTGGCTGCCGCCTCGCGCGCGGCCGGCAGGCGATCATAAAAGCCCTTGGCCAACGGGCAGGAGTCGCCGTGACA
GGCCTTGTCCAGGTGTTCGCACGCCTTGTCCCGCGCCACCAACTCCAACACGTGCAAAGGCAGGTCGGCACTGGCG
TGCAGTATCTGCACCGCGTCCAGCGCCAGCTTGCGGCCAGGGGTCTTGGCCGTCAGAAAGAACAGCTTGTCCAGTT
GCTGCGGCGCCATGGCCTTGAGCATCGGAAACACCGTGCCAATGGTCTTGCCGATCCCCGTGGGCGCCTGTGCCAT
CAGGCAGCGCCCGGTGCTGACGGCCTTGTACACCGACTCGGCGAGGGTACGCTGGCCCAGACGAAAGGCCGCGTGA
GGAAAAGCCAGAGCGGTGCTGGCGGCGTCGCGGGCCAGTCGCTGTTGCGCCTGCTGCTCGGCCCAGCCGAGAAACA
ACGCACACTGGCGATTGAAGAACGCCTCCAGGTCATCCGCCGCACACACCTGGCTGATCACGGTTTCGCGCTCGCT
GACGATATCGAAATACACCAGCGCCAGCGCGATCTCAGGCAACCCCAGCTTGCTGCACATCAGCCAGCCATAGACC
TTGGCCTGGGCCCAGTGCAGTTGCCGATGGTTGGCCGGTTGGGCATCCAGGTCGCCACGGTAGGTCTTGACCTCTT
CCAGCACATTGCGATCCGGATCATAGCCATCGGCGCGACCGCGTACGGTCAACTGCTGGTAGAGGCCTTCCAGGGC
CACTTCACTCTGGTAGTGCGCGCTGCGGCGGGACGCCACGGTACGGTGGCCGAGCATGCCTTCCTGGGCACTGGGT
GAGGGGGTAAACCGCAGATCGAGGTCACCCACCTTGGCCGTGAACTCGCACAATGCCCGTACAGCGACGGTGTAGC
TCAAGCGCCCTGCTCCGCCCAGCGTACATAACACACGGTAACCGGCATCTGGTGCTCGCTGCAGAACTCCAGCCAG
CGCAGTTGGTTGTCCTGCAGGCGGTCGCCCGGGCCTTTGACTTCGATCATGCGGTAGGTTTTTTGCGCGGGCCAGA
ACTGGATCAGGTCTGGCATGCCGGCGCGATTGGCGCGGATATCCAGCAACAGCCGGTCGAACCAGTGCCGCAGGTG
CGCGGCAGGCAGGCAATCGAGCGCCTGCTCGAGCAGTGTTTCGCTCAATACATTCCAGAACACAAAGGGTGACTGG
ATCCCCCACTTTTCGGCGTAGCGCTGGCGGATCGTGGTTTTGTAGCGCTGGTCGTGCAACTGGTCGAGGCAGGCGG
CGAACAGCTCGGCGCGGCGCTGCTGGAAGTCTTCACTGTGCAAATCCACCGGCCCGCGCTGGAATGGGTGAAAGAA
CGAACCGGGCAACGGCGCGAAGATCGCCGGCCAGCACAGCAGGCCGAACAGCGAGTTGATCAGGGTGTTCTCGACG
TAGTGCACCGGCCCCCCATCATCCCCCAGGTGCGCCTGCACGCAGAACTCAACGCTGGCCACCGGTTCCGGGAACG
GCAGTTCCAGGTCCAGGCGCTGAATCACCTGGGGTTTGGGCTTGGGCAGCGCCGGCTCGCCCAGCTTGCGCCGCAA
CCTCGGCAGGACCCGCAACAGGTGTTGCTGCTCGGCCGCGCTTTGTGGCGCGGCCTGGGCCGCCTGGGCCAGGGCC
ATCGCCTGGGCGAAATCCTCCTGGCGCTCCAGCACCCGAATCAGCCGGGCGCGGGCGCCGGGGTAGGCACAATCGC
GGTAGATCACCTCGGCCAGGGCCAGTTCGGCGAGCCGCTCGCAATGCTGGCCAACCTGGAACAGCAGCTTGGCCCG
ACGCTTCTCCAGCCAGGGGTTATCGGTGCTCAGCGTGGCGATCTGCGCCAGCACCTCTTCCAGCGGTTCGCCCGCC
TCGAAGGCCTGCTGGCATTGATGCAGGAACAGCAGGCCATACACATCGTCACGATGGCGCAGCCCTCGGGACTCGG
CACAGAACTCGACCTTTTCGTAGGTGTAGATCCCCAGGTCCGCCAGTACAAACTCCGACCAGTCCTGGTACAGGTT
GCCGAAGAACATCAGGCGCAGGCGGTCACACAGGTCCATCACCGCCAGGCTGTAAAGACGCTCGTCCAACTGCGGG
CACCATTGCGCAAACGTACGTGGCTCGGTGAACCGGGCCGATAAACCGGGCAGCCAATCGGATTTCTTGCCCCTGG
GCTGCTCGATCCAGGGCTTGAACGCCTGCAGCGCCTCGCCTTTTTGCAACTGCCCCCACACCTCATCCAGGGACAG
CAGGCCCTGCTCACTGACCCAGCCCAGGGCCAGCAATGGTTGCACGGCCTGGTCAGCCGGGCCAATCTCGAGGTAG
TTGAGCTTGCTCGCACGAAAATGCACCCCTTTGCGCATCACCATGCGCACCAGCAACGCTTGGGCCGGTTGCGGCA
CTTGGCTGAACTGCGCAATGAAGCGCTGTTCTTCATCATCCAGCACGTCGGCATAGCGCTGCCCCAGCCAGTCGAG
CACTTGCTGGAAGTTATGCAGGTAGTAGAACGGGTCTTCGAGGGGGGTTGGACATGACGGGCACTTGGGCAAACACT
GGTTATGCATACAGAGTGCCGTCGCCAGGCCTGCCTTGCAATCGCTAATAGATCAGTGGCACACGCAACTTTTTGC
ATAAAAGTGATCAGATGGGGGTGGATCCGCGTAACATTTGTGCCCCGCAGCCGTTGAGCCCGGGGTTTTCGAAGGA
TGAGAGTAAAGGTTATGAACATGAAGAACTGGGGCCTGCCGCTGGCTGTACTGACGTTTCTGGCATTGGCCGGTTG
CTCGACGCCGACCGTCGTGACCTTGCAGAACGGTACTCAGTACCTGACCACCGATACGCCGAATGCCAATACGCCT
GATGGTTTTTACGAATTCACCGATATCGCCGGCAAGCGCGTACGGGTCAAGGCGGCGGATGTCGCTACCGTCCGCA
AGGAAAAAATGACCAACCACTGTAGGAGCCGGCTTGCCGGCGATGGCGGCCTTGGAAGCAACCAAGAGCCTCATCGC
TGGCAAGCCGGCTCCTACAGTTTTAGGTGATGACGAAGGCCTCTGCCGACAGGCTGCCAAGTCCCACTCCCACCAG
CGTCACGGAATTATCGCCGAACGTCAGCAACGTATCGCTGCCCACTGCCTTGGCATGATCGAGGTAGTTGTACTGC
CCGCCCACCCCCGCCACCCCCATGAACACCAACTTGTCCGTCGACTGGTAGCCCAGGATCCGGTCCTGGCCGAAGT
CGCCGTCGAACAGGAAGGTATTGCGCCCGCCGCCCGCTTCGAGCAGGTCATTGCCCGCGCCCCCTACCAGCACATC
GTTGCCTTTGCCGCCGATCAGGTGATCATCGCCCTGCAGCCCAAACAACCACTGCCCGCCATCGCCGGCCTTGAGC
ACATTGGCGCCGGCATCGCCCTTGAGCGACGCGGCGTAGGCGGTCAGGTCCTTGCCCGCCAGCAGCCCCTTGTCGG
TCACGCTGTGGGTCACGTTCTTGCTGAACAACAACAGATAGCTTTCCTTGCTCACTAGCGCGCCCATGTCGCGCGT
CATGCTGATGCCGCCCTGCCCGTCGCGGATATACAGGGTACCTGCGCCATCGTTGGCCACCTCGACGTTTTTCAGC
GACTGCTGCAAGTCCAGGGTATTGAACCCCGTGCCACCGAGCAGGATGTTGTAGCCACCGCCATCGCGGAAAGTAT
CGTTGCCGGCACGTCCTTCCAGGTAGTCATTGCCGCGCCCGCCCTGGATCAGGTCGTTGCCGTCGCTGCCGATAAT
GAACGTGCTGCCCTGGTGGACCTCGGCGTTGCGATTGAGGTCCTGCACCCAGGTAGTGGCACGCGCAGAGTCAGAC
AGGTTGGCGACGATGATCGTCGAATCGCGGCTGGTCAGGTCATAGAACTTCGACTCGAGGATGCGGCTCATGCCAT
CGCCATACCCCGTGGGTAAATGGGAAATCCACGTTGGCAGGTTGAGGATGGAAAACGGCAGGATGTTCCACAGCGT
CGAGGCGTAATGGTCGTTGAAGCTTGCGATGTTATCGGCGCTGGATTCGTGCGGTTGGTCGTGCACGCCCAGGGAA
GACAGATTGAACGCCGAACCGTCGAGGGCGCGATAGACCGGGTCGTTTTCGTAGCCGATGTTCAGCACCTTGTCGC

CACTGCTCTGGGTCGGCGAGGCGTAGGCCACATAGTTGGCGTCCTGGTAGAACCCCGCCCAGGTGTGGTTGCTCAA
GTCCGCCATGCTGTTGACCGCCATGCCACCGAGGCTGTGGCCGCTGACCAGTACATCCTGGCCGCTCAGCCCCTGC
GCGCTGGCATACGCGGCGACTTTGCCAAGCAAGGTGCCAAACGCCTCGCCCGCGTAGTTTTTTGCATAGTCCGCCG
GCCCGAACGCGGCCAGTAAATCACTGATCACATCGCCAATAGAGTCGACGATCAGGTTCTCCCTGGGGCCGGATGT
GCCACGGAAGGCGATGCCGATTTCCAGCAGCTTGCCGGCGTCATCGTATTTGCCCAGGACCTCGGCCTGGGCACTG
GTGTAGCCGGTTTTCTCGCCGAAGAAGGTGCCCCGCGCATCGACCTTGCCACCATAGTCCAGGGTGCTGGCGCTGA
TGGGCGTCCAGCCGGCCTTTTGCACGGCGTCCAGGGCGGCCTTTTCCGAGTCCGGGTTCCACGGGATACCGGGAAT
CACCCCCTGGGAGTCGCTGCTGCCCAGCAACGCCTGCACCAGGGTGGCCGGCAAACCCAGGCCGAAGCCGTTTTGC
TGATACCCCACGGCAAAGCCGTTATCCAGGTTGTGGTAGGAATACAACGTGATCGCCATGGCGTCGGCGAACAGGG
CTTTGGAGTCCTCAGTGGTTAGGTTTTTGTAGTCATAGACACCCATGGTATTGCCTCTCTTTTGTTGGAATTGTCG
AGAAAGCTAAAAATCCTGAACATCACACCGCCCCTTGTAGGAGCCGGCAAGCCGGCGCCTACAGGGTAAAACTCAG
AACGCCCAGTCGAGGCTCAAGCCCACGCCGTGGATTTTCTCCTTGCTGGCCAGCAGGCCGTTGTAGTCAAAGTTCA
ACCGCGCATCCTTGCTCAGCGCCAGGCTGGCCCGTGCGCCGACCAATGCGGCATCGCGTACCATCGGTGCGCTTTG
CACGGCAAACGCGCTACTGCCTGAGGCAAACGCCAGGTGGCGCGCGGAATCGGTGGCGCTGAGGTTGTGCTGCCAC
CCCAGGGTGCCGGAAACGTCCAGAGCCTGATGGTCGGTGAGGTTGAAGGTCTTCAAGGCACGCACGCCGAGGGTGC
TCAGCACCAGGTCGCGTGTGTCATCGCCGCCCTTGAGCGCCGCCGCGTCGCCTTTCTCGCTGAAGCCATCGCTGTC
CAGGTGCACATAGGCCAGGTTGGCGAATGGTTCCAGCGCCAGCGGTTGCAGGTTCAGGCGATAAGCCGCTTCGGTG
AAGAGCTGGGTGCTGCGGGCATCGACCTTGGCTTTCTGCTTGCCGGCGACTTCGCCGTATTGCAGCTCACGCTTTA
CATCGGCGCGATGCCAGCTGTAGGTCGCCCCACCGCTCAGGCGCCAGGCGCCGATTTCGTGCCCGGCATAGGCGCC
CAAGTGGTAGCTGTCCACCGATGCCCGCGAGTGGGTGCCATCGCCCATGTTCAGCGAGGTATCGCTGTAGCCGGCG
ACCAGCCCCAGGCGCGTGTCTTCATCCAGCGCACCGTCCACACCCGCGAGCAATCCGCCGATGGAAGTGGTGTAGC
CCGCCGTGTCGCTGCTGCTGTCGGTTTTACCCCAGACACCAAGCGCCTTGACCCAGACATTGCCGCGGGTGTCGAT
GGTCTGTGGCGAGGAGCCCATTTCGCCCTGCTGCAGGCGCTCGCCTACCGCTTCGCGCAGGTAGCGGTTGTCATTG
ACCAGGGCGGTTTCCAGGGCCGGGTAGATTTCCCCCGACAACTGTTGGAACGCCCCTTGCGCCGAAGCCGCACTCG
GCGCCAACAGCAGGCTTTCATACACGCCATTACCCGCGCCCAGTTGTTCGGCAGCGCCCGCCACGGCACGCTGGTT
ATCGGTCAATGCCACGCTGGCGAAGCTGTTGGCAGTTCGCGCGACATCCAGTTGCACACCGTTGGCGCCATAGTTC
AGGTTGCCGCCCACAAACAGGTAGTTGGGCAGTACCTGCGCGAAGCTACCGGTGACGCCCCCTGCCGCCTGCAGGA
TGGTGTACTGGCGGCCGATCAGGCTTTGCGCCTCGGGCTGGCTGAGCAGTGTCGGGCTGTTCTCCAGGGCCAGGGT
CACAGTGCCGCCGTTGAGGGTGGCCTTGCCGCCGGCGACGATGCGGTCACTGCTGCTGTTCGACAGCTCTACCGCA
TAGGTGGACCCGGCGTCGAAGTTGAGATCCCCGGCCACGTTCAACGTCCCAATGGAGTTGCCCGGCGCTACCGTAC
CGCCGCTTTTCGCGTGCAGCGCGCCGATGCTGCCGGAGCCGCCCAACACGCCGCTGTTGCTCACCGTGACCTGGGA
TACCAGCGAGCCATTGACCGCCAGCAGGCCCTGATTGACCAGGGTCGGCCCGCTGTAGGTATTGCTGCCGGTCAGC
ACCAGCGTGCCGATGCCTTGCTTGGTCAGGCCGCCATGACCGGAAATGTCGTTGCTCCATACATCCCGCCCGCAGT
GCAGATCGTCGCATACACGCTCGGTGGGCTTGCCGGCGTCGACCACCGCCCCTACCCCCGGCAAGTCCGCGACAAA
CTGGCCGCTGCCGTAGCCGCCCTCGACGCGGAACTCGGCCGGGATGTCCTCGGCGGTGACCAACATGCCCGGCCCG
CTGACCGCCTTGCCCAGGTTGATCATGCCCCAGCCGTACAGCGCGTCGATGCCCGGTGCACCGAGGTCGGTGGCGG
TGGTCTTGAGCAGCGTGGAGATCTGCTCGCCACTCATATACGGGAAACGCTCCATCAAGACGGCGGCGGCGCCTGC
CACGTGGGGAGCGGCCATGGAGGTGCCGTTTTTATTGTTCCAGCCCAGGGTCATGTTATCGACCGTGGTGCCCACC
ATCACCGAGCTGTAGATCGCCGTGCCCGGTGCTGACACACAGAAACTGGCGGCATAGCCGCAACGGGACGAGAAGG
TGCTGATCACATAAGGGTTGGGGCTGTTGGTGTCCGGGTTGCGTTGCAGTGCGGCCACCGACAGCCAATTGGGCGT
GATCTCGGGTACGAAGTAAGCCAGGCCGGAAATCGCATCAGGGTTGTTCAGGTTGTAGTCGTTGCCGGCCGCGAAG
ATCGTCAGCACCCCGCTGCGCGCCGCGTTGATCGCCCCGGTGTAGGCGCCACCTGGCGCGGTACCCAGCAACGGCT
TGATCTGGTCGAACTGGGCCTGCGCGTCGCTCAGGTCAAAATGCGCGAACGCAGGGTCTTTGCCGCCCTTGGCAAA
CTGCTCGCCAATGCCGATCCCCCAGCTGTTATTGATGATGCGTGCGCCATTGGCCACCAGGCTGTCCCAGCCGGCC
TGGTACACCGCACCGTCGTTGCCCAGGATGATGCCGTCTTCGGGGCCCGGGTCGCCGTTCTCGGCACTGAGGATCT
GCGCGTTATAGGCCACGCCGTGCATCGGCCCGCCGTTGCGGTCGCCAGCCGCGATGCCACCGACATGGGTGCCGTG
GTCGCCCAGCTTGCCGTTGGAGCCCTGGGACGGTGTACCGTCATAGCGGAACGGGTCACCGGCCTTCACCGGGATA
TAGGGGTCGGTGTATTGGCGGATCCCCTCAGTGACCAGGGTCTGCACCTTGCCCGGGCTGGAGAACTCGGGATGCT
GGGCATATACCGGCTGATCGAAAATCCCCAGCTTCACGCCCTTGCCACTGTAACCGGCGGCATACGCCACATCGGC
ATTGACCGCCCCCAGTCCCCAATCGGCCTTGAACTCATTGCTGCGCCAACTGGAGGCATCCCCCAGTTTGCCGGCT
TCCACGTAGGGTGCGGCCTGGGCGGTACCCAGGCTGGCCAGGCAACACAGCAACGCGCCGTATGGCACGCGACTCA
AGGCTTTGAGCGGGTAGCCCGGGCCTGTTGGGATGGCGGAGTGAAACCCTCGTTGTTTTGCGTTCACGGTGACCTT
CCTTAGTTTTGTTGCGTTTGTTGTTCTTATTCACCCGCCAGCAAGAGCCGGCGTACCGGCTCCTGCCGCAAGTTTA
GAATTGCCAGTCGACGCTCAGGCCCACCCCTTGGTTGCTCTCCTTCGAGCCCAGTTGGCCGCTGTAATCGAGGTTG
ACCCGGGTGGAGTCACTCAATGCCAGGCTGGCCTTCAAGCCCACCAGCGCGGCGTCGCGCTGCAACGGTGTGCTGC
GCACGGCAAACGAGGGGCCGCCAGCGACAAAGGCCAGATGTTCCTCGGACTCAACCGGCGTAAGGCTGTGCTGCCA
GCCCAGGGAACCGGAGAGTTCCACGTGCTGCTGGTCATTCAGAGACAGGGTTTTGAGCGCGCGCAGGCCCAGGGTG

CCGAGGGTCGCGTCACGCCGATCACTGCCGCGTTGCAAGGCCGCGCCATCACCTTTTTCATGCAATCGCTCGCTGT
CCAGGTGCACGTAGGCCAGGTTGGCGAACGGTTCCAGGGCCAGGGCTTGCAGGTCCAGGCGGTAGGCCGCCTCGGT
GAACACCTGAAGGCTGCGCGCATCCAGTTTGGTTTTTTGCTTGGCGCTGACGTCACCGTATTGCAGGTCGCGCTGC
ACATCGCCGCGATGCCAGCTATAGGCACCGCCCACGCTGACGCGCCAGTTGCCCAGTTCACGCCCGGCGTAGGTGC
CCAGGTGATAGCTGTCGACATCGGCCGACGAATGCGTGCCATCGCCCATGTTCAGCGAACTGTCGCTATAGCCCGC
CATCACACCCACGCGGGTCTGCTCGTCCCACTGGCCATCGACGCCCAGCAACATCCCGCCGATAGAGCGGGTGTAA
CCGGCATATTCGCTTTGGCTGTCGGCCTTGCCCCAGGCACCGAGGGCCTTGATCCACAGGTTGCTTTCGCCCGTCA
CAGGCACATGGCGCAGGCGCTCACCGACGGCATCGCGTACATGGCGGCTGTCGTTGATCAGCATGGCGCCCACCGC
CGGGTAGATCTCGCCAGATAACTGCTGGAAGGCCTGGCGGGCAACCCCGCTGGAGTCGCTGCTCAGCACGCTTTCA
TACACAGGGTTGCCAGCGCCCAGCTGCTCGGCCGCAGCGGCCACGGCGCGCTGGTTACGGGTTGCGGCGACGCTGG
CAAAGCTTGCGTCATTGCGCGCGATAGCCAGTTGCACGCCGTTGCCGCTGTAGTCGAGGTCGCCACCGAGGAACAG
GTAGTTGGGCAATACCGCACCAAAACTGCCGGTGACCCCGCCCGCCGCCTGCAGGATGGTGTACTGACGGCCGATC
AGGCTTTGCGCCTGGGGCTGGCTGAGCAAGGTCGGGCTGTTTTCCAGGGCCAGGGTCACGGTGCCGCCGTTGAGGT
TGGCCTTGCCGCCGGCGACGATGCGGTCACTGCTGGCGTTCGACAGCTCCACTGCATAGGTGGAACCGGCGTCGAA
GCTCACATCCCCGCTCACATTCAAGGTGCCGATGGAATTGCCCGGCGCAACCACACTGCCGCTGTTGGCCTGCAAC
GCGCCAATGCGCCCGGAACCACCCAAGGTCCCGCCATTGTTGACCGTGACCTGCGATGCCAGCGAACCATCGACCT
TCAACACCCCACCATTGACCGTGGTCGGGCCGCGATAGGTGCTGTCGCCGCTGAGCACCAGGCTACCGGCGCCGGA
CTTGATCAGGCTGCCTTGGTAAATGCGCTGGGCCGCGGCCGCATCCCGGGCCATGCCCACGGCGTAGTCAGTCTGT
TCCTGCTGGCTGGCGCCGGCGCCGATGCCATTTTCCCAGCCTTTGTCCTGCAAGGTCTGTTGCCAGGCCAGGTGCT
CGGCGCTGTCTTCGGCCTGGCGCTGCAGCAGCGCCTTGTCGGAGATGCCATTGCTCCATACATCGCCCTGCCCGGC
CGCCAGATTGACGTCCATGGCGCCCAGCAACTGTCCCGGCCCATGCATCGCCCGGCCAAGGTCCGGCACGCCCCAG
CCGATCTTGGCGCTCGGCGCATCGGTCAGTGAACCGTCCAGTTGCGTGGCCGTTGTCAGCAACACCTGCAAGGCTT
GCTCGTTGTTCAGGTACGGGTAGCGTTCCATCACCAGGGCCAAGGCCCCCGTGGCATGGGGCGCCGACATCGAGGT
GCCGGACTTGACCGCATAACCGCCGCCGGGAACGGTGCTGTCGATCTTCGCGCCGGGGGTGGAGACGCACCAATAC
TTGGCGATGCCGCACTGGTTGTACTTCTGGTTATTGGCCTGGTCCAGGCCCGAGACCGCCAGCCAGTGGCCTTCCA
GCTCTGGCTCAAAGTACGGCAAGGCCGAGCGCACGCTGGCGTTGGCATAGCCGCTGTTACCGGCGCTGAACACATT
GATCACCCCGGCTCGCGCCACATTGGCGGCGGCGTCCAGCCAGGTGCCCTGGTTGAAGTGCTGGGCATAGGCTGCG
TGCAGTTCATTCAAGGTCTGGTAGCTGACATCCTTGGGCTGGCTGCCCCAGCTGTTGTTGATCGCGCGCACCCCGG
CATCGACCATGGCGCTGTACACCGCCTTGAAGTACTTAGGGTCGGGCGTCGGGCCAAACAGAAAGCTGTCGTTGGC
GTTGGTGTTGCCCACATAGAGCTGCGCGTTGTAGGCCACGCCGTGCATGCCGGTGCCGTCACGCGCAGCGGCCATG
GTGCCGGTGACATGGGTGCCGTGGGAGTCGTTATTGGGGTTCAGCGCACCGGTAGTGGAGAACGGGCTGCCATCCA
CATCGGTGCCGTTGGCGGTCACCGGGTGGAAGCGCGCCGGGGAGGCTTCCGGGTGGCTGGCATCGAAGCCCGAGTC
CAGGGCGCCGATCTTCACCCCGGCACCGCTGATTCCCGCGGCGTAAGCCTGATCGGCCTGCATGCGCCCCAGGCCC
CAGTCGCGCTGGAACTCCGCCGAGCGCCAACTGGCGGCGTCGCCCGGCTTGCCCACTTCCACGTAGGGCGCGGCCT
GGACGGTACCCAGGCTGGCCAGCCCACACAGCATGACGCAGTGATAGCCTCGTTGTTTCGCATTCACGGTGACCTT
CCTCATTTTTATTGTGTGGGTTATCCCCTGTAGGAGCCGGCTCCCGCAGGGTCAGCGTTGGGCTGGGCCGAAGGCT
TCGTCGACCTTGGCCAGGTCGGTGTCACGCAGATTGCCCGCGTAGTAGTGCAACTTCGTCCAGGCCATCAGGTAGT
CGTAGCGGGCCTGGGCCAGGTCGCGACGGGTGCTGTACAACTGCTGTTCGGCGTTGAGGGCATCCAGGTTGACCCG
TTCGCCACCGAGGATGCTCTGCTTGGTCGACACCACCAGCGCCTCGGCGGAGACCAAGGCCTTTTGATAGGCCCGC
AGCTTGCTCACCCCCGACAGGCAGGCGCTGAACTGGCGACGCAACTCGATCAGGGTTTCGCGGGTCTTGCCCTCCA
GTTCGTACTCGGCCTGCTCCATGGAGCGGGCGGCCTGGCGGGTGGACGCCGAGACGCCGCCCCCGGCATACAGCGG
CACGCTGACTTCAATACCGATGGTGTTGGTGTCGTAGCGCTGGTTGTAGGTGTTACCGCTGTCGGACTCTTGCTGG
CGCGAGCTGGCGTACGCGGTGATCCGCGGCAAGTGCCCGGCGCGGTTGCGCTCCACCTCATAACGCGCCACTTCCA
CGGCCTGGCGCTGGGAAGCCAGGGTCGGGTTGTTGGTGATTGCCAATTCGTGCCAGCTGTCGTAGTTGGCCGGGGC
CAGGGTGAAAGCGGCAAAGTTCTGATTCAACGGTGCCAGGTCGGCAATATCGACACTCTGTACGCCGAGCAACGCC
CCTAGTTCGCGTAGCGACGCGTCCTGCTCGTCGAGCGCCTGGATCTCCTCGGCGGTCGCCAGTTCATAACGGGATT
CGGCCTCCAGGATGTCGGTGCGCGTGCCCTCGCCCTGCTGGAACAGATGCTGGTTCTGCTGGAACTGTTGCTCGTA
GGCTTTCTTTTTTGCCCGGGCGATATCGATCTGGTCCTGGGCGAACAGGGCCTGGGTGTAATACGTCAGCACCCGC
ACCAACAGCGCCTGGCTCTTGTCGCGGAAACTCTCGTCGGCAAACAGCGCCTGGGCCACGCCCTTGCGGTAGTTGG
CATAGGCTTCGTAGTCGAACAGCGGCTGTTGCAGGGTGAAGGTCGAGCCGAAGCTGTTGTAGTTGCGGTCGTCGTG
ATAGTGGCCGCCACGGCCATCGGGCAGGTTGGCCTGGGAGTTGTTGCGGCCCTTGTTGTAGTTGTACGACAGCCGC
GGCAACAGACCGGCGCGGCCAATGGTGCGGTTTTCCAGGCCGGCGTCACGCTCCTTGATTGCGCCGAGGAACACCG
GATCGTTACGCAGGGCCTGTTCATACACATCGAACGGGCCCATGGCGGCCTGGGCGCTGCTGCAGGCGAGGAGCAA
GGCGATGAATACGGGCTTCATGCTCATTCCTCGGTCAACGCCGAGCCGGCCCGGTCGAGCAGCGGCTTGAACAGGT
AGTTGAGCAGCGAGCGCTCGCCGGTACGCACAAACATCTCGGCGGGCATGCCCGGCTTGATCACCAGGCCCTGGAG
CTTTTCCAGGGAAGCATCGCTGACGGTGGTGCGCAGCACGTAATATGGCGCGCCGGTTTTTTCGTCGAGCAACTGG
TCGGCGGAGATCAGGCTCACTTCTCCCGGCACCCGAGGCGTGCGGCTCTGGTTGAAGGCGGTGAACAGGATATCTA

CCGGCAGATGGCTGCCGACCTTGTCCACCAGGTTCACCGGCAAACGCCCCTCCACTTCCAGGCGCGTGTCCTGGGG
CACGATTTCCAGCAGGGTTTCCCCGGCCCGCACCACTGCGCCCTCGGTATGCACCCCGAGGTTCACCGCAATGCCA
TCGGCCGGCGCGTTGATTTCACTGTGTTGCAGGTCAAAGCCGGCGGAGGTCAACTGCTGCTCCAGGGTCAGGCTGC
GCAGCTGTGCGTCGGCCAGTTGGCTGCGCACTTCCTTCTGGTACTCCTCGCTGTGCTGTTGCAACTTGAGGCGCGA
TTCGAGGATGCCTTGCTCGATACGCCCACTTTCACCGGTGTTCTGCGCCAGGTCCTGCTGCACCTGGGACAGTTGG
CGCTGGTATTCCAGCAGGCGATTGCGCGGGATATAGCCGTTGTCGGCCAAGGGTTGCAGGTTGGCCAGTTGATCAC
GTAGCGATTGCGCCTGGGCGCTCAGGTCAGTGCGGGCGCGGCGCATGCCGTTGAGTTGGGCGCTGGCGCCTTCAAT
ACTGGCGCGGATCCCCGACTGCTCGCGGGCAAAGGCATCACGCCGGCTGCTGAACAGCTGACGCTGGCCTTCAAGT
ACCAGGGCCAGCGCAGGATCGGCGGCGCTACTTAGTTCAGCGGGAAAGACCACCGCCGGTAAGTTGTCGCGCTCGC
TCTGCCAGCGGGCGACGCTGGCCCAGGCCATGCGGTATTGCGCTTGCAGGGAATGCACGTCGGCCTGGCTTTGGGT
CTGGTCGAGGCGAAACAGCGGCTGGCCCTGTTTGACCACCTCGCCTTCGCGCACCAGGATTCGGCTGACCACGCCG
GGGCTCAGGGTTTGCACGGCCTTGCGCTTGCCGGACACCACCACCGTGCCCTGTACAGGAATGCCCTGGTCCAGGG
GCGCCAGGCTGGCCCAGAGGAAGAACCCTCCGGCGCCGACCACCGTCATTACCCAACCCAGGCGGGCGAAAAAGCG
CGCATCGCGCTCGGGGAAGATCTGTTCGTTTGGCAGGCTGTTATCGGTACTCATTGGCCAGGATTCCTTGCCGCAG
GATATTGACGGCTGAGGCTGACGCCGGCCTTTTCACGGGGTGGTTCCGCTTGCCCGGACAGCGCCCGCAGCACCTC
CTGGCTGGGGCCGAATGCTTGCAGGCGACCTTCGTTGAGCACCAGCAGCTTGTCGGCCTGGGCCAGCGCCGAAGAG
CGGTGGGTGACCAGGATCACGCTGCTGCCCTGGGCTTTCATCTGCATGATGGCGCTGGCCAAAGCCGCCTCGCCCA
CGGTGTCGAGGTTGGAATTGGGCTCGTCGAGCACGATCAGGCGCGGCCCGCCATACAGCGCGCGCGCCAGGGCCAC
CCGTTGTTTCTGCCCTCCGGACAAACCGCTGCCGTCGTCGCCCAACACGGTGTCGTAGCCCTGCGGCAGGCGCAGG
ATCAGGTCGTGCACCCCAGCTTGCTGGGCGGCCTGTACCACCCGCTCCGGGTCTGCCTGGCGAAAACGCGCGATGT
TGTCGGCGATGCTGCCGCTGAACAGCTCGATGTCCTGGGGCAAGTAACCGACGTGCGGACCAAGGTCGTCGCGGTC
CCAGCGATGGATATCGGCGCCGTCCAGGCGCACCGTGCCGGCCAGGGTCGGCCACACGCCCACCAGCACGCGCGCC
AAGGTCGATTTGCCGGACCCGGACGCCCCCAATACCCCCAACACTTCACCGGCCGCGAGGTTGAAACTGACCTGGT
GCAAAGTGGCCACCCGCCGCCCCGGAGGGCCGGCGCTGACCTGCTCGAAACTCACCTGGCCCTTGGGCGCCGGCAA
CGCCATGGCCTGCGCCCCCGGCGGGAACTCCTGCAACAGCTCATCGAGGCGCTGGTAGGCCAGCTTGGCCGAGCTC
CATTGCTTCCACACCGCGATCAACTGGTCGATCGGGCTGAGCACCCGCCCCATCAGAATGGTCCCGGCAATCATCA
TCCCGGCGGTCATCTGGCCCTTGATCACCAACAAGGCACCGAGCCCCAGCACCAGCGATTGCAGGAGCAGGCGCAA
GGACTTGCTCAGGGAGCTGATCACCGAACCGGTGTCGCTGGCCTGGTTCTGCAAGCCGAGAAACCGCGAATGCACC
TGGAACCAACGAGCGCGCAGCGCCCCCAGCATGCCCATGGCCTGGATGGTCTCGGCGTTGTGCAAGTGGCTGGTGG
CCAATTGGGTGGACTGTTGCGAGTAGCCGCTGGCTTCGCGCAACGGTTTTTTGGTCAGGTATTCGTTCAGGCATGC
CAGAGCGATCAACAGCACGGCCCCAGCCGTGGCCAACACCCCAAGCCACACGTTGAACAGGAAGATCACCAGCAAG
TAGATGGGGAACCACGGCGCATCGAAGAACGCAAACAACGCCGGCCCGGTGAGGAACTGGCGGATATGGGTCAAGT
CGCCCAAGGATTGCCCGGCGTGGCCTTCGCCCCGTTGCAGGTTGCGCTCGAAGGCGGCCTTGTACACCTGCAGGTT
GAACCGGCGCTCCAATTGGCTGCCGATGCGGATCACGATAAAGCTGCGCACCACTTCCAGGGTGCCGATGAAGGCA
AAGAACCCCACCACCATCAGGGTCAACATCACCAAGGTGGTTTCGTTCTGCGATGACAGGACCCGGTCATAAACTT
GCAGCATATAAATGGACGGCACCAGCATCAGCAAATTGATCAGCGCAGTGAAACAACCGACGCTGATCAGGATACT
TTTATAATCGCCGAGGGCTTTAAACAGTGGTGCAGTGGGAGTGGTCTTCGCCATGTCGCCTGATCTTCCCTGGACT
GAAATACGTGCGATAGTCTGCCGAGCGCCCCACTAAAGGGGGCGGGCAACTATTTATAAGGTGTTTAATACGTTGG
AAGTGACAGGTTGAGATAGGTCACGCCCAGTAAATACCCCTATATCCGGCGGTTATAAATCTATAACCGCATGACT
TTATTATTGTGTACGTTGCAGTAACAGCTCCAACCCAGTAGCAGAACGCGCGGTATATTCGCCTTCTTTCTGCCGG
TTCAAATGGGTTATTCCGGTGCCCTCGGCATTCATCAGCCAGATACCATCCGGGGTGGGCAGCCAGGTCAGGGGTG
TTTGCCCCAGCCACTGCTGCGCACAATCAATGTCACCGGCCAGGCTGTTGGGTTGAGCGATCAGATCCAGCTTGCA
CACCTGCTCTTGCCGGCGCAGTTGCCAGTGCCCGGCCAGTTGGGCGGTGCTGGGTAAAACAGGAATCCTTGCCATT
GCGTTGGCTCCTACCGACACGAGCAACGCCGGGATCAACCAGGCGATTGCGTTACGAAAAATAGTCATCAACAGTA
CCCGGTTTGTGGTGAGCAGCACTTGTTGTTGTGTGGGGGGTTGCTGTGGCGAGCGGGCTTGGCCGGGAGCTGAAAA
ATGGGACTGCTGCGCAGTCCAACGGGGGACAAGCCCCCTCGCCACGACAAGCTCCACCACCAAAGCAAACGTTTAC
CAAAGTAAGCCCCTACCACAACAAGCAAGCCCCGCTCACCACAGGTCCTACGCCTTGATCAGGCCACGATGTCGCT
GTAGGCCGCCTGGCCCACGGTGGTGACGAGGAAGTCCGCCACGCCGTGCCCGGCGAAGTCCACCGCCAACGTGCCC
AGGTTGGTACCCGAGGCGTACGTCAGCACGGCATCGCCGGCAGCGCCGGTAAAGGCGTTGACGAAATGCAGGCCGC
TGCCTTTGGTGATGCCGGTCAGGTCGATCTTGTCCGAACCCGAGGTGAAGTCAAGGATCTGGTCCGCCGCACCTGG
GCGGGAGTCGGAGCTGGCACCGAACACAAAGGTGTCCGAGCCCGCGCCGCCCCACAGTTTGTCGGCCCCCGCCACCG
CCGTAGATCAGGTCATTGCCGGCCCCGCCCTTGAGGGTGTTGGCCACTGCGTTGCCGATCAACAGGTCGTTGCCCG
AACCACCAAAGGCGTTTTCCACGGTCACGCCCTGGGCGATCGACACGTTACCCACCAGGCCGCCCACATCGGAGAA
CGACGTCTCGTTGAGGTTGATTTTCTGGTTCTGGGTAAAGCCGGAGAAATCCAGGGTGTCGTTGCCACCACCGTCC
CATACCGAGAACACCAGCTTGTCGGCATTGGAGGTGGCGCTGAGGTGATCACGCCCGGTGTTGGAGTTGAACCCGT
AGGTGGTATCGCCGGCACGGGTGTTGTAGTTGGCACCGTAGAGCTTCTGGATCGCCGCGATATCGTCGATCAGCGG
GCCGGACGCATAAGCCTCTACCCCGCCTTTGGTGAAGTTCTGGTTGGTGTTGTTTTCGCCCCAGTAGCTCATCACG

CTGTAGCCACGGGTGTCCTGGCCGTAGTCCGCGTCTCGGTAAGACGGGTTGCCGGTCCCGGCGTTGTAGTCGCCAG
GGTGATCCAGGCCCAGGGTATGGCCGATCTCGTGGGTCAGGGTCTGACGCCCGTAGTTGTTCAGGCCCGGGTTGAT
GTTGGCGGTGTAGCTGTTGTTGATCAGGTACCACGAGGTGCCATCCGTGCCGCTCGTGTGGTACTTCTCATTGGTG
CCGGGCAAGTAGGCAAAGGCAGCGGCACCATCCTGGCCGGCGCTGTAGTTGCCGAAGGTCATGTGGAAGTCGCCGC
CCGAGGCTCTCTCGGTGAAGGTGACATTGGCCACATCGGCCCAGGATTGCATGGCCAGTACGGCCTGGCCTTTTTG
CAGGTTGCTGAACTGGCTGAACCCGGAGATGCCATGCTTGTTCATGGTCGCCGAAGAGGCCGACGTCAGGAAGGTG
TAGGTCAGATCGATCCTGCCGTTGCCGTCAACGTCCCGGTATGCCGCGTTTTCGCGCAGCAAATGGTCGGCTGCCT
GGTCGACAGAGAACGAAGGTTTGCCATTGACCGTGAGGTTGCCGCCGCGGTCGTACAAATGGCTAAAGCTATCGAT
TTGTGAATAGGCACTACTTGCTTGAGCTGCCGAGACAATAGCTTTGTCTTTTACTTTCGACATAAACGTACTTCCT
TGTTTGCAAGTGGAACAGTTTTTGTCCGATATGACGCTCTCTGGCGAGATCGTCCTATCACTCGCCTCTTATGAAG
GCGAAAGAAAACTGACACAAACTTAAATCACACGTCCAGTATTTTTTTTGCTGGGAAAGTGCTCGAAAGGAATAAC
GCCTGCAAACATTGGGGAGGAAGTGTTTATATTATTTGTTTTGGGCTGCTTGGCGGGAAGTTGGTTATTTAAATAT
TAAATAGTGCTAAATAATTAAATGCCAGACAGGTCACGTTTTAATATTGAATGGATCCAATTATTTGATTGGGTTG
CATTTTAAAAGTGTCACCGTGTCATTAAACACAATACATCCTGTGGCGAGCGGGCTTGCCCCGCGCTGGGGTGCGT
AGGAGCCCTAAAAGCAGGCACTGCGGTTTACCTGAACGTCCGTGGAAGTCCTATTGGGGCTGCTGCGCAACCCAGC
GCGGGGCAAGCCCGCTCGCCACAACAAGCCCGCTCGCCACAGACGCAGGCGCCCCCTCAACCCTTTAGCGCCATCC
CGTCTGGCGCATTGCCTCCAGCAGCGTCTGCCCATCCAACCCCGGCACCAGCACGCCATCGGCAGTGGTCATGTCT
TCCCCGGCGAGGATCGCATTAATGATCGCCTCTTCCACCGCCTCGGCCGCGGCACTGAACAATGGCGAAATATGAT
CATTGTTGACCATGTTCAATGCCGTACTGAGGGGCAAGTCCTTGCGCCCGTAATCCGCAGGCGGCAGGTCATGGTT
GCCGGTGGCGAACGCCAGGAACAGATCGCCACTGGAATCCTCGGTGCCGCCGCCGGTGCGGGCAATACCGATGGAG
GCGCGTTGTGCCAGGCGCTGGCATTGGTGCGGCAGCAACGGCGCATCGGTGGCGATGATCACCACGATGGAGCCCA
TGCCCGGGGTGCCACGGTCGGCAAACGGCGAGGCAATTTCCATCAAATGGCGCCCCACCGGATACCCATCCACCCG
CAGTTCCTGGCGCTTGCCGTGGTTGGCCTGCACCAGCACGCCAACGGTCCAGCCGCCCTGCTCCGCCGGCAACTTG
CGCGAAGCGCTGCCAATGCCGCCCTTGAACTCATGGCAGATCATCCCGGTGCCGCCGCCCACCGCGCCTTCCTGCA
CCGGGCCGGATTCGGCATTGGCCATGGCCTGGAGCACATGCTCCGGCCCGATGTGCTGGCCCCAGATATCGTTGAG
CAGGCCGTCATAGGTCTCCATCACCACCGGCATGCACCAGTACACCGCCGGGTCAGCCAGGCTGTCGCGCTCCAGG
GCAATCAGGGTGTCACGCACAATGCCAATACTGTGGGTGTTGGTGATTGCCATGGGCGTGGTCAGCAGCCCGGCTT
CGCTGATCCATTCCAGGCCAGTGGCGTCGCCATTGCCGTTGAGCACGTGATACCCGGCAAAACACGGCTGCAGCCG
CGCAGCACCGGCCCGCGGCTGGATCAGCGTGACCCCGGTGCGAACCTGCTTGCCATCGATCTGCTCCTTGAGCGTG
CTGTGGCCGACCCGAACCCCGGGGACATCGGTAATCGCATTCAATTCGCCGGGTGTGCCCAACCCCAACGTAATGC
CCAATTGACGTGCGCGCATAACCACTCCTTACAGTTTCTGGAACGCCGGACTCGACCGGCCGCTGATGCAATACAG
GATCACCGACAAGGCCAGCAGGCCGATGATAATCATGATGTCGCGCATGGACGCGGCGATAAACAAGGTAAACAAC
AGATATCCCGCGCCCGCCACAGCCAGCAAGGCCGGCACAGGCCACAGGGGCATGCGGTACGGGTGCTCGCGGTCAC
GCAGCAGCACCCGGCTCATCAGCGCGCTGAGGGCGACGATCAGGTACACCAGCATGATCAACAACACGCTGAAGGA
CGTCAGGTCCGCCAGGTTGGAGCTGAAGCTGAGCAGTGCCGAAGGGATCGCCAGGAACAGCGTGGCCAACCATGGC
GAATCCCAACGCGGGTGGATACGGGTGAAGAGTTTGTTGATGGTCGGTGTCCACAGCGCATCGCGGCCACTGCTGA
ACACGACCCGGCCGATCTGGATCACGATGGCGACGATGGCGTTGAACACGGAGAGGAAGATCCCGGCACTGACCAG
GCGTGACAGGGTTTCATTGCCATGACTGGTCAGCAGGTAACCGATGGGGTCCGGGCTGCTGATCATCTCGCGCAGG
GATGGGGCGCCAATCAACAGTGCGGTGATTGGCACCAGTTCGATCACCACCACCAGGCCCAGGGACCACAGCACCG
CCTTGTGCACGCCCTTGCCGCCGCATTTCATGTCTTCGGCCAACAACACCGCCGGGCCATAGCCGTTGAACGAGAA
CAGGCCGATGCCCACGGCGCCGATCACCAGGGCCCAGGGCGCCAGGTGCAATACGCCGTTTTCGACGATCTGCGGC
TGGAACAGCACGCTGGCCGGCTGCACCGGGTTGCCAAAGCCGATAAACACAATCACTAGCAACGCCGCCACTTCCA
GCAGCAGGCAGGTACCGGTGATCCACGCATTGAGCTTGATATTGAGGATGCCCAGGGCGTAACTGCAGACCACGAT
CACCAGGGCCACGGTTTGTGAGTCGAATTGCGTGCCCAGGGCGTTGTTGAGGTAGGTCGCCGCGCCAGTGGCCAAT
ACCGGCGGGATAAACAGCAGCATTACCAGTACGGTGAGGAAGGTGGCATAGCCCGCCATCCCGCCGAACACTCGCT
TGGCGTACACATACTCGCCACCCGCGCTGTTATGGGCACGCCCCAGTTCGGCGTAGCAAAGGCGAACATCAGCGC
CAGTAGCCCGGCCATCACAAACGCCAGGAACACCCCACTGCCGGCTTGCTGGATGGCAAACGGCGCAATGACAAAC
ACGGAACTGGCTGGGGTCACCGCCGAAACGGTAATGGCCACTACATCGAACACACTCAAGGTTGGTTTCAGCACGC
CATCAGGCGTGGGGGTGGTGCTCATATCGTTATCCTCTTGTTTATTCTTTTTATTGGAAAGCTGGTTTGAGGCAGA
AACGAAAAAGAAGGAGCGATCAATTTTTTTATTGTAGGAGCGAGCTTGCTCGCGAAAGACGTCAACGCAAACGCGT
GCTGGCTGGGCAACCGCAGCGCGCCCAGGTTTTTCGCGAGCAAGCTCGCTCCTACAGGGAATGTAGGGGTTGCCAC
TGCACCGGCCAATTCCCCTATTGGAGTACTCCCCTTGACGGGGCTGGGTAAAAACCGAACCCTTGGCCGATCTTTC
AGCCACCGGAACCCATCACATGAGCCTGTTCAGGGAAATCAGCATGCATGCGGGCCTGGGCCGTACCGTGTCGCAA
ATTGGCACCGAGCGCTTCTGGAAGCAATTGGTGCTGTTGCTGCACCAGAACCTCGCCTTTGATAACGCCCTGGCGA
TCTTCTACCCCGTGGATGGCCCGCCCCAGGCGCTGGAAGAGTACGACGCGCAGCCCACCAGCAAACCAGCGTCGAT
GCTGGTGTACCTCAATGGCTTGTACTTGCTCGACCCGTTCTTCCAGGCGTGCCGCGAAGGCTACGCCAGTGGCGTA
TACCGCCTGGAAGAAGTGGCGCCGGACCATTTCCGCCAGAGCGAGTACTTCCTCAATTACTTCCACGACAACGTGC

TGGAGGATGAAGTGCAATTCATCCTGCAACTGCCTGGGGCGGGCACCTTGTCGTTGTCATTGGGGATGCAGCGGGT
ATTTAGCGCGACGGAAACCGGGCTGCTGTCGCTGCTGGCGGCGTGGGTGCTGCCGTTGATGCAACAGCATTGGCAG
CAGAGTACCCAGCGCGCGCCGGCCGGCGAGGCAATGGCCAGCCAGATACGCGATGCGTTGAGCCATTTCGGCAGCG
GGGTGCTGTCCGAGCGCGAACTGGAAATCGCCCGCCTGGTCCTGCGCGGGTTCTCGTCCAAGGCCATGGCCGAACG
CCTGAGCATCTCGCCGGACACGGTCAAGGTGCATCGACGCCACCTTTACGCCAAGCTGGATATCTCGTCGCAGCCG
GAGTTGTTCTCGTTGTTCATCCAGTCGCTGGGGCATGACCTGGATAACCCATAGGGCAGGCATGTGGGAGTGGTGG
ACCAAGTAAGGATGTGTCGACTGGCCTACCGCCATCGCAGGCAAGCCAGCTCCCACATTTGACTTGTGGTGTGGTC
ACAATTTTCTGCTCGCTGAGTTACCCCTGTGGGAGCGGGCTTGCCCGCGATAGCGGTGTGTCAGTTACATTTATCT
AACCTGACACTCCAGCCCCCACCAGGGTAAGCCTGCTGCCTTAGCGCCCGGTGCGGATGGTGTTCCAGATCCGTGT
GCGAATGCGGTCGATCTTCATCGGCATGGCTTCGAGGGCGAACAGCTTGCCCATCATCTCTTCGCTCGGGTACACC
TTGGTGTCGCTCTTGATCACCGGGTCTACCAGGCTGTCGGCAGCGCTGTTGCCGTTGGCGTAGTGCACGAAGTTGG
TGATACCGGCCATCACGTCCGGGCGTAGCAGGTAGTTCATGAAGGCATAGCCGGCTTTTTCATCCGGGGCGTCGGC
GGGCATGGCGACCATGTCGAACCAGATCGCGGCGCCTTCCTTGGGGATGTTGTAGCCGATTTTCACGCCATTCTTG
GCTTCCTTGGCCCGGCTTTCGGCCTGCAGCACGTCACCCGAGAAGCCTACCGCCACGCAGATATCGCCGTTGGCCA
GGTCGCCGGTGTATTTGGAGGAATGGAAGTAGGCGATGTAGGGCCGCACCTTGATCAGCAGCGCCTCGGCTTTTTT
GTAGTCCTCGGGGTTCTTGCTGTGGTGCGGCAAGCCCAGGTAGTTCAGGGCGATCGGCAGCAGTTCCGGACCGTTA
TCGAGGATCGCCACCCCGCATTTGCTGAGTTTTTCCATGTACTCGGGCTTGAAGATCAAGTCCCAGGAATCGACCG
GCGCATCGTCGCCCAGTACGGCCTTGACCTTGTCGATGTTATAGCCGATGCCAGTGCTGCCCCACAGGTACGGGAA
GCCGTGTTCATTGCCCGGATCGTTGGTTTGCAGGGCCTTGAGCAGCACCGGGTTGAGGTTCTTCCAGTTCGGCAAC
TGGCTCTTGTCGAGTTTTTCAGGGCCCCACCCTGGATCTGCCGGGCCATGAAGTGGTTGGACGGGAAAACCACGT
CGTAACCCGAATTGCCGGTCATCAACTTGCCATCCAGGGTCTCGTTGCTGTCGTACACGTCGTAGCTGAAACCGAT
CCCGGTTTCCTTCTGGAAGTTCTTGGTGGTATCCGGTGCGATGTAGCTCGACCAGTTGTAGATCTTCACCGTTTCG
GCGGCCTGGGTCAGGGACGCCACCAGCATCAAGGGCAACAGGGCAATGGGTCTCATGGTTCGATTTCCTGGCGGTT
ATAGGGCTGTTTTTATGGCAGGCGATCAAGGATCAAAAGATTAGAGGATCAACACATAGGTCTTGCGCACGGTCTC
CTGGATATCCCAGATCCCCGTGCTGTTGGCCGGCAACATCAGTGCATCACCGGCTTCGATCTGCAGGGTGTCACCG
TCATCGGGGGTGAAGGTGCAGCGGCCCCGGATAAAGTGGCAGAACTCCTGGGCCACGATCTGGCGCCGCCAGCGGC
CTGGGGTGCATTCCCAGATGCCGGTTTCGACGCCATCGCTGCGCTCCACGCTGAGGGTGGACGCCACGGCAATCGG
GGTGCCGAGGGGCACAGCGACCGGGCTTGAGTCCGGCAGGTGGCGGTTAAGTGTGTCTTTGAACTGGGTAATGCTC
ATGGTCATACCTGTGGTGATTGAAGCGACTTAGTGCATGAAACCTTCCATGAAGCCGGCCATGCCGCTGGCCAGCT
TGCGCCGCCAGGGCGCGGTGTTCGGATCCGCCAGGGTCTGGTCCTCGTGGACAAAGCTGCGGATGATCGCGTTGTA
GCCCAGCCAGCGGCACGGCTCCGGTTCCCAGCCCTTGAGGGTGTTCAGGCCGCCATCGCGCAGCACCCACGGTTGC
TGGACCAGCGGTGTGTCGCGCCCAAGGATCAGGTCAGCCAGGGTTCGCCCGCCCAGGTTGCTGGCGCCGACGCCCT
CCCCGCCGTACCCGCCCGACAAGGCGATGCCGCTCTGTTGGTCGCACAGCATGTGCGGCCGAAAGCGCCGCGACAT
GCCGAGGTTGCCCCCCCAGGAGTGGGTGACGCGCACATGCTGCAGTTGCGGGAACAGCTCGCAGAACAGGTAGCGG
CGCAACTCCACCTCGCTTTCGGTGAGGTCGAAGTTGTGCCGCAACTTGCCGGCAAACTGATAACCACCGCGGGCCC
CGAATACCAGGCGGTTATCCGCGGTGCGCTGGCCATAGGTGACCTGGCGGCTGCTTTCACCAAAAGCCTGGCCCTG
ATCCAGGCCGATTTCCTCCCAGGTACTGGCAGGCAAGGGTTCGGTCGCCACGATCAGGCTTTGCACCGGCAGTTGA
TAGCGGCCCAGGGGCTTGAGCGTCGGGGCATAGCCCTCCACCGCTGGCACCACCCATGAGGCCCGGACGTGGGCCT
TGGCGGTGCGCAGGCTGGCGGCCTGCCAGTGGGTGACCGGACTGTTCTCGTAGATCGCCACGCCCATGGCCTCGAC
CACCCGCGCCAACCCGCGCACCAGCTTGGCCGGATGGATCGTCGCAACGTGGGGGGTATAGACCGCGCCATAAGCC
GAGGCGATACGGATCTGCCCGGCCAGTTGCTCGGGGCTGAGCCAGCGGTAGTCAGCGTCGCTCAGGCCTTGGCCGT
GGAGCTTGGCCAGGTATTCGCGCAGGCTGGCTTCTTGCTCGGGGTAGCGGGCGGCACAGTGCAACGCCCCGCCCTT
GCGGTAGTCGCAGTCGATGCCTTCGCGCTGCAGCACCTGCCCGACTTCATCGGGGATGCCGTGCAGCAGGTCAAAC
GAGGCCCGGCGTTGCTCGGCAGGTAAACCGGCGAGCAAGCGGTCTTCGCCCAAGAGGTTGCCCATCAACCAACCAC
CATTGCGCCCGGAAGCGCCGAAACCGGCAGTCTGGGCTTCGATGATCACCACCTTGAGCTGCGGTGCGTGGCGCTT
GAGGTAATAGGCAGTCCACAACCCGGTGTAGCCGGCGCCAATAATGGCCACATCGACATCCAGGTCATGCTCCAGG
GACGCGCGCGCCTCCAGCGGCTCTTGCAGCTGGTCCATCCACAAACTGATCTTGCGCCATGCCGGCATGCCCAGAC
TCCACCACCAAACGTCGATGGCGTGATCCTAGTGCCTGGCTTCAGTCGTCGTCTTGCGCGCGTGCACGCAGAGAAA
TTTGTTTCACGTAGGCCTTGGGTGACAGGCCGGTGTGTTGGCGGAAACAGCTGTAGAACGCCGATAACGAGTTGAA
TCCGGCGGAAAATGCCAGCTCATCAATGCGTATCGGCGGCGTGGCGCCATCCAGTGCCGCGAGCAAATGCTGCAGA
CGGGCCTGGTTGACGTAGCGGTAGAAGCTCTGCCCCAGCACCTGGTTGAGCAGGTAGGAAATCTGGTTGCGACTGT
AGCCACACTCCTTGGCTACCCGTTGCAGGTCCAGTTCAGGGTCGAGATACGGTTGCTGACGCTGGAAGTACTGCTG
CAAATCGTTGGCCAGGTAGCTCAGTTGCTGGGGCGACAAGCCCAGGCGGCTGACGGTCGGTCGCGTGGCCTGGGAC
CGGGAGCGGCTGGTGGCGGGTTCGTGTACCAGGGATGCGTATTCGTTGACACGCCAGATCAGCCCGTCGCGCACGG
TGATCGCCTCACTGGTACGAAACGACACCAGGCCCTGGCCGCCACGCAAGGTCACGCGATACTGGATAAATGCAGT
ATCGCCATCGGTGCGGATACGATCAGTGTGCTCGATAGCCTCATCGGGGTCGCGGGGCATGCTGGCTTGCAGGTAC
TCACGCAGTTGGTCGAAGCACACCACACGGTTCTGGAAGAAGTCGTGGTACTGGATATCTGGGTGATAGTGCGCCA

TGACCCCGTCCAGATCGCGGTGCTTCCAGCACAGGTGATGGCGCAACACTACGTCGGCAGTCGCTTGCGTCTGTTG
CGGGCTATCGGCCGGGACGGTCTGTGGCATACAACACTCGCAAGCGGCTAAAGCCTGGAGCTTGCACAACTTTACC
GAGTGCTTCAATGGCAAAGTGACAGTTTCAAATAATGGCCTTTTGCCTAAGCAGTTGTAAGACATGACCCACGTCA
AACTTCTTGCAAGCAACTGCCGAACGGACCGAAAAAAACCCGATACCTATTGCGAGTGAGTGCTATTTTTTAAGCT
TCACGGTCAGGACCATTGATGGACCTGACGCCCTTGCCGCGAGCTAAAGGAAGCGCTCAATGAACAAGGTTGGTCA
CATGAACAAAGTGACATTCCCCAATGCCTGCCAGTTGATGCGCTGGCATTTTCACCCGGTAGGCTTTGAAGGCAGC
ATGGACGCGCCCGGCAGCATGATCGCGCGATTGTTTGACCGAGAGAGTGGCGAAACCCTGATCGCCATTGCTGGCA
TTCCCTGTGCCACGGTGATGAACGCCGCGGATGTGGAACGCATTATCGAAGCGATTGAGGCAGAGCTCGAATCGTT
TATACGCCCGCAGTTGCGGCAGGCGTAGTTGCAGGGCTTGCTGTGGCGAGCGGGCTTGTTGTGGCAGCGGCCTTCT
TGTGGCGAGCGGGCTTGCCCCNNNNNNNNNNCCCATGCGCGCCGCGATATCTGTGCGCTTCTTCCCGCAAGGAGTC
TCTTCAATGTCCAACTTGCCGATGGATCCTTCAAACGAACCCGCCGACGATCAGGCGCCCCCCCAGCCGTTGTCGC
AGGATATGGTGCTCTACCACACCCCTGAACTCCCCCCAACGCGCGTCGAAGACCTCGACCCGCCTTACTCGGCTTA
CCTCGCCGTGCCGCTGGAGCAGGCGCTTTATCCGGAAAACGCGGTCTACGCCCTGGGTATTCGTCAGGTGCAGCCC
TATTCGTATTGGTTCTTTGAAAGATGGTTCACATGGAGCTCCGGCGACTACTACGCGATTTATATCGATAACCTGG
TAGAGGCTAAGCAAAGCGGTATCGCGTACGAAGACAAGCCTCGCTACGGGCTGATCGTACCGGAAGAGACGATGCC
CAGAGGCGAAGTGACCCTGTACGGCCGGGTGTTGCGCGCCGGCTCGTTTCAGGAAAGCACCTCACCGCTGCAGACC
ATTCTGATCAAGACCGACCGCCCCGGCGGGACTGATCAGGACCCAGGCGCCACGTGGCATACCCGCTTGATAATGT
CCATCGAAGGCTTTCCAGAAAACTCGGTGATCAATCAGGAAAACTCTGCTGGCGGCGTGTGGTGCCTGATCGAGCC
CTATCCCTATATCCGCAAGAATGACAAGATCGAATTCTCCTGGGACGGCGTGATCGTCATCCATATTGTCAGCCCC
GAGGAGGCTGCCGGCTCCGGGCCGATTCGCATCTGGGTGAGTAAATCGGTCATTGATCAGGGCGGTCTGAATGGTA
AGTTGATCCTGCGTTTTCGCGTGCAGGACGTTGCAGAAAATATCTCCGGGGATAAATACCAGTATTCCCAGCACTA
CCTCCTTCAGAGCGAACTGGATCCCTCCCTGAGCCCCGCACCGTTTTTCCTGATCGACGGGGTCGAGTCCAGCCAG
GTGGACTTCGATACCGATAGCGAAAAAACCTTCAGTCTGCAGTGCTACACCGACCGGGAGGTACCGGCGCCCAACC
CGCCGTATCAGGTGGTTGTCACGCTGTATGCCACGCTGATCGACGGCTCGACCCGCACCTTTAGCCTGCCGCCGGT
AAACGCGAGCAACCTGTTTCTGACCGAAGTGCCAGTGCCGCGCGAGATCATCGAACAAACCGTCGGCGGCTCGTTC
CGCGCGTCGTTCCAGTTGCTTACCGCTGGGGGGGCTTTCCTCAAGCAGTCGGGCAGCGTGACCGTCACCGTGGTCG
GAACGCCCGTGAGCATGCCGGGCGTGACCATCGTGCCGATCGAGCTGGGCCTGATTGATCCGGACAGCGACATTGA
AATCACCATCCCCTTCTACGAACCCCATGACCCGGACTGGCTGGAAACCCTGGTGATCGAGTCCCGCGAGCCGGGT
GGGGGCAGCGCAACCTACACCGACGCGCAACTGGCGGGGCCCCAGGGTGGCAGTTATCGGGTCACTGCGCAGGACT
TGCAGCAGTTCCGTGGACACAACATTTACGTTTACTACTTGGTCAATGACGGCGCCGTGCATATCTTCGGCGGCCA
GACGCAAGCGATCCGCAAGTCCCTCGAAGTCGGCGCGCAGGTCGGAGAACGTTTGGCCAGCCTGCCTGCGCCGCAT
CTGCAATACGCGCAAGGCAACAATATCAACCCGGCCCACGTACCCGGCGCGAATCTGTTGCTGACCCTACCGTACA
CCGGCACGCAAAGCGGCGATGAGGTGTACTGGGTGATCTTCGGTTCTGCGGTTGGGGGTTCGGCGAACGGCACCAT
CACCGTCAACGGTGCGACGGCCGGCCAGCCGCTGGTCTTTGCGATCAATCGCAACCTGGTCGACTCCAACCTCAAT
GGCAGCCTGCGCATCAGCTACAGCCTGCAACGCGGCGGCCCGGGCGGCACGATCTTGCGCTCGGAAATCCTCGACC
TCACGGTCGGCGTCGGTGTGCAACTTGAACGGCCGATCATCGAAGGCGCCTCCCTCTTGCCGGACCGGCTCAATCC
GCTGGCGGCCGTGGCCGGTGCCTGGGTCATCGTGAGGTTTATACCCATGCACGCCACCGATCAGATTGACGTCGAC
TGGCTGAGCAGCGACGGCATCGGCAGTTACACCGCGAAAGTCCAGGGCAACCCCGGCACCAACGAAGTGCGCGCGT
TTATCCCGCCGGAACTCATCGCCAAAGGTGTACGCCCCGATGGCAACCAAATCAGCGTGCAATACCGCTTCATGCG
TGGCTCGTTCCTGTATGAATCGCAGGTCGTCGACCTCGAACTGCAACCGCTGACCGGCCTGCCTACACCGAGCATC
GACGGCATCGGCGACAACATGGTGCTGGAATTGTCGCAATTGAACGAAGAAGCGCGCACGCGGATAGCCAAGTGGA
GCTTTATCGCCCCCAATCAACGGATGTGGATGACCTACGAGGGCACCTTCGAGGATGGCACGCCTTATGTCGAAAA
TACCTACACCGCCAACCTGGTCACGGCAGCCGGGGTGCTCAACGGTATTTCCCCGCCCACCCCGGTGGACAAGCTG
CGCCGTCTCAAAGATGGCTCGCAACTGACCATTCATTTCTGGGTCACCCTGTCGGAAAGCGCCGATAAGAACTCGG
CGGTGCTATTCGGCGTGCGTGTGCACAGCATTCAAGCAATCCCATCGACCTTGCCACGGCCGCAATTTGCCAACCT
GCCAGGTCAAGCCATCACCATCGACCCGTTGACCTATGAAAACAACGCCTCGGTAGTGGTGGCCTACCCCGGTATG
AACTCGACACATCGAATCAAACTGCTGTGGCTGTTGCCCAATGGTGATATCCCGTACATCGCCGATAAAGACGGCC
TGGCCGGTGGCCGCGTGGACTTTTTGATCAGCCCGGCGATCATGGCCGCCAGCGTGGGCAAGAAAGTGACGGTGCG
GTATATCGCGACGATTAACGGGGTGGATGTCGACTCGTTTACTCAAGAGGTCAATGTACAAGTTATTCAGGCTGCC
AATTTGCCACGGCCGGTGATTAACAATATTGCCAATGGTGGTACGTTGGATCTCAATACCTTTACCGGCAATGCCA
CTGCGTCTGTCGCCAAGTGGCGCTTGAGTGCAGCCGGTCAGCGGGTGTGGCTGACTTGCAGCAGTGCCGGAGTGAG
CGATTTGTATGTAATCAATGGGGTAGCCATTACTGCGACGGAAGCCGCGAATGGGTTGATCAATAAAACCGTGTGG
CGTACCTGGTTGGAGGCGTTGCCGACAGGCCGGCAAATTACTGTGACATGCAAGGTGACCTTTGATGGTTCAACCA
AGGAAGTCGATGCCATTGCTTTCACCACAACCACTTATTTGATTAGTGCTTCTCCAGGATTCATAGACCAAATATT
TTTAGGCTTTGGACAGTTTGACATTGTCGCAAGTTTGACTAGAGCAGAAGTGTATGTGAGCTACCCCGGCCGCATT
AGTGTAATAGACACGACTCAAAATCGCATAATTCGTAGCGTGCCCGCCTCGGACGCAAAGGAGCGCATGGCCCTTC
ACCCCAATGGCTCTAGTATGTACATCCCCCTCAAAAATGATAACAGCATTGAAATTGTCAATATCAACACTGGCGC

AAGATCTAGAATAATCAACATCCCATCTCCAAGCAATCCAACATTCAATGCAACAGGCAGCAGGCTTTATGTTTCA
AATAGCACTGACAACTTTGTCTATGTTATAGATACTTTTACCGCAAAAATCATCCATTCGATTAGTATAACAGGCC
CCAGCCATTCGATGCTCGGTTCGAACAATAGGCTCTATGTATCTACCCGACTATCACAAATCGCTGTCATTGACGT
TAACACCTACCAAATTCTTGCATATATACCTATTACGACCCCCGGCAGTTTCTTGGCCCAGAGCCCTCACCGTCCA
GTGTTTTACCTAGGACATTACGATAAAAAGGTTTCGGTGATCAATATGAATACTAACATGGTTGAAAAAGTCATAC
CTATCAATGGGGAGGTTGCAGCCATCGCCTTTAGTAATATCATTGAAAAAGCCTGCGTTGCTGGCTATTACACCAA
CTCTATAGATATCATAAACACTCAGACCGACTCAACTTCAAGCATCCTACAACCAGCATATAGGCCATTAGGCATA
GCCATTCCACCAGAAAGTTCAGTGGCGTATGTCTGCAGCAATAGTGACCATGTCGTATATGTAGTACAACTCTAAA
AAATCTACAAACTAAAACCGGGACAGATCTATTTAAAAGACCTGTCCCGTTTTCACTTTATTAGACCCGCCACCAC
CCAAGACCCGACCGACAACTAGCACCACCGAGCCCTTGACCGCCAGCGCCTTAGCCGCAATGCTTAAAACCAAGCA
CAGAGGGCTGTAGTGGCGAGCGGGCTTGCCCCGCATTGGGACCCGCCTCTACGGAGTTTCAGATACACAGCATTCG
GCTTACTGGGGCCGCTTCGCAGCCCAGCGCGGGCGATGCGGCGTTCCGACAAACCCGCTCGCCACAGGGAAACTCT
TCAATTTCTGGAAGTTGTGTAGATACCTTTGGACATCGCAGGCAAGCCAGCCCTCACACAGGTAAACTCCCCTCCT
TTCCCTAATTCCCACGGAGTTCGCCTTGCGTCTGTTCCACACCTCCGACTGGCACCTCGGCCAAAACCTCCACGGC
CAGGAACGCGACTTCGAGCACGCCTGTTTCCTCGATTAAAAAAACGGGACAGATCTATTTAAAAGACCTGTCCCGT
TTTCACTTCATTAGACACACCAAAAAACGGGAAAAATTTACTCCGGCCATGACTGTGTATCAGCCAGCCCATCTTC
GACTGACACGCCGCCATCCCAGGCAAGCCAGCTCCCTCCCTTGATCTACTTCATCGGGAATACCCCGTCATGCATT
GTCAAAGGCCAGCAAACCAAATCCCTTTAATTTGCAGGACGTTTCCTAGACAATCCTTCGGCCTTGCGCCTGCTCA
CACCACTGGCTAGCCTCTGCTCCGTCGCTGCACATCAGCGATCGGGGGTGAGATCCCGCAAACCAGTAGGCGCACT
GCGCCACAACCGTCGCAACATGCTCTATGGCGGCTGTGTGCGGGCAGACTTCGGTCTGGCCGGGTCCTACTGTCCG
GTATCTCACCCCGCACATAGCTGCCACCTTTCGTCGAGTGAGATCGGCAAAAGATGGCTTCCAACCCAGTAGGAGC
TTACGCATGAACAAAATCGTCCCCGACCCACCCCGCCTCCCCTCCGACCGCGAGTGTTTTGACCGCGCCTTGAGCC
ACTACCTGCAACCCGCCTCGCCGCCCTCCTTCACCGCGCACCAAGACCTCAGCTTCGAAGACGCTCTGGCGCAAAT
CTGTGACCTGCTGCGCTGCGCAGCCGCCACCGCAGCCGGCACCACCCAGGGGCTGATCGGCGACCAACGCCACATA
GCCGGCGCCACTGAACACTTGATCGACATGGCCAAAACCCTGGCCGACCTCGCGCTGGACTGCCTGCAACCCAAGG
TATAACCCCACCTCACAAACATCACAAAACCACAGTGGGAGCGGGCTTGCTCGCGAAAGCGGTGTGTCAGCTGGCA
GCTATTTCAACAGACCCACCGCTTTCGCAGGCAAGCCAGCTCCCACATTTGATTTGTGCAGCCCTGAAGAGAAAGC
GGTGTGTCAGCTGGCAGTTTTTTCAACTGACCCACCGCCTTCTCAGGCACGCCAGCGCCTACACAGGTAAACTCCT
CCCCTCTTTACCTCTTCCCCACGGAGTTCGCCTTGCGTCTGTTCCACACCTCCGACTGGCACCTTGGCCAAAACCT
CCACGGCCAGGAACGCGACTTCGAGCACGCCTGTTTCCTCGATTGGCTGCTGGGCCAACTGAAACTGCATGGCCCC
GATGCGCTGCTGATCGCCGGCGACATCTTCGACACGGTCAACCCGCCGCTCAAGGCTCAGGAGCGCCTGTATGACT
TCATCATCAGCGCCCATGAACAAAACCCGACGCTGACCATCGTGATGATCGCCGGCAACCACGATTCCGGCTCGCG
CATCGAACTGCCTGCGCCGTTGATGCGCCGTTTGCGTACCCATGCGCTGGGCCGCGTGTTGTGGCTGGACGATGGG
CAATTGGATGCCGAGCGCCTGCTGATCCCGTTGCCGGATGCCAAAGGCAAGATCGCCGCGTGGTGCCTGGCATTAC
CGTTCTTGCGCCCGGCAGAAGTGACCGGTGCGCACCTGGGTGACGACTACCTGCGCGGCATCGGCCAGGTCCATGA
ATGGCTGATTGCCGCCGCCAACGCCAAACGCAAAAAAGGCCAGGCGCTGATTGCCATCAGCCATGCGCATATGGCC
GGTGGTTCGGTATCGGAAGACTCCGAGCGCAGCCTGATCATCGGCAATGCCGAGGCGCTGCCGGCCAACCTGTTCG
ACAAGAGTGTCGGCTATGTTGCCCTCGGCCATTTGCACAAGCCGCAAAAGGTGAATGGCGAAGAGCGGATTCGCTA
CAGCGGCTCGCCGATTCCGCTGTCGTTCTCCGAAATCGGCTACAAGCACCAGATTCTCGACGTGACATTCCAGGGC
CAAATGCTGGTCAGCGTCGAACCCCTCCTGATTCCGCGCTCGGTCAACCTGCAACGCCTGGAAGCCGCGCCTTTGG
CCGATATCCTCAAGGCCCTGGCCGATCTGCCGGATATCGACCTGCTCGCCGAAACCCAGCGCCACCCTTGGCTCGA
AGTGCGGGTGCGCCTCGACGAGCCGCAACCGGACCTGCGCCAGCAAATCGAAACGGCCCTGCTGGGCAAGGCCGTG
CGCCTGGTGCGCATCGCCGCCGAGTACGCCGGCCAACGCGGCAGCGACGGCAGTGATGAAGACACCCTGATCGAAC
TCGACCAGCTCACCCCCCAGGAACTGTTCAGCCGCGCCTGGCAGGACAGCTATGGCAGTGAAGTGGATGAACAGAC
CCTGAAGGATTTCGCCGTGCTGCTCCAGGACGTGCAACAGGAGGGCGAACAGCCATGAAGATCCTCGCCATCCGCC
TGAAAAACCTCGCCTCGCTGGCCGGGCCATTCGAGATCGACTTTACCGCCGAGCCCCTGGCCAGCGCCGGGTTGTT
TGCGATTACCGGGCCCACCGGCGCCGGTAAAAGCACCTTGCTCGACGCCCTCTGCCTGGCGCTGTTTGGCGCCGTG
CCACGCCTGGGCGATACCGGCCAGGCGAAAATGCCCGATGCCGATACCGATATCTCCATTGGCGACCCACGCACCC
TGATCCGGCGTGGCACCGGTGGAGGTTATGCCGAGGTCGACTTTATCGGCGTCAACGGGCGCCGCTACCGTGCGCG
CTGGGAAGCCAACCGCGCCCGGGACAAGGCCAGCGGCAAGCTGCAACACAGCCGCCAGAGCCTGATCGACCTGGAC
AGCGAACAACTGCTGGCCAGCCAGAAAACCGAATACAAGACCCAGCTGGAACTGGCCCTGGGCCTGAATTTCGAGC
AGTTCACCCGGGCTGTGCTACTGGCGCAAAGTGAGTTCAGCGCGTTTCTCAAGGCCAACGACAACGAGCGCAGCGA
ACTGCTGGAAAAACTCACCGACACCGCGCTCTACACCCAACTGGGGCAGCGCGCGTTCGTCAAGGCCCGGGAGGCC
AAGGACGCCCACAAGCTGCTGCAGGACCAGGCCACAGGTGTCACGCCGCTGCCGGCCGAGGCCCGCGCCGAGCTGG
ATCAACAGTTGAGCGCGGCTCAGCAGCAGCTCAAGACCCAGCAGGCCCAGCTCAAACAGCTCGAACTGCAACACAC
CTGGCTCAAGGAACTGCGCGAGTGGCAAGAGCGCCAGCACAGTGCGGTCGAGCAACTGCAACAGGCCGAACAGGCG
TGGCACAGCCAGGGCCCGCAACGCCTGGACTTGAGCCGCCTGGAGCAACTGGCGCCGCAGCGTCATCATTTTGCGC

GGCACAGCGAGTTGACCGGCCAGTTGGAGCCCCTGGCCGCGCAGATCCGCCAGCACCTAGAGCAGCAGACAGCCCT
GCACGCGCGTCAGGAACAACTGCAGCAGCAACAAGCCAGCGCGCACGGCGCCTTGGCCGTGGCCCTGCAGCAACAG
ACCGAGGCTGCGCCGTTGCTGCGCCAGGCCTTCGAGGAACACAGCACCCTCACCCGCCTGACCCGCGAGCTGGCCC
AAAGCAGCGAGCAACTGCAGCTTGGGCAAACCGCCTGCACCGAGGGCCAGGCCAGCCTGGATGCCCTGCACACCCA
GCAACAGCAGGTTGCCAGCCGCCTGCAACAGCTGGCCGCACAGCTTGAACGCAGCGCCGCCCTCGCGCCCCTTGAGT
GATGCCTGGAACGCCTACCGCGACCGCCTGCAGCAACTGATGCTGATCGGCAATCGCCTGAACAAAGGCCAGGCCG
AACTGCCGCAGCTTGAGGAACGGGCCAACCGCGCCAGCGAGCAATGGACCCAACAGCGCACTGCTCTGGACCTGCT
GTACCAGGAGGCCGGCGCCGAACCCCAGGCCGTGGCCGAGCAGATCCAGTTGCTCGACAGCCTGCTCAAGGACAAT
CGCAAGCAACAACGGGCCTTCGAGGAGTTGACGCGGCTCTGGGACAGCCAGCAGCAATTGGACCAGCAACAGCAGG
AACTGGCCCGCAAGCAAGCCACCGCCCAACAACAGCGCGAGCAACTGAACCAGACCGGCCTGCAAGCCAAGGCCGA
GCTGGCGGTGGCCGAGCAGACCCTGAGCGTGACCAAGCAGTTGCTGGAACGCCAGCGCCTGGCGCGCAGTGCCAGT
GTCGAAGAGTTGCGTGGGCAATTGCAGGATGAGCAGCCCTGCCCGGTGTGCGGCAGCGTCGAGCATCCGTATCACC
AGCCCGAAGCCCTGTTGCACAGCCTCACGCGCCATGATGAAAACGAAGAAGCCCGTGCGCAGCAAGCCGTCGATAG
CCTCAAGGAAAAGCTCACCGAACTGCGCGGTGAAGTCGGCGGTTTGATCGCCCAGCAAAAGGAGTTCCTGCAACAA
CAGGAACAACTGACGAGCCAGCTCCAGGCCCTGGCGCCGAGCCTCGAGGCCCATCCGTTGAGCGCCGCCCTGTTCA
ACCAGGACAGCGGCAAACGCAGCGCCTGGCTGGCGCAGCAACTGGGCCAGCTCAACCAGAGCATCACCCAGGACGA
ACAGCGCCAGGGCGCCCTGCTCAACCTGCAACAAAATGCCGGGCGCCTGCAGCAGCAGTTGCAAGCCGCCCAGGAC
GCCAGCCAGCAAGCCCGCCAACAACTGATCGACCAGCAACGGGAACTGGCCAGCGACCGCGAACGCCTGGACGAAG
AACTCAATGCCTTCGCCAGCCTGCTACCGGCCGAGACCCTGGCCGGCCTGCGCACGGAGCCAGCGGCAACGTTCAT
GCTGCTGGACCAGCAGATCAGCCAGCGCCTGGAGCAGCTTGGCTACCAGAAGGATGAACTGGCCGAGCAACAACAA
CGCCAGCAAGCCATCGAAAAAGAACAGGACCGCCAGCAACATCGCCAGCAACAGTTGGCCGTGCTGCAGCAGCAGT
TGACCGAGCTGACCCACCAGCAGCAGGCCGCACAGGAAAAACTCAGCGCGCTGCTGGGCACACATAGCAGCGCCGA
GCACTGGCAGCAGCAACTCGAGCAGGCCGTGGAACAGGCCCGCCACAGTGAGACCCAGGCCAATCAACAGTTGCAG
GAAACCCGCAACGAACTGATCCAACTGGCCGCCGACCTCAAGTCCCGCCAGGAACGCCAGCAAGCCCTGGACACCG
AACTGCAGGCGCTGCAAGGGCGTATCAGCGACTGGCGTGCCCAGCATCCGGAGTTGGACGACACAGGCCTGGCCCA
TCTGTTGGCCTTCGACGAGGCCGGCGTCAACCAGCTGCGCCAACAGGTGCAGCACAGCGAAAAGGCCATCGAACAG
GCCAAGGTCCTCCTGCAGGAACGCGAGCAACGCCTCAAGGACCACCAGGCCTTGCACAATGGCAACCTCGACGCCG
AACAGCTGGACAGTGCCCTGGCCGATCTCAACCAGCAATTGGACAGCGGCGAAAAACACTGCGCCGAACTGCGCGC
CCAACAAGCCGAAGACCAGCGACGCCAGGACGCCAACCAGACACTGGCGCTCACCACCGCCAAGGCCTATGAAGAG
TGGCAGCGCTGGGCCCGACTCGATGCGCTGATCGGTTCGGCCACCGGCGACAAGTTCCGCAAGTTTGCCCAGGCTT
ACAACCTCGACCTGCTGGTGCACCACGCCAACGTGCAACTGCGTCAACTGGTGCGCCGCTACCGCCTCAAGCGCGG
CGGCAGCATGCTGGGCCTGCTGGTGCTGGACACGGAGATGGGCGATGAACTGCGCTCGGTGCACTCGCTGTCCGGT
GGCGAGACGTTCCTGGTCTCCCTGGCCCTGGCGCTGGGGTTGGCGTCGATGGCCTCCAGCACCTTGAAGATCGAGT
CGTTGTTTATCGACGAAGGCTTCGGCAGCCTGGATCCCGAATCCTTGCAACTGGCGATGGACGCCCTGGACGGCCT
GCAGGCCCAGGGGCGCAAGGTGGCGGTGATCTCCCACGTGCAGGAAATGCACGAGCGCATCCCCGTGCAGATCCAG
GTCAAGCGCCAAGGCAATGGCTTGAGCACCCTGGAGGTGAAGTGAGCGAGGCACTGCTGTATTCGTTTCGCCGCTG
CCCGTACGCCATGCGCGCACGCATGGCGCTGCGCTATTGCGCGGTGGCGGTAGAGATTGTCGAGGTCAGCCTCAAG
GCCAAGCCGGCCCAAATGTTGGCGCTCTCACCCAAGGGCACGGTGCCGGTGTTGAATGTGGGCGGCCAGGTGATCG
ACGAGAGCCTGGCGATCATGCGCTGGGCGCTGGCGCAGAACGACCCCGAGGACTGGTTGCTCAAGGATGATCCCGC
GGCGCAGGGGCTGATGGCAGGGTTGATTGAAGAAAATGACCTGGTCTTCAAGGTGCACTTGAATCGCTATAAATAC
GCCGAACGTTATCCAGAGCAGCCGCTGGAACACTATCGCGCCGAGGGCGAAGTATTTTTGCGCAGGCTGGAGGCGT
TGCTGGCGCAGCGCGACTACTTGCTGGCCGATCACCTGAGCCTGGCGGATGTGGCGCTGGCACCGTTTGTGCGGCA
ATTTGCCCATGTGGATCGGGAGTGGTTTGCTGGCGCGCCTTATCCGCGTTTGCAACAGTGGCTGGAGCGGTTTTTG
GCGTCGGCACTGTTTACCGGGGTCATGGCCAAGGGCTGAGCCTGTGGTCAGGAGAGCTTTTTGTGGTGATGGGGCC
TTTTGTGGCGAGGGGGCTTGTCCCCCGCTGGGCTGCAAAGCAGCCCCAAAACCAGCCCCCTCGGTGTGCCTGATAC
TACTCGGCAGTCCTTACTGGGGCTGCTGCGCAGCCCAGCGGGGGACAAGCCCCCTCGCCACAACTCAGGACTGGAT
CTTGTGGTCCAGAGCCGCATAGAAGTTGGCACTTTGCTGCAGGTATTTCTGGGTGTAGGCGCTGGTGCTGGATTTT
TTGCTGCTGATTTCCACGCTGGCGCTGGCTGGCAAGGCAACGGTGGCGGAAACGGCGGAAGCGGCGATGATGGAGA
AGAGATTGAAGTTCATGGCGGTAACCCTTGTGTTCGGTTGGTCAGGTGGCCCGTGGAGGCCTTGGAACCCAACTTA
CGCCGAGGGGGTTGAACGCAGAAATGCTTTGAAACAGTAGCTGTTATCACCACGATTAATACAACACCACAGGCCC
CGAACCACGGAGCCTGTGGCGGATTGTCACATCAGGAACTTGAGGTCGCTGGGCGCATCGATCGCCAGTTTCTGCA
CGGTTTTACCCAACAGGCCGGTCTGGGTATCGCGTTCGATTACCACGATCTGGTTGCTTTTCTGGTTGGCGACCAA
CAGGAACCTGCCGCTTGGATCGAGGCTGAATTCACGGGGGTGATCGCCTTCCACGGAGCGGCGCTGGATTTCCTTC
AATTGGGCTGTCGCCGGGTCGATGCTGTAGACCAGCAACGTGTTGGCCGTGCCCCGGTTGCTGACATACAGGAACT
TGCCGTCAGCAGAGGCATGCAACGCCGCGCCGGCCTTGTCCGACGTCGGTTGCCCGACAGCCATGTCCACCAGTTG
GGTTCGGGTCAGTTGGCCGTCCTGATAGTCGAAGACGGCAATCTGCGCGCTCATCTCCATGGTCAGCCAGGCGTGC
TTGCCGTCGGCGCTGAACAGCAGGTGACGTGGACCGCTGCCCGCCGGCAGCTCTACCACGGCCGCCGGCACCAGTG

GGCGCTCGGGATTGGCCTTGGGGTCATAGCGATAGACAAAGGTCTTGTCGGCCCCCAGATCATTGGCAAACACAAA
CTTGCCATCGGGCGAAGACACCACTGAATGCACATGGTTGGACGCCTGGCGCTCGGGGTTGACCCCACTGGCGGGA
TGGGCACTCAGTTGCACCGGTGCCGCCAACCTGCCCTCGGCATCTACGGGCAGGATCGCCAGACTGCCACCCGGAT
CTTCCAGCACGCTGTAGTTGGCCACGAACAGAAACCGGCCATCGGCTGCCAGGCTGGAGTGAGTCGGTTCGTTGCC
CAGGCTCTGCACCTGATTGATAAGGTGCAACTGGCGGCTGTCCGGGTCGATGCGGTAGCTGCTGACGCGCCCGACC
GTGTCCTGCTGGCCGGGGCCGTTTTCATTGACCACGAACAGGCGCTTGCGGTCGCTGGACACCGTCAGCCACGACG
GGTTTTGCGTCTTCGCGGCCAGCACCGCAGGGCCGGCGAAGTGCCCGGTACGGCTGTCGAACTGCACCCGATAGAT
ACCTTCGCTGGCACCTGCGGTGTAGCTGCCCACCAGCAGTTCGTGGGTCTGGGCCGGCGCGGCCTGGACTGACATC
GCCCCCACACTGCCGGCCATCAGCAAAGGCCAGAATTTACGCATCAGCATCGTCTTCATCCTCGTCGTGGTTGCTG
CCACTGATCCCACTCATGCACACCAGGCGGTGCTGGCCCGAGTCGCTAGTGAGGGTCCAGCTTTGCAAGGTTTCAT
CGAAGACGGCGGCCAATACCTGTTCTGGAGTAAAGCGCCAGCGCTTCTTGTCCCGGCCATCCATGCACTCAATGTG
CAAGCACCCTTCCAGCTTGAAATCAAAGGTATACAGGCCATCGATTTCGAGCATGTTGCAGGTTTCAAGGGCAGTC
GGCAGGTTAACGGCAGTCATCACAATCAGTCATCTGTTGGAAAAAAACAGATGATAGGCCAATCCCTGGGTTCTTT
CTCGTTCCACGTGGGGTTGGGGTAGCCCGCTACCACCTCTGCCCCGACGACAACCGCCACTGTTGGGTTCTATCAA
CCCCACAGAACGGAATCTGCCATGCCCCCTGTTTCCCTGGCCGCCCAGGCGGTCAGCCAGCAATTTGCCAGCCGTC
AGCCGCTCGCCACCACCGTCCAGGCACTGTTGCAGCGCAACCTCAGCGAGCGTTACCCCAACCTGGTCTTTGATCT
GCAGCAAATCCAACTTGCCGAGCCACAACCCAGCCGCAGTTGGCGGTTCAAGCCACTGATGAGCGTGGTTCAGGAC
TATTTGGGCGGTGGCGCGGACCTGGACTTCAGCGAACGTCACGGGCTGTTGCCTTACCTCTCGCAGACGCCACCCA
AGCGCTTGAAGCTGCCCGAACTCAATCAGAACATCGACATGCAGGTGATCGAAGGGCTGATCCGCGAGCTGCCCGC
CCTGCTGCCCATCGCCTTGCAAGACGCGCTGGTCGATGACTGGAATGCCAGTGGCCCCAGTGGCGTCACCCGCTGG
CGCTGGCTCGGCGACTGGCTGATGAACACCCTGCGCACCAGCGGCCTGAAGCAGTCGGGACTGGACGCGGCCTCGC
GGCAAACCCTCGACCAGCTCGTGAGCTTTCCCGAACGCGCCCAGCGCATCGCCCGCCACGGTGAGCAGGCGGTGAT
TGCCTACAGCCTGGAGACGGTGGTGGAACACAACGGGCAAACCACCCGCCTGTTGTCGGAGTATCTGCTCCTGACG
CGCCCCATCAATGGCCGGGCCCCGGTGCTGCTGTGTGCGCCCAACGGGCATGTCGAGTGCTTTGCCTCCATGGGCG
AGTTCAATACCGCCTGGAGCCAGCGCCTGGATGCACGCTTTGTGGCGCAGACCCTCACCACCCACCGCTACGAGCC
GCAGGGTTCGATCTTCGAGCATCAGGCCAGCCTGCTGCTCAATCAACAACTGGAACACCTCGCCGCCCTCAAGCTG
CCCCTGGGCAAGGACTGGCGCAGCCTGCAAACCCTGTACACCGAACTCACGGAGCCGGCGCTCTACTTCCTTGATG
CCGACCAAACCCCCTACGCCCACCTGGACAGCCTGCGCGCGCACGTACCGGATCCTTTGCGCCACGCCACGCCCTC
GGAGCGTTACCGCTATAGGCAATTAAGCCTGGAGCTGGCCAGCGCGCGGCAGCGCGCCGGCGATGAAACTTACCTG
AGCGGCATCGACGACCTGCGCACCTATACGGTCAACGCCTTGCAGCGTGAGTTACAGCGACTGGCCGCCCAGGCGC
AGGCCCCCTTGACCACCCTGCCCCACCCCGATGAGGTGATGCTGACCTTTACCGTCGCCGCGGGCTATCCCGGTGG
GGCCGGCATTGTCGAACGGCTGCCCATCAGCCTCACGGACCTGGCGATCCGCAACCTCGCCGCACGCCCCAGCGGC
CAGGTGGCGCTCACTCATCGCCTGGCACACACCCTGCCGGCCTGGCTGACCACCGATGCGGTGATGGGCAGTGATG
GCCTGATCGAGCGGGTCGACATTGGCAAGCACTATCCCCTGCTGCTCAAGGACGCACTGCTGAGCGACAGCCCCGA
GGCGCTGCGGCGCGAACGGCTGTTCAGCGAGCAACAGGTGCTGCAATTGCCGCTGCTGGCCCTGGAGCTGAGCCTC
AAGCACGAGCTCGGCATCAGCGCAGAAGGCGCACGCATGGTCGCCGCGCTGATGCAGCCCGACCCCAGCAACCAGC
ACGTCCACCAGCGCCCCGTGGTGATCCGGCACCTGGCCCTGCTGCGCAGTCCCAATGCGGCAGCAGACAAAGTCGA
GAACATGTACCTGATCGAATACCAGGACATTTCCCCAGGCCCGCTGTTGCTGTACCGGCCGCTCTACGCCACGTCC
TTGCAGCAATACCCCAGCCGCGCGGCGCTGTTCGAGGCCATGGCGCAGCCCGGCGCATTGCAGGCCAGCGTGCTGA
CGTGGCTGTCGGACAGTGCGCGACCGATCTACGATCACGGAGGGTTCCGCGAGCCCCACTATCTGCGGTTTGGCCT
GGGCAGCGAGTTCGCTCCGGTACGCACGCCACCGCCGGCAACCCTGGGCAGCGATGGGATCAATGACGAGTTGCAA
CAGTGCCTGGTGACCGGGCGGCTGATGCAGTACCTGTTCAGCGAGCATAGCCGCGCCCTGGTCGACCAGGCCGAAC
GTGAATCGACCTCCAACCGTGAGAGCCGTTGGCAAGTGTTGATGGAGGGCGCCAGCCTGTTGTTCGGCAGCCTGCT
GCAAGGCTTGCGCGGCCCGGCGATGCTCGTGGGTTGGTTGTTGACGATGACTGCCAGCTTCACCCAGGCCATCCAG
ACCATCGTCAGCGACGACACCAGCGCCAAGGAGCAGGCAACGATCGATCTGTTGCTCAACGTCGCGATGTTGCTGC
TGGACTTTGCGCCGGCAAAGGCTTGGTCAAGCCTCGATCCGACGCTCAGGCAGCAAGCCCTGAGCACATCGCTGCG
GCGCCGTGAAAGTGACGTATGGCCCGTGCCGCCTGCAGCGCAGGTGCGCCAGGGCACGGTAGGACTGCCGGGGGAA
ATGGCACGCGGCGACAACGCAGCGCTGGATTTCAGCTTCACCCAGGCCCGTCATCGCCTCACCCCCAACCAGCTGT
CACGCCTGCAGGCCTTCAAAGTTGACGCGCCTCCCAAACTGCCGGCGCCGCAATCGAGCGGGCCATACAAAGGCTT
GTATACGGTCGAAGACCGCTGGTACGCCGTGGTCAATGGCGACCTGTATCGGGTCAGCCTGGAGGATGACGCCAGC
GTGGTGCTTGTCGATGCTGCCGACAGCACGCGGCGCGGCCCTTATCTGTGGGTCGATGAACGTGGGGCCTGGACAG
TCGATACGCGGTTGCGCCTGCGCGGCGGCATGCCGCCCAAACGCATCGCTGCCGAACGCCAGCGCAAGGCGCAGCG
CATCGAGGCGCTGACCGCTGCTTATCAGCGCATGCAGTTGCGCCAACGGGCCTTGATCGCCGCCACGGACCAGGCG
CAGCAGACCATGCTGAACCTGGAACAACTGCCCCGGCCCAACGAGGTGCAATTGGCCAATGCCCGCCGCCATTTCG
ACACCACGCTCAACGAGGAAACCACCGAGTACCAACAGATCCTCGATAGCCTCAAGGAACGGGAGGAACTGCGCAT
CTCGCCACCGCTCAAAGACACCCTGGCGCTCCTGGAAAACACCATCAAAAATAGCCGTAAACACGTGATCATCGCG
GAAAAGGATCGGCTGGCGCTGTACCGCACCTATCAGCGTTTTGGCATCGAGGGCCCCGCGTTGTCGCTGGCAGTGC

TGCGCGATCAAGCGGGCTATCAGCAGTTCCTCGTGCACTTGCTGGAGATCAACGAGCGCACCATCCATTGGCTCGA
ACTGCAGGAGCGTTTGCTGGAGCAATTATTCGGCCTGGGCACCAGTGGCGCTGCCGTGTACGAGCGCCTGACCGAG
GGCCGGCCGAACGAAATCACCGCCCTGGCCGTCAAGGACCTGCAGATCCGTGGTTTGAAGCGCATGATCGTCAAGG
ACTTTGCCCACCCGTTGTTCAACGCCCTGGACAGTATCGTCTCGCCCTTGCAGCAACATGTACGCACCCACGGTGA
GCTCAACAGCCTGGAGCTTTCAGCGGGCGATCGCCTGAGTGTGCTGGAGAGCCTGATGGAGCACTACGGCAGTGCA
TTGGACGGCCTGCGGGGGTTGAACATTGTCGATGCCGAAGTGCTCGACCCGATGTACACCGGCAAGCTCATCGGGG
TGATCGAGGCGCTTTACCAGAACGCCGCCCAGCGCCTGGCCAACGAAGTCAAACCCATTGCGCAACCTGCAAGGCG
CCCACCCAAGCGTCCCCCCGTCAGCGCCGGAAGGCCGCTGAAACGGGTCATAAAAACCCAGAGAAAAGGCACGTTT
ATCGGCCAGGTCAGCTCCGTCGGCAGCCTGGAAGTGGTGGAGGTACGAGACGAGGTCAGCAACCAGTTGCTGGGCA
TGTACTCGCAGCGTGGCGACGAATGGGTCGAATTTATCGAAGCCCCACCGCCGGCGCCGGCCCTTACCACCCGTGC
CGTCAGCGTCGTCAAGGGCGAGGCGCGCAAGTTGCTGGCAATGCTGGACGACCACCTGCGCCGGGCCGAGGACTAC
CGGAAAATCTCCCGGCATCCGCAAGAGGTGGAAGAGGTGCTGCAGTACGAGGCTTCACGCTACGACAAACTGGCCA
GCGAGCTGGACCGGGCGATCCAGGCCCAGGCCGAAAGCGCGCGCATCGCCGCCGACCAGAAGCTGGCCAGCGATCT
GCACCTGGCCTCAGCGCGCTTGAGCACCCGGGGCCAGGCACTGCGCCGCCAATTGTGCCTGGAACTGCCGCCGAAC
CACGGCAACCTGGAGTACCTGATCACGCAAGGCCAGGTCAGCGTGGCGAAACTGGGCGAGCGTCTTCAATTGAGCG
GTGAGCGACGGGACTTCATCCAGGAATACGCGATCAACGACAATGCAGGTTACCCGGTGTGGTACGCGCACTTTCA
CTACCCCGCCGCGACTACCCCCAAGACCGAGTACAGCACCGCGCACTTGAAAACCCGCGAGCAACGCCGGCAAAAC
TACTACACCCTGCTAGACAAAGCGCAGGGAACCCAGGCGGTGGTAGACATACACCGCGGGTTGATTGGCAAGGCGT
TGGCTGAGCGTTGGTTTCTGTCGTNNNNNNNNNNTGTTTTGTGGCGTGTCCAAAATTCCCTGCCTCGCCGCGATCC
CCTGTGGGAGCGGGCTTGCCCGCGATGAGGCCACAACAGCCCCACATTTCTCCAGGCATAAAAAAACGCCGCGTAC
CTTGCGATACGCGGCGTTTTTTGTGTCAGCAGACCTTAGGCTTGAACCACCGGAATGTTGGCACTTGCAGCGATTT
TACGGAACTCGGCGATCTGGTCGAAGTTCAGGTAGCGGTAGACATCACTGGCCATGGTGTCCAGTTTGGCCGCGTA
GCCCATGTACTCTTCGACGGTCGGCAGGCGACCCAGCGTGGAAGCGACTGCCGCCAGCTCAGCCGAGGCCAGGTAG
ACGTTCGCACCATCACCCAGACGGTTCGGGAAGTTACGGGTCGAAGTCGACACTACGGTGGAGTTCGGCTCTACAC
GTGCCTGGTTACCCATGCACAGCGAGCAGCCCGGCATTTCCATGCGCGCGCCAGCCTTGCCGTAGATGCCGTAGTA
GCCTTCTTCGGTCAGTTGGTGAGCGTCCATCTTGGTCGGCGGCGACAGCCACAGACGGGTTGGCAGCTGGCCCTTG
ACCTGCTCCAGCAACTTGCCGGCAGCGCGGAAGTGACCGATGTTGGTCATGCACGAACCGATGAACACTTCGTCGA
TCTTCTCGCCAGCAACGCTGGACAGCAGACGGGCGTCATCCGGGTCGTTCGGCGCGCAGAGGATTGGCTCGTTGAT
TTCGGCCAGGTCGATTTCGATGACTTCGGCGTATTCGGCGTCGGCATCGGCTTCCATCAGCTCAGGGTTGGCAACC
CAGGCTTCCATCGCTTGGGCACGACGTTCCAGGGTACGCGCATCACCGTAGCCTTCACCGATCATCCAGCGCAGCA
GGGTGATGTTGGAGTTCAGGTACTCGGTGACGGAGTCTTTCGACAGCTTGATGGTGCAACCGGCAGCCGAACGTTC
GGCCGAGGCGTCGGACAGCTCGAAAGCCTGCTCCAGCGTCAGGTCGTTCAGGCCTTCGATTTCCAGGATGCGACCA
GAGAAGGCGTTTTTCTTGCCTTTTTTCTCAACGGTCAGCAGGCCGGACTGGATCGCGTAGTAAGGAATGGCATGAA
CCAGGTCACGCAGGGTGATGCCAGGTTTCATTTTGCCTTTGAAGCGCACCAGGATCGATTCCGGCATGTCCAGTGG
CATTACGCCAGTGGCTGCGGCGAACGCGACCAGGCCAGAACCGGCCGGGAACGAGATGCCCATCGGGAAACGGGTG
TGGGAGTCACCGCCGGTACCTACGGTGTCCGGCAGCAGCATGCGGTTCAGCCAGCTGTGGATGATGCCGTCGCCTG
GACGCAGCGATACACCGCCACGGGTCATGATGAAGTCAGGCAGGGTGTGGTGGGTGGTCACGTCGATCGGCTTTGG
ATAGGCCGCGGTGTGGCAGAAGGACTGCATCACCAGATCGGTGGAGAAGCCCAGGCACGCCAGGTCTTTGAGTTCG
TCACGGGTCATAGGACCGGTGGTGTCCTGGGAGCCGACGGTGGTCATCTTCGGTTCGCAGTACGTACCTGGACGAA
CGCCTTTGCCTTCTGGCAGGCCACAGGCCTTGCCGACCATCTTCTGGGCCAGGGTGAAACCCTTGCCAGTGTCGAC
AGGTGCTTCAGGCAGCTTGAACAGGTCGGTAGGGCCCAGGCCCAGCTCGGCGCGGGCCTTGTCGGTCAGGCCACGG
CCGATGATCAGTGGGATACGGCCGCCGGCACGTACTTCGTCCAACAGGACCGGGGTCTTCATTTCGAAGGTGGTCA
GGACTTCATCGGTGCCGTGTTTGCAGACTTTGCCAGCATGCGGGTACAGGTCGATCACGTCGCCCATGTGCATGTT
GGTAACGTCGAATTCGATTGGCAGTGCGCCAGCATCTTCCATGGTGTTGTAGAAGATTGGAGCGATCTTGCTACCG
AAGCAGAAGCCGCCAGCGCGCTTGTTAGGCACGTAGGGAACGTCGTCGCCGAAGAACCACAGTACCGAGTTGGTTG
CCGACTTACGGACGAACCGGTACCGACTACGTCACCGACGTAGGCGATCGGGAAGCCCTGGCCGCGCATTTCTTC
GATCTGCTTCATCGGGCCGGTCTTGCCTTGCTCGTCCGGCACGATGCCTTCACGGGCCATTTTCAGCATGGCCAGG
GCGTGCAGCGGGATGTCAGGACGGGACCAGGCATCGGGAGCAGGGGACAGGTCGTCGGTGTTGGTTTCGCCGGTGA
CCTTGAATACACGCAGGCTGATCTTGTCGGCCAGGGTCGGGCGGTTCTTGAACCACTCGCCGTCGGCCCAGGACTG
GATCACTTCCTTGGCGTGCTGGTTGCCGTTCTTGGCTTTTTCCGCGACGTCGTGGAAAGCATCGAACATCAGCAGG
GTGTGCTTGAGCTGGGCAGCTGCAACGGCGGCCAGTTCGGCGTCGTCCAGCAGTTCGACCAGCGTCACGATGTTGT
AGCCGCCTTGCATGGTGCCGAGCAGTTCAACAGCGCGCTTTTTGTCGATCAGGGGAGAAGAAACTTCGCCCTTGGC
CAGGGCAGACAGGAAACCGGCCTTGACGTAGGCAGCTTCGTCAACGCCTGGTGGAATGCGGTTGGTGATCAGGTCA
ACGAGGAACTCTTCTTCGCCAGCCGGGGGATTTTTCAGCAGCTCGACCAGGCCTGCGGTTTGTTCGGCGTTTAGCG
GCTGGGGAACGATACCCAGGGCTGCACGCTCTTCGATATGTTTGCGGTAGGCTTCAAGCACAGTTATTACCCTCAT
CAGTGGTCCCACGGGACGCTCATCCAGACATGCACGGCACGCATGCGCTCGAGGGCTTTTTGAGCCGTAGAGCCAG
CGCTGCCGGCATTTCTCACAGAAGCTGCTTTCAAAGTTTTACGCCTGCAGAACGGAGCTGATGAGGGTTGGTGCTG

GTTATTTAAAGGTTAAACAACCAGCGCCAACACCGTTCTGAAGGAACGACTGTGCTCGTGACGCTTTGAAAACAGC
TTCCAACGGATTATTTGGCGCCTTACAAGGCCGGATGATTCTACGGCAAAAATTTTCTAAAGGTAAGTTGCCGCAG
CAAGTTTGCAGGGTGATGAACCTTAGACAAAGGGCTAACATGGGCCACTGTTTCGCTGATTTGTGCGCTGCCACCC
ATGTCCAACCAAACCATCAAGACCCCTTGCATCGGCCTGTGCTCCACGGTTTACGGCGATCTCGTGTGCCGGGGCT
GCAAGCGTTTCCACCACGAAGTGATCCAGTGGAACGGCTATGGAGAGGAAGAAAAACGTGCCGTATGGCTGCGGCT
TGAGCAACTGCTGGTGCAGGTGATGGCGGGCAAACTGGAGGTTTTCGATCCCAAACAGCTGCGTTGGCAGTTGGAG
CAGCGCAAAATCCGCTTTGTGCCGCACCAATCCGAATACTGCTGGGCCTACCAGTTGATCGCCCGGGGGGCGCGGG
TGATCAACAACCTGGAAGCCTATGGGATGGTCCTGATGCCGGAGTTTCGCGACTGGAACCTGCCGGAACTGCGCGA
TGCCATTGATCGGGAATTTTTTATCCTGTCCGAGGCGCATTACCAGCGCTACATCGCCCCTGGGTTCCTCAAGGAT
GCACTTTCTGGCTGACGACACCGATCAAAATGTGGGAGCGGGCTTGCCCGCGATGGCGTCCGTTCAGGCAGAGATG
TATCAACTGACCCACCGCCATCGCAGCGGTGCGGCGATCCGACAAGCCAGCCCCTACAATTGATTTTCATTGGCCG
AGGGATGGGTATCAGGGCTTATTGGCCAGTTCCTCCAGGTGATCGATGATGCTCTGGGGCTTAAGCACCAGCACAT
CGCTCTCCAGCGCATCCAGCACCACTTCTGCCGTGTTGCCGATCAGCGCCCCGGAAATCCCGGTACGCGCCACGGT
GCCAATGATTGTGACCGCCGCGCGCAATTTATGGGCGGCATGGGGAATCAGTACATCGGCCGGGCCTTCTTCGATG
TGCAGGCGGTTGTCATCGATGTCGAATTCGGCCTGGAAGGCCTTGCACTGCTCGCGATAGCGGGCTTCGATGGTTT
CTTTGAGTTGGAACACAGGATCAGCGGCCGACAGCATGGGCGACGGATGGGCGCTGATCACATGCAATTGGCCCTT
GGCCAGGCTGGCAATGTCAAAGCCATGATCGACAATGGCGGCGTGCAGGGATTTATGCTCGTTGTCGAGGTTGCCC
ACGTCGATGGCCGCCAGGATCACCCCGTCGGTCCACGGGGTCGCGGTCTTGACCAACAGCACTGCCGCCGGGCACA
AGCGCAGCAGCTTCCAGTCGTCGGGTGTCAGCAGGGCCTTTTTCAGCGGGCTGTCGGGGTAGTGCTGCTTGATCAC
CAGTGCGCACCCTTCGGCTTGCTGCACGGCGATGATGGTTTCGTGCCGATTGTTTTTCCAGGCCAGTTCGGTGCTG
ACGCTATAGCCGTCTGCCAACAGTTGTTCCTTCAGCAAGCTCAACAATGCCGAATGCTCATGTTTTGGATCGCATA
CCAGCAAATGCAAGTGTGCGTCGGTCACCCCGGCGATCAGCTTGGCGCGCTTGAGGGCCAGGCTCTCCGAGTGTTC
GGGCTCGATGATCACGAGGATTCTGCCAATATCGTTCATGAGCGGGATCTCCAAGGGTGGAAAGGACAGGGCGTGG
AACAACTATAGTTGTTGCCCGCCGTACGGCATGTTGATGCACATCAAGGCCCGTGACTGGTGGTCTGCAGCGGGGT
CGGTATAATCGGCGGCCTTTTGTGATCCTTTGCCCGTGAGCCCGATGAACCTGCCCGAAATCCATGAATTCCTCGG
TTGCCGCACCCCCGATGCCTGGGTCCAGGCCGCACTGGCCGACCAGGAAACCCTGCTGATCGACCACAAGAACTGC
GAGTTCAAGGCCGCCAGCACTGCCCTGAGCCTGATTGCCAAGTACCACTCCCATGTCGACCTGATCAACATGATGT
CGCGCCTGGCCCGCGAAGAACTGGTGCACCATGAGCAAGTCATGCGCCTGATGAAAAAGCGCAAGATCGAGCTGCG
CCAGTTGCATGCCAGTCGCTATGCCTCGGGTCTACGCAAGGTGGTGCGCAGTCACGAACCGGTCAAGCTGGTGGAC
ACCCTGGTGGTAGGCGCTTTTATCGAAGCGCGCAGTTGCGAGCGTTTCGAAGCACTGGTGCCACATTTGGACGAAG
AGCTCGGCAAGTTCTACTTTGGCCTGCTGAAAAGCGAGGCGCGGCATTTCCAGGGTTATCTGAAGCTGGCTTATCA
GTATGGTGATGCCAAGGACGTTGCCCAGGTGATCGAGCGGGTCCGCGCCGCCGAGCAGGAGTTGATCGAATCGCCG
GACGTCGAGTTCCGTTTCCACAGCGGCGTACCGGCCTGATCGTGAAAGACTGCGGGCTATTTGTAAAAAACTCTTA
AAAACATGAAGTTTGCGTCGAAGCCCCGCAGTAAGGGGCTCTGCATGCGCTTGTGGAGCGCGGTTGCCCACTATAA
TGCGCGCACTTCAATCGGCGCGCGCAGTGGAAACTTATGGAAAACTTGGGTCTGGGCAAGGTCTTGCTCGTTGAGG
ACGATGAGAAGCTGGCAGGGCTGATCGAGCATTTCCTGGCACAGCATGGCTTTGACGTGCGCGTGGTGCACCGGGG
CGATGAAGCCTTGGCGGCGTTCCTGGCCTTCAAGCCGAAGATCGTCGTGCTCGACCTGATGCTGCCGGGCCAGAGC
GGCCTGCATGTGTGCCGCGAGATCCGCAATGTGTCCGACACACCCATCGTGATCCTCACCGCCAAGGAGGATGACC
TGGACCATATCCTCGGCCTGGAATCCGGCGCCGATGACTACGTGATCAAACCGATCAAACCACCCGTACTGCTGGC
ACGCCTGCGCGCCTTGCAGCGCCGCCATCTGCCGGAGCCGACGGTGCGTGGCTCGCTGGCGTTCGGCCAACTGGCG
ATTGACCGCAGTTGCCGGGGCGTGAGCCTGGGTGATGAGAAGATCGACCTGACCACCATGGAGTTCGAGCTGCTGT
GGTTGCTGGCCAGCAGCGCGGGGAAGATCCTCTCGCGCGACGATATCCTTAACCGCATGCGCGGGATTGCCTTTGA
TGGGCTCAACCGCAGTGTCGATGTGTATATCAGCAAGCTGCGCAGCAAACTCAACGACAACCCCCGAGAACCCGTG
TGCATCAAGACCATCTGGGGCAAGGGCTACCTGTTCAATCCGTTTGCCTGGGAGGTCTAGCCAGATGCTGCGCCTG
TTTCTGCGCCTGTACATCATCCTGGCCCTGGGGTTGGCGGGGGCGATCTGGCTGGTGAACTACACCTTCGACGAAT
TGCTGCCCGAAGCCAACGAAACCTACAACCGTGAAGCCATGCGAGGCCCGGCCTACGGCTTGGTGGAGCAGTTGCG
TCCGTTGCCGCCCGGGCCGGCCCGCGAGGCGCGGCTGGCCGAGCTGCAAAAGCACTATGGCTTGCGCCTGGGGTTT
ATTGACCGCGACGCAGCCGGCCTTGACCCCCGCGAGCAACAGTTGCTGGCTGATGGCAAGTTGGTGGTGCGCGGCG
ATTTCATGGAGTTCATCACTGAAATCGACGGCGGCGCGCAGTTGCTTGAGGTCAAGCTGCCGGTGGAACCACGCTG
GCTGTATCTGTGGGCCTACAGCTTGTTGGGCTTGTGCCTGGCCATCGTCCTGTATTTCTGGGTGCGCCCGCACTGG
CGTGACCTTGAGCACATCCGCCTGGCGGCCCAGCGTTTTGGTGACAATGACCTGGGCTCGCGCATCCTGTTGCCGC
GTCGCTCCACCGTGCGTGAGTTGGCCGGGCACTTCAACCAGATGGCCGAGCGCATCGAGCACTTGATCGCCAACCA
GCGTGAGCTGACCAACGCGGTGTCCCATGAATTGCGCACACCGATTGCGCGCTTGTCGTTTGAGCTCGATCAACTC
AAGCAGGAAGCGGACCCGCGCCAGAGCCGCGCGCTGATTGGCGACATGTACGCCGACCTTGGCGAGCTGGAAGACA
TGGTTTCCGAGCTGTTGACCTACGCCAGCCTTGAGCGTGGTGCCACCCAAGTCACCCGCGAGAACATCGAGGCCCA
AAGCTGGCTCGACAGTGTGATCGGCAGCGTCGCCCTGGAAGCGGAAGCGGAAGGCGTGCAGTTGTCACTGCGTACC
TGTGAAGTGGAGTTCATCCAGATCGAGCCACGGTTTATGGCGCGCGCCGTGATCAACCTGCTGCGCAATGCCATTC

GTTATGCCGAGCATCGGGTGGAGGTGTCGTTGGTCAAATTCGGCAGTGGCTATGAGGTACGCGTCTGCGATGACGG
CCCCGGCGTGCCCGAGGATGGCCGTGAGAGAATCTTCCAGCCGTTTACGCGCCTGGACGCCAGCCGCGACCGCCGC
ACCGGCGGCTTTGGCCTGGGCCTGGCGTTGGTCCAGCGTGTCAGCCAGTGGCATGGCGGGCAGGTGCAGGTGCTGG
ACTCGCAGTGGGGCGGGGCGTCGTTTCGCATGACTTGGGCCTACGCCGATCTGTAGATGCCGACCGGCCGATTGTG
GCAAACGGGCTGTTGTGGCGAGCGGGCTGTTGTGGCGAGCGGGCTTGCCCCGCGCTGGGCTGCGAAGCAGCCCCAA
TAAGTCCGCCGCGTTTCTTCAGATATACGGCGGTGACTGGGTTTGGGGCTGCTCCGCAGCCCAGCGCGGGGCAAGC
CCGCTCGCCACAGGGCAAATCGTCAAAAGGTGTAGTTCAGCACGCCCATCACCGAAGTCTGCATCCTGCGCTCCAC
AATCGGGCTTTTACCCGCATCACCCGACAAGTACTGCACATCCAGCAAGGTCGAGAATGTGGTGTGCTCGCCAAGG
GGCACGCTCCAATCGAGGTTCAGCCCCCGGCTGACCATCCCACCCTTGGCGTCATACGCGCGAAAGCGGCTGCGCT
GGGCTTGGCCGCTGGTCACGCCATACCACGTCTGTACATAGTTGCTATCGCCAAAGCGGCTGTTGAGGCTGGCGTC
CACGGTGCCGTAGTCGCCGTCATACAGGTTGGTGCCGATGCTCAACTCCAATGAAGTGAAGGCCTTGCCGCTGTCT
TTTTTATCGTCTTGCTCCAAGGCATGTTCCAGGGTTGCGCCCAGGATCGCGCTGCCCAGGGTATAGCTGGCACTCA
CCCCAAACTGTGGCCGCGACTTGATGTCGCCCATGCCCTTGAGCCGGTCGGAACCCAGGTGGGTACTTTTCTTCTG
GTCCTTGCGCGCGGTGCTGGCGCCGACATAGGCGCTGAAGCTCAACTGGTCATCTTCATAACCCCAGCCCAACCCC
TTGTTGGTATCGAGAAAAATCCCCCATGGGCTGACGACGTTTACGCCCAGCAGGGGCGCGGTCATCCGTTCATTGC
TGCCGCTGTAGCGCGGCAGGTTGGCCGCCCCGGCTTGCAGGCTGTATTGCCAGTCGGTGGCGACTGACAGTTGGGT
GCAGGTGAGCAGGCACACAGAGGTGGCAGCGAGGTAGATTGTTCTTGAACGCATGATGGCCTTCGTTGTTCCATGG
GAGGCTCGAATCCTAGGGACGGCGTGGGCAGGGGTCTTTGACAGCTTTGTCGGGAAACTGTCAAAGACTGTTAAGC
CGGGGCTGCCTGCCCAGCGCCCAAACGCCGCACCGCCCCCAGACTTGATTTCAATCAAGGGCTCACTGGCCACCTG
CCACCTACCATCGGCTGCATATTCCGGCCGTTGAAGTCAGTGAGCCCATGAACCGCACCAAGCCCTCGACCCTCGC
TACCTTGGCGGCAAGTCCCGTCACCTGGTTGGTTGTCCTGGTTTTACTGTTCAGCCTGGCCGGCCCGGCCCAGGCC
AAGTCCTACGGCGCGATTGAACAGCAGCGCATCGACAAGATCTTCCCCCAGGCAGACGCCATTTCCGCCCCTGAAG
GCAAGTTCAAGGTGCGCACGCTGTCGGCCAAAGGCGCGGTGATCGGCTACGTGTTCCAGAGCCTGGATGTGGTGGA
TATTCCGGCTTACTCCGGCAAACCGATCAACACCCAAGTGATCCTCGACCCGGCCGGGGTGATCCGTGATGCCTAT
GTGCTGGAGCACCACGAGCCGATCCTGCTGATCGGCATTCCCGAGGCCAAGTTGCATGACTTCACGGCGCGCTACA
GTGGGATCAACGTCAGGCAGCGGGTAGTGGTGGGGCACTCCAGCGACAGCCAGGCGGTGACCGTGGATGCGGTGGC
CGGTGCCACCGTTACGGCCATGGTGGTCAATGAAGTGATCATGCGTGCCGCCCACGAAGTGGCGGTGTCGCTCAAA
TTGATCGAGGACAAGGCCAGCACCGCGCAGAAAGTCGCGCTGGTGCGCCCTGATCTGTTCGAGCCCGCCAACTGGG
CGCAATTGACCGGCAATGGCGCAGTGCGCCGCCTGCACCTGACACGCGGCCAGGTGGACGCTGCCTTCAAGGGCAC
CGAGGCCGAAGGTGTGGAAGTGGCGGGGCCGGGGCAGGCCGACGAGACATTTATCGACTTGTATGTGGCGGATCTC
AATGCGCCGACCATCGGCCGCAACCTGTTGGGCGAGGCGCAATACCGGGTGCTGATGCAGGGCCTCAAGCCTGGCG
AACAGGCCATTGCGGTGCTGGGGCGCGGGGTTTTTTCGTTCAAGGGCTCGGGCTACGTGCGCGGCGGGATTTTTGA
TCGGGTGCAGTTGCGCCAGTTCGGCAACGTCATCAGCTTTCGCGATATGGACCACCAGCGCCTGTATGACGTATTC
GCCGAGGGCATGCCCGAGTTCGAGGAAATGTCGGTGTTTATCGTGCGCCCCCAGGCCGCGTTCGACCCAGGCTCGG
CGTGGACCCTGGAGTTGCTGGTGCGGCGCCAGACCGGCCCGGTCAGCGGCACCTTCACCAGCTTTGAACTGCCTTA
CCAGATGCCCGAGGCCTACCTGGAACGACCCCTGCCGACCGCCGCGGAACAGGCCGCCATCGACGAAGCCAACCGG
CCGATGTGGATGACGATCTGGTACCAGAAACAGGTGCAGGTCCTGGTGCTGGGCGCCGCCCTGGTGCTGTTGACGG
TGATTCTGTTTTTGCAGGACAGCCTGGCCCGGCGCCCGCGTCTGCTGCACTGGGTGCGTCGCGGTTATCTGCTGTT
TACCGTGGTGTTTATCGGCTGGTACGCCCTGGGCCAGTTGTCGGTGGTCAATGTGCTGACCTTCGTGCATGCGCTG
TTCCAGCAGTTCCGCTGGGAGCTGTTCCTCACCGACCCGCTGATCTTCATGCTCTGGGTATTCACCGCCGCCAGCA
TCCTGTTGTGGGGGCGTGGGGTGTTCTGTGGTTGGTTGTGCCCGTTCGGGGCGTTGCAGGAGTTGATCAACGAGGC
GGCGCGCAAGCTCAAGGTGCGCCAGTTCGAACTGCCGTTTGCCGTGCATGAGCGGCTGTGGGCGATCAAGTACCTG
ATCCTGCTGGGGCTGTTTGGCCTGTCCCTGGAGTCGATGGCCACTGCCGAACGGTTTGCCGAAGTGGAACCGTTCA
AGACCGCCATCACCCTGCATTTCGACCGCCAGTGGTGGTTCGTCGCCTATGCCTTGGTGCTGCTGGTGATCAACAT
TTTCACGCGCAAGGTCTATTGCCGCTATCTCTGCCCGCTGGGCGCGGCGCTGGCAATCCCGAGCAAGTTCCGCCTG
TTCGACTGGCTCAAGCGGCGCAAGGAATGTGGCGATCCGTGCCAGTTGTGCGCCAAGGAATGCGAGATCCAGGCGA
TCCACCCCGATGGCCATATCAACGCCAATGAATGCCACTACTGCCTCGATTGCCAGATGACCTGGCACAACGACCA
CAAATGCCCGCCACTGATCAACAAACGCAAGAAACGCAACAAGGCCGCACCCACCCCTGGGCAATTGATCCCGGTG
GTGCAGGTGGCGGTGCCTGAATGACGGCCTTGGGCACCCACTTTTCCCATCTGGAGCACATCATGAACGACAGCGA
ATCGAAAAAAACCGGGGCCACACCGCAACCCGAAGGCATGAGCCGGCGTGGCTTCCTCGGCACCGGCGCCGTGACC
GGCGCGGTGCTGGCAGGCGCCTCGGCCATCGGCGGTACGGTGTTTACCCGCGAGTCCTTTGCGGCGGCGGCCAAGG
AAGCGAAGTCGAAGATCCATGTGGGCCCGGGTGAGCTGGACCAGTACTACGGTTTCTGGAGCGGCGGCCACCAAGG
CGAAGTGCGGGTGCTGGGGGTGCCGTCGATGCGCGAGCTGATGCGCATCCCGGTATTCAACGTCGATTCGGCCACC
GGCTGGGGCCTGACCAACGAGAGCAAGCGCATCCTCGGCGAGGGCGCCAAATACCAGAACGGTGATTGCCATCACC
CGCACTTGTCCACCACCGATGGCCGCTACGACGGCAAATACCTGTTTATCAACGACAAGGCCAACACCCGCGTCGC
GCGGATCCGCCTGGACATCATGAAGTGCGACAAAATCGTCACCGTGCCCAACGCCCAGGCGATCCACGGCCTGCGC
CTGCAAAAGGTGCCGTACACCAAGTACGTGTTCTGCAACGCCGAGTTCATCATTCCGCACCCCAATGACGGCCACA

CCTTCGACCTGCAAGACAAAAACAGCTTCACCATGTTTAACGCCATCGACGCAGAAAAAATGGAAATGGCCTTCCA
GGTCATCGTCGACGGCAACCTCGACAACGCCGACATGGACTACACCGGCAAATACGCTGCGGCCACTTGCTACAAC
TCGGAAAAGGCCTCCGACCTGGGCGGCATGATGCGCAACGAGCGCGACTGGGTGGTGGTGTTCAACATCCCGCGCA
TCGAGGCGGCGATCAAGGCCGGCAAGTTCATCAACCTCGACGGGGTCAAGGTGCCGGTGGTGGACGGGCGCAAGGG
CGACGATGGCAAGGACAGTGAATTCACCCGCTATATCCCGGTGCCGAAGAACCCCCATGGCTGCAACACCTCGCCG
GACGGCAAGTACTTTATCGCCAACGGCAAGCTGTCGCCGACCTGCACCATCATCGCCATCGACAAGCTCGACGACC
TGTTCAGCGACAAGATCAAAGACCCGCGCGGCGTGGTCGTCGGCGAGCCGGAGCTGGGCCTGGGCCCGTTGCACAC
CACCTATGATGGCCGGGGTTTTGCCTACACCACGCTGTTTATCGACAGCCAGGTGGTGAAGTGGAACATCGCCGAA
GCCATCCGCGCCTATGGCGGCGAGAAGGTCAACTACATCAAGCAGAAGCTCGATGTGCACTACCAACCGGGCCACA
ACCACGCCTCGCTCACCGAAACCCGGGATGCCGACGGCCAATGGCTGGTGGTGCTCAGCAAATTCTCCAAGGACCG
CTTCCTGCCCACCGGCCCGTTGCACCCGGAAAACGATCAGTTGATCGATATCTCGGGCGATGAAATGAAACTGGTC
CACGACGGCCCGACCTTCGCCGAACCCCATGACTGCGTGATGGCGCGGCGTGACCAGATCAAGACCAAGAAAATCT
GGGAGCGCAACGACCCGTTCTTTGCCGAAACCGTGGCCATCGCCGCCAAGGACGGGATCAAGCTGGAGACCGATAA
CAAGGTGATCCGCGACGGCAAGAAAGTACGGGTGTACATGACCTCCATGGCGCCGACCTATGGCCTGACCGAGTTC
ACCGTCAAGCAGGGCGATGAGGTCACGGTGACCATTACCAACATCGACCAGATCGAGGACGTGACCCACGGCTTTG
TCATGACCAACCATGGCGTGAGCATGGAAATCAGCCCGCAGCAAACGTCGTCCATTACCTTCATCGCCGACAAGCC
GGGGCTGCATTGGTACTACTGCAGCTGGTTCTGCCATGCGCTGCATATGGAGATGGTAGGGCGCATGTTGGTCGAG
CGGGCTTAACGGCGAGCACCAACCAATGTGGGAGCGGGCTTGCCCGCGATAGCGGCGGTTCAGCCACTATTTTTGG
CGGCTGGTATACCGCCATCGCAGGCAAGCCAGCTCTCACACTAGATCTGTGTCGCTCCACAGATTGGGTTTTGATC
AAAAGGAATGCTCCGTGGTTCAACCCCGCACGTTATTGATCGGGTGTGTGTGGCTGCTATTTTCGGCCACCGCCAG
CGCCGCTTTACTGCCCATCACCGACTTGCCGTTGGTAGCCGCAGGCCCGCAGCACTGGCGCCTGCCCGCCGGCGAC
TACCTGGGCTCCTTCAGCATCGACCGACCCCTGCAACTCACCTGCGAACCCGGTGCGGTGATCCACAGCCAGGGCT
TGGGCAACGGCTTGCTGATCAGCGCTGCCGACGTCACCGTCGAGGGCTGCACCTTCCTCGACTGGGGCCATGACCT
CACCGCGATGAACGCGGCGATCTTCATCCAACCCAAGGCCCACGGCACCTTGATCAAGGGCAACCATATGCGCGGC
CAGGGTTTTGGGGTGTGGGTCGATGGCACGGCGGACGTGAGCGTGATCGACAACCAGATCCAGGGCGACCCCACCC
TGCGCTCCCAGGACCGGGGCAACGGCATCCACCTGTATGCGGTCAAGGGCGCGCGAGTGATCGGCAACCACGTGCG
CGACGCCCGCGACGGCATCTACATCGACACCTCCAACGGCAACCTGCTGCAAGGCAACACCCTGGAAGACCTGCGC
TATGGCATCCACTACATGTTTGCCAACGACAACCAGGTACTCGACAACATCACCCGTCGCACCCGTACCGGCTACG
CCTTGATGCAAAGCCGCAAGCTGACGGTGATCGGTAATCGCTCCGAAGAGGATCAGAACTACGGGATCCTGATGAA
CTACATCACCTATTCCACCCTGCGCGACAACGTGGTCAACGATGTGCGCGACGGCACCACGGGCGACACCATGATC
ACTGGTGCCGAAGGCAAGGCACTGTTTATCTACAACTCGCTGTTCAACAGCATCGAACACAACCACTTCGGGCGCA
GTGCGGTGGGCATTCACCTCACGGCCGGCTCCGAAGACAACCGGATTGCCGGCAACGCCTTTGTCGGCAACCAGCG
CCAGGTCAAGTACGTGGCCACACGTTTGCAGGAGTGGTCGGCGGATGGCCGGGGCAATTACTGGAGCGACTACCTG
GGCTGGGACCGCAACAGCGATGGCCTGGGGGATGTGGCCTACGAGCCCAACGATAACGTCGACCGCCTGCTGTGGC
TGTACCCCCAAGTGCGCCTGTTGATGAACAGCCCCGGCATCGAGTTGCTGCGCTGGGTGCAGCGGGCGTTTCCGGT
GATCAAGTCGCCGGGAGTGATGGACAGCCATCCGCTGATGAAAGACCCCACGCTTTCCCTGCTCAAGGAGCCCGCA
TGATGCCTGTCGTCGAAATCCAAGGCGTGACCCAGCGTTATGGGCACGCCACTGTGCTGCATGACCTGAGCCTGAG
CCTGGCGCCCGGGGAGGTACTGGGCCTGTTCGGCCATAACGGCGCGGGCAAGACCACCAGCATGAAGCTGATCCTC
GGTCTGTTGCAGGCAAGCAGCGGCCAGGTGCGGGTGTTCGGCCGGGCGCCCAGCGATCCGCAGGTACGGCAGATGC
TCGGGTACCTGCCGGAAAACGTGACCTTCTACCCACAACTCAGCGGCCTGGAGACCCTGCAGCATTTTGCCCGGCT
CAAGGGCGCGCCGATGAGCCAGGTCGCGACGCTGCTCGAAGACGTCGGCCTGACCGCGGCGGCGCGGCGGCGGGTC
AAGACCTATTCCAAGGGCATGCGCCAGCGCCTCGGCCTGGCCCAGGCCCTGCTTGGCGAGCCGCGCCTGCTGCTGC
TCGACGAGCCCACCGTAGGCCTCGACCCGATCGCCACCCAGGATTTGTATCGCTTGCTCGACCGCCTGCGCGGGCA
GGGCACCAGCATCATCCTTTGCTCCCATGTGCTGCCGGGGGTGGAAGCCCATATCAACCGCGCCGCGATCCTCACC
CAGGGCCGCCTGCTGGCCCTCGGCACCCTGGCGGCGTTGCGCGAAGAAGCCGGGCTGCCGACCCTGATCCGCAGCA
GTGGCTTGCAACGGGCGGCGCTTTTGCAAAAACGCTGGAGCGAGGCCGGCCACGTGACCCGGCGCTGGGGCGCGGA
GGGCCTGCAAGTGGCTGCGTTCAATGGCAACAAGCTGGACCTGTTGCGTCAGTTGCTCGACCAGGAAAACCCCAGC
GATGTGGAAATCGTCGTGCCGTCCCTGGAGGACCTGTACCGCCATTACATGGAAAGCCCTGCGGAGCGTGCCCTAT
GACGCCGATCTGGAACATGGCCCGCAAGGAATTCAGCGATGGCCTGCGCAATCGCTGGTTGTTAGCGATCAGCCTG
TTGTTCGCGGTGCTGGCCATCGGCATCGCCTGGCTCGGTGCGGCGGCGTCCGGGCAACTGGGCTTCACCTCGATCC
CGGCGACCATCGCCAGCCTGGCCAGCCTTGCGACTTTCCTGATGCCGTTGATTGCGCTGTTGCTGGCCTATGACGC
GATTGTGGGCGAGGACGAAAGCGGCACCTTGCTGCTGTTGCTCACTTATCCCCTGGGGCGCGGGCAGATCCTGCTG
GGCAAGTTCGTGGGGCATGGGTTGATCTTGGCCCTGGCGACCTTTATCGGATTTGGCTGCGCCATGCTCGCCATTG
CGCTGCTGGTGGATGACGTCGAGTTCAGCCTTCTGCTGTGGGCGTTTGGTCGATTCATGCTGACGTCGACTTTGCT
CGGCTGGGGCTTTCTGGGGCTGGCGTATGTGTTGAGCGCCGTCTCGGCAGAGAAGTCCACGGCGGCCGGGCTGGCC
CTGGGCGTGTGGTTCTTTTTTGTCCTGGTGTTCGACCTGGCGTTGCTGGCGCTGCTGGTCATCAGCGAGGGCCAGT
TCAGCCCGACGCTGCTGCCCTGGTTGTTGCTGTTCAACCCGGCGGATGTGTACCGGCTGATCAACCTGTCGGGCTT

CGATACCGGCCCGGCGTCGGTGGGCGTGATGGCATTGGCGGGCGACCTGGCAGTACCGACGGCGGTGTTGTGGCTA
TGCCTGGCTTTATGGGTCGTAGGGCCACTGGCCTGGGCCTATTGGCTGTTTGGGCGGCGCGCAGCCTGAGTTTTTA
TTACTGGAGCGGATGAGCATGAAAATCGGGTTGGTATTGCTGTTGAGCCTGCTGTTGGTCGGGTGTGACAAACCGG
TGGCGGCGGGCGTGGGTGATGGGCCGGTGGCGTTTCATCCGGCCGATGAGTGCCATGTGTGCGGCATGGTGATCAG
CGAATTCCCCGGGCCCAAGGGCCAGGTGGTGGAGCAGGGCGTGGCGAAGAAGTTTTGCTCGACCGCAGAAATGCTC
GGCTGGTGGTTGCAGCCGGAAAACCATCATGCCCAGTCAAAGCTGTATGTGCATGACATGGGGCGCAGTCATTGGG
ACACCCCGGATGATGCCCATCTGATCGACGCCAAGACGGCGTACTACGTGCTTGGCACCGGGCTCAAGGGCGCCAT
GGGCGTGGTGCTGGCCTCGTTTGCCGAGCAGGCGGTGGCACAGAAAGTCGCGGCGGATACCGGCGGGCGGGTGTTG
CGCTTTGAGGAGATTGACCTGGCACTGTTGCAGCAGCCGAGTGGCATGGCCCATCGCGGTCATTGAGGCTGCTCTC
CTGTGGGAGCGGGCTTGCCCGCGATAGCGGCAGTTCAGTCAGCATTTCTGGCGGCTGACACACCGCTATCGCAGGC
AAGCCAGCTCCCACAGCCTAGCGGTTGACCAGCTTCGCCGGCAAGGCGATCACCAGCAAGCAACTGAGCACCAGGC
TCCCGGCAAAGAACCACATCGCCCCGGCGCTGCTGCCCGTCAGGTCAATGGCCAGACCCATCAGCGAGTTGCTGAC
CAAGCCGGCGATGTTCGCCAGGGAACAGGCCAGGGCAAAGCCAGTGGCTGCCGCGGTGCCGGTGAGGAAGGTCGCT
GGCAGGCTGAAAAACACCGGCACCGCGCCAAGGATCGCCGCCGAAGCCACGGCAAACAGGGCCACGGTGGCGACCA
CGTTATGGGTGAAGAAGGTGCTGCTGGCCATGGCCACGGCGCCCACCAGAAAGGGCACGATAATGTGCCAGCGGCG
CTCACGGTATTTGTCGGAGCTGGCGCCGATCAGCAGCATGCCGGCCAGCGCGGCGACGCTGGGCAGGGCGGTGAGC
AGGCCGATATGGAAGGTGTCGCTGATCCCCGCGTTACGGATCAGTGTCGGCATCCAGAAACCCATGGCATAGGCAC
TGAGCAGGATCGAAAAATCGATCCCACCGAGCATCCACACCTTAATGTTGAAGAACCCATCACGAAAGCGCCGCGG
GCTGTGGCTGGCCTGGCTGGCGTCGGCGTGCAATTGCTGTTGCAGCAGGGCCTTTTCGTCGGCAGACAACCATTTG
GCCTGGTGATAGGTATTGGGCAGCGCCCAGAACGCCAGCACCCCCAACAGCACGCTGGGCAATGCTTCAAGCAGGA
ACAGCCATTGCCAACCGCGCAGGCCGGCCAGTTGGTCGAAGTGGCTCATGATCCAGCCAGACAACGGCCCACCAAT
CACACTGGACAGCGGCAGGCCGATCATGAACAGCGCAATCACCCGCGCCCGGCGATGGGTGGGGAACCAGGTGGTC
AGGTAGTACAACACCCCCGGCAGGAACCCCGCCTCGGCAACGCCGAGCAAAAAGCGCAGGACATAGAACTGGGTAG
TGGAGGTGACCAGCAGCGTGCAGGCCGAGAGCAGGCCCCAGGTGATCATGATGCGGGCAATCCAGATCCGCGCGCC
GACCCGCTCCAGGATCAGGTTGCTCGGCACTTCAAACAGGATATAGCCGACAAAAAACAGGCCGGCGCCCAGGCCG
AACGCGGTTTCGCTGAACTGCAACTGGTCTTGCATCTGCAGCTTGGCAAAACCGATGTTGATCCGGTCCAGATAGG
CGGCCAGGTAGCAGAAACACAGGAAGGGAATCAGCTTCCAGGTGATCTTGTGGTACAGCGCATGGATCGGGTCGGC
GACCGTGGGCATCGGTGAGTCTCATGGGCAAGGAGTGGGGCGGGGCGAATATAGCATTCCGACACCGACAAGCTCT
GTCTGAAACGCGCCACCTGACTGGGGTTGGCGTGCGAAAGCAGCTACGTTTAATGCTTCTAATAAAACAGAGTATC
CACGGATGAACTACCAACCCTTTACCCGAACCTTGATCGCGACGGCCCTGGTCCTGACATTCAGCGGTGTGCAGGC
GGCCTCCCAGGCCCCGGTGGCCGGTGAAAACGGCATGGTGGTGACGGCCCAGCATTTGGCGACCCATGTGGGCGTC
GATGTGTTGAAGGCCGGCGGCAACGCCGTGGATGCGGCTGTCGCCGTGGGTTACGCCCTGGCGGTGGTTTATCCGG
CGGCGGGTAACCTGGGGGGCGGTGGGTTCATGACCGTGCAACTGGCGGACGGGCGCAAGACCTTCCTCGACTTCCG
CGAAAAAGCCCCATTGGCGGCCACCGCCGACATGTACCTGGACAAGGACGGCAACGTCGTCCCCGGCCTCAGCGCC
AAGGGCCACCTGGCCGTCGGCGTACCCGGCACCGTCTCCGGCATGGAGCTGGCCCTGAGCAAATACGGCACCCTCA
AGCGCGCCCAAGTCATCGCGCCGGCAATCAAGCTCGCAGAAAACGGCTTCGCCCTCGACCAGGGCGATATCGACAT
GCTGCATAGCGCCACCGAAGAGTTCAAAAAAGACCAGGACCTGCGCGGCATCTTCCTCAACAAGGGTGAGCCACTG
CAGGTCGGGCAGAAACTGGTGCAAAAAGACCTGGCCCGCACCCTGCGGGAAATCTCCGCCAAGGGCACCGATGGCT
TCTACAAAGGCTGGGTGGCCAAGGCCATTGTCGATTCCAGTCAGGCCGGCAAGGGCATCATCGCACAGGCCGACCT
GGACAAATACCAGACCCGCGAACTGGCGCCCATCGAGTGCGACTATCGCGGCTACCACGTAGTCTCGGCGCCACCA
CCCAGCTCGGGCGGCCTGGTGATCTGCCAGATCATGAACATCCTCGAAGGCTACCCCATGGCCGAACTGGGCTACG
GCTCGGCCCAGGGTACCCATTACCAGATCGAAGCCATGCGCCACGCCTACGTCGACCGCAACAGCTATCTGGGCGA
CCCGGACTTTGTACAGAACCCGGTGGAGCACCTGCTGGACAAGAACTACGCCGCCAAACTGCGCGCCTCCATCGAG
CCGCAAAAGGCTGGCGACTCCACGGCCATCAAGCCCGGCGTGGCGCCCCATGAAGGCAGCAACACCACCCATTACT
CCATCGTCGACAAATGGGGCAACGCAGTCTCGGTGACCTACACCCTCAACGACTGGTTCGGCGCCGGCGTCATGGC
CAGCAAGACCGGGGTGATCTTGAACGACGAGATGGACGACTTCACCGTCAAGGTCGGCGTGCCCAACATGTACGGC
CTGGTCCAGGGCGAAGCCAACGCCATCGCCCCAGGCAAGGCGCCACTGTCGTCCATGAGCCCGACCATCGTCACCA
AGGACGGCAAGGCTGTGATGGTCATCGGCACACCGGGCGGTAGCCGCATCATCACCGCCACTTTGCTGACCATCCT
CAACGTCATCGACTACAAGATGAACATCCAGGAAGCCGTCAACGCCCCGCGCTTCCACCAACAGTGGATGCCCGAC
AGCACCAACCTCGAAACCTTCGCCCTGAGCCCCGACACCCAGAAAATCCTCGAAAGCTGGGGCCACAAATTCGCCG
GCCCCCAAGACGCCAACCACCTGGCCGCCATCCTCGTCGGCGCCCCATCCCTGGACGGCAAACCCGTGGGCAACAA
CCGCTTCTACGGCGCCAACGACCCACGGCGCAACACCGGGCTGTCATTGGGCTACTAACCCCAGGCAGGGCAGGGG
CCAGCCGCCCTTGCTCTGCTTCACAGAAGAATATTTTTTGAAATCAAGGGGTTGCCAGTCCCGGCAAATCAGTACA
TAATTGCCGCCATCGAACGCACTGAGGCATAAAAAACTTCAATGTTTTCAATGAGATAGAGTAGAGGCAAGCTTCA
CATAGCCCACTCAAGTTTATATGCGGTGAATGTCGGTGGTTTTGGCATTGATCGTTTGAGGCCGAGTAGCAAAATG
GTTATGCAGTGGATTGCAAATCCACCTACGCCGGTTCGATTCCGACCTCGGCCTCCACTCTTGAAAGCCCCGTAGA
TCAATGGTCTACGGGGTTTTTTATTGTCTGGGATTTGTGATCGTTTCCGCAACCTCTACATTTTTGACGTTCGCTA

ACCTTGTGAAATATCACACATTGACTCTGATGTAGGTGCTAAATTTCTCCTTGACCACTCTGCACTATCACGCCGG
TGGGCAGACAAGGAACGCGATTATGGCAATGAATCCTGCAACGGCTGTTTCACTAAGTACTGTCCGAATTGAATCT
TATTCAGAGGCTGGGAAAAGTGTTGGCACTGGTTATTTTTATAATATAGAAACCCCGACTACAGAGTCGGGATTTG
TTGTAACCCCTCTGATTGTTACTAATAAGCACGTGGTGTGTGGGTCTGAGTTAATTAAAGTTACTTTGGATATTGC
AAAAAGAAGCTCGCATATAAATGAAGATGGTAGTGTGGATTGGCAGGAAAGGCATACCTTTGAGTTGGATCCGCTT
GATGGATCTATTTTGATGCATCCTGATGATGAAGTAGATTTGTGTGTGATACAGATGGGGCAAATATTAGGTCGAA
TTACTGAGGGTTACACTCCAAAAAATAATTTCTTGGATAAGCGATGGCGACTTGGTGTGGATGATTTTCCGCACAT
AAGACCAATCGAGTCAGTGGTGATGGTTGGATATCCAAATGGAATGTGGGATAAGCATAATAATAAGCCTATTGTA
CGGAAGGGAATGACCGCAACCCATTGCTTAACTAGGTGGGATAACAACAGATATTTCGTAATAGACTGCGCTTGTT
TTCCAGGCTCAAGTGGATCGCCTGTATTTCTTTACGAAGATGGAATTTATCGAGTGAGTGGAGGCAAATATGCCCC
CGGCTTGGGTGCTAAGCTCATAGGTACGTTATGGGGAGGGCCGACATATTCAACTAAAGGTAAGCTCATTCCAAAG
CCGGTTCCATCTCTTTTGGCTACTGACGCAGGCGCTATTCCTGTTATAGAAGTAATGATGAATTTGGGGTTTGTAA
TTCATGCTGATGCGCTGGATGATTTTATTCCCATAATTAGTGGTCTGATTGAAAATAACCAGTGATGGGGATGCCC
GTCTTTATTTTTAATCGATTTAGGTGTCTTTTATGCGGCGGAATACGCCGAACATTTTTTATATTTTATTTTAAGA
GGAAAGATGGTTTTATTTTGTTGGCTTCACTATCTCACCTATTCTTCTATACACTTTTTTGGTCATTTCTTCTATG
GAATGGCCGAGAAGCCGACTGGCATGAGTTAACTCGATTTCACTAGCAGCTTTTGGGCGAATGTCTTTGAACTGGA
ACTGACGGATCAGCACGGCTAGGGCCGTGTCACCGTCGGCACCGGCCTTAATGGCGGCTTTTTCTCGAGCTTCGTC
CCAGCGGTTGCGTAACATTTGCGCGCTCATGCGAAGGCCGGATGTGTTGGTGATCAGTCTCGACGTTTTGATCCCT
GCGAGGGTCCGGCGCTCCTGTAGATTGCTGATAAAAGCACTGAGCCCGGATTGCACTCCGTCGTCCTCCAGTAGAA
GGCGCAGCTTTTTTTCGGTTTTTCCCTGTTTGACCATCAAAAACCCTGCGTTCAAGTCGGTGACGGCCACTTTGAG
CACATCGGCAGGACGCTGGCCAGTGAGATAGGCCATGTCCATCGCGTCCTTTAGTTCCTGCACGGCCTCGGTGTAC
ACCGCAACCCGCCGCACCCATTCAGCGGCGCAGCTCCACAAGCGGATATTTAAAGAGGCGTTAGGTTTGAGGGAGG
CCAATCAAAGGGGCGAACAGGCGAGCACCAAGGCCAAGGGCTTCGCCCACAAGCCCGTTGAAAATCTCCTTGCCCC
CTTCTTTGCCGGTAGCGGCAAGTCGTTCACCAATAGAGGGGCCGCTTTCGAGACTGGACGGTGTTGCTTTTAAAAC
TTCCAAACCTTTGGCAGTCAGGACGGCTTCTTCGATTCCATCTCTTGTTTCGCTAAAGCTGATGTATCCAGAAGTA
GCCAGCCAACGAGCTGTTGCATAGAACATCTTGACTGCTTGGACTCGCTCTGGGTCTTGCGGAGACGGTTTACTCA
AAATGCTATCAGCTATGAAGTGGCGGGACTGGAGATGTGTAGGAATGGGAAAGCTGGTGTAGAGCTTGCCAAAAAT
TTGTCCTGTCAATTCGTCGAAGCGTTCGATGTTGGAGGCTGTCATGCCTTGAACTCCTTGTAATTCAGCCAGGTGT
GGAAATGCGATCAAACTCAGGTTTGGCTGCGTTTATAGCATATGGCTACCACCTTTAGAGAACCGTCATGTCGTTT
ACTCCGAAGCACCTCGAAGCCATCGAGCGCGCCATTGCGCGCGGCGAAAAGACCGTGCGCTACAGCGACCGCACGG
TGGAGTACCGCTCCATCGACGAGCTACTCAAAGCCCGCGACCAGATCCGCACGTCGCTGACCGATTCCGCCGGGCC
ACGCTCTCGCGTGATCCGGCTCATTCACGGAGGCAAGGGGATCTAATGGCCCGACATTTTCCGACGCTCTCGCGTG
TGCAATGCCAACCACCACCGCCACCGGCCGCGTGGCTCATGGAGCCACAAGAGCCCAACTTGACCCAGCGCATGCT
CAACGAATTATCGGCATCACCGATGTCGGTGACCAAAACGGAAAAGGGGACGGATCTATTTTCAAAATAAATCCGC
CCCCTTTTTACCTCGCGCCCCCTTTTTACTGTAGAGCGCTGGAGCGCCAATCAGCGAATACTCCTGTCACCTTCAA
CCCACCTGCTCGAACCTCCGCCGCTTCAGGAACAACCCCACCCCCAGTGCAACGGCAAAGATCGACAGCAACCCCA
GGTCAAACCCAAACAAGCGGGTTTGGCTCGCGGCAACGCAGCCGACTGCACCTATCGCTATCAGCAGTACCCCTAA
CCATTTGCGAAGGGGGTACGGCTTAACAAATCGGTCGAGCAGGAAGAACAGCACCAGTGCAACGACCAGTTGGGTA
ATGTCAGACATGTGTTTCTCCCGTCAGGTCTTGATGATCAGTGTCGAGGCGCTGCGTTGCGGGCCGATGCCGCCAT
TGACCTGCGCCTCAATGCTCACCAGTACGTCGCCTGCGTGATAGGTGGGCAGGGTTTCATTCACGTACATGCGCAC
ACCGGCGCCGACAGGGGCGCCCACGTAGGGGGAGGCAACATAAGAGGTGGCGCCAGCGAACGCCGAGGCTGCAAGG
TTGGCGATTTTGGTGCGTTGAGTGTTGAGAACATCGCCCTGTTGCCGGGTCAGTGGCTTGGAGATGCCGATCATCA
TCAGGCACGGAAGGTTCTTGGCCAGCATCTTGTCGTACACCTCGACGACCTTGCGGTTGACCTCGTAGTGAGCGCT
TTTCGCCTCGCCAAAATGCAGCTCGCGCAGGCGTCCGCGTTCGCTGTCGGTGAGGGTGACCTGCGAGACGTTGAAC
ACGATCCCGTGATTGCCCATGTATTGGAAAGTCCCTTCGAACTTCATCGCACGCATCCTTTCGAGCCAAAGGCGGG
GATTGTCTAGAAAGCACGCGCTGGATTCAAATCATTTCACCTGGCCGATCAGCCTGGATGCCCGGCCAGGAACACG
TTAGATTCAGCCTCTAAAGGATGACTCACCCCCGTGGAGTAGAATTTTGCGCAAACTGATTATGGTGCTCATGCTC
GGGTTCCCGTGCAGTTTTCCGGTAATCGCGGGTGATGGCAGGGCGGTGGAAGTCTCGGTCTTTGGAGCGGTTGTTT
CGTCGAGTCTTGCGGTGACTCTTTTCCCCGTCACGCTGACTGAAGACAGTCAAGGAAGTAGCCGCAATAGCTCAAA
GGGAGGCGATTCCAAGATAGTTGCCGCTCGCAATGATGCTGCCGCTTTCGTCGCGAGTGATGGGGTTATTAGAGGC
GCGAGCCTGGAGGCGGCGTTCGAGGTTTTGCGGATTGGGCATGAGCCGGCCACAGCCAGCGACATGATGCTGGCTG
AAGCCGTTTTGGTTTACGAATCGCGCTAGCGAAATAGGGCCTCGACCACTTTTAGCCAATCTGCCCACTGGGTTTG
TGCGTCCTGCAGTCCCGTCGTGCCGTGGGGTTAAATCTTGAATTGCTTCAACAGACCTTCAAGGCGATGACCTATG
GCGTATAGTTCATCGACGGTTTGCACGCCGTTTTGGGTCTGGTCGGCGACGCTTTCAATCGCGCGGGCTATTTGAT
GGACGCTTCGGTTGATTTCCTCGGCCACTGACGTCTGCTCCTGCGCGGCACTGGCGATGTGTGCGTTCATCGCATT
GATGGTAACGATCAGTTCGACCGCTTTGCCCAACGACTGGCCAGCCTGTTCTGCGTGCAGGCTGGCGCCATTGCCC
GCTTCGCCGGAGTGGCTCATCGCGGTCACGACCTTAGTGGTGGCCTGGTTGAGCCGCTCGATCATGGCTTGGATCT

CCTGGGTGCTGAGCTGCGTCCGGCTGGCCAATGCGCGCACTTCATCGGCCACCACGGCAAACCCGCGCCCGGCGTC
GCCGGCCCGTGCCGCTTCGATCGCGGCATTGAGTGCCAGCAGGTTGGTTTGCTCGGCGATCGAGCGGATGACATCC
AGCACGGTGACGATGCCTTCTACATCGCCTTGCAGGCTTTTCAGCGATACGCCGCCCGTGGACAGGTCTGCGGTCA
GCGTGTCCAAGCGGGCGATGCTTTCATTGACGACCTGCCTGGCATGCTGGGCCTGTAGATCGGTTTCGGCGGCTGC
ATCTGAAGCGCTCTGGGCACTTACGGCCACCTCATGCGCAGCGGATGACATTTGATTGATTGCTGCGGCGACCTGA
TCGGTTTCCTGGCGTTGCTGCGCCATGACGTGGTCGATGCATTTGCTCTGTTCGGCCACTTGGGTGACGGACTCAT
TCAACTCACGGGCCATGCTGACGATCTGGCTGACCAGCCCATGAATCTTGTCGCAAAAGGCATTGAACGACACGGC
TACCTGTCCGAACTCATCGTGGCTGGTGGGTAGGCGCCGCGTCAGATCGCCTTCGCCAGCGGCGATTTCATCGAGG
TGCTCGCGAATCCGTGACAGGGGGCGCAGGCTGTTTTTCACCAATACCCAGCCCAGCCCGCCGATGATGCCCAGTG
CAATCAAGGCGATGACGGTTATCCCATAGACGAGGCTGCGCACCCGTTGATGGATTTCGGCGCGCACCTGGGCGAC
CTGGCTTTCGATGCTGTCGAGGTTGATCGCGGTGACTACCACCATGTCCCAGGCGGGCAGGTAAAAACTGTAGGCC
AGTTTCGCAACCACATTGTCGGTATTGATCAAGGCCCCTGAATACGTGACGAAGTGGCTGCCGTTCTTCGCGGCGG
CGACCATTTCCCGGTTCAGGTAAACCCCGTTCTTGTCTTTGCGGTCACTCATGTTGGTGCCGACGCCTTCGCTGCT
GTCGCCACGAAACAGGCGCACGACCTGGCTGTCGTAACCGATAAAATAGCCGTCGTCGCCGTACTTCATCTTGGAC
AGTCGTGCGATCGCCAGCGCACGTGTGGCCTTTTCGCTCGTCTGGGCCTGCGCATAAAGGTCCGCCACCGAGGTTT
GCGCAAGGGAGGCGTAGTCCTTCAAGCGTTCCCGACTTTCATCGAGCAAACGCTCGCGGGTCACCGTGACTTCCTG
CTCGGCGCTCGCCAGCAGGTTGGCGCTTGTTGTCAGGGCCAGCACCGTGGACAGCAGCACCACGGGGGCCAGCACC
AGGATCAGTACTTTGTTCTTGAACAGCATGTGGGGATGTCCTTACCTGAACTGGTGGGAAGGCGGCCGCAAGGGCC
GCGATCGTCGACTTACCAGAGGGCAATGTCATAGGTGAGGTAGAGGCGGTTGCTGTCGCGCCCGCGGGAAAACTCA
GAGCGGTAGACATAGTTGCGCATGCGCACACCCAGCCCCTTGAGCGTGCCGGCCTGCACCACGTAGGCCAGTTCGG
TATCGCGCTCCCACTCCTTGACCGTACTGGTGGACTTGCCGTCATTGCCACTCAGGTAGCGCGCAGCGAAGGTCAG
GCCGGGCACGCCGACGGCGGCAAAGTTGAAGCCGTAGTTGAGCATCCAGGTCTTTTCGTCTTCCTCGATGAACTTG
CCAATGCCGGCGTTGCTGAACGAGTACACGGTGGCGCCGCTGATGTAGGGCAAGCCGGCATCGCCGCGCAGGGTCT
GGTAGCCCCCGCCGACGGTATGCCCGGCGATCGCGTAGGACAGTTGCCCGCTGAGCATGTCGTTATCGATTTTGCC
TGCATAGGCCGAGCCGCTGTCTACGCTGTTGAAGTAGCGCAGGTCGCCGGTCAGTACGCCACCGGCCAGCGGCAGA
TCGTGCTGGATGCCGGCGAAGTTCTGCCGGTAAAAATTCTCCAGCTCCCCGTAGTAGTAGTTCAAGCGGGTGTTCT
TCCCCCATTTGTACTCGGCCCCGGCATAGTTGAAGTCTCCCGATTTTCCCCCGCTGTAGCCGTCAGGCACGATGGG
TACGCTGTCGGTGGAGTCGCGTAATTTGAAACGGTCCAGGTGCCCGCCAGTGATGGTCAGGTTTTCAATGTCGTTG
CTGCTGATCTGGGTGCCCTGGTAGGTCTGGGGCAACAAGCGTGCGTCGTTGTAAATCAGCACCGGCGTTTTGGGCA
GCAGGGTGCCGTACTTGACGGTGGTGTTGGCGAACCTCGCTTTTGCGGTGGCGCCCGCGCTGGCAAATTCGCCGGC
TGCGCGGCCGTCGTCATGCACCGGCATCAGCCCGGTGCCGCTGCGACCGCGTCCTGAGTCAAGCTTTACGCCGTAG
AGCCCCAGGGCGTCTACGCCGAAGCCAAGAGTGCCGGCGGTGAACCCGGACTGATAGTCGAGCAGCAATCCCTGGG
CCCACTCGGTGCGCTCGCTTTTTGCCGTTCTCGCGGCGCGTGCGCTCATGCCGTGTTCGTCGCGAAAGTTCTCGTT
GAAATACACATTGCGCAGTTGCAGCTTGAGTTTGCTGTCATCAATAAAGCCTTGGGCATTGGCACCGGCGATGGGT
ACCAGGCCGAACGCGCAAAAAAGGGCCCGACGGGTATACATAGGGTTCATGGCAAACGTCTCGTTGTTGTTATTGG
GCGTGCAGGGCAGCACGCGCCCCGTGAGCGAGGCGCGTTGGAACGCAGCAGGAAGATGAATCAGGTAAACAGGCTG
ATAGCCCCGGTCAGCAGGGCCAGCGCGGTAACGACCAGGGAGGTCAGCACCGCCCATTTGACGGTGGCTTTCTGGA
AGTCGCCGATGTCACGGTCGACCATGCCCACCAGCAACAGGGTCGAGGCCACCAGTGGGCTCATCAGGTGCACCGG
TTGCCCGAGGATTGAGGCTCGGGCGATTTCCACTGGATCAATGCCGTAGGCCGCGGCGGCATTGGCCAGGATCGGC
ACCACGCCGAAGTAGTAGGCGTCATTGGACAGCACGAAGGTCAGCGGCATGCTGGTGACCGCAACCACCAGTGGGA
ACAGATGACCCCAAGACGGCGGGATCCAGTCGACCAGGGTCTGCGCCAGGGCGTCGACCATCTTGGTGCCGGAAAA
AATCCCGGCAAAAATCCCTGCGGCGAACACCAACAGCACGACCGTCATAGCGTTGCCCGAGTGCGCCAGGATGCGT
TCTTTCTGAATGTCCAATTGCGGGTAGTTGATCATCAGCGCGAGCACAAAGCCGATCAGGAACAGGACCGCCGAGT
GCATCAGACCCATCACCAGGGCGATCAATACCGCAATCACCAGCACCAGGTTGACGTAGGCCAGCTTTGGACGTTT
GTGTGGGGTGTCTTCAAGAATCGCGTTGATGTAGCAATCTCCGCCACCGCTTTGCAGTTGCACGTTGCCAATCCGC
TTGCGCTCGGCACGCCCCAGTATGAAGGCGGTGAAGACCACCCACAGGGCGCCGCCGATCATGGTTGGCAGCAACG
GCACAAAGTATTCGCCGGCATCCAGGCCCAGTGCGGCGATGGCCCGTGTCGCGGGGCCGCCCCAAGGGGTCATGCC
GCTCATGATGCTCAACGACAGCATGGAGATGGTCGCCAGGATCATCGGGTTCATGCCGATGCGTTTATACAACGGC
AACATTGCGGCGCAGGTAATCATGTAGGTGGTGGTGCCGTCGCCGTCGAGGGCGACCAGCAATGACAGCAGGGCGG
TGCCGACCGCGATTTTCATCGGGTCGCCATTGACGCGCTTGAGGATCTTGCGGATCAGCGGATCGAACAACCCCGC
GTCGATCATCAGGCCAAAGAACAGGATGGCAAACAGCAGCAAGGCGGCAGACGGTGCGACCATTTTCAGGCCGTCG
AGCATCATTTTGCCGGTGGTGCCGCCGAAGCCGCCAATAACCGCGAAGACGATAGGCACCACGGTGAGTGCGACGA
TCGGTGACAAGCGCTTGGTCATGATGAGGAAGGTGAATACGACCACCATGGCCAAGCCAAGGAAAGCGAGCATAGG
ACGATTCTCTTGTTGTTATCGTAAGAATGGCTGACGCCGAGGCGCACGAGCCCTGACCCGCCGGGCGGCACAATGG
CGCCTGGCGGAGAAGGGGTTCAGTCAAGTCAGGGGTAGGTGTTTGTGGTTTGCGCTAGACGCGGCTGGCGACCTTC
TGGTCTTCCCAGGCTTGTGCCTGTGCCGCACCGGGTTTTGCGTCGAGCATCCGCGCCAGCGTTTCGCGGTGGCAGG
CATTTTCGGACATCGAAATCACCACCGTCGCGACCGCGTTGCTGGCCAGGCTGGTCAGCGCCCGCGCTTCCGACAT

GAAGCGGTCAACGCCGATCAGCAGCGCCAGGCCGGCCAACGGGATGTCGTGAATGACCGTCAGGGTCGAGGCCAGT
GCCACGAACCCGCTGCCGGTCACGCCGGCCGCACCTTTGGAGGACAGCAGCATGATCGCCAGCATGGTGATGATTT
GCGTCAGGCTCAGGTCAATGTTGCAGGCCTGGGCAATGAAAATCGCCGCCAACGACAGGTAGATGGCCGTGCCGTC
CAGGTTGAACGAGTAGCCGGTGGGCAAGACCAGGCCCACGACGCCTTTTTTGCAGCCCAGCGCTTCGAGTTTTTCC
AGCATGCGCGGCATGACCGGTTCCGTGGAGGAGGTGCCGAGCACCACCAGGAACTCTTCGCGAAAGTAGCGCAGCA
GTTTCCACAGGCTGAAACCATTGGCGCGGCAAATGCTGCCCAAGACGACGAACACGAAGAAGCCGCAGGCGATGTA
CAAGGTCATGATCAGTTTCGCCAGCGAGCCCAGCGAGGTGATGCCGTACTGGCCGACCGTGAACGCCAGGGCCCCG
AAAGCGCCGATCGGTGCAAAACGCATCAGGTACGCGAAGATCTTGAACACCATGTGCGAGGCGGATTCGAGTACAT
CGAGCACCGGCTTGCCACGTTCGCCCAGGGATGACAAGGCGAAGCCGGACAACACCGCGATAAACAGGACCGGCAG
CACTTCCCCTTTACTGAAGGCACCGATAAAGGTGTCCGGGATGATGTGCATGAAAAACTCCACAACCCCAAGCTTC
GCCGCCGACTCGGTGTACTGGGCCAGGCCTTTGGTGCTCAGTTGGGTGGGGTCGATGTTCATGCCGGCACCCGGCT
GGAACACGTACACCGCCACCAGGCCAATCACGAGGCTGATCACCGTGAGGCCGAAAAACAGCAGCATGGTTTTGCT
GAGCAGGCGGCCGAGCGAGCGTTTGTCGCTCATCCCGGCGATGCCGGTGACGATGGTGCAGAACACCACGGGAGCA
ATCATCATCTTGATGAGCTTGATGAACGCATCGCCCAGCGGCTTGAGTGCAACGGCCTGCTGGGCCCAGAAGTGCC
CGACCACGATGCCCAGCACCACGGCGCATAGAATCTGGAAGTAGAGCGATTTTACAACTTTCATGGCATCACCCAT
TTTTAGTATTGTGCGGATGCAAAAATGCCAACGACCGCCGGAATTATCCGGCGAGGCGCTGAGAGTCCGTGGCATA
GACGTAGCCGGAAAAAAGCCTGCGCGCGGTGCGAACGACCGAGGCCTGAATTGTCTCACCCTTGACGCCGGTGTAG
GACAATACGACATCAATTCCTCCGCTTGGGTGCTCGATACGCACCGCTTCAAGGCGCGGCGTACTGATCCCGCCGA
GCATCTGCGCGACGATGCTGCCTTCAACTACGCACGCGGTGGCCAAACCAATCGAGCCGGTAATGGCCAGCGCGCG
GTGGCAGTTATGTGGCATGAAATAGCGAACCTGGATCGTACCGCCGGCTTTGGCGGGCGAGACTAGCACCGGCTTT
GGAATCACTTTGTCGCTGACGTCACCCAGGCCCATGGCCCGGCCGGCCTGAAGTCGCAATGACTCAAGGCGTCGCA
GGAAGTCTTTGTCGGCGTCCAGTTCGGCCGGGCTTTCATCGCCACGCTTGCCCAGTTGGCTGGCCTCGATCAGCAC
CATGGGCATTGCCATATCGATGCAGGTCACCGCAATACCGTCGATCACGTCTTGTGTATTGCCGGTCGGGAAGAGT
TTGCCGGTCTTGCTGCCGGCCGCATCGAGGAATGTCAGTTGTACCGGCGCGGCGGTACCCGGCACGCCATCGATCG
CGGTGTCGCCCTCGTAACTGACCTTGGCATCCGGTGTGTACACGTCGGAGTTGATGAAGGTCCCTGTGTTTACGTT
GCGGATGCGGACCCGGGTGCGGCCCGGCGTGCCTTTGACCAGGCCCTGTTCGATGGCGAACGGGCCGACGGCGCAC
AGCATGTTGCCGCAGTTGGGCGCGCTGTCGACCCGACGTTGCGAAACCATCACTTGCACGAACAGGTAGTCAACGT
CGGCGTCCGGGTGCAGCGATGGGCTGACAATCGCAACCTTACTGGTTTGTGGGCTGCCACCGCCGATTCCGTCGAT
CTCCAGTTCGTGGCCGGAGCCCATCAGGTTGAGCAGCAGTTCGTCACGGGCCTCAATGGCTGTCGGCAAATCCCAG
TCGAGAAAGACTGGGCCTTTGGAGGTGCCACCGCGCATCAGCACACAGGGAATTCTTTGCATGATACTGACTCTTG
TTGATCAATCGACGTAATTGATGTTCTTGGAGCAAGAGTCTCAAGCTTTAAAAAATGTATCAAATGCAAAAATTGA
AGAGATTATTGCGTTTTTCGAATGATGAATTTACGCTGAGCCTTCTTCTGGCGCCTGCTGCTGATGAGATAAGACA
TGGACTATGAGCTGAACGATATTCGATCTTTCGTTAAAATCGCCGAACTTGGCAGCTTTCACGAAGCAGCTGATGC
CTTGCATCTGTCCCAGCCTGCACTCAGTCGCCGGATAAAAAAACTGGAAGAGGGGTTGGGCACCTCTTTGCTTGAT
CGCACCACACGTAAGGTCAGCCTCACGAGCGTCGGGCGTGACTTTCTTCCCAAGGCGCGTCGTTTGTTGGATGACT
TCGATGAGTCGATTCTCAATATCCGTGAACTGGCCGAACGCCAGATTGGCCGGGTAACGCTGGCATGTATCCCCAC
TGCCGCCTTCTATTTCTTGCCTTCAGTCATTCGCCTGTATAACGAACGCTACCCAAAAATCCGCATCCGACTGCTG
GACTTGAGTGCCAATGAGGGGCTTGAAGCGGTACTGCGGGGGGAAGCCGATTTCGGTATCAACATGATGAGTGGGC
AGCACCCGGACATTGAGTTCGTACCATTGGTCAGCGAACCTTTTGTATTGGCGTGCCGGCGTGATCATGAATTGGC
AGGGCGCAGTTCGGTCACCTGGTCTGAATTGAGCGATTACCGTCTGATCGGGGTAGGGCGCCTGAGCGGCAACCGC
ATGTTGCTCGACCACGCCCTGTCGGGCTTGAGCTGGCGGCCGCAATGGTTCTACGAAGTCCAGCACCTCTCTACTT
CGTTGGGCCTGGTGGAGGCTGGTTTAGGGGTTTCGGCGATGCCGAGCTTGGCCATGCCGGCTGAAGATCATCCCAC
CTTGGTCAGCGTCCCTTTGATTGAGCCGGTTGTGAACCGCTCTTTGGGCTTGGTCTACCGCCGCGGCTCATCGCTT
TCACCAGCGGCGGAAAAATTCGTCTCGATACTGCTTGAGCAATGGCCGCAGTGAGCCAGGGCTAATCGAGCACAAA
GGGGCAGATTTTTTATCTAAAAAGGGGCCCGCTCTGCGAGCGTAAATCGGTCCCTCATGTTGTCCGTTTTTGTATT
GTCGATGATGATCAGGCGCGCCGCGCCATCAATAGCATCGAGGCCGCAGCGGACAACAAACCGGCACAGCAACGAT
TAAAAATCTTCTTGCCGCGAGGCTGCGTGAACCAGCGGCGCATGTGCAATCCGATGTATGCGTAGATGCCAATCGC
AATCCACTCCAGTGCCAGGAACAGCAATCCAAGCAGTGTGAATTGGGAGCCGACATCTACCGTGGGGTCAACGAAT
TGAGGCAAGAACGCGGTAAAGATCAGAATGGCTTTGGGGTTGCCAGCAGCGACCAGGAATTCCTGCCTAGCCAGCC
CCCACAGCCCCACCCGAGCATTTACTGATTCGGCCTCTGCTTGCGGTGCGGCGTTCCACAGTTGATACGCCAGGTA
GAAGAGGTACGCCGCGCCGACTATTTTGATCCCGTAGAAGACAAGTTCTGAGGTTTGCAACACGACGGCCAAGCCC
GCCGAGGCAAGTGCGATCATGATCGTGAATGCCAGCAAACGACCGACGCCTGCCATGCAGGCTGTGCGATATCCGT
AGCGTGTTGCATTGCTGATGGACAGCAGGTTGTTGGGCCCTGGCGCCATATTCAGCGCAAAGCAGGCCGGCAGGAA
TAACGCGAGTGTTGCCAAGTCCATATGCATCTCCAGAGTTGCTGGGCATCAATCCACAGCCTGTAACCATTCAAGC
AGCTCCATTTCCACACTACCCGGCCTGGGCGCTGTAGGCGACAGCAGGCAGTACCGCGAACCATCTTCAACAAAGC
CCAATGGTGCGGCCAGCACGCCGCTTTGGATATCGTTGCGCACCAGGTGCCAGGGGCCGATGGCTACGCCAAGGCC
AGCAACGGCTGCCTGGATGCTGAAATAGAAGTGTTCGAACGTTTGCATTTCCCTATCGGGAGTGGGTAGTCCCTTT

GCGTAAGCCCATTCCTGCCATGCCGTAGGCCGGCTGGCTGTCTGCAGCAGCGGGGCAGAGGCGAGCAGGTTTTTGC
CGTTTTTGCCAAACCACTGACCTGCCTTTTCTGGCCGGCAAACGGGGCCGACCTTCTCTGGGAATAGCGTCTCGGT
ATGGTAGCCGGGAGGGCAGGGGAAATCGTCGCGCCGGATGGCCAGGTCGATACCCTGGTTGAATGAGAACGGGCCA
CCGCCGGCAACCAAGTGAATATCCAGACCTTTGTGATGGGTTTGGAAGTCGCTCCAACGAGGGATCAGCCAGCGCA
TCAGCAGCGTTGGCTCGCAGGACAAGACAAGCTGGCGAGCTCGACGGGCGTCGCTGCGTATCTCGCGAACGGCGTG
GCTCATCAACCCCAGCCCGTCACTCACGGCTTTTGCCAGGCGTCTTCCGGCATCGTTCAAAAACACTTTTCGGTTA
CGCCGCTCGAACAGCTCAACGCCCAAATCCTCTTCAAGCAGACGCACTGCGCGGCTAATGGCGCTGGGTGTCAGAT
GCAGCTCGTCAGCCGCCCGGGCGAAATTTTCCAGGCGTGCGGCTGATTCGAAGCAGCGCAAGCCAAGCAGCGATGG
CAGGCGAGCCTCAGAAATCAGTGATTTATAGTCACTATTTTCTTCAGGAATCATCGTTATTACCCAAGCAAAGGAT
CTGTAAACATGGCCTATGCGACCGATAATTCAAAGGGTACAACATGAGCGAATGGATAGCAGTAATCACCATCACA
CTACTGGCGTGCATCAGCCCAGGGCCGGATTTCGCCATGGTCTCGCGCAACGGCTTGCTCCTGTCTCGAAGGGCTG
GCGTGCTCACCGCTGTGGGAATTGGCCTAGGGGTGCTGGTTCACGTCTGCTACACACTGCTCGGCTTGGGGTTGGT
GCTGCAACAGACACCTTGGTTGTTCAATGTGTTGAAGCTGGCTGGAGCGGCCTATCTTGTGTACTTGGGGATCAAG
ATGCTGCGCTCCAAGCCGGCCGTTGAACAGCTGGACGCGCCACCTCCTGCCCTGTCGGATCTGGAAGCGCTGCGTA
CGGGGTTTCTGACAAATGCGTTGAACCCTAAGACATCGATCTTCATCGTCAGCCTATTTATGGGCGTCGTAAGGCC
GGACACTGGTTGGTCCGTACAAATCGGTTATGGGCTATTTATCGCCGGGGCACATGTAGTGTGGTTCAGCCTTATC
GCGCTGTGTTTCTCCGCTGGGGCAGTGCGCGATAGGTTATTGGCCGCACGACAATGGATCGACCGGATTTTTGGTG
CCTTGCTGGTGAGTTTCGGCGTACTGCTGATAATGGCTTGAAGGCGCGCGCGTTGGGGAAGCCAGCTGACGCTTGA
GGTTCTATACGGGATTGGCGGGTCTGCCATTGCTTCCGCGCTGGGTGCCTGGATCGCCTGGCGCTACAGCCGCGGC
TCAGGCGTTTATGGGAAGCGTCGACAAGGAAGTGGCCGATGATCGGGAAGTTGCTAGTGAGCGTTGGTATCGTGTT
TTACGCAGTAGTAGTGCCGGTTGTTGTGCTGCTAGATATCGCCGCGATGCACGATATGCACTTTGTTGCCGTCCGG
ATCGCGCAGGTAAGCGCCGTAGTAACCGTCGCCATAGTGTGGGCGCGGGCCGGGCTCGCCGTCGTCGATTCCTCCA
GCTAAAACACCCGCGGCATAGGCTACATCGACGGCTTCTGGCGAGGGTGCGGAGAAAGCCAGCATGCTGCCATTGC
CCACTGTGGCGTCCTGGCGGTCGTAGGGGGCGTAGACGTAGAAGCGCGGCAATGGCCTGCCTGGGGTAATCCAGCA
TGCAGAAGACGGTCCGCCATCCGGCGTCACATGGCGCCGGCACAGACCCAGTGGGATCAGTATGGCGTCGTAGAAC
ATGGCGGCTTTGTCCAGATCGTTGGTTCCGACAGTGACGTGGCTGAACATACCTACTCCTTGTGGGCTGTTGTGCA
ACAGCCGGGTTTTCAGATGACCGTACCCTAGCAGAGTAGGCGGTGACGCATTGCATCTTCGGTAGTAATCCCATTT
ACCCAAGCCCATTCTCTTTGGTATCCCGCGACCTGCTTACGTTGGACACCGGTATGCCCGGCTGCAATTCTTGGGC
TCTACCCTCAAGGGGTTACTCTCAAGGATAAGGAATCGCCTGATGCTCGGTTACTACGCGCAATACAGCCAGTACT
ACATCACTACGCTGATGATCGTCACCACCCTGTTTTTTGCCTTGCCGATCTTTATCGCGCCGATTGCCTGGGCCCG
CGCGATGCGCTGGACGATCCCCGAACATCAGCATCTGGCAATCTATTTTGGGCGCTGTTTGGGGGCGTTTATTCTG
GTGGTGGAAGTGGCGATGTTGCGCTCTGCAACCACGGGCACCAGCTATAGCTATGCCTTCGATATGCTGTTCGTGG
TTTTCTTGTTGATGTTTGTGGTGCATGTGTACGGCGCGTTGAAACGTATCCAGCCTATTACGGAAACCCTGGAGAT
CGGCTTCTGGATGATTCTGTTTGTGCTCAATATGCTCTTTTATCCTGCGACGGTAATTACCCTTTGAGAAGCACGC
CTTGCAAAGCTCCCGTGCGCCTTGTAAGTGCTGTCGAGCCTACCTTTTTTCATCTGGAGTACTACGCTTATCGCAG
CATAGGTATCTACACAACGTTCAAAAACTAACGAAAGTACCTGTGGCGAGCCCGCTCGCCACAACATCTCGATCTA
CTTGGATGTATGCGTTGCGGCAAAATTGTGTGGATGCCGATGCTCAACGCAGGACGACTCTGACTAGCTGTTCGAC
GTGATGCCGTCTGCTTATTCACTTGTCTCAAGGAGTTGTCCAATGAAACTCAAATACTTGGCGTGGTGTGGCCTCT
GTCTCGCTTCGCTTGATGCTTATGCTGATATTGCCTTGCACACTGAAAAGCTGGACTCCGAGCAGTATGCGGTTGA
AATCGTCTTGGGAAATACCTGTTCGAGCGCTGGCGGCAGGACGGTTTTGCTCCAGCCGGCGCAGGGATCCAAAGAT
AATGGCAGCGTGATCGTCTGCGAGGTGAAACATACTTCTGCGGGTAAAAGTCGTAGCTTTCTGCCTTTTTCTGTAA
ATGCCAAGGATGCCCCTCAACCATTAGGGTGTTCCATATCTAATGGGTCAAGGTCAGAAAACCAGGTTATTTGGCA
ACATAAGGGCAGTTATCGACCTGACGATCCGAAAAAACCTGAAGACACGCTCGTCAGACAGCTCTCAGGACATAAC
ACGGCAATCATAAGAAACTACAATAAATTCAAATCGGTATGGATCAAGTACGACTATAACGGCCAAGTCCGTCAGG
AGTTGATCGTACCTGAAGGGCGGCTGACTACAAGGCAGAGCGCGAAAATCCTGCAGGTCTGGTACCCCCATCCCTA
TGGTGATTTTGACTGCAGGGAGATGTAGGAAAAGGCGACTCTACGTCGCACGGCCAAAAACCTTTGGTTGCCCGTG
AACAGGGCAGCCCTAGGCTGGCAGGCGCTCATGGCGTTAAGCTGGCCCTTTGCAATCATGGAGGGCTTTTTCTTGG
GGATACTTCTCGTCACCTTAGTGATGGTTGGGGTCAGCTACTTCTTCCTTCATTGGATAGGTAAGGTTTGCCCACC
ACTGGGCAACTTCATATTCAAGTACAGCAAGCTCATCGGGCCGCTTCTTGTGGCCTATTGGTTGTATTACATGTTG
CATAAGGACACCAACGGGTCTGCCATGCTGATGTTGGCCGTAGGTATCGTTTTGACGTTGATGCCCAATAAGAAGA
GGTTTGCCTGACACCCTTACGAGTAGACAAATAGCACTGCGCATTATCCGGATTCACCGGAATATGTTTCCCGGAT
CGGGGCGATTATCAAAAAATCCCTGGTAGATATCCTGTGGTCATCGAAACCACTGACTATCTACGAGGCGACACTG
ATGTCCAGACTTTTCACCCCAGGTATTGTTGGCCAACTGAACACGCCCAACCGCGTGGTCATGGCGCCCATGACCC
GATCCCGCAGTACCCAACCTGGCGATGTGCCGAACGCCATGAACGCGGTCTACTACGCACAGCGGGCTTCGGCCGC
TTTTATTGTTACTGAAGCCACGCAGATTTCGCCCCAGGGCAAGGGCTATTCCTTTACCCCGGGGATTTACAGTGAC
GAACAGGTCGCCGGCTGGCGCCTGGTGACCGATGCGGTGCATGCCGCGGGAGGGCGAATTTTCCTGCAGCTGTGGC
ACGTAGGGCGTATGTCGCACCCTGACTTCCACAATGGTGATTTACCTGTCGCGCCGTCTGCGATCCCTTTCGATGG

CAGCATCTGGAAAGTGGATGCCCAATCCGGTGTCGGCAGCATGGCCCCATGCCCGACACCACGGGCACTGGAGCGC
GACGAAATCCGCGCGATTGTCGACGACTACCGGACGGCTGCGCGTAATGCCATGACGGCGGGTTTCGACGGTGTTG
AAGTGCATGGGGCCAACGGCTATCTGATTGACCAGTTCCTGCGTACCACCTCTAACGTTCGTACGGATGAGTACGG
CGGGTCGCGTGAGAACCGTCTGCGCTTTCTCAAGGAAGTCATGGACGCGGTGATTGATGAAGTGGGCGCCGACCGT
ACGGCGATCCGTGTGGCGCCGTTTCTCACAGCGCGTGGCATGGCGTGCCCGGACATTCTGCCCACTTTGTTGGAAG
CGACCAGCTATCTGCAGGGCAAGGGCATTGCCTACCTGCACTTGGTGGAAGCGGACTGGGATGATGCCCCCAAGTT
TCCCGAGGATTTCCGCCAAGCCGTGCGCGAGCGCTTTCGCAATGCGATTGTGGTGGCCGGCAAGTACGACGTGGAG
CTCGCAGAATGGGTCCTGGAAAAAGGCTACGCCGACTTCGTGGCATTCGGACGTAAGTTTGTAGCCAACCCGGATC
TGCCGTTGCGCCTGGAGAAGGGGTATCCATTGGCCGGCCTGGAAGGTGCCGAGTTGTTTGGTGGTACCGAGCGCGG
TTACTCGGATTTTTCTGCCTGGGCCCAGTAGGGCGCAAACCTATAAGCCATAGCCAGGAACATCTCCTTGAAGCGC
ACTCAGGTCGCCAGCTTCAAGGAGATTTCGTCCATCAGCAGTTTGACCCGACGCGGCACCGCGTGGCCTGAAGGGT
AGACAATCTGCAAATCCACGCCCTGCAACGAGTAGCTTTGCAGCACCTCCACCAGCCGACCTGCCTGCAAATCATC
ACGTACATCAATGTAGGACTTGATACAAATGCCATGCCCGTCCAGGCACCACTGGCGCACCTGCTCGCCATCATTG
GCAATACGCCTGCCCGATACCAGAACCTTGAACGTACGGCCCTCGGCACGGAACTGCCATTCCCGGTGGATGTTGC
TGCCAAAGCGCATCAGGATGCACTGCTGTTGTGCCAGGTCGGATGGATGCCGGGGCGTCCCGTGGGCGGCAATGTA
GGCGGGGGATGCACATACGACCCTACGGCCCTCGCTGAGTTTTCTGACACGCAGGGTGCTGTCGGACAAGGCGCCG
TATCGAATAGCGAAATCAATCCCTTGCCCCGCGAGGTCCAGGTAGCCATCGGTCAGGTTCAGGTCGAGGCTTACGT
TGGGATGTTCTTGCAGGAAATCGTCGAGGATTGGCACCACACGGTTGCGGCCCAGGTCTACGGGGGCGCTCAGGCG
TATCAGGCCTGAAATGCGTTCGGTGCCCAGCTTGATATTGCTCTCGATATCTTCCGCTTCTGCCAGCAGCCGCCGT
GCGCCGTCCATCAGCATCCGCCCTTCATCCGTCAAACTGATCGAGCGTGTGGTACGGGTCAGCAAGGTTGCCCCGT
AATGCCTTTCCAAGGCCCCGAGCCGTTCCGACACAGACGTAGGCGAAAGCCCGACCTCGCGGCCTGCGGCCGACAA
TCCGCCTTTTTCGACAATCAGTAGAAACAACGCGAGGTTCTCGAAAAGCATTGTCCGGACTTCCCTGAATATGGCT
GGGCTTTTCGCAGGATTCTACGTCAGTACTGGATAGCGCTCATGGCATTTATGGCGCAGGCGAAACCATGAGTGCG
CGCCTGACGAATTGCGCGGTTTTCTCCAGGGTTATTTCCAGGTCGGGATGCTTGAGCTGATAGCGGCTCTCCAGCT
CCCGGCTCTGAGACATATCGAATCGCGCAGTCTGGATGAAGTTCAGGGACTCCGGGCTGGTACCCAAGTGCGTTGC
CACCCCCGGTATTTTCAGTAGCCATTTGAGCAGGCCCAGGGGCAGATGGTGGCGGGGCGCTTGCACTTGCAGGGTG
CCGGCGATATGCGTGAGCAGGCCTTGCAGGTTCGGGGTGCGCTCGTCCAGTGCCAAGGCTTGTTGGTTTGCCATTG
CCGGGTCGAAGGCTGCTGCGACCATCAACGCAACCAAGTAATCAACACTGACCAAGGGCAGCCAGTGGCGTGGCGA
CCCTGGAATCGCTGTCAACTTGCCTGTCGCCAGGCCGCGAATCAGCTCGGCTATGGGCTGGCCCGCGAGGATATGC
CCGCTTTCGCTATGCCCGCACACCGTTGCAGGGTGCACGATGGTGTAGTCGGCCTGCTGCGCCTGCATGTCGCGGA
TGACCGCAAAGTGTGCCTCCAGCTTGCTCGCCTCGTAGCCACCCACGCGCTGGTACACGGCGGGCCAATCGGTGGT
TTCGGGGCGTTGGTGGTCAATGCCAATACTGTTCAGGTGGACGATGTTCTGCAGCATATAGCCGCCGACCATCAAC
AGCCGGCTACGCTGGCTTGCGGCCAATCGGGCGACCGCCAGGGCACCGTCCACGTTGACCGCGCGGGCTTGGTCCA
AAGGCAGGCCCCAGGCGAATTGTGCGGCGAGGTGAAATACCACGCTGGCCGCGGCGATGCGCTGGCGGTCGGCCGC
ATTGAGCCCCAGTTCCTGCTGGTTAAGGTCGCCCTGAACGGCATGTATACGCGAAGGTTCTCCCCCCAGTTGCCGC
ACCTGTTCCCTGAGCGTCTCAAGGTTTGAAACGTGGCGCATCAGCACCCAGACCTTGTGCCCCGAAAAGGTCAGGC
GAGCCAGCAAGTGTTGCCCGATAAATCCGCTGCCGCCGGTGACAAAACATTCCACGCTCATAGCGCACTCCTTGGT
TGAATAAGGGGTGCAGGCTAAACTGTAGAGCTACCTCTACAGTCAAGGAGAATTTACCGTGAAAGTCGCAGAGCTG
GAGCGTAGGAGCGGCCTCGGACGCCATACCTTGCGCTACTACGAGGAGCTGGGGTTGATCGCTGTACAGCGGGGCG
CCAATAACTATCGCGCTTACAGCGAACAGACGGCACAGGATCTGGAGTTTATTCAGATGGCGCAGAAGATGGGATT
TTCCCTGGCCGAAATCGGCGAGATCCTGGAGGCCAAACGGCAAAGTACGATCGACTGCGCCCAGGGTGCCGCGATG
GTGGCCGATAAAATGGCTGAGATTGAACTCAAGATCAGCCATCTCAAGGTATTGCATGGCTTTCTGGATAAGGAGC
GTCTGCGGCTTGAGGCCAGTGCCCTGGCCCTGGGGCAGACCGTTACCTATCGTCAGGCGCCGATGCCGGTTTAAGT
GCCAGTCCTTTCAACGCCTCCCTTCAGGTACAGCTGACTTTGATCGTAAATTGTATCTCTCGATCTACCTCTAGCT
GTACGGGGCGGCGCGTCACAGAACCCTCTACTGTCAGGGCGACAATAACTCGCCGGATGACGCACCATGTTTATGT
CCACCAGCCTGCTTTCAGCCTTTGCGCTGTTTGCCTTCGTGTCTTCGATTACCCCAGGCCCCAATAACACCATGCT
GTTGGCATCGGGCGTGAACTTCGGTTTTCGCCGCTCGATTCCCCATGCCCTGGGGATTAGCGTGGGCTTTATGGTG
TTGGTGATTTCGGTTGGCTTGGGCCTGGGGGAAGTGTTCAAGGCGTTACCTTGGGCATACACCGCGCTGCGTTACA
TCGGCGCGGCGTACTTGCTGTACCTGGCGTGGAAAATTGCGACTTCCAGCGCGATGTCAGACGACACCGACCGCAA
GAGCAAGCCCATGACGTTTTTTGCCGCCGCGGTGTTCCAGTGGGTCAACCCCAAGGCCTGGGTCATGGCGTTGGGG
GCGATCACCACCTACACACCGGCGCAAGGTTACTTCACTAACGTGCTGGTCATTGCCGTGGTGTTTGCCGTGGTCA
ACCTGCCAAGTGTGTGCGTGTGGGCCGGGTGCGGCACCGCTTTGCGCAATGTCTTGCGTGAGCCGCGCTGGTTGAA
GCTATTCAACTGGTCGATGGCGGCGCTCTTGGTGCTTTCGTTGTACCCGATGTTTTTTCTCGGGTGAGAGGTTCGG
GATATAAACCCCCGGACCTATAGTGAAACACTCTAATGAACTGTGAAAATTGACAGTATCGGACAGTAAAGTGCTT
GCGCACTATTAACCGCTGTTCACGGTTTTCGGGCCTTCCAAAAAATGCTGTTTTATTCGGGGCATCGCTAAACCAT
CAATCTTGAGTCAATGGAGTGTCACCCCATGTCAAAGGAATTAATCAACCCGCCCGGCACACAAGCGTTGCAAACC
AATCCTGAAGTCGGAGGTGCTGCTGAGGCGTTGAAAGCCGCACCCTCTCAGTTGGCCTACCTCAAGGACAACTCCG

TTAACCCGCCCAGATATTTGATGATGTCTAACGGATACGTCATTACCACCAAGGATGTTAACCAGGCCTCGAAATT
CCTGGTTATCAGTTACGGCGACGGGAATTGGTACATCATGACCGTCGATACCGCAGAGTATCTGTCGTTTACCGAT
CGCAGCTATCTCTATGCCTATTCGGCGTGGAGCAATGCCAGGTACGTGACCCTTGATCCGGTGAGTTTTAAAAAAT
ACCCCGGGCTCTACCTGCATGATGGCTACGTCTGTTGCAATGGGATGAAGGACAAGCCAGACAGCCTGATGAGCGT
CCATCTCGAATTGATCCGTTAACGCCTTGAAAGACGCCAAACCGTGCGCTGGGTAACGCAATATCAGAAGTACCAT
TGTGCATCACGCGCACCTTGCCCCACCTACCGGTATCAGACCGGTGAAATCGTGGCCAGCAGGCCGATTCCAACGG
TCAGTGCCAGAAACCCTAAAAGAAAAAATGCCATCTTAGCCATGGGACCTCCAAAAGGCGCAAGGGCGGCGGGAGA
GTTCCTGCCGATAAGCCATTGTCGGGGCTATGGTCTGTTCGGTACAGATGCAGGTGGTAAAGAAAAAAGCGTATCA
GATGCAGCTTGCAGGCTGGTATGCCGGGGTTAGGGCTGGCGCCTGGAGGTACTGATCAGACTGTACGCGGCACCTT
CGGCGCTCTTGGGGCTGGCTCAACTGCGGGCAGTACATATTTGAGCCATTCTGTTCGGGGTGAGCCGGGCAGGGCC
GGTATCGGGTCGGCGGCTGTCGATTCAGCTTCATCGATTGGGTAGCAGTTGTCGCTGCTGCGCCTGCCGTCTGGGT
AGACGATGAAGCCGCCGCGACCGGTTTCACATTCGTGCAGATACACCGGCGACAGTTCCCGCTGCTTATCAATGCG
CGCATTCAGATGGACGATATAGGTCCCCATGGCAATCGACACCCCCATAATCAGGCCCAGGCACACCAGTAACTGC
CAGGTGTAACTGGCGCCCGCGTTATCGCAACGCACTGGCGACACAGCACGCAGCTGCCTGCTCGATACTCTGGCCC
ACCAGGAACCGACTGCCGCCGCCCAGGTTTACCAATTGCCCGCGAGACGGCAGGATGCTGGCCATCAGGCCGAAGA
GAATCAAGGCCAGCAGGACTCTTTTGACCATCGCGTAAAGGTTCATGAGGGTGATGACCTCTGGATATGAGAGCCC
CAGCTTAGAGAGTGCCGGGTTAACGCCAGGTAAACATCGGCGGGAAATTGCTCGACTTCCGTTATCCTTCAGACCT
CTGCGCAACACCTGTCCGAGGGGACTTTTCCGTGCATATTCACCTGCTGCTGGTCGAGGACAACCTTGACCTGGCC
ACCACCGTCATCGAGTACCTGGAGATCAGCGGCATGGTCTGCGACCACGTCAGCAATGGCCAGGCCGGCCTCAACC
TGGCACTCGACCAGCATTACGACGTCATTCTCCTGGACATCATGCTGCCGCGCCTCGACGGCCAGCAATTGTGCGC
GCGGCTACGCCAGCAAGGCAGCCAGACACCGATCTTGATGCTCACTTCACTGGATGCCCTTTCGGACAAGCTCGCA
AGCTTTGCCGCGGGCGCTGACGACTACCTGGTCAAGCCATTCGAACTGGCCGAACTGGTGGCCAGAATTCGTGCCC
TGAGCATGCGTCGCAGCAGCCAGAGCAGCCGCCTGCAGGTGGACGACCTGGTGATGGAACTGACCACCCGCAAAGT
CAGCCGTGCCGGGCAGGAGCTGCACCTGTCGCCGATCTGCTGGACCCTGTTGGAATACCTGATGCGCGCCAGCCCT
GAACCCGTCCCTCGGGAGCGCCTGGAAACCACCATCTGGGGCGACGAGCCACCAGACAGCAATACCCTTAAAGTCC
ACATGCACCGGCTGCGCAAGGCCGTAGACAAACCCTTCAGCCGGCCGCTGATCCACACCCTGCCTGGGGTCGGTAT
ACAGGTGAAGTCGCATGCGTAATGGCCTGAGCCTGAAGTGGGTGATCAGCGGCAGCTTCCTGTTGCTGATTGCCGT
GGTGTTACTGATCTACAGCCAGCTGCTGCCGGTGTACACCGTACGCGGCCTGCTCTACACCGCCAGCGCGATGATG
GAAGAAGAGGCCCAGTACTTCGTCAGGCACTACAAGAAGGATCCGACCACGCCACCCCCTCATAACTACTTCTTCG
AAGCGGTCATCGGCAAGGAAGCCCTGCCGCCAAGCGTGCGACAGATGCTGGAGGCCCCGCCGAGCATGTCCTTCGG
TGCCGTTCAGGTGTTCGGTGATACAGACTCCGATGACGAAGACGCTGAAGTCCGGGCGGTCCTCTCCCAGCCCTTG
GGGGACGGCAAGACCCTTTACATGTATGACATCGACCATGACCACGAAGAGGGCGGCGAGGTCGAGACGCCGCTGT
CCGATGCCTACTTTGACCGGGTGCTGCAGGGCGTTGCGTTCATCAGCCTGGCGGTGTTCGTGCCGGCGCTGATCAT
CATCGCCCTGTTGGTCTGGTGGTTGGTGCGGCCATTGGGGCGCCTGGCGCAATGGTCCAGCACCCTGCAGAACCCG
GATGCCATGGCCAGTGAGCGGCCTGATTTCAGCTTCAAGGAGTTCAACATGCTGGCCGATGCCCTGGCCCAGAGTG
TGGAGCAGGTGCAGGCCGCCAGCCAGCGTGAAGGTCGCCTGTTGCGCTACACCAGTCATGAGCTGCGCACCCCACT
GGCAGTGCTCAAGGCCAATATCGAACTGCTGACCCTGCAGGCCGGCGGTGTCCTGCCGTCCTCCCTGCAGCGCATC
GAACGTTCGGTGCTGAACATGCAGCTTATCGCCGAGACCTTGCTGTGGATGAGCCGCGAGCGTCCCGAACCGTTGC
CTGAGGAAGACATTGACCTGAGCGAACTGGTGGGCGAGCTGATTGACAAACATCGCTACCTGATCGGCAACCGGGA
TATCGAACTGCTTGTCGACATCCACAACCAACCTTGCCGACTGCCCCCCACCGCGTGCCGCATCGTGATCGGCAAC
TTCCTGCGCAACGCGCTGCAATATGCCGAAGAAGGCCAGGTGGAAATCAGCTTCAAATATCCCCGACTACTGATTG
CCAACCACATCAGGGAAGCCAAGCCACGGGAGGATTCCAGCGATTTTGGTTACGGCCTGGGGCTGCAGTTGATGCG
TCAGTTATGCGCGAAATTGGGCTGGCAGATTGACGTGATTCTTGAGCAGCAGCGTTTTACGGTGGTTCTTGAGTTT
ACGAGTTTGCGGGACGCCGAGAGTCTGGGGGCGGGGGAATTGCGCAACAGCTAGAGCGTTGCGGCGCACATAAGAC
AAGGGCGCCCCCATGCCCAAACCAATGCTTTAGATTGCATCGATGTGCTGAAGGCTGGCGTGACTGTAGTTTTTGA
CTTGCCAGCCAGCACAAAAAGTAAGTTTTCACAGATATGAAAAACCATCGGTGTCGTTAAACTTCATAACTATATT
TGACCACCCTATACGCAATAAGCAAATAGTCCTGGGAAAGACGTGTCTTTATATTACAAAGCGAGGCGCGAATAAT
TTAGTTAAGCTGGTCTTGTTTTTAGCGTATATAACAGGATGTGCCGTGTGAAGCGCCCAGCGACAAACCCAAGATT
TACCATTGTTTTAGCTATTTTTTATACGCTGTCAATGGTTCTTGCGGTCAAGATAACTTATTTGATGATTTTTTCT
CATGAGTTTCTGAGTAGCGAGGGTCGTAAACGGTCAACGCGCGTGACTGAAACAGTCCCCGTTCGCGGGACTATAT
TCGATAGATTCAATCAACCCTTGGCAGTTTCCACGCCGGTCAGCACCTTATGGGTAAACCCTAAGCAATTTGCGCC
AACAAATCAGCAACAAGCAATATTGAAAAAACATCTTGATGTTGATGATTCGCTGGCCAGAAGTCTAATGCTGAAC
ACTCAAGATAAATCCTTTGCCTATTTGGCCAGAAAAATTGATCCCGAACTCGCGAACAAGGTAATAAAATTATCCA
TTCCTGGGGTTTATGAGCGTCAAGAATACAAACGATATTACCCTCAGTCTTCGATGCTTGCTCAGTCAATAGGCTT
TACTAATATTGACCAGAAAGGTATCGAGGGCATCGAATTAATGTACAACCATAAACTTTCTGGTAAGCCTGGCGTA
GAAGAGCGCACCTTGAACCTGCACAGGCGAGTCACCCGTGAAGTTCTTGTGCACAAGCCCATTACTCCAGGGGACA
ATCTGTATTTAACAATTGACTCGACGCTGCAGCATTACGTTGAAAAAGCACTACGCAAAGCACTCATAAACAACGC

CGCCAAGTCTGCAAGCGCGATACTCATACACGCTAAGACAGGTGAGATATTGGCATTGTCGACAATGCCCTCATTC
AATCCCAATGCTCGTAAAAGCTTCAACGCGGACCTCATTCGAAACCGGGTTTTTACTGATGTTTATGAGCCTGGCT
CAGTGATAAAACCATTCAGTATGGCCGCTGTGCTAACCGCAAAAATAATTGACAAAAACGCGATTATCCAAATAGC
ACCAGGCTTCGTAAAAATAAATGGATATACCATTAGAGATGTAGGCAGGGACAATCAGTTGGATTTGGCGGGGATT
ATCCGCCGATCTTCAAATGTTGGGATGAGCAAGGTCGCCATTCCAACTGGGGGAGAAGTAATTTCGCAATTACTTA
CTGAGTTGGGATTCGGAATCAACACGTCTATCCACTTTCCGGGGGAAAGCGTTGGGACCGTTCCAGTCAATAGGGT
GTGGTCAGATATTGCTACAGCGAGCCTCTCATACGGATATAGTTTGTCTGTCAACTTAGCGCAACTGGTTCAAGCA
TATACCGTCTTCGCGAATGAGGGCGATCTAGTTCCATTGTCAATAATCAAAGGCGAGCCAAACCCGCCACGCAATG
TCATAAGCCCGGAGGTCGCCAACAGTGTTCTAGACATGATGGTGCAGGCAGTTGATGGCCCTGGCGCAGGTGGAAA
GCGCGCAGCCATCGACGGCTATGTAATTGCGGGCAAAAGCGGGACGTCACGCAAGGCATCAAGTGGCGGATATGAA
AGCAACAAATATAGAGCGATGTTCGTAGGTATCGCTCCGGCCAACGCGCCAGAATTCATCATGGCTGTCATCGTAG
ATGAACCAACTGCAGGAGGCTATTTCGGGGGGGCTATTGCAGCACCCATTTTTAAGGAAGTTCTCGAAGATGTTCT
TCGGATCAGAAATATTCCTAGTGATGAAAGTCAATTCTCGCCAACAGCCACTATTTACTGATTGCCTTGGGTCGAC
AGTAGCTTTTCAGGCATGAAGGGCGTGCCGAGCGTGCCAAGTTTCGGTGTGACGGATAGCTCGTAACTGACGGCTG
CGCATAAGCAGCTTCCTTGGTAAATCTCTGTCATACGCTTTTGCCCGTTTGCGAAAAACGCCAGCGTAACCGTCCC
ACTCTAATCAGTCGATCGACAAACTCTGGATGAGCGCGACCATACGATGAGTCTCAGGGCTCGTGGCCTGTCCGGT
AGTTTGCGTTCGCTCGGGGGAGGGGTAATTGCGCAGCAGAAAAGATAAGGGCCTGCATGAAGTTCATCATGCAAGC
CCTTGATATCTATGGTGCCCGAACCCGGAATCGAACCGGGACGCCCTTACGAGCGGGGGATTTTAAGTCCCATGCG
TCTACCAGTTTCGCCATTCGGGCGGCAGCGCGATGAAGCAGCTCAGTGGGGCTTTGCCTGGCAAAGCGCTTGAGCG
GTGCAGCAGGCTAGGGAATATATAGATCAAGGCACCTTGGCGCAAGTTTGCGTGCGGTCTTTTTCTGCTTGATCAC
GTTTGTCCGTTTGTAAAAAAAGCCCGTCATATCAGTGGTCTACATTTGCTTGGCAGGGGCGTGCGACTCGCTGGCG
CACCCATTGCTGCCCCAGGATGCTTGCCGATACGGCCTGGTTCATGCAAGTCGAATGCACTAAGTCCAACCCAGGC
TGTCAGTGTTGTTTCAGGCATTCGACCCTATTCGCAGGAGAGAAACATGGCTACTACACAGAGTGATGACAAGCGC
CCAACTCCAGATCCTGCAGAAGACAATGCGTTTTTCCCTTCTCCCTATTCGCTCAGTCAATTTACTTCCCCCCAAT
CTGATCTCAGCGCTGCGGATTACCCAAACCCCTACACGGGCGGCCGCTGGAAGTTCTTGATGATCGGTGCCGATGA
GCGCTACTTGCCTACGGACAACGGAACGCTGTTCTCCACGGGTAATCACCCGGTCGAAACGCTGCTGCCGATGTAT
CACCTCGACAAGGCCGGCTTTGCTTTTGATGTGGCGACCCTGTCCGGCAATCCGGTGAAGTTCGAGTTCTGGGCCA
TGCCGTCGGAAGACGCCGAGGTCAAAGGGTTCTATCGCAAGTACCGCGATCAGTTCAGGAGCCCGCTTAAACTGGC
CGATGTGATTGAGCAGGCGTTGGGTGAAAACTCGGATTACATCGGTGTGTTCATCCCAGGAGGGCATGGGGCGCTG
ATCGGTTTACCGCACAGTGCTGATATGAAGAGGCTTTTGCAGTGGGCCATCGCCAAGGACAAGTTTGTGGTTTCGT
TGTGCCACGGCCCTGCGGCGCTGCTGGCGGCGGGCGTTGGTGAGTCAAAGGATTCCTATATCTTCAACGGTTACAG
GATTTGTGCGTTTCCCGATGCGCTCGACGCGACTACGCCCGATATCGGCTACATGCCCGGGCACCTGACCTGGAAG
TTCGGCGAGCAGTTGCAGGCGCTGGGTGTTGAGATCGTCAATCAGGATATCGACGGGGCGACGCTACAAGACCGAA
AACTGCTGACCGGCGACAGCCCGTTGGCGGGTAACAATCTGGGCAAGTTGGCGGCGAGCGTGCTCTTGAAGGCTGT
CTGAACGAGTCGAGCCGCAGCGAGGCTGCGGCTCGACAGCGGCTACTAAAGCCAGGCTTTGATCCGCGCGCTGACC
GCCTCGGTCGAGATGCCGTAACGATCATGCAGTGTCGGCAACGCGCCGGCATCAAGGAAGGCATCCGGCAGGGCGA
TTTGCCGGAAGGTCGGCGTCACGCCGTTGCGTAACAGGACGCCTGCCACCGCTTCGCCAAGCCCGCCGATGATCGA
ATGGTTTTCCGCCGTGACCACCAGCCGTCCCGGCTTCCTGGCCTGCTCCAGAATGGTCTGCTCATCCAGCGGTTTG
ATGGTGGGTACATGCAACACCGCCACATCCACGCCATCGGCGCGCAGCTTTTCGGCGGCTTCCAGTGCCCGCATGG
TCATCAGGCCGGTGGAGATGATCAGCACATCGTTGCCGGTTCGCAGGGTTTTCGCCTTGCCAATTTCAAACGTGTA
GCCATATTCATCCAATACCACCGGTACGTTGCCACGCAGCAAGCGCATGTAGACGGGGCCCTGGTGGGCGGCGATG
GCAGGCACGGCCTGTTCGATCTCCAGGGCATCGCAGGGGTCGATGATCATCAGGTTGGGCATGGCCCGGAAGATTG
CCAGGTCGTCCGTGGCCTGGTGGCTTGGGCCGTAGCCGGTGGTCAGACCCGGCAAGCCGCAGACGATCTTGACGTT
GAGGTTTTCCTCGGCAATCGCCATGCAGATGAAGTCGTACGCACGACGAGAGGCGAATACCGCGTAGGTGGTGGCA
AAGGGTACGAAACCTTCACGGGCCATGCCGGCCGCGGCGCTCATCAGCAGTTGCTCGGCCATGCCCATCTGGTAGA
AGCGATCGGGGTGGGCCTTGGCGAAAATATGCAGGTCGGTGTACTTCGACAGGTCAGCGGACAAGCCGACAATGTC
CTGGCGCTGCTCAGCCAGCGCGGCCAGGGCATGGCCAAAGGGCGCTGAGCGAGTGGCTTGGCCTTCAGAGGCAATC
GAGGCAATCATTGCGGACGTGGTCAGGCGTTTTTTACCCGTTTGCAAGGCTGTGTGCTTGACGCTGCTCATGCGGT
TTTCTCCATCTGAAGATTGCTCAATGCCAGTTCCCACTCGTTCTCGTCGACACGGATGAAGTGGGTTTTTTCCCGG
GATTCCAGAAAGTCCACGCCCTTGCCCATGCGTGTGTCGCAAATGATCACTCGCGGCTGGCTACCGGTGTGGTTGC
GGGCGTTGTCGAAGGCGGCGACCAACGCTTGCAGATCGTTGCCATCCACGCGCTGGGTGAACCAGCCAAAGGCCTG
CCAGCGATCGACGATGGGCTCAAAGGCGAGAATTTGGCTGGAATGCCCATCGGCCTGCTGGTTGTTCACATCGATG
ATTGCAATCAGGTTGTCGAGCTGCCAGTGGGACGCAGACATCGCCGCTTCCCAGGTCGAGCCCTCGTTCAGCTCGC
CGTCGGACAGCAGGTTATAAACCCAGGATTGCGAGTTCTTACGCTTGAGACCCAGGCATGCGCCGACGGCGATGCC
CAAGCCATGACCCAGCGAACCGCCGGTGATTTCCATGCCCGGGGTGTAGGCCGCCATGCCCGACATCGGTAGGCGG
CTGTCGTCCGAACCGTAGGTTTCCAGCTCCTCGAGTGGGATTACCTGGGCTTCGATCAAGGCGGCATATAGCGCGA
TGGCGTAGTGACCGATCGACAGGTAAAAACGATCACGCTCTTCCCACTCCGGGTTATGGGGCTGATAGCGCAGGGC

ATGGAAATACGCCACCGCCAGCAGGTCAGCCGCGCCCAGGGCTTGCCCTACATAGCCTTGCCCTTGGACCTGGCCC
ATGCGCAAGGCATGGCGACGGATGTTGTAGGCGCGTTCTGCCAGGGACAGCGATTCAGTGTGTGATGCAGCAGTCA
TGATAAAGACTCCGTTAACTCAACGATTGACCAAGGCGGCCGGGACTCGCAGCACCAGCAGTGCTCCGGCGCACAG
CACGGCGGTGATCAGGTACATGCCGATGGCGCTGGAGCCTGTCGTGGTGGTGATCCAGCCGATCAAATAGGGCGAG
CAGAACCCGGCCAGGTTGGCAAAGCTGTTGATGCCAGCGATGCCTGCTGCGGCCGATACACCGCCGAGCAAGGTGG
TGGGCAGCATCCAGAACAGCGACGAAGCAGACAGCACTCCGGCAGCGGCCAGGCACAGGCTGAGCATCGACAGGAG
CAGGTTGCCGCCCATCAGTGCGGCCAGGGTGAGGCCGATAGCGCCGGCAATCATCGGGATGATCAGGTGCCAGCGA
CGCTCTTGATGTTTGTCGCCACTGCGGCCCATCAGCAGCATGGCGACGATGGCGCACATGTACGGCAGGCTGGTCA
GCAGGCCGATATGCAGTGGGTCGGACACCCCGGCATTGCGGACCAGGGTCGGCAGCCAGAAGGTGATTGCGTACTG
GCCCATGACCACGCAGAAGTAGATGCCGGCCAGCAGCCACAAGCGTCGATCGCGAATGAACTCGCCGACCGAGGCG
TGGGTGACTTTGCAACTGTCGTCCTCGGCCAGCTCTTTTTTGATCAGCCCCTTTTCGTCGTCCGTGAGCCAGGTGG
CCTGGTCGACGCCGTCTTTCAGGTAGCTGAGCACCATCAGCCCGACGATCACCGTAGGGATGGCCTCGATCACGAA
CATCCATTGCCAGCCATGCCAGCCGTGTACTCCGGCAAACGCATTCATGATCCAGCCCGAGAGTGGGCCGCCGATC
ATGCCCGACAGGGGGATGGCCACGAACCACAGCACGGTCATCCGCGCCCGGCGATAGGAAGGGAACCAGTAAGTGA
GGTACAGCAGCAGGCCCGGTGCCAGGCCCGCCTCGGCAATGCCCAGCAAAAAGCGCAGGGCATAGAACTGCCAGGC
GGTTTCAACAAAGGCGAAGAGGGCCGAGATGATGCCCCACGAGATCATGATGCGGGCGATCCAGCGGCGTGCGCCG
ACGCGGTGCAGGATGATGTTGCTCGGCACCTCGCAAAGGAAATAGCCGATGAAAAACATCCCGGCACCCAAACCAT
AGACGGCCTCGCTGAGCGCCAGGTCGTTCATCATCTGCAGCTTGGCGAAGCCAACGTTGACTCGGTCCAGATAAGC
GCACAGGTAGCACAGCATCAGGAACGGCATCAGCCGCCAAGCAGTCTTGCGGTAGGCGTTGGCGCGAACGGTCGCG
CCCGCGTCCAAGGATAAGGTGGTCATTGTGGTCTGATCTCTTATTTTTATTGACAGCTGACGGCTGCAACGTCGAT
TCAGAACAGCGCTCGCCGTGACGGCGGGCGCAAGATCCATCAGTGAATGAGCATGCCACCATTCACATCAAGGGTA
ATGCCGGTCAGGTAGGACGAGAGGTCGCTGGCCAGGAACAGCGCGGCGTTGGCCACGTCCTGCGCGGCGCCCAGGC
GGCCCAGTGGAATGCCATCGATGATCGCGTGACGGCGCTCGTCCTGCATCAGGCCGCCGGTGATGTCGGTGTGGAT
CAGGCCCGGGGCAATCGAGTTGACCCGAACGTTGTCCGGCCCCAGTTCCCGCGCCATGGCTTTGGCCAGGCCCAGC
ACGCCGGCCTTGGCGGCGCTGTAGTGCGGGCCGCCGAAGATGCCGCCGCCGCGCTGCGCCGACACCGACGACATGC
AGACGATGCTGCCGGATGCCTGTTGACGCATCAACGGGATCACCGCCTGGGACATCAGCAGCGTGCCGCGTAGGCT
CACATCCAGCACTTTGTCGTAGTCGGCGTGGCGAATATCGAGGGTCTTCAGGGGTTGGGTGATGCCTGCGTTGTTG
ACCAGCACATCAATGCGACCGAAGTGCTCGATTACCCTGGCAATGGCGGCATTGACCTGGGTTTCGTCCGCCACGT
TGGCGGCCAGGCCGAGATGACCTTCGCCCAGTGCGGCAGCGGCGTCACGTGCCGCCGACTCATCCAGGTCGAGGAT
GACCACCCGCGCTCCGTGTTGGGCGAAGGTGGAAGCAGTAGCGCGGCCGATGCCACGTGCAGATGCGGCGCCAGTG
ATGATTGCGACTTTGCCTTGAAGCAGCATGTGAAAGACCTCTGATGATTGTTTTTATGGTTCGACCCTGACGAATC
GATGACTCAGCTTGGGCTGCTGATTGACCCTGAGCAATAACGCAAAAGTCTTTGGGTGCTGAAAAAAATTCAGCAC
TCGTCAGTGCCAGGCATTGATGATTGAATGGTGAACACCAATAAATACACCGGTACGACCGGGAGGTTTTGTCGAT
GCGTGGCGATCTCGAAACATCTGCTGTGCTGCCACCCTTGAGAGCAATTCAGGCCTTTGAGCAAACCGCTCGCTTT
GGAAACGTCGCCAGGGCGGCCGAGGTCCTGGATTTGACGCCGTCCGCGGTCAGCCATCAACTGGCCAAGCTCGAGG
CCATGATCGGTCGACAGCTGTTCTTGCGTAATGCCCGCGGGGTGTCGCTCACGCCGGTGGGGGAGCAGTATCTGAA
AGAGGTGTCCGGCGTTCTGCACAGCTTGGCCGTGGCCACCGAGCGCGCAGCCAGCGACCTGAGCCTGGAATGCCTG
CATCTGCATTCGGCGCCCAGTTTTGGCCTGCTATGGTTGATGCCCAGGCTGGAAGCGTTCCGTTTGCAGCACCCGG
ATATCCAGATCAACCTGTCGTGTTCGTACGAGTCGTTGCACTTTAGTCGTGACAAGATAGACGTGGACATCCGCCA
CGGTTTTCCCAACTGGCCAAGCTATGAAGTGCGCACCGTGCGCAACGAAACCTTTGCGGTACTGGCCTCGCCCAAG
CTGTTGGGCTTGCGCCCAGTCAGAAGCGCGGCCGATTTGCTCGACAGGGAGCTGATCCTGTCGCAAGCCACGTTGC
TAAAATGGCCGCAATGGTTCGCTCAGCATGGCCTGGCGCGACCTGAAAACCCCTATGCGTTGAGCTTTGATCGCTC
GTACATGAGCCTGGAGGCCGCCAGTCATGGGTTGGGATTTGCATTGGAAAGCACCTTGCTGGCGCAGAAGTACCTC
ACCGCTGGAACGCTGGTCGAAGTTGCGCCGCAAACGCTCAGTGCCCCGGTGGCGGCGCACCATCTGGTTTTTCCAA
AAGCCCATTCCAGTTTTCCTCGAGTCAGGCGTTTTCTGGAGTGGATGGAAAGTGAGCTCGGGCACAGTTTCAATTA
TTGAGTGTTAGTCGGCGCGCCGCTGTTTCTTGCATTGCAAACAGGGCGCGCAAATCCACCAAACCCGACCAGACGC
TTACTCTCGCAAGCGCCGAATAATCTCCTCGTACCCGACCGCCCGCGACAGCCGCTGCAGTGCATCCAGGCGGCGG
GAATAGGCTTTTGCGTCACCATAAGTCAGCTCGACATGCAGGCTGTCGATCGTCACCAGATAGGCCTCTGCCAGCA
CTTCACTGGCCTGGTCCTCTACGCCTGGATGGGCTTTAAGTAACCCTTTCTGGAAGTGACTGCGGATCGTGGCCAT
GTAGTCATTGAACCCGGACATGACCACGTGCCTTAGCGGCGTCGGTGGGTAAAAGGCGGCCCTGAGCAAAAAGCGC
AGGGACTCATGCTGGTTGTAGCGGCGTTGCAGGTTGTCGATGTAGACCTCGCCGGGGGCCTTGATCGATCGAGAGT
TTTTCGCAAATTGGCTTTGCACGTAGTCCAGTTCGGCGCGCAGTGCGAGTTCGAGTGCGACCACGTACAGCGCATC
CTTGCTCGCAAAATGTGCGTACAGCGAGGCCTTTTTGATGCCTGCGCGCACGGCGATATCATTCAGCGAAGCGGCG
TCGTAACCGTGCTCGGCAAATTGGTCCAGTGCCAGATACGCGATGCGTTCGGCAGCCGAGGTCAGGATGGTGGCTG
TCATGTGGTTCTTCCTTAATCAGTGCCCGGCCAGGTCAAAGGTTTCGCCCGCTCCTCGGGGGAGGCTGATTGTCGT
CAGCAGGCAGGTCAGCAATGCACTGGCCAGCAGCAGTGCGGTCCAAGCCACCGGTCCATAGGTCAGCGCCAATGCC
GACAATGGCGTCGCGCTGGCGGAAACGGACAGTTGGAACGCGCCCAGCAGTGCAGCGGTGGAGCCCAGTGCGCGCT

TTTGCGAGGACATCACCAGTGACATCAGCGTGGACTCGGCGATGCCCAGGCCAAACAGGGCAAGGGCCATGCCCGC
CACTATACCGGGCAGACCCACGTAGGTCAGGGTGGATGCCACCGCGATTGCCGAGCCAGCGGCCATGCTAAACACG
CCGATTAGTGACAGGCGGCCCAAGCCAAGCCGGCTGACCAGTTTCCCGGCGCTCATTGCCCCGATCAGGATGGCTA
CCCCGGTGACGCCGAACAGCATGCCAAAGGCTTGCGGGCTGAGGCCGTAGGTCTGCTGGTAGACCAGTGTCGCCCC
GCCGATGTAGGCAAACAGAAAGAAGAAAACTGCGGCCACGGCCAGGGTCGGACGCAAGAAGTGTCGGTCTCTGGCG
ATCGTCAGGTACGTGCGGCAGGCGGCGCCCAGGCGTAACGGCTCGCGTTGGCTGGCGGGCAGGGTTTCCGGCAGCG
ACAGCCAACTGTTGCCCAGCACCGCCAGGCCAAATGCAGCGAGCACCAGCATCACCGCACGCCAGCCGTAGTGAGT
GTCGATAACCCCGCCCAGTGCCGGGGCGAAGATGGGCGCGACGCCTTCGATGGTCATCAGCAATGCGAAGAGCTTG
GCCGCCGCCACGCCTTCGCTGACGTCCCGCACCATGCTCATGATCACCACCAGCGTCAGGGCGCTGCCAAGCCCCT
GCACGAAGCGCAGGGCGATCAGCGTTTCAAGTGTCGGCGCCCATGCGGCACCCAATGAGCACATGGTAAACACCAC
AAGGCCGGCCAGCAGGGGCCGGCGGCGCCCATAGGCGTCGACGATGGGCCCGAAGATCAACTGGCCGGCACCCATG
GCCAGAAGGAAGAACGTCAGGGTCAACTGCACGCTGGCGAAGCTGCTCTGATATTCGCGGGCAATTTCGGGAAGGC
TCGACAGGTACATATCGACAGCCGAAGGGCCAAGGGCGCCGATCAGTCCCAGGCCAATAGCCAGCCTCAGGGCGAC
CCTGTGGGTGGGTTGCGAATTCATAGCGTCTCCAACGAAAAAAAACAGCCTACCGGTCGGTAGGTTTTCGCATAAT
AGCGACACCTCTTAAATCGGTCAACGCCCCAGTACTCGGGAGGATTTGCACATACACAGTGCAATCCGAGCGAGCG
AATGAGAGTTGTTATCAATTATGTTTCTTTAGTTTTTTGATTGGATTAGACTCCCGCCAGCTGTGCACGAGCAGCG
TTCTGGGCATTAGAACAAACAAAAAAAAACGGCGTAGGGAGCAGGACGTGAATTTCAAGAAAACGACAATGGCAGTG
GCTGTTGGCTCGGCGATCTACCTGTGCAACGGTGCGTGGGCCGCAGAACCTGCGAGCGCGCTGGAGATTGCACCGA
TCACCGTCACCGGTGAAAAGATCAACCGCAGCCTGGAGCAGACCCAGTCGAGCGTGGTGGTGGTCACCGACCAGCA
ACTGCGTGAAAAGCAGGACCGTGACCTGGTGGACGTATTCGCGCGTACGCCAGGCGTGTACAACCAGTCCGGCAAC
GAAAACTGGGGTATTCGCGGCGTGCCCGTGTCCGGTTTCGACGACCAGGGGCCCGCCACGCTCAACGGTGCGGTTT
CAGTATTTGTCGACGGCGCCGTGCAACCCAACCGCGCCCTGACCCTCAGCCCGATGCCGCTGTGGGACGTCGACCA
GATCGAAGTGTTCCTCGGCCCGCAATCCACCACCCAGGGCCGTAATTCCCTGGCCGGTGCGGTGGTAATCCAGACC
CGCAACCCCACCTTCGAGCCGAGTTTTTCTGCACGCACCAACATGGGCAGCTATGGCGAGCGCGGTGCGGCAGTGG
CCGGTGGCGGGGCGTTGGTCGATGACAAGATCGCCGGGCGTATTGCCGTGGATTACCAAGAGGGTGACGGCTATAT
CGACAACGTCGCCCTGCATGACGACGCCAACCCCACACGCACCGGCAATGCCCGGGGCAAGCTGTTGATCCTACCC
AACGATGACCTGGATGTGCTGCTGACTTACGCCCACAGTGAAAGCCGCAAGGGCGATAACTCGGCGATGCGCCAGA
ACGACAAGGTCCGCTATTACAAGATGACCTCCAACACCAAGGCCTATGACAAGCTTGGGCAGGACACGCTCAGCGC
CAAGGTGGACTATCGCCTGGACGACAACTGGTCGCTGACCAGCCTGACCGCCAATACCCGCTCGGACTACGACGCG
CGCCTGGATTTCGACCAGTCGGCCGACGCCAACCAAGTGGTATTGCGCAAACAGGACGGCGACCTGTTCAGCCAGG
AACTGCGCCTGAACTACAGCGGCGATACGGTGAAGAGTTTTGTCGGTGCCTACTACGGCCACAACACCAACAACTT
CCACGACCGCCTGTTGTTCAACAACCTTCTGTTCGGTACCGCCAAGGGCGATACGACCATTGAAAGCAAGGCCGTG
TTTGGTGAAATCAACTGGACATTCGCCCCGCGCTGGACCTTGATCACCGGCCTGCGCTACGACCACGAGACCAACG
ACACCGATATCAAGCAAGACGATTTCTCCAGCCCCGGCAAGCTCAGCAAGTCGTTTGATGCGCTCCTGCCGAAACT
GGGCCTGGACTATGAACTGGCCGCCAACCAGTACCTGGGCGTGATGGTGCAGAAGGGCTATCGCGGAGGCGGCGTC
AACGTGCGTGCCGGTGGCGGCCACGAAGGTTATGACCCGGAATACACCACCAACTACGAGCTGTCCTACCGTGGCT
CGTTCTTCGACAAGACCCTGCGCACCCGCGCCAACCTGTACTACACCGACTGGAAAGACCAGCAGGTCAGCGCGCT
CGAGCGCAACACCGACTTCGTCCAGGTGTTCAACGCCGGCAGCAGCGATATCAAGGGCCTGGAAGTATCTGTCGAA
AAAGACCTCGGCGAGCAGTTGACCCTGACGGCGGGTGGCTCTATCACCGCGGGCAAGTACAAGGACTTTGTGACAG
GTGATGGTCGCGATATGAGCGGCGAGGAGTTCCTCTATTCACCCAAGTACAAACTGTCCCTGGGTGGCATCTACCG
TTGGAATGACCGCCTGACCCTGAACACCGACCTGGTGTACCAGAGCACCGCGCCCTCGGAGTATGAGTTCGATACC
GCAGGCCAGGTCACGGGCGAGCGCCGGAGCGACAGCTACTGGCTGGTCAACTTCAACACCGAGTACAAAGTCACCA
AGAATGTTGCGGTTTCCGGCTTCGTGAAAAACGCTTTTGACAAGGAGTACATCACCAACAACCGCAGCGGCGATAT
CGTCGATGTGGGAGCGCCACGTACGGTTGGGCTGGTGTTGCGCTACGACATGTAGTGACAAGGAAGACAGGCTACT
CCCTCACTCCCTCAGGGATCGACGGCGTAGCCTGTTTTTTCAGCACATACAAAGTTCCGACGCGTGCATCGATCAG
TGGGCGATGCAATTTCGCCCATGCGTTCTCTTTTCAGCAGGGCGGCAAGGAAATTTCCGAGACTTTTGTCGGAAGC
GTTCCGTGGGCTGCCCGATCGGTGGTGTTTAGCATTCTCCAGGCAAAGAAAGCTCCTGCGACAAGTCACGGTGTTG
ACACACAACGACCTGCGCAAGAGGCCTTGTTCATAGAGAAAGCGGAGCCCCACCTTGAAAACTTTAAGCAAAGTGT
TATTGCCAATGGCCGGCCTGTTGATTGCCGGCTCGCTGCAGACTGCTAGCGCTGCCAACGGCACCGTGAATTTCAC
AGGGGAGATTGTCCAGTCGACCTGCGCGGTTGTTTCCGGTGATCAAACCAAAACCGTGGTCTTGGGCAAATACCCG
ACCAGTGCCTTCCCCAAGACCGGTGCAACCAGTGGCGCCAAGGCGTTCACCATCAGCCTGGAAAAATGCGAGGCCG
GTGACTACTCCCTGCGTTTTGACGGCAATACCCCGACCGGTAATCCGGACCTGCTGGCTGTTACTGGGGGCGCGAA
GGGCGTAGGCGTGGAAATTCTCGATAACAACAGCGCCATCGTCCCCATCACCCAGGACGTGGCTGCCCCGGCCAGT
GTCACCATTGCGGCCACCGGCACCAGCCCGGGGGCTGCGACATTCAACCTGCGGGCCCGTTACCGCTCATTCCAGG
ATGTAGTGACAGCGGGCCAGGCCAACTCCAACGCCACGTTTACCATTCAATACAAGTAAATCGCTGGTTCTGATGG
GGCGTGAAAGGCTCATCGTCATCATGAGTAGTTGTTATGAAAGTTTTAACTTGTTTTCCCCTGCTTATGTTTTCGA
TGAGCGCTTATGCTGGCATCCAGGTTGATGCAACACGCGTAATTTATAACGGTGGCAATCAGTCTTCCTCGCTGTC

GATTAAAAACGACAGTGACGATACCTATATGGTGCAAAGCTGGTTGGATACTGGTGATTCAACGCAAAATCCCAAA
GGGTTGCCAATAGTGGTAACGCCGCCCATCTTAAAGTTGGCTTCGAAAAAGGAGGCTGTCCTGCGGTTTATCTACT
CCGGCCAGGGCTTGCCGCAAGATAAAGAGTCGTTGTTCTGGATTAATGTTCAGGAGATACCACCGGCACCGAAAGT
GGAGAACGTGTTGCAGGTTGCCATTCGCACACGGATCAAGCTGTTCTACCGCCCGGCAGCGCTGAAGATTGATTTG
TACAAGCAGGCGCAAGCCCTTAAGTGGCAGCGGCACGGTGACGGCGTCGTGGTGACTAACAACGGGCCGGCCCATG
TCACTCTGGGGACTCTGAACTTCGCGAAAAACGGTCAGCTCGGGTGTTCCCTCAATGGTGACATGTTGATGCCTCT
GGGCAACTTGCGTATTGCATTGCCGCCCGCATGCCGAATGGCCCGCGAACTTTCATTCAGTTTTATCAATGACTAC
GGTGGTCACACCGAAATCAAAGACATTAGGCTCGATCGTTAAGTCTGTCATGAGGTAGTGGTCGTGGTGTTTATCT
CAACGCGCATAAGTGTGGCCGGTCGTCAGGCGGGGTTATGTTTGACTTGTCTGTTACTGAGCAAAATTTCCGCTGC
GCAAGAGCAATATAATGCCTCTTTTATACGGGGTGATGCCGCGGTTGAAATGGTCAGCCAGTTGGCTGCCGGCGAT
GACATTCTGCCCGGCACTTACTTGTTTGATATTTATCTTAACGACCAGCAGATCGATCAACGTGAACTGACGTTTG
CCCGTCGCGAGAAGGACTCGGCAGTTGCCCCATGCCTGAGCATGGAGGATTACCGCGAGTACGGCGTGCGGTTGCC
AGTGGCCAATGAGCAGCCAGGTTGTTATGACTTGGTGAGTACCATGGGCGAGGTGAAAATGGCGATTGATGCCGGG
GTTCACCGCCTTGACCTATATGTTCCGCAGACCCATCTGATCGCACGTCCGCAGGGGGCGGTGTCTACGAAGCTGT
ACGACCAGGGCATCAACGCTGGCTATCTGAACTACAACCTCAATGGCACCCAAAGCCGTAACCGCTTCAACGGAAC
CAATGCCAAGGCGGATTACTATTTCGCCAGCGTCAACAGTGGCATCAACCTGGGGCCATGGCGTCTGCGCAATGAT
TCTACGGTTGATCGGCAACCTGAAGCAGGTACGCAGTGGCGTAACATCGCGACGTGGGCAGAGACCGATATTGTGT
CCTGGCGCAGCCGCTTGATGATCGGCCAGGGCAGTACCGGTAACGTCATTTTCGATAGCTTCTCGTTCAGCGGCGT
GCAGTTGGCAAGCAGCAGCGAAATGCTGCCGGAAAGCCTGCGTGGCTATGCCCCGGTGATTCGCGGCGTGGCGGCC
AGCAATGCCCGGGTCGAAATCCGTCAGAACGGCTACACCGTCTACAGTACACAAGTGCCTGCTGGCCCCTTTGCCT
TGAACGATGTGTATCCCGGCACCCTCAGCGGTGACTTGCAGGTCAGTGTGATCGAGGCCGACGGTACGAAAAAGGT
GTTTACCGTACCTTACTCGGCCGTGCCCAACATGCTGCGCGACGGTATCACCGACTACCAACTGAATCTGGGCCGT
TACCGTGACGGGCGTAGCCAGTACCAGCCGAACTTTATCCAGGCAGGCGTGGCCCGAGGCTTATCGCGAGATATCA
CGCCCTATGGTGGTGTGCTGGTGGCGCAGAACTACCGGGCCGGGGTTGTGGGCGTCGGCAAGAACCTGGGCGCCTG
GGGGGCGTTGTCCCTCGACCTGTCGTTGTCGGATACGGATCTTGCCAGCGGGGATAACAAGCGCGGGCAAAGCGTG
CGCTTTCTGTACTCCAAGTCCTTGAACACTTTGGGCACCGAGTTCCGCCTGGCCGGCTACCGCTATTCCACGGCCG
GCTACTACGACTTCAACGACGCTGTAGCCGAGCGTGACCGCTGGGACTCGGGTGTATACCGCCATGACTACCTGGA
CGACAGCCAGAACTACCGTGGCGTGCCCGAATGGACCGAGGCGCGCCAGCGCAGCTACTACGCCAGCAGTTTCAAC
AACAAACGCCAGCGTCTGGACCTGTCGGTGAACCAGAGAATCGGTGATGTTTCCAGTCTCTACCTCAATATCAGCA
ATCAGAGTTACTGGGGCGAGGTCGGGCAGGACCGGACTGTGCAGGCTGGTTTCAATAGTGCCTATAAAAACATCAG
TTACGGCCTGTTCGTCCAGGACACCCGCAGCAATATGGTCTATGGCGAGCGCAGTGTGAATCTCACGCTGTCGCTG
CCATTAGGAACGGGTGGGCGCTACATCAACTCCAGTTCCAGCGTCAGCCATACCAAGCGCACAGGCACGACCTACA
ACACCGGCATCAATGGCACGCTGCTTGATGACAGTCGTTTGAACTATGGCGCCCAGGTCAGCCATAACGCGCAGGC
AGGCAGCAACAGTTCGGTGAACCTCGGTTACCAGGGCAGCAAAGGAAACGTGGACTTCAACCACAGCTACGGTCGC
CAATACCAGCAGACATCCCTGGGTATGGCGGGCGGTCTGGTGGTACATGAAGGCGGTGCGACTTTGACCCAGCCGT
TGCATAACACCCTGGTGCTGGTGGAGGCCAAAGGTGCCGAGGGCGTGGGCCTGGACAACCAGGGTGGCGCCGCTAT
CGACAAATCCGGCTTTGCAGTGATGACATCCGCGATGCCTTACCGGCAGAACCGCGTGGGCCTGCGCACCGAAGAT
ATCGGTGCGGGCCTGGAAATCCCCATGCCAGCACGAGATGTGGTGCCCACCCGCGGTGCGATCGTACGCGTCAAGT
TTGATACCCACCAAGGCCGTAACGTGTTGGTGCACAGCAAGCGTCCCGACGGCTCGGTCCCGCCGATAGGCGCTAC
CGTGTTTGGCCATGACGGCAGAAGCAACGGCGTGGTGGGCACTGATGGCGAAATCTTTATTTCCGGTGTCGCCGAT
GGCGACCGCCTGCTGGTGAAGTGGGGCAGCGGGGCAGGGGACGCCTGTTCATTAGTGTTGCCGGGCAATCCCGGCA
ACATGGCGCAACCTACCCAGGGCTATGACACCGTTTCACTGATCTGCAACACCGTCCCCGAGGCACCGCTGCCATG
AAGTCTACGTTTGAAAATACCCTGAAGTTGCTGGCTCTGTTATTGGCTATGTGCTGGTCCCAAGTGGCTTTGTCCA
TGACCTGCAGCACTGCTCTCGGCGGCTCCACCGAGGTACTTAACATGGGTGCGATAAAGGTCACGCCAGCCCAGGT
GAGGGCCGGTGCGGAGATCTGGCGCTCGGCAACGATAAGTCGGCAGTTTATGTGTTCTGAACGGCCAAACAGTCTG
AATGAAGACACACTCAAGGCGTTTTTTTTTCTAGATCCAATCAGCACGGTGTCAGGGCTGGATCGGTCCCTGGAAG
TCGGTGTGACTTATCTGGGGCGCAACACCAAGGCTTCCCACGGCTCCAGAATCAACCTTGGGACGGCCGTAAACTG
CACGCCGTTCTGCGAGCCAGGCAGCACAATGACTGGTTACAACCGTTACTACGCGCTACCCATCAGTGTGTCATTT
CAGGTGCACGTTAAGTTGACCGGAATAACCCCGCCGGGCAGCGGCAAAATAGCCAACCCTGGCACCCTGACAGTGC
TCAGGCTGGCAGGAGGCAACGATACGTTTGGCAACTTACCGGCCAGCAATTTTGCAACCACACTGTCAGGCCTCAA
CACTGTGTCGTTCACGTCCTGCCAGCCGACCATCACCGTCACCGGAAACAGCGGTGCAACCGTTGGCTTTGGGTCT
ATCTCACAGAAGAACGCGGTGCTCAACAAGATAGAAAAACAGGTGCCATTCTCGGTCAACGTGAACATGAGCAGTG
TCGATAATGGCCAGGCCTGCCCTGGCGCGACGATGCAGGCGGCGTTCAGTACCACTTACACAGTTCGCGACCAGAC
CACCATCCTGCCTGCCAGCAACAGTGGCTTTGGCATCGTGTTGGCGCGGGCGACGGCACCCACTGTGCCGATTGTC
ATGAACCGTATCGTCGACCTGGGCCTGGTCAATGGCACCGTGGTGCAAAACCGTTTCACGGCCGGGCTAAAATGGC
TCAGTACAACCCCCAGGGTGGGGCCTTTCACGGCCTCCGCGACCGTGGATATCACCTTCAAGTAATCGGCCTGATC
TGTCTGGAGTGCGTTCAATGATGTCGTTGCGAGTACTGGTGCTCGATGCCCAGCTGTCTTCGCGCAAGCTCAAGGT

TGCATCACTCGGCGCGCTGGGCACGCGGGCGGTCTATCAGGCCAGCCACTGCGATAAGGCGATGGCGATCCTGCGC
CAGGTTGACGGTGTGGACATTGTAGTATGTGAACTCAACAACGACACATTGGCGCGTTTCGACTTTCTGCTGGCGG
CTGCCAGGGACGGTCTGGTCAGTGCCGTGGTGCTGTGCTCGGCGCTTGCACCGCAACTGCATCGCGCCTTGGAGCG
GATCAATCTATTCGCCAGGATCAGCCTGGCGGGTGTGGTCGGGCCGGATGCACCCGTCCAGCAGTGGCATTTGATT
TTGACTCACTATATCCGCCGTAAAACCATGTCCCATACGCTCCCAGCGATCCTGTTCAAGCTGCCCACCGAGTACG
AAGTCAGGCAAGGCCTTGCCGCCGGGCAGTTCAACGCCTGGTTCCAGCCCAAGTTTGATCTGCGGCGCGAGGCATT
GTGTGGCGTCGAGGCGCTGGTGCGTTGGGAACACCCCAGTCGCGGGGTGTTGTTGCCTAGGGATTTTCTCTCGGCG
GTGCTGGCGTACGACTTGATCGATGACATGTTCAAGCAGGTGTTTAGCCAGGGCCTGGATTTGCTCGATGCCTTGG
GGCAGGGCGGTAGGCATCTGGAAGTGGCGTTTAACCTGCATGCTTCGCAGTTGGCCAGCTTCGATCTGCCGAGCTA
CGTCGAGGCCGCCTTGATCGAGCGCAAGTTGCCGGGCAGTGCGGTGTCGTTTGAGATAGCCCAAAACGGTTTGCTG
GACATGAGCCTGGCGACCATGGCCAGCCTATTGCGTCTAAAGGGGTTGGGGTGTGGTTTGTCCATCGATGATTTCG
GCGTGGGGTTTTCTTCCCTGACCTTGCTCTGCCAACTGCCGTTCAACCAACTCAAACTCGACGCTTCATTGGTTGA
GGAGCTGTCGGACGAAAACAGTCGGGCGATGCTGGCAACCAGCGTTGCCCTGTCCAGGGCCGTTGGCATGAGCCTG
ATGGTCGAAGGTGTCAGCAGCCAAGCTATCCAGGATACTGTGATTGCCATGGGCGGCACATTTGCCCAGGGGTTTC
ACCTAGCCAAGCCGATGACGGCCAAACGGCTCCTGCACTGGTTGGGCAGTACCGCACCCGCTGTTGAATAAAAATA
ATAGGGCCGATGTGGAGCGGACTATGCTTGTACGGCATTCCCGTCACTTGATATTCCTCCCTCGACGTCTTTGTCA
CTTTGGCAAATTGGATAAGTGAGGTAACTATGCGTAAAGCGTTGGTCGTGGATGATCATCCCATCGTGCGTGCAAC
CGTGAAAATGGTCTTGCAGTACGAGCATTTTGACGTGATCGTCGAGGCCGCCGACGGTGCGGATGCCGTGCAGCAG
GCTCGCCAGTTGCAGCCTGATCTGGTTGTGCTGGATATCTCGATGCCCAAGCTCGATGGTCTGGAGGCCATACGCC
GGATTGTCGACTTGAATATTGGCACGCGGATTCTGGTCCTCACTTCCCTGGAACCTGCTTTGTACCTCAAGCGCTG
CATGGTGGCCGGTGCGGCGGGCTTTCTGTCCAAGGTCGATGATCTGGATGACTTACAGCGTGCGGTCAATGTGCTG
ATGTCTGGTTACACCCATTTTCCCAACCTGACCATCGGTTCGGTGCGCCGCAGCGACAATGAGATTGATGAGGCTC
GGGTCATCGACAGCCTGTCGGACCGCGAGTTGGTGATTTTCAAGCACCTGGCGCAGGGCCGCAGCAACAAGGAAAT
AGGCGATTCAATGCTGCTCAGCAACAAAACCATCAGCACCTATAAAACCCGGCTGCTGGAGCGGTTTGAGGTCACC
TCAGTAGTTTCGCTGGCGGAAGTGGCCAAGCGCAACAGTATTGTGTGATGAGCCGCGTCAGGTTTTTTCTCGCCGC
CACACTGCTGGCCAGTCTGCCTTTGGCCCAGGGCGAGACGCTGTCAGCGTCGGTCGGCCCCCATGAATTTGATTTG
AGCTTCAAAGTGCACACCGGCGCCGACACAGGGTTCTACCTGCTCATGGCGTTGACCAGTCTTGTCTCGATAGTTT
GCTGGCATGGTGCCTGTGTGTTCTGGCGACGGCGCAGGCTGGATGGCAACCGAGTACTGGCCGCCCAAGTGGCCGC
AATGCGATCAATGCTCGATAACACACCACAGCCGGTATTTATCCGTGACGCTCAGGGGCGGCTGGTTGCGTGCAAC
GACAGCTATCTAGATTTCCTGTCCACGGACCTGGATCAGTTGCTGGGCAAGAAGGTAACCGACGGCAAACACATGG
CCCCGGATGAGGCCGCGCAATATCATGCGTTCTACCTCAAAGTGATGCGCAATCGCTTGCCTGAAATCGGCGGCGG
GATCCTGGTCGCACCCTTTGGTCAGACGGTAGCGATTTTTCACTGGATATTTCCGTGCTTTGATACCCAAGGCAAC
GCGATCGGTGTGATGGCGGGGTGGATCGATGTCAGCGACCGTCAGCAATTGGTTGACCAGCTCAAAGCCTCGCAAA
AGGAAGCCGAGGATGCCAACAAGGCGAAGACGACTTTCCTGGCAACCATGAGCCATGAGATCCGCACGCCGATGAA
CGCGGTGCTGGGCATGCTGGAAATGGCATCCAAACGTGCCGAACAGGGGATTGTCGACCAGGTTTCGCTGGACGTC
GCGTCTAGCGCCGCCAACGGCCTGGTGGACCTGATCGGCGATATCCTCGACATTGTGCGCATTGAGTCCGGGCAAT
TGAACCTGGCTCCGGGGCGGGTGAATCTGCAGGAGTTGTCTCGTTCGGTGGCACGAATTTTCGACGGGGTGGCACA
GCAAAAATTCCTGGACCTGCGGGTCGAACTCGACAGTCGCGCCGACTGCGACGTGTGGGTGGATTCGCTGCGCCTC
AAGCAAATCCTCTCGAACCTGCTGAGCAATGCGATCAAGTTCACCGTTATCGGCGAAGTGCGCTTCTCACTACGTT
GTGTCGTGGTCGGGGGCGGCGAGGAGTTGGACCTGATCTTCAACGTCATGGACACCGGGATCGGTATTTCCCAGGA
CGATCAGGCCCGATTGTTCAGCCCATTCAGCCAGGTGGGCAACCCTGCTGATACGATGCGTACTGGCTCCGGTCTG
GGTCTGACGATTACCCGTACCTTGTGCGAGATGATGCATGGCCAGTTGTCCCTAACCAGCAGCCTGGGAGGGGGTA
CCGATGTCTCGGTGGCGCTAAGGTTGCCGATCCTTGAGCCGGTAGCACCGGCAGCTGTACCTCTGGTTGAGCCGGT
GCAACCCGCCAGACGTCTGCGGGCGCTGGTGGTAGATGATTACCCGGCCAACCGTGTGCTGCTGTCCCAGCAACTG
ACTTACCTGGGGCACGATGTCAGTGATTCCGAGGACGGCGCCCATGGGTTGCGCGCTTGGCGCAAGGGCGCCTTTG
ATGTGGTGATCTCCGATTGCCATATGCCGATAATGAACGGTTATGACCTGGCTAGCGCTATCCGCGCAGAAGAGGC
GCGCTTGCAGTTGCCCCGTTGCCTGATTATCGGCCTTACCGCTAATGCGCTGCCGGAAGAGAAACAGCGCTGCCTG
GATTCCGGCATGGACGACTGCCTGTTCAAGCCCATCAGCCTTCGCGATCTGAGCGAGCGCCTGGTATCGGCTCGCC
CCAGGACACTGGCGGCCGAACTTCAGGTGGCGCAGTTGGGACAAGGCCAGACACTTGATCTGAGTACCTTGAGGCA
ATTGGCTAGAGGCGACGAACAGGCAATCAGCACGCTGGTGCGGGACCTGGCAGACAGCCTGGACAGCGACAGGGTG
CGATTGCTCGAAGGCTATGGCACAGGAGATCTTAAAGTGCTTGCCGAGCTGGCTCATCGGGTCAAGGGGGGGGGCA
GGATCGTGGGTGGGCAAAACGTCATCATCCACTGCACCCGCCTGGAGGCGGCTTGCCAAATCAGGGAGCCGGCCCA
GCTTGAAACCGCCGTCACGGCCTTGACACAAGCCATGGTGACACTGAGCGCGGAGCTCAAAAGATTAGGCTGAAGT
CGGTTTTGGAGAACTTTCCATCCATCACCGGAAACTTCCGATAAATGCATGGAGTTTTCTATTTCCATTTAGGAGT
CTTCCTAGATACGCCGGCAAGAGGCGCCGTGAGAATGGGCGCATTCCCATCTCATGTGTGATTTGTCATGTCCTTT
CTTCCCATCCGCGTTCTGGTCGTCGAGGAGCATCCCTTCAAGCAACTGGTAGCTACTCAGGCATTTAAGGAGGCGG
GGTGTGATTGGGTGATGGCTGCGGCCGATCTGGCCGCTGCGCTCGCGTTGCTTGATCGTGCAGGGCCGGTGGATAT

CGTGCTCTGCACCTTGAAGAAAGAGGGTATGCAAGGGTTGACGGTCCTTGAAGTGCTCAGTCGCACTCTTCAGGTC
AAGTCCGCCATCATCTGCAGTGGGCACACCCAGGATCTGCATGACGCCATCGAACGCATGATCGGCCTGCTTGGGG
TCGGCGTCCTGGGTTATGTCGATGTCCCGGTGCGAGCGGGTGCGATTGCTTCGATGTTGACGCGCTATCTGGAAAC
GACGGTGGCTCCTGCGTCTCAAGTCGCCAGGGAGGCGGGGGATGTACTCGCCAGCAAGGCTTGCCTGGAGCGCGCT
ATTGCACGTTCTGAAGTTCAGGCTTTCCTTCAACCCAAGTTCAATTTACTGACGGGCGAAGTCGGCAGCCTTGAAG
TGCTGGCGCGCTGGCAACATCCACAGCACGGCGTATTGTGCCCGGCGGATTTCCTGCCGTTGATCAGCCGGTTCGA
CTTGATGGATGAGTTGTTTTTTGCCCTACTCGAGCAAGCGCTGACGTTCTTGCGGGAGGCACAGGATCAAGGGTAC
ATGCTGTCGCTGGCGTTCAATCTGGAAGCCGCGCAACTGGCTCACGAATCCTTGCTCCCCCGCCTGTGCGAAGTGC
TGGAGCGCCACGCCATCCCGGCGTCGCGGCTGACTTTCGAAATCACCGAAAGCGGCTTGCTGGAGGTCTCGCCCGG
CGTGCTTGAAACCCTGATCCGCTTGCGGATGATGGGTGCCGGCCTGTCCATTGATGACTTCGGCATCGGCTACTCC
TCGCTGGAGCGTCTGTGCCAGTTGCCGTTTACCGAAATCAAGCTAGACGCCAGTTTTGCCCGCGATCTGGATTCCA
GCCCCCGTAACCGCGCAGTGATCAGCAGCACCTTGGCATTGGCGACCGCGCTGGACATGATGGTCGTGGTCGAAGG
TGTCGAGCACGACAGCCAGCGCCAACAACTGCTCAAGCTGGGCTGCCAACAAGGCCAGGGTTATCTCTGTGCACGC
CCCATGAGCACTGCATCTGTGTTGCCCTGGCTGGCAACGAAGCTGGCGTCGCACAACACTCTATGATGATTCGCAA
GTAGGGGCTTCCCATGTCAAATCCGCGTTTGCGCATCGTGCTGGTCGATACCGATCCAGGGCGGCGAATGAGCATC
GAGAAAGACCTCGGTTCTTTGGGATATCACCGGATCGTACCGGTCTCTTCGTTGCCAGAATTGATCGCGTTGCTGG
ACAACGCGCTGGATGTGTTTGACCTGCTGGTCATCAATGCAGAGACCGTTGGTGACGCAGGAACAGGATTCAATCA
GTTGATGGATGAATACTCATGCGTCAGACACTCGCTGATCTATCAAGGTTCGGTCCTTCAGATGCTTCCATTTACC
AACATACGCTTCAAGCGCTATGGTTTTCTGGCTTCTGGCGTTCCTGATCGGTCTAACTTGGAGCACGTTATGAGCC
GGGTTGATGCACCGCTCCAGCCGATCAGGCCATCCTGCGAACAACGCAGGTGTGAAGGGGATTGCAGGGGCTAGAC
TGAAACAGTCAATTGCCTTCTTGGAGCATCCCGATGGCCTACAAAAACACCATCTGCCTCTGGTTCGACGGCACAG
CTCTGGAGGCTGCCAGATTCTACGCCGCCACCTTCCCGGACAGCACCGTAGATGCCGTGCACACAGCGCCGGGCGA
TTATCCCGCGGGCAAGCAAGGCGATGTCCTGACCGTGGAATTCACCGTCATGGGCATCCCGTGCCTGGGCCTCAAT
GGCGGGCCGGGTACCCAGCACAACCAGGCGTTCTCATTCCAGGTGGCCACTGACGACCAGGCCGAAACGGATCGCC
TGTGGCAAGCGATTGTCGGCAACGGCGGCGAGGAGGTTGCCTGTGGCTGGTGCCGTGACAAGTGGGGCCTGTCCTG
GCAAATCACCCCGCGCGTGCTCACGGCAGCGATCATCCATCCCGACCCCGCGGCGGCCAAGCGCGCATTCAATGCA
ATGATGGGCATGGTGAAAATCGACATCGCCGGCATTGAGGCGGCGCTCAAGGGTTGATGAGACTCTGGTATAACCT
GCGTTTTCAACATGCAGTAACGGCAAGGGAATGCGTATGAAATTGACGGCCCAGAGGCTGGTCGAACTGGCGATGG
TCAACCCCATCAACGCTGAACTGGTGGCGCGCCTGCCAGCCCTTGGCCTGGACCAATGCCTGCTGACGGCAGGTTG
CCTGTTCCAGGCGGTGTGGAATCATCAGGCCGGGCTGAGCCCCGATTGGGGGGTGAAGGATTACGACGTGTTCTAC
TTTGACACCGATATGTCCTGGGAAGCCGAGAATGAGGTGATTGAGCGCGCCAGCGAGCTCTTGCGCGATCTGGATG
TAAACATTGAAGTGAAGAACCAGGCGCGTGTACACCTCTGGTATCCGCAGCGGTTCGGCATGCCTTACCCGCAATT
GCACACAGCCCGGGGTGGGGTAGATCGCTACTTGGTGGCGGGCACTTGCATCGCCTTGGCCGTGGACACGGGGGAA
ATCTATGCGCCCTACGGCCTGGCCGACGCCGAGCAGGGAATACTTCGGCTCAACCCCATCACCCCGCAGCCAGAGC
TGTTCGAGCACAAGGCCAGGAGCTATCAGGCTCGCTGGCCATGGCTCAGGATTGAAGACCCACACATTGCGTAGAG
ACGGACTCGTTCACGGCTACGACGGTTGTACGCGTTTCTCGAGGGCCTCTTCCTCATAGCGCCTTATTGCTTCATT
CACGATGCTGTCGATTTGTGGTGAGTAATTCTCGATATTGGCCTTGCCGCTTTTCAGATAAAGCGCGTGGTACATC
AACTGGTCGCTCAAGGTTGAGTACCAGTTTTCATCATCCCCCACTAGCGGCAAGATCGATTCGGCGGAGTACAGCA
AGAACCCAACAAAGTACTCATATTGTTCATATTCCTCGCTGCCCGGCTTAAAGCTCTCGAATCGCGGAGACTCAAT
GGGGTAGGAGGCAGCGGAAAATTTCGGGTTTGCAAAGGCCAGGGAAATGTAGTCTTTGTAGATGGACTTGGCCGAA
GCCAGTTTCGATTCATTCAACTGATCGTAGGCAACGAATGCCGCACTAAAGGCAACCGTCAATGTGAAAAAAGATG
AGACTGCGCTGAATTTTTCATACTTTTGCTCTGATGGGACTGCGGCCCATAGCTTGTAAAAAACAAGCAGTGCCCC
AACGGCCGCGATAACCAGAAAAAATCATTTGGAACTATCCTCTTCAATACAGAAATATCGACTGTTCTTAATACTTC
GGATACGGCTGATTCAATCAGCGCAGATCGAAGTGGCTGTTCTTGAACCGCTCGTAGTTATCCAGGGCCAGTGCCA
CGATCCTCTCCCTGACATGCCCTGTGTAAGCCTCCCATTGAGCGCGGTTGTAACGTTTGATAGCCCGCAGCACATA
CCATTCAATGTTGTTCATTGCATCGTCTGGACGCCGGGGAGTGCAGGCTTTGAGCAATGTGTAATAAGTGTGGTAG
TCGACTTTTTCGCTTTCAACCGAAGCACTGTTCGGATTGAAGAGGGTCTTCGAGTACGCAATGACCTCCAGCAACG
TTTCGATCTCATGCTGCGCGATTAAGCGAAGCAGAGCGTCAGGCTGCTCCAGCGCGGTTTTATGTTGTTCGCGAAT
CATTGGTTCGGTAATTTCTTCGAACCAAAGCTTGTGGTTGTACTCGGGAGAGGCTTTGATTTCTTCGCCGTTCGGG
CCAAGTAAATGCGCGAGCGTGAACTGCGTGGTGTGTGCCCTGATTTTTTCCTTGATGGTAGCCTGGTTCTCGTTGA
GACTGCCCTTGATCACCCCATCTAGTCGCTGATCCAGTTGTTTTTGGGGCACGTCGCCGTCATTCACTGCCAACGC
CGAAAAGAATGGAATTTGGTACGTCCCTGCCAACTCGTTACCGTAAGTGTAGAAACCATACATGCACGCAGCCTGA
TTGAGCCAGGATCCAGAAACAACGCACTTGACCGAAGACTTTGGCCTATCGCTGGAATTCTGATCTACGACTAGGT
CGATAATTTTTTCTGCCAGTTTGTCTTCAACGAAAACATAGATGAAGGGAATGAACTTCCCTACCATTGAGCCTGT
GCGCGTGACTGTTTTTGTGCCGATGGTACCGATACTCATCCTATTGCCTTTTGCAAGTGACAGACCTTACGAGTGA
AGCGTTGTCAGGATTTCGCTATCGGCAGATGAACGTGAGTCTTTAGGCTCGCAGCCCACACTCACTTATCCCGACG
CCCTGGAATCGCCACCCGGCTGGTGGTCTTCACCTCGCGCAGCGCCAGGCTCGACTGGATCGAGGCAATCCCCAAC

TGGCGCCGCAGCACGCTTTCGACAAAGTCGCTGTAGCTATCCAGATCTTCGGCCAATACCTGCAACAGATAATCGG
CATCGCCGGTGATTTTGTGGCAGGCCTGCACCTGCGGCAGTTGCTTGATCACGGCCTCGAAGGCCTCGGGCGTGTG
GTCGGTATGGGTGGAGAAGCGGATGTGGACAAATGCCATGATATCCAGCCCCAGCTTGCGACGGTCCAGGTTGGCC
TGGTAATCCTTGATCACGCCTTCTTCCTCCAGGCGTTTGCGTCGGCGCCAGCACGGCGTGAGGCTCAAGGACAGGC
GTTCGCTCAATTCAGCGTTGGAAATGCTCGCGTCTTCTTGCAGCAGGGCAAGAATGGCCAGGTCGGTGTCGTCAAG
CGTGGGGCGGTTGGATAGATTTTTCTTCATTGTCTTTTTTTTGAGGAAATATTCCCGATAAAGCTAGCATTGCGAG
AAGTAAAAGCAAAGAAAGCCCCTCAAGCCCCGAACAAAATAGACGCCTGAACCTCATTAGGCGGATATCTTCCCAT
GTTGACTGTTTTCAGCGATTCCCACCGTTTGCACCATGGCACCGAACTTAAAGATGGTGTCCTCAAGCCGTCCTTC
GAACAGCCGAGCCGGGCCGATACCGTGCGTGATCGGGTCAGGCATGTCGGCTTGGGCGATATCATCGTGCCGCGTA
CGTTTGATCGTGCCTGCTACGTCAACGCCCACAGCGAGCGCTACGTATCCTTCCTTGAAACCGCCTGGGCACAGTG
GACAGCCGTAGGCCGCAGCCATGACGCTTTGCCCCTGGTCTGGCCGGTACGTGACTTGGCCAATCAACAGGTGCCG
GACTTTATCGATGGCAAGCTCGGCTTCTTTGCCATGGACGCCGGTTCGCCGATCACCGCCAACACCTGGGACGCGG
TGAAGACCAGCGCCGATATTGCGCTTACAGGCCTGGCCTTGATTGATGAAGGCCACGACAGCGCCTTCGCCCTGTG
CCGCCCACCTGGGCATCATGCTGCGCGCGAATACATGGGCGGTTATTGCTACCTCAACAACGCCGCCATCGCCGCG
CAACAGGCAATTACCCAGGGCGCGCAGCGGGTGGCAGTGCTGGATGTGGACTTTCACCATGGCAATGGCACACAGA
ACATCTTCTATGACCGCCCGGATGTGATGTTTATCTCCCTGCACGGCGAGCCTTCGGTGTCTTATCCGTACTTCTC
AGGTTTCCATACGGAGCAGGGCACGGGTGCCGGTGAAGGGTTCAACCTGAACTATCCATTGCCCAAGAACACCAGT
TGGCAGGCCTACAAAAGCGCGTTGCTCGATGCCTGTAAAAAACTGCGCGACTTTGCCCCGCAAGTATTGGTGATCT
CCCTGGGTGTCGATACGTTCAAGGACGATCCCATCAGCCATTTCCTGTTGGAAAGCCATGACTTTCTCGGCATGGG
CGAGATCATCGCCAGCGTCGGCGTGCCCACGCTGTTTGTGATGGAGGGTGGCTATATGGTCGACGAGATCGGCATC
AATGCGGTCAATGTGCTGCACGGCTACGAGAGACAACGCGCCTGAGGATTCAACGCCCAAAGTCCGAACCACGTTG
ACCCGAACAATAAAAAACCTTCGATATTTTTCTGCCAAAACTCAAAAACATAAGAGCAAGAGGTTTTGTGATGAAA
ACATCTGCACCCGGGCTGGAAAAGCCTGCGCTTCAACGCACCCTCAGTAATCGTCATATTCAACTGATGGCCATGG
GCGGCGCGATTGGTACGGGCCTGTTCATGGGCTCGGGAAAGATCATTGCCTTGTCTGGCACCTCGATCATCCTGAT
CTACATGATCATTGGCCTGTTCGTGTATTTCGTCATGCGGGCCATGGGCGAGTTGCTGTTATCCAACCTGAACTTC
AAGAGCTTCGCGGACTTTGCCGGTGCGTACCTGGGGCCGCGCGCGGCGTTTTTTCTTGGCTGGTCCTACTGGTTGA
GCTGGAGCGTGGCGGTGGTCGGGGACGCGGTGGTAGTGGGCGGGTTCTTCCAGTACTGGTTCCCCAATGTGCCGGC
CTGGCTGCCGGCCATAGGCATGATGCTGACGCTGTTTGCCTTGAACGTGCTGACCGTAAAGTTGTTCGGTGAGGTT
GAGTTCTGGTTTGCGATCATCAAGCTCATCGCGGTTGTGACGCTGATCGGTGTGAGTGTGTTGCTGATCGGCAGTT
CATTTGTTTCCCCCACGGGCGTAACGGCGTCGCTGGGCAACCTCCTGGATAAACAGGCGGTGTTTCCCAACGGCTT
GCTCGGGTTCTTCGCCGGCTTCCAGATGGCGATCTTTTCCTTTGCCGGCACCGAGCTGATTGGCACAGCGGCAGCG
GAAACGCGCTCGCCGGAAAAAACCTTGCCCAAGGCCATCAACTCGATTCCGCTGCGCATCATCCTGTTTTATGTGC
TGGCCCTGGCGTGCATTATCGCGGTCACCTCGTGGCAGGCGGTGTCACCGACCAAGAGCCCGTTTGTGGAGTTGTT
CCTGATCGCCGGCTTTCCGGCAGCCGCCGGTATCGTCAACTTTGTGGTGCTGACCTCGGCGGCGTCCTCGGCCAAC
AGTGGGGTGTTTTCCGCCAGCCGCATGCTGTTCGGCCTGGCGGACCTGGGCAGCGCGCCAGGGGTGTTCAAGCGAT
TGTCGAAAAATAGCGTTCCGTTTGTCAGTCTGGCGTTTACCACTTTCCTGATGCTGCTGGGCGTATTGCTGCTGTT
CATCGTCCCGGAGGTCATGACAGCGTTTACCATCGTTTCCACCGTGTCCGCGATTCTGGTGATTTTTACCTGGTCG
ACCATCCTGGCGTCCTACATCGCCTACCGTAAGAAACGCCCTGACCTGCATGCCGCGTCACACTACAAGATGCCGG
GTGGCGTGCCGATGGCGTGGTTTTCCCTGGCGTTCCTTGGGTTCGTCCTGTGCCTGCTGGCGCTGCGTCCTGATAC
CCGATTGGCACTGTGTGTCATGCCGGGGTGGTTTATCTGGCTGGCGATTGCCTATCAGCTCTCGACGTTCAAAAAA
CGCTGGTCCTTGTCGCACTCCTTGTAAGGACCTGCTGCAGCGATCGCCAGCAAGCCGCCTCCTGTGGTAGCCCGAT
GGCTTTAACTCGGGCTTTTACCGCGTACCTGCAACGAATTTCGCTGTCCTGAGTGCTGGCAGGGTGCCACAATGCC
ATTTTGTCGCAGCACTAGGATCAGGCGAACCAAATGAATGGCCTTGGGCGTGAGCAGGACCAGGATTTTTTCATCG
AAGAGCAGGTGCGCACTGATCGCCTGCGTCAGTTGTTTAGCCAATCATTCTCTGCGGTTTTTGGCAGTTATCTGGC
CGCGGGCATGCTGTGCTGGCTGTGTTGGGACCGGCTTGATCCCCGCCTGATGATCGGTTGGCTGGTATTGCTGGCA
GGCTCGTCGCTCCTGCGTGTGTCGATGTTCCTCCAGTATTTTCGCAGCCCCCGCAGCGAGCGCACGCCCCAGCGCT
GGGAGCGTCATTACTGGATCACCCTGGTGCTGTCGGCCGGGATCTGGGGGGCAGGGGCCTTTGCCGTGATGCCCGC
CGATGACCGGCTGACCCAGGCCCTGGTCATGCTCTTTGCCGTGGGAATGTCGGTCAGCGCGGTGTCGTGCTACTCG
GCCTACCGTTACATGACCATAGTGTCCATGGCCCTGGTGCTGTTGCCGTGCACCCTGTGGCTGTTGTTTCAACCCT
CCAAGATGCAGATGGGCATGGCTGTTGCCGTGCTGGTGTTCTCGTCATTTGTCGTGGGGGCGTCGCGTAAATTGTC
CGAGGCCCTGGAGACGGCGTTTCGTCTGACCCGCGAAATAGAGCGCGCCCACAGTATTTCAACGCGCGCCGCCCAG
ACCGATGAACTCACCGGTTTGATGAACCGCCGCGCGTTCTTCGAAAATGCCCAGGTGCTCTATGACCAATGCAAGC
GCACTCACCAGCCGCTATGCGCGTTGATGCTGGACATGGATCACTTCAAGGCGATCAACGACACCTACGGCCACCA
GGCCGGCGATCAGGTGTTGCGCCAGATTGGCCAGGTGATCAGAGCGTCCTTTCGCCAGTCGGATATCTACGGTCGC
CTGGGGGGCGAGGAGTTTGCGGTGCTGCTACCCAATACCACCCTGGAAGTGGCACAGGACATCGGCGAACAGTTGA
TCCGCGCCATTGCGCGCTTGACCATCGACCCGGTACAGGGATTGAGTGCGAGCCTGGGCGTAGCCCCGAACTATGC
TGCGGGTGATGACTTGCAGCACTTGATGAATGCCGCTGACAAAGCCCTGTACCGCGCCAAGGCCATGGGGCGCAAC

CAGGTGGCGGTTGCGTCCTAGCTTCAATCCTTGCCCATGATCTTGGCCAGCACCCGGCAGATTTGGTCGGCGGAGT
AGGGTTTGGCCAAGAACTCAAAGCCGTCGTAGCCACAATTGGCCAGCTCCTCGCTGTAGCCAGACGTCAGGATCAC
CGGCAGGTCGGGCCGGCGTCGGCGCAGCTCACGGGCCAGCGCCACGCCCCCCATGCCCGGCATCACCACGTCGGAA
AACACCCCGTCGAAGGCCCTGGCATCCGGGCCGATCATCTCCAGGGCTTCTTCGGCATGGGTGGCCCAGACGGTCT
GGTAACCCAGGTCCTGCAGGATCTGGTTGGCGAAGCGGCCTACTTCCAGATTGTCTTCCACGATCAGGATGCGCCG
CCGCGCGGCGTCCGGATCCAGCTCCAGTGGTGCCTGGACCGTGTTGTCGAGGATCGACTCGGGCTCCACTTGTGGC
AGGTACAACGTAAACACGGTGCCCTGGCCGAGCACACTGGCCACATCCACGTTGCCCCCCGATTGCTTGGCGAACC
CGAACACCTGCGACAGCCCCAGCCCGGTGCCCTTGCCGACCTCCTTGGTGGTAAAGAACGGCTCGAAGATGCGTTC
AAAGGTCGCCTGGGCAATCCCGCTGCCGGTGTCTGCCAGGGAGATTGCCACGAAGGGTTTGCCTGACCCGCCATGC
CCGCGTATGCACGGCAATGGCTGGTCCCGGTCCAGGCGCAGCACCAGCGTGCCCTGGCCGTCCATCGCATCCCGGG
CATTGAGGGCCATGTTGATCAAGGCCGTTTCAAATTGGCTGGAATCGGCGCAGACGTAACAGGCCAGTTCCGGCAA
TTGGACGCTGACATGGATTCGCGCGCCGGTGACCGTCTCCAGCATGTCGCCGATGTTCTGTACGCGCTGGCCCACA
TTGAACACTTGCGGGTTCAGCGGTTGGCGCCGGGCGAAGGCGAGCAACTGGCTGGTCAGCTTGCTGGCCCGCTCTA
CGGTATCGGTCACGGCGCTCATATAGCGCTGGCGGCGTTCTTCGGGCAGGTTGGGCATGCGCAGGAAGTCGACTGA
CGAGCGGATGATCGTCAGCAGGTTGTTGAAGTCGTGGGCCACGCCACCGGTCAACTGGCCGATGGCTTCAAGCTTC
TGCGACTGGCGCAACGCCGCTTCAGTCTGGTTGAGCAGGGTGGTGCGTTCCACGACCCGCTGTTCCAGGGTCGCAT
TCAAATCGGCCAGTGCCGACAAGCCCTCGCGGACGATGGCATCGGCGCGCACCCGTTCGATATGCGCCCAGGAGCG
CTCCGTGACCTCACCCAGCAGCGCCAGGTCGTAGGAAGACCAGCGGCGCGGCATGCGGTCGTGAATGGCCATCAGC
GCGGTCAAGCGTCCGGACTTGATCAGCGGCATGCAAATCGTCGCCGTCACACCCAGAGCCTGGAAGGTCGCGGCTT
CCTCGGGCGTCAGTTCCGTCAGGTTGTCATTAATGATCAACGGCTCACCGGCCTGCAGGCGCTCGACTGCCAATTG
GCCGAAGTCGGCCAGGCGGTAGTGCCCGACAATGCTGGGCGAACCTTCTGCAGCCCAGTTGCCGCGAATGGTAAAG
CCGTCTTCATCGGCCTCCATGTCGGCATAGGCGCAGTTGGACAGGTTCAGGTGGGTCGCCAGTATCTGTGTGGTGG
CCGCCATGATCGCTGCAGGATCGGTGGCATTGGCTACACCGTTGCTGATCGCATCCAGCACGGCGAGGCGGCGGGC
CATGAACACCGAGCCGGTAGTTTCGGTGACGGTATCGAGAATGCCCACGACGTTGCCGCAGGCATCGCGGATCGGG
CTGTAGCAAAACGTGAAATAGGCCTGGTCCGGCGCATCACCACGCTCGACGATCAATGGGAAGTCTTCGATAAACG
TTGCGTGGCCTGCAAATGCGGCATCGGCGATCGGGCTGATCTGGCTCCAGGCTTCCTGCCAGATGGTACTGAAGGG
CTGGCCGAGTGCATCGGGTTTGCTACCAAGGATCGGGACGAAGGCGTCGTTGTACAGGGTGGTCAACTCGGGGCCC
CACACCACCGCCTGGGGAAAGTGGGAGGCCAGGCTCAAGGACACGGTGGTCTTGAGCACATCCGGCCATTGGTCCA
TCGGCCCCAGGGGCGTGTTGGTCCAGTCGTGGCGGCGAACCCGTTCGGCCATTTCACCGCAGCCTTGTAGCCAGTC
TGTCATCTACTCAACACCTAGTTCGTCCCACGTTGCGATATGGCACCGCAATATCAAACAACAGACCCGGGCAATG
TCCGGCAGTTCAGCGCTACTGCACGCCCAGAAGTGCCGTCCAGTAAATCCCGGCGTCGCTTTTCGGGTCCAGCCCG
TAGGCGGCACCCAATTCGCGAAACTGCGGGTTCATCAAGTTGGCGCAGTGCCCGGGGCTGGCGAGCCAGCCATCGA
CCACTTTGCGCGGCGTATCCAGGCCCGCTGCAATATTTTCACCGACCTGCTGGGCCACGTAGCCAGCCAGCTCCGC
GCGGTCGCCCGGGGTGCGGCCGTCGCGGTCCAGATGGTCGAAGAAGTTGTGGTTGGCCATGTTGCGTGCATGACTG
TCGGCGGCGCTTGCCAGTTGCGCGTTCCAGGCCAGTGGCGTGGTGGCGGCGAAAGCCTGCTCGCCACATTGGCGCG
GTTGCTTGCGGGCGGCGTTGACCATCTCCAGCAGTTGCGGGCCGGCCTCCTGGGAACTGGCCAGGCCACGGCTCAG
CAGCGGACGGGCGAGGACAATGCGCCAGTCCTGGCCATTGTTGCTGACGCCGATATCGACGAACTGCGGGTCCAGC
ACCACGCGGCAGAAACTCTCGCGCACCGCTTTCATCGCCGATTGCGCGTCGCGAGGCCCGGACAGGCTGATCGCCT
GGACATTCACCATCGGGTAGGCCGCCTGGGCCAGGTCCTGCTGCAAATCGCCGGCATTATTGACCGGCAATACCAG
GCGTGGGTCGACCGCCAGCGGCGGCAGTTCCTGGGAACCCTGGTCGCCACAGCGTTGCAGTTGGCTGCGGTACTGG
TTGATTGAGTCGATCAATTGACTCTCTTCGCTCGCCGACGCCGTGGCGCAAAACACCAGGCTGGCGGCCAGCGTCG
TAACACCCATCATCAATGACAGAACGCGCATGGACGTTTCTCTTGAGCATGAAAGTGCCCATGATGCGCGATCAAG
GGGCGCAGATGCACCCCTTGGGCAACATTTTGCCCTCAGTTCTTGAATGCCGAGTGCTTGCGGCCCTGGTCACACA
GGGCAAACACCACCACCAGCACCGAGGCGATAGCGAGGATGATCACCACACCGTCCGTCGAGTGCGTGGTCGAAGC
GGCCACCATCGACAAGGCACCCAGCGGCGCCAAGCCACCGGCGATGGCCGTACCGATTTCACGCCCGGTACCAAAG
CCCGAAGAGCGAGTCTGGGTCGGGAACTGGCGGCTGAGGAACGAACCTTGGGGGGCGAACATCATCGGCGCGAGGA
TCCCGGTGCCGATCCCGATGGCCAGGTAGATCATCAGGCTTTCACCGGTGTTCAGCAGCGCCAGGAACGGGTAGGC
GAACAGCAGCGACAGCACGCCGCCCAGCACCAGTACCGATTTGCTGCTCCATTTGTCACACAGCCAGCCGAAGCAC
GGCACGGCGACAATGGCGACCAGGCTGGCAATGGTCACCGACAGCGAAGTGACATGCACGTCGACACCCTTGAACT
GGGTGAGGTAGGCCAGGGAGAAGGTCTTGAAGATGTAGCTCAGGGCGTTGTAGCCAATAGCCACGAAGAACACCAC
CGCCAGGCCCTTGAGGTCGTTCTTGAACAGCGCCTTGAGCGGCGAGACCTTGGCCTTGGCCGGCTCCTGGCTCAAC
TCCTTGAACTCCGGGGTTTCCGGGATGCTGTTACGTACCCACAGGCCCACACCCACCAGGGCGATGCTGCAGATAA
ACGGGATACGCCAGCCACCGGCCAGCAGGAACTCATTGCCATTGATGGTCAGCAGGTACACGGTCAGCGATGACAG
CAGCAGGCCGAGGTTCAAGCCCAGCGCCGGCCAGGCACCCTGGCTGCCGCGCTTGCCTTCTGAAGCATGCTCGTAG
GAAGTGACCGCCGCGCCCGACAGCTCGGCGCCGGCGCCCAGGCCCTGGATGATGCGGATAAACACCAGAATGATCG
GCGCCCAGATGCCAATCGAGGCGTAGCTGGGGATCAGGCCGATCAGCGTGGTGCACACGCCCATCATGCAGAATGT
CAGCACCAGCACATGTTTGCGGCCGAACTTATCGCCCAGGTAGCCGAACAGAATGCCGCCGAAGGGGCGGGCGATA

AAACCGATGGCGAAGGTGGAAAACGCCATCAGGGTCGCGGTCTTCGGGTCACTGTTATCAAAGAAGATCTTTGAAA
AGACAATCGCCGCCATGGTCGCGTAGAGGTAGAAGTCATACCACTCCAGCATCGAGCCGAAGATGGTGGCGGCAGC
GACCTTGCGCAGGCGTTTCTTCGTTTGTGCCGGGGTTTCCACCGTGGCGGGGTTATGGGCTTTTGCCGTCATGCTG
TACTTCCTTCTCAGTCATTATATTTATTAGGGTGTTGCGAACGCGTTCAACTGTGGGAGCTGTCGAGCCTTGGCGA
GGCTGCGATAGCGGTGGGTCAGGCGATATTTTTCTTGCCTGTGCCGCCGTCATCGCAGCCTCGCGGGGCGAGACAG
CTCCCACAGGGGGTATTCAGTTGGGGGTGAACATGCGGTCGGCGATCATTTCGCCAATCGGAATGGCAGAGGTGGC
CGCCGGTGACGGCGCGTTGCACACGTGCAACATGCGCGGCGTCTGGGCGAACAGAAAGTCGTGTACCAAAGTGCCG
TCACGCATCACCGCCTGGGCGCGGATCCCGGCTTCGTAGGGCAGCAGATCGTCGAGTTCCAGGGATGGGCAATACT
TGCGGCACTGCTCCAGGTAGCCGGACTTGAACAACGAGTTCTTCATTTCCACGCTGCCGGAGCCGAGGTTCTGCCA
GATGGTTTTCCAGAAGCCGGGGAAGCGCGCGTACTCGGCCACATCCTTCCAGTTGACGGAAAACTTCTTGTAGTTT
TCCCGACCCAGGCCCAATACGGCATTGGGGCCGACGGTGACGCTGCCATCGATCATGCGGGTCAGGTGCACCCCCA
GGAACGGCAGCTCAGGGTCGGGGATCGGGTAGATCAAGTGGTTGACGATGTTGTTCTTCGACGCCGGCAAGCGGAA
ATACTCGCCACGGAACGGGATGATCTGGTGGTCGATATCAATCCCGGCCAGGGTCGCCAGGCGGTCCGATTGCAGG
CCGGCGCACACCACCAAGTGCCGTGCCTGCCAGGCGCCATCGAGGGTGTTGACGGTGACTTTGTCGGTGTCTTCGG
CAATCGCGGTGACGGTTTTTTCCAGGCAGATCTCACCGCCCTTGCGACGAATCACTTCGGCCATGGTTTCGCAGAC
CTGGCGGTAGTCGACGATCCCGGTGGCATCGAGGAACAACCCGCCCAGGCCGACGATATTCGGCTCGCGGGCGCGC
AGTTGCTCGGCGTCCAGGCGTTCGACTTTCATCCCGTTGAGTTGCGAGCGCGCATACAGCGCTTCCATGCGCTGCA
CTTCCAGGGCCGTGGAGGCCACCAGTAACTTGCCGCACACCTCGAACTTGATCCCATGTTCGCGGCAGAACTGTTT
GGTGGCCTCGGCGCCGCGCTTGCACAGGTCGGCCTTGAGGCTGCCGGGTGCGTAGTAGATGCCGGCATGGATCACG
CCGCTGTTATGGCCGGTCTGGTGCTTGGCCAGCACCGGTTCCTTTTCCAGGATCACCAGTGATGCGTTGGGCTGGC
GCTTGAGCAGCTCCATCGCGGTGGCCAGGCCGACAATGCCGCCGCCGATGATGCAAAAGTCATAAATCATGTGGGG
GCTACCTAAATCAATACATTTCAGTCTGCTTATCAGGTTGTCAGACTAATGTTTTTGTTAAAAAAACGGCGCGCCC
GCAGGCACGCTTTATCGGTATGGGGCGATCAGTGGGTCGCGGGCAGCTCCAGTTGCAGGCGTTTGGCGGCAGCACG
CAAATGCGCTTCGGCGGCAGCGGCGGCGCCTTGAGGGTCGGCGTCGACAATGGCCTGGTACAGCGCCTGGTGCTCC
TGGGTGGCATCGGCCGAGCCGCTGGCAAAGCGCGAGGCCGAGTTTTCCCAGGCGGTCTTGCGCGCGCTGGCCAACT
GGCTGCGCAAAAAGTCGTGGAAGGCGACGAAGTATTCGTTTTTGCTGGCGTCGGCGATGGCGCGGTGAAACTCCAC
GTCGGCGAGGGCGGCGGCTTCAAAGTCTGCGCGCTTGTCGTGCATTTCCTGCAGGGCCCGCTTCATGCGCTGCAGG
TCGCCCGCGTCACGGCGTTGGGCGGCAATGGAGGCTGCCTGGGTTTCGATCCACAAGCGCACTTCGAACATTTGCG
CGAGGTCAGGGCGCCGGCCGTTCTTTTCTGGAAAGCGGAACACCGTACCGGCCGGGGTCTGCGAAATAAACGAGCC
CAGGCCACGCCTGGCGGTCAGTACGCCATCGGCCTTGAGCTGGGCAATCGCTTCGCGCACCACCGAACGGCTGACA
TTCAACTGTTCGGCCAGTTGCTGCTCGGTCGGCAGGCGCGACTCGGGCGCCAGTTGTCCGGAGTCAATCTCGGCCC
GTATGGCGCTGACTACACGCACGACGAGGGAATCTGGACGCTGGAGCTCAAGCATGGCAAAACCTAGGTGATCAGG
TTGTCAGACAATAGCTGTTACGATTTTCCCTTGTCAAGGCGCTGTGCTGGCCTGCAGCGTTTTTACCCGGCGCGCG
CAGGCCAGCAGCAGAGCCAGTGCGACCAGCCCCGAGGCGACCCAGAACGCTAGGCGAAACCCTCGGCCAACCGCGG
CAGGCAGCGCCGCATGGACATCGCCCATGCCCCAGGCAAACACCGCTCCCAGCGCCCACGCGCCGACAATCAAGCC
CAGGTTGCGCGACAGGTTCAGCCAGCCGGCAATCGTCCCGCGTCGATCCGCCGGCACATTGCCCATTACCGCCGTG
TTGTTGGCCGCCTGGAATTGCGCGTAGCCCAAGGTGAGAATCCCCAGCGCACCGATGTAACCCGTGACGCCCAGAG
GCATCAACGCCAGCACCAGGCAGCCGGCCAGCATGCACAGCAGGCCGCCCAGCGTGGTGGCGTAGCTGCCGCAGCG
GTCGGTCAGGCGCCCGGCCGGTATGCCCGCCAGCGCCGCGATACACGGCCCTACCGCCATCGCCAGGCCCATCTGC
GCGGGATCGAGTCCTATGCCATGGCTCAAGTAAAACGGCCCGGCGACCAGGGTGGTCATCATCACCGCGGCCACCA
GCGTACTCATGCCCAGCCCGGCGCCCAGGGCCCGGTCATGGAGTGTCGCGAAGACACTCAAGCGGCCCGCACCTGC
TGGTTGCGGGTCTCTGTCGGCCTGTGTGCGTTGCACCAGCACAAAGGCGAGGGCGGCCAACGGCAGCATCACCAGA
AACAGCGCACGCCAGCCAGACGCCGCAATCAGTGCGCCGCCGATGGCCGGGCCCAGGCCAGTGCCCACCGCCGACA
GCGTCCCCAGCAAACCCATGACGCGACCGGTCTGCGCTTTGGGGACGGTGGCCGCGACCAGTGCCATGGTCATTGC
CAGCATGATCGCCGCGCCAAACCCTTGCAGCGCCCGCGCCGCGACCAGCACGCCAAGGGAGGGCGCGGCACTGCAT
AGGGCACAGGCCACCGCGAAGACCAGCACGCCGGCCAACAGCAGGCGGCGATGGCCGAAGCGATCCCCGAGGCGAC
CGACACTCACCAGCACAGCGGTGATTGCCAGCAAATAGCTCAGCACCACCCACTGCACCGCCGCAAAGCCTGCCGC
AAAGGTCTCGGCCATTTGCGGCAGGCCGACATTGGCAATGCTGGTGCCCAGCGAAGCCAGGAGCATGCACAGCGAC
AAACCGGCGAGCGCGCCGCGTGGGGGAGGGGATGTCATGGGGTTTCCTTGTTCTGCACCGTGGTCGTTGCCCGCAC
TCTAGGCTGTCGCCTAACATGGCGGAAGACGCATCAGTTGCACTCTATGTGTGCGTCAAACGCCTGATAAGGATTG
CCGCCGTGTCGCTGCCTGACTTCAACCTGCTGATCACCCTGGACGTGCTGTTGGCCGAAGGCAGCGTCGCCCGTGC
CGCCCAACGCCTGGGCCTGAGCCCCTCAGCCATGAGCCGTGCGTTGGCGCGGCTGCGTGAAACCACCGGCGATCCG
CTGCTGGTGCGTGCCGGACGCGGCCTGGTGCCGACGCCCCGGGCCCTGGAACTGCGCGAACGGGTCGGCCAACTGG
TGCAGGAGGCCGAAGCGCTGCTGCGTCCGCTGCAGGTCCTGGACCTCAAGGGCCTGGTGCGGACCTTCGTGCTGCG
CACCAGCGAAGGGTTCGTCGAAAGTTTCGGTCCTGCGCTGATCGCCCATGTGGCCGAGCAGGCACCCGGCGTGCGC
CTGCACTTTGTGCATAAGCCCGGTGCGCCGCTGCGCGACAGCAGCGTGGACCTGGAAACCGGCGTCGTCGATAGCA
GCGCCCACCCCGAAGTCCTCACCCAGTTGCTGTTTCGCGACCGCTTTATCGGTGTGGTGCGCGCCGGCCACCCATT

AAGCCAAGGCCCCATCACCACCACGCGCTACGCCGCCGGCCGGCATATCTACGTGTCTCGGCGCGGGCGCGAACGT
GGGCAGATCGACCACGCACTTGAGGCCTTGGACCTGACGCGAGACGTGGCAACTATCGTCACCGGGTTCTCTACGG
CCGTGGCCCTGGCGCAACGGACCGACCTGATCGCCAGCGTTCCCGAGCGCTATACCAGCGGCCTGCGTGAGGGCCT
GTTCAGCTTTGCCCTGCCGGTCGAGGTGCCGCCGTTCAGCGTGGCGATGCTCTGGCATCCACGGCTGGATGCCGAC
CTCGCGCATCGTTGGTTGCGCGGCTGTTTGCGCCAGGTGTGCGGCGCAGAACAGGTACAATCCCCGCTGCCTTGAT
TGCGAAATACCAGGAAACAGGAATTCGATGTTCACCCTTACCCACTACACCAGCCCATGCCCCGAGCCCGTCAACA
GCCAGATCTTGCAGATGGTGGTCGACTACCTGACCGACATCAGCTCGGTCGCCTTACCGCCGAGCAACCTGCTGTA
CAACATCTACCAATATGCAATCGGCTACGAAGTGCACCTGTACCTGGAGGCGCTGGGTGGGGCCAAGGGCATTGCG
GTCGAGCTGATTGTGGCGACGGATGAGCAGGATCCGGCCAAGGTCGTCGGCTTCTTGCTGTACCTGCCGGTCAAGG
ACGATCCCGAGGCCTGCGGCGTGGCCTATATGGCCGTGCATGCCAGCCACCGCCGCCAGGGCGTGGCGCGGGCGAT
GATGGATGAGATGCTCAGCCGTTACCCCCATGGCGAATTGACCTGCACCGTGGAAAAAGTCCCGGCCTTTGAATCC
ATGGGCTTCCAGGTGCGCGGCGTGCGCGGGACTCAGGTGTTGATGAATACACGCGACTACAGCACCGATGGCCTGA
TGGGCGTGTTGGATGTGGCATCGATCTATAGCTCGCTGGAAGTGCGGCAAATCCACACCTACCTGCTACAGAAACA
CGGCAAGCGCGCGATGATTGATGCCGAGAAACAGCGCGACCGGCATTTCGACCAGATGACCCGCAAGGCCCAACTA
TTTGTACAAGTCCGGCAGGAGTAACTTTCTGCTCTTTGCATAGATGTCCTCAGTTGCGGTAGGCACTGGACTCAAT
GGCAATTAACAGAGCGCGCCCGGATATATAGGAACTGTTTTTGTTTATCGGCCACTACTGGTGACCAGCCCGCACA
AGCTGCAGTAGAGATGCCGCCGAGGTTGGTGAAACTATTACCAATGGAGAAAAACATGTTTTCGATTTCCGCCAAT
ACCCCAGGAATACTTGCCCCCACCATCGATACCTCACAGCAAAAACCCACTGCATTCGATGCAAGGCTGGAGACGT
TGGATAATGTTCAAAAACACAAATTAGAAAACGAGCAATCGGCGAACAATAACGAGGGGCAAGACGCTGCCAAGCA
AAATGCATTTACATTTAAAACGCCGGGTGGCGGCAGTTATACTTGGTAAATCATGCATTTGACTCCCGTCACCGGT
TTATCCGGTGGCAGGCCTAGTTTTCTCGAAACAAGTCATGGGCATCGAGCAACCGGTACGCAATCTCCGGCCGTTT
CTCCAGCCCTTTGCGAATCGCTGCCGGAATCGACTGCCGCGTCTTGCGGCACAGCCCCGGCAGTTCATCGATCACG
ATCTGGATCCCGCGCATGCTTTTGACTTCGGTATGCCCTGGGGCGATGTGCACGCGGATGCCCAATTGCTGGTGCA
TCAAGTGTTGCATGCGTTCCAGGTCTGCCAGGCTTTGCAGGTTTTCCAGGCGTTCGAGCAGTTTTTTTCTTCCTG
GCGGGTCAATTGCAGGATGCGCTGGTCGGTACCGGGCGCATCGAGCAATTGCTCGCGCTCGCAATCACACACAGCA
GGTGGGCAGGGTTGGCGAATTGTCATGATCACTCCCTCCCCACGCTGGTGCTGACGGCTCAGTAGGTCAGTTGCAC
GCCCAGGCTGCCCAGGGCGTTCCAGTACTCAGGATAGGTCTTGCCCACGCAGTCCGGGTCCTGGATCCGGATGCCC
GAGACTTTCAGGCCCGCCAGGGCAAAGCACATGGCGATGCGGTGGTCGGCGTGGGTGTCGATCAGCGCGGTGCAGG
CCGTGCCCGCCAGCGCTGGATCGCTGGCCACCAGCAAGTCATCACCGATAATGGTCGCCAGGCCTGGGCGGATCTC
GTTGAGGCCGTCATGCAGGGCCTGGACGCGGTCGCATTCCTTGACGCGCAGGTTGGCCAGTTCGGTGAAGCGCACC
GGGGTGTTGTTGAACGCCGCCAGCACCGCCAGGGTCGGGATGGCGTCCTGCATCTGCGAGCCGACCACCGTGGCCG
GCATCTGCGGGAATTGCGCGATCACGGCCTGGGCCTTGGCGTCGGGCTGGGTGAAATCCTGTGCGGCGACGCCCAG
GTCGATGCTGCCCCCGGTCAGCACTTCGGCAGCCCACAAGTAGGTGGCGGCAGACGCGTCGGGTTCGATCAGGTAA
TCATGGGCGATGTAGCCGGTAGGGGCCACGCGCCAGGTGCTGTCGTTGACCACGTCCACCTGGGCGCCAAAGGCGC
GCATGCAATCCAGGGTCAGGTCGACGTAACCACGGGCACCGATGTCTTTGCCGGTCAACGCCACTTCGATCGGCGC
TTCGCCACAGGCGGCAAGCATCAGCAATGCCGAGACGTATTGGCTGGACAGGCCGCCATCGATCTCGAAGCGCTTG
GCCTGCACGCGGCCAGTGCCGTGCACGGTCACAGGCGGGCAGCCGGTGGGGCTTTCAACCTTGATGCCATTGTGGT
TCAGGGTCGTCAGTAGCGGGCCGATGGGGCGCTTTTGCATGTACTCGTCGCCGTCCAGTACCACGGTGCCTTCCAC
GGTCGCCACGGCGGCTGTGAGAAAGCGCATCGCGGTGCCGGCGTTGCCGAGGAACAGCGGTTGCGCCGGCAACTGC
AGCTTGCCATGGCCGGTGACAACAAAGGTCACGTCATCCGGCTCATCGATGGTCACGCCCATCTGGCGCAAGGCCA
CGGACATATGCCGGGTGTCATCGCTTTTCAACGCGCCGCTCAGGCGGCTGGTGCCCTTGGCCAGGGCTGCCAGCAG
CAGGGCGCGGTTGGTAATGGATTTGGAGCCGGGAGGCGCTACCTTGCCGTGCAATGGAAAGTTGGGCGGTGTCACG
GTCACGGTTTTCTGCGAACTCAAGGTACAAGGCTCCTGATTCAAGGCGCGGTGGCTGGGAGTGGCCGGCTGGCGGA
GACGAATAATCGACCATCCGGCACGCTAGGGTCAAATCATGGCGCAGGTTTAACCGCGCAGGCTCAACCAATAGGC
ATGCAAGGCCCTGGCCGCCGGTTCGATGCGGGCCACCTGTTGTTGATGGGCGGCCACATCCAGGTCGGCCGGCAAT
TGGAAGTTGTCCTGCTCGGCGCACCAGGAGATGGCTTCCAACTGCGCTGCCTGCTCGGTCGGCAGGGCTGGCCATT
GGCTGATCGCGGCGCCACGGATATAGCCAAAGCACCATTCCTCGGCGAGGGTTAGCGGCAGGCCCTGGTGTTCGGT
TTGTTCGAAGCGCGCCTGAAAGCCCTGGGCATCACTGGCCAACTGCTGGGCGATGCCAGTGAGATGACGTGTGCAC
AGGTCGATGAACTGCTCCAGTTCGGCCTTGCTTTCCCATGCCGGGATCTGGTCGCCCCAGATGGCCGGGAACCAGA
TGGCAACATCCACCGGCGCCGGGCCGGACACCAGGGCGGTGAAGTAGCCATCGAACTCGGCCAGGTTCAGTACCGA
GTTGTCGTCGCCGTACTTGAGCAGAATGTCTTCGATGAACTCGAAATCGGCAGGGGCGAGGGCTGGGTCGTGCATG
AAAATATCCTTTGAGCGTCAAGCGCCGCAGATAAGGCGGCGTAAAACGCGGAGGATGGCGCGAGTCGGCGTTTTAA
TCCAGTGCTAGATCACCTTCACCGCCCGCCCAATCGCCTGGATCGGCCCGGTTGGCTTACCGGTCGGCGAGCCATT
GGGGCCTTCCAGGGTCATCTCGAACAGTTGGTTTGGCGCCAGCGGCGGCAGCTTGTCCAGGGGGATCGACAGGCTC
TGCCCCGGCTTGACCAGCCCGAGGGAAACCGGGCCTTGCCAGTCATCACCCTTGGTCCAGAACTCCAGTGCCTTGT
CCGCCGGTATCTGCACGACGCCCAGGGGGATCAACTGGATTTGCTGATCGTTGCTGGCCTGGATTACCCAGCCTGG
GGCCTGGCTTTGTGGCGCCGTCAGCACCACCACGTAGGCCGTCGGGTTGATGGCGGCGGGGCGGGTCAGCAGCAGC

GATGCCAGCACCAAACTGGCCAGCAAGCCGGCACCGGCCAGGCCACGCCACAGCGCGAGCAGGTTCCACCAGGGCA
CCGGCGGCGTGTCGCCCACAGCGTGATGGCCCAGGCTGCGTTCAATGCGCCGCCACAGGTAGGGTGAAGTCGCCAC
CGGCTCGGCCAGCTCGGTCAGCGGCAACAAGCGCTGTTCCCAGGCCTCCACCGCCGCGCGCAAGGGCGCATCGTGT
TCCAGGCGTTGCTGCACGTGCAGGCGTTGCTCGGCCGCCAGGGTGCCCAGTACGTATTCGCCGGCCAGGTCGTTCA
ATTCTTCGGGGGTCATGCCATGCACTCACGCAATGCCGCGAGGCTGCGTTTGATCCAGGCTTTGACAGTCCCCAGC
GGGGTGCCCAGTTTCTGTGCGATTTCACTGTGGGACAGGCCGTCGACATACGCATGCAGGATGCAACTGCGACGCG
ACGGTTCCAGGTGTTCCAGGCACTGATAAACCTTGCCGCTACGGGCACGGTATTCGAAATGCTCGGTGCTGGGGTC
AGCCGCCAAGGTCTGTTCATGCTCATCACTGAGCGCAACTTCGCGGCCCTTTTTGCGCATGACGTTGAGCGCCAGA
TGCCGGGTCACGCTATACACCCAGCCACGGGCCGAACCCCTACGCGGGTCGAAACCGCCGGCGCCGTTCCAGATAT
TGATAAACGCCTCATGCACGATGTCCTCCGCCAGGGCCGTATCCCGCGCAATGCGCTTGGCCACGCCGAGCAGCCG
GGCGCAATCCTGTTCGTACAACTGACGCAAGGCTTGCTGTTCGCCGCGGGCGCAGGCCAACAGGCAGGCTTCATAG
TCAAAGAGAGGTTCGGGCAAGGATTGTGGCCGCCTTTGAGGGTCAAGCCCCGGCCGCGATTGGCCGGGGTGCTAGC
GCAGCAGCTTAGACGACTTTTTTTGCCAGGCGCATCATTTGGCCGCCCAGAAGATGTAATCCGCCTGGTACTTCAC
CACTTCTTGCTGGCCTTTGTTGGCGGTACTGCATTCGGCACTTGGCGCCACGCCACCCTTGAGCGCGACGCGCTGG
ATATAGCTGACCCCGCTCATTGCACCCTTGCCTTCGGCCGGGTTGGCCTTGACCAGTTGATAGGGCAGGTTGCCCG
CGCTGGACGGCGCCACGGCCAATTGCGTGCCGGTGACTTTCGAACCGTCCTTGGCCTGCCAGGTGGCGGGCGGGCC
GAAGTAGGTGCCGACCTGCTTGCCGCTGCGATCATTGAGCACTGCCTTGGGGCCGACGAAGACCCATTCGGTCTGG
CCGGCAGCGTTGGCCTTGTCGCGGCACTCGTAGGTGATTTCGCCGACACCGGTGGTTTCCATGGTCACCTTGTGGC
CGTCCGGCACCTTGATGCTGTCGGGCAGGCTGGTCTGGGCAAAGGCCGCGGGCGCCGCGAGCAGCAGGCAGGTCAG
GCAGAGCAGGGGTTTAGTGTTCATCGGTGTCTCTCCGTAGGGGTGAACAACAGAGCAGCGTTATGGCTACTACCTG
TACTACTCACCGCACGGCTGACTGGATGCACGGCGCGACAAATAAATCCCTGAGACGTAAAAGGAATAAAGTCAGA
TCGTATTGATATAAGTCGTTATAAGAAAAATCGCTATGCTGCCGCCAGAATTTTATAAGGCCGCAGGTAGTTGTAA
TGGATGTGGGTAATTTTGGTTTTGTAGTGGCAGGCCTGATCGTTGGTTTTATCGTCGGCATGACCGGCGTGGGTGG
CGGGTCGCTCATGACCCCGATCCTGTTGTGGTTCGGCATCAACCCCGCCACGGCGGTGGGCACCGACTTGCTGTAT
GCGGCCATCACCAAGTCCGGTGGGGTGCTGGTGCATGGCAAGAACAAGAACATCGACTGGACCATCACCGGCTGGC
TGACCCTGGGCAGCGTGCCGGCAGTATTGTTGACGCTGTGGTTCCTCAAGAGTCTGCACACCGACCCCAGCGCGAT
GAACGAGGTGATCAAGCAGGCCCTGGGCGTGGTACTGCTGCTCACGGCCCTGGCGATTCTGTTCAAGAAAAAACTG
TTGGCCTTTGCCCAGCACCATGCGGGCGACAACTACCATATGAGCCCGCGCAACCTCAACGGGCTGACAGTCTTCA
CTGGCGCGATCCTCGGCACCATGGTCGCCCTGACCTCCATCGGTGCCGGCGCGCTGGGCACCGTGGCGTTGTTTAT
CCTGTATCCGTTCCTGGCCACCAAGCGCCTGGTGGGCACCGAAATCGCCCACGCCGTACCGCTGACCCTGGTCGCA
GGCCTAGGGCATGCGAGCATGGGTAACATGGACTGGCATTTGTTGGGGTTCTTGCTGATGGGGTCGCTGCCGGGGA
TCTACGTTGGCAGCCATATGTCAGGCCGGGTGCCGGATGAACTGTTGCGCCCGTGCCTGGCGGTGATGCTGGGGTT
GATTGGTTTCAAGCTGGCGTTCTGACTGCGGCATGCTGTGGCAGTGGTGCCGTTGTGGCTGTGGTGCTTTTGTGGA
GAGGGGCTGCTGCGCAGCCCAGCGCGGGGCAAGCCCGCTCGCCACAACAAGCCCGCTTCCCACAACAAGTCCGCTC
GCCACGATAAGCCTTATGCCTTACGCGATTTATCCAACCACTCCACCGCCGTGCGCCAGATACAAATCCCCAGGAA
ATACGCCGACATCAACAACCACAGGCCCATGGTCAGCTTGTTGGTATACGGCTGGTCGATCACCAGCAACAGGCTG
CACAGCAGCCACACCGTGGTCACCGCAATGTTGATCGGCATAAAGCGCTTGACCCGGAATGGGTGCAGGAACTTCA
TCGGGGTCACTGTCAGCAGCGCCAGGCCAATCACCGTGGCCAGGGTGATCCAGGCGTCCGGGTCAATGATGTAGAC
ACACAGCGCCACCACGTTCCAGGCCGCGGGGAAGCCCTGGAAGTAGTTGTCCTTGCTCTTCATGTTGACGTTGCAG
AAGCAGAACAGCGACGACACCAGGATCACCGAGACGGTGAACAGGTGGGTGAAGTCCGGCAGGTCGATGTAGCGGT
AGATAAACAGCGCCGGGATAAACACATACGTCAGGTAGTCGATCACCAGGTCCAGCACCGAGCCATCAAAGCTTGG
CAGCACGGTCTGCACATTGACCCGTCGCGCCAGCGACCCATCCACGCCATCCACAATCAATGCCAGGCCCAGCCAC
AGCAGGCAGGCCTTGGGTTGGTTCTCCAGCAGCGCCAGCGTCGCGAGGAAGGCCAGCACCACGCCGGTGGCGGTAA
AACCATGGGCGCCCCAGGCTTTGAGTCTGGCTACATGCAGGGTCGATATCACAGGGGCGTTCTCCAAAAGGTGAAA
CACGGCAGCCGACGGCGATTGAGCGTCGAATCTGGCCAAGGGATGCAGGTATCGACCGGAAAGGTGGGGATAAGGT
TCACCACCACGGCGTTAGGTTTAGCGTAGCAAGCGTTGACGGATTCGGCATCAGCCCTGACGGAACACCAGGGCCT
TCAAGCCGCTTTGCGGGTCGATATCGGGAAATTCCGGCGGGTTTGCCAGGCGCTGCTCAAAGCGCAGCCCCGGCGC
TTCGCGGGTCACGCCTTCGATCAGGAAGTCTTCGCCAAACGCCGGGTCGTTCATGCAGGCCAATACTGTGCCCTGG
GGCGTCAGCAAATCTGGCAGGCGGCGCAGTACGCGCTGATAGTCCTTGGTCAGCAGGAAGCTGCCTTTCTGGAAGG
ACGGCGGATCGATGATCACCAAGTCATAGGGACCGTTGCCGGTGACCTTGGCCCAGGACTTGAACAAGTCGTGCCC
CAGAAAACTCACCTTGCCCAGGTCATGGCCATTGAGCCGGTGATTGTCGCGGCCCCGACTCAGTGCGCCACGGGCC
ATATCCAGGTTGACCACATGCTCGGCCCCGCCCTCGATGGCGGCCACGGAAAACCCGCAGGTATAGGCAAACAGGT
TCAGCACCCGCAGGCCACTGGCGTGTTCGCGTACCCAATTGCGGCCGTAGCGCATATCCAGGAACAGCCCGCTGTT
CTGTTTTTTGCCCAGGTCGATGCGATACAGCAGGCCGCCTTCGGTGATGGTCAGCTCCTCGACCATCTGCCCCCAC
AACCATTCGGTGGTGCTTTGCGGCAGATAGCGATGTTGCAACGCCAGGGTGTGGGCGCCGGCCTGCTGCCATTCGT
CGCTGGTGCAGAGCTTGCGCAGTAATTGCTGCAAGGCATCCAGTTGCACCGCTTCGGGCTCCTTGAACAAAGCAAC
CAGCACCACGCCTTGCAGCCAATCCACAGTCAGTTGTTCCAGGCCCGGCCAGCAGCGGCCCCGGCCGTGGAACAGG

CGCCGGGTTTCTGCAGGTACGTCGCGCAGGGCGGCGAGCAAATGGGTGTGGAGCAGGGCGAGGGCGTCAGGGGTCA
TCGGGCAGCGTCGATCATTAAACGGGCGGCATTTTAAACACAATTGCGGTGTTTCAGGTGGTTGAAGCACAACGTG
CTTTTAATTGGCTGTGTTTAAGCATTTTGTGCTGCAAATCGCCCGGCAACCTATCCAGAACAGTCAAAAAATCCTT
TTGGGCAGACATAAACTGGAAAAAACAGCTCGGAGGTGCCCATGAACGGTTTCGTAAAGACAGTCAATCAAACAGC
CATTGACCAGCAGGAGCTCACCGAGTGGCGCGACGCCCTGGCATCCCTGGTACGCCACGCCGGTCCTGAGCGCGCG
CGGGAGATCCTCGATATGCTGGCCGACGCCGGCAACACCCCGGCCATCGGCTGGAAGCCGCGCCATGGAACGCCGT
ACATCAACAGTATTGACGTGGATCAACAGCCCCACTTTCCTGGCGACCTGGCCACCGAAGAACGCCTGGCCTCGCT
GGTTCGCTGGAATGCCCTGGCCATGGTGGTGCGGGCCAATCAGGCTTATGGCGAACTGGGCGGCCATATCGCCAGC
TATGCCAGCGCGGCGGACCTGTTCGAAGTGGGCTTCAACCATTTCTTCCGTGCGCGCCACAACAACGAGGGCGGCG
ACCTGGTGTTCTACCAGCCGCATTCCGCGCCCGGCATCTACGCGCGGGCGTTTCTTGAAGGCCGCTTGAGCGAGCA
GGACCTGGCCCATTACCGCCAGGAACTGGGCGCCGTCAAGGCGGGCGCCCGTGGCCTGTCCAGCTATCCGCACCCG
TGGTTGATGCCGGACTTCTGGCAGTTCCCCACCGGCTCCATGGGCATCGGCCCGATCAGCTCGATTTTCCAGGCGC
GCTTTATGCGCTACCTGCACCATCGCGGCTTGCAGGACACCACCGACCGGCACGTCTGGGGCGTGTTTGGCGATGG
CGAGATGGACGAGCCGGAAAGCATGTCGGCCCTGACCCTGGCCGCTCGCGAAGGCCTGGACAACCTGACCTGGGTG
GTCAACTGCAACCTGCAACGCCTCGATGGCCCGGTGCGCGGCAACGGGCGAATCATCGACGAACTGGAAGCATTGT
TTGCCGGTGCCGGCTGGAACGTGATCAAGCTGGTCTGGGGTTCGGACTGGGACGCCTTGCTGGCCAAGGATGAAGA
CGGTGCGTTGGTGCGCACCTTGTCCCAGACCGTGGACGGGCAGTTCCAGACCTTTGCCGCCAAGGACGGTGCCTAC
AACCGTGAGCATTTCTTCGGCCAGAGTGAATCCCTGGCCAAGCTGGCGGCGGGCTTGAGTGATGAGCAGATCGACC
GCCTCAAGCGCGGCGGCCACGACATGCTGAAAATCCATGCCGCCTACCATGCTGCGCGGCGGGTCAAGGGACGCCC
CACGGTGATCCTGGCCCAGACCAAGAAAGGCTTCGGCATGGGCGAGGCCGGCCAGGGCAAGATGACCACCCACCAG
CAGAAAAAACTCGACCGCGAGGCGTTGATCGGCTTTCGCAACCGCTTCCAGTTGCCCTTGACCGACGAGCAGACCG
AATCCCTGAGCTTCTACAAACCCGCCGCCGACAGCCTGGAGATGCGCTACCTGCACCAGCGCCGTGCTGCCCTCGG
CGGCTATGTGCCCAGCCGCAGCCAGAGCGCCGAGCCGGTACCCGTTCCGCCCGTGAGTGGCTACGCCGGTTTCGCC
ACCCAGGCCGAAGGCAAGGAAATGTCCACCACCATGGCCTTTGTGCGCATGCTCAGCAACCTGCTCAAGGACAAGG
CCCTGGGCCCGCGGATCGTACCGATTGTCGCCGACGAGGCGCGCACGTTTGGCATGGCCAACCTGTTCAAGCAGAT
CGGTATTTATTCCAGCGTCGGCCAGCGCTACGAGCCGGAAGATATCGGTTCGATCCTCAGCTATCGCGAAGCCACC
GACGGGCAAATTCTGGAAGAAGGCATCAGCGAGGCCAGCGCGATCAGCTCCTGGGTCGCGGCGGCCACCAGTTATT
CGGTGCATGGCTTGCGCATGCTGCCGTTCTACATCTACTACTCGATGTTCGGTTTCCAGCGCGTGGGCGACCTGAT
CTGGGCTGCCGCCGACCAGCGCGCCCGTGGCTTTCTGCTCGGTGCCACGGCGGGGCGTACCACCTTGGGCGGCGAG
GGCTTGCAGCACCAGGACGGCAGCAGCCACTTGATGGCTGCGACCGTGCCCAACTGCCGCGCCTACGACCCGGCAT
TCGCCGGCGAGTTTGCGGTGATTCTCGACCACGGCATGCGCCAGATGCTTGAGCACGACGTCGACGAGTTCTACTA
CGTGACCCTGATGAACGAAAACTACCCGCAGCCGAGCCTGCCGGCGGGCGTTGAAGCCGCGATCATCAAGGGCATG
TACCGCCTCGACGGCCATGCCGGCGCCAAGGTGCGGCTGCTCGGCTCGGGCACCCTGGTGCGCGAGGCCCAGGCTG
CCGCGCAGTTACTGGCTGACGATTGGCAGATCGACAGCGAGGTGTTCAGCGTGACCAGCTTCAGCGAACTGGCCCG
CGAAGCGCGGGAGGTGGAGCGCTACAATCGCCTGCATCCGCTGCAAAGCCCACGGCACAGCCACCTGGCGACCTGC
CTGCCACCAGGCGCGCCAGTGGTTGCGGTGTCGGACTACGTGCGCGCAGTGCCACAGATGATCGGCTCGTACCTGG
GGTCGAGCTATACCGTGCTGGGGACTGATGGGTTCGGGCGTAGCGACACCCGTGCGGCGTTACGCGACTTCTTTGA
GGTAGACCGCCACCACATCGTCCTTGCCGCGCTGGCGGCCCTGGTGGAACAAGGCAGCCTGCAGGCGCAGGTGTGC
CAGCAGGCCATCGACCGCTACGGCCTGCACACCGAGCGCGGGGGGTCCTGGACCCACTGACGAGGACTCACTGTAG
GAGCGGGCTTGTGTGGGAGCTGGCAAGCCAGCTCCCACACAAGCCAGCTCCCACAGGGTTTTTCGGTGTGTTCTAG
AGCGTGGCAATCAACGCCGGCCCAAATGCCTCATGCAAAAACTGCGGCGCGATGTAGCGCTGGTAATGCGCCTCCG
ATAGCAGGAAAAACTCCCGATCAATCGCATCACGCAAATCAGCCAGGTGCCGCTCACGAAACTCCGGCAGCAGCGC
CAGGCCATAGGCATCGAGCCTGGAAATCACCCGCGCACCGCGGGCAATCAACTGATAGGCCCAGCAGTAAGGTGAC
TGGTGCGGCATAAAGCGGATCTTGCGCGCTTCCAACTGCTGGCGCAGGCGCGGGGCATCCAGCACTTGCAGCTTGC
TGGCCATGACCTGCACCAGCAACTGTTCGAGGCGTAGCCAGACCGCTTGTTTTTCCTCGGCGCTATAACCGTTCCA
CTGAATGACTTCATGGTGATAACGCTTGCAGCCACGGCACACCAGGTCGCCATACACGGTGGAGCACAGGCCGACG
CACGGCGTCTTGATGGTTTGATTTGGCATAAAAACCGCGTCTTTCCTAATCCACGACAAAAGGGTTCATCGGCGCT
CCAGGGCGCTGAACTGCTGGTTCAACCACTGATACAACTGGGTGACTGCCCGTGACATCTGCCCACGGTGGGGGCA
GGCGAAACTCAAGGGGTACACATCACCGGGAACCTCGGGCAACAGCACCACCAAACGCTCGGCCTGCACATCGGCG
TACACGTCCAGCCAGCATTTGTAGGCGATGCCTTCACCCGCCACCGCCCAGCGCCGCACCACGTCGGCGTCGTCAC
TGAACAAGGGGCCGGCAACCTGCACGGTCTGCTCACCCAGTTCCCAGCGGTCATGCAGACGGCCGTTTTGCAGGTA
CAGCAGGCATGGGTGGCGGGTCAGGTCGTTGACCGATGCGGGGGCACCGTGGCGCGCCACGTAATCGGGCGATGCC
ACCAGCACCCGGCGATTCCAGGGCGCCAAGGGCAGGGCGATGTAGTTGGCATCCTCGTTCCAGCCATAGCGCAGGG
CCACGTCCACCGGGTCGCGAAACAGGTTGGTGACCTGATCCGATACCAGTAAGCGCAAGGTCAGCTCAGGGTGTAG
GCGGCGGAACTCGGTGAGCATCGGCAGCAGGATATTGCGCCCCAGGTCCGAAGGCGCGGCGACCTGCAATGTGCCC
TGCAGCGGCGCGTCATCGCCGCGCAAGCGTTCGCGGCCCAGGGCCAACTGCTCCAGCACGCCACGGGCGGTGGGCA
GGTACTGTTCGCCCTCGGCACTCAACCGCAGGCTGCGGGTGGTGCGGGTAAACAGGCGCACCTGCAGTTCCCGCTC

CAGGCGCTTGATGGCTGCGGCGACTTGCCCCGGCAACAGATTGGCCTCAACCGCGGCGGCGGTGAAGCTGCCCAGG
GCGGCGCTGCGCAGGAACAGCCCAAGATCATCGATGCGGATCATTTTCACTCCAGGGATGAAAGTGTTGCTGCATT
TTGTCGATTTTTCTTCGCGCTGGGAACGGTAAAGATATAAGCCATCGAGGGCCATCGCCCCTCATCAACCGTGAGT
TCCCATGAAAGCAATCTCCTTTACCCAACATGGCCTGCCCATTGACGACCCGCGCTCGCTGATCGACGTGAACATC
GATGCTCCCCAGCCAGGCCCCCGGGACCTGCTGGTAGAAGTGCAGGCCGTGGCGGTCAACCCGGTCGATACCAAGG
TGCGCGCCGGCACTTTCGCCAAGGAGCCGAAAATCCTCGGTTGGGATGCGGCGGGCATCGTGCGCCAGGTGGGCGC
CGACGTCACCCTGTTCAAACCGGGCGACGCGGTGTACTACGCAGGCTCCTTGATCCGTTCCGGCAGCTACAGCGAG
TTCCAGGTGGTTGATGAGCGCATCGTCGGTCACCGGCCGCGCACAGTGGATGCGGCCCACGCCGCCGCCTTGCCGC
TGACTTCGATCACTGCCTGGGAGTTGCTGTTCGATCGCCTTGGAGTGACCGAAGGCGGCGGCGAGGGCGAGGTGCT
GTTGGTGGTCGGTGCCGCTGGTGGGGTGGGTTCGATCCTGGTGCAACTGGCCCGCCAACTCACCCGCATGACCGTG
ATCGGCACCGCCTCGCGGCCCGAGACCATCGAGTGGGTCCAGCGCATGGGAGCCCATCACGTGATCGACCATCGCC
GGCCCATGCTCGCCCAGCTCAAGGCACTGGGGGTTGATGGGGTCAGCCATGTCGCCAGCCTCACCCACACCGAGGA
GTATCTGCCGCAACTGGTAGAAGTGCTGCGCCCCCAAGGGCGGCTGGGGTTGATCGACGATCCGGCCAGCCTGGAT
GTCATGCCACTCAAGCTCAAATCCCTGTCCTTGCACTGGGAACTGATGTTCACCCGCTCGCTGTTTGAAACGGCGG
ACATGATCCAGCAACACCATCTGCTCAACCGCGTGGCCCAACTGATTGACCAGGGGGTGTTGCACACCACCCTCGG
CGCGCATTTTGGTGCGATCAACGCGGCCAACATGCGCAGCGCCCATGCCCTGGTGGAAAGCGGCAAGGCGCGGGGC
AAGATTGTGCTTGAAGATTTCCAATGAACACCCTGATCAAAGCCCTGGGCTTATCGCTGTGCGTAATGCTTGCCGG
CTGCGCCAGCCAACCTACCAATGGAGTCGCCAGCATGACCAAACCGTTGATATCCATCGCCGTACTCAAGGCCAAA
CCGGGCCAGGAACAAGCGCTCAAGGCCGGCCTGCTGGCCTTGGTAGAACCGACCCGCACCGAGCCAGGCAACCTGG
ACTACGTGCTGTTCGAACTGCGTGACGAGCCCGGCACGTTCTATATGCGTGAAGCGTTCAAGAACCAGGCGGCCCT
CGATGCGCATTTTGCGATGCCGTACTTCCAGCGCTTTGCCGCCACGGCAGACGACTTGCTCCAGGAACCGCTGAAA
CTGATCTTCCTTGAGCAAGTGTCCAACTGAGGCCGTACACCGCAACTGATTGTAGGAGCAAGCTTGCTCGCGAAAA
ACGCCTAGGCGCCGCGTTTATCCGGGGGGCCTGGGTTATCGTTGACGATCTTCGCGAGCAAGCTCGCTCCTACAAA
AAGTGTGGGGCGATCAATCCCAGCTCAACGCCCCGCCAGTCTGATACTCGATCACGCGGGTCTCGAAGAAGTTCTT
CTCTNNNNNNNNNNGCTTCGTTGAGGCCGCGAAATTTACGCAGATAGCGGACCTAAGTCAATAGCCTGCTTGAAGT
TTTTCTGAAAAAGACAAAATCACGTCAAGACAGCTACCCGCCCTACTCGCTGTAAATCATCTTCTTGCTCATGCCG
CCATCCACCACGAACTCTTGCCCCGTCACAAACCCAGCGCTGCGCGACAGCAGCCACGCCACCATGGCGGCCACAT
CCTCAACCGTCCCAACCCGGCCCGCAGGATGCTGCGCATGATCGGCGTCACTCAACGGCTCTGCGCGGCGCGCGGC
GGGATCACGGGCATCGATCCAGCCAGGGCTGACAGCATTGACCCGAACCTCCGGCCCCAGGCTGATGGCCAGCGCA
TGGGTCAGGGCCAACAGGCCACCCTTGCTCGCCGCATAGGCCTCGGTGTCCGGCTCCGACTGCCTGGCCCGGGTAG
AAGCCAGATTGACGATAGAACCGCCGTGGGCACGCAGATACGGTGCACAGTGCTTGGCCAGCAACATCGGCCCACT
GAGGTTCACCGCCAGCACCCGATTCCAGTAAGCCAGGTCCAGGCTATCCAACGTGATATTGCGCGGGTCGGCCACT
GCGGCATTGCACACTAGGGCATCCAGGCGCCCGAACTGGCCCAGCACTTCAGCAACCCCAAGGGCCACCTGCTGCT
CATCGGCCACGTCCATGGTGATAAACCAGGCGTTATCGCCCAGCACCTTGGACACTTTAGCGCCCCGCTCGCGATC
AAGATCGGCCAACACCACTTGCCAGCCTTCGCTGATCAGCCAGGCAGCGATCCCCAGGCCGATACCCCGTGCAGCG
CCCGTTACCAACGCGACCCGGCCATTACTGGCGCCAGCCGCCTCCTGGGACCACTCGATCACAAGGCAGCCAGCCC
GCGGGCCAGATCGGCCTGCAAGTCAGCCACGTCTTCCAGCCCCACGGCAACCCGGATCAGGCTGTCGCGGATACCG
GCCGCTTCACGCTCCTGCGGGGCCAGGCGCCCATGGGAGGTGGTGCTTGGATGGGTAATGGTGGTTTTGCTGTCAC
CCAGGTTGGCGGTGATGGAAATCAGGCGCGTGGCGTCGATAAAGCGCCATGCGCCCTCTTTACCACCCTTGACCTC
AAAACTGACCACCGCCCCAAACCCGCGCTGCTGACGCTGGGCCAACTCATGTTGCGGGTGGCTCTTCAAGCCGGCG
TAGTGGACTTTTTCGATGCCGTCCTGCTGTTCCAGCCACTCGGCCAGGGCCTGGGCGTTGGCGCAATGGGCTTTCA
TCCGCAGGCTGAGGGTTTCCAGGCCCTTGAGGAAAATCCAGGCGTTGAACGGGCTCAGGGTCGGGCCTGCGGTGCG
CAAGAAACCCACTACTTCTTTCATCTGCTCGCCACGACCGGCCACTACACCGCCCATGCAACGGCCCTGGCCATCG
ATGAACTTGGTAGCCGAATGCACCACGATATCTGCGCCCAGCTTCAGCGGTTGCTGCAGGGCGGGCGTGCAGAAGC
AGTTATCCACTACCAGCATTGCACCTTTGGCGTGCGCTACTTCGGCGAGGGCGGTGATGTCTACGAGCTCTGCCAG
CGGATTTGACGGTGACTCGACGAACAGCAATTTGGTATTGGCCTTGATTGCCCCATCCCAACCTGAAAGGTCTGCC
AGCGGCACGTAGTCGACTTCAATGCCGAAACGCTTGAAGTACTTCTCGAACAGGCTGATGGTCGAACCAAACACGC
TACGCGAGACCAATACATGGTCCCCGGCGCTGCACAGGCTCATGACCACCGCGAGGATGGCCGCCATGCCCGTTGC
CGTAGCGACGGCCTGCTCTGCACCTTCCAGGGCCGCGATACGCTCTTCGAAGGCACGAACGGTCGGGTTGGTATAA
CGGGAGTAGACGTTACCCGGCACTTCGCCAGCAAAGCGAGCCGCCGCGTCCGCCGCGGTGCGGAACACGTAGCTGG
AGGTGAAGAACATCGGGTCACCGTGCTCACCTTCCGGCGTGCGGTGCTGGCCGGCGCGCACAGCCAGGGTATCGAA
AGCCACGCCATCGAGGTCGCTGTCCAACCGACCGGCATCCCATTCCTGACTCATGCTGCCACTCCTTCACTCAATT
CTTTATTTAAGATACAAAACCGGCCCCTCAGGGCCGGTAGTCAATCAGTTGTTGTACAGATCGATGATCGCGCTGA
CCGCCTGGGTCTTGATCTTCGAGGAGTCGTTGCGCGCCTGCTCGATCTTGTTCAGGTAAGCCTCATCGACATCACC
GGTCACGTACTTGCCATCGAAGACCGCACAATCAAACTGCTCGATCTTGATCTTGCCGCCGCCAACCGCCTCGATC
AAGTCAGGAAGATCCTGATAGATCAGCCAATCAGCGCCAATCAGATCAGCCACATCCTGGGTCGAACGATTGTGCG
CAATCAGTTCGTGGGCGCTCGGCATGTCGATACCGTACACGTTCGGGTAACGCACGGCAGGCGCAGCGGAACAGAA

GTACACGTTCTTCGCCCCGGCTTCACGGGCCATCTGGATGATCTGCTTGCAGGTGGTGCCACGCACGATGGAGTCA
TCCACCAACATCACGTTCTTGCCGCGGAACTCCAGCTCGATGGCGTTGAGCTTCTGGCGTACCGACTTCTTGCGAG
CCGCCTGGCCCGGCATGATGAAGGTGCGGCCGATGTAGCGGTTCTTGACGAAACCTTCGCGGAACTTGACGCCCAG
GCGGGTGGCCAATTCCAGCGCAGCGGTGCGGCTGGTGTCCGGGATCGGGATCACCACGTCGATGTCGTGCTCTGGA
CGCTCGCGCAGGATCTTCTCGGCGAGCTTCTCGCCCATGCGCAGGCGCGCCTTGTACACCGAGACGCCATCGATGA
TCGAATCCGGGCGCGCCAGGTACACGTGCTCGAAAATGCACGGTGTGAGCTTGGGGTTGATCGCGCACTGGCGGGT
GTGCAGCTTGCCATCTTCAGTGATGTACACCGCTTCGCCCGGGGCCAGGTCGCGCACCAGGGTGAAGCCCAGTACA
TCCAGGGATACGCTTTCGGAGGCAATCATGTACTCGACGCCTTCGTCGGTGTGACGCTGGCCGAACACGATAGGAC
GGATGCCATGCGGGTCGCGGAAACCAACGATCCCGTAGCCGGTGATCATTGCCACGACCGCGTAGCCACCCACGCA
ACGGTTATGCACGTCGGTCACAGCGGCGAACACGTCTTCTTCGGTTGGCTGCAACTTGCCGCGCTGGGCCAGTTCG
TGGGCAAACACGTTGAGCAGCACTTCCGAATCGGAACTGGTGTTGACGTGGCGCAGGTCAGATTCGTAAATCTCCT
TGGCCAACTGTTCAACATTGGTCAGGTTACCGTTGTGCGCCAAGGTAATGCCGTAGGGCGAGTTGACGTAAAACGG
TTGAGCTTCGGCCGAAGTCGAGCTACCGGCAGTCGGATAACGCACATGGCCAATACCCATATGCCCGACCAGGCGC
TGCATGTGACGCTGATGGAACACGTCACGTACCAGGCCATTGTCCTTGCGCAGGAATAACCGGCCGTCGTGGCTGG
TCACAATACCGGCAGCGTCCTGGCCGCGGTGCTGGAGGACGGTTAGCGCGTCATACAGCGCCTGATTGACGTTCGA
CTTACCGACGATACCGACGATGCCACACATGCGACGCAACCCCTACTTAATGAATCTTGACTGAACACTACTTACT
GCGGCGCGTTGCCCGGCAAGAGGTGCTCCTTGAACGGAATATCAGCGGGTACGCTGATACCGCTGGCCAGCCACTG
ACTACTCCACCCCAATATGAGGTTCTTGGACCAGTCTGCAACCAATAGAAATTTTGGCACGAGACTCGACTCCTGC
CACCAAGCATCCTGCTGTACCGGGCCCAGGCTCAACAGCCCGACCGCAACGACCACCAGCAACGCGCCACGCGCGG
CACCGAAGGCCATGCCGAGAAATCGATCGGTCCCGGAGAGGCCGGTGACACGTATCAATTCGCCGATAAGATAATT
GACCATTGCTCCCACCAGCAGCGTGGCGATGAACATGATGGCGCAGCCCGCGATGACACGAGCCGAAGGTGTTTCG
ATGTAGCCGGCCAAGTAAACGGCCAGGGATCCGCCGAACATCCAGGCGACGACTCCTGCAACGATCCAGGTCAGCA
GCGATAATGCTTCTTTGACGAAGCCGCGGCTTAGACTGATCAATGCGGAGATGGCGATGATGGCTACGATCGCCCA
ATCAACCCAGGTAAATGGCACAGTGCAGCCTACAGACGGATAAGGCGGCGCATTTTAGCAGAGCGCTGGGCTATCG
GTAAGCGGTGATTGGCGGTCCATTGCAAATCAATGGGTTGTGGCAAGCTGCGGCCATCTCAGACGGGCATAAACCA
ATGTGGGAGCGGGCTTGCCCGCGATAGCGGCGTGTCAGTCAACTTTAATATCGACTGATGCACCGTCATCGCGGGC
AAGCCCGCCCCCACACAAGCCTGCACACACATTTGGAGCCAGAATCAGCCGCGTTCTGGCTGGAAGCGCACCACAA
AACCCTTCAGATTCTGCTGACGCCCCAGCAAGTCACGCAGGCGGTCAGCCTCGGCACGCTCAATCAGCGGCCCGAC
AAACACCCGGTTCTTGCCATCGGCAGTACGGATATAAGCGTTATAGCCCTGGCTGCGCAGGGTTTTCTGCAAGGCT
TCGGCACTTTCGCGGTTGGACAGACTGGCAAGCTGCACCGACCAACTGATCGGCAGGCCATTGGGATCAATACGGC
TTTGACCGACATCCGGCTTGCCTGGCGCAGCAGGCTGCGGCGCGACAGGTTTTGGCGCTGGAGCAGGCGCCGCAGG
CTTGGCGGCCACGACTGGCGCTGGCTTGACCACGGGAACGCTGGGTTGAATTGGCGCCGTGGGTGCCTGCTGCTGG
GCAATTTCTGCATCCGTCGGCACTGGCTCTTCCTTGGGCAGCGCCTGCGGCTCAGGCACCACCACCGGCTCGACCT
GAACCTGCGGCATTACCGGGGCCTGGGGCGCAGCCGGCGCGTCAACCGTGACCTGGCGCTGCTCGTCCTGACGGGA
AAACAGCATCGGCAGGAATATCACCGCCAATGCCACCAGCACCAGGGCTCCGACCATTCGCTGCTTGTATGCGCTA
TCCAGTAATGCCATGTGCAGCTTCCTCCGTGGAGCGCCGGGCCAACCACTCAAGCGCCTCGGCAACACAATAAAAT
GATCCGAACAACAGAATCTCGTCCTCGGCCGTTGCTACCGCGCATTGAGCCTCCAATGCAGCGGTCACGCTTGCAT
GGGACGTCACCGTCGCACCAAGGTTCTGCAGCAACACCTGTACATCATCGGCCGAACGGCTGCGAGGTGTGTCCAG
CGGCGCTACCGCCCACGCCTGCACACTGCTCAGCAAGGGTGCGACCACACCTTCCAGGTCTTTGTCCGACAGCAAG
CCGAACACCGCCAGGCGTCGACCCGTCGGCACATGGCTGGCCAAACGGCGCGCCAAGTACTGCGCAGCATGCGGGT
TATGACCAACGTCCAGCAACAGGTCAAGGCGCTTGCCTTGCCACTCGAACGCGCGGCGATCCAGGCGCCCGACCAC
CCGTGTACGTTGCAAGGTATCGGCAATCTGCCCGGCATTCCATGGCAGATCCAGCAACAGGTAGGCTTGCAGGGCG
AGTGCGGCGTTTTCCATGGGCAGGTTCAGCAGTGGCAGGCCGTGCAACTCGACGCCCAGACCCCGCGCATCGACAC
CACGCCACGACCAATGATCGTCTGCCACGTCCAGGGAAAAATCCCGGCCCCGCAGGAAGAAGGGGCAATCCAGCTC
GCGCACCTTGTCCAGCAAGGTATGCGGCGGATCCAGGTCACCACACAGTGCCGGCTTGCCTGGGCGCAGGATCCCG
GCCTTTTCATAGGCCACCGATTCGCGGGTATCACCCAGGTAATCGGTATGGTCCACGCCAATGCTTGTGATCAACG
CCAGGTCGGCGTCCACCACATTGACAGTATCCAGGCGACCACCCAGGCCCACTTCCAGCACCACGGCATCCAGCTG
TTCGCGCTCGAACAACCAGAACGCAGCCAGGGTGCCCATCTCGAAATAGGTCAGGGAGGTATCGCCGCGCCCGGCA
TCCAGCGCCGCAAAGGCTTCGCACAATTGCTCGTCGGTGGCTTCAACACCGTTGAGTTGCACGCGCTCGTTGTAAC
GCAGCAAGTGCGGCGAGCTGTAGACACCCACCTTCAAGCCCTGGGCCTGCAGCAATGCAGCGACAAAGGCGCAGGT
AGAGCCCTTGCCGTTGGTGCCCGTGACCGTAATCACTCGCGGCGCGGGCCGACCCAACCCGAGGCGGGCCGCTACC
TGTTGCGAGCGCTCCAGGCCCATGTCGATGGCTGACGGATGCAACTGCTCAAGGTAGGCGAGCCACTCGCCCAGGG
TTCGTTGGGTCATAGGTTTGCAGGCACCGGCGGAACGACGATAGGTTCGACTTTAGGCGCGACGTATTCTGGTGTC
GGCAAGCCCATCATCTGCGCCAGCAGGTTACCCAAGCGTGGACGCAGTTCGGAACGGGCGATGATCATGTCAATCG
CCCCGTGCTCCAGCAGGAACTCGCTGCGCTGGAAGCCTTCCGGCAGCTTTTCGCGCACGGTCTGCTCGATCACACG
TGGGCCGGCAAAGCCGATCAGTGCCTTGGGCTCGCCAACGATTACGTCGCCGAGCATCGCCAGGCTGGCGGAAACG
CCGCCATAGACCGGGTCGGTCAGCACGGAGATGAACGGGATGCCTTCCTCGCGCAGACGCGCCAGTACCGCAGAGG

TCTTGGCCATTTGCATCAGCGAGATCAACGCTTCCTGCATCCGCGCACCACCCGAGGCGGCGAAGCAGATCATTGG
GCAACGGTTTTCCAGGGCGTAGTTGGCGGCACGTACAAAGCGCTCACCGACAATGGCGCCCATCGAACCACCCATG
AAGGAGAATTCAAACGCCGACACCACCACCGGCATGCCCAGCAGCTTGCCGCTGATCGAGATCAGCGCGTCTTTTT
CACCGGTCTGCTTCTGCGCAGCGGTCAGGCGGTCCTTGTACTTCTTGCCGTCGCGGAACTTGAGACGGTCCACCGG
CTCCAGGTCAGCGCCCAGCTCGGCACGGCCATCGGCGTCGAGGAAGATGTCGATACGGGCGCGGGCGCCGATACGC
ATGTGGTGGTTGCACTTGGGGCAAACGTCCAGGGTCTTTTCCAGCTCTGGGCGGTACAACACCGCGTCGCAGGATG
GGCACTTGTGCCACAGACCTTCAGGGACCGAGCTTTTCTTCACCTCGGAACGCATGATCGAGGGGATCAGTTTGTC
TACTAACCAGTTGCTCATGCTTTCTTTCTCCAGTACCGGTGGCTTGAACACAGCCCCGCGTATGCCCTTGAGCTAA
ATTCATTGATGTGGCGATAACAGCTGCAGGACGGGGTCAGACGTTCGACCAGCCATTTCCTGCACCTGCCCTCAGC
CTTCCAGACAACCCGCCAACGCGTGGCTGCCACTTCATTGGCGCCGCCCGAGGGCAACGCCGGCCTGTTTCACACA
GTGGTAGTTACTGACGGCGGCAGACTGCCAGCCGTCACATCGCGCCACTGCTGCTGCGCACTGCCTGCATGAATGC
GCGAATCTTGGCAGAATCCTTGATGCCCTTGCCCTGCTCCACCCCGCCGCTGACGTCCACCGCATACGGGCGAACC
TGGGCGATGGCCTGCGCCACGTTTTCGGCACACAGGCCGCCGGCCAGGATGATCGGCTTGCTCAAGCCCTGGGGAA
TCAGCGACCAGTCAAAGGCCTCGCCCGTACCACCAGGAATGCCCTCGACATAGGCATCGAGCAAAATCCCGCTGGC
CCCGGCGTAGGCGGCGCAACTGGCGGCGATATCGTCCCCGGCCTTGACCCGCAAGGCCTTGATATAAGGGCGATGG
TAGCCGGCGCACTCATCCGGGGTTTCGTCGCCATGGAACTGCAGCAGGTCCAGGGGGACGGCATCCAGGGTTTCGT
TGAGCTCACAGGCGCTGGCGTTGACGAACAGCCCCACGGTGGTCACGAACGGCGGCAAGCCGGCAATGATCGCCCG
TGCCTGCTGCACCGTCACCGCGCGCGGGCTCTTGGCATAAAACACAAAACCAATGGCGTCAGCCCCCGCCTCGACT
GCCGCCAGCGCGTCTTCCATGCGGGTAATCCCGCAAATCTTGCTGCGAACGCCTGACATATCGTCGGAACCTCAGG
GGGCTGTCCAGGAAAGTCCCGGATGGTAACAAATGCTTTTCCAGGCGTCAGCCGTCAAGTTCAGTGAAACCTGTGA
GGAAGTGTGGCCCGATGAAACGCTGCGGCAACTCGAACTCATCGCGGTATTCCACATCCACCAGATACAGGCCGAA
CGGGTGCGCCGTCACCCCGCCGGTACGACGCACTCGACTCTCCAGCACTTCCTTGGCCCATTCCACCGGGCGCTCG
CCGGTGCCAATGGTCATCAGTACACCGGCGATATTGCGCACCATATGGTGCAGGAACGCACCGGCACGGATATCCA
GGACAATCATCTTGCCGTGGCGCGTGACGCGCAGGTGGTGGACTTCCTTGATCGGCGACTTGGCCTGGCACTGGCC
AGCCCGGAACGCGCTGAAATCATGGATTCCCACCAGGTGCTGGGCCGCCTCAGCCATACGCTCGGCGTCCAGCGGG
CGGTGGTTCCAGGTAATTTCTTCGTTCAAGTGTGCCGGACGGATCTGATCGTTATAGATCACGTAGCGGTAGCGCC
GGGCAATCGCCTTGAACCGCGCATGGAAGTGCGCCGGCATGACCTTGGCCCAACTGACGCTAACATCGTGGGGCAG
ATTGATATTGGCGCCCATGACCCAGGCTTTCATCGAACGCTCGGCCTGGGTGTCAAAATGCACCACTTGCCCGCAG
GCATGCACGCCGGCGTCGGTACGCCCGGCGCACATCAATGAAACCGGCGAGTCGGCGACCTTCGACAGGGCCTTCT
CCAGGGTTTCCTGTACGGTCGGCACGCCCGAGGCCTGGCGCTGCCAGCCGCGATAGCGCGAGCCTTTGTATTCAAC
GCCCAGGGCGATTCTGAAAAAGCCAGCGGCCGCCATTTCGGCGGCCGCGTTATCTATATTTGCCAAGAGCTGAGGG
CCTGATGAGTTGCGCAAAGGCAGCCATTATATATGGCTTGAGGATTTTTGTAGGAGCGAGCTTGCTCGCGAAGATT
GTCAACGATAACGCGCCCATCCTGGAAGAACGCGGTGCCCTTGAGTTTTTCGCGAGCAAGCTCGCTCCTACAGGAG
TTGCGTAATGGCTTTAGGCCAGGTTGCTGAGCATTTCCTTGGCTTCATCCCGTTGCTTTTCATCGCCTTCGGTAAG
GACTTCGGCCAGGATGTCGCGCGCACCGCCCTCATCCCCCATGTCGATATAAGCCTGGGCCAGGTCCAGCTTGGTG
GCGACTTCGTCGGTGCCCGACAGGAAGTCGAACTCCGGCTCATCGTCGCCCTTGGCGGCGTCTTCGGCAGTGAAGC
TCGGCTCGATCGACGGGCTTTCCAGGCTCTGGGACAGACGCTCCAGTTCGGCGTTGACATCGTCAAGCTCCGAAGC
GAATGCGTCCGGCTTGGCTGACGCCGCGGGTTCGTCCGCCAGGGACAGATCAAAGTCTTCCGGCAGGTCAAAATCT
TCCAGCGCGGCCGGTGTCAGGGTGGGCGGCTCTACCGGCGGCAGGTCCTTCATCTGTTCTTCCAGGCCCGAGAGGA
AGTCGTCATCGGACGCAGAGGCTGGCGCATCCAATTGCAAGTCGAGGTCAAAATCCGATAGATCGTCCTCGCTGGC
CTTGGCTTCATTCTGCTGCTGCAATACCGACTCGAAGCTCAGATCATCGATCTCGGGCTCTGGCGCAACCACGGCC
GGCGAAGCGGCTTCCAGGTCATCCAGGCTCAGGTCGAAGTCGGTGTCGAACCCATCGGCCTCAGGCGTGGCGGCGG
GCGCCGGTTCTTCCTGGAGCAGATCCTTGACGTACTGCGCATCCAGCGCAGCGGCGGCGACGGCAGCACTGACACC
CGCTGCCAGCACGGCCATGGCCGGGTAGCGGTTTTTCAACAGTTCGACCTGGGCGTGATTTTCGCCATTGGCGACC
AGTTGGCGCTCCTGGGTTACGAAGCCATCTTTGTCGCCCTGCTGACCATAGACTTCCAGGAGCTTGAGACGCAGAT
CGCTGCGCTTGGGCTCCAGCTTGATCGCTTCTTCCAGCACGGCGGCCGCCTGGTTCAGGTGACCACGATCCAGGTG
GGACTGAGCGAGCGCCAAAGCGTCGGAGGAGCCGGGTTCGATGATCGGCTTGCTCGGCGGCGCCATGCTGGTCACC
GGTGCAGGCACAGGCGCCACGGCAACCGGCGCTGAAGCTGGAGCTGGAGCTGGAGCTGGAGCCAGCTTGACGTTTG
CCGCAGGGACTTCAAGGCCTTCGAAACTGTCCGATGGCATGTCCTGGTCAAGGCTCGAGGAGAACTCCGGTTCCTC
GGCCAGCGCCCGCGCCATGCGCAGGTGTTTTTCCTCTTCCAGGCGCGCATTGCGATGACGCGCCCATAGCAGCAGG
AGCAGCAATACCAGCAGGCCAGCGGCACCGCCAATCACCCCGAGAACGATGGGATTGGTCAGCAGGTCATTGAACT
TGCCTTCGGTGCTGGCCACGGGCGCGGGCTCAGCCGGGTTTTCCACAGGCGCAGCCACGGGCGCTTCGGCGGCTGG
CGCCGGGGTAGCAGGTGCTGGTGCAACGGGCGCCGCCGCCAACTCCGCAGGCATGGCCGCCGGCTGCGCCGGTGCG
CCACCCTCGGCCTGCAACTTGGCCAATTGGTTGTTCTTCAGTTCAATCAGCCGCTGCAACTTGTCCAACTGACTTT
GCAGGTCAGTCATGCGGCTTTTCAGTTCTTCATTTTCGCGGCGGGTCGAATCCAGGCTTTCCTGGGTCACCGCCAG
CTTGTTGTTCAAAGCCTTGCTATCCCCTGCGGCGCCTTTGACTCCAGCCTTGGCGGCCTCGGCCGAGACCAGGCTG
AGGTTGTCCTTGGCAGCCGTTTGCGACGGCGCGGCACCGGCCTGGGTGCGCTTGGTGGCGTCCAGTTGCTGACGCC

CCGCTGCCTGATTAGTGGCACCGCGACGCCCCTGACGCCAGGCCGCGTTCTGCGCAGTCACTTCGGCAATCGCCTG
AGGTTGCGGCAGGCTGGTGCTTTGTACCGGGTCAGGCAGGCGCAGTACCTGGCCGGTTTTCATGCGGTTGATATTG
CCGTCGACAAAAGCCCCAGGGTTCAGCGCCTGGATCGCCAGCATCGTCTGCTGGATCGAGCCGCCATTGCGATTTT
TAGCGGCGATTTCCCACAGGGTGTCGCGGGAAGTCGTAGTGTGTTCAGCCGCCTTGCTGACGCTGCTGGCCGCAGG
CGGGGCAAGGCGCGGTGCCGGGGCAATGGCCTCCGGGGCCTTTTGCTCGAACTTGGCCGGGTCCAGCAAGACGCTG
TAGTCGCGCATCAATCGGCCATTGGGCCATTGCACCTGCAACAGGAAGCGCACATAGGAGTCGGGCAAGGGCTTGC
TGGAGGTAACACGCACCACGCTGCGACCGCTGGGGTTGAGCACCGGGGTAAAGCTCAGTTCATCAAGGAACGCTTG
GCGATCCACGCCCGCATCCACAAAGGCCTGGGAAGACGCGAGGCTGGGTACGATGTCGGAGGCGCTCAGCCCGCCT
ACATCGAGCAGCTCGATCTCCACCGACAACGGCTGGTTCAACTTCGACTTGAGTGTCATCTCCCCAAGTTGCAGCG
CTTGCGCCATACCGGAGGACAGCGCCGAGGCGGCCGCTATTGCTAACACCAGTTTGCGAACTTGAACCATAGCCTC
ATCCTTTGTTTGAACACTCCTCGGCCCGCGAGAAGGTTGGGTACCGCTGCCATTCGGCATGGCGAGCCGATAGGTC
ACCGACGCGCAACTTTTCGTGCATGGGGCCAAGCATAGCGCTTAGCTAGAATCAATCTACAAATTGCCGCCAAGTA
TCTTTTACGCAAGGTCTTTTATCAACAACTGCGCCAGTTGCACCGCGTTAAGCGCAGCACCTTTGCGTACGTTATC
TGACGTCAGCCACAAGTTTAGTTCCGCTGGGTCGTCAATGCCCGCTCGCACCCGGCCCACGTAGACCACATCCTGA
CCGACCGCATCCCCAACAGCGGTAGGGTAATCGCCCTCTTCCACCAGTTCGACGCCCGGCGCCGACTCCAGCACGC
GATTGACTGCGCCAAGGTCCACAGGGCTCGCCAGTTGCAGGGACACGCTCAGGCTATCGCCAAAAAACACCGGAGC
TTGAACGCAGGTCGCGGAAATCTTCAGCAACGGTAAATCAAGCAAGACGCGCAGTTCGTGGACCAGGCGTTTTTCC
AGGGCCGTGTGGCCCTGGGCGTCCGGCGTACCGACCTGGGCCAGCAGGTTGAAGGCCATTTGCCGATCGAAGAACT
TTGGCTCCAGGGGGCGCACATTCAACAACTCGGCGGTTTGCCGGGCCAGCTCACTCACGGCTTCACGGCCCTGGGC
AGACACGGCCAGGCTGGCGGTGACACTCACGCGCTGAATGCCCAGCAAGCCCCGCAACGGCGCCAGGACCACCGCC
AGATGAGTGGCGCACGGGCTCGGGCTGCCGACCTGGAAGGGCTTTCTCAGACCTTTCAGTACGTAGGCGTTGGCTT
CCGGCACCACCTGAGGTGCCTGCTCGGCCGGCAAGGCCCCAGACAAGTCGATCAGCGAGCACCCCGCCGCAGTGGC
GCGCGGGGCAAAACTCAAGGTGACCGCCGGGCCAGCCGCGAAAAAAGCCAGTTGCACCTTGCTGAAATCAAACTCG
TCGACTTCCCGCACCCGCACGTTCTTGCCGCGAAATGGCACCGAAGCGCCGGCCGACTCACTGCTGGCCAGCAAGT
GCAGGGTGCCGACCGGGAAATCCAGCTCTTCGAGGATCTGTACCAGGGTTTCGCCAACGGTGCCGGTGGCGCCGAT
CACGGCGATATCAAAGGTCTGGGTCATGGAGGCTGCCTCGGGCATTGCGGGGGGAGCGGCACTTTACCGGGTGGTG
GGGGGTGAGGCAATTAACCTGGGCTTTGTGGTGTGATGGGGGCCGTTATCGCGGGCAAGCCCGCTCCTACATTTTG
ATTTGTGAACACATTCAAGTGTAGGAGCGGGCTTGCCCGCGATGACGGCCCCAGGCGCAATAAAAAACCCGCGCCT
GTCACCAGGGCACGGGTTTGTTCAACACCAAAAAGCGATCAACGCTCCAGCAGGATCCGCAACATCCGGCGCAACG
GCTCGGCCGCGCCCCACAGCAGTTGGTCACCCACGGTGAAGGCGCCCAGGTACTGGGTGCCCATGTTCAGCTTGCG
CAGACGCCCCACCGGAACATTCAGGGTGCCAGTGACCTTGGTCGGGCTCAGCTCCTGCATGCTGATATCGCGGTTG
TTCGGCACCAGCTTGACCCATGGGTTGTGCTGGCTGATCAGCCCTTCGATATCGGCAATCGGTACGTCTTTGTTCA
GCTTGATGGTCAGCGCCTGGCTGTGGCAGCGCATGGCGCCGATGCGCACGCAGATACCGTCCACCGGGATCGGGTT
CTTGAAGCGCCCCAGGATCTTGTTGGTCTCGGCCTGGGCCTTCCACTCTTCGCGGCTCTGGCCGTTCGGCAGTTCT
TTGTCGATCCACGGGATCAGGCTACCGGCCAATGGCACGCCGAAGTTCTCGGTCGGATAGGCGTCGCTGCGCATGG
CCTCGGCCACACGACGGTCGATGTCGAGGATCGCGCTGGCCGGGTCGGCCAGTTGATCGGCGACGGCCGCGTGGGT
CGCGCCCATCTGTTTGATCAGCTCACGCATGTTCTGCGCACCGGCACCGGAGGCCGCCTGATAGGTCATGGCGCTC
ATCCACTCCACCAGCCCGGCTTCGAACAGGCCACCCAGGCCCATCAGCATCAGGCTGACGGTGCAGTTGCCGCCGA
CATAGTTCTTGGTGCCGGCGTCGAGCTGCTGGTCGATGACCTTGCGGTTGACCGGGTCGAGGATGATCACGGCGTC
GTCCTGCATGCGCAGGCTCGAGGCGGCGTCGATCCAGTAACCCTGCCAGCCGGCTTCACGCAGCTTGGGGAAGACT
TCGCTGGTGTAGTCGCCGCCCTGGCAGGTCAGAATGACGTCGAGGGTCTTGAGCTCTTCAATGTTGTAGGCGTCCT
TGAGCGGGGCGATATCCTTGCCCACGGACGGCCCTTGGCCACCGACATTCGAAGTGGTGAAAAACACCGGCTCAAT
AAGATCGAAATCCTGCTCTTCCAGCATCCGCTGCATGAGCACGGAACCGACCATACCGCGCCAACCGATCAGACCT
ACACGTTTCATCGCAACTACACCTTGTTAAAAAGTGGGCCGCCTTGCAGCAACAACTGCAAGCGGGCCCGAGAGAT
TACAGATTCCGCAGCGCGGCGACTACTGCGTCGCCCATTTCCTGCGTCCCGACCTTGGTGCAACCCTGCGACCAGA
TGTCGCCGGTACGCAGGCCCTGGTCCAGCACCAGGCTCACCGCCTTCTCGATCGCGTCCGCCGCTTCGCTCAGGTT
GAAGCTGTAACGCAGCATCATCGAGACCGACAGGATGGTCGCCAGCGGGTTGGCAATGCCCTGGCCCGCGATATCC
GGCGCCGAACCGTGGCACGGCTCGTACATGCCCTTGTTGTGGGTGTCCAGGGACGCCGACGGCAGCATGCCGATGG
AACCGGTGAGCATCGAGGCCTGGTCGGACAGGATGTCGCCGAACAGGTTGTCGGTGACGATCACGTCGAACTGCTT
GGGTGCACGCACCAGTTGCATCGCGGCGTTATCGACGTACATGTGGCTCAACTCGACGTCCGGGTAGTCCTTGGCC
ACTTCTTCGACGATCTCGCGCCACAGCTGGCTGGAGGCCAGGACATTGGCCTTGTCCACCGAGCAGACCTTCTTGC
CACGTACGCGGGCCATGTCGAAACCGACACGGGCGATACGGCGGATTTCGCTCTCGCTGTACGGCAGGGTGTCATA
GGCCTGGCGCTCGCCATTTTCCAGCTCACGCACACCCCGTGGCGAGCCGAAATAGATACCGCCGGTCAGCTCGCGC
ACGATCAGGATATCCAGGCCCGCCACCACTTCCGGCTTGAGGCTCGACGCGTCGGCCAGTTGCGGATAGAGGATCG
CCGGGCGCAGGTTGCCGAACAGGCCCAGTTGCGCACGGATTTTCAGCAGGCCGCGCTCCGGGCGGATATCACGTTC
GATCTTGTCCCATTTCGGCCCACCCACGGCGCCGAGCAGCACGGCGTCGGCAGCACGGGCACGGTCCAGGGTCTCG
TCGGCCAGGGGCACGCCATGCTTGTCGATGGCCGCGCCGCCGATCACGTCGTGGCTCAATTCAAAGCCCAGGCTGT

ACTTGCTGTTGGCCAATTCCAGGACCTTGACCGCTTCGGCCATGATTTCCGGACCAATGCCGTCGCCAGGGAGAAT
CAGAATCTGCTTGCTCATGCGTTCCTCATTTCATCAAGCGACCCGCCCTCGGGCAGGTCGGGAAAATTATTAGCGT
TCGGCCCAGACCACCAGCACGTCGGTGCTGAAGGTGCCATCGGCTTGAATCTCAAAATAATCGCGCACTTCCTGGC
CCATCGCCTTTTGCAGTGCCAGGATCGCTGCGCACAATACCTCTGGCGTGCGCATGCGCTCGACCCACGAGGTGTA
TTCCAGGCGCAGGCGCTGGCGACTGCTATTGCGTACATGCAAGCCGGCTTCGCTGAGCTGGCGCATCCACTCGCCA
GCGGAGTAATCGCGCACATGGCTGGTGTCGCGCAGCACTTCGACGGTTTGCAGGTAAGTGTCCAACAGCGGGCTGC
CCGGTGACAAGACGTCGACAAATGCCGCCACGCCACCCGGCTTGAGCACCCGGCGCACTTCGCGCAGCGCCAGGCC
CAGGTCGCTCCAGTGATGGGCCGAGTAGCGGCTGAACACGAAGTCGAACTCGCCATCGGCGAACGGCAGGCGCTCG
GCGGCCCCGTGCAGGGTGCGGATATTGTCCAGCCCACGGTCGGCGGCAGCCGCGGCGACCACGTCGAGCATTTGCT
GCGACAGGTCGTAGGCCACCACTTCCTTGACCAGCGGTGCTACGTGGAAACTCACGTGACCGGCGCCACAACCCAG
GTCCAGCAGGCGTGCCGCGCCCTGCCCGGCCAGTTCGGCCTGCAGCAGTGCGAATTCGGTGCCTTGGGCGTGCACG
GCACTGCTCAGGTAGGCCGAGGCCTGTTCACCGAATTGTTTTTGCACGACTTGGGTGTGGGCGGTGCTGGTCATGG
AAGTTTCCTTGGGTTTTGTGGTGTTGGTACGGGCGCTATCGCCGGCAAGCCAGCTCCCACATTTGACCGTGTTCAC
AGATCAAAATGTGGGAGCGGGCTTGCCCGCGATGGGGCCCGCACGGTTTACCGCAAATCAGGCATCACGAAACAAC
CAAGGCTGGCTGGCCCGGTGCTTGGCTTCAAACGCCGCAATGGCATCGCCGTCCTGTAAGGTCAGGCCGATATCGT
CCAGGCCGTTGATCAGGCAGTGCTTACGGAACGCATCCACTTCAAAGTGGTACACCTTGCCGTCCGGACGGGTCAC
GGTTTGCGCCGCGAGGTCGACGGTCAATTGGTAGCCGACATTGGCTTCCACTTGCTGGAACAACTCGTCCACTTCG
GCATCGCTCAAGATGATCGGCAACAAACCGTTCTTGAAGCTGTTGTTGAAGAAGATATCGGCGTAGCTCGGCGCGA
TGATGCTGCGAAAGCCATATTCTTCCAGGGCCCAAGGCGCGTGTTCACGGCTGGAACCGCAACCGAAGTTCTCCCG
GGCCAGCAACACACTGGCGCCCTGGTAACGCTCGGCATTGAGCACGAAGTCTTTGTTCAGCGGGCGCTTGGAGTTG
TCCTGGTAGGCGTAGCCCACGTCCAGGTAGCGCCACTCGTCGAACAGGTTCGGGCCGAAACCGGTGCGCTTGATCG
ACTTCAAGAACTGCTTGGGGATGATCTGGTCGGTGTCCACGTTGGCACGGTCCAACGGTGCGACAAGACCTGTGTG
TTGGGTAAAAGCTCTCATCGGGTATTCCTCAGATCAATTCGCGAACGTCGATGAAACGACCGTTGACGGCAGCCGC
AGCGGCCATGGCCGGGCTCACCAGGTGGGTGCGGCCACCGGCGCCCTGACGCCCTTCGAAGTTACGGTTGGAGGTG
GACGCGCAATGCTCGCCGGACTCCAAACGGTCCGGGTTCATCGCCAGGCACATGGAGCAACCCGGCTCGCGCCATT
CAAAACCGGCTTCGAGGAAGATCTTGTCCAGGCCTTCGGCTTCGGCCTGGGCCTTCACCAGGCCCGAACCTGGCAC
CACAATGGCTTGCTTGATGGTCGCGGCCACTTTGCGGCCCTTGGCGATCACCGCCGCGGCGCGCAGGTCTTCGATC
CGCGAGTTGGTGCAGGAACCGATAAACACCCGGTCCAACTGGATGTCGGTGATCGCCTGGTTGGCTTTCAAGCCCA
TGTACTTCAAGGCCCGCTCGATGGAACCGCGTTTGACCAGGTCGGCTTCCTTGGCCGGGTCCGGCACGTTCTGGTC
GACGGCCAGGACCATTTCCGGCGAAGTGCCCCAGCTGACTTGTGGCTTGATCTGGGCGGCGTCGAGTTCGACCACG
GTGTCGAACACCGCATCTTTGTCAGAAACCAGGTCTTTCCAGGCCTCCACGGCGGCATCCCAATCGGCGCCTTGCG
GAGCGAATGGACGGCCCTTGACGTACTCCACGGTTTTTCATCCGCCGCCACCATGCCCACACGGGCACCGGCTTC
GATGGACATGTTGCAGATGGTCATGCGGCCTTCGATGGACAAATCACGAATCGCGCTACCGGCGAACTCAATGGCA
TGGCCGTTACCGCCAGCGGTGCCAATTTTGCCGATCACGGCCAGCACGATGTCCTTGGCGGTGACGCCAAAGGGCA
AGGTGCCTTCGACCAGCACCAGCATGTTCTTCATTTTCTTGGCGACCAGGCACTGGGTGGCGAACACATGTTCCAC
CTCGGAGGTGCCGATACCGTGGGCCAGGGCGCCGAACGCACCATGGGTGGAGGTATGGGAGTCGCCGCAGACCACG
GTCATGCCCGGCAAGGTTGCGCCCTGCTCCGGGCCGATAACGTGGACGATGCCCTGGCGCACGTCATTCATCTTGA
ATTCGGTGATGCCGTACTCGTCGCAATAGTCGTCGAGGGTCTGGACCTGCAAGCGCGAGACCTGGTCGGCAATGGC
TTCGATGCCGCCCTTGCGCTCCGGGGTGGTCGGCACGTTGTGGTCCGGGGTCGCGATGATCGAGTCGACGCGCCAA
GGCTTGCGCCCGGCCAGTCGCAGGCCTTCGAACGCTTGGGGCGAGGTCACTTCATGGATGATGTGACGGTCGATGT
AGATCAGCGATGACCCATCATCGCGCCGTTTCACTTCATGGGAATCCCAGAGTTTGTCGTAAAGCGTTTTGCCGGC
CATCAGTCGGTCCTCATCAGCGTCTTTCTATGCCCTGGGCGTTGAACGATTCAATAACCCTTTGGCTTGTGAGGCT
GATGCTATGGCGTTACATTAAATAACTCAAATTCATATTTTTCATGCTTTGGATAACTCACTGGAATACAACCATG
GATCTGGCCAACCTCAATGCCTTTATTGCTATCGCCGAAACCGGCAGCTTCTCCGGCGCCGGGGAGCGCCTGCACC
TGACGCAACCGGCAATCAGCAAGCGTATCGCCGGCCTTGAGCAGCAACTGAAGGTGCGCCTGTTTGATCGACTGGG
CCGTGAAGTGGGCCTGACAGAAGCCGGGCGCGCACTGCTGCCCCGCGCCTATCAGATCCTTAATGTGCTGGATGAC
ACTCGTCGCGCCCTGACCAACCTGACCGGCGAAGTCAGCGGGCGCCTGACTCTGGCCACCAGCCATCACATCGGCC
TGCACCGCCTGCCGCCGGTATTGCGCACGTTTACCCGGGAATACCCGAACGTGGCCCTGGATATTCAGTTCCTGGA
TTCGGAAGTCGCCTACGAGGAAATCCTCCATGGCCGCGCCGAAGTCGCCGTCATCACCCTGGCGCCGGAGCCCCAC
AGCCTGGTGCGGGCCACCCCGGTATGGGATGACCCCCTGGATTTCGTGGTGGCGCCCGAACACAGCCTGGCCAATA
ACGGCACTATCAGCCTTGCCGACATTGCCCGCCATCCGGCGGTTTTCCCTGGCGGCAATACCTTTACCCACCATAT
CGTGCACCGGCTGTTCGAGGCCCAGGGCCTGACGCCGAACATCGCCATGAGCACCAACTACCTGGAAACCATCAAG
ATGATGGTTTCGATCGGCCTGGCCTGGAGCGTGTTGCCGCGCACTATGCTTGATGATCAAGTGGCCAGCATCGCTT
TACCGGGCATACAGCTCAGTCGCCAGCTAGGCTATATCGTGCACACCGAAAGGACGTTGTCGAACGCTGCGCGGGC
TTTCATGAGCCTATTGGATGCACAGGTTGATCCGGCGGAGATGCCGGCATGAGGCCGTGCGGCCTGTAGACCCTGC
TCCCGCGCCGAATGCCCATCGAGCCAAGGCCCCTTGATAATGCCCAACCCTGTCGATCCCGTGCCGCCGCTGCCTC
GCATTCACGCCCTGGACCCCCAGCAGGCGGAACAAAGCTGGGAGAACGCCCCGCAGCTGCTGGCGGCGCTCAATGC

CGCACGGCTCGGCGCCTGGAGCTGGGATATCGACAGCGGGCGAATCAGCTGGTCGCGCGGCACCCAGGCGCTGTTT
GGTTTTGACCCCCGCCAGCCATTGCCCAAGGACCTGGATTACCTCGACTTGCTGGCCGCCGAGGACCGCGGCCGCG
TGATCCGGGCCTTTCACGCCGTCCTGGCGGGCGCGCCCTTCGAGCAAGCCATGCACCACCGCATCCAGTGGCCGGA
TGGCAGCCTGCACTGGCTGGAAATCAACGGCAGCCTGCTGCCGGACGCGTCCGGGCGGCGGCGCATGATCGGGGTG
ATTCGCGAAACCACCCAGCAACGCGAGCGCGAACATGCCCTGGGCCACTCGGAAAAACGCTTCGCCACCTTGTTTC
ATCTGTGCCCGAACATGGTCCTGCTGACTCGCCAGGAAGACGGCCTGATCAGTGAGGCCAACCAGTATTTTGAAAG
CCTGTTCGGCTGGCCGGTGGCCGATGCCATTGGCCGCACCACTCTGGACCTGGGGCTGTGGGTGCATCCTGAGCAA
CGCGCCTTGCTGGTCAAGGCGACCCAGCGCAAGGGCGAACCCATCACCATGGAAGTGCAGTTTCGCGCCAGCAATG
GGCAAATCCATGACGGCACCCTCAGTGCCCAAAAGGTCGAACTCGACGGCCAGGCCTACCTGATCAGCACTTTCCT
CGACACCACCGAGCGCAAGAACGCCGAACAGGCCCTCAAGGACAGCCAGGAACGCCTGGACCTGGCCCTGGATTCG
GCCCAACTGGGCACCTGGGACTGGCATATCCCCAGCGGCATGCTCTACGGCTCAGCCCGGGCGGCGCAGTTGCACG
GCCTGGAGCCGCATCCGTTCCATGAATCCTTCGATGCGTTTTTTGAAGGTATGCCCGATGAGGAGCGCGCCAGCAT
GCGCGACGCCTATCGCACCCTGCGTGAAGGCCCGGCAGGCAATTACCAACTGACCTACCGCGTGCAACTGGAGGAC
GGCAGTTCGCGCTACCTGGAAAGCCGCGCCCGCCTTTACCGTGACGCCCAGGGCGCGCCCCTGCGCATGGCCGGCA
CGCTGCTGGATATCACCGACCAGGTGGAACGCGAGCAGCGTCTGAGTGCCTCTGAAGAGAAGTTCGCCAGCCTGTT
CCTCGCCAGCCCCGACCCGATCTGCGTTACCCGCCTGTCGAGCGGCGAATTCATCGAAATCAATCCCAGCTTCTGC
CAGACCTTTGGCTGGACCGCCGCCGAAGTCATCAATCACACCGCCGAGCAGATCGGCTTGTGGGATGAGCCGACCC
AACGCCTGCAGCGCATCGAGCAGGTGATCCGCGAGCAAGCGCTGAACAACGTGGCGATCATCGTCCACCACAAGAA
TGGCCAGCGTCTGACCTGCATGATTTCCAGCCGGCTGATCACCGTCGGCAACCAGCCCTGCGTGGTCACCACCCTG
CGCGATATCACCCAGCAACAGCGCTCGGAAGCGGCGCTCAAGGCCAGCGAGGAAAAATTCGCCAAGGCCTTTCACT
CAAGCCCCGACGCAGTGTCCATTACCGAGCGTGACACTGGGCGCTACGTGGAGGTCAACGACGGCTTCTGCCGCCT
GACCGGCTACCGCGCCGAGGAAGCCATTGGCCTGACCCTGTACCAGATCGGCATCTGGGCCGATGAGAACCAACGC
TCGGCATTGCTCGCCGAACTGCAGATCAAGGGCCGCATCCACCACCTGGACATGCTCTGGCACAACAAGCGCGGCG
AGTTGCTGGCAGTGGAAGTGTCGGTCGAGCCCATTACCCTCAATGAAACGCCATGCCTGCTGCTGACCGCACGGGA
TGTGAGCCTGCTGAAAAGCGCGCAGGCGCAGATCCGCCACCTGGCCTATCACGACCCGCTGACCAACCTGCCCAAC
CGCGCCTTGCTGATGGACCGCCTGAGCCAGCAGATTGCCCTGCTCAAGCGGCATAACCTGCGAGGCGCGCTGCTGT
TTCTGGACCTGGATCATTTCAAGCACATCAACGATTCCCTCGGGCACCCGGTGGGCGATACCGTGCTGAAAATCGT
CACCGCGCGCCTGGAAGCCAGCGTGCGCATGGAGGACACCGTGGCGCGCCTGGGTGGCGATGAGTTTGTCGTGCTG
CTCAGCGGCCTGGAAGGTTCGCGAATGGAGGTCAGCAAGCAGGTGCAGCTGCTGTCCGACACCCTGCGCGAGCTGC
TGTCGGAACCGATGTTCCTCGACGGCCAGCGCTTACAGGTCACCCCCAGTATTGGTGTTGCGCTGATTCCCGATCA
CGGCTCGACCCCGGCCGACCTGCTCAAGCGCGCGGATATTGCCCTGTACCGGGCCAAGGACTCCGGGCGCAACACC
ACGCAGATGTTCCATAACAGCATGCAACGCACCGCCAGCGAGCGCTTGCGCATGGAGACCGACCTGCGCCTGGCGC
TGTCGCGGGGCGAGTTCAGCGTGCATTACCAACCCCAGGTCGATGCCCGGGGCAATCGCATTGTCGGCGCCGAAGC
CCTAGTGCGCTGGCAGCATCCGCAACTGGGCGCACAATCACCGACAGAGTTCATCAAGGTCCTGGAGGACAGCGGT
CTGATCCTCGAGGTGGGCACCTGGATCCTCGATGAAGCGTGCGCCACCTTTGCCAGGCTGATTGCCGAAGGCCTGA
TTGAGCCCAAAGGTTTCAGCCTGTGCGTGAACATCAGCCCCCGGCAGTTTCGCCAGAACGACTTTGTCGAACGGGT
GGAACGCAGCCTCAAGCAGCACCAACTGCCATTTAGCCTGCTGAAGCTGGAAATCACCGAAGGCATCGTGATCCAG
AACCTGGACGACACCATCGGCAAAATGCGCCGCCTGAAAAAACTCGGGGTCAGCTTTGCCATGGACGACTTCGGTA
CCGGGTATTCGTCATTGACCTATCTCAAGCGCCTGCCGGTCGATGCCTTGAAAATCGATCAGTCATTTGTGCGCGA
TGCCACCCACGACCCCAACGATGCAGAGATCATCCGCGCGATCGTAGCCATGGCGCGCAGCCTCAACCTGGAAGTG
ATTGCCGAAGGCGTGGAAACCTGCGAGCAACTGGCGTTCCTGCAAGGCTTGGGGTGCCATCTGTATCAGGGCTATT
TGCATAGCCGGCCACTGCCGGTGGACGCGTTCACGCAACTTTTGAGGAGCGCTGCCGGAGGCTGAAAACAATCCGC
CAGATGAACCGCCAGGCCCAGCACGGGGCATCAGACCAATTTCAACGCTCGCACGAAGTAGCCCTTCCAGTTGGAG
GGCGCCAGGCTGCGCTTTTCTCCATACATGTCGAACATCCCATCAACGACCGTCACTGAATGGTGGGTGTCCGCCA
GCGTTCCAATGGCCCCGTCAGCCAGTTCTTGCACCGTGCTTTCCCGGATGTAGGCATACAGCCCCAGCCTGCGCAG
CGCCGATCCGGGATACTCACCGTCGTTCAAGGTATCCATGGCGACCTCATAACTCTGGTTTCCCCGGCCGTCGTTG
TTCTCCAATTGCGCCCGCTTGGCACTTACGGCGTACAAAAAATTGGCATCGTCCAAAAGGGATTGATTGCTGCCCT
GCAAACCGGAGTGCACCTTGGCCTTGTTCAATTCGTCGTGGGTCAACGTCAGTTTGTAGCCATCGCGCATGACCAC
TTCGTATCCGTCAGCGGTGGCGGAGATTTTTTTATAGATGCCTTGCGGATTCTGGCCAAACTTCATCATGGCGGCC
TTGATCGCTGACACTGTGACACAGTTACCTTCTACACCCTGGTAAAAACCACTCCAGATATTAGCGGGGCGGATAC
CCACTGGCACACTGGAAAGAGTCGGGGCCTTTTCATCAACTATTTCGGGGGATACATCAGGCGTTTTTTTATCATT
GAACAACATGAACCCATAACTTTTATCTACCTTGCGCTGCTCCGAATAAAGATGTTGGGTGTTGTCCATCACAAAA
GCCGAGCCGAAGTTATGGGTCTCCACGACACCCAAAGCGCCACGCGCCGCCATATAGGTTGCAGGCACAAACTGGG
CAAAACCCATCAGGCCCATACCACGCAAGAAGTTCTGGGTAGTTTCACCTTCTAGACTCTTGAGCAGGACGTCTTC
GAAACGCTCATGATTGCCGATCAGCTGCTTGCGCTTGATAAATGCCGCGAGCACTAAGTTGGCGTCCGATACCGCC
ATGCCATCGTTCCCGGCAAACCGTGATGCCTGCGCGGTCATCCTTAGTTCCTCGGCAGACACATGAACCCTGAACT
CATCTTTCATCGTGATCTCATAACCGTCGCCCACCGCCTTGACCTTTTCAAACATATCATGTGGGCTTTGACCGAA

GGTCATCATCATCAATTTAATGACTGCGGCATGGGTACTATTATCAAAACACTCACCAAAGTTACGTCGGGTGGCG
GAAAATGCATTCAAAATATCGCGGGGTTTGTTCCCCCCGGCCGCCGACAATGTGACTGTCGATAAACGCCGGACAG
ATTCATGAGCCACACTCAAACCATCAAACAACATCCGACCGAACATACCGATACTCATAGGAGTTTCCTACCCAAA
AATATTATTAAAGACACGACAAACAACACACAGCCGATAAGTGACGAATTCAAACTTAAAAATACAATGACGAATA
AAAACCTGCGCAGGATAAAAACAACCACTTTTATTCTTAAATCAACATCTAGTGTAAAAACCGCTATTTACCTACC
AACACCCCCCATCAGTCGACTAACGCGACTGCGTGCCCCTGGCTGGGCGCCCTTCCTTTACGGCCCCACAACTCTT
CCTTGCCACCAATCACCGCAACCGAATGCCCGGTGCGGTTGACCATGCCGACTTGCCCCCTGGCCAACACACTTAC
CGCCACACGCTTCATGTGCTTCTTCAAGCCCAACCGCAGAAAACCCTCTCCCGCCCCTCTCTCATCCTCACCATCA
TTCAGGCTGCGGATTGCCGCGCTAAAACTTCTAGCCGCTCGGCCATCGTTGTTTTCCATCTGGGCGCGCTTGGCGC
TGACGGCAAACAGGAACTGAGCATCCTTGAGCATTCCCTTGTCTTTTCCAACAAAACGCGCGCCCTGTGCCGCTTG
CTTCAACTCCTCATGAGTCAGCGTCAAGCGAAAGCCATCTCGCATCGCAACGTGATAACCGCGCTCGGTTTTGATG
ACTTTTTTGTAAATATCAGTAGGGCTCTGTCCGAATTTATGCATCGCCGCCTTGATTGCCGATACCGTTACGCAGT
TACCATCCGGGCCCTGACGAAAACCGCTCCAGATATCATCGGCCTTGGCCCCGTTGCGCTTTTCCGAAAGCCCCTC
AATCCTGACCGGGTGTGAATCGGGCGTAGGCACTGCCGGTGTAGGCTTGTCAGGAATCACATCGGTTTGAACGCGT
TTCTCCTTATCCCGTCCTGACAACAACGCTGATAGCTGACTCAGCAAATTCATGAACATTGAAACAAGACTTTTTA
ATTCCACTTCCTTAGTTGCAACGTCACCCCTGAAATCCGTGAAACTGTTAGATAGGTCAACGACGCCCGCGGTCTG
CCTAAGACGCTGTGGCTGCAGAGAGTTAAAGTTCCCCTCTACCCTACCCGAACAACCACATGGCACGTTTGTAGCA
CTGTTAATAGTTAAGTTAGACATTAGTTTTCCTGCCACAAAGTGCCCCTCCGGCCTAAACTTTGCCGAAGAGGTCA
CTGATTGAAATCAAGAACTTTATTGGGTGTTTACTGATGTAGCGGGCGCATTAGAACCGGCGAGACAAACCCCGCG
CCCAGCTTCTTTCACCCGGGCGGCAATCAGGGCCGGGCGACGTCAGGTCTGGCTCAGGCTTGGGGTCGTGCGACGG
CATCACTGGGGTTGGCCGGGGACGAACAGCAGGCGGCACCTGCGCGTCGGTTTCCGGTACTGCCAGATCAGGCACC
CGATCAGGTTTTACCTGGCGGTCGGTCAACGACACTGTTGGCCTGTGCACCCAGTCGGGCCGGGCTTGCGGGTCGA
TCGTCGGGAGCGGCCTCGGATGCGGCCGCACACGAGTGTCGACCTTTGGCACAGCAGGATGAGAGTCCTCCGGGCA
GTGGCAATTGTTCACATTGAGATCGACGTTTACCCGCACAACCGTGTCAGACGATGCCTTGGAATCGGTACCAGGA
ACCCTCGGTGTAATTGTGATATTGGGTAGCACCACGGGGTGCTTGCCCACATCCGAATTCACTTTCGTATCTGGTA
TCACGGGCTTGCCATCGGTCTGCGCCTTCTTCTCCTGTGATTTTTCAGCCTCGAGCTGCACCTTGGCGTCATTGCC
GAGTATCAGGGGCTTGCTGTCGATCTGTGCCTTCTTCTCCGGTTGTTTGCCAGCGTCAACCTGCACTTTGCTGTCC
AGGCCTGTAGTTACCGTTTTATCCCCCGGTGTAGGTTCGGGTAAGGGCTTTTTGCCAGACAACAGATCGCGCATCG
CCTTGAAAACAAGCTCAAACATGTCGAACAGTTTTGCAAGAGAATCAACACTCACAACAATGTTGTCGCGTCCGAC
GCCTGTAGCCCGGTTATATTCCGGCAACACATTCAACTTATTACCCAGCAACTTGCTCGCATCGTTCCCCCCAGGT
GGAGTGGGTACCGGTGTCGGCAAATCAAACTGGCGATTAGCGACATTCGCGCCTGGAGGATTGTTTATAGATAGAT
AAGACATGCGTCATTCCTCTTCTCAAGACAGCACAGGGCGCTCAGGCCCCCATAACGAACAGTGTTTACTTATGAC
TTGAAACGGCAGCCACCGATTCGAAGTCCCTAGTTTCGTGGAGTAAAAAACGTTCGAGTTCCAGAAATCGCGGAAA
ACATAAGGACATCAAAAGACAATTTCGGACTTTTACCTATAGACTTCTGCAAAGGCCTGCCTTACAAGGCCGTCCT
TTGAAAAAAACAGGTCATACTCGCTAATAAACTGCGCCATGGAAAAAGAACGTCACGATTGTTTGAACTGTCTTTG
CATCGCCCATGATATTTCCCGCGCGCTTAATACTGCTGCCACGCCAAGCAAAATCAAAAAACAAAAAACCGCTGCA
ACACTCACAGTTGACTAGAACTACAATAAAGTTACACACACCTACTCTCAAAATGCTCAAAATATACAATCAAACC
AAACATGAATTTAACTGACGGCATCTGAATTACTTGCCCGACCAACAGAGTCTTAAACAAAAAAAGGGCGCCAGAG
GCGCCCTTTTCAGAGGAGGTGAAAAACTTAAATACATGGACTCAATGTTGCAAAGCTGGCTTCTGCGCGCCGTTGA
TTGGAATGCGCTTGGCCTTGGCTTCTTCCGGCACCACCCGCAACAGATCGATACTCAACAAGCCGTTGCTCAGGTC
CGCCGCCTTGATTTCAATGTGGTCCGCCAAGCGGAACGACAGTTTGAAGGCGCGCTGGGCAATACCTTGGTGCAGA
TAGGTAACGCTCTCAGCCTTGGTGTCGCGCTTGCCGCCACTGACGGTCAATACGCCTTTCTCCACCTGCAGCTCCA
GATCGTCTTCCTGGAAGCCGGCAGCCGCGATGACAATGCGGTACTCGTCTTCACCATGCTTCTCGACGTTGTAGGG
TGGGTAGCTGCTGCCCGGCTCATTGCGCAGTGCGGTCTCGAACAGATCGTTGAAACGATCAAAACCGACGGAGGAG
CGGAACAGCGGCGCCAGGGAAAATGCAGTACTCATGGTCAAATCTCCTGAAATATCAGCAAGGTTCTTTATCTCCA
CGACCCGAATTCGGCATCGTGTAATCCCTAGATAAGGACCGCCAATTACATTTCAAGAGTGGCTGTAGGAAAAATT
TCAGGCCACTTGCGGATGGGGCATTCCGAGTAGACGGCTAACCTGCTGAAGATCGGTTTCCCTGCGCAACGCGGTA
AACAGCTCCACGGCTTGCGGGTAATTTCGCGTCAGCATGGCCAGCCACTGCTTCAGTCGGCCCGGCGCCTGGCGTT
CCGTCAATTGCGCCACGGACTGCCTCCAGAACTCCTGGAGCATGGGCTGCATGTCCTCCCAGGTCATCTCCACCAC
GTCCTGACCGGCGCGCGCGGCGGCGATTTGCCGGGCCAGGTCGGGACGCGCTACCAGGCCGCGACCGAGCATGATG
TCTTCGACCCCGCTGATCTCACGGCAGCGCCGCCAATCTTCGACACACCAGATATCACCATTGGCAAATACCGGGA
CCTTGACCACGTCCTGCACCCGCGGGATCCACTCCCAGTGTGCAGGCGGCTTGTAGCCATCGGCCTTGGTCCGCGC
ATGCACCACAATATGCGCGGCGCCGCCTTCAGCCAGGGCCGTGGCGCACACCAGCGCGCCGTCCGGGCTGTCAAAG
CCCAGGCGCATCTTGGCGGTCACTGGGATATGCGCAGGCACGGCACGGCGCACGTGTTCGACGATCTGGTTGAGCA
GCTCCGGCTCCTTGAGCAGCACCGCGCCACCCCGGGACTTGTTGACGGTCTTGGCCGGGCAGCCGAAGTTCAGGTC
GATAACCTCAGAGCCCAACTCACAGGCCAGTGCGGCGTTTTCCGCCAGGCACACCGGGTCGGAACCGAGCAACTGC
ACGCGCAACGGCACGCCCGCAGCGGTCTTGGCACCGTGCAGCAGTTCTGGGGCAAGCTTGTGAAAATAGGCCGGGG

TCAGCAGGCGGTCGTTGACCCGAATAAATTCGGTCACGCACCAATCGATACCACCCACCTGGGTCAACACGTCCCG
CAGGATGTTGTCGACCAACCCCTCCATGGGCGCCAAAGCAATTTGCATGGAAAACACTCAACGAAAAACGTGGCGC
AGTTTACTGGGTTTCCCACAGCAACCGTTAACCCTACCGCCCCCTGTAGGAGCGAGCTTGCTCGCGAAAAACGCCC
AGACGACACGGTAAAACTGATCGACCGCGTTATCGTTGACGTTCTTCGCGAGCAAGCTCGCTCCTACATGGACAAT
GCCGTAAGCGCGACGCCATAGCCCTCGATAAACTCCGCCGGCATGCGCTTGGGCTTGCCACTGGAGAGCTCGATGC
AGACAAAGGTGGTTTGCGCACGCAACAAGGTGGTGCCATCGCTGGGGCGCATCAGTTGGAAGCGTCGGGTCATTTT
CAGGCGCTGGTCCCAATCGACGATCCAGGTTGCCAGCTGCAGCTCATCGCCTTCGTAGCCGGCCGCCAGGTAATCG
ATTTCGTGGCGCACCACCGCCATGGCCCGGTCCAGGCGCCGGTACTCGGTGAGGTCCAGGCCCAGGCGCTGGGAAT
GGCGCCAGGCACAACGCTCCAGCCAAGACACGTAGACGGCATTGTTGGCATGCCCCAGGCCGTCGATGTCCTCAGG
GGCCACCTGCAGATCGATGATAAACGGCTTTGCCAAGTCCCAGCCCATGCCCTGCTCCCGGTCGATTAATGTCGGC
GGGGAGCAGTGTAACCTATCGCCTGGCTCAAGCGGTTTGCCGGGCCGGCTCACGGCAGCCGGCGAGCAGCGCCAGC
ACACCTTCGACCACCCTTGGGTCGGCCAGGACTTTTTGATGGCCGCCCTGCTCCAGGCGTAAAAGGCGGCTGTCGA
ACCAGGCCTCGTGGATCATCTGCGAAGCTTTGACCGGGACGAAGGTATCGTCCTCGGCATGCACGATCAGCCCGGG
GATATTCATTTGATAGTGGGCCACATCCAGGTGCTTGAGCGGAATGCCCAGGGAGTACTCGACCTCCTGGATAAAC
GCCGCACGCGCCCGCGCCGGCATGCCGACTACTCCAGCGAAGCCGCGCAATACATCGAGCAAGCGTGAAGGGGCAG
CAATGCTCACCAGCGCATTGGTGCGCAGGCCCAACTGGATGGCCAGCATCGCACTGGCGCCACCCATGGAATGGCC
AACCACCGCGTGCAAGGGCGGCAATTCAGCGGCAGCCTCCAGCATCGCCCGGGCAAACAGCACCACATGGGCTTCG
CGGCCCGGCGAACGACCATGGGCCGGGCCATCGAGGGCAATCACCGAGTAACCCGCGCCCACCAGTGCGTCGATCA
GGCTGGCGAACTGGGTCGGGCGCCCTTCCCAGCCATGCATCATCAGCACCGCTGGGCCCTGGCCCCAGCGCAGGGC
CGACAGGCCAAAGCGCAAGGTGACGCGCTCGGCCTGCGCCAGCAATGGCAACTCCCAATCACGCGGCGGCAAGTCT
CGTGGCGTCATGAAGGCACGCCGCATCTGGTTGGCGATGGTTTGCGGCGCCAGCCTGCCCACGGTGCCGTTGACTC
GGCGAATCCAGCTCAAGGTGCTCATCCACTCACTCCCCAGGCTCACAAGCCTTGTTCAGCGCACAGCCGACTTGGA
GGCGCGTAATACACGATCCGATAATTCGCCCGGCCCCAGAGCCCTGGCCAATGCCAGGCCACCAATCATCAAGGCC
ATGTCGGCCAGGGCCTTGTCGGTGTCTTCCGGGCTGGCGGCCAGCTGTGCGGCCATCAACTCGACGTGCTGGTTCA
ATACATCGCGAAACTCATCCGGCAGCCGGCTCATCTCGCCGACCGTGGCCGGGATCGGGCAGGCCTGGGCGGTAGA
ATCGCGGTGTTTGCGCGAGAGGTAGAACGCGGCTACCAGGGCTCGGCGCTCTTCGCCTGTCAGGCTGGGGTCCATA
TCGTCGATAGATGCCCGACGCCGCGCCAAGAGCTGCTCGAACGCCTCCAGCATCAGAGCGTCCTTGCTTTCAAAGT
GGGCGTAGAAGCCACCCACGGTCAGCCCCGCGGCGCCCATGACTTCACCCACACTCGGTTCGGCCGGGCCACGCTG
GACCAGCGCGGAACTGGCGGCTTGCAGAATACGTTCACGGGTTTGCGCTTTTTTATCGCTCATGACGGCCTCCGAA
TATTACGGTTAAAATATTATCCGCATAATACTTTTTAGCAAGCGGTGAATTCGACCGCTGGTCAGAAGAGGTTTTT
TGAAGAGGGAGGGAGAAAACCCAAACGCCAGACAAACAAAAGGGCCATTCAATAATTGAATGACCCTTAAAAATCC
CGCAGAGCGGGTAATCGTGGCGTCCCCTAGGGGACTCGAACCCCTGTTACCGCCGTGAAAGGGCGGTGTCCTAGGC
CACTAGACGAANNNNNNNNNNNCGAAGAGTGGGTCAATGTCGACAAACCGAGATCAATAATTTACACGCCGGCCTGG
ATTAATATTCTGTATGACTCTGCGCGTAGCAGCGAGGAACGGATTTATCGTCGGCGCCACTCATCTATCGACTCGC
CCAGTTAAAGCCCTCTCGGCACTGCTACAGGAGCTTATCTGCGGAAAGCATCCAATACCGTAACAAACTTGAGTAC
ACCGGCGTCGCCAGACTGAAACAGGCTATTCACCTATCAGGCGACACCGGCAAACTTTATAACTAACGAGATCAAG
AAATGGCAATTACAGCGCCCAGTACGACCCAGCCCCTGCTGCAATCAAATGAATCCCCACAAACATCTGCAGCAGA
ATTATCAGCCCAGGCTAAACCTTACGAGACTATGACTAACAAAGAACTCGTCGGCCAACTATTGACTCATTCCAAG
GCTATTGATCTGTATAAGGAAGACAGGTTCTCAACTCATACCCTAAAGCAATTATCACAAGGGACTCAAGCAAACG
GCACAGACGCTACACCGGAGCAGAAGGATCTTGCGAAAGAACTATTGAAACGCCCGGAACTGCTTGACTACCTCGA
CCTATCTCCCAATGGCGGTGTCGAAGATGGACAGATAACCTGGGACGCCGTCGCAGCCATGGCTGGAGAACCTTCT
CGCCTCAGCGATCGGGGACTGTTAATGGAGGCCAAAAGATATCACCAGGAGTTCGATTCAACCGGCAATGGCTACG
TGAACTTTAAAGAGTTGAAAGAAGCAGCCGGATTATTACCCAGTGATCGAACGTTTTCCGAGGGGGCACGACAAAC
TGCCCGCGAACTACTGAAGCGCCCTAGCCTTCTCAAGACGTTGGATATCGGTGTCGGTTTCTTAGGGCTGCCTGGC
AAACAAGACGAAAGGTATGATGTCACCAATCTAGACCATACAATCGCTAAGAGCTCACGCACCCATAATCATCCTT
TTAGAAGCAAGACGGACTGATATTGACCTTGCCGTGCAGCGAGCAAGGGGCGCGAACAGCGGCTTTGCTCACTGCT
TTGGATTTCAATTCGTCAGGTGTCCGCGCCTGCGCCACCCTTGATTATGTTGTCCGCGTGGTTGCCGATGATCACG
TCATTGCCCGAGCCCAACCAACCATTGCGCGAAGACCCGCTGCAGTTACATATAGGCTCAGAAGATATTTTTGTAT
TACGGCAACAGATCAATACCTGTTCATGTTTTTTGATATAGATCGGCATGAGTTTTCTCAGCGCGCGCACTCACTC
AATTGACGGGATCCCCCTGACCATGCTCTCCTACACATCTTGTTTCAGGTGCCCTTCACAGGGTGAAACGGGAAAC
CGGTGACCCATGCGTGCATGACAGTCCGGTGCTGCCCCCGCAACGGTAGGCGAGCGAAGTGTCAGATCCACTGTGC
CTGTACGGCATGGGAAGGTGATGCTTTTAGGCCCAGAGCCCCTCGCAAGCCCGGAGACCGGCCCGAACAATCAGAT
AACAAACCCGCGGTGGGCGGGCGCTGTTCAGAACCCTGCGTGCCCGGCTCGCGGGGTTTTCATGCGCTCGTATCAC
CCTGCCACTCGAAAGGGACGTGCCATGTCGATCATCAGCAGCACCTCACACACCGCCAGCAGCTCCGCCACCTTGA
GCCAGCGCCTGACCGCAGCCATTGGCGCGTCGATCCTGGGTGCGTGCCTGGTGTATTTCGCCGGGTTCTCCCATAT
CGAGGCCGTACACAACGCCGCCCACGATACGCGCCACAGCGCTGCCTTCCCGTGCCATTGAGACCTGCCGCCATGA
TCAAGCGAATTGCGCAAACCGCAGGTTTCAGCGGCCTGCTGGCCGCCCTGCTGCTGACGCTGCTGCAGAGTTTCTG

GGTAGCCCCGCTGATTCTGCAGGCGGAAACCTTCGAAAACGCCCCGGCCGCAACCGAGGTACATGAACATGCCTCA
GGTGGCGCAGCCCACAGTCACGACGCCGAGGCCTGGGAACCGGAAGACGGCTGGCAACGCGTGCTGTCCACCACCG
GCGGCAACCTGGTGGTCGCTGTCGGTTTTGCGCTGATGCTCGCGGGCCTATATACCCTGCGCGCTCCGACCCGTAC
TGCCCAGGGCTTGCTCTGGGGCCTGGCCGGCTACGCCACCTTTGTGTTGGCGCCGACCCTGGGCCTGCCACCGGAA
CTGCCCGGCACCGCAGCGGCAGACCTGGCGCAACGGCAGATCTGGTGGATTGGTACCGCAGCCTCCACGGCTGCCG
GCATTGCGCTGATCGTGTTTGGCCGCAACTGGCTGTTGAAGATCCTCGGCGTGGCGATCCTGGCGGTGCCCCATGT
GGTTGGCGCGCCACAGCCGGAAGTTCACTCGATGCTGGCTCCCGAAGCACTGGAAGCACAATTCAAGATCGCCTCG
CAGCTGACCAACGTCGCGTTCTGGCTGGCGCTGGGGCTGATTAGCGCCTGGTTGTTTCGCCGCAATCGTGACGATC
AATACGCCGCATGATTTTGACGGTTGGCCTGGGTTGCCAGCGTGGCTGTGAGGTTGAAGCCTTGATGGAACTGTTC
AACAGTGCGCTGGCCGAGGCCGGTATCGACCCCGGCCAAATCTGCGCTTTGGCAAGTATTGCTCACAAGTGCGATG
AGCCAGGCATAGTCGCCCTGGCCAACGCGTTGAACCTGCCATTGCAGTGCTTTGATGCACAACAACTGCAGGTCTA
CGAAGGACGATTGAGCCATAAGTCGGATGTGGTTTTTGCCCATACGGGGTGCTACGGCATCGCCGAAAGTGCCGCT
TTGGCATTGGCAGAACAGCTTGCCAAGGCCCCTGCCCACCTGCTGATCACCCGCAAAAAAGCCCCCAGAGCCACGT
TTGCATTGGCCTGCGCTGGCTAAAGTCTGATAATTGCCCTTCTCGATCATGAGCCGTACTCATGCGCGCCCGCTTT
TGCACAGGATTTTTCCATGACCGTCTTCTTCATCGGCGCTGGCCCCGGCGACCCGGAACTGATTACCGTCAAAGGC
CAGCGGCTGATCCGCGATTGCCCGGTAATCGTCTACGCTGGCTCCCTGGTGCCGGCTGCCGTGCTTGAGGGGCACC
GGGCCGAACAGGTGGTCAACAGCGCCGAACTGAACCTGGAGCAGATCATTGGCTTGATCAAGGCCGCCCATGAAAA
AGGCCAGGATGTGGCGCGGGTACATTCTGGTGATCCGAGCCTGTATGGGGCGATTGGCGAGCAGATCCGCCATTTG
CGCGAGCTGGGCATCCCCTTCCAGATCATCCCCGGTGTTACCGCAGTCGCCGCTTGTGCCGCGCTGCTGGAGACCG
AGTTGACCCTGCCGGATATCGCCCAGAGCGTGATCCTGACTCGCTATGCCGATAAAACCACGATGCCGGCCGGTGA
AGCCTTCGCCAGCCTGGCCAGCCATGGCACGACCATGGCGGTGCATTTGGGGGTCAATCATCTGCAGAAAATCGTC
GCCGAGTTACTGCCCCATTATGGTGCCGACTGTCCGATTGCCGTGGTGCATCGGGCGACGTGGCCGGATCAGGATT
GGGCCATTGGCACCTTGGCGGATATTGTTGAAAAGGTTGCTGCGAAGGGGTTCAGACGGACTGCGCTGATTGTGGT
GGGCCGGGTGTTGGCCAGTGACAGCTTTAGCGAGTCCTCGCTGTATCGCGCAAGCCATGCCCACCTCTACCGGCCC
TGACACCACGCAGACACTGTGTGGGAGCTGGCTTGCCTGCGATAGCGGTAAAACAGTCGATAGATAAGTCGCCTGG
CGCACGGTCATCGCAGGCAAGCCAGCTCCCACAGTTGATTCTCTTTGTTAGATAGCTCGTGTAACTCATTAATTTA
AATCAATAAAAAACGGCGCTCACGGGCGCCGTTTTTTGTTTGCAGCGAACGCCTTAGTAGTAGGCGTTTTCTTTCT
GCGTATGGTCGGTCACATCACGTACGCCCTTGAGCTCGGGGATGCGCTCGAGCAACGTACGCTCGATACCTTCACG
CAAGGTCACGTCAGCCTGGCCACAGCCCTGGCAGCCGCCACCGAACTTGAGCACGGCGATGCCGTCATCCACCACG
TCGATCAGGCTGACCTGGCCGCCATGGCTGGCCAGGCCCGGGTTGATCTCGGTCTGCAGGTAATAATTGATGCGCT
CGTTGACCGGGCTGTCGGCATTGACGTTCGGCACCTTGGCGTTGGGGGCCTTGATGGTCAGTTGGCCGCCCATGCG
ATCAGTGGCGTAGTCGACCACGGCATCGTCAAGGAACGCTTCGCTGAAGGCATCGATGTACGCAGTGAAGCTCTTG
AGCCCCAGCGCGGTATCTTCAGGCTTCTCTTCCCCCGGCTTGCAGTAGGCAATGCACGTCTCTGCGTACTGGGTGC
CCGGCTGGGTGATGAAGACGCGGATGCCGATGCCCGGGGTGTTCTGCTTGGACAGCAGATCGGCCAGGTAATCGTG
GGCGGCGTCGGTAATGGTTATGGCAGTCATGGGAACTCCTCACAGGCTTGCCGGCAGTTTACGCCAATCGCCCCGG
CGGCACAAAGTCCTAGTATTTTTGTCAGGCAAATCCACATCTACAGGTTCTCGTAGCGGTTCATATCCAACACACC
CTCTTCCACCGGCGTGGTTTCGTGCAGGTACTGCGACAGATCGTGGAAGTACTCCCAGAACTGCGGGTGACTGCGG
CGGATGCCCCAGCGCTCGACAATCCGCTCAAAACGCTTGGTATCCCTGGCCAACTCCATGTCCTCGACGAACTCAG
GCACATCCTCGGCCGGCACGTTGAAGATGAAGTTGGGGTAGCTGCTGAGTACGCCCGGGTAGATCGTCAGGGTATC
CAGGCCCGGCTGGTAGCGATAGGCCTCGCCCAGCATAAACGCCACGTTGCTGTGGGCACGGTTGCGCAGCAGGCTG
TAGACCTCGCGCTTGCCACTGCGGGTCTGGATGCGCAGCAAGGTGGCTTCCGGCAATTGCTCGATCACCTTGAGCC
CGGCCGCCGGCCTGGAAGTCAGGCGGCTGAGGGTCTGTTCGGCATCGCGCAGTTGCGGATCGATACCGGGACGCGA
GCAATAGGCGCTGATACAGCGGTTGATCGGGTCGGGGCTGGCGTTCAAATTGCCATAACGGGCGAGCAACTGGTTG
GCAAAGTCGCGTTTCGGGTCCTTTTCATCCAGCTTCAGGCCCGTTGGCTTGTCGTTGTCGATGGCTTCATAGTCCA
GCCACAGCTTGACCTTGCCGCTGTTCTGGTACCAGTCATCCAGGTATCCATCGCGCGAATCCGCCGGCATCAAGCG
CAGGAAGTTCTGTTCGGCGCCATTGCGGATCAGGTCGAAATACAGGCGGGTCTGGGCCTGGTGCGAGACATTGCCG
AATACGTCGAAGTTGACCGCCAACTGGTAATAGGTGCGCTCCAGCAACGGGAAATCGAACAACCACATCGTCTGCG
GCACTTCGCCAATCAAGCCCTTGGTCACCGAGGCGCTATCGAAATGGCGGAAGATGCTCAGCAAGGCGTTGTCATT
GCCTGCCCACAGGCTCGGCCAGCCAGGCGCCGGTAAGTCAGCATAGTTGTCGCGGCGCAGGGCCTCGTATTGATTG
CGTTTGTCGCGGTAGGCCAGCCACAGGCTCAACACACTGCCCACGTCATCGTTCTGCCCCGGCATGGCAAGCAACG
GCGTGGCCTGGCCGCGATAACGGGCATCGGTGATGTACAGGTCGTGGTCCGGATCCTGGAACAGGGTCCAGAAGTT
GTCGCGGATCACATCCGTGGCAATCTGCCCCCGGCACACCGGCCCACGGATAAAGGTGCGCACGAAGTATTCGGCG
TTATCGAGCATGAACTGATAGCGCGCCTTGGCCGGGATCGCCTCGAAGGTTTCGAACGGGTTGGCCCGACGGCCAG
GGCCGTAGCCCGGCAGCGCCATGGCCTGCCAGTCGCCGCTGTAGAACAGCGACTTGATCCGCGCCATCTTCGCCGC
GCTGAACGGGTAGGTAATGTGGGTCTTGTGCACGATCACCCCTTGTACCGGCCACAGGCGGTAGTACACCTGGGTG
CCCGGGTCGTCATTGGGGCGACGGGTGTTGATCAGGTCGATCGGCTGGCCGCTGGGCGTACGCGAGCGCACCCATT
GGAAGTAATGCCCCGGCTCACCGCCTTCGAAATAGATGTGGGCAAGGAACAAATGCTCGAACAACCAGCGCGCCAC

CAGGCTTTCGCGGGCACCGGGCTGGTTCAGCAGGTTTTCCCATTGCTGCACCTGCAAGGCTTCCTGGGCATTGGGC
GCCAGGCCTTGCTCATCAATCGGCGCGCCGGACGCGAGCCAGCGCTGCAAGGTCTGGTATTGCTGGTCGGTCAGCC
CAGTGACGGCCAGGGGCATGCCTTCCTTGGGATGCGCGCCTGCGTAGGCGTCGAACTCCCCCGGCAGCGGGCACAT
GTTCTGGCGGTTCAGGCCCAGCACAATGTCATCGGGCAACTTGGCATTGGGTGCCAAGGGGGTGTTATGGCCCAGC
TCCAGCATGCGCGCCATCAACGCAGCCTGGGAGCCCTGGGCATCCAGTACGGAGGCAAAGCCCTGGCGTTGCCAGG
CGGCCTTGCCAAAGGCGTCATAAAACAACCGGGTCGGCTGCGACGCCTGGCTGCGCTCGCCGTCATACACCGGCGC
CTTGCTTGCGCCGCGTGCCGCGCCTTCGCCACTGCCCAGGTTGAGCTGGCAGGCAGAGTCATAGCAGGCATGGCAA
GCCACGCATTTCTCGGTGAAGATCGGTTGAATGTCGCGGGTATAGGAAATCGCCGGGGCGGGTCCGTTGGCCTGTG
CGATACAGGCGACCAGCGCCAAGGCGGCGCTGGTAATGAGGCGAAGTGACATGTATCCGGTCCCGATTCAATGCGT
GCGCTGAAAAATTGCCGCGATTCTACCGATGTCGGCACCCCATCAACATGAGCGATATTCATGCAAAACCAGGGCA
TGCTCTAAAAGCGCACAGGTTTGCTATGATCCACGCCCTCCGTAATGCCTGATTAGAGTAGTCCCATGTCCGATCG
TAGCACTCGCCTGCAAGCCCTCCAGCAAGCCCTCAAGGAACGCATCCTGATCCTCGATGGCGGCATGGGCACGATG
ATCCAGAGCTACAAGCTGGAGGAAGAGGACTACCGCGGCAAACGCTTTGCCGACTGGCCAAGTGACGTCAAGGGCA
ATAACGACCTGTTGATCCTGACCCGCCCGGACGTAATCGGCGGGATCGAGAAGGCGTATCTGGATGCCGGCGCCGA
TATCCTCGAAACCAACACCTTCAACGCCACCCAGGTGTCCCAGGCCGACTACGGCATGGAGGCGCTGGCCTATGAG
CTGAACCTGGAAGGCGCGCGCCTGGCGCGTAAAGTCGCCGACGCCAAGACCCTCGAGACCCCGGACAAACCGCGCT
TTGTCGCCGGCGTGCTCGGCCCGACCAGCCGTACCTGCTCGCTGTCGCCCGACGTGAACAACCCCGGCTACCGCAA
CGTCACCTTCGATGAACTGGTGGAGAACTACACCGAGGCCACCAAAGGCCTGATCGAAGGCGGTGCGGACATGATC
CTCATCGAAACCATCTTCGACACCCTCAACGCCAAGGCTGCAATCTTCGCCGTACAAGGCGTATATGAAGAGCTTG
GCGTCGAGCTGCCGATCATGATTTCCGGGACCATCACCGACGCCTCCGGGCGCACCCTGTCGGGCCAGACCACCGA
GGCGTTCTGGAACTCCATCGCCCACGCCAAACCGATTTCCGTGGGTTTGAACTGCGCCCTGGGCGCCAGTGAGTTG
CGGCCGTACCTCGAAGAGCTGTCGAACAAGGCCGACACCTACTTGTCCGCGCACCCCAACGCCGGCCTGCCCAACG
AATTCGGCGAGTACGACGAGTTGCCCTCGGAAACCGCCAAGGTCATCGAAGAGTTTGCCCAGAGTGGCTTCCTCAA
TATCGTCGGCGGCTGCTGCGGCACCACGCCGGGGCATATCGAAGCCATCGCCAAGGCCGTGGCCGGCTACGCGCCG
CGCCCGATCCCGGACATCCCCAAGGCCTGCCGCCTGTCGGGCCTGGAGCCGTTTACCATCGATCGCCAGTCGCTGT
TCGTCAACGTCGGCGAGCGCACCAACATCACCGGCTCGGCCAAGTTCGCCCGGTTGATCCGCGAAGACAACTACAC
CGAAGCCCTGGAAGTTGCCCTGCAGCAGGTGGAAGCCGGCGCCCAGGTGATCGATATCAACATGGACGAGGGCATG
CTCGATTCGAAGAAGGCCATGGTGACCTTCCTCAATCTGATTGCCGGCGAACCGGATATCTCGCGGGTACCGATCA
TGATCGACTCCTCCAAGTGGGAAGTGATCGAGGCCGGCCTCAAATGCATCCAGGGCAAGGGCATCGTCAACTCCAT
CAGCATGAAGGAAGGCGTCGAGCAGTTCATCCATCACGCCAAGCTGTGCAAGCGCTACGGCGCGGCGGTGGTGGTG
ATGGCGTTCGACGAAGCCGGCCAGGCCGACACCGAGGCGCGCAAGAAAGAAATCTGCAAGCGCTCCTACGACATCC
TGGTCAATGAGGTGGGCTTCCCGCCGCAAGACATTATCTTCGACCCGAACATCTTCGCCGTGGCCACCGGTATCGA
AGAGCACAACAACTACGCCGTGGACTTTATCAACGCCTGTGCCTATATCCGCGATGAGCTGCCCTACGCGCTGACC
TCTGGCGGCGTGTCCAACGTGTCGTTCTCGTTCCGTGGCAACAACCCGGTGCGCGAGGCGATCCACTCGGTGTTCC
TGCTGTATGCGATCCGCAACGGCTTGACCATGGGCATCGTCAACGCCGGCCAGTTGGAGATCTACGACCAGATCCC
CGCCGAGTTGCGCGACGCCGTCGAAGACGTGGTACTCAACCGCACGCCGGAAGGCACCGATGCCCTCCTCGCTATC
GCCGACAAGTACAAGGGGGATGGCAGCGTCAAGGAAGCCGAGACCGAGGAATGGCGTGGCTGGGACGTCAACAAGC
GCCTGGAACACGCGCTGGTCAAGGGCATCACTACCCATATCGTCGAAGACACTGAAGAGTCGCGGCTGTCCTTCGC
GCGCCCGATCGAGGTGATTGAGGGCCCGCTGATGTCCGGCATGAACATCGTCGGCGACCTGTTCGGCGCCGGCAAA
ATGTTCCTGCCCCAGGTGGTCAAGTCCGCCCGGGTGATGAAGCAGGCCGTGGCTCACCTGATCCCGTTCATTGAAC
TTGAAAAGGGCGACAAGCCGGAAGCCAAGGGCAAGATCCTCATGGCCACGGTCAAGGGTGACGTGCACGATATCGG
CAAGAACATTGTCGGTGTGGTACTCGGTTGCAACGGCTACGACATCGTCGACCTGGGCGTGATGGTACCGGCGGAA
AAAATCCTGCAGGTGGCCAAGGAGCAGAAGTGCGACATCATCGGCCTGTCCGGGCTGATCACGCCGTCCCTGGATG
AAATGGTGCACGTGGCCCGCGAAATGCAGCGCCAGGACTTCCACCTGCCATTGATGATCGGCGGCGCCACCACCTC
CAAAGCGCATACGGCGGTGAAGATCGAACCCAAGTACAGCAACGATGCGGTGATCTACGTGACCGACGCCTCGCGT
GCGGTGGGCGTGGCCACACAGCTGCTGTCCAAGGAACTCAAGGCCGGCTTTGTCGAGAAGACCCGCCTGGAATACA
TCGACGTGCGTGAGCGCACTTCGAACCGCAGCGCCCGCACCGAGCGCCTGAGCTACGCGGCGGCCATCGCCAAGAA
GCCCCAGTTCGACTGGGGCACCTACACCCCGGTCAAGCCGACGTTTACCGGGGCCAAGGTGCTGGACAATATCGAC
CTCAAGGTCCTGGCCGAATACATCGACTGGACGCCGTTCTTTATTTCCTGGGATCTGGCCGGCAAATTCCCGCGCA
TCCTCACCGATGAAGTGGTGGGTGAAGCCGCCACCGCGCTGTACGCCGACGCCCAGGAGATGCTCAAGAAGCTGAT
CGACGAAAAACTCATCAGCGCCCGCGCGGTGTTCGGCTTCTGGCCGACCAACCAGGTGCAGGACGATGACCTGGAA
GTGTACGGCGACGATGGCCAGCCGATTGCCAAGTTGCACCACCTGCGCCAGCAGATCATCAAGACCGACGGCAAGC
CGAACTTCTCCCTGGCCGACTTTGTGGCGCCCAAGGACAGCGGCGTGACCGACTACATCGGCGGGTTTATTACCAC
CGCCGGCATCGGCGCCGAAGAGGTGGCCAAGGCCTACCAGGATGCGGGCGACGACTACAACTCGATCATGGTCAAG
GCCCTGGCCGACCGCCTGGCCGAGGCCTGCGCCGAGTGGCTGCACCAGCAGGTGCGTAAGGACTACTGGGGTTACG
CCAAGGACGAAGCGCTGGATAACGAGGCTCTGATCAAAGAGCAGTACAGCGGCATCCGCCCTGCCCCAGGCTACCC
GGCGTGCCCGGATCACACCGAGAAGGCCCAATTGTTCCAGTTGCTGGACCCTGAGGCCCGCGAAATGCACGCTGGG

CGCAGCGGTGTGTTCCTCACCGAGCACTACGCGATGTTCCCGGCAGCGGCGGTCAGCGGCTGGTACTTCGCCCACC
CGCAGGCGCAGTACTTCGCCCGTGGGCAAGATCGACAAGGACCAGGTGACGAGCTACACCGCACGCAAAGCGCAAGA
CCTGAGCGTGACCGAACGCTGGCTGGCGCCGAACCTGGGATACGACAACTAAGGGCTGCATGGGTCCCTCTGGCTA
GAGGGACAAGCCCCCTCGGTGTATCTGGCAGGACTTGGTGAGGCTATTGGGGCGGCTTTGCCGCCCAGCGCGGGGC
AAGCCCGCTCGCCACAAGAAAAGCCAGTGTTGGCAGCGAGNNNNNNNNNNTTTGGCGGCTACTTCGCCTTCTTCTT
CTTTGTCGAATTCGTCCCAGCTCAGCATGACGTGTCGTCTCCTGCGTGAGGGCCAATGGTGCCCGTGTGAAACCGG
ATGGTTGGGTGTTCACACGGCCCAAGGGCCGCGGTGGATCTTTAAGGAATCGTTTGTTGCAACAGCTCGCGCAGGC
ATTAAACGGAATAGGGCTACGGGTACTCTGCATGATGCCTGAGGGGCGGAGCGACGGTGTCGTACTCCTGCGGGGC
CTCAGGTCTGGCTCGTTACCCCCGTGTAAAGGGATATTGCAGGCCCGATTTACCCGCGCATTATAGGGAAAAATTT
GGCTTTGTGTTGCGGCGGATAGCCTCAGCCAAGGGCCAAAATGAGGGTATTTCCGGCCGGGGTGCGGGTTTACAAG
GGTTTGCCGGGGTAGGATTTTTTTGACGGGCGGTGAGGTATTTTCGGAGCAGGGGGCAAGGCGAGGATTTTGCGGG
TGTTTCTTTGCCGCAAGGACAATTCCTTGCGGCGAACATTGCCAGGATAGGTTTCAACTAACTAATTAATTATCCG
TTAGAGCAACCGTTACCCATGACTTTGTAATTAAGGATATGCCGCTGGCCTTGGTGATCTTCATATTCCATGCGCG
CTGGAACAACTTCGCAGACATTCGGAATGGAACTCATGGATACCACCTTGGCAATATCCAGATCAGTGGCGTAGGT
GTATTCCTCAACAACGGCGGTGCTCTGGCTGGCTACGTCAGTCGGGATCTCGTCGGCCAGGGCTGCGGTAGCGCAC
AGGCTGGCAAGGGCCATAACTAATAAAGCTTTCATTTCCAATTTACCTTTTTACAGTCGAAGGGGGTCACGGGGCC
CTTGTGAGGCCACGTGTGTAACTTGAGTAGTGAGTTCGGATTAACGTGCCTTCGTGGGGGCTGCAACTTGGTTAAT
CACGGTGCCTTGTTGGCGAGGCCGATTTTAGGCGCCTGGGTTGCATTCATATACCCGTGCTTTTGATAAACACTAT
TGGTGGTTTTCGTAACAATCCACGACAGAGGTTTTTTGCAGTGTTGCAAAATGCGCTACAGGCGCCGAGCCAGAGC
GCTGCAAGGGGATATCAGGGTAATTTGTAGGGGCTTGTTACTACCATCGTCGAATGGTTCTATAGGCCTACCCCGG
CTAAAACAGGGGCATACAACAACAACTATTGTCACCGAGGTAAGAAAGATGAGTGCGGCTTCCCTGTACCCCGTTC
GCCCCGAAGTAGCAGCCAACACGCTGACCGACGAGGCGACCTACAAGGCCATGTACCAGCAGTCGGTGGTCAACCC
CGACGGCTTCTGGCGCGAGCAAGCCAAGCGTCTTGACTGGATCAAGCCTTTCACCACGGTCAAGCAGACCTCGTTC
GACGACCACCATGTGGATATCAAATGGTTCGCTGACGGCACCCTCAACGTTTCCTACAACTGCCTGGACCGTCACC
TCGCCGAGCGTGGCGACCAGGCAGCGATTATCTGGGAGGGCGACGACCCTTCCGAGAGCCGCACCATCACCTACCG
CGAACTGCATGAGCAGGTCTGCAAATTCGCCAACGCCCTGCGTGGCCAGGATGTGCACCGCGGCGACGTGGTGACC
ATCTATATGCCAATGATCCCCGAAGCCGTGGTCGCCATGCTGGCCTGTACCCGCATCGGCGCGATCCACTCGGTGG
TGTTTGGCGGTTTCTCCCCTGAAGCCCTGGCCGGTCGCATCATCGACTGCAAATCCAAAGTGGTGATCACTGCCGA
CGAAGGTATTCGTGCCGGCAAGAAGGTGCCGCTCAAGGCCAATGTCGACGACGCGCTGACCAACCCGGAAACCAGC
AGCATCCAGAAAGTCATCGTGTGCAAGCGCACCAATGGCCAGATCAAGTGGAACCAGCATCGCGACATCTGGTACG
AAGACCTGATGAAAGTGGCGGGCACCGTCTGCGCGCCAAAAGAGATGGGCGCCGAAGAAGCGCTGTTCATCCTTTA
TACCTCCGGTTCCACCGGCAAGCCCAAGGGCGTGCAGCACACCACCGGCGGCTACCTGTTGTACGCGGCCCTGACC
CACGAGCGCGTGTTCGACTACCGTCCGGGCGAAGTCTACTGGTGCACCGCCGACGTCGGCTGGGTCACTGGCCACA
CCTACATTGTCTACGGCCCGCTGGCCAATGGCGCGACCACGCTGCTGTTCGAAGGCGTGCCGAACTACCCGGATAT
CACGCGGGTCGGGAAAATTGTCGACAAGCACAAGGTCAATATCCTCTACACCGCGCCGACCGCGATCCGCGCGATG
ATGGCCTCTGGCACCGCCGCCTGCGAAGGCACCGATGGCAGCAGCCTGCGCCTGTTGGGTTCGGTGGGTGAGCCGA
TCAACCCGGAAGCGTGGGACTGGTACTACAAGAACGTCGGTCAATCCCGTTGCCCGATTGTCGATACCTGGTGGCA
GACCGAAACCGGCGCAACCCTGATGAGCCCATTGCCGGGCGCCCACGCGCTCAAGCCGGGTTCGGCGGCACGTCCG
TTCTTTGGCGTGGTGCCAGCGTTGGTGGACAACCTGGGCAACATCATCGAGGGCGCTGCCGAAGGCAACCTGGTAA
TCCTCGATTCGTGGCCGGGCCAAGCGCGTACGCTGTATGGCGACCATGACCGTTTCGTCGACACTTACTTCAAGAC
CTTCCGTGGCATGTACTTCACCGGCGACGGTGCGCGTCGCGATGAAGATGGCTACTGGTGGATCACCGGCCGCGTG
GATGACGTGCTCAACGTTTCCGGGCACCGCATGGGTACTGCCGAAATCGAAAGCGCCATGGTTGCCCACCCGAAAG
TCACCGAGGCCGCCGTGGTCGGCGTACCGCACGACATCAAGGGCCAGGGCATCTATGTGTATGTCACCCTGAAAAA
CGGTGAGGAGCCGACCGAAGCGCTGCGCCTGGAGCTGAAGAACTGGGTGCGCAAGGAGATCGGCCCGATTGCATCG
CCGGACGTGATCCAATGGGCACCTGGTTTGCCAAAGACCCGTTCGGGGAAAATCATGCGCCGTATCCTGCGCAAGA
TTGCCACGGCCGAGTATGACGGGTTGGGGGATATCTCCACCCTGGCCGATCCAAGCGTGGTGCAGCATTTGATTGA
TACCCACAAGACCATGAACGTCGCGTAAGCGGGTTTATGGGACCAAGCCCCATTCGGCGTAGGCCGGGTGGGGCTT
TTTTGTGGGAATTTGAATATGAAATAGGGTCGATGTGGGAGCTGGCTTGCCTGCGATAGCGGTGGGTCAGTTTCAA
ATGGGGTGGGGAAGCTGACAGACCGTTCTCAGCACCAAGCCAATAAATATCGGTTTATCTAGTAGGACGTTTCTGA
ATGTTACCCCGCGGTGCAAATGTGTAACCTTGCGCCCAAACATGAGGCAAAGTGCTACATCCATGCCCCGAAAACG
CCCGTTTTAGACGCCCGGTAAAATTAGATCAAACGCTACACTTGCCGGAATAGAAGGGTTTGCGAATAATAGGCCC
GCAATTTGCAGCGTTAATAGGTTTAATATCTTTTGCCTCTGCATAAAAGTCAGAGGCTGTCAATGTGTTGGGGTCG
CTTTCTCGGTGCTTCTGTAAATTGTTGTCGCATTGAGGAAATATCGGCTTCCGGCCTGTCGTTAGAATGCCGATCA
CTCGCTCGTCGTCGCCATGTTCAACTGGTGTAGGACGCAGCACCGCTATTCGGTTTCACTCAGTCGCATCGTGGGC
CATGGCTCATACTGCTGTTTTTGTCCTATACCGATGGAGTCCCAAGATGAAGAAACTCGTGCTGTTGGGCGCCCTG
GCGCTGTCCGTGCTGTCCATGCAGGCCTTCGCTGAAGAGAAGCCGCTGAAAATTGGTATCGAAGCAGCCTACCCTC
CGTTTGCCTCGAAGGCGCCGGATGGCAGCATCGTCGGTTTTGACTACGACATCGGCAACGCCCTGTGCGAAGAGAT

GAAGGTCAAGTGCACCTGGGTCGAGCAAGAATTCGACGGTCTGATCCCGGCCCTCAAAGTACGCAAGATCGACGCG
ATCCTGTCGTCCATGTCCATCACTGACGACCGCAAGAAGTCCGTGGACTTCACCAACCGCTACTACCTGACCCCAG
CCCGCTTGGTGATGAAGGACAGCGTAACCGTCAGCGACAGCCTGGTTGAGCTCAAGGACAAGAAGATCGGCGTACA
GCGTGGCTCGATCCACGACCGTTTCGCCAAGGAAGTCCTGGCGCCTAAAGGTGCGACCATCGTTCCTTACAGCACC
CAGAACGAAATCTACCTGGACGTGGCTGCCGGTCGCCTTGATGGCACCGTGGCCGATGCGACCCTGCTCGAAGACG
GTTTCCTGAAGACCGACTCTGGCAAAGGCTACGCGTTCGTAGGTCCTGCGTTCACCGACGCCAAATACTTCGGTGA
CGGCATCGGTATCGCAGTACGTAAAGGCGACAAGGCCAACCTGGACCGCATCAATGCTGCCATCGCCGCGATCCGC
GCCAATGGCAAGTACAAAGAAATCGAGAAAAAGTATTTCAACTTCGATATTTACGGCGCTGACGCCAAGTAAACCT
TCGCAGTTGTCTGTCCAGAATGGCGCAAGCACCAGGGTCTCTGAAGTTTGCGCCATTTTTTCATCCGCCTTTTCGA
GGACCTGAATCATGTTGAAAGGCTACGGGGCCGTTATCCTCGATGGCGCATGGTTGACGCTTCAGCTCGCCTTGTC
GTCCATGGCCTTGGCCATTGTTCTGGGTCTGATCGGGGTTGCATTGCGCCTGTCGCCGGTACGCTGGCTGGCTTGG
CTGGGTGACCTGTACTCCACGGTGATCCGCGGGATCCCCGACCTGGTGCTGATCCTGCTGATTTTCTACGGCGGCC
AGGACCTGCTCAACCGCGTCGCGCCGCTACTGGGCTACGACGACTATATTGACTTGAACCCCTTGGCCGCCGGCAT
CGGCACCCTGGGTTTCATCTTTGGCGCCTACCTGTCGGAAACGTTCCGCGGTGCGTTCATGGCCATCCCCAAGGGG
CAGGCCGAAGCGGGCATGGCCTATGGCATGAGCCCGTTCCAGGTGTTTTTCCGGGTGATGGTGCCGCAGATGATCC
GGCTGGCAATCCCCGGCTTTACCAACAACTGGCTGGTATTGACCAAGGCCACTGCGTTGATTTCGGTGGTGGGCCT
GCAAGACATGATGTTCAAGGCCAAGCAGGCGGCAGATGCCACCCGCGAACCTTTTACCTTCTTCCTCGCAGTGGCG
GCGATGTACCTGGTGATCACCAGCGTCTCGTTGCTGGCCCTGCGTTATCTTGAGAAGCGCTACTCGGTAGGCGTAA
AGGCGGCTGATCTATGATCTTCGACTACAACGTCATCTGGGAGGCCATGCCGCTGTACCTTGGCGGCCTGCTGACC
ACCCTCAAGCTGCTGTTGATTTCGCTGTTCTTCGGCTTGCTCGCTGCCGTCCCCCTGGGGCTGATGCGGGTGTCCA
AGCAGCCGCTGGTCAACGGCGCGGCCTGGCTCTACACCTACGTGATCCGCGGTACACCGATGCTGGTGCAACTGTT
TTTGATCTACTACGGCCTGGCCCAGTTCGAAGCCGTGCGCGAGAGTGTCCTCTGGCCGTGGCTGTCCAGCGCAACG
TTCTGTGCGTGCCTGGCCTTTGCCATCAACACCAGCGCCTACACCGCCGAAATCATTGCCGGCAGCCTCAAGGCTA
CGCCCCACGGCGAGATCGAAGCAGCCAAGGCCATGGGCATGTCGCGCTACAAGCTGTACCGCCGCATCCTGCTGCC
TTCGGCCCTGCGCCGGGCGCTGCCGCAGTACAGCAACGAAGTGATCATGATGCTGCAGACCACCAGCCTGGCCTCC
ATCGTCACCCTGATCGATATCACCGGTGCCGCACGTACGGTCAACGCCCAGTACTATCTGCCGTTCGAAGCCTATA
TCACCGCTGGCCTGTTCTACCTGTGCCTGACGTTCATCCTGGTGCGCCTGTTCAAATTGGCCGAGCGTCGCTGGTT
GAGCTACCTGGCTCCACGGAAGCACTGATATGGAACGCATCGATCACGTACTGCCCTGGGGCCACTTGGGCTGCGA
GCGCCAACTGACGGTGTTCCGTTTCGGCCACGGCGAGCGCAAGGCGTATATCCAGGCCAGCCTGCACGCCGATGAA
TTGCCGGGAATGCGCGCGGCCTGGGAGCTGAAAAAGCGCCTGGTCGAACTGGAGCGGCAGGGCGCCCTCAACGGGG
TGATCGAGCTGGTGCCGGTGGCCAACCCGATGGGCCTCGGCCAACTGCTGCAAGGCAGCCACCAGGGGCGTTTCGA
GATTGGCAGCGGCAAGAACTTCAACCGTGACTTCGTGGAGTTGAGCGAGCCGGTTGCGGCCTTGCTGGAGGGCAAG
CTGGGGGATGATCCCCACGCCAACGTGCGCATGATCCGCCAGGCGATGAGCGACACGCTCAACGCGCTGCCTGAGC
CGAGCAGCCAGCTGCAAGGCATGCAACGGGTATTGCTGAGCCACGCCTGCACCGCCGATGTGGTCCTCGACCTGCA
CTGCGACGCCGAAGCCGCGCTGCACATGTACGCGCTGCCCCAGCACTGGCCGCAATGGCGTTCGTTGTCGGCGCAC
TTGAATGTAAAAGTCGGCCTGTTGGCGGAAGACTCCGGTGGCAGTTCGTTTGATGAAGCCTGCTCGCTGCCCTGGC
TGCGTTTGTCGCGGGCGTTTCCCGAGGCGCAGATTCCCCTGGCGTGCCTGGCGACAACCCTGGAGCTGGGTGGCCA
GGCCGATACCGGACGTGACGAGGCCATCTTCCATGCCGAAGGCATCCTCGCGTTCCTCGCCGAGCAGGGCCTGATC
AACGGTGAATTTGCGGCGCCGCAATACGAGCCGTGTGAAGGCGTGCCCTTCGAGGGCACCGAATTGCTGTTTGCAC
CCCATGCCGGAGTGATCAGTTACCTGCGTAAGGCCGGTGACTGGGTGGAAACCGGCGAACCGATTTTTGAAGTGAT
TGACCCTGTGGCCGACCGCGTCAGCATTGTCTGTGCCGGCACCTCGGGGGTGTTGTTTGCCGTTGAACGGCTACGT
TATGCCCAAGCGGGTTTCTGGCTGGCCAAGGTGGCGGGGCGCGAAGCGCTGCGTCACGGGCGCTTGCTCAACGACT
GACCAACTGTTTTTGTGAGAACCGACCGCATGTACAAACTTGAAGTCCAAGACCTGCATAAACGCTATGGCAGTCA
TGAAGTGCTCAAAGGGGTGTCCCTGGCCGCCGCGGCCGGCGATGTGATCAGCATCATCGGCTCCAGCGGTTCGGGC
AAAAGCACCTTTTTGCGCTGCATCAACCTGCTGGAGCAGCCTCACGCCGGCAAGATCCTGCTCAATAACGAAGAAC
TGAAGCTGGTGGCCAACAAGGACGGCGCGATGAAAGCTGCCGACCCCAAGCAGTTGCAGCGCATGCGTTCGCGCCT
GTCGATGGTGTTCCAGCATTTCAACCTGTGGTCGCACATGACCGCGCTGGAAAACGTCATGGAAGCACCGGTGCAC
GTATTGGGCATGTCGAAAAAAGACGCCCGTGAAAAGGCCGAGCACTACCTGGCCAAAGTTGGCGTGGCCCACCGCA
AGGACGCTTTCCCCGGCCATATGTCCGGTGGCGAGCAGCAGCGCGTGGCGATTGCCCGGGCGCTGGCGATGGAGCC
TGAGGTGATGCTGTTTGACGAGCCGACCTCGGCCCTCGACCCGGAGCTGGTCGGCGAAGTCCTGAAAGTCATGCAG
GACCTGGCCCTGGAAGGCCGCACCATGGTGGTGGTGACCCACGAAATGGGCTTTGCCCGCGAGGTGTCGAACCAGT
TGGTGTTCCTGCATAAAGGCATTGTCGAAGAGCGCGGCAACCCGCGTGAAGTGCTGGTCAACCCGCAGTCCGAGCG
GTTGCAGCAGTTCCTGTCCGGTAGCTTGAAATAATCAAGGCCGCTGCACCCCCTGTAGCAGCGAGCTTGCTCGCAA
AGATCGTCAACGAAAACGCGGGAAGCCTGATACCCCGTGGTGTTCTCAGGTTTTTCGCGAGCAAGCTCGCTCCTAC
AGGAAAGCAGGTCTTTATCGTTTTTAAGCTTTGTGTTGGTTTACGGGCTAGCATTGGCCTTTGTCTTCATTACCGT
TACTCGCTTCGGATAGCACTCCATGACCGCCCATAAAATTGGTTTCCTGATTTGGCCCAGCACAAAAGCACTCACG
CTTGCGCTGGCTGAGGAGGCCTTGCGCGTTGCTCAGCGTGTGCATCCGGATGTGGTGTATGAACTGTCGTTCCTGC

AGGCCGAGCCGCAAACCGAAGGCGCCTGGCAACTGCCGGGCGAACCCTGGGCCGGCAAGCTCGAAGGCTTCCAGAA
ACTGTTCCTGTTGGCCGACGAACCGCCGACCGTGATCGCCTCGCAACTAAGCTCGGCGCTCAAGCAGCTCGTTCGT
TCCGGTTGCGTGATCGGCGGCCTGTCGGCAGGCGTGTATCCGCTGGCGCAACTTGGCCTGCTCGACGGCTACCGCG
CCGCCGTGCACTGGCGCTGGCAGGACGATTTCGCCGAGCGTTTTCCCAAGGTCATCGCCACCAGCCATTTGTTCGA
CTGGGACCGTGATCGCCTGACGGCCTGCGGTGGCATGTCGGTGCTCGACCTGCTGCTGGCGGTGCTGGCCCGTGAC
CACGGCGCCGAACTGGCTGGTGCGGTCTCCGAAGAACTGGTGGTGGAGCGCATCCGCGAAGGCGGCGAGCGCCAAC
GAATCCCCCTGCAAAACCGCCTGGGCTCCAGCCATCCGAAGCTGACCCAGGCCGTGTTGTTGATGGAAGCCAATAT
CGAAGAGCCGCTGACCACCGATGAAATTGCCCAGCACGTATGCGTGTCCCGACGACAACTGGAACGGATCTTCAAG
CAATACCTCAATCGCGTTCCCAGCCAGTACTACCTGGAGCTGCGCCTGAACAAGGCGCGGCAGATGTTGATGCAGA
CCAGCAAGTCGATCATCCAGATCGGCCTGTCTTGCGGCTTCTCCTCGGGGCCGCACTTCTCCAGCGCCTACCGCAA
CTTCTTTGGCGCCACCCCCCGTGAAGATCGCAACCAGCGCCGTAGCAGCAGCCCGTTCGAATTGTCGTCGGTACCC
TCCGAGCGCGGCTGACAAGGCGCCGCTTGTTGTGGAGAGGGGACAAGCCCCCTGGCCACAACAAGCCTTCAACACC
ACTGCCTCCCTCACCGCAGCAGGCTCACTCTTCCATAAGCTCTGGCGTGCTGGGCGGTGTCAGTGCGGCACGGTTT
TCGTCGAACATGGCTTCTTCCACGCCCAACTCATAACCGGTCTGCCGATAGTAAGTGCGCAGTATCGTCATGCTTG
CGGCGGACAAGGGGTTGCCGCTCATGTCTGAGTCGTCGTTGAGTGGCAGGTGCATTTCCAGCAGGTCTGTCGGCAG
GTGCTGGATCAGGTTGTCGCTCAAATCCAGGGCCTGCAAGCGCGTCAGTTTGAATACTCCCGGCGGCGGCTCGCTC
AACCCGCAATTGTGCAGGTACACCATGACCAGGCGTGTGAGGTTGCGCAGGTCCGGGCTCAGTGTGAGCGGGTTGT
CGCTCAAGTCCAGGAAGGTGAGCTCGTTCAGGCGCGACAGCTGTTCGATGCTGTCTGGTGTCAGGTGGATTGAGTT
TTCGTTGAGTTTCAGGTGCTGCAGGTGATTGAGATCAAACACGCGCTCGGGAATGCGCTGCAGGGCATGGCGCTCC
ACCGTCAGGCTTGTCAGGTTGGGGAAAGCGCGCAGGAACTCGCCTACACGTTTATGGTTACCGTCGCCCATCAGTT
CCAATAGCGTGACATGTTCCAGGCGCACATTCAGCGACGGCAGATCGCCGAGCAATGGGTTGTTGAAGATCAGCTT
ATAGCTGCGCCGAGTCGGCTCTTGAGTTTCGTCCAGTTCCGTCTCTCGCCGCCAGCAGCGCAGGAGTAACTCCTTG
AATGCCAGGCGGCGAACCTGCTCCTCGGCACGCGCGTTTTCGTTAAGCACCGGGACTTGCAGTTGATCCATGAGCG
GCACGTTCAAGGCGAACTCCTGCAGCTGGGTGTCCAACTCTGCGTACTCGGCGCTCAGGCGATTGATATGGGTGTC
GGCGTCCTCCAGTTCGCCCGGCAGTTCGAAGAAGATGCGCTTGCTGTCACGCTCCGAAAGCCTGGGGTAAAGGGCC
TTGAGGCGCTGCAGATCGGTTGTGGGAATATCAGCGTTCCAATGCTCTCCCTTGGTTTGGCAATAGCCTTTCACAC
GTCGCAGGGTGGAGGGCGACAAGGGGTTGGTCGACAAGTCAAACGCGGCGCTGACGGCCGGGGGCAAGAGGAAGGC
CTGGTCTGGCAGCTCGCGCAGCAGGTTTTGGCTCAAGTCCACGCGAGCGCGCACCGTGGCGGGTTGAATCAGTGAG
TTGGGCAGTTGCGTCAAGCCGGTGTTGCGCAGTGACAGGCTGATCAGTTGGGGCAATCGGCTGAAGTCCGGCAGTG
TCGTCAAAGGGTTGTTGTTCAGGTTCAGGTGTTTGAGTTCGGCCAGGCGCTGCAAGGCTTGCGTGCTGGTGGGCGA
TAAGCTCAGGGCGCAGCGGTCCAGCACCAGGTAGGAGAGTTGCGGCAAGTGGCTGATCTGCGGCAATTCACCCAGT
GCGTAGTCCTCGATATGCAGCCCGGTCAGCGCGGGAAAGCTCTGCAGGAACGCATCCAGATTCAAGTGTGTCCCGC
GACCTTTGAGCGCCAGCACCGACACGTGATTGAACGATGCACGCAGGGCGGGCAGTGTGCCGCTCACGGTGATGAA
TAGGCTGAATTCGTAGCTGGATGTAGGTTGCCCCTCGGGTGGCTCGCCCGACGTCACATCCCGGCGCCAGCCTGTC
TCCAAGGCATCAATGAAGCGGTGGCGCAAGGCTTGTTCTGCCGCATCGACGCGGCTGTCGTTGGCCCAGTCGAGGG
TGTCCAGGCGGATGAGGGGAAACTCGCTTTCCAGCCGGTTCATCTCGGTGCGGCCGGTAGCCAGGTCGCCCGGCAG
GGCAAAAATGAAGCGGTTGATTTGATCGCCGTTGAATGAGGGGAACAACCGTTTGGCCTGCTCCACGTCGGGCTGG
GGCGGGGTGGCTTCCAGATAGTGATGGGTACGCTGGAAGTAGATTTTGATGCGCTCCAGCGCCTCGTTTGAAAGCG
GGTTGTCGGACAGGTCGAAGTGCCGCGTGATGTCGGTGGGCAGGTCGAACAACTGCGCAGGCAGTTCGCTGATCTG
GTTGTTGGACAGCATCACGTATATCAGATGAGTGCGGCTCAACAGACCAGGAGGCAGTTGGCTGATGCCGGTGCTG
GCCAAGTCCACGTATTGCAACTGCGGCATCTGTTCCAGGTTGGGCAGCAAGGTCAGGGGGTTGTTGTACAGGTCAA
GGGTCAATAGGCGCGTCATGCCGTTGACGACGGCCAGGCTTTGCTCGGTCAGGCTGATGTTGCAACCGCTCAGGAT
CAGTTCGTTGAGGCGAGTGAGCGAGGCCAGGCAAGGCGGCAGTTCGTTCAAGGCTACATTGCGGATGCTCAAGTAG
CGCAGCCTTGGAAAAAGCTGCAGGAAACCGTCGACGTCGATGCGCTGGTGCGCACCTTGCAGTTCCAGGTGCGAGA
TATGGTCGAAGTTTGCAGCCAGTGCGGGCAGCTCGCCGAGCAGTGGGCCGGACAGTCGCAGCATGTGGCCGTTGGT
GGCCGGTGCCTCGTAGAAATTGTCGATTGCGGTTTCCTGGCGCCACGCCCGGCGCAACTGGTGGGCGAACAGGCTG
CGATCACGGCGCAGGTACTCCTTGTCAGTCGCACTGAGCGGCGCCCGAGTGAGAGGGTGCAGGTCGGGCGTTTGAG
ATTCCCAGACCGTCAGGGCGAGGTCCAGCCGGATGTATTCGTTCCTCAGGCGCGTCAGCACTGGCTGGGCACCGCC
AAAGGTGTTTTGCAGGGTGGCGATCATTTCGCGGACTTTTGCCGGCGACAGTTGCGGGCGCAGCGTCTGCAGTTGC
TGCTCCAGTGAAGGGCCGCGGCTTGGCCCTGGCTGTTCGGGAAGGGCCGGCGGGTAGCCGTCCATGCCGCCGCGCA
GGCGCATGACAGTCGGATCGTAGGCGGGTTTGCGCGGCGGGTCGGTATCAAGCAGCGTACGCAGGCTCGGGCGGTC
CAGTGGGTGTTCGCCGATTGCCTGCTTGAGGCGCGGGCCCTGGCCGATATGGATCTGCAGGGCATCCCGGGCACTG
TCAGGCAGGGCTTGCAGGATGGCGGTGTAAAAATCCGTGCTGCCGAACAGCGCGCCTTGGCCGTTTTCCGGGATGT
ATTGGCCGTCGGGTGTCGCCACCAGGGTTCTTTTGGTCAACGCATAGGTGGGGCCGATTGCATCGAGCAGCGTACC
TTCGGCGCGGTACTCCCTGAGTTCCAGGCGTATCCACGTCGGCCAGCCGCTGAGGTTTTCCAGGGAGTGCAGGGCC
AACCGGCAAGTGTCCGGGTTCTCCACGGTGTCCAGGTACAGCCCTTCATAGGCGCGGGTGGTGCGCACTTCATGCA
GCGACCACTCGGCCAGTTCCGCCAGGCGCGTGGGTACAGTACCGGCGTCGATGGCCTTGAGTTCCTCGCCACTGGC

GCTGGACAGCAGTTCCTCGGCCACCGGCGCGGGCAGGCCGGGACGCGAGGCACTGTCGATGATTTTCTGGACCCGG
GCATTGGGCGCGCGGTCCAGGCCGCGGTAGCGTGACTCGAATAACGAGAAACGCTTGTCCTGGGCGATCCGCGCCA
ATTGCTTGCGCAAGGCGGTGGCGCGGGTGCGGCTGGAAGAGGGCGGCAGGCCGAACTCTTCTTCCAGCAGGTGTTT
GCGCTCCGGCTCACTCAAGCCCTCGATCAGGGTCGACAATAGATCGCCGTTGTGCAGTTGTGCTTCCAGCACCTGT
ACGGTGCTGGCCCCCTCGTCACCGGGGAATTGCCAGAGGGTTCTGCCGCTGGCATTGACTACCCGCAGGGTCTTGC
TGGAGGGCCACAGTCCGTAGCTGATGAGTAACTGCAACAGGGTCTGCGGATCGGCCTTGGCGTAGACCGCCGGATC
GTCACTGTTCAGTTGCGCCAGGAAGTCCTGCAACTGCTGGTCGATGCCAAAGCGCTTGAGGGTGTCAGCCAGCAGT
GGCGGGGGTGTCTGCTGGGTGGCATGCACTTTTCGCAGGGTGTTGTCGTGGGTGGCGCTGCAGTCGCGGACCTGGG
CAAGTTGCTCATCGCTGAAGCTATCGGTCTGGTAACCGAGGCGGCGCATCAGGGTGGCGCGGTCCCAGCCCAATGG
GTTCTCCAGCTCTGTGACCCAGGCGCCCTGGCCATTGGTCATGACCTGCGGGCGGTAGGCTCCAGGGCGCGTGGGG
TGCTGGATGAAATAGCGCCCGGCGTCTTGTTCTACGTGGTAATGCTCGCCGTTGAGGGCAAGGATGGGTTTGCCGT
TGTGCCGGTGCAGGCCTTGCGCGTCAGGGCGGGTATCGGCCGGCAGCGTCACCTCCTGGCGATAGGGGCTGAGGTC
CGGTTGCCACAGGCGGTGTTGACCATCGGGGCGCTTGACGGGCAGCAGACTTTCGAAAAAATCCAGCATTTGTTGG
GGTAAAGCCTTGCGCAGCAACTCGGTGGCGATGGGAATGCCGGTGGCAAACAGGCCCAGTTGCGCCAGTTGTTCCA
TCAAGCCCAAGGCATGGCCCAGGGCTTCGCGTTGCAGGCCTTGGGCCCAGTCGACAACCCCTTCGAAGGCTTCGTC
GAGGACTTGATAAGCGGTGTAGGCGAGCATCAGCGCGCCCAGGGGCGGGATGAATGGCAGGGCCACAAAGGCGATA
ACTTGCAAAACACTTTCGGCGATCTTCTGAATGAACGCCCAGCGTCGCCAACGGGCGTTCTGGTCGACGCGCGCGG
TGGCGACAGCGCGGCTCTGGCTGTCGGCTTGCAGCTTGCTCAGTTGCATCTGGAACAGATGAGCGAACAGATCACC
GCTGATTTTAAGCCCGCGAAACTGCAGGTTGGGGCGCTGTGTGGGGCTCTCGCGCCAACTGGGCAGCGGATCGCCT
GCGGTGTGCGGATGCCACGAGACCTGGCCGAGGCGTTGGTTGAGGTTGGCAAAAAATACCGCGGCGTCTTCGTGGC
GCACAAAACGGCTGAAAAACTGTTGATAGTCGGTGGCGCGCAGCCTGGCGACCAGGCTTTGCATGAACGCCGCGCT
GCTGGGGTAGTGTTTGACCGGGGTTTGCGGGTCGTCGGGAATGTAGGCGATCAAGGGCACCACCTCGCGGCTGGCC
TCCAGGTCCGGAGCAAACAGCACAATGCCAGTCAGGCGGCAGTCCATTATCAGCAGTTCATGGGCCACCAGCGGCG
GGCAGCCGTTGTGTGCTGCATGGCCATTGTGGAGCAGGTTGAGGACGGCATCATGGCTGGCGGCAGGCAGGTCGCC
CTTCAGCAAGGCCATGTACAACGCCAGCTGCAGCGCCCCCAGGTGGCTTTGTTTGAGCCAGTATTCCAGGCTGGTG
CGGGCCACGGGATTGTCCAGGTCCAGGTATTCATCCAGATAGCGCCGATAGGCCGCGCCGATATCCAGGCGGCGGC
ACAGTTGGGTGAATTGGGCGATGCTGATCTTCGCGTCCAGGGCCGGCAGTTGGGTGAACTGGCCCGCCGGCGTTGG
CTGGGTGCTGAATCCGGAGTGCTGCTCGTAGGGCTCGTCGGTTTCGAAATTATGCAGGGCCGCGTCCAGCAACGAG
ACCGACCATGTGCGCGCCGCCCCCGGCTTGACGGTGAACCCGGCGAGGGTCAGCGGTACATACAGTTGCAGGTAGG
TCGTGCGCACGTCGGCGTCGACGCCGAACTCATCCTTGAGCGCCGCTTGCAGGCGTGCGGCGCCAAAGGTCTCGGG
GCTCTGCAGGGCGGCGAGGGCTTTGTCCACGCGGTTTTGTGCGGTCCAGGCGGCGGCATTCAGGTGCTTGAGATGC
GCGTGCTGCTGCTCGGTGGCGCGCAGGTACCAGGCGGCAATGGCCGGCGTACCCTCGGCCAGCGCCGCACGCTTGG
CGGCTGATGCAGTGCCCAGCCAGGAGGGGATGGCGTTGCTGAGGATGTCGAGGTGCGGGGATTGGGTTGCAAGCGG
TTGCGCCATGGTGCCTGTCCTTGATGGCTCTTGAAGTTCCTTCAGCAAACCAGTGCGCAAGGCGGTGCAGGCGGTA
GACAGTTAGTGCGAGCGTGTCTTGCGGATTTGCGAAGATCCCGTCATCCCCTGCCCACAAAGCCGTTGCACGGTTT
AAACTGCGCCTTTGCGACGCTATTTGTCGCATTGGCGTAAACCCGCGCAAAACACGGGTTGGCGCTATAAGAAGTT
GTCGCTTGGCGGCAAGGCCGCTCTGAAAACTGTCCTTACAATCCCTGCATCGCCCGCCAGTTCCAGGCAGGCGTTC
CTCTTCAGGAGACTCCGATGTCCGTTGAGCAAGCCCCGGTGCAACGTGCCGATTTCGACCAGGTCATGGTGCCTAA
CTACGCACCTGCCGCTTTCATCCCTGTGCGTGGCGAAGGTTCGCGCGTGTGGGACCAGGCGGGTCGCGAGCTGATC
GACTTTGCCGGCGGCATCGCAGTGAACGTGCTGGGCCACGCCCACCCGGCGCTGGTCGGTGCACTGACCGAACAGG
CCAACAAGCTGTGGCACGTCTCCAACGTCTTCACCAATGAGCCGGCCTTGCGCCTGGCCCATAAGCTGATCGATGC
GACCTTTGCCGAGCGCGTGTTCTTCTGCAACTCCGGCGCCGAAGCCAACGAGGCCGCCTTCAAGCTGGCCCGTCGC
GTGGCGTTCGACCGTTTCGGCAGCGAGAAATACGAGATCATCGCCGCCCTGAACAGCTTTCACGGCCGCACCCTGT
TTACCGTCAACGTCGGTGGCCAGTCGAAATACTCCGACGGTTTCGGTCCTAAAATCACCGGTATCACCCACGTTCC
GTTCAACGACCTGGAAGCGCTCAAAGCCGCCGTGTCGGACAAGACCTGCGCGGTGGTCCTGGAGCCGATCCAGGGC
GAGGGCGGTGTATTGCCGGCCGAACTTGCCTACCTGCAAGGCGCCCGTGACCTGTGCGATGCCAACAATGCGCTGC
TGGTGTTCGACGAAGTGCAGACCGGCATGGGCCGCAGCGGCCACCTGTTTGCCTACCAGCACTACGGCGTGGTGCC
CGACATCCTGACCAGCGCCAAGAGCCTGGGCGGGGGTTTCCCGATTGCCGCGATGCTGACCCGTGAAGACCTGGCC
AAGCACCTGGTGGTCGGTACCCACGGCACCACCTACGGCGGCAACCCGCTGGCGTGTGCGGTAGCCGAAGCGGTGA
TCGACGTGATCAACACTCCTGAAGTGCTGGCAGGTGTCAACGCCAAGCATGACCTGTTCAAGGCCCGCCTGGAGCA
GATCGGCAAGCAGTACGGGATCTTCACCCAAGTCCGTGGCATGGGCCTGCTGCTCGGTTGTGTATTGAGCGACGCG
TTCAAAGGCAAGGCCAAGGATGTGTTCAACGCTGCCGAGAAAGAAAACCTGATGATCCTGCAAGCCGGCCCGGACG
TGGTGCGTTTTGCCCCGAGCCTGGTGGTGGAAGACGCGGATATCACCGAAGGCCTGGATCGTTTTGAACGTGCTGT
AAAAACGCTGACGCAAGCCTGATAGAGACACATTTGTCTAACGAACCGGATCAAATGTGGGAGCGGGCTTGCCCGC
GATAGCGATCTGTCAGTCGACGTTGTTGTGACTGGCCCACCGCAATCGCGGGCAAGCCCGCTCCCACATTGGGACC
TGGGTGATTCCAGGTTCAGTGTCCGGCATTTTTTTATGAAGTTTTCGAGTTAAGGAGTGACACCATGCTGGTGATG
CGCCCCGCGCAAATGGCTGATCTGGGCGAGGTACAGCGTCTGGCTGCGGACAGCCCGATTGGTGTCACTTCCCTGC

CGGATGACGTTGAACGCCTGAGCGACAAGATCGCCGCCAGCGAAGCGTCCTTCGCCGCCGAAGTGAGTTTCAATGG
TGAGGAAAGCTATTTCTTTGTCCTTGAAGACACCGTCGCCGGCAAGCTGGTGGGCTGTTCGGCCATCGTCGCTTCG
GCCGGGTATTCCGAGCCGTTCTACAGCTTTCGCAACGAGACCTTCGTCCACGCGTCCCGCGAACTGAAGATCCACA
ACAAGATCCACGTCCTGTCCCAATGCCACGACCTGACCGGCAACAGCTTGCTGACCAGTTTCTACGTGGTGCCGGA
ACTGGTGGGGTCGGCCTGGTCGGAACTCAATTCCCGTGGCCGCCTGCTGTTTGTCGCCAGCCACCCCGAGCGCTTC
GCGGACTCGGTGGTGACCGAGATTGTCGGCTATAGCGACGAGAACGGTGATTCGCCGTTCTGGGATGCCATCGGCC
GCAACTTCTTCGACCTCAACTACGCCGCCGCCGAGCGCCTGTGTGGGTTGAAGAGCCGCACGTTCCTCGCCGAACT
GATGCCCCATTACCCGATCTACGTGCCGCTGTTGCCCGACGAAGCCCAGGAAGCCATGGGCCAGGTGCACCCGCGT
GCGCAGATCACCTTCGACATCCTGATGCGCGAAGGTTTTGAAACCGACCATTACATCGACATCTTCGACGGTGGCC
CGACCCTGCATGCACGGGTTTCTGGCATCCGTTCGATTGCCCAGAGCCGCGTGGTGCCGGTGAAGGTCGGCGAAAT
GGTCAAGGGCGTCGGCCGCCAGTACCTGGTGAGCAACGCGCAGTTGCAGGATTACCGTGCGGTGATGCTGGAACTG
GACTACGCGCCAGGCAAGCCAGTGACCCTGGACCTGGAAGCAGCCGAAGCCCTGGGCGTCGGCGAAGGCGCCAGTG
TGCGTCTGGTAGCGGTTTAAAGCAGAGTTTAACGGGTGGCCTGTAACAGGCGGCCCGTCCGAGGAGATAGCATGAT
CGTTCGTCCCGTACGCAGCAGCGATTTACCGGCCCTGATTGATCTGGCGCGCAGCACCGGCACCGGCCTTACCACC
TTGCCGGCCAACGAAGAGCGCCTGACCCACCGGGTTGGCTGGGCTGAAAAGACCTTTCGCGGCGAAGCCGGGCGTG
GTGATGCCGACTACCTGTTTGTGCTGGAAAACGACGAAGGCCGCGTGGTGGGGATTTCGGCGATCGCCGGTGCGGT
CGGCCTGCGTGAGCCTTGGTACAACTTCCGGGTCGGCCTGACGGTCAGCGCCTCCCAGGAGCTGAATATCTACCGC
GAGATCCCCACGCTGTTCCTGGCCAACGATCTGACGGGCAACTCCGAGCTGTGCTCGCTGTTCCTGCATGCCGATT
ACCGCAACGGCCTCAACGGCCGCATGCTGGCCAAGGCGCGGATGCTGTTCATTGCCGAGTTCCCGCAACTGTTCGG
CAACAAGATCATTGCCGAGATGCGCGGTGTGTCCAACGAGGCCGGGCGTTCGCCGTTCTGGGAAAGCCTGGGCCGG
CACTTCTTCAAGATGGAGTTCAGCCAGGCCGACTACCTCACCGGCGTGGGCAACAAAGCGTTTATTGCCGAACTGA
TGCCCAAGTTCCCGCTGTACACCTGCTTCTTGTCCGAGGACGCGCGCAACGTGATCGGCAAGGTCCATCCCGACAC
CGAGCCGGCCTTGAGCATGCTCAAGAGCGAAGGCTTCAGCTACCAGGGCTACGTCGATATCTTCGACGCAGGTCCG
GCGGTGGAGTGTGAGACCGGCAAGATCCGTGCGGTGCGTGACAGCCAGGCGCTGGTGCTGGCCATCGGCACGCCGG
GGGACGACGCCACGCCGTTCCTGATGCATAACCGTAAACGTGAAGATTGCCGCATCACCGCCGCACCGGCGCGCCT
GGCGGCTGGCACCCTGGTGGTCGATCCGCTGACCGCCAAGCGCCTGCAACTGGTGGCCGGTGATCAAGTGCGTGCC
GTATCGTTGTCTGCTGCCCGGGAGTCGAAATAATGAATTCGCTGTATATCGCAGGGAGCTGGCTGGCTGGCCAGGG
TGAACGCTTTGAGTCGCTGAACCCGGTGACCCAGCAAGTGGTGTGGGCCGGCAATGGCGCCACCGTCGAGCAGGTT
GAGTCCGCCGTGCAGGCCGCCCGCCAGGCCTTCCCGGCCTGGGCCATGCGCCCGCTGGAAGAGCGTATCGGCGTGC
TGGAAACCTTCGCCGCGACCTTGAAAAGCCGCGCCGATGAAATCGCCCGTTGCATCGGCGAAGAAACCGGCAAGCC
GCTGTGGGAGTCGGCTACCGAAGTCACCAGCATGGCTAACAAAATCGCGATCTCGGTGCAGAGCTACCGCGAACGT
ACCGGCGAGAAGAGCGGCCCCCTGGGCGACGCCACCGCCGTGTTGCGCCACAAGCCGCACGGCGTGGTCGCGGTGT
TCGGCCCCTACAACTTCCCCGGCCACTTGCCCAACGGGCATATCGTGCCGGCCTTGCTCGCGGGCAACACCGTGTT
GTTCAAGCCGAGCGAGCTGACCCCTAAAGTGGCCGAGCTGACCGTGCAGTGCTGGGTCGAAGCCGGCCTGCCGGCG
GGCGTCTTGAACCTGCTGCAAGGCGCGCGGGAGACCGGGATTGCCCTGGCCGCCAACCCGGGGATCGACGGTTTGT
TCTTCACCGGCTCCAGCCGCACCGGCAACCACCTGCACCAGCAATTCGCCGGGCGCCCGGACAAGATCCTGGCGCT
GGAAATGGGCGGCAACAACCCGCTGGTGGTCGATCAAGTGGCCGATGTCGATGCGGCGGTCTACACCATCATCCAG
TCCGCTTTCATTTCGGCCGGCCAGCGCTGCACCTGCGCCCGGCGCCTGCTGGTGCCGGAAGGCGCCTGGGGTGATG
CACTGCTGGCGCGCCTGGTTGCCGTCAGCGCGACGATTGCCGTGGGCGCCTTCGACCAGCAACCGCCACCCTTCAT
GGGTTCGGTGATTTCCCTGGGCGCGGCCAAGGCCTTGATGGATGCCCAGGAGCTGATGCTGGCCAACGGCGCCGTG
GCGCTGCTGGAAATGACCCAGCCCCAGGCGCAGGCAGCCTTGCTGACGCCTGGCATCATCGACGTGACCGCCGTGA
CCGAGCGTGAGGACGAAGAGCTGTTCGGCCCGTTGCTGCAAGTGATTCGCTACGCGGATTTCGAAGCGGCGATCCG
TGAAGCCAACAACACCCAATACGGTTTGGCCGCAGGGTTGCTGTCGGACTCCGAGGCGCGCTACCAGCAGTTCTGG
CTGCAAAGCCGTGCGGGTATCGTCAACTGGAACAAACAGCTGACCGGCGCGGCGAGCACCGCGCCATTTGGTGGGG
TAGGGGCGTCGGGCAATCATCGCGCCAGTGCCTATTACGCGGCGGATTACTGCGCGTACCCGGTGGCATCCCTGGA
GACACCGAGCCTGGTCTTGCCAGCCGCGCTGACGCCGGGTGTAACGCTGAACTAATGTGGGAGCGGGCTTGTGTGG
GAGCTGGCTTGCCTGCGATGGCATCCCCTCGATCTGGCTGAAAGACCGAGGTGCCCGCATCGCAGGCAAGCCAGCT
CCCACACACAAGCCAGCACCTGCAGTTGATCGGGTTTGCAAAGACAGATTTGTGGTGTTTTGAAACCTTTGATGCC
TATAAAAACAGATGTTCGTGGAGCCTCGCCGATGAAATCCTGTGAAGTCAATTTTGACGGTCTCGTGGGGCCGACC
CATAACTACGGCGGTTTGTCCTACGGCAACGTCGCGTCCCAGAGCAACAGCCAGCAATCCTCCAACCCCAAGGAAG
CGGCGCTGCAGGGCCTGGCGAAAATGAAAGCCCTGATGGACATGGGTTTTGTGCAAGGTGTACTGGCGCCCCAGGA
ACGTCCGGATGTGGCCGCGCTGCGCCGCCTTGGCTTTGCCGGCAGCGATGCCCAGGTGATCCAGCAGGCGGCCAAG
CAAGCCATGCCGTTGCTGGTTGCCAGTTGTTCCGCATCGAGCATGTGGGTGGCCAATGCCGCCACCGTCAGCCCGA
GCGCCGACACCGTTGACGGGCGTGTGCATTTCACTGCCGCGAACCTCAACTGCAAATACCACCGCAGCATCGAACA
CCCGACCACCAGCCGCGTGCTGGGGGCGATGTTTGCCGATCAGCAGCACTTCGCCCACCACGCCGCGTTGCCGGCG
GTGGCGCAGTTCGGTGACGAAGGCGCGGCCAACCACACGCGTTTCTGCCGCGAATATGGCGAGGCGGGCGTGGAGT
TTTTCGTGTTTGGCCGCAGTGCGTTCGACACGCGCTACCCGGCGCCGCAAAAGTACCCGGCGCGCCAGACCCTCGA

AGCGTCCCAGGCAGTTGCGCGCCTGCACGGCCTGCAGGATGACGGGGTGGTCTACGGCCAGCAGAACCCGGCGGTG
ATTGATGCCGGGGTGTTCCACAACGACGTGATCGCTGTGGGCAACGGTGAAGTGCTGTTCTATCACGAGGATGCGT
TCCTCAATACCGACCAGATGCTCGCTGAACTGCAGGCCAAGCTGGGCAAGGTCGGCGGCAACTTCCAGGCGATCTG
CGTACCCCGCGCCCTGGTCAGTGTCGAAGACGCCGTGCGCTCCTACCTGTTCAACAGCCAGTTGCTGAGCCGTGCT
GACGGGTCGATGCTGCTGATCGTGCCCGAAGAGTGCCGCAACAACGAGCGCGTCTGGCACTACCTGCAAAGCCTGA
CCAGCTCCGGCGGGCCGATCGGCGAAGTCAAAGTCTTCGACCTCAAGCAAAGCATGCAGAACGGCGGTGGTCCGGC
CTGCCTGCGTTTGCGCGTAGCGTTGAAGGAGAGTGAACTGGCGGCGGTCAATCCAGGGGTTATCATGACTGCACCG
CTGTATGAAACGTTGACCCAATGGGTCGACAAGCACTACCGCGACAGCCTGCGCGAAACCGACCTGGCTGACCCGC
AGTTGCTGCTCGAATGTCGCACGGCATTGGATGAACTGACGCAAATCCTTAAACTGGGCGCGGTTTATCCTTTCCA
GATCAATTAATACCCCACGGCTGAGACCTATTTATGACCACCGATACCCTGAAGCTGATCCTTGAAGACACCGACG
GCACCCAGCTGGAAACCTCCTGCACTCGCCTGGCCGTGGTATGGCAGGGCAAGGAAATCTGGATCCAGCACGACGG
CCGTGGCCAACTGCTGATCGGCGTGGATGTGGAAGAAGGTGATGCCGAATACGCCAACCTGCTGATGCGTCCCCTG
GCCACCAATCTGATGAGCCTGCAGCTGGAAATGGAACCGGCCGATGTTGAAGGTGATGACGACGATCACGTCCACG
GCCCAGGCTGCAACCACTAAGGAAGCTGCTCTATGCTCGCCCTCGGCAAACTGCTTGAACTGACCCTCGCCGGTCG
TGAACCGGCGCAAAAAATTCAACTGACTGTCGACGGCGTGCAGATGCGCTGGCTCAGTGAGGGCGCACTCGAAGTG
CGGCCTCCCCAAGCCCGGGACAACGGCGCCGACGTGCTGCTGTCGTCCGGCATCCATGGCAACGAAACCGCGCCGA
TCGAATTGGCCGACCGCTTGCTGCATGGCATCGCCCGTGGGGAGATCAAGCCCCGCACCCGTATTCTGTTCCTGTT
CGGTAACCCCGAGGCCATGCGTCGCGGCGAGCGTTACCTCGAACAGGACGTCAACCGGCTGTTCAATGGCCGTCAC
GAACAAAGCATCGGCCCGGAAGCGATGCGCGCGGCCGAGCTTGAACAGTTGGCCCGTACCTTCTTCAGCGTGCCTG
ACCGTAGTCGTTTGCACTACGACCTGCATACGGCGATTCGTGGCTCGAAGATCGAGCAGTTCGCGCTTTATCCGTT
CAAAGAAGGGCGCCAGCACTCCCGCGCCGAACTGGCCCGCCTGCGGGCGGCAGGGATGGAAGCGGTGCTGTTGCAG
AACAAGACCTCCATTACCTTCACCGCGTTTACCTACGAGCAACTGGGCGCTGAGTCGTTCACCCTGGAGTTGGGCA
AGGCCCGGCCATTCGGGCAGAACCAGGGGGTGGATGTATCGCGCCTGGAAACCCGCCTCATACAGATCATCGAAGG
CAACGAGCCGGCCACCGAGGGCCTGGACGGCCTGAAGTTGTTCAGCGTGGCCCGGGAAGTGATCAAGCACAGCGAC
GCCTTCACCCTGCATTTGCCGGCGGACGTGGAGAATTTTTCGGAGTTGGAGAAGGGCTATCTGCTGGCCGAGGACA
TTGCCAAGACCCGCTGGGTGATCGAGGAGGAGGGCGCGCGCATCATCTTCCCCAACCCCAAGGTCAAGAATGGCTT
GCGGGCAGGTATCCTGATTGTGCCGACCACAGACGCCGGCCTGGCCTAGATCCAATTGTGGGAGCGAGCAAGCCCG
CTCCCACAATTGGTTTTGCGGTGCGGCTTAGACTGCTAAGGCGCGTGGCTCATTGCGGCGGATCGCACGGATCTTG
TGCAGGGTATCGGCACAGGTCTGCGCGGCTTCCTTGCCCTTGTGCACAAAGTGCTCAAAGAAGAAGTTGTGATGCT
CGCTGCCCGCGTGGAAATGATGCGGGGTGAGGGACACCGAGAACACTGGCACTTCAGTTTCCAACTGCACCTGCAT
CAGGCCGCTGACCACCGATTGCGCGACGAATTCATGGCGGTAGATCCCACCGTCCACCACCAGCGCGGCGGCGACG
ATGCCGGCGTAGCGGCCGGACTTGGCCAGCAGCTTGGCGTGCAGGGGCATTTCAAAGGCGCCGCCGACTTCAAAGA
AATCGATATCCGATTCCTGGTAGCCCTGGGCGATCATTTCGGCGAGGAAGCCTTTACGGCTCTGATCAACAATATC
TTTGTGCCAGCAAGCCTGGATAAACGCGACGCGCTCACCGTGATGGTTTTTGCTCTTGCTGTCGATTGCGGTGGGT
TGCATGTTCTGACTCCTGTTTATGAAAAAAACAGGGCGTTATGAATCGAATGGGATTTAAGGGTACGGCCGAATCG
ATCATGCCAGGCATGATTTCTCGCACACGGCCCTTAGGCGCCAATCCCGTTCTCTCTTCATCCGGACTATGACCGT
CGGCCCCGGGATCACACCGGGTCTGCTGACCTTGCCGCCGTCCTGCATAAGCAGTTCGAGGCGCCAAGCGCTCGCG
GGCTATGCGTATTGCACGCAATTACCGCCGGTGGGGAATTACACCCCGCCCTGAGAACGTTGCCACCAGAACCCTG
GCAGCGGAGAGTTTTTACCATACATTTCGAAAAACTGCATAGGGTGCTTGTTGATATCGACTATCGACAGGTTTGC
GGGCAGCCCCTGTAGGAGCCGGCAAGCCGAATCCTACAAGGGCAGGGGTTGATATTTGCCCGTGATACCCGCAGTA
ATCGGTTACAACAGGGAACTCCCCACACGCAGAGGCCAGCTTGATGTCCGTGATTGATCTTCGTAGCGACACCGTG
ACCCAACCCACCCCAGGCATGCGCGATGCCATGGCCAGCGCGCCGAGCGGCGATGATGTCTACGGTGAAGACACCA
GCGTCAACCACCTTGAGGCCGAACTGGCCAAGCGCCTGGGCTTTGCCGCAGCGCTGTTCGTGCCTACCGGTACCAT
GAGCAACCTGCTGGCGCTGATGGCTCACTGCGAGCGGGGCGAGGAATACATCGTCGGCCAGCAAGCCCACACCTAC
AAATACGAGGGCGGCGGCGCGGCGGTGCTGGGCTCGATCCAGCCGCAGCCCCTGGAAGTCCAGGCCGACGGTTCGC
TGGACCTGGCCCAGGTCGCAGAAGCGATCAAGCCGGATGACTTCCACTTCGCCCGCACCCGGCTGTTGGCCCTGGA
AAACACCATGCAGGGCAAAGTGCTGCCCATCAGCTACCTGGCTGCCGCACGCGCGTTTACCCGTGAGCATGGCCTG
GCCCTGCACCTGGACGGCGCGCGCCTGTACAACGCAGCGGTCAAGCTGGGTGTGGATGCGCGGGAGATTACCCAGC
ATTTCGATTCGGTCTCGGTATGCCTGTCCAAGGGCCTTGGCGCGCCGGTGGGCTCGGTGCTGTGCGGTTCCAGTGC
GTTGATCGCCAAGGCTCGCCGTTTGCGCAAGATGGTCGGCGGTGGCATGCGCCAGGCCGGTTCGCTGGCAGCGGCG
GGGCTGTATGCCCTGGACCATCAGGTGGCACGCCTGGCCGAAGATCACGCCAATGCCTTGTGGTTGGGCGACGAGC
TGCGCAAGGCCGGTTATAGCGTGGAGCCGGTACAGACCAACATGGTCTACGTGCAGATCGGCGAGCAGGCCCAGGC
GCTGAAAGCCTTTGCCGAGGAGCGCGGGATCAAGTTGAGTGCGGCGCCGCGCCTGCGCATGGTCACGCACCTGGAC
GTCAGCCGGGCACAGATCGAACAAGTGTTGGCGACATTTGTCGAGTTTTCCCGGAAATGACAGTGCAGACCGTCTA
ATTGACTGTTTCTATCGTATAAACACGCTGTACCACGGGCAAAGGGCCGATATAATGCGGCCCTTTGCCGTCGCTC
CGTCTGATTGATGTTGCGCACTGGCCTTTGGCCGCAGCCTCCGTGGAAGAACCTAATGAAAAGCGCAGAAATCCGT
GAAGCCTTCCTTCGCTTCTTCGAAGAGCAAGGTCACACCCGTGTCTCCTCCAGCTCTTTGATCCCAGGCAATGACC

CAACCCTGCTGTTCACCAACGCGGGGATGAACCAGTTCAAGGACTGCTTCCTGGGCCAGGAAAAACGCGCCTACAC
CCGTGCTGTCAGCAGCCAGAAGTGCGTGCGCGCCGGTGGTAAGCACAACGACCTGGAAAACGTCGGCTATACCGCC
CGTCACCACACCTTTTTCGAAATGCTCGGCAACTTCAGCTTTGGCGACTATTTCAAGCAAGACGCGATCAACTTCG
CCTGGACCTTCCTCACTGGCGTGCTGAAACTGCCGAAGGAAAAACTCTGGGTCACGGTCTACGCCAGCGATGACGA
GGCCTACGACATCTGGACCAAGGAAGTCGGCGTGCCGGCCGAGCGCATGGTGCGTATCGGCGACAACAAAGGCGCC
CCGTACGCCTCCGACAACTTCTGGACCATGGGCGATACCGGCCCGTGCGGCCCGTGCACCGAGATCTTCTACGATC
ACGGCGCCGACATCTGGGGCGGCCCACCGGGCTCGCCAGAGGAAGACGGCGACCGCTACATCGAGATCTGGAACAA
CGTGTTCATGCAGTTCAACCGCACCGCCGATGGCGTGTTGCATCCGTTGCCAGCGCCGTCGGTCGACACCGGCATG
GGCCTTGAGCGGATCAGTGCGGTGATGCAGCACGTGCACTCCAACTACGAAATCGACCTGTTCCAAAGCCTGCTGG
CGGCGGCGGCCAAGGCCATCGGCTGCGCCAACGACGACCAGGCATCGCTCAAAGTGGTGGCTGACCACATTCGTTC
CTGCGGCTTCCTGATTGCCGATGGCGTGCTGCCATCCAACGAAGGCCGTGGCTACGTGCTGCGTCGCATCATTCGT
CGCGCCTGCCGTCACGGCAACAAGCTGGGCGCCAGCGGCAGCTTCTTCTACCAGATCGTCGCGGCCCTGGTGGCCG
AGATGGGCCAGGCGTTCCCCGAGCTCACCGAGAACCAGGCGCACATTGAGCGCGTGCTCAAGGCTGAAGAAGAGCA
GTTCGCCAAGACCCTGGAGCAGGGCCTGAAAATCCTCGAGCAAGACCTCGCCGAGTTGAAAGGCGACGTGGTGCCG
GGTGATGTGGTGTTCAAACTCTACGACACCTACGGCTTCCCGATGGACCTGACCGGCGATATCGCCCGCGAGCGCA
ACCTGACCCTCGACGAGGCCGGTTTCGAGCGCGAGATGGAAGCCCAGCGCGTGCGCGCACGTTCGGCCAGCTCCTT
TGGCATGGACTACAACAGCCTGGTGAAGGTTGACGTGGCCACCGAATTTACCGGTTACACCGCCACCAACGGTTCG
GCCAAGGTGGTTGCCCTCTATAAAGAAGGCCAATCGGTTGATGTGTTGAATGAAGGCGATGAGGGCGTGGTGGTCC
TTGATCAGACGCCGTTCTACGCAGAATCCGGTGGCCAGATTGGCGACTGTGGTGTGCTCAAGGCAGCCTCCGGTCG
TTTTGATGTGCGTGACACCACCAAGACCGGCGGCGCGTTCCTGCACCACGGTGTACTGGCGTCGGGCAGCCTGCTG
GTGGGCGCGCCAGTGGAAACCCAGGTGGACGCCGACGTGCGCCACGCCACTGCGCTGAACCACTCGGCCACCCACT
TGCTGCACGCCGCGCTGCGCCAGGTACTGGGCGAGCACGTCCAGCAGAAGGGTTCGTTGGTCGACAGCCAGCGCCT
GCGCTTCGACTTCAGCCACTTCGAAGCGATCAAGCCTGAGCAGATCAAGGCCCTGGAAGATATCGTCAACGCCGAG
GTGCGCAAGAACACCCCGGTGGAAACCGAAGAAACCGACATCGAGACCGCCAAGGCCAAAGGCGCCATGGCGCTGT
TCGGCGAGAAATACGGCGACAACGTACGCGTACTGAGCATGGGCGGCAGCTTCTCCGTGGAGCTGTGCGGTGGTAT
CCATGCCAAGCGCACCGGCGACATCGGCCTGCTGAAAATCATCAGCGAAGGCGGTGTGGCTTCCGGTGTACGGCGT
ATCGAGGCAGTGACCGGTGCTGCGGCCCTGGCCTATCTCAATGCGGCCGAAGAACAACTCAAGGAAGCGGCGACCC
TGGTCAAGGGCAGCCGTGACAATCTGATCGACAAGCTCTCTGCCGTACTGGAGCGCAACCGCCTGCTGGAAAAACA
GCTGGAGCAGTTGCAAGCCAAGGCGGCCAGCGCAGCGGGCGACGACTTGTCGGCCTCGGCACTGGACATCAAGGGC
GTGAAGGTTCTGGCCGCACGCCTGGACGGTCAGGACGGCAAGGCGTTGTTGGCCTTGGTTGATCAGTTGAAGAACA
AGCTCGGCCGCGCAGTGATCCTGCTCGGCAGTGTCCATGAGGAAAAGGTCGTACTGGTTGCCGGTGTAACCAAGGA
CCTGACTGGCCAACTCAAGGCCGGTGATTTGATGAAGCAAGCTGCTGCGGCAGTGGGCGGCAAGGGCGGCGGTCGT
CCGGACATGGCGCAAGGCGGTGGTGTAGACGCTGGAGCCCTGGACGCGGCGCTGGCCCTGACCGTGCCATTTGTCG
AAGCCGGTATTTAAGGCGCTGTCGGCGGGCCCATGCTTAAGGCATGGGCTTGCCTAGGTACTGGAAGATTTGTTTA
TTGGGCGCCCCTTTACGGGCTGAGGCGGCTTAGAAATGGCTTTGATCGTACAGAAATTTGGAGGCACCTCGGTCGG
CTCTGTCGAAAGAATCGAGCAGGTCGCCGACAAGGTTAAGAAATTCCGCGATGCGGGCGACGACCTGGTTGTGGTG
CTGTCGGCCATGAGCGGCGAGACCAATCGCCTGATCGAGCTGGCCAAGCAAATCAGCGGTGATCAAGCTCCGCTGG
CACGTGAGCTGGATGTGATCGTGTCCACGGGCGAGCAAGTGACCATTGCCCTGTTGGCCATGGCGCTGAACAAGCG
TGGCGTTCCGGCGGTGTCCTACACTGGCACTCAGGTACGGATCCTGACAGACAGCGCGCATACCAAGGCGCGCATC
CTGCAGATTGACGACCAGAAGATTCGTGGTGACCTGAAGGCCGGTCGCGTAGTGGTTGTCGCCGGTTTCCAGGGCG
TTGACGAGCACGGCAATATCACGACCTTGGGCCGTGGTGGCTCCGACACCACTGGTGTGGCGCTGGCGGCAGCCTT
GAAGGCTGACGAATGCCAGATCTACACAGATGTGGACGGCGTCTACACTACCGATCCGCGCGTGGTGTCCGTGGCC
CAGCGCCTGGACAAGATCACCTTCGAAGAGATGCTGGAAATGGCCAGCCTCGGCTCCAAGGTGCTGCAGATCCGTG
CGGTGGAGTTCGCCGGCAAGTACAACGTACCGCTGCGCGTATTGCACAGCTTTAAAGAGGGTCCGGGCACCCTCAT
TACTATTGATGAAGAGGAATCCATGGAACAGCCGATCATTTCCGGCATCGCCTTTAATCGCGATGAAGCCAAGCTG
ACCATCCGTGGCGTGCCAGACACCCCGGGCGTGGCATTCAAGATTCTCGGGCCGATCAGTGGCGCGAACATTGAAG
TCGACATGATCGTGCAGAACGTTGCGCACGATAACACCACCGACTTCACCTTCACTGTGCACCGCAACGAGTACGA
CGCGGCCGAACGTATCCTGCAGAACACTGCCAAGGAGATCGGCGCCCGTGAAGTGGTGGGCGACACCAAGATCGCC
AAGGTGTCGATCGTCGGTGTGGGCATGCGCTCCCATGCGGGTGTTGCCAGCCGCATGTTCGAATCCCTGGCCAAGG
AGACCATCAATATCCAGATGATCTCCACCTCGGAGATCAAGGTGTCGGTGGTGATCGAAGAGAAGTACCTAGAGCT
GGCTGTGCGTGCACTGCATACCGCTTTCGAACTGGACGCGCCGGCTCGACAGGGCGAGTAACGCGTTGCCAGGAAG
GCGCGGTTGATCCGCGCCTTTCTTTTTTGTCGGGTGAGGGTGCAGGCCTGTTCTTTTGTCCGTGCTCGACAATACT
TAGGCATGTAGGGTCATGGCCGCTGGGTCATGGGGCTAAGGCCTTTTTTTGCAGACTGTTGTTCCCGAAATGAAAT
GCGTGAGGAGAAAGGTATGCTGATTCTGACTCGTCGATGCGCAGAAAGCCTGATTATTGGTGATGGCGAAATCACC
GTGACCGTGCTTGGCGTCAAAGGCAATCAAGTGCGGATTGGGGTCAATGCCCCCAAAGAGGTGGCCGTCCACCGTG
AGGAAATCTACCTGCGGATCAAGAAAGAGAAGGACGACGAACCAAGCCATTAATTTTTATCGTTTTTTATGTTTGC
AAACGGGGATGAAGCTGGTTAATATACGCCCCGTGTTGCGGAGAGCTGGCCGAGTGGCCGAAGGCGCTCCCCTGCT

AAGGGAGTACACCTCAAAAGGGTGTCGGGGGTTCGAATCCCCCGTTCTCCGCCATTATTTGCTTAGTACGTTGCAA
TCTGGTTTTTTCGCTAAGTTGTTGAAATTAAACGAAAAAATAGCTTTACATAGAGATTGAACGGCCTATAATGCGC
GGCAACAAATGCACTCGTAGCTCAGCTGGATAGAGTACTCGGCTACGAACCGAGCGGTCACAGGTTCGAATCCTGT
CGAGTGCACCATACAAACAAAAAGCCCGCCTAGTGCGGGCTTTTTGCCGTCTGGGGTTTTTATTTTCCTGTTGCGG
GTCGAGTGTTTTTTGTTGGGCCTATCTCGGTCGCGCATGTTTTTCTCTCGCCTATGTGTTTTCTTTTCTGTCGGTG
TCGTCGCAGTTTCATACGTGCGGCCAATACTCAGCTTTGCTGCGCAAGCGCTTGTTCTTTCCAGTTTTTTAACGTT
TAAAGCGGGCGCAACGTGTATCATTGCGCCCGTCAGCCCCGCCGGGGCTTGTGGAATACCTCCATGGACTTACCCA
GTAGTTACTCAGTACCCCGTTTTACCAATCATGAATTGACTGATTGATCCCTCCGGCGTGCCCCGCTGCTGGGAGT
GGAGTTCGCCTATGACCGAAGTAGAAGTAAAGAAAACACAAGAAAGCCTGCAGGATCGCCTGGCGCAAGTCGTCGA
GCTGCTGCATCGCCAGCGTGTGGTCGAAGATCTGACCCATCGCCAGGAAGGTCCTCATCATGACCGGGTCGAGAAC
CTGGTCCACCGGCAGAATCTCGTCGAATTGCAGCGCAAGCTCGATGACCTGCACTCTGCCGACGTTGCCTACATCC
TTGAAGCCCTGCCGCTGGACGACCGTCTGACGCTTTGGCAGTTGGTCAAGGCTGATCGCGATGGCGACATCCTCCT
TGAAGTATCCGACTCGGTGCGTGAGACCCTGATCGCCGACATGGACGATCACGAGCTCCTGGCTGCGGCCAAGGAG
ATGGACGCCGACGAGCTGGCCGACCTGGCCCCTGAACTGCCCCGTGACGTTGTCCATGAGCTGATGGAGACCCTCG
ACGGCCAGCAGCGTGAGCGAGTGCGCTCCGCGTTGTCCTATGACGAGGAGCAGGTCGGTGCGCTGATGGACTTCGA
AATGGTGACCATCCGTGAGGATGTCAGCCTGGAAGTTGTCCTGCGTTACCTGCGGCGCCTCAAGGAACTGCCGGGG
CACACAGACAAGCTGTTTGTGGTCGACTATGAAGGCATCCTCAAGGGCGTGCTGCCGATCAAGCGCCTGCTGGTCA
ATGATCCAGACAAGCAGGTGGCCGAGGTCATGGCCAGCGACCCGGTCAGTTTTCATCCAGATGAAGATGCCTATGA
TGCCGCCCAGGCATTTGAGCGTTATGACTTGATCTCGGCCCCGGTGGTCGACAAGAACGGCAAGCTTATCGGCCGT
TTGACCATCGACGAGATGGTCGACCTGATCCGTGAAGAAAGCGAAAACGAAGTCCTCAACATGGCGGGTCTGCGTG
AAGAGGAAGATATTTTTGCGTCAGTCTGGCGATCCCTGAGCAACCGTTGGGCGTGGCTGGCGGTCAACCTGGTCAC
TGCGTTCATTGCATCGCGGGTGATTGGCCTGTTTGAGGGCTCTATCGAGAAGCTGGTGGCGTTGGCAGCGCTGATG
CCCATCGTGGCCGGTATTGGCGGCAACTCGGGTAATCAGACCATCACCATGATCGTACGGGCGATGGCCCTGGATC
AGGTGAGTACGGGCAATACTTCGCGCTTGATGCGCAAGGAATTGGCGGTGGGTCTGATCAATGGCCTGGTGTGGGG
TGGTGTCATTGGTGTGGTGGCCTATCTGCTGTATGGCAGTTGGTCCCTGGGGGTGGTCATGACAGCGGCCATGACG
CTCAATCTGTTGCTGGCAGCGTTAATGGGGGTATTGATCCCCATGACCCTGGCACGCCTTGGGCGCGACCCTGCGA
TGGGCGCCAGTGTGATGATCACCGCCATGACCGATAGTGGTGGCTTCTTCATCTTCCTGGGCTTGGCGACGATCTT
CCTGCTCTGACCCGCAGCTCGCTCCCACTTCTGTAGGAGCGAGCTTGCTCGCGAAAATCGTTAACGATAACGCCGG
GCACCAATAGAAAATCGGTCCCCTTGGTTTTCGCGCGTCGTCCCTAAATTCCAGGCAAAAAAAAGCCAGCGCAAGG
CTGGCTTCAGCTTTCAGTTGCCGATTATTCGGCTTCTGCGGCTGCCTCGACGTCATGGGCAATCAGCGACACAAGC
GCATTTTGCTGACGGTGGGACAGTTGGCGAAACCGCTGCAGCAGCTCGCGTTCGTGCAGTGACAGCTCCGGGCTGT
CCATGCGCATGCTCAGTTCGTCACCCAACGCGCCTTCCTGAATAAGGCTTTGCTCCATGCGCGCGATGATTTCAGA
ATTCATGCTGCGGTGATGATTGCGAGCCACCTCGGCAATGCGTTCCCGCATTCCGTCTGGCAGACGTACGACGAAC
TTGTCAGCCGTACGGCTGGAATAAATTGCCTGTTTCAATGGGCGCATATATTTAACCGGTTAGTTCAGGGGAGCGG
TTTTTGGAATTGGCCGCAAGATGTGTGTTAGGACAAGGCTTGCGAACAAATGTTCAACCTGAATTGCAAAGAGGCC
GCATCATGCCTCAAATTTGCCAGTTCCTTGGCGTCAATTCTGTGACAAATATTGAACTTACTAAAGGCGTTATGCC
AGTACTCATTTTCAAATTTGCGGACTGGTTGGAAAACTTTTCCACGCCGTAAACGCGGCGCGGGCTCTCGACGCGA
GAGGGTTTTCGGGCGCACATCCTATAGCAATGTGGCCTTTTGCCCTTAATTTTAAGACTAGTGGCAAATGGCCGAT
TTGCGAGCCGAGGCGACATAAGGTCGGGCGATAGGCGGGGCTGGAGCGAGGAGGGTGAACCGGCTATTCAGCGTAG
GACCGGGTCGAATTTGAAGCGGCGCCCGACAATCGCGGCAATCACCCATGCGCTGGCGAAGACGGCGCAGGCGCTC
AGAGAAAGGATCTGCCAATAATTCGACGTTGCGTTGATCCCCAGGATACCGGTGGCGATTGTCAGGGAAAGAAGCA
ACCAGGTGGTCTTGGTCATGATCGGCTCGCTTAGAAAATCCAGTGTTCAGAAGTGCTGGCGGCCTGCGCACCTTGT
GCCAGTTGGACAGAGGGTTGGCCATTGGCTGTCATCACCGCCAATTGTGCTCGTTGCTGGGTCTGGTGGCTGCCTG
TGTGGCTGAGCTGTGCCGCATCGCTGTCGCTACTGTTCTGAAACTGGAACATCACGAGGACGGCCAGGGCCAGTGC
GTTCAGAAGCAGGAGGGTGCTGTTCATGGGTGTTTCCCCGTGTTCATGTGCAGGACAGATTGATAAGGTAAAGAGT
GCAGTCATCGTGCCAGCTTCTAAGTTGATAAAAATCCTTTAAAAACAATAGTTTATAAACGATTCGCAAGTGCTGC
TCGTTGCAAGATGCAATGATGGCCTTTTGTGTTAGTGCATTTTGCACGATGAGACTTGGCAGCCCTGTGGACCTGG
GCCGGCCGTGGGTCTGGAGTGGTGGAGTCTTACGCCTAAAAAGCTGACGGACGACATGACAAAACTCACCTTGGCC
GTTAACATGCACGCCGTCTGTCGCAGTGCAATTTGGCTCTGCGCCTGTCATTAGTCCCAGTAGCTCAATTGGATAG
AGCATCCCCCTCCTAAGGGGAAGGTTGGCCGTTCGAACCGGCCCTGGGACACCACATAAATAGGGCCTCTTAACTA
GATCCGCTGCCAGCTCACTTTTATCGTGACAGCAGGGTGACAGCAGCAGGCGTTTAAAGCCCCGTACAGGTTCACC
TTGCGGGGCTTTTATCTAGGCAATAGAAAACGTTAAAAATATTGACTGATTGCACTGGTGTACTGTCGTTGATATA
GAAGATACTCATCCCAATCTCACGGATGAGCTAGTAATGGACCGCAAATCTCATGGCTATATTTCTCATCGTTCCA
ATCGCGAACCCTCACTTGATCGAGCGAGAGCTTGAAACCTTGAAAAACAGCGCAAAGCTCGATTTCATCAAGCTTC
CGACCTCCGGCTTCGCAGTCAGCTACTCCGGTACCAGCCAAGAACTGTCGAATATCGTTGGCATCTCAGAGGGCAC
CACCGGTACTGGTGTGGTTGCGTCAATCGGTTCGTATTTCGGCCGCGCGCCAACACATATCTGGGACTGGATGAAG
TCGCGGTGGGAGGCGTAATGGCTAAACATCGGCGTGAGGATGACACGACACCATCATCACCAATATCAACACCTCC

AAGATCTGGCCCTGATTGGGCTATGCAGGCCTTTACCGACTTGAAGGTTTCCGTAGTCTCAATGGAAGCAAGCATC
AAAGCGCTCACAGACACAGTAAAAGAGCTAAAGGACGAGCATGCTAAGGTCAAAGGCAAACTCTCGACCGTTGAGA
AGCAGATTTATGCAGCCTTAGCTGTGCTGGCAATAGTCGGCTATGTCGGCAATAAAGCGATCGATTTCGGCATGGA
TATGGCTAAGCGATCGGTGTACAGCCAGACAGTGCCTGCTACTGCACTGCCTCAACTTATTCAGCAGCCGGTTCAG
CAGGTGCAGCCGGTGCAACCGCAACAAGCACCGCAAACACGCTAGAGTCCCTCTCTAATTCGCAAGACTGTAGTAG
TTGAGCAGTTAGCGTGCCTAGCTGTTGCTCGGATACCTAGCCCAGCGCTTAGCAACTCCCTCACGCGCTTGTGCAG
ATCGACGTCCACAGGGCGTCCCTGGTATTTGCCTGCGGCCTTCGCTTTTTCAATACCCTGGGCCTGGCGATTGCGG
CGCTGCTCGTAATCCTTGCGCGCGATTGCGGCCATCATTTCCACCAGCATTGAGTTGATTGCTCCCAGCATCCGCC
CGGTGAACTCATCGCCCTTGGTGTCCTGCATTCCCTGGTGGCTGGTCGGCAGGTCAAGCGCGACGATGCGCAGGCC
CTTGGAGTCGATCGCAGCCTTCAGCTTTTGCCAGTCCTCCACCGGTAAGCGGGAGAGGCGGTCTATCGACTCCACC
AGCAACACGTCACCCTTGCGGGCATCCTTGAGCAGGCGCATTAGCTCAGGCCGGTCGGCTGTGGCGCCGCTGGCGT
TCTCCAGGTACACGCTTGCAATGACCTTGTTATGGTCGCTGGCGAACTGCTCGAGCGACGCCCGGGCACGGCCGGC
GTCTTGCTCTTCGGTGGAGGCTCGGAGGTATGCGCGCATGAACATGATGGGTGCCTGTATCAGTTAGGGCGTTCTA
CTAATAATGTTGCACTATGGGTGTTACTTATCAAGCGAAAAGCACGGAAAAGGCAAAATAAGCCTGTATCTGGATA
GGCATACCCTAAAGGATGGCACTCTGATATCTTTCGTTTTCAATTGGAAAGTAGGTTATGGAGTGACTTAGATGGC
TAGCTCGAAATGCGTAAAATGCGACGGTACAAGCTTTGAGATGAAGGAGGCCAAAATTGCAGGGTCTAATTTCAGA
ATGATGTTTGTTCAGTGCTCGCGTTGCGGTGGGGTGGTCGGCGTCACCGAGTTTATGAATGCGGGCTCTATGCTGG
AGCGCATAGCTAGAAAGCTTGGAGCCTAAGGCTTAGGGCTGGAACAGACTGGCGAAAGCCACACAGTGTGTAGGTT
TCAACACACTGTGTTGTTTTCGCCACGATGGTAGGTCAATGCGAGCTGGGGCGAATATCCGTTCCGTTCTGCGTGC
CAGAGATTCGGTACTTCTGAACCTCACCCGCCCCCACCACTGATGCCGTTTCCTTGACTGGCTGGCCGATCTGCAT
CGCGCCAAGGCCGTTGCCCTCATAATCACCACCGATGCCGATAATATGACGGCCCGGCTCGATCTGAAGTTTCACC
ACTTCGCCAGTGTTGATGCGCGCGACCTTTTTCCCGTCAATGAGTACGGTCGCGATACAGCCACCTGAAGCCCAAA
CCCCGTTATCCCGGCTTACCAGCAACGTAGCGCCCCCTGGGACGGCGGCCTGGTAGGCATAGACCCGATCGGCAGG
CACGCGCTTGGGCAGTTAGGCCGCTTCGGATTGATCAGGAGACGCTTTTGCGGAGGTGCGTGTGGGCATTTTCGCC
AGCCATTCGCGAAGTGCTTCGGAGCCCTCACAGCCCGATCGCCACTTCTCAAACCGGCCGGTGTACCCATTCGAAT
TGGCGACCATACCGCTCAGTGCGACACCAGCATTGGAGGCGGTGGCCAGCACGTCATCATAGATCGCTAGCATCTG
CTTGAGCCCTTCTATGTCTTCGGCGCGGGACGCGGCAATACCTGCACGCGAAAGGCTGTCAGCCGCCTCTGGCGAT
AGCCCGGAGAGCATTGGGTTTGCCCTGAGCACCGCCTCTACAACGTCGGGATGACTACCTGCACGCGCCTCAGCGA
CCAGTCGCTGGCGCTCTTCCGGCTTAAGGCTCCGCACGATGGCTCGTAGCTCCTGATCAGCCAGGAAGCCAACAAC
GTCCGAGCTTGCGCGAGGCGTGACCGGAGCAAAGCCGTTGACCACTGTTTGGGCCAGCGTCAGCATATCCTTTGCC
GCCTTTTCCATAGCCGGCATCAAGGCAGGAACGATCCCGTCGATCTGTTCCTTGCCCTTGCGCTCGATGGCTGCGG
CGTTGAGGTCTTTGTTTCCCCGAATCGAGAGAATTTCTTCTCCAAGGTCCTTGAAAGTCTCCAGCGCCAAGCCCTG
CAAATGGCGAACCGACTTCAGCAAGCCGCCGGCCTGCCATGTGCCTTTGGTGCCGTCGGGGCGCTGAATATTGAAT
GTTCGGGCCTCTACCCGGGAGTGCTCGTATTCGGAGTAATGGAGGTTGGCCAAGCCAACTTTGAGGACGGAGCGTT
TGCCGATTTTCATTGAGTTACCTTTTGATTGTCTGGCCGCGGTTCGGCCGGCTTTGGTCTGAGGCTTCGCTCAGGA
TCTCGAGTAGGGAAGCGTTCTCCGGGGAGTCGCCGCCGGTTGCTGCTGCGTGCAACTCGTCCATAAGGGCGCTGAT
CTCTCGCACGTCACGATTGAACTGAGTGAGCCCGGATTGCTGGCGCAGTTTGGCCACCTGGTTACGGATGTTCATG
GCGGGCCTCGATGATCAGTTGGTACTGTTCTGCGACGTGCTCTTTGATGTTTTGCCCATCAGCCAGCGGCGGGAAG
ATCCAGGCACTAGAGCTTGGGAATGGCCGCATATCCATCACGCCTACCGGAGCGGGACGGCGGAGGCTTTCAATTG
CGCGGACGTGGAGAATGAACGCCTCTGCTAGATTGGCCGCGTCGTTGCTGTTCATGGTGCACAGTGGGAAGTGCAT
CACCGTTTGCACGCCCAGGACGGGATAGTTGAAGGAGTGGCCCACTGTCTTGGGGTAGTCCATCTCCTCCTCAACC
GCGTCAACGCTGCAGTCCACTACCGTCAACATAACGAGCACTGTCTCTTCGTCTTCGGCGCCGATCTTTTCCGCAA
TTCGCTTGAGCTCGGCGCGCATCCACTTAATGCTCATTGCCGCATTCCCTCCATCTGTTCGCGAAGTTCTTCGACG
GCCTTCTGTAGCTCGGCAACTTCGATTACCCGGGCAACAGCGCCTAGCCCTTCGATCAACGCTTTGCCCTGGTCGG
GCGGTAGTTCGCCGGCGGCAACTGCGCGAAGGATTGATTTGGCTGCGCTGGGTAGGTCGGTGTCATCGAGTTCGAA
GCACACGGTCTCGGCAGTTGGTTTGATTGGCGGTACCAGGCGCTCGAGGATCAGTCGGCAAGCCTGCATATCGCCC
TTCTTGGCCGCTGTCAGAACCTTCTTTGTCACGGCATCTGCACCTTCCGCGAGGCGGGCGCGCAGCTCCTGCGTTT
GGCCAGAACGACCACCGGGATTACCTGACTGCCCCGGCTTCCATTTGCCGGCTTTATCGCGATCAGTCATGCGGTG
GGCGATTCCATAGCGATGACCGGTTTGTTTTCAGCGAGCGCTATCAGCAGCGCGGATGCTTGAACGCTTCCGTCTG
CTGCGGCTTCGCGAAGGCGGCCCCAGGCGGCAGCAACTTCGGAGCGAGTTGGTTTGTGCTGGCGTCCTCGGGCGGG
CTTAGCTGTGTTCATAGGTGCTGCCTCGAATGGGTTTTGAATGGCTCAAAAACTCACATTAGGCATGATCTGATCA
TTTATCCAGTGCTTTTTAGGTGTACTCTAGATGATACACGTAACCGGATTAAACGGGACTGAAATAGTTGGTTTTG
GCAGGATTGGAGAGGGTGTTTTTGTTGCAGTAAGTCGAGGATAGGCAAAGTGATACAGGGCTGGAATTTCAGCTCA
GTCCGGCCTCAGATTGAGGGTGGGGCGGGGGGGCTGAACTACCGTTTTGGGATGGCAGGTTTTGTCAGATTCTCGC
GCCACGTTTTGCGAGATATGAAAACGTGGCGCGGATTTGATGCGTCCAGCTTTGGACGCATCTTCCACCAGTGGTG
GTAAACCTACACTGCTACCAGGTATTCCGATTGGGCTTTGCGGGGACTGTCGAAAACTTATGCCCTTTTTTGGACA
AGTAGCGATCCTGGGCTAACGTCATGGCCACAGGCCAGATAGCCGGCCTGCTCACGAGCTACCGTACAAAGGAAAG

CTAATGGAGCGTTATAGGAACTTAGGGGGAGACTCAAATGTGGCCGCATTTGAGATAGGGAATGGTTCAATCACTG
TCGAGTTTAACGACGGTGCGCAGTACCTCTATACCAATGAAAGCGCTGGGCCGGGATCTATTGCTGAAATGCACCG
GCTGGCTAGGGCTGGCCAAGGGCTGAATAGCTATATTGGGCGCGTGGTCAAGAAAGGCTACGCGAGAAAAATCCGC
TAAGAGGCAGATGCGGCACGCTTTCGCATTTTGAAGGCGTGCCCTGGTTTAAGTGGGCCAAGATCACCTTTATCTG
AAGGTGATGCTGTCATCGCAACACCTGCTCCAGCCTGCTGTCAGGAGCGACGGCCGGCAAGCAGACCAAACGGTCC
GAACCGGCTCATTTGGTCTGTCTCGACGGGGAGGCAGCGGCTCCATCCTGTGCCTTGCGGCGATGGTATGGTGATG
TTGACCACCCCACCGTCTGTTGTTCGCCACACTCTGTGGCGTTTCCGCCACGCTGAGTGAGACCGAACCGTCTCAG
TGAGATGAAATTGTCTTACAGAGCACGAGGCGTCACACTACGCATCAGTCCAATTTCAGCAGGGAAGCACCGCGCT
GAGGACAATGCTTCATATTGTACGAGTAATTAACAGGGAGAGGGCGTGGAATTGCGTAAGGTTATAAACAGTCCTA
TTGGGACAGCACTTCTTGTTGTGCTAGTTGCTTTTATAGTTATCTTTACAATTAGTTCGGCTTGGGAGTTTATAAG
TACTACTATTTTTCCAAATACTACTCTAGGTGCCTATAATAGAGGGTTCTGGGAAAATGTGCTTGTTGAGCTGCAC
GGGATGCTGATAGAGCTTGCCGTTGTCGGTGTGCTTTTATTGTGGCTGGATGGACGACGAGAGCATGCAAAATCTA
TATTGCAGAGTAAGGAGGAGCTTGCTGATTACGCGGATCTTGATTTTCCGGAGGCTCACTTGAGAAAGATTGGAGC
TCTAAAAAGATTAAGTGTGGCCGGAAATACAAAATTCATTGTAAGAGATTTGCATTTGGTGGGGAGAGAGCTGAAA
GGCCTGAAGTTAAAGGGTTGTGCTGTGATTGGCCTGAAGTTAACTGGTGGGAAGATAACTTCTACCACTTTTGATG
ATGTTGATATGAGGTCTTCAAATTTTGTTGATTGCACAATTAGAAATACGGATTTTATGTCCGTAAAGATGTATAA
GTGCAACTTTCAGGGTGCCACTCTGGCTGGCGTTAAGTTTGAAGGCGTGGATATAAATCAGGCGGAATTTATTAAT
TGTACGATGCCTAATACGATTTTTAAAAAGGTTAGCTTGGCGGGCGTGAGGTTTGATGGTGCCGATTTGAGTCGAT
GCAGTTTCTTGGGTGCACGAGATATAGATGTGGCCCAATTGGCTAAAGCTAAGAAATTAGATTATATATCAATTTC
GAAAGAGTTATTAACGGATTTAATAGTGCTGCGGCCAAATATTAAATTTCAACGTGATACTCGTCGGCCTTGAGGG
AATTGAACCCTCTCACTAGTAAACTAGCTGACCCGCATCTGGCCACGAGCGTTGACAATACGTCAGCACTGCATTT
TCGTCAAGCCAGCAGCATACGCCCGCGCGTAATAGCCGCAGGCAGTTCAGGTGGCTCGACTCTCGACTCATGGGGA
ATGTGCAGAGCAGGAATTTTTATCGGGATGACGAGCTACGTTGGCGGCCCTTATCTGTTCTCTGTCGTGAGGTTGA
GGCAAGCAGACCTGATGAGTCGGCTCACTAGGTCTGTCTCGTCGATGTGAGAGCGATTACCGATCGGCCAGATAGA
CCAAAACGTCAAACTGTGAGGTATTCCACTGGTGAGATAATTGCCGCGCTCTGAATCTGCCGTAGAGGCGGCGTGA
TGTTAGCCCTGTTCGATTCATCAAAAACTATGCGCTGACTTCTCTTATTCAGAGAAGATCAGTACCTAGAGTAATT
TCTAGAAATTTATATGAGGCTACCAACTAATATGATGGGCAATTGCTCTAAAATTTTTAGGGAATAGTTCGAAAAG
ATCTATATCCTTTCCTGCAAATCCTGCCTCCAACAGAATACTTTTAAATGGATCGGATTCAACTTCCGCCAAAATT
CTGCTTGCGTTGCTAGAGTGCCATCCAGATCTGCCGACTACCATCCATGCCCAGCCATTGCGGGGATTCTTTGCTA
AGGATTCTTGAATGGCATCTTTATTTTCAGCTTCGCTTTCAAAGAAAGCAAGAGATCCTAATATTTCGAAGCGCTC
ATAAAGTAGTTCAAAGTCAGATACGAGGCCCATAAAAGGCTTGCTATAATCAGAGAAAAGACTTAGTAGGTAGTCA
CTCAGCGCCGTTTTACGATCTTTGTAGCCCTCCATATTTTTCCATTGGTCATTTTCAGCTCCCTTCCACTTCCATA
AGAAAAGGGTATCAATTATTCGGGCTGGGCCTCGATACGTTAAGTGCAGCTGAGCGGTGAATAGCTTGTGAAGAGT
TCCCCATCGTTCACTTCGGGTTAATCCCAGTCCGTAGGCAGTAAAGATAAGTAAGGCTGGATATGAGCGTAAGCCA
ATATAGCCGGTATTTCCGCTTCCCACACTTTCAGCATGAGTGTGCAGTGCGCGAATAAGTTCAAGAACAAGAGGGA
GTTCACTCCCTTCTTTGTCGCCCCACCTGCCAAGGACTCCGCATATTTTTATCAGTGCCTCGCTCACAGCTTCGTA
TCTTAAAGCATAGGACTGTAATTCTTTAGAAGTCCATTGGTACGGCTGTGAAAAGTCTGCACTTCCTATCTGTGAT
AAGAGTCGCTCGCTTTCTTGGTGTACTAAGTCGTCGAGCTGTATTCGATACTCTTGTTTTGCAAGGAAGCGCTTTG
CGCTACTAACGAGAAGTTCGATGCTTAGAGGGTTTTGGCGCTGACTCTGTGCGAGCGTATCCACGTATTTCTGAAG
TGTGGTGAATAAAGTGTCAGCATCTCCTGCTGAAACAACTCGTGCCTTACGGTGATCAACTAGCTCCTGCGCACCA
CTGCCGAGGCGGCCGCGAGATGCCCAGTAAGTGGTGTAACGACGGTTTGGGGCACGAATAAACGCTGCTCGGAGTG
CATGATCCCATTCCCCTGACCATCCGCTAACAATAAGTCCGTGCTCATCCAGGATACGATCCAAGAGAACATTGTA
CTGTTCTGGATACCCGCTCAATTCAGTCTCTGTGTTGAGTATGCGGGCGTCTTTATAGTCTCCGTGGAGCTTCAAA
ATGTAGCAAGAGCTATGGGTAATAGGTTCCGCTCCCGCTAATACATCCACAGAAGAAACCACGGTCGGCTCAATGC
CTTTCTCGCGTAAAGCATTTTCCATCAACCGGTCGAAGTTGGTGGTTATGATTACGCGTATGAAACCGCCTGCGAC
GAGGTTAGCAATAGCGTGATGAGCAGCAGTAGGTAATTTTTTGCCTGCTTCTCGGTCGTCAGCGTTTGGCTCGATG
TAGCGGTGCATTATCGCGCGGCGCTCATCGGGGGATGATGCGAGCTTTTCCAGCAGTGTCGAGTAGTTGGGTTCTT
CCCCAGTAGTCTCGCGATACCAGGCAGCCCAATCTGCCTGCTCCTCAATTCCTTGGGCTAAGGCGGCGCGTTTCAC
CAAGTCTAGGGTGATTTCCCAACCCGTCGGTATCTCGGCCGCCCGCGAGAGGCCTGAACCAAGCAAAAGCGCAAAG
ACACCTTTGCTTTCGTAAACAGAAAACGCGAGCTGAGTTACGTCATCCTTAAGTAGCGCCATAGCAATAGTCCCTA
TTCAATTTACATAGCCGCATGCTAAGCCATTAGCCACTATGAAGTCTTCGCCTCCCACCACTCATTCCAACCTCGT
TTATGGCTGCCGCTCCTGTTGCCCACCGATAAAGCGACAGAGGTCTGCAAATATTTTTCTCGCCACAGTTGTGTTA
CCACTCACGATGACGCATGATTAGTGTCATCGTGAGTGGTAACGAGGTGCGACATGAAAGAGCTGGAAAAAACAAA
GCCATTATCCGCAGCGGATCGACAAAGGCTCTTTAAGGAGCGTCAGCGCGAAGCAGGTTTCAGGCACACAACGGTA
TGGCTTCACACGGCGACGGAAGAGGAGGGCAAGCAGGCGGCTCGGGACGGGAAGCCACTCAAACCGATGGGCACGA
GAGACCCGCTGAGCTGGGCAGCGGGATGGATCAGTGAGAAGGGTAAGCAGTAACCGGCGGAATATTTCGCCAATTG
ATCGGCAGAAATTAGCCGATCAGGCCGATGGTCCAGATTTTGGCCATCTCAGAAGGGGGGCCAAGCCGCTGCGCTA

ACAGCGGCTTGGCGATCACACGACGAAACTTGAAGGTCGACGTCATGCAACTTTACCAAAATATCAGTAACGCACT
GGAAGGCCAAGATAAGTCCGAATTCGAGGGGGAGGGCTCGGTAGGCAATGAGTCGGGCGACTCTACCTCCGTACTT
CTGCCTCTCTTCACCGACAAACAACGAACCTTAGTGGTTGAGCTCATCCTGGCAGAGCGTCGTCACTCTACTCGAT
GGTGGACGCACCTGAGCGAGATGCGGCGGCGTAGGGAGCTCCCTGAGTGGGTGGCGGCGGACATTGGCACTCATGA
TGAGCATGACCGTCTGCTTGAGAGTAGGAAGGCCGTCAATCAGGTTCTGTTCGGCTCAGATGACCTGGGCGGCGAT
CAGCAATACCGCGCGGTGGTGTTGGAGTAGGGCTGATAAGCAACGAAACCTTCGCGGCATCGAGGGCACTACCCCG
AAATAGGGTGGTGCCCTTTTTCATTTGTTGGCGGTCGGTGCCTTCTCAGCAAAGTAGATCAGGTTGAGCTGTATGC
CTCCAACCACTCATCAACACTCTGGTCCTGTGGTGGCTTAGGTGTGTGTATGGGGGAGTAAGGAAAGGTCTTGTAA
CCGTCCTCCCTTATTACCTTGTTTCCGTAGTTTGAAACACACGTAGTTGGAAACGGCTGTAACCCTCGTGTTTCAT
GGTCTGTAGGCATTTCCTTGTCCGGCACTTTTGTCCGGGAGTTGTCCGGCGCTTGTCCGGAAATTGTCCGGATGTT
GTCCGGCAAAATGGCTGCTCTGAATTGCTCTGCGGTCAGACTGACAGGCACTAGACTGCCGACTTTGAAGCGGTCA
TCAGTCACCAATCCAGTCACCAGGACGCTACGTAGCTTCCCCATGTAATCGTGCATAGCCGCCAGCCGCCGAGGAA
AAGGCAGGCCGGTCCCCATCCAGAACTTGTAGAAGTGGCTGCGCTTGCCGATGCGGAATTGTCCTTTTGGTAGCAG
AGCGCCAGCCTTAGCCGTAGCGGTGTTCCGCTGACGCGTCAGATCGGCGTTGAACACTGCGTAGACAGATTGGCCG
GACAGCATCGAGACAAACTGAAGCGTGATACCGGCGCTCCGGGTTTTGCCGTAGTGCCCGGTTGAATAGCCTACGT
ACTCGAATGCATCCAGGCCCACCGACATGAGCACTTCACGGCAAATTCTGGCCCGGTTGATACTGATCCACTCCGG
CGGTACGGGCTTGCCGCTCGCCGGTTGAATGGCGAGCTGCCCACGCTCAATGCTAACCGCGTCGCCACGGCTAAGT
AGTTTTGTCGCTAGGCATTCCACTGAATCGACCTCAGCGTAATTCGGCTGGATAACCAACGGGCCGAAGCCCACCG
GCAGCTCACAGGCAGGCGCAAGAGGTCGCCGCAAAACTGCGGAGACCTCTACGACCTCTGGATTACGACGCTTCGC
GCATCAGAATGTACTTGGCCACGCGGTGAGGTGGCCGGCCTGGCTCGGTGGACTGGTGCGTCCAATAGGTGCGGAT
CTCATGACCCTTTTTCCTGAGGCGGCGGATGGTGTTGGGGGGCTGGACGATATCCAGTTCCTTGGCGGCTTCGATG
GTCGATACCGGCCCGTTTCGCAGGGCCTCCATCATCTTGCAGTCTTGCTCTGTTGAAGAATGACGTGTCATAGTTT
GCCCTTCACGTTGGTGCTATCCAGTGTGTTTTCGCTAAGGCGGTGTTCGAGCACCGCCTTGGCAACCTCTCGCGCA
GCCATGCGCAACCCCCAATCCTTATTCCTTTCGCACTTCACCGTGCGCCGACCTTTGCTGCGCGCTCGATAAAAGC
CTTCAGCTCGGATGCCAGTGCCAGGCGTCGGCGGCCGATTTTGAAAGTTTTGAGTTCACCTTGAGCGATCAGCTCG
TAGGTGGCCGAGCGCGAGATACCTAAAATGCGGGCAGATTCTTCGACGCTCACCGACAGCGGCGCCATTTGTTGCT
GTGCTTGTTCCATGGTGGTCTCCCAAAAAACGAAGCCAGAGAGGCGTCGTGTTTCGCTAGCCTACATCGTGCAAAA
CTATATTTGCAAGCAAAAGCTAAATTGACTTGTACGCGCATGGATTGACAGGGGTTTGCGCGTCGCGCAAGCTGTG
GCAATCAGGTCGGTTAGGCTGACCTAATCAGAGCAGATGGAATGTATGGCCACTAAGACAAGTAAGCGGACTGGCG
AATCGAGCACCACGGTCGCCGTGGGCATTAGGATTGACCCAAAAATTAAATTTGCTCTGGATATAATGGGCCGACT
GCAGAAGCGCTCTCTAACCGCAGTGATTGAATGGGCGATTTCCCAGGCAATCGCGCATCAAAATGTGGACGGTTCG
AAAGCTGCGCTTGGTGAGGTAATTGACGGGATTTGGTCTACTGATGAGTCGATCAGGTTTGTGAAGCTTTGTTTTG
ATTTGCCCCAGGCGCTGACTTACGACGAGCTGAGGACATGGGAAACGATAAAAGTAAGCCCGGTTTTTTGGATTGC
GGATGAGGTGGGTTGCTATGAGGTCAACCGTGAATCTATATCGTTGGATGCGGTAAGTATGGGATGGAGCTCTTTG
CTTGCTCATGTTGAAAAACATAAAACGAGTTCTACCGTCGTCCCCATGCGCGATCTCGATTACGTTCCTTTTAAAT
AAGCTCCACCGCGGCGGCCTTGGTCCCTGGCGCAAGGTGCGCATAGCGCAGCGTCATTTTTATATCGGCGTGCCCC
AGCAGATCCCGTACTGTGTTGAGCGGTACGCCGGCCATTACCAGGCGTGAAGCGAAGTCGTGCCGCATGTCGTGCC
AGCGGAATTCGTCGATCTGCGCCCGCTCAAGCAACTTGAGCCAGGCGCTTTTGACGTCCTCAAAGCGACCGCCGCC
CTGACCTGGGAAGACATACGGCGACTTGCTCACCTGCTCCTTCCAGGCTTCCAGCACACCAATGGTCTCCTTGTTC
ATCGGGATGTGGCGCGTATCGCTTGTTTTGGTGGTGGCCCCGGCGACTGTGATTGTCTTCGTGTCGAAGTTCACCG
CTGACCATTTCAGGTCGAATAGCTCCCCGCGCCTCATGCCGGTATTCAACGATACCAACACCATTGGCTTCAGATG
ATCGGTGAAGGGTAGTTGCAGGAGGCTTGGCAGTGGTTCTCTGTGGCGGTCGGTCCGCCAGGTGTTTGCACTCTCA
CGCTCGGCCCTCATTTCATCCTGTCGAGCGTCCAGGGCATCGCGTAGACGCTTGGTCTCGTCAGCAGCGAGGTAGC
GAATCACCCCCTTTGAATCGACTTTGAGCTGTTTCAGCTTGGCCAGCGGGTGTGTGTCGATGTACTCCCACTCAAC
GGCACGACTGAAAACACCACTGATCGAGCCCATTTTTCTATTCACCGTCGATGCTTTGTTGCCGGCTTGCATCCAG
GCGGTACGGATTTGCTCCAGGTCTCGCCCGGTGATCGCGTCCAGGCGCTGGGACATGATTGCTTCAAAGTTATTGC
TGAGGGTGTGTTGTGTCTTCTCGTGGCCTTTGTGATGAGCCTTGAACCACGGCATATAGGTGTCGTCGATGAAATC
GCGCAGCGAGGGCAGGGCTGACCCCTTGCGCCCCTGGGCGATGGCCAGGGGCTCGCCGTGGGCGTGCGCATCGGCT
AAGTACTGGGCGGCCTCGGTTCGGGCTTGGTCAAGGGTAAGCACGCCGACACGCCCGAGGGTGGCGTTCTTGTTTC
GGCCCCAAGTGACCATGTAGGACTTGTGACCGCTGGGCAGCACGCGAATGAAGAAGCCGGGTTGCACTGTGTCATG
CACGCGGTATGCTTTCGCCTCGGCGGCTAGGCTGCCAATTAGCTTCTGAGTGATTTTGGATTTCAAGGGTTCGTCC
GTGACAGCAGGGTGGCAGCAAAACGCACTATGCCGCTCGATGTTCACGGACTCAATGGGACTAAAAGGCCCAGTAA
TACTGGGCTTGTGGCTGTGCCTGCTGGGTCGAAATTGACCCTCCTAAGGGGAAGGTTGGCCGTTCGAACCGGCCCT
GGGACACCATCTGCAGTTGCGCCGCCTAACCGGCCGTGACGTAAAAGAACCCCGACCAGATCGCTCTGGCGGGGTT
TTTTTGTGGGGATCAGTTTTCTTAGCCGTCTGGCCAGTGCCTTCGGGCGGGTTGGCGAGATATATTGTCAAAACCT
TGTACCTCGCCCCCTGCCTGGATGCGTCTGACACGTATCTGAACAGCGAAGGAACTGTTGGGTCTCGAGTTCATCG
AAGGCTGCATGCTTCGACGATCGTGAGGTTTGCCGGATCAAAATACCCCTGGGTGCCGATGTAGTTGGGTGCCTGG

GTTTAACGACGCTTCGCCCTCAGGCTAGTCTGAATAGCCTGACACCCACTGCAAGGAGCCGCTCGGACCGCCGATG
CGCCAGATCTGGAAATCTTTTCGAGCCCTTTATTTCGCTTCCTTGATGATGCTGATCGGCTCCGGCCTGCTCAGTA
CTTACCTGGCCCTGCGCCTGGCGGCGGATAACGTCGACAGCCTGTGGGTCGGTGCGCTGATGGCCGCCAACTATTT
TGGCCTGGTGCTGGGTGGCAAGATCGGGCATCGCCTGATCGCCCGGGTCGGGCATATCCGTGCCTATGCCACTTGC
GCCGGGATCGTCGGCGCGGCAGTGCTCGGTCATGGTCTGGTGGATTGGCTGCCGGCCTGGATCGTCCTGCGGATCA
TTGTCGGCCTGGGCATGATGTGCCAGTACATGGTCATCGAGAGCTGGCTCAACGAGCAGGCGGATGCCAAGCAGCG
CGGCGTGGTGTTCAGCGGTTATATGATCGCGTCGTACCTGGGCCTGGTGCTGGGCCAGTTGATTCTGGTCATGCAC
CCTCAGTTGGGGCTTGAGCTGCTGATGCTGGTGGCATTGTGTTTTGCGCTGTGCCTGGTGCCGGTCGCCATGACCC
GGCGTATTCACCCTGCGCCTTTGCATCCGGCGCCGATGGAGCCGCGCTTCTTTATCAAGCGCGTGCCGCAGTCCCT
GAGTACGGTGTTGGGGGCAGGTCTGATCGTCGGTTCCTTCTACGGCCTGGCGCCACTGTATGCGGCTCAGCAGGGG
CTGAGTACCGAGCAGGTCGGTCTGTTCATGGGTAGCTGCATTTTTGCCGGCCTGTTGGTGCAATGGCCGTTGGGCT
GGCTGTCGGATCGCTATGACCGTGCGTTGCTGATTCGCTGTTTCGCCCTGGCCCTGGCGGTGGCTGCGTTGCCGCT
GGCGATCATGACCCAGGTGCCGCTGGAAGTACTGTTTATCGCCGGCTTCCTATGTTCATTGGTGCAGTTCTGCCTC
TATCCACTGGCCGTGGCGTTTTCCAACGACCATGTCGAAGGTGATCGCCGGGTGTCCCTCACGGCAATGTTGCTGG
TGACCTACGGTGTGGGGGCGAGTATCGGGCCGTTGCTGGCGGGTGTGGTCATGAAGCTGTTTGGCAACCAGATGCT
CTATGCGTTCTTCAGCTTGTGCGCATTGATCCTGGTCTGGCGCATTCGACCCAAGGCTGTGACCAACCTGCATCAG
GTGGACGACGCGCCGCTGCATCACGTGGCGATGCCCGACAGCATGTCCAGCTCGCCATTGGTAGCCGCCCTCGACC
CGCGTGTCGATGAACAGGTGGTCCAGGACCAGATGCAAACCGCCGTGCCTGAGCCTGAGCCCGAGGCTGAGCCTGA
AGTTGCGCCGCAACCCGAGCCCGAGCCGCCGGAGCATCCCGACCCGGATGATCACCCGCATGACCTGAGCCGCGCC
CGCCCTTGAAGCAAAAAAAACGGGCAGTTCCCAAAGGAGCTGCCCGTTTTTTATGCCGATGGTTCGACGTTTACAT
GTCGTCGTCTTTATCGAACCGTCGCGCCTCGCGCTGCAGTTGGTAGACAAAACGCTCGACCTGGCGCTGCACCAGG
CCGCTCATGTTGTGGAAGCGCACGCCGGCAAAGGTGGTGTTGATCCTTTCTTCAAAGTGCAGGTAGCGCAACTCCA
CCGCCGTGGTCATGCTGCCAAAGGGCAGGGCGGCGATGAAACGGTCGTAGACCTGGCCCAGTTGCAGGCGCTCGGA
GATGTCGCCTTCAAAGCGCAGCTTGCAGCCGGTGGCGGAGATATCCAGCAACTTGCCGCTGATGGGCGCCTTGAGT
TTCTCGCCGCCCAATTCGATATTCACCAGTTGTGCCAGCTTCAATGCCGCACGAAAGGCATTGCGCCGCTGATGGT
AGACCACTTCTTCCGGCAGGCTGCCCTTGTAGATGCGGTGGCCGTCCTTTGCGCCAATGGTCAGGGTGCCATTGCT
GTCCCAGGCCACGCGTACACCTTCATGGAAGCCTTCGATACGAAACGCTTCGCCATTCTCCAGGTAGCGTTCACCT
TCGGGCGGGATCATTTCGTCCAGGCTCAGGGTCTTGCTGTCCTTGTCGACATCCACCAGGTAGCTCTGGAAGCGCT
GGTTGCGTTCGTGGAAGGTAATGATCAGCGGGTCGTGGCTCTCTTGCAGCAGCCGCAGGGTGCTGGCAATTTCCAG
AGGAGTGGTGAGGACCTTGGGTGGCTGCGGAGCATCTTCCGCGCTAAGGGTGTTGAACACGGTTCTTCCATCTCCA
GACAAAATACGACTACACGCAAGAACCGGCATTTTGCCAGCATGTGGCGTGCCTTGATAGGGCGCGCGATAAACCG
GCGTCAGGCTTGGCTGAGGGTGCTGGGCTTGACCAGCCGTGAGGTGGAGCCTTGGGCGTTATACAGTGCTGGAGGT
TCGCCACCATGCAGGATGCGCAACTGGTTGGCGGTGGTAGCTTGCTGCAGTTGGATCGACTGGCCATTGAGCACAT
TGGCCTCTTGGCAACTGATCAACAACTGATTGAGCGCTGCGCTCTGGGCCAGCAACTGGTCGCCGACCGACGAATG
GCTGGCCAACTGTTCCAGGCCGCTGTGGTCAGCGGGCAGGCCCAGGCTGAGCAGGATCTGGCTGCGCTTCTGGCCG
TGCTGTTCGAGCAGGATGATCAGCGATTGCTTGCGTGCCAGGATATCTTCCAGCAGGCGCATATCGCGGCCATAGA
GGGCCACGAATTCTTCTTGAAGCAGCTCCAGCAGGTGTTGCGCCGGAGCCAGGTCTTCGTTGATCAGTTGCAGCAG
ATTTTCGTCGTGCATGGCTGGCCTTGGGGTTTAAGCGTCCAAAAGCCTGGCGCAGGCCTTGGCCTAGCGCTCGGCT
TTGATGATTTTGCCGGCTAAACGGTCGTTGTCGATCTGATAGCTGCCATCAGCAATCGCCTGTTTCAATTCGGCCA
CGCGGGCTTTATTGACAACCGGCTGATCGCGCAGCGAGTCCGTGATCTTCTGCAACTGTTGAGCCTCGTTGCTCAA
ATGCACCGATTCGCCACTTTGGGTCGAAGCGGTTTGTTCTGCCTTGGCAGGCAGTGCCGGGGCCTTGGCTTCAACG
CTGTCCTTGGCCGCGTTGGTACGCGTGCCGCCGGGCGTTGCCTGGGCGCCGTTCAATCGGCTGAAATCAATGACCA
TGTTAAAAAACCTCTGGGTATTTGGACGCTTGCCATGTTTTCGGCCATACCCGGACAAACTTTAGGCTCGATCGAC
AATAAACCTGTGCATAGCGAGATCATGCGCACAGTGTAGGAAAAGCCAGCGTCCCACGCCAGTGATCTATAAAGCC
ACCTCCACCTGGCCAGGGGCTGTCACCCGGGCCTTGATCACGCGCTTGGAGTTGAGGTTCTTCACGCGAATCTGTT
CGCTCATCCCGCCGTTGGCCAGGGCTTCCCCCGGCATTTTCACGTTCAGGCCGCCGCTGCTGGCGGATATCACCAC
ATGGTCACCCTTGCGCACCACTTCGGCCTGTTCCAGGTGAACCAGGGTAATGACCTGGTCGGTGACCATTGGTCGG
GTCAGTCTCTGCCCAACGGCCTGGTCCACGGAGGTGAGGTAACCCTGGCTGATCTGGCTGATGTCCCGCTCACGCA
GGGCTACGTCTTCAAAACCGATGATGCCGGTACGCTTGAGCGGGCGTGTGACGGTCACTACGTCGCGAAACAGGCG
GACCTGGGCCGGCACAAACACCGTCCAGGGCGAGCTGCCGTCGCAGCGCACGCGCACGGTCACGCGGCCGATGGGC
TGGGCGGGGCTTTCCAGGGAGCCTGTCAATTCCTTGTCGCACATGGGCATGCGCAAACGCGGGTCCAACTGGTTGA
CCTGGATTTCATAACGGCCCGGTGTCTGGGTGGTCGCCAGATAATCTTCTACAGTGAACTCAAGAAAGCCTTGAGT
GACGCCGATAAGCAGATCAGGCAAGGTGACGTTGTCGGCACGGGCCTGATGGCCTGCGCCCAAGGCAAGCAACGCC
ATCCAGGCACAGAGCAATCTGCGGTACTGTGGCGAGCGAAGGCGTCGGGAAACTGTCGTTTTGATGTCCATGGCCA
ATAAAAAGCAAAGCGCGTGCCGTTTCGTGTTGGGTGCGCGTCGTACCCGTAAGTTTTGTAGCGTAGGAGTCTGGGC
ATGGCGGGTGTAATGGATTCAGTGAACCAGCGCACACAGCTGGTAGGGCAGAATCGCCTGGAGTTGTTGCTGTTTC
GACTCGATGGCAAGCAGCTATATGGCATCAACGTCTTCAAGGTGCGGGAGGTGCTGCAATGCCCCAAACTCACCAT

AATGCCCAAGTCCAACCCGGTGGTGTGTGGCGTGGCCAACATCCGTGGCTCGACGATTCCGATTCTTGACCTGGCG
ATGGCTACCGGTTCTGCCGGTTTGCAGGACCGCGAGAGTCCGTTTGTGATCATCACCGAGTACAACACCAAGACCC
AGGGTTTCCTGGTGCGCTCGGTGGAACGTATCGTCAACATGAACTGGGAAGAGATCCATCCACCACCCAAGGGCAC
CGGGCGTGATCACTACCTCACGGCAGTGACGCGGGTCGATAACCAGTTGGTGGAAATCATCGACGTGGAGAAGATC
CTCGCAGAGGTGGCGCCGACTTCGGAGCATATTTCGGTGGGTGTGGTGGATGAGCAGACCCAGCAGAAGGCCGTGT
CCCTGCGGGTGTTGACGGTGGATGACTCCTCTGTGGCACGCAAGCAGGTGTCCCGTTGCCTGCAGACCGTTGGTGT
CGAAGTAGTGGCACTCAATGACGGCCGCCAGGCCCTGGACTATCTGCGCAAGCTGGTGGACGAAGGCAAGAAGCCG
GAAGAAGAATTCCTGATGATGATTTCCGACATCGAGATGCCGGAAATGGACGGCTACACCCTCACGGCCGAGATAC
GTAACGACCCACGCATGCAAAAATTGCATATCATCCTGCATACTTCATTGTCCGGTGTATTCAACCAGGCAATGGT
CAAGAAGGTCGGTGCCGATGACTTCCTGGCCAAATTCCGTCCTGATGACCTGGCTTCCCGGGTCGTCGAACGGATC
AAATCTGCAGATCACGGCTAGGGGCTTTCTCCCTGGCGGTCACACGAGTTAAGAGGCGGTTTCATTGTCTACGGGT
AATTTGGATTTCGAACAGTTCCGGGTATTCCTGGAAAAAGCCTGTGGCATATTGCTCGGTGAAAACAAGCAATACC
TGGTATCAAGCCGTCTCAACAAATTGATGGAACAGCAGGGCATCAAGTCCCTGGGCGAGTTGGTGCAGCGCATCCA
GGGTCAACCGCGCAGCGGGTTGAAGGAGATGGTGGTCGATGCCATGACCACCAACGAAACCCTGTGGTTTCGTGAT
ACCTACCCCTTCGAGGTGCTCAAGAACAAGGTATTGCCCGAGGCCATCAAGGCCAGCCCCGGCCAGCGCTTGCGCA
TCTGGTCGGCGGCGTGTTCATCGGGGCAGGAACCCTATTCGCTGTCGATGTCCATCGATGAGTTCGAGCGGACCAA
CATGGGCCAGTTGAAAGCAGGTGTGCAAATTGTTGCCACTGATTTGTCCGGCACCATGCTGACCAACTGCAAGACC
GGCGAATACGACAGCCTCGCCCTGGGTCGCGGCCTGTCCCAGGAGCGCCTGCAGCGTTACTTCGACCCCAAGGGCG
CCGGGCGCTGGGCGGTCAAGGCGCCGATCAAGAGCCGTGTGGAGTTCCGCTCGTTCAACCTGCTGGACAGCTACGC
CAGCCTCGGCAAGTTCGACATCGTGTTTTGCCGCAACGTATTGATCTACTTCTCGGCTGAAGTGAAGAAGGACATC
CTGCTGCGCATCCACGGCACGCTCAAGCGCGGTGGCTATCTGTTCCTCGGCGCGTCCGAAGCGTTGAACGGCTTGC
CGGACCATTACCAGATGGTGCAGTGCAGCCCCGGTATTATCTACCAGGCTAAATAACACCTCACCCAACATTTGGA
GTGGGGCTTGTGTGGGAGCGGGCTTGCTCGCGAAGGCGGTAGTGAAGTTGGTTGATAGCTGACTGACACACTGAAG
TGCGCCCTTCGCGAGCAAGTCGAACCGTCGCACCGCCGCTCCCACATTTTTTGTGTCAGACCTTGGAAAGCGGCA
ACTCCCCATTGCCGCTTTCCTGACGTTTCGCGGAAAGCGCTTGCCGCTTTTCCGCCATTGCCGGCGGCAACCTCTA
CCTAACCCCTTGATTTATTGGGCTTGATAAACCTGGCATGGCGCTTGCTATATCCCTGTTACGAAAAGCAGGTCAG
CCCCTAAAGGTTTCCGCCATGAGCATCAGCTTCGATAAAGCGCTCGGTATCCACGAACAGGCCCTGGGCTTCCGCG
CCCAGCGTGCCGAAGTCCTGGCTAACAACATCGCCAACGCCGACACCCCGAACTACAAGGCTCGGGACCTGGACTT
TGCCGCCGTGCTCGCCGCACAGAGCGACAAGACCAAGAACGGCTCCTTCGCCTTGAACATGACCAATAACCGTCAT
ATCGAAGCGCAGGGCCTGAGCAGTGGCGATGAGTCGCTGATGTATCGCACGCCGATGCAGCCGTCGATCGACCAGA
ACACCGTCGATGCGCAGTTGGAGCAATCGAACTACGCCGAGAACTCGGTGAACTTCCAGGCCAGCTTCACCCTGCT
CAACAGCAAATTCAAAGGGCTGATGTCAGCCCTGCGCGGAGAGTAAGCCATGTCTCTATCCAGTGTTTTCAATATT
GCCGGTAGTGGCATGAGCGCCCAGACCACGCGTCTGAACACGGTCGCCAGTAACATTGCCAACGCCGAGACGGTCT
CGTCGAGCATCGACCAGACCTACCGCGCCCGGCATCCGGTGTTCGCCACCATGTTCCAGGCCGGTCAGAACGGCGG
CAGCGACTCGTTGTTCCAGAGCCAGGATGCTGCCGGCCAGGGCGTACAGGTGTTGGGTGTGGTCGAAGACCAGAGC
AACCTCGATGCGCGCTACGAACCCAATCATCCTGCCGCGAACGAAAAGGGCTACGTGTACTACCCCAACGTCAACG
TGGTCGAGGAAATGGCAGACATGATTTCCGCCAGTCGTTCGTTCCAGACCAACGCGGAAATGATGAACACCGCCAA
AGCCATGATGCAGAAGGTCCTGACCCTCGGTCAGTGATAAGGGGCGCTAAAGAATGTCCGTCACTAACGATGTGTC
GAGTCAATCGACGATCCAGAGTCTGTTCGACTCCAAGGTCAAGGATGCCAAGTCCAGCCAGGGCAACCTGGCCGAT
ACCGCCAAGGCGGCCTCGGGCAACCAGTCCCTGGGCAAGGATGCATTCCTGCAACTGCTGGTCACCCAGCTGAAGA
ACCAGAACCCGCTGTCGCCCCAGGACAACGGCGCGTTCGTGGCGCAACTGGCGCAGTTCAGCAGCCTGGAAGGCAT
CAACACCTTGAACGATTCGGTGAACACCATCACCGGTAACTACAAGTCGTCGCAGGCGCTGCAAGCCTCGTCGCTG
GTGGGGCGTTCGGTAATTATTGAAACCAACAAGGCCATGGTCGACACCAGCAAGAGCTTTACCGGCTCGGTCGCCG
TGGGTGCGTCGGTGGGCAACGTTACCGTCAAGGTCACCGACAAGGACGGCAACCTGGTCAAGACCATCGATCTGGG
CGCCCAGAGCGCCGGCAAGTCGGACTTTATCTGGGACGGCAAGAACGAAGCGGGCGAGCAGCTTGATTCTGGCACC
TACACCTTCGCGGCCAGCACCAAGAATGACGCCGGTGATCCGGTGGCCTTGAAAACCTCGCTGCCGGCCACGGTCA
CCAGCGTGACCTTGAGCCAGACCGGCGGCGAAATGCTGCTCAATCTTTCTGGCGGCATGGGCAGCATCAAGCTGTC
GCAAATTCAGACTATCGGTACATAGAGCCGGCTAAATACGGCAAGAGGAGAGAAACATGTCTTTTAATATCGGCCT
TAGCGGCCTCTATGCGGCCAACAAACAACTGGACGTGACCGGCAACAACATCGCCAACGTCGCGACCACCGGCTTC
AAGTCGTCCCGCGCAGAGTTCGGCGACATTTATGCGGCGTCCCGCCTAGGCACAGGCAATAAGACCATCGGTACCG
GCGTGAACCTGGCGGCGGTTTCCCAGCAATTCACCCAGGGTGAAGTCAACGGCAGCGGTGGCGTGTTGGACATGGG
CATCCAGGGCGGCGGCTTCTTCGTACAGAAGGGCAGCGACGGTTCGCTGGAGTACACCCGTAATGGCTCGTTCCAG
GCCAACAAAGACGGTTACATCACCAACAACACCGGCACCTCGCGCCTGCAGGGTTATGCAGCCGATGAAGATGGCA
AGATCTCCAAGAGCGGCCTGACCGACCTGCGGATCAACCTGTCCAACTTGCCGCCTAAAGCGTCGACCAAGGTTGA
CTCCAGCAGCAACCTGAACTCCTCCAAGCCGGTGATCGACCAGGCGACCAGCCCGTTTGATCCGACGGTAGCGAGC
TCGTTTACCGAGCAGTACGGCACCACTTTGTATGACACTCAAGGCAATGCCCATGAGATGGTGCAGTACATGGTGA
AAACCGGCTCCAATACCTGGAAGTCCTACACCCTGATCGATGGGCGTAACCCTGACGGTACGCCGGTCAAGGGAAC

GGGCGCGGTTGCCCCGGTGGCCTCCACGGTGACATTCGATACATCAGGCACCCTGAAGGGGATCATCACCCCGCCG
AGCACTGTATCCAATACCACCCTGACCATCAGCAACTGGAAGCCGGGCGTCATGGACAACGGCGTGTGGAACCCTG
ACAACGGGGCGGCGGCCAACCCGGGCGGTATCGCGATCAACATGTCCGATATCACTCAGTACAACTCGGCCAGCTA
CCGCAACCCGCCGATCACCGATGGCTACGCCACCGGCGAAATCACCGGGCTGAAAATCGACGGCAGCGGTGTACTG
TTCGCCACCTTCAGCAACCAGCAGAGCAAGGCCATTGGCCAGATCTCCCTGGCCAGCTTCAACAACGAGCAAGGCT
TGCAGCCGGCCGGTGGCACCACCTGGAAAGAAACCTTCGCTTCGGGCCAGCCTGGCTACGATGCACCAGAAGCCGG
GACCCTGGGTTCGATCGTGGCCAACTCCCTGGAGAACTCCAACGTCAACCTGACCAACGAGCTGGTGGACCTGATC
AAGGCCCAGAGCAACTACCAGGCAAACGCCAAGACCATCTCCACCCAGAGCACCATCATGCAGACCATCATTCAGA
TGACCTGATGCGTTAGCGCTGCACAAGAAGCCCCTCGTCAGAGGGGCTTTTTTGTGGGCGTAGGAAAAGCACCGGT
GCCGCATTGGGTTACGCACTGAAATGCTTGGGAAAGGAACGCTTCAATCGGCCCGCACCACTGATCGGGGCAAGAG
CCCAGGGGGCTCATCGATCAATGGGAGCTGAAAATGTGGAACTCCGGGGTGTCCGCGCCAGGTAATGGGCGCATGG
CTTTGGTGCCGACTGTTACGCGTCGGGAGCCTGCTCCCATCGAACCCGTCAGTGACGTGTCGGCGGTCAAGACGTT
GTTCAACCAGTTGTTCGAGGCCGAGCAAACCTCCAAGACCCAATTGGCTCGCGATCTGCGTGACAAGCTCAATGCA
AAGCGCGAGGAGATCAGTGATGAAAAACTCAAGGATATTCTCAATCAAATGATTGAGTCGGATGAAACGCCTCTGT
CGTTTCTTGAGTTTCTCAGGCGCCTGCGGGATGAGTGGTTCCCAGCATCTGATTCAGCCTTGGGAAATGGTGGTAC
TGGGAATGCCGGTGGGGGCTCGGGTGTCGGTGGAGGAAGGAGAGGGGGAGGAGGGGGAGGAGGGGGTGGTGGCGGC
AATGCCAATGTAGGTGGCGGCCAAAGAAGTGCTGAGCGTATGAGCGACAAGCCTCTGTCAGTAGGGGCGGGTTATA
CCGATTATCGACCTGACTCCAGTGCCGAAAACGCAGGAAAAAAGCCCAAAAATATCTGGAGTGGATTCAGTCAGGG
ACAGGAGGGTAATTGTGGAACAGTCTCTGCGATCAAAGCGTCAATGATGCATTTTGGCCAGAAGCCAACTGACATT
TATAAGGAGGTCAAGGAGGCGGGTAACGGTTATGACGTGACAATGCGCGATGGATATAAGCTTCACCTTAGTCGAG
ATGAGTTGAAGCAGGCGGCCAGGGGAGCAAACTTTAAGGGTGACGATCCGGAAATGATGACGGATGCCAACTTTAT
GTTTGCTGTAAGTGCCAAGCGTGCACAGGAAGAAAATAACGACGGCAGGGTGGGGATGAGTTACGGACAGGCTATT
GATTCGTTAAATGATGGCGAGTGGAGTAAGGAGGCCTTTGACCGCCTGGGTTTGAAGAATCATGTTAAACAGGTGA
GTTTAAGTGACTTGGCGAGGGGAGCAGTCGGTACGGCGGATACGAATATTCATTCGGTGGCCGTGGTTAATGGCAG
GAAGGAACTTTGGGGAGGGCGTGGTGGCTATCCAACAGAGGGTATAGCTTTTGGCTTTGTTTAACCTTTGGTTCTA
CGGCAAAAGAAAACCCCCGATACACCATAGGCCTATCGGGGGCTTTTCATCCTTGGGGGGTTAAACCCAAGGTGCT
GTCAGCTTACTGGCAGGCTTCGCAATCCGGCTCGTCGATCGCGCAGGCTTTAGGCACTGGCGCTGGGCCGGCCGGC
GCTGCGAGAACCGAATCATCACCGTGGTTACCGCTGGATACAGCGTTCAGCTTGCCGGTGTTGATGGTCGACTTCT
CGGTGCTGGTCGCGGCCAGGGCACGGAGGTAGTAAGTGGTTTTCAGGCCACGGTACCAAGCCATGCGGTAGGTCAC
GTCGAGCTTCTTGCCCGATGCGCCGGCGATGTACAGGTTCAGGGACTGCGCCTGGTCGATCCACTTCTGACGACGG
CTGGCGGCGTCAACGATCCACTTGGTGTCCACTTCGAACGCGGTCGCGTAGAGTTCTTTGAGTTCTTGCGGGATGC
GCTCGATCTGCTGTACCGAACCGTCGTAGTACTTAAGGTCGTTGATCATCACCGAGTCCCACAGGCCGCGGGCTTT
CAGGTCGCGGACCAGGTACGGGTTGATCACGGTGAATTCGCCCGACAGGTTCGATTTCACATACAGGTTCTGGTAG
GTCGGTTCGATCGACTGCGACACGCCAGTGATGTTGGCGATGGTCGCGGTCGGTGCGATGGCCATGATGTTGGAGT
TACGAATGCCTTTCTGCACACGGGCACGTACCGGCGCCCAGTCCAGGGATTCGTTGAGGTCAACATCGATGTACTT
CTGGCCACGGGCTTCGATCAGGATCTGTTGCGAGTCCAGCGGCAGGATGCCCTTGGACCACAGCGAACCCTGGAAC
GTCTCGTAGGCGCCGCGCTCGTCGGCCAGGTCGCAGGAAGCCTGGATCGCGTAGTAGCTGACCGCTTCCATGGACT
TGTCGGCGAACTCGACCGCAGCATCCGAACCGTAGGGAATGTGCTGCAGGTACAAAGCGTCCTGGAAGCCCATGAT
GCCCAGGCCGACCGGACGGTGCTTGAAGTTGGAGTTCTGCGCCTGTGGCACCGAGTAGTAGTTGATGTCGATAACG
TTATCGAGCATGCGAACGGCGGTGTTGACAGTGCGTTCCAGCTTGGCGGTGTCCAGCTTGCCGTTGACGATGTGGT
TCGGCAGGTTGATCGAGCCCAGGTTGCAAACGGCGATCTCGTCCTTGTTGGTGTTCAAGGTGATCTCGGTGCACAG
GTTCGAGCTGTGGACCACGCCCACGTGCTGCTGCGGGCTGCGCAGGTTGCACGGGTCCTTGAAGGTCAGCCATGGG
TGGCCGGTTTCAAACAGCATGGACAGCATTTTGCGCCACAGGTCTTTGGCCTGGATGGTCTTGAACAGCTTGATCT
TGCCTGGGTACTGGGACAGGGCTTCGTAGTACTCGTAGCGCTCTTCGAAGGCCTTGCCGGTCAGGTCGTGCAGGTC
TGGCACTTCCGATGGCGAGAACAGGGTCCACGGGCCGTCATCGAAGACGCGCTTCATGAACAGGTCAGGGATCCAG
TTGGCGGTGTTCATGTCGTGGGTACGACGACGGTCATCACCGGTGTTCTTGCGCAGCTCGATGAACTCTTCAATGT
CCATGTGCCAGGTTTCCAGGTAGGCACACACAGCGCCTTTGCGCTTGCCACCCTGGTTGACCGCGACAGCGGTGTC
GTTCACGACTTTGAGGAACGGCACAACGCCCTGGGACTTGCCGTTGGTGCCCTTGATGTACGAACCCAGTGCACGG
ACCGGCGTCCAGTCGTTGCCCAGGCCGCCAGCGAATTTGGACAGCATGGCGTTGTCGTGGATCGCGTGGTAGATGC
CCGACAGGTCATCCGGCACGGTGGTCAGGTAGCAGCTCGACAGCTGTGGACGCAGGGTGCCGGCGTTGAACAGGGT
CGGAGTCGACGACATGTAGTCGAAGGACGACAGCAGGTTGTAGAACTCGATGGCACGGTCTTCTTTGTGCTTCTCT
TCGATCGCCAGGCCCATGGCCACACGCATGAAGAACACCTGCGGCAGTTCGAAACGGATCCCGTCCTTGTGGATGA
AGTAACGGTCGTACAGGGTCTGCAGGCCCAGGTAGGTGAACTGCTGGTCACGCTCGTGGTTGATCGCCTTGCCGAG
TTTTTCCAGGTCGAAGGTGGCCAGGATCGGGTTCAGCAATTCGAATTCGATACCCTTGGCGATGTAGGCAGGCAGG
GCCTTGGCGTACAGGTCGGCCATTTCGTGGTGGGTGGCGCTATCGGCCACGCCCAGGAACCCCAGGCCTTCGGCAC
GCAGGGTGTCCATCAGTAGGCGCGCGGTCACGAACGAGTAGTTCGGCTCACGCTCAACCAGGGTACGGGCGGTCAT
CACCAGGGCGGTGTTGACGTCGGTCAGGGCCACGCCGTCGTACAGGTTCTTCAGGGTTTCACGCTGGATCAGGTCG

CCGTCGACTTCTTCCAGGCCTTCGCAGGCTTCGGTGATGATGGTGTTCAGGCGGCCCAGGTCCAGCGGCGCGAACG
AACCATCAGCCAAGGTAATGCGGATCGACGGGTGGGCTTGCACGGCTTCGGCGGACGGTGCGTGTACGGCACGTTC
CTTGGCGCGCGAGTCGCGGTAGATCACGTAGTCGCGGGCCACTTTCTGCTCGCCGGCGCGCATCAGGGCCAGTTCG
ACCTGGTCCTGGATTTCTTCGATGTGGATGGTGCCGCCCGAAGGCATGCGACGCTTGAAGGTCGCGGTGACTTGTT
CGGTCAGGCGGGCAACGGTGTCGTGGATACGCGACGAAGCGGCAGCGGTGCCGCCTTCAACTGCAAGAAACGCTTT
GGTGATAGCGACGGTGATTTTGTCGTCGGTGTAAGGAACGACAGTGCCGTTACGCTTGATCACGCGCAATTGGCCG
GGCGCGGTGGCGGACAGATCCATATTCGAATCAGCGCCCTGCGGCACGGAGCCTTGCGGGTTCTCGCGAGTTGTGT
CGGTTTGCATGGGGGGTTGTCTCCACATTCTATATTTATTTGGGCACCATCACGGTGCCCACCGTTCCGTCCTGAA
GCACTTACAACAGGCCTTGGCCTGGCATAACAACCTCGGGACAGGAGGAAGGGAGCTGTTCGCGCCGCTTCCATGC
CGAAGTCTTCGGTTCCAAGCCCTGGGCTCTTTACCGTATTCCTGTGTGGGTGTCGTTTGTATTGCAACACCCGTAC
CGCTTTCTCCCGATGGGGCTTGAAAACAGCAGCCATCCGCGTGCGCGGTTTGGGCTTTCGAAAAAGGTTTAAGTTC
CACCAAACAGGAGCAAGAAAGCACTTGAGTTTCTTGTCTGATTTGTGTTTGGTCTTGTCGCTTGAAACCCTACATG
TAGGGTTTTTTTTGCGACGAGGTACAAGATAATGCGTTTTGGGGGGCTTAGCAACGCACTACCTGTGGATAACGCT
GTGGGTAAAATGTGTATGAAAGGTGGAACCGCCGTGTAGGCCGCAGTACTGCTGCATCGCACCGTTTGTCACCGAA
TATTCCAAGCTGAAAACACCCGGTTGGATTTTTGCGGGCGCGAACCCTATCACAAAAAATCGCGATCACCGAATGC
ATTTCCTGGCTTGTGTTGCAGGGTCAGGCCATGGCTACAATCGGCCTTTATTCCTACACTGTTATGCCCTTTATGC
ATGACAAATTTGGTACGGCTGGCGAAGATCAAAATGTGGGAGCGGGCTTGCCCGCGATAACGGTGGATCAGCAGTA
GGTATGTTGCCTGACACACTGCTATCGCGGGCAAGCCCGCTCCAACATTTGAACCTGCGCAGTCCTGACAAATCCC
TTATAAAAACGAGGACCACCCGTGGAGCAAGAAGCCTGGCAGGTACTGATTGTCGAGGACGACCAGCGGCTGGCTG
AGTTGACCCGTGACTACCTGGAAAGCAACGGCCTGCGGGTCTCCATCGAGGGCGACGGAGCCCTGGCGGTGGCGCG
GATCATTGCCGAGCAGCCGGACCTGGTAATCCTCGACCTGATGCTGCCCGGCGAAGACGGCCTGAGCATCTGCCGC
AAGGTGCGCGAGCGCTACGACGGGGTAATCCTGATGCTCACCGCGCGTACCGACGACATGGACCAGGTTCTGGGCC
TGGACATGGGGGCCGATGACTATGTGTGCAAGCCGGTGCGCCCGCGCCTGCTGCTGGCGCGGATCCAGGCGCTGTT
GCGGCGCAGCGAAAGCGCCGAGCCTGCCGCCCCGGAAAAACAGCGGCGCCTGCAATTCGGCCCCCTGGTGGTCGAC
AACGCCTTGCGCGAAGCCTGGCTGCACGATGGCGGCATCGAACTGACCAGCGCCGAATTCGATCTGTTGTGGCTCT
TGGTGGCCAATGCCGGGCGCATCCTGTCCCGGGAAGAGATCTTCACCGCCTTGCGCGGTATCGGCTACGACGGCCA
GGACCGCTCCATTGACGTACGCATCTCGCGGATCCGCCCGAAAATCGGCGACGACCCCATCCACCCGCGCCTGATC
AAGACCATTCGCAGCAAAGGCTACCTGTTCGTCCCCGAAGCCGCCGCCGATATGCTGTCGTGAACTCGATCTTCCT
GCGTATCTATGGCGGCATGTGCGCGGCGCTGATCCTCGTGGCCCTGCTTGGCGTATTGGCGCTGCACCTGCTCAAT
GAGGTGCGCAGCGGCCAGTACCGCGAGCGGCTGGCCCACGGCACCTTTGCCCTGATGGGCGACAACCTGCAGCCGA
TGAGCAGCATCGAGCGCCAGCGCGCCCTGGCGGTGTGGGAGCGATTGCTGGGGATTCCCTTGCAATTGCAGACGGT
GGCGGATGCCCGCCTGGACCTGAGCCAGCGCAATCGCCTGCAGCGCGGCCAGGTCCTGGTGGAGCAGACCGGGCCC
CATGCGGCCCGGGTGTTGCGCCTGGTCAGTGAGAAGGAGCAACTGCTGCTCACCGGCGAAGTGCAGCAGATCAGCG
AGCAGTTGGCCCGGGCCACCATTTATTTACTGGCCGACGAACTGGTGCGGTTTCCAGTGGCCGAGCAGCCGCAGCG
GTTGGCCGACCTGAAAGACGCCAAGGGCTTCGGCTTTGAAATGCACCTGCTCAAGCTCGACGAAGCGGACATGGAC
GAGGACCAGCGGCGTCGCGTGGCCGAAGGCGACACGGTGATGGCCCTGGGCAAGGGCGGCGACTCGATCCGTGTGT
TTGCCGGCATGGTCGGCACGCCGTGGGTCCTGGAAATCGGCCCGCTGTACCAGATGAATCCTTACCCGGCGCAATG
GCTGGTGCTGATTGCATTGATTGGTCTGACCCTGATCGGCTTGATCGTGTATTTGCTGGTACGCCAACTGGAGCGC
CGCCTACGCGGGCTCGAAGCGGCCGCCACGCGCATCGCCAAGGGCAACCTGGAAGCGCGGGTGCCGGCGCGCGGTG
CCGACTCGGTAGGGCGCCTGGCGGCGGCCTTCAACGGCATGGCCGAGCACTTGCAGCGCCTGTTGGCGATTCAGCG
TGAACTGGTGCGCGCGGTGTCCCATGAGTTACGCACCCCGGTGGCCCGCCTGCGTTTTGGCCTGGAGATGATCGGC
GACGCCGCGACGCCCGAAGCCCGGCGCAAATACATGGAAGGCATGGACAGTGATATCCAGGACCTCGACGGCCTGG
TGGATGAAATGCTCACCTATGCGCGCCTGGAGCAGGGTTCGCCGGCCCTGAGTTTCCAGCGGGTCGACCTCAGTGC
CTTGCTCGACCAGGTGATCGGCGAGCTGGCACCCTTGCGGCCAGAGATCGAGGTGGCGCGGGGCGTGTGTCTGTCA
TCGGCGCACCTGGATGCGGCCTGGGTCGACGCCGAGCCGCGCTACCTGCACCGCGCCCTGCAAAACCTGGTGAGCA
ATGCCATGCGCCATGCGCAGTCGCGGGTGCTGATCAGTTACCAGGTGGGGCAGGTGCGTTGCCGTATCGACGTCGA
AGACGATGGCCCAGGCGTGCCGGAAAGCGCCTGGGAGCGGATCTTTACCCCATTCCTGCGCCTTGATGACAGCCGT
ACACGCGCCTCGGGTGGGCATGGCCTGGGTTTGTCGATTGTGCGGCGGATTATCTACTGGCACGGCGGCCGGGCAT
TGATCAGCAAGAGCAACAACCTGGGCGGGGCGTGTTTCAGCCTGAGCTGGCCGCGGGATCAGGACAAGACTTGAGG
TGGTCATCGCAGGCAAGCCAGCTCCCACAGTTGACTGCATTCCAAAGGATGTACTCGGTCAACTGTAGGAGCTGGC
TTGCCGGCGATAGCGCTATATCAGGCACCGATGGCCACGAGGCTCAACAACTGCCCGTCATCCACCGCAAACTGCC
CCTCCAGCTCCTTGCCATAACACCACTCACTGGACAAATCCGTCAGCAATCGCAAGCGCAAAAATCCCGAATCGGA
CCACTCCACCACCTCGGCATGCTCAAAGTAGAAACGCTTCTGCACAATCGGGTAGAGCGCCTTGAACAGACTCTCC
TTGGCCGAAAACGTCAGGGTCACCAGTTGCGCAACCTGCTCCCTTGGCCCTGTGGCCATGCGTTGCATCTCATCCG
GAGTAAGGATTTCCCCGGCCAGGCGCTCGGCCCGCTCAAGGGGCAATAGGTTTTCCAGGTCCATGCCCAGCCCGCG
CCACTGGGCCTTGTGGCCGACGATGGCGGCGGCATGCCCGGTGCTATGGGTGATCGAGCCGCTGATATGCGAGGGC
CACACCGGGGCGCGGTCCTCACCGATGGCGGGGATAAAGTCCAGGCCTTCTAGCTGTTGCAAGGCGCTGCGGGCGC

ACAGCCGGCCCGCCAGAAACTCCGCCTGCCGCTTGGCCACCGAGCGTTGGATACTGGCCGGCGGCGGCACGGCGCA
GCGCTGGAAGTCGCCGGGTTCGAGTAATTTGGGCTCGAAACGGGTGCTCAGGAACACCGTGCCGGGCAAGGTGGCG
GGCAGCGGCCAATGGTCATCAAGTGGGGTGCAGCAGGCGGGTATCGGGTTCATGCCGGCTATTCTGCACGGGGCGG
GACCTGCCTGGGTAGCCCGCCGGTTCAGACTGTGTTCAGTTCAACCGCCGTAAACTGCGCAAGCATACCTTCAGGG
CCGCTATGATGGTTCCCTGCGGGACGGTGGATTGGCCTTGGAGAGTGTTATGAAATTGCTGGTTGTCGAAGATGAG
GCGTTGTTGCGCCATCACCTGCTGACCCGTCTTGGCGACAGCGGCCACGTGGTCGAAGCCGTGGCTAACGCCGAAG
AAGCGCTGTACCAGACCGGGCAATTCAATCATGACCTGGCGATCATCGACCTGGGCCTGCCGGGCATGGGCGGCCT
GGACCTGATCCGCCAACTGCGGGCCCAGGCCAAGACCTTCCCGATCCTGATCCTCACCGCGCGCGGCAACTGGCAG
GACAAGGTCGAAGGCCTGGCCGCTGGGGCCGATGACTATGTGGTCAAGCCGTTCCAGTTCGAAGAGCTGGAGGCGC
GGATGAACGCCCTGTTGCGCCGCTCCAGCGGTTTTACCCAATCGACCATCGTCGCCGGCCCCTTGCTGCTGGACCT
CAACCGCAAGCAGGCGTCCCTGGACGAGCAGCCCCTGGCGCTGACGGCCTATGAGTACCGGATCCTCGAATACCTG
ATGCGTCATCACCAGCAAGTGGTGGCCAAGGATCGGTTGATGGAACAACTCTATCCCGATGATGACGAGCGTGATC
CGAACGTGATCGAAGTGCTGGTCGGCCGCCTGCGTCGCAAGCTCGAAGGCCCGGCCGGGTTCAAGCCCATCGACAC
CGTGCGTGGCCTGGGCTACATGTTTAACGAGCGCTGCCGTTGATTCGTTCGCTGCGTGTGCGCCTGATGCTGGCGG
CCACCACCTTGGCCGTATTGTTCATGCTCGGGCTGTTACCGGCGATGCAGGGCGCGTTCAGCCTGGCGTTGCAGGA
TTCCATCGAGCAGCGCCTGGCGTCCGATGTGACCACCCTGATCTCTGCCGCGCGGGTCGATAACAACCGCTTGCTG
ATGCCGGCGCAGTTGCCGGACGAGCGCTTCAACCTGACCGATAGCCGCCTGCTGGGCTATATCTACGACCGTGAAG
GCCATCTGGTTTGGCGTTCGCGGGCGACCAGGGAAGAAAACATCAACTACCGGCCGCGCTACGACGGGCGCGGCAA
CGAGTTTGCGAAGATCCGCGAAGCCAACGGCCAGGAATTCTTCGTCTATGACGTCGAGGTCAAGCTGCTGGGGGGC
AAGAGCGCGGCGTTCAGTATTGTCGCCCTGCAACCGGTGCGCGAATACCAAGTGACCCTTGAAGGCCTGCGGGAGA
ACCTCTACCTGGGGTTTGGCGCCGCCTTGCTGGTGCTGCTGAGCCTGCTGTGGATCGGCCTGACCTGGGGCTTGCA
GGCCTTGCGCCGGCTGAGCCAGGAGCTGGATGAAATCGAAGGCGGCACCCGGGAAAGCCTCAGCGAGCAACACCCG
CGGGAGCTGCTGCGCCTGACCGGTTCCCTCAACCGCCTGCTCCATAGCGAGCGCGAGCAGCGCACCCGTTACCGCG
ACTCCCTGGACGACCTGGCCCACAGCCTGAAAACCCCGCTGGCGGTGTTGCAGGGGGTGAGTGAAGACATGGCCCA
GCGCCCCCAGGACCTTGAACAGGCACGGGTCCTGCAGTCGCAGATCGAACGCATGAGCCAGCAGATCAGCTATCAG
TTGCAGCGCGCCAGCCTGCGTAAAAGCGGCCTGGTACGCCACCAGGTGCGCCTGCGCCCGGTGCTGCAAAGCCTGT
GCGACACCCTCGACAAGGTCTACCGCGACAAGCGCGTGCGCGTGTCTTTCGCGCTACCAGAGCAGTGCGACGTGCC
CATCGAGCAAGGCGCCTTGCTGGAGTTGCTCGGCAACCTGCTGGAAAACGCCTACCGCCTGTGCCTGAGTGAGGTG
CGCGTGACCCTGGATGAAAGTGCCGATGGCGTGCGCTTGTGCATCGAAGACGACGGCCCCGGTGTGCCTCCCGACC
AACGTGCGCGGATCCTGCAGCGCGGTGAGCGGTTGGACCGCCAGCATCCGGGGCAGGGGATCGGGTTGGCGGTGGT
CAAGGACATTATTGAAAGCTACGGCGCACGGTTGACCCTGGGGGATTCGCCTCTAGGGGGCGCGGCGTTCAGGATT
CATTTTCCGGCTGTTTGATCTTTATTGGATTGACCGGCCGCATCGCAGGCAAGCCAGCTCCCACATTTTGATTGGT
GAATACAGTCAAATGTGGGAGCTGGCTTGCCTGCGATGGCGCCCGTCCAAACAATACATCCTCAACTCTCCTGCGC
TCGATAAGCCCCCGGCGTCACCCCCGTCCACTTCTTGAACGCTCGGTGAAACGCCGACGGCTCGGAAAACCCCAAC
TGCTCGGCAATCTCCTGCAACGACAGATCCGCCCGCCCCAAATGAAAAATCGCAATATCCCGCCGCAGCTCATCCT
TGAGTGCCTGGAAGCTGGTGCCTTCCTCACGCAAATGCCGGCGCAGGGTCTGTGGGCTGATGTGCAGGTGCTGCGC
CACGGCTTCCAGGTCGGGCCAGGGTGTGCGGTCGCGGCTTAACAGGCGCCGCAACTGGCTGCTCAGGCTGTCGCCT
TCGTCCGGTCGCGACAGCAGGTCGGCGGGGGAGCGTTCGAGGAAATGCTTGAGGGTGCGCTCGTCCTGCAGCAGCG
GCAGGCTCAGGTAGCGGCTATGGAACACCAGGCTGCTGCGGGCTGCGCTGAACACCAGGGGGCAAGGGAACAGCAG
GTCGTACTCACTGGCATGGGCCGGCGCCGGGTAGCTGAACGTCGCTTGCTCCAGGCGGATACGCTGGCCAATCAAC
CAACTGCCGAGGCGATGCCAGATCACCAGCAGGGACTCGCTCAGAAAGTGATCCGGGTCCCACAGTTGCGAGTCAT
CCAGGCTCAGGCAGGCCATATCGCCTTCGCGGCTCAGGCGCCAGCGCGGCCCCTGGGGGAACAGGCTATAGAACAA
CAGGCCGCGCTCCAGCGCCTTCTCCAGGGTGCGGCAGTGAATCAGCGCGTGGCACATCATGGCGAAGGTCCCACGC
TTGCTCGGCCCATCGGCGAAACCCAGGTACTCGTCATCCAGGGCCAGCCACAGCCTCTGCAACAGCTGCGTGAACT
GCTCCGGCGCGATGCGTGCGCGGGGCTCTGCCAGCAATTGCGGGGTAATGCCCAGTTGCTGCAGCAGCGTCGAGCA
GTCGTACCCGGCCCGGTGCGCACCGCCCAAGGCAGCGCGGGCGTAATGACTGGCGATGGTGCGTTCGCGCATGCAG
GGGCCTCGTCCATTGAGTGGCCGATGGTAGCCATCATGTCTGGGCTGGACAAGGCGGATATCCGCCAAATTGTAGG
ACGAGATTGGGCGGGTGGGCGGATATCCGCCACCTGCGGCCCCCTCTGAAACAGCCATGAATTTCCGTCAGCCCCC
ATGAGCCAAGGGTTACAGGGTTTTTCAGGAAAGTGGCACGCGGTTTGCGATAAGGAGTACAGACCTGCCAGCCAGC
AGATCGCCAAAACAAATCCCTCCAGTGCAGGAGGGTTCGCAATTCAAGTGTCGTGGGCAATGGCGGATATTTGCCC
CACGGGACTCTTGAGGAAACTTTGCAATGACGACTCGTCAGCCGTTCTACAAAACCCTGTACTTCCAGGTAATCGT
TGCCATCGTTATCGGTATCCTGCTCGGTCACTTCTACCCGCAGACCGGTGTGGCCCTCAAGCCATTGGGTGACGGC
TTTATCAAGCTGATCAAAATGGTCATCGCCCCGATCATCTTCTGTACCGTTGTCAGCGGCATCGCCGGCATGCAGA
GCATGAAGTCGGTGGGCAAGACCGGCGGCTACGCGCTGCTGTACTTCGAAATCGTCTCCACCATCGCCCTGTTGAT
CGGCCTGGTCGTGGTCAACGTGGTACAGCCAGGTGCTGGCATGCACATCGACGTCGCCACCCTGGACGCTTCGAAA
GTGGCTGCTTATGTCACCGCCGGTAAAGACCAGAGCATCGTCGGCTTTATCCTCAACGTGATCCCGAACACCATCG
TCGGCGCCTTCGCCAACGGCGATATCCTGCAAGTGCTGATGTTCTCGGTGATCTTCGGTTTCGCCCTGCACCGCCT

GGGTGCCTATGGCAAGCCGGTGCTGGACTTCATCGATCGCTTCGCCCACGTGATGTTCAACATCATCAACATGATC
ATGAAGCTCGCGCCAATCGGTGCCCTGGGCGCCATGGCGTTCACCATCGGTGCCTACGGTGTTGGCTCGCTGGTGC
AACTGGGCCAGTTGATGATCTGCTTCTACATCACCTGCATCCTGTTCGTGCTGGTAGTACTGGGTGGCATCTGCCG
CGCCCACGGCTTCAGCGTCCTGAAACTGGTGCGCTACATCCGTGAAGAACTGCTGATCGTACTGGGTACTTCCTCC
TCTGAATCCGCACTGCCGCGCATGCTGATCAAGATGGAACGCCTGGGCGCGAAGAAGTCGGTGGTTGGCCTGGTTA
TCCCGACTGGCTACTCCTTCAACCTCGACGGTACTTCGATCTACCTGACCATGGCTGCCGTGTTCATCGCCCAGGC
GACTGACACCCACATGGACATCACCCACCAGATCACCCTGTTGCTGGTGCTGTTGCTGTCCTCCAAAGGCGCTGCA
GGCGTGACCGGCAGTGGTTTCATCGTACTGGCCGCAACCTTGTCGGCTGTTGGCCACCTGCCGGTTGCCGGCCTGG
CGCTGATCCTCGGCATCGACCGCTTCATGTCCGAAGCCCGCGCCCTGACCAACCTGGTGGGTAACGCCGTAGCAAC
CATCGTCGTTGCCAAGTGGGTCAAGGAACTGGACACCGACAAGCTGCAAAGCGAGCTGGCTTCCGGCGGTACCGGT
ATCTCCGAAACCCGCGAACTGGATGACCTGGGCGTAGCCGAAGGCCCGGCGCCAGCGATCAAGTAAACGCTGAGGC
ACACTAAAAAACCCATCTTCGGATGGGTTTTTTATGGGCGCACGAGCACAACGGTCACTGTCGATGATGGTTACG
GTAATTGCCGCTGGCGCCCAAGGCTGACTACTCTGTGCCCCATCGAACTGGAATCGGAGCTCCCATGTCAGGCCCC
TTGGCGTCCCTCAAAGTGCTGGATTTCTCGACCTTGTTGCCGGGGCCTTTTGCCTCGTTGCTGTTGGCGGACATGG
GCGCCGAGGTACTGCGCATCGAATCCCCGACGCGCATGGACCTGTTGCGCGTGTTGCCGCCCCACGACCGTGGCGT
GTCGGCCAGCCATGCCTACCTCAACCGCAACAAGCGCAGCCTGGCCCTGGACCTCAAGCAGCCCGAGGCGCTGGAG
ATCGTGCGGCAACTGGTGGCCGACTACGACATCGTGCTGGAGCAGTTCCGCCCCGGGGTCATGGAGCGCCTGGGCT
TGGGTTATGAGGCGCTGAAGGCGATCAACCCGAAGCTGATCTATGTGTCCATCACCGGCTACGGCCAGACGGGCCC
CTACAAGGATCGCGCCGGGCATGACATCAATTACCTGGCACTGTCTGGCGTCGCCAGCTACACCGGGCGTGAGGAC
AGCGGCCCGCTGCCCCTCGGCGTGCAATTGGCCGATGTGGGCGGTGGCTCGTTGCACGCCGTGGTCGGCCTGCTCG
CGGCGGTGATTGCCCGGCAGCACAGCGGGGTGGGCCAGTACCTGGATGTGAGCATGACCGACTGCGCCTTCAGCCT
CAACGCCATGGCCGGCGCGGCCTACCTGGCCTGTGGCGTGGAGCCCGGGCGCGAAGACCAGGTGCTCAATGGCGGC
AGCTTCTACGATTACTACCGCTCGCGGGACGGCCGCTGGCTGTCGGTGGGCAGCCTGGAACCAGGGTTTATGCAGC
AACTGTGTGGCGCGCTGGGGCGCCCGGAACTGGCGGCCCTGGGGTTGTCGCCCAAGCCCGAGCAGCAAAAGGCACT
CAAGCAGGCATTGCAGGTCGAGTTTGAACGGCACAGTTTTGAGGCGCTGTGTGCGTTGTTCGCTGGGGTGGATGCC
TGCGTGGAACCGGTCCTCAGTTTGGGCGAAGCCCTTGAACACCCGCAGTTACAGGCCCGGGAGCTGGTGAGCCAGG
TGCCCCGCGGGGATGGTTCGTTCCAGGCGCAGATGGCGTGCCCGCTGAAGTTTTCCGAGGGGTTGCCGGCGCCGAG
GCATATTGGGGGGGCGGTGGGGGCGCACAGTGATGAGGTGTTGGGGGAGTTGGGTTTCAGTCTTCAGCGGATTGCC
GAACTGCGGGGGGCCAAGGTGGTGGGGTAGTGGCTGTTCTGGCCCCATCGCGGGCAAGCCCGCTCTCACATTTTGA
TTTGTGAATACATTCAGAATGTGGGAGCGGGCTTGCCCGCGATGGGGCCAGAACAGGCACCCCAAAACCTCACTCC
ACCCGCGTCTCCCCAGTAAACACCAACGTCTCTCGACAGCGCCGGCACAAATACCGCCGCCCCTGGTTCACCAACC
CATGGCGCTGGGCCGAGAACGCAAAGTCACTGTCGGCACACGGGCACCGGTAGATATAACGGGTCACGCGCCGTCG
CTGGATCTCATAGGTGTGGCACCGATCAGGCGGCAGTTCGTAGACCCCGCGCATGATCAGTTGCCATTCCTCGCCA
TGGGGCTGGATGCGCTCGCCGAACAATTGGTGGGCGATCAGGTGCGCCACTTCGTGGGCCACCGTCTGTTTGAGGA
AGTGTTGGCTGTTTTCTCGGTACAACTGGGGGTTGAAGCGCAGCAGGTTCTCGTGCAAATGCGCGACACCGGCTTT
CTGCCCACGCAGCTTGAGGCTGACCTGGGGGCGTTTGAAACTTCGTTTGAAAAAGGATTCGGCTTGCAGGAAACAA
TCTTCGACGCGGGTATTGAGTTGCTCGGGCATGCTGTACATATCTCCAGAGACGTCCAGTATGCCGCATCCAAGGG
TATTTCCGAATCTGTCAGGCGCCGAATGGTCATGCATAACGCACAAAGCCACCTTGCGGTGGCTTTTCCTGCGGTT
GAAAGCGCGTCAGTTGGTGTAGATCGGCCCCACGCCCAGGCCCCAGACAATCACGGTAAAGGCCATGATCGCCACC
AGTACCACCAGGCCTACCGCGAGCACCGAGCTGGAAAACAGGAAGCCTTCGTCCGGGTCGATGCTCATGAAGGTCG
GCAACCCCACGTACAGCAGGTAGACGGTGTAGCAAATCGCCGCCGTGCCCACCACCATGCCCAGCCACATATGTGG
ATACAACGCCGCCAGCCCGCCGATAAATAGCGGCGTGGCGGTGTAGGTGGCGAACGCGACGCAGCGCGCCATGCTT
GGATTGGCGTCATAGGTGCGCGCCATCCAGTGAATGAAGGCGCCCATCACCGCGACGCCGCCGAGCATCGCCAGGT
ACGACATGATGGTCATCCACAACGCACTGTCCTGGGTCAGCATGACCGGGGAGCGATTGCCGATGACCCAGCCAAC
CTGGGTGGTGCCGATAAAAGCCGATATGGCGGGGATCGCCGCGAGAATCAGCGTGTGAGTGAGGTACATGTGGCCG
ATGCTTTCTTCTTTATCGCCACGAATTTCCCGCCATTCCTGGTCGGGATGGGTGAAAAGCCCCACGACGTGATGGA
TCATGCCAGTCACTCCTGTCGTTATTACCATCGCCCCCCAAGCGGAGCGCCCGCCGGCCAAGTGGCCTGAAAGGTC
TGGATATATGTGCGACCTCAAGTCGCAGTATAGGAAGTGATGACCGGAAAAAGTGTGTGGCTTTAGAGCAAATTGC
ACTGTAAACACTGGGCTTAATCGCGCCTGGGTCTGTGACGTGGAATGCAGTCCCCTGTGGGAGCGGGCTTGCCCGC
GATAGCGGTAGGTCAGGCAGCGGATTTATTGATCGATCCACCGCCATCGGGAGCAAGCCCCCTCCCACATTGGAAC
TCGGTGCTCCATACCGCTAAAATACCCGGCTTTTCGTCACACACCTTTTCGCGGATCCAAGCGCCATGGGCACCCT
TACCGTCAATCAGAACAAACTGCAAAAACGCCTGCGGCGCCTGGCCGGTGAGGCTGTCGCTGACTTCAACATGATC
GAGGAGGGCGACAAGGTCATGGTCTGCCTGTCTGGCGGCAAAGACAGCTACACCATGCTGGACGTGCTGATACACC
TGCAGAAGGTCGCGCCGATCAAGTTCGACATCGTTGCCGTCAACATGGACCAGAAACAACCCGGGTTCCCCGAGGA
TGTGCTGCCGGCCTACCTCAAGACTCTCGGCATCGAATACCACATCGTCGAGAAAGACACCTATTCGGTGGTCAAG
GAACTGATCCCGGAAGGCAAGACCACCTGCTCGCTGTGCTCGCGCCTGCGTCGTGGCACCCTGTACACCTTTGCCG
ATGAAATCGGCGCGACCAAGATGGCCTTGGGTCATCATCGTGATGACATCGTCGAGACCTTCTTCCTCAATATGTT

CTTCAACGGCTCCCTCAAGGCCATGCCGCCCAAGCTGCGTGCCGATGACGGGCGCAACGTGGTGATCCGCCCGCTG
GCGTATTGCAACGAGAAGGACATCCAGGCCTACTCCGACTTCAAGCAGTTCCCGATCATCCCCTGCAACCTCTGCG
GCTCCCAAGAGAACCTGCAGCGCCAGGTGGTCAAGGACATGCTCCAGGAGTGGGAGCGCAAGACGCCGGGGCGCAC
CGAAAGCATCTTCCGCAGCCTGCAGAATGTGATCCCGTCGCAGTTGGCCGACCGCAACCTGTTCGACTTCACCAGC
CTGCGCATCGATGAAACCGCCGCTTCGCGCTTCGTCAACATTGTGAACCTCTGAGCCTTTTCCAGGCTCTGACAGA
CGGCGCTCATGGGCGCCGTTTTCATTTCAACCCGTAGGAGAGGGCATGCGCGATTACAAATGGCTGCACGAATACT
GTCTGAACCGCTTTGGTTCGGCGGCCGAGCTGGAAGCCCACCTGCCGGTGCCCAAGACGCCAGCGCAATTGCGCCA
GATCAGCGATGACCGCTACCTGTCGACCCTGTCGCTGCGGGTATTCCGCGCCGGGCTCAAGCACAGTGTGGTGGAC
GCCAAGTGGCCGGCGTTCGAGCAGGTGTTCTTTGGTTTTGATCCAGAAAAAGTCGTGTTGATGGGCGCCGAGCACC
TTGAGCGCCTCATGCAGGACACGCGGATCATCCGCCACCTGGGCAAACTCAAGAGCGTGCCACGCAATGCGCAGAT
GATTCTCGACGTCGCGCAGGAAAAGGGCAGCTTCGGCGCACTGATCGCCGATTGGCCGGTGACCGATATCGTTGGA
CTGTGGAAATACCTGAGCAAACACGGCCACCAGTTGGGCGGGTTGTCCGCGCCGCGCTTCTTGCGCATGGTAGGCA
AGGACACTTTTGTGCCGAGCTATGACGTGGTCGCGGCGCTCAATGCGCAGAAGATTGTCGACAAGGCCCCCACCAG
CCTGCGGGACCTGGCGACGGTGCAGGCGGCGTTCAACCAGTGGCACGCCGAGAGCGGGCGGCCGATGTGCCAGTTG
TCGATGATGCTGGCCTATACCGTCAACCATTGACCTGTAGGAGCGAGCTTGCTCGCGAAAAACCTGAGGGCGCCGC
GTGCATTCTGAATGCCCGCGTCATCGTTAACGTTTTCCGCGAGCAAGCTCGCTCCTACATGGCCAACCGCCGATTG
AGCTGGAACCGCCAACGCACATACAGCAGCGCCGTGCAGAACACTGCCACGCTCGCCAGCATCTCCAGCACGCCAA
ACCACTGGCGGTTGGGGTCATAGGCCGCCAGGGCGCCCTTGATGAAATACAGGTTCACCACAAAACACATCCACGA
ATGCCCGCGCGCACTGCCCATGAGCATGCCTGGCGCGAGGATCAGCAGCGGCACCAGTTCGATCAGCAGGATCACC
CAGGGCCGGGCGCCATGCAGGTCGGCGACCAGCAGGTAGTAAACGCAGAGCAGGCCTACCAGGCCAAAAAACGCCA
GCAGGCTCAAGGCCCGTGCCACCTTGACCCGGGGTTCGAGCCAGGCCTGGGGCGGCAGGACTTTAGGCTTCTTGGC
CACGGCCACTCTCCAGTTTCGCTGCCGTCTTCGCCAGCCGCAGGCCGAGGGCGCGGCACAGGGCGATTTCATGGGG
GTCCAGGGCACGCTTGCCGTCAGCGCCGGAATGGTGGCTGGCGCCGTAGGGCGTACCGCCGCCCTGGGTGTCGAGC
AACGCCTGTTCGCTGTAGGGCAGGCCGGTGATCAGCATGCCGTGGTGCAGCAGTGGCAGCAGCATCGACATCAAGG
TGGTTTCCTGGCCGCCGTGCAGGCTGGCGGTCGAGGTGAACACCCCGGCTGGCTTGCCGACCAGGGCGCCGGTCAG
CCACAGGTTGCTGGTGCCGTCGAGAAAATACTTCAACGGCGCGGCCATGTTGCCAAAGCGCGTCGGGCTGCCCAGG
GCCAGGCCCGCGCAGTTCTTCAGGTCGTCGAGGCTGGCGTACAACGCACCTTCGGCCGGGATGCTCGGCGCTACGG
CCTCACACTCGCTGGAGATTGCCGGTACCGTGCGCAGGCGCGCCTCCAGGCCGCCTTGCTCGATGCCTCGGGCAAT
CTGCCGGGCCATTTCGCTGACCGAGCCGTTGCGGCTGTAATACAACACCAGGACGTAGGGCGCGCTCACGGCAGGA
TCTCCAGGATATTTTCCGGCGGGCGGCCAATCACGGCCTTGTCGGCGGTGGCCAGGATCGGCCGTTCCATCAACTT
GGGGTGGGCGGCGATGGCAGCAATCAATTGCGCCTCGCTGAGGCTGGCGTCGGCCAGGTTGAGGGTCTTGTATTCG
TCTTCGCCGGTGCGCAGCAGTTGCCGGGCGCTGATCCCCAGTTTGCCGAGCAGGGCTTCAAGTTGCGCGGCGTCCA
GCGGGGTTTCCAGGTAACGCACGACGTTGGGCGCCAGGCCACGGGCTTGCAGCAGTTCGAGCGCACCGCGGGATTT
CGAGCAGCGCGGGTTGTGATAAAGCGTCAGATCGGTCATGTGCGGGTCGCATCTTGCGTAAGGTGGCGGGTATTCT
ACCTGCGCTGCGGCTTGTGCTTAAACCGTAAGAGGCGATCGGCCGCAGCCCACGTTCATTTTTGCTAAGGATTACC
CCATGACACGGCGACTGATAGGCGCACTGGCAGTTATCACCACCCTCCTGCTCAGTGGCTGCGGCAATGATTATGG
CGTCGACCAATACGGTCAGAAAGTCGCGTCCGAACGCGTCGACAAGCAATGGCTGGTGGTCAATTACTGGGCCGAG
TGGTGTGGTCCGTGCCGCATCGAAATCCCTGAGCTCAACGCCCTGGCCGAGCAGCTCAAGGGCCAGCCGGTGAGTG
TGTTCGGCGTCAACTTCGACAACGTGCAGGGTGAAGAACTCAAGAGCGCGAGCGAGAAGCTGGGCATCAAGTTCAC
CGTGTTGGCACAGAACCCCGAGGCGATTTTTGATATCCCGCGCAGCGAAGCGTTGCCGGTGACCTACATCATCGAT
GACAAGGGCAAGGTGCGGGCGCAGATGTTGGGTGAGCAGACGGCGGAAGGTGTACTGGCCAAGCTCAAAGAGTTGC
GCGGCTGAAGCTTCGCGATCTCTGTAGGAGCGAGCTCGCTCGCGAAGCACTCACAGGCACCGCGTTCATCCTGGAT
GCACGGGTTTTTGTTGACGTTCTTCGCGAGCAAGCTCGCTCCTACAGGCGTTGGGTCAGCCTTCTTCCAGCCACAG
GCGCAGAGGCTTGCCCTCGGCAGGCCAGAAGCGGGTTTGCTCGATGGGCGAGATATCCCAGCGCTGCACGGTTTCC
AGGGCCTTGAAGAAGCGCTGCTCCTGCTCCATCAGGGCTTCGGCACACAGCTTGCGGGTACTGCCGATCTTGCCGA
AGCTGAGCTTGTCACCGTCCAGGCTGTAGGGCGCGAACCAGTGGTTGCAGCCGCCATTGCCATAGGCCCGGCCATC
GGCGCCGAGGGTCACGGTCAGGTGGGCGTAATCCATCAGCGGCCGTTCGCCGATCCATTCGACGATGTAGCTGTGA
TCCTGCTTGAGCTTGACCTCATCGCTAGCGCAGCCGGCGAGGCCGACGCCCAGCGCGGCGAGCACCAGCAGGCGCT
TCATTGCGCGGCCTTCTGGCATTTCGGGCAACGGTGTTTATCACCGACAGTGGCCCAGCCCAGCTCGGCAATGCGC
GCGGTGGCCGCCGGTTGCAGTGGCTCCTTGGTCAGCTTGGTCTCCACCGAGAACTCAAACTCCAGCACCGCGTCGC
AGCTGTCGCAGTTGACCTTCCAGGTGTGAATTTCCAGCTCGCCGAACTTCGGCCCTTGCGCCATGGCGACCCATTG
CCCGGCCGGGCGGATGGAGTAGCGCGCATCGCCTTCGACGGTCAGGCGCATCGACAGGGTCTTGCTGCCGCGCAAG
GTGACGATCAGCACGTCGCCATGCTTGATCGAGCCACCATTGCCGGTGACTTGGTAACGACCCGGCACCAGGGCGC
GGCATTCGATCAGGGTGTGTTGTGGGCTCAGCAAACTGAAGCGGAAATCGTGTTCGGCCATGGATCCTCCAAATAG
GCGCGGCATCCTATCACGCTCGCCTGTTTACCATGCGCGCGGTATGTGACCGTTGATCATCCGTGCACCAGGTTTT
GCGGTTGACCGCTGGCCCAGGCCTGGATATTGGCCAGGGTGGTCCCGGCAATCGCCGCCAGCGCTTCGCGTGTCAG
GAATGCCTGGTGCGCGGTGATGATTACATTGGGGAAGGTCAGCAAGCGCGCCAGCACGTCGTCCTGCAACGGCAGG

TCCGAGCGGTCCTCAAAAAACAGTTGGGCTTCTTCCTCGTACACATCCAGGCCCAGGTAGCCCAATTGCCCGTCCT
TGAGCGCTTCGATCAGCGCCGGGGTATCCACCAGGCCGCCGCGCCCGGTATTGATCAGCATCGCCCCGGGCTGCAT
ATGGGCCAGGGATTGGGCATTGATCAAGTGCTTGCTGTCGGCGGTCAGCGGGCAGTGCAGGCTGATGATCTGCGCC
TGGGCCAATAACTGGGGCAGGTCGACATAGCGCGCGCCAAGGGCTTCGACCTGCGGGTTGGGATAGGGGTCGTAGG
CCAGCAAGGTGCAGCCAAAACCGGCCATGATCTTGGCAAAGGTCGCGCCGATCTGCCCGGTGCCCACTACGCCAAC
GGTCTTGCCTACCAGGTCGAAACCGGTCAGGCCGTGCAGGCTGAAGTCGCCGTCGCGGGTGCGGTTGTAGGCGCGG
TGCAGGCGACGATTGAGGGCCAGCACCAGGGCCACGGCATGTTCGGCCACCGCGTGGGGCGAGTAGGCAGGCACCC
GCACGATGGTCAGGCCCAGGCGTTTGGCGGCGGCCAGGTCCACATGGTTGTAGCCGGCCGAGCGCAGGGCGATCAG
GCGCGTGCCACCGGCGGCGAGGTGTTCGAGCACCGCTGTACTGAGGTCGTCGTTGATAAAGGCACAGACCACTTCG
TGTTTGTCGGCCAGGGCCACGGTATCGAGGTTCAGGCGTGCCGGTTGGAATTGCAGCTCCATGCCTGCGGGCAGCG
GTTCGGCGAGAAAGCTGTCGCGGTCATAGGTCTGGCTGCTGAATACAATGATGCGCATCAAAGGGCTCCCTGCAAG
ATAGCGTGTGGTACCTGTAGGCGCCGGCAAGCCGGTTCCTACAGGTATGTTGCCCGTCATGGGGGGTTGGCGTGTT
GTTCTGTGTCAGGCGTTGACCCGGGCTTCGCTGGCAAGGCGGGCAATCGCCATGTCCAACTCATCAAGGGCGGCGA
TGGCCTTGGCGTCATCTTGCTTGAGCAAGGTTTCGGCGCGCTGGCAGGCGGCGCGTAATTGCGGCACGCCGCAGTA
GCGGGTGGCGCCGTGCAGGCGGTGGACCCGTTCGATCAGCGCGTTATTGTCGTTGGCTTCGCGGGCGATGCACACC
GCCTCGCGGTCGGCTTCCAGGGACGCCAGCAGCATGGCCAGCATGTCGGCGGCCAAGTCTGCCTTGCCGGCCGCCA
GGCGCAGGCCTTCCTCATGGTCCAGCACTGGCAGTTGGGCGGCGGGGCCGTGGCCTTCGACGGCGCGCTCCGGGCC
TTGATTGCGCAAGGCCAGGCCGGTCCATTTCAGCACCACTTGGGCCAGTTGCCGCTCGCTGATGGGTTTGGTCAGG
TAGTCATCCATGCCGCTTTGCAGCAGGGCGCGTTTTTCATTGGCCATGGCATGGGCCGTCAGCGCGACGATAGGCA
GCGGTGTGCCATGGCGCTCGCTTTCCCACTGGCGAATCGCCTCGGTGCTCTGGCGCCCGTCCATACCGGGCATCTG
CACATCCATCAGCACCAGGTCGAAGGCTTCTTTTTTCACCGCTTCGATGGCGGTATAGCCGCTTTCCACGGCCTGG
ACCTTGGCGCCCATGTCTTCGAGCAGGGTCTGCACCAGCAGCAGGTTGGCCGGGTTGTCATCGACACACAGCACCC
GTGGTGCGCGGCTGGACAACGGTTCGCCGGGCTCGCTGCGCAACGGCCGCGGGCTGATCAGGTCGGACAGTGCCCG
GCGCAATTTGCGGGTGCAGGCCGGTTTGGCCTGCAGCTGGCTGTTGGGGTTGGGCACCGACAGGTTGAACAGCAAT
TGCTCGGTGGTCGGGCACAGCACCAGGACTTTGCAGCCCAGGTGTTCGAGGTCCCACAGGTGTTGGTTGAGGCGTT
CCGGCGGAATGTCATTGGCGGTGATGCCCAACACCGCCAGATCGATGGCATGTTCGGTCTGGTGCGCGCTGGTGAT
GCCGTTGGTGAGGTTCTCCAGGCTGTTGAACGGCGTGACCTCCAGGCCACAGTCTTCCAACTGGTGTTGCAGGGCC
TGGCGCGCCAGTTCGTGATTCTCCAGCACCGCCACGCGCCGGCCCAGCAGCGGCGGGGAGGGCAGGTCTTCGATAT
CGTCGCGGGTCTTGGGCAGGTTGAGGCTGATCCAGAACTCCGAACCCTGGCCCGGCGTGCTGTCGACGCCGATTTC
ACCGCCCATCTGCTCGATCAGGCGCTTGGAGATCACCAGGCCCAACCCCGTGCCGCCGGGCTGGCGGGACAGTGAG
TTATCGGCCTGGCTGAAGGCCTGGAACAGGGCGCGTACATCCTGGTTGGACAGGCCGATGCCGGTGTCCTGCACGC
TGATGCGCAGTTGTACGCTGTCTTCCTGCTCGTCTTCAAGCATGGCCCGGGCCACGATAGTGCCCTCGCGGGTGAA
CTTGATGGCATTGCTCACCAGGTTGGTGAGGATCTGCTTGAGACGCAACGGGTCGCCCACCAGGGACAGGGGCGTA
TCGCGGTAGACCAGGCTCACCAGCTCCAGCTGTTTGGCGTGGGCGGCCGGGGCGAGAATGGTCAGGGTGTCTTGCA
GCAGGTCGCGCAGGTTGAACGGAATATTGTCGAGTACCAGCTTGCCGGCTTCGATTTTCGAGAAGTCGAGAATTTC
GTTGATGATACCCAGCAGGTTGTCGGCGGATTTTTCGATGGTGCCCAGGTAATCCAACTGACGGGGCGACAGTTCG
CTTTTCTGCAGCAGGTGGGTAAAACCGAGGATGCCGTTGAGCGGCGTGCGGATTTCATGGCTCATATTGGCCAGGA
ACTCCGACTTGATGCGGCTGGCTTCCAGGGCCTCCTTGCGGGCCAGGTCCAGCTCGATGTTCTGGATCTCGATGGT
TTCCAGGTTCTGGCGCACATCTTCGGTGGCCTGGTCGATACTGTGTTGCAGTTCTTCCTGGGCGTTTTGCAGGGTT
TCGGCCATGCGGTTGATACCGGACGCCAGTTGATCCAGCTCCTGGCTGCCCAAGGGCGGCAAACGGGTTTCCAGGT
TGCCATCCTTGAGCTGCGCCACCGCTTGCTTGATCTGCCCGATGGGTGAGTTGATCGTGCGGCTGATGCGCAGTGC
CAGGGCTGCGTTGCAGATCAGGCCGACGGCGATCAACAGGAGGCTGGCGAACAGGCTGCGATAGCCGCGTAGCAAC
ATGCCGTTGTGCGACAGTTCCAGCTCGACCCAGCCCAGCAGGCGGTCGGCTTCGTCGGGAATCAGCTCGCCGGCGA
GGTTGCGATGGCGGCCGAACACGGGCAGCAGGTAGCGGGTGGCGTCATTGTCGGTGCGTTGCAACAGGTGGGAGCT
GTTGCCGATGGGCGGTGGGTTGAGCATGGTCGGGCCGGCATGGGCCAGGGACGAGCGGTCGGGCGCCAGGAAGGAC
ACGGCCCGCACATCGACTTGTTCCAGGGACTGGGTAGCGATGCGCTCCAGCAGATCGATGTTCTTGCTGCCCAGTG
CCGGTGCCACCAGCGGCGCCAACTGTTCGGCGATCATCTCGCCGCGATTGAGCAGCTGGGTTTGCAGGTCCGAAAG
CTGCATCCAGGTGAAATAACCGCCCAGCAGCAACGCCATCAGGCTGGTAGGCAACAAGGTCAGCAACAGTACGCGG
CCTTTTATCCCCATTCTTCTTAGCACGCCACTCTCCCGCTACCACACTGATGTCGACATTCTACGCAGGCATCCGG
CCCGCGCTACCTGCGCAGTGTAGCCATATGAGGGGCTTGGAATCTTGCAAAAAATGCTTGAGAGGGCGATGGCGGG
ATTATTGAGCGGCAGTAACGGCGGGTATCGCTGCCTCCCTGATCGTCAAAGCGTACGCGTACTTCGGTAGGCGCTG
GCTTGCTCGCGATGGCGCCCGCCGGGACATCTCTGCTCCTGAACTGAGGGGCCGCCTTCTATCGATTACGCTGCTG
GCGCTTTTTCCATTGATGACCCACCCACCACCGCCAGTACACCATCGTCAGGCAGTACGCCAGCGCGCCCAAGACC
AGCCCCATCACCACCGAGCCCAGCAGGAACGGCTGCCACATGGTCGACAGTTGCCCACTAATCCATTCCCAGGTCA
GTTCGTCGGGCAGGGTGCGCGGTGGGATGTTCATCAGCCAGGTGCCGGTCATGTAGGTGCAGAAGAACACCACCGG
CATGGTGATTGGGTTGGTTAGCCATACCAGGCTCACGGCAATGGGCATATTGCCGCGCACGCAGATGGCGAGGACG
GCCGCCAGCAGCATCTGCAAGGGGATGGGAATGAAGGCGGCAAACAGGCCGACAGCCATGGCCCGGGCAACCGAGT

GCCGGTTGAGGTGCCAAAGGTTGGGGTCATGGAGCAGCGTCCCGAGAAAGCGTAAGGACTTGTGTTCCCGAATGCT
GGTCGGATCGGGCATGTACCGTTTGAATAAGCGCCGTGGCATAGGGGGTCCAGGTCAGTTCGAGGGGCAAGTATGC
CCGTATTCTACGAACGGGAAATTCAGACTTTGTGACAAAAAATCATAGTCCGCCCGGAACCACTCGGCTAACACTC
AATGGGTCACTTTCAAGGAATGATCCATGCGTACAGGGATGTTTGCGCTGGCGTTGGGGCTGCTGGCGTTGCGCCT
GCTGCCTGCCTTGCCGCCGGTCGGTTGGTTGCTGGTGTTGCCGGTGGTGGCGTTGATGCTGCTGCCATTTCGAACT
TATCCGCTGGCATTTTTCCTGTTGGGCCTGGGGTGGGCCTGTTTCAGCGCGCAACGAGCCCTCGACGATCGGCTCA
GCCCCGCGCTGGACGGCCAGACCCGCTGGCTGGAAGGGCGCGTCAGCGGGCTGGCGCAGCAGGCCAATGGGGTGGT
GCGCTTCGAGTTGAGCGACAGCCGGTCACGTCAGGACCGGCTGCCCCAGCGTATCCGCGTGAGCTGGCAGGGTGGC
CCCGCAGTGCGCAGCGGTGAGCGCTGGCGTTTGGCGGTGACCCTCAAGCGTCCCGCCGGCCTACTGAATTTCCATG
GCTTCGACCAGGAGGCCTGGCTGTTGGCCCAGCGTGTGGGGGCGATCGGTTCGGTCAAGGATGGCCAGCGCCTGGC
GCCGGCCCGGCATGCCTGGCGCGATGGGATCCGCCAGCAGTTGTTGGCAGTCGATGCCCAGGGCCGCGAAGCCGGG
CTGGCTGCACTGGTGCTGGGCGACGGCTCCGGTCTGGCCGCCGAGGACTGGCGCATTTTGCAGGCCACTGGCACCG
TGCATCTATTGGTCATCTCCGGTCAACATATCGGCCTGTTGTCGGGCTTGATCTACGCCCTGGTGGCCGGGCTGGC
CCGCTACGGCGGCTGGCCCAGGCGCTTGCCCTGGCTGCCTTGGGCCTGCGGCCTGGCATTTGCCGGGGCGCTGGCC
TATGGCGTGCTGGCCGGTTTTGAAGTGCCGGTGCAGCGGGCCTGTGTAATGGTGGGGCTGGTGCTGCTGTGGCGCT
TGCGGTTTCGCCATCTGGGGATCTGGTGGCCACTACTGCTGGCCCTGAATGCGGTGTTGGTCATCGAGCCGCTGGC
CAGCCTGCAACCGGGGTTTTGGCTGTCGTTTGCCGCCGTGGCCGTGTTGGTGCTGGCCTTCGGCGGCCGCCTGGGG
CCATGGCGCGCCTGGCTTGCCTGGACCCGTGCCCAATGGCTGATCGCTATTGGTCTGTTCCCGCTGCTGCTGATCC
TCGGCCTGCCCATCAGCTTGAGCGCGCCGCTGGCCAATCTGTTTGCCGTGCCGTGGATCAGCCTGCTGGTGCTGCC
GTTGGCGCTGGCGGGCACGCTGTTGCTGGCGCTGCCGTGGCTGGGCGGCGGCCTGCTGTGGCTGGCGGGTGGCGCA
CTGGATTGGCTGTTCATGGCCCTGGCCATGCTGGCCGAGCGCGTACCGGCCTGGATGCCTGCCGAGGTGCCCGTTG
GCTACTGGTTGCTGAGCCTGCTGGGGGCACTGCTGTTATTGATGCCCAAGGGCATTCCCTTTCGCCTGCTGGGTTG
GCCCATGTTGCTGCTGGCGGTGTTCCCGCCCCGCGAATCGATCCCGTTGGGGCAGGTAGCGGTGGAGCAACTGGAT
GTCGGCCAGGGCCTGTCGATAGTGCTGCGCACCCGCAACCACGTGCTTTTGTATGACGCCGGTCCGCGGCTTGGGG
AGTTCGACCTCGGTGAGCGCGTGGTACTGCCGGCGCTGCGCAAGCTGGGGGTTGGCCAAGTGGACTTGATGCTGCT
CAGTCACGCCGACGCCGACCATGCCGGGGGCGCTGCGGCGGTGGCGCGGGGGTTGCCGGTCAAACGGGTGGTGGGC
GGGGAAACCCAGGGCCTGCCAGCGTTCCTCCAGACCCAGGCCTGTGTCAGCGGTGAGCAATGGGAATGGACGGTG
TGGCGTTCCAACTGTGGCAATGGGCGGATGCCAGCAGCGGCAATGCGAAGTCCTGCGTGCTGCAAGTCCAGGCCAA
TGGTGAGCGCCTGCTGCTGACCGGCGATATAGACCGCGAGGCCGAGCGGGCCTTGCTCGGCACGCCCCTGGCGGTC
GCCACCGATTGGCTGCAAGCGCCCCATCATGGCAGTCGCAGTTCCTCGTCATGGCCGTTCGTCCAGCGAGTCGCCC
CCAAGGCCGTGCTGATCTCCCGCGGGCGTGGCAACTCCTTTGGCCATCCGCACCCGCAAGTACTGGCGCGTTACCG
GTATTTGCGCAGCGCCATTTATGACAGCGCCGAGCAGGGCGCCATCCGCCTGCGCCTGGGGGCTTTCCAGGCGCCG
GTCATGGCACGCAGCCAGCGTCGTTTCTGGCGCGCGCCTGTCATTGACAGTTACCAAAGGCCGGTGCCTGCGACAT
GATGGCTGTCGGCGGGCAGGCCCTTCCTCGTCAGCCTATATGCTAAAGTGGCGCACTTTTTCGAGGGGACATTCAC
TGTGTGGGAATTGGTCAAATCCGGCGGCTGGATGATGTTGCCGATCATTCTGAGCTCCATCGCCGCACTCGGCATC
GTTGCCGAACGCCTGTGGACCCTGCGTGCCAGCCGCGTGACCCCTGATCATCTGCTGGGGCAGGTCTGGCGCTGGA
TCAAGGACAAGCAACTGGACAAGGAAAAACTCAAGGAATTGCGGGCCAATTCCCCCCTGGGCGAGATCCTCGCCGC
CGGCCTTGCCAACTCCAAGCACGGTCGCGAGATCATGAAGGAATGCATTGAAGAAGCCGCCGCCCGGGTCATCCAC
GAGCTGGAGCGCTATATCAATGCCCTGGGCACCATCGCCGCGATGGCACCGTTGCTCGGCTTGCTTGGCACGGTGC
TGGGCATGATCGATATCTTCAGCGCCTTTACCGGTTCGGGCATGACCACCAACGCCTCGGTATTGGCCGGGGGGAT
TTCCAAGGCCTTGATCACCACGGCTGCGGGCTTGATGGTGGGTATCCCGGCGGTATTCTTCCACCGCTTCCTGCAG
CGCCGCATCGATGAACTGGTGGTGGGTATGGAGCAGGAGGCGATCAAGCTGGTGGAAGTGGTCCAGGGCGACCGTG
ACGTCGACCTGGTCGAGGGCAAAGCGTGAAATTTCGCCGCAAGCCTCGGGAAACCGTCGATATCAACCTGGCGTCG
TTGATCGATGTGGTGTTTATCCTGTTGTTGTTTTTTGTCGTCACCACCACCTTTACCCGGGAAACCCAACTGCGGG
TCGAACTGCCGGAAGCCGTCAGCGGCTCGCCGGCCGAAGACCAGCAGCTCAAGCAGTTGGAGATCACCATCAGCGC
CGAAGGGGTGTTTTCGGTGAACAACCAGTTGCTTGAGAAGAACGACGTGGCCAGCGTAATGGACGCGCTGCAGAAG
GAGTCCGGCGGTGATACGCAAATGCCGCTGTCCATCAGCGCCGATGGCAAGACCCCGCACCAGGCCGTGATCACCG
CAATGGATGCTGCCGGCAAGCTCGGTTTCAGCCATTTGCGCATGACCACCGTCGAGGCGGCGGGGCAGCCTTGATG
GCCTTGTCCGATCGTTTGCTCAAGGCCTGGTACGAGGGCCATCCGGCGCTCAAGCTTCTGCAACCGTTGGAGGCGC
TCTATCGCCGGGTGGTCAAGCGCAAGCGCGACCGGTTCCTGGCGGGCGAGGGGACGATCTACCAATCACCGGTGCC
CATCGTCGTGGTCGGCAATATCACCGTGGGCGGCACGGGCAAGACGCCGTTGATCCTGTGGTTGATCGACCATTGC
CAGCGCAGTGGCCTGCGGGTTGGCGTGGTCAGCCGTGGCTACGGGGCCAAGCCGCCGCAGTTGCCCTGGCGGGTGC
GAGCCAGCGATAGCGCGCAAGTGGCCGGTGACGAGCCCTTGCTGATCGTGCAGCGCAGTGGCGTCCCCCTGGTGAT
CGACCCTGATCGCAGCCGTGCGGTGCAGGCCCTGCTCGCCAGCGACACCCTGGACCTGATCCTGTCCGACGATGGC
CTGCAGCATTACCGCCTGGCCCGTGACCTTGAACTGGTGCTGATCGACAACGCCCGCGGCCTGGGCAATCGCCGCT
GCCTGCCCGCCGGGCCGCTGCGCGAGCCGGTCGAGCGCCTGCGCAGCGTCGATGCGCTGCTCTACAACGGCGCCGG
GGCTGATCGCGAGGATGGCTTTGCCTTCCGCCTGCTACCCACCACCCTGGTCAACCTGCTGACCGGCGAGCGCCAG

CCACTGGACCATTTTCCGCAGGGGCAGCGGGTGCATGCGGTCGCCGGCATCGGCAACCCGCAACGTTTCTTCAATA
CCCTTGAAACGCTACACTGGCTGCCGATACCCCATGCGTTTGCCGACCATGCGCCCTACAGCGCCGAGGTCCTGAA
TTTTACGCCGTCACTGCCGCTGGTCATGACCGAAAAGGACGCGGTGAAGTGCCGCGCCTTTGCCCAGGCACACTGG
TGGTACCTTGCAGTCGATGCGGCACCGTCACCGGCGTTTATCGCCTGGTTCGACACGCAACTGATGCGCCTGCTGC
CGGCTCGACTTTTGCCTTAAAACGCTTTTATCCAGGAAACCCCCATGGACACCAAACTGCTCGATATCCTCGCTTG
CCCGATCTGCAAAGGCCCGCTCAAGCTCAGCGCCGACAAGACCGAGCTGATCAGCAAAGGCGCAGGCCTTGCGTAC
CCGATCCGCGACGGCATCCCGGTGATGCTGGAAAGCGAAGCCCGTACCCTGACGACCGACGAGCGCCTGGATAAAT
GACCACTGCCTTCACTGTTGTCATCCCGTCGCGCTATGCCTCCACCCGCCTGCCCGGCAAACCTTTGCAGATGATC
GGCGGCAAGCCGATGATCCAACTGGTCTGGGAACAGGCCTGCAAAAGCAGCGCCCAGCGCGTTGTGGTGGCCACTG
ATGACCTGCGGATCATCGAGGCCTGCAAGGGTTTTGGCGCCGAAGCGGTGCTGACCCGTGAAGACCACAACTCCGG
CACCGACCGCCTGGCGGAAGTCGCCACGCACCTGGGCCTGGCGCCGGACGCCATCGTGGTCAACGTGCAAGGCGAC
GAGCCGCTGATCCCGCCGTGTGTCATCGACCAGGTCGCCGCCAACCTGGCCGCCCATGCCGAAGCGCGCATGGCGA
CCCTGGCCGAACCGATCGAAGACATTGAGACCTTGTTCAACCCCAATGTGGTCAAGGTGGTCAGTGACATCAATGG
CCTGGCCTTGACCTTCAGCCGTTCGACCCTGCCCTGGGCGCGCGATGAATTCGCCAGGCAACGTGACGTCTTGCCG
GCCGGCGTGCCGTATCGCCGCCATATCGGCATCTACGCCTACCGCGCCGGTTTCCTGCATGACTTCGTCAGTTGGG
GCCCATGCTGGCTGGAAAACACCGAGGCCCTGGAGCAACTGCGGGCCCTGTGGCACGGCGTGCGCATTCATGTCGA
CGATGCCATGGAAGCCCCGCCGGCCGGCGTCGATACGCCGGAAGACCTTGAGCGCGTGCGTCGGTTGCTGGGGGCT
TGATGGAAGTCCTGTTTGTTTGCCTGGGCAATATCTGCCGCTCACCCACCGCCGAAGGCGTCCTGCGCCATAAATT
GCGGGCTGCCGGGCTGGCCGACCAGGTCGAGGTGGCCTCCGCCGGCACCGGCGAATGGCATATCGGCAAGGCGCCG
GACAAGCGCAGCCAGCAAGCGGCGCTGCGCCGTGGCTACGACTTGTCGGCCCAGCGTGCCCAGCAGGTGTCGCGCG
CCGACTTTGCGCGTTATGACCTGATCCTGGCCATGGACCAGAGCAACCTGCGTAACCTCAAGGCCATGCAACCGGC
CCAGGGCAAGGCAGAGTTGGACCTGTTCCTGCGCCGCTACGACTCGGCGGTGGATGAAGTGCCGGATCCGTATTAC
GACGGCGAGCAAGGTTTTGAGCAGGTCCTGGACCTGATCGAGGCCGCCTGTGATTTGCTGGTGATCGAATTGAAGG
GGCGTTTATGAGCCTGGATGTACAGCCCCAGGTTTCCCTGAAGGCCTTCAACAGTTTTGGCATTGATGTGCGTGCG
CAGTTGTTCGCCGAAGCCCATAACGATGCCGATGTGCGTGAGGCCCTGGCCTATGCGGCGGCGCAACAGCTGCCGT
TGCTGGTGATCGGGGGCGGCAGCAACCTGCTGCTGACCCAGGACATCCCGGCGTTGGTATTGCGCATGGCAACCCA
GGGTATCCGGGTGCTGCACGACGACGGCCAGCAGGTTGTGGTGGAAGCCGAGGCGGGGGAGGCCTGGCATCCCTTT
GTGCTGTGGACCCTTGCACAAGGGTTCTCTGGCCTGGAAAACCTCAGCCTGATTCCCGGTACGGTCGGTGCGGCAC
CGATGCAGAACATTGGCGCCTACGGGGTCGAGATCAAGGATGTGTTCGCCGGCCTGACCGCCCTCGACCGCCAGAC
CGGCGAGTTGCGGGATTTCAGCCTGGCAGAATGCAACTTCGCCTACCGCGACAGCCTGTTCAAGCACGAAGTGGGG
CGCTGGCTGATCCTGCGCGTACGCTTTACCTTGAGCCGCGTGGCGCACTTGCAACTCGGCTATGGCCCGGTGCAAC
AACGCCTGGCTGAGCAGGGCATTACCCAGGCGACACCGAGCGATGTCAGCCGGGCCATTTGCAGCATTCGCAGTGA
GAAACTGCCAGACCCTGCGGTCTTGGGCAACGCCGGTAGTTTCTTCAAGAACCCGCTGGTGCCCCAGGTGCTGGCG
GCGCAATTGAAGCTGCAGTATCCCGATCTGGTGGCTTACCCCCAGGCCGATGGCCAAGTGAAACTGGCGGCCGGCT
GGTTGATTGAAAAGGCCGGCTGGAAAGGCTTTCGCGACGGTGATGCTGGTGTGCATCGCTTGCAGGCGCTGGTGCT
GGTCAACTACGGCGCGGCCACGGGCCTGGAGATTTCCAGCCTGGCCCAACGCATCCAGCGCGATATCGCCGAGCGC
TTCAAGGTCGATCTGGAAATGGAGCCCAATCGTTATTGAGCTACGCTTCTAGCGTGTTGAACAAAGCCCTGCATTG
CAGGGCTTTTTTGTTAATGCTGGGTTAACTTAGCAGGCTAACGATGCCTGTTTATCGTTTTATAGATACTGTTTAT
CAAAGGCCCGGTGAAAGGTCGGTGTTGTTCGTGCAGGGAATGGTAGGAGCGAGCTTGCTCGCGAAGATCGTCAACG
ATGACGTGGGCGCTCTGAATCAACGTGGTGTCCTGGCGTTTTTCGTGAGCAGGCTCGCTCCTACAGTGATGCACAT
TCCCTGACGCAACACCTGTCTTCACAAGAGAGCCCGATTAGCCTGAGCCATCTGCGTGAACCAACTCCGCTCCCCT
GAAAGCGGCAGATAGCTCGACCCCATAATCCAATCACTATGCGGGCGTGCCCATGATTACTCTCAAACTCAACGGT
CAAGATCATCCGCTGGATGTCACCGAGGACATGCCCCTGTTGTGGGCGATCCGCGATGTGGCGGGTTACAACGGCA
CCAAATTCGGTTGCGGCATGGGCCTGTGCGGCGCCTGCACTATCCATATCGATGGCTCGCCGGCCCGCAGTTGCAT
CACGCCTATTGGCTCGGTCAAAGGGCAGAACGTCAGCACCATCGATAACCTGCACAATGACCCGGTTGGCCAGGTT
GTGCAGCAAGCGTGGCTGGACACGGCAGTGGCCCAGTGTGGTTACTGCCAGGGTGGGCAGATCATGTCGGCTACGG
CACTGCTGAAAACCAACCCCAATCCCAGCGATGCGCAGATTGAAGAGGCCATGGTCGGCAACATCTGCCGCTGCGG
CACGTACAACCGTATCAAGACAGCGATCCGCCAGGCCGCGACGCATCTGCAGGGGGACAAGGCATGAGCCAGTTGC
CAAGCAATTTCGCCCTGAACAACCTGAGCCGTCGTGGTTTCCTCAAGGGCGTCGGTGCGACCGGTGCGCTGGTGGT
GGCCGCCAGTTGGGGCCTGCCCCAGGCTTTTGCCGAGGAGGTGAAGCAATACGGCGGGGCAGCCATGCCCAACGGC
GTGATCGATGACCCCAAGGTGTATGTCAGCATCGCCACCGATGGCACTGTCACCGTGATCTGCAACCGCTCCGAAA
TGGGACAAGGCGTGCGCACCAGCCTGACCATGGTGGTGGCCGACGAGTTGGAGGCCGATTGGGCGCTGGTGAAGGT
TGCGCAGGCCCCGGGCGATGAAGTGCGTTTTGGCAACCAGGATACCGACGGTTCGCGCAGCATGCGCCACTGGTAT
GAACCGATGCGCCGTTGCGGCGCAGCCGTGCGCAGCATGCTGGAACAGGCCGCCGCCGAGCAGTGGAAAGTGCCGC
TGGGCGAGTGCCGTGCGCAGTTGCATAAAGTGATTCACCAGCCCAGCGGCCGTGAGCTGGGCTATGGCGCCCTGGC
TGCAGCCGCCGGTGCGCTGGCGGTGCCGGCGCGGGACAGCCTACGGCTCAAGCAGCCATCGGAGTTTCGCTATATC
GGCAAGGAGGCCACCAAGGCCATCGACGGTGCCGATATCGTCAACGGCCGGGCGGTGTACGGCGCCGATGTGCATT

TTGACGGCATGTTGTTTGCGACCATTGCGCGGCCCAAGGTGTATGGCGGCAAGGTCAAGTCGTTCGATGCCAGCGC
GGCGTTGAAAGTCCCCGGGGTGATCAAGGTCCTGCAAATCGAAAGCCGGCCCGTGCCCTCGGAGTTCCAGCCCTTG
GGCGGTGTAGCGGTGGTTGCCAGCAATACCTGGGCGGCGATCAAGGGGCGTGAAGCGCTGCAGATTGTCTGGGAGG
ACGGCGTCAATGCCGGCTACAACTCCATCGACTACCGCAAGACCCTGGAAGCCGCGGCCCTTGAGCCCGGCAAAGT
GGTGCGCAATACCGGCAGCATCGACCAGGCCCTGAGCGATGCCGACAGCACCCTGGAAGCCGCGTATTACTTGCCA
CACCTGGCGCAGTCGCCCATGGAGCCGATGGTTGCCGTGGCCCGTTATCAAGACGGGCAATGCGAGGCCTGGGCGC
CGAGCCAGGCGCCGCAGGTCACGCGGGAGCGGGTCGCCGAGCGCCTTGGTTTGCCATTCGATAACGTCACGATCCA
CGTCACCCTGTTGGGCGGCGGCTTTGGGCGCAAGTCCAAGCCGGACTTCATCATTGAAGCTGCGGTGCTGGCCAAG
GAGTTTCCCGGCAAGGCCGTGCGCGTGCAGTGGACCCGTGAAGACGATATCCATAACTCCTACTTTCACACCGTGT
CTGCCGAGTACCTCAAGGCCGGCCTGAACAAGGACGGCATGCCCTCCGCCTGGCTGCACCGCACCGTGGCGCCGAG
CATCACCGCGCTGTTTGCGCCGGGCATGAACCATGAGGCCGCGTTTGAATTGGGCATGGGCTTTACCAACATGGCC
TACGCCATTCCCAATGTGCGCCTGGAAAACCCCGAGGCCGCCGCCCACACCCGTGTGGGTTGGTACCGTTCGGTGT
CGAACATCCCCCATGGTTTTGCCATCCAGAGCTTTGTCGATGAGCTGGCGCACAAGGCTGGCCAGGATCCGCTGAA
ATATCAGCTCAAGCTGCTGGGCCCGGATCGTCAGATCGATCCACGCACTTTGAGTGAAGAGTGGAACTACGGCGAG
TCACCCGAGCGTTACCCGATTGATACGGCGCGCATGCGTGGCGTGCTCGAGACCGCGGCCAAGGCTGCCGGCTGGG
GGCGCACGCTGCCCAAGGGGCGCGGCCTGGGGCTGGCGGTGCACTACAGCTTTGTGACGTATGTGGCGGCGGTGAT
CGAGGTGGAGGTCAAGGGCGATGGCACGTTGATCGTGCACAAGGCGGATATCGCCGTGGATTGCGGCCCGCAAATC
AACCCGGAGCGCATCCGCTCGCAGTTCGAGGGCGCTTGCGTCATGGGCTTGGGGAATGCGGTCCTGGGAGAAATCA
GCTTCAAGGATGGCAAGGTCCAGCAGGACAACTTCCATATGTACGAAGTGGCGCGCATGTCCCTGGCTCCCCGGGA
AATTGCCATACATTTGGTCACACCGGCAGGCGAGGTCCCCCTCGGCGGTGTGGGTGAGCCGGGCGTGCCGCCGATT
GCGCCGGCGCTGTGCAATGCGATATTTGCCGCCACCGGCAAGCGCATCCGTAACCTGCCGGTGCGTTATCAGTTGC
AGGGTTGGCAGCAGGAGGCGCAGGCCTGATGGACAGCGTGGATCTGAATGTCCTGCGCAGCGTGCTGGAGTGGCGC
CGCGCAGGGCAACAGGTGGTGCTGTACAGCGTGGTCCAGACCTGGGGCAGCGCACCACGGCCGCCGGGGGCGATGC
TGGCCCTGCGTGGCGACGGGGTGGTGATCGGCTCGGTATCCGGTGGCTGCATCGAAGATGACCTGATCGCCCGTCT
GCACGATGGCCGCCTGCCAGACAATGGGCCGCCGGTGCAGTTGGTGACCTACGGCGTCACCCGCGACGAAGCGGCG
CGCTTTGGTCTGCCATGCGGCGGTACCTTGCGCCTGACGGAGGAGCGGGTGGAGGAATATGCCTGGGTGGCGGAGT
TGCTGGCGCGTTGTGAGGCCCATGAAATTGTGGCGCGGGAGCTGGATTTGGCGACGGGCAGTGTGCGCTTGAGCGT
GGCGAGCAAGTCCGACATCGTCAGTTTTGACGGTGAGCGTTTGCGGGCCATCTACGGTCCGCGCTGGCGCCTGCTG
CTGATCGGTGCGGGCCAGTTGTCGCGTTATGTGGCAGAAATAGCGCGGCTGTTGGATTTTGAAGTACTGATCTGCG
ATCCCCGTGCGGAGTTTGTGCATGGCTGGGAAGAACAGCACGGGCGCTTTGTGCCGGGAATGCCCGATGAGGCGGT
GCTCAATATCCAGACGGACGAGCGCACGGCCATTGTTGCCCTGACCCACGATCCGCGCCTGGACGATATGGCGCTA
CTGACCGCGTTGAACTCCCGGGCGTTCTACATCGGTGCCCTGGGTTCACAGCTCAATAGCCGCAAGCGTCGGGAAA
ACCTCGCGTTACTGGGCCTGGGGGCCGAGGCAATTGCGCGGCTGCACGGCCCGATCGGTCTTCATATTGGCAGTCA
TACGCCGGCAGAGATCGCCTTGTCCCTGATGGCGCACATCGTCGCGATCAAGAACGGTGTGGAAGTGTTGCAAGCC
AAACCCGTACGGGAGGCGGTCAGTTGACGAGTGCGATCACCGCAATCGTCCTGGCGGCGGGGCAGGGCAGCCGGTT
CCGTGCGCAAAGCGGGCAGGACAAACTGCTGGTTCATTGTGTGGGGCGTGATGGAGTGACACGGCCGGTCTTCGAG
CATGTGCTGCTCAATCTGCCGGCCAGCATCACTCGGCGGTGGGTCGTTACATCGCCAGCGTGCAGCGAGGTCATCC
GTCTGGCCACGGATCATGGCTGCGAAGTGTTGCTGCTGGAGTCGGCAGGCATGGGGGACAGCATCGCGGCAGCGGT
GGCGGCCAGTGCCCCGGCAGCGGGTTGGCTGGTGGTGTTGGGGGATATGCCGTTTATTCAGGCGTCGAGCATTGAG
CGGGTGATAGAGCGGGTGGATGGGATCAGTGTGCCGGTGCACGCCGGCCGGCAGGGCCATCCCGTTGCATTTGGCC
AGGCATTGGGGCTCGCCTTGATGGAACTGACGGGGGATCGTGGTGCCAAGGCCCTGTTTGCCCAGGCTGATGTTCG
GCAGGTGCCGGTGGATGATCCCGGGGTGTTGTGGGATGTGGATGTGCCGCAAGCACTGGAATTCAGGTAGGGGTTG
GCTTGGCAGCGATGGCGGCCTCAAGCTCACCGCGCCTCTCTCTACCGCCATCGCGGGCAAGCCCGCTCCCACATTT
GATTTCTGTCGTTGAATGGAACGCATAAAAAAGCCCCGCCTGATTGCTCAGGCGGGGCTTTTTAGTTGCCGTTGGA
ATCAGGCGAGGGGTTTAGGCTCGTGCTCTTCTTCCAGGGCTTTGGGGTTGTGCTCTACCACTTCTTCAACGGAACG
CAGGTTCTCGTCGATGACCGGGGCAGTGTGCTCAACTTCAGCTTCGATGGCCGGTGCTTGCGCAACGGCCTCGACT
ACGGCCGGGGCCGCTGCCGCTGCTTCTTCGGCTTCTTTCTGCAGGCGCTCGGCTTCACGCTTGCGACGACGCACTT
CACGCGGGTCGTTAGGCGCACGGCCGTTTTCGGTGAGGGCGCTGGCAGGGGCTTCGACCACTGGGGCGGCCACTTC
GGCTACCACTGGGGTTTCCACCACCACCGGAGTCGGCTCGGCAACCACGACTGGTTCTGCAACCACCGCAGGTTCG
GCGACAGGTGCTGGAGCTTCCTCTACCGGAGCCGGGGCTGGTGCTTCGGCGACGACGGCTGGCTCGGCAGTCCACT
GGAAGGCGGTCTGTTCTTCGCGAACTTCATGCACTTCTGGGGTTGGCTCGGCAGCTGGCGCTTCGACAACCGCTTC
AACCGCAGGCTGTGGCTCAACCACGACCGCAGGTGCGGCCACGACTTCGGCTTCAGCTTCAGGCTGGGCTTCGTGA
ACCGGCGCAACTTCCACTTCTGGAGCGGCAGGTGCTTCGACCACGGTGGTGGCTTCGACAACCGGTGCTTCGACGA
CGGCAGTTTCCTGCACGGCAGCGGTGGCACGTTCGGCCTGCTGGTTGGCTTCGGCTTCGGCAGGGGCGCTGATGAT
CGAGCTGGCAACCGCTGCGGTAACGGCCAGGCCGGCAGCCAGTTCGGTGCTGGTCGGCTCGTTGTTGGCTGCTTCT
TCAGTGGCTTCGCCGGTCTCTTCCGAGCCTTCGATCACATTGCCGTTGGCATCACGTTGACGCTCACGACGGTTGC
TGCGACGACGCTGGCCACGGGAGCGGCGGCGTGGACGATCGCCTTCGGCGTTGTCCTGGCCATCTTCCTGTGGTTG

TTCGTCGTTGCTCAGCAGTTCTTCTTCACTGGCGGCGGCAGCTTGCTCGGCACGTGGTTGACGCTCTTCACGCGGT
GGGCGTGGGGCGCGTTCTTCACGGGGCTGGCGGGCCGGACGTTCTTCGGTCGCGGTATCCACTGCAACAGCACCAG
CGGCGGCCACGGCTGGAGCGGCGTCCAGTGGCTCGCGCAGTTCACGTACACGTTCTTCACGCTCGCCACGCGGCTT
GCGATCTTCACGTGGGGCGCGAGGCGCGCGCGGTGCGCGTTCTTCACGTGGCGCACGCTCTTCACGCGGAGCACGT
TCTTCACGGGCTACGGCCGGAGTTTCTTCACGGGCTTCGCGCGGTTGACGCTCTTCACGTGGTGCGCGTTCTTCAC
GCGGTGCACGCTCTTCGCGAGGCTTGCGTTCTTCGTCACGGCGACCGTTGCGGTTACGGCTCTGCTGGCGACCGTT
GCGACGCTCTTCGTTGCGTGCAGGCCGTTCAGCAGCAGGCTTCTCGACCACGGCTGGAGCAGCAGGCTCTTCCTTG
GTGGCGAACAGGCTGACCAGCGACTTCACCAGGCCCTTGAACAGGCTTGGTTCCGGCAGGGAAGCAGGCGCGGCAA
CCGGGGCAGCGGCTTCAGCCGGCACGGGTGCATTGGCACGGGCCGGCGCGGTCTTGACGGCAGCTTCCTGGCGAAC
CAGGGTGCGGGTCGCGGCGGCTGGCTGGACTTCTTCGACTTCGGCAGCCGCCGCGGCGATTTCGTAGCTGGACTGG
CCGCTGTGGGCTTCCGGGCTGTCGTCACGCAGGCGCTGGACTTCGAAGTGCGGCGTCTCGAGGTGATCGTTCGGCA
GGATCACGATGCGGGCACGGGTGCGCAGTTCGATCTTGGTGATCGAGTTGCGTTTTTCGTTGAGCAGGAACGCGGC
GACCGGGATCGGCACTTGCGCGCGGACTTCGGCGGTGCGGTCTTTCAGGGCTTCTTCTTCGATCAGGCGCAGGATC
GCCAGGGACAGCGATTCAACGTCACGGATGATGCCGGTGCCGTTGCAACGCGGGCAGACGATGCCGCTGCTCTCGC
CCAGGGATGGACGCAGGCGCTGACGGGACATTTCCAGCAGGCCGAAGCGCGAGATGCGGCCAACTTGCACGCGGGC
ACGGTCGGCTTCCAGGCATTCGCGGACTTTTTCTTCCACGGCGCGCTGGTTCTTGGCTGGGGTCATGTCGATGAAG
TCGATCACGATCAGGCCGCCGATATCACGCAGGCGCAGTTGGCGAGCGATCTCTTCGGCTGCTTCCAGGTTGGTCT
GCAGGGCGGTTTCTTCGATGTCGCTGCCTTTGGTGGCGCGCGCCGAGTTGATGTCGATGGACACCAGGGCTTCGGT
CGGATCGATGACGATGGAGCCACCGGAAGGCAGTTCGACTACGCGCTGGAAGGCGGTTTCGATCTGGCTTTCGATC
TGGAAACGGTTGAACAGCGGTACGCTGTCTTCGTACAGCTTGATCTTGCTGGCGTACTGCGGCATCACCTGGCGGA
TGAAGGTCAGGGCTTCGTCCTGGGCTTCAACGCTGTCAATCAGCACTTCGCCGATGTCCTGGCGCAGGTAGTCGCG
GATGGCGCGGATGATCACGTTGCTTTCCTGATAGATCAGGAATGGCGCGGAGCGGTCCAGGGATGCTTCTTTAATA
GCAGTCCACAGTTGCAGCAGGTAATCGAGGTCCCACTGCATTTCTTCGCTGCTGCGGCCAAGGCCGGCAGTGCGCA
CGATCAGGCCCATGTCGGCCGGGGCGATCAGGCCGTTCAGCGCTTCACGCAGTTCGTTGCGCTCTTCGCCTTCGAT
GCGACGGGAGATGCCGCCGGCACGCGGGTTGTTCGGCATCAGGACCAGGTAACGGCCAGCCAGGCTGATGAAAGTG
GTCAGGGCTGCGCCCTTGTTGCCACGTTCTTCTTTTTCGACCTGGACGATGACTTCCTGGCCTTCGCTCAGGACGT
CCTTGATGTTCACGCGGCCTTCAGGGGCTTTCTTGAAGTATTCGCGGGAGATTTCCTTGAGGGGCAGGAAGCCATG
GCGCTCAGAGCCAAAATCGACAAAGGCAGCCTCAAGGCTTGGCTCGATGCGAGTAATCCGGCCTTTATAGATGTTG
GCCTTCTTCTGCTCGCGTGCACCGGACTCGATGTCCAGGTCGTAGAGGCGTTGGCCATCTACCAGTGCAACACGCA
ACTCTTCGGGTTGAGTTGCGTTAATCAGCATTCTTTTCATGTTGTACCGTCGGTTTCCGGGCTGCCGGAAACGGCG
TTCGGCACACACGACTTCTCACGGTCGGTGTCAGGTGCGTCAAGAGTGGTTGGCCACTCCAGTGTCCAGCAATACC
GGCCAATTGGGCTGGTATCGCGACTTACGCGTCCTGCTTGCTGTGGTGACTTAATAAGCACTCAGTCAGGAGGAGG
AATCAACCGGCGACTGTGGACGAGATGAAGCGTCTAAATATAAGCCTAGTGCTACACAGTCCGACGGTTGTGCATC
TCCACCCTACACGTATCCCTGATAATTCGGGTGCTGCCGCGCGCAGAATCCGCAGCGGGTTGGCATTTACCGTGTT
CTCCAATGGGGAGTTCACGCTCATGGCTAATAACAGGCGTTGTTTCCGAAGCATTCGCCGTGGTCTGGTACAAGAC
TGACTGCACTTTGTGAACTGGCCGTAAATATCGGCGCAATAGGCGAGTAATCACTCTGCTTATCGCACTCGCTTCA
GGCCTCATGTCACCTGCGCTTGTTACAATCTTGAGCCTTCCAGTGTGGCAAGAGCCCCGTAGGACGGCCTCGCGTC
CTGATGAATTGCGATGGTCAGGGGCGGCAGTTTGCCGCGAGTCCTCTGTCCAGGCCGCTTTTGGCGGCGTTCGCGA
CTATAGCAGCAATGATTAAGTGCTTCAATTCCATAAAAAATTGTTATCATTCCCGCCATGACGACTACCACCCCCC
CGACCCCCAGCGTCCAGCTGCTTGAGGTCTCGCCGGAATATGCCGGCCAACGCATCGACAATTTTCTTCTGGCCAG
GCTCAAAGGCGTGCCCAAGACCTTGATTTATCGCATTTTGCGTAAAGGCGAAGTGCGGGTGAACAAGGGTCGGATC
AAGCCCGAGTACAAGCTGCAGGCGGGCGATATCGTCCGGGTGCCGCCTGTGCGGGTGCCTGAGCGTGACGAGCCCG
TGCCGCTGGCCCAGGGCCTGTTGCAACGCCTCGAAGCCTCGATTGTCTTCGAAGACAACAAGCTGATCGTGATCAA
CAAGCCGTGTGGCATTGCGGTTCATGGCGGCAGCGGCCTGAATTTCGGGGTGATCGAAGCCTTTCGTCAGTTGCGT
CCGGATGCCAAGGAGTTGGAGTTGGTCCATCGCCTGGATCGCGATACCTCCGGCCTGCTGATGATCGCCAAGAAGC
GCAGCATGTTGCGCCACCTGCATACCGCCTTGCGCGGCGATGGCGTGGACAAGCGGTATATGGCCCTGGTGCGTGG
CAATTGGGCATCGTCGATCAAGAGCGTGCGTGCGCCGTTGCAGAAGAGCAACCTGCGCTCCGGCGAGCGTATGGTC
GAGGTGGACGAGGAGGGCAAGGAGGCGCTGACCCTGTTCAAGGTGCTACGGCGTTTCGGCGACTTTGCGACCATGG
TCGAGGCCAAGCCGGTGACCGGGCGTACCCACCAGATTCGCGTACATACCTTGCACGCCGGCCACTGTATTGCTGG
TGATACCAAGTATGGCGACGAGGGGTTCTCCAAGGAAATCCGTGATCTGGGCGGTAAGCGCCTGTTCCTGCACGCC
TACATGCTGACGGTGCCGTTGCCAGATGGTGGTGAATTGAAGTTACAGGCGCCCGTCGATGAGATGTGGGCCAAGA
CCGTGGAGCGCCTGAGTGTCGCGCCCTGACTATAAACTGCTGATTTTTGACTGGGATGGCACCCTGTGCGATTCCA
TTGGCCGGATTGTCGAGTCCATGCATATGGCCTCGACCCGCTCCGGCTTTGAGCTGTGCAGTGACCTGGCGGTCAA
GGGCATCATTGGCCTGGGTTTGCCCGAAGCCATTCGTACCTTGTACCCGCAGATATCCGACAGTGAACTGGTAGCG
TTCCGCGAGCACTACGCCGATCACTACATTGCCCTTGAGTCCGAACCTTCGCCTTTGTTCGATGGGGTGGTGCAGT
TCCTGGCGGCATTGCGTGAGCAGGGCTATCACCTGGCAGTGGCGACCGGCAAGGCCCGGCGCGGGCTGGATCGGGT
GCTCAAGGCCAATGGCTGGGAAGATTACTTCGACGTCACACGGGCCGCCGATGAAACCGCGAGCAAGCCTCATCCG

TTGATGCTGGAGCAGATCATGGCCCATTGCGGCGTTTCTGCCCGTGAGTCGTTGATGGTCGGCGATGCCTCGTTTG
ACCTGCTGATGGCGCGCAATGCCGGCATGGACAGTGTTGCCGTCAGTTATGGCGCCCAGTCCCCGCAAGCGCTGCA
GGCTTACGAGCCGCGCCTGACGATTGACAGGTTTTCCCAATTGCACGCCTGGCTCAACCAGGCCCGTTAAGTTTTT
GCTGGGGTAGATGGCATGACCGATGAGTGGAAGGCGCCGACCAAGGCCGCTGCCGACAATGGCGATGATAAAAGCT
GGAAACTGCTGGAAAAAACCCTGCTGGCGGGTGTCCAGGAGCAGCGTCGTGCGCGGCGCTGGGGGATCTTCTTCAA
GCTCCTGACCTTTACCTACCTGATCGGCATGTTGGTGCTGTTCAGTCCCTTGGTGGATATGGAAAAAAGCGCCGGC
CGCAGCGGCAGCTACACCGCGCTGATCGAGGTGCGCGGGGTGATTGCCGACAAGGAGTCTGCCAGCGCCGACAATA
TCGTCAGCAGCCTGCGCACCGCCTTCGAGGATCCCAAGGTCAAGGGTGTGGTGCTGCGCATCAATAGCCCGGGTGG
CAGTCCGGTGCAATCGGGCTATGTCTATGACGAAATCCGCCGCCTGCGCGCGTTGCATCCTGAGATCAAGCTGTAC
GCGGTGATCTCTGATCTGGGGGCTTCGGGCGCCTATTACATTGCCAGTGCCGCCGACCAGATTTACGCCGACAAGG
CCAGCCTGGTGGGTTCCATTGGCGTGACGGCGGCCGGCTACGGGTTTGTCGGGGCCATGGAGAAGCTGGGGGTTGA
GCGGCGTACCTACACCTCGGGCGAGCATAAGTCGTTTCTCGACCCGTTCCAGCCGCAAAAGGCTGACGAAACCCAG
TTCTGGCAGAGCGTGCTCGACACCACCCATCGCCAGTTCATTGCGAGCGTCAAGCAGGGGCGCGGTGAGCGGCTCA
AGGATAAAGAACATCCGGAGTTGTTCTCCGGGCTGATCTGGTCTGGCGAACAGGCCTTGCCATTGGGCTTGATCGA
TGGCCTGGGCAGTGCCAGTTCGGTGGCGCGGGATGTGATTGGCGAGAAGGAGTTGGTGGACTTCACCGTCCAGGAA
TCGCCGTTCGATCGCTTCTCCAAGCGTCTGGGTGCCAGTGTGGCGGAGAAGCTGGCGCTGTATATGGGCTTCCAGG
GCCCGACCCTGCGCTGATCTCAGGTTGGCATAGCTCAAAATGTGGTGCGGGCTTGCTCGCGAATGTGCAGTGTCAG
TTACTGGCTGTGTATCTGACACGCCGCATTCGCGAGCAAGCCCGCTCCCACATAGGTTTTGTGGTGGTCTACGGTA
CCTGCACACCTTCGGCCAGCAGCATATCTACCAGGCGAATCAGCGGTAGGCCGATGAGGCTGGTGGCGTCCGGGCC
TTCGGTGCTTTGAAACAGGCTCACCCCCAGCCCTTCAGCCTTGAAGCTGCCAGCGCAATCGTAGGGCTGTTCAATC
CGCAGGTAGCGCTCGATCTGCGCCGGCTCCAGTTGGCGCATTGTGACGGTGAAGGGGACGCAGTCCACCTGGCAGC
GCCCGGTCTCGCTATTGAGCAATGCCAGGCCGGTGAGGAAGCTGACCCGTCGGCCGCTGGCCGCCAGCAATTGTTC
ACGGGCCTTTTCAAAGGTGTGGGGCTTGCCGATGATCTGGCCATCCAGCGCCGCGACCTGGTCCGAGCCGATGATC
AGGTGCCCTGGGTGGCTGTTGGCGAGGGCGCGGGCTTTCTCTTCGGCCAGGCGCTTGACCAGGTCGACCGCGGACT
CATTTGCACGATGGTTTTCGTCGATATCCGGAGAGCTGCAGATGAACGGCAGCTGCAGGCGATTCAACAGTTCCCG
GCGATAAACCGAGCTGGAAGCGAGTAATAAAGGCAACATGTGGCTCTCCTCAAGGCAGTCGCGGATTCTAGCGAGT
GGGCAGTCTGGCGCACAGGGCTGAATTTCCTTTGACATGGGCGGGTGCATCCCTATAATGCTGCGCCTATGTTGAA
TGACCCGATTCCACCTCACGTTGACCCGCGCAAATTGGCTGATCGTGGCACCACCCTTCAAGGTGAAGTGTTGCTG
GCCGATTTGGAGAGACTCTGCGACCCGCTTTCCGACAATGTCGGTACGGTGCAGGCTAAATTCGTTTTTGAACGAG
ATGAACGTAAATCTGTGGTAATTCACAGCTTTATCGACACCGAGGTCAAAATGGTTTGCCAGCGTTGTCTTGAGCT
GGTCACCCTGCCGATCCACAGCGAATGCAGTTATGCTGTGGTGAAAGAGGGTGCGAATACCCAGTCGTTGCCGAAA
GGTTATGACGTGCTGGAACTGGGCGAAGATCCTTTGGATCTGCATGCACTGATCGAGGAGGAGCTTCTGCTCGCCT
TGCCCATTGTGCCTGCTCATCATCCGGAAGAATGCCAGCAGCCGGCGGGGCTCGATGACGAGTCCGAACCGAGCGA
GGACGAGGTAACGCGGTCCAACCCGTTCAGTGTATTGGCGCAGTTAAAGCGTGACCCAAACGTTTAGGAGTTAATC
AATTATGGCTGTTCAGCAGAACAAAAAATCCCGCTCTGCCCGTGACATGCGTCGTTCCCACGATGCCCTGACGGCA
AGCACTCTGTCTGTAGAAAAAACCACCGGTGAAATTCACCTGCGTCACCACGTATCGCCAGAAGGCGTATACCGTG
GCCGTAAAGTGATCGACAAGGGCGCTGACGAGTAATCACTTGTCTGCTCAAGTCATCGCGATTGACGCAATGGGCG
GGGACTTCGGTCCCCGCAGCATTGTTCAGGCGTGCATTGCCAGCCTGTCCGCTACGCCCTCGCTGCACCTGACCCT
TGTCGGTCAACCTTCCCTACTTGAAGAACTGATTGCCAGCCATCCGGCTGTGGATCGCGCGCGCCTGACGATAACG
CCAGCCAGCGAAACCATCGGCATGGATGAAAAGCCAGCCCAGGCCCTGCGTGGCAAGCCCGATTCCTCCATGCGCG
TAGCCCTTGAGTTGCTGCGTGATGGCAAGGTCCAGGCCTGTGTCAGTGCCGGCAACACCGGGGCCTTGATGGCCTT
GTCCCGTCATGTGCTGAAAACCCTGCCGGGTATTGATCGGCCGGCGATGGTCGCGGCGATCCCGACACAGCGTGGC
TACTGTCAGTTGCTGGACCTGGGAGCGAATGTCGATTGCAGCGCCGAGCACCTGTTGCACTTTGCCCTGATGGGGT
CGGTTGCGGCCGAAGCGCTGGGTGTGGTGCGTCCGCGCGTGGCGTTGCTGAACATCGGTACCGAGGACATCAAGGG
CAACCAGCAGGTCAAGTTGGCCGCCAGCCTGTTACAACAGGCGCGGGGCTTGAACTACATCGGGTTTGTCGAGGGC
GATGGGTTGTATCGCGGCGAGGCCGATGTGGTGGTTTGTGACGGTTTTGTCGGAAATATTCTGCTCAAGTCCAGTG
AAGGCCTGGCAACGATGATTGCCGCGCGGATCGAGGCGTTGTTCAAGCGCAACCTGGCTTCCCGGCTGGTGGGTGC
CCTGGCGCTACCCTTGATGCGGCGCCTGCAGGCGGACCTGGCCCCGGCGCGGCATAATGGCGCAAGCTTCCTCGGG
TTGCAGGGCATTGTGGTAAAAAGCCACGGTTCGGCAGGGGTCCAGGGCTTTCAGAGTGCCATTGCGCGCGCGCTGA
TCGAGATCCAGGAGAACCTGCCGCAGCGCTTGCATGGCCGTCTTGAGGATCTGTTGCCTTAGGCGAATCGGGCCGG
GAATGCTTAAATGTGACCGGCCAGTTCAATTGACCATCCAATCTGTCAGTTTCTTGCGCTCCCAGCTGTGGGGCGT
CAATTCACCGACGACCAGATCATTAGGGGCTTGTTACATGTCTACATCCCTCGCATTCGTCTTTCCAGGGCAGGGT
TCGCAGTCCCTCGGCATGTTGGCCGAGCTGGGCGCGCAATACCCGCTCGTCCTGGAAACATTCAAGGAAGCTTCCG
CTGCCTTGGGCTACGACCTTTGGGCATTGACCCAGGACGGGCCGGAAGAACTGCTCAACCAAACCGATAAAACCCA
GCCGGCGATCCTGACGGCTTCTGTCGCGCTGTGGCGTGTCTGGCTAGCTGAAGGCGGCGCACGCCCGGCTTTTGTC
GCCGGCCACAGCCTGGGCGAGTACAGCGCCTTGGTCGCCGCAGGCAGCCTGACCCTCGCTGAGGCGGTCAAGCTGG
TAGAGCGCCGTGGTCAACTGATGCAAGAGGCTGTTCCGGCCGGGCAGGGCGGCATGGCCGCTATTTTGGGCCTGGA

CGACGCCGTGGTGATCGAAGCCTGTGCCGAAGCGGCCCAGGGCGACGTGGTCAGCGCGGTGAACTTCAACTCCCCT
GGCCAGGTGGTGATCGCCGGTGCCAAGGCTGCGGTTGAGCGTGCCATCGAAGGTTGCAAGGCCCGCGGTGCCAAAC
GTGCATTGCCACTGCCGGTCAGCGTGCCATCCCATTGCGAGTTGATGCGCCCGGCGGCCGAGCGTTTCGCCGAGTC
CATCGCAGCCATCAACTGGCAGGCGCCGCAGATCCCGTTGGTACAAAACGTCAGCGCCGACGTGGCTCCCGACCTC
GAAACCCTCAAGCGTGACCTGCTGGAGCAACTGTACAAGCCGGTGCGCTGGGTTGAGTCGGTTCAGACCCTGGCTG
CCAAGGGCGCTACTCAACTGGTTGAGTGTGGTCCGGGCAAAGTCCTGGCCGGCCTCAACAAGCGCTGCGCCGAAGG
CGTGTCGACTTCCAATCTCAATACCCCGGATGGCTTCGCAGCCGCCCGCGCCGCACTGGCCTGAACTAAGGAGAAG
CCTGCATGAGTCTGCAAGGTAAAGTTGCACTGGTTACCGGCGCAAGCCGTGGCATTGGCCAGGCGATTGCCCTGGA
ACTGGGCCGTCAGGGCGCGGTAGTGATCGGTACTGCGACCTCCGCCTCCGGCGCCGAGCGTATCGCGGCAACCCTC
AAGGAAAACGGCGTGCAGGGCACTGGCCTGGAACTCAACGTGACTAGCGATGAGTCCGTGGCTGCCGTGCTGGCGC
AGATCACTGCACAGTTCGGGACGCCAGCCATTCTGGTGAACAACGCCGGTATTACCCGCGACAACCTGATGATGCG
CATGAAAGACGACGAGTGGTACGACGTGGTCGATACCAACTTGAACAGTCTGTTTCGCCTGTCCAAGGGTGTTTTG
CGCGGCATGACCAAGGCGCGTTGGGGTCGAATTATCAATATTGGCTCCGTAGTGGGTGCCATGGGCAACGCAGGCC
AAGTAAACTACGCCTCTGCCAAGGCCGGCCTGGAAGGTTTCAGCCGTGCCCTGGCGCGTGAAGTCGGCTCGCGGTC
GATTACGGTCAACTCGGTGGCTCCAGGGTTTATCGATACCGATATGACCCGCGAATTGCCTGAAGCGCAGCGTGAA
GCGTTGCTGACGCAAATTCCGCTGGGCCGTCTGGGCCAGGCCCAAGAGATCGCCAATGTGGTCTCTTTCCTGGCGT
CTGACGGTGCGGCATACGTGACTGGGGCTACAATCCCGGTGAACGGCGGGATGTACATGAGTTAAATTGTGACGGA
TCGCTTCAAAAAAATGTCATACGAGCTGTCTAAAATCCGTTATAAAGCTGCAATCTATTTATAGACAGGTGGTCGG
TAGGGTACGAGGGTGAAGCTTTCAGTTGAAAAGCTGAAAAGCCTTTCTATACACTTACCCACTGGCCAGCTGCCTG
AATTTGTCCATTAGGAGTTAAACAAGGTATGAGCACCATCGAAGAGCGCGTCAAGAAAATCGTCGCCGAGCAACTG
GGCGTTAAAGAAGAAGAAGTGACCAACACTGCTTCCTTCGTAGAAGACCTGGGTGCCGATTCCCTTGACACCGTTG
AGCTGGTGATGGCTCTGGAAGAGGAATTCGAGACCGAAATCCCGGACGAAGAAGCTGAAAAAATCACTACTGTTCA
AGCTGCTATCGACTACGTTACTAGCCACCAGGCGTAATAGTTTGTAATCGTCGCTTGCTGTTATGGAAAAACCGCA
CTGCTATAACGGCGTGCGGTTTTTTCATTAGCTCTGATGAAAAGTCAGCTCAAGTGTCGTCATCAAGAAAAAGGAG
AGTGCTGTGTCGCGTAGACGCGTCGTAGTCACCGGTATGGGTATGTTGTCGCCACTGGGTACGGATGTGCCGAGCA
GTTGGCAGGGCATTCTGGCTGGCCACAGTGGTATTGGTCTGATTGAACATACGGACCTTTCTGCCTATTCCACCCG
TTTTGGCGGCTCGGTAAAGGGCTTCAATGTCGAGGAATATCTCTCGATCAAAGAATCCCGCAAACTCGACCTGTTC
ATTCAATACGGCCTGGCAGCCGGTTTTCAGGCGGTGCGTAACGCCGGCCTGGAAGTGACCGATGCCAACCGTGAGC
GCATTGGCGTGGCCATGGGCTCGGGTATTGGCGGTTTGACCAATATCGAAGAAACCAGCCGTACCTTGCACGAATC
CGGCCCTCGTCGAATTTCACCGTTCTTCGTCCCGGGCTCGATCATCAATATGATTTCCGGCTTCCTGTCCATCCAT
CTGGGTGCCCAGGGGCCTAACTACGCCATTTCCACGGCGTGCACCACGGGCACTCACTGCATCGGCATGGCGGCGC
GCAATATCGCCTATGACGAGGCTGACGTGATGATCGCCGGTGGCGCCGAAATGGCCGCCTGCGGCCTGGGCATGGG
TGGCTTCGGCGCGTCCCGTGCGCTGTCGACGCGCAATGAGGAGCCGACCCGCGCCAGTCGTCCGTGGGACAAGGGC
CGTGATGGCTTTGTACTGTCCGACGGTGCCGGTGCCCTGGTCCTCGAAGAGCTGGAGCACGCCAAGGCCCGTGGCG
CGACGATCTATGCCGAACTGATCGGTTTTGGCATGAGTGGCGATGCGTACCACATGACCTCGCCCCCTGCCGATGG
TGCGGGCGCCGCGCGTTGCATCAGCAATGCCCTGCGCGACGCGAAGATCAACGTCGATCAAGTGCAGTACATCAAC
GCCCACGGCACCTCGACGCCGACGGGCGACCTGGCGGAAGCCCAGGCCATCAAGTCGGTATTCGGCGAGCACGCCT
ACAAGCTGGCGGTCAGCTCCACCAAGTCCATGACCGGTCACCTGTTGGGTGCGGCAGGTGCGGTCGAAGCGATTTT
CAGCGTGCTGGCAATCAAGGATCAGGTCGCGCCGCCGACCATCAACCTTGATGAACCAGATGAGGGCTGCGACCTG
AACTTCGTGCCTCACGAGCCGCAGAAAATGCCGATCGACGTGGTGTTGTCCAACTCGTTCGGCTTTGGCGGTACCA
ACGGTTCCCTGGTGTTCCGCCGGTTCGCCGAGTGATGCACAGCTGGGTCGACGGTCAGCCAGCGGACGCCGTGCCG
CTGAAAGATCGCGGCCTGGCCTACGGCGATGGTCTGTTTGAAACCATCGCAGTCAAGGCCGGGCAGCCCGTGCTGC
TTGATCGTCACCTGCAACGCCTCGACGAGGGTTGCAGGCGCCTTGCGCTTGCCGCAGACCAGGAACTGGTCCGCAG
CGAAGTGCTGGCCTTTGCCGCAGCCCTGGGCGATGGCGTACTCAAGTTGATCCTCACCCGTGGCGATAGCCAGCGG
GGTTATGGCATCAACGCTGGCGCCTCGGTGCGCCGTATTCTGCAAGGCAGCCCGCCTGCCACCTATCCCCAGGCTC
ATGGTTCTTCAGGCGTTCGCCTGTTCCCATGCGCTACCCGTTTGTCCGAGCAACCTTTGCTCGCAGGTCTCAAGCA
CCTAAACCGCCTCGAACAGGTCATGGCTCGCGCCGAGTGGCAAGATCCCGATCACGTGGAAGGATTGATGCTGGAT
ATGTCCGGGCGCGTCATCGAAGGCGTGTTCAGCAACCTGTTCCTGGTGCATGACGGCCAGTTGCTGACGGCCGACC
TGAGCCGTTGCGGCGTGGCCGGCGTCATGCGTGCCGAGCTGTTGGCCAGGGCCGAGGGGTTGGGCATTGTCACCAC
CGTTGCCGATATCAGCCTTGCGCAGTTGCAGCAGGCTGATGAAGTGTTCGTCTGCAACAGCGTATATGGCATTTGG
CCGGTGCTTGGATGTGGGCCAATGAGCTGGTCGGCTGGGCCGCTCACCCGTAAACTGCAGGGCATTGTTCGCGCGC
TATTGGGTGTTTGATTTGAGACGTAAAATTGTATTGCTGCTGGAGATCGGTCTGGTCCTGGCGGGTTTGCTGCTGG
GCTTTTCGGCCTGGAAGCTGGACTCGGCCTTGAAGCAGCCGCTAAATCTGTCCCAGGAGCAATTGCTGGATGTAGC
GGCTGGTGCGACCCCTACCGGTACTTTCAATCGTCTGGAAAACGACGGTGTACTTGAGGATGCCTTCTGGCTGCGC
CTTTACTGGCGCTTCAATCTTGAGGGTCAGCCACTGCACAGTGGCGAGTACCGCATGACGCCAGGCCTGACGGCCC
AGGGCCTGATCGGCCTGTGGCAACGCGGTGAAGTGGTGCAGTACAGCCTGACGCTGGTGGAGGGCTGGAACTTCCG
CCAGGTGCGCAGTGCGCTGGCCAAGCACGAAAAGATCGAGCAGACCCTGACGGGCTTGAGTGACCGCGAGGTGATG

GCAAAAATCGGCCATCCCGACGTCTTCCCGGAAGGGCGGTTCTTCCCTGATACCTACCGTTTCGTGCGCGGCATGA
CCGATGTCGAGTTCCTGAAGAAAGCCTACAACCGCCTGGACGATGTACTTGAGCAGGAATGGAGCCGACGTGCCCC
GGATGCGCCCTATACCGACCCCTACCAGGCGCTGATCATGGCATCGCTGGTCGAGAAGGAAACCGGCGTGCCTGAA
GAGCGTGGGCAGATTGCCGGGGTGTTTGTGCGGCGCCTGAAGATCGGCATGCTGCTGCAGACCGACCCCACCGTGA
TCTACGGCATGGGCGAGCGCTACAACGGCAAGTTGACGCGCGCGCACCTGAAAGAAGCCACGCCCTATAACACCTA
CATGATCAGCGGCTTGCCACCGACGCCGATTGCCATGGTCGGCCGCGAAGCGATCCATGCCGCGTTGAATCCGGTA
CCGGGCAGCAGCCTGTATTTCGTGGCGCGGGGCGATGGCAGCCATATTTTCTCCGATAGCCTGGATGCCCATAATG
CGGCGGTGCGCGAGTACCAACTCAAGCGTCGCGCCGATTATCGCTCCAGCCCTGCGCCGGCCACACCTGCGACCGT
TGAGCCTGTCGCGCCTGAAGCTGCGCCCGCGCAAAGCCCGCAATGACTCTGATTAAGGACTGCCTGTGACTGGCTT
GTTTATTACCCTGGAAGGCCCGGAAGGCGCCGGCAAGAGTACCAACCGTGAATACCTGGCCGAGCGCTTGCGGGCC
GCAGGGATCGAGGTGCTGCTGACCCGCGAGCCCGGCGGCACGCCCCTGGCGGAGAAGATCCGCGAGGTGTTGCTGG
CCACTGGCGACGAGGTCATGAACCCGGACACCGAGTTGTTGCTGGTGTTTGCCGCGCGTGCCCAGCACCTGGCCGA
AGTGATCCGGCCGGCGCTGGCCCGTGGTGCGGTGGTGATCTGCGACCGCTTTACCGATTCTACCTACGCCTACCAA
GGCGCCGGCCGTGGCTTGTCCCTGGAGCGCATCGCGACCCTGGAGACCTTTGTCCAGGGCGATCTGCGCCCGCACC
TGACCCTGGTCTTCGACCTGCCGGTGGACGTCGGCCTGGCGCGGGCCAGCGCCCGTGGTCGCCTGGACCGCTTCGA
ACTGGAAGGCCGGGAATTTTTTGAAGCGGTGCGCGCGGCGTACCTCCAGCGTGCCGAAGCTGCACCGTCGCGCTAT
TGCTTGCTGGATGCCGCGCAACCGCTGGAGCAAGTGCAACAGGCGATCGATGCGCTGCTCCCGAAACTGTTGGAGC
TTTACCGTGGCTGAAGCCTATCCGTGGCAGGACAGCCTATGGCAGCAATTGGCCGGACGTGCCCAGCATGCCCATG
CCTATCTGCTCCATGGGCCGGCAGGCATCGGCAAGCGCGCCTTGGCCGAGCGCTTGATGGCTCGCCTGCTGTGTCA
GCAGCCAGCAGGGCTGGAGGCGTGCGGAAAATGCAAATCGTGCCTGCTGCTCAAGGCCGGCAGCCATCCGGACAAC
TACGTGCTGGAGCCTGAAGAGGCCGACAAGGCGATCAAGGTCGACCAGGTGCGTGACCTGGTGAGCTTCGTGGTGC
AGACCGCGCAGATGGGCGGGCGCAAGGTGGTGCTGATCGAGCCGGTGGAGTCGATGAACATCAACGCCGCGAACGC
CTTGCTCAAGAGCCTTGAGGAGCCCTCGGGCGACACCGTGCTGCTGCTGGTCAGTCATCAGTCCAGCCGTTTGCTG
CCGACGATTCGCAGTCGTTGCGTGCAACAGGCCTGCCCGCTGCCGAGCGAGTCCATGAGTTTGCAATGGCTGGGCC
AGGCCTTGCCGGAGTGCAGTGAGCAGGAACGGGTTGAATTGCTGACCCTGGCGGCCGGCTCACCCCTGGCGGCGGT
CAGCCTGCAGGCCCAGGGCGTACGCGAGCAGCGTGCGCTGGTGGTCGATGGGGTGAAGAAGCTGCTCAAGCAAGAG
CAGTCTGCCACGCAACTGGCCGAGGGCTGGAAAGACGTGCCGCTGTTGCTGCTGTTTGACTGGTTCTGTGATTGGT
CCAGCTTGATCCTGCGCTATCAACTGACCCAGGACGAGAGCGGCCTGGGCTTGATGGACATGCGCAAGGTCGTGCA
GTACCTGGCGCAGAAGAGCGCCCAGGACAAGGTTTTGAACATTCAGGACTGGATCCTCGCCCAGCGCCAGAAGGTC
CTGGGCAAGGCCAACCTGAACCGGGTGCTGTTGCTTGAGGCACTGTTGGTGCAGTGGGTCGGGTTACTCGGCCGAC
GTTGATATTCTCGGGGCCGACGGATGTCTCGTTGGCCAGACACTTCCTGTGACGCAAGTTGTAACTTCTTATGCTC
GTAGATTCCCATTGCCACCTTGATCGCCTCGACCTCGCCGCCCACGGCGGCTCCCTGGATGCCGCCCTCGACGCCG
CACGCCAGCGTGGCGTCGGGCATTTCCTGTGCATCGGTGTCAGCGCCGAGAACGCCGCCGACGTCAAGGCCCTGGC
TGAGCGCTATGCCGACGTGGATTGCTCGGTAGGCATCCATCCCCTGGACCTCAAGCCTGGCGAAGCCCCGGCCCTC
GACTGGCTGCTGGGCGAACTCAATCACCCGCGAGTGGTGGCCATTGGCGAGACCGGCCTGGATTACCACTACGAGC
CGCAAGCCGCCGATTTGCAGCAGGCCTCGTTCCGCCTGCACCTGCAAGCGGCGCAGCAGACCGGTAAACCAGTGAT
CGTCCATACCCGTGGCGCCCGTGCCGACACCCTGGCCTTGCTGCGCGAGGCGGCACTGCCCCAGGCGGGCGTGTTG
CATTGTTTTACCGAAGACTGGGAGATGGCCAAGGCGGCGCTCGACCTGGGGTTCTATATTTCCCTGTCGGGGATTG
TCACCTTCCGCAATGCTGATGCCCTGCGGGATGTGGCGCGCCAGGTGCCGGCGGATCGTTTGCTGGTGGAAACCGA
TTCGCCTTACCTCGCGCCAATCCCCCATCGCGGTAAACCGAACCTCCCGGAATATGTGCGGGATGTGGCGGATTAC
CTGGCGATGCTGCGCGGCGAGTCTGTCGAGCGTTTTGCCGAGCAGACCACCGAGAACTTCAAGCGCCTGTTCCCGT
TGGCGCACGTTCGCTGAATCCAAGGCAAAAAAAACCCGGGTTCTGGGGGGTGAATCCGGGTTAAGACCATTAGGAG
TAAATAAAGGTACGCGGTCCATTGGTACCCCTATCGGTGTTTCACTTGGGGGAGATGTCACACCGACAGATGAAGT
ATTGATCAGTATCCCGTGCAGTCCAGCCGCATCTGATCGTTTTTAAACAGATTTGGAATACGTGCGCTTCGCTTG
AGTTCTCATTGAGCGGCAATACATTGCGCGAACCTGTGTTTTATTTCTGACCCATATCGCTTGATTCGTTGGCGGG
CTGTAAGTTTAGTGTATGGGTTGTTCATATAGACGTTGCCCAACGACCGCCCAGTCGGGATAATCCCTCGCACCGG
GTAAAACGTTCCGTCATCGGGGCATTCAACCCTCCCAATACTGACCTCTGCAGACCTCTTTCTATGCACAAAGAAC
CCCGTAAGGTCCGTGAGTTTCGCCGCCGTGAGCAGGAAATTCTCGACACCGCGCTCAAATTGTTCCTCGAACAGGG
TGAAGACAGCGTCACCGTCGAGATGATTGCGGATGCCGTGGGTATCGGCAAAGGCACGATCTACAAGCACTTCAAG
TCCAAGGCCGAGATCTACTTGCGCCTGATGCTCGATTACGAGCGCGATTTGAACGAGCTGCTGCACTCGGCCGATG
TCGACAAAGACAAGGAAGCGCTGTCCCGGGCTTACTTTGAGTTCCGCATGCGTGACCCGCAGCGCTACCGCCTGTT
CGACCGCCTCGAAGAAAAGGTGGTCAAGGGCCACCAAGTGCCGGAAATGGTCGAGGAGTTGCACAAGATCCGTGCT
TCGAACTTCGAGCGCCTGACCCTGCTGATCAAGGGCCGGATCAGTGAAGGCAAGCTGGAAGATGTGCCGCCTTACT
TCCACTACTGCGCCGCCTGGGCGTTGGTGCATGGTGCCGTGGCGCTGTACCACTCGCCATTCTGGAGCAATGTGCT
GGAAGATCAGGAAGGCTTCTTCCAGTTCCTGATGGACATCGGCGTACGCATGGGCAACAAGCGCAAGCACAGCACC
GACCTGCCGAACCCCGAGACGCCTGCCACATAAGGCATTCATCCGGACAATGGCCGGTTGATTTACTGCCGTATGT
AGTAACCCAGGAATATACTCAGGCAAGGACCTTGCTAAAATCTGATTTATGAGTCAGGTTTTAGCGCGCCCGAATT

ATCCGCTGCCGGAGTGATCCATGATCGTTGATCGTCAAGGCAGGCGTTTTCGCAATTTGCGGATCAGCCTGACCTC
AGCCTGCAATTACGCCTGTACCTACTGCGTGCCCAACGGCAAGCGGTTGGTGGCTGCGCAGGACGAGCTCTCGGCC
GAGGCCATGGCCCGTGGGGTGGCCTACCTGATCGAGGCTGCGGGCATCGATCGTCTGCGCATCACCGGCGGCGAGC
CGCTGGTCAGTCCCAAGCTCGAAACCTTTATGCAGGCCGTCGGGCAGATGGGCTTGAGCGATATCAGCTTGACCAC
CAATGGCCAGTTACTAGCGCGCAAGTTGCCATTACTGGTCGATGCCGGTATTCGCCGTATCAATGTTTCCCTCGAC
ACCCTCGATGCCGCTGCCTTTCGCGGGATCGCCAGGGGGGGCGACCTGGCCACGGTCCTCGATGGCATGGACCAGG
CGCGTGCGGCGGGAATCAAGATCAAGGTCAATATGGTGCCGTTGCGCGGGCAGAACCTCGACCAGGTCATGCCCCT
GCTGGAGTATTGCCTGGAGCGCGGCTATGAACTGCGTTTCATCGAGTTGATGCGCATGGGCCACCTTGCCAGTGAC
TCCAACGCGTTCCTGCAACAGTTCGTCAGCCTGCAGCAACTGTTGAGCCTGATTGGCGAACATCACGAATACCTGC
AGGCTGACGCTCCGGTAGATGCCACGGCCGTGCGCTATGAAATTCCCGGTCAGGGCTTCTTCGGCGTGATCGCCAA
TGAAAGCGTGCCGTTTTGCCGTACCTGTTCGCGCCTGCGCCTCTCCTCGACCGGCTGGTTGCACGGCTGCCTGTCG
TCGAGCAACCGCCACTATGTCGGCGACCTGTTGGACAAGCCGCGCCACCAGGCCCTGCCGGCGTTGCAGCGTCTGC
TGGTCAAGGCCTTGGGCGACAAGCAGGAAGTGGCGTTTTCCGGCGGGGCGACCGTGATGAAGATCATTGGCGGGTA
GCCAGGCAAGCTGGCGCAAAATCTGCATCTGGTGCCCATTCGCCGGTTTTCCGTCACCGGCTCCTGGAGGATAGGA
TGCGTAGTCTGGTTTTGTTGCTGGCGTCGTTCGCGCTGGGTGGCTGCATGACAGTCAGCGATATGGCCGAAGGCAC
TCGCTATCAGATGAGCGATGCCGGTTTGCTGGATCACAGCGACACCCGTCGTTCCAACTCGGTTCGCATACAGCCC
GATTCGTTTGTATTCATCGCCCAGGGCGCGTTCACGCCGCCGGGCAGCGCCTATCCGCGTCCCAATGTGGTGGCCG
AGGAAGCCTTCAATGGCTTTATCGAATACTTCCCCATGGTCCGCCGCGCCCGCGCCCCGCAAGGGCTGGAGCAGGC
CATGGCCGAAGCCCGCGAGGTAGGCGCCCATTACCTGTTGTATACGCGGTTCGCCAAGGCCGATGATCGTATCGGC
AATGCAGATGAGTGGGCCGATCAGGAAGCCCTGGACCGACTGGGCGTCGACAGCGGGGTCATCCAGATCATGTTGA
TCGAGACCAGCACCCAATATTTGATCGATACTGCACGGATTCGCAGTCGTGGCGGTTTACTGACGTTCCATGACAA
CAAACCCGAAGATCTGCTTGGCCCACCGCTGCGCCAGTACGCCCGCAGCCTGTTGGGCATGAGCGACCAGTAATTC
AGAGGAGAACAGCATGACGGATTCGGCCAAGGCCAATGATTTGTTGGCGCAGTTGCCCAAGGGCAAGGGACCGGCG
CCGGTTCATCTGTGGAACCCGGATTTCTGTGGCGATATCGACATGCGCATCGCCCGTGATGGCACCTGGTATTACC
TGGGCACGCCTATCGGGCGCAAACCGATGGTCAAGCTGTTCTCCAACATCATCCGTCGCGATGGCGATGATTACTT
CTTGATCACGCCGGTGGAAAAGGTCGGGATCAAGGTGGACGACGCGCCCTTTGTGGCCGTGTCGCTGGAGGTCGAG
GGCGATGGCGAGAACCAGCTCCTACGGTTTACCTCCAATGTCGAGGACCTGATCGAAGCCGGCCCGGCGCATCCGT
TGCGGGTGGTGATTGATCCAGACACTCAGGAGCCTTCGCCCTACGTGCTGGTGCGCAACAACCTTGAGGCGCTGGT
CCACCGCAACGTGTTCTACCAGTTGGTGGAACTGGCAGTAACACGCCGGATCGACGGCGAGAGTTGGCTGGGCGTC
TGGAGTGGCGGGGTGTTCTTCCCGATAGGCCTGGAGCCCAGGTACTGATACCGGCAAGCCGGCTCCTGCATAGGTT
TGGAATGTTTATAGGAACTGGCTTGCCAGCGATGACGGCCTGCCTAGCGCTAGATCATCACCAGGTACACCGCTAT
CGCCGGCAAACCGTTTATTACGCGTCTTCGAGCTTGCGCGCCGCGTCCACGCCGGATGCTGTCAGTTTGATCGGCA
GGTGCAATGCGTGTTCGCCGGCCTCGTTACAGGTCACATGGCCTTCCTTGATCAACCCGCGGTCCTGAAGAATCGC
CAGGGTACTGGCCACGACTTTTTCGCCCCGGTAGTTATCCAGCACCTCCTTGCCCAAGCCATTCGGGTGCGCGTGC
AGCAAGCGGGTAAGGACTTCTTTCTCAAGGTTTTTATCGACGTTCATGCATACCTCTTCAGGGTCAGTGGTGGGGT
AGTGTTCGTCTGTCGGCCAGTGGCTCAAGGCTGTTTTGGCGCGCCGGGCAGGTTGTCATCGTTGCCGCTTGGGGTG
GCATTCTTGCCGGGGCCGATCTTGCCGTTCTCGACATCGGTGCCCAGGCCCTGGCGGTTGCCGTCATTGCCTCGGG
TGCGTGGGTCGGCGCCGTCCTGGATGATGGCTCCGCCATTGGTATCGAGCCCGGTGCCCATGGAGTTCTCGGTTTC
TTGGGTGGCGGGGTTGGGCGGGCTGCCAATAGGGTCGGTCTTGCCGGTGGAAGGGTCGGTTGCCGCAACACTGGCC
AGGGACACCGCAGACAGCAAGCCAGCGAACGCGAGGGCAGTCACTTTGGAAGTATTCATGGTGGAGCCTCCATAGG
AATAAAGTCCTTACCTTAGGTTGGTCAGCCCACTCTTATTGTTGGTGCCTGGATCACGACGAACGGTGCTAGGTCA
CCTGTAAGATTTTGTGTTTCGACACCCGGCGCCAAATCAGGCGCAGCACTGTGATCAGCCAGTTGAGCAAGGCGAT
GCCCAATACACTGGCGAGCAGGGTTGGCACGCCATGCTCGACAATCAGCATGCCCGCCAGCAGCGGAAACCCGAAC
ACCCCGATAAAGTACGACAGGCTGAACAGCAGCAAGGCTTGGGGCGTGCTGGCAGTTGGCGCTTCGTTGGCGGCCA
GGCCGTTGATCACTGAATAGGTCAGGCCGTAACCCACTCCCAAGGTGATAGCCGCCAGCAAGTAGCTGAAGCTGTC
ATGCACGCCGAAGCTGAACATCAGGATCGACACCAGCATCAATCCGGATAACACACAGGCCGACCAGTACGCATCC
CGCTTGACCACCACGCTGGCAATCAACAATCGACTGGTGATCGCCGCGCCCATGAAACCCAGGAAAAACAGCGAGT
AATCCAGCCCCTGACTTGCCGCGTAGCTGGTCTGGAAACTCGACAGCCCGCCAAACACACAGCCGCCCAGGCCAAC
CATGATGATCGCGAATGCCGCCTTCGACCCCAGTACGCGCCGGGTGCTGGCCCAGGAGATTTTCGACACGGTGCCA
GTCCCCAGCAAGTTGGGTTGCTGCTTGAGATAGCTGCCCAGGCGCCAGAACATCAGCACGCCCACCAGGCTGGCGA
GAGCTGCAATATAAAATGCGCTTTCCAGAGGCAGGCCCATGGCGCTGGCCGCCCGCCCCAACAGGGGGCCCGAACC
GATGCCGGTCATCATGCTTCCCGACAGCCAGGCGAAATACTTGGCCCTGTGGCTTGGCGCCACCAGCATCGCCACG
ATGATCGGGCCCAGGGTGTAGAACACGCCCCAGCCCAGCCCGAGGGTCAGGCCGAACAGCATCAGGCCCTGGCCAA
AACCCGGCGTCAGGGCAAAGCCCAGGCAGGCCACCACCAGCAGGATCCCCAGGCAGGCAATTGAGCGCGCGGCACC
CAGGGCATCGGCCAGGTGCCCGGATGCAATCACCGCGATAAACGTACTGAGCATCGCCATGGAAATCACGCTGCCG
GCGTCGTGTTCATTGCCGCCTCTGGAATCGATCAGCAGCGACAACAAAAACGTCGAGCCATACGACAGTGACAGCA
GGTAGCTGGCAAGGCAGAACAGGGCGAAGAGCGTTCCGCCAACTGGAGCAGTGGGGCGCATGGGCAAGCCTTGGGG

TTTGTTGGATTTATGGCGGACTTTTTACCACGGGCCACCCCGTGCTTTGTTCTGTGGTTACTGGCGTCAGCATTGC
TGCGCAGCGGAGTAATCGCGTGCATAAATCCCTGCAAGCGGTCATCAAAGCGCCTTAGTATGTGTCCTTTCTTTTT
TAGCCGCAGGCCTGTTCATGACCATCGAGATCCGTCCCGCCGTACCCAGCGATGCTGCGCAGATCCTGACCTTTAT
TACTGAACTGGCCGAGTACGAAAAAGCCCGCCACGAAGTGATCGCCAGCGTGGTGGATATCGAGCGCAGCCTGTTC
AGCGAAGGTGCCACCGCCCATGGCCTGATCTGCCTGCGCGATGGCTTGCCGATTGGTTTTGCGGTGTTCTTCTTCA
GCTACTCCACATGGCTTGGCAGCAACTGCCTGTATCTGGAAGACCTGTACATCAACCCGGAGCAGCGCGGGGGTGG
GGCGGGCAAGAAGTTGCTGCGCCACCTGGCGAAGATCGCCTACGACAATGGCTGTGGGCGCTTTGAGTGGAGCGTG
CTGGACTGGAACGAACCGGCCATTGCGTTCTACAAATCCATCGGCGCCCAACCCCAGGAAGAGTGGGTGCGCTATC
GCATGGAAGGCGATGCGTTGCGTGAGTTTGCCCAAGGCTGAGATCGGCCCTGTTATGAGGCCCGGCATTTGCCATA
ATGGCCGCCGCTTTCGCATTTCCAGATAGGCTCCGACATGCAGGAAAACCCACCGACGCCCGCCAAAGACTCCGCG
CCCACGGGCACCCAGACCCTATTGCGCGGCCTGGGCGTGGTGCAGGCGGTGGCGTCCGGGGCGCGGGATCTCAAGG
AGATTGCCCGGCGCATCGGCACCACTCGCAGCACCACGCATCGCCTGGCCAGTTGCCTGGTGGACGAGCGCTACCT
GCGGGTAGTGCCGCAAGTCGGCTACCTGCTGGGCCCCAAGCTGATCGAGTTGGGCTTCCAGGCCCGGGAAGAACTG
CCGCTGGTGACGCTGGCGATGCCTTATCTGGATGAGCTGTCGGCGCTGACTGGCGACACCATCCACCTGGCGATCC
GTGAATACGACGATGTGCTGTACCTGCACAAGAATCCTGGGCGCAACGGCCCGGAAATGCGCTCGCGGGTCGGCCA
CCGCATGCCCCTGGCACGTACCGGGATCGGCAAGGCCTTGTTGCTCGATGATTCGCAAGAGGAATGGCAGCGCCTG
TATCAGGCCAGCTTGCCGGTAGGCGGGAAAAACCTGCACTGGCCGCAGCACCCGGAACAATCCTGGGAGCAATTCG
AGCAACGCATGCGCGAATATGTCGCGGGCGGCTATGCGTTCGACCTGGAAGACAACGAGCCGTCGATCCGCTGTGT
GGCCGCGCCCGTGCGCGATGCCAGCCGGCGGATTGTCGCCGGCATCAGCATCGCCAGTACGGTGCCCTACATGCCG
CTGGAAAAAATGGCCGAGCTGATTCCTGTGGTCAAACAGGTTGCAGCACGGCTGTCAGCGGAGTTGGGGGCGAAGG
CCTGATCAAGCCTTCAGGGTGGTCATGTCGATCACGAAGCGGTACTTCACGTCACCAGCAATCATGCGTGCATAGG
CTTCGTTGATCTGGCGGATATCGAGCATTTCGATGTCGCAGGTAATGCCATGCTCGGCGCAGAAATCCAGGACTTC
CTGGGTTTCGGCAATGCCGCCGATCAGTGAGCCGGCCAGCACTTTACGGCCCAGCACCAGCTTGGCGGCGTTGACC
GGAGGGTCTACCGGCTCGATCAAACCCACCAGGATATGCACGCCGTCAAAGCGCAGGGTATCGAGGTAGGGGTTGA
GGTCGTGCTGCACCGGAATGGTGTCCAGCAGGAAATCGAAATGCCCGGCGGCGGCTTTCATCTGCTCGGCATCGGT
TGACACGATCACGTGATCGGCGCCCTGGCGCCGGCCTTCTTCTGCCTTGCTCGCGGAGCGGGTGAACAGCGTCACT
TCAGCGCCCATGGCCTTGGCGAACTTGATGCCCATATGGCCCAGGCCGCCCATGCCGAGAATCCCGACCTTGTCGC
CGGCCTTGACGCCATAGTGCTTGAGCGGCGAGTAGGTAGTGATGCCTGCGCAGAGGATCGGTGCGGCGCTGGCCAG
GTCGAGCTTGGCCGGAATCTTCACCACGAAGTGCTCGCTGACCACGATGCTATCGGAGTAGCCGCCCATGGTGTTG
CTGCCGTCCACGCGATCCGGGGTGGCATAGGTCATGGTCGGGCCTTCGAGGCAGTATTGCTCCAGGTCCGATTTGC
AGGCTTCGCAGTGGCGGCACGAGTCCACCATGCAGCCCACGCCGACCAGGTCGCCGACTTTATGCGCGCTGACGCT
GGCGCCGACGGCCGTGACCTTGCCGACAATCTCATGGCCCGGCATCAGCGGGTAGACGGCGATGCCCCATTCGTTG
CGAGCCTGGTGGATGTCGGAGTGGCAGACGCCGCAGTACAGAATCTCGATTGCCACGTCATCGGCCCGCGGGCTGC
GGCGTTCGAAAGACATGGGGGCGAGGGGAGTGGTGGCCGACTGGGCGGCGTAACCGATGGCGGTGTACATGGTGAA
AACCTCGCAAAAGCTTGGACGGGAAAGGTGGCGAATTCTGCGCGCGGACCCGGTGGGCGAACATGGCAAATGCTCC
GAGTCTCATGCCTATTCGTCCGGGAGTGCCCCTGGATTGGATTCATTGGCCGCCAGACCTGCGATGATGTTCTCAT
CCCTTTTTCGCGAAGTTTTTGCCATGTTGTTGACTCGCCATCTTGATGCCAACGCCACGCTGGTTTCCTTGATTGA
GGGGCTGACGCCCCGCGATGGTTTTTCCCCGACTCACCTGGCGGGTGTGAAGGTGTTGCGCGCCAGTTGCGACGTA
GCACGCGGCCCGCAGATCTACGAGCCGAGCCTGATGTTCGTGGCCCAGGGTAGCAAGGTCGCCTACCTGGGCCCCA
GGACGCTGGAGTACGGCGCCGGGCATTACCTGATCCAGGCAATGCCGGTGCCGTTCGAGTGCGAGACGTTTGCCAT
GGCCGCCGATGCCCCACTGTACGGGGTGACTGTGGGTATTGATCGGGTGGTGTTGGGCGAGTTGGTGATGGCCATG
GGCATGCAGGCTGGCCCACCGCCGACGGCGCAGACCTTGGAGTCGATGAGCTCGGTGGTACTGGATGACGCGATGC
GCGGTTGCGTCGAGCGCCTGTTGCAGTGCCTGCACGATCCATTGGAGGCCCGGATCATGGGGCCGGCGCGGGTGCG
GGAGTTGTTGTTCACTGCGCTGCGTGGGCCTCAGGCCGACGTACTGCGCGCCCTGGTGGAGCAGCAGGGGCAGTTC
TCGCGGATTGCCACGTCCTTGAACCACCTGCATGCCCATTACGCCGAACCCCTGAACATCGAGACCTTGGCCGGTT
ATGCGCATATGAGTGCGTCGACGTTCCATGAGCACTTCAAACGTTGCACCTTGTTGTCGCCGGTGCAGTACCTCAA
GCGTCTGCGCCTGCTCAAGGCCCAACAGCTGTTGCTGGTGGACGGCATGGGCGTGGCGCAGGCGGCGCACAACGTG
GGGTATCAGAGTACGTCGCAGTTCAGTCGGGAATATAAACGCTATTTCGAGCGTAACCCGGGTGAAGAACGCGCCG
CATAAATCCCGTAGATGCCTCTATCTAAATGTGGGGGCTTGCTCGCTCCCACATTAGATTTGTGTCGGCCATAAAA
AAAGGCTCTCCAGTGGAGAGCCTCATGTTCAAGCAGATGGCTTACATATTCGGGTATGTCGGCCCGCCCGCGCCTT
CCGGTGTCACCCAGGTGATGTTCTGCGAAGGGTCCTTGATATCACAAGTCTTGCAGTGCACGCAGTTCTGGGCGTT
GATCTGGAAGCGCTTCTCACCGTCTTCCTTGGTGATGACTTCGTACACGCCGGCCGGGCAGTAACGCTGCGCCGGT
TCATCGTAAAGCGGCAGGTTGGTGCCGATCGGGATGCTCGGGTCCTTGAGTTTCAAGTGGCACGGCTGTTCTTCTT
CATGGTTGGTGCTGGAGAGGAACACCGAGCTCAACTTGTCGAAGCTCAACTTGCCGTCGGGTTTTGGGTAGTCGAT
CTTTTGGCTGTCCTTGGCCAGCTTGAGGCAGGCGTAGTCCGGCTTGGTGTCATGCAGGGTGAACGGCATTTTGCCG
CCGAGGATGTTCTGGTCGAACCAGTTGAAGCCAGCACCGATGATCGGGCCGAACTTGTGCATCGCCGGGCCGAAGT
TGCGGGTGGCGAACAGTTCTTCGTAGAGCCAACTGGCTTTGAAGCTGTCGACGTAGGCGGTCAGTTCATCGCCGCC

TTCAGACTCGGCGAACAGGCGATCAGCCACGGCGTCGGCGGCGAGCATGCCGGACTTCATTGCGGTATGGCTGCCC
TTGATCTTGGCCACGTTCATGGTGCCCAGGTCACAACCGATCAAGGCGCCGCCTTTGAATATCATCTTCGGCAGCG
AGTTGATGCCGCCCTTGGCCAGGGCGCGCGCGCCGTAGCTGATGCGCTTGCCGCCTTCCAGGTACTGGGCCAGCAC
CGGGTGATGCTTGAGGCGCTGGAACTCATCGAACGGCGACAGGAAGGTGTTGCTGTAGGACAGGTCGACGATCAGG
CCTACGACCACCTGGTTGTTTTCCAGGTGATAGAGGAAAGAGCCACCGGTGTTCTCGTTGCTCATGATGTCCAGCG
GCCAGCCGGCGGTGTGGACCACCAGGCCTGGCTGATGCTTGGCCGGGTCGATTTCCCAGATTTCCTTGAGGCCGAT
GCCGTAGTGCTGCACGTCAGCGTCGCTGTCCAGGTTGAAGCGCTGGATCAGTTGCTTGCCCAGGTGCCCACGGCAG
CCTTCGGCGAACAGCGTGTACTTGCCGCGCAGTTCCATGCCGGGGGTGTACAGGCCGTCTTTTGGCTGGCCTTCAC
GGTCCACGCCAAGGTCGCCGGTGATGATCCCACGCACTACGCCGTTTTCGTCGAACAACACTTCCTGGGCGGCGAA
GCCTGGGTAGATTTCCACGCCCAGGTTCTCGGCCTGCTGGGCCAGCCAGCGGCACAGGTTGCCCAGGGAGATGATG
TAGTTGCCTTCGTTGTGCATGGTCTTGGGCACAAAGAAATCTGGAACCTTGGTGGAGCTGTCGCTGCTGCGCAGTA
CATAGATGTCGTCGCGCACGACTGGGGTATTGAGCGGGGCGCCCAGCTCTTTCCAGTCTGGGAACAATTCATTCAG
GGCGCGTGGCTCAAACACGGCACCGGACAGGATGTGTGCGCCGACTTCGGAGCCTTTTTCGACCACGCAGACGCTG
ATTTCCTTACCGGCTTCGGCGGCCTTCTGCTTCAGTCGGCAGGCGGCAGACAGGCCCGACGGCCCTGCGCCGACGA
TGACCACGTCGAATTCCATGTATTCGCGTTCCACAGGCTATCTCCTACTCAAGGCTCAACAGTTTTTTTTCTAATG
GGTGGAGGTTCGGTGTTTTATCTGTCGTCTTCTGCGCCAATTTCTTACAAAAGGGCAGGGGACGACCCACCTTTCT
CTCTAGGTGGCGCATTATATCTACACCACTCTCAGCGTCCAATACAAACGTTTGTTTGAATTGCCCGCGAGCTAGG
TAAATCAAAGCAACGCGGCTTATATCTGACCATTTTGCTGTATTGACCGGAAAAGGTGTTCCGGTCAATATACGGT
CGGTTTTGCGCTTCTCGTAGGCGGACTGCCGGTTTCAAGGCCACGTCCAAAGACAAGGCACTGCTTCGACAGGTTG
ACGCGCACGTAATGCAGGCGCGCAGTTTACACGTTGCGACGATGAATGACTTGTCAGTCACCGCTGACGAACGGTC
TTTATTATTGAGTACGTGCAACGTTACCTGTCATGACGGCCTGCGTTTTTAGAGGTGCCCTTGTGCGCCGTTGAGC
ATCTACCGCCAGGTTTATCCGGGCGGCTTCCTTTTCACCGGAGAGTAACGAGGAATCCATGAAGGTTCTTGTAGCT
GTCAAACGCGTTGTCGATTACAACGTGAAAGTTCGCGTCAAGGCGGACAATTCCGGCGTCGATCTGGCTAACGTCA
AAATGTNNNNNNNNNNGACCTTCAAATGCCGCGCATACCACGGCCGCTTGGGCACCTTGCGGAACAAGTCCTGCAG
TTTGTCGTCATCGGCACCGAAGGTGATGCGCAATGCCAGTTTCATGGTTTCCGGGTCCATTTCCACGGTGCGGCCC
ATCTGCAGTCCCGGGGTGGTGCTGCAACCATGGGTATCCGGCCCCAGCCAAGGATCACCCACCTCAACCCAGCGCC
CAGGCGCAAACCACGGCACACCATTGACCTCCAGGCGGTTCAGCTCACCCGGATGAAAGCGCTGATGGGCGCGAAA
CCAATCCTCAAGGCGATCATCGATCCAACCGTGAAAATGCCAGAACACCGGGTTTACATGGGAGGAAAACGGGTCG
CCCAAAAAGTCATTCTCCGGCTCATACCAACGCGCCGCAAAATCCGCCGGATCCCGGGCAAAGGGCACCGGCGCGC
CGTTGGACGGGTCGCGCGGTACCGACGCCCAGCGCATATGCAACCAATCGTGCAGCCCCAGCTCAACCTCAGAGCC
AAACTGGCCCAGGGTCAACTTGGCCAGGTAACGCGGGTCGCGGTACTGCGACTCCCACACCTGGAAGTTGCTGTGA
TAGGTCTCGGCCGCCTTGATATCACTGACCCACTGGCTGTACTGCTCGTCGCCACTGGCCGTCCAGGTCGGCGGCA
GTGCGAAGCCATCGTGATTGTCGAAGTAGCGCGCAAAGCCCTGGCGATCACGCTCAAGCTCTGGCTGGGGCTGCGG
GAAGCGCGGCCAGGACGGTAAGTCCTGCAGGGAACGCGCGGTGCCAAGCATGTGCCGGTGCATGAAGAAAAAGTCG
ATACCCGAGCCATTGCGATCCTTGCGCGGGCCACGGGCGTCGCGCTCGTTATCCCGTGGCCCGGGCTGCCAACCGA
TACCGCGCAGGGCATTGCGCTTGTCTTCGGGCAGCTTGTGCCACTGGTCCCGCGAGGCGTGCCAGAGCTGGTGGAA
CAGGCGGTGCTCGGGGGCTATCAACCAGGCCAGTAGCGCAGGGTTCAGGCCGATTCGTTCGCGGGCTTCGGGGAAC
AACTGCTTGGTGGCGACAAACCGGTTATCCAGCTCCAACGCTGCCAGGGGCCGGTCGAGGCTGAGGATCTGCCCGC
TGAGGGTGCCGCTGCCAGCATTGCCGAAACTGTCCCAGACTTCGTCCAGGCTCATCTTGAATTCATAGGCCGGCAT
GCCATCTGGCGAGTCGCGGTCGATCAGGCGCCAGTACAGCAGCGCTGCATTGCCCGGAACCAGATCACCCAGGACC
TGATAGCGCGGCTCATCCGGCCCCCGCAGGTGCCCAGCGGTGTCCAGGTAGCCGCGCACGCCACGGCCACGCGGTG
CGATATCAATAAACAGTTGCAGATCGTCGAGGGGCAAGCCTTGCAACCCGGCATCACGCCCTTCCAGGCGCAGGCT
CCAGATCCCGCGCAGGCTGTTCGCCAACTGCTGGCCGGCGGCGTCCGCCAGGTCCACCGTGGCTTCCTCGGGCGTG
ATGGGGAACTCCTCACGCGTCAACTCGCGATGGGCATAAAAGGCTGCAGGTACGGCGGCGCCGGTCAGCGCCAGGC
CTGCGATAAACCCTCGTCGAGAAATCGTCATGTTCCTACCTGTGTCAGCCTTGGAGCAGGCTTTATCCAAGCTAGA
ACGTTTTGCCGGACAGCAAATTTACTGTGGGAGCCGGCTCCCACCGTTGATGGTGGGGATACTTAAATTTTCCGGG
CCTGCGCTCGTTCTTCCCAGATAGCAAAGGCCCCTTGCGCCTGCACCTCAAAGGCGAACCGACTGAGATGGCAATG
ACAAAACCACGTTCGAAAAAGGCTCTATACATCGGTCTGCCCTTGGCCCTGGCCATCGCTGCCGGCGCAGGCTTTG
CCTGGGACCACTGGTTTCGCGGCAACGCCGGCTATTCGCTGGAAGTGATCAAGCAGGCCAATGAACTGCAGGACCG
TCTGCTTTCGTTCGACAGCCATATCACCGTGCCCCTGGACTTCGGCACCGCCGGCCATGAAGCAGACAAGGACGGC
AGCGGCCAGTTCGACCTGGCCAAGGCCAGTCGCGGGCGCCTGTCGGGGGCGGCGCTGACGGTGTTCGGCTGGCCCG
AACTGTGGAATGGCCCCAACGCGCCGCACAAACCCACCGCAGGCTTTGTCGACGAGGCCCGAAACCAGCAAGAGGT
GCGCTACAAGATCATTACCGGCATGGTTCGTGACTTTCCCAACCAGGTGGGCATTGCCTACACCCCGGCCGATTTC
CGCCGCCTGCATGGCGAGGGCAAGTTTGCGATCTTTATCAGCATGCTCAACGCCTACCCCCTGGGCAATGACCTGA
ACCTGCTGGACATGTGGACGGCGCGGGGCATGCGCATGTTCGGCTTCAGCTACATCGGCAATAACAGCTGGGCCGA
CTCTTCGCGCCCGCTGCCGTTTTTCAATGACTCGCCGGACGCCCTCGGCGGCCTCTCACCCATCGGCAAGCAAGCG
GTGCAGCGCCTGAACGACCTGGGCGTGATCATCGACGTGTCGCAAATGTCCACCACCGCCCTGGAGCAAGTGGCGC

AGTTGAGCCGCACGCCCATGGTCGCGTCCCACTCCGCGCCCCGCGCCTCAGTGGATATCCCGCGCAACCTCAGCGA
CAAAGAACTGCAACTGATCAAGAACAGCGGCGGCGTGGTGCAAGTGGTGGCGTTCGGCGGGTATCTGCGCCCGCTG
AGCCAACCGATCCAGGACAAGCTCAACGCACTGCGCGCGCGTTTCGACCTGCCGCCCCTGCCCAACCTGGCGATGG
CCCTGATGCCCGGCGACCCGATCATTGCCGCCTGGCCGGAACAGAAGTTCGGCGAATACGCCAGCGAGCTCTACGC
CATTCTCGAAGAGGAGCCCAAGGCCAGCCTCAAGGACCTGGGAGATGCCATCGACTACACCGTGCGCAAGATTGGT
ATCGACCACGTCGGTATCGCCTCGGACTTCAACGAAGGCGGCGGCGTCAAGGGCTGGAACGATGTGAGTGAGGTGC
GCAACGTGACCGCCGAACTGCTCACCCGTGGCTACTCCGAAGCCGATATCGCCAAGCTGTGGGGCGGCAACTTCCT
GCGGGTGTGGGAGCAGGTGCAACAGTCCGCCAAGCCCGGCGTGCCGCCAAGCGAGCAGATGCCATGAGCGACCGCC
GCACCTTTCTCAAACAGGCTGGCATCCTCGCCGCCAGCCTGCCGTTGGGGGCCAGCCTGGACGTTCAGGCCGCCAG
CGCCAAGGCACCCGCCGATAAGTGGGCCAGCCTGCGCCAACTGTTCGACCAGGACCCGAACTACATCCACTTCTCC
AACTTCCTGATCACCTCCCACCCAAGGCCGGTACGCGAGGCCATCGAGCGCCACCGCGCCGCCATTGACCGCAACC
CCGGCCTGACCATGGATTGGGACCTGCAGGAACCCTGGAAACGCGAAGGCCAGGTGCGTGAATGGGCCGCGCGTTA
TCTGCAGGCCAAGCCCGGCCAGATCGCCCTCACCGGCAGCACCACCGAAGGGCTGGCGATGATCTACGGCGGCCTG
CGCGTGCGCCCCGACCAGGAAATCCTGACCACCGAGCACGAACACTTTTCCACTGACAGCATCCTCGCGTTCCGCA
CCCAGCGTGACGCCACCCAAGTGCGCAAGATCAAACTGTTCGCCAACGCCCACCACGTGTCGGTCGATGAAGTGCT
GACGAGCATCGAGCGCAGTATCCGCCCCAATACCCGGGTGCTGGGCATGACCTGGGTCCAGTCGGGCAGTGGCGTG
AAACTGCCGATCGGCGAAATCGGCCAACTGGTCAAGCGCCACAACCAGGGGCGTGACGAAAAGGACCGCATTCTCT
ACGTGGTGGATGGCGTGCATGGCTTTGGCGTCGAAGACCTGGCCTTCCCGGACATGCACTGTGACTTCTTTATTGC
CGGCACCCATAAGTGGATGTTCGGGCCACGGGGCACCGGCCTGGTATGCGCCGCCTCGGAGCAAGTGACCAACCTC
ACCCCGAGCCTGCCGACGTTCTCCGAAGACACTGACTTTGCTACTTCCTTCTCCCCCGGCGGCTATCACGCCTTTG
AGCACCGCTGGGCGCTGGATGAGGCCTTCAAGCTGCACCTGCAACTGGGCAAGGCCGAGGTCCAGCAGCGCATCCA
CAGCCTCAATACCTACCTCAAGGAACGCTTGCAGGCACGCCCGAACATCGAGCTGGTCACCCCCGCCAGCCCGCAA
CTGTCGGCCGGTTTCAGCTTCTTCCGGGTCAAGGGCCAGGACAGCGACAAGGTGGCCGCCTACCTGATGCGCAACC
GGGTCATCAGCGACGCGGTGAACCGCGATGTCGGCCCGGTGGTGCGCACTGCGCCGGGCCTGCTCAATACCGAAGA
TGAAGTCGACCGCTTCCTGGCGCTGCTCGACAAAACCCTTTGACACCTTTTTCTTGTTATCGAGACGACTCATGAA
CGATGAACCGCTCACCCCAACGCTGCTCAAGTCCCTGGCCCTGACGGCATTGATGGCCTGCCTGGCGCCAGCCGCC
CAGGCTGCAACACCGCCCAAGCCCGGCACTGTGTTCAAGGACTGCAAGCACTGCCCGGAAATGGTGGTGCTGCCCA
CCGGCACCTTCAAGATGGGCACCCCGGACGATGAAGTCGGCCGCCAGCCCGATGAGGGGCCGATCCATGACGTGAC
CTTCGCTAAACCCGTGGCCATCAGCCGCTTCCAGGTACTGGCCGGCGAGTGGGACCGTTTTATACGAGAGACCGGC
TATAAAATGCCCGACGGCGACGACCGACCAGGGCGCAAATGCACGGCTGGCAAGCCCAGCTACCCCATCACCGCCA
AGCATCCGGCCGTGTGCATGGACTTTGCCGAAGCCAATGCCTATGTCGCCTGGCTGTCGAAGAAAACCGGCAAGCA
CTACCGCCTGGTCAGCGAGTCCCTGCGCGAGTACGCCGCCCGGGGCGGTACCTCCGGGCCCTTCCCGTTCCCGTTC
GACGAGGGTGAGAAATACGGCATCGCCAAGCACGCCAATACCTACGGGCCGGCGGATGGCTACAGCTACACCTCGC
CCGCCGGCAGCTATGCGCCCAATGCGTTCGGTGTGTATGACATGCACGGTAACGTTTATGAATGGGTCGAAGACTG
TGAACACGACAGCTATGTGGGCGCGCCCAGCGATGGCAGCGCCTGGCTGACCGGGCAATGCGAGGTGCGGCAGATT
CGCGGCAACGACTGGGGCGAAGCGCCGATTTTTTCCCGTTCCGGCAACCGCAACAGCCTCTACTCCAATGTGCGTG
GCGACTGGCTGGGCTTGCGGGTGGCACGGGACCTGTGAGCCTGCGCAACTCGGCGCGAGGCTAAATCGCGCCCCAC
CCGACTCGTCCTCCTACTATCGGCCTGCTAGCGGTAAACCTGCGCACGCAGGCCTTCACTTGCCCACAGCACGTAG
CCACGTCGCGATCGAGGTAGTTTCATGACCGCAGCACCGCGCAGCGCCATTCGCGAACTCTTGAGCCTGCTCAAGC
CCTATCGACGGATTGTCATTTCTTCCATCACCCTGGGCATCATCGGCGGCCTCAGCGTCACCGCGCTGCTGGCCAC
GGTCAACCGCGGCCTGCACAGCGCCGACGGCATGGGTACCGGCGTGGTCCTGGCCTTCGCCGGGCTGTGCCTGCTG
GCCTTGCTCAGCAGTATCGGCGCCGATATCGGCACCAACTTCGTCGGGCAGAAGGTGATTGCCAAACTGCGCAAGG
ACCTCGGCGCCAAGGTGCTCGCCGCGCCCATCGAGCAGATCGAGCGCTATCGCACCCACCGCCTGATCCCGGTGCT
GACCCACGACGTCGACACCATCAGCGACTTCGCCTTCGCTTTCGCCCCGTTGGCGATTTCCACCACAGTGATCCTT
GGCTGCCTGGGCTACCTGGCGGTGCTGTCCTGGCCGATCTTCCTGATGACCCTGCTGGCCATCGGGATCGGTTCGG
CGGTGCAGTTCGTGGCCCAAAGCAAAGGCATCAAGGGCTTCTACGCGGCCCGCGATGCCGAAGACAACCTGCAAAA
ACACTACTCGGCGATTGCCGAGGGCGCCAAGGAACTGCGCATCCACCGCCCGCGCCGGCACGCCATGCACACCCGC
AATATCCAGGGCACTGCCGACCATATCTGCGATACCCATATCCGCTCGATCAATATTTTCGTGATCGCCAAGAGCC
TGGGCTCGATGCTGTTTTTTGTGGTGATTGGCCTGGCGCTGACCCTGCAACAGTTCTGGCCCAGCTCGGACCCGGC
GGTGATGAGCGGCTTTGTCCTGGTGCTGCTGTACATGAAAGGCCCGCTGGAAAACCTGATCGGCAACCTGCCGATC
ATCAGCCGCGCGCAAATTGCCTTCCGCCGCATTGCCGACCTGTCGGAGCGCTTCTCCTCGCCGGAACCGCACCTGC
TGCTCGATGACGCCGACAAAAGCGTGCCGGTGATGAAGCACCTGGAACTGCGCAACGTGCAGTACGCCTTCCCGCC
CGTGGAGGGCAGCGAGCCGTTCAAGCTGGGCCCGGTCAATTTGAGCATCGAGCCTGGGGAAATCCTGTTTATCGTC
GGCGAAAACGGCTGCGGCAAGACCACCCTGATCAAGCTGCTGCTGGGCCTGTACACCCCGCAGCAAGGCGAAATCC
GCCTCAACGGCCTGCCCGTGGACGCACTGGGCCTGGACGACTACCGCCAGATGTTCACCACGATCTTTGCCGACTA
CTTCCTGTTCGACGACCTGGTGCAGAACGACAAGGCCCTGCCCGACGACGCCACCCAATACCTGGAGCGCCTGGAG
ATCGCGCACAAGGTCAGCGTGCGCGACGGTGCGTTCACCACCACCGACCTGTCCACCGGGCAACGCAAGCGCCTGG

CCCTGGTCAACGCCTGGCTCGATGAGCGCCCAGTGCTGGTGTTTGACGAGTGGGCGGCCGATCAGGACCCGACCTT
CCGGCGGATTTTCTACACCGAGTTGCTGCCGGACCTCAAGCGCCTGGGCAAGACCATCATCGTCATCTCCCACGAC
GACCGTTACTTCGATGTGGCTGACCAACTGGTGCGCATGCAGGCCGGGCGCGTCATCAGCGAAAAAGCCTTTGCCT
GATACCTGAGGCAGGCCTTCTGTGGCAATGAGCTTGCCTGTGGCGAGCGGGCTTGTCTGTGGCGAGCGGGCTTGCC
CCGCGCTGGGCTGCGTAGCAGCCCCCCCTAAACGCCCCGCGTTTATTCAGACAAACCGAGGCGCCTGGTTTTAGGG
CCGCTTCGCAGCCCAGCGCGGGGCAAGCCCGCTCGCCACGGACAAGCTCCCAGCACCGCAGACAATCTCCAGCAAC
CACAGACAAGCTCCCTAGCCACAGGCCATTGCGCCTAAATCTCATCGATCGATCTTCGTCTTCCTGACACAGCTTA
TTTCCTCTGCCGACGGCGACGAGATCGATCATGAACCAATTGAACCAACTTCCCGACGACGACCTGCTGGCCCTGT
TGCTGGCCGATGAAGCCGGCGCCGGGCAGTCCATCCAGAGCCTCGCCGGGCATGACCCGGTACCGCTGTCGTTCGC
CCAGCAGCGCCTGTGGTTCCTGCAACAGTTCGATCCCCAGAGCCATGCCTACAACCTGCCACGGGCGATTGCCCTC
AAGGGCCCGGTGGACGCGCGCGCCCTGGAAGCGGCCCTGAATCGGGTGATCGATCGCCACGACATCCTGCGCACGG
CCTTTCGCGACACCGACGGCACACCCCTGCAAGTCATCGAACCTGGCGCGCGCCTGACCCTGCTGGAAGAGGACCT
AAGCACCCTGGATAACCAGCAGCGCGACCTGCAACTGCAACAGCGCATCCGCCAGCACGCCACCCAACCCTTCGAC
CTGCGCCAGGCGCCGCTGATTCGCAGCACCCTGCTCAAACAGGCCGAGCAGCACTACGTGCTGCTGCTCAACATGC
ATCACATCGTCTCGGACGCGTGGTCCAACCCGATCCTGATCCACGACCTGGCCAGCGCCCTGCGCGAACCGGGCCA
GGCCCTGCCGCGCCCGGCCATCCAGTACGCCGACTACGCCCAGTGGCAGCGCCAGGACTACCCGCTGACGCCGCAA
CACAGCTTAGCTGCGCAGTATTGGAAAGCCTGCCTGGGCGACGAGATCCCCACCCTGGAGCTGCCCCGCGAGCGCC
TGGAACAACACGCAGGCGCCGGCCATCATGGGCACGCGCTGCCCGCCCTGTTGAGCGAGCGTTTGCAGACTTTTTG
CCAGCAACAGGGCGTGACACCCTTCGTGGTGCTCCTGGGTGCCTGGCAGCTGCTGCTGGGGCGCTACAGCGGCCAA
GTCGACTTCACCGTCGGCGTGCCCAACGCCACCCGCAACCAGGCGCAGATCCAGGACCTGGTGGGCTTTTTTGTCA
GCAGCCAGGTGTATCGCGCGCGACTGGCGCCCGAACAAACCTGCAGCGCGTTCCTCCAGGGCCTGCGCCAGCAATC
CCTCGCTGCCCTGGAACATGCCGACTACCCCATCGAGCTGATCCTCGAAGACCTGCACCTGCAACGCAGCCCCCAG
GCCAACCCGCTGTTCCAGACCCTGTTCAACTGGCGTGTCGGCGCCGTGCAGGAGGCGGCCCTGCACCTGGGCCCGC
TGCAATTGAGTTTTATGGACAGCCACCCGCTGCAGGCCAAGTTCGACCTGTCGCTGGATGTGGAATACAGCCAGCA
GCAGATCACTGCGCATTTTGAATACGACGCCGCTTGCTTCGCCCCAGCGACCATTGCCACCCTGGCCGGGCACTTC
CAGAACCTGGTGCAGGCGATGCTCGAGCAGCCCCAGGCACGGATCGGCGAACTGCCGATGCTCAGCGCCGAGGAGC
GTGGCCAGCAGTTGGCGCAGTGGAATGCCACGGCGGTCCATTACCCCAACGCCGCACCGGTGCATGTCTTGCTCGC
GGAACAAGCCGAACGCCTGCCCCATGCCGTTGCCGTGGAGTTCGGCAACCAGCAACTGACCCTGGACCAGCTCAAC
CGCCGCGCCAACCAACTGGCCCATAAGCTGCTGGAACTGGGCGTCGGCCCCAATACCTGCGTGGGCCTTGGCCTGG
AACGCAGCCTGGAAATGGTGATCGCCCTGCTGGCCGTGCTCAAGGCCGGCGGCGCCTATGTCCCGCTGGACCCGGC
CTACCCCGCCGACCGCCTGGCCTACATGATCAATGACAGCGGCATCGGCCTGCTGCTGACCCAAAGCAGCCTGGCA
TTGCCCGTCCCGGACCGCCTGCCGTCCCTGAGCCTGGACCAGGACAGCGACTGGCTGGAAGGTTATGACAGCGCCA
ACCCACCGGTCACGGTCAGCGCCGATCACCTGGCGTACATGATCTACACCTCCGGCTCCACCGGGCAGCCCAAGGG
CGTGCAAGTGCGCCATGGCGCCCTGACCAATCATATGCTCTGGATGCAGGGCGAATTGGGCCTGAGCGCTGCGGAC
CGGGTCTTGCAAAAAACCGCCTTCAGCTTCGACGCCTCGGTGTGGGAATTCTGGCTGCCATTGCTCAACGGTGCGC
AGCTGGTCCTAGCGTCCAGCGAACTGTCCGCAGACCTGTCGATGCTCTGGTCGCAAGTGGCCGCCCAGCGCATCAG
CGTGTTGCAAATGGCCCCTTCGCTGCTGCAAGCCTTGCTTGCCGATGAACAGGGCGCGCAGATGCAATTCTTGCGT
TTGCTGCTGCTCGGCGGCGAAGCCCTGGGCACCGCGTTGGTGGCACAATTGCGCGAGCGCTGGAACGGCCGCCTGT
GCAACCTCTACGGCCCAACCGAAGCCACCATCGACACCACGTTGTTTGATGTCGACCGCCTGCCAGAGGGCGCCAT
CGCCCCTATCGGCCGCCCCATCGCCAACGTGCGCACCTACCTGCTCGATCATGACCTGCAGTTGTGCCCGGTGGGC
ACCCACGCCGAGCTGCTGATCGGCGGCGACTCCCTGGCGCAGGGCTACCACCAGCGCCCGGGCCTGACCGCCGAAC
GCTTTATTCCCGACCCGTTTGCCGGCAATGGCGCGCGCCTGTACCGCAGCGGCGACCTGACCCGCTACCGCGCCGA
TGGGGTCATCGACTACATCGGGCGCATCGACCATCAGGTCAAAATCCGCGGTTTGCGCATCGAACTGGGGGAAATT
GAAGCGCAACTGCTGCAACACCAGACGGTGCGCGAAGCCGTGGTCATCGCCCACCCAGCCCCGGGCGGCGCGCAGT
TGGTGGGCTATGTGGTGCCAACAGAACCGGCAACGGACATCACCCGCCAGGACGCCTTGCGCGACACCCTCAAGGC
CCACCTCGGCGAACACCTGCCGGACTACATGGTCCCCGGCCACCTGCTGATCCTGGCGCAATTACCCCTGACTCCC
AACGGCAAGCTCGACCGCAAGGCCCTGCCGGCCCCCGATGCCAACCAGGCCCTGCACGCCTACGTGGCCCCGCAAA
ACGAAGTGCAACAACGCATCGCAGGCATCTGGCAAGACGTGCTCAAACTGGAAAAAATCGGCCTGAACGACAACTT
CTTCGAACTGGGCGGCGACTCCATCATCTCTATCCAGGTCGTCAGCCGCGCGCGCCAGGCCGGCATCCACTTCACC
CCCAAGGACCTGTTCCAACACCAGACCATCCAGGGCCTGGCCAGCGTGGCGAAAAACCAGGGCCAGGGCCTGGCGA
TCGACCAGGGCCCGGTCACGGGTTTGGCACCGCTGCTACCGATCCAACACCTGTTCTTCGACACCGACATCCCCGA
GCGCCACCACTGGAACCAGTCCGTGCTGCTCAAACCCGCCACAACCCTGGACCCGCACCTACTGGAACAGGCCCTC
AACGCCCTGATCCAACACCACGACGCCCTGCGCCTGCGCTTCACCCAGACCGACTCTGGCTGGAACGCTGAATACC
GCCCGCAAACCGCGTCGGTGCTGTGGCAAACCCGCATCGACGACGCCACACAGTTGCAATCTTTGGGCGACGAAGC
CCAACGCAGCCTCGACCTGCAACATGGGCCGCTACTGCGCGCGGTACTGGTGGGTTTGAAGGACGGCAGCCAGCGG
TTGCTGTTGGCTATTCACCACCTGGTGGTGGATGGGGTGTCGTGGCGGATTTTGTTTGATGATTTGCAGATGGCTT
ATCGTCAACTGCAAGCGGGTCAGACGATCGGGCTGCCGGAAAAGACCAGCGCCTACAAGACCTGGGGTGAGAAGTT

GCTGGGGTATGGGCAGAGGGCTGAGTTGCAACAGGAACTGACGTACTGGACGCAGCAACTAGACGACACCAGCCAC
GACCTGCCACACGCAAACCCTGAAGGCAGCCAGCGGCACACCCAGGCGACGACTGTCGATATCCAACTCAGCAAAA
CCCNNNNNNNNNNNTGCCTGGCGTGCCAAGTTACTTCGTGACGAATCCTGCTTGGATGCTACAAACACGCTGCCGGG
GCGTTTGTAGAAAGCAAGCCCCATAATGGGCTTCTAACTTCCTGTCCGGCAGTAGCAAAACCTAGATAGGGCTTGA
GTCGCAGGCGCGCACCCGCCGTAATGACAGGTGCGACGCTCAAGGGTTAAGGCATCACGGGCGGCATGTACTTCAA
CGTCGTCCCCAGTGCCCACAGCAAAAACAGCACCAACGGCGTGTGCACCAGCAACTGCACAAACGAGAAGCCAATC
AGGTCCCGCGCCTTGAGCCCCAGCACACCGAGCAGCGGCAGCATGTAGAACGGGTTGATCAGGTTCGGTAGCGCCT
CGGCGGCGTTGTAGATCTGCACGGCCCAGCCCAGGTGATATTGCAGGTCATTGGCCACCTGCATCACATACGGCGC
CTCAATGATCCACTTGCCACCGCCCGATGGGATAAAGAACCCCAGTACCGCCGAATACACGCCCATCAGCAGCGCA
TAGGTGTCGTGTGAAGCGATGGAGGTGAAGAAGGTCGAGATATGGTGGGCCAGGGTCTGGCCATCGCCGCCCTTGA
CCACGGTCATCAGCGCCGCGATGGAGCCATACAGCGGGAACTGGATCAACACCCCGGTGGTGGTCGGCACCGCGCG
AGCCACGGCATCGAGGAAGCTGCGCGGGCGCCAGTGCAGCAAGGCGCCGACCATGATGAACAGGAAGTTATAGGTG
TTGAGCCCCGAGATAGCGCTGATCGCAGGTTTGGTCGAGAACTCATGGAACAGCCAGCCGGCAGCCAGCAGCGCCA
GCAAAATGGTGAGCAACGGGCTGTGTTCCAGCCATTCTCCCGGACGGCTTGGTGGCTGTGGCTTGGGCAGGTTGAA
TGCCGGGTCCACACCGCAGGCCTTGGCGTCACGCGCCGAGTTGGGGCCCGGTGCGGTGACGTAGGCGATGACCAGC
GACACCACGATCAGCGCCAGCAACAGCACGCCCGATTGCCACAAAAAGATCGTATCGGTGAAGGGAATAGTGCCGG
TGATCGCCAGGATCGACGGCGGCAAGCTGGCCGGGTTGGCCTGCAGTTGCGCGGCCGATGACGACAACCCCAGCGC
CCACACGGCACCCAGGCCCAAATAGGCCGCCGCGCCGGCAGCGCGATAGTCCATGCGCAAATCCGTGCGGCGGGCC
AGCGCCCGCACCAGCAACCCGCCGAACACCAGGGACAGGCCCCAGTTGAGCAGCGAGGCAACCATCGAGATCAACG
CCACCCACGCCACGGCCGAGCGGCCGTTTTTCGGGATGCGCGCCAGGCGGTCGATCAACTTCACGGCAGGCGGCGA
GCTGGCGACCACATAGCCACCAATCACCACGAAGGCCATTTGCATGGTGAACGGTATCAGGCTCCAGAAACCGTCG
CCAAAGGCCTTGGCGGCGTCGGTGGGCGCTGCACCCATGCCCAGCGCGGCCACGGCGACAATGATTACGGCGAGGG
CGGCAAACACCCAGGAGTCAGGAAACCAGCGTTCGGCAAAGTTCGAGCAACGCAGGGCAAAGCGGGCGTAGCGGCT
TTCTTCGATAGGATCGGCCACGTTATTTACCTCTTGTATTTATTATGGGGCGGATTGCGGGCATCTTGGCCCACGG
CCATTTATTTGGGAATGTTGTTATTTTCATGGATCAATGAGTAAACCGAATATATCCGCTGCGATTGCCAGGATCC
GGCCCAGCTCATAACCTGCACGCCATTTTGTCTGTCTGCCGAGCTTCTCCATGACTGCCACCGCTTATCCACAGGC
GCAGCGCTTTTCCCGCTCCGACTATAAAACCCTGGGCCTGGCCGCCCTGGGCGGTGCCCTGGAAATCTACGACTTC
ATCATTTTCGTATTTTTCGCCCTGACCCTGAGCCAGTTGTTCTTCCCCCCGGAAATGCCCGAATGGCTGCGCCTGC
TGCAGAGTTTCGGAATTTTCGTCACCGGCTACCTGGCGCGGCCGCTGGGCGGCATCCTGATGGCGCACTTCGCCGA
TCACCTGGGGCGCAAGCGGGTGTTCAGCCTGAGCATCCTGATGATGGCCTTGCCGTGCCTACTGATCGGGATCATG
CCGACCTACGCGCAAATCGGCTATTTCGCACCGCTGATCCTACTGGCGCTGCGGGTCTTGCAGGGCGCCGCGGTGG
GCGGCGAAGTGCCCAGCGCCTGGGTCTTTGTCGCTGAGCACGCGCCGGCCAACCATCGCGGTTATGCCTTGGGGTT
CCTGCAAGCCGGGCTGACCTTCGGTTACCTGCTGGGGGCATTGACGGCGACCTTGCTGGCGCAGATGTTCACCGCT
GAACAAATCCTCGATTACGCCTGGCGCTTTCCGTTTCTATTGGGCGGTGTGTTTGGTGTGATCGGTGTCTGGCTAC
GCCGCTGGCTCAGTGAAACCCCGGTGTTCCTGGCCATGCAGGCGCGCCGCCAGGCCGACGCCGAACTGCCGCTGCG
CACGGTGTTGCGCGACCATCGCCAGGCGTTGTTGCCGGCCATGTTGCTGACCTGTGTGCTCACCTCGGCGGTGGTG
GTGTTGGTGGTGATCACGCCAACCATGATGCAGAAAACCTTCGGCATGAGCCCCAGCCACACCTTCGCCCTGAGTG
CATTGGGCATCGTCTTCCTGAATATCGGTTGCGTATTGGCGGGCCTGTTGGTGGATCGCATCGGCGCCTGGCGCGC
GGTGCTGGTCTACAGCCTGCTGCTGCCGGTGGGCATCGCCGTGCTTTACGCCAGCCTGATTGCCGGCGGCGTGTGG
CTGGGCGCAGCCTATGCCATTGCCGGGTTGAGTTGCGGGGTGGTGGGCGCGGTGCCTTCGGTGATGGTCAGCCTGT
TCCCGGCGCCGGTGCGGGTGTCGGGGATTTCCTTTACCTACAACATCGCCTACGCGCTGTGGGCGAGTACCACGCC
GTTGATGCTGATTGCATTGATGCCCGCCAGCCCATGGATTTGCGTTTTTTATTGCATGGTGATGGGTGCGGTCGGC
GTGGCCAGTGCCGTGCGGTTTGCCAATCGACCGGTTTTTTAAAGCCACCACAATCTCCCTGTAGGAGCGGGCTTGC
CCGCGATGGCGGTGGATCAGTCACTGTTTGGATCGCTGGTGTACCGCTACTCGCGCTCTTCCAGCACGTAGCCCAC
CCCGCGCAGGGTGTGGATCAACTTGCGCTCGAACGGGTCGTCGATCTTTGCGCGCAGGCGGCGGATCGAGACTTCG
ACCACATTGGTGTCGCAATCGAAGTTCATGTCCCACACAAACGAGATGATTTGCGTACGTGACAGCACCACGCCGC
TCTGGCGCATCAGCAAGTGCAGCAGGGCGAACTCCTTGGTGGTCAGGTCAATGCGCTGGCTGCCGCGAAAGGCCCG
GTGGCGACCCTGGTCCAGTTCCAGGTCGGCCACCCGCAGCACGTCCGGTACCGCCGCCTCGACGCTGCGGCGCATC
AGGGTGCGGACCCTGGCCAGCAGTTCGGGAAACTCGAAAGGCTTGACCAGGTAGTCGTCGGCGCCCAGTTCCAGGC
CTTTGATTTTGTCGGCCAGGCGGCCGTGGGCGGTCAGCATCATGATCCGGGTGTTGCTGGTCTGGCGGATCCGCTG
CAACACATCCCAACCGTCCATCAGCGGTAGGTTCACATCCAGAATCACCAGGTCATAGGCGTGTTGCCGCGCCAGG
TGAACGCCGTCCGCGCCACTGGCTGCGCAGTCGACCACATAGCCACTCTCGCTCAGTCCCTGGTGCAAGTACTCGG
CCGTTTTCAGTTCATCTTCGATAACCAGGATTCGCATGTCGAGGGTGCCTCGGTGCAAGGGGAAGTCGCTGAAATT
GCCGCGAAGGTTAAAGCAAAAGCCCGGGCCAGAGCCTTTGCTAACGGATTTGTAATGTTCTGGCCACCCCCCTGAT
AAGTAGCGAAAGTTACATTGCCCCTGCTTGGATTTGTCCGCCAACTCATGGGTGTGGAACGCTTGTTATGCCAATG
TTTTCAAGAACAATTGTGTGCTCCGTAGTGGGGTTATTGGCGATGGCCCAAGTGGCAAGCGCCCAGACCCTGACCC
TCGAATCGGCCCTGCAAAGTGCGTTTGCCGGCAACCCGGACCTGGCCGCTGCGCAATGGGAAATCGACATCGCCCA

GGGCGGTCGTCAGCAGGCCGGGCTGATCCCCAACCCGGTGGCCTCCTGGGATGCCGAAGACACCCGCCGCAGCACC
CGCACCACCACCGTCAAGCTGAGCCAGACCCTGGAGTTGGGCGGCAAACGCGGGGCGCGCATCGAGGTGGCCTCGC
GGGCCCAAGATGCGGCCGCCCAGGAACTGGAACAGCGGCGCAACCTGCTGCGAGCCGAGGTCATCGATGGCTTTTA
TGGTGCGCTGTGGGCCCAGGAGCGCCTGGAACTGGCCCAGCGTTCAATGGCGCTGGCCGAGCGCGGCCTGGTGATT
GCCAATGGCCGGGTCACGGCGGGCAAGGCGTCGCCGGTCGAAGCCACCCGCGCCCAGGTAGAGGTCTCGCAAATCC
GCCTGGAGCGCAGCCGCGCCGAGATCGGCGTAAGTGATGCCTATCGCCGCCTGGCGGCCATCACTGGCAGCGCCAC
CATCGGTTTTGAACGTGTCGAGGCAGGTGCCGTGAATACGCCGGCGCTGCCTTCGGTCACCCAACTGCTGGCGCGC
CTGGAGAGCACCGCCGAGCTGCGCCTGGCGCAGCTGCAAATCCAGCAACGGGACGCCGGCCTGGGCCTGGAGAAGG
CCCAGCGGATTCCCGACCTGGATATCTCCATCGGCAGCCAGTACGACGCCAGCGTCCGCGAGCGGGTGAATCTGGT
GGGCGTCTCGATGCCGATCCCGCTGTTCAATCGCAACCAGGGCAATGTGCTGGCGGCCAGCCGCCGAGCCGATCAG
GCGCGGGACCTGCGCAACGCCACCGAACTGCGCCTGCGCACCGAAACCCGCCAGGCCCTGGACCTGTGGGCAACCG
CCAACAGTGAGGTGCGCGCCTTCAACCAAGTGATCCTGCCCGCCGCCCAAAGCGCGGTGGACAGCGCCACCCGTGG
CTTTGAGATGGGCAAGTTCAACTTCCTCGATGTGCTCGACGCCCAGCGCACCTTGATCGCCGCCCGCACCCAATAC
CTCGCCGCACTCGCCCAGGCCACCGATGCCTGGGTGCGCATCGAACGGATTTACGGCGACCTTGCCCGCATTTGAT
CTTTCAGGAGTTCCCCATGAACAAACAACAAGGCCTGGCTTTGGCTGTTGCAGCGGCTATTGCCCTCGCGGCGATG
GTGTTTTGGCCGGCCACCAGCGTGGCGCCCCAGGCACCGGTGGCGAGCGTCAAAGCGCCGGAGGAAGAAGAGGAAG
AGGGCGTGCTGGTGCTCAATGAGCAGCAGATCCAGGCCGCCGGCATTCAACTGGCCAAGGCCCAGCCCCGACAAAT
CAGCACCCTGCTGTCGTTGCCGGGCGAGGTGCGATTCGACGAAGACCGCACCTCCCATATCGTGCCCCGTGCGGCG
GGGGTGGTGGAGTCGGTGAAGGTCAACCTTGGGCAGAAGGTCAAGCAAGGCGAGCTGTTGGCGGTGATCGCCAGCC
AGCAGATTTCGGATCAGCGCAGCGAGTTGGCGGCCAGCCAACGCCGGGTCGAACTGGCCCGCACCACCTTCCAGCG
CGAGCGCCAGTTATGGCAGGACAAGATCTCCGCCGAACAGGATTACCTGCTGGCCCGCCAGAGCCTGCAAGAAGCC
GAAATTGCCCTGAACAACGCGCGGCAAAAGATGAACGCCCTGAGTGGCAGCGCGGTCCTGGCCGGCGGCAATCGCT
ATGAACTGCGTGCGCCGTTTGCCGGGGTGGTGGTGGAAAAACACCTGGGCGTTGGCGAAGTGGTGAGTGAGACCAG
CGCCGCTTTCACCCTGTCGGACCTGTCCCAGGTGTGGGTCACGTTTGGTGTGTTCCCCAAGGATCTGAACAAGGTC
CAGGTCGGTCGGCCGGTGAAGGTCAGCTCGGCGGAAATGGGTACCGACGTGATGGGCACCGTGGCCTATGTCGGCA
ACTTGCTGGGCGAGCAGACGCGTACCGCCACGGTGCGGGTCAGTGTGGCCAACCCCGACGACTCGTGGCGACCTGG
CCTGTTTGTCGCGGTGCAGGTGGCCACGGATAGCTATTCGGCGCCGGTCACTGTGCCGGTGGAGGCGATCCAGACC
GTGGAAGACAAACCCTCGCTGTTTGTGCGCACCGCCGAAGGCTTCGTCACCCGCCACGTTGAACTGGGTGTCAGTG
AAAACGGCTTTGTCGAGGTGCGCCAGGGCCTGGAGGCTGGCGCCGAGGTGGCGACCGTTGGCAGCTTCGTCCTCAA
GTCCGAACTGGGCAAGGCTTCGGCCGAGCACGCCCATTGATCCTGCGAGTGATTCCCCATGTTTGAACGTCTGATT
CAATTTGCCATCGAGCAGCGCATCATCGTGTTGCTGGCGGTGTTGTTGATGGCCGGTGTCGGCATCGCCAGCTACC
AGAAACTGCCGATCGACGCGGTACCGGACATCACCAACGTCCAGGTGCAGATCAACTCGGCGGCGCCCGGCTTTTC
ACCGCTGGAAACCGAACAGCGCATCACCTTTCCTATTGAAACCGCCATGGCCGGTTTGCCGGGCTTGCAGCAGACC
CGTTCGCTGTCGCGTTCGGGGCTGTCCCAGGTCACGGTGATCTTCAAGGATGGCACCGATCTGTTCTTCGCCCGGC
AACTGGTCAACGAGCGTTTGCAGGTGGCCCGCGAACAATTGCCGGACGGTATTGAAACCGCGATGGGGCCGATTTC
CACTGGCCTCGGGGAAATCTTCCTGTGGACCGTGGAGGCCGAGGAGGGGGCGCGCAAGGAAGACGGCTCGGCCTAC
ACGCCGACGGACCTGCGGGTGATCCAGGACTGGATCATCAAGCCGCAGTTGCGCAACGTGCCTGGCGTGGCCGAGA
TCAACACCATTGGCGGCTTTGCCAAGGAATACCAGATCGCCCCGGACCCCAAGCGCCTGGCCGCCTACAACCTGAC
CTTGAGCGACCTGGTGACGGCGCTTGAACGCAACAACGCCAACGTCGGCGCCGGTTACATCGAGCGCAGCGGCGAG
CAATTGCTGATCCGCGCGCCGGGGCAGGTGGCGTCCATCGATGACATCGCCAACATCGTGATCAACACCGTCGACG
GCACGCCGATCCGCGTGCGCAATGTGGCCCAGGTCGATATCGGTCGCGAGTTGCGCACCGGCGCGGCCACCGAAGA
TGGCCGTGAAGTGGTACTCGGCACGGTGTTCATGTTGATCGGCGAAAACAGCCGCAGTGTGTCCCGGGCGGTGGCG
AAAAAACTCGAAGAGATCAACCGTTCGCTGCCCGAAGGCGTGGTCGCGGTTACGGTCTATGACCGCACCAACCTGG
TGGAAAAAGCCATCGCCACCGTGAAGAAAAACCTCTTCGAAGGTGCGCTGCTGGTCATCGTGATTCTGTTCCTGTT
CCTGGGCAACATCCGCGCGGCGTTGATCACCGCGATGGTGATCCCGCTGGCCATGTTGTTCACCTTTACCGGGATG
TTCACTAACAAGGTCAGCGCCAACCTGATGAGCCTGGGGGCCTTGGATTTCGGGATTATCGTCGATGGCGCGGTGG
TGATCGTCGAGAACGCCATCCGCCGCCTGGCCCATGCACAGCAACGCCACGGCCGCCTGCTGACCCGCAGCGAGCG
CCTGCACGAAGTGTTCGCCGCTGCCAAAGAAGCGCGCCGCGCGCTGATCTTCGGGCAACTGATCATCATGGTGGTG
TACCTGCCGATCTTCGCCCTGACCGGGGTGGCCGGGAAAATGTTCCACCCCATGGCGTTCACCGTGGTCATGGCCT
TGCTGGGCGCGATGATTCTGTCGGTGACTTTCGTCCCGGCGGCGATTGCGCTGTTCGTCACCGGCAAGGTCAAGGA
GGACGAAAACTGGGTGATGCGCAACGCGCGCCGTGGTTATGCGCCGGTGCTGGATTGGGTGATGGCGCACCGTACC
TGGGCGTTCGGCCTGGCGCTGTTGACCATCGTGATGTCTGGCGCCGTGGCCAGCCGCATGGGCAGCGAATTTATCC
CCAGCCTCAGTGAAGGTGACTTCGCCCTGCAAGCCTTGCGCGTTCCGGGCACCAGCCTCACGCAATCGGTGCAGAT
GCAGCAGCAACTGGAAAAAAACTTGATGGCGCAGGTGCCGGAAATCCAGCGGGTATTTGCCCGCACCGGTACTGCA
GAAATTGCCTCCGACCCGATGCCGCCGAATATTTCCGACGCCTACTTGATGCTCAAGCCCAAGGAGCAGTGGCCGG
ACCCGAAAAAGTCCCGCGAAGCGCTGATCGCCGATATCCAGCGCGCCAGTGCGATTGTGCCGGGCAGCCAGTACGA
GTTGTCGCAACCGATCCAACTGCGTTTCAACGAGCTGATTTCCGGGGTGCGCAGTGACGTGGCGGTGAAAGTATTT

GGCGATGATCGGGATGTGCTGAACAACACCGCCGGGGAAATCGCCGAAACCTTGCGCAAGCTTGATGGCGCGTCGG
AAGTGAAGGTGGAACAGACCTCCGGCCTGCCGGTGCTGACTATCAATATCGACCGAGACAAGGCCGCGCGCTTTGG
CCTGAATGTGGGAGATGTGCAAGACACCATCGCCGTCGCCGTGGGCGGGCGCCAGGCCGGCACGATGTACGAAGGC
GACCGGCGTTTTGACATGGTGGTGCGTTTGTCGGAAACCCTGCGCACCGATATCGAGGGCTTGTCGCGGCTACTGA
TCCCGATCCCCGCGCTGGCCGGCAATACCACGGGGCAACTGGGCTTTATCGCCCTGTCGCAAGTGGCCAGCCTGGA
CCTGGTGTTGGGCCCGAACCAGATCAGCCGCGAAAACGGCAAGCGTCTGGTGATTGTCAGTGCCAACGTGCGCGGG
CGGGATATTGGCTCGTTTGTCGCTGAGGCCGAACGGGTGATGGCAGAGCAGGTGAAGATCCCCGCCGGCTACTGGA
CAACCTGGGGCGGCCAGTTCGAACAGCTCAAGGAAGCCTCCGAGCGCTTGCAGATCGTGGTCCCGGTGGCGTTGCT
GCTGGTGTTTGGTTTGTTGTTCATGATGTTCAACAACCTCAAGGATGGGCTGCTGGTATTCACCGGCATTCCCTTT
GCGTTGACCGGTGGGATCATGGCGCTGTGGTTGCGCGATATTCCGTTGTCGATTTCTGCCGGGGTGGGGTTTATCG
CGTTGTCGGGGGTGGCGGTGCTCAATGGCCTGGTGATGATTGCGTTTATCCGCAACCTGCGTGAGGAGGGGCGTTC
GTTGTCGGTGGCGATCAATGAAGGAGCCTTGACCCGCTTGCGCCCGGTGTTGATGACGGCGTTGGTGGCGTCCCTC
GGCTTTATCCCCATGGCCCTGGCCACCGGCACCGGCGCTGAAGTACAGCGGCCGCTGGCGACGGTGGTGATTGGCG
GGATTATCTCGTCGACTTTGTTGACCTTGCTGGTACTGCCAGCGCTTTACCACTGGGCCCATCGGCGCGATGAAGA
GCCGGTAGCGTCCTGACCTCGCGCTGGATGGGCTTACGGTGGAGAAGGGGCTTGTTGTGGAGAGGGGGCTTGTCCC
CCGTTGGGTGGCGAAGCCGCCCTAAAACCTGCTTGCTCGGTGTGCCTGGGGTAAGTGGGGTGGGTTTGTTGGGGCT
GCTGCGCAGCCCAGCGCGGGGCAAGCCCGCTCGCCACGACAAGCCCGCTCGCCACGACAAGCCCGTTCTCCACAAC
GAACCTGCTTGCCATAGCAAGCCCGTTTGCGGCAATAAGACTGCTGAGTGCGGCAAGGGAGTCGGTAGCTACGGGA
CTTGCGGGGTGGCGGGCAGGGTGGGGAATATCAGTCGGAACTGGGTCACGTCGCCCGGCACGCAGTTGGCCTCGGC
CTGGCCCTGGTGCAGGCTCATGATCGAGCGCACGATCGCCAGCCCCAGCCCGGTGCCGCCTTCGGTGCGGGCGCGG
CTGTTGTCGGCGCGGTAAAAACGCTCGAACAGGTGCGGCAGGTGTTGCGCTTCAATTCCCGTGCCCGGGTTGCTGA
CGGTCAGCGATACACGCTGGTGATAGGTGTCGACCTGCAATGAAATTCTAGAGCCGTGCGGGCTATGGCGGATTGC
GTTGGACAGCAGGTTGGAAATGGCCCGTTGAATCATCAGCCGATCACCCAGGACCCAGGCGTCGCCCGCCTGGCTC
AGCTTCAACGCTTTGTCTTCGGCGCTCAACGCAAACAGCTCCATGACCCGTAGCGCTTCATCGCGCAGGTTCACTG
CGGTAAACGAAGCCCGCGCCGCCGGATGGCTGACCTGGGCCAGAAACAGCATGTCGGCGACGATCCGCGCCAAGCG
GTCCAGTTCTTCGGTGCTGGATTCCAGAGCCGATTTGTACTCGTCGGGCGCCCGGGCACGGGACAAGGTCACCTGG
GCCTTGCCCATCAGGTTGGTCAAGGGCGCGCGCAGTTCGTGGGCCAGGTCGTCGGAGAATTGCGACAGTTGCTGTA
CTCCGGCATCCAGCCGATCGAGCATCACGTTGATGCCCCGCGCCAGTTCGTTGAGTTCCCTGGGCAGGTTGTCGAC
GGCCAGGCGATGGCTCAGGTCCTGGGTAGTGACTTTGGCGGCGACGCGGCTGAATTGCTGCAATGGGGCGAGCCCG
CGCTGCACCAGCCACCACGCACCCATGCCAATCAGGATCAACAACAGCGGCAGGGCGATGACCGTGGATTGCAGGT
AGGCACTGAGCAGTGCCTGATCGTCGGAACGATCCAGGGACAGCAACACCCGCACATTCTCCCCGGTGTGCAGGCG
CATCACGCTCGATGCGCTGAGGATCTGGTTGTCACGGCCATCGGTCCAGTTCAGAAACCCCAGGGTTTGCCTCGCG
GCAAAGTCCAGCAGCAAGGGCTCCTGGGGCCTGGCACCGACGCTCAGCAACACCGCATTGGCGGGCGCGGGGCCGA
TGATGGTCAGGTAGAAGTTGTCATGGCCCATCACCAGGTCCAGCAATGAATGCGGCCGGGCACTGATGTCGCGGGT
GTTGAGGTCTTGCAGCAGGCTGTGCTGGATCTGCTCCATCTTGCTTTCCAGGCCCTTGCGCGCGAGGTTTTCCAGC
TCATGGGTGAGGGCCAGGTACGCCAGGCTCGCCAGCAACAGCACCAGGCCGGCGCCCATCAGGCTGACGGTGAGCC
CCAGGCGCATGGATAGACTGCCGGTTCTCATTGGCGTGTTTCCAGTACGTAGCCCACGCCACGAATGGTGTGGATC
AACTTGACCTCGCTCTGGTCATCGACCTTGGCCCGCAGCCGGCTGATCGAGACCTCTACCACGTTGGTGTCGCAGT
CGAAGTTCATGTCCCAGACCAGCGAGATAATCTGCGTGCGCGTCAGCACCTCGCCACTCTGGCGCATCAATACCTG
CAACAGCGCGAACTCCTTGGTGGTCAGGTCAATGCGCCGCGTACCCCGGTATGCCCGGTGGCGACGTGGGTCCAGC
TCCAGGTCGGCGACGCGCAATATTTCCGGCAACGCAATCGGCTCACTGCGCCGCAGCAACGAGCGCACCCTGGCCA
GCAACTCGGGAAACTCGAAAGGCTTTACCAGATAGTCATCGGCGCCCATGTCCAGGCCTTTGATCTTATCGGCCAG
GCGGCCACGGGCGGTGAGCATCATCACCCGTAAGGTGTTGTCGCGGCGCAACTGTTGCAGCACCTGCCAGCCGTCC
TGGCCGGGCAGGTTGACGTCAAGGATCACCAGTTCATAGCGGTGCTGGCCGGCCAGGTGCAGGCCGTCGATGCCGC
TGGCGGCGCAATCGACGACATAACCACTCTCACTCAAGCCTTGATGCAAGTAGTCGGCAGTTTTTGGCTCGTCCTC
AACCACAAGGATACGCATGCAGATTGACCTCTTACTGATAAGCGAATAGGCGACGGGAGCTACACGGGATAACGGC
TGTAGTCCACAAAGTAGGAAAATTCTGAGCCTTGAAGTAGCTTCAGGGTCAAGGGCGGCGTTGGATGGCGTCGAGC
GGGCAAAAAAAAGCGCCCGTGAGGGCGCTAAAAATTCATCTCCTTCCAAAGGAGCTCCACAAAAAAAGCTTCAAGA
GATGAATGTGCTCGGGGTGCGCCGCTCAGATGGCGGTGGGGGCCGATGTATCCGAGGGCGCTGGATTTTGCTCGGC
CACGGCTTGGCTGCCGTGGATCTGTTGCTGCTGCTGGCGAAACTCGGCCAGCCACTGGCGGGAGCGATCACCGCCG
TCATCGGCCAGGGCTGCGGTGGAGGCAGCACTGATTAAAACGCTGAATAAAATGTGCAGTGGCTTCATTAACTTTC
TCCGCAAAAGACCCGTGCCGTTAGTTCGCGGTCGGTCGAGGCAGGCAGTATATGAAGGGCAACTTAACGAGAACGT
GAGGCGCATATTACAGTTCTGACAGGCTGCAAATTGTGAAGAAGTGCTAGTCAACGGCAAGTGAATGTAAGCACTT
GCCGTTGAGTGTTATCAGAGGATGGCCAGCGGGTATTCCATGATCAGCCGCAATTCTTCCAGGTCCGATTCAAAGG
CGCTGGAGCGTACCGTCGCCTGGCGAATGCGTATCGACAGGTCTTTCAGGCTGCCGCCCTGGAACACGTACTTGGC
CTCGATATCGCGCTCCCAGCGGCGCTGGTCGGTGAGTGGGTCACCCAAGGCGTCGCGACGCATATACACCGAGTTG
GCGTTGCTGTAGTCAGCGCCAGTGCCCTTGCCATAGCGGGTCATGAACGACAGGCCGGGGATGCCCAGGGTCTGCA

TGTCCAGGTCATATCGCAGGGCCCATGAACGTTCCTTGGGCGAGTTGAAGTCGCTGTATTGGATCGAGTTGTCGAG
GAAGATCGAGTCGGACTGGCGCAGGTAATCGAAGTCATCGTTGCCGTTGTTGCGCTGGTGGGACAGCGCCAGGCGA
TGGGCGCCGACCTTAACCCCAAGCTTGGCACTCCAGATATTGTTGTTGAGCTCGCCCAGCAACCGCTTGCCCTCGT
CCTGGGCGTTGTAATAGTTGAAACCGGCAAACATCGCCACCTCGTCCGTCAGCGGATAATTGACCGCTGTACCGAA
GTAGTACTGATCCCAGGCGTCCTTGAGGCGGCTGCTGTAGAGGCTCAGGCTCCAATGCTGGGCCTGGTAGTCGCCG
CCGAAGTAACCGACCCAGGGCGCATTCACATTGCCTGCGTAAAACGTCGCAAAACCCTCACGCATGCCGCTTTGGG
TTGGCTGGCTCATGGCGTGCAAGCGTCCACCTTGCAAGGTCAGGCCTTCCAGGCTGGTGTTTTCCAGGGTCACACC
ACGAAAGCTTTCCGGCAGCAGGCGTGAGTCGCCGTAGTGCACCACCGGGGTTTGCGGGAAGACGTCGCCGACTTTG
ACCACCGTGTCCAGCAAACGCATTTTTACTGCACCGCCGAGCTTGCTGTAGTCGTCTTCGGGACGGTTGTCGCTGT
CTACCGGCAACATGTCGAATGAGCCCCGGCCGCCGCTGCGCCCGGTGCCGGAATCGAGCTTGAGGCCATACATCGC
AAACGCATCCAGGCCGAACCCCACGGTGCCCTGGGTGAACCCGGACTCGAACTTGCCGATCAGGCCCTGGGCCCAC
GCTTCTGAATAGCCGTTTCCGGTCGGGCTTGACTGGCCTTTGCGGTCATCGCGATTGAAGTAAAAGTTGCGCGCCA
GTACGTTCAGCGTACTGCCTTCGATAAACCCCTCGGGTTTATCTTCTGCGGCCATGCCCGCCAGCGGTGTTGTGCT
GGCCAGTGCTGCGATGATGGAAAGGGAAAGTGTGCGGTTAGTACGCATGTATCGCTCCTCGGTGTTGGCAGCTATG
AGCTTTTTAGTCGCCTGGGGCGTTCTTATTGGAGAGCTATCCCTGATGCGGGGAGCCCGGCTCATAGTTGCAAACA
GCGGATAACGCCGAGGTGGCCTGCACATTACAATTTTGCCAGTTCAACGGTTTATGACGGATACGTAATCCGCCCG
TGAAGCTCCTGACAGGAAGCGTTAGTTACCCTCGATCCTGAACTTTCAACGACTTGAAATCAGACGAGGACACTTT
AATGAAGCATTTGACACGAGCCCTGGGCGTGGTGGTTTTTTCTCTGTCCATGGGGGCGATGGCCGAAGGTGGCGGG
GACCGCGCCATGGCGCAGTTGATGCAAAAAAACCAGGAGGCCATGGCGCGCTATGCCACCGCGCAGGGCAAGCCGG
TGCCTGAAGTCCAGGCTTATCGCTACGGGATGAAGCTGGATATCGCCCGGGTGGTGAATGTCACGCCGCCGGTGCG
AACCTGCAAGGTGGTGCCCTCGCGCATGACCTATGAAGATTCCAGCGGTCAGCTCAAGACCCTTGAGTATCAGGTC
TTGGGCTTGTGCCGTAATAACGGCAGTTGAGGCACTGAAAAAATAATTGTATCAGGGGGCTCAAGCCCTCTTCAGA
AAACGCCGGCAGCGCCGATGGTTAGACAATCCACTATGACTGCCCTCCGGTGTTCCCCATGTTCAATACCCGCTTG
AAGCAGGAGCTGTCCGCTCTTCGCGAAGAACTGTCCAGCCTGCAGCAGGTCAAGGAAAGCCTGGAGAGCGAGATGC
TGGCATTGAGCCTCGACCCTGAGGGCCGGATCGAGTCGGTCAATCAGAACTTCCTCGACGAAATGTTCTACAAGTC
CAGCGATCTGCTGGGCCATGCCATCCATGGGTTTGTGCCGGAGCATGTGAAGCGCGACGAATTCCAGCAGCGCTTT
GCCAACGCCTTGAGCCGTGGCGAACACTTTGCCGGCACCGTGCGCCTGCTGCGCGGCAATGGCAAGGAAGCGTGGT
TGCGCTCCATCGTGCAGCCGGTGCGGAGCGCGGACGGGCGCATCAAGCGGTTTTCGGTGTACTCCAGCGACCTGAC
CCGCACCATTGAGGCGTCCCGTGAGCATGAAAACCTGATCACCGCGCTGGTGCGCTCCACGGCGGTGATCGAGTTC
GACCTCAATGGCAATGTGCTGGGCGCCAACGAGCGTTTCCTGCAGGGCATGGGTTACAGCCTGGCGCAGATCCAGG
GCAAACATCACCGTATGTTCTGTGAGCCCCAGGATTACAACAGCGCTGACTACCAGGCCTTCTGGAAACGCTTGAA
CAGCGGTGAGTTTGTCGCCGGCCGCTTCAAGCGCGTGGATGCCCATGGCCGCGAAGTCTGGCTGGAAGCCTCCTAT
AACCCGGTGCTGGACGCCAACGACCGGCTGTACAAAGTGGTCAAGTTCGCCACGGTAATCACCGACCAGATCAATC
AGGAACGCGCGGTGGCCGAGGCCGCCAACATTGCCTACAGCACCTCATTGCACACCGACAGCAGTGCCCAGCGCGG
TACCGCTGTGGTTACCCAGGCGGTGGAGGTGATGCGCGACTTGGCCAAGCACATGGAACAGGCCGGCGAGGGCATC
GAGGCGCTCAACGCCCAGTCCCAGGTGATTGGCACTATCGTCAAGACCATCAGCGGGATTGCCGAGCAGACCAACC
TGCTGGCGCTCAACGCCGCGATTGAAGCGGCCCGTGCCGGCGAGCAGGGTCGCGGGTTTGCGGTGGTGGCCGATGA
AGTGCGCCAGTTGGCCTCGCGTACAAGCAAAGCCACGGAAGAAATCGTCGGCGTGGTGCGCCAGAACCAGGACATG
GCCCGCGATGCGGTGGCGCTGATGACCGATGGCCGGTTGCAGGCCGAGCAGGGTTTGTCGCTGGCGGCAGAGGCGG
GCACGGTGATCGTCGAGATCCAGGACGGCGCGCAGAAAGTGGTCAGTGCGGTGGGCCAGTTTGCCAACCAGTTGTC
CACCTAGACTCAAGGCCGGCCAATCAGGGGGCATGAACCTCGAACAGCAGCAGGTCGAACCATTGTCCATCGAGCA
GGAACGCCTTGCGTGAATGCCCGTACTGGGTAAAGCCGTTGCGCTGCAACACCCGCAGCGAGCCTGGGTTCTGCGG
GCGTACGGTGGCTTCGATGCGCTTGAGGGCATGCTCGCCAAAGGCTTTTTGCAGGCCCAGCGCCACGGCCTGTCGT
GCCAGGCCACGCCCGGTGAACTGGGCCCCCATGCGGTAGCCCAGGGACGCCGAGTGATAGTAGCGGCGGCGCACTT
GGGTAAAGTTGATCCGCCCCACCAGCACGCCGTTTTCGCGGATCAGATACTGATAGGCGCGGTCTTCGCTGGCCTC
CAGCAGTGCGCTCTCGATGGCGCGCTGAACGCCTTCGACGCTGTAGTACGCCTCGGGCCGGGCATTGATCCAACTT
TCGAAAAAGTCCCGATTGGCCAGCTCGAACGCCAGTAACTCGTTCACGTCATCCCATTGTGGGGGCGTCAATGTCA
GGTTCATCCCTGGAAAACCTCTAGGTGGTGGCCGCGCATCCTTTCTTCTCACACTCGGGCCGCTGGATTCAAGCGC
TGCCTACCATTGCGCCTGTGTCAGTGCGTCGGCAAAGGCGATCAGCTTCGGCGAGTATTGGCGCGAGGGTGGATAC
ACCAGGGATATTGCGCGGCTGCGCCCGGCGAAGCTTTCCAGGATCGGCACCAGGCGCCCGGCAGCGAGGTCGTCGC
GCACGGCAAAGTCCATCACCTGGGCAATCCCGAAGCCGCCCACCGCGCCGTCCACCAGGGCATCGCCAATGTCGAA
GATCAGCCGCCCTTCGACGCTGACATCGCGCACCTTGCCGTCGAGCATGAACTGCCAGTCCACCATCCGCCCGCTG
CGCAGATTGCGCACGGTCAGGCAGCTATGGTCGTGCAATTCTTCGACGGTTCGCGGGGTGCCAAAGCGCGCCAGGT
AGGTGGGGGAGGCGACGGTCACCCAGCGCAGGGGCGTCAAGGCCCGGGCGATCAGGCGCTGGTCCTGGATCTCGCC
GGTACGCAGCAGGGCGTCGAAACCCTCATCGACGATATCCACGAGGCGGTCGGTCATCACCGCTTCGATGCGCAAC
TCGGGGTAGCGTAGGGTCAGCTCGCCAATCACCGGCATCACCACTTTGCGCCCGAACAGCGAGGGGGTGCTGATTT
TCAGTAAACCCGAGGGCGTGCTGCGACGGTCGAGCAGCAGCTTTTCGGTTTCGGCCAGCTCGGCCAGCAACGGTGC

GCTGCGTTCATACAGCATTTGCCCATCCGGGGTCAGGCTGACGCTGCGGGTGTTGCGTTGCAGCAGGCGTACGCCC
AGCTCGGCCTCAAGGCGTGAAATGGCGCGCGACAAGCCTGATTGGGTGAGCCCCAGATCCCCGGCGGCGCGGGTGA
AACTGCGGGTTTCGGCGACGCGAACCAGCAAGCGAACGGCGTTCAGATCCATGATTAAAGTCATAACTGAAAGATT
GATATGGCTATTTATCATCTGCTGGGCATGAAAGACACTGGCCGCACTCTTTTTTAAACAGGATGTTGTGATGCCC
GCCATTCCCTCAAGTCGTCCCTCGGGCTGGATGCTGATGTTGCTGGCCACGGCCCAACTGATCATCGCCCTGGACG
CGACCATTGTGTTTGTCGCCTTGCCGCAGATCGGCAGCCACCTGGGCTTTTCTGCCCAACAGTTGCAGTGGGTCGT
CAGCGCGTACACCGTGGCGTTTGGCGGTTTTCTATTGCTCGGCGGCCGGGCCACGGACCTGCTGGGCAAACGCCGT
ATGTACCGGGTGGGCCAGTCGTTGTACGCGCTGTCATCGTTGGCGGCGGTACTGGGCGGCAGCGCGTTGCTGCTGG
TGCTGGCGCGCGCCGTACAAGGGGTGGGCGGTGCGCTGCTGTTTCCGGCGACCTTGGCGTTGATCAACACGCACTA
TGCGCAAGGGCCGCAGCGCAACCGGGCCTTTGCGGTGTGGAGTGCGGCGTCGGCCGCCGGGCTGGCGTTGGGTGCG
TTGTTGGGCGGCGTGCTGACCCAATGGTGGGGCTGGGAGGCGGTGTTTCTGGTCAACGTGCCCTTGGCTGGCGGCT
GTGCGCTGGCGGCGCGCTACTGGATCCCCGCCGATGGCGAGCGCAGCCGAGGTCGCAGTTTTGATGTGAGTGGCGC
GTTGACCGTTACCGTCGGCGGCACCCTGCTGGTGTTCGCCCTGGTGCAGGGGCCTGAATGGGGTTGGACGGCACCC
GCGACCCTGGGCTGCATGTTGCTGGCGCTGGCCTTGCTCGGGCTGTTCGCCTGGATCGAGCACCGTGGCCGTGACC
CGTTGATGCCACTGCGCCTGCTGGGTTACCGCGAGTTGCGCATGGCCATGGTGCTGACCGCTGTCTTTATGAGCAG
CTTCGGCGTGCAGTACTACTTCCTCGCGCTGTACTACCAGCAAGTCTACGGCTACAGCGTGCTGCAGGCCGGGCTG
GCGTTCTTGCCGGCGACACTGGTGTGCACCCTGGGCATCTGGCTGGCCGAGCGCGCGCTGGTCAAGATCGGCTTGC
GCAACACACTGGTCAGCGGCCAGTTGGCGGGGGCGCTGGGGATTGCCCTGGTGTGCCTGGCGTTGCCCACAGGGGT
GGGGTTCTGGTCGTTGCTGCCGGGGATCTTTATCTTGAGCCTTGGCCAGGGCATGACCTGGACCGCGATGTGGGTG
GCCGCAGGCCTTGGCGTCAAGCCGGGAGAGCAGGGCGTGGCGGCGGGCATGGCCTCGACCACGCAACAGATCGGCG
GGGCACTGGGGTTGGCGGTGCTGGTGTCGCTGGCCAACGCCGGTGGCGCGACGGGCGCGGCTCAGGCCCAGGGGAT
CGAGTTGGCGTTGTGGTGGAGCGCGGGCATCGCGTTGGTGGGAGCGCTGGTGGCATTGCGACTGAGGTCATCGCGG
CCCGGACCGGTTCTGGAGGCGGCTTAGGGTTTGTGTAGGAGCGAGCTTGCACGCGAAGCGCTTAACTGCACTGCGT
TTATCCTGGATAAACGTGGCGTTCTCAGGTTTTTCGCGAGCAAGCTCGCTCCTACCGTTTAGCGGCCGAGCATCTT
CACCCAACGTGACGGTGGGATACCGAAGGTGCTGCGAAATTGCCGAGTCATGTGGCTCTGGTCGGTGAAGCCGGCG
ATCAGCGCCGCATCCACCAGTGACTGGCCCTGGCCCAGCAGGTTGCGCACCAGATCCAGGCGGCGCATGGTCAGGT
AGCGGTAGGGGCTGGTGCCGAACAACAGGCGAAAATCCCGCGACAAGGCCCAGCGATCCCGCCCGCAGTGCGCGGC
CATCTCATCGAGGGTGATGCTGCGCCCCAAGGCGCTGTGGATAAATTCCCGGGCCCGCTCGGCGGCCTGGTAATCA
AAGGTCTTGCGGTTGGCGCTGATACCCGATGCGTTGTTCAGGGCATGGGCCAGGTCGAACAATGCGTCTTGCTCCT
GCATCGGGTCCAGCGGGCAATCGAGTTGCTGCACAAAGGCCTGGCAGGCTCGGTGCAGCCACGGGTCGTTGGACAG
CCCGTTGTGGATAAAGGGCAGCGGCTTGCCGCCGAGAACCTGCTGGATCAGCGCCGGCTCTACATAAATCATCCGG
TACTTGAAGCCTTCATCGCTGCCGGCCCAGCCATCGTGGACCTCATCAGGATGAATCACCATGGTCGTGCCTGGCA
GGCTGTGGGTCTGGCTGCCGCGATAGTGAAAGCTCTGCACACCAAACAAAGTGTGGCCAATGGCGTAGGTATCGTG
GCGATGAGGGTCGAAGCCAAACCCGGAAAAATACGCTTCGATCCGCTCCAGGCCGCTGCCATGGGGCGCGCGGTGC
AGCCAGTCGATATTCGCGGTGAGTTTGCCCATGGAATGTTTTTCGAAAGATGTCGGATGGAGTTACGTTAACTGAG
TCTGGGGCGGCTGTCTGCCGGGGTCTTGTACGATTGTGCGCGGTCGCAACGCTGGCGAAGCGCTGACGGCCCGTGG
TAATAAGCCTTACTTTCTACAGTGAGGCCGGGTCATGAGTCAGTTGCGGGTAGGGATTATTTTTGGTGGCCGTTCG
GCCGAGCACGAAGTGTCGTTGCAGTCGGCACGCAACATCGTCGACGCCCTCGACCGGACGCGCTTCGAGCCGGTGC
TGATCGGCATCGACAAGGCTGGCCACTGGCACCTCAATGACACCTCGAACTTCCTGCTCAACCAGGAGAACCCTGC
GCTGATCGCCCTCAACCAGTCCAACCGCGAACTGGCGGTAGTGCCAGGCAAGGCCAGCCAGCAACTGGTGGAAACC
TCGGGCCAGGGCCTGCTGGAACACGTCGATGTGATCTTCCCGATTGTCCACGGCACCCTTGGCGAAGACGGTTGCC
TGCAAGGTTTGCTGCGCATGGCAGACCTGCCGTTCGTGGGTTCCGATGTGCTGGGCTCGGCGGTGTGCATGGACAA
GGACATCAGCAAACGCCTGCTGCGCGATGCCGGCATTGCCGTGGCGCCCTTTATCACCCTCAACCGTGGCAACGCG
GCGCGCACAACCTTCGACCAGGCACGGCAAAAGCTCGGTTTGCCGCTGTTCGTCAAGCCGGCCAACCAAGGCTCCT
CCGTTGGCGTGAGCAAAGTCGCTAATGAAGCCGAGTATCTGGCCGCTGTTGAGCTGGCGTTGGGCTTCGATGAAAA
AGTGTTGGTGGAATCCGCCGTCAAAGGCCGGGAGATCGAGTGTGCGGTACTGGGCAATGAGCGTCCCATTGCCAGC
GGTTGTGGCGAGATCGTGGTCCGTGACGGCTTCTATTCCTACGACAGCAAATACATCGACGACCAGGCCGCGCAAG
TGCTGGTGCCGGCCGACATCAGCGTCGAGGCCAGCGAGCGCATCCGGGCCCTGGCCGTCGAGGCGTTTGAAGTGCT
GGGCTGCGCCGGGCTGGCGCGGGTGGATGTGTTCCTGTGTGAAGACGGTGAAGTCCTGATCAACGAAGTCAACTCA
CTGCCCGGTTTCACCCGCATCAGCATGTACCCCAAGTTGTGGCAGGCGGCGGGGATGAGTTACAGCGAACTGGTCA
GCCGCCTGATCGAACTGGCGCTGGAACGGCACACGGCGCGCAAGGGCTTGAAGATCAGCCGCTAAGGGACTGACCA
AGGACTGTGGCGAGGGGGCTCGTCCCCTCGCCACCAGGGCTGGCTCTCAATCCCCCAGCGCCTCACCCTCACGCCG
CGGATCCGCGCCGCCACTCCACGTCGCCTTGCCCTGAGCATCGCGCACTCGCACGATGGCCTGGGTGCCGCTGGTC
ATATCGATCTCGCTCAAGGCGTGCCCACGGTCCTTGAGTGCCGCTTTCAATTGCGCGCTGAACAGCCCTCGTTCCA
GCTCGGTCGCGCCGTTGCGGCTACCGAAGTTGGGCAGGCTGATGGCGGCTTGTGGGTCGAGGTTCCAGTCGAGCAT
GCCCACCAGGGATTTGCTCACGTATTCGATGATCTGCGACCCGCCGGGGGAGCCGACCGTGGCCAGCAACTCGCCG
CTTTGGCGGTCGAAGACCAGGGTCGGCGCCATGGACGAACGCGGGCGTTTGCCCGGCTCGATTCGGTTGGCCACCG

GTTGGCCGTTTTCTTCGGGGATAAAGGAAAAGTCCGTCATCTGATTATTCAGTAGGAAGCCCTGGACCATCACGTG
GGAGCCGAACGCGGCTTCGATGGTGGTGGTCATCGACACCGCGCCGCCCTGGTCATCCACAGCGACCACTTGCGAG
GTGGAAATACGCAGTGGCGAGCGATCTGGCGCGTAAGCCACCTCAACCCCCGCCGGTTGGCCGGGCTTGGCTACGC
CCATGCTGCGCTCGCCAATCAATGCTGCGCGGCTGGCCAGATAGCCGGGGGCGACCAGGCCCGCGACCGGCACGGC
GACGAAGTCTTGATCGGCCACATATTGTGCACGGTCGGCGTAGGCCAGGCGATCGGCCTCGGCAATTATGTGCACG
GCGCTGGGCGCAGGTTCCAGGCCAGCGGCAGTCGCGGTCTTCACGGGTTTGAGCGGCGCCAGGGACCAGCGCGGGT
CGCGGGCTTCGAGGGCCTGCAAGGTGCCGAGGATCTGCGCAATGGCCACCCCACCCGAAGACGGCGGCGGCATGCC
GCATACCTGCCAGCGCTTGTAGTCGGTGCACAGCGGCGCACGCTCTTTGGCGCGGTAGGCCTGGAGATCGTTCAGC
GCAAGGCGGCCAGTGTTGTGATGGCCCTGGACCTTGCGTGCAATCTCCTCGGCGATCGGGCCGTGGTAAAGCGCGT
CGGGCCCTTCCTTGGCGATACGTTTGAATACATTGGCCAGCGCCGGGTTTTTCAACAGGGTGCCAGTGGCCTTTGG
GCTGCCGTCGGCGTTGAGGAAGTACGCCGCCATGTCCGGTGACTGGCTGATGTAGCGGTCGGCGGCGATCAGGCTG
TGCAGGCGCGCAGAAATCGCAAAACCTTGCTCGGAGAGCTGGATTGCCGGCGCAAACAATTGCGCCCAGGGCAGTC
GGCCGCTTTGTTCGTGGGCCATCTTCAGCGCGCGCAGTACACCGGGGGTGGCGACCGAACGGCCCCCGATCTGCGC
CTGGGCAAACGCCATCGGTGTGCCGTCGGCATCGAGCAGCATATCGGGCGTGGCGCTGGCCGGTGCGGTTTCACGG
CCGTCATAGGCCCGTACCTGCTTGCCATCCCACAGCATGATGAATGCGCCACCGCCGATCCCCGAGGACTGCGGCT
CCACCAACGTCAGCACCGCCTGCATGGCGATGGCCGCATCGATGGCCGAGCCACCCTGGCGCAGGATGCTTCGCCC
GGCTTCGGCCGCCAGCGGGTTGGCTGCCGCTGCCATATGGCGCTCGGCGTGGCGTACGTGCATGTCGGTGCGATAG
CCCGAACCCAGCTCCGGGGCGGGCGGCAAATCGGGGCTGCTGGCGTTGTGGCAGGCGCCGAGGCTGAGGGCGGCGG
CGATCACCGAAAGGCTGGTAAGGCGTTGCAAGCGTAGGGTGAAAAGCACGCGCTGAACTCCGTTCATTTTGAGAAT
GATCTGTTGTCCCTGGGGTGATGCTTTGCGGGTAAGTCGCACCGGGCAGGTATCTGCGGGGAAGGAGGGCGGACAC
TAACAGTGGGATGCAGGAGCGTCAAAAGCTGAGCGCGATCAACCTGACGGTGGACTTCTGGTGCTTCAGTCAACCG
GTGAAGGTCAAGCAGGGCAACTGTCGTTTAACTGGCCATTCGGGGGCGGTGAGCAGAGCATTATTTCGACTTTTTA
GTAAATATTTTAAAGTTGCCAAATATCTAGTTATTGGTGGGTTTTCCAGATTTTCGACGGATTTTGCAGAAAATAA
ATCTGCTTGAAATGTATGGAGTTGTATCGGACTTGTCATAAATGCTTTTCTAAATGATAAGGATTTGCATTACACT
CCGCAGCCTTTAGTGCTCCGTCTTGGTGCATGCCGGCTCAACAAGGACTGTTTGCAATGAATAATCCCCCCTATCT
TTTCAGTTCTCTTTTCCTGGCCGTCGGCATGCTGATCGCCGCCCCGGCGTTGCAAGCAGAAGAGATTGGTGAGGTC
GATAAACACGCCGTCACAGAACTCGACACCTCGTTTGTCACCGCCACCGGCGGCGCCACCGACCTGCGCGATGCGC
CGGCCAGCGTCAGCGTCATTACCCGCGAAGAAATCGAGCGCCAGCCGGTGTACGACCTCAACACCCTGCTGCGGCG
TGTGCCAGGTGTCACCGGTGGCTTCGGGCCGGTGGGCGAGCAGTCGAAAATCAAACTGCGCGGCCTGGACGACAAG
TACACCTTGATCCTGGTGGATGGCAAACGCCTCGGCGGCTCGGGGGATTTGAGCTATCGCCGTGACCTGGCGCGCC
AGGACCTGAACTGGATCACCCCGAACATGATCGAGCGCATCGAGATCGTGCGCGGGCCTATGTCTTCGCTGTACGG
TTCGGATGCCATGGGCGGCGTGATCAACATCATCACCCGCAAGGTGTCGCGCACCTGGAGCGGGTCGGCCAACGTC
AACACCACGATTCCCGAAGACTCCGACCGCGGCCAGACTACCCAGACCAGTCTCAACCTGTCCGGGCCTCTGACCG
AGTCGGTGGGCCTGCGCCTGGGTGCCAACCTCACCCGCCGTGCGGCTGATGAGGTCACCGCGCGCCTGGATAAGGA
TGGCGAGTTCATGTACGGCGACGGCGCGGGCGGCGCCAAGGACCACAGCGTCAACGCCTTGCTCGATTGGAAGATC
AATGACGAGCAAAGCCTGTCGTTCGAGGCTGTGCATGGCGTCGAGCGGTCCTGGTCGAGCAAAAAGACTTTTGGTG
ACTGGGGTGAAACCCTGGGTGAGGGCTTCGGTCCCGGCCGTTTGATTCGCGACAGCTATTCCGTGTCCCACACGGG
CGACTGGAGTTTCGGTACGTCGAAAGTGGACCTGTACCTGAACAAGTTCAAGAACGACATCGACTGGGGCAAATCC
AGCGCCGAAGAAAAAATCGCCGAAGGCAGCCTGAACATCCCGTTTGAATTCCTTCTCGATCAGCGTTTGACGGTGG
GCGGGCAGTGGAAGCGTGAAGAGCTGACCAACAGCGACACCCTGGGCACGGTGCCCGTGGACTATCAAGGTGCTGC
AGTCTCCGGTTCTTCGCTCAAGGGCGACTACTCGGCCGCGTTTATCGAAGATGAGTTGTACCTGCTCGACAACCTG
GCGCTGACCCTGGGTAACCGCTTCGACCACAGTCAAGACTATGGCAACCACAACAGCCCGCGGGCGTACCTGGTCT
ACCATCCGCATCCGGACTGGACCGTGCGCGGCGGTGTGTCCAAGGGCTTCCGTGCGCCCAGCCTGAAAGAGGGCAG
CGCCGGTGCCGCCACCGAGTCCGGCGGTCGCGGTTGCGGTTCGTTGAAACCCCTGGGTTACGTGAGCGGCAGTTGC
TGGATGGCCGGTAACCCGAACCTGTCGCCAGAAACCAGCGTCAACAAGGAGATTGGCGTGTCCTTCGACCGTGACG
GTTGGGAGGCCAGCCTCACCTACTTCCACACCGATTTCGAAGACAAGATCGAATACGCGCCACTGGGCCAGTTCCA
GGGCCGCTGGTGGACCATGCTGGAAAACGTCGAAGAAGCCCGGACCCGTGGCTGGGAAGGCTCGTTTCGCGTCCCG
CTGACCGACTCCCTGGACTGGCGCACCAACGCCACCTACATGCTGGAGAGCAAAAACCTGTCCACCGGCCAGGACC
TGATCAGCGCGCCCAAGTTGTCTGCCTTCAGTGCGCTCAACTGGCAAGTCACCGACAAGCTCAACACCGAACTGTC
GGCGCAGCATGTCGGCAAACAGCGGGGTATTGGCAATGACTTCGTAAAGTCGTACACCACCTACGACCTGACGGCG
AACCTGGCTGTGACCAAGTGGTTGACGTTGAATGGCGGCGTGCAGAACTTGCTGGATGAAGATGCGCGGGATGGTT
CGACGACGTTCTATGTGCCGGGCCGTGCGTTCTTTGCCGGGGCGACCACTTACTTTTGAGGGGTAGCCAAGCCAGG
GCTCGCGAGCGTCAGTTGTTCGCGGGCTTGTTCTTGAAGATTCGCGCAGGCATAAAAAAACGGCCTACCTTTCGGT
AAGCCGTTTTTTTACGCCTGATGGTGCGCAGTCCTCGGTGTTCGGCTGAAGCGGGTGCGGGTAGCTGTGCGCCGCG
CGCATCTCCATGGCCCTCTGTTCAGTCAGTCCATGGTCGAGCCAATCTGCCGGTCAGCAGCTTCTGTTCTGCGCTC
GGCCAGGCAGATAAGCAGAGACAGCAGCAAGGCAAGAGTGGCGAACCCACCGGCCATCCAGAGATTAGCTGTCAGG
CCAACCTCATCTAAAACCAGGCCGCCAGCCCATGCGCCTAATGCAATGGCGACATTGAAAACGCCTACATACAGCG

CCGTCGCAATTTCCATGGCGCGCGGTGCGGCTTTCATCATCCACGTCATTAGGCCGACCGACACACCACCGTAGGC
TAGCCCCCAAACCAGCAGCACGATGCCGCCGAGGTTGGAGTCGCCGACGATGAGGAACAGCGCTGGAGTAATTAGC
AGGCCTGATGAGATAGTCATCAAGGTTTGCGCTGTGCGCTGCGCTGCGATGACCCCCGCAAGGAAGTTGCCGACAA
TGCCCGCGAAGCCATAGGCGAACAGCAGTGCGCCAACCCATTGGGTATCGAACCCCGAAACCGACACCAGTAACGG
ACGCACGAAGGTAAAAACCATGAAGTGCCCGGCAACCAGTGCCAGTGTCAGAAGCAGCCCGACTTGAAGCTTCCGG
TTGGCTAGCTGTTCGCCAAACTGGCGCAAGGTCACTGAGCCAGCAACAGGTAGTCCGGGGATAACAGCAAGATGCA
ACGCCAATACCAATCCGCAGAATAGCGCCATGCCGCCAAAGGCCCAGCGCCACCCAGCAAAGTCACCGATCAACGC
GCCCAGTGGAACCCCCAGCACTGAAGCCGCTGCCACTCCTCCGAAGATGATGGAGGTAGCCAGGCCTACAGAGTGG
TCAGGAACAAGCCGACAAGCCAGCCCCCCGGCGATAGCCCATATACCCCCCATGCAAAATCCGACCAGGACACGGG
CGGCCAGCATCCAAGCCATGCTGGGTGCCAATGCCGACGCAATGTTGGCAACCACCAACAGCCCTAGCAACCCGCA
TAGAATTTTGCGCCGGTCTATGCCGCCAGATGCGATCACTACCAGCGGTGCGAACAACGCCGCCAGCAACGCAGGC
AGAGAGATCATCAGTCCCGCTGTTCCTGTGGAGGTGTCCAGCGTTTCGGCAATCGGTGTCAGCAATCCGACAGGGA
GCATTTCAGTGGTCACAACGGAAAAGGTGGCAAGCCCCACTGCAGTCACTGCCATCCATGGATGCTGAGCAGAGGT
TGCTGCGGGGTTGGGTGAAAACGTGCTCATGGTTCAGATCCTTGGAATAGAGGAGCAAGCAATGCCGTCGGTAAGT
GTCATTGCGGTGAGGAGCGGTTGTTGGTTTTCCTCGGTGCGACTGCGACGCATGGCGGTAAAATATGTGAAGATAC
ATACGCAATGTATGAATAGGACTGTATATTCATACGCAGAGAATGTAAATAATCATACGGATAGTATGTAAGAGGG
GTGGTAATGGCTCGTCGCACGCGTGCTGAAATGGAAGAAACTCGAGCCACATTGCTGGTAACTGCCCGTAAGGTCT
TTAGTGATCGCGGCTACGCCGATACGTCTATGGACGACCTCACCGCTCAAGCGAACCTGACACGGGGTGCGCTCTA
TCACCACTTCGGCGACAAGAAAGGGTTGCTGGCAGCGGCGGTGGAGCAAATCGATGCTGAGATGGACCAACGCCTG
CAAGCCATCTCCAACGCCGCCGATGATGAGTGGGAAGGTTTCCGCAGCCGCTGTCGGGCTTATCTGGAGATGGCGC
TTGAGCCGGAAATCCAGCGCATCGTGCTGCGCGACGCCAGGGCGGTGCTAGGGGGGGCGTCGCCTGACTCACAGCG
TCATTGTGTTGAATCTAAGCGGCGCCTGATCGACAGCCTCATGCAGAGCGGCATCGTTGCTCCGGCCGATCCGCAG
GCCTTGGCATCGATGATCTACGGCAGTCTGGCGGAAGCCGCGTTCTGGATCGCCGAGGGTGAAGATGGCAACACTC
GACTGGCCCAGGGCGTCGTAGCGCTGGAGTTGCTTCTGCGTGGGATGCTGATTAAACAGTAAGGGCAACGGCGAGA
GGGAGGCGACGTTTTTTCGAGCCCGGCCAGGTTGCCAGTGGAAATCACGGCGCATTCTTTAGGCTCTTGCTGGTTC
AGGGAATGCGCGGAGAAGGTACAGGGGGATGCAGGGAGGGATAGCCAGAAACAAGAAAGCCCGCACGAGGCGGGCT
TCTTGAGGTGATTCATAGACTGCTGTAGACATCTATGAATCGGTACTTGGTGGCTACACAGGGACTTGAACCCCGG
ACCCCAGCATTATGAATGCTATGCTCTAACCAACTGAGCTATGTAGCCAAGTGGCGCGCATTATTCGCTCAGAACG
AAGAAGCGTCAAGCGCTTTTTTTGAAAATTTATCTACGCTATCAACTGTTTAAGCAAAAACGCCGGGTAAGGCGGC
CATTGGCTGGGTTTTGCGCAGGTCCCAAAGCCAAAGATTAAAGTGTGGGAGCGGGCAAACCCGCTCCCACATTTGA
TCTGGGGTGTTCTACCGTCGGCGTACTAGCTTGCTGGCCCTTTACAAGACATGACCGCTGCATCGATTCACCTACT
CGTTAGCCTGCTAACGGGATAGCCTTCCTGCCCCAATAAGTGGCACATTGTCACACCTGCACCTGTGCATCCATCT
GCTGTACGGAAACGTTCCTCATGGCTCTTACCAATTCCCAGCCTTCAGTGGCTACGGCGCCGGCCGCCGGGCAATC
CAGCCCGTTGGTCATGTCTATCATCGGCGCCGTAGCGCTGGCGCACTTGATCAATGACCTGATTCAGGCGGTGTTG
CCGTCGATCTATCCGATGCTCAAGGAAAGTTATGGCCTGACCTTTACCCAGGTCGGCCTGATTACCCTGACGTTCC
AACTCACGGCGTCCTTGCTCCAGCCGTGGGTGGGTTACTACACCGATCGCCATCCCAAGCCGTTCCTGCTGCCGTG
CGGGATGATCTGCACGCTGATCGGGATTCTGATGATGTCCCAGGTCGGCAGTTTTCCACTGATCCTGCTGGCGGCG
GCCTTGATCGGTATCGGTTCCTCGACCTTCCACCCGGAAGCATCCCGCGTGGCGCGCCTGGCATCGGGTGGCCGCT
ACGGCCTGGCGCAGTCGACCTTCCAGGTGGGCGGTAACGCCGGCTCGGCCTTTGGCCCGTTGCTGGCGGCGGCGAT
CATCATTCCGTTTGGCCAGGGCAATGTGGCCTGGTTCGGTTTGTTCGCGGTGTTCGCCCTGGGGGTGCTGTACGCG
ATCAGCCGCTGGTACCGCAACCACTTGAACCTGTTCAAGCTCAAGCAAGGCCAGGCCGCTACCCATGGCCTGTCGA
AAAACCGCGTACTGGGTGCGCTGGTGGTGCTGGGGTTGTTGGTGTTTTCCAAGTACTTCTACATGGCCAGCTTCAC
CAGCTACTTCACCTTTTACCTGATCGAGAAGTTCGACCTGTCGGTGGCCAGCTCCCAACTGCATCTGTTCCTGTTC
CTTGGGGCCGTGGCGGCGGGCACGTTCTTTGGTGGGCCGATTGGCGACAAAATCGGCCGTAAGGCGGTGATCTGGT
TCTCGATCCTGGGCGTGGCCCCGTTCACCCTGTTGCTGCCCCATGTCGATCTGTTCTGGACCAGCGTGCTCAGTGT
GGTGATCGGTTTTATCCTGGCCTCGGCGTTCTCGGCGATTGTGGTGTACGCGCAGGAATTGGTACCGGGCAATGTG
GGGATGATCGCCGGGGTATTCTTTGGCCTGATGTTCGGGTTTGGCGGGATTGGTGCGGCGTTGCTGGGCTATCTGG
CGGACATCCACGGCATCGAATACGTGTACACGCTGTGCTCGTTCCTGCCGCTGCTCGGGGTGCTGGCGATTTTGTT
GCCACGTTCGAAAAAAGCCTGAAACCGGAAGGTTGGCCCTATCCCTGGGCGCTCCCTCTCCTACTGGAACGGGTGA
GAGTTTGTCGCGCGTTTCAGTTGTTCCCGTGCCCGGGACAGGCGGGAACGGACGGTACCGATCGGAATCGCCAGGG
CATCGGCGGTGTCCTGGTAGCTGCCGTCGGTTTCCAGTGAGGCATACAAGGTGTTGCGCATTTCCAGGGGCAGGTG
CTGGATCGCATTGAGGGTGTTTTCCAGCCGGCGGCTGATCTCGAATTGGCGCGCCAGGTCCTGCTCTTCGTTGTGT
CCGTGGGACAACGATTCATCGAACTCGCAATGCACTGGCTTGGCATAGAGCCGGCGAAAGTGATTGCGGATCAAAT
TCAGCGCGATCCCGCACATCCAGGTGCTCAGGGACGCCTGCGCGCTGAAGTGATGGCGGTTACGCCATGCCTCCAG
ATACGTCAGTTGCAGGATATCGTCCGCATCCTCCGGGTTCAGTACGCGTTTATGAATGAACCGGCGCAGGCGTTTG
TGGTGCTCGTCGGACAGATCTTGGATGGATTGGCTAGGGTCGCGGGGAAGATTGACTAAAACACTGATTGGGATAT
CCATGATTTTTCCTCAGGGTAAAAACGGTCGAAATGGTTTCGACGAGAACCCTGTTAGCACCGCTTGTGCCAAACG

GAAAAGATCGATATCTATTTGATAAATATAAGGAAATATCTTGTTTTAGGTGTTTTTTGTGCAATCGCTTGCGCAG
AGCATCGTGGATTTGTGCGGGTATTTTCATGCGCCATGGGAGGCGCATGAAGATGGAACCGGATCGGGCCTTCCAG
CCACACAAGGATACGAATCTCTTGCTATGGCATTTCCATGAAAGTCGATCCCAACCTAGATGTGCAAAATTCCGCG
CACCTGAATCAACCGGCACCGGCACGGATTGAAAGTGGGCTGTCTTCGCCGATAAGCGCCCCCGTTGCGGACGATC
TCAGTGTGCTGTTCAGCCAAGAGGTCGAACTCAACGCCCGGGCGTTGGGGCAGCGCCAACTAGGCTTGCGCATCCC
GGCGGTCGAACAACTGACCCAGCTCTATGAGCAATTGGGTCACCCGGCCCAGGCGAAAATGGCGACCATCGCCCGC
AATGTCAGGGTCCAACTGCTGCTCGGTGCCGGGGTCGGCAAGATGATGGACATCACCGGCAACGACCCCGCGCGGG
CCTATGTGGTGCTCAAGCACGTGGCGCTGCAGGCGGATGCCGAGGGCCGCAGCGCAGAGGCAAGCCTGGCGCGCAC
TTCCCTGGCTGGCCTGGAGGCCGATTACAAGCCGCAGATCCAGGCGGGGCTGAATACCGCGTTGGCGCTGCAGGCG
GGCACTGATGACCCGCAGCTGCGCCAGGCTGTACGCAGCATGTATTACACCAGCGTGGTCACGAGCCAATCGCTGG
CGACCCTGATGCAATCATTGCTGGGGCTGTTCGGTGAAGGGGACTTTGCGTCCGGGTTGAAGCTGATACGCCGGGC
CCTGGCCGATGATGTTGCGGCACAGACCTCGTCGGTCGACCGGGGCCAGTTGCGGACCTTGTTGCTGGGTCTCAAT
GACTGCAGTCGATTGAGTGGTGTGCTCGCGCGCTGCAAGGCGTTGATCGGGCAATCCCTGCTTCTGTACCCCGAAC
TGCAGCTGGATCCGGTGGGCTTGTTGCAACGCTTGCTGGGGTATGCGGCCGGTGGTCTTTCCTCCCAGGAAATCAA
ACATCTGGCCCGGGATTTGGGCGGCAGTCAGCTGGCTCATCAGTTGAGCGGCCTCAGTGCCTTTTTCCCGGAACTC
AAGGCCCTGCCCCTGGCCTGGTGGTGTGATGCGAGACGCCGCGAAGAAGCGTTGAACAGCTACAAGCACGTGCTGC
TTGAATACGCCGCGCAGGAGCGTGGCTCGCGCCATACCCACACGTGGCCGGGATTGAAGGCATGAGTGCGTTGCGG
GCAATCAATGCGTTGCTGCTAAAGGTTGCACAACGCGCCGAGGTGCTGGGCGCGGTGGTGGTCATGGCTATCGTGT
TTATCTTTATCGTGCCGTTGCCCACCTGGCTGGTGGATATCCTGATTGCCCTCAATATTTGTATTTCGTGCCTGTT
GATCGTGCTGGCGTTGTATTTGCCGGGGCCGCTGGCCTTTTCTTCGTTTCCATCGATTCTGCTGTTGACCACGATG
TTTCGCCTGGCGTTGTCGATTGCGACCACACGCCTGATCCTGCTGGAGCAGGACGCCGGTGACATTGTCGAGGCGT
TCGGCAACTTTGTGGTGGGCGGCAATCTGGCGGTGGGGTTGGTGATCTTCATGATCCTGACCCTGGTCAACTTCCT
GGTCATTACCAAGGGCTCGGAACGGGTCGCGGAAGTGGCGGCACGCTTTAGCCTGGACGCGATGCCCGGCAAGCAA
ATGTCCATCGACAGCGACCTGCGTGCCGGGCTGATCGACGGCAACCAGGCCCGGGACAAGCGTGAGCAGCTGTCGC
GCGAAAGCCAGCTGTTCGGGGCCATGGACGGCGCGATGAAGTTCGTCAAGGGAGACGCCATCGCGGGGTTGATCAT
TGTGATGATCAACTTGCTGGGTGGTTTTGCCACCGGCATGTTCCAGCATGGCATGAGTGCCGGCGACTCCATGGCG
TTGTACTCGGTATTGACCATTGGTGATGGCCTGATCGCGCAGATTCCGGCCCTGCTGATCTCGCTGACTGCCGGCA
TGATTATCACCCGTGTGGCCCCGTCCGGGCGCAGCAAGGGCAGCAACAACATGGGCGCCGAAATTGCCCGGCAAAT
GACCAGCGAACCCAAGAGCTGGATGATCGCTTCGGTCGGCATGCTTGCTTTTGCGGCGTTGCCGGGGATGCCCACG
GTGGTGTTTATCCTGATTTCGCTGATGACTGGCAGCCTGGGTTATTACCTGATGCGCCAGCGCCAGCGTGACGAAA
AATCACAGGAGCCGGTTGCCGATACCGTCCCGCCTGAGCAAAACGGGGTGGAGGATCTGCGCGGTTTTGATCCGTC
GCGCCCCTACCTGTTGCAGTTTCCGCTGGCCCTGCAGAACAGCCCGCTGGTGACGGAGGTCATCCACGGCGTGCGC
CAGAGCCGCAACAGCCTGGTGACCCAGATTGGCTTGACGTTACCGCCATTCGAAGTCGAGTTTGCGCCGGCGTTGG
CCGACGATGAATTTCGCTTCTGCGTGCACGAAGTGCCCCTGTTGAAGGCCACGCTCGGTGACTGGGTTGGCGTGGA
GCTTGCGGCACTGGCAGCGGATCCAGAAAGCGGGAGCAGGGGCCTGGCCGAGCGCGATGAACAGGACTGGGTGTGG
TTTGCGCCCGACGACCCTGTGCTTGCCGATGAGCACTTGGCGCGCTCGACGGCGTACAGCCTGCTTCAGGAGCGGA
TGACCCGGGCCATGTTGCTCAGCGGCCCGCAGTTCCTCGGTATCCAGGAGAGCAAGGCGATTCTCAGTTGGCTGGA
GTTCAATCAGCCGGAGTTGGTGCAGGAACTGCAGCGGATCATGCCGCTTTCGCGGTTCTCATCGGTGTTGCAGCGC
CTGGCCGGCGAAGGGGTACCGCTGCGGGCGGTGCGGCTGATTGTCGAGGCCCTGATCGAACACGGTCAGCATGAAC
GTGAACCGGAAGCCCTGACCGACTATGCGCGCATTGCCCTCAAGTCGCAGATTCTTCACCAGTACAGCGAAGCCGA
CGGCCTGCATGCCTGGCTGTTGTCGCCCCATACCGAAAACGTGTTGCGCGATGCCCTGCGCCAGACCCAGACGGGC
GTGTTCTTTTCCCTGGATAACGAAAGCAGTGCGGCGCTGGTCAGCCTGCTCAAGCAAGCCTTTGTCGTGCGCCCCA
AGAGCAAAAGTGTGCTGTTGGTTGCACAGGATTTGCGCAGTCCCATGCGCACCTTGCTGTTGGAAGAGTTCAACCA
TGTACCGGTCCTGTCGTTCGCCGAGCTCAGCGCTTCGTCCAAGGTCAAGGTGTTGGGGCGCTTTGATCTTGCCCAG
GACAATCTGTTGCGCGAGGAGGTGGCATGAGCACGTCTGGGGTGGTTGTGCAGGCTGTTGAACACGTCACTGTCAT
TGCAACGTTCATGAGGCCTGGCGATGTTTGAGCTACGTGTGCTTGATGGTTCGCGCCAGGGCGCGGCGTTGCCGTT
GTTCGGCGGGCAATGGAGTATTGGTGCGCATCCGGATGCCGACCTTTCGCTGTATGACGAAGGGATTGCCGAGCGC
CATGTGCTGCTACGGTGTATTGAGCAGCACTGGTCGGTCCAGGCTCAAGAAGGGTTGGTGCGGGCCAGTGACGGTC
AGCTCCTGGCACAGATTGCCGACCTGGCACTGGGCGTTCCTTTTGCCCTGGGCAATGTTCGACTGTGCGTCACCCT
GGCCGATCAGCCCTGGCCGCAAGCCCCGGCTGCCATGCCCATCGCGCCAGCGGCCAGGGCGGTGAATGAGGCTGCA
CCGGGGCTGATGTTGTCGTCGATATCCGGGGCCCAGCAGAAGCGTCTGATCAGCCTGGTGGTGGTGATCGCGGTCA
TCATCATGGTAGTGGGCCTGGCGACCACCGGAGAGCATGAGGCCCAGGCGTCGTTGATGGCCGCGCCAACTCAGAA
AAACGAGCTGGGTTCGGCCTACGAAGTACGCCAGCAACTGCTGAAAATGCTCAACGAACGTGAACTGGGCAATCGC
ATCCGCCTGCAGGTGATCAATGACCAGGTGACATTGGAAGGGGACGTTGCCCAGGAAGAGATGGGATTGGTGTCGC
GCATGCTCAATCGTTTTGGGGAGCAGTTCGATACGTCTGTGCCAGTGATCAGCCGGGTCAAGGAACGTAGCACGGC
ACTCCCTTTCAAGATTCTGCAGATCGTCGGCGGGCCCAATGGCCATGTGGTCCTGGATGAAGGCAATCGGCTGTTT
GTGGGAGATGAAGTGGATGGCTTGCGATTGGTCCTGATCGATAACACCAAGGTGGTGTTCGACGGCGTACAGCGTT

ATGAGGTGCGTTGGTGAGTGCCGACCTGCAGGCGCGCCTGGAGGCCTGGCAGCAGGACCGGGCGCAGGCGCTGGCC
AGCGCGTCAACGGCATGTTGCTGCAATGCCGTTTGCCCCAGGCGCGTATCGGTGATTTGTGCCAGGTTGAAAAGTC
CACCGGCGATTACATGCTGGCCGAGATCATCGGTTTCGATCAGCAGGATGCGGTGCTCAGTGCCCTGGGCAATCTT
GAAGGCGTGCGGGTGGGCGCCAGTGTCGAACAGTTGGGCGTCTCGCACCGGGTTCGTGTCAGTGATGAACTGTTGG
GCCAGGTGCTGGATGGCTTTGGGCGCCCGATTGCGGGGGAGGGCGCCAGCGCCTTCGTCGAGGCGCACGCCGAGGA
CACCAGCCTGGTGTTGTGCGAGGCGCCCTTGCCCACCGAGCGGCCGCGTATCCACCGGGCACTGGCCACCGGGGTG
CGCTCGATCGATGGCCTGTTGACCCTGGGCGAAGGCCAGCGCGTTGGTTTGTTTGCGGGCGCCGGCTGTGGCAAGA
CCACCTTGCTCGCGGAGATCGCGCGTAACGTTGACTGTGATGTGATTGTGTTCGGCCTGATTGGTGAGCGTGGCCG
CGAGCTGCGCGAGTTTCTCGACCACGAACTGGACGATCAACTGCGCGCCAAGGCGGTGCTGGTGTGCGCGACGTCC
GACCGCTCAAGCATGGAGCGCGCCCGCGCCGCCTTCACCGCCACGGCATTGGCTGAAGGCTATCGACGCCAGGGCA
AACGGGTCCTGCTGCTGATCGACTCCCTGACCCGTTTTGCCCGGGCCCAGCGTGAAATCGGCCTGGCCGCTGGCGA
GCCGCTGGGGCGTGGCGGTCTGCCGCCTTCGGTATACAGCTTGCTGCCACGTCTGGTGGAACGTGCCGGGTTGACC
CGCGAAGGGGTGATCACGGCGATCTACACCGTACTTATCGAGCAGGACTCCATGAGCGATCCGGTGGCCGATGAAG
TGCGCTCGCTGCTTGATGGCCATATCGTCTTGTCGCGCAAACTGGCCGAGCGCGGCCACTACCCGGCGGTGGATGT
GCTGGCCAGCCTGTCGCGGATTCTGAGCAACGTCGCCGAGCCCGCGCATATCCAGGCCGGCACCGCGCTGCGCCGC
TTGTTATCGGCGTATCAGCAGATTGAGTTGATGCTCAAGTTGGGCGAGTACCAACCGGGCAGCGATGCCCTGACTG
ATATGGCGGTGGACAGTCGCCAGGCGGTGGACGGCTTTCTGCGCCAGGACTTGCGCGAACCCGCGCCGATGCCGTT
GACACTCGAGCAACTGATGAGCCTGACCACCCATGTCCCTTTCTGAAATCGACACCTTGCGGCGGCTGCGTCGGCA
TCGGGCAGACCGCGCCGAGCGCGCGTTGCGTGAAGCCAAGCGGTATCAGCAAGCCTTGTTGGTGCAAATCCAGCAG
GCACAGGCGGCGCTGGAGCAAACGCGGCTGCAGGAGATCGAGAAAACCGCCGAGTTGCTGGATAAGCATCAAGGCC
AGGTGCTCTCGTTGCAGCAACTCAAGGCCTGGAGCCTCCAGGAGCGCACCTTGTCCGCAGGGACCCGGCGCGAAGA
AGGCCAGTTGGGTCAATTGCATGACCAGCGGGAGGAGCACGTGGTGCAGATCGCCAGCGCGCAGAAACAGGTGAGC
CAATGCCTGCGAGAGGTCGAGAAACTCCAGGAGTTGTCGGTTTTATTGAGGCAGGAAGAGGTACAGGAAGAACTAT
GACCCAAGTCCAAGCAAGCAAACCTGAACGCCCGCGCCCGCGCGATCCACGAGCGGAACAGGAGCCTCAACCGTCG
GGGGTCGTACCTTGGGAGCAGGGGCGGTTGTTTGCGCAACTGCTGGACAGTGATGGCGAGGGGGCAGGCTACGGCA
CCTCGGCAACGGCCGCCGGGGTGGCGGGCGATACCTTGATGATTGAGGCGATGACGGCGCAATTGGCCCCCCGGAT
TCATGGCAGCGCGCAATGGCCGCTGCAGGCGGTGCTTTACTTGCCGCGCCTGGGGCGGATCAGCGCCAGTGTGCGC
CGTGAACAGGGGCTGTGGAACATCGAGCTGGCGGCCGAGCAGGAGCTTGCCGCGCGTTGGCTGGGCGGTGTGCGCG
AGCGCTGTGAGGAGCGTCTGGCCGATGCCCTGGGCGCGCCTGTCGCGTTACACCTGGTCCATGTGGGCGCGGCATG
ATCATGCCGGCACTCGCGATTCCCTTGGTCAAAAGCGCCACGGTTGCGGCACGCCTGCGCCTGGGGCGGGGCCTGC
GTATGCCGTTTCAGGTCACAGGCCAGCACGGTGAGCTGCTGCTTGAGCCTGGTCGTGCGCCCAGGAGTGCCGCGCC
TCTGTATTTCGAAACCGCCTGTGGGCTGTTGGCCTTCAGCGAGCCAGGGCCGATGTTCAGTCTTCTGGGGGAGTGC
CCGGTGACGCTGGCCGAGGCCGGCAATGATCCGGACTCCTGGTTCTGGGAACTGTTCCAGCACCACTTGAGCCCTC
AGGTGCGCGCGTTGTTTGGTTATCTGCGGCTGCGGGCCTCCCCTGGAAAACTGAATTTCGGCTGCCGGCTCACGGT
GACATTGGGTGCCTCCAGGGTGGTGGGCTACCTGTTGCTGGCTCCCGAAAGTCTGTTGGCATTGTGTGATGCGGGG
CGCTGGCAACCCACGGCCCTGCCATTGCCGCCCTCGTTTCAACTGACGATTGCCGCGACGTTGGGGCGTTTGAAGC
TGCCCCTGAGCCAAGTTCGCGGCCTGCGTGCCGGTGATGTGGTGATGCTGGAGCAGGCTTTTTTTGATGTGCATGG
CAATGGTCATTTACGGATTGGCAAGCATCGCCTGCATGGGCGCATTGACGATGAATCAGGGCCGATGGGCCTGACA
CTTATATCTACTGAGGAAACGTCTGTGGACGAGGATCTTTCAGCACAACACTACTCGGGGTATGAAGAGCATGACC
TTGATGAACCCGTGGTGGATGTTTTTGGCCATGAGCCTTTCGATGAGTTGAGCATGGCCTTGAATGTGCGCTGTGG
CACCCTGAACCTGACGCTCGGGGAACTGCGCAACCTTGCGCCCGGTGCGGTGTTGAGCATCGCGGGCTACGCCCCC
GGCATGGCGGGGCTCTACTATGGCGATCGGCCGATTGGCCAGGGCCAGTTGGTGGAGATGGACGGACGCCTGGGCT
TGCAGCTGTCCCGTGTGATTTTTGCGCGATGACGTTCCAGGGGGTCGAGCCCGTAATCCTCGCGTTATTCATCGGC
GCACTGGCCTTGATGCCGATGCTGTTGATCGTCTGCACCGCCTTTCTGAAAATTATCATTGTGCTGATGATCACCC
GTAACGCCATCGGCGTGCAGCAGGTGCCGCCGAGCATGGCGCTCAACGGCATCGCCCTGGCGGCGACTCTGTTTAT
CATGGCGCCGGTAGGGTATGCCATCGCCCAGAACGTCAAGCAAGCCCCGGTGGACTTGAGCAGCGTGCAACGCTTC
CAGGAAACGGGGCTGGTGGCCATCGAGCCGCTGCGCCTGTTCATGACCCGCAATACCGACCCGGACGTCCTCACGC
ACCTGCTGGAAAACACCGCACGCATGTGGCCGCCGGAAATGGCGCAGAACGCCCAGCGCGACGACCTCATCTTGCT
GATTCCGGCCTATGTGATTTCACAATTGCAGGCGGGGTTCGAAATCGGTTTCCTGATCTATATCCCCTTTATCGTC
ATCGACCTGATTGTCTCCAACCTGCTGCTGGCCCTGGGCATGCAGATGGTCTCGCCGATGACCATCTCCCTGCCCC
TCAAGTTGCTGCTGTTCGTCTTGGTTTCCGGTTGGTCGCGGCTGCTCGACAGTCTGTTCCTTTCTTATCTGTGAGT
GACCTATGGAACCGATCGTACTGTTCAAGCAAGGCATGCTGTTGGTGGTGGTGCTCTCGGCGCCGCCGCTGATTGT
CGCGGTGATCGTCGGGGTGCTGACCTCCCTGGTGCAGGCCCTGATGCAGGTACAGGACCAGACATTGCCCTTTGGC
ATCAAGCTGGTGGCCGTCGGCATCACCCTGATCCTGACCGGGCGCTGGATTGGCGTGGAGTTGATTCAACTGATCA
ACATGACATTCGACATGATTGCCCGCTCGGCACTGAACTGAGGCAGACGCATTGCTCCTTTACCTTGAGTTCTTGC
CCAGCCTGTTGATCAGCATGGCGCGCATCTACCCGTGCATGTTCCTGGTGCCGGCCTTCTGTTTCCAGCATGTGCG
CGGGATGACCCGGCACACGATCGTGATGGTCGTGGCCCTGTTTCCCACACCCGGCATTCATGCCGCATTGATGGGC

AAGGACTACTCCGCATTGATGCTGGGCGGGCTGGTGCTCAAGGAGGCGGCCCTGGGGTTCTTGCTCGGGGTTTTGC
TGTTCATGCCGTTCTGGATGTTCGAGTCGGTAGGCGCGTTGCTCGATAACCAGCGTGGCGCCCTGGCGGGCGGGCA
ACTCAACCCTTCGCTGGGGCCGGATGCGACACCGGTCGGGCACCTGTTCAAGCAGTTGGCGATTTTTCTGCTGATC
TCGACCCTTGGGTTGGGCGTGCTGACGCAAGTGATCTGGGACAGCTACCTGATCTGGCCACCCACGGCGTGGGTGC
CGCTGCCGGCGGTCAATGGCTTCAGCGAGTTCCTTGGCCTGCTCGGCGATACCTTCGTGCACATGATGCTCTACGC
GGCGCCGTTTATTGCGGTGCTGTTGCTGCTGGAGTTCGGTTTCGCCTTGCTCGGGCTCTACAGTCCGCAGCTACAG
GTGTCCACCCTGGCCATGCCGGCCAAGAGCCTGGCGGGCCTGGCTTTCCTGTTGCTGTACTTTGCCCTGTTGGAGG
ATTTGATCGTCGGGCGCATGGGCTTGCTGACGGACCTCAAGCATGCTCTTGGCCTGATGGTTCGGGGCCCGGGGCA
ATGAGTGACTCCGGCGAAAAAAAACACGCGGCGAGCCCCAAGAAACTCAAGGACCAACGCAAGAAAGGCCAGGTTG
CCCAAAGCCAGGACGTGGCCAAACTGTTGGTGCTCACCGCGATCAGCGAGATCGCTCTGTTCACCGCCGAAACCAG
CCTGCAGCGCTTCCAGCAGATGATGGCATTGCCTTTTGCGCGCGTCGGCCAGCCGTTTGTGCGCGCCCTGGAAGAA
GTGCTGATGGATGGCCTGGTGGTGTTTTTTTCGTTCGGCCTGTTGATGTGCGGGGTGGCGATCGCGGTGAAGCTGA
TAAGCAGCTGGATGCAGTTCGGCTTGTTGTTCGCCCCGGAAAGCCTGAAGCCGGACTTCAATCGCCTCAACCCGCT
CAGCCAGCTCAAGCAAATGTTCTCTGCGCAGTCGGTGATCAATCTCTTGATGGGACTGGGCAAGGCGTTGTTGCTC
TCGCTGATCCTCTATGTAGTGATAACCCCCTCGCTACAGGTGTTGATCGACTTGGCCACCAGCGATCTGCAAAGCT
ACATCGTGGCGCTGATCGCCCTGTTCCGCCACTTGCTGCATGCCTGCCTGGGGTTGCTGCTGGTGCTCGCGCTGAT
CGACTGGAGCATGCAGAAGTATTTCTTTGCCAAGCGCATGCGCATGACCCAGGTGGAGGTGGTCAAGGAGTACAAG
GACATGGAGGGCGACCCTCACGTCAAGGGCCAGCGTCGCTCCCTGGCCTACCAACTCGCCCAGGAAGAGCCAAAGG
TCAAGCTGCCCAAGCTGGAAGAGTCCGACATGCTGGTGGTCAACCCGACGCACTTTGCCGTGGCACTGTACTACCG
CCCCGGCAAAACGCCGCTGCCGATGTTGGTCGACAAAGGCACAGACGCCCAGGCGCGCCAGTTGATTGGCCGGGCC
AAGGCCGCCGACGTGCCGGTGATCCAGTGTGTGTGGCTGGCGCGCATGCTGTATGAGCGAAAGCTGGGGGCGCCGA
TTCCACGGGAGAGTTTGCAGGCGGTGGCGCTGATCTATCGCACCCTGCGTGAGCTGGATGATGAATCCAAGCGCCA
GACCCTTGAGTTACCGGAGCTTGCGCGCCGCTAGGGCGCGCGTTGTACATCCAGGGCTTCGAGCTTGGCCAGTGAG
AGCAGGCGGCTGACGATGTCATCGAGCGGCTGGCCGTTGGGCACCAGCGCATGCCAGATCACCAGGTTGCCCTTGG
CATCGAGGTACACGAAGCATCTATCGAACACCATGGCGTGGACAAAGCGCCGCTCCAGCACCCGCTGCACCTGGCC
GGCCTGCAGCGCCTCAGGTGCAATCTTCAGGGCCAGGCCGGGCTTCTGGCCGGCGTTCAACCGGCACAGGCTGATG
CCCGGGCACAACGTGTGGTGAGCGACGTCGCCGCTCACCAGTTTGTCCAGAGGCACCTGGCGAGGCGCATCAGTGT
CGGGTAAGCCCATGGCGGTCACTGTTCCAACGAGATCGTCTGATAGTCCGCCCTTTCGCCTGATGGGCCGGGTTCG
ACGCGCATTTGCGGCGGCCACCAGACCACCAGCTTGCCAGCGCTCGATTGCGGCCGGGAGCAGGAGTTGCCTTTGC
ACGTCGGGGTACAGCCGGCCATAAGCAGCACTAGGCCCAGGAGGGTGATGCAGGAGAGTCGCTGTTTCATGGCGCG
GTCTTCCGTGTTGGCGTGAGCAGGGAGGGGCGGGTTACACCGATATGTTCATCCTTCACCACCGGGCGCATGGCGA
TGAACACCTCCGCCTCTTCACCCGGCTTGAGCCAGGCCCGTGGCCAGACGCTGACGGCCAGGGTCTGGGAATTGCT
GCATTCGTTCTCGTCGATGCGCACGTTGCGATTGAACTGATTGCGCATCACCACCACCGCCACGTTGAACTGCGGG
CCGGCGTACCACTGGCTGCGTTCGGTGTTGAGCGCCAGCAGGTCACGGGTTTTGCACAAGGTGTCCAGGCCCAGCG
GCATGGGGGCGGCTTTGAAGGCGTCAGGTACTTGCCCGGTGACGAGGTGGGCGAGGGCGCTGGTGATCGCGCTGCG
CTTGATCACCGGCGGGTGCTCGGCATAGCGCCTGGCCAACGGTTTGAGGGCCGCCTGCAGTTCTACCTGGTCGTCC
GGTGGCAAGTAGCGTGACGGGTCGGCCTGGTCGCCGATAACACGAGGGGTGAGGATAAACAGGCGTTCACGTCGGT
TGTTCTGCCGCTCGGTGGAAGAGAACAGTGCCTTGCCCAGCAACGGTATATCGCCCAGCAACGGCACCTTGCTTCT
TTTATCGGTGCTTTCGGTGACATGGAACCCGCCGACCACCAGCGAACGTTTTTCCGCCATCACCGCCTGGGTGCTG
ACTTTGCCCCGGCGTACATCGGGGCCTATGCGTTCGGGGTTGGACTCGTCGAAGTTGCCGTCTTCGATATCCACCG
CCAGATGGACCTGGTGCCTGCCACGGGTGGTAATGACCCGTGGCACCACCTGGAAACTGGTGCCCACGGTGATCGG
CAGGATGGTCGCGTTTTCGACGCCGGCCGTCAGGTATTGAGTGCGGTTGAAGTCAATCACCGCAGGTTGGTTTTCC
AGGGTCAGCACCGAAGGGTTGGACACCATGGTCGCCAGGCCCTTGGCCTCCAGGGCCCGTACATCGGCGTAGAAAC
GGTCGCGCTTTTCGATCGACAGTTGCGCCGATGTCCCAGGCGCCATGTTCACACCGCCGAGAAAGCGGCTGTTCTG
AAAACCCCAGTTGACCCCGAACTCTTGCAGTTGGGTGCGCTCGATATCCAGGATGATTGCGTCGATCTCCACCAGT
TTGCGTGCCACATCGAGTTGGGCGATCAGCTCGCGGTACATTGCCTGGCGCTCTGGCAAGTCATAGATCAAGACGG
CGTTGTTGCGCACATCCGCCTCCACCCGAATTCGCCCTCTTGCAGTGGGGGCTCTGGAGGTGAGCGACATGCCGGG
CTCCGTCTGCCCCAGGGGCGCCTGTGCGCCGAGCAACTGGTTCAGCATCGGGTTGCCCAGCCGGGGAAGGATCGGG
GTGATGGGCGAGGGCTGCGCTGCCGAGGCCGGGCGTGGGCTCATCGACGACAGGACAAAGTTGTCCCGGGGCTCCA
ACAGCCCGCGTAGCATGCTGGCAACACCCGCGATGACCAGCTTTTCTCCGCGATAATCAATCGGGCGGTCCGCCGC
GTTGGCGAATCTCAGCGGGAACGTCAGCACGCTCTGTTTTTCATCGGGGGATTTTCGCTGGCTGCTGAATTGCCTG
ATCTGTTCGATGTAACGCTTGGGGCCGGATATCAGCACCACGCCATCGTCAGGGAGTTCCCCCCAACCGAAACGGT
TGTCGAGCAGGCCAATGTCAGTCAATGCCTGTTTGAGGTCCGCAACGGTTTGCGATGAGACCTCCAGGCGCGCGGA
CTGCTGCTGGTCCAGGGTGCTGATGTACAGCATGTTGTTGTACATGTACCACTGGAAGCGATGCTCTACTGCCAGC
CGATCAAGCAACGACTGCGGGGTGTTGGCGCGTATCTTGCCGTTGACGGTGCCTTCCAGGTGGCCGTCGAATTGAA
TCTGCGTGCCAAAGGTCTGGGCGAAATCCTCCAGCACCTCACGCACCTGCTTATGGTCAGCCTCATAGGCGTAGGC
GGTATTTTTCCATTCGGCGGGGATCGCGGCGAGCGAGCTTTGCATGGGCAAAAGCAACCAGGGCAGTACCAGCAGG

TAGCGCCAATGAGGGCCTTTTTGAAGGGCCGCGGGCGAGACGGAGCGCAAGCGGGTGCGCTCGTGATTCTTGTTGG
GCATGGGGTGTTCTCTGATTAGTGCGGCAGCGAGCGCAGTGACGTGGGTTGGAACCGTGGGGCCTGTTTTGCAAGG
CGCGCGATCATTGCGCGCCAGGTATCGCGCTGGTTGAGCAGGGCTTGGAGACTGTCCAGCAAAGGCTTCTCGCTGG
TGCTTTGCTGTAGACACTGAGTCAACCAGAGCGCACCCGTTGCAGGCGCCTGTGCGAGTGCACCTTGAAAGTGACT
CAGGCTTGCGTTACCCAGGAGCATCCAGGTGTTGAGCTGTGATTTGTCAGGGCGGCCCTGGGTCAGTTGGACCTGC
AACAAGAGGGTGTCAGGGCCGTGGCGCAGGAGTATTTCGTCATGGTCTTCGATCAGCCTTAATTCTTCGGCGCGGC
TGTTCAACCAATTGGTAATGAGCGCCTTCACGTTATTGTCCGCTGTCCAGGCCGGACTTCAGTAGCATATGCCTGG
CCCTGATATGTTCAGTGAAGGCGTATTCGGATTTACGGTTGGTCATCACCAGTTCATAGAACAAGTCCATGTCCGC
CTCGGATCCAGAGTTCTGCTCCATCAAGTCGCTGACATCGTTTCTGCTGCGCTTCGCATCGCTAAGAAGTTGCTCT
CTGTAACCCGAGTAGTAGTTCATGGATTTGCTCCTTTTTTGTGTTTCCTCTCAGTGGTTTTGTATGTCGAATTGGT
TCCATGTGTAGCTGTTTATTTGGCGCGTGTGCGTGGTGGAAAATCAGGCATCCAACCGCTTGCTGCCTGGCTCTAG
AAAGGTGTCGCGCATGGCGGCCCAATCAATGCAAAAGTCGCCCTCTTCGGTTTGTAACAGCAACGTCTGGGGTTTG
ACGGTATCGTCGGCGCGTAGCGCCCAGAGCGTGGTGTTGTGGTCGGCGAGAGCCTGGCGTGCGGCCTCCAGCTCCT
GGGGATGGCACAGCAATGCCGCCTTGACCATCGGCATCTGGCTGGCCATCAATTGTCTGACCAACGCGGCCATGCG
TTGTGGCAGGGGCGTATCTTCCAGCAGCAGACGAATCGCCTGGTTGGCGATTGAAGAGGCATATCGTTCAAGGTTG
TCGTACATCGCCTGGCGTTCGCCTTCCCAGCGTTTGAACTGCGCGTCGGCACGCCGCCAGATTTCGAGGCCGGCCT
GCTCCAGCAGTTTTTCCCGCTGCTTTTCTGCCCGCTTCAGCAATTGCCTGGCCTGGGCCTTGGCATGCTCCAGTGT
GTTGTGGGCATGGGCGTAATTGGCCAGGGTTTCCCGGGGGATCAGGGTTTCGGCAAAGGCGGTGGAGCCTTTGGGC
AAGTCGATTTTATGTCGGCAAAACATCGTGTTCAGTCTCCATGGTCCTTGGCGCCAGGTGCTTGAGCGTCGCTGTG
GATTGTCGCAATACGCCAGACCACGGCGTGCCAAAGGGTATCGAGGCGGCTGTAGGAGTCCCCAAGCGAAAGGGCT
TTTTTTTCCAGTTCCAGAACGCGCTGGCGAGGGAATCTCAACCTGATGCGTTGCCAGGTGGCGGGGCTGACCCAGG
TGCGTAGCAGCTGCAAGGGGTCTTCATCGGGCTGCAGCATATCTGGGGGCAATGCCTTGGACAGTCTGTTGCACCA
CTGGTGGAGGTTTTCATTCAACGCGCTTGCGTAGGCCGGGCGACACGCGCTGTCAATCAACGCCAGCACCAGGTCC
AGTTGCTCCGGCGAGGCGAGCGCCAGTTGCAGCAATGTGGGCTGAGGCGTGGGCGGCAGGCAGGGCGTGATCCCAT
AGAGTCTGGCTACCCACAGGTGTCGACTGTGAACAAGCGCAGTCAGTGCCGCGTGCTCATCAACCGTTCTCCAGCC
TTCGCGTGAATGCAGCCAGGGGGTTGCCCACCACTGGACCCATGCCAGGTTACTCATCGTGCGAGGATGGGCCGGC
CGTTTGGGCATGCGGCAGCGCTCGCTGGCCCCTGATCCGCCAGAATGTCACGGCGATAATGAGCACCACTAAAACG
ATCATGGTGAAGGTGACGCTGGCCCGCCAAAAGCCAATGTCATCGCCTGGCACCAGGAACGGACCGAAATAGGCCA
GGCGTTGCTGCTCCAAATAGGCCGTAGCGGGAACGAATACCACCGTCAGTTTTTGCGGATTTTCGACGGCGTTGGT
CATGCCAGGAATACTGCTGGAGACCATCCGGCGCACACGGGCCTGGATGTTGTCGGGGTCAAGCCTGGGATCGTGC
TTGATGAACACGGACGCGGAGGCCGGTTGTACCGGTTCGCCCGGTGCAACACGCTCGGGCAACACTACGTGTACGC
GGGCGACAATCACGCCGTCGATGTTCGATAACGTGGTTTCCAGTTCCTGGGACAAGGCATAGATATAGCGCGCGCG
CTCCTCGAGCGGAGTTGAGATAACCCCTTCCTTGCGAAAGGTGTCGCCCAGGGTGGCGCGGGCGGTCTTGGGCAAT
CCAACAGCTTCCAGCGTGCGTACGGCCCGCCCGATATCGGAGGCGTTGACCACGACGACCACGCCGTCTTTGGCGG
GGACTTTGGTGGCGCGTATATGCTTGTCTGCCAACTCCGCGATGACTTCATTGGCTTCCTGCTCGGAGAGTTGGCG
GTGAAGTTCGACGCGGTCACTGCAGGCATTGAGCATGAATGACAACACTAGCGTTAACGTAATAAACCGTAGGCGA
GTCATTTTTATCTACTGAAGGTTGCAGATTTTATCGATGCCTTGTGCGGTTTTACCGACACTTTTAACGAGCATTT
GCGTCAGCAATAACGTATCGGACAGTTGCTCAGGATACTTTCGCGCTTCATCAAACTTATTCTCGCTGCTCAAGGC
TCGCAGACTTTTGGTCATGCGCTGGGTGACAGAAGTCAGCCGCTCGGATGCTTCGGACAGCATGCTCTGTGGTGTC
GTGTAATGGGTCCCTGGGCTGGGCTGGGCTGACGAGTCCATGATTGAGGCGAAATAATTGACATCGGTGTGGGAGA
TCGAGGACTCCTGTGCGCTGGAGGACCCATTGAGATCCTCATGTTCAGCAGAGCGTGCTCTTCCTATAAACATATG
GCGCCTATTACGTAAGTATCGCGGGGAGGGTTTGTTATTGATGAGGCCATTCGTTGTTAGATTGGCGAAGTGACCT
TCCCGCCACCCAGTGGCAGGAAGGTCAGAGAGGGCCAGGCTGATTACAAACGGAGTGCGTCAGACGCGGTTTTGAT
GCCTGCGGTATTTTGGTTTACCGCAGTTTTCCATGCGTTTTTCACACCGTCAGCCCTCTTTTTCGCTTCTGCGGCT
TCAAGGCCTGCGTCTGCCTGCTCTGCACTCATACCACCAGCGCTGCTACCGTAAGGGTTGGAGCCGGAAGAGCCAA
CTGAACCAATACTCATATTGTTTACTCCGTATTTAACGTTGATTTCACTGGACTCTAGCGATGCTGAGAGAAGCAT
TTATCACATGCAGTTTCGCCGTTCCATCATTTATGTGGCGCAGGAAATGTATGCGTTCCAATTGTGTCTGCAGGGT
ATGTTTCAAAAAATGTAATTTTTAAGTTTGTTGCCAGATCTTTATAGTGCGCAAGTTATGTCAGTGATATTTCCAG
TTGCTTCATTCGGTAATAGAGGGTGCGCTTGGGCACGCCTAACTCGATGGCGACGCCATCAACATTATGGGCATGT
CGGTTCAGCGACTCTTCAATCAGAACTTTCTCGATTTGCTGTAAACGTTCCTTGAGCGGCATCTCCCAGTCCTGGC
GTTCTTTTACCGCAGCACAGAGGGGAGGGAGCCCCAGGACGAAACGCTTGGCTGTCGAGCGCAGTTCGCGCACATT
CCCTGACCAGGGATGGCAAAGCAATTGTGAAAGCAGAGCGTTGGGAGGCGAGGGTGCTGGGCACTTGAAGTACTCA
GCCTCCTGCCGGATCATTTTCACGAACAGCGGGATGATGCGTTCTGGTTGCTGCCTGAGTGGCGCCAGGTTGATGT
TGACCACATTCAGGCGAAAGTACAGGTCGCGCCTGAAGGACCCTTGAGCGACCATCTCATGCAAGGACTGCTGGGC
AGAGGCAATGACGCGCATGTCTACGGGGATAAACCGGGTCGAGCCCAAGCGTTCGACGCCACGGACTCCAATACC
CGCAGCAACTTGGCTTGCAGGGGAAGCGGCATGCTGTCTATTTCGTCAAGGTACAACGTGCCCAGGTGCGCGGCTT
CGACAGAACCTGCACGCGATTGCATGGCCCCGGTGTAGGCGCCGCTGTTGACTCCAAAGAGCTGGCTTTCGGCAAG

GCTCTCCGGTATCGCCGCACAGTTGACCGCGACGAAGTTCCCCCGTCGTCCTGACAGGCAATGAATACGTTGCGCT
AAAGTGTCCTTGCCAGTGCCTGTTTCCCCAAGCAGCAGAATGTCGATGTTGAGCGCGGCAGTGTTATTGATGGTTG
TTTCTATGTCTGGGCAATCATTGAACGGGGTGTCTGTTTCAACTTCACTTTGATAGGAGGCCGACATGTTGCTATT
GCTCCAATTCCATTAGAGTTGTCGCGTCACTATTGTTTGATTTTTCTAGTGATAGGGATCAGTAGACCGGAAAATG
CCCATGATGTCATTGCGAAAGGATGTTGGGATTGCTCACTGTTTTGCCGGTGTTGTTAAAGAACTTGAATTGGAAA
AATATTCAAGTTTTTGTAGGAGTGGTATTTGCTGTAGCGCGTAGGAGCAATCCTAAATATTTTATGATTGTTATTT
AATTGGCGGGCTGTGCGCTTGAAGTGTCAGTTTTCTGCTCTGCCTGTTCATGTTATTAAAATACATAACAGTGTCT
TAATATTTCCCCCGCTGGAAAGTCCCTGTTGTAAATGAAGCGCCCACAAAAAAACCGATGCAGGGCATCGGCTTTT
TTGTGGGCGGCAGGTGCCGCAGGTCAAGCGTTACACGTTGAAGCGGAAGTGCATTACATCGCCATCTTTGACGATG
TATTCCTTGCCTTCCAGGCGCCATTTACCGGCTTCCTTGGCCCCGGCTTCACCCTTGAACTGGATGAAGTCGTTGT
AGGCGATCACTTCGGCGCGGATAAAGCCTTTTTCGAAGTCGGTGTGGATCACGCCGGCAGCCTGTGGCGCGGTGGC
GCCGACCTTGACGGTCCAGGCGCGGACCTCTTCGACACCAGCGGTGAAGTAGGTTTGCAGGTGCAGCATTTCGTAG
CCTGCACGGATCACACGGTTAAGGCCGGGCTCTTCCAGGCCCAGGGCCTCGAGGAACATGTCTTTTTCTTCGCCGT
CTTCCAGTTCGGCGATTTCCGCTTCGATCTTGTTGCACACCGGAACAACCATTGCGCCTTCTTCTTCGGCAATGGC
CAGGACCACGTCGAGCAGCGGGTTGTTCTCGAAGCCGTCTTCAGCCACGTTGGCGATGTACATCACAGGCTTGGTG
GTCAGCAGGTGGAAGCCCTTGATCACGGCCTTTTCGTCGACGCTCATGTTCTTCATCAGGCTGCGTGCAGGCTTGC
CCAGGGTGAAGTGAGCAATCAGTTGCTCAAGCAGGGCCTTCTGCACCACGGCGTCCTTGTCACCGCCTTTGGCATT
GCGCGTAACCTTTTGCAGTTGCTTCTCGCAGCTGTCGAGGTCGGCGAAGATCAGTTCCAGGTCGATGATCTCGATG
TCGCGTTTCGGGTCGACACTGTTGGAGACGTGAATCACGTTCTCGTCTTCAAAGCAGCGGACCACGTGAGCGATGG
CATCGGTCTCACGGATGTTGGCCAGGAACTTGTTGCCCAGGCCTTCACCTTTCGACGCACCGGCTACCAGGCCTGC
GATGTCGACGAACTCCATGGTGGTCGGCAGGATGCGCTTGGGATTGACGATGGCGGCCAGGGCCTCCAGGCGCGGA
TCGGGCATCGGCACGATACCGGTGTTGGGTTCGATGGTGCAGAAGGGGAAGTTCTCCGCCGCAATACCGGACTTGG
TCAGGGCGTTGAACAGGGTGGACTTGCCGACGTTAGGTAGGCCGACGATGCCGCAATTGAATCCCATGGTGTTTCC
CCTCGGAAAAAAGTCAGGCCTTCTGGCTGTGCAGGTTTTTCATCGCGCGGTTCCATTCACCGGCGAAGATATCCGG
CAGCACGCCGAGGGCAAAATCGATGCTGGCATCGAGTTTTTCCTGTTCGGCGCGTGGCGCACGACCCAGGACGAAA
TTTGAAACCATACTGGCAACGCCTGGGTGGCCAATGCCGAGCCGCAGACGGTAAAAGGTATTCTGATTGCCCAACT
GCGCAATGATGTCGCGCAGGCCATTATGACCGCCATGGCCGCCGCCTTGCTTGAGCTTGGCAACGCCGGGTGGCAG
GTCCAGTTCGTCATGGGCCACCAGGATTTCTTCGGGTTTGATCCGGAAGAACCCCGCAAGCGCCGCGACAGCCTGG
CCGCTGCGGTTCATGTAGGTGGTGGGAATCAGCAGACGAACATCCTGACCCTGATGCGAGAAGCGTCCGGTCAGGC
CAAAATATTTGCGATCGGCCACAAGATTGACGCCTTGGGCGTGGGCGATGCGCTCAACAAAAAGGGCCCCCGCGTT
ATGCCGGGTCTGTTCGTATTCGGCGCCTGGATTTCCCAGGCCAACGATCAGTTTGATGGCAGTCACGACAGGGGCC
CTTCCTTTGGAGTTGTGGATAACATCGCCGCAACCTCGTTGCGGCGAAAGTGGACGACAGGAGCTCACTTATCCAG
AGATAAACTTCGCGTTATCGCCCGCTTTCTCGCTACGTTTCGGTCCGCGATGTTTCCTCAAGCTCCGACGCTGCAG
AGAGTATTACTCGGCAGCGGTTTCGCCAGCTTCATCTTCTGCTACAACACGTGGCGCGTGGACGTTGGCAACAGCC
TTGTCGTCGTTGTGAGCCAGTGCAACGAACTCAACGCCTTTAGGGGCCTTGAGGTCGGACAGGTGAATGATGGTGC
CGATTTCAGCAGCCGACAGGTCGACTTCGATGAACTCAGGCAGATCTTTCGGCAGGCAGCTTACTTCCAACTCGGT
AGTGGTGTGGGAAACAACGCCACCTTTCTTGACCGGAGCTTCTTCACCAACAAAGTGCACAGGCACGATAGCGGTC
AGTTTCTGACCAGCAACAACGCGTACGAAGTCAGCGTGCAGTACGTGACCTTTGGCCGGGTGGCGTTGCAGAGCCT
TGATGATAACGTTCTGCTTTTTGCCGCCAACGTTCAGTTCGATAACGTGGCTGTAGGCAGCATCGTTTTCGAGCAG
TTTGGCAACTTCTTTAGCCAGCATGCTGATGGATTCAGGGGCTTTTTCGCCACCGTAAACTACAGCTGGAACCAGG
CTTGCGAGACGACGCAGGCGGCGGCTCGCACCTTTCCCCAGGTCGGAACGCAGTTCAGCATTCAAAGTAAAATCGT
TCATGTTGTATCTCCAAAATAACCACATTCGCCCCAGCGTTTGCGACCAGCGCTAAAGGCGATATGGGCAAAAAAG
CCCCGCCCCAGCATGGTGCTGGGGCGGGGCGCTTTTCGTCAGGTTGATGCCACCAAGGTTTGACCCTTAGCGGAAC
ATCGCACTGATCGATTCTTCATTGCTGATGCGGCGAACCGCCTCAGCAACAACCGGTGCGATATCCAGTTGACGGA
TACGCGCACAGGCTTGAGCAGCGGCGGACAACGGGATGGTGTTGGTCACCACCAGTTCGTCCAGCACGGAATTCTC
GATGTTCTCGATCGCTCGGCCCGACAGCACAGGGTGTGTGCAGTAGGCGAAGACTTTAGCCGCACCGTGCTCTTTC
AAGGCTTTAGCCGCGTGGCACAGGGTGCCGGCGGTATCGACCATGTCATCGACCAGAATACAGGTACGCCCTTCGA
CATCACCGATGATATGCATCACTTCAGAGTGATTGGCTTTCTCACGGCGTTTGTCGATGATCCCGAGGTCCACGCC
CAGGGATTTGGCAACAGCCCGTGCACGCACGACGCCACCAATGTCCGGGGACACGATCATCAGGTTTTCAAAGCGC
TGGTCTTCGATGTCATCCACAAGAACTGGGGAGCCGTAGATGTTATCTACCGGAATATCGAAGAACCCCTGGATTT
GGTCAGCGTGCAGGTCAACCGTGAGAACACGATCGATGCCTACCACGGTCAGCATGTCAGCGACGACTTTCGCGCT
GATAGCCACACGTGCGGAACGCGGACGGCGATCCTGACGGGCATAACCAAAGTAAGGGATTACAGCTGTGATTCGA
GTCGCTGAGGAGCGGCGGAAGGCATCAGCCATCACGACGAGTTCCATCAGGTTATCGTTGGTCGGAGCGCAAGTCG
GCTGAATAATGAAAACATCTTTACCGCGGACATTTTCATTGATCTCGGCTGTAATCTCGCCGTCGGAAAATTTACC
GACTGAGATGTCACCGAGAGGGATATGCAGCTGACGTACAACACGCCGAGCCAGATCGGGGTTAGCATTCCCCGTA
AAGACCATCATCTTGGACACGCGCAGTACCTAGAGGCTGAGGGTAACCTGGATGAGTATAGAAAATGGCAGGGGCG
GCTGGATTCGAACCAACGCATGGCAGGATCAAAACCTGCTGCCTTACCGCTTGGCGACGCCCCTGTATCTGTTGCA

ACGAGTACCCAGTACTCGGTTCCTTTAGAGCAGACTTTGCAGCTTGCGATGCAACATCGAAACGTTGCTCCCCTTT
GCTACAAACCCTGTAAGGGTCTCTGTAAGAAGGGCCGAGACTTTATCAGCTTCAGCTTTGTTTGGGAAGCCCCCAA
ACACACAACTTCCAGTTCCGGTGAGTTTTGCTTCGGTAAATTTACCTAACAAATTCAATGCGTTACGTACATCTGG
ATAACGCATTGCTACCACCGGTAAGCAGTCATTTCGACTGTTTCCCTTGGGAACGGGGCGCACTTTAATGGGAGAA
GAGTTACGTGTCAACAACGGATCTGAAAAAATTTCTGCTGTACTTACAGATACTTGCGGAACAAGCACGACATACC
ACGGCTCTTCGGGGTCTACGGGGGTGAGTTTTTCCCCGACACCCTCAGCAAATGCGGCGTGCCCACGCACAAAAAC
CGGGACGTCGGCGCCCAGTGTCAGGCCCAGCGCGGCCAGGCGATCTTCATCCCAGCCCAGTTGCCACAAGTGATTA
AGACCGAGCAGGGTGGTTGCGGCATTCGAGCTGCCACCGCCGATGCCGCCGCCCATTGGCAGGATTTTATCGATCC
AGATATCAATACCCAGCGAACAGCCGGATTGTTCCTGGAGCTTTTTTGCCGCCTTCACGATCAGATTGCTGTCATG
GGGCACATTGGCAAATTCGGTGTGCAGTTGAATCACCCCATCGTCGCGTACGGCGAAGGTCATCTCATCGCCGTAG
TCGAGAAATTGAAACAGCGTCTGCAACTCGTGATAGCCGTCTTCACGACGACCGAGGATGTGCAGCATCAAATTGA
GCTTGGCCGGGGAGGGCAGGGTCAGGCGTTCGGTCATGTCACTGCCCCAGCTTGCGCGGTTGCCAGTCCTTGATCA
CCAGCGTGACGTCAAGGTTGCTGCCGTGCAGCTTGATGCGCTCGGGCAGCCAATAGCCGCTTTGCTCCACATAACT
GAGGTACTCAACCTGCCAGCCATCCTGTTCCAGGGTGGCCAGGCGGCTATCGCCATTGAGGCTCAAGCGGCTCTTG
CTGTCAGGCGCGGGCAGGCCGCGAACCCACCAGACCAGGTGCGAGACCGGCAGCTTCCAGCCGACCTGCTCTTCAA
GCAATGCCTCCGGTGATGGCGCTTCATAGCGGCCCTGGTTGGCCACTTCGAGGCTCACTTGCCCCGGTCGCCCGGT
CAGGCGTGCAGCGCCACGGCCCAGGGGGCCAGAGAGGCGGATATCGTAATAATCCTGGCGTTGCAGCCAGAACAAG
GTGCCGCTGCCCGAATCCTTCGGTGCACGCACGCCGATCTTGCCTTCGATCTGCCAGCCATCAATGCTGCTGAGCT
GATCCTTGTGCTGGCGCCATTGCGCCGGGTTGCCCTGGCCTTCGACGGATTCACGGGCACCGAAGCCCGCACAACC
GGCGAGCAGGGCGATAAAACTGAAAACGATGGCGTGGCGCAAAAACATAGAATTAAAGAGTCTCGGATCCGGTCAG
GCGCTTGATGGTGCTGCGCAGGATAGGGCTGTCGGGTTGTTCCTTGAGGAACTTGGCCCAGATTTGTCGGGCTTCG
CGTTGGTTGCCCTTGGCCCAGAGCACTTCGCCCAGGTGTGCGGCCACTTCCTGGTCGGGGAACCGCTCCAGGGCTT
GGCGCAACAGGCGTTCGGCTTCGTCGAGGTTGCCCAGGCGGTAATTCACCCAGCCCAGGCTGTCCAGCACTGCCGG
GTCTTCCGGGTTGAGCTGGTGCGCCTGTTCGATCAGGACCTTGGCCTCGGCATAGCGCGTTGTACGGTCTGACAAG
GTGTAGCCCAGGGCGTTCAGTGCCATGGCATTGTCCGGGTCGCGCTTGATGATCAAGCGCAGGTCCTTTTCCATCT
GCGCCAGGTCATTGCGTTTCTCGGCCTGCATGGCGCGGGTGTACAGCAAGTTCAGGTCGTCGGGGTATTGCAGCAG
GGCCTGGGCGAGGATTTTCCAGGCACGCTCCCCCTGATTGTTGGCCGACAAGGTCTCGGCCTGGATCAGATACAAC
TGGATGGCATAGTCCGGCTCGGCATCGCGGGCAGCGGCCAGGCGTTTTTCGGCTTCGTCGGTGCGGCCGTTGTTCA
TCAGGATATCGGCCTGGCGCAGTTGCGCCGGCAGGTAGTCGTTGCCGGGGCCGACCTGGGCGTACTCGATCAGCGC
GCCTTGCGGGTCATCCCGTTCCTCGGCGATGCGCCCCAGGTTCAGGTGGGCCGAGTCGACGTGGCTTTCCCGGGCG
ATCAGTTCTTCCAGGTAACCCTTGGCCTCTTCCCAGGCCTTGGCTTCCAGGCAGACCAGCGCCAGGGAGTAACGTA
GTTCGTCATCATCCGGGTATTGCTGGACCAGGCTGGCGAACTCCACCTTGGCGTCTTCCATGCGGTCCTGTTCCAC
CAACATGCGCGCGTAAGTCAGGCGCAGGCGCTTGTCGTCCGGATACTTCTTGATGCTCTTTTGCAGCAGCGGCAAG
GCTTCCTTGCCCCGGTTGAGGCTTTGCAGCAGGCGTGCGCGCAACAGCAGCGGGGCTATTTCACCTTCTTCCGGCG
GGTTTTGCTCCAGCAGCTTGAGGGCGGCTTGCGGTTCGTCATCCTGTTGCAGCAGTAGCGCCTTGCCAAATACCAG
CTGGCCGTTGCTCGGGTGCTTTTGCAGCAGGCGGTCGAAACTCTTCATCAGGCCATCGCGGGTGTCCTGGTCGGTA
TCGGCCGCGGAGAGGGCGAGGAAGTCAAAGTGGGTATCGCCCTTGCCCTGCAAGACTTTCTCCATATAGACCATGG
AGTCGTCGTAGCGCCCGGCCCGCGCCAGCTGGATCGCCGCGGCGCGCAGGGCCTCCAGGTCGTCCGGGGCGTTTTT
CGCCCAGATCAACGCGGTATCCAGGGCTGCCTGGTCGGCGCCCAGGTATTCGGCGATGCGAAACGCTCGCTCGGAG
ATCCCCGGGTCCTGGGTGTTGACGGCCTGGGTCACGTAGTTATCCAGGGCAATGTCGAAACGATTGCGCTGGCCGG
CGAGCTCCGCGCTCAGCAGGCTGAACACCGTTTCTTCGCTGAACGAGGAATAAACCTTGGGCTTTTCAGGGGCGGG
GGTGCTGTCTTCCACCGGCGCAGGACTCTGCGACGATACGGGGGCCATGGACTGGCAGCCCCCGAGGAAGGCAAGG
GCAAGGAGCAACGCGGAAGATCTATTCATATAGGAAGAGGACGACTAACCTGCGGTCGGATCATCATGACACAAGC
CCTCGGTCAAACATAACCGGGGCACTCGTATTGCCTTAAATGCCTTCTATGTAGCAGCGAGCTTGCTCGCGAAAAA
CGCCAAGACGCCGCGTTTACCCAAGGTGCCAGCGTTTTCGTTGACGATTTTTGCGAGCAAGCTCGCCCCTGCAAAG
GGTCTTTATAGACACAAGGCATTAGGACAATAGACGACGGTGGTTGTTCTGGATGAGTCGAAGTAGGACAATTGTC
GGCTTCTCGACATCATCAGCGATATTGAATGGCCTTCCTTGCTCTGGGTATCAACCACAAGACTGCTTCTGTAGAC
GTCCGCGAGCGCGTGGCGTTTACCCCGGAGCAATTGGTTGAGGCTTTGCAGCAGCTGTGCCGGCTCACCGACAGCC
GCGAAGCTGCGATCCTTTCCACCTGCAATCGCAGCGAGCTCTATATAGAGCAGGAACATCTTTCGGCGGATGTCGT
GCTGCGCTGGCTGGCTGATTACCATCATTTGAGCCTGGATGACCTGCGCGCCAGTGCTTATGTGCATGAAGATGAT
GCGGCAGTTCGTCACATGATGCGTGTTGCATCCGGGCTCGATTCGCTGGTGCTGGGCGAACCGCAAATCCTCGGCC
AGATGAAGTCGGCCTACGCCGTGGCCCGCGAAGCCGGGACCGTGGGGCCGTTGCTGGGGCGTTTGTTCCAGGCCAC
CTTCAATTCGGCCAAGCAGGTGCGCACCGATACGGCGATCGGTGAAAACCCGGTCTCCGTGGCTTTTGCTGCCGTC
AGCCTGGCCAAGCAGATCTTCAGCGACCTGCAGCGCAGCCAGGCCCTGCTGATCGGCGCGGGCGAGACCATCACCC
TGGTCGCCCGGCACCTGCATGACCTGGGCGTCAAGCGTATCGTGGTAGCCAACCGAACCCTGGAGCGCGCCAGCAT
CCTGGCCGAAGAGTTCGGTGCCCACGCGGTATTGCTGGCTGACATCCCGGCCGAGCTGGTGCGCAGCGATATCGTG
ATCAGCTCCACCGCCAGCCAGTTGCCGATCCTGGGCAAGGGGGCGGTGGAAAGCGCCTTGAAGCTGCGCAAGCACA

AGCCGATTTTCATGGTGGATATCGCCGTTCCCCGGGATATCGAGCCAGAAGTCGGCGAGTTGGACGACGTTTACCT
CTATAGCGTCGACGACCTGCATGAAGTGGTCGCCGAAAACCTCAAGAGCCGCCAGGGTGCTGCCCAGGCGGCCGAG
GAAATGGTCGCGATCGGCGCCGATGACTTTATGGTGCGTCTGCGCGAGCTGGCTGCCGTGGATGTGCTCAAGGCCT
ATCGCCAACAGGGCGAGCGCTTGCGTGACGAGGAATTGCTCAAGGCCCAGCGTCTGCTGGCCAATGGCGGTAACGC
TGAAGATATTTTGATGCAATTGGCCCGTGGCCTCACCAACAAGCTGTTGCATGCTCCCAGCGTACAGCTTAAAAAA
CTGACAGCCGAAGGCCGCCTCGATGCGCTGGCCATGGCCCAGGAACTCTTTGCCCTCGGTGAGGGCGCGTCAGACA
GCTCTTCGGATAAAAAACTGCAATGAAAGCGTCACTGCTCAATAAACTGGACGTGCTCCAGGACCGTTTCGAAGAA
CTGACCGCCTTGCTTGGCGATGGCGAGGTTATTTCCGATCAAACCAAGTTCCGCGCCTATTCCAAGGAATACGCCG
AAGTTGAGCCGATCGTGACCACCTACTCGCAACTGCTCAAAGTGCAGGCCGACCTCGAAGGCGCCCAGGCGCTGCT
CAAGGACAGCGACCCGGACATGCGTGAAATGGCCGTGGAAGAAGTCCGCGAAGCCAAGGAAAAGCTCGTTGAGCTG
GAAGGCGACCTGCAACGCATGCTGCTGCCCAAGGATCCTAACGATGGGCGCAACGTCTTCCTGGAAATCCGCGCCG
GTACCGGCGGCGACGAGGCGGCGATCTTCTCCGGCGACCTGTTCCGCATGTATTCGCGCTATGCCGAGCGTCGCGG
CTGGCGTGTCGAAATCCTCTCCGAGAACGAAGGGGAGCACGGCGGCTACAAGGAAATCATCGCCCGGGTCGAAGGC
GAGAACGTCTACGGCAAGCTGAAGTTCGAATCCGGCGCCCACCGCGTACAGCGCGTTCCAGCCACCGAGTCCCAAG
GCCGCATCCACACCTCGGCCTGCACCGTGGCGGTATTGCCCGAGCCGGACGAGCAGGAAACCATCGAAATCAATCC
GGCAGATTTGCGTGTCGACACCTATCGTTCGTCCGGCGCCGGTGGCCAGCACGTCAACAAGACCGACTCGGCGATC
CGCATCACTCACTTGCCGTCCGGCATTGTGGTGGAGTGCCAGGAAGAGCGGTCCCAGCACAAGAACCGTGCGCGGG
CCATGTCCTGGCTGTCGGCCAAGCTCAACGACCAGCAGACCAGCGCCGCCGCCAACGCGATTGCCAGCGAGCGCAA
GTTGCTGGTGGGCTCGGGTGACCGGTCCGAGCGCATTCGCACCTATAACTACGCCCAGGGCCGGGTCACCGACCAT
CGGGTCAACCTGACCCTGTATTCCCTGGACGAAATCCTTGCCGGTGGCGTGGACGCAGTGATCGAGCCGTTGCTTG
CCGAATACCAGGCCGACCAACTGGCCGCGATAGGTGAATAAATGACCATTATTGCCAGCTTGTTGCGCGCCGCCGA
GCTGCCCGACTCGCCGACTGCCCGTCTGGATGCGGAGTTGCTGCTGGCGGCTGCCCTGGGCAAGTCCCGCAGCTAC
CTGCACACCTGGCCAGAAAAAATCGTCAGCAGCGAAGACGCGCTACGTTTTGCCGGCTACCTGCAACGTCGTCGCA
GCGGTGAGCCGGTGGCCTACATCCTCGGCCAGCAGGGCTTCTGGAAGCTCGACCTGGAAGTCGCGCCGCACACGTT
GATCCCGCGCCCCGACACCGAGCTGCTGGTGGAAGCGGCCCTGGAATTATTGCCCGCCAGCCCGGCCAACGTCCTC
GACCTGGGCACCGGCAGCGGCGCCATCGCCCTGGCCCTGGCCAGCGAGCGCCCGGCCTGGCGCGTGACCGCGGTGG
ATCGCGTGCTCGAAGCCGTGGCCCTGGCCGAGCGCAATCGCCAGCGCCTGCACCTGGATAACGTCACGGTGCTCAA
CAGCCACTGGTTCAGTGCCCTGAAAGATCACACCTATCACCTGATCATCAGCAATCCCCCCTATATTGCCGACAAC
GATCCGCACCTGGTGGCGGGCGATGTGCGCTTCGAACCCGCCAGCGCATTGGTCGCAGGTCACGATGGCCTGGACG
ACCTACGGCTGATCATCGCCCAGGCGCCGCTGCACCTGGAGGCCGGTGGCTGGTTGCTGCTGGAGCATGGTTACGA
CCAGGCGCCGGCCGTGCGTGACCTGTTGCTGGCGCAAGGTTTTGCCGAGGTCCACAGCCGTATCGACCTCGGCGGC
CATGAGCGCATCAGCCTGGGACGCCGGCCGTGCTGACCGATCAGGAGCTGTTGCGCTATAGCCGACAGATTCTGTT
GCAACAGGTCGATATCGACGGCCAGTTGCGCCTGAAAAACAGCCGTGTATTGATCGTCGGCCTGGGTGGCCTCGGT
GCTCCGGTGGCCCTGTACCTGGCAGCCGCCGGTGTGGGTGAGCTGCATCTGGCGGACTTTGACACGGTCGACCTGA
CCAACCTGCAACGCCAGATCATCCACGACACCGACAGCGTCGGCCTGAGCAAGGTCGATTCGGCTATCCGCCGCCT
GAGTGCGATCAACCCCGAGATCCGGCTGGTTGCCCACCGCGCTGCGCTGGACGTTGATTCGTTGGCCGCTGCTGTA
GCCGGCGTGGACCTGGTGCTCGATTGCTGCGACAACTTCGCGACCCGCGAAGCGGTGAACGCGGCGTGCGTCGAAG
CGGGCAAGCCTTTGGTCAGCGGTGCGGCGATTCGCCTGGAAGGCCAACTCTCGGTGTTCGACCCGCGGCGCCCGGA
AAGCCCGTGCTACCACTGTTTATATGGGCACGGCAGCGACACCGAACTGACCTGTAGCGAAGCCGGCGTCGCCGGT
CCGCTGGTAGGCCTGGTCGGCAGCCTGCAAGCCTTGGAAGCCCTGAAGCTGCTGGCCGGTTTTGGCGAACCCCTGG
TCGGCCGGCTGCTGCTGATCGACGCCCTGGGCACACGTTTTCGCGAGCTGCGGGTCAAGCGTGATCCCGCTTGCAG
CGTCTGCGGTCGCCAGCATGGTTAAGGATGCACCGATTGGTGTATTCGACTCCGGGGTCGGCGGCCTGTCGGTGCT
CGACGAAATTCATCGTTTGTTGCCCCATGAGTCGCTGCTGTACGTGGCGGATTGCGGGCATATCCCCTACGGCGAG
AAAACCCCGGCATTCATTCGCGAACGTTCGCGGGTGGTTGCAGAGTTTTTCCGCGAGCGGGGCGCCAAGGCCTTTG
TGATTGCCTGCAACACGGCAACTGTGGCGGCGGTCGCGGATTTGCGCAGTGATTATCCGGATTGGCCGCTGGTGGG
CATGGAGCCGGCGGTCAAACCTGCGGCTGCCGCGACGCGCAGTGGTGTGGTCGGGGTACTTGCCACCACGGGCACC
TTGCAAAGCGCCAAGTTCGCCGCGCTGCTGGATCGCTTCGCCACCGATGTGCGGGTGGTGACGCAACCTTGCCCGG
GGCTGGTAGAGCTGATCGAAGCCGGCGACCTCAACAGCCCGGCGCTGCGCCAGATGCTGCAAGGGTATGTCGCGCC
GCTGATCGAGGCCGGGTGCGACACCATTATTCTGGGCTGCACTCACTATCCTTTCCTCAAGCCCTTGCTGGCGACG
CTGCTGCCACCGTCAATCATCCTGATCGACACCGGCGCGGCAGTGGCGCGTCAGTTGCAGCGCCTGCTGGGCGAAC
GCCAACTGCTGGCCGAGGGCAATCCTCGGGCGGCGCAATTCTGGACCAGCGGTGATCCGCAACATCTCAGAAATAT
CCTACCCACACTATGGAAATTCAGCGGCGTTGTGCGAAGCTTCGCCCTGTGAAAAATTCGTGAAATAGCGGGTTTT
TCAGCTGAACTTCTGCGTTGTCCGTGATTTCTATAGCTCTTGATGAGCAATACAAAACCATACAACTTCGTTTGGA
AAGGATGTTTCTTATGAAGCGCTTGTTCTGTTTGGCCGCGATTGCGGCCGTTTTGGTAGGGCACTCTGTTTCTTCC
CAGGCTGCCGGCCTGGAATTTGGGGTGGGGCATACAAGCGATTCGACCATGACCTATCGCCTGGGCCTGCAATCGG
ACTGGGACAAGAGCTGGTTGCAGAGCGATGTCGGGCGCCTGACCGGCTACTGGAGCGGCGCCTATACCTTCTGGGA
TGGTGACGAACGCGCCAGTGTTAGCAGCTTGTCGTTTTCCCCGGTGTTTGTGTATGAATTCGCCGGTGAGTCGGTG

AAACCCTATATCGAAGCGGGGATCGGCGTGGCGTTGTTCTCACGCAGCAAGGTGGAAGACAACAACATTGGCGGGG
CTTTCCAGTTCGAAGATCGGCTGGGCTTTGGCTTGCGCTTCGCCGGCGGGCACCAGGTGGGGATCCGTGCCACGCA
CTATTCCAACGCGGGCATCAGCAGCCACAACGATGGTATCGAGAGCTATTCGTTGAACTACACAATGCCACTCTGA
TACTCCCCAAGCACAGCCCGACCCATGTAGGAGCGGGCTTGTGTGGGAGCCGGGCTTGCCCGCGATGCAGACGACT
CGGTGCTTCAGGCAAACCGCAGTGATGCTATCGCAGGCAAGCCAGCTCCCACACAAGCCAGCTCCCACATGTGACC
ACCACCGTTTTCAGAATCAGCGGTAGACCGTTGCAATCCCCTGGCGCTCTTCAAGGCACTCCGGCGCGCCCATCTC
GAATTCCCGGCAAATCAGCGGTCGTTTTTCATAGATCGTGCACATCATCGTGTCGCGATCCAGTGCGGCGCACCAG
CCGTCGTCCAGGCGCAGCATGACTTCGCCTCCCCAATCGTCCGTGTCGATATAGCGATCAGGCACGCCGGTGTCGG
TGATCAGCATGACTTCCAATTGACAGCAGCAGGCTGCGCACGTGGAACAGGTGACGGCGGGTTCGGTGATTTGGGT
GTGGGGGATGTTGGTCATAGACGCGCAGTGTAAGGCAAGCCGGTCGAATGCGTGTGAAAGCTCTGACGGACGTCAG
CGCGCGAGCCGGTGCAGGCCGGGAAATACCACGGCCCAAAGCAGCGCGATTGCCACCAGGGTCGGGGCTGTGGCAA
ATCCGAAGCTCACGCCTGCCAACTGGCTGCCGGCGTAGTACGACAGCGGCCCGCCCACGGCCCCCAGCAGGCTTGC
GCGCCACCAGGGCCGCGCGCTCCAGGCCAGGCAATGGCGCAAGGTGGTCGCCAGCAACGCCCACAGCAAGATCAAC
CACCCAGGAATCAACGGGCCGGGTTCACTGAACTCGAACACGCCTAACGTGCGCAATAGCGTGTCGAGCAGGGTAC
CGAGCACGGTCACGCTCAAGATCAACCGCCCCTCAGCGGCCCATGAAGTTATCCACAGCAGGTGAATTGCCAGGAC
GACCAGCCCGAGCAATAACCACAGGCTGTCGCCCCCCATCACACAGGCAAACCAGCCGCACTGGAACAGCGCGGCA
TTGGCCAGGTTTTTAAGCATTGAAACGCCCGAGCAGCGGTTGTGTCACCGCGCTGGGCTTGGCCAGCAGCAATTGC
GCAGTGCCGATGGTGCGCTCCATAAAGCCGCCCTCGCAGTAGCACAGGTAGAACTCCCACAACCGCAGGAAGTATT
CGTCGTAGCCCAATTCCGTGAGCCGGCCATGGGCGCGGCGAAAGTTCTCATGCCACAAGCGCAGGGTTCGCGCGTA
ATGCAGGCCGAAGTCTTCCATGTGCAGCAGGTTCATATCGGTGTCGCGGCTGACGATCTGCAACATTTTCTGCACA
CAGGGCAGGGCGCCGCCGGGGAAGATGTAGCGCTGGATGAAGTCGACGCTGCTTTTGGCCTGTTCGTAGCGTTGTT
CGCGAATGGTGATGGCCTGCAGCAACATCAGCCCGTCGCTCTTGAGCAAGTGTGCGCATTGCTTGAAGTAGGTGGG
CAAGAAACGATGGCCAACCGCTTCGATCATTTCAATGGACACCAGCTTGTCGTACTGCCCGCTCAGGTCGCGGTAG
TCCTGCAGCAGCAGCGTCACCTGATTCTGCAAACCCAAGGCCTGGATGCGCTGTTCGGTGTAGGCAAATTGCTCTT
TGGACAAGGTAGTCGTGGTAACCCGGCAGCCGTATTGCTGGGCTGCGTAGAGGGCCATGCTGCCCCAGCCAGTACC
GATTTCCAGCAAGTGGTCCGAGGGCTTGAGTGCCAGTTTCTGGCAGATGCGCTCCAGCTTGTTGAGCTGTGCCTGC
TCCAGCGTATCGTCGGGGGTAAGGAACTGCGCCGCCGAATACATCATGGTCGGGTCGAGAAACTCCTGGAACAGGT
CGTTACCCAGGTCGTAGTGGGCGGCGATGTTTTTTTGCGAGCCCTTGCGGGTATTGCGGTTGAGCCAGTGCAAGCC
TTGGGTGAAGGGGCGGGCCAGCTTGGCCAGGCCACCCTCGAGGGCGTCCAGTACATCAAGGTTGCTGACCATCACG
CGTACCACGGCAGTCAGGTCCGGGCTGCTCCAGAAACCATGAATGAACGCCTCGCCCGCGCCGATGGAACCGTTGG
CAGCCACCAGGCCCCACACGGCCGGGTCGAGGATCTGGATCTCACCCAGCAAATGCGCTTCCCGTGCCCCGAACAC
CTGGCGTTCACCGTCCTCGATCACCACCAGTTGGCCATGGCGCAGCTTGCCGAGTTGGCGTAGCACGGCTTTGCGC
AACAGCGCGCCGGTCAGGCCGTTGATGTTCAGGCGCTGGGATTTGATCGATAAGCTAGGGGATTTCATGGTGGCGA
TCCTTGGGATACCCAATTGCACAGGCGTCATCGGCCGCCTGATGGGGGAAAATCGGTGTGCGTTTAAGGAGCAGGC
GCAGGGCTTGCCAGTAGATAGCCAGGCAGGTCTTGGCGGTCATCCACGGAAAGCGCCGCAAGTAGCGATGCAGGCT
GGCGCGGTCGAGGGCTTCGCGTTGCAGGCTCAAGGTGGCGTCGAACACCTTGCTCTCGCCTTGCCAGTCGGCCATA
TGCACCCCCAGGCGCTGGCCGGGCGGGCTGAAGCTCATGCGGTATTGCAGGTCCCGGGGCAGGAAGGGCGATACAT
GAAATGCCTTGGCCACCGCGAAATGCTGATGCTCGCCGCCCGCCAGGGCCCGGGCGGGCAGCACATAGTGATAGCG
CTCGCGCCACGGGGTGTTGGTCACTTCACAGACAATCGCCGCCAGTTGCCCGTCGCCCTCGAAGCAGTAGAAGAAA
CTCACCGGGTTAAACGCCAGGCCCCAACTGCGGGCCTGGGTCAGCAGGCAGATATTGCCTTGGGGGGTATGCCCAA
GGGTCGTGCCGACCAGTTGGCGCACGGCGTCGCTCAAGCAGGTGCCATCGCGGGTCAACTCACGCAGGTAATCCTG
CTGGCGAAACGCAAAGGGCGCGAAGCGGCCACTGCCGGCCAACGGTGACAGCCCCAGCACCTGCTCCTGTTCGCTG
AGGTCCAGGTACAGCAGGCCGATGCGATAGCGAAAGGCATGCCCCTTGGGGGTAAACCGCCGATGGGCAATCCAGC
CGCTGTAGAGGGCGCTGTTCACAGGGTTTCCCCAAACGCCAGGGCCACGCGCAGGGCGCTGACCACGCCGTCTTCA
TGAAAGCCGCTGGCCCAGTAGGCCCCGCAATAATAGGTGTGCTGGGCGCCATGCAATTCTTCCCAGCGTGCCTGTG
CCGCCACCGCCGCCAGGCTGTATTGCGGATGGGCATAGGTGTAGCGGGCAAGGATTTTCAGCGGGTTGATAATCGC
TGTCTGGTTGAGGCTCACACAAAAGGTGGTGTCGCTGTCGATCCCTTGCAGGATGTTCATGGCGTAGGTGACGGCT
GCCGGCTTGTGCGTGCTGGCGTCCAGCCGATAGTTCCAACTGGCCCAGGCCAGGCGGCGGTCGGGCAGCAAGCGCG
TATCGGTGTGCAGGACCACGTCATTGTCGGCGTAGGTTATCGCCCCCAGGATCTGCTGTTCGGCCAGGCTGGGATC
GTCCAGCAAAGCCAAGGCCTGGTCGCTGTGGCAGGCGAAGATCACTTTGTCGAAGATTTCGCGGCCGTCGGCACTG
TAGATCACTGCGCCTTCTGGCGTGCGCTCGACTTTCTGTACCGGGCAGTTGAGGCGGATGTGTTGGCGAAACGAGT
GGGTCAGTGGCTCGATATAGCGGCTGGACCCGCCTTCGATCACGCACCATTGCGGCCGATTATTCACCGACAGCAA
GCCATGGTTCTTGAAGAACCGCACGAAGAACTGCAGCGGGAAGGCCAGCATGTCCGCCAGGGACATCGACCAGATC
GCCGCGCCCATGGGCACGATGTAGTGGCGGATAAACCGCGGGCCATAATTGCCCGCCTGCAAGTATTCACCCAGGG
TCATCTGCGCGCTGATGCGTTGTTCCTGCAAATCCAGGGGCGCCTGGCGATTGAAACGCAGAATGTCGCGCAACAT
GCCCCAGAAACCCGGAGACAGGATATTGCTGCGCTGGGCGAACAGGCTGTTGAGGTTATTGCCGTTGTACTCGACG
TTTTCCTCCGGGGCGCAGACCGAGAAGCTCATTTGGGTCGGCTTGAACGGCACGCCAATTTGCTGCAGCAGGCGGA

TGAAGTTGGGGTAGGTCCAGTCGTTGAACACGATAAAGCCGGTGTCCAGCGCGTACTGTGCGCCGTCGACGGTCAC
GTCTACCGTGTGGGTATGGCCGCCAATCCGGTCGGCGGCTTCGAACAGCGTGATGTCATGGCGGCGGCTGAGCAGA
TAGGCGCTGGTCAGCCCGGAAATTCCGCTGCCGATAATCGCGACTTTCACAGGTTGTCCTTGTGCGGCGGCGGGCT
GCGCAGCATGCGTTTGCCGATGATCAGTTGCGCGCGGTTCGGCAGTTTCGACAGCGGCCACAGGGTGGCGATGAAC
AGGGCTGGGAAGGCGATCTCCAGTGGGCGTTTTTCAAGTTTGCCGAAGATATGCCGTGCGGCTTTGTCGGCCGGCC
AGCTCAAGGGCATAGGGAAATCGTTGCGTTCGGTCAGTGGGGTGTCGACGAAACCGGGGCTCACTACCGTGACGTC
GATGCCCTCGGGCGACAGGTCGATGCGCAGGGATTCGAACAAGTAGCGCAGGCCGGCCTTGGACGCGCCATAGGCT
TCTGCGCGGGGCATCGGCAGGTAGGTGACGGCACTGGCGACCCCCACCAAATGCGGGGTTTGCCCGGCACGCAGCA
GCGGCAGCGCGGCTTCGATGCAATAACTGCTGGCCAGCAGGTTGGTGCGCACCACGTGTTCGATAATCGAGGCATC
GAACTGCTTGGCATCCACATATTCGCAGGTGCCGGCATTGAGGATCACGGTGTCCAGGGCGCCCCAGGTTTGCGCG
ATCTGTTCGCCGATTTCCCGTACCACCTGGCTGTTGGTCAAGTCGCCGGCGACCACCAGCACTTGCCCGGGGTAGC
GTTGGGCCAGTTGTTCCAGTGGCGCCTTGGAGCGCGCACTCAGCGCCACCTGCGCACCACTGCCCAGCAGCTCTTC
GGCCAATGCGGCACCGATGCCACTGCTTGCCCCTGTCAGCCAATAACGCCGTGGCGATTTCAGGCTCATGCCATTC
TCCCTTTCAACCAACGGACCAGCCGGCCAAGGACCGGTAGGTGTTCGTAAAGCAGCGCGCCAGCATCAAAGTAATC
GCGGTGCCGGTAAACCTTGTCGTGCCATAGCAGATGGGAGCAGCCCTCGACCTGAATCTGTCGGCCACCCTCCAGG
CGCGGGTGGCAGAAACTCATGGTCCAGCGCAGGTAACCCTCGCCATCGGCCACGTGGTCGAAGGCGTGGAAGTCAA
AACGCAGTTGCTCGACGTTGCTATAAAGCTCGGCGAAGTACGCTTGCAGCGCGCTGATACCGTGGACCTGATGCAG
TGGGTCGGTAAACGATATGTCCTGGCTGTACAGGCTGTCGAGCAGGTGCAGGTTGTGCTTGTCCAGGCGGGCGAAG
GACTGGGCGAACTGGCGCAGGAAGTCACTCATACACGCCTCCCACCGCCTGGCGTGAGGGCAGGGCACGAAAGGCC
GCCAACGCACGCTCACGGGAATGTTTGAGGTCGACGATGGGCCGCGGGTAGTCGGCCACGCCAAACAGCCCGCCAA
CGGCGTCGGGGTTGTGTACTTCCTTCTTGTTCAAGCCCGCCAGTTGCGGCAGCCATTGCTTGATAAAGCGCCCTTC
GCCGTCGAACTTTTCAGACTGGCTCAGCGGGCTGAAAATGCGGAAGTACGGCGCCGAGTCAGTGCCGGTGGACGAA
CTCCACTGCCAGCCGCCGTTGTTGGCCGCCAGATCGCCGTCGATCAAGTGCCGCATAAAAAAGCGCTCGCCTTCGC
GCCAGTCGATCAGCAGGTTCTTGGTCAGGAACATCGCCACCACCATGCGCAGGCGGTTATGCATCCAGCCGGTTTC
CAGCAGTTGGCGCATCGCGGCGTCGATGATCGGCAGGCCGGTGCGCGCCTCTTGCCAGGCTGCAAGGTCAGCGGGG
GCATGGCGCCAGGCCACGGCTTCGGTTTCCGGGCGAAACGCACGGTGGCGCGACACCCGTGGGTAGCCCACCAGGA
TATGTTTATAGAACTCGCGCCACAGCAACTCGTTGATCCAGGTGATGGCGCCGATATCGCCGCTTTCGAATTCACC
CTGGTTGCTTTGCAGCGCCGCATGCAGGCACTGGCGCGGCGAAACCACCCCGGCTGCGAGGTATGCCGACAGTTGG
CTGGTGCCGGGCTTGGCCGGGTAGTCGCGCTCGTCCTTGTAGTAGCTGATCTGAGCGTCGGCAAACTGCTCAAGGC
GCCGCTGTGCCTCGTCTTCACCGGCCGGCCAAAGTGCGCGCAGGGCGTCGCTGGGCGGGGCAAACCCCTTGACCTG
CTCGGGAACCGCATCACTGGCCAATGGCAGCGCGGCCTGGGCCTTGGGGGTTGGCACTCGACGTGGCAGGGCGCTG
TGCAGGCGGTTGTAGCAGACCTTGCGAAACTGGCTGAACACTTGGAAGTAGGTGCCCGTCTTGGTCAGCACGCTGC
CGGGCTGGAACAGCAATTGGTCCAGATAGCGGTAAAAACCCAGGCCTTGGGCTTCCAGGGCCTGGGCCACGGCAGC
GTCACGCCGGCTCTCGTGGATCCCGTACTCTTCGTTGACGTGCACGGTCTGGACCGACAACTCCTGGCACAACTGC
AGCAATACCTGTGGGACTTGGTCCCACGTATCCGCGTGGCGGATCAGCAATGGGATATTCAGCTCACCCAGTGCCT
GGCCCAGGCACTTGAGGTTGCGCAGCCAGAAGTCGATTTTGCACGGTGCGTCGTCATGGGCCAGCCATTGCCCAGG
CGTGATCAAGTACACCGCCGCCGTCGGGCCACGCTGGCAGGCGGCCGCGAGGGCGGTGTTGTCATGCAGGCGCAGA
TCGCTGCGCAGCCAGATCAGGTGCATTTAAAGAAGCCCACGCTGGATCAGGAGTTGATGAGCCGACAACGGGTCTT
CGGCCAGCAACAACTGAGGAATGTCTTGGGTTAATACCGCCAACTCGGCCTGGTGGATGCACACCGTTGGTCCGGC
GATAAGGGTCGGGCAGTTGGTGCCGCTCAAGAGTTTCGGCAGGGCCGCCAGATGGATGGCCTTGCTTGAGTACAGC
AGCACGGCGCGCGGCTGCAAATGCTCAACGGCCAAGGCCAGTTCGCCGTTGGGCAACGGCCAGTCGAAGACCTCCA
CCGGGCAATCGGCGCTGCTGGCCAGCCAGGCGCTCAGCCACAGGTGCGGCTCCAGCGGCAGGTCGGAATGATTGAT
CAACAGCAGCGGCGGGCCATTGAGCTGGCGATTGTTGTGATACATCCGCGCGCCAAATTTGCTGCGCAGCCAGGAG
TGGAAAAACACCCGCTCCATCTGTGCGCCGAACTGGCCCTGCCAGCGCTGTTCCAGTTCCTTGAGCAGCGGCAACA
ACAGCTGCTCGCACAGCGTGCGCGGTGGGTAGAGGGCCATGGCCTGGTTGAACACATCGTCCACCCGGCGTTCGGC
CAGTTGAGCGATTGCCTGCACCAGCGTCTGGCGCAGGCTCTGCCATTCGTTTTCGATGCTGTCGTCCACCGGCTGG
GCGTTGTCGATCAACTGCTTGACCTGGCTGACCGGCACGCCACGGTTGAGCCAGGTCAGGATGGTCTGGATGCGCT
GCACATGTTCGGCGTTGAACAGCCGGTGGCCCTTGGGGGTGCGCTGGGGCACGATCAAGCCATAGCGCCGCTCCCA
CGCGCGCAAAGTCACCGCGTTGACCCCGGTTTGGCGCGCCACTTCGCGAATCGGCAGCCAGCCTTGTTCCAGGGCC
TGGGCCACGTCTTCGACGGGGGGGGTTGGGTGTTGCAGGGAAACATTCATGGGTTAGATCGCATTACGCAGGCTGA
GATTTTCCGGATGGGGCTGCAGGTACGCCTGTTGCGCGATGTAGCGGTCCGGGTGTTGGCGAAAGTGATGTTTGAG
CAGGGTCAGCGGCACCACCAGCGGCACGATGCCGTGACGGTATTGGCCGATCAGGGTCTGCATTTCTTGTTTGTCG
TCGGCGCTGAGGGCTTGCTTGAGGTAGCCGCTGATGTGTTGCAGCACGTTGGAATGGGTGCCACGGGTCGCGCACT
TTTTCAGTGCGCTCATCAGCTCAGTGAAATAGTCGCTAGCCAGGGCTTGCAGGTCGCAGCCCTTGCCCATGCTGCC
CAGCAGATGACCCAGGCTCTTGTAGTGGGCCGGGCTGTGGGCCATCAGCAGGTATTTGTAGCGCGAGTGGAAGGCC
AGCAGGCGGTGACGTGTCAAGCCCTCTGCCAGCAGTTGCTGCCAGGTGGCATACACAAACACCCGCGTCAGGAAAT
TTTCGCGCAGCACCGGGTCGTGCAGGCGACCGTCTTCTTCCACCGGCAGGTTGGGGTGGCGGGCACAGAAGGCCTG

GGCATAGATGCCGCGCCCGCCGCCATCCACCGGGGTGCCGTTGTCGCGGTAGACCTTGACCCGTTCCAGGCCACAG
GACGGCGACTTCTGCATGAAGATGTAGCCGCACAGGTCGGTATGCTCGGCGGCCATTTGTTGACCATAGGCGTCCA
GGGGCGCGGTTACATTCAATTCACGGTGCACAGTGCCCACGGCCTGCGGTTGCTGCGGATCGCCCACCAGGCGAAT
CGGCTCGCGGGGGATGCCCAGGCCAATGGCGACTTCGGGGCACAACGGCACGAAGTCAAAGTATTCACCGAGTGTC
TGGCTGCACAGGGGGGGATTGCTTGTGCCCGCCGTTGAAGCGCACGTTTTCGCCAAGCAGGCAGGCGCTGATGGCGA
TCTTGGGTTTGCCGGTGGCGGACACAGGGGTGGACATGGCAAAACCTCTACAAGATTCCTGTACAGAATTTAATTT
CTGTACAACTTAATCTCATCATAGGTCTGAGGCTGTACAAGTCAAATTATTTGTACAGGTTTTTACGTCCAGCCGA
TTTTCCAGCGCTCGGCATCTTGCAGGCTTTGCCAGGCCAGGCGCTCTGAACGGCGGGTGAGGACGGCTTGCAGTTC
AATCTCCAGCACGCTGCCTTCGTCATCGCGAAACAATTCGGTGACCAGGAAATGTTTTTCCTTGTGCCGAGGTTGC
GTGGCTGTCCATTTCGAGAGCAGCAATTTCGCCGGGTTGATGCGGTTCATTGCAGGTGTTCGAGCAGGCGCCGCGC
CGCTTCCTGGCCGCTGAGCCACGCGCCTTCGACCCTGCCGGACAGGCACCAGTCGCCACACACGTACAACCCCAGG
TCGGCGTCCGCCAGCACGCCCCATTCATGGCTGCCAGCCGGGCGGGCGTACAGCCAGCGGTGGGCGAGGCTGAAGG
AGGGCGCCGGCATGCTGCAGTGCAGCAATTCGGCAAAGGCGCCGTGCAGCAATTCAATAATGGCGTCCTTGGGCAG
GTCCAGGTGCTTTTTACTCCAGGCACTGCTGGCATGCAGGATCCAGGTGTCGGGCGTAGCATCGCGCCCGGGTTTG
CTGCGGTTGCGGGCCAGCCAATCCAGGGGGCTGTCCTGTACAAAGCAACCTTCCATCGGTGTATCCAGCGGCGTGT
CGAAGGCCAGGGCGATGGCCCAGGTCGGGTCCATTTTCACCCCGGCGGCAGTGCTCGCCAGTTTCGGCGCTGCCGC
CAGCAGAGCGGTGGCCTGGTGCGCCGGGGTGGCGATGATCACGTGGCTGAAGGGGCCGTGGTTGCCGCCATCGGCG
TCCAGCAGGTTCCAGTGTTGCTGGCCCTGGAACACTTCGGTGATACGGCAGCCAAACTCCACCGGCAGGTCATCGA
GCAGGGCGCGGGTGATGGCGCTCATACGCGGGGTGCCGACCCAGCGGGTCTGTTCGTCCGGGGAGGGGCTCAGTTG
GCCGGATTGGCAATTGTAGAGTTGCGGCTCCCACTCGGCAGCCCAGCCATTGGCTTGCCAGCGTTGGACTTCATTG
ACGAAACGTCGGTCGCGCGCGGTGAAATACTGCGCGCCCAAGTCCAGTGCACCGGCTTCGCTGCGCTTGCTGGACA
TGCGCCCGCCACTGCCGCGGCTTTTATCGAAGAGTTGTACAACGTGCCCGGCATCTTTTAGCGCTCGGGCGGCCGA
AAGTCCGGCGATGCCGGTACCGATGATCGCGATAGGAACAGTCATGGGAGGCCTCGTTTTACCGTTGGGTACAGAC
TACGCCGACACCAATAGCTGTACAATATTGTTTTTTGGTATAAGTTTCGGCATGCCTGAATGTGTCGCGTGACCTA
TGGTTAAACCTAGGTTGCGCCGATTAAAAAGATCCCTGCTTATAAAATAGACCAGCGACGCTCGGCCAACGTTACA
GGAGGAAGATCTCATGCACATTTTGCTGACCGGCGGTACGGGTCTGATCGGCCGCCAGCTCTGCCAACACTGGAGC
GCCCAGGGGCATCGGTTGACGGTCTGGAGCCGCAACCCCACTCAGGTCGCCGCGCTGTGCGGTGCGCAAGTGCAGG
GCGTCGCCCGTTTGCAGGATGTAACGCAGCCGGTGGATGCGGTGATCAACCTGGCCGGCGCGCCGATTGCCGACCG
CCTCTGGACCCACAAACGCAAGGCGTTGCTGTGGAGCAGCCGGATCAGCCTGACGGAAACCCTGCTGGCCTGGCTC
GAGGGCCTGGCAGTCAAACCTGCGGTATTGATCAGCGGCTCGGCGGTGGGTTGGTACGGCGATGGCGGTGAGCGTG
AGCTGACCGAAGCCAGCGGCCCGGTGCAGGACGATTTCCCCAGCCAGTTATGCATCGCCTGGGAAGAAACCGCCCA
GCGTGCCGAAGCTTTTGGCTTGCGGGTGGTGCTGGTGCGTACTGGCTTGGTGCTAGCGGCTGAGGGCGGCTTTTTG
TCGCGGCTGTTGCTGCCCTTCAAGCTGGCGCTGGGCGGGCCTATCGGCAATGGTCGGCAGTGGATGCCGTGGGTGC
ATATCAAGGATCAAATCGCCCTGATTGATTTTCTTCTGCATAAACCTGACGCCAGCGGTCCTTATAATGCCTGCGC
GCCACAGCCGGTGCGTAACCGCGAGTTCGCCAAGACCCTGGGCCAGGTCCTGCACCGCCCGGCGTTCATGCCCATG
CCGGCGTTTGCCCTGCGGCTGGGGCTGGGCGAGTTGTCCGGGTTATTGCTGGGGGGGCAGAGGGCAGTACCCGAGC
GGCTGTTGGCTGCCGGTTTCATTTTTCAGTTCACTGATTTGCACGCGGCCCTGGACGACTTGTCCAGCCGCCTCTA
GAGATAGGATGTTGCATGACGGATCACGCGTTGTTACTGGTCAACCTGGGTTCACCGGCGTCCACTTCGGTGGCCG
ATGTGCGCAGCTACCTCAATCAGTTTTTGATGGACCCCTATGTGATTGACCTGCCGTGGCCGGTCCGGCGCTTGCT
GGTGTCGCTGATCCTGATCAAGCGCCCCGCGCAGTCGGCCCATGCCTACGCCTCGATCTGGTGGGACGAAGGCTCG
CCGCTGGTAGTCCTCAGTCGCCGCCTGCAGCAGCAGATGACCGCCCAGTGGACGCAGGGTCCGGTGGAGCTGGCGA
TGCGCTATGGCGAACCCTCGATCGAAAGTGTACTCACCCGCCTGGCCGGGCAGGGCATCAGCAAGGTCACGCTGGC
GCCGCTGTATCCGCAATTTGCCGACAGCACCGTGACCACGGTGATTGAAGAAGCGCGACGGGTGGTGCGGGATAAG
CAACTCGACCTGCAGTTCTCGATCCTGCAACCGTTCTACGACCAGCCGGAATACCTCGACGCCCTGGTGGCCAGTG
CCAGGCCGCACTTGCAGCAGGATTACGATCACCTGCTGTTCAGCTTCCATGGCCTGCCGGAACGGCACCTGAACAA
ACTCAACCCTGGGCATTCGCTTGAAGGCAGTGGTGACTGCTGCGCCAATGCCTCGCCCGAGGTGCGCACGACGTGT
TATCGCGGGCAGTGCTTCAGCGTCGCCCGTGACTTTGCTGCGCGCATGGGCCTACCGGACGATAAATGGTCGGTGG
CGTTCCAGTCGCGCCTGGGCCGGGCGAAGTGGATCGAACCCTACACCGAGGCACGCCTGGAGGCATTGGCCCAGCA
AGGGGTGAAGAAGTTGTTGGTGATGTGCCCGGCGTTTGTCGCCGATTGCATCGAGACCCTGGAAGAGATCGGTGAT
CGCGGGCTGGAGCAGTTCCGCGAAGCCGGGGGCGAGGAGTTGGTGTTGGTGCCGTGCCTCAATGATGATCCGCAAT
GGGCGGTGGCGCTCAACACCTTGTGCGAAAGAGCGCCTGTCAGCCTTTAGCGTGGCAGCGGGCTGGTGTGGGAGCT
GATGTGGAAGCTGGCTCCCACCCAAGCCAGCCCCTGCACAAGCCAGCTCCCAGATGGGTTCACTGGGGGTTATTCG
TGGTGCGGTTGGCCGAGGAAGGTCAGTGAGCTGAACAGCGCACGGGTCTGGATCTCGCTATCGCCTTGGACCGGTA
CTTCCACCTGCGCCGCAATCACATTCAACGCCTCGTTGCGCACCAGTACCTGGGCCCGGCCATCCTTGTCGGTCTC
GGTGCTCAAGGTGTTCGGCGCGCTGCGGTAGTCGCCAATCAACTTGATCCCCGCTGCAGGCTTGCCATCGATCAAC
ACTCGCACCGGCAGGGATTTGCCAGGGCCAACGGTGAGCGGATCGACCTCGGGCACGATCACCATCCTCAATTGAT
CCAGCTTGGGCAACTTGGCCCCGGCCTGATAGATCGCCAGGCTGTACTTAAGGGCCTGGGTGGTTTCGAGGGCGCC

GGGTACTTGGCTGCGGCCCTTGTTCACCCACTTCTTGTCCGGGGTTTGCGACCACAAGCCATTATCAAACGCCACC
GCCAGCACCGCAGGCGGCTTGAGCGGTTGCAGGCGCGCCTGGTCGTCCAGGCGTTGCACCGTCACCGGGATCATCC
GGCCACTGGGGTCATAGGCCCAGGCACCGCTGACCTTTTTCGCCTTGAAGGCTTCGTCTTCGGCACCGTGGCCATA
GACCACCTCGATATGGCCCCGGCGTTGCTCGGTCCACAGGCCATGGGCCGAGACCTGGGTGGCGAACAGCAGACCG
AGCACGGCCAATGATTTGAGCGCTTGCATGGGGACGTCCTTTTACAGGTTGAGGGTCAGGCTGACGGTGAAGTTGC
GCGGCTCTCCGGGGTTGACCCAGTAGTTGCTGTAGGAGCGCTCGTAGTACTTTTCGTCGAACAGGTTGTTGAGATT
CAGGCCCACGGTGACGTTATCGCTGGCTTTGTAATGGGCCAGCAGGTCGACGGTGTGGTAGGCCGGCAACTCGAAG
CTTTTGCCAGCCTCGCCCGAACGGTCGCCGACATAAGTAAACGCCGCGCCCACGTCAGAGCCGCGCAGGGCGCCAT
CCTGGAACTCATACACGCCCAACAGGCTGCCACTGCGCTTGGCCACGCCGAGAATGCGGCTGCCGGTGGGCACGTC
TTGGTCGCCCTTGGTTACTTCTGCGTCGATGTAGGCAAAGGCGCCAATCACCCGTACGGCGTCAGTCAGTTGCCCG
GTCAATTGCAGGTCGAAACCCTGGCTGCGGGCCTTGCCGATGGCGCGCTTGGTGTCGGTGGCCGGGTCCGTGGCCA
GGGTGTTTTCCTTCTCGATATGGAAGGCGGCGAGGGTGGTGCTCAGGCGGTCGTCGAACAGCTCGCTCTTGATGCC
CACTTCATAACCGACGCCTTCCTCGGGCTTGAGGAAATTGCCGGCGGCATCCAGCTCACTTTGTGGTTTGAACGAG
GTGGAGGCGTTGGCAAACAGGCCCACTTGCGGGGTCAGTTGGTACAACAGGCCGGCCCGCTGCGTCATGACGTCGT
GGGTTTTCTGGGTCTTGGCCCCGTTACGGGTGAAGTCCTCTGAACGTTGCTCTACGTGCTCCAGGCGCATGCCAAC
CACACCGCGCAGGCGCTCGGTAAAGACGATCTGGTCTTGCAGGTTCAGCGCCTGGCTTTTCACCTGGGTGAAAAAA
TCAGTGCCCGAGCGCTGGCCATTGGGCTTGGGCTGGCCATAGACCGGCCGGTAGATATCAATCGGGTAACTGCCGG
CGATGGCGGTCACCCGTTCTTTCTTGCGGTAGTCCTCGTACTCGGTGCCGATCAGCAGTTCATGCTGCCAACTGCC
CACCTCGAACAGGCCGCGCAGTTCCAGTTGGGTGATGCTGTCATGCCACCCCATCGAGCGTTGGCGGTAGCGGCGG
TTGACGGTATGGCCATCGGCATTCAGCGCACGGCTCTCCGAGGCTTCGCCCCACAGGCTGCCCTGTTTGTAATGGC
TGGCCAGGCGCAGCTTCCAGGCATCGTTCAAGTGATGCTCGAGGGTGGCTTGCAGACGGTTGTTGTGGTTGTCGAT
ATCGCCGTCGTTGGGCTCGCCAAGGAAGGTCGAGCGCGACATGCCGCTCCAACGGTTGTTGGGGGCCACGATGCCC
CGGTCGAAGGTCGAACTGTGGCGCACGAACTCACTCTCCACCAGCAGGCTGGTGTCCGGGTCCAGTTGCCAGCTGA
AGGAGGGCGCAACAAACACCCGCTTGCTCTGCACGTGATCGCGGAAGCTGTGGTTGTCTTCCACCGCCAGGTTGAC
CCGCGATAACACCTGGCCTTCGCTGTCCAGCGGGGTGTTGATATCGACGGCGGTGCGGTAGCGATCCCAACTGCCG
GCGCTGGTCTGTACGGTGGTGAAGGCTTCGGCTTGGGGTTTCTTGGTGACGATGTTTACGGTACCGCCGGGATCGC
CACGGCCATACAGGCTGGCGGCGGGGCCCTTGAGCACTTCAATGCGTTCGATATTGGCCGTGTCCGGCGTACTGGG
GTAGCCGCGGTTGGCGCTGAAACCGTCCTGGTAGAACTGTGAGGTGGTGAAGCCGCGAATGCTGTACTCGTAAAGG
GTCAGGCCGCCGAAGTTGTTCTGCTTGGACACGCCGCCGGCAAACTCCAGGGCGCGCTCGACGTTGTTGCTGCCCA
GGTCCTTGAGCACTGTGGCGGGAATCACACTGATCGATTGTGGGATATCGCGCAGGGCCGTGTCGGTCTTGGTCGC
GCTGGCCGAGCGGGTCGCGCGGTAGCCCTGGACCGGGCCGGTGGCGGATTCATACGCCTCGCCGGTGATGCTGGTG
GTGCCCAACTCAAGGGTGTTGGTGTCTTCTGCAAACACCGGGTCCACCAGCAATCCCAACGCGAGACCGGCCAGGG
GCGCAAACTTTCGAGACGTCATGGTCTAACATTCCAATATCAGTATTGAAATAATATAACATGACTGTTGAGAGTG
TATCTCTTTAAGATCGCCCTTTATTCTTGCTCTTTAACCGGTGCTGGCGGCGGCCGCAGCCCCACTTCGGCCAGGA
GTTTGATCTCTTTGCCGTTGCGCATGACCTGGATCGCGACCTTTTCCGTAGGCTTGATCCGCGCCACCTGGTTCAT
CGAACGACGGCCATCGCCAGCCGGCTCGCCGTCGATGCTGAGGATCACATCGCCCACCTGCATCCCGGCTTTTTCC
GCTGGACCGCCGCGCAGGATGCCGGCAATGACAATGCCTGGGCGCCCGGTCAGGCCAAAGGACTCCGACAGCTCCT
TGGTCAGCGGCTGCACTTCAATCCCCAGCCAGCCACGGATCACCTGGCCGTGCTCGATGATCGACTTCATCACTTC
CATCGCCAGCTTTACCGGGATCGCGAAACCAATGCCTTGGGAGCCACCGGACTTGGAGAAAATCGCGGTGTTGATG
CCCGTCAGGTTGCCGCTGGCATCCACCAGCGCGCCGCCGGAGTTGCCTGGGTTGATTGCTGCGTCGGTCTGGATAA
AGTCTTCGTAGCTGTTGAGGCCCAACTGATTACGCCCGGTGGCGCTGATGATGCCCATGGTCACGGTCTGGCCGAC
GCCGAACGGGTTGCCGATGGCCAGCGCCACATCGCCGACGCGCAGGCCTTCGGAGCGGCCGATGGTAATGGAGGGC
AGGTTCTTCAGGTCGATTTTCAGCACCGCAAGGTCGGTTTCCGGGTCGCTGCCCACCACCCGCGCCAGGGTTTCGC
GGCCATCGCGAAGGGCCACCACAATCTGGTCGGCGCCGGTGCTCACATGGTTGTTGGTCAGGATGTAGCCTTCCGG
GCTCATGATCACCCCGGAGCCGAGGCTCGATTCCATGCGGCGCTGCTTGGGCGAGTTGTCACCGAAAAAGCGCCGG
AACTGTGGGTCTTCAAACAGCGGATGCGCCGGTTTATTGATGACTTTGGTGGTGTACAGGTTGACCACCGCAGGCG
CCGCGACCACTACCGCATCGGCGTAGGTCACCGGGCCCTGTTGCACCGCGCTGGTTTGCGGCGCCTGTTGCAGGTT
GACGTCCAGGCTTGGCAGGCCGACCCATTGCGGATAACGCTGAATAATCAGCATCGCGATCAGCACGCCGGCCAAC
AATGGCCATCCAAAAAAACGCAGCGCCTTGAGCATCAAGTAAGTCCTGACAGGTTGCAGGGGGCGCGGGAGCGCCC
ATAATGTCGCGCATTATACGAGGCCGCGAGCGCCTCTGAACGGGATATTTAGGAGTCTTTTATGGCCGTCGCCCTG
AGCACCCTGGTTGAAGAAGCAGATCGCTACTTGAACAGCGCGAAAATTGCCGATTACTGCCCCAATGGCCTGCAGG
TCGAAGGGCGGCCGCAGGTGTTGCGTATTGTCAGTGGCGTGACTGCCAGCCAGGCGCTGCTCGATGCAGCCGTCGA
GGCCAAGGCCGACCTGGTGCTGGTGCATCACGGCTATTTCTGGAAAGGCGAAAGCCCGTGCATCACCGGCATGAAG
CAGCGCCGCCTCAAGACCCTGCTCAAGCACGACATCAGCCTGCTGGCCTACCACCTGCCGCTGGACCTGCACCCGG
AAGTGGGCAACAACGTGCAATTGGCCCGTCAGTTGGACATCACCGTCGAAGGCCCGCTGGACCCGGACAACCTCAA
GGTTGTGGGCCTCGTGGGCTCCCTGGCCGAGCCGCTGTCGGCGCGCGATTTCGCCCGTCGTGTGCAGGAAGCCATG
GGCCGCGAGCCGCTGCTGATCGAAGGCAGCGCGATGATCCGCCGCGTGGGCTGGTGCACCGGCGGCGGTCAGGGCT

ACATCGACCAGGCCGTGGCGGCCGGAGTCGACTTGTACCTGAGCGGCGAAGCTTCAGAGCAGACGTTCCACAGCGC
CCGTGAAAACGACATCAGCTTCATCGCCGCCGGCCATCACGCCACCGAGCGTTACGGCGTGCAGGCCCTGGGCGAC
TACCTGGCCCGGCGCTTTGCCCTGGAACACCTGTTCATCGACTGCCCCAACCCGATCTAAGCCTCCATTGCGTCCA
CTGTAGGAGCCGGCTTGCCGGCGATGAGGCCCTTGAGTCATGCGTTGATTTCAAGGACGCCATCGCCGGCAAGGCG
GCTCCTACGGGGCGATGTACGGCCAAACGAGGCGGTATATCCATATACCGTTTCGATCTAGCTGGCTCCCTGAATA
GAAGAAGGTGCTGTGCTAGCATGCCCCGCTCGAACACGGCCCGCAGGCCGTCCAAAAAGAATCGTTTTTCCGTGAG
TAGCCATGGTCGACAAACTGACGCATCTGAAACAGCTGGAGGCGGAGAGCATCCACATCATCCGTGAGGTTGCCGC
CGAGTTCGACAACCCGGTAATGCTCTACTCCATCGGCAAAGACTCCGCCGTGATGCTGCATCTGGCGCGCAAGGCA
TTCTTCCCGGGCAAGCTGCCGTTTCCGGTGATGCATGTGGACACCCAGTGGAAATTCCAGGAGATGTACAAATTCC
GCGACCGCATGGTCGAGGAACTGGGCCTGGAACTGATCACCCACGTCAACCCGGATGGCGTCGCGCAGGGCATTAA
CCCGTTCACCCACGGCAGCGCCAAGCACACCGACATCATGAAGACCGAAGGCCTCAAGCAGGCCTTGGACAAGTAT
GGTTTCGACGCAGCATTCGGCGGCGCCCGTCGCGATGAAGAGAAATCCCGTGCCAAAGAGCGCGTGTACTCGTTCC
GCGACAGCAAGCATCGCTGGGACCCGAAAAACCAGCGCCCGGAGCTGTGGAACGTCTACAACGGCAACGTCAACAA
GGGCGAGTCGATCCGCGTGTTCCCGCTGTCCAACTGGACCGAACTGGATATCTGGCAGTACATCTACCTCGAAGGC
ATCCCCATCGTGCCACTGTACTTCGCCGCCGAACGGGAAGTGATCGAGAAGAACGGTACGCTGATCATGATCGACG
ACGAGCGCATCCTCGAGCACCTGTCCGACGAAGACAAAGCCCGTATCGTCAAAAAGAAAGTACGTTTCCGTACCCT
TGGCTGCTACCCGTTGACGGGCGCGGTGGAGTCCGAAGCCGAGACGCTGACGGACATCATCCAGGAAATGCTCCTG
ACGCGAACTTCCGAGCGCCAGGGCCGTGTCATCGACCACGATGGCGCCGGCTCCATGGAAGACAAAAAACGTCAGG
GTTATTTCTAAGGGGTTGTCATGTCGCACGTATCTGATTTGATCAGCGAGGACATCCTCGCTTACCTGGGCCAGCA
CGAACGCAAGGAAATGCTGCGCTTTCTGACCTGTGGCAACGTCGATGACGGCAAGAGCACCCTGATCGGGCGCCTG
CTGCACGACTCGAAGATGATCTACGAAGATCATCTGGAAGCCATCACCCGCGATTCGAAGAAAGTCGGCACCACCG
GTGACGACATCGACCTGGCATTGCTGGTCGACGGCCTGCAGGCCGAGCGTGAGCAGGGCATCACCATCGATGTGGC
CTACCGCTATTTCTCCACCGCCAAGCGCAAGTTCATCATCGCCGACACCCCTGGCCATGAGCAGTACACCCGCAAC
ATGGCCACCGGTGCTTCCACCTGTGACCTGGCGATCATCCTGGTGGATGCGCGCTACGGCGTACAGACCCAGACCC
GTCGCCACAGCTTTATTGCTTCGCTGTTGGGCATCAAGCACATCGTCATCGCGATCAACAAGATGGACCTCAACGG
TTTCGACGAGAGCATTTTCGAATCGATCAAGGCCGACTACCTGAAGTTTGCCGATGGCATCGCGTTCAAGCCGAGC
ACCATGGCCTTTGTGCCGATGTCGGCGCTCAAGGGCGACAACGTGGTGAACAAGAGCGAGCGTTCGCCCTGGTACA
CCGGCCAGTCGCTAATGGAGATCCTCGAAACCGTCGAGATTGCCAACGACCGCAACTACACCGACCTGCGGTTCCC
GGTGCAGTACGTCAACCGTCCGAACCTGAATTTCCGTGGTTTCGCCGGCACCCTGGCCAGCGGCATCGTGCACAAG
GGCGACGAAGTCGTGGTGCTGCCGTCGGGCAAGAGCAGCCGCGTCAAATCCATCGTCACCTTCGAAGGTGAACTGG
AACACGCAGGCCCAGGCCAGGCCGTGACCCTGACCATGGAAGACGAGATCGACATCTCCCGTGGCGACCTGCTGGT
GCATGCCGACAACGTGCCGCAAGTCACCGACGCCTTCGACGCCATGCTGGTGTGGATGGCCGAGGAGCCGATGCTG
CCGGGCAAGAAATACGACATCAAGCGCGCCACCAGCTACGTGCCGGGTTCCATCACCAGCATTGTGCATCGTGTGG
ATGTGAACACGCTCGAAGAAGGCCCGGCCAGTGCCTTGCAGTTGAACGAGATCGGCCGGGTCAAGGTCAGCCTCGA
TGCGGCCATCGCCCTGGACGGTTACGCCAGCAACCGCACCACCGGCGCCTTTATTGTGATTGACCGGTTGACTAAC
GGCACCGTGGCAGCGGGCATGATTATCGCGCCGCCGGTCAGCCATGGCAGCGCAGCGCAGCACGGCAAGTTGGCCC
ACGTGGCCACCGAAGAACGCGCCCAGCGTTTCGGCCAGCAGCCAGCCACCGTGTTGTTCAGTGGCCTGTCGGGTGC
TGGCAAGAGCACCCTGGCCTATGCGGTCGAGCGCAAGTTGTTCGATATGGGGCGTGCGGTGTTTGTGCTCGATGGG
CAGAACCTGCGCCATGACCTCAATAAAGGTCTGCCGCAGGATCGTGCCGGGCGTACCGAGAACTGGCGTCGTGCTG
CCCACGTGGCGCGTCAGTTCAACGAAGCCGGCTTGCTGACCCTGGCGGCCTTTGTGGCGCCGGACGCCGAAGGCCG
TGAACAGGCCAAGGCGTTGATCGGCAGTGATCGTTTGCTCACCGTATACGTGCAGGCATCGCCGCTGGTATGTGCC
GAGCGTGACCCGCAAGGCCTGTACGCTGCCGGTGGGGATAACATCCCTGGCGAGTCGTTCCCCTACGACGTGCCGT
TGAATGCCGACCTGGTGATCGACACCCAGGCCCTGTCGTTGGAAGACAGCGTCAAGCAAGTGCTGGAACTGTTGCG
TCAGCGCGGCGCGATCTAAACCTCGCCTCCATAAAAAGCCCGCCACCGATCACTCGGTGGCGGGCTTTTTTTCACA
CTTTAAAGGTCAACACCGTCAATGTGGGAGCTGGCTTGCCTGCGATTGCAGGAGGCAGGCAATGCACTTACCTCTG
ACAGACCGCTATCGCAGGCAAGCCAGCTCCCACATCGTGATCACCTATTGCCTGGGTACTCGATGTGAAGTCGGCT
CAGCAGAGCATCCTTGTCTTCCCATAACTGGTTGATCCAGCCCTGGAACGCCAGCCGATACTCCCCATCCTGCTCA
TAATTCTTGCCAATGAACTCGGCCGGGATCTGCACCTCTTCAAACTGCACAACCACGTCTTTGACATTGCCGCACA
GCAAGTCCCAGTACCCCGGACGCCCGCCAGGGTAGTGGATCGTCACGTTCACCAATGACTTCAACTGCTCGCCCAT
GGCATCCAGCACAAACGCAATGCCACCGGCCTTGGGCTTGAGCAGGTAACGAAACGGCGACTGCTGCTGCGCATGT
TTGCCTGGGGTAAAGCGTGTGCCTTCGGCAAAGTTGAAAATGCCCACCGGGTTGTCACGGAACTTTGCACAGGTCT
TGCGGGTGGTTGCCAGGTCCTTGCCTTTCTTCTCCGGGTGTTTTTCCAGGTAAGCCTTGGTGTAGCGCTTCATGAA
GGGGAAGCCCAGCGCCCACCAGGCCAGGCCAATCACCGGCACCCAGATCAGTTCCTGTTTGAGGAAAAACTTCAGC
GGGCGGATCCGCCGGTTGAGCACGTACTGCAACACCATGATATCGACCCAGCTCTGGTGGTTGCTGGTCACCAGGT
ACGAGTGCTGGTAGTCCAGGCCTTCCAGGCCCTTGATGTGCCAGCGTGTGCGGCGCACCAGGTTCATCCAGGTGTT
GTTATTGCTGATCCAGGCCTCGTGGGTCTGGTTCATCAGCCATTCGCTGAAGCGCTTGGCGAACGGCAGCGCCTTG
AACAACGCGACGATAAACAGGAATGAACACAACAGGATGGTGTTCAACGCCAACAGCAGCGAGGCGATCACCCCGC

GCAGGGGGGCAGGCAGAAAGTCCAGCATTTAGGCATCCATAGGTCGGTTGGCGGCTTGAATCGCGGTCAGGGCGAT
GGTGTAGACGATGTCATCTACCTGGGCCCCACGGGGCAGGTCGTTCACGGGTTTGCGCAGGCCTTGCAGCATCGGG
CCGAGGCTGACGCAATCGGCACTGCGCTGCACCGCCTTGTGGGTGGTGTTGCCGGTATTGAGGTCGGGGAACACAA
AGACCGTGGCCTTGCCCGCCACCAGGCTGTTGGGCGCCAGTTGCCGGGCGACGTTTTCGTTGGCCGCAGCGTCGTA
CTGCAACGGGCCGTCGATCAGCAGCGAGCTTTGCTGTTCGTGGGCCAACAAGGTGGCCTCGCGGACTTTTTCCACT
TCCTCGCCACTGGCCGAGTCACCGCTGGAATAGCTGATCATCGCCACGCGCGGGGTAATGCCGAAGGCAGCGGCCG
AGTCGGCGCTTTGCAGGGCGATTTCCGCCAGCTCCGCGGCGCTGGGGTGCGGGTTCATCACGCAGTCGCCGTAGAC
CAGCACCTGCTCGGGAAACAGCATGAAAAACACCGACGACACCAGGGTGCAACCCGGCGCCGTTTTAATCAGCTGC
AATGCAGGGCGGATGGTATTGGCGGTGGAGTGGATCACCCCGGACACCAGCCCATCCACCTCATCCAGCGCCAGCA
TCATGGTAGCGATCACCACGGTGTCTTCCAGTTGCTGCTCGGCCATCGGCGCGTTGAGGCTTTTGCTCTTGCGCAG
CGCCACCATCGGCTCGACGTAGCGCTGGCGGATCAGGTCCGGGTCAAGAATTTCCAGGCCTTCGGGCAGTTCGATG
CCATGGGCGCGGGCGACGGCCTCGACGTCGGCCGGCTTGGCCAGCAGCACGCAACGGGCAATGCCGCGGGCCTGGC
AGATGGCGGCGGCTTGCACGGTCAACGGCTCGCTGCCTTCGGGCAGCACGATGCGCTTGTTGGCGGCCTGGGCGCG
CTGGATCAACTGGTAGCGGAACACCGCCGGCGACAGGCGCATCTCCCGTGGTGTACCGCAACGCTGGTGCAGCCAG
CGCGCATCCAGGTGACTGGCGACGAAATCGGTGATGATCTCCGCACGTTCGCGGTCATCAATCGGGATTTCCTTGT
TCAGGCTGTTGAGCTGGTTGGCGGTGTCATAGGACCCGGTGCTCACCGACAGCACCGGCAACCCGGCCTGGAATGC
ACCACGGCACAGGTCCATGATCCGTGGGTCAGGCAGGGTGTCGCTGGTCAGCAGCAGGCCGGCCAAAGGTACGCCG
TTGATCGCGGCCAGGCTGACGGCGAGGATGATGTCGTCGCGATCCCCCGGTGTCACCACCAGCACGCCGGGCTTGA
GCAGCTCCACGGTGTTGCGCATGGTGCGCGCGCAGATGATGATTTTGCTCATGCGCCGGGTTTCATAGTCGCCTGC
GTTGAGCACTTGCGCGCCCATCAGGTCCGCCACGTCACGGGTGCGCGGGGCGTTGAGCTCGGGCTGGAACGGAATG
CAGCCGAGCAGGCGGAAATCGCCACTGCGTAGCAGCGGCGAATGCTCCTTGAGACGGGCGGCGAAGGCCTCCATGC
TCTCGTCGGTCTGCACCTTGTTGAGAATCACGCCGAGCACTTTCGGGTCTTTCGGGCCACCGAACAGTTGCGCCTG
CAATTCCACACGCCCGGACAGTTCGGTCAGCACTTCGTTTTCCGGGGCCGAGACCAGGATGACTTCGGCATCCAGG
CTTTTTGCCAGGTGCAGGTTGACCCGTGCCGCATAGCTGGCGCTGCGGGTCGGCACCATGCCCTCGACAATCAGCA
CGTCCTTGCCCACGGCGGCCTGTTGGTAAAGGGTGATGATCTCTTCGAGCAATTCATCCAACTGGCCATCGCCGAG
CATCCGCTCGACATGGGCCAGGCCCAGTGGCTGCGGTGGTTTCAGACCATGGGTACGGGCCACCAGCTCGGTGGAG
CGCTCAGGCCCGGTGTCGCCGGGATGGGGCTGGGCAATGGGCTTGAAAAACCCGACTTTCAGCCCGGCCCGCTCAA
GGGTACGCACCAGCCCAAGGCTGATGGAGGTCAGACCCACACCAAAGTCGGTGGGCGCGATAAAAAAAGTCTGCAT
GCGAATTCTCTGAAGGTGCATGGCTTAGGTGGTGCGCTATGGCTGCGCGCCGACCGGGAATCAGCTGCCAAGGGTA
TCGTTATCCGGGCGCTGCGCACACCAGCCACAGGCAAAGGGCTGGCCTATTTTTGCCAGGCGTTGCGCCGCATCCA
GCACCCAGCCCCGGGATTGCCAGGGCGGTTGGTGCCGTAGGTGCTGCGTATGGCCGCAGGACAGCGTGGCGACCCA
GTGGCCGTCCGCGTCCTGATGAAATCCGACGACCGTCGAGTCACTGGATCGACTCCGTCTGTCCGGGTTGTGTTCG
CTTTCGGGCAAATCCTTGTTTAGACTTGTCCGTTCTTCATTCTTATGCAAAAGGTCTCGCCCCATGCCGATCGCCG
CCAACAAGGCTGTCTCCATTGACTATACCCTGACCAACGACGCTGGTGAGGTCATCGACAGCTCCGCCGGCGGCGC
GCCGCTGGTCTACCTGCAAGGCGCAGGTAACATCATCCCGGGCCTGGAAAAGGCCCTGGAAGGCAAAGAGGTTGGT
GATGAGCTGACCGTTGCCATCGAACCTGAAGATGCCTATGGCGAGTACTCGGCCGAACTGGTCAGCACCCTGAACC
GCAGCATGTTCGAAGGCGTCGACGAACTGGAAGTGGGCATGCAGTTCCACGCCTCCGCGCCGGACGGCCAGATGCA
GATCGTGACCATCCGCGACCTGGATGGCGACGACGTCACCGTCGACGGCAACCACCCGCTGGCCGGCCAGCGCCTG
AACTTCCAGGTCAAGATCGTTGCCATCCGCGACGCATCCCAGGAAGAAGTGGCTCACGGCCACGTGCACGGCGAAG
GTGGTCACCACCATTGATTGACGGGCTACGGCCGGTCAGTTGAAAAGAAAGGCGCCTTCGGGCGCCTTTTTTTGATT
GGCGGCTATTCTTGCCGACTTGGCGGCTGTAATCTCGAAATGCCGTTAACCAGAATATGGGAAACCTGGAGTGTGT
CATGAGTGCTTTCCACGACCTTAAACTCAAAGCCCTGGATGGTCAGGAGCTGCCTCTGGCGCCCTATAAAGGGCAA
GTCGTGCTGGTGGTCAATGTCGCCTCCAAATGTGGGCTGACCCCGCAATATGCGGCGCTGGAAAACCTCTACCAGC
TATACAAGGATCAGGGGTTTACCGTGCTGGGCCTGCCGTGCAATCAGTTTGCTGGCCAGGAGCCGGGAACGGAGGA
AGAAATCCGCGAGTTTTGCAGCCTCAACTACGGGGTGACCTTTCCATTGGGCAGCAAGCTGGATGTGAACGGCCCC
GAGCGCCATCAGTTGTATCGCTTGCTCGCGGGCGAGGGCGCCGAGTTTCCCGGGGACATCACCTGGAATTTCGAGA
AGTTCCTGTTGGGCAAGGATGGGCGGGTTTTGGCGCGTTTCTCCCCGCGCACTGCGCCAGATGACCCGACGATCAT
CCAAGCCATCGAAAAAGCCCTGGCCTGATCCATGTAGGAGCCGGCTTGCCGGGTCCTGCAATGTGTTGTGTAGCTC
CCGGCTTTTGCATGTTAATCACTGAAATCAATAGTGCTACCCAGCACCGCCCGCACTCCATATTATCCGCATCATA
AAATCCCTCTCGTGCGGAGTGCTCCCATGCCTGTCCAAGCCTTGTTCAAACCTTTCCAGCTCGGTGCCCTGCAACT
GCCGTCCCGTGTGGTCATGGCGCCGATGACCCGCTCGTTTTCCCCGGGTGGCGTACCCAATTCCAAAGTGATCGAG
TACTACCGTCGCCGCGCCGGGGCCGGCGTAGGCCTGATCATCACCGAAGGCACGGTAGTGGGCCATCAGGCTTCCA
ACGGTTACCCGAATGTCCCGCATTTCTACGGTGCAGCGGCATTGGCTGGCTGGAAGAAAGTGGTCGATGCGGTGCA
TGCCGAAGGCGGCAAGATCGTCCCGCAGTTGTGGCACGTCGGCAGCGTACGGCGTATCGGCACCGAGCCGGACGCC
AGCGTGCCGGCCTACGGCCCGATGGAAAAACTCAAGGATGGCACCGTGCTGGTCCACGGCATGACGGTCCAGGATA
TCCAGGAGGTGATCGCCGCCTTCGCCCAGGCGGCCAAGGATGCCCAGGATATTGGCATGGACGGTGTGGAAATCCA
CGGCGCCCACGGTTACCTGGTCGACCAGTTCTTTTGGGAAGGCAGCAACCAGCGTACGGACGAATACGGCGGCAGC

CTGGCCAACCGTTCGCGGTTTGCCATTGAATTGATCCAAGCCGTGCGGGCTGCCACAGGCCCCGATTTCCCGATCA
TTTTCCGTTTCTCCCAGTGGAAGCAGCAGGATTACACCGCGCGTCTGGTGCAAACCCCCGAGGCCCTGGGGGAGTT
CCTCAAGCCGTTGGCCGACGCCGGTGTGGATATTTTTCACTGCTCCACCCGTCGGTTCTGGGAGCCGGAGTTCGAG
GGCTCCGAGCTGAACCTGGCCGGTTGGACCCGTAAGCTCACCGGCAAGCCGACCATCACCGTGGGCAGTGTGGGCC
TGGATGGTGAGTTCCTGCAGTTCATGGTCAATACCGACAAAATCGCTCAGCCAGCGAGCCTGGAAAAACTGCTGGA
GCGCTTGAACAACGATGAGTTCGATCTGGTGGCCGTGGGCCGTGCGCTGCTGGTCGACCCGGACTGGGCGGTGAAG
GTGCGTGAAGGCCGTGAGGGGGATATCTTGCCCTTCAGTCGCGAGGCGCTGACCACTCTGGTTTAGGGGGGAATTG
ACGGGCGCCATCGCGGGCAAGCCCGCTCCCACAGGTTGATTGCATTTCAATGTGGGAGCGGGCTCGTCAGGTCAGC
TTCGAACCGCCCGTCTCCATCACATCCTCACCACACGCCCCGCGCAATTGCTGTTCAAACCGCTCGATAATCGCCG
GCCAGCCCTGGCGACTGGCGTGCTGGCGCGCGTTCAGGCGCACCCGACGCAAGCTCTCGGGCTCTTCCAGCAACCA
ACAGGCGGCATCGCAAAACGCATCCTCATCCCCCGGCATCGCCACGGCACCACTGTAGCCATGGCGGATGTGCTGG
GTGGCGGCCGCCTGGTCGTAGGCCACCACGCCCAATCCCGATGCCATCGCTTCGAGCACCACATTGCCGAAGGTCT
CGGTGAGGCTGGGGAACAGAAACAGGTCCCCCGACGCATAGTGGCGCGCCAGTTCCTCACCGCGTTGTGTGCCGCA
GAAAATTGCGCCGGGGAGGGCCTTGGTCAGTGCCTCGCGTTGCGGCCCATCGCCGACGACGATCAGCGTCAATCGG
CGCTGTGGATAAGTCGCCTGCAAGGCCTCGAGGCAGCGTTTGAGCACACCGAGGTTTTTCTCCGGTGCCAGGCGGC
CTACATGCATGACGGCAATGTCCTCAGTGCCCAGCCCCCACTGTTCACGTAGGACGTTGTCACGTCTGGCTGGGTG
GAACAGCTGGCTGTCGACACCGCGCGATAACAGGTCCAGGCGTTCGAAGTGGCGGCGTTCCAGTTCCAGGCGCTGG
CTGACGCTGGGCACCAGGGTCAGGCTGGAGCGGTTATGGAACCAGCGCAGGTAGTGGGTCAGGATGCGGCTGAGCA
TGCCCAGGCCGTATTGGCTGGAATACTGCTGGAAGTTGGTGTGGAAACCGCTGACCACGCTGATCCCCAGGCGCCG
TGCCGCCCGCAAAGCCGACAAGCCCAGCGGGCCTTCGGTGGCGATGTACAGCACGTCTGGACGCTGGCGTTTCCAG
CGCCGCAGCAGTTTGTGCATCGAGGACTGGCCCCATTGCAGGCCGGGATAACCCGGTAGAGGCCAGCCGCGACACA
GCAGCAGGCCGTCGTCGCTCTGGCGACTCAAGTCGCCAGTCTGGCGCGGGCGCACCAGTTCCACCTGATGTCCACG
TGCGCGCAGGCCTTCACACAGGCGGCCAAGGGTGTTGGCCACGCCGTTGATTTCCGGTGGGAAGGTTTCGGTAATC
AGGGTGATATTCAGGAAAGCTGTCGTCATGCAGCCAGTGTCGGCGCGGGCCATGTCGCCATTGTGTCGTCGCAATG
ACGGATTTATGACGACCGGCCCGCTACCGGCTTAGTTGAACGGCGCAGGACGCGGGGTCTTGGCCGAGGAGGGCGG
GAGGATCGGCAGCTTGAAGTCCGGCTGGTCGCCGGTCAGGGTGTCGAGGAACGCGACAATCTGGCTGACCTCATCG
GTGCTCAACTTGCGCCCCAATTGCAGGCGGGCCATGACGTCTACCGCTTCGCTCAGGTTCCAGTAGGCACCGTCGT
GGAAGTAGGGGTAGGTCAGGGCGACGTTGCGCAAGGTCGGTACCTTGAACATCATCCGGTCCTGATCCTTGCCGGT
CAGGCCTGCCACGCCTTGGGCCGGGTTCTTGGTGGCATAGGGTTCGACCATGCCCATTTTCTGGAAGGTGCTGCCA
CCGACCGCCTCGCCGTTATGGCAGGCCACGCAGCCGATGCTCTTGAACAACTGGTAGCCCTGCGCTGCCGTGGCGC
TGATGGCTTTTTTATCGCCCAGCAGCCAGCGATCGAACGGGGCGTTGGGGGTGACCAGGGTTTCTTCGAAGGCCGC
GATGGCGTTGGTCACATCGTCGAACTTGATGCCGTCATCGCCATACACTTTCTTGAACGAGGCCTGGTATTGCGGG
ATCGAGCGCAGCACGTCCACGGCCAGATCATGGGTGAAGCCCATTTCGCCCGGGTTGGAAATCGGGCCGCCGGCCT
GCTCTTGCAGGGTTGCCGCGCGTCCATCCCAGAACTGCGCAACGCTCATGCTGGAGTTGAGCACCGTCGGTGAGTT
GATCGGCCCCTGGTGCCAGTTATGGCCGATGGAGGTGGGCAGATTGTCGCTGCCGCCCATGCTCAGGTTGTGGCAC
GAGTTGCAGGAAATGAAGCCGGACTTGGACAGGCGCGGGTCGAAGAACAATTGCTTGCCCAACTCGACCTTGGCGG
CGTCAGTGATTTTCGCCGGCTGCACCGGCAAGATCGGCTCGTTGGCGGCAGCGGCCCCCCAGGCCTCGCCGCCCAA
CATCAACCCAAACATCAGCATCAACGGTCTGTACATGGCTTGCTCCCCACAGGCTTATAGTTTTAATAACCATGTG
CTTTATGTGGGAAAATCGTCGTGATCCAGGTCAATCGAACAGATTAGGGCTTTTGCGCCAGCATGGTTTCGGCGCC
GCGCTCACGCACCCAGAACAACGTCGCCCCGGCCACCGCCGCGGGCATCATCAGCAGGTTGACCACCGGCACCAGC
AACACCAGGTAAACAATCCCACCGAAGCTCATGCTCTGCCAGCGCTTCTGCCGCAGCCAGGCGAGCATTTCGTTCC
AGCCCAGCTTGTGGTTATCGGCTGGGTAGTCGATGTACTGGATCGCCATCATCCATACCCCGAACAGCAGCCACAG
GGGGGCGGCGATCAGGTTGACCACGGGAATGAACGACAGGATAAACAGGCCGATGGCGCGTGGCAGGAAGTAGCCG
AGCTTGCGCATTTCCCGCGCCAGTGTGCGCGGGACCATGGCGAACAGTTCGGCCCAACTGAAGGCCGGGAAGTCAT
CGGTACCGCGCACCACCACTTCGACCTTCTCGGCGAGAAAACCATTGAACGGCGCAGCGATGATACTGGCGAGCAT
GGTGAAGCTGAAAAACACCATAAAAGCCACCAGCAGCACGAACAGCGGCCACAGGATGTAGCTGAGGAAACTCAGC
CAACTGGGCAGCGTTGGCATCAAGGTGTCGACCCACAGGCTGAACTGATGGCCAGCAAAATAGATCAATCCGACGA
ACAGTATCAGGTTGATCGCCAGGGGCAGCAGGACGAACAGGCGCAGGCCTGGGCTCAAGACCAGTTTCAGGCCTTC
GCGCAGGTATTGTGGGCCTGACAGAACGGGGGCGGGCATGGGTAACTCCGGGTAAAGGGGCAAACGCGCCGACCTT
ACCGGCTTTGCGAAAGCGGTGAAAGCACGGTCAGCGAGTCGACATTAACGGTAACAAACATGTCGATTAATAGGGG
GCACAAGATAGAGACCACCTATGAGCTGGATTGTTATTCCGTATTTCCTTAATCTTGCCCCCCTCTATACGCTGCA
CCCATTATTTTCAGGGCCTGCGAGTTCAAGCCTTCCCCAAGTGCTTCGCGGTCCTTTTTTATTCCAGCCGCTCTGT
AGTCCGGGCGGTCGATAGGAGTGAGTCATGTCTGATACCCGTCATTCGCGAGTGATTATTCTCGGTTCCGGCCCTG
CCGGTTACAGCGCTGCGGTCTACGCGGCCCGGGCCAACCTCAAGCCGTTGCTGATCACCGGCATGCAGGCGGGTGG
TCAACTGACCACCACCACTGAAGTCGACAACTGGCCGGGCGACGTCCATGGCCTGACCGGCCCGGTGCTGATGGAA
CGCATGAAAGAACACGCCGAGCGCTTCGAGACCGAAATCGTCTTCGACCACATCAATGCCGTGGATTTCTCGAAAA
AGCCCTACAGCCTGACTGGCGACAGCGGCGTCTACACCTGTGACGCGCTGATCATCGCCACTGGCGCCAGCGCCCG

TTACCTGGGCCTGCCATCGGAAGAGGCCTTTATGGGCAAGGGCGTTTCCGCCTGCGCGACCTGCGATGGTTTCTTC
TACCGCAACAAGCCGGTGGCGGTGGTCGGTGGTGGTAACACCGCCGTGGAAGAAGCACTGTACCTGGCCAATATCG
CCAGCACCGTGACCCTGGTTCACCGTCGCGAAACCTTCCGCGCCGAGAAGATCCTGATCGACAAGCTGCACGCGCG
GGTTGCCGAAGGCAAGATCATCCTCAAGCTCAACGCCAACCTGGACGAAGTCCTGGGCGACAACATGGGCGTGACC
GGTGCGCGTTTGAAGAACAACGATGGCAGCTTCGACGAGCTGAAAGTCGACGGCGTATTCATTGCTATCGGCCACA
CCCCGAACACATCGTTGTTCGAAGGCAAGCTGACCGCCAAAGACGGTTACCTGGTGGTGCAGGGCGGTCGTGAAGG
CAATGCCACCGCGACCAACATCGAAGGCATCTTTGCCGCTGGCGACGTGGCCGACCACGTCTACCGCCAGGCCATC
ACCTCGGCCGGCGCCGGTTGCATGGCGGCCCTGGATGCCGAGCGTTACCTCGATGGTTTGAAAGACGCTTCTTTCT
AAACCGTTGCAATAAAAAAACCGGCCGCGCGGCCGGTTTTTTTATGTGCTTGATTTGACGAACACCGCAATCTCAA
TCTGGCAATCCAATCAATGTGGGAGCTGGCTTGCCTGCGATGGCGGTGTGACAGCCACCACCGCCATCGCGGGCAA
GCCCGGCTCCCACATTGACCGGGTTTGCAAGCTGGATCAGCGGCGGGTCAAAGGCGGCTGGACAAACTTCACTCCC
GCCAACCCATGCTCGATCAACGCGCGGATGTTGCTGTGATCTGTGCCCTCAGGCGTAGCCAGCACCGAACGGTAAT
GCTCGCCGAACGCCAGCAAAGCCTGCTGGTCGCTCAAGCCTTCGAGCAGCGCCAGGCCCAGGGTCTTGCACGAGCC
TTCGTTCTGCCCAGCGGCGTTTTCTACGCTGCCATTGGTAAAGGCCTGTGGCTGGTAGTCGTAGCCAGCGGCGATG
AAGGCCAGGGTATCGGCAAACTGGTGTTCGCCGCTGTCGAGGCTGGTGTGCAGGGTGTTCAGGTCAGTCATGGGTT
TTTCCTTTGGCAAACGCCGCTTGTTGTTCGGCGCTGGCTTCTTGCTGGTACTGGGCTTTCCACTCGGCGTACGGCA
TGCCGTACACCATTTCGCGGGCGTCATCGAGGCTGACTTCGATCTGGCGTTCGTCGGCCTCGGCCTTGTACCACTT
GGACAGGCAGTTGCGGCAGAACCCGGCAAGGTTCATCAGGTCGATGTTCTGCACATCCTTGCGGCTGTCCAGGTGC
GCAACCAGGCGGCGGAAGGCAGCAGCTTCGAGTTCCAGGCGTTGTTGATCGGTCATAGGGTTCACGCAGATTCAGG
GGGGTTAACGGCTGGCGGCGAGCGTAATCGATACCGATTCGGCAAAGCGCAGCGCATGGGGCTTGTCGACTTCGAC
TTCGGCATACAGCACCGACTCATTGCTCATCACCAGGTCCAGCACTTCCTGGGTCAGGCGTTCGAGCAGGGCAAAG
CGGTTGCTTTCGACGTGGGCGATGATGGCCTTGGTGATGGTGCGGTAGTTCAAGGCGTGGTCGATATCGTTGTCGC
GCACGGCGTCTTGTGCGGCATACAGGATGGTCAGGTTGATCAACACATCCTGCTTGTTGAGGATTTCATCCTCGTT
GATGCCAATAAAGGTACGCAGACACAGGTCCTTGACCCGGATGCGCGCCATACCTGGTTGAAGTTGTGGCATTGCT
ACTTGCTCCGTCCAATCAGTTGCAGGAACCCCAGGCGGCTGGTGTTCGGGTTTACTTGACATGGCGCCCGCCATTG
ACGGTCAGGGTGGTGCCGGTCACATAAGGGTTGTCCAGCAGGTAACGCACGCTCTGGTAAATCACCTCAGGCCCGG
GTTCGATACCCAGTGCCGACTTGGCCAGGACCTTGGCGCGATAAGCCGCGTCGTCGCCTTCGTTGAACATCACCAT
GGCTGGCGCGATGCCATTGACCTTGATCCGAGGCGCGTATTGGGCGGCGAAGGACAGGGTCAGGCTGTCGAGCCCG
GCCTTGGTGGCGCAGTAGGCGATGTGCTGGCGGCTGCCCTTGCGGGTTACGTCGTCGCTGATATGCACAATGTCGG
CCGGCGTCGAGCCTCTGAGCAAGTCAGCACAATGCAGGTTGATCAGGTACGGCGCAAGCATGTGCACGCTGAACAT
ATCGATAAAGGCGCTGGCCTCGTCACCAGGTGTCTCTGCGGCCCAGGTCGAGGCGTTGTGGATGATTGCCCGCAGG
CTGTCGGTATGGGTCTTGAGTTCGCTGATGAACGCCAGGATACCGGCCTCGGACGAGAAATCGGCAAATACGCCCA
CCGCGCCACGCTCGCGCAAGGCCTGCACACCGGGACGTTCACTGCGATAGCTGAAGATCACCGGTTGCTGTTCATC
CAGCAGGCGCTGGGCGCAGTGCAGGCCGACACGTTGGCCGGCGCCGGTGATGAGGATCGGGGCGGGGGTACAGGTC
ATGGGAAGCTCGGGTCGCGGGCAAGCTAAAACTATACCAGCGACCGGCCTGTAGTGGATCGGCTCAAACGGTTGCT
TCGCCAGGTTTTACCCGGCGCGGGGCAGGATTGAGCCAGCCCGCCAGCAAATGGGTCGACAAGGGAATAAACAGAT
AGACCATCAAGGGCGTCAGGGCCAGGGTGCTGACCAGCACGCGGCTGAACAAGCCCAACTCGGCCAACAACGGTTC
CAGGCCGACGTTGAACAGCAGTGACACCGGGAAAAATGCCAGCCAGATCGCCACGGCCTGTTTCCAGCGCGGTGGG
CGCTGGCCCGCCGCGCCAAACCAGCCCTCCATGCCGCTGACACGATGCTCCATGGGGTCGGCGAACAATTCGCTGC
CGCGCCCCAGCCAGGCACTGCGCGAGGCGGAGAACTCCCAGGCGTGCAGGGTCTGCTCGTCGGCGAAGCGGAAGAT
GATTTGGAATTCGCCACCGTCGGCAGGCGGTGCAAGCACGCCTGAACCCAGGTAGCCGGGGAAGTCAGTGGCCAGT
TGCTCGCCTTCGCGCAGCCAGCTCATCATGTCTTGATAACGACCTTGGGCGGCGCGGCGCGCAACCATCAAGGTGA
CGGGTAGGGTAGACATTGTGTATCTCCGAATGCATGGGAGCGCAGCACCGGGTAGGTGGCGTCCCTGGCGAAACCG
CAGGGTAGGGCGGCGACGCAGAAAAACAGGCAAGGATTATTCCTGATTTGACGAAATACGCCAGCGGCATTGGACG
GCTGGGCAGGCCCCTTGAAAAAGACAGGTATACAAGCGTTAAATGGGATCCAACTTCGTATAAGTTTCTGGTCATT
TTATGCCTGTAATCACAGCCCCTTCCCCCGGTCTTGAGCATGACGGCCTATCCGCTGCGGATGAGTTGTTCCCGAT
TCGCGAAGTCGCAAGATTGACAGGTATCAACCCGATTACCCTGCGTGCGTGGGAGCGCCGTTATGGTTTGATCCAG
CCGGTGCGCACCGAAAGTGGGCATCGCCTGTACACGCAGAAGGACATCGAGACCGTTCATCGCATTCTGGATTGGA
TCGAGCGGGGCGTGGCAGTCAGCAAAGTCGGCAAGATTCTCGCCCGGGATGATGCGCAACTGTCTTCACTGCAACA
CCCACAAGAAGGCAGCGGTGAGCTTGAGTGGGCGCAGTGGCAGCAGCAGTTGCAACTGGCCATCGGTGCGTTTGAT
GAGCTGCAACTGGAGCGCCTGTACGGGCAGGTCTTTGCGGCCTACCCCGCTTCAGTGGCGTTTCAGGACATCCTGA
TGCCCCTTTGGCGCCAATTGCTGCGGCATCAGGGCCGTTTTGGCCAGGCCAGCGAATGGCTGTTTTTCGACAGCTT
CTTGCGCGCGCGAATTTCCCAGCGCCTGCAACTGGCCCGCGCCGGCCGTACGCCGCAGGTACTGTTGGCAGCCATT
GCCGGTGAATGCCGAGAACTGGAGTTGCTGGTGGCGGGGCTCATGCTCAATGGTGATGAACTGGCCGTGCATGTGC
TGAACGTGGGCCAGCCCCTGGATGAGTTGACACTGATCTGCGAGAAGACCCGCCCGCAGGCGCTGGTGCTGTTCTC
CAACCGTTCACCCACCCATGAGTTGCCCAAGCGGTTGGCCCGATTGGCCCTGACCCTGGAGTGCCCGCTACTGCTG
GCCGGTGATGCCTCGGACATGGCCGAGCAGCTACTGGCCGGTACTGCCATTGGCTGCCTGGGCAATGAGGGAAGGC

TGATGCAGCGGCGCTTGCAGCAGTTTTTAAGGGGAACCCTGGATACCTGAGATCAGGCATGCACCTCAGGGTGGGC
CAGGCGGTGCTGTTGCAGGATGAACTGGCGCAGGCGCTCGGTTTCATCCACATCACCCTGGCTCAAGCGGTAGGCA
AACAGCCCGCGAGCGGTCTCGCGCTCCAGCGTGCCACGCACCGCAATCCTCTCGTAGCCGGACGGGCTGAACCACA
TCGAGAAAGTCCTGGGTGGTTTGCTACGGCCACGGGACTGCACCAGCAAACCCTTGAACGATACTTCATGTACCCA
TAGCGCCCCAGGCTGGCCCTTGACGTTTTCCAGGGCGACCGGCTCTTCGAGGACCAGGCGCCAAGGCCGGATCATT
GGCCCGTCCTCGAAAATGCTCGGTACTCCCAGGCGCAGGTGCAGGGCATGAAACTCGTCCTCCACCAATTGCAGGG
GGAAGGTCATCTGCTGGTTATCGAACTGCGCCTGGATCGTCACCTGTTCATGGGCCGCAAGGCGCGTGAGCAAGTC
GCGGATCTGCGCGCCGCCATTGACCGTCAGGCTCGACGACACATCCCGCACATTCAGTTGCGGGTTGTGGTGCATG
TTCTGGATGAAGTCCAGTTCGTCCTGGGTCAAGAGCGTGTCGCGTTGCATTGATGTGGCTCAAGGAAAGGCGTGAT
TAAACTGATTATCACCCTAAAGACCGTGTTTTCTAAAGTTTGTTAGATCCGCTCGTCGCCCGGAGTGCTGCCAGCT
CGGCTGTAAGCTGCGCCACCTGGGCTTCAAGCTGGGCTACACGCTGCTGGGCCTTGACCTGAACCGTTACATCTTT
TTGCACGCCAACGAAGTACGTCTGCTGATCGGCCTGGTTGTAAACCGTCGATAATGACAGCTCATTCCAGAAGTGC
GTGCCGTCCTTGCGGAAGTTGCGCAGGATTTCCCGGCACGCGCCGCCAGTGTCCAGGGCTTCGCGGATGGCCATCA
GGGCCGGCTGGTCACGGTCACCCGATTGCAGGAAGCGGCAATCCTGGTAAAGGATGTCGTCCAGGCTGTAGCCGGT
CATGCGTTCGAAGGCCGGGTTCACGTAAATCAGGGGCTTGTCCTTGCCCTCCCTCTCAGCCACGCAAATCCCGTCG
TTAGAGGCATTGATCACCATCTGCATCAGCTTGGCGTTGATCATTGAGCGATCCATAGCGTGTTTGTGGATTTCGT
AGTGTAAGACAACTCACGCGGCTGCCAACTGCTAATATCCCAGGTTTTCACTCAACTACAGGATCAGATTGATGAA
AGTCGCCATCCTTTCCGGCTCGGTCTATGGCACGGCCGAAGAAGTTGCCCGCCACGCCATGGGTCTCCTCAACGCA
GCCGGGTTCGAGGCCTGGCATAACCCGCGGGCGACCCTGGCCGATGTGCAGGCCTTCGCCCCGGACGCCTTTCTGG
CCGTAACCTCCACCACCGGCATGGGCGAATTGCCAGACAACCTGATGCCGCTGTATTCGACAATCCGCGATCAACT
GCCCGCCGCCTGGCGCGGTTTGCCAGGTGCGGTGATCGGCCTGGGTGATGCCAGCTATGGCGACACATTCTGTGGT
GGCGGGGAGCAGATGCGCGAGCTGTTCAGCGAGTTGGGCGTACGCGAAGTGCTGCCGATGCTGCGCCTGGACGCCA
GCGAAAGCGTCACCCCGGAAGCGGACGCCGAGCCCTGGTTGGCAGAGCTGGTCACGGCACTGCGCGGCTGATCGGG
TACTCGCGTAGTAACGCCAGCCAGGCCAGGGCGGCTTTCGACAGATAGGCGCCCTGGCGCCAGATAAAAGCGATGT
CCCAGCGTAGGAATTCGGGCGCCCTGAGTGTCAGGCGCACCACGCCAGGGCGCTCCAGGCCGCGCGCGACCACACT
GGGCAGCAGCACCACGCCCTGGCCTGCAGCCACCAGTGCCACGAGAAAATCCGCCTGGCCGCTGCGTCCGCTTTCC
CGGGGTGTGAAGCCCACTTGCTGGCAAGCCTGGAGCAGCCGGTCATTGAGCACAAAGCTGCGCTGATACATCAAAA
ACGGCGTGTCGGCCAATTCCGCCAAGCCAATCTGCCCACGCTGCGCCAGCGGGTGGTCCACGGGCAGCAGCGCATC
CAGTGGTTCATCACAGAAGGCTTGCCAGGCAAACGCCGGATCGGTGGGCAACAAGCTGCCGCCTACGTCCAGTTCC
CCGGCGAGGATCGCCTGCTCGATATTGCGACTGCCGCCCTCAAGCAATTGGATGCTGACATTGGGATAGCGTCGCC
GATATTCGGCAAACAACTCGGCAAACAGTGTGTCGCCCCCCAGTTGCGGCAGGCCCATGCGCAACTCGCCACGGGT
CAACTGATTCAAATCGTCCAACTCGCTCAACAATTCGGTTTGCAGACGCAGCATTGCCTCGGCCCGTTGCACCACC
ACGTGCCCGGCGGCGGTCAGGCGCACCTGCGAGCCAATGCGGTCGAGCAGCGGCGTACCCAGGCTTTGCTCCAGTT
GCGCCACTTGCTTGCTCACCGCGGACTGGCTGATATGCAGGGTTCTCCCGGCCTGGGTAAAGCCGCCACGGTGAAC
CACTTCGATAAAACTGCGCAGCTGTTTGAATTCCATTACGGTAATTCCAGATTGGAATGGCTTGCAGTCTAACAAT
TCGCTTTGGGGATGCCAGGGGGCTCTTTAACATGGGGGCCTGCGAGGAACCCATGTCTACTAAACGTTTCACCTTC
AAATATGTGTGCCGGTTGCTGGTTGAGTTGCTGGTATTGCTGGCGATTTACCTGCTGGGTTGCCAAGTAGCCCTGT
GGCTGGCCTGGCCGATTCCAGGCGGGGTCATTGGCCTGGGCCTGCTGTTGCTGGTGTTCGCCAGTGGCCTGATCAA
GCCCGCAACCCTGCAATTGGGCGCTGGCATGTTGATGGCGGAAATGCTGCTGTTCTTTATTCCGGCGTTGATGAGC
CTGCTCGATTACGGTGGGCTGATGCGCCATGACGGTTGGCGCATCATGTTGGTGATCGCCCTTAGCACCCTGACGG
TGATGTTGGTCACGGCATTTACCGTTGAACTCGTCTGCCGCTGGAAGCTGCGCCATGAAGCTTGAACTGATGCCAT
TGTTCTGGCTGGCCCTGACCCTTGTGGCCTACGGTTTCAGCCGCTGGATCTACCGGCGAACCGGGCGTTACCTGCT
ATCGCCGCTGATCCTGGTGCCCGCGCTGCTGCTGGCGGTGGCCGTGCCGCTGAACACCGCCTATGCCGAGTACGCC
ACCGACACCCATTGGCTGATGCTGGTGCTGGGACCGGTGACGGTGGCGTTCGCCATCCCGATCTGGCAGCAGCGCC
AGTTACTGATGCGCCACTGGTCGGCATTGCTGCTGGGCATGCTGGCGGGTAGCGCGGCGTCCATCGGGACTTCTTT
CGGCTTGGCCAAGGCGTTGGCCTTGGACAGTTCGGTCACCCTGTCATTGGTGCCGCGCTCGATCACTACGCCCTTT
GCCATGCCCCTGGCCCACGACCTGGGCGGCGTGCCGGAACTCACGGCGGTATTCGTGATGTTCACCGGCGTGTTCG
GCGCCCTGCTTGGCGGGGTGCTGCTCAAGTGGCTGCCGCTGCGCAGTGCCCTGGCGCGGGGCGCGTTGTTTGGCGT
GGGCGCCCACGGTGCTGGCGTCAGTCGTGCCCATGAAGTAGGCGGCGAAGAGGGCTCGGTGGCGGGCTTGGTGATG
GTGCTTACGGGCTTGCTCAACCTGTTCGCCGCACCTTTATTGGCGTCGCTTCTCTGACACCACGTTGTACGAATCC
GAACTATCCTCACGGTTGACTCGCTCGGTCATCAAGCTGGCTGCCAACGCAACTACGGCGTGCCCGAGGTCTGACT
AGACTGGCCCTTCAGTCTAAAAATAATAAAAACACCACAGCGTCGAGGTCATTGCCGTGAGCCTAGCCCCCGTTCA
ATCGACACAGAGTGTTAAAGACCAGGTCAGCGCTGCCGAATGGCAGGCCCGCATAGACTTGGCCGCGTGCTATCGC
CTGGTGGCCATGCATGGTTGGGATGATCTGATTTTCACCCACATCTCGGCCAAGGTACCCGGCACTGAAGACTTCC
TGATCAACCCCTTCGGGCTGATGTTCCACGAGATCACCGCCTCCAGTCTGGTCAAGGTCGACCAGGCCGGTAACAA
ACTGATGGACAGCCCCTACGAGATCAACCCGGCGGGCTACACCATTCACAGCGCGGTACACGAAGTACGTCACGAC
GTGGTCTGCGTGCTGCACACCCATACCGCAGCCGGCGTGGCGGTGTCGGCGCAGAAGCAGGGCGTGTTGCCCATCA

GCCAGCAGTCGCTATTCGTGCTGTCGAGCCTGGCCTATCACGCCTACGAAGGCGTCGCGCTGAACCACGAGGAAAA
GGCCCGCCTGCAAGCCGACCTGGGGCCGAACAATTTTCTGATGCTGCACAACCATGGCCTGCTGACCTGCGGGGCG
ACCATCGCCGATACCTTCCTGATGATGTTCACCTTCCAGCGCGCCTGCGATATCCAGGTACTGGCGCAAAACGGCG
GGGCCGAATTGATCGCCATCGAGCCGCAGATTCTGGCGGGGGCCAAGGCGATGATCGCTGGCGTAACCAAAAGCGC
CCAAGGCATGGGTGGTGCATTGGCCTGGCCGGCGCTGTTGCGCAAACTCGATAAACAAGACCCTGGATACCGACTC
TAATGCCACTGGCCGAGATTCCGCTGCGTGCCTGGCGCAAACGCGGGCAGAGTTTTGTTTTTCGCGGGCAGACCAT
CCGTTACTGGGTGGCCGGCCAGGGCGAACCATTGTTGCTGATCCACGGCTTCCCCAGCGCCAGTTGGGACTGGCAT
TACCTCTGGCAGCCCCTGGCCCAGCGCAACCTGGTGATTGCCTGCGACATGCTCGGGTTTGGCGACTCGGCCAAGC
CCCTGGACCACACCTATTGCCTGCTGGAGCAGGCTGATCTGCAACAGGCCCTGCTGGCCCATCTGCGCGTGCAGCA
GCCGGTGCATATCATGGCCCATGACTACGGCGACAGCGTGGCCCAGGAACTGCTCGCCCGGCACTACGAGGGCAGC
TTCCAGCTGGCCAGTTGTGTCTTTCTCAATGGCGGGCTCTTCCCCGAGACCCACCGCGCGGCGCTGGTGCAAAAAC
TATTGCTCAGCCCCTTGGGTTGGATGATCGGCCGTGCATTCGGGCGCAACGCCCTGGCTGACAGTTTCAAGCAGAT
CTTCGGCCCCGGCACCCGCCCCAGCGAAAGCGCTCTGGATGACTTCTGGAGCCTGATCGAAAACAACGACGGCCCA
CGGATCCTGCACAAACTCATCGCCTACATCCCCCAGCGCCGTCAACAACGCGAGCGGTGGGTGGCGGCCATGCAAC
GCGGCGAAGTACCGTTGCGGGTGATCGATGGCGAGGTAGACCCGATTTCCGGCGGGCATATGGTCGCGCGCTACCG
CGAGCTGATAGCGGATGCCGACACGGTATTGCTGGCCAATATCGGCCACTACCCGCAGGTGGAGGCGCCGGTCCAG
GTGCTCAAGCATTACCTGGCGTTTCGCGAGGGCATTGAAAGCAACCAGGATCGCCTGTGGGAACTGGCTTATGCAG
GAGCAGGTTTGCCTGCGATGCGGGTACCTCGGTAAGTCAGTTGCACCGAGGTGAGGCAATCGCAGGCAAGCCAGCT
CCCACACAAGCCAGCTTCTACACGAGCCGATCATCCCCGTACCTTATTGCGCACCATTCACCCCCGCCCGTAATTA
TTGTGACCACAAGCTCCGTGCCTGACACTCAACCCATCGTCCACTGTCTTCGCTGGAGTTCCCTATGAGTGAGTCT
GTGCAGTTCCAAGATAAAGTCGTGATCGTTACCGGGGCCGGTGGTGGCTTGGGTCGTGCCCATGCGCTGCTGTTCG
CCAGGCACGGGGCCAAGGTACTGGTCAACGACCTTGGCGGCTCGACTCAGGGCGAAGGCGCCAACGCCTCGGCCGC
CGACCGCGTAGTGGCCGAGATCCGCGAGGCCGGGGGCATTGCCGAGGCCAACCATGATTCGGTGACCGACGGCGAA
AAGATAGTGCAACACGCCCTCGATGTATTTGGCCGCATCGACGTGGTGGTGAACAACGCCGGGATCCTGCGCGACA
AGACCTTTCATAAAATGGAGGACGGCGACTGGGACCTGGTTTACCGCGTGCACGTCGAAGGTGCCTACAAAGTCAC
CCGCGCTGCCTGGCCGCATATGCGCGAGCAGGGTTATGGCCGGGTGATTTTCACCGCGTCGACGTCGGGTATCTAC
GGCAACTTCGGCCAGTCCAACTACGGCATGGCCAAGCTCGGCCTGTATGGCCTGACCCGCACCCTGGCGTTGGAAG
GGCGCAAGAACAACATCCTGGTCAATGCCATCGCGCCCACGGGCGGTACGCGCATGACCGAAGGCCTGATCCCGCC
ACAGGTGTTCGAGCGCCTCAAGCCCGAGCTGGTCAGCCCGCTGGTGGTGTACCTGGGCAGCGAGGCGTGCCAGGAA
ACTTCCGGGCTGTTCGAAGTGGGGGGCGGCTGGATCGGCAAGACCCGTTGGGAGCGCAGCCTGGGCGTGGGCTTTG
ATCCCGAGGCCGGCTTCTCGCCAGAGGATGTGGCGGCGCACTGGCAGCAGATCTGCGACTTTGAAGGGGCGGCCCA
TCCCAAGGACAACATCGAAGCCCTGAAAGAGATGATGGACAACCTGCAGAAATACAGCCTCTGATCGTTACCTGAA
AAACATTCAATCCTGCGGGGCGCTGATGGCGCCCCGCTTTGTTTCAGGCATAAAAAAGGCCGCTGCATACGCAGCG
GCCGAAGTAAGACGTTGGATCAAGGAGCTACAAATCAACGTCAGTGAACACGGGTAACAGACTGAAAATTCGCTTC
AATCTGTTGAAGACTGGCTTCCAGGAAACGGATGGTTTGTTCTGAAAGCCCGGTAAGAATAGTCCCTTGTAGCGGA
ATGAAAAATACTGATTCAGGACAAGGACTATTACCAAAACAACAACAGTGACTACCCCCTGTAGGAGCGAGCTTGC
TCGCGAAAAACGTGAACGATGACGCGTTTTGTCTGGTTTAACGCGGCGTACTCACGTTCTTCGCGAGCAAGCTCGC
TCCTACAGGGGGCGTGGGGTTATCCATTCTCTTGATAACCCTTCATCCACTACGTCGATAGCCCCGCCAATACCCG
CGCTAGAGCCTGCGCTAATCCTTTCGAGCGACAAAACGAATACCTCCTTGGTGCGCTTATCGCTCCTGATGGCGGG
CAGTAGGGTGGGGCCTTCTGTCACACCACCGGGGAAGACGCATGACAAAAACAACAATGCGCGCCATCTTCAGGCC
ACAGGCGCTGGCCGCTGCGGTTGCTTTGGGTTGCTGCGTTCAGGCGCAGGCTGTTTCATTCAACATCGGCGAGATC
GAGGGGCAATTCGATTCGTCGCTGTCGGTAGGTGCCAGTTGGGGCATGCGCGATGCCGACCGCAAGCTGGTGGGCA
CGGTCAATGGCGGCAGTGGCCAGGCCTCCACTGGGGATGACGGGCGCTTGAACTTCAAGAAAGGCGAGACCTTCTC
GAAAATCTTCAAGGGCCTGCATGACCTGGAGCTCAAGTACGGCGATACCGGTGTGTTTGTGCGGGGCAAATACTGG
TACGACTTCGAACTCAAGGACGAAGACCGCGAGTTCAAGCCCATCAGCGACCACAACCGCAAGGAAGGCTCCAAAT
CCAGCGGGGCGCAGATCCTCGATGCCTTCGTCTATCACAACTACTCCCTGTGGGATTTGCCGGGCACCGTGCGCGC
CGGTAAACAGGTGGTCAGCTGGGGCGAAAGTACCTTCATCGGCAACTCCATCAACAGCATCAACCCTATCGACGTC
TCGGCCTTCCGCCGGCCCGGCGCCGAGATCAAGGAAGGCCTGATTCCGGTGAACATGCTGTTCGCCTCCCAGGGCC
TGACCAACCAACTGACCGTGGAAGGCTTCTACCAACTGGAATGGGACCAGACGGTGCTGGATAACTGCGGCACCTT
CTTTGGTGGCGACGTGGCGGCCGATGGCTGCACCGACAACTACACCGTCGGCAGCCCGGCGATCCGCCCGCTGCAA
CCGGTGGCCGCCGCGTTCGGCCAGGGGTTTGGCGTGACCGATGAAGGGGTGATCGTGCGCCGCGCCGCCGACCGTG
ATGCACGGGATTCCGGGCAGTTCGGCATGGCGTTGCGCTGGCTCGGCGATGACACCGAGTACGGCCTGTACTTCAT
GAATTACCACAGCCGCACGCCGACCGTGGGCACGCTCACGGCCAACACTAACCTTGCGACCATCGGCAATATCATC
AATACCGCCAACGGCATTGCCCCCGGCAGTGGCGCCGGCCTGGCGCAGAGCGTGATGCTCGGACGCGGCCAGTACT
ACCTCGATTACCCGGAAGACATCCGCCTGTACGGCGCCAGCTTTTCCACCACCTTGCCCACCGGCACGGCCTGGAC
CGGGGAAGTCAGCTACCGTCCCAACGCGCCAGTGCAACTCAACACCACAGACCTGACCCTGGCTCTGGTCAACCCG
ATTGCCGGTAACACCGTGTCACCCATTCGCAGCTCATTCGGTGCCGACAACACCGGCTACCGGCGCAAGGAAATCA

CCCAGTTCCAAACCACCATGACCCAGTTCTTCGACCAGGTGCTGGGCGCCGAACGCCTGACTGTAGTGGGCGAGGC
GGCCATCGTGCGGGTGGGCGGGCTGGAGAACAAAACCAAGCTGCGCTACGGCCGCGATTCGGTCTACGGCCAGTAC
GGCTTTGGTGGTGATACCGACGGCTTCGTCACGGCGACGTCCTGGGGTTATCGCGCCAGGGCCATTCTTGATTACA
ACAACGTGATCGCCGGGATCAACCTCAAGCCCAACCTGTCCTGGTCCCATGACGTCAAGGGCTATGGCCCCAACGG
CCTGTTCAACGAGGGGGCCAAGGCGCTCAGCGTCGGGGTCGATGCGGACTACCGCAACACCTACACCGCGAGCTTG
AGCTACACCGACTTTTTTGGCGGTGACTACAACACCCTGACCGACCGTGACTTCCTCGCCCTCAGCTTTGGCGTGA
ACTTCTGATTCGGCTAAAAAAGAACAAGGACAATAACCATGCGTAAGATGATTTTGCAGTGCGGCGTACTGGCCCT
CAGCCTGTTGGCCGCCAACGTGATGGCTGCCGTGTCAGCGGAAGAAGCCGCCAAGCTTGGGACCACCTTGACTCCG
GTCGGCGCGCAAATGGCCGGTAATGCCGATGGCTCGATCCCGGCCTGGACCGGTGGCATCCCGAAAAACGCCGGTG
CAGTCGACAGCAAGGGCTTTTTGGCCGACCCGTTCGCCAATGAAAAACCGCTGTTTGTGATCACCGCCGCCACGGT
CGACAAGTACAAGGACAAGCTGTCGGATGGCCAGGTGGCGATGTTCAAACGTTACCCCGAGACTTACAAAATCCCG
GTCTACACCACCCACCGCACGGTCAACCTGCCCGACGATATCTACGAGTCGATCAAGCGCAGCGCACTCAACGTCA
AGTCGATCAACGACGGCAACGGCCTGGAGAACTTCACCGGCAACCGTTACTACGCCTTCCCGATCCCGAAGACTGG
CGTCGAGGTGTTGTGGAACCACATCACCCGTTACCACGGCGGCAACCTGCGGCGCATCATCACCCAGGCCACACCG
CAGACCAACGGCAGCTACACGCCGATCCGCTTTGAAGAAGAAGTGGCGGTGCCGCAACTGATCCCCGACATGGACC
CGGCCAAGGGCGCCAACGTGCTGACGTTCTTCAAACAGTCGGTCACCGCCCCGGCGCGCCTGGCGGGCAACGTGTT
GCTGGTACACGAAACCCTCGACCAGGTGAAGGAACCGCGCCTGGCGTGGATCTACAACGCCGGCCAACGCCGCGTA
CGCCGCGCACCGCAAGTGGCCTACGACGGGCCGGGCACCGCGTCCGACGGCCTGCGTACCTCGGATAACTTCGACA
TGTTCTCCGGTGCGCCAGATCGCTACGACTGGAAGCTGGTGGGCAAGAAGGAAATGTACATTCCCTACAACGCCTA
CAAGCTCGACTCGCCTTCGCTCAAGTACGACGACATCATCAAGGCCGGGCATATCAACCAGGACCTGACCCGCTAC
GAACTGCACCGGGTCTGGGAGGTGGTCGGCACCGTCAAGCCGAGCGAGCGGCACATCTACGCCAAGCGCCATATGT
ACATCGATGAAGACAGCTGGCAGGTGGCGCTGGTGGATCACTACGACGGTCGTGGCCAACTGTGGCGCGTTGCCGA
AGGTCACGCGCAGTTCTACTACAACCACCAGACCCCGGCCTACACCGTCGAGACCCTGTACGACATCATCGCCGGG
CGCTACATCGCCCTGGGCATGAAGAACGAGGAGAAGAGCAGTTTTGTGTTCGGCTTCAATGCCAAGGCGGCGGACT
ACACCCCCTCGGCCCTGCGCGCCTCGGGCGTCCGCTGATCCAAAGTTGTAGACCCAATCATTGTGGGAGCTGGCTG
ACCTTTGATCGTGCCGGGCTTGTTTTGAAGCCCGGCTTTTTTATGCCCGGCAATCAGCCCGCTGAATACTTCTTCA
ACAACGCCCATCGAGGGGACTACGGTAGGCCGCACGCCGCATAAAAATAAAAAAGGGACGCTGTAATGACCGCCAT
GACCCGTCGCCTGGATCGTCCTGGATTCATGCCACGTTTGTCTTCCCACCATTTGCTGCGCCCCCGCCTGGCCGAC
CCGTTGCTAGGGACGCAGGCGCGGGTAAGGTTGCTGTGCGCGCCGGCCGGCAGCGGCAAGAGCGCCTTGTTGGCTG
AATGCGCCCAGCAGGCACCGGCGAGCTGCCAGGTGTATTGGTTGGCGCTCAACGGTACGGCATTGGACCCTCATGC
TCTGTGCCAGCGCCTGGCACAAAGCCTGGGCCTGGCGTTTGTCGATGAAGCCAGCTTGTTGCTGGACCTGAGCCGC
TGGCAAACGCCAGCCTGGCTGTTTCTCGATGACTATTGCCGGGTGGCGGCGCCCGCATTGGATGCTTTGCTCGATC
GGCTGCTGGCCTCGAGTAGCCCGGCCTTGACCTGGTGGCTGGGCGCGCGGCGTCGGCCGGCGTGCAATTGGCCGCG
GTTGCTGCTCGATGATGAGCTGTGGGAATGGGGCGGCACCGAGTTGGCGTTCGATCAGCGCGAGGTCCAGCAACTG
CTCGATCACCACGCCGCTGCTGGGCAGTCTGCCGACAAAGTCCTGCAATTTTCTGCGGGCTGGTGCGCAGGCGTGC
GTATCGCCCTGCTCGAAGGCGACAACCATCCCGACAGCGGCGCCTGGCATGGGCGTTCGCAAACCTTGCTCGATTA
CCTGCAGCACGAGTTGTTCAGCACGTTGCCCGAAGAATTGGAGGAGGCCTGGCGCGTCCTGGCACATCTGCCGCGT
TTCAACCCGGGACTGTGCGAGCACCTGTTCGGGTTTGGCGATGGCGCCGGCTACCTGCGCGACCTTCAGGCCCTGG
GTGCGTTCATCGAGCCCTGGGACGACTCGCCAGACTGGCTGCAGGTCTTCCCGCCCCTGGCCCAGTTGCTGCGCGA
CGAACCGTGGCCGGCCCGGCGCTCCTGGCATCGGCGCGCCTGCCAATGGTTCACCGCGGCCCAGGACTGGCAGGCG
GCGTTTGAGCAGGCGCTGTTGGCTGAAGAATACGAAGTGGCGGTCAGCCTGTTGGAGCACTTCAGCTTTGAAGACT
TGTTCCGCCAGCAAAACGCCGTGCTGTTGTTGCGCCTGCATGAGCAACATGGCGACGAGTTGATGCTCGGCTCGGC
GCAACTGGTGGGGCTGGTCACCGCCGCCTTGCTGTTCGCCGGGCGTTTCGACCAGGCCGCGCAATGTATCGACCAA
CTGGCGCGCTTCACCCCGCAACCTACGGCCGCCCTGCAACGGCATCTGCTGGCGCGCTGGCAGGCGCAGTGGGGGT
GGTTGTTGCATCTTGGGGGGGACGCGGTACCTGCGCGCGGGCATTTTCTGGAAGCGTTGAATGACTTGCCGGACAG
CGCCTGGACATCGCGGTTAATGTGCTTGTCGGGCCTGACCCAGCAAGCGTTGCTGCGCGGCGAACTGGACGTGGCC
CAGGCCCTCAACCGTGAGGCGTTGTGCCTGGCGCGGGCCAACGGTTCGTTGCTGTTGGAGGCGGTGCTGGAACTTG
ACCATGCGCAGTTGCTGGAACAGCGAGGCGCACCGTACCGGGCCCAGAGCCTGTTGGAAGGTGTGCAGGCGATGCT
GGTGCAGCAGCGCCTCAAGGCGGGGCCATTGATGGGCCGGATAGCCTTGCGCCGTGGCCACCTGGCCTTGCGCCAG
GGGCATGATGCGTTGGCCACCGAGTGCCTCGAAGCCGGCCTGGGCATGTGCTTGCACAGCCAGGACAAGCGTGTGC
TCTATGGTTTCCTTGGGATGGCGATGCTGGCGGCCAATCGTGGTGATTACGCCCAGGCGTTTATCCAGCTACGTGA
CGCCGAACGCCTGATGCAACGCCGGCACGTGCCCGACACGGTGTACCGGGCGGTGTTGCTCTTGCTCAGCGGGCAT
TTCTGGTTACAGCAGGGGCGGGCCGAACTGACCCAGGAAGCGGTCAGCCGGGTGCTGCGCCACTATCACGGGCCCC
AGGCCAAACAGGCACCGCCGGCGACCCTGGAACTGATCCCCCGGCTGGAATACCTGTTGGTGCTGGCCCAAGTCAA
ACTGCGCCACGCCGAGCAGCCCATCCAGCGGCTCACAGCCTTGCTCGACACTGCCCGCGAGCGCGGCATGCAATGC
CTGGAGGTGGAACTGCACTTGGTGTTGGGCGAGGTGGCCTGGCAACTGGGCGAGCGAGCGAACGCGGTGCGCTCGC
TGCAAACCGGGCTGGCCCTGGCGGCGCGCTGCCAGGTGCAGCAGGCGGTGCGTGAACTGCGCCTGCGCCAGCCGGG

GTTGCTCTGTGAGTTGGGGCTTGAACCTCAAGAGGCGGTGGCGGCACCTGGAGAAAACCCCCTGAGCCAGCGGGAA
TTGGAGGTGCTGCAACTGATTGCGCTGGGCAACTCCAACCTGGAAATTGCCGATCTGTTGTTTATCTCGCTGCACA
CCGTAAAGACCCATGCCCGGCGGATCCACAGCAAGCTGGGGGTGGAGCGCCGCACCCAGGCGGTGGCCAAGGCCAA
GACCCTGGGCCTGATGGCCTGATTTTCAAGCAAGCCAGCTCCCACATTTGCCCACGTGCGTGCAGTCAGGGCTGAT
ACCCCATCCGCCAACTCACCGCCTGGCTCGCCGCAAGCAATTGCCTGGCCGCCGGCCCATCTTCATCGGCATGGAA
CAACGAGGTCGGCCCCACCACGGTCATCACCGCCACCACCTGCCCCATCGCGTTGAACACCGGCGCCGACAGCGCA
TCGACCCCCGGCATCAGCAAGCCATGCACAAAGTGCAGGCCACGCTGGCGAATCTGCTCACAGGCCTGCCTATACG
CCTGGTCGTCCGCCAGCGCATGGGCGGTGCCGGCCTCGATCTCCTGCGCGCGCAGGTCTACGGTTTCGCGCTCTGG
CAAGAAGGCGCTGAACACCAGCCCGGTTGAGGAACTGAGCAGCGGCAGCACCGACCCCAATTGCGTCACCACCGTG
ACCGCGCGTACGGCCGGTTCGATATGCACCACGGTCGCGCCGTGGTTGCCCCACACCGCGAGAAAGCAGGTTTCAT
TCAACTCATCGCGCAACTGCGCCAGGGGCAGGGTGGCGACTTTCAGCACATCCATGCTGCCCAGCGCCGCCAGCCC
CACGCGCAGGGCCTCGCGACCCAGGCCGTAATGGTTGGTGGCGGTGTTCTGCTCGGCAAAGCCCGAGGCAATCAGG
GCCTGCAAGTAGCGGTGGACCTTGCTGGCGGGCATCTGCACATGTTCGGCCAGGCGCGACAGGGAGGTGGCCGGCG
ACAGTTCGGCCAGGGCCTTGAGGATGTCGGTGCCGACTTCGGCCGAGCGGACTTTCTGTTTACCGGTGTCGCGGGG
GGCGGGCGGCTTTTCCATGGAGAACGTGTGTCCCAAAGACGAATGGCGGTCTTTATAGCTTGACGGTCGATACGGA
GCAAATTACGTTATGCGTAACTGGATTACGATAAAAACAACTTCCTTACTGAGGACGATCCATGACCCTCGATTAT
CAATCGGGCTTTGGCAATGAATTCGCCAGCGAAGCCTTGCCAGGTGCACTGCCGGTCGGCCAGAACTCCCCGCAAA
AAGCGCCCTACGGGCTGTACACCGAGCTGTTCTCCGGCACCGCCTTCACTATGGTGCGCAGCGAAGCCCGGCGCAC
CTGGATGTATCGCATCCAGCCGTCGGCCAATCACCCGGCATTCGTCAAACTGGAACGGCAACTGGCCGGTGGCCCG
CTGGGTGAGGTCACGCCTAACCGCCTGCGTTGGAACCCGTTGGATGTTCCCGCAGAACCCACCGACTTTATCGATG
GGCTGGTGGCCATGGTGGCCAACTCCGGTGCCGAAAAGCCCTCAGGCATCAGCATCTATCATTACCGCGCCAACCG
TTCCATGGAGCGCGTGTTCTTTAACGCCGATGGCGAACTGTTGATCGTTCCCGAGCAAGGCCGCCTGCGCATCGCC
ACCGAGTTGGGCGTGTTGGAGGTCGAGCCGCTGGAAATCGTCGTGCTGCCTCGGGGCCTGAAATTCCGCATCGAAC
TGCTGGATGCCCAGGCGAACGGCTACGTTGCCGAAAACCACGGCGCACCGCTGCGCCTGCCGGACCTGGGGCCGAT
CGGCAGCAATGGCCTGGCCAACCCGCGGGACTTCCTGACCCCGGTCGCCCACTACGAAGAGCTGTCCCAACCCACG
CCCCTGGTGCAGAAGTTCCTCGGCGAGCTGTGGGGCTGCACCCTGGACCACTCACCGCTGAACGTGGTGGCCTGGC
ACGGCAACAACGTGCCGTACAAATACGACCTGCGCCGCTTCAACACCATCGGCACGGTGAGTTTTGATCACCCGGA
CCCGTCGATCTTTACCGTGTTGACCTCGCCCACCAGCGTCCATGGCCTGGCCAACCTCGACTTCGTGATCTTCCCG
CCGCGCTGGATGGTGGCCGAGAACACCTTCCGGCCACCGTGGTTCCACCGCAACCTGATGAACGAATACATGGGCC
TGATCCAGGGCGCCTACGATGCCAAGGCCGAAGGCTTCCTGCCGGGCGGCGCGTCGCTGCACAGCTGCATGAGCGC
CCACGGCCCGGATGGCGAGACCTGCACCAAGGCCATCGCCGTGGACCTGGCGCCGCACAAGATCGATAACACCATG
GCCTTCATGTTCGAGACCAGCCAGGTGCTGCGCCCGACCCGGTTCGCCCTGGACTGCCCGCAACTGCAAAACACTT
ACGACGCTTGCTGGGCCACGTTGCCGGCAACCTTCAACCCGAATCGGAGATAAACCATGACTCAGTCCACTCTCAC
CCGTAGTTGGGTGGCCTCGGCCAACGGTCACACGGATTTCCCCCTGCAGAACCTGCCCCTGGGCATCTTCAGCCTC
AACGGTTCGGCGCCGCGCAGTGGCGTGGCGATTGGCGAGCATATTTTTGATCTGCAGGCCGCCCGTGAGCTGTTTG
ATGGTGAAGCGCGCCGCGCCATCGACGCCATGGCAGGCGGCCAATTGAACGCCTTCTTCGAACTGGGCCGCGGCCC
GCGGGTTGCCCTGCGCGAACGCCTGCTGGAATTGCTGGCCGAGGGCAGCGACCAGCAGGCCAAGTTGCAAGCCCTG
GGCGCCCAGGTGTTGCCCCTGGCCAGCGATTGCCAGATGCACCTGCCGGCGAAAATCAACGACTACACCGACTTCT
ACGTCGGCATCGAGCACGCGCAGAACGTCGGCAAACTGTTTCGCCCCGACAACCCCTTGCTGCCCAACTACAAGTA
CGTGCCGATCGGTTATCACGGTCGTGCGTCAACCATCCGCACCTCCGGCACCGAGGTGCGTCGGCCCAAGGGCCAG
ACCCTGCCAGCCGGCCACAGCGAACCGACCTTCGGCCCGTGCGCACGCCTGGACTATGAATTGGAACTGGGCATCT
GGATCGGCCAGGGCAATGCCATGGGCGACTCGATTGCCATTGGCGACGCCGCCGAGCATATCGCCGGTTTCTGCCT
GCTCAACGACTGGTCGGCGCGGGATATCCAGGCCTGGGAATACCAGCCGCTGGGGCCGTTCCTGTCCAAAAGCTTT
ATCACCAGCATCTCGCCGTGGGTGGTCACGGCCGAAGCCCTGGAACCGTTCCGCAAGGCCCAGCCCGCGCGGCCGG
CCGGTGATCCACAACCGCTGCCGTACCTGCTGGACAGCCGCGACCAGGCCGCTGGTGGCTTTGATATCGAACTGGA
AGTACTGCTCACCACCGCGGCGATGCGCGAGCAGAACCTGCCGGCCCATCGCCTGACCCTGAGTAACACCCAGCAC
ATGTACTGGACCGTGGCGCAAATGGTTGCGCACCACAGTGTCAACGGCTGCCAATTGCAGGCGGGCGACCTGTTTG
GTTCGGGTACTTTGTCGGGCCCGGCGGCCGGCCAGTTCGGCAGCTTGCTGGAAATCACCGAAGGCGGGAAAAAGCC
GATTGAACTGGCCTCGGGCGAGGTGCGCAAGTTCCTTGAAGACGGCGACGAAATCATCCTGCGTGCCCGCTGCCAG
CGTGATGGATTTGCCTCCATCGGCTTTGGCGAATGCCGTGGCACCGTAGTCGCGGCGCGCTAAGAGGGCAGGGGCA
TGGAACTCTATACCTACTACCGTTCCACGGCGTCCTATCGGGTGCGCATTGCCCTGGCCCTCAAGGGCCTGGCGTT
CACCGCGCTGCCGGTCAACCTGCTGGTACCCAAGGGCGGCGCCAATCGCCAGCCCGAATACCTGGCGATCAACCCG
CAGGGCCGTGTCCCGGCCTTGCGCACCGATGAAGGCGAGCTGCTGATCCAGTCGCCGGCGATCATCGAGTACCTGG
ATGAACGTTATCCACAGCCCGCGCTACTGGCCGAGGACCTGGCGACCCGCGCCCATGAGCGCGCGGTGGCCGCGAT
CATCGGCTGTGACATTCACCCGCTGCACAACTCCAGCACCCAGAACCTCCTGCGCCAGTGGGGGCATGACGAAGCG
CAGTTGCTGGCATGGATCGGGCATTGGATCAGCCAGGGCCTGGGCGCTGTCGAGCAATTGATCGGCGACCAGGGTT
ACTGCTTTGGCGAGCAGCCTGGCTTGGCGGATACCTTTCTGATCCCTCAGTTGTACGCGGCGGAGCGCTTCAAAGT

GGCGCTGGACAGCTATCCACGGATCCTGCGCGTCGCAGCCTTGGCTGCCAAACATCCTGCATTTATCCAGGCCCAT
CCCTCCAACCAACCTGACACCCCCTAGATCAATGTGGGAGCTGGCTTGCCTGCGATGACGGTGTTGACTGACACAC
CGCCATCGCAGGCAAGCCAGCTCCCACAAGTGATACCCCATAAAAATAAAATAGGTACCTTGCGATGCACAATCAG
ATTGCCAGCTTTCGCGCGGCACTCGACGCCCGTCCGGTGTCGCGCTATCAGTGGTTGATTCTTCTGTTGTTGGCGT
TGTTGCTGGTGACCGATGGCTACGATGCCCAGGTGCTGGGTTACGTGGTCCCGGCCCTGGCCCAGGACTGGGGCCT
GGAAAAAGCCGCCTTCGGCCCTGTGTTCAGTGCCAACCTGCTGGGGCTGACCCTCGGCTCGCTGGCCGTGACGCCC
TTGGCTGATCGCTTCGGCGTGCGCCGTATTTTGCTCGCTTGCGTACTGATCTACGCCAGCCTCACGGTGTTGATGG
TGTTCGCCAATTCCCTCGAAACGCTGATGGTCGCGCGTTTTATCTGCGGCATTGGCATGGGCGGCGCGATGCCCAG
CGCCATGGCGTTGATGTCGGAATACTCGCCACCGCGTATGCGCACCCTGATGGTGACGTTGGCGGCCTGTGGCTTC
TCGTTCGGCGGGGCGGCGGGTGGTTTTGTCGCGGCGGGCTTTATCGACAGCTTCGGCTGGCAGGCGGTGTTCCTCG
CCGGTGGCGTGACGCCATTGTTGTTGTTCCCGTTTCTGGCATGGCTGCTGCCTGAGTCCTTACCGCGTTTGTTGCG
CGATGCGCCGCCCTATGCGCGCCTGCAGAAAGTCACGGAGCGCATGCTGCCCGGCTGGCAGGCACCGGCGGCCAGC
GAACAACAGAATCGCCAGGAGCAGGGCAGCAAACTGACGGTGGTGGAGTTGTTCCGCAATGGTTATGCACGGCCAA
CGTTGCTGATCTGGTCGACCTTTTTCGTCAGCCTGATCCTGCTGTATTTCATGATCAGTTGGTTGCCGTCACTGTT
GCTGGAAAGTGGCATGGCCTTGAATAAAGCCAACCTGGTGACCTCGATGTTCCTGTTCGCCGGCACCTTGGGCGCG
ATTGGCATGGCCTGGTTTGCCGACCGCCTGAAAAGCAAAGTGCGCTTGCTCTCGGGTGTGCTGGCGGCAGCGGCGC
TGTGCACGATTCTGCTGGGGCTCAATCACGACAACCCGCGTTACCTGGTGGCATTTGTGTTTGCGGCGGGCTTTTG
CATCATTGGCGGCCAGTTGACCCTCAATGCGTTTGCCAGCAACTTCTATCCAGCCCATGTGCGGGCCACTGGCACG
GGATGGGCGTTGGGTGTCGGGCGCTTTGGTTCGATCCTGGGGCCACTGTTTGGCAGCATGCTGCTGGCGATGCATG
TACCGGTGCAGCAGATTTTCTTCTTCTGTGCGATTCCGGCGGTGATTGCCGCGTTACTGATCATTCAGGTGCGTTC
GCCTGAGGCCAAGGCGCCAAAAGATTCACTGCGTGGCGATATCCTCAAAACCTGAAGAACACCATGCTTACCTGTG
GGAACTGTCGAGCCCGGCGCGGCTGCGATGGGGGCGACGCGGTCTAACGGTCGCCCCCATTCAATGCACCACCGCG
CCAGGTGGCAGGTGGCCCAGGCGTTCAGTCAGTCTGAGTCGCTGCACCGGATCCTCGCTGAGCAACAGCGCATGCT
CAAGGTCGAAGCGCTCGGCATTCGGGCAATCCAGATGCTTGTACAGGCTGGCCCGTGCCAGATAGTCGGCGGCATT
GGCGTCGCCCAGTTCCAGCACCCGTTCGGCATCGATCAGCGCCGCAAGGTGATTGTCATTGGCCACGTACAGTTGC
CGCAGGTTGCGCGACAGCCGTTGCAGGATCAGCCGCGGTTCGGCGCTGCGCAAGTGTTCGGCTTGCAGCTTGAGAT
TGGGGCCGTACTGGCGTTGCAGCAATTCGCGGCAGTCGTTGGGGTACAGCCGTCGCCCGCCACAGGGATCGAGCAA
ATGATCGGCACCGCTCACCCGTAGCAGGAAGTGCCCTGGAAAGTTGACCCCGGCCATGGGAATCCCCAGGCGCCGC
GCCAACTCCAGGGCAATCAGACCCAGGGCCAGGGGTTGCCCGCGTCGGCGCTGCAACACCTTGTCCAACAGCGCGG
CGGCGGGGCGCAAGGGGGTGAAGTCGTCCTGGGCGAACCCCAGGTCATTCATTATCCGCAGCAACGGCTGGCCCAG
TTCATCGGCCGGCAGCAGCGGCATGCCCAGGCTGACCTGCTGGTGCAGCACAGTGAAGTCAGCCAGGATCGCCCGT
GGCTCTACGGTAGGGTCGTGCTCGGCAGCGATCCATAAGGCGGCTTCAAACAGCGCCGGAGGCGAGCGGTGCAGGC
AGTCGAAAAAGGCTTGGCGGGGATTCATTGCATTCTCCGTCGGATGTCCCGTTTTAGCCTTGCCGAGAACTTTCGT
CCAGTCCCTTAGGACCTGGCCTACCTGGTTATGTCGCAAAGCCTGTAATTACGGGCTGCTTATTCCTGCGCGCTCC
TGCAACCTTTTCCCGGCGGCCTATACTTGGCGACTACAAGCAGTCATTCGGGAGCTCGACGATGTTTGCTCTCATG
CACAGCACCCGCGTTGAATCCCTGCACCTGAGCGTTGACCCGGTGACCGGGTTGAAGGCGGTCATTGCCATTCACA
GCAGTCGCCTGGGGCCGGCCCTGGGGGGCTGTCGCTACCTGGCTTACCCCGACGACGAAAGCGCCGTGGCCGACGC
CGCACGCCTGGCCCAGGGCATGAGCTACAAAGCCGCGCTGGCCGGTTTGCCGGTAGGCGGCGGCAACGCGGTGATC
ATCCGTCCCGCCCATGTCGAAAGCCGCGCCGCGTTGTTCGAAGCCTTCGGCCGGTGTATCGAGCAGCTCGATGGCC
GCTACATCACTGCCATCGACAGCGGCACTTCCGTGGCCGACATGGATTGCATCGCCCAGCACACCCGTTTTGTCAC
CAGCACCACCGCCGCCGGCGACCCTTCGCCCCATGCGGCCATGGGCGTATTCGCCGGCATCCGCACCGCCGCCATG
GCCCGCCTGGGCAGCGACAACCTGGAAGGCCTGCGAGTGGCAATCCAGGGCCTGGGCAACGTCGGCTACGCCCTGG
CCGAACAACTGCACGCCGCCGGCGCCGAATTGCTGGTGAGTGATATCGACCCTGGCAAAGTGCAACTGGCCATGGA
GCAACTGGGCGCCCACCCGATTGCCAACGATGCGTTGCTCAGCACCCCGTGCGACATCCTCGCGCCCTGCGGCCTG
GGCGCCGTGCTCAATCGCCAGAGCGTCGCCCAACTGCGCTGCGCCGCCGTGGCCGGCTCGGCCAGCGCACAACTGA
CCCACCTGCAAGTGGCCGACCAGTTGGAGGCACGTGGCATCCTCTACGCACCGGATTACGTGATCAACTCCGGCGG
CCTGATCTACGTCGCCCTCAAGCACGGTGGCGCGGACCTGGCGACTATCACCGCACACCTGTCCAACATCGGTACA
CGCCTGACCGAAATCTTCGCCCACGCCCAAGCCGAAAAACGCTCACCCGCGCGGGTCGCCGATGAACTGGCCGAGC
GCCTGCTGTACGCCTAGCACTTATTAAAAAGGCCCTGAATCATCGATTCAGGGCCTGTTCAATTCGGGCTTTATTC
CGCCGCGGGGTTCAGCAACTCGGACAGCGCGTCCGGCTGGCTCTTGAACGCCTTGGCAAACACATCGCGATTCTTC
GCCATGTAGATCCCGGCTTCCTCCACCTGCTGCTCACTCAGGGACGGCACGGCTTTTTGCAACACTTCTGCGAGCA
ACTCAGCGAGTTCAAGCATCTTGTCATGACGGTCAGCTTCGGCTTTATCCATGAACAAACGCTCCAGATCTCGGCT
GCTGCGGTATACCACTTCGACGGCCATTCACCACCTCACATGCCTTCACGATAGTTGTCTATTGCGACTACTGTAT
TTATATACAGCTAAAAGGATAAGCCAATCCAGGGGGTTTGGGTAGCAGCTTTTTAATGTAGTCGGGAAATGGGGCT
TTGCTACAAAGGAAAATTTCTGACAACTTGCCGGGGTTGCGCTGGATTTGTCCGGTGGCTTACCTTCCTCGGGCGC
TGTGTATGCAGCGATTGGGCTTGTCGGCCCGGTAGAAGATAGCGCAACAACCCCCAACAAGAACGCTGGTGTTTTT
ACGCCTGCTTTCCTGTGTCGAGGCGGGGTGCTGGGTTTCTTCTTACCGGTCCGTCACCCAATCTGCTTGGCGGCAG

ACGGTGCTGGATATCCATTCGATCAAGAAGAGATACGCCATGCCCAACCCCCCAGACTCACTTCATCGCTTTGAGC
ACGACAGTCAAAACAGCAACGACTAGACTCCTTCTTCATCAACAATGGAGGCTTTTATGGACACTTCCTTTTCTTC
TGCTACCCCCGCAGAGACTGAAGAGCAGGCCGACAGCTATGACCGGTGGTTTCGCGCCAAGGTGCAAGCCGCTCTT
GATGACCCAAGGCCAGGTATTCCTCATGATGAGGTGATGGCTGAGATGCGCGCCCTGATCGCATCCAAACGCAAGA
AAGGCGATGCTGATTGAGTGGGCGCCCGCAGCGCGGGAGCAACTCCAGCAAATTATTAGTTACATCAGCGACCGCA
ATTTAGTCGCAGCACTTGAGCTAAACCAGGCAATAGAAGCCTCTGTACTGGCTCTGTCCCGTAGGCCCCATCTTTA
CCGACCCGGACGTGTTATCGGCACGCGGGAGATGGTGGTTCATCCAAATTATCTGGTTGTTTATCAAGTGACGGAC
TACATCCGGGTACTCTCGGTCTTGCATGCGCGCCAACGGTATCCCTGATAAACCGGCCTCTCGGAGGCTCAGAGTT
AATGTGTTTTTCTGTATGCGTGTCTCCTGTCATTCCACCATCATCTCATTCTGCAATCTCTGGCCTTCTTTCTGCA
TGCAGAGAAATCGTCATGCCAAACGGGCATTATCCCCGGGCCAAAGCATGGCTTAGGCAATGGCAAACAGTTGGCT
ATTTTTTAAACAGTGGTGGTGAGTAATGACAGAAATGAAGTGGGCCGACAAAGTGCGCAAAAGCTTGCACGCAAAC
GCAGATTCCCTGGGTAATCTGGCGGTGGAGGCGTTTCACTACCTAGCGCTGTTTGGCATCGGTGCAATCACCGCCT
ATGCGGCTGTGCTGACCTTTATCGACATGTTGCAAAAAGGTGGCATCAGCGTTGATGACATCCTGTTGCTGTTTAT
CTACCTGGAGCTGGGCGCCATGGTGGGGATCTATTTCAAGACCAACCACATGCCCATCCGCTTCCTGCTTTACGTA
GCAATCACCGCATTGACCCGCCTGCTGATCGGCGACGTGTCTCACCACAATGCCCCGGACCTGGGCATCATTTACC
TGTGCGGTGGGATTCTGCTGTTGGCGTTTGCCATCCTGGTGGTGCGCTTTGCGTCGTCGCGTTACCCATCGGTCAA
GGAAAAGTCCGAGTCCTGAGTCAGAGCCCGGCCGTGATCGGCGGCCGGTTGCTCGGTGGGCGCTGCAGCAGTTTGT
GACTGTCGCCGTCGGTCATCACCGCCAGGATCTCCATGGCGCTGTGCCCCTGTTCAATGGCAATGCCGAATTGCAC
GCTTTGCACCAGGCGTTTGAGGCGTTGTGGGTCGTTGCGCTGTTCGGCGCTGATCATGCGTTTGGCAACCACGCCG
CTGTCGTCGGAGAGGGTCAGCATGATGCTGCCGTCCAGGCGCTGGATGCTCAGGTTGACCCGGTACTTTGGGGAGA
ACGTGTCGGTGATCAGCTGAAAAGGATTGTCCATGGTGCGTTACCGCCTGATAAGAACATGCACTGATTGACGACC
GGTATCGGGATTGGTTCGTGTTGCCTGACCACCGGTCACAAGCTCCGTTTTCCTTCAGTGGGTGTACAGCAAGGGG
CGTGCCGTACAGGCATCTTTCTGACGACGAGCTGCCCCTGTGGGAGCGGGCTTGCCCGCGATTGCGGTGGATCAGC
CAACCATTTAGTGACTGACATACCGCCATCGCGGGCAAACCCGCTCCTACAGGGTGATCTGTTTCAGGGCACTAGC
GAGAAAATGACCGCAGACAGCGCAATCAACCCCAGCAGCACCACAAATACATTCGATACCTGGCCCGAATACTGGC
GCAGCGATGGCACGCGGCGGATGGCGTACATCGGCATCAGGAACAGCAAGCACGCGATGATCGGCCCGCCCATGGT
TTCGATCATGCCCAGGATGCTTGGGTTGAAGGTGGCGACGGCCCAGCAACTGAGGATCATGAACACAGCGGTGCAA
CGTTCCAGCCAGCGCGCCGACAGGGTGCGGTTGCGCCCGCGCAGGGATTTGACGATCAGGCCCTGGAAGCCTTCGC
TGGCGCCGATGTAGTGGCCGAGGAAGGATTTGGTGATGGCCACCAGCGCGATCAAAGGCGCAGCGTAGGCGATCAC
CGGGGTCTGGAAGTGGTTGGCCAGGTAGGACAGGATCGAGATGTTCTGTGCCTTGGCGGCCGCGAGGTCGGCGGGT
GACAGTGCCAGTACGCAACTGAAGCAGAAGAACATCACGGTGAGCACCATCATGCCGTGGGCCATGGCCAGGATGC
CGCTGCTCTTGCGTTCGGCTTGGGCGCCGTACACGCGTTTCTGGTCCACCGCGAAGGCCGAGATGATCGGCGAATG
GTTAAACGAAAACACCATCACCGGAATCGCCAGCCACAGGGTCTTGAAGAATGCCGGCAGGGGCATGCCCTCGCTG
GCCGAAGCGAAGAACGCACCGTTCCAGTTGGGGATCAGGCTCAGCGCCAGCAACAGCAGGGCGGCGACAAACGGGT
AGACCAGCACGCTCATGGCCTTGACGATCACGCCCTGGCCGCAACGCACAATGGCCATCAGGCCGAGGATCAGTAC
CAGGGACAGAATCGCCCGTGGGGGCGGGGTCATGTGCAGTTGGTGTTCCATGAAACTGCCGAGGGTGTTGGTCAGC
GCGACGCTGTACACCAGCAGGATCGGGAAGATGGCGAAAAAGTACAGCAGGGTGATCAGCTTGCCGGCGCCAACGC
CGAAATGTTCTTCCACGACGTCCGTAATGTCGCCGGATTTGCCCGACAGCACAAAGCGCGTCAGCCCACGGTGGGC
GAAGAAGGTCATGGGGAAGGCCAGCAGCGCCAGGATGATCAATGGCCAGAAGCCACCGACCCCGGCGTTGATCGGC
AGGAACAGGGTGCCGGCACCGATGGCGGTGCCGTAGAGGCCGAGCATCCAGGTGGTGTCGTGCTTGGTCCAGCCGG
TGGTTACGGTTTGCGTGACAGAAGCGGGATTTTCGGCAGCAGGTGTACGTACATCGGTCATCGTTATTGCCTCGTT
ATTATTTTTGCGCGGGCTCACGTATGGGGCGGGTAGTCGTGCTCCTCAGCACTCCACCCAACTGACAGCCAGGCCG
CCCCGTGAAGTTTCTTTGTATTTGTCATGCATGTCGGCGCCGGTATCGCGCATGGTGCGGATCACCCGGTCGAGGG
AGATAAAGTGCTTGCCGTCGCCGCGCAGGGCCATCTGCGTGGCGTTGATGGCTTTGACGGCGGCGATGGCATTGCG
CTCGATACAGGGCACCTGGACCAGGCCACCGACTGGATCGCAGGTCAGGCCGAGGTTGTGCTCCAGGCCGATTTCG
GCGGCGTTTTCCAGTTGCTCGGGGGTGGCGCCGAGAATGTCCGCCAGACCTGCGGCGGCCATGGCGCAGGCCGAGC
CGACTTCGCCCTGGCAGCCGACTTCGGCGCCGGAGATCGAGGCGTTTTTCTTGCACAGGATGCCGACGGCGGCGGC
AGCCAGAAAGAAATTGACCACATCATCATCAGACGCGTCGGCGTTGAATTTCATGTAGTAGTGCAGCACTGCCGGA
ATGATCCCGGCCGCGCCATTGGTCGGCGCGGTGACCATGCGCCCACCGGCGGCGTTTTCTTCGTTGACGGCGAGGG
CGAACAGGTTGACCCATTCCATGGCCGACAACGTTGAAGTGATCACATTCGGTTTGCCTATTTCCAGCAGGCTACG
GTGCAGCTTGGCGGCGCGGCGCGGTACATCCAGGCCGCCGGGCAAGATGCCCTCATCACGCAGGCCCTGCTCGACG
CACTCGCGCATCACCGACCAGATATGCAGCAGGCCGCTACGGATGTCTTCGTCACTGCGCCAGGCGCGCTCGTTGG
CCATCATCAATTCGGAAACCCGCAGGCCGTGCTTGTTGCACAGGGCCAGCAGTTCGACGGCGCTGGAAAAGTCGTA
GGGCAGTTCTACATCGCTGGTGGGAGCAATACCCGACTCGGCTTCCGCGGCCTCGATGATGAAACCACCGCCCACC
GAGTAGTAGGTCTGTTCGCTCAACAGGCCGTCTGCCCCGTAGGCATGCAGGGACATGGCGTTGGGGTGGTAGGGCA
GGCTTTCCTCCAGCAGCAGGAGGTCGTGCTGCCAGTTGAAGGAGATTTCTTGGGTACCGGCAAGCAACAATCGGCC
TGATTCGCGCAACTGCTGGATACGCGGCCCGATCGAGGTAGGGTCAACCCGATCCGGCCATTCGCCCATCAGCCCC

ATCACGCAGGCGCGGTCGGTGGCATGGCCCACGCCGGTGGCAGACAGAGAACCGTACAGGCGCACTTCCACGCGCT
GGGTTTGCTCCAGCACTTGTTGGTCTGCGAGGGATTGGGCGAAGGTGGCCGCCGCCCGCATGGGGCCAACGGTATG
GGAGCTGGACGGGCCGATGCCCACCTTGAATAGATCAAAAACGCTGATAGCCATGCTAAAGCCTTACAAGCTATGG
AGTAGGAAATCGCTGCCATTGTTTGTAGGACAAGCGGAAGTGGCGCGATACTGCCCCTCTAGATCGGCACTGACCA
ACGAATTATCCTAAGACACCCTTTAGCAGGACTAAACTCTATGGCCCGCCCCCTGCATGCCCAGACGTATGTCTGG
TTGCACGTATTTGCCTGCGCTGCACGGCATCTGTCCTTTACCCGTTGCGCCGAAGAACTGCACATAACACCCGGTG
CAGTCAGCCAGCAGATTCGCCAATTGGAAGAGCGCCTGGGCTTTCGCCTGTTTCATCGACGGGCGCGCGGCGTGGA
GTTGAGTGCCGAAGGGCAGCGGCTGGCGATCACGGTGAGCGAGGCCTACGGCAGTATCGATGCCGAGTTGCAGCGC
CTGGATGCGGGGATGATCAGCGGCACCTTGCGCCTGCGCTCCATTCCGTCATTCCTCGGCAAGTGGTTGACGCCGC
GCCTGCCGCGTTTGCAGCAGCGTTTTCCGGATATCCAACTGCGATTGGTGGCCGAAGACAGCAGCATCGCCTTGCA
CGAAGGCGACTTCGATCTGGCCATCGACCTTAACGACGGCAGTTATCCGGGTTTGTTATCCACAGCCTTGCTGGAT
GAGCAGATATTCCCGGTGTGCGCGCCGAGCCTGTTGCGCGGTCGGCCACCGCTGCACGGCCCGGCCGACCTGGCCC
ATTTCCCGTTACTGCACGACATCACCGCCTGGCGTGGGAGTTATGAGTATGCGGAGTGGGAGTTTTACCTGAATGC
TATCGGCTATCACGACGCGGACGTGCGGCGCGGCCACACCTTCAACCGTAACCACCTGACCATCGAGGCCGCCATT
GCCGGCATGGGTGTGGCGATTGCCCGGCGCACCCTGCTCAATGATGAATTGGAGCGTGGCACCCTGATCGTGCCGT
TCGGGCTCGCGGTTGCGAATCACAAACGCTACGTGCTGCTGTATGCGCCAGGCGCGCTGAACCATCCCGGCGTACG
TGCGGTGCATGACTGGCTGGTGGAGGAGGCGGAGATTTTTCGCGGATTGCACCCCTTGGGAGAGGGGCAATTGTGA
GGATTTACGCAGGTGAGCAGGTGTACCCAACTCCCGACCATAACAGCGCTTTGGGCGGTTGTCAGACTTAATTTGT
AATGTAGGATTTATCTTTGCGTAGGGGTTGAATTGTTCTCTCAGGTGCTCAATTGTGTAATCAAGAGGTCATGGTT
TCACCGCATCGGTGTTGCAGTTGTGATCTCTGGCGAGCTAGCTGAAATAAGGGAAACACTATGCAAATCCTAGTCA
ACAGCGATAACCATATTGAAAGCAGCATCCGACTGGAGGAGTGGGTACGTACTACCATTGAGAGCACGCTCGAACG
TTATGAAGAAGACTTGACCCGCATCGAAGTCTTTTTGAAGGATGAGAACGGCGACAAGCCGGGCCCCCACGATTTA
AGTTGCCGCCTGGAAGCGCGGCCAAAGGGGCATCAACCGATTTCGGTTATCCATAAAGCCGACACCCTGGAACAAG
CGATCGACGGGGCGGCCACCAAACTGGATAACGCGCTGGAACATCTGTTCGGCAGGCTGCAAGGCAAGCCGCGTGC
AGAGAAAAGCACGCCAAACAACCAAGTCGATGAAGCCGGGCTTGAAGAGGAATTCCTGGAAAACGAACAGGCTGCG
CTTAACAGCTGATTGCTGAGTGTTTTTCAACCCCATACAAAACGGGCCTGCAGATGCAGGCCCGTTTTTTTGTGTC
TGATGAACGCTTCACTGTGGGAGCGAGCTTGCTCGCGAAGGGCGTAAAGGATAACGCGCAGATCCTGCGGGCACGC
GGTGTTCTTAAGCTTTTCGCGAGCAAGCTCGCTCCTACAGTAAGCCGGTAGTCCGGCCGTCAGAATGCGACCGAGG
TCTGTACGTACACCGTGCGCGGTTCGCCCACGTATTTGCCCTTGTTGTTGTCGTCAAACGAGCGGGTGTAGTACTG
GGTGTTGAAGATGTTCTTCACCCCCACCGCCACATTCAAGTCCGACAATTGCGGGCCGAAGTCATAGGCCGCGCGG
CTGCTGAACAGCATGTAGCCAGGAATGCGCCCGGTGCTGCCGTCCACGCTTTCAGCCCGGGTGTTGGCGTTGTCGG
CGAATTGGCTGCTCTGGTAGGTGCTGTCCAGGTTCAACTTCCATGCACCTTCGGTGTAGCCCACGCCCAGGGTGCC
TTTGTGCTTGGACGAAAACGGCACGCGGTTGCCTTTGTTCGGGCCGTCTTCACGGATGGTTGCATCGACGTAGGCG
TAGCTGGCGTACACATCAAAACCGGCCAGCGCCGGGCTCAAGCCATCGAGAGCATAATTGAGGCTGGACTCGATGC
CCTGGTGCCGGGTTTCGCCCCGGGCGATCACCGAGTCGTTGGTCTGGTTGCTTTCATACTGGTTGTCGAAGTTGAT
CAGGAACGCACCGATTTCCGCGCGCAGCGCGCCGTTGTCGTAGCGGGTGCCGACTTCCCAGGTGCGGGCCTTTTCC
GGCTTGACCGCGCCGCTGCTCACACGGTTGGGCATCTGGCTGTACTGCACGCTGCCGAAGGAGCCTTCGGTGTTGG
CGTACAGGTTCCAGTCGTCGGTCAGGTGATAGAGCACGTTCAACGCCGGCAAGGCGGTGTTGTAGTCGCCCTTGTA
TTTGACGTTGGTCAGGTTGTTGGTCTGTTGCGACTTGATCATCTCGTAGCGGATGCCAGGGGTTATGGTCCACTTG
CCGATATCGATCCGGTCGTCGATAAAGAATGCATTGGCTTCGGTGCCACCCCGGGTGTCGCGGTCGTTGCGGCTGT
CGGTGTTGGGCAGTTGCTGGTTGGCCGCAATCGGCGTGCGGTAGCGCAATTCATGCCCGGCTTCGTTGATGTAGCG
GTAGCCCACACCGACTTCGTGGCTGGTCGGGCCGAGGTCGAAGCCTTGGGCCAGGCGCGTTTCCAGGCCGCGTACC
CAATACTCGCGCGGTGACAGCGAAAGGAAGCTGCCCTGGTCCAGATAACCGCTGCGCAGGGTCTTGGTAAAGAAGC
TGTTGACGGTGAATTCCCGGCGATCTTCCTGGTAGCGGTAGCCGACGTTAAACATCGTACGGCGGCCCCAGAATTT
GTCGTAGGGGCGGGTGGACTGATACGGGTCGGCCTTGTAGGCCGCAGTGCTCAAGCCACCGGGCATATCGGCCTGA
CCTTCGTAGTACTGGGCCATGGCATTGAAGCTGTTGGCTTCGTCCAGTTGGTAGTTGCCCTTGAGGATCAGGTCGT
CGATTTGTGTGTCGCTGTGTTCGCGCCAGTCACCGCCGCGGGTGCCGGAATACAGGATCGCGCCGCCCAGGCCGTT
GTCGGCAGTGCCGCCCGCCAGCAGATTGCCGGTGGTCTTGAAGCCATCATGGCTGGAGGATGGGCTGGTCTCGGTC
TGCAGGCCACCCTTGACCGTCGGTGCATCCGGAATCGCCCGGGTCACGAAGTTGACTACGCCGCCGACGTTTTGCG
GCCCGTAACGCACGGCGCCACCGCCGCGCACCACGTCTACTGCATCCATGTTGCCCATGCTGACCGGCGCGAACGA
CAGTTGCGGTTGGCCATACGGGGCGAAGGGCACCGGAATCCCGTCCATCAATACCGTCGAGCGTGACGCCAGGCGC
GGATTGAGCCCGCGAATGCCGAAGTTCAGCGCCATGTCGTGGCTGCCGGTGCCGTTGTTTTCTGGAGCGTTGACCC
CGGGAATGCGGTTGAGCACGTCCCTGGCCTGGGTTGCGCCCTGGCGCTCGAACTCTTCGCGACGGATCACATCACG
GGCGCCGGGGTGTTCGAAGACGTTGGTTTGCGCGGCATCGCCCAGCCAGTCTCCCACGACATTGGACGTGCCCAGC
TCCAGGGTGGCCGGTGCGTTCAGCGGTTGCAGGCTGTAGGCGTTGTTACCTTCGGCGCGGGCTTGCAGGCCGGTGC
CCTTGAGCAATGCGTTCAAACCTTGCTCGGTGGTGTAGTTGCCTGTCAGGCCCTGGCTTTGTACGCCGGCGGTCAC
TTCCGAGCCAAAGGAAATCAGCACGCCGGCTTCACGGCCAAACTGATTCAGCGCGGCCTCCAGGGACGTCGGGGCG

ATCTGATAAGGCTTGGCCTCGGCCGCCATGGCGTAGGGCAGGGCGCTGAAACTGAGGCTCGCGCCCAGCAGCAAGT
GGCGCAGCGTGCGGGTAAGTGGCGTCAGGCGAGTCGGTTGCTGGGGCATGAAGAACGTTCCTCAGAGGTGGGAAAC
TGGGTGGCTTTCCCTCTCTGTCACGCGAGGTGCGGAAAACGGCTCAAGTTTGCCGAAAATAATTTAAACGCGGGCT
TCGACGCTGACCCAGTAACGGGTAAAACGTCGCACTTTGACCGGCAGGCTGATTTGCAGCAGGTCGAGGATGCGTT
CGCTGTCGTCCAACGGGTAGCTGCCGGAGATCAGCAAGTTCGCCACCTGTGGATCACACTGCAATTGGCCGCGGCG
ATATCGGCTCAGTTCCTCGAGGAAATCTGCCAGGCGCATGTGCGACGCCACCAGCATGCCGTCGGCCCAGGCGCCC
GTATTGGCATCGGTGGGGCGCGGCATATCCCAGCCTTTGCGGGTGAGGTTGGCCAGGTGGGATTTTTCCAGGGTCA
GGGGCAGACCGCTGTAGGACTTGGGTGACATTTGCACCTGGCCATCCAGCACCGCGACCTGGGTGTGATCGTTGAA
CTGGCGCACATTCACCTGCGCCGCCTGGGTGGTGACGATGCCCTGGCTGGTGATGATCTGCAGTGGGGTGTTGGCG
CGCAGCAATATCTCGCCTTCGAGCAGGCGGATCAGCCGTTGTGGGCCATCGAATTGCACATCCACCGCACTCGCGG
TATTGAGTTGCAGTTGGCTGCCATCGGCCAGCTGTACCTTGCGCCGCTGGCCGAGGGGGCTGCGGTAGTCGGCGGT
CAATGGCGGGATCAAGTGCTGCTGGCGCAAGCCCCAGGTCGCTGCCGAACCGGCGCCCAGAATCAACAACAGCTTC
AGGGCCTGGCGCCGGCTACTCGATTGCGGTGCGTTCAACGTCGCATGGGCCAGGGGCGAGGGCATCCCGCGCAAGC
GCTGGTTGACCCGCTGTATATGCTCCCACGCCCGTTGATGTTCGCTGTGGGCATCCAGCCAATGCTGCCAGTTTGC
CTGTTGGCGCGGGTTGAGCGGGCCTTGCTGCATTTGCATCAGCCACTGCACCGCCTGCTCGGCGACTTGGGTCGAG
AAGTTCGCGGTGGGGTTCATAGGGCGAAATAGCAGCGCATGGCGGCTTTATTCAGATGACGTTTGACGGTGGCGAT
GGAGATCCCCAGTTCGGCAGCGATTTGCCCGTAGGTCAGGCCATCGAGTTGGGCCAGCAGGAAAGCGCGCTTGACC
AGCACTGGCAAACCGTCGAGCAACTGGTCCAGCTCCATCAGGGTTTGCAGGATGATCGCCCGCTCTTCTTCCGACG
GCGCAACGAACTCGGGCATTTGCGCCAGGGCATCGAGGTAGGCGCGTTCCAGGTCCTGGCGGCGGTAGTGGTTGAA
CAGCACGCGCTTGGCGATGGTGGTGAGGAAGGCGCGGGGCTCGATCAGCGTCGGTGTCTCGCGGGCGGTCAACACC
CGGATAAACGTATCCTGGGCCAGGTCTGCGGCACTTTGCGGGCAGCCGAGCTTGCGTCGCAACCAGCCGGTCAGCC
AGTGGTGATGGTCGTGATACAGGACTTCGACGGTATTGGACGGCCGCAACGCGAACACTCCGGCAGCATCTGCATG
CTTTAGGTAACGAGAATTGTTCGCATTGTAGGCGGCGAGATGGCATTCAGCAATCGGCAGAAGACCGATAGCTGCT
TTTATAGCCGCCAGTCACCTGTGGGAGCTGGCTTGCCTGCGATAGCGCTGTGTCAGTCACCTATGCGTTAACTGAC
CCACCGCTATCGCAGGCAAGCCAGCTCCCACATTTGTTTTGTGGCGTGTCCAAAACTTCCTGCCTCGCCGAGATCC
CCTGTGTGGGCTTGCCCGCGAAGGCGGTGGTTCAGCCACCTGTATATCGACTGCCCCACCGCCATCGCAGCCAAGG
TACATTTGATGTTCGTCGTTCTCTTTTGCTTATGAGAATATTTATCATTAAACTCCCGCCCCTGATAAACCAGGCG
CCACTGGCATGAAAAGCAAAGCCTCACTTCCTCTTTCCTATCGCCTCGGTGTGACGTCGCGCCTGCTGGCGGCTGT
CGTCGGCGGCTATGTGTTGGCGTCCCTGGCCAGCGTGTGCCTGAGCTTCTGGCTGCCACTCTCGCGCGCCGATGCG
GTGGTCAGTGGCATGCTCAGTTCCTTTGTGTTCTACCTGCTGGCGGTGATCTGGTGCTTTGCCTGCCGCAGTGCGG
CCCGCGCCTGGTTTGGCGTGCTGGTGCCGTGCGCGATATTGGCCACCCTGGCTGGCCTGGCCTATTGGATGGGGCG
CCCATGAAAGAGGGTTTCCGTCAGGCAATGGCCTGGTTGCACACCTGGGCCGGGTTGATTTTCGGCTGGTTGCTGT
TCGCGATTTTCCTCACCGGCACCCTGTCTTACTTCAAGGGCGAAATCACCCACTGGATGCAGCCGGAAGTACCCGC
CCATGCCGTGGACGATGCGCGCAGCGTGGCCGTGGCGCAACGCTACCTGGAACAGCACGCACCCACGGCGGCCCGT
TGGTTTATCGGCTTGCCCGATAGCCGCGACCCCGGTTTATCGGTGATGTGGCAAGACAAGATCGACCCTGGCCGGC
GCGGCAATTTCATCGAGAAAATGCTCGACCCGGTCTCCGGCGAACAGCTCCAGGCCCGGGAAACCATGGGCGGCGA
GTTCTTCTACCGGTTCCACTTCCAGTTGCAGATGCCCCATCCGTGGGGGCGCTGGTTGTCGACTATCGCCGCGATG
GTGATGTTTGTGGCGCTGATCACCGGGATCATCACCCACAAGAAAATCTTCAAGGACTTCTTCACCTTCCGTCCGC
GCAAGGGCCAGCGCTCCTGGCTCGATGGGCACAATGCGGTCGGCGTGCTGGTGTTGCCGTTCCACCTGATGATCAC
CTACAGCAGCCTGGTGATTTTCATGACCATGGTCATGCCCGCCAGCATCATCGCGTCCTATGACGGCGACACCCGC
GCCTTCTTCAATGAATTGTTCCCCGCCACCGACAACGCGCCGGCCCTCGGCCAGCCGGGCAAACTGCAGCCGTTGG
CGCCGCTGGTGGAACAGGCGCGGGCGCAGTGGGACGGTGGTCATGTGGGGCGGCTGGCGGTGAACAACCCTGGCGA
TGTGAATGCCTCGGTCAATGTCTCCCGCGCCGGCTCTGACCGGGTGGTGCATGATTTCGGCAGTACCGTGTCGTTT
AATGGCAACACTGGCCAGCTGCTACGGGTCAGTGCCGAGCAGTCGCTGCCGATGGCGATTGGGGGCAGCTTCTATG
GCCTGCACATGGGGCATTTCGCCGGCCCGCTGCTGCGGTGGCTGTACTTTATCTGCGGCCTGGCGGGCACGGCGAT
GATTGGCACCGGCCTGGTGATCTGGCTGGGCAAGCGGCAGCTCAAGCACGCAAAAACCGGGGTGATGCCGTTTGAA
TTGCGCCTGGTTGAAGTGCTGAACCTGGCCAGTATGTCCGGGCTGCTCATCGCCATTGCAGTGTTTTTCTGGGCCA
ATCGCCTGCTGCCTGCGACCTTCGCCGAGCGTTCGGACTGGGAAGTGCAGAGCTTCTTTATCGCCTGGGGCCTGAC
CTTGCTCCATGCCATGCTGCGCCGTGGCCGCCGCGCCTGGATCGAGCAACTGGGCCTGGGCGCCGTGCTGTTCATG
GCCGTGCCGCTGCTCAATGCGCTGACCACTTCCGAGCACCTGGGCGTGTCGCTGGCCAAGGGTGATTGGGCCATGG
CCGGTTTTGACCTGACGTGCCTGGGCAGCGGCCTGTTCCTGGCCTGGGCTGCCTGGAAAATGCAGCACCGCAGCGC
CGAGCAGCCCCGGGCCGAGCGTGCCCGCCCACTGACTCTCAAAGGCGAGGTGCACTGATGTTGCTGGCCTTGTTGC
TGTGCTACGCAGGCTTTGTTGCGCTGTGCCTGTCCCTGGATCGTCACCACGGCGAGTTGCTGCACAGCAAACCTTC
GCCACGGCGGCGCCTGGGGTTGCGTGTGGGCGGATGGCTGCTGTTGGCGCTGTCGATCTGGCCGGCGGTGCACATG
GCCGGCTGGAGCCGGGGCCTGGTGGACTGGTGCGCGGTGCTGATGCTCAGCGGGCTGTTGCTGGTGTTGCTGTTGC
CGTATCGGCCAAGGCTGGCCTTGATCCTCGCGGGCATCGGCCTGCTGGCCAGCCCCGTTGCGGCATTCGCGACCCT
CTGAGCCCTGTCGATGATCAGCACACCGCCCGAACCCCAGGATCACCCCCACGCAGAATCGTCGGGCGGGCGTGCA

CATTTCCTGCAGGTATTTTTGTCCCAGCGCTCACAGATGGAGGCGTTGGTCAGCCGCCGCGTAGGCTGCCGGGCGA
CGGCCGCGGACCTGGTGCAGGACTTGTTCCTGCGCTTCTGGCGCCGGCCCCTGGTGCAGGTCGAAGAACTCAGCAC
CTACCTGCTGCGCTGCGCCGGCAATATCGCCATCGACCACTTGCGCAGCGAAGGCGCGCGGGTGCGCGGCAATGAA
GGCTGGTTGCCGGAACATCAGGATCATCACGGCTCCGAGCCCCAGGCCGCGCTGGAAGCGGGCAACGATCTGCGCC
ATGTGGAAGCGGCGCTGCGCAGTTTGCCGGAGCGTACGCGGCAGATCTTTTTGCTCAACCGCATCCACGGGCGCAA
ATACGCAGAAATCGCCAAGGCCATGGGCCTGTCCCAAAGTGCCGTGGAAAAACATATGATGCGCGCCCTCGAAGCC
TGCAAAGCCAGCCTTCGTGAACCCCCACCACCACGCTCGCCAGGGAAAGCACCGTGAACGTCACCCCGACGCCCGC
CCAGGAACAGGCCGCCCTGGCCTGGCTCAGCCTGCTGCACGATGCGCCGAGCAGTGGCGACCAGGCCACGTTCAGC
CGCTGGCTGCGCGCCGACCCGGCCCATGTCGAGGCGTATGCCCAGGCCCAGGTGTTGTGGGAGTTGAGCGAAGTGC
CGGCGAGCACCCTGGCCGACGAAGATGCCTTGGCCTTGCAGCGTTACCTCAAGGCCATGGATGGCGCGAAACCCGC
CCGTGTGCGCCGCTGGTCTGCGGCACTGGCGATGGCGGCTTGCCTGGTGTTGATGGTGTCGATGGGCGCCGGTTGG
CAGCCGCAGCGCTGGGCCGACGACCTGGGCGCCGATTACGTCAGCGCGCCGGGTGAAGTCAAAACCGTGACCCTGG
CCGATCACAGCCAAATCACCCTGGATGCCGACAGCGCGATTGCCGTGGATTTCAGCCAGGGCGAGCGGCATATCCA
ACTGCGTCGTGGCGCCGGTTTTTTCAATGTCGCCCACACAGGGCAAGCCTTTGTGGTGCACGCCGACAGCGGCGAA
GTGCGGGTGCTGGGCACGCAGTTTGAAGTGCGCCTGCAACCGGCGGGCGCCCAGGTAACGGTGTTGTCGGGGCGGG
TAGGGGTGACGCCGTCTGCCCAGGGTACTCAGCAGATTCTGACGGCCGGCCAACAACTGGCCTACGTCGAGGGTGT
GGCTGATGAGCGGCATGCCGTGGACAGTGAATCACGCCTGGGGTGGCGCGACGGTTGGCTCAACTACTACAAGGCC
CCATTGGCCGATGTGGTCAAGGACCTTGGCCGTTACTACCCCGGCCGCATCCTGCTGCTCAATGACCAACTCGGCG
CCCGCCGGGTCAGCGGCAGCTTCCCGAGCAAGGACCCCGAGGCGGTGCTCAAGGCATTGCAAGCAGTGCTGGGTTT
TGAGCAGCACAATGTGCTGGGCCGGCTGATCGTCGTTCGCTAAAAACTATCCACACCCCTGTAGGAGCCGGCCGGC
GATGGCGTCGTCAAGAGCAGCGTAGGTCTTGGGGGCCTCATCGCTGGCAAGCCAGTTCCTACAGGGAATGGGTGGG
TGAGAGAGAGTTGATAACTGTTCTTATTTGTATTTATGATATGTTATTACATTCAGCGAAGGAGCCTCCCCCATGA
AAGTCCTATCCTCACTTAAAGAAGCCAAAAACCGTCACCGCGACTGCCAGATCGTCAAGCGCCGTGGGCGGATCTA
TGTGATCTGCAAATCCAACCCACGCTTCAAGGCCCGCCAGGGTGGGGCAAAAAACAAGAATAAAGGCTGAGGGTGA
AATTATTTTCAATTTCACCTGAGGTAAAACCCCACGCCATCCGTGTAGTGCTTGAAACTGCGATAAATTCGCATTA
ACGGCGTTTTCTACACGGGTTCTCAAGCATGAAGTCCAGGGCAAAGTCGGCGGCAGGCGGTTCGGTTAAACAGTGG
CTGGGAGCCTCCCTTCTGGCCGCTTCAGGTCTGGCGGTATTGCCGCTGAGCCTGGCGCAGGCGGCGGATGCGCAAC
AGCAAAGCACGGTCTTCAACTTTGCACTCCAGGCCAAACCGTTGCCCCAGGCACTGAGCGATTTCAGTCGCGTGAC
CGGCATCAGCGTGGTCTACACCGACGAAGCGCCCTACACCCTCAATGCCCCGGCCGTCACTGGGCAGATGAGTGCG
GCCCAGGCCCTGCAGCGCCTGCTGGGCAGTTCCGGCTTGACCTTCCGCCAGATCGACGCGCGCACCCTGGCCCTGG
AGCCGCTGCCTACCGAAGGCGCGGTCAACCTCGGCGCCACGACCATCAGCGGCGTGAACCCGCAGCAAGCCACCAG
CTACCAGCCACCGCCCACCAGTTCGGTGATGCGCTCCCAGGGCTTGCTGCTGGAAACACCGCAAACCGTGAACATC
GTGCCGGCCCAGGTGATGCGCGACCAGGTGCCGCGCAACCTCGACGATGCATTGACCAACATCAGCGGCGTCACCC
AAGCCAACACCTTGGGCAGCACCCAGGATGCGGTGATGCTGCGTGGCTTTGGTGATAACCGCAATGGCTCGGTCAT
GCGCGACGGCATGCCGGTGGTGCAGGGGCGGGCGTTGAACGAAACCGCCGAGCGCGTCGAAGTGCTCAAGGGCCCG
GCGTCGTTGCTGTATGGCATCCAGGACCCGGGCGGGGTGATCAATATCGTCAGCAAGAAACCCGAGCTGACCCAAT
CCACCGCTCTTACTGTGCGCGGCTCGAGCTATGGCAGCGGCAAGAACGGCAGCGGCGGCAACCTCGATACCACCGG
CCCCATCGGCGACAGCGGCCTGGCTTACCGCTTGATCGTCGATCATCAGGATGAAGACTACTGGCGCAATTTTGGC
ACCTACCGCGAAAGCCTGATCGCACCGTCCCTGGCCTGGTACGGCGACAACACCAAAGTGTTGCTGGCCTACGAGC
ACCGCGAATTCCTCTCGCCGTTCGACCGTGGCACAGCCATCGACCCGAATACCAACCACCCACTGAACATCCCCGC
CACGCGCCGCCTGGACGAGTCGTTCAACAATATGGAAGGCCGCTCCGACCTGTATCGCTTCGAGGTTGACCACGAC
CTGAATGACGACTGGAAGGCCCATTTCGGCTACAGCTGGAACCGCGAAACCTACGACGCCAGCCAAGTGCGCGTGA
CCAAGGTCAACCCCAATGGCACCCTGAACCGCAATATGGACGGCACCCAGGGCGCGCTGACCACCGACCGCTTCAC
CACCGTCAGCCTCGAAGGCAAAGTGGATGTGGCCGGCATGCGCCATGACCTGGTGTTCGGCCTGGATGACGAGTAC
CGCAAAATCTACCGCGCCGACCTGATCCGCCAGGCCAGCCGCAGTGTGTTCAATTACAACGACCCGGTGTATGGCC
GCGAAGTGGCGGGCACCACGGTCAGCGCGCCGGACAGCAACCAGACCGATCTGCTGCGCAGCGACTCGGTGTTTTT
CCAGGATGCCATCCACCTCACCGACCAGTGGATCCTGGTCGGTGGCGCCCGCTTCCAGGAGTACGACCAGTACGCC
GGCAAAGGCCGGCCGTTCACCGCCAACACCGACAGCAACGGCCAGAAGTGGGTGCCCCGCGCCGGTCTGGTGTACC
GCTATACCGATGCGCTGTCGTTCTACGGCAGCTACACCGAATCGTTCAAACCCAACTCCACCATCGCGCCGTTGGA
CAACAAGATGGTGATCGACGGCAGCATCGCCCCGGAAGAATCCAAGTCCTGGGAGCTGGGGGCCAAGCTCGATATG
CCGGGGCGCATTACCGCCAACGTGGCGTTGTTTGATATCCACAAGCGCAACGTGCTGGTGTCGATCACCGAGGGCG
CGACCTCGATCTACAGCGTCGCCGGCAAGGTACGTTCCCGTGGTCTGGAAATGGACCTGAGCGGGCAATTGACCGA
GCAGTGGAGCCTGATCGGCAGCTATGCCTATACCGATGCCGAAGTCACCGAAGACCCGACCTACAAAGGCAAGGGC
CTGCAAAACGTGGCGAAGAATTCCGGCTCTGTTTCAGCCGTGTACGACTTCGGCACTATCGTCGGCGGCGATCAAC
TGCGCGTCGGTGCCGGTGCGCGTTATGTCGGTGAGCGTGCCGGCGATGCCCTCAACAGCTTCGACTTGCCGGGCTA
TACCGTGGCGGATGCGTTTGCCACCTATGACACCAAGGTCGACGGGCAGAAGGTCAAGTTCCAGCTCAACGTAAAG
AACCTGTTTGACCGTACTTACTACACCTCGGCCGTCAGCCGTCTGTTTGTGTCCATGGGCGATGCGCGCCAGGTGA

CGTTGTCCAGTACCCTGGAGTTCTGATCATTGGCGATGACGCTGGCGGCGTGAGCTGCTAGCGTTGCGCTCTTTGA
TGATTGCCGGGACGGTTTGATGCAGTTGGGACGATGGATACACGCCAGTGCCATGCTGGTGGGCTTGAGCCTGTTG
TTGGGTGGCTGTGCCACGTCTTCGAAAACCAGCGAGCAGGCACTGGACCAATTGCTGGCCGATCCGGCCCTGGCCG
GTGCCAGCGTTTCCTTGATGGTGCGCGATGCCCGCAGCGGCAACACGCTGTACCAGCACAACCCACGCACGCGGCT
GGTGCCGGCGTCCAGCATGAAGCTGCTGACCACGGCGGCGGCCATGGATGTGCTGGGCCCGCAGTATCGGTTTTCT
ACGCAGTTGCTGAGTAATGGCAGCCAACAAGGCACACGCCTGATGGGCAATCTGTACCTGCGCGGCCTGGGCGACC
CGACGATCCAGTGGGCCGATTACCAGGCGCTGGCGGCGAGCCTGGCCGGGCAGGGCATCCAGCAGATCCAGGGCGA
TTTGGTGTTCGATGACACTTGGTTCGACGCCGAACGCCTGGGCGTGGATTGGGCCCATGACGACGAAGACAAGTAT
TACGGCGCGCAGATTTCCGCGCTGACGGTGTCACCCAATGCCGATTTTGATGCGGGCACCTTGATCGTCACGGCCA
AGGCGCCTGTGGCGGTCGGCCAACCGCTGAGCGTCAGCCTCAGCCCACCTACCGATTATGTGCAACTGAGCAATCT
GGCCGTCAGTGGCCCAGGCAACAGCTATGGGCTCAATCGCCAGCATGGCAGCAACCTGCTGCGCCTCAGTGGCACC
CTCAAGCCGGGGACGCAAAGCCGCCAGTGGGTCAGCGTGTGGGAGCCGACGCAACTGGTGGCCAATCTGTTTGCCC
AGGCCCTGGCGGAGCAGGGGATCAGCGTGCAGGGCCGGCGGGTGATCGGTGGTGTGAGCCCGGCGAACGCCACTGT
GCTGGCGACACACCAGTCGGCGCCGTTGCAGGAACTGATCAGGCCGCTGCTCAAGCTGTCCAACAACAGCATGGCC
GAAGCGCTGCTCAAGGCCATGGGGCGACAACAGGCGAATGCCGGGACAGCGGCGGCGGGTGTGGCAGCGGTTGCGG
ATTTTCTGCGCCGCCAAGGCCTGGACCCGGCAGCGCTGAACCAGGTGGATGGCTCCGGTTTGTCACGACGTAACCT
GGTGTCGGCACAGAACTTCACCGACCTGCTGCTGGCCATGGGCAAGCAGCCGTGGTTCAACGCCTGGTACGACGCG
CTGCCGATTGCCGGCAACCCGGATCGCCTGAGCGGCGGCAGCCTGCGTTACCGCCTGCGCGGACCGCGGCACAAG
GCAACCTGCAGGCCAAGACGGGTTCGATGGGTGGGGTGTCTTCGTTGACGGGCTACATCACCGATGCGAGTGGGCG
GCGGTTGGCGTTTTCGATGCTGACCAATAACTACGTGGTGGAGGGTAGCCGGATCAAGGCTGTGGAAGACCGTGTG
GCGACAGTCCTTTGTAGGAGCGAGCTTGCTCGCGAAGGTCGTTAACGCTAACGCGGGGAGCCTGACGCCCCGCATT
GGCCTCAGGTTTTTCGCGAGCAAGCTCGCTCCTACAGGGGGCTGGGTTGGAGCGCCTGGCGAAACCAGTTGGCAAC
GGCCCCGGCCCAAAGCCCCTTGTAGGAGCGAGCTTGCTCGCGAAGGTCGTTAACGCTAACGNNNNNNNNNNAAAAC
CCGGAGCCCAGAAGTATCAGAGAGTTGTGTAGATACTCATGCTGCGATGAGTGCCTTACAGGCACTGCATGATTCA
GCCCGCCAGCAACCAAACCCCAGTCGCCGCCGAAGCCGCCCCCGCCACCCGCACCAGCGGTGCCGCTGCAGCCGGC
AGGAACCGCACCAGCGCATAACCGGCCGCGTGCAATACCGCCGTCGCCCCCACGAACCCGGCGGCATACGCCCAAG
GGCTGGTCATGTCCGGCAGCTCCAGGCCATGGGCCACACCATGGAACAGGGCAAACACGGCTGTTGCGGCCACGGC
CACCGCCACCGGTGGGCGAATCGCCAAGGCCACCGCCAGGCCCAGGGCCAGTACCGACGCGGCAATCCCGCTTTCC
AGGGCTGGCAATGCCAATCCTTCAAAACCCAGCATGCCGCCCAGCAGCATGGTGCCGACAAAGGTACACGGCAGCG
CCCAGCGCGCCGCGCCTTGTTGCTGCGCCGCCCACAGGCCGACCGCCAGCATCGCCAGCAAATGGTCCAGCCCGCC
GAGTGGATGGCTGATACCGGCCACCAGGCCATTGTCGCCATGACCCGGATGGGCAAAGGCCAGGGCCGGGGCCAGC
AGCAGGGTGGCGGCGGCAAACAGTTTGTTGAGGCTCATAAACAGCTCCTTGGTAAGGGGATCAGGCTGCGCTCAGC
AGGCCTTGGCGTTCGATAAAGGCGACGATTTCTTCGAGGCCGACCCCGGTTTTCTGGTTGCTGAACACGAACGGCT
TGCCGCCGCGCATGCGCTGGGTGTCGCTGTTCATCAGTTCCAGCGAGGCGCCGACCAACGGCGCGAGGTCGATCTT
GTTGATCACCAGCAGGTCCGACTTGCAGATCCCCGGCCCACCCTTGCGCGGCAGCTTGTCGCCGGCCGACACATCG
ATCACGTAGATCGTCAGGTCCGACAGTTCGGGGCTGAAGGTGGCCGACAGGTTGTCGCCGCCCGACTCCACCAGAA
TCAGGTCGAGGCCTGGAAAACGCCGGTTCAACTGGTCTACGGCTTCCAGATTGATCGACGCGTCTTCGCGAATGGC
CGTGTGCGGGCAGCCGCCGGTTTCCACGCCGATGATGCGCTCGGGTGCCAGCGCCTGGTTACGCACCAGAAAGTCG
GCATCTTCGCGGGTATAGATATCGTTGGTGACTACCGCCAGGTTGTAGCGATCACGCAGGGCCAGGCACAGCGCCA
GGGTCAGGGCGGTCTTGCCGGAGCCAACGGGGCCGCCGATACCGACGCGCAGGGGTTGTGTGTTCATGGGGCTCTC
CTAGGAACGGAACAGGCGGCTGTACTGGCGCTCGTGGGCCATACATGCCAGGGACAGGCCGAACGCGGCGCTGCCA
AAATGATGGGGATCGCGCTGGCGGGCGTCCTGCTGGGCCTGTTGCAGCAACGGCAGCAGTTCGCTGGTCAGGCGTT
GGGCGGCTTGCTGGCCCAGGGGCAGGGTTTTCATCAGCACCGCCAATTGGTTTTCCAGCCAGCTCCAGAGCCAGGC
GGCCAGGGCATCGTCGGGGCTGATTGCCCAGGCGCGTGCGGCCAGTGCCCAGCCCAGGGCCAGGTGGGGTTCGTGG
GTGGCGTCGAGAAAACGGCGGGCCGGCTCATCCAGCTCCGGCAGGCCGCTGAGCAGTTGTTGCAGGGAGTAGCCCA
TTTGCCGGCTCTCCTGGAACAGCTCGCGGGTTTCGCGGCTGGCGCGGTGTTCTTCACACAGGTGGCGCAGCGCCTC
CCAGTCGTGGTCCGCAGCGGCCTGGCAATGGGCAAGCAGCAGCGGCGCTTCGAAACGCGCGAGGTTGAGCAGCAGT
TGGTCGCTGATCCAGCGTCGGGCGCTGGCGGCATCGCACACACGGGCATTGTCCACGGCCATTTCCAGGCCCTGGG
AGTAGCTATAGCCGCCAATCGGCAGTTGCGGACTGGCCAGACGCAGCAGCGCCCAGGCCGGGTTCACAGGCGCACG
CCGAACTGGTGCAGTTTGGGCGGATAGTTGAAGTCTTCATCACCGTGGCGCGAGTGATGATGACCGCCACCATAGG
CCCCGTGCTCGGGCTGGAAGGGCGCTTCGAGGGTTTGGGTATGGGCGCCCAGTTGCTCGAGCATGGCCTTGAGCAC
GTAGTCATCGAGCAGACGCAGCCAGCCGTCACCCACTTGCAATGCTACGTGGCGGTTGCCCAGGTGATAGGCCGCG
CGGGTCAACTCGAAGGCGCTGCTGCAGGTGACGTGCAGCAGTTGTTCAGGGCGCGCGCAGACACGTACGACACGTC
CGTCCTCGGCCTGTAGGAATTCACCATCATGCAGCGGCGGCTGGCCGCGCTCCAAAAACAGGCCGACGTCTTCGCC
CTCGGCACTGAAACAGCGCAGACGGCTTTTGCTGCGGGCTTCGAAGTTCAGCAACAACTCCGCAGCCCAGGTGGAT
TGGGGCGCAATTTTGCGGTGGATCACCAGCATCGGAAAGCTTCCAGCTATAAGCGTTAAACAGGCTAGAGCAAGGG
GCTTGCCAACCAGGCTGGATGTAGGAAAACGCCGTAGGTTCTGGGCGTTACGCCACTAAACGGGGCGAGCGTTCTG

GAAGGGGGTGATCCAATTTGGTGCGCGATGGGGTGTGCAAGCACTCGAATTGGGCGATTTGAACGTGGGGTATTTA
TCGGTGGTGATGTTTGGGATTTGTCCTACGAGAATCTTGGATTTTCCGTTCATCATGCTACGCGTCGTTTTACTAG
GATTTCGCCCCGTGTTTAATCCATTGCGACGTTCATCATGTTCAAGTCTTTTTTATGTGTAGTTCTTGTCGGTCTG
TTCGGCATCGTTTCATCGGCCTCGGCCAACTTCACCAAGCGCAACAACACTGGCCTGGCCCCGTTCGGCTCTTCGC
TGCAGGCCGTGCCGCAACCGACCATCGACAATGTGATCGACCGCGCCCATCAATTGCTGGGCACCGCTTACACCTG
GGGCGGTACCTCCCAGGAGCAGGGGTTTGATTGCAGCGGCTTGCTGGTCTATCTGTTCAAGACTGAAGCCAACATC
GTCATCCCGCGCACCACGGCAGCGATGCACCGTTCAACGGCGGCGACGATCAAGCGCAACGCACTGCAACCCGGCG
ATGCGGTGTTTTTCCGGGCCAATGGCAGTGGCCAGGTCGGTCATGTCGGCCTGTATGTGGGGGAGGGTAAATTCAT
TCACTCGCCACGCAAGGGCAAGAGCGTGCGCATCGACTCGCTGGCCAACAATTACTGGAAGAAGAACTACACCACG
GCCAAGCGTTTCCACGCGGTGCGTTGAGCGGCTACTCGGTGCTGCCCAGGCCTTGCCAGTGCTTGAGGCCAATAAA
GATAAAGCGCAGTTGCTGGGTGATCTTGGCCTGGGGCGTGAGGTGCGCCGGCAGGGCAGCCGCGGGCGGGTCGATG
ATGTCGGGCAGGGTGGCGAACACGCTCTTGACGATCAGGTCGGCCATCACATGCAGGCCCTCGGCATCCAGGTGCT
GGAGCTTGGGCATCAGCGTCAGGTCGGCCGCCAGGTCCGAGGTGATGTCTTCGCGCAAGGCGCCGATGGCTTGGCG
CACGGCCAGGCAGCCGCCGTATTGCTCGCGGGCCAGGAATAAAAACTGCGAACGGTTGGCCGCCACCACATCGAGA
AAGATGCGCACTGAGGCATCGATGATGCCACCCATGACAAATTCGTTGTGGCGTACCAGGCGGATGGTGGCGCGAA
AGGTCTGGCCGACCTCACTGACGAGCACCAGGCCCAGTTGGTCCATATCGGCAAAGTGACGGTAAAAACCGGTGGG
CACGATGCCGGCGCTCTTGGCCACTTCCCGCAGGCTCAGGCTGCCAAACCCTCGGCCACCCTCCATCAGATGGCGG
GCTGCGTCCATCAAGGCAAGGCGGGTCTGTTGCTTCTGTTCGGCGCGGGGCAGCATGGGCGGGTGGGCTTTGTAGA
CAAGGACAGCGACGCACTCTAGCAAAACAACTTTACCGGCGTCGAACTTGAAGCAGGACAAAGGTGGTGCGGATCG
TACAAAGTCAAAGCCCGATCTCAGGGGATCGGGCTTTTTTCAGGGCCAGCACCGGCTTAGCTCGTTGCTTTATGCA
TTTCTTCCAGACGGTCGGAACCGCCTTCGGCGACGAATTCGCGTTGTTGCAGACGGTCCGAGCCACCTTCAACCAG
GCCTTTCTGTTCCAGGCGATCACGGCCGCCTTCAGTGACGAATTCGCGTTTTTGCAGACGGTCCGAACCACCTTCA
ACCAGGCCTTTCTGTTCCAGGCGATCACGACCCCCTTCGGCAACGGTCTGGCCGATGTGCGAAGCGTCAGCCTTGG
CTTGTGGGATGGCTTGTTCGGTAGCTGGCAGGGCAAAAGCACTGGTGGCCAGGACAGAGAGTGCAACACTGAGCAG
TAATTGGCGTTTCATGATGAGTTGCTCCTTGGGAGGGCGATAAAGTGGGTACGGAGGCAATGCTACTCTCGATATC
TCGATATAAAAGTTCATAAACACAATGGTAATAATCAACAGAATTGATTGTTCTGCGCCGAGGCTCTAGATCGCGC
CTCTCAAGCACGCGCTTTTGCACCGGGGTGGGTATTTTCGATACGGGCATGGGTAACAACGGTGCGCTGGCCATGA
GACAAACTGTCGGATCAGTCAGAAAAAGCGCTTTTCACCGCTAATTGCCATGTGCGGTTAAACCCCCAGGCCATTT
GCCAGTCGTAGCCATATAAGCTTTAGTCCTTGCGCGCGTTGCCGGTGGCACGCGCAGCCGTAATCAGGAGCCCTGT
GCAATGACGCGCACTCGTAAAATTCTCGCCTGGAGCAGTGCCTGCTTCGTTCTGTTAATCGCCGCCGTGGTGTTGA
TCCTGGTGTTCTTCGACTGGAACCGGATCAAGCCGCAAATCAATGCCAAGGTCTCTGAAGAGCTGCACCGCGCCTT
TGCCATCAATGGCAACCTGGCTGTGGTCTGGCAGCGCGAGCCGGACGAGGGCGGTTGGCGGGCCTGGGTGCCGTGG
CCCCATGTGGTGGCCGAGGACCTGAGCCTGGGCAACCCCGAGTGGTCGAAAAAACCGCAGATGGTCACCCTCAAAC
GCGTCGAACTGCGCATCTCGCCCTTGGCGTTGCTGGCCCAACGCGTGGTGATCCCGCGCATCGACCTGACCGAGCC
GAGCGCCCAATTGCAGCGTCTGGCCGATGGTCGCGCCAACTGGACCTTCACCTTTGATCCCAAGGATCCGAATGCC
GAACCCTCCAATTGGGTGGTAGACATCGGCGCCATAGGCTTCGATAAAGGCCATGTGACCCTCGACGACCAGACCC
TCAAGACCCGTCTCGATGTGCTGATCGACCCCCTGGGCAAGCCGATTGCGTTCAGTGAGATCGTCGGCGAGCGCGA
TGCTAAAAAAGCCCTGGAGCAGGGCGCCACGCCCCAAGACTATGCGTTTGGCCTCAAGGTCAGCGGCCAGTACCAC
GGGCAGAAACTGGCGGGTAGCGGCAAGATCGGCGGGTTGCTGGCCCTGCAAGACGCGGCCAAGCCATTCCCCTTGC
AGGCCCAGGCGAAGGTCGGTGATACCAGCATCGCCCTGGCCGGCACCCTGACTGACCCCCTGAACCTCGGCGCTCT
GGATCTGCGCCTGAAACTGTCCGGCACCAGTCTTGGCAACCTTTACCCCCTGACCGGCGTGACCCTGCCGGACTCG
CCGGCCTATTCCACCGATGGTCGCTTGATCGCCAAGTTGCATGAAGCCGGCGGTGCGTCGTTCCGCTATGAAAACT
TCAACGGCAAGATCGGCAACAGCGATATCCACGGCGACCTGGCCTATGTCGCCAGCCAGCCACGGCCCAAGCTCAG
TGGCGCGCTGGTGTCCAACCAACTGCTGCTGGCCGACCTGGCGCCGCTGATCGGTGCCGACTCCAACGCCAAGCAA
AAGGCCCGTGGCGGTGAAAGCAAGCAACCGGCGACCAAAGTGCTGCCGGTGGAGGAGTTCCGCACTGAGCGCTGGC
GCGATATGGACGCCGACGTCGAGTTCACCGGCAAGCGCATCGTGCACAGCGCCGAACTGCCCTTTACCGACCTCTA
CACCCACCTGGTGCTCAACGATGGCCAATTAAGCCTCGAACCCCTGCGCTTCGGCGTGGCCGGTGGGAAGCTCGAT
GCGCAGATCCGCCTCAATGGCCGCATCACGCCGCTGGAAGGCCGGGCGAAGCTGACGGCGCGCAACTTCAAGCTCA
AGCAGCTGTTCCCAACCTTTGAACCGATGAAAACCAGCTTCGGCGAGCTTAACGGCGACGCCGATATCGCCGGGCG
CGGCAACTCGGTGGCGGCGCTGTTGGGCAGTGCCAATGGCGATCTGAAAATGCTCATCAACGACGGCGCGATCAGC
CGCAGCCTGATGGAAATTGCCGGGTTGAACGTGGGCAACTATGTGATCGGCAAGTTGTTTGGCGACAAGGAAGTGA
AGATCAACTGCGCAGCGGCGGACTTCGGGATCAAGACCGGCCTGGCCACCAGCCGGCTGTTTGTGTTCGATACCGA
GAACGCGATCATCTACATCGATGGCACCGCGAACATGGCCAGCGAACAACTGGACCTGACCATCACGCCAGAGTCC
AAGGGCTTTCGCTTGTTCTCACTGCGTTCGCCGTTGTACGTCAACGGGCCGTTCATCAAGCCCAATGCCGGGGTCA
AAACGGTGCCGCTGTTGCTGCGTGGCGCGGGGATGGTGGCGCTGGGTGTGATCGTCGCACCTGCGGCGGGCTTGCT
GGCGCTGGTGGCGCCCAGTGGGGATGAGCCGAATCAGTGTGTGCCGTTGTTGCAGCAGATGAAGGAAGGCAAGGCG
CCGAAGACTGTGAAGGGCTAACTGACTGAACCACAACCAAATGTAGGAGCTGGCTTGCCTGCGATAGCATCGCCTC

GGGTTTACTGAAAAACCGAGGTGCATGCATCGCAGGCAAGCCAGCTCCTACATTGGTTTGTAGTGTGGCTTACAGG
TCTTTGAGAATGTCCGCCATATCGTCAGCGTGCTCTTCTTCCTGGGCCAGGATGTCTTCAAAGATCCGGCGGGTGG
TTGGGTCTTTCTCGCCGATGTACTGAATGATCTCGCGGTAGCTGTCCACTGCGATGCGCTCGGCCACCAGGTCTTC
GTAGACCATTTCCTTCAAGGTCTTGCCGGCCACGTATTGTGCATGGGAATTCTTGGACAGCAGGTCCGGATTGAAC
TCCGGCTCGCCGCCCAGTTGTACGATGCGTTCGGCGAGTTTGTCGGCGTGTTCCAGTTCCTGGTTGGCGTGCTCCA
GGAACTCATCGGCAGCCACGCTGGCTTTCAGGCCGCTGGCCATGAAGTAGTGGCGCTTGTAGCGCAAGACGCAGAC
CAGTTCGGTGGCCAGCGACTCATTGAGCAGGCGCAGGATCTCTTCGCGGTCGGCACTGTAGCCTTCTGTCACTGCG
CCGTTTTCCACATTCTTGCGCGCACGTTCGCGAAGGGTGCTTACGTCAGACAAATGCATGTCGGTCATTTCAATCT
CCTGAGGCTAATCCGGTTTTGCGCCACACTCTGGTGGTGTGATCGCTCAAAGTTGTGAGTGCTGCGCGGCGCAAAA
AGTTTTATCGGATGTTGCCGGCTGTCATATCGATTACCCAGGAGCTGGTCTACGATCAGCTCAGTCGCCCCTTTTC
AAGGATGTTCGCCGCCATGGCGCAGACATTGGCTACACGTTATCCCCTGGTGTTGGTGCCGGGCATGCTCGGGTTT
GTAAGGCTGGTGCTGTACCCCTACTGGTACGGCATCGTCCCGGCCTTGCGTGCCGGTGGGGCGCAGGTGTTTGCGG
TGCAGGTGGCGCCGCTCAACTCCAGTGAGGTGCGCGGCGAGCAGTTGCTGGTGCAGATCGAGCAGATACGCCGCCA
GACCGGCGCCGACAAGGTCAACCTGATCGGCCACAGCCAGGGTTCGCTCACCGCCCGTTACGCGGCGGCCAAACGT
CCGGAATGGGTGGCTTCGGTCACTTCGGTGGCCGGGCCCAACCACGGGTCGGAACTGGCGGACTACCTGCACACCC
ATTACCCGGCCGACAGCGCCAAGGGCCGCATCATGAGTACGCTATTGCGCCTGGTGGGCGTGGTGATGGGCCTGCT
CGAAACCGGCTATCGCGGCCCGCGCTTTGCCGCGGATATCCATGCCTCCCATCAATCCCTGACCAGCGCGGGGGTG
GCGCTGTTCAATCGCCAATACCCCCAAGGCCTGCCACACACCTGGGGCGGGCAGGGCGCCGAGGAAGTGAACGGTG
TGCGCTTTTACTCCTGGTCTGGCACCCTGCAGCCGGGCAAGACCGATCGCGGGCGCAACCTGCTGGACGGCACAAA
CCGCAGTTGCCGGCTGTTTGCGCGGACCTTCGTCAAGGAGAAGGGCCAGTGCGATGGCATGGTCGGGCGCTACAGC
TCACACCTGGGTACGGTGATCGGCGATGACTATGCGCTGGACCATTTCGATATCGTCAACCAGTCCCTGGGCCTGG
TGGGCAAGGGCGCCGAGCCCGTTCGGCTATTTGTCGAACATGCGCAGCGGTTGAAGGCGGCCGGGGTCTAGGCGGT
AGCAATCGGTCAGGCGACGCGCCTGACCGGTGTCGTCCACCGCTCCGACAGAATCACCCCACCCAACGTCAACACA
CCCCCCACCAGGTGATACAGCGCCAACTGCTCCTTGAGCACCACCGCTGCAATCAGTGCGGTGATCAACGGCAGCA
GATTGAAAAACAGCGTGGTCCGGCTCGGTCCCAGGTTTTTCACCGAATGCATCCACGCCAGTGGCGCGATCATCGA
GGCCAGCAGGCAGGCATACACCACCAGGCCAATATTCGACGGGCCCAGGCCGACCTTGTCCGAGGCCAGGAACAGC
GGAAACAGCACCACCACCGCCACCAGCACCTGCAAATACAGCAGCACCAACGGTGGCAGGCGCAGCTGCCATTTCT
TCAGCAGGGTGCTGTAGATCGCATAGGCCAGGGTGGCTACCAGCATCATCGCGTCGCCCAGGTTCACCCCGTGTTG
CAGCAACGCGCCGAGGCTGCCGGACGAGACGACCACCGCGACCCCGGCAAACGACAGTACCGCACCGGTCAGGGCG
CCAAAGGTCAGGCGCTGGCCGAGGCTGATAATGGCCGCGGTCAGGGCCATCAACGGCATCAGCGACAGGATGATCC
CCATATTGGTGGCGCTGGTCAGGCTCGCGGCGTAGTACGCCAGGCTCTGGTAGACCGCCATGCCGAGCACGCCAAG
CACGGCGATCTTGCCCAGGTTGGGGCGGATCAGCGCCCAGTTGGCGATGACCGGCTTGAGCATGAACGGGGTAAAC
AGGATGGCAGCCAGCAGCCAGCGATAAAAGCCGATCTCGGCCGGGAAAATCGAACCCACGGCGAGTTTGTTGACCA
CGGTATTGCCGGCCCAGATAAAGATGGCCAGCAGCGGATAAGCGTATTGCATTGCAAGGAACCAGGAACGTTAGTG
AAGGCTGATTATCCTTTGTCTGGATCGAAGCCTATACTTCGATCCGGCCAAGCCACCTTTGGATCCAGACAACATG
TCCCAGACCTACATCAACCTGCCCGATTTCGAAGTGCTGCCGGCCCCGGTGTACTTTCGCTATTCGGAATTTGCCG
CCGATAGCCGCGCCTCCAGCCATCAGCATGCCTGGGGCTCGCTGGATTATTCGGCCCGTGGCGTGATGCGCTTTGA
AGTGGGCGGCAACCGGTTTATGTCGCCACCGCAATACGCGGTGTGGATCCCGCCCAACACCCAGCACAGTTCCTAC
AACGCCCAGGCGATTGTCTACCGCTCGGTGTACCTGGCGCCGTCTTTGTGCGAGCAATTGCCCTCGCAGCCCTGCA
CCCTGACCATCAGCAACATCCTCAAGGCCATCCTCAGTGACTTTGCCGAGCGCGATGTCAACATCCCCGCCAACGA
GGCCGACGTGCGCCTGGCCCAAGTGCTGGTGGACCAACTCCGGCAGGCGCCGGTGCATGATTGTTTCCTGCCCTAT
GCCAGCAGTCCCGGCCTGCTCAGCGTGCTTGAAGGCATGCAGGTGGATCCTGGAGACAACCGCGCGCTGGCGCAGT
GGGCACAGCAGGTGCACGTCAGCGAACGCACCCTGGCCCGGCAATTTGCGCGGGAACTGGGCATGAGTTTTGGCGA
ATGGCGCCAGCGCCTGCGCTTTCTTGCGGCCATCGAAGCCCTGGAAAGCAGCCGCAGCGTGCAGGAAGTGGCCTTT
GACCTGGGCTACAGCACCGCCTCGGCGTTTATTGCGATGTTCCAGCGCCAGGCCGGCAGCACACCGGAGCAGTATC
GGCGGACGAATCTACGTAGCAGGTGAAGGTGTAACAGGCTTTGTCTACACTGCAGGGGAGGCCGCGCCCCTCGGCG
TGGTAACAGGGAGAAAACTCCATGAAGATGTTGCGTATTCCGTTGTTGATGATCGGTCTGCTGCTCTGTTCCCAAG
GGTTTGCGGCCACTGCCCAGCAAAACAAAATGACCACCTGCAACGCCGAAGCCACCACCAAGACCTTGAAGGGCGA
CGAGCGTAAGGCCTTCATGAAAACCTGCCTCTCGGCCCCGGCAGCCAACGACGCCAAGACCCTGACCCCGCAGCAG
CAAAAAATGAAGGACTGCAACGCGTCGGCGAAAACCAAGGCGTTGACTGGCGATGCGCGTAAAACCTTTATGAGCA
CCTGCCTGAAGAATTAATAGCCCCGGACTACCTAAGGCTGCGACACTCGCACAGCCCTTACACCTGTAGGAGCGAG
CTTGCTCGCGAAGAACGTGAACGGTATCGCGCTCATCCTGAATGCACGCGTTGCCCGTGAGTTTTTCGCGGGCAAG
CCCGCTCCTACATGTCCTTTAACGCCGTTCGTTTAGAGGCTGTATGCCAACGTTTTCTTCGCGTCATGTGTTGTTG
CTGGCCAGCTACATCATCATCTTCGGCGGGTTGCTGCTGGTCCTGCCGTTAAAACTGCTGCCTAGCCTGTTGGCCG
GCCTGTTGGTTTTCGAACTGGTCAACATGCTCACTCCACAGCTGCAACGCCTGATCGAAGGCCGCCGCGCGCGTTG
GCTGGCGGTGGCATTGCTCGGTACCGTGATTGTCAGCGTGCTGACCCTGATCTTCGCCGGCGCTATCAGCTTTTTG
CTGCACGAAGCGGAAAACCCCGGGGCCTCCCTGGATAAATTCATGGGCGTGGTCGACCGCGCGCGCGCGGCCAGTTGC

CGCCGTTCCTCGACGCCTACCTGCCGGCCAGCGCCGCCGAGTTTCGCGTAGCCATCGGCGAATGGTTGAGCAAGCA
CCTGAGCGACCTGCAACTGGTGGGCAAGGACGCGGCGCATATGTTCGTGACCCTGCTGATCGGCATGGTCCTGGGG
GCAATCATCGCCCTGCAGCGGGTCCCCGACCTGACCAAACGCAAACCCCTGGCCGCCGCGCTGTTCGACCGCCTGC
ACCTGCTGGTCCAGGCTTTTCGCAACATCGTTTTCGCGCAAATCAAAATCGCTGCCCTCAACACCGTGTTCACCGG
GATATTCCTCGCGGTGGTGCTGCCGTTGGCGGGGATCCATCTGCCGCTGACCAAGACCCTGATCGTCCTCACATTC
TTGCTGGGCCTGCTGCCGGTGATCGGCAACCTGATGTCCAACACCCTGATCACTATCGTCGCGTTGTCGATGTCGA
TCTGGGTGGCGGTGGCGGCGCTGGGTTATCTGATCGTGATCCACAAGGTCGAGTACTTCCTCAACGCACGCATCGT
CGGCGGGCAGATCAGTGCCAAGTCGTGGGAGTTGCTGTTGGCGATGTTGGTATTTGAAGCCGCATTCGGCCTGCCG
GGCGTGGTGGCGGGGCCGATCTACTATGCGTATTTGAAGAGTGAGTTGAAGCTGGGCGGGATGGTCTGAGGGTTCA
GGGTGCCTGGACTGGCCCTTTCGCGAGCAAGCCCGCTCCCACATTTGGACTGCATTCCCCAGGCGGAACTCGATCT
AAATGTGGGAGCGGGCTTGCTCGCGAAGGCCGCACCACGGTCTTAACTAAAGATCAGTAGCCGTAACGCTTACTGG
CCTCAATCGCCAACCCGCTACCAATGCTGCCGAAGATGTTGCCTTCTACATGTTGGGCATTGGGCAGCATCGCCGA
AATGCTCTNNNNNNNNNNNGCTGGGGCTCCAGAACGCTTGCACCACCAGGGTCGACTGTGAGATCCGTGTGACCAGG
GCATCCAGTTCGTCGCCATCCACCGAGTTGGCCGCCAGGGTGGCCTCAATCTCGACCTCGTCACTGCCAAAGGGCC
TTACGTCGACATCACTGGCTGGGTAGTTACAGCGCGCCAGTTCCGACTCCAGCAACGCCATCACCGCTTGCTGCTG
GGTACGCCGGGCGATCACGTAGAGGATGTTGGTGACCTCGGCAGACACCACATCCAGCGGCTGGCGGTTGATGTTA
TTGACGATCGGGCGCAGCAGCGTGTTGGCCGCCAGCACAAACATCGTGCCCAGGAACGCCTCGAGGATCAGGTCTG
CACCGGCGCAGGCGCCCACGGCGGCGGACGTCCACAGCGTGGCTGCGGTATTGAGCCCGCGCACGTTGCCCTCCTC
GCGCATGATCACCCCGGCGCCGAGAAAGCCAATACCCGACACCACATACGCCACCACTCGCACGGCGCCTTCGGCC
CCGGCCAGGCGGTTGGCCATGTCGACAAAGATCGCCGCCCCCACCGCCACCAGTACGTTGGTGCGCAGCCCCGCCG
TGCGCTGGCGGAACTGGCGCTCAAAGCCGATCAGCCCGCCCAGAATAAAGGCGGCACTAAGACTGACCACGGTGTC
GATCAGGGAATCCAGATTGATGTTGTTGAGGGCTTGCATGATGAATCTCCCTTTGATTGCCCGCAGAATCAGCGTC
GAAAAAAGGGGGATAAGGCAGGAACCAGGGAGGTTCGCCACCGAAATACCGCTTCTCGCTTTAACGGCGAGGCAAC
GGCACGAAGATGCGCGTCAGCTGGCCAAGCCTGGTCGACAACTGTCACTCGAAAGTCTGTTCACGAAAGTCTGCGC
TATGGTTGAAAACCGGCGCAGCTTACGCCCGCAGTCTGCGCGAGCAAAGTGACAGTGCGGCGAATCGGCGGGTTCT
TCAGCAAGGGTTCAGGCAAGGGTCGCTCCCGCGGGCACGGGAACGACCCTCCAGGCTGTCAGCTCGCGGTGGCCAG
CAGCACGTCACGCACCGAACGCCCCTGGCCCAGGTGCCGATCATGTTGATGCAGCGACGCGGCAATCTCATCGGCA
CGAATCGGCAGGATCGACAGCAAGGTATCGCTCAAGCCATGGCTGGCCTGGCTGAAACCCTGCATGTAGATACCGG
CCTTGCAGCGCTCGTCGGTGACCACGCGGTAGTCGCGGCTCACTTCAAACTCACCCATGTAGGCTTCGAGCGGCGC
CAGCAACTGGCGATGCATCTGCCGCTCATAACCTGTGGCCAGGACCACCGCATCGTAGTGATTGATCACGGTCTCG
CCGGTAGCGTTGTGACGCACCACCAACTCGATGCCCAAGGGCCCGGCACTGGCTTTTTCCACGGTGGTCAGGGTAC
GGAAGGCGTGACGGGCGATGCCCGAGACTTTCTGCCGGTAGAAAATCCCGTAGATGCGCTCGATCAGGTCCAGGTC
CACCACCGAATAGTTGGTGTTGTGGTACTCGTTGACCAGGCGTTCGCGCTCGCTGCTGGGCTGCTGGAACACCAGG
TCGGTGAACTCCGGGGAGAACACTTCGTTGACGAACGGGCTGTCATCGGCAGGCTTGAGGGCCGAACCGCGCAGGA
TGATATCCACCTGCACCGAAGGGAAGCTGTCGTTCAGATCGATAAACGCCTCCGCCGCGCTCTGCCCGCCGCCGAT
GATCGCAATGCGCATTGGCTTGCCCTCGACACAGGGCTGCTGGGCCATGCGCGCCAGGTACTGGGAATGGTGGAAC
ACCCGCCCGTCATCCTTGAGCGCCTTGAACGCCTCGGGGATACGTGGCGTGCCACCGGCACTGACCACCACCGAGC
GCGCGGTGCGCACCAGTTGCTCGCCGTGGGCATCCCGGGAGATCACCCGCAGCGCCTCGACCTGCTGCTGATGCAG
GATCGGCTCGATGGCCAGCACTTCTTCGCCATAGCGGCTCTGCTCGGTGAACTGCCCGGCGACCCAGCGCAGATAG
TCGTTGTACTCCATGCGGCACGGGTAGAACGTGCCCAGGTTGATAAAGTCCACCAGGCGCCCGTGGGCCTTGAGGT
AATTGACGAAGGAATAGGGGCTGGTGGGGTTGCGCAGGGTCACCAGGTCCTTGAGGAAGGAAATCTGCAACTCGCT
CTGGCTCACCAGGGTGTTGCCGTGCCAGCGGTAGTCGGCCTGCTTGTCGAGAAACAGTACATCGAGCTTGCCCTGG
GCTTTCTCGCGCTCTTGCAGGGCGATGGCCAGCGCCAGGTTCGAAGGACCGAAACCAATACCGATCAGGTCGTGAA
CCGCGGGCGATGCAATTGCCTGTGTCATTCCAGTGTCCTCTGGATAAGCCCCGTGGCGGTGGGGCGGAAAGCCTGG
GCGACCTGCTGAACAACAGGCCATGTGTTGATAGGAAACGAGGACAATGAAAAAAAATTTAAGGCCCGCACCTGAT
TCACTCCAGCAGCCACGAAGGTAGCTGCCGTGGCCGCACAGTGATGGCAGTTTGGGCTCTTCTAATTTATAGTAAT
TCTCATCTAGATTTGAATTGCCTCACCCTCGCCGTCCAATACTGGCGTTTCGCCCAGCTGTAACGGCATATGTAAG
CAGTTAAATTTCAGAGCAAATACCTCGTTTACCTGACAGCTTCCCGCGCCTGTCTCTGCCCTTGCGGGCGATTGAG
TTGCGGTCCGAACTTTCCTGGCTGGAACCCGTGCATGAACCGTCCCCGACACACCCGGCGCGCTCTGCTGGCAGCA
CTTTGCCTGATCCCCGTGGCCGCCTTCGCCGCCTGGCAACTGATCCCGCCCGGTCGGGATAAATTTGCCACCCTGC
AAGTACACCGGGGCGATATCGAAAGCAGCGTGACCGCCCTCGGCACCCTGCAGCCGCGGCGCTACGTCGATGTCGG
CGCCCAGGCGTCGGGGCAGATCCGCCGGATTCACGTCGAAGCCGGCGCCCAGGTCAAGGAAGGCGACCTGCTGGTC
GAGATCGATCCCGCCACCCAACAAGCCAAGCTCGACGCCACTCGCTACTCCATCGCCAATCTGCAAGCCCAACTGC
AGGAGCAGCACGCGCAAAACCGCCTGGCGCAGCAGAAATTCCAGCGCCAGCAACGTCTGGCCGCAGGTGGCGCCAC
CCGCGATGAAGACGTGCAGACTGCCCAGGCCGAACTGGACGCCACCCAGGCACGCATCAACATGTTCAAGGCCCAG
ATCAGCCAGGCCCAGGCCACGCTGCGGGTCGACCAGGCCGAGCTGGGCTACACGCGCATCTACGCGCCGATCTCCG
GCACGGTAGTCGCCGTGGATGCCCGCGAAGGCCAGACCCTCAACAGCCAGCAGCAGACGCCGCTGATCCTGCGGAT

CGCCAACCTGTCGCCCATGACCGTCTGGGCGGAAGTTTCGGAAGCTGATATCGGCCATGTCGAACCTGGCATGCAG
GCCTATTTCACAACCCTGAGCGGTGGCAACCGGCGCTGGACCAGCACCGTGCGCCAGATCCTGCCGGTCCCCCCA
AGCCCCTGAACGAAACCAGCCAGGGCGGTGGCAGCCCCAGCAGTTCGAGCAAAAGTGGCAGCGGCCGCGTGGTGCT
CTATACCGTGCTGCTGGATGTCGACAATGCCGATAACGCTTTGATGGCGGAAATGACCGCCCAGGTGTTTTTCGTC
GCCAGTCGCGTCACCAATGCGCTGACTGCGCCGGTCGCAGCCCTGCAAGGCGGCACCCGCCCCGACATGCAATTGG
CCCGCGTGGTGGCCAAGGACGGCAGCATCGAAGACCGCGAGGTGCGGGTCGGTATCAGTGATCGCCTGCGCATCCA
GATCCTCGATGGCCTCAACGAAGGCGATCACCTGCTGATCGGCCCCGCCCAAAGCAGCGGAGGCTGAATGTTGACG
CCGCTGATCGAACTTCGGGACATCCGCAAATCCTACGGTGGCGGTGATGCGCCTCAGGTCGACGTGCTGCGCGGGA
TCGACCTGTCGATCCATGCCGGGGAATTCGTGGCGATTGTCGGTGCCTCGGGCTCCGGCAAGTCCACCTTGATGAA
TATCCTCGGCTGCCTCGACCGCCCCACCGTCGGCCAGTACCTGTTCGCCGGGGAAAACGTTGCTCAACTAGACACC
GACGAACTGGCCTGGCTGCGCCGCGAGGCCTTTGGCTTTGTATTCCAGGGCTACCACCTGATCCCCTCCGGCTCAG
CCCAGGAAAACGTCGAGATGCCGGCGATCTATGCCGGCACGCCCGCCGCCGAGCGCCACGCCCGCGCCGCCGCACT
GCTGACCCGCCTGGGGCTGGCCGGGCGCACCGGCAACCGCCCGCACCAACTGTCCGGCGGCCAGCAGCAACGGGTG
TCGATTGCCCGGGCCTTGATGAACGGCGGCCATATCATCCTCGCCGACGAACCCACCGGCGCCCTCGACAGCCATA
GTGGCGCCGAGGTCATGACCCTGCTCGACGAGCTGGCCAGCCAGGGTCACGTGGTGATCCTGATTACCCATGACCG
CGAAGTGGCGGCCCGGGCCAAGCGCATCATCGAAATCCGCGACGGCCTGATCATCAGCGACACCCCCAACACCCAC
GACCAACCTGCCGCCGAGCCCAACCCCAAGGCCCTGCAAGCGGTGGACCTGCGCCAACGCCTGGCCGATGGCGCCG
AGCACAACGGTGCGTGGAAAGCCGAACTGCTGGACGCGGTGCAGGCAGCCTGGCGGGTGATGTGGATCAACCGCTT
CCGCACGGCCCTGACCCTATTGGGGATTATTATCGGCGTAGCCTCGGTGGTGGTGATGCTTGCGGTGGGTGAAGGC
AGCAAACGCCAGGTCATGGCGCAGATGGGCGCCTTTGGCTCCAATATCCTTTATGTCAGCGGCTCGGCGCCCAACC
CGCGCACACCGCCCGGCATCATCACCATGAACGACGTCGCCGCGATGGCCGCGCTGCCACAGGTCAAGCGCATCAT
GCCGGTCAATGGCGCGGAGGCCGGAGTGCGTTTCGGCAACGTCGACCACATGGCCTACGTGGGTGGCAACGACACC
AACTTCCCGGCGATCTTCAACTGGCCGGTGGTCGAGGGCAGCTACTTCACCGAGGCTGACGAACGCGCCGCCGCGA
CCGTCGCGGTCATTGGCAAGCGGGTGCGCGACAAGCTGTTCAAGGACCTCCCCAGCCCTATCGGCCAATACATCCT
GATCGAAAACGTGCCGTTCCAGGTGATCGGTGTGCTCGCCGAAAAAGGTGCCAGTTCCGGCGACAAGGACAGCGAC
GACCGTATCGCCATCCCCTACTCCGCCGCGAGTATCCGCCTGTTCGGCAGCTACAACCCGGAATACGTGGTGATTG
CCGCCGCCGACGCGCGCAAGGTGCATGAGGCAGAAAAAGCCATCGACCAGTTGCTGCTGCGCTTGCACAACGGCAA
GCGCGATTTCGAACTGACCAACAACGCCGCGATGATCCAGGCCGAGGCGCGGACCCAGAACACCCTGTCGCTGATG
CTGGGTTCGATTGCCGCCATCTCGCTGCTGGTGGGCGGCATCGGGGTGATGAACATCATGCTGATGACCGTACGCG
AACGCACCCGGGAAATCGGCATCCGCATGGCCACCGGCGCTCGACAGCGGGACATCCTGCGCCAGTTCCTCACCGA
AGCGGTGATGCTCACGGTGGTTGGCGGGGTGGCCGGGATCGGCCTGGCCCTGCTGGTGGGTGGCGCGCTGATCCTC
AGCAGCGTGGCTGTCGCCTTCTCGCTGCCTGCCGCCTTGGGCGCCTTCGCCTGCGCCCTGGTCACCGGCGTCATCT
TCGGCTTTATGCCCGCCCGCAAAGCAGCCCGGCTCGACCCGGTCACGGCCCTTACCAGTGAATGATCGCCCTATGA
AAGCGCACTTGACCCTTGTCGCCGCCAGCGTGCTGCTGGCCGCATGCAGCAGCCCGGTGCAGCGCCCGGACAGCGG
CCTGCAGCCACCCCCTGCCTGGGCCACCGCAAACCAGGCCAGCGCCCTGCACGCCAACCAGCAATGGTGGACAGCC
TTCGCCAGCCCTCAGCTCGACCAACTGATCGAACAGGCTCGCAACGCCAGCTTCGACCTGGCCAGCGCCGTGGCGC
GAGTGCGTCAGGCCCAGGCCGGCGCCGTGGTGGCCGGCGCACCGCTGCTGCCCGAACTCAAGGGCGGACTCAACGC
CAACCGCCAGAAACTGCTGCGCGGCAAGGGCTACAGCGAACTGGACGCCGACAACAACAACGACGCGGTGGACTAC
TTCAATGCCAGCCTGACGGCCACCTACGAAATCGATTTCTGGGGTGGCCAGCGCGCCGCGCGCGACAGTGCCGAAC
TGGCGGTGCGCGCCAGTGAATTCGAGCGTGCCACGGTGGAGTTGACCCTGCTCAGCGGCGTGGCCACCACCTACAC
CCAGCTCCTGGCCCTGCACGAGCAACAACGCATCGCCGAACTGAACCTGGCCAATGCGCAGAACGTCCTTAAGCTG
GTGCAGACCCGTTATGAAGCCGGCTCTGCCACCCAGCTTGAGCTGGCCCAGCAAAAGAGCCTGGTCGCCGCGCAGC
AACGCCAGTTGCCAGAAGTACGCCAGCGTGCCGAGGCTGCCCGAATCAGCCTCGCCGCCCTGCTCGGCCAACCGGT
GCAGGCGCTGCAACTGACGCCACAGGCCTTTGATCAACTGCGCTGGCCGAGCATCGCTGCCGGCGTGCCCAGCGAC
CTGCTCAGCCGCCGCCCGGACATCGCCAGTGCCGAAGCACAACTGGCGGCCGCCCAGGCCGACATCATTGTCGCCC
GCGCCGCCATGCTGCCCAGCATCACCCTTACCGCCAACCTGGGCTCCGGCGCCAACCGCGCCGCGGATATGCTGCG
CAACCCGTTCTACAACCTCACGGGCGGGCTGATCGCACCGATTTTCAACAACGGCCGCCTCAGCGCCGAACGTGAC
AAGGCCACGGCACGCCAGGATGAACTGCTGCAAACCTATCGCGCAGCGATCATCAATGGCTTTGCCGATGTGGAAA
AAGCCCTGAGCAGCATTCGCCGGCTGGACGAACAACGCCAGTGGCAGCGCGAGGAGCTGGACCAGGCGCAGACCGC
GTTCCGCATCGCCCAAAGCCGCTACGAGGCTGGCGCGGAGGACTTGCTTACCGTACTGGAAACCCAGCGCACGCTG
TATGCCGCCCAGGATTTGAACGTGCAACTGCGCCAGGCGCGGTTGGCGGCGAGTATTGCGCTGTACAAGGCGCTGG
GCGGCGGTTGGCAGGCGTCCGATATCTAACAGGTAGCGAGCTTGCTGTGGGGAGCGGGCTTGTTGTGGCGAGCGGG
CTTGCCCCGCGCNNNNNNNNNNCTTGCGCTACTTGTGCCAAAGCTGCCTAATGGCGCACCCGGCAGGACTCGAACC
TGCGACCATCCGCTTAGAAGGCGGATGCTCTATCCAGCTGAGCTACGGGCGCCTGATTAATCTGTACTCTTGGAGG
ACTACAAACTAAATGCTTCCAGCCTTACACAATAAAACGACTATTGCGCTCGACCTTCTTAACCAGTGCTAGGCTG
TGCCCGACAAGTGCGACGAATAGTATAGACGCCCCCAGGGGCCGTCAAATCTTTTTTGAAAAAAAATTCATTTTATT
TAAGGGGTTAGGGGAATTTGCAGACCAAGCGCCTTTGCCCTCACGTCCTGGCGTGCGAGAATGCGCGCACTTTTCC

TCCCCCTCTCGATGGTTAATCACGCGTAATGACTGCACAACTTATCGACGGCAAATCAATCGCCGCCAGCCTGCGC
CAGCAGATCGCCAAACGTGTCGCCGAGCGTCGCAAGCTTGGCCTGCGCACGCCGGGGCTCGCGGTGATCCTGGTCG
GCAGCGATCCTGCCTCCCAGGTTTATGTCTCGCACAAGCGTAAAGACTGTGAAGAGGTCGGCTTTATTTCCAAGGC
CTACGACTTGCCTTGCGATACCACCCAACAGGCCCTCACCGACCTGATCGATGGCCTCAACGACGATCCGAACATC
GACGGCATCCTCTTGCAATTGCCGCTGCCGGAGCATCTGGACGCCTCGCTGTTGCTGGAGCGCATCCGCCCCGACA
AGGACGTCGATGGTTTCCACCCTTATAACGTTGGCCGCCTGGCCCAGCGCATCCCCCTGCTGCGCCCCTGCACGCC
AAAGGGCATCATGACCCTGCTGGAAAGCACCGGCGTCGACCTTTATGGCCTCGATGCAGTGGTCGTGGGTGCGTCG
AACATTGTCGGTCGCCCAATGGCCATGGAATTGCTGCTGGCCGGCTGCACCGTGACCGTCACCCACCGCTTCACCA
AGGACCTGGCAGGCCACGTCGGGCGTGCGGACCTGGTCGTGGTCGCCGCGGGCAAGCCGGGCCTGGTCAAGGGTGA
GTGGATCAAGGAAGGCGCCATCGTGATTGACGTCGGCATCAACCGCCAGGACGACGGCAAACTGGTGGGCGACGTG
GTGTATGAAACGGCCCTGCCCCGTGCCGGCTGGATTACCCCGGTGCCAGGTGGTGTGGGCCCGATGACTCGTGCCT
GCCTGCTGGAAAACACCCTGTACGCAGCCGAAACCCTGCACAGCTAATAACCCGCCGCACTAAAAAAGCCCTGCCT
TATGGCGGGGCTTTTTATTGCCCGCAAAAAAATCGGTTTTTTCTTTGTTTTTAAGAGCTTTCGTCTGGTTTCAGAA
GAACATTCGACAGTTTCTAGCCCATACTAATAATATGTAACGTCTTTTTTCATAAAGCCGGCCACACCCGGTATTT
TTCTACCTTTTAGCGAGTTCATCCGCGTGAATATTCGTCTTTCTCTTGTCAGCCTGTTCTTCGCACTCGCAGGCAC
TGTCGCCAACGCCAGTGAAACCACCCTGGCCCCTCGAGACACCTCGCAGCTGCACATCGCCTCGGGCAGCGCGATG
CTGGTGGACTTGCAGACCAACAAGGTGATCTACGCCAGCAACCCCGACGTAGTAGTGCCGATCGCGTCCGTCACCA
AATTGATGACGGGTATGGTGGTGCTGGACGCCAAGCAGAATATGGACGAGTACATCTCCATTAACATCAGCGACAC
CCCGGAAATGAAAGGCGTGTTCTCACGGGTCAAGCTCAACAGCGAGATGCCGCGCAAGGAGATGTTGCTGATCACC
CTGATGTCCTCGGAAAACCGTGCTGCCGCCAGCCTGGCCCACCACTATCCGGGTGGCTATGCAGCTTTTATTGCCG
CGATGAACGCCAAAGCCAAGGCCCTGGGCATGAACAGCACCCGTTACGTCGAGCCCACCGGCCTGTCGATCCATAA
CGTGTCCACCGCCCGCGACTTGAGCAAGCTGGTGCAGGCCGCGCATAAATACCCGCTGCTCACGCAATTGAGCACC
ACCAAGGAAAAAACCGTGTCGTTCCGCAAGCCCAGCTACACCCTGGGCTTTTCCAACACCGATCACCTGGTCAACC
GCGCCAACTGGGATATCAAGCTGACCAAGACCGGCTTCACCAACCAGGCTGGTCATTGCCTGGTGCTGGTGACGAG
CATGGGCAACCGCCCCGTGTCGCTGGTGATCCTCGATGCCTTCGGCAAACTCACGCACTTTGCCGATGCCAGCCGT
ATTCGCAAGTGGGTCGAAACCGGCAAGAGCGGCTCGGTGCCGGATGTAGCCCTGCAATACAAGGCCGACAAGAACC
TCAAGGCCCGCCAGACCGGGGTTGCCCAGTCCCATTGATTTGACTGGCAGGCATAAAAAACGGCGCCCTCGGGCGC
CGTTTGCTTTATGGCTGCTTCTGCCTTTGGGCCATGACCTTCAGGGCCTGGGCTGCCGCCTGCTCCTGGCCGGCCT
GGGCAGTTGCGTTGGCGGCCTGCTGCCAGCGCGCCGCATCCAGATTGGCCGGCAACTGGCTCGGGCGCTGGACCAA
CACGGCCCACTGCCCGGCGCTTTTCCAGGCGGACTCGAAGTCGCTGAACGCTACTGGGCGGCGCCGGTCCCTTGCG
ACTCGCAGCAACACCGTACTGTTCTGCTGGTTAAAGCCCACCAACACCGCATAACGCGGCTCGGCCCATAGCGTCG
AGCCCTCGGTAATCCGCACCATCACCGGGTACCCCGCCGCCACCTGCGCCAACAGCGCCGTCAGCTTGGCATCCAG
CGGGTACACCAGCATCCCGTACTCACGCGCCAGGACCTGCATGTTGTGCTCAAGGTCAGCTTCCCCACCCGGCAGG
TGCAAGGGTTTATCCAGCAAGCCCGGTGTAATCACAATGCCTTGTTGCGACAGCATGCTGGCCAAGGCCGCCGGGC
CACCCTGGTAGGTGTCACCACGAAACGCCGGCACACCGTTGAGCTCTACACGCTCGGGCAGGCCCTGGATTTTCGA
GGGTGTGGGATGCCCCGCACATGCAGCCAACCCGAGGACGCAGGCAGCCACCAACAAGGTTTTTTTGAGATTCGAC
CGTAAACGCAACTTCTTCACTCTCTTGATCATCTGCGGGCTGGGGCGTTGATCATAGGGTGCTCTTGGACGCCGGT
ATAGCCTTGGACAGCAGTAAAAAGCCCGGGATAGAGCAAACAAGTTGGAAGCACACGACCATTGGTCAATAGCACG
CAACCGGGAGGCAGCTAGACTGTCCATTGCAAAGACAGTGTGTGCCCTGCACAGGGCAAAAGGAGGCACTCATGAG
CCTGACAACGACGATTTTCCTGCTGGTACTTGGCTGGCTGGCCGTGGCCGGCGCCATGTTGTGGGGGGTGTTGCGC
GTGACGCGCCGGCATCATCACCCGCAGCCAAAGACTGCTACACGGGCCAAAGCCCACAAGCCTGTCGCGCATCACG
CCTGAGCACATGCAGGAGCCGGCGAACCGGCTCCCACCGTTTTACGCCACCTGCTTGCGGGCCACCGCAGCCGAGG
CCGCCAGCATCGCCCGCAGCAACACCGCGCATCCTGCCGCCAGGTCATCGGGTGCCGCATTTTCGATTTCGTTATG
GCTGATCCCGCCCTCACACGGCACAAATATCATCCCCGCCGGCCCCAACTCAGCGACGAAGATTGCGTCGTGCCCG
GCGCCGCTGACGATATCCATGTTCGACAGGCCCAGACCCGTGGCCGCCTCACGCACGGCATCGACACAGCCCTTGT
CGAAATACAGCGGCGGAAAGTCTGCAGTAGGCAGCATCTCGAAGGTCAGGCCGTGCTTGGCGCAGGTGTCGTCAAT
CACCTTGCGCACCTGGGCAATCATCGAGTCCAGGCGCGCCGGCTCCAGGTGCCGGAAGTCCAGGGTCATGCGCACT
TCGCCAGGGATCACATTGCGCGAGCCAGGATAGGCCTGCAGGCAACCCACCGTGCCACAGGCATGGGGCTGATGGC
CGAGGGCGGCAGCGTTGACTGCGGCAACCACCGCCGAGGCACCGACCAAGGCGTCCTTGCGCAGGTGCATCGGTGT
CGGCCCGGCATGGGCCTCGACGCCGCGCAGGGTCAGGTCGAACCATTTCTGGCCGAGGGCCCCCAGCACCACGCCG
ATGGTCTTGCGTTCGTCTTCAAGGATCGGGCCTTGTTCGATATGGGCTTCGAAGTAAGCCCCCACCTTATGCCCGC
TGACCGGCCGAGTGCCCGCATAGCCAATCGCATTCAACGCCTCGCCCACGCTGACGCCCTCGGCGTCGGTCTTGGC
CAGGGTATCTGCCAGGCTGAATTTCTCGGCAAACACCCCGGAGCCCATCATGCATGGCGGGAAGCGCGAGCCTTCT
TCGTTGGTCCACACCACCACCTCCAGCGGCGCCTCGGTTTCGATCTTGAGGTCGTTGAGCGTGCGCAACACCTCGA
CACCCGCCAATACGCCGAAGCAGCCATCGAACTTGCCACCCGTCGGCTGGGTGTCGATATGGCTGCCCGTCATCAC
CGGCGGCAATGCCGGATTGCGCCCCGGGCGACGGGCAAAGATATTGCCCACCCCATCCACTGTCACGGTGCAACCC
GCCGCCTCGCACCACTGCACGAAAAGATCGCGAGCCTGGCGATCGAGATCCGTCAGGGCCAGGCGACACACACCGC

CCTTGGGCGTGGCGCCGAGCTTTGCGAGGTCCATCAGCGACTGCCACAGGCGGTCGCGGTTGATGTGCTGATGGGT
GGATTGCAGAACGTCGATGGCAGCGTTCATGGTGATCTCCTCAGGCAATATTTCTTATGATTGACGCGGTCCAACT
GTGGGAGCGGGCTTGCTCGCGAAGACGGTGTATCAGTCAATATATCCATCACTGACCCACCGCCTTCGCGAGCAAG
CCCGCTCCCACAGTTGATTGCATTCCACATCGCTTACAGGGATGTCTTGGCAGCGACCGGGCTGGTTTTGCGCTTG
CTGCACAGGCCGTAATACAACACCCCGCCCAGCGCCGACCCGGTGAACCAGCCATAGCTGTAGAACCAACTGAACG
CATCGCTACCCAGCGACAACAGGGTCAGCACCACCGGCACGCCAAACGCGATAAATCCCGCCACATTCCACGCCGG
ATACACGTCATCGCGGTACAACCCCGCCAGGTCCAGTTGCTGGCGGCGGATCAGGAAATAATCCACCACCATGATC
CCGGCAATCGGCCCCAACAGGCTCGAATAGCCGAGCAACCAGTTGGAATAGACCGTTTCCAGGCTGACATCGGAAA
CAATCAGGCCCAGTTTTTTCAGCAGCTCGTGGGCCATCAGCGCCAGCCCCACCAAGCCAGTCAGAATCACCGCCGT
GGTGCGGTTGATCAGCTTGGGCGCGATGTTCTGGAAGTCGTTGGTGGGCGAGACAATATTCGCCGCGGTGTTGGTC
GACAGCGTGGCAATGATGATCAGCGCCATCGCCACCGCCACCCACACCGGGCTCTGGATATGCCCGATCAGGGTCA
CCGGGTCGGAGACGCTGACGCCTACCAGTTTCACCGACGCGGCGGTCATGATCACGCCCAGGGCTGCGAACAGGAA
CATGGTCAGCGGCAGGCCGAAAATCTGCCCCAGGATCTGGTCCTTCTGGCTTTTGGCATAGCGGCTGAAGTCGGGA
ATATTCAACGACAAGGTGGCCCAGAAACCGACCATTGCCGTCAGCCCGGCCATAAAGTAACCCGTCAGGTTCGCGC
CTTCGGGACGCTTGGGCGGGATCGCCATCAATTCGCTGAGCGACACATTGGGCAACGCCCACACCAGCAAGCCGAT
GCCCACGGCTACCAGCAGCGGCGCCGACAGGGTTTCCAGCCATTTGATCGACTCGGCACCACGCAGCACCACCCAG
AGGTTCAGCACCCAGAAGATCATGAAACCGATCACCTCGCCGGTTCCGCCCAGGCTCTTCCAGCCCTCGAAAATCG
AGCCGAGGAACAAATGGATCGCCAGCCCGCCGAACATGGTCTGGATACCGAACCAGCCGCAGGCCACCAGGGCGCG
GATCAGGCACGGCACATTGGAGCCCAGAATGCCGAAGGACGAGCGCAACAGCACCGGGAACGGAATGCCGTACTTG
GTGCCGGGAAAGGCGTTAAGCGTCAGGGGGATGAGCACCACGATATTGGCCAGCAGGATCGCCATCAGCGCCTCGC
CCACCGACAACCCGAAATACGCGGTGAGCACCCCGCCCAGGGTGTAGGTCGGCACGCAGATCGACATGCCGACCCA
CAGTGCGGTGATGTGCCACTTGTTCCAGGTTCGCTCGTGCACCTTGGTCGGTGCCATATCGTGGTTGTAACGGGGG
CTGTCGAGGACGTCGCTGCCGGCGTCGAGTTCATACAGGCCGTCGCGCTCTATGACTTGCGATCTGTTCTGTTGCA
TGGCCGCTCCACGGTTTTTTAAAATTATTGTTGCTCATCCGGCGCTTGACGCTGGATGGCCGTAGTCCCGCATGA
ACATGACATCACGGACCCGGCCAACCGGCAGCGCACTCAATTACCGTGCCGCACGCCGTCACCCCGTCCGTACCGC
TTATATAACTAGCTGATGTTTATCAGCTTTATTTTTTCTACCGAAAAGTCCCACGGTACTTTCAACGCTACTCAGG
CCAAATAACGCAGAAGTTGCCCGAACAATCAGGACTCAATCTGGTGCAACACAATTATTTTTATTACTGCCCGCCG
TCAAGAGGCAACTCTCAAGCGTCTGATTTGCAATAGGAAATGTTGCTCATATTGGGAACCTGTCAAGTGCGTCAAA
ACGGTGAGAGGGTGCTACATTTTGGTGATTTTCATTTTTTATCCTTATAAATCAACAAGATATGAAATATATAAGC
TTATAAAAAATAATCTTGATCAATCCAAAAACTCGGGCTAGTTTCTATTCCTGTCACCCGTGACAAGAATAGATCT
TGCACCGTGAGCTGCACTACAACACTTAGAACTGGCATAAGCCGGTCAAGCCTCTGAGGAACTCGGCATGTCTCTG
TTGATCCGTGGCGCTACCGTTGTTACCCATGACGAAAGTTATAAAGCCGATGTCTTGTGCGCAGACGCTCTAATCC
GCGCCATTGGCACTAACCTGGATGTTCCCGCCGGCACCGAGATACTTGATGGCAGCGGCCAATACTTGATGCCCGG
CGGGATCGATCCCCATACCCACATGCAATTGCCCTTCATGGGTACGGTGGCCAGCGAGGACTTCTTCAGCGGCACG
GCAGCGGGCCTGGCGGGCGGACCACATCGATCATCGACTTCGTGATTCCCAACCCGCAGCAGTCGTTGATGGAGG
CCTTTCACCAGTGGCGCGGCTGGGCCGAGAAGTCGGCGGCCGACTACGGGTTTCATGTGGCGATTACCTGGTGGAG
CGAACAGGTGCGCGAGGAAATGGCCGAGTTGGTGACCCATCACGGCATCAACAGCTTCAAGCACTTCATGGCCTAC
AAGAACGCGATCATGGCCGCCGACGACACCCTGGTCGCCAGCTTCGAGCGCTGTCTGGAACTGGGCGCGGTGCCCA
CCGTGCACGCAGAGAACGGCGAACTGGTCTATCACCTGCAACGCAAGCTGCTGGCACAGGGCATCACCGGCCCCGA
GGCCCACCCGCTGTCGCGCCCTTCGCAAGTGGAAGGCGAAGCCGCCAGCCGCGCGATCCGCATTGCCGAAACCATC
GGCACGCCGCTGTACCTGGTGCACGTGTCCACCCAGGAAGCCCTCGACGAAATCACCTACGCCCGCGCCAAGGGCC
AGGCGGTCTATGGCGAAGTCCTGGCCGGGCACCTGCTGCTAGACGACAGCGTGTACCAACACCCCGACTGGCAAAC
CGCCGCAGGCTACGTGATGAGCCCGCCCTTCCGCCCGCGCGGGCATCAAGAGGCACTCTGGCGTGGCCTGCAATCA
GGCAACCTGCACACCACCGCCACCGATCACTGCTGCTTCTGCGCCGAACAAAAGGCCGCCGGGCGTGACGACTTCA
GCAAGATCCCCAATGGCACCGCCGGCATCGAAGACCGCATGGCGCTGCTATGGGACGAAGGGGTCAACACCGGGCG
CCTGTCGATGCAGGAGTTTGTCGCGCTGACCTCCACCAACACCGCAAAGATCTTCAACCTCTACCCGCGCAAGGGC
GCGATCCGTGTCGGTGCCGATGCCGACCTGGTGCTGTGGGATCCCCAGGGCACACGGACCATCTCGGCCAAGACCC
ACCATCAGCAGGTGGACTTCAACATCTTCGAAGGCAAGACCGTACGTGGCGTACCCAGCCACACCATCAGCCAGGG
CAAGCTGGTGTGGGTCGACGGCGATCTGCGCGCCGAGCGTGGTGCCGGGCGCTATGTCGAGCGGCCGGCGTATCCG
GCGGTGTTCGAGCAGTTGCGCAAGCGTGCGGAGCGTTCCAAGCCAGCCGCGGTGAAACGCTGACATCGCCGATCGC
AGGCAAGCCAGCTCCCACACTTGATCGGGTTCACACAGTCAAATGTGGGAGCGGGCTTGCCCGCGATGAGGCCAGT
GCAAACCACAAAAAAACCAATGCCCGCCAGAGGCAAAAAAAACCGTGAGGCCACTACCGTGATCCAGACCCTGACC
CACCTTCCCCACCCCCATGAAGACGGCGCAACCCTCGCCCGCCATTTCACCGACCTGGCACCGCCGCTCAACGCCC
GCCAGGCGCACCTGGAGGCCTCGCGCTGCCTGTATTGCTACGACGCACCCTGCGTCAATGCATGCCCCAGCGAGAT
CGATATCCCATCGTTCATCCGCAATATCCACACCGACAACGTGCAGGGCGCGGCGCAGAAGATTCTCTCGGCCAAC
ATCCTGGGCGGCAGTTGTGCCCGGGTCTGCCCGACCGAAATCCTCTGCCAGCAAGCCTGTGTGCGCAACAACAGCG
AAGAATGCGCGCCGGTGCTGATCGGCCTGCTGCAGCGCTACGCCGTAGACAACGCGCACTTTGACCAACACCCCTT

CCAGCGCGCCGCCCCTACTGGCAAACGCATCGCGGTGGTGGGCGCCGGGCCAGCGGGTTTGTCCTGCGCCCATCGC
AGTGCCTTGCACGGGCATGACGTGGTGATTTTCGAAGCCCGGGACAAGGCCGGTGGCCTCAATGAATACGGGATCG
CCAAATACAAACTGGTGGACGACTTCGCCCAGAAGGAGCTGGATTTCCTCCTGCAGATCGGCGGCATCGAAATCCG
CCACGGCCAACGCCTGGGCGACAACCTGAGCCTGAGCGACCTGCACCAGCAATTTGATGCGGTATTCCTCGGCCTG
GGCCTGGCCGCCAGCAAACACCTGGGCCTGCCCCACGAGGATGCCCCCGGCCTGCTCGCTGCCACTGACTACATCC
GCGAACTGCGCCAGGCCGATGACCTCAGCAAACTGCCCCTGGCCGACCGCTGCATCGTGCTGGGTGCCGGCAACAC
CGCCATCGACATGGCCGTGCAAATGGCCCGCCTGGGCGCGCGCGACGTCAACCTGGTCTACCGCCGTGGCCTCGCC
GATATGGGCGCCACCGATCACGAGCAGGATATCGCCAAGGCCAACCAGGTACGCCTGCTGACCTGGGCCCAACCCG
AAGCCGTGCTGTTGGACGACCAAGGCCAGGTGCGCGGTATGCGCTTTGCCCGCACGCGCCTGGAAAACGGCCGCCT
GCACACCACCGGCGAAACCTTCGAGCTGGCGGCAGATGCAATCTTCAAGGCCATCGGCCAAGGCTTCGACGAGCAG
GCCTTGCATGACCCACTGGCCCAGCAACTGCAACGCCAGGGCGAGCGGATCTTTGTCGATGCGCAGCTGCAAACCA
GTGTCCCCGGGGTGTATGCCGGTGGCGACTGCGTCAGCCTCGGGCAGGACCTCACCGTCCAGGCCGTGCAACACGG
CAAGCTGGCCGCCGAGGCCATGCACGCTCAACTCATGCTCAATGTGGAGGCTGCGTAAATGGCCGATCTATCGATT
GTGTTCGCCGGCATCAAAGCCCCCAACCCGTTCTGGCTGGCCTCCGCGCCCCCCACCGACAAGGCCTACAACGTGG
TGCGCGCGTTCGAGGCCGGCTGGGGTGGCGTGGTCTGGAAAACCCTTGGCGAGGACCCGGCAGCCGTCAACGTCTC
GTCCCGTTACTCGGCGCACTTTGGCGCCAACCGTGAAGTCTTGGGTATCAACAATATCGAGCTGATCACCGACCGT
TCCCTGGAGATCAACCTGCGGGAAATCACCCAGGTGAAAAAGGACTGGCCGGACCGCGCCCTGATCGTGTCGTTGA
TGGTGCCATGCGTCGAAGAATCCTGGAAAAACATCCTGCCGTTAGTCGAAGCCACCGGTTGCGACGGCATCGAGCT
GAACTTCGGCTGCCCCCACGGCATGCCCGAACGCGGCATGGGCGCAGCAGTGGGCCAGGTGCCGGAATATGTGGAA
CAGGTCACGCGCTGGTGCAAGACCTATTGTTCGCTGCCGGTGATCGTCAAGCTCACGCCCAATATCACCGATATCC
GCGTGGCCGCCCGGGCCGCCTATCGCGGCGGCGCCGATTCGGTATCACTGATCAACACCATCAACTCCATCACCAG
CGTGGACCTGGAGCGCATGGTCGCCCTGCCGATCGTCGGCACCCAAAGCACCCACGGCGGCTACTGCGGTTCGGCG
GTCAAGCCGATTGCCTTGAACATGGTCGCTGAGATCGCCCGCGATCCCCAGACCCAGGGCCTGCCGATCTGCGGGA
TTGGCGGCATCGGCAACTGGCGCGATGCCGCAGAATTCGTGGCCCTGGGCTGTGGCGCGGTGCAGGTGTGCACGGC
GGCGATGCTGCATGGTTTTCGGATTGTCGACGAGATGAAGGATGGCTTGTCGCGATGGATGGACAGCCAGGGCTAC
AGCAGCTTGCAGGAGTTCTCGGGGCGCGCCGTGGGCAATACCACGGACTGGAAATACCTGGACATCAACTACCAAG
TGATCGCCAAGATCGACCAGGCCGCCTGCATTGGCTGCGGACGCTGCCATATTGCCTGCGAGGACACGTCGCACCA
GGCGATTGCCAGCCTCAAGCAGGCGGACGGTACCCATGTGTACCAAGTGATCGACGACGAATGCGTGGGCTGCAAC
CTGTGCCAGATCACCTGCCCGGTGGCGGACTGCATCGAGATGGTGCCGATGGACACGGGCAAACCGTTCCTGAATT
GGACGCAGGATCCACGCAACCCCTACCGAGAGGCGGTGTAGCCTTTTCAATCGCTATCGCCGGCAAGCCAGCTCCC
ACACTTGATCGGGTTCACACAATCAAAATGTGGGAGCTGGCTTGCCTGCGATGAGGCCCTCAACCACACCCCAAAC
TCATGGCTCCAACCCGATCCCCCGCAAAATCACACTGGTCACCGTCTGCACCGCCCGTTCGAACTGCATGTCCGAC
AACGCCTGATGATCGTTGAGGATCATCACCTGGTGATCAAAGTCGGCATAGTGCTGGGTCGAGGCCCAGATCATAT
ACAGCAGGCTCGACGGTTCCACCGCGAGGATGCGTTTGTCTTCCACCCACTGGCGGATTTTCGCTTCCTTCATTTT
GGCCCAGTCGTACAGGCTCGCATCCAGCGCTTCGCCGAGGGTCGGCGCGCCGTGGATGATTTCGTTGGCCCAGACC
TTCGAGCCATACGGGCGGCTGCGCGAATGCTGCATCTTCGCCCGGATGTAGCTGCTGAGGACCACGCGCGGGTCAT
CGAACATCTCGAAGCACAGGGCGTCCTGCTTCCACACCTCCAGCAGGTCGAACAACACCGCGCTGTACAACTCGCT
TTTGGTGCTGAAGTAGTAATGCAGGTTGGAGCGCGGCAGTTGCGCCTGTTCGGCAATGTCGGCCATGGCGGTGCTG
CCATAGCCCTTCTCGGCGAAGACCTTTTCAGCCGCCAGCAGAATCTTTTCGACGTTGACCCGACGGATACCGATCT
TGTGATTGCCCATAGGGGCTCCCTGACAACGACATGCCTGGAAGACTACCATCCGCTCTAGATCGGGGCGACAGCC
TGAGTGGAAACTTCGTACACTGCCCCCCTCTCCCATTGATAGGTAGTGCACGATGTTGATCAAGAAAACCCTGACT
ACGGTGACCTTGCTGGCCTCGCTGCTGGGGGCTTCGACCGCCTTTGCCCATGCCCACCTGAAAACCACTGCGCCGG
CCGCCGACAGCACCGTCGCCGCGCCCGCCGACCTGCGCCTGGTGTTCTCCGAAGGTGTCGAGGCCACGTTCACCAA
GGTCTCCCTGAGCAAGGACGGCACCGAAGTGGCGATCAAGGGCCTGGAAACTCCAGACGCCGACAAGAAAACCCTG
GTTGTGACCCCTGCCGCACCGTTGGCCGCCGGCACCTATAAGGTCGAGTGGCAAGCCGTCTCGGTCGACACCCACA
AAAGCGCAGGCAATTACAGCTTCAAGGTCGGCCAATAACCCATGGCAACCCTGCTGGTGCTGTGCCGTTTCCTGCA
CTTCATGGTGGTTTTGCTGATGTTCGGGGCCTGTGTATTCAGGCCCTGGTTATTGGGCACAAGCCCGCGGCCGGCC
CTGGATCGGCAGTTGTCACGCATCACCACGCTGCTGGCCTGGCTGGCCCTGGGGTCGGGTGTGAGCTGGTTGCTGT
TGATCAGTGCCAGCATGGCCGGCAGTGAACTGGCGGCGCTGGATCTGGCGACGGTGCAGTTGGTGCTGGGCAAAAC
GTTCTTCGGCAAGGTCTGGAGCCTGCATCTGTTGCTCAATGGCTTGCTGGTGCTGAGCCTGCTGACACCCTGGCAT
CACGTGCGTCTGCTCCTCAGTGCGCTGCTGCTGGCTACCCTGGCCCCCGTGGGGCACGGCGCGATGCTCAGTGGCC
TCAGCGGGCAATTGCTGATTCTCAACCAGATGGTGCACCTGCTGTGCGTCGGTGCCTGGCTTGGCGGGTTGCTGCT
GCTGGTGTTGATCCTGCGTCAAGCGGCCCTGTACCCGCTGGAGAAAATCCTGCAGCGCTTCAGCGGTGTCGGTTAT
CTGTTGGTGGCCGGTTTGCTGGTCACCGGGCTGATCAATATCCGTGTGCTCACCGGGCAGTTCTGGCCGACTCCAC
TGTTCAGCGGGTTTGCCTTGATCCTGTTGATCAAGGTGCTGCTGGTGGCGGGCATGCTGGGGTTGGCATTGCTCAA
TCGGCTGCGGATCAACAATTGCCAGCAGCGCGTGGGGCAACTGCAGGCCAGTGTGAAGCTCGAATGGTTGTTAGGC
GTGGCCGCTGTGGCGGCGGTGTCCCTGCTGGGCACCCTGCCACCGATGCTGTAGGAGCGAGCTTGCTCGCGAAAAA

CTTACCGACAACGCGTTTACCCAGGCAACCCGCGTTATCGTTGACGACTTTCGCGAGCAAGCTCGCGCCTACTCGC
GGTGTGTGGAATGTCAGGGCGGGCTGTTTTTCAGGCGGCCTGCCGTGTCGATGCTGACCACCACCGACAACCCCGG
TCGCAGGCGTTCGCTCTGCTCCTGATCCGGGTCCACGGTGATCCGCACCGGCACCCGCTGGGCGATCTTGACGAAG
TTGCCGGTGGCGTTATCGGCCTGCAACAGGCTGAACTCGGCGCCCGTGGCCGGCGAGATACGCTGTACCGTGCCAT
GGAATCTACGGTGGTTCAGGGCATCGACGGTGAACGTCACCGGCTGGCCCACCTGCACGTGGTCCATCTGGGTTTC
CTTCATATTGGCGATCACCCACAGCTGGTTCGGCACCAGCGCCATCAACTGCGCGCCGGAGTTGACGTAGGCCCCC
AAACGCACGCCGATCTGCCCCAACTGACCGTCCCGGGGCGCCAGCACGCGGGTGTTGGACAGGTCGATGCGCGCCA
GCTCCACCGCGGCCTCGGCGCTGGCGACGGCGGCTTCCAGTGAACCGCGATTGACGATCACCGTCTGCAAGTCCTG
GCGGGCGATTTCCAGGCTGGCCTGGGCCTGGGCCACGGCGGCGATGGTCTGCGCGTTGGCGGCGCGGGTCACGTCC
AGTTCACGGCGCGACACCGAGCCATCGCTGATCAGTTCTTCGTTGCGCCGCAGGTCGGCCGTGCTCTTGCGGGCCT
GGGCCTGGCTGTCGGCCAGGGCGGCCTGGCGCAGCTTGATGGTCGCCTCGGCGCTGTTGCGCTGTTGCACCACATT
GGCCAGGGACGCCTGCTGCACCGCCAATTGCGCTTGGGCCTGGTCCAGGCGCTGCTTGTAGATGCGGTCGTCGAGC
CGCACCAACAGATCCCCGGCCTTGACGTGCTGGAAGTCCTGCACCGGCACCTCGAACACATAACCGCTCAGTTGCG
GGCCGATGATCGTCACCTGGCCGCGCACCAAGGCGTTTTCGGTGGTTTCCACGGCGCTGCTGAAGGGTGGCAATTG
CCAGGCGTACAACACAATCAATACGCCGACGATAGCAATCGCGGCAAAGCCCAAAGACGAGATGATGCGCACGCGC
AGCGAGCGCTGTTCGGTGACCGTGGCTCCGGGCGGCGTGGTGCCCTCCGGGGTGGAAGCAATGGCATTGGTGGTCG
TGGTGGTAGCCGTAGGTTCGCTCATGAAGAAAGTGCGCCGCTAGGTTGAACGGAAGGCGCTGCACTGGCGGCAGCT
TTTTTGGTGGTGCTCATCAGCCACAGGCTGCGGGTAAAGATCCACAACATGGTCAGTATCGCGATAATCGCGATCA
GCATGAACACGTCGTTATAGGCCATCACATTGGCTTCACGGGTGGCCGCCGTGGCCAGGCTGCGGATGCCCAGCAG
GTTGCGCAGCTCCGGGTCGGCGATCACCGAGCCATAAGCCGAGCCGCCGCTCTGCACGCGGGCGGCCACGCGCGGG
TCCAGCAACGTCAGGTGTTCGACAATCATGCTGGAGTGAAACTTCTCGCGCACGATCTGGAACGTGCCAAGCAACG
CCGCGCCCAGCAGCCCGCCAAGGTTCTGGCAGATACCAAACAGCACCGAAAAACTCACCAGGTTGCGCGGGTTGCT
CAGCACATTGCGCATGCCCAGCACCATGGTCGGGCCGAGGAAGAAAGTACCGCCAAACCCCAGCAGAAACTGGCTA
ACGTACATGTTCTCTGGTCGGGTCAGGTTGCTGGAGAAGCTGTCCATCACCGAGCCTGTGGCCATCAGGGCCAGGG
AAATCACCAGGGGCATCAGCAAGTGCGCCGGGTTGATGGTCAATGCGCTGATCACCAAGCCACTGATCGCTCCCAG
CAGCATCACCAGATACAAGGTGCGCATCTGCTCGCTGCTCATATTCAGTGCCTGCAAAAAGCCCACGGCGCCGGTG
GACTGCTCGGACAGCACCATGCGGATCAGGATCACTGCCAGGGCCAGGCGAATCATCACCCCGCCGCCCAGCCAAC
GGGTCATCAGCAGCGGGTTGGCGCGGTTATGCTCGATGGCCAATCCGGCCATGATCAGCACCAGCGAACAGGCGGA
GGCGACGCCGATCCACGGTGCTTCCAGCCACCAGTCGATACGCCCCAGGGACAGCACCGCACACAGCAATGCCACG
CCCGAGGCGAGCAAGGCGAAGGTCAGGAAGTCGAGTTTTTCAAAGGTCTTGAAGCGATCACCCGGCGGCAGCTTGA
GCAGGAACACACAGCCCAGGCACGTCAGCGCCATGCCCAGCTCAAACAGGTACAGGCCGCGCCACTCGGCAATCTG
CAGCAAGTCTTCGGAAAACAGCCGCGCCATCGGCAGGGCCAGTTGTGCGGTACCCAGGCCCAGCACCAGGGCCTTG
AGCCGCCACTTTGCGGGGAACGCCTGCACCATGTAATACAGGCCCAGGGAACTGAGGGCCGCGCCAACCATGCCGT
GGGCCGCACGCACGGCAATCGCCGAACTGAGGTCGTTGACAAACAAGTGGCCGAAGGTCACCAGCGCGTACAGCAC
CAGGAACACTTCGGTAAACGCCCGCAGCCCGAACTGCTGGCGGAATTTCACCAGCAGCAGGTTCATCGACACGTTG
GTCATGACATACGCCGCAGGCAACCAGGCCATTTCGGCGGTGGTCGCGCCCAATGCGCCTTGCAGGTACGGCAGGT
TGGCGATCACCAGGGCGTTGCCGAGGCCGCCGGTGATTGCTACCAGCACGCCAACCAGGGCAAAGGCCCAGCGCTT
GAGCGTGGGGTGATGGGGCGTCGATGGCGACCCCGGCAAGCTTGGACGCTCGTGGGGTTGCCAGGTGTGAGGGGTG
TATTTATCCATTCGTGACCTTGATGACCGACGCACGAGGCCGGTAAAACCCTGACTCAGAGCGTAGTAGACCTGAG
CGGGGTTGATCTTGTCATCTTTGTAGGGGCTGGCTTTTGTGGGAGCTGGCTTTTGTGGGAGCTGGCTTTTGTGGGA
GCTGGCTTCTGTGGGAGCTGGCTTGCCTGCGATGCAGGCGCCGCGGATTGGGGGGAGGTGTACATATCCGTTGCTG
CGGTCACGGCCACTTAGGGTTCCGCCCTGACGGCGGGTCACTTTCGAAGAGCCGGAGTGCCGGCCCAGTCCAAAGT
AACCAAAGCGCTCTTGCCCCACCACTCGGTGCCTCGCCTAGGCTCGGCATGCCCGCAGTCAGGCANNNNNNNNNNG
CCCCTATTCCCCCCTTCAGCACAACAGCAGGCTTGCCAAGATCCACCTAAGGCACCATCCCGCAATTACGCGCATA
GGCGAACAAATCCACATCGGTGGAAATGCACAGGCGTTGCATCGCGGTACTTTTCTGTTTGCTGATCGTGGAAATA
CTGCGATTGACCCGTGCGGCGATCTGGCTGACGGTCAAGCCGCTGGCCAGCATCCGCACCACTTCCCGCTCTTTGC
CCGACAACTGCGGCAGTTGTGACTGGTCCCCCGTCCCGGCTTCCACCAGTTGCACCCGCAGGCACTCGCTGGCAAA
GGTCTGGCGGCCGATCACGGCGTTGATGGCCTGTGGCAACTCCCTGGCCGAAGCGCTCTTGGCAACGATGGCGCGG
GCGCCATGGGCGAGGGAGGCCCGCAGGGTGGCGACATTGGCGAACATGGTCACCAGGATGACCGGCAAGTTGGGAT
ACTGCCGAGCCAGCAGGCTCAACAAACCATAACCATCTGCCTGCTGCCCTCCAGGCATGGCGAAGTCGGTGACCAG
GACATCGCACGGCGTGGTACTCAATAACCTGAGCAGCGCGTCGGGGCCGTCGGCCTCCCCCACCACTTTGCAGCGA
CCATTGGCCTCGATCACCACTCTCTGCCCGATACGCACAATGGGATGATCGTCAGCAATAATTACTCGAAGCATAG
ATACCCACGGATGATGGCAACTTGAATCCGCGCAAAATAACCCCGCTGGCGATCCTCAACAACCGTCCAGGATAAG
CCCCACAAGGCGGCGCCCCCTGTTTGGTAGTTACCAGTTCTTACCCTACATAACAAAAGGAAAACCCCTACAGCCA
AAAACCAGACAGCCAGATTGCCAAATGCATCTCGACACAAGTCACTCCGTCCACTGCGCTTACACACTGCGCAAAT
ATTCCAGACACCGCCGCAAGTCCGCCCGGAATGCCTGCAACGCCGGAGCGTGGGCCAATACGCCATTGGCGTGAAC
CAGCTCAATCAACTTCACCCCTCTTAACTCAAGGTCCGTCCCGCCCAAAAACGCCAGGCTGCCTACCAGGCGATGC

AAGCGCTGCGCGGTCAATAAAGTACTCAGGGTGCAACAGGCTTGTTCCAGGATCGCGAAGTCTTCCCAGGCTTCGT
CCAGCAGGCCGGGGAGCATCTGGTCGACGACCTGCTCATTGCCGAAGGTTTGCACAAGCTGCGCACGCGTCGGCCA
TGTCCCGGCAGAGCGCCGGCCCCATGCCGAAAAAGTACCCGGCAGCCAGCGCACCAACACCGCATGCAATTGCTCC
AGCGCCAGCGGCTTGAGCAACCAGGCATCCATGCCGGCGTCGCGGCAACGTTGTGGGTCGTCGTTTTGCAGATTGG
CGGTCAAGGCAATGATCGGCACGCGCTTGAGCCCCCTGCGCAGTTCATGCTGGCGAATGGCGCGAGCCATGCGGTA
ACCGTCCATCTCCGGCATTTGGCAGTCGCTGATAATCAGGTCCGAAGGCCGGTTGATAACGGCCAACAGGCCCTGG
CGGCCATCGGCCACGGACTGATGGGCAACACCCAATTTTTGCAGGAAGCAGCCCAGCATCGCGCGATAGGCTGAAT
GATCATCCACGACCAGTACGTTCAGGTGCGGTGCGGGGGGTGGCTGCGACGCTCGGCCCTCGCCGGTGGTTGAACG
TACCACAGAGCCCATGGGCACCCCTTGTGCGCGATCGGACTGGAGGCAGGCAGCCCCGAGTCACCTCCGGCAGCGC
TGGCGTAATAGTCAGGAGGGCACTGTGCAGCGCCTTAAGTGACACCGGTGACTATCATCGCGGCAAAAATATATCC
TTTTGGATACTCCGACGCCGATACAACAACTACGAAAAACAACTTTCCTACGTCACTGAAAACCTATTCTTACGGA
CTTGCCGCGGATTTCCGTACGACCGCCCATTGTCGTATTTTTTGTATTTGCCCCCCGCCCTGCCCTCGCTAAATTG
CGCCCCACCCCAGAGAGGGACGGCTCGCCACTCGGCGTGCCAGCCACAACAAACAACGCCTCGCACACTGCCAGCC
CATTGGGGGCCAGGCATTGCCGTGCATCTTTTTTATCGATCAGCCTTGCAATGGACAATTGAAATGAACGCCTCCC
GACACCTGATGAGCACCACCTCCCAAGCCTTCCCAACAGCCGCAAATCAGGTCCTGTAACCATGAAAAACTTTGCC
TCGCGCCTCATGCTCACCTTGCTGTTCACGCCTTCGGTATTTGCCGCAAGCTCCATCGAACTGGCCGTCAGGGGGA
CTATCACCCCGAGTGCCTGCACACCCGCATTCGCCGGTGGCAACGTCGTCGAGCACGGCAAAATATCGGCCAAGGA
TCTAGTACAGAACGGCTCTACACACCTGCCTAAAGCCACCATGCAATTGTCGATCACTTGCGAAGCCAGCACGCTT
TTCGCGATCAAGCCCATCGACAATCGTAGCGGTACCTCCACCAGTTCCAGCGATTTCGGCCTGGGATTTGTCAACG
GCAACAAAAGACTCGGCGAGTTCTATCTGACCGCCCAGAACATGCTTGCCGACGGCGTCAGTGCACAGCCCATCGC
TTCGATGGACGGGGGTAGTAACTGGCATGCCGACATGTCATGGGAACTGGGAACCCTGTGGGGCGCTGGAGCCATG
GACGACGCGACCACCCTGCTGCCGTCCAGGCTGCTGGTAATCGACCTTGACGTCGGTACCACCATCGCCCGCGCCG
ATCAATTTGACTTGAGCGATGAGGTTGCCATCGACGGCTCCGGCACCCTGGAAGTGCTGTACCTGTAGTTCCCGTT
TCACCCACAAGGAAGGTCCATCGAATGAAACTCTCCAGCGCAGTACCTCTGGTTGCACTGTTGTACGCCACCGCCT
CAGTGGCGTTGGCCGCCAGTGACACAGAGCTGGCCATCAAGGGCTCGATCACCCCCAGCGCTTGTGAGCCGCTGGT
CTCCAGTGATGGGATGGTCGACTTCGGAAAAATATCTGCCAAGCAACTCAACGGCAGTTCTTACACCTCCCTGCCC
GATGAAAGGCTGCTGTTGAATGTGCACTGCGATGGGCCGACCTTTTTTACGCTCAACAGCATTGATAACCGCTCGG
GCTCCTCGGCTCACCACGACATGTATCACGGCCTGGGCATGACCCCCGACGGTGAAAAGCTGGGGGGCACAGGTTT
TGGTTTCTACGAACTGATGGCCGACGGCGTAAGCCGGCACTCGATTATTTCAACCGACGGCGGCGTGACCTGGAAA
CCCGCCGCCGTGATAAGCCCGATGTCGCTGACGGCCGTCGCAGCAGGCCACGATTGGGTCCCCATTGCCGTGCAGA
ATCTGGACGCGAAATTGACGCTTTACACCCACATCGCCCCCGCCGACGGCCTGACGCTTATTGACGAAGTCCCTCT
GGATGGCCACGTCACCGTGCAAGTCAATTACCTCTGATGGCTTGAACAGGCCCATTTAAAGGAGCTCCTCCGATG
AAAATCCTACTGACCACGCTTGCCACCAGCGCCCTGCTGCTGGGTAGCGCCACCGCCTTTGCCGCCTCGACCGTCG
ACCTGGTCGTCAGGGGCAAGATTATTCCTGCCGCCTGCGTTCCCGGCCTCTCCAGTAGCGGGGTGATCGATCACGG
CAAGATTTCCGCCAAGGACTTGCGAGCGGACAACCCAACGCTGATCGGCACGCACCTGCTGAACATGACGGTCAAC
TGCGACGCCCCCGTCTTGTTCGCCCTGCATGCCATCGACAACCGCGCCGCCTCGGCAGCGATGCCCAGTGACTACG
GCCTGGGCTTTATCAACGATAACCAGAAACTCGGTTGGTACACCCTGAGCCTGCTCAACCCGGTGGCTGAAGACGG
CGTGGTGGTGCAACCGATTGCCTCCGGCGATCAGGGCAAGACCTGGTACCGGGAAAGTGCCTGGGAGCCGACCCTG
TACATGTCGGTCGCCTCGATGGACGACGACACCCAGCCGCTGCCGGTCAAGGAGCTGCGGGTAGGCCTGGAAGTAC
GCACGTCGATCGCCCGCACCGACTCCCTGGACTTGAGCAATGAGGTGACCATCGACGGCTCGGCCACCCTCGAAGT
GAAGTACCTGTAACCATGGCCGCCCATGCCCTGGACACTTGCGGGCATGGGCTGCGCGTACCTGCGCCAGTGATCG
CCCACGTTGCAGCCGTGCTGCTGTGTATGGCAATCACGACACAGGCCAATGCGCTGTCCCATGTCGACCTCACGGT
GACCGGTCGCTTGACGCCGGATGCCTGCCATATCGAGCTGTCCGACCTAGGCACCATCGAGCATGGAGAGATCCCG
ACCCACCAGCTAAATACCCACGAACCCACGGTACTGCCCAGCCAGAAGCTGGACCTGACGGTGATCTGCGAAAGGC
CGATGCTGTTTGCGCTGGTGGGCATCGACAACCGCCAGGACTCCGCGCCGGCCCCCGACACGTTCGGCCTGGGCGT
CAACATTTATGCCCCGGACCAGATGCTGGGGGCCGTGAAGCTTTCCTATCGCGCAGCCCTCGGCGATTTACAGCCG
ATGCAGGTGCTGGCCTCCAGCGACAACGGCGAAACCTGGACACCCCAGGCTGGCGCCCATCCCCACGCGTACATGG
GCTTTGCCCTGCCGGGCGACCGCCAGCCGGATTTCATCAGCCAGCTGTTCACCCAGTTGTGGGTCGACACCTCCAT
CAATGCCGCACGCCTCCTGACCCTGGATCAGGAAGTGCCGCTGGATGGCTCCATCGTCCTGGATCTGCGCTACCTC
TGATTCGCGCCCTTTTTGTTGCACCAACGGAACCTGCTGTCATGACCACGACCTTCTCAACAACCGCTGTCGGTCT
CATTGCCCTGCTGCTTGTACTCGGCAATCACGTCAAAGCCGACGGCATGGTGCCCGACACCTCGGTGGTGATCGTT
CACGAAGCCGATGGCGAAGTCGCCGTGTCGGTGACCAATACCGACAGCAAGCTGGCGCTGCTGCACGTCACCTTGC
AAGACATCCCCGAAGACACCGAGCCGTTGCTGGTAGTGACACCGCCGTTATCGCGGGTGGAAGCGTCCAAGTCGCA
GCTGGTGCGTTTCATCCTGCAAAACCAGACACCGCTGCTCACCCAACGGCTCAAGCGCGCGGTGTTCGAAGGCATG
CCCCAGGGCCGCGCCGCCCCTGCTGCCGGGCACGCCCGCGTCGGCGTGACCGTGCGCCAGAACCTGCCGGTGATCG
TGCATCCCAAGGGCCTGCCACTCAACCGCACGCCCTGGACCGACCTGACCTGGACCCTGCGCGACGGCAGGTTGCA
CGTGCGCAATGACACCCCCTATGTGGTGCGCCTGGCCCAGGATCTGCGGCTGCTGCCCGGCGACGGCAAGGCGATG

CTGCCGCGTACCTATGTGTTGCCGGGCGAAAACCTGAGCGTCCCGGCCACTGGCGGCCCGGCCACTAAAGTCAGGC
TGCAACCGGCCACGGTGTACGGCTTTGCCGTCAAGGCCTACGAAGCACCTCTGAGCCTCTGATTGGTCACCTGCAG
GCGCCTGCCACGAAGCGGCGCCTGGCTTAAGCAAGTGACGGCGCATCGACCGTCACCGCAATGCCCGCAAGCACCG
GAACAACCGGGCCGGGACGTTGAACATTTGAGTGCCTTGCGTGAAAACAGTATTGAATTACCGTGACGGCGTAGCC
GTGCCCGCAGCACCTGCAATGGCGTTGCTGCTGTGGGGGCTACTGCCGAGTCAAACCTGGGCCAGCGGCTTCGACG
CCCAGACCCTGCATCAGCGTGGCATTGATCCGCAGTTGGCCAGCCTGCTGCTGGACGCCCCGCGTTTTGCCGCCGG
GCAGCATGCAGTCAGCCTGCGGGTCAACGGCCAGGGGCGTGGCCGGCTGGAGGTCAGTTTCGATCAACAGGGCAAC
CTGTGCTTCGACCGGGCCTTGCTCGACGCCGCAAACCTGAGAATCCCGGACGACGCCGAGCGCTGCCACGCCTTCC
TCGCGCAATTTCCCCAGACAGTGGTGGAACCGGACCCGGCCACACTCAGCGTGGCGCTGGTGGTGCCCACCGAAGC
CTTGCGTCCGGTGCTGCAAGACATATCAGGCTATGAAACCGGCGGCTTCGCAGGCTTGCTCAACTACGACCTCACG
GGCTTCTACAACCGCTATGGCGATGACGCCAGCCGTTTTGGCTCGGCCAACACCGAAGTGGGATTCAACGCCGGCG
ACTGGATCGTACGCAGCCGCCAAGTGCAGACCTGGCAGGAGGGCCAGTCCCGCAGCACGCACCTGGAGGCCTACGC
CCAACGCACGTTCGCCAGCCATCAGGCGGTGCTCCAGGCCGGGCAGATCAGCCTGTACAACCCAGTGTTATCCGGG
GCGCAGATCACCGGGGTCCAGGTCCTCACCGAGCACGCCCTTCAAGAGCAAGGCCAGAGCGCCGTGATCGAAGGTA
TCGCCAACAGCCCGGCGCAAGTGGAGGTGCGGCAGAACGGCGTGCTGATTCATTCCACCGTAGTGCCCGCCGGGCC
TTTTTCGCTGAACGATGTGCGCCGTCTCAATACCCGTTCGGACGTCGAGGTCACGGTCAAAGAAAGCGTCGGTGGC
GAGCGGCGCTTCAACGTGCCGGCGGCCATGCTCGGCCTCGGGTTGCCGGCGCCGGGCTACTCGGTGGCCGCCGGGC
GGGTTCGCAATGTCGGCGATGCCCAGGGTGACGACCCGTGGGTGGTGAGCGCCGGCTGGACAGCGGCCGTGCATCC
GCAACTGACCCTGGGCAGCGGCGTCTTGGCTGCCAGCGAGTACCGCTCCGCAGGCTTGAGCCTGGGCTGGCTACCG
TGGCTGGACAGCCAGGTCCAGCTGTCGACGCAACTGTCCAATACCAATGGGCGGGAAAAGGTGTCCGGGGTGCAGA
CGGATCTTGCCTGGGCCCAGCGCCTGAATGACCAGTGGTCCTTCAGCACCAGTAACTCCTGGCGCAGCGCAGGCTA
TCGCGAGCTGGAAGAAAGCACCTACGCGCGCAACCAGCACTCGCAACGCTCACGCTATCGCGATCAACAAAGCGTC
AACCTGGGCTGGTCCCATCCCTGGCTGGGCGCATTCAGCGCCGGACTGTCGCGCTCGGCCAACTTTGCCGGGGACA
GCAGCAGCCGGGCCCTGATGTCCTGGGGCAGCAACATCGGCGCAGTGTCGCTATCGGCCAGTGCCGAATGGCAAAT
GGGCGGGGCCCAGCAACAAGACAACGCGGTGTACCTGAACATCAGCGCACCGCTGGGCGAGCAGCGCCGGGTGCGC
GGCTGGGTACGCCACAGCGCTGGCGAAAACCGCACCGGCCTGGGGCTGACCGAACAGATCGACGAGCAACTCAGCT
ACCGGGTCAGCGCCGAGCGCGACTCGCGCGATCGTCAGGTCGAAACCAGCGTTGGCCTCTCGGCATTGCCGCGCTA
CAGCCAGTTGGATTTGAGCTACAGCCGCTCCGATGCACAGCGTTCCAGCTACCAGGGCGGCGCGCGCGGTGGGGTG
GTGATGCATGACGGTGGCGTCACGTTTTCCCCGTATCCGGTGCGCGATACCTTCGCCCTGGTGACGGTGGGCCAGA
TGAGCGGCATCCGCATCAGTACCCCCAGCGGCCCGGTGTGGACCGACTGGCAAGGCCAGGCCGTGGTACCGCAAGT
CAGCGCCTATGGCCGCAGCCCTGTGGAAGTCCAGACCCGCTCGTTGCCGCGCAACGCCGACATCAGTAACGGCCTG
GCGATGATCCAGGCCGGGCGCGGCGCAGTGGACCGGGTGGACTTTGGCGTCAGCCTGACCCGCCGCGTCTTGCTGA
CCGTCAGCACTGACCACGGCGCGCCACTGCCCTCGGGCGCGTCAGTGAGTACTGCCGAGGGCGAGTTCGTCACCCT
GGTCCAGGAAGGCAGCCAAGTATTCCTGCCCAATGTGCTCGATCAACCGCCCCTGTGGATAACCTCGCCGGGAGGT
CAACGCTGCCTTTTGCGCTACACGCTGGCGCAAAAGGCCGACCCCTCGGTGTACTTCGAAACTGCCCCGGCCCGTT
GCCACGCGCCTTGAGGACGACTGCCATGCCCCTTCCCCGCTTGCACCACCTGAGTTTATGGCTATTGATCGGCATT
GCCGGCGCCGCCCATGGGCTGGAGGAATGCCAACTCAACCTCAGCGAACCCACGGTGGATTTTGGCTTGATGAGCC
GCCTGGCCCAAAACGACAGCGCGCCCGAACGCCTGCTGGGCGAGCGGCGGCTGAACTTGACCTTCAGTTGCCCCCA
GGCCGACGACTTGAGCCTGTTCTACCGTGCCCTGGCGGCCTCCGCTGAACGCCTGCAGTTTACCGAGCACGGCAGC
TACGAAATCGAGGTCAGTCACGGCGTACTCGACGGACAGGCAGTGGAGCTGGGGCTGATTGCTGCCATGGGCCAGC
CGCCAGTGAGTAGCGCCAGCACGCTCACCTGGCGCACCGGGCATGGCATCGCGCCGATAGAACACGGTGCCGTGCG
CATGGGCAAAACCCTGTCGCTGCAATTGACCTTGCGCGCCTGGGCCGACCCCGAAGCCACCCGGGTGCGCGATGCC
ACCACCTGGGAAGTCCCCGGCATGCTCTACAGCGCCAGTCATGGGCGTTACCGCGAGATGACGCTGCGTGCGCACT
TTGCCCCCGTCGCCTGCCAGCCGGCGTTGTCGGACCAGGGAGTAGTGGACTACGGCACCCTGTTTGCCAAGAACCT
CAACCCCACCACCGAAACGCCATTGCCCATCCGCACCCTGCGCTTGAGCGTCACCTGCGATGCACAGACGCGCTTC
GCCCTGCGCATGCATGACAACCGCGACGGCTCGGCCACCGGCGGCACCGATGAAACCGCCTATGGCCTGGACCTGG
ACGCCAGCCATAACAAAATCGGGCGTTTTTACCTGACCATCGACCCCGCCGAGTTCAGCGCCGATACCCTGCGCAC
CTTGTACCGGACCGACTCCACCAGCAACGGCGCCGCCTGGAGCAACTCCAGCGCACGCCAGATCCCGATGGCGGCC
AACAGCTACATGGGCTTTACCGACAAAAGCGGCCTGAGTAGCGGCCCAGTGGCTATCCAGACCCTGGCCGGCACCG
TGCGGATCAAGACTTACCTGGCTCCCATGCAGTCGCTGGACCTGCGCCAGGTGGTCCATATCAACGGCTCCGGCAC
CATCGAAATCATCTACCTGTGACTTGCCCCTGCGAGGTACGCGCCATGAAAACCGTTGCCCTTTCCAGCCTGCTGT
TTCTGACCTGCGCTCAATCGGCGGTGGCCGTGTCGATGATCGACCTGAGCGTCCAGGGCCAGATCATTCCCCGCGC
CTGTACGCCGCAGTTATCCGGCAGTGGCCTGATCGACTACGGCAAGATTTCCCGCCAAGACTTGAATCTGGAGCAA
GGCACGCGTCTGCCGCTCAAAACCCTGCAGGTCAGCATCGACTGCAACGGGCCTAGCCGCTATGCCTTGCGCATGC
GCGACAACCGCGACGGGTCGGCCACGGTCAACAGCGAAATCTACTACGGCCTGGGATTCGACACGGCGGGCAATCG
GCTCGGCCTCTACTCCATGAGCTTCGACCCGCGCCAGACCCAGGCGGACAGCAGCGCGCAGATTTACGGCACCGAG
TCCACCACCGGCGGCCTGGGCTGGCGCACGGCCAACCTGAACCCTGTCGACATCGGCGCCACCAGCTACCTGGGTT

TCACCGACACCGAGGGCAGTACCGCCGGGCCGACGGCAATCGCCACCCTCAACACCACCGTGCAGATCCGCACGGT
TATCAATGCCCGGCAGAACCTGGACTTGAGCGTTGAAACCGACCTCGATGGCTCGGCGACTCTGGAAGTGGTGTAC
CTCTAGAGTCTCTCGCTGGCGACGTGAACATAACGGGCGCGCCCGGCACCGAGGCCAGCGATGATCGCCCCCACAC
CCAGCAGCGCGAAAATCCAGCCGGTTGCCGCCCAGCCACCGGTCCAGTCGTGTACCAGACCGACCGCCAGCGGGCC
GAGGGACGCCAGGGTGTAGCCGACGCCCTGGGCCATGCTCGACAGGTTGGCGGCCACATGGGCGTCGCGTGAGCGC
AGCACGATCAGGGTCAGCGCCAGGCTGAACGTACCGCCCTGCCCCAGGCCCAAGACAATCGCCCAGCCCCACAGGC
CGTCGAGCGGCGCATACAGGCAACCGAACAGGCCGCCGAGGGTCAGCAGCATCACCACCACAATGGCCAGGCGCTG
ATCCTTGCCGCGGGTGGCCAGCCACGGTGCCGCAAGGGAACTGGCCAGTTGCACGATCACCGATCCCGACAGCACC
AGCCCGGCCTGGGTCGCGCTCAAGCCACGGTCGATCAGGATCGACGGCAGCCAGCCAAACACGATGTAGGCCAGGG
AGGATTGCAGGCCCATGTACAACGTCACTTGCCAGGCCAGGGGATCACGCAGCAAACCGCGCACCCGATAGGCCAC
TTGATGGCTACCCTGTTTCTGTCCGACCTGAGGCAACCAACATAGCGCCGCCAGCAGTGCCGGCACCATCCAGAAG
CCCAGGCCGATTTTCCAGCTGTCGCCAAAATGCTGGCTCAACGGCACCGTAGCCCCGGCGGCGAGCGCCGCGCCCA
GGCACAGGGCCATAGTGTAGACACCGGTCATGACCCCTGCATGCCGGGCGAAATCACGCTTGACGATCCCGGGCAA
CAGCACGCCGATGATGCCGATGCTGGCCCCGGCCAGCAGGCTGCCGGCGAACAGCCCGACAGCGCCCAACGAACTG
CGCAGGATGATCCCCGCCGCCAGTGTCAGCAAAATCCCCAGCACCACCCGTTCGGCGCCGAAGCGCCGGGCCAGGA
ACGGCGCCAGCGGCGCAAACAGGCCCAGGCACAGGACCGGCAGCGTCGTCAGCAACCCAGCCCTGGCCGCCGAGAG
GCCGAGGCTTTCGGACACGTCGTTGAGCAACGGCGCCATGCTCGACAATGCCGGGCGCAGGTTCAACGCCACCAGC
ACCAACCCCAGCAGCAACAGCCAGGCCCGGTTGACGACCGGCTGACTGTGCTGCACCTGGTCATCATCGGCTTCGG
CGTCGATCAGCAATTCTTCAAGTGCGTTTTCTGGGCGGGGCATGGTGCTCTCGGGTTCAAGGCGGGATTGTGCGCG
AGAGAGCCATGGTAATGCCCTGCACCACCTTGTGGCGAGCAGGCTCACCACAGTGGGGGGGCGGGTTAATGCAGGA
TCTGGCTCAGGAACAACCGGGTCCGGTCGTTCTGCGGGTTGTCGAAGAAATCGTTCGGCGCCGCCTGTTCGACGAT
TTCGCCCTTGTCCATGAAGATCACGCGGTTGGCCACGGTGCGCGCAAAGCCCATCTCGTGGGTCACGCAGAGCATG
GTCATGCCGTCTTCGGCCAGGCCGATCATGGTGTCGAGGACTTCCTTGACCATCTCCGGGTCGAGGGCCGAGGTCG
GCTCATCGAACAGCATGATCTTGGGCTTCATGCACAACGCCCGGGCAATCGCCACGCGCTGTTGCTGGCCGCCGGA
CAATTGCCCCGGAAACTTGTGGGCCTGCTCCGGGATGCGCACGCGCTCCAGGTAGTGCATGGCAATTTCCTCGGCC
TTGCGCTTGGGCATCTTGCGCACCCACATCGGCGCCAACGTGCAGTTCTGCAGGATGGTCAGGTGTGGGAACAGGT
TGAAGTGCTGGAACACCATCCCCACTTCGCGGCGCACGGTTTCGATCTGCTTGAGGTCGTTGGTCAGCTCCACGCC
ATCGACCACAATGCGCCCCTGCTGGTGCTCTTCCAGGCGGTTGAGACAGCGGATGGTGGTGGACTTGCCCGAACCC
GACGGGCCGCACAGCACGATGCGCTCGCCCTGCTTGACGTTGAGGTTGATGTCTTTCAGTACATGGAACTGGCCGT
ACCACTTGTTGACGCCCTGCATCTGGATAATGCCTTCAGGGCTCATCGGCTGTTTGATCGCTTCGCTCATAACAAC
AACTCCTAACGCTTGTGGCCAGTGTCGAGCTTGTGTTCCAAATGAATGGAATAGCGCGACATGCCAAAACAGAAAA
TCCAGAACACCAGGGCCGCGAACACATAGCCCTCAGTAGCCATGCCCAGCCATTTCGGGTCGGCGGCGGCTTGCTT
GACGCTGTTGAGCAGGTCGAACAGGCCGATGATGATCACCAGGCTCGTATCCTTGAACAGCGCGATAAAGGTGTTG
ACGATGCCCGGTATCACCAGCTTCAACGCTTGCGGCAGGATCACCAGGCCCATGCTGCGCCAGTAACCCAGGCCCA
TCGCCGCCGCGGCTTCGTACTGGCCTTTGGGAATGGCCTGCAAGCCACCGCGCACCACTTCTGCCACGTAGGCCGA
CTGGAACAGGATCACGCCGATCAACGCCCGCAACAGCTTGTCGAAGTTCATGCCTTCAGGCAGGAACAGCGGCAGC
ATCACCGACGCCATGAACAGCACCGTGATCAACGGCACGCCGCGCCAGAACTCAATGAAGGTCACGCAGATCACGC
GAATCGCCGGCATATTGGAGCGCCGCCCCAACGCCAGCACGATACCCAGCGGCAAGGCGCCTGCGATACCGACGGT
GGCGATCACCAAGGTGAGCATCAGGCCGCCCCATTGGCTGGTCGCGACGTTGGTCAGGCCAAAGATCCCGCCGTGC
AGCAGGAAGAAGGCAATGATCGGGTACATCACCAGAAAGCCCAGGCCATAGATCGCCTTGGGTTTAAAACGCGAGA
TGAACAACGGCGCCACGCCGACGATCGCCAGCCACACGGTCAGGTCTACGCGCCAGCGCAGGTCGCCGGGGTAATA
GCCGTACATGAATTGCCCGAAGCGCTGCTGGACAAACACCCAGCAGGCGCCCTCCTTGGTGCAATCGGCCTGGGTG
GTGCCGACCCAGTTGGCGTCGAGGATCGCCCAATGCAAAATCGGCGGCACCACCAGATAGACCAGGTAGATCGCCA
GCAACGTCAGCAGGGTGTTGAACCAACTGGAGAACAGGTTGGCGCGCATCCACGCCATCGGCCCGAACACTTTGTT
CGGTGGCGGCATGTCAGGTTTGAAAATATGGGAAGTCATGCAGGTTTCCTCACCGCTCGATCAGCGCAATGCGCTT
GTTGTACCAGTTCATCAACAGGGAAATGCTGATGCTGATCGCCAGGTACACGCTCATGGTGATGGCAATGACCTCA
ATGGCCTGGCCGGTCTGGTTGAGCACCGTGCCGGCAAACAGCGAGACCATTTCCGGGTAGCCGATGCCGGCGGCCA
GGGATGAGTTTTTCGCCAGGTTCAGGTATTGGCTGGTCAGCGGCGGGATGATCACCCGCAGCGCTTGCGGGATGAT
GACCTTGCGCAGCGTAGGCCCCGGACGCAGGCCCAGGGAGCGCGCGGCTTCGGTCTGGCCGTGGCTCACAGACTCA
ATGCCCGAGCGCACGATCTCGGCAATAAACGCGGCGGTGTAGACGGTAAGCGCCAGGGTCAGTGCCAGCAGTTCGG
GGATCAATACCCAGCCACCGACAAAGTTGAAACCCGCAAGCCTTGGCATTTCCCAGTGCAGCGGCACACCGAAGAT
CAGGACGCACAACGCCGGAATCACGATGAACAGCGCCAGGCCCGCCCAGAACTTATGGAACGGTACGCCGGTGGCT
TCAAAGCGCTTGTTAGCCCAACGCGCCATCAGCACGATTGCCACGATGGCCGCCCCGACACTGGCCACAAACGGCC
AGAAACCGTCAGCCGCCAGCGCCGCCGGCATATTCAGGCCCCGGGCGCTCATAAAGAATGTATCGCCCAAGTTGTG
GCTGTTGCGCGGCAACGGCATGGTCAGGAAGACCGCGAAGTACCAGAACAGGATCTGCAACAACGGCGGAATATTG
CGGAACACTTCCACATACACCGTCGCCAGTTTGTTGATCATCCAGTTCGGTGACAGACGCGCCACCCCAATGATGA
AACCGAGCAGCGTCGCCAGGACCACACCGATCACGGTCACCAGCAACGTGTTGAGCAGGCCGATCACAAAGACCCG

GGCATAGCTGTCCGATTCGGTGTAGTCGATCAAATGCTGAGCGATGCCAAACCCGGCACTGCGCTCCAGGAAGTCA
AAGCCGGAGGTAATGCCCCGGTGCTGAAGATTGGTTTGCGTGTTGTTGAACAGATACCAGCCCAACGAAACCACCG
CGACGATGGTGATGATCTGAAATAGCCACGCGCGCACTTTGGGATCGCTGAAGCTGAGCTTCTGCTTGGGTGCGCC
GATTTGAGTTTGCATTGAGTGCCCCAGAAATAATGGAACAGAACATCACCCGGCGGTTGGCCCACCGGGTGATAGA
ACCATCAGCGATCAGCGCACAGGGGGTGCGTATTGAATGCCACCGTTGTTCCACAGCGCGTTCAGGCCACGGTCGA
TTTCCAGGGGAGTGCTCTTGCCCAGGTTGCGCTCGAACACTTCGCCGTAGTTACCGACCTGCTTGACGATCTGTAC
GACCCAATCCTTCTTCACTTTCAGGTCTTTGCCGTATTCGCCGTCAGCACCGAGCAGACGCGCGACGTCCGGGTTC
TTGGTGGCCTTGGCTTCGGCTTCAACGTTTTTCGAGGTGATACCGGCTTCTTCAGCGTTGAGCATGGCGTAGCCTA
CCCAGCGCACGATGGCCAGCCACTCGTCGTCGCCATTACGCACGACCGGGCCCAGGGGCTCCTTGGAGATGGTTTC
CGGCAGCACCACGTAGTCCTTCGGCGCGGCCAGCTTGCTGCGCTGGGCGAACAGTTGCGACTTGTCGGAGGTCAGC
ACGTCGCAGCGCCCGGACTCCAGGGACTTGGCGCTTTCATCGGAGGTGTCGAAGGTGATCGGGGTGTACTTGAGGT
TGTTGCCACGGAAGTAGTCGGAAACGTTCAGCTCGGTGGTGGTACCGGCCTGGATGCAGATGGTTGCACCGTCAAG
TTCCTTGGCACTTTTTACGCCCAGCTTGCTGTTGACCAGAAAGCCGATGCCGTCGTAGTAGGTGATAAAGCCCGGG
AATTTCAGGCCCATGCCCGCATCGCGGGAGCTGGTCATGGTGGTGTTGCGCGACAGGATGTCGACTTCGCCCGATT
GCAGCGCGGTGAAACGCTCCTTGGCATTCAACTGGCTGAACTTGACCTTGGTCGCATCGCCAAACACGGCTGCGGC
CACGGCGCGGCAGTAGTCAGCGTCGATCCCGAGGATCTTGCCGCTGGCATCCGGCACCGAGAAACCCGGCAGGCCG
TCGCTGACGCCACATTGCACAAAACCTTTCTTCTGCACTGCATCCAGGGTGGCGCCGGCCTGGGCCAGGCCACTGA
TCCCTAATACGGTCGCCGCGGTCACGATGGCCAGGGTGGACTTCAATACCTTCATTCAAACCTCCAGTTGCTCTTG
TTGTGTCGGAGCTCGAACCCCTGCCCACCCTTATGAGGCGATATCGACCCGTGTTGGCTTTTTTTGGGGTCAAGCA
GCAGAGGCTGTCGCTGTCATTGCAGCGGCTATCCCAGAGGCGGCAGTCACTGATAGTGTTACCGTCCCGGATAGTT
CTGACATCGACCTACACATAGCAAGCGCCGTACCAGAGTGTCGGCTGAGCCGTTTGCGCCCAGGATCTGTCGCAAA
ACAGTCAACCATGCGACATCCTGTTAACTGATCCACCGTCCACGCACTGCCCCGACGCCTCCCATCGGCGCGCACG
CACATTTTTGGAGCACACATGACCGACCCCTTGATTCTTCAGCCCGCCAAGCCCGCAGATGCCTGCGTCATCTGGT
TGCATGGCCTGGGTGCCGATCGCTACGACTTCCTGCCGGTGGCCGAAGCGCTGCAGGAAACCTTGCTGTCCACACG
CTTCGTATTGCCCCAGGCACCGACCCGCGCGGTGACCATTAATGGGGGCTACGAGATGCCAAGCTGGTACGACATA
TTGGCCATGAGCCCGGCGCGGGCCATCAGCCGTGAGCAGCTGGATGAATCGGCCAAAATGGTCATCGATTTGATCG
AGCAACAAAAAGCCAGCGGGATAGACGCCTCGCGAATTTTCCTCGCCGGTTTTTCCCAGGGTGGCGCCGTGGTCCT
GCACGCCGCGTTTGTGAAATGGCAAGGGGCCCTGGGTGGCGTGCTTGCCCTCTCCACCTATGCACCCACTTTCAGC
GATGAGCTGGAACTGTCCGCCAGCCAACAGCGCATTCCGGCCCTGTGCCTGCACGGCCAACACGATGAAGTGGTGC
TCAACGCCATGGGCCGCAGCGCCTATGAGCATTTAAAGCAGCGTGGTATCACCGTGACATGGCAGGAATACCCAAT
GGGTCACGAAGTGTTACCCGAAGAAATCCGCGACATCGGCACCTGGTTGGCCGGCCGCCTGGGCTGAAAACCGACC
ATTGTGTAGCCGCATGACCAACGCACTACGCCGCGCCCGATTCTTGCATTACACTGGCCGGCGTACATTCCTTAAC
CAATTGATGAGATGACCGTGCTCAAAGCACTCAAGAAGATGTTCGGTAAAAGCGAGGCTGAACCACTCGCCCCTGT
TCCCAGCGCCCCTGTCCAGACGTCAGGCAGCCGCAACGACGGTAAACAGCCTGGCCGAACAGCGCCTGTTGCCTCG
CCAAAATCGCCGCCCGTAAAGCCTGCTGTCCCGGCACCCGTCGCCGCCCAGCCCGCAGAAGAACCCGCGCGTGTGG
AAAAGCCGCGCCGCGAACGCGCGCCAAAACCGGTGGTAAAGCCGTGGAAGCTGGAAGACTTCGTGGTCGAGCCCCA
AGAGGGCAAGACGCGCTTTCACGACTTCAAGCTGGCCCCGGAACTGATGCACGCCATCCAGGACCTGGGCTTCCCG
TACTGCACGCCGATCCAGGCACAGGTATTGGGCTTCACCCTGGCCGGCAAAGACGCCATCGGCCGCGCCCAGACCG
GTACCGGCAAGACCGCTGCGTTCCTGATCTCGATCATCACCCAACTGCTGCAGACCCCGCCGCCCAAAGAGCGCTA
CATGGGTGAACCTCGGGCCCTGATCATCGCCCCGACCCGGGAGCTGGTGGTACAGATCGCCAAGGACGCCGCCGAC
CTGACCAAGTACACCGGCCTCAACGTCATGACGTTCGTGGGCGGCATGGACTTCGACAAACAACTCAAGCACCTGG
AAGCGCGGCACTGCGACATCCTGGTCGCCACCCCGGGCCGTTTGCTGGACTTCAACCAGCGCGGCGACGTGCATCT
GGACATGGTCGAGGTGATGGTGCTGGACGAAGCCGACCGCATGCTCGACATGGGTTTTATCCCGCAAGTGCGCCAG
ATCATTCGCCAGACCCCACCGAAAAACGAGCGCCAGACCCTGCTGTTCTCCGCGACCTTCACCGAAGACGTGATGA
ACCTCGCCAAGCAGTGGACCACCGACCCGTCCATCGTCGAGATCGAAGCGCTGAACGTCGCCAGCGAAAACGTCGA
ACAGCATATCTATGCCGTGGCCGGTGCCGACAAATACAAGCTGCTCTACAACCTGGTCAACGATAACGGCTGGGAG
CGGGTCATGGTGTTTGCCAACCGCAAGGACGAAGTGCGGCGCATTGAAGAACGCCTGGTGCGCGATGGCGTCAATG
CTGCGCAGTTGTCCGGTGATGTGCCGCAGCACAAGCGGATCAAGACCCTGGAAGGCTTTCGCGAAGGCAAGATCCG
CGTGCTGGTGGCCACCGATGTGGCAGGCCGTGGGATTCATATCGACGGCATCAGCCACGTCATCAACTTCACCCTG
CCGGAAGTACCGGACGATTACGTGCACCGGATCGGCCGTACCGGGCGTGCCGGCGCGGCGGGTGTGTCGATCAGCT
TTGCCGGGGAAGATGACTCGTACCAGTTGCCATCGATCGAGACGCTGCTGGGCCGCAAGATCAGTTGCGAAACGCC
GCCGACGCACCTGTTGCGGGCCGTGGAGCGCAAACGCCCTTAAAACGGGTGCAGGAGCGGGCTTATGTGGGAGCGG
GCTTGCCCGCGATAGCGGTGTGACAATCACTGAAGTGATTGACTGACACACCGCATTCGCGAGCAAGCCCGCTCCC
ACATTTGCCTTCAGCGCTTTCAAATTCAAGGAGGACCACATGAACCAGGCTGATTACCTCATCATCGGCGGCGGTA
TTGCCGGTGCCAGCGCCGGTTACTGGCTGTCCCGACACGCCCGCGTCATCGTGCTGGAGCGTGAGTCCATGCCGGG
CTATCACTCCACTGGCCGCTCCGCCGCCCTCTATATCGCCGCCTATGGCACGCCACAAGTGCGCGCCCTGACCCTG
GGCAGCCGCGACTTCTTCGATCACCCACCTGCAGGTTTTACCGAGCACCCTTTGCTCACGCCCCGTGGCGAAGTGC

TGGTGGACCTGCTTGGCGACCCAGAAGAGCTGCAGCGCCAATACCTGAGCGCCAAGGCGCTGGTACCCGAAACACG
CCTGCTGGACACCGAAGAAGCGCTGCAGATGCTGCCGATCCTGCGCCGGGAAAAAGTCCATGGCGCGATCTACGAC
CCGACCGTCTGCGATATCGACACCGATGCGTTGTACCAGGGCTATTTGCGCGGCATTCGGCGTAACGGCGGGGCTA
TTCATACCGACAGTGAAGTGCAAAAGCTCAACCGTGACAGTCAGGGCCAGTGGCAAGTGCGCACCTCACAACAGCG
CTACAGTGCGCCAGTGATCATCAACGCCGCCGGGGCCTGGGCCGATACCATCGGCGAGCTGGCCGGTGCACAAAAA
ATCGGCCTGCAGCCCAAGCGCCGCAGTGCCTTTGTGTTCGCCCCGCCTGCCGAAATGAATATCCATGGATGGCCGG
AGTTGGCCGCCCTCGACGGTTCGTTCTATATGAAGCCCGACGCGGGGATGTTCCTCGGCTCACCGGCCAACGCCGA
CCCAGTCGAGCCCCACGACGTGCAGCCCGAAGAGCTGGATATCGCCACGGGCATCTATCACATTGAAGAGGCCACG
ACCCTGACCATCCGCCGCCCAGCCCGGACCTGGGCGGGGCTGCGCAGTTTTGTCAGCGACGGCGATCTGGTCTGTG
GCTTTGATCCGCAGGTAGAGGGTTTTTTCTGGATCGCGGCCCAGGGCGGCTACGGGATCCAGACCTCGCCGGCGAT
GGGTCAGGCCAGCGCGGCGCTGGTGCGTGGGCTCGGATTGCCCGAGCCTTTGCAGCGCGCCGGGCTGACCGAGGCG
ATGTTATCGCCTGGTCGCCTGGGTTGATCGTGCTGCTGGACTCGTCCAATACAGCGGCCAGACGATAGGGTGTGAC
GGGCACACGGAAGGCCACTCACGCCTTCCAGCGATCAGCCGCCCTATGATCACTGGCCCGCCCTTCCACCCAGCGC
GCCCCTTCGCTGGTATTTTCCTTCTTCCAGAACGGCGCTCGGGTCTTCAGGTAGTCCATCACAAACGCACAAGCAT
CAAACGCGGCCTGTCGATGGGCACTGGCGGCGGCGACGAACACAATAGGTTCGCCGGGCTCCAGGGCGCCGATGCG
GTGCAACACCTCGAGCTTGAGCAACGGCCAGCGTTGCTCGGCCTCAACCGCGATCTTGGCCAGGGCCTTTTCGGTC
ATGCCGGGGTAGTGCTCAAGGAACATTCCCGCCACGTCGAGGCCATCGTTGAAGTCGCGCACATAGCCAACAAAAC
TCACCACGGCGCCCACACCCACATTGGCGGCGTGCATGGCGTTGACTTCGGCGCCGGGATCGAACGGTTGGGTTTG
TACACGAATGGCCACGGTCAGCCTCCGGTCACGGGCGGGAAAAATGCCACTTCGTCGCCGGCCTGCAGAGGCTCGT
CGAGGCCACACAACTCCTGGTTGCGCGCGCACATCAGGCTGGTCTCGTTGAGCACCGCAAAGTCTGGGTCACTGGC
CAGCGCCAGGCGTACGGCGTCGACCGTGGCGAAGTCGCCTTCGACCTCCAGGGAATCGAGGCCCACCGCTTCGCTG
TAACGGGCAAAAAACAAAACGTTGATACTCATGCCTGGTCCGCCTTGAAATGCCCGCTCTTGCCACCGAGTTTTTC
AAGCAGGCGAATGTTTTCGATGGTCATGCCACGGTCCACCGCTTTGCACATGTCGTAGATGGTCAGCGCGGCGACA
CTGGCCGCCGTCAACGCTTCCATCTCCACGCCGGTTTGCCCGGACAGCTTGCAGCGCGCGACGATATGCACGGTAT
TTTCCCCTTCGGCGGCCAGCTCGACCTTGACCCCGGTCAGCATCAGCGGATGGCACAGGGGGATCAGATCACTGGT
CTTTTTCGCGGCCTGGATCCCGGCAATGCGGGCCACGGCGAACACGTCGCCCTTGGGGTGGGCGCCGTCGACGATC
ATTTGCAGGGTCTGGGGCAGCATGCGCACCCGCGCTTCGGCCACGGCTTCACGGAACGTCACGCTTTTGTCGGTGA
CGTCGACCATATGGGCACGGCCCTGGGAATCGAGATGAGTCAGCACGGGGATACTCCTGATCAGAAGCCCCGATTG
TAAACCCAACGCCGATCAAAATGTGGGGGCTGGCTTGCCTGCGATAGCGCTGTGTCAATCAACCTATCATCGACTG
AACCACCACTATCGCGGGCAAGCCCGCTCCCACACTGAACGCGGTTACAGATGGGATTCGGCGTATTCGGCCAGAA
TCGAGCGTGGTACCCCTTGCAGCGCAATGTGCACGCCGTTCGGGAAGTCCTTGAAGCGTTCCGTCAGGTAGGTCAG
CCCGGAGCTGGTCGCGGACAGGTAAGGGGTGTCGATCTGTGCCAGGTTGCCCAGGCACACCACTTTGGAACCGGCG
CCGGCACGGGTGATGATGGTTTTCATCTGGTGCGGTGTGAGGTTCTGGCATTCATCGATCAAAATCAGGCTCTGCT
GGAAGCTGCGACCTCGGATGTAGTTGAGGGATTTGAACTGCAACGGCACTTTGCTGAGGATGTAGTCGACGCTGCC
ATGGGTGTTTTCGTCATCCATATGCAGGGCTTCGAGGTTGTCGGTGATCGCCCCCAGCCACGGCTCCATTTTTTCT
GCTTCGGTGCCGGGCAAAAAGCCGATTTCCTGGTCCAGGCCCTGCACGCTGCGGGTGGCAATGATGCGGCGATAGC
GCTTGGTGACCATGGTCTGCTCGATGGCAGCCGCCAGGGCGAGGATGGTTTCCCCGAGCCGGCAGCCCCCGTCAG
GTTGACCAGGTGGATGTCCGGGTCGAGCAGCGCGTACAGCGCCAGGCTCTGGTAGATATCACGCGGTTTCAGGCCC
CAGGCTTCCTGGTGCAGCAGGGGTTCCTGATGCAGGTCGAGGATCAGCAGCTTGTCGACCTGAATCTCTTTGATCC
AGCCCACAAAGCCCTGTTCGTCGATGATGAATTCGTTGATGTGCACGGCCGGCAGGTTGTCGGTCAGCTGCACCTG
GTGCCAGGTGCGGCCATGGTCCTGGCGGGTCTCGACCTTGCTGACGCGGTCCCAGAACGAGCCGGTCATGGTGTGA
TAACCACGGGACAGCATCGACACGTCGTCGACCAGTTGGTCGGTACTGTAGTCCTCGGCAGCGATCCCACACGCTC
GTGCCTTGAGGCGCATATTGATGTCTTTGGTCACCAGCACCAGGCGCAGGTCCTTGTCGCGCGCATGCAGGTCGAT
CAATTGGTTGATTATCTTGTTGTCGTTGAGGTTTTCCGGCAGCAGGCTGTTGGGTTCGCTGCGCTTGCTCATCAGG
ATCGACAGCAAGCCCTTGGGCCCGCTCTTGCCACGCTGGATCGGTACGCCGACCTCAACATCCTCGGGCGAGGCTT
CACCCAGGGTCTTGTCGATCAGGCGGATCGCCTGGCGGCATTCGGCGGCGACGCTGTGGTGCCCACTTTTGAGTTT
GTCGAGTTCCTCAAGCACGATCATCGGGATGGCGACGTGGTGTTCTTCGAAATTGAGCAACGCGTTTGGATCGTGG
ATCAAAACATTGGTATCAAGCACATAAAGGATTGGCTGGTCGGAAGAAGGGGTACGTCCATGATCATCCATACTCG
GTCACCTTTGTGGGAGCCAGTCGACGCAATACCACAACAGTGCTGCGCCTCGATTCGACCACCGAATGCACCTGAG
GGACGTCAAGTGCATTGCCCACCTGAGGAGTCTGGGAAGACGCCACCTGTGGTGCAGGTTTCGGCGATCTGTCTTC
GTAATACCGCAAAACCCATGACAGGAAAAAGCACTTTGACGCTTTTTTGAAGTTTATTTTTCAGGATGACGAATAG
CACTGGCGGGGCGCCACGCACACCGCTAAAGTCGGAAGTCCGCCTGCATCGAAATGTCGCAGCAAATCACCCCGAA
AAACCCTCATCCCCAGCAGGGCCGGGCACTTCAGCGAAATGCCTCGCGACACTCCTGCCAACCCAAGCCACTCTGC
GCCGTTGCCTGCAACAACATCGGCAATGCCACCCGCGCCTTGTCCTGGGTGTCGTGGAACACAATCACCCCTTTGC
GCCACAACAACATCAGCGTCAGCACCCGCTGGGCCGACTCGTCGGCCTTGAGCTGGCCGGGCTCATCCTGGGAGTC
GATATCCCACAACGCCACTTGCAGGCCCTGGACCTGGAAGAAGCCCTGGCTGTCGGCCCGGCGCTGGCCATAGGGC
GGACGGAACAGCGGCACATAGTTTTCCGGCAGCAGGTTCTGGGTCAGTGATGCACTGCGGGTGATCGAACTCTGCC

AGTCGACCCAATGGCTATGGGAGCGATACTGCCAGCCCTGGATGCCCACGCACTGGCCTTGATACAACGCCTGCAC
ATCAGCGGCCGACGTTTTCTCCAGGCGGGTTTGCAGGCTGTTGCCCAGGGCAAAGAAGGTGGCCGTCATTTTCTGC
TTGCGCAGGTAATCGGCCAGCCAGCCGGTTTGGCCGTTAAGTGGCGCCGGGCCGCCATCGAAGGTCAATAGAAACA
GGCGGTCGTTGAGTTCATCGCCATTGCGCTCGAAATCACCAAACCGTGCGACTTCGCTGCTGATCTGCGGGAACAG
AGCCGCCTTGCGCAGCAACTCGTCCAGATAGCGCTGGTGAAAGGTGCGGCTAGGGCCGGCCCAGCCTATGTAGAAG
GATTGGTCGCTGACCTCGAATTTGCCGGCCTGCTCGCGCAGGTCGTCCATGTTGTCCACCAGGTAGCAGAACGAGG
CGTCCTGTTCGCAACTTTGCTGAGCAAATCCGTAGTTCTCCAGCAACCGTTGCCAGAGGTGGCGACGCAGGTCGTC
GATGGCGCCCTGGTTGATGATCTTCAGCCCCAGGCGTTGCTTGAGGGCCGCCTCATCCAAGGCCTCGCTGGCCAGC
AGCGTGTGGGCGAACATGAGGATTTCGGCGCGTGACGCCACATCGAACAGCGCCGGGCTGCTGAGTTTTTCCGGCC
AGGTGCCACGGTCGAGGCTCGCGACATCTACCGGGGCGGCGTGGACATTCAGGCACAACAGGCAAACCGATAATAA
AAGCGTTATTCGCACGCGGGGTCTTCCTCTGCAGAATTACCGGCCATGATAGCGGACTGTCACCGTTGCCGTTGTC
CTGGCTTGGAATCGCTTCGTTAAACACATTGGCCGAACTCAAGCGCATTGCTGATTTGTAACGACGCCGCAAAAGT
ATCAAAGGCGTGTTCCGTCATACACCTACCTAGCCGAACGGACAGTTGTGCAGTCTGCGCTGCCTATGCAACAAAT
AAATACGATGTATTTATCGCAACCCGAACAAAATAAAAGAATAGCAAGCACAGACCCAAAGAACGCGAGACAGGTG
TCAAGGGGGATTAATGAATATAAGTAAAATAGAATCCTACTTAACAAGCGCAGAGATAGAATCAGCCCACAGATGT
GCTACGAGGCTAATTTTATCACTGCCGAATAAAGTGAAAATGAATAACAATCGAGTACTCCTTGGCTATGGTGGAG
GAAAAGACAGCAGCTACACTGTAGCCTGGGTCAGATATATACAACATATCACGCTGACGGCATCTCTAACCCCCTG
CACACCGATAATATATTACGCGATGGGAGCGGCTCAGGACTTGCCGTCACCGCAATCAAACACCAACTAAGTTGTG
TCGTTTGCCTACAAGAATGCACTCAAGGCGGCAGGCTGCGTATCCATCAAAAAATATGGGACCCGTCTCATGAGCG
TTTTAAAGTTAAAGGGGAGCTGGGATATCGTGAAGATGTGATCAGCGGTAGCAATGCCTGTGACTACTCCCCTAGG
CGAGGCGATATCTACGCATTCAATCCCAATCATTACCATCAAATCCTTGCTGTTTCAGGTACGACCCGTATCACAA
TGGGGTTTTTCCTAGGCAACGTTGATGACGATATGAAAAAAGCTATTGCGTGGAGTTAGGCAGCAGGTACTTACGC
CCACCCTGCAGTACTGAGGAAGTCAACATGTTCATTAAGGATATTTTTTTAGCGATCAGCGTAATGCTCATTTGGG
GGCTGAATTTTGTTGTAATCAAAATAGGCCTAAACCATATGTCACCCTTTTTTTTGGCCGGTTTGAGATTTGCGCT
TGTCGTATTCCCGGCCGTTTTCTTCATACCGATGCCCAGAGCCCCCCTCAAATGGCTAATCATTTATGGGGTAACG
ATCAGCTTTGGCCAGTTTGCGTTACTATTTTTAGCAGTCCAGTTAGGCATGTCTGTTGGCGTAGCATCACTGGTGA
TACAGAGCCAAGCATTTTTTACAATGTTTCTCGGTGTTTTATTGTTATCTGAGAAACCAGGAAAGAGCCAGATGAT
CGGTGCGTTGGTTGCGTGCATGGGTATAGCATGTCTGGCCAAAGACGCCGTGGGCGGTGATTTTCGAGTTGGAAAC
GTCTCTCTATTGACATTATTTTTAACGATTGGCGCAGCGGCGTGTTGGGCAATGGGAAATATCGCCAACAAGATAA
TTCTACGCCGATGGCAAGTGCCCACTCTTTCGTTAGTAGTGTGGAGTGCATGGGTGCCTATTGTTCCATTTTTTGT
CTGCTCATGGATGTTCGAAGGGGATATTTCTATGCTGACTCGCGTAATGGAAATAAATACTCAGACGATTCTGTCA
CTGATCTACTTATCTTTTTTCGCGACGATCATTGGCTATGGTATCTGGGGAGGGCTGCTCAAGCGTTATGAAACTT
GGCGCGTTGCACCCTTTTCACTATTGGTCCCAGTGGTCGGCCTGACGAGTGCGTCGTTGATATTGAAGGAAGTGCT
ATCGGCAACCCAGATGCTCGGAGTGGCAATTGTCGTGATCGGCCTACTCATCAATACTTTTGGTGGATATTTACAT
ATAAGTAAAAAATCTTAACCAACTAGCGAGACCTCATAAACTTTTTACGACATCTGCCCCCCCCCCAACTAAAGCT
TCAGGTATTAACATGTTAATCCCCAACTGCATGTCCTCTTGGCTACCGAACTGAGCTTTGAAAAGGTCCGTCGGCT
AGTAGGGCAGAACCTGATCGGAGAGATGACCTGACCCTCCTTTTGCCGACGCCTGATTTGGAAACTCTTGGCGCTT
TCGAATTGATCCAAATTCCGTCGGCTCGAACGCCGCTATCGCAGCATAAATACACGCCACAAGAGCTGGCGAGGTT
TGCAAAAGAGGCCATCGGCTGGGTAGCCAGCCGGTGACTCAAAGATGCGTAGATACCTATGGCTACACAGGCAAGC
CAGCCATATCAATCCGTGTCACGCCAGTTTTGGTGCCTATCGCCATCATAGGTGGAGTCAAACCGCCCTACCCCCT
AGAATCCCCCGACGATTACAGGAGACGACTTCATGCTGATGGTGATTTCCCCCGCCAAAACCCTCGATTTCGAGAC
TGCGCCTGCGACCCAGCGCTTTACCCAGCCGCAATACCTCGACCACTCCCAGGAATTGATCGAGCAGTTGCGCGAA
CTGAGCCCGGCGCAGATCAGTGAACTGATGCATGTTTCCGACAAGATCGGCGGCCTCAACGCCGCACGTTTCGGCA
GCTGGAGCCCGGCCTTCACCCCCGCCAATGCCAAGCAGGCGCTGCTGGCGTTCAAGGGCGACGTGTACACCGGCCT
GAATGCCGAAACCTTCAGCGATGCCGACTTCAGTTACGCCCAGGACCACCTGCGCATGCTCTCCGGCCTGTACGGC
CTGCTGCGCCCGCTGGACCTGATGATGCCGTACCGCCTGGAGATGGGCACCAAGTTGGCCAACGCCCGTGGCAAGG
ACTTGTACGCATTCTGGGGCACACGGATCAGCGAGTGGCTCAATGAAGCCCTGGCTGAGCAAGGCGATGATGTACT
GCTCAACCTGGCGTCCAACGAGTACTTCTCCGCGGTCAAGCGCCCGGCCCTCAACGCGCGGATCATCAACACCGAG
TTCAAGGACCTGAAGAACGGCCAGTACAAGATCATCAGTTTCTACGCCAAAAAGGCCCGGGGCATGATGAGCCGCT
TCGTGATCGAAGAACGCATCAACGACCCGGCCCAGCTCAAGCAATTCGATGTGCAGGGCTACCGCTTCAATGCCGA
GCAGTCCAAGCCGGATAACCTGGTGTTTTTGCGCGATCACGCACCGGAATAGGTCGACAGCACAACGCAAGTCCCA
TGTAGGAGCTGGCTTGCCTGCGATAGCGGTGGGTCAGCTGCACATTCAGCAACTGGCAGACCGCTATCGCAGGCAA
GCCAGCTCCCACATTTTTATCCGCATTCCCCCAATGAAATCGTCATTATTTTGGCGCCAAAAATATTTCTTCAGAA
TTCATAACGTTTCTTTCACTCGACATCCGTCAGTTTTTTTCGTAGTGGCACCACTTTTTTCAAACAGTAGTGGCAT
AAAACTATATCCCCGCGCCAACTCCCTATATCCACTAGCCCAATCCGCAAGTGCTATCAATATAGTACTAGTGCCA
TCTTTGCTTATATTTCAAGAAATTTCGAGAAACAGGATGAGCGGCCAGGAACTATGTCTGCGAGTGCCGCTCATAC
CACCGGTAACGATTATCTCTGTTCTTGTGCGATATGACTTACACAGCGACCAACGAAAGTAGCAAAGTTGTCATAT

ATAACTTTGACTGCTCGGTCTAATTACCAACTGTTTAGTTGAGGGCGGGCGATCAGGTTGCATGACCATCCCCTAC
AAGGCCAGGGCTAAAGGCTACCTAAATGGGTCAGTGAGACAACCCAAGGCATTGATAGATAAAGGCTAAATGCCAA
TATCAAGGCTTTGTGGCACTGACGCCAGACCCTTCTCACAGAAGACTGGCTGCATTTCAGGGCAAGCCCCAACAAC
AAGGCCAAGTTGCGCACAACTTGAGTGCATTAGTTAGTCACTCGACTTTTAACATGCCTGTACATGGCAATGCAGT
GTCTATTCACGGCATTTCCTAGTGCATGAGTGACGCTTGAAAACATGAACTCCGGCCATTTAATGGCGTGATTAAG
ATAGAGGTAAATGCGATGCGCATCAGCATATTTGGTTTGGGTTACGTGGGCGCAGTATGTGCCGGTTGCCTGTCTG
CACGGGGCCATGAAGTGGTCGGTGTAGACATTTCCAAGGATAAGATCGACCTGATCAATGCGGGCAAATCCCCCAT
CGTTGAACCGGGCCTGGGCGAACTATTGAGCCAGGGCATTGCCAGCGGCCGGTTGCGCGGCACCACCAACTTCGCC
GACGCCATCCGCGATACCGACCTGTCGATGATTTGCGTCGGCACGCCCAGCAAGAAAAACGGCGACCTGGAACTCA
ACTACATCGAAGCGGTGTGCCGCGAAATCGGTTTTGTCCTGCGTGACAAGACCACCCGCCACACCATCGTGGTGCG
CAGCACCGTGCTGCCCGGCACTGTGGCCAACGTGGTAATCCCGATCCTCGAAGACTGCTCCGGCAAAAAAGCCGGC
GTCGACTTCGGTGTCGCGGTCAACCCGGAGTTCCTGCGCGAATCCACCGCCATCGCCGACTACGACCTGCCACCGA
TGACCGTAATCGGTGAACTGGACAAAGCCTCGGGCGACGTCCTGCAGTCGCTGTACGAAGAACTCGACGCCCCGAT
CATCCGCAAGGACATCGCCGTTGCCGAGATGATCAAGTACACCTGCAACGTGTGGCACGCCACCAAAGTCACCTTC
GCCAACGAAATCGGCAACATCGCCAAGGCCGTGGGCGTCGATGGTCGCGAAGTGATGGAAGTGGTCTGCCAGGACA
AGACCCTCAACCTGTCCCAGTACTACATGCGTCCGGGCTTTGCCTTCGGCGGCTCGTGCCTGCCCAAGGACGTGCG
CGCCCTGACCTACCGCGCCAGCTCCCTGGATGTGGAAGCACCGCTGCTCAACTCGCTGATGCGCAGCAACGAATCC
CAGGTGCAGAACGCCTTCGACATCGTCTCCGGTCACGACAAACGCAAGGTCGCCCTGCTGGGCCTGAGCTTCAAGG
CCGGCACTGATGACCTGCGCGAAAGCCCACTGGTGGAGCTGGCGGAAATGCTGATCGGCAAGGGCTTCGACCTGAG
CATCTACGACAGCAACGTCGAATACGCCCGTGTGCATGGGGCAAACAAGGATTACATCGAAGGCAAGATCCCCCAT
GTGTCGTCCCTGCTCAACTCGGACTTCGACGATGTAATCAACAACAGCGACGTGATCATCCTCGGCAACCGTGACG
AGAAATTCCGCGCCCTGGCGCAGAACGCCCCGCACGGCAAGCAAGTGATCGACCTGGTGGGCTTCATGTCCCAGGC
CACCAGTGTCAATGGCCGTACCGAAGGCATTTGCTGGTAACCGCGCTGATCGCCGGCAAGCCGGCTCCTACAGGTC
GCGCATGTCATGTAGGAGCCGGCTCGCCGGCGATGGCGCCCCACTGTAAGCCTGCCACAACCCCATCGGGCCACCC
CACAGGCTCGCCCGAGACTCCAGACGGATGCAGATTATGCACAGGCTAAAGCACGGCCTCCTTCAGGCCGCCGGTT
GGCTGTTGTTTTTAAGCGTGTTGATGTGCCTGGCCCTGGCGTTGCCGGCGAGCACATTCGACTCGCAGTCGAGAAA
TTTCATCTTCCTGATTGGCGCCGTCGGTATCTGGCGCTACTCCATGGGCGCCACGCACTTTGTACGCGGCATGCTG
TTCCTGTACGTGGTCTACCCGCACCTGCGGCGCAAAGTGCGCAAGCTGGGCAAGGCGGCGGACCCCTCCCACGTAT
TCCTGATGGTCACCAGTTTTCGGATCGACGCGCTGACTACCGCCCAGGTCTACAGCTCGGTGATTCGCGAGGCCAT
CGAGTGCGGCTTCCCTACCACCGTGGTCTGCTCCCTGGTGGAAATGTCCGATGAGCTGCTGGTCAAGAGCCTCTGG
GAAAAGATGAACCCACCGGATCACGTCAAGCTCGACTTCGTGCGCATCGCCGGCACCGGCAAGCGCGATGGCCTGG
CCTTCGGCTTTCGTGCGATCTCCCGCCACCTGCCGGACGACCGCGCCGTGGTTGCGGTAATCGACGGCGACACCGT
GCTGGCCGAGGGCGTGGTGCGCAAGACCGTGCCGTGGTTCCAGCTGTTCGGCAATGTCGGCGGCCTGACCACCAAC
GAGTTTTGCGAAGTGCGCGGCGGCTACATCATGAGCGAGTGGCACAAACTGCGCTTCGCCCAGCGCCACATCAACA
TGTGCTCCATGGCCTTGTCCAAGCGCGTGCTGACCATGACCGGGCGCATGTCGGTGTTCCGGGCCAGCGTCGTCAC
CGACCCCGGCTTTATCGCCGACGTGGAAAGCGACTCGCTGCAACACTGGCGCCTGGGCCGCTTCAAGTTCCTGACC
GGGGATGACAAGTCCAGTTGGTTCAGCCTGATGCGCCTGGGCTACGACACCTTCTACGTGCCCGATGCAGCGATCA
ACACCGTGGAACACCCCCCTGAAAAGAGCTTCATCAAGGCCAGCCGCAAGCTGATGTTCCGCTGGTACGGCAACAA
CCTGCGCCAGAACTCCCGAGCCCTGGGCCTGGGTATGCAGCGCCTGGGCCTGTTCACCAGCGTGGTGCTGTTCGAC
CAGCGTGTGTCGATGTGGTCTTCGCTGCTGGGCCTGACCGTGGCGATCCTCGCCACCTTCAAGTACGGCGGTGCGT
TCATCCTCGCTTACCTGCTGTGGATTGGTATCACGCGCCTGATCCTGACCCTGTTGCTGTCGTGCTCGGGCCACAA
GATCGGCCCCGCCTACCCGGTGATTCTCTATTACAACCAGATCATGGGCGCGCTGGTGAAGATCTACGTGTTCTTC
CGCCTCGATCAACAGTCCTGGACCCGCCAGGACACCAAACTGACCCGCGATTTGGCCAGCTTTCAACGTTGGTTCA
ACACCTGGTCGTCTCGGACCATGACCTTCTCCGCCGGCAGCATTTTCGTCGCCGTGTTGCTGATGATGGTCTGACC
CTGCCAAGCCTGAATTAACCTAGGAATTAGTACGCCATGAACAGCCAAGTAAACGCCAACGTTGTCCACGAATCCG
AAGCCCAGCGCCAACACGCCCGTGTCAAAATCCCGGCCAAGCTGCGCTTTTTCGGCGCCGACCGCACGCCCATGGA
AGTGCGGGTCATCGACCTCTCCGCCGGCGGCCTGGCCTTCAATGCCGCCCAACAACCGTTGAAAGTCGGTGATGTG
CACAAGGCACGCCTGCAATTTGTGATCGACAATCTGGGCCTGGCGATGGACGTGGAGCTGTTGATCCGCTCCCACG
ACCGCCAGACCGGCCGCACCGGTTGCCAGTTCCAGAACCTGGATGCCCAGGATATTTCCACACTGCGTCACCTGAT
CACCTCGCACCTGTCCGGCGACATCGTGACCATGGGTGACGTGCTGGCCACCTTGCAGCGCGACAACTTCACCAAG
GCGCGCAAGGTCAAGGACAGCGGCAGCGGCATGAGTGCCTTTGGCCGCCTGCGCGCCGTGACCTTCAGCCTGGCGA
TCTTCCTGGTGGGCCTGGCGGCGTTCGGGTTTGTGTTCAAGTCGGTGTACGGCATGTACTTCGTCAGCCACGCCCA
GGCCGGCCTGGTCAGCGTGCCGGGGATGAACGTCACGATGCCCCGCGACGGCACCGTGCAAAGCCTGATCAAAGAC
AACGGCGTTGCCGCCAAAGGCGCGCCACTGGCGACCTTCAGCACCAGCATGCTTGATGTGCTCAAGGGCCACCTGG
ACGAAGACCAACTGCAACCGGCCAAGGTCGAAGAGCTGTTCGGCAAACAAATGACCGGCACCCTGACCTCGCCTTG
CGACTGCATCGTCGCCCAGCAGTTGGTGGCCGACGGTCAATATGCGAGCAAGGGTGATGTGATCTTCCAACTGGTG
CCGCGCGGCAGCCAGGCCAACGTCGAGGCGCGTTTCTCCTATCGCCAGTTCGGTGATGTGCGCCCAGGCACGCCGG

TGAGCTTCCAGGTCGCCGATGAAGAACAGACCCGCACCGGCACCATCGTCAGCAGCACCAGCCTCAACAGCGCCGA
CCTGTCTTCGGATATCCGTGTGCAGATCAAGCCCGACGCCCCCCTGGACAGCGCCTACGCCGGCCGCCCGGTGGAA
GTGACCAGCGACCGTGGCCCGTCCCTGAACTGGCTGATCGATAAAGCCATGGCTGCTGGTTTGTAAGCGAGGACAT
GCCTGTGAACCCTATTTCAAGAACAACACAATCCCTGTGGGAACCGCCTGTTGTGGGAGCCGGGCTTGCCAGCGAT
GCAGGCGACGCGATGCATCAGACAAACCGCGACGATGCCATCGCAGGCAAGCCAGCTCCCACAAAAGCGCGCTCCC
ACATCCGCCCTGTGGCGCTGCTGGCATTGGCGGTCAGCCTGGCCGGTTGCGCCGGCCTGCCCGACCAGCGCCTGGC
CAATGAAGCGCTCAAGCGTGGCGACACCGCCACCGCTGCGCAGAACTATCAGCAACTGGCAGACCTGGGTTACAGC
GAGGCCCAAGTCGGCCTCGCGGATATCCAGGTCGGCAGCCGCGACCCGGCGCAGATCAAGCAGGCCGAGGCCACCT
ATCGCGCGGCGGCCGATGTATCGCCACGCGCCCAGGCCCGCCTCGGTCGCCTGCTGGTGGCCAAGCCCGGTGCCAC
CGAGGCCGAGCACCGCGAAGCCGAAAGCCTGTTGAAAAAAGCCTTTGCCAATGGCGAGGGCAACACCTTGATCCCG
CTGGCGATGCTGTACCTGCAATACCCCCACGATTTCCCCAGCGTCAACGCACAGCAGCAGATCGATACCTGGCGCA
CTGCCGGCTACCCCGAAGCGGGCCTGGCGCAGGTGCTGCTGTATCGCACCCAGGGCACTTACGACCAGCACCTGGA
TGACATCGAGAAAATCTGCAAGGCCGCGCTCAGCAGCACCGATATCTGCTACGTCGAGCTGGCCACCGTCTACCAG
AAGCGCGAGCAACCGGAGCAACAGGCCGAACTGCTCAAGCAGATGGAGGCCGGCTACAGCCGTGGCACCGTCACCG
CCCAGCGGGTCGACAGTGTGGCCCGTGTGCTGGGCGATGCCTCCCTGGGCAAGACTGATGAAAAAACCGCCCAGGC
CCTGCTGGAAAAAATCGCCCCGGGCTACCCGGCGTCCTGGGTCAGCCTCGCGCAGTTGCTCTACGACTTCCCGGAA
CTGGGCGACGTCGAGCAGATGATGAAGTATCTGGACAACGGCCGTGCCGCCGACCAGCCCCGCGCCGAATTGTTGC
TGGGCAAGCTGTACTACGAAGGCAAGTGGGTGCCGGCCGATGCCAAGGCCGCCGAAGAGCACTTCCAGAAAGCCCA
GGGCCAGGAAGTGGCTGCCGATTACTACCTCGGCCAGATCTACCGCCGGGGCTACCTGGGCCAGGTCTACTCGCAA
AAGGCCCTGGACCATTTGCTGACCGCGGCGCGCAACGGCCAGAACAGTGCCGACTTCGCGATCGCTCAATTGTTTT
CCCAGGGTAAGGGCACCCGGCCCGACCCGCTCAACGCCTATGTGTTCAGCCAACTGGCAAAGATCCAGGACACCCC
GCAGGCCAATGAGCTGGCCGCGCAACTTGAACAACAACTGCCGCCCGCCCAGCGCGCCGAGGGCCAGCGCCTGCTG
CAACAAGAGCTGGCCGCCCGTGGCGCCTTGAGCCAAAGCACGCTGCAACTGCACGCCCTGCAAGAAGAAGACGGCG
AGGAATCCCTATGAAACTCAATCCATTCGTCAAGGCCGGCATTGGCCTGACCTTCGCCCTGCTGTGGTCATGCCCG
ACCCTGGCCGCGCTGACCGAAGACAAAAACTTTGGCCTGGAAGTGAAAGTCACCGGCCAGTCCGAAGATGACCGCG
ACCTGGGCACGCAAAAAGGCGGCGACGTCAACGGCATCGGCCTGGATGTGCGCCCGTGGATCTACGGCGAAAGCGG
CGCCTGGAGCGCCTACGCCATGGGCCAGGCGGTGGCGTCCAGCGACATCATCGAGACCGACACCCTGCAACAGTCC
GATGGCGACACCACCGAGCAGTCGAGCAACAACGACCGCAAGACCAAGAAGAACTACCTGGCCCTGCGCGAGTTCT
GGGTCGGCTACAGCGGTTTCACGCCCTACCCTGGCGAGATCCTCAAGCTCGGCCGCCAGCGCCTGCGCAATAACGA
TGGGCAATGGCGCGACACCAATATCGAAGCGCTGAACTGGACCTTCGACACCACCCTGCTCAAGGCCAATGTCGGC
GCTGCCGAACGCTTCAGCGAGTACCGCACCGACCTCAAGGAACTGTCTCCCGAAGACAAGGACCGCCAGCACCTCT
ATGCCGATGCGGCCTACCAGTGGACTCCCGGCCATTGGGTCGGCGTGCGCGGGCATCACACCCACGACGACGGCAA
GCTCGACTACCCACAACCGGGCGTGGCTGCCGACCCACTGGACAAGCGCCAGAACGGCGACCTGACCTGGCTCGGC
CTGGAAGCCAACAGTGACGCCTACAACTGGCGCAACACCAATACCGTCAACTACTGGGCCAGCGTCACCGGTATGC
AGGGCGACCGCAGCAACGTCAACGCGTTGAACGCCGACGGTAGTCGCCCCACCAACGCCAAGCGCAACACCGATGT
AGACGGCTGGGCCACTGACCTCGGCGTGCGCCTGCGCCTCGACCCGCAGTGGCAAGTGGGCGCGGCTTATGCCCGC
GCCAGCGCCGACTACGAACAGAACGGCCTGCAAAGCAACCGCTCGAACTTCACCGGCACCCAGTCGCGGGTGCATC
GTTTTGGCGAGGCGTTTCGCGGCGAGATGAACAATATGCAGTCCATGAGCCTGTTCGGTTCCTGGCAGCTGCGCGA
CGACTACGACGCCAGCCTGGTCTACCACAAGTTCTGGCGCGTGGACGGCAACAAGCCGGTGGGCAGCAACGGCATC
AATGCCGTGCAGAACAACTACGACGATGTCACCGGTGCCGTGTTGTCCAGCGCATCCCTGCCGCTGGTAGACGGCA
ACAAGGACCTAGGCCAGGAGATGGACCTGGTGGTCACCAAGTACTTCAAGAAAGGCCTGCTGCCCGCAGCCCTCAG
CCAGTCGATCGACGAACCGTCGGCCCTGGTGCGCTTTCGTGGCGGCGTATTCAAACCGGGCGATGCCTATGGCAAG
CAAGTCGACTCGTACATGCACCGCGCCTTTGTCGACGTAGTCTGGCGCTTCTGATGGGAGCCTGCGCAATGAATCC
TCACGCCCTCAAAGGCTCGGCCCTGCTCGCTGCGGCAATGCTGCTGGCGTGCGCCCCGGCCTTTGCCAATGTCGAG
CCGCAGGTCAAGGCGCCCACCATCGCCAAGGAACTGCAACAGGCCAAGACCTACACCATCACCAGCCCGCCCACCG
CGCCGCTGGAGATGCCCAAGCCGGCGCTGCCGGACCTCTCGGGCTATACCGCACAAGCCGTGGCGAAGAAAATCGT
GCGCAGCAAGGCCGGCAAGGTCAGCGTGCGCCGGATGATGCAGGAAAACGCCCTCAAGGACTTTATCGGCGGCGAC
AACAAGATGGCCGAATGGGTGGTGCGCCAGCATGGCATCCCCCAGGCGATCTTTATCGACGACGGCTACATGAACC
TCAAGGACCTGCTCAAGAAGGTGCCCAAGCAATACCTGAGCGAAACCTCGCCCGGGGTATTCCTGGCCAGGCTGCC
GATTGTGGTCGGCGAAAAAGGCATCCTCGAAATCGATAACCAGACCCAGGAATTGCGCCTGTCCCAGGAGGCCGGT
TCGTTCCTGGTCAACGACGGCCAGCTGTTTGTGCGCGACACCAAGATCACCGGCTGGCGCGAGAAGAGCAACGGCC
CGGCGACCTTCCGCTCGCCCAAGGAATTTCGCCCGTTCCTGCTGGCCTGGGGCGGCACCCAGACCTATATCGTCAA
CAGCAAAATGGCCAGCTTCGGCTACGCCAACAGTAAGTCCTATGGCGTGAGTATTTCCCAGTACACACCGAACATG
GCCAAGGTCCTCAAGCGCCCGGAACCCACCGGCTGGATTGTCGATTCCGAGTTCTCCGACATGTGGTACGGCTTCT
ACTGCTACGAGACCACCGGCTTTGTGATCAAGGGCAGCACCTACAAAGACAACATCGTCTACGGCATCGACCCCCA
CGACCGTTCCCACGGCCTGATCATTGCCGACAACACCGTCTACGGCACCAAGAAAAAGCACGGGATCATTATTTCC
CGTGAGGTCAACGACAGCTTCATCTTCAACAACCGCAGCTACGACAACAAGCTCTCGGGCCTGGTGATCGACCGTA

ACAGCGTGAACAACCTGATCGCCGATAACGAGATTTACCGCAACCACACCGACGGCATCACCCTCTATGAGAGCGG
CGACAACCTGCTGTGGGGCAACAAGGTGATCAGCAACCGTCGCCACGGCATTCGGATCCGTAACAGCGTGAATATC
CGCCTCTACGAGAACCTCGCCATGGCCAACGGCCTGACCGGCGTCTACGGCCATATCAAGGACCTGACCGACACCG
ACCGCGACATTGAGCTCGACCCGTTCGACACCAAAGTCTCGCTGATCGTGGTCGGCGGCGAACTGGCGGCCAATGG
CAGCGGGCCGCTGTCCATCGACTCGCCGTTGAGCATCGAGCTGTACCGCGTGTCGATGCTCGCGCCGACCAAATCC
AGTGGCATCAGCTTCTCCGGCGTTCTTGGTGAGCGCCAGGAAGAGATTCTCGACTTGCTGGTACGCCAGAAAAAAG
CCGTGCTGATCGACCCTGTCGAACGCCAGACCCAATTGCAGGACTGAGGATGACCTTTATGCACCCACACATGATC
AAGCTGCTCAGCCTCTCGGGTTTGACCCTCGGCATCCTGGCCGCCAGCAGCGGCGTGCGCGCCGATGAAGCCAAGG
CGCCGAGCTTTACCGCCGAGCCGTGCTGCAACCTGTGCCCCGAGGCTCACGACGCAAAGAACTACACCACTCGCTA
CCAGCAGAACTTCACCACCCTGGTACAGGCCCAGGGCGACTGGCTGTTCCGTACCCAGGAAGACCTGCGCACCGAA
TTCGACACCACCCCGGCCGGCTACAAGCGCATGCAGCAACTGCACGATGCGTTCAAGAGCAAGGGCGTGGAGTTGG
TGGTGGTCTACCAGCCGACCCGTGGCCTGGTAAACCGCAACAAGCTCAACCCGGCAGAGAAAGCGCGCTTTGATTT
CGACAAGGCCCTGGGCAACTACAAAACCATGCTTGGGCGTTTTGCCAAGATGGGCTACGTGGTGCCGGACCTGTCG
CCGCTGACCAATGAACAACTGCCGGACGAACTGCCGGCCCACGACTTCTACTTCCGCGGCGACCAGCACTGGACCC
CCTATGGCGCCCAGCGCACCGCGAAAATCGTCGGGGCCAAGGTGCGCGCCATGCCTGAATTCGCCGGGATCCCCCA
GCGTGAATTCGAGACCAAGAAATCCGGACGCATGGGCAAGACCGGCACCCTGCACAACATGGCAGGGCAGTTGTGC
GGCACCAGCTACGCGATCCAATACATGGACCAGTTCAGCACCGAGCCCAAGGGCGAGGCCGCGGACGGCGACCTGT
TCGGCGATTCCGGCAACCCGCAGATCACCCTGGTGGGCACCAGCCACAGCGGCAAGAACTACAACTTCGCCGGCTT
CCTGGAACAGGAAATCGGTGCCGACATCCTTAACGTCGCCTTCCCCGGCGGTGGCCTGGAAGGCTCGATGATCCAG
TACCTGGGCAGCGAAGAATTCCAGAAAAGCCCACCGAAGATTCTGATCTGGGAGTTCTCGCCGCTGTATCGCCTCG
ACCAGGAAACCATCTACCGCCAGATGATGGCCCTGCTCGACAACGGCTGCGAAGGCAAGCCGGCGCAGATGAGCGC
CAGCACCACCCTCAAGCCCGGCAAGAACGAGCTGATGGTTAACAGCAAGAACATGGACCTGCGCAATAGCCGCCAT
CAGGTCGACATTCGCTTCGCCGATCCTTCGGTGAAAACCCTGCAAGCCACCCTCTGGTACATGAACGGTCGCCACG
AGGACATCAAGATCGACAAACCCGAAACATCCGAGACAGACGGGCGTTTCGCCTTTGAACTGCGCACCGATGAAGA
CTGGGCCTCGCAGAACCTGCTGGCCGTGGAAGTGCAGGGCCCCGAAGCGGGCGCTGCCGCGCAGAAAGTCGAAGCG
AAAATTTGCACACGCAACGTGTTCCCAAGCGGCGGTCAGCAGACCGCGCAACTTGGACAATGAGGTTACCTATGCA
CAAGTTACTGATTCCAACGCTGCTGGGCCTGGCGATGTTTGCAGGCCAAGTCAACGCCGCCGCGCCGCTGCGCCCG
CCCCAGGGCTATTTCGCGCCGATTGAAGCGTTCAAGACCGGCGACTTCAAGAACGACTGCGACGCCATGCCGACGC
CCTACACCGGCTCACTGCAGTTTCGCAGCAAATACGAAGGTTCCGATAAAGCCCGTTCGACCCTGAACGTGCAGTC
GGAAAAAGCCTTTCGCGACAGCACCGCCGATATCACCAAACTGGAAAAAGACACCAGCAAGCGGGTGATGCAGTTC
ATGCGCGACGGTCGCCCGGAGCAATTGCAATGCACCCTGAACTGGCTGGTGAGCTGGGCCAAGGCCGATGCGCTGA
TGTCCAAGGACTTCAACCACACCGGCAAGTCCATGCGCAAATGGGCGTTGGGCAGCATGGCCTCGGCCTATGTGCG
CCTGAAGTTCTCCGAGTCGCAACCGCTGGCCAAGTACCCGCAGGAGTCGCAACTGATCGAAGCCTGGTTCAACAAA
CTGGCCGAGCAAGTGGTCAGCGACTGGGACAACCTGCCCCTGGAAAAAACCAACAACCACTCGTACTGGGCCGCCT
GGTCGGTGATGGCCACGTCCGTCGCCACCAACCGTCGCGACCTGTTCGATTGGGCCGTCAAGGAATACAAGGTCGG
CGCCAATCAGGTCGACGCCCAGGGCTACCTGCCCAACGAATTGAAGCGCCAGCAACGGGCCTTGGCCTACCACAAC
TACGCCCTGCCGCCGCTGGCGATGATCGCCAGTTTTGCTCTGGTCAATGGCGTGGATGTGCGCGGTGAGAACAACG
GCGCGCTCAAGCGCCTGGGCGACAAGGTGCTGGCCGGGGTGAAGGATCCGGACATCTTCGAGCAGAAGAACGGCAA
GGAGCAGGACATGCATGACCTGGAGCAGGACTCCAAATTCGCCTGGCTCGAACCCTTCTGCACCCTCTACACCTGC
GCCCCGGAAGTGATCGAACGCAAGCACGACATGCAACCGTTCAAGACCTTCCGCCTGGGCGGCGACCTGACCAAGG
TCTACGACCCGGCGCACGAAAAAGGCCGTTGATGGAACGGGCTCCCAATGTGGGAGCGGGCTTGCTCGCGAAAGCG
GTCTGACATTCAGCATTGATGTCACCTGACACACCGTTATCGCAGGCAAGCCAGCTCCCACATTTCGAGCCGGTTT
CGACAGTGAAAAAGTAGTACCGCCCTCCCCGTTTTTTCGTGGGGGGTTTGGGGGGGCCGTTGGCCCTTGACTGTTG
GTTAAACATGGAGAGATCGGGATGGTTTTCTCGTCCAATGTGTTCCTGTTTCTGTTCTTGCCGATCTTTCTCGGCT
TGTACTATTTGAGCGGGCAACGCTATCGCAATCTGCTGCTGCTGATTGCCAGTTACGTGTTCTACGCCTGGTGGCG
GGTGGACTTCCTGGCGCTGTTCGCCGCCGTGACCCTGTGGAACTACTGGATCGGCCTCAAGGTCGGTGCAGCAGGC
GTGCGCACCAAGCCGGCGCAGCGCTGGCTGCTGCTCGGGGTGGCGGTCGACCTGTGCATCCTGGGCTACTTCAAGT
ACGCCAACTTCGGCGTCGACAGCATCAACCTGATGATGAAATCGGCGGGGTTGGAGCCGTTTATCCTGACCCACGT
GCTGTTGCCGATCGGGATCTCGTTCTACATCTTCGAGTCCATCAGCTACATCATCGACGTGTACCGCGGCGACACT
CCGGCGACCCGCAACCTCATCGACTTTGCGGCGTTCGTGGCGATCTTCCCGCACCTGATCGCCGGCCCCGTGCTGC
GCTTTCGCGACCTGGCCGACCAGTTCAACAACCGCACCCACACCCTGGATAAATTCTCCGAGGGCTGCACGCGGTT
TATGCAGGGCTTTATCAAGAAGGTGTTTATCGCCGACACCCTGGCGGTGGTGGCCGACCATTGCTTCGCCCTGCAA
AACCCCACCACGGGCGATGCCTGGCTCGGCGCACTGGCGTATACCGCGCAGCTGTATTTCGACTTCTCCGGCTACA
GCGACATGGCCATCGGCCTGGGCTTGATGATGGGTTTCCGCTTCATGGAAAACTTCAAGCAGCCGTATATCAGCCA
GTCGATCACCGAGTTCTGGCGGCGCTGGCATATCAGCCTGTCTACCTGGTTGCGCGACTACCTGTACATCACCCTG
GGTGGCAACCGCAAAGGCACGCTGACCACCTACCGCAACCTGTTCCTGACCATGTTGCTCGGCGGCCTGTGGCACG
GCGCGAACATCACCTACATCGTATGGGGCGCCTGGCACGGCATGTGGCTGGCGATTGAAAAAGCTATCGGCCTGAA

CACCTCGCCACGCAGCTTCAACCCGGTGCGCTGGGCCTTCACCTTCCTGCTGGTGGTGATGGGTTGGGTGATCTTC
CGCGCCGAAAACCTGCACGTTGCCGGGCGCATGTACGGCGCGATGTTCAGCTTTGGCGAGTGGTCGCTGTCGGAAC
TCAACCGCGCCAACCTCACCGGCCTGCAAGTGGCAACCATGGTGGTGGCCTACGCCACACTGGCGTTCTTCGGCCT
GCGCGACTTCTACAGCAATCGCCCGGCCGACAAGGCCAAGCCGGCTGACCCGAGCCTGATCAAGGCGGTGCCTGGC
GATACCCCCGGCAGCATCCAGGAACCCGGCTTCAGCGTCGGCAGCAACGCCGCCGTGCAACCGGCCTACTGGACTG
CCGACTGGCCACGCTATGCCATGCGCGCGGTGATCCTGCTGCTGTTCGTGGCTTCGATTCTGAAACTCTCGGCGCA
AAGCTTCTCGCCGTTCCTTTACTTCCAATTCTGAGGGAGCCGACCATGACCCGTTCATTCCGCATCCTCTACGCCG
CGCTGTTCATGGCCCTGCTGCTGGCCCTGGGCGCGTGGTCGGTGCGCAGTTTCTTCGGTTTCAATACCAACGCCGA
CGCCACCGTGCTCAACGGGCGCTGGGCCAAGGCGGTCGAAACCCATTACGACGATGAGTTCCCGATCAAACGCCTG
GGCACCAACCTCTGGGCGGCCCTGGACTACAAGCTGTTCAACGAGGGCCGTCCCGGCGTGGTGCTGGGTCGCGATC
ACTGGCTATACAGCGACGAGGAGTTCAACCCCGCCGTCAACGAAGAGCAGAACCTGCAAGACAACTACGCCCTGGT
CGAAGGCGTGCGCCAGACCCTCAAGGCCAAGGGTGTGCAATTGGTGATGGCAATTGTCCCGGCCAAGGTGCGCCTG
TACCCCGAGCACCTGGGTGAAGTGCAACCAGCGAGTATTCATGCCGGGCTCTACCAGGACTTCCATGCCCGGGTGG
CCGCCGACAAGATCATCGCCCCCGACCTGCTGGGCCCGCTGCAACAGGCCAAGCTCCACGGCCAGCAAGTGTTCCT
GCGCACCGACACCCACTGGACCCCGGATGGCGCCGAAGTCGCCGCCAAGCAACTGGCCAGCGTGATCAACGCCAAG
ACCCTGCTCAGCGGCGAGCCCCAGCGGTTTGTCACCGACGCCGAAAGCATCGCCCCCCACAGCGGTGACCTGCGCC
TGTTCCTGCCCCTGGACCCGCTGTTCGAAAACCTGATGCCGCCCAAGGAACCCCTGCAAAAACGCGTGACCCACGC
GGTGCAGGCCCAGGCGGCGGGCGATGATGCGTTGTTTGCCGACAGCGAGATGCCGGTGGCCCTGGTGGGCACCAGC
TACAGCGCCAACCCCAACTGGAACTTCGTCGGTGCCCTCAAGCAAGCCCTGGGCAGTGAAGTGGTGAACTACTCCG
AGGACGGCCACGGCCCGATCCTGCCGATGCTCAGCTACCTGAAAAGCGATGACTTCAAGAACAGCCCGCCGCAGGT
GCTGATCTGGGAGTTCCCGGAACGCTATCTGCCGGTGAACAACGAAATCGGCGACGCCGACCCCAAGTGGGTTGCG
CAACTCAAGCAAGCCGGTACACGCCAACAGAACATGGCAATCAACACACCCGTTAAAACCCAGAAATCCGAGACGC
CCGACCGGGCGCAAAACTGAAAGAGAGGTAACTCACATGACTTTCACTACTACTCCTCGTCGTCTCGCCAAGACCC
TGGCCCTGGCCGCCGGCATGAGCTTCGTGTCGATGTCCGCCTTCGCCGGCGACGCCGCCCTCTACGGGCCCATCGC
GCCGAAAGGCTCGAGCTTCGTGCGCCTGTTCAACGCCAGCAACCAGGAAGTCAGCGCCAGCGTCGGCAACACCGCG
TTGAGCGATGTGGCGCCACTGGCCAGCAGCGACTTCAGCTTCCTGCCGGGCGGTGACTACAGCGCCAAGGTCGGCA
GCCAGAACGTCACGGTCAAGCTGGCCGCCGATCACTATTACACCCTGGTCAACAGCGGCACCGGCCAGCCGCAACT
GATCGAAGAACCACCGTTCAAGAACAAGCAGAAATCCCTGGTGCGCGTGCAGAACCTCAGCGACAAGGCCCTGACC
CTGAAGACCGCCGACGGCAAGACCGACGTGGTCAAGGCCGTGGCCGCCAAGGGCCGTGGCGAGCGCGAGATCAACC
CGGTGAAGGTCAGCCTGGCGCTGTATGACGGCGATAAAAAAGTCGGCGACGTGAAGCCGGTGGCGCTGGAGCGTGG
TGAGGCGGCGGTGCTGTATGTCACCGGTTCCGGTTCGAGCCTGTCGCCAGTGTGGGTCAAGCGCCCCGTGTCGACC
CGCTAATTATTTCCTGAACTGACGGCTACCCACTGTAGGAGCGAGCTTGCTCGCGAAAATCGCCAACGATAACGCG
AGCATTCTGAATGAACGCGGTGTCTGGAAGTGCTTCGCGAGCAAGCTCGCTCCTACAGGGGATCAAGGCGTCAGTT
CGGACCGAGACAAAAACAAGAGTGAAACGACTGAATTTCGTTGCTCTAACCCATACAGATTCAAGGAGAAACCCAT
GATCCCAGTAATCCTTTCCGGTGGTAGCGGCTCACGTCTTTGGCCGCTTTCGCGTAAACAGTTCCCTAAGCAGTTC
CTGGCCCTGACCGGCGAGCACACGCTGTTCCAGCAAACCCTGGAGCGCCTGGTGTTCGAAGGCATGGACAGCCCGA
TCATTGTCTGCAACAAAGACCACCGCTTCATCGTCAACGAGCAGTTGAGCGCCCGCAAGCTGGAAAGCCAGCGCAT
CCTGATGGAGCCATTTGGCCGCAACACCGCGCCGGCCGTGGCCCTGACCGCGATGATGCTGGTCAATGAAGGCCGT
GACGAACTGATGCTGGTCCTGCCCGCCGACCATGTACTGGACGACCAGAAAGCCCTGCAACGCGCCCTGGCCCTGG
CCACCGTGGCCGCCGAGCGTGGCGAGATGGTGCTGTTCGGCGTACCGGCCACCCGCCCGGAAACCGGCTACGGCTA
CATCAAGTCCACCAACGACTCACTGCTGCCCGAAGGCGTCAGCCGCGTGCAGCAGTTCGTCGAGAAGCCGGATGAA
AAACGTGCGGTGGAGTTCGTCAAAAGCGGTGGTTATTTCTGGAACAGCGGCATGTTCCTGTTCCGCGCCAGCCGCT
TCCTCGAAGAGCTGAAAAAACACGACCCGGACATCTACGACACCTGTGTGCTGACCCTCGAGCGCAGCGAGCATAC
CGCTGATACCGTGACCTTCGACGAAGCCACCTTCGCCTGCTGCCCGGACAATTCCATCGACTACGCGGTGATGGAA
AAAACCCAGCGCGCCTGCGTGGTGCCGCTCAGTGCCGGCTGGAGCGACGTGGGCTGCTGGGCCTCGCTGTGGGCGG
TCAATGACAAAGACGCCAACGGCAACGTGACCAAGGGCGACGTGGTGATCCAGGACAGCAAGAACTGCATGATCCA
CGGCAACGGCAAGCTGGTGTCGGTGATCGGCCTGGAAAACATCGTGGTGGTGGAAACCAAGGACGCGATGATGATT
GCCCACAAGGACAAGGTCCAGGGCGTCAAGCAGATGGTCAACACCCTCAACGAACAAGGGCGCAGCGAGACCCAGA
ACCACTGCGAAGTCTACCGCCCGTGGGGCTCCTACGACTCGGTGGACATGGGCGGGCGCTTCCAGGTCAAACACAT
CTCGGTCAAGCCGGGTGCGTGCCTGTCGCTGCAGATGCACCATCACCGCGCCGAACACTGGATCGTGGTCAGTGGT
ACCGCTGAAGTAACGTGCGACGAGAACGTGTTCCTGCTGTGCGAGAACCAGTCCACCTACATCCCGATTGCCTCGG
TGCATCGCCTGCGCAACCCGGGCAAGATCCCCTTGGAGATTATCGAAGTGCAATCGGGCAGCTATCTGGGCGAGGA
TGATATCGAGCGGTTTGAAGATATCTACGGGCGCTCGACGCCGGTTGAGCGTGGCGTGTCGGTGAAAACTATCGCG
CAGTAGACCCCGCAAAAAACAGCCGCCTATCACCCTCTGCGTCCCCTATCCGCAGCGAGTGATGGGCGGTTTGTCT
ATTGGCGGTCAAGCGGTGTACGGCGTCTCGCCGCGAGAAATGACGCGACTCACCAGTTCACCGCTGGCGCCATCCC
TGTATTGCAACGGAATGGAGAAAGGGGTATCCACATAAAAAATATGTGGATGTCCGGGGACTTCCACCAACTTCAG
ACCGGTGGTGTTTATCTCAAAGTAATCGGTCATCTTATTCAGCGTTTTGCCATATTTGTTACGCCCGAAAATCATC

TGGGATATGACCTCCGTAGGCTTATCTTCAGGCGCCAAATCGGTGAAGTAATGCCTGCCCTGCCCTCTAGGCAGCG
GGCCACCTTTTAAATCGGTAGAGGACGGTGAAAAAGAGCGGTCAGCGACGAAAGCGGAATGACGACTTCGGTTGTT
GTATTTGTAGAAGACGTTCTTTCCTTTGATGGGAGGTTCCAGGGTGGCTTTTTCACCGGGAAAGGCTTCAGGTAAC
TTGGGGCGTTTTACGGTGGATTGCCCACCGGAAGTGGATGGACCCGAAGAACGGTCTGGCTCAGCAAGGCGTTTTC
GACCCGTGCTCACATCCCTATCGGTACTTTGGACCCCGCCCAGCATTCTGCTCGAACGCCATTGGCCATTAGCCAC
AGCGTCGACTGTCAATACAGGCTTGCCGCTCTGGGGATCGATAATCTGGACATAGTCGTTACTGAGTTTGAAATCA
CTCTTGATCTCATACGTGTTGGCGATACCTGTCTGGTCGGTATAACGGATAAACCAACGGTCTTTTCCCAGCTCAT
CCTTGAATTGATAAATACCTTTGGCATTAACTGTACTTTTGGCAAGTAATGACTCGCCGTCCGCAACAGCGTAGAT
GCTGATATTGCCCGCTTGTGACGGCAGCAGGCGATTGGGTATGTCGAAACTAGCGTATTCAGGGCCGGATGCCGGC
TCAAGGATTGGCGGCTCTTTTTCCGGCAGTACGAACGGATCTTCAGCTTTTGAGCCGACACCTACAGAAGAGAACA
ACACGTTGAGCATTCCATCGAATACCTTCGCCGCCCCGTCATGACGTTCAGCCTGAGTATCGCCCGTGATCTCTTT
TTCAGTGCCCAGCGCCACTTCGGTGAGCCCCGTGATAACTGCGGCTGCGGCGATCGGAGCCGCCAGCGGGGCCAGC
TTGGTCAGCAACCCTGCGCCGACCGAGATGTCATTGAGCCAAATATCACGAGTGACCTCGCTGTTGGACTTGATCG
CGGTGTCTGCATCACTTTTCATGCGCTGTTGGCTTAGATGCGCCATGTGGCTGAACACATCGCCCTTGATCGCAAA
GTTGCCGCGGTCGATGACCCTGCCCTCTTGATTACTGACGGTTCCTGCACCGAGGTATTCCAACGATGTATCGACG
CCGTCTTTCGGACGGACTTCAGCCAGGAAGCTTAGCTGCACTATTGTGTTAAATAATACGGCCCCCGCCCCGGTTT
CACTGTTCTGCCGATCAGCCAGCGAGAAGTGCGACTCCAGGGCTTTACGCAAGGAGGGCGTGCTGGCCTGATCGCT
GACCCATCGATCCATTTTTTCCAACGAGTCGAATTTCAGAAAGGCTGGCTGGGCGTCAGGAATGTAGAGGATTTGC
ACGCCGTCCAGACGACCGTGGACGTATTTTTTGGGCAGCTTGCCAGGTAGGGTAAAACGCACAATGTCATTTGACG
GGAAGCCATTGATATCGAAAGCATGGGCCTGGACAGCACCTGTTACCGGGGATGTTGCCTGCAATTGTGCAAAGGT
CAGGGGCTGGTTTTCAGATACGCCCTCACCACAAACAGCCGTCATCAGCTGTTTATAGTCGCTCCTGGTGAGCTGA
TGCTCGGGCGGCTGCAGCGCTCGTTCAGCAGGTAACTTGGCCTCCTGGGACTTGAGTTGTTCACTTGCCTTATGGA
TGAACACCCCCTTCTGGACAGTCCGATAGTTATCGGCGTGGTGTTCCCAGAAGTCGTCGATTTTTTCCGACATTAC
CGTTTGGAAGTCGACTTTCCAAAGAGCCTTCATAAACTGGGAAGGCGCCAGGGGAATTTGGTTATGAGCGCCGTAG
CCTGCTTTTTTGCTTTGTCCGGGGCCGTCTTTATACAAGCCCGCTTCCGAGTCCAGATTGCCGGGAACCCCGTCGT
TTTCGGAGAAGTTCTTCAATACCGCATCGGTCAAAGTGATTGAAGAGGAAGGTTCTTCCTTCATATGCTCCCAACC
GGTAGCGGTTGTAGCAGATTGCGTGCCGTTAAAACGATTCAGATACGTCTTATCGGCGTCTACATGGATGCCGGTC
AACTTGAACACGATGTCTTGCGCGCACTTTTTGGCTTCTGCGCGCACATCTGGAAATGCCGCAGCCGTGCCTAAGG
CAATATTGTTGAATTTGGCTTTCTCGACATTGGCCTTGGCCACTTGCTCGGTAATGACTGCATAGGCATTGGAAGT
GCTTTCCGGTGCCTGTGAAGACACGATGTGCCTGAGTTGGGTGAGTTCTTCACGATTTTTCGGAATGTTCCAGCCG
TTGGCTGTGATGTATTGCCATAGGCTGACTTCTATGACCGGGCCTGCATCTTTCGTATCGGGCTCCAGTTGGATCT
TCGTGGTTTGCAAGACCGGAAAAATAGGCTCATTGGTTGTGCGACCCGTTGTGATGCCTTGCTCCAGGCCTACCAG
TGTCTGAGTCAGCGAGTTACGGTTGTCCAGATCACTGGAGCCTGCGCCGGTGGTTGGTTGCGTATTGGATGTGGGC
TTTATAGAGCTGCTCTCTTGGGTAACCTCGTATTCGTAGGCTCCGGAAGAAAGATTCTGTATCGATATACTCATGA
TGTTTATCCCCGACAGATACAAGAAAGCCCGCAGAACAACAGTTATCAGTCTGTGGCTGTATCTATGTAAGTGATG
CGAACAACGCCATCCATTCCAGTGAAAAGGTAACGGGGGATATCTCCGGAGCGTACTCGCAACCCGATAGGCAACC
GTACCCCGCCAACGGTACGATGGTTCCCCCATCCGCGAGGCTGCCTCCATGTTCTTCGGCGTTCTACTGATCATCA
CCTGGCTGATCCTGCTGCTGCGCTATCCCGCCAAGGCGTTGCCTGTATCCCTCGTAGCAGCAGTGGGTCTGGGTTT
CGTGGCGATGTGGGTGGTATGGCTGGACAACCGCGAAGCCGCGCAACTGGCGCGCCTGGAGCTACGCATCAGCTAC
GCCCCCGAGCAATGCCCTGCCGATCGCCCCTTGCAGCTCAAACTCAACAATGGCAATGACGTCCCCCTCACCGAAT
TGCGCTGGCGCGTCGCCGCCTATGCGCCGGGCGATACGGTCAACCTGGCCGACAACGTCTATGCTGCCCCACGCTA
TCGCGGCCCCGGCGAATTGCAGGCCGGCGCCACCTGGGAAGATTGCCTGCCGATGCCGCCGCTGCGCCCCGGCTAT
CGCCCGCAAACCCTGGAGTTTCGTGCCGAACGCTTGCAAGGCAGCTTCTGCGACTAATTAATAACGACAAAGGATT
GAGCCATGCCCAACGTGCTTATCACCGGTTGCTCCAGCGGCATCGGCCGCGCCCTGGCCGATGCCTTCAAGGCTGC
CGGCTATGACGTGTGGGCCAGCGCCCGCCGTCCCGAAGACGTCGCGACCCTGGCGGCAGCCGGCTTCAAGGCCGTG
CAACTGGATGTCAACGATGGGGCGGCCCTGGCGCAACTGGCCGGGCAAGTGGGCGAGCTGGATGTGCTGATCAACA
ACGCCGGCTACGGCGCCATGGGCCCGTTGCTCGATGGCGGTACCGCGGCGATGCAGCGGCAATTCGAGACCAATGT
GTTCTCCATTGTCGGCGTGACCCAGGCGTTGTTTCCCGCGTTGCGCCGTAACAAAGGGTTGGTGGTCAATATCGGC
AGCGTGTCCGGGGTATTGGTCACCCCGTTTGCCGGCGCCTACTGCGCCTCCAAAGCCGCCGTCCATGCCCTGAGCG
ATGCCCTGCGCATGGAGCTGGCGCCGTTTGGCGTGCGGGTCATGGAAGTGCAGCCGGGGGCAATCAACACTAGTTT
CGCCAAGAACGCCGGCACCCAGGCCGAACGGCTGATCAACGAACAATCGCCATGGTGGCCGTTGCGCGAAAGCATC
CGTGCGCGCTCCCAGGCGTCCCAGGACAAGCCAACCCCAGCCAGCGTGTTTGCGGCGGACGTGCTCAAGGCCGCGC
AACAACCTGAACCACCGCGCCTGTTGCGCTCAGGCAATGGCAGCCGCGCGTTGCCGTGGATGGCGGCGCTGTTGCC
CAAGGCGTTGCTGGAGAAGGTGTTGAAGAAGCGGTTTGGGTTGGGTGGCAAGTTGTAGGTTTATTCCGAGACCGAG
GCGCCTGTATCGCGAGCAAGCCCGCTCCCACACTTACAGAGTACATCCTTGAAATACGGTCAACCTGTGGGGGCGG
GCTTGCTCGCGATGGCGTCCCAGCCAGCAACATAACCCTCCTTCATGAAATCAATAAACGCCCGCACCTTGAGCGG
CAAATGCCGCGTGTCCGGATACAGCGCATACACCCCTTGGGGTACGAAGCGATGATCCGGCAACAGGTGTACCAAT

CGCCCGGTGTCCAGATCCTCCTGTACCAGCCACTGCGGCAGGATTGCCACGCCATGGCCGCGCACGGCAAATGCCT
GGAGCACGGCGGCACTGTCAACCATAAGCGCACTGGGGCCGGAGCGATATTGATGCTCGCGACCAGCCGGGTCGAT
CACACTCAACTCGTTCAATCGGCCGTGCCCCAGCTTGGGCAGTTGTTCAAGTTCATCCAGCGTATGGGCGCTACCA
AAACCAGGCGCCGCGACGGCGAATATCGGGTACGTGGATAACTGCACCGCACGATGATTGGAATCCAGCAGGCGCC
CGAGCCGAATCGCCACGTCAAAGCGTTCGGAGATCAGATCGGCATGGGTGGAGGATGTGGATAAGTGGACATTCAG
ATCCGGGTGCAAACGGCCAAAGGCCTCCACCGCAGGGACGACCACGGCCAGCGCATATTCCACGGTGGTGGTGATG
CGTAGGGTGCCTTTGAGCTGGGTGTGCTCCGAACGCGCCTCTTCCACCGCCAACTGCGCTTCTTCGAGCATGCGTG
TACAGCGCAGGTAAAACCGCTCGCCGGCATCGGTCAACGCCAATTGCCGGGTGTTGCGCGTCAGCAGGGTGACACC
GAGTTCAGCCTCCAGGCGCTTGAGATTAAAGCTGACCACGGCGCGGGTCTGCCCCAGTCTATCGGCCGCGGCAGTC
AGCGAGCCGGCCTCCACCACCGTCTTGAACGTCTCGAAACGATCCAGGCTGACCATGGGCCACCGCCTCCACTTGT
CAAAATATTTTTGACAAACTAACAGTCAAACCAGCGTTCCCCAAGTATCCGCAGCACTCTACCCTGCCGGGATTGA
ATGCAGGAACGCCCCAATGACCTACCGCTCGAAAATCGCCTGGATCTTTTTACTGGGTTTCGCCCTCGATCTGGTG
AACATGTTTGTCGCCACCGTCGCCTACCCCGATATCGCCCACGAACTGCAGGCTTCGGTCACGCAATTGGCGTGGG
TCAGCAATGCCTACCTGCTCGGCCTGACGCTGATCATCCCCTTGAGTGTGTGGCTGGCGGCGGTCATGGGGGAGCG
CCGCCTGATCACCGCCAGCCTGCTGATCTTTGCCGGTGCTTCGGTGCTGGTGGGCCAGGCGGGCTCCATCGAAACC
CTGATCGGCTGGCGCGCCCTGCAAGGGCTGGGAGGCGGCTTGCTGATTCCCGTGGGCCAGGCCATGGCCTACCGAC
ACTGCCCAGCAGCAGAGCGCAGCCAATTGACCGCTCGTGTGATGTCCGTGGCCCTGCTGGTGCCGGCGCTGTCACC
GGCGTTAGGCGGGTTGCTGGTCGATAGCGTGTCCTGGCGCTGGATCTTCTACGCCAACTTGCCGTTGGCGTTGTTC
ACCCTGCTGCTGGCGTGGCTGTGGATAAAACCGGACGCGCCGGCAACTGCGCGCCCCTCTCTGGAGATGGGCAACC
TGTTCAAACAGATCGGCAACCCGATGCTGCGCATCGCGATGCTGATCTACTTGTGCGTCCCCGGTGTGTTTATCGG
CACCAGCCTGATCGCCATCCTCTACCTGCGCGGCCTGGGCTATGACGCCGCGCACACCGGCGCGCTGATGCTGCCC
TGGGCGCTGGCATCGGCGCTGGCCATCTTCCTCAGCAAGACCTTGTTCAACCGCTACGGCCCCAAGCCCTTGCTGC
TGGCGGGCATGGGGCTGCAATGCAGCGGTATTGTGTTGCTCAACGAGCCTTCCCTGATCATTCTCGCCTACGGCCT
GATGGGCCTGGGCGGCAGCCTGTGCAGCAGCACTGCGCAGACCCTGGCCTTTCTCGATATCCCCGCCCCGCGCATG
GGCCACGCCAGCGCGCTGTGGAATATCAATCGGCAGCTGAGCTTTTGCCTCGGTGCAGCGGCACTCAGCGCCGTGC
TATCAGCCTCGGATTCCTTCACCATGACCTTCTTGATCGCGGCAGCCCTGACACTACTGCCGCTTTTGGCGGTGCT
GAGCCTGGATACCTCCAGGGTTCGCGCACTGCTTCACCCAGCCAGCGAGCCTCAATGATGATTGATTACAGCGACT
TTTTTGAAGAAGTGATCCAGACCCACATCGAGATCGAGCAATGGTTTGCCGGCGTCGCGCCGGATGGCACCTTGCA
AAACCTGCTGGCGCGCTTCTCCCCGGAGTTCAGCATGATCGCCCCCGCCACCGGCGCACGGGTCAACACAGCCGGG
GTCAACGCCCTGTTCACCCGCCTGGGCGGCATGCGTCCTGGGTTGAAAATCACCTTGAGCGAACTGACCGGCATTG
ACCGACATGCACGGGGCGCCACGGTGACCTACCGTGAACACCAGATCGATGACAGCGGCACCCAAACGGACCGCCG
CGCCACCGTGGTGTTCGAGAAACAGGCAAGCGGCGCGTTGCTGTGGCGGCATTTGCATGAGACCTATATCCAGGGC
CAATAACTCTCCCCTGTAGGAGCCGGCTTGCCGGCGAAAATCGTCAACGCTAACGCGGAGCATCAGGCTCCCCGCG
TTGCTCTCAGGTTCTTCGCCGACAAGCCGGCTCCTACAGTAAACCGCGAACTAGCTGACATCATTCGACGGACTCA
GTCTTCAGGCTCTTTCACCTCTGCCCACGGGAAGGTGAATGGATGCTCAAGGATTTCCTCAACAGCGGCGTGCCCA
ACGCTCATACAACCACCCGCCAGCGACTGACCAGCCTGCTGGATTTGCCAAAACTCTATCGAACCATTGACGCCAA
CCCTGCCATCGTCGGCGCCGGCGTGGTGCACATCGGCAGTGACTATCGGGTAACGGTGCTGCGCGAGTTTTTGCCG
TTGTGCAGTCTCATGCCCAAGCGGGTGATATTGCGGGAAGTGGCGGGGGCAATGACAGCCAATCAATATGCTGAGC
TGGCGACCAGTTCGCCTCGTGAGTCAAAGCTACTTGTAGAAGCAACGGGGATGACTTTCGCTTGCGCAGGAGCCGT
TATCGGAATAGCCGTCGCAGCTACCGGTGTTGCATCCACGCCATTAAGTGGGGGCGCCAGTCTTGCAGTTACCCGT
ATCGCCTATACAGCTGCAGCCGCCAGCGGCTTGCAGTGCATCAACGGTGCCTGGAGGACTTATACAGAATTAACTA
ACCCTGCTGAGAATGACTATTACGATTCGCTGAGTTGGTACCAATCCATGAGCGTTGCGCTGGATGGTATTTCATT
GGTCGGCGCAGGTAGCACTACGTTTGTCACGGTCAGAACAGTGATGACGATCAAAAAGTCGACGGGCAGAAACCTG
GCAGACATCCTGAGGAGCCTAAGCCGCCGAGAGCGTGCAAAGTTGACGGATGAAGTCCTGCGACTGAAAAACCCTC
GCCATCCCCGCGAAATACTCCGGTTAAAACAGCTAGCTGGAACACTTCCAAAACGCTATAGCAACAGCCAATTACG
CCAAGACATTCTCACTCAAATAGCAGATCAGGTGGGTGCAGCTCTCGCACTCACAGGCAGCAGCATTTCCGGAAAC
ATTAACCACATTGTCATCGCGATTTATGAAGAGTTCAGCAACCATGACCAATAACGGCAATCCCGCGCGCGCGCCG
CGAACAGTGCGTAGTTACACCCACCGTTTTGCTTTCACGGTGGGCACCGCAGTATCAGTGAGCTTAGTGAGTATCT
CAGGCAACCTGTCACACAGCCTGTCGTATTACGCCCGAATTAACTATCTGACTAGCGAATACGCTGTGATGGCGAG
CCTGGTACTGTTGTTGGCCATGTGTATCGGGTTGGCTCTGCTGGTGGCGGGATTTCGTTCAGGCCCCGCTGTACTT
GTAGGCGTCGCTGCTCTGAATGCTCTGCTTGCGACAGGCTACTTGGTTAGTCACCCCGTGACTCTGTTTGCGCTAT
TTCCTCTGTTTCAATCACTGATCTGCCTGATCATGCTCAATACAAAAAACCACCGCCGCTACACCAGCATGCTAAG
GGTCAAACGACGGCGCAAAATCAGGGCAAGAACACCCACCGCTGGTTAGTTTTGAGCCTGGTTGACGATCACGGTG
CAGGTAATCCCCGCCGCCAACAACACGCCCTCCGGCACTTCATCGATATGGATCCGCACGGGCACGCGCTGGGCCA
AACGCACCCAGTTGAAGGTCGGGTTCACATCGGCAATCAGTTCGCGGCTCTCGGGGTTGTCGCGGTCGTAGATGCC
ACGGGAGATGCTTTCTACGTGGCCCTTTAACGTCTCGCCGCTCATCAGTTGCATATCGGCTTTGTCGCCAACTTTG
ACGTGGGGCAGCTTGGTTTCTTCGAAAAAACCGTAGACCCAGAACGAGTTCATATCGACCACGGCCATCTTGGCTT

CGCCTATGCGTGCGTAGTCGCCGCGATGCACGTTGAGGTTGGTGACGTAACCATCCACCGCAGCCAGCACTTGCGT
GCGTTTCAAATTCAACTCCGCCGCTTCCAACTGCGCCAGCGCGTGTTGGTAATCAGCCAGAGCGGAATCAGCAATG
TTGCTCGCGTCGTCGCGGTTTTCCTTGGAGATCACCAGTGCATCAAGGTCCGCACGGCGATGGGCGTTGACCTTGC
GCATTTCCCAGGTGGCTTTGCGCGAGGCCACCAATGATTGCGCCTGCTTGACTGCGATGCGGTAGTGCTCGGGGTC
GATCTGCATCAGCAGGTCGCCCTTTTTCACCAACTGGTTATCCCGCACCGGCACGTCGACCACTTCGCCGGTGACG
TCGGCGGCGACGTTGATGATATCGGCGCGTACCCGGCCATCGCGGGTCCACGGGGTTTGCATGTAATGCACCCACA
ACGTACGGCCAATCCAGATCGCCAGGGCCAGCACCAGCAGGGTTGCGAGCAGGCTGAAAAACTTTTTCATCGGGGC
ATTCTCAACGGTAGACGGTCAGCGCCATGGCGCCGAAAAGACAGGTGAACAGGCTCAGGCGCAGCAACGCCGGGTG
CCAGAAAAAACGGTACAGGTCAAAGCCGGCCAGGAAGCGATCCAGGGCCCAGGCCAGTCCCGCGGCGATAAAAAAC
ATCAGGGTCATGGTCGGCATGTAGATGCCATGGAAGGCGATTTCACGCGGCATGAACGGTTCCTTCAAGCTTGAGA
GCAGCCAGGGGCGATTGCGGGTCCAGCAGGGACGTGCGGATAAAGTGCAGGTAGCTTTTGACGCGGCGCAGGGCCG
AGGTATCGAAGTGCGGGGCGAAGGGTTCGTCGGTGGCCTGTACACGGCTGATGGCGTGGTCCACCGCAATCAAGGC
GCGCTCAAGGTTGCTCTGGCTCGGTTGCAGGAACAGCCGCACCAGCGAACGCCCCATCACCCGGATCGCCTGGCGC
CACGATTGGGACTCGGCATACGCCGGATGCACCGGCAGGATCGCCTGCTCTTTGCGCAATTCAATAATGGCGTGGC
CGACTTCCAGCACCACGAACATCCAGCGCAACAGGTTGCGTTGCACCTGCGGCTGGCCGGCGGCCAGGCCATAGGC
CTGGTGCAGCAGGTCTCGGGTGCGGCTCTCGAACCCCGAGGCCAGGCCCTTGAGCTTGCCGCTGATGGCATACACC
ACTTGCTCGCGCAGGTCGCGCTCCAGGCGGCGCCACAGCCAGGGGCTGTTGGGCGGCAGGATGATCGCCCCCGCCG
CTGCGCACACCAGCATGCCGATGATCATGGCGATGTAGTCGTTGATAAAGGTGTAGGGGTTGTAGATCGTCAGGTT
ATCCGGCACCGAACCGGTGCTGAAGAAAATCAGCAGGCCCAGGCCGACCCCAGCGTATTGCGGGCGCGAGGACAGG
AACGCGCCGAGCACGATCACCGGGGCGAGCATCACACAGAGCAAAGGGAACCCGTCGATCCTGGGAAACACGAAGA
ACATCTCGACGAACCCCACCAGGGCGCCGATCAAGGTGCCACAGGCCATTTGAAACGCCATGCGCTTGGGGTTCGG
CGTGGCAGCCGACAGGCCAACGGTGGCCGCCGCGATCAGGGTCATGGTGGCGCCACTGGGCCAGGCGGTGGCCACC
CAGTAACTGCCCAGCACCACCAGAATGAACGACGCGCGAATCCCCGACGCGGCGCAGGCCAGCCAGTTGGTTTTCG
GCGTGTAGGCCTCATCCCAGTGCTCACGCGCGTGGCTGTGTTCGGCCAGGGAGGCGTGGGTCTGCGCGTAGTTGTG
CAGGTCGTCGACAAAGCGGTACAGCAGCTCATAAGCGGTGTGGAAATCCAACTGCTCGGCATCGCTGGGGCTGCTC
TGCTGGAACTGCGCCCGCAGGCTGCGGACCTTGGCGGGCAGGCTCTCTTTATAGGCCGTCAGTTGGCTGACCAGGC
GCGTGGCATCGGGGCTGGTCAGGGCGCGCCCGCTGAAGCCATCGAGCAACTCGGCCAAGTCCTGCAGGCCGGGTTT
GATCGCGGCCACCACATGGTCGGCAGCATCGCTGCGCAGGCGTTCAAGCAACTGGTGCAGGGCGTTGAAGCGCGTG
GTGATGCCCATGAATTCGCTGTTCAAGCGGCTGAGCCGGCCATTGCGCCGACGCATATGCGGGTCTTCGAACACGG
TGACACTGCGCAGCCCTTCAAGGCCCACGGCTTCGGCGATAAAGCGCACGTTGCTGCTTTCAAAACCTTCGCGCTG
GCTACGGCCACGCAGGCCGTCGGTAACGAACAGGGCGAACGTGCCGAAACGCTGGTACAAGGCGTTGCGCATGGCC
GCGCTGCTGGTCTGCGGGAGGATCGCGGCGCTGACCAGGGTCGCGCAGAGAATCCCCAGGGAGATTTCCAGCACCC
GCCACACCGCCGCCATAAAGGCGCCGTCCGGGTGGGCCAGCGCCGGCAGGCCCACCATCGCCGCCGTGTAGCCCGC
CAGTACAAAACCATAGGCGCGGAAGTTGCGGTTGCGCGTGGCACCGGCCGTGCACACGCCCACCCAGATCGCCAGC
GCGCCCAAAAACAACTCGGTGTTCTGCGCAAACAGCGCGATCAACAACACCATCACCGCCGACCCCGCCAGGGTGC
CGAGGAAGCGGTAGAAGCTCTTGGCAAACACCTGGCCGCTTTGCGGTTGCATCACGATAAACACGGTGATCATCGC
GGTACGCGGTTGCGGCAGCTCCAGGCGCATGGCCAGCCACAGGGTGAGGAACGCGGCGATCAGCACTTTGAAGATG
TAGACCCAGGTCACGCCGTCACTGCGCGCCCAGTCGAAGAAGCCACGGCGCCATTCCAGGGAATGCAGCCAACGCA
GCGGTGCAGGCAAGGGAGTCATGGGAAACCTGATCAGTGATCGAATACGGCCAACGCCGCCGGGGTCTTGCTCGCG
AGGGTGGTGCCTGCTGCCGGCGCATCGTTGCCCGCGCCCAGACCGCCGCCCAGGGCAGTGACCAGTTCGGCATGGG
CACTCAGGCGCGCGGCCTGGACCTGCTGCTCGACCTGTTGCTGGCGAAACAGCAAGGTCTGGGCATTGAGTACGTT
GAGGTAATCGGTCAGGCCGCGCTGGTAGGCGATCATCGCGATGTCATAGGTTTTCTGCGCCGAGGCCACCGATTCG
GCGGCGAAGGTCTGCTGCTTGGCCATGGACTCACGGCGGATCAGTTGGTCGCTGATGCCCTTGAGCGCATTGACCA
GGGTCTGGTTGTAGCGGGCCACGGCAATGTCGTAGCCGGCGCTGGCTTCGCCCAATTGCGCGCGCAGGCGGCCACC
GTCGAAGATCGGCAGGGTGATCGCCGGGCCGACGCTGTAATTGAGCTTCTTGCCGGTAAGGAATTCCAGCATGCCG
CCACCGGTAGCGACGTAGCCGAGGCTGCCCACCAGGTCGACATTGGGGTAGAAACCGGCGTGGGCCACGTCGATGC
CGCGGGCCTGGGCCGCCACTTGCCAGCGGCTGGCGACCACATCCGGACGCTGGCCGAGCAGTTGCGCGGGCAGTGC
CGATGGTAATTTCAGGGGCGCAGCGAGGGCCAGGGTCGGCCGTTGCAAGGTTGCGCCCTCCCCCGGGCCCTTGCCG
GCCAAGGCCGCCAGTTGGTTGCGGGTCAGGGCGATCTCTTCGTCGAGGGCGTCCAGTTGCCGATGGGTTTCGGGCA
ATGGGGTCTGGGCCTGGCTGACTTCAAAGTGGGTGCCGATGCCGCCATCCAGGCGCTTTTGCGCCAGGTCGAGGAT
CTGCTGTTGCTGGGCCAGGGTGGCGGCGACGATATCGCGCTGGGCGTAATGCAGCGACAGTTGGATGTAGGCGCGT
ACCACGTTGTTCTGCAGCTCCAACTGGGCCTGGCGCGCCTGGGCGACGCTCATATGGGCCAGGTCGAGCGCGCGCT
CAGTGGCGTTGCGCTCACGCCCCCACAGATCGAGGGCGTAGCTCAGGCCCAGGGCGGCGTTGTTGTCCCAGGTCGT
GGAGTTGTCCAGTTTTCCAGGGCCGTAGAACTGATCGCTCGGCCAGTTGTGGCGCTTGAGCGTGGCATCCCCGTTG
ATCTGCACAGCCTCGGCAGACTCGGCCAGCCCAGCCATGGCCCGCGCTTCACGTACCCGGGCGGCGGCTTCGGCCA
TGCTTGGGCTGCCTTGAACGGCGAGGTCGACCCAAGCATTGAGTTGCGGATCGCCGTAGGCTTGCCACCACTGGGT
GGTCGGCCAGTGGGCATCCCGGGCGGCGCTCTGGATTGCGTCGTCGGTGGCCAGGTTATTGGCGGCGAGGCTCTTG

CCTTGGGGGGCAATTCCACCGGTTCCGATGCAGCCGCTGATTGCTAACGATAAAGCCCAAACACTGAGAGTCTTCA
GCTCTCTGCTGATGCGACGCGGCACTGCTGCGAATTCCTGAGGTGGTGTTGCGGGGACGGTGCGGCAATTCTAGGG
GGCGCGAGGAATGGCGATAAGGTGGGATTCCTGTGAATCTTTGTTACCGTTCAGGCGATAATCCATTGGTCGGGGT
CACTCGCCCCCGTAACTTCGTGTCACAATTTGCCATCTCCCTTGAGAGCACCCCATGGACACTTTGCAAAACATGC
GCGCCTTCAGTTGTGTCGCCGAAGCCGGCAGCTTCACCGCCGCCGCGGTGCAACTGGACACCACCACAGCCAACGT
CTCGCGCGCGGTCTCCAATCTGGAGGCCCATCTGCAAACCCGCTTGCTCAACCGCACCACCCGCCGCATCGCCCTG
ACCGAGGCCGGCAAGCGCTATTTATTGCGTTGCGAGCAAATCCTGGCCTATGTCGAGGAAGCCGAAGCCGAGGCCA
GCGACGCCCATGCGCGCCCCGCCGGGCAGCTCAAAGTGCATACCATGACCGGCATCGGCCAGCATTTCGTGATCGA
TGCGATTGCCCGCTACCGCCGCACCCACCCCGATGTGACCTTCGACCTGACCCTGGCCAACCGCGTGCCGGACCTG
CTCGACGAGGGCTACGACGTGTCCATCGTGCTCGCCAGCGAGTTGCCGGATTCGGGGTTTGTGTCCCAGCGCCTGG
GGATCACCTACAGCATCGTCTGTGCCTCGCCGGCCTACGTCAAAGCCAACGGCTGCGCGCAGCGGCCCAGTGATTT
GCTCAACCATGCGTGCCTGCGCCTGGTCAGCCCGGTGATCCAATTGGACAAATGGGTGTTCAACGGCCCCGATGGC
CAGGAAAGCGTGAGCATCAACAGTTCGCCATTTTTGGTGAACTCCGCCGACGCGATGAAGACCGCGATCATCAGCG
GCATGGGCGTTGGCCTGCTGCCGGTCTACGCGGCCATCGAAGGCCTGCGCAATGGCACCCTGGTGCGGATGATGCC
GACTTACCGCTCCCAGGAACTGAACCTGTACGCCATCTACCCGTCGCGCCAGTACCTGGATGCGAAGATCAAGACC
TGGGTGGAATACCTGCGTGGCTCACTGCCAGAGATCCTGGCGGCGCACCAGGCGGAGCTGGCGGCGTATGAGTTAA
GCGGCAGTTTGGGAGCGGTGCGCCTTACCACCTGATTTGGAACACGGCCCCCAAATATAGAAAGTCCCCTGTAGGA
GCGGGCTTGCCCGCTCCCACATTGGTGATGCGGTGTCCTGACAAGTGGGTGCAGGAATGTTAGCTTGCTTGGCATT
CTTCCCTGTCGATGAGCCCCACTTGCGATGAAAAAGACTGTCCTCGCCTTCAGCCGTGTCACCCCCCAGATGATCG
AACGCCTGCAACAGGACTTCGAGGTCATCGTTCCCAATCCCAAGCAAGGCGATATCAACGCGCAGTTCAATGAAGC
GCTGCCCCACGCCCACGGTTTGATCGGGGTGGGCCGCAAGCTGGGCCGGGCGCAGCTCGAAGGTGCGAGCAAGCTT
GAGGTGGTGTCCAGCGTCTCGGTGGGTTATGACAACTACGACGTGGACTACTTCAACGAACGCGGGATCATGCTCA
CCAATACCCCCGATGTGTTGACCGAAAGCACCGCCGACCTGGCCTTCGCCCTGTTGATGAGCAGCGCCCGCCGCGT
GGCCGAACTGGATGCGTGGACCAAGGCCGGGCAGTGGAAGGCCAGCGTCGGCGCGCCGTTGTTCGGCTGTGATGTG
CACGGCAAGATCCTGGGCATCGTCGGCATGGGCAATATCGGCGCAGCCATCGCCCGCCGTGGGCGCCTGGGCTTCA
ATATGCCGATCCTCTATAGCGGCAACAGCCGCAAGACCGCAGTGGAACAGGAACTGGGCGCGCAGTTTCGTAGCCT
GGACCAGTTGCTGGCCGAGGCGGATTTTGTGTGCCTGGTGGTGCCCTTGAGTGAAAAGACCAAGCACCTGATCAGC
CATCGCGAGTTGGGCCTGATGAAATCCAGTGCGATCCTGGTGAATATCTCCCGGGGCCCTGTGGTGGACGAGCCGG
CACTGATCGAGGCCCTGCAAACCCAGCGCATTCGCGGCGCCGGCCTGGATGTGTATGAGCGCGAGCCCCTGGCTGA
GTCGCCGCTGTTCCAACTGAGCAATGCCGTGACCTTGCCGCATATCGGTTCGGCCACCCATGAAACCCGCGAAGCC
ATGGCCAACCGTGCCCTGGATAACCTGCGCAGCGCCCTGTTGGGCCAACGCCCGCAGGATCTGGTGAATCCGCAAG
TCTGGAAAGGCTAAGACACAACACAACCCACTGTAGGAGCGAGCTTGCTCGCGAAAAACATTAACGATACGCGTCT
ATCCTGAATAAACGCGGCGCCCTCGGTTTTTCGCGAGCAAGCTCGCTCCTACAGTTTTTTGGGCCAGCGTCGATAA
CCTGCCATAGAAGATTGTTATCGCTGATCCACAGAAGGTTTTTATGTCCACCACCAAAGCCCGCGCAGATTCACTC
TCGCTTCTGCTCTTTACCTTGCGCAGCGGCAAGCTGATGGCCATCAACCTGCTGAAAGTCAGCGAAATCATTGCCT
GCCCGCCGCTGACCAAGCTGCCGGAGTCCCATCCACACGTCAAAGGCATCGCGACCCTGCGTGGCGCGGCCCTGTC
GGTGATCGACCTCAGCCGGGCACTGGGCGAAATGCCCCTGAAAGACCCCGACGGCGGCTGCCTGATTGTTACCGAT
GTCAGCCGGTCCAAGCAGGGCCTGCACGTGCAGGCGGTGAGCCGGATCGTGCATTGCCTGACCACTGACATCCGCC
CGCCACCCTTTGGCTCCGGCGGCACGCGCTCGTTTATCACCGGCGTGACCCAGGTGGACGGCGTGCTGGTGCAAGT
GCTGGACATCGAAAAAGTCATCCACGCCATTGCCCCCGCGCAAATCGAAGTCGCGCCCACCGACCTGACCATGGAA
GAAGCCGAAGTGCTCGGCAATGCGCGCATCCTGGTGGTGGACGACAGCCAGGTGGCCCTGCAGCAATCGGTGCACA
CCCTGCGCAACCTCGGCCTGACCTGCCACACCGCACGCAGTGCCAAGGAAGCCATTGATGTGCTGCTGGAGCTGCA
AGGCAGCGCCGGGCAGATCAATGTGGTGGTGTCTGACATCGAAATGTCGGAAATGGACGGTTACGCCCTCACCCGT
ACCCTGCGTGAAACCCCGGACTTCCAGGACCTCTACGTGCTGCTGCACACCTCCCTGGACAGCGCGATGAACAGCG
AAAAAGCGCGCTTGGCCGGGGCCGACTCGGTGCTGACCAAGTTCTCCTCGCCCGAGCTGACCAAATGCCTGGTGGT
CGCGGCCCAGACCGTGGCGCAAAAAGGCCTGTAAGTGACAGCGTATTACCAACTGCTGCGCCGCGACCTCACCAAG
AGCCTGCCCGCGCCGCAGTGGCCAGCCGACACCCGTCTGGACCATTACCGCGATGAGCTCGCCCCGGCGATCCATG
CGGTATTGCGCATGACCCAGGCCCAGGGCGGCGGCCACGTGCCCAACCTGAAGACCTGGCAGCAGCAATTCATCAC
CGATGCGGAATTTGACCCCACGCTGTGCCTGGTGGCGAGTAATGCCGACGGTATTCTTGGCGTGGCGCAATGTTGG
ACCAGTGCGTTTATCAAGCATCTGTGCATCCACCCTTGCGCCCAGGGCCAGGGACTGGGGCGCGCCTTGCTGCTAC
ATAGCTTCCAGGTGTTCAAGCAGCGCGGCGAACCCTACGTGGACCTCAAGGTGCTGGAAAGCAATCAGCGCGCACG
CCAACTGTATGAGAGCGCCGGCATGGTCTTGGTGCTGCGTGACGTGGTCAGCGAAGACTGAGCGGCGCTGGCGCAT
AACTTGCTTCCAGAGTCTGCGAGCGGCGGATAGAGGTAAAAACCCGTCACCGCTCAAGAGAGCCCTCTGACCTGGC
CTGGTAGCAGATCCTCCATGAATACCCATTTTTCCTGCGTCGGTTGCGGCAAATGCTGCACCGACCATCATGTCCC
CCTGACCCTCGACGAAGCCCGCCAGTGGGCGGCAGATGGCGGCAACGTCATCGTGCTGGTGGAAGGTTTCCTGGGC
AATGGCCTGGGCCTGCCTCTGCAACAGCGCGAGCATGCCGAACGGCGTTCAGTGGTGGTGCCCAGCGGCAATACCC
AGGCGTTCGTAGCGATCACCTTTGCCGCCTACAACGCCGGGCGCTGCCGGAATCTTGACGAAGACAACCGTTGCGG

CATCTATGAGCGCAGGCCGCTGGTCTGTCGCATCTACCCGATGGAGATCAACCCGCATATCCCGCTGAACCCGGCC
GCCAAGGATTGCCCGCCGCAATCCTGGGAACAAGGCCCGGCGCTGATCGTCGGCGGTGAACTGATGGACCGGGAAC
TGGCAGACCTGATCCGCCGCTCGCGCCAGGCCGACCGTGACGATGTACAGACCAAGGAGGCGGTGTGCGCCCTGCT
GGGGATTCGGACCACGGCGCTCAAAGGCGATGGGTTTACCGCTTACCTGCCGGACATGGGCGCCTTTGCCCAGGCC
ATTGAGTTGGCCGCGACCCAGCCAGCCGGGGCCAATGAATGGGTGTTCCATGTCTCCGGGATGGACATTGCCGAGC
AGTTGCTGGACGCCGGGGCGCAGATCGCCACCGAAGTGCCGGCCAACTACGCGTTCATCTCCCTGCGCGCTGCCTG
AGGGCCGTCAGAGGCGTAAGCCTGTCGCTGTATCGAGCACGCTTGTAGTGGCGAGCGGGCTTGTTGTGGCGAGCGG
GCTTGTTGTGGCGAGCGGGCTTGCCCCGCGCTGGGCTGCGTAGCAGCCCCCCCAGGCACCGCAGTTGTTTCAGACA
TACCGAACCAGCAGGTTTTGGGGCGGCTTCGCCACCCAGCGCGGGGCAAGCCCGCTCGCCACAGGGAGGGCGGTGG
CACGTCGGGAGTTTGGGGGGCTGCAGCACAGCAGTTTTCTTGGACTGTAGATTTCTTGCGGCCAGCGCGCTTGTGG
TGGCGAGCGGGCTTGCCCCGCGCTGGGCTGCGGAGCAGCCCCACTCCAGGCACCGCAGTTGTTTCAGATGTACCGA
GCCAGCAGGTTTTGGGGCGGCTTCGCCACCCAGCGCGGGGCAAGTCCGCTCGCCACGACAAGCCCGCTCGCCACTG
GGAGGGCGGCGGCAGGTTAGCGGCGTTTGCCCCAGAACTCTTCTGCCAAGCGCCGCAGGTCTTCCGCCAGCGCGGC
GACGTCGCCCGAGCTGAGCTGGCTCTGTTGGCCGGCGCTGACGGTCGCCTCGCCCGCATTGCGGATGCCGACGATA
TTGCGATTGATGTCCTCGCTCACCGCGCTCTGTTCTTCTACCGCCGCCGCAATCTGCACACTCATGGCCGTGATCT
GGTTGACCCGCTGGCTGATGCCATCCAGGGCCGACGCGGCGCGCTGGGCATGGTCGACGCTGACTTCGACATGCTG
GCTGCTTTGCTCCATAGCCATCACCGCATCACGGGCGCCGCCCTGCAAGGTGCTGATCATGCGTTGGATTTCATTG
GTGGATTGCTGGGTACGTTGCGCCAGGCCGCGCACTTCGTCGGCCACCACCGCAAAGCCACGGCCCTGCTCGCCAG
CCCGCGCCGCCTCGATGGCCGCGTTGAGGGCCAGCAGGTTGGTCTGTTCGGCAATCGCCCGGATCACTTCCAGTAC
GCTGGAGATATCCCCGCTGTGGCTTTCCAGTTGATGGATCACGGTGGTGGCCCTGGCCAGTTCCTGGGACAGGCGC
AGCACCGCATCGCGGCTTTCGTCCACGCGCAGGTGGCCTTCACGGGTCTCGCTGCCGGCCAGGTCTGCCGCTTTCG
AGGCTTGTTGGGCGTGGCTGGCAACTTGCGCCACGCTGGCCGCCATCTGCTGGATGGCGGTCGCCACCTGGTCGGT
CTGGCTCTGCTGGCCCAGGGTGCTGGTGTGGCTGCTGTCCAGTTGCTGGACCAGGGCGGCGGCGTGCCCGGATAAA
CGCTGCGAGGCATCGCCAATCCGGCCCACCACCGCGCCCGCCTGGGCTTCGAGCATTTGCATGGCAAACTCGATTT
GCCCCAGCTCGTCCGCGCGCCCGGTGTAGATCGCCTGGCTGACGGGGTTGTCGGCAATCTGCCGCGCCCGCTCGCT
CAATTGCCTCAGGGGCCGCAACAACAGATTGATGCCCAGCACGCCAACCGCGCCCACTGCCGCCAGGCCCAGCACT
TGCAACGCCCATGAATAACTGGCCAGCCCGGTCCCCACGCCCCAGGTCAGCGCGCACGTCGCCCCAGTGACCAGCG
ACAACTTCATCGGCATCTCCAGCACCGGCCGCATGCGCCGGGAGCCTGCGCGCAACCGCATATAGGCGCGCTCTGC
CGCTCTCACTCGGCGTGCGTCGGGCTTGGTGCGCACCGACTGGTATTCCACCGTCGCCCCGTCACGGGTGACCGGC
GAGGCATAGGCGCTGACCCAGTAGTGATCACCGTTCTTGCAGCGATTCTTCACCATGCCCATCCACGAGCGACCAC
TTTTGAGGGTCTGCCACATATGGGCAAACGCCGCCGACGGCATGTCGGGATGGCGTAGCAGATTATGTGGCGCGCC
CAGCAGCTCCTCACGGCTGTAGCCACTGATGTCGATAAAGTCCTGGTTGGCGTAGGTAATCGAACTACCTAGGTCA
GTGGTCGAGAGGATGTTGGCGTCTTGGGCAATGTCCACACTTCGACCCGTGACCGGGAGATTGATCTTCATGGAAA
GCGCGCTCGGGGTTGTTGAGGGAGTCGGCAGGGGCACCGACTCTAAGCGCACCGGTTGAGCAAATACTGATCCAGC
TCATGTTCCACGCATTTAGTTACCCGCTGCCCCCGTCAACGATCCGGGAGGGTCGCCGACCACGGCCACGCATTCG
ATCGCCACCCGTGCGCCGCGCGGCAGCCGGCGCACGGCCAGCACACTGCGGGCGGGCAGCCGGCCTTGGGCGAAGT
AGCCCAGGTACACGTCGTTGACCAGCTTCAGGTCCTCCAGCTCGACCAGCATCAAGGTGCACTTGACCACGCGGTC
CAGCGACGACTCATGACGCACCAATAGTTCGTGCAGCGTATTCATCAGCTGTTTCATCTGCGCGCCCGTGCCGCCC
TTCACCAGGTTGAGGTTGGCATCCACCCCGGCAATGCTGGAGGTGTACAGCGTGTTACCGACCCGCACGGCCTCGG
AAAACGGCAGCTCCGGGTCAGCGGGGTAAAACTGCGGCCCGAGCGGGTCCTGAGCACTGGCGGTGTCGTGTGCCTC
CTGTGGATAACCCTGGAGAATGCGCTGGTACTCGCCGCTCTGCCTGAGCTGCTCCAGTGCGCGGTTCAGCTCACTG
CGCAACTCGGGGTCGGACTTGCGCACGCCGATAGCCACGGCAGTGCCCATCAGCTGGCCGGCCACCGGTGGGCCGA
CGAAGTCAAACGCCCGGCCCTGCTCGGTGTCGAGCAACTCCTGGCGGATTTCTGCGGTGCCTTGCAGCGCCGCGTC
GATATCGCCGGCTACCAGGCTGCGGATCAACTGGTCGTTGAGCCAGAAGCTCTTGATGATCACCCCGGCCGGCGCC
CATTGCGCCAGGGCGAAGGCTTCACGATTACTGCCGGCCAGCACCCCGACGCGCTTGCCCTTGAGCGCACGCACGG
TCGGCAGCAGCCCGGAATACTTGCGGGCCACCAACTGGGTGGTAAGGGGATAGAGGTCGTCGGTGAAATCGATCAG
TTGCCGACGCTGGGGGGTGGATGCCATGCCCATGATCGCATCGAAACGCCGGCGTTCCAGGGCATCAATGCTGTGG
GAAAATACCTGGTCGACCCAGGTGCAGCGCACATCGAGTTGGGCACACAGGGCATTGCCCAGTTCGATGTTCAAGC
CCACCAATCGCCCTTGGGCATTGCGGCTTTCGTAGGGCGGGAACTGCGGGGAGATCGCGAAGCGGATCTCGCTGCG
CGGCGCAGTGCCGGCCACCGCCCCCATAAAAAAGCCGCCCGACAGCAGCAGCCCAATGGATAACACGAAGATCTTT
GCCAAAGCCATGCAGACATCCCTTTCCTGAAAGACCCACACCCTCAACCCAAACTGCTGCCAATCAGGCGCGGCGA
AGGGGTATCAGGCAAGCTTTCAAGAAAAGGTCGTGGACCGACAAGACGAAAAGTCAGCAATGGCTGTAGGCACAAC
TGCCGTCAGCGTTTACCCATCGAGCGGCGGGTGCCTGGCGGCGCCATACCAGGGGTTTTGGTGTGGCCGTTCTTCG
CGCCGTTTTTGTACCACAGCTGATTGCTGTCTTTCGCCCCGGCCAGTTCACCCGGTTTGAAGGGGAATTTGAAGGC
CGGGATCACCGGCTTGGCCTCACTGTCGAGGCTGTCTGGATCAGCGAGTTCGGCGCTTTCAACAGGCGGTTGTGTC
GGGCAATTCATGGAAGCTCCGGAGCGTGCAAAAAATGACGCGGGCCCGACTTGCGGGCCACACTGGCGCGCAGTAT
ACCTGCGCGCCCTGGTTCTTTAACCACTGCCCGTACGAATGGTCGACCTTTGCTGTCAGCGCTGTCAGTTAACGGT

CACTTTGCTGACCGGGTTTGCCGGCTATAAAGAGCCTTCACTCTTCCCATTCTCTGTCCGGACGCTGCGAGCCCAT
GCACATTCAACGCAAAACCACCTTGATTGTGCTCGCCGTCGTGGCCCTGGCGGTCGTGGCCTGGGTCGCGACCCGG
CCGGCCAAGACCAAGCTGTCCGCCTCCACTGCGATCCCGGTGCGGGTGGTCAGTGTCGCCCAGCAGGACATCCCGC
GGTTTGTCAGCGGCATCGGCTCGGTGCTGTCGCTGCACAGTGTGGTGATTCGCCCGCAAGTCGACGGCATCCTCAC
CCGATTGCAGGTCAAGGAAGGGCAACTGGTCAAGGCCGGCGACCTGCTGGCGAGCATCGACGACCGGGCGATCCGC
GCCAGCCTCGACCAGGCCAGGGCCCAGTTGGGCGAAAGCCAGGCGCAGTTGCAGGTGGCCCAGGTCAATCTCAAGC
GCTACAAGGAGCTGAGCATCGACGACGGCGTGTCGAAGCAGACTTACGACCAGCAGCAAGCCCTGGTCAATCAGCT
CAAGGCCACGGTCCAGGGCAACCAGGCGGCGATTGATGCGGCCCAGGTCCAGCTTTCCTACACGCAGATTCGCTCC
CCCGTCAGCGGTCGCGTCGGGATTCGCAATGTGGATGAAGGCAACTTCCTGCGCACCAGCGACACCCAAGGCCTGT
TCTCGGTGACGCAGATCGACCCGATTGCGGTGGAGTTTTCCCTGCCACAACAGATGCTGCCGACCCTGCAAGGCCT
GATCGCCGCGCAACACCCGGCCAGTGTCGATGCGTTCCTGGGGGCCGACACCGAGAGCCCGGCCGCGATCCTGCTC
GGCGAGGGCCGCCTGAGCCTGATCGATAACCAGATCAGCGCCACCACCGGCACCATCCGCGCCAAGGCCGAGTTCA
GCAATGCCGCGCAAACGCTGTGGCCAGGGCAACTGGTCACGGTAAAGATCCAGACCGCCCTCGACAAGGCCGCCCT
GGTGGTGCCACCGACCGTGGTCCAGCGCGGCATGGACTCGCACTTTGTGTACCGCCTCAATGGCGACAAAGTGGAC
GTGGTGCCCGTGCAGGTGACCTACCAGAACAGCGACCTGACGATTGTCACCGGCGTGCAGCCCGGTGATGTGCTGG
TCAGCGACGGCCAGTCACGCCTCAAGGCCGGCGCCCAGGTGGAGGTGCTCAAGGAGCCGCCGCAAGTGATCCAGAC
CGCCCAAGCGCAGGCCCGGCCATGAAGGGCCGTGGCTCGGTATCGGCGTGGTGTGTCGACCATCCGGTCGCCACCC
TGCTGTTGACCTTTGCCCTGGTGCTGCTCGGGCTGATCGCCTTCCCGCGCCTGCCGGTCGCGCCGCTGCCGGAGGC
GGAGTTCCCGACGATCCAGGTCACCGCCCAACTGCCCGGCGCCAGCCCCGATACCATGGCGTCCTCGGTGGCAACG
CCCTTGGAAGTGCAATTCAGCGCCATCCCCGGCATGACCCAGATGACGTCCAGCAGCGCCCTGGGTTCCAGCCTCC
TGACCCTGCAATTCACCCTCGACAAGAGCATCGATACCGCCGCCCAGGAAGTCCAGGCAGCGATCAACACCGCGGC
CGGCAAGTTGCCCAGCGATATGCCCAACCTGCCGACTTGGCGCAAGGTCAACCCGGCGGACAGCCCGGTGCTGATC
CTCAGTATCAGCTCCAACAACATGCCCGGCACCGAACTGAGCGACTACGTGGAAACCCTGCTGGCCCGGCAGATCA
GCCAGATCGACGGGGTCGGCCAGATCAGCATCACCGGCCAGCAACGCCCGGCGATCCGCGTGCAGGCCTCGCCGGA
CAAGCTCGCCGCCATCGGCCTGACCCTGGCCGACATCCGCCTGGTGCTCCAGCAATCGAGCCTGAACCTGGCCAAG
GGCGCGCTGTATGGACGCGACAGCGTGTCGACGCTGTCGACCAACGATCAACTGTTCCACCCAGAGGAATACGGCG
ACCTGATCGTTTCCTACAAGGACGGCGCGCCGGTGCAGTTGCGCGATGTGGCGCGGGTGATCAATGGTTCGGAGAA
CGCCTACGTGCAGGCCTGGTCCGGTGATACGCCGGGGGTCAACCTGGTGATTTTCCGCCAGCCGGGGGCCAATATC
GTCGACACCGTGGACCGCATCCAGGCCGAGTTGCCGCGCCTGCAGGCAATGCTGCCGGCGTCGGTGGACGTCAGCT
CGTTGTCGGACCGCACCAAGACCATTCGCGCCTCGCTGCATGAAGTGGAAATCACCCTGTTGATCGCCGTGTTGCT
GGTGGTGGCGGTGATGGCGCTGTTCCTGCGCCAATGGTCGGCAACCCTGATTGTTTCCAGCGTGCTGGGAGTATCG
CTGGTGGCCACTTTTGCCTTGATGTACGTGATGGGCTTCAGCCTGAATAACCTGACCCTGGTGGCCATCGTCATCG
CCGTGGGTTTTGTGGTGGACGATGCGATTGTGGTGGTGGAGAACATCCACCGCCACCTTGAGGCCGGCCTGGACAA
GCGTGAAGCAGCAATCAAGGGCGCCGGCGAGATCGGGTTTACCGTGGTGTCCATCAGCTTCTCGCTGGTGGCGGCG
TTTATCCCGTTGCTGTTCATGGGCGGCGTGGTCGGGCGCCTGTTCAAGGAATTTGCCCTGACGGCGACCTCGACCA
TCCTGATTTCGGTGGTGGTGTCCCTGACCCTGGCGCCGATGCTGGCCGGGCAGTTCATGCGCGCCCCTAGCCATCA
TCCCCACGACAAGCCGGGCTTTGGCGAACGCCTGCTGGCCGGCTATGCGCGGAACCTGCAACGGGCACTGGCCCAT
CCGCGCACGATGCTCGCTGTCTTCACCGTGACCCTGGGCCTGGCGGTGGCCGGCTACGTGTTTATTCCCAAGGGGT
TTTTCCCGGTACAAGACACCGGCTTTATCCTCGGCACCAGCGAGGCGGCGGCGGATGTGTCCTACCCGGATATGGT
GGCCAAGCACCAGGCCCTGGCCGAGATCATCAAGGCTGACCCGGCGGTGGAAAACTTCTCTCATTCGGTGGGTGTC
ACCGGCAGCAACCAGACCATCGCCAACGGCCGCTTCTGGATTGCCTTGAAGGATCGGGGTGAGCGTGATGTGTCGG
CCAGCCAGTTTATCGACCGGATCCGCGCCAAACTGGCCCAGGTGCCGGGGATCGTGCTGTACCTGCGCGCCGGCCA
GGATATCAACCTCAGTTCCGGGCCCAGCCGCAGCCAGTACCAGTACGTGCTCAAGAGCAACGATGGCGCGACCCTG
AACACCTGGACCCAGCGCCTGACCGAAAAACTGCGCGCCAACCCGGCCTTCCGCGATCTGTCCAACGACCTGCAAC
TGGGCGGCAGCATCACCCACATCAGCATCGACCGCCAGGCGGCCGCACGGTTTGGACTGACGGCCACCGATGTCGA
TCAGGCGCTGTACGATGCGTTCGGCCAACGCCAGGTCAATGAATTCCAGACCGAGACCAACCAATACCAAGTGGTG
CTGGAGCTGGACAGCCAGCAACGGGGCAAGGCCGAGAGCCTGAATTATTTCTACCTGCGCTCGCCGCTCAGTGGCG
AGATGGTGCCGTTGTCGGCGCTGGCCAAGGTCGACCCGCCGACGGTCGGGCCGTTGTCCATCAGCCATGACGGCAT
GTTCCCGGCGGCCAACCTGTCGTTCAACCTGGCACCGGGCGTGGCCCTGGGTGACGCCGTAATCATGCTCGACCAG
GCGAAGAACGAAATCGGCATGCCGAGCACCATCATCGGTAACTTCCAGGGCGCGGCCCAGGCGTTCCAGAGTTCAT
TGGCCAGCCAGCCGTGGTTGATCCTCGCGGCGCTGGTGGCGGTCTATATCATCCTCGGCGTGCTGTATGAGAGCTT
CGTGCACCCGCTGACGATCATCTCGACCTTGCCCTCGGCCGGCCTGGGCGCGCTGATCATGCTCTGGCTGCTGGGC
CAGGACTTTTCCATCATGGCGTTGATCGGCCTGGTGCTGTTGATCGGCATCGTCAAGAAAAACGGCATCCTGATGA
TCGACTTTGCCCTGGAAGCCCAGCGCGTGCGTGGGCTGGCACCTGGCGAGGCGATTTACGAAGCCTGTGTCACGCG
GTTCCGGCCGATCATCATGACCACCCTCGCCGCCCTGCTGGGCGCGGTGCCGCTGATGCTCGGCGCAGGCCCCGGC
GCGGAGTTGCGCCAGCCCCTGGGGATCGCGGTGGTCGGCGGGTTGCTGGTGAGCCAGGCGCTGACGTTGTTCACCA
CGCCGGTCATATACTTGTACCTTGAGAAACTTTTCCACAGACCCAAACCGGCCCTCGGGTTGGCGACCACACATTG

AGGCGGGGTCATGCGCGTCCTGATTATTGAAGACGAAGAAAAAACCGCCGACTACCTGCATCGCGGGCTGACGGAA
CAAGGCTACACCGTGGACGTGGCCCGCGACGGCATCGAGGGCCTGCACCTGGCCCTGGAAAGCGACTACGCGGTGA
TCGTGCTCGATGTGATGCTGCCCGGCCTCGACGGCTTTGGCGTGCTGCGCGCACTGCGGGCCCGCAAGCAGACGCC
AGTGATCATGCTCACCGCCCGCGAGCGTGTGGAAGACCGCATTCGCGGCCTGCGCGAAGGCGCCGACGATTACCTG
GGCAAGCCGTTTTCGTTCCTCGAACTGGTGGCGCGCCTGCAAGCCCTGACCCGGCGCAGCGGTGGGCATGAGCCGG
TACAGGTGAGCATCGCCGACCTGTGGATCGACCTGATCAGCCGCAAGGCCAGCCGCGCCGGCCAGCGCCTGGACCT
GACCGCCAAGGAGTTCTCGCTGCTCAGCGTCCTGGCCCGGCGCCAGGGCGAAATCCTGTCGAAGACGGCGATTGCG
GAAATGGTCTGGGACATCAATTTCGACAGCGATGCCAATGTGGTCGAAGTGGCGATCAAGCGCCTGCGGGCCAAGC
TCGACGGGCCATTTGAGCACAAGCTGCTGCACACCATCCGCGGCATGGGCTATGTGCTGGAGAATCGCAGTGTCGA
GTAACTCGATTGCCCTGCGCCTGAGCGGCATGTTCACCCTGGTGGCGCTGGTGATTTTCCTGCTGATCGGTGGCGC
GTTGTACCAGCAGGTGGACCGTGGCCTGGGCTTGTTGCCGGGGGCCGAGTTGGATGCGCGCTACAGTGTGCTGGAG
TCGTCGATCACGCGTTTCGGCACGCCGGATCATTGGGTGAAGATGGCCAGCAAACTCAAGCTGCTGGGGGAAGAGG
ACACGCGCATTCGTTTCTGGGTGGTGAGCAGTGACCCGACCTATGAGTACGGCAAACCCGACGCACGTATCCGCGA
ATTCGCCGCCGGCCCCACGGGCAAGCGTGATCTGTACCTGCCGGGCCACGCCTATCCATTCAAGGTGCTGGTCAGT
CAGTTCCCGGCCAAGGACCAGCGCCCGCCGCTGCGCTTTATGATCGCCATCGACACCGCCAACTTCCGCGCCACCC
AGCACCATCTGCTGATGGCGTTGATCAGCCTGTCGTTTGTCGGCGTGGTACTGGCTTCGCTGCTGGGCTTCTGGGT
CGCGCGCATCGGTCTCAAACCGTTGATCAAGCTGTCGGATGAGGCGCAAAAACTGGCACCGCCCAAACTGTCGGGG
CGCTTGCAGTTGTCGCCGTTGCCGCCGGAGCTCAAGCAGTTCGTCAACTCCTTCAACTCGACCCTGGAGCGGGTGG
AAACCGCCTACTCACGCCTGGAGTCCTTCAATGCCGACGTGGCCCACGAACTGCGCTCGCCACTGACCAACCTGAT
CGGCCAGACCCAGGTGGCGCTGACCCGTGGCCGCTCGGCCGAGCACTACTTCGAAGTGCTGCAATCGAACCTGGAA
GAGTTGGAGCGCCTGCGCTCGATCATCAATGACATGCTGTTCCTGGCCAGCGCCGACCAGGGCAGCAAGGCCACCA
AACTGATTGAGTGCTCCCTGGCCGATGAGGTGGCGACGACCCTCGAGTACCTGGACTTTATCCTCGAAGACGCCCA
GGTGCGGGTCACCGTGGTCGGCGATGCCCAGGTCCAGATCGAAAAGGCTCATTTGCGCCGGGCGCTGATCAACCTG
CTGAACAATGCCGTGCAGCACACCTCGCCGGGGCAGGAGATCCAGGTACGGATTGAAACGCAGGAGCAGCAGGTGC
TGATTGGCGTGACCAACCCAGGGGAGCTGATCGCCAGCGAACATCTGCCGCGCTTGTTCGAGCGCTTCTACCGCGT
CGACGCCTCGCGCAGTAACAGCGGGGCCAATCATGGGTTGGGGCTGGCCATCGTCAAGGCGATTGCCTTGATGCAT
GGCGGGGATGTGTTTGTGCGCAGTGAACACGGTGGCAATACCTTTGGTATCACCCTGCCCATCTAATTTTTTTAAA
CCACTACACAATCAAAATGTGGGAGCGAGCAAGCCCGCTCCCACACAAGCTCATCCCCATAGGTAGATCGCCGTCT
CGACTGTTGCGATTTGGGCAACAGTCATTATCTTGTTACACTCTGTACCGTACCGCTCGCCTGCGCCAATATGTCG
CATCGACGAGCGAGACGGCAAAGACAGCCGACCCGCCCTGCTATTTCCCTCGACTTATTTCCAGGGCTCTACACTG
GGAATCTGTCTTAAAGCCGCTGACTTCAGCGGCTTTTTTTTGCCGTAAAAAAGGCCAGGAACACCCCACAACATTG
GCCGTTCAGACTCATCCAAAGGAGCTTCCCCGGTGAAACCCGCGCTGACATTCACCCCGTTATTCCTCGCGATTGC
AGCAACTATCTCCCCTCTCGCCCATGCCGCAGATGCCCCTCCCACCGACGGATTCGTCGAAGGCTCCAGCCTCACT
ATCAACACCCGCAACTACTACATGAACCGCGACCGCCGCGACATCCACACTGACGACAGCAAAGAGTGGGGCCAAG
GCTTCCTCGGGGTCTTCGAGTCTGGCTACACCCAAGGCACCGTGGGTTTTGGCCTGGACGCTCATGCCATGCTCGG
CCTGAAACTGGACGGCGGTGGTGGCACCGACGGCTCCAGCATCCTGCCGTACGGCAGCGGCAACGGCAAAGCGCCG
GGCTCGTTTTCTACCGGTGGCGGCACCCTGAAAATGCGCGCCTTCGACACCGAACTCAAAGCCGGCGACCTATTCC
TCAACAACCCGGTGATTGCCGGTGGCATGACGCGCATGCTGCCCGAGACCTTTCGTGGCGTCAGCCTGACCAACCA
CAGCCTTGACGGCTGGATGTTCGAAGGTGGCCAGGCGAGTTTCGCCAAGCTCTATAACCAGAGCGGCCATGGGCGT
ATCGGCACCAGCTACGGCGCGCTGCCCAAGGGCCAGGACAGCCAGCACATGAACTGGGCCGGCGTGGCCTGGAGCG
GCCTGCCAGGGCTGACCAGCAACCTGTACGCCTCTGAACTCAAGGACGTGTGGAACCAGTACTACTACGACCTGGA
CTACACCTACGCGCTCAATGACCTGGTCAGCCTCAACCCCGGCCTGCACTTCTACCACACCCAAGACACCGGCCAT
GCCCTGCTGGGGGATATCGACAACAACACCTACAGCCTGCATTTCACCGTGGGCGTGGGCAATCACAGCGTCACGG
CCGCGTACCAGCGGGTCAATGGCAACACGCCGTTCGACTACATCGCCCAGGGCGACAGCGTGTACCTGGACAACTC
CCAGCAATACTCGGACTTCAACGGGCCCAACGAGCGCTCGTGGAAGCTCAAGTACGCCTACGACTTCGCAGGCCTT
GGCGTGCCCGGTCTGACCTCGGCGGTGTCGTACATCAGCGGCAAGACCGACCTGACCAAGGTCGACCCCAACAGCC
GCGGCTACTCAGCCTGGTACAGCGCCGAGGGCAAGAATGCCAAGCACTGGGAACGGGATATCGACCTCAAATACGT
GGTACAGGGCGGCAAGGCCAAGGACTTGGCGGTGCGCCTGCAATGGGCGACCAACCGTGGCAGCAATGGCTACTCG
GCGGTGGACCGTGATGTGGATGAGTACCGGGTAATCGTCGACTACCCGATCAATGTGTTCTGACGGCGAATTATCT
TTTTCGCCGATTGCTTAATTTCAGGCGCGCGGAATTAAACTGCCATCATCGTCACCGCCGAAGTCAGTCCGATGAG
TCAACGCCGCGCCCTTAAACTGCCTGCACGCCCGGAGCTTTTGCTGATTCTGGGCAGTGGCCTCACCGTTGTGCTG
ATTCTGATCATCGTCGCCGTGCTGCTGATCCGCGAGCACGCCAGCACGTTACAGACCGCCCAGCGCTCCACCAGCA
ATATCACCCAGTTGATCAATGCCGATGTGCTGCGCAACGTCGAGCTGTATGACTTGGCGCTGCAAGGGCTGATTGC
TGCTGCAGGTCGGGATTTGTCCGGACTGCCGGGCGATGTGCGGCACCAACTGCAATTCGACCAATCCACCGCAGCG
CCCTACAAAGGCGAAGTCCTGCTGTTGGACGCCCAGGGCACGGTGCTGGCCGACTCGTCGACGCAGACCCCCACCC
TGCGCAACTTCGCCGACCGCGACTACTTCCAAATCCATCAGCAAAACCCCGAGACCGGCCTGTTTATCAGCCGCCC
GTTCAAGATCCACTGTGACTGCGAGCAAATCTGGCGCATCGCTTTCAGCCGCCGCGTGACCGGGCCCAATGGCGAA

TTTACCGGGGTAGCGGTGGCGACCATGCGCCTGGCGTATTTCGACCAGTTGTTCAGCAGCCTGACCATTGGTAGCG
GCAACACCATCAACCTGCTGAACAATCAGGGCATCCTGCTGGCCCAGCAGCCCCTGCTCGAACGGGACATGATCGA
CAAGGACCTGAGTTCGCGCCCCAACTTCAAGCGCATGCTGCAAGAGGGCAGCGGCAGCTTCCGGGCGATCTCTGCG
ATCAGTGGTACCCCCCGGCTTTACACCTTCACCAATGTCGGCCAGTTGCCATTGATCGTGGTGGTGGCCTTGGCCA
GCGACGATGTATTCGCCGCCTGGCAACGCGCCGCATGGCTGACGGCAGGCGCCACCGGGGTGTTGTGTGTGGGCCT
GTTATGGTTGAGCTGGATGTTGCGCCTGGAACTACGGCGGCGTTACCGCGCCGAGAAGGTGCTGTCAGAACTGGCA
GCCACCGACGGCCTGACCGGCCTGGCCAACCGCCGTATCCTCGACCAGCGCCTGACCCTGGAATGGGACCGCGCCC
GCCGCTCCGCCGAGCCCTTGGCGCTGCTGATGATCGACGTCGACCACTTCAAGGCCTTCAACGACCGCCATGGCCA
CCTGGGCGGCGACGAAGCGCTGCGCAGCGTGGCACAGGTGATCGGCAGCCAGATCCGCCGCCCGGCCGACCTGGCG
GCGCGTTATGGCGGCGAAGAGTTTGCGGTGATCCTGCCCAATACCGATGCCAGCGGTGCCCGGGCGATTGCTGAGC
GTATCCGCCAGGGGATCCAGCACTTGCCGCCGGTTGCGGGTGATAGTGAGCCAGTTACGGTGAGTATCGGCTTGAG
CACCTGGGACAAACGCAGCAGCGCGTCATTGGAGCAGTTGCTGCTCAGTGCTGACCGGGCGTTGTATGAGGCGAAA
AATAGCGGGCGCAACCGCGTGGTCGATGCGGCCACACTGCTGGCGCCTTGAATGTAGGAGCCGGCTTGCCGGCCCC
TATAGGCGGATATTTGCCAATAAAAAAAGGCCACCCGAAGGCAGCCTTTAAAAACTAAAGAAAGAGAGTTGTTACT
TACACTGCCGCAACGGGACGCATGTAAGAGATCGGTGCGGTGCTGGCATCTTCGAAGGTCACGACTTCCCAAGCAT
CTTTCTGCTCAATCAATTTGCGCAGGAGCTGGTTGTTCAGTGCATGGCCCGACTTGAAGCCTTTGAACTCGCCTAT
CAGGCTATTGCCCAGCAGGTAGAGGTCACCAATTGCATCGAGGATCTTGTGCTTCACGAACTCGTCTTCATAGCGA
AGGCCGTCTTCGTTCAGTACACCATCCGCGTCGACCACGATGGCGTTTTCCACGCTGCCGCCGAGTGCGAGGTTGT
GCTTGCGCAGGTACTCGATGTCACTCATGAAACCAAAGGTACGGGCGCGGCTGACTTCTTTTACGAACGAAGTGCT
GGAAAAATCCACGCTTGCACTTTGGGTGCGGTCACGGAATACCGGGTGATCGAAATCGATCTCAAAGCTCACTTTA
AAGCCTTCGAACGGGACGAAGGTGGCGCGCTTGTCGCCGTCTTCTACTGTCACTTCCCGCAGAATGCGAATGAACT
TCTTGGCTGCGTCCTGTTCTTCCAGGCCGGCAGATTGAATCAGGAATACGAAAGGTCCAGCGCTACCATCCATGAT
CGGGACTTCAGACGCGGAGAGCTCGACGTAGGCGTTATCGATGCCCAGGCCAGCCATGGCCGAGAGCAAGTGCTCC
ACCGTGTCCACTTTGACGTCACCGTTGACCAATGTGGTCGACATGGTGGTTTCGCCAACGTTTTCCGCGCGAGCAG
GAATCTGCACCACAGGGTCCAGGTCGGCACGAACAAACACGATGCCGGTGTCGACAGGTGCGGGCTTGAGGGTCAG
GTATACCTTCTCCCCGGAGTGCAGACCTACACCTGTGGCACGGATAATATTCTTCAGGGTGCGTTGTTTAATCATG
GCTTGGACCGCTTCAGCGCAAATTGCGAACTGGTATCAACAAAGGCTGGCGATAATAACAGACCAGACCTTTGCTG
AACACCAATCACCTTCATAGCCCTGATACATTCCATCAATCGGCCTGACGACGCAGGAAAGCCGGGATGTCCAGGT
AATCCAGATCATCTTGCGGATTCGGGCTACGGGACGCAGCAGCACCGGCCTGAGCCTGGTTGCGCATCACGGTCGG
ACGATCCAGGTCACGGTAGTTCACCGACGGCATTTCCGGGCGCACAGGCGCTGGAGCGGCAGTCGAAGACGCCTGG
CTGGTGTGAACGGTATTGTCGATGACCTTCACAGGCTTCTCGATTTTAGCGCCCAGGCCAGTGGCAACCACGGTCA
CATGCAGTTCGTCGCGCATGTCCGGATCGATAACGGTACCGACCTTGACCATCGCGTGCTCGGAAGCGAAGGCTTC
GATGATGCTACCCACGTCGGAGTACTCACCCAGGGACAGGTCAGGACCGGCGGTGATGTTCACCAGGATGCCGCGT
GCACCTTGCAGGTTCACGTCTTCGAGCAACGGGTTGCGGATGGCCGCTTCGGTCGCTTCACGTGCACGGTTAGGAC
CGCTGGCGCAGCCAGTGCCCATCATCGCCATGCCCATTTCGCTCATGACAGTGCGTACGTCGGCAAAGTCGACGTT
GATCATGCCCGGGCGCTTGATGATGTCGGAGATACCGCGAACGGCACCGGCCAGTACGTCATCAGCCTTGGCGAAA
GCCGACAGCAGGCTTGCGTCTTTACCGAGGATGGTCAGCAGTTTCTCGTTGGGGATGGTGATCAACGAGTCAACGC
TTTCAGACAGCAGACGGATACCTTCGTCGGCGATCTGCATGCGCTTGCGGCCTTCGAACGGGAACGGACGGGTCAC
GACAGCAACCGTCAGGATCCCCATTTCCTTGGCCACTTCGGCAATGATCGGCGCTGCACCGGTACCGGTACCGCCG
CCCATGCCAGTGGTGATGAACACCATGTTGGTGCCTTGCAGCACTTCGGCAATACGCTCGCGGTCTTCCAGGGCGG
CTTGACGGCCGACTTCCGGATTGGCGCCAGCGCCCAGGCCCTTGGTCACCGCAGTGCCCAATTGCAGGATGGTCCG
CGCGCCAATGCTCTTGAGCGCCTGGGCATCAGTGTTGGCGCAGATGAATTCAACGCCTTCAATGTTGCTCTTGACC
ATATGGTTGACAGCGTTGCCGCCGCCACCGCCGACACCGATTACTTTGATAACCGGGCTTGCGGGGATGTTGTCTA
CGAGTTCGAACATTTTCCCTCTCCTTTCATTTCTCTAGTTTTTTCGCCTACTACTTGAAACGGTGTTGCGGTAAAT
CTTTAGAAATTGCCCTTCACCCAGGCTTGCAGGCGCTCCAGCAACGGCGCTTTAGGCTCGTCACTGCTGTAGCTTT
CGCGGCTGCCAATGCCCGACAGGGAAATGCCGTCGGTCTGCTTTTGCAGGCCGTACAACAGCAAGCCCACACCGGT
GGAATAAATCGGGTTGCGCACCACGTCGCCCAGGCCCTTGACGCCATGGGGCACGCCCAGGCGGACCGGCATGTGG
AAGATTTCCTCGGCCAGTTCGACCGCGCCTTCCATCTTCGAGGTGCCACCGGTCAGCACGATGCCGGCCGGGATCA
AATCTTCGTAGCCACTGCGGCGCAGTTCAGCCTGGATCAGGGTGAACAGTTCGTCGTAGCGCGGCTCGACCACTTC
GGCCAGGGCCTGGCGCGACAGTTCGCGCGGTGGACGATCGCCCACGCTTGGCACCTTGATGGTTTCACCGGCACCG
GCCAGCTTGGCCAGGGCGCAGGCGTAGCGGATCTTGATTTCTTCGGCGTACTGGGTCGGTGTACGCAACGCCATGG
CGATGTCGTTGGTCACCTGGTCGCCGGCAATCGGGATCACGGCGGTGTGACGGATCGCACCCTCGGTGAAGATTGC
GATGTCGGTGGTGCCGCCGCCGATGTCCACCAGGCACACGCCCAGTTCTTTTTCGTCGTCGGTCAGTACCGAGTAG
GCCGAGGCCAGTTGCTCAAGAATGATGTCGTCGATTTCCAGGCCGCAGCGGCGCACGCATTTTTCGATGTTCTGTG
CAGCATTCACCGCGCAGGTCACTACGTGGACCTTGGCTTCCAGACGTACGCCCGACATGCCCAGGGGCTCACGTAC
GCCTTCCTGGTTATCGATCACGTAGTCCTGCGGCAGGGTGTGCAACACGCGCTGGTCAGCCGGGATCGCCACGGCC
TGGGCGGCATCAAGGACGCGCTCAAGGTCGGCCGAGCTGACTTCACGGTCGCGGATCGCCACGATGCCGTGGGAGT

TCAGGCTGCGGATGTGATTGCCCGCCACGCCGACGAACGCCGAGTGAATCCGGCAGCCGGCCATCAGTTGCGCTTC
TTCGATGGCGCGCTGGATCGATTGCACGGTGGATTCGATATTGACCACCACGCCCTTCTTCAGGCCCCGGGACGGA
TGCGTGCCGATTCCGACGATTTCCAGCGTGCCGTCGTCCGCGACCTCGCCTACCAGCGCCACCACCTTGGAGGTGC
CGATATCGAGACCGACGATCATTTTGCCGCTTTGCACGTTTGCCATGGGTCCTGCCTCTTCTTAATTCTTCGCGAC
AGCGGGTTGCGCTGCCGTGGGCGCAGCCGGTTCGCGCCAGCTAACGGCCAGGCCGTTGGCATAACGCAGGTCGATG
CTCGCAATGTTCGTAATCTGTTCTTTCAGGGTTTTGTCGTAGATGGCGATAAAGCGGCGCATCTTCTCCACCAAGT
GGTCGCGTCCCAGCAACAACTCGATGCCCGGGCCGGAACTGCCTGCCCCGGTGGTCAGGAACCAGCTGCCCCGCTC
GCGCAATTCCAGGCGCGCAATGGAGAAGCCCAGTGGCCGCAGCATCTGGCTCAAGGCCTGGTACTGCTGCATCACT
TGCTGCTGTGCCCGTTGCGGTCCGAACAACTGCGGCAAATGCTCGTAGTTGGCCAACTCGCGCGGGTTGAATGCCT
GGCCCTGGTTGTTCAACAGCGCCTCGTCGCCCCAACGGGCCACGGGCAGTTGTTCTTCCAGGCGGATCGTCACTTG
ATCCGGCCACACGCGCCGTACTTCGGCGTGGGCAATCCACGGCATCTGTTCCAGTTCGGCACGCATGCCTGCCAGG
TCGATGGTGAAGAAGCTCGCGGCCACGAAAGGCGCGATCCGCTGCTGCACCGCTTGCTGGCTGATGTAGCTCAAGT
CGCCCTGCACGCTGATCTTGGTGATCGGGCGGTCGGCATACGGCAACAGCCGCTGCGCACCTTCATAGGTACCAAA
GCCCAGCGCTACCAGCAGTACCGGCCAGAACAAGGCCTTGAGAAAACCAAAGTTGGCTTTCGGCAGGCGCGCCGAC
ATCGGCTCTTTAGCCACCATTCGACTGGCACCACGCGGCACCGGCTTGCGGCCGGGAGCGGGTTGCTGATGACGAA
GCGATGCGCCTTGCATGGTCTTAACCTCGTGCCTCTGTATAGCTAGCCGCCAGAATTGCCAGCACCAACTGCTGGA
AGTCCAGGCCGGCTGCCCGTGCAGCCATGGGTACCAGGCTATGGTCGGTCATGCCCGGTGCAGTGTTGACTTCCAG
GAACCAGAAATTCCCTTGGTCATCCTGCATCACGTCTGCCCGCGCCCAACCAGCGATACCCAACGCCTCACAGGCT
TTCGCCGTGAGGTCCATCAATTCCTGCTCTTTGGTTGCATCGAGGCCGCATGGGATCCGGTACTGGGTATCGGAAG
CCACATACTTGGCGTCGTAGTCGTAAAAGGTGTGGGGCGTGCCCAATGCGATGGGCGGCAATACCTGGCCACGCAG
GGTGGCGATGGTGTACTCGGGACCCTGGATCCACTGTTCCACCAACACTTGCGAATCGTAGGTACTTGCCGCTTTC
CATGCGTCGATCAATTCGGCGGCCGAGTTCACTTTAGCCATGCCGATACTGGAGCCTTCATGGGCTGGTTTGACGA
TCAAAGGCAGGCCCAGTTCCTTGGCTGCAGAAATACAATCGTCTTCGCTGCACAGCACACTGTGACGCGGCGTCGG
AATGCCCAGGCTGTGCCACACCTGCTTGGTGCGCAACTTGTCCATGGCCAGTGCCGAAGCGAGGATGCCGCTGCCG
GTGTAAGGAATACCGGCGCACTCCAGCAGGCCCTGCATGCTGCCGTCTTCACCGCCACGGCCGTGCAGGATGATGA
AGGCGCGGTCGATCTTCTCGGCCTGCAGGCGGGCGAGGAAGTCATCGCCTACGTCGATGCCGAAGGCGTTCACACC
CGCGCTTTGCAGCGCTTGGAGCACGGCGTTGCCAGACTTGAGCGACACTTCGCGCTCAGCGCTCTTGCCGCCGAAC
AGCACGGCCACGCGGCCGAAGTCGGCAGGCGCGATGGTGGAAAACAGGGAGCCGTAGTTCGTGGTCATTTCGACTT
CCCCTGCACAGCAGCGGCCGCAAACAGCGGGCTCGCCAGCAATTTAGGGGCAAGGCCGCCGATATCACCGGCACCC
TGGCACAGCAGGATGTCGCCGGCACGCAGCAGCGGCTTGACGATCGGCGCGAGGTCGACGCCACGCTCGATATAGA
TCGGGTCCAGTTGCCCGCGCTGGCGGATGCTGTTGCACAGCTTGCGGCTGTCGGCGCCGGGGATCGGTTCTTCACC
GGCCGGGTAGACTTCCATCAGCAGCAACACGTTGGCATCGGCCAATACATTGACGAAATCGTCGTACAGGTCGCGG
GTGCGGCTGTAGCGGTGCGGCTGGTAGACCATCACCAGGCGGCGCTCCGGCCAACCACCGCGCACGGCCTTGATCA
CGGCGGCAACTTCGGTCGGGTGGTGACCGTAGTCGTCCACCAGCATCACGTCGCCGCCGTCTACCGGCAACTGGCC
GTAGACCTGGAAGCGCCGGCCCACGCCAGCGAAGCCCGACAGGCCTTCGACGATGGCTTCATCGCTGACACCCTCA
TCGGTGGCGATGCAGATGGTCGCCAGGGAATTGAGCACATTGTGGTTGCCCGGCATGTTCACCGACACATCCAGTG
GTTCGCGATCCGGGCGCAGCACGGTGAAGAAGGTCTGCATGCCTTGCTGACGTACATTGATCGCACGCACGTCGGC
GTCTTCGCTGAAGCCGTAGGTCACGGTCGGACGCTTGACCAGCGGCAGAATTTCCCGCACCACCGGATCGTCCAGG
CACACCACGGCCAGGCCGTAGAACGGCAGGTTGTGCAGGAATTCGACGAAGGTTTTCTTCAGTTTGTTGAAGTCAC
CGTCGTAGGTCGCCATATGGTCGGCGTCGATGTTGGTGACCACGGCCACCAGCGGCTGCAGATGCAGGAAGCTGGC
GTCGCTTTCATCGGCTTCGGCGATCAGATAGCGGCTGGTACCCAGTTGTGCATTGGTACCTGCTGCATTCAGCCGG
CCACCGATCACGAAGGTCGGGTCCAGGCCGCCGGCGGCGAACACCGAAGCGATCAGGCTGGTCGTGGTGGTCTTGC
CGTGGGTACCGGCGACGGCGATGCCGTGGCGGTAGCGCATCAGCTCAGCCAGCATCTCGGCGCGGGGCACCACGGG
AATACGGCGCTCAAGGGCGGTGGCCACTTCCGGGTTGGAGGTGTTCACGGCACTGGAAACCACCAGTACATCGGCT
TGCGCGGCGTTCTCGGCACGGTGGCCGATAAAGATCTGCGCGCCGAACGACTCCAGGCGCTCAGTGACCGGCGAGG
TCTTGAGGTCGGAACCGGAAACCTGGTAACCCAGGTTCAGCAACACTTCGGCGATCCCGCACATGCCCACGCCGCC
GATACCGACGAAGTGGATGCGACGGATGCGGCGCATTTCCGGTTGCGGCATGGCTTTTTGATTCTCAACCATGGGC
CACCTCCAGGCAGATATCGACCACGCTGCGGGTGGCGTCGGGTTTGGCCAGGCGGCTTGCGGTGCTCGCCATGCTG
TTCAGTCGTTCGGGTTGCATCAAAACCTCAGTCAGGCGTGCGGCCAGATCGGCGGCGCCAGTCGTTCTTTGCGGCA
GCAGGAAGGCAGCGCCCTCCCCGGCCAAATATTCGGCGTTGCGGGTCTGGTGGTCGTCAATCGCATGGGGCAAAGG
CACCAGCAAGGACGGCAGACCGGCAGCAGCCAGTTCACTGACGGTCAACGCACCTGCGCGACAGACCACCAGGTCG
GCCCAGCCATAGGCATGGGCCATGTCTTTGATAAAGGGCTCTACCGTCGCCTCGACACCGGCCTCGCGATAGCGGG
CGGCAGTCACTTCATCGTGGTTCTTGCCCGCCTGGTGGAAGATCTCGGGACGCAGTTCCACAGGCAGTTGCGCCAG
CGCTTCTGGCAGCAATTTGTTCAACGGCTCGGCGCCCAGGCTTCCGCCCAGGATCAGCAGATGCGCCTTGCGCCCG
GCCAGGGCTTGGCGGGCAATGTCCATGAACAATTCGGTACGCACCGGATTGCCGGTGGTACGCCGCTTGTCCATGG
CCGCAAACGTATTCGGGAACGCCTCGCAGACCCGCGCCGCCAATGGCGCCAACAGACGGTTGGCAGTACCGGCCAC
GGCATTCTGTTCGTGGACGATCACCGGCACACCCGCCAACCGCGCGGCCACACCGCCGGGACCGGTGACATAGCCA

CCAAAACCCAGCACGCATACCGGCTTCAACTCACGGATGACCTTGCGCGCCTGCCACACCGCCTTGAGCAATACGA
ACGGCGCCTTGAGCAGGGACAATTTGCTCTTGCCGCGCAGGCCGCTGACGTTGATCAGGTGCAGGGGCAAACCGGC
AGCCGGAACCAATTCATTTTCAATGCCACGGGGCGTACCCAGCCAATGCACGGTGTAGCCACGGGCCTGGAACTCA
CGGGCACAGGCCAGCGCCGGGAACACGTGGCCGCCGGTGCCGCCGGCCATGATCAGCACGTTAGCGCCCATGGGTC
GGCTCCTCGGCGAAATCGCTCTCGCTGAATTCCATCTCTTCGCTGCCCAGATGGGTTCGACTCTCCCATTCGATAC
GCAGCAACAAGCCCAGACAGGCACAGCAGATCACCAGCGAGCTGCCGCCGTAGCTGAGGAACGGCAAGGTCAGGCC
CTTGGTCGGCAGCAGGCCCACGTTTACGCCGATGTTGATCAGGAACTGGCCGATCCACAGGAAGGCCAGGCCGTAC
GCGACATAGGCCGCAAAGAATTGCTTGGCTTTCTCCGCCCACAGACCGATGTACATGGCCCGCACGCAGACGAACA
CGAACAGCCCGACAGTGGCCAGGGAACCGACCACGCCCAACTCTTCGGCGAGCACCGAGAACACGAAGTCGGTATG
CGCTTCCGGCAGGTAGAACTGCTTCTGCACGCTGTTGCCCAGGCCCACGCCCAGCCACTCGCCGCGACCGAAGGCG
ATCAGCGCCTGGGTCAACTGGTAGCCGGAACCGAACTGGTCGGACCACGGGTCGGTAAAGGTGATCAGTCGCGCCA
TCCGGTAGGGTTGCGCCTGCACCAGGATCGTCACAGCGGCCACCGCCAGCCCGACCATCAGGGCAAAGCGGAACAG
GCCCACACCGCCAAGGAACAGCATCGCCGCAGCGGCGCCCATCATTACCACGGTGGCGCCGAAGTCCGGCTCCATC
AGCAGCAGGCCAGCCATGGGCAGCAGCACGATGAACGGCTTGAAGAAGCCCATCCAGCTTTCGCGCACTTCTTTCT
GTCGGCGCACCAGGTAACCGGCCAGGTAAATCACCACGAAGACCTTGGCGATTTCCGAAGGCTGCACGTTGAACGC
GCCGAAGCCGATCCAGCGCATCGACCCGTTCACTTCGCGGCCGATACCGGGGACGATCACCATCACCAGCAAGCCG
AAGGCACCGATCAGCATCAGCCAACCCAGGCGCTGCCAGGTGGCGATAGGGATCATCATGGTGACGATGCAGGCAC
CGAGACCAATCACCAGGTAAATCAGGTGGCGAATCATGTGGTAAAGGGTGTTGCCCGACTGTACCGCGGCCACTTC
CGAGGAGGCCGAGGTGATCATCACCAGGCCCAGGCCCAGCAGCGCCAGGCAACCGGCGAGCATCGGGAAGTCGAGG
TCGATCCCGCGCCCGGTAATGATCGGCGACGGGTACGGCTTGATGATGTTCTTCAGGTCCAGACTCATGCCAAGCC
CTCCACGGCGCGGGCGAACAACTGGCCGCGCTCTTCGTAGTTCTTGAACATGTCGAAACTGGCGCACGCCGGCGAC
AGCAGCACGGCATCGCCGGGCTGGGCCAGGGCACGGCATTGGGCGATGGCGTCGTCCAGGGAGGTGGCACGCACTT
GCGGCACCGCGTCGCCCAGGGCGGCGGCGATCAAGTCGCTGTCACGGCCCATCAACACCACCGCGCGGCAATGGGA
AGCCACCGGGCCCTTGAGGTCCTTGAAGTCGGCGCCCTTGCCATCGCCACCGGCGATCAGCACCAGCTTGCCATCG
ATATCGGCGCCCAGGCCTTCAATGGCGGCCAATGCCGCACCGACGTTGGTGGCCTTGGAATCGTTGTAGTAGCTCA
CGCCATCCAGGTCGCGCACCCACTGGCAGCGGTGCTCAAGCCCGGCAAAGGTGCGCAGGCTCGACAACATCGCGTC
GAACGGCAGGCCAACCGCGTGCCCCAACGCCAAGGCCGCCAGGGCATTGGACTGGTTATGGGCGCCACGGATCTTC
AGTTCACGCACCGGCATCAGGTTCTGGAATTCGAAGGCCAGGTATTTCTCGCCATTCTCTTCACGGATACCAAAGG
CCTTGAAATCGGGTTTGCTCAAGCCAAACGTCCAGCATGGCAGGCCTTCGCCCATCAACGGACGGCTCAGGGCATC
CTGGCGATTGACCACCACTTGCCGCGCGCCCCGGAAGATCCGGTGCTTGGCCAGGTGGTAAGCCGGCAGGCCGCTG
TAGCGGTCCATATGGTCTTCGCTGACGTTCAACACGGTGGCCACTTCGGCGCCGAGTTGATCGGTGGTCTCCAGTT
GGAAGCTCGACAGCTCCATCACGTACAGTTCGATATCGTCACTGAGCAGGTCCAGCGCCGGCGTACCGAGGTTGCC
GCCCACGGCCACGCGCTTGCCGGCCGCAGCCGCCATTTCGCCGACCAGGGTGGTGACGGTGCTCTTGGCGTTGGAG
CCGCTGATCGCCACGATCGGCGCCTTGGCGTTACGCGCAAACAGCTCGATATCGCCAGACAATTTCACCCCACGCG
CCGCGGCCGCTTGCAGCGCCGGGGTCGCCAGGGCCAGGCCGGGGCTCACGTAGAGTTCGTCGGCACGGCACAGAAA
CTCGACATCCAACTCGCCACAACGCACTTCCACGTGCGGGTAGTCACGGCGCAGCGTGACCAGCTCCGGTGGATTT
TCCCGCGTATCGGCCACGGCAAACGACGTGCCCCGGTTCGCCAGGAAGCGAACCAGGGACATGCCGCTCTTGCCGA
GGCCGACAACGATGCGGAAGTGGTCTGAAGCGATCAGGGACACTCGTTTCTACCTCAGTTTCAGGGTGGCAAGGCC
AACCAGCACAAGAATCACGGTGATGATCCAGAAACGGACGATCACCCGCGGCTCAGGCCAGCCCTTGAGTTCAAAG
TGGTGATGAATCGGCGCCATGCGAAACACACGGCGGCCAGTCAACTTGAAGGACGCAACCTGGATCACCACCGACA
GGGTCTCCATCACGAACACACCGCCCATGATGAACAGGACGATTTCCTGGCGCACGATCACGGCGATGGTGCCCAG
GGCCGCACCCAGCGCCAGGGCGCCGACGTCACCCATGAACACTTGGGCCGGATAGGTGTTGAACCACAGGAAGCCC
AGGCCGGCACCGATCAGCGCGCCGCAGAACACAATCAATTCACCGGCGCCCGGTACATAAGGGATCAGCAGGTATT
CAGCGAATTTCACGTTACCCGACAGGTAGCAGAAGATGCCCAGGGCGCCGCCCACCATCACTGTCGGCATGATCGC
CAGGCCATCGAGGCCATCAGTCAGGTTGACCGCGTTGCTGGAGCCAACGATCACGAAGTAGGTCAGCACCACGAAA
CCAATGCCCAACGGGATGCTGGCATCCTTGAGCATCGGGAAAATCAGGGTGGTTTCCACCGCGCTCGGCGCTGTCA
TATAAAGGAAGATCGCCGCGCCCAGGCCGAACACCGACTGCCAGAAATATTTCCAGCGGCTTGGCAGGCCCTTGGA
GTTCTTCTCGATCACCTTGCGATAGTCATCGACCCAGCCGATGGCGCCGAACAAGAGGGTTACCAGCAACACCACC
CACACGTAGCGGTTGGCCAGGTCGGCCCAGAGCAAGGTGCTGATGCCGATGGACGACAGGATCAACGCGCCGCCCA
TGGTCGGTGTGCCGGACTTGGACAAGTGCGACTGCGGGCCGTCATTGCGCACAGACTGGCCGATCTGCAGGTTTTG
CAGGGTGCGGATCATCCATGGCCCGAGAAACAGCGACAAACACAACGCGGTCAGCACACCCAGGATCCCGCGCAGG
GTCAGGTACTGAAAGACCGCGAAGCCTTTGTGGAACTGTTGCAGATACTCAGCCAGCAGCAGCAGCATTAATGTTT
CTCCGTACTTGAGCCACACAAGGCCGCGACGACGTTTTCCATCACCGCGCTGCGCGATCCCTTGATCAAAATGGTT
GTGTGTTTGTCTTGTTCGGCCATACCCAGCGCCTGGATCAATTCGGCCTGGGTCGCGAAATGCCGCGCGCCTGCAC
CGAACGCGGCGACGGCGTGGGCCATGTTCGGGCCGACGGCGTAGAGCGCGTCGACTTTACCGCCGGCGTAGGCGCC
GACTTCACGATGGGACTGTTCGGCCCAGTCGCCCAGTTCGGCGATATCGCCCAGCACCAGGACCTTGCGACCGTCA
AAGCCCTTGAGCAGGTCAATGGCAGCACAGACCGAGGACTGGTTGGCGTTGTACGTGTCATCAATCACGCGCATGC

CGTTGGTCGCCAACTGCGCCACGGTGCGGCCCTTGACCGGCTGCACGGCGCCAAGGCCGGTGGCGATGCCAAACAG
CGACACGCCCAGGGCGTAGGCGGCAGCCGCAGCGGCCAAGGCGTTGGCAACGTTATGGTTGCCCAGCAGGTTCAGT
TGCACAGGCTCGTCACCTTGCGGGCAGTGCAAGGTGAAAGCCGGGCAGCCACGGGCATCGAGCGTGATGTTCGAAG
CATGGAAATCAGCCGCAGCGTTGTGCACGGCGAAGCTCAGGACCTTGCGCCCGGCGGCACGTACACGCCAGGTTTC
GAAGGCTTTGTCATCGAGGTTCAGGACTGCAGTGCCGGAAGCATCCAGGCCCTCAAGGATTTCACCCTTGGCCTGG
ACGATCTTCTCAGGGCCACCAAACTCGCCAACATGGGCGGTACCCGCGTTGTTGATAATGGCGACGTGCGGCCGAG
TCATGGCCACGGTGTAGGCAATTTCGCCAATGCGCGACGCGCCCAACTCGATGACCGCCGCCGTGTGTTCCGGGGC
CAGTTCGAGCAGCGTCAGCGGTGCGCCAAAATCGTTGTTGAGGTTGCCACGGGTGGCAAGCACCGGCCCGCGCGTG
CGCAGGATGCCAGCGAGCAATTCCTTGACCGTGGTTTTGCCGCTGGAACCTGTGATCGCCGCAACCGGGTTGGTAA
ACGCTGCACGGTTAAGGGCTCCCAGTTGACCAAGGGCCAGGCGAGTGTCAGCCACCAGCAACTGCGGCAGTGTCGA
GTCAGCGACTTCACGCTGTACCAAGGCGCCGACGGCACCTTTGGCAGCTACTTCGTTCAGGTAGTCGTGCCCGTCG
AAACGCGGGCCGGCCAAGGCAACAAACAGTTGCCCCGGCTTGATGTTGCGACTGTCGATGCTGACGCCGTCAAAGC
TGCAATCGCTCGACAGGACGCGGGCCGCCAGGGCCTGGGTCAGCTCGCTGAATTTCATGGCCTTAAGCATGGGCAA
CCTCCCATGCAGTCAAGGCGTGATCGGCCTCGACCAGATCGGAAAAAGCGTGGCGCTCGCCGTTGATTTCCTGGTA
GTCCTCATGGCCTTTACCGGCCAGGACCACCACGTCGTCTGCGCTGGCGCTGGCGATCAATTGGGCAATGGCAGCA
CCGCGACCGGCAACAAAGGTCACTTGGGAGGCGTCTTTGAAGCCTGCACGAATATCATCGAAAATCTGGCTCGGGG
CTTCGCTGCGCGGGTTGTCGTCGGTGACCAGCACGCCATCGGCCAGGCGTTCGACGATCTCGGCCATCAGCGGGCG
TTTGCCGCGGTCACGGTCGCCACCGCAGCCGAACAGGCACAGCAACTTGCCCTTGGCATGCGGGCGCAGGGCTTCG
AGCACTTTTTCCAGGGCATCGGGGGTGTGGGCGTAATCAACCACCACCAGCGGTTGCGTCCCGCCACCCAGGCGCT
GCATGCGCCCGGCCGGGCCTTCGAGCTTGGGCAACACGCGCAGGATTTCGTCCAGGGCGTAATCCAGGCCCAGCAA
GGCGCCGATGGCTGCAAGGACGTTGCTCAGGTTGAAGCGGCCCAGCAAGGTGCTGCGCAAGTGGTGCTCGCCCTGG
GGCGTGACCAGCGTTGCGAGCACGCCTTCATCATTGAACTGTGCTTCGCGGCAATACAGGTGGGCGCTGGAGTCTT
CCAGGCTGTAGCTGATCAGGCGTGCTTCGCTTTTTTCTGCGGCCAGCTGGCGACCGAATGCATCGTCCAGGTTCAC
CACCCGGCACTTGAGGTCATTCCAGCCGAACAGCTTGGCCTTGGCGGCAGCGTAGGCCTCCATGCTGCCGTGGTAA
TCCAGGTGGTCGCGGGACAGGTTGGTCATCACCGCCACGTCGAACGCCAGCGCGGTCACCCGGCCCTGATCCAGGC
CGTGGGACGAAACTTCCATGGCGACGGCCTTGGCCCCGGCCTTTTTCAGGTCGGCCAAGGTCGCCTGCACGGCAAT
CGGATTCGGCGTGGTATGCAGGCCGCTTTGCAGCGCGCCGTAGAAACCGGTGCCCAGGGTCCCGACGATGCCGCAG
TGCTGACCCAGCAGGTCCAGCGCCTGGGCCACCAGTTGGGTGACGCTGGTCTTGCCGTTGGTGCCAGTGACACCAA
TCAGGTTGAGCTGGCGGCTCGGGTCGCCATAAAAACGCCCGGCGATATCCGACAACTGCGCCGCCAGGCCCTTGAC
CGGGATCAGCGGCACATCGGTGATTGGCAGGACGGTGGCGCCTTCAACTTCATAGGCCACCGCTGCCGCACCGCGC
TGCAAGGCGTCGGCAATATGAGCACGGCCATCGAGTTTGCCACCCGGCACGGCCAGGAACAGGTCGCCGGCACGTA
CATTGCGGCTGTCCAGGCTCAGTTCGCGAATCAACAGATCGCGGCCAGCGTGGGCGAAAATCTTGTTCAGGCTAAG
AGACATCAGCCGCGCCCTCCATTGGCTTTTACCGCAGCGGCCGGTGGTCCGGCGTTGGCTTGTTGGGTCGGCGGCA
GGTTGTCCGGCGTGATGTTCATCAAGCGCAGGGTGCCGGACATGACTTTGCTGAATACCGGTGCCGAGACCAGGCC
ACCGAAGTAGCCGGCCTTGCTCGGTTCATCGATGACGACAACGATGGCGTAGCGCGGATCGCTCATCGGGCCGAAG
CCAGCAAACAGCGAACGGTAGGAGTTTTCGGCATAACCCTTGGTGCCCACCGACGTCTTGCGCGCGGTACCGGATT
TGCCGGCCACGTGATAGGCCGGGACCTGGGCACGGAACACACCACGCGGCGCCTCGATCACCTGTTGCAGCATGGT
CTGCATGGTCTTGGCGACGCTTTCCGGGATGACCTGTTCGGATTTCGGCGCTTCGTCCACGTGGATCAGGCTCAAC
GGCGTCATGCGGCCATTGTTGGCCAGCACGGAGAAGGCATGGGCCAATTGGATCGCGGTCACCGACAGGCCGTAGC
CGTAGGAGAGGGTCGCCGTCTCGGCCTTGCGCCATTCACGGTAGTTGGGCAGGTTGCCGACGCGCTCACCCGGGAA
GTCCAGGCCAGTGGGCTGGCCCAGGCCGATCTTCTGCGCCAGGTGGTAGATGGTCTCGCCGCCGATATCAAAGGCG
ACTTTACTCATGCCGACGTTACTGGAGTTGATCAGGATGCCGGTCAGGTCCAGCACCGGCCCTTCGCTGCGGGATA
CGTCGCGGATGGTGTACTTGCCCAGTTGCAGGGTGCCGGGATAAACCTCGACCTTGTCGGTCGGCTTCCAGCGCCC
GGATTCCAGGGCCGCACTCATGGAGATGGCTTTCATGGTCGAACCCGGTTCGAACACGTCGATCATCGCGCGGTTG
CGCATCATCGCCGGTTGCAGGTTGCGACGGTTGTTGGGGTTGTAGGTCGGCTGGTTGACCATCGCGAGGATCTCGC
CGGTCTTCACGTCCATGATCACCAGGCTGCCGGCCTTGGCACCGTTCTCGATGATGGCGTTGCGCAGCTCGCGGTT
GGCCAGGTATTGCAGGCGCAGGTCAATCGACAACGCCAAGGGCTTACCGGCCTTGGCGTTTTTGGTGACCTGGACA
TCCTTGATCAGTCTGCCGCGACGATCCTTGATGACCTGTCGTTTGCCGGGAACCCCGGCCAACCACTCGTCGTAGG
CAAGTTCCACCCCTTCGCGGCCATGATCATCAATGTCGGTGAAGCCAACCATATGGGCGGTGGTTTCACCGGCCGG
GTAGAAACGTCGGAATTCCTCGATGCCATAGACACCGGGGATTTTCAGGTCGAGCACACCTTGACCCTGTTCAGGG
GTGAGGCCGCGAACCAGATAGATGAATTCTTTATTGGCCTGGGCCTCAAGACGCTCCTTGAGGGCCTTGGGGTCCT
GGCCGAGGGCATGGGCCAGCTCGGTCCATTTGTCCTTGGCGACTTGCAGTTCCTTGGCGTTGGCCCACAGGGTGGT
AACCGGGGTACTGACAGCCAGGGGTTCACCGTTACGGTCGGTGATCAGGCCGCGGTGCGCAGGAATCGGGATATGC
CGCAGGCTACGGGCGTCGCCCTGGCCGATCAGGAAGTCGCGGTCTACCACTTGCAGGTCAATGATGCGCCAGGCAA
TCGCGCCCACCATCAATGCGAGCAAGCCCAGCACGACGCGGAAGCGCCACGGGTAGAGAGCGCCTTCGAGTTTCAT
CATGGCGCCACCATGCGGACTTCTGCTGCGCCAGGAATATGCATCTTCAGTTGCTCAGCGGCGAGCACTTCGATGC
GGCTGTGGGCCGTCCAGGTGCTCTGCTCCAGGATCAACCGACCCCATTCCGCCTGCGCCTTGTCACGCACGCTCAA

TTCGCCGTACAGGCTATTGAGCAACTGACGATTCCAGTGGGCGCTGTAGGACACCGCTATCGCGGACACCAGCACG
CCGACATACAGCAGCATCATAAAGAAGCTGCCGCCCGGAAGGGGCTTGGCGAAAAGCTTGCTCACCGCAGCTTCTC
CGCAACGCGCATGACGGCGCTGCGAGCACGTGGGTTGGCCTTGAGTTCGGCTTCGGAAGCGAACTGCGCTTTGCCA
TGGATTTTGATTTTCGGCACAAAGGCTTCGAAACGTACCGGCAGGTTGCGCGGCAGATTATCGGTTTCGCCCTTGA
CCAGACGACGCATGAACAGTTTGACGATGCGGTCTTCCAGGGAGTGGAAGCTGATCACTACGAGACGACCGCCCAC
TTCCAGGGACTCCAGGGCAGCCTCGAGGCCAGCCTCCAGGTCACCCAGTTCGTTGTTGACGTGAATGCGCAGGCCC
TGGAACGCGCGAGTTGCCGGGTTCTTGCCCTTTTCCCACGCCGGGTTGGCGACTTTCAGTACTTCAGCCAGGTCGG
CGGTGCGTTCGAACGGCTGGATTTCCCGGCGCTCGACCACGGCACGGGCCATGCGGCCGGCGAAACGTTCTTCGCC
ATATTCCTTGAATACTCGGGTGATTTCTTCGTGGGGTGCGGTGGCGATGAACTGCGCGGCACTGATGCCACGGCTC
GGGTCCATGCGCATGTCCAGCGGGCCGTCGTTCATGAAGCTGAAGCCGCGTTCAGGGTCGTCCAGCTGCGGCGAGG
ACACGCCCAGGTCCAGCAAAACCCCGGCCACCTTGCCCGCCATGCCGCGTTCGGCGACTTCTGCGCCCAGCTCGGC
AAAGCTGCGCTGCACAACGACAAAGCGGCCGTCTTCGGCCGCTAGCGCTTGCCCGGTGGCAATCGCCTGGGGATCC
TTGTCGAACCCGAGGAGTTTGCCCTCCGGACCGAGCTGGCTGAGGATCAACCGGCTGTGCCCCCCGCGCCCAAACG
TGCCATCCAGATAGCAGCCATCAGGGCGCACGGCGAGAGCCTCGACGGCTTCGTCAAGCAGCACGGTGATGTGGTT
AAAGCCGCTATCAATAGTCACAGGATCAAATCACGCAGTTCATCAGGCATGGCGCCCGGTTGTTGAATAGCAGCCA
GGTCAGCTGCAGAAACAGCATCCCAGGCATCTTCGTCCCACAATTGGAACTTGTTCAGTTGGCCTACCAGCATCGC
GCGCTTATCCAGCTTGGCGTATTCGCGCAAACGCGGCGGAACCAGGAAACGACCACTGCCATCGAGTTCGAGGTCG
ACGGCATTACCAATCAGCAAACGTTGCAGACGGCGGTTTTCTTCACGTAACGAAGGCAGAGCGCGCAACTTGGTTT
CAATCAACTCCCACTCATCGAGGGGGTAAACACACAAACATGGATCAACGGCATCAATCGTGATGATTAACTGGCC
TGAACTTCGCGAAATGAGCTCGTCACGATACCGGCTCGGCATGGCGAGACGGCCTTTTGCATCGAGACTTATAGCG
TTAGCTCCGCGAAACACAGATGCGTTTCTCCAAATTTTAGCGTTTTACGTTCAAAAAACCCACTTTGTGCCACTTT
CCGCCACTTGCGCACACTATAGGAATGCGCCCACCACACCGTCAAGGCGCGGATTAAAGGAAAAGCCTTACAGAAC
GGAGATTTAGGAGCGCAAAAGGAGGAGAAACGAGAGTTCGGTGATGTTTTTGACTCAACAACCCCAAATAGCTCAA
AGAGCTAGAGCTTGAACTTAAAGTAATTTATTAAGAGTAAGATTTTTTCGGTATTACGAAGACGCATCTGCCAATG
ATTTGGCAGGGAGGGATTCTTGCGTTACTACCTGGTTTCTGCCCGAGACTCGCGCAGAAGGAAAAAGGTGGAGAGT
CGATCTATAAGCCGGGTTCTGTCTTGAACAGTCATTCGTCTACGATGGCCATCACTGGACATCTTTAGCAACCTAC
CCGGTTCCAGCGCGGGCCACGCCTTGGAACCCTATTTGGTCTTGCTCCAAGTGGGGTTTACCTAGCCACGAACTGT
TGCCAGTCGTGCGGTGCGCTCTTACCGCACCTTTTCACCCTTACCGGCACCGAAGTGCTTAGGCGGTTATTTTCTG
TGGCACTTTCCGTAGGCTCACGCCCCCCAGGCATTACCTGGCACTTCGCCCTATGGAGCCCGGACTTTCCTCCCCC
CCCTAATTTTCATAGAGGGCAGCGACTGTCCAATCGACTCTCCGCCGCGAAGGTTAACGGCACGGCGGCCTTAGAA
CAAGTATTAAAAGACAGCATTGCCATCATGTTCCGACGTCGTGCACGTTAAACCCAGGAACTATTCGCTCTTTTGC
TTATCAAGCGCGGCCTGATACAACACGTTCTTGCGCACTCCGGTTATTTCCGCCGCCAGGGCCGCGGCACGCTTGA
GGGGCATCTCCTTGAGCAACAGGTCCAGCACGCGCATGGCTTCGCTACCCACCGCGTCTTCGCTTTGCGGTGCAGT
CCAACCCGCAACCAGTACCACGCACTCGCCACGCTGCTGATTGCTGTCACCCTCGACAAAGGCGCGCAACGCTTCC
AGAGGCAGGCCCTTGAGGGTTTCAAAGGTCTTGGTCAGTTCCCGCGCCAGCAGCGCCAGGCGCTCGCCACCGAAGA
CCAGTTCCATGTCTTGCAGGCACTCAAGGATACGGTGCGGTGCTTCGTAGAAGATCAGGGTGCGCGGCTCTTCCTT
CAATAACTCCAGGCGCGCCCGCCGCCCCACGGCCTTGGCCGGCAGGAAACCTTCGAAGATAAAGCGGTCGGACGGC
AGGCCCGCCGCCGACAACGCGGCAATCAACGCGCACGCACCGGGAACCGGGACTACATTGATACCAGCGGCACGTG
CCTGGCGCACCAGGTGGTAGCCAGGGTCGGAGATCAGCGGCGTACCGGCGTCGGAAATCAACGCCACGTCGTCGCC
CGCAAGCAGGCGGGTAATAAAGCGGCTGCCTTCGTCGCGCTCGTTATGTTCATGGCAGGCGGCCAATGGCGTGGCA
ATACCAAAATGCTGCATCAAGCGCAGGGAGTGCCGGGTGTCTTCGGCAGCAATCAATTTGACGTCACGCAACACTT
TCAGCGCCCGTGCACTGATGTCGTCCAGGTTGCCAATGGGCGTCGCCACCACAAAAAGCGAGCCAGCAGTGGAATT
CAAAGGACCTGGAGCAGTCAAAGCGCACACCTCGAAGATTGGCAAAAGCCATATTGTAGCGCGTAGACGTCAAGAA
AATGCGCCGACGAGGTTGCGTCCAGAAGGTCTGTTTGCGGCGGTTACCGACGCCCTGCAACATTTAAACGATCTAA
ATAGACGGTTTCACGCCACTAACATCGCGCCCCGGCCAGTGCTTGGGTACAATTCCACGCTAATTTGATCGGTATC
AGGAACACTTACATGATCGCTTGCCTGCGGCTGTTCTCTGCCCTTTGCCTGGCTGCCCTGTTGGCGGCTTGCGCAA
GCTCGCCCTCGTCAAGCCTTGGCGAACTCCCACGGACCCCGGATGCCACCATCGAGCAACTGCTCGAACAGGCGAC
TTCGGCCAAGACCCCGGAAAAAGCTGCACTGCTGCGCCTGAGCGCGGCAGACATGGCCTTTCACCAGAACAACCCC
GGCCGCTCTGTGCAGATCCTTGCGCAAGTGCCACTGGACAGCCTCAAGCCGGCCGCCCAGGTCTTTGCCAGCACCC
TCACGGCAGAACTGGCCATGACCCGCAACCAGCCGAAGGCTGCGTTGACGGCGCTGGGCCATCCAAGCCTGCAAAA
CCTCAAGGAATTGCCGCCGGAGCAACAGATCCGCACTGGCACCGTGCATGCTCGTGCTTATGAAGCCGACGGCCAG
ACCCTGGCCGCCGCCCGTGAACGCGTAGCCATGGCGCCGCTACTCACCGGCGATGCCGCCGCCAGCAACCACGACG
CCATCTGGAGCCTGATTGCCGCCGTGCCTGGCGAGCAGTTGCAGGCCAGTGGCAACCCGACTCTCGATGGCTGGAT
CACCCTGGCGCAGGCCGTGAAAAACGCCGGCACCCTGGAGCAGCAGCAAGCCGCCATCGACACCTGGCGCGCGCAG
AACCCTGCGCACCCGGCTGCGGTGCAACTACCAACGCCGCTGACCAAGCTCAAGGAGCTGGCCAGCCAGCCGCTGA
ATAAAATCGCCCTGCTGCTACCACAGCAAGGTCCGCTGGCCAGCGTGGCCAAGGCCCTGCGCGAAGGTTTCATGGC
CGCCCATTACCAGGCGCAGCAGGCCGGGCAGAAGCCGCCGGTCATCGAGGTCTATGACAGCTCACGCGTCAGCTCC

ATCGATGAGTTCTACAAGCAGGCCCAGGCTGACGGCGTGCAATTGGTTGTCGGCCCACTGGAAAAACCGCTGGTCA
AGCAACTCAGCGCCCGCCCGCAATTGCCTATCACCACCCTGGCCCTGAACTACAGCGAAGGCGACCAAGGCTCGGC
CCAACTGTTCCAGTTCGGCCTCGCGGCCGAGGACGAAGCCCGTGAAGTGTCGCGCCGCGCCCGCGCTGACGGCCTG
CACCGCGCCGCTGCCATGGTGCCCAAAGGCGAATGGGGCGAGCGCGTGTTCAAGGCCTTCCGCCAGGACTGGGAAG
CCAACGGCGGCACCCTGATCGGCGTCGAATATGTCGACCAGCCGGTGGCCCTGGCCCAGCAAATCGCCGACCTGTT
CAAGCTGCGCCAGAGCGAAGGTCGCGCCAAGAGCCTGCAAAGCACGGTTGGCGCTGACGTCGCGGCACAGCCATCG
CGTCGCCAGGATATCGAGTTCATCTTCCTCGCTGTGACACCGCAACAGGCCCAGCAGATCAAGCCGACGCTGAACT
TCCAGTACGCGGGTGATGTGCCGGTGTACGCCACGTCCCACGTGTTCAGCGCCAGCGGCGACAAGAACCAGTACAA
CGACATGAACGGCATTATGTTCTGCGAGACGCCGTGGCTGCTCAACACCACCGACCCGCTGCGCAACCAGGTCGCG
ACACAATGGCCACAAGCCAATGGCAGCCTGGGCCGCCTGTACGCGATGGGTGTTGATGCCTACCGCCTGGCACCGC
GCCTGGGTCAGCTCAAGGCGCTGCCTGATACCCGTATCGAGGGTCTTTCCGGCAACCTGGGCATGAGCCCGAGCCA
GCGGATCGAACGCCAGATGCCGTGGGCCAAGTTTGTCAGTGGCCAGATCGAACGCCTGCCCGATACCCCGCGCTGA
TGCCCCCACGCTCCAGCGCACAAAGCGGCAAGGACGCCGAACAACAAGCACTTGAACACCTGCAGCAACAGGGTCT
GCGCCTACTGGCGCAGAACTGGTTGTGTAAACGCGGCGAGCTTGATCTGGTCATGCTTGACGGCGATACAGTAGTA
TTTGTCGAAGTCCGCTACAGAAAATACGCACAATGGGGTGGCGCACTCGCCAGTATCGACGAGCGCAAACGCCAAA
AGCTGATACTCGCCGCGCAGTTTTTCCTGCTCAGTGAACGTCGCTGGGCTGACTCACCCTGCCGTTTCGATGTGGT
CGCCATAGAAAGCACCGCCCACGAAACCGCTGATCTGAACTGGCTGCGCAACGCCTTCGATGGTTGAGCGCCACCG
CTACGGATCGGACATCTTCACCGCACACTTTTGCTCTTTGCTTTGCGGGCTGCACATTCGTGTGCCATACAGCCGC
GCTACTTAAGGTCACACAGATGGACATGCAATCCCGAATTCGCCAGCTTTTCCAGGCCAGTATCGATACCAAGCAA
CAGGCGATGGACGTACTTGCACCGCACATCGAGCAAGCCAGCCAGGTCATGGTCAACGCCCTGCTCAACGAAGGCA
AAATGCTTTCGTGCGGCAACGGTGGTTCGGCCGGCGACGCCCAGCATTTCTCTTCGGAGCTGCTCAACCGCTTTGA
GCGCGAGCGCCCGAGCTTGCCGGCGATCGCACTGACCACCGACTCGTCGACGATCACCTCGATCGCCAACGACTAC
AGCTACAACGAAATTTTTTCCAAACAAATCCGTGCCCTCGGCCAACCGGGCGATGTGCTGCTGGCTATTTCAACCA
GCGGCAACTCGGCGAACATTATTCAAGCGATCCAGGCCGCACATGATCGCGAAATGATTGTCGTAGCATTGACCGG
GCGCGACGGCGGCGGCATGGCCTCGCTGCTATTGCCTGAAGACGTGGAAATCCGCGTCCCGGCCAATGTCACTGCA
CGTATTCAGGAAGTCCATCTGCTGACGATCCACTGCCTGTGCGATCTGATCGACAGCCAACTGTTCGGGAGTGAAG
AATGACCGTTAACCGCCTCAGCCTTATGGCCATCACGCTGTGCCTCGCTATCAGCGGCTGCACCTCGGCCATTACT
GCCACCCGCGACACACCGATCCAGGACGACCGGGGCACACGCACCTTTGGCAGCAAGATCGATGACTCGCTGATCG
AAACCAAGGTCGGCGTCAACGTGGCCAAGGCCGCGCCCGACCTGGGCAACGGTGCATCGCGCATTGTCGTGACCAG
CTTCAACGGCATCGTGCTGCTCGCCGGGCAAACCCCGCGCGCCGACCTCAAGACCCAGGCTGAACAAGCCGCCTCG
ACCGTACAACGGGTCAAGAAGGTCCACAACGAATTGCAGGTCGCACAACCCATCACCCTGCTGGCTATCAGCAACG
ATGCGGTGCTGACCACCAAGATCAAGGCGCAGATGCTGACCGACAGCGCGATTCCCGGCTCTCGGATCAAGGTCGT
GACCGACAACGGCATCGTCTACCTGATGGGCCTGTTGACCCAGCAGGAGGCCACCCGCGCTACCAACCTGGTACAG
GGTGTGTCCGGAGTACAGAAAATCGTGAAGCTGTTCGAATACATCGACTGATGTAATCACCAGCCACAAAAAAGGG
CGCCGTGCATTGACTGCACGGCGCCCTTTTTATTGGCTGGTGGTTACTGGTACTGCTGGTATGGATTGCCCTGGAA
CTGGTTGTTCTGCTGCTGTTGCTGAGCAGGCTGGCCATACTGCTGGCCGGGAATCGGACCGAGGTTGACCTCCACG
CGACGGTTCTGCGCGCGACCATTGACGTCGGCGTTGCTGGCAATCGGGTTATCCGGGCCGGCACCACGGGTGGTCA
GGTTGGTCGGGCTGACACCTTGGGAGGTCAGGTAATTGGCCACGCTTTGCGCACGACGCTGGGACAGGTCCATATT
GAGCTGGCGGCTGCCGGTGCTGTCGGTATAACCGACGATCTGGATGGTGTTCTGATTGAACTGCTTGAGCGAACCG
GCCAGGTTATTCAGTGGGGTATAGAAGCTGCTGGCGATAGCATCGGAGTTGGTGGCGAACGTGATGTTGCCCGGCA
TGATCAACTTGATCTGATCACCCTGGCGCTGCACCTCGACGCCGGTGTTGGCCATGCTTTCGCGCAGTTTCTTTTC
CTGCTGGTCAGCGTAATAGCCATAACCCGCAGCACCGATACCCGCCACTGCTGCGCCGATCAGCGCGCCCTTGCCA
CGGTTGTTATGGTCAATGGCCGCACCGGCCAGCGCACCCGCCAGCGCGCCGAGGCCGCCGTACTTGGCGGTTTTGC
TCATGCCTTGTGAACCGTTGTCCGCCTGTCCCTGGCTGCCATCGTAAGGATTAGGGGAAGCACAGCCCGACAATAG
GGCTACCGCGGTAGCAACAACAAGCAGACGACGCGAAGTGAACATGAAGGAAGCTCCTACTTTTGCATTCTGTGAT
GCAGAGGACATTGGCAATGGACCTGTGCCGAGTTTGGAACGCGACAAACGGCAAAAATTCCATGACCGGCCTCTGT
CCTGTAACAAAAGATGGAGGCCGGTGCAGCTACCTAAACCCTAGCAGAGCCTCGCGATTTCACGCCCTGACGAACG
GATTCTCACGCATTTCATCCCCCAGGCGTGTATCCGGGCCATGCCCCGTCACCACGGTCGCGTCTTCGTCCAGGGT
ATACAGGCGCTGCTGGATCGAACGCACGATGGTTGCCTGGTCGCCACCCCACAAATCCGTACGCCCTACCCCGCGC
CGGAACAGGGTGTCCCCGGCGATCAGCAGCTTGGCATCGGCAAACCAGAAACTCATGGAACCCGGCGTATGCCCAG
GCGTGTGCAAAGCCACGCCACAGCCACAGGCCAACTCCTCATCATCGGCCAGCCAACGATCCGGCGACGGCACCGG
GGTGTAGGGCACACCGAACATCTGGCACTGCATTTCCAGGTTATCCCACAGGAACTGATCTTCCTTGTGCAGGTGC
AGCGTGGCGCCGGTCTTTTCTTTCAATTGGCCGGAAGCCAGGAAGTGATCCAGGTGCGCATGGGTATGAATGATGC
TCACCACCTTCAAGCCCAGCGCATCCAGGCGTGCCAGGATCAGCTCATGGTTGCCGCCCGGATCGACCACGATCGC
CTTCTTACTGATCGGGTCGCCGATGATCGTGCAGTTGCACTGCAACGGGCCGACGGGAAAGGTTTCGCGGATCAGT
CGGGGAGTTGTCGCGTGCATAAGGAATCCCTTCTAGCTGTTGGCCCGTGCGGCGTGCAGTTTCTTGTAGCTGTCGA
TCAACCGCAGGTGTCGGTCCAGGCCTTCGAGCTTCATGCTGGTCGGCGTCAAGCCATGGAACCGCACGCTGCCCTG

CACCGAACCGATGGCCGCATCCATGCGCTCGTTGCCAAACATCCGGCGGAAGTTGGCCTCGTAGTCTTCCAACTCC
AGGTCTTCGTCCAGTTGCATCTCCAGCACCGCATTCACCGCCTGGTAGAACAGGCCGCGCTCGACGGTGTTGTCGT
TGTATTGCAGGAACATCTCTACCGCCTCTTTCGCCTCTTCATACTGCTGCAGGGCGAGATAGATCAACAGCTTCAA
CTCCAGGATGGTCAACTGGCCCCATGCGGTGTTGTCGTCGAACTCGATACCGATCAGCGTGGTGATATCGGTGTAG
TCATCCAGCTCGCTTTCCACCAGGCGCTCGACCAGCGCTTGCAGGCCTTCCTCATCCAGGCTGTGCAAGTTCAGGA
TATCGGCGCGGAAGAACAACGCCTTGTTGGTGTTATCCCAGATCAGGTCGTCAACCGGATAGATTTCCGAATAGTC
CGGCACCAGGATGCGGCAGGCCTTGGCGCCGATATGCTCGTAGACCGCCATATAGACCTGCTTGCCCATGCCTTCG
AGAATGCCGAACAACGTGGCGGCCTCTTCGGCGTTGGAGTCTTCGCCCTGGCCGGAGAAGTCCCATTCCACGAATT
CGTAATCGGACTTGGCACTGAAGAAGCGCCACGACACCACGCCACTGGAGTCGATGAAGTGTTCGACAAAGTTGTT
CGGCTCGGTGACCGCATGACCTTCGAAGGTCGGCTGCGGCAAGTCGTTCAGGCCTTCGAAACTGCGGCCCTGGAGC
AGCTCGGTCAAGCTGCGCTCCAGCGCCACTTCCAGGCTTGGGTGGGCACCGAACGAGGCAAACACCCCACCGGTAC
GCGGGTTCATCAGGGTCACGCACATCACCGGGAATTCGCCGCCCAGGGAGGCGTCCTTGACCAGCACCGGGAACCC
CTGTTCTTCCAGGGCCTGGATACCGGCGAGGATGCCCGGGTATTTGGCCAGCACGTGGGCCGGGACATCCGGCAGG
GCGAATTCGCCTTCGATGATTTCGCGTTTCACCGCGCGCTCGAAGATCTCCGACAGGCACTGCACCTGGGCTTCGG
CCAGGGTGTTGCCGGCACTCATGCCGTTGCTCAGGTACAGGTTTTCGATCAGGTTGGAGGGGAAATACACCACCTC
GCCGTCCGACTGACGCACGAACGGCAGCGAGACGATGCCACGCTCCTCGTTGCCGGAGTTGGTGTCGATCAGGTGC
GAACCACGCAATTCGCCATCGCGGTTGTAGATCTTCAGGCAGTACTCGTCGAGGATCTCAGGCGGCAGCGCATCTT
TGCGGCCCGGCTTGAACCAGCGCTCGTCCGGGTAATGCACAAACGCCGCGTTGGCGATGTCTTCACCCCAGAACTG
GTCGTTGTAGAAGAAGTTGCAGTTCAGGCGTTCGATAAACTCGCCCAGGGCCGATGCCAGTGCGCCTTCCTTGGTC
GCGCCCTTGCCGTTGGTAAAGCACATCGGCGAATGGGCATCGCGGATATGCAGCGACCAGACATTGGGCACGATAT
TGCGCCACGAGGCGATTTCAATCTTCATGCCCAGGCCCGCCAGAATCGCCGACATGTTGGCGATGGTCTGCTCCAG
CGGCAGGTCCTTGCCTGCGATATAGGTGCCCGCGTCTGAAGCAGAAGCCGGCATCAACAATGCCTGGGCGTCGGCG
TCGAGGTTTTCCACCTCTTCGATGACAAACTCAGGCCCGGCCTGGACCACCTTCTTGACCGTGCAGCGATCAATGG
AGCGCAGGATGCCTTGGCGATCCTTGTCGGAAATATCCGCGGGCAACTCGACCTGAATCTTGAAGATCTGGTTGTA
GCGGTTTTCCGGGTCGACAATGTTGTTCTGCGACAGGCGGATGTTGTCCGTGGGGATATTGCGCGTGCTGCAGTAC
AACTTCACAAAGTAAGCCGCGCACAACGCCGACGAAGCCAGGAAGTAGTCGAACGGACCGGGGGCCGAGCCATCGC
CCTTGTAGCGAATCGGCTGGTCGGCCACCACGGTGAAGTCATCGAACTTGGCTTCAAGCCGGAGGTTGTCGAGAAA
ATTGACCTTGATTTCCATGCGGGTTTACCAGAATAGCGGAGCAAACGAATTGGCCGCCATTATCCGGCTTTTGCAG
CGCAAGTCTCCCCCTTACCCCAACAACCCCATCTCCTGTGCCCGCGCGACCGCCTGAGTGCGGCGCTCGACCCCCA
GTTTGCTGTTGATATGACTGGCGTGGGTCTTGACGGTGTGCAGCGAGATAAACAGCTGGTTGCTGATTTCCTGGTT
GGAGCACCCTTGGGCGATCAACTGCAGCACCGCCAACTCACGGGCGCTCAAGGTTTCGCTGGAGTGGCTGGTTTCG
ACCGGCTCACGCGGGGCAACCGCCGGCAGCAGGGCCCACAGGCTTTGCCGTAGCAAGTCACGCGGCGCCTGGCCCA
GTTGCTCGCGCATCCACTCGGGATAACGCTCCAACAGGCGCTGGAAGGGTTGCAACGCGCCGCCGCTCGCCATTTG
CAGGGCCCGGGCAAAAACCGGCCGGGCTGACGCTGCCTGGCCATCATCCAGCAACAACAGGGTGTGCTGGTTCAGA
GCCATCACCGCGATCATCTGCTGGCCCTTGCCTTCAGCCTGCCGGGCGAGCGCATCGAGCCGACGCAGGGCGCTTT
CGGAGTGACCACGAATGGCCTCCAGGGCCGCCTGTTGCAATTCGATATGTTGCGGCAGATGCGGATGAAACTCCGG
GGCCGCCGCTGCCTGGTCGCCGTTATAGGTCTGCCCCAGGCGTACCAGCCAGGCATCGGCCAGGTCAGTACGGCCC
TGGGCCAGCCACAACTCGCATTTGATCAGGGTAATCATGGCCAGGTAGTAAATCGGCGGCACATCCCAGATATGCA
TCAGCCGCTCGGCTTCGGCCAGTTCGGCAAAGGCCTTGGGGAAGTCGCCACGGCGCCCCTCAAGGTTGGCGATGAC
ACAGTGGCCGATCAGCACGCTGATATCCCGGCAGGCGCGGGCCTCGGCAAGCCCCGCGCGCAGGCGCAACAGGCCG
GTTTCGGGCTGGCAACGCATCAACAACAGGAACCCTTCGTACAGCGCAAGTCGCGCGCGCACCGCATACAGGCGCT
GGGACGCCAGGCCCTGCAAACGTTGCAGGCCGTCACGCACTTCCTCCAGGGAACGCAGGATTTCCCCACGCGCCTG
CAGCACCCGCGCCCGATCGTAATGGGCCAGGGCCTCGAACAGCGGGTTGCCGACGCGTTGCGCCAGCTCCAGGGAT
TCGCGATTCAAGCCCCGCGCGCGCCACAGGTCGGCATCGACGATGGCCAGGTTCGACAAGGTCGACAGGCACATCA
GGCGCTGGCCGTAACGCTTGTGCGGCAGGCTTTCCAGGGCTTCGCTGCAATACAACTGGGTCAACTCGCGATTGCC
GCGCCCCCGGGCAATGATGCCGCTGAGGGCCAGCCATTGGGCAAGCATCGACTTCTGCGCCGTGGCGGACGGTGCC
GGCAAGAACCGGCTGAGATGGCTGGACAATTCCTCTGCGGCGTCCAACTGACAGGCCAGCCCCAGTGCCCAGCTGT
AGAGCACGATCAGGCGTGGCGTGCTGATCAACAGGCTGTCGGGCAAGTCCATCTTCCAGCGCAGGAGCATGCCGAC
GTTCTGTTCGGCCAGCAGTTGCTCTTCGGAGAGGTTCTGCACCAGGTTCGCCGCTACATCCAGATGCCCCGCGCGC
AGCGCCTGTTCGACCGCCTCATCGAGCAAGCCCTGGGCATTGAACCAGCGACAAGCGCGCAAGTGCAGGCTGGCGG
CCGGCAGCATCGCGTTCGGTGAGCGCCGGGTGCGCAGCAAATCGGAAAACAGGTGGTGATAGCGATACCAGTGGCC
CTGCTCGTCCAGTGGCACCAGGAACACTTGGTGGGCTTGCAGGTAACGCAGGATCTCCGCACTGTCATGGGCCTCG
CGCACGGCGTCGCACAGCTCGCTGCAAAAACGCTCCTGGGAAGCGGTGTCATAGAGGAAGGCCTGGACCTCCGGCG
GCAGGCAGTCGATCACCTCTTCCAGCAGATAGTCACGGATCAGCCCTTCCCCACCATGCAGGCCTTGGGGCAGGTC
GGCCTCGCTGCCGGCTTCGGAGGCCGCCAGCAACCAAAAACGCAGGCCGGCAACCCAGCCTTCGCTACGTCGGATC
AGGTTGTCCAATGCCTCACCGCGCAACGAGCTGCTATGGCGGTCGAGCAAGGCCAGGGATTCATCGTTGGTCAGGC
GCAGGTCCTGCTCGTGCAACTCCAGCAAATGCCGGGACAAACGCAACCGCGCCAGATGCCAATCGGACGCTGGCG

GCTGGTGACCAGCACCAACAAGCCATCGGGCAAATGGTTGAGAAAAAACTGCAGGCAGCGATCCAGCACCGGGCCC
TGGGCCAGGTGATAGTCATCCAGCACCAGCAACAAGGGCGCGCGGGTGGAAAGGCAGACGGTCAGTTCATCGAGCA
GGCCATCCAGCCACTCTTCAAAGGCAAACGGTTGATGGCGCTGGCGCATTTTCAACAGGCCCAGGGCCTGGTTGCC
TACCTGCGGGAAATACTGTTGCAGACCTTCCAGCAGACGCTCAAGGAAACGTCCCGGATCACTGTCCCGCGAACTT
AGACCCAGCCACAGGCTTTGCCAGTGATCGGGCAGGCTTTGGCAAAACTCCACCGCCAGCGAGCTCTTGCCAAACC
CGGCTGGCGCGCTGACTAGCAGCAAACGGCCATCCAGGCCCGCGCTCAAACGCTCGCACAGGCGCGGGCGCAGCAC
ATAGCCATCAGGCAGCGGGGGTCGATAGAAGCGCCCTTCCAGCGTGGGGATTACCGCACTGGCAGGCCCTTGGATT
CGGGACAGATCAGTCATCGCCGGGCTCTTGTAGTAGGCTGTGGTCGGCATTGCAGATGTCGGCAGACTAGCGTTAA
AAGCGGCAGCTTTGTAGATCTTTGCAACATTTGTATGAAAAAGACTACAACAAGAAATAACCAGCGATGTTCAGGC
CCCCCAAACAAATGTAGGAGCTGGCTTGCCTGCGATTGCATCGTCTCGGTATGTCAGATAGACCGAGGTGATGCCA
TCGCTGGCAAGCCAGCTCCTACAGAGGCCAACGTGTTTACTGCACGTTAGCGAACACCCTCTTGGCGCAGGGCTGC
CGGAGTGAAGTCTTTGGTAGTGGCCGTAAAGCCGAAGTCATAGGCCTTCTTCTCTTCGTTCTTCATGCCCAGTGCC
AGGTAACGGCCGGACTGCAGGTCGTAGAGGGTTTCGAGGGCGTACCACGGCACTTGCTTGTCGTAGTAGTTCTCGC
CATGGGCTTCGGCCACGCGCCACAGTTGGCCACGACCGTCGTAGTGGTCGATCACGGCTGCTTGCCAGGTGTCTTC
GTCGATATAGAAGTCACGTTTGGCGTAGATATGACGCTGGCCTTCCTTCAGGGTCGCGACCACGTGCCAGACGCGG
CGCAGTTCATAACGTGCCAGGTCCTGGTTGATGTGGCCGGCCTTGATGATGTCGGCGTACTTGAGCTTCGGATCGT
CGAGCTTGTAGCTGTTGGAGGCGATGTAGATTTCCTTCTTGCCTTCCAGCTTCCAGTCGTAACGATCCGGCGCGCC
GTTGTACATGTCGAGGTTGTCGGAGGTACGCAGGCCATCGGCAGCGGTGCCCGGCCCGTCATAGGACACTTGCGGT
GCACGACGCACACGGCGCTGGCCGGCGTTGTAGACCCATGCCGCGCGTGGCTCCTTCACCTGGTCCAGGGTCTCGT
GTACCAGCAATACACCACCGGCCAGACGCGCCGGTGCAGTCACTTGCTGCTTGAAGTAGAACAGGATGTTGCCCGG
ATTGGCCGGATCGTAGTCCTTCATCTTGTCGCGGAACACGAACTGATCCTGGAAGTACACCAGGCTGTACGAGCCG
TTGGCCTGCGGCGTGGCCTGGGTCACCAGGCGGGTCACGCTGCCGCCACGATAACGGGTGATGTGGTTCCAGATCA
CCTCGACCCCGCTTTTAGGGATCGGGAACGGAATCGCGGTCTCGAAGTTTTCCAGGCCGTTACCGCCACCTACCAG
GTTGGTGCTGGTGGCGTTTTTCTTGATGGAGGCGAACACTTCGGCCGGCACAGTGGCGCCGCGATGGGTGGTGTAC
ACCGGCATCTTGAAGGTTTCGGGGTAGCGCTTGAACATCGCGTACTGCCCCGGCGCCAGCTTGTCTTTGTACTGCT
CGACGTTCTGCGCGGTGATGGTGAACTGCGGTTGCTCGCTGGCATAAGGGTTGGCCAGGAAGCCCTTGGCATCCAC
GGCGCCGGCGTTGGTTGGCAGGGCTTTCCAGGCGGGGATGGTGCCGGCAGCGTTGCCGGCCATTTCCGCGCCCATC
GGGGTCAGCGTGGTGCCCAGCTTGGCGGCTTCATCCGCCGAGACGGCCGCCATGACGCTGCTGGCCATGATCGACA
GTCCCAGCACACCCACGTGCAACAGACTTTTGGTTATTTTCATGTTCTGGTCTTCCTGAAATGCAGTGTGCTTAGA
AGTTCGCGCCGAAACTCAGGGCCACGAAATCGCGGTCAACCGAGGTGTTGTAGTCGCCACCAAAGAAGTTGGTGTA
GGCCAGGCTCGCGGTGTAGGTGTTCTGGTATTCCGCGTCCAGCCCCAGGCTCACCGCTTTGCGACCTTCTTCGAAG
TTGCCGCCAGGACCTGGCGAGTAACCGCTCACGTCATGGGACCAAGCCACACTAGGCTTGAGGTTCACACCAGCGA
ACACATCCGGGTATTCCCAGATGGCGCGGCCGCGATAGCCCCAGGATGTGGAGGTGGTGAAGCCGTCGTTGTTGCA
GTTTTTGGAAAGGTTACTTGCGTCTGTTCCCGGACCCGCGCCACCGATAGTGCCGGTGTTGAGGATTTGCGAGCAG
GTATTCGCGGCGCCCGTGTTAGGCAGCGCACCCGGGCCATAGACCGGGTCACGGCCATAACGAATCTTCGACGAGC
TCTCAAGCCCACCCACGTGCGTGACTCCCACTTCACCCACCAATGTCAGTCGACTGGCGCCCATCACCTGATCGAA
GAAGTGGGTCAGCGTGGTCTGGAACTGGGTAATTTCCTTGCGACGGTAGCCGTGCATGTCCTGCCCTGGAACACCA
TTGAGCAGCGAGGCATTATCGTAAATGCCCTTCAATGGTTTAACTGCGGAGTACAGGATATCGGTGGTGTTGAGCT
GTACCGGAGCATTCGGGCGATAGCTGACCTCACCGCTCCATGCGGTGCCGGTAGGCAGTGTGGTGGAGAAGCTCAA
GCCATACAGACGGATGTCTTCCGGGTACTCGATGAAGTACTTGGAGTTACCCGCAATAAACACCGGAGCCAATGAT
CCGAAGTTAGGTGCCATCGCATAAGCCGACGCAGGCGCAGCCGTGGCACTGAAGATCGGTGCCCGGCTGTGATAGT
TCATGAAATACGCGCCGAACTCGGTATCCAGCGGCTCGAAGTTATAGTGCAACGCCGCGCCGAATTGACCACTGTC
GCGCGCATCCCTGTCGGCACTGCGGCGAACCAGTGTGCCCTCGTTATTGATATCCATGCCCATGGCGTCGAATGCC
GCCAATGCACCACGGCCCGGGGCCGATGCAGCAATAGTCGAACGCTTGGCCAACAGGCGCAGGTTATCGCTGCAAC
CATCGGACACCACATCCGGCTGGGAGAAGAATGTCCCGCAGTTGTCGGTAACCGTCTGGTCCCATTCCAACTGGTA
GAACGCTTCTGCCGACAGGTTATCGGTCAAGGATTGCGACACGTAGAACATGTTGACCGGAATCAGGCCTTCCTTG
ATTTCGGCGCCCGGACGACGGAACGCAGACACGTCGATCGGGTTGACGGAGTTGATACCGCCGCCGATGAAAGTAC
TTTCACCCCAGCTCACGACTTGCTTACCGAACCGCACCGAACCCGGCGCATCGGCAATGGAGTAGTTGTGGTAGAT
GAACGCATCGAGGATCTGCCCGCCAGAAGACTTGGCGCCCTCTTTGCGGTTGTGATCGCTGATGTCCTTGAACTCG
CGGCTCTCATCCTTGAGTTCAAAGTCATACCAATACTTGCCACGCACAAACACACCGGTATCGCCGTACTTCAGCT
CCAGGTCATGGATACCCTTGAAAATCTTCGAGAACGTCTCCCCACGCTTGAAGTTCAAGTGACCGTCATCGGAGGT
CTGCGACAGGCCCTTGCCACCGTTGTTGACACCAATCAGGTCGCGATCGGGCTTGGCCGTGCTCCAGCTGGCCCCC
ACCGACAGGGATGAGTCGAAGCTGCCTTCGACTTCACCGATGTTGAAACTGACGCCGAATGCGGGCCCGGCGAGGG
[truncated: 1,187,119 more chars]
